# Identifying Potential Tumor Antigens and Antigens-Related Subtypes in Hepatocellular Carcinoma for mRNA Vaccine Development

**DOI:** 10.1155/2022/6851026

**Published:** 2022-08-29

**Authors:** Weiran Liao, Zhitian Shi, Haoren Tang, Tiangen Wu, Cheng Zhang, Yutao He, Renchao Zou, Lin Wang

**Affiliations:** Department of Hepatobiliary Surgery, The Second Affiliated Hospital of Kunming Medical University, Kunming 650101, Yunnan, China

## Abstract

**Background:**

The mRNA vaccine has become a promising platform for cancer therapy. Lots of studies have been focusing on discovering novel potent cancer-associated antigens to develop mRNA vaccines against cancers. Besides, immunotyping shows the immune status, and immune microenvironment of immunotyping is related with therapeutic reaction. However, potential antigens for mRNA vaccines and immunotyping of liver hepatocellular carcinoma (LIHC) remain far from being understood.

**Methods:**

In this study, we collected gene expression data and clinical information data from ICGC and TCGA databases. Using GEPIA2, we calculated differential expression genes and prognostic indices. We applied TIMER to calculate the correlation coefficient between immune infiltrating cells and each gene. Consensus cluster was used for immunotyping of LIHC.

**Results:**

We uncovered four most potential candidates including PES1, MCM3, PPM1G, and KPNA2, which were all related with antigen-presenting cell (APC) infiltration and poor survival in LIHC in two independent datasets. Furthermore, three immune-related subtypes (IS1-IS3) of LIHC were identified. All these results were validated in two independent datasets. Furthermore, we validated our results in *vitro*.

**Conclusions:**

The above candidates will be expected to be potential antigen genes for developing anti-LIHC mRNA vaccine, and furthermore, patients with IS2 and IS3 tumors are supposed to be appropriate for mRNA vaccine in LIHC.

## 1. Introduction

Globally, liver cancer is the fourth leading cause of cancer-related death, ranking sixth in incidence rate. According to the World Health Organization, more than one million patients will die of liver cancer in 2030, from 2000 to 2016, the mortality rate of liver cancer in the United States increased by 43% (the number of deaths per 100000 people increased from 7.2 to 10.3) [1]. In recent years, the treatment of advanced liver cancer has made rapid progress. New chemotherapy, targeted therapy, and immunotherapy were organically combined with traditional surgery, radiotherapy, and interventional therapy which have improved the therapeutic effect of liver cancer patients [2, 3]. However, the five years survival rate of liver cancer is only 18%. Liver cancer is the second leading cause of death in various cancer types, after pancreatic cancer [4].

Compared with other therapies, the cancer mRNA vaccine introduces the mRNA encoding the cancer-specific antigen, and uses the protein synthesis mechanism of host cells to produce antigen, thus triggering the immune-related response. Cancer mRNA vaccine has the advantages of high specificity, nontoxicity, lasting nontoxicity, and immunological memory [5, 6]. In fact, researchers are trying to dig out potential tumor antigens from various cancer types including pancreatic cancer and cholangiocarcinoma as the target of mRNA vaccine. At the same time, in order to explore the suitable population of mRNA vaccine, immune profiles and immune-related subtypes of the abovementioned cancers were performed [5–7]. Though the improvement of the treatment in liver cancer is urgent, there is a lack of specific tumor antigens and immune-related subtypes analysis in HCC.

To identify the HCC-specific tumor antigens and immune-related subtypes in HCC, we collected gene expression profiles and clinical information of LIHC samples from ICGC and TCGA databases. Then, we identified four most potential candidates associated with poor survival as well as antigen-presenting cell infiltration in LIHC, including PES1, MCM3, PPM1G, and KPNA2. Then, according to the clustering analysis of immune-related genes, we divided the patients into three subtypes. Each subtype has diverse clinical, molecular, and cellular characteristics, respectively. We performed the immune profiles of LIHC by analyzing the distribution of the relevant gene signatures for LIHC patients. Our findings will provide new knowledge to develop mRNA vaccines as well as select appropriate therapeutic schedules for patients of LIHC.

## 2. Methods

### 2.1. Patient Dataset Collection

The gene expression, clinical data, and mutation of LIHC were downloaded from ICGC (https://dcc.icgc.org/) (Table S1). A total of 294 LIHC samples with the information of gene expression data, copy number variation (CNV) data, and mutation data, from ICGC database were used in this study. Concurrently, a total of 424 LIHC samples with the information of gene expression data, CNV data, and mutation data, from TCGA database were used as validation (Table S2). The cBioPortal database can provide the interactive exploration of multidimensional cancer genomics data sets [8, 9]. Thus, the gene expression (level 3), clinical data, and mutation data for validation were downloaded from TCGA (https://tcga-data.nci.nih.gov/) and cBioPortal Database (https://www.cbioportal.org/) (Table S3) [10].

### 2.2. Selection of DEGs and Survival Analysis

The DEGs (differently expressed genes) between cancer and normal tissues were calculated using Gene Expression Profiling Interactive Analysis version 2 (GEPIA2, http://gepia2.cancer-pku.cn) using false discovery rate (FDR) cutoff of 0.05 [11]. GEPIA2 is an open-access online tool collecting tumor and normal samples from TCGA and GTEx databases, which can be applied to perform the differential expression analysis in LIHC and calculate the prognostic index of each selected antigen. GEPIA2 web server was also used to evaluate gene expression from the gene level to transcript level, and supports analysis of a specific cancer subtype, and comparison among these subtypes [12]. Besides, overall survival (OS) and relapse-free survival (RFS) were compared by the log rank test. We considered *Q* values < 0.05 as statistically significant.

### 2.3. Immune-Related Subtypes Analysis

TIMER is an open-access resource for comprehensively investigating molecular characterization of tumor-immune interactions [5, 6]. As described by Xing et al. in previous research [13], we used Tumor Immune Estimation Resource (TIMER, https://cistrome.shinyapps.io/timer/) to perform the relationship between immune infiltrates and expression level of potent antigens we identified. In this study, *P* values < 0.05 were deemed as statistically significant.

We found 76 intersection genes of differently expressed genes and survival-related genes. Using the Database for Annotation, Visualization, and Integrated Discovery (DAVID) v6.8 (https://david.ncifcrf.gov/) and Metascape (https://metascape.org/gp/index.html#/main/step1) analysis, we found that these genes were immune-related genes. The DAVID and Metascape program were used to functionally annotate these genes by Gene Ontology (GO) analysis [14].

### 2.4. Discovery and Validation of the Subtypes

Due to low t-expressed genes can not to be used to develop potential mRNA vaccines, TCGA tumor samples lacking clinical information, and genes with 0 Transcript Per Million (TPM) in >50% of the samples were excluded. A total of 76 immune cell-related genes with log-transform data were used for the subsequent analysis. Using the “Consensus Cluster Plus” package of R software, consensus clustering analysis of immune-related genes was conducted to determine the number of clusters in LIHC from the ICGC database. The parameters were: “pItem” = 0.8, “reps” = 100, “pFeature” = 1, “maxK” = 10, and “distance” = “spearman.” We used “ward.D2” method as hierarchical clustering and distance function method as reported [11]. We then validated the immune-related subtypes in independent cohort (TCGA) with the same settings [15].

### 2.5. Immune Score Evaluation of Immune-Related Subtypes

The *t*-test was used to define the association between immune-related subtypes with immune-related cellular and immune-related molecular characteristics. We performed single-sample GSEA (ssGSEA) from ImmPort database, which provides publicly available clinical trial and immunology research datasets and bioinformatics tools for analyses to calculate the immune enrichment scores for every sample, which can calculate separate enrichment scores for each pairing of a sample and immune-related gene set [16]. Each ssGSEA enrichment score represented the degree to which the genes are coordinately up- or down-regulated within the sample.

### 2.6. Immunoblotting

HepG2, Huh7, SK-Hep-1, MHCC97H, and LX2 cell lines used in this study were obtained from the Conservation Genetics CAS Kunming Cell Bank (Kunming, Yunnan, China), which performs authentication on its cell lines. All human cell lines were cultured in Dulbecco's modified Eagles' medium with 10% fetal bovine serum. A total of 4 HCC patients, who underwent hepatectomy, were recruited at the Department of Hepatobiliary Surgery, the Second Affiliated Hospital of Kunming Medical University from January 2022. Those patients with HCC were diagnosed, according to the AASLD practices guidelines on the management of HCC (2005 version). Tumor specimens and corresponding adjacent liver tissues (2 cm from the tumors) from the 4 patients were used for immunohistochemistry. Written informed consent was obtained from individual patients. The experimental protocol was established, according to the ethical guidelines of the 1975 Declaration of Helsinki, and was approved by the Ethics Committee of Kunming Medical University. The cells and tissues were collected and lysed with the lysis buffer containing protease inhibitors and the protein concentration was determined using BCA method (Beyotime Institute of Biotechnology, Shanghai, China). 20 *μ*g whole-cell lysates of cells and tissues were separated using sodium dodecyl sulfate-polycrylamide gel electrophoresis. Then we tried to transfer these to polyvinylidene fluoride (PVDF) membranes. The blots were probed using PPM1G antibody (Proteintech, 15532-1-AP), a KPNA2 antibody (Proteintech, 10819-1-AP) and a beta Tubulin antibody (Proteintech, 10094-1-AP), as well as horseradish peroxidase-labeled goat antirabbit secondary antibody (Proteintech, SA00001-2). Afterward, the blots were illuminated with chemiluminescent detection reagents (Tanon).

## 3. Results

### 3.1. Identification of Potential Tumor Antigens of LIHC

**Figure 1:**
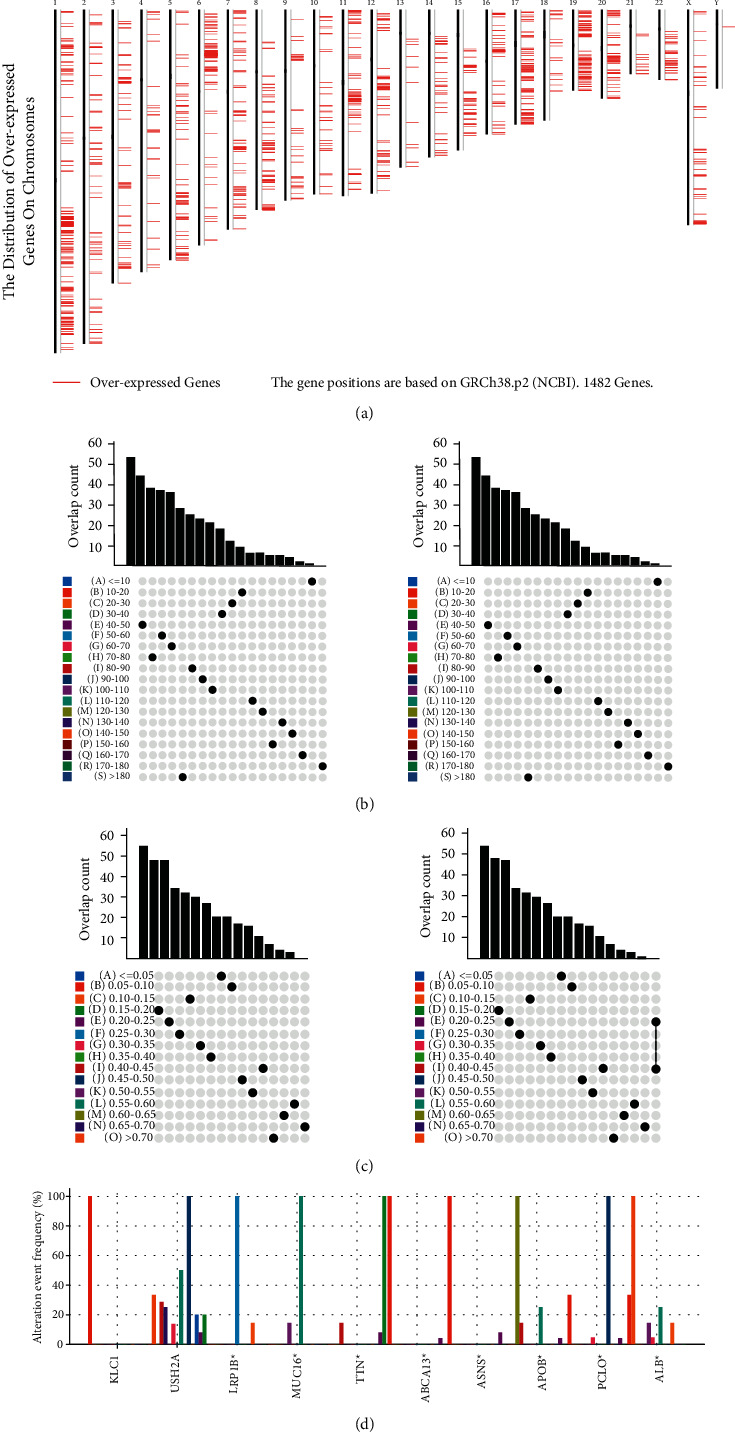
Potential tumor antigens of LIHC. (a) The distribution of overexpressed genes on chromosomes. Samples overlapping in (b) fraction genome altered and (c) mutation count. (d) Genes with high frequency in fraction genome-altered groups.

Firstly, we screened differentially expressed genes to identify potential antigens of LIHC. Then, there were 2213 overexpressed genes which were identified as tumor-associated antigens in LIHC (Figure 1(a)). By calculating mutation counts and altered genome fraction in patients, 10462 mutated genes encoding tumor-specific antigens were screened (Figures 1(b) and 1(c)). As shown in Figure 1(d), genes including KLC1, USH2A, LRP1B, MUC16, TTN, ABCA13, ASNS, APOB, PCLO, and ALB with the highest alteration frequency are in the fraction genome altered group. Finally, 763 frequently mutated and high-expressed tumor-specific genes were determined. We performed KEGG and GO enrichment analyses and found that these genes were significantly associated with cancer-related pathway and GO terms (Supplementary Figure.  1A, 1B).

### 3.2. Identification of Tumor Antigens Related with Survival and Antigen-Presenting Cells in LIHC

**Figure 2:**
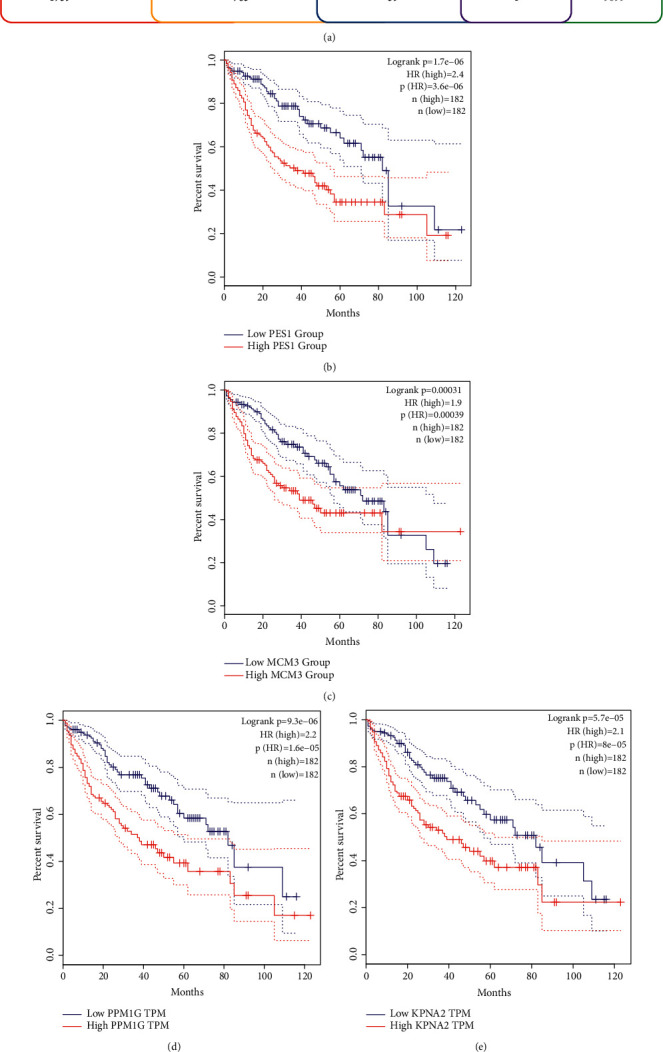
Tumor antigens related with prognosis in LIHC. (a) 763 high expressed and mutated potential tumor antigens in LIHC, and 4 candidates significantly related with survival. (b–e) Overall survival of LIHC patients stratified on the basis of (b) PES1, (c) MCMC3, (d) PPM1G, and (e) KPNA2 expression levels.

**Figure 3:**
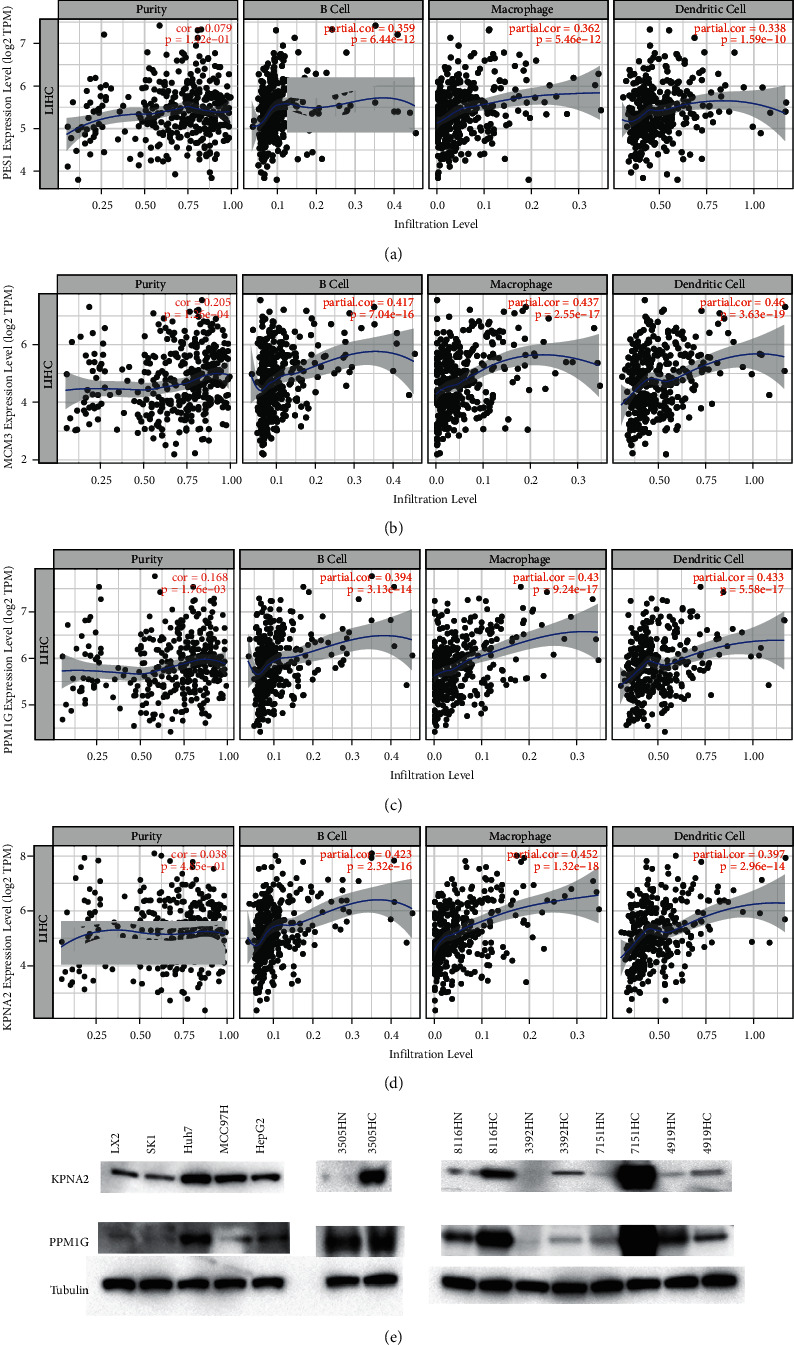
Tumor antigens related with APC. Relationship between the expression levels of (a) PES1, (b) MCM3, (c) PPM1G, (d) KPNA2 and infiltration of B cells, macrophages. and dendritic cells of LIHC. (e) Western blot analysis was performed with liver cancer cell lines and tissue samples from liver cancer patients.

We subsequently used these frequently mutated and high-expressed genes to intersect with prognosis-related tumor antigen genes. Among the 763 overexpressed and frequently mutated tumor-specific genes, we found that a total of 39 genes are associated with the OS of LIHC patients, and 4 genes of which are significantly correlated with the RFS (Figure 2(a)). As shown in Figures 2(b)–2(h), the four most potential candidate genes included are:PES1, MCM3, PPM1G, and KPNA2. Besides, their overexpression levels were significantly related with increased tumor infiltration of B cells, macrophages, and DCs (Figure 3). The abovementioned results illustrated that these 4 tumor antigens could be directly recognized and presented to T cells by APCs (antigen-presenting cells), and activated immune responses upon recognition by B cells. Therefore, these genes might be candidates for developing mRNA vaccine against LIHC. We further verified our conclusion on liver cancer cell lines and liver cancer patient samples and found KNPA2 and PPM1G are high expressed in liver cancer cell lines and tissues from liver cancer patients (Figure 3(e)).

### 3.3. Identifying Potential Immune-Related Subtypes in LIHC

**Figure 4:**
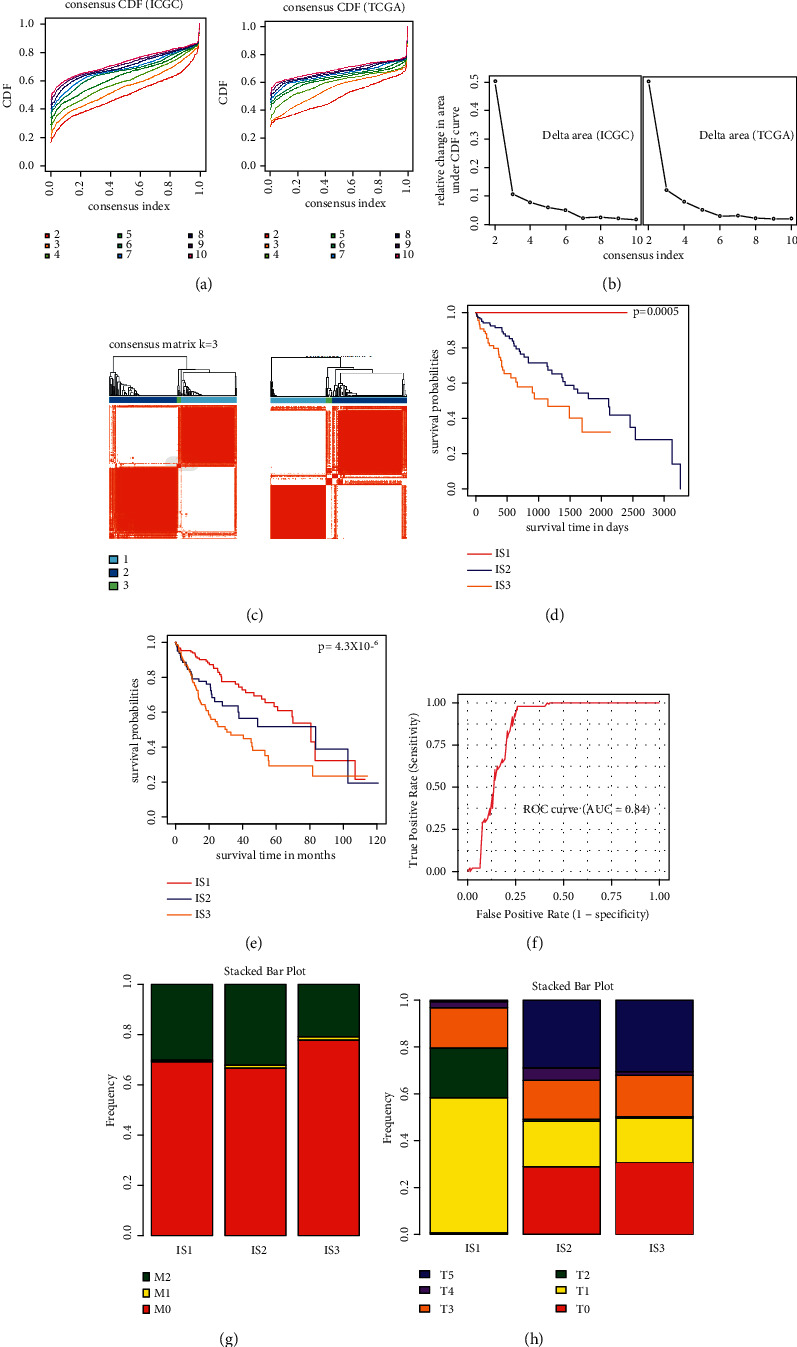
Immune-related subtypes in LIHC. (a) Cumulative distribution function curve, (b) delta area of cluster for immune-related genes in ICGC and TCGA. Clustering heatmap using ICGC (left) and TCGA (right), respectively (c). Overall survival of LIHC immune-related subtypes in ICGC (d). (e) OS survival curves of LIHC immune-related subtypes in TCGA. (f) Receiver operating characteristic (ROC) curves for evaluating the stability of the prediction model. (g, h) Distribution of IS1-IS3 across LIHC. (g) Metastasis staging and (h) stages in TCGA.

Immunophenotyping can reflect the immune status of tumor and its microenvironment, and help to determine the appropriate vaccination for patients [17]. Here, we analyzed the gene expression landscape of immune-related genes in 294 LIHC samples downloaded from ICGC and 424 LIHC samples from the TCGA database to construct consistent clustering, respectively. We selected *k* = 3 as the optimal number of clusters, in which immune-related genes were stably aggregated according to their function delta area and cumulative distribution function (Figures 4(a) and 4(b)), and obtained three immune-related subtypes designated as IS1-IS3 (Figure 4(c)). To further demonstrate each subtype has the same expression pattern in 2 cohorts, samples from TCGA and ICGC datasets were merged as an integrated set to show the clustering (Supplementary Figure 2). Interestingly, IS1 was associated with the best prognosis, while IS3 and IS2 had poor survival probability (Figure 4(d)). We also analyzed the gene expression landscape of 396 immune-related genes in 424 LIHC samples from the TCGA database to construct consistent clustering. Consistent with the results obtained in the ICGC cohort, three immune-related subtypes were obtained in the TCGA cohort and correlated with prognosis (Figure 4(e)). We also plot receiver operating characteristic (ROC) curve to show the true positive rate (sensitivity) for these risk models (Figure 4(f)). The result revealed that our risk model has very good predictive efficiency in patients. We investigated the subtype distribution of different metastasis and stage status (Figures 4(g) and 4(h)), while IS1-IS3 was irregularly aggregated in metastasis and stage status (Figure 4(g)). As shown in Figure 4(h), the T1 and T2 subtypes mainly included IS1. While the T5 subtype mainly included IS2 and IS3 subtypes. Thus, immunophenotyping could be used to predict the prognosis of LIHC patients and typical subtypes.

### 3.4. The Association of Immune-Related Subtypes with Tumor Mutational Burden and Mutational Status

**Figure 5:**
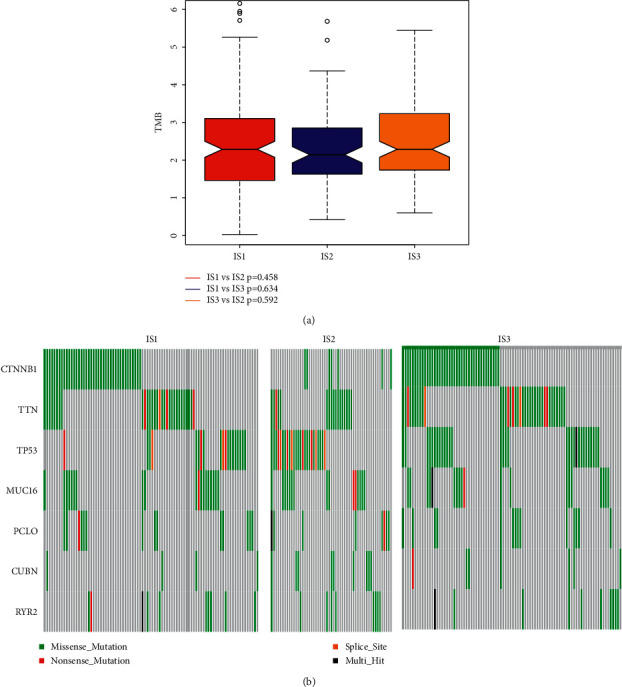
Relationship between immune-related subtypes and TMB status, mutation status. (a) TMB in IS1-IS3 of LIHC. (b) Mutation profiles of seven highly mutated genes in LIHC immune-related subtypes.

Higher tumor mutation burden (TMB) is related to stronger anticancer immunity [18]. In our research, we used the mutect2-processed mutation data set of TCGA in IS1-IS3 to calculate the TMB of each patient [19]. No significant difference in TMB was identified among the four immune-related subtypes (Figure 5(a)). Seven genes including RYR2, CUBN, PCLO, MUC16, TP53, TTN, and CTNNB1 were most frequently mutated in these subtypes (Figure 5(b)). These findings indicated that there was no significant difference in tumor antigens among our immune-related subtypes.

### 3.5. Association between Immune-Related Subtypes and LIHC-Related Tumor Marker

**Figure 6:**
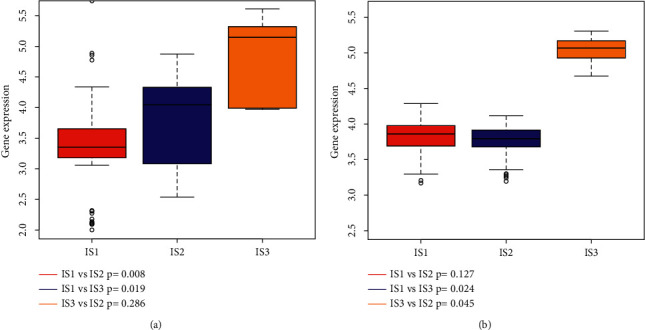
Correlation between these immune-related subtypes as well as expression level of CA125. (a, b) Expression level of CA125 in LIHC immune-related subtypes in TCGA and ICGC database.

CA125 is the most commonly used prognostic tumor biomarker of LIHC, and its high value indicated tumor progression, poor prognosis, or tumor recurrence. In our study, the gene expression level of CA125 was significantly different among immune-related subtypes in ICGC (Figures 6(a) and 6(b)), and IS1 showed lower CA125 expression in the ICGC queue (Figure 6(a)), and also TCGA queue displayed a similar trend in the CA125 expression level (Figure 6(b)). However, these results suggested that immune-related subtypes were superior to CA125 in predicting the prognosis of LIHC patients.

### 3.6. Cellular and Molecular Characteristics of Immune-Related Subtypes

**Figure 7:**
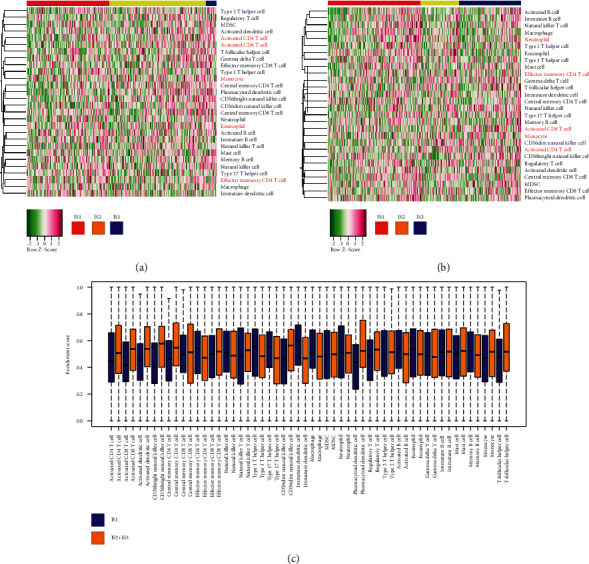
Molecular and cellular characteristics of immune-related subtypes in LIHC. (a, b) Differential enrichment scores of twenty-eight immune cell signatures in LIHC immune-related subtypes of TCGA and ICGC database. (c) Enrichment index of survival-related immune cell signatures in (c) TCGA.

Because tumor-immune status would affect the adequacy of mRNA vaccine [20, 21], we used ssGSEA to score 28 marker genes in ICGC and TCGA cohorts, and further described the resistant cell segments in four immune-related subtypes. The immune cell composition of each subtype in the ICGC cohort was significantly different (Figure 7(a)). Most immune cells were more abundant in IS2 and IS3 subtypes rather than IS2 subtype. As shown in Figures 7(b) and 7(c), the scores of activated CD4 T cells, activated CD8T cells, and monocyte in IS2 and IS3 were significantly higher than those in IS1. Therefore, IS2 and IS3 were immune “hot” types, while IS1 is immune “cold” type. TCGA queue presented a similar trend. These results indicated that the immune-related subtypes could reflect the immune status of LIHC and screen the suitable patients for mRNA vaccination. The mRNA vaccine with these antigens could induce immune infiltration in patients with immune “cold” IS2 and IS3 tumors.

## 4. Discussion

According to the above results, we systematically explored the potential vaccines in liver cancer and found four most potential candidates including PES1, MCM3, PPM1G, and KPNA2. These potential candidates were all associated with antigen-presenting cell (APC) infiltration and poor survival in LIHC in two independent datasets. We also identified three immune-related subtypes (IS1-IS3) of LIHC for further exploring the applicability of mRNA vaccines. Last, we validated our results in two independent datasets and in *vitro*.

Although the vaccine is very promising for the treatment of liver cancer, there is a lack of basic research on vaccine for liver cancer [22]. Cytotoxic T lymphocyte (CTL)-mediated immune response is an effective way to kill cancerous cells. Theoretically, if we can find the key sequences in the process of cell malignant transformation, we can perform tumor CTL vaccine. Compared with traditional vaccines, mRNA vaccines have lots of advantages. It can not only trigger a comprehensive immune response, but also induce local immune response and immune memory [5–7]. Cancer vaccines, including viral vector-based cancer vaccines, immune cell-based cancer vaccines, peptide-based cancer vaccines, and nucleic acid-based cancer vaccines are promising for cancer therapy. Of them, mRNA vaccine has now been an attractive alternative to others for anticancer treatments because mRNA vaccine shares some basic features with DNA vaccine.

At present, the successful application of mRNA vaccine in the field of COVID-19 makes the mRNA vaccine of liver cancer see the dawn [23, 24]. Meanwhile, the identification of genome-wide potential antigens in pancreatic cancer, cholangiocarcinoma, and renal clear cell carcinoma for the development of mRNA vaccine was carried out [5, 6]. However, it is pity that no mRNA vaccine against LIHC antigens has been developed to date. Following the methods used to identify potential antigens in pancreatic cancer, cholangiocarcinoma and renal clear cell carcinoma, our study explored the possibility of liver cancer mRNA vaccine for the first time. It is conducive to the development of mRNA vaccine in liver cancer.

Only a small number of patients with HCC achieved clinical benefit from ICPI therapy [23, 24]. As reported previously, high-throughput studies on HCC samples have identified many patients to harbor transcriptomic hallmarks of adaptive or exhausted immunity [9, 25, 26]. It is urgent to translate this transcription-related knowledge into clinically predictive relationships of response and survival to spare patients from potentially ineffective mRNA vaccination therapies. Subpopulation of patients suitable for vaccination will be identified.

## 5. Conclusion

In this study, we identified four most potential candidates including PES1, MCM3, PPM1G, and KPNA2 related with poor prognosis and APC infiltration in LIHC, which would be very helpful to develop mRNA vaccines. We defined 3 robust immune-related subtypes based on immune-related genes, which could be used to select suitable patients for vaccination in LIHC. Eventually, we performed the immune landscape of all LIHC samples by performing the relevant gene signatures in LIHC patients. This study promotes the research of the mRNA vaccine of LIHC, and helps people to develop the mRNA vaccine of LIHC in the future.

## Data Availability

The datasets used and analyzed in the current study are available from the ICGC and TCGA databases.

## Supplementary Materials

Supplementary Materials Table S1: clinical data from ICGC. Table S2: Differently Expressed Genes Analysis from cbioptrol. Differential Expression Analysis from GEPIA. Table S3: mutation Analysis of LIHC from cbioptrol. Supplementary Figure 1: KEGG and GO enrichment analyses of these candidate genes. Supplementary Figure 2: heatmaps of antigens in TCGA and CGGA cohort with cluster annotations.
