# Supplementary material for: Identifying Potential Tumor Antigens and Antigens-Related Subtypes in Hepatocellular Carcinoma for mRNA Vaccine Development
Source: J Oncol. 2022 Aug 29;2022:6851026. doi: 10.1155/2022/6851026 (PMC9444406; doi:10.1155/2022/6851026)
Supplement: Supplementary Materials — Table S1: clinical data from ICGC. Table S2: Differently Expressed Genes Analysis from cbioptrol. Differential Expression Analysis from GEPIA. Table S3: mutation Analysis of LIHC from cbioptrol. Supplementary Figure 1: KEGG and GO enrichment analyses of these candidate genes. Supplementary Figure 2: heatmaps of antigens in TCGA and CGGA cohort with cluster annotations. [file 6851026.f1.zip › Table S1 (1).pdf]

Table 1: clinical data from ICGC.

sampleID	cancer_his	cancer_typ	disease_sta	donor_age	donor_age	donor_age
DO44923	unknown			85		85
DO44883	unknown			75		75
DO52238	unknown			58		58
DO22851	unknown			73		73
DO52193	unknown			72		72
DO22966	unknown			62		62
DO52298	unknown			83		83
DO48486	unknown			75		75
DO23043	unknown			62		62
DO219539	unknown			70		70
DO219549	unknown			65		65
DO52320	unknown			67		67
DO49694	unknown			70		70
DO49696	unknown			65		65
DO52299	unknown			53		53
DO52301	unknown			68		68
DO23002	unknown			50		50
DO49695	unknown			78		78
DO52232	unknown			64		64
DO22941	unknown			35		35
DO23006	unknown			80		80
DO44903	unknown			67		67
DO52216	unknown			63		63
DO44994	unknown			75		75
DO52203	unknown			69		69
DO52322	unknown			56		56
DO23014	unknown			40		40
DO44899	unknown			65		65
DO48592	unknown			72		72
DO22962	unknown			66		66
DO52295	unknown			51		51
DO52201	unknown			68		68
DO44887	unknown			60		60
DO52199	unknown			48		48
DO44915	unknown			57		57
DO52192	unknown			81		81
DO49717	unknown			68		68
DO23048	unknown			23		23
DO44982	unknown			67		67
DO52230	unknown			53		53
DO22956	unknown			54		54
DO23073	unknown			52		52
DO52202	unknown			52		52
DO44878	unknown			45		45
DO219529	unknown			59		59
DO223919	unknown			33		33
DO222354	unknown		relapse	39	39	43
DO222355	unknown		relapse	24	24	34
DO222357	unknown		complete r	27	27	28
DO222358	unknown		relapse	50	50	53
DO48492	unknown			64		64
DO22837	unknown			75		75
DO44954	unknown			43		43
DO45001	unknown			37		37
DO219535	unknown			85		85
DO52307	unknown			58		58

DO44990	unknown		77		77
DO44889	unknown		75		75
DO223923	unknown		74		74
DO22838	unknown		80		80
DO22908	unknown		76		76
DO219505	unknown		58		58
DO219515	unknown		64		64
DO22869	unknown		52		52
DO22802	unknown		43	43	
DO22804	unknown	relapse	67	67	78
DO22810	unknown	complete r	42	42	48
DO22812	unknown	relapse	32	32	34
DO228155	unknown		43	43	
DO228157	unknown		45	45	
DO228186			50	50	
DO228187			66	66	
DO228188			52	52	
DO228189			58	58	
DO228190			39	39	
DO228191			45	45	
DO228192			55	55	
DO228193			58	58	
DO228194			39	39	
DO228195			73	73	
DO228196			55	55	
DO228197			41	41	
DO228198			68	68	
DO228199			65	65	
DO228200			44	44	
DO228201			35	35	
DO228202			63	63	
DO228203			45	45	
DO228204			53	53	
DO228205			65	65	
DO228206			43	43	
DO228207			57	57	
DO228208			61	61	
DO228209			38	38	
DO228210			52	52	
DO228211			45	45	
DO228212			30	30	
DO228213			48	48	
DO228214			69	69	
DO228215			39	39	
DO228216			26	26	
DO228217			42	42	
DO228218			35	35	
DO228219			33	33	
DO228220			42	42	
DO228223			32	32	
DO228224			55	55	
DO228225			48	48	
DO228226			62	62	
DO228227			40	40	
DO228228			63	63	
DO228229			39	39	
DO228230			47	47	
DO228232			52	52	

DO228234			61	61	
DO228235			46	46	
DO228237			37	37	
DO228239			54	54	
DO228241			44	44	
DO228243			71	71	
DO228244			54	54	
DO228245			34	34	
DO228252			49	49	
DO228261			52	52	
DO228264			65	65	
DO228270			62	62	
DO228275			61	61	
DO228278			43	43	
DO228280			44	44	
DO228284			46	46	
DO228288			39	39	
DO228297			39	39	
DO228299			38	38	
DO22830	unknown	complete r	83	83	86
DO228301			53	53	
DO228311			39	39	
DO228313			49	49	
DO228327			56	56	
DO228330			45	45	
DO228333			53	53	
DO228337			40	40	
DO44942	unknown		70		70
DO228342			29	29	
DO228348			39	39	
DO219559	unknown		29		29
DO228357			47	47	
DO228359			57	57	
DO22926	unknown		69		69
DO228362			46	46	
DO228364			36	36	
DO22944	unknown		76		76
DO228374			45	45	
DO228377			57	57	
DO219517	unknown		82		82
DO228380			37	37	
DO228385			55	55	
DO44970	unknown		56		56
DO228390			58	58	
DO228393			48	48	
DO228401			49	49	
DO228405			45	45	
DO228408			49	49	
DO228416			61	61	
DO44888	unknown		64		64
DO228420			46	46	
DO228422			46	46	
DO228432			48	48	
DO228434			63	63	
DO228436			44	44	
DO228445			57	57	
DO22887	unknown		72		72
DO228456			45	45	

DO228459		49	49	
DO228461		41	41	
DO228464		59	59	
DO228470		61	61	
DO228474		50	50	
DO52302	unknown	90		90
DO228487		50	50	
DO228501		37	37	
DO228508		61	61	
DO22938	unknown	42		42
DO228512		56	56	
DO228520		57	57	
DO228522		56	56	
DO228524		43	43	
DO228531		60	60	
DO44882	unknown	80		80
DO228540		39	39	
DO228546		64	64	
DO228552		52	52	
DO228553		51	51	
DO228555		59	59	
DO228556		41	41	
DO228559		51	51	
DO228560		44	44	
DO228562		49	49	
DO228566		58	58	
DO219521	unknown	58		58
DO228570		49	49	
DO228574		40	40	
DO228577		49	49	
DO228585		51	51	
DO228589		49	49	
DO228592		40	40	
DO228597		55	55	
DO219511	unknown	64		64
DO228601		46	46	
DO228608		46	46	
DO228609		53	53	
DO228617		51	51	
DO228623		37	37	
DO48518	unknown	76		76
DO228634		61	61	
DO228642		26	26	
DO228646		49	49	
DO228650		59	59	
DO228652		53	53	
DO228654		72	72	
DO228655		64	64	
DO228659		42	42	
DO52334	unknown	73		73
DO228664		46	46	
DO228667		59	59	
DO228672		39	39	
DO228677		47	47	
DO228682		46	46	
DO22842	unknown	81		81
DO228694		47	47	
DO228702		60	60	

DO228711			41	41	
DO22834	unknown		74		74
DO228726			52	52	
DO228733			36	36	
DO228743			44	44	
DO228749			36	36	
DO23010	unknown		57		57
DO228753			62	62	
DO228766			48	48	
DO228770			58	58	
DO228773			51	51	
DO228779			60	60	
DO219507	unknown	complete r	55		59
DO228784			65	65	
DO228786			63	63	
DO228793			39	39	
DO228796			52	52	
DO228802			61	61	
DO228806			50	50	
DO228807			49	49	
DO228809			36	36	
DO23063	unknown		68		68
DO228813			44	44	
DO228815			62	62	
DO228816			51	51	
DO228819			60	60	
DO228821			62	62	
DO228823			56	56	
DO228825			52	52	
DO228827			45	45	
DO228828			65	65	
DO228832			48	48	
DO228833			65	65	
DO228835			56	56	
DO228837			41	41	
DO219506	unknown		51		51
DO228841			48	48	
DO228844			57	57	
DO228846			39	39	
DO228848			28	28	
DO228850			47	47	
DO228852			53	53	
DO228856			58	58	
DO228860			49	49	
DO228862			48	48	
DO228863			48	48	
DO228864			33	33	
DO228866			61	61	
DO228867			64	64	
DO52198	unknown		61		61
DO228870			55	55	
DO228871			49	49	
DO228874			50	50	
DO228877			72	72	
DO228878			59	59	
DO228879			53	53	
DO228881			65	65	
DO228883	unknown		41	41	

DO228884		60	60	
DO228886		48	48	
DO228887	unknown	69	69	
DO228890		47	47	
DO228892		44	44	
DO228893	unknown	68	68	
DO228894	unknown	64	64	
DO228895		26	26	
DO228896	unknown	62	62	
DO228897	unknown	74	74	
DO228898	unknown	76	76	
DO228899	unknown	30	30	
DO44879	unknown	78		78
DO228900	unknown	60	60	
DO228901	unknown	57	57	
DO228902	unknown	57	57	
DO228903	unknown	32	32	
DO228904	unknown	79	79	
DO228905	unknown	49	49	
DO228906	unknown	81	81	
DO228907	unknown	81	81	
DO228908	unknown	44	44	
DO228909	unknown	53	53	
DO228911	unknown	30	30	
DO228912	unknown	53	53	
DO228913	unknown	48	48	
DO228914	unknown	42	42	
DO228915	unknown	37	37	
DO228916	unknown	55	55	
DO228917	unknown	45	45	
DO228918	unknown	46	46	
DO228919	unknown	67	67	
DO228920	unknown	45	45	
DO228921	unknown	49	49	
DO228922	unknown	48	48	
DO228923	unknown	32	32	
DO228924	unknown	86	86	
DO228925	unknown	28	28	
DO228926	unknown	64	64	
DO228928	unknown	53	53	
DO228929	unknown	35	35	
DO48589	unknown	71		71
DO228931	unknown	64	64	
DO228933	unknown	48	48	
DO228934	unknown	49	49	
DO228936	unknown	46	46	
DO228937	unknown	76	76	
DO228938	unknown	51	51	
DO228939	unknown	56	56	
DO228940	unknown	65	65	
DO228941	unknown	46	46	
DO228942	unknown	40	40	
DO228943	unknown	65	65	
DO228944	unknown	61	61	
DO228945	unknown	48	48	
DO228946	unknown	63	63	
DO228947	unknown	70	70	
DO228948	unknown	64	64	

DO228949	unknown	56	56	
DO228950	unknown	48	48	
DO228952	unknown	16	16	
DO228955	unknown	50	50	
DO228957	unknown	72	72	
DO22947	unknown	51		51
DO228960	unknown	46	46	
DO228961	unknown	38	38	
DO228963	unknown	26	26	
DO228965	unknown	41	41	
DO228967	unknown	40	40	
DO228969	unknown	67	67	
DO228971	unknown	62	62	
DO228972	unknown	28	28	
DO228974	unknown	77	77	
DO228976	unknown	56	56	
DO228978	unknown	46	46	
DO228981	unknown	42	42	
DO228983	unknown	42	42	
DO228985	unknown	67	67	
DO228986	unknown	67	67	
DO228988	unknown	53	53	
DO44881	unknown	76		76
DO228991	unknown	68	68	
DO228993	unknown	56	56	
DO228998	unknown	77	77	
DO229004	unknown	72	72	
DO229006	unknown	65	65	
DO229008	unknown	56	56	
DO229009	unknown	73	73	
DO229012	unknown	41	41	
DO229015	unknown	62	62	
DO229018	unknown	64	64	
DO229019	unknown	26	26	
DO45017	unknown	61		61
DO229022	unknown	57	57	
DO229024	unknown	73	73	
DO229025	unknown	49	49	
DO229026	unknown	59	59	
DO229027	unknown	46	46	
DO229028	unknown	53	53	
DO229029	unknown	41	41	
DO229030	unknown	49	49	
DO229031	unknown	68	68	
DO229032	unknown	70	70	
DO229033	unknown	56	56	
DO229034	unknown	56	56	
DO229035	unknown	58	58	
DO229036	unknown	50	50	
DO229037	unknown	57	57	
DO229038	unknown	76	76	
DO229039	unknown	50	50	
DO229040	unknown	53	53	
DO229041	unknown	62	62	
DO229042	unknown	47	47	
DO229043	unknown	45	45	
DO229044	unknown	74	74	
DO229045	unknown	45	45	

DO229046	unknown		50	50	
DO229047	unknown		62	62	
DO229048	unknown		65	65	
DO229049	unknown		74	74	
DO219509	unknown		54		54
DO229050	unknown		44	44	
DO229051	unknown		28	28	
DO229052	unknown				
DO229053	unknown		79	79	
DO229054	unknown		70	70	
DO229055	unknown		45	45	
DO229056	unknown		62	62	
DO229057	unknown		47	47	
DO229058	unknown		37	37	
DO229059	unknown		59	59	
DO229060	unknown		64	64	
DO229061	unknown		53	53	
DO229062	unknown		34	34	
DO229063	unknown		45	45	
DO22911	unknown		53		53
DO22890	unknown		70		70
DO44884	unknown		66		66
DO44875	unknown		72		72
DO219513	unknown	progressio	68		74
DO219519	unknown	complete r	49		59
DO219523	unknown	complete r	64		68
DO219525	unknown	complete r	45		48
DO219527	unknown	complete r	68		71
DO219531	unknown		68		68
DO219533	unknown	progressio	81		84
DO219537	unknown	progressio	70		71
DO219541	unknown	complete r	66		66
DO219543	unknown	progressio	65		65
DO219545	unknown	complete r	57		57
DO219547	unknown	complete r	17		17
DO219551	unknown	progressio	69		69
DO219552	unknown	complete r	45		45
DO219553	unknown	complete r	55		55
DO219555	unknown	complete r	57		57
DO219557	unknown	complete r	68		68
DO219560	unknown	progressio	32		35
DO219561	unknown	progressio	40		44
DO219563	unknown	complete r	69		72
DO219565	unknown	progressio	53		56
DO219567	unknown	progressio	61		64
DO219569	unknown	progressio	50		52
DO219571	unknown	complete r	70		72
DO219573	unknown	complete r	50		50
DO219575	unknown	progressio	72		75
DO219577	unknown	complete r	55		59
DO219578	unknown	progressio	67		67
DO219579	unknown		61		61
DO219580	unknown	complete r	58		58
DO219581	unknown	complete r	53		54
DO219582	unknown	complete r	68		68
DO219583	unknown	complete r	61		62
DO223911	unknown	progression			
DO223912	unknown	complete r	56		62

DO223913	unknown	complete r	59	60
DO223914	unknown		24	24
DO223915	unknown		74	74
DO223916	unknown		20	20
DO223917	unknown		75	75
DO223918	unknown	progressio	64	65
DO23083	unknown			
DO23084	unknown			
DO23086	unknown			
DO23088	unknown			
DO23090	unknown			
DO23092	unknown			
DO23094	unknown			
DO23096	unknown			
DO23098	unknown			
DO23100	unknown			
DO23102	unknown	relapse	67	
DO23104	unknown	relapse	71	
DO23106	unknown	relapse	46	
DO23108	unknown	no evidenc	69	
DO23110	unknown	relapse	53	
DO23112	unknown	relapse	65	
DO23114	unknown	relapse	57	
DO23116	unknown	relapse	56	
DO23118	unknown	no evidenc	58	
DO23120	unknown	no evidenc	47	
DO23122	unknown	relapse	78	
DO23124	unknown	no evidenc	75	
DO23126	unknown	no evidenc	68	
DO23128	unknown	relapse	69	
DO23130	unknown	relapse	62	
DO23132	unknown	no evidenc	63	
DO23134	unknown	relapse	65	
DO23136	unknown	no evidenc	76	
DO23138	unknown	no evidenc	64	
DO23140	unknown	relapse	62	
DO23142	unknown	relapse	57	
DO23144	unknown	relapse	41	
DO23146	unknown	relapse	58	
DO23148	unknown	relapse	73	
DO23150	unknown	no evidenc	74	
DO231512	unknown	no evidenc	75	
DO231515	unknown	no evidenc	66	
DO231518	unknown	no evidenc	58	
DO23152	unknown	relapse	63	
DO231520	unknown	no evidenc	77	
DO231524	unknown	no evidenc	67	
DO231527	unknown	no evidenc	77	
DO231531	unknown	no evidenc	77	
DO231534	unknown	no evidenc	74	
DO231538	unknown	no evidenc	60	
DO23154	unknown	relapse	68	
DO231541	unknown	no evidenc	65	
DO231545	unknown	no evidenc	70	
DO231549	unknown	no evidenc	58	
DO231553	unknown	no evidenc	56	
DO231557	unknown	no evidenc	59	
DO231559	unknown	no evidenc	72	

DO23156	unknown	relapse	56
DO231563	unknown	no evidenc	83
DO231566	unknown	no evidenc	63
DO231569	unknown	no evidenc	69
DO231573	unknown	no evidenc	60
DO231577	unknown	no evidenc	70
DO23158	unknown	relapse	65
DO231580	unknown	no evidenc	74
DO231583	unknown	no evidenc	80
DO231587	unknown	no evidenc	70
DO231590	unknown	no evidenc	61
DO231593	unknown	no evidenc	75
DO231596	unknown	no evidenc	63
DO231598	unknown	no evidenc	56
DO23160	unknown	relapse	58
DO231602	unknown	no evidenc	80
DO231605	unknown	no evidenc	74
DO231607	unknown	no evidenc	56
DO231610	unknown	no evidenc	77
DO231613	unknown	no evidenc	68
DO231618	unknown	no evidenc	75
DO23162	unknown	no evidenc	67
DO231620	unknown	no evidenc	74
DO231622	unknown	no evidenc	78
DO231625	unknown	no evidenc	77
DO231628	unknown	no evidenc	73
DO231631	unknown	no evidenc	48
DO231636	unknown	no evidenc	71
DO231638	unknown	no evidenc	75
DO23164	unknown	relapse	77
DO231641	unknown	no evidenc	60
DO231645	unknown	no evidenc	59
DO231646	unknown	no evidenc	80
DO231649	unknown	no evidenc	49
DO231652	unknown	no evidenc	63
DO231655	unknown	no evidenc	66
DO231658	unknown	no evidenc	74
DO23166	unknown	relapse	72
DO231663	unknown	no evidenc	72
DO231666	unknown	no evidenc	38
DO231669	unknown	no evidenc	75
DO231672	unknown	no evidenc	72
DO231673	unknown	no evidenc	59
DO231677	unknown	no evidenc	51
DO23168	unknown	relapse	59
DO231681	unknown	no evidenc	56
DO231683	unknown	no evidenc	69
DO231685	unknown	no evidenc	67
DO231690	unknown	no evidenc	75
DO231691	unknown	no evidenc	32
DO231694	unknown	no evidenc	63
DO231697	unknown	no evidenc	74
DO23170	unknown	relapse	59
DO231700	unknown	no evidenc	58
DO231702	unknown	no evidenc	58
DO231704	unknown	no evidenc	56
DO231706	unknown	no evidenc	49
DO231708	unknown	no evidenc	56

DO231710	unknown	no evidenc	78	
DO231712	unknown	no evidenc	73	
DO231713	unknown	no evidenc	54	
DO231716	unknown	no evidenc	52	
DO231718	unknown	no evidenc	69	
DO231719	unknown	no evidenc	78	
DO23172	unknown	relapse	77	
DO231722	unknown	no evidenc	72	
DO231724	unknown	no evidenc	73	
DO231726	unknown	no evidenc	64	
DO231728	unknown	no evidenc	57	
DO231730	unknown	no evidenc	0	
DO231732	unknown	no evidenc	66	
DO231734	unknown	no evidenc	63	
DO231736	unknown	no evidenc	64	
DO231737	unknown	no evidenc	55	
DO231739	unknown	no evidenc	70	
DO23174	unknown	relapse	77	
DO231742	unknown	no evidenc	58	
DO231744	unknown	no evidenc	75	
DO231745	unknown	no evidenc	76	
DO231747	unknown	no evidenc	78	
DO231750	unknown	no evidenc	72	
DO231752	unknown	no evidenc	72	
DO231754	unknown	no evidenc	65	
DO231756	unknown	no evidenc	56	
DO231758	unknown	no evidenc	58	
DO23176	unknown	relapse	67	
DO231760	unknown	no evidenc	69	
DO231762	unknown	no evidenc	75	
DO231764	unknown	no evidenc	61	
DO231766	unknown	no evidenc	79	
DO231768	unknown	no evidenc	63	
DO231770	unknown	no evidenc	58	
DO231772	unknown	no evidenc	64	
DO231774	unknown	no evidenc	63	
DO231776	unknown	no evidenc	75	
DO231778	unknown	no evidenc	63	
DO23178	unknown	no evidenc	76	
DO231780	unknown	no evidenc	64	
DO231782	unknown	no evidenc	69	
DO231784	unknown	no evidenc	69	
DO231786	unknown	no evidenc	74	
DO231788	unknown	no evidenc	81	
DO231790	unknown	no evidenc	59	
DO231792	unknown	no evidenc	70	
DO231793	unknown	no evidenc	73	
DO231795	unknown		69	69
DO231797	unknown	no evidenc	72	
DO231799	unknown	no evidenc	54	
DO23180	unknown	relapse	75	
DO231801	unknown	no evidenc	77	
DO231803	unknown	no evidenc	58	
DO231805	unknown	no evidenc	62	
DO231806	unknown		49	49
DO231808	unknown	no evidenc	74	
DO231810	unknown	no evidenc	74	
DO231813	unknown	no evidenc	73	

DO231816	unknown	no evidenc	50	
DO231819	unknown	no evidenc	68	
DO23182	unknown	no evidenc	64	
DO231821	unknown	no evidenc	70	
DO231824	unknown	no evidenc	59	
DO231827	unknown	no evidenc	60	
DO231830	unknown	no evidenc	83	
DO231832	unknown	no evidenc	76	
DO231835	unknown	no evidenc	61	
DO231837	unknown	no evidenc	68	
DO231839	unknown	no evidenc	74	
DO23184	unknown	no evidenc	76	
DO231844	unknown	no evidenc	47	
DO231848	unknown	no evidenc	67	
DO231851	unknown	no evidenc	71	
DO231854	unknown	no evidenc	60	
DO231856	unknown	no evidenc	53	
DO231858	unknown	no evidenc	74	
DO23186	unknown	relapse	77	
DO231862	unknown	no evidenc	75	
DO231866	unknown	no evidenc	81	
DO231868	unknown	no evidenc	53	
DO231871	unknown	no evidenc	61	
DO231875	unknown	no evidenc	66	
DO23188	unknown	no evidenc	76	
DO231880	unknown	no evidenc	51	
DO231885	unknown	no evidenc	69	
DO231887	unknown	no evidenc	64	
DO231890	unknown	no evidenc	80	
DO231893	unknown	no evidenc	74	
DO231895	unknown	no evidenc	70	
DO231897	unknown	no evidenc	62	
DO23190	unknown	relapse	40	
DO231900	unknown	no evidenc	79	
DO231902	unknown	no evidenc	63	
DO231904	unknown	no evidenc	76	
DO231906	unknown	no evidenc	72	
DO231909	unknown	no evidenc	68	
DO231910	unknown	no evidenc	63	
DO23192	unknown	no evidenc	85	
DO231933	unknown		67	67
DO231934	unknown		62	62
DO231935	unknown		42	42
DO231936	unknown		45	45
DO231937	unknown		65	65
DO231938	unknown		64	64
DO231939	unknown		55	55
DO23194	unknown	relapse	68	
DO231940	unknown		47	47
DO231941	unknown		53	53
DO231942	unknown		61	61
DO231943	unknown		41	41
DO231944	unknown		53	53
DO231945	unknown		72	72
DO231946	unknown		45	45
DO231947	unknown		33	33
DO231948	unknown		58	58
DO231949	unknown		46	46

DO231950	unknown		36	36
DO231951	unknown		67	67
DO231952	unknown		36	36
DO231953	unknown		39	39
DO231954	unknown		62	62
DO231955	unknown		37	37
DO231956	unknown		58	58
DO231957	unknown		55	55
DO231958	unknown		57	57
DO231959	unknown		47	47
DO23196	unknown	relapse	67	
DO231960	unknown		64	64
DO231961	unknown		45	45
DO231962	unknown		52	52
DO231963	unknown		71	71
DO231964	unknown		43	43
DO231965	unknown		48	48
DO231966	unknown		24	24
DO231967	unknown		66	66
DO231968	unknown		52	52
DO231969	unknown		48	48
DO231970	unknown		53	53
DO231971	unknown		67	67
DO231972	unknown		34	34
DO231973	unknown		54	54
DO231974	unknown		60	60
DO231975	unknown		58	58
DO231976	unknown		50	50
DO231977	unknown		55	55
DO231978	unknown		56	56
DO231979	unknown		77	77
DO23198	unknown	relapse	50	
DO231980	unknown		68	68
DO231981	unknown		73	73
DO231982	unknown		75	75
DO231983	unknown		71	71
DO231984	unknown		66	66
DO231985	unknown		62	62
DO231986	unknown		77	77
DO231987	unknown		62	62
DO231988	unknown		54	54
DO231989	unknown		67	67
DO231990	unknown		62	62
DO231991	unknown		66	66
DO231992	unknown		47	47
DO231993	unknown		55	55
DO231995	unknown		56	56
DO231998	unknown		45	45
DO23200	unknown	no evidenc	81	
DO232000	unknown		67	67
DO232002	unknown		72	72
DO232004	unknown		58	58
DO232005	unknown		48	48
DO232008	unknown		62	62
DO232010	unknown		47	47
DO232012	unknown		61	61
DO232014	unknown		80	80
DO232019	unknown		59	59

DO23202	unknown	no evidenc	77	
DO232020	unknown		57	57
DO232021	unknown		54	54
DO232022	unknown		53	53
DO232023	unknown		43	43
DO232024	unknown		48	48
DO232025	unknown		46	46
DO232026	unknown		67	67
DO232027	unknown		70	70
DO232028	unknown		62	62
DO232029	unknown		53	53
DO232030	unknown		64	64
DO232031	unknown		59	59
DO232032	unknown		53	53
DO232033	unknown		64	64
DO232034	unknown		48	48
DO232035	unknown		58	58
DO23204	unknown	no evidenc	83	
DO23206	unknown	relapse	84	
DO23208	unknown	relapse	72	
DO23210	unknown	no evidenc	72	
DO23212	unknown	relapse	67	
DO23214	unknown	no evidenc	75	
DO23216	unknown	relapse	71	
DO23218	unknown	relapse	62	
DO23220	unknown	no evidenc	36	
DO23222	unknown	no evidenc	76	
DO23224	unknown	relapse	70	
DO23226	unknown	no evidenc	67	
DO23228	unknown	no evidenc	46	
DO23230	unknown	relapse	58	
DO23232	unknown	relapse	74	
DO23234	unknown	relapse	65	
DO23236	unknown	relapse	51	
DO23238	unknown	no evidenc	69	
DO23240	unknown	no evidenc	60	
DO23242	unknown	relapse	62	
DO23244	unknown	relapse	72	
DO23246	unknown	relapse	77	
DO23248	unknown	no evidenc	81	
DO23250	unknown	relapse	73	
DO23252	unknown	no evidenc	57	
DO23254	unknown	relapse	68	
DO23256	unknown	no evidenc	58	
DO23258	unknown	no evidenc	67	
DO23260	unknown	relapse	69	
DO23262	unknown	no evidenc	75	
DO23264	unknown	relapse	61	
DO23266	unknown	no evidenc	71	
DO23268	unknown	relapse	85	
DO23270	unknown	relapse	61	
DO23272	unknown	relapse	72	
DO23274	unknown	relapse	75	
DO23276	unknown	relapse	79	
DO23278	unknown	relapse	57	
DO23280	unknown	relapse	74	
DO23282	unknown	no evidenc	81	
DO23284	unknown	no evidenc	68	

DO23286	unknown	no evidenc	62
DO23288	unknown	no evidenc	60
DO23290	unknown	no evidenc	68
DO23292	unknown	relapse	69
DO23294	unknown		
DO23296	unknown	relapse	66
DO23298	unknown	relapse	66
DO23300	unknown	relapse	71
DO23302	unknown	relapse	72
DO23304	unknown	relapse	50
DO23306	unknown	relapse	57
DO23308	unknown	relapse	66
DO23310	unknown	no evidenc	54
DO23312	unknown	relapse	73
DO23314	unknown	no evidenc	29
DO23316	unknown	relapse	70
DO23318	unknown	relapse	56
DO23320	unknown	relapse	60
DO23322	unknown	no evidenc	78
DO23324	unknown	no evidenc	70
DO23326	unknown	relapse	66
DO23328	unknown	no evidenc	74
DO23330	unknown	no evidenc	75
DO23332	unknown	no evidenc	61
DO23334	unknown	relapse	45
DO23336	unknown	relapse	62
DO23338	unknown		
DO23340	unknown	relapse	56
DO23342	unknown	relapse	70
DO23344	unknown	relapse	72
DO23346	unknown	relapse	72
DO23348	unknown	relapse	77
DO23350	unknown	relapse	65
DO23352	unknown	relapse	58
DO23354	unknown	no evidenc	29
DO23356	unknown	relapse	62
DO23358	unknown	relapse	71
DO23360	unknown	no evidenc	71
DO23362	unknown	relapse	59
DO23364	unknown	relapse	51
DO23366	unknown	relapse	64
DO23368	unknown	relapse	52
DO23370	unknown	relapse	59
DO23372	unknown	relapse	23
DO23374	unknown	relapse	62
DO23376	unknown	relapse	58
DO23378	unknown	relapse	57
DO23380	unknown	relapse	54
DO23382	unknown	no evidenc	65
DO23384	unknown	relapse	61
DO23386	unknown	relapse	47
DO23388	unknown		72
DO23390	unknown	relapse	66
DO23392	unknown	relapse	70
DO23394	unknown	no evidenc	59
DO23396	unknown	no evidenc	72
DO23398	unknown	relapse	69
DO23400	unknown	relapse	41

DO23402	unknown	relapse	37	
DO23404	unknown	relapse	68	
DO23406	unknown	relapse	52	
DO23408	unknown	relapse	73	
DO23410	unknown	relapse	70	
DO23412	unknown	relapse	68	
DO23414	unknown	relapse	62	
DO23416	unknown	no evidenc	73	
DO23418	unknown	relapse	69	
DO23420	unknown	no evidenc	58	
DO23422	unknown	no evidenc	70	
DO23424	unknown	relapse	51	
DO23426	unknown	relapse	57	
DO23428	unknown	relapse	76	
DO23430	unknown	relapse	29	
DO23432	unknown	relapse	66	
DO23434	unknown	relapse	70	
DO23436	unknown	relapse	51	
DO23438	unknown	relapse	36	
DO23440	unknown	relapse	52	
DO23442	unknown		70	
DO23444	unknown		77	
DO23446	unknown		47	
DO23448	unknown		69	
DO23450	unknown		70	
DO23452	unknown		71	
DO23454	unknown		69	
DO23456	unknown		67	
DO23458	unknown		63	
DO23460	unknown		77	
DO23462	unknown		62	
DO23464	unknown		25	
DO23466	unknown		73	
DO23468	unknown		48	
DO23470	unknown		76	
DO23472	unknown		70	
DO23474	unknown		69	
DO23476	unknown		53	
DO23478	unknown		59	
DO23480	unknown		65	
DO23482	unknown		58	
DO23484	unknown		59	
DO23486	unknown		74	
DO23488	unknown		73	
DO23490	unknown		79	
DO23492	unknown		64	
DO23494	unknown		69	
DO23496	unknown		58	
DO23498	unknown		68	
DO23500	unknown		78	
DO23502	unknown		68	
DO23504	unknown		72	
DO23506	unknown		75	
DO23508	no		79	79
DO23509	no		60	60
DO23510	yes		80	80
DO23511	no		83	83
DO23512	no		60	60

DO23513	no			76	76	
DO23514	no			64	64	
DO23515	yes	C16		71	71	
DO23516	no			69	69	
DO23517	yes			62	62	
DO23518	no			67	67	
DO23519	no			65	65	
DO23520	unknown			59	59	
DO23521	no			63	63	
DO23522	no			82	82	
DO23523	yes			52	52	
DO23524	no	C50		71	71	
DO23525	no			71	71	
DO23526	no			66	66	
DO23527	no			72	72	
DO23528	yes			45	45	
DO23529	no			72	72	
DO23530	no			53	53	
DO23531	no			78	78	
DO23532	yes			71	71	
DO23533	no			61	61	
DO23534	no			60	60	
DO23535	no			67	67	
DO23536	no			56	56	
DO23537	no	C16		79	79	
DO23538	no			67	67	
DO23539	unknown	C62		77	77	
DO23540	no			73	73	
DO23541	no			65	65	
DO23542	yes			66	66	
DO23543	no			63	63	
DO23544	no			67	67	
DO23545	no	C16		67	67	
DO23546	unknown			63	63	
DO23547	no			74	74	
DO23548	yes			78	78	
DO23549	no			74	74	
DO23550	no			69	69	
DO23551	no			73	73	
DO23552	no			80	80	
DO44627	unknown		relapse	74	74	74
DO44628	unknown		relapse	35	35	37
DO44630	unknown		relapse	63	63	64
DO44632	unknown		relapse	30	30	30
DO44634	unknown			35	35	
DO44636	unknown		relapse	64	64	69
DO44638	unknown		relapse	67	67	69
DO44642	unknown		relapse	38	38	39
DO44644	unknown		relapse	56	56	57
DO44648	unknown		complete r	56	56	60
DO44650	unknown		complete r	61	61	62
DO44652	unknown			69	69	
DO44654	unknown		relapse	40	40	42
DO44656	unknown		relapse	68	68	70
DO44658	unknown		complete r	76	76	76
DO44660	unknown		relapse	56	56	59
DO44664	unknown		relapse	68	68	71
DO44666	unknown			73	73	

DO44668	unknown		69	69	
DO44670	unknown	complete r	78	78	81
DO44672	unknown		75	75	
DO44674	unknown		74	74	
DO44676	unknown		68	68	
DO44678	unknown	complete r	66	66	69
DO44680	unknown		79	79	
DO44682	unknown	complete r	65	65	68
DO44684	unknown	relapse	77	77	83
DO44686	unknown	complete r	78	78	78
DO44688	unknown	relapse	60	60	61
DO44692	unknown	complete r	44	44	56
DO44694	unknown	relapse	60	60	69
DO44696	unknown		53	53	
DO44698	unknown	relapse	68	68	74
DO44700	unknown	relapse	40	40	42
DO44704	unknown	relapse	63	63	66
DO44706	unknown	relapse	73	73	79
DO44708	unknown		67	67	
DO44710	unknown	relapse	42	42	43
DO44712	unknown	complete r	68	68	75
DO44714	unknown		62	62	
DO44716	unknown	relapse	60	60	63
DO44718	unknown	complete r	71	71	78
DO44724	unknown	relapse	67	67	72
DO44726	unknown	relapse	62	62	64
DO44728	unknown	complete r	65	65	67
DO44740	unknown		73	73	
DO44744	unknown	relapse	46	46	47
DO44748	unknown	relapse	80	80	81
DO44752	unknown	relapse	69	69	70
DO44754	unknown		69	69	
DO44756	unknown	complete r	42	42	42
DO44764	unknown	complete r	66	66	70
DO44766	unknown	complete r	26	26	27
DO44768	unknown	complete r	21	21	22
DO44772	unknown	relapse	70	70	75
DO44774	unknown	relapse	63	63	64
DO44776	unknown	relapse	34	34	35
DO44780	unknown		67	67	
DO44782	unknown	relapse	62	62	64
DO44784	unknown	complete r	56	56	63
DO44786	unknown	relapse	78	78	82
DO44788	unknown		72	72	
DO44790	unknown	complete r	74	74	74
DO44792	unknown		77	77	
DO44794	unknown		68	68	
DO44796	unknown	complete r	54	54	59
DO44798	unknown		69	69	
DO44800	unknown		43	43	
DO44802	unknown		71	71	
DO44804	unknown	relapse	69	69	72
DO44806	unknown	complete r	65	65	65
DO44808	unknown		74	74	
DO44810	unknown	complete r	49	49	53
DO44812	unknown		63	63	
DO44818	unknown	relapse	26	26	31
DO44822	unknown	relapse	67	67	70

DO44824	unknown	relapse	28	28	28
DO44828	unknown	relapse	65	65	70
DO44830	unknown	relapse	50	50	51
DO44832	unknown		70	70	
DO44834	unknown	relapse	71	71	77
DO44836	unknown	relapse	59	59	61
DO44840	unknown	relapse	77	77	78
DO44846	unknown		60	60	
DO44848	unknown		61	61	
DO44854	unknown		61	61	
DO44856	unknown		73	73	
DO44858	unknown	complete r	73	73	74
DO44862	unknown		73	73	
DO44864	unknown		70	70	
DO44868	unknown		57	57	
DO223920	unknown	progressio	43		46
DO223921	unknown	complete r	49		51
DO223922	unknown	progressio	16		18
DO223924	unknown	complete r	58		60
DO22835	unknown		67		67
DO22836	unknown	progressio	72		77
DO22839	unknown	complete r	61		61
DO22845	unknown	complete r	80		80
DO22848	unknown	complete r	52		52
DO22854	unknown	complete r	70		70
DO22857	unknown	progressio	73		73
DO22860	unknown	complete r	61		61
DO22863	unknown	complete r	63		66
DO22866	unknown	complete r	68		68
DO22872	unknown	complete r	71		71
DO22875	unknown	complete r	44		44
DO22878	unknown	complete r	53		53
DO22881	unknown	complete r	48		48
DO22884	unknown	complete r	45		48
DO22893	unknown	complete r	54		54
DO22896	unknown	complete r	75		76
DO22899	unknown	progressio	68		68
DO22902	unknown	complete r	76		76
DO22905	unknown		77		78
DO22914	unknown	complete r	81		81
DO22917	unknown	complete r	69		70
DO22920	unknown	progressio	55		59
DO22923	unknown	progressio	68		74
DO22929	unknown	complete r	62		65
DO22932	unknown	complete r	46		46
DO22935	unknown	complete r	73		73
DO22950	unknown	complete r	62		62
DO22953	unknown	complete r	67		67
DO22959	unknown	complete r	46		46
DO22970	unknown	complete r	79		80
DO22974	unknown	progressio	73		73
DO22978	unknown	complete r	38		38
DO22982	unknown		20		21
DO22986	unknown	progressio	23		25
DO22990	unknown	complete r	66		66
DO22994	unknown	progressio	71		73
DO22998	unknown	complete r	68		72
DO23018	unknown	progressio	64		64

DO23023	unknown			52		52
DO23028	unknown		complete r	50		50
DO23033	unknown		complete r	73		76
DO23038	unknown		complete r	24		24
DO23053	unknown		complete r	71		71
DO23058	unknown		complete r	61		61
DO23068	unknown		complete r	36		36
DO23078	unknown		complete r	58		58
DO44872	unknown		complete r	70		72
DO44873	unknown		complete r	54		55
DO44874	unknown		complete r	51		51
DO45029	yes		no evidenc	73		
DO45031	no		relapse	66		
DO45033	unknown		relapse	62		
DO45035	yes		relapse	58		
DO45037	no		relapse	67		
DO45039	yes		relapse	58		
DO45041	unknown		no evidenc	69		
DO45043	yes		relapse	56		
DO45045	yes		relapse	57		
DO45047	yes		relapse	40		
DO45049	yes		relapse	73		
DO45051	unknown		relapse	57		
DO45053	no		no evidenc	47		
DO45055	no		relapse	60		
DO45057	yes		relapse	65		
DO45059	no		relapse	49		
DO45061	no		relapse	53		
DO45064	unknown		relapse	66		
DO45065	no		relapse	41		
DO45067	no		relapse	66		
DO45069	unknown		relapse	58		
DO45071	yes		relapse	58		
DO45073	no		relapse	50		
DO45075	unknown		no evidenc	66		
DO45077	no		relapse	66		
DO45079	unknown		no evidenc	59		
DO45081	yes		relapse	63		
DO45083	no		relapse	62		
DO45085	no		relapse	68		
DO45087	yes		relapse	56		
DO45089	yes		relapse	45		
DO45091	no			56	56	
DO45092	no			62	62	
DO45093	no			71	71	
DO45094	no			76	76	
DO45095	no			66	66	
DO45096	no			69	69	
DO45097	no			69	69	
DO45099	unknown			46	46	
DO45101	unknown			63	63	
DO45103	yes			47	47	
DO45105	unknown			61	61	
DO45107	yes			63	63	
DO45109	no			63	63	
DO45111	yes			74	74	
DO45113	yes			58	58	
DO45115	no	C67		81	81	

DO45117	yes		73	73
DO45119	yes	C15	74	74
DO45121	no		61	61
DO45123	yes		78	78
DO45125	no		58	58
DO45127	no		73	73
DO45129	yes		61	61
DO45131	yes		53	53
DO45133	yes		64	64
DO45135	yes		77	77
DO45137	yes		38	38
DO45139	yes		63	63
DO45141	no		73	73
DO45143	no		69	69
DO45145	no		78	78
DO45147	yes		57	57
DO45149	no		70	70
DO45151	no		64	64
DO45153	no		77	77
DO45155	yes	C67	69	69
DO45157	no		62	62
DO45159	no		65	65
DO45161	no	C18	74	74
DO45163	yes		76	76
DO45165	yes		75	75
DO45167	yes		67	67
DO45169	no		74	74
DO45171	unknown		56	56
DO45173	yes		63	63
DO45175	yes		74	74
DO45177	no		81	81
DO45179	yes		78	78
DO45181	yes		84	84
DO45183	no		72	72
DO45185	unknown		89	89
DO45187	unknown		32	32
DO45189	yes		85	85
DO45191	yes		61	61
DO45193	no	C16	68	68
DO45195	no		62	62
DO45197	no		51	51
DO45199	no		86	86
DO45201	yes		49	49
DO45203	no		71	71
DO45205	yes		67	67
DO45207	no		68	68
DO45209	yes		70	70
DO45211	no		76	76
DO45213	yes		58	58
DO45215	yes		65	65
DO45217	no		69	69
DO45219	no		78	78
DO45221	no		75	75
DO45223	no		67	67
DO45225	no		69	69
DO45227	no		57	57
DO45229	no		74	74
DO45231	no		84	84

DO45233	no	C67		83	83	
DO45235	no			68	68	
DO45237	no			58	58	
DO45239	no			42	42	
DO45241	yes			49	49	
DO45243	no			74	74	
DO45245	unknown	C16		75	75	
DO45247	no			31	31	
DO45249	yes			57	57	
DO45251	no			57	57	
DO45253	yes			50	50	
DO45255	yes			81	81	
DO45257	no			46	46	
DO45259	no			65	65	
DO45261	no			74	74	
DO45263	no			67	67	
DO45265	no			73	73	
DO45267	yes			73	73	
DO45269	no			59	59	
DO45273	no			62	62	
DO45275	no			65	65	
DO45277	no			64	64	
DO45279	no			67	67	
DO45281	no			83	83	
DO45283	unknown	C15		60	60	
DO45285	no			59	59	
DO45287	no	C20		74	74	
DO45289	no			78	78	
DO45291	no			69	69	
DO45293	no			68	68	
DO45295	no			76	76	
DO45297	no			61	61	
DO45299	unknown			70	70	
DO45301	unknown			56	56	
DO45303	unknown			62	62	
DO45305	no			66	66	
DO45307	no			54	54	
DO45309	no			73	73	
DO44876	unknown		complete r	61		66
DO44877	unknown		complete r	52		52
DO44880	unknown		complete r	69		69
DO44885	unknown		progressio	77		78
DO44886	unknown		complete r	60		60
DO44890	unknown		complete r	51		51
DO44892	unknown		complete r	35		35
DO44895	unknown		complete r	60		60
DO44907	unknown		progressio	65		66
DO44911	unknown		complete r	78		83
DO44919	unknown		complete r	80		80
DO44927	unknown		complete r	64		64
DO44930	unknown		complete r	67		69
DO44934	unknown		complete r	46		50
DO44938	unknown		progressio	59		59
DO44946	unknown		complete r	45		45
DO44950	unknown		complete r	72		73
DO44958	unknown		progressio	32		39
DO44962	unknown		complete r	69		69
DO44966	unknown		complete r	84		84

DO44974	unknown		complete r	47		47
DO44978	unknown		complete r	70		70
DO44986	unknown		progressio	56		61
DO44998	unknown		complete r	58		58
DO48672	yes	C34		81	81	
DO48674	no			80	80	
DO48677	unknown			58	58	
DO48679	no			72	72	
DO48681	no			57	57	
DO48682	yes	C25		72	72	
DO48684	no	C50		71	71	
DO48686	yes			75	75	
DO48687	no			73	73	
DO48689	no			63	63	
DO48691	no	C67		71	71	
DO48692	no			77	77	
DO48693	yes			71	71	
DO48694	yes			71	71	
DO48695	no	C34		77	77	
DO48697	no			64	64	
DO48700	yes			78	78	
DO48701	yes			78	78	
DO48703	yes			64	64	
DO48704	yes			68	68	
DO48706	no			72	72	
DO48709	no			74	74	
DO48712	yes			50	50	
DO48715	no			58	58	
DO48716	yes			71	71	
DO48717	yes			54	54	
DO48719	no			80	80	
DO48720	no			62	62	
DO48721	yes			58	58	
DO48723	yes			73	73	
DO48725	no			66	66	
DO48727	no			81	81	
DO48728	yes	D11		74	74	
DO48730	no			73	73	
DO48732	yes			73	73	
DO48733	yes			72	72	
DO48736	yes			76	76	
DO48737	no			52	52	
DO48738	no			83	83	
DO48741	yes			64	64	
DO48742	no			76	76	
DO48743	no	C50, C05		69	69	
DO48746	no			76	76	
DO48747	no			62	62	
DO48751	no			68	68	
DO48753	no			55	55	
DO48757	no			68	68	
DO48759	no			73	73	
DO48760	no			70	70	
DO48761	yes			55	55	
DO45005	unknown		complete r	58		58
DO45009	unknown		complete r	76		76
DO45013	unknown		progressio	65		70
DO49660	unknown			64	64	

DO49661	unknown		83	83	
DO49662	unknown		29	29	
DO49663	unknown		46	46	
DO49664	unknown		24	24	
DO49665	unknown		30	30	
DO49666	unknown		47	47	
DO49667	unknown		33	33	
DO49668	unknown		29	29	
DO49669	unknown		30	30	
DO49670	unknown		32	32	
DO49671	unknown		42	42	
DO49672	unknown		34	34	
DO49673	unknown		67	67	
DO49674	unknown		23	23	
DO49675	unknown		43	43	
DO49676	unknown		40	40	
DO49677	unknown		36	36	
DO49678	unknown		21	21	
DO49679	unknown		34	34	
DO49680	unknown		48	48	
DO49681	unknown		38	38	
DO45021	unknown	complete r	74		74
DO49683	unknown		27	27	
DO49684	unknown		29	29	
DO49685	unknown		31	31	
DO49686	unknown		46	46	
DO49687	unknown		40	40	
DO49689	unknown		47	47	
DO49691	unknown		50	50	
DO49692	unknown		35	35	
DO45025	unknown	complete r	67		67
DO48473	unknown	complete r	73		81
DO48475	unknown	progressio	44		45
DO48477	unknown	complete r	20		20
DO48478	unknown	complete r	71		71
DO48481	unknown	complete r	60		60
DO48483	unknown	complete r	66		66
DO48496	unknown	complete r	29		29
DO48505	unknown	complete r	58		58
DO48528	unknown	complete r	68		68
DO48532	unknown	complete r	61		61
DO48552	unknown	complete r	25		26
DO48555	unknown	complete r	50		50
DO48558	unknown	complete r	59		59
DO48559	unknown	progressio	65		65
DO48562	unknown	complete r	53		53
DO48587	unknown	complete r	77		79
DO48593	unknown	complete r	65		65
DO48599	unknown	complete r	48		48
DO48602	unknown	complete r	66		66
DO49657	unknown	progressio	66		66
DO50736	unknown	relapse	59	59	59
DO50737	unknown	relapse	66	66	66
DO50738	unknown	relapse	72	72	72
DO50739	unknown	relapse	71	71	71
DO50740	unknown	relapse	59	59	59
DO50741	unknown	relapse	73	73	73
DO50742	unknown	relapse	51	51	51

DO50743	unknown		relapse	59	59	59
DO50744	unknown		relapse	60	60	60
DO50745	unknown		relapse	79	79	79
DO50746	unknown		relapse	53	53	53
DO50747	unknown		relapse	75	75	75
DO50748	unknown		relapse	67	67	67
DO50749	unknown		relapse	73	73	73
DO50750	unknown		relapse	78	78	78
DO50751	unknown		relapse	67	67	67
DO50752	unknown		relapse	66	66	66
DO50753	unknown		relapse	61	61	61
DO50754	unknown		relapse	62	62	62
DO50755	unknown		relapse	78	78	78
DO50756	unknown		relapse	72	72	72
DO50757	unknown		relapse	61	61	61
DO50758	unknown		relapse	81	81	81
DO50759	unknown		relapse	53	53	53
DO50760	unknown		relapse	79	79	79
DO50761	unknown		relapse	61	61	61
DO50762	unknown		relapse	59	59	59
DO50763	unknown		relapse	47	47	47
DO50764	unknown		relapse	64	64	64
DO50765	unknown		relapse	56	56	56
DO50766	unknown		relapse	49	49	49
DO50767	unknown		relapse	61	61	61
DO50768	unknown		relapse	63	63	63
DO50769	unknown		relapse	51	51	51
DO50770	unknown		relapse	72	72	72
DO50771	unknown		relapse	63	63	63
DO50772	no			70	70	
DO50773	unknown		relapse	50	50	50
DO50774	no			68	68	
DO50775	unknown		relapse	71	71	71
DO50776	no	C61		75	75	
DO50777	unknown		relapse	75	75	75
DO50778	yes			60	60	
DO50779	unknown		relapse	63	63	63
DO50780	no			70	70	
DO50781	unknown		relapse	70	70	70
DO50782	unknown		relapse	74	74	74
DO50783	no	C66		62	62	
DO50784	unknown		relapse	69	69	69
DO50785	no			37	37	
DO50786	unknown		relapse	63	63	63
DO50787	no			63	63	
DO50788	unknown		relapse	61	61	61
DO50789	yes	C16		73	73	
DO50790	unknown		relapse	62	62	62
DO50791	yes			79	79	
DO50792	unknown		relapse	62	62	62
DO50793	yes	C16		76	76	
DO50794	unknown		relapse	66	66	66
DO50795	unknown		relapse	63	63	63
DO50796	no			69	69	
DO50797	unknown		relapse	62	62	62
DO50798	no			77	77	
DO50799	yes			66	66	
DO50800	yes			74	74	

DO50802	yes	C18		49	49	
DO50803	no	C18		62	62	
DO50804	unknown	C66		81	81	
DO50805	no	C61		72	72	
DO50806	yes			70	70	
DO50807	yes			67	67	
DO50808	no	C67		68	68	
DO50809	yes			56	56	
DO50810	unknown			53	53	
DO50811	no			62	62	
DO50812	unknown		complete r	62	62	65
DO50813	no			56	56	
DO50814	no			83	83	
DO50815	no			62	62	
DO50816	no			63	63	
DO50817	no			75	75	
DO50818	no			65	65	
DO50819	no			72	72	
DO50820	no			77	77	
DO50821	unknown		complete r	60	60	65
DO50822	no			66	66	
DO50823	unknown		complete r	53	53	59
DO50824	unknown		complete r	49	49	53
DO50825	no			70	70	
DO50826	unknown			57	57	
DO50827	no			48	48	
DO50828	unknown			69	69	
DO50829	yes			78	78	
DO50830	unknown		relapse	63	63	63
DO50831	no			52	52	
DO50832	yes			73	73	
DO50833	unknown		relapse	63	63	72
DO50834	unknown			72	72	
DO50835	unknown			65	65	
DO50836	unknown		complete r	52	52	58
DO50837	no			53	53	
DO50838	unknown			60	60	
DO50839	yes			47	47	
DO50840	no			49	49	
DO50841	unknown		complete r	56	56	62
DO50842	no			75	75	
DO50843	unknown		relapse	58	58	66
DO50844	yes			75	75	
DO50845	no			72	72	
DO50846	unknown		relapse	72	72	73
DO50847	unknown			60	60	
DO50848	no			74	74	
DO50849	unknown		relapse	66	66	67
DO50850	no			74	74	
DO50851	no			80	80	
DO50852	unknown			44	44	
DO50853	unknown			32	32	
DO50854	unknown			64	64	
DO50855	no			47	47	
DO50856	unknown		relapse	78	78	78
DO50857	yes			72	72	
DO50858	unknown			67	67	
DO50859	no	C25		77	77	

DO50860	unknown		45	45	
DO50861	unknown		77	77	
DO50862	unknown	relapse	54	54	62
DO50864	unknown	relapse	68	68	71
DO50865	unknown		71	71	
DO50866	unknown	complete r	84	84	90
DO50867	unknown		60	60	
DO50868	unknown	complete r	69	69	74
DO50869	unknown		76	76	
DO50871	unknown	complete r	74	74	81
DO50872	unknown	relapse	66	66	69
DO50873	unknown	relapse	41	41	41
DO50874	unknown		76	76	
DO50875	unknown		69	69	
DO50877	unknown	complete r	75	75	78
DO50879	unknown		71	71	
DO50880	unknown		78	78	
DO50881	unknown		56	56	
DO50882	unknown	complete r	75	75	75
DO50883	unknown		77	77	
DO50884	unknown	complete r	59	59	61
DO50885	unknown		75	75	
DO50886	unknown		64	64	
DO50888	unknown	complete r	63	63	66
DO50889	unknown		43	43	
DO50890	unknown	complete r	53	53	57
DO50891	unknown	complete r	60	60	61
DO50892	unknown	complete r	72	72	76
DO50893	unknown		72	72	
DO50894	unknown		50	50	
DO50895	unknown	complete r	57	57	60
DO50897	unknown	complete r	80	80	81
DO50898	unknown		83	83	
DO50899	unknown		55	55	
DO50900	unknown		49	49	
DO50901	unknown	complete r	76	76	81
DO50902	unknown		58	58	
DO50903	unknown		73	73	
DO50904	unknown	complete r	68	68	74
DO50905	unknown		55	55	
DO50906	unknown		57	57	
DO50907	unknown		67	67	
DO50908	unknown	relapse	64	64	68
DO50909	unknown		50	50	
DO50910	unknown		69	69	
DO50911	unknown		75	75	
DO50912	unknown		54	54	
DO50913	unknown		66	66	
DO50914	unknown	relapse	52	52	60
DO50915	unknown	relapse	52	52	55
DO50916	unknown		65	65	
DO50917	unknown		34	34	
DO50919	unknown		73	73	
DO50920	unknown		41	41	
DO50921	unknown		75	75	
DO50922	unknown		65	65	
DO50923	unknown	complete r	74	74	76
DO50924	unknown	relapse	80	80	81

DO50925	unknown	relapse	69	69	72
DO50926	unknown	complete r	21	21	24
DO50927	unknown		65	65	
DO50928	unknown	relapse	61	61	62
DO50929	unknown	complete r	85	85	86
DO50930	unknown	relapse	73	73	74
DO50931	unknown		57	57	
DO50932	unknown	complete r	58	58	59
DO50933	unknown		56	56	
DO50934	unknown		48	48	
DO50935	unknown		61	61	
DO50936	unknown		75	75	
DO50937	unknown	relapse	56	56	59
DO50938	unknown		53	53	
DO50939	unknown	complete r	57	57	57
DO50940	unknown		74	74	
DO50942	unknown	relapse	48	48	48
DO50944	unknown		90	90	
DO50946	unknown		53	53	
DO50947	unknown	relapse	37	37	39
DO50948	unknown	complete r	33	33	33
DO50949	unknown	complete r	65	65	66
DO50950	unknown	relapse	62	62	63
DO50951	unknown		68	68	
DO50952	unknown		65	65	
DO50953	unknown		49	49	
DO50954	unknown		70	70	
DO50955	unknown	complete r	75	75	81
DO50956	unknown	complete r	17	17	23
DO50957	unknown	complete r	55	55	61
DO50958	unknown	relapse	65	65	69
DO50960	unknown		77	77	
DO50961	unknown	relapse	54	54	55
DO50962	unknown		76	76	
DO50963	unknown	relapse	65	65	72
DO50964	unknown	complete r	78	78	82
DO50966	unknown	complete r	58	58	64
DO50967	unknown	relapse	80	80	84
DO50968	unknown		75	75	
DO50969	unknown		71	71	
DO50970	unknown		72	72	
DO50971	unknown	relapse	61	61	66
DO50972	unknown		71	71	
DO50973	unknown	complete r	73	73	77
DO50974	unknown		78	78	
DO50976	unknown	relapse	74	74	74
DO49658	unknown	progressio	66		66
DO49659	unknown	complete r	63		63
DO49682	unknown	complete r	54		54
DO49693	unknown	complete r	65		65
DO49697	unknown	complete r	64		64
DO49698	unknown	progressio	61		62
DO49699	unknown	complete r	79		79
DO49701	unknown	complete r	48		48
DO49703	unknown	progressio	68		68
DO49705	unknown	complete r	64		64
DO49707	unknown	complete r	61		61
DO49709	unknown	complete r	63		64

DO49710	unknown	complete r	77	78
DO49712	unknown		61	61
DO49714	unknown	complete r	34	34
DO49715	unknown	complete r	74	75
DO49716	unknown	complete r	54	54
DO49718	unknown	complete r	64	65
DO49720	unknown		79	81
DO49722	unknown	progressio	64	70
DO52163	unknown	complete r	58	58
DO52166	unknown	complete r	70	70
DO52168	unknown	complete r	61	61
DO52169	unknown	complete r	75	76
DO52170	unknown	complete r	62	62
DO52171	unknown	complete r	50	50
DO52172	unknown	progressio	26	26
DO52173	unknown	complete r	63	63
DO52174	unknown	complete r	48	48
DO52175	unknown	complete r	57	57
DO52177	unknown	complete r	59	59
DO52179	unknown	complete r	47	47
DO52181	unknown	complete r	58	58
DO52182	unknown	complete r	54	54
DO52183	unknown	complete r	56	56
DO52184	unknown	complete r	59	59
DO52185	unknown	complete r	61	61
DO52186	unknown	complete r	18	18
DO52187	unknown	complete r	74	74
DO52188	unknown	complete r	39	39
DO52189	unknown	complete r	51	51
DO52190	unknown	complete r	52	52
DO52194	unknown	complete r	51	51
DO52196	unknown	progressio	65	70
DO52197	unknown	complete r	74	78
DO52200	unknown	progressio	62	67
DO52205	unknown	progressio	69	73
DO52206	unknown	progressio	75	80
DO52207	unknown	complete r	70	70
DO52208	unknown		66	66
DO52209	unknown	progressio	48	52
DO52210	unknown		64	65
DO52211	unknown	progressio	50	51
DO52212	unknown	complete r	39	43
DO52213	unknown	complete r	59	62
DO52214	unknown	complete r	43	46
DO52215	unknown	progressio	66	66
DO52217	unknown	progressio	73	73
DO52218	unknown	progressio	51	52
DO52219	unknown	complete r	66	67
DO52220	unknown	complete r	43	45
DO52222	unknown	progressio	54	56
DO52224	unknown	complete r	66	67
DO52226	unknown	complete r	73	75
DO52227	unknown	complete r	66	68
DO52229	unknown	complete r	51	53
DO52231	unknown	complete r	51	53
DO52233	unknown	complete r	43	45
DO52234	unknown	complete r	70	72
DO52235	unknown	complete r	68	70

DO52237	unknown	complete r	58	59
DO52239	unknown	complete r	59	60
DO52241	unknown	complete r	55	55
DO52243	unknown	complete r	45	45
DO52244	unknown	complete r	59	59
DO52245	unknown	complete r	58	62
DO52247	unknown	complete r	63	63
DO52248	unknown	complete r	60	60
DO52249	unknown	complete r	48	48
DO52251	unknown	complete r	55	55
DO52253	unknown	complete r	45	45
DO52254	unknown	complete r	52	52
DO52256	unknown	complete r	51	51
DO52258	unknown	complete r	50	50
DO52260	unknown	complete r	49	49
DO52261	unknown	progressio	59	59
DO52262	unknown	complete r	72	73
DO52263	unknown	complete r	45	45
DO52264	unknown	complete r	69	70
DO52266	unknown	complete r	65	66
DO52267	unknown	complete r	60	61
DO52268	unknown	complete r	51	52
DO52270	unknown	progressio	55	56
DO52271	unknown	complete r	59	60
DO52272	unknown	complete r	73	74
DO52273	unknown	complete r	51	53
DO52275	unknown	complete r	48	54
DO52276	unknown	complete r	38	44
DO52278	unknown	complete r	44	50
DO52279	unknown	complete r	56	60
DO52281	unknown	complete r	46	51
DO52282	unknown	complete r	35	40
DO52283	unknown	progressio	38	42
DO52284	unknown	complete r	56	60
DO52285	unknown	complete r	38	42
DO52286	unknown	complete r	54	58
DO52288	unknown	progressio	69	73
DO52289	unknown	complete r	69	72
DO52290	unknown	complete r	76	76
DO52291	unknown	progressio	60	61
DO52292	unknown	complete r	31	31
DO52293	unknown	complete r	48	48
DO52294	unknown	complete r	58	58
DO52296	unknown	complete r	50	50
DO52297	unknown	complete r	58	58
DO52300	unknown	complete r	38	38
DO52303	unknown	progressio	64	64
DO52304	unknown	complete r	66	66
DO52305	unknown	complete r	57	59
DO52308	unknown	complete r	48	48
DO52309	unknown	complete r	47	48
DO52311	unknown	progressio	66	67
DO52312	unknown	progressio	42	43
DO52314	unknown	complete r	59	60
DO52316	unknown	complete r	69	71
DO52317	unknown	progressio	62	63
DO52318	unknown	progressio	24	25
DO52319	unknown		59	60

DO52321	unknown	complete r	51		52
DO52323	unknown	progressio	60		60
DO52324	unknown	complete r	69		69
DO52325	unknown		68		68
DO52326	unknown	complete r	71		71
DO52327	unknown	complete r	62		62
DO52328	unknown	complete r	59		59
DO52329	unknown	complete r	66		68
DO52330	unknown	complete r	72		73
DO52331	unknown	complete r	67		68
DO52332	unknown	complete r	56		56
DO52333	unknown	progressio	51		53
DO52336	unknown		55		55
DO52393	unknown	complete r	53	53	59
DO52394	unknown	complete r	49	49	53
DO52395	unknown		56	56	
DO52396	unknown		68	68	

donor_diag	donor_inte	donor_rela	donor_rela	donor_sex	donor_surv	donor_tum	donor_tum	donor_tum
C22.0				female	9			
C22.0				male	11			
C22.0				male	12			
C22.0				male	14			
C22.0				male	16			
C22.0				male	19			
C22.0				female	27			
C22.0				female	31			
C22.0				female	34			
C22.0				male	36			
C22.0				female	46			
C22.0				male	52			
C22.0				male	56			
C22.0				male	65			
C22.0				female	65			
C22.0				male	67			
C22.0				male	91			
C22.0				male	91			
C22.0				female	115			
C22.0				male	129			
C22.0				male	153			
C22.0				male	171			
C22.0				female	171			
C22.0				male	194			
C22.0				male	195			
C22.0				male	214			
C22.0				male	223			
C22.0				male	233			
C22.0				female	283			
C22.0				female	308			
C22.0				male	359			
C22.0				male	365			
C22.0				male	373			
C22.0				male	381			
C22.0				female	394			
C22.0				female	410			
C22.0				male	412			
C22.0				male	415			
C22.0				male	419			
C22.0				male	425			
C22.0				male	438			
C22.0				female	452			
C22.0				male	469			
C22.0				male	535			
C22.0				female	555			
C22.0				female	556			
C22.0	1729	456	local recur	female	1729			
C22.0	1826	748	local recur	female	1826			
C22.0	412	412		female	412			
C22.0	1239	503	local recur	female	1239			
C22.0				female	558			
C22.0				male	581			
C22.0				female	601			
C22.0				male	612			
C22.0				female	633			
C22.0				female	639			

C22.0				male	643			
C22.0				female	660			
C22.0				male	693			
C22.0				male	695			
C22.0				male	711			
C22.0				male	724			
C22.0				female	757			
C22.0				female	770			
C22.0				male		T1N0M0	BCLC:A	TNM 7th e
C22.0	1826	1386	local recur	female	1826	T1N0M0	BCLC:A	TNM 7th e
C22.0	1826	1826		female	1826	T1N0M0	BCLC:A	TNM 7th e
C22.0	697	380	local recur	female	697	T3bN0M0	BCLC:C	TNM 7th e
D13.4				female				
D13.4				female				
				male				
				female				
				male				
				male				
				female				
				male				
				female				
				male				
				male				
				male				
				male				
				male				
				male				
				male				
				female				
				male				
				male				
				male				
				male				
				male				
				male				
				male				
				male				
				male				
				male				
				male				
				male				
				female				
				male				
				female				
				male				
				female				
				male				
				male				
				male				
				female				
				female				
				male				
				male				
				male				
				male				
				male				
				male				
				male				

			male				
			male				
			male				
			male				
			male				
			male				
			male				
			female				
			male				
			male				
			male				
			male				
			female				
			male				
			male				
			male				
			male				
			male				
			male				
C22.0	1353	1353	male	1353	T1N0M0	BCLC:A	TNM 7th e
			male				
			male				
			male				
			male				
			female				
			male				
			male				
C22.0			male	785			
			male				
			male				
C22.0			female	827			
			male				
			male				
C22.0			male	837			
			male				
			male				
C22.0			male	837			
			male				
			male				
C22.0			female	848			
			male				
			male				
C22.0			female	899			
			male				
			male				
			male				
			male				
			male				
			male				
C22.0			female	931			
			female				
			male				
			female				
			male				
			male				
			female				
C22.0			female	1135			
			male				

	male	
	male	
	male	
	male	
	male	
C22.0	female	1147
	male	
	male	
	female	
C22.0	male	1149
	male	
	male	
	male	
	male	
	male	
C22.0	female	1210
	male	
	male	
	male	
	male	
	male	
	male	
	male	
	male	
	male	
	male	
C22.0	male	1229
	male	
	male	
	male	
	male	
	male	
	male	
	male	
C22.0	male	1271
	male	
	male	
	male	
	male	
	male	
C22.0	male	1372
	male	
	male	
	male	
	female	
	male	
	male	
	female	
	male	
C22.0	male	1386
	male	
	male	
	male	
	male	
	male	
C22.0	male	1423
	male	
	male	

		male	
C22.0		female	1490
		male	
		male	
		male	
		male	
C22.0		female	1560
		male	
		female	
		male	
		male	
		male	
C22.0	1569	female	1569
		male	
		male	
		male	
		male	
		male	
		male	
		male	
		male	
C22.0		male	1622
		male	
		male	
		female	
		male	
		male	
		male	
		male	
		male	
		female	
		male	
		male	
		male	
		male	
C22.0		male	1624
		male	
		male	
		male	
		male	
		male	
		male	
		male	
		female	
		male	
		male	
		male	
		female	
		male	
C22.0		male	1685
		male	
		female	
		male	
		male	
		female	
		male	
		female	
C22.0		female	

	male				
	male				
C22.0	male				
	male				
	male				
C22.0	male				
C22.0	male				
	male				
	male			I	TNM
C22.0	male				
C22.0	female				
	male			II	TNM
C22.0	female	1694			
	male			II	TNM
C22.0	male				
	male			I	TNM
	male			I	TNM
C22.0	male				
	female			I	TNM
C22.0	male				
C22.0	male				
	male			I	TNM
	male			II	TNM
	female			III	TNM
	male			I	TNM
	male			II	TNM
	male			III	TNM
	male			I	TNM
	male			I	TNM
	male			I	TNM
	male			III	TNM
	male			I	TNM
	male			III	TNM
	male			I	TNM
	male			I	TNM
	male			I	TNM
	male			I	TNM
	male			II	TNM
	male			I	TNM
	male			II	TNM
	male			I	TNM
C22.0	male	1791			
	female			I	TNM
	male			I	TNM
	male			I	TNM
	male			III	TNM
	male			I	TNM
	female			I	TNM
	male			I	TNM
	male			I	TNM
	male			III	TNM
	male			IV	TNM
	male		T1N0M0	I	TNM
	male		T2N0M0	II	TNM
	male		T1N0M0	I	TNM
	male		T1N0M0	I	TNM
	male		T1N0M0	I	TNM
	male		T1N0M0	I	TNM

	male		T1N0M0	I	TNM
	male		T2N0M0	II	TNM
	female		T2N0M0	II	TNM
	female		T1N0M0	I	TNM
	female		T1N0M0	I	TNM
C22.0	female	2116			
	female		T2N0M0	II	TNM
	male		T2N0M0	II	TNM
	male		T1N0M0	I	TNM
	male		T1N0M0	I	TNM
	female		T1N0M0	I	TNM
	male		T2N0M0	II	TNM
	male		T1N0M0	I	TNM
	male		T3aN0M0	IIIA	TNM
	male		T1N0M0	I	TNM
	male		T1N0M0	I	TNM
	male		T3aN0M0	IIIA	TNM
	male		T1N0M0	I	TNM
	male		T1N0M0	I	TNM
	male		T2N0M0	II	TNM
	male		T1N0M0	I	TNM
	male		T2N0M0	II	TNM
C22.0	female	2131			
	male		T1N0M0	I	TNM
	male		T1N0M0	I	TNM
	male		T1N0M0	I	TNM
	male		T2N0M0	II	TNM
	male		T2N0M0	II	TNM
	male		T1N0M0	I	TNM
	male		T1N0M0	I	TNM
	male		T3bN0M0	IIIb	TNM
	male		T1N0M0	I	TNM
	female		T1N0M0	I	TNM
	male		T1N0M0	I	TNM
C22.0	female	2456			
	male		T1N0M0	I	TNM
	female		T1N0M0	I	TNM
	male		T2N0M0	II	TNM
	male		T2N0M0	II	TNM
	male		T1N0M0	I	TNM
	male		T2N0M0	II	TNM
	female		T2N0M0	II	TNM
	male		T1N0M0	I	TNM
	male		T1N0M0	I	TNM
	male		T1N0M0	I	TNM
	male		T1N0M0	I	TNM
	male		T1N0M0	I	TNM
	male		T1N0M0	I	TNM
	male		T1N0M0	I	TNM
	male		T3bN0M1	IIIb	TNM
	male		T1N0M0	I	TNM
	male		T1N0M0	I	TNM
	male		T2N0M0	II	TNM
	male		T1N0M0	I	TNM
	male		T1N0M0	I	TNM
	male		T1N0M0	I	TNM
	male		T1N0M0	I	TNM
	male		T1N0M0	I	TNM

		male		T2N0M0	II	TNM
		male		T1N0M0	I	TNM
		male		T1N0M0	I	TNM
		female		T1N0M0	I	TNM
C22.0		female	2532			
		female		T2N0M0	II	TNM
		male		T3N0M0	III	TNM
		female		T1N0M0	I	TNM
		male		T3N0M0	III	TNM
		male		T2N0M0	II	TNM
		male		T1N0M0	I	TNM
		male		T2N0M0	II	TNM
		female		T4N0M1	IVb	TNM
		male		T1N0M0	I	TNM
		female		T1N0M0	I	TNM
		male		T1N0M0	I	TNM
		male		T2N0M0	II	TNM
		female		T1N0M0	I	TNM
C22.0		male	2542			
C22.0		male	2759			
C22.0		female	3125			
C22.0		female	3258			
C22.0	2442	male	2442			
C22.0	3675	male	1210			
C22.0	1582	female	1339			
C22.0	1257	male	0			
C22.0	1270	male	2415			
C22.0	357	female	12			
C22.0	1133	female	334			
C22.0	456	male	265			
C22.0	158	male	0			
C22.0	243	male	9			
C22.0	63	male	527			
C22.0	61	female	470			
C22.0	289	male	120			
C22.0	0	male				
C22.0	0	male				
C22.0	0	male				
C22.0	0	male				
C22.0	1292	male				
C22.0	1485	male				
C22.0	1291	female				
C22.0	1210	male				
C22.0	1140	male				
C22.0	918	male				
C22.0	808	male				
C22.0	230	male				
C22.0	1233	male				
C22.0	1644	male				
C22.0	266	male				
C22.0	113	male				
C22.0	19	male				
C22.0	588	male				
C22.0	23	male				
C22.0	553	male				
C22.0	0	male				
C22.0	2455	male				

C22.0	697			male				
C22.0	0			male				
C22.0	22			female				
C22.0	15			female				
C22.0	9			male				
C22.0	473			female				
				female				tumor stag
				female				tumor stag
				female				tumor stag
				male				tumor stag
				female				tumor stag
				female				tumor stag
				male				tumor stag
				female				tumor stag
				female				tumor stag
				female				tumor stag
	1940	288	local recur	male	1940	4A		tumor stag
	120	0	distant rec	male	120	4B		tumor stag
	711	94	local recur	male	711		2	tumor stag
	1817			female	1817		2	tumor stag
	1746	313	local recur	male	1746	4A		tumor stag
	1833	806	local recur	male	1833	4A		tumor stag
	578	118	local recur	male	578		3	tumor stag
	1718	282	local recur	male	1718		3	tumor stag
	1792			male	1792		3	tumor stag
	1494			female	1494		2	tumor stag
	339	87	local recur	male	339	4A		tumor stag
	557			male	557		2	tumor stag
	1586			male	1586		2	tumor stag
	616	575	local recur	male	616		2	tumor stag
	92	6	local recur	male	92	4A		tumor stag
	1553			female	1553		2	tumor stag
	1605	1130	local recur	female	1605		1	tumor stag
	253			female	253		2	tumor stag
	1461			male	1461		3	tumor stag
	775	440	local recur	female	775		1	tumor stag
	1404	707	local recur	male	1404		2	tumor stag
	740	200	local recur	male	740		3	tumor stag
	1357	188	local recur	male	1357		3	tumor stag
	480	53	distant rec	male	480	4A		tumor stag
	1339			male	1339		2	tumor stag
C22.0	487			male	487	G1		neoplasm_
C22.0	1121			male	1121	G2		neoplasm_
C22.0	771			male	771	G1		neoplasm_
	904	260	distant rec	male	904		3	tumor stag
C22.0	1086			female	1086	G2		neoplasm_
C22.0	1103			male	1103	G1		neoplasm_
C22.0	1049			female	1049	G2		neoplasm_
C22.0	977			male	977	G2		neoplasm_
C22.0	926			male	926	G1		neoplasm_
C22.0	309			male	309	G1		neoplasm_
	603	265	local recur	male	603		3	tumor stag
C22.0	361			male	361	G2		neoplasm_
C22.0	357			male	357	G2		neoplasm_
C22.0	351			male	351	G2		neoplasm_
C22.0	248			male	248	G1		neoplasm_
C22.0	400			male	400	G1		neoplasm_
C22.0	419			male	419	G2		neoplasm_

	770	31	local recur	male	770	4A		tumor stag
C22.0	386			male	386	G1		neoplasm_
C22.0	351			male	351	G1		neoplasm_
C22.0	375			male	375	G2		neoplasm_
C22.0	842			male	842	G2		neoplasm_
C22.0	873			female	873	G1		neoplasm_
	893	175	local recur	male	893		3	tumor stag
C22.0	858			male	858	G1		neoplasm_
C22.0	599			male	599	G2		neoplasm_
C22.0	647			male	647	G1		neoplasm_
C22.0	734			female	734	G1		neoplasm_
C22.0	718			male	718	G2		neoplasm_
C22.0	727			male	727	G1		neoplasm_
C22.0	469			male	469	G1		neoplasm_
	1258	1132	local recur	male	1258		3	tumor stag
C22.0	550			male	550	G1		neoplasm_
C22.0	538			female	538	G2		neoplasm_
C22.0	524			male	524	G2		neoplasm_
C22.0	460			female	460	G1		neoplasm_
C22.0	114			female	114	G2		neoplasm_
C22.0	417			female	417	G2		neoplasm_
	1315			male	1315		3	tumor stag
C22.0	1338			male	1338	G1		neoplasm_
C22.0	1305			male	1305	G2		neoplasm_
C22.0	1336			male	1336	G1		neoplasm_
C22.0	1312			male	1312	G1		neoplasm_
C22.0	564			female	564	G3		neoplasm_
C22.0	1280			male	1280	G2		neoplasm_
C22.0	1299			male	1299	G1		neoplasm_
	1166	201	local recur	male	1166		3	tumor stag
C22.0	1233			male	1233	G2		neoplasm_
C22.0	99			male	99	G2		neoplasm_
C22.0	144			male	144	G2		neoplasm_
C22.0	1203			male	1203	G3		neoplasm_
C22.0	1080			male	1080	G1		neoplasm_
C22.0	1219			male	1219	G1		neoplasm_
C22.0	1217			male	1217	G1		neoplasm_
	620	291	local recur	female	620	4A		tumor stag
C22.0	1174			male	1174	G2		neoplasm_
C22.0	1168			male	1168	G1		neoplasm_
C22.0	202			female	202	G2		neoplasm_
C22.0	433			male	433	G2		neoplasm_
C22.0	430			male	430	G1		neoplasm_
C22.0				male		G2		neoplasm_
	1187	990	local recur	male	1187		2	tumor stag
C22.0				male		G2		neoplasm_
C22.0				female		G2		neoplasm_
C22.0				female		G1		neoplasm_
C22.0				male		G2		neoplasm_
C22.0				male		G2		neoplasm_
C22.0				male		ND		neoplasm_
C22.0				male		G1		neoplasm_
	1248	79	local recur	male	1248	4A		tumor stag
C22.0				male		ND		neoplasm_
C22.0				male		G2		neoplasm_
C22.0				female		G2		neoplasm_
C22.0				male		G2		neoplasm_
C22.0				male		G2		neoplasm_

C22.0				male		G2		neoplasm_
C22.0				female		G2		neoplasm_
C22.0				male		G2		neoplasm_
C22.0				male		G2		neoplasm_
C22.0				male		G2		neoplasm_
C22.0				male		G2		neoplasm_
	1198	419	local recur	male	1198	4A		tumor stag
C22.0				male		G2		neoplasm_
C22.0				male		G2		neoplasm_
C22.0				male		G2		neoplasm_
C22.0				male		G2		neoplasm_
C22.0				female		G2		neoplasm_
C22.0				male		G2		neoplasm_
C22.0				male		G2		neoplasm_
C22.0				female		G2		neoplasm_
C22.0				female		G1		neoplasm_
C22.0				female		G2		neoplasm_
	305	151	distant rec	male	305		3	tumor stag
C22.0				male		G2		neoplasm_
C22.0				male		G2		neoplasm_
C22.0				female		G2		neoplasm_
C22.0				female		G2		neoplasm_
C22.0	326			female	326	G2		neoplasm_
C22.0	211			male	211	G3		neoplasm_
C22.0	742			male	742	G2		neoplasm_
C22.0	57			male	57	G1		neoplasm_
C22.0	726			male	726	G2		neoplasm_
	1133	191	local recur	male	1133		3	tumor stag
C22.0	694			female	694	G2		neoplasm_
C22.0	662			male	662	G1		neoplasm_
C22.0	687			male	687	G2		neoplasm_
C22.0	673			male	673	G2		neoplasm_
C22.0	26			male	26	G2		neoplasm_
C22.0	261			male	261	G1		neoplasm_
C22.0	151			male	151	G2		neoplasm_
C22.0	578			male	578	G3		neoplasm_
C22.0	640			male	640	G2		neoplasm_
C22.0	39			female	39	G1		neoplasm_
	1126			male	1126		2	tumor stag
C22.0	148			male	148	G3		neoplasm_
C22.0	611			male	611	G3		neoplasm_
C22.0	432			male	432	G2		neoplasm_
C22.0	544			male	544	G2		neoplasm_
C22.0	564			male	564	G2		neoplasm_
C22.0	524			male	524	G2		neoplasm_
C22.0	283			female	283	G2		neoplasm_
C22.0	493			male	493	G2		neoplasm_
C22.0				male				
C22.0	544			male	544	G2		neoplasm_
C22.0	544			male	544	G1		neoplasm_
	1111	477	local recur	male	1111		2	tumor stag
C22.0	9			female	9	G2		neoplasm_
C22.0	172			male	172	G3		neoplasm_
C22.0	92			male	92	G2		neoplasm_
C22.0				male				
C22.0	458			female	458	G1		neoplasm_
C22.0	20			male	20	G2		neoplasm_
C22.0	472			male	472	G3		neoplasm_

C22.0	138			female	138	G1		neoplasm_
C22.0	176			male	176	G2		neoplasm_
	9			male	9		3	tumor stag
C22.0	447			male	447	G2		neoplasm_
C22.0	414			male	414	G2		neoplasm_
C22.0	423			male	423	G2		neoplasm_
C22.0	377			male	377	G2		neoplasm_
C22.0	122			male	122	G2		neoplasm_
C22.0	380			male	380	G2		neoplasm_
C22.0	394			male	394	G2		neoplasm_
C22.0	377			male	377	G1		neoplasm_
	1055			female	1055		3	tumor stag
C22.0	57			female	57	G2		neoplasm_
C22.0	292			male	292	G2		neoplasm_
C22.0	335			male	335	G2		neoplasm_
C22.0	331			male	331	G3		neoplasm_
C22.0	80			male	80	G1		neoplasm_
C22.0	355			male	355	G3		neoplasm_
	771	523	distant rec	male	771		3	tumor stag
C22.0	342			male	342	G2		neoplasm_
C22.0	22			male	22	G2		neoplasm_
C22.0	360			male	360	G1		neoplasm_
C22.0	337			male	337	ND		neoplasm_
C22.0	368			female	368	G1		neoplasm_
	1053			male	1053		3	tumor stag
C22.0	309			male	309	G2		neoplasm_
C22.0	267			male	267	ND		neoplasm_
C22.0	290			male	290	G2		neoplasm_
C22.0	335			female	335	G1		neoplasm_
C22.0	305			male	305	G1		neoplasm_
C22.0	306			male	306	G1		neoplasm_
C22.0	236			female	236	G2		neoplasm_
	548	200	local recur	female	548	4A		tumor stag
C22.0	260			female	260	G2		neoplasm_
C22.0	275			male	275	G2		neoplasm_
C22.0	247			male	247	G2		neoplasm_
C22.0	154			male	154	G2		neoplasm_
C22.0	152			male	152	G2		neoplasm_
C22.0	256			female	256	G2		neoplasm_
	1044			male	1044		2	tumor stag
C26.8				female	49		IIIA	TNM
C26.8				male	2067		II	TNM
C26.8				female	364		IIIA	TNM
C26.8				female	2477		II	TNM
C26.8				male	384		IVA	TNM
C26.8				female	980		I	TNM
C26.8				male	241		IIIA	TNM
	240	201	local recur	male	240		2	tumor stag
C26.8				male	658		IIIA	TNM
C26.8				male	262		II	TNM
C26.8				male	1586		IIIA	TNM
C26.8				male	2662		IVA	TNM
C26.8				male	673		IIIA	TNM
C26.8				female	677		II	TNM
C26.8				male	1602		IIIA	TNM
C26.8				female	189		IIIA	TNM
C26.8				female	1632		IVA	TNM
C26.8				male	2266		IIIA	TNM

C26.8				male	70		IVA	TNM
C26.8				female	420		II	TNM
C26.8				male	48		IVA	TNM
C26.8				male	3362		IIIA	TNM
C26.8				male	0		IIIA	TNM
C26.8				male	350		II	TNM
C26.8				female	327		IIIA	TNM
C26.8				female	3312		IIIA	TNM
C26.8				male	166		IIIA	TNM
C26.8				male	611		II	TNM
	1015	332	local recur	male	1015	3		tumor stag
C26.8				male	3612		II	TNM
C26.8				male	140		IVA	TNM
C26.8				male	2718		II	TNM
C26.8				male	1543		II	TNM
C26.8				female	30		IVA	TNM
C26.8				male	136		II	TNM
C26.8				female	3935		II	TNM
C26.8				male	4014		IIIA	TNM
C26.8				male	4053		II	TNM
C26.8				male	3696		II	TNM
C26.8				male	1602		IIIA	TNM
C26.8				male	411		II	TNM
C26.8				male	1664		IIIA	TNM
C26.8				female	1847		III	AJCC
C26.8				male	1894			
C26.8				male	1956		II	TNM
C26.8				male	2516		III	TNM
C26.8				male	2493		II	TNM
C26.8				male	1516		II	TNM
C26.8				female	1750		II	TNM
	1029	344	local recur	female	1029	4A		tumor stag
C26.8				male	58		IVA	TNM
C26.8				male	1546		II	TNM
C26.8				male	189		IVA	TNM
C26.8				male	570		II	TNM
C26.8				male	1692		II	TNM
C26.8				male	459		IIIA	TNM
C26.8				male	168		IIIA	TNM
C26.8				female	243		I	TNM
C26.8				male	55		IVA	TNM
C26.8				male	84		II	TNM
C26.8				male	1922		II	TNM
C26.8				male	129		II	TNM
C26.8				male	1793		IVA	TNM
C26.8				female	320		IIIA	TNM
C26.8				male	1207		IIIA	TNM
C26.8				male	1913		IIIA	TNM
	976			male	976	3		tumor stag
C26.8				male	1562		IVA	TNM
C26.8				male	45		II	TNM
C26.8				male	2127		II	TNM
C26.8				male	132		IIIA	TNM
C26.8				male	479		IVA	TNM
C26.8				male	81		IVA	TNM
C26.8				male	1667		II	TNM
C26.8				male	203		IIIA	TNM
C26.8				male	104		IVA	TNM

	91			male	91		3	tumor stag
C26.8				male	392		IVA	TNM
C26.8				female	1999		IVA	TNM
C26.8				male	2332		II	TNM
C26.8				male	2353		IIIA	TNM
C26.8				male	73		IIIA	TNM
C26.8				male	2456		II	TNM
C26.8				male	6		II	TNM
C26.8				female	390		IIIA	TNM
C26.8				male	2330		II	TNM
C26.8				male	192		IIIA	TNM
C26.8				male	1116		IIIA	TNM
C26.8				female	2428		II	TNM
C26.8				male	911		II	TNM
C26.8				male	2724		II	TNM
C26.8				male	143		II	TNM
C26.8				male	2482		II	TNM
	606			female	606		2	tumor stag
	211	22	local recur	male	211		3	tumor stag
	969	847	local recur	female	969		1	tumor stag
	862			male	862		3	tumor stag
	889	261	local recur	male	889		2	tumor stag
	771			male	771		2	tumor stag
	911	155	local recur	male	911	4A		tumor stag
	204	189	distant rec	male	204		2	tumor stag
	757			female	757		2	tumor stag
	293			male	293		3	tumor stag
	881	89	local recur	male	881		2	tumor stag
	254			male	254		3	tumor stag
	824			female	824	4A		tumor stag
	773	279	local recur	male	773	4A		tumor stag
	860	277	local recur	female	860		3	tumor stag
	841	127	local recur	male	841		3	tumor stag
	685	132	local recur	male	685	4A		tumor stag
	697			male	697		2	tumor stag
	677			male	677		3	tumor stag
	837	809	local recur	male	837		2	tumor stag
	833	635	local recur	female	833		2	tumor stag
	829	644	local recur	male	829		3	tumor stag
	795			male	795		3	tumor stag
	423	330	local recur	male	423		3	tumor stag
	712			male	712		3	tumor stag
	278	228	local recur	male	278			tumor stag
	92			male	92		2	tumor stag
	642			male	642		2	tumor stag
	278	74	local recur	male	278		3	tumor stag
	690			female	690		3	tumor stag
	605	238	local recur	female	605		2	tumor stag
	608			female	608		1	tumor stag
	175	79	local recur	male	175		3	tumor stag
	600	244	local recur	male	600	4A		tumor stag
	580	121	local recur	male	580	4A		tumor stag
	560	209	local recur	male	560		3	tumor stag
	487	396	local recur	female	487		2	tumor stag
	547	153	local recur	male	547	4A		tumor stag
	340	141	local recur	male	340		3	tumor stag
	513			male	513		3	tumor stag
	435			male	435		3	tumor stag

472			female	472		2	tumor stag
99			male	99		3	tumor stag
408			male	408		3	tumor stag
437	251	local recur	male	437		1	tumor stag
			male				tumor stag
4926	3576	local recur	male	4926		2	tumor stag
3898	241	local recur	male	3898		2	tumor stag
3677	560	local recur	female	3677		3	tumor stag
3328	2667	local recur	male	3328		2	tumor stag
406	135	local recur	male	406	4A		tumor stag
3228	434	local recur	female	3228		2	tumor stag
2441	641	local recur	male	2441		3	tumor stag
2970			male	2970		3	tumor stag
447	370	local recur	male	447	4A		tumor stag
1951			male	1951		2	tumor stag
2067	1775	local recur	female	2067		2	tumor stag
2040	209	local recur	male	2040	4A		tumor stag
1251	607	local recur	female	1251		2	tumor stag
1739			male	1739		2	tumor stag
320			female	320		2	tumor stag
308	131	local recur	male	308		3	tumor stag
330			male	330			tumor stag
349			male	349		3	tumor stag
295			male	295		3	tumor stag
343	85	distant rec	female	343	4A		tumor stag
306	215	local recur	male	306		3	tumor stag
			male				tumor stag
2769	1607	local recur	male	2769	4A		tumor stag
4901	4901	local recur	female	4901		2	tumor stag
1932	214	local recur	male	1932		3	tumor stag
481	435	local recur	female	481		3	tumor stag
1490	207	local recur	female	1490	4A		tumor stag
5077	2173	distant rec	male	5077			tumor stag
842	281	local recur	male	842		3	tumor stag
1214			male	1214		2	tumor stag
1495	368	local recur	male	1495		2	tumor stag
2970	1178	local recur	male	2970		2	tumor stag
3437			male	3437		3	tumor stag
552	129	local recur	male	552		2	tumor stag
289	105	local recur	male	289		3	tumor stag
389	251	local recur	male	389		2	tumor stag
962	237	local recur	female	962	4A		tumor stag
1648	1292	local recur	male	1648		2	tumor stag
1633	36	local recur	female	1633	4A		tumor stag
1114	595	local recur	male	1114		2	tumor stag
1096	535	local recur	male	1096		2	tumor stag
930	208	local recur	male	930	4A		tumor stag
4453	4427	local recur	male	4453		2	tumor stag
37			female	37		2	tumor stag
482	178	local recur	male	482	4A		tumor stag
285	67	local recur	female	285		2	tumor stag
			female				tumor stag
1021	318	local recur	female	1021		2	tumor stag
3649	2760	local recur	male	3649		3	tumor stag
1353			female	1353	4A		tumor stag
2786			male	2786	4A		tumor stag
791	634	local recur	male	791	4A		tumor stag
95	65	local recur	male	95	4B		tumor stag

	770	139	local recur	female	770	3	tumor stag
	742	614	local recur	male	742	4A	tumor stag
	975	383	local recur	male	975	4A	tumor stag
	742	293	local recur	female	742	3	tumor stag
	2275	433	local recur	male	2275	3	tumor stag
	1022	364	local recur	male	1022	3	tumor stag
	525	149	local recur	male	525	3	tumor stag
	424			male	424	2	tumor stag
	1040	656	local recur	male	1040	3	tumor stag
	3623			female	3623		tumor stag
	3781			male	3781		tumor stag
	423	346	local recur	male	423	3	tumor stag
	1969	303	local recur	male	1969	4A	tumor stag
	1057	606	local recur	male	1057	4A	tumor stag
	3436	1189	distant rec	female	3436	3	tumor stag
	235	211	local recur	male	235	4A	tumor stag
	551	188	distant rec	male	551	4A	tumor stag
	1004	294	local recur	male	1004	4A	tumor stag
	721	183	local recur	male	721	4A	tumor stag
	135	67	local recur	female	135	4A	tumor stag
	3534			male	3534	3A	tumor stag
	1041			male	1041	2	tumor stag
	1011			male	1011	2	tumor stag
	1229			male	1229	2	tumor stag
	983			male	983	1	tumor stag
	1141			male	1141	1	tumor stag
	2257			male	2257	3A	tumor stag
	2431			female	2431	2	tumor stag
	614			male	614	1	tumor stag
	1272			male	1272	2	tumor stag
	1970			male	1970	3A	tumor stag
	2141			male	2141	2	tumor stag
	1188			male	1188	2	tumor stag
	1983			female	1983	1	tumor stag
	178			female	178	2	tumor stag
	1782			male	1782	1	tumor stag
	1752			male	1752	1	tumor stag
	1752			male	1752	1	tumor stag
	1451			male	1451	1	tumor stag
	1539			male	1539	1	tumor stag
	1467			male	1467	2	tumor stag
	1029			male	1029	2	tumor stag
	1436			male	1436	2	tumor stag
	657			male	657	2	tumor stag
	1438			male	1438	1	tumor stag
	1233			male	1233	2	tumor stag
	1193			male	1193	3A	tumor stag
	1187			male	1187	2	tumor stag
	699			male	699	2	tumor stag
	8			male	8	2	tumor stag
	90			male	90	3A	tumor stag
	617			male	617	1	tumor stag
	570			male	570	2	tumor stag
C22				female	390	2	TNM by LC
C22				male	780	3	TNM by LC
C22				female	870	2	TNM by LC
C22				female	180	3	TNM by LC
C22				male	1260	1	TNM by LC

C22				male	1200	3		TNM by LC
C22				male	1290	2		TNM by LC
C22				male	1230	2		TNM by LC
C22				male	20	3		TNM by LC
C22				male	1200	2		TNM by LC
C22				male	1320	2		TNM by LC
C22				male	630	3		TNM by LC
C22				male	1410	4		TNM by LC
C22				male	1110	3		TNM by LC
C22				female	1320	1		TNM by LC
C22				male	1080	3		TNM by LC
C22				female	1050	2		TNM by LC
C22				male	1050	4		TNM by LC
C22				male	1050	3		TNM by LC
C22				male	1050	1		TNM by LC
C22				female	1020	2		TNM by LC
C22				male	930	4		TNM by LC
C22				male	180	4		TNM by LC
C22				female	900	3		TNM by LC
C22				female	960	1		TNM by LC
C22				male	900	2		TNM by LC
C22				male	840	2		TNM by LC
C22				male	960	1		TNM by LC
C22				male	930	2		TNM by LC
C22				male	900	2		TNM by LC
C22				male	450	2		TNM by LC
C22				male	1110	1		TNM by LC
C22				male	750	1		TNM by LC
C22				male	930	2		TNM by LC
C22				male	960	3		TNM by LC
C22				male	1020	2		TNM by LC
C22				male	960	2		TNM by LC
C22				male	570	2		TNM by LC
C22				male	1050	2		TNM by LC
C22				male	1020	1		TNM by LC
C22				female	840	1		TNM by LC
C22				male	990	3		TNM by LC
C22				male	990	1		TNM by LC
C22				male	150	3		TNM by LC
C22				female	810	1		TNM by LC
C22.0	215	47	local recur	female	215	T1N0M0	BCLC:0	TNM 7th e
C22.0	465	243	local recur	male	465	T2N0M0	BCLC:A	TNM 7th e
C22.0	476	181	local recur	male	476	T2N0M0	BCLC:A	TNM 7th e
C22.0	159	92	local recur	male	159	T2N0M0	BCLC:A	TNM 7th e
C22.0				female				
C22.0	1826	1213	local recur	male	1826	T2N0M0	BCLC:A	TNM 7th e
C22.0	781	477	local recur	male	781	T1N0M0	BCLC:0	TNM 7th e
C22.0	200	139	local recur	male	200	T3bN0M0	BCLC:C	TNM 7th e
C22.0	704	368	local recur	male	704	T2N0M0	BCLC:A	TNM 7th e
C22.0	1745	1745		male	1745	T1N0M0	BCLC:A	TNM 7th e
C22.0	387	387		female	387	T3bN0M0	BCLC:C	TNM 7th e
C22.0				female				
C22.0	491	491	local recur	male	491	T3bN0M0	BCLC:C	TNM 7th e
C22.0	829	643	local recur	male	829	T3bN0M0	BCLC:C	TNM 7th e
C22.0	32	32		female	32	T1N0M0	BCLC:A	TNM 7th e
C22.0	1125	398	local recur	male	1125	T3aN0M0	BCLC:B	TNM 7th e
C22.0	923	321	local recur	male	923	T1N0M0	BCLC:A	TNM 7th e
C22.0				male				

C22.0				male				
C22.0	1013	1013		male	1013	T2N0M0	BCLC:0	TNM 7th e
C22.0				male				
C22.0				male				
C22.0				male				
C22.0	876	876		male	876	T1N0M0	BCLC:A	TNM 7th e
C22.0				female				
C22.0	1103	1103		male	1103	T2N0M0	BCLC:A	TNM 7th e
C22.0	1826	1210	local recur	male	1826	T3bN0M0	BCLC:C	TNM 7th e
C22.0	85	85		male	85	T2N0M0	BCLC:A	TNM 7th e
C22.0	547	487	local recur	male	547	T3bN0M0	BCLC:C	TNM 7th e
C22.0	1826	1826		male	1826	T3bN0M0	BCLC:A	TNM 7th e
C22.0	1826	84	local recur	male	1826	T3aN0M0	BCLC:A	TNM 7th e
C22.0				male				
C22.0	1826	836	local recur	female	1826	T2N0M0	BCLC:A	TNM 7th e
C22.0	565	298	local recur	male	565	T2N0M0	BCLC:A	TNM 7th e
C22.0	1312	609	local recur	male	1312	T3bN0M0	BCLC:C	TNM 7th e
C22.0	1826	935	local recur	male	1826	T1N0M0	BCLC:A	TNM 7th e
C22.0				male				
C22.0	146	137	local recur	male	146	T3bN0M0	BCLC:0	TNM 7th e
C22.0	1826	1826		female	1826	T1N0M0	BCLC:A	TNM 7th e
C22.0				female				
C22.0	953	619	local recur	male	953	T1N0M0	BCLC:A	TNM 7th e
C22.0	1826	1826		male	1826	T1N0M0	BCLC:A	TNM 7th e
C22.0	1490	500	local recur	female	1490	T1N0M0	BCLC:A	TNM 7th e
C22.0	608	495	local recur	male	608	T1N0M0	BCLC:A	TNM 7th e
C22.0	619	619		male	619	T2N0M0	BCLC:A	TNM 7th e
C22.0				male				
C22.0	275	63	local recur	male	275	T1N0M0	BCLC:A	TNM 7th e
C22.0	513	188	local recur	male	513	T2N0M0	BCLC:A	TNM 7th e
C22.0	365	365	local recur	male	365	T3bN0M0	BCLC:C	TNM 7th e
C22.0				male				
C22.0	30	30		male	30	T3bN0M0	BCLC:C	TNM 7th e
C22.0	1399	1399		female	1399	T1N0M0	BCLC:A	TNM 7th e
C22.0	335	335		female	335	T3aN0M0	BCLC:B	TNM 7th e
C22.0	382	382		female	382	T2N0M0	BCLC:A	TNM 7th e
C22.0	1786	1482	local recur	male	1786	T1N0M0	BCLC:A	TNM 7th e
C22.0	408	287	local recur	male	408	T1N0M0	BCLC:0	TNM 7th e
C22.0	157	157	local recur	male	157	T3bN0M0	BCLC:C	TNM 7th e
C22.0				male				
C22.0	442	255	local recur	male	442	T3bN0M0	BCLC:C	TNM 7th e
C22.0	1826	1826		male	1826	T1N0M0	BCLC:A	TNM 7th e
C22.0	1525	1344	local recur	male	1525	T2N0M0	BCLC:B	TNM 7th e
C22.0				male				
C22.0	13	13		male	13	T2N0M0	BCLC:A	TNM 7th e
C22.0				male				
C22.0				male				
C22.0	1826	1826		male	1826	T2N0M0	BCLC:A	TNM 7th e
C22.0				female				
C22.0				female				
C22.0				male				
C22.0	994	994	local recur	female	994	T2N0M0	BCLC:A	TNM 7th e
C22.0	44	44		male	44	T2N0M0	BCLC:B	TNM 7th e
C22.0				male				
C22.0	1520	1520		male	1520	T2N0M0	BCLC:A	TNM 7th e
C22.0				male				
C22.0	1703	1041	local recur	female	1703	T3bN0M0	BCLC:C	TNM 7th e
C22.0	1119	804	local recur	male	1119	T2N0M0	BCLC:A	TNM 7th e

C22.0	507	332	local recur	male	507	T3aN0M0	BCLC:B	TNM 7th e
C22.0	1826	690	local recur	female	1826	T2N0M0	BCLC:A	TNM 7th e
C22.0	255	255	local recur	male	255	T3aN0M0	BCLC:B	TNM 7th e
C22.0				male				
C22.0	1826	786	local recur	female	1826	T1N0M0	BCLC:A	TNM 7th e
C22.0	740	398	local recur	male	740	T1N0M0	BCLC:A	TNM 7th e
C22.0	234	172	local recur	male	234	T1N0M0	BCLC:A	TNM 7th e
C22.0				male				
C22.0				male				
C22.0				male				
C22.0				male				
C22.0	481	481		male	481	T1N0M0	BCLC:A	TNM 7th e
C22.0				female				
C22.0				male				
C22.0				male				
C22.0	1339			female				
C22.0	1030			female				
C22.0	925			male				
C22.0	867			male				
C22.0	79			male				
C22.0	2017			female				
C22.0	27			male				
C22.0	254			male				
C22.0	136			male				
C22.0	111			male				
C22.0	204			male				
C22.0	0			female				
C22.0	1231			female				
C22.0	69			male				
C22.0	180			male				
C22.0	12			male				
C22.0	193			male				
C22.0	0			male				
C22.0	1175			female				
C22.0	4			male				
C22.0	421			female				
C22.0	252			female				
C22.0	97			male				
C22.0	693			female				
C22.0	0			female				
C22.0	662			male				
C22.0	1678			female				
C22.0	2415			male				
C22.0	1144			female				
C22.0	60			female				
C22.0	13			male				
C22.0	129			male				
C22.0	0			male				
C22.0	183			male				
C22.0	391			female				
C22.0	352			male				
C22.0	4			female				
C22.0	602			female				
C22.0	1036			male				
C22.0	8			female				
C22.0	887			female				
C22.0	1629			male				
C22.0	37			male				

C22.0	111			female			
C22.0	0			male			
C22.0	1359			male			
C22.0	-8			female			
C22.0	0			male			
C22.0	2			male			
C22.0	3			male			
C22.0	269			male			
C22.0	987			female			
C22.0	458			female			
C22.0	7			female			
	2305			female	2305		tumor stag
	3004	457	local recur	male	3004		tumor stag
	775	426	local recur	female	775		tumor stag
	1954	1613	local recur	male	1954		tumor stag
	879	274		female	879		tumor stag
	951	639	local recur	male	951		tumor stag
	2188			female	2188		tumor stag
	1634	274	local recur	male	1634		tumor stag
	1768	700	local recur	male	1768		tumor stag
	1133	152	local recur	female	1133		tumor stag
	1905	1431	local recur	male	1905		tumor stag
	812	152	local recur	female	812		tumor stag
	1494			female	1494		tumor stag
	2991	2405	local recur	female	2991		tumor stag
	3100	122	local recur	male	3100		tumor stag
	4935	883	local recur	female	4935		tumor stag
	3275	2161	local recur	male	3275		tumor stag
	3064	1187	local recur	male	3064		tumor stag
	740	183	local recur	male	740		tumor stag
	4097	3074	local recur	male	4097		tumor stag
	1137	335	local recur	male	1137		tumor stag
	1679	183	local recur	male	1679		tumor stag
	5378	1278	local recur	male	5378		tumor stag
	3683			male	3683		tumor stag
	5337	2070	local recur	male	5337		tumor stag
	5456			male	5456		tumor stag
	904	244		male	904		tumor stag
	1653	1644	local recur	male	1653		tumor stag
	603	244	local recur	male	603		tumor stag
	770	761	local recur	male	770		tumor stag
	4010	3409	local recur	male	4010		tumor stag
C22				male	1320	2	TNM by LC
C22				male	1200	4	TNM by LC
C22				male	720	2	TNM by LC
C22				male	1650	3	TNM by LC
C22				male	1620	2	TNM by LC
C22				male	1290	2	TNM by LC
C22				male	1620	3	TNM by LC
C22				male	1590	3	TNM by LC
C22				male	1680	2	TNM by LC
C22				male	450	3	TNM by LC
C22				male	1680	1	TNM by LC
C22				female	1710	2	TNM by LC
C22				male	1590	2	TNM by LC
C22				male	1590	2	TNM by LC
C22				male	1560	3	TNM by LC
C22				male	840	2	TNM by LC

C22	female	1110	2	TNM by L(
C22	male	510	3	TNM by L(
C22	male	1530	3	TNM by L(
C22	female	570	2	TNM by L(
C22	male	1680	3	TNM by L(
C22	male	420	3	TNM by L(
C22	male	1470	3	TNM by L(
C22	male	360	4	TNM by L(
C22	male	660	3	TNM by L(
C22	male	1410	1	TNM by L(
C22	male	1320	1	TNM by L(
C22	male	1320	3	TNM by L(
C22	male	1320	2	TNM by L(
C22	male	1290	3	TNM by L(
C22	male	60	3	TNM by L(
C22	male	720	2	TNM by L(
C22	female	1260	2	TNM by L(
C22	male	1230	3	TNM by L(
C22	female	1380	2	TNM by L(
C22	male	2160	2	TNM by L(
C22	male	1980	2	TNM by L(
C22	male	1470	3	TNM by L(
C22	male	540	2	TNM by L(
C22	male	960	2	TNM by L(
C22	male	690	2	TNM by L(
C22	male	1440	2	TNM by L(
C22	male	1350	3	TNM by L(
C22	male	600	3	TNM by L(
C22	male	1170	3	TNM by L(
C22	male	1140	2	TNM by L(
C22	female	1260	3	TNM by L(
C22	female	600	2	TNM by L(
C22	female	1200	2	TNM by L(
C22	female	570	2	TNM by L(
C22	male	600	2	TNM by L(
C22	female	1260	1	TNM by L(
C22	female	1200	2	TNM by L(
C22	female	1200	2	TNM by L(
C22	female	120	4	TNM by L(
C22	male	390	1	TNM by L(
C22	male	1170	2	TNM by L(
C22	female	1350	2	TNM by L(
C22	male	900	1	TNM by L(
C22	female	1170	2	TNM by L(
C22	female	90	2	TNM by L(
C22	male	1050	1	TNM by L(
C22	male	1350	4	TNM by L(
C22	male	240	3	TNM by L(
C22	male	1200	1	TNM by L(
C22	male	1170	2	TNM by L(
C22	female	270	3	TNM by L(
C22	male	1080	2	TNM by L(
C22	female	390	4	TNM by L(
C22	male	1020	4	TNM by L(
C22	male	1050	1	TNM by L(
C22	female	1020	2	TNM by L(
C22	male	990	1	TNM by L(
C22	male	930	2	TNM by L(

C22		male	900	3	TNM by LC
C22		female	900	1	TNM by LC
C22		male	900	2	TNM by LC
C22		male	870	2	TNM by LC
C22		male	1080	1	TNM by LC
C22		female	930	3	TNM by LC
C22		female	240	2	TNM by LC
C22		female	210	2	TNM by LC
C22		male	630	1	TNM by LC
C22		female	900	2	TNM by LC
C22		male	870	2	TNM by LC
C22		male	720	3	TNM by LC
C22		male	810	3	TNM by LC
C22		male	330	3	TNM by LC
C22		male	990	3	TNM by LC
C22		male	270	3	TNM by LC
C22		male	1020	3	TNM by LC
C22		male	570	3	TNM by LC
C22		male	750	3	TNM by LC
C22		male	480	2	TNM by LC
C22		male	10	3	TNM by LC
C22		male	600	3	TNM by LC
C22		male	1290	3	TNM by LC
C22		male	750	4	TNM by LC
C22		male	510	3	TNM by LC
C22		male	480	3	TNM by LC
C22		male	390	1	TNM by LC
C22		female	360	3	TNM by LC
C22		male	570	3	TNM by LC
C22		male	450	3	TNM by LC
C22		male	300	2	TNM by LC
C22		female	1260	4	TNM by LC
C22		female	1440	1	TNM by LC
C22		male	1440	2	TNM by LC
C22		female	990	3	TNM by LC
C22		male	300	4	TNM by LC
C22		female	240	4	TNM by LC
C22		male	30	3	TNM by LC
C22.0	2018	male			
C22.0	12	male			
C22.0	16	male			
C22.0	445	male			
C22.0	5	female			
C22.0	1	female			
C22.0	6	male			
C22.0	6	female			
C22.0	433	female			
C22.0	1836	male			
C22.0	0	male			
C22.0	120	male			
C22.0	1008	female			
C22.0	1711	male			
C22.0	364	male			
C22.0	3	male			
C22.0	428	male			
C22.0	2849	male			
C22.0	289	male			
C22.0	10	female			

C22.0	7	male			
C22.0	243	male			
C22.0	2141	female			
C22.0	133	male			
C22		male	450	2	TNM by LC
C22		male	1260	2	TNM by LC
C22		male	1140	3	TNM by LC
C22		male	1200	2	TNM by LC
C22		male	900	2	TNM by LC
C22		female	120	3	TNM by LC
C22		female	810	3	TNM by LC
C22		male	990	3	TNM by LC
C22		female	120	2	TNM by LC
C22		male	900	2	TNM by LC
C22		male	900	2	TNM by LC
C22		female	900	3	TNM by LC
C22		male	900	2	TNM by LC
C22		male	840	2	TNM by LC
C22		male	720	2	TNM by LC
C22		female	810	2	TNM by LC
C22		female	810	2	TNM by LC
C22		male	780	2	TNM by LC
C22		male	750	2	TNM by LC
C22		female	750	3	TNM by LC
C22		male	870	2	TNM by LC
C22		male	900	1	TNM by LC
C22		male	810	2	TNM by LC
C22		female	690	2	TNM by LC
C22		male	720	2	TNM by LC
C22		male	180	4	TNM by LC
C22		male	690	2	TNM by LC
C22		female	570	2	TNM by LC
C22		female	900	1	TNM by LC
C22		male	600	1	TNM by LC
C22		male	780	1	TNM by LC
C22		female	390	2	TNM by LC
C22		female	150	2	TNM by LC
C22		female	630	2	TNM by LC
C22		male	630	3	TNM by LC
C22		male	600	2	TNM by LC
C22		female	600	1	TNM by LC
C22		male	720	1	TNM by LC
C22		male	780	4	TNM by LC
C22		male	240	3	TNM by LC
C22		male	600	2	TNM by LC
C22		female	570	2	TNM by LC
C22		male	570	3	TNM by LC
C22		male	540	3	TNM by LC
C22		male	480	2	TNM by LC
C22		male	510	3	TNM by LC
C22		female	480	3	TNM by LC
C22		male	720	3	TNM by LC
C22		female	690	2	TNM by LC
C22		male	870	2	TNM by LC
C22.0	302	male			
C22.0	6	female			
C22.0	1869	male			
D13.4		male			

D13.4				male			
D13.4				female			
D13.4				female			
D13.4				female			
D13.4				female			
D13.4				female			
D13.4				female			
D13.4				female			
D13.4				female			
D13.4				female			
D13.4				female			
D13.4				female			
D13.4				female			
D13.4				male			
D13.4				male			
D13.4				female			
D13.4				female			
D13.4				female			
D13.4				female			
D13.4				female			
D13.4				female			
C22.0	0			male			
D13.4				female			
D13.4				female			
D13.4				female			
D13.4				female			
D13.4				female			
D13.4				female			
D13.4				male			
D13.4				male			
C22.0	328			male			
C22.0	3071			female			
C22.0	447			male			
C22.0	6			female			
C22.0	334			male			
C22.0	6			female			
C22.0	44			male			
C22.0	6			male			
C22.0	6			male			
C22.0	334			female			
C22.0	6			male			
C22.0	391			male			
C22.0	6			female			
C22.0	6			female			
C22.0	236			male			
C22.0	6			female			
C22.0	843			female			
C22.0	230			female			
C22.0	6			female			
C22.0	122			male			
C22.0	216			male			
C22.0	196	145	local recur	male	196		BCLC:A
C22.0	1826	875	local recur	male	1826		BCLC:A
C22.0	1715	364	local recur	male	1715		BCLC:A
C22.0	1142	244	local recur	male	1142		BCLC:A
C22.0	189	119	local recur	female	189		BCLC:A
C22.0	1826	417	local recur	male	1826		BCLC:A
C22.0	1036	379	local recur	male	1036		BCLC:A

C22.0	985	740	local recur	male	985		BCLC:A	
C22.0	1826	1717	local recur	female	1826		BCLC:A	
C22.0	636	163	local recur	female	636		BCLC:B	
C22.0	871	371	local recur	male	871		BCLC:A	
C22.0	619	244	local recur	male	619		BCLC:A	
C22.0	684	251	local recur	male	684		BCLC:A	
C22.0	226	110	local recur	male	226		BCLC:A	
C22.0	1325	77	local recur	male	1325		BCLC:A	
C22.0	1826	610	local recur	male	1826		BCLC:A	
C22.0	751	725	local recur	male	751		BCLC:A	
C22.0	905	193	local recur	male	905		BCLC:A	
C22.0	1826	89	local recur	male	1826		BCLC:C	
C22.0	1826	235	local recur	female	1826		BCLC:0	
C22.0	495	283	local recur	male	495		BCLC:A	
C22.0	1826	300	local recur	male	1826		BCLC:A	
C22.0	297	120	local recur	female	297		BCLC:A	
C22.0	1541	393	local recur	male	1541		BCLC:A	
C22.0	314	125	local recur	female	314		BCLC:A	
C22.0	1369	87	local recur	male	1369		BCLC:A	
C22.0	590	247	local recur	male	590		BCLC:A	
C22.0	875	325	local recur	female	875		BCLC:A	
C22.0	114	50	local recur	female	114		BCLC:B	
C22.0	788	357	local recur	female	788		BCLC:A	
C22.0	352	241	local recur	male	352		BCLC:A	
C22.0	323	124	local recur	male	323		BCLC:B	
C22.0	580	308	local recur	male	580		BCLC:B	
C22.0	1303	254	local recur	male	1303		BCLC:A	
C22.0	976	694	local recur	male	976		BCLC:A	
C22.0	706	298	local recur	male	706		BCLC:A	
C22				male	660	1		TNM by L(
C22.0	1030	429	local recur	male	1030		BCLC:A	
C22				female	720	2		TNM by L(
C22.0	1528	363	local recur	female	1528		BCLC:A	
C22				male	690	1		TNM by L(
C22.0	1105	312	local recur	male	1105		BCLC:A	
C22				male	630	4		TNM by L(
C22.0	984	546	local recur	male	984		BCLC:A	
C22				male	660	4		TNM by L(
C22.0	454	250	local recur	female	454		BCLC:C	
C22.0	1826	606	local recur	male	1826		BCLC:A	
C22				male	480	3		TNM by L(
C22.0	1324	298	local recur	male	1324		BCLC:A	
C22				male	90	4		TNM by L(
C22.0	671	329	local recur	male	671		BCLC:A	
C22				male	480	3		TNM by L(
C22.0	616	287	local recur	female	616		BCLC:A	
C22				female	570	2		TNM by L(
C22.0	694	43	local recur	male	694		BCLC:A	
C22				female	480	1		TNM by L(
C22.0	187	39	local recur	male	187		BCLC:A	
C22				male	540	4		TNM by L(
C22.0	217	126	local recur	male	217		BCLC:C	
C22.0	879	606	local recur	male	879		BCLC:B	
C22				male	540	2		TNM by L(
C22.0	255	137	local recur	male	255		BCLC:A	
C22				male	30	3		TNM by L(
C22				female	540	2		TNM by L(
C22				male	510	2		TNM by L(

C22				male	930	2		TNM by LC
C22				female	540	1		TNM by LC
C22				male	720	2		TNM by LC
C22				male	690	2		TNM by LC
C22				male	540	4		TNM by LC
C22				male	540	1		TNM by LC
C22				male	570	2		TNM by LC
C22				male	720	3		TNM by LC
C22.0				female				
C22				male	450	1		TNM by LC
C22.0	1147	1147		male	1147	T2N0M0	BCLC:A	TNM 7th e
C22				male	420	2		TNM by LC
C22				male	420	3		TNM by LC
C22				male	150	3		TNM by LC
C22				male	390	2		TNM by LC
C22				male	390	2		TNM by LC
C22				male	510	2		TNM by LC
C22				female	510	2		TNM by LC
C22				female	510	3		TNM by LC
C22.0	1510	1510		male	1510	T2N0M0	BCLC:A	TNM 7th e
C22				male	510	2		TNM by LC
C22.0	1826	1826		male	1826	T2N0M0	BCLC:A	TNM 7th e
C22.0	1312	1312		male	1312	T2N0M0	BCLC:A	TNM 7th e
C22				male	390	2		TNM by LC
C22.0				male				
C22				female	480	2		TNM by LC
C22.0				male				
C22				male	570	3		TNM by LC
C22.0	144	113	local recur	male	144	T3bN0M0	BCLC:C	TNM 7th e
C22				male	510	2		TNM by LC
C22				male	90	3		TNM by LC
C22.0	1826	970	local recur	male	1826	T1N0M0	BCLC:0	TNM 7th e
C22				male	540	2		TNM by LC
C22.0				male				
C22.0	1826	1826		female	1826	T2N0M0	BCLC:A	TNM 7th e
C22				female	480	1		TNM by LC
C22.0				male				
C22				female	330	3		TNM by LC
C22				male	330	2		TNM by LC
C22.0	1826	1826		male	1826	T2N0M0	BCLC:A	TNM 7th e
C22				female	300	4		TNM by LC
C22.0	1826	1826	local recur	male	1826	T1N0M0	BCLC:A	TNM 7th e
C22				male	300	4		TNM by LC
C22				male	210	3		TNM by LC
C22.0	352	49	local recur	male	352	T3bN0M0	BCLC:C	TNM 7th e
C22.0				male				
C22				male	480	3		TNM by LC
C22.0	560	384	local recur	male	560	T2N0M0	BCLC:A	TNM 7th e
C22				male	720	3		TNM by LC
C22				male	540	2		TNM by LC
C22.0				female				
C22.0				female				
C22.0				male				
C22				male	570	2		TNM by LC
C22.0	295	284	local recur	male	295	T2N0M0	BCLC:A	TNM 7th e
C22				male	210	2		TNM by LC
C22.0				male				
C22				male	180	2		TNM by LC

C22.0				male				
C22.0				male				
C22.0	1826	986	local recur	female	1826	T2N0M0	BCLC:A	TNM 7th e
C22.0	890	159	local recur	male	890	T2N0M0	BCLC:A	TNM 7th e
C22.0				male				
C22.0	1826	1826		male	1826	T3bN0M0	BCLC:C	TNM 7th e
C22.0				male				
C22.0	1826	1826		female	1826	T2N0M0	BCLC:0	TNM 7th e
C22.0				female				
C22.0	1826	1826		male	1826	T3bN0M0	BCLC:A	TNM 7th e
C22.0	1077	483	local recur	male	1077	T1N0M0	BCLC:A	TNM 7th e
C22.0	192	110	local recur	male	192	T3bN0M0	BCLC:C	TNM 7th e
C22.0				female				
C22.0				male				
C22.0	1092	1092		male	1092	T1N0M0	BCLC:A	TNM 7th e
C22.0				male				
C22.0				male				
C22.0				male				
C22.0	74	74		male	74	T1N0M0	BCLC:A	TNM 7th e
C22.0				female				
C22.0	757	757		male	757	T2N0M0	BCLC:A	TNM 7th e
C22.0				male				
C22.0				male				
C22.0	1214	1214		male	1214	T2N0M0	BCLC:A	TNM 7th e
C22.0				male				
C22.0	1217	1217		male	1217	T3aN0M0	BCLC:B	TNM 7th e
C22.0	195	195		male	195	T2N0M0	BCLC:B	TNM 7th e
C22.0	1357	1357		male	1357	T2N0M0	BCLC:A	TNM 7th e
C22.0				male				
C22.0				male				
C22.0	1085	1085		male	1085	T1N0M0	BCLC:A	TNM 7th e
C22.0	423	423		male	423	T2N0M0	BCLC:A	TNM 7th e
C22.0				female				
C22.0				female				
C22.0				male				
C22.0	1668	1668		male	1668	T2N0M0	BCLC:B	TNM 7th e
C22.0				male				
C22.0				male				
C22.0	1826	1826		female	1826	T1N0M0	BCLC:A	TNM 7th e
C22.0				male				
C22.0				male				
C22.0				male				
C22.0	1612	1026	local recur	male	1612	T2N0M0	BCLC:A	TNM 7th e
C22.0				male				
C22.0				female				
C22.0				male				
C22.0				male				
C22.0				male				
C22.0	1826	611	local recur	male	1826	T2N0M0	BCLC:A	TNM 7th e
C22.0	1193	1096	local recur	male	1193	T3aN0M0	BCLC:B	TNM 7th e
C22.0				male				
C22.0				male				
C22.0				male				
C22.0				male				
C22.0				male				
C22.0				male		T2N0M0	BCLC:A	TNM 7th e
C22.0	1050	1050		male	1050	T2N0M0	BCLC:B	TNM 7th e
C22.0	449	261	local recur	male	449	T2N0M0	BCLC:C	TNM 7th e

C22.0	953	934	local recur	male	953	T1N0M0	BCLC:A	TNM 7th e
C22.0	1063	1063		female	1063	T1N0M0	BCLC:A	TNM 7th e
C22.0				male				
C22.0	331	30	local recur	male	331	T3bN0M0	BCLC:B	TNM 7th e
C22.0	412	412		male	412	T1N0M0	BCLC:A	TNM 7th e
C22.0	308	30	local recur	male	308	T2N0M0	BCLC:C	TNM 7th e
C22.0				male				
C22.0	420	420		male	420	T2N0M0	BCLC:A	TNM 7th e
C22.0				female				
C22.0				female				
C22.0				male				
C22.0				male				
C22.0	690	30	local recur	male	690	T3aN0M0	BCLC:B	TNM 7th e
C22.0				female				
C22.0	223	223		female	223	T3bN0M0	BCLC:C	TNM 7th e
C22.0				male				
C22.0	227	30	local recur	female	227	T3bN0M0	BCLC:B	TNM 7th e
C22.0				male				
C22.0				male				
C22.0	560	560	local recur	female	560	T4N0M1	BCLC:B	TNM 7th e
C22.0	140	140		male	140	T1N0M0	BCLC:A	TNM 7th e
C22.0	383	383		male	383	T1N0M0	BCLC:A	TNM 7th e
C22.0	444	214	local recur	male	444	T3aN0M0	BCLC:A	TNM 7th e
C22.0				male		T3bN0M0	BCLC:C	TNM 7th e
C22.0				male		T2N0M0	BCLC:B	TNM 7th e
C22.0				male		T2N0M0	BCLC:A	TNM 7th e
C22.0				male				
C22.0	1826	1826		male	1826	T1N0M0	BCLC:A	TNM 7th e
C22.0	1826	1826		female	1826	T1N0M0	BCLC:A	TNM 7th e
C22.0	1826	1826		male	1826	T2N0M0	BCLC:A	TNM 7th e
C22.0	1499	1410	local recur	male	1499	T3aN0M0	BCLC:B	TNM 7th e
C22.0				male				
C22.0	349	165	local recur	male	349	T3bN0M0	BCLC:B	TNM 7th e
C22.0				male				
C22.0	1826	394	local recur	female	1826	T2N0M0	BCLC:A	TNM 7th e
C22.0	1429	1429		male	1429	T1N0M0	BCLC:A	TNM 7th e
C22.0	1826	1826		male	1826	T1N0M0	BCLC:A	TNM 7th e
C22.0	1392	1392	local recur	male	1392	T2N0M0	BCLC:A	TNM 7th e
C22.0				male				
C22.0				male				
C22.0				female				
C22.0	1514	420	local recur	male	1514	T3bN0M0	BCLC:C	TNM 7th e
C22.0				male				
C22.0	1440	1440		male	1440	T1N0M0	BCLC:A	TNM 7th e
C22.0				male				
C22.0	213	135	local recur	male	213	T1N0M0	BCLC:A	TNM 7th e
C22.0	227			female				
C22.0	0			male				
C22.0	6			male				
C22.0	182			male				
C22.0	360			male				
C22.0	407			female				
C22.0	15			female				
C22.0	180			male				
C22.0	130			male				
C22.0	291			male				
C22.0	155			male				
C22.0	433			male				

C22.0	391	male
C22.0	265	male
C22.0	52	male
C22.0	498	male
C22.0	135	male
C22.0	395	female
C22.0	1066	female
C22.0	2258	male
C22.0	328	male
C22.0	322	male
C22.0	20	male
C22.0	409	female
C22.0	146	male
C22.0	20	male
C22.0	0	female
C22.0	0	male
C22.0	0	male
C22.0	0	male
C22.0	0	male
C22.0	0	male
C22.0	0	male
C22.0	0	male
C22.0	0	male
C22.0	0	male
C22.0	0	male
C22.0	0	male
C22.0	0	male
C22.0	0	female
C22.0	0	female
C22.0	0	female
C22.0	347	male
C22.0	2000	male
C22.0	1800	female
C22.0	1931	male
C22.0	1618	male
C22.0	1828	male
C22.0	9	male
C22.0	58	female
C22.0	1769	male
C22.0	400	male
C22.0	429	male
C22.0	1475	male
C22.0	1240	male
C22.0	1157	male
C22.0	170	male
C22.0	137	female
C22.0	564	female
C22.0	658	male
C22.0	1029	male
C22.0	1037	male
C22.0	672	male
C22.0	959	female
C22.0	930	female
C22.0	890	male
C22.0	864	male
C22.0	763	female
C22.0	730	female
C22.0	734	female

C22.0	636	male
C22.0	527	male
C22.0	92	male
C22.0	120	male
C22.0	161	male
C22.0	1742	male
C22.0	216	male
C22.0	252	male
C22.0	203	male
C22.0	294	female
C22.0	300	female
C22.0	293	male
C22.0	288	male
C22.0	300	male
C22.0	259	female
C22.0	141	female
C22.0	382	male
C22.0	344	male
C22.0	386	male
C22.0	450	male
C22.0	370	male
C22.0	470	male
C22.0	498	male
C22.0	476	female
C22.0	476	male
C22.0	1067	male
C22.0	2405	male
C22.0	2406	male
C22.0	2198	male
C22.0	1823	male
C22.0	1882	male
C22.0	1867	male
C22.0	1718	male
C22.0	1788	male
C22.0	1699	male
C22.0	1644	male
C22.0	1505	male
C22.0	1451	male
C22.0	0	male
C22.0	366	female
C22.0	0	male
C22.0	342	male
C22.0	260	male
C22.0	295	male
C22.0	279	female
C22.0	92	male
C22.0	240	male
C22.0	120	male
C22.0	741	male
C22.0	62	male
C22.0	640	female
C22.0	579	male
C22.0	468	male
C22.0	472	male
C22.0	734	male
C22.0	455	female
C22.0	526	male
C22.0	438	male

C22.0	486	male	
C22.0	253	male	
C22.0	139	female	
C22.0	31	female	
C22.0	30	female	
C22.0	122	male	
C22.0	142	male	
C22.0	782	female	
C22.0	706	male	
C22.0	717	female	
C22.0	21	female	
C22.0	743	male	
C22.0	250	male	
K74.69	1826	male	1826
K74.69	1312	male	1312
K74.69		female	
K74.69		male	

donor_vita	prior_malig	project_co	study_don	submitted_	_primary_s	_primary_disease
deceased	unknown	LIHC-US		TCGA-DD·	Liver	Liver cancer : Hepatocellular carc
deceased	unknown	LIHC-US	PCAWG	TCGA-DD·	Liver	Liver cancer : Hepatocellular carc
deceased	unknown	LIHC-US		TCGA-DD·	Liver	Liver cancer : Hepatocellular carc
deceased	unknown	LIHC-US		TCGA-DD·	Liver	Liver cancer : Hepatocellular carc
deceased	unknown	LIHC-US		TCGA-DD·	Liver	Liver cancer : Hepatocellular carc
deceased	unknown	LIHC-US		TCGA-EP-	Liver	Liver cancer : Hepatocellular carc
deceased	unknown	LIHC-US		TCGA-G3-	Liver	Liver cancer : Hepatocellular carc
deceased	unknown	LIHC-US		TCGA-GJ-	Liver	Liver cancer : Hepatocellular carc
deceased	unknown	LIHC-US		TCGA-BC-	Liver	Liver cancer : Hepatocellular carc
deceased	unknown	LIHC-US		TCGA-2Y-	Liver	Liver cancer : Hepatocellular carc
deceased	unknown	LIHC-US		TCGA-5R-	Liver	Liver cancer : Hepatocellular carc
deceased	unknown	LIHC-US		TCGA-UB-	Liver	Liver cancer : Hepatocellular carc
deceased	unknown	LIHC-US		TCGA-G3-	Liver	Liver cancer : Hepatocellular carc
deceased	unknown	LIHC-US		TCGA-KR-	Liver	Liver cancer : Hepatocellular carc
deceased	unknown	LIHC-US		TCGA-G3-	Liver	Liver cancer : Hepatocellular carc
deceased	unknown	LIHC-US		TCGA-GJ-	Liver	Liver cancer : Hepatocellular carc
deceased	unknown	LIHC-US		TCGA-BC-	Liver	Liver cancer : Hepatocellular carc
deceased	unknown	LIHC-US	PCAWG	TCGA-HP-	Liver	Liver cancer : Hepatocellular carc
deceased	unknown	LIHC-US		TCGA-DD·	Liver	Liver cancer : Hepatocellular carc
deceased	unknown	LIHC-US		TCGA-CC-	Liver	Liver cancer : Hepatocellular carc
deceased	unknown	LIHC-US		TCGA-BC-	Liver	Liver cancer : Hepatocellular carc
deceased	unknown	LIHC-US		TCGA-DD·	Liver	Liver cancer : Hepatocellular carc
deceased	unknown	LIHC-US		TCGA-DD·	Liver	Liver cancer : Hepatocellular carc
deceased	unknown	LIHC-US	PCAWG	TCGA-FV-	Liver	Liver cancer : Hepatocellular carc
deceased	unknown	LIHC-US		TCGA-DD·	Liver	Liver cancer : Hepatocellular carc
deceased	unknown	LIHC-US		TCGA-UB-	Liver	Liver cancer : Hepatocellular carc
deceased	unknown	LIHC-US		TCGA-DD·	Liver	Liver cancer : Hepatocellular carc
deceased	unknown	LIHC-US		TCGA-DD·	Liver	Liver cancer : Hepatocellular carc
deceased	unknown	LIHC-US		TCGA-DD·	Liver	Liver cancer : Hepatocellular carc
deceased	unknown	LIHC-US		TCGA-BC-	Liver	Liver cancer : Hepatocellular carc
deceased	unknown	LIHC-US		TCGA-G3-	Liver	Liver cancer : Hepatocellular carc
deceased	unknown	LIHC-US		TCGA-DD·	Liver	Liver cancer : Hepatocellular carc
deceased	unknown	LIHC-US		TCGA-DD·	Liver	Liver cancer : Hepatocellular carc
deceased	unknown	LIHC-US		TCGA-DD·	Liver	Liver cancer : Hepatocellular carc
deceased	unknown	LIHC-US		TCGA-DD·	Liver	Liver cancer : Hepatocellular carc
deceased	unknown	LIHC-US		TCGA-DD·	Liver	Liver cancer : Hepatocellular carc
deceased	unknown	LIHC-US		TCGA-YA-	Liver	Liver cancer : Hepatocellular carc
deceased	unknown	LIHC-US	PCAWG	TCGA-DD·	Liver	Liver cancer : Hepatocellular carc
deceased	unknown	LIHC-US	PCAWG	TCGA-DD·	Liver	Liver cancer : Hepatocellular carc
deceased	unknown	LIHC-US		TCGA-DD·	Liver	Liver cancer : Hepatocellular carc
deceased	unknown	LIHC-US	PCAWG	TCGA-ES-	Liver	Liver cancer : Hepatocellular carc
deceased	unknown	LIHC-US	PCAWG	TCGA-G3-	Liver	Liver cancer : Hepatocellular carc
deceased	unknown	LIHC-US		TCGA-DD·	Liver	Liver cancer : Hepatocellular carc
deceased	unknown	LIHC-US		TCGA-DD·	Liver	Liver cancer : Hepatocellular carc
deceased	unknown	LIHC-US		TCGA-2Y-	Liver	Liver cancer : Hepatocellular carc
deceased	unknown	LIHC-US		TCGA-WX	Liver	Liver cancer : Hepatocellular carc
deceased	unknown	LICA-FR		CHC255	Liver	Liver cancer : Hepatocellular carc
alive	unknown	LICA-FR		CHC334	Liver	Liver cancer : Hepatocellular carc
alive	unknown	LICA-FR		CHC442	Liver	Liver cancer : Hepatocellular carc
deceased	unknown	LICA-FR		CHC906	Liver	Liver cancer : Hepatocellular carc
deceased	unknown	LIHC-US		TCGA-DD·	Liver	Liver cancer : Hepatocellular carc
deceased	unknown	LIHC-US		TCGA-FV-	Liver	Liver cancer : Hepatocellular carc
deceased	unknown	LIHC-US		TCGA-DD·	Liver	Liver cancer : Hepatocellular carc
deceased	unknown	LIHC-US		TCGA-DD·	Liver	Liver cancer : Hepatocellular carc
deceased	unknown	LIHC-US		TCGA-2Y-	Liver	Liver cancer : Hepatocellular carc
deceased	unknown	LIHC-US	PCAWG	TCGA-PD-	Liver	Liver cancer : Hepatocellular carc

deceased	unknown	LIHC-US		TCGA-DD·	Liver	Liver cancer : Hepatocellular carc
deceased	unknown	LIHC-US	PCAWG	TCGA-DD·	Liver	Liver cancer : Hepatocellular carc
deceased	unknown	LIHC-US		TCGA-XR-	Liver	Liver cancer : Hepatocellular carc
deceased	unknown	LIHC-US	PCAWG	TCGA-ES-	Liver	Liver cancer : Hepatocellular carc
deceased	unknown	LIHC-US		TCGA-BC-	Liver	Liver cancer : Hepatocellular carc
deceased	unknown	LIHC-US		TCGA-2Y-	Liver	Liver cancer : Hepatocellular carc
deceased	unknown	LIHC-US		TCGA-2Y-	Liver	Liver cancer : Hepatocellular carc
deceased	unknown	LIHC-US		TCGA-BC-	Liver	Liver cancer : Hepatocellular carc
d	unknown	LICA-FR		CHC1915	Liver	Liver cancer : Hepatocellular carc
deceased	unknown	LICA-FR		CHC361	Liver	Liver cancer : Hepatocellular carc
alive	unknown	LICA-FR		CHC464	Liver	Liver cancer : Hepatocellular carc
deceased	unknown	LICA-FR		CHC469	Liver	Liver cancer : Hepatocellular carc
	unknown	LIAD-FR		CHC2432	Liver	Liver cancer : Hepatocellular ade
	unknown	LIAD-FR		CHC2444	Liver	Liver cancer : Hepatocellular ade
	no	LICA-CN		LICA-CN-I	Liver	Liver cancer : Hepatocellular carc
	no	LICA-CN		LICA-CN-I	Liver	Liver cancer : Hepatocellular carc
	no	LICA-CN		LICA-CN-I	Liver	Liver cancer : Hepatocellular carc
	no	LICA-CN		LICA-CN-I	Liver	Liver cancer : Hepatocellular carc
	no	LICA-CN		LICA-CN-I	Liver	Liver cancer : Hepatocellular carc
	no	LICA-CN		LICA-CN-I	Liver	Liver cancer : Hepatocellular carc
	no	LICA-CN		LICA-CN-I	Liver	Liver cancer : Hepatocellular carc
	no	LICA-CN		LICA-CN-I	Liver	Liver cancer : Hepatocellular carc
	no	LICA-CN		LICA-CN-I	Liver	Liver cancer : Hepatocellular carc
	no	LICA-CN		LICA-CN-I	Liver	Liver cancer : Hepatocellular carc
	no	LICA-CN		LICA-CN-I	Liver	Liver cancer : Hepatocellular carc
	no	LICA-CN		LICA-CN-I	Liver	Liver cancer : Hepatocellular carc
	no	LICA-CN		LICA-CN-I	Liver	Liver cancer : Hepatocellular carc
	no	LICA-CN		LICA-CN-I	Liver	Liver cancer : Hepatocellular carc
	no	LICA-CN		LICA-CN-I	Liver	Liver cancer : Hepatocellular carc
	no	LICA-CN		LICA-CN-I	Liver	Liver cancer : Hepatocellular carc
	no	LICA-CN		LICA-CN-I	Liver	Liver cancer : Hepatocellular carc
	no	LICA-CN		LICA-CN-I	Liver	Liver cancer : Hepatocellular carc
	no	LICA-CN		LICA-CN-I	Liver	Liver cancer : Hepatocellular carc
	no	LICA-CN		LICA-CN-I	Liver	Liver cancer : Hepatocellular carc
	no	LICA-CN		LICA-CN-I	Liver	Liver cancer : Hepatocellular carc
	no	LICA-CN		LICA-CN-I	Liver	Liver cancer : Hepatocellular carc
	no	LICA-CN		LICA-CN-I	Liver	Liver cancer : Hepatocellular carc
	no	LICA-CN		LICA-CN-I	Liver	Liver cancer : Hepatocellular carc
	no	LICA-CN		LICA-CN-I	Liver	Liver cancer : Hepatocellular carc
	no	LICA-CN		LICA-CN-I	Liver	Liver cancer : Hepatocellular carc
	no	LICA-CN		LICA-CN-I	Liver	Liver cancer : Hepatocellular carc
	no	LICA-CN		LICA-CN-I	Liver	Liver cancer : Hepatocellular carc
	no	LICA-CN		LICA-CN-I	Liver	Liver cancer : Hepatocellular carc
	no	LICA-CN		LICA-CN-I	Liver	Liver cancer : Hepatocellular carc
	no	LICA-CN		LICA-CN-I	Liver	Liver cancer : Hepatocellular carc
	no	LICA-CN		LICA-CN-I	Liver	Liver cancer : Hepatocellular carc
	no	LICA-CN		LICA-CN-I	Liver	Liver cancer : Hepatocellular carc
	no	LICA-CN		LICA-CN-I	Liver	Liver cancer : Hepatocellular carc
	no	LICA-CN		LICA-CN-I	Liver	Liver cancer : Hepatocellular carc
	no	LICA-CN		LICA-CN-I	Liver	Liver cancer : Hepatocellular carc
	no	LICA-CN		LICA-CN-I	Liver	Liver cancer : Hepatocellular carc
	no	LICA-CN		LICA-CN-I	Liver	Liver cancer : Hepatocellular carc
	no	LICA-CN		LICA-CN-I	Liver	Liver cancer : Hepatocellular carc
	no	LICA-CN		LICA-CN-I	Liver	Liver cancer : Hepatocellular carc
	no	LICA-CN		LICA-CN-I	Liver	Liver cancer : Hepatocellular carc
	no	LICA-CN		LICA-CN-I	Liver	Liver cancer : Hepatocellular carc
	no	LICA-CN		LICA-CN-I	Liver	Liver cancer : Hepatocellular carc
	no	LICA-CN		LICA-CN-I	Liver	Liver cancer : Hepatocellular carc

	no	LICA-CN		LICA-CN-I	Liver	Liver cancer : Hepatocellular carc
	no	LICA-CN		LICA-CN-I	Liver	Liver cancer : Hepatocellular carc
	no	LICA-CN		LICA-CN-I	Liver	Liver cancer : Hepatocellular carc
	no	LICA-CN		LICA-CN-I	Liver	Liver cancer : Hepatocellular carc
	no	LICA-CN		LICA-CN-I	Liver	Liver cancer : Hepatocellular carc
	no	LICA-CN		LICA-CN-I	Liver	Liver cancer : Hepatocellular carc
	no	LICA-CN		LICA-CN-I	Liver	Liver cancer : Hepatocellular carc
	no	LICA-CN		LICA-CN-I	Liver	Liver cancer : Hepatocellular carc
	no	LICA-CN		LICA-CN-I	Liver	Liver cancer : Hepatocellular carc
	no	LICA-CN		LICA-CN-I	Liver	Liver cancer : Hepatocellular carc
	no	LICA-CN		LICA-CN-I	Liver	Liver cancer : Hepatocellular carc
	no	LICA-CN		LICA-CN-I	Liver	Liver cancer : Hepatocellular carc
	no	LICA-CN		LICA-CN-I	Liver	Liver cancer : Hepatocellular carc
	no	LICA-CN		LICA-CN-I	Liver	Liver cancer : Hepatocellular carc
	no	LICA-CN		LICA-CN-I	Liver	Liver cancer : Hepatocellular carc
	no	LICA-CN		LICA-CN-I	Liver	Liver cancer : Hepatocellular carc
	no	LICA-CN		LICA-CN-I	Liver	Liver cancer : Hepatocellular carc
	no	LICA-CN		LICA-CN-I	Liver	Liver cancer : Hepatocellular carc
	no	LICA-CN		LICA-CN-I	Liver	Liver cancer : Hepatocellular carc
alive	unknown	LICA-FR		CHC917	Liver	Liver cancer : Hepatocellular carc
	no	LICA-CN		LICA-CN-I	Liver	Liver cancer : Hepatocellular carc
	no	LICA-CN		LICA-CN-I	Liver	Liver cancer : Hepatocellular carc
	no	LICA-CN		LICA-CN-I	Liver	Liver cancer : Hepatocellular carc
	no	LICA-CN		LICA-CN-I	Liver	Liver cancer : Hepatocellular carc
	no	LICA-CN		LICA-CN-I	Liver	Liver cancer : Hepatocellular carc
	no	LICA-CN		LICA-CN-I	Liver	Liver cancer : Hepatocellular carc
	no	LICA-CN		LICA-CN-I	Liver	Liver cancer : Hepatocellular carc
deceased	unknown	LIHC-US		TCGA-DD	Liver	Liver cancer : Hepatocellular carc
	no	LICA-CN		LICA-CN-I	Liver	Liver cancer : Hepatocellular carc
	no	LICA-CN		LICA-CN-I	Liver	Liver cancer : Hepatocellular carc
deceased	unknown	LIHC-US		TCGA-DD	Liver	Liver cancer : Hepatocellular carc
	no	LICA-CN		LICA-CN-I	Liver	Liver cancer : Hepatocellular carc
	no	LICA-CN		LICA-CN-I	Liver	Liver cancer : Hepatocellular carc
deceased	unknown	LIHC-US		TCGA-BC-	Liver	Liver cancer : Hepatocellular carc
	no	LICA-CN		LICA-CN-I	Liver	Liver cancer : Hepatocellular carc
	no	LICA-CN		LICA-CN-I	Liver	Liver cancer : Hepatocellular carc
deceased	unknown	LIHC-US		TCGA-BC-	Liver	Liver cancer : Hepatocellular carc
	no	LICA-CN		LICA-CN-I	Liver	Liver cancer : Hepatocellular carc
	no	LICA-CN		LICA-CN-I	Liver	Liver cancer : Hepatocellular carc
deceased	unknown	LIHC-US		TCGA-2Y-	Liver	Liver cancer : Hepatocellular carc
	no	LICA-CN		LICA-CN-I	Liver	Liver cancer : Hepatocellular carc
	no	LICA-CN		LICA-CN-I	Liver	Liver cancer : Hepatocellular carc
deceased	unknown	LIHC-US		TCGA-DD	Liver	Liver cancer : Hepatocellular carc
	no	LICA-CN		LICA-CN-I	Liver	Liver cancer : Hepatocellular carc
	no	LICA-CN		LICA-CN-I	Liver	Liver cancer : Hepatocellular carc
	no	LICA-CN		LICA-CN-I	Liver	Liver cancer : Hepatocellular carc
	no	LICA-CN		LICA-CN-I	Liver	Liver cancer : Hepatocellular carc
	no	LICA-CN		LICA-CN-I	Liver	Liver cancer : Hepatocellular carc
	no	LICA-CN		LICA-CN-I	Liver	Liver cancer : Hepatocellular carc
deceased	unknown	LIHC-US	PCAWG	TCGA-DD	Liver	Liver cancer : Hepatocellular carc
	no	LICA-CN		LICA-CN-I	Liver	Liver cancer : Hepatocellular carc
	no	LICA-CN		LICA-CN-I	Liver	Liver cancer : Hepatocellular carc
	no	LICA-CN		LICA-CN-I	Liver	Liver cancer : Hepatocellular carc
	no	LICA-CN		LICA-CN-I	Liver	Liver cancer : Hepatocellular carc
	no	LICA-CN		LICA-CN-I	Liver	Liver cancer : Hepatocellular carc
	no	LICA-CN		LICA-CN-I	Liver	Liver cancer : Hepatocellular carc
deceased	unknown	LIHC-US	PCAWG	TCGA-BC-	Liver	Liver cancer : Hepatocellular carc
	no	LICA-CN		LICA-CN-I	Liver	Liver cancer : Hepatocellular carc

	no	LICA-CN		LICA-CN-I	Liver	Liver cancer : Hepatocellular carc
	no	LICA-CN		LICA-CN-I	Liver	Liver cancer : Hepatocellular carc
	no	LICA-CN		LICA-CN-I	Liver	Liver cancer : Hepatocellular carc
	no	LICA-CN		LICA-CN-I	Liver	Liver cancer : Hepatocellular carc
	no	LICA-CN		LICA-CN-I	Liver	Liver cancer : Hepatocellular carc
deceased	unknown	LIHC-US		TCGA-HP-	Liver	Liver cancer : Hepatocellular carc
	no	LICA-CN		LICA-CN-I	Liver	Liver cancer : Hepatocellular carc
	no	LICA-CN		LICA-CN-I	Liver	Liver cancer : Hepatocellular carc
	no	LICA-CN		LICA-CN-I	Liver	Liver cancer : Hepatocellular carc
deceased	unknown	LIHC-US		TCGA-DD-	Liver	Liver cancer : Hepatocellular carc
	no	LICA-CN		LICA-CN-I	Liver	Liver cancer : Hepatocellular carc
	no	LICA-CN		LICA-CN-I	Liver	Liver cancer : Hepatocellular carc
	no	LICA-CN		LICA-CN-I	Liver	Liver cancer : Hepatocellular carc
	no	LICA-CN		LICA-CN-I	Liver	Liver cancer : Hepatocellular carc
	no	LICA-CN		LICA-CN-I	Liver	Liver cancer : Hepatocellular carc
deceased	unknown	LIHC-US		TCGA-DD-	Liver	Liver cancer : Hepatocellular carc
	no	LICA-CN		LICA-CN-I	Liver	Liver cancer : Hepatocellular carc
	no	LICA-CN		LICA-CN-I	Liver	Liver cancer : Hepatocellular carc
	no	LICA-CN		LICA-CN-I	Liver	Liver cancer : Hepatocellular carc
	no	LICA-CN		LICA-CN-I	Liver	Liver cancer : Hepatocellular carc
	no	LICA-CN		LICA-CN-I	Liver	Liver cancer : Hepatocellular carc
	no	LICA-CN		LICA-CN-I	Liver	Liver cancer : Hepatocellular carc
	no	LICA-CN		LICA-CN-I	Liver	Liver cancer : Hepatocellular carc
	no	LICA-CN		LICA-CN-I	Liver	Liver cancer : Hepatocellular carc
	no	LICA-CN		LICA-CN-I	Liver	Liver cancer : Hepatocellular carc
	no	LICA-CN		LICA-CN-I	Liver	Liver cancer : Hepatocellular carc
deceased	unknown	LIHC-US		TCGA-2Y-	Liver	Liver cancer : Hepatocellular carc
	no	LICA-CN		LICA-CN-I	Liver	Liver cancer : Hepatocellular carc
	no	LICA-CN		LICA-CN-I	Liver	Liver cancer : Hepatocellular carc
	no	LICA-CN		LICA-CN-I	Liver	Liver cancer : Hepatocellular carc
	no	LICA-CN		LICA-CN-I	Liver	Liver cancer : Hepatocellular carc
	no	LICA-CN		LICA-CN-I	Liver	Liver cancer : Hepatocellular carc
	no	LICA-CN		LICA-CN-I	Liver	Liver cancer : Hepatocellular carc
	no	LICA-CN		LICA-CN-I	Liver	Liver cancer : Hepatocellular carc
deceased	unknown	LIHC-US		TCGA-2Y-	Liver	Liver cancer : Hepatocellular carc
	no	LICA-CN		LICA-CN-I	Liver	Liver cancer : Hepatocellular carc
	no	LICA-CN		LICA-CN-I	Liver	Liver cancer : Hepatocellular carc
	no	LICA-CN		LICA-CN-I	Liver	Liver cancer : Hepatocellular carc
	no	LICA-CN		LICA-CN-I	Liver	Liver cancer : Hepatocellular carc
	no	LICA-CN		LICA-CN-I	Liver	Liver cancer : Hepatocellular carc
deceased	unknown	LIHC-US	PCAWG	TCGA-DD-	Liver	Liver cancer : Hepatocellular carc
	no	LICA-CN		LICA-CN-I	Liver	Liver cancer : Hepatocellular carc
	no	LICA-CN		LICA-CN-I	Liver	Liver cancer : Hepatocellular carc
	no	LICA-CN		LICA-CN-I	Liver	Liver cancer : Hepatocellular carc
	no	LICA-CN		LICA-CN-I	Liver	Liver cancer : Hepatocellular carc
	no	LICA-CN		LICA-CN-I	Liver	Liver cancer : Hepatocellular carc
	no	LICA-CN		LICA-CN-I	Liver	Liver cancer : Hepatocellular carc
	no	LICA-CN		LICA-CN-I	Liver	Liver cancer : Hepatocellular carc
	no	LICA-CN		LICA-CN-I	Liver	Liver cancer : Hepatocellular carc
deceased	unknown	LIHC-US		TCGA-ZS-	Liver	Liver cancer : Hepatocellular carc
	no	LICA-CN		LICA-CN-I	Liver	Liver cancer : Hepatocellular carc
	no	LICA-CN		LICA-CN-I	Liver	Liver cancer : Hepatocellular carc
	no	LICA-CN		LICA-CN-I	Liver	Liver cancer : Hepatocellular carc
	no	LICA-CN		LICA-CN-I	Liver	Liver cancer : Hepatocellular carc
	no	LICA-CN		LICA-CN-I	Liver	Liver cancer : Hepatocellular carc
deceased	unknown	LIHC-US		TCGA-BC-	Liver	Liver cancer : Hepatocellular carc
	no	LICA-CN		LICA-CN-I	Liver	Liver cancer : Hepatocellular carc
	no	LICA-CN		LICA-CN-I	Liver	Liver cancer : Hepatocellular carc

	no	LICA-CN	LICA-CN-I	Liver	Liver cancer : Hepatocellular carc
deceased	unknown	LIHC-US	TCGA-BC-	Liver	Liver cancer : Hepatocellular carc
	no	LICA-CN	LICA-CN-I	Liver	Liver cancer : Hepatocellular carc
	no	LICA-CN	LICA-CN-I	Liver	Liver cancer : Hepatocellular carc
	no	LICA-CN	LICA-CN-I	Liver	Liver cancer : Hepatocellular carc
	no	LICA-CN	LICA-CN-I	Liver	Liver cancer : Hepatocellular carc
deceased	unknown	LIHC-US	TCGA-DD	Liver	Liver cancer : Hepatocellular carc
	no	LICA-CN	LICA-CN-I	Liver	Liver cancer : Hepatocellular carc
	no	LICA-CN	LICA-CN-I	Liver	Liver cancer : Hepatocellular carc
	no	LICA-CN	LICA-CN-I	Liver	Liver cancer : Hepatocellular carc
	no	LICA-CN	LICA-CN-I	Liver	Liver cancer : Hepatocellular carc
	no	LICA-CN	LICA-CN-I	Liver	Liver cancer : Hepatocellular carc
alive	unknown	LIHC-US	TCGA-2Y-	Liver	Liver cancer : Hepatocellular carc
	no	LICA-CN	LICA-CN-I	Liver	Liver cancer : Hepatocellular carc
	no	LICA-CN	LICA-CN-I	Liver	Liver cancer : Hepatocellular carc
	no	LICA-CN	LICA-CN-I	Liver	Liver cancer : Hepatocellular carc
	no	LICA-CN	LICA-CN-I	Liver	Liver cancer : Hepatocellular carc
	no	LICA-CN	LICA-CN-I	Liver	Liver cancer : Hepatocellular carc
	no	LICA-CN	LICA-CN-I	Liver	Liver cancer : Hepatocellular carc
	no	LICA-CN	LICA-CN-I	Liver	Liver cancer : Hepatocellular carc
	no	LICA-CN	LICA-CN-I	Liver	Liver cancer : Hepatocellular carc
deceased	unknown	LIHC-US	TCGA-DD	Liver	Liver cancer : Hepatocellular carc
	no	LICA-CN	LICA-CN-I	Liver	Liver cancer : Hepatocellular carc
	no	LICA-CN	LICA-CN-I	Liver	Liver cancer : Hepatocellular carc
	no	LICA-CN	LICA-CN-I	Liver	Liver cancer : Hepatocellular carc
	no	LICA-CN	LICA-CN-I	Liver	Liver cancer : Hepatocellular carc
	no	LICA-CN	LICA-CN-I	Liver	Liver cancer : Hepatocellular carc
	no	LICA-CN	LICA-CN-I	Liver	Liver cancer : Hepatocellular carc
	no	LICA-CN	LICA-CN-I	Liver	Liver cancer : Hepatocellular carc
	no	LICA-CN	LICA-CN-I	Liver	Liver cancer : Hepatocellular carc
	no	LICA-CN	LICA-CN-I	Liver	Liver cancer : Hepatocellular carc
	no	LICA-CN	LICA-CN-I	Liver	Liver cancer : Hepatocellular carc
	no	LICA-CN	LICA-CN-I	Liver	Liver cancer : Hepatocellular carc
	no	LICA-CN	LICA-CN-I	Liver	Liver cancer : Hepatocellular carc
	no	LICA-CN	LICA-CN-I	Liver	Liver cancer : Hepatocellular carc
deceased	unknown	LIHC-US	TCGA-2Y-	Liver	Liver cancer : Hepatocellular carc
	no	LICA-CN	LICA-CN-I	Liver	Liver cancer : Hepatocellular carc
	no	LICA-CN	LICA-CN-I	Liver	Liver cancer : Hepatocellular carc
	no	LICA-CN	LICA-CN-I	Liver	Liver cancer : Hepatocellular carc
	no	LICA-CN	LICA-CN-I	Liver	Liver cancer : Hepatocellular carc
	no	LICA-CN	LICA-CN-I	Liver	Liver cancer : Hepatocellular carc
	no	LICA-CN	LICA-CN-I	Liver	Liver cancer : Hepatocellular carc
	no	LICA-CN	LICA-CN-I	Liver	Liver cancer : Hepatocellular carc
	no	LICA-CN	LICA-CN-I	Liver	Liver cancer : Hepatocellular carc
	no	LICA-CN	LICA-CN-I	Liver	Liver cancer : Hepatocellular carc
	no	LICA-CN	LICA-CN-I	Liver	Liver cancer : Hepatocellular carc
	no	LICA-CN	LICA-CN-I	Liver	Liver cancer : Hepatocellular carc
	no	LICA-CN	LICA-CN-I	Liver	Liver cancer : Hepatocellular carc
	no	LICA-CN	LICA-CN-I	Liver	Liver cancer : Hepatocellular carc
deceased	unknown	LIHC-US	TCGA-DD	Liver	Liver cancer : Hepatocellular carc
	no	LICA-CN	LICA-CN-I	Liver	Liver cancer : Hepatocellular carc
	no	LICA-CN	LICA-CN-I	Liver	Liver cancer : Hepatocellular carc
	no	LICA-CN	LICA-CN-I	Liver	Liver cancer : Hepatocellular carc
	no	LICA-CN	LICA-CN-I	Liver	Liver cancer : Hepatocellular carc
	no	LICA-CN	LICA-CN-I	Liver	Liver cancer : Hepatocellular carc
	no	LICA-CN	LICA-CN-I	Liver	Liver cancer : Hepatocellular carc
	no	LICA-CN	LICA-CN-I	Liver	Liver cancer : Hepatocellular carc
	unknown	LICA-FR	CHC2539	Liver	Liver cancer : Hepatocellular carc

	no	LICA-CN	LICA-CN-I	Liver	Liver cancer : Hepatocellular carc
	no	LICA-CN	LICA-CN-I	Liver	Liver cancer : Hepatocellular carc
	unknown	LICA-FR	CHC2199	Liver	Liver cancer : Hepatocellular carc
	no	LICA-CN	LICA-CN-I	Liver	Liver cancer : Hepatocellular carc
	no	LICA-CN	LICA-CN-I	Liver	Liver cancer : Hepatocellular carc
	unknown	LICA-FR	CHC2691	Liver	Liver cancer : Hepatocellular carc
	unknown	LICA-FR	CHC2697	Liver	Liver cancer : Hepatocellular carc
	no	LICA-CN	LICA-CN-I	Liver	Liver cancer : Hepatocellular carc
	unknown	LICA-CN	LICA-CN-(	Liver	Liver cancer : Hepatocellular carc
	unknown	LICA-FR	CHC2443	Liver	Liver cancer : Hepatocellular carc
	unknown	LICA-FR	CHC2538	Liver	Liver cancer : Hepatocellular carc
	unknown	LICA-CN	LICA-CN-(	Liver	Liver cancer : Hepatocellular carc
deceased	unknown	LIHC-US	TCGA-DD-	Liver	Liver cancer : Hepatocellular carc
	unknown	LICA-CN	LICA-CN-(	Liver	Liver cancer : Hepatocellular carc
	unknown	LICA-FR	CHC2132	Liver	Liver cancer : Hepatocellular carc
	unknown	LICA-CN	LICA-CN-(	Liver	Liver cancer : Hepatocellular carc
	unknown	LICA-CN	LICA-CN-(	Liver	Liver cancer : Hepatocellular carc
	unknown	LICA-FR	CHC2707	Liver	Liver cancer : Hepatocellular carc
	unknown	LICA-CN	LICA-CN-(	Liver	Liver cancer : Hepatocellular carc
	unknown	LICA-FR	CHC2448	Liver	Liver cancer : Hepatocellular carc
	unknown	LICA-FR	CHC2449	Liver	Liver cancer : Hepatocellular carc
	unknown	LICA-CN	LICA-CN-(	Liver	Liver cancer : Hepatocellular carc
	unknown	LICA-CN	LICA-CN-(	Liver	Liver cancer : Hepatocellular carc
	unknown	LICA-CN	LICA-CN-(	Liver	Liver cancer : Hepatocellular carc
	unknown	LICA-CN	LICA-CN-(	Liver	Liver cancer : Hepatocellular carc
	unknown	LICA-CN	LICA-CN-(	Liver	Liver cancer : Hepatocellular carc
	unknown	LICA-CN	LICA-CN-(	Liver	Liver cancer : Hepatocellular carc
	unknown	LICA-CN	LICA-CN-(	Liver	Liver cancer : Hepatocellular carc
	unknown	LICA-CN	LICA-CN-(	Liver	Liver cancer : Hepatocellular carc
	unknown	LICA-CN	LICA-CN-(	Liver	Liver cancer : Hepatocellular carc
	unknown	LICA-CN	LICA-CN-(	Liver	Liver cancer : Hepatocellular carc
	unknown	LICA-CN	LICA-CN-(	Liver	Liver cancer : Hepatocellular carc
	unknown	LICA-CN	LICA-CN-(	Liver	Liver cancer : Hepatocellular carc
	unknown	LICA-CN	LICA-CN-(	Liver	Liver cancer : Hepatocellular carc
	unknown	LICA-CN	LICA-CN-(	Liver	Liver cancer : Hepatocellular carc
	unknown	LICA-CN	LICA-CN-(	Liver	Liver cancer : Hepatocellular carc
	unknown	LICA-CN	LICA-CN-(	Liver	Liver cancer : Hepatocellular carc
	unknown	LICA-CN	LICA-CN-(	Liver	Liver cancer : Hepatocellular carc
	unknown	LICA-CN	LICA-CN-(	Liver	Liver cancer : Hepatocellular carc
	unknown	LICA-CN	LICA-CN-(	Liver	Liver cancer : Hepatocellular carc
	unknown	LICA-CN	LICA-CN-(	Liver	Liver cancer : Hepatocellular carc
deceased	unknown	LIHC-US	TCGA-NI-	Liver	Liver cancer : Hepatocellular carc
	unknown	LICA-CN	LICA-CN-(	Liver	Liver cancer : Hepatocellular carc
	unknown	LICA-CN	LICA-CN-(	Liver	Liver cancer : Hepatocellular carc
	unknown	LICA-CN	LICA-CN-(	Liver	Liver cancer : Hepatocellular carc
	unknown	LICA-CN	LICA-CN-(	Liver	Liver cancer : Hepatocellular carc
	unknown	LICA-CN	LICA-CN-(	Liver	Liver cancer : Hepatocellular carc
	unknown	LICA-CN	LICA-CN-(	Liver	Liver cancer : Hepatocellular carc
	unknown	LICA-CN	LICA-CN-(	Liver	Liver cancer : Hepatocellular carc
	unknown	LICA-CN	LICA-CN-(	Liver	Liver cancer : Hepatocellular carc
	unknown	LICA-CN	LICA-CN-(	Liver	Liver cancer : Hepatocellular carc
	unknown	LICA-CN	LICA-CN-(	Liver	Liver cancer : Hepatocellular carc
	unknown	LICA-CN	LICA-CN-(	Liver	Liver cancer : Hepatocellular carc
	unknown	LICA-CN	LICA-CN-(	Liver	Liver cancer : Hepatocellular carc
	unknown	LICA-CN	LICA-CN-(	Liver	Liver cancer : Hepatocellular carc
	unknown	LICA-CN	LICA-CN-(	Liver	Liver cancer : Hepatocellular carc
	unknown	LICA-CN	LICA-CN-(	Liver	Liver cancer : Hepatocellular carc
	unknown	LICA-CN	LICA-CN-(	Liver	Liver cancer : Hepatocellular carc

	unknown	LICA-CN	LICA-CN-(	Liver	Liver cancer : Hepatocellular carc
	unknown	LICA-CN	LICA-CN-(	Liver	Liver cancer : Hepatocellular carc
	unknown	LICA-CN	LICA-CN-(	Liver	Liver cancer : Hepatocellular carc
	unknown	LICA-CN	LICA-CN-(	Liver	Liver cancer : Hepatocellular carc
	unknown	LICA-CN	LICA-CN-(	Liver	Liver cancer : Hepatocellular carc
deceased	unknown	LIHC-US	TCGA-BC-	Liver	Liver cancer : Hepatocellular carc
	unknown	LICA-CN	LICA-CN-(	Liver	Liver cancer : Hepatocellular carc
	unknown	LICA-CN	LICA-CN-(	Liver	Liver cancer : Hepatocellular carc
	unknown	LICA-CN	LICA-CN-(	Liver	Liver cancer : Hepatocellular carc
	unknown	LICA-CN	LICA-CN-(	Liver	Liver cancer : Hepatocellular carc
	unknown	LICA-CN	LICA-CN-(	Liver	Liver cancer : Hepatocellular carc
	unknown	LICA-CN	LICA-CN-(	Liver	Liver cancer : Hepatocellular carc
	unknown	LICA-CN	LICA-CN-(	Liver	Liver cancer : Hepatocellular carc
	unknown	LICA-CN	LICA-CN-(	Liver	Liver cancer : Hepatocellular carc
	unknown	LICA-CN	LICA-CN-(	Liver	Liver cancer : Hepatocellular carc
	unknown	LICA-CN	LICA-CN-(	Liver	Liver cancer : Hepatocellular carc
	unknown	LICA-CN	LICA-CN-(	Liver	Liver cancer : Hepatocellular carc
	unknown	LICA-CN	LICA-CN-(	Liver	Liver cancer : Hepatocellular carc
	unknown	LICA-CN	LICA-CN-(	Liver	Liver cancer : Hepatocellular carc
	unknown	LICA-CN	LICA-CN-(	Liver	Liver cancer : Hepatocellular carc
	unknown	LICA-CN	LICA-CN-(	Liver	Liver cancer : Hepatocellular carc
	unknown	LICA-CN	LICA-CN-(	Liver	Liver cancer : Hepatocellular carc
deceased	unknown	LIHC-US	TCGA-DD-	Liver	Liver cancer : Hepatocellular carc
	unknown	LICA-CN	LICA-CN-(	Liver	Liver cancer : Hepatocellular carc
	unknown	LICA-CN	LICA-CN-(	Liver	Liver cancer : Hepatocellular carc
	unknown	LICA-CN	LICA-CN-(	Liver	Liver cancer : Hepatocellular carc
	unknown	LICA-CN	LICA-CN-(	Liver	Liver cancer : Hepatocellular carc
	unknown	LICA-CN	LICA-CN-(	Liver	Liver cancer : Hepatocellular carc
	unknown	LICA-CN	LICA-CN-(	Liver	Liver cancer : Hepatocellular carc
	unknown	LICA-CN	LICA-CN-(	Liver	Liver cancer : Hepatocellular carc
	unknown	LICA-CN	LICA-CN-(	Liver	Liver cancer : Hepatocellular carc
	unknown	LICA-CN	LICA-CN-(	Liver	Liver cancer : Hepatocellular carc
	unknown	LICA-CN	LICA-CN-(	Liver	Liver cancer : Hepatocellular carc
	unknown	LICA-CN	LICA-CN-(	Liver	Liver cancer : Hepatocellular carc
deceased	unknown	LIHC-US	TCGA-DD-	Liver	Liver cancer : Hepatocellular carc
	unknown	LICA-CN	LICA-CN-(	Liver	Liver cancer : Hepatocellular carc
	unknown	LICA-CN	LICA-CN-(	Liver	Liver cancer : Hepatocellular carc
	unknown	LICA-CN	LICA-CN-(	Liver	Liver cancer : Hepatocellular carc
	unknown	LICA-CN	LICA-CN-(	Liver	Liver cancer : Hepatocellular carc
	unknown	LICA-CN	LICA-CN-(	Liver	Liver cancer : Hepatocellular carc
	unknown	LICA-CN	LICA-CN-(	Liver	Liver cancer : Hepatocellular carc
	unknown	LICA-CN	LICA-CN-(	Liver	Liver cancer : Hepatocellular carc
	unknown	LICA-CN	LICA-CN-(	Liver	Liver cancer : Hepatocellular carc
	unknown	LICA-CN	LICA-CN-(	Liver	Liver cancer : Hepatocellular carc
	unknown	LICA-CN	LICA-CN-(	Liver	Liver cancer : Hepatocellular carc
	unknown	LICA-CN	LICA-CN-(	Liver	Liver cancer : Hepatocellular carc
	unknown	LICA-CN	LICA-CN-(	Liver	Liver cancer : Hepatocellular carc
	unknown	LICA-CN	LICA-CN-(	Liver	Liver cancer : Hepatocellular carc
	unknown	LICA-CN	LICA-CN-(	Liver	Liver cancer : Hepatocellular carc
	unknown	LICA-CN	LICA-CN-(	Liver	Liver cancer : Hepatocellular carc
	unknown	LICA-CN	LICA-CN-(	Liver	Liver cancer : Hepatocellular carc
	unknown	LICA-CN	LICA-CN-(	Liver	Liver cancer : Hepatocellular carc
	unknown	LICA-CN	LICA-CN-(	Liver	Liver cancer : Hepatocellular carc
	unknown	LICA-CN	LICA-CN-(	Liver	Liver cancer : Hepatocellular carc
	unknown	LICA-CN	LICA-CN-(	Liver	Liver cancer : Hepatocellular carc
	unknown	LICA-CN	LICA-CN-(	Liver	Liver cancer : Hepatocellular carc
	unknown	LICA-CN	LICA-CN-(	Liver	Liver cancer : Hepatocellular carc
	unknown	LICA-CN	LICA-CN-(	Liver	Liver cancer : Hepatocellular carc

	unknown	LICA-CN		LICA-CN-(	Liver	Liver cancer : Hepatocellular carc
	unknown	LICA-CN		LICA-CN-(	Liver	Liver cancer : Hepatocellular carc
	unknown	LICA-CN		LICA-CN-(	Liver	Liver cancer : Hepatocellular carc
	unknown	LICA-CN		LICA-CN-(	Liver	Liver cancer : Hepatocellular carc
deceased	unknown	LIHC-US		TCGA-2Y-	Liver	Liver cancer : Hepatocellular carc
	unknown	LICA-CN		LICA-CN-(	Liver	Liver cancer : Hepatocellular carc
	unknown	LICA-CN		LICA-CN-(	Liver	Liver cancer : Hepatocellular carc
	unknown	LICA-CN		LICA-CN-(	Liver	Liver cancer : Hepatocellular carc
	unknown	LICA-CN		LICA-CN-(	Liver	Liver cancer : Hepatocellular carc
	unknown	LICA-CN		LICA-CN-(	Liver	Liver cancer : Hepatocellular carc
	unknown	LICA-CN		LICA-CN-(	Liver	Liver cancer : Hepatocellular carc
	unknown	LICA-CN		LICA-CN-(	Liver	Liver cancer : Hepatocellular carc
	unknown	LICA-CN		LICA-CN-(	Liver	Liver cancer : Hepatocellular carc
	unknown	LICA-CN		LICA-CN-(	Liver	Liver cancer : Hepatocellular carc
	unknown	LICA-CN		LICA-CN-(	Liver	Liver cancer : Hepatocellular carc
	unknown	LICA-CN		LICA-CN-(	Liver	Liver cancer : Hepatocellular carc
	unknown	LICA-CN		LICA-CN-(	Liver	Liver cancer : Hepatocellular carc
	unknown	LICA-CN		LICA-CN-(	Liver	Liver cancer : Hepatocellular carc
	unknown	LICA-CN		LICA-CN-(	Liver	Liver cancer : Hepatocellular carc
deceased	unknown	LIHC-US		TCGA-DD-	Liver	Liver cancer : Hepatocellular carc
deceased	unknown	LIHC-US	PCAWG	TCGA-DD-	Liver	Liver cancer : Hepatocellular carc
deceased	unknown	LIHC-US		TCGA-DD-	Liver	Liver cancer : Hepatocellular carc
deceased	unknown	LIHC-US	PCAWG	TCGA-DD-	Liver	Liver cancer : Hepatocellular carc
alive	unknown	LIHC-US		TCGA-2Y-	Liver	Liver cancer : Hepatocellular carc
alive	unknown	LIHC-US		TCGA-2Y-	Liver	Liver cancer : Hepatocellular carc
alive	unknown	LIHC-US		TCGA-2Y-	Liver	Liver cancer : Hepatocellular carc
alive	unknown	LIHC-US		TCGA-2Y-	Liver	Liver cancer : Hepatocellular carc
alive	unknown	LIHC-US		TCGA-2Y-	Liver	Liver cancer : Hepatocellular carc
alive	unknown	LIHC-US		TCGA-2Y-	Liver	Liver cancer : Hepatocellular carc
alive	unknown	LIHC-US		TCGA-2Y-	Liver	Liver cancer : Hepatocellular carc
alive	unknown	LIHC-US		TCGA-2Y-	Liver	Liver cancer : Hepatocellular carc
alive	unknown	LIHC-US		TCGA-2Y-	Liver	Liver cancer : Hepatocellular carc
alive	unknown	LIHC-US		TCGA-3K-	Liver	Liver cancer : Hepatocellular carc
alive	unknown	LIHC-US		TCGA-5R-	Liver	Liver cancer : Hepatocellular carc
alive	unknown	LIHC-US		TCGA-5R-	Liver	Liver cancer : Hepatocellular carc
alive	unknown	LIHC-US		TCGA-BD-	Liver	Liver cancer : Hepatocellular carc
alive	unknown	LIHC-US		TCGA-CC-	Liver	Liver cancer : Hepatocellular carc
alive	unknown	LIHC-US		TCGA-CC-	Liver	Liver cancer : Hepatocellular carc
alive	unknown	LIHC-US		TCGA-CC-	Liver	Liver cancer : Hepatocellular carc
alive	unknown	LIHC-US		TCGA-CC-	Liver	Liver cancer : Hepatocellular carc
alive	unknown	LIHC-US		TCGA-DD-	Liver	Liver cancer : Hepatocellular carc
alive	unknown	LIHC-US		TCGA-DD-	Liver	Liver cancer : Hepatocellular carc
alive	unknown	LIHC-US		TCGA-DD-	Liver	Liver cancer : Hepatocellular carc
alive	unknown	LIHC-US		TCGA-DD-	Liver	Liver cancer : Hepatocellular carc
alive	unknown	LIHC-US		TCGA-DD-	Liver	Liver cancer : Hepatocellular carc
alive	unknown	LIHC-US		TCGA-DD-	Liver	Liver cancer : Hepatocellular carc
alive	unknown	LIHC-US		TCGA-DD-	Liver	Liver cancer : Hepatocellular carc
alive	unknown	LIHC-US		TCGA-DD-	Liver	Liver cancer : Hepatocellular carc
alive	unknown	LIHC-US		TCGA-DD-	Liver	Liver cancer : Hepatocellular carc
alive	unknown	LIHC-US		TCGA-DD-	Liver	Liver cancer : Hepatocellular carc
alive	unknown	LIHC-US		TCGA-G3-	Liver	Liver cancer : Hepatocellular carc
alive	unknown	LIHC-US		TCGA-K7-	Liver	Liver cancer : Hepatocellular carc
alive	unknown	LIHC-US	PCAWG	TCGA-MR	Liver	Liver cancer : Hepatocellular carc
alive	unknown	LIHC-US		TCGA-RC-	Liver	Liver cancer : Hepatocellular carc
alive	unknown	LIHC-US		TCGA-T1-	Liver	Liver cancer : Hepatocellular carc
alive	unknown	LIHC-US		TCGA-WX	Liver	Liver cancer : Hepatocellular carc
alive	unknown	LIHC-US		TCGA-2V-	Liver	Liver cancer : Hepatocellular carc
alive	unknown	LIHC-US		TCGA-DD-	Liver	Liver cancer : Hepatocellular carc

alive	unknown	LIHC-US	TCGA-GJ-	Liver	Liver cancer : Hepatocellular carc
alive	unknown	LIHC-US	TCGA-RC-	Liver	Liver cancer : Hepatocellular carc
alive	unknown	LIHC-US	TCGA-RC-	Liver	Liver cancer : Hepatocellular carc
alive	unknown	LIHC-US	TCGA-RC-	Liver	Liver cancer : Hepatocellular carc
alive	unknown	LIHC-US	TCGA-RC-	Liver	Liver cancer : Hepatocellular carc
alive	unknown	LIHC-US	TCGA-WX	Liver	Liver cancer : Hepatocellular carc
ɪe	unknown	LINC-JP	CCC1	Liver	Liver cancer : Hepatocellular carc
ɪe	unknown	LINC-JP	CCC2	Liver	Liver cancer : Hepatocellular carc
ɪe	unknown	LINC-JP	CCC3	Liver	Liver cancer : Hepatocellular carc
ɪe	unknown	LINC-JP	CCC4	Liver	Liver cancer : Hepatocellular carc
ɪe	unknown	LINC-JP	CCC5	Liver	Liver cancer : Hepatocellular carc
ɪe	unknown	LINC-JP	CCC6	Liver	Liver cancer : Hepatocellular carc
ɪe	unknown	LINC-JP	CCC60	Liver	Liver cancer : Hepatocellular carc
ɪe	unknown	LINC-JP	CCC64	Liver	Liver cancer : Hepatocellular carc
ɪe	unknown	LINC-JP	CCC67	Liver	Liver cancer : Hepatocellular carc
ɪe	unknown	LINC-JP	CCC68	Liver	Liver cancer : Hepatocellular carc
alive	unknown	LINC-JP	HCC1	Liver	Liver cancer : Hepatocellular carc
deceased	unknown	LINC-JP	HCC2	Liver	Liver cancer : Hepatocellular carc
deceased	unknown	LINC-JP	HCC3	Liver	Liver cancer : Hepatocellular carc
alive	unknown	LINC-JP	HCC4	Liver	Liver cancer : Hepatocellular carc
alive	unknown	LINC-JP	HCC5	Liver	Liver cancer : Hepatocellular carc
alive	unknown	LINC-JP	HCC6	Liver	Liver cancer : Hepatocellular carc
deceased	unknown	LINC-JP	HCC7	Liver	Liver cancer : Hepatocellular carc
alive	unknown	LINC-JP	HCC8	Liver	Liver cancer : Hepatocellular carc
alive	unknown	LINC-JP	HCC9	Liver	Liver cancer : Hepatocellular carc
alive	unknown	LINC-JP	HCC11	Liver	Liver cancer : Hepatocellular carc
deceased	unknown	LINC-JP	HCC12	Liver	Liver cancer : Hepatocellular carc
alive	unknown	LINC-JP	HCC13	Liver	Liver cancer : Hepatocellular carc
alive	unknown	LINC-JP	HCC14	Liver	Liver cancer : Hepatocellular carc
alive	unknown	LINC-JP	HCC15	Liver	Liver cancer : Hepatocellular carc
deceased	unknown	LINC-JP	HCC16	Liver	Liver cancer : Hepatocellular carc
alive	unknown	LINC-JP	HCC17	Liver	Liver cancer : Hepatocellular carc
alive	unknown	LINC-JP	HCC18	Liver	Liver cancer : Hepatocellular carc
alive	unknown	LINC-JP	HCC19	Liver	Liver cancer : Hepatocellular carc
alive	unknown	LINC-JP	HCC20	Liver	Liver cancer : Hepatocellular carc
deceased	unknown	LINC-JP	HCC21	Liver	Liver cancer : Hepatocellular carc
alive	unknown	LINC-JP	HCC22	Liver	Liver cancer : Hepatocellular carc
deceased	unknown	LINC-JP	HCC23	Liver	Liver cancer : Hepatocellular carc
alive	unknown	LINC-JP	HCC24	Liver	Liver cancer : Hepatocellular carc
deceased	unknown	LINC-JP	HCC25	Liver	Liver cancer : Hepatocellular carc
alive	unknown	LINC-JP	HCC26	Liver	Liver cancer : Hepatocellular carc
alive	unknown	LINC-JP	THCC_100	Liver	Liver cancer : Hepatocellular carc
alive	unknown	LINC-JP	THCC_105	Liver	Liver cancer : Hepatocellular carc
alive	unknown	LINC-JP	THCC_106	Liver	Liver cancer : Hepatocellular carc
deceased	unknown	LINC-JP	HCC27	Liver	Liver cancer : Hepatocellular carc
alive	unknown	LINC-JP	THCC_107	Liver	Liver cancer : Hepatocellular carc
alive	unknown	LINC-JP	THCC_108	Liver	Liver cancer : Hepatocellular carc
alive	unknown	LINC-JP	THCC_109	Liver	Liver cancer : Hepatocellular carc
alive	unknown	LINC-JP	THCC_118	Liver	Liver cancer : Hepatocellular carc
alive	unknown	LINC-JP	THCC_119	Liver	Liver cancer : Hepatocellular carc
alive	unknown	LINC-JP	THCC_12-	Liver	Liver cancer : Hepatocellular carc
alive	unknown	LINC-JP	HCC28	Liver	Liver cancer : Hepatocellular carc
alive	unknown	LINC-JP	THCC_12-	Liver	Liver cancer : Hepatocellular carc
alive	unknown	LINC-JP	THCC_12-	Liver	Liver cancer : Hepatocellular carc
alive	unknown	LINC-JP	THCC_12-	Liver	Liver cancer : Hepatocellular carc
alive	unknown	LINC-JP	THCC_12-	Liver	Liver cancer : Hepatocellular carc
alive	unknown	LINC-JP	THCC_12-	Liver	Liver cancer : Hepatocellular carc
alive	unknown	LINC-JP	THCC_12-	Liver	Liver cancer : Hepatocellular carc

deceased	unknown	LINC-JP		HCC29	Liver	Liver cancer : Hepatocellular carc
alive	unknown	LINC-JP		THCC_12-	Liver	Liver cancer : Hepatocellular carc
alive	unknown	LINC-JP		THCC_12-	Liver	Liver cancer : Hepatocellular carc
alive	unknown	LINC-JP		THCC_12-	Liver	Liver cancer : Hepatocellular carc
alive	unknown	LINC-JP		THCC_124	Liver	Liver cancer : Hepatocellular carc
alive	unknown	LINC-JP		THCC_125	Liver	Liver cancer : Hepatocellular carc
alive	unknown	LINC-JP		HCC30	Liver	Liver cancer : Hepatocellular carc
alive	unknown	LINC-JP		THCC_126	Liver	Liver cancer : Hepatocellular carc
alive	unknown	LINC-JP		THCC_127	Liver	Liver cancer : Hepatocellular carc
alive	unknown	LINC-JP		THCC_130	Liver	Liver cancer : Hepatocellular carc
alive	unknown	LINC-JP		THCC_132	Liver	Liver cancer : Hepatocellular carc
alive	unknown	LINC-JP		THCC_134	Liver	Liver cancer : Hepatocellular carc
alive	unknown	LINC-JP		THCC_135	Liver	Liver cancer : Hepatocellular carc
alive	unknown	LINC-JP		THCC_150	Liver	Liver cancer : Hepatocellular carc
alive	unknown	LINC-JP		HCC31	Liver	Liver cancer : Hepatocellular carc
alive	unknown	LINC-JP		THCC_151	Liver	Liver cancer : Hepatocellular carc
alive	unknown	LINC-JP		THCC_155	Liver	Liver cancer : Hepatocellular carc
alive	unknown	LINC-JP		THCC_156	Liver	Liver cancer : Hepatocellular carc
alive	unknown	LINC-JP		THCC_159	Liver	Liver cancer : Hepatocellular carc
deceased	unknown	LINC-JP		THCC_161	Liver	Liver cancer : Hepatocellular carc
alive	unknown	LINC-JP		THCC_167	Liver	Liver cancer : Hepatocellular carc
alive	unknown	LINC-JP		HCC32	Liver	Liver cancer : Hepatocellular carc
alive	unknown	LINC-JP		THCC_28	Liver	Liver cancer : Hepatocellular carc
alive	unknown	LINC-JP		THCC_31	Liver	Liver cancer : Hepatocellular carc
alive	unknown	LINC-JP		THCC_34	Liver	Liver cancer : Hepatocellular carc
alive	unknown	LINC-JP		THCC_36	Liver	Liver cancer : Hepatocellular carc
deceased	unknown	LINC-JP		THCC_50	Liver	Liver cancer : Hepatocellular carc
alive	unknown	LINC-JP		THCC_52	Liver	Liver cancer : Hepatocellular carc
alive	unknown	LINC-JP		THCC_53	Liver	Liver cancer : Hepatocellular carc
alive	unknown	LINC-JP		HCC33	Liver	Liver cancer : Hepatocellular carc
alive	unknown	LINC-JP		THCC_61	Liver	Liver cancer : Hepatocellular carc
deceased	unknown	LINC-JP		THCC_65	Liver	Liver cancer : Hepatocellular carc
alive	unknown	LINC-JP		THCC_76	Liver	Liver cancer : Hepatocellular carc
alive	unknown	LINC-JP		THCC_82	Liver	Liver cancer : Hepatocellular carc
alive	unknown	LINC-JP		THCC_83	Liver	Liver cancer : Hepatocellular carc
alive	unknown	LINC-JP		THCC_85	Liver	Liver cancer : Hepatocellular carc
alive	unknown	LINC-JP		THCC_86	Liver	Liver cancer : Hepatocellular carc
deceased	unknown	LINC-JP		HCC34	Liver	Liver cancer : Hepatocellular carc
alive	unknown	LINC-JP		THCC_91	Liver	Liver cancer : Hepatocellular carc
alive	unknown	LINC-JP		THCC_98	Liver	Liver cancer : Hepatocellular carc
deceased	unknown	LINC-JP		THCC_99	Liver	Liver cancer : Hepatocellular carc
alive	unknown	LINC-JP		THCC_12-	Liver	Liver cancer : Hepatocellular carc
alive	unknown	LINC-JP		THCC_12-	Liver	Liver cancer : Hepatocellular carc
histologic_	unknown	LINC-JP		HCC-JP-7	Liver	Liver cancer : Hepatocellular carc
alive	unknown	LINC-JP		HCC35	Liver	Liver cancer : Hepatocellular carc
histologic_	unknown	LINC-JP		HCC-JP-1	Liver	Liver cancer : Hepatocellular carc
histologic_	unknown	LINC-JP		HCC-JP-1	Liver	Liver cancer : Hepatocellular carc
histologic_	unknown	LINC-JP		HCC-JP-1	Liver	Liver cancer : Hepatocellular carc
histologic_	unknown	LINC-JP		HCC-JP-1	Liver	Liver cancer : Hepatocellular carc
histologic_	unknown	LINC-JP		HCC-JP-1	Liver	Liver cancer : Hepatocellular carc
histologic_	unknown	LINC-JP		HCC-JP-1	Liver	Liver cancer : Hepatocellular carc
histologic_	unknown	LINC-JP		HCC-JP-1	Liver	Liver cancer : Hepatocellular carc
alive	unknown	LINC-JP		HCC36	Liver	Liver cancer : Hepatocellular carc
histologic_	unknown	LINC-JP		HCC-JP-1	Liver	Liver cancer : Hepatocellular carc
histologic_	unknown	LINC-JP		HCC-JP-1	Liver	Liver cancer : Hepatocellular carc
histologic_	unknown	LINC-JP		HCC-JP-1	Liver	Liver cancer : Hepatocellular carc
histologic_	unknown	LINC-JP		HCC-JP-3	Liver	Liver cancer : Hepatocellular carc
histologic_	unknown	LINC-JP		HCC-JP-3	Liver	Liver cancer : Hepatocellular carc

histologic_	unknown	LINC-JP	HCC-JP-3:	Liver	Liver cancer : Hepatocellular carc
histologic_	unknown	LINC-JP	HCC-JP-3:	Liver	Liver cancer : Hepatocellular carc
histologic_	unknown	LINC-JP	HCC-JP-3:	Liver	Liver cancer : Hepatocellular carc
histologic_	unknown	LINC-JP	HCC-JP-3:	Liver	Liver cancer : Hepatocellular carc
histologic_	unknown	LINC-JP	HCC-JP-3:	Liver	Liver cancer : Hepatocellular carc
histologic_	unknown	LINC-JP	HCC-JP-3:	Liver	Liver cancer : Hepatocellular carc
alive	unknown	LINC-JP	HCC37	Liver	Liver cancer : Hepatocellular carc
histologic_	unknown	LINC-JP	HCC-JP-3:	Liver	Liver cancer : Hepatocellular carc
histologic_	unknown	LINC-JP	HCC-JP-3:	Liver	Liver cancer : Hepatocellular carc
histologic_	unknown	LINC-JP	HCC-JP-3	Liver	Liver cancer : Hepatocellular carc
histologic_	unknown	LINC-JP	HCC-JP-3!	Liver	Liver cancer : Hepatocellular carc
histologic_	unknown	LINC-JP	HCC-JP-3!	Liver	Liver cancer : Hepatocellular carc
histologic_	unknown	LINC-JP	HCC-JP-3(	Liver	Liver cancer : Hepatocellular carc
histologic_	unknown	LINC-JP	HCC-JP-3(	Liver	Liver cancer : Hepatocellular carc
histologic_	unknown	LINC-JP	HCC-JP-3	Liver	Liver cancer : Hepatocellular carc
histologic_	unknown	LINC-JP	HCC-JP-3	Liver	Liver cancer : Hepatocellular carc
histologic_	unknown	LINC-JP	HCC-JP-3{	Liver	Liver cancer : Hepatocellular carc
alive	unknown	LINC-JP	HCC38	Liver	Liver cancer : Hepatocellular carc
histologic_	unknown	LINC-JP	HCC-JP-3{	Liver	Liver cancer : Hepatocellular carc
histologic_	unknown	LINC-JP	HCC-JP-3{	Liver	Liver cancer : Hepatocellular carc
histologic_	unknown	LINC-JP	HCC-JP-3{	Liver	Liver cancer : Hepatocellular carc
histologic_	unknown	LINC-JP	HCC-JP-3!	Liver	Liver cancer : Hepatocellular carc
alive	unknown	LINC-JP	HCC-JP-4(	Liver	Liver cancer : Hepatocellular carc
deceased	unknown	LINC-JP	HCC-JP-4(	Liver	Liver cancer : Hepatocellular carc
alive	unknown	LINC-JP	HCC-JP-4(	Liver	Liver cancer : Hepatocellular carc
alive	unknown	LINC-JP	HCC-JP-4:	Liver	Liver cancer : Hepatocellular carc
alive	unknown	LINC-JP	HCC-JP-4:	Liver	Liver cancer : Hepatocellular carc
alive	unknown	LINC-JP	HCC39	Liver	Liver cancer : Hepatocellular carc
alive	unknown	LINC-JP	HCC-JP-4:	Liver	Liver cancer : Hepatocellular carc
deceased	unknown	LINC-JP	HCC-JP-4:	Liver	Liver cancer : Hepatocellular carc
deceased	unknown	LINC-JP	HCC-JP-4:	Liver	Liver cancer : Hepatocellular carc
alive	unknown	LINC-JP	HCC-JP-4:	Liver	Liver cancer : Hepatocellular carc
alive	unknown	LINC-JP	HCC-JP-4:	Liver	Liver cancer : Hepatocellular carc
deceased	unknown	LINC-JP	HCC-JP-4:	Liver	Liver cancer : Hepatocellular carc
alive	unknown	LINC-JP	HCC-JP-4:	Liver	Liver cancer : Hepatocellular carc
alive	unknown	LINC-JP	HCC-JP-4:	Liver	Liver cancer : Hepatocellular carc
alive	unknown	LINC-JP	HCC-JP-4:	Liver	Liver cancer : Hepatocellular carc
alive	unknown	LINC-JP	HCC-JP-4:	Liver	Liver cancer : Hepatocellular carc
alive	unknown	LINC-JP	HCC40	Liver	Liver cancer : Hepatocellular carc
alive	unknown	LINC-JP	HCC-JP-4	Liver	Liver cancer : Hepatocellular carc
alive	unknown	LINC-JP	HCC-JP-4	Liver	Liver cancer : Hepatocellular carc
deceased	unknown	LINC-JP	HCC-JP-4	Liver	Liver cancer : Hepatocellular carc
alive	unknown	LINC-JP	HCC-JP-4	Liver	Liver cancer : Hepatocellular carc
alive	unknown	LINC-JP	HCC-JP-4!	Liver	Liver cancer : Hepatocellular carc
alive	unknown	LINC-JP	HCC-JP-4!	Liver	Liver cancer : Hepatocellular carc
deceased	unknown	LINC-JP	HCC-JP-4!	Liver	Liver cancer : Hepatocellular carc
alive	unknown	LINC-JP	HCC-JP-4!	Liver	Liver cancer : Hepatocellular carc
	unknown	LICA-FR	CHC2557	Liver	Liver cancer : Hepatocellular carc
alive	unknown	LINC-JP	HCC-JP-4!	Liver	Liver cancer : Hepatocellular carc
alive	unknown	LINC-JP	HCC-JP-4!	Liver	Liver cancer : Hepatocellular carc
deceased	unknown	LINC-JP	HCC41	Liver	Liver cancer : Hepatocellular carc
alive	unknown	LINC-JP	HCC-JP-4!	Liver	Liver cancer : Hepatocellular carc
deceased	unknown	LINC-JP	HCC-JP-4!	Liver	Liver cancer : Hepatocellular carc
alive	unknown	LINC-JP	HCC-JP-4(	Liver	Liver cancer : Hepatocellular carc
	unknown	LICA-FR	CHC2207	Liver	Liver cancer : Hepatocellular carc
alive	unknown	LINC-JP	HCC-JP-4(	Liver	Liver cancer : Hepatocellular carc
alive	unknown	LINC-JP	HCC-JP-4(	Liver	Liver cancer : Hepatocellular carc
alive	unknown	LINC-JP	HCC-JP-4(	Liver	Liver cancer : Hepatocellular carc

alive	unknown	LINC-JP		HCC-JP-4	Liver	Liver cancer : Hepatocellular carc
alive	unknown	LINC-JP		HCC-JP-4	Liver	Liver cancer : Hepatocellular carc
alive	unknown	LINC-JP		HCC42	Liver	Liver cancer : Hepatocellular carc
alive	unknown	LINC-JP		HCC-JP-4	Liver	Liver cancer : Hepatocellular carc
alive	unknown	LINC-JP		HCC-JP-4	Liver	Liver cancer : Hepatocellular carc
alive	unknown	LINC-JP		HCC-JP-4	Liver	Liver cancer : Hepatocellular carc
alive	unknown	LINC-JP		HCC-JP-4	Liver	Liver cancer : Hepatocellular carc
deceased	unknown	LINC-JP		HCC-JP-4	Liver	Liver cancer : Hepatocellular carc
alive	unknown	LINC-JP		HCC-JP-4	Liver	Liver cancer : Hepatocellular carc
alive	unknown	LINC-JP		HCC-JP-4	Liver	Liver cancer : Hepatocellular carc
alive	unknown	LINC-JP		HCC-JP-4	Liver	Liver cancer : Hepatocellular carc
alive	unknown	LINC-JP		HCC45	Liver	Liver cancer : Hepatocellular carc
alive	unknown	LINC-JP		HCC-JP-4	Liver	Liver cancer : Hepatocellular carc
alive	unknown	LINC-JP		HCC-JP-4	Liver	Liver cancer : Hepatocellular carc
alive	unknown	LINC-JP		HCC-JP-4	Liver	Liver cancer : Hepatocellular carc
alive	unknown	LINC-JP		HCC-JP-4	Liver	Liver cancer : Hepatocellular carc
deceased	unknown	LINC-JP		HCC-JP-4	Liver	Liver cancer : Hepatocellular carc
alive	unknown	LINC-JP		HCC-JP-4	Liver	Liver cancer : Hepatocellular carc
alive	unknown	LINC-JP		HCC46	Liver	Liver cancer : Hepatocellular carc
alive	unknown	LINC-JP		HCC-JP-4	Liver	Liver cancer : Hepatocellular carc
alive	unknown	LINC-JP		HCC-JP-4	Liver	Liver cancer : Hepatocellular carc
alive	unknown	LINC-JP		HCC-JP-4	Liver	Liver cancer : Hepatocellular carc
alive	unknown	LINC-JP		HCC-JP-4	Liver	Liver cancer : Hepatocellular carc
alive	unknown	LINC-JP		HCC-JP-4	Liver	Liver cancer : Hepatocellular carc
alive	unknown	LINC-JP		HCC47	Liver	Liver cancer : Hepatocellular carc
alive	unknown	LINC-JP		HCC-JP-4	Liver	Liver cancer : Hepatocellular carc
alive	unknown	LINC-JP		HCC-JP-4	Liver	Liver cancer : Hepatocellular carc
alive	unknown	LINC-JP		HCC-JP-4	Liver	Liver cancer : Hepatocellular carc
alive	unknown	LINC-JP		HCC-JP-4	Liver	Liver cancer : Hepatocellular carc
alive	unknown	LINC-JP		HCC-JP-4	Liver	Liver cancer : Hepatocellular carc
alive	unknown	LINC-JP		HCC-JP-5	Liver	Liver cancer : Hepatocellular carc
alive	unknown	LINC-JP		HCC-JP-5	Liver	Liver cancer : Hepatocellular carc
alive	unknown	LINC-JP		HCC48	Liver	Liver cancer : Hepatocellular carc
alive	unknown	LINC-JP		HCC-JP-5	Liver	Liver cancer : Hepatocellular carc
alive	unknown	LINC-JP		HCC-JP-5	Liver	Liver cancer : Hepatocellular carc
alive	unknown	LINC-JP		HCC-JP-5	Liver	Liver cancer : Hepatocellular carc
alive	unknown	LINC-JP		HCC-JP-5	Liver	Liver cancer : Hepatocellular carc
alive	unknown	LINC-JP		HCC-JP-5	Liver	Liver cancer : Hepatocellular carc
alive	unknown	LINC-JP		HCC-JP-5	Liver	Liver cancer : Hepatocellular carc
alive	unknown	LINC-JP		HCC49	Liver	Liver cancer : Hepatocellular carc
deceased	unknown	LICA-CN		LICA-BGI-	Liver	Liver cancer : Hepatocellular carc
alive	unknown	LICA-CN		LICA-BGI-	Liver	Liver cancer : Hepatocellular carc
deceased	unknown	LICA-CN		LICA-BGI-	Liver	Liver cancer : Hepatocellular carc
alive	unknown	LICA-CN		LICA-BGI-	Liver	Liver cancer : Hepatocellular carc
alive	unknown	LICA-CN		LICA-BGI-	Liver	Liver cancer : Hepatocellular carc
deceased	unknown	LICA-CN		LICA-BGI-	Liver	Liver cancer : Hepatocellular carc
alive	unknown	LICA-CN		LICA-BGI-	Liver	Liver cancer : Hepatocellular carc
deceased	unknown	LINC-JP		HCC50	Liver	Liver cancer : Hepatocellular carc
alive	unknown	LICA-CN		LICA-BGI-	Liver	Liver cancer : Hepatocellular carc
deceased	unknown	LICA-CN		LICA-BGI-	Liver	Liver cancer : Hepatocellular carc
alive	unknown	LICA-CN		LICA-BGI-	Liver	Liver cancer : Hepatocellular carc
alive	unknown	LICA-CN		LICA-BGI-	Liver	Liver cancer : Hepatocellular carc
deceased	unknown	LICA-CN		LICA-BGI-	Liver	Liver cancer : Hepatocellular carc
alive	unknown	LICA-CN		LICA-BGI-	Liver	Liver cancer : Hepatocellular carc
alive	unknown	LICA-CN		LICA-BGI-	Liver	Liver cancer : Hepatocellular carc
deceased	unknown	LICA-CN		LICA-BGI-	Liver	Liver cancer : Hepatocellular carc
alive	unknown	LICA-CN		LICA-BGI-	Liver	Liver cancer : Hepatocellular carc
alive	unknown	LICA-CN		LICA-BGI-	Liver	Liver cancer : Hepatocellular carc

deceased	unknown	LICA-CN	LICA-BGI-	Liver	Liver cancer : Hepatocellular carc
alive	unknown	LICA-CN	LICA-BGI-	Liver	Liver cancer : Hepatocellular carc
deceased	unknown	LICA-CN	LICA-BGI-	Liver	Liver cancer : Hepatocellular carc
alive	unknown	LICA-CN	LICA-BGI-	Liver	Liver cancer : Hepatocellular carc
deceased	unknown	LICA-CN	LICA-BGI-	Liver	Liver cancer : Hepatocellular carc
alive	unknown	LICA-CN	LICA-BGI-	Liver	Liver cancer : Hepatocellular carc
alive	unknown	LICA-CN	LICA-BGI-	Liver	Liver cancer : Hepatocellular carc
alive	unknown	LICA-CN	LICA-BGI-	Liver	Liver cancer : Hepatocellular carc
deceased	unknown	LICA-CN	LICA-BGI-	Liver	Liver cancer : Hepatocellular carc
deceased	unknown	LICA-CN	LICA-BGI-	Liver	Liver cancer : Hepatocellular carc
alive	unknown	LINC-JP	HCC51	Liver	Liver cancer : Hepatocellular carc
alive	unknown	LICA-CN	LICA-BGI-	Liver	Liver cancer : Hepatocellular carc
deceased	unknown	LICA-CN	LICA-BGI-	Liver	Liver cancer : Hepatocellular carc
alive	unknown	LICA-CN	LICA-BGI-	Liver	Liver cancer : Hepatocellular carc
alive	unknown	LICA-CN	LICA-BGI-	Liver	Liver cancer : Hepatocellular carc
deceased	unknown	LICA-CN	LICA-BGI-	Liver	Liver cancer : Hepatocellular carc
deceased	unknown	LICA-CN	LICA-BGI-	Liver	Liver cancer : Hepatocellular carc
alive	unknown	LICA-CN	LICA-BGI-	Liver	Liver cancer : Hepatocellular carc
alive	unknown	LICA-CN	LICA-BGI-	Liver	Liver cancer : Hepatocellular carc
alive	unknown	LICA-CN	LICA-BGI-	Liver	Liver cancer : Hepatocellular carc
alive	unknown	LICA-CN	LICA-BGI-	Liver	Liver cancer : Hepatocellular carc
alive	unknown	LICA-CN	LICA-BGI-	Liver	Liver cancer : Hepatocellular carc
alive	unknown	LICA-CN	LICA-BGI-	Liver	Liver cancer : Hepatocellular carc
alive	unknown	LICA-CN	LICA-BGI-	Liver	Liver cancer : Hepatocellular carc
alive	unknown	LICA-CN	LICA-BGI-	Liver	Liver cancer : Hepatocellular carc
alive	unknown	LICA-CN	LICA-BGI-	Liver	Liver cancer : Hepatocellular carc
alive	unknown	LICA-CN	LICA-BGI-	Liver	Liver cancer : Hepatocellular carc
alive	unknown	LICA-CN	LICA-BGI-	Liver	Liver cancer : Hepatocellular carc
alive	unknown	LICA-CN	LICA-BGI-	Liver	Liver cancer : Hepatocellular carc
alive	unknown	LICA-CN	LICA-BGI-	Liver	Liver cancer : Hepatocellular carc
alive	unknown	LICA-CN	LICA-BGI-	Liver	Liver cancer : Hepatocellular carc
alive	unknown	LINC-JP	HCC52	Liver	Liver cancer : Hepatocellular carc
deceased	unknown	LICA-CN	LICA-BGI-	Liver	Liver cancer : Hepatocellular carc
alive	unknown	LICA-CN	LICA-BGI-	Liver	Liver cancer : Hepatocellular carc
deceased	unknown	LICA-CN	LICA-BGI-	Liver	Liver cancer : Hepatocellular carc
alive	unknown	LICA-CN	LICA-BGI-	Liver	Liver cancer : Hepatocellular carc
alive	unknown	LICA-CN	LICA-BGI-	Liver	Liver cancer : Hepatocellular carc
alive	unknown	LICA-CN	LICA-BGI-	Liver	Liver cancer : Hepatocellular carc
deceased	unknown	LICA-CN	LICA-BGI-	Liver	Liver cancer : Hepatocellular carc
deceased	unknown	LICA-CN	LICA-BGI-	Liver	Liver cancer : Hepatocellular carc
deceased	unknown	LICA-CN	LICA-BGI-	Liver	Liver cancer : Hepatocellular carc
deceased	unknown	LICA-CN	LICA-BGI-	Liver	Liver cancer : Hepatocellular carc
alive	unknown	LICA-CN	LICA-BGI-	Liver	Liver cancer : Hepatocellular carc
alive	unknown	LICA-CN	LICA-BGI-	Liver	Liver cancer : Hepatocellular carc
deceased	unknown	LICA-CN	LICA-BGI-	Liver	Liver cancer : Hepatocellular carc
deceased	unknown	LICA-CN	LICA-BGI-	Liver	Liver cancer : Hepatocellular carc
alive	unknown	LICA-CN	LICA-BGI-	Liver	Liver cancer : Hepatocellular carc
alive	unknown	LICA-CN	LICA-BGI-	Liver	Liver cancer : Hepatocellular carc
alive	unknown	LINC-JP	HCC53	Liver	Liver cancer : Hepatocellular carc
deceased	unknown	LICA-CN	LICA-BGI-	Liver	Liver cancer : Hepatocellular carc
deceased	unknown	LICA-CN	LICA-BGI-	Liver	Liver cancer : Hepatocellular carc
alive	unknown	LICA-CN	LICA-BGI-	Liver	Liver cancer : Hepatocellular carc
deceased	unknown	LICA-CN	LICA-BGI-	Liver	Liver cancer : Hepatocellular carc
deceased	unknown	LICA-CN	LICA-BGI-	Liver	Liver cancer : Hepatocellular carc
deceased	unknown	LICA-CN	LICA-BGI-	Liver	Liver cancer : Hepatocellular carc
alive	unknown	LICA-CN	LICA-BGI-	Liver	Liver cancer : Hepatocellular carc
deceased	unknown	LICA-CN	LICA-BGI-	Liver	Liver cancer : Hepatocellular carc
deceased	unknown	LICA-CN	LICA-BGI-	Liver	Liver cancer : Hepatocellular carc

alive	unknown	LINC-JP	HCC54	Liver	Liver cancer : Hepatocellular carc
alive	unknown	LICA-CN	LICA-BGI-	Liver	Liver cancer : Hepatocellular carc
alive	unknown	LICA-CN	LICA-BGI-	Liver	Liver cancer : Hepatocellular carc
alive	unknown	LICA-CN	LICA-BGI-	Liver	Liver cancer : Hepatocellular carc
alive	unknown	LICA-CN	LICA-BGI-	Liver	Liver cancer : Hepatocellular carc
deceased	unknown	LICA-CN	LICA-BGI-	Liver	Liver cancer : Hepatocellular carc
alive	unknown	LICA-CN	LICA-BGI-	Liver	Liver cancer : Hepatocellular carc
deceased	unknown	LICA-CN	LICA-BGI-	Liver	Liver cancer : Hepatocellular carc
alive	unknown	LICA-CN	LICA-BGI-	Liver	Liver cancer : Hepatocellular carc
alive	unknown	LICA-CN	LICA-BGI-	Liver	Liver cancer : Hepatocellular carc
deceased	unknown	LICA-CN	LICA-BGI-	Liver	Liver cancer : Hepatocellular carc
deceased	unknown	LICA-CN	LICA-BGI-	Liver	Liver cancer : Hepatocellular carc
alive	unknown	LICA-CN	LICA-BGI-	Liver	Liver cancer : Hepatocellular carc
alive	unknown	LICA-CN	LICA-BGI-	Liver	Liver cancer : Hepatocellular carc
alive	unknown	LICA-CN	LICA-BGI-	Liver	Liver cancer : Hepatocellular carc
deceased	unknown	LICA-CN	LICA-BGI-	Liver	Liver cancer : Hepatocellular carc
alive	unknown	LICA-CN	LICA-BGI-	Liver	Liver cancer : Hepatocellular carc
alive	unknown	LINC-JP	HCC55	Liver	Liver cancer : Hepatocellular carc
alive	unknown	LINC-JP	HCC56	Liver	Liver cancer : Hepatocellular carc
alive	unknown	LINC-JP	HCC57	Liver	Liver cancer : Hepatocellular carc
alive	unknown	LINC-JP	HCC58	Liver	Liver cancer : Hepatocellular carc
alive	unknown	LINC-JP	HCC59	Liver	Liver cancer : Hepatocellular carc
alive	unknown	LINC-JP	HCC60	Liver	Liver cancer : Hepatocellular carc
alive	unknown	LINC-JP	HCC61	Liver	Liver cancer : Hepatocellular carc
deceased	unknown	LINC-JP	HCC62	Liver	Liver cancer : Hepatocellular carc
alive	unknown	LINC-JP	HCC63	Liver	Liver cancer : Hepatocellular carc
deceased	unknown	LINC-JP	HCC64	Liver	Liver cancer : Hepatocellular carc
alive	unknown	LINC-JP	HCC65	Liver	Liver cancer : Hepatocellular carc
alive	unknown	LINC-JP	HCC66	Liver	Liver cancer : Hepatocellular carc
alive	unknown	LINC-JP	HCC67	Liver	Liver cancer : Hepatocellular carc
alive	unknown	LINC-JP	HCC68	Liver	Liver cancer : Hepatocellular carc
alive	unknown	LINC-JP	HCC69	Liver	Liver cancer : Hepatocellular carc
alive	unknown	LINC-JP	HCC70	Liver	Liver cancer : Hepatocellular carc
deceased	unknown	LINC-JP	HCC71	Liver	Liver cancer : Hepatocellular carc
alive	unknown	LINC-JP	HCC72	Liver	Liver cancer : Hepatocellular carc
alive	unknown	LINC-JP	HCC73	Liver	Liver cancer : Hepatocellular carc
alive	unknown	LINC-JP	HCC74	Liver	Liver cancer : Hepatocellular carc
alive	unknown	LINC-JP	HCC75	Liver	Liver cancer : Hepatocellular carc
alive	unknown	LINC-JP	HCC76	Liver	Liver cancer : Hepatocellular carc
alive	unknown	LINC-JP	HCC77	Liver	Liver cancer : Hepatocellular carc
deceased	unknown	LINC-JP	HCC78	Liver	Liver cancer : Hepatocellular carc
alive	unknown	LINC-JP	HCC79	Liver	Liver cancer : Hepatocellular carc
deceased	unknown	LINC-JP	HCC80	Liver	Liver cancer : Hepatocellular carc
alive	unknown	LINC-JP	HCC81	Liver	Liver cancer : Hepatocellular carc
alive	unknown	LINC-JP	HCC82	Liver	Liver cancer : Hepatocellular carc
deceased	unknown	LINC-JP	HCC83	Liver	Liver cancer : Hepatocellular carc
alive	unknown	LINC-JP	HCC84	Liver	Liver cancer : Hepatocellular carc
alive	unknown	LINC-JP	HCC85	Liver	Liver cancer : Hepatocellular carc
alive	unknown	LINC-JP	HCC86	Liver	Liver cancer : Hepatocellular carc
deceased	unknown	LINC-JP	HCC87	Liver	Liver cancer : Hepatocellular carc
alive	unknown	LINC-JP	HCC88	Liver	Liver cancer : Hepatocellular carc
alive	unknown	LINC-JP	HCC89	Liver	Liver cancer : Hepatocellular carc
alive	unknown	LINC-JP	HCC90	Liver	Liver cancer : Hepatocellular carc
alive	unknown	LINC-JP	HCC91	Liver	Liver cancer : Hepatocellular carc
alive	unknown	LINC-JP	HCC92	Liver	Liver cancer : Hepatocellular carc
alive	unknown	LINC-JP	HCC93	Liver	Liver cancer : Hepatocellular carc
alive	unknown	LINC-JP	HCC94	Liver	Liver cancer : Hepatocellular carc
alive	unknown	LINC-JP	HCC95	Liver	Liver cancer : Hepatocellular carc

alive	unknown	LINC-JP	HCC96	Liver	Liver cancer : Hepatocellular carc
alive	unknown	LINC-JP	HCC97	Liver	Liver cancer : Hepatocellular carc
alive	unknown	LINC-JP	HCC98	Liver	Liver cancer : Hepatocellular carc
alive	unknown	LINC-JP	HCC99	Liver	Liver cancer : Hepatocellular carc
ıe	unknown	LINC-JP	HCC100	Liver	Liver cancer : Hepatocellular carc
alive	unknown	LINC-JP	HCC101	Liver	Liver cancer : Hepatocellular carc
alive	unknown	LINC-JP	HCC102	Liver	Liver cancer : Hepatocellular carc
alive	unknown	LINC-JP	HCC103	Liver	Liver cancer : Hepatocellular carc
alive	unknown	LINC-JP	HCC104	Liver	Liver cancer : Hepatocellular carc
alive	unknown	LINC-JP	HCC105	Liver	Liver cancer : Hepatocellular carc
alive	unknown	LINC-JP	HCC106	Liver	Liver cancer : Hepatocellular carc
deceased	unknown	LINC-JP	HCC107	Liver	Liver cancer : Hepatocellular carc
alive	unknown	LINC-JP	HCC108	Liver	Liver cancer : Hepatocellular carc
alive	unknown	LINC-JP	HCC109	Liver	Liver cancer : Hepatocellular carc
alive	unknown	LINC-JP	HCC110	Liver	Liver cancer : Hepatocellular carc
alive	unknown	LINC-JP	HCC111	Liver	Liver cancer : Hepatocellular carc
alive	unknown	LINC-JP	HCC112	Liver	Liver cancer : Hepatocellular carc
alive	unknown	LINC-JP	HCC113	Liver	Liver cancer : Hepatocellular carc
alive	unknown	LINC-JP	HCC115	Liver	Liver cancer : Hepatocellular carc
alive	unknown	LINC-JP	HCC116	Liver	Liver cancer : Hepatocellular carc
alive	unknown	LINC-JP	HCC117	Liver	Liver cancer : Hepatocellular carc
alive	unknown	LINC-JP	HCC118	Liver	Liver cancer : Hepatocellular carc
alive	unknown	LINC-JP	HCC119	Liver	Liver cancer : Hepatocellular carc
alive	unknown	LINC-JP	HCC120	Liver	Liver cancer : Hepatocellular carc
alive	unknown	LINC-JP	HCC121	Liver	Liver cancer : Hepatocellular carc
alive	unknown	LINC-JP	HCC122	Liver	Liver cancer : Hepatocellular carc
ıe	unknown	LINC-JP	HCC123	Liver	Liver cancer : Hepatocellular carc
alive	unknown	LINC-JP	HCC124	Liver	Liver cancer : Hepatocellular carc
alive	unknown	LINC-JP	HCC125	Liver	Liver cancer : Hepatocellular carc
alive	unknown	LINC-JP	HCC126	Liver	Liver cancer : Hepatocellular carc
deceased	unknown	LINC-JP	HCC127	Liver	Liver cancer : Hepatocellular carc
deceased	unknown	LINC-JP	HCC128	Liver	Liver cancer : Hepatocellular carc
alive	unknown	LINC-JP	HCC129	Liver	Liver cancer : Hepatocellular carc
deceased	unknown	LINC-JP	HCC130	Liver	Liver cancer : Hepatocellular carc
alive	unknown	LINC-JP	HCC131	Liver	Liver cancer : Hepatocellular carc
alive	unknown	LINC-JP	HCC132	Liver	Liver cancer : Hepatocellular carc
deceased	unknown	LINC-JP	HCC133	Liver	Liver cancer : Hepatocellular carc
deceased	unknown	LINC-JP	HCC134	Liver	Liver cancer : Hepatocellular carc
deceased	unknown	LINC-JP	HCC135	Liver	Liver cancer : Hepatocellular carc
deceased	unknown	LINC-JP	HCC136	Liver	Liver cancer : Hepatocellular carc
deceased	unknown	LINC-JP	HCC137	Liver	Liver cancer : Hepatocellular carc
deceased	unknown	LINC-JP	HCC138	Liver	Liver cancer : Hepatocellular carc
deceased	unknown	LINC-JP	HCC139	Liver	Liver cancer : Hepatocellular carc
deceased	unknown	LINC-JP	HCC140	Liver	Liver cancer : Hepatocellular carc
alive	unknown	LINC-JP	HCC141	Liver	Liver cancer : Hepatocellular carc
deceased	unknown	LINC-JP	HCC142	Liver	Liver cancer : Hepatocellular carc
deceased	unknown	LINC-JP	HCC143	Liver	Liver cancer : Hepatocellular carc
alive	unknown	LINC-JP	HCC144	Liver	Liver cancer : Hepatocellular carc
alive	unknown	LINC-JP	HCC145	Liver	Liver cancer : Hepatocellular carc
alive	unknown	LINC-JP	HCC146	Liver	Liver cancer : Hepatocellular carc
deceased	unknown	LINC-JP	HCC147	Liver	Liver cancer : Hepatocellular carc
ıe	unknown	LINC-JP	HCC148	Liver	Liver cancer : Hepatocellular carc
alive	unknown	LINC-JP	HCC149	Liver	Liver cancer : Hepatocellular carc
deceased	unknown	LINC-JP	HCC150	Liver	Liver cancer : Hepatocellular carc
deceased	unknown	LINC-JP	HCC151	Liver	Liver cancer : Hepatocellular carc
alive	unknown	LINC-JP	HCC152	Liver	Liver cancer : Hepatocellular carc
alive	unknown	LINC-JP	HCC153	Liver	Liver cancer : Hepatocellular carc
deceased	unknown	LINC-JP	HCC154	Liver	Liver cancer : Hepatocellular carc

alive	unknown	LINC-JP		HCC155	Liver	Liver cancer : Hepatocellular carc
alive	unknown	LINC-JP		HCC156	Liver	Liver cancer : Hepatocellular carc
deceased	unknown	LINC-JP		HCC157	Liver	Liver cancer : Hepatocellular carc
alive	unknown	LINC-JP		HCC158	Liver	Liver cancer : Hepatocellular carc
deceased	unknown	LINC-JP		HCC159	Liver	Liver cancer : Hepatocellular carc
alive	unknown	LINC-JP		HCC160	Liver	Liver cancer : Hepatocellular carc
alive	unknown	LINC-JP		HCC161	Liver	Liver cancer : Hepatocellular carc
alive	unknown	LINC-JP		HCC162	Liver	Liver cancer : Hepatocellular carc
alive	unknown	LINC-JP		HCC163	Liver	Liver cancer : Hepatocellular carc
alive	unknown	LINC-JP		HCC164	Liver	Liver cancer : Hepatocellular carc
deceased	unknown	LINC-JP		HCC165	Liver	Liver cancer : Hepatocellular carc
alive	unknown	LINC-JP		HCC166	Liver	Liver cancer : Hepatocellular carc
deceased	unknown	LINC-JP		HCC167	Liver	Liver cancer : Hepatocellular carc
alive	unknown	LINC-JP		HCC168	Liver	Liver cancer : Hepatocellular carc
alive	unknown	LINC-JP		HCC169	Liver	Liver cancer : Hepatocellular carc
alive	unknown	LINC-JP		HCC170	Liver	Liver cancer : Hepatocellular carc
alive	unknown	LINC-JP		HCC171	Liver	Liver cancer : Hepatocellular carc
deceased	unknown	LINC-JP		HCC172	Liver	Liver cancer : Hepatocellular carc
deceased	unknown	LINC-JP		HCC173	Liver	Liver cancer : Hepatocellular carc
deceased	unknown	LINC-JP		HCC174	Liver	Liver cancer : Hepatocellular carc
alive	unknown	LINC-JP		BN01	Liver	Liver cancer : Hepatocellular carc
alive	unknown	LINC-JP		BN02	Liver	Liver cancer : Hepatocellular carc
alive	unknown	LINC-JP		BN03	Liver	Liver cancer : Hepatocellular carc
alive	unknown	LINC-JP		BN04	Liver	Liver cancer : Hepatocellular carc
alive	unknown	LINC-JP		BN05	Liver	Liver cancer : Hepatocellular carc
alive	unknown	LINC-JP		BN06	Liver	Liver cancer : Hepatocellular carc
alive	unknown	LINC-JP		BN13	Liver	Liver cancer : Hepatocellular carc
alive	unknown	LINC-JP		BN15	Liver	Liver cancer : Hepatocellular carc
deceased	unknown	LINC-JP		BN17	Liver	Liver cancer : Hepatocellular carc
deceased	unknown	LINC-JP		BN19	Liver	Liver cancer : Hepatocellular carc
alive	unknown	LINC-JP		BN20	Liver	Liver cancer : Hepatocellular carc
alive	unknown	LINC-JP		BN22	Liver	Liver cancer : Hepatocellular carc
deceased	unknown	LINC-JP		BN23	Liver	Liver cancer : Hepatocellular carc
alive	unknown	LINC-JP		BN24	Liver	Liver cancer : Hepatocellular carc
alive	unknown	LINC-JP		BN26	Liver	Liver cancer : Hepatocellular carc
alive	unknown	LINC-JP		BN27	Liver	Liver cancer : Hepatocellular carc
alive	unknown	LINC-JP		BN28	Liver	Liver cancer : Hepatocellular carc
alive	unknown	LINC-JP		BN30	Liver	Liver cancer : Hepatocellular carc
alive	unknown	LINC-JP		BN31	Liver	Liver cancer : Hepatocellular carc
alive	unknown	LINC-JP		BN32	Liver	Liver cancer : Hepatocellular carc
alive	unknown	LINC-JP		BN33	Liver	Liver cancer : Hepatocellular carc
deceased	unknown	LINC-JP		BN34	Liver	Liver cancer : Hepatocellular carc
alive	unknown	LINC-JP		BN36	Liver	Liver cancer : Hepatocellular carc
deceased	unknown	LINC-JP		BN37	Liver	Liver cancer : Hepatocellular carc
alive	unknown	LINC-JP		BN38	Liver	Liver cancer : Hepatocellular carc
alive	unknown	LINC-JP		BN40	Liver	Liver cancer : Hepatocellular carc
alive	unknown	LINC-JP		BN41	Liver	Liver cancer : Hepatocellular carc
alive	unknown	LINC-JP		BN42	Liver	Liver cancer : Hepatocellular carc
alive	unknown	LINC-JP		BN43	Liver	Liver cancer : Hepatocellular carc
deceased	unknown	LINC-JP		BN44	Liver	Liver cancer : Hepatocellular carc
deceased	unknown	LINC-JP		BN45	Liver	Liver cancer : Hepatocellular carc
alive	unknown	LINC-JP		BN49	Liver	Liver cancer : Hepatocellular carc
alive	unknown	LINC-JP		BN50	Liver	Liver cancer : Hepatocellular carc
alive	no	LIRI-JP	PCAWG	RK028	Liver	Liver cancer : Hepatocellular carc
deceased	no	LIRI-JP	PCAWG	RK030	Liver	Liver cancer : Hepatocellular carc
alive	no	LIRI-JP	PCAWG	RK038	Liver	Liver cancer : Hepatocellular carc
deceased	no	LIRI-JP	PCAWG	RK040	Liver	Liver cancer : Hepatocellular carc
alive	no	LIRI-JP	PCAWG	RK043	Liver	Liver cancer : Hepatocellular carc

alive	no	LIRI-JP	PCAWG	RK053	Liver	Liver cancer : Hepatocellular carc
alive	no	LIRI-JP	PCAWG	RK057	Liver	Liver cancer : Hepatocellular carc
alive	yes	LIRI-JP	PCAWG	RK060	Liver	Liver cancer : Hepatocellular carc
deceased	no	LIRI-JP	PCAWG	RK074	Liver	Liver cancer : Hepatocellular carc
alive	no	LIRI-JP	PCAWG	RK075	Liver	Liver cancer : Hepatocellular carc
alive	no	LIRI-JP	PCAWG	RK076	Liver	Liver cancer : Hepatocellular carc
alive	no	LIRI-JP	PCAWG	RK085	Liver	Liver cancer : Hepatocellular carc
alive	no	LIRI-JP	PCAWG	RK087	Liver	Liver cancer : Hepatocellular carc
alive	no	LIRI-JP	PCAWG	RK088	Liver	Liver cancer : Hepatocellular carc
alive	no	LIRI-JP	PCAWG	RK090	Liver	Liver cancer : Hepatocellular carc
alive	no	LIRI-JP	PCAWG	RK093	Liver	Liver cancer : Hepatocellular carc
alive	yes	LIRI-JP	PCAWG	RK095	Liver	Liver cancer : Hepatocellular carc
alive	no	LIRI-JP	PCAWG	RK101	Liver	Liver cancer : Hepatocellular carc
alive	no	LIRI-JP	PCAWG	RK102	Liver	Liver cancer : Hepatocellular carc
alive	no	LIRI-JP	PCAWG	RK104	Liver	Liver cancer : Hepatocellular carc
alive	no	LIRI-JP	PCAWG	RK105	Liver	Liver cancer : Hepatocellular carc
alive	no	LIRI-JP	PCAWG	RK110	Liver	Liver cancer : Hepatocellular carc
deceased	no	LIRI-JP	PCAWG	RK113	Liver	Liver cancer : Hepatocellular carc
alive	no	LIRI-JP	PCAWG	RK116	Liver	Liver cancer : Hepatocellular carc
alive	no	LIRI-JP	PCAWG	RK117	Liver	Liver cancer : Hepatocellular carc
alive	no	LIRI-JP	PCAWG	RK118	Liver	Liver cancer : Hepatocellular carc
alive	no	LIRI-JP	PCAWG	RK120	Liver	Liver cancer : Hepatocellular carc
alive	no	LIRI-JP	PCAWG	RK124	Liver	Liver cancer : Hepatocellular carc
alive	no	LIRI-JP	PCAWG	RK125	Liver	Liver cancer : Hepatocellular carc
deceased	yes	LIRI-JP	PCAWG	RK128	Liver	Liver cancer : Hepatocellular carc
alive	no	LIRI-JP	PCAWG	RK131	Liver	Liver cancer : Hepatocellular carc
alive	yes	LIRI-JP	PCAWG	RK134	Liver	Liver cancer : Hepatocellular carc
alive	no	LIRI-JP	PCAWG	RK135	Liver	Liver cancer : Hepatocellular carc
alive	no	LIRI-JP	PCAWG	RK140	Liver	Liver cancer : Hepatocellular carc
alive	no	LIRI-JP	PCAWG	RK143	Liver	Liver cancer : Hepatocellular carc
alive	no	LIRI-JP	PCAWG	RK145	Liver	Liver cancer : Hepatocellular carc
alive	no	LIRI-JP	PCAWG	RK150	Liver	Liver cancer : Hepatocellular carc
deceased	yes	LIRI-JP	PCAWG	RK151	Liver	Liver cancer : Hepatocellular carc
alive	no	LIRI-JP	PCAWG	RK152	Liver	Liver cancer : Hepatocellular carc
alive	no	LIRI-JP	PCAWG	RK153	Liver	Liver cancer : Hepatocellular carc
alive	no	LIRI-JP	PCAWG	RK155	Liver	Liver cancer : Hepatocellular carc
alive	no	LIRI-JP	PCAWG	RK156	Liver	Liver cancer : Hepatocellular carc
alive	no	LIRI-JP	PCAWG	RK164	Liver	Liver cancer : Hepatocellular carc
deceased	no	LIRI-JP	PCAWG	RK169	Liver	Liver cancer : Hepatocellular carc
alive	no	LIRI-JP	PCAWG	RK172	Liver	Liver cancer : Hepatocellular carc
deceased	unknown	LICA-FR		CHC008	Liver	Liver cancer : Hepatocellular carc
deceased	unknown	LICA-FR		CHC012	Liver	Liver cancer : Hepatocellular carc
deceased	unknown	LICA-FR		CHC013	Liver	Liver cancer : Hepatocellular carc
deceased	unknown	LICA-FR		CHC014	Liver	Liver cancer : Hepatocellular carc
	unknown	LICA-FR		CHC018	Liver	Liver cancer : Hepatocellular carc
deceased	unknown	LICA-FR		CHC028	Liver	Liver cancer : Hepatocellular carc
deceased	unknown	LICA-FR		CHC031	Liver	Liver cancer : Hepatocellular carc
deceased	unknown	LICA-FR		CHC033	Liver	Liver cancer : Hepatocellular carc
deceased	unknown	LICA-FR		CHC034	Liver	Liver cancer : Hepatocellular carc
alive	unknown	LICA-FR		CHC043	Liver	Liver cancer : Hepatocellular carc
alive	unknown	LICA-FR		CHC046	Liver	Liver cancer : Hepatocellular carc
	unknown	LICA-FR		CHC051	Liver	Liver cancer : Hepatocellular carc
deceased	unknown	LICA-FR		CHC059	Liver	Liver cancer : Hepatocellular carc
deceased	unknown	LICA-FR		CHC060	Liver	Liver cancer : Hepatocellular carc
deceased	unknown	LICA-FR		CHC081	Liver	Liver cancer : Hepatocellular carc
deceased	unknown	LICA-FR		CHC097	Liver	Liver cancer : Hepatocellular carc
alive	unknown	LICA-FR		CHC1035	Liver	Liver cancer : Hepatocellular carc
	unknown	LICA-FR		CHC1040	Liver	Liver cancer : Hepatocellular carc

	unknown	LICA-FR		CHC1041	Liver	Liver cancer : Hepatocellular carc
alive	unknown	LICA-FR		CHC1044	Liver	Liver cancer : Hepatocellular carc
	unknown	LICA-FR		CHC1052	Liver	Liver cancer : Hepatocellular carc
	unknown	LICA-FR		CHC1053	Liver	Liver cancer : Hepatocellular carc
	unknown	LICA-FR		CHC1055	Liver	Liver cancer : Hepatocellular carc
alive	unknown	LICA-FR		CHC1060	Liver	Liver cancer : Hepatocellular carc
	unknown	LICA-FR		CHC1061	Liver	Liver cancer : Hepatocellular carc
alive	unknown	LICA-FR		CHC1062	Liver	Liver cancer : Hepatocellular carc
deceased	unknown	LICA-FR		CHC1065	Liver	Liver cancer : Hepatocellular carc
alive	unknown	LICA-FR		CHC1069	Liver	Liver cancer : Hepatocellular carc
deceased	unknown	LICA-FR		CHC1146	Liver	Liver cancer : Hepatocellular carc
alive	unknown	LICA-FR		CHC1154	Liver	Liver cancer : Hepatocellular carc
deceased	unknown	LICA-FR		CHC1162	Liver	Liver cancer : Hepatocellular carc
	unknown	LICA-FR		CHC1185	Liver	Liver cancer : Hepatocellular carc
alive	unknown	LICA-FR		CHC1190	Liver	Liver cancer : Hepatocellular carc
deceased	unknown	LICA-FR		CHC1192	Liver	Liver cancer : Hepatocellular carc
deceased	unknown	LICA-FR		CHC1199	Liver	Liver cancer : Hepatocellular carc
deceased	unknown	LICA-FR		CHC1201	Liver	Liver cancer : Hepatocellular carc
	unknown	LICA-FR		CHC121	Liver	Liver cancer : Hepatocellular carc
deceased	unknown	LICA-FR		CHC123	Liver	Liver cancer : Hepatocellular carc
alive	unknown	LICA-FR		CHC126	Liver	Liver cancer : Hepatocellular carc
	unknown	LICA-FR		CHC129	Liver	Liver cancer : Hepatocellular carc
alive	unknown	LICA-FR		CHC130	Liver	Liver cancer : Hepatocellular carc
alive	unknown	LICA-FR		CHC137	Liver	Liver cancer : Hepatocellular carc
deceased	unknown	LICA-FR		CHC154	Liver	Liver cancer : Hepatocellular carc
alive	unknown	LICA-FR		CHC155	Liver	Liver cancer : Hepatocellular carc
alive	unknown	LICA-FR		CHC158	Liver	Liver cancer : Hepatocellular carc
	unknown	LICA-FR	PCAWG	CHC197	Liver	Liver cancer : Hepatocellular carc
deceased	unknown	LICA-FR	PCAWG	CHC205	Liver	Liver cancer : Hepatocellular carc
deceased	unknown	LICA-FR		CHC208	Liver	Liver cancer : Hepatocellular carc
deceased	unknown	LICA-FR		CHC211	Liver	Liver cancer : Hepatocellular carc
	unknown	LICA-FR		CHC218	Liver	Liver cancer : Hepatocellular carc
alive	unknown	LICA-FR		CHC226	Liver	Liver cancer : Hepatocellular carc
alive	unknown	LICA-FR		CHC235	Liver	Liver cancer : Hepatocellular carc
alive	unknown	LICA-FR		CHC237	Liver	Liver cancer : Hepatocellular carc
alive	unknown	LICA-FR		CHC239	Liver	Liver cancer : Hepatocellular carc
deceased	unknown	LICA-FR		CHC242	Liver	Liver cancer : Hepatocellular carc
deceased	unknown	LICA-FR		CHC245	Liver	Liver cancer : Hepatocellular carc
alive	unknown	LICA-FR		CHC250	Liver	Liver cancer : Hepatocellular carc
	unknown	LICA-FR		CHC253	Liver	Liver cancer : Hepatocellular carc
alive	unknown	LICA-FR		CHC254	Liver	Liver cancer : Hepatocellular carc
alive	unknown	LICA-FR		CHC258	Liver	Liver cancer : Hepatocellular carc
deceased	unknown	LICA-FR		CHC301	Liver	Liver cancer : Hepatocellular carc
	unknown	LICA-FR		CHC302	Liver	Liver cancer : Hepatocellular carc
deceased	unknown	LICA-FR		CHC303	Liver	Liver cancer : Hepatocellular carc
	unknown	LICA-FR		CHC304	Liver	Liver cancer : Hepatocellular carc
	unknown	LICA-FR		CHC306	Liver	Liver cancer : Hepatocellular carc
alive	unknown	LICA-FR		CHC307	Liver	Liver cancer : Hepatocellular carc
	unknown	LICA-FR		CHC309	Liver	Liver cancer : Hepatocellular carc
	unknown	LICA-FR		CHC313	Liver	Liver cancer : Hepatocellular carc
	unknown	LICA-FR		CHC314	Liver	Liver cancer : Hepatocellular carc
deceased	unknown	LICA-FR		CHC317	Liver	Liver cancer : Hepatocellular carc
deceased	unknown	LICA-FR	PCAWG	CHC320	Liver	Liver cancer : Hepatocellular carc
	unknown	LICA-FR	PCAWG	CHC322	Liver	Liver cancer : Hepatocellular carc
alive	unknown	LICA-FR		CHC326	Liver	Liver cancer : Hepatocellular carc
	unknown	LICA-FR		CHC327	Liver	Liver cancer : Hepatocellular carc
deceased	unknown	LICA-FR		CHC339	Liver	Liver cancer : Hepatocellular carc
alive	unknown	LICA-FR		CHC399	Liver	Liver cancer : Hepatocellular carc

deceased	unknown	LICA-FR		CHC402	Liver	Liver cancer : Hepatocellular carc
alive	unknown	LICA-FR	PCAWG	CHC429	Liver	Liver cancer : Hepatocellular carc
deceased	unknown	LICA-FR		CHC430	Liver	Liver cancer : Hepatocellular carc
	unknown	LICA-FR	PCAWG	CHC433	Liver	Liver cancer : Hepatocellular carc
alive	unknown	LICA-FR		CHC434	Liver	Liver cancer : Hepatocellular carc
deceased	unknown	LICA-FR		CHC437	Liver	Liver cancer : Hepatocellular carc
alive	unknown	LICA-FR		CHC441	Liver	Liver cancer : Hepatocellular carc
	unknown	LICA-FR		CHC609	Liver	Liver cancer : Hepatocellular carc
	unknown	LICA-FR		CHC614	Liver	Liver cancer : Hepatocellular carc
	unknown	LICA-FR		CHC793	Liver	Liver cancer : Hepatocellular carc
	unknown	LICA-FR		CHC794	Liver	Liver cancer : Hepatocellular carc
deceased	unknown	LICA-FR		CHC798	Liver	Liver cancer : Hepatocellular carc
	unknown	LICA-FR		CHC891	Liver	Liver cancer : Hepatocellular carc
	unknown	LICA-FR		CHC909	Liver	Liver cancer : Hepatocellular carc
	unknown	LICA-FR		CHC961	Liver	Liver cancer : Hepatocellular carc
alive	unknown	LIHC-US		TCGA-XR-	Liver	Liver cancer : Hepatocellular carc
alive	unknown	LIHC-US		TCGA-XR-	Liver	Liver cancer : Hepatocellular carc
alive	unknown	LIHC-US		TCGA-XR-	Liver	Liver cancer : Hepatocellular carc
alive	unknown	LIHC-US		TCGA-XR-	Liver	Liver cancer : Hepatocellular carc
alive	unknown	LIHC-US		TCGA-DD	Liver	Liver cancer : Hepatocellular carc
alive	unknown	LIHC-US	PCAWG	TCGA-DD	Liver	Liver cancer : Hepatocellular carc
alive	unknown	LIHC-US	PCAWG	TCGA-G3-	Liver	Liver cancer : Hepatocellular carc
alive	unknown	LIHC-US		TCGA-G3-	Liver	Liver cancer : Hepatocellular carc
alive	unknown	LIHC-US		TCGA-G3-	Liver	Liver cancer : Hepatocellular carc
alive	unknown	LIHC-US	PCAWG	TCGA-EP-	Liver	Liver cancer : Hepatocellular carc
alive	unknown	LIHC-US		TCGA-DD	Liver	Liver cancer : Hepatocellular carc
alive	unknown	LIHC-US	PCAWG	TCGA-CC-	Liver	Liver cancer : Hepatocellular carc
alive	unknown	LIHC-US		TCGA-G3-	Liver	Liver cancer : Hepatocellular carc
alive	unknown	LIHC-US	PCAWG	TCGA-G3-	Liver	Liver cancer : Hepatocellular carc
alive	unknown	LIHC-US		TCGA-G3-	Liver	Liver cancer : Hepatocellular carc
alive	unknown	LIHC-US	PCAWG	TCGA-CC-	Liver	Liver cancer : Hepatocellular carc
alive	unknown	LIHC-US		TCGA-G3-	Liver	Liver cancer : Hepatocellular carc
alive	unknown	LIHC-US		TCGA-CC-	Liver	Liver cancer : Hepatocellular carc
alive	unknown	LIHC-US	PCAWG	TCGA-G3-	Liver	Liver cancer : Hepatocellular carc
alive	unknown	LIHC-US		TCGA-CC-	Liver	Liver cancer : Hepatocellular carc
alive	unknown	LIHC-US	PCAWG	TCGA-BC-	Liver	Liver cancer : Hepatocellular carc
alive	unknown	LIHC-US		TCGA-BC-	Liver	Liver cancer : Hepatocellular carc
alive	unknown	LIHC-US		TCGA-EP-	Liver	Liver cancer : Hepatocellular carc
alive	unknown	LIHC-US		TCGA-DD	Liver	Liver cancer : Hepatocellular carc
alive	unknown	LIHC-US	PCAWG	TCGA-FV-	Liver	Liver cancer : Hepatocellular carc
alive	unknown	LIHC-US		TCGA-DD	Liver	Liver cancer : Hepatocellular carc
alive	unknown	LIHC-US		TCGA-DD	Liver	Liver cancer : Hepatocellular carc
alive	unknown	LIHC-US		TCGA-DD	Liver	Liver cancer : Hepatocellular carc
alive	unknown	LIHC-US	PCAWG	TCGA-BC-	Liver	Liver cancer : Hepatocellular carc
alive	unknown	LIHC-US	PCAWG	TCGA-EP-	Liver	Liver cancer : Hepatocellular carc
alive	unknown	LIHC-US	PCAWG	TCGA-EP-	Liver	Liver cancer : Hepatocellular carc
alive	unknown	LIHC-US		TCGA-EP-	Liver	Liver cancer : Hepatocellular carc
alive	unknown	LIHC-US	PCAWG	TCGA-CC-	Liver	Liver cancer : Hepatocellular carc
alive	unknown	LIHC-US	PCAWG	TCGA-DD	Liver	Liver cancer : Hepatocellular carc
alive	unknown	LIHC-US	PCAWG	TCGA-G3-	Liver	Liver cancer : Hepatocellular carc
alive	unknown	LIHC-US		TCGA-BC-	Liver	Liver cancer : Hepatocellular carc
alive	unknown	LIHC-US	PCAWG	TCGA-FV-	Liver	Liver cancer : Hepatocellular carc
alive	unknown	LIHC-US		TCGA-DD	Liver	Liver cancer : Hepatocellular carc
alive	unknown	LIHC-US	PCAWG	TCGA-DD	Liver	Liver cancer : Hepatocellular carc
alive	unknown	LIHC-US		TCGA-BC-	Liver	Liver cancer : Hepatocellular carc
alive	unknown	LIHC-US	PCAWG	TCGA-DD	Liver	Liver cancer : Hepatocellular carc
alive	unknown	LIHC-US	PCAWG	TCGA-DD	Liver	Liver cancer : Hepatocellular carc
alive	unknown	LIHC-US	PCAWG	TCGA-G3-	Liver	Liver cancer : Hepatocellular carc

alive	unknown	LIHC-US	PCAWG	TCGA-EP-	Liver	Liver cancer : Hepatocellular carc
alive	unknown	LIHC-US	PCAWG	TCGA-CC-	Liver	Liver cancer : Hepatocellular carc
alive	unknown	LIHC-US		TCGA-G3-	Liver	Liver cancer : Hepatocellular carc
alive	unknown	LIHC-US		TCGA-CC-	Liver	Liver cancer : Hepatocellular carc
alive	unknown	LIHC-US		TCGA-CC-	Liver	Liver cancer : Hepatocellular carc
alive	unknown	LIHC-US		TCGA-CC-	Liver	Liver cancer : Hepatocellular carc
alive	unknown	LIHC-US		TCGA-CC-	Liver	Liver cancer : Hepatocellular carc
alive	unknown	LIHC-US		TCGA-G3-	Liver	Liver cancer : Hepatocellular carc
alive	unknown	LIHC-US	PCAWG	TCGA-FV-	Liver	Liver cancer : Hepatocellular carc
alive	unknown	LIHC-US		TCGA-DD·	Liver	Liver cancer : Hepatocellular carc
alive	unknown	LIHC-US		TCGA-ED-	Liver	Liver cancer : Hepatocellular carc
alive	no	LINC-JP	PCAWG	HX4	Liver	Liver cancer : Hepatocellular carc
deceased	no	LINC-JP	PCAWG	HX5	Liver	Liver cancer : Hepatocellular carc
deceased	unknown	LINC-JP	PCAWG	HX9	Liver	Liver cancer : Hepatocellular carc
alive	no	LINC-JP	PCAWG	HX10	Liver	Liver cancer : Hepatocellular carc
deceased	unknown	LINC-JP	PCAWG	HX11	Liver	Liver cancer : Hepatocellular carc
deceased	no	LINC-JP	PCAWG	HX12	Liver	Liver cancer : Hepatocellular carc
alive	no	LINC-JP	PCAWG	HX13	Liver	Liver cancer : Hepatocellular carc
deceased	no	LINC-JP	PCAWG	HX14	Liver	Liver cancer : Hepatocellular carc
alive	yes	LINC-JP	PCAWG	HX15	Liver	Liver cancer : Hepatocellular carc
deceased	no	LINC-JP	PCAWG	HX16	Liver	Liver cancer : Hepatocellular carc
alive	no	LINC-JP	PCAWG	HX17	Liver	Liver cancer : Hepatocellular carc
deceased	no	LINC-JP	PCAWG	HX18	Liver	Liver cancer : Hepatocellular carc
alive	no	LINC-JP	PCAWG	HX19	Liver	Liver cancer : Hepatocellular carc
alive	no	LINC-JP	PCAWG	HX20	Liver	Liver cancer : Hepatocellular carc
deceased	no	LINC-JP	PCAWG	HX21	Liver	Liver cancer : Hepatocellular carc
alive	no	LINC-JP	PCAWG	HX22	Liver	Liver cancer : Hepatocellular carc
deceased	no	LINC-JP	PCAWG	HX23	Liver	Liver cancer : Hepatocellular carc
deceased	no	LINC-JP	PCAWG	HX24	Liver	Liver cancer : Hepatocellular carc
deceased	no	LINC-JP	PCAWG	HX25	Liver	Liver cancer : Hepatocellular carc
alive	no	LINC-JP	PCAWG	HX26	Liver	Liver cancer : Hepatocellular carc
alive	no	LINC-JP	PCAWG	HX27	Liver	Liver cancer : Hepatocellular carc
alive	unknown	LINC-JP	PCAWG	HX28	Liver	Liver cancer : Hepatocellular carc
deceased	no	LINC-JP	PCAWG	HX29	Liver	Liver cancer : Hepatocellular carc
alive	no	LINC-JP	PCAWG	HX30	Liver	Liver cancer : Hepatocellular carc
alive	no	LINC-JP	PCAWG	HX31	Liver	Liver cancer : Hepatocellular carc
alive	no	LINC-JP	PCAWG	HX32	Liver	Liver cancer : Hepatocellular carc
deceased	no	LINC-JP	PCAWG	HX33	Liver	Liver cancer : Hepatocellular carc
alive	no	LINC-JP	PCAWG	HX34	Liver	Liver cancer : Hepatocellular carc
alive	no	LINC-JP	PCAWG	HX35	Liver	Liver cancer : Hepatocellular carc
deceased	no	LINC-JP	PCAWG	HX36	Liver	Liver cancer : Hepatocellular carc
alive	no	LINC-JP	PCAWG	HX37	Liver	Liver cancer : Hepatocellular carc
deceased	no	LIRI-JP	PCAWG	RK001	Liver	Liver cancer : Hepatocellular carc
deceased	no	LIRI-JP	PCAWG	RK002	Liver	Liver cancer : Hepatocellular carc
deceased	no	LIRI-JP	PCAWG	RK003	Liver	Liver cancer : Hepatocellular carc
alive	no	LIRI-JP	PCAWG	RK004	Liver	Liver cancer : Hepatocellular carc
alive	no	LIRI-JP	PCAWG	RK005	Liver	Liver cancer : Hepatocellular carc
deceased	no	LIRI-JP	PCAWG	RK006	Liver	Liver cancer : Hepatocellular carc
alive	no	LIRI-JP	PCAWG	RK007	Liver	Liver cancer : Hepatocellular carc
alive	no	LIRI-JP	PCAWG	RK010	Liver	Liver cancer : Hepatocellular carc
alive	no	LIRI-JP	PCAWG	RK012	Liver	Liver cancer : Hepatocellular carc
alive	no	LIRI-JP	PCAWG	RK014	Liver	Liver cancer : Hepatocellular carc
alive	no	LIRI-JP	PCAWG	RK015	Liver	Liver cancer : Hepatocellular carc
alive	no	LIRI-JP	PCAWG	RK016	Liver	Liver cancer : Hepatocellular carc
alive	no	LIRI-JP	PCAWG	RK018	Liver	Liver cancer : Hepatocellular carc
alive	no	LIRI-JP	PCAWG	RK019	Liver	Liver cancer : Hepatocellular carc
alive	no	LIRI-JP	PCAWG	RK020	Liver	Liver cancer : Hepatocellular carc
alive	yes	LIRI-JP	PCAWG	RK021	Liver	Liver cancer : Hepatocellular carc

deceased	no	LIRI-JP	PCAWG	RK022	Liver	Liver cancer : Hepatocellular carc
deceased	yes	LIRI-JP	PCAWG	RK023	Liver	Liver cancer : Hepatocellular carc
alive	no	LIRI-JP	PCAWG	RK024	Liver	Liver cancer : Hepatocellular carc
deceased	no	LIRI-JP	PCAWG	RK025	Liver	Liver cancer : Hepatocellular carc
alive	no	LIRI-JP	PCAWG	RK026	Liver	Liver cancer : Hepatocellular carc
deceased	no	LIRI-JP	PCAWG	RK027	Liver	Liver cancer : Hepatocellular carc
alive	no	LIRI-JP	PCAWG	RK029	Liver	Liver cancer : Hepatocellular carc
deceased	no	LIRI-JP	PCAWG	RK031	Liver	Liver cancer : Hepatocellular carc
deceased	no	LIRI-JP	PCAWG	RK032	Liver	Liver cancer : Hepatocellular carc
alive	no	LIRI-JP	PCAWG	RK033	Liver	Liver cancer : Hepatocellular carc
alive	no	LIRI-JP	PCAWG	RK034	Liver	Liver cancer : Hepatocellular carc
alive	no	LIRI-JP	PCAWG	RK035	Liver	Liver cancer : Hepatocellular carc
alive	no	LIRI-JP	PCAWG	RK036	Liver	Liver cancer : Hepatocellular carc
alive	no	LIRI-JP	PCAWG	RK037	Liver	Liver cancer : Hepatocellular carc
deceased	no	LIRI-JP	PCAWG	RK041	Liver	Liver cancer : Hepatocellular carc
deceased	no	LIRI-JP	PCAWG	RK042	Liver	Liver cancer : Hepatocellular carc
alive	no	LIRI-JP	PCAWG	RK044	Liver	Liver cancer : Hepatocellular carc
alive	no	LIRI-JP	PCAWG	RK046	Liver	Liver cancer : Hepatocellular carc
alive	no	LIRI-JP	PCAWG	RK047	Liver	Liver cancer : Hepatocellular carc
alive	yes	LIRI-JP	PCAWG	RK048	Liver	Liver cancer : Hepatocellular carc
alive	no	LIRI-JP	PCAWG	RK049	Liver	Liver cancer : Hepatocellular carc
alive	no	LIRI-JP	PCAWG	RK050	Liver	Liver cancer : Hepatocellular carc
deceased	yes	LIRI-JP	PCAWG	RK051	Liver	Liver cancer : Hepatocellular carc
alive	no	LIRI-JP	PCAWG	RK052	Liver	Liver cancer : Hepatocellular carc
alive	no	LIRI-JP	PCAWG	RK054	Liver	Liver cancer : Hepatocellular carc
deceased	no	LIRI-JP	PCAWG	RK055	Liver	Liver cancer : Hepatocellular carc
alive	no	LIRI-JP	PCAWG	RK056	Liver	Liver cancer : Hepatocellular carc
alive	no	LIRI-JP	PCAWG	RK058	Liver	Liver cancer : Hepatocellular carc
alive	no	LIRI-JP	PCAWG	RK061	Liver	Liver cancer : Hepatocellular carc
alive	no	LIRI-JP	PCAWG	RK062	Liver	Liver cancer : Hepatocellular carc
alive	no	LIRI-JP	PCAWG	RK063	Liver	Liver cancer : Hepatocellular carc
alive	no	LIRI-JP	PCAWG	RK064	Liver	Liver cancer : Hepatocellular carc
alive	no	LIRI-JP	PCAWG	RK065	Liver	Liver cancer : Hepatocellular carc
deceased	no	LIRI-JP	PCAWG	RK066	Liver	Liver cancer : Hepatocellular carc
alive	no	LIRI-JP	PCAWG	RK067	Liver	Liver cancer : Hepatocellular carc
alive	no	LIRI-JP	PCAWG	RK068	Liver	Liver cancer : Hepatocellular carc
alive	no	LIRI-JP	PCAWG	RK069	Liver	Liver cancer : Hepatocellular carc
alive	no	LIRI-JP	PCAWG	RK071	Liver	Liver cancer : Hepatocellular carc
deceased	yes	LIRI-JP	PCAWG	RK072	Liver	Liver cancer : Hepatocellular carc
alive	no	LIRI-JP	PCAWG	RK073	Liver	Liver cancer : Hepatocellular carc
alive	no	LIRI-JP	PCAWG	RK079	Liver	Liver cancer : Hepatocellular carc
alive	no	LIRI-JP	PCAWG	RK080	Liver	Liver cancer : Hepatocellular carc
alive	no	LIRI-JP	PCAWG	RK082	Liver	Liver cancer : Hepatocellular carc
alive	no	LIRI-JP	PCAWG	RK083	Liver	Liver cancer : Hepatocellular carc
deceased	no	LIRI-JP	PCAWG	RK084	Liver	Liver cancer : Hepatocellular carc
alive	no	LIRI-JP	PCAWG	RK086	Liver	Liver cancer : Hepatocellular carc
alive	no	LIRI-JP	PCAWG	RK089	Liver	Liver cancer : Hepatocellular carc
alive	no	LIRI-JP	PCAWG	RK091	Liver	Liver cancer : Hepatocellular carc
alive	no	LIRI-JP	PCAWG	RK092	Liver	Liver cancer : Hepatocellular carc
alive	no	LIRI-JP	PCAWG	RK096	Liver	Liver cancer : Hepatocellular carc
deceased	no	LIRI-JP	PCAWG	RK098	Liver	Liver cancer : Hepatocellular carc
alive	no	LIRI-JP	PCAWG	RK099	Liver	Liver cancer : Hepatocellular carc
deceased	no	LIRI-JP	PCAWG	RK100	Liver	Liver cancer : Hepatocellular carc
alive	no	LIRI-JP	PCAWG	RK103	Liver	Liver cancer : Hepatocellular carc
alive	no	LIRI-JP	PCAWG	RK106	Liver	Liver cancer : Hepatocellular carc
alive	no	LIRI-JP	PCAWG	RK107	Liver	Liver cancer : Hepatocellular carc
alive	no	LIRI-JP	PCAWG	RK108	Liver	Liver cancer : Hepatocellular carc
alive	no	LIRI-JP	PCAWG	RK109	Liver	Liver cancer : Hepatocellular carc

alive	yes	LIRI-JP	PCAWG	RK112	Liver	Liver cancer : Hepatocellular carc
alive	no	LIRI-JP	PCAWG	RK115	Liver	Liver cancer : Hepatocellular carc
alive	no	LIRI-JP	PCAWG	RK126	Liver	Liver cancer : Hepatocellular carc
alive	no	LIRI-JP	PCAWG	RK130	Liver	Liver cancer : Hepatocellular carc
alive	no	LIRI-JP	PCAWG	RK133	Liver	Liver cancer : Hepatocellular carc
alive	no	LIRI-JP	PCAWG	RK137	Liver	Liver cancer : Hepatocellular carc
deceased	yes	LIRI-JP	PCAWG	RK138	Liver	Liver cancer : Hepatocellular carc
alive	no	LIRI-JP	PCAWG	RK141	Liver	Liver cancer : Hepatocellular carc
alive	no	LIRI-JP	PCAWG	RK142	Liver	Liver cancer : Hepatocellular carc
alive	no	LIRI-JP	PCAWG	RK146	Liver	Liver cancer : Hepatocellular carc
alive	no	LIRI-JP	PCAWG	RK147	Liver	Liver cancer : Hepatocellular carc
alive	no	LIRI-JP	PCAWG	RK148	Liver	Liver cancer : Hepatocellular carc
alive	no	LIRI-JP	PCAWG	RK157	Liver	Liver cancer : Hepatocellular carc
alive	no	LIRI-JP	PCAWG	RK159	Liver	Liver cancer : Hepatocellular carc
alive	no	LIRI-JP	PCAWG	RK165	Liver	Liver cancer : Hepatocellular carc
deceased	no	LIRI-JP	PCAWG	RK166	Liver	Liver cancer : Hepatocellular carc
alive	no	LIRI-JP	PCAWG	RK167	Liver	Liver cancer : Hepatocellular carc
deceased	no	LIRI-JP	PCAWG	RK170	Liver	Liver cancer : Hepatocellular carc
alive	no	LIRI-JP	PCAWG	RK177	Liver	Liver cancer : Hepatocellular carc
alive	no	LIRI-JP	PCAWG	RK180	Liver	Liver cancer : Hepatocellular carc
deceased	no	LIRI-JP		RK182	Liver	Liver cancer : Hepatocellular carc
alive	no	LIRI-JP	PCAWG	RK184	Liver	Liver cancer : Hepatocellular carc
alive	no	LIRI-JP	PCAWG	RK194	Liver	Liver cancer : Hepatocellular carc
alive	no	LIRI-JP	PCAWG	RK204	Liver	Liver cancer : Hepatocellular carc
alive	yes	LIRI-JP	PCAWG	RK208	Liver	Liver cancer : Hepatocellular carc
alive	no	LIRI-JP	PCAWG	RK226	Liver	Liver cancer : Hepatocellular carc
alive	yes	LIRI-JP	PCAWG	RK269	Liver	Liver cancer : Hepatocellular carc
alive	no	LIRI-JP	PCAWG	RK272	Liver	Liver cancer : Hepatocellular carc
alive	no	LIRI-JP	PCAWG	RK279	Liver	Liver cancer : Hepatocellular carc
alive	no	LIRI-JP	PCAWG	RK298	Liver	Liver cancer : Hepatocellular carc
alive	no	LIRI-JP	PCAWG	RK303	Liver	Liver cancer : Hepatocellular carc
deceased	no	LIRI-JP	PCAWG	RK307	Liver	Liver cancer : Hepatocellular carc
alive	no	LIRI-JP	PCAWG	RK308	Liver	Liver cancer : Hepatocellular carc
deceased	no	LIRI-JP	PCAWG	RK309	Liver	Liver cancer : Hepatocellular carc
alive	no	LIRI-JP	PCAWG	RK310	Liver	Liver cancer : Hepatocellular carc
alive	no	LIRI-JP	PCAWG	RK312	Liver	Liver cancer : Hepatocellular carc
deceased	no	LIRI-JP	PCAWG	RK316	Liver	Liver cancer : Hepatocellular carc
deceased	no	LIRI-JP	PCAWG	RK317	Liver	Liver cancer : Hepatocellular carc
alive	unknown	LIHC-US		TCGA-DD·	Liver	Liver cancer : Hepatocellular carc
alive	unknown	LIHC-US	PCAWG	TCGA-FV-	Liver	Liver cancer : Hepatocellular carc
alive	unknown	LIHC-US		TCGA-BC-	Liver	Liver cancer : Hepatocellular carc
alive	unknown	LIHC-US	PCAWG	TCGA-DD·	Liver	Liver cancer : Hepatocellular carc
alive	unknown	LIHC-US		TCGA-CC-	Liver	Liver cancer : Hepatocellular carc
alive	unknown	LIHC-US	PCAWG	TCGA-FV-	Liver	Liver cancer : Hepatocellular carc
alive	unknown	LIHC-US		TCGA-ED-	Liver	Liver cancer : Hepatocellular carc
alive	unknown	LIHC-US		TCGA-ED-	Liver	Liver cancer : Hepatocellular carc
alive	unknown	LIHC-US		TCGA-DD·	Liver	Liver cancer : Hepatocellular carc
alive	unknown	LIHC-US		TCGA-FV-	Liver	Liver cancer : Hepatocellular carc
alive	unknown	LIHC-US	PCAWG	TCGA-FV-	Liver	Liver cancer : Hepatocellular carc
alive	unknown	LIHC-US		TCGA-BC-	Liver	Liver cancer : Hepatocellular carc
alive	unknown	LIHC-US	PCAWG	TCGA-DD·	Liver	Liver cancer : Hepatocellular carc
alive	unknown	LIHC-US		TCGA-DD·	Liver	Liver cancer : Hepatocellular carc
alive	unknown	LIHC-US		TCGA-G3-	Liver	Liver cancer : Hepatocellular carc
alive	unknown	LIHC-US		TCGA-CC-	Liver	Liver cancer : Hepatocellular carc
alive	unknown	LIHC-US		TCGA-DD·	Liver	Liver cancer : Hepatocellular carc
alive	unknown	LIHC-US		TCGA-DD·	Liver	Liver cancer : Hepatocellular carc
alive	unknown	LIHC-US		TCGA-BC-	Liver	Liver cancer : Hepatocellular carc
alive	unknown	LIHC-US	PCAWG	TCGA-FV-	Liver	Liver cancer : Hepatocellular carc

alive	unknown	LIHC-US	PCAWG	TCGA-ED-	Liver	Liver cancer : Hepatocellular carc
alive	unknown	LIHC-US	PCAWG	TCGA-G3-	Liver	Liver cancer : Hepatocellular carc
alive	unknown	LIHC-US	PCAWG	TCGA-DD-	Liver	Liver cancer : Hepatocellular carc
alive	unknown	LIHC-US		TCGA-G3-	Liver	Liver cancer : Hepatocellular carc
alive	yes	LIRI-JP	PCAWG	RK059	Liver	Liver cancer : Hepatocellular carc
alive	no	LIRI-JP	PCAWG	RK070	Liver	Liver cancer : Hepatocellular carc
alive	no	LIRI-JP	PCAWG	RK077	Liver	Liver cancer : Hepatocellular carc
alive	no	LIRI-JP	PCAWG	RK081	Liver	Liver cancer : Hepatocellular carc
alive	no	LIRI-JP	PCAWG	RK111	Liver	Liver cancer : Hepatocellular carc
deceased	yes	LIRI-JP	PCAWG	RK119	Liver	Liver cancer : Hepatocellular carc
deceased	yes	LIRI-JP	PCAWG	RK121	Liver	Liver cancer : Hepatocellular carc
alive	no	LIRI-JP	PCAWG	RK122	Liver	Liver cancer : Hepatocellular carc
alive	no	LIRI-JP	PCAWG	RK123	Liver	Liver cancer : Hepatocellular carc
alive	no	LIRI-JP	PCAWG	RK136	Liver	Liver cancer : Hepatocellular carc
alive	no	LIRI-JP	PCAWG	RK139	Liver	Liver cancer : Hepatocellular carc
alive	no	LIRI-JP	PCAWG	RK144	Liver	Liver cancer : Hepatocellular carc
alive	no	LIRI-JP	PCAWG	RK149	Liver	Liver cancer : Hepatocellular carc
alive	no	LIRI-JP	PCAWG	RK154	Liver	Liver cancer : Hepatocellular carc
alive	yes	LIRI-JP	PCAWG	RK162	Liver	Liver cancer : Hepatocellular carc
deceased	no	LIRI-JP	PCAWG	RK163	Liver	Liver cancer : Hepatocellular carc
alive	no	LIRI-JP	PCAWG	RK171	Liver	Liver cancer : Hepatocellular carc
alive	no	LIRI-JP	PCAWG	RK175	Liver	Liver cancer : Hepatocellular carc
alive	no	LIRI-JP	PCAWG	RK176	Liver	Liver cancer : Hepatocellular carc
alive	no	LIRI-JP	PCAWG	RK178	Liver	Liver cancer : Hepatocellular carc
alive	no	LIRI-JP	PCAWG	RK181	Liver	Liver cancer : Hepatocellular carc
alive	no	LIRI-JP	PCAWG	RK183	Liver	Liver cancer : Hepatocellular carc
alive	no	LIRI-JP	PCAWG	RK185	Liver	Liver cancer : Hepatocellular carc
deceased	no	LIRI-JP	PCAWG	RK186	Liver	Liver cancer : Hepatocellular carc
alive	no	LIRI-JP	PCAWG	RK187	Liver	Liver cancer : Hepatocellular carc
deceased	no	LIRI-JP	PCAWG	RK188	Liver	Liver cancer : Hepatocellular carc
alive	no	LIRI-JP	PCAWG	RK190	Liver	Liver cancer : Hepatocellular carc
alive	no	LIRI-JP	PCAWG	RK191	Liver	Liver cancer : Hepatocellular carc
alive	no	LIRI-JP	PCAWG	RK193	Liver	Liver cancer : Hepatocellular carc
alive	no	LIRI-JP	PCAWG	RK195	Liver	Liver cancer : Hepatocellular carc
alive	no	LIRI-JP	PCAWG	RK196	Liver	Liver cancer : Hepatocellular carc
deceased	no	LIRI-JP	PCAWG	RK197	Liver	Liver cancer : Hepatocellular carc
alive	yes	LIRI-JP	PCAWG	RK198	Liver	Liver cancer : Hepatocellular carc
alive	no	LIRI-JP	PCAWG	RK199	Liver	Liver cancer : Hepatocellular carc
alive	no	LIRI-JP	PCAWG	RK200	Liver	Liver cancer : Hepatocellular carc
alive	no	LIRI-JP	PCAWG	RK201	Liver	Liver cancer : Hepatocellular carc
alive	no	LIRI-JP	PCAWG	RK202	Liver	Liver cancer : Hepatocellular carc
alive	no	LIRI-JP	PCAWG	RK205	Liver	Liver cancer : Hepatocellular carc
alive	no	LIRI-JP	PCAWG	RK206	Liver	Liver cancer : Hepatocellular carc
alive	no	LIRI-JP	PCAWG	RK207	Liver	Liver cancer : Hepatocellular carc
alive	no	LIRI-JP	PCAWG	RK210	Liver	Liver cancer : Hepatocellular carc
alive	yes	LIRI-JP	PCAWG	RK211	Liver	Liver cancer : Hepatocellular carc
alive	no	LIRI-JP	PCAWG	RK212	Liver	Liver cancer : Hepatocellular carc
alive	no	LIRI-JP	PCAWG	RK213	Liver	Liver cancer : Hepatocellular carc
deceased	no	LIRI-JP	PCAWG	RK214	Liver	Liver cancer : Hepatocellular carc
alive	no	LIRI-JP	PCAWG	RK215	Liver	Liver cancer : Hepatocellular carc
alive	no	LIRI-JP	PCAWG	RK216	Liver	Liver cancer : Hepatocellular carc
alive	no	LIRI-JP	PCAWG	RK217	Liver	Liver cancer : Hepatocellular carc
alive	no	LIRI-JP	PCAWG	RK219	Liver	Liver cancer : Hepatocellular carc
alive	no	LIRI-JP	PCAWG	RK220	Liver	Liver cancer : Hepatocellular carc
alive	unknown	LIHC-US		TCGA-G3-	Liver	Liver cancer : Hepatocellular carc
alive	unknown	LIHC-US	PCAWG	TCGA-FV-	Liver	Liver cancer : Hepatocellular carc
alive	unknown	LIHC-US		TCGA-DD-	Liver	Liver cancer : Hepatocellular carc
	unknown	LIAD-FR		CHC976	Liver	Liver cancer : Hepatocellular ade

	unknown	LIAD-FR		CHC917	Liver	Liver cancer : Hepatocellular ade
	unknown	LIAD-FR		CHC845	Liver	Liver cancer : Hepatocellular ade
	unknown	LIAD-FR		CHC687	Liver	Liver cancer : Hepatocellular ade
	unknown	LIAD-FR		CHC674	Liver	Liver cancer : Hepatocellular ade
	unknown	LIAD-FR		CHC622	Liver	Liver cancer : Hepatocellular ade
	unknown	LIAD-FR		CHC603	Liver	Liver cancer : Hepatocellular ade
	unknown	LIAD-FR		CHC575	Liver	Liver cancer : Hepatocellular ade
	unknown	LIAD-FR		CHC517	Liver	Liver cancer : Hepatocellular ade
	unknown	LIAD-FR		CHC471	Liver	Liver cancer : Hepatocellular ade
	unknown	LIAD-FR		CHC469	Liver	Liver cancer : Hepatocellular ade
	unknown	LIAD-FR		CHC464	Liver	Liver cancer : Hepatocellular ade
	unknown	LIAD-FR		CHC462	Liver	Liver cancer : Hepatocellular ade
	unknown	LIAD-FR		CHC361	Liver	Liver cancer : Hepatocellular ade
	unknown	LIAD-FR		CHC340	Liver	Liver cancer : Hepatocellular ade
	unknown	LIAD-FR		CHC1915	Liver	Liver cancer : Hepatocellular ade
	unknown	LIAD-FR		CHC1854	Liver	Liver cancer : Hepatocellular ade
	unknown	LIAD-FR		CHC1666	Liver	Liver cancer : Hepatocellular ade
	unknown	LIAD-FR		CHC1665	Liver	Liver cancer : Hepatocellular ade
	unknown	LIAD-FR		CHC1488	Liver	Liver cancer : Hepatocellular ade
	unknown	LIAD-FR		CHC1439	Liver	Liver cancer : Hepatocellular ade
	unknown	LIAD-FR		CHC1432	Liver	Liver cancer : Hepatocellular ade
alive	unknown	LIHC-US		TCGA-ED-	Liver	Liver cancer : Hepatocellular carc
	unknown	LIAD-FR		CHC1425	Liver	Liver cancer : Hepatocellular ade
	unknown	LIAD-FR		CHC1424	Liver	Liver cancer : Hepatocellular ade
	unknown	LIAD-FR		CHC1382	Liver	Liver cancer : Hepatocellular ade
	unknown	LIAD-FR		CHC1337	Liver	Liver cancer : Hepatocellular ade
	unknown	LIAD-FR		CHC1328	Liver	Liver cancer : Hepatocellular ade
	unknown	LIAD-FR		CHC1315	Liver	Liver cancer : Hepatocellular ade
	unknown	LIAD-FR		CHC1124	Liver	Liver cancer : Hepatocellular ade
	unknown	LIAD-FR		CHC1023	Liver	Liver cancer : Hepatocellular ade
alive	unknown	LIHC-US		TCGA-DD-	Liver	Liver cancer : Hepatocellular carc
alive	unknown	LIHC-US		TCGA-DD-	Liver	Liver cancer : Hepatocellular carc
alive	unknown	LIHC-US		TCGA-G3-	Liver	Liver cancer : Hepatocellular carc
alive	unknown	LIHC-US		TCGA-ED-	Liver	Liver cancer : Hepatocellular carc
alive	unknown	LIHC-US		TCGA-DD-	Liver	Liver cancer : Hepatocellular carc
alive	unknown	LIHC-US		TCGA-ED-	Liver	Liver cancer : Hepatocellular carc
alive	unknown	LIHC-US		TCGA-DD-	Liver	Liver cancer : Hepatocellular carc
alive	unknown	LIHC-US		TCGA-ED-	Liver	Liver cancer : Hepatocellular carc
alive	unknown	LIHC-US	PCAWG	TCGA-ED-	Liver	Liver cancer : Hepatocellular carc
alive	unknown	LIHC-US		TCGA-DD-	Liver	Liver cancer : Hepatocellular carc
alive	unknown	LIHC-US		TCGA-ED-	Liver	Liver cancer : Hepatocellular carc
alive	unknown	LIHC-US	PCAWG	TCGA-DD-	Liver	Liver cancer : Hepatocellular carc
alive	unknown	LIHC-US		TCGA-ED-	Liver	Liver cancer : Hepatocellular carc
alive	unknown	LIHC-US		TCGA-ED-	Liver	Liver cancer : Hepatocellular carc
alive	unknown	LIHC-US		TCGA-G3-	Liver	Liver cancer : Hepatocellular carc
alive	unknown	LIHC-US		TCGA-ED-	Liver	Liver cancer : Hepatocellular carc
alive	unknown	LIHC-US		TCGA-DD-	Liver	Liver cancer : Hepatocellular carc
alive	unknown	LIHC-US		TCGA-DD-	Liver	Liver cancer : Hepatocellular carc
alive	unknown	LIHC-US		TCGA-ED-	Liver	Liver cancer : Hepatocellular carc
alive	unknown	LIHC-US		TCGA-K7-	Liver	Liver cancer : Hepatocellular carc
alive	unknown	LIHC-US		TCGA-4R-	Liver	Liver cancer : Hepatocellular carc
deceased	unknown	LICA-FR		BCB109	Liver	Liver cancer : Hepatocellular carc
deceased	unknown	LICA-FR		BCB111	Liver	Liver cancer : Hepatocellular carc
alive	unknown	LICA-FR		BCB151	Liver	Liver cancer : Hepatocellular carc
alive	unknown	LICA-FR		BCB157	Liver	Liver cancer : Hepatocellular carc
deceased	unknown	LICA-FR		BCB167	Liver	Liver cancer : Hepatocellular carc
deceased	unknown	LICA-FR		BCB231	Liver	Liver cancer : Hepatocellular carc
deceased	unknown	LICA-FR		BCB301	Liver	Liver cancer : Hepatocellular carc

deceased	unknown	LICA-FR		BCB307	Liver	Liver cancer : Hepatocellular carc
deceased	unknown	LICA-FR		BCB325	Liver	Liver cancer : Hepatocellular carc
deceased	unknown	LICA-FR		BCM229	Liver	Liver cancer : Hepatocellular carc
deceased	unknown	LICA-FR		BCM257	Liver	Liver cancer : Hepatocellular carc
deceased	unknown	LICA-FR		BCM265	Liver	Liver cancer : Hepatocellular carc
deceased	unknown	LICA-FR		BCM269	Liver	Liver cancer : Hepatocellular carc
deceased	unknown	LICA-FR		BCM275	Liver	Liver cancer : Hepatocellular carc
deceased	unknown	LICA-FR		BCM321	Liver	Liver cancer : Hepatocellular carc
alive	unknown	LICA-FR		BCM325	Liver	Liver cancer : Hepatocellular carc
deceased	unknown	LICA-FR		BCM329	Liver	Liver cancer : Hepatocellular carc
deceased	unknown	LICA-FR		BCM337	Liver	Liver cancer : Hepatocellular carc
alive	unknown	LICA-FR		BCM339	Liver	Liver cancer : Hepatocellular carc
alive	unknown	LICA-FR		BCM371	Liver	Liver cancer : Hepatocellular carc
deceased	unknown	LICA-FR		BCM375	Liver	Liver cancer : Hepatocellular carc
alive	unknown	LICA-FR		BCM397	Liver	Liver cancer : Hepatocellular carc
deceased	unknown	LICA-FR		BCM399	Liver	Liver cancer : Hepatocellular carc
deceased	unknown	LICA-FR		BCM423	Liver	Liver cancer : Hepatocellular carc
deceased	unknown	LICA-FR		BCM439	Liver	Liver cancer : Hepatocellular carc
deceased	unknown	LICA-FR		BCM455	Liver	Liver cancer : Hepatocellular carc
deceased	unknown	LICA-FR		BCM483	Liver	Liver cancer : Hepatocellular carc
deceased	unknown	LICA-FR		BCM489	Liver	Liver cancer : Hepatocellular carc
deceased	unknown	LICA-FR		BCM501	Liver	Liver cancer : Hepatocellular carc
deceased	unknown	LICA-FR		BCM529	Liver	Liver cancer : Hepatocellular carc
deceased	unknown	LICA-FR		BCM531	Liver	Liver cancer : Hepatocellular carc
deceased	unknown	LICA-FR		BCM543	Liver	Liver cancer : Hepatocellular carc
deceased	unknown	LICA-FR		BCM545	Liver	Liver cancer : Hepatocellular carc
deceased	unknown	LICA-FR		BCM565	Liver	Liver cancer : Hepatocellular carc
deceased	unknown	LICA-FR		BCM567	Liver	Liver cancer : Hepatocellular carc
deceased	unknown	LICA-FR		BCM617	Liver	Liver cancer : Hepatocellular carc
alive	no	LIRI-JP	PCAWG	RK221	Liver	Liver cancer : Hepatocellular carc
deceased	unknown	LICA-FR		BCM643	Liver	Liver cancer : Hepatocellular carc
alive	no	LIRI-JP	PCAWG	RK222	Liver	Liver cancer : Hepatocellular carc
deceased	unknown	LICA-FR		BCM671	Liver	Liver cancer : Hepatocellular carc
alive	yes	LIRI-JP	PCAWG	RK223	Liver	Liver cancer : Hepatocellular carc
deceased	unknown	LICA-FR		BCM683	Liver	Liver cancer : Hepatocellular carc
alive	no	LIRI-JP	PCAWG	RK224	Liver	Liver cancer : Hepatocellular carc
deceased	unknown	LICA-FR		BCM689	Liver	Liver cancer : Hepatocellular carc
deceased	no	LIRI-JP	PCAWG	RK225	Liver	Liver cancer : Hepatocellular carc
deceased	unknown	LICA-FR		BCM695	Liver	Liver cancer : Hepatocellular carc
deceased	unknown	LICA-FR		BCM703	Liver	Liver cancer : Hepatocellular carc
alive	yes	LIRI-JP	PCAWG	RK227	Liver	Liver cancer : Hepatocellular carc
deceased	unknown	LICA-FR		BCM711	Liver	Liver cancer : Hepatocellular carc
deceased	no	LIRI-JP	PCAWG	RK228	Liver	Liver cancer : Hepatocellular carc
deceased	unknown	LICA-FR		BCM723	Liver	Liver cancer : Hepatocellular carc
alive	no	LIRI-JP	PCAWG	RK229	Liver	Liver cancer : Hepatocellular carc
deceased	unknown	LICA-FR		BCM735	Liver	Liver cancer : Hepatocellular carc
alive	yes	LIRI-JP	PCAWG	RK230	Liver	Liver cancer : Hepatocellular carc
deceased	unknown	LICA-FR		BCM739	Liver	Liver cancer : Hepatocellular carc
deceased	no	LIRI-JP	PCAWG	RK232	Liver	Liver cancer : Hepatocellular carc
deceased	unknown	LICA-FR		BCM759	Liver	Liver cancer : Hepatocellular carc
alive	yes	LIRI-JP	PCAWG	RK233	Liver	Liver cancer : Hepatocellular carc
deceased	unknown	LICA-FR		BCM769	Liver	Liver cancer : Hepatocellular carc
deceased	unknown	LICA-FR		BCM783	Liver	Liver cancer : Hepatocellular carc
alive	no	LIRI-JP	PCAWG	RK234	Liver	Liver cancer : Hepatocellular carc
deceased	unknown	LICA-FR		BCM791	Liver	Liver cancer : Hepatocellular carc
deceased	no	LIRI-JP	PCAWG	RK235	Liver	Liver cancer : Hepatocellular carc
alive	no	LIRI-JP	PCAWG	RK236	Liver	Liver cancer : Hepatocellular carc
alive	no	LIRI-JP	PCAWG	RK237	Liver	Liver cancer : Hepatocellular carc

alive	yes	LIRI-JP	PCAWG	RK241	Liver	Liver cancer : Hepatocellular carc
alive	yes	LIRI-JP	PCAWG	RK243	Liver	Liver cancer : Hepatocellular carc
alive	yes	LIRI-JP	PCAWG	RK244	Liver	Liver cancer : Hepatocellular carc
alive	yes	LIRI-JP	PCAWG	RK245	Liver	Liver cancer : Hepatocellular carc
alive	no	LIRI-JP	PCAWG	RK254	Liver	Liver cancer : Hepatocellular carc
alive	no	LIRI-JP	PCAWG	RK256	Liver	Liver cancer : Hepatocellular carc
alive	yes	LIRI-JP	PCAWG	RK257	Liver	Liver cancer : Hepatocellular carc
deceased	no	LIRI-JP	PCAWG	RK258	Liver	Liver cancer : Hepatocellular carc
	unknown	LICA-FR		CHC1010	Liver	Liver cancer : Hepatocellular carc
alive	no	LIRI-JP	PCAWG	RK259	Liver	Liver cancer : Hepatocellular carc
alive	unknown	LICA-FR		CHC1028	Liver	Liver cancer : Hepatocellular carc
alive	no	LIRI-JP	PCAWG	RK260	Liver	Liver cancer : Hepatocellular carc
alive	no	LIRI-JP	PCAWG	RK261	Liver	Liver cancer : Hepatocellular carc
alive	no	LIRI-JP	PCAWG	RK262	Liver	Liver cancer : Hepatocellular carc
alive	no	LIRI-JP	PCAWG	RK263	Liver	Liver cancer : Hepatocellular carc
alive	no	LIRI-JP	PCAWG	RK264	Liver	Liver cancer : Hepatocellular carc
alive	no	LIRI-JP	PCAWG	RK265	Liver	Liver cancer : Hepatocellular carc
alive	no	LIRI-JP	PCAWG	RK266	Liver	Liver cancer : Hepatocellular carc
alive	no	LIRI-JP	PCAWG	RK267	Liver	Liver cancer : Hepatocellular carc
alive	unknown	LICA-FR		CHC1079	Liver	Liver cancer : Hepatocellular carc
alive	no	LIRI-JP	PCAWG	RK268	Liver	Liver cancer : Hepatocellular carc
alive	unknown	LICA-FR		CHC1081	Liver	Liver cancer : Hepatocellular carc
alive	unknown	LICA-FR		CHC1085	Liver	Liver cancer : Hepatocellular carc
alive	no	LIRI-JP	PCAWG	RK270	Liver	Liver cancer : Hepatocellular carc
	unknown	LICA-FR		CHC1137	Liver	Liver cancer : Hepatocellular carc
alive	no	LIRI-JP	PCAWG	RK275	Liver	Liver cancer : Hepatocellular carc
	unknown	LICA-FR		CHC1148	Liver	Liver cancer : Hepatocellular carc
alive	no	LIRI-JP	PCAWG	RK277	Liver	Liver cancer : Hepatocellular carc
deceased	unknown	LICA-FR		CHC1152	Liver	Liver cancer : Hepatocellular carc
alive	no	LIRI-JP	PCAWG	RK278	Liver	Liver cancer : Hepatocellular carc
alive	no	LIRI-JP	PCAWG	RK280	Liver	Liver cancer : Hepatocellular carc
alive	unknown	LICA-FR		CHC1177	Liver	Liver cancer : Hepatocellular carc
alive	no	LIRI-JP	PCAWG	RK282	Liver	Liver cancer : Hepatocellular carc
	unknown	LICA-FR		CHC1180	Liver	Liver cancer : Hepatocellular carc
alive	unknown	LICA-FR		CHC1182	Liver	Liver cancer : Hepatocellular carc
alive	no	LIRI-JP	PCAWG	RK283	Liver	Liver cancer : Hepatocellular carc
	unknown	LICA-FR		CHC1183	Liver	Liver cancer : Hepatocellular carc
alive	no	LIRI-JP	PCAWG	RK284	Liver	Liver cancer : Hepatocellular carc
alive	no	LIRI-JP	PCAWG	RK285	Liver	Liver cancer : Hepatocellular carc
alive	unknown	LICA-FR		CHC1186	Liver	Liver cancer : Hepatocellular carc
alive	no	LIRI-JP	PCAWG	RK287	Liver	Liver cancer : Hepatocellular carc
alive	unknown	LICA-FR		CHC1191	Liver	Liver cancer : Hepatocellular carc
alive	no	LIRI-JP	PCAWG	RK289	Liver	Liver cancer : Hepatocellular carc
alive	no	LIRI-JP	PCAWG	RK297	Liver	Liver cancer : Hepatocellular carc
alive	unknown	LICA-FR		CHC1205	Liver	Liver cancer : Hepatocellular carc
	unknown	LICA-FR		CHC1207	Liver	Liver cancer : Hepatocellular carc
alive	no	LIRI-JP	PCAWG	RK304	Liver	Liver cancer : Hepatocellular carc
deceased	unknown	LICA-FR		CHC1209	Liver	Liver cancer : Hepatocellular carc
alive	no	LIRI-JP	PCAWG	RK305	Liver	Liver cancer : Hepatocellular carc
alive	no	LIRI-JP	PCAWG	RK306	Liver	Liver cancer : Hepatocellular carc
	unknown	LICA-FR		CHC1210	Liver	Liver cancer : Hepatocellular carc
	unknown	LICA-FR		CHC1211	Liver	Liver cancer : Hepatocellular carc
	unknown	LICA-FR		CHC1530	Liver	Liver cancer : Hepatocellular carc
alive	no	LIRI-JP	PCAWG	RK326	Liver	Liver cancer : Hepatocellular carc
deceased	unknown	LICA-FR		CHC1531	Liver	Liver cancer : Hepatocellular carc
alive	no	LIRI-JP	PCAWG	RK337	Liver	Liver cancer : Hepatocellular carc
	unknown	LICA-FR		CHC1534	Liver	Liver cancer : Hepatocellular carc
alive	yes	LIRI-JP	PCAWG	RK338	Liver	Liver cancer : Hepatocellular carc

	unknown	LICA-FR	CHC1539	Liver	Liver cancer : Hepatocellular carc
	unknown	LICA-FR	CHC1545	Liver	Liver cancer : Hepatocellular carc
alive	unknown	LICA-FR	CHC1556	Liver	Liver cancer : Hepatocellular carc
deceased	unknown	LICA-FR	CHC1566	Liver	Liver cancer : Hepatocellular carc
	unknown	LICA-FR	CHC1568	Liver	Liver cancer : Hepatocellular carc
alive	unknown	LICA-FR	CHC1569	Liver	Liver cancer : Hepatocellular carc
	unknown	LICA-FR	CHC1591	Liver	Liver cancer : Hepatocellular carc
alive	unknown	LICA-FR	CHC1592	Liver	Liver cancer : Hepatocellular carc
	unknown	LICA-FR	CHC1594	Liver	Liver cancer : Hepatocellular carc
alive	unknown	LICA-FR	CHC1595	Liver	Liver cancer : Hepatocellular carc
alive	unknown	LICA-FR	CHC1596	Liver	Liver cancer : Hepatocellular carc
deceased	unknown	LICA-FR	CHC1597	Liver	Liver cancer : Hepatocellular carc
	unknown	LICA-FR	CHC1598	Liver	Liver cancer : Hepatocellular carc
	unknown	LICA-FR	CHC1600	Liver	Liver cancer : Hepatocellular carc
alive	unknown	LICA-FR	CHC1601	Liver	Liver cancer : Hepatocellular carc
	unknown	LICA-FR	CHC1602	Liver	Liver cancer : Hepatocellular carc
	unknown	LICA-FR	CHC1603	Liver	Liver cancer : Hepatocellular carc
	unknown	LICA-FR	CHC1604	Liver	Liver cancer : Hepatocellular carc
deceased	unknown	LICA-FR	CHC1611	Liver	Liver cancer : Hepatocellular carc
	unknown	LICA-FR	CHC1616	Liver	Liver cancer : Hepatocellular carc
alive	unknown	LICA-FR	CHC1624	Liver	Liver cancer : Hepatocellular carc
	unknown	LICA-FR	CHC1626	Liver	Liver cancer : Hepatocellular carc
	unknown	LICA-FR	CHC1629	Liver	Liver cancer : Hepatocellular carc
alive	unknown	LICA-FR	CHC1700	Liver	Liver cancer : Hepatocellular carc
	unknown	LICA-FR	CHC1704	Liver	Liver cancer : Hepatocellular carc
alive	unknown	LICA-FR	CHC1708	Liver	Liver cancer : Hepatocellular carc
alive	unknown	LICA-FR	CHC1712	Liver	Liver cancer : Hepatocellular carc
alive	unknown	LICA-FR	CHC1714	Liver	Liver cancer : Hepatocellular carc
	unknown	LICA-FR	CHC1715	Liver	Liver cancer : Hepatocellular carc
	unknown	LICA-FR	CHC1717	Liver	Liver cancer : Hepatocellular carc
alive	unknown	LICA-FR	CHC1719	Liver	Liver cancer : Hepatocellular carc
deceased	unknown	LICA-FR	CHC1720	Liver	Liver cancer : Hepatocellular carc
	unknown	LICA-FR	CHC1725	Liver	Liver cancer : Hepatocellular carc
	unknown	LICA-FR	CHC1731	Liver	Liver cancer : Hepatocellular carc
	unknown	LICA-FR	CHC1732	Liver	Liver cancer : Hepatocellular carc
alive	unknown	LICA-FR	CHC1734	Liver	Liver cancer : Hepatocellular carc
	unknown	LICA-FR	CHC1736	Liver	Liver cancer : Hepatocellular carc
	unknown	LICA-FR	CHC1737	Liver	Liver cancer : Hepatocellular carc
alive	unknown	LICA-FR	CHC1738	Liver	Liver cancer : Hepatocellular carc
	unknown	LICA-FR	CHC1739	Liver	Liver cancer : Hepatocellular carc
	unknown	LICA-FR	CHC1741	Liver	Liver cancer : Hepatocellular carc
	unknown	LICA-FR	CHC1742	Liver	Liver cancer : Hepatocellular carc
deceased	unknown	LICA-FR	CHC1743	Liver	Liver cancer : Hepatocellular carc
	unknown	LICA-FR	CHC1744	Liver	Liver cancer : Hepatocellular carc
	unknown	LICA-FR	CHC1745	Liver	Liver cancer : Hepatocellular carc
	unknown	LICA-FR	CHC1746	Liver	Liver cancer : Hepatocellular carc
	unknown	LICA-FR	CHC1747	Liver	Liver cancer : Hepatocellular carc
	unknown	LICA-FR	CHC1749	Liver	Liver cancer : Hepatocellular carc
alive	unknown	LICA-FR	CHC1750	Liver	Liver cancer : Hepatocellular carc
deceased	unknown	LICA-FR	CHC1751	Liver	Liver cancer : Hepatocellular carc
	unknown	LICA-FR	CHC1753	Liver	Liver cancer : Hepatocellular carc
	unknown	LICA-FR	CHC1754	Liver	Liver cancer : Hepatocellular carc
	unknown	LICA-FR	CHC1756	Liver	Liver cancer : Hepatocellular carc
	unknown	LICA-FR	CHC1757	Liver	Liver cancer : Hepatocellular carc
	unknown	LICA-FR	CHC1763	Liver	Liver cancer : Hepatocellular carc
d	unknown	LICA-FR	CHC1774	Liver	Liver cancer : Hepatocellular carc
alive	unknown	LICA-FR	CHC2029	Liver	Liver cancer : Hepatocellular carc
deceased	unknown	LICA-FR	CHC2034	Liver	Liver cancer : Hepatocellular carc

alive	unknown	LICA-FR		CHC2039b	Liver	Liver cancer : Hepatocellular carc
alive	unknown	LICA-FR		CHC2043	Liver	Liver cancer : Hepatocellular carc
	unknown	LICA-FR		CHC2048	Liver	Liver cancer : Hepatocellular carc
deceased	unknown	LICA-FR		CHC2052	Liver	Liver cancer : Hepatocellular carc
alive	unknown	LICA-FR		CHC2098	Liver	Liver cancer : Hepatocellular carc
deceased	unknown	LICA-FR		CHC2099	Liver	Liver cancer : Hepatocellular carc
	unknown	LICA-FR		CHC2103	Liver	Liver cancer : Hepatocellular carc
alive	unknown	LICA-FR		CHC2110b	Liver	Liver cancer : Hepatocellular carc
	unknown	LICA-FR		CHC2111	Liver	Liver cancer : Hepatocellular carc
	unknown	LICA-FR		CHC2112	Liver	Liver cancer : Hepatocellular carc
	unknown	LICA-FR		CHC2113	Liver	Liver cancer : Hepatocellular carc
	unknown	LICA-FR		CHC2115	Liver	Liver cancer : Hepatocellular carc
alive	unknown	LICA-FR		CHC2127	Liver	Liver cancer : Hepatocellular carc
	unknown	LICA-FR		CHC2128	Liver	Liver cancer : Hepatocellular carc
alive	unknown	LICA-FR		CHC2134	Liver	Liver cancer : Hepatocellular carc
	unknown	LICA-FR		CHC2141	Liver	Liver cancer : Hepatocellular carc
deceased	unknown	LICA-FR		CHC2202	Liver	Liver cancer : Hepatocellular carc
	unknown	LICA-FR		CHC2206	Liver	Liver cancer : Hepatocellular carc
	unknown	LICA-FR		CHC2208	Liver	Liver cancer : Hepatocellular carc
alive	unknown	LICA-FR		CHC2211	Liver	Liver cancer : Hepatocellular carc
alive	unknown	LICA-FR		CHC2213	Liver	Liver cancer : Hepatocellular carc
alive	unknown	LICA-FR		CHC2215	Liver	Liver cancer : Hepatocellular carc
alive	unknown	LICA-FR		CHC2216	Liver	Liver cancer : Hepatocellular carc
d	unknown	LICA-FR		CHC2351	Liver	Liver cancer : Hepatocellular carc
d	unknown	LICA-FR		CHC2358	Liver	Liver cancer : Hepatocellular carc
d	unknown	LICA-FR		CHC2362	Liver	Liver cancer : Hepatocellular carc
	unknown	LICA-FR		CHC432	Liver	Liver cancer : Hepatocellular carc
alive	unknown	LICA-FR		CHC451	Liver	Liver cancer : Hepatocellular carc
alive	unknown	LICA-FR		CHC510	Liver	Liver cancer : Hepatocellular carc
alive	unknown	LICA-FR		CHC703	Liver	Liver cancer : Hepatocellular carc
deceased	unknown	LICA-FR		CHC734	Liver	Liver cancer : Hepatocellular carc
	unknown	LICA-FR		CHC736	Liver	Liver cancer : Hepatocellular carc
deceased	unknown	LICA-FR		CHC789	Liver	Liver cancer : Hepatocellular carc
	unknown	LICA-FR		CHC796	Liver	Liver cancer : Hepatocellular carc
alive	unknown	LICA-FR		CHC799	Liver	Liver cancer : Hepatocellular carc
deceased	unknown	LICA-FR		CHC801	Liver	Liver cancer : Hepatocellular carc
alive	unknown	LICA-FR		CHC805	Liver	Liver cancer : Hepatocellular carc
deceased	unknown	LICA-FR		CHC879	Liver	Liver cancer : Hepatocellular carc
	unknown	LICA-FR		CHC884	Liver	Liver cancer : Hepatocellular carc
	unknown	LICA-FR		CHC889	Liver	Liver cancer : Hepatocellular carc
	unknown	LICA-FR		CHC892	Liver	Liver cancer : Hepatocellular carc
alive	unknown	LICA-FR		CHC896	Liver	Liver cancer : Hepatocellular carc
	unknown	LICA-FR		CHC898	Liver	Liver cancer : Hepatocellular carc
alive	unknown	LICA-FR		CHC902	Liver	Liver cancer : Hepatocellular carc
	unknown	LICA-FR		CHC912	Liver	Liver cancer : Hepatocellular carc
deceased	unknown	LICA-FR		CHC923	Liver	Liver cancer : Hepatocellular carc
alive	unknown	LIHC-US		TCGA-BC-	Liver	Liver cancer : Hepatocellular carc
alive	unknown	LIHC-US	PCAWG	TCGA-BW	Liver	Liver cancer : Hepatocellular carc
alive	unknown	LIHC-US		TCGA-ED-	Liver	Liver cancer : Hepatocellular carc
alive	unknown	LIHC-US		TCGA-G3-	Liver	Liver cancer : Hepatocellular carc
alive	unknown	LIHC-US		TCGA-KR-	Liver	Liver cancer : Hepatocellular carc
alive	unknown	LIHC-US		TCGA-KR-	Liver	Liver cancer : Hepatocellular carc
alive	unknown	LIHC-US		TCGA-LG-	Liver	Liver cancer : Hepatocellular carc
alive	unknown	LIHC-US		TCGA-LG-	Liver	Liver cancer : Hepatocellular carc
alive	unknown	LIHC-US		TCGA-LG-	Liver	Liver cancer : Hepatocellular carc
alive	unknown	LIHC-US		TCGA-MI-	Liver	Liver cancer : Hepatocellular carc
alive	unknown	LIHC-US		TCGA-MI-	Liver	Liver cancer : Hepatocellular carc
alive	unknown	LIHC-US		TCGA-MI-	Liver	Liver cancer : Hepatocellular carc

alive	unknown	LIHC-US		TCGA-MI-	Liver	Liver cancer : Hepatocellular carc
alive	unknown	LIHC-US		TCGA-MI-	Liver	Liver cancer : Hepatocellular carc
alive	unknown	LIHC-US		TCGA-MR	Liver	Liver cancer : Hepatocellular carc
alive	unknown	LIHC-US		TCGA-NI-	Liver	Liver cancer : Hepatocellular carc
alive	unknown	LIHC-US		TCGA-O8-	Liver	Liver cancer : Hepatocellular carc
alive	unknown	LIHC-US		TCGA-ZP-	Liver	Liver cancer : Hepatocellular carc
alive	unknown	LIHC-US		TCGA-ZS-	Liver	Liver cancer : Hepatocellular carc
alive	unknown	LIHC-US		TCGA-ZS-	Liver	Liver cancer : Hepatocellular carc
alive	unknown	LIHC-US		TCGA-5C-	Liver	Liver cancer : Hepatocellular carc
alive	unknown	LIHC-US		TCGA-5C-	Liver	Liver cancer : Hepatocellular carc
alive	unknown	LIHC-US		TCGA-5C-	Liver	Liver cancer : Hepatocellular carc
alive	unknown	LIHC-US		TCGA-BD-	Liver	Liver cancer : Hepatocellular carc
alive	unknown	LIHC-US		TCGA-BD-	Liver	Liver cancer : Hepatocellular carc
alive	unknown	LIHC-US	PCAWG	TCGA-BW	Liver	Liver cancer : Hepatocellular carc
alive	unknown	LIHC-US	PCAWG	TCGA-BW	Liver	Liver cancer : Hepatocellular carc
alive	unknown	LIHC-US		TCGA-CC-	Liver	Liver cancer : Hepatocellular carc
alive	unknown	LIHC-US		TCGA-CC-	Liver	Liver cancer : Hepatocellular carc
alive	unknown	LIHC-US		TCGA-CC-	Liver	Liver cancer : Hepatocellular carc
alive	unknown	LIHC-US		TCGA-CC-	Liver	Liver cancer : Hepatocellular carc
alive	unknown	LIHC-US		TCGA-CC-	Liver	Liver cancer : Hepatocellular carc
alive	unknown	LIHC-US		TCGA-CC-	Liver	Liver cancer : Hepatocellular carc
alive	unknown	LIHC-US		TCGA-CC-	Liver	Liver cancer : Hepatocellular carc
alive	unknown	LIHC-US		TCGA-CC-	Liver	Liver cancer : Hepatocellular carc
alive	unknown	LIHC-US		TCGA-CC-	Liver	Liver cancer : Hepatocellular carc
alive	unknown	LIHC-US		TCGA-CC-	Liver	Liver cancer : Hepatocellular carc
alive	unknown	LIHC-US		TCGA-CC-	Liver	Liver cancer : Hepatocellular carc
alive	unknown	LIHC-US		TCGA-CC-	Liver	Liver cancer : Hepatocellular carc
alive	unknown	LIHC-US		TCGA-CC-	Liver	Liver cancer : Hepatocellular carc
alive	unknown	LIHC-US		TCGA-CC-	Liver	Liver cancer : Hepatocellular carc
alive	unknown	LIHC-US		TCGA-CC-	Liver	Liver cancer : Hepatocellular carc
alive	unknown	LIHC-US		TCGA-DD-	Liver	Liver cancer : Hepatocellular carc
alive	unknown	LIHC-US		TCGA-DD-	Liver	Liver cancer : Hepatocellular carc
alive	unknown	LIHC-US		TCGA-DD-	Liver	Liver cancer : Hepatocellular carc
alive	unknown	LIHC-US		TCGA-DD-	Liver	Liver cancer : Hepatocellular carc
alive	unknown	LIHC-US		TCGA-DD-	Liver	Liver cancer : Hepatocellular carc
alive	unknown	LIHC-US		TCGA-DD-	Liver	Liver cancer : Hepatocellular carc
alive	unknown	LIHC-US		TCGA-DD-	Liver	Liver cancer : Hepatocellular carc
alive	unknown	LIHC-US		TCGA-DD-	Liver	Liver cancer : Hepatocellular carc
alive	unknown	LIHC-US		TCGA-DD-	Liver	Liver cancer : Hepatocellular carc
alive	unknown	LIHC-US		TCGA-DD-	Liver	Liver cancer : Hepatocellular carc
alive	unknown	LIHC-US		TCGA-DD-	Liver	Liver cancer : Hepatocellular carc
alive	unknown	LIHC-US		TCGA-DD-	Liver	Liver cancer : Hepatocellular carc
alive	unknown	LIHC-US		TCGA-DD-	Liver	Liver cancer : Hepatocellular carc
alive	unknown	LIHC-US		TCGA-DD-	Liver	Liver cancer : Hepatocellular carc
alive	unknown	LIHC-US		TCGA-DD-	Liver	Liver cancer : Hepatocellular carc
alive	unknown	LIHC-US		TCGA-DD-	Liver	Liver cancer : Hepatocellular carc
alive	unknown	LIHC-US		TCGA-DD-	Liver	Liver cancer : Hepatocellular carc
alive	unknown	LIHC-US		TCGA-DD-	Liver	Liver cancer : Hepatocellular carc
alive	unknown	LIHC-US		TCGA-DD-	Liver	Liver cancer : Hepatocellular carc
alive	unknown	LIHC-US		TCGA-DD-	Liver	Liver cancer : Hepatocellular carc
alive	unknown	LIHC-US		TCGA-DD-	Liver	Liver cancer : Hepatocellular carc
alive	unknown	LIHC-US		TCGA-DD-	Liver	Liver cancer : Hepatocellular carc
alive	unknown	LIHC-US		TCGA-DD-	Liver	Liver cancer : Hepatocellular carc
alive	unknown	LIHC-US		TCGA-DD-	Liver	Liver cancer : Hepatocellular carc
alive	unknown	LIHC-US		TCGA-DD-	Liver	Liver cancer : Hepatocellular carc
alive	unknown	LIHC-US		TCGA-DD-	Liver	Liver cancer : Hepatocellular carc
alive	unknown	LIHC-US		TCGA-DD-	Liver	Liver cancer : Hepatocellular carc
alive	unknown	LIHC-US		TCGA-DD-	Liver	Liver cancer : Hepatocellular carc

alive	unknown	LIHC-US		TCGA-DD	Liver	Liver cancer : Hepatocellular carc
alive	unknown	LIHC-US		TCGA-DD	Liver	Liver cancer : Hepatocellular carc
alive	unknown	LIHC-US		TCGA-DD	Liver	Liver cancer : Hepatocellular carc
alive	unknown	LIHC-US		TCGA-DD	Liver	Liver cancer : Hepatocellular carc
alive	unknown	LIHC-US		TCGA-DD	Liver	Liver cancer : Hepatocellular carc
alive	unknown	LIHC-US		TCGA-DD	Liver	Liver cancer : Hepatocellular carc
alive	unknown	LIHC-US		TCGA-DD	Liver	Liver cancer : Hepatocellular carc
alive	unknown	LIHC-US		TCGA-DD	Liver	Liver cancer : Hepatocellular carc
alive	unknown	LIHC-US		TCGA-DD	Liver	Liver cancer : Hepatocellular carc
alive	unknown	LIHC-US		TCGA-DD	Liver	Liver cancer : Hepatocellular carc
alive	unknown	LIHC-US		TCGA-DD	Liver	Liver cancer : Hepatocellular carc
alive	unknown	LIHC-US		TCGA-DD	Liver	Liver cancer : Hepatocellular carc
alive	unknown	LIHC-US		TCGA-DD	Liver	Liver cancer : Hepatocellular carc
alive	unknown	LIHC-US		TCGA-DD	Liver	Liver cancer : Hepatocellular carc
alive	unknown	LIHC-US		TCGA-DD	Liver	Liver cancer : Hepatocellular carc
alive	unknown	LIHC-US		TCGA-DD	Liver	Liver cancer : Hepatocellular carc
alive	unknown	LIHC-US		TCGA-DD	Liver	Liver cancer : Hepatocellular carc
alive	unknown	LIHC-US		TCGA-DD	Liver	Liver cancer : Hepatocellular carc
alive	unknown	LIHC-US		TCGA-DD	Liver	Liver cancer : Hepatocellular carc
alive	unknown	LIHC-US		TCGA-DD	Liver	Liver cancer : Hepatocellular carc
alive	unknown	LIHC-US		TCGA-DD	Liver	Liver cancer : Hepatocellular carc
alive	unknown	LIHC-US		TCGA-DD	Liver	Liver cancer : Hepatocellular carc
alive	unknown	LIHC-US		TCGA-DD	Liver	Liver cancer : Hepatocellular carc
alive	unknown	LIHC-US		TCGA-DD	Liver	Liver cancer : Hepatocellular carc
alive	unknown	LIHC-US		TCGA-DD	Liver	Liver cancer : Hepatocellular carc
alive	unknown	LIHC-US		TCGA-DD	Liver	Liver cancer : Hepatocellular carc
alive	unknown	LIHC-US		TCGA-DD	Liver	Liver cancer : Hepatocellular carc
alive	unknown	LIHC-US		TCGA-DD	Liver	Liver cancer : Hepatocellular carc
alive	unknown	LIHC-US		TCGA-DD	Liver	Liver cancer : Hepatocellular carc
alive	unknown	LIHC-US		TCGA-DD	Liver	Liver cancer : Hepatocellular carc
alive	unknown	LIHC-US		TCGA-DD	Liver	Liver cancer : Hepatocellular carc
alive	unknown	LIHC-US		TCGA-DD	Liver	Liver cancer : Hepatocellular carc
alive	unknown	LIHC-US		TCGA-DD	Liver	Liver cancer : Hepatocellular carc
alive	unknown	LIHC-US		TCGA-DD	Liver	Liver cancer : Hepatocellular carc
alive	unknown	LIHC-US		TCGA-DD	Liver	Liver cancer : Hepatocellular carc
alive	unknown	LIHC-US		TCGA-DD	Liver	Liver cancer : Hepatocellular carc
alive	unknown	LIHC-US		TCGA-DD	Liver	Liver cancer : Hepatocellular carc
alive	unknown	LIHC-US		TCGA-DD	Liver	Liver cancer : Hepatocellular carc
alive	unknown	LIHC-US		TCGA-G3-	Liver	Liver cancer : Hepatocellular carc
alive	unknown	LIHC-US		TCGA-G3-	Liver	Liver cancer : Hepatocellular carc
alive	unknown	LIHC-US		TCGA-G3-	Liver	Liver cancer : Hepatocellular carc
alive	unknown	LIHC-US		TCGA-G3-	Liver	Liver cancer : Hepatocellular carc
alive	unknown	LIHC-US		TCGA-G3-	Liver	Liver cancer : Hepatocellular carc
alive	unknown	LIHC-US		TCGA-G3-	Liver	Liver cancer : Hepatocellular carc
alive	unknown	LIHC-US		TCGA-G3-	Liver	Liver cancer : Hepatocellular carc
alive	unknown	LIHC-US		TCGA-G3-	Liver	Liver cancer : Hepatocellular carc
alive	unknown	LIHC-US		TCGA-K7-	Liver	Liver cancer : Hepatocellular carc
alive	unknown	LIHC-US		TCGA-K7-	Liver	Liver cancer : Hepatocellular carc
alive	unknown	LIHC-US		TCGA-KR-	Liver	Liver cancer : Hepatocellular carc
alive	unknown	LIHC-US		TCGA-QA	Liver	Liver cancer : Hepatocellular carc
alive	unknown	LIHC-US		TCGA-RC-	Liver	Liver cancer : Hepatocellular carc
alive	unknown	LIHC-US		TCGA-RC-	Liver	Liver cancer : Hepatocellular carc
alive	unknown	LIHC-US		TCGA-RC-	Liver	Liver cancer : Hepatocellular carc
alive	unknown	LIHC-US		TCGA-RC-	Liver	Liver cancer : Hepatocellular carc
alive	unknown	LIHC-US		TCGA-RG-	Liver	Liver cancer : Hepatocellular carc
alive	unknown	LIHC-US		TCGA-UB-	Liver	Liver cancer : Hepatocellular carc
alive	unknown	LIHC-US		TCGA-UB-	Liver	Liver cancer : Hepatocellular carc
alive	unknown	LIHC-US		TCGA-UB-	Liver	Liver cancer : Hepatocellular carc

alive	unknown	LIHC-US	TCGA-UB-	Liver	Liver cancer : Hepatocellular carc
alive	unknown	LIHC-US	TCGA-UB-	Liver	Liver cancer : Hepatocellular carc
alive	unknown	LIHC-US	TCGA-UB-	Liver	Liver cancer : Hepatocellular carc
alive	unknown	LIHC-US	TCGA-WJ-	Liver	Liver cancer : Hepatocellular carc
alive	unknown	LIHC-US	TCGA-WÇ	Liver	Liver cancer : Hepatocellular carc
alive	unknown	LIHC-US	TCGA-WÇ	Liver	Liver cancer : Hepatocellular carc
alive	unknown	LIHC-US	TCGA-ZP-	Liver	Liver cancer : Hepatocellular carc
alive	unknown	LIHC-US	TCGA-ZP-	Liver	Liver cancer : Hepatocellular carc
alive	unknown	LIHC-US	TCGA-ZP-	Liver	Liver cancer : Hepatocellular carc
alive	unknown	LIHC-US	TCGA-ZP-	Liver	Liver cancer : Hepatocellular carc
alive	unknown	LIHC-US	TCGA-ZP-	Liver	Liver cancer : Hepatocellular carc
alive	unknown	LIHC-US	TCGA-ZP-	Liver	Liver cancer : Hepatocellular carc
alive	unknown	LIHC-US	TCGA-ZS-	Liver	Liver cancer : Hepatocellular carc
alive	unknown	LIHM-FR	CHC1081	Liver	Liver cancer : Hepatocellular carc
alive	unknown	LIHM-FR	CHC1085	Liver	Liver cancer : Hepatocellular carc
	unknown	LIHM-FR	CHC1097	Liver	Liver cancer : Hepatocellular carc
	unknown	LIHM-FR	CHC2351	Liver	Liver cancer : Hepatocellular carc

:inoma (Secondary to alcohol and adiposity)
:inoma (Secondary to alcohol and adiposity)
:inoma (Secondary to alcohol and adiposity)
:inoma (Secondary to alcohol and adiposity)

cinoma (Secondary to alcohol and adiposity)
cinoma (Secondary to alcohol and adiposity)
cinoma (Secondary to alcohol and adiposity)
cinoma (Secondary to alcohol and adiposity)

:inoma (Secondary to alcohol and adiposity)

:inoma (Secondary to alcohol and adiposity)

cinoma (Secondary to alcohol and adiposity)

cinoma (Secondary to alcohol and adiposity)
cinoma (Secondary to alcohol and adiposity)

cinoma (Secondary to alcohol and adiposity)
cinoma (Secondary to alcohol and adiposity)

cinoma (Secondary to alcohol and adiposity)

cinoma (Secondary to alcohol and adiposity)

cinoma (Secondary to alcohol and adiposity)
cinoma (Secondary to alcohol and adiposity)

:inoma (Secondary to alcohol and adiposity)

:inoma (Secondary to alcohol and adiposity)

cinoma (Secondary to alcohol and adiposity)
cinoma (Secondary to alcohol and adiposity)
cinoma (Secondary to alcohol and adiposity)
cinoma (Secondary to alcohol and adiposity)
cinoma (Secondary to alcohol and adiposity)
cinoma (Secondary to alcohol and adiposity)
cinoma (Secondary to alcohol and adiposity)
cinoma (Secondary to alcohol and adiposity)
cinoma (Secondary to alcohol and adiposity)
cinoma (Secondary to alcohol and adiposity)
cinoma (Secondary to alcohol and adiposity)
cinoma (Secondary to alcohol and adiposity)
cinoma (Secondary to alcohol and adiposity)
cinoma (Secondary to alcohol and adiposity)
cinoma (Secondary to alcohol and adiposity)
cinoma (Secondary to alcohol and adiposity)
cinoma (Secondary to alcohol and adiposity)
cinoma (Secondary to alcohol and adiposity)

cinoma (Secondary to alcohol and adiposity)
cinoma (Secondary to alcohol and adiposity)
cinoma (Secondary to alcohol and adiposity)
cinoma (Secondary to alcohol and adiposity)
cinoma (Secondary to alcohol and adiposity)
cinoma (Secondary to alcohol and adiposity)
cinoma (Secondary to alcohol and adiposity)
cinoma (Secondary to alcohol and adiposity)
cinoma (Secondary to alcohol and adiposity)
cinoma (Secondary to alcohol and adiposity)
cinoma (Secondary to alcohol and adiposity)
cinoma (Secondary to alcohol and adiposity)
cinoma (Secondary to alcohol and adiposity)
cinoma (Secondary to alcohol and adiposity)
cinoma (Secondary to alcohol and adiposity)
cinoma (Secondary to alcohol and adiposity)
cinoma (Secondary to alcohol and adiposity)
cinoma (Secondary to alcohol and adiposity)
cinoma (Secondary to alcohol and adiposity)
cinoma (Secondary to alcohol and adiposity)
cinoma (Secondary to alcohol and adiposity)
cinoma (Secondary to alcohol and adiposity)
cinoma (Secondary to alcohol and adiposity)
cinoma (Secondary to alcohol and adiposity)
cinoma (Secondary to alcohol and adiposity)
cinoma (Secondary to alcohol and adiposity)
cinoma (Secondary to alcohol and adiposity)
cinoma (Secondary to alcohol and adiposity)
cinoma (Secondary to alcohol and adiposity)
cinoma (Secondary to alcohol and adiposity)
cinoma (Secondary to alcohol and adiposity)
cinoma (Secondary to alcohol and adiposity)
cinoma (Secondary to alcohol and adiposity)
cinoma (Secondary to alcohol and adiposity)
cinoma (Secondary to alcohol and adiposity)
cinoma (Secondary to alcohol and adiposity)
cinoma (Secondary to alcohol and adiposity)
cinoma (Secondary to alcohol and adiposity)
cinoma (Secondary to alcohol and adiposity)
cinoma (Secondary to alcohol and adiposity)
cinoma (Secondary to alcohol and adiposity)
cinoma (Secondary to alcohol and adiposity)
cinoma (Secondary to alcohol and adiposity)
cinoma (Secondary to alcohol and adiposity)
cinoma (Secondary to alcohol and adiposity)
cinoma (Secondary to alcohol and adiposity)
cinoma (Secondary to alcohol and adiposity)
cinoma (Secondary to alcohol and adiposity)
cinoma (Secondary to alcohol and adiposity)
cinoma (Secondary to alcohol and adiposity)
cinoma (Secondary to alcohol and adiposity)
cinoma (Secondary to alcohol and adiposity)
cinoma (Secondary to alcohol and adiposity)
cinoma (Secondary to alcohol and adiposity)
cinoma (Secondary to alcohol and adiposity)
cinoma (Secondary to alcohol and adiposity)
cinoma (Secondary to alcohol and adiposity)
cinoma (Secondary to alcohol and adiposity)

:inoma (Secondary to alcohol and adiposity)
:inoma (Secondary to alcohol and adiposity)
:inoma (Secondary to alcohol and adiposity)
:inoma (Secondary to alcohol and adiposity)
:inoma (Secondary to alcohol and adiposity)
:inoma (Secondary to alcohol and adiposity)
:inoma (Secondary to alcohol and adiposity)
:inoma (Secondary to alcohol and adiposity)
:inoma (Secondary to alcohol and adiposity)
:inoma (Secondary to alcohol and adiposity)
:inoma (Secondary to alcohol and adiposity)
:inoma (Secondary to alcohol and adiposity)
:inoma (Secondary to alcohol and adiposity)
:inoma (Secondary to alcohol and adiposity)
:inoma (Secondary to alcohol and adiposity)

cinoma (Secondary to alcohol and adiposity)
cinoma (Secondary to alcohol and adiposity)
cinoma (Secondary to alcohol and adiposity)
cinoma (Secondary to alcohol and adiposity)
cinoma (Secondary to alcohol and adiposity)
cinoma (Secondary to alcohol and adiposity)
cinoma (Secondary to alcohol and adiposity)

cinoma (Secondary to alcohol and adiposity)
cinoma (Secondary to alcohol and adiposity)
cinoma (Secondary to alcohol and adiposity)
cinoma (Secondary to alcohol and adiposity)
cinoma (Secondary to alcohol and adiposity)
cinoma (Secondary to alcohol and adiposity)
cinoma (Secondary to alcohol and adiposity)
cinoma (Secondary to alcohol and adiposity)
cinoma (Secondary to alcohol and adiposity)
cinoma (Secondary to alcohol and adiposity)
cinoma (Secondary to alcohol and adiposity)
cinoma (Secondary to alcohol and adiposity)
cinoma (Secondary to alcohol and adiposity)
cinoma (Secondary to alcohol and adiposity)
cinoma (Secondary to alcohol and adiposity)
cinoma (Secondary to alcohol and adiposity)
cinoma (Secondary to alcohol and adiposity)
cinoma (Secondary to alcohol and adiposity)
cinoma (Secondary to alcohol and adiposity)
cinoma (Secondary to alcohol and adiposity)
cinoma (Secondary to alcohol and adiposity)
cinoma (Secondary to alcohol and adiposity)
cinoma (Secondary to alcohol and adiposity)
cinoma (Secondary to alcohol and adiposity)
cinoma (Secondary to alcohol and adiposity)
cinoma (Secondary to alcohol and adiposity)
cinoma (Secondary to alcohol and adiposity)
cinoma (Secondary to alcohol and adiposity)
cinoma (Secondary to alcohol and adiposity)

cinoma (Secondary to alcohol and adiposity)

cinoma (Secondary to alcohol and adiposity)

cinoma (Secondary to alcohol and adiposity)

cinoma (Secondary to alcohol and adiposity)

cinoma (Secondary to alcohol and adiposity)
cinoma (Secondary to alcohol and adiposity)

cinoma (Secondary to alcohol and adiposity)

cinoma (Secondary to alcohol and adiposity)

cinoma (Secondary to alcohol and adiposity)

cinoma (Secondary to alcohol and adiposity)

cinoma (Secondary to alcohol and adiposity)

cinoma (Secondary to alcohol and adiposity)
cinoma (Secondary to alcohol and adiposity)

cinoma (Secondary to alcohol and adiposity)

cinoma (Secondary to alcohol and adiposity)

cinoma (Secondary to alcohol and adiposity)

cinoma (Secondary to alcohol and adiposity)

cinoma (Secondary to alcohol and adiposity)
cinoma (Secondary to alcohol and adiposity)

cinoma (Secondary to alcohol and adiposity)

cinoma (Secondary to alcohol and adiposity)

cinoma (Secondary to alcohol and adiposity)

cinoma (Secondary to alcohol and adiposity)

cinoma (Secondary to alcohol and adiposity)
cinoma (Secondary to alcohol and adiposity)

cinoma (Secondary to alcohol and adiposity)

cinoma (Secondary to alcohol and adiposity)

cinoma (Secondary to alcohol and adiposity)

cinoma (Secondary to alcohol and adiposity)
cinoma (Secondary to alcohol and adiposity)

cinoma (Secondary to alcohol and adiposity)

cinoma (Secondary to alcohol and adiposity)
cinoma (Secondary to alcohol and adiposity)
cinoma (Secondary to alcohol and adiposity)

cinoma (Secondary to alcohol and adiposity)

cinoma (Secondary to alcohol and adiposity)

cinoma (Secondary to alcohol and adiposity)
cinoma (Secondary to alcohol and adiposity)
cinoma (Secondary to alcohol and adiposity)
cinoma (Secondary to alcohol and adiposity)
cinoma (Secondary to alcohol and adiposity)
cinoma (Secondary to alcohol and adiposity)
cinoma (Secondary to alcohol and adiposity)
cinoma (Secondary to alcohol and adiposity)
cinoma (Secondary to alcohol and adiposity)
cinoma (Secondary to alcohol and adiposity)
cinoma (Secondary to alcohol and adiposity)
cinoma (Secondary to alcohol and adiposity)
cinoma (Secondary to alcohol and adiposity)
cinoma (Secondary to alcohol and adiposity)
cinoma (Secondary to alcohol and adiposity)
cinoma (Secondary to alcohol and adiposity)
cinoma (Secondary to alcohol and adiposity)
cinoma (Secondary to alcohol and adiposity)
cinoma (Secondary to alcohol and adiposity)
cinoma (Secondary to alcohol and adiposity)
cinoma (Secondary to alcohol and adiposity)
cinoma (Secondary to alcohol and adiposity)
cinoma (Secondary to alcohol and adiposity)
cinoma (Secondary to alcohol and adiposity)
cinoma (Secondary to alcohol and adiposity)
cinoma (Secondary to alcohol and adiposity)
cinoma (Secondary to alcohol and adiposity)
cinoma (Secondary to alcohol and adiposity)
cinoma (Secondary to alcohol and adiposity)
cinoma (Secondary to alcohol and adiposity)
cinoma (Secondary to alcohol and adiposity)
cinoma (Secondary to alcohol and adiposity)
cinoma (Secondary to alcohol and adiposity)
cinoma (Secondary to alcohol and adiposity)
cinoma (Secondary to alcohol and adiposity)
cinoma (Secondary to alcohol and adiposity)
cinoma (Secondary to alcohol and adiposity)
cinoma (Secondary to alcohol and adiposity)
cinoma (Secondary to alcohol and adiposity)
cinoma (Secondary to alcohol and adiposity)
cinoma (Secondary to alcohol and adiposity)
cinoma (Secondary to alcohol and adiposity)
cinoma (Secondary to alcohol and adiposity)
cinoma (Secondary to alcohol and adiposity)
cinoma (Secondary to alcohol and adiposity)
cinoma (Secondary to alcohol and adiposity)
cinoma (Secondary to alcohol and adiposity)
cinoma (Secondary to alcohol and adiposity)
cinoma (Secondary to alcohol and adiposity)
cinoma (Secondary to alcohol and adiposity)
cinoma (Secondary to alcohol and adiposity)
cinoma (Secondary to alcohol and adiposity)
cinoma (Secondary to alcohol and adiposity)
cinoma (Secondary to alcohol and adiposity)
cinoma (Secondary to alcohol and adiposity)
cinoma (Secondary to alcohol and adiposity)
cinoma (Secondary to alcohol and adiposity)
cinoma (Secondary to alcohol and adiposity)

cinoma (Secondary to alcohol and adiposity)
cinoma (Secondary to alcohol and adiposity)
cinoma (Secondary to alcohol and adiposity)
cinoma (Secondary to alcohol and adiposity)
cinoma (Secondary to alcohol and adiposity)
cinoma (Secondary to alcohol and adiposity)
cinoma (Secondary to alcohol and adiposity)
cinoma (Secondary to alcohol and adiposity)
cinoma (Secondary to alcohol and adiposity)
cinoma (Secondary to alcohol and adiposity)
cinoma (Secondary to alcohol and adiposity)
cinoma (Secondary to alcohol and adiposity)
cinoma (Secondary to alcohol and adiposity)
cinoma (Secondary to alcohol and adiposity)
cinoma (Secondary to alcohol and adiposity)
cinoma (Secondary to alcohol and adiposity)
cinoma (Secondary to alcohol and adiposity)
cinoma (Secondary to alcohol and adiposity)
cinoma (Secondary to alcohol and adiposity)
cinoma (Secondary to alcohol and adiposity)
cinoma (Secondary to alcohol and adiposity)
cinoma (Secondary to alcohol and adiposity)
cinoma (Secondary to alcohol and adiposity)
cinoma (Secondary to alcohol and adiposity)
cinoma (Secondary to alcohol and adiposity)
cinoma (Secondary to alcohol and adiposity)
cinoma (Secondary to alcohol and adiposity)
cinoma (Secondary to alcohol and adiposity)
cinoma (Secondary to alcohol and adiposity)
cinoma (Secondary to alcohol and adiposity)
cinoma (Secondary to alcohol and adiposity)
cinoma (Secondary to alcohol and adiposity)
cinoma (Secondary to alcohol and adiposity)
cinoma (Secondary to alcohol and adiposity)
cinoma (Secondary to alcohol and adiposity)
cinoma (Secondary to alcohol and adiposity)
cinoma (Secondary to alcohol and adiposity)
cinoma (Secondary to alcohol and adiposity)
cinoma (Secondary to alcohol and adiposity)
cinoma (Secondary to alcohol and adiposity)
cinoma (Secondary to alcohol and adiposity)
cinoma (Secondary to alcohol and adiposity)
cinoma (Secondary to alcohol and adiposity)
cinoma (Secondary to alcohol and adiposity)
cinoma (Secondary to alcohol and adiposity)
cinoma (Secondary to alcohol and adiposity)

cinoma (Secondary to alcohol and adiposity)
cinoma (Secondary to alcohol and adiposity)
cinoma (Secondary to alcohol and adiposity)
cinoma (Secondary to alcohol and adiposity)
