# Supplementary material for: Identifying Potential Tumor Antigens and Antigens-Related Subtypes in Hepatocellular Carcinoma for mRNA Vaccine Development
Source: J Oncol. 2022 Aug 29;2022:6851026. doi: 10.1155/2022/6851026 (PMC9444406; doi:10.1155/2022/6851026)
Supplement: Supplementary Materials — Table S1: clinical data from ICGC. Table S2: Differently Expressed Genes Analysis from cbioptrol. Differential Expression Analysis from GEPIA. Table S3: mutation Analysis of LIHC from cbioptrol. Supplementary Figure 1: KEGG and GO enrichment analyses of these candidate genes. Supplementary Figure 2: heatmaps of antigens in TCGA and CGGA cohort with cluster annotations. [file 6851026.f1.zip › Table S3 (1).pdf]

Table 3.Differently Expressed Genes Analysis from cbioptrol.Differential Expression Analysis from GI

Gene nam	gene	Nomal	Cancer	FC	P
AADAT	ENSG0000	1.83	11.5	-2.143	8.81E-52
AASS	ENSG0000	3.24	11.16	-1.52	9.03E-20
AATF	ENSG0000	29.171	10.64	1.374	3.60E-73
ABCA8	ENSG0000	3.33	13.07	-1.7	4.52E-35
ABCA9	ENSG0000	0.63	3.27	-1.389	1.21E-33
ABCB6	ENSG0000	39.371	18.615	1.041	8.28E-19
ABCC6P2	ENSG0000	8.95	3.735	1.071	3.34E-29
ABCC9	ENSG0000	2.25	6.825	-1.268	3.07E-26
ABHD12	ENSG0000	35.671	12.07	1.488	3.60E-75
ABLIM3	ENSG0000	12.69	37.135	-1.478	3.05E-29
AC004538.	ENSG0000	0.14	2.04	-1.415	9.74E-43
AC004540.	ENSG0000	0.17	1.654	-1.182	1.94E-25
AC005077.	ENSG0000	0.52	6.74	-2.348	2.36E-51
AC005255.	ENSG0000	3.45	0	2.154	6.77E-37
AC005943.	ENSG0000	1.38	0	1.251	8.76E-11
AC005943.	ENSG0000	2.24	0	1.696	5.01E-03
AC006128.	ENSG0000	1.15	7.035	-1.902	1.11E-20
AC007318.	ENSG0000	4.63	1.58	1.126	1.13E-24
AC009065.	ENSG0000	1.34	0	1.227	9.02E-31
AC009120.	ENSG0000	1.99	5.625	-1.148	4.51E-07
AC009963.	ENSG0000	12.75	43.499	-1.694	7.81E-18
AC012146.	ENSG0000	5.99	2.17	1.141	8.25E-30
AC016292.	ENSG0000	2.15	0.485	1.085	2.75E-23
AC016739.	ENSG0000	12.83	4.745	1.267	4.16E-35
AC016747.	ENSG0000	4.32	1.61	1.027	9.53E-37
AC068535.	ENSG0000	1.62	9.54	-2.008	3.12E-22
AC092171.	ENSG0000	2.23	0.56	1.05	2.10E-36
AC093673.	ENSG0000	5.26	2.085	1.021	1.12E-24
AC099668.	ENSG0000	2.33	10.819	-1.828	3.09E-21
AC104534.	ENSG0000	5.81	0.42	2.262	6.03E-12
AC104809.	ENSG0000	0.04	3.375	-2.073	1.11E-34
AC132217.	ENSG0000	21.11	365.062	-4.049	3.36E-12
AC138035.	ENSG0000	0.6	3.465	-1.481	7.14E-22
AC239868.	ENSG0000	22.92	4.91	2.017	4.33E-23
AC239868.	ENSG0000	22.92	4.91	2.017	4.33E-23
ACACB	ENSG0000	8.47	23.61	-1.378	2.85E-25
ACAD11	ENSG0000	12.74	40.844	-1.607	7.07E-44
ACADL	ENSG0000	2.9	8.555	-1.293	3.27E-23
ACADS	ENSG0000	39.17	97.48	-1.294	1.55E-37
ACADVL	ENSG0000	359.313	861.913	-1.26	2.35E-22
ACAP3	ENSG0000	7.86	17.3	-1.046	7.95E-04
ACBD6	ENSG0000	24.71	9.845	1.245	2.08E-59
ACCS	ENSG0000	6.22	14.189	-1.073	6.11E-12
ACLY	ENSG0000	20.91	8.174	1.256	5.83E-28
ACOT12	ENSG0000	13.96	29.518	-1.029	2.17E-12
ACP5	ENSG0000	22.77	9.84	1.133	2.02E-08
ACP6	ENSG0000	18.6	8.69	1.016	6.14E-32
ACSL1	ENSG0000	129.984	406.58	-1.638	4.55E-31
ACSL4	ENSG0000	33.02	2.355	3.342	4.63E-30
ACSM1	ENSG0000	8.56	1.085	2.197	2.43E-16
ACSM3	ENSG0000	18.45	58.215	-1.606	1.69E-24
ACSM5	ENSG0000	28.829	79.263	-1.428	1.12E-22
ACTN4	ENSG0000	102.749	47.2	1.106	1.82E-31
ADAM15	ENSG0000	42.241	16.735	1.286	1.78E-44
ADAMTS1	ENSG0000	3.15	7.81	-1.086	8.91E-16
ADAMTS1	ENSG0000	2.32	14.475	-2.221	1.49E-97

ADAMTS2	ENSG0000	1.37	4.445	-1.2	2.95E-16
ADAMTSL:	ENSG0000	3.15	11.359	-1.574	3.48E-25
ADAT2	ENSG0000	1.23	3.685	-1.071	1.97E-11
ADCK2	ENSG0000	14.49	5.585	1.234	6.84E-62
ADCY1	ENSG0000	0.4	4.115	-1.869	5.43E-29
ADGRA3	ENSG0000	8.9	21.525	-1.186	8.54E-24
ADGRG7	ENSG0000	0.72	4.425	-1.657	4.94E-46
ADH1B	ENSG0000	360.135	884.396	-1.294	5.15E-11
ADH4	ENSG0000	154.032	549.147	-1.827	2.35E-13
ADIPOR1	ENSG0000	66.791	32.8	1.004	1.88E-43
ADM2	ENSG0000	2.8	0.75	1.119	3.37E-20
ADRA1A	ENSG0000	0.75	16.38	-3.312	2.62E-82
ADRA1B	ENSG0000	1.21	3.855	-1.135	8.39E-31
ADRA2B	ENSG0000	0.41	1.885	-1.033	4.15E-50
ADSL	ENSG0000	31.91	14.58	1.079	1.69E-44
AF064858.	ENSG0000	0.81	3.285	-1.243	3.50E-13
AFG3L1P	ENSG0000	3.2	8.22	-1.134	2.33E-10
AFM	ENSG0000	50.38	143.334	-1.49	1.15E-19
AFP	ENSG0000	11.46	4.685	1.132	2.19E-11
AGAP4	ENSG0000	2.28	5.63	-1.015	5.30E-08
AGAP5	ENSG0000	1.11	3.235	-1.005	3.29E-11
AGAP6	ENSG0000	3.38	12.338	-1.607	1.98E-13
AGAP9	ENSG0000	2.16	10.75	-1.895	3.89E-19
AGBL5	ENSG0000	8.21	3.445	1.051	2.57E-39
AGER	ENSG0000	1.41	4.07	-1.073	5.63E-10
AGPAT1	ENSG0000	46.821	21.794	1.069	7.90E-49
AGXT2	ENSG0000	13.63	35.03	-1.3	1.27E-23
AHSA2	ENSG0000	10.42	24.35	-1.15	2.24E-02
AIDA	ENSG0000	24.519	10.875	1.104	1.27E-46
AIM1L	ENSG0000	1.81	0.17	1.264	1.32E-33
AIMP2	ENSG0000	27.651	13.26	1.007	1.33E-32
AKAP17A	ENSG0000	10.16	25.474	-1.246	3.02E-09
AKIP1	ENSG0000	12.78	5.335	1.121	7.69E-50
AKR1B10	ENSG0000	177.158	3.015	5.472	9.84E-30
AKR1C1	ENSG0000	481.068	212.636	1.174	9.13E-08
AKR1C2	ENSG0000	299.123	125.148	1.25	8.98E-05
AKR1C3	ENSG0000	351.236	89.588	1.959	3.03E-46
AKR1C8P	ENSG0000	0.26	1.74	-1.121	2.75E-20
AKR1D1	ENSG0000	10.96	40.685	-1.801	1.01E-11
AKR7L	ENSG0000	4.43	14.072	-1.473	1.15E-28
AL158801.	ENSG0000	2.19	0.275	1.323	4.13E-33
AL161668.	ENSG0000	1.63	5.785	-1.367	3.53E-22
AL162151.	ENSG0000	202.151	43.611	2.187	1.42E-02
ALDH1A1	ENSG0000	458.284	217.994	1.068	2.39E-10
ALDH3A1	ENSG0000	2.17	0.46	1.119	7.82E-12
ALDH8A1	ENSG0000	27.029	64.679	-1.229	3.10E-22
ALDOA	ENSG0000	289.595	130.929	1.139	1.02E-26
ALG1	ENSG0000	19.83	8.77	1.092	3.96E-57
ALG1L	ENSG0000	7.27	0.325	2.642	9.46E-43
ALG3	ENSG0000	57.289	26.005	1.11	3.84E-51
ALG8	ENSG0000	45.711	19.841	1.164	3.48E-66
ALKBH2	ENSG0000	25.259	11.915	1.024	4.09E-37
ALMS1	ENSG0000	5	1.79	1.105	1.28E-57
ALOX12P2	ENSG0000	1.39	6.128	-1.577	2.52E-25
ALPL	ENSG0000	10.67	40.58	-1.833	1.71E-26
ALYREF	ENSG0000	37.749	13.9	1.379	1.14E-52
AMDHD1	ENSG0000	23.48	51.479	-1.1	6.39E-15
AMN	ENSG0000	9.84	21.844	-1.075	1.73E-06

ANAPC11	ENSG0000	192.35	76.061	1.327	1.35E-65
ANGPTL6	ENSG0000	1.67	11.6	-2.238	1.35E-96
ANK3	ENSG0000	1.01	3.435	-1.142	6.39E-15
ANKRD29	ENSG0000	3.48	0.61	1.476	6.70E-29
ANKRD39	ENSG0000	14.36	6.27	1.079	4.26E-64
ANLN	ENSG0000	2.26	0.175	1.472	2.75E-40
ANO10	ENSG0000	10.95	4.33	1.165	1.20E-42
ANXA10	ENSG0000	10.59	34.34	-1.608	2.59E-18
ANXA11	ENSG0000	86.99	38.454	1.157	3.81E-43
ANXA13	ENSG0000	7.52	2.45	1.304	2.16E-08
ANXA2	ENSG0000	254.866	63.065	1.998	1.96E-44
ANXA2P2	ENSG0000	4.42	0.755	1.627	1.14E-37
AP000349.	ENSG0000	1.28	0.03	1.146	1.78E-25
AP000355.	ENSG0000	0.63	2.28	-1.009	1.62E-16
AP003391.	ENSG0000	2.06	0	1.614	1.08E-03
AP006285.	ENSG0000	0.47	2.525	-1.262	4.77E-13
AP006285.	ENSG0000	0.06	1.265	-1.095	2.02E-45
AP1M2	ENSG0000	2.83	0.56	1.296	4.23E-15
AP1S1	ENSG0000	45.54	17.135	1.36	4.79E-61
AP3B1	ENSG0000	16.49	6.325	1.256	6.45E-72
APIP	ENSG0000	18.439	8.705	1.002	1.29E-36
APLN	ENSG0000	1.59	0.1	1.235	3.06E-34
APOA1BP	ENSG0000	120.684	38.634	1.618	9.18E-90
APOA2	ENSG0000	31278.85	10880.07	1.523	9.24E-04
APOA4	ENSG0000	3.03	26.19	-2.754	1.79E-07
APOA5	ENSG0000	97.267	211.834	-1.115	5.78E-13
APOBEC3E	ENSG0000	2.34	0.22	1.453	2.35E-29
APOC2	ENSG0000	2260.615	475.779	2.246	3.11E-13
APOE	ENSG0000	5880.405	2771.689	1.085	8.27E-14
APOF	ENSG0000	17.701	113.668	-2.616	2.62E-38
APOLD1	ENSG0000	4.3	1.215	1.259	1.19E-25
AQP3	ENSG0000	24.599	60.915	-1.274	4.29E-21
ARF1	ENSG0000	269.025	132.775	1.013	3.51E-42
ARF3	ENSG0000	30.759	14.79	1.008	1.65E-30
ARGLU1	ENSG0000	17.771	38.953	-1.09	4.10E-06
ARHGAP1(	ENSG0000	1.86	5.19	-1.114	4.07E-22
ARHGEF39	ENSG0000	1.81	0.4	1.005	1.83E-36
ARL2	ENSG0000	24.729	8.94	1.372	6.90E-39
ARPC1A	ENSG0000	87.772	39.045	1.148	8.56E-59
ARPC1B	ENSG0000	99.181	38.33	1.349	1.13E-27
ARPC3	ENSG0000	174.634	81.089	1.097	8.44E-55
ARPC5	ENSG0000	86.762	29.82	1.51	3.85E-56
ARRDC2	ENSG0000	15.5	7.085	1.029	1.11E-14
ARSE	ENSG0000	49.9	23	1.085	9.66E-07
ARSEP1	ENSG0000	2.5	0.09	1.683	1.24E-10
ARV1	ENSG0000	17.67	8.02	1.049	3.26E-45
ASF1B	ENSG0000	3.27	0.28	1.738	7.91E-48
ASMTL-AS	ENSG0000	3.23	11.1	-1.516	3.17E-06
ASNA1	ENSG0000	43.761	17.964	1.239	1.73E-56
ASPDH	ENSG0000	33.751	71.634	-1.064	4.55E-11
ASPG	ENSG0000	7.77	75.475	-3.124	8.33E-41
ASPH	ENSG0000	41.04	16.18	1.291	4.24E-21
ASPM	ENSG0000	2.96	0.125	1.816	8.72E-54
ATAD2	ENSG0000	6.91	1.855	1.47	1.16E-38
ATF3	ENSG0000	14.94	36.796	-1.246	2.39E-11
ATF5	ENSG0000	145.946	767.753	-2.387	6.51E-30
ATHL1	ENSG0000	9.89	26.485	-1.336	7.22E-03
ATIC	ENSG0000	66.201	32.119	1.021	1.34E-34

ATOH8	ENSG0000	4.42	18.95	-1.88	2.47E-30
ATOX1	ENSG0000	347.29	114.178	1.596	6.63E-63
ATP1A1	ENSG0000	136.342	51.872	1.377	1.61E-31
ATP1B1	ENSG0000	97.951	37.295	1.37	1.12E-29
ATP1B3	ENSG0000	21.14	8.2	1.267	1.30E-18
ATP5E	ENSG0000	77.471	33.549	1.184	5.86E-58
ATP5G1	ENSG0000	239.967	89.216	1.417	2.90E-65
ATP5G2	ENSG0000	428.751	189.499	1.174	3.40E-74
ATP5H	ENSG0000	378.119	168.709	1.16	5.68E-70
ATP5J2	ENSG0000	584.111	238.425	1.289	2.71E-73
ATP5J2-PT	ENSG0000	3.96	1.075	1.257	3.69E-31
ATP5L	ENSG0000	304.183	126.381	1.261	4.57E-59
ATP6AP1	ENSG0000	65.639	24.015	1.414	1.58E-72
ATP6V0B	ENSG0000	142.339	68.669	1.041	8.52E-40
ATP6V0E1	ENSG0000	207.691	99.105	1.06	2.92E-53
ATP6V1C1	ENSG0000	16.8	6.985	1.157	5.23E-30
ATP6V1D	ENSG0000	38.409	18.475	1.017	3.53E-39
ATP6V1E1	ENSG0000	67.568	28.939	1.196	3.07E-53
ATP6V1F	ENSG0000	102.047	36.464	1.46	5.33E-67
ATP6V1H	ENSG0000	23.62	11.17	1.016	1.12E-44
ATRAID	ENSG0000	74.109	34.835	1.068	2.28E-55
ATRN	ENSG0000	27.481	10.28	1.336	3.10E-24
AURKA	ENSG0000	7.35	0.99	2.069	2.38E-56
AURKB	ENSG0000	5.25	0.53	2.03	3.98E-50
AUTS2	ENSG0000	3.86	9.375	-1.094	2.28E-16
AVPR1A	ENSG0000	0.47	5.405	-2.123	1.46E-24
AZGP1	ENSG0000	569.518	1221.805	-1.1	5.97E-16
AZGP1P1	ENSG0000	6.43	19.275	-1.448	8.77E-28
B3GAT1	ENSG0000	0.11	1.63	-1.245	5.52E-11
B3GNT3	ENSG0000	2.33	0.495	1.155	1.36E-10
B3GNT5	ENSG0000	2.3	0.45	1.186	1.39E-19
B9D1	ENSG0000	10.26	3.88	1.206	3.33E-37
BAG6	ENSG0000	179.805	88.398	1.016	1.81E-55
BAMBI	ENSG0000	13.81	6.22	1.036	1.44E-05
BANF1	ENSG0000	152.165	69.61	1.117	1.06E-68
BAX	ENSG0000	78.71	24.245	1.659	1.38E-59
BBOX1	ENSG0000	2.69	11.375	-1.746	1.48E-11
BCAM	ENSG0000	57.46	21.915	1.351	2.50E-22
BCAP31	ENSG0000	202.657	70.416	1.512	1.98E-72
BCAS4	ENSG0000	1.85	0.41	1.015	2.00E-31
BCHE	ENSG0000	17.321	38.95	-1.125	8.65E-13
BCL2L12	ENSG0000	19.401	8.03	1.176	9.33E-50
BCL3	ENSG0000	31.12	75.011	-1.243	2.34E-15
BCL6	ENSG0000	10.78	24.681	-1.124	5.88E-13
BCO2	ENSG0000	2.42	31.444	-3.246	8.54E-76
BEX2	ENSG0000	2.48	0.685	1.046	1.26E-11
BGN	ENSG0000	56.79	133.176	-1.215	1.42E-13
BHMT	ENSG0000	58.582	197.176	-1.734	1.51E-11
BIRC5	ENSG0000	6.81	0.505	2.376	1.57E-54
BLOC1S1	ENSG0000	213.441	94.87	1.161	2.56E-73
BLVRA	ENSG0000	19.46	3.145	2.303	3.46E-46
BMPER	ENSG0000	0.06	1.515	-1.246	1.97E-87
BMS1P8	ENSG0000	1.92	0.11	1.395	1.45E-28
BOLA1	ENSG0000	26.79	11.355	1.169	3.78E-58
BOLA2	ENSG0000	10.8	2.74	1.658	3.31E-53
BOLA2B	ENSG0000	84.67	20.26	2.011	1.17E-74
BOLA3	ENSG0000	36.339	13.22	1.393	4.46E-65
BOP1	ENSG0000	28.041	9.04	1.532	7.46E-50

BPGM	ENSG0000	8.15	3.535	1.013	3.91E-40
BPNT1	ENSG0000	13.67	6.01	1.065	1.24E-31
BRIX1	ENSG0000	13	5.58	1.089	2.97E-39
BRMS1	ENSG0000	30.751	11.85	1.305	3.54E-69
BROX	ENSG0000	10.95	4.735	1.059	1.51E-45
BSG	ENSG0000	301.329	101.335	1.563	1.20E-64
BTF3	ENSG0000	338.66	168.376	1.004	8.92E-46
BTG3	ENSG0000	8.55	3.095	1.222	8.12E-29
BUB1	ENSG0000	1.72	0.11	1.293	3.44E-40
BUB1B	ENSG0000	1.6	0.16	1.164	1.37E-34
BUD31	ENSG0000	64.382	29.16	1.116	6.44E-52
BX842568.	ENSG0000	0.4	2.485	-1.316	3.16E-30
BYSL	ENSG0000	12.53	4.635	1.264	2.49E-42
BZW2	ENSG0000	15.3	6.63	1.095	3.77E-34
C10orf35	ENSG0000	3.84	1.375	1.027	1.80E-18
C11orf96	ENSG0000	6.79	26.472	-1.818	1.04E-24
C11orf98	ENSG0000	63.592	30.65	1.029	1.59E-48
C12orf45	ENSG0000	19.5	8.885	1.052	3.14E-58
C12orf73	ENSG0000	7.32	2.955	1.073	9.38E-59
C12orf75	ENSG0000	6.37	1.335	1.658	2.87E-18
C14orf105	ENSG0000	5.67	19.307	-1.606	5.36E-20
C14orf166	ENSG0000	107.94	52.815	1.017	2.38E-65
C14orf180	ENSG0000	0.01	1.21	-1.13	2.10E-53
C15orf39	ENSG0000	4.46	1.5	1.127	5.00E-32
C15orf40	ENSG0000	15.5	7.065	1.033	4.08E-49
C15orf48	ENSG0000	3.02	0.55	1.375	7.15E-16
C16orf59	ENSG0000	2.19	0.37	1.219	1.35E-39
C17orf49	ENSG0000	55.241	23.97	1.171	1.71E-42
C17orf58	ENSG0000	10.87	4.22	1.185	4.39E-59
C17orf89	ENSG0000	74.47	35.49	1.048	1.48E-45
C19orf48	ENSG0000	26.67	11.515	1.145	7.86E-38
C19orf53	ENSG0000	130.861	56.883	1.188	5.48E-67
C19orf66	ENSG0000	42.679	100.513	-1.217	3.13E-30
C1orf106	ENSG0000	1.81	0.26	1.157	1.60E-19
C1orf168	ENSG0000	3.31	11.729	-1.562	6.25E-32
C1orf198	ENSG0000	12.46	4.765	1.223	7.64E-27
C1orf228	ENSG0000	1.79	6.188	-1.365	4.05E-27
C1orf35	ENSG0000	12.7	5.775	1.016	1.91E-48
C1orf43	ENSG0000	182.214	81.021	1.159	4.33E-62
C1orf54	ENSG0000	7.23	2.655	1.171	3.55E-24
C1QA	ENSG0000	81.492	39.245	1.035	1.34E-09
C1QTNF1	ENSG0000	4.54	17.865	-1.768	5.91E-25
C1QTNF5	ENSG0000	11.43	5.08	1.032	2.00E-19
C1R	ENSG0000	414.692	877.009	-1.079	1.34E-22
C1RL-AS1	ENSG0000	1.81	5.113	-1.121	6.51E-12
C20orf27	ENSG0000	22.11	9.443	1.146	4.31E-49
C2orf54	ENSG0000	6.44	2.305	1.171	4.81E-06
C3P1	ENSG0000	11.36	51.089	-2.075	5.67E-42
C4A-AS1	ENSG0000	2.72	10.738	-1.658	2.36E-24
C4B-AS1	ENSG0000	2.72	10.738	-1.658	2.36E-24
C6	ENSG0000	47.422	120.137	-1.323	5.41E-20
C6orf1	ENSG0000	20.87	9.29	1.088	3.18E-43
C6orf47	ENSG0000	10.63	4.24	1.15	4.29E-35
C6orf48	ENSG0000	78.378	31.529	1.287	4.33E-40
C6orf89	ENSG0000	20.281	9.085	1.077	1.24E-38
C7	ENSG0000	5.88	49.485	-2.875	7.22E-31
C7orf50	ENSG0000	47.619	20.535	1.175	3.79E-50
C8A	ENSG0000	73.338	173.446	-1.231	4.02E-19

C8orf33	ENSG0000	18.379	7.305	1.223	4.89E-48
C8orf4	ENSG0000	12.81	58.63	-2.11	1.62E-36
C8orf46	ENSG0000	1.62	5.51	-1.313	2.86E-14
C8orf59	ENSG0000	87.069	34.17	1.324	3.10E-61
C8orf76	ENSG0000	19.81	8.225	1.174	8.54E-53
C9	ENSG0000	23.051	263.854	-3.461	3.80E-35
C9orf16	ENSG0000	50.801	23.865	1.059	2.63E-38
CACNA1H	ENSG0000	1.51	5.3	-1.328	2.70E-03
CACNB2	ENSG0000	0.74	2.575	-1.039	1.27E-15
CACYBP	ENSG0000	43.419	15.51	1.428	6.64E-39
CADM1	ENSG0000	40.07	14.41	1.414	1.01E-20
CALM3	ENSG0000	104.213	50.399	1.033	1.35E-34
CALR	ENSG0000	813.782	337.302	1.268	2.50E-47
CAMK2B	ENSG0000	0.24	2.31	-1.416	1.36E-26
CANT1	ENSG0000	14.81	6.46	1.084	2.58E-39
CANX	ENSG0000	179.83	85.843	1.058	6.23E-29
CAP2	ENSG0000	8.86	1.345	2.072	3.05E-52
CAPG	ENSG0000	26.6	3.725	2.546	7.53E-36
CAPN2	ENSG0000	26.33	11.385	1.142	1.95E-25
CAPN3	ENSG0000	3.25	13.15	-1.735	2.72E-26
CAPNS1	ENSG0000	328.51	144.006	1.184	3.25E-61
CAPRIN1	ENSG0000	35.17	17.019	1.005	9.85E-33
CASC5	ENSG0000	3.48	1.025	1.146	7.01E-22
CASK	ENSG0000	8.9	3.435	1.159	1.41E-26
CAV1	ENSG0000	11.49	4.81	1.104	7.21E-21
CBFA2T3	ENSG0000	0.57	2.535	-1.171	1.29E-39
CBR1	ENSG0000	181.056	75.123	1.258	1.46E-21
CBS	ENSG0000	4.02	13.905	-1.57	8.68E-05
CBX1	ENSG0000	14.33	5.675	1.2	2.02E-35
CBX8	ENSG0000	4.54	1.57	1.108	1.07E-43
CCBE1	ENSG0000	0.06	1.185	-1.044	3.49E-54
CCDC107	ENSG0000	40.179	15.745	1.298	1.88E-61
CCDC167	ENSG0000	49.179	20.844	1.2	8.38E-48
CCDC28B	ENSG0000	3.8	1.31	1.055	2.88E-26
CCDC34	ENSG0000	5.74	1.22	1.602	1.03E-51
CCDC64	ENSG0000	4.25	1.51	1.065	1.30E-13
CCDC80	ENSG0000	4.3	1.31	1.198	5.83E-10
CCDC84	ENSG0000	6.33	15.905	-1.206	2.85E-05
CCDC86	ENSG0000	12.32	4.795	1.201	1.91E-38
CCHCR1	ENSG0000	11.72	4.52	1.204	6.93E-41
CCL14	ENSG0000	7.25	46.812	-2.535	1.04E-34
CCL15	ENSG0000	29.421	9.305	1.562	1.09E-23
CCL18	ENSG0000	2.04	0.51	1.01	1.72E-11
CCL19	ENSG0000	2.7	8.89	-1.418	1.65E-06
CCL20	ENSG0000	22.42	1.5	3.228	7.05E-19
CCL21	ENSG0000	7.73	17.895	-1.114	1.21E-05
CCL23	ENSG0000	0.22	2.155	-1.371	1.11E-67
CCNA2	ENSG0000	2.96	0.455	1.445	1.85E-36
CCNB1	ENSG0000	14.8	0.9	3.056	1.24E-71
CCNB2	ENSG0000	4.81	0.27	2.194	3.46E-57
CCNE1	ENSG0000	1.55	0.22	1.064	5.34E-30
CCNL1	ENSG0000	21.84	60.633	-1.432	8.69E-22
CCNL2	ENSG0000	33.029	101.006	-1.584	1.99E-10
CCT3	ENSG0000	185.734	51.09	1.842	3.78E-87
CCT4	ENSG0000	57.519	25.355	1.151	2.01E-54
CCT5	ENSG0000	82.509	34.429	1.237	3.35E-42
CCT6A	ENSG0000	83.169	34.536	1.244	1.14E-46
CCT7	ENSG0000	105.829	49.213	1.089	1.59E-57

CD151	ENSG0000	152.704	70.965	1.095	6.23E-29
CD163L1	ENSG0000	2.63	0.49	1.285	1.64E-30
CD1D	ENSG0000	1.31	5.54	-1.501	6.84E-32
CD24	ENSG0000	50.85	6.745	2.743	5.43E-13
CD248	ENSG0000	3.79	1.195	1.126	1.40E-25
CD2BP2	ENSG0000	24.97	9.89	1.254	4.12E-56
CD320	ENSG0000	20.33	9.475	1.026	1.42E-40
CD34	ENSG0000	14.17	1.625	2.531	8.03E-94
CD36	ENSG0000	16.32	7.178	1.083	7.68E-10
CD3D	ENSG0000	4.12	1.295	1.158	2.24E-16
CD5L	ENSG0000	1.35	3.725	-1.008	2.12E-11
CD63	ENSG0000	685.588	273.832	1.321	1.84E-54
CD74	ENSG0000	753.727	185.354	2.018	1.25E-25
CDA	ENSG0000	15.42	32.819	-1.042	2.27E-14
CDC123	ENSG0000	33.16	16.075	1	6.77E-45
CDC20	ENSG0000	9.32	0.31	2.978	3.93E-63
CDC25C	ENSG0000	1.84	0.055	1.429	3.83E-53
CDC45	ENSG0000	3.07	0.32	1.624	7.92E-45
CDC6	ENSG0000	2.44	0.365	1.334	8.64E-29
CDCA3	ENSG0000	5.67	0.685	1.985	4.65E-50
CDCA5	ENSG0000	3.89	0.275	1.939	6.90E-56
CDCA8	ENSG0000	2.69	0.29	1.516	7.21E-44
CDH13	ENSG0000	3.25	0.32	1.687	2.95E-66
CDHR2	ENSG0000	1.82	16.165	-2.606	1.02E-28
CDK1	ENSG0000	6.32	0.33	2.46	7.38E-61
CDK11A	ENSG0000	11.85	26.269	-1.086	7.63E-07
CDK4	ENSG0000	47.33	19.38	1.246	1.22E-41
CDK5	ENSG0000	12.8	4.67	1.283	5.06E-63
CDKN2A	ENSG0000	10.79	0.315	3.164	9.92E-63
CDKN2AIP	ENSG0000	16.91	6.3	1.295	5.74E-71
CDKN2B	ENSG0000	2.03	0.37	1.145	2.24E-30
CDKN2C	ENSG0000	7.91	1.415	1.883	4.49E-54
CDKN3	ENSG0000	10.96	0.45	3.044	4.14E-74
CDT1	ENSG0000	3.68	0.265	1.887	1.30E-49
CENPA	ENSG0000	2.09	0.12	1.464	6.70E-44
CENPF	ENSG0000	6.14	0.295	2.463	1.12E-53
CENPH	ENSG0000	3.04	0.59	1.345	7.84E-51
CENPK	ENSG0000	1.51	0.24	1.017	2.37E-30
CENPL	ENSG0000	1.82	0.34	1.073	8.02E-41
CENPM	ENSG0000	5.97	0.33	2.39	8.75E-58
CENPN	ENSG0000	6.1	2.08	1.205	4.90E-38
CENPU	ENSG0000	4.69	0.885	1.594	4.48E-42
CENPW	ENSG0000	8.1	1.1	2.115	3.78E-62
CEP131	ENSG0000	5.98	2.185	1.132	3.42E-49
CES3	ENSG0000	7.32	16.224	-1.05	1.23E-13
CES4A	ENSG0000	1.93	10.081	-1.919	1.34E-22
CETN2	ENSG0000	20.93	9.26	1.096	3.54E-44
CFHR3	ENSG0000	33.541	174.664	-2.346	3.33E-15
CFL1	ENSG0000	437.093	184.272	1.242	1.62E-54
CFP	ENSG0000	2.32	8.934	-1.581	3.12E-36
CGREF1	ENSG0000	9.8	4.105	1.081	7.12E-12
CH17-13I2	ENSG0000	1.3	0.105	1.058	2.82E-10
CH17-340	ENSG0000	9.38	4.11	1.022	8.62E-42
CHAF1A	ENSG0000	5.12	1.39	1.357	4.33E-53
CHCHD3	ENSG0000	24.45	10.515	1.144	7.26E-34
CHCHD6	ENSG0000	8.61	3.59	1.066	5.59E-48
CHEK1	ENSG0000	2.38	0.48	1.191	1.77E-38
CHMP2A	ENSG0000	166.352	74.886	1.141	3.08E-69

CHMP4B	ENSG0000	57.181	27.285	1.041	1.47E-42
CHRD	ENSG0000	9.55	72.098	-2.793	1.16E-30
CHRNA4	ENSG0000	0.33	12.095	-3.3	9.74E-29
CHST4	ENSG0000	0.03	1.585	-1.327	1.28E-18
CIDEB	ENSG0000	62.721	137.997	-1.125	1.22E-23
CITED2	ENSG0000	16.549	36.75	-1.105	3.51E-15
CKAP2	ENSG0000	3.09	0.855	1.141	2.09E-33
CKAP4	ENSG0000	32.45	11.11	1.466	9.61E-39
CKB	ENSG0000	26.379	10.115	1.301	4.73E-13
CKLF	ENSG0000	29.829	9.115	1.608	2.26E-62
CKS1B	ENSG0000	71.878	24.695	1.504	3.76E-61
CKS1BP3	ENSG0000	5.44	0.475	2.126	1.92E-51
CKS2	ENSG0000	31.349	8.62	1.75	3.56E-45
CLASRP	ENSG0000	20.34	45.469	-1.123	2.99E-03
CLDN10	ENSG0000	0.1	1.76	-1.327	6.13E-06
CLDN15	ENSG0000	24.411	8.805	1.374	1.73E-16
CLDN2	ENSG0000	5.48	16.855	-1.462	1.92E-05
CLDN7	ENSG0000	35.24	14.474	1.228	2.16E-08
CLEC14A	ENSG0000	7.72	3.055	1.105	1.10E-24
CLEC1B	ENSG0000	0.03	8.658	-3.229	1.07E-97
CLEC4G	ENSG0000	0.12	23.634	-4.459	5.34E-114
CLEC4M	ENSG0000	0.02	18.949	-4.29	1.36E-146
CLGN	ENSG0000	2.2	0.6	1	5.64E-11
CLIC1	ENSG0000	143.867	43.331	1.708	1.91E-47
CLN6	ENSG0000	19.69	9.18	1.023	2.95E-51
CLRN3	ENSG0000	4	14.575	-1.639	1.60E-12
CLTA	ENSG0000	122.402	57.029	1.089	1.99E-50
CMSS1	ENSG0000	13.65	5.695	1.13	5.50E-47
CMTM7	ENSG0000	8.1	3.55	1	1.06E-15
CNDP1	ENSG0000	0.49	17.989	-3.672	1.82E-56
CNIH4	ENSG0000	32.81	10.014	1.618	1.20E-64
CNPY2	ENSG0000	165.673	75.695	1.12	4.93E-71
CNTFR	ENSG0000	0.49	3.555	-1.612	1.21E-06
CNTN3	ENSG0000	0.08	2.035	-1.491	2.99E-40
COA3	ENSG0000	130.119	62.989	1.035	3.77E-45
COA6	ENSG0000	53.471	13.715	1.888	2.18E-88
COCH	ENSG0000	1.42	0.18	1.036	1.03E-21
COL15A1	ENSG0000	3.03	0.17	1.784	4.85E-52
COL1A1	ENSG0000	28.3	10.504	1.349	2.14E-09
COL1A2	ENSG0000	29.91	12.124	1.236	1.68E-09
COL27A1	ENSG0000	6.03	16.685	-1.331	4.27E-10
COL4A1	ENSG0000	26.299	4.465	2.321	3.62E-43
COL4A2	ENSG0000	30.359	7.015	1.968	1.19E-37
COLEC10	ENSG0000	0.39	12.865	-3.318	1.21E-96
COLEC11	ENSG0000	18.71	53.509	-1.468	2.49E-20
COMMD4	ENSG0000	110.17	35.546	1.605	1.73E-97
COMMD5	ENSG0000	24.38	9.165	1.32	5.36E-65
COPA	ENSG0000	47.009	19.744	1.211	6.92E-30
COPG2	ENSG0000	6	2.18	1.138	8.90E-34
COPS6	ENSG0000	83.707	38.084	1.116	9.69E-69
COPZ1	ENSG0000	81.882	40.161	1.01	1.86E-49
CORO1B	ENSG0000	47.55	23.209	1.004	1.49E-42
COX20	ENSG0000	40.969	18.425	1.111	2.80E-44
COX4I2	ENSG0000	3.1	0.33	1.624	4.93E-71
COX6A1	ENSG0000	639.1	304.795	1.066	4.56E-61
COX6A2	ENSG0000	2.46	6.19	-1.055	4.48E-09
COX6B1	ENSG0000	407.285	148.543	1.449	4.41E-65
COX6B1P4	ENSG0000	0	1.055	-1.039	8.51E-76

COX6C	ENSG0000	582.17	237.502	1.29	3.06E-60
COX7A2	ENSG0000	317.364	149.805	1.078	3.75E-45
COX7B	ENSG0000	168.51	73.211	1.192	4.09E-56
COX7C	ENSG0000	750.183	309.093	1.276	2.01E-63
COX7CP1	ENSG0000	0	1.532	-1.34	6.12E-13
COX8A	ENSG0000	396.56	187.065	1.08	2.35E-54
CPD	ENSG0000	16.511	6.295	1.263	8.17E-23
CPE	ENSG0000	15.74	4.685	1.558	2.12E-24
CPEB3	ENSG0000	1.11	3.71	-1.158	7.09E-40
CPED1	ENSG0000	3.01	7.33	-1.055	2.93E-23
CPQ	ENSG0000	47.94	14.525	1.656	4.21E-58
CPSF3	ENSG0000	13.29	5.94	1.042	1.43E-58
CPT1B	ENSG0000	5.21	13.954	-1.268	5.88E-07
CPVL	ENSG0000	14.5	2.755	2.045	3.69E-20
CRHBP	ENSG0000	0.56	16.829	-3.515	3.43E-75
CRIP1	ENSG0000	26.359	7.01	1.772	1.06E-23
CRNDE	ENSG0000	4.62	0.5	1.906	2.52E-27
CRNKL1	ENSG0000	13.85	5.9	1.106	4.78E-63
CROCCP2	ENSG0000	2.91	7.045	-1.041	5.78E-21
CRP	ENSG0000	251.671	2792.323	-3.467	1.62E-13
CRTAP	ENSG0000	47.31	22.299	1.052	1.27E-22
CSAD	ENSG0000	14.78	44.387	-1.524	5.27E-29
CSE1L	ENSG0000	21.24	8.83	1.178	3.31E-55
CSF3R	ENSG0000	1.43	6.905	-1.702	3.81E-27
CSNK2B	ENSG0000	182.771	79.873	1.184	4.67E-67
CSRNP1	ENSG0000	7.19	23.779	-1.597	3.50E-31
CSTB	ENSG0000	62.012	30.3	1.009	3.64E-26
CSTF2	ENSG0000	4.47	1.56	1.095	1.37E-34
CTB-25B1	ENSG0000	9.2	3.654	1.132	2.22E-49
CTB-50L1	ENSG0000	1.12	5.205	-1.549	6.55E-24
CTB-63M2	ENSG0000	136.882	29.105	2.195	1.57E-20
CTB-79E8.	ENSG0000	0	2.14	-1.651	3.13E-21
CTB-89H1	ENSG0000	1.4	3.94	-1.041	5.23E-13
CTC-246B.	ENSG0000	5.76	1.865	1.238	2.29E-25
CTC-505O	ENSG0000	2.9	11.22	-1.648	1.94E-11
CTC-524C	ENSG0000	0.71	3.425	-1.372	4.47E-21
CTC-529I1	ENSG0000	0.66	3.14	-1.318	2.44E-37
CTD-2240	ENSG0000	0.3	2.59	-1.465	3.83E-54
CTD-2537	ENSG0000	1.46	8.73	-1.984	1.04E-19
CTD-2540	ENSG0000	125.4	57.014	1.124	2.20E-18
CTD-2619.	ENSG0000	1.41	6.11	-1.561	1.97E-20
CTD-2619.	ENSG0000	1.39	8.145	-1.936	1.05E-25
CTD-2619.	ENSG0000	1.95	6.439	-1.334	7.86E-19
CTD-3080	ENSG0000	0.44	3.79	-1.734	1.40E-29
CTD-3092.	ENSG0000	2.47	9.255	-1.563	2.53E-27
CTHRC1	ENSG0000	2.35	0.245	1.428	3.20E-24
CTNNA1	ENSG0000	88.812	42.721	1.039	1.10E-43
CTNNBL1	ENSG0000	45.379	20.99	1.077	2.39E-33
CTSA	ENSG0000	181.974	53.563	1.746	1.71E-70
CTSC	ENSG0000	35.961	14.13	1.289	1.43E-20
CTSD	ENSG0000	769.138	382.084	1.007	1.02E-31
CTSH	ENSG0000	163.46	77.63	1.065	5.46E-19
CTSS	ENSG0000	37.281	13.98	1.354	5.87E-11
CUEDC1	ENSG0000	7.88	3.19	1.084	6.86E-26
CUTA	ENSG0000	288.894	108.548	1.404	1.34E-74
CXCL10	ENSG0000	11.72	1.145	2.568	3.36E-23
CXCL12	ENSG0000	11.66	60.325	-2.276	7.53E-40
CXCL14	ENSG0000	0.19	14.215	-3.676	7.94E-78

CXCL2	ENSG0000	18.181	119.784	-2.655	1.51E-45
CXCL9	ENSG0000	4.18	0.765	1.553	5.77E-14
CXorf36	ENSG0000	2.25	0.32	1.3	1.83E-66
CYB5R1	ENSG0000	47.698	20.285	1.194	2.90E-52
CYC1	ENSG0000	157.324	75.334	1.052	5.84E-48
CYP1A1	ENSG0000	0.97	4.71	-1.535	1.72E-04
CYP1A2	ENSG0000	0.48	44.13	-4.93	3.50E-37
CYP21A1P	ENSG0000	2.25	6.15	-1.137	7.39E-04
CYP21A2	ENSG0000	5.8	14.85	-1.221	6.93E-07
CYP26A1	ENSG0000	0.28	4.96	-2.219	5.10E-42
CYP2A6	ENSG0000	132.862	592.285	-2.148	3.83E-11
CYP2A7	ENSG0000	1.97	29.83	-3.376	7.48E-14
CYP2B6	ENSG0000	15.26	76.219	-2.248	3.21E-25
CYP2B7P	ENSG0000	5.24	14.939	-1.353	1.85E-03
CYP2C19	ENSG0000	0.33	6.81	-2.554	3.42E-45
CYP2C8	ENSG0000	85.017	509.168	-2.568	5.79E-27
CYP2C9	ENSG0000	134.596	470.368	-1.798	1.33E-17
CYP2D7	ENSG0000	19.04	65.65	-1.734	1.53E-12
CYP2E1	ENSG0000	500.525	2526.544	-2.333	3.98E-21
CYP39A1	ENSG0000	3.25	17.415	-2.115	3.87E-28
CYP3A4	ENSG0000	31.139	386.278	-3.591	1.22E-16
CYP3A43	ENSG0000	0.75	2.875	-1.147	1.02E-13
CYP3A5	ENSG0000	100.148	212.607	-1.078	1.23E-05
CYP4A11	ENSG0000	108.217	299.559	-1.46	1.21E-15
CYP4A22	ENSG0000	23.29	77.329	-1.689	1.47E-19
CYP7A1	ENSG0000	10.65	1.939	1.987	7.80E-10
CYP8B1	ENSG0000	34.489	127.326	-1.854	2.61E-21
CYR61	ENSG0000	14.11	53.815	-1.859	8.70E-30
CYSTM1	ENSG0000	72.781	21.57	1.709	6.22E-58
CYTH2	ENSG0000	23.18	9.76	1.168	2.22E-48
DAD1	ENSG0000	207.245	96.907	1.089	1.94E-61
DAP3	ENSG0000	95.378	39.351	1.256	2.69E-68
DAPK2	ENSG0000	4.37	1.105	1.351	2.77E-40
DARS2	ENSG0000	10.48	3.105	1.484	3.52E-50
DAXX	ENSG0000	23.62	10.495	1.099	5.71E-40
DBH	ENSG0000	0.56	10.785	-2.917	5.04E-99
DBH-AS1	ENSG0000	5.1	20.855	-1.841	1.48E-21
DBN1	ENSG0000	4.76	1.49	1.21	5.55E-22
DBNDD1	ENSG0000	11.41	2.12	1.992	7.68E-36
DBNDD2	ENSG0000	8.35	3.465	1.066	1.79E-24
DBP	ENSG0000	18.81	6.13	1.474	2.74E-34
DCAF13	ENSG0000	17.179	6.265	1.323	4.36E-45
DCDC2	ENSG0000	2.38	0.62	1.061	3.39E-11
DCK	ENSG0000	3.95	1.415	1.035	8.38E-27
DCN	ENSG0000	14.87	101.289	-2.688	1.21E-29
DCTN2	ENSG0000	55.341	26.62	1.028	6.03E-34
DCTPP1	ENSG0000	22.201	9.24	1.18	5.91E-53
DDAH2	ENSG0000	45.041	19.78	1.148	3.31E-31
DDOST	ENSG0000	104.22	48.816	1.079	1.42E-35
DDX3Y	ENSG0000	3.68	8.605	-1.037	3.27E-10
DDX41	ENSG0000	59.511	26.05	1.162	1.64E-69
DEFA1	ENSG0000	0.09	2.755	-1.784	7.86E-44
DEFA1B	ENSG0000	0	2.35	-1.744	8.92E-47
DEFA3	ENSG0000	0	1.345	-1.23	2.14E-31
DEPTOR	ENSG0000	9.3	3.985	1.047	4.12E-28
DERL1	ENSG0000	56.191	26.628	1.05	5.19E-37
DES	ENSG0000	0.19	2.725	-1.646	3.52E-34
DHFRP1	ENSG0000	2.31	0.635	1.018	4.52E-10

DHODH	ENSG0000	14.04	49.561	-1.749	3.99E-44
DHRS7	ENSG0000	80.309	37.9	1.064	5.29E-22
DHX9	ENSG0000	41.55	19.855	1.029	8.48E-40
DIO3OS	ENSG0000	0.41	4.344	-1.922	2.75E-26
DIRAS3	ENSG0000	0.25	2.305	-1.403	1.29E-38
DLGAP1-A	ENSG0000	7.94	2.765	1.248	3.17E-30
DLGAP5	ENSG0000	1.41	0.07	1.171	3.76E-38
DLL4	ENSG0000	3.79	1.165	1.146	6.49E-35
DNAJB11	ENSG0000	145.411	63.964	1.172	4.51E-58
DNAJC12	ENSG0000	6.66	21.085	-1.528	1.18E-18
DNAJC19P	ENSG0000	2.02	0.425	1.084	5.55E-20
DNASE1L3	ENSG0000	6.64	39.306	-2.399	3.21E-36
DNASE2	ENSG0000	32.07	13.445	1.195	5.48E-27
DNHD1	ENSG0000	1.45	6.529	-1.62	1.38E-17
DNPH1	ENSG0000	125.6	57.579	1.112	3.92E-46
DOCK5	ENSG0000	2.04	5.98	-1.199	5.82E-19
DOLK	ENSG0000	10.08	4.42	1.032	8.50E-57
DPCD	ENSG0000	7.67	3.15	1.063	3.19E-37
DPH1	ENSG0000	10.36	22.265	-1.034	1.87E-17
DPM3	ENSG0000	133.61	60.894	1.121	1.08E-40
DPP3	ENSG0000	21.58	10.23	1.008	6.32E-37
DPP4	ENSG0000	19.36	5.345	1.682	3.61E-16
DPT	ENSG0000	0.55	6.09	-2.194	5.77E-25
DPY30	ENSG0000	58.651	25.86	1.151	3.29E-71
DRAP1	ENSG0000	101.391	38.361	1.379	4.29E-83
DRG1	ENSG0000	40.159	18.46	1.081	7.30E-57
DSN1	ENSG0000	7.32	2.66	1.185	1.72E-48
DTD1	ENSG0000	10.72	4.72	1.035	4.28E-20
DTL	ENSG0000	2.23	0.145	1.496	1.04E-41
DTNA	ENSG0000	6.14	0.99	1.843	4.13E-27
DTNBP1	ENSG0000	8.48	3.67	1.021	1.58E-32
DTX1	ENSG0000	4.03	11.335	-1.294	1.17E-21
DTX4	ENSG0000	3.77	8.57	-1.005	1.15E-10
DTYMK	ENSG0000	19.68	7.14	1.345	3.89E-55
DUSP1	ENSG0000	76.452	213.619	-1.47	6.33E-21
DUSP23	ENSG0000	131.497	63.76	1.033	3.93E-33
DUSP9	ENSG0000	2.14	0.27	1.306	1.47E-24
DUXAP8	ENSG0000	1.89	0.225	1.238	1.59E-27
DYNC1H1	ENSG0000	65.922	29.81	1.119	3.19E-53
DYNLL1	ENSG0000	246.304	98.267	1.317	1.33E-55
DYNLRB1	ENSG0000	145.139	51.949	1.465	1.38E-80
E2F1	ENSG0000	5.49	0.28	2.342	2.90E-57
EBF4	ENSG0000	2.55	6.54	-1.087	8.38E-07
ECHDC2	ENSG0000	148.549	458.109	-1.618	1.73E-42
ECM1	ENSG0000	2.51	17.755	-2.418	1.30E-63
ECT2	ENSG0000	3	0.61	1.313	4.23E-34
EEF1A1P5	ENSG0000	63.341	26.253	1.239	2.14E-14
EEF1A1P6	ENSG0000	11.7	3.45	1.513	1.01E-30
EEF1A2	ENSG0000	7.09	0.45	2.48	9.75E-14
EEF1D	ENSG0000	621.107	280.43	1.144	2.20E-43
EEF1G	ENSG0000	721.421	356.435	1.015	1.20E-44
EFCAB11	ENSG0000	4.05	1.51	1.009	6.94E-49
EFHD1	ENSG0000	5.26	17.155	-1.536	2.61E-09
EFNA4	ENSG0000	8.27	2.885	1.255	2.00E-46
EFTUD2	ENSG0000	32.139	14.38	1.107	2.23E-57
EGR1	ENSG0000	18.64	44.085	-1.199	1.97E-11
EHD4	ENSG0000	9.79	3.77	1.178	6.04E-22
EHMT2	ENSG0000	23.36	8.135	1.415	1.27E-58

EIF2D	ENSG0000	30.789	13.13	1.17	1.04E-57
EIF3CL	ENSG0000	33.16	14.54	1.136	9.35E-38
EIF3D	ENSG0000	84.829	35.248	1.244	9.48E-55
EIF3E	ENSG0000	194.253	69.545	1.469	1.00E-45
EIF3H	ENSG0000	142.754	54.924	1.362	7.81E-48
EIF3K	ENSG0000	205.429	85.005	1.263	5.18E-70
EIF3M	ENSG0000	133.963	61.922	1.101	2.64E-48
EIF4BP7	ENSG0000	1.68	0.34	1	3.21E-40
EIF5AP4	ENSG0000	3.95	0.16	2.093	9.78E-37
EIF6	ENSG0000	180.305	77.275	1.212	5.07E-65
ELOVL1	ENSG0000	37.6	17.105	1.092	1.59E-39
ELOVL2	ENSG0000	17.6	4.335	1.802	7.73E-15
EMC2	ENSG0000	31.99	14.01	1.136	6.04E-41
EMC3	ENSG0000	74.279	28.66	1.344	1.83E-55
EMC4	ENSG0000	77.299	36.715	1.054	5.22E-67
EMCN	ENSG0000	3.89	1.22	1.139	2.32E-25
EMP1	ENSG0000	7.72	3.295	1.022	2.12E-06
ENAH	ENSG0000	10.65	2.805	1.614	2.54E-29
ENGASE	ENSG0000	5.9	13.544	-1.076	1.47E-04
ENO3	ENSG0000	11.36	58.1	-2.258	4.36E-29
ENPP2	ENSG0000	14.99	3.555	1.812	3.56E-22
ENSA	ENSG0000	109.949	44.47	1.287	1.18E-60
FUCA2	ENSG0000	34.951	16.73	1.02	1.28E-29
SEMA3F	ENSG0000	5.44	1.655	1.278	1.43E-50
LASP1	ENSG0000	64.408	28.585	1.145	4.22E-38
SLC7A2	ENSG0000	14.44	46.174	-1.611	3.88E-24
PLXND1	ENSG0000	36.579	15.975	1.147	2.23E-29
LAMP2	ENSG0000	125.756	60.959	1.033	4.88E-20
TMEM98	ENSG0000	30.38	7.44	1.895	2.53E-21
TMEM132/	ENSG0000	2.42	0.575	1.119	1.31E-17
TNFRSF12/	ENSG0000	46.019	15.247	1.533	8.77E-15
GGCT	ENSG0000	31.26	13.475	1.156	8.64E-56
GTF2IRD1	ENSG0000	7.07	2.745	1.108	1.25E-33
IL32	ENSG0000	748.417	295.25	1.339	1.34E-21
RPS20	ENSG0000	1550.372	610.077	1.344	9.99E-54
TENM1	ENSG0000	0.12	1.3	-1.038	5.58E-26
MLXIPL	ENSG0000	98.7	263.982	-1.41	2.76E-10
METTL13	ENSG0000	18	7.735	1.121	6.04E-57
SEMA3G	ENSG0000	1.93	0.435	1.03	6.60E-27
SLC6A13	ENSG0000	2.29	11.89	-1.97	1.19E-28
NME2	ENSG0000	15.899	5.455	1.389	5.15E-22
SYT7	ENSG0000	6.73	21.18	-1.521	2.42E-13
PSMC4	ENSG0000	71.401	32.394	1.116	7.36E-60
SLC25A39	ENSG0000	125.382	59.779	1.056	2.03E-60
TACC3	ENSG0000	10.26	3.13	1.447	2.48E-40
POLA2	ENSG0000	7.17	2.465	1.238	1.69E-50
NPC1L1	ENSG0000	4.68	13.435	-1.346	1.66E-15
IGF1	ENSG0000	4.77	12.475	-1.224	9.57E-14
RUFY3	ENSG0000	6.88	14.95	-1.017	7.58E-15
SLC11A1	ENSG0000	0.59	2.335	-1.069	5.08E-20
MARCO	ENSG0000	0.39	19.3	-3.868	4.03E-61
HGF	ENSG0000	0.79	6.615	-2.089	6.68E-35
SERPINB1	ENSG0000	26.379	10.953	1.196	4.83E-24
RRAGD	ENSG0000	8.76	3.255	1.198	1.36E-28
NCAPH2	ENSG0000	23.71	10.6	1.091	1.53E-48
VIM	ENSG0000	130.273	52.559	1.293	1.76E-19
RNASET2	ENSG0000	42.86	17.46	1.248	9.02E-33
GRN	ENSG0000	187.857	59.719	1.637	6.44E-71

TMSB10	ENSG0000	859.646	208.556	2.038	1.53E-37
MYOM2	ENSG0000	0.86	4.635	-1.599	2.62E-37
RPL26L1	ENSG0000	43.989	19.929	1.104	1.27E-65
FAM65C	ENSG0000	0.33	7.74	-2.716	8.69E-74
ZIC2	ENSG0000	1.42	0.01	1.261	2.17E-31
TPR	ENSG0000	20	9.455	1.006	7.29E-31
NEDD4L	ENSG0000	25.941	11.405	1.119	1.58E-29
HEXB	ENSG0000	156.096	62.003	1.318	5.25E-66
HOMER3	ENSG0000	7.3	2.49	1.25	2.80E-23
RAD51	ENSG0000	1.75	0.235	1.155	6.48E-39
THOC3	ENSG0000	22.43	9.25	1.193	6.88E-48
PRSS8	ENSG0000	3	10.11	-1.474	3.16E-09
LAMA3	ENSG0000	3.81	0.88	1.355	1.87E-22
PHPT1	ENSG0000	99.787	48.509	1.026	2.25E-41
TARBP1	ENSG0000	14.1	5.62	1.19	5.14E-36
SPAG4	ENSG0000	6.14	2.21	1.154	1.70E-20
POLD1	ENSG0000	13.28	4.905	1.274	6.07E-49
SPA17	ENSG0000	2.84	0.75	1.134	2.96E-32
NGFR	ENSG0000	1.25	7.695	-1.95	2.04E-36
RFXANK	ENSG0000	44.099	17.625	1.276	1.95E-58
OAT	ENSG0000	9.78	26.915	-1.373	7.28E-09
ZNRD1	ENSG0000	25.93	10.03	1.288	1.05E-59
PKM	ENSG0000	38.791	13.675	1.439	7.36E-21
RRP15	ENSG0000	4.32	1.655	1.003	5.88E-26
RHOA	ENSG0000	154.396	75.585	1.021	1.12E-41
IFI35	ENSG0000	35.901	15.2	1.188	3.78E-32
GPKOW	ENSG0000	14.82	6.73	1.033	2.64E-56
PRR11	ENSG0000	1.37	0.14	1.056	2.78E-35
UFD1L	ENSG0000	57.039	23.485	1.245	3.98E-58
PABPC1	ENSG0000	653.57	181.313	1.844	9.41E-59
RPL31	ENSG0000	1529.028	743.015	1.04	2.85E-40
LMCD1	ENSG0000	10.45	3.68	1.291	2.04E-21
WBSCR22	ENSG0000	56.441	25.895	1.095	4.57E-64
TRIP13	ENSG0000	1.55	0.21	1.075	3.15E-33
FAM50A	ENSG0000	61.922	25.359	1.255	1.46E-43
HMMR	ENSG0000	2.54	0.2	1.561	4.19E-43
P4HA2	ENSG0000	15.899	5.81	1.311	1.88E-35
FCGR2B	ENSG0000	1.64	9.1	-1.936	7.25E-22
XRCC1	ENSG0000	13.98	4.955	1.331	6.31E-57
MCM2	ENSG0000	6.75	0.735	2.159	1.21E-49
SMARCE1	ENSG0000	49.05	20.464	1.221	1.03E-43
GSDMB	ENSG0000	9.1	23.562	-1.282	5.33E-06
IGF2BP2	ENSG0000	2.68	0.68	1.131	2.89E-15
NOTCH3	ENSG0000	4.32	0.925	1.467	5.02E-30
TEAD2	ENSG0000	7.46	3.145	1.029	1.47E-13
HACD3	ENSG0000	47.261	19.23	1.254	2.71E-30
TUBE1	ENSG0000	4.17	14.245	-1.56	2.56E-55
NUP37	ENSG0000	10.3	3.445	1.346	1.67E-59
SEC31B	ENSG0000	1.22	5.16	-1.472	4.60E-23
KIFAP3	ENSG0000	9.63	3.71	1.174	1.70E-37
MCM6	ENSG0000	7.88	1.485	1.837	2.31E-49
MCAM	ENSG0000	14.76	3.97	1.665	3.84E-36
UBE2T	ENSG0000	8.81	0.695	2.533	9.85E-75
IL4R	ENSG0000	17.09	36.155	-1.038	2.59E-17
EXOSC5	ENSG0000	22.461	8.788	1.261	1.15E-60
UBE2A	ENSG0000	25.029	11.63	1.043	4.00E-40
FBLN1	ENSG0000	8.62	3.655	1.047	4.88E-07
MAP2	ENSG0000	3.05	0.965	1.043	8.47E-17

PMS2P1	ENSG0000	5.08	2.01	1.014	4.84E-48
PAFAH1B3	ENSG0000	14.81	3.225	1.904	2.54E-43
KCNN2	ENSG0000	0.25	4.35	-2.098	1.91E-53
NDC80	ENSG0000	2.91	0.18	1.728	3.06E-52
JMJD4	ENSG0000	14.75	6.215	1.126	8.15E-59
HSPB11	ENSG0000	21.91	10.425	1.004	5.79E-49
PLOD1	ENSG0000	76.388	36.346	1.051	2.95E-32
SLC27A5	ENSG0000	248.413	497.947	-1	5.86E-10
FAT1	ENSG0000	33.42	11.8	1.427	3.61E-20
HAL	ENSG0000	20.48	54.784	-1.377	3.89E-14
GCKR	ENSG0000	22.119	98.499	-2.106	1.31E-37
IGSF9	ENSG0000	1.64	7.91	-1.755	1.31E-18
TTC39A	ENSG0000	2.76	0.555	1.274	3.15E-21
FTL	ENSG0000	11550.76	5723.571	1.013	5.51E-19
GNAO1	ENSG0000	0.68	7.76	-2.382	4.76E-52
PIR	ENSG0000	23.02	9.2	1.236	1.28E-27
TPX2	ENSG0000	6	0.765	1.988	5.60E-45
SMOX	ENSG0000	4.76	1.82	1.03	1.40E-09
FKBP1A	ENSG0000	88.56	40.05	1.125	5.76E-48
TESC	ENSG0000	7.24	2.13	1.397	5.28E-16
RPL6	ENSG0000	779.064	371.739	1.065	3.44E-53
RPLP0	ENSG0000	1398.049	655.112	1.092	1.85E-44
IGBP1	ENSG0000	20.49	8.45	1.185	2.05E-47
FXYD5	ENSG0000	29.699	13.652	1.067	1.43E-14
GRAMD1A	ENSG0000	18.69	8.595	1.037	7.23E-16
LYZ	ENSG0000	81.413	9.505	2.972	1.14E-24
KIF4A	ENSG0000	1.87	0.07	1.423	4.49E-43
ITGA6	ENSG0000	10.25	2.74	1.589	1.72E-41
ESR1	ENSG0000	0.53	4.47	-1.838	3.35E-35
TMEM101	ENSG0000	23.46	9.185	1.264	7.51E-62
PSME1	ENSG0000	221.797	100.384	1.136	5.05E-56
HAUS4	ENSG0000	21.44	9.115	1.15	4.55E-25
WDR76	ENSG0000	2.02	0.295	1.222	2.31E-41
TBX15	ENSG0000	2.27	7.885	-1.442	1.26E-14
MYL6	ENSG0000	1006.338	476.208	1.078	8.54E-53
UNC13D	ENSG0000	1.63	5.01	-1.192	1.65E-13
SUCO	ENSG0000	6.03	2.25	1.113	2.38E-32
MSH2	ENSG0000	5.18	1.415	1.356	2.42E-46
HOOK2	ENSG0000	8.22	22.89	-1.374	5.02E-07
TMEM38B	ENSG0000	8.39	3.005	1.229	1.28E-35
SMIM24	ENSG0000	1.55	6.257	-1.509	9.69E-03
TREM2	ENSG0000	3.51	0.245	1.857	9.01E-33
TMEM14A	ENSG0000	50.499	22.89	1.108	1.53E-28
HSP90AB1	ENSG0000	400.622	139.516	1.515	4.29E-45
SCD	ENSG0000	205.457	85.176	1.26	1.12E-05
TSPAN15	ENSG0000	5.6	2.11	1.086	1.23E-19
MZF1	ENSG0000	4.09	11.175	-1.258	1.24E-10
IGFALS	ENSG0000	2.83	40.84	-3.449	2.19E-47
GADD45B	ENSG0000	66.472	152.783	-1.189	2.93E-20
SERPIND1	ENSG0000	344.389	146.027	1.232	4.45E-04
MMP11	ENSG0000	5.24	0.35	2.209	9.60E-39
GGT5	ENSG0000	8.17	19.87	-1.186	5.36E-16
SNRPD3	ENSG0000	99.401	46.269	1.087	1.58E-47
PES1	ENSG0000	40.87	19.35	1.041	2.02E-45
LRP5L	ENSG0000	2.81	7.535	-1.164	3.77E-13
PIK3IP1	ENSG0000	7	2.74	1.097	1.30E-17
NHP2L1	ENSG0000	81.379	35.463	1.176	1.60E-59
KDELR3	ENSG0000	9.84	3.98	1.122	1.90E-13

TOMM22	ENSG0000	38.84	17.85	1.08	5.91E-53
MCM5	ENSG0000	21.45	4.025	2.16	2.99E-56
PDGFB	ENSG0000	4.05	1.06	1.294	4.44E-36
ZMAT5	ENSG0000	25.2	10.63	1.172	6.35E-63
SYNGR1	ENSG0000	6.5	2.715	1.014	5.92E-08
SAMM50	ENSG0000	30.89	14.73	1.02	2.94E-36
FOXRED2	ENSG0000	6.45	2.045	1.291	1.30E-29
PVALB	ENSG0000	0.11	1.22	-1	2.77E-41
KCTD17	ENSG0000	4.51	1.625	1.07	2.73E-18
TIMM9	ENSG0000	33.88	14.115	1.206	4.79E-61
GSTZ1	ENSG0000	29.67	81.055	-1.42	1.10E-26
SLC10A1	ENSG0000	33.819	69.567	-1.019	2.91E-07
VRK1	ENSG0000	4.99	1.65	1.177	7.51E-52
PSMB5	ENSG0000	103.421	47.67	1.101	6.32E-71
PSMA6	ENSG0000	123.974	57.615	1.092	8.22E-62
PSME2	ENSG0000	253.755	120.229	1.071	5.98E-45
REC8	ENSG0000	2.28	7.14	-1.311	1.43E-14
PNN	ENSG0000	14.91	32.62	-1.079	5.10E-11
MMP9	ENSG0000	3.01	0.8	1.156	1.18E-15
PYGB	ENSG0000	14.47	5.205	1.318	6.43E-36
GINS1	ENSG0000	1.72	0.2	1.181	2.40E-37
IFT52	ENSG0000	7.39	3.06	1.047	2.12E-37
MYBL2	ENSG0000	4.07	0.19	2.091	3.56E-43
NDRG3	ENSG0000	7.09	2.73	1.117	1.16E-41
PABPC1L	ENSG0000	11.35	31.315	-1.388	3.95E-02
PFDN4	ENSG0000	21.78	8.525	1.258	3.44E-54
PRPF6	ENSG0000	40.029	14.02	1.45	6.06E-85
SLC17A9	ENSG0000	29.769	90.333	-1.57	4.91E-07
HM13	ENSG0000	160.162	70.709	1.168	1.15E-45
MANBAL	ENSG0000	32.901	15.21	1.064	1.33E-61
MAPRE1	ENSG0000	14.98	6.11	1.168	5.40E-28
OXT	ENSG0000	0.13	5.293	-2.477	6.22E-25
PXMP4	ENSG0000	10.38	3.335	1.392	3.71E-58
FAM83D	ENSG0000	4.5	0.855	1.568	1.65E-36
PIGU	ENSG0000	16.75	4.875	1.595	1.06E-81
LIPG	ENSG0000	8.01	21.904	-1.346	2.07E-20
PSMD10	ENSG0000	20.941	8.67	1.182	5.29E-39
SRPX	ENSG0000	0.84	5.53	-1.827	1.85E-35
F9	ENSG0000	42.659	138.457	-1.675	2.08E-23
PLP2	ENSG0000	24.319	8.734	1.379	1.90E-17
SMS	ENSG0000	25.701	11.905	1.049	2.98E-37
HTATSF1	ENSG0000	19.28	8.46	1.1	9.73E-37
TIMP1	ENSG0000	274.601	132.577	1.045	2.16E-02
MAGED2	ENSG0000	63.711	22.045	1.49	1.27E-57
RBM3	ENSG0000	79.539	36.115	1.118	1.99E-23
GLA	ENSG0000	19.061	7.165	1.297	6.62E-36
RGCC	ENSG0000	11.91	2.12	2.049	1.35E-36
MAPK3	ENSG0000	24.54	9.99	1.217	2.16E-53
NUTF2	ENSG0000	55.861	25.134	1.121	1.43E-51
HCFC1R1	ENSG0000	44.622	21.699	1.007	2.88E-40
TAF1C	ENSG0000	6.94	14.985	-1.01	1.46E-07
TCEB2	ENSG0000	295.517	138.164	1.091	2.37E-51
PYCARD	ENSG0000	16.68	4.764	1.617	1.09E-21
MAZ	ENSG0000	114.332	43.406	1.377	1.78E-68
NOMO1	ENSG0000	31.299	13.774	1.128	4.10E-47
OIP5	ENSG0000	1.88	0.17	1.3	3.71E-42
RIPK2	ENSG0000	6.29	2.515	1.052	6.24E-25
LAPTM4B	ENSG0000	34.179	7.56	2.039	7.12E-31

NIPAL2	ENSG0000	5.82	2.35	1.026	1.57E-22
SQLE	ENSG0000	36.309	9.635	1.811	2.09E-25
SLC39A14	ENSG0000	54.681	160.457	-1.536	6.14E-37
MCM4	ENSG0000	11.24	2.725	1.716	1.41E-37
FGL1	ENSG0000	714.652	1526.116	-1.093	5.42E-08
MAN2B1	ENSG0000	55.591	17.704	1.597	1.74E-60
RELB	ENSG0000	13.58	4.209	1.485	2.92E-31
RNASEH2A	ENSG0000	16.151	2.735	2.199	4.56E-77
OLFM2	ENSG0000	31.021	12.169	1.282	6.92E-10
PDCD5	ENSG0000	85.241	41.33	1.027	2.56E-49
RPS16	ENSG0000	1328.515	622.443	1.093	5.50E-47
FBL	ENSG0000	123.391	47.738	1.352	7.33E-50
TBCB	ENSG0000	58.351	26.444	1.113	4.50E-42
RPS19	ENSG0000	1703.043	813.895	1.064	4.40E-40
PTPRS	ENSG0000	0.58	2.84	-1.281	8.57E-09
LIG1	ENSG0000	16.18	6.24	1.247	6.01E-44
PLA2G4C	ENSG0000	16.41	5.25	1.478	3.54E-30
PPP2R1A	ENSG0000	101.412	44.099	1.183	2.18E-57
LILRB5	ENSG0000	1.45	4.175	-1.079	5.91E-27
TFPT	ENSG0000	11.74	4.665	1.169	2.46E-54
PIK3R2	ENSG0000	16.35	6.97	1.122	1.80E-33
TMEM147	ENSG0000	69.05	28.196	1.263	1.04E-50
HAMP	ENSG0000	6.75	669.968	-6.436	3.63E-66
NAMPT	ENSG0000	38.001	86.288	-1.162	1.32E-14
H2AFV	ENSG0000	78.302	31.871	1.271	2.69E-68
TAX1BP1	ENSG0000	42.499	18.625	1.148	4.00E-42
GARS	ENSG0000	59.231	24.455	1.243	2.68E-45
HSPB1	ENSG0000	644.572	168.996	1.925	2.04E-43
PDAP1	ENSG0000	72.978	35.554	1.017	1.75E-36
SNX8	ENSG0000	17.73	5.43	1.542	3.64E-55
NUDT1	ENSG0000	15.59	2.595	2.206	2.77E-77
SERPINE1	ENSG0000	20.831	46.29	-1.115	3.42E-05
PLOD3	ENSG0000	60.601	23.364	1.338	1.88E-53
EZH2	ENSG0000	3.65	1.275	1.031	7.56E-30
LHX2	ENSG0000	0.36	2.33	-1.292	3.44E-33
NPM3	ENSG0000	17.14	5.595	1.46	2.79E-27
UBE2S	ENSG0000	19.39	3.845	2.073	5.29E-67
RPL28	ENSG0000	1238.089	565.741	1.129	5.32E-43
PBLD	ENSG0000	29.59	69.384	-1.202	6.28E-19
KRT23	ENSG0000	2.85	0.21	1.67	2.75E-10
RPL19	ENSG0000	1450.263	637.353	1.185	4.15E-61
RAD51C	ENSG0000	13.68	5.425	1.192	5.70E-52
TRIM16L	ENSG0000	7.1	2.925	1.045	6.52E-17
RASD1	ENSG0000	14.23	43.902	-1.56	8.63E-11
ICAM2	ENSG0000	12.49	5.214	1.118	1.55E-25
SYNGR2	ENSG0000	83.169	40.321	1.026	2.46E-38
LGALS3BP	ENSG0000	160.385	54.264	1.546	2.76E-08
VAT1	ENSG0000	49.37	16.41	1.533	1.56E-44
TMEM97	ENSG0000	38.62	18.5	1.023	7.48E-16
SULT1E1	ENSG0000	1.41	5.65	-1.464	1.08E-08
HGFAC	ENSG0000	11.26	117.783	-3.276	5.22E-35
NCAPG	ENSG0000	2.59	0.115	1.687	1.63E-46
HTATIP2	ENSG0000	77.831	32.464	1.236	1.48E-26
FNBP4	ENSG0000	9.22	20.065	-1.043	2.32E-05
SNX15	ENSG0000	9.93	4.415	1.013	8.06E-43
HPX	ENSG0000	1194.589	2403.131	-1.008	3.35E-11
MDK	ENSG0000	132.339	3.805	4.794	7.57E-81
RPS13	ENSG0000	649.415	322.018	1.01	6.76E-48

NDUFS8	ENSG0000	131.06	64.625	1.009	9.08E-47
VWF	ENSG0000	14.41	1.715	2.505	7.16E-57
PTGES3	ENSG0000	137.758	65.443	1.062	8.02E-42
TNS2	ENSG0000	24.931	67.367	-1.399	2.42E-23
MAGOHB	ENSG0000	17.95	7.98	1.077	7.20E-53
TRPV4	ENSG0000	0.77	2.83	-1.114	2.01E-14
FOXM1	ENSG0000	5.72	0.91	1.815	2.70E-41
RAD51AP1	ENSG0000	1.72	0.29	1.076	1.24E-31
MGP	ENSG0000	34.7	15.225	1.138	2.51E-06
SPSB2	ENSG0000	6.75	2.24	1.258	4.55E-54
SLCO1B3	ENSG0000	1.89	30.546	-3.448	4.57E-31
GYS2	ENSG0000	7.64	28.294	-1.761	1.57E-25
TMEM14C	ENSG0000	101.159	43.904	1.186	2.44E-59
MCM3	ENSG0000	20.53	5.435	1.742	9.00E-46
VNN1	ENSG0000	17.96	39.013	-1.077	3.43E-09
GMNN	ENSG0000	31.111	6.19	2.159	2.77E-59
SLC39A7	ENSG0000	107.157	45.144	1.229	2.79E-43
VEGFA	ENSG0000	53.05	116.073	-1.115	3.43E-07
SLC29A1	ENSG0000	46.571	22.445	1.021	4.68E-19
LAMA4	ENSG0000	4.83	1.38	1.293	2.58E-36
GHR	ENSG0000	7.3	26.178	-1.711	1.09E-29
KIF20A	ENSG0000	2.79	0.13	1.746	9.12E-45
SPARC	ENSG0000	214.881	65.26	1.704	8.74E-29
IK	ENSG0000	56.141	25.39	1.115	5.67E-66
THBS4	ENSG0000	3.68	0.15	2.025	6.22E-40
LMNB1	ENSG0000	7.73	2.21	1.443	2.22E-27
SUB1	ENSG0000	184.144	73.124	1.321	2.45E-61
LIFR	ENSG0000	0.71	3.515	-1.401	4.82E-49
H2AFY	ENSG0000	56.539	24.299	1.185	1.46E-44
PDGFRB	ENSG0000	10.1	4.26	1.077	1.10E-16
USP9Y	ENSG0000	0.4	2.55	-1.342	1.78E-14
RPL24	ENSG0000	884.733	404.767	1.126	5.84E-52
SLC41A3	ENSG0000	18.379	6.215	1.425	5.16E-66
PLSCR4	ENSG0000	5.39	13.155	-1.147	8.18E-31
RRP9	ENSG0000	12.34	5.515	1.034	3.72E-45
VIPR1	ENSG0000	0.56	11.848	-3.042	4.67E-92
SSR3	ENSG0000	89.059	40.774	1.108	4.02E-38
NKTR	ENSG0000	4.61	13.3	-1.35	1.96E-10
SPCS1	ENSG0000	167.846	78.948	1.079	7.46E-50
STEAP3	ENSG0000	19.849	62.63	-1.61	3.11E-40
TP53I3	ENSG0000	20.09	5.15	1.778	2.13E-35
FNDC4	ENSG0000	22.841	53.096	-1.182	9.26E-18
PSMD14	ENSG0000	27.46	11.975	1.133	1.07E-37
PPM1G	ENSG0000	39.769	17.025	1.177	6.71E-53
STAT1	ENSG0000	38.62	17.54	1.096	1.35E-16
PDCL3	ENSG0000	11.29	4.515	1.156	1.68E-53
IL1RL1	ENSG0000	0.08	1.33	-1.109	2.80E-34
UXS1	ENSG0000	15.49	5.405	1.364	1.89E-47
RND3	ENSG0000	9.27	21.98	-1.162	3.53E-24
FAM20B	ENSG0000	8.97	3.605	1.114	6.50E-49
ERRFI1	ENSG0000	67.672	160.379	-1.233	7.82E-19
SCAMP3	ENSG0000	85.793	25.355	1.72	5.81E-83
LAMTOR2	ENSG0000	162.263	71.28	1.176	2.33E-64
UCHL5	ENSG0000	15.85	7.27	1.027	2.61E-26
SRSF11	ENSG0000	25.31	68.614	-1.404	8.39E-11
OLFML3	ENSG0000	2.83	8.29	-1.278	3.70E-14
TMEM9	ENSG0000	78.102	28.145	1.44	6.19E-64
HAO2	ENSG0000	11.13	111.712	-3.216	4.03E-33

GNPAT	ENSG0000	27.72	11.88	1.157	9.80E-51
TSNAX	ENSG0000	14.6	6.555	1.046	5.62E-45
TBCE	ENSG0000	16.95	7.4	1.096	1.50E-51
KMO	ENSG0000	7.17	20.115	-1.37	7.00E-22
HSD11B1	ENSG0000	76.531	189.827	-1.299	4.75E-11
IRF6	ENSG0000	21.24	9.51	1.081	3.04E-07
RSRP1	ENSG0000	16.95	36.53	-1.064	9.46E-03
STMN1	ENSG0000	56.668	6.28	2.986	1.65E-75
MAN1C1	ENSG0000	4.64	13.085	-1.32	1.14E-21
NEK2	ENSG0000	3.21	0.11	1.923	2.38E-51
NENF	ENSG0000	81.311	29.561	1.429	4.92E-71
NSL1	ENSG0000	13.84	6.095	1.065	1.05E-34
TXNDC12	ENSG0000	31.04	14.5	1.048	4.23E-55
MYL12B	ENSG0000	310.295	142.938	1.113	5.47E-42
RPN2	ENSG0000	201.829	74.294	1.43	1.68E-75
SPP1	ENSG0000	75.378	8.524	3.004	2.34E-15
ITGB1BP1	ENSG0000	32.839	12.855	1.288	1.65E-48
PHF19	ENSG0000	6.2	1.995	1.265	3.53E-35
NDUFA8	ENSG0000	57.432	27.36	1.043	3.50E-55
IFI27L2	ENSG0000	12.91	4.625	1.306	1.37E-20
NPC2	ENSG0000	121.456	52.019	1.208	5.03E-45
PPP1R3C	ENSG0000	10.36	28.32	-1.368	1.33E-15
HELLS	ENSG0000	1.83	0.405	1.01	1.27E-29
SFRP5	ENSG0000	0.05	4.03	-2.26	1.01E-24
MTHFD1L	ENSG0000	4.12	1.275	1.17	1.52E-27
LRP11	ENSG0000	7.82	3.315	1.032	1.44E-31
PDZD11	ENSG0000	30.37	11.065	1.379	4.14E-71
ENY2	ENSG0000	127.009	62.88	1.003	1.36E-46
TGFBI	ENSG0000	114.213	51.15	1.144	5.76E-22
EPHX2	ENSG0000	47.891	106.244	-1.133	3.89E-18
ZNF706	ENSG0000	102.992	38.45	1.398	4.72E-59
SCPEP1	ENSG0000	25.849	11.89	1.059	5.18E-11
NCAPH	ENSG0000	2.55	0.17	1.601	2.29E-45
MND1	ENSG0000	2.58	0.17	1.613	4.58E-64
PILRB	ENSG0000	7.6	26.129	-1.657	3.78E-11
RPL5	ENSG0000	560.899	273.187	1.035	3.44E-40
FKBP9	ENSG0000	17.64	8.315	1.001	5.14E-27
PLAU	ENSG0000	2.82	0.77	1.11	1.07E-23
ZWINT	ENSG0000	9.69	0.865	2.519	7.03E-58
PKN1	ENSG0000	44.609	13.205	1.683	1.92E-45
ZC3H13	ENSG0000	4.81	15.4	-1.497	1.80E-30
OPTN	ENSG0000	50.569	22.641	1.125	2.32E-36
SPATS2	ENSG0000	5.64	2.025	1.134	7.78E-35
ORMDL2	ENSG0000	48.691	19.455	1.281	9.75E-68
NR4A1	ENSG0000	8.57	25.707	-1.481	1.62E-17
TUBA1B	ENSG0000	234.996	72.433	1.684	8.76E-30
HJURP	ENSG0000	2.22	0.245	1.371	2.04E-40
G0S2	ENSG0000	46.051	133.107	-1.511	9.21E-13
SLC12A5	ENSG0000	2.37	0.515	1.153	6.52E-24
PIGT	ENSG0000	65.05	22.78	1.474	1.20E-72
PTGIS	ENSG0000	0.37	1.815	-1.039	1.91E-16
PCK1	ENSG0000	84.799	405.426	-2.244	1.54E-23
SNRPC	ENSG0000	118.742	39.239	1.573	4.96E-80
RPS10	ENSG0000	973.069	427.846	1.184	1.26E-42
TBCC	ENSG0000	11.02	4.93	1.019	3.26E-45
KLHDC3	ENSG0000	61.12	29.215	1.04	5.23E-54
GNMT	ENSG0000	30.38	90.336	-1.541	8.24E-10
MEA1	ENSG0000	62.691	22.585	1.433	2.41E-84

SOX4	ENSG0000	3.07	0.71	1.251	5.22E-16
SSR1	ENSG0000	43.609	20.554	1.049	4.69E-23
RPP40	ENSG0000	7.11	3.025	1.011	2.19E-36
MT1G	ENSG0000	40.209	2587.601	-5.973	3.31E-51
MT2A	ENSG0000	261.977	2324.572	-3.145	5.54E-41
RAP2A	ENSG0000	5.38	1.86	1.158	1.82E-31
6-Sep	ENSG0000	22.06	9.535	1.13	9.56E-18
NDUFA1	ENSG0000	183.101	75.371	1.269	2.97E-71
SOX9	ENSG0000	4.95	1	1.573	3.90E-17
MIF4GD	ENSG0000	16.58	7.17	1.106	5.98E-40
PPDPF	ENSG0000	190.982	72.223	1.391	1.61E-16
PLGLB2	ENSG0000	65.348	195.78	-1.568	4.11E-23
RPL23	ENSG0000	944.625	439.386	1.102	1.17E-46
TNFSF14	ENSG0000	2.84	14.71	-2.032	5.52E-16
FOSB	ENSG0000	1.62	4.38	-1.038	3.59E-11
SNRPD2	ENSG0000	193.165	70.121	1.449	8.88E-60
RTN2	ENSG0000	5.1	1.68	1.187	3.26E-19
TGM3	ENSG0000	1.68	0.19	1.171	3.54E-22
SNRPB	ENSG0000	126.473	35.895	1.789	1.23E-70
MKKS	ENSG0000	22.899	10.375	1.071	4.69E-37
SNRPB2	ENSG0000	28.699	12.59	1.128	1.39E-52
NECAB3	ENSG0000	21.341	8.84	1.183	1.03E-53
ID1	ENSG0000	15.63	47.12	-1.533	3.47E-20
RALY	ENSG0000	63.039	26.163	1.237	1.08E-57
ERGIC3	ENSG0000	192.324	94.039	1.024	4.39E-64
ROMO1	ENSG0000	249.154	86.933	1.508	3.79E-69
PROZ	ENSG0000	8.74	32.433	-1.779	3.48E-29
PDCD2L	ENSG0000	6.35	2.57	1.042	1.95E-47
RBM42	ENSG0000	52.83	21.78	1.241	5.56E-66
HCST	ENSG0000	12.42	5.039	1.152	2.51E-17
PRDX5	ENSG0000	278.165	128.23	1.111	2.65E-66
IFI6	ENSG0000	95.742	36.279	1.376	2.97E-10
TIMM17B	ENSG0000	46.659	22.065	1.047	2.67E-61
PZP	ENSG0000	0.18	5.14	-2.379	1.20E-55
TRAF2	ENSG0000	10.5	4.355	1.103	2.86E-43
TSPAN8	ENSG0000	52.008	7.16	2.7	2.31E-22
PKMYT1	ENSG0000	5.72	0.695	1.987	1.95E-56
SMARCA4	ENSG0000	29.769	13.425	1.093	2.20E-50
VIL1	ENSG0000	14.91	4.055	1.654	1.06E-07
SHFM1	ENSG0000	305.07	97.133	1.641	1.47E-81
STYXL1	ENSG0000	31.679	14.21	1.103	1.62E-37
STEAP4	ENSG0000	0.77	4.24	-1.566	4.38E-36
ZFP36	ENSG0000	53.742	127.304	-1.229	2.91E-21
SRD5A3	ENSG0000	15.24	6.015	1.211	1.74E-50
GNAZ	ENSG0000	2.38	0.15	1.555	1.68E-35
LSM8	ENSG0000	21.201	8.135	1.281	6.01E-51
PODXL	ENSG0000	5.7	1.355	1.508	8.20E-32
SMO	ENSG0000	16.5	7.11	1.11	2.25E-16
KNSTRN	ENSG0000	7.78	2.795	1.21	1.60E-38
RNASE1	ENSG0000	56.531	18.8	1.539	4.55E-29
NEDD8	ENSG0000	160.385	77.409	1.041	5.01E-71
INS-IGF2	ENSG0000	0	4.03	-2.331	9.78E-65
PRKCSH	ENSG0000	198.376	98.155	1.008	8.08E-72
THEM6	ENSG0000	35.921	16.975	1.038	1.03E-27
TOMM40	ENSG0000	36.99	16.13	1.149	8.57E-43
RPL36	ENSG0000	739.803	336.869	1.133	1.54E-46
PLVAP	ENSG0000	43.03	2.464	3.668	7.99E-127
NSUN5	ENSG0000	17.259	7.33	1.132	4.49E-59

LSM7	ENSG0000	71.848	34.91	1.021	2.95E-46
EPO	ENSG0000	0.19	3.185	-1.814	6.95E-16
MAP1S	ENSG0000	14.04	5.995	1.104	2.83E-44
GDF15	ENSG0000	44.51	15.795	1.438	1.80E-15
LSM4	ENSG0000	102.757	45.219	1.167	4.90E-61
H19	ENSG0000	98.988	321.895	-1.691	1.02E-02
PNPLA7	ENSG0000	1.7	5.1	-1.176	7.03E-45
UBE2M	ENSG0000	40.19	17.905	1.123	1.37E-56
TRIM28	ENSG0000	143.419	66.495	1.097	4.58E-45
SLC6A8	ENSG0000	3.29	1.05	1.065	4.62E-12
HIGD1B	ENSG0000	2.2	0.19	1.427	6.54E-69
GINS2	ENSG0000	2.4	0.41	1.27	1.15E-44
PPT1	ENSG0000	19.291	7.325	1.285	1.05E-31
RAB11FIP4	ENSG0000	5.22	1.225	1.483	3.08E-36
TUBG1	ENSG0000	24.47	8.475	1.427	1.90E-43
RPL27	ENSG0000	1270.164	553.58	1.197	2.01E-54
RAMP2	ENSG0000	17.27	5.77	1.432	1.02E-32
NDUFA2	ENSG0000	139.111	64.451	1.098	2.33E-63
EXOC4	ENSG0000	11.34	5.17	1	6.07E-48
TOP2A	ENSG0000	9.54	0.405	2.907	3.35E-65
PEX11B	ENSG0000	23.079	10.54	1.061	3.73E-58
GCH1	ENSG0000	11.76	29.19	-1.242	1.20E-26
LGALS3	ENSG0000	30.231	6.845	1.993	1.84E-25
RAMP1	ENSG0000	103.679	31.471	1.689	1.78E-15
RAN	ENSG0000	208.441	81.905	1.337	2.40E-41
PNISR	ENSG0000	11.81	31.325	-1.335	2.01E-08
SEC61G	ENSG0000	167.195	79.423	1.064	8.75E-38
JCHAIN	ENSG0000	2.83	8.089	-1.247	1.53E-07
ITGB4	ENSG0000	5.13	1.67	1.199	9.14E-26
XAF1	ENSG0000	7.13	29.705	-1.917	1.11E-22
PCNA	ENSG0000	48.09	13.71	1.739	1.99E-57
NES	ENSG0000	5.32	1.63	1.265	6.86E-33
LPIN3	ENSG0000	4.75	13.924	-1.376	5.67E-11
PEMT	ENSG0000	50.53	119.908	-1.23	5.18E-26
SPG20	ENSG0000	1.55	5.41	-1.33	4.11E-21
RFC3	ENSG0000	3.4	1.165	1.023	5.19E-32
GSTT2B	ENSG0000	3.38	10.82	-1.432	6.07E-07
SEC14L4	ENSG0000	2.04	5.92	-1.187	1.11E-14
SWAP70	ENSG0000	5.09	1.985	1.029	7.00E-23
LYVE1	ENSG0000	0.83	7.055	-2.138	1.12E-41
TTC9	ENSG0000	2.24	0.43	1.18	1.69E-22
LOXL2	ENSG0000	3.68	1.03	1.205	1.84E-25
GSTM1	ENSG0000	1.28	70.601	-4.973	2.84E-03
SORT1	ENSG0000	7.08	1.66	1.603	2.69E-34
PTGFRN	ENSG0000	10.95	4.845	1.032	4.96E-18
FKBP11	ENSG0000	70.438	29.725	1.217	5.20E-22
TMEM106(	ENSG0000	44.05	11.765	1.819	6.54E-59
SAA2	ENSG0000	105.36	2489.516	-4.549	4.99E-25
FST	ENSG0000	51.929	24.758	1.039	7.56E-05
FOLH1B	ENSG0000	0.43	4.13	-1.843	2.53E-28
TPGS2	ENSG0000	20.93	8.2	1.253	2.54E-30
TIMM10	ENSG0000	60.48	29.36	1.018	4.07E-50
TMEM258	ENSG0000	160.841	73.84	1.113	4.55E-62
PDGFRA	ENSG0000	0.92	3.855	-1.338	3.43E-19
STT3A	ENSG0000	76.882	32.829	1.203	1.44E-42
NREP	ENSG0000	17.25	7.315	1.134	3.45E-19
GOLM1	ENSG0000	22.4	5.695	1.805	7.37E-18
SDS	ENSG0000	58.318	343.496	-2.538	1.03E-11

GLS2	ENSG0000	7.55	64.115	-2.929	2.16E-23
TROAP	ENSG0000	6.69	0.325	2.537	3.39E-57
PAN2	ENSG0000	13.25	46.264	-1.73	5.37E-17
HNRNPA1	ENSG0000	363.698	146.569	1.305	6.58E-44
GNS	ENSG0000	30.759	13.59	1.122	3.96E-38
NTPCR	ENSG0000	54.161	16.505	1.656	2.67E-68
GLUL	ENSG0000	323.024	144.037	1.16	5.79E-11
PIGC	ENSG0000	22.07	6.605	1.601	1.92E-71
LAMC1	ENSG0000	26.829	5.775	2.038	2.21E-43
STAB2	ENSG0000	0.08	3.505	-2.06	1.98E-103
GPNMB	ENSG0000	12.74	3.525	1.602	4.77E-17
RAC1	ENSG0000	116.994	54.913	1.077	8.53E-51
RTP4	ENSG0000	5.18	1.725	1.181	3.68E-22
MRPL47	ENSG0000	30.3	14.075	1.054	8.16E-49
EPRS	ENSG0000	37.111	13.56	1.388	2.11E-61
UGGT1	ENSG0000	14.17	5.615	1.197	1.62E-43
TXN	ENSG0000	347.458	121.873	1.504	1.17E-50
RPS6	ENSG0000	1117.066	553.925	1.011	2.75E-32
YIPF3	ENSG0000	100.002	47.349	1.063	3.77E-55
TMEM14B	ENSG0000	75.572	32.725	1.183	2.85E-60
TUBB2A	ENSG0000	60.17	28.979	1.029	8.62E-16
LRRC1	ENSG0000	2.35	0.34	1.322	2.45E-27
UQCC2	ENSG0000	98.373	32.169	1.583	7.45E-74
HMGA1	ENSG0000	36.319	6.9	2.24	8.96E-51
TCF19	ENSG0000	4.27	0.44	1.872	6.76E-47
NRM	ENSG0000	8.47	2.63	1.383	1.91E-38
MYO7A	ENSG0000	4.79	10.68	-1.012	2.15E-13
MRPL15	ENSG0000	50.699	24.155	1.039	2.07E-40
GGH	ENSG0000	121.852	51.186	1.235	1.99E-17
SORL1	ENSG0000	6.55	15	-1.084	1.74E-23
NUSAP1	ENSG0000	8.31	1.675	1.799	2.72E-40
KIF23	ENSG0000	1.48	0.19	1.059	7.83E-31
RPLP1	ENSG0000	1491.242	599.659	1.313	5.50E-68
ITPKA	ENSG0000	3.46	0.555	1.52	3.00E-30
RPL7P9	ENSG0000	28.391	9.16	1.533	1.79E-48
SLC3A1	ENSG0000	0.66	2.48	-1.068	4.67E-03
LOXL4	ENSG0000	3.58	0.72	1.413	6.30E-08
OIT3	ENSG0000	2.66	15.075	-2.135	1.80E-52
RPS24	ENSG0000	1646.514	799.358	1.042	1.33E-38
METTL5	ENSG0000	34.82	15.849	1.088	4.15E-61
OLA1	ENSG0000	40.941	16.146	1.291	2.43E-44
UBL7	ENSG0000	29.531	12.445	1.183	4.75E-68
FAM13A	ENSG0000	3.1	15.24	-1.986	1.03E-33
RASGEF1B	ENSG0000	4.63	11.45	-1.145	8.14E-25
LEF1	ENSG0000	2.67	0.29	1.508	8.05E-29
PAPSS1	ENSG0000	6.5	2.495	1.102	1.33E-25
MAPK8IP3	ENSG0000	5.62	18.72	-1.575	5.30E-08
ZCRB1	ENSG0000	33.481	15.205	1.089	1.97E-48
RPL14P1	ENSG0000	9.55	3.85	1.121	3.84E-40
SNRPF	ENSG0000	27.971	13.225	1.026	4.38E-44
FAM222A	ENSG0000	4.25	1.125	1.305	9.68E-36
SLC46A3	ENSG0000	11.35	23.705	-1	1.20E-15
SLC39A5	ENSG0000	52.399	108.891	-1.041	3.77E-11
RDH16	ENSG0000	39.609	193.762	-2.262	4.68E-32
NABP2	ENSG0000	19.38	7.77	1.217	5.42E-57
N4BP2L1	ENSG0000	12.22	28.05	-1.136	5.56E-34
VPS33A	ENSG0000	7.39	3.075	1.042	3.20E-40
SLAIN1	ENSG0000	0.63	2.575	-1.133	7.08E-22

FITM1	ENSG0000	1.31	7.18	-1.824	3.99E-55
SLC38A6	ENSG0000	6.79	2.24	1.266	5.55E-61
SLC25A47	ENSG0000	13.85	213.567	-3.853	3.91E-45
GTF2A2	ENSG0000	61.158	26.395	1.182	1.28E-61
SRP14	ENSG0000	253.474	113.165	1.156	1.94E-66
UBE2Q2	ENSG0000	5.68	2.01	1.15	1.86E-21
NEIL1	ENSG0000	8.76	20.924	-1.168	1.85E-11
FANCI	ENSG0000	3.08	0.565	1.382	1.22E-44
OSGIN1	ENSG0000	89.492	40.993	1.108	1.48E-08
SKAP1	ENSG0000	7.37	17.729	-1.162	9.64E-12
PTRH2	ENSG0000	27.851	13.168	1.026	6.31E-49
SLC13A5	ENSG0000	60.4	161.954	-1.408	6.58E-20
SECTM1	ENSG0000	5.34	2.085	1.039	4.35E-19
RNF157	ENSG0000	3.3	0.58	1.444	5.21E-26
RNF165	ENSG0000	0.16	1.53	-1.125	1.29E-60
P3H4	ENSG0000	3.91	1.29	1.1	3.48E-29
SAMD1	ENSG0000	17.89	7.82	1.099	1.55E-40
SLC39A3	ENSG0000	29.3	11.91	1.231	6.69E-58
SIK1	ENSG0000	1.57	4.18	-1.011	6.29E-07
SAE1	ENSG0000	33.261	12.505	1.343	1.26E-52
TM4SF5	ENSG0000	200.31	92.641	1.104	3.90E-09
RPS11	ENSG0000	1591.31	734.489	1.114	3.62E-54
SH3BGRL3	ENSG0000	70.629	32.565	1.094	7.00E-26
FCN3	ENSG0000	2.32	67.3	-4.363	1.37E-68
HSPG2	ENSG0000	22.611	8.685	1.286	9.63E-24
ITGB3BP	ENSG0000	8.56	3.155	1.202	6.97E-46
KIF2C	ENSG0000	2.21	0.17	1.456	6.33E-43
SLC44A3	ENSG0000	6.79	1.63	1.567	4.44E-26
TIPRL	ENSG0000	13.95	6.325	1.029	5.64E-42
MPC2	ENSG0000	279.053	110.929	1.323	4.12E-61
UCK2	ENSG0000	18.22	6	1.457	5.06E-48
TMCO1	ENSG0000	124.94	41.106	1.581	1.55E-60
RFWD2	ENSG0000	30.789	12.06	1.283	9.26E-68
UFC1	ENSG0000	97.497	33.145	1.528	7.85E-76
NUF2	ENSG0000	2.48	0.13	1.623	8.54E-46
RGS5	ENSG0000	18.01	4.01	1.924	2.20E-34
PFDN2	ENSG0000	104.959	42.964	1.269	3.23E-57
PRCC	ENSG0000	41.28	15.355	1.37	7.36E-76
MRPL24	ENSG0000	161.735	54.922	1.541	2.07E-75
LYPLAL1	ENSG0000	36.221	14.405	1.273	2.19E-48
SF3B4	ENSG0000	35.839	10.79	1.644	1.44E-68
ZNF687	ENSG0000	11.88	4.36	1.265	3.11E-45
RFX5	ENSG0000	10.55	3.49	1.363	4.80E-36
MRPL9	ENSG0000	49.261	19.215	1.314	1.94E-66
GOLPH3L	ENSG0000	9.31	3.605	1.163	6.65E-38
SMYD2	ENSG0000	30.199	12.82	1.175	1.71E-33
SUSD4	ENSG0000	3.92	1.295	1.1	1.23E-08
TP53BP2	ENSG0000	12.89	4.945	1.224	1.25E-22
S100A8	ENSG0000	3.42	29.025	-2.764	1.36E-39
TPM3	ENSG0000	124.154	46.16	1.408	3.43E-50
SNAPIN	ENSG0000	32.479	15.445	1.026	1.25E-54
SLC39A1	ENSG0000	72.098	29.34	1.269	8.80E-45
HAX1	ENSG0000	107.328	46.864	1.178	6.03E-70
ILF2	ENSG0000	80.991	27.02	1.549	3.65E-67
SRP9	ENSG0000	168.685	79.5	1.076	2.63E-44
PARP1	ENSG0000	36.99	15.685	1.187	1.12E-34
PSEN2	ENSG0000	14.1	6.11	1.087	1.08E-31
PYCR2	ENSG0000	40.649	18.06	1.128	1.26E-48

EPHX1	ENSG0000	1532.848	505.37	1.599	2.23E-17
PDIA6	ENSG0000	159.155	77.753	1.024	3.64E-27
FBLN7	ENSG0000	6.2	2.045	1.242	1.89E-27
TRPM8	ENSG0000	4.06	10.085	-1.131	5.06E-12
FANCD2	ENSG0000	2.68	0.595	1.206	8.55E-35
FAM198A	ENSG0000	0.36	3.95	-1.864	4.73E-34
RPL32	ENSG0000	1338.312	560.083	1.255	1.99E-50
RP11-977(	ENSG0000	1.01	0	1.007	1.70E-11
NFKBIZ	ENSG0000	3.85	18.334	-1.995	1.07E-34
NR1I2	ENSG0000	9.26	30.295	-1.609	2.22E-27
PLAC8	ENSG0000	1.03	5.975	-1.781	4.43E-28
NDUFS6	ENSG0000	89.12	35.605	1.3	6.13E-68
RPL37	ENSG0000	1566.576	594.465	1.396	2.08E-66
TSLP	ENSG0000	0.33	2.1	-1.221	2.45E-42
NHP2	ENSG0000	99.332	37.635	1.377	6.68E-71
TBC1D7	ENSG0000	15.92	6.43	1.187	8.27E-60
ZMAT2	ENSG0000	48.509	23.279	1.028	1.50E-47
HIGD2A	ENSG0000	145.804	67.002	1.11	1.48E-68
FAM193B	ENSG0000	17.13	46.951	-1.403	3.06E-04
TNFRSF21	ENSG0000	5.45	2.02	1.095	5.04E-13
PRIM2	ENSG0000	4.04	1.06	1.291	2.40E-57
NFKBIE	ENSG0000	11.85	4.04	1.35	4.40E-28
RSPO3	ENSG0000	0.06	1.57	-1.278	2.81E-43
IGFBP3	ENSG0000	49.428	164.471	-1.714	1.52E-24
PSPH	ENSG0000	8.69	2.705	1.387	2.07E-35
TMEM209	ENSG0000	5.27	2.015	1.056	2.42E-34
NCAPG2	ENSG0000	2.93	0.895	1.052	3.71E-28
TMEM27	ENSG0000	1.03	7.24	-2.021	5.51E-33
GPC3	ENSG0000	148.158	0.92	6.28	6.38E-59
SLC25A37	ENSG0000	4.4	12.924	-1.367	8.04E-34
RPL7	ENSG0000	1178.47	467.589	1.332	4.79E-43
POLR2K	ENSG0000	55.819	19.015	1.505	8.99E-80
NDUFB9	ENSG0000	377.752	144.862	1.377	2.15E-64
TATDN1	ENSG0000	22.869	8.72	1.296	7.80E-54
RP11-35N	ENSG0000	3.71	1.305	1.031	1.27E-21
GSN	ENSG0000	85.882	37.655	1.168	6.32E-15
LCN2	ENSG0000	38.101	5.525	2.583	1.58E-13
MKI67	ENSG0000	2.9	0.17	1.737	6.18E-46
RGS10	ENSG0000	7.6	1.915	1.561	4.08E-31
SAA4	ENSG0000	251.479	770.846	-1.612	7.75E-21
PAMR1	ENSG0000	0.7	2.855	-1.181	6.04E-24
GLYAT	ENSG0000	35.37	138.764	-1.942	3.66E-18
SERPINH1	ENSG0000	37.121	15.61	1.199	5.16E-20
ST14	ENSG0000	8.05	2.715	1.285	2.33E-11
PLCH2	ENSG0000	0.57	2.5	-1.157	6.00E-03
TMEM25	ENSG0000	2.85	7.29	-1.107	1.37E-21
FAU	ENSG0000	796.206	363.206	1.13	2.00E-62
TM7SF2	ENSG0000	103.407	41.026	1.313	3.57E-16
TMCO3	ENSG0000	15.33	5.335	1.366	1.16E-24
LYPD1	ENSG0000	2.34	0.225	1.447	3.30E-24
GPM6A	ENSG0000	0.16	1.64	-1.186	9.84E-45
PRSS53	ENSG0000	0.23	4.55	-2.174	2.46E-52
MAT1A	ENSG0000	220.907	484.649	-1.13	7.57E-19
THRSP	ENSG0000	6.27	72.531	-3.338	4.00E-20
NDUFC2	ENSG0000	323.853	140.872	1.195	2.15E-56
ITIH2	ENSG0000	623.739	212.342	1.55	1.81E-09
TMEM45B	ENSG0000	29.9	8.1	1.764	7.97E-11
TDO2	ENSG0000	76.478	213.996	-1.472	1.71E-17

GEMIN6	ENSG0000	11.22	5.08	1.007	7.82E-55
HAUS1	ENSG0000	8.63	3.675	1.043	4.37E-37
SPC25	ENSG0000	2.11	0.16	1.423	3.18E-47
SPARCL1	ENSG0000	19.1	4.845	1.782	1.87E-22
GJA1	ENSG0000	4.23	1.415	1.115	3.08E-18
GGPS1	ENSG0000	12.93	5.865	1.021	4.77E-50
RASSF3	ENSG0000	6.27	2.21	1.179	1.48E-21
SLC25A27	ENSG0000	2.23	11.03	-1.897	7.15E-23
FAM49B	ENSG0000	17.579	7.295	1.163	1.50E-28
LINC00467	ENSG0000	10.37	4.585	1.026	1.15E-42
THY1	ENSG0000	20.42	1.995	2.838	1.97E-62
FAM134B	ENSG0000	2.32	9.124	-1.609	1.44E-17
TCEB1	ENSG0000	67.911	26.575	1.321	2.54E-55
LY96	ENSG0000	8.45	3.375	1.111	7.04E-14
SKA1	ENSG0000	1.33	0.04	1.164	7.50E-40
ZCCHC10	ENSG0000	18.15	8.21	1.056	1.41E-57
RHOC	ENSG0000	174.755	63.284	1.451	4.75E-51
KDM8	ENSG0000	5.66	45.724	-2.811	9.08E-45
VBP1	ENSG0000	18.29	8.53	1.017	3.50E-39
NAT2	ENSG0000	2.28	27.324	-3.11	1.07E-60
SLC28A1	ENSG0000	8.97	30.939	-1.68	7.70E-22
UQCRB	ENSG0000	349.269	129.912	1.42	4.17E-69
RPL30	ENSG0000	1964.708	654.091	1.585	1.70E-64
HKDC1	ENSG0000	8.11	1.01	2.18	3.33E-23
UTP14A	ENSG0000	7.05	2.75	1.102	2.94E-44
WDYHV1	ENSG0000	8.96	3.605	1.113	2.23E-42
FBXO32	ENSG0000	4.23	1.22	1.236	3.93E-29
NSMCE2	ENSG0000	20.92	5.565	1.739	4.91E-85
PDE6D	ENSG0000	11.73	5.08	1.066	9.55E-46
NRG1	ENSG0000	0.45	2.87	-1.416	1.20E-10
MMP14	ENSG0000	15	5	1.415	6.88E-21
SLC35B2	ENSG0000	32.739	13.99	1.17	4.34E-43
TMEM164	ENSG0000	2.59	0.78	1.012	5.00E-28
PSMG3	ENSG0000	31.519	11.92	1.332	7.20E-62
KRTCAP3	ENSG0000	1.41	6.48	-1.634	1.47E-04
MRPL17	ENSG0000	33.91	14.065	1.212	5.09E-61
FANCC	ENSG0000	2.86	7.004	-1.052	1.14E-24
TAGLN2	ENSG0000	252.545	76.164	1.716	3.34E-43
SLAMF8	ENSG0000	2.05	0.415	1.108	2.94E-20
OR10J6P	ENSG0000	0.62	5.62	-2.031	6.99E-38
FAM160B2	ENSG0000	9.28	22.195	-1.174	1.12E-07
MIS18A	ENSG0000	4.36	1.595	1.047	1.36E-38
STC1	ENSG0000	2.25	0.325	1.294	2.19E-22
RCAN1	ENSG0000	17.179	48.371	-1.441	5.50E-43
SNF8	ENSG0000	88.101	39.81	1.127	1.69E-58
PSMD4	ENSG0000	242.576	78.784	1.61	2.53E-79
PSMB4	ENSG0000	347.073	134.526	1.361	3.11E-83
UROC1	ENSG0000	3.89	38.638	-3.019	9.88E-33
SPON2	ENSG0000	94.851	45.069	1.057	1.97E-17
TPPP3	ENSG0000	4.09	1.46	1.049	3.36E-13
G6PD	ENSG0000	9.9	2.875	1.492	1.33E-28
SPATC1L	ENSG0000	3.81	0.41	1.77	3.21E-38
FCN2	ENSG0000	0.28	30.658	-4.628	2.54E-103
GPSM1	ENSG0000	3.67	1.165	1.109	1.63E-18
TLCD1	ENSG0000	16.11	4.87	1.543	4.12E-47
FLAD1	ENSG0000	34.291	12.85	1.349	4.75E-73
SHC1	ENSG0000	58.189	27.29	1.065	5.31E-27
UBE2Q1	ENSG0000	39.719	15.774	1.279	3.97E-40

FDPS	ENSG0000	291.63	108.698	1.416	2.85E-38
RUSC1	ENSG0000	10.76	4.85	1.007	2.44E-33
FAM189B	ENSG0000	12.53	4.445	1.313	1.79E-47
PMF1	ENSG0000	61.158	26.235	1.191	2.11E-61
LMNA	ENSG0000	175.52	45.859	1.913	4.12E-85
PTH1R	ENSG0000	1.32	23.575	-3.405	1.44E-102
GPATCH4	ENSG0000	18.181	7.33	1.203	2.74E-34
HK3	ENSG0000	1.21	4.105	-1.208	1.17E-20
LY6E	ENSG0000	54.53	259.294	-2.229	6.44E-29
VPS28	ENSG0000	192.071	76.699	1.313	3.18E-66
RECQL4	ENSG0000	8.65	1.435	1.987	6.01E-51
SQSTM1	ENSG0000	460.225	152.9	1.583	1.68E-40
RPL8	ENSG0000	1876.722	569.775	1.718	6.14E-59
RACGAP1	ENSG0000	4.61	0.705	1.718	1.73E-50
SPC24	ENSG0000	5.15	0.33	2.209	5.18E-66
IP6K3	ENSG0000	0.36	2.394	-1.319	3.14E-09
PAQR4	ENSG0000	3.82	0.8	1.421	7.08E-32
FLYWCH2	ENSG0000	14.31	4.85	1.388	1.74E-39
UBXN1	ENSG0000	75.378	36.076	1.043	4.46E-62
RPL29	ENSG0000	1049.145	487.919	1.103	1.66E-49
PDZK1IP1	ENSG0000	15.74	1.505	2.74	1.12E-16
FAM151A	ENSG0000	0.45	2.274	-1.175	1.09E-05
RBP7	ENSG0000	17.259	2.635	2.329	3.32E-59
TMEM82	ENSG0000	5.08	21.11	-1.863	2.33E-21
LAPTM5	ENSG0000	28.489	13.69	1.005	3.00E-11
RBBP4	ENSG0000	30.969	14.58	1.037	1.25E-12
KIAA1522	ENSG0000	11.99	3.85	1.421	4.79E-36
GBP2	ENSG0000	45.972	15.32	1.525	3.30E-36
IGSF8	ENSG0000	46.529	21.859	1.056	1.58E-26
PEA15	ENSG0000	45.819	12.42	1.803	3.81E-58
NCSTN	ENSG0000	62.552	26.736	1.196	1.96E-34
OLFML2B	ENSG0000	2.78	0.275	1.568	3.12E-45
FLVCR1	ENSG0000	3.43	0.705	1.378	3.91E-51
MT2P1	ENSG0000	0	7.11	-3.02	1.10E-64
MRPL55	ENSG0000	113.739	49.114	1.195	2.74E-56
H3F3A	ENSG0000	397.496	139.034	1.509	9.56E-73
INHBB	ENSG0000	10.18	4.304	1.076	1.55E-09
SCNM1	ENSG0000	33.21	10.825	1.533	9.85E-72
VPS72	ENSG0000	28.489	8.295	1.666	7.94E-78
S100A11	ENSG0000	77.321	36.376	1.067	7.15E-13
SPRR3	ENSG0000	0	1.315	-1.211	5.13E-29
S100A9	ENSG0000	11.29	46.889	-1.962	1.32E-14
S100A12	ENSG0000	0.19	3.425	-1.895	2.35E-62
PMVK	ENSG0000	77.262	35.51	1.1	2.50E-58
PBXIP1	ENSG0000	38.369	13.51	1.44	5.51E-53
PYGO2	ENSG0000	15.31	6.875	1.05	1.25E-47
TMEM183/	ENSG0000	27.02	12.51	1.052	5.56E-52
KRTCAP2	ENSG0000	342.651	102.454	1.732	1.10E-93
SSR2	ENSG0000	259.267	82.939	1.633	6.01E-67
HDAC11	ENSG0000	8.49	2.1	1.614	4.48E-54
SERPINI1	ENSG0000	3.56	0.94	1.233	6.41E-31
RPL22L1	ENSG0000	61.469	28.814	1.067	3.95E-20
THOC7	ENSG0000	41.271	19.789	1.024	1.66E-54
GMPS	ENSG0000	19.41	6.88	1.373	9.06E-52
MTHFD2L	ENSG0000	3.57	9.69	-1.226	7.44E-37
RPN1	ENSG0000	160.362	76.362	1.061	9.65E-46
RFC4	ENSG0000	12.42	3.89	1.457	1.42E-48
RPL39L	ENSG0000	5.42	1.46	1.384	1.23E-15

TKT	ENSG0000	113.921	35.77	1.644	3.14E-33
LRPAP1	ENSG0000	95.259	43.646	1.108	2.91E-40
S100P	ENSG0000	6.05	2.36	1.069	1.30E-08
H2AFZ	ENSG0000	99.808	32.785	1.577	4.21E-53
PLXNB1	ENSG0000	30.89	67.29	-1.099	9.51E-09
TEX264	ENSG0000	62.651	29.615	1.056	3.68E-67
POC1A	ENSG0000	4.81	1.72	1.095	4.36E-44
HMGB2	ENSG0000	34.871	11.52	1.519	1.95E-43
HAND2	ENSG0000	0.4	5.02	-2.104	1.16E-45
MAD2L1	ENSG0000	3.9	0.535	1.675	1.61E-47
HPGD	ENSG0000	14.55	40.456	-1.415	6.59E-12
NPY1R	ENSG0000	0.4	2.24	-1.211	1.79E-37
HHIP	ENSG0000	0.06	1.475	-1.223	2.21E-43
NDUFAF2	ENSG0000	25.06	11.515	1.058	6.33E-43
NDUFS4	ENSG0000	82.081	38.374	1.077	4.28E-45
SPINK1	ENSG0000	187.155	23.855	2.92	1.47E-06
ESM1	ENSG0000	3.22	0.09	1.953	1.59E-52
NSA2	ENSG0000	56.629	26.857	1.049	7.30E-45
RPS14	ENSG0000	955.822	419.331	1.187	1.83E-54
PTTG1	ENSG0000	20.589	0.87	3.529	5.59E-80
KCNK5	ENSG0000	1.67	4.375	-1.009	3.47E-14
FABP5	ENSG0000	16.66	2.88	2.186	1.76E-44
RAD21	ENSG0000	33.499	14.025	1.199	1.17E-29
TMEM184/	ENSG0000	5.88	13.27	-1.052	3.29E-07
YWHAZ	ENSG0000	107.38	40.699	1.378	1.29E-36
KIAA1429	ENSG0000	32.019	13.155	1.222	4.60E-66
KIAA0196	ENSG0000	15.47	6.095	1.215	2.18E-52
NUDT2	ENSG0000	20.569	9.42	1.05	1.35E-43
FBP1	ENSG0000	116.403	296.902	-1.343	4.11E-20
WBSCR27	ENSG0000	3.28	0.965	1.123	2.17E-25
NDUFB6	ENSG0000	61.601	29.35	1.044	8.17E-52
MELK	ENSG0000	2.23	0.1	1.554	1.08E-44
SPTSSA	ENSG0000	24.609	11.13	1.078	1.28E-21
FOLR2	ENSG0000	10.76	4.165	1.187	2.82E-09
MBL2	ENSG0000	12.81	37.715	-1.487	4.36E-18
HEPACAM	ENSG0000	0.07	1.355	-1.138	2.67E-04
SKA3	ENSG0000	1.16	0.07	1.013	1.35E-39
KIAA1462	ENSG0000	3.31	0.52	1.504	9.40E-51
NDRG2	ENSG0000	83.239	176.349	-1.074	4.03E-23
PRAP1	ENSG0000	676.102	259.618	1.377	4.48E-05
PSMC3	ENSG0000	107.507	52.736	1.014	1.17E-59
IFI27L1	ENSG0000	14.55	4.515	1.495	1.39E-60
IFI27	ENSG0000	145.209	26.64	2.403	3.73E-19
TPP1	ENSG0000	67.568	32.47	1.035	2.90E-23
MOGAT2	ENSG0000	5.55	49.036	-2.933	1.71E-41
RIC3	ENSG0000	0.07	1.775	-1.375	1.08E-35
WDR72	ENSG0000	1.77	8.21	-1.733	1.72E-23
TRIM66	ENSG0000	0.84	3.18	-1.184	1.30E-17
RPL27A	ENSG0000	1599.05	693.57	1.204	5.94E-51
MFAP4	ENSG0000	3.46	14.005	-1.75	2.29E-16
MCM7	ENSG0000	32.919	12.145	1.368	6.89E-41
TMED3	ENSG0000	20.71	6.7	1.495	1.13E-09
SEC11C	ENSG0000	180.33	89.39	1.004	4.71E-25
NGFRAP1	ENSG0000	82.928	34.27	1.251	1.48E-06
NNMT	ENSG0000	269.342	1779.553	-2.719	8.31E-23
PPIB	ENSG0000	710.208	316.299	1.164	2.58E-57
KIAA0101	ENSG0000	9.86	1.51	2.113	9.70E-50
PLIN1	ENSG0000	1.84	8.99	-1.815	7.43E-29

MESP1	ENSG0000	3.86	0.96	1.31	6.00E-48
GLYATL1	ENSG0000	44.609	140.035	-1.629	8.95E-25
PLK1	ENSG0000	3.77	0.765	1.434	6.79E-36
PDIA3	ENSG0000	370.671	173.452	1.091	2.37E-44
PHB	ENSG0000	120.25	54.451	1.129	2.57E-52
SNRPD1	ENSG0000	42.22	14.7	1.461	1.13E-60
UGT1A6	ENSG0000	70.37	21.275	1.68	2.54E-05
IGF2	ENSG0000	10.29	89.75	-3.007	3.53E-10
TBC1D16	ENSG0000	5.07	1.81	1.111	6.32E-26
RRM1	ENSG0000	13.68	4.735	1.356	1.14E-46
FN3K	ENSG0000	54.059	25.804	1.039	7.59E-25
TRAPPC2L	ENSG0000	54.651	23.875	1.162	1.09E-53
TUBA1A	ENSG0000	15.45	6.205	1.191	8.89E-18
TUBA1C	ENSG0000	72.028	27.1	1.378	5.52E-19
GPD1	ENSG0000	15.39	46.359	-1.531	1.03E-17
LENG8	ENSG0000	19.07	47.91	-1.285	1.03E-05
YIF1B	ENSG0000	47.891	22.32	1.068	4.91E-35
HDGFRP2	ENSG0000	27.739	12.485	1.092	3.29E-62
PLIN4	ENSG0000	4.27	22.554	-2.16	1.51E-25
SLC43A2	ENSG0000	4.48	1.57	1.092	9.52E-20
TK1	ENSG0000	17.25	2.335	2.452	2.99E-58
SRRM2	ENSG0000	123.442	265.349	-1.098	9.48E-06
FTH1	ENSG0000	3615.3	1496.627	1.272	3.64E-49
POLR2G	ENSG0000	53.772	23.625	1.153	1.79E-62
RPSA	ENSG0000	680.38	312.941	1.118	2.42E-39
SAC3D1	ENSG0000	13.78	4.47	1.434	1.33E-70
SCARA3	ENSG0000	3.69	1.065	1.183	2.01E-20
PBK	ENSG0000	2.1	0.1	1.495	8.04E-41
RAB4A	ENSG0000	37.219	17.995	1.009	1.56E-41
RNF187	ENSG0000	55.749	23.695	1.2	3.40E-56
NT5DC2	ENSG0000	11.39	3.31	1.523	4.62E-26
SMIM4	ENSG0000	34.9	9.335	1.796	1.83E-59
MFSD2A	ENSG0000	3.81	77.691	-4.032	5.52E-45
TAP1	ENSG0000	22.079	9.69	1.11	1.05E-22
STIP1	ENSG0000	52.57	22.525	1.187	2.85E-33
FEN1	ENSG0000	12.59	2.9	1.801	7.26E-62
RNF181	ENSG0000	192.417	83.178	1.2	5.57E-67
VAMP5	ENSG0000	90.88	33.767	1.402	1.87E-43
PPIC	ENSG0000	23.64	11.08	1.028	2.60E-31
STXBP6	ENSG0000	6.17	1.34	1.615	9.91E-40
LGALS9	ENSG0000	20.79	8.12	1.257	1.77E-17
SLC25A6	ENSG0000	110.063	52.654	1.05	5.00E-32
NSMCE1	ENSG0000	51.61	24.375	1.052	2.32E-56
RP11-231(	ENSG0000	6.94	27.937	-1.866	3.68E-16
LMAN2	ENSG0000	226.866	97.544	1.209	4.84E-65
SLC50A1	ENSG0000	65.289	24.135	1.399	1.38E-50
NPIPB3	ENSG0000	8.82	24.769	-1.392	1.05E-07
PTK2	ENSG0000	22.721	9.61	1.161	3.37E-38
SDC2	ENSG0000	176.557	68.859	1.346	1.43E-24
FAM103A1	ENSG0000	15.7	7.285	1.011	9.60E-51
SPNS1	ENSG0000	31.6	13.335	1.185	4.26E-64
FASN	ENSG0000	217.896	108.112	1.004	2.11E-08
MT1E	ENSG0000	86.228	797.532	-3.194	1.59E-32
RAC3	ENSG0000	14.47	6.25	1.093	3.17E-22
ROBO1	ENSG0000	9.19	1.07	2.299	1.09E-32
MUC3A	ENSG0000	1.62	5.13	-1.226	5.18E-04
TM4SF4	ENSG0000	430.001	95.683	2.156	2.86E-18
TM4SF1	ENSG0000	41.469	14.024	1.499	5.03E-16

HNRNPA3	ENSG0000	124.077	57.412	1.098	4.79E-50
GLB1	ENSG0000	33.079	15.252	1.068	3.66E-39
FABP4	ENSG0000	7.47	2.645	1.216	2.62E-10
FOS	ENSG0000	15.56	80.208	-2.294	5.31E-30
HSD17B13	ENSG0000	6.73	74.827	-3.294	2.68E-16
PA2G4	ENSG0000	52.57	23.56	1.125	6.70E-46
NUDCD2	ENSG0000	15.3	6.895	1.046	8.99E-32
HSPA4	ENSG0000	68.029	27.19	1.292	1.13E-52
SYT9	ENSG0000	0.01	1.26	-1.162	8.39E-44
GSTA4	ENSG0000	18.01	6.855	1.275	2.86E-28
TPT1-AS1	ENSG0000	3.42	8.05	-1.034	1.23E-12
ZNF160	ENSG0000	1.91	4.945	-1.031	1.05E-15
FPR1	ENSG0000	0.53	2.29	-1.105	7.81E-18
LRG1	ENSG0000	121.229	339.166	-1.477	1.29E-15
KCND3	ENSG0000	0.84	3.235	-1.203	7.35E-28
KRT13	ENSG0000	0	2.74	-1.903	6.91E-36
ZNF581	ENSG0000	9.69	4.23	1.031	1.32E-44
MCC	ENSG0000	1.22	3.825	-1.12	2.64E-38
ETFDH	ENSG0000	27.81	58.846	-1.055	2.86E-33
FGB	ENSG0000	3258.516	6850.767	-1.072	1.51E-09
RP11-443I	ENSG0000	1.78	0.02	1.447	1.02E-27
LGALS4	ENSG0000	224.27	46.12	2.257	2.48E-06
RHNO1	ENSG0000	6.63	1.77	1.462	2.76E-52
RRM2	ENSG0000	9.26	0.595	2.685	1.36E-50
RPS21	ENSG0000	1270.692	486.517	1.383	9.69E-56
RPS7	ENSG0000	895.903	394.121	1.183	1.42E-54
PPIH	ENSG0000	16.36	6.63	1.186	8.15E-51
QARS	ENSG0000	101.447	45.099	1.152	6.28E-61
MRPL13	ENSG0000	47.431	19.64	1.23	8.48E-57
ISG20	ENSG0000	19.96	9.17	1.043	7.63E-16
STARD5	ENSG0000	6.45	19.359	-1.45	3.13E-42
TTC36	ENSG0000	4	63.26	-3.684	2.66E-42
MYEOV2	ENSG0000	94.693	41.148	1.183	1.77E-53
FIBP	ENSG0000	36.801	17.76	1.011	8.92E-57
RPL38	ENSG0000	1230.73	457.934	1.424	8.63E-66
MIR4435-2	ENSG0000	33.1	4.45	2.645	4.40E-53
TRMT112	ENSG0000	215.358	83.786	1.352	3.43E-75
MTX1	ENSG0000	58.838	20.895	1.45	1.55E-82
SNCG	ENSG0000	6.78	1.72	1.516	1.21E-23
PPP1R3B	ENSG0000	10.71	24.785	-1.139	9.18E-19
NAA20	ENSG0000	71.858	29.014	1.279	1.48E-58
SAA1	ENSG0000	287.157	4468.423	-3.955	3.44E-23
MST1	ENSG0000	205.486	472.132	-1.196	7.93E-24
SULT1B1	ENSG0000	1.05	3.175	-1.026	2.81E-14
UQCRH	ENSG0000	217.594	83.829	1.366	1.14E-57
MUC13	ENSG0000	5.82	0.22	2.483	6.99E-25
TOMM20	ENSG0000	65.871	21.944	1.543	3.13E-69
SPTBN2	ENSG0000	8.04	18.735	-1.126	2.26E-16
PHLDA3	ENSG0000	6.77	2.53	1.138	4.69E-10
PODN	ENSG0000	1.39	3.865	-1.025	7.70E-11
EXO1	ENSG0000	1.81	0.1	1.353	1.41E-45
RPL4	ENSG0000	1141.566	557.933	1.032	2.02E-40
RPL15	ENSG0000	755.139	377.046	1	1.90E-44
HRAS	ENSG0000	32.271	15.19	1.039	3.00E-48
SEZ6L2	ENSG0000	2.88	0.205	1.687	3.02E-23
SLC22A1	ENSG0000	56.102	495.588	-3.12	2.77E-29
UBE2C	ENSG0000	13.24	0.6	3.154	1.56E-68
MARCKSL1	ENSG0000	29.07	13.709	1.032	3.51E-18

PSMD2	ENSG0000	81.48	38.55	1.06	4.01E-29
PHYHD1	ENSG0000	14.71	34.515	-1.177	9.58E-19
PHYKPL	ENSG0000	20.569	47.765	-1.177	1.10E-27
UCP2	ENSG0000	14.85	5.555	1.274	9.18E-13
RMI2	ENSG0000	3.39	0.47	1.578	1.38E-41
RUVBL1	ENSG0000	17.349	7.765	1.066	1.18E-37
SFN	ENSG0000	9.74	0.605	2.742	1.61E-31
ETV4	ENSG0000	3.97	0.46	1.767	9.97E-26
KBTBD11	ENSG0000	0.26	2.645	-1.532	7.57E-57
RNF152	ENSG0000	2.87	7.37	-1.113	7.43E-30
TTTY14	ENSG0000	0.41	2.95	-1.486	2.34E-05
TYMS	ENSG0000	16.4	3.05	2.103	2.21E-48
TYMSOS	ENSG0000	2.58	0.44	1.314	1.16E-36
TALDO1	ENSG0000	149.955	65.534	1.182	8.91E-51
ZFAS1	ENSG0000	81.002	27.78	1.511	7.16E-40
ERICH5	ENSG0000	18.92	3.41	2.175	1.43E-14
RPLP2	ENSG0000	879.597	343.329	1.355	2.02E-51
GBA	ENSG0000	73.48	12.61	2.452	2.46E-103
POLR2L	ENSG0000	155.448	74.78	1.046	6.25E-51
SOX12	ENSG0000	4.26	1.51	1.067	6.01E-28
MAMDC4	ENSG0000	2.66	11.405	-1.761	4.27E-16
RPS27	ENSG0000	3269.149	1428.86	1.193	1.73E-47
IMPDH2	ENSG0000	67.662	29.13	1.188	2.28E-39
GRAMD1C	ENSG0000	2.24	5.925	-1.096	1.36E-19
STAP2	ENSG0000	66.68	31.891	1.041	2.07E-21
EXOSC4	ENSG0000	41.63	15.195	1.396	1.23E-55
RRS1	ENSG0000	16	4.47	1.636	1.32E-34
MAGED1	ENSG0000	64.44	31.6	1.005	1.20E-12
HLA-DQB1	ENSG0000	22.92	5.189	1.95	3.59E-16
LINC0017	ENSG0000	1.05	3.635	-1.177	4.56E-19
SHARPIN	ENSG0000	59.272	24.24	1.256	4.45E-61
MAF1	ENSG0000	56.469	23.51	1.229	1.43E-55
TPPP2	ENSG0000	0.76	3.525	-1.362	1.01E-29
PCBP1-AS	ENSG0000	8.9	20.05	-1.088	1.30E-02
PUF60	ENSG0000	112.259	47.77	1.216	3.24E-54
TMEM150	ENSG0000	6.44	0.645	2.177	3.44E-36
GREM2	ENSG0000	0.93	6.41	-1.941	4.38E-16
FAM83H	ENSG0000	14.96	5.055	1.398	1.52E-39
MRPL14	ENSG0000	79.71	35.33	1.152	9.91E-59
LSMEM1	ENSG0000	0.64	3.055	-1.306	5.49E-22
NQO1	ENSG0000	11.72	1.285	2.477	2.02E-29
NPM1	ENSG0000	485.355	170.378	1.505	2.36E-47
TMEM45A	ENSG0000	11.38	32.179	-1.422	4.51E-14
RPL24P4	ENSG0000	9.02	3.245	1.239	7.20E-36
MRPS23	ENSG0000	28.859	9.985	1.443	1.07E-47
PHLDA2	ENSG0000	4.54	1.015	1.459	2.33E-12
SNRPE	ENSG0000	137.548	38.494	1.811	5.11E-83
RP11-69E1	ENSG0000	0.83	3.935	-1.431	4.93E-20
TDRKH	ENSG0000	4.09	1.165	1.233	1.56E-35
MRPS16	ENSG0000	81.452	35.44	1.178	4.09E-69
YBEY	ENSG0000	17.97	8.19	1.046	9.18E-35
KPNA2	ENSG0000	28.43	6.31	2.009	1.03E-62
MFSD5	ENSG0000	14.31	5.98	1.133	6.51E-58
SATB1	ENSG0000	1.65	4.965	-1.17	2.49E-22
SKA2	ENSG0000	14.76	6.375	1.096	2.07E-41
RPL35A	ENSG0000	803.914	353.091	1.185	3.04E-53
SLC25A18	ENSG0000	21.47	46.775	-1.088	1.48E-13
HMGN4	ENSG0000	13.39	5.36	1.178	1.88E-18

PYCR1	ENSG0000	6.32	2.035	1.27	4.77E-11
RABIF	ENSG0000	6.21	2.225	1.161	5.55E-69
RUVBL2	ENSG0000	50.822	22.285	1.154	9.46E-65
PLGLB1	ENSG0000	29.8	74.586	-1.295	1.09E-20
TMEM50A	ENSG0000	50.779	20.175	1.29	2.01E-54
TRAIP	ENSG0000	1.87	0.385	1.051	1.16E-40
NUDT14	ENSG0000	19.94	9.41	1.008	7.68E-22
FAM3B	ENSG0000	2.72	0.6	1.217	4.04E-11
IQGAP3	ENSG0000	2.35	0.12	1.581	4.48E-45
UQCR10	ENSG0000	135.532	66.201	1.023	7.94E-53
NR2C2AP	ENSG0000	14.33	4.33	1.524	1.09E-71
IRAK1	ENSG0000	36.75	13.79	1.352	1.22E-39
RPL23AP8:	ENSG0000	9.59	4.1	1.054	1.44E-41
WDR27	ENSG0000	2.94	8.715	-1.302	8.39E-10
PTP4A3	ENSG0000	8.63	2.31	1.541	1.56E-32
SOCS3	ENSG0000	6.84	20.916	-1.483	7.50E-13
NDUFA12	ENSG0000	82.681	39.89	1.033	1.74E-63
TMED9	ENSG0000	183.304	91.034	1.002	6.92E-43
IMMP2L	ENSG0000	17.52	8.165	1.015	8.77E-32
LCN12	ENSG0000	6.83	17.9	-1.271	7.33E-14
MUC6	ENSG0000	0.02	1.58	-1.339	2.73E-09
MANEAL	ENSG0000	9.16	3.035	1.332	1.01E-29
NOMO2	ENSG0000	83.609	35.69	1.205	5.96E-48
WBP5	ENSG0000	20.89	9.55	1.053	1.10E-06
UBALD2	ENSG0000	27.02	12.254	1.08	6.24E-33
IL3RA	ENSG0000	2.95	0.6	1.304	7.64E-59
SMYD3	ENSG0000	13.12	3.06	1.798	2.36E-59
PARPBP	ENSG0000	1.5	0.19	1.071	4.70E-39
OLFML2A	ENSG0000	1.48	0.175	1.078	1.57E-41
NDUFA4L2	ENSG0000	14.62	3.17	1.905	4.81E-41
NTF3	ENSG0000	0.23	2.445	-1.486	1.37E-78
SLC52A2	ENSG0000	14.78	5.38	1.307	2.94E-44
NPIPB4	ENSG0000	4.31	16.34	-1.707	4.23E-13
NDUFA13	ENSG0000	306.702	131.338	1.217	6.06E-60
POLR3C	ENSG0000	10.38	4.26	1.113	5.62E-43
SLC51B	ENSG0000	3.87	0.415	1.783	6.74E-27
TOR3A	ENSG0000	17.179	7.93	1.026	1.02E-27
MST1P2	ENSG0000	9.95	74.23	-2.78	5.13E-30
RPS23	ENSG0000	1376.605	648.044	1.086	5.30E-45
MST1L	ENSG0000	10.36	64.444	-2.526	5.43E-21
MPPED1	ENSG0000	1.34	5.769	-1.532	5.30E-26
TNFRSF4	ENSG0000	2.97	0.32	1.589	4.05E-65
NAP1L1	ENSG0000	87.462	40.215	1.102	1.73E-30
MT1X	ENSG0000	145.29	1597.997	-3.45	8.62E-43
EPOR	ENSG0000	2.57	7.27	-1.212	1.33E-17
PTMA	ENSG0000	508.498	243.57	1.059	1.61E-41
ISG15	ENSG0000	71.55	30.819	1.189	9.07E-13
HAPLN4	ENSG0000	0.31	1.81	-1.101	1.90E-25
GABRD	ENSG0000	1.66	0.06	1.327	1.49E-62
HIST1H1C	ENSG0000	128.818	48.314	1.396	2.82E-24
PALM3	ENSG0000	7.61	18.025	-1.144	2.18E-17
TPRG1	ENSG0000	1.7	4.69	-1.075	2.30E-23
MORN2	ENSG0000	12.46	5.615	1.025	9.02E-40
LAMTOR4	ENSG0000	128.079	55.479	1.192	3.09E-57
PLA2G2A	ENSG0000	23.811	207.166	-3.069	5.77E-07
ZP3	ENSG0000	2.71	0.66	1.16	1.18E-29
H2AFX	ENSG0000	14.94	4.97	1.417	8.39E-40
HBA2	ENSG0000	22.15	162.612	-2.821	4.20E-38

CLN3	ENSG0000	58.809	19.355	1.555	2.78E-68
SUMO2	ENSG0000	146.859	49.28	1.556	7.57E-57
IDO2	ENSG0000	0.11	2.54	-1.673	3.37E-33
PARVB	ENSG0000	16.31	6.394	1.227	3.72E-13
RBM34	ENSG0000	44.711	15.919	1.434	4.00E-70
PRELP	ENSG0000	1.25	4.32	-1.241	4.30E-16
RPL14	ENSG0000	706.917	314.714	1.165	1.55E-49
NDUFA4	ENSG0000	120.508	59.309	1.011	1.15E-48
FAM111B	ENSG0000	1.57	0.09	1.237	5.95E-33
H1F0	ENSG0000	142.438	60.62	1.219	4.46E-28
HN1	ENSG0000	64.208	19.065	1.7	8.00E-54
S100A13	ENSG0000	75.29	27.839	1.403	6.17E-19
FHIT	ENSG0000	11.09	4.22	1.212	4.78E-39
FAM150B	ENSG0000	0.13	1.28	-1.013	5.92E-11
FAM180A	ENSG0000	0.22	3.43	-1.86	4.66E-57
KAZN	ENSG0000	0.34	1.91	-1.119	1.53E-33
IL1RAP	ENSG0000	6.41	25.615	-1.845	5.13E-43
KIAA0895L	ENSG0000	2.04	7.16	-1.424	1.66E-10
HLA-DRB1	ENSG0000	66.431	27.555	1.24	4.58E-10
SERPINA3	ENSG0000	370.748	888.913	-1.259	9.53E-13
S100A4	ENSG0000	29.25	13.083	1.103	9.94E-12
HRCT1	ENSG0000	4.34	0.48	1.851	2.27E-36
TUBB	ENSG0000	207.015	88.763	1.212	3.10E-32
PPIA	ENSG0000	1363.405	530.68	1.36	1.65E-75
EVL	ENSG0000	25.86	54.749	-1.054	8.84E-15
XRCC6	ENSG0000	110.598	53.725	1.028	4.22E-48
ZNF775	ENSG0000	8.49	3.35	1.125	1.01E-34
MYL6B	ENSG0000	27.219	9.079	1.485	1.42E-52
NACA	ENSG0000	953.903	425.79	1.162	3.21E-62
MME	ENSG0000	0.91	7.135	-2.091	2.01E-06
LINC00238	ENSG0000	0.19	3.295	-1.852	2.37E-34
TOMM7	ENSG0000	327.397	128.181	1.346	3.43E-57
PDXDC2P	ENSG0000	2.91	11.375	-1.662	3.44E-20
HLA-DQA	ENSG0000	12.49	3.505	1.582	1.17E-09
GM2A	ENSG0000	16	4.965	1.511	1.23E-64
ZNF585A	ENSG0000	5.72	2.155	1.091	7.45E-34
LAGE3	ENSG0000	36.59	11.005	1.647	2.33E-67
FAM163B	ENSG0000	0.09	2.415	-1.648	2.16E-47
SRC	ENSG0000	8.85	3.365	1.174	1.05E-12
SND1	ENSG0000	82.521	38.05	1.097	2.44E-57
MRPL21	ENSG0000	61.23	29.245	1.041	5.43E-45
OGDHL	ENSG0000	18.45	41.286	-1.12	1.77E-17
PDGFA	ENSG0000	9.08	1.965	1.765	1.02E-27
NMB	ENSG0000	3.43	0.835	1.272	8.27E-32
S100A10	ENSG0000	241.168	54.345	2.129	2.01E-41
RPL37A	ENSG0000	2021.064	985.047	1.036	9.48E-37
GPAA1	ENSG0000	115.511	39.639	1.52	7.85E-66
UGT2B17	ENSG0000	0.75	3.04	-1.207	1.79E-03
HIST1H2BI	ENSG0000	106.042	36.82	1.501	4.03E-23
S100A6	ENSG0000	69.07	28.38	1.254	2.49E-12
RPL12	ENSG0000	922.752	451.379	1.03	9.95E-36
MPZL1	ENSG0000	21.41	8.445	1.247	1.17E-30
RPS4X	ENSG0000	486.365	194.576	1.317	6.51E-48
GRK6	ENSG0000	10.62	4.71	1.025	1.04E-47
PRIM1	ENSG0000	5.97	1.56	1.445	1.73E-42
RP11-347(	ENSG0000	0.37	1.89	-1.077	4.93E-25
ZNF544	ENSG0000	9.53	3.565	1.206	2.66E-35
SULT1C2	ENSG0000	4.09	0.35	1.915	3.66E-19

RPL23A	ENSG0000	690.213	342.33	1.01	5.01E-38
UBL5	ENSG0000	409.522	183.329	1.155	1.28E-70
MT1F	ENSG0000	7	191.386	-4.588	6.81E-49
TXNRD1	ENSG0000	30.751	11.718	1.32	1.14E-22
FLVCR1-A:	ENSG0000	2.93	0.71	1.201	1.73E-32
HLA-DRB5	ENSG0000	14.2	5.88	1.144	1.50E-05
ZNF28	ENSG0000	3.54	0.65	1.46	1.30E-20
PPIAP22	ENSG0000	165.466	34.006	2.25	9.13E-79
STK39	ENSG0000	2.72	0.525	1.286	7.44E-24
TAT	ENSG0000	98.25	345.525	-1.804	4.06E-16
LPA	ENSG0000	1.21	8.365	-2.083	6.63E-44
MT-CO2	ENSG0000	13276.62	27014.57	-1.025	1.28E-24
GLMP	ENSG0000	123.023	36.071	1.742	4.49E-67
MT-CYB	ENSG0000	6751.535	15334.04	-1.183	2.38E-28
F5	ENSG0000	206.614	92.446	1.152	8.08E-13
EPS8L3	ENSG0000	4.76	0.13	2.35	2.06E-30
MT-ND2	ENSG0000	6531.958	14680.36	-1.168	2.13E-23
MT-ND5	ENSG0000	1410.997	3065.91	-1.119	9.28E-17
SCAMP5	ENSG0000	3.87	0.69	1.527	2.60E-24
HMGN2	ENSG0000	225.736	103.877	1.112	3.38E-42
SELM	ENSG0000	33.981	12.185	1.408	9.10E-14
MT-ND3	ENSG0000	9254.247	19245.72	-1.056	5.56E-24
TSEN15	ENSG0000	11.54	5.17	1.023	1.19E-41
MT-ND4	ENSG0000	9684.084	23104.72	-1.254	1.08E-34
MT-ND1	ENSG0000	5574.782	11534.36	-1.049	3.52E-24
SHISA4	ENSG0000	11.06	3.83	1.32	1.95E-21
MT-ATP6	ENSG0000	11214.67	31942.73	-1.51	1.16E-41
PRC1	ENSG0000	10.68	1.12	2.462	3.32E-64
RPL39	ENSG0000	1924.809	846.93	1.183	1.93E-44
SOWAHA	ENSG0000	2.79	0.565	1.276	1.14E-34
MFAP3L	ENSG0000	1.98	7.005	-1.426	9.41E-28
TGM2	ENSG0000	49.931	107.727	-1.094	3.91E-12
RNU4-1	ENSG0000	2.49	0	1.803	8.85E-02
RNU4-2	ENSG0000	13.67	5.598	1.153	5.34E-06
TATDN3	ENSG0000	7.83	3.265	1.05	3.12E-45
SAMD5	ENSG0000	0.48	2.47	-1.229	5.79E-26
SOX18	ENSG0000	4.61	1.78	1.013	5.70E-29
FAM127B	ENSG0000	33.109	15.435	1.053	8.59E-12
NEU4	ENSG0000	9.57	24.385	-1.264	4.71E-12
GGTA1P	ENSG0000	3.88	1.105	1.213	7.57E-28
PFDN6	ENSG0000	118.569	41.236	1.501	1.97E-73
HNRNPCP	ENSG0000	7.96	0.895	2.241	1.00E-63
HLA-DMA	ENSG0000	37.32	12.255	1.532	1.92E-20
TAP2	ENSG0000	17.289	7.24	1.15	1.36E-20
HLA-DRA	ENSG0000	171.468	42.669	1.982	3.47E-16
FKBPL	ENSG0000	5.26	2.025	1.049	4.76E-60
NELFE	ENSG0000	58.618	14.75	1.92	4.92E-73
NEU1	ENSG0000	55.299	15.57	1.765	3.02E-68
LSM2	ENSG0000	38.869	13.895	1.42	2.80E-64
VARS	ENSG0000	36.521	13.985	1.324	3.20E-47
GPANK1	ENSG0000	16.02	6.145	1.252	4.25E-59
NFKBIL1	ENSG0000	18.87	8.68	1.038	1.09E-51
HLA-C	ENSG0000	499.658	223.828	1.155	7.73E-16
LRCOL1	ENSG0000	1.04	7.855	-2.118	2.13E-29
TRIM31	ENSG0000	2.55	0.295	1.455	7.90E-21
PPP1R11	ENSG0000	42.579	19.67	1.076	8.00E-46
GNB2L1	ENSG0000	891.937	427.994	1.058	4.11E-47
HLA-F	ENSG0000	95.709	30.939	1.598	5.53E-21

GABBR1	ENSG0000	0.68	2.665	-1.125	3.18E-12
ST8SIA6-A	ENSG0000	3.81	0	2.266	2.87E-27
FAM83A-/	ENSG0000	0.45	4.95	-2.037	2.15E-08
PRSS1	ENSG0000	0	1.17	-1.118	6.84E-25
PSENEN	ENSG0000	80.968	34.785	1.196	1.95E-59
PSMB10	ENSG0000	59.112	28.645	1.02	1.14E-29
KRT17P4	ENSG0000	0.05	1.34	-1.156	1.80E-35
MT1H	ENSG0000	2.16	332.67	-6.722	2.39E-61
MT1DP	ENSG0000	0.59	4.235	-1.719	2.76E-18
MT1A	ENSG0000	15.19	81.693	-2.353	1.74E-18
MT1M	ENSG0000	1.78	112.018	-5.345	8.93E-60
EXOC3L4	ENSG0000	17.59	43.365	-1.255	2.97E-16
SERF1B	ENSG0000	18.65	6.19	1.45	1.62E-60
HMGN1	ENSG0000	69.42	32.281	1.081	3.30E-36
LCMT1	ENSG0000	12.62	5.335	1.104	5.15E-53
SMIM11	ENSG0000	4.77	1.32	1.314	3.18E-14
FAM99B	ENSG0000	0.23	3.835	-1.975	1.97E-44
FAM99A	ENSG0000	1.1	37.31	-4.189	1.13E-52
HBA1	ENSG0000	10	98.975	-3.184	2.99E-47
HLA-H	ENSG0000	36.931	6.175	2.402	1.66E-34
HLA-A	ENSG0000	714.504	165.214	2.106	3.22E-46
IGKC	ENSG0000	145.713	295.005	-1.013	2.58E-03
IGKV4-1	ENSG0000	1.76	6.825	-1.503	1.78E-02
IGLV1-51	ENSG0000	1.47	5.9	-1.482	1.29E-04
IGLV1-47	ENSG0000	1.11	3.538	-1.105	5.66E-02
IGLV1-44	ENSG0000	1.35	3.99	-1.086	7.36E-02
IGLV1-40	ENSG0000	1.33	6.649	-1.715	1.26E-03
IGLV3-25	ENSG0000	0.84	3.975	-1.435	9.68E-04
IGLV3-19	ENSG0000	1.17	3.615	-1.089	4.90E-02
IGLV2-14	ENSG0000	2.14	11.483	-1.991	2.77E-06
IGLV2-11	ENSG0000	1.12	4.605	-1.403	9.43E-05
IGLC2	ENSG0000	39.769	93.769	-1.217	5.45E-03
IGLC3	ENSG0000	24.69	52.18	-1.05	1.49E-03
TRBC2	ENSG0000	11.13	5.035	1.007	3.92E-09
IGHA2	ENSG0000	2.83	10.167	-1.544	5.71E-12
IGHG4	ENSG0000	4.9	13.65	-1.312	2.88E-03
IGHG2	ENSG0000	10.09	28.674	-1.42	3.72E-04
IGHA1	ENSG0000	21.08	103.5	-2.243	3.01E-16
IGHM	ENSG0000	8.76	27.634	-1.553	1.81E-10
IGHV2-5	ENSG0000	0.23	1.725	-1.148	3.21E-04
IGHV3-15	ENSG0000	0.45	1.995	-1.046	6.26E-03
IGHV3-33	ENSG0000	0.74	2.745	-1.106	4.21E-03
IGHV5-51	ENSG0000	0.64	2.455	-1.075	1.92E-03
RPL15P3	ENSG0000	8.69	3.015	1.271	6.17E-38
MT-ND4L	ENSG0000	5341.391	15483.03	-1.535	6.04E-27
LPAL2	ENSG0000	1.31	6.39	-1.678	4.47E-39
RP11-641[	ENSG0000	174.126	60.058	1.52	2.02E-49
SUPT4H1	ENSG0000	66.192	30.304	1.102	4.01E-58
GSTM2	ENSG0000	4.74	20.76	-1.923	4.67E-22
LCAT	ENSG0000	32.421	153.649	-2.21	2.18E-42
GBP7	ENSG0000	6.71	14.798	-1.035	7.89E-07
RPLP0P6	ENSG0000	13.89	4.415	1.459	7.08E-44
SLX1A-SU	ENSG0000	2.04	6.45	-1.293	6.72E-12
HEXA	ENSG0000	76.68	33.93	1.153	2.55E-44
SLC35F6	ENSG0000	16.66	7.18	1.11	4.73E-44
UGT2B11	ENSG0000	3.8	0.615	1.572	4.59E-20
UBD	ENSG0000	334.949	7.468	5.31	1.60E-66
LTB4R	ENSG0000	2.01	5.365	-1.08	3.40E-08

TAX1BP3	ENSG0000	26.981	11.99	1.107	2.37E-17
TSPAN4	ENSG0000	40.52	18.425	1.096	2.62E-21
LYRM4	ENSG0000	16	6.435	1.193	1.35E-52
PTMAP5	ENSG0000	2.79	0.765	1.103	9.23E-26
PLIN5	ENSG0000	18.75	68.379	-1.813	8.12E-20
RPL7P1	ENSG0000	20.549	5	1.845	1.49E-45
MEG3	ENSG0000	2.21	22.203	-2.854	6.21E-17
TOMM6	ENSG0000	230.017	86.413	1.402	1.25E-72
RP11-480I	ENSG0000	3.39	0.955	1.167	3.16E-25
GOLGA8B	ENSG0000	7.7	19.393	-1.229	3.83E-03
GCGR	ENSG0000	4.47	57.661	-3.423	2.38E-39
TSTD1	ENSG0000	79.181	35.715	1.127	6.37E-20
IFI30	ENSG0000	140.769	51.639	1.429	1.31E-30
RPS2P55	ENSG0000	2.26	0.33	1.293	2.92E-51
RP11-475(	ENSG0000	15.42	7.145	1.011	2.34E-28
FTH1P8	ENSG0000	9.88	1.33	2.223	4.15E-60
RP11-40C(	ENSG0000	389.91	0	8.611	3.30E-78
TRIM16	ENSG0000	6.27	2.35	1.118	7.03E-22
HEPN1	ENSG0000	0.21	5.295	-2.379	5.40E-09
UBA52	ENSG0000	852.821	414.275	1.04	8.44E-57
TIAF1	ENSG0000	0.48	2.72	-1.33	2.07E-39
LINC0015Z	ENSG0000	35.26	4.54	2.711	1.71E-59
RP11-632I	ENSG0000	1.68	5.815	-1.346	2.31E-26
HLA-DPB1	ENSG0000	59.821	17.4	1.725	2.74E-14
UBE2SP2	ENSG0000	3.54	0.085	2.065	3.79E-71
SNRPGP15	ENSG0000	0.25	1.765	-1.145	4.66E-19
HNRNPA1	ENSG0000	7.86	3.015	1.142	1.06E-33
RP11-51O	ENSG0000	21.64	8.325	1.28	1.06E-44
SH3BP5-A	ENSG0000	0.9	3.05	-1.092	1.24E-18
RP11-228I	ENSG0000	0.38	2.354	-1.281	1.39E-25
RP11-415J	ENSG0000	0.74	3.8	-1.464	3.31E-31
RP11-390I	ENSG0000	1.26	4.439	-1.267	5.45E-28
RPL35P5	ENSG0000	5.17	1.38	1.374	7.81E-45
MTND2P2	ENSG0000	211.541	441.922	-1.059	2.46E-12
FAM195B	ENSG0000	68.721	30.14	1.163	3.78E-40
FAM229A	ENSG0000	1.52	4.93	-1.235	6.43E-12
RP11-384I	ENSG0000	1.46	4.205	-1.081	2.87E-23
NOL7	ENSG0000	38.55	15.44	1.266	1.50E-59
RP4-706A	ENSG0000	22.42	9.355	1.177	2.60E-30
RP11-419(	ENSG0000	2.46	11.16	-1.813	2.07E-18
RP11-61N	ENSG0000	6.12	1.49	1.516	1.21E-19
FTH1P20	ENSG0000	13.07	1.48	2.504	1.81E-84
RPL39P40	ENSG0000	1.67	0	1.417	4.39E-02
RP11-128I	ENSG0000	1.61	0.24	1.074	4.26E-37
TBCAP1	ENSG0000	1.64	0.26	1.067	8.15E-27
RP3-417G	ENSG0000	2.94	0.94	1.022	5.27E-29
LINC00511	ENSG0000	1.86	0.105	1.372	2.19E-23
RPS28P7	ENSG0000	722.222	182.518	1.979	1.96E-74
WASH7P	ENSG0000	1.2	4.41	-1.298	1.11E-15
HPN-AS1	ENSG0000	0.59	3.69	-1.561	1.59E-26
LTB	ENSG0000	7.99	2.18	1.499	1.25E-15
SPAG5-AS	ENSG0000	0.5	2.125	-1.059	1.73E-24
RP11-452I	ENSG0000	11.78	4.12	1.32	1.81E-47
MT-ATP8	ENSG0000	11020.48	37358.53	-1.761	1.62E-29
LINC0132(	ENSG0000	0.46	2.05	-1.063	1.73E-09
ORM2	ENSG0000	1430.198	3502.573	-1.292	8.12E-14
OST4	ENSG0000	273.879	129.4	1.076	7.70E-69
RP3-342P2	ENSG0000	0.1	3.96	-2.173	5.87E-47

RP1-241P1	ENSG0000	3.77	0.74	1.455	9.14E-15
SPCS2P4	ENSG0000	30.96	12.14	1.282	2.97E-41
RP11-556E	ENSG0000	1.14	0	1.098	1.21E-22
LINC00659	ENSG0000	1.39	4.703	-1.255	2.20E-14
PGA4	ENSG0000	0.05	1.7	-1.362	3.57E-43
MT1P3	ENSG0000	0	1.41	-1.269	1.22E-09
HMGN1P3	ENSG0000	1.59	0	1.373	5.96E-38
ORM1	ENSG0000	4575.773	15121.36	-1.724	3.35E-19
HLA-DRB6	ENSG0000	4.28	0.965	1.426	1.57E-14
PET100	ENSG0000	68.322	29.18	1.2	8.36E-63
PGA3	ENSG0000	0.06	2.469	-1.71	8.52E-49
GOLGA6L5	ENSG0000	0.93	3.465	-1.21	1.80E-18
RP4-631H	ENSG0000	0	1.5	-1.322	1.76E-07
HLA-DPA1	ENSG0000	49.969	17.045	1.498	2.45E-11
RPS18	ENSG0000	2144.461	874.278	1.293	5.72E-48
FTH1P7	ENSG0000	25.5	2.38	2.971	2.02E-72
GOLGA8N	ENSG0000	1.07	3.665	-1.172	2.69E-20
LINC00665	ENSG0000	2.16	0.485	1.089	3.09E-18
GTF2IP4	ENSG0000	23.86	8.225	1.43	1.57E-28
MTND4P2	ENSG0000	4	25.358	-2.398	3.83E-27
RP11-295	ENSG0000	20.34	5.555	1.703	9.96E-37
MT1XP1	ENSG0000	0	2.03	-1.599	2.90E-28
RP11-250E	ENSG0000	0.5	2.48	-1.214	3.11E-40
H2BFS	ENSG0000	7.35	2.365	1.311	1.07E-27
MAGI2-AS	ENSG0000	1.53	8.76	-1.948	1.65E-24
HMGN2P5	ENSG0000	42.659	13.404	1.6	2.32E-34
RP11-465N	ENSG0000	4.77	1.63	1.134	3.17E-22
RP11-166E	ENSG0000	0.87	3.453	-1.252	1.57E-17
GAS5	ENSG0000	169.388	80.573	1.063	1.93E-27
HLA-B	ENSG0000	788.298	281.004	1.485	1.15E-16
RPS3AP6	ENSG0000	12.82	5.885	1.005	1.09E-27
RPL23AP4	ENSG0000	644.751	238.359	1.432	1.41E-52
SOX9-AS1	ENSG0000	2.87	0.72	1.17	9.13E-19
FABP5P7	ENSG0000	8.22	1.645	1.801	1.41E-32
TECRP1	ENSG0000	6.04	1.785	1.338	4.63E-39
HGH1	ENSG0000	22.409	10.215	1.062	1.99E-38
RPL39P3	ENSG0000	319.151	110.798	1.518	2.15E-18
RP4-763G	ENSG0000	5.4	19.574	-1.685	9.76E-20
SUMO2P1	ENSG0000	1.16	0	1.111	1.18E-06
FAM103A2	ENSG0000	6.95	2.265	1.284	2.92E-28
RP11-575L	ENSG0000	0.44	2.52	-1.289	9.72E-23
SNRPGP10	ENSG0000	1.81	5.694	-1.252	3.22E-15
TOPORS-A	ENSG0000	9.65	3.685	1.185	1.22E-48
RPL6P27	ENSG0000	12.27	4.93	1.162	9.28E-36
H3F3AP4	ENSG0000	144.256	34.374	2.038	4.70E-16
PNMA6A	ENSG0000	9.82	4.305	1.028	2.33E-07
RP11-215A	ENSG0000	2.32	0.17	1.505	1.69E-56
HSPB1P1	ENSG0000	5.31	0	2.658	1.66E-49
RP11-175E	ENSG0000	1.41	4.86	-1.282	6.52E-12
RPL13AP5	ENSG0000	68.802	31.064	1.122	1.31E-37
PITPNA-A	ENSG0000	8.52	3.245	1.165	5.67E-37
LINC00106	ENSG0000	0.56	2.26	-1.063	1.09E-12
LINC01554	ENSG0000	1.42	69.249	-4.859	4.99E-26
NDUFA6-A	ENSG0000	5.28	12.819	-1.138	9.18E-15
RP4-669L1	ENSG0000	3.09	8.316	-1.188	1.19E-09
HAND2-A	ENSG0000	0.12	2.285	-1.552	3.44E-58
HLA-DQA	ENSG0000	2.19	0.425	1.163	1.62E-08
UBE2Q2P6	ENSG0000	655.975	307.128	1.092	1.39E-15

KIFC1	ENSG0000	4.37	0.205	2.156	2.31E-55
LINC0137(	ENSG0000	0.53	6.555	-2.304	1.99E-14
LINC0084Z	ENSG0000	5.59	37.984	-2.565	5.09E-17
PTENP1	ENSG0000	1.98	0.39	1.1	5.57E-32
OR2I1P	ENSG0000	25.71	1.305	3.535	2.25E-34
SCARNA1Z	ENSG0000	0.37	1.865	-1.064	5.67E-19
RP11-464I	ENSG0000	2.61	0.35	1.419	1.55E-43
RP11-295I	ENSG0000	3.58	10.585	-1.339	6.20E-08
NME1	ENSG0000	100.099	29.344	1.736	3.98E-60
MRPS17	ENSG0000	16.48	7.29	1.076	5.73E-45
ITIH4-AS1	ENSG0000	0.58	3.97	-1.653	4.22E-33
IGKV3-20	ENSG0000	6.35	17.302	-1.316	2.63E-03
LY6G5B	ENSG0000	1.49	4.07	-1.026	3.60E-05
PSMB9	ENSG0000	50.011	17.194	1.487	6.35E-29
RP11-499I	ENSG0000	2.1	7.765	-1.499	3.20E-16
IGKV1-17	ENSG0000	0.59	2.365	-1.082	1.24E-02
PLGLA	ENSG0000	4.32	22.95	-2.171	6.68E-46
FLJ22763	ENSG0000	0.76	6.25	-2.042	3.65E-27
IGKV2-24	ENSG0000	0.37	2.074	-1.166	5.29E-08
RPL36A	ENSG0000	883.691	296.337	1.573	5.82E-59
IGKV3-11	ENSG0000	8.89	19.145	-1.026	2.15E-03
RPP21	ENSG0000	44.901	18.17	1.26	2.39E-62
RPL37P6	ENSG0000	2.03	0	1.599	8.18E-40
PSMC1P1	ENSG0000	13.25	5.405	1.154	3.47E-19
INMT	ENSG0000	1.57	7.73	-1.764	6.84E-36
IGKV1-9	ENSG0000	0.57	2.357	-1.097	1.74E-03
HOGA1	ENSG0000	7.79	21.02	-1.325	6.56E-23
PRR34-AS	ENSG0000	7.78	3.39	1	3.51E-24
IGKV1-33	ENSG0000	1.17	5.51	-1.585	1.25E-03
LINC0096S	ENSG0000	15.97	33.294	-1.015	1.63E-03
PEG10	ENSG0000	5.21	0.71	1.861	3.12E-13
STAG3L5P	ENSG0000	5.22	13.125	-1.183	5.08E-08
UGT1A10	ENSG0000	1.21	0.1	1.007	4.13E-17
HLA-DMB	ENSG0000	14.22	4.74	1.407	5.56E-17
MBL1P	ENSG0000	0.22	3.06	-1.735	3.31E-49
FTH1P23	ENSG0000	2.4	0.63	1.061	2.57E-49
IGKV1-5	ENSG0000	1.45	5.679	-1.447	3.65E-03
NME1-NM	ENSG0000	398.434	162.714	1.287	1.52E-54
RP11-274I	ENSG0000	4.96	12.679	-1.199	4.58E-10
RP11-6B4.	ENSG0000	0.17	4.765	-2.301	2.21E-48
RP5-966M	ENSG0000	6.86	66.16	-3.095	4.32E-35
PLA2G4B	ENSG0000	1.46	4.7	-1.212	7.46E-13
NPIPB5	ENSG0000	9.71	48.205	-2.2	8.53E-16
RPS12P21	ENSG0000	0.07	1.17	-1.02	2.74E-46
RPL23AP6!	ENSG0000	2.83	0.525	1.329	2.76E-26
RP11-404(	ENSG0000	0.35	2.57	-1.403	1.16E-22
IGKV2-28	ENSG0000	2.52	8.89	-1.49	2.38E-04
IFITM10	ENSG0000	1.82	8.635	-1.773	3.62E-14
XXbac-BP(	ENSG0000	5.75	1.324	1.538	9.66E-05
LINC0129€	ENSG0000	2.28	0.15	1.512	2.92E-34
IGKV3-15	ENSG0000	1.61	5.659	-1.351	1.39E-02
RP11-10G.	ENSG0000	2.99	0	1.996	3.34E-66
IGKV1-27	ENSG0000	0.4	1.94	-1.07	5.39E-03
HBB	ENSG0000	15.86	167.277	-3.319	2.55E-49
NEAT1	ENSG0000	124.507	481.719	-1.943	1.13E-11
SNHG6	ENSG0000	110.921	47.349	1.211	2.29E-36
H2AFJ	ENSG0000	110.208	42.105	1.367	5.74E-43
UBAP1L	ENSG0000	0.45	1.93	-1.015	5.99E-24

TWF2	ENSG0000	30.83	12.465	1.241	1.10E-36
MTND4P1	ENSG0000	39.65	0.81	4.489	5.49E-17
RRN3P1	ENSG0000	1.24	5.655	-1.571	1.25E-33
TRIM52-A	ENSG0000	9.2	3.35	1.229	7.07E-50
MTATP6P1	ENSG0000	1387.526	3715.008	-1.42	1.04E-23
RP11-328	ENSG0000	3.02	13.645	-1.865	2.86E-17
RP11-510	ENSG0000	0.27	1.56	-1.011	8.48E-13
RP11-290	ENSG0000	3.19	20.87	-2.384	1.44E-36
LINC0109	ENSG0000	0.55	23.3	-3.971	3.78E-87
NPM1P27	ENSG0000	5.95	1.935	1.244	5.04E-35
RP11-434	ENSG0000	1.32	11.964	-2.482	2.64E-30
RP11-423	ENSG0000	0.19	1.485	-1.062	2.96E-15
ECSCR	ENSG0000	6.8	2.27	1.254	1.22E-32
PVT1	ENSG0000	6.01	1.035	1.784	7.25E-20
GBA3	ENSG0000	8.22	31.277	-1.808	1.37E-18
LINC0101	ENSG0000	5.58	19.171	-1.616	8.20E-12
YJEFN3	ENSG0000	0.55	2.535	-1.189	4.56E-10
RP11-622	ENSG0000	5.56	23.667	-1.911	1.49E-28
HULC	ENSG0000	307.319	57.466	2.399	1.54E-20
F11-AS1	ENSG0000	2.75	8.87	-1.396	2.92E-32
IGKV1D-3	ENSG0000	3.94	11.967	-1.392	4.79E-03
MALAT1	ENSG0000	13.79	31.885	-1.153	4.83E-04
RP11-119	ENSG0000	4.61	10.829	-1.076	2.11E-05
RNA5SP21	ENSG0000	0	7.302	-3.054	1.59E-19
TRNP1	ENSG0000	5.14	1.415	1.346	8.41E-12
LINC0160	ENSG0000	8.22	3.345	1.085	3.98E-34
PRKDC	ENSG0000	22.389	9.685	1.13	4.97E-16
RP11-115	ENSG0000	0.56	3.09	-1.391	1.34E-09
RP11-115	ENSG0000	1.93	7.789	-1.585	9.26E-04
RP11-496	ENSG0000	0.65	2.6	-1.125	9.34E-08
RNF185-A	ENSG0000	3.09	0.212	1.755	6.30E-05
RP11-326	ENSG0000	0.96	7.133	-2.053	1.31E-07
SAA2-SAA	ENSG0000	2.75	17.052	-2.267	1.56E-11
PIGY	ENSG0000	8.94	0.005	3.306	6.41E-13
RP11-111	ENSG0000	4.52	1.645	1.061	4.45E-37
RP11-196	ENSG0000	0.57	2.975	-1.34	2.77E-47
MT1JP	ENSG0000	0.05	4.62	-2.42	1.61E-49
TRAPPC2B	ENSG0000	10.94	3.775	1.322	5.49E-35
RPL41P2	ENSG0000	37.139	17.225	1.065	2.90E-18
RP1-102E	ENSG0000	0.27	2.495	-1.46	2.12E-46
RP11-73M	ENSG0000	1.31	0	1.208	1.45E-21
RP11-680	ENSG0000	3.77	1.305	1.049	1.13E-16
SNRPEP2	ENSG0000	7.97	1.61	1.781	2.82E-64
HP	ENSG0000	8497.954	26421.96	-1.636	2.98E-17
RP11-203	ENSG0000	1.67	4.945	-1.155	4.29E-11
RP11-579	ENSG0000	1.95	0.33	1.149	5.42E-28
RP11-620	ENSG0000	5.08	1.055	1.565	8.54E-53
RP11-114	ENSG0000	17.64	3.005	2.219	4.02E-19
RP11-256	ENSG0000	0.83	3.32	-1.239	5.22E-20
RP11-386	ENSG0000	69.622	32.095	1.094	7.95E-18
RP11-161	ENSG0000	58.602	23.37	1.29	4.29E-30
RP11-164	ENSG0000	0.77	2.985	-1.171	1.98E-22
RP11-66N	ENSG0000	1.31	5.55	-1.504	6.60E-14
LINC0159	ENSG0000	0.99	6.505	-1.915	1.21E-14
RP11-316	ENSG0000	78.699	24.778	1.628	2.61E-19
RP11-830	ENSG0000	0.04	1.39	-1.2	2.41E-51
SLX1B-SU	ENSG0000	0.99	3.245	-1.093	4.32E-11
MRC1	ENSG0000	1.92	5.135	-1.071	2.95E-07

RP11-345J	ENSG0000	2.12	0	1.642	1.56E-04
MT1L	ENSG0000	5.34	45.263	-2.867	1.56E-33
PECAM1	ENSG0000	11.39	3.285	1.532	2.92E-39
SMG1P7	ENSG0000	1.05	4.145	-1.328	1.69E-23
RP11-345J	ENSG0000	18.04	5.385	1.576	5.76E-58
RP11-676J	ENSG0000	0.11	1.625	-1.242	6.65E-29
RP11-667I	ENSG0000	6.33	0.655	2.147	5.25E-22
GDF2	ENSG0000	0	4.84	-2.546	1.40E-115
RP11-334E	ENSG0000	6.87	0.46	2.43	1.24E-44
RP11-159I	ENSG0000	1	3.94	-1.305	8.45E-12
SNRPGP2	ENSG0000	9.32	2.87	1.415	5.66E-38
GJA5	ENSG0000	2.24	0.385	1.226	1.02E-31
RBM8A	ENSG0000	83.152	34.049	1.264	3.29E-57
RP11-138I	ENSG0000	198.074	98.4	1.002	6.64E-21
RP11-524F	ENSG0000	0.37	2.685	-1.427	1.64E-41
RP11-452I	ENSG0000	7.38	2.75	1.16	3.59E-14
RP5-890E1	ENSG0000	4.32	0	2.411	2.20E-14
MRPS21	ENSG0000	63.999	22.47	1.47	1.96E-74
FXYD1	ENSG0000	79.34	237.14	-1.568	2.63E-12
RP11-49K2	ENSG0000	3.59	1.135	1.104	3.04E-08
SMUG1P1	ENSG0000	0	1.055	-1.039	1.88E-58
POLR2J2	ENSG0000	5.5	2.205	1.02	1.64E-14
NDUFA7	ENSG0000	96.837	42.579	1.167	9.36E-70
RP3-461F1	ENSG0000	7.65	1.78	1.638	3.70E-34
ZSCAN16-	ENSG0000	8.45	3.54	1.058	4.19E-41
PTOV1-AS	ENSG0000	2.39	6.895	-1.22	4.51E-08
RP11-394C	ENSG0000	0.48	2.335	-1.172	1.21E-17
RP5-940J5	ENSG0000	20.359	2.015	2.825	1.22E-06
RP11-34P1	ENSG0000	0.85	2.82	-1.046	5.09E-08
RP1-232P2	ENSG0000	0.06	1.595	-1.292	1.26E-34
NBPF8	ENSG0000	2.65	12.719	-1.91	1.69E-33
HIST2H4A	ENSG0000	8.54	3.165	1.196	6.77E-22
RP11-238F	ENSG0000	0.14	2.04	-1.415	1.53E-04
SRXN1	ENSG0000	30.141	10.395	1.45	1.06E-28
RP11-125S	ENSG0000	0.32	2.325	-1.333	1.81E-26
SNX29P2	ENSG0000	0.59	3.925	-1.631	4.19E-40
RP11-42O	ENSG0000	4.66	11.395	-1.131	6.70E-13
HIST2H2A	ENSG0000	29.59	9.48	1.545	1.62E-12
RP11-284F	ENSG0000	1.99	0.06	1.496	3.11E-29
RP11-713I	ENSG0000	10.17	3.25	1.394	3.38E-32
STAG3L5P	ENSG0000	14.78	42.15	-1.451	4.67E-08
LL22NC03	ENSG0000	1.96	0.29	1.198	1.07E-26
RP3-508I1	ENSG0000	0	1.745	-1.457	4.80E-18
RP11-458F	ENSG0000	1.31	3.715	-1.029	1.74E-12
RP4-583P1	ENSG0000	2.09	7.12	-1.394	1.02E-32
RP11-756A	ENSG0000	2.13	0	1.646	2.60E-20
TAF9	ENSG0000	33.171	15.97	1.01	1.93E-35
Metazoa_S	ENSG0000	343.769	129.247	1.404	1.20E-14
uc_338	ENSG0000	0	1.365	-1.242	4.08E-27
RP4-568C1	ENSG0000	1.02	3.25	-1.073	3.07E-10
RP4-564F2	ENSG0000	0.54	5.865	-2.156	5.41E-43
RP13-516M	ENSG0000	0.38	1.96	-1.101	1.50E-25
PRSS2	ENSG0000	0.03	1.25	-1.127	1.60E-22
HULC	ENSG0000	127.221	34.625	1.848	6.45E-12
UHRF1	ENSG0000	1.79	0.115	1.323	6.25E-35
HERC2P2	ENSG0000	4.35	13.429	-1.431	8.98E-06
MARCKS	ENSG0000	15.05	5.1	1.396	5.08E-28
TRAC	ENSG0000	7.94	2.665	1.286	3.38E-12

PSMB3	ENSG0000	225.627	100.068	1.165	7.70E-57
SRD5A2	ENSG0000	1.17	6.605	-1.809	1.59E-20
RP11-452I	ENSG0000	37.569	5.96	2.47	9.36E-52
IGLV2-8	ENSG0000	0.55	2.785	-1.288	3.27E-06
RP11-132/	ENSG0000	1.1	6.998	-1.929	2.20E-45
RP11-609I	ENSG0000	1.08	3.6	-1.145	9.69E-08
RP11-369J	ENSG0000	1.73	0.33	1.038	8.97E-05
RP11-258I	ENSG0000	0.23	1.655	-1.11	9.13E-37
RP11-715I	ENSG0000	0	1.02	-1.014	1.02E-18
RPL41	ENSG0000	2353.512	831.227	1.5	7.63E-52
RP11-394I	ENSG0000	0.23	1.495	-1.02	2.61E-32
RP11-43N	ENSG0000	0.69	3.215	-1.318	1.67E-22
RP11-286I	ENSG0000	0.06	1.165	-1.03	5.25E-29
RP11-361I	ENSG0000	20.53	65.36	-1.624	1.61E-03
LINC0134	ENSG0000	1.2	8.645	-2.132	5.14E-42
U91328.1	ENSG0000	1.96	0	1.566	1.88E-03
RP11-817J	ENSG0000	0.04	1.375	-1.191	3.70E-21
WASH5P	ENSG0000	5.2	12.53	-1.126	3.52E-05
LINC0100	ENSG0000	3.89	14.567	-1.671	1.84E-11

:EPIA2 (TCGA)
