# Supplementary figures and images for: Identifying Potential Tumor Antigens and Antigens-Related Subtypes in Hepatocellular Carcinoma for mRNA Vaccine Development
Source: J Oncol. 2022 Aug 29;2022:6851026. doi: 10.1155/2022/6851026 (PMC9444406; doi:10.1155/2022/6851026)

A

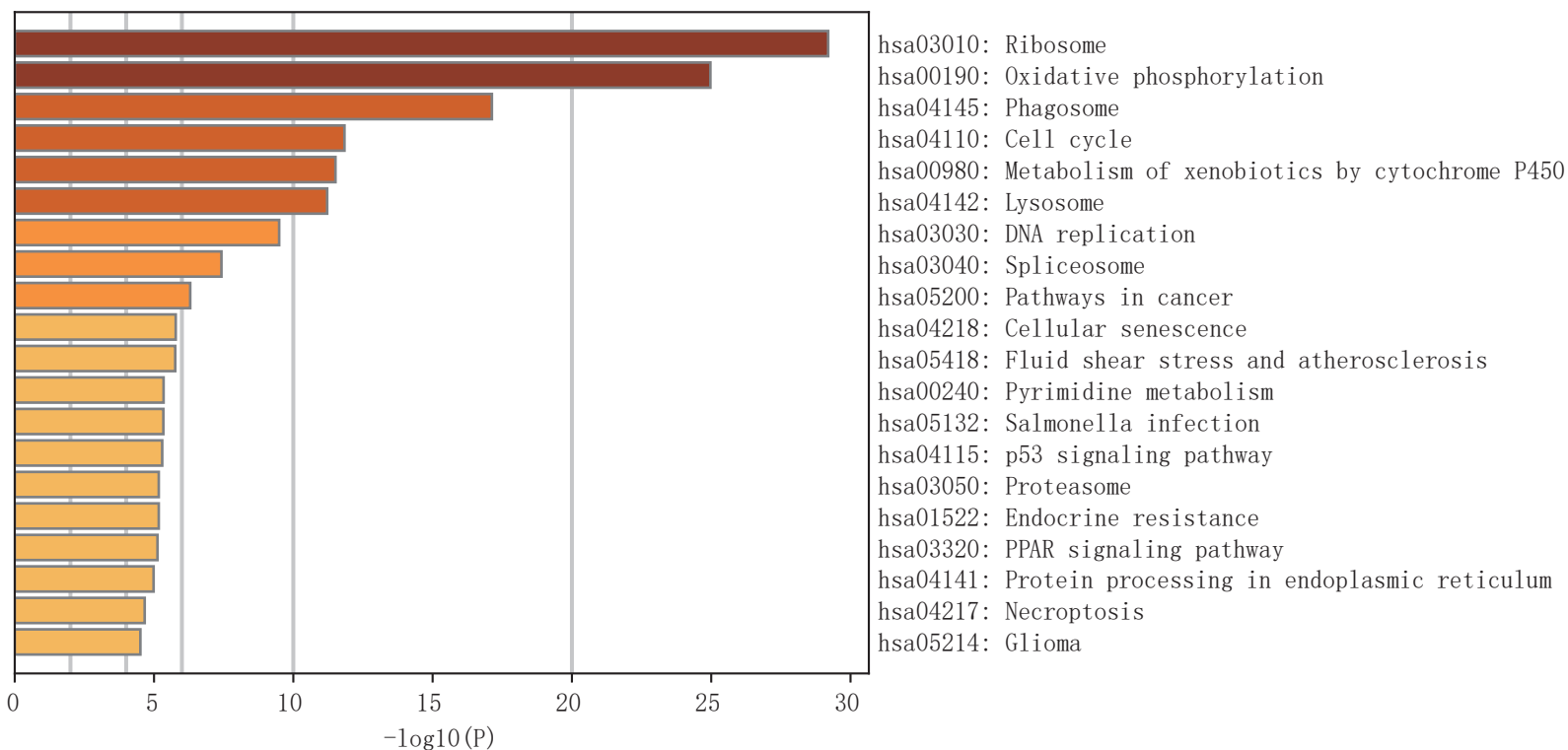

B

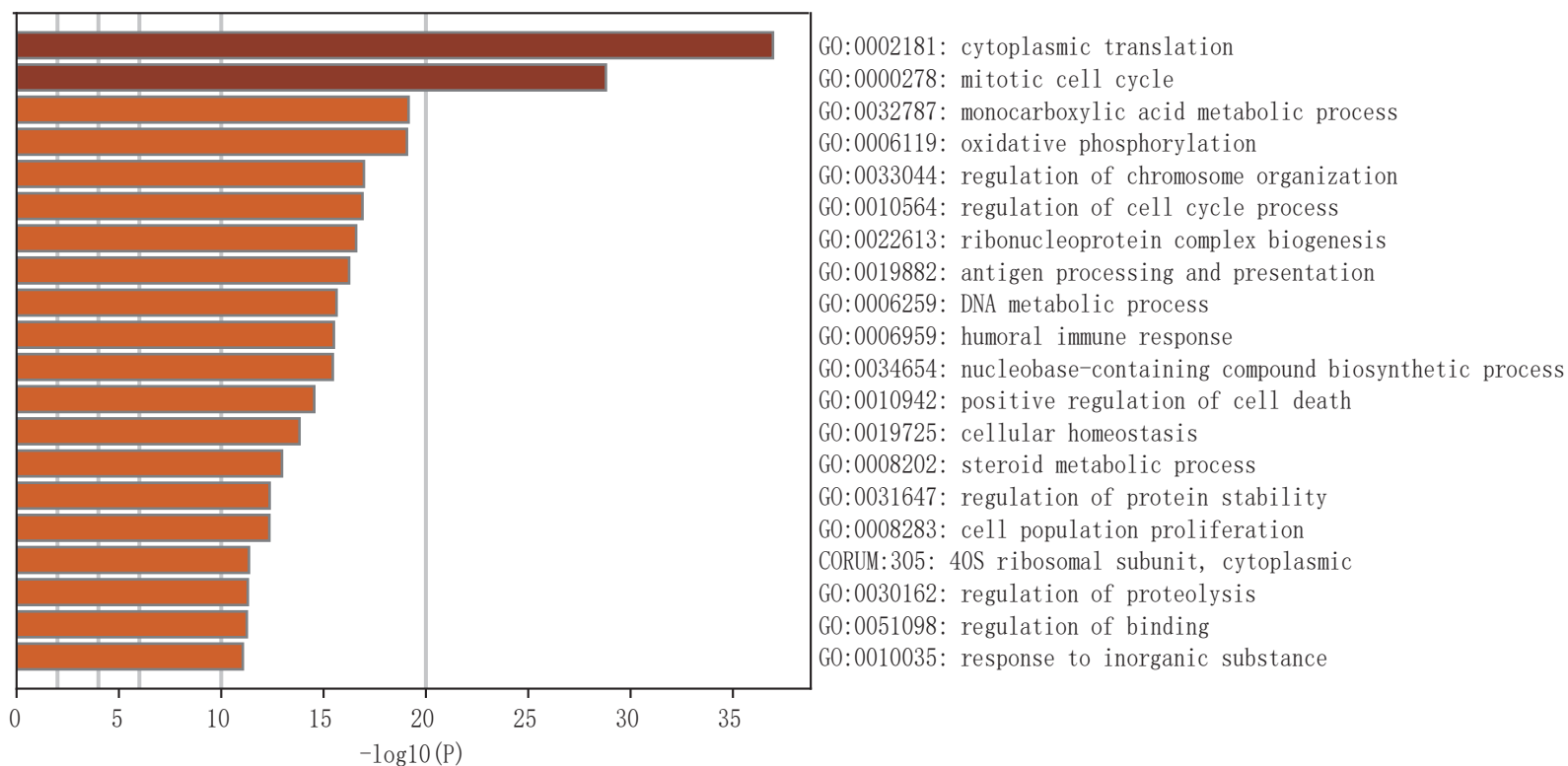

Supplement: Supplementary Materials — Table S1: clinical data from ICGC. Table S2: Differently Expressed Genes Analysis from cbioptrol. Differential Expression Analysis from GEPIA. Table S3: mutation Analysis of LIHC from cbioptrol. Supplementary Figure 1: KEGG and GO enrichment analyses of these candidate genes. Supplementary Figure 2: heatmaps of antigens in TCGA and CGGA cohort with cluster annotations. [file 6851026.f1.zip › Supplemental figure1.pdf]

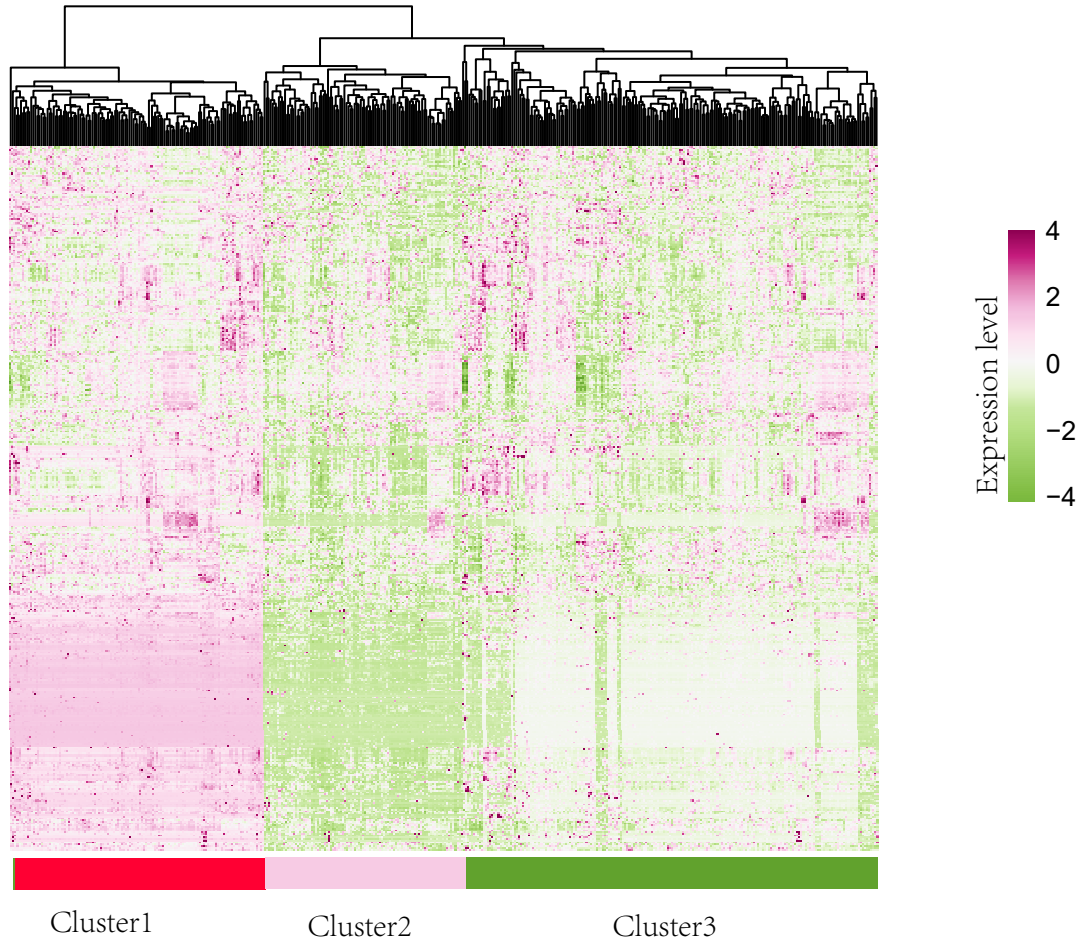

Supplement: Supplementary Materials — Table S1: clinical data from ICGC. Table S2: Differently Expressed Genes Analysis from cbioptrol. Differential Expression Analysis from GEPIA. Table S3: mutation Analysis of LIHC from cbioptrol. Supplementary Figure 1: KEGG and GO enrichment analyses of these candidate genes. Supplementary Figure 2: heatmaps of antigens in TCGA and CGGA cohort with cluster annotations. [file 6851026.f1.zip › Supplemental figure2.pdf]
