# Supplementary material for: Identifying Potential Tumor Antigens and Antigens-Related Subtypes in Hepatocellular Carcinoma for mRNA Vaccine Development
Source: J Oncol. 2022 Aug 29;2022:6851026. doi: 10.1155/2022/6851026 (PMC9444406; doi:10.1155/2022/6851026)
Supplement: Supplementary Materials — Table S1: clinical data from ICGC. Table S2: Differently Expressed Genes Analysis from cbioptrol. Differential Expression Analysis from GEPIA. Table S3: mutation Analysis of LIHC from cbioptrol. Supplementary Figure 1: KEGG and GO enrichment analyses of these candidate genes. Supplementary Figure 2: heatmaps of antigens in TCGA and CGGA cohort with cluster annotations. [file Table S2 (1).pdf › Table S2 (1).pdf]

Table 1: Gene expression data from ICGC.

	DO22834	DO22835	DO22836	DO22837	DO22838	DO22839	DO22842	DO22845
A1BG	0.000275	0.000925	0.001934	0.002445	0.004345	0.001283	0.002596	0.001333
A1CF	4.70E-05	6.10E-05	7.42E-05	3.24E-05	1.30E-05	4.70E-05	2.71E-05	5.05E-05
A2BP1	0	0	0	0	0	0	0	0
A2LD1	3.39E-06	8.64E-06	8.37E-06	4.37E-06	5.28E-06	5.45E-06	8.42E-06	7.40E-06
A2M	0.006141	0.000293	0.001051	0.002665	0.001183	0.00056	0.001143	0.002772
A2ML1	0	7.40E-09	7.88E-08	0	0	0	6.41E-09	0
A4GALT	1.01E-05	2.63E-06	1.06E-06	2.92E-06	1.80E-06	1.13E-06	4.19E-06	2.33E-06
A4GNT	1.65E-07	0	5.77E-08	0	3.54E-08	0	0	0
AAA1	4.97E-07	1.68E-07	0	0	0	1.44E-06	0	0
AAAS	2.40E-05	1.89E-05	1.24E-05	1.66E-05	1.22E-05	2.13E-05	1.33E-05	1.49E-05
AACS	9.51E-06	5.99E-06	2.28E-06	4.07E-06	9.10E-07	4.66E-06	2.95E-06	3.03E-06
AACSL	1.20E-07	0	0	0	0	0	0	0
AADAC	7.82E-05	0.000267	0.000344	0.000356	0.00022	0.000865	0.000202	0.000165
AADACL2	0	2.29E-08	0	0	0	0	0	0
AADACL3	0	0	0	0	0	0	0	1.74E-08
AADACL4	0	0	0	0	0	0	0	0
AADAT	2.31E-06	8.71E-06	3.44E-05	7.36E-06	2.60E-06	6.44E-06	2.44E-06	2.27E-06
AAGAB	1.47E-05	1.17E-05	5.39E-06	9.44E-06	5.13E-06	1.24E-05	1.20E-05	1.08E-05
AAK1	8.05E-06	4.87E-06	1.28E-06	1.85E-06	1.14E-06	3.20E-06	1.71E-06	2.19E-06
AAMP	0.000116	7.72E-05	6.03E-05	5.71E-05	4.29E-05	8.01E-05	5.08E-05	4.98E-05
AANAT	0	0	0	0	0	2.84E-08	2.89E-08	0
AARS	5.85E-05	0.000104	5.07E-05	3.33E-05	1.73E-05	8.74E-05	3.53E-05	8.03E-05
AARS2	7.30E-06	1.26E-05	2.92E-06	2.98E-06	2.35E-06	5.67E-06	3.48E-06	4.83E-06
AARSD1	4.86E-05	4.82E-05	1.43E-05	2.08E-05	2.57E-05	3.97E-05	3.40E-05	2.39E-05
AASDH	5.65E-06	1.94E-06	2.86E-06	3.15E-06	6.72E-07	4.15E-06	2.31E-06	3.23E-06
AASDHPP	2.22E-05	1.66E-05	1.64E-05	1.20E-05	4.05E-06	1.92E-05	9.04E-06	1.54E-05
AASS	3.24E-05	4.16E-05	1.90E-05	1.16E-05	9.82E-06	3.84E-05	2.46E-06	9.95E-06
AATF	5.19E-05	4.99E-05	1.30E-05	2.05E-05	2.28E-05	2.97E-05	2.63E-05	2.47E-05
AATK	1.21E-05	4.05E-07	1.06E-07	5.40E-07	7.88E-08	1.74E-07	2.16E-06	1.76E-07
ABAT	6.00E-05	0.000345	0.000238	0.000126	2.57E-05	0.000143	7.55E-05	8.27E-05
ABCA1	1.09E-05	4.77E-06	1.76E-05	7.04E-06	4.00E-06	1.36E-05	3.98E-06	1.75E-05
ABCA10	1.49E-06	4.09E-07	5.04E-07	6.69E-07	3.05E-06	3.12E-07	2.96E-07	5.98E-07
ABCA11P	2.85E-06	1.13E-06	6.68E-07	1.08E-06	3.35E-07	9.02E-07	8.82E-07	1.27E-06
ABCA12	9.59E-08	1.53E-08	1.40E-08	2.68E-08	0	2.54E-09	5.36E-09	0
ABCA13	2.73E-07	1.01E-07	3.21E-09	2.46E-07	0	1.19E-08	4.89E-09	5.31E-08
ABCA17P	2.40E-07	0	1.46E-08	1.71E-08	1.48E-07	0	2.68E-08	4.10E-08
ABCA2	1.43E-05	7.11E-06	8.01E-06	5.56E-06	1.82E-06	5.00E-06	7.09E-06	1.16E-05
ABCA3	5.10E-06	2.32E-07	4.76E-07	7.47E-07	6.14E-07	2.43E-07	7.32E-07	1.70E-06
ABCA4	1.66E-07	1.57E-06	2.94E-08	4.82E-08	1.02E-07	2.04E-06	5.04E-08	7.56E-08
ABCA5	5.43E-06	1.55E-05	8.63E-06	3.90E-06	2.57E-06	7.88E-06	5.13E-06	6.12E-06
ABCA6	2.15E-05	4.06E-05	3.73E-05	2.41E-05	1.40E-05	4.35E-05	1.06E-05	2.35E-05
ABCA7	7.00E-06	5.08E-06	6.54E-07	2.55E-06	1.06E-05	2.29E-06	2.98E-06	3.67E-06
ABCA8	1.02E-05	5.24E-06	1.48E-05	1.37E-05	5.85E-06	1.50E-05	9.61E-06	3.31E-06
ABCA9	6.49E-07	3.01E-07	1.26E-06	2.24E-06	3.39E-07	1.15E-06	5.98E-07	8.63E-07
ABCB1	5.25E-05	3.17E-05	1.36E-05	2.71E-05	1.86E-05	3.25E-05	1.46E-05	1.09E-05
ABCB10	9.93E-06	1.35E-05	8.68E-06	4.75E-06	2.02E-06	1.10E-05	5.58E-06	9.09E-06
ABCB11	1.68E-06	4.20E-05	2.10E-05	2.45E-05	1.06E-06	4.23E-05	6.05E-06	1.12E-05
ABCB4	5.74E-06	3.88E-05	3.96E-05	0.000104	4.69E-05	5.71E-05	4.27E-05	6.71E-05
ABCB5	3.16E-08	8.30E-07	0	7.82E-08	0	0	0	7.21E-08
ABCB6	2.05E-05	2.60E-05	1.22E-05	6.73E-06	2.56E-05	4.33E-05	1.46E-05	8.50E-06
ABCB7	2.05E-05	1.45E-05	1.04E-05	1.06E-05	7.65E-06	1.34E-05	6.75E-06	1.64E-05
ABCB8	7.14E-06	9.05E-06	1.14E-05	9.04E-06	9.57E-06	1.93E-05	1.26E-05	2.43E-05
ABCB9	3.97E-06	2.53E-06	6.08E-07	5.62E-07	4.30E-07	6.62E-07	6.25E-07	8.93E-07
ABCC1	7.58E-06	4.68E-07	9.47E-07	2.35E-06	2.29E-07	1.38E-06	9.99E-07	9.89E-07
ABCC10	5.74E-06	1.78E-06	1.03E-06	1.59E-06	9.96E-07	1.80E-06	1.79E-06	3.00E-06
ABCC11	2.11E-07	2.33E-06	1.59E-06	9.60E-07	7.87E-08	1.11E-06	6.57E-07	2.62E-06

ABCC12	0	0	0	0	0	0	4.73E-09	7.12E-09
ABCC13	0	2.74E-08	0	1.03E-08	0	0	0	0
ABCC2	2.78E-05	5.44E-05	3.57E-05	1.61E-05	6.82E-06	7.37E-05	1.26E-05	2.18E-05
ABCC3	7.36E-05	4.29E-05	5.14E-05	4.18E-05	6.83E-05	0.000101	5.70E-05	4.50E-05
ABCC4	3.77E-05	1.31E-05	1.27E-06	1.93E-06	6.18E-08	3.63E-06	8.44E-07	2.44E-06
ABCC5	1.26E-05	2.57E-06	1.40E-06	3.62E-06	2.11E-06	3.67E-06	2.65E-06	3.05E-06
ABCC6	6.18E-05	8.91E-05	9.26E-05	4.69E-05	5.50E-05	7.85E-05	5.35E-05	6.66E-05
ABCC6P1	1.07E-05	7.89E-06	1.16E-05	6.11E-06	4.25E-06	8.73E-06	4.31E-06	5.20E-06
ABCC6P2	3.40E-05	2.34E-05	1.53E-05	1.02E-05	6.53E-06	2.25E-05	5.98E-06	3.31E-05
ABCC8	2.72E-07	0	9.29E-09	5.53E-09	0	2.52E-08	0	1.26E-07
ABCC9	7.71E-07	7.50E-06	1.65E-05	5.91E-06	2.58E-06	8.42E-06	4.91E-06	5.45E-06
ABCD1	9.41E-06	1.29E-05	6.85E-06	6.81E-06	9.31E-06	1.14E-05	7.21E-06	2.36E-05
ABCD2	2.06E-07	0	7.77E-08	9.77E-08	0	8.57E-08	1.23E-08	6.16E-09
ABCD3	3.32E-05	6.54E-05	4.43E-05	3.60E-05	1.00E-05	7.46E-05	1.50E-05	2.72E-05
ABCD4	1.69E-05	1.35E-05	1.68E-05	1.66E-05	1.76E-05	1.34E-05	1.44E-05	1.43E-05
ABCE1	1.71E-05	1.03E-05	1.27E-05	1.24E-05	2.85E-06	1.70E-05	9.29E-06	1.48E-05
ABCF1	3.31E-05	5.27E-05	1.86E-05	2.55E-05	2.40E-05	3.26E-05	3.10E-05	4.21E-05
ABCF2	9.38E-06	1.78E-05	5.28E-06	9.55E-06	5.15E-06	1.49E-05	1.09E-05	1.51E-05
ABCF3	3.63E-05	3.78E-05	2.23E-05	2.45E-05	2.58E-05	3.41E-05	2.14E-05	3.54E-05
ABCG1	3.64E-05	2.40E-06	6.13E-06	4.12E-06	7.77E-07	1.67E-06	4.63E-06	6.30E-05
ABCG2	2.95E-06	1.31E-05	9.77E-06	7.67E-06	4.18E-06	2.05E-05	5.61E-06	2.75E-06
ABCG4	8.25E-08	7.11E-09	1.96E-08	3.10E-08	1.09E-08	0	3.70E-08	9.30E-09
ABCG5	2.47E-05	3.00E-05	2.45E-05	2.49E-05	2.48E-05	1.91E-05	1.30E-05	1.02E-05
ABCG8	3.27E-05	6.81E-05	3.56E-05	2.50E-05	1.65E-05	6.18E-05	2.56E-05	2.57E-05
ABHD1	9.07E-07	4.89E-06	3.00E-06	6.45E-07	5.01E-07	7.61E-06	7.99E-07	5.92E-07
ABHD10	1.46E-05	1.57E-05	1.66E-05	1.09E-05	1.41E-05	1.55E-05	1.14E-05	1.30E-05
ABHD11	1.31E-05	3.67E-05	5.64E-06	1.65E-05	7.66E-06	5.60E-06	1.70E-05	1.71E-05
ABHD12	7.28E-05	6.79E-05	1.73E-05	2.86E-05	5.52E-05	2.06E-05	4.31E-05	3.60E-05
ABHD12B	3.57E-08	1.84E-07	3.81E-08	2.20E-08	0	4.98E-08	3.24E-08	0
ABHD13	4.76E-06	5.06E-06	4.53E-06	4.76E-06	1.50E-06	3.65E-06	3.20E-06	3.70E-06
ABHD14A	1.26E-05	1.48E-05	1.50E-05	1.58E-05	3.15E-05	1.93E-05	2.04E-05	2.26E-05
ABHD14B	0.000111	5.53E-05	0.000128	0.000122	0.000243	0.000183	0.000152	7.62E-05
ABHD15	1.10E-05	2.25E-05	1.59E-05	6.41E-06	8.85E-06	3.33E-06	1.17E-05	1.59E-06
ABHD2	8.26E-05	4.74E-05	5.62E-05	6.14E-05	1.86E-05	2.90E-05	6.11E-05	4.12E-05
ABHD3	7.44E-05	2.42E-05	1.39E-05	1.83E-05	4.88E-06	1.67E-05	1.30E-05	1.94E-05
ABHD4	6.99E-05	2.90E-05	6.92E-06	1.07E-05	4.75E-06	3.13E-05	9.62E-06	3.12E-05
ABHD5	3.46E-06	5.46E-06	6.81E-06	4.65E-06	1.02E-06	5.66E-06	5.96E-06	5.05E-06
ABHD6	1.03E-05	2.31E-05	3.40E-05	2.13E-05	1.12E-05	2.46E-05	2.33E-05	1.11E-05
ABHD8	1.07E-05	8.63E-06	9.03E-06	7.68E-06	1.08E-05	7.31E-06	1.84E-05	1.05E-05
ABI1	2.16E-05	1.68E-05	9.56E-06	1.39E-05	3.96E-06	1.43E-05	1.03E-05	9.89E-06
ABI2	7.40E-06	4.04E-06	2.03E-06	3.27E-06	1.42E-06	2.53E-06	1.85E-06	1.53E-06
ABI3	9.76E-06	1.71E-06	4.53E-06	5.81E-06	2.94E-06	4.67E-06	5.52E-06	2.79E-06
ABI3BP	5.86E-06	1.91E-06	1.53E-06	3.23E-06	2.27E-07	5.76E-08	1.75E-06	9.28E-07
ABL1	1.58E-05	4.70E-06	4.01E-06	9.95E-06	2.56E-06	9.86E-06	6.98E-06	9.20E-06
ABL2	2.60E-06	1.68E-06	8.77E-07	2.05E-06	5.58E-07	3.34E-06	1.41E-06	1.91E-06
ABLIM1	2.37E-05	8.01E-06	6.03E-06	1.15E-05	3.17E-06	1.94E-05	1.29E-05	6.43E-06
ABLIM2	1.60E-05	2.90E-07	5.89E-07	2.55E-06	6.97E-07	4.11E-06	2.13E-06	1.67E-06
ABLIM3	2.17E-05	1.50E-05	2.27E-05	2.67E-05	1.05E-05	2.92E-05	4.59E-05	3.97E-05
ABO	0	3.94E-08	1.28E-07	6.37E-08	1.48E-08	0	1.65E-08	1.53E-07
ABP1	2.06E-06	1.20E-07	9.20E-08	7.12E-08	2.45E-06	2.69E-08	1.76E-06	1.42E-08
ABR	1.35E-05	1.28E-06	2.78E-06	8.04E-06	2.08E-06	2.12E-06	3.69E-06	2.19E-06
ABRA	1.69E-08	0	0	0	0	1.57E-08	0	0
ABT1	2.43E-05	2.05E-05	1.21E-05	1.44E-05	8.24E-06	1.66E-05	1.66E-05	2.12E-05
ABTB1	1.76E-05	1.87E-05	1.16E-05	2.21E-05	1.72E-05	1.19E-05	1.78E-05	1.30E-05
ABTB2	5.33E-06	1.07E-05	7.84E-06	4.54E-06	1.56E-06	8.19E-06	6.09E-06	1.06E-05
ACAA1	0.000121	0.000338	0.00035	0.000193	0.000383	0.000278	0.000276	6.66E-05
ACAA2	0.000277	0.000238	0.000533	0.000359	0.000195	0.00024	0.00034	0.000124
ACACA	2.17E-05	3.54E-05	7.73E-06	4.80E-06	6.52E-06	9.78E-06	8.20E-06	1.96E-05

ACACB	1.50E-05	2.78E-05	4.11E-05	9.78E-06	1.96E-05	1.20E-05	1.12E-05	1.10E-05
ACAD10	1.97E-05	4.53E-05	1.48E-05	1.58E-05	6.66E-06	2.00E-05	8.25E-06	1.18E-05
ACAD11	2.86E-05	4.38E-05	4.87E-05	2.76E-05	1.95E-05	5.19E-05	2.29E-05	6.08E-06
ACAD8	2.23E-05	2.03E-05	1.71E-05	1.63E-05	7.62E-06	1.88E-05	1.08E-05	2.19E-05
ACAD9	3.20E-05	3.80E-05	2.49E-05	2.38E-05	2.81E-05	2.96E-05	3.31E-05	2.35E-05
ACADL	5.66E-08	1.33E-05	1.30E-05	1.16E-05	0	2.64E-06	4.91E-06	2.32E-06
ACADM	5.64E-05	0.000155	0.000124	7.42E-05	1.63E-05	0.000139	5.06E-05	5.63E-05
ACADS	3.51E-05	6.60E-05	0.000147	8.74E-05	3.98E-05	4.29E-05	7.48E-05	3.78E-05
ACADSB	3.23E-05	5.85E-05	0.000158	4.95E-05	2.16E-05	6.34E-05	3.20E-05	3.63E-05
ACADVL	0.000153	0.000233	0.000285	0.000357	0.000158	0.000286	0.000405	0.000291
ACAN	5.92E-07	5.59E-08	5.30E-09	0	3.32E-08	5.77E-08	1.58E-07	5.69E-08
ACAP1	4.67E-06	1.37E-07	1.55E-06	3.79E-06	1.37E-07	1.91E-06	1.72E-06	8.79E-07
ACAP2	6.58E-06	4.74E-06	4.33E-06	3.42E-06	7.74E-07	4.34E-06	2.59E-06	4.18E-06
ACAP3	1.30E-05	7.71E-06	4.35E-06	6.76E-06	4.23E-06	5.94E-06	1.12E-05	8.38E-06
ACAT1	0.000116	0.000133	0.000441	0.000298	0.000176	0.000201	0.000225	0.000295
ACAT2	5.72E-05	0.000217	8.76E-05	4.27E-05	1.58E-05	6.26E-05	2.99E-05	4.58E-05
ACBD3	2.21E-05	1.89E-05	9.15E-06	1.58E-05	2.36E-06	1.86E-05	9.80E-06	2.90E-05
ACBD4	3.84E-05	4.36E-05	4.93E-05	4.31E-05	9.34E-05	5.22E-05	5.56E-05	1.01E-05
ACBD5	2.22E-05	4.25E-05	3.10E-05	2.38E-05	8.82E-06	2.33E-05	1.85E-05	1.39E-05
ACBD6	1.39E-05	9.05E-06	5.76E-06	7.23E-06	5.73E-06	1.15E-05	1.32E-05	1.16E-05
ACBD7	3.93E-07	3.06E-07	1.41E-08	1.42E-07	1.82E-07	3.22E-07	8.62E-08	1.00E-07
ACCN1	1.74E-08	9.77E-09	0	2.34E-07	0	8.04E-09	6.73E-08	0
ACCN2	3.92E-06	1.44E-08	7.94E-08	8.62E-08	2.53E-07	9.68E-07	3.74E-08	2.26E-07
ACCN3	1.14E-07	7.38E-07	4.00E-08	1.12E-07	1.32E-06	4.06E-07	2.66E-07	1.31E-07
ACCN4	0	0	2.09E-08	0	0	0	9.72E-09	0
ACCN5	0	0	0	0	0	2.68E-08	1.39E-08	0
ACCS	4.10E-06	2.38E-06	2.96E-06	3.39E-06	2.67E-06	1.06E-05	3.09E-06	3.74E-06
ACCSL	0	0	0	0	0	1.23E-08	0	0
ACD	1.06E-05	6.29E-06	3.20E-06	4.74E-06	3.62E-06	5.94E-06	6.13E-06	5.83E-06
ACE	1.03E-05	1.82E-06	1.32E-06	1.99E-06	5.42E-07	2.03E-06	2.31E-06	3.74E-06
ACE2	1.79E-06	2.92E-06	1.75E-06	1.24E-06	6.84E-08	2.69E-05	9.67E-08	4.86E-08
ACER1	0	1.11E-07	1.05E-07	5.95E-08	6.25E-08	6.29E-07	0	0
ACER2	3.42E-07	5.77E-07	4.33E-07	4.58E-07	7.00E-09	9.45E-07	1.74E-07	2.03E-07
ACER3	4.09E-06	1.94E-06	1.46E-06	1.89E-06	4.92E-07	9.78E-07	1.54E-06	2.67E-06
ACHE	9.39E-06	3.07E-05	9.87E-07	3.31E-06	6.08E-07	1.54E-07	8.19E-06	2.25E-07
ACIN1	4.91E-05	2.31E-05	1.63E-05	2.77E-05	1.51E-05	2.34E-05	2.60E-05	2.43E-05
ACLY	7.01E-05	5.75E-05	1.21E-05	2.08E-05	5.02E-06	3.35E-05	1.56E-05	6.48E-05
ACMSD	0.000103	0.000214	9.07E-05	5.22E-05	7.96E-05	7.05E-05	7.79E-05	8.40E-05
ACN9	3.72E-06	8.85E-06	5.58E-06	3.80E-06	3.80E-06	6.82E-06	3.47E-06	4.81E-06
ACO1	1.95E-05	0.000248	7.55E-05	5.25E-05	1.72E-05	5.69E-05	3.34E-05	5.33E-05
ACO2	0.000224	0.000213	5.36E-05	4.85E-05	3.35E-05	5.58E-05	5.03E-05	9.48E-05
ACOT1	2.56E-05	3.00E-05	0.000103	4.27E-05	5.35E-05	3.85E-05	7.53E-05	6.35E-05
ACOT11	2.20E-06	8.37E-07	2.80E-07	3.53E-07	5.43E-08	4.00E-08	2.27E-07	0
ACOT12	3.09E-05	2.48E-05	3.62E-05	3.44E-05	1.30E-05	4.51E-06	2.48E-05	1.89E-05
ACOT13	2.29E-05	4.27E-05	2.29E-05	1.19E-05	1.20E-05	2.56E-05	1.60E-05	1.97E-05
ACOT2	9.51E-06	2.22E-05	4.32E-05	2.15E-05	1.69E-05	2.00E-05	4.75E-05	3.02E-05
ACOT4	9.01E-06	8.11E-06	9.88E-06	9.68E-06	7.62E-06	7.28E-06	1.17E-05	1.25E-05
ACOT6	7.99E-07	1.76E-06	1.08E-06	9.56E-07	1.59E-06	5.97E-07	9.14E-07	1.15E-06
ACOT7	3.84E-05	2.10E-05	1.47E-05	2.27E-05	1.90E-05	1.22E-05	1.89E-05	1.41E-05
ACOT8	2.92E-05	4.38E-05	2.04E-05	2.09E-05	4.16E-05	2.52E-05	2.17E-05	2.40E-05
ACOT9	1.98E-05	8.55E-06	7.15E-06	1.96E-05	6.65E-06	1.52E-05	1.44E-05	2.13E-05
ACOX1	6.72E-05	8.35E-05	6.41E-05	4.86E-05	3.41E-05	6.02E-05	5.63E-05	1.72E-05
ACOX2	1.22E-05	0.000106	0.00011	5.53E-05	2.66E-05	9.20E-05	3.23E-05	5.45E-05
ACOX3	9.04E-06	1.22E-05	1.36E-05	1.17E-05	5.29E-06	1.08E-05	1.93E-05	1.29E-05
ACOXL	4.97E-08	5.60E-08	0	1.22E-08	8.54E-09	0	2.89E-08	0
ACP1	0.00013	0.000117	5.81E-05	4.86E-05	5.18E-05	7.78E-05	5.32E-05	4.12E-05
ACP2	6.91E-05	7.50E-05	4.96E-05	4.78E-05	3.77E-05	4.20E-05	3.43E-05	0.000103
ACP5	9.51E-05	3.59E-06	3.48E-05	6.87E-05	3.28E-06	3.58E-06	5.02E-05	3.56E-06

ACP6	7.09E-06	4.73E-06	7.64E-06	3.68E-06	1.20E-05	1.69E-05	3.91E-06	5.75E-06
ACPL2	4.90E-06	7.44E-07	1.78E-06	2.07E-06	1.33E-06	2.33E-06	1.00E-06	2.22E-07
ACPP	4.54E-08	2.49E-08	4.59E-08	1.54E-07	2.28E-08	1.23E-08	3.78E-08	1.09E-08
ACPT	0	8.57E-08	0	2.30E-08	0	0	0	0
ACR	9.19E-08	9.04E-07	3.64E-07	1.58E-07	9.51E-08	3.36E-07	1.24E-07	1.90E-07
ACRBP	6.31E-07	4.59E-07	1.61E-07	7.37E-07	6.29E-07	4.51E-07	1.10E-06	6.00E-08
ACRC	3.56E-07	1.96E-07	6.75E-08	1.86E-07	3.80E-07	4.03E-07	7.75E-08	2.45E-07
ACRV1	4.17E-08	4.62E-08	0	9.26E-08	1.29E-07	0	2.57E-08	0
ACSBG1	1.13E-07	2.33E-07	5.37E-08	5.04E-08	2.66E-08	9.55E-09	8.97E-08	4.54E-08
ACSBG2	1.89E-08	2.12E-08	0	0	0	0	0	0
ACSF2	4.02E-05	6.38E-05	4.40E-05	3.75E-05	0.000151	0.000109	2.18E-05	1.81E-05
ACSF3	1.28E-05	4.90E-06	1.34E-05	1.72E-05	1.46E-05	2.32E-05	1.71E-05	1.10E-05
ACSL1	3.50E-05	0.000357	0.000967	0.000201	9.32E-05	0.000146	0.000282	0.00061
ACSL3	4.00E-05	3.58E-05	2.40E-05	1.82E-05	1.76E-05	2.40E-05	2.40E-05	6.94E-05
ACSL4	7.68E-05	8.10E-06	3.08E-06	8.90E-06	1.21E-06	1.26E-06	3.36E-05	0.000345
ACSL5	3.37E-05	3.58E-05	4.60E-05	4.72E-05	1.53E-05	0.000208	4.73E-05	1.29E-05
ACSL6	6.15E-06	6.03E-06	1.08E-06	4.70E-07	4.65E-08	9.02E-07	1.07E-07	1.46E-07
ACSM1	8.15E-08	0.000144	2.51E-06	4.22E-07	1.06E-06	2.89E-05	4.82E-07	4.02E-06
ACSM2A	6.78E-05	0.000239	0.000279	0.000139	0.000285	0.000126	0.000287	0.000151
ACSM2B	9.03E-05	0.000104	0.000257	0.000127	0.000131	0.000158	0.00013	0.000171
ACSM3	2.21E-06	0.0001	7.15E-05	2.24E-05	8.55E-06	5.86E-05	2.40E-05	1.97E-06
ACSM4	0	0	0	0	0	1.30E-08	0	0
ACSM5	1.61E-06	0.000114	0.000138	8.42E-05	1.18E-05	8.45E-05	8.63E-05	4.76E-05
ACSS1	9.26E-06	1.19E-06	3.30E-06	3.43E-06	2.36E-06	1.17E-06	2.49E-06	5.27E-06
ACSS2	5.84E-05	0.000248	5.57E-05	2.57E-05	2.87E-05	2.89E-05	3.32E-05	3.86E-05
ACSS3	1.17E-05	1.98E-05	3.43E-05	3.51E-05	4.41E-06	6.09E-05	2.43E-05	1.88E-05
ACTA1	2.50E-07	3.80E-08	1.77E-07	2.05E-08	5.75E-08	1.55E-08	0	4.92E-08
ACTA2	0.000241	6.26E-05	2.97E-05	8.08E-05	0.000133	0.000113	0.000122	0.000104
ACTB	0.002499	0.002833	0.001325	0.002154	0.000843	0.001513	0.003105	0.002183
ACTBL2	8.33E-09	0	0	1.12E-07	0	0	0	2.45E-08
ACTC1	3.10E-08	0	0	7.57E-09	1.06E-08	2.87E-08	0	2.72E-08
ACTG1	0.000977	0.001533	0.000767	0.001493	0.000539	0.000681	0.00115	0.000707
ACTG2	1.31E-05	5.94E-06	2.03E-07	1.81E-06	6.63E-06	1.48E-05	7.47E-06	1.05E-06
ACTL6A	1.96E-05	9.73E-06	8.41E-06	1.20E-05	1.74E-06	1.20E-05	9.10E-06	9.01E-06
ACTL6B	0	1.95E-07	0	3.46E-08	0	0	0	0
ACTL7A	0	0	0	0	0	0	0	0
ACTL7B	0	0	0	0	0	0	0	5.27E-08
ACTL8	0	0	0	0	0	1.69E-07	0	0
ACTL9	0	0	0	0	0	0	0	0
ACTN1	6.81E-05	3.10E-05	2.67E-05	4.39E-05	1.28E-05	7.65E-05	5.07E-05	3.21E-05
ACTN2	2.69E-08	6.78E-08	1.39E-07	2.11E-07	9.80E-08	4.14E-06	3.26E-08	5.47E-06
ACTN3	1.63E-08	1.83E-08	2.53E-08	4.98E-08	0	1.51E-08	0	0
ACTN4	0.000182	0.000168	7.70E-05	0.00013	0.000159	0.000109	0.000136	8.68E-05
ACTR10	2.81E-05	1.10E-05	1.27E-05	1.56E-05	1.11E-05	1.52E-05	1.37E-05	1.31E-05
ACTR1A	4.73E-05	2.44E-05	2.14E-05	3.26E-05	2.88E-05	3.82E-05	3.63E-05	4.48E-05
ACTR1B	4.53E-05	3.30E-05	2.45E-05	2.40E-05	2.08E-05	3.11E-05	3.24E-05	2.19E-05
ACTR2	0.000112	0.000112	4.13E-05	4.12E-05	1.05E-05	4.83E-05	2.97E-05	4.49E-05
ACTR3	8.08E-05	5.03E-05	3.08E-05	4.12E-05	1.35E-05	4.69E-05	3.33E-05	3.59E-05
ACTR3B	1.97E-06	3.28E-06	2.04E-06	2.16E-06	2.81E-06	3.92E-06	3.64E-06	5.30E-06
ACTR3C	4.84E-06	1.05E-05	8.19E-06	5.76E-06	3.06E-06	3.22E-06	2.85E-06	1.14E-05
ACTR5	6.22E-06	4.17E-06	3.87E-06	2.46E-06	1.70E-06	3.01E-06	2.93E-06	2.34E-06
ACTR6	1.13E-05	1.34E-05	9.19E-06	1.03E-05	3.73E-06	1.93E-05	1.20E-05	7.78E-06
ACTR8	8.38E-06	6.26E-06	3.01E-06	4.14E-06	1.87E-06	4.29E-06	2.80E-06	3.47E-06
ACTRT1	0	0	0	0	0	0	0	0
ACTRT2	0	0	0	0	0	0	0	0
ACVR1	2.79E-05	1.23E-05	1.43E-05	1.60E-05	3.56E-06	1.52E-05	8.95E-06	1.32E-05
ACVR1B	2.94E-05	1.19E-05	9.67E-06	1.01E-05	3.21E-06	1.05E-05	8.16E-06	1.69E-05
ACVR1C	1.44E-07	4.48E-07	6.95E-07	1.27E-07	1.10E-08	1.17E-07	3.09E-08	3.86E-08

ACVR2A	6.12E-06	3.21E-06	2.72E-06	2.77E-06	1.30E-06	1.50E-06	2.83E-06	2.12E-06
ACVR2B	9.26E-07	3.35E-07	5.35E-07	3.29E-07	3.41E-07	4.95E-07	3.47E-07	4.39E-07
ACVRL1	7.44E-06	2.28E-06	1.73E-06	2.52E-06	3.73E-06	2.38E-06	8.88E-06	2.90E-06
ACY1	0.000227	0.000322	0.00028	0.000193	0.000255	0.000314	0.000285	0.000145
ACY3	3.11E-05	8.39E-06	2.52E-05	3.04E-05	6.44E-06	1.42E-05	9.61E-06	1.47E-05
ACYP1	9.29E-06	1.77E-06	9.89E-07	2.28E-06	2.64E-06	4.31E-06	2.83E-06	2.45E-06
ACYP2	6.85E-06	1.27E-05	1.13E-05	9.04E-06	5.81E-06	1.42E-05	6.95E-06	8.26E-06
ADA	1.16E-05	9.94E-07	2.52E-06	4.48E-06	7.80E-07	2.51E-06	4.35E-06	4.11E-06
ADAD1	0	0	0	0	0	0	0	0
ADAD2	2.32E-08	6.56E-08	1.21E-08	3.77E-08	3.99E-08	5.38E-08	1.67E-07	0
ADAL	7.90E-06	5.67E-06	3.52E-06	2.83E-06	1.46E-06	4.47E-06	2.17E-06	2.07E-06
ADAM10	2.51E-05	2.66E-05	1.10E-05	1.37E-05	1.74E-06	1.31E-05	5.73E-06	1.41E-05
ADAM11	6.02E-07	1.15E-07	3.69E-08	5.44E-08	7.64E-08	5.77E-08	8.62E-09	3.92E-08
ADAM12	3.65E-06	1.20E-07	4.23E-08	2.31E-07	4.25E-08	1.39E-07	7.86E-07	5.92E-07
ADAM15	2.92E-05	4.21E-05	1.08E-05	1.35E-05	2.36E-05	1.86E-05	2.58E-05	3.69E-05
ADAM17	8.76E-06	7.98E-06	2.07E-06	4.10E-06	2.59E-07	2.61E-06	2.37E-06	2.78E-06
ADAM18	0	0	0	0	0	0	0	0
ADAM19	7.46E-06	1.21E-06	5.98E-07	2.98E-06	6.51E-07	1.66E-06	2.15E-06	5.85E-06
ADAM2	0	1.24E-08	0	0	0	0	0	0
ADAM20	5.00E-08	0	0	2.04E-08	1.43E-08	0	0	3.67E-08
ADAM21	1.96E-08	2.21E-08	2.04E-08	2.40E-08	0	2.36E-07	1.90E-08	2.88E-08
ADAM21P	1.17E-08	0	0	1.43E-08	0	9.74E-08	1.13E-08	1.72E-08
ADAM22	5.61E-07	4.45E-07	2.11E-08	4.99E-08	0	6.39E-09	1.34E-08	0
ADAM23	1.85E-07	1.17E-05	0	1.91E-08	3.75E-08	1.78E-08	1.12E-07	7.20E-08
ADAM28	3.44E-06	7.50E-08	8.48E-08	4.58E-07	2.81E-08	4.19E-08	1.28E-07	1.28E-07
ADAM29	0	0	0	9.00E-09	0	0	0	0
ADAM30	0	0	0	0	0	0	0	0
ADAM32	2.79E-06	0	0	2.09E-08	1.87E-07	9.75E-09	1.62E-08	0
ADAM33	1.20E-06	3.94E-08	1.46E-07	6.19E-07	1.64E-07	1.47E-07	2.38E-07	2.92E-07
ADAM3A	0	0	0	0	0	0	0	0
ADAM5P	0	0	0	0	0	0	0	0
ADAM6	1.35E-05	3.07E-08	7.44E-06	2.44E-05	1.35E-07	1.54E-06	2.90E-06	7.61E-07
ADAM7	0	0	0	0	0	0	0	0
ADAM8	2.77E-06	6.63E-07	1.67E-06	3.49E-06	6.49E-07	8.21E-07	9.69E-07	1.02E-06
ADAM9	1.80E-05	1.98E-05	4.21E-06	1.36E-05	5.42E-06	2.34E-05	7.11E-06	8.76E-06
ADAMDE(	8.49E-06	3.60E-08	5.55E-08	2.13E-07	0	2.33E-07	1.55E-06	1.88E-07
ADAMTS1	1.10E-05	1.11E-05	8.85E-06	4.66E-05	1.52E-06	3.77E-06	5.69E-06	9.23E-06
ADAMTS1	1.79E-06	3.46E-07	7.19E-07	6.76E-06	1.56E-06	4.78E-07	3.88E-06	4.26E-06
ADAMTS1	7.07E-07	1.48E-07	1.38E-07	5.41E-07	0	1.99E-07	2.42E-07	2.93E-07
ADAMTS1	7.42E-07	1.56E-06	2.64E-05	1.82E-05	2.87E-06	1.15E-06	2.09E-06	5.24E-06
ADAMTS1	3.29E-07	1.91E-08	3.07E-08	7.32E-08	3.31E-08	2.31E-07	2.17E-07	2.67E-07
ADAMTS1	9.21E-07	1.37E-07	8.44E-09	5.48E-07	0	6.79E-08	2.37E-08	9.56E-08
ADAMTS1	0	5.07E-09	9.85E-08	2.33E-07	0	8.39E-09	0	0
ADAMTS1	2.27E-07	4.10E-06	1.21E-06	6.03E-07	1.23E-07	5.88E-08	1.55E-06	9.78E-07
ADAMTS1	3.79E-09	5.93E-08	0	1.31E-08	0	1.40E-08	4.06E-08	3.88E-08
ADAMTS1	0	0	0	0	0	0	0	0
ADAMTS2	3.67E-06	3.72E-07	2.29E-06	8.48E-06	4.80E-07	5.45E-07	2.01E-06	9.01E-07
ADAMTS2	3.46E-08	1.89E-08	0	0	7.09E-09	0	0	0
ADAMTS3	4.05E-07	1.29E-08	4.73E-08	1.41E-07	6.61E-09	1.43E-08	2.25E-08	1.01E-07
ADAMTS4	2.93E-06	1.06E-05	5.09E-07	2.82E-05	1.33E-06	4.41E-06	5.37E-06	7.04E-06
ADAMTS5	9.92E-07	6.63E-07	3.80E-07	3.13E-07	8.45E-08	2.99E-07	2.97E-07	2.81E-07
ADAMTS6	1.88E-06	4.71E-08	3.98E-08	1.65E-07	5.57E-09	1.50E-08	6.33E-09	1.37E-07
ADAMTS7	1.29E-06	7.13E-07	3.44E-07	5.15E-07	2.95E-07	2.61E-07	1.51E-06	4.85E-07
ADAMTS8	5.18E-08	7.13E-08	6.59E-08	3.55E-07	4.61E-08	1.18E-07	1.12E-07	1.29E-07
ADAMTS9	4.23E-06	1.36E-06	3.31E-07	3.19E-06	3.23E-07	3.64E-06	5.69E-07	1.78E-06
ADAMTSL	9.87E-07	9.96E-08	3.77E-07	1.30E-06	3.22E-07	1.26E-06	1.72E-07	9.06E-07
ADAMTSL	4.81E-06	2.56E-06	1.86E-05	3.64E-05	3.51E-06	4.37E-07	4.22E-06	3.02E-06
ADAMTSL	3.98E-06	2.27E-06	5.25E-06	4.04E-06	5.73E-08	4.89E-07	1.18E-06	3.31E-06

ADAMTSL	1.30E-05	1.74E-05	2.70E-05	2.53E-05	1.47E-05	2.56E-05	1.15E-05	3.33E-05
ADAMTSL	7.51E-06	5.14E-06	9.68E-07	1.48E-06	2.47E-07	3.47E-07	2.36E-06	6.34E-07
ADAP1	1.09E-05	3.22E-07	1.67E-06	3.56E-06	4.25E-07	8.78E-07	1.72E-06	1.92E-07
ADAP2	1.77E-05	2.74E-06	4.27E-06	6.02E-06	9.03E-07	2.73E-06	4.97E-06	1.13E-06
ADAR	5.00E-05	5.05E-05	3.03E-05	2.32E-05	1.10E-05	5.89E-05	2.41E-05	8.57E-05
ADARB1	3.53E-06	1.32E-06	1.61E-06	2.57E-06	1.85E-06	2.19E-06	2.34E-06	2.32E-06
ADARB2	8.69E-08	4.21E-08	1.37E-07	0	2.83E-08	1.45E-08	1.58E-08	1.84E-08
ADAT1	5.50E-06	3.41E-06	2.42E-06	1.84E-06	1.54E-07	4.36E-06	9.86E-07	3.05E-06
ADAT2	2.81E-06	5.47E-07	3.36E-07	3.19E-07	2.48E-07	8.11E-07	5.28E-07	5.96E-07
ADAT3	2.05E-06	2.05E-06	4.80E-07	2.19E-06	1.71E-06	1.29E-06	2.92E-06	5.07E-07
ADC	7.70E-07	3.12E-07	3.96E-07	7.56E-07	1.68E-07	1.52E-07	7.44E-07	9.50E-07
ADCK1	6.50E-06	2.83E-06	1.61E-06	3.16E-06	5.40E-06	5.22E-06	4.70E-06	6.82E-06
ADCK2	9.41E-06	1.54E-05	8.52E-06	9.20E-06	5.81E-06	1.06E-05	1.71E-05	1.81E-05
ADCK4	1.60E-05	8.24E-06	8.46E-06	1.13E-05	1.11E-05	3.22E-05	1.02E-05	7.11E-06
ADCK5	8.50E-06	7.31E-06	5.17E-06	5.07E-06	1.29E-05	6.94E-06	1.61E-05	4.93E-06
ADCY1	1.38E-06	1.92E-06	2.89E-06	9.97E-07	2.19E-06	2.52E-06	4.82E-06	1.71E-06
ADCY10	2.57E-07	3.64E-06	8.86E-07	2.40E-07	1.23E-07	3.26E-06	9.12E-07	2.09E-06
ADCY2	5.42E-09	1.21E-08	0	0	0	5.01E-09	1.05E-08	1.59E-08
ADCY3	1.30E-05	3.34E-06	1.37E-06	4.20E-06	1.32E-06	2.43E-06	2.82E-06	2.80E-06
ADCY4	2.95E-06	1.52E-06	6.53E-07	6.50E-07	1.69E-06	1.20E-06	2.23E-06	2.59E-06
ADCY5	4.31E-06	7.99E-08	1.02E-06	1.04E-06	4.12E-08	1.08E-07	8.25E-08	4.96E-07
ADCY6	8.99E-06	3.27E-06	2.22E-06	3.17E-06	2.30E-06	4.07E-06	6.27E-06	6.16E-06
ADCY7	4.82E-06	5.17E-07	1.43E-06	2.25E-06	3.07E-07	1.16E-06	1.12E-06	5.33E-07
ADCY8	0	0	0	2.51E-07	0	0	5.99E-09	0
ADCY9	1.14E-05	1.05E-05	5.73E-06	7.64E-06	2.83E-06	6.72E-06	6.36E-06	7.41E-06
ADCYAP1	7.31E-09	2.46E-08	1.66E-07	1.06E-07	6.28E-09	4.06E-08	6.33E-08	8.58E-08
ADCYAP1I	0	1.79E-08	1.65E-08	3.83E-08	0	1.46E-08	1.51E-08	0
ADD1	8.72E-05	7.07E-05	4.78E-05	6.42E-05	3.27E-05	6.05E-05	5.52E-05	6.69E-05
ADD2	1.74E-07	0	7.94E-08	5.03E-08	3.30E-08	1.02E-07	1.49E-08	6.75E-08
ADD3	1.77E-05	1.68E-05	7.36E-06	8.40E-06	2.23E-06	1.06E-05	3.33E-06	1.62E-06
ADH1A	0.00022	0.000127	0.001772	0.000447	0.001192	0.001188	0.000822	0.000669
ADH1B	4.52E-05	0.00014	0.004456	0.001403	0.000881	0.003197	0.001956	0.000685
ADH1C	1.50E-05	8.74E-06	0.001123	0.000635	0.000209	0.001655	0.000439	0.000863
ADH4	2.85E-05	2.19E-05	0.002569	0.000328	0.000804	0.001889	0.000785	5.09E-05
ADH5	0.000186	0.000114	0.000129	0.000125	7.20E-05	0.000159	0.000119	0.000186
ADH6	4.07E-05	7.82E-05	0.000343	6.74E-05	0.000174	0.000346	0.00016	4.03E-05
ADH7	0	0	1.50E-07	1.36E-08	1.91E-08	1.85E-07	2.14E-08	1.63E-08
ADHFE1	3.20E-05	4.98E-05	2.75E-05	1.60E-05	2.66E-05	2.64E-05	5.19E-05	1.29E-05
ADI1	0.000166	0.000965	0.000442	0.000318	0.000399	0.000521	0.000515	0.000203
ADIG	1.49E-07	0	0	0	1.94E-07	1.01E-06	0	0
ADIPOQ	0	0	0	0	0	0	0	0
ADIPOR1	9.93E-05	8.51E-05	6.37E-05	5.47E-05	2.78E-05	0.000109	5.64E-05	0.000126
ADIPOR2	4.34E-05	6.61E-05	5.74E-05	3.24E-05	1.40E-05	5.41E-05	3.18E-05	5.61E-05
ADK	1.48E-05	1.34E-05	2.89E-05	1.84E-05	2.23E-05	3.49E-05	1.79E-05	1.51E-05
ADM	9.61E-06	9.87E-06	1.73E-05	0.000113	4.81E-06	1.51E-05	2.18E-05	1.24E-05
ADM2	6.33E-06	4.29E-06	1.30E-06	5.31E-07	6.69E-07	3.71E-07	3.35E-06	5.55E-06
ADNP	3.21E-05	1.75E-05	8.76E-06	1.06E-05	1.99E-06	1.33E-05	7.18E-06	8.03E-06
ADNP2	5.69E-06	2.88E-06	2.57E-06	3.08E-06	5.73E-07	2.15E-06	1.37E-06	3.39E-06
ADO	6.60E-06	5.86E-06	3.59E-06	5.30E-06	1.34E-06	2.85E-06	4.55E-06	3.48E-06
ADORA1	3.59E-07	1.00E-08	3.76E-07	7.49E-07	2.30E-08	2.63E-07	4.83E-07	1.05E-07
ADORA2A	4.39E-06	2.21E-06	1.63E-06	1.93E-06	1.57E-06	1.76E-06	1.92E-06	2.72E-06
ADORA2B	1.74E-06	4.35E-08	9.40E-08	1.02E-06	3.30E-08	2.26E-07	9.88E-08	0
ADORA3	3.14E-06	4.88E-07	7.70E-06	9.68E-06	4.43E-07	1.60E-06	4.64E-06	8.27E-07
ADPGK	2.17E-05	1.01E-05	9.10E-06	1.39E-05	6.84E-06	1.24E-05	1.04E-05	1.19E-05
ADPRH	4.58E-06	1.07E-06	1.12E-06	3.58E-06	8.37E-07	1.21E-06	2.38E-06	1.40E-06
ADPRHL1	6.14E-06	2.65E-05	3.25E-07	5.53E-07	2.88E-07	4.07E-07	2.14E-06	1.20E-06
ADPRHL2	2.71E-05	1.22E-05	9.22E-06	1.85E-05	1.06E-05	2.08E-05	1.69E-05	1.57E-05
ADRA1A	0	3.39E-06	2.27E-05	5.89E-06	3.12E-07	6.41E-06	4.04E-07	1.83E-06

ADRA1B	2.91E-06	9.37E-07	6.71E-06	7.76E-06	7.50E-07	5.11E-06	2.71E-06	3.54E-06
ADRA1D	6.13E-08	1.97E-08	0	6.11E-07	1.51E-08	8.93E-08	4.25E-08	1.54E-07
ADRA2A	9.91E-06	5.94E-08	2.91E-07	9.00E-07	1.52E-07	1.25E-07	4.76E-07	3.19E-07
ADRA2B	4.38E-07	4.36E-07	2.01E-06	2.24E-06	7.83E-07	6.15E-07	6.17E-07	9.63E-07
ADRA2C	8.47E-07	4.59E-07	2.19E-07	5.12E-07	5.39E-07	1.48E-07	5.57E-07	7.77E-07
ADRB1	4.39E-07	1.19E-07	2.19E-07	8.94E-08	5.65E-07	6.02E-08	1.89E-07	0
ADRB2	1.53E-06	2.73E-06	6.00E-06	6.99E-06	1.66E-06	4.61E-06	5.55E-06	9.95E-06
ADRB3	1.77E-08	0	0	0	0	8.19E-09	8.57E-09	0
ADRBK1	3.42E-05	2.08E-05	1.57E-05	1.91E-05	8.81E-06	1.82E-05	2.04E-05	2.41E-05
ADRBK2	1.43E-06	2.14E-07	9.90E-07	1.47E-06	9.91E-08	3.05E-05	5.21E-07	1.03E-06
ADRM1	0.000132	0.000124	9.28E-05	8.93E-05	0.000119	9.83E-05	7.85E-05	0.000119
ADSL	3.71E-05	3.06E-05	2.22E-05	2.68E-05	1.76E-05	2.80E-05	3.31E-05	3.44E-05
ADSS	1.17E-05	1.81E-05	8.37E-06	1.14E-05	2.40E-06	1.92E-05	8.31E-06	2.28E-05
ADSSL1	2.63E-05	1.92E-05	2.05E-05	8.33E-06	2.27E-06	3.70E-06	9.58E-06	1.29E-05
AEBP1	3.50E-05	3.88E-06	1.00E-05	0.000124	2.01E-05	5.33E-06	4.89E-05	9.26E-06
AEBP2	5.02E-06	3.60E-06	2.89E-06	3.34E-06	6.52E-07	2.50E-06	2.24E-06	1.98E-06
AEN	5.43E-06	6.29E-06	4.70E-06	1.48E-05	7.00E-06	2.06E-05	1.29E-05	1.13E-05
AES	0.000222	0.000102	0.000165	0.000154	0.000223	0.000149	0.000161	0.000145
AFAP1	4.18E-06	4.85E-07	6.59E-07	1.58E-06	2.03E-07	5.17E-07	9.84E-07	7.22E-07
AFAP1L1	5.23E-06	2.41E-06	2.18E-06	1.69E-06	1.26E-06	1.26E-06	4.74E-06	2.52E-06
AFAP1L2	1.07E-05	7.67E-07	6.48E-07	6.27E-07	4.15E-07	9.30E-07	1.24E-06	1.08E-06
AFARP1	4.58E-07	1.53E-07	1.02E-06	3.06E-07	4.95E-08	2.84E-07	2.57E-07	8.48E-08
AFF1	1.41E-05	8.11E-06	1.06E-05	7.76E-06	2.67E-06	5.10E-06	4.87E-06	6.77E-06
AFF2	3.59E-09	9.15E-08	5.48E-09	1.97E-09	1.38E-09	1.49E-09	3.50E-09	0
AFF3	1.05E-05	4.55E-07	9.98E-07	7.67E-07	8.96E-07	1.73E-06	1.24E-06	2.13E-06
AFF4	1.84E-05	1.17E-05	1.55E-05	1.30E-05	3.85E-06	8.84E-06	9.55E-06	1.26E-05
AFG3L1	7.69E-06	1.33E-06	1.79E-06	2.15E-06	1.20E-06	2.21E-06	2.22E-06	3.22E-06
AFG3L2	4.35E-05	4.93E-05	2.56E-05	2.56E-05	9.18E-06	2.93E-05	2.89E-05	3.18E-05
AFM	0.000153	4.74E-05	0.000389	0.000145	0.000212	5.28E-05	4.87E-05	8.64E-05
AFMID	0.000252	4.54E-05	9.87E-05	7.82E-05	8.53E-05	0.000148	5.37E-05	5.09E-05
AFP	0.000257	1.76E-06	8.43E-07	3.26E-06	5.82E-06	3.50E-06	2.38E-06	4.64E-06
AFTPH	2.94E-05	3.25E-05	1.08E-05	1.05E-05	3.33E-06	9.24E-06	8.37E-06	1.19E-05
AG2	7.54E-06	9.58E-06	6.20E-06	0.000162	7.50E-06	3.33E-06	3.92E-05	1.02E-05
AGA	2.36E-05	1.13E-05	9.56E-06	1.41E-05	3.82E-06	2.61E-05	1.01E-05	2.70E-05
AGAP1	8.57E-06	1.20E-06	1.04E-06	1.60E-06	5.76E-07	1.48E-06	1.93E-06	1.33E-06
AGAP11	2.85E-07	9.04E-08	4.54E-08	1.16E-07	2.51E-08	4.07E-08	3.55E-08	4.29E-08
AGAP2	1.58E-06	8.50E-07	4.97E-07	7.90E-07	8.50E-07	1.11E-06	1.65E-06	1.50E-06
AGAP3	2.00E-05	2.97E-05	2.07E-05	2.80E-05	2.89E-05	2.54E-05	3.90E-05	4.63E-05
AGAP4	4.72E-06	1.50E-06	9.74E-07	1.52E-06	1.51E-06	1.79E-06	1.38E-06	2.30E-06
AGAP5	1.07E-06	3.06E-07	3.15E-07	2.31E-07	6.67E-08	2.70E-07	1.23E-07	3.56E-07
AGAP6	1.91E-06	6.05E-07	2.96E-07	7.30E-07	7.44E-07	9.34E-07	5.22E-07	1.21E-06
AGAP7	2.92E-07	6.58E-08	7.09E-08	5.12E-07	8.37E-08	5.41E-08	1.32E-07	1.29E-07
AGAP8	7.69E-07	1.10E-07	2.33E-07	2.38E-08	1.46E-07	9.69E-08	1.32E-07	2.86E-07
AGBL1	0	0	0	0	0	0	0	0
AGBL2	7.57E-07	3.82E-06	4.90E-07	9.45E-08	1.97E-07	4.21E-07	5.90E-08	2.25E-07
AGBL3	8.45E-08	5.17E-07	2.68E-08	1.03E-07	3.35E-08	1.26E-07	1.26E-07	5.71E-08
AGBL4	1.49E-07	8.80E-09	2.19E-07	9.48E-09	1.35E-08	2.52E-07	1.50E-08	0
AGBL5	9.79E-06	1.58E-05	4.54E-06	5.34E-06	2.73E-06	1.20E-05	2.82E-06	5.47E-06
AGER	2.96E-06	1.64E-06	5.35E-07	6.43E-07	1.52E-06	4.87E-07	7.92E-07	7.74E-07
AGFG1	1.47E-05	7.87E-06	4.50E-06	6.95E-06	1.82E-06	9.28E-06	4.55E-06	5.60E-06
AGFG2	2.77E-05	3.28E-05	2.01E-05	1.76E-05	1.88E-05	2.74E-05	2.14E-05	2.45E-05
AGGF1	1.11E-05	1.06E-05	6.27E-06	5.53E-06	1.87E-06	8.17E-06	4.69E-06	7.49E-06
AGK	7.22E-06	2.88E-06	4.71E-06	5.78E-06	1.06E-06	1.03E-05	6.49E-06	1.02E-05
AGL	8.64E-06	1.51E-05	1.98E-05	7.98E-06	2.68E-06	1.20E-05	2.97E-06	2.63E-06
AGMAT	3.31E-05	3.51E-05	6.57E-05	2.71E-05	9.58E-06	4.76E-05	1.95E-05	2.78E-05
AGPAT1	5.58E-05	5.93E-05	3.29E-05	3.42E-05	2.64E-05	4.78E-05	3.81E-05	4.99E-05
AGPAT2	0.000107	5.33E-05	0.000128	0.000133	0.000133	3.23E-05	0.000136	7.94E-05
AGPAT3	2.97E-05	2.71E-05	2.56E-05	1.75E-05	1.74E-05	2.43E-05	2.75E-05	4.16E-05

AGPAT4	3.85E-06	1.82E-06	1.66E-07	9.06E-07	1.08E-07	1.69E-07	7.06E-07	4.78E-07
AGPAT5	4.53E-06	1.10E-05	6.43E-06	5.48E-06	1.92E-06	3.97E-06	5.34E-06	2.28E-06
AGPAT6	2.20E-05	1.95E-05	9.42E-06	1.31E-05	1.26E-05	2.89E-05	1.07E-05	1.57E-05
AGPAT9	1.45E-05	8.20E-06	3.70E-06	6.35E-06	2.02E-06	9.73E-06	2.29E-06	1.70E-05
AGPHD1	2.78E-06	2.27E-06	2.40E-06	1.45E-06	4.98E-06	4.96E-06	1.54E-06	2.57E-06
AGPS	1.09E-05	7.59E-06	5.90E-06	7.43E-06	3.65E-06	8.23E-06	6.05E-06	4.88E-06
AGR2	0.000179	9.53E-07	0	0	0	0	0	0
AGR3	0	0	0	0	0	0	9.11E-07	0
AGRN	6.28E-05	1.02E-05	1.36E-05	2.50E-05	9.50E-06	1.46E-05	1.94E-05	1.12E-05
AGRP	0	0	4.27E-08	4.69E-08	6.55E-08	0	0	3.40E-07
AGT	0.001027	0.001331	0.001366	0.000787	0.000557	0.000767	0.000658	0.001749
AGTPBP1	5.93E-06	1.57E-06	1.58E-06	2.68E-06	4.04E-07	1.89E-06	1.35E-06	1.45E-06
AGTR1	6.67E-06	2.64E-05	4.46E-05	4.12E-05	7.90E-06	1.24E-05	2.46E-05	2.57E-05
AGTR2	0	0	0	0	0	0	7.77E-09	0
AGTRAP	2.93E-05	2.85E-05	1.28E-05	2.42E-05	9.39E-06	2.63E-05	2.17E-05	1.74E-05
AGXT	8.98E-05	0.001448	0.001642	0.001076	0.002304	0.000787	0.000672	0.001239
AGXT2	2.16E-05	2.35E-05	7.13E-05	3.15E-05	2.40E-05	0.000112	2.76E-05	1.46E-05
AGXT2L1	1.42E-05	9.25E-05	4.34E-05	1.70E-05	2.27E-05	4.35E-05	1.28E-05	0.000122
AGXT2L2	1.48E-05	1.28E-05	2.70E-05	2.15E-05	7.07E-06	1.89E-05	2.51E-05	1.37E-05
AHCTF1	5.86E-06	9.67E-06	4.79E-06	3.61E-06	1.08E-06	9.67E-06	3.06E-06	8.03E-06
AHCY	0.000292	0.000237	9.88E-05	0.000107	0.000176	0.000103	0.000126	9.73E-05
AHCYL1	6.89E-05	5.22E-05	3.18E-05	2.70E-05	1.19E-05	8.54E-05	2.24E-05	2.62E-05
AHCYL2	3.76E-06	4.68E-06	3.22E-06	2.36E-06	7.68E-07	3.33E-06	1.67E-06	4.32E-06
AHDC1	5.25E-06	2.72E-06	1.64E-06	2.21E-06	1.02E-06	1.60E-06	1.86E-06	1.71E-06
AHI1	3.90E-06	7.07E-07	8.65E-07	1.06E-06	2.24E-07	1.54E-06	6.57E-07	1.21E-06
AHNAK	6.97E-05	4.39E-05	2.53E-05	4.59E-05	3.03E-05	2.49E-05	3.94E-05	2.10E-05
AHNAK2	2.70E-06	5.37E-09	6.13E-08	1.09E-07	4.55E-08	1.61E-07	1.07E-07	5.35E-07
AHR	2.18E-05	2.01E-05	2.01E-05	1.32E-05	3.32E-06	3.58E-05	1.16E-05	1.95E-05
AHRR	6.78E-08	8.37E-07	1.40E-07	1.54E-08	1.39E-08	5.19E-08	1.09E-07	1.42E-07
AHSA1	0.000111	6.73E-05	4.07E-05	4.91E-05	4.10E-05	8.36E-05	7.18E-05	5.59E-05
AHSA2	1.94E-05	4.93E-06	1.73E-06	2.86E-06	3.06E-06	4.14E-06	2.24E-06	2.96E-06
AHSG	8.09E-05	0.002738	0.004922	0.003858	0.005534	0.001527	0.002342	0.002706
AHSP	0	1.71E-07	2.58E-07	8.66E-08	6.02E-08	0	6.26E-08	0
AICDA	2.71E-08	0	0	2.21E-08	0	0	0	0
AIDA	1.94E-05	1.85E-05	1.09E-05	1.34E-05	8.75E-06	1.84E-05	1.22E-05	1.91E-05
AIF1	8.61E-05	1.42E-05	3.81E-05	8.40E-05	2.19E-05	4.49E-05	8.52E-05	1.67E-05
AIF1L	2.47E-06	2.64E-06	7.56E-07	1.24E-06	1.61E-06	2.23E-06	2.36E-06	2.73E-06
AIFM1	7.42E-05	0.00011	7.20E-05	6.11E-05	8.71E-05	7.86E-05	6.33E-05	0.000156
AIFM2	2.35E-05	1.86E-05	9.49E-06	8.48E-06	8.15E-06	2.66E-05	6.37E-06	1.56E-05
AIFM3	3.02E-06	8.91E-07	2.89E-07	7.81E-07	3.00E-07	8.65E-07	2.66E-07	1.11E-06
AIG1	0.000115	0.00016	0.000132	0.00012	5.68E-05	0.000122	0.000121	0.000106
AIM1	1.14E-05	6.18E-06	7.51E-06	6.73E-06	1.99E-06	9.25E-06	3.01E-06	3.69E-06
AIM1L	6.96E-06	6.56E-07	3.08E-08	2.03E-06	2.03E-07	2.12E-06	1.44E-06	5.50E-07
AIM2	1.69E-06	0	4.09E-07	6.17E-07	0	3.58E-07	1.45E-07	9.90E-08
AIMP1	3.30E-05	1.67E-05	1.21E-05	1.39E-05	7.27E-06	2.74E-05	2.06E-05	2.17E-05
AIMP2	5.81E-05	8.77E-05	2.67E-05	2.27E-05	1.82E-05	7.08E-05	3.52E-05	5.08E-05
AIP	6.02E-05	5.74E-05	3.49E-05	4.76E-05	5.96E-05	4.15E-05	6.18E-05	5.77E-05
AIPL1	0	0	1.66E-08	0	0	0	0	0
AIRE	7.74E-08	0	0	0	0	0	0	4.01E-08
AJAP1	2.31E-08	0	1.07E-07	4.56E-07	5.08E-08	2.21E-08	3.85E-08	0
AK1	1.54E-05	1.54E-05	1.34E-05	1.90E-05	2.48E-05	2.26E-05	2.48E-05	2.69E-05
AK2	0.000123	9.41E-05	8.07E-05	7.10E-05	6.04E-05	8.54E-05	7.64E-05	8.32E-05
AK3	3.84E-05	7.29E-05	0.000114	7.68E-05	2.69E-05	0.000118	6.87E-05	5.11E-05
AK3L1	7.53E-05	3.31E-05	3.01E-05	1.17E-05	7.01E-06	2.75E-05	1.02E-05	7.11E-06
AK5	7.11E-08	0	7.34E-09	1.22E-07	0	6.58E-09	2.76E-08	1.04E-08
AK7	2.86E-06	7.63E-07	4.95E-07	7.77E-07	4.62E-08	2.74E-07	3.42E-08	7.12E-07
AKAP1	6.34E-05	5.56E-05	2.80E-05	2.34E-05	2.32E-05	3.46E-05	2.34E-05	2.22E-05
AKAP10	3.69E-06	3.39E-06	3.52E-06	3.36E-06	1.98E-07	4.12E-06	1.67E-06	2.57E-06

AKAP11	4.78E-06	3.63E-06	3.99E-06	3.17E-06	4.67E-07	3.80E-06	1.72E-06	2.35E-06
AKAP12	2.17E-05	2.55E-05	4.87E-06	1.95E-05	1.29E-06	4.72E-06	4.54E-06	3.81E-06
AKAP13	2.69E-05	1.75E-05	9.65E-06	1.65E-05	2.71E-06	1.13E-05	8.56E-06	1.57E-05
AKAP14	0	9.82E-08	1.00E-07	0	0	1.47E-07	1.41E-07	7.96E-07
AKAP2	8.60E-06	3.38E-06	3.60E-06	6.58E-06	4.44E-08	2.29E-06	1.14E-06	2.20E-06
AKAP3	4.60E-08	3.45E-07	5.31E-07	2.44E-07	1.58E-07	3.98E-07	9.67E-08	1.91E-07
AKAP4	0	0	0	0	0	0	0	0
AKAP5	7.31E-07	1.73E-07	1.30E-07	2.02E-07	1.18E-07	7.14E-07	6.38E-08	4.53E-08
AKAP6	8.81E-07	1.36E-06	6.10E-07	3.22E-07	1.02E-07	1.08E-06	3.96E-07	5.05E-07
AKAP7	1.32E-05	4.31E-06	6.06E-06	1.49E-05	8.93E-07	1.66E-05	7.58E-06	3.04E-06
AKAP8	1.16E-05	4.02E-06	4.94E-06	6.67E-06	3.64E-06	7.42E-06	4.42E-06	5.05E-06
AKAP8L	2.49E-05	1.69E-05	8.79E-06	1.48E-05	1.60E-05	1.20E-05	2.65E-05	1.19E-05
AKAP9	0.000311	3.07E-05	1.77E-05	1.36E-05	5.01E-06	1.10E-05	8.25E-06	1.02E-05
AKD1	2.66E-06	7.12E-07	5.85E-07	8.00E-07	1.32E-07	8.15E-07	5.71E-07	8.27E-07
AKIRIN1	3.39E-05	2.99E-05	1.84E-05	3.04E-05	2.31E-06	1.73E-05	1.57E-05	2.42E-05
AKIRIN2	2.09E-05	5.19E-05	1.58E-05	2.93E-05	7.56E-06	2.48E-05	3.78E-05	1.22E-05
AKNA	5.31E-06	1.14E-06	3.10E-06	5.95E-06	7.23E-07	3.11E-06	2.66E-06	1.98E-06
AKNAD1	2.12E-08	1.29E-07	0	1.62E-08	6.91E-09	0	0	0
AKR1A1	0.000104	0.000225	0.000222	0.000202	0.000187	0.000207	0.000209	9.05E-05
AKR1B1	0.000117	1.41E-05	9.53E-06	3.09E-05	4.62E-06	7.22E-06	2.69E-05	5.82E-06
AKR1B10	5.96E-05	0.000129	2.92E-05	3.03E-05	0.000175	0.001119	0.000304	0.000284
AKR1B15	9.87E-07	1.89E-06	4.39E-07	2.82E-07	0	8.87E-06	2.25E-06	2.49E-06
AKR1C1	0.000923	0.000317	0.000467	0.000256	0.000541	0.001688	0.000667	0.000166
AKR1C2	0.00029	0.000173	0.000152	5.08E-05	0.000238	0.000904	0.000173	5.75E-05
AKR1C3	0.000591	0.000322	0.00012	9.81E-05	0.000156	0.000369	0.00019	0.000239
AKR1C4	0.000299	0.00017	0.000258	0.000145	0.00058	0.000426	0.000451	0.000216
AKR1CL1	1.12E-07	2.53E-07	1.64E-07	6.86E-08	1.35E-07	4.88E-07	2.60E-07	1.65E-08
AKR1D1	7.89E-07	1.80E-06	0.000135	2.36E-05	2.95E-05	3.44E-05	5.23E-05	4.16E-05
AKR1E2	4.92E-08	1.24E-07	4.70E-08	2.00E-07	8.34E-08	1.80E-08	1.81E-07	5.72E-08
AKR7A2	5.95E-05	5.81E-05	4.58E-05	4.81E-05	3.65E-05	4.87E-05	4.88E-05	4.81E-05
AKR7A3	5.92E-05	0.00014	0.000146	0.000235	4.59E-05	7.31E-05	0.000139	0.000113
AKR7L	9.02E-06	5.52E-06	5.48E-06	8.91E-06	5.24E-06	1.69E-06	6.45E-06	3.15E-06
AKT1	7.73E-05	5.10E-05	8.24E-05	5.73E-05	3.87E-05	4.67E-05	7.64E-05	6.09E-05
AKT1S1	4.33E-05	3.41E-05	2.32E-05	2.39E-05	5.33E-05	4.11E-05	4.23E-05	2.74E-05
AKT2	2.88E-05	3.23E-05	2.98E-05	2.26E-05	1.92E-05	3.90E-05	2.93E-05	2.30E-05
AKT3	5.40E-06	3.35E-06	3.04E-06	3.84E-06	7.54E-07	1.83E-06	2.53E-06	3.83E-06
AKTIP	3.11E-05	6.72E-06	1.06E-05	1.13E-05	5.14E-06	1.26E-05	1.10E-05	1.28E-05
ALAD	6.02E-05	5.45E-05	0.000114	6.20E-05	2.95E-05	0.00011	7.98E-05	4.24E-05
ALAS1	6.13E-05	0.000294	0.000315	0.000252	0.000107	0.000536	0.000257	0.000228
ALAS2	0	1.42E-07	7.91E-08	4.05E-07	2.16E-08	4.66E-08	0	0
ALB	0.050602	0.058067	0.086642	0.074672	0.000401	0.006576	0.04229	0.035072
ALCAM	0.000127	3.29E-05	2.98E-05	3.94E-05	3.61E-05	5.76E-05	2.66E-05	3.20E-05
ALDH16A1	1.16E-05	8.81E-06	9.48E-06	1.02E-05	1.63E-05	6.33E-06	5.49E-06	1.16E-05
ALDH18A1	3.24E-05	1.62E-05	1.23E-05	1.13E-05	4.79E-06	1.78E-05	1.44E-05	2.21E-05
ALDH1A1	0.000542	0.001583	0.000579	0.000562	0.000599	0.001488	0.000419	0.000465
ALDH1A2	4.29E-07	1.19E-06	5.01E-07	5.66E-07	5.05E-08	2.72E-08	3.14E-07	1.51E-06
ALDH1A3	1.34E-06	1.82E-07	1.69E-06	8.88E-06	2.12E-07	1.25E-07	3.63E-07	2.63E-06
ALDH1B1	3.05E-05	5.12E-05	0.000186	7.66E-05	2.73E-05	0.000169	3.30E-05	5.07E-05
ALDH1L1	4.35E-05	0.00025	0.000321	0.000123	0.000236	0.000935	0.000143	3.07E-05
ALDH1L2	3.00E-07	4.31E-08	4.57E-08	1.92E-07	2.56E-08	8.52E-08	1.19E-07	1.30E-07
ALDH2	0.000204	0.000907	0.001628	0.000875	0.000373	0.000807	0.00059	0.000399
ALDH3A1	1.65E-07	7.07E-08	2.05E-06	2.07E-07	8.26E-08	0.001689	3.93E-06	1.61E-07
ALDH3A2	2.03E-05	0.00021	0.000111	8.79E-05	8.56E-05	0.000293	0.000103	6.47E-05
ALDH3B1	2.18E-05	5.07E-06	3.31E-06	4.93E-06	1.12E-06	2.81E-06	4.62E-06	3.04E-06
ALDH3B2	1.68E-07	2.12E-08	1.27E-07	2.07E-08	0	4.69E-08	1.38E-07	1.11E-07
ALDH4A1	0.000153	0.000247	0.00028	0.000189	0.000111	0.000472	0.000177	0.000185
ALDH5A1	1.66E-05	7.40E-05	5.19E-05	2.44E-05	1.81E-05	2.64E-05	3.14E-05	3.00E-05
ALDH6A1	8.20E-05	0.000183	0.000209	9.22E-05	1.88E-05	0.000224	2.56E-05	2.38E-05

ALDH7A1	4.51E-05	5.26E-05	8.45E-05	5.83E-05	1.37E-05	4.52E-05	3.65E-05	8.84E-05
ALDH8A1	0.000108	0.000102	0.000171	8.63E-05	5.64E-05	6.83E-05	6.98E-05	6.19E-05
ALDH9A1	3.96E-05	0.000149	0.000123	6.77E-05	2.52E-05	9.71E-05	5.97E-05	7.90E-05
ALDOA	0.000626	0.00052	0.000104	0.000214	8.11E-05	0.000196	0.000222	0.00029
ALDOB	0.002483	0.003175	0.005318	0.002003	0.001657	0.00142	0.001101	0.002141
ALDOC	1.87E-05	2.14E-05	8.59E-05	3.39E-05	4.16E-07	2.88E-05	1.32E-05	0.000112
ALG1	9.97E-06	1.28E-05	5.08E-06	8.51E-06	3.08E-06	9.57E-06	8.53E-06	9.80E-06
ALG10	1.99E-06	3.24E-06	1.05E-06	8.31E-07	1.97E-07	1.26E-06	7.25E-07	8.89E-07
ALG10B	1.21E-06	1.46E-06	1.02E-06	6.44E-07	1.75E-07	8.79E-07	2.71E-07	2.98E-07
ALG11	5.21E-07	5.07E-07	2.03E-07	1.63E-07	1.67E-07	2.92E-07	2.93E-07	3.57E-07
ALG12	3.10E-05	2.91E-05	1.33E-05	1.22E-05	2.29E-05	1.63E-05	1.44E-05	2.22E-05
ALG13	2.05E-05	2.20E-05	1.17E-05	1.13E-05	4.96E-06	1.22E-05	9.84E-06	2.38E-05
ALG14	1.59E-05	2.45E-05	1.27E-05	1.73E-05	1.33E-05	3.53E-05	1.41E-05	1.53E-05
ALG1L	1.40E-05	2.54E-05	3.32E-07	7.82E-07	1.58E-05	2.39E-07	4.36E-07	5.74E-06
ALG1L2	0	0	3.16E-08	0	0	0	2.65E-08	0
ALG2	2.15E-05	1.14E-05	1.64E-05	1.60E-05	9.73E-06	1.93E-05	1.24E-05	1.66E-05
ALG3	4.82E-05	5.65E-05	3.14E-05	3.17E-05	6.49E-05	5.10E-05	5.00E-05	6.86E-05
ALG5	6.03E-05	3.71E-05	2.99E-05	3.54E-05	2.92E-05	4.40E-05	4.18E-05	4.30E-05
ALG6	4.91E-06	5.52E-06	2.25E-06	2.71E-06	1.85E-06	3.34E-06	1.79E-06	1.48E-06
ALG8	3.45E-05	5.02E-05	2.43E-05	2.99E-05	2.34E-05	4.31E-05	2.42E-05	4.90E-05
ALG9	6.38E-06	3.53E-06	4.86E-06	5.01E-06	2.93E-06	7.68E-06	5.56E-06	1.41E-05
ALK	1.05E-08	2.36E-08	1.09E-08	1.15E-07	0	9.71E-09	7.31E-08	0
ALKBH1	5.91E-06	3.29E-06	2.78E-06	3.91E-06	1.36E-06	4.91E-06	3.77E-06	4.27E-06
ALKBH2	2.03E-05	2.04E-05	2.36E-05	1.10E-05	4.25E-05	3.67E-05	3.12E-05	4.31E-05
ALKBH3	5.72E-06	6.76E-06	6.60E-06	9.80E-06	4.52E-06	8.05E-06	1.10E-05	1.26E-05
ALKBH4	4.40E-06	8.79E-06	3.32E-06	4.31E-06	7.36E-06	7.11E-06	5.40E-06	6.10E-06
ALKBH5	3.19E-05	5.61E-05	4.09E-05	3.98E-05	2.97E-05	4.16E-05	4.51E-05	3.88E-05
ALKBH6	1.08E-05	2.47E-05	5.48E-06	6.39E-06	1.01E-05	8.70E-06	1.17E-05	7.79E-06
ALKBH7	4.73E-05	7.63E-05	6.57E-05	6.87E-05	0.000128	5.50E-05	8.29E-05	4.56E-05
ALKBH8	1.63E-06	1.80E-06	1.67E-06	1.83E-06	9.65E-07	1.64E-06	1.29E-06	2.56E-06
ALLC	2.59E-08	1.98E-08	8.15E-08	2.06E-07	2.98E-08	1.20E-08	1.66E-08	2.56E-08
ALMS1	2.97E-06	1.10E-06	1.07E-06	1.26E-06	4.53E-07	1.66E-06	8.07E-07	9.72E-07
ALMS1P	4.64E-08	8.81E-08	1.15E-07	1.14E-07	6.67E-08	5.75E-08	1.49E-08	2.28E-08
ALOX12	1.03E-07	9.03E-08	1.04E-08	1.06E-07	8.60E-09	2.78E-08	3.45E-08	9.22E-08
ALOX12B	1.34E-07	0	7.80E-08	1.15E-08	0	0	9.09E-09	0
ALOX12P2	1.35E-07	1.11E-06	8.64E-07	1.05E-06	1.66E-07	8.22E-07	1.19E-07	2.10E-07
ALOX15	1.74E-07	7.90E-07	2.71E-08	0	2.25E-08	8.08E-09	2.45E-08	2.56E-08
ALOX15B	2.08E-06	1.77E-07	8.17E-08	7.28E-07	4.06E-08	1.14E-07	4.51E-07	1.03E-07
ALOX5	1.74E-05	4.69E-07	2.00E-06	3.86E-06	1.82E-07	8.62E-07	2.57E-06	2.62E-07
ALOX5AP	2.19E-05	8.15E-07	4.16E-06	1.84E-05	7.69E-07	3.20E-06	5.41E-06	2.79E-06
ALOXE3	4.99E-08	0	4.42E-08	1.19E-07	0	6.60E-09	6.92E-09	0
ALPI	5.52E-08	1.97E-07	9.56E-09	0	1.58E-08	0	0	0
ALPK1	6.13E-06	1.68E-06	1.55E-06	2.16E-06	3.99E-07	4.44E-06	1.88E-06	2.06E-06
ALPK2	3.92E-07	9.87E-07	4.36E-06	6.04E-06	3.03E-06	1.82E-06	6.26E-06	1.83E-07
ALPK3	1.26E-05	6.48E-06	4.13E-07	1.72E-06	7.56E-07	1.64E-07	1.51E-06	4.12E-07
ALPL	2.65E-06	2.93E-05	5.04E-05	4.12E-05	6.59E-06	2.96E-05	2.36E-05	5.94E-05
ALPP	0	9.40E-08	0	0	0	0	1.57E-08	0
ALPPL2	0	5.31E-08	0	0	0	0	0	0
ALS2	2.03E-05	1.02E-05	1.08E-05	6.17E-06	2.64E-06	6.22E-06	6.74E-06	8.16E-06
ALS2CL	8.48E-06	4.39E-06	2.32E-06	4.10E-06	2.97E-06	1.56E-06	3.61E-06	9.20E-06
ALS2CR11	3.55E-08	8.81E-09	1.21E-08	1.53E-08	1.19E-08	3.65E-09	0	2.31E-08
ALS2CR12	2.42E-06	5.60E-08	0	4.55E-08	2.92E-08	0	1.15E-08	1.82E-08
ALS2CR4	1.78E-05	5.53E-06	1.03E-06	3.31E-06	7.64E-07	3.11E-06	2.25E-06	1.34E-06
ALS2CR8	1.84E-06	5.73E-07	8.96E-07	8.16E-07	1.94E-07	1.14E-06	4.00E-07	3.93E-07
ALX1	0	0	0	0	0	0	0	3.21E-07
ALX3	1.18E-06	2.11E-07	1.07E-07	2.07E-07	1.45E-08	2.66E-07	1.62E-07	2.74E-07
ALX4	1.55E-08	1.77E-08	0	0	0	0	0	0
AMAC1	0	0	0	0	0	0	1.24E-08	1.90E-08

AMAC1L2	8.57E-08	4.93E-08	0	2.64E-08	0	1.99E-08	4.10E-08	3.18E-08
AMAC1L3	0	0	0	0	3.72E-08	0	0	0
AMACR	2.50E-05	3.83E-05	6.32E-05	4.90E-05	2.70E-05	0.000148	3.33E-05	2.10E-05
AMBN	0	0	0	0	0	9.02E-07	0	1.61E-07
AMBP	0.002887	0.003706	0.009395	0.006064	0.012296	0.004311	0.00475	0.005666
AMBRA1	1.02E-05	1.07E-05	5.08E-06	6.69E-06	2.24E-06	1.86E-05	5.71E-06	1.09E-05
AMD1	2.50E-05	1.26E-05	1.09E-05	1.74E-05	2.84E-06	1.24E-05	1.61E-05	1.51E-05
AMDHD1	9.53E-06	0.000385	7.97E-05	8.07E-05	2.72E-05	2.56E-05	1.99E-05	4.78E-05
AMDHD2	1.30E-05	3.06E-05	8.87E-06	1.31E-05	1.06E-05	1.27E-05	1.52E-05	1.36E-05
AMELX	0	0	0	0	0	0	0	0
AMELY	0	0	0	0	0	0	0	0
AMFR	8.43E-05	7.59E-05	7.38E-05	5.85E-05	5.12E-05	6.70E-05	8.85E-05	9.40E-05
AMH	3.56E-08	0	1.24E-08	4.36E-08	1.12E-07	0	2.29E-08	1.75E-08
AMHR2	0	1.48E-08	5.75E-07	1.28E-07	0	0	2.52E-08	0
AMICA1	1.39E-05	9.88E-07	3.28E-06	8.75E-06	1.93E-05	3.41E-06	6.87E-06	5.45E-06
AMIGO1	1.99E-06	3.37E-06	6.83E-07	9.90E-07	5.40E-07	2.47E-06	7.74E-07	1.20E-06
AMIGO2	2.42E-06	7.48E-07	3.05E-06	2.67E-06	1.81E-07	4.24E-07	5.52E-07	2.06E-06
AMIGO3	2.15E-06	1.87E-06	3.55E-07	6.39E-07	1.56E-06	1.49E-06	2.38E-06	2.81E-06
AMMECR1	2.97E-06	2.19E-06	1.33E-06	1.95E-06	9.50E-07	1.81E-06	1.20E-06	2.52E-06
AMMECR1	7.88E-06	4.59E-06	3.62E-06	4.33E-06	1.74E-06	5.84E-06	5.09E-06	4.34E-06
AMN	5.78E-05	1.07E-05	5.29E-05	0.000101	5.55E-05	1.07E-06	0.000107	5.76E-07
AMN1	3.06E-06	4.78E-06	1.73E-06	1.78E-06	2.13E-06	3.28E-06	2.84E-06	2.73E-06
AMOT	1.28E-05	6.40E-07	1.93E-06	1.01E-06	1.12E-07	1.33E-06	4.19E-07	1.45E-07
AMOTL1	7.68E-06	1.40E-06	2.20E-06	1.96E-06	2.30E-06	4.34E-07	2.42E-06	9.72E-06
AMOTL2	2.10E-05	8.65E-06	1.12E-05	1.82E-05	4.34E-06	1.17E-05	1.30E-05	1.44E-06
AMPD1	3.04E-08	2.29E-08	7.39E-08	1.48E-07	0	0	0	0
AMPD2	1.60E-05	1.86E-05	1.99E-05	2.03E-05	2.46E-05	6.39E-05	2.13E-05	2.38E-05
AMPD3	3.52E-06	2.70E-07	4.94E-07	1.21E-06	2.01E-07	8.25E-07	6.04E-07	1.57E-07
AMPH	2.52E-07	1.65E-08	6.83E-08	2.99E-07	8.38E-08	1.36E-08	3.57E-08	1.07E-07
AMT	2.86E-05	6.38E-05	3.56E-05	2.53E-05	2.87E-05	4.53E-05	2.47E-05	1.70E-05
AMTN	0	0	0	0	0	0	0	0
AMY1A	4.09E-08	3.09E-08	2.87E-08	8.36E-08	7.04E-08	5.06E-08	5.26E-08	2.01E-08
AMY2A	0	1.20E-07	0	5.56E-08	0	1.09E-07	3.09E-08	0
AMY2B	5.45E-06	7.52E-06	2.39E-06	3.31E-06	2.15E-06	3.23E-06	2.48E-06	1.18E-06
AMZ1	1.41E-06	6.38E-08	8.93E-07	8.15E-08	0	1.06E-08	5.00E-08	4.74E-07
AMZ2	4.67E-05	3.64E-05	1.93E-05	1.85E-05	1.76E-05	4.92E-05	2.33E-05	1.85E-05
AMZ2P1	2.40E-06	1.81E-06	7.53E-07	1.14E-06	1.09E-06	1.59E-06	1.52E-06	1.00E-06
ANAPC1	4.93E-06	2.55E-06	2.25E-06	1.39E-06	6.63E-07	4.16E-06	1.27E-06	1.66E-06
ANAPC10	1.24E-05	5.43E-06	4.76E-06	8.83E-06	2.58E-06	7.86E-06	9.40E-06	7.35E-06
ANAPC11	0.000244	0.000237	9.37E-05	9.63E-05	0.000389	0.00022	0.000122	9.49E-05
ANAPC13	5.70E-05	5.33E-05	3.76E-05	4.94E-05	3.95E-05	6.50E-05	5.54E-05	3.61E-05
ANAPC16	3.56E-05	3.62E-05	3.03E-05	2.32E-05	2.27E-05	2.90E-05	3.04E-05	1.80E-05
ANAPC2	2.47E-05	1.40E-05	1.39E-05	1.53E-05	1.36E-05	2.17E-05	1.11E-05	2.53E-05
ANAPC4	1.37E-05	5.90E-06	2.87E-06	5.07E-06	2.32E-06	9.58E-06	3.78E-06	8.05E-06
ANAPC5	4.16E-05	6.57E-05	3.16E-05	3.59E-05	3.03E-05	5.58E-05	3.49E-05	3.67E-05
ANAPC7	1.89E-05	1.64E-05	7.09E-06	8.77E-06	7.16E-06	1.40E-05	7.98E-06	7.78E-06
ANG	0.000293	0.000447	0.001675	0.001331	0.001315	0.000898	0.000605	0.00145
ANGEL1	1.14E-05	5.83E-06	3.24E-06	2.99E-06	1.56E-06	3.98E-06	4.32E-06	4.38E-06
ANGEL2	5.51E-06	9.91E-06	4.47E-06	4.13E-06	1.69E-06	7.18E-06	3.84E-06	8.24E-06
ANGPT1	2.52E-06	4.67E-07	2.87E-07	1.08E-06	2.12E-07	8.92E-07	3.69E-07	1.12E-06
ANGPT2	2.10E-06	6.28E-06	4.18E-07	3.82E-07	2.46E-07	1.82E-06	1.49E-06	1.14E-06
ANGPT4	0	4.26E-08	2.86E-08	1.66E-08	0	0	0	2.00E-08
ANGPTL1	9.65E-06	9.09E-07	6.61E-06	8.21E-06	1.64E-06	1.27E-06	1.84E-06	1.38E-06
ANGPTL2	8.28E-06	5.86E-06	5.64E-06	1.47E-05	1.23E-06	4.54E-06	9.40E-06	1.05E-05
ANGPTL3	0.000182	0.000204	0.000479	0.000241	0.000187	0.000432	0.000147	0.000393
ANGPTL4	0.000125	6.86E-05	4.22E-05	0.000248	0.000193	7.67E-05	0.000129	3.31E-05
ANGPTL5	0	0	0	0	0	0	0	0
ANGPTL6	5.21E-06	1.91E-06	2.01E-05	1.56E-05	5.24E-06	3.07E-06	5.11E-06	2.82E-06

ANGPTL7	0	0	2.21E-08	1.04E-07	0	0	0	0
ANK1	4.35E-07	6.02E-08	6.68E-08	8.02E-08	1.50E-08	4.83E-08	2.48E-08	6.42E-08
ANK2	2.33E-06	6.78E-07	4.03E-07	6.65E-06	2.22E-07	5.36E-07	6.24E-07	5.26E-07
ANK3	3.82E-05	1.75E-06	3.85E-06	9.73E-06	3.19E-07	2.70E-07	5.31E-07	5.46E-07
ANKAR	9.31E-07	4.66E-07	1.71E-07	3.33E-07	3.00E-07	3.52E-07	2.76E-07	2.16E-07
ANKDD1A	1.32E-06	4.76E-07	3.04E-07	8.16E-07	3.57E-07	2.33E-07	3.33E-07	3.73E-07
ANKFN1	0	0	0	0	0	7.10E-07	0	0
ANKFY1	6.20E-06	5.73E-06	7.59E-06	5.31E-06	3.91E-06	9.72E-06	4.60E-06	6.06E-06
ANKH	1.91E-05	1.50E-05	1.28E-05	9.56E-06	6.43E-06	7.57E-06	1.16E-05	2.06E-05
ANKHD1	3.27E-05	3.00E-05	2.41E-05	2.15E-05	7.66E-06	2.65E-05	1.79E-05	2.79E-05
ANKHD1-	1.11E-05	6.51E-06	4.72E-06	5.03E-06	1.06E-06	9.92E-06	5.38E-06	4.80E-06
ANKIB1	8.45E-05	9.46E-06	5.02E-06	4.96E-06	1.43E-06	6.18E-06	2.90E-06	4.13E-06
ANKK1	6.45E-08	2.60E-07	9.58E-09	5.64E-08	1.66E-07	3.42E-08	0	0
ANKLE1	1.53E-07	9.08E-08	3.35E-08	8.33E-08	7.16E-08	2.90E-08	7.05E-08	4.74E-08
ANKLE2	2.65E-05	1.67E-05	1.09E-05	1.22E-05	7.23E-06	1.04E-05	9.29E-06	1.07E-05
ANKMY1	3.83E-06	1.97E-06	1.99E-06	2.34E-06	3.57E-06	4.62E-06	4.29E-06	3.83E-06
ANKMY2	1.25E-05	1.11E-05	3.94E-06	3.56E-06	1.18E-06	9.02E-06	3.58E-06	8.33E-06
ANKRA2	1.04E-05	7.82E-06	5.47E-06	5.49E-06	1.61E-06	1.25E-05	4.19E-06	7.76E-06
ANKRD1	1.20E-05	2.24E-06	1.15E-07	6.24E-05	5.96E-07	1.31E-06	4.80E-06	1.25E-07
ANKRD10	3.96E-05	1.73E-05	1.62E-05	1.98E-05	7.25E-06	2.47E-05	1.31E-05	1.80E-05
ANKRD11	3.14E-05	1.02E-05	9.28E-06	1.23E-05	4.64E-06	1.63E-05	7.92E-06	1.39E-05
ANKRD12	5.75E-06	2.39E-06	4.23E-06	3.00E-06	5.42E-07	2.68E-06	1.68E-06	2.16E-06
ANKRD13,	3.42E-05	1.93E-05	8.45E-06	1.31E-05	5.81E-06	9.76E-06	1.24E-05	1.23E-05
ANKRD13	2.55E-06	6.66E-07	1.35E-07	4.40E-07	7.35E-07	3.33E-07	4.89E-07	4.83E-07
ANKRD13	4.50E-06	4.70E-06	3.22E-06	3.42E-06	6.90E-07	5.60E-06	1.87E-06	3.19E-06
ANKRD13	1.19E-05	6.78E-06	3.08E-06	7.05E-06	4.22E-06	3.44E-06	4.70E-06	8.38E-06
ANKRD16	2.52E-06	1.05E-06	2.22E-06	2.74E-06	1.36E-06	3.00E-06	6.33E-06	4.10E-06
ANKRD17	1.83E-05	9.71E-06	7.78E-06	7.11E-06	1.79E-06	1.52E-05	4.34E-06	9.54E-06
ANKRD19	2.16E-06	3.25E-07	4.41E-07	5.14E-07	1.88E-06	5.06E-07	3.41E-06	5.08E-07
ANKRD2	3.73E-08	5.89E-08	0	3.67E-07	1.61E-08	6.95E-08	3.31E-08	2.76E-08
ANKRD20,	1.41E-07	1.22E-07	6.51E-08	6.33E-08	4.94E-09	2.13E-08	1.75E-08	1.18E-07
ANKRD20,	1.96E-08	1.46E-08	1.34E-08	0	0	0	6.34E-09	1.91E-08
ANKRD20	8.79E-08	1.16E-07	5.14E-08	1.48E-08	8.81E-09	5.82E-08	1.41E-08	1.18E-07
ANKRD22	2.47E-06	9.23E-07	1.94E-07	5.38E-07	0	3.76E-07	6.68E-08	6.70E-08
ANKRD23	1.86E-06	5.62E-07	3.16E-07	3.23E-07	3.95E-07	3.03E-07	3.25E-07	3.94E-07
ANKRD24	1.65E-06	1.03E-06	8.03E-07	1.39E-06	1.48E-06	2.69E-06	2.54E-06	7.29E-07
ANKRD26	1.92E-06	6.98E-07	5.24E-07	6.57E-07	1.27E-07	7.35E-07	2.71E-07	5.04E-07
ANKRD26	4.34E-09	9.69E-09	0	0	0	0	0	0
ANKRD27	6.94E-06	6.84E-06	2.25E-06	3.03E-06	9.97E-07	2.56E-06	6.30E-06	2.72E-06
ANKRD28	8.63E-06	1.23E-05	8.19E-06	7.78E-06	5.01E-06	8.15E-06	7.77E-06	1.03E-05
ANKRD29	1.11E-05	1.02E-05	5.64E-07	1.71E-06	1.88E-07	5.84E-06	4.48E-07	2.22E-06
ANKRD30,	0	0	0	0	0	0	5.01E-09	0
ANKRD30	6.54E-09	0	0	7.99E-09	0	0	6.89E-08	0
ANKRD31	1.99E-08	1.12E-08	0	0	0	1.11E-07	0	1.46E-08
ANKRD32	1.44E-06	1.11E-06	8.65E-07	1.02E-06	3.78E-07	1.08E-06	6.59E-07	1.19E-06
ANKRD33	2.45E-06	1.40E-07	1.57E-08	1.82E-08	1.01E-08	1.38E-08	1.43E-08	0
ANKRD34,	5.33E-07	4.26E-08	2.61E-08	5.42E-08	3.27E-08	1.35E-07	8.62E-08	1.86E-07
ANKRD34	0	0	0	0	0	0	0	0
ANKRD34	0	0	0	0	0	9.97E-08	0	0
ANKRD35	1.14E-06	2.91E-07	1.19E-06	7.44E-07	9.75E-07	1.75E-06	7.64E-07	1.70E-07
ANKRD36	2.48E-06	3.63E-07	4.96E-07	9.44E-07	1.40E-07	4.85E-07	2.93E-07	5.81E-07
ANKRD36	5.03E-07	6.46E-08	9.78E-08	8.16E-08	0	1.21E-07	7.22E-08	4.54E-08
ANKRD36	2.26E-06	3.50E-06	1.17E-06	6.25E-07	0	2.45E-06	1.39E-07	1.71E-06
ANKRD37	4.40E-06	3.11E-06	2.48E-05	1.52E-05	2.48E-06	6.61E-05	7.22E-06	9.98E-06
ANKRD39	4.52E-05	1.92E-05	1.10E-05	9.98E-06	1.09E-05	1.25E-05	1.47E-05	2.30E-05
ANKRD40	1.44E-05	1.68E-05	7.42E-06	1.00E-05	3.17E-06	1.40E-05	9.32E-06	9.09E-06
ANKRD42	1.96E-06	7.18E-07	1.81E-06	3.03E-06	9.67E-07	3.09E-06	1.25E-06	1.37E-06
ANKRD43	3.31E-06	1.28E-06	1.02E-06	1.55E-06	7.81E-07	2.18E-06	2.74E-06	9.05E-06

ANKRD44	2.96E-06	5.19E-07	7.92E-07	1.39E-06	5.87E-08	2.48E-06	1.67E-07	2.00E-07
ANKRD45	6.99E-08	2.08E-06	0	5.35E-08	2.25E-08	4.05E-08	0	1.03E-07
ANKRD46	1.25E-05	1.25E-05	1.81E-05	8.35E-06	7.35E-06	7.85E-06	1.15E-05	8.14E-06
ANKRD49	8.76E-06	3.88E-06	2.98E-06	3.47E-06	1.91E-06	4.01E-06	2.57E-06	5.12E-06
ANKRD5	3.17E-06	5.15E-06	2.45E-06	1.82E-06	1.93E-07	3.17E-06	1.02E-06	1.66E-06
ANKRD50	2.49E-06	1.34E-06	2.16E-06	2.99E-06	8.59E-07	3.07E-06	2.14E-06	2.15E-06
ANKRD52	6.25E-06	2.37E-06	9.56E-07	1.98E-06	1.06E-06	2.86E-06	2.20E-06	4.84E-06
ANKRD53	1.47E-06	1.03E-07	8.82E-08	4.97E-07	8.68E-08	1.15E-07	5.81E-08	0
ANKRD54	1.28E-05	9.38E-06	6.17E-06	6.51E-06	6.55E-06	1.13E-05	8.81E-06	9.04E-06
ANKRD55	1.13E-07	2.12E-08	2.32E-06	8.70E-07	1.06E-07	1.32E-07	3.64E-08	1.67E-07
ANKRD56	7.15E-06	8.57E-06	8.90E-06	5.91E-06	7.37E-07	3.79E-06	4.89E-06	6.00E-06
ANKRD57	1.25E-05	2.77E-05	1.78E-05	1.68E-05	4.30E-06	1.51E-05	1.09E-05	3.54E-06
ANKRD58	1.02E-06	1.23E-07	3.77E-07	5.14E-07	1.20E-07	1.30E-07	8.06E-07	6.86E-08
ANKRD6	3.64E-06	2.35E-06	1.97E-06	2.21E-06	5.81E-07	7.15E-07	1.49E-06	9.17E-07
ANKRD7	5.58E-08	0	0	0	0	0	0	0
ANKRD9	8.61E-06	2.38E-06	9.42E-07	3.28E-06	5.91E-07	2.85E-06	7.59E-06	2.58E-06
ANKS1A	7.54E-06	4.82E-06	5.95E-06	4.13E-06	9.57E-07	2.86E-06	2.19E-06	2.25E-06
ANKS1B	9.50E-08	1.12E-07	2.86E-08	4.28E-08	0	1.70E-06	1.39E-08	2.29E-07
ANKS3	5.51E-06	3.47E-06	1.52E-06	3.68E-06	3.71E-06	2.74E-06	5.32E-06	2.53E-06
ANKS4B	3.22E-05	2.82E-05	1.41E-05	1.28E-05	3.46E-06	1.00E-05	1.19E-05	6.50E-06
ANKS6	5.38E-06	5.58E-08	6.63E-07	1.51E-06	3.84E-06	7.72E-08	2.97E-07	3.08E-06
ANKZF1	3.15E-05	9.54E-06	6.67E-06	7.72E-06	1.01E-05	1.18E-05	1.04E-05	7.04E-06
ANLN	1.15E-05	3.75E-06	1.10E-07	1.92E-07	2.76E-07	1.18E-06	2.01E-07	9.33E-07
ANO1	5.10E-06	9.10E-06	4.07E-05	2.43E-05	1.14E-05	3.29E-07	1.67E-05	3.20E-05
ANO10	1.25E-05	8.20E-06	5.33E-06	6.86E-06	5.98E-06	8.78E-06	1.07E-05	7.13E-06
ANO2	4.31E-08	3.10E-07	6.33E-09	3.01E-08	5.28E-08	9.79E-07	2.33E-07	9.01E-09
ANO3	9.28E-06	1.34E-07	2.59E-07	4.34E-07	1.75E-08	0	1.61E-08	0
ANO4	1.75E-06	2.04E-08	1.07E-08	3.03E-07	8.81E-09	2.18E-08	3.97E-08	1.51E-08
ANO5	1.96E-06	1.26E-06	1.25E-06	7.12E-07	5.75E-09	1.28E-06	4.94E-07	7.26E-07
ANO6	1.94E-05	3.29E-05	2.65E-05	1.92E-05	1.02E-05	3.56E-05	9.65E-06	2.68E-05
ANO7	1.19E-06	6.41E-07	3.57E-07	1.41E-07	1.36E-06	1.63E-07	3.21E-07	1.86E-07
ANO8	5.53E-06	1.15E-06	1.88E-06	2.22E-06	4.37E-07	2.34E-06	1.57E-06	2.34E-06
ANO9	4.78E-06	8.69E-08	2.41E-07	1.20E-06	1.89E-08	1.81E-07	1.98E-07	4.23E-07
ANP32A	7.85E-05	4.28E-05	3.15E-05	3.89E-05	3.47E-05	3.74E-05	4.89E-05	3.25E-05
ANP32B	8.45E-05	3.59E-05	4.34E-05	7.03E-05	6.40E-05	7.18E-05	8.61E-05	6.04E-05
ANP32C	1.01E-06	1.00E-07	6.28E-07	5.24E-08	1.46E-07	7.89E-08	1.18E-07	0
ANP32D	2.08E-07	0	2.97E-07	0	0	0	0	0
ANP32E	1.91E-05	9.83E-06	1.12E-05	1.05E-05	5.10E-06	1.86E-05	1.18E-05	1.30E-05
ANPEP	0.0005	0.000113	0.00024	0.000238	0.000137	0.000109	0.000209	0.000119
ANTXR1	7.52E-06	1.69E-06	1.76E-06	7.37E-06	8.04E-07	1.33E-06	4.34E-06	3.01E-06
ANTXR2	9.37E-06	3.28E-06	9.64E-06	1.29E-05	3.59E-06	4.03E-06	9.12E-06	8.35E-06
ANTXRL	0	0	0	0	0	0	0	0
ANUBL1	1.57E-06	2.74E-06	2.49E-06	1.60E-06	9.21E-07	2.59E-06	1.84E-06	1.60E-06
ANXA1	4.87E-05	7.74E-06	1.07E-05	8.57E-05	5.19E-06	1.17E-05	2.44E-05	1.47E-05
ANXA10	7.17E-07	1.82E-05	6.19E-05	5.24E-05	2.61E-05	1.73E-05	3.38E-05	5.36E-05
ANXA11	0.000115	7.74E-05	3.12E-05	8.29E-05	3.26E-05	4.95E-05	6.87E-05	4.86E-05
ANXA13	4.68E-05	1.49E-05	2.83E-06	1.79E-05	1.42E-05	5.32E-06	6.98E-06	1.51E-06
ANXA2	0.000667	9.75E-05	7.47E-05	0.000333	0.000127	0.000372	0.000254	0.000197
ANXA2P1	7.96E-06	5.38E-07	8.04E-07	1.06E-06	1.78E-07	2.22E-06	1.10E-06	9.45E-07
ANXA2P2	0.000299	4.26E-05	4.32E-05	0.000137	2.34E-05	0.00016	8.05E-05	8.10E-05
ANXA2P3	2.11E-06	2.14E-08	3.39E-07	1.84E-07	4.83E-08	1.04E-07	1.08E-07	5.53E-08
ANXA3	2.65E-05	1.74E-06	1.62E-06	1.14E-05	1.26E-07	8.19E-07	2.50E-07	4.31E-07
ANXA4	0.000656	6.19E-05	8.20E-05	0.000112	3.64E-05	5.42E-05	6.07E-05	5.95E-05
ANXA5	0.000353	4.14E-05	5.04E-05	0.000194	3.38E-05	0.00015	0.000129	0.00017
ANXA6	6.80E-05	6.23E-05	9.95E-05	0.000134	5.44E-05	6.87E-05	5.73E-05	2.45E-05
ANXA7	0.000102	9.87E-05	7.45E-05	6.99E-05	2.95E-05	0.000107	6.69E-05	9.53E-05
ANXA8	9.67E-07	1.18E-07	1.74E-06	6.03E-06	4.49E-08	1.21E-08	1.29E-08	5.76E-08
ANXA8L1	0	0	0	0	0	0	0	0

ANXA8L2	2.62E-06	2.32E-07	5.10E-07	3.29E-06	8.32E-08	1.12E-07	4.67E-07	1.78E-08
ANXA9	2.15E-05	1.86E-05	2.40E-05	1.56E-05	1.35E-05	2.42E-05	1.69E-05	2.82E-05
AOAH	5.69E-06	4.47E-07	2.66E-06	5.12E-06	6.05E-07	1.87E-06	5.00E-06	6.25E-07
AOC2	5.03E-07	1.03E-06	1.75E-07	4.21E-07	5.28E-07	6.91E-07	2.82E-07	2.86E-07
AOC3	5.99E-06	5.63E-06	5.63E-06	7.42E-06	2.14E-06	4.68E-06	6.84E-06	6.61E-06
AOX1	0.000168	0.000337	0.000495	0.000221	9.29E-05	0.000787	0.000277	0.000223
AOX2P	0	0	0	3.40E-08	0	0	7.84E-08	0
AP1AR	1.50E-05	9.62E-06	7.65E-06	7.81E-06	1.67E-06	1.82E-05	6.10E-06	9.66E-06
AP1B1	4.74E-05	4.09E-05	2.63E-05	3.05E-05	2.83E-05	2.97E-05	3.94E-05	3.51E-05
AP1G1	2.78E-05	1.38E-05	1.28E-05	1.31E-05	3.93E-06	2.15E-05	8.12E-06	1.52E-05
AP1G2	2.16E-05	9.56E-06	3.67E-06	9.27E-06	3.08E-06	3.69E-06	8.66E-06	1.41E-06
AP1M1	4.72E-05	3.66E-05	4.10E-05	3.82E-05	4.31E-05	5.34E-05	5.32E-05	5.09E-05
AP1M2	3.86E-06	3.57E-05	8.05E-07	2.31E-06	0	1.11E-06	3.48E-07	2.72E-06
AP1S1	2.99E-05	8.52E-05	2.49E-05	2.86E-05	3.61E-05	6.48E-05	3.32E-05	6.19E-05
AP1S2	8.06E-06	2.03E-06	4.14E-06	6.65E-06	8.71E-07	2.93E-06	5.94E-06	2.86E-06
AP1S3	7.56E-06	7.39E-07	5.50E-07	1.00E-06	9.56E-08	7.28E-07	3.30E-07	1.27E-06
AP2A1	5.17E-05	4.31E-05	3.64E-05	4.42E-05	5.33E-05	6.09E-05	5.37E-05	5.58E-05
AP2A2	2.32E-05	2.51E-05	2.34E-05	1.63E-05	9.06E-06	2.20E-05	1.49E-05	1.88E-05
AP2B1	5.07E-05	2.51E-05	1.54E-05	2.19E-05	8.84E-06	5.22E-05	1.77E-05	2.28E-05
AP2M1	0.000174	0.000103	9.50E-05	0.000111	9.62E-05	0.000144	0.000124	0.000101
AP2S1	0.000103	7.26E-05	7.92E-05	8.73E-05	0.000207	0.000117	9.90E-05	9.79E-05
AP3B1	2.08E-05	1.03E-05	5.32E-06	6.65E-06	2.40E-06	1.52E-05	5.65E-06	1.26E-05
AP3B2	3.13E-07	1.00E-08	9.24E-09	2.18E-08	1.90E-08	5.71E-09	0	0
AP3D1	4.98E-05	4.59E-05	3.28E-05	3.65E-05	3.89E-05	5.15E-05	5.06E-05	4.36E-05
AP3M1	1.60E-05	1.19E-05	7.91E-06	7.17E-06	3.54E-06	1.24E-05	6.78E-06	6.36E-06
AP3M2	3.18E-06	2.50E-06	8.32E-07	1.39E-06	5.63E-07	2.13E-06	1.50E-06	1.74E-06
AP3S1	5.16E-05	5.37E-05	3.25E-05	3.83E-05	1.69E-05	8.00E-05	2.58E-05	3.61E-05
AP3S2	1.58E-05	1.06E-05	1.11E-05	1.16E-05	8.62E-06	1.24E-05	1.69E-05	1.34E-05
AP4B1	7.30E-06	4.79E-06	3.08E-06	4.77E-06	3.13E-06	1.20E-05	3.06E-06	5.43E-06
AP4E1	3.97E-06	2.33E-06	2.13E-06	2.20E-06	8.32E-07	2.62E-06	1.73E-06	2.49E-06
AP4M1	1.25E-05	6.43E-06	2.91E-06	4.35E-06	3.28E-06	3.86E-06	3.86E-06	5.82E-06
AP4S1	1.57E-06	2.65E-06	1.43E-06	3.18E-06	1.10E-06	2.90E-06	2.64E-06	6.86E-07
APAF1	5.98E-06	3.11E-06	2.32E-06	2.85E-06	9.67E-07	2.15E-06	1.91E-06	1.94E-06
APBA1	1.05E-05	5.16E-06	4.29E-06	3.41E-06	1.32E-06	5.37E-07	3.65E-06	5.59E-06
APBA2	4.93E-07	5.68E-08	3.68E-07	6.30E-07	4.87E-08	5.04E-08	4.64E-07	2.22E-07
APBA3	6.69E-06	5.75E-06	5.58E-06	6.60E-06	7.36E-06	8.12E-06	7.21E-06	6.49E-06
APBB1	2.45E-05	3.38E-05	3.16E-06	8.51E-06	2.87E-06	5.98E-06	7.77E-06	6.19E-06
APBB1IP	3.33E-05	1.06E-05	5.49E-06	1.06E-05	5.36E-06	4.46E-06	4.06E-06	1.68E-05
APBB2	5.33E-06	6.03E-06	3.61E-06	4.07E-06	1.85E-06	6.14E-06	3.67E-06	3.17E-06
APBB3	1.15E-05	6.00E-06	4.61E-06	1.19E-05	1.42E-06	5.75E-06	5.25E-06	1.15E-05
APC	5.07E-06	3.12E-06	2.45E-06	2.09E-06	3.96E-07	2.16E-06	1.13E-06	2.39E-06
APC2	6.76E-08	2.73E-08	0	1.50E-08	5.43E-08	7.58E-09	1.06E-07	6.28E-08
APCDD1	1.29E-05	1.85E-07	1.25E-06	1.11E-06	7.86E-08	2.03E-07	7.44E-07	2.55E-07
APCDD1L	7.42E-09	8.34E-09	7.67E-09	0	0	0	2.88E-08	1.09E-08
APCS	0.00183	0.000778	0.002253	0.001571	0.000852	0.004949	0.000934	0.002908
APEH	5.44E-05	5.67E-05	4.25E-05	5.09E-05	4.35E-05	5.20E-05	5.31E-05	4.43E-05
APEX1	0.000116	7.96E-05	7.00E-05	7.77E-05	3.59E-05	0.000109	8.77E-05	8.15E-05
APEX2	1.68E-05	2.21E-05	8.55E-06	1.35E-05	5.32E-06	1.73E-05	1.55E-05	3.87E-05
APH1A	0.000122	0.000122	9.49E-05	0.000107	6.31E-05	0.00015	0.000113	0.000159
APH1B	4.00E-06	9.36E-07	1.21E-06	2.70E-06	3.94E-07	1.53E-06	1.37E-06	1.88E-06
API5	3.21E-05	4.09E-05	2.34E-05	1.97E-05	7.41E-06	2.98E-05	1.59E-05	2.68E-05
APIP	2.46E-05	2.22E-05	1.09E-05	1.62E-05	6.09E-06	2.34E-05	9.72E-06	3.39E-05
APITD1	1.64E-05	7.61E-06	5.40E-06	3.98E-06	2.26E-06	1.01E-05	2.53E-06	4.17E-06
APLF	1.68E-06	7.42E-07	5.31E-07	8.84E-07	1.25E-06	6.36E-07	4.04E-07	1.16E-06
APLN	1.70E-06	6.46E-06	1.53E-08	3.63E-07	3.38E-07	6.59E-07	1.16E-05	3.59E-07
APLNR	7.40E-06	9.08E-07	7.58E-06	5.36E-06	1.21E-05	1.94E-06	5.83E-06	5.88E-06
APLP1	3.14E-06	1.34E-07	3.38E-08	5.72E-07	3.06E-07	3.00E-08	2.61E-07	4.13E-07
APLP2	0.00032	0.000168	0.000266	0.000308	0.000129	0.000217	0.000184	0.000345

APOA1	0.006415	0.009466	0.017081	0.014954	0.032046	0.00219	0.013468	0.001544
APOA1BP	0.000109	0.000166	6.80E-05	7.37E-05	0.000123	0.000102	9.44E-05	0.000185
APOA2	0.000397	0.044363	0.028909	0.022755	0.088296	0.033048	0.03137	0.078139
APOA4	0.00024	6.62E-06	5.02E-05	3.47E-05	1.49E-05	1.10E-05	1.03E-06	8.50E-06
APOA5	0.000237	0.000116	0.000442	0.000276	9.05E-05	0.000115	0.000109	0.000132
APOB	0.000522	0.000963	0.000738	0.000448	0.000316	0.000702	0.00032	0.000533
APOB48R	5.86E-06	6.64E-07	1.72E-06	4.53E-06	5.36E-07	1.01E-06	1.96E-06	1.20E-06
APOBEC1	0	0	0	0	0	0	0	0
APOBEC2	1.05E-07	7.76E-08	5.43E-08	5.53E-08	2.93E-08	0	8.18E-08	5.03E-08
APOBEC3/	5.70E-07	3.91E-07	4.46E-07	1.64E-06	7.79E-08	1.93E-07	9.47E-08	3.20E-07
APOBEC3I	5.69E-06	1.20E-05	2.52E-07	6.73E-06	1.02E-06	2.07E-06	1.35E-06	4.55E-06
APOBEC3(	1.23E-05	1.09E-06	3.02E-06	7.46E-06	2.65E-07	1.25E-06	3.95E-06	1.47E-06
APOBEC3I	4.36E-06	1.26E-06	7.08E-07	1.40E-06	2.21E-07	7.08E-07	8.68E-07	6.81E-07
APOBEC3I	5.60E-06	1.74E-06	2.11E-06	2.66E-06	2.07E-06	3.25E-06	1.39E-06	2.27E-06
APOBEC3(	1.76E-05	2.14E-06	2.83E-06	5.65E-06	7.22E-07	4.73E-06	2.83E-06	1.65E-06
APOBEC3I	3.42E-06	8.63E-08	3.88E-07	9.50E-07	1.82E-07	6.48E-07	5.62E-07	5.67E-07
APOBEC4	8.40E-09	0	0	0	0	0	0	0
APOC1	0.009186	0.018064	0.018569	0.019172	0.064698	0.014807	0.027812	0.024106
APOC1P1	2.84E-05	6.69E-06	0.000316	0.000279	0.000618	1.75E-05	5.38E-05	0.000125
APOC2	0.007331	0.005021	0.003937	0.00552	0.009561	0.003248	0.009408	0.006187
APOC3	0.007297	0.010413	0.020942	0.011481	0.02736	0.007513	0.009846	0.016208
APOC4	9.17E-07	0.00025	0.000667	0.000459	0.000707	0.001181	0.000481	0.001197
APOD	2.76E-07	3.32E-06	1.50E-07	2.96E-06	1.47E-06	8.80E-07	1.66E-05	3.42E-07
APOE	0.004647	0.012649	0.005907	0.006117	0.006111	0.003533	0.008088	0.006029
APOF	1.73E-07	6.26E-05	0.00031	0.000209	1.11E-05	3.53E-05	7.58E-05	0.000207
APOH	0.000733	0.008954	0.007384	0.005375	0.009667	0.007481	0.005865	0.003106
APOL1	0.000277	3.38E-05	0.00016	0.000135	4.62E-05	9.00E-05	8.09E-05	0.00013
APOL2	8.64E-05	1.99E-05	4.15E-05	2.82E-05	2.75E-05	3.24E-05	2.53E-05	3.96E-05
APOL3	5.25E-05	8.41E-06	3.38E-05	2.55E-05	6.16E-06	2.29E-05	1.66E-05	4.76E-05
APOL4	5.91E-06	2.45E-07	1.03E-07	1.17E-06	3.93E-07	7.85E-07	9.63E-07	1.22E-06
APOL5	0	5.37E-07	0	1.71E-07	1.50E-08	1.62E-08	3.35E-08	1.03E-07
APOL6	2.08E-05	1.56E-05	2.59E-05	1.97E-05	5.06E-06	1.38E-05	9.00E-06	1.75E-05
APOLD1	9.52E-06	1.22E-05	1.70E-06	8.45E-06	4.07E-06	1.21E-05	1.19E-05	7.97E-06
APOM	0.000288	0.00027	0.000537	0.000733	0.000602	0.000595	0.000968	0.000749
APOO	1.88E-05	1.97E-05	7.84E-06	1.01E-05	4.74E-06	1.40E-05	1.02E-05	2.63E-05
APOOL	6.35E-06	7.20E-06	4.40E-06	2.10E-06	3.00E-07	6.21E-06	6.78E-07	4.66E-06
APP	0.000255	0.0002	0.000157	0.000233	0.00011	6.85E-05	0.000129	0.000174
APPBP2	9.78E-06	7.15E-06	4.62E-06	3.70E-06	1.08E-06	1.14E-05	2.79E-06	2.92E-06
APPL1	8.58E-06	5.78E-06	5.64E-06	5.21E-06	1.63E-06	6.90E-06	4.30E-06	5.11E-06
APPL2	4.28E-05	1.77E-05	5.81E-06	8.55E-06	2.66E-06	1.10E-05	5.83E-06	5.58E-06
APRT	0.000265	0.0001	0.000106	0.000129	0.000161	0.0002	0.00014	0.000131
APTX	1.18E-05	1.59E-05	9.35E-06	1.07E-05	6.10E-06	2.14E-05	1.10E-05	1.16E-05
AQP1	5.41E-05	2.45E-05	2.06E-05	7.69E-05	1.63E-05	1.41E-05	2.06E-05	3.82E-05
AQP10	0	1.89E-07	0	1.66E-08	0	0	0	0
AQP11	1.10E-05	1.15E-05	1.35E-05	6.17E-06	1.08E-05	2.33E-05	3.28E-06	5.29E-06
AQP12A	0	0	0	0	0	0	0	0
AQP12B	0	0	0	0	0	0	0	0
AQP2	0	0	0	0	0	0	0	0
AQP3	0.000142	8.24E-06	6.34E-05	0.000151	2.91E-05	7.73E-06	5.65E-05	3.19E-05
AQP4	5.24E-07	1.03E-06	4.33E-07	5.64E-07	4.91E-08	7.33E-08	7.28E-08	5.41E-07
AQP5	1.33E-08	3.01E-08	4.19E-08	4.89E-08	4.58E-08	1.11E-07	1.03E-07	0
AQP6	1.94E-07	1.14E-08	1.19E-07	6.18E-08	6.42E-07	4.21E-06	0	0
AQP7	2.16E-06	7.28E-06	1.55E-05	1.01E-05	4.22E-06	2.43E-06	2.28E-05	5.88E-06
AQP7P1	5.30E-07	4.91E-06	3.34E-05	1.54E-05	3.89E-06	9.45E-07	6.85E-05	1.74E-06
AQP7P3	3.19E-07	7.92E-07	5.46E-06	3.56E-06	1.29E-06	4.84E-07	1.03E-05	3.93E-07
AQP8	0	6.73E-06	4.15E-08	7.16E-08	7.03E-07	0	2.68E-06	3.65E-06
AQP9	2.73E-06	0.000109	0.000352	0.00023	0.000113	0.000642	0.000187	0.000231
AQPEP	4.30E-07	2.84E-07	5.16E-09	1.72E-08	0	0	0	1.50E-08

AQR	7.97E-06	4.30E-06	4.38E-06	4.38E-06	8.64E-07	6.22E-06	2.81E-06	4.65E-06
AR	3.59E-06	6.11E-06	1.37E-05	3.61E-06	1.27E-06	1.31E-05	2.19E-06	4.77E-06
ARAF	6.02E-05	6.33E-05	5.04E-05	3.92E-05	4.72E-05	4.12E-05	5.01E-05	8.22E-05
ARAP1	3.10E-05	1.58E-05	1.26E-05	1.93E-05	1.32E-05	2.16E-05	2.79E-05	1.90E-05
ARAP2	3.00E-06	1.52E-06	1.88E-06	3.21E-06	1.56E-07	1.20E-06	7.46E-07	1.85E-06
ARAP3	5.14E-06	1.46E-06	1.33E-06	1.66E-06	2.16E-06	1.31E-06	3.36E-06	2.70E-06
ARC	1.58E-08	1.59E-07	4.08E-08	2.16E-06	6.77E-09	2.92E-08	1.76E-07	4.62E-08
ARCN1	6.82E-05	3.75E-05	5.19E-05	6.55E-05	2.48E-05	6.64E-05	4.60E-05	8.13E-05
AREG	2.55E-05	1.33E-06	3.11E-07	9.88E-06	1.78E-08	4.62E-07	6.14E-07	9.19E-08
ARF1	0.000324	0.000323	0.000187	0.000221	0.000118	0.000295	0.000238	0.000416
ARF3	7.16E-05	4.56E-05	2.15E-05	3.07E-05	8.49E-06	3.81E-05	2.41E-05	3.00E-05
ARF4	0.000222	0.000106	8.51E-05	0.000133	3.99E-05	0.000145	0.000106	0.000108
ARF5	0.00014	0.000157	0.000114	0.000133	0.000181	0.000133	0.000166	0.000185
ARF6	4.21E-05	2.94E-05	2.36E-05	4.59E-05	1.15E-05	2.63E-05	4.05E-05	2.83E-05
ARFGAP1	2.76E-05	2.08E-05	9.93E-06	1.62E-05	3.52E-05	1.56E-05	1.96E-05	1.85E-05
ARFGAP2	5.38E-05	5.70E-05	3.61E-05	3.38E-05	4.10E-05	4.30E-05	4.64E-05	5.53E-05
ARFGAP3	5.73E-05	2.33E-05	1.69E-05	1.89E-05	8.15E-06	3.95E-05	1.88E-05	2.64E-05
ARFGEF1	2.66E-05	2.47E-05	1.04E-05	9.30E-06	6.32E-06	1.09E-05	1.93E-05	1.19E-05
ARFGEF2	2.04E-05	1.89E-05	9.94E-06	7.08E-06	1.96E-06	1.27E-05	5.37E-06	1.29E-05
ARFIP1	1.47E-05	6.44E-06	8.41E-06	1.08E-05	3.16E-06	9.59E-06	9.28E-06	1.58E-05
ARFIP2	4.68E-05	2.01E-05	1.03E-05	1.44E-05	1.40E-05	2.06E-05	9.66E-06	1.97E-05
ARFRP1	2.01E-05	1.64E-05	9.21E-06	1.47E-05	3.17E-05	1.61E-05	2.33E-05	1.17E-05
ARG1	2.76E-06	0.001024	0.000625	0.000342	5.08E-05	0.000417	0.000405	0.0003
ARG2	3.68E-05	1.19E-05	2.46E-06	3.56E-06	1.21E-06	1.25E-05	1.87E-06	1.29E-05
ARGFX	0	0	0	0	0	0	4.33E-09	0
ARGFXP2	6.42E-08	4.92E-08	0	0	0	0	2.05E-08	3.17E-08
ARGLU1	4.72E-05	2.07E-05	1.41E-05	1.71E-05	4.21E-06	1.27E-05	1.22E-05	1.55E-05
ARHGAP1	3.69E-05	2.68E-05	1.61E-05	2.57E-05	8.58E-06	2.59E-05	2.33E-05	3.35E-05
ARHGAP1	6.92E-06	3.82E-07	9.45E-06	5.91E-06	3.98E-06	1.15E-06	1.21E-05	1.37E-06
ARHGAP1	4.01E-06	1.64E-06	2.28E-07	3.34E-07	2.24E-07	8.77E-07	3.01E-07	3.07E-07
ARHGAP1	2.48E-06	4.51E-07	2.58E-07	8.54E-08	0	1.71E-07	7.48E-08	1.96E-07
ARHGAP1	1.54E-05	1.57E-05	8.75E-06	1.17E-05	4.45E-06	2.12E-05	7.44E-06	9.67E-06
ARHGAP1	5.19E-06	2.92E-07	1.91E-06	7.17E-06	1.08E-06	1.73E-06	4.34E-06	1.19E-06
ARHGAP1	2.98E-05	2.28E-05	1.00E-05	1.55E-05	7.02E-06	1.38E-05	1.77E-05	1.37E-05
ARHGAP1	1.34E-05	5.32E-06	2.12E-06	3.14E-06	6.00E-07	4.85E-06	3.38E-06	1.44E-06
ARHGAP1	3.06E-06	1.22E-06	1.06E-06	1.15E-06	7.58E-07	1.18E-06	1.81E-06	1.28E-06
ARHGAP2	1.52E-07	7.65E-08	5.02E-07	3.30E-07	3.59E-08	2.14E-08	7.78E-08	3.34E-08
ARHGAP2	1.50E-05	8.31E-06	5.29E-06	6.48E-06	2.75E-06	5.61E-06	5.06E-06	5.78E-06
ARHGAP2	1.31E-06	2.35E-07	1.16E-07	1.09E-06	2.04E-07	2.74E-07	9.46E-07	2.48E-07
ARHGAP2	7.40E-06	1.06E-06	8.54E-07	2.91E-06	7.10E-07	1.22E-06	2.16E-06	1.80E-06
ARHGAP2	4.16E-06	7.34E-07	4.11E-06	7.09E-06	1.82E-06	9.39E-07	2.62E-06	3.25E-06
ARHGAP2	9.46E-06	4.36E-06	4.63E-06	6.09E-06	1.07E-06	4.50E-06	6.32E-06	1.64E-06
ARHGAP2	2.30E-06	4.53E-07	1.40E-06	1.09E-06	2.65E-07	9.92E-07	1.16E-06	1.34E-06
ARHGAP2	1.73E-05	2.46E-06	2.63E-06	5.17E-06	1.12E-06	2.27E-06	3.91E-06	1.66E-06
ARHGAP2	5.40E-07	2.29E-07	6.33E-08	1.00E-07	5.66E-08	1.53E-08	1.73E-07	5.38E-07
ARHGAP2	1.27E-05	1.04E-05	6.01E-06	5.42E-06	1.07E-06	1.15E-05	3.88E-06	3.85E-06
ARHGAP3	8.64E-06	1.44E-06	3.69E-06	7.36E-06	5.45E-07	4.35E-06	4.58E-06	1.28E-06
ARHGAP3	2.31E-06	1.64E-06	1.12E-06	1.31E-06	3.21E-07	7.12E-07	1.04E-06	9.57E-07
ARHGAP3	2.04E-06	8.10E-07	1.73E-06	9.81E-07	3.74E-07	1.75E-06	6.88E-07	2.24E-06
ARHGAP3	1.80E-06	1.01E-06	5.92E-07	8.60E-07	4.93E-07	9.64E-07	7.07E-07	8.83E-07
ARHGAP3	0	0	0	0	0	0	1.86E-08	0
ARHGAP3	1.26E-06	1.00E-06	1.43E-07	5.05E-07	1.32E-07	5.42E-07	1.18E-06	3.60E-07
ARHGAP4	3.50E-05	2.43E-06	4.08E-06	1.03E-05	2.36E-05	7.41E-06	8.00E-06	5.27E-06
ARHGAP4	4.23E-06	3.13E-06	2.79E-06	2.53E-06	3.70E-07	2.58E-06	9.92E-07	1.29E-06
ARHGAP5	1.04E-05	1.08E-05	7.74E-06	7.15E-06	1.94E-06	8.92E-06	4.34E-06	4.38E-06
ARHGAP6	5.09E-07	2.98E-07	1.70E-07	3.87E-07	1.97E-07	2.30E-07	3.39E-07	5.12E-07
ARHGAP8	5.77E-06	5.14E-07	4.84E-07	7.21E-07	2.19E-07	4.36E-07	3.31E-07	0
ARHGAP9	1.12E-05	5.57E-07	3.04E-06	7.86E-06	6.83E-07	3.19E-06	2.83E-06	1.83E-06

ARHGDIA	0.000311	0.000203	0.000134	0.00021	0.000172	0.000154	0.000212	0.000171
ARHGDIB	0.000148	2.47E-05	5.04E-05	0.000124	4.26E-05	6.48E-05	0.0001	5.74E-05
ARHGDIG	7.74E-08	0	0	1.78E-08	0	0	0	0
ARHGEF1	4.51E-05	2.04E-05	1.56E-05	3.39E-05	2.11E-05	2.51E-05	2.78E-05	2.42E-05
ARHGEF1(	2.64E-06	9.74E-07	5.14E-07	1.41E-06	4.59E-07	6.09E-07	8.04E-07	6.39E-07
ARHGEF1(	2.62E-05	2.05E-05	3.00E-05	2.43E-05	1.82E-05	1.78E-05	1.66E-05	7.35E-05
ARHGEF1:	9.65E-06	9.60E-06	4.79E-06	5.54E-06	1.84E-06	2.77E-05	7.43E-06	1.50E-05
ARHGEF1:	3.11E-05	2.27E-05	2.99E-05	2.30E-05	4.94E-06	3.50E-05	1.51E-05	3.63E-05
ARHGEF1!	3.25E-06	2.66E-06	1.33E-06	7.39E-07	3.42E-06	1.77E-06	4.37E-06	2.77E-06
ARHGEF1(	2.86E-05	2.32E-06	2.03E-06	5.25E-06	1.98E-06	8.69E-06	2.56E-06	4.81E-06
ARHGEF1	7.51E-06	1.56E-06	1.31E-06	2.42E-06	9.76E-07	1.23E-06	1.89E-06	2.08E-06
ARHGEF1{	1.49E-05	7.07E-06	5.96E-06	1.11E-05	6.14E-06	7.91E-06	9.64E-06	6.91E-06
ARHGEF1!	2.78E-06	9.59E-07	3.51E-07	6.57E-07	1.85E-07	2.58E-07	4.63E-07	4.41E-07
ARHGEF2	1.16E-05	9.87E-06	3.55E-06	7.39E-06	4.49E-06	3.96E-06	1.23E-05	4.59E-06
ARHGEF3	9.14E-06	5.32E-07	1.59E-06	3.68E-06	1.32E-06	1.76E-06	1.62E-06	2.84E-06
ARHGEF3:	5.37E-08	9.78E-09	6.31E-08	4.25E-08	3.87E-09	7.07E-08	2.20E-08	1.32E-08
ARHGEF3!	1.04E-05	1.62E-05	5.46E-06	1.10E-05	1.40E-07	1.82E-05	5.10E-06	1.42E-05
ARHGEF3	4.12E-06	1.53E-06	3.90E-07	6.97E-07	8.60E-07	2.25E-06	1.75E-06	3.80E-07
ARHGEF3{	1.56E-07	0	0	2.14E-08	0	0	1.67E-08	0
ARHGEF4	2.03E-06	8.94E-07	9.92E-08	3.81E-08	0	1.44E-08	1.51E-08	3.43E-08
ARHGEF5	6.45E-06	7.51E-06	3.11E-06	7.62E-06	1.55E-06	1.14E-05	7.65E-06	1.34E-05
ARHGEF6	4.69E-06	1.01E-06	2.38E-06	3.10E-06	5.60E-07	2.89E-06	1.60E-06	9.28E-07
ARHGEF7	1.24E-05	1.09E-05	8.00E-06	6.19E-06	2.24E-06	7.00E-06	6.04E-06	7.03E-06
ARHGEF9	3.47E-06	3.74E-06	1.83E-06	1.73E-06	2.16E-06	3.48E-06	1.48E-06	4.17E-06
ARID1A	2.77E-05	1.88E-05	1.35E-05	1.69E-05	5.03E-06	1.83E-05	1.24E-05	1.31E-05
ARID1B	6.14E-06	4.34E-06	4.52E-06	5.09E-06	7.95E-07	5.21E-06	3.73E-06	3.99E-06
ARID2	3.42E-06	2.47E-06	1.80E-06	1.80E-06	6.00E-07	2.33E-06	1.40E-06	9.25E-07
ARID3A	2.35E-06	5.99E-07	4.65E-07	3.56E-07	1.29E-07	2.85E-07	6.45E-07	1.50E-06
ARID3B	2.47E-06	1.02E-06	9.45E-07	1.30E-06	3.13E-07	6.94E-07	8.08E-07	1.27E-06
ARID3C	4.34E-07	2.25E-06	2.24E-06	1.09E-06	3.57E-08	2.89E-07	5.94E-07	3.06E-07
ARID4A	7.06E-06	2.80E-06	5.89E-06	5.60E-06	1.26E-06	3.92E-06	4.48E-06	2.03E-06
ARID4B	7.73E-06	4.13E-06	4.68E-06	5.95E-06	1.27E-06	9.38E-06	2.86E-06	7.98E-06
ARID5A	4.29E-05	2.45E-05	1.66E-05	2.52E-05	1.96E-05	8.12E-06	2.48E-05	5.24E-06
ARID5B	8.25E-06	2.09E-06	1.80E-06	6.79E-06	4.25E-07	2.51E-06	1.43E-06	1.44E-06
ARIH1	1.93E-05	1.27E-05	8.99E-06	8.93E-06	2.58E-06	1.45E-05	7.78E-06	1.00E-05
ARIH2	2.54E-05	1.65E-05	8.20E-06	1.27E-05	9.23E-06	1.33E-05	1.20E-05	1.39E-05
ARL1	3.57E-05	3.40E-05	2.16E-05	2.16E-05	7.40E-06	3.31E-05	2.04E-05	3.03E-05
ARL10	4.31E-07	2.85E-08	1.77E-07	3.10E-07	1.32E-08	8.07E-08	1.02E-07	1.17E-07
ARL11	8.53E-07	1.39E-06	4.69E-07	6.83E-07	1.26E-07	3.30E-07	5.56E-07	1.23E-07
ARL13A	0	0	0	2.85E-08	0	0	0	0
ARL13B	3.27E-06	3.57E-06	1.50E-06	2.32E-06	2.76E-07	1.68E-06	1.07E-06	1.66E-06
ARL14	6.72E-06	6.80E-08	1.27E-07	6.45E-06	1.71E-08	1.10E-07	3.41E-07	2.63E-06
ARL15	7.95E-06	8.09E-06	2.91E-06	3.21E-06	4.05E-07	4.47E-06	2.31E-06	6.79E-06
ARL16	2.74E-05	1.63E-05	6.01E-06	1.15E-05	1.41E-05	1.65E-05	1.10E-05	1.34E-05
ARL17A	8.54E-06	2.68E-06	2.52E-06	1.69E-06	1.92E-06	8.02E-06	1.86E-06	1.55E-06
ARL17B	1.05E-07	8.42E-08	5.16E-08	3.75E-08	2.82E-08	2.38E-07	5.42E-08	5.00E-08
ARL2	2.61E-05	2.77E-05	1.21E-05	2.34E-05	3.94E-05	2.07E-05	3.05E-05	2.76E-05
ARL2BP	4.03E-05	1.48E-05	1.75E-05	2.76E-05	6.05E-06	2.34E-05	2.87E-05	2.36E-05
ARL3	5.28E-06	2.21E-06	2.93E-06	3.30E-06	2.17E-06	4.19E-06	3.93E-06	2.66E-06
ARL4A	7.53E-06	2.70E-06	2.78E-06	7.77E-06	8.03E-07	1.89E-05	3.30E-06	8.71E-06
ARL4C	2.10E-05	1.43E-06	5.67E-06	2.46E-05	4.92E-07	4.76E-06	5.48E-06	3.40E-06
ARL4D	3.11E-05	5.65E-05	2.72E-05	9.03E-06	5.39E-05	2.72E-05	2.43E-05	1.26E-05
ARL5A	2.14E-05	1.09E-05	1.30E-05	1.08E-05	3.72E-06	1.63E-05	8.49E-06	8.32E-06
ARL5B	8.22E-06	1.17E-05	9.82E-06	1.11E-05	3.45E-07	6.48E-06	4.50E-06	1.19E-05
ARL5C	0	0	2.35E-08	0	1.88E-08	0	4.17E-08	0
ARL6	5.92E-07	2.23E-06	8.66E-07	7.96E-07	1.37E-07	1.57E-06	4.85E-07	1.58E-06
ARL6IP1	0.000234	0.000394	0.000181	0.000214	7.71E-05	0.000118	0.000139	0.000101
ARL6IP4	0.000153	0.000181	8.88E-05	0.000187	0.000316	0.000197	0.000274	0.000154

ARL6IP5	7.98E-05	2.73E-05	3.53E-05	4.79E-05	2.36E-05	5.64E-05	2.45E-05	2.27E-05
ARL6IP6	9.31E-06	2.34E-06	2.16E-06	2.30E-06	6.75E-07	2.55E-06	2.12E-06	1.79E-06
ARL8A	1.95E-05	9.96E-06	2.12E-05	3.16E-05	1.25E-05	3.33E-05	3.39E-05	4.78E-05
ARL8B	3.31E-05	3.58E-05	1.95E-05	2.25E-05	1.05E-05	2.35E-05	1.97E-05	1.60E-05
ARL9	1.14E-06	7.86E-08	3.75E-08	1.66E-07	2.91E-08	0	0	5.01E-08
ARMC1	2.71E-05	2.17E-05	1.04E-05	7.83E-06	2.63E-06	1.15E-05	1.26E-05	1.48E-05
ARMC10	5.79E-06	1.91E-05	8.22E-06	7.56E-06	4.44E-06	1.01E-05	7.50E-06	1.08E-05
ARMC2	2.96E-07	2.14E-07	8.42E-08	2.30E-07	7.52E-08	4.38E-07	2.09E-07	2.86E-07
ARMC3	3.87E-08	0	0	2.50E-08	0	0	0	1.42E-08
ARMC4	9.12E-09	9.95E-07	7.53E-08	4.11E-07	0	8.46E-09	0	0
ARMC5	4.28E-06	2.42E-05	9.40E-06	7.84E-06	1.35E-05	8.42E-06	1.50E-05	3.93E-06
ARMC6	2.10E-05	6.40E-05	4.65E-05	2.64E-05	1.64E-05	3.03E-05	1.10E-05	1.17E-05
ARMC7	1.40E-05	1.45E-05	3.25E-06	9.94E-06	1.37E-05	8.35E-06	1.43E-05	6.68E-06
ARMC8	8.82E-06	6.55E-06	4.96E-06	6.33E-06	1.77E-06	7.57E-06	4.89E-06	5.27E-06
ARMC9	1.97E-06	3.35E-07	1.73E-07	1.46E-06	2.16E-07	1.69E-06	6.17E-07	2.96E-07
ARMCX1	6.29E-06	9.98E-07	1.28E-06	2.41E-06	5.00E-07	6.33E-07	1.28E-06	9.86E-07
ARMCX2	1.72E-06	8.48E-07	8.70E-07	2.09E-06	3.69E-07	3.83E-07	1.20E-06	7.61E-07
ARMCX3	3.38E-05	1.18E-05	9.63E-06	1.26E-05	5.66E-07	1.07E-05	2.41E-06	1.72E-05
ARMCX5	3.72E-06	2.77E-06	3.27E-06	2.63E-06	1.63E-06	4.70E-06	3.63E-06	5.70E-06
ARMCX6	6.34E-06	1.64E-06	1.49E-06	2.53E-06	4.86E-07	1.94E-06	9.12E-07	1.93E-06
ARMS2	2.10E-07	0	0	4.35E-08	9.13E-08	0	3.31E-08	0
ARNT	1.66E-05	1.79E-05	1.99E-05	1.28E-05	8.20E-06	2.19E-05	1.68E-05	2.58E-05
ARNT2	1.64E-06	7.68E-09	1.46E-07	1.56E-06	2.09E-08	1.29E-08	6.11E-08	1.53E-07
ARNTL	1.14E-05	1.48E-05	9.77E-07	6.04E-06	1.61E-06	7.89E-06	2.54E-06	2.24E-05
ARNTL2	1.60E-05	2.88E-06	9.54E-07	2.73E-06	4.21E-07	2.02E-07	1.09E-06	2.88E-06
ARPC1A	0.000204	0.000185	4.55E-05	4.61E-05	6.94E-05	7.15E-05	7.51E-05	8.83E-05
ARPC1B	0.000435	0.000128	5.18E-05	0.000103	5.90E-05	5.92E-05	0.000131	0.0001
ARPC2	0.000244	9.18E-05	6.34E-05	0.000106	4.99E-05	0.000102	9.28E-05	8.65E-05
ARPC3	0.000341	0.000202	0.000127	0.00018	0.000118	0.000181	0.000194	0.000168
ARPC4	0.000143	7.27E-05	5.24E-05	9.33E-05	9.67E-05	8.59E-05	0.000111	6.48E-05
ARPC5	0.000104	0.000114	5.20E-05	7.36E-05	4.01E-05	0.00013	8.65E-05	0.000108
ARPC5L	4.38E-05	2.86E-05	2.05E-05	3.80E-05	3.33E-05	3.41E-05	4.39E-05	4.42E-05
ARPM1	6.58E-07	8.26E-07	1.45E-06	2.19E-06	5.87E-07	1.17E-06	6.71E-07	1.64E-06
ARPP19	4.16E-05	1.94E-05	1.50E-05	1.70E-05	6.02E-06	2.79E-05	1.36E-05	1.63E-05
ARPP21	9.40E-06	4.99E-07	7.95E-08	4.73E-07	4.66E-07	3.55E-08	1.22E-07	3.37E-08
ARR3	1.94E-08	0	0	0	0	0	0	0
ARRB1	2.68E-05	1.93E-05	7.51E-06	5.37E-06	1.59E-06	1.59E-06	5.23E-06	9.93E-07
ARRB2	4.58E-05	1.55E-05	1.87E-05	3.78E-05	3.25E-05	2.15E-05	5.02E-05	1.98E-05
ARRDC1	2.60E-05	1.52E-05	2.16E-05	2.45E-05	1.67E-05	1.45E-05	1.84E-05	2.02E-05
ARRDC2	3.01E-05	1.22E-05	4.46E-06	2.30E-05	5.14E-05	7.81E-06	4.05E-05	1.15E-05
ARRDC3	2.91E-05	1.57E-05	1.76E-05	2.70E-05	1.49E-06	1.80E-05	2.10E-05	1.04E-05
ARRDC4	5.28E-06	5.52E-06	1.15E-05	1.22E-05	1.15E-06	6.51E-06	5.89E-06	6.54E-06
ARRDC5	1.20E-07	0	6.33E-08	3.68E-08	0	1.53E-07	5.77E-08	4.42E-08
ARSA	4.93E-05	6.04E-05	4.00E-05	3.39E-05	6.54E-05	7.72E-05	3.96E-05	2.78E-05
ARSB	2.99E-06	1.89E-06	3.05E-06	3.13E-06	1.26E-06	4.46E-06	2.59E-06	6.08E-06
ARSD	3.24E-05	1.85E-05	3.37E-05	1.60E-05	6.42E-06	2.40E-05	9.81E-06	1.97E-05
ARSE	5.84E-05	0	0.000101	6.24E-05	6.70E-05	4.94E-05	2.40E-05	6.30E-05
ARSF	4.76E-08	0	1.80E-06	7.44E-07	1.43E-07	1.10E-08	2.30E-08	3.22E-05
ARSG	1.55E-06	3.57E-07	1.24E-06	1.13E-06	6.71E-06	2.47E-06	2.10E-06	3.90E-06
ARSH	0	0	0	0	0	0	0	0
ARSI	6.27E-07	0	1.28E-07	1.37E-06	1.25E-08	4.72E-08	7.07E-08	7.48E-08
ARSJ	4.94E-06	2.54E-07	6.62E-07	3.73E-06	6.22E-08	1.34E-07	4.47E-07	1.22E-06
ARSK	3.98E-06	2.92E-06	2.13E-06	1.82E-06	4.08E-07	4.67E-06	1.49E-06	1.85E-06
ART1	0	0	0	0	0	0	0	2.86E-08
ART3	8.23E-08	0	6.51E-08	1.71E-07	0	5.13E-08	0	0
ART4	1.00E-05	1.09E-05	1.82E-05	1.43E-05	3.83E-06	1.30E-05	1.20E-05	1.18E-05
ART5	2.16E-08	8.08E-08	2.20E-08	1.04E-07	0	0	8.56E-07	3.03E-08
ARTN	5.16E-07	1.28E-07	2.34E-08	6.91E-08	1.12E-07	1.49E-07	1.25E-07	9.66E-08

ARV1	2.01E-05	2.39E-05	1.29E-05	1.09E-05	3.54E-06	2.43E-05	1.08E-05	1.85E-05
ARVCF	7.34E-06	8.63E-06	5.96E-06	1.28E-05	4.23E-06	4.08E-06	9.14E-06	6.46E-06
ARX	0	0	0	0	0	0	0	0
AS3MT	0.000123	1.88E-05	4.95E-05	2.44E-05	8.86E-05	2.77E-05	2.81E-05	6.09E-05
ASAH1	7.56E-05	4.87E-05	3.37E-05	5.55E-05	6.89E-06	2.72E-05	3.20E-05	2.78E-05
ASAH2	3.07E-08	1.04E-07	4.36E-07	2.50E-07	2.64E-08	3.83E-06	2.97E-08	2.40E-07
ASAH2B	1.86E-06	2.59E-06	2.55E-06	3.87E-07	0	1.47E-06	4.00E-07	6.42E-07
ASAM	1.92E-06	3.00E-08	2.77E-08	2.29E-07	7.65E-09	8.24E-09	1.12E-07	1.31E-08
ASAP1	2.42E-05	4.42E-06	2.40E-06	4.63E-06	6.46E-07	2.03E-06	3.18E-06	4.01E-06
ASAP1IT1	2.19E-08	0	0	0	0	0	0	0
ASAP2	7.11E-06	2.68E-06	1.18E-06	1.12E-06	4.11E-07	2.77E-06	1.07E-06	1.28E-06
ASAP3	1.75E-05	9.94E-06	2.44E-06	4.90E-06	2.46E-06	4.90E-06	4.09E-06	5.69E-06
ASB1	4.70E-06	1.02E-06	8.24E-07	3.16E-06	8.81E-07	1.29E-06	2.49E-06	2.98E-06
ASB10	0	0	0	0	0	0	0	0
ASB11	0	0	0	3.67E-08	0	0	0	0
ASB12	7.39E-08	3.49E-08	8.04E-08	1.29E-08	0	2.15E-08	2.20E-08	0
ASB13	3.18E-05	8.94E-06	2.95E-05	1.85E-05	1.92E-05	2.65E-05	3.18E-05	1.20E-05
ASB14	2.05E-07	9.29E-08	2.25E-08	3.97E-08	6.29E-09	4.30E-08	1.20E-07	6.35E-08
ASB15	1.72E-08	0	0	0	1.35E-08	2.40E-08	8.36E-09	5.06E-08
ASB16	4.60E-07	2.24E-07	6.87E-08	4.85E-08	0	1.95E-07	6.35E-08	1.56E-07
ASB17	0	2.94E-08	0	0	0	0	0	0
ASB18	0	0	0	0	0	0	0	0
ASB2	1.91E-06	3.79E-08	1.86E-07	4.03E-07	2.46E-07	4.98E-07	1.24E-07	1.90E-07
ASB3	9.98E-06	8.16E-06	4.82E-06	4.54E-06	2.78E-06	8.35E-06	4.03E-06	3.27E-06
ASB4	1.85E-07	7.78E-07	7.91E-08	2.25E-07	0	8.35E-07	1.84E-07	6.03E-07
ASB5	1.58E-08	3.56E-08	0	9.68E-09	0	0	0	1.16E-08
ASB6	8.24E-06	3.37E-06	2.71E-06	4.98E-06	2.91E-06	3.44E-06	4.87E-06	7.28E-06
ASB7	8.90E-06	5.28E-06	2.88E-06	2.97E-06	1.41E-06	4.02E-06	2.84E-06	2.99E-06
ASB8	1.68E-05	1.81E-05	1.45E-05	9.90E-06	7.16E-06	1.76E-05	1.57E-05	1.59E-05
ASB9	1.01E-05	2.88E-06	8.78E-06	3.55E-06	1.59E-06	8.86E-06	5.01E-06	4.85E-06
ASCC1	1.91E-05	1.42E-05	8.94E-06	5.52E-06	3.20E-06	1.28E-05	6.92E-06	1.50E-05
ASCC2	3.36E-05	3.52E-05	2.44E-05	2.41E-05	3.17E-05	3.13E-05	3.27E-05	3.47E-05
ASCC3	1.30E-05	5.23E-06	5.57E-06	5.66E-06	9.30E-07	9.23E-06	4.50E-06	7.69E-06
ASCL1	3.79E-08	6.41E-08	3.79E-05	1.16E-08	0	1.76E-08	0	1.95E-07
ASCL2	6.28E-07	8.89E-08	4.12E-07	2.89E-07	5.63E-08	1.58E-07	3.53E-07	1.93E-08
ASCL3	0	1.13E-07	0	0	0	4.41E-08	0	0
ASCL4	1.05E-08	0	0	0	0	0	2.03E-08	0
ASF1A	6.34E-06	5.96E-06	6.71E-06	5.39E-06	3.07E-06	1.54E-05	4.86E-06	5.98E-06
ASF1B	1.35E-05	4.92E-06	4.75E-07	6.74E-07	1.76E-06	2.50E-06	9.91E-07	1.31E-06
ASFMR1	1.53E-08	1.42E-07	0	0	0	5.66E-08	9.61E-09	0
ASGR1	0.000151	0.000183	0.000668	0.000605	0.000419	0.000635	0.000623	0.000627
ASGR2	0.000373	0.000142	0.000958	0.000953	0.000617	0.000694	0.000937	0.000875
ASH1L	4.77E-06	5.27E-06	2.84E-06	2.23E-06	3.44E-07	5.32E-06	1.21E-06	3.05E-06
ASH2L	1.20E-05	2.15E-05	1.39E-05	1.35E-05	3.40E-06	1.90E-05	7.63E-06	1.19E-05
ASIP	1.09E-07	2.67E-07	0	6.88E-07	3.84E-07	0	1.63E-06	0
ASL	0.000155	0.000165	0.000188	0.000183	0.000104	5.25E-05	0.000129	0.000341
ASMT	5.69E-08	0	0	0	8.13E-08	4.81E-08	0	0
ASMTL	1.76E-05	2.93E-05	2.54E-05	2.32E-05	2.49E-05	1.87E-05	2.81E-05	5.76E-05
ASNA1	5.79E-05	3.54E-05	2.66E-05	4.18E-05	4.03E-05	7.03E-05	4.51E-05	4.95E-05
ASNS	5.57E-06	6.56E-07	7.48E-07	2.71E-06	2.64E-06	2.39E-07	9.06E-07	9.80E-07
ASNSD1	2.38E-05	2.17E-05	1.76E-05	1.53E-05	9.57E-06	1.95E-05	1.45E-05	1.69E-05
ASPA	2.30E-06	8.88E-07	2.01E-06	1.89E-06	4.03E-07	1.43E-06	3.39E-07	1.83E-07
ASPDH	3.09E-06	6.52E-05	0.000117	8.45E-05	0.000267	8.00E-05	7.82E-05	6.87E-06
ASPG	1.48E-05	1.98E-05	6.72E-05	1.80E-05	1.86E-05	2.22E-08	1.91E-05	1.21E-05
ASPH	5.38E-05	3.95E-05	3.28E-05	2.37E-05	1.16E-05	0.00011	5.19E-05	4.09E-05
ASPHD1	4.27E-05	1.15E-05	1.42E-07	2.36E-06	1.16E-07	6.89E-07	5.02E-07	1.24E-07
ASPHD2	1.36E-06	2.65E-07	1.58E-07	1.70E-07	6.56E-08	2.89E-07	2.29E-07	4.28E-07
ASPM	5.61E-06	2.71E-06	1.46E-07	1.59E-07	2.22E-07	2.11E-06	1.69E-07	1.11E-06

ASPN	1.72E-05	1.87E-06	1.04E-05	1.85E-05	2.88E-06	2.44E-06	7.55E-06	7.63E-06
ASPRV1	6.01E-07	1.51E-06	1.28E-06	6.62E-07	5.60E-07	8.64E-07	8.75E-07	4.18E-07
ASPSCR1	2.11E-05	2.89E-05	2.08E-05	2.10E-05	5.11E-05	0.000156	2.24E-05	2.29E-05
ASRGL1	8.85E-05	1.17E-05	9.93E-07	1.28E-06	1.16E-06	4.07E-07	1.06E-06	3.74E-06
ASS1	0.000402	0.004001	0.001606	0.000745	0.000758	0.000471	0.000453	0.000393
ASTE1	2.68E-06	2.44E-06	2.08E-06	2.41E-06	6.01E-07	2.70E-06	2.27E-06	1.59E-06
ASTL	3.92E-08	0	2.10E-08	2.41E-08	0	0	0	2.90E-08
ASTN1	8.91E-09	2.31E-07	2.49E-08	5.20E-08	3.97E-08	4.23E-08	4.51E-08	3.56E-08
ASTN2	4.25E-06	1.23E-06	2.18E-06	2.91E-06	1.61E-06	7.15E-06	2.00E-06	5.76E-06
ASXL1	2.69E-05	9.68E-06	4.12E-06	6.68E-06	5.72E-06	9.57E-06	7.49E-06	7.84E-06
ASXL2	6.01E-06	2.84E-06	1.56E-06	1.06E-06	6.33E-08	2.73E-06	4.91E-07	1.33E-06
ASXL3	2.91E-09	4.54E-08	1.22E-07	4.62E-08	0	2.69E-09	1.42E-08	3.40E-08
ASZ1	0	0	0	0	0	0	0	0
ATAD1	2.28E-05	1.75E-05	1.49E-05	1.10E-05	5.82E-06	1.54E-05	9.26E-06	1.62E-05
ATAD2	3.09E-05	1.63E-05	2.57E-06	2.44E-06	2.54E-06	6.33E-06	5.05E-06	1.95E-05
ATAD2B	4.02E-06	3.15E-06	1.55E-06	1.75E-06	5.09E-07	1.53E-06	7.69E-07	1.59E-06
ATAD3A	1.75E-05	3.00E-05	9.73E-06	1.30E-05	8.23E-06	1.62E-05	9.30E-06	1.62E-05
ATAD3B	1.17E-05	1.25E-05	3.54E-06	6.49E-06	6.09E-06	5.50E-06	5.76E-06	8.19E-06
ATAD3C	7.27E-07	3.36E-06	2.01E-06	3.97E-07	1.22E-07	2.54E-06	1.84E-07	1.30E-07
ATAD5	1.15E-06	7.64E-07	3.30E-08	1.01E-07	8.30E-08	1.82E-07	9.08E-08	9.83E-08
ATCAY	0	0	0	0	3.84E-09	0	0	8.29E-09
ATE1	7.26E-06	4.90E-06	5.04E-06	3.83E-06	9.23E-08	7.10E-06	1.61E-06	2.63E-06
ATF1	1.09E-05	1.12E-05	6.61E-06	6.88E-06	8.67E-07	1.06E-05	5.18E-06	6.01E-06
ATF2	1.75E-05	1.64E-05	8.36E-06	6.26E-06	2.72E-07	1.53E-05	2.53E-06	7.43E-06
ATF3	1.67E-05	0.000177	7.97E-05	0.00021	1.83E-06	1.76E-05	0.000116	3.06E-05
ATF4	0.000198	0.000192	0.000165	0.000251	0.000179	0.000126	0.00036	0.000185
ATF5	2.88E-05	7.27E-06	0.001063	0.000191	0.000234	8.61E-05	0.000509	0.000273
ATF6	7.41E-05	7.64E-05	2.55E-05	2.33E-05	4.54E-06	4.69E-05	2.22E-05	5.46E-05
ATF6B	4.03E-05	2.38E-05	2.30E-05	3.20E-05	1.06E-05	4.37E-05	2.41E-05	3.51E-05
ATF7	1.06E-05	6.22E-06	5.62E-06	5.85E-06	1.23E-06	8.08E-06	4.59E-06	6.00E-06
ATF7IP	1.04E-05	7.04E-06	4.98E-06	4.38E-06	8.71E-07	6.07E-06	2.69E-06	4.25E-06
ATF7IP2	2.84E-06	8.97E-06	3.96E-06	4.27E-06	7.26E-06	4.35E-06	6.28E-06	4.84E-06
ATG10	4.30E-06	5.48E-06	1.50E-06	2.41E-06	8.08E-07	2.30E-06	2.17E-06	4.14E-06
ATG12	1.69E-05	1.42E-05	8.00E-06	9.02E-06	2.65E-06	1.18E-05	6.09E-06	1.43E-05
ATG16L1	1.75E-05	7.87E-06	6.93E-06	9.24E-06	3.39E-06	1.16E-05	7.69E-06	9.89E-06
ATG16L2	1.29E-05	2.25E-06	4.27E-06	6.47E-06	5.03E-06	3.89E-06	3.27E-06	8.44E-06
ATG2A	1.89E-05	2.48E-05	1.61E-05	1.94E-05	1.75E-05	1.45E-05	2.17E-05	2.71E-05
ATG2B	7.99E-06	2.11E-06	3.65E-06	2.91E-06	7.96E-07	3.62E-06	2.37E-06	3.23E-06
ATG3	4.75E-05	4.65E-05	2.49E-05	2.97E-05	1.63E-05	3.49E-05	3.01E-05	2.75E-05
ATG4A	1.06E-05	1.24E-05	9.69E-06	8.04E-06	9.95E-06	1.29E-05	8.25E-06	2.23E-05
ATG4B	3.17E-05	1.43E-05	9.47E-06	1.35E-05	1.67E-05	1.12E-05	1.30E-05	1.19E-05
ATG4C	6.93E-06	7.10E-06	4.31E-06	4.16E-06	1.17E-06	8.02E-06	3.05E-06	3.71E-06
ATG4D	2.35E-05	7.54E-06	7.05E-06	1.04E-05	1.49E-05	6.09E-06	1.34E-05	1.71E-05
ATG5	1.31E-05	1.42E-05	8.86E-06	1.09E-05	3.54E-06	1.21E-05	9.28E-06	8.48E-06
ATG7	9.60E-06	4.30E-06	3.07E-06	5.59E-06	5.10E-06	5.37E-06	5.92E-06	4.58E-06
ATG9A	5.40E-05	2.41E-05	1.93E-05	1.82E-05	3.75E-05	2.64E-05	1.93E-05	2.62E-05
ATG9B	1.56E-07	0	2.74E-08	6.95E-08	5.37E-08	3.89E-08	7.52E-08	2.39E-08
ATHL1	2.39E-05	6.48E-05	6.60E-06	1.04E-05	1.62E-05	6.03E-06	3.72E-06	1.60E-05
ATIC	6.59E-05	3.58E-05	2.75E-05	2.41E-05	2.15E-05	3.99E-05	3.70E-05	3.14E-05
ATL1	2.52E-06	1.70E-07	3.60E-07	9.46E-07	1.76E-07	5.44E-07	4.57E-07	3.13E-07
ATL2	2.96E-05	4.57E-05	1.83E-05	1.40E-05	5.67E-06	2.14E-05	9.85E-06	1.97E-05
ATL3	1.50E-05	1.13E-05	6.76E-06	6.13E-06	2.57E-06	1.31E-05	4.64E-06	1.59E-05
ATM	8.66E-06	3.19E-06	4.14E-06	3.38E-06	3.84E-07	3.61E-06	1.57E-06	2.42E-06
ATMIN	1.08E-05	6.14E-06	7.70E-06	8.02E-06	3.79E-06	1.18E-05	6.26E-06	9.13E-06
ATN1	2.60E-05	1.44E-05	8.87E-06	1.40E-05	4.33E-06	1.19E-05	1.08E-05	1.44E-05
ATOH1	0	0	0	0	0	0	0	0
ATOH7	1.18E-07	1.93E-08	1.80E-07	2.91E-07	2.04E-07	6.29E-08	3.91E-07	7.50E-08
ATOH8	5.82E-06	4.89E-07	6.19E-06	1.00E-05	2.46E-06	2.69E-06	2.34E-06	1.98E-06

ATOX1	0.000258	0.000237	0.00019	0.000407	0.000485	0.000657	0.000423	0.000379
ATP10A	3.62E-06	5.62E-08	1.81E-07	3.36E-07	1.33E-07	1.35E-07	2.77E-07	3.03E-07
ATP10B	2.04E-08	0	4.11E-08	1.31E-08	0	0	0	2.12E-08
ATP10D	7.29E-06	3.33E-06	1.18E-06	2.19E-06	1.86E-07	9.62E-07	1.01E-06	3.50E-06
ATP11A	1.54E-05	5.73E-06	4.54E-06	4.63E-06	8.69E-07	9.45E-06	4.02E-06	7.27E-06
ATP11B	1.24E-05	8.02E-06	7.44E-06	7.77E-06	2.75E-06	4.87E-06	4.94E-06	7.24E-06
ATP11C	6.18E-06	3.44E-06	2.06E-05	6.54E-06	4.30E-06	1.12E-05	4.38E-06	4.72E-06
ATP12A	0	0	0	0	0	0	0	0
ATP13A1	2.98E-05	2.56E-05	1.28E-05	1.74E-05	3.29E-05	1.78E-05	2.47E-05	2.20E-05
ATP13A2	1.56E-05	3.57E-06	4.09E-06	6.08E-06	3.56E-06	7.23E-06	1.13E-05	5.40E-06
ATP13A3	5.86E-05	3.47E-05	5.05E-05	3.29E-05	1.89E-05	4.25E-05	2.49E-05	3.63E-05
ATP13A4	1.20E-06	0	1.95E-07	1.43E-07	0	0	7.01E-09	0
ATP13A5	0	0	0	0	0	0	0	0
ATP1A1	0.000629	0.000197	0.000114	0.000274	0.000163	0.000232	0.000192	0.000296
ATP1A2	4.74E-07	1.71E-07	8.25E-08	1.34E-07	1.93E-07	8.70E-07	1.95E-08	2.42E-07
ATP1A3	3.70E-06	1.33E-08	6.87E-08	1.28E-07	0	4.60E-08	2.32E-08	9.75E-09
ATP1A4	3.65E-08	0	0	3.86E-08	0	0	0	4.65E-08
ATP1B1	0.000585	0.000476	9.84E-05	0.000116	4.01E-05	0.000418	8.97E-05	0.000485
ATP1B2	1.29E-06	9.45E-07	1.03E-06	1.27E-06	2.31E-07	2.30E-07	4.42E-07	4.15E-07
ATP1B3	6.65E-05	4.18E-06	3.85E-06	5.49E-05	6.26E-06	8.18E-06	8.66E-06	1.53E-05
ATP1B4	0	0	0	0	0	0	0	0
ATP2A1	2.42E-07	8.37E-07	2.25E-08	6.42E-08	4.43E-07	1.72E-07	1.48E-07	3.85E-08
ATP2A2	6.37E-05	4.58E-05	3.06E-05	2.82E-05	3.32E-05	4.94E-05	4.00E-05	5.50E-05
ATP2A3	1.20E-05	2.39E-06	2.92E-06	6.45E-06	3.69E-06	2.58E-06	3.39E-06	4.13E-06
ATP2B1	1.89E-05	8.37E-06	4.76E-06	6.41E-06	1.63E-06	3.59E-06	4.03E-06	8.84E-06
ATP2B2	1.77E-05	2.22E-05	6.45E-06	7.83E-06	5.99E-06	4.11E-06	5.10E-06	2.26E-05
ATP2B3	3.48E-09	0	0	0	5.16E-09	7.83E-09	0	0
ATP2B4	1.37E-05	1.70E-05	6.22E-06	8.65E-06	2.39E-06	1.41E-05	7.64E-06	3.53E-05
ATP2C1	2.62E-05	2.53E-05	1.15E-05	1.21E-05	4.37E-06	1.64E-05	1.19E-05	1.08E-05
ATP2C2	8.34E-08	1.35E-08	8.08E-09	5.29E-07	2.05E-08	2.21E-08	7.08E-08	2.29E-08
ATP4A	8.86E-08	0	0	0	0	0	0	0
ATP4B	0	0	0	0	2.89E-08	0	0	7.65E-08
ATP5A1	0.000667	0.000325	0.000303	0.000288	0.000136	0.000338	0.000238	0.000344
ATP5B	0.000902	0.00104	0.00036	0.000386	0.000146	0.000499	0.000408	0.000441
ATP5C1	0.000391	0.000321	0.000169	0.000178	0.000171	0.000223	0.000194	0.000201
ATP5D	0.000187	0.000185	0.000113	0.000128	0.000614	0.000133	0.000255	0.000118
ATP5E	0.001532	0.001358	0.000482	0.000753	0.001651	0.00094	0.001228	0.000595
ATP5EP2	3.90E-05	2.59E-05	2.04E-05	6.76E-06	3.20E-05	1.27E-05	9.38E-06	8.71E-06
ATP5F1	0.000145	0.000172	6.79E-05	6.63E-05	5.43E-05	0.000141	5.81E-05	7.85E-05
ATP5G1	0.000483	0.000432	0.000131	0.000128	0.00028	0.000337	0.000164	0.000149
ATP5G2	0.000459	0.000385	0.00019	0.000251	0.000338	0.000355	0.000344	0.000243
ATP5G3	0.000363	0.000275	8.61E-05	9.26E-05	8.91E-05	0.000109	0.000117	0.000124
ATP5H	0.00069	0.000723	0.000252	0.000273	0.000585	0.00049	0.000305	0.000371
ATP5I	0.00084	0.000705	0.000324	0.00048	0.001254	0.000663	0.000708	0.000651
ATP5J	0.000308	0.000187	0.000165	0.000185	0.00034	0.000193	0.000272	0.000191
ATP5J2	0.000685	0.000734	0.00021	0.000202	0.000458	0.000359	0.000281	0.000345
ATP5L	0.00021	0.000111	7.86E-05	0.000102	0.000134	0.000111	0.000105	0.000142
ATP5L2	3.55E-08	4.18E-08	3.20E-07	4.41E-08	6.17E-08	3.32E-08	0	0
ATP5O	0.000414	0.000279	0.000198	0.00022	0.000274	0.000226	0.00022	0.000248
ATP5S	5.46E-06	3.55E-06	4.41E-06	3.70E-06	1.87E-06	5.06E-06	2.96E-06	1.45E-06
ATP5SL	3.86E-05	2.16E-05	2.07E-05	2.20E-05	1.65E-05	2.34E-05	2.21E-05	2.32E-05
ATP6AP1	0.0001	9.24E-05	3.51E-05	5.54E-05	6.27E-05	4.33E-05	5.97E-05	0.000117
ATP6AP1L	1.80E-06	2.78E-06	1.21E-06	1.33E-06	6.24E-07	1.58E-06	1.11E-06	1.17E-06
ATP6AP2	0.00015	5.76E-05	4.85E-05	5.64E-05	5.21E-05	5.25E-05	5.40E-05	9.41E-05
ATP6V0A1	4.97E-05	4.64E-05	1.85E-05	2.63E-05	5.21E-05	4.61E-05	3.34E-05	3.74E-05
ATP6V0A2	2.49E-06	5.12E-06	2.10E-06	2.19E-06	8.82E-07	2.47E-06	1.84E-06	3.09E-06
ATP6V0A4	0	0	0	0	0	2.12E-08	7.41E-09	0
ATP6V0B	0.000215	0.000178	8.76E-05	0.000145	0.000111	0.000144	0.000163	0.00017

ATP6V0C	0.000213	0.000206	7.52E-05	0.000135	7.34E-05	0.000116	0.000211	0.000112
ATP6V0D1	0.000102	4.34E-05	4.32E-05	6.53E-05	4.08E-05	6.11E-05	5.47E-05	0.000112
ATP6V0D2	5.47E-07	4.15E-07	3.11E-08	8.52E-07	0	9.22E-09	1.93E-08	2.92E-08
ATP6V0E1	0.000291	0.000123	0.000147	0.000208	0.000121	0.000311	0.000186	0.000305
ATP6V0E2	9.78E-06	5.29E-05	2.92E-05	1.04E-05	4.58E-05	0.000159	2.66E-05	3.56E-05
ATP6V1A	3.77E-05	3.24E-05	1.78E-05	1.17E-05	4.74E-06	2.23E-05	1.08E-05	1.41E-05
ATP6V1B1	1.70E-06	8.72E-09	0	1.85E-08	8.80E-08	1.44E-08	4.03E-07	5.99E-08
ATP6V1B2	2.96E-05	3.06E-05	2.36E-05	3.13E-05	4.31E-06	2.61E-05	1.63E-05	1.72E-05
ATP6V1C1	3.41E-05	1.94E-05	6.55E-06	6.76E-06	5.92E-06	1.18E-05	2.02E-05	1.53E-05
ATP6V1C2	1.35E-06	1.57E-06	6.11E-07	6.96E-07	3.07E-07	7.40E-07	7.08E-07	1.21E-06
ATP6V1D	5.33E-05	3.02E-05	2.20E-05	2.75E-05	1.49E-05	4.30E-05	2.61E-05	3.61E-05
ATP6V1E1	0.000151	0.000122	4.43E-05	7.12E-05	6.00E-05	9.42E-05	7.66E-05	9.16E-05
ATP6V1E2	2.44E-06	4.59E-06	5.20E-07	8.88E-07	5.32E-07	2.00E-06	1.18E-06	9.40E-07
ATP6V1F	0.000162	0.000105	5.91E-05	0.000131	9.41E-05	0.000122	0.000111	0.00016
ATP6V1G1	0.000106	3.54E-05	4.69E-05	7.22E-05	2.59E-05	9.33E-05	6.38E-05	5.98E-05
ATP6V1G2	4.61E-07	7.56E-07	5.45E-07	3.80E-07	1.05E-07	7.62E-07	4.27E-07	2.05E-07
ATP6V1G3	0	0	0	0	0	0	0	0
ATP6V1H	5.17E-05	2.32E-05	1.31E-05	1.50E-05	1.08E-05	2.09E-05	3.34E-05	2.13E-05
ATP7A	1.28E-06	8.95E-07	6.21E-07	8.17E-07	3.60E-07	7.46E-07	4.46E-07	9.44E-07
ATP7B	1.40E-05	7.62E-06	7.26E-06	3.74E-06	2.02E-06	1.10E-05	6.02E-06	4.88E-06
ATP8A1	2.06E-06	2.05E-06	6.10E-07	1.16E-06	1.40E-07	6.55E-07	3.42E-07	3.23E-07
ATP8A2	1.43E-08	0	0	5.81E-09	0	8.81E-09	4.63E-09	1.39E-08
ATP8B1	9.81E-06	7.46E-06	5.12E-06	5.62E-06	1.47E-06	5.29E-06	5.32E-06	9.57E-06
ATP8B2	1.01E-05	5.09E-06	1.16E-06	2.88E-06	2.30E-07	1.40E-06	1.17E-06	1.28E-06
ATP8B3	4.74E-06	7.25E-08	1.40E-07	6.22E-07	1.35E-07	3.35E-07	5.46E-07	6.90E-07
ATP8B4	1.17E-06	5.00E-07	1.06E-06	1.63E-06	2.38E-07	1.35E-06	1.45E-06	7.27E-07
ATP8B5P	1.03E-06	9.98E-07	4.47E-07	9.14E-07	1.86E-06	6.50E-07	5.71E-07	8.34E-07
ATP9A	2.20E-05	1.05E-05	5.39E-06	7.13E-06	3.61E-06	6.42E-06	7.81E-06	9.00E-06
ATP9B	8.63E-06	3.78E-06	5.30E-06	5.96E-06	1.61E-06	5.80E-06	5.14E-06	5.46E-06
ATPAF1	2.13E-05	2.28E-05	2.17E-05	1.69E-05	6.93E-06	2.69E-05	1.18E-05	1.81E-05
ATPAF2	1.02E-05	2.51E-05	1.21E-05	1.11E-05	1.29E-05	1.67E-05	1.35E-05	1.25E-05
ATPBD4	4.32E-06	4.38E-06	1.38E-06	1.59E-06	1.15E-06	2.50E-06	2.43E-06	2.32E-06
ATPIF1	0.000412	0.000397	0.000223	0.000222	0.000367	0.000292	0.000296	0.000171
ATR	5.12E-06	3.29E-06	1.72E-06	2.05E-06	6.52E-07	5.90E-06	9.53E-07	1.81E-06
ATRIP	5.53E-06	2.83E-06	2.15E-06	2.49E-06	2.79E-06	4.18E-06	3.91E-06	3.56E-06
ATRN	4.81E-05	5.37E-05	3.21E-05	2.91E-05	2.16E-05	2.81E-05	3.09E-05	3.68E-05
ATRNL1	1.37E-07	0	5.03E-07	3.81E-07	3.71E-08	7.65E-08	4.10E-08	3.18E-07
ATRX	8.25E-06	5.05E-06	3.28E-06	3.10E-06	7.64E-07	4.07E-06	1.62E-06	4.33E-06
ATXN1	6.93E-06	3.39E-06	3.54E-06	4.52E-06	1.02E-06	6.04E-06	2.88E-06	2.67E-06
ATXN10	4.93E-05	4.76E-05	3.10E-05	3.40E-05	3.06E-05	3.45E-05	3.05E-05	4.54E-05
ATXN1L	8.75E-06	2.79E-06	2.70E-06	3.52E-06	6.27E-07	5.15E-06	2.66E-06	4.84E-06
ATXN2	1.40E-05	1.47E-05	8.48E-06	8.80E-06	4.60E-06	1.52E-05	1.09E-05	9.88E-06
ATXN2L	3.65E-05	5.33E-05	1.51E-05	1.99E-05	7.94E-06	2.82E-05	2.05E-05	2.36E-05
ATXN3	7.20E-06	2.38E-06	3.05E-06	3.47E-06	1.30E-06	5.80E-06	4.47E-06	4.64E-06
ATXN3L	1.81E-07	0	0	0	0	0	0	0
ATXN7	5.63E-06	3.41E-06	3.74E-06	3.91E-06	6.20E-07	4.28E-06	1.78E-06	3.13E-06
ATXN7L1	2.62E-06	3.05E-06	1.84E-06	3.02E-06	9.13E-07	7.16E-06	1.49E-06	2.79E-06
ATXN7L2	2.31E-06	9.14E-07	1.11E-06	1.07E-06	1.62E-06	1.68E-06	1.06E-06	1.41E-06
ATXN7L3	2.88E-05	1.55E-05	6.22E-06	9.77E-06	7.93E-06	1.78E-05	1.31E-05	1.41E-05
ATXN7L3E	3.66E-05	2.77E-05	2.29E-05	1.80E-05	7.38E-06	5.25E-05	1.63E-05	1.84E-05
ATXN8OS	0	0	0	0	0	0	0	0
AUH	2.26E-05	1.68E-05	1.19E-05	1.36E-05	3.36E-06	1.81E-05	1.27E-05	1.74E-05
AUP1	0.000233	0.000184	9.29E-05	0.000106	0.000148	0.00011	0.00011	0.000143
AURKA	4.02E-05	1.77E-05	8.97E-07	1.14E-06	2.62E-06	5.43E-06	1.70E-06	1.58E-05
AURKAIP1	0.000158	0.000216	0.000134	0.000128	0.000277	0.000142	0.000154	0.000138
AURKAPS1	7.88E-07	2.38E-07	3.41E-08	7.91E-08	5.55E-08	8.97E-08	1.39E-07	2.14E-07
AURKB	1.12E-05	4.15E-06	1.69E-07	7.14E-07	1.62E-06	2.16E-06	1.07E-06	2.45E-06
AURKC	6.37E-07	8.16E-08	9.21E-07	8.15E-07	5.91E-07	7.01E-07	3.21E-07	4.21E-07

AUTS2	8.22E-07	8.17E-06	8.50E-06	5.60E-06	4.42E-06	4.10E-06	3.78E-06	1.21E-06
AVEN	6.79E-06	5.51E-06	7.39E-06	8.44E-06	6.27E-06	8.97E-06	7.61E-06	1.08E-05
AVIL	2.46E-06	4.69E-07	2.32E-07	3.89E-07	1.73E-07	4.76E-06	5.89E-07	3.95E-07
AVL9	9.07E-06	1.19E-05	3.36E-06	3.11E-06	1.01E-06	7.01E-06	1.99E-06	8.25E-06
AVP	3.53E-07	0	0	0	0	0	0	0
AVPI1	2.92E-05	2.10E-05	4.71E-05	2.75E-05	3.83E-05	3.98E-05	2.71E-05	4.76E-05
AVPR1A	0	9.37E-07	3.46E-06	1.22E-06	4.70E-09	1.57E-07	1.33E-07	4.98E-06
AVPR1B	1.37E-08	0	0	0	0	0	2.64E-08	0
AVPR2	7.56E-08	2.92E-07	1.60E-08	9.28E-08	2.27E-07	5.62E-08	5.34E-07	4.46E-08
AWAT1	0	0	3.83E-08	0	0	0	0	0
AWAT2	1.12E-08	0	0	0	0	0	1.08E-08	0
AXIN1	1.18E-05	1.13E-05	4.08E-06	8.63E-06	2.92E-06	5.66E-06	1.03E-05	8.08E-06
AXIN2	6.75E-07	5.35E-08	6.67E-07	1.02E-06	6.62E-08	1.61E-05	8.39E-07	5.54E-07
AXL	1.78E-05	7.30E-07	5.59E-06	1.03E-05	9.35E-07	2.60E-06	4.77E-06	1.68E-06
AZGP1	0.001139	0.002708	0.002415	0.001405	0.002866	0.00123	0.001108	0.000929
AZI1	1.02E-05	5.97E-06	7.33E-07	2.59E-06	4.18E-06	3.67E-06	3.43E-06	2.77E-06
AZI2	1.36E-05	1.28E-05	9.21E-06	9.08E-06	3.03E-06	1.17E-05	8.13E-06	1.30E-05
AZIN1	6.21E-05	4.12E-05	3.07E-05	2.18E-05	7.23E-06	2.93E-05	6.36E-05	4.91E-05
AZU1	0	0	6.63E-08	3.71E-08	2.60E-08	0	0	0
B2M	0.007329	0.001667	0.002959	0.004268	0.000535	0.004456	0.003742	0.006845
B3GALNT1	3.41E-06	6.01E-07	1.77E-07	1.27E-06	2.09E-07	6.59E-07	4.86E-07	5.25E-07
B3GALNT2	1.18E-05	3.31E-06	2.21E-06	2.64E-06	1.65E-06	3.88E-06	2.80E-06	5.63E-06
B3GALT1	2.19E-08	9.91E-08	0	0	0	3.05E-08	0	0
B3GALT2	1.40E-05	2.17E-08	7.33E-08	2.61E-07	5.55E-09	1.79E-08	1.19E-07	1.89E-08
B3GALT4	2.03E-06	8.07E-07	1.77E-06	2.61E-06	7.11E-07	8.61E-07	2.01E-06	8.33E-07
B3GALT5	9.34E-09	0	4.85E-08	8.00E-08	0	0	1.81E-08	0
B3GALT6	1.14E-05	4.05E-06	5.02E-06	7.55E-06	1.27E-05	9.63E-06	1.07E-05	5.23E-06
B3GALTL	3.65E-06	1.56E-06	1.39E-06	2.04E-06	6.28E-07	2.37E-06	9.57E-07	1.58E-06
B3GAT1	8.13E-07	5.11E-08	2.92E-06	2.21E-07	2.80E-08	1.27E-07	6.32E-08	3.15E-08
B3GAT2	1.27E-07	4.23E-08	2.68E-08	7.11E-08	0	2.91E-08	6.43E-09	1.87E-08
B3GAT3	4.54E-05	5.58E-05	2.11E-05	3.21E-05	3.34E-05	2.98E-05	2.96E-05	7.40E-05
B3GNT1	1.94E-05	3.05E-05	1.50E-05	1.56E-05	1.20E-05	1.83E-05	1.34E-05	2.13E-05
B3GNT2	2.19E-05	1.63E-05	1.01E-05	9.66E-06	1.77E-06	4.97E-06	6.08E-06	7.79E-06
B3GNT3	3.96E-05	1.19E-07	9.21E-07	7.07E-06	0	2.44E-08	4.36E-06	1.92E-06
B3GNT4	2.64E-07	0	0	0	1.56E-07	3.50E-08	1.82E-07	0
B3GNT5	1.00E-05	1.66E-06	5.00E-07	3.57E-06	7.59E-08	7.20E-07	1.02E-06	5.17E-07
B3GNT6	0	0	0	1.15E-08	0	0	0	0
B3GNT7	2.57E-05	3.40E-07	8.14E-07	1.29E-06	4.88E-08	4.39E-07	6.88E-07	1.76E-07
B3GNT8	7.21E-07	1.17E-07	6.63E-07	9.95E-07	2.99E-07	1.43E-07	7.21E-07	2.09E-07
B3GNT9	2.94E-06	1.05E-06	1.54E-06	3.30E-06	1.02E-06	3.74E-07	1.16E-06	5.53E-07
B3GNTL1	7.19E-06	4.38E-06	1.19E-06	1.88E-06	2.98E-06	8.32E-06	3.00E-06	3.53E-06
B4GALNT1	8.40E-06	7.88E-07	1.30E-07	6.80E-08	3.50E-07	1.47E-06	2.31E-08	3.60E-06
B4GALNT2	8.64E-07	0	0	0	0	0	0	5.38E-06
B4GALNT3	6.44E-06	9.24E-09	1.42E-07	4.95E-07	7.06E-09	7.15E-07	7.63E-08	0
B4GALNT4	1.78E-06	0	0	1.38E-07	0	6.16E-09	3.87E-08	0
B4GALT1	3.18E-05	7.01E-05	6.04E-05	6.31E-05	2.29E-05	6.42E-05	5.89E-05	8.44E-05
B4GALT2	2.11E-05	1.76E-05	1.58E-05	1.54E-05	2.26E-05	2.66E-05	2.44E-05	2.18E-05
B4GALT3	2.14E-05	3.92E-05	1.02E-05	1.88E-05	7.65E-06	2.49E-05	1.94E-05	3.55E-05
B4GALT4	2.04E-05	1.30E-05	7.55E-06	9.18E-06	3.48E-06	9.93E-06	7.31E-06	5.79E-06
B4GALT5	3.06E-05	1.97E-05	8.01E-06	2.28E-05	3.49E-06	9.19E-06	9.62E-06	1.28E-05
B4GALT6	2.45E-06	8.07E-07	4.96E-07	5.64E-07	9.20E-08	1.07E-06	8.05E-07	6.36E-07
B4GALT7	1.75E-05	9.01E-06	9.55E-06	1.40E-05	1.88E-05	1.61E-05	2.00E-05	3.16E-05
B9D1	5.55E-06	5.16E-06	4.29E-06	7.09E-06	1.05E-05	9.68E-06	1.03E-05	7.47E-06
B9D2	5.40E-06	1.22E-06	2.11E-06	5.02E-06	2.07E-06	4.30E-06	3.53E-06	1.38E-06
BAALC	1.12E-05	6.27E-07	8.53E-07	2.30E-06	8.35E-07	5.54E-07	1.49E-06	7.41E-06
BAAT	0.000483	0.000264	0.000508	0.000182	0.000218	0.000378	0.000315	0.00047
BACE1	1.22E-05	1.01E-05	1.33E-05	9.83E-06	6.36E-06	1.37E-05	9.17E-06	1.95E-05
BACE2	3.06E-05	2.31E-06	2.31E-06	2.74E-05	1.09E-06	1.01E-06	5.96E-06	9.26E-06

BACH1	1.51E-05	6.96E-06	7.39E-06	9.38E-06	1.60E-06	1.39E-05	4.89E-06	6.11E-06
BACH2	1.91E-07	2.10E-07	5.74E-07	9.59E-07	9.88E-08	4.60E-07	3.18E-07	7.16E-07
BAD	5.81E-05	0.000103	4.81E-05	4.94E-05	8.26E-05	3.31E-05	4.79E-05	5.91E-05
BAG1	9.28E-06	1.89E-05	1.28E-05	1.48E-05	1.12E-05	1.29E-05	1.41E-05	1.89E-05
BAG2	1.68E-05	2.35E-05	6.38E-06	4.37E-06	2.58E-06	1.65E-05	4.66E-06	4.15E-06
BAG3	1.99E-05	2.05E-05	7.95E-06	2.82E-05	5.98E-06	1.34E-05	1.86E-05	1.55E-05
BAG4	2.79E-06	4.56E-06	4.35E-06	2.85E-06	1.22E-07	3.79E-06	1.10E-06	2.99E-06
BAG5	8.29E-06	5.99E-06	5.07E-06	6.99E-06	2.77E-06	6.69E-06	6.03E-06	7.32E-06
BAGE	4.06E-07	0	0	0	0	0	0	1.04E-08
BAGE2	1.12E-06	6.31E-08	7.77E-08	2.86E-08	0	1.56E-07	1.34E-08	1.46E-07
BAHCC1	2.40E-06	4.36E-07	6.21E-07	7.28E-07	3.66E-07	7.59E-07	8.82E-07	1.39E-06
BAHD1	8.42E-06	7.86E-06	4.69E-06	4.37E-06	5.10E-06	5.74E-06	6.24E-06	4.77E-06
BAI1	3.66E-08	9.08E-09	2.50E-08	4.46E-08	1.05E-08	7.52E-09	1.19E-08	1.78E-08
BAI2	1.06E-06	8.47E-08	1.42E-08	1.77E-07	3.12E-08	2.24E-08	1.09E-07	3.52E-08
BAI3	8.78E-08	8.89E-09	1.35E-07	2.13E-07	0	0	0	7.30E-08
BAIAP2	2.83E-05	3.64E-05	3.46E-05	3.17E-05	3.57E-05	5.85E-05	3.91E-05	1.64E-05
BAIAP2L1	0.000171	6.63E-05	1.54E-05	1.98E-05	1.41E-05	1.18E-05	1.80E-05	2.03E-05
BAIAP2L2	2.99E-05	1.22E-06	6.85E-07	1.83E-06	2.33E-06	3.02E-07	1.12E-05	2.87E-06
BAIAP3	8.89E-06	6.04E-06	3.03E-06	2.69E-06	2.16E-06	2.39E-06	4.13E-06	2.13E-06
BAK1	3.12E-05	1.60E-05	3.24E-06	8.99E-06	3.42E-06	4.87E-06	7.53E-06	1.02E-05
BAMBI	5.34E-05	2.89E-07	1.27E-05	3.30E-05	9.50E-06	7.61E-06	2.22E-05	1.58E-05
BANF1	0.000139	0.000146	5.92E-05	8.40E-05	0.000102	0.00012	0.000148	0.000129
BANF2	0	6.26E-08	0	0	3.21E-07	1.84E-06	0	0
BANK1	5.00E-06	9.67E-08	3.71E-08	1.67E-07	0	9.31E-08	8.11E-08	1.11E-08
BANP	9.47E-06	7.18E-06	4.51E-06	5.38E-06	4.24E-06	7.30E-06	4.33E-06	8.30E-06
BAP1	5.43E-05	3.39E-05	1.95E-05	1.93E-05	4.07E-05	3.55E-05	3.89E-05	3.88E-05
BARD1	1.43E-06	8.67E-07	1.37E-07	2.12E-07	0	1.25E-07	1.12E-07	1.99E-07
BARHL1	0	0	0	0	0	0	0	0
BARHL2	0	0	0	0	0	0	0	0
BARX1	3.83E-08	7.02E-07	6.15E-08	1.18E-07	2.88E-06	0	5.51E-08	8.51E-08
BARX2	1.79E-07	0	1.45E-08	1.01E-07	0	3.83E-08	7.96E-08	2.03E-08
BASE	0	0	0	0	0	0	0	0
BASP1	1.05E-05	5.38E-07	5.72E-06	1.54E-05	5.25E-07	2.87E-06	6.81E-06	2.04E-06
BAT1	0.000168	9.02E-05	6.63E-05	7.64E-05	7.13E-05	9.28E-05	8.45E-05	8.83E-05
BAT2	2.55E-05	3.71E-05	1.34E-05	1.64E-05	5.60E-06	2.09E-05	1.63E-05	1.89E-05
BAT2L1	2.55E-05	6.80E-06	7.15E-06	8.28E-06	2.75E-06	1.57E-05	1.05E-05	1.90E-05
BAT2L2	4.25E-05	3.15E-05	1.63E-05	2.23E-05	1.72E-05	3.33E-05	2.39E-05	3.13E-05
BAT3	0.000102	0.000112	4.95E-05	5.26E-05	3.44E-05	6.39E-05	6.16E-05	7.29E-05
BAT4	2.04E-05	1.85E-05	7.90E-06	9.53E-06	9.64E-06	1.82E-05	1.47E-05	1.83E-05
BAT5	6.34E-05	6.99E-05	2.76E-05	3.37E-05	1.80E-05	5.23E-05	3.47E-05	4.55E-05
BATF	1.11E-05	3.39E-07	1.51E-06	1.91E-05	7.32E-07	1.76E-05	2.38E-06	1.96E-06
BATF2	8.86E-06	2.36E-06	3.80E-06	5.30E-06	1.14E-06	3.75E-06	3.15E-06	5.07E-06
BATF3	3.23E-06	6.36E-08	7.52E-07	2.17E-06	5.22E-07	1.18E-06	1.20E-06	8.57E-07
BAX	5.86E-05	3.23E-05	3.42E-05	8.52E-05	7.18E-05	8.56E-05	6.27E-05	5.11E-05
BAZ1A	6.90E-06	5.68E-06	3.85E-06	5.48E-06	1.76E-06	3.71E-06	2.79E-06	7.67E-06
BAZ1B	1.64E-05	1.67E-05	9.34E-06	9.14E-06	3.20E-06	1.21E-05	9.10E-06	1.16E-05
BAZ2A	1.82E-05	1.11E-05	5.79E-06	5.49E-06	2.34E-06	7.78E-06	5.75E-06	6.07E-06
BAZ2B	1.02E-05	2.76E-06	3.38E-06	2.94E-06	6.20E-07	3.90E-06	1.41E-06	1.93E-06
BBC3	9.60E-06	5.93E-06	3.63E-06	7.61E-06	4.66E-06	4.48E-06	1.56E-05	1.03E-05
BBOX1	5.81E-07	2.66E-05	4.10E-05	2.79E-06	2.31E-05	1.97E-05	1.80E-06	4.17E-07
BBS1	6.07E-06	5.52E-06	3.60E-06	5.58E-06	7.48E-07	8.36E-06	3.32E-06	2.83E-06
BBS10	7.60E-06	7.14E-06	3.35E-06	3.05E-06	1.20E-06	6.94E-06	1.91E-06	1.53E-06
BBS12	6.54E-07	3.03E-07	3.16E-07	4.86E-07	6.71E-08	8.95E-07	3.86E-07	1.98E-07
BBS2	9.70E-06	1.40E-05	5.00E-06	7.17E-06	1.23E-06	1.32E-05	3.42E-06	5.90E-06
BBS4	1.38E-05	7.96E-06	3.21E-06	3.74E-06	3.02E-06	1.38E-05	3.16E-06	4.06E-06
BBS5	1.64E-06	2.01E-07	8.50E-07	1.63E-06	1.54E-07	9.00E-08	4.06E-07	3.51E-07
BBS7	5.53E-06	9.72E-07	9.22E-07	1.67E-06	3.52E-08	2.90E-06	1.11E-06	1.74E-07
BBS9	2.70E-06	1.72E-06	1.07E-06	2.98E-06	1.23E-06	3.89E-06	2.44E-06	1.16E-06

BBX	9.44E-06	6.98E-06	4.44E-06	5.25E-06	7.54E-07	1.25E-05	2.33E-06	2.56E-06
BCAM	9.09E-05	8.70E-05	2.42E-05	4.64E-05	8.77E-05	8.20E-05	0.000131	3.15E-05
BCAN	1.13E-07	3.44E-07	1.37E-08	3.98E-08	1.14E-08	2.27E-07	1.17E-07	2.68E-07
BCAP29	2.29E-05	2.43E-05	1.00E-05	1.31E-05	7.85E-06	1.70E-05	1.17E-05	2.40E-05
BCAP31	0.000249	0.000252	0.00014	0.000191	0.000598	0.000403	0.000259	0.000425
BCAR1	3.63E-05	2.20E-05	1.15E-05	4.26E-05	9.00E-06	1.33E-05	2.15E-05	1.87E-05
BCAR3	3.77E-05	1.68E-05	7.79E-06	8.74E-06	3.54E-06	1.36E-05	7.89E-06	6.25E-06
BCAR4	0	0	0	0	3.97E-08	0	4.26E-08	0
BCAS1	5.39E-06	1.24E-07	4.01E-07	4.24E-07	1.84E-07	0	8.48E-08	9.89E-08
BCAS2	4.49E-05	4.18E-05	2.59E-05	2.75E-05	1.04E-05	4.23E-05	2.28E-05	2.95E-05
BCAS3	7.15E-06	2.30E-05	8.02E-06	1.03E-05	1.70E-05	2.95E-05	1.32E-05	7.13E-06
BCAS4	6.70E-05	4.91E-06	4.81E-06	3.18E-05	1.28E-05	1.10E-05	4.36E-05	9.72E-06
BCAT1	2.04E-06	3.59E-07	3.18E-07	1.14E-06	1.44E-07	5.11E-07	1.21E-06	2.46E-07
BCAT2	3.26E-05	2.55E-05	4.77E-06	7.92E-06	3.96E-05	9.88E-06	7.38E-06	1.27E-05
BCCIP	5.63E-05	3.79E-05	2.95E-05	3.57E-05	3.11E-05	5.13E-05	4.21E-05	4.03E-05
BCDIN3D	3.15E-06	2.85E-06	1.86E-06	1.90E-06	1.63E-06	3.05E-06	2.18E-06	1.76E-06
BCHE	7.28E-06	4.98E-05	0.000102	4.82E-05	8.35E-05	2.32E-05	4.93E-05	4.02E-06
BCKDHA	2.70E-05	7.81E-05	7.78E-05	5.81E-05	5.89E-05	9.37E-05	7.67E-05	3.94E-05
BCKDHB	1.92E-05	3.84E-05	3.38E-05	1.89E-05	5.42E-06	2.61E-05	1.46E-05	1.28E-05
BCKDK	3.25E-05	6.66E-05	5.69E-05	3.96E-05	2.51E-05	5.01E-05	4.52E-05	5.11E-05
BCL10	6.95E-06	5.53E-06	4.11E-06	5.54E-06	7.23E-07	5.84E-06	3.96E-06	3.10E-06
BCL11A	1.17E-07	4.65E-08	1.76E-07	1.43E-07	1.58E-08	4.33E-08	1.07E-07	7.53E-08
BCL11B	9.50E-07	2.61E-08	2.45E-07	6.11E-07	1.00E-08	2.22E-07	2.06E-07	1.28E-07
BCL2	1.70E-06	6.10E-07	8.21E-07	2.30E-06	7.24E-07	9.39E-07	1.40E-06	8.82E-07
BCL2A1	9.86E-06	8.36E-07	1.66E-06	1.06E-05	1.08E-07	1.13E-06	2.35E-06	1.39E-06
BCL2L1	0.000119	4.82E-05	3.95E-05	0.000103	3.93E-05	0.000112	6.18E-05	5.99E-05
BCL2L10	8.31E-06	2.91E-07	2.77E-06	2.86E-06	9.26E-06	2.91E-08	2.95E-08	1.86E-07
BCL2L11	1.76E-05	7.10E-06	1.04E-05	5.66E-06	1.03E-06	2.96E-06	5.26E-06	5.22E-06
BCL2L12	1.16E-05	6.86E-06	2.54E-06	6.59E-06	1.31E-05	6.14E-06	1.46E-05	6.01E-06
BCL2L13	3.03E-05	3.40E-05	2.62E-05	1.96E-05	8.88E-06	2.73E-05	1.68E-05	2.05E-05
BCL2L14	3.86E-06	7.38E-07	8.04E-08	4.07E-07	8.49E-07	3.75E-07	6.16E-07	1.49E-07
BCL2L15	3.22E-07	1.61E-07	1.51E-08	8.58E-08	8.40E-09	4.50E-07	3.33E-08	2.66E-08
BCL2L2	9.21E-06	4.08E-06	4.40E-06	4.70E-06	1.90E-06	5.75E-06	6.53E-06	5.94E-06
BCL3	4.46E-05	6.22E-05	2.23E-05	7.73E-05	3.08E-05	2.53E-05	5.47E-05	5.96E-05
BCL6	2.16E-05	8.35E-06	1.04E-05	1.43E-05	4.66E-06	3.64E-05	1.27E-05	1.02E-05
BCL6B	4.27E-06	4.19E-06	5.44E-07	2.25E-06	3.50E-06	1.77E-06	7.96E-06	4.12E-06
BCL7A	7.06E-06	3.96E-06	4.08E-06	4.87E-06	1.37E-06	4.07E-06	4.45E-06	5.90E-06
BCL7B	2.91E-05	5.72E-05	2.06E-05	3.59E-05	1.79E-05	3.32E-05	4.42E-05	5.55E-05
BCL7C	3.83E-05	3.43E-05	1.97E-05	3.47E-05	6.18E-05	2.53E-05	5.56E-05	2.59E-05
BCL8	0	9.43E-08	0	1.87E-08	0	0	6.30E-08	1.84E-07
BCL9	5.29E-06	2.66E-06	1.18E-06	2.01E-06	6.06E-07	3.80E-06	1.38E-06	1.80E-06
BCL9L	1.64E-05	1.81E-06	2.53E-06	4.87E-06	1.23E-06	3.84E-06	3.54E-06	6.47E-06
BCLAF1	2.38E-05	1.11E-05	1.14E-05	1.18E-05	3.28E-06	1.39E-05	8.23E-06	1.03E-05
BCMO1	1.62E-06	1.97E-07	2.98E-07	5.04E-08	0	2.61E-06	0	4.29E-07
BCO2	4.25E-06	3.67E-07	2.90E-05	1.96E-05	6.99E-08	4.51E-06	2.29E-06	4.37E-06
BCOR	9.97E-06	5.08E-06	5.17E-06	7.67E-06	1.68E-06	5.47E-06	7.09E-06	7.32E-06
BCORL1	1.13E-05	4.81E-06	1.71E-06	2.46E-06	1.81E-06	1.36E-06	2.87E-06	1.37E-06
BCORL2	0	2.17E-08	0	6.11E-08	0	3.07E-08	1.86E-08	3.89E-08
BCR	2.00E-05	6.01E-06	5.84E-06	7.37E-06	8.00E-06	9.97E-06	1.13E-05	1.05E-05
BCS1L	2.59E-05	1.82E-05	1.20E-05	1.17E-05	1.15E-05	2.02E-05	1.56E-05	1.10E-05
BCYRN1	0	0	0	0	0	0	0	0
BDH1	3.82E-05	5.80E-05	0.000101	7.03E-05	0.000101	8.06E-05	0.000101	4.91E-05
BDH2	2.34E-05	6.57E-06	2.12E-05	1.28E-05	1.13E-05	6.62E-06	1.29E-05	3.11E-06
BDKRB1	2.78E-06	9.05E-08	4.23E-08	5.73E-07	8.52E-08	1.84E-08	1.70E-07	8.77E-08
BDKRB2	2.07E-05	9.95E-07	9.04E-08	3.71E-06	8.64E-07	1.88E-07	8.64E-07	6.40E-07
BDNF	7.01E-08	2.73E-08	0	2.68E-07	0	1.21E-07	2.98E-08	9.02E-09
BDNFOS	6.93E-07	2.30E-06	9.37E-07	1.40E-06	2.45E-07	2.40E-06	1.31E-06	1.77E-06
BDP1	9.26E-06	4.34E-06	3.78E-06	4.31E-06	6.77E-07	4.03E-06	2.16E-06	5.08E-06

BEAN	6.57E-07	3.79E-08	1.09E-07	2.16E-07	7.41E-08	7.11E-08	2.04E-07	3.71E-08
BECN1	6.87E-05	7.81E-05	2.36E-05	2.98E-05	1.64E-05	6.22E-05	2.92E-05	2.87E-05
BEGAIN	1.46E-06	4.34E-06	4.53E-08	4.17E-07	1.73E-07	3.20E-08	5.75E-08	8.97E-08
BEND2	0	0	0	0	0	0	0	0
BEND3	6.91E-07	3.40E-07	1.40E-07	3.89E-07	8.34E-08	2.36E-07	4.22E-07	2.65E-07
BEND4	1.64E-06	2.53E-08	6.70E-08	6.06E-08	0	1.17E-08	2.46E-09	1.11E-08
BEND5	1.43E-06	2.18E-08	3.47E-07	2.11E-07	1.53E-07	3.17E-06	1.46E-07	1.13E-07
BEND6	9.58E-08	0	6.46E-09	1.28E-07	9.82E-09	5.80E-09	3.92E-08	1.68E-08
BEND7	7.16E-06	4.70E-06	4.17E-06	6.19E-06	3.32E-06	4.95E-06	4.71E-06	4.59E-06
BEST1	1.29E-06	6.59E-07	3.10E-07	5.12E-07	5.68E-08	2.54E-07	4.25E-07	4.87E-07
BEST2	0	2.86E-08	0	0	0	0	0	0
BEST3	4.74E-08	0	8.36E-09	7.29E-08	0	0	0	0
BEST4	8.77E-07	2.06E-07	0	1.40E-08	9.80E-09	3.17E-08	4.40E-08	3.35E-08
BET1	9.31E-06	1.07E-05	7.30E-06	5.38E-06	2.69E-06	1.21E-05	5.68E-06	7.30E-06
BET1L	3.87E-05	2.87E-05	2.03E-05	2.12E-05	1.35E-05	2.71E-05	2.41E-05	4.16E-05
BET3L	1.48E-07	7.85E-08	2.29E-07	4.15E-08	0	6.25E-08	0	0
BEX1	2.46E-05	7.54E-08	1.51E-06	2.08E-06	8.38E-08	0	1.22E-07	4.82E-08
BEX2	2.30E-05	7.90E-07	1.10E-06	3.59E-06	0	5.96E-08	1.21E-07	0
BEX4	2.28E-05	2.41E-06	5.16E-06	1.19E-05	1.62E-06	2.53E-06	7.45E-06	2.43E-06
BEX5	1.17E-05	4.05E-07	2.00E-06	4.23E-06	6.28E-07	1.61E-07	2.64E-06	0
BEYLA	0	0	0	0	0	0	0	0
BFAR	4.26E-05	3.59E-05	1.47E-05	2.06E-05	5.47E-06	2.19E-05	1.69E-05	2.19E-05
BFSP1	1.47E-06	5.71E-07	1.82E-07	3.11E-07	5.28E-07	6.23E-07	3.13E-07	1.19E-07
BFSP2	9.05E-08	0	0	3.71E-08	0	2.80E-08	7.27E-08	8.91E-08
BGLAP	2.79E-06	3.61E-06	1.79E-06	5.03E-06	6.20E-06	3.88E-06	8.66E-06	2.98E-06
BGN	0.000167	1.94E-05	0.000168	0.000372	6.83E-05	1.84E-05	0.000128	0.000108
BHLHA15	1.26E-06	2.80E-06	3.87E-07	5.77E-07	3.66E-08	1.78E-06	1.42E-06	1.27E-07
BHLHB9	1.76E-06	1.97E-06	8.91E-07	5.38E-07	5.52E-07	1.50E-06	7.65E-07	3.19E-07
BHLHE22	1.36E-07	1.53E-08	1.55E-07	5.42E-07	2.34E-08	1.26E-08	2.58E-07	2.00E-07
BHLHE23	0	0	0	0	0	0	0	0
BHLHE40	6.76E-05	0.000105	8.68E-05	0.000321	6.80E-06	4.61E-05	0.000129	0.00011
BHLHE41	1.05E-06	9.48E-08	5.04E-07	2.10E-06	1.56E-08	4.48E-08	7.58E-07	3.81E-07
BHMT	3.66E-05	0.001047	0.000367	0.00013	0.000167	7.84E-05	0.000433	1.72E-05
BHMT2	9.03E-05	0.000266	0.000251	0.000126	3.52E-05	0.000215	6.08E-05	0.000155
BICC1	1.57E-05	5.45E-08	9.49E-07	6.32E-06	3.57E-08	2.51E-08	1.77E-06	4.13E-07
BICD1	2.77E-06	1.22E-06	2.39E-07	7.80E-07	1.46E-07	1.01E-07	3.98E-07	6.91E-07
BICD2	8.85E-06	4.05E-06	4.24E-06	5.06E-06	1.29E-06	4.42E-06	3.26E-06	4.33E-06
BID	3.14E-05	4.42E-05	2.07E-05	1.82E-05	2.10E-05	2.02E-05	2.38E-05	3.20E-05
BIK	9.97E-07	1.33E-07	6.28E-08	1.98E-06	2.22E-07	2.24E-06	1.38E-06	0
BIN1	7.16E-05	5.36E-05	2.23E-05	2.27E-05	3.79E-05	1.02E-05	3.28E-05	1.95E-05
BIN2	7.65E-06	8.70E-07	2.96E-06	6.05E-06	2.91E-07	2.12E-06	4.41E-06	1.05E-06
BIN3	9.85E-06	1.42E-05	6.27E-06	1.26E-05	5.76E-06	1.04E-05	6.49E-06	4.39E-06
BIRC2	4.81E-05	1.88E-05	1.62E-05	2.18E-05	4.36E-06	2.32E-05	1.30E-05	1.87E-05
BIRC3	4.53E-05	9.33E-06	5.79E-06	2.59E-05	2.48E-07	1.40E-05	9.54E-06	0.000103
BIRC5	7.40E-06	4.37E-06	8.56E-08	1.16E-07	5.71E-06	1.16E-06	2.89E-07	7.49E-07
BIRC6	1.15E-05	8.27E-06	4.99E-06	3.37E-06	9.32E-07	5.68E-06	2.18E-06	3.40E-06
BIRC7	7.91E-07	0	0	3.77E-07	5.59E-08	0	3.86E-07	3.20E-08
BIRC8	0	0	0	0	0	0	0	0
BIVM	1.91E-05	9.24E-06	9.35E-06	5.76E-06	4.42E-06	8.95E-06	6.15E-06	4.92E-06
BLCAP	7.29E-05	3.73E-05	5.40E-05	4.74E-05	3.35E-05	9.97E-05	3.46E-05	4.65E-05
BLID	3.22E-08	0	0	0	5.59E-08	6.03E-08	0	0
BLK	1.68E-07	2.69E-07	1.09E-07	2.78E-07	1.06E-08	2.50E-06	1.58E-07	6.57E-08
BLM	2.51E-06	2.60E-06	7.10E-08	3.02E-07	3.40E-07	4.27E-07	3.67E-07	1.11E-06
BLMH	2.33E-05	8.29E-06	4.34E-06	9.10E-06	8.31E-06	1.37E-05	1.13E-05	6.32E-06
BLNK	1.69E-05	8.40E-06	1.77E-05	1.49E-05	8.53E-06	1.88E-05	1.38E-05	1.41E-05
BLOC1S1	0.000205	0.000256	0.000177	0.000204	0.000375	0.000286	0.00026	0.00023
BLOC1S2	2.19E-05	2.06E-05	1.16E-05	1.69E-05	1.11E-05	2.23E-05	1.71E-05	1.41E-05
BLOC1S3	9.10E-06	5.59E-06	1.91E-06	2.65E-06	6.11E-06	4.91E-06	6.13E-06	5.89E-06

BLVRA	7.16E-05	1.89E-06	7.78E-06	1.86E-05	2.24E-06	1.56E-05	1.66E-05	4.48E-05
BLVRB	0.000161	0.000325	0.000176	0.000186	0.000352	0.000455	0.000245	0.000224
BLZF1	9.75E-06	1.38E-05	5.68E-06	5.79E-06	1.21E-06	1.10E-05	3.97E-06	1.13E-05
BMF	9.50E-06	4.78E-07	1.94E-06	3.25E-06	1.52E-06	1.09E-06	4.95E-06	2.17E-06
BMI1	2.56E-05	3.74E-05	1.31E-05	1.30E-05	3.95E-06	1.68E-05	1.52E-05	2.48E-05
BMP1	8.25E-06	1.30E-05	8.14E-06	1.49E-05	4.93E-06	1.78E-05	6.52E-06	3.93E-06
BMP10	0	0	1.46E-06	6.68E-07	0	0	0	0
BMP15	0	0	0	0	0	2.04E-08	0	0
BMP2	2.39E-05	1.15E-05	2.97E-06	7.85E-06	7.99E-07	1.72E-06	1.05E-05	6.91E-06
BMP2K	6.29E-06	1.14E-06	2.46E-06	3.00E-06	9.09E-07	1.71E-06	1.36E-06	2.69E-06
BMP3	0	4.37E-09	0	1.19E-07	0	0	0	0
BMP4	2.17E-06	1.61E-06	2.12E-06	3.17E-06	1.13E-06	1.09E-05	3.80E-06	1.10E-06
BMP5	2.32E-08	2.63E-08	7.79E-07	1.26E-06	0	0	6.36E-08	8.54E-08
BMP6	1.44E-06	3.94E-07	1.33E-06	1.12E-06	8.48E-07	3.75E-07	1.75E-06	4.40E-07
BMP7	3.39E-08	6.67E-09	6.13E-09	0	0	0	2.32E-08	8.72E-09
BMP8A	2.58E-06	1.52E-07	8.59E-08	1.46E-07	3.08E-08	7.02E-08	2.02E-07	2.39E-07
BMP8B	7.07E-06	2.71E-08	9.97E-08	4.14E-07	1.36E-08	1.68E-08	4.12E-08	2.66E-08
BMPER	6.76E-09	7.59E-09	2.94E-06	2.45E-06	2.32E-08	3.76E-08	6.57E-09	9.91E-09
BMPR1A	1.00E-05	4.87E-06	3.68E-06	5.85E-06	2.46E-06	4.73E-06	3.54E-06	6.12E-06
BMPR1B	1.97E-06	0	7.33E-08	2.82E-07	7.22E-09	3.83E-09	4.10E-09	2.46E-08
BMPR2	8.09E-06	4.94E-06	5.64E-06	5.08E-06	1.39E-06	4.62E-06	2.62E-06	3.32E-06
BMS1	6.75E-06	4.29E-06	3.42E-06	4.62E-06	2.22E-06	5.74E-06	4.72E-06	5.22E-06
BMS1P4	1.20E-06	3.71E-07	1.87E-07	2.65E-07	9.75E-08	2.05E-07	1.12E-07	4.26E-07
BMS1P5	9.02E-07	3.55E-07	6.07E-08	5.24E-07	1.34E-07	4.17E-07	3.60E-07	4.64E-07
BMX	1.18E-07	2.35E-07	1.06E-07	3.30E-07	8.00E-08	1.47E-07	2.25E-07	1.86E-07
BNC1	6.39E-08	5.50E-09	2.52E-08	1.32E-07	4.22E-09	9.09E-09	3.83E-08	2.88E-08
BNC2	1.58E-07	9.00E-08	8.88E-08	2.58E-07	6.15E-09	5.42E-08	1.04E-07	7.29E-08
BNIP1	1.19E-05	4.73E-06	5.94E-06	6.54E-06	5.46E-06	9.29E-06	1.06E-05	1.01E-05
BNIP2	2.78E-05	7.52E-06	1.53E-05	1.66E-05	7.97E-06	1.52E-05	1.39E-05	1.55E-05
BNIP3	7.04E-05	9.67E-05	0.00011	0.000106	8.19E-05	0.000182	0.000117	8.09E-05
BNIP3L	1.40E-05	1.62E-05	1.45E-05	2.24E-05	2.42E-06	1.62E-05	8.96E-06	5.40E-06
BNIPL	3.71E-07	3.73E-08	1.04E-07	1.52E-08	1.61E-07	1.94E-07	7.61E-08	1.82E-08
BOC	6.42E-07	7.84E-07	7.65E-08	1.97E-06	8.95E-07	5.66E-07	9.64E-07	1.14E-06
BOD1	1.72E-05	1.03E-05	8.99E-06	1.26E-05	6.79E-06	1.61E-05	1.73E-05	1.43E-05
BOD1L	5.53E-06	2.90E-06	2.34E-06	2.50E-06	3.69E-07	4.45E-06	1.24E-06	2.66E-06
BOK	4.84E-05	6.59E-05	7.00E-05	7.59E-05	0.000238	0.000116	7.71E-05	6.73E-06
BOLA1	2.58E-05	1.87E-05	1.84E-05	1.28E-05	1.81E-05	3.17E-05	1.46E-05	3.58E-05
BOLA2	9.36E-05	5.90E-05	1.46E-05	2.20E-05	3.36E-05	3.19E-05	3.38E-05	4.51E-05
BOLA3	9.34E-05	0.000103	2.49E-05	1.94E-05	2.17E-05	3.88E-05	3.41E-05	2.83E-05
BOLL	0	0	0	0	0	0	0	0
BOP1	9.47E-06	1.20E-05	2.12E-06	5.10E-06	1.39E-05	7.74E-06	2.07E-05	9.24E-06
BPESC1	6.53E-09	1.46E-08	0	0	0	0	0	0
BPGM	5.66E-06	1.23E-05	5.65E-06	6.55E-06	3.65E-06	8.84E-06	7.72E-06	1.54E-05
BPHL	2.58E-05	0.000171	7.80E-05	5.46E-05	5.26E-05	6.92E-05	6.22E-05	8.08E-05
BPI	2.62E-08	8.91E-08	8.26E-08	6.43E-08	4.51E-08	0	0	0
BPIL1	2.54E-08	2.88E-08	8.00E-08	1.56E-08	6.56E-08	3.54E-08	0	0
BPIL2	1.28E-08	0	1.33E-08	0	0	0	0	0
BPIL3	0	0	0	0	0	0	0	0
BPNT1	1.85E-05	2.27E-05	1.24E-05	1.13E-05	6.21E-06	2.56E-05	1.15E-05	1.72E-05
BPTF	1.81E-05	9.40E-06	5.89E-06	6.52E-06	3.96E-06	1.09E-05	5.56E-06	5.00E-06
BPY2	0	5.46E-07	0	0	0	1.16E-07	0	0
BRAF	1.79E-06	2.06E-06	1.58E-06	1.52E-06	3.79E-07	1.69E-06	1.15E-06	2.06E-06
BRAP	7.27E-06	7.07E-06	3.65E-06	4.21E-06	2.55E-06	5.07E-06	4.53E-06	4.75E-06
BRCA1	6.77E-06	6.66E-06	4.37E-06	7.21E-07	1.56E-06	2.66E-06	8.81E-07	1.05E-06
BRCA2	8.09E-07	3.00E-07	7.69E-08	5.67E-08	3.32E-09	1.33E-07	3.59E-08	1.25E-07
BRCC3	1.13E-05	8.91E-06	4.73E-06	4.76E-06	1.68E-06	6.34E-06	3.55E-06	6.56E-06
BRD1	8.35E-06	6.44E-06	6.12E-06	7.12E-06	2.63E-06	4.70E-06	5.17E-06	5.12E-06
BRD2	5.97E-05	4.88E-05	2.38E-05	3.92E-05	1.18E-05	4.04E-05	3.43E-05	5.43E-05

BRD3	5.48E-06	3.41E-06	5.08E-06	4.24E-06	1.01E-06	7.30E-06	4.78E-06	9.54E-06
BRD4	1.82E-05	1.13E-05	1.19E-05	1.12E-05	8.30E-06	1.11E-05	1.39E-05	9.19E-06
BRD7	2.30E-05	1.85E-05	1.18E-05	1.60E-05	5.13E-06	2.04E-05	1.31E-05	1.77E-05
BRD7P3	0	0	0	1.17E-08	0	0	0	0
BRD8	3.54E-05	1.26E-05	6.24E-06	8.62E-06	3.64E-06	1.57E-05	7.44E-06	9.54E-06
BRD9	1.33E-05	6.59E-06	5.37E-06	9.34E-06	3.70E-06	9.25E-06	1.04E-05	8.74E-06
BRDT	0	6.91E-08	0	0	0	0	0	0
BRE	0.000156	4.87E-05	1.61E-05	2.37E-05	2.80E-05	3.48E-05	2.99E-05	2.63E-05
BREA2	1.33E-07	0	2.92E-08	3.30E-08	1.62E-07	2.49E-08	1.02E-07	2.39E-07
BRF1	1.62E-05	6.57E-06	5.77E-06	7.59E-06	9.89E-06	6.98E-06	1.09E-05	1.07E-05
BRF2	8.33E-06	1.24E-05	2.72E-06	4.37E-06	2.35E-06	6.09E-06	4.67E-06	3.84E-06
BRI3	0.000385	0.000214	0.000158	0.000143	0.000274	0.000111	0.000245	0.000171
BRI3BP	1.03E-05	5.99E-06	3.27E-06	1.95E-06	1.71E-06	3.50E-06	2.65E-06	4.15E-06
BRIP1	1.99E-06	6.06E-07	1.78E-07	1.09E-07	1.28E-08	6.29E-07	9.65E-09	1.23E-07
BRIX1	1.75E-05	2.64E-05	1.07E-05	1.75E-05	5.40E-06	1.79E-05	1.83E-05	2.27E-05
BRMS1	4.44E-05	5.04E-05	1.57E-05	3.05E-05	3.67E-05	2.71E-05	2.98E-05	5.18E-05
BRMS1L	4.46E-06	2.85E-06	2.91E-06	2.25E-06	8.26E-07	2.64E-06	1.63E-06	2.05E-06
BRP44	0.00031	0.000968	0.00023	0.000161	0.00022	0.000323	0.000277	0.00031
BRP44L	0.000351	0.000112	0.000268	0.000257	0.000125	0.000117	0.000117	0.000123
BRPF1	4.75E-06	3.35E-06	1.58E-06	3.67E-06	9.91E-07	2.17E-06	3.35E-06	2.66E-06
BRPF3	1.76E-05	2.22E-05	8.25E-06	7.34E-06	3.08E-06	1.17E-05	7.66E-06	1.67E-05
BRS3	0	0	0	0	1.53E-08	0	0	0
BRSK1	3.96E-07	9.34E-08	2.44E-07	3.15E-07	1.81E-07	1.21E-07	3.45E-07	4.12E-07
BRSK2	6.46E-06	5.74E-07	1.40E-08	1.63E-08	1.14E-08	8.75E-09	1.28E-08	1.27E-07
BRWD1	1.02E-05	8.35E-06	1.35E-05	1.03E-05	4.17E-06	1.39E-05	1.24E-05	8.19E-06
BRWD3	3.88E-06	1.07E-06	1.04E-06	1.07E-06	3.11E-07	1.57E-06	6.10E-07	1.39E-06
BSCL2	2.93E-05	2.34E-05	2.21E-05	3.27E-05	1.75E-05	4.10E-05	2.26E-05	5.50E-05
BSDC1	4.28E-05	2.41E-05	4.70E-05	4.67E-05	1.24E-05	5.69E-05	4.16E-05	5.30E-05
BSG	0.00053	0.000231	0.000128	0.000174	0.000223	0.000156	0.00026	0.000324
BSN	2.48E-07	4.13E-07	4.19E-08	7.55E-08	2.34E-07	1.69E-07	2.59E-07	6.63E-08
BSND	1.49E-06	6.30E-08	0	0	0	0	1.76E-08	0
BSPH1	0	0	0	0	0	0	0	0
BSPRY	5.46E-06	1.20E-08	5.73E-07	8.15E-07	1.65E-07	6.93E-08	1.11E-06	3.14E-07
BST1	2.38E-06	9.81E-07	1.70E-06	4.42E-06	6.25E-07	1.24E-06	2.28E-06	3.75E-07
BST2	0.000372	0.000132	0.000285	0.000146	0.000813	0.000343	0.000508	0.000332
BSX	0	0	0	0	0	0	0	0
BTAF1	1.77E-05	4.32E-06	3.01E-06	2.84E-06	5.52E-07	4.25E-06	1.31E-06	3.14E-06
BTBD1	3.10E-05	2.18E-05	1.26E-05	1.44E-05	5.99E-06	1.58E-05	9.83E-06	1.24E-05
BTBD10	1.06E-05	5.32E-06	3.55E-06	6.40E-06	8.73E-07	5.73E-06	4.59E-06	8.28E-06
BTBD11	2.62E-07	1.53E-07	1.25E-06	3.02E-07	7.32E-08	2.94E-06	3.23E-07	6.37E-07
BTBD12	1.57E-06	8.38E-07	4.06E-07	5.15E-07	2.38E-07	1.30E-06	7.87E-07	6.65E-07
BTBD16	3.97E-06	1.78E-06	2.67E-06	1.62E-06	5.62E-06	1.13E-06	1.45E-06	2.25E-06
BTBD17	0	0	0	0	0	0	0	2.15E-08
BTBD18	7.88E-09	0	0	0	6.77E-09	2.19E-08	7.64E-09	1.16E-08
BTBD19	5.73E-07	2.68E-07	4.32E-07	5.59E-07	6.76E-08	2.77E-07	1.51E-07	2.08E-07
BTBD2	3.04E-05	1.67E-05	1.74E-05	2.06E-05	2.63E-05	3.09E-05	2.55E-05	1.79E-05
BTBD3	1.02E-05	5.06E-06	2.71E-06	3.00E-06	1.18E-06	2.82E-06	3.21E-06	5.51E-06
BTBD6	5.11E-05	1.05E-05	1.75E-05	1.72E-05	1.01E-05	1.88E-05	1.41E-05	1.24E-05
BTBD7	6.01E-06	1.92E-06	2.78E-06	3.46E-06	1.80E-06	3.87E-06	1.98E-06	3.03E-06
BTBD8	5.72E-07	5.93E-07	3.21E-07	2.35E-07	0	3.22E-07	1.80E-08	2.78E-08
BTBD9	2.68E-06	3.78E-06	8.15E-07	1.15E-06	4.69E-07	1.54E-06	8.19E-07	1.26E-06
BTC	4.40E-07	1.09E-07	1.64E-07	2.82E-07	0	3.56E-08	3.67E-08	6.79E-07
BTD	3.15E-05	4.43E-05	4.57E-05	3.10E-05	4.72E-05	3.51E-05	1.99E-05	2.13E-05
BTF3	0.000494	0.000291	0.000246	0.000305	0.000121	0.000411	0.000334	0.000318
BTF3L1	0	5.79E-08	5.64E-08	0	0	0	0	0
BTF3L4	1.97E-05	1.02E-05	9.96E-06	1.00E-05	2.44E-06	9.63E-06	9.89E-06	8.95E-06
BTG1	0.000156	5.89E-05	4.20E-05	4.39E-05	2.59E-05	4.29E-05	3.97E-05	3.28E-05
BTG2	8.79E-05	1.29E-05	1.73E-05	3.74E-05	2.68E-06	1.06E-05	9.92E-06	9.70E-06

BTG3	1.48E-05	7.51E-06	3.70E-06	1.29E-05	1.69E-06	1.36E-05	7.42E-06	5.55E-06
BTG4	0	0	0	0	0	0	0	0
BTK	3.97E-06	9.80E-07	2.24E-06	4.53E-06	7.10E-07	1.40E-06	3.10E-06	8.64E-07
BTLA	5.40E-07	0	6.67E-08	1.57E-07	6.47E-09	1.94E-07	3.66E-08	5.26E-08
BTN1A1	4.08E-08	0	8.45E-09	0	0	0	7.91E-09	0
BTN2A1	1.30E-05	9.18E-06	3.62E-06	5.64E-06	1.27E-06	5.22E-06	3.75E-06	1.10E-05
BTN2A2	1.36E-05	7.01E-06	1.81E-06	2.96E-06	9.70E-07	6.92E-06	2.06E-06	4.99E-06
BTN2A3	2.00E-06	1.60E-07	2.16E-07	1.43E-07	1.09E-08	3.76E-07	1.53E-07	4.39E-07
BTN3A1	2.08E-05	9.64E-06	5.49E-06	6.88E-06	1.16E-06	1.34E-05	5.34E-06	2.19E-05
BTN3A2	2.36E-05	1.32E-05	1.07E-05	1.14E-05	1.74E-06	1.28E-05	1.41E-05	2.52E-05
BTN3A3	1.75E-05	5.01E-06	7.54E-06	8.59E-06	1.23E-06	1.00E-05	4.49E-06	1.70E-05
BTNL2	0	0	0	0	0	0	0	0
BTNL3	0	0	2.44E-07	1.60E-07	3.73E-08	0	0	2.13E-08
BTNL8	1.25E-07	3.60E-07	3.25E-08	2.67E-07	5.99E-08	1.14E-07	5.34E-08	4.54E-08
BTNL9	6.41E-07	7.50E-06	1.16E-06	1.24E-06	1.68E-06	2.89E-06	3.57E-06	2.29E-06
BTRC	3.76E-06	1.72E-06	2.19E-06	2.48E-06	1.19E-06	3.35E-06	1.82E-06	2.32E-06
BUB1	8.61E-06	3.05E-06	5.51E-08	1.94E-07	1.55E-07	1.06E-06	2.75E-07	4.60E-07
BUB1B	5.73E-06	1.18E-06	5.03E-08	6.72E-08	1.42E-07	8.43E-07	1.37E-07	3.04E-07
BUB3	5.92E-05	2.22E-05	1.74E-05	2.49E-05	1.61E-05	4.10E-05	2.48E-05	3.09E-05
BUD13	1.18E-05	4.07E-06	4.64E-06	6.51E-06	3.98E-06	7.27E-06	6.25E-06	7.85E-06
BUD31	0.000166	0.000136	3.76E-05	6.34E-05	9.13E-05	7.07E-05	8.86E-05	7.21E-05
BVES	2.68E-07	4.96E-08	6.35E-08	1.31E-07	0	4.59E-08	8.86E-08	1.45E-07
BYSL	1.17E-05	1.59E-05	5.36E-06	1.04E-05	5.64E-06	1.26E-05	1.24E-05	1.36E-05
BZRAP1	1.71E-06	2.57E-07	1.02E-06	1.61E-06	1.39E-06	4.82E-07	1.77E-06	4.60E-07
BZW1	8.91E-05	0.000106	6.38E-05	6.69E-05	3.71E-05	7.35E-05	8.62E-05	6.07E-05
BZW2	2.87E-05	2.70E-05	8.91E-06	1.10E-05	4.87E-06	3.24E-05	1.64E-05	1.89E-05
C10orf10	9.07E-05	8.26E-05	0.000102	0.000206	3.47E-05	9.49E-05	7.57E-05	0.000102
C10orf105	3.53E-07	4.30E-08	1.03E-07	1.88E-07	4.09E-08	1.85E-07	2.25E-07	2.00E-07
C10orf107	1.43E-07	0	2.20E-08	5.03E-08	0	1.90E-08	3.92E-08	0
C10orf108	1.15E-05	1.90E-06	4.76E-06	4.53E-06	5.67E-07	3.04E-06	1.40E-06	1.92E-06
C10orf11	5.65E-06	2.82E-06	8.51E-06	1.22E-05	8.76E-06	5.72E-06	1.82E-05	9.52E-06
C10orf110	1.54E-06	1.18E-06	2.95E-07	2.54E-07	2.08E-07	4.54E-07	2.06E-07	2.12E-07
C10orf111	1.14E-07	0	1.50E-08	1.92E-07	9.81E-08	7.93E-08	8.23E-08	8.39E-08
C10orf113	0	0	0	0	0	0	0	0
C10orf114	3.50E-07	6.92E-06	7.64E-06	6.52E-06	2.12E-05	5.47E-06	7.33E-06	2.32E-06
C10orf116	4.94E-05	1.81E-05	3.02E-05	7.67E-05	7.30E-05	7.07E-06	4.20E-05	5.28E-06
C10orf118	3.63E-06	1.06E-06	1.94E-06	1.71E-06	8.12E-08	1.78E-06	5.43E-07	1.38E-06
C10orf119	1.56E-05	8.11E-06	1.02E-05	1.11E-05	3.88E-06	1.15E-05	9.43E-06	7.21E-06
C10orf12	7.01E-07	1.09E-06	3.99E-07	8.42E-07	2.57E-08	5.52E-07	5.72E-07	7.78E-07
C10orf120	0	0	0	0	0	0	0	0
C10orf122	0	0	0	0	0	0	0	0
C10orf125	5.39E-05	0.000149	0.00028	0.000195	0.000537	0.000219	0.000224	0.000153
C10orf128	3.05E-06	5.37E-07	5.51E-07	7.13E-07	1.00E-07	2.24E-07	3.37E-07	1.31E-06
C10orf129	0	2.04E-08	1.90E-08	0	0	3.57E-07	0	0
C10orf131	4.40E-08	0	0	0	0	2.05E-08	4.21E-08	0
C10orf137	6.65E-06	2.17E-06	2.46E-06	2.97E-06	1.79E-06	3.35E-06	2.70E-06	2.52E-06
C10orf140	2.25E-08	4.43E-07	6.92E-07	5.58E-07	8.60E-07	5.78E-07	5.58E-07	2.48E-07
C10orf18	6.58E-06	3.20E-06	4.75E-06	3.82E-06	1.29E-06	5.35E-06	3.52E-06	4.68E-06
C10orf2	4.00E-06	5.06E-06	1.98E-06	3.58E-06	2.71E-06	5.60E-06	7.79E-06	3.41E-06
C10orf25	1.61E-06	3.99E-06	1.97E-06	2.05E-06	1.71E-06	4.10E-06	1.48E-06	1.13E-06
C10orf26	2.46E-05	1.05E-05	1.96E-05	1.45E-05	1.23E-05	1.81E-05	1.57E-05	1.65E-05
C10orf27	0	0	0	0	0	0	1.59E-08	0
C10orf28	1.27E-05	4.09E-06	3.20E-06	5.40E-06	5.00E-06	5.50E-06	7.92E-06	4.53E-06
C10orf32	4.52E-05	1.26E-05	3.57E-05	2.25E-05	3.34E-05	4.30E-05	2.15E-05	2.83E-05
C10orf35	1.27E-05	1.97E-06	1.02E-06	5.78E-06	0	6.90E-08	2.54E-06	5.54E-06
C10orf4	8.77E-06	8.63E-06	4.88E-06	5.99E-06	7.25E-06	9.28E-06	7.83E-06	4.06E-06
C10orf40	0	0	0	0	0	0	0	0
C10orf41	1.44E-06	7.53E-07	9.06E-07	6.19E-07	9.19E-07	1.48E-06	1.19E-06	5.99E-07

C10orf46	7.87E-06	6.06E-06	4.73E-06	6.63E-06	3.98E-06	6.01E-06	8.02E-06	6.87E-06
C10orf47	1.21E-05	9.73E-06	7.51E-06	1.81E-05	9.11E-06	1.35E-05	2.12E-05	8.35E-06
C10orf50	1.21E-07	8.42E-08	2.64E-08	3.00E-08	6.30E-08	0	0	3.61E-08
C10orf53	0	0	0	0	0	0	0	0
C10orf54	3.02E-05	4.12E-06	4.35E-06	1.96E-05	2.41E-06	4.75E-06	1.25E-05	2.34E-06
C10orf55	6.96E-08	2.11E-08	0	4.58E-08	8.05E-09	8.68E-09	1.03E-08	4.13E-08
C10orf57	1.80E-05	2.26E-05	3.13E-05	2.35E-05	1.86E-05	5.31E-05	4.18E-05	3.09E-05
C10orf58	6.90E-05	0.000259	0.000128	0.000104	0.000125	6.89E-05	0.00011	0.000101
C10orf62	2.13E-07	2.45E-07	2.29E-08	7.87E-08	2.02E-07	5.95E-08	1.43E-07	0
C10orf67	0	0	4.08E-08	0	0	0	0	0
C10orf68	1.09E-07	1.99E-08	0	1.02E-07	8.75E-09	3.30E-08	3.79E-08	3.75E-08
C10orf71	0	0	8.06E-09	0	0	0	0	0
C10orf72	1.76E-06	2.61E-06	2.03E-06	2.00E-06	1.40E-06	5.47E-06	1.10E-06	1.92E-06
C10orf75	6.72E-06	1.17E-05	1.38E-05	5.03E-06	3.69E-06	4.33E-06	3.35E-06	4.34E-05
C10orf76	1.52E-05	5.10E-06	6.59E-06	8.52E-06	5.60E-06	1.19E-05	8.14E-06	8.56E-06
C10orf78	4.21E-06	1.99E-06	1.38E-06	1.90E-06	7.70E-07	1.44E-06	1.83E-06	1.47E-06
C10orf79	6.67E-08	2.95E-07	5.17E-08	3.24E-07	6.45E-08	2.94E-07	5.89E-08	3.85E-08
C10orf81	9.26E-09	3.79E-08	9.62E-09	6.55E-08	0	0	5.43E-08	4.68E-08
C10orf82	1.35E-08	0	0	1.66E-08	0	0	7.79E-08	0
C10orf84	1.04E-05	4.73E-06	6.10E-06	7.48E-06	2.69E-06	8.85E-06	6.64E-06	7.17E-06
C10orf88	3.52E-06	2.55E-06	1.99E-06	1.97E-06	1.13E-06	2.78E-06	1.95E-06	1.79E-06
C10orf90	1.26E-07	0	8.74E-08	2.25E-08	0	0	0	2.33E-08
C10orf91	2.37E-07	0	4.51E-08	4.92E-08	3.44E-08	0	0	1.19E-07
C10orf93	2.21E-08	0	4.78E-08	0	5.73E-08	2.06E-08	0	0
C10orf95	1.26E-07	1.38E-07	0	6.30E-08	4.39E-08	1.13E-07	0	2.51E-08
C10orf96	0	0	0	0	0	0	0	0
C10orf99	0	0	8.37E-07	0	0	0	0	0
C11orf1	1.99E-05	1.68E-05	1.39E-05	1.47E-05	3.72E-06	2.23E-05	1.41E-05	1.16E-05
C11orf10	0.000311	0.000243	0.000158	0.000257	0.000212	0.000366	0.000278	0.000329
C11orf16	4.30E-08	0	0	0	1.23E-08	0	0	2.11E-08
C11orf17	1.86E-05	1.08E-05	7.81E-06	9.30E-06	8.18E-06	1.50E-05	1.21E-05	2.21E-05
C11orf2	3.95E-05	5.81E-05	3.55E-05	4.55E-05	4.02E-05	3.18E-05	3.94E-05	5.58E-05
C11orf20	1.60E-06	5.09E-07	0	4.87E-08	0	0	1.47E-07	0
C11orf21	2.95E-07	7.40E-08	1.82E-07	3.21E-07	9.00E-09	2.73E-07	1.27E-07	1.12E-07
C11orf24	4.95E-05	3.36E-05	3.92E-05	4.80E-05	2.55E-05	3.16E-05	3.95E-05	4.63E-05
C11orf30	5.11E-06	2.39E-06	2.32E-06	2.53E-06	5.21E-07	3.23E-06	1.16E-06	3.24E-06
C11orf31	4.62E-05	6.59E-05	3.30E-05	4.54E-05	8.90E-05	6.72E-05	3.45E-05	5.53E-05
C11orf34	1.60E-08	0	0	1.97E-08	0	0	0	0
C11orf35	2.90E-06	6.75E-06	1.93E-06	2.39E-06	6.17E-07	2.79E-06	2.57E-06	2.27E-06
C11orf36	0	0	0	0	0	0	0	0
C11orf40	0	0	0	0	0	0	0	0
C11orf41	1.08E-07	0	9.64E-09	2.54E-08	0	5.58E-09	2.04E-08	0
C11orf42	9.14E-08	0	0	0	0	0	0	3.40E-08
C11orf45	1.11E-06	6.43E-08	2.21E-07	2.48E-07	1.12E-08	3.80E-07	3.77E-07	3.72E-07
C11orf46	1.37E-05	1.44E-05	1.40E-05	1.19E-05	6.84E-06	1.04E-05	1.11E-05	1.28E-05
C11orf48	3.65E-05	3.73E-05	1.12E-05	1.99E-05	4.40E-05	2.14E-05	2.63E-05	2.84E-05
C11orf49	7.22E-06	5.96E-06	2.70E-06	5.98E-06	6.85E-07	2.21E-06	5.25E-06	5.34E-07
C11orf51	3.28E-05	1.56E-05	1.87E-05	2.67E-05	2.88E-05	3.27E-05	2.81E-05	2.72E-05
C11orf52	8.10E-05	2.21E-05	1.49E-05	1.74E-05	2.21E-05	3.20E-05	1.89E-05	2.45E-05
C11orf53	2.27E-07	0	0	0	2.18E-08	0	0	0
C11orf54	0.000215	3.15E-05	0.000113	5.55E-05	3.41E-05	3.91E-05	2.52E-05	5.26E-05
C11orf57	9.94E-06	9.15E-06	5.61E-06	6.17E-06	3.27E-06	9.14E-06	5.41E-06	9.90E-06
C11orf58	4.02E-05	3.21E-05	2.92E-05	2.98E-05	8.06E-06	4.40E-05	2.66E-05	3.69E-05
C11orf59	0.000109	0.000111	5.24E-05	6.20E-05	5.95E-05	9.16E-05	7.70E-05	0.000103
C11orf61	2.90E-06	7.29E-07	6.34E-07	1.57E-06	4.89E-07	1.08E-06	9.75E-07	1.87E-06
C11orf63	2.39E-06	7.17E-08	1.16E-07	5.28E-07	6.85E-09	1.45E-07	1.08E-07	1.52E-07
C11orf64	0	0	0	0	0	0	0	0
C11orf65	5.86E-07	3.57E-07	2.25E-07	1.96E-07	6.31E-08	6.02E-07	9.81E-08	3.08E-07

C11orf66	2.10E-07	1.48E-07	5.28E-07	5.11E-07	1.60E-06	7.90E-07	1.20E-06	3.00E-07
C11orf67	2.54E-05	2.76E-05	3.38E-05	6.21E-05	9.73E-05	6.59E-05	5.46E-05	6.02E-05
C11orf68	3.78E-05	1.88E-05	1.53E-05	2.10E-05	2.45E-05	2.90E-05	1.66E-05	3.17E-05
C11orf70	3.15E-07	0	2.76E-08	3.12E-08	2.19E-08	1.89E-08	7.24E-08	3.76E-08
C11orf71	2.99E-05	9.23E-06	2.20E-05	1.07E-05	1.56E-05	2.36E-05	4.31E-06	1.92E-05
C11orf73	2.22E-05	1.16E-05	1.06E-05	1.49E-05	1.13E-05	1.37E-05	1.36E-05	2.22E-05
C11orf74	1.53E-05	1.70E-05	7.22E-06	8.72E-06	1.29E-05	2.06E-05	8.41E-06	1.75E-05
C11orf75	8.29E-05	2.74E-05	4.84E-05	5.34E-05	4.75E-05	2.22E-05	4.74E-05	9.74E-05
C11orf80	7.78E-06	1.83E-06	9.60E-07	2.19E-06	2.17E-06	3.98E-07	1.88E-06	1.65E-06
C11orf82	1.58E-06	8.57E-07	1.88E-07	2.99E-07	2.14E-07	4.70E-07	2.11E-07	9.13E-07
C11orf83	6.22E-05	4.63E-05	3.33E-05	3.32E-05	0.000116	3.40E-05	3.26E-05	6.31E-05
C11orf84	9.97E-06	6.53E-06	1.55E-06	3.23E-06	1.26E-06	1.69E-06	2.74E-06	2.25E-06
C11orf85	1.92E-07	0	1.06E-07	4.18E-08	1.79E-07	0	3.93E-07	2.94E-07
C11orf86	1.61E-06	2.26E-07	2.35E-08	2.69E-07	3.76E-08	6.09E-08	1.46E-07	0
C11orf87	9.06E-09	0	4.64E-09	0	3.89E-09	0	0	0
C11orf88	4.55E-08	0	0	0	0	6.91E-09	7.24E-09	0
C11orf9	1.91E-05	7.03E-06	5.20E-06	1.39E-05	3.82E-06	7.08E-06	4.85E-06	1.75E-05
C11orf90	0	0	0	0	0	0	0	0
C11orf92	1.54E-06	1.11E-07	1.52E-07	1.21E-07	1.70E-07	1.38E-07	4.43E-08	6.65E-08
C11orf93	9.93E-06	6.27E-07	1.70E-07	3.27E-07	1.68E-07	3.13E-07	1.70E-07	5.76E-07
C11orf94	0	0	0	0	0	0	2.11E-07	0
C11orf95	4.95E-06	3.88E-06	3.83E-06	4.15E-06	3.27E-06	2.70E-06	3.31E-06	2.60E-06
C12orf10	3.19E-05	4.32E-05	2.21E-05	3.06E-05	2.78E-05	3.54E-05	3.26E-05	3.26E-05
C12orf11	1.19E-05	1.26E-05	7.47E-06	7.50E-06	2.58E-06	1.12E-05	6.36E-06	5.45E-06
C12orf12	0	0	0	0	0	0	7.59E-09	0
C12orf23	2.49E-05	1.29E-05	1.28E-05	1.86E-05	8.50E-06	1.23E-05	1.49E-05	1.89E-05
C12orf24	3.59E-06	3.52E-06	2.47E-06	2.86E-06	1.50E-06	3.70E-06	1.90E-06	3.47E-06
C12orf26	2.36E-06	2.03E-06	1.10E-06	1.82E-06	6.08E-07	2.56E-06	1.46E-06	8.24E-07
C12orf27	5.29E-06	1.65E-06	3.59E-07	3.40E-07	1.30E-06	5.59E-07	5.19E-07	3.52E-07
C12orf29	5.06E-06	6.92E-06	6.78E-06	5.37E-06	2.20E-06	5.78E-06	4.55E-06	4.13E-06
C12orf32	9.53E-06	5.20E-06	1.79E-06	5.60E-06	2.37E-06	7.35E-06	4.77E-06	4.00E-06
C12orf34	1.55E-05	6.20E-06	1.20E-06	1.69E-06	3.07E-06	5.93E-07	2.95E-06	4.76E-06
C12orf35	2.89E-05	3.34E-05	2.14E-05	2.31E-05	7.89E-06	2.72E-05	2.63E-05	1.44E-05
C12orf36	7.01E-06	0	0	4.15E-08	0	0	8.21E-09	0
C12orf39	1.44E-06	3.20E-06	2.14E-07	6.90E-08	8.62E-07	3.59E-06	3.52E-07	2.22E-06
C12orf4	3.46E-06	1.94E-06	1.49E-06	3.11E-06	1.23E-06	3.46E-06	2.72E-06	1.87E-06
C12orf40	0	0	0	0	0	0	0	0
C12orf41	1.14E-05	9.33E-06	4.81E-06	8.61E-06	3.24E-06	7.50E-06	8.55E-06	6.63E-06
C12orf42	7.64E-08	2.19E-08	4.09E-08	4.70E-08	3.92E-08	0	0	0
C12orf43	9.37E-06	1.05E-05	5.62E-06	6.28E-06	3.17E-06	7.25E-06	7.54E-06	6.58E-06
C12orf44	2.77E-05	3.13E-05	2.78E-05	5.29E-05	4.66E-05	3.01E-05	4.84E-05	3.44E-05
C12orf45	1.17E-05	2.06E-05	1.43E-05	1.94E-05	2.35E-05	1.68E-05	2.53E-05	2.41E-05
C12orf47	1.53E-05	1.19E-05	5.10E-06	7.06E-06	1.16E-05	7.06E-06	1.43E-05	4.75E-06
C12orf48	3.17E-06	1.15E-06	2.06E-07	4.16E-07	2.43E-07	9.71E-07	4.76E-07	1.09E-06
C12orf49	9.00E-06	3.31E-06	2.03E-06	4.45E-06	1.21E-06	2.88E-06	4.16E-06	1.92E-06
C12orf5	1.80E-06	7.59E-07	5.43E-07	1.59E-06	1.88E-07	9.27E-07	9.91E-07	1.48E-06
C12orf50	0	1.32E-08	0	0	0	0	0	0
C12orf51	4.47E-06	2.54E-06	1.55E-06	1.25E-06	3.60E-07	2.29E-06	1.07E-06	1.45E-06
C12orf52	1.90E-05	2.90E-05	1.91E-05	1.82E-05	2.12E-05	1.92E-05	2.69E-05	2.00E-05
C12orf53	4.61E-08	3.12E-08	0	0	1.88E-08	0	1.06E-08	0
C12orf54	0	0	0	0	3.82E-08	0	0	6.62E-08
C12orf56	3.60E-07	3.70E-06	3.23E-08	0	2.53E-08	0	0	0
C12orf57	0.000159	0.000177	0.000124	0.000181	0.000324	0.000156	0.000295	0.000152
C12orf59	9.18E-08	2.82E-08	4.33E-08	1.74E-07	0	6.42E-07	2.43E-08	0
C12orf60	2.83E-06	1.55E-06	1.83E-06	1.74E-06	1.02E-06	2.31E-06	1.24E-06	1.65E-06
C12orf61	6.96E-07	5.28E-07	2.91E-07	2.85E-07	2.00E-07	1.75E-07	5.17E-07	1.50E-07
C12orf62	0.000161	0.000137	8.80E-05	8.63E-05	0.00016	0.000138	7.72E-05	7.26E-05
C12orf63	2.48E-08	1.39E-08	1.92E-08	7.58E-09	0	1.15E-08	0	1.82E-08

C12orf65	1.74E-05	1.15E-05	7.07E-06	6.88E-06	7.76E-06	1.21E-05	1.15E-05	9.30E-06
C12orf66	2.85E-06	6.45E-06	3.13E-06	2.06E-06	3.87E-07	4.40E-06	1.55E-06	2.48E-06
C12orf68	3.93E-07	2.61E-07	1.68E-07	6.29E-07	2.42E-07	1.96E-07	7.02E-07	1.48E-07
C12orf69	6.57E-07	4.35E-07	6.28E-07	7.49E-07	4.68E-07	8.72E-07	2.85E-07	2.00E-07
C12orf70	4.81E-07	0	0	4.94E-08	3.46E-08	0	5.76E-08	2.97E-08
C12orf71	2.79E-08	3.25E-08	0	6.91E-08	2.42E-08	0	2.66E-08	0
C12orf72	2.22E-06	3.54E-06	3.43E-06	1.79E-06	1.05E-06	5.05E-06	2.30E-06	1.33E-06
C12orf73	5.15E-06	5.09E-06	2.36E-06	2.80E-06	3.10E-06	2.63E-06	5.29E-06	3.74E-06
C12orf74	7.59E-08	2.09E-07	5.84E-08	8.98E-08	0	2.38E-07	0	2.70E-08
C12orf75	0.000146	1.63E-05	1.02E-06	8.72E-06	7.39E-07	4.63E-06	1.71E-06	8.45E-07
C12orf76	2.66E-05	3.74E-06	2.98E-06	3.55E-06	3.88E-06	3.55E-06	2.60E-06	1.86E-06
C12orf77	0	0	0	0	0	0	0	0
C13orf1	7.96E-06	1.06E-05	7.14E-06	6.23E-06	4.56E-06	1.33E-05	1.10E-05	7.26E-06
C13orf15	1.16E-05	1.06E-05	1.48E-06	3.29E-05	1.35E-05	1.27E-05	2.17E-05	1.10E-05
C13orf16	3.33E-07	0	4.79E-08	2.08E-07	1.45E-07	0	3.92E-08	6.29E-08
C13orf18	8.39E-07	8.30E-08	2.39E-07	9.91E-07	1.55E-07	6.00E-07	7.98E-07	1.83E-07
C13orf23	6.81E-06	3.48E-06	2.12E-06	2.49E-06	8.42E-07	2.64E-06	1.92E-06	2.76E-06
C13orf26	2.68E-08	0	5.89E-08	6.64E-08	0	0	0	0
C13orf27	3.35E-05	1.97E-05	2.26E-05	2.51E-05	7.63E-06	2.22E-05	1.95E-05	1.54E-05
C13orf28	0	0	0	0	0	0	0	0
C13orf29	3.19E-07	6.10E-06	9.04E-08	1.81E-07	5.46E-08	1.28E-07	1.34E-07	1.34E-07
C13orf30	0	0	0	0	0	0	0	0
C13orf31	5.97E-06	9.29E-07	2.05E-06	3.07E-06	2.86E-07	3.50E-06	9.35E-07	1.98E-06
C13orf33	2.11E-06	8.56E-07	9.24E-08	2.21E-06	5.09E-08	1.37E-07	4.11E-07	5.65E-07
C13orf34	2.54E-06	1.44E-06	8.23E-07	6.16E-07	2.09E-07	9.63E-07	6.43E-07	5.86E-07
C13orf35	0	1.19E-08	0	0	0	9.80E-09	0	0
C13orf36	0	0	0	0	0	6.66E-09	0	0
C13orf37	6.52E-06	4.70E-06	2.36E-06	2.87E-06	1.07E-06	4.51E-06	3.04E-06	2.58E-06
C13orf38	6.11E-07	0	0	0	0	0	0	0
C13orf39	0	0	0	0	0	0	0	0
C14orf1	6.09E-05	8.09E-05	6.43E-05	5.09E-05	5.91E-05	9.36E-05	6.81E-05	5.42E-05
C14orf101	7.39E-06	4.13E-06	2.98E-06	3.51E-06	3.54E-06	3.66E-06	3.07E-06	2.77E-06
C14orf102	9.62E-06	2.70E-06	4.12E-06	4.90E-06	5.42E-06	8.24E-06	6.23E-06	3.56E-06
C14orf104	7.60E-06	6.83E-06	5.79E-06	5.41E-06	1.34E-06	6.21E-06	3.52E-06	4.71E-06
C14orf105	1.71E-05	1.73E-06	5.89E-06	8.82E-06	9.44E-07	2.56E-06	6.13E-06	4.81E-06
C14orf106	3.95E-06	1.32E-06	1.06E-06	1.29E-06	2.60E-07	1.69E-06	6.31E-07	8.16E-07
C14orf109	2.82E-05	1.18E-05	9.20E-06	1.14E-05	1.54E-05	9.98E-06	1.00E-05	9.86E-06
C14orf115	0	7.55E-09	0	0	5.78E-09	0	0	0
C14orf118	2.99E-06	1.57E-06	1.62E-06	1.56E-06	1.85E-07	1.52E-06	7.46E-07	1.14E-06
C14orf119	2.86E-05	1.40E-05	8.12E-06	1.05E-05	8.47E-06	1.08E-05	1.23E-05	1.17E-05
C14orf126	4.94E-06	6.57E-06	4.94E-06	3.77E-06	1.75E-06	3.76E-06	3.73E-06	4.69E-06
C14orf128	3.66E-06	2.94E-06	1.71E-06	1.49E-06	9.43E-07	3.98E-06	1.66E-06	7.95E-07
C14orf129	2.47E-05	7.71E-06	8.23E-06	7.95E-06	3.53E-06	9.68E-06	7.62E-06	3.81E-05
C14orf132	2.88E-06	2.98E-08	4.85E-07	3.44E-07	1.61E-07	4.95E-08	1.22E-07	6.04E-07
C14orf135	1.35E-05	3.10E-06	4.64E-06	5.81E-06	1.42E-06	6.64E-06	4.27E-06	3.25E-06
C14orf138	5.33E-06	2.89E-06	1.86E-06	3.37E-06	2.72E-06	2.29E-06	1.97E-06	1.80E-06
C14orf139	1.15E-06	3.24E-07	4.95E-07	8.06E-07	1.72E-06	4.12E-07	1.86E-06	5.20E-07
C14orf142	2.07E-05	1.04E-05	7.71E-06	8.04E-06	8.01E-06	1.24E-05	7.17E-06	1.08E-05
C14orf143	5.19E-06	1.22E-06	9.17E-07	3.22E-06	1.85E-06	3.51E-06	1.98E-06	2.34E-06
C14orf145	4.74E-07	2.98E-07	1.71E-07	2.96E-07	7.50E-08	1.41E-07	1.52E-07	2.53E-07
C14orf147	4.46E-05	4.34E-05	1.44E-05	3.50E-05	1.80E-05	0.000147	5.97E-05	5.82E-05
C14orf148	3.39E-07	1.39E-07	2.88E-08	1.51E-07	1.53E-07	1.82E-07	7.58E-08	6.04E-08
C14orf149	1.02E-05	2.22E-06	4.98E-06	4.28E-06	1.49E-06	2.22E-05	4.29E-06	3.37E-06
C14orf153	2.41E-05	1.72E-05	1.27E-05	1.33E-05	2.06E-05	1.83E-05	2.46E-05	1.10E-05
C14orf156	0.000277	0.000115	0.000112	0.000122	0.000194	0.000196	0.000193	0.000136
C14orf159	2.46E-05	1.58E-05	1.23E-05	1.29E-05	3.73E-06	1.83E-05	1.52E-05	1.39E-05
C14orf162	0	1.08E-05	1.16E-07	6.68E-08	1.56E-08	1.97E-06	1.74E-08	6.59E-06
C14orf165	0	0	0	0	0	0	0	0

C14orf166	0.000157	6.45E-05	6.90E-05	0.000102	0.000149	0.000134	0.000114	0.000101
C14orf166	0	0	0	0	1.29E-08	0	0	0
C14orf167	1.48E-05	2.96E-05	1.94E-05	6.94E-06	4.84E-06	1.24E-05	9.23E-06	3.06E-06
C14orf169	5.66E-06	2.52E-06	1.90E-06	3.51E-06	1.71E-06	3.62E-06	5.63E-06	3.76E-06
C14orf174	7.09E-08	1.34E-08	7.42E-08	1.88E-07	0	7.67E-08	3.43E-08	1.74E-08
C14orf176	1.22E-06	3.33E-07	6.89E-08	2.79E-07	9.79E-08	5.88E-07	1.09E-07	5.51E-07
C14orf177	0	0	0	0	0	0	0	0
C14orf178	6.17E-08	0	3.42E-08	0	4.30E-08	0	9.17E-08	0
C14orf179	5.11E-05	1.67E-05	1.34E-05	2.82E-05	2.98E-05	3.13E-05	4.30E-05	3.56E-05
C14orf180	0	0	8.55E-07	3.00E-06	3.38E-08	0	0	0
C14orf181	3.07E-06	2.88E-07	5.05E-07	6.23E-07	2.42E-06	6.68E-07	5.03E-07	4.99E-07
C14orf182	2.43E-07	1.08E-07	2.70E-08	8.50E-08	4.48E-08	4.83E-08	1.94E-07	3.83E-08
C14orf183	0	6.41E-08	0	0	0	0	0	0
C14orf184	9.01E-08	0	0	1.10E-08	0	0	0	2.65E-08
C14orf19	7.83E-07	2.05E-07	2.70E-07	1.41E-07	0	1.06E-07	5.19E-08	8.55E-08
C14orf2	0.000189	0.000102	7.78E-05	8.80E-05	0.000262	0.000123	0.000144	8.49E-05
C14orf21	1.13E-05	7.56E-06	5.88E-06	4.07E-06	3.36E-06	7.10E-06	6.27E-06	9.28E-06
C14orf23	0	0	0	0	0	0	0	0
C14orf28	1.54E-06	1.22E-06	2.36E-06	1.44E-06	1.64E-06	2.06E-06	1.98E-06	1.57E-06
C14orf33	8.28E-07	2.98E-07	3.83E-07	4.05E-07	2.52E-07	1.36E-06	4.61E-07	3.41E-07
C14orf34	0	0	0	0	0	0	0	0
C14orf37	2.40E-06	1.57E-07	3.50E-07	4.29E-07	8.13E-08	8.80E-08	8.93E-08	1.98E-07
C14orf39	1.08E-08	0	0	0	0	0	0	0
C14orf4	9.51E-06	6.28E-06	8.83E-06	1.25E-05	1.75E-06	5.68E-06	6.97E-06	7.06E-06
C14orf43	7.27E-06	2.86E-06	3.40E-06	5.46E-06	3.56E-07	1.63E-06	4.20E-06	2.50E-06
C14orf45	1.79E-06	2.21E-06	7.03E-07	1.06E-06	2.58E-07	1.39E-06	8.62E-07	6.80E-07
C14orf48	0	0	0	0	0	8.59E-08	0	0
C14orf49	4.94E-07	1.98E-07	5.00E-07	3.37E-07	6.78E-08	2.80E-07	1.87E-07	3.40E-07
C14orf50	2.29E-06	0	1.14E-07	1.10E-07	4.61E-08	8.29E-08	1.71E-08	2.64E-08
C14orf53	0	8.80E-08	8.30E-07	6.26E-08	3.29E-07	2.46E-06	2.18E-07	0
C14orf64	2.24E-07	1.31E-07	6.16E-08	5.71E-07	0	4.77E-08	6.53E-08	5.05E-08
C14orf68	1.12E-06	3.50E-05	0.000312	0.000611	0.000347	1.00E-05	0.000355	3.32E-07
C14orf70	0	0	0	0	0	0	0	0
C14orf72	1.25E-06	5.20E-07	0	4.20E-07	0	4.51E-08	8.96E-08	1.45E-07
C14orf73	4.75E-06	3.12E-06	1.29E-05	1.25E-05	5.61E-06	6.53E-06	1.56E-05	1.08E-06
C14orf79	3.24E-06	1.50E-06	1.16E-06	2.84E-06	1.52E-06	5.31E-06	2.48E-06	2.69E-06
C14orf80	6.62E-06	2.35E-06	3.19E-06	3.06E-06	4.87E-06	1.75E-06	6.88E-06	2.48E-06
C14orf86	0	0	0	0	0	0	0	0
C14orf93	4.95E-06	5.85E-06	1.90E-06	2.56E-06	2.74E-06	3.18E-06	3.15E-06	3.39E-06
C15orf17	1.06E-05	3.75E-06	3.07E-06	4.15E-06	3.57E-06	4.72E-06	6.20E-06	3.96E-06
C15orf2	0	0	2.82E-09	1.01E-08	0	0	5.38E-09	0
C15orf21	2.62E-06	5.08E-07	1.17E-07	8.09E-07	3.95E-07	9.15E-07	1.28E-06	5.28E-07
C15orf23	1.14E-05	1.03E-05	2.77E-06	2.87E-06	1.61E-06	4.97E-06	3.59E-06	5.61E-06
C15orf24	0.000139	9.63E-05	5.81E-05	7.77E-05	3.28E-05	8.25E-05	8.80E-05	0.000108
C15orf26	1.22E-07	1.30E-07	1.66E-08	8.58E-08	0	0	0	5.83E-08
C15orf27	5.12E-07	2.91E-08	2.55E-08	1.06E-07	1.69E-07	1.26E-08	1.76E-07	3.20E-07
C15orf28	4.21E-08	2.36E-08	7.25E-09	0	0	0	6.81E-09	1.03E-08
C15orf29	7.46E-06	5.28E-06	3.62E-06	4.12E-06	2.64E-06	3.35E-06	4.94E-06	3.21E-06
C15orf32	0	0	0	0	0	0	0	0
C15orf33	3.74E-07	2.59E-07	2.41E-07	4.01E-07	4.61E-08	1.65E-06	4.12E-07	3.27E-07
C15orf34	4.89E-07	5.89E-08	9.05E-08	7.47E-08	8.25E-08	7.84E-07	1.44E-07	3.84E-08
C15orf37	2.97E-06	1.63E-06	2.20E-06	1.62E-06	4.61E-07	2.66E-06	1.65E-06	1.95E-06
C15orf38	9.46E-06	9.65E-06	3.29E-06	3.38E-06	2.72E-06	4.72E-06	3.77E-06	3.49E-06
C15orf39	7.91E-06	3.69E-06	2.26E-06	6.25E-06	3.76E-06	4.55E-06	5.00E-06	3.98E-06
C15orf40	2.11E-05	7.50E-06	4.25E-06	6.99E-06	7.76E-06	1.90E-05	8.63E-06	6.73E-06
C15orf41	5.37E-06	7.34E-06	1.25E-06	2.71E-06	1.64E-06	5.46E-06	2.43E-06	2.57E-06
C15orf42	1.55E-06	3.77E-07	1.40E-08	5.69E-08	9.32E-08	8.97E-08	8.32E-08	6.37E-08
C15orf43	0	0	2.58E-07	1.23E-07	5.19E-08	7.84E-07	3.46E-07	0

C15orf44	1.89E-05	1.48E-05	1.24E-05	1.56E-05	6.89E-06	1.54E-05	1.67E-05	1.41E-05
C15orf48	9.60E-06	8.55E-07	6.60E-07	9.11E-06	1.84E-07	4.02E-07	1.01E-05	2.06E-06
C15orf5	1.66E-08	1.89E-08	1.76E-08	0	0	1.54E-08	1.60E-08	0
C15orf50	0	8.71E-08	4.07E-08	0	0	0	1.83E-08	0
C15orf51	1.95E-07	2.00E-08	3.07E-08	9.19E-09	1.94E-08	0	2.32E-08	4.40E-08
C15orf52	1.86E-06	1.10E-06	7.84E-07	3.99E-06	4.77E-07	1.32E-06	1.18E-06	2.53E-06
C15orf53	2.34E-08	0	0	0	0	2.17E-08	1.13E-08	0
C15orf54	1.50E-08	0	7.76E-09	0	0	1.39E-08	0	2.20E-08
C15orf55	0	0	0	0	0	0	0	0
C15orf56	6.27E-07	1.27E-08	5.86E-08	1.37E-08	0	0	1.08E-08	0
C15orf57	1.82E-05	7.94E-06	5.75E-06	1.14E-05	3.35E-06	8.04E-06	7.46E-06	6.00E-06
C15orf58	4.00E-06	3.76E-06	1.44E-06	1.54E-06	5.00E-07	1.84E-06	2.21E-06	2.98E-06
C15orf59	2.16E-06	8.61E-08	3.22E-07	2.55E-07	6.49E-08	0	9.75E-07	1.11E-07
C15orf60	3.33E-08	0	0	0	0	0	0	4.99E-08
C15orf61	7.67E-06	4.36E-06	4.25E-06	3.84E-06	7.37E-06	6.01E-06	9.96E-06	3.98E-06
C15orf62	1.78E-06	2.78E-06	4.22E-07	3.93E-07	7.30E-07	5.16E-07	9.05E-07	4.85E-07
C15orf63	5.95E-05	6.02E-05	2.97E-05	4.28E-05	5.37E-05	5.76E-05	9.36E-05	4.11E-05
C16orf11	0	0	0	0	0	0	0	0
C16orf13	4.79E-05	9.63E-05	2.86E-05	3.76E-05	7.34E-05	3.76E-05	6.00E-05	4.69E-05
C16orf3	2.69E-07	2.50E-07	1.48E-07	1.66E-07	1.63E-07	5.02E-08	0	1.20E-07
C16orf42	5.39E-05	7.74E-05	3.12E-05	3.52E-05	5.50E-05	5.19E-05	5.33E-05	3.97E-05
C16orf45	4.85E-06	9.34E-06	1.62E-05	1.32E-05	2.89E-06	9.39E-06	1.10E-05	1.32E-05
C16orf46	6.76E-07	3.28E-07	1.07E-06	8.67E-07	4.34E-07	2.52E-06	5.84E-07	9.63E-07
C16orf48	7.34E-06	5.07E-06	6.56E-06	8.99E-06	6.28E-06	7.03E-06	8.80E-06	4.70E-06
C16orf5	3.90E-05	5.15E-05	1.54E-05	1.82E-05	8.69E-06	2.59E-05	3.12E-05	2.23E-05
C16orf52	6.95E-06	7.58E-06	7.77E-06	5.55E-06	9.68E-07	9.10E-06	3.46E-06	7.71E-06
C16orf53	1.35E-05	1.14E-05	6.59E-06	6.61E-06	5.96E-06	9.03E-06	1.09E-05	8.95E-06
C16orf54	1.66E-06	2.84E-07	6.18E-07	2.66E-06	3.87E-08	5.35E-07	7.08E-07	4.23E-07
C16orf55	1.92E-06	9.91E-07	7.49E-07	1.17E-06	1.28E-06	1.41E-06	1.38E-06	1.00E-06
C16orf57	4.43E-05	1.49E-05	1.21E-05	1.96E-05	6.33E-06	2.01E-05	1.33E-05	2.60E-05
C16orf58	4.65E-05	0.000142	5.49E-05	5.00E-05	2.27E-05	5.91E-05	5.03E-05	5.77E-05
C16orf59	3.13E-06	5.73E-06	2.06E-07	3.44E-07	1.69E-06	9.55E-07	1.73E-06	1.11E-06
C16orf61	8.55E-05	4.63E-05	3.78E-05	4.17E-05	3.70E-05	4.19E-05	5.19E-05	5.28E-05
C16orf62	6.40E-06	7.11E-06	8.20E-06	9.09E-06	2.41E-06	1.71E-05	8.59E-06	1.94E-06
C16orf63	1.27E-05	2.90E-05	1.44E-05	1.19E-05	5.35E-06	1.88E-05	1.31E-05	9.95E-06
C16orf68	5.18E-06	6.19E-06	3.98E-06	4.60E-06	5.54E-06	5.74E-06	4.60E-06	3.44E-06
C16orf7	9.74E-06	4.98E-06	4.06E-06	5.49E-06	4.35E-06	5.93E-06	5.08E-06	7.02E-06
C16orf70	1.42E-05	1.38E-05	2.53E-05	2.18E-05	8.95E-06	3.38E-05	2.46E-05	2.58E-05
C16orf71	1.47E-07	2.91E-07	1.43E-07	1.27E-07	4.45E-08	2.00E-07	1.34E-07	8.86E-08
C16orf72	1.60E-05	1.99E-05	9.49E-06	1.46E-05	4.33E-06	1.31E-05	1.11E-05	9.84E-06
C16orf73	5.77E-08	2.94E-08	1.51E-08	3.28E-08	0	1.33E-08	5.05E-08	1.05E-07
C16orf74	2.15E-06	2.87E-07	2.72E-07	1.03E-06	2.66E-07	2.01E-07	9.61E-07	1.83E-07
C16orf75	1.20E-05	6.65E-06	6.50E-07	2.15E-06	3.28E-06	4.45E-06	1.54E-06	1.86E-06
C16orf78	0	0	0	0	0	0	0	0
C16orf79	1.35E-06	1.93E-06	3.06E-07	5.16E-07	1.90E-06	4.19E-07	2.44E-07	5.82E-07
C16orf80	3.87E-05	2.39E-05	2.59E-05	3.13E-05	9.26E-06	3.77E-05	2.48E-05	2.34E-05
C16orf81	3.17E-08	1.19E-08	1.10E-08	1.29E-08	0	6.86E-08	1.02E-08	0
C16orf82	0	0	0	0	0	0	1.01E-08	0
C16orf86	4.02E-06	1.66E-06	2.27E-06	4.10E-06	6.01E-06	4.76E-06	2.91E-06	6.10E-07
C16orf87	1.62E-05	1.52E-05	1.18E-05	8.44E-06	1.39E-06	1.82E-05	5.95E-06	7.25E-06
C16orf88	8.57E-06	1.19E-05	3.82E-06	3.82E-06	1.74E-06	5.57E-06	5.43E-06	4.96E-06
C16orf89	4.44E-08	2.92E-08	1.74E-08	1.05E-06	1.13E-07	1.44E-07	1.58E-08	7.26E-08
C16orf90	0	0	0	3.76E-08	0	0	0	0
C16orf91	2.76E-05	2.37E-05	8.97E-06	1.38E-05	1.29E-05	1.80E-05	2.07E-05	1.63E-05
C16orf92	0	0	0	0	0	0	0	0
C16orf93	8.31E-07	3.23E-07	7.48E-08	5.13E-07	2.19E-07	5.99E-07	1.64E-07	3.95E-08
C17orf10(	2.96E-06	4.33E-07	2.67E-06	3.21E-06	9.43E-07	2.12E-06	2.15E-06	6.09E-07
C17orf101	1.64E-05	1.10E-05	1.63E-05	1.63E-05	3.19E-05	3.06E-05	2.40E-05	2.42E-05

C17orf102	0	6.38E-09	5.86E-09	6.96E-09	0	0	0	0
C17orf103	1.60E-06	1.53E-06	1.79E-06	2.15E-06	1.74E-06	4.12E-06	4.00E-06	3.08E-06
C17orf104	9.37E-08	2.09E-07	9.51E-08	2.26E-08	3.44E-08	1.66E-07	8.83E-08	7.23E-08
C17orf105	0	0	0	0	0	0	0	0
C17orf106	2.78E-05	2.10E-05	2.34E-05	2.85E-05	3.99E-05	2.94E-05	3.05E-05	2.35E-05
C17orf107	2.99E-06	7.82E-07	1.39E-06	2.43E-06	2.86E-06	1.78E-06	4.08E-06	1.69E-06
C17orf108	4.19E-06	1.37E-05	6.54E-06	6.09E-06	8.34E-06	6.05E-06	6.27E-06	8.04E-06
C17orf28	2.31E-05	1.01E-06	8.31E-07	3.11E-06	1.79E-06	6.67E-07	2.46E-06	4.65E-06
C17orf37	0.000158	0.000119	4.87E-05	5.30E-05	6.46E-05	0.000112	6.66E-05	7.58E-05
C17orf39	1.55E-06	3.95E-06	2.75E-06	1.87E-06	1.03E-06	3.61E-06	2.60E-06	2.52E-06
C17orf42	1.05E-05	1.07E-05	4.62E-06	3.93E-06	4.30E-06	6.10E-06	5.05E-06	2.78E-06
C17orf44	1.80E-06	6.18E-07	2.01E-06	1.19E-06	6.47E-07	3.42E-06	1.18E-06	1.47E-06
C17orf46	1.98E-08	1.27E-07	0	3.06E-08	0	3.69E-08	8.45E-08	2.93E-08
C17orf47	1.38E-07	2.73E-07	1.20E-08	1.41E-08	1.98E-08	1.07E-08	1.11E-08	5.07E-08
C17orf48	3.01E-06	2.84E-06	4.48E-06	6.76E-06	2.31E-06	1.10E-05	6.10E-06	6.07E-06
C17orf49	5.05E-05	2.78E-05	3.51E-05	5.72E-05	5.94E-05	7.51E-05	7.96E-05	7.90E-05
C17orf50	2.58E-08	0	0	0	0	0	0	0
C17orf51	1.03E-06	1.69E-06	4.98E-07	7.64E-07	7.93E-07	3.93E-07	2.21E-06	1.11E-06
C17orf53	2.03E-06	2.94E-06	2.24E-07	3.17E-07	3.04E-07	3.44E-07	3.56E-07	4.69E-07
C17orf54	0	0	0	0	0	0	1.54E-08	0
C17orf55	6.77E-06	2.54E-07	3.71E-07	1.01E-06	2.63E-06	3.54E-07	1.95E-06	1.99E-06
C17orf56	1.28E-05	1.19E-05	4.19E-06	6.71E-06	9.71E-06	8.78E-06	8.75E-06	7.28E-06
C17orf57	3.64E-06	1.85E-06	4.27E-07	3.83E-07	8.31E-08	5.76E-07	3.23E-07	7.96E-08
C17orf58	1.61E-05	2.87E-05	6.57E-06	6.77E-06	8.09E-06	1.61E-05	1.00E-05	1.08E-05
C17orf59	4.45E-06	6.04E-06	3.49E-06	4.26E-06	4.39E-06	6.88E-06	6.71E-06	6.26E-06
C17orf60	2.40E-06	5.92E-07	4.04E-07	2.64E-06	7.29E-07	1.25E-06	2.56E-06	2.23E-07
C17orf61	0.000141	0.000138	0.000201	0.000146	0.000154	0.000219	0.000235	0.000105
C17orf62	4.89E-05	4.66E-05	2.03E-05	2.52E-05	1.71E-05	4.23E-05	2.58E-05	3.35E-05
C17orf63	1.20E-05	6.47E-06	3.69E-06	5.14E-06	2.63E-06	4.38E-06	3.56E-06	5.77E-06
C17orf64	2.81E-07	3.27E-08	0	0	1.71E-07	7.88E-08	0	0
C17orf65	4.20E-06	1.57E-06	1.17E-06	1.59E-06	1.29E-06	3.96E-06	1.62E-06	1.44E-06
C17orf66	2.47E-08	0	1.72E-08	1.60E-08	0	2.43E-08	0	3.85E-08
C17orf67	1.33E-06	1.02E-06	4.69E-07	6.59E-07	5.31E-07	3.76E-07	8.40E-07	5.87E-07
C17orf68	1.96E-06	1.91E-06	2.44E-06	2.01E-06	5.75E-07	1.68E-06	2.05E-06	2.11E-06
C17orf69	1.86E-06	4.70E-07	1.31E-07	4.83E-07	4.10E-08	2.69E-08	1.22E-07	8.42E-08
C17orf70	1.30E-05	1.56E-05	9.23E-06	1.13E-05	1.29E-05	1.58E-05	1.28E-05	1.23E-05
C17orf71	1.11E-05	8.32E-06	3.62E-06	5.10E-06	1.46E-06	9.86E-06	4.25E-06	5.25E-06
C17orf72	4.11E-07	3.45E-07	3.43E-08	2.35E-07	4.07E-07	1.49E-07	4.57E-07	4.01E-07
C17orf73	5.21E-08	6.04E-08	0	0	0	0	0	0
C17orf74	0	0	0	0	0	0	0	0
C17orf75	1.12E-05	1.57E-05	3.80E-06	4.02E-06	2.36E-06	8.31E-06	3.45E-06	5.42E-06
C17orf76	5.88E-07	1.18E-07	1.16E-07	3.28E-07	7.52E-08	7.15E-08	1.61E-07	7.55E-08
C17orf77	0	0	0	1.14E-08	0	0	0	0
C17orf78	1.30E-08	1.47E-08	0	0	0	1.20E-08	1.25E-08	0
C17orf79	0.000101	8.92E-05	3.45E-05	5.57E-05	0.000112	3.21E-05	8.13E-05	4.23E-05
C17orf80	9.81E-06	8.64E-06	2.99E-06	3.33E-06	2.66E-06	7.73E-06	3.64E-06	2.68E-06
C17orf81	7.53E-05	1.65E-05	1.84E-05	1.96E-05	3.21E-05	3.35E-05	6.56E-05	5.24E-05
C17orf82	1.03E-06	9.21E-08	6.86E-08	9.93E-08	4.60E-07	1.07E-06	3.11E-07	4.78E-08
C17orf85	4.24E-06	3.27E-06	3.42E-06	5.08E-06	1.71E-06	5.63E-06	2.79E-06	3.56E-06
C17orf86	2.75E-06	1.83E-06	6.30E-07	9.67E-07	1.48E-06	1.83E-06	1.16E-06	6.13E-07
C17orf87	3.38E-06	3.11E-07	2.76E-06	3.49E-06	1.85E-07	1.47E-06	1.81E-06	4.09E-07
C17orf88	3.66E-08	0	0	0	0	0	0	0
C17orf89	9.82E-05	6.39E-05	3.48E-05	3.87E-05	0.000255	0.000144	8.60E-05	2.76E-05
C17orf90	3.31E-05	4.21E-05	1.51E-05	1.67E-05	5.26E-05	4.48E-05	2.29E-05	1.49E-05
C17orf91	7.81E-06	4.41E-06	5.63E-06	1.69E-05	4.53E-06	5.55E-06	5.12E-06	8.91E-06
C17orf93	0	0	0	0	0	0	0	0
C17orf95	5.67E-05	5.74E-05	1.74E-05	1.88E-05	2.92E-05	4.20E-05	2.24E-05	1.54E-05
C17orf96	1.60E-05	1.28E-07	1.18E-06	2.90E-06	1.35E-07	3.34E-06	2.94E-06	6.39E-07

C17orf97	5.03E-07	5.79E-06	7.96E-07	9.92E-07	5.18E-07	4.45E-06	1.81E-06	4.95E-07
C17orf98	5.73E-08	0	0	0	0	0	1.06E-07	0
C17orf99	2.94E-08	0	0	0	0	1.57E-07	0	1.44E-08
C18orf1	1.02E-06	1.48E-06	5.50E-06	3.18E-06	7.50E-07	1.99E-06	3.52E-06	3.13E-06
C18orf10	4.74E-05	1.22E-05	8.93E-06	1.79E-05	4.74E-06	2.06E-05	9.56E-06	1.34E-05
C18orf16	2.37E-07	4.57E-08	3.08E-08	0	1.02E-07	0	6.68E-08	7.57E-08
C18orf18	1.12E-05	4.03E-06	8.32E-06	7.34E-06	5.03E-06	1.40E-05	9.11E-06	5.55E-06
C18orf19	8.46E-06	1.14E-05	7.39E-06	5.04E-06	1.43E-06	4.51E-06	4.72E-06	4.25E-06
C18orf2	4.23E-07	0	2.68E-07	5.13E-08	1.03E-06	0	3.56E-07	0
C18orf20	0	0	0	5.72E-08	0	0	4.28E-08	0
C18orf21	1.81E-05	1.38E-05	7.30E-06	9.40E-06	5.50E-06	1.38E-05	1.18E-05	1.12E-05
C18orf22	2.83E-05	9.23E-06	1.40E-05	1.63E-05	1.09E-05	2.87E-05	3.19E-05	1.45E-05
C18orf25	6.39E-06	2.73E-06	3.07E-06	3.89E-06	6.18E-07	3.64E-06	2.65E-06	3.27E-06
C18orf26	0	0	0	0	0	3.50E-08	0	0
C18orf32	0.000153	9.95E-05	6.24E-05	7.54E-05	3.32E-05	8.33E-05	7.02E-05	7.81E-05
C18orf34	0	1.48E-07	0	0	0	0	6.28E-07	0
C18orf45	2.00E-06	1.60E-06	1.14E-06	1.33E-06	4.32E-07	2.19E-06	1.58E-06	1.16E-06
C18orf54	7.66E-07	1.05E-07	8.61E-08	9.32E-08	6.16E-08	4.21E-08	1.09E-07	2.09E-07
C18orf55	1.93E-05	1.69E-05	1.33E-05	9.75E-06	5.27E-06	1.39E-05	1.23E-05	1.45E-05
C18orf56	5.67E-06	7.77E-07	1.11E-06	1.97E-06	4.66E-06	4.34E-06	1.13E-06	8.93E-07
C18orf62	0	0	0	0	0	0	0	0
C18orf8	1.73E-05	1.47E-05	1.13E-05	1.19E-05	3.88E-06	1.23E-05	9.95E-06	1.23E-05
C19orf10	0.000184	8.48E-05	8.39E-05	0.000146	0.000219	0.000153	0.000246	0.000181
C19orf12	1.15E-05	1.21E-05	1.46E-05	1.57E-05	9.44E-06	1.02E-05	1.33E-05	1.21E-05
C19orf18	2.69E-07	2.09E-07	3.64E-07	3.34E-07	3.01E-06	2.80E-08	1.14E-07	3.13E-07
C19orf2	1.99E-05	2.24E-05	2.31E-05	2.23E-05	1.49E-05	3.15E-05	2.09E-05	1.72E-05
C19orf20	2.49E-06	6.17E-06	2.98E-06	8.48E-06	4.79E-06	4.46E-06	2.07E-05	2.72E-06
C19orf21	3.19E-05	3.39E-07	4.22E-08	4.98E-08	0	0	8.70E-08	0
C19orf22	5.67E-05	2.39E-05	1.87E-05	3.72E-05	4.04E-05	2.64E-05	3.62E-05	3.67E-05
C19orf23	1.38E-06	4.04E-07	2.78E-07	8.47E-07	6.74E-07	8.99E-07	2.14E-07	3.58E-07
C19orf24	0.000108	8.17E-05	6.99E-05	7.34E-05	0.000285	8.85E-05	8.16E-05	0.000117
C19orf25	1.99E-05	4.24E-06	5.73E-06	8.72E-06	2.96E-05	1.22E-05	1.29E-05	1.19E-05
C19orf26	3.47E-07	2.16E-07	0	5.25E-07	0	2.54E-08	1.77E-08	2.68E-08
C19orf28	2.97E-05	2.68E-05	1.24E-05	3.53E-05	3.68E-05	1.85E-05	5.95E-05	2.16E-05
C19orf29	7.43E-06	5.58E-06	4.67E-06	6.20E-06	5.50E-06	6.08E-06	7.96E-06	5.84E-06
C19orf30	0	0	0	0	3.26E-08	0	0	0
C19orf33	2.73E-05	1.12E-06	5.05E-08	7.16E-08	0	0	1.23E-06	7.61E-08
C19orf34	0	0	0	0	0	0	0	0
C19orf35	3.37E-07	3.08E-08	1.33E-07	7.81E-08	3.92E-08	5.91E-08	2.21E-07	5.35E-08
C19orf36	3.05E-06	1.70E-06	2.45E-06	1.96E-06	2.27E-06	3.97E-06	3.59E-07	1.09E-06
C19orf38	1.88E-06	1.39E-06	1.86E-06	2.11E-06	5.41E-07	1.28E-06	1.20E-06	8.23E-07
C19orf39	2.94E-06	1.61E-06	2.67E-06	2.62E-06	6.09E-06	3.98E-06	3.90E-06	3.76E-06
C19orf40	2.71E-06	1.43E-06	6.46E-07	5.67E-07	3.18E-07	1.11E-06	8.34E-07	9.55E-07
C19orf41	0	0	0	0	0	0	0	0
C19orf42	5.79E-05	3.16E-05	4.76E-05	4.73E-05	5.24E-05	5.29E-05	5.31E-05	6.76E-05
C19orf43	0.000142	6.41E-05	0.000102	0.000161	0.000253	0.000126	0.000289	0.00015
C19orf44	1.65E-06	1.27E-06	1.23E-06	2.22E-06	1.16E-06	1.98E-06	2.47E-06	1.25E-06
C19orf45	3.29E-08	0	0	0	1.00E-07	0	1.40E-08	0
C19orf46	6.77E-06	1.60E-05	5.11E-06	3.90E-06	1.14E-05	4.04E-06	9.24E-06	5.62E-06
C19orf47	5.31E-06	5.49E-06	4.22E-06	6.00E-06	4.98E-06	4.56E-06	6.12E-06	5.28E-06
C19orf48	2.95E-05	1.56E-05	1.17E-05	1.27E-05	4.47E-05	2.07E-05	3.51E-05	1.45E-05
C19orf50	6.41E-05	4.56E-05	2.86E-05	3.52E-05	3.20E-05	4.14E-05	5.00E-05	4.42E-05
C19orf51	3.82E-07	7.87E-08	2.06E-07	6.60E-07	2.45E-07	1.93E-07	6.55E-08	1.44E-07
C19orf52	9.12E-06	5.33E-06	4.92E-06	6.31E-06	3.22E-06	7.95E-06	9.31E-06	7.78E-06
C19orf53	0.000162	7.30E-05	5.99E-05	7.93E-05	0.000145	0.000101	0.00011	0.000105
C19orf54	7.33E-06	4.87E-06	2.91E-06	2.57E-06	3.74E-06	5.79E-06	3.37E-06	3.57E-06
C19orf55	1.15E-06	2.88E-07	3.88E-07	4.48E-07	5.52E-08	7.60E-07	3.38E-07	3.94E-07
C19orf56	0.000147	7.86E-05	9.86E-05	0.000134	0.000242	0.000176	0.000191	0.000101

C19orf57	9.36E-07	3.00E-06	2.91E-07	2.64E-07	1.13E-06	9.94E-07	5.85E-07	6.45E-07
C19orf59	1.30E-06	0	1.25E-07	7.16E-07	0	0	1.86E-08	0
C19orf6	6.27E-05	4.52E-05	4.48E-05	5.52E-05	0.000107	5.18E-05	8.50E-05	5.25E-05
C19orf60	2.32E-05	3.13E-05	2.10E-05	3.28E-05	7.84E-05	3.39E-05	5.42E-05	2.52E-05
C19orf61	1.81E-05	7.18E-06	3.53E-06	5.68E-06	1.23E-05	8.39E-06	8.84E-06	5.76E-06
C19orf62	5.38E-05	3.03E-05	3.32E-05	4.11E-05	6.37E-05	5.68E-05	5.17E-05	3.93E-05
C19orf63	0.000107	3.28E-05	6.94E-05	0.000102	6.23E-05	4.70E-05	9.14E-05	0.00014
C19orf66	0.000186	2.84E-05	5.72E-05	4.64E-05	4.66E-05	8.24E-05	6.29E-05	7.08E-05
C19orf69	0	0	0	0	0	0	2.73E-08	0
C19orf70	6.61E-05	5.45E-05	4.44E-05	6.08E-05	0.000206	8.30E-05	7.82E-05	6.64E-05
C19orf71	3.15E-07	4.85E-07	2.61E-07	3.94E-07	3.92E-08	1.69E-07	2.95E-07	0
C19orf73	3.24E-06	2.13E-06	2.40E-06	1.36E-06	5.44E-06	2.75E-06	2.32E-06	1.00E-06
C19orf75	0	0	0	0	0	0	0	0
C19orf76	1.92E-07	6.37E-08	8.26E-08	2.35E-07	3.01E-07	8.37E-08	4.25E-07	2.16E-07
C19orf77	5.80E-05	5.59E-06	4.94E-06	6.87E-05	5.22E-08	1.41E-07	1.15E-05	0
C1D	2.28E-05	1.83E-05	1.38E-05	1.27E-05	1.56E-05	1.77E-05	1.34E-05	1.15E-05
C1GALT1	2.26E-05	1.53E-05	6.79E-06	6.76E-06	4.03E-06	2.94E-05	6.11E-06	7.41E-06
C1GALT1(	3.03E-05	1.72E-05	1.87E-05	2.18E-05	2.04E-05	2.18E-05	1.49E-05	2.55E-05
C1orf100	0	0	0	0	0	0	0	0
C1orf101	5.06E-08	1.32E-07	8.66E-08	1.27E-07	5.43E-08	4.23E-07	2.96E-08	8.12E-08
C1orf103	1.27E-05	1.91E-05	8.69E-06	4.14E-06	3.91E-06	9.42E-06	4.15E-06	1.01E-05
C1orf104	1.71E-06	3.97E-06	1.95E-07	3.00E-07	1.55E-07	4.13E-07	1.89E-07	8.47E-07
C1orf105	6.58E-07	7.33E-07	1.20E-07	3.39E-08	0	1.38E-06	2.35E-07	2.86E-07
C1orf106	2.21E-05	1.11E-07	2.89E-07	3.47E-06	1.42E-08	5.10E-09	3.64E-07	4.27E-07
C1orf107	2.10E-06	3.25E-06	1.76E-06	1.92E-06	3.54E-07	9.47E-06	2.54E-06	3.97E-06
C1orf109	6.02E-06	6.06E-06	3.21E-06	2.54E-06	8.12E-07	5.98E-06	2.48E-06	3.90E-06
C1orf110	0	0	0	0	0	0	0	0
C1orf111	6.61E-08	1.52E-07	0	1.63E-07	0	2.05E-08	4.22E-08	6.54E-08
C1orf112	2.23E-06	3.84E-06	1.32E-06	1.55E-06	4.93E-07	2.27E-05	1.27E-06	1.69E-06
C1orf113	1.39E-06	1.18E-06	2.22E-07	6.18E-07	8.90E-08	2.44E-07	4.46E-07	1.82E-07
C1orf114	1.16E-07	1.47E-08	1.36E-08	3.33E-07	1.11E-08	0	0	3.81E-08
C1orf115	4.86E-05	5.43E-05	7.81E-05	5.64E-05	4.23E-05	7.57E-05	5.46E-05	8.43E-05
C1orf116	4.94E-06	1.40E-07	3.31E-07	3.87E-07	0	1.17E-08	3.71E-08	4.95E-08
C1orf122	3.27E-05	4.49E-05	1.78E-05	2.67E-05	3.02E-05	6.21E-05	2.96E-05	1.64E-05
C1orf123	3.50E-05	2.62E-05	2.14E-05	3.13E-05	2.55E-05	2.90E-05	3.53E-05	2.51E-05
C1orf124	3.35E-06	3.32E-06	1.90E-06	2.31E-06	4.25E-07	2.93E-06	1.31E-06	2.60E-06
C1orf125	4.17E-08	2.24E-08	0	0	0	0	0	0
C1orf126	1.51E-07	7.25E-09	1.96E-08	1.92E-07	6.33E-09	1.39E-08	2.67E-08	0
C1orf127	1.16E-07	5.98E-08	1.20E-07	2.31E-07	3.64E-08	8.84E-08	2.73E-07	0
C1orf128	2.95E-05	1.99E-05	2.15E-05	2.63E-05	1.16E-05	2.77E-05	2.98E-05	2.13E-05
C1orf129	0	1.19E-08	0	0	0	0	0	0
C1orf130	8.30E-07	4.08E-06	2.05E-06	1.36E-06	7.82E-07	8.54E-08	1.64E-06	1.27E-07
C1orf131	1.16E-05	2.02E-05	5.49E-06	6.47E-06	2.84E-06	2.35E-05	6.87E-06	1.04E-05
C1orf133	3.90E-07	8.35E-08	0	1.32E-07	2.16E-07	1.33E-07	4.35E-07	2.66E-07
C1orf135	8.57E-07	7.21E-07	1.82E-07	1.56E-07	1.99E-07	1.29E-07	1.46E-07	1.70E-07
C1orf14	0	0	0	0	0	0	0	0
C1orf141	0	0	0	0	0	0	0	0
C1orf144	6.13E-05	2.25E-05	1.96E-05	2.60E-05	1.12E-05	3.73E-05	3.15E-05	3.03E-05
C1orf146	0	0	0	0	0	0	0	0
C1orf150	1.79E-08	1.01E-08	9.29E-09	3.28E-08	0	0	0	5.25E-08
C1orf151	3.28E-05	2.28E-05	8.82E-06	1.60E-05	1.45E-05	1.91E-05	1.46E-05	1.21E-05
C1orf152	1.30E-06	4.01E-07	9.67E-07	6.29E-07	7.32E-08	3.55E-07	3.95E-08	1.27E-06
C1orf156	4.12E-06	7.74E-06	2.23E-06	2.43E-06	2.30E-06	2.98E-06	5.65E-06	5.84E-06
C1orf157	1.53E-08	0	0	0	0	0	1.57E-08	0
C1orf158	5.23E-08	0	0	0	0	0	0	1.17E-07
C1orf159	3.90E-06	2.10E-06	1.74E-06	3.02E-06	1.79E-06	2.22E-06	2.42E-06	3.15E-06
C1orf161	1.57E-07	7.93E-07	3.10E-07	6.72E-07	2.45E-08	8.46E-07	4.16E-08	2.07E-06
C1orf162	9.26E-06	1.56E-06	1.39E-05	2.88E-05	3.49E-06	1.23E-05	1.83E-05	4.63E-06

C1orf163	9.88E-06	1.37E-05	5.62E-06	4.47E-06	8.92E-07	1.04E-05	4.81E-06	1.13E-05
C1orf168	1.31E-06	5.65E-06	7.44E-06	6.43E-06	2.14E-06	3.52E-06	4.24E-06	1.53E-06
C1orf170	1.83E-06	7.42E-08	2.70E-08	9.01E-07	9.13E-08	2.36E-07	5.87E-07	2.51E-07
C1orf172	5.20E-06	3.48E-06	2.95E-06	4.07E-06	1.83E-06	8.20E-07	2.18E-06	1.20E-07
C1orf173	0	7.99E-08	1.05E-07	2.66E-08	8.01E-09	1.73E-07	1.21E-08	2.28E-08
C1orf174	1.91E-05	1.27E-05	9.10E-06	9.96E-06	4.59E-06	9.92E-06	1.00E-05	1.00E-05
C1orf175	1.98E-07	1.37E-07	5.16E-07	1.83E-07	3.79E-08	3.78E-08	2.11E-07	6.69E-08
C1orf177	7.09E-07	2.58E-07	4.54E-08	7.04E-08	8.65E-08	7.99E-08	1.52E-07	1.81E-07
C1orf180	0	9.19E-09	0	0	0	2.98E-08	0	0
C1orf182	3.17E-07	2.40E-06	9.72E-08	2.81E-07	9.71E-07	6.88E-06	1.41E-06	9.57E-07
C1orf183	1.77E-06	6.66E-07	1.41E-07	1.11E-06	2.87E-07	7.57E-07	1.05E-06	3.84E-07
C1orf185	0	0	0	0	0	2.81E-08	0	0
C1orf186	1.03E-06	6.68E-08	6.21E-08	2.89E-07	2.53E-07	2.73E-08	5.66E-08	8.67E-08
C1orf187	0	0	1.44E-08	1.68E-08	2.36E-08	1.27E-08	1.32E-08	0
C1orf189	0	0	0	1.41E-07	9.72E-08	1.05E-07	9.59E-08	0
C1orf190	9.21E-07	1.31E-07	1.62E-08	6.75E-08	0	3.22E-07	1.02E-06	1.07E-07
C1orf192	1.52E-06	2.80E-06	1.89E-06	8.76E-07	1.93E-07	1.18E-06	7.00E-07	1.06E-06
C1orf194	0	0	0	0	0	3.75E-08	0	1.46E-07
C1orf198	4.41E-05	2.50E-05	3.32E-06	1.89E-05	2.44E-06	1.01E-05	1.55E-05	1.50E-05
C1orf200	2.66E-08	0	5.59E-08	1.63E-08	0	4.93E-08	2.56E-08	5.87E-08
C1orf201	1.86E-06	1.88E-06	6.77E-07	2.71E-06	1.10E-06	1.12E-06	2.12E-06	9.71E-07
C1orf203	2.89E-06	5.54E-06	1.63E-06	1.56E-06	1.59E-06	7.09E-06	9.32E-07	9.88E-07
C1orf204	7.56E-07	5.12E-08	2.59E-08	1.91E-07	0	1.67E-07	4.48E-08	2.27E-08
C1orf21	2.38E-06	1.38E-06	3.23E-06	3.07E-06	7.02E-07	5.14E-06	1.26E-06	6.73E-07
C1orf210	6.80E-06	6.01E-06	3.68E-06	7.83E-06	1.34E-06	7.69E-07	2.71E-06	3.82E-06
C1orf212	2.64E-05	2.22E-05	1.80E-05	2.46E-05	1.45E-05	2.50E-05	1.69E-05	2.15E-05
C1orf213	7.05E-07	2.45E-07	1.26E-07	2.10E-07	2.24E-07	4.61E-07	2.11E-07	1.16E-07
C1orf216	6.59E-06	3.02E-06	2.31E-06	3.40E-06	3.67E-06	2.68E-06	2.96E-06	4.23E-06
C1orf220	4.18E-07	1.53E-07	7.55E-08	4.45E-08	2.73E-07	8.42E-08	0	2.67E-08
C1orf223	1.52E-07	6.14E-08	0	6.37E-08	0	1.43E-07	9.46E-08	1.47E-07
C1orf226	1.99E-06	1.25E-05	3.44E-06	2.21E-06	1.62E-06	1.13E-05	6.60E-06	9.44E-06
C1orf227	1.38E-07	6.04E-07	0	0	6.08E-08	3.93E-07	5.06E-07	2.13E-07
C1orf228	7.83E-07	9.06E-07	5.81E-07	6.23E-07	4.00E-07	5.52E-07	2.10E-07	1.07E-06
C1orf229	4.51E-07	1.18E-07	7.66E-08	1.54E-07	9.02E-09	1.65E-07	1.12E-07	2.47E-07
C1orf230	8.09E-08	0	0	0	0	4.64E-07	0	0
C1orf25	7.37E-06	1.09E-05	6.59E-06	4.26E-06	1.40E-06	1.28E-05	3.98E-06	7.65E-06
C1orf26	1.90E-06	1.75E-06	9.06E-07	8.53E-07	3.57E-07	2.26E-06	7.84E-07	9.35E-07
C1orf27	1.55E-05	1.17E-05	7.21E-06	6.42E-06	3.08E-06	1.29E-05	2.81E-06	1.10E-05
C1orf31	5.42E-05	4.72E-05	2.26E-05	2.71E-05	2.54E-05	0.000135	3.19E-05	6.11E-05
C1orf35	1.20E-05	1.39E-05	4.14E-06	6.24E-06	8.72E-06	1.15E-05	1.29E-05	1.42E-05
C1orf38	1.72E-05	2.36E-06	7.05E-06	2.00E-05	1.29E-06	7.74E-06	1.01E-05	3.90E-06
C1orf43	0.000196	0.000322	0.000152	0.000151	0.000157	0.000334	0.000198	0.000282
C1orf49	3.93E-08	1.54E-08	0	0	0	0	0	0
C1orf50	9.56E-06	6.83E-06	6.91E-06	9.10E-06	6.98E-06	1.19E-05	9.84E-06	8.43E-06
C1orf51	6.81E-07	3.68E-06	5.22E-06	2.18E-06	1.83E-06	6.28E-06	1.01E-05	5.53E-06
C1orf52	7.32E-06	1.25E-05	3.73E-06	6.40E-06	1.10E-06	6.35E-06	4.43E-06	5.16E-06
C1orf53	4.31E-06	1.71E-05	9.57E-06	1.03E-05	8.90E-06	2.47E-05	2.06E-05	1.12E-05
C1orf54	2.90E-05	2.45E-06	8.23E-06	1.16E-05	7.15E-06	1.04E-05	2.12E-05	9.49E-06
C1orf55	1.14E-05	1.15E-05	6.64E-06	9.52E-06	8.19E-07	1.52E-05	4.42E-06	1.47E-05
C1orf56	6.43E-06	7.86E-06	6.15E-06	4.38E-06	6.10E-06	8.86E-06	6.86E-06	7.34E-06
C1orf57	3.88E-05	3.07E-05	2.98E-05	3.12E-05	1.57E-05	6.24E-05	2.81E-05	7.31E-05
C1orf58	1.09E-05	1.25E-05	4.45E-06	2.96E-06	5.60E-07	1.09E-05	2.19E-06	5.80E-06
C1orf59	1.54E-06	1.96E-07	6.14E-07	2.12E-06	4.46E-07	3.30E-06	1.09E-06	4.50E-07
C1orf61	3.61E-06	2.61E-06	0	7.35E-08	2.03E-07	1.42E-07	1.51E-08	2.15E-08
C1orf63	2.31E-05	5.55E-06	4.16E-06	4.73E-06	5.73E-06	7.02E-06	5.09E-06	7.84E-06
C1orf64	0	1.04E-07	3.29E-08	4.79E-07	0	1.16E-05	2.83E-08	0
C1orf65	1.14E-08	0	0	2.78E-08	9.78E-09	0	0	5.01E-08
C1orf66	1.36E-05	2.44E-05	1.37E-05	1.14E-05	8.11E-06	2.28E-05	1.48E-05	1.88E-05

C1orf68	0	0	0	0	0	0	0	0
C1orf69	1.53E-06	1.37E-06	1.34E-06	1.38E-06	9.63E-07	1.87E-06	2.59E-06	2.61E-06
C1orf70	2.80E-07	1.35E-07	1.77E-07	2.89E-07	1.01E-07	0	8.94E-08	1.39E-07
C1orf74	3.54E-06	2.30E-06	1.14E-06	1.18E-06	3.27E-07	5.14E-06	1.16E-06	2.68E-06
C1orf77	6.25E-05	5.63E-05	2.36E-05	3.84E-05	2.22E-05	4.09E-05	4.25E-05	3.30E-05
C1orf83	2.14E-06	1.05E-06	8.04E-07	6.92E-07	2.10E-07	1.72E-06	9.36E-07	8.31E-07
C1orf84	5.91E-07	5.44E-07	2.64E-07	2.37E-07	9.38E-08	1.51E-07	2.33E-07	2.63E-07
C1orf85	7.12E-05	0.000131	5.41E-05	6.53E-05	6.05E-05	0.000141	6.55E-05	0.000105
C1orf86	2.75E-05	3.42E-05	2.22E-05	3.00E-05	5.75E-05	2.45E-05	3.84E-05	1.70E-05
C1orf87	0	0	0	2.92E-08	0	2.21E-08	1.15E-08	7.70E-07
C1orf88	4.72E-07	0	4.40E-08	3.27E-07	0	1.01E-08	9.80E-08	3.20E-08
C1orf89	1.57E-06	9.01E-07	1.04E-06	5.83E-07	3.41E-07	1.76E-06	1.22E-06	1.47E-06
C1orf9	1.67E-05	6.99E-06	2.71E-06	4.85E-06	1.11E-06	9.26E-06	4.16E-06	1.02E-05
C1orf91	1.12E-05	4.15E-06	2.90E-06	5.28E-06	2.86E-06	7.54E-06	2.97E-06	6.42E-06
C1orf92	2.43E-08	1.38E-08	0	1.49E-08	0	1.13E-08	1.18E-08	0
C1orf93	1.69E-05	1.08E-05	3.12E-06	8.68E-06	2.11E-06	4.11E-06	8.59E-06	1.29E-05
C1orf94	0	0	0	0	0	0	0	0
C1orf95	1.17E-07	3.12E-08	5.73E-09	6.80E-09	4.78E-09	5.16E-09	1.08E-08	7.34E-08
C1orf96	2.84E-06	1.05E-06	7.49E-07	7.20E-07	2.68E-07	7.95E-07	5.96E-07	1.26E-06
C1orf97	1.92E-05	3.99E-05	1.02E-05	7.94E-06	6.18E-06	2.54E-05	8.80E-06	2.53E-05
C1QA	0.000361	4.18E-05	0.000221	0.000427	9.96E-05	0.000225	0.000418	6.62E-05
C1QB	0.000443	4.73E-05	0.000271	0.000459	5.75E-05	0.000237	0.000426	6.14E-05
C1QBP	5.96E-05	7.41E-05	5.33E-05	5.97E-05	1.57E-05	0.000119	0.000116	7.05E-05
C1QC	0.000284	2.96E-05	0.000193	0.000303	2.57E-05	0.000164	0.000294	4.15E-05
C1QL1	0.00013	0	1.76E-07	1.53E-06	1.14E-07	7.69E-08	4.78E-08	7.34E-07
C1QL2	0	1.32E-08	0	0	1.00E-08	2.16E-08	0	0
C1QL3	1.04E-07	5.94E-07	2.35E-07	2.30E-07	4.05E-08	1.66E-07	1.00E-07	3.60E-07
C1QL4	2.75E-06	0	1.21E-08	2.83E-08	2.09E-07	0	1.12E-08	3.23E-07
C1QTNF1	8.11E-06	1.71E-06	2.25E-05	5.99E-05	4.61E-06	1.98E-06	5.52E-06	5.46E-06
C1QTNF2	5.63E-07	8.91E-08	1.03E-07	3.14E-07	2.55E-07	1.01E-07	1.15E-07	2.18E-07
C1QTNF3	1.43E-06	4.46E-07	3.96E-07	6.96E-07	2.34E-07	1.13E-05	5.16E-07	6.42E-07
C1QTNF4	3.54E-08	2.02E-08	0	6.53E-08	2.29E-07	6.58E-08	9.19E-07	2.62E-08
C1QTNF6	8.34E-06	5.32E-06	2.24E-06	3.49E-06	1.08E-05	4.99E-06	2.62E-06	5.12E-06
C1QTNF7	2.62E-07	8.76E-08	3.81E-07	4.72E-07	4.30E-08	1.93E-08	5.08E-08	6.88E-08
C1QTNF8	1.35E-08	0	0	0	0	0	0	0
C1QTNF9	5.31E-08	1.72E-07	1.04E-07	1.14E-07	9.15E-08	1.93E-07	7.69E-08	3.91E-08
C1QTNF9I	0	1.06E-07	1.86E-07	5.05E-08	0	0	0	0
C1R	0.000522	0.000919	0.00148	0.001428	0.000797	0.001489	0.001354	0.00083
C1RL	5.10E-05	6.66E-05	0.000128	7.90E-05	3.71E-05	9.35E-05	6.90E-05	7.68E-05
C1S	0.000877	0.001592	0.001871	0.001743	0.000697	0.002847	0.001269	0.00136
C2	0.000324	0.000897	0.000448	0.000447	0.000457	0.000336	0.000563	0.000717
C20orf103	2.13E-06	4.65E-08	4.32E-08	9.73E-07	2.57E-08	0	1.45E-07	4.40E-08
C20orf106	1.35E-07	0	0	1.12E-07	3.92E-08	0	1.26E-07	0
C20orf107	0	0	4.97E-08	0	4.12E-07	4.18E-08	0	4.03E-07
C20orf108	3.68E-05	6.26E-05	4.83E-05	3.19E-05	2.48E-05	7.72E-05	3.08E-05	3.27E-05
C20orf11	2.29E-05	1.41E-05	1.07E-05	1.03E-05	1.11E-05	1.09E-05	1.64E-05	1.31E-05
C20orf111	5.18E-05	2.21E-05	1.75E-05	1.50E-05	1.12E-05	3.89E-05	1.61E-05	2.66E-05
C20orf112	9.50E-06	1.72E-06	1.65E-06	2.04E-06	1.55E-06	1.68E-06	2.18E-06	1.92E-06
C20orf114	8.46E-08	3.20E-08	2.50E-08	0	0	1.96E-07	5.44E-08	0
C20orf117	4.00E-06	2.03E-07	2.89E-07	5.67E-07	7.30E-07	3.26E-07	9.80E-07	3.44E-07
C20orf118	1.98E-05	6.77E-08	1.73E-07	2.33E-07	4.19E-08	2.90E-07	1.83E-06	3.52E-08
C20orf12	6.88E-07	3.99E-07	1.22E-07	5.00E-07	1.29E-07	3.54E-07	2.41E-07	1.12E-07
C20orf123	2.34E-08	0	0	1.43E-08	1.01E-08	3.26E-08	0	0
C20orf132	3.33E-07	7.76E-07	9.41E-07	6.03E-07	5.88E-07	1.11E-06	7.28E-07	1.16E-07
C20orf134	5.19E-07	1.34E-08	9.89E-08	3.62E-07	4.06E-08	1.75E-07	5.13E-07	5.21E-08
C20orf135	3.35E-06	1.12E-06	1.63E-06	2.45E-06	3.31E-06	3.76E-06	1.53E-06	1.59E-06
C20orf141	0	0	0	0	0	0	0	0
C20orf144	0	0	0	0	0	0	0	0

C20orf151	0	8.43E-08	1.73E-08	1.02E-08	0	0	1.62E-08	0
C20orf152	1.35E-07	6.97E-08	3.59E-08	3.37E-08	2.12E-07	1.57E-08	8.14E-08	4.99E-08
C20orf160	2.40E-06	1.93E-06	8.30E-07	6.61E-07	2.16E-06	1.49E-06	3.45E-06	1.54E-06
C20orf165	3.35E-06	1.56E-06	5.16E-07	4.59E-07	9.98E-07	8.45E-07	1.15E-07	6.79E-07
C20orf166	0	0	0	2.59E-08	0	0	0	0
C20orf173	0	0	0	0	0	0	1.09E-08	0
C20orf177	4.76E-06	1.12E-06	6.99E-07	4.55E-07	4.92E-09	1.47E-06	3.04E-07	8.10E-07
C20orf185	0	0	0	0	0	0	0	0
C20orf186	0	0	0	0	0	1.01E-08	0	0
C20orf191	0	1.23E-06	0	0	0	9.01E-08	0	0
C20orf194	5.44E-06	5.95E-07	1.16E-06	2.10E-06	4.64E-07	1.50E-06	1.66E-06	8.55E-07
C20orf195	4.71E-08	2.72E-08	5.11E-08	2.80E-08	2.04E-08	2.20E-08	4.50E-08	0
C20orf196	2.77E-06	1.17E-06	4.62E-07	1.67E-06	1.46E-06	3.69E-06	1.10E-06	1.34E-06
C20orf197	5.12E-08	0	2.65E-08	2.09E-08	7.33E-09	4.74E-08	1.65E-08	3.76E-08
C20orf199	0.000107	2.81E-05	2.99E-05	6.41E-05	5.82E-05	7.77E-05	0.000118	4.46E-05
C20orf20	2.64E-05	9.49E-06	5.51E-06	7.10E-06	1.14E-05	1.00E-05	1.31E-05	1.12E-05
C20orf200	3.92E-08	9.18E-07	0	1.26E-08	7.78E-09	8.39E-09	8.78E-09	0
C20orf201	1.28E-07	3.73E-08	0	0	0	0	3.02E-08	0
C20orf202	6.29E-07	9.78E-07	1.30E-07	1.13E-07	7.93E-08	4.28E-08	1.03E-07	9.06E-08
C20orf203	0	0	0	0	5.06E-09	0	0	8.64E-09
C20orf24	0.000278	0.000277	6.83E-05	9.60E-05	8.54E-05	0.000151	0.000117	0.000133
C20orf26	0	4.56E-08	0	1.64E-08	0	0	5.42E-09	0
C20orf27	3.50E-05	2.77E-05	1.34E-05	1.21E-05	2.99E-05	2.66E-05	3.13E-05	1.63E-05
C20orf29	1.87E-05	6.95E-06	6.55E-06	6.07E-06	8.74E-06	9.66E-06	7.63E-06	9.34E-06
C20orf3	0.000147	0.000157	0.000193	0.00017	0.000104	0.000341	0.00013	0.00029
C20orf30	0.000179	0.000169	0.000103	8.35E-05	7.81E-05	0.000134	9.73E-05	7.07E-05
C20orf4	3.31E-05	3.02E-05	1.47E-05	2.05E-05	1.97E-05	2.28E-05	2.15E-05	2.04E-05
C20orf43	0.000107	8.56E-05	4.09E-05	5.23E-05	3.82E-05	7.15E-05	4.78E-05	4.73E-05
C20orf46	1.12E-05	5.19E-06	1.40E-06	2.03E-06	1.57E-06	9.17E-07	1.75E-06	6.64E-06
C20orf54	5.54E-06	1.27E-07	8.55E-08	5.75E-07	1.04E-07	5.13E-07	2.54E-07	8.11E-07
C20orf56	5.07E-05	2.41E-05	7.20E-05	3.41E-05	2.47E-05	2.66E-05	3.89E-05	2.24E-05
C20orf7	2.17E-05	1.56E-05	5.64E-06	8.64E-06	9.22E-06	9.42E-06	1.60E-05	1.36E-05
C20orf70	7.53E-08	2.91E-08	0	0	0	0	4.79E-08	7.47E-08
C20orf71	0	0	0	0	0	0	0	0
C20orf72	2.58E-05	9.65E-06	5.81E-06	5.76E-06	4.54E-06	1.14E-05	8.18E-06	4.90E-06
C20orf79	0	0	0	0	0	0	0	0
C20orf85	0	0	0	0	0	0	0	0
C20orf94	1.11E-06	8.76E-07	3.61E-07	1.97E-07	9.23E-08	5.14E-07	3.94E-07	3.69E-07
C20orf96	5.49E-06	4.24E-06	1.01E-06	2.06E-06	2.13E-06	1.88E-06	2.62E-06	1.61E-06
C21orf119	8.50E-06	5.78E-06	6.55E-06	4.96E-06	1.26E-05	1.44E-05	4.23E-06	2.00E-06
C21orf121	1.57E-07	0	4.07E-08	0	4.05E-08	2.19E-08	1.53E-08	2.31E-08
C21orf122	5.57E-07	3.68E-07	4.93E-07	3.99E-07	6.14E-07	1.37E-06	3.96E-07	5.10E-07
C21orf125	7.52E-06	1.96E-06	3.99E-08	1.89E-07	1.06E-07	3.72E-07	1.50E-08	4.30E-07
C21orf128	0	0	0	0	0	0	1.07E-08	1.63E-08
C21orf129	1.18E-08	0	2.46E-08	1.44E-08	1.01E-08	4.37E-08	4.55E-08	5.19E-08
C21orf130	0	1.34E-08	1.24E-08	5.81E-08	2.04E-08	0	1.15E-08	0
C21orf131	0	0	0	0	0	0	0	0
C21orf15	9.95E-08	0	2.14E-08	4.90E-08	1.72E-08	1.85E-08	3.82E-08	2.06E-07
C21orf2	6.41E-06	1.35E-05	1.00E-05	1.12E-05	1.75E-05	7.60E-06	1.14E-05	9.48E-06
C21orf29	5.74E-09	4.57E-07	5.91E-08	2.10E-08	4.44E-08	1.91E-07	1.68E-08	2.52E-08
C21orf33	0.000103	0.000111	0.000158	0.000102	0.000143	0.000178	0.000145	0.000178
C21orf34	1.28E-06	1.58E-07	6.11E-07	3.12E-07	2.33E-07	3.08E-07	5.52E-07	2.74E-07
C21orf45	7.86E-06	5.67E-06	2.30E-06	3.48E-06	2.06E-06	3.37E-06	3.93E-06	2.74E-06
C21orf49	3.04E-07	2.02E-07	3.28E-07	2.41E-07	2.06E-07	3.64E-07	1.14E-07	4.46E-08
C21orf54	0	0	0	0	0	0	0	0
C21orf56	8.82E-06	1.46E-06	5.45E-07	1.86E-06	1.23E-06	3.70E-06	1.09E-05	3.60E-06
C21orf57	3.12E-05	2.13E-05	7.98E-06	1.50E-05	5.32E-05	2.50E-05	3.68E-05	1.46E-05
C21orf58	2.02E-06	4.34E-07	1.97E-07	1.01E-07	3.01E-07	9.53E-07	1.60E-07	2.96E-07

C21orf59	3.22E-05	1.79E-05	1.65E-05	1.60E-05	1.51E-05	2.76E-05	2.46E-05	2.30E-05
C21orf62	1.69E-08	1.26E-08	1.42E-07	1.40E-08	0	0	0	0
C21orf63	5.08E-06	3.19E-06	1.41E-06	1.09E-05	2.46E-06	7.30E-06	1.37E-05	2.11E-06
C21orf67	1.56E-06	2.69E-06	1.82E-06	2.37E-06	3.66E-06	3.42E-06	2.82E-06	1.65E-06
C21orf7	6.37E-06	2.41E-06	1.20E-06	2.81E-06	3.04E-06	7.72E-06	4.74E-06	3.85E-06
C21orf70	1.45E-05	9.05E-06	1.17E-05	1.81E-05	3.28E-05	1.38E-05	2.24E-05	1.35E-05
C21orf71	1.66E-07	2.13E-07	0	0	7.35E-08	1.58E-07	3.75E-07	1.29E-07
C21orf81	9.18E-09	1.03E-08	0	0	7.89E-09	0	0	1.35E-08
C21orf82	1.47E-07	6.91E-08	2.06E-07	5.89E-07	1.80E-07	0	2.22E-07	2.97E-08
C21orf84	9.25E-05	2.03E-07	2.00E-07	7.67E-07	6.99E-06	1.05E-07	5.14E-07	2.03E-06
C21orf88	3.59E-08	0	1.65E-07	2.45E-07	0	0	0	0
C21orf90	2.77E-08	2.26E-07	1.22E-07	6.86E-08	4.39E-07	1.04E-06	7.92E-08	1.24E-07
C21orf91	2.60E-06	9.30E-07	5.87E-06	2.54E-06	3.34E-07	1.36E-06	1.41E-06	1.36E-06
C21orf94	0	0	0	0	0	0	0	0
C21orf96	1.67E-08	0	0	0	0	4.65E-08	0	0
C21orf99	0	0	0	0	0	0	0	9.85E-09
C22orf13	9.02E-05	3.50E-05	5.09E-05	7.34E-05	3.96E-05	0.000106	8.68E-05	0.000113
C22orf15	1.24E-07	1.21E-07	3.72E-08	4.74E-08	3.87E-08	6.59E-08	0	5.23E-08
C22orf23	8.82E-07	2.06E-07	3.45E-07	4.65E-07	1.24E-07	3.68E-07	5.71E-07	2.93E-07
C22orf24	0	0	0	5.05E-08	0	0	2.11E-08	0
C22orf25	2.17E-05	1.35E-05	5.69E-06	9.33E-06	5.35E-06	1.09E-05	1.05E-05	9.35E-06
C22orf26	2.63E-07	1.49E-07	4.15E-08	1.45E-07	5.67E-08	2.20E-07	1.91E-07	1.36E-07
C22orf27	3.06E-06	2.01E-06	1.82E-06	2.30E-06	2.14E-06	3.55E-06	2.42E-06	2.55E-06
C22orf28	6.18E-05	5.04E-05	3.88E-05	5.16E-05	2.54E-05	6.50E-05	5.14E-05	6.85E-05
C22orf29	4.20E-06	2.10E-06	9.56E-07	1.24E-06	6.28E-06	3.49E-06	3.76E-06	2.07E-06
C22orf30	4.15E-06	2.69E-06	1.42E-06	1.61E-06	6.34E-07	2.54E-06	1.68E-06	3.13E-06
C22orf31	2.46E-08	5.69E-08	0	9.11E-08	2.13E-08	0	0	0
C22orf32	2.91E-05	4.12E-05	2.55E-05	2.49E-05	4.09E-05	3.53E-05	3.10E-05	2.89E-05
C22orf33	0	0	0	0	0	2.41E-08	0	0
C22orf34	3.19E-08	1.32E-07	1.80E-08	0	1.46E-08	1.97E-08	0	2.50E-08
C22orf36	1.22E-05	1.10E-05	1.46E-05	1.59E-05	3.32E-05	9.22E-06	1.38E-05	1.32E-05
C22orf39	1.55E-05	2.22E-05	1.03E-05	1.18E-05	2.21E-05	1.37E-05	1.73E-05	7.59E-06
C22orf40	8.05E-06	8.02E-06	4.32E-06	3.83E-06	1.05E-05	6.72E-06	7.50E-06	6.71E-06
C22orf41	1.27E-06	2.25E-06	1.37E-06	2.33E-07	1.86E-06	2.44E-06	5.71E-07	1.57E-06
C22orf42	0	0	0	0	0	0	0	0
C22orf43	1.19E-07	0	0	4.90E-08	5.16E-08	0	5.73E-08	0
C22orf45	1.00E-05	2.95E-05	2.16E-05	1.20E-05	1.85E-05	2.23E-05	1.94E-05	4.78E-06
C22orf46	3.84E-06	2.99E-06	1.56E-06	2.05E-06	1.52E-06	4.69E-06	2.35E-06	3.16E-06
C22orf9	1.65E-05	1.42E-05	6.92E-06	1.35E-05	9.19E-06	6.79E-06	1.84E-05	1.49E-05
C2CD2	6.00E-06	2.08E-06	2.72E-06	3.64E-06	1.48E-06	5.89E-06	3.87E-06	4.63E-06
C2CD2L	3.15E-06	2.09E-06	2.93E-06	3.75E-06	1.35E-06	3.46E-06	3.43E-06	6.47E-06
C2CD3	5.10E-06	2.37E-06	1.36E-06	2.92E-06	6.80E-07	3.50E-06	1.60E-06	3.46E-06
C2CD4A	1.54E-06	2.25E-07	3.65E-07	1.57E-06	1.72E-08	8.04E-08	4.80E-07	3.91E-08
C2CD4B	1.17E-05	3.74E-07	1.49E-07	2.77E-06	3.50E-07	7.26E-08	1.37E-06	9.23E-08
C2CD4C	8.43E-07	9.14E-08	1.68E-07	1.18E-07	1.27E-07	5.48E-08	1.22E-07	0
C2CD4D	3.84E-07	4.85E-08	3.00E-08	5.24E-08	7.35E-08	6.61E-08	1.90E-07	0
C2orf14	1.47E-07	1.57E-08	0	0	0	0	0	0
C2orf15	2.15E-06	3.66E-07	3.04E-07	3.26E-07	1.54E-07	4.43E-07	1.37E-07	7.90E-08
C2orf16	3.39E-07	7.62E-07	1.37E-07	1.76E-07	1.42E-07	4.51E-07	1.55E-07	2.59E-07
C2orf18	3.14E-05	4.90E-05	1.11E-05	1.52E-05	1.05E-05	1.15E-05	2.44E-05	3.54E-05
C2orf24	9.12E-05	2.57E-05	2.79E-05	3.43E-05	2.52E-05	3.85E-05	4.45E-05	3.28E-05
C2orf27A	9.47E-07	9.67E-08	1.59E-07	2.07E-07	2.54E-07	1.96E-07	2.22E-07	3.12E-07
C2orf27B	0	0	0	0	1.65E-08	0	0	0
C2orf28	0.000196	0.000292	4.36E-05	7.36E-05	8.21E-05	0.000124	8.39E-05	8.88E-05
C2orf29	4.73E-05	2.26E-05	1.14E-05	1.42E-05	9.23E-06	1.59E-05	1.53E-05	1.49E-05
C2orf3	9.05E-06	7.69E-06	6.59E-06	4.42E-06	2.68E-06	8.59E-06	4.32E-06	4.34E-06
C2orf34	3.61E-06	1.73E-06	1.91E-06	1.70E-06	1.52E-06	3.09E-06	1.03E-06	2.33E-06
C2orf39	4.71E-08	2.44E-08	0	2.79E-08	8.10E-09	1.14E-07	1.83E-08	0

C2orf40	3.33E-07	3.93E-07	8.38E-08	2.03E-06	9.65E-08	2.08E-07	2.10E-07	1.67E-07
C2orf42	5.13E-06	5.07E-06	4.97E-06	4.07E-06	4.11E-06	6.59E-06	4.36E-06	3.54E-06
C2orf43	1.41E-05	1.67E-05	3.91E-06	4.42E-06	2.98E-06	9.10E-06	6.07E-06	4.03E-06
C2orf44	4.38E-06	3.93E-06	1.43E-06	1.15E-06	1.21E-06	3.78E-06	1.79E-06	1.96E-06
C2orf47	3.41E-05	5.33E-05	3.37E-05	2.02E-05	2.34E-05	2.86E-05	2.61E-05	2.35E-05
C2orf48	2.53E-08	2.73E-07	0	4.66E-08	4.36E-08	9.40E-08	3.67E-08	0
C2orf49	2.16E-05	6.81E-06	3.35E-06	5.77E-06	1.19E-06	6.69E-06	5.09E-06	7.43E-06
C2orf50	3.48E-08	0	0	2.13E-08	0	0	2.39E-08	0
C2orf51	0	4.21E-08	0	0	0	0	0	5.36E-08
C2orf52	1.78E-07	9.10E-08	0	9.77E-08	0	2.95E-07	9.51E-08	2.06E-07
C2orf53	0	0	0	0	0	0	0	0
C2orf54	0.000124	3.33E-06	5.77E-06	4.02E-06	2.25E-05	2.80E-06	8.77E-06	2.89E-07
C2orf55	9.02E-07	2.31E-07	1.09E-07	5.62E-07	6.12E-07	2.29E-07	1.01E-06	7.08E-07
C2orf56	1.42E-05	7.43E-06	5.74E-06	5.18E-06	6.90E-06	8.78E-06	5.72E-06	6.66E-06
C2orf57	0	0	0	0	0	0	0	0
C2orf58	1.25E-07	6.30E-08	4.39E-08	1.02E-07	1.20E-08	5.16E-08	5.36E-08	4.09E-08
C2orf60	3.39E-06	2.46E-06	1.37E-06	1.26E-06	7.08E-07	1.55E-06	1.51E-06	9.17E-07
C2orf61	3.09E-08	1.26E-08	0	0	0	0	0	0
C2orf62	1.75E-07	1.36E-07	0	1.08E-07	0	2.20E-07	1.31E-07	0
C2orf63	2.87E-07	1.13E-07	1.20E-07	9.57E-08	8.62E-08	1.02E-07	1.19E-07	1.18E-07
C2orf64	3.72E-05	2.61E-05	1.47E-05	1.22E-05	1.85E-05	2.05E-05	1.08E-05	9.04E-06
C2orf65	3.15E-06	3.95E-08	1.84E-08	4.26E-08	2.99E-08	3.22E-08	2.45E-07	7.68E-08
C2orf66	1.81E-08	2.07E-08	0	1.12E-07	0	1.69E-08	0	0
C2orf67	8.08E-06	4.35E-06	3.04E-06	3.23E-06	1.10E-06	3.08E-06	2.53E-06	1.68E-06
C2orf68	1.74E-05	1.28E-05	5.40E-06	5.82E-06	7.05E-06	8.40E-06	5.36E-06	5.89E-06
C2orf69	7.90E-06	4.26E-06	4.49E-06	3.81E-06	1.99E-06	4.71E-06	2.87E-06	3.48E-06
C2orf7	3.15E-05	7.34E-05	2.90E-05	2.53E-05	6.21E-05	3.66E-05	4.35E-05	3.08E-05
C2orf70	2.31E-06	0	0	5.35E-08	0	0	0	0
C2orf71	0	0	0	0	0	2.93E-09	0	0
C2orf72	0.000121	4.46E-05	5.38E-05	4.62E-05	6.66E-05	5.10E-05	7.42E-05	7.17E-05
C2orf73	0	0	0	1.89E-08	0	1.43E-08	1.48E-08	0
C2orf74	4.73E-05	3.51E-05	1.87E-05	1.08E-05	1.90E-05	1.50E-05	1.17E-05	2.08E-05
C2orf76	8.54E-06	7.74E-06	4.36E-06	3.97E-06	2.37E-06	4.34E-06	9.53E-06	3.83E-06
C2orf77	5.20E-07	5.98E-07	7.39E-07	2.68E-06	2.44E-07	2.10E-07	7.77E-07	2.70E-06
C2orf78	0	1.11E-07	3.93E-08	0	0	5.63E-08	6.64E-08	5.57E-08
C2orf79	5.93E-05	7.94E-05	3.92E-05	4.97E-05	3.94E-05	5.65E-05	2.66E-05	2.18E-05
C2orf80	0	0	0	0	0	0	0	0
C2orf81	6.05E-07	7.20E-08	1.22E-07	3.62E-07	3.28E-07	2.82E-07	5.92E-07	2.06E-07
C2orf82	2.11E-06	4.87E-06	8.13E-07	1.59E-06	8.85E-06	1.12E-06	4.10E-05	4.86E-07
C2orf83	0	0	0	0	0	1.27E-07	0	0
C2orf84	2.74E-08	1.55E-08	1.44E-08	1.51E-07	8.25E-08	2.54E-08	2.64E-08	1.61E-07
C2orf85	1.35E-07	0	2.84E-08	1.65E-07	1.16E-08	1.75E-07	0	0
C2orf86	5.55E-07	7.88E-07	3.39E-07	5.88E-07	1.70E-07	4.64E-07	6.33E-07	3.06E-07
C2orf88	3.77E-06	1.63E-06	1.54E-06	7.51E-07	2.21E-07	8.46E-08	1.92E-07	8.36E-08
C2orf89	1.93E-05	1.80E-05	9.58E-07	3.48E-06	2.63E-05	1.40E-07	2.99E-06	4.26E-07
C3	0.004332	0.003671	0.007193	0.008188	0.007844	0.003026	0.007571	0.007104
C3AR1	1.52E-05	2.27E-06	4.59E-06	1.08E-05	1.15E-06	4.57E-06	8.21E-06	1.41E-06
C3orf1	5.60E-05	6.10E-05	2.71E-05	2.58E-05	2.69E-05	3.87E-05	2.63E-05	2.63E-05
C3orf10	0.000172	8.23E-05	7.96E-05	0.000114	0.000116	0.000128	0.000127	8.06E-05
C3orf14	6.71E-06	1.33E-07	5.97E-07	2.90E-06	1.31E-07	3.87E-07	6.03E-07	1.13E-07
C3orf15	3.95E-08	1.45E-06	1.03E-07	4.28E-07	1.06E-07	3.22E-07	8.72E-07	6.78E-08
C3orf16	1.00E-07	2.25E-08	0	0	0	0	0	0
C3orf17	8.47E-06	6.76E-06	4.26E-06	5.31E-06	1.08E-06	5.53E-06	4.89E-06	4.35E-06
C3orf18	8.40E-06	8.18E-06	1.58E-06	1.77E-06	2.41E-06	3.52E-06	2.70E-06	4.52E-06
C3orf19	1.15E-05	6.50E-06	3.69E-06	4.80E-06	2.93E-06	5.22E-06	4.98E-06	6.34E-06
C3orf20	0	9.83E-08	0	0	0	1.82E-08	0	0
C3orf21	5.60E-06	9.79E-07	1.28E-06	2.11E-06	1.48E-06	3.03E-06	2.23E-06	3.04E-06
C3orf22	0	0	3.15E-08	0	0	2.67E-08	0	0

C3orf23	3.14E-05	3.38E-05	2.39E-05	1.83E-05	9.86E-06	4.37E-05	2.61E-05	1.71E-05
C3orf24	0	0	0	0	0	0	0	0
C3orf26	1.99E-05	2.35E-05	7.22E-06	8.61E-06	7.65E-06	1.18E-05	1.81E-05	1.10E-05
C3orf27	0	0	0	0	0	0	0	0
C3orf30	0	0	0	0	0	0	0	0
C3orf31	4.57E-06	1.20E-06	3.13E-06	3.12E-06	1.13E-06	5.05E-06	2.01E-06	2.36E-06
C3orf32	2.57E-06	1.71E-06	1.47E-07	3.54E-07	5.80E-06	3.34E-08	1.70E-06	1.58E-06
C3orf33	2.87E-06	1.87E-06	1.58E-06	2.47E-06	1.95E-06	2.01E-06	2.00E-06	1.66E-06
C3orf34	1.20E-06	5.47E-07	3.45E-07	4.32E-07	6.63E-08	4.80E-07	2.88E-07	2.59E-07
C3orf35	4.80E-07	1.71E-07	0	1.74E-07	7.41E-08	8.91E-08	9.26E-08	4.04E-08
C3orf36	7.19E-07	3.34E-08	3.10E-08	1.26E-07	1.77E-07	1.64E-07	8.49E-08	4.33E-08
C3orf37	4.48E-05	4.62E-05	2.46E-05	3.33E-05	1.24E-05	2.74E-05	3.45E-05	3.11E-05
C3orf38	7.42E-06	6.09E-06	4.63E-06	5.19E-06	1.93E-06	5.52E-06	4.48E-06	4.95E-06
C3orf39	9.84E-06	9.19E-06	4.66E-06	4.81E-06	4.88E-06	4.71E-06	5.81E-06	9.53E-06
C3orf42	4.57E-07	1.31E-07	2.04E-07	4.46E-07	1.81E-07	2.13E-07	5.12E-07	1.13E-07
C3orf43	0	0	0	0	0	1.24E-08	1.29E-08	0
C3orf45	5.33E-08	2.76E-08	0	3.23E-08	4.32E-07	6.96E-08	1.78E-07	1.12E-07
C3orf47	3.36E-07	4.16E-07	2.15E-07	4.24E-07	3.72E-07	8.13E-07	5.24E-07	2.07E-07
C3orf48	3.06E-08	0	0	0	0	0	0	0
C3orf49	0	0	0	0	0	0	1.89E-08	0
C3orf50	0	1.83E-07	0	0	0	0	0	1.81E-08
C3orf51	0	0	0	0	0	1.95E-08	0	0
C3orf52	3.89E-06	1.34E-08	4.95E-08	2.44E-06	0	2.19E-08	1.50E-07	2.75E-08
C3orf54	1.93E-06	4.72E-07	4.45E-07	4.72E-07	4.91E-06	8.08E-07	2.67E-06	1.71E-06
C3orf55	0	3.87E-08	0	0	0	0	0	0
C3orf57	7.24E-06	2.08E-07	1.50E-07	2.26E-07	3.09E-07	3.80E-08	1.09E-07	6.03E-08
C3orf58	1.08E-05	1.64E-05	8.13E-06	6.96E-06	7.44E-07	1.74E-05	4.84E-06	4.95E-06
C3orf59	9.91E-07	4.29E-07	2.47E-07	1.54E-06	1.76E-07	3.10E-07	5.44E-07	8.51E-07
C3orf62	4.47E-06	1.90E-06	4.87E-07	1.06E-06	1.58E-06	1.24E-06	2.57E-06	1.47E-06
C3orf63	1.45E-05	1.16E-05	5.17E-06	5.70E-06	2.21E-06	6.83E-06	4.62E-06	4.24E-06
C3orf64	1.08E-05	3.23E-06	1.12E-06	2.47E-06	1.02E-06	1.65E-06	2.01E-06	2.30E-06
C3orf65	1.22E-08	6.92E-08	0	4.98E-08	0	0	0	1.80E-08
C3orf66	9.30E-09	5.24E-08	1.93E-08	0	0	3.22E-06	1.80E-08	0
C3orf67	1.01E-05	2.35E-06	4.07E-07	6.73E-07	1.55E-07	1.81E-06	2.05E-07	6.88E-07
C3orf70	3.35E-07	2.29E-07	1.21E-07	8.81E-08	9.45E-08	1.93E-07	3.59E-07	2.72E-07
C3orf71	1.79E-06	6.31E-07	5.86E-07	3.98E-07	1.06E-06	9.17E-07	5.07E-07	5.45E-07
C3orf72	0	0	0	6.82E-09	0	0	5.43E-09	0
C3orf74	0	0	0	0	0	0	7.44E-09	0
C3orf75	2.01E-05	1.70E-05	1.01E-05	1.44E-05	1.98E-05	2.07E-05	1.67E-05	1.36E-05
C3orf77	0	0	0	0	0	0	0	0
C3orf79	0	0	0	0	0	0	0	0
C3P1	5.25E-05	3.98E-06	3.89E-05	2.59E-05	3.30E-05	3.40E-05	2.82E-05	2.25E-05
C4A	0.002431	0.003026	0.003011	0.003354	0.005633	0.004779	0.006584	0.004743
C4BPA	3.78E-05	0.000389	0.000934	0.000746	0.000231	0.002252	0.000557	0.001794
C4BPB	5.31E-05	0.000199	0.000247	0.000202	0.000398	0.000796	0.00039	0.000311
C4orf10	7.24E-06	4.21E-06	2.05E-06	2.71E-06	1.24E-06	3.98E-06	2.45E-06	1.90E-06
C4orf11	0	0	0	0	0	0	0	0
C4orf12	9.43E-07	3.04E-07	4.42E-07	1.93E-07	2.71E-08	6.25E-07	1.51E-08	9.28E-08
C4orf14	1.05E-05	1.07E-05	6.11E-06	5.79E-06	2.34E-06	1.00E-05	7.58E-06	9.66E-06
C4orf17	0	0	0	0	0	2.87E-08	1.49E-08	0
C4orf19	1.53E-05	1.33E-05	1.04E-05	8.57E-06	8.11E-06	5.29E-06	6.43E-06	1.32E-05
C4orf21	2.54E-06	4.67E-07	1.63E-07	1.71E-07	2.65E-07	4.73E-07	3.21E-07	6.53E-07
C4orf22	3.13E-08	0	0	0	0	0	0	0
C4orf23	5.14E-06	5.12E-06	3.52E-06	4.22E-06	1.43E-06	5.18E-06	4.27E-06	3.79E-06
C4orf26	0	0	0	6.09E-08	0	3.93E-08	0	0
C4orf27	1.46E-05	7.77E-06	9.06E-06	1.26E-05	9.71E-06	1.50E-05	1.13E-05	2.07E-05
C4orf29	7.26E-06	4.51E-06	5.69E-06	2.24E-06	1.97E-07	5.86E-06	1.45E-06	4.46E-06
C4orf3	5.14E-05	1.89E-05	5.67E-05	4.88E-05	2.67E-05	7.00E-05	4.49E-05	2.60E-05

C4orf31	8.09E-08	0	5.87E-08	6.93E-08	0	7.49E-09	2.67E-07	3.56E-08
C4orf32	7.47E-06	7.09E-06	4.12E-06	7.08E-06	1.45E-06	1.17E-05	8.02E-06	2.23E-06
C4orf33	1.26E-05	5.10E-06	3.55E-06	6.59E-06	1.24E-06	5.21E-06	4.49E-06	5.20E-06
C4orf34	2.27E-05	7.32E-05	7.02E-05	4.04E-05	1.83E-05	6.12E-05	4.15E-05	4.60E-05
C4orf35	0	0	0	0	0	0	0	0
C4orf36	2.41E-07	7.04E-08	1.00E-07	3.36E-07	2.62E-08	8.46E-08	4.01E-07	4.51E-08
C4orf37	0	1.69E-08	0	1.83E-08	0	1.38E-08	0	0
C4orf38	6.10E-07	2.71E-07	4.10E-07	1.74E-07	1.41E-07	7.18E-07	2.00E-07	1.76E-07
C4orf39	6.56E-08	0	1.14E-08	4.02E-08	0	1.01E-08	1.06E-08	0
C4orf40	0	0	0	0	0	1.25E-07	0	0
C4orf41	1.20E-05	6.95E-06	8.56E-06	6.79E-06	1.87E-06	1.12E-05	4.98E-06	7.53E-06
C4orf42	9.45E-06	8.43E-06	4.00E-06	3.56E-06	1.82E-06	9.73E-06	2.48E-06	7.06E-06
C4orf43	9.31E-06	5.79E-06	4.98E-06	6.06E-06	2.79E-06	9.74E-06	7.47E-06	6.91E-06
C4orf44	2.05E-07	1.33E-07	3.09E-08	7.20E-08	6.31E-08	6.80E-08	1.27E-07	4.32E-08
C4orf45	3.45E-08	0	0	0	0	0	0	0
C4orf46	2.20E-06	7.51E-07	5.21E-07	6.52E-07	3.54E-07	7.64E-07	5.49E-07	8.25E-07
C4orf47	2.22E-07	1.59E-07	1.89E-07	5.53E-08	9.56E-08	4.16E-07	0	0
C4orf48	6.97E-06	0	0	3.63E-06	7.63E-07	2.24E-07	7.05E-06	2.44E-07
C4orf49	4.52E-07	8.63E-08	6.05E-08	5.80E-07	1.14E-07	8.77E-08	2.35E-07	5.58E-08
C4orf50	1.45E-08	0	0	1.77E-08	6.23E-09	6.72E-09	0	0
C4orf51	0	0	0	0	0	0	0	0
C4orf52	4.83E-05	3.71E-05	2.18E-05	2.03E-05	4.53E-06	5.00E-05	2.53E-05	1.93E-05
C4orf6	3.18E-08	4.47E-07	3.54E-08	5.92E-07	0	8.92E-08	7.24E-07	1.09E-06
C4orf7	1.87E-07	1.54E-07	0	0	0	5.89E-07	5.74E-08	4.11E-06
C5	0.000161	0.000134	0.000189	0.000131	6.96E-05	0.000263	0.000106	0.000298
C5AR1	5.78E-06	4.32E-06	4.28E-06	2.75E-05	7.45E-07	3.67E-06	5.38E-06	3.14E-06
C5orf13	4.73E-05	1.37E-05	3.63E-05	7.05E-06	4.18E-06	7.57E-06	7.11E-06	8.72E-06
C5orf15	9.25E-05	3.17E-05	3.97E-05	3.38E-05	1.11E-05	4.80E-05	1.92E-05	2.40E-05
C5orf20	1.33E-06	8.27E-09	1.37E-07	1.62E-07	1.90E-08	1.36E-07	2.07E-07	6.48E-08
C5orf22	8.05E-06	1.26E-05	4.57E-06	4.95E-06	1.12E-06	6.27E-06	5.28E-06	1.11E-05
C5orf23	3.72E-06	1.29E-05	1.62E-06	3.18E-06	3.82E-07	8.38E-07	7.13E-07	1.60E-06
C5orf24	2.18E-05	1.33E-05	1.62E-05	1.19E-05	1.67E-06	1.44E-05	9.31E-06	1.36E-05
C5orf25	6.25E-06	4.47E-06	9.52E-06	2.84E-06	5.95E-07	1.77E-06	4.80E-06	5.27E-06
C5orf27	1.28E-07	5.27E-05	3.28E-05	7.33E-06	8.66E-08	3.61E-07	6.37E-05	0.000136
C5orf28	4.35E-06	4.05E-06	2.26E-06	2.37E-06	9.28E-07	3.04E-06	2.15E-06	3.51E-06
C5orf30	3.41E-06	2.46E-07	3.66E-07	4.61E-07	6.61E-08	1.21E-07	2.91E-07	1.02E-07
C5orf32	0.000199	0.000162	4.49E-05	7.95E-05	2.07E-05	6.93E-05	5.40E-05	6.82E-05
C5orf33	4.02E-05	4.18E-05	0.000132	7.08E-05	3.75E-05	9.53E-05	8.80E-05	6.98E-05
C5orf34	8.91E-07	1.35E-06	4.40E-08	1.87E-07	6.49E-08	2.68E-07	1.87E-07	4.02E-07
C5orf35	5.88E-06	6.79E-06	4.19E-06	5.59E-06	2.68E-06	2.94E-06	4.10E-06	4.94E-06
C5orf36	1.65E-07	1.07E-07	3.57E-07	3.33E-07	1.02E-07	3.48E-07	1.87E-07	2.07E-07
C5orf38	0	0	0	0	0	0	0	0
C5orf39	1.68E-06	2.85E-07	5.18E-07	6.41E-07	8.14E-07	9.79E-07	1.82E-06	1.16E-06
C5orf4	1.57E-05	1.21E-05	0.000101	6.21E-05	4.57E-05	1.95E-05	7.50E-05	7.21E-05
C5orf40	2.24E-08	0	0	0	0	1.04E-08	0	3.29E-08
C5orf41	3.99E-06	1.85E-06	3.71E-06	1.86E-06	7.80E-08	4.75E-06	6.96E-07	3.03E-06
C5orf42	4.08E-06	1.83E-06	7.49E-07	1.36E-06	5.36E-07	1.92E-06	7.72E-07	1.85E-06
C5orf43	3.33E-05	2.40E-05	1.83E-05	1.63E-05	4.75E-06	2.09E-05	1.65E-05	2.26E-05
C5orf44	1.03E-05	9.93E-06	5.61E-06	5.62E-06	1.25E-06	9.11E-06	4.99E-06	9.43E-06
C5orf45	7.45E-06	2.58E-06	2.67E-06	3.62E-06	4.98E-06	4.53E-06	4.47E-06	2.92E-06
C5orf46	2.98E-06	4.07E-07	0	1.89E-06	1.04E-06	5.02E-06	2.95E-06	6.30E-06
C5orf47	1.11E-08	1.26E-08	1.16E-08	5.45E-08	2.87E-08	2.06E-08	0	8.18E-08
C5orf48	0	0	0	1.02E-07	0	0	0	0
C5orf49	4.93E-07	0	1.99E-07	1.19E-06	2.25E-08	1.23E-05	3.71E-07	0
C5orf51	1.12E-05	1.07E-05	4.75E-06	4.10E-06	6.92E-07	8.87E-06	3.11E-06	7.90E-06
C5orf52	0	0	0	0	0	0	0	0
C5orf53	9.27E-06	4.92E-06	5.14E-06	3.30E-06	2.14E-06	5.18E-06	4.42E-06	4.88E-06
C5orf54	1.36E-06	6.43E-07	7.30E-07	3.24E-07	4.63E-07	7.75E-07	3.21E-07	4.13E-07

C5orf55	4.15E-06	2.70E-06	3.17E-06	1.44E-06	2.73E-06	4.23E-06	7.18E-07	2.45E-06
C5orf56	8.33E-06	2.99E-06	3.55E-06	3.87E-06	1.96E-06	7.27E-06	3.44E-06	6.52E-06
C5orf58	6.92E-06	1.87E-06	9.63E-08	1.13E-07	0	4.15E-08	2.13E-08	1.04E-07
C5orf60	2.16E-08	2.48E-08	4.66E-08	5.32E-08	0	0	0	0
C5orf62	9.21E-06	2.43E-06	2.59E-06	9.82E-06	1.20E-06	2.31E-06	4.12E-06	2.68E-06
C6	3.44E-05	8.31E-05	0.00032	0.000172	5.22E-05	0.000191	0.000154	9.00E-05
C6orf1	1.48E-05	3.71E-05	1.03E-05	1.43E-05	1.65E-05	1.73E-05	2.25E-05	3.12E-05
C6orf10	0	0	1.34E-08	0	0	0	0	0
C6orf103	0	0	0	0	0	0	7.30E-09	0
C6orf105	2.96E-07	4.27E-07	2.69E-07	1.19E-06	1.16E-08	0	1.03E-06	6.71E-07
C6orf106	6.71E-05	0.000128	4.20E-05	2.94E-05	4.21E-05	5.40E-05	5.06E-05	6.58E-05
C6orf108	0.000118	0.000154	0.000102	0.000112	0.000202	0.000162	0.000117	0.0001
C6orf114	3.00E-07	4.85E-07	3.95E-07	3.48E-07	4.12E-07	4.11E-07	3.57E-07	1.57E-07
C6orf115	3.66E-05	1.67E-05	2.35E-05	3.39E-05	9.60E-06	1.45E-05	6.14E-05	2.92E-06
C6orf118	0	0	0	0	0	0	1.27E-08	0
C6orf120	1.50E-05	1.08E-05	1.39E-05	8.92E-06	3.12E-06	1.69E-05	7.16E-06	8.14E-06
C6orf122	1.56E-06	4.66E-07	1.11E-06	8.06E-07	4.67E-07	2.02E-06	7.45E-07	5.82E-07
C6orf123	8.72E-06	1.12E-06	3.30E-06	1.20E-06	2.11E-06	3.20E-06	3.87E-07	2.35E-06
C6orf124	7.46E-07	5.54E-07	3.94E-07	4.26E-07	1.23E-07	6.92E-07	1.88E-07	1.65E-07
C6orf125	0.000107	0.00013	4.94E-05	6.29E-05	0.000171	9.13E-05	0.000121	9.17E-05
C6orf126	5.85E-07	6.45E-07	0	0	0	0	0	0
C6orf127	0	0	0	0	0	0	0	0
C6orf129	5.65E-05	7.33E-05	2.63E-05	3.00E-05	0.000122	6.07E-05	4.77E-05	5.13E-05
C6orf130	3.62E-05	3.63E-05	1.93E-05	2.00E-05	1.67E-05	4.00E-05	2.32E-05	1.86E-05
C6orf132	2.58E-06	1.42E-08	1.05E-07	5.68E-07	2.16E-08	0	3.63E-08	3.69E-08
C6orf134	6.85E-06	2.39E-06	1.12E-06	2.11E-06	1.30E-06	3.70E-06	2.07E-06	3.57E-07
C6orf136	2.35E-05	2.48E-05	1.05E-05	1.12E-05	1.86E-05	1.33E-05	1.19E-05	3.01E-05
C6orf138	4.53E-08	1.88E-07	5.96E-08	5.54E-07	4.58E-08	5.02E-06	2.28E-07	2.54E-07
C6orf141	2.68E-06	3.13E-07	1.52E-08	5.06E-07	5.75E-07	0	2.80E-07	3.88E-08
C6orf142	2.07E-07	3.93E-06	2.40E-05	1.78E-05	1.45E-06	1.79E-05	1.07E-05	1.96E-05
C6orf145	4.67E-05	8.78E-05	6.93E-05	9.72E-05	5.57E-05	8.01E-05	8.49E-05	4.20E-05
C6orf146	8.65E-08	6.11E-08	0	0	0	0	0	0
C6orf147	7.26E-07	1.97E-08	3.68E-08	2.12E-08	0	1.61E-08	0	0
C6orf15	0	0	0	0	0	0	0	0
C6orf150	1.92E-06	2.14E-07	1.84E-07	8.08E-07	3.47E-08	3.00E-07	1.82E-07	1.59E-07
C6orf153	3.69E-05	4.83E-05	1.53E-05	2.39E-05	1.40E-05	2.82E-05	2.01E-05	3.91E-05
C6orf154	1.28E-06	8.86E-07	5.87E-08	2.23E-07	5.01E-08	6.66E-07	1.25E-07	4.88E-07
C6orf155	1.50E-07	3.55E-08	9.82E-08	1.93E-08	2.04E-08	1.17E-07	6.90E-08	0
C6orf162	3.59E-06	5.26E-06	2.42E-06	2.33E-06	1.25E-06	2.67E-06	1.68E-06	1.33E-06
C6orf163	3.27E-07	2.04E-07	2.36E-08	4.15E-08	6.80E-08	0	4.37E-08	1.66E-08
C6orf164	4.24E-07	3.75E-07	1.02E-07	1.99E-07	6.64E-08	9.11E-08	1.60E-07	9.25E-08
C6orf165	5.46E-08	1.23E-08	0	2.41E-08	0	2.03E-08	4.23E-08	1.61E-08
C6orf167	1.24E-06	5.56E-07	1.38E-07	2.23E-07	1.12E-07	2.68E-07	1.56E-07	1.60E-07
C6orf168	7.60E-07	0	0	4.42E-08	0	1.26E-08	0	3.31E-09
C6orf170	6.50E-07	1.77E-07	3.60E-08	1.47E-07	1.88E-08	4.03E-07	1.15E-07	8.99E-08
C6orf174	1.51E-06	3.93E-08	#######	1.99E-07	6.61E-09	6.47E-09	4.01E-08	5.99E-08
C6orf176	0	7.46E-07	0	5.07E-07	3.44E-08	1.24E-08	0	3.45E-08
C6orf182	3.52E-06	1.50E-06	1.79E-06	1.88E-06	4.99E-07	2.55E-06	1.51E-06	2.02E-06
C6orf186	1.78E-06	1.10E-07	3.16E-08	5.57E-07	0	6.65E-08	0	0
C6orf191	0	0	0	0	0	0	0	0
C6orf192	1.35E-05	8.03E-06	4.06E-06	6.89E-06	2.71E-06	9.38E-06	4.95E-06	4.77E-06
C6orf195	1.38E-07	0	0	1.41E-08	0	0	0	0
C6orf201	6.22E-07	9.03E-08	9.48E-08	4.42E-08	9.55E-08	2.19E-07	1.66E-07	7.86E-08
C6orf203	3.02E-05	1.85E-05	1.74E-05	1.46E-05	1.67E-05	2.33E-05	1.89E-05	2.19E-05
C6orf204	5.69E-07	1.54E-07	1.21E-07	2.44E-07	0	1.05E-07	6.58E-08	9.53E-08
C6orf208	1.94E-06	7.46E-07	1.54E-06	7.86E-07	2.61E-07	1.39E-06	3.09E-07	5.67E-07
C6orf211	1.86E-05	1.35E-05	1.55E-05	1.25E-05	4.06E-06	3.77E-05	1.22E-05	9.04E-06
C6orf217	3.20E-07	0	5.60E-08	3.92E-08	0	4.44E-08	7.80E-08	4.71E-08

C6orf218	0	2.59E-08	0	0	0	0	0	0
C6orf221	0	0	0	0	0	0	0	0
C6orf222	2.45E-08	6.86E-09	0	0	0	0	1.19E-08	0
C6orf223	4.76E-06	2.90E-05	3.49E-08	1.13E-07	0	7.66E-09	8.15E-09	0
C6orf225	1.69E-06	4.13E-06	2.72E-06	3.57E-06	5.49E-06	3.20E-06	2.06E-06	9.24E-07
C6orf226	1.72E-05	1.28E-05	1.15E-05	1.18E-05	3.09E-05	1.98E-05	8.57E-06	8.53E-06
C6orf227	0	0	0	0	0	0	0	0
C6orf25	0	6.51E-08	6.40E-08	3.95E-08	0	0	0	0
C6orf26	6.08E-06	4.03E-06	7.45E-07	1.28E-06	1.93E-06	1.53E-06	9.46E-07	1.92E-06
C6orf27	9.39E-06	6.03E-06	6.93E-07	4.19E-07	2.74E-08	1.11E-06	4.48E-07	2.15E-06
C6orf35	3.56E-06	2.69E-06	3.58E-06	4.13E-06	2.14E-06	5.81E-06	5.72E-06	1.74E-06
C6orf41	2.48E-07	1.15E-06	4.23E-07	5.79E-07	1.31E-06	4.88E-07	1.05E-06	1.11E-06
C6orf47	2.03E-05	1.56E-05	7.35E-06	9.00E-06	8.18E-06	8.94E-06	8.45E-06	2.15E-05
C6orf48	5.61E-05	8.29E-05	4.24E-05	6.02E-05	5.50E-05	2.03E-05	6.87E-05	6.60E-05
C6orf52	5.83E-07	3.08E-07	1.78E-07	3.86E-07	1.14E-06	6.61E-07	8.42E-06	2.23E-07
C6orf57	1.66E-05	2.36E-05	9.40E-06	8.62E-06	2.88E-06	1.44E-05	1.25E-05	6.23E-06
C6orf58	0	4.95E-08	0	0	0	2.00E-08	2.06E-08	3.19E-08
C6orf59	1.58E-07	5.95E-08	0	1.13E-07	0	1.22E-08	8.87E-08	3.87E-08
C6orf62	8.30E-05	7.87E-05	3.42E-05	4.03E-05	3.00E-05	6.82E-05	5.57E-05	5.50E-05
C6orf64	1.19E-05	1.09E-05	6.46E-06	6.73E-06	6.50E-06	9.46E-06	8.82E-06	1.83E-05
C6orf70	1.24E-05	6.75E-06	8.79E-06	8.55E-06	4.75E-06	1.37E-05	9.79E-06	8.46E-06
C6orf72	3.66E-05	2.40E-05	2.56E-05	2.79E-05	1.01E-05	3.24E-05	2.53E-05	2.47E-05
C6orf81	8.77E-07	8.50E-07	7.20E-08	1.96E-07	1.54E-07	3.62E-07	1.06E-07	2.02E-07
C6orf89	1.88E-05	2.99E-05	1.05E-05	1.07E-05	1.31E-05	2.28E-05	1.44E-05	2.10E-05
C6orf94	0	0	0	0	0	2.52E-08	0	0
C6orf97	1.79E-07	7.62E-07	2.53E-07	3.43E-07	1.35E-07	9.68E-06	7.09E-07	4.43E-07
C7	7.86E-06	3.15E-06	5.86E-05	0.000194	1.40E-05	1.32E-06	1.93E-05	1.63E-05
C7orf10	1.02E-05	2.66E-05	1.94E-05	1.57E-05	3.20E-05	1.68E-05	3.37E-05	3.45E-05
C7orf11	5.81E-05	0.000157	5.58E-05	5.03E-05	3.28E-05	6.43E-05	6.02E-05	2.29E-05
C7orf13	1.91E-06	5.84E-06	3.39E-06	2.14E-06	5.62E-06	2.49E-06	4.86E-06	3.75E-06
C7orf16	0	0	0	0	1.28E-08	0	0	0
C7orf23	1.40E-05	4.12E-06	3.97E-06	8.77E-06	1.46E-06	3.98E-06	3.38E-06	1.46E-06
C7orf25	1.55E-05	9.84E-06	4.81E-06	4.97E-06	3.75E-06	8.15E-06	5.58E-06	1.04E-05
C7orf26	1.19E-05	1.52E-05	7.76E-06	1.32E-05	1.03E-05	9.83E-06	1.27E-05	1.62E-05
C7orf27	2.93E-05	4.21E-05	1.47E-05	1.68E-05	1.58E-05	2.15E-05	1.74E-05	2.06E-05
C7orf28A	4.60E-05	4.10E-05	1.32E-05	9.11E-06	4.57E-06	2.38E-05	1.59E-05	2.17E-05
C7orf28B	6.72E-06	2.56E-05	3.51E-06	1.39E-05	3.97E-06	7.96E-06	3.87E-06	1.48E-05
C7orf29	4.30E-06	8.88E-06	1.52E-06	3.02E-06	6.49E-06	7.72E-06	5.88E-06	2.10E-06
C7orf30	5.44E-05	0.00012	4.03E-05	3.70E-05	3.88E-05	5.76E-05	4.68E-05	4.89E-05
C7orf31	1.80E-06	3.26E-06	5.71E-07	6.77E-07	3.68E-07	7.37E-07	9.21E-07	5.29E-07
C7orf33	0	0	0	0	0	0	0	0
C7orf34	0	0	0	0	0	0	0	0
C7orf36	1.25E-05	1.48E-05	5.44E-06	8.31E-06	4.97E-06	8.70E-06	9.21E-06	6.67E-06
C7orf4	0	0	0	0	0	4.83E-08	4.77E-08	0
C7orf40	1.46E-05	4.30E-06	3.60E-06	1.48E-05	5.75E-06	4.11E-06	4.65E-06	4.04E-06
C7orf41	6.43E-06	1.67E-06	2.19E-06	2.02E-06	8.61E-07	6.38E-06	8.96E-07	8.31E-07
C7orf42	4.00E-05	5.40E-05	3.31E-05	3.12E-05	2.10E-05	3.12E-05	2.80E-05	3.75E-05
C7orf43	1.28E-05	7.49E-06	3.71E-06	8.19E-06	2.75E-06	6.49E-06	1.02E-05	1.28E-05
C7orf44	4.24E-05	2.44E-05	1.36E-05	1.67E-05	9.02E-06	2.14E-05	1.58E-05	2.52E-05
C7orf45	0	0	0	2.77E-08	0	0	0	0
C7orf46	7.14E-06	7.56E-06	4.13E-06	3.04E-06	8.74E-07	4.17E-06	2.16E-06	3.95E-06
C7orf47	3.99E-05	4.03E-05	6.42E-06	1.40E-05	1.44E-05	1.14E-05	1.98E-05	1.95E-05
C7orf49	1.23E-05	1.29E-05	6.90E-06	9.20E-06	4.75E-06	1.53E-05	1.12E-05	2.31E-05
C7orf50	0.000108	8.24E-05	2.43E-05	3.64E-05	9.92E-05	4.82E-05	7.52E-05	8.32E-05
C7orf51	9.07E-08	8.69E-08	7.26E-08	1.95E-07	1.99E-08	8.44E-08	1.88E-07	1.63E-07
C7orf52	3.17E-08	3.91E-07	7.07E-08	0	7.60E-07	3.67E-08	3.84E-08	0
C7orf53	6.14E-07	6.03E-07	1.99E-07	3.77E-07	1.01E-06	3.33E-07	6.30E-07	1.13E-06
C7orf54	9.12E-09	1.03E-08	1.89E-08	0	0	8.45E-09	8.83E-09	1.34E-08

C7orf55	5.18E-05	5.66E-05	5.99E-05	4.69E-05	0.000119	7.52E-05	3.35E-05	2.74E-05
C7orf57	2.52E-08	0	1.32E-08	1.55E-08	0	0	0	0
C7orf58	1.12E-06	9.83E-07	1.63E-05	7.50E-06	5.93E-07	6.12E-06	4.44E-06	5.74E-06
C7orf59	0.000313	0.000213	6.68E-05	0.000114	0.000354	0.000134	0.000156	0.000159
C7orf60	1.53E-06	1.22E-06	1.02E-06	1.19E-06	4.12E-07	1.11E-06	8.26E-07	8.64E-07
C7orf61	2.13E-07	4.01E-07	1.16E-07	9.86E-08	9.21E-08	9.92E-08	2.53E-07	7.92E-08
C7orf63	1.72E-06	7.65E-07	6.22E-07	6.32E-07	2.75E-07	1.05E-06	3.63E-07	2.40E-07
C7orf64	4.71E-05	6.76E-06	4.61E-06	3.93E-06	1.58E-06	4.46E-06	3.77E-06	6.29E-06
C7orf65	0	1.79E-08	0	0	0	7.38E-09	0	0
C7orf66	0	0	0	0	0	0	0	0
C7orf68	2.60E-06	3.59E-06	1.13E-06	1.15E-05	1.24E-06	2.28E-06	4.79E-06	2.31E-06
C7orf69	4.14E-08	0	2.05E-08	0	0	0	0	1.45E-08
C7orf70	1.37E-05	1.37E-05	3.15E-06	3.91E-06	2.26E-06	5.46E-06	5.15E-06	9.28E-06
C7orf71	0	0	0	0	0	1.16E-08	0	0
C7orf72	0	0	0	0	0	0	0	0
C8A	4.09E-05	0.000112	0.00038	0.000256	6.68E-05	0.000236	0.000113	0.000232
C8B	0.000141	0.000157	0.000469	0.00039	0.000151	0.000177	0.000319	0.000341
C8G	0.000176	0.000341	0.000343	0.000365	0.000525	0.000394	0.000551	0.000859
C8orf12	0	0	0	0	0	0	0	0
C8orf22	0	0	2.17E-08	0	0	0	0	0
C8orf30A	2.44E-05	1.99E-05	1.03E-05	1.06E-05	2.12E-05	1.61E-05	5.68E-05	3.39E-05
C8orf31	1.64E-07	2.77E-07	1.36E-07	3.81E-07	2.00E-08	4.80E-08	7.45E-08	0
C8orf33	3.96E-05	3.20E-05	7.46E-06	1.13E-05	1.31E-05	1.95E-05	4.99E-05	2.44E-05
C8orf34	0	1.40E-08	6.61E-09	0	0	2.02E-08	1.19E-08	0
C8orf37	3.07E-06	1.09E-06	7.34E-07	6.69E-07	1.72E-07	1.31E-06	7.22E-07	7.10E-07
C8orf38	2.81E-05	6.30E-06	5.58E-06	3.42E-06	6.03E-06	3.98E-06	1.54E-05	9.64E-06
C8orf39	2.29E-06	9.91E-08	1.43E-07	2.08E-07	1.81E-07	2.73E-07	3.91E-08	3.14E-07
C8orf4	3.50E-05	1.69E-05	0.000182	0.000155	1.09E-05	2.35E-05	4.51E-05	8.12E-05
C8orf40	2.20E-05	8.08E-05	6.96E-05	6.12E-05	6.58E-05	3.96E-05	3.34E-05	6.15E-05
C8orf41	3.69E-06	7.96E-06	4.18E-06	4.47E-06	1.73E-06	5.33E-06	3.44E-06	2.49E-06
C8orf42	5.83E-06	2.00E-05	6.62E-06	6.07E-06	1.80E-06	4.92E-06	5.47E-07	5.12E-06
C8orf44	1.98E-06	7.77E-07	4.79E-07	1.89E-07	2.67E-07	3.14E-07	6.41E-07	1.15E-07
C8orf45	3.67E-07	1.78E-06	2.28E-07	8.97E-08	1.85E-07	1.03E-06	2.25E-07	4.32E-07
C8orf46	5.22E-06	6.36E-06	2.51E-07	1.24E-06	7.58E-07	1.17E-06	1.22E-06	5.48E-07
C8orf47	0.000163	1.87E-05	8.36E-06	1.70E-05	2.61E-05	1.48E-07	1.22E-05	6.48E-05
C8orf48	1.55E-07	1.97E-08	2.39E-07	3.40E-07	1.49E-08	4.82E-08	1.49E-07	5.10E-08
C8orf51	1.31E-06	6.60E-07	3.36E-07	1.31E-07	2.05E-08	3.97E-07	5.29E-07	3.68E-07
C8orf55	3.54E-05	6.44E-05	3.24E-05	1.98E-05	3.95E-05	1.62E-05	4.95E-05	5.90E-05
C8orf56	2.44E-07	0	0	0	0	2.01E-07	1.94E-07	2.18E-07
C8orf58	1.63E-06	2.17E-06	1.20E-06	2.02E-06	1.52E-06	8.01E-07	2.89E-06	1.05E-06
C8orf59	0.000162	7.23E-05	2.00E-05	2.96E-05	8.23E-05	3.38E-05	0.000161	4.56E-05
C8orf71	0	0	0	0	0	0	0	0
C8orf73	1.04E-05	1.64E-05	1.41E-06	7.13E-07	1.35E-06	4.86E-07	2.52E-06	3.02E-07
C8orf74	0	0	0	0	0	0	0	0
C8orf75	1.80E-08	0	0	2.21E-08	0	1.67E-08	0	2.66E-08
C8orf76	6.13E-05	2.96E-05	1.04E-05	1.09E-05	2.04E-05	1.50E-05	2.60E-05	2.55E-05
C8orf77	3.49E-07	8.00E-08	7.36E-09	1.74E-08	1.22E-08	5.28E-08	5.53E-08	4.18E-08
C8orf79	1.28E-06	2.76E-07	2.21E-07	7.80E-07	1.84E-08	1.76E-08	2.32E-08	1.39E-08
C8orf80	2.99E-07	1.85E-06	1.00E-05	1.03E-05	5.34E-07	1.78E-05	1.27E-06	1.56E-06
C8orf83	2.81E-05	1.23E-05	1.23E-05	1.27E-05	4.45E-06	1.38E-05	2.73E-05	1.46E-05
C8orf84	1.55E-06	9.43E-08	1.67E-07	1.69E-07	1.50E-07	2.50E-05	5.85E-08	8.81E-08
C8orf85	0	1.54E-08	0	0	0	1.26E-08	6.56E-08	0
C8orf86	0	0	0	0	0	0	0	0
C8ORFK29	3.18E-07	1.77E-08	3.91E-08	6.61E-08	4.07E-07	7.53E-08	1.42E-07	5.21E-08
C9	3.96E-05	0.000704	0.000312	0.000428	7.50E-06	0.00064	0.000372	0.000478
C9orf100	1.67E-06	1.66E-06	8.50E-08	2.60E-07	5.24E-07	1.15E-06	3.65E-07	5.38E-07
C9orf102	2.10E-06	8.24E-07	1.50E-06	1.11E-06	4.57E-08	1.68E-06	2.30E-07	8.41E-07
C9orf103	2.92E-05	4.63E-05	3.42E-05	2.42E-05	3.81E-05	2.35E-05	1.36E-05	6.97E-06

C9orf106	2.32E-07	0	7.40E-08	0	0	0	3.14E-08	9.92E-08
C9orf109	6.58E-08	3.45E-08	3.56E-08	5.19E-08	9.95E-09	2.15E-08	2.64E-08	2.26E-08
C9orf11	5.71E-08	0	0	5.10E-08	0	0	0	0
C9orf110	2.17E-07	6.47E-08	9.10E-08	2.17E-07	6.63E-09	8.58E-08	4.14E-08	4.52E-08
C9orf114	2.05E-05	1.84E-05	1.25E-05	1.44E-05	1.52E-05	1.58E-05	1.33E-05	2.27E-05
C9orf116	3.53E-07	1.44E-06	8.04E-07	2.51E-06	8.92E-07	4.18E-06	1.15E-06	1.73E-06
C9orf117	1.19E-07	3.21E-08	1.49E-08	5.21E-08	4.87E-08	9.20E-08	9.55E-08	7.81E-08
C9orf119	2.27E-05	2.02E-05	1.29E-05	1.81E-05	5.46E-05	4.83E-05	2.13E-05	2.15E-05
C9orf122	9.32E-07	0	4.17E-07	6.07E-07	0	0	0	0
C9orf123	9.87E-06	3.40E-06	7.76E-06	1.24E-05	1.10E-05	1.99E-05	1.09E-05	4.60E-06
C9orf125	1.56E-05	2.32E-07	1.16E-06	2.13E-06	5.90E-08	5.30E-08	7.07E-07	4.76E-06
C9orf128	1.57E-08	5.74E-08	0	0	0	7.29E-09	0	0
C9orf129	4.76E-06	3.16E-06	2.82E-06	1.89E-06	3.89E-08	4.44E-06	7.31E-07	2.84E-06
C9orf130	2.37E-06	1.67E-06	2.46E-06	1.60E-06	2.17E-06	3.72E-06	5.38E-07	1.05E-06
C9orf131	1.40E-08	3.26E-08	1.50E-08	1.77E-08	0	6.71E-09	0	0
C9orf135	4.09E-07	0	1.21E-07	0	0	0	0	0
C9orf139	5.99E-08	0	1.94E-08	3.80E-08	5.14E-09	2.77E-08	5.82E-09	8.77E-09
C9orf140	6.11E-06	4.01E-08	7.97E-08	1.24E-07	9.72E-08	2.37E-07	1.97E-07	1.31E-07
C9orf142	3.26E-05	1.43E-05	1.87E-05	2.75E-05	3.43E-05	2.25E-05	4.09E-05	4.79E-05
C9orf144	0	0	0	0	0	0	0	0
C9orf144E	0	1.77E-08	0	0	0	4.89E-09	0	0
C9orf150	1.24E-05	2.79E-05	2.85E-05	2.75E-05	6.42E-06	1.12E-05	6.42E-06	3.71E-05
C9orf152	5.28E-08	2.38E-07	3.65E-07	3.45E-07	6.05E-08	9.41E-06	1.71E-08	2.58E-08
C9orf153	9.80E-08	4.39E-08	1.36E-08	0	2.22E-08	5.99E-08	6.23E-08	3.80E-08
C9orf156	9.58E-06	6.23E-06	5.55E-06	8.01E-06	2.94E-06	6.90E-06	5.06E-06	7.12E-06
C9orf16	3.85E-05	4.63E-05	3.10E-05	4.74E-05	8.45E-05	0.00011	7.65E-05	8.73E-05
C9orf163	3.19E-07	9.23E-08	9.46E-08	2.23E-08	7.04E-08	1.60E-07	2.65E-08	6.68E-08
C9orf167	1.27E-05	8.71E-07	1.32E-06	2.97E-06	4.66E-07	1.23E-06	2.20E-06	9.01E-07
C9orf169	4.52E-07	2.23E-07	8.16E-07	4.28E-07	1.10E-06	1.60E-06	2.82E-07	6.59E-07
C9orf170	1.35E-08	0	0	8.23E-09	0	0	0	0
C9orf171	0	0	0	1.79E-08	0	0	0	0
C9orf172	2.69E-07	2.49E-07	1.64E-08	2.90E-08	0	1.47E-08	1.31E-07	4.06E-07
C9orf173	1.30E-07	2.96E-08	0	0	0	3.09E-07	0	0
C9orf21	6.99E-06	3.73E-06	2.28E-06	7.95E-06	9.41E-07	6.96E-06	4.96E-06	3.76E-06
C9orf23	4.35E-05	4.83E-05	4.16E-05	4.37E-05	7.66E-05	4.59E-05	4.63E-05	3.09E-05
C9orf24	1.57E-07	3.09E-06	8.15E-08	9.85E-08	8.21E-07	1.14E-06	3.45E-07	4.44E-08
C9orf25	4.76E-06	5.70E-06	1.65E-06	3.34E-06	1.55E-06	2.88E-06	2.61E-06	4.69E-06
C9orf27	0	0	0	0	0	0	0	0
C9orf3	4.36E-05	1.10E-05	2.11E-05	2.49E-05	3.45E-05	2.41E-05	3.17E-05	1.65E-05
C9orf30	1.11E-05	6.20E-06	6.26E-06	1.57E-05	2.62E-06	7.92E-06	5.06E-06	1.16E-05
C9orf37	4.40E-06	3.06E-06	3.03E-06	3.74E-06	6.49E-06	1.02E-05	4.30E-06	4.04E-06
C9orf4	4.33E-08	0	0	0	0	2.02E-08	0	0
C9orf40	1.92E-06	2.58E-06	1.08E-06	1.22E-06	1.86E-06	1.62E-06	7.67E-07	6.87E-07
C9orf41	3.45E-06	4.10E-06	4.75E-06	1.91E-06	9.32E-09	2.71E-06	7.34E-07	2.20E-06
C9orf43	4.01E-07	5.99E-07	2.77E-07	2.06E-07	1.55E-07	1.02E-06	2.90E-07	1.24E-07
C9orf44	0	0	0	1.47E-08	1.03E-08	0	1.16E-08	0
C9orf45	9.25E-07	1.05E-07	1.26E-07	2.01E-07	1.93E-07	1.00E-07	3.39E-07	1.92E-07
C9orf46	1.65E-05	2.54E-05	1.19E-05	1.89E-05	1.06E-05	1.97E-05	2.02E-05	2.75E-05
C9orf47	2.34E-08	1.57E-08	1.44E-08	1.72E-08	4.01E-09	4.32E-09	0	0
C9orf5	4.62E-05	1.85E-05	9.20E-06	9.30E-06	2.97E-06	3.67E-05	5.90E-06	1.40E-05
C9orf50	0	0	0	4.14E-08	2.90E-08	0	0	0
C9orf53	0	1.23E-07	0	0	4.44E-08	3.83E-07	0	1.54E-07
C9orf57	1.80E-08	0	0	0	0	0	0	2.74E-06
C9orf6	1.11E-05	5.70E-06	4.43E-06	4.49E-06	1.85E-06	1.14E-05	4.41E-06	5.32E-06
C9orf64	2.11E-05	1.38E-05	1.23E-05	1.20E-05	3.51E-06	1.07E-05	1.13E-05	1.01E-05
C9orf66	6.94E-07	9.40E-09	4.24E-07	3.48E-07	7.18E-09	4.65E-08	1.30E-07	2.45E-08
C9orf68	6.29E-07	7.10E-07	6.79E-07	1.35E-06	3.51E-07	4.36E-07	9.57E-07	1.19E-06
C9orf69	1.69E-05	8.10E-06	6.84E-06	8.90E-06	1.10E-05	1.64E-05	1.66E-05	1.89E-05

C9orf7	2.80E-05	2.66E-05	2.69E-05	3.21E-05	5.06E-05	2.54E-05	3.26E-05	8.16E-05
C9orf70	4.01E-08	0	0	0	0	0	0	0
C9orf71	1.50E-10	2.37E-08	1.12E-06	5.77E-06	0	0	0	0
C9orf72	3.12E-06	4.62E-06	4.85E-06	4.14E-06	3.75E-07	9.41E-06	2.84E-06	3.34E-06
C9orf78	6.70E-05	2.75E-05	4.70E-05	3.80E-05	3.65E-05	4.72E-05	4.34E-05	5.36E-05
C9orf79	0	0	0	0	0	0	0	0
C9orf80	8.25E-06	5.01E-06	4.30E-06	3.89E-06	4.89E-07	4.92E-06	2.17E-06	3.29E-06
C9orf82	9.39E-06	9.59E-06	1.03E-05	7.60E-06	2.77E-06	1.52E-05	4.37E-06	7.86E-06
C9orf84	1.01E-08	0	1.03E-08	0	4.31E-09	9.30E-09	4.78E-09	7.36E-09
C9orf85	8.04E-06	3.39E-06	3.66E-06	3.82E-06	1.34E-06	6.35E-06	4.68E-06	3.64E-06
C9orf86	1.57E-05	4.74E-06	5.68E-06	1.02E-05	1.17E-05	6.95E-06	1.08E-05	1.24E-05
C9orf89	2.31E-05	1.54E-05	9.28E-06	1.80E-05	8.66E-06	1.05E-05	1.59E-05	1.45E-05
C9orf9	2.45E-06	1.18E-06	1.37E-06	2.08E-06	5.17E-07	4.78E-06	9.33E-07	2.26E-06
C9orf91	4.16E-06	1.44E-06	2.75E-06	2.69E-06	9.22E-07	3.58E-06	2.69E-06	4.70E-06
C9orf93	2.61E-07	2.98E-07	1.19E-07	2.59E-07	1.40E-08	5.10E-07	7.60E-08	2.40E-07
C9orf95	2.16E-05	1.32E-05	2.86E-05	2.42E-05	1.48E-05	2.33E-05	2.64E-05	1.52E-05
C9orf96	1.24E-07	4.35E-07	6.37E-08	6.71E-08	2.88E-08	2.07E-07	2.67E-08	3.02E-08
C9orf98	5.64E-08	0	0	8.32E-08	4.87E-08	6.43E-08	1.09E-07	0
CA1	9.08E-07	4.63E-08	4.33E-08	4.97E-08	0	0	1.94E-07	1.79E-07
CA10	4.65E-08	2.63E-08	0	0	0	0	0	1.20E-07
CA11	2.38E-06	1.62E-06	7.67E-07	2.36E-06	3.32E-07	6.19E-07	7.56E-07	3.93E-07
CA12	5.41E-05	3.56E-06	6.02E-07	3.65E-07	2.61E-08	1.29E-06	1.24E-06	2.43E-06
CA13	8.78E-06	1.26E-06	9.71E-07	1.80E-06	6.82E-07	7.58E-07	9.29E-07	2.03E-07
CA14	5.80E-08	4.14E-07	4.56E-06	3.43E-06	6.09E-07	2.64E-06	3.46E-06	8.56E-08
CA2	0.000125	0.000163	9.87E-05	4.18E-05	4.56E-05	1.58E-05	5.15E-05	4.15E-05
CA3	6.28E-06	2.82E-07	3.48E-07	2.51E-06	1.51E-07	1.81E-07	1.13E-07	8.62E-08
CA4	2.93E-07	8.37E-06	0	2.22E-07	8.56E-07	2.85E-06	2.50E-06	0
CA5A	0	1.12E-05	2.66E-05	2.68E-05	1.71E-05	2.31E-05	1.27E-05	1.88E-05
CA5B	1.54E-06	1.95E-07	2.84E-07	5.43E-07	2.09E-07	6.55E-06	3.14E-07	5.96E-07
CA5BP	1.53E-05	4.52E-06	5.12E-06	4.51E-06	1.55E-06	5.54E-06	6.00E-06	4.73E-06
CA6	0	0	0	0	0	0	2.15E-08	0
CA7	0	0	0	0	0	0	0	0
CA8	7.55E-08	2.15E-07	5.41E-08	3.23E-08	9.24E-08	4.22E-08	2.71E-07	3.05E-08
CA9	4.41E-06	3.70E-08	7.43E-07	3.37E-06	4.20E-08	3.37E-07	7.41E-08	2.40E-08
CAB39	4.44E-05	2.51E-05	2.12E-05	1.87E-05	9.03E-06	2.29E-05	1.85E-05	1.90E-05
CAB39L	1.39E-06	1.58E-06	1.33E-06	2.80E-06	4.53E-07	1.43E-06	2.17E-06	1.33E-06
CABC1	3.11E-05	7.34E-05	5.50E-05	4.09E-05	5.08E-05	0.000159	5.12E-05	3.96E-05
CABIN1	1.77E-05	1.37E-05	7.79E-06	1.21E-05	1.56E-05	1.76E-05	1.28E-05	1.67E-05
CABLES1	3.26E-06	2.33E-06	4.30E-06	5.30E-06	1.91E-06	3.68E-06	6.15E-06	7.67E-07
CABLES2	2.56E-06	2.48E-06	1.17E-06	1.20E-06	7.10E-07	2.03E-06	1.48E-06	3.11E-06
CABP1	9.39E-07	9.86E-07	2.94E-08	0	4.22E-07	4.26E-07	4.65E-07	1.60E-07
CABP2	0	0	0	0	0	0	0	0
CABP4	3.32E-07	8.57E-07	3.27E-07	1.68E-07	1.53E-08	1.27E-07	3.75E-07	2.62E-08
CABP5	0	7.03E-08	0	0	0	0	0	0
CABP7	2.18E-07	1.03E-07	3.39E-08	6.18E-08	1.07E-07	3.86E-08	2.87E-07	2.45E-08
CABYR	7.78E-06	8.09E-07	7.18E-08	2.72E-07	4.86E-07	3.76E-07	1.50E-07	2.00E-07
CACHD1	3.05E-06	2.78E-06	1.53E-06	1.49E-06	2.99E-07	3.44E-07	1.27E-06	2.75E-06
CACNA1A	3.39E-07	2.57E-08	1.18E-08	2.10E-08	5.42E-08	3.72E-08	1.34E-07	5.05E-08
CACNA1B	0	0	0	0	0	6.21E-09	0	0
CACNA1C	8.93E-07	1.19E-07	7.54E-08	3.96E-07	1.88E-07	1.22E-07	2.68E-07	2.29E-07
CACNA1D	5.75E-06	7.80E-07	9.40E-07	1.01E-06	1.91E-07	3.93E-07	3.83E-07	2.73E-06
CACNA1E	1.12E-07	0	0	0	0	0	2.94E-09	3.11E-06
CACNA1F	7.43E-09	8.29E-09	0	4.53E-09	3.18E-09	0	0	0
CACNA1G	1.34E-08	0	0	1.58E-08	0	0	3.17E-09	0
CACNA1H	4.52E-05	2.84E-07	1.23E-05	9.64E-07	7.15E-07	2.19E-07	1.63E-05	5.61E-06
CACNA1I	8.95E-09	0	2.28E-09	1.64E-08	3.83E-09	8.72E-08	1.96E-08	2.29E-08
CACNA1S	3.63E-09	4.05E-09	0	0	3.11E-09	3.36E-09	0	0
CACNA2D	2.04E-07	2.28E-08	5.73E-08	1.12E-07	1.34E-08	1.11E-08	5.81E-09	4.58E-08

CACNA2D	1.07E-06	6.30E-08	2.14E-07	1.15E-07	4.68E-08	1.89E-08	2.99E-07	2.67E-08
CACNA2D	5.76E-08	6.98E-09	1.92E-08	3.80E-08	5.35E-09	7.38E-08	3.03E-08	1.82E-08
CACNA2D	6.13E-07	3.40E-07	5.60E-07	5.84E-07	3.93E-07	4.96E-07	4.39E-07	1.08E-07
CACNB1	1.06E-06	5.56E-08	1.73E-07	2.70E-07	1.53E-07	1.03E-07	2.11E-07	2.83E-07
CACNB2	3.79E-06	5.54E-07	1.99E-07	3.26E-07	6.67E-08	1.51E-07	1.14E-07	6.35E-07
CACNB3	2.89E-06	3.61E-07	2.19E-07	1.27E-06	5.20E-07	2.46E-07	7.30E-07	7.55E-07
CACNB4	5.14E-08	9.55E-09	1.46E-08	6.96E-09	2.36E-09	9.42E-07	3.17E-08	1.61E-08
CACNG1	0	2.32E-08	0	0	0	1.88E-08	1.94E-08	0
CACNG2	0	0	0	0	0	0	0	0
CACNG3	0	0	0	2.11E-08	0	1.60E-08	0	0
CACNG4	4.06E-08	3.04E-08	2.09E-08	1.57E-07	0	1.25E-08	3.15E-07	9.91E-09
CACNG5	0	0	0	0	0	0	0	0
CACNG6	0	0	0	3.68E-08	0	0	2.89E-08	0
CACNG7	0	0	0	0	0	0	0	0
CACNG8	1.77E-08	2.02E-08	0	0	0	0	1.70E-08	0
CACYBP	1.99E-05	3.95E-05	7.64E-06	7.52E-06	4.74E-06	1.36E-05	1.15E-05	1.24E-05
CAD	1.29E-05	1.18E-05	1.72E-06	1.90E-06	1.79E-06	7.58E-06	2.56E-06	3.57E-06
CADM1	3.44E-05	8.42E-05	2.61E-05	4.59E-05	1.57E-05	6.21E-05	2.66E-05	7.89E-05
CADM2	0	1.26E-06	1.43E-07	2.78E-07	0	1.54E-07	0	4.05E-08
CADM3	3.16E-07	3.09E-08	4.65E-08	2.60E-07	0	5.80E-08	1.69E-07	9.43E-08
CADM4	1.93E-05	5.55E-07	4.56E-06	3.45E-06	2.54E-07	1.13E-06	8.54E-06	1.24E-05
CADPS	1.33E-06	0	2.36E-08	5.24E-09	2.72E-08	1.05E-08	0	0
CADPS2	4.10E-06	3.52E-06	2.77E-06	3.67E-06	1.54E-06	1.02E-05	2.60E-06	7.10E-06
CAGE1	2.12E-08	7.30E-08	0	0	0	0	0	0
CALB1	5.12E-07	1.19E-08	0	0	1.59E-08	0	1.02E-08	0
CALB2	1.85E-08	0	1.97E-08	9.09E-08	0	0	1.77E-08	2.73E-08
CALCA	6.75E-05	0	8.36E-08	2.51E-07	0	0	0	2.22E-07
CALCB	1.24E-05	0	0	6.36E-08	0	0	0	3.83E-08
CALCOCC	2.98E-05	2.99E-05	1.78E-05	3.35E-05	8.17E-06	3.75E-05	1.57E-05	1.35E-05
CALCOCC	7.47E-05	7.62E-05	3.77E-05	3.64E-05	1.42E-05	5.71E-05	3.07E-05	3.10E-05
CALCR	5.86E-08	2.82E-08	8.67E-09	1.02E-08	0	0	8.92E-08	7.48E-08
CALCRL	1.00E-05	4.83E-06	3.35E-06	3.43E-06	2.81E-06	3.66E-06	2.31E-06	1.02E-05
CALD1	7.77E-05	0.000146	0.000119	0.000127	4.68E-05	8.49E-05	7.26E-05	0.000118
CALHM1	7.57E-09	0	0	0	0	0	0	0
CALHM2	7.56E-06	1.85E-06	1.95E-06	4.92E-06	2.08E-06	1.57E-06	3.87E-06	3.65E-06
CALHM3	0	0	0	0	0	0	0	0
CALM1	0.000108	3.91E-05	4.39E-05	5.85E-05	4.20E-05	3.49E-05	7.62E-05	4.39E-05
CALM2	0.00064	0.000356	0.000168	0.000347	0.000107	0.000259	0.000247	0.00022
CALM3	0.000156	8.51E-05	7.61E-05	8.03E-05	5.74E-05	0.000144	0.000114	5.36E-05
CALML3	0	2.23E-08	3.32E-06	1.92E-07	3.36E-08	1.27E-07	3.92E-07	1.73E-07
CALML4	6.93E-06	2.14E-06	2.74E-06	2.55E-06	3.34E-06	2.83E-06	4.79E-06	1.40E-06
CALML5	0	0	3.12E-06	0	0	0	0	4.72E-08
CALML6	1.66E-07	1.65E-07	0	1.17E-07	2.05E-07	2.22E-08	1.82E-07	1.06E-07
CALN1	2.39E-09	0	2.04E-07	5.82E-09	0	0	2.28E-09	0
CALR	0.000786	0.000568	0.000433	0.0006	0.000713	0.00078	0.000755	0.001172
CALR3	3.96E-08	1.13E-07	0	2.44E-08	5.12E-08	1.10E-07	1.90E-08	5.86E-08
CALU	3.95E-05	2.75E-05	1.83E-05	2.68E-05	6.62E-06	2.29E-05	1.96E-05	4.52E-05
CALY	2.73E-07	1.90E-07	0	0	0	5.09E-08	0	0
CAMK1	3.23E-05	1.86E-05	2.03E-05	1.99E-05	2.28E-05	1.76E-05	2.62E-05	8.15E-06
CAMK1D	4.56E-06	3.35E-07	2.74E-06	1.58E-06	3.91E-07	4.63E-06	1.60E-06	4.86E-06
CAMK1G	1.43E-07	4.29E-08	6.93E-08	2.45E-07	6.54E-08	3.53E-08	6.45E-08	1.26E-07
CAMK2A	3.57E-08	6.66E-09	2.45E-08	2.90E-08	5.10E-09	1.10E-08	0	8.70E-09
CAMK2B	5.74E-06	2.58E-08	3.34E-06	2.33E-07	1.27E-06	3.75E-08	9.19E-07	4.55E-08
CAMK2D	1.06E-05	5.39E-06	8.19E-06	7.98E-06	1.32E-06	5.72E-06	6.17E-06	5.42E-06
CAMK2G	1.38E-05	6.78E-06	3.89E-06	4.28E-06	1.95E-06	6.87E-06	5.82E-06	5.83E-06
CAMK2N1	0.00011	1.98E-05	3.75E-05	5.66E-05	3.56E-05	2.94E-05	4.63E-05	5.27E-05
CAMK2N2	5.16E-06	1.92E-05	6.33E-07	4.56E-07	1.02E-06	1.24E-06	4.91E-06	8.58E-06
CAMK4	1.01E-06	8.33E-08	1.80E-07	5.86E-07	3.17E-08	1.13E-07	1.07E-07	7.22E-08

CAMKK1	1.42E-06	1.23E-07	4.85E-07	5.34E-07	3.66E-07	3.13E-07	8.69E-07	2.89E-07
CAMKK2	1.61E-05	2.26E-05	9.00E-06	9.37E-06	1.05E-05	1.32E-05	1.81E-05	1.23E-05
CAMKV	3.94E-08	0	0	0	0	0	0	0
CAMLG	2.23E-05	9.88E-06	1.01E-05	1.19E-05	6.18E-06	1.31E-05	1.10E-05	1.67E-05
CAMP	8.24E-08	0	4.73E-08	2.06E-07	3.59E-08	0	0	0
CAMSAP1	3.87E-06	1.94E-06	1.38E-06	3.35E-06	5.60E-07	2.75E-06	1.68E-06	4.30E-06
CAMSAP1	5.86E-06	2.53E-06	1.78E-06	2.43E-06	7.82E-07	4.30E-06	1.95E-06	2.94E-06
CAMTA1	3.25E-05	4.76E-05	3.04E-05	3.59E-05	2.22E-05	3.36E-05	3.63E-05	2.09E-05
CAMTA2	7.70E-06	3.59E-06	4.79E-06	6.15E-06	6.45E-06	7.30E-06	1.01E-05	8.24E-06
CAND1	1.83E-05	1.61E-05	1.15E-05	1.15E-05	4.22E-06	1.50E-05	8.07E-06	1.15E-05
CAND2	3.51E-07	5.96E-07	6.63E-07	4.26E-07	3.54E-07	1.20E-07	3.53E-07	1.18E-07
CANT1	2.66E-05	2.34E-05	8.31E-06	1.27E-05	1.13E-05	2.33E-05	1.36E-05	1.18E-05
CANX	0.000267	0.000194	0.000116	0.000116	3.03E-05	0.000195	9.21E-05	0.000229
CAP1	0.000131	7.21E-05	5.41E-05	8.21E-05	1.84E-05	8.53E-05	5.38E-05	6.58E-05
CAP2	7.87E-06	2.88E-05	1.84E-06	7.26E-06	1.26E-06	1.94E-05	5.75E-06	1.39E-05
CAPG	9.25E-05	1.67E-05	6.96E-06	6.60E-05	1.09E-05	5.22E-06	2.82E-05	4.19E-06
CAPN1	8.27E-05	8.57E-05	3.87E-05	4.50E-05	1.08E-05	3.39E-05	3.52E-05	5.19E-05
CAPN10	9.41E-06	3.80E-06	2.50E-06	4.30E-06	4.94E-06	3.14E-06	5.49E-06	4.17E-06
CAPN11	2.05E-07	8.65E-08	5.32E-08	5.23E-08	1.47E-07	5.57E-08	1.99E-07	1.63E-07
CAPN12	8.19E-05	1.40E-05	5.06E-06	8.14E-06	2.29E-05	3.70E-06	5.29E-06	1.37E-05
CAPN13	1.66E-08	5.67E-08	0	0	0	0	8.48E-09	0
CAPN14	1.21E-08	2.70E-08	0	7.37E-09	0	1.11E-08	0	1.77E-08
CAPN2	7.05E-05	3.11E-05	1.77E-05	4.18E-05	1.53E-05	3.04E-05	3.01E-05	3.57E-05
CAPN3	3.31E-06	2.21E-06	3.19E-06	3.89E-06	3.38E-06	3.69E-06	2.79E-06	2.87E-06
CAPN5	2.72E-05	3.93E-05	2.58E-05	1.18E-05	1.17E-05	3.12E-06	1.38E-05	2.97E-05
CAPN6	1.71E-05	1.61E-08	2.76E-07	1.11E-05	0	7.81E-08	1.18E-07	0
CAPN7	7.99E-06	9.93E-06	6.80E-06	5.78E-06	2.31E-06	9.65E-06	3.39E-06	4.98E-06
CAPN8	2.04E-07	1.80E-07	0	2.78E-08	9.77E-09	0	5.49E-08	0
CAPN9	6.99E-08	3.57E-07	2.04E-08	0	0	0	0	0
CAPNS1	0.000244	0.000197	9.17E-05	0.000187	0.000241	0.000195	0.000222	0.000159
CAPNS2	7.92E-08	9.19E-08	1.45E-07	3.27E-08	0	2.47E-08	1.51E-07	3.94E-08
CAPRIN1	3.84E-05	4.24E-05	1.74E-05	2.07E-05	6.97E-06	2.98E-05	1.68E-05	2.27E-05
CAPRIN2	4.87E-06	4.31E-06	2.18E-06	2.84E-06	1.15E-06	4.41E-06	1.66E-06	2.63E-06
CAPS	5.67E-06	1.62E-06	2.83E-06	3.37E-06	1.16E-05	3.85E-06	3.73E-06	2.73E-06
CAPS2	7.78E-07	5.10E-07	2.12E-07	3.05E-07	1.62E-07	3.40E-07	8.84E-08	1.24E-07
CAPSL	5.54E-08	0	0	0	0	0	0	0
CAPZA1	6.91E-05	5.71E-05	2.73E-05	3.50E-05	6.18E-06	5.22E-05	2.07E-05	3.47E-05
CAPZA2	4.07E-05	5.02E-05	3.61E-05	4.00E-05	1.85E-05	4.41E-05	3.65E-05	5.22E-05
CAPZA3	0	1.13E-07	7.96E-08	1.81E-07	4.22E-08	0	2.10E-07	3.63E-08
CAPZB	0.000154	0.000101	7.08E-05	9.83E-05	5.53E-05	0.000111	9.56E-05	9.00E-05
CARD10	2.34E-05	1.06E-05	6.25E-06	9.99E-06	8.11E-06	1.06E-05	1.19E-05	9.04E-06
CARD11	4.88E-06	1.51E-07	8.90E-07	1.82E-06	2.23E-08	6.87E-07	8.07E-07	4.02E-07
CARD14	2.24E-07	4.17E-07	1.26E-07	7.91E-08	7.34E-08	2.51E-07	5.62E-08	3.91E-08
CARD16	2.07E-05	2.81E-06	5.22E-06	2.35E-05	4.14E-06	1.51E-05	1.70E-05	8.19E-06
CARD17	7.77E-08	0	0	0	0	0	0	0
CARD18	1.65E-06	0	0	0	0	0	0	2.88E-07
CARD6	2.36E-06	1.46E-06	1.52E-06	3.07E-06	4.10E-07	2.16E-06	9.29E-07	7.00E-06
CARD8	7.81E-06	5.67E-06	4.52E-06	5.62E-06	4.65E-06	8.34E-06	3.24E-06	1.16E-05
CARD9	1.84E-06	2.03E-07	6.33E-07	1.62E-06	2.12E-07	5.10E-07	9.98E-07	9.12E-08
CARHSP1	5.96E-05	6.81E-05	4.28E-05	4.32E-05	2.94E-05	1.79E-05	4.30E-05	4.15E-05
CARKD	4.46E-05	5.28E-05	3.29E-05	2.83E-05	2.19E-05	4.46E-05	3.45E-05	4.82E-05
CARM1	2.83E-05	1.28E-05	1.35E-05	1.52E-05	1.69E-05	1.98E-05	2.52E-05	1.82E-05
CARNS1	4.88E-07	1.18E-07	1.11E-07	2.49E-07	8.94E-08	1.56E-07	5.06E-08	1.88E-07
CARS	1.92E-05	2.08E-05	2.51E-05	2.22E-05	1.29E-05	1.97E-05	1.98E-05	2.96E-05
CARS2	3.14E-05	3.69E-05	1.58E-05	2.12E-05	1.38E-05	2.24E-05	2.48E-05	2.09E-05
CARTPT	0	1.76E-07	0	0	0	0	0	0
CASC1	2.04E-08	2.31E-08	1.84E-08	1.54E-07	0	6.64E-08	2.03E-08	0
CASC2	1.61E-07	1.47E-07	1.47E-07	2.35E-07	1.21E-08	2.27E-07	7.74E-08	8.95E-08

CASC3	2.79E-05	2.32E-05	8.98E-06	1.04E-05	6.59E-06	2.47E-05	1.30E-05	1.15E-05
CASC4	3.43E-05	2.32E-05	2.68E-05	2.34E-05	5.61E-06	2.48E-05	2.11E-05	2.54E-05
CASC5	1.72E-06	1.43E-06	4.39E-07	7.85E-07	3.01E-08	1.41E-06	1.68E-07	5.00E-07
CASD1	1.23E-05	9.64E-06	3.67E-06	3.89E-06	9.75E-07	5.20E-06	3.65E-06	4.44E-06
CASK	5.70E-06	2.93E-06	2.73E-06	4.66E-06	1.15E-06	7.83E-06	3.60E-06	6.74E-06
CASKIN1	2.09E-07	2.98E-08	0	0	1.05E-08	4.08E-09	1.83E-08	0
CASKIN2	1.23E-05	1.47E-05	3.25E-06	4.52E-06	7.49E-06	5.76E-06	5.92E-06	6.96E-06
CASP1	2.34E-05	3.04E-06	6.91E-06	1.93E-05	1.35E-06	1.50E-05	8.78E-06	8.04E-06
CASP10	7.99E-06	3.05E-06	2.32E-06	2.73E-06	1.18E-06	2.24E-06	2.70E-06	1.75E-06
CASP12	1.33E-07	1.34E-07	1.11E-07	0	3.68E-08	6.21E-08	1.87E-07	1.90E-07
CASP14	0	2.96E-08	2.79E-08	0	0	0	0	0
CASP2	5.84E-06	4.04E-06	2.13E-06	2.98E-06	1.97E-06	3.47E-06	2.58E-06	4.60E-06
CASP3	3.05E-05	7.85E-06	6.81E-06	9.31E-06	4.62E-06	1.03E-05	1.16E-05	1.17E-05
CASP4	8.22E-05	2.12E-05	2.36E-05	5.26E-05	7.35E-06	5.30E-05	2.60E-05	5.97E-05
CASP5	4.55E-07	0	3.55E-08	1.11E-07	0	8.39E-08	8.53E-08	0
CASP6	1.82E-05	5.80E-06	1.24E-05	9.28E-06	5.75E-06	9.60E-06	7.75E-06	7.22E-06
CASP7	1.92E-05	1.50E-05	1.47E-05	1.40E-05	4.90E-06	7.17E-06	1.37E-05	8.71E-06
CASP8	2.96E-05	1.64E-05	7.89E-06	7.69E-06	1.38E-06	1.18E-05	3.24E-06	8.08E-06
CASP8AP2	3.11E-06	2.35E-06	1.48E-06	1.01E-06	1.42E-07	1.35E-06	5.24E-07	1.02E-06
CASP9	3.10E-05	1.09E-05	5.23E-06	6.54E-06	2.27E-06	9.87E-06	4.08E-06	1.31E-05
CASQ1	9.82E-08	6.95E-08	0	4.51E-08	0	1.14E-08	2.37E-08	1.81E-08
CASQ2	1.51E-07	1.37E-06	2.95E-08	2.54E-07	4.05E-07	4.56E-07	4.08E-07	5.58E-07
CASR	7.44E-07	1.23E-07	8.48E-08	3.92E-08	0	8.50E-09	0	6.72E-09
CASS4	8.65E-07	2.83E-07	5.71E-07	9.76E-07	4.51E-08	1.31E-06	1.94E-07	4.18E-07
CAST	7.61E-05	6.09E-05	4.78E-05	6.63E-05	1.14E-05	4.47E-05	5.52E-05	6.72E-05
CASZ1	3.81E-06	1.10E-06	6.20E-07	1.83E-06	1.28E-07	6.15E-07	6.54E-07	1.42E-06
CAT	0.000133	0.0004	0.00055	0.000196	0.00016	0.000455	0.000279	0.000158
CATSPER1	3.57E-08	0	0	1.09E-08	0	0	1.73E-08	2.62E-08
CATSPER2	1.79E-06	2.87E-07	2.08E-07	2.88E-07	9.14E-07	4.06E-07	3.64E-07	2.51E-07
CATSPER2	1.89E-06	1.28E-06	8.13E-07	6.18E-07	4.29E-07	1.17E-06	5.43E-07	1.17E-06
CATSPER3	3.59E-07	3.46E-07	1.62E-07	5.81E-07	4.89E-07	2.28E-07	3.26E-07	3.42E-07
CATSPER4	0	0	0	0	0	0	0	0
CATSPERE	5.95E-07	0	2.26E-08	1.33E-08	0	0	0	1.02E-07
CATSPERG	2.16E-07	2.00E-07	1.57E-07	3.88E-08	3.27E-08	1.77E-07	1.05E-07	1.86E-08
CAV1	1.87E-05	3.36E-05	8.83E-06	1.69E-05	6.52E-06	8.84E-06	2.44E-05	1.26E-05
CAV2	2.57E-05	1.22E-05	1.27E-05	2.67E-05	9.85E-06	2.02E-06	2.07E-05	1.87E-05
CAV3	0	0	0	0	0	0	0	0
CBARA1	4.15E-05	3.47E-05	5.71E-05	4.40E-05	4.70E-05	9.33E-05	6.00E-05	5.49E-05
CBFA2T2	8.38E-06	3.88E-06	9.78E-07	1.29E-06	6.74E-07	1.48E-06	1.79E-06	1.71E-06
CBFA2T3	1.42E-06	4.49E-07	3.38E-06	3.32E-06	7.57E-07	8.01E-07	1.83E-06	3.98E-07
CBFB	1.56E-05	6.14E-06	5.16E-06	9.14E-06	9.07E-07	6.31E-06	5.64E-06	6.95E-06
CBL	3.23E-06	7.60E-07	8.34E-07	1.10E-06	1.58E-07	7.07E-07	8.41E-07	1.16E-06
CBLB	8.92E-06	1.17E-05	2.31E-06	9.71E-06	8.96E-07	4.63E-06	4.03E-06	4.87E-06
CBLC	7.13E-06	5.23E-06	1.14E-05	1.60E-05	1.98E-05	4.72E-06	1.94E-05	1.97E-05
CBLL1	4.37E-06	7.84E-06	3.65E-06	3.71E-06	1.70E-06	4.85E-06	3.40E-06	9.44E-06
CBLN1	5.91E-08	1.22E-06	2.72E-07	2.61E-07	5.79E-07	3.97E-06	3.61E-06	1.93E-06
CBLN2	0	0	2.04E-07	1.20E-08	0	0	0	0
CBLN3	1.55E-06	9.89E-07	1.22E-06	7.28E-07	3.73E-07	8.69E-07	5.95E-07	7.55E-07
CBLN4	1.22E-07	5.27E-08	7.20E-07	1.37E-07	6.44E-08	1.41E-06	4.53E-08	1.37E-08
CBR1	0.000167	0.000238	0.000228	0.000168	0.000387	0.001503	0.000178	0.000235
CBR3	1.62E-06	1.52E-06	4.60E-07	8.71E-07	1.99E-06	4.61E-07	6.52E-07	8.74E-07
CBR4	1.98E-05	8.73E-06	2.07E-05	1.30E-05	8.77E-06	1.60E-05	1.19E-05	1.22E-05
CBS	0.000161	0.000239	0.000195	0.000102	0.000147	0.000173	0.000117	6.42E-05
CBWD1	2.61E-05	1.38E-05	1.03E-05	1.25E-05	8.29E-06	2.12E-05	8.26E-06	1.38E-05
CBWD2	2.42E-05	1.51E-05	9.44E-06	1.33E-05	8.25E-06	2.20E-05	7.96E-06	8.57E-06
CBWD3	4.40E-05	3.61E-05	2.68E-05	2.41E-05	8.10E-06	2.24E-05	8.64E-06	7.29E-06
CBWD5	0	0	4.88E-07	2.32E-06	0	0	0	0
CBWD6	3.63E-05	7.14E-06	4.94E-06	1.71E-05	1.84E-06	2.41E-05	1.34E-05	1.23E-05

CBX1	2.74E-05	2.23E-05	7.34E-06	1.06E-05	5.21E-06	1.63E-05	1.03E-05	1.13E-05
CBX2	1.50E-06	3.35E-07	1.51E-07	7.97E-08	5.00E-07	1.34E-06	4.60E-07	6.45E-07
CBX3	0.000136	9.98E-05	3.44E-05	4.80E-05	2.25E-05	4.74E-05	4.44E-05	6.31E-05
CBX4	1.71E-05	1.92E-05	1.19E-05	8.19E-06	5.26E-06	2.30E-05	1.11E-05	1.69E-05
CBX5	6.18E-06	5.15E-06	2.52E-06	2.86E-06	8.67E-07	4.72E-06	3.93E-06	3.50E-06
CBX6	4.20E-06	1.32E-06	1.40E-06	2.07E-06	5.41E-07	1.03E-06	5.09E-07	4.07E-06
CBX7	6.31E-06	5.76E-06	6.63E-06	6.28E-06	4.76E-06	9.98E-06	1.12E-05	1.21E-05
CBX8	1.46E-05	1.01E-05	2.95E-06	2.58E-06	3.78E-06	9.62E-06	3.84E-06	1.05E-05
CBY1	1.93E-05	1.16E-05	1.15E-05	1.89E-05	2.06E-05	1.84E-05	2.33E-05	1.41E-05
CC2D1A	1.04E-05	9.51E-06	9.19E-06	1.32E-05	1.24E-05	1.96E-05	1.10E-05	9.30E-06
CC2D1B	2.24E-05	1.51E-05	8.71E-06	1.00E-05	8.22E-06	1.15E-05	8.65E-06	1.71E-05
CC2D2A	2.86E-06	8.92E-07	1.16E-06	1.80E-06	1.04E-06	1.74E-06	8.85E-07	5.31E-07
CC2D2B	1.56E-07	0	0	4.14E-08	2.26E-08	2.43E-08	1.27E-08	8.36E-08
CCAR1	2.53E-05	8.93E-06	6.10E-06	8.80E-06	4.90E-06	1.58E-05	6.47E-06	1.10E-05
CCBE1	6.56E-08	1.05E-08	1.67E-06	3.10E-06	8.00E-09	8.63E-09	1.53E-07	4.12E-07
CCBL1	2.04E-05	8.06E-06	6.08E-06	6.12E-06	5.62E-06	6.33E-06	3.75E-06	1.14E-05
CCBL2	1.96E-05	7.36E-06	6.55E-06	1.01E-05	2.92E-06	2.30E-05	5.04E-06	9.74E-06
CCBP2	9.95E-07	7.15E-06	3.53E-06	4.42E-06	1.75E-06	2.33E-05	8.87E-06	1.96E-06
CCDC101	1.44E-05	2.99E-05	1.62E-05	1.48E-05	1.99E-05	1.21E-05	2.27E-05	1.49E-05
CCDC102/	3.64E-06	7.32E-07	6.76E-07	2.79E-06	2.33E-07	8.54E-07	1.35E-06	5.98E-07
CCDC102I	3.07E-06	1.00E-06	4.00E-07	1.49E-06	3.14E-07	1.00E-06	9.12E-07	1.29E-06
CCDC103	1.81E-06	1.12E-06	1.43E-07	1.03E-06	3.54E-07	8.46E-07	2.67E-07	2.78E-07
CCDC104	2.54E-05	1.46E-05	5.46E-06	1.32E-05	5.82E-06	1.32E-05	9.07E-06	6.31E-06
CCDC105	0	0	0	0	0	0	0	0
CCDC106	7.01E-06	1.60E-05	9.72E-06	9.73E-06	2.12E-05	1.15E-05	1.17E-05	5.43E-06
CCDC107	2.91E-05	3.63E-05	1.79E-05	4.35E-05	6.57E-05	3.80E-05	6.93E-05	7.96E-05
CCDC108	1.42E-07	2.03E-08	0	0	3.89E-08	3.56E-07	0	5.51E-09
CCDC109/	1.43E-05	6.02E-06	4.23E-06	7.23E-06	2.63E-06	5.36E-06	5.47E-06	1.08E-05
CCDC109I	1.70E-05	2.76E-07	1.32E-06	4.35E-06	4.85E-07	1.08E-06	1.23E-06	1.61E-06
CCDC11	1.19E-07	1.35E-07	4.19E-08	1.14E-07	9.14E-08	6.65E-07	2.05E-07	3.91E-08
CCDC110	4.46E-07	4.51E-08	1.83E-07	1.27E-07	7.18E-09	1.97E-07	2.43E-08	1.18E-07
CCDC111	5.07E-06	3.89E-06	2.81E-06	2.88E-06	6.84E-07	3.64E-06	2.39E-06	2.86E-06
CCDC112	2.33E-06	9.96E-08	4.16E-07	1.28E-06	1.43E-07	2.44E-07	4.21E-07	4.18E-07
CCDC113	8.17E-07	3.70E-07	2.85E-07	6.61E-07	2.80E-08	2.48E-06	3.87E-07	4.11E-07
CCDC114	5.49E-08	3.56E-08	1.24E-08	6.22E-08	8.16E-08	3.52E-07	6.72E-08	1.75E-08
CCDC115	2.75E-05	1.80E-05	1.55E-05	1.80E-05	4.81E-06	1.66E-05	1.29E-05	1.31E-05
CCDC116	1.17E-07	2.45E-08	0	2.65E-08	0	2.45E-08	0	5.13E-08
CCDC117	1.98E-05	2.20E-05	1.15E-05	7.05E-06	6.95E-06	1.69E-05	1.10E-05	1.10E-05
CCDC12	4.34E-05	2.66E-05	2.35E-05	4.67E-05	8.69E-05	4.45E-05	5.47E-05	3.93E-05
CCDC120	3.80E-06	3.04E-06	1.47E-06	3.40E-06	8.93E-07	2.07E-06	3.61E-06	3.68E-06
CCDC121	2.48E-06	2.31E-06	1.26E-06	6.60E-07	2.78E-07	9.48E-07	5.48E-07	9.65E-07
CCDC122	1.56E-06	9.46E-07	9.64E-07	1.01E-06	1.95E-07	1.16E-06	7.73E-07	2.65E-07
CCDC123	5.74E-06	1.88E-06	1.26E-06	3.15E-06	1.49E-06	4.40E-06	2.39E-06	2.60E-06
CCDC124	9.12E-05	6.85E-05	3.90E-05	5.69E-05	0.000142	6.62E-05	8.09E-05	5.43E-05
CCDC125	8.12E-06	6.56E-06	7.25E-06	5.52E-06	2.93E-06	1.06E-05	4.83E-06	6.57E-06
CCDC126	5.32E-06	6.69E-06	3.89E-06	2.42E-06	6.80E-07	3.92E-06	2.06E-06	4.84E-06
CCDC127	2.07E-05	1.16E-05	7.47E-06	8.30E-06	7.99E-06	1.04E-05	9.04E-06	1.39E-05
CCDC129	0	0	0	0	0	0	0	0
CCDC13	5.16E-07	2.24E-05	9.59E-08	4.65E-07	1.22E-07	1.67E-07	2.01E-07	5.50E-07
CCDC130	1.97E-05	8.96E-06	6.63E-06	1.15E-05	1.21E-05	9.19E-06	1.22E-05	1.12E-05
CCDC132	8.20E-06	8.63E-06	3.14E-06	3.72E-06	1.32E-06	5.53E-06	3.50E-06	4.78E-06
CCDC134	4.09E-06	1.53E-06	8.19E-07	1.01E-06	2.50E-06	6.47E-07	9.00E-07	5.29E-06
CCDC135	0	3.14E-07	3.56E-08	9.75E-08	3.69E-08	5.42E-07	5.41E-08	1.64E-07
CCDC136	4.72E-07	1.64E-08	4.10E-08	9.01E-08	2.56E-08	8.70E-08	3.18E-08	2.76E-08
CCDC137	1.86E-05	1.66E-05	4.65E-06	8.55E-06	1.06E-05	1.30E-05	1.06E-05	9.55E-06
CCDC138	2.32E-06	1.03E-06	6.42E-07	3.84E-07	1.17E-07	4.83E-07	2.25E-07	1.27E-07
CCDC14	1.53E-05	4.83E-06	1.45E-06	2.56E-06	1.28E-06	2.30E-06	1.88E-06	1.89E-06
CCDC140	0	0	0	0	0	0	0	0

CCDC141	6.10E-08	2.99E-07	5.16E-08	1.13E-07	1.23E-08	6.16E-08	5.86E-09	0
CCDC142	6.16E-06	3.04E-06	1.08E-06	9.10E-07	5.81E-07	1.90E-06	1.56E-06	1.15E-06
CCDC144/	4.52E-08	4.30E-09	4.18E-08	1.97E-08	0	0	7.50E-09	0
CCDC144I	6.11E-08	5.10E-08	5.97E-08	3.11E-08	0	1.67E-08	0	0
CCDC144(	5.41E-09	1.81E-08	5.55E-09	0	0	5.00E-09	0	0
CCDC144I	0	3.45E-07	0	0	0	0	0	0
CCDC146	3.25E-06	9.16E-07	1.73E-06	8.46E-06	6.36E-07	1.78E-06	2.65E-06	1.26E-06
CCDC147	2.79E-08	0	2.16E-08	8.51E-09	1.79E-08	1.94E-08	0	2.04E-08
CCDC148	2.41E-06	7.16E-08	1.33E-07	1.36E-07	5.21E-08	1.77E-07	3.39E-08	0
CCDC149	5.90E-06	2.72E-06	2.72E-06	4.28E-06	1.62E-06	7.73E-06	2.52E-06	7.19E-07
CCDC15	5.10E-07	6.96E-07	2.47E-07	2.36E-07	1.21E-07	7.32E-07	2.28E-07	4.38E-07
CCDC150	2.00E-06	8.18E-07	8.25E-07	3.64E-07	1.71E-06	7.31E-07	1.64E-07	1.20E-07
CCDC151	3.27E-07	5.63E-07	6.37E-08	2.83E-07	8.52E-08	5.08E-07	3.40E-07	5.36E-08
CCDC152	5.65E-06	1.02E-05	1.07E-05	7.40E-06	1.79E-05	6.54E-06	6.91E-06	6.86E-06
CCDC153	7.14E-07	4.44E-08	4.69E-07	6.08E-07	6.54E-07	2.47E-07	5.32E-07	5.65E-08
CCDC154	7.86E-08	3.75E-07	1.14E-07	1.27E-07	3.23E-07	4.02E-08	3.09E-07	2.99E-07
CCDC155	4.62E-08	0	0	0	7.62E-07	3.13E-08	0	0
CCDC157	2.78E-07	1.22E-07	6.39E-08	2.55E-07	1.72E-07	1.86E-07	2.18E-07	1.78E-07
CCDC158	3.54E-07	6.46E-07	3.69E-07	2.81E-07	1.63E-08	1.88E-07	1.29E-07	9.25E-08
CCDC159	1.32E-05	4.96E-06	8.79E-06	1.02E-05	1.66E-05	1.40E-05	1.17E-05	1.28E-05
CCDC160	0	0	6.29E-08	0	0	0	1.88E-08	0
CCDC163I	2.38E-06	1.14E-06	1.53E-07	1.99E-07	7.11E-08	3.82E-07	7.68E-08	6.28E-07
CCDC17	1.18E-06	1.26E-07	3.23E-07	2.23E-07	6.76E-07	5.29E-07	7.04E-07	1.25E-07
CCDC18	3.69E-06	9.14E-07	9.54E-07	1.30E-06	3.73E-07	3.12E-06	5.51E-07	6.77E-07
CCDC19	9.73E-08	2.10E-08	7.86E-08	1.16E-07	3.83E-08	8.09E-08	1.30E-07	1.20E-07
CCDC21	6.67E-06	4.28E-06	2.55E-06	1.83E-06	3.65E-07	3.72E-06	1.53E-06	1.71E-06
CCDC22	1.16E-05	1.06E-05	6.25E-06	7.20E-06	5.92E-06	9.98E-06	8.95E-06	1.45E-05
CCDC23	2.04E-05	5.28E-06	9.01E-06	1.85E-05	8.13E-06	4.17E-05	1.18E-05	1.16E-05
CCDC24	5.35E-06	3.45E-06	2.23E-06	2.05E-06	1.18E-06	6.63E-06	2.78E-06	3.00E-06
CCDC25	1.11E-05	2.95E-05	1.36E-05	1.51E-05	4.70E-06	2.46E-05	6.53E-06	6.37E-06
CCDC27	0	0	0	0	0	0	0	0
CCDC28A	1.58E-05	2.32E-05	2.15E-05	2.88E-05	1.71E-05	4.94E-05	2.69E-05	1.44E-05
CCDC28B	1.00E-05	1.51E-06	7.33E-07	2.36E-06	2.80E-06	9.38E-07	1.92E-06	1.44E-05
CCDC3	1.09E-05	2.46E-06	8.65E-06	2.34E-05	4.55E-06	3.40E-06	8.54E-06	2.86E-06
CCDC30	2.05E-07	6.16E-08	2.91E-08	3.23E-07	3.16E-08	1.62E-07	5.81E-08	7.94E-08
CCDC33	0	0	0	0	0	0	0	0
CCDC34	7.72E-06	1.10E-05	1.96E-06	2.91E-06	5.14E-06	9.93E-06	3.38E-06	3.04E-06
CCDC36	5.03E-08	9.74E-09	8.98E-09	1.06E-08	1.33E-08	3.06E-08	0	2.31E-08
CCDC37	0	2.64E-08	0	0	0	0	0	1.72E-08
CCDC38	0	1.11E-07	1.29E-08	1.51E-08	0	1.14E-08	1.19E-08	7.24E-08
CCDC39	1.67E-07	2.65E-08	4.88E-08	2.89E-08	5.08E-09	3.84E-08	0	3.47E-08
CCDC40	4.41E-07	8.38E-07	1.37E-07	5.56E-07	1.50E-07	5.85E-07	3.55E-07	2.31E-07
CCDC41	3.54E-06	1.85E-06	5.87E-07	1.10E-06	3.31E-07	1.56E-06	1.34E-06	1.04E-06
CCDC42	1.82E-08	5.16E-08	5.83E-08	5.52E-08	0	3.39E-08	4.28E-08	0
CCDC42B	1.41E-07	7.03E-08	2.13E-08	1.13E-07	7.86E-08	3.91E-08	2.91E-08	0
CCDC43	1.32E-05	8.63E-06	3.13E-06	5.25E-06	2.56E-06	9.48E-06	3.83E-06	4.86E-06
CCDC45	1.77E-05	8.03E-06	3.56E-06	6.23E-06	2.21E-06	7.19E-06	5.06E-06	4.18E-06
CCDC46	2.29E-06	1.61E-06	3.13E-07	1.44E-06	7.27E-07	2.32E-06	1.21E-06	7.52E-07
CCDC47	0.000106	0.000111	4.13E-05	3.93E-05	1.13E-05	6.63E-05	3.01E-05	4.23E-05
CCDC48	2.78E-07	3.61E-07	5.67E-07	4.78E-07	5.30E-07	9.17E-07	9.76E-07	5.22E-07
CCDC50	1.46E-05	8.11E-06	8.96E-06	1.22E-05	3.72E-06	9.95E-06	9.00E-06	1.01E-05
CCDC51	2.26E-05	1.98E-05	8.03E-06	7.14E-06	7.45E-06	1.67E-05	1.16E-05	1.08E-05
CCDC52	3.44E-06	7.33E-07	8.05E-07	1.25E-06	1.94E-07	8.63E-07	7.45E-07	8.62E-07
CCDC53	3.05E-05	2.47E-05	1.65E-05	2.41E-05	1.34E-05	0.000108	2.07E-05	2.61E-05
CCDC54	7.85E-08	2.25E-08	0	2.42E-08	0	3.65E-08	0	2.91E-08
CCDC55	1.85E-05	1.65E-05	7.99E-06	1.07E-05	3.91E-06	1.07E-05	8.82E-06	1.07E-05
CCDC56	0.0002	0.000449	0.000102	0.000107	0.000261	0.000289	0.000181	8.83E-05
CCDC57	8.11E-06	5.06E-06	7.77E-06	4.30E-06	2.42E-06	5.48E-06	3.12E-06	3.99E-06

CCDC58	3.45E-05	3.66E-05	1.82E-05	1.93E-05	2.02E-05	2.55E-05	2.82E-05	1.55E-05
CCDC59	1.50E-05	1.51E-05	8.61E-06	1.25E-05	5.80E-06	1.14E-05	1.32E-05	9.66E-06
CCDC6	8.09E-06	7.31E-06	4.36E-06	5.11E-06	1.54E-06	5.23E-06	3.75E-06	4.02E-06
CCDC60	0	0	0	0	0	0	0	0
CCDC61	5.54E-06	1.32E-06	2.85E-06	4.57E-06	5.77E-06	5.68E-06	7.90E-06	4.40E-06
CCDC62	3.72E-08	0	3.85E-08	1.16E-08	1.28E-08	3.52E-08	0	0
CCDC63	0	0	0	0	0	0	0	0
CCDC64	9.31E-06	7.77E-06	6.25E-07	1.75E-06	1.88E-07	5.61E-06	6.75E-07	1.33E-05
CCDC64B	5.40E-07	2.89E-08	3.54E-07	1.67E-06	0	0	6.13E-08	0
CCDC65	2.27E-07	3.18E-07	5.62E-08	8.19E-08	4.60E-08	2.60E-07	6.44E-08	1.97E-08
CCDC66	2.51E-06	2.24E-06	1.02E-06	1.34E-06	6.98E-07	1.20E-06	1.14E-06	1.50E-06
CCDC67	1.29E-08	0	0	0	0	1.62E-08	8.50E-09	0
CCDC68	1.12E-05	1.92E-06	1.93E-06	5.15E-06	1.07E-07	4.55E-07	2.13E-06	1.05E-06
CCDC69	8.31E-06	5.50E-06	1.16E-05	7.89E-06	3.32E-06	1.11E-05	4.66E-06	1.37E-05
CCDC7	9.69E-07	9.45E-07	4.06E-07	4.70E-07	2.28E-07	4.23E-07	3.90E-07	3.79E-07
CCDC70	0	0	0	0	0	0	0	0
CCDC71	1.30E-05	1.30E-05	7.95E-06	1.07E-05	6.47E-06	1.48E-05	1.36E-05	7.92E-06
CCDC72	0.000409	0.000195	9.88E-05	0.000184	0.000295	0.000237	0.000359	0.000113
CCDC73	2.68E-07	2.73E-07	2.39E-07	4.23E-07	3.67E-07	3.58E-07	5.22E-07	4.58E-07
CCDC74A	2.19E-06	5.86E-08	1.53E-07	6.52E-07	7.38E-08	9.45E-08	7.14E-07	2.06E-07
CCDC74B	1.59E-07	1.90E-08	3.49E-08	1.03E-07	1.73E-08	3.72E-08	9.59E-08	8.89E-08
CCDC75	3.08E-06	3.52E-06	1.77E-06	1.00E-06	1.23E-07	2.08E-06	2.53E-07	1.65E-06
CCDC76	3.88E-06	3.21E-06	1.39E-06	1.96E-06	7.20E-07	5.68E-06	1.51E-06	2.02E-06
CCDC77	3.50E-06	1.11E-06	1.05E-06	1.77E-06	9.71E-07	5.91E-06	1.31E-06	1.51E-06
CCDC78	1.94E-06	7.33E-07	5.77E-08	2.75E-08	0	6.15E-09	1.07E-07	5.84E-08
CCDC79	1.22E-08	0	0	0	0	0	0	0
CCDC8	6.01E-07	7.74E-08	3.63E-07	9.53E-07	3.50E-07	6.39E-08	4.49E-07	1.92E-07
CCDC80	8.09E-06	8.61E-07	9.61E-07	8.58E-06	2.49E-06	1.37E-06	1.86E-05	7.53E-06
CCDC81	2.54E-07	7.58E-08	1.41E-08	6.56E-08	3.45E-08	9.40E-08	1.93E-07	3.94E-08
CCDC82	1.21E-05	5.15E-06	6.45E-06	7.08E-06	1.42E-06	5.75E-06	2.80E-06	7.11E-06
CCDC83	0	0	0	0	0	0	0	0
CCDC84	6.79E-06	2.07E-06	1.56E-06	3.09E-06	2.16E-06	3.54E-06	3.48E-06	4.61E-06
CCDC85A	2.40E-07	7.30E-07	5.30E-08	6.99E-08	3.93E-08	1.48E-07	3.50E-07	1.68E-07
CCDC85B	7.28E-06	5.50E-06	4.21E-06	1.29E-05	2.09E-05	6.55E-06	5.09E-05	6.94E-06
CCDC85C	1.14E-05	4.72E-06	2.38E-06	2.94E-06	3.30E-06	3.19E-06	8.94E-06	1.00E-05
CCDC86	1.30E-05	2.28E-05	5.43E-06	1.17E-05	3.59E-06	2.55E-05	1.09E-05	1.63E-05
CCDC87	2.23E-07	1.20E-06	9.07E-08	5.84E-08	2.74E-08	4.20E-07	3.09E-08	1.52E-07
CCDC88A	2.61E-05	8.93E-06	3.02E-06	3.93E-06	2.15E-06	3.98E-06	2.64E-06	1.98E-06
CCDC88B	2.03E-05	5.35E-06	4.06E-06	9.64E-06	5.51E-06	5.79E-06	5.94E-06	1.22E-05
CCDC88C	5.30E-06	5.08E-07	2.07E-06	2.48E-06	5.83E-07	1.04E-06	1.69E-06	1.06E-06
CCDC89	2.91E-08	1.09E-08	5.04E-08	1.19E-08	8.33E-09	4.49E-08	5.63E-08	4.27E-08
CCDC9	1.10E-05	1.48E-05	4.43E-06	1.18E-05	3.39E-06	8.80E-06	1.01E-05	1.12E-05
CCDC90A	1.15E-05	2.02E-05	1.35E-05	1.33E-05	1.10E-05	2.07E-05	1.87E-05	1.37E-05
CCDC90B	2.11E-05	1.39E-05	2.22E-05	2.05E-05	7.88E-06	2.66E-05	1.66E-05	1.62E-05
CCDC91	9.06E-06	1.25E-05	4.80E-06	5.49E-06	2.44E-06	6.79E-06	5.89E-06	6.92E-06
CCDC92	2.09E-05	2.33E-05	1.91E-05	2.46E-05	1.39E-05	2.31E-05	2.41E-05	1.25E-05
CCDC93	7.71E-06	2.66E-06	7.80E-07	1.58E-06	7.78E-07	2.29E-06	2.19E-06	2.38E-06
CCDC94	2.18E-05	8.75E-06	1.23E-05	1.91E-05	2.42E-05	1.40E-05	2.22E-05	1.62E-05
CCDC96	7.95E-07	5.99E-07	1.50E-07	1.07E-06	2.66E-07	5.12E-07	5.45E-07	6.01E-07
CCDC97	1.32E-05	7.44E-06	4.20E-06	7.29E-06	3.61E-06	6.88E-06	7.39E-06	5.53E-06
CCDC99	5.67E-06	2.46E-06	8.36E-07	6.86E-07	5.06E-07	1.50E-06	9.02E-07	1.83E-06
CCHCR1	1.15E-05	2.06E-05	4.40E-06	5.07E-06	9.70E-06	9.19E-06	9.51E-06	1.08E-05
CCIN	3.71E-08	0	0	1.52E-08	0	6.89E-08	5.98E-08	3.65E-08
CCK	0	0	3.79E-08	4.62E-07	0	3.17E-08	0	5.07E-08
CCKAR	0	0	0	0	0	0	0	0
CCKBR	1.15E-08	1.31E-08	0	0	0	0	0	0
CCL1	6.11E-08	0	0	0	0	0	0	0
CCL11	1.47E-07	0	3.25E-08	8.02E-07	2.30E-07	5.50E-08	7.83E-07	4.39E-07

CCL13	3.60E-07	3.84E-08	2.56E-07	1.22E-07	2.56E-07	1.23E-07	9.00E-07	2.94E-07
CCL14	1.62E-05	6.04E-05	0.000228	0.000173	1.00E-04	2.88E-05	0.000175	8.24E-05
CCL14-CC	7.14E-06	2.65E-05	1.12E-05	1.20E-05	8.59E-06	1.28E-05	1.40E-05	8.56E-06
CCL15	6.56E-05	2.22E-05	3.80E-05	2.61E-05	6.13E-05	4.27E-05	1.82E-05	2.96E-05
CCL16	4.23E-06	7.16E-05	4.25E-05	7.87E-05	0.000112	7.85E-05	0.000111	4.69E-05
CCL17	7.10E-07	6.14E-08	1.80E-07	2.55E-07	8.88E-08	3.35E-07	7.57E-07	1.53E-07
CCL18	3.62E-05	7.94E-07	3.17E-06	4.78E-05	4.87E-07	2.03E-06	4.81E-05	6.96E-06
CCL19	4.34E-06	6.60E-08	1.54E-05	0.000128	8.11E-07	3.34E-06	7.00E-07	7.74E-05
CCL2	1.57E-05	6.32E-06	1.48E-05	0.000567	1.37E-05	1.49E-05	5.31E-05	2.17E-05
CCL20	0.000222	5.18E-05	1.22E-06	9.74E-05	4.92E-07	3.51E-06	6.56E-05	0.00021
CCL21	3.90E-05	3.32E-07	9.64E-06	0.00021	8.70E-06	7.61E-06	8.04E-06	2.92E-05
CCL22	7.39E-07	9.02E-09	2.16E-07	9.52E-07	2.76E-08	1.04E-07	1.01E-07	1.06E-07
CCL23	4.96E-08	0	2.27E-06	2.86E-06	3.52E-07	4.67E-08	2.31E-07	0
CCL24	3.65E-06	0	1.35E-06	1.66E-07	0	3.68E-07	1.98E-06	0
CCL25	6.18E-08	6.86E-07	1.03E-07	1.57E-07	3.81E-06	5.78E-08	2.55E-07	3.15E-05
CCL26	5.79E-08	0	0	0	1.52E-07	5.48E-08	3.75E-07	8.86E-08
CCL27	0	0	0	0	0	0	0	0
CCL28	1.79E-05	1.56E-06	1.01E-06	2.39E-06	4.83E-08	5.40E-07	2.08E-06	2.76E-08
CCL3	1.70E-05	5.31E-06	1.70E-05	6.30E-05	4.45E-06	4.93E-06	2.68E-05	1.34E-06
CCL3L1	3.59E-06	3.44E-07	3.93E-06	7.24E-06	4.83E-07	5.19E-06	3.73E-06	1.09E-07
CCL3L3	1.85E-07	0	3.85E-07	7.42E-07	3.83E-07	5.48E-07	1.75E-07	0
CCL4	4.07E-05	4.34E-06	1.13E-05	9.26E-05	2.27E-06	1.03E-05	1.70E-05	2.19E-06
CCL4L2	2.39E-05	4.89E-07	7.01E-06	5.22E-05	7.81E-07	2.39E-05	9.45E-06	8.05E-07
CCL5	5.55E-05	5.57E-06	2.87E-05	6.53E-05	3.39E-06	3.67E-05	2.66E-05	1.38E-05
CCL7	0	0	0	1.18E-06	0	0	0	6.58E-08
CCL8	5.12E-07	7.05E-07	2.64E-07	1.59E-06	4.42E-07	1.60E-06	3.20E-06	5.76E-07
CCM2	6.47E-05	6.08E-05	3.38E-05	4.61E-05	8.77E-05	4.90E-05	5.97E-05	7.95E-05
CCNA1	0	0	0	1.69E-08	0	0	0	2.03E-08
CCNA2	1.28E-05	3.87E-06	1.74E-07	4.51E-07	1.02E-06	2.30E-06	5.54E-07	1.29E-06
CCNB1	3.09E-05	8.21E-06	1.09E-06	1.77E-06	1.55E-06	4.65E-06	2.26E-06	6.03E-06
CCNB1IP1	6.14E-05	4.05E-05	3.44E-05	3.19E-05	4.37E-05	1.77E-05	4.54E-05	1.81E-05
CCNB2	1.74E-05	5.49E-06	1.39E-07	4.43E-07	3.01E-06	2.77E-06	1.06E-06	1.79E-06
CCNB3	1.64E-07	1.44E-06	2.30E-07	2.70E-07	3.08E-07	6.08E-07	1.30E-07	1.07E-06
CCNC	3.13E-05	9.07E-05	2.75E-05	2.88E-05	9.14E-06	3.80E-05	2.74E-05	2.19E-05
CCND1	3.05E-05	2.02E-05	2.98E-05	4.61E-05	4.44E-05	5.38E-05	3.59E-05	2.39E-05
CCND2	3.72E-06	6.54E-07	5.04E-07	2.30E-06	3.12E-07	9.32E-07	2.35E-06	1.10E-06
CCND3	2.95E-05	2.17E-05	1.08E-05	2.48E-05	7.36E-06	1.34E-05	2.00E-05	1.81E-05
CCNDBP1	1.92E-05	1.31E-05	1.02E-05	1.76E-05	3.44E-06	1.90E-05	1.24E-05	1.30E-05
CCNE1	1.73E-06	1.47E-06	1.58E-07	3.37E-07	8.00E-07	8.08E-07	9.19E-07	7.72E-07
CCNE2	8.12E-06	3.69E-06	2.22E-07	3.81E-07	2.11E-07	4.49E-07	5.28E-07	1.31E-06
CCNF	5.29E-06	2.00E-06	3.86E-07	2.99E-07	5.69E-07	5.60E-07	7.61E-07	6.85E-07
CCNG1	6.29E-05	5.31E-05	6.21E-05	4.44E-05	1.76E-05	6.11E-05	3.48E-05	4.68E-05
CCNG2	7.51E-06	3.18E-06	1.98E-06	2.05E-06	5.96E-07	4.45E-06	2.12E-06	1.99E-06
CCNH	3.22E-05	1.72E-05	1.66E-05	2.57E-05	6.15E-06	1.82E-05	1.61E-05	2.08E-05
CCNI	9.30E-05	3.84E-05	4.65E-05	5.63E-05	1.99E-06	2.88E-05	3.56E-05	2.53E-05
CCNI2	3.15E-07	0	1.04E-08	3.74E-08	9.00E-09	5.57E-08	2.98E-08	1.48E-08
CCNJ	2.75E-06	1.97E-07	5.59E-07	1.09E-06	2.33E-07	9.67E-08	2.43E-07	2.17E-07
CCNJL	2.24E-06	6.63E-08	1.68E-07	3.36E-07	9.51E-08	3.42E-08	2.43E-07	1.51E-07
CCNK	2.28E-05	9.52E-06	8.61E-06	9.94E-06	5.59E-06	1.33E-05	1.33E-05	1.21E-05
CCNL1	4.14E-05	1.86E-05	1.77E-05	2.90E-05	7.88E-06	1.19E-05	1.95E-05	1.70E-05
CCNL2	4.52E-05	9.82E-06	9.35E-06	9.58E-06	8.88E-06	1.46E-05	7.66E-06	2.21E-05
CCNO	2.54E-06	5.03E-07	1.12E-07	1.93E-07	2.56E-07	0	8.40E-08	1.87E-06
CCNT1	2.60E-06	1.79E-06	1.01E-06	8.58E-07	8.42E-09	1.20E-06	2.37E-07	6.01E-07
CCNT2	1.48E-05	7.86E-06	4.37E-06	3.84E-06	5.72E-07	5.46E-06	2.53E-06	4.57E-06
CCNY	2.35E-05	2.42E-05	2.12E-05	2.09E-05	1.55E-05	3.69E-05	2.60E-05	2.40E-05
CCNYL1	1.35E-05	1.12E-05	5.43E-06	4.79E-06	3.06E-06	4.32E-06	4.88E-06	2.78E-06
CCPG1	2.51E-05	2.33E-05	3.35E-05	2.54E-05	4.50E-06	3.33E-05	1.28E-05	2.82E-05
CCR1	4.60E-06	6.09E-07	4.33E-06	8.92E-06	1.80E-07	2.46E-06	3.26E-06	6.02E-07

CCR10	2.88E-07	4.02E-07	8.85E-08	4.31E-07	3.38E-07	1.34E-07	5.13E-07	9.15E-08
CCR2	1.56E-06	1.46E-07	9.15E-07	1.06E-06	6.76E-08	1.47E-07	5.55E-07	2.20E-07
CCR3	1.42E-07	0	1.36E-08	0	0	2.08E-07	0	1.91E-08
CCR4	1.34E-06	2.52E-06	1.87E-07	1.12E-06	0	1.92E-07	8.54E-08	2.62E-07
CCR5	6.34E-06	1.13E-07	1.16E-06	3.19E-06	1.04E-07	1.52E-06	7.70E-07	5.61E-07
CCR6	3.62E-06	3.42E-08	1.85E-07	1.01E-06	4.37E-08	2.60E-07	4.68E-07	5.57E-07
CCR7	2.29E-06	4.95E-08	4.12E-07	3.46E-06	6.59E-08	1.36E-06	5.08E-07	6.60E-07
CCR8	1.92E-06	0	2.06E-08	0	0	3.67E-07	0	2.85E-08
CCR9	9.55E-08	0	6.95E-08	3.50E-08	0	0	8.93E-09	0
CCRL1	5.98E-07	2.05E-07	2.23E-07	2.68E-07	1.84E-08	1.00E-07	1.14E-07	6.28E-08
CCRL2	9.46E-06	1.33E-06	5.20E-07	4.48E-06	3.28E-07	9.81E-07	2.21E-06	6.28E-07
CCRN4L	2.27E-05	9.07E-06	8.93E-06	8.23E-06	2.96E-07	1.41E-06	2.05E-06	7.48E-06
CCS	4.70E-05	0.00013	6.99E-05	5.87E-05	8.33E-05	7.41E-05	7.17E-05	6.56E-05
CCT2	7.06E-05	8.51E-05	3.12E-05	5.08E-05	1.27E-05	6.73E-05	5.05E-05	3.71E-05
CCT3	0.000241	0.000255	7.13E-05	9.23E-05	7.61E-05	0.000237	0.000135	9.13E-05
CCT4	9.62E-05	0.000169	3.47E-05	4.27E-05	3.88E-05	6.47E-05	5.93E-05	3.75E-05
CCT5	8.21E-05	6.48E-05	2.22E-05	3.70E-05	1.21E-05	4.80E-05	3.78E-05	3.60E-05
CCT6A	0.000117	9.78E-05	2.69E-05	3.88E-05	2.09E-05	6.50E-05	5.11E-05	5.35E-05
CCT6B	3.85E-07	2.63E-06	1.53E-06	1.15E-06	1.07E-06	2.46E-06	2.58E-07	1.07E-07
CCT6P1	2.35E-06	1.55E-06	6.77E-07	8.22E-07	2.93E-07	6.78E-07	9.84E-07	1.50E-06
CCT7	0.000216	0.000171	7.28E-05	8.73E-05	9.33E-05	0.000137	0.000118	8.46E-05
CCT8	0.000111	5.97E-05	4.41E-05	5.46E-05	3.84E-05	9.97E-05	6.27E-05	6.18E-05
CCT8L2	0	0	0	0	0	0	0	0
CD101	9.05E-07	3.22E-07	1.88E-07	2.80E-07	5.94E-09	3.08E-07	4.40E-08	2.27E-07
CD109	4.04E-06	2.29E-06	6.12E-07	7.12E-07	1.01E-07	2.27E-07	5.98E-07	1.01E-06
CD14	0.00011	0.000146	0.000821	0.000683	0.000228	0.000277	0.000607	0.001145
CD151	0.00034	0.000191	0.000115	0.000257	0.000104	8.58E-05	0.000214	0.000207
CD160	2.08E-07	2.07E-07	1.33E-06	1.48E-06	1.64E-07	1.86E-07	9.12E-07	2.34E-08
CD163	1.01E-05	3.23E-06	2.96E-05	3.47E-05	1.95E-06	1.02E-05	2.83E-05	2.19E-06
CD163L1	3.42E-06	1.00E-06	4.82E-07	1.21E-06	3.60E-07	1.13E-06	2.26E-06	6.44E-07
CD164	0.00024	0.000155	0.000121	8.92E-05	2.23E-05	8.76E-05	7.27E-05	4.88E-05
CD164L2	0	0	0	0	0	0	1.55E-08	0
CD177	5.51E-07	7.91E-08	1.04E-08	3.68E-08	0	3.72E-08	0	0
CD180	1.49E-06	2.71E-07	1.74E-06	3.07E-06	1.99E-07	6.05E-07	2.07E-06	2.40E-07
CD19	2.47E-07	0	2.93E-08	1.51E-07	0	1.16E-07	8.34E-08	2.05E-08
CD1A	1.65E-06	0	1.19E-08	2.79E-08	0	0	1.10E-08	1.68E-08
CD1B	7.74E-07	4.99E-08	5.76E-08	6.00E-08	0	9.09E-08	0	6.32E-08
CD1C	1.17E-06	4.07E-08	7.41E-07	1.25E-06	7.73E-08	1.59E-07	1.69E-07	7.96E-08
CD1D	2.90E-06	2.64E-07	5.92E-06	5.46E-06	2.11E-07	2.30E-06	1.03E-06	5.16E-06
CD1E	1.57E-06	8.56E-08	4.17E-07	9.40E-07	4.27E-08	5.75E-08	1.13E-07	8.15E-08
CD2	2.48E-05	5.71E-07	3.61E-06	9.36E-06	4.20E-07	6.76E-06	5.54E-06	3.89E-06
CD200	4.34E-06	1.76E-06	1.87E-07	9.04E-07	5.20E-07	1.07E-06	2.39E-06	3.37E-06
CD200R1	1.09E-06	1.39E-07	4.50E-07	7.60E-07	4.16E-08	2.86E-07	6.37E-07	2.46E-07
CD200R1L	0	0	0	0	0	0	0	0
CD207	2.12E-06	5.86E-08	4.75E-07	7.29E-07	0	3.60E-08	4.99E-08	3.81E-08
CD209	3.42E-06	7.51E-07	6.04E-07	1.56E-06	4.24E-07	2.03E-06	3.95E-06	5.35E-07
CD22	2.65E-06	2.92E-08	2.40E-07	8.97E-07	3.22E-07	3.95E-07	4.05E-07	2.52E-07
CD226	6.37E-07	1.10E-07	4.42E-07	9.61E-07	5.34E-08	1.46E-07	1.46E-07	6.51E-08
CD24	0.000183	9.54E-05	2.01E-05	6.37E-05	6.00E-05	2.59E-06	1.33E-05	7.44E-06
CD244	6.02E-07	1.50E-07	1.57E-06	1.64E-06	5.61E-08	4.16E-07	3.27E-07	1.23E-07
CD247	6.14E-06	5.80E-07	1.72E-06	4.61E-06	3.14E-07	2.48E-06	3.63E-06	1.57E-06
CD248	9.43E-06	3.72E-06	1.59E-06	1.01E-05	3.92E-06	3.28E-06	1.25E-05	7.05E-06
CD27	1.56E-05	4.33E-07	1.25E-06	5.15E-06	5.45E-08	5.73E-06	2.37E-06	1.59E-06
CD274	1.48E-06	2.32E-07	4.74E-07	1.74E-06	3.87E-08	2.67E-07	1.65E-07	2.14E-07
CD276	3.53E-05	1.94E-05	2.15E-05	3.10E-05	2.65E-05	1.07E-05	2.50E-05	2.20E-05
CD28	1.73E-06	3.70E-07	2.55E-07	1.29E-06	9.78E-08	9.42E-07	9.75E-07	2.75E-07
CD2AP	1.66E-05	1.34E-05	8.16E-06	7.06E-06	2.13E-06	8.18E-06	7.78E-06	8.05E-06
CD2BP2	3.44E-05	2.74E-05	6.68E-06	1.62E-05	1.04E-05	9.57E-06	1.64E-05	1.59E-05

CD300A	1.21E-05	1.08E-06	6.64E-06	1.36E-05	1.17E-06	2.88E-06	5.11E-06	1.94E-06
CD300C	1.47E-06	1.68E-07	1.27E-06	3.03E-06	1.41E-07	6.68E-07	9.74E-07	1.69E-07
CD300E	0	9.33E-08	2.69E-07	9.81E-07	0	1.11E-07	3.71E-08	0
CD300LB	2.27E-07	4.65E-08	2.65E-07	1.54E-06	3.54E-08	2.87E-08	1.30E-07	1.06E-07
CD300LD	4.75E-08	0	0	0	0	0	0	0
CD300LF	5.64E-06	3.37E-07	8.19E-07	2.60E-06	2.43E-07	4.62E-07	1.58E-06	3.87E-07
CD300LG	2.54E-08	7.08E-06	3.42E-07	7.26E-07	1.01E-07	1.53E-06	1.86E-06	4.97E-08
CD302	3.94E-05	5.04E-05	0.000113	7.37E-05	3.24E-05	6.76E-05	4.44E-05	6.82E-05
CD320	3.43E-05	1.88E-05	1.57E-05	1.75E-05	4.31E-05	2.00E-05	2.54E-05	2.54E-05
CD33	3.20E-06	7.60E-07	3.13E-06	3.51E-06	5.01E-07	3.15E-06	4.18E-06	7.81E-07
CD34	2.33E-05	1.93E-05	2.36E-06	4.39E-06	1.91E-05	1.61E-05	3.08E-05	2.56E-05
CD36	3.23E-05	5.28E-05	1.84E-05	1.02E-05	8.81E-06	0.000131	2.02E-05	6.30E-06
CD37	1.51E-05	2.37E-06	5.13E-06	1.81E-05	1.58E-06	5.31E-06	1.50E-05	2.52E-06
CD38	5.48E-06	3.94E-08	1.80E-06	3.01E-06	7.18E-08	1.69E-06	5.56E-07	1.95E-07
CD3D	3.36E-05	9.30E-08	4.41E-06	1.32E-05	4.78E-07	8.87E-06	6.28E-06	4.31E-06
CD3E	2.54E-05	7.47E-07	5.92E-06	1.65E-05	6.22E-07	9.39E-06	8.08E-06	5.71E-06
CD3EAP	3.65E-06	3.61E-06	1.70E-06	3.90E-06	8.25E-07	3.70E-06	2.83E-06	3.00E-06
CD3G	3.15E-06	1.11E-07	5.38E-07	1.02E-06	0	7.73E-07	1.85E-07	5.72E-08
CD4	3.92E-05	8.27E-05	5.24E-05	3.88E-05	4.72E-06	2.57E-05	2.81E-05	6.89E-06
CD40	1.11E-05	2.39E-05	1.67E-05	1.20E-05	2.71E-06	1.19E-05	7.32E-06	2.50E-05
CD40LG	9.11E-07	1.56E-08	3.61E-07	8.26E-07	4.73E-08	1.53E-07	7.95E-07	5.46E-07
CD44	1.74E-05	1.84E-06	6.87E-06	2.62E-05	5.85E-07	1.74E-05	1.17E-05	2.99E-06
CD46	8.49E-05	0.000118	5.87E-05	4.68E-05	1.04E-05	9.16E-05	3.01E-05	6.88E-05
CD47	2.41E-05	1.13E-05	7.53E-06	1.54E-05	2.91E-06	6.65E-06	6.04E-06	8.43E-06
CD48	2.08E-05	1.97E-06	9.25E-06	2.07E-05	1.09E-06	6.50E-06	1.01E-05	4.53E-06
CD5	2.88E-06	9.04E-08	6.95E-07	4.04E-06	3.14E-08	1.07E-06	1.14E-06	1.46E-06
CD52	6.74E-05	5.14E-06	1.55E-05	8.24E-05	4.23E-06	2.06E-05	3.63E-05	2.01E-05
CD53	4.65E-05	4.09E-06	1.39E-05	3.87E-05	1.35E-06	1.53E-05	1.82E-05	5.99E-06
CD55	2.76E-05	1.42E-05	1.01E-05	1.26E-05	6.28E-07	3.29E-05	4.67E-06	5.70E-06
CD58	2.08E-05	4.24E-06	3.57E-06	8.72E-06	1.68E-06	4.55E-06	3.55E-06	6.98E-06
CD59	6.56E-05	4.16E-05	2.89E-05	6.95E-05	3.13E-05	5.63E-05	6.37E-05	7.68E-05
CD5L	3.61E-07	9.58E-08	4.74E-05	4.83E-05	1.09E-06	2.35E-05	1.74E-05	3.24E-06
CD6	4.06E-06	1.01E-07	1.13E-06	4.87E-06	2.14E-07	1.28E-06	2.13E-06	9.38E-07
CD63	0.00104	0.000577	0.000352	0.000833	0.000712	0.00043	0.000974	0.000395
CD68	0.00016	5.51E-05	8.28E-05	0.000175	2.17E-05	8.42E-05	0.000166	4.95E-05
CD69	3.79E-06	4.48E-07	3.50E-06	1.83E-05	2.14E-07	1.57E-06	3.68E-06	1.29E-06
CD7	1.54E-05	9.34E-06	4.50E-06	8.36E-06	5.78E-07	6.84E-06	3.93E-06	3.30E-06
CD70	1.28E-06	3.50E-08	3.32E-08	1.11E-07	1.30E-07	2.52E-07	1.69E-07	1.79E-07
CD72	5.17E-06	1.63E-07	1.64E-06	6.39E-06	1.09E-06	3.37E-06	5.25E-06	9.46E-07
CD74	0.004353	0.000355	0.000753	0.002405	0.000336	0.001262	0.001681	0.001424
CD79A	3.53E-06	7.85E-08	1.54E-06	4.39E-06	2.95E-07	1.42E-06	1.77E-06	3.31E-07
CD79B	3.45E-06	1.36E-06	2.36E-06	2.33E-06	4.15E-06	3.09E-06	4.16E-06	2.39E-06
CD80	1.42E-06	4.70E-08	2.32E-07	4.15E-07	3.50E-08	3.27E-07	2.97E-07	9.12E-08
CD81	0.000347	0.000598	0.000949	0.001002	0.000927	0.000452	0.000783	0.000225
CD82	5.33E-05	4.71E-05	3.57E-05	3.94E-05	4.46E-06	2.09E-05	2.73E-05	4.64E-05
CD83	5.01E-06	2.86E-06	2.26E-06	1.87E-05	3.02E-07	1.89E-06	2.91E-06	8.65E-07
CD84	3.38E-06	5.92E-07	1.34E-06	1.41E-06	2.05E-08	5.18E-07	6.16E-07	1.39E-07
CD86	7.56E-06	6.79E-07	2.61E-06	7.42E-06	4.29E-07	2.40E-06	3.10E-06	6.00E-07
CD8A	1.26E-05	3.39E-07	3.10E-06	1.11E-05	1.37E-07	8.40E-06	1.70E-06	1.48E-06
CD8B	5.57E-06	1.09E-07	2.65E-06	5.33E-06	1.17E-07	3.96E-06	8.08E-07	5.44E-07
CD9	6.44E-05	0.000175	2.90E-05	9.32E-05	1.79E-05	2.67E-05	5.62E-05	2.52E-05
CD93	1.26E-05	8.76E-06	3.01E-06	6.66E-06	2.51E-06	6.20E-06	1.11E-05	7.78E-06
CD96	3.62E-06	1.14E-07	5.78E-07	2.68E-06	2.19E-08	1.18E-06	9.84E-07	7.78E-07
CD97	4.30E-05	6.05E-06	8.38E-06	3.14E-05	4.14E-06	6.26E-06	1.73E-05	1.16E-05
CD99	8.44E-05	8.49E-05	0.000129	0.000154	0.000106	0.000302	8.04E-05	0.000264
CD99L2	1.43E-05	4.41E-05	1.86E-05	1.52E-05	2.92E-05	2.28E-05	3.61E-05	9.87E-05
CDA	3.61E-05	2.13E-05	5.67E-05	3.33E-05	1.66E-05	2.20E-05	3.58E-05	8.24E-05
CDADC1	6.64E-06	3.52E-06	4.08E-06	2.47E-06	1.27E-06	8.45E-06	3.81E-06	6.24E-06

CDAN1	5.50E-06	2.50E-06	3.02E-06	1.99E-06	4.73E-06	3.27E-06	3.56E-06	3.49E-06
CDC123	3.82E-05	2.88E-05	2.08E-05	2.61E-05	1.57E-05	4.52E-05	2.67E-05	2.65E-05
CDC14A	1.54E-06	2.01E-06	4.37E-07	8.51E-07	7.76E-08	5.96E-07	3.98E-07	4.36E-07
CDC14B	1.18E-05	8.55E-06	1.27E-05	6.45E-06	1.31E-06	3.01E-05	4.25E-06	6.34E-06
CDC14C	3.37E-07	3.07E-07	5.48E-07	1.18E-07	0	8.77E-07	5.45E-08	2.12E-07
CDC16	2.91E-05	3.13E-05	1.92E-05	2.25E-05	9.07E-06	2.87E-05	2.32E-05	1.86E-05
CDC20	2.18E-05	5.25E-06	4.32E-07	3.59E-07	3.58E-06	2.58E-06	1.50E-06	1.10E-06
CDC20B	4.12E-06	4.62E-08	0	9.62E-08	0	0	0	3.91E-07
CDC23	1.59E-05	1.49E-05	7.79E-06	7.69E-06	2.55E-06	1.71E-05	5.80E-06	1.18E-05
CDC25A	1.93E-06	1.32E-06	1.97E-07	3.24E-07	6.58E-07	3.37E-07	3.61E-07	3.07E-07
CDC25B	8.70E-05	1.78E-05	1.06E-05	1.91E-05	2.53E-05	9.37E-06	3.91E-05	9.90E-06
CDC25C	4.25E-06	2.30E-06	1.33E-08	6.23E-08	3.94E-07	7.99E-07	1.58E-07	5.48E-07
CDC26	1.56E-05	9.72E-06	6.41E-06	7.65E-06	5.68E-06	9.62E-06	9.99E-06	6.42E-06
CDC27	2.07E-05	1.56E-05	6.79E-06	6.60E-06	3.44E-06	1.38E-05	6.35E-06	7.09E-06
CDC34	5.90E-05	3.55E-05	4.60E-05	6.94E-05	9.81E-05	5.08E-05	0.000104	5.53E-05
CDC37	0.000135	5.70E-05	5.12E-05	7.22E-05	7.75E-05	9.70E-05	9.64E-05	8.11E-05
CDC37L1	9.40E-06	1.57E-05	4.07E-05	2.34E-05	8.63E-06	2.68E-05	1.52E-05	3.90E-05
CDC40	1.08E-05	1.12E-05	7.46E-06	5.82E-06	1.43E-06	1.05E-05	5.35E-06	4.30E-06
CDC42	0.000196	8.96E-05	6.39E-05	0.000106	3.45E-05	9.93E-05	8.48E-05	9.68E-05
CDC42BPA	7.11E-06	8.25E-06	6.40E-06	6.16E-06	2.14E-06	1.37E-05	5.85E-06	8.89E-06
CDC42BPB	3.14E-05	1.93E-05	1.54E-05	1.43E-05	7.05E-06	2.04E-05	1.49E-05	1.52E-05
CDC42BPG	2.05E-06	9.55E-07	2.16E-07	1.00E-06	7.84E-09	1.27E-08	2.36E-07	1.14E-07
CDC42EP1	0.000114	8.22E-05	4.72E-05	0.000104	8.19E-05	3.44E-05	0.000145	0.00013
CDC42EP2	8.38E-06	3.23E-06	1.55E-06	5.48E-06	5.72E-06	2.44E-06	3.60E-06	2.21E-06
CDC42EP3	1.96E-06	2.03E-06	1.48E-06	3.26E-06	3.73E-07	1.71E-06	3.24E-06	1.63E-06
CDC42EP4	2.67E-05	3.24E-05	1.27E-05	1.59E-05	1.35E-05	2.47E-05	1.89E-05	1.11E-05
CDC42EP5	1.96E-06	1.51E-06	9.83E-07	3.92E-06	1.92E-06	1.06E-06	1.08E-05	1.46E-06
CDC42SE1	3.00E-05	1.70E-05	1.05E-05	2.41E-05	2.19E-06	1.48E-05	1.39E-05	2.79E-05
CDC42SE2	2.60E-05	1.44E-05	1.37E-05	1.66E-05	2.34E-06	1.79E-05	8.38E-06	2.13E-05
CDC45	9.57E-06	4.91E-06	7.22E-08	5.74E-08	2.05E-06	1.23E-06	7.17E-07	1.47E-06
CDC5L	1.94E-05	3.53E-05	1.32E-05	1.40E-05	3.51E-06	2.12E-05	1.24E-05	1.63E-05
CDC6	2.29E-05	8.48E-06	3.45E-07	2.87E-07	9.44E-07	2.62E-06	5.07E-07	1.71E-06
CDC7	3.30E-06	1.45E-06	2.22E-07	6.05E-07	3.96E-07	1.55E-06	3.64E-07	1.07E-06
CDC73	9.11E-06	6.02E-06	4.85E-06	4.80E-06	9.26E-07	6.96E-06	2.56E-06	8.02E-06
CDCA2	1.74E-06	1.00E-06	4.43E-08	1.11E-07	1.15E-07	3.74E-07	8.16E-08	1.62E-07
CDCA3	1.51E-05	3.78E-06	1.72E-07	4.69E-07	2.37E-06	2.06E-06	6.60E-07	1.18E-06
CDCA4	6.62E-06	1.63E-06	1.08E-06	1.81E-06	1.11E-06	1.78E-06	1.54E-06	1.97E-06
CDCA5	1.19E-05	1.04E-05	3.30E-07	3.89E-07	2.10E-06	1.99E-06	9.42E-07	2.88E-06
CDCA7	8.33E-07	5.41E-08	4.52E-08	9.41E-08	2.48E-08	2.07E-07	9.31E-08	2.43E-07
CDCA7L	9.87E-06	1.61E-06	2.22E-06	3.23E-06	1.66E-06	2.95E-06	1.86E-06	4.36E-06
CDCA8	1.11E-05	3.90E-06	2.27E-07	2.90E-07	7.41E-07	1.39E-06	6.32E-07	1.04E-06
CDCP1	5.74E-06	6.25E-08	3.21E-07	1.44E-06	2.37E-08	8.63E-08	2.65E-07	1.24E-07
CDCP2	1.71E-08	0	8.88E-09	1.05E-08	0	0	0	0
CDH1	9.34E-05	5.17E-05	5.24E-05	6.25E-05	1.33E-05	1.62E-05	3.04E-05	4.01E-05
CDH10	0	0	0	0	0	0	1.44E-08	0
CDH11	6.01E-06	1.68E-06	2.73E-07	2.03E-06	9.45E-08	3.90E-07	1.13E-06	1.94E-06
CDH12	4.31E-08	2.07E-08	0	1.50E-08	4.48E-09	5.70E-09	3.59E-08	2.70E-08
CDH13	2.97E-06	5.61E-06	9.14E-08	3.79E-07	1.73E-06	1.89E-06	4.63E-06	1.73E-06
CDH15	1.63E-08	3.67E-06	5.89E-08	2.28E-06	1.40E-08	4.59E-06	1.42E-07	7.04E-07
CDH16	6.46E-08	2.03E-07	9.59E-09	1.58E-07	2.38E-08	0	1.45E-07	0
CDH17	1.49E-05	0	2.45E-08	0	0	7.30E-09	0	1.16E-08
CDH18	3.80E-07	0	0	0	0	0	0	0
CDH19	3.31E-08	9.29E-09	6.27E-07	2.18E-06	5.84E-08	0	1.04E-07	3.64E-08
CDH2	6.47E-05	4.28E-05	2.79E-05	4.43E-05	1.17E-05	2.49E-05	2.97E-05	2.15E-05
CDH20	1.63E-08	9.15E-09	0	9.95E-09	0	1.51E-08	7.89E-09	0
CDH22	0	0	0	5.35E-08	0	1.80E-07	0	0
CDH23	5.42E-06	8.95E-06	3.73E-06	4.75E-06	9.80E-07	6.03E-06	2.50E-06	6.23E-06
CDH24	3.13E-06	1.35E-06	1.76E-07	6.03E-07	1.71E-06	6.40E-07	4.71E-07	4.98E-07

CDH26	1.20E-07	1.59E-07	7.49E-09	1.63E-07	0	4.89E-08	5.71E-08	1.06E-08
CDH3	6.90E-08	7.13E-08	7.30E-08	6.00E-08	4.69E-09	1.01E-08	1.06E-07	9.60E-08
CDH4	1.51E-08	8.49E-09	0	9.24E-09	6.49E-09	0	7.33E-09	0
CDH5	1.18E-05	1.41E-05	8.38E-06	7.06E-06	4.34E-06	8.19E-06	1.67E-05	1.30E-05
CDH6	2.97E-05	6.53E-07	4.15E-07	6.69E-06	1.02E-07	4.45E-07	3.68E-07	4.15E-07
CDH7	0	0	0	0	0	0	0	0
CDH8	0	2.87E-08	8.20E-09	0	5.03E-08	2.64E-08	0	3.72E-08
CDH9	9.82E-07	0	0	0	0	0	0	0
CDHR1	6.71E-07	2.05E-08	1.58E-07	3.42E-07	5.23E-09	7.04E-08	7.69E-08	7.12E-08
CDHR2	7.10E-05	1.47E-05	1.25E-05	1.82E-05	4.12E-07	6.53E-07	3.84E-05	0
CDHR3	3.58E-07	6.22E-07	1.61E-07	2.10E-07	1.31E-07	4.65E-07	8.64E-08	4.55E-07
CDHR4	0	0	0	1.17E-08	0	0	0	0
CDHR5	0.000189	0.000231	0.000111	6.91E-05	7.50E-05	9.64E-05	0.00011	4.81E-05
CDIPT	0.000134	0.000212	4.86E-05	8.20E-05	3.85E-05	8.04E-05	0.000101	6.32E-05
CDK1	2.61E-05	1.03E-05	3.50E-07	4.49E-07	1.82E-06	3.33E-06	9.73E-07	2.20E-06
CDK10	4.70E-05	2.32E-05	3.17E-05	4.11E-05	5.82E-05	4.16E-05	3.85E-05	4.06E-05
CDK11A	3.36E-05	1.90E-05	8.98E-06	1.10E-05	1.03E-06	1.89E-05	8.27E-06	1.00E-05
CDK11B	6.97E-05	3.08E-05	1.77E-05	2.76E-05	8.93E-06	3.07E-05	1.99E-05	2.29E-05
CDK12	1.37E-05	9.11E-06	4.24E-06	3.88E-06	1.00E-06	7.13E-06	2.62E-06	4.11E-06
CDK13	1.97E-05	1.34E-05	7.89E-06	9.12E-06	4.73E-06	1.07E-05	7.10E-06	9.28E-06
CDK14	4.46E-05	1.44E-05	4.04E-06	4.05E-06	1.40E-06	3.46E-06	3.44E-06	6.20E-06
CDK15	1.52E-08	0	0	1.87E-08	0	0	0	0
CDK16	3.52E-05	1.78E-05	7.55E-06	1.17E-05	5.16E-06	1.17E-05	1.25E-05	2.66E-05
CDK17	9.24E-06	7.61E-06	4.05E-06	6.64E-06	1.21E-06	4.08E-06	3.65E-06	4.11E-06
CDK18	6.11E-06	2.08E-05	5.87E-06	1.56E-05	1.42E-06	1.32E-05	2.51E-05	2.81E-06
CDK19	5.99E-06	3.26E-06	1.82E-06	2.44E-06	9.83E-07	2.89E-06	1.88E-06	2.38E-06
CDK2	2.70E-05	1.24E-05	6.43E-06	7.20E-06	1.87E-06	7.31E-06	7.01E-06	5.14E-06
CDK20	3.14E-06	2.64E-06	1.10E-06	2.05E-06	3.53E-07	1.75E-06	1.49E-06	2.63E-06
CDK2AP1	5.38E-05	4.25E-05	2.08E-05	3.84E-05	1.53E-05	3.27E-05	5.13E-05	2.13E-05
CDK2AP2	8.32E-05	0.000136	5.22E-05	6.31E-05	8.88E-05	7.53E-05	7.43E-05	0.000269
CDK3	6.74E-06	1.43E-05	2.83E-06	5.00E-06	9.26E-06	3.08E-06	4.76E-06	1.92E-06
CDK4	6.89E-05	5.77E-05	2.87E-05	4.50E-05	2.95E-05	4.81E-05	7.10E-05	3.95E-05
CDK5	1.25E-05	2.63E-05	8.85E-06	1.08E-05	1.55E-05	2.95E-05	1.52E-05	2.37E-05
CDK5R1	1.11E-06	4.01E-07	1.95E-07	3.68E-07	9.15E-08	9.43E-07	2.53E-07	1.99E-07
CDK5R2	1.41E-06	0	0	1.16E-08	0	0	0	0
CDK5RAP1	1.94E-05	1.34E-05	7.96E-06	9.88E-06	1.20E-05	1.33E-05	1.37E-05	1.43E-05
CDK5RAP2	1.27E-05	1.06E-05	9.23E-06	1.30E-05	9.35E-06	2.83E-05	1.18E-05	1.75E-05
CDK5RAP3	9.51E-05	7.93E-05	2.94E-05	4.77E-05	7.86E-05	0.000112	4.73E-05	4.17E-05
CDK6	2.11E-05	2.57E-06	2.36E-06	3.07E-06	5.70E-07	4.50E-06	1.62E-06	1.08E-06
CDK7	1.45E-05	7.60E-06	6.56E-06	7.38E-06	3.09E-06	1.09E-05	8.82E-06	1.24E-05
CDK8	1.05E-05	4.71E-06	3.69E-06	3.56E-06	2.09E-07	4.33E-06	2.69E-06	4.92E-06
CDK9	3.63E-05	1.45E-05	2.04E-05	2.72E-05	1.50E-05	2.58E-05	3.09E-05	3.26E-05
CDKAL1	7.49E-06	5.82E-06	3.64E-06	5.81E-06	1.93E-06	6.39E-06	5.67E-06	6.80E-06
CDKL1	7.90E-07	7.58E-08	1.19E-07	1.35E-07	0	8.18E-08	3.56E-08	1.30E-07
CDKL2	2.74E-07	5.36E-09	3.44E-08	1.76E-08	0	4.22E-09	9.33E-09	7.02E-09
CDKL3	5.61E-07	7.98E-07	2.56E-07	5.26E-07	3.27E-07	4.71E-07	7.44E-07	3.73E-07
CDKL4	1.40E-07	0	0	2.82E-08	0	2.75E-07	0	0
CDKL5	4.83E-07	6.02E-08	8.29E-08	1.15E-07	0	1.92E-07	6.51E-09	1.18E-07
CDKN1A	6.40E-05	0.000112	7.60E-05	0.000215	3.28E-05	9.78E-05	6.21E-05	0.000148
CDKN1B	6.16E-05	1.48E-05	2.94E-05	1.88E-05	1.12E-05	3.46E-05	1.91E-05	1.51E-05
CDKN1C	1.56E-05	7.77E-07	4.10E-06	5.08E-06	2.36E-06	7.62E-07	2.35E-06	8.96E-07
CDKN2A	1.75E-05	1.38E-05	1.45E-07	2.57E-06	8.42E-06	1.21E-05	1.01E-05	2.34E-06
CDKN2AIF	1.06E-05	4.66E-06	5.71E-06	6.75E-06	1.07E-06	7.84E-06	5.92E-06	9.42E-06
CDKN2AIF	2.42E-05	2.79E-05	9.03E-06	1.07E-05	1.12E-05	1.71E-05	1.42E-05	1.80E-05
CDKN2B	4.43E-06	5.28E-06	4.32E-07	4.22E-06	3.35E-07	2.04E-06	2.16E-06	9.53E-07
CDKN2BA	4.76E-07	6.22E-07	0	1.35E-07	2.21E-07	3.70E-07	1.05E-07	3.61E-08
CDKN2C	4.50E-05	8.67E-06	1.84E-06	2.91E-06	6.85E-06	1.59E-05	5.34E-06	3.50E-06
CDKN2D	7.06E-06	4.31E-06	1.67E-06	4.98E-06	3.45E-06	2.73E-06	3.48E-06	3.61E-06

CDKN3	1.44E-05	9.62E-06	2.54E-07	1.35E-06	3.96E-06	3.57E-06	1.42E-06	4.02E-06
CDNF	2.52E-06	4.61E-06	3.86E-06	1.43E-06	1.26E-06	2.85E-06	8.57E-07	1.24E-06
CDO1	2.63E-05	0.000153	0.000283	0.00013	7.40E-05	0.000267	0.000128	0.000166
CDON	2.03E-06	2.50E-07	5.08E-07	7.69E-07	6.02E-07	2.56E-06	5.47E-07	5.24E-07
CDR1	1.10E-08	0	8.37E-08	0	0	0	7.50E-08	2.89E-08
CDR2	3.85E-05	2.31E-05	7.05E-06	2.33E-05	2.37E-06	5.68E-06	1.11E-05	1.12E-05
CDR2L	1.71E-05	3.86E-07	5.69E-07	3.07E-06	6.02E-07	5.05E-07	1.54E-06	1.33E-06
CDRT1	2.44E-07	7.87E-08	1.30E-07	1.03E-08	6.45E-08	1.24E-06	2.42E-08	1.71E-07
CDRT15	0	0	0	0	0	0	0	0
CDRT15P	3.09E-07	2.47E-07	7.74E-08	5.87E-08	4.11E-08	1.11E-07	4.54E-08	2.47E-07
CDRT4	1.46E-06	1.24E-06	6.79E-07	1.72E-06	3.49E-07	2.52E-06	1.49E-06	7.16E-07
CDS1	3.09E-06	2.23E-08	1.03E-06	1.01E-06	4.28E-09	1.62E-07	9.71E-08	1.46E-08
CDS2	2.90E-05	1.69E-05	2.04E-05	1.88E-05	1.01E-05	1.57E-05	1.27E-05	1.30E-05
CDSN	2.04E-06	0	7.26E-07	3.04E-07	3.95E-08	1.70E-08	8.02E-08	4.05E-08
CDT1	6.05E-06	4.28E-06	2.08E-07	3.10E-07	1.31E-06	1.21E-06	6.35E-07	1.04E-06
CDV3	5.19E-05	5.58E-05	3.27E-05	5.09E-05	1.50E-05	3.10E-05	5.89E-05	3.04E-05
CDX1	1.51E-07	0	0	1.35E-07	5.90E-08	5.09E-08	5.29E-08	6.06E-08
CDX2	1.37E-06	2.58E-08	0	0	0	0	1.10E-08	0
CDX4	0	0	0	0	0	0	0	0
CDY1	0	0	0	0	0	0	0	0
CDY1B	0	0	0	0	0	0	0	0
CDY2B	0	0	0	3.00E-08	0	0	0	0
CDYL	8.55E-06	3.84E-06	2.65E-06	3.60E-06	8.58E-07	3.62E-06	2.81E-06	3.72E-06
CDYL2	2.75E-06	1.22E-06	8.08E-07	5.98E-07	2.74E-08	1.73E-06	2.98E-07	1.25E-07
CEACAM1	5.29E-05	2.50E-05	2.28E-05	3.32E-05	1.27E-05	2.58E-05	2.75E-05	2.73E-05
CEACAM1	5.78E-08	3.28E-08	0	0	0	0	0	0
CEACAM1	0	0	0	0	0	0	0	0
CEACAM1	1.53E-06	2.35E-06	1.49E-06	3.09E-06	6.96E-06	1.02E-06	5.13E-06	1.72E-06
CEACAM2	6.14E-07	4.14E-05	3.53E-08	2.04E-08	0	9.44E-06	1.94E-07	4.91E-08
CEACAM2	6.39E-07	4.26E-08	6.41E-07	9.32E-07	3.38E-07	6.58E-07	4.19E-07	1.56E-07
CEACAM2	7.68E-09	1.38E-07	7.94E-09	2.82E-08	0	7.82E-08	0	2.25E-08
CEACAM3	6.45E-07	1.84E-08	1.71E-07	2.22E-07	0	4.20E-08	3.10E-08	1.00E-07
CEACAM4	5.23E-07	3.56E-08	2.76E-07	7.89E-08	9.21E-08	5.96E-08	1.43E-07	4.56E-08
CEACAM5	9.43E-08	2.96E-07	2.69E-08	0	0	0	2.04E-08	0
CEACAM6	8.09E-07	2.00E-08	4.61E-08	1.09E-08	0	0	4.31E-08	2.61E-08
CEACAM7	1.03E-08	0	1.08E-08	0	0	0	0	0
CEACAM8	2.05E-08	0	1.07E-08	1.26E-08	0	0	0	0
CEBPA	7.38E-05	0.000148	8.04E-05	8.65E-05	0.000197	4.26E-05	0.000172	7.46E-05
CEBPB	6.58E-05	0.000129	6.64E-05	0.000133	0.000124	7.08E-05	0.00012	0.0001
CEBPD	0.000155	0.000204	0.000101	0.000191	0.000115	5.66E-05	0.000556	0.000103
CEBPE	8.57E-08	2.46E-08	1.15E-07	2.64E-08	1.85E-08	1.99E-08	1.64E-07	0
CEBPG	1.98E-05	3.49E-05	2.13E-05	2.94E-05	1.32E-05	1.48E-05	3.56E-05	2.98E-05
CEBPZ	2.08E-05	2.74E-05	1.19E-05	1.47E-05	6.30E-06	1.70E-05	1.03E-05	1.41E-05
CECR1	2.35E-05	8.64E-06	1.37E-05	2.66E-05	2.09E-06	8.47E-06	1.55E-05	1.25E-05
CECR2	3.80E-08	3.08E-06	1.79E-06	9.73E-07	5.70E-07	1.94E-06	2.09E-06	1.99E-06
CECR4	1.25E-06	3.17E-07	2.28E-07	5.60E-07	4.79E-06	1.87E-06	4.63E-08	0
CECR5	3.24E-05	4.31E-05	2.61E-05	3.27E-05	4.23E-05	4.84E-05	4.40E-05	4.80E-05
CECR6	2.14E-07	1.29E-08	5.92E-08	4.92E-08	7.41E-08	5.32E-08	1.68E-07	4.21E-08
CECR7	2.57E-08	1.05E-06	1.77E-08	0	0	0	8.29E-09	0
CEL	3.95E-08	0	3.09E-08	6.05E-08	1.61E-07	1.10E-06	1.91E-08	8.71E-08
CELA1	0	0	0	7.03E-08	0	0	0	0
CELA2A	0	0	0	0	0	0	0	0
CELA2B	0	0	3.27E-08	0	0	0	0	0
CELA3A	7.26E-07	0	0	0	0	0	0	9.04E-08
CELA3B	0	0	0	0	0	0	0	7.95E-08
CELF1	4.01E-05	4.34E-05	3.37E-05	2.65E-05	4.39E-06	4.27E-05	1.95E-05	3.08E-05
CELF2	2.72E-06	3.88E-07	8.64E-07	1.96E-06	1.25E-07	7.57E-07	8.46E-07	4.84E-07
CELF3	2.46E-08	2.79E-08	0	0	0	1.14E-08	0	0

CELF4	8.10E-08	6.48E-09	7.14E-08	4.95E-08	1.49E-08	0	1.12E-08	2.54E-08
CELF5	4.34E-08	2.23E-06	1.05E-07	6.90E-08	6.30E-09	0	7.61E-07	7.79E-09
CELF6	5.36E-07	2.10E-06	1.97E-07	3.23E-07	3.22E-07	3.55E-06	4.82E-07	2.01E-06
CELP	0	0	0	3.37E-08	0	0	0	0
CELSR1	3.43E-06	6.98E-07	6.33E-07	1.26E-06	7.49E-07	2.31E-06	1.00E-06	1.13E-07
CELSR2	3.03E-06	1.61E-07	9.09E-07	5.04E-07	1.83E-07	1.62E-06	1.39E-06	1.11E-06
CELSR3	7.02E-06	1.05E-06	6.31E-08	1.87E-07	1.38E-06	9.47E-07	1.56E-06	3.96E-07
CEMP1	1.13E-05	1.10E-05	5.95E-06	7.24E-06	1.72E-05	1.06E-05	5.00E-06	8.37E-06
CEND1	2.16E-06	7.62E-07	4.72E-08	3.66E-08	3.85E-08	8.30E-08	2.87E-08	8.78E-08
CENPA	7.62E-06	2.30E-06	2.00E-08	1.72E-07	6.70E-07	3.82E-07	3.56E-07	3.27E-07
CENPB	4.73E-05	4.52E-05	2.10E-05	2.61E-05	3.28E-05	2.78E-05	3.84E-05	3.04E-05
CENPBD1	4.63E-06	5.03E-06	2.97E-06	3.53E-06	2.84E-06	6.25E-06	3.41E-06	4.13E-06
CENPC1	4.30E-06	2.26E-06	3.60E-06	2.51E-06	5.79E-07	3.89E-06	2.00E-06	3.27E-06
CENPE	3.51E-06	6.25E-07	5.49E-08	5.36E-08	8.31E-08	4.80E-07	6.56E-08	2.13E-07
CENPF	4.73E-06	3.04E-06	1.03E-07	1.83E-07	1.58E-07	1.29E-06	2.03E-07	9.41E-07
CENPH	5.93E-06	2.75E-06	5.79E-07	1.22E-06	9.79E-07	1.80E-06	1.20E-06	1.28E-06
CENPI	1.35E-06	8.06E-07	0	2.24E-08	4.18E-08	1.27E-07	1.89E-08	8.58E-08
CENPJ	2.33E-06	1.04E-06	2.80E-07	8.54E-07	1.82E-07	1.42E-06	5.70E-07	9.10E-07
CENPK	3.94E-06	9.33E-07	9.20E-08	8.92E-08	1.98E-07	1.06E-06	2.24E-07	5.09E-07
CENPL	2.84E-06	4.29E-06	3.65E-07	6.10E-07	2.86E-07	1.43E-06	4.80E-07	1.00E-06
CENPM	1.80E-05	8.65E-06	1.65E-07	1.01E-06	1.07E-05	2.54E-06	1.74E-06	3.60E-06
CENPN	1.05E-05	3.17E-06	2.14E-06	2.16E-06	2.17E-06	5.20E-06	3.05E-06	4.53E-06
CENPO	6.90E-06	1.97E-05	7.56E-07	1.07E-06	2.98E-07	9.24E-07	6.84E-07	9.48E-07
CENPP	1.62E-06	9.39E-07	2.50E-07	2.65E-07	2.20E-06	5.00E-07	6.61E-07	8.03E-07
CENPQ	7.45E-06	1.16E-05	8.47E-07	1.63E-06	8.25E-07	2.05E-06	1.49E-06	1.23E-06
CENPT	1.33E-05	7.29E-06	4.23E-06	7.27E-06	5.36E-06	6.93E-06	6.04E-06	8.94E-06
CENPV	1.37E-05	3.45E-05	1.29E-05	1.76E-05	2.67E-05	1.34E-05	4.41E-05	1.28E-05
CENPW	3.14E-05	1.43E-05	1.76E-06	5.22E-06	4.16E-06	5.00E-06	8.76E-06	6.99E-06
CEP110	7.36E-06	1.89E-06	2.51E-06	2.84E-06	4.36E-07	3.34E-06	1.44E-06	3.06E-06
CEP120	4.54E-06	3.09E-06	4.13E-06	3.74E-06	1.30E-06	3.64E-06	1.58E-06	2.90E-06
CEP135	1.68E-06	4.03E-07	2.55E-07	6.31E-07	6.45E-08	5.01E-07	2.08E-07	3.75E-07
CEP152	1.71E-06	5.60E-07	1.35E-07	2.43E-07	2.66E-08	3.20E-07	1.76E-07	1.80E-07
CEP164	5.82E-06	1.80E-06	1.37E-06	2.33E-06	2.35E-06	3.15E-06	2.07E-06	2.87E-06
CEP170	1.05E-05	4.24E-06	3.18E-06	5.40E-06	6.70E-07	6.25E-06	2.24E-06	5.07E-06
CEP170L	3.45E-07	8.55E-08	2.68E-08	1.22E-07	0	2.30E-08	0	0
CEP192	5.58E-06	3.62E-06	2.05E-06	2.84E-06	1.15E-06	3.36E-06	2.28E-06	2.51E-06
CEP250	5.80E-06	2.87E-06	1.46E-06	1.52E-06	9.57E-07	3.03E-06	1.97E-06	2.24E-06
CEP290	2.46E-06	1.09E-06	4.85E-07	1.05E-06	1.59E-07	1.40E-06	4.62E-07	1.10E-06
CEP350	7.22E-06	6.11E-06	4.66E-06	3.48E-06	1.20E-06	5.72E-06	2.32E-06	7.23E-06
CEP55	5.52E-06	1.07E-06	1.26E-07	1.94E-07	2.89E-07	6.92E-07	2.25E-07	3.12E-07
CEP57	6.11E-05	2.06E-05	1.98E-05	1.37E-05	6.94E-06	1.85E-05	1.47E-05	4.89E-05
CEP63	9.21E-06	7.34E-06	6.19E-06	5.77E-06	1.37E-06	8.24E-06	5.21E-06	5.99E-06
CEP68	6.51E-06	4.55E-06	1.88E-06	3.36E-06	2.41E-06	5.72E-06	3.46E-06	3.04E-06
CEP70	3.16E-05	1.18E-05	6.57E-06	7.74E-06	6.70E-06	2.35E-05	6.64E-06	2.51E-06
CEP72	4.30E-06	1.93E-06	7.42E-07	7.89E-07	1.93E-07	6.15E-07	9.55E-07	2.72E-06
CEP76	3.71E-06	3.21E-06	2.14E-06	1.43E-06	2.68E-07	4.63E-06	8.80E-07	1.97E-06
CEP78	7.25E-06	4.61E-06	1.99E-06	2.14E-06	1.13E-06	1.90E-06	1.50E-06	1.93E-06
CEP97	1.17E-06	9.03E-07	3.83E-07	4.06E-07	3.28E-08	6.88E-07	1.20E-07	3.28E-07
CEPT1	2.43E-05	2.29E-05	2.24E-05	2.01E-05	1.21E-05	3.12E-05	1.73E-05	1.14E-05
CER1	0	0	0	2.63E-08	0	0	0	0
CERCAM	1.31E-05	7.89E-07	1.18E-06	6.86E-06	7.77E-07	4.59E-07	3.06E-06	1.70E-06
CERK	2.35E-05	7.08E-06	1.09E-05	1.47E-05	9.78E-06	1.67E-05	1.51E-05	2.22E-05
CERKL	1.79E-05	3.78E-07	7.76E-07	1.27E-06	2.17E-07	7.22E-07	1.00E-06	6.75E-07
CES1	0.000374	0.001329	0.001763	0.000509	0.001396	0.011462	0.00077	0.000678
CES2	4.29E-05	0.000274	0.000163	0.000262	0.000189	0.000211	0.000223	6.72E-05
CES3	1.00E-07	3.70E-06	2.12E-05	9.91E-06	2.36E-05	5.29E-06	3.57E-05	4.44E-06
CES4	3.28E-06	3.46E-05	1.18E-05	2.29E-05	2.40E-06	0.000157	1.45E-05	4.99E-06
CES7	3.55E-08	2.45E-07	3.78E-06	9.60E-07	5.94E-07	3.63E-06	2.10E-06	1.92E-06

CES8	7.23E-07	8.23E-06	6.88E-06	2.36E-06	1.15E-06	4.97E-06	2.26E-06	2.91E-07
CETN1	0	0	0	0	0	0	0	0
CETN2	5.31E-05	5.05E-05	1.82E-05	2.92E-05	2.76E-05	4.35E-05	2.84E-05	4.73E-05
CETN3	1.91E-05	8.44E-06	7.83E-06	8.85E-06	3.06E-06	1.43E-05	1.12E-05	9.32E-06
CETN4P	1.47E-07	5.93E-08	5.79E-08	0	0	1.85E-07	9.17E-08	1.49E-07
CETP	6.10E-07	1.19E-05	2.26E-05	1.07E-05	4.51E-06	6.64E-06	4.97E-06	2.34E-06
CFB	0.000947	0.00324	0.002137	0.002519	0.001791	0.001422	0.002044	0.002534
CFC1B	0	0	0	0	0	0	0	0
CFD	4.90E-06	3.29E-06	5.52E-06	2.69E-05	2.05E-06	2.66E-06	3.26E-05	7.68E-07
CFDP1	4.30E-05	1.32E-05	1.95E-05	4.33E-05	2.47E-05	3.93E-05	3.71E-05	3.56E-05
CFH	0.000639	0.00048	0.000784	0.000635	0.00042	0.000843	0.000339	0.001655
CFHR1	0.000384	0.000285	0.000893	0.001118	0.000359	0.00146	0.000811	0.001459
CFHR2	4.43E-05	0.000727	0.000586	0.000558	0.000441	0.001099	0.000729	0.001034
CFHR3	6.46E-06	2.84E-05	0.000366	6.59E-05	1.04E-05	0.000241	0.00048	8.72E-05
CFHR4	5.00E-06	2.49E-05	3.93E-05	3.10E-05	1.17E-05	9.90E-05	7.55E-05	6.60E-05
CFHR5	1.15E-07	0.000133	5.56E-05	4.46E-05	1.10E-05	0.000505	5.90E-05	0.000203
CFI	0.000206	0.000164	0.000475	0.000335	0.000132	0.000652	0.000296	0.00044
CFL1	0.000775	0.000483	0.000244	0.000459	0.000355	0.000513	0.000518	0.000686
CFL2	2.05E-05	4.24E-05	4.36E-05	3.82E-05	1.42E-05	3.10E-05	2.91E-05	3.16E-05
CFLAR	5.59E-05	1.94E-05	2.33E-05	2.38E-05	8.69E-06	3.08E-05	1.77E-05	9.80E-06
CFLP1	1.13E-08	3.82E-08	3.53E-08	0	0	2.09E-08	0	0
CFP	2.05E-06	3.92E-07	3.59E-05	1.92E-05	2.49E-06	6.33E-06	2.58E-06	1.54E-06
CFTR	6.69E-06	4.08E-09	8.49E-07	6.76E-06	3.14E-09	0	8.55E-08	1.07E-08
CG030	5.22E-07	1.03E-07	7.58E-08	9.58E-08	5.15E-08	8.97E-08	7.19E-08	1.28E-07
CGA	4.20E-08	3.25E-06	4.35E-07	5.24E-08	0	7.89E-08	0	6.34E-06
CGB	7.32E-08	0	0	0	0	0	0	0
CGB1	3.99E-08	0	0	0	0	0	0	0
CGB2	6.78E-08	0	0	0	5.98E-08	0	6.23E-08	0
CGB5	6.28E-08	0	0	0	0	0	0	0
CGB7	8.92E-08	0	0	0	0	0	4.30E-08	4.69E-08
CGB8	3.11E-08	0	0	1.72E-08	0	0	0	0
CGGBP1	2.65E-05	1.86E-05	1.57E-05	1.32E-05	6.00E-06	1.55E-05	1.05E-05	1.19E-05
CGN	4.02E-05	4.81E-05	2.89E-05	4.81E-05	1.17E-05	9.41E-06	2.66E-05	4.46E-05
CGNL1	4.38E-06	1.32E-05	2.11E-05	1.15E-05	1.03E-05	2.16E-05	1.37E-05	1.77E-05
CGREF1	5.21E-05	3.12E-06	4.54E-06	5.21E-06	5.76E-05	1.90E-05	1.34E-05	1.35E-05
CGRRF1	1.05E-05	6.73E-06	4.27E-06	7.33E-06	2.60E-06	5.37E-06	6.91E-06	7.36E-06
CH25H	6.39E-07	9.61E-07	1.37E-06	1.28E-05	3.47E-07	2.68E-06	1.09E-06	7.30E-07
CHAC1	4.89E-06	8.39E-06	4.58E-06	1.70E-06	1.28E-06	2.11E-06	7.17E-06	2.62E-06
CHAC2	3.61E-06	2.52E-06	2.25E-06	2.61E-06	8.96E-07	2.90E-06	1.32E-06	2.69E-06
CHAD	4.88E-06	1.46E-05	1.34E-05	9.52E-06	1.54E-05	1.95E-05	7.83E-06	9.85E-06
CHADL	5.37E-07	2.32E-06	1.05E-06	1.16E-06	6.07E-06	3.37E-06	2.02E-06	1.36E-06
CHAF1A	9.26E-06	3.41E-06	1.55E-06	2.09E-06	3.95E-06	4.53E-06	1.88E-06	3.51E-06
CHAF1B	6.26E-06	4.69E-08	1.08E-07	5.21E-07	3.30E-07	5.01E-07	3.32E-07	1.08E-06
CHAT	0	1.37E-08	0	0	0	0	0	0
CHCHD1	6.38E-05	4.46E-05	3.65E-05	3.76E-05	6.13E-05	5.99E-05	4.48E-05	4.73E-05
CHCHD10	0.000182	0.000439	0.000126	0.000112	0.000585	0.000191	0.000428	0.000108
CHCHD2	0.000499	0.000688	0.000228	0.000265	0.000366	0.000267	0.000321	0.000257
CHCHD3	3.33E-05	5.23E-05	1.07E-05	1.87E-05	7.88E-06	2.92E-05	2.16E-05	3.20E-05
CHCHD4	2.01E-05	2.72E-05	9.11E-06	9.84E-06	5.80E-06	1.41E-05	1.24E-05	1.32E-05
CHCHD5	4.68E-05	5.23E-05	3.48E-05	4.30E-05	8.30E-05	4.54E-05	7.65E-05	2.98E-05
CHCHD6	6.97E-06	4.31E-06	3.47E-06	6.32E-06	1.31E-05	7.20E-06	1.01E-05	5.68E-06
CHCHD7	5.38E-05	3.59E-05	9.20E-06	1.26E-05	1.25E-05	1.16E-05	2.02E-05	2.44E-05
CHCHD8	0.000161	7.77E-05	6.80E-05	7.32E-05	8.53E-05	0.000147	7.66E-05	4.78E-05
CHD1	1.39E-05	7.88E-06	5.34E-06	7.75E-06	1.05E-06	7.93E-06	4.13E-06	7.87E-06
CHD1L	4.34E-05	1.48E-05	2.34E-05	1.31E-05	7.85E-06	4.03E-05	1.74E-05	1.79E-05
CHD2	1.38E-05	6.07E-06	5.11E-06	8.17E-06	3.89E-06	7.98E-06	5.82E-06	7.20E-06
CHD3	1.08E-05	4.61E-06	1.99E-06	7.41E-06	4.27E-06	2.43E-06	8.44E-06	6.22E-06
CHD4	4.14E-05	2.51E-05	1.49E-05	2.52E-05	8.70E-06	3.60E-05	1.97E-05	1.82E-05

CHD5	6.89E-09	0	0	2.80E-09	5.64E-09	0	0	5.16E-09
CHD6	9.59E-06	5.12E-06	3.61E-06	4.11E-06	1.61E-06	4.11E-06	2.56E-06	2.64E-06
CHD7	7.57E-06	2.26E-06	1.27E-06	1.69E-06	8.85E-07	2.38E-06	2.32E-06	1.37E-06
CHD8	1.10E-05	6.57E-06	3.80E-06	5.06E-06	1.55E-06	6.71E-06	4.44E-06	5.05E-06
CHD9	8.62E-06	6.34E-06	5.46E-06	4.66E-06	9.49E-07	9.27E-06	2.01E-06	5.59E-06
CHDH	4.00E-05	2.29E-05	1.87E-05	2.22E-05	9.57E-06	3.12E-05	3.86E-05	1.27E-05
CHEK1	7.08E-06	1.47E-06	6.06E-07	7.87E-07	1.40E-06	2.76E-06	6.44E-07	1.46E-06
CHEK2	7.68E-06	1.24E-05	1.64E-06	5.51E-06	4.17E-05	5.96E-06	8.21E-06	1.93E-06
CHERP	1.70E-05	1.37E-05	2.21E-05	1.74E-05	1.61E-05	4.05E-05	2.32E-05	1.53E-05
CHFR	8.74E-06	3.88E-06	3.33E-06	4.98E-06	2.18E-06	6.74E-06	1.59E-06	6.56E-06
CHGA	2.41E-08	5.45E-08	0	0	0	1.41E-07	1.70E-07	5.17E-08
CHGB	2.97E-08	0	0	3.63E-08	0	9.17E-09	1.72E-08	2.91E-08
CHI3L1	1.52E-05	2.96E-06	6.31E-05	0.000127	1.46E-06	0.000892	2.19E-06	0.000609
CHI3L2	2.53E-06	4.16E-08	3.33E-07	1.22E-07	6.28E-08	2.16E-07	1.43E-07	8.07E-08
CHIA	0	2.48E-08	2.33E-08	7.98E-08	0	0	0	0
CHIC1	1.30E-06	1.86E-06	3.53E-07	5.25E-07	6.15E-08	1.57E-07	1.88E-07	1.95E-07
CHIC2	1.04E-05	7.79E-06	9.68E-06	1.98E-05	2.47E-06	9.58E-06	1.36E-05	8.34E-06
CHID1	3.09E-05	3.96E-05	5.27E-05	3.92E-05	6.35E-05	4.98E-05	4.58E-05	7.20E-05
CHIT1	5.23E-06	3.39E-08	2.67E-07	1.94E-05	0	1.23E-07	5.28E-07	1.55E-08
CHKA	2.90E-05	3.06E-05	5.38E-06	1.41E-05	6.71E-06	1.41E-05	1.38E-05	1.53E-05
CHKB	1.16E-05	4.98E-06	4.57E-06	9.87E-06	4.53E-06	6.97E-06	7.23E-06	1.26E-05
CHKB-CP	1.27E-05	4.59E-06	1.92E-06	2.83E-06	6.32E-06	2.72E-06	3.55E-06	4.33E-06
CHL1	1.22E-08	0	1.06E-07	6.86E-07	5.23E-09	8.93E-06	2.97E-09	6.69E-08
CHM	9.69E-06	3.89E-06	2.90E-06	3.09E-06	9.57E-07	5.02E-06	1.57E-06	4.12E-06
CHML	2.91E-06	2.18E-06	6.61E-07	1.13E-06	5.22E-07	5.08E-07	4.74E-07	3.85E-06
CHMP1A	4.82E-05	2.72E-05	2.48E-05	3.33E-05	2.23E-05	3.71E-05	3.10E-05	3.40E-05
CHMP1B	4.13E-05	1.70E-05	2.04E-05	3.45E-05	3.12E-06	6.16E-05	1.45E-05	2.89E-05
CHMP2A	0.000206	0.000206	0.000123	0.000145	0.000408	0.00019	0.000165	0.0002
CHMP2B	2.44E-05	2.28E-05	1.51E-05	1.52E-05	3.04E-06	1.27E-05	1.22E-05	1.52E-05
CHMP4A	6.57E-05	1.86E-05	2.00E-05	3.67E-05	2.93E-05	4.59E-05	2.98E-05	1.13E-05
CHMP4B	0.000146	8.08E-05	3.74E-05	8.92E-05	7.59E-05	6.76E-05	7.26E-05	6.22E-05
CHMP4C	2.09E-05	9.13E-06	3.13E-06	6.00E-06	2.22E-06	4.36E-06	1.05E-05	8.25E-06
CHMP5	4.16E-05	5.17E-05	3.37E-05	3.94E-05	1.37E-05	5.25E-05	3.06E-05	3.39E-05
CHMP6	2.43E-05	2.80E-05	1.67E-05	2.19E-05	3.30E-05	3.17E-05	2.50E-05	2.35E-05
CHMP7	1.38E-05	2.23E-05	1.09E-05	1.38E-05	5.74E-06	1.91E-05	8.10E-06	8.61E-06
CHN1	2.98E-06	1.36E-06	5.72E-07	9.75E-07	3.34E-07	1.52E-06	1.07E-06	1.56E-06
CHN2	1.18E-05	2.85E-05	1.76E-05	1.91E-05	1.27E-05	2.08E-05	1.46E-05	1.53E-05
CHODL	1.11E-08	0	1.16E-08	4.06E-08	0	9.65E-08	2.04E-08	1.63E-08
CHORDC1	9.68E-06	9.38E-06	3.15E-06	3.41E-06	1.15E-06	3.83E-06	2.73E-06	4.85E-06
CHP	0.000221	0.000161	0.000176	0.000139	0.000174	0.000127	0.000221	0.000116
CHP2	0	0	0	0	0	0	0	1.46E-08
CHPF	8.04E-05	4.17E-05	3.81E-05	5.41E-05	2.51E-05	4.11E-05	2.04E-05	8.49E-05
CHPF2	1.40E-05	1.62E-05	1.07E-05	1.61E-05	7.82E-06	1.31E-05	1.15E-05	2.28E-05
CHPT1	5.53E-05	0.000179	8.37E-05	8.50E-05	4.59E-05	8.91E-05	9.13E-05	6.07E-05
CHRAC1	1.18E-05	2.12E-05	8.22E-06	1.12E-05	1.14E-05	1.18E-05	1.81E-05	9.25E-06
CHRD	6.69E-05	6.09E-06	6.15E-06	8.01E-06	8.33E-06	1.78E-05	5.86E-06	1.25E-05
CHRDL1	1.23E-07	1.36E-08	1.95E-07	2.48E-06	0	0	5.62E-09	2.66E-08
CHRDL2	1.85E-06	4.65E-07	2.72E-07	3.19E-06	1.70E-07	5.62E-07	1.59E-06	9.16E-07
CHRFAM7	1.17E-07	1.06E-06	0	1.44E-07	0	7.72E-09	5.65E-08	2.44E-08
CHRM1	0	0	8.54E-09	0	0	7.63E-09	0	0
CHRM2	0	0	2.26E-07	3.56E-07	0	0	9.48E-09	0
CHRM3	2.04E-08	3.23E-07	1.62E-07	7.08E-08	7.17E-08	8.66E-08	6.92E-08	4.17E-07
CHRM4	7.12E-07	3.29E-07	3.61E-08	4.17E-08	0	3.15E-08	4.89E-08	2.51E-08
CHRM5	4.35E-08	1.96E-08	6.32E-08	1.06E-08	6.54E-08	2.42E-08	1.69E-08	2.55E-08
CHRNA1	1.21E-08	2.63E-08	3.81E-08	9.20E-07	1.04E-08	0	8.99E-08	1.78E-08
CHRNA10	2.35E-07	1.12E-07	1.30E-07	2.13E-07	1.60E-07	1.50E-07	2.04E-07	2.55E-07
CHRNA2	1.16E-08	5.84E-08	5.96E-09	7.08E-09	4.98E-09	5.36E-09	5.63E-09	0
CHRNA3	8.61E-08	0	0	0	0	1.43E-08	1.50E-08	3.40E-08

CHRNA4	1.64E-08	1.29E-05	2.96E-06	3.15E-07	4.37E-06	2.65E-08	2.52E-06	0
CHRNA5	2.10E-06	6.56E-08	1.01E-08	5.94E-08	1.58E-07	2.70E-08	1.13E-07	0
CHRNA6	1.42E-07	0	0	0	1.02E-08	3.29E-08	0	1.74E-08
CHRNA7	0	1.47E-08	0	1.59E-08	0	0	1.22E-07	0
CHRNA9	0	0	0	0	0	0	0	0
CHRNB1	7.79E-06	5.98E-06	6.19E-06	3.96E-06	1.39E-06	7.21E-06	4.32E-06	6.18E-06
CHRNB2	1.18E-08	1.40E-07	4.02E-09	2.87E-08	1.35E-08	1.82E-08	1.91E-08	0
CHRNB3	0	0	0	0	0	0	1.19E-08	0
CHRNB4	1.69E-08	0	9.99E-09	0	1.46E-08	9.79E-08	1.63E-08	1.48E-07
CHRND	0	2.70E-07	0	0	0	0	0	0
CHRNE	2.44E-06	5.23E-07	8.71E-07	1.25E-06	1.36E-06	5.46E-07	1.58E-06	3.63E-07
CHRNG	1.13E-08	0	0	0	0	0	0	0
CHST1	2.19E-06	5.02E-07	5.25E-07	5.04E-07	2.88E-07	4.93E-07	8.06E-06	1.83E-06
CHST10	6.77E-06	1.39E-07	2.74E-07	9.30E-07	2.49E-07	2.07E-07	7.62E-07	3.15E-07
CHST11	9.05E-06	5.39E-07	1.30E-06	3.22E-06	1.40E-07	1.05E-06	1.48E-06	1.31E-06
CHST12	4.80E-06	5.59E-06	2.64E-06	5.52E-06	2.69E-06	3.66E-06	6.06E-06	3.45E-06
CHST13	1.88E-05	2.16E-05	1.64E-05	2.21E-05	6.42E-05	1.22E-05	4.73E-05	1.48E-05
CHST14	1.32E-05	8.94E-06	5.23E-06	8.84E-06	2.20E-06	8.53E-06	8.93E-06	6.42E-06
CHST15	6.49E-06	3.98E-06	3.68E-06	1.09E-05	3.28E-06	2.80E-05	1.01E-05	7.90E-06
CHST2	4.82E-06	1.26E-06	5.51E-07	1.94E-06	5.75E-07	8.60E-07	2.02E-06	7.70E-07
CHST3	2.17E-06	3.28E-07	5.62E-07	1.92E-06	4.44E-07	4.35E-07	7.64E-07	5.42E-07
CHST4	9.94E-06	0	7.28E-06	9.77E-06	0	2.32E-08	1.21E-08	3.69E-08
CHST5	0	5.58E-07	0	1.34E-08	9.32E-09	1.02E-08	1.05E-08	0
CHST6	1.15E-07	0	3.31E-09	1.19E-08	2.61E-08	0	3.72E-08	0
CHST7	4.90E-06	1.41E-05	5.06E-06	4.83E-06	3.14E-06	6.73E-07	6.58E-06	1.25E-06
CHST8	3.34E-08	1.26E-08	8.15E-08	1.09E-07	6.71E-08	0	5.39E-08	1.64E-08
CHST9	1.12E-05	1.15E-06	1.37E-06	5.38E-06	7.49E-07	8.66E-07	5.65E-07	4.03E-06
CHSY1	1.01E-05	3.69E-06	3.39E-06	1.55E-05	7.25E-07	4.94E-06	5.30E-06	5.31E-06
CHSY3	3.94E-07	4.64E-07	4.47E-07	9.88E-07	3.39E-08	1.42E-06	6.38E-07	4.72E-07
CHTF18	5.22E-06	1.72E-06	4.17E-07	1.09E-06	1.46E-06	1.06E-06	2.14E-06	1.11E-06
CHTF8	3.95E-05	2.63E-05	3.46E-05	2.75E-05	1.25E-05	4.39E-05	3.13E-05	2.31E-05
CHUK	9.94E-06	1.20E-05	7.01E-06	6.69E-06	2.83E-06	9.74E-06	5.78E-06	8.10E-06
CHURC1	2.57E-05	1.07E-05	1.35E-05	1.38E-05	7.29E-06	1.42E-05	1.20E-05	1.22E-05
CIAO1	4.33E-05	3.98E-05	2.40E-05	2.04E-05	1.46E-05	2.68E-05	1.98E-05	1.86E-05
CIAPIN1	6.51E-05	2.00E-05	1.20E-05	1.62E-05	6.53E-06	1.93E-05	1.55E-05	2.10E-05
CIB1	0.00023	0.000183	0.000104	0.000175	0.000196	0.000155	0.000218	0.000109
CIB2	7.25E-06	5.13E-07	1.52E-07	1.14E-06	9.35E-07	9.14E-08	2.37E-06	1.04E-06
CIB3	4.78E-08	0	0	0	8.36E-08	0	6.12E-08	0
CIB4	0	0	0	0	0	0	0	0
CIC	1.26E-05	8.43E-06	5.55E-06	7.50E-06	4.28E-06	9.96E-06	9.79E-06	1.16E-05
CIDEA	0	2.16E-05	2.75E-08	0	0	0	0	0
CIDEB	0.000185	0.000134	0.000293	0.000246	0.000182	9.34E-05	0.000266	4.61E-05
CIDEC	8.87E-06	1.30E-06	9.03E-07	9.43E-06	3.80E-07	2.32E-06	2.18E-06	1.52E-07
CIDECP	1.34E-05	1.78E-05	3.67E-06	6.02E-06	6.83E-06	3.91E-06	9.43E-06	6.68E-06
CIITA	5.55E-06	4.70E-07	1.34E-06	2.32E-06	1.66E-07	1.77E-06	1.07E-06	1.41E-06
CILP	2.54E-08	5.67E-08	1.05E-06	1.88E-06	1.13E-07	4.22E-08	2.12E-07	1.78E-07
CILP2	1.62E-07	7.88E-08	5.01E-08	1.12E-06	1.39E-08	2.00E-08	4.11E-07	7.13E-08
CINP	4.57E-05	1.69E-05	2.27E-05	2.46E-05	2.31E-05	2.27E-05	3.13E-05	3.22E-05
CIR1	5.07E-05	1.55E-05	1.51E-05	1.87E-05	1.07E-05	2.05E-05	1.60E-05	1.98E-05
CIRBP	9.72E-05	3.22E-05	7.22E-05	0.000129	9.79E-05	0.000139	0.000128	0.000113
CIRH1A	2.78E-05	1.28E-05	1.03E-05	1.21E-05	3.42E-06	1.58E-05	1.03E-05	1.11E-05
CISD1	4.80E-05	2.96E-05	1.19E-05	1.34E-05	2.08E-05	2.02E-05	1.48E-05	1.39E-05
CISD2	3.50E-05	2.62E-05	2.53E-05	2.30E-05	1.87E-05	3.67E-05	2.42E-05	4.35E-05
CISD3	5.21E-05	7.84E-05	3.60E-05	3.47E-05	0.000108	4.12E-05	6.27E-05	2.83E-05
CISH	1.11E-05	1.76E-05	6.12E-05	4.30E-05	2.39E-05	6.14E-06	1.15E-05	1.53E-05
CIT	2.08E-06	1.13E-06	3.44E-07	1.34E-06	3.35E-08	3.84E-07	4.14E-07	1.17E-07
CITED1	1.03E-07	2.02E-07	3.85E-08	1.28E-07	1.49E-07	3.84E-08	6.49E-08	5.15E-07
CITED2	2.07E-05	3.74E-05	0.000102	4.75E-05	2.53E-06	1.15E-05	2.75E-05	2.45E-05

CITED4	1.36E-05	7.43E-06	1.18E-05	4.69E-05	5.34E-05	2.81E-05	5.86E-05	1.21E-05
CIZ1	1.12E-05	7.25E-06	7.62E-06	1.38E-05	6.79E-06	1.80E-05	1.31E-05	1.55E-05
CKAP2	9.21E-06	4.45E-06	1.48E-06	1.31E-06	1.96E-06	4.13E-06	1.04E-06	1.29E-06
CKAP2L	3.50E-06	2.62E-06	8.87E-08	6.72E-08	4.81E-08	4.56E-07	5.28E-08	2.89E-07
CKAP4	7.42E-05	3.73E-05	1.44E-05	2.79E-05	1.59E-05	3.27E-05	4.23E-05	3.74E-05
CKAP5	2.68E-05	2.03E-05	7.58E-06	9.04E-06	2.66E-06	1.20E-05	7.44E-06	1.87E-05
CKB	0.000479	2.11E-05	5.37E-06	2.27E-05	6.35E-06	1.40E-06	8.61E-06	8.78E-07
CKLF	7.17E-05	1.48E-05	1.62E-05	2.95E-05	1.86E-05	2.93E-05	3.36E-05	2.55E-05
CKM	1.59E-08	5.42E-08	0	5.85E-08	0	1.47E-08	7.64E-08	0
CKMT1A	1.92E-05	6.40E-07	0	1.92E-08	1.70E-06	0	4.51E-08	0
CKMT1B	2.20E-05	1.37E-06	1.22E-07	9.52E-08	7.28E-07	0	1.35E-07	0
CKMT2	1.43E-05	9.30E-06	7.16E-08	8.28E-07	1.29E-08	0	4.51E-08	4.98E-08
CKS1B	5.98E-05	4.81E-05	2.02E-05	2.34E-05	2.23E-05	5.16E-05	6.11E-05	4.51E-05
CKS2	9.32E-05	3.78E-05	1.22E-05	1.39E-05	1.82E-05	1.92E-05	1.86E-05	1.95E-05
CLASP1	2.01E-05	1.03E-05	8.64E-06	7.20E-06	2.47E-06	1.42E-05	5.56E-06	6.31E-06
CLASP2	7.49E-06	5.56E-06	3.39E-06	4.72E-06	1.27E-06	4.47E-06	3.09E-06	2.58E-06
CLC	4.91E-08	0	1.16E-07	6.16E-08	0	0	0	7.46E-08
CLCA1	0	0	0	0	0	0	0	0
CLCA2	0	0	1.51E-07	6.91E-09	0	0	1.10E-08	1.33E-07
CLCA3P	0	0	0	0	0	0	0	0
CLCA4	0	1.77E-08	0	0	0	0	0	3.16E-08
CLCC1	1.30E-05	1.20E-05	9.49E-06	1.09E-05	3.71E-06	2.00E-05	6.11E-06	1.45E-05
CLCF1	6.14E-06	4.07E-06	3.93E-07	1.73E-05	3.75E-06	1.58E-06	4.15E-06	3.64E-06
CLCN1	3.15E-08	8.84E-09	3.09E-08	2.89E-08	1.35E-08	0	1.53E-08	2.19E-07
CLCN2	6.67E-06	3.74E-06	5.79E-07	7.52E-07	5.09E-07	1.02E-06	9.87E-07	8.74E-07
CLCN3	2.16E-05	9.11E-06	8.39E-06	6.80E-06	1.07E-06	1.47E-05	3.66E-06	1.10E-05
CLCN4	1.67E-06	1.30E-06	1.53E-06	1.37E-06	5.02E-07	3.09E-06	1.39E-06	3.99E-06
CLCN5	7.80E-06	5.43E-06	6.95E-06	2.72E-06	2.16E-06	5.48E-06	2.39E-06	4.09E-06
CLCN6	9.09E-06	4.83E-06	2.48E-06	2.32E-06	5.68E-07	3.31E-06	1.93E-06	2.16E-06
CLCN7	2.45E-05	5.75E-05	1.57E-05	1.95E-05	2.21E-05	2.04E-05	3.18E-05	3.38E-05
CLCNKA	2.25E-06	1.01E-07	1.02E-08	3.59E-08	0	0	9.48E-09	3.89E-07
CLCNKB	3.36E-08	2.53E-08	0	1.37E-08	0	0	0	4.94E-08
CLDN1	0.000181	0.000127	0.000115	0.000204	8.60E-05	5.41E-05	0.000186	7.51E-05
CLDN10	2.73E-05	1.15E-08	2.55E-06	1.59E-06	0	4.35E-07	0	6.77E-08
CLDN11	1.37E-06	2.49E-07	6.12E-07	5.88E-06	2.75E-07	1.79E-08	2.06E-07	1.42E-07
CLDN12	0.000132	5.16E-05	2.22E-05	1.91E-05	8.78E-06	3.49E-05	1.59E-05	2.73E-05
CLDN14	1.11E-05	3.13E-05	2.83E-05	1.85E-05	1.66E-05	1.34E-05	1.30E-05	1.04E-05
CLDN15	1.29E-06	9.76E-05	1.57E-05	1.57E-05	1.46E-05	2.71E-05	2.28E-05	1.90E-05
CLDN16	2.49E-07	1.14E-06	7.77E-07	4.09E-07	0	3.25E-07	5.44E-07	2.46E-07
CLDN17	0	0	0	0	0	0	0	0
CLDN18	7.11E-09	8.47E-08	1.47E-08	1.68E-08	0	0	6.90E-09	0
CLDN19	8.13E-09	1.67E-06	3.47E-08	3.11E-08	1.40E-08	1.51E-08	1.58E-08	0
CLDN2	3.75E-05	7.36E-06	3.19E-05	4.23E-05	6.69E-06	0.000168	2.63E-05	1.05E-05
CLDN20	6.39E-08	0	0	0	0	5.95E-08	0	0
CLDN22	0	0	0	0	9.61E-07	0	0	0
CLDN23	1.60E-05	1.54E-05	5.10E-06	1.03E-05	1.59E-06	8.85E-06	4.92E-06	2.72E-06
CLDN25	0	0	4.99E-08	0	0	0	0	6.52E-08
CLDN3	2.36E-05	0.000117	1.67E-05	6.10E-05	7.39E-05	7.97E-06	9.16E-05	1.45E-06
CLDN4	7.38E-05	3.70E-05	1.81E-06	6.24E-05	1.28E-07	3.80E-06	1.61E-06	7.83E-08
CLDN5	9.30E-06	8.21E-06	6.23E-06	5.59E-06	2.04E-05	4.66E-06	4.98E-05	1.02E-05
CLDN6	3.67E-08	2.10E-08	5.89E-08	5.43E-07	3.17E-08	0	1.76E-08	5.44E-08
CLDN7	4.72E-05	1.57E-05	8.48E-06	5.11E-05	5.69E-06	5.07E-06	7.09E-05	3.87E-06
CLDN8	0	0	3.79E-08	1.48E-08	0	0	0	0
CLDN9	3.96E-07	1.52E-06	6.59E-07	2.69E-06	1.80E-06	1.08E-07	1.67E-06	6.86E-07
CLDND1	3.16E-05	3.59E-05	1.78E-05	2.45E-05	9.61E-06	1.82E-05	1.97E-05	2.04E-05
CLDND2	1.10E-07	0	1.52E-07	2.73E-07	1.36E-06	1.93E-06	9.20E-07	4.11E-08
CLEC10A	6.03E-06	9.14E-07	3.79E-06	6.20E-06	5.56E-07	8.74E-07	3.78E-06	6.37E-07
CLEC11A	5.81E-06	6.93E-07	2.81E-06	1.40E-05	4.77E-06	6.96E-07	7.15E-06	2.69E-06

CLEC12A	2.83E-06	9.22E-08	9.76E-07	1.90E-06	7.56E-08	8.92E-07	2.19E-07	1.04E-07
CLEC12B	1.24E-07	8.61E-09	1.59E-08	2.45E-08	0	2.13E-08	0	2.25E-08
CLEC14A	1.68E-05	1.10E-05	4.55E-06	6.23E-06	1.35E-05	5.93E-06	2.12E-05	1.32E-05
CLEC16A	2.60E-05	1.24E-05	2.88E-06	3.81E-06	2.23E-06	5.86E-06	7.24E-06	7.31E-06
CLEC17A	2.40E-08	0	2.52E-08	7.36E-08	0	1.11E-08	4.64E-08	4.86E-08
CLEC18A	5.00E-07	4.51E-07	6.18E-08	1.34E-07	1.02E-07	2.17E-07	8.06E-08	1.61E-07
CLEC18B	6.88E-08	9.03E-07	4.04E-08	1.13E-08	1.13E-07	8.43E-08	3.00E-08	5.55E-08
CLEC18C	1.39E-08	3.82E-07	0	1.62E-08	5.68E-08	0	2.38E-08	0
CLEC1A	1.39E-06	1.35E-06	1.11E-06	5.67E-07	5.13E-07	5.96E-07	1.57E-06	1.22E-06
CLEC1B	0	7.46E-08	2.12E-05	1.04E-05	1.35E-07	0	0	0
CLEC2A	0	0	0	0	0	0	0	0
CLEC2B	4.76E-06	1.38E-06	2.26E-06	4.93E-06	1.52E-06	1.83E-06	4.19E-06	1.61E-06
CLEC2D	5.20E-06	8.52E-07	1.20E-06	2.31E-06	6.30E-07	2.64E-06	1.59E-06	1.81E-06
CLEC2L	1.97E-08	4.75E-07	0	0	0	0	0	0
CLEC3A	0	0	0	0	0	0	4.80E-08	0
CLEC3B	1.91E-05	2.01E-05	4.11E-05	2.78E-05	9.45E-05	1.48E-05	2.49E-05	2.80E-05
CLEC4A	5.10E-06	5.01E-07	9.58E-07	2.64E-06	4.71E-07	5.85E-07	1.35E-06	9.20E-07
CLEC4C	2.68E-08	2.41E-08	2.94E-08	3.32E-08	0	3.91E-08	0	0
CLEC4D	2.36E-07	1.41E-08	1.31E-08	7.63E-08	0	0	1.20E-08	1.83E-08
CLEC4E	2.71E-06	1.12E-07	6.22E-07	1.31E-06	1.04E-07	8.17E-08	1.49E-07	3.24E-07
CLEC4F	8.18E-07	4.51E-08	1.81E-07	1.05E-07	4.90E-08	4.93E-08	1.84E-08	2.79E-08
CLEC4G	1.30E-07	4.24E-08	4.61E-05	3.40E-05	3.36E-07	6.89E-08	2.14E-07	0
CLEC4GP1	1.33E-08	1.50E-08	4.19E-08	1.63E-08	1.14E-07	0	0	5.86E-08
CLEC4M	7.45E-09	0	2.29E-05	7.83E-06	1.17E-08	0	3.12E-08	0
CLEC5A	3.28E-07	8.72E-08	1.26E-07	6.13E-07	6.07E-09	0	1.03E-07	0
CLEC6A	1.52E-07	0	0	1.87E-08	0	0	0	0
CLEC7A	7.51E-06	3.84E-07	1.82E-06	4.39E-06	4.42E-07	9.66E-07	1.26E-06	6.35E-07
CLEC9A	2.92E-07	0	2.01E-07	1.08E-07	1.26E-08	1.36E-07	2.82E-08	1.51E-07
CLECL1	1.75E-06	0	5.68E-07	1.28E-06	8.49E-08	1.01E-06	4.77E-07	0
CLGN	3.60E-06	3.11E-06	1.40E-06	1.56E-07	3.29E-07	1.58E-08	3.08E-06	1.21E-05
CLIC1	0.0003	0.000145	5.71E-05	0.000207	7.09E-05	0.00013	0.000182	0.000114
CLIC2	4.96E-06	2.20E-06	3.37E-06	3.22E-06	5.83E-07	2.19E-06	2.60E-06	2.46E-06
CLIC3	2.53E-06	3.67E-07	3.12E-07	6.90E-07	2.41E-07	1.30E-07	1.94E-06	1.56E-07
CLIC4	2.14E-05	3.15E-05	2.15E-05	1.76E-05	4.79E-06	1.22E-05	1.23E-05	1.22E-05
CLIC5	2.02E-06	1.45E-06	7.64E-08	3.45E-07	9.85E-08	6.77E-07	2.61E-07	6.57E-07
CLIC6	1.27E-05	8.08E-08	5.51E-07	1.40E-05	0	8.34E-08	4.26E-07	8.80E-08
CLINT1	6.79E-05	3.12E-05	3.57E-05	3.03E-05	5.44E-06	2.74E-05	1.62E-05	2.33E-05
CLIP1	4.26E-05	4.96E-05	2.23E-05	2.24E-05	1.11E-05	2.03E-05	1.99E-05	1.79E-05
CLIP2	1.64E-05	4.84E-07	1.43E-06	3.84E-06	6.20E-07	7.68E-07	1.75E-06	1.82E-06
CLIP3	9.63E-06	4.78E-07	5.31E-07	1.92E-06	6.49E-07	2.94E-07	1.56E-06	8.55E-07
CLIP4	6.81E-06	1.12E-07	1.02E-06	2.58E-06	3.80E-08	8.67E-06	3.45E-07	2.11E-07
CLK1	6.16E-05	2.78E-05	1.57E-05	3.64E-05	1.73E-05	2.66E-05	2.58E-05	2.45E-05
CLK2	1.68E-05	1.19E-05	7.71E-06	6.90E-06	4.48E-06	1.50E-05	8.83E-06	1.32E-05
CLK2P	6.41E-07	2.73E-07	1.97E-07	1.48E-07	8.05E-08	3.60E-07	1.42E-07	3.15E-07
CLK3	2.36E-05	1.42E-05	1.16E-05	1.75E-05	1.04E-05	1.65E-05	1.54E-05	1.63E-05
CLK4	9.90E-06	3.11E-06	3.67E-06	5.98E-06	2.00E-06	6.51E-06	2.97E-06	4.87E-06
CLLU1	1.04E-08	2.76E-08	2.56E-08	1.49E-07	1.05E-08	0	0	0
CLLU1OS	4.35E-08	0	1.01E-07	0	0	0	0	0
CLMN	3.65E-06	3.28E-06	5.73E-06	5.21E-06	2.01E-06	8.07E-06	4.22E-06	5.66E-06
CLN3	4.28E-05	6.72E-05	2.42E-05	2.58E-05	3.19E-05	3.00E-05	3.05E-05	4.15E-05
CLN5	2.22E-05	2.41E-05	1.40E-05	1.37E-05	6.75E-06	1.03E-05	9.72E-06	1.01E-05
CLN6	2.62E-05	1.45E-05	1.16E-05	1.41E-05	1.94E-05	1.64E-05	2.53E-05	1.98E-05
CLN8	2.42E-06	6.80E-06	2.10E-06	3.03E-06	6.32E-07	1.22E-06	1.89E-06	1.42E-06
CLNK	1.46E-08	0	1.54E-08	4.64E-08	0	0	1.41E-08	2.15E-08
CLNS1A	6.74E-05	5.40E-05	4.72E-05	4.58E-05	2.28E-05	0.000116	6.71E-05	9.22E-05
CLOCK	1.84E-06	1.99E-06	1.32E-06	9.20E-07	3.70E-08	2.33E-06	2.94E-07	1.57E-06
CLP1	9.54E-06	7.69E-06	5.91E-06	5.99E-06	3.76E-06	9.38E-06	4.99E-06	1.21E-05
CLPB	1.12E-05	8.27E-06	7.72E-06	5.98E-06	7.49E-06	1.20E-05	4.11E-06	1.18E-05

CLPP	6.53E-05	9.68E-05	4.73E-05	5.63E-05	0.000121	7.62E-05	8.01E-05	5.26E-05
CLPS	0	0	0	0	0	0	0	0
CLPTM1	0.000115	0.000157	6.71E-05	5.93E-05	6.35E-05	6.81E-05	7.08E-05	8.88E-05
CLPTM1L	5.72E-05	8.16E-05	8.50E-05	8.24E-05	3.29E-05	7.10E-05	7.29E-05	7.76E-05
CLPX	3.37E-05	3.29E-05	3.66E-05	2.77E-05	6.28E-06	6.04E-05	2.27E-05	2.92E-05
CLRN1	0	2.26E-08	0	0	0	0	0	0
CLRN1OS	5.71E-08	2.18E-08	3.15E-07	0	0	1.06E-07	0	0
CLRN2	3.41E-08	3.61E-07	0	0	0	0	0	0
CLRN3	8.02E-05	2.09E-06	2.52E-05	3.10E-05	0	1.69E-07	8.69E-07	3.37E-08
CLSPN	1.52E-06	4.61E-07	3.19E-08	5.31E-08	1.40E-08	1.45E-07	2.11E-08	1.29E-07
CLSTN1	9.03E-05	9.03E-06	9.03E-06	1.49E-05	9.42E-06	7.27E-06	1.09E-05	1.55E-05
CLSTN2	7.09E-07	2.07E-08	1.97E-07	7.40E-07	2.87E-08	6.42E-08	9.01E-08	4.06E-08
CLSTN3	6.62E-05	2.83E-05	1.22E-05	1.72E-05	1.45E-05	1.64E-05	1.71E-05	1.50E-05
CLTA	8.58E-05	0.000134	6.04E-05	0.000107	0.000116	8.91E-05	0.000144	9.38E-05
CLTB	7.14E-05	4.32E-05	4.57E-05	6.78E-05	7.06E-05	4.57E-05	7.30E-05	8.74E-05
CLTC	0.000109	0.000103	3.78E-05	3.07E-05	1.71E-05	7.77E-05	2.55E-05	3.79E-05
CLTCL1	7.30E-06	2.15E-05	1.39E-06	5.32E-06	4.71E-06	4.54E-06	2.06E-06	5.61E-06
CLU	0.001116	0.005258	0.002464	0.002071	0.001911	0.003383	0.001314	0.000932
CLUAP1	7.00E-06	1.01E-05	4.40E-06	7.05E-06	1.42E-06	9.04E-06	7.38E-06	4.01E-06
CLUL1	7.78E-08	1.87E-07	1.36E-08	7.95E-08	0	1.20E-08	1.25E-08	3.82E-08
CLVS1	6.44E-08	7.57E-08	1.65E-08	4.02E-08	2.73E-06	6.87E-07	7.78E-07	3.78E-08
CLVS2	0	0	0	0	0	0	0	0
CLYBL	3.10E-05	4.98E-05	4.92E-05	3.74E-05	1.66E-05	3.43E-05	2.13E-05	3.31E-05
CMA1	3.64E-08	8.59E-08	3.29E-07	0	0	6.82E-08	1.72E-07	0
CMAH	2.21E-06	9.02E-06	1.58E-06	1.91E-06	3.56E-07	7.20E-07	7.57E-07	2.14E-06
CMAS	4.39E-05	5.24E-05	3.28E-05	1.96E-05	1.61E-05	3.81E-05	2.02E-05	3.15E-05
CMBL	2.02E-05	0.00011	9.28E-05	2.04E-05	1.84E-05	9.36E-05	3.48E-05	1.62E-05
CMC1	5.78E-05	4.58E-05	2.07E-05	2.21E-05	1.59E-05	4.00E-05	3.08E-05	2.73E-05
CMIP	1.71E-05	5.75E-06	7.93E-06	9.16E-06	1.63E-06	8.78E-06	6.96E-06	1.01E-05
CMKLR1	5.59E-06	6.07E-07	2.27E-06	2.55E-06	1.01E-06	1.74E-06	4.27E-06	7.49E-07
CMPK1	8.62E-05	7.52E-05	9.01E-05	8.75E-05	3.60E-05	8.17E-05	6.79E-05	7.71E-05
CMPK2	4.08E-06	1.22E-06	2.29E-06	1.97E-06	1.27E-07	2.43E-06	1.10E-06	6.81E-06
CMTM1	2.76E-06	1.05E-06	2.27E-07	6.04E-07	3.41E-07	5.26E-07	2.42E-07	4.31E-07
CMTM2	4.87E-07	1.07E-07	1.69E-07	1.13E-07	1.32E-07	5.69E-08	5.78E-08	4.55E-07
CMTM3	1.51E-05	4.00E-06	4.28E-06	1.21E-05	4.75E-06	4.27E-06	1.27E-05	4.97E-06
CMTM4	4.32E-06	1.69E-06	9.22E-07	1.53E-06	1.73E-06	7.27E-07	3.21E-06	1.78E-06
CMTM5	0	5.85E-08	2.56E-08	3.51E-08	0	2.65E-08	0	0
CMTM6	5.74E-05	3.62E-05	5.33E-05	3.75E-05	1.11E-05	3.49E-05	2.68E-05	2.30E-05
CMTM7	1.58E-05	1.62E-06	3.95E-06	1.27E-05	3.40E-06	5.05E-06	1.21E-05	2.20E-06
CMTM8	2.03E-05	2.48E-05	2.26E-05	1.23E-05	2.15E-05	2.71E-05	2.29E-05	1.78E-05
CMYA5	2.99E-07	5.34E-07	8.93E-07	3.85E-07	3.66E-08	3.32E-08	1.10E-07	9.73E-08
CN5H6.4	1.63E-07	0	1.73E-08	0	2.11E-07	1.67E-07	6.29E-08	1.21E-07
CNBD1	0	0	0	0	0	0	0	0
CNBP	0.000109	0.00012	9.73E-05	9.42E-05	5.53E-05	9.04E-05	9.71E-05	5.91E-05
CNDP1	1.08E-08	2.54E-07	6.79E-05	4.90E-06	9.02E-07	2.74E-06	4.74E-08	1.24E-06
CNDP2	0.000159	8.67E-05	0.000102	0.000108	3.74E-05	0.000174	0.000182	9.27E-05
CNFN	1.76E-05	2.39E-07	2.95E-06	2.36E-06	4.52E-07	0	7.10E-07	0
CNGA1	9.68E-07	3.61E-05	6.76E-06	1.30E-05	9.05E-06	1.55E-06	7.74E-06	8.08E-06
CNGA2	0	0	0	0	0	0	0	0
CNGA3	0	0	0	0	0	0	0	0
CNGA4	9.98E-08	1.43E-08	1.33E-08	2.46E-08	4.36E-08	5.87E-08	0	1.86E-08
CNGB1	4.61E-06	2.23E-08	8.17E-09	1.31E-07	0	0	0	3.50E-08
CNGB3	0	7.11E-08	0	3.53E-08	0	0	0	0
CNIH	0.000105	7.62E-05	9.50E-05	7.60E-05	5.60E-05	0.000135	7.45E-05	9.21E-05
CNIH2	4.74E-07	1.27E-06	2.03E-08	2.10E-07	6.23E-07	1.69E-07	7.30E-08	8.44E-08
CNIH3	8.94E-08	1.01E-07	9.31E-08	3.16E-07	5.98E-08	2.95E-07	8.66E-08	4.38E-08
CNIH4	6.30E-05	4.66E-05	2.42E-05	3.76E-05	2.20E-05	6.49E-05	2.63E-05	8.96E-05
CNKSR1	5.64E-06	1.16E-07	1.06E-07	4.74E-06	0	5.19E-08	9.04E-08	2.74E-08

CNKSR2	1.19E-06	6.50E-07	9.71E-08	1.63E-07	0	1.77E-07	9.74E-08	1.06E-08
CNKSR3	1.54E-05	9.98E-06	1.37E-05	1.81E-05	5.77E-06	1.54E-05	1.81E-05	1.37E-05
CNN1	1.03E-05	1.48E-06	3.62E-07	3.41E-06	3.49E-06	3.32E-06	3.15E-06	2.75E-06
CNN2	5.58E-05	4.17E-05	2.45E-05	5.86E-05	1.32E-05	1.51E-05	3.69E-05	1.53E-05
CNN3	7.02E-05	0.000133	6.80E-05	6.01E-05	1.53E-05	0.000165	5.04E-05	8.15E-05
CNNM1	6.16E-06	2.54E-07	2.94E-07	3.26E-07	2.88E-06	4.49E-08	1.39E-06	2.05E-07
CNNM2	6.81E-06	3.01E-06	3.21E-06	2.14E-06	3.93E-06	3.61E-06	3.24E-06	2.43E-06
CNNM3	3.12E-05	1.62E-05	1.50E-05	1.29E-05	1.57E-05	1.11E-05	1.56E-05	8.38E-06
CNNM4	8.83E-06	3.08E-06	1.41E-06	2.15E-06	3.43E-07	1.88E-06	1.58E-06	2.82E-06
CNO	1.71E-05	1.64E-05	4.90E-06	7.74E-06	7.11E-06	1.01E-05	7.74E-06	9.65E-06
CNOT1	3.60E-05	1.96E-05	1.87E-05	1.74E-05	1.11E-05	2.70E-05	1.84E-05	1.03E-05
CNOT10	1.17E-05	1.12E-05	6.20E-06	7.94E-06	3.94E-06	1.00E-05	7.63E-06	6.81E-06
CNOT2	2.00E-05	1.44E-05	1.04E-05	1.24E-05	4.63E-06	1.38E-05	1.28E-05	9.76E-06
CNOT3	1.44E-05	1.28E-05	8.17E-06	9.60E-06	7.73E-06	1.17E-05	1.31E-05	8.24E-06
CNOT4	4.27E-06	7.40E-06	5.17E-06	3.81E-06	3.18E-06	5.70E-06	4.15E-06	6.13E-06
CNOT6	7.10E-06	1.03E-06	1.69E-06	2.66E-06	4.55E-07	1.53E-06	1.44E-06	2.93E-06
CNOT6L	6.14E-06	2.13E-06	3.96E-06	5.51E-06	1.18E-06	5.46E-06	3.01E-06	4.20E-06
CNOT7	2.05E-05	4.17E-05	1.99E-05	2.03E-05	3.27E-06	2.75E-05	1.05E-05	1.13E-05
CNOT8	2.46E-05	8.68E-06	1.49E-05	1.33E-05	3.71E-06	1.60E-05	1.21E-05	1.48E-05
CNP	2.17E-05	2.64E-05	1.50E-05	1.87E-05	1.03E-05	2.70E-05	2.09E-05	1.66E-05
CNPY1	0	1.20E-08	0	1.30E-08	0	0	0	0
CNPY2	0.000114	0.000111	5.72E-05	7.21E-05	0.000129	8.87E-05	4.87E-05	7.80E-05
CNPY3	4.11E-05	6.28E-05	4.11E-05	3.82E-05	3.06E-05	3.70E-05	3.49E-05	4.87E-05
CNPY4	1.28E-05	6.11E-06	2.73E-06	6.87E-06	5.04E-06	3.27E-06	5.02E-06	7.66E-06
CNR1	1.26E-08	1.40E-07	3.74E-08	4.09E-08	3.59E-09	4.87E-08	1.63E-08	2.53E-08
CNR2	1.37E-08	3.11E-08	8.65E-08	1.01E-07	1.18E-08	1.27E-08	1.32E-08	0
CNRIP1	3.57E-06	2.12E-06	2.12E-06	4.93E-06	1.72E-06	1.83E-06	5.40E-06	2.21E-06
CNST	6.61E-06	1.01E-05	9.24E-06	6.37E-06	2.13E-06	1.38E-05	4.53E-06	8.28E-06
CNTD1	8.61E-07	6.50E-07	1.21E-07	1.43E-07	1.89E-07	4.59E-07	2.19E-07	2.71E-07
CNTD2	2.00E-07	1.34E-06	4.89E-07	6.54E-07	5.74E-07	2.23E-07	5.79E-07	3.96E-06
CNTF	3.35E-07	1.88E-07	5.35E-08	1.41E-07	1.10E-08	2.48E-07	1.35E-07	5.91E-07
CNTFR	1.07E-05	6.40E-06	8.79E-06	8.35E-06	5.80E-07	1.16E-08	6.03E-08	0
CNTLN	2.79E-06	5.40E-06	3.83E-06	2.58E-06	1.50E-06	3.54E-06	2.15E-06	5.10E-06
CNTN1	6.77E-09	1.08E-06	1.09E-07	8.27E-08	0	0	0	9.92E-09
CNTN2	9.09E-09	2.01E-08	0	3.32E-08	1.04E-08	5.60E-09	1.18E-08	4.42E-09
CNTN3	2.10E-06	2.02E-08	7.59E-07	8.32E-07	0	0	2.15E-07	3.96E-08
CNTN4	1.08E-06	4.02E-07	8.79E-07	1.41E-06	1.49E-07	2.12E-07	2.70E-07	2.53E-07
CNTN5	1.12E-08	1.40E-07	8.05E-09	1.24E-07	0	0	0	0
CNTN6	6.78E-09	0	0	0	0	0	1.97E-08	0
CNTNAP1	3.40E-06	7.30E-07	6.77E-08	6.86E-07	7.09E-08	2.68E-07	5.84E-07	3.27E-07
CNTNAP2	8.57E-06	8.24E-09	3.27E-06	2.59E-06	7.59E-08	4.45E-06	6.83E-08	2.32E-06
CNTNAP3	4.74E-08	3.13E-07	1.28E-07	7.40E-07	5.25E-08	4.38E-06	9.11E-08	1.09E-06
CNTNAP4	0	1.13E-08	5.39E-09	0	0	3.40E-08	0	3.23E-07
CNTNAP5	0	0	0	0	0	0	4.15E-09	0
CNTROB	7.92E-06	4.22E-06	4.41E-06	6.08E-06	2.91E-06	7.71E-06	8.56E-06	7.28E-06
COASY	9.09E-05	0.00011	3.09E-05	2.96E-05	6.37E-05	8.66E-05	5.06E-05	2.45E-05
COBL	2.32E-05	2.32E-05	1.04E-05	1.12E-05	6.71E-06	7.40E-06	1.18E-05	1.59E-05
COBLL1	1.67E-05	1.60E-05	3.40E-05	1.42E-05	7.03E-06	4.28E-05	1.22E-05	1.06E-05
COBRA1	3.32E-05	1.53E-05	1.19E-05	2.15E-05	1.87E-05	2.21E-05	2.62E-05	2.96E-05
COCH	1.26E-05	3.97E-08	2.69E-07	3.06E-07	4.70E-08	6.08E-08	4.95E-06	9.08E-07
COG1	1.98E-05	1.60E-05	5.45E-06	7.52E-06	3.84E-06	1.41E-05	7.25E-06	7.42E-06
COG2	1.69E-05	1.90E-05	4.75E-06	5.27E-06	2.09E-06	1.34E-05	5.26E-06	7.02E-06
COG3	1.90E-05	1.89E-05	8.92E-06	1.01E-05	3.79E-06	3.56E-05	1.21E-05	1.20E-05
COG4	3.58E-05	1.79E-05	1.79E-05	2.46E-05	2.39E-05	3.32E-05	2.31E-05	3.14E-05
COG5	4.86E-06	5.76E-06	2.42E-06	3.44E-06	1.10E-06	5.80E-06	1.59E-06	4.65E-06
COG6	5.67E-06	5.02E-06	4.17E-06	3.45E-06	9.64E-07	4.72E-06	1.80E-06	3.14E-06
COG7	9.44E-06	1.27E-05	6.55E-06	6.02E-06	2.15E-06	8.54E-06	4.50E-06	8.80E-06
COG8	1.26E-05	7.15E-06	9.50E-06	8.50E-06	2.14E-06	1.24E-05	7.16E-06	1.07E-05

COIL	1.38E-05	1.43E-05	5.51E-06	5.39E-06	4.42E-06	1.17E-05	5.38E-06	5.36E-06
COL10A1	1.58E-06	7.86E-09	7.23E-08	6.42E-07	6.01E-09	1.30E-08	3.94E-07	1.44E-07
COL11A1	1.39E-06	0	7.22E-09	6.15E-08	0	0	2.41E-08	1.81E-07
COL11A2	7.12E-07	1.28E-07	2.73E-07	9.49E-08	2.76E-07	1.18E-07	8.16E-08	6.02E-08
COL12A1	3.97E-06	1.85E-06	5.97E-07	2.05E-06	3.37E-07	7.41E-07	1.56E-06	2.23E-06
COL13A1	2.16E-07	5.80E-08	1.60E-07	2.88E-07	1.02E-07	2.30E-08	2.33E-07	7.27E-08
COL14A1	7.60E-06	1.40E-06	5.17E-06	2.45E-05	1.06E-06	8.99E-07	3.00E-06	5.34E-06
COL15A1	3.85E-06	3.15E-06	1.31E-07	7.34E-07	2.36E-06	3.59E-06	5.04E-06	4.56E-06
COL16A1	4.33E-06	7.37E-08	7.84E-07	6.51E-06	1.12E-06	1.93E-07	1.18E-06	1.09E-06
COL17A1	1.49E-07	1.46E-07	3.68E-07	2.94E-07	1.04E-07	1.85E-08	1.52E-08	4.10E-08
COL18A1	0.000136	0.000218	0.000296	0.000439	0.000424	0.00035	0.000465	0.000219
COL19A1	1.02E-08	3.55E-08	1.04E-08	9.30E-09	0	1.02E-08	0	3.71E-09
COL1A1	0.000247	1.14E-05	9.71E-06	9.37E-05	1.24E-05	1.37E-05	0.000134	5.43E-05
COL1A2	0.000176	1.11E-05	1.05E-05	6.80E-05	1.96E-05	1.33E-05	7.84E-05	4.20E-05
COL20A1	0	0	6.48E-09	2.30E-08	0	0	0	0
COL21A1	2.33E-07	6.97E-07	3.59E-07	5.40E-07	9.55E-07	1.15E-06	1.17E-06	4.22E-07
COL22A1	2.05E-07	1.40E-05	7.21E-09	6.84E-08	0	2.33E-08	7.17E-08	1.59E-07
COL23A1	2.69E-07	2.25E-08	8.30E-08	6.22E-07	1.54E-07	1.39E-07	1.25E-07	1.90E-07
COL24A1	1.35E-07	3.44E-08	3.93E-09	4.69E-09	3.30E-09	1.07E-08	1.50E-08	9.00E-08
COL25A1	5.57E-09	1.00E-07	3.74E-07	3.72E-07	1.43E-08	2.06E-08	1.62E-08	8.15E-09
COL27A1	2.17E-05	4.67E-06	7.50E-06	7.31E-06	2.26E-07	2.01E-06	2.80E-06	3.58E-06
COL28A1	1.31E-08	3.33E-07	4.04E-08	7.97E-09	0	0	3.95E-08	0
COL29A1	0	0	0	2.03E-08	0	0	7.31E-09	3.79E-08
COL2A1	0	0	2.41E-08	0	0	0	1.85E-08	2.85E-08
COL3A1	0.00018	1.26E-05	3.48E-05	0.000125	1.16E-05	1.48E-05	5.00E-05	5.12E-05
COL4A1	0.000165	3.98E-05	4.86E-06	3.45E-05	1.27E-05	3.11E-05	5.38E-05	3.30E-05
COL4A2	0.000185	3.35E-05	6.46E-06	4.61E-05	1.95E-05	2.93E-05	6.66E-05	3.51E-05
COL4A3	4.16E-06	3.49E-08	4.40E-08	3.44E-07	1.12E-08	5.54E-08	1.20E-07	4.04E-08
COL4A3BI	1.34E-05	7.32E-06	8.53E-06	7.23E-06	1.55E-06	7.56E-06	4.50E-06	7.27E-06
COL4A4	2.86E-06	2.27E-07	2.59E-07	1.72E-06	6.42E-08	1.25E-07	3.66E-07	1.16E-07
COL4A5	5.40E-06	3.81E-06	3.34E-07	7.42E-07	5.12E-08	2.77E-08	1.02E-07	1.04E-07
COL4A6	1.15E-07	4.33E-07	2.87E-08	5.70E-08	0	6.24E-08	1.11E-08	0
COL5A1	1.83E-05	1.24E-06	1.59E-06	1.33E-05	1.76E-06	1.23E-06	9.87E-06	5.29E-06
COL5A2	3.39E-05	7.44E-06	3.88E-06	1.06E-05	5.25E-06	5.98E-06	6.40E-06	5.31E-06
COL5A3	1.06E-05	1.18E-05	1.45E-06	5.18E-07	1.44E-05	1.32E-05	7.14E-06	3.68E-06
COL6A1	0.000106	1.70E-05	3.72E-05	7.69E-05	2.91E-05	1.22E-05	8.28E-05	3.93E-05
COL6A2	0.00012	1.70E-05	4.25E-05	0.000128	5.53E-05	2.02E-05	0.000126	5.58E-05
COL6A3	3.68E-05	5.13E-06	5.70E-06	1.94E-05	4.70E-06	6.93E-06	1.47E-05	2.54E-05
COL6A4P2	4.84E-08	2.43E-08	0	2.95E-09	4.15E-09	0	2.36E-09	1.77E-08
COL6A6	2.19E-08	8.35E-09	5.76E-07	1.21E-07	9.45E-09	0	0	3.83E-09
COL7A1	1.29E-05	3.69E-06	1.48E-08	9.72E-08	2.67E-07	2.17E-06	9.43E-08	3.18E-08
COL8A1	3.74E-06	1.28E-06	3.16E-07	1.03E-06	7.75E-08	5.43E-07	1.37E-06	3.48E-07
COL8A2	3.32E-06	5.32E-08	3.68E-07	1.72E-06	2.72E-08	1.18E-07	5.45E-07	3.32E-07
COL9A1	5.97E-07	1.13E-06	0	1.08E-08	1.29E-07	9.15E-08	1.71E-08	1.43E-07
COL9A2	1.08E-05	1.59E-07	1.70E-07	1.55E-06	2.82E-08	1.52E-07	1.04E-07	8.43E-08
COL9A3	5.80E-07	1.03E-06	1.01E-06	1.30E-06	4.67E-08	1.72E-06	1.49E-07	3.92E-07
COLEC10	1.02E-06	1.31E-07	1.91E-05	2.48E-05	1.71E-06	1.34E-07	1.82E-06	2.33E-07
COLEC11	1.62E-05	8.26E-05	7.79E-05	0.000133	2.47E-05	7.22E-06	2.83E-05	2.22E-05
COLEC12	1.99E-07	3.56E-07	3.05E-08	4.05E-07	8.23E-08	3.89E-07	1.50E-06	7.56E-08
COLQ	1.53E-07	3.51E-07	2.15E-07	5.35E-07	3.50E-07	6.22E-07	4.59E-07	3.75E-07
COMMD1	4.25E-05	7.03E-05	2.70E-05	4.04E-05	4.61E-05	4.82E-05	5.08E-05	3.64E-05
COMMD1	1.36E-05	1.10E-05	7.18E-06	7.90E-06	1.14E-06	1.27E-05	6.90E-06	1.16E-05
COMMD2	2.65E-05	3.07E-05	8.69E-06	1.18E-05	5.56E-06	1.37E-05	1.02E-05	1.05E-05
COMMD3	5.46E-05	4.86E-05	2.98E-05	4.16E-05	3.87E-05	4.41E-05	5.16E-05	3.01E-05
COMMD4	8.61E-05	8.98E-05	4.41E-05	7.31E-05	0.000194	0.000112	0.000108	0.000108
COMMD5	3.87E-05	2.19E-05	1.37E-05	1.73E-05	1.98E-05	1.89E-05	4.30E-05	2.69E-05
COMMD6	4.52E-05	2.76E-05	1.97E-05	3.83E-05	6.22E-05	5.01E-05	4.14E-05	1.77E-05
COMMD7	2.22E-05	2.23E-05	1.71E-05	2.06E-05	2.02E-05	1.74E-05	3.13E-05	2.04E-05

COMMD8	1.67E-05	1.47E-05	6.19E-06	7.55E-06	2.89E-06	7.15E-06	6.09E-06	1.04E-05
COMMD9	1.57E-05	1.81E-05	9.57E-06	1.09E-05	8.50E-06	1.89E-05	9.87E-06	1.68E-05
COMP	5.67E-07	0	0	3.82E-06	1.77E-08	9.56E-09	5.03E-06	4.55E-08
COMT	3.75E-05	3.31E-05	0.000121	7.68E-05	4.88E-05	0.000116	9.11E-05	8.65E-05
COMTD1	1.94E-05	2.06E-05	1.00E-05	1.74E-05	4.86E-05	8.99E-06	4.24E-05	9.79E-06
COPA	6.53E-05	0.000121	2.98E-05	3.34E-05	7.10E-06	7.02E-05	2.58E-05	6.18E-05
COPB1	7.38E-05	4.39E-05	2.85E-05	3.46E-05	7.58E-06	5.00E-05	1.98E-05	4.70E-05
COPB2	6.54E-05	4.84E-05	3.16E-05	3.47E-05	1.06E-05	6.25E-05	2.22E-05	3.53E-05
COPE	0.000147	0.000126	8.42E-05	0.000118	0.000186	0.00015	0.000143	0.000123
COPG	8.45E-05	8.43E-05	4.35E-05	5.02E-05	2.85E-05	8.53E-05	5.96E-05	6.57E-05
COPG2	4.64E-06	2.23E-06	3.69E-06	5.67E-06	3.96E-06	8.97E-06	5.22E-06	7.31E-06
COPS2	1.92E-05	1.61E-05	1.19E-05	1.17E-05	3.33E-06	1.84E-05	1.04E-05	1.12E-05
COPS3	1.86E-05	3.06E-05	1.66E-05	2.19E-05	1.51E-05	3.52E-05	2.96E-05	2.45E-05
COPS4	2.05E-05	1.18E-05	1.30E-05	1.42E-05	6.59E-06	2.29E-05	1.47E-05	1.91E-05
COPS5	0.000114	0.000102	3.95E-05	4.03E-05	4.29E-05	5.17E-05	9.96E-05	6.64E-05
COPS6	0.000144	0.000116	4.84E-05	5.63E-05	6.24E-05	7.17E-05	5.94E-05	8.79E-05
COPS7A	6.50E-05	3.96E-05	3.45E-05	3.43E-05	2.97E-05	5.75E-05	4.47E-05	4.43E-05
COPS7B	1.93E-05	9.46E-06	5.85E-06	9.33E-06	5.66E-06	1.21E-05	8.73E-06	1.01E-05
COPS8	3.89E-05	1.79E-05	1.03E-05	1.31E-05	5.65E-06	1.56E-05	1.27E-05	1.38E-05
COPZ1	9.08E-05	8.24E-05	4.91E-05	6.91E-05	4.81E-05	0.0001	8.18E-05	7.56E-05
COPZ2	1.78E-05	4.29E-05	1.83E-05	2.08E-05	5.12E-05	1.70E-05	2.76E-05	2.79E-05
COQ10A	1.88E-05	1.74E-05	1.35E-05	6.97E-06	1.09E-05	1.10E-05	7.29E-06	2.27E-05
COQ10B	3.66E-05	2.32E-05	2.24E-05	2.78E-05	3.66E-06	2.60E-05	2.11E-05	4.29E-05
COQ2	1.70E-05	2.57E-06	7.83E-06	7.99E-06	4.41E-06	8.96E-06	7.32E-06	7.40E-06
COQ3	1.08E-05	1.85E-05	1.04E-05	7.33E-06	4.98E-06	1.05E-05	6.83E-06	7.38E-06
COQ4	2.48E-05	2.73E-05	3.61E-05	5.42E-05	4.83E-05	7.54E-05	6.00E-05	5.62E-05
COQ5	4.80E-05	4.21E-05	3.10E-05	2.67E-05	2.23E-05	5.28E-05	3.19E-05	2.82E-05
COQ6	2.10E-05	1.79E-05	1.59E-05	1.48E-05	1.00E-05	2.24E-05	1.45E-05	1.87E-05
COQ7	1.33E-05	1.28E-05	5.27E-06	5.83E-06	3.41E-06	7.93E-06	5.23E-06	4.97E-06
COQ9	0.000136	7.70E-05	5.45E-05	6.06E-05	3.16E-05	6.52E-05	4.18E-05	9.94E-05
CORIN	9.60E-08	4.32E-08	4.96E-08	3.35E-08	8.29E-09	8.20E-07	1.41E-08	3.54E-08
CORO1A	5.16E-05	4.87E-06	1.77E-05	3.40E-05	3.30E-06	2.23E-05	1.99E-05	1.05E-05
CORO1B	9.05E-05	0.000105	3.28E-05	4.39E-05	1.75E-05	5.41E-05	3.54E-05	6.40E-05
CORO1C	6.30E-05	2.67E-05	1.50E-05	3.12E-05	3.52E-06	1.47E-05	2.29E-05	1.40E-05
CORO2A	9.86E-06	3.43E-06	6.39E-07	1.12E-06	4.63E-07	2.03E-06	2.49E-06	1.54E-07
CORO2B	2.94E-06	1.10E-07	1.66E-07	3.94E-07	3.59E-08	1.16E-07	2.51E-07	2.15E-07
CORO6	4.35E-07	1.06E-07	6.22E-08	1.46E-07	1.39E-07	2.74E-08	5.20E-08	3.20E-07
CORO7	1.04E-05	7.81E-06	4.52E-06	9.49E-06	2.52E-06	5.69E-06	9.12E-06	7.33E-06
CORT	3.39E-07	2.82E-07	1.12E-07	2.52E-07	5.88E-07	6.80E-07	2.15E-07	2.99E-07
COTL1	7.72E-05	5.52E-06	2.96E-05	6.60E-05	9.02E-06	2.24E-05	3.35E-05	1.28E-05
COX10	5.37E-06	4.03E-06	3.57E-06	5.00E-06	8.44E-07	1.02E-05	3.54E-06	7.28E-06
COX11	4.86E-05	2.86E-05	2.65E-05	2.21E-05	1.45E-05	4.28E-05	1.97E-05	2.02E-05
COX15	8.92E-06	1.10E-05	6.62E-06	5.12E-06	5.14E-06	9.59E-06	4.42E-06	6.25E-06
COX16	3.24E-05	1.21E-05	1.99E-05	2.25E-05	2.99E-05	2.78E-05	3.66E-05	2.53E-05
COX17	0.000211	0.000315	0.000126	0.000146	0.000234	0.000125	0.000204	0.000122
COX18	6.75E-06	4.52E-06	6.29E-06	4.34E-06	1.60E-06	5.76E-06	4.32E-06	5.63E-06
COX19	4.20E-06	1.59E-06	8.67E-07	1.44E-06	2.56E-06	2.53E-06	2.98E-06	1.36E-06
COX4I1	0.000849	0.000584	0.000399	0.000582	0.000573	0.000525	0.000487	0.000598
COX4I2	1.44E-06	3.24E-06	1.52E-07	3.28E-07	2.04E-05	4.74E-06	1.08E-05	3.44E-06
COX4NB	1.56E-05	1.13E-05	7.63E-06	8.58E-06	5.39E-06	1.25E-05	1.13E-05	8.46E-06
COX5A	0.000287	0.000253	0.000117	0.000135	0.00017	0.000163	0.000219	0.0002
COX5B	0.00067	0.000981	0.000294	0.000335	0.000474	0.000365	0.000525	0.000304
COX6A1	0.000815	0.001082	0.00041	0.000374	0.000663	0.000473	0.000463	0.00039
COX6A2	9.07E-06	0	2.54E-05	7.19E-06	3.92E-05	1.85E-05	5.59E-06	4.79E-06
COX6B1	0.000907	0.000728	0.000318	0.000452	0.001121	0.000461	0.000666	0.000535
COX6B2	5.91E-08	1.68E-08	9.91E-09	0	0	1.37E-08	0	2.18E-08
COX6C	0.00208	0.001533	0.00042	0.000448	0.001574	0.00057	0.001502	0.000814
COX7A1	3.96E-06	1.47E-06	1.37E-06	8.14E-06	1.38E-05	2.02E-06	8.02E-06	1.83E-05

COX7A2	0.000761	0.001133	0.000304	0.000286	0.00016	0.00046	0.000356	0.000362
COX7A2L	0.000111	0.000197	7.07E-05	7.30E-05	7.58E-05	0.000105	8.48E-05	4.77E-05
COX7B	0.000985	0.001025	0.000309	0.000329	0.000704	0.000334	0.000329	0.000724
COX7B2	1.47E-05	1.14E-06	0	0	0	1.74E-07	0	1.66E-05
COX7C	0.001568	0.001646	0.000815	0.000919	0.00094	0.001018	0.000853	0.001288
COX8A	0.000707	0.000995	0.000344	0.000397	0.000494	0.000764	0.00032	0.000541
COX8C	0	0	2.34E-07	0	5.55E-08	0	0	0
CP	0.00051	0.001628	0.001294	0.001004	0.000225	0.00069	0.000505	0.000841
CP110	1.73E-06	1.67E-06	1.42E-06	1.71E-06	2.02E-07	2.37E-06	6.38E-07	7.78E-07
CPA1	0	2.00E-08	0	5.42E-08	4.13E-08	0	3.36E-08	0
CPA2	0	2.18E-08	0	0	0	0	0	0
CPA3	3.63E-07	4.95E-08	1.06E-06	1.80E-06	2.50E-08	2.70E-07	2.24E-07	6.43E-07
CPA4	3.60E-08	0	7.48E-08	5.05E-08	1.55E-08	1.29E-06	2.44E-07	0
CPA5	3.20E-08	2.83E-08	0	0	0	2.42E-08	0	0
CPA6	4.56E-07	4.30E-08	0	0	0	3.10E-08	1.60E-08	0
CPAMD8	4.81E-07	2.20E-07	8.21E-07	2.76E-07	2.77E-07	2.09E-07	4.01E-07	5.84E-07
CPB1	0	5.85E-08	5.45E-08	1.47E-07	4.42E-08	0	9.53E-07	7.58E-08
CPB2	0.00028	0.000728	0.000756	0.000437	0.000348	0.000695	0.000468	0.000471
CPD	4.61E-05	2.59E-05	1.11E-05	1.53E-05	1.02E-05	2.49E-05	2.24E-05	4.61E-05
CPE	0.000122	6.01E-06	4.46E-06	9.88E-06	5.40E-06	4.72E-06	9.79E-06	1.62E-05
CPEB1	1.01E-07	0	0	3.55E-08	0	0	0	2.20E-08
CPEB2	7.07E-06	7.83E-06	1.13E-05	4.99E-06	7.28E-07	6.59E-06	4.34E-06	6.03E-06
CPEB3	2.25E-06	3.00E-06	1.15E-05	6.95E-06	7.05E-07	2.17E-06	1.82E-06	2.05E-06
CPEB4	1.28E-05	1.72E-05	1.87E-05	1.68E-05	1.96E-06	1.97E-05	7.52E-06	2.67E-05
CPLX1	9.78E-06	4.19E-06	1.70E-06	2.45E-07	8.41E-07	3.32E-06	6.09E-06	3.72E-07
CPLX2	2.08E-07	6.06E-07	0	0	0	0.000188	0	4.25E-08
CPLX3	2.38E-08	0	0	0	1.02E-08	1.10E-08	1.15E-08	0
CPLX4	0	1.38E-07	1.28E-08	3.00E-08	0	4.54E-08	0	1.80E-08
CPM	6.32E-05	1.92E-05	7.14E-06	1.22E-05	9.06E-06	3.41E-06	1.27E-05	1.63E-05
CPN1	3.99E-05	4.02E-05	6.87E-05	4.83E-05	5.38E-05	4.09E-05	7.35E-05	5.10E-05
CPN2	3.10E-05	0.000117	0.000176	9.75E-05	4.87E-05	0.000158	8.93E-05	6.44E-05
CPNE1	8.44E-05	4.23E-05	2.49E-05	3.61E-05	3.38E-05	3.48E-05	5.02E-05	2.38E-05
CPNE2	1.98E-05	8.75E-06	7.32E-06	9.24E-06	6.14E-06	1.33E-05	1.11E-05	6.97E-06
CPNE3	3.74E-05	3.27E-05	2.05E-05	1.60E-05	9.06E-06	1.78E-05	2.93E-05	2.47E-05
CPNE4	4.62E-08	0	0	0	0	0	0	1.10E-08
CPNE5	1.13E-06	1.79E-07	1.78E-07	6.41E-07	4.41E-07	1.32E-07	6.21E-07	4.81E-07
CPNE6	3.58E-08	3.14E-07	3.39E-08	0	9.32E-09	0	1.15E-08	0
CPNE7	5.25E-06	1.09E-08	6.04E-08	3.55E-07	9.98E-08	1.26E-07	4.03E-07	6.25E-07
CPNE8	7.26E-06	7.39E-06	4.45E-06	4.38E-06	7.41E-07	3.62E-06	3.36E-06	2.33E-06
CPNE9	7.01E-08	0	2.45E-08	4.30E-08	0	6.51E-08	1.13E-08	1.72E-08
CPO	0	0	6.87E-08	5.24E-08	0	3.96E-08	0	3.15E-08
CPOX	1.02E-05	1.49E-05	1.47E-05	7.15E-06	2.32E-06	1.10E-05	8.48E-06	6.72E-06
CPPED1	2.01E-05	2.02E-05	1.07E-05	8.92E-06	5.14E-06	3.08E-05	7.16E-06	1.34E-05
CPS1	0.000328	0.00078	0.000716	0.000281	0.000176	0.000659	0.000213	0.000183
CPSF1	5.34E-05	1.56E-05	1.39E-05	1.07E-05	1.77E-05	1.61E-05	2.70E-05	2.57E-05
CPSF2	1.59E-05	8.09E-06	6.97E-06	6.02E-06	3.54E-06	1.03E-05	5.76E-06	9.32E-06
CPSF3	4.18E-05	4.20E-05	1.25E-05	1.52E-05	2.38E-05	2.38E-05	2.11E-05	2.02E-05
CPSF3L	4.39E-05	2.90E-05	2.50E-05	3.10E-05	2.83E-05	3.99E-05	3.24E-05	3.01E-05
CPSF4	2.10E-05	2.43E-05	7.45E-06	7.58E-06	1.12E-05	1.16E-05	1.25E-05	1.12E-05
CPSF4L	4.72E-08	0	0	5.91E-08	4.12E-08	1.33E-07	0	0
CPSF6	1.07E-05	6.51E-06	3.58E-06	5.84E-06	2.11E-06	6.56E-06	5.57E-06	5.08E-06
CPSF7	3.14E-05	2.14E-05	1.20E-05	1.51E-05	5.34E-06	2.09E-05	1.18E-05	1.86E-05
CPT1A	9.49E-05	0.000159	3.31E-05	5.99E-05	2.81E-05	5.04E-05	4.35E-05	7.58E-05
CPT1B	2.46E-06	2.09E-06	1.00E-06	3.33E-06	8.10E-06	1.44E-06	3.55E-06	2.88E-06
CPT1C	5.79E-07	1.73E-07	1.57E-07	2.92E-07	4.07E-07	3.90E-07	6.61E-07	6.93E-07
CPT2	2.74E-05	3.58E-05	5.51E-05	5.64E-05	1.55E-05	6.92E-05	2.75E-05	5.19E-05
CPVL	4.63E-05	4.62E-06	1.44E-05	3.33E-05	3.44E-06	1.84E-05	2.87E-05	2.67E-06
CPXCR1	0	1.80E-08	0	0	0	0	0	2.33E-08

CPXM1	2.21E-06	4.45E-08	0	1.75E-06	4.92E-07	6.40E-07	5.06E-06	2.63E-06
CPXM2	1.34E-06	1.44E-07	8.85E-08	6.40E-06	5.96E-07	6.53E-07	7.70E-07	7.01E-07
CPZ	2.05E-06	7.53E-08	5.88E-07	9.68E-06	5.58E-08	6.18E-08	7.09E-07	1.63E-07
CR1	2.59E-07	5.45E-08	5.88E-07	3.72E-07	2.21E-08	6.21E-07	5.46E-07	5.27E-08
CR1L	1.26E-07	0	2.63E-07	4.85E-08	0	2.77E-08	0	0
CR2	8.97E-07	0	6.22E-09	1.48E-08	0	2.24E-08	1.76E-08	8.85E-09
CRABP1	2.00E-06	0	0	0	0	0	3.50E-08	0
CRABP2	2.60E-05	1.11E-06	3.70E-07	2.18E-06	4.61E-07	2.62E-08	1.44E-05	2.09E-07
CRADD	2.46E-05	3.19E-05	2.16E-05	1.79E-05	9.19E-06	2.99E-05	1.73E-05	2.09E-05
CRAMP1L	4.10E-06	1.38E-06	5.48E-07	8.82E-07	4.01E-07	1.15E-06	1.70E-06	1.14E-06
CRAT	6.54E-05	7.05E-05	9.40E-05	7.20E-05	4.93E-05	0.000132	7.22E-05	7.87E-05
CRB1	4.45E-09	3.29E-08	2.87E-08	1.47E-08	0	4.74E-08	5.52E-08	1.01E-08
CRB2	1.19E-08	7.01E-09	0	2.91E-07	1.37E-08	1.47E-08	1.55E-08	1.16E-08
CRB3	3.02E-05	2.24E-05	1.58E-05	2.07E-05	1.05E-05	1.43E-05	2.50E-05	1.78E-05
CRBN	1.40E-05	1.30E-05	1.50E-05	1.16E-05	3.90E-06	1.93E-05	1.40E-05	1.28E-05
CRCP	1.71E-05	3.54E-05	1.74E-05	1.43E-05	3.85E-06	1.55E-05	9.84E-06	5.50E-05
CRCT1	4.03E-06	5.11E-08	2.47E-07	2.46E-06	0	0	0	0
CREB1	8.01E-06	3.44E-06	2.49E-06	2.47E-06	7.83E-07	2.46E-06	1.65E-06	2.10E-06
CREB3	2.33E-05	2.66E-05	1.37E-05	2.10E-05	1.08E-05	1.74E-05	1.65E-05	2.26E-05
CREB3L1	1.00E-05	4.17E-07	1.77E-07	2.05E-06	1.92E-07	3.86E-07	3.47E-06	7.21E-07
CREB3L2	7.82E-06	5.51E-06	5.85E-06	7.01E-06	3.39E-06	1.04E-05	6.41E-06	1.30E-05
CREB3L3	0.000271	6.62E-05	6.34E-05	0.000133	0.000127	0.000208	0.000116	0.000228
CREB3L4	9.33E-06	1.69E-05	5.41E-06	6.39E-06	4.99E-06	1.86E-05	1.06E-05	1.01E-05
CREB5	1.88E-06	5.65E-07	1.66E-07	3.11E-06	4.91E-08	2.56E-07	1.28E-07	3.93E-07
CREBBP	1.33E-05	1.34E-05	6.79E-06	7.07E-06	2.35E-06	7.60E-06	6.29E-06	5.44E-06
CREBL2	2.94E-05	1.79E-05	2.58E-05	2.70E-05	5.42E-06	2.31E-05	2.24E-05	1.69E-05
CREBZF	1.09E-05	4.73E-06	3.56E-06	3.00E-06	1.32E-06	3.81E-06	2.10E-06	5.04E-06
CREG1	7.39E-05	0.000164	0.000142	0.000138	5.45E-05	0.000302	0.000111	0.000307
CREG2	2.63E-08	0	0	8.04E-09	0	0	6.39E-09	0
CRELD1	2.33E-05	3.37E-05	1.18E-05	1.70E-05	1.98E-05	2.79E-05	2.20E-05	1.93E-05
CRELD2	3.32E-05	5.98E-05	1.89E-05	2.59E-05	5.23E-05	5.90E-05	3.65E-05	6.11E-05
CREM	1.83E-05	2.34E-05	1.37E-05	3.03E-05	7.93E-06	1.74E-05	1.65E-05	1.60E-05
CRH	0	2.28E-08	0	0	0	0	0	0
CRHBP	1.84E-07	3.73E-07	6.73E-05	3.00E-05	1.88E-06	1.22E-07	4.32E-07	7.76E-08
CRHR1	0	0	0	0	0	0	0	0
CRHR2	5.33E-08	1.78E-08	0	6.71E-08	0	0	1.59E-07	0
CRIM1	2.13E-05	5.95E-06	4.75E-06	1.61E-05	6.90E-07	9.16E-06	5.28E-06	2.43E-06
CRIP1	0.000202	1.16E-05	7.16E-06	7.06E-05	7.93E-05	1.62E-05	4.72E-05	3.18E-05
CRIP2	7.64E-05	4.15E-05	2.00E-05	8.73E-05	0.000111	2.91E-05	0.000127	5.57E-05
CRIP3	3.33E-06	3.98E-06	1.83E-06	2.92E-06	2.08E-06	1.40E-05	2.10E-06	2.82E-05
CRIPAK	2.39E-06	1.41E-06	6.44E-07	1.08E-06	1.42E-06	1.33E-06	2.22E-06	2.06E-06
CRIPT	2.01E-05	1.73E-05	7.11E-06	8.16E-06	5.18E-06	9.17E-06	8.05E-06	6.08E-06
CRISP1	0	0	0	0	0	0	0	0
CRISP2	3.75E-08	2.15E-08	0	0	0	0	0	0
CRISP3	1.13E-08	0	1.18E-08	0	0	0	0	3.83E-07
CRISPLD1	5.20E-07	0	2.56E-07	2.16E-07	1.44E-07	1.45E-07	1.17E-08	4.21E-07
CRISPLD2	4.66E-06	1.00E-06	3.22E-06	4.17E-05	1.68E-06	1.46E-06	3.23E-06	9.38E-06
CRK	2.14E-05	1.68E-05	1.84E-05	1.93E-05	1.03E-05	2.14E-05	1.87E-05	2.11E-05
CRKL	3.07E-05	1.69E-05	9.06E-06	9.94E-06	1.02E-05	2.09E-05	1.40E-05	1.92E-05
CRLF1	2.44E-07	5.68E-07	2.85E-08	7.64E-07	8.27E-07	1.00E-07	2.28E-06	1.99E-08
CRLF2	0	4.40E-08	0	1.85E-07	0	6.98E-08	4.26E-08	1.70E-05
CRLF3	6.26E-06	4.32E-06	2.11E-06	2.63E-06	3.93E-07	2.41E-06	1.87E-06	1.80E-06
CRLS1	2.64E-05	3.45E-05	2.33E-05	2.18E-05	2.88E-05	6.53E-05	3.80E-05	1.70E-05
CRMP1	5.70E-06	5.02E-07	1.97E-07	4.65E-07	4.83E-07	3.29E-07	7.82E-07	1.46E-06
CRNKL1	1.48E-05	1.03E-05	5.37E-06	6.37E-06	3.25E-06	8.65E-06	6.55E-06	5.69E-06
CRNN	0	0	2.77E-07	0	0	0	0	0
CROCC	4.65E-06	1.04E-06	9.46E-07	3.05E-06	2.32E-06	1.37E-06	2.17E-06	1.42E-06
CROCCL1	2.91E-05	1.47E-05	2.00E-05	1.35E-05	7.34E-06	1.80E-05	1.80E-05	1.19E-05

CROCCL2	6.74E-07	3.86E-07	3.41E-07	3.11E-07	7.95E-08	3.19E-07	2.77E-07	3.14E-07
CROT	3.25E-05	2.16E-05	7.68E-06	7.65E-06	1.14E-05	1.18E-05	6.02E-06	8.12E-06
CRP	0.006792	3.39E-05	0.000728	0.0004	0.002706	0.000191	0.003506	0.00644
CRTAC1	3.13E-08	3.54E-08	8.71E-08	2.05E-07	4.49E-08	0	0	0
CRTAM	8.05E-07	1.48E-07	3.29E-07	1.46E-06	4.50E-08	1.84E-07	3.32E-07	1.92E-08
CRTAP	2.00E-05	1.33E-05	1.01E-05	1.71E-05	5.32E-06	2.25E-05	1.80E-05	1.41E-05
CRTC1	1.50E-06	9.95E-07	8.91E-07	1.32E-06	1.73E-06	1.66E-06	1.56E-06	1.92E-06
CRTC2	2.02E-05	2.75E-05	1.19E-05	1.56E-05	7.49E-06	2.30E-05	2.18E-05	2.66E-05
CRTC3	7.41E-06	3.99E-06	2.99E-06	4.93E-06	3.07E-06	4.25E-06	4.34E-06	4.41E-06
CRX	0	0	5.21E-09	1.24E-08	4.79E-08	4.70E-09	0	0
CRY1	6.83E-06	6.87E-06	2.06E-06	7.64E-06	1.26E-06	4.29E-06	5.51E-06	6.05E-06
CRY2	2.65E-05	2.15E-05	2.78E-05	1.59E-05	1.39E-05	4.89E-05	1.91E-05	6.43E-05
CRYAA	4.45E-07	2.27E-05	2.36E-05	3.84E-05	0.000112	7.42E-07	5.05E-05	3.26E-05
CRYAB	0.000457	3.33E-05	4.18E-06	2.71E-05	5.52E-06	3.59E-06	1.97E-05	3.45E-06
CRYBA1	0	0	0	0	0	0	0	0
CRYBA2	4.07E-06	0	0	0	0	0	0	0
CRYBA4	0	0	7.84E-08	0	2.73E-07	0	0	0
CRYBB1	2.07E-07	3.45E-08	2.29E-07	4.77E-07	7.70E-08	5.53E-07	6.75E-07	1.33E-07
CRYBB2	1.51E-07	1.78E-07	8.56E-08	9.39E-08	2.62E-07	0	3.56E-07	0
CRYBB3	4.24E-07	0	1.46E-07	4.05E-08	1.30E-06	3.66E-07	3.71E-07	9.77E-08
CRYBG3	3.04E-06	2.29E-06	1.09E-06	1.82E-06	1.88E-08	1.40E-06	5.83E-07	1.89E-06
CRYGA	0	0	0	0	0	0	0	0
CRYGB	0	0	0	0	0	0	0	0
CRYGC	0	0	0	0	0	0	0	0
CRYGD	0	0	0	0	0	0	0	0
CRYGN	8.12E-08	0	0	7.69E-08	0	0	0	0
CRYGS	2.22E-06	1.95E-07	3.71E-08	3.71E-07	2.68E-06	3.11E-07	8.81E-07	2.49E-07
CRYL1	4.29E-05	0.000141	0.000172	0.000191	0.000106	0.000281	0.000193	9.12E-05
CRYM	2.07E-05	5.72E-05	1.35E-05	9.46E-06	1.17E-05	8.91E-06	1.47E-05	1.47E-05
CRYZ	0.000149	9.53E-05	9.73E-05	0.000103	2.66E-05	0.000412	6.52E-05	0.000103
CRYZL1	1.18E-05	4.28E-06	4.54E-06	5.54E-06	3.06E-06	5.79E-06	7.00E-06	7.88E-06
CS	6.12E-05	4.56E-05	1.57E-05	2.23E-05	1.07E-05	3.27E-05	2.39E-05	3.13E-05
CSAD	8.98E-06	2.67E-06	1.45E-05	1.82E-05	1.10E-05	2.54E-05	2.12E-05	8.71E-06
CSAG1	3.23E-07	1.00E-06	0	0	0	8.70E-08	8.53E-08	7.00E-08
CSAG2	0	7.29E-07	0	0	0	0	0	2.83E-07
CSAG3	2.67E-07	3.41E-06	9.65E-08	4.71E-07	0	0	0	7.31E-06
CSDA	5.77E-05	6.75E-06	1.11E-05	2.27E-05	3.43E-06	3.35E-06	1.34E-05	6.80E-06
CSDAP1	1.56E-06	4.71E-08	3.06E-07	2.54E-07	5.95E-08	1.28E-08	2.13E-07	2.04E-08
CSDC2	7.87E-06	5.98E-06	7.71E-08	2.95E-07	3.11E-07	3.44E-08	0	4.08E-08
CSDE1	0.000107	0.000151	9.53E-05	9.45E-05	3.96E-05	0.000221	6.72E-05	7.25E-05
CSE1L	3.46E-05	2.68E-05	1.53E-05	1.63E-05	1.31E-05	2.90E-05	1.63E-05	1.37E-05
CSF1	1.72E-05	4.65E-06	5.39E-06	1.48E-05	3.56E-07	8.51E-06	6.31E-06	4.38E-06
CSF1R	2.15E-05	4.02E-06	1.36E-05	2.41E-05	4.50E-06	1.35E-05	2.30E-05	4.23E-06
CSF2	3.66E-08	4.32E-08	0	0	0	0	0	5.50E-08
CSF2RA	4.62E-06	1.75E-06	7.67E-07	2.71E-06	2.75E-07	3.45E-07	1.87E-06	5.57E-07
CSF2RB	4.47E-06	4.72E-07	9.52E-07	1.44E-06	1.73E-07	8.07E-07	6.80E-07	4.67E-07
CSF3	0	2.67E-07	0	7.88E-07	0	1.53E-08	1.74E-07	0
CSF3R	1.24E-05	8.62E-07	3.64E-06	8.10E-06	4.85E-07	1.26E-06	2.80E-06	1.76E-06
CSGALNA	2.51E-05	1.70E-06	8.51E-07	2.67E-06	5.74E-07	1.54E-06	2.62E-06	1.41E-06
CSGALNA	9.08E-06	6.80E-06	4.25E-06	5.85E-06	1.00E-06	4.02E-06	4.14E-06	6.54E-06
CSH1	0	0	0	0	0	0	0	0
CSH2	0	3.65E-08	0	0	0	0	0	0
CSHL1	0	0	0	0	0	0	0	0
CSK	3.23E-05	1.93E-05	3.11E-05	2.62E-05	2.18E-05	2.66E-05	4.09E-05	2.70E-05
CSMD1	2.56E-07	7.50E-08	0	0	0	7.47E-09	5.88E-09	7.82E-08
CSMD2	3.62E-07	1.40E-07	0	1.64E-08	2.88E-09	1.24E-08	1.82E-08	2.94E-08
CSMD3	2.05E-09	0	6.26E-09	0	0	0	4.00E-09	0
CSN1S1	0	0	0	0	0	0	0	0

CSN1S2A	0	0	0	0	0	0	0	0
CSN2	0	0	0	0	0	2.21E-06	0	0
CSN3	0	0	0	0	0	2.89E-06	0	0
CSNK1A1	6.50E-05	3.86E-05	4.41E-05	5.51E-05	1.47E-05	6.00E-05	3.84E-05	4.69E-05
CSNK1A1L	1.17E-07	3.31E-08	1.32E-07	3.60E-08	8.42E-09	9.08E-09	1.90E-08	1.44E-08
CSNK1A1P	2.89E-08	0	3.05E-08	7.10E-08	1.25E-08	1.34E-08	4.18E-08	2.13E-08
CSNK1D	0.000142	0.000105	2.95E-05	6.27E-05	3.18E-05	6.30E-05	6.91E-05	6.19E-05
CSNK1E	3.54E-05	1.42E-05	9.32E-06	2.41E-05	1.09E-05	1.46E-05	2.07E-05	1.91E-05
CSNK1G1	2.92E-06	1.67E-06	6.37E-07	1.38E-06	1.14E-06	2.13E-06	1.38E-06	1.37E-06
CSNK1G2	2.22E-05	1.38E-05	9.99E-06	1.44E-05	9.73E-06	1.25E-05	1.41E-05	1.08E-05
CSNK1G3	8.21E-06	7.04E-06	6.87E-06	4.97E-06	1.78E-06	9.20E-06	4.06E-06	4.27E-06
CSNK2A1	5.13E-05	1.98E-05	1.17E-05	1.12E-05	3.30E-06	1.47E-05	9.42E-06	1.08E-05
CSNK2A1P	3.13E-05	1.03E-05	6.51E-06	5.05E-06	3.62E-07	8.69E-06	3.54E-06	5.79E-06
CSNK2A2	1.36E-05	7.79E-06	7.88E-06	9.25E-06	6.61E-06	1.34E-05	1.09E-05	9.56E-06
CSNK2B	0.000178	0.000236	8.43E-05	0.000107	0.000138	0.000142	0.000159	0.000142
CSPG4	3.41E-06	2.45E-06	9.29E-08	3.72E-07	1.58E-06	9.46E-07	1.95E-06	1.74E-06
CSPG4PY2	0	0	0	0	0	0	0	0
CSPG5	5.22E-06	3.33E-08	3.58E-08	1.68E-07	1.71E-07	1.33E-07	8.83E-08	8.40E-08
CSPP1	1.20E-05	5.18E-06	2.39E-06	3.49E-06	2.44E-06	3.88E-06	3.15E-06	3.04E-06
CSRNP1	2.31E-05	3.57E-05	6.10E-05	7.30E-05	2.92E-06	1.12E-05	3.40E-05	3.36E-05
CSRNP2	5.72E-06	3.41E-06	1.78E-06	4.10E-06	4.18E-07	2.18E-06	3.40E-06	3.47E-06
CSRNP3	1.58E-08	5.34E-08	9.84E-08	1.94E-08	0	7.34E-09	7.68E-09	0
CSRP1	0.000118	0.000139	5.53E-05	0.000122	6.39E-05	4.32E-05	8.71E-05	2.04E-05
CSRP2	4.19E-05	2.51E-05	1.60E-05	2.01E-05	1.21E-05	2.64E-05	2.10E-05	8.87E-06
CSRP2BP	1.67E-05	9.85E-06	5.45E-06	6.38E-06	5.77E-06	1.03E-05	7.53E-06	4.60E-06
CSRP3	0	0	0	0	0	0	0	0
CST1	6.07E-07	0	0	4.73E-08	0	0.00018	3.22E-07	5.71E-08
CST11	0	0	0	0	0	0	0	0
CST2	2.57E-07	0	0	0	1.87E-07	1.21E-07	3.22E-07	0
CST3	0.000709	0.000329	0.000471	0.000703	0.000456	0.001139	0.000644	0.000445
CST4	7.91E-08	4.69E-08	0	0	0	3.19E-06	3.73E-08	0
CST5	3.86E-08	0	4.40E-08	9.63E-08	0	0	0	0
CST6	1.71E-06	1.25E-07	6.13E-08	4.31E-07	2.98E-08	4.86E-08	0	0
CST7	1.37E-05	2.15E-06	5.99E-06	2.46E-05	5.08E-07	1.27E-05	6.02E-06	4.03E-06
CST8	0	0	0	0	0	0	0	0
CST9	0	0	0	0	0	0	1.40E-08	0
CST9L	0	0	0	0	0	0	0	0
CSTA	4.63E-06	5.89E-05	7.48E-06	1.05E-05	3.91E-05	2.14E-05	3.54E-05	2.26E-05
CSTB	0.00034	0.000192	0.000135	0.00027	0.000213	0.000313	0.000359	0.000386
CSTF1	2.70E-05	1.75E-05	1.09E-05	1.08E-05	7.23E-06	1.92E-05	1.01E-05	1.52E-05
CSTF2	9.22E-06	8.53E-06	2.68E-06	3.38E-06	1.67E-06	6.02E-06	2.96E-06	5.95E-06
CSTF2T	1.01E-05	9.15E-06	6.80E-06	7.37E-06	2.15E-06	8.25E-06	5.74E-06	8.00E-06
CSTF3	2.41E-05	2.06E-05	1.49E-05	1.18E-05	1.06E-05	2.55E-05	7.93E-06	1.44E-05
CSTL1	0	0	0	0	0	7.42E-08	0	0
CSTT	0	0	0	0	0	0	0	0
CT45A1	0	6.14E-08	0	0	0	0	0	0
CT45A2	0	3.07E-08	0	0	0	0	0	0
CT45A3	0	1.54E-08	0	0	0	0	0	0
CT45A4	0	2.00E-07	0	0	0	0	0	0
CT45A5	0	0	0	0	0	0	0	0
CT45A6	0	1.84E-07	0	0	0	0	0	0
CT47A1	0	0	0	0	0	0	0	0
CT47A10	0	0	0	0	0	0	0	0
CT47A11	0	0	0	0	0	0	0	0
CT47A2	0	0	0	0	0	0	0	0
CT47A6	0	2.26E-08	0	0	0	0	0	0
CT47A7	0	0	0	0	0	0	0	0
CT47A9	0	0	0	0	0	0	0	0

CT47B1	0	0	0	0	0	0	0	2.82E-08
CT62	0	4.49E-08	0	0	0	0	3.82E-08	0
CTAG1B	1.20E-06	6.71E-06	0	0	0	0	0	0
CTAG2	3.01E-06	2.95E-07	0	0	0	0	0	0
CTAGE1	3.89E-07	2.30E-07	4.48E-07	1.61E-07	9.03E-09	1.46E-07	2.05E-08	2.00E-07
CTAGE4	1.12E-06	8.43E-07	5.18E-07	5.12E-07	3.83E-08	1.54E-06	5.53E-07	1.50E-06
CTAGE5	4.36E-05	4.86E-05	4.87E-05	3.36E-05	2.33E-05	4.20E-05	2.86E-05	3.23E-05
CTAGE6	9.66E-08	9.88E-08	5.47E-08	3.22E-08	0	1.22E-07	0	1.29E-07
CTAGE9	3.70E-06	2.07E-06	1.50E-06	2.24E-06	6.25E-08	5.76E-06	1.59E-06	3.36E-06
CTBP1	8.15E-05	7.04E-05	4.81E-05	5.97E-05	6.91E-05	6.28E-05	7.04E-05	7.11E-05
CTBP2	1.57E-05	1.30E-06	2.17E-06	5.58E-06	2.10E-06	1.10E-06	2.33E-06	1.81E-06
CTBS	1.41E-05	1.74E-05	1.93E-05	7.72E-06	3.05E-06	1.96E-05	6.55E-06	7.43E-06
CTCF	2.48E-05	1.48E-05	1.20E-05	1.46E-05	6.51E-06	1.61E-05	1.49E-05	1.68E-05
CTCFL	2.91E-06	1.39E-06	4.08E-07	5.39E-07	1.88E-07	6.86E-07	6.02E-07	9.86E-07
CTDP1	6.62E-06	4.43E-06	4.55E-06	7.12E-06	2.25E-06	6.80E-06	5.91E-06	5.90E-06
CTDSP1	0.000121	6.09E-05	9.31E-05	9.42E-05	8.64E-05	9.05E-05	0.000121	0.000103
CTDSP2	6.22E-05	2.49E-05	1.91E-05	1.68E-05	1.12E-05	3.37E-05	3.79E-05	2.21E-05
CTDSPL	1.74E-05	6.40E-06	6.17E-06	7.77E-06	9.35E-06	2.96E-06	8.04E-06	5.76E-06
CTDSPL2	6.22E-06	3.57E-06	2.66E-06	2.39E-06	5.29E-07	3.81E-06	2.09E-06	2.68E-06
CTF1	7.37E-07	1.51E-07	4.83E-07	1.77E-06	1.02E-07	8.22E-08	3.13E-07	1.96E-07
CTGF	3.73E-05	2.39E-05	6.11E-05	0.000177	7.80E-06	2.53E-05	8.10E-05	1.85E-05
CTH	7.57E-05	0.000176	5.94E-05	2.45E-05	8.70E-06	0.00023	9.34E-06	0.000132
CTHRC1	6.16E-05	6.29E-07	4.54E-08	3.12E-06	9.24E-07	1.11E-06	1.40E-05	2.61E-06
CTLA4	5.99E-06	0	1.21E-07	8.48E-07	1.41E-08	1.79E-06	2.54E-07	3.32E-07
CTNNA1	0.000163	9.39E-05	5.31E-05	7.43E-05	2.57E-05	0.000126	5.94E-05	9.55E-05
CTNNA2	1.29E-08	7.72E-07	1.36E-08	4.34E-07	0	6.14E-06	0	0
CTNNA3	2.62E-08	3.25E-07	1.08E-06	1.34E-07	1.83E-08	1.14E-07	4.94E-08	2.43E-07
CTNNAL1	1.87E-05	7.19E-06	8.49E-06	2.85E-05	7.87E-06	2.47E-05	7.38E-06	4.16E-06
CTNNB1	0.000109	9.36E-05	6.49E-05	7.72E-05	2.52E-05	0.000139	5.45E-05	5.15E-05
CTNNBIP1	5.41E-06	8.50E-06	6.32E-06	5.66E-06	4.83E-06	1.21E-05	9.53E-06	6.57E-06
CTNNBL1	5.34E-05	4.26E-05	2.88E-05	2.74E-05	5.80E-05	0.000268	4.83E-05	2.74E-05
CTNND1	7.67E-05	6.42E-05	3.51E-05	4.46E-05	9.89E-06	4.91E-05	2.65E-05	3.70E-05
CTNND2	9.38E-06	0	2.14E-07	5.79E-07	0	4.68E-09	2.26E-08	7.40E-09
CTNS	3.83E-06	6.76E-06	3.29E-06	4.51E-06	5.36E-06	5.79E-06	5.65E-06	5.86E-06
CTPS	1.21E-05	9.22E-06	4.38E-05	1.40E-05	8.85E-06	3.71E-05	2.08E-05	4.15E-05
CTPS2	1.26E-05	2.98E-06	3.54E-06	3.95E-06	1.61E-06	4.56E-06	3.86E-06	5.79E-06
CTR9	1.97E-05	1.93E-05	1.18E-05	1.34E-05	3.58E-06	2.80E-05	7.77E-06	2.70E-05
CTRB1	0	3.72E-08	0	0	0	0	0	0
CTRB2	0	0	0	3.84E-08	0	0	0	0
CTRC	0	0	0	0	0	0	0	0
CTRL	1.22E-06	4.33E-07	0	3.28E-07	1.91E-07	1.03E-07	1.49E-07	1.64E-07
CTSA	0.00025	0.00022	8.70E-05	0.000148	0.00016	0.000141	0.00016	0.000352
CTSB	0.000507	0.001239	0.000478	0.000739	0.000162	0.00043	0.000355	0.000157
CTSC	0.000171	5.96E-06	2.35E-05	4.44E-05	6.82E-06	6.57E-05	3.90E-05	2.92E-05
CTSD	0.000922	0.000745	0.000791	0.000986	0.000733	0.001007	0.000702	0.001509
CTSE	3.51E-07	7.06E-08	3.33E-08	5.81E-07	0	2.96E-08	0	1.84E-08
CTSF	2.05E-05	9.02E-05	7.53E-05	5.70E-05	7.85E-05	9.60E-05	9.15E-05	0.000163
CTSG	0	0	1.34E-06	1.46E-07	2.56E-07	8.27E-08	5.33E-07	0
CTSH	0.000183	0.00015	7.85E-05	0.00017	0.00018	0.000187	0.000116	0.000102
CTSK	1.17E-05	7.33E-07	1.92E-06	1.47E-05	1.72E-06	2.29E-06	5.07E-06	4.17E-06
CTSL1	0.000165	0.00015	0.000151	0.000197	5.76E-05	8.48E-05	9.76E-05	0.000261
CTSL2	4.81E-06	1.01E-06	1.06E-07	1.43E-07	2.87E-08	3.09E-08	4.80E-08	0
CTSL3	0	0	0	0	0	0	0	0
CTSO	3.42E-05	4.70E-05	4.17E-05	3.97E-05	9.84E-06	4.55E-05	2.64E-05	5.26E-05
CTSS	8.22E-05	2.28E-05	6.49E-05	9.71E-05	2.34E-06	5.25E-05	3.47E-05	6.14E-05
CTSW	4.82E-06	1.23E-06	3.78E-06	7.21E-07	1.51E-07	2.84E-06	6.13E-06	1.27E-06
CTSZ	0.000261	0.000251	0.000261	0.000427	0.000256	0.000195	0.000297	0.000144
CTTN	5.51E-05	6.84E-05	2.85E-05	4.04E-05	1.87E-05	5.52E-05	3.31E-05	5.39E-05

CTTNBP2	3.34E-07	2.26E-07	5.30E-07	1.42E-07	1.61E-08	1.15E-07	5.12E-08	3.29E-08
CTTNBP2N	7.04E-06	1.73E-06	1.21E-06	2.83E-06	4.17E-07	1.87E-06	1.23E-06	1.34E-06
CTU1	1.13E-06	1.78E-06	5.09E-07	9.29E-07	1.66E-06	1.08E-06	4.72E-06	6.33E-07
CTU2	1.06E-05	5.41E-06	6.49E-06	6.50E-06	6.53E-06	9.10E-06	6.87E-06	9.91E-06
CTXN1	1.13E-06	2.32E-07	4.34E-08	1.54E-06	6.63E-07	1.88E-07	3.87E-07	1.80E-07
CTXN2	0	0	0	0	0	0	0	0
CTXN3	0	0	0	0	0	0	0	0
CUBN	8.32E-08	6.99E-08	1.69E-08	2.48E-08	1.11E-08	6.83E-09	5.95E-08	4.86E-08
CUEDC1	9.23E-06	1.94E-06	1.96E-06	4.71E-06	9.56E-07	5.58E-06	4.25E-06	4.91E-06
CUEDC2	5.37E-05	3.82E-05	2.71E-05	4.14E-05	5.00E-05	4.96E-05	4.28E-05	3.48E-05
CUL1	2.12E-05	3.39E-05	2.46E-05	2.84E-05	1.29E-05	3.59E-05	2.28E-05	3.81E-05
CUL2	1.22E-05	1.61E-05	8.75E-06	8.41E-06	3.84E-06	1.47E-05	6.10E-06	8.65E-06
CUL3	1.56E-05	9.77E-06	7.98E-06	8.18E-06	3.36E-06	8.23E-06	8.63E-06	7.61E-06
CUL4A	3.08E-05	4.28E-05	2.06E-05	1.90E-05	7.81E-06	3.05E-05	2.20E-05	1.85E-05
CUL4B	2.25E-05	1.98E-05	9.56E-06	9.94E-06	7.29E-06	1.22E-05	9.07E-06	1.71E-05
CUL5	8.32E-06	5.31E-06	5.27E-06	4.20E-06	1.78E-06	6.77E-06	3.47E-06	5.84E-06
CUL7	1.43E-05	1.31E-05	4.09E-06	5.51E-06	7.04E-06	6.04E-06	7.56E-06	1.39E-05
CUL9	8.40E-06	6.96E-06	4.23E-06	5.71E-06	1.34E-05	9.70E-06	6.54E-06	7.48E-06
CUTA	0.00017	0.000229	0.000136	0.000145	0.000328	0.000225	0.000221	0.000216
CUTC	1.55E-05	1.63E-05	1.56E-05	1.25E-05	1.26E-05	2.30E-05	1.55E-05	1.06E-05
CUX1	1.88E-05	3.23E-05	2.76E-05	2.51E-05	4.27E-05	3.95E-05	3.11E-05	3.19E-05
CUX2	8.68E-06	1.08E-05	8.28E-06	6.82E-06	3.12E-06	2.56E-05	7.39E-06	2.08E-06
CUZD1	1.82E-07	6.65E-08	2.67E-08	2.11E-08	1.29E-07	1.62E-08	5.11E-08	8.69E-08
CWC15	3.48E-05	2.61E-05	2.10E-05	2.53E-05	1.27E-05	3.25E-05	2.75E-05	3.55E-05
CWC22	1.60E-05	9.95E-06	5.83E-06	9.18E-06	2.84E-06	7.51E-06	6.46E-06	6.65E-06
CWC25	1.82E-05	9.07E-06	5.72E-06	7.33E-06	2.94E-06	6.37E-06	6.32E-06	6.70E-06
CWC27	1.19E-05	5.84E-06	3.31E-06	6.06E-06	1.87E-06	7.40E-06	4.66E-06	6.20E-06
CWF19L1	1.17E-05	6.63E-06	5.56E-06	6.13E-06	8.00E-06	7.05E-06	7.80E-06	4.69E-06
CWF19L2	3.88E-06	3.43E-06	2.68E-06	4.16E-06	1.89E-06	4.52E-06	2.42E-06	5.10E-06
CWH43	0	1.11E-08	1.45E-06	7.25E-08	0	0	7.65E-08	0
CX3CL1	2.04E-05	1.16E-05	9.25E-06	3.19E-05	1.50E-05	7.77E-06	2.85E-05	4.18E-06
CX3CR1	9.30E-07	9.19E-07	1.44E-06	6.36E-07	1.34E-07	4.61E-07	1.20E-06	6.54E-08
CXADR	2.50E-05	2.89E-05	1.46E-05	1.16E-05	1.78E-06	2.77E-05	6.45E-06	1.65E-05
CXADRP2	2.87E-08	1.08E-08	9.94E-09	1.17E-08	0	1.77E-08	2.78E-08	1.40E-08
CXADRP3	1.07E-07	1.22E-07	9.70E-08	0	0	4.26E-08	2.94E-08	2.26E-08
CXCL1	6.08E-05	1.37E-07	2.63E-06	2.88E-05	4.51E-07	1.11E-07	1.59E-06	1.41E-05
CXCL10	0.000151	1.09E-06	1.31E-05	4.11E-05	3.05E-07	4.76E-05	3.14E-06	3.26E-05
CXCL11	1.41E-05	6.50E-07	1.03E-06	2.27E-06	1.45E-08	4.89E-06	5.16E-07	1.04E-06
CXCL12	1.26E-05	3.03E-06	0.000116	0.000257	8.88E-06	8.04E-06	2.32E-05	9.83E-06
CXCL13	1.16E-05	3.94E-07	2.47E-06	1.85E-07	3.70E-08	4.37E-06	1.44E-07	1.27E-07
CXCL14	2.80E-07	6.89E-08	3.37E-05	3.04E-05	1.26E-07	1.02E-07	7.17E-07	2.33E-07
CXCL16	0.000121	2.99E-05	3.05E-05	6.64E-05	1.73E-05	2.11E-05	5.07E-05	0.000116
CXCL17	3.26E-08	3.45E-07	7.29E-08	1.62E-07	3.12E-07	1.53E-07	9.29E-08	1.35E-05
CXCL2	0.000101	2.78E-05	0.000118	0.000159	4.03E-05	1.93E-05	4.27E-05	0.000142
CXCL3	2.59E-06	1.04E-07	1.46E-07	4.89E-06	7.79E-08	6.30E-08	1.72E-07	2.34E-07
CXCL5	1.86E-05	6.47E-08	1.99E-08	9.84E-07	0	0	1.85E-08	1.41E-08
CXCL6	0.000124	1.34E-07	1.51E-06	4.15E-05	1.27E-07	1.37E-08	3.17E-06	6.90E-06
CXCL9	0.000126	9.33E-07	1.11E-05	2.11E-05	2.22E-07	2.22E-05	2.94E-06	2.29E-05
CXCR1	1.20E-06	1.28E-07	4.92E-07	5.55E-07	1.63E-08	4.38E-08	1.65E-07	5.83E-07
CXCR2	2.03E-06	1.20E-07	5.23E-07	3.64E-07	7.64E-09	4.12E-08	1.12E-07	2.48E-07
CXCR2P1	6.12E-06	6.30E-08	4.47E-06	2.85E-06	8.63E-08	2.60E-06	5.17E-07	6.72E-07
CXCR3	4.12E-06	6.37E-07	6.42E-07	1.70E-06	9.12E-08	1.00E-06	1.14E-06	7.10E-07
CXCR4	5.58E-05	1.21E-05	1.93E-05	0.000103	2.09E-06	1.72E-05	4.42E-05	1.76E-05
CXCR5	2.63E-07	0	5.03E-08	3.32E-07	7.11E-09	1.52E-07	1.36E-07	2.41E-08
CXCR6	8.15E-06	1.39E-07	2.08E-06	4.45E-06	1.16E-07	4.03E-06	2.06E-06	1.77E-06
CXCR7	6.87E-05	1.19E-05	3.59E-06	1.00E-05	9.29E-07	1.62E-06	1.01E-05	2.44E-05
CXorf1	0	0	0	0	0	0	0	0
CXorf21	1.59E-06	2.43E-07	5.36E-07	1.59E-06	0	3.23E-07	5.04E-07	1.38E-07

CXorf22	0	0	6.74E-09	0	0	0	0	5.58E-08
CXorf23	3.46E-06	1.02E-06	9.40E-07	1.69E-06	6.33E-07	2.17E-06	1.21E-06	2.25E-06
CXorf26	2.40E-05	1.77E-05	1.27E-05	2.53E-05	2.05E-05	1.80E-05	2.40E-05	2.42E-05
CXorf27	0	0	0	0	0	0	0	0
CXorf30	0	9.13E-09	8.41E-09	9.93E-09	0	0	0	8.34E-08
CXorf36	3.34E-06	4.39E-06	2.40E-07	7.91E-07	1.68E-06	2.55E-06	7.63E-06	2.61E-06
CXorf38	1.23E-05	2.56E-06	4.15E-06	3.97E-06	5.56E-07	4.27E-06	3.37E-06	6.98E-06
CXorf40A	1.67E-05	1.95E-05	8.44E-06	9.68E-06	1.53E-05	1.31E-05	1.13E-05	2.77E-05
CXorf40B	1.78E-05	1.80E-05	1.20E-05	1.22E-05	1.42E-05	1.62E-05	1.31E-05	2.74E-05
CXorf41	0	0	0	0	0	0	0	0
CXorf42	6.33E-07	1.21E-07	1.92E-07	4.41E-07	2.34E-07	3.43E-07	2.83E-07	2.86E-07
CXorf48	0	1.46E-08	2.70E-08	0	0	0	0	1.90E-08
CXorf49B	0	0	0	0	0	0	0	0
CXorf50B	7.10E-08	0	0	1.45E-08	1.02E-08	0	1.14E-08	0
CXorf51	0	0	0	0	0	0	0	1.38E-07
CXorf56	1.24E-05	1.01E-05	5.91E-06	7.71E-06	8.93E-07	9.93E-06	5.52E-06	1.16E-05
CXorf57	1.58E-06	1.68E-06	3.68E-07	3.72E-07	4.85E-07	5.16E-07	3.57E-07	5.41E-07
CXorf58	3.21E-08	5.46E-08	5.10E-08	0	0	1.49E-08	4.63E-08	4.74E-08
CXorf59	0	0	2.57E-08	0	0	0	0	1.26E-07
CXorf61	0	0	0	0	0	0	0	0
CXorf64	0	0	2.25E-08	0	1.45E-07	0	0	0
CXorf65	6.33E-07	0	0	7.66E-08	0	5.74E-08	5.60E-08	0
CXorf66	0	4.57E-08	1.71E-07	7.35E-08	1.55E-07	2.04E-07	0	8.85E-08
CXXC1	1.64E-05	6.84E-06	8.27E-06	1.14E-05	8.77E-06	1.12E-05	1.23E-05	8.42E-06
CXXC4	2.78E-07	8.86E-07	9.74E-07	6.97E-07	0	3.64E-08	1.83E-07	1.17E-07
CXXC5	9.14E-05	4.31E-05	4.60E-05	4.04E-05	6.58E-06	1.62E-05	3.49E-05	4.21E-05
CYB561	2.07E-05	1.00E-05	6.69E-06	6.83E-06	5.53E-06	1.03E-05	3.94E-06	1.92E-05
CYB561D1	1.70E-06	7.73E-07	3.33E-07	8.13E-07	1.00E-06	4.01E-07	1.18E-06	7.68E-07
CYB561D2	3.77E-05	1.81E-05	1.63E-05	2.30E-05	3.21E-05	2.41E-05	3.12E-05	4.12E-05
CYB5A	0.000217	0.000871	0.001049	0.000808	0.000477	0.001449	0.000986	0.000931
CYB5B	1.87E-05	1.40E-05	1.06E-05	1.02E-05	3.30E-06	2.30E-05	9.54E-06	7.50E-06
CYB5D1	3.95E-06	3.58E-06	4.71E-06	5.04E-06	5.66E-07	6.49E-06	3.39E-06	3.73E-06
CYB5D2	2.03E-05	6.62E-06	2.73E-05	2.26E-05	4.49E-05	3.22E-05	1.59E-05	1.95E-05
CYB5R1	4.09E-05	5.61E-05	3.05E-05	2.80E-05	2.30E-05	4.59E-05	2.72E-05	9.85E-05
CYB5R2	6.01E-06	1.33E-06	7.11E-06	4.60E-06	4.55E-07	4.88E-06	2.98E-06	2.42E-06
CYB5R3	0.000308	8.46E-05	0.000145	0.000174	9.43E-05	0.000152	0.000218	0.000231
CYB5R4	1.02E-05	3.67E-06	4.18E-06	6.20E-06	6.35E-07	8.72E-06	4.27E-06	5.80E-06
CYB5RL	9.68E-07	4.51E-07	2.34E-07	2.47E-07	3.90E-08	3.07E-07	2.77E-07	4.57E-07
CYBA	0.000703	1.44E-05	6.28E-05	0.000146	2.73E-05	4.13E-05	0.000101	8.29E-05
CYBASC3	2.10E-05	2.27E-05	1.52E-05	1.75E-05	1.64E-05	3.09E-05	1.62E-05	3.43E-05
CYBB	2.21E-05	2.03E-06	8.90E-06	1.25E-05	7.99E-07	6.94E-06	6.24E-06	1.10E-06
CYBRD1	2.09E-05	2.13E-06	7.66E-06	1.56E-05	8.42E-07	1.28E-06	5.87E-06	3.44E-06
CYC1	0.000405	0.000401	0.000127	0.00011	0.000142	0.000125	0.000394	0.000233
CYCS	9.09E-05	0.00012	1.46E-05	1.82E-05	7.41E-06	1.59E-05	1.99E-05	3.24E-05
CYCSP52	0	0	0	0	0	0	0	0
CYFIP1	3.97E-05	4.23E-05	2.13E-05	2.94E-05	9.10E-06	2.00E-05	1.85E-05	1.88E-05
CYFIP2	5.80E-05	1.79E-05	8.27E-06	8.61E-06	1.58E-06	7.18E-06	5.25E-06	4.21E-06
CYGB	8.97E-06	8.29E-06	1.51E-05	1.74E-05	1.03E-05	1.20E-05	1.21E-05	1.55E-05
CYHR1	6.63E-05	3.27E-05	2.26E-05	2.13E-05	5.25E-05	2.53E-05	7.16E-05	2.28E-05
CYLC1	0	0	0	0	0	0	0	0
CYLC2	1.09E-08	0	0	0	0	0	0	0
CYLD	7.69E-06	4.75E-06	5.05E-06	7.37E-06	7.91E-07	7.16E-06	3.35E-06	7.28E-06
CYMP	0	0	0	0	0	0	0	0
CYorf15A	0	4.71E-06	0	3.84E-06	1.03E-06	9.79E-06	2.31E-06	2.72E-06
CYorf15B	0	1.04E-06	0	7.55E-07	4.17E-07	1.36E-06	6.68E-07	9.47E-07
CYP11A1	8.77E-06	1.08E-05	2.15E-06	5.46E-06	4.80E-06	1.19E-05	9.16E-06	4.76E-06
CYP11B1	0	0	0	0	9.47E-08	0	0	0
CYP11B2	0	0	0	0	6.79E-09	0	0	0

CYP17A1	7.46E-05	4.10E-07	1.43E-06	3.28E-07	3.94E-06	4.10E-06	3.74E-08	6.71E-06
CYP19A1	3.43E-08	1.73E-08	0	1.26E-08	0	0	0	5.06E-08
CYP1A1	5.03E-07	1.10E-06	3.56E-05	2.16E-06	6.56E-07	8.79E-05	1.28E-06	0.000165
CYP1A2	0	1.05E-06	0.00033	2.54E-05	1.47E-06	1.10E-05	1.90E-05	4.12E-05
CYP1B1	1.00E-05	3.58E-06	2.63E-06	3.38E-06	2.03E-07	2.05E-06	7.53E-07	1.62E-05
CYP20A1	3.38E-06	1.94E-06	2.16E-06	2.24E-06	1.50E-06	1.72E-06	2.31E-06	1.60E-06
CYP21A2	4.46E-06	3.55E-06	2.49E-06	6.01E-06	5.49E-06	1.38E-05	8.99E-06	3.35E-05
CYP24A1	8.46E-08	1.94E-08	0	0	0	0	0	0
CYP26A1	6.68E-07	2.62E-07	1.87E-06	1.47E-06	2.39E-06	1.60E-06	2.50E-07	1.72E-06
CYP26B1	1.61E-06	1.53E-07	1.63E-07	3.99E-07	2.26E-08	3.38E-07	3.54E-07	1.90E-06
CYP26C1	0	0	0	9.74E-09	0	0	1.51E-08	0
CYP27A1	6.27E-05	0.000255	0.000482	0.000344	0.000662	0.000546	0.000518	0.000247
CYP27B1	3.74E-06	2.58E-07	2.84E-08	1.50E-07	1.06E-07	7.38E-08	3.20E-07	1.69E-07
CYP27C1	1.54E-07	3.72E-07	6.93E-08	8.25E-08	0	2.68E-08	2.82E-08	1.91E-07
CYP2A13	1.39E-08	3.00E-07	2.29E-06	2.91E-07	1.01E-06	0	0	2.20E-06
CYP2A6	2.82E-07	0.000368	0.001764	0.000778	0.00342	5.60E-06	0.000383	0.000886
CYP2A7	2.24E-08	3.25E-06	3.35E-05	1.44E-05	7.20E-05	8.31E-08	3.25E-06	2.79E-05
CYP2B6	3.29E-06	6.51E-05	0.000146	4.78E-05	8.12E-05	6.96E-06	3.66E-05	1.71E-05
CYP2B7P1	3.94E-06	3.76E-05	3.17E-05	2.59E-05	3.91E-06	2.96E-08	1.81E-06	2.49E-05
CYP2C18	0.000308	1.21E-05	8.45E-05	3.65E-05	6.93E-05	2.49E-06	5.95E-05	5.16E-05
CYP2C19	1.09E-05	2.30E-06	5.96E-05	4.19E-06	8.79E-07	1.26E-06	5.05E-06	3.80E-06
CYP2C8	0.000535	9.21E-05	0.000682	0.000367	0.000446	8.66E-05	0.000188	0.00043
CYP2C9	0.000742	0.000375	0.000774	0.000469	0.000344	0.000128	0.000618	0.000252
CYP2D6	8.83E-06	5.38E-05	0.000322	5.75E-05	0.000492	0.000585	0.000217	0.000118
CYP2D7P1	1.38E-06	2.27E-05	1.47E-05	1.98E-05	1.36E-05	7.13E-05	1.43E-05	1.65E-05
CYP2E1	5.88E-05	0.000213	0.005865	0.001349	0.003393	0.00751	0.000141	0.000868
CYP2F1	0	1.56E-08	0	1.69E-08	0	0	0	0
CYP2J2	1.68E-05	6.38E-05	7.94E-05	6.31E-05	4.02E-05	4.81E-05	6.29E-05	5.49E-05
CYP2R1	1.17E-05	6.71E-06	3.19E-06	3.80E-06	3.16E-06	6.45E-06	4.20E-06	1.08E-05
CYP2S1	3.60E-06	8.85E-08	9.37E-07	9.63E-07	1.64E-07	1.82E-07	3.46E-07	1.73E-06
CYP2U1	3.41E-06	1.70E-06	2.73E-06	2.73E-06	1.33E-06	2.94E-06	1.75E-06	2.85E-06
CYP2W1	4.10E-08	0	0	0	1.76E-08	5.71E-08	9.93E-09	0
CYP39A1	3.05E-06	9.87E-07	4.83E-05	2.20E-05	2.69E-06	1.33E-05	3.59E-06	1.85E-05
CYP3A4	1.99E-06	5.31E-05	0.002051	0.001054	3.62E-06	0.000955	0.000634	2.22E-05
CYP3A43	2.49E-06	3.60E-06	6.94E-06	9.43E-07	2.15E-07	4.84E-07	1.32E-06	5.04E-07
CYP3A5	0.000175	0.000303	0.000109	5.95E-05	9.21E-05	3.79E-05	6.26E-05	0.000137
CYP3A7	0.000284	0.000484	0.000104	0.000238	6.34E-05	5.53E-06	1.38E-05	2.88E-05
CYP46A1	9.31E-08	1.32E-07	1.62E-07	2.00E-07	9.89E-09	1.22E-07	5.29E-07	1.03E-07
CYP4A11	0.00019	9.19E-05	0.000548	0.000514	0.000382	1.21E-05	0.000555	7.08E-05
CYP4A22	6.89E-06	6.79E-06	4.47E-05	2.61E-05	5.48E-06	8.91E-06	3.12E-05	1.18E-05
CYP4B1	8.59E-08	6.90E-08	2.56E-08	2.15E-08	0	1.40E-07	1.30E-07	3.59E-08
CYP4F11	4.85E-05	1.38E-05	5.24E-05	2.40E-05	4.30E-05	8.92E-05	2.11E-05	2.97E-05
CYP4F12	4.44E-06	3.17E-05	3.62E-05	2.53E-05	3.72E-05	7.82E-06	6.52E-06	3.77E-06
CYP4F2	2.17E-05	6.50E-05	0.000189	6.00E-05	0.000167	3.65E-05	6.85E-05	3.15E-05
CYP4F22	5.38E-08	1.20E-06	6.49E-06	2.81E-06	3.62E-06	1.66E-08	4.75E-06	2.16E-05
CYP4F3	6.85E-05	6.52E-05	8.96E-05	4.66E-05	2.12E-05	0.000102	2.24E-05	2.98E-05
CYP4F8	0	1.95E-08	1.82E-08	0	0	0	0	0
CYP4V2	6.34E-05	4.19E-05	9.83E-05	5.51E-05	3.48E-05	2.23E-05	3.08E-05	2.50E-05
CYP4X1	4.42E-07	4.74E-07	2.48E-06	3.51E-06	5.97E-07	1.27E-06	8.90E-07	1.09E-06
CYP4Z1	1.26E-08	2.86E-08	3.98E-08	1.86E-07	8.69E-08	1.17E-08	4.88E-08	3.72E-08
CYP4Z2P	1.98E-08	2.27E-08	0	0	0	3.69E-08	1.74E-08	0
CYP51A1	0.000969	0.000417	8.08E-05	4.07E-05	2.92E-05	3.90E-05	4.10E-05	6.34E-05
CYP7A1	7.11E-07	8.93E-05	1.35E-06	5.93E-08	7.83E-06	1.83E-05	9.75E-06	2.02E-07
CYP7B1	2.96E-06	1.37E-05	7.75E-06	5.54E-06	3.36E-06	4.73E-06	8.75E-06	4.33E-06
CYP8B1	6.64E-07	0.000132	0.000282	0.000147	0.000111	0.000384	0.000128	2.77E-06
CYR61	2.82E-05	2.62E-05	0.000137	0.000212	7.11E-06	4.49E-05	9.58E-05	1.99E-05
CYS1	2.27E-05	5.79E-08	1.38E-06	1.49E-06	6.63E-08	3.18E-08	2.08E-07	8.81E-08
CYSLTR1	9.99E-07	2.00E-07	4.87E-07	6.04E-07	6.05E-08	3.35E-07	3.78E-07	3.87E-07

CYSLTR2	1.25E-06	1.95E-07	1.18E-07	2.52E-07	0	5.97E-08	0	3.26E-07
CYTH1	2.86E-05	2.53E-05	1.63E-05	1.88E-05	1.72E-05	2.77E-05	1.86E-05	1.91E-05
CYTH2	9.25E-06	7.37E-06	3.83E-06	1.32E-05	3.62E-06	9.69E-06	1.04E-05	7.58E-06
CYTH3	8.12E-06	2.83E-06	1.29E-06	2.82E-06	1.35E-06	1.90E-06	3.22E-06	3.36E-06
CYTH4	9.44E-06	1.14E-06	3.37E-06	6.45E-06	1.52E-06	4.47E-06	8.13E-06	1.91E-06
CYTIP	6.99E-06	5.94E-07	2.80E-06	1.02E-05	1.75E-07	2.07E-06	1.99E-06	1.15E-06
CYTL1	5.35E-08	1.55E-07	0	2.32E-07	4.64E-08	2.00E-07	1.86E-06	3.99E-08
CYTSA	9.05E-06	9.64E-06	5.17E-06	9.03E-06	5.96E-06	6.95E-06	1.47E-05	1.46E-05
CYTSB	8.33E-06	5.42E-07	3.48E-07	4.00E-06	4.82E-07	7.64E-07	1.75E-06	3.89E-07
CYYR1	5.32E-06	3.79E-06	1.80E-06	2.01E-06	2.22E-06	3.07E-06	3.51E-06	5.26E-06
D2HGDH	1.63E-05	8.32E-06	1.43E-05	1.35E-05	2.09E-05	2.26E-05	1.12E-05	1.09E-05
D4S234E	1.49E-07	4.81E-08	1.11E-07	1.57E-07	7.33E-08	4.94E-08	4.12E-08	1.41E-07
DAAM1	6.63E-06	2.42E-06	6.13E-06	5.68E-06	7.62E-07	1.22E-05	3.76E-06	6.39E-06
DAAM2	1.33E-06	1.86E-06	2.34E-06	3.30E-06	2.62E-07	5.59E-06	1.14E-06	1.04E-06
DAB1	3.47E-06	7.84E-07	1.97E-06	1.87E-07	2.06E-08	2.19E-07	0	2.40E-07
DAB2	3.12E-05	8.11E-06	7.23E-06	1.17E-05	1.98E-06	1.25E-05	1.16E-05	8.20E-06
DAB2IP	1.59E-05	4.72E-06	5.01E-06	9.30E-06	6.84E-06	7.02E-06	8.95E-06	9.02E-06
DACH1	2.21E-07	2.25E-07	6.89E-07	6.72E-07	1.68E-08	7.80E-08	1.76E-07	1.26E-08
DACH2	0	0	0	0	0	0	0	0
DACT1	3.14E-06	2.33E-07	1.24E-06	1.53E-06	1.24E-07	1.73E-07	6.45E-07	3.61E-07
DACT2	3.71E-07	8.86E-08	4.72E-06	1.55E-06	2.03E-07	3.40E-06	2.30E-08	6.48E-07
DACT3	1.75E-06	1.66E-07	4.25E-07	2.14E-06	9.24E-07	1.82E-07	4.99E-07	5.78E-07
DAD1	0.000273	0.000372	0.000156	0.000216	0.000215	0.000274	0.000272	0.000247
DAD1L	0	0	0	0	0	0	0	0
DAG1	4.30E-05	6.37E-05	2.35E-05	3.07E-05	1.94E-05	2.74E-05	2.17E-05	5.78E-05
DAGLA	4.29E-06	5.66E-08	2.04E-07	1.39E-06	5.02E-08	1.73E-07	7.41E-07	2.01E-06
DAGLB	2.07E-05	1.80E-05	5.92E-06	7.68E-06	7.78E-06	6.14E-06	8.52E-06	1.07E-05
DAK	1.78E-05	3.23E-05	0.000115	3.56E-05	4.80E-05	6.34E-05	1.92E-05	3.25E-05
DALRD3	1.12E-05	1.15E-05	4.78E-06	9.01E-06	4.21E-06	1.01E-05	1.03E-05	5.82E-06
DAND5	4.22E-08	4.79E-08	0	1.72E-08	2.42E-08	0	0	0
DAO	1.94E-06	3.42E-05	0.000115	4.82E-05	2.42E-05	3.01E-05	3.33E-05	4.75E-05
DAOA	0	0	0	0	0	0	0	0
DAP	0.000148	6.82E-05	8.63E-05	9.52E-05	2.78E-05	0.000103	7.51E-05	0.000104
DAP3	7.97E-05	0.00014	4.72E-05	4.54E-05	2.73E-05	0.000124	5.21E-05	0.000142
DAPK1	3.26E-05	1.85E-05	1.51E-05	1.86E-05	3.18E-06	7.60E-06	7.50E-06	7.03E-06
DAPK2	5.75E-06	3.00E-06	1.12E-06	5.08E-06	1.05E-06	2.20E-06	4.56E-06	9.02E-06
DAPK3	2.70E-05	1.14E-05	8.90E-06	3.11E-05	2.25E-05	1.68E-05	4.61E-05	1.66E-05
DAPL1	6.13E-08	0	1.51E-07	0	0	0	0	9.41E-08
DAPP1	1.78E-06	7.10E-08	4.91E-07	1.04E-06	4.75E-08	3.67E-07	3.60E-07	1.51E-07
DARC	1.76E-06	3.04E-07	3.94E-07	1.78E-06	1.21E-06	1.67E-06	1.75E-06	2.28E-06
DARS	5.68E-05	4.52E-05	2.71E-05	2.39E-05	8.73E-06	4.20E-05	2.45E-05	2.26E-05
DARS2	1.42E-05	3.35E-05	6.02E-06	5.33E-06	2.31E-06	1.62E-05	3.79E-06	8.13E-06
DAXX	2.15E-05	2.53E-05	1.11E-05	2.96E-05	1.32E-05	1.99E-05	3.65E-05	2.14E-05
DAZ1	0	8.70E-08	0	1.79E-07	0	0	0	8.48E-07
DAZ2	0	0	0	0	0	0	0	0
DAZ3	0	0	0	0	0	0	0	9.94E-09
DAZ4	0	0	0	0	0	0	0	0
DAZAP1	4.16E-05	3.72E-05	2.57E-05	3.50E-05	2.96E-05	3.37E-05	4.01E-05	2.68E-05
DAZAP2	0.000204	0.000186	0.000121	0.000137	4.74E-05	0.000146	0.000132	0.000102
DAZL	0	0	2.36E-08	9.29E-09	0	0	0	0
DBC1	1.62E-06	8.10E-09	2.83E-07	6.88E-07	0	0	0	3.17E-08
DBF4	2.63E-06	2.19E-06	3.84E-07	7.34E-07	4.16E-07	6.13E-07	7.59E-07	1.40E-06
DBF4B	3.70E-06	1.15E-06	2.51E-07	8.36E-07	2.39E-07	1.35E-06	3.12E-07	6.96E-07
DBH	1.38E-06	1.09E-06	4.34E-05	2.12E-05	3.84E-07	2.74E-07	1.23E-07	2.35E-07
DBI	0.001564	0.000715	0.000589	0.000436	0.000804	0.000342	0.000413	0.000217
DBN1	3.81E-05	8.90E-06	1.34E-06	1.17E-05	4.00E-06	1.83E-06	5.69E-06	2.93E-06
DBNDD1	1.97E-05	3.05E-05	4.10E-06	6.21E-05	1.14E-05	2.74E-06	2.40E-05	6.42E-06
DBNDD2	6.80E-05	7.21E-06	3.73E-06	1.29E-05	5.59E-06	6.37E-06	1.03E-05	6.90E-06

DBNL	6.66E-05	3.04E-05	2.89E-05	3.54E-05	3.17E-05	3.59E-05	3.55E-05	5.73E-05
DBP	6.57E-06	9.31E-06	1.92E-05	4.77E-06	2.14E-05	4.09E-06	2.33E-05	9.24E-06
DBR1	5.82E-06	4.89E-06	3.19E-06	4.49E-06	1.15E-06	4.22E-06	3.68E-06	3.78E-06
DBT	7.78E-06	1.09E-05	8.53E-06	3.62E-06	1.83E-06	1.01E-05	2.14E-06	3.39E-06
DBX1	0	0	0	0	0	0	0	0
DBX2	0	0	0	0	0	0	0	0
DCAF10	3.77E-06	9.55E-06	5.76E-06	4.17E-06	1.70E-06	6.27E-06	4.43E-06	5.79E-06
DCAF11	5.60E-05	7.41E-05	6.35E-05	3.83E-05	4.44E-05	4.52E-05	4.23E-05	3.19E-05
DCAF12	1.15E-05	2.13E-05	1.17E-05	1.18E-05	2.81E-06	1.70E-05	7.16E-06	9.96E-06
DCAF12L1	3.05E-06	0	7.00E-09	0	0	0	0	0
DCAF12L2	6.86E-07	2.01E-08	0	0	0	0	0	0
DCAF13	1.95E-05	2.13E-05	6.76E-06	8.72E-06	5.92E-06	1.55E-05	2.19E-05	1.38E-05
DCAF15	1.32E-05	5.94E-06	6.48E-06	1.01E-05	1.16E-05	7.43E-06	1.06E-05	7.10E-06
DCAF16	9.18E-06	1.39E-06	1.64E-06	2.18E-06	8.16E-07	2.69E-06	2.02E-06	1.36E-06
DCAF17	3.76E-06	2.69E-06	1.75E-06	2.00E-06	7.31E-07	3.38E-06	1.76E-06	1.68E-06
DCAF4	3.43E-06	2.76E-06	2.12E-06	2.85E-06	1.66E-06	3.85E-06	5.45E-06	3.61E-06
DCAF4L1	5.21E-08	1.11E-07	1.46E-08	2.31E-08	2.03E-08	6.13E-08	5.99E-08	1.25E-07
DCAF4L2	8.31E-08	7.77E-09	0	8.46E-09	0	1.41E-07	0	1.09E-05
DCAF5	1.36E-05	7.27E-06	8.18E-06	7.77E-06	4.24E-06	1.00E-05	7.77E-06	8.60E-06
DCAF6	5.70E-05	9.39E-05	4.99E-05	3.67E-05	1.67E-05	7.50E-05	6.63E-05	5.17E-05
DCAF7	3.41E-05	1.37E-05	9.21E-06	1.20E-05	6.64E-06	2.34E-05	1.21E-05	1.42E-05
DCAF8	3.04E-05	5.62E-05	3.29E-05	2.86E-05	1.47E-05	6.88E-05	1.84E-05	5.37E-05
DCAF8L1	7.30E-08	0	0	0	0	0	6.45E-09	0
DCAF8L2	4.76E-08	0	1.25E-08	0	0	2.21E-08	0	2.52E-06
DCAKD	2.48E-05	2.99E-05	1.24E-05	1.13E-05	1.01E-05	3.21E-05	1.14E-05	1.07E-05
DCBLD1	6.77E-06	2.98E-06	6.34E-06	6.05E-06	1.79E-06	7.19E-06	8.24E-06	4.73E-06
DCBLD2	9.15E-06	6.92E-06	4.61E-06	5.33E-06	1.85E-06	3.56E-06	3.47E-06	3.14E-06
DCC	1.05E-08	1.05E-07	0	0	0	5.40E-07	1.02E-08	0
DCD	0	0	0	0	0	0	0	0
DCDC1	4.93E-08	6.47E-09	0	0	0	1.33E-08	0	0
DCDC2	5.81E-05	1.08E-06	1.33E-06	1.24E-05	2.88E-06	6.56E-06	1.04E-06	3.25E-06
DCDC2B	0	0	0	0	0	0	0	0
DCHS1	4.00E-06	8.33E-07	1.08E-06	1.13E-06	3.93E-07	6.29E-07	1.58E-06	6.75E-07
DCHS2	2.10E-07	0	6.35E-08	1.52E-08	0	4.60E-09	1.78E-08	0
DCI	0.000105	0.00021	0.00015	0.000127	0.000184	0.000161	0.000233	0.000138
DCK	1.41E-05	4.26E-06	3.64E-06	3.87E-06	5.02E-07	4.61E-06	3.48E-06	4.64E-06
DCLK1	3.13E-07	4.55E-08	2.62E-08	1.75E-07	2.32E-08	5.28E-08	1.05E-07	1.40E-07
DCLK2	7.62E-07	2.56E-07	1.77E-07	8.58E-07	1.42E-07	2.30E-07	3.15E-07	2.61E-07
DCLK3	8.42E-08	9.88E-08	3.45E-08	1.03E-08	3.61E-08	7.40E-08	1.11E-07	0
DCLRE1A	3.86E-06	3.23E-06	3.40E-06	1.99E-06	8.05E-07	4.77E-06	1.94E-06	2.70E-06
DCLRE1B	2.59E-06	2.00E-06	8.49E-07	9.31E-07	2.32E-07	2.04E-06	1.28E-06	1.85E-06
DCLRE1C	2.97E-06	1.91E-06	5.71E-07	1.30E-06	7.58E-07	1.32E-06	1.90E-06	7.29E-07
DCN	6.20E-05	5.00E-06	0.000136	0.000361	1.33E-05	2.30E-06	4.61E-05	1.81E-05
DCP1A	7.15E-06	3.53E-06	2.65E-06	3.53E-06	8.91E-07	3.61E-06	3.91E-06	2.09E-06
DCP1B	7.06E-06	5.71E-06	4.65E-06	5.00E-06	3.10E-06	5.43E-06	5.06E-06	5.44E-06
DCP2	6.07E-06	2.95E-06	2.23E-06	3.32E-06	1.57E-06	3.49E-06	2.54E-06	2.32E-06
DCPS	9.95E-05	2.31E-05	2.66E-05	4.75E-05	2.07E-05	2.70E-05	2.29E-05	9.75E-05
DCST1	1.10E-08	4.21E-08	0	0	0	0	0	0
DCST2	1.27E-07	5.60E-07	7.09E-08	2.38E-08	1.17E-07	1.90E-07	5.66E-08	1.57E-07
DCT	0	6.27E-07	1.07E-08	0	3.54E-08	0	1.99E-08	0
DCTD	4.61E-05	2.03E-05	5.03E-05	3.60E-05	1.81E-05	4.23E-05	2.32E-05	3.27E-05
DCTN1	5.15E-05	5.30E-05	2.16E-05	2.60E-05	3.28E-05	2.47E-05	2.81E-05	3.52E-05
DCTN2	0.000114	7.30E-05	2.91E-05	4.55E-05	2.53E-05	6.00E-05	4.83E-05	4.26E-05
DCTN3	3.67E-05	8.85E-05	4.13E-05	5.47E-05	5.12E-05	6.61E-05	5.65E-05	5.27E-05
DCTN4	2.01E-05	1.82E-05	1.15E-05	1.05E-05	2.63E-06	1.88E-05	9.71E-06	1.93E-05
DCTN5	1.14E-05	1.27E-05	5.65E-06	9.08E-06	2.90E-06	9.41E-06	7.94E-06	9.03E-06
DCTN6	2.10E-05	3.47E-05	2.01E-05	2.65E-05	5.08E-06	3.45E-05	1.25E-05	1.66E-05
DCTPP1	5.73E-05	5.92E-05	1.75E-05	2.13E-05	1.40E-05	2.24E-05	3.19E-05	2.12E-05

DCUN1D1	1.48E-05	7.23E-06	1.06E-05	5.94E-06	1.47E-06	8.29E-06	3.77E-06	7.01E-06
DCUN1D2	5.29E-06	4.83E-06	1.95E-06	1.73E-06	1.60E-06	1.53E-06	2.59E-06	2.26E-06
DCUN1D3	3.60E-06	3.81E-06	4.04E-06	6.84E-06	1.03E-06	2.54E-06	4.16E-06	1.78E-06
DCUN1D4	1.03E-05	1.37E-05	1.08E-05	6.89E-06	3.29E-06	1.26E-05	7.21E-06	1.08E-05
DCUN1D5	1.16E-05	1.00E-05	6.36E-06	9.66E-06	3.10E-06	1.18E-05	8.61E-06	1.03E-05
DCX	8.57E-08	1.36E-08	2.49E-09	4.48E-08	2.10E-09	2.94E-08	9.55E-09	0
DCXR	0.000176	0.001426	0.001677	0.000718	0.002951	0.00338	0.0011	0.000483
DDA1	1.22E-05	4.18E-06	5.10E-06	7.38E-06	5.27E-06	8.31E-06	9.54E-06	5.69E-06
DDAH1	1.56E-05	5.44E-05	4.60E-05	2.73E-05	1.46E-05	4.03E-05	1.85E-05	2.32E-05
DDAH2	2.75E-05	2.68E-05	2.50E-05	5.96E-05	2.22E-05	2.73E-05	4.48E-05	2.44E-05
DDB1	6.92E-05	5.71E-05	3.59E-05	3.94E-05	1.86E-05	7.80E-05	2.98E-05	3.44E-05
DDB2	1.84E-05	9.94E-06	1.49E-05	2.32E-05	1.19E-05	2.04E-05	1.02E-05	1.80E-05
DDC	5.36E-05	5.47E-05	3.05E-05	1.07E-05	1.25E-05	4.62E-05	1.58E-05	1.92E-06
DDHD1	1.33E-06	2.62E-07	3.81E-07	6.62E-07	1.47E-07	4.89E-07	5.25E-07	5.24E-07
DDHD2	4.32E-06	6.09E-06	3.68E-06	4.08E-06	3.91E-07	4.51E-06	1.59E-06	2.93E-06
DDI1	0	0	0	0	0	0	0	0
DDI2	6.69E-06	6.84E-06	5.88E-06	2.50E-06	1.61E-08	8.15E-06	7.34E-07	3.37E-06
DDIT3	4.38E-05	9.90E-05	1.73E-05	3.06E-05	1.84E-05	1.82E-05	0.000113	5.75E-05
DDIT4	5.12E-05	7.44E-05	9.78E-05	8.59E-05	3.68E-05	4.50E-05	0.000107	0.000124
DDIT4L	8.13E-08	1.73E-07	1.03E-07	4.42E-08	1.55E-08	5.02E-08	4.38E-08	6.63E-08
DDN	1.79E-07	2.74E-08	0	7.46E-09	0	0	5.93E-09	0
DDO	9.04E-06	1.59E-05	8.87E-06	5.44E-06	1.38E-06	1.06E-05	6.36E-06	3.26E-06
DDOST	0.000166	0.000122	6.50E-05	8.48E-05	4.87E-05	0.000165	7.99E-05	0.000111
DDR1	2.90E-05	8.26E-06	4.12E-06	1.28E-05	5.23E-07	2.27E-06	7.21E-06	3.68E-06
DDR2	6.81E-07	8.96E-07	5.44E-07	6.08E-07	0	3.66E-07	1.21E-07	6.22E-07
DDRGK1	0.000127	9.26E-05	5.59E-05	7.02E-05	0.000168	7.30E-05	9.77E-05	0.000105
DDT	0.000433	0.000422	0.000675	0.000368	0.00153	0.000366	0.000442	0.000191
DDTL	4.13E-05	5.93E-05	9.04E-05	8.54E-05	0.000133	4.06E-05	6.28E-05	1.37E-05
DDX1	6.26E-05	7.05E-05	2.58E-05	3.11E-05	1.62E-05	4.78E-05	2.72E-05	3.49E-05
DDX10	1.16E-05	6.95E-06	3.75E-06	4.88E-06	3.02E-06	9.33E-06	6.09E-06	1.10E-05
DDX11	8.09E-06	3.20E-06	4.39E-06	3.60E-06	1.48E-06	1.93E-06	1.79E-06	2.99E-06
DDX11L2	1.44E-06	1.68E-07	4.05E-07	6.52E-07	1.27E-08	2.05E-07	4.83E-07	5.65E-07
DDX12	3.84E-06	6.72E-07	2.86E-07	2.63E-07	4.01E-07	3.85E-07	5.08E-07	6.74E-07
DDX17	0.000114	5.85E-05	4.66E-05	8.11E-05	3.20E-05	5.34E-05	7.28E-05	5.56E-05
DDX18	3.18E-05	3.17E-05	1.49E-05	1.60E-05	3.51E-06	2.44E-05	1.22E-05	1.18E-05
DDX19A	2.77E-05	1.18E-05	1.21E-05	1.25E-05	5.48E-06	1.68E-05	1.11E-05	1.50E-05
DDX19B	3.84E-05	1.81E-05	2.68E-05	2.90E-05	1.07E-05	2.83E-05	2.92E-05	1.97E-05
DDX20	4.87E-06	5.05E-06	2.44E-06	2.73E-06	8.99E-07	5.06E-06	2.06E-06	3.02E-06
DDX21	1.64E-05	1.88E-05	1.17E-05	1.76E-05	2.82E-07	1.78E-05	7.25E-06	1.34E-05
DDX23	4.47E-05	3.61E-05	2.10E-05	2.13E-05	1.26E-05	3.31E-05	2.36E-05	2.53E-05
DDX24	4.47E-05	2.99E-05	2.98E-05	4.66E-05	1.84E-05	6.27E-05	3.68E-05	5.02E-05
DDX25	0	1.81E-07	6.99E-08	5.62E-08	5.72E-08	6.17E-08	1.28E-08	0
DDX26B	3.71E-06	1.77E-07	1.63E-07	9.65E-07	1.10E-07	3.46E-07	1.78E-07	4.99E-07
DDX27	5.88E-05	2.82E-05	9.47E-06	1.82E-05	1.01E-05	1.70E-05	1.71E-05	1.41E-05
DDX28	6.20E-06	5.46E-06	4.37E-06	6.64E-06	3.04E-06	1.02E-05	8.05E-06	8.99E-06
DDX31	2.79E-06	2.65E-06	2.06E-06	1.95E-06	9.43E-07	3.58E-06	1.77E-06	4.95E-06
DDX39	6.08E-05	3.22E-05	9.87E-06	2.02E-05	3.40E-05	1.52E-05	2.22E-05	3.71E-05
DDX3X	9.15E-05	4.50E-05	5.93E-05	5.51E-05	1.07E-05	4.31E-05	3.87E-05	7.04E-05
DDX3Y	0	1.71E-05	0	1.21E-05	2.80E-06	1.22E-05	8.00E-06	6.83E-06
DDX4	2.01E-08	0	0	0	0	0	0	0
DDX41	3.80E-05	2.61E-05	1.98E-05	2.46E-05	1.57E-05	3.07E-05	2.82E-05	2.88E-05
DDX42	5.21E-05	4.02E-05	2.49E-05	3.03E-05	2.62E-05	5.25E-05	3.24E-05	2.89E-05
DDX43	1.23E-06	7.98E-08	0	3.29E-07	2.24E-08	2.41E-08	0	2.85E-07
DDX46	1.64E-05	9.10E-06	6.54E-06	6.63E-06	1.11E-06	1.08E-05	3.65E-06	7.05E-06
DDX47	2.80E-05	1.75E-05	1.69E-05	2.03E-05	8.42E-06	2.58E-05	2.30E-05	1.77E-05
DDX49	3.18E-05	3.52E-05	1.56E-05	1.49E-05	3.34E-05	3.06E-05	2.62E-05	2.68E-05
DDX5	0.000238	0.000172	7.00E-05	0.000168	4.59E-05	0.000173	9.61E-05	0.000102
DDX50	1.26E-05	1.06E-05	7.21E-06	9.58E-06	4.76E-06	1.21E-05	9.96E-06	9.23E-06

DDX51	5.49E-06	4.70E-06	2.25E-06	3.17E-06	2.12E-06	2.29E-06	3.72E-06	4.29E-06
DDX52	1.57E-05	1.20E-05	3.85E-06	5.87E-06	2.23E-06	6.58E-06	4.85E-06	4.34E-06
DDX53	6.75E-08	0	0	0	0	2.09E-08	0	2.33E-06
DDX54	1.52E-05	2.39E-05	1.41E-05	1.66E-05	1.31E-05	1.80E-05	2.24E-05	1.64E-05
DDX55	9.50E-06	7.55E-06	4.03E-06	5.37E-06	3.17E-06	6.25E-06	6.34E-06	4.55E-06
DDX56	6.97E-05	6.49E-05	2.60E-05	3.37E-05	3.71E-05	5.27E-05	4.86E-05	4.66E-05
DDX58	6.61E-06	5.27E-06	4.79E-06	4.01E-06	1.17E-06	7.81E-06	2.90E-06	7.09E-06
DDX59	8.36E-06	6.46E-06	4.94E-06	6.13E-06	8.03E-07	1.08E-05	3.73E-06	1.17E-05
DDX6	2.54E-05	9.14E-06	1.28E-05	1.48E-05	5.54E-06	1.77E-05	1.43E-05	1.56E-05
DDX60	4.89E-06	1.98E-06	4.41E-06	3.54E-06	3.33E-07	6.34E-06	2.36E-06	1.07E-05
DDX60L	3.82E-06	1.27E-06	2.84E-06	1.63E-06	2.63E-07	2.22E-06	1.13E-06	3.01E-06
DEAF1	1.33E-05	9.41E-06	5.06E-06	6.16E-06	7.68E-06	8.84E-06	5.95E-06	8.49E-06
1-Dec	0	0	0	0	0	0	1.96E-08	3.03E-08
DECR1	0.000429	0.000281	0.000315	0.000206	0.00021	0.000199	0.000321	0.000249
DECR2	3.47E-05	0.000114	7.46E-05	4.18E-05	3.47E-05	0.000139	0.000128	0.000113
DEDD	1.66E-05	2.75E-05	7.88E-06	1.23E-05	6.66E-06	1.82E-05	1.13E-05	2.83E-05
DEDD2	2.60E-05	1.07E-05	1.34E-05	1.39E-05	1.90E-05	1.72E-05	1.71E-05	1.42E-05
DEF6	6.20E-06	1.43E-06	1.88E-06	4.26E-06	1.19E-06	2.42E-06	3.01E-06	1.38E-06
DEF8	2.98E-05	7.66E-06	7.99E-06	1.20E-05	9.76E-06	1.70E-05	1.24E-05	1.94E-05
DEFA1B	2.09E-07	0	3.85E-06	3.13E-06	2.56E-07	0	0	5.44E-07
DEFA4	1.22E-07	0	2.25E-07	1.55E-07	0	0	0	0
DEFA5	0	0	0	0	0	0	0	0
DEFA6	0	0	0	0	0	0	0	0
DEFB1	0.001225	2.91E-06	0.0002	0.001151	9.03E-05	0.002091	4.35E-05	0.000304
DEFB103B	0	0	0	0	0	0	0	0
DEFB104A	0	0	0	0	0	0	0	0
DEFB105A	0	0	0	0	0	0	0	0
DEFB106A	0	0	0	0	0	0	0	0
DEFB107A	0	0	0	0	0	0	0	0
DEFB108B	0	0	0	0	0	0	0	0
DEFB109P	0	0	0	0	0	0	0	0
DEFB109P	2.63E-07	0	0	0	0	0	0	0
DEFB110	0	0	0	0	0	0	0	0
DEFB112	0	0	0	0	0	0	0	0
DEFB113	0	0	0	0	0	0	0	0
DEFB114	0	0	0	0	0	0	0	0
DEFB115	0	0	0	0	0	0	0	0
DEFB116	0	0	0	0	0	0	0	0
DEFB118	0	0	0	0	0	0	0	0
DEFB119	0	0	0	0	0	0	0	0
DEFB121	0	0	0	0	0	0	0	0
DEFB122	0	0	0	0	0	0	0	0
DEFB123	0	0	0	0	0	0	0	0
DEFB124	0	0	0	0	0	0	0	0
DEFB125	0	0	0	0	0	0	0	0
DEFB126	0	0	0	0	0	0	0	0
DEFB127	0	0	0	0	0	0	0	0
DEFB128	0	0	0	0	0	0	0	0
DEFB129	0	0	0	0	0	0	0	0
DEFB130	0	0	0	0	0	0	0	0
DEFB131	0	0	0	0	0	0	0	0
DEFB132	1.80E-07	7.63E-08	4.70E-08	0	8.71E-08	5.01E-07	1.30E-07	1.03E-06
DEFB134	0	0	0	0	0	0	0	0
DEFB135	0	0	0	0	0	0	0	0
DEFB136	0	0	0	0	0	0	0	0
DEFB4A	0	0	0	0	0	0	0	0
DEGS1	4.59E-05	2.27E-05	1.66E-05	1.95E-05	7.18E-06	1.52E-05	1.47E-05	2.65E-05
DEGS2	3.33E-06	1.79E-06	3.23E-07	4.82E-07	3.08E-07	1.16E-07	1.54E-07	1.58E-07

DEK	3.72E-05	4.23E-05	2.36E-05	2.29E-05	1.08E-05	3.12E-05	2.00E-05	2.51E-05
DEM1	3.81E-06	2.02E-06	2.81E-06	2.80E-06	1.04E-06	2.46E-06	1.55E-06	2.66E-06
DENND1A	1.39E-05	7.82E-06	7.34E-06	7.76E-06	9.03E-06	1.06E-05	7.82E-06	1.01E-05
DENND1B	1.91E-06	8.07E-07	8.80E-07	3.24E-07	1.62E-08	1.09E-06	2.01E-07	1.21E-06
DENND1C	1.02E-05	4.46E-06	3.55E-06	4.45E-06	3.30E-07	2.24E-06	3.42E-06	2.59E-06
DENND2A	2.82E-06	7.19E-07	1.12E-06	2.81E-06	1.04E-06	8.90E-07	1.50E-06	2.35E-06
DENND2C	1.55E-06	1.66E-06	5.28E-07	4.97E-07	4.43E-08	8.63E-07	2.68E-07	5.97E-07
DENND2D	2.98E-05	1.17E-05	8.51E-06	7.86E-06	1.70E-06	5.65E-06	3.97E-06	2.86E-06
DENND3	1.51E-05	6.86E-07	1.95E-06	2.31E-06	1.23E-06	1.26E-06	2.15E-06	1.94E-06
DENND4A	5.45E-06	3.37E-06	3.88E-06	5.08E-06	9.67E-07	8.18E-06	1.98E-06	4.36E-06
DENND4B	7.34E-06	4.90E-06	2.48E-06	3.86E-06	2.35E-06	5.43E-06	4.68E-06	7.22E-06
DENND4C	5.83E-06	7.54E-06	6.07E-06	5.38E-06	1.48E-06	6.58E-06	2.91E-06	3.98E-06
DENND5A	1.54E-05	7.68E-06	4.64E-06	7.34E-06	1.47E-06	1.31E-05	5.91E-06	1.24E-05
DENND5B	5.72E-06	5.83E-06	4.33E-06	3.61E-06	8.61E-07	5.23E-06	2.59E-06	4.48E-06
DENR	2.06E-05	2.28E-05	1.11E-05	1.22E-05	7.15E-06	1.58E-05	1.29E-05	1.34E-05
DEPDC1	3.26E-06	1.19E-06	2.87E-08	4.77E-08	1.92E-07	5.24E-07	7.30E-08	2.61E-07
DEPDC1B	1.96E-05	1.10E-06	7.80E-08	4.13E-07	1.58E-06	5.04E-07	1.73E-07	6.61E-07
DEPDC4	3.62E-07	2.56E-07	2.38E-07	2.25E-07	9.82E-08	4.34E-07	1.78E-07	9.08E-08
DEPDC5	2.06E-05	9.72E-06	5.46E-06	6.59E-06	5.04E-06	1.34E-05	3.81E-06	5.84E-06
DEPDC6	3.00E-06	2.50E-05	8.79E-06	1.08E-05	5.70E-06	8.71E-06	1.11E-05	5.30E-06
DEPDC7	4.14E-05	1.58E-05	4.12E-05	1.63E-05	1.68E-05	6.19E-05	1.46E-05	1.70E-05
DERA	4.46E-05	8.15E-05	3.96E-05	3.15E-05	1.14E-05	3.51E-05	2.92E-05	1.93E-05
DERL1	7.87E-05	5.21E-05	3.53E-05	3.28E-05	2.52E-05	4.01E-05	5.93E-05	6.69E-05
DERL2	2.31E-05	2.73E-05	2.86E-05	3.07E-05	1.26E-05	7.40E-05	3.84E-05	4.41E-05
DERL3	4.16E-06	1.85E-06	1.34E-06	4.26E-06	2.86E-06	1.14E-05	1.11E-06	2.17E-07
DES	1.63E-06	1.19E-08	6.60E-08	1.50E-06	3.08E-07	3.91E-08	1.02E-08	1.09E-07
DET1	2.49E-06	3.36E-06	1.84E-06	1.95E-06	1.29E-06	2.95E-06	1.85E-06	2.21E-06
DEXI	2.07E-05	4.33E-05	2.48E-05	2.08E-05	3.48E-05	2.98E-05	4.97E-05	2.96E-05
DFFA	1.41E-05	9.29E-06	6.61E-06	5.48E-06	2.18E-06	1.20E-05	4.17E-06	7.66E-06
DFFB	1.57E-06	3.45E-07	7.66E-07	1.14E-06	3.15E-07	8.21E-07	7.05E-07	6.74E-07
DFNA5	2.19E-05	4.86E-07	2.13E-06	5.82E-06	3.57E-07	2.18E-06	1.92E-06	1.08E-06
DFNB31	6.47E-06	2.16E-06	7.42E-07	2.52E-06	8.46E-08	2.51E-06	6.71E-07	2.08E-06
DFNB59	1.54E-06	3.70E-07	1.73E-07	4.76E-07	6.50E-07	8.84E-07	2.06E-07	3.02E-07
DGAT1	2.75E-05	2.64E-05	1.51E-05	8.97E-06	3.52E-05	9.90E-06	8.19E-05	1.72E-05
DGAT2	1.56E-05	4.57E-05	0.000213	4.43E-05	9.14E-05	3.94E-05	7.36E-05	8.39E-05
DGAT2L6	0	0	0	0	0	0	0	0
DGCR10	3.23E-07	1.03E-07	0	0	1.27E-07	1.10E-07	0	4.39E-08
DGCR11	1.14E-06	9.31E-07	3.58E-07	7.74E-07	2.21E-07	5.96E-07	7.77E-07	1.56E-06
DGCR14	4.97E-06	4.96E-06	1.57E-06	2.62E-06	2.46E-06	3.20E-06	3.52E-06	4.25E-06
DGCR2	5.65E-05	3.35E-05	1.48E-05	2.32E-05	2.71E-05	2.42E-05	3.76E-05	3.07E-05
DGCR5	8.02E-06	6.28E-06	1.86E-06	1.13E-06	4.84E-06	2.00E-06	1.87E-06	1.40E-06
DGCR6	6.44E-05	0.00012	2.51E-05	2.03E-05	7.29E-05	3.10E-05	3.56E-05	1.46E-05
DGCR6L	4.38E-05	0.000282	7.51E-05	5.79E-05	0.000162	6.73E-05	9.42E-05	6.84E-05
DGCR8	1.14E-05	6.44E-06	4.90E-06	6.75E-06	4.58E-06	7.61E-06	6.70E-06	5.85E-06
DGCR9	6.44E-07	3.30E-07	4.06E-08	9.55E-08	1.09E-07	3.98E-07	0	1.86E-07
DGKA	1.25E-05	7.43E-07	8.08E-07	3.31E-06	6.73E-07	1.29E-06	1.65E-06	1.70E-06
DGKB	1.34E-08	2.61E-08	5.12E-08	8.15E-09	2.87E-09	9.27E-09	3.26E-09	2.44E-08
DGKD	1.18E-05	2.07E-06	1.42E-06	2.65E-06	1.94E-06	2.12E-06	3.03E-06	2.42E-06
DGKE	1.54E-06	3.33E-07	1.07E-06	6.67E-07	2.01E-07	7.11E-07	6.24E-07	2.68E-07
DGKG	1.09E-06	6.47E-07	2.14E-07	4.48E-07	2.02E-07	5.10E-07	5.06E-08	2.14E-07
DGKH	6.65E-07	3.10E-07	1.47E-07	3.07E-07	1.91E-07	3.55E-07	2.27E-07	4.80E-07
DGKI	3.89E-08	3.26E-08	0	1.19E-08	1.25E-08	8.75E-09	9.46E-09	7.12E-09
DGKK	1.17E-08	0	0	0	0	5.42E-09	0	0
DGKQ	5.62E-06	3.55E-06	1.98E-06	2.69E-06	1.18E-06	3.41E-06	4.32E-06	8.24E-06
DGKZ	1.52E-05	4.53E-06	3.25E-06	5.63E-06	5.60E-06	5.10E-06	6.46E-06	7.00E-06
DGUOK	0.000117	3.92E-05	2.62E-05	3.29E-05	2.81E-05	4.15E-05	4.58E-05	3.27E-05
DHCR24	0.00019	0.000597	0.000408	0.000246	0.00019	0.000456	0.000316	0.00081
DHCR7	7.76E-05	0.000276	5.73E-05	7.40E-05	4.18E-05	2.26E-05	3.91E-05	0.000106

DHDDS	2.32E-05	1.32E-05	7.27E-06	8.65E-06	3.56E-06	1.14E-05	6.78E-06	1.23E-05
DHDH	1.70E-06	7.30E-07	1.59E-07	1.80E-07	6.30E-08	6.80E-08	0	3.61E-08
DHDPSL	2.26E-05	3.87E-05	2.80E-05	1.64E-05	2.29E-05	3.33E-05	1.51E-05	7.12E-08
DHFR	7.33E-06	4.41E-06	7.34E-06	5.04E-06	1.70E-06	4.47E-06	2.00E-06	3.00E-06
DHFRL1	5.21E-06	5.73E-06	3.55E-06	2.36E-06	1.70E-06	3.02E-06	2.20E-06	1.42E-06
DHH	4.23E-07	2.96E-07	1.31E-08	7.63E-08	1.39E-07	1.04E-07	6.01E-08	2.02E-07
DHODH	4.15E-06	8.77E-06	4.63E-05	3.21E-05	1.18E-05	5.24E-05	3.37E-05	5.38E-05
DHPS	3.76E-05	3.16E-05	3.67E-05	3.43E-05	3.19E-05	4.53E-05	4.00E-05	4.73E-05
DHRS1	3.37E-05	2.00E-05	8.49E-05	4.58E-05	0.000118	2.08E-05	9.75E-05	9.34E-06
DHRS11	7.35E-06	1.59E-05	5.79E-06	7.16E-06	1.23E-05	1.19E-05	8.88E-06	4.87E-06
DHRS12	1.42E-05	2.23E-05	1.96E-05	2.20E-05	1.60E-05	2.11E-05	2.69E-05	1.04E-05
DHRS13	1.17E-05	1.22E-05	1.75E-06	2.76E-06	5.10E-06	2.94E-06	3.80E-06	6.80E-06
DHRS2	1.57E-06	0.000412	5.86E-07	1.11E-06	4.64E-05	0.000246	3.59E-05	4.30E-07
DHRS3	0.000203	0.000205	0.000184	0.000228	0.000231	0.000211	0.000274	0.000309
DHRS4	3.42E-05	7.88E-05	0.000109	7.41E-05	0.00013	9.98E-05	9.47E-05	6.21E-05
DHRS4L1	8.23E-07	2.63E-06	3.55E-06	2.05E-06	4.53E-06	3.32E-06	2.93E-06	1.15E-06
DHRS4L2	2.01E-05	2.93E-05	4.59E-05	2.96E-05	0.000143	3.07E-05	5.91E-05	2.85E-05
DHRS7	0.000145	2.79E-05	6.23E-05	6.68E-05	0.0001	4.57E-05	6.84E-05	0.000106
DHRS7B	2.23E-05	3.50E-05	1.53E-05	2.36E-05	2.46E-05	1.71E-05	3.01E-05	2.30E-05
DHRS7C	0	0	0	2.68E-07	0	0	0	0
DHRS9	1.47E-06	2.04E-06	9.58E-07	8.86E-06	1.17E-07	4.74E-07	4.28E-06	3.34E-07
DHRSX	1.37E-05	1.41E-05	1.57E-05	1.50E-05	2.00E-05	9.94E-06	2.12E-05	2.97E-05
DHTKD1	3.90E-05	0.000122	8.72E-05	4.07E-05	1.69E-05	4.38E-05	2.79E-05	6.66E-05
DHX15	4.92E-05	3.96E-05	1.79E-05	2.51E-05	4.43E-06	2.62E-05	1.42E-05	2.31E-05
DHX16	1.71E-05	2.67E-05	1.08E-05	1.25E-05	1.09E-05	1.86E-05	1.61E-05	1.58E-05
DHX29	1.23E-05	6.85E-06	6.78E-06	6.31E-06	1.45E-06	1.09E-05	4.50E-06	1.56E-05
DHX30	2.70E-05	3.36E-05	1.59E-05	1.94E-05	1.47E-05	2.42E-05	1.78E-05	1.90E-05
DHX32	1.97E-05	5.41E-06	6.79E-06	8.48E-06	3.10E-06	8.81E-06	6.37E-06	1.14E-05
DHX33	2.17E-06	3.35E-06	2.23E-06	1.75E-06	2.92E-08	4.56E-06	8.53E-07	3.21E-06
DHX34	1.27E-05	1.04E-05	3.47E-06	5.33E-06	6.28E-06	8.23E-06	6.68E-06	7.92E-06
DHX35	7.01E-06	5.01E-06	2.53E-06	2.56E-06	8.46E-07	4.00E-06	3.54E-06	2.47E-06
DHX36	1.45E-05	9.89E-06	5.71E-06	6.98E-06	1.43E-06	1.36E-05	3.64E-06	5.98E-06
DHX37	5.84E-06	1.16E-05	3.16E-06	4.77E-06	3.83E-06	5.83E-06	5.56E-06	4.57E-06
DHX38	1.60E-05	1.28E-05	9.95E-06	1.16E-05	8.07E-06	1.70E-05	1.19E-05	1.19E-05
DHX40	1.76E-05	1.82E-05	7.69E-06	1.09E-05	3.26E-06	2.16E-05	6.60E-06	7.46E-06
DHX40P1	0	0	2.50E-08	0	0	0	0	0
DHX57	5.08E-06	5.00E-06	1.38E-06	2.46E-06	1.51E-06	4.96E-06	1.92E-06	3.10E-06
DHX58	8.06E-06	6.93E-06	1.72E-05	5.74E-06	4.54E-06	2.11E-05	8.11E-06	7.89E-06
DHX8	1.53E-05	1.51E-05	6.12E-06	7.25E-06	3.44E-06	1.74E-05	7.54E-06	9.34E-06
DHX9	6.24E-05	4.73E-05	2.46E-05	4.37E-05	1.93E-05	5.21E-05	3.54E-05	4.59E-05
DIABLO	3.56E-05	2.62E-05	1.61E-05	2.07E-05	1.58E-05	2.31E-05	3.02E-05	1.94E-05
DIAPH1	0.00011	0.000122	6.89E-05	5.95E-05	2.92E-05	6.95E-05	7.63E-05	8.57E-05
DIAPH2	3.15E-06	1.29E-06	3.51E-06	2.86E-06	1.00E-06	3.04E-06	1.70E-06	3.51E-06
DIAPH3	3.84E-06	2.60E-06	1.15E-07	3.45E-07	5.54E-07	1.05E-06	3.62E-07	4.94E-07
DICER1	8.67E-06	3.72E-06	9.76E-06	6.72E-06	2.84E-06	6.34E-06	3.24E-06	7.12E-06
DIDO1	2.89E-05	1.66E-05	9.07E-06	9.98E-06	4.30E-06	9.67E-06	9.54E-06	8.48E-06
DIMT1L	7.76E-06	7.81E-06	7.50E-06	8.89E-06	1.53E-06	1.20E-05	8.61E-06	8.78E-06
DIO1	0.000183	0.000107	0.000142	6.69E-05	5.84E-05	1.96E-05	3.85E-05	4.56E-07
DIO2	2.20E-07	3.68E-08	1.79E-08	4.30E-08	3.14E-09	1.02E-08	4.64E-08	3.76E-06
DIO3	9.54E-07	0	1.19E-07	1.03E-06	2.34E-07	0	1.10E-07	0
DIO3OS	1.33E-07	4.11E-07	4.82E-07	4.38E-07	3.81E-07	0	0	4.06E-08
DIP2A	8.33E-06	3.21E-06	2.75E-06	3.43E-06	1.52E-06	3.69E-06	3.93E-06	2.85E-06
DIP2B	1.55E-05	7.97E-06	5.29E-06	4.97E-06	1.78E-06	4.14E-06	3.37E-06	5.34E-06
DIP2C	1.15E-05	1.75E-06	4.27E-06	3.32E-06	2.32E-06	4.00E-06	3.04E-06	1.91E-06
DIRAS1	2.33E-06	3.79E-08	6.98E-09	5.78E-08	6.39E-08	1.25E-08	1.38E-07	1.98E-08
DIRAS2	8.86E-08	0	1.65E-07	4.06E-08	0	1.03E-08	5.39E-09	2.43E-08
DIRAS3	3.31E-07	1.03E-07	4.90E-06	3.05E-06	2.85E-07	2.77E-06	1.60E-07	2.67E-07
DIRC1	0	0	0	0	0	0	0	0

DIRC2	6.66E-06	2.96E-06	3.08E-06	4.14E-06	1.13E-06	4.07E-06	3.47E-06	4.05E-06
DIRC3	1.28E-06	6.54E-09	1.20E-08	5.71E-08	0	2.16E-08	1.14E-08	1.71E-08
DIS3	8.43E-06	1.06E-05	6.40E-06	6.14E-06	1.40E-06	7.80E-06	3.61E-06	5.06E-06
DIS3L	8.28E-06	7.46E-06	7.13E-06	7.12E-06	3.75E-06	9.42E-06	6.20E-06	5.12E-06
DIS3L2	9.41E-06	4.10E-06	3.69E-06	6.12E-06	6.79E-06	8.21E-06	6.28E-06	3.85E-06
DISC1	8.20E-07	2.27E-07	4.40E-07	5.72E-07	9.85E-08	2.77E-06	4.99E-07	2.73E-07
DISC2	1.18E-08	0	0	0	0	0	0	0
DISP1	5.20E-06	4.00E-06	2.29E-06	2.61E-06	1.23E-06	6.60E-06	2.80E-06	7.86E-06
DISP2	9.91E-07	5.20E-06	2.20E-07	2.24E-07	6.92E-08	6.63E-08	2.75E-07	1.26E-06
DIXDC1	2.02E-06	1.98E-06	3.11E-06	3.40E-06	6.26E-07	5.23E-06	2.18E-06	5.70E-06
DKC1	1.75E-05	1.94E-05	9.41E-06	1.31E-05	8.08E-06	1.77E-05	1.43E-05	2.09E-05
DKFZP434	0	0	0	0	0	0	0	0
DKFZp434	0	0	0	0	0	2.57E-07	1.83E-07	0
DKFZP434	8.71E-08	1.69E-07	0	0	0	0	0	0
DKFZP434	2.67E-08	1.04E-07	0	0	2.12E-08	0	0	0
DKFZp434	0	0	0	0	0	0	0	0
DKFZp566	0	0	1.58E-07	8.08E-08	1.12E-07	0	1.18E-07	0
DKFZP586	4.75E-06	5.76E-06	2.66E-06	2.63E-06	2.85E-06	3.53E-06	3.62E-06	4.44E-06
DKFZp686	0	2.75E-08	0	0	0	0	0	0
DKFZP686	1.24E-06	2.32E-07	2.66E-07	4.82E-07	2.57E-07	7.44E-07	5.29E-07	3.71E-07
DKFZp686	2.80E-06	7.83E-08	1.43E-07	2.35E-07	3.01E-09	5.19E-08	5.81E-08	2.05E-08
DKFZp761	5.41E-06	7.09E-06	2.34E-06	3.46E-06	9.19E-07	1.94E-06	3.49E-06	8.35E-06
DKFZp779	5.46E-06	4.36E-06	5.07E-06	5.55E-06	1.75E-05	4.83E-06	3.83E-06	1.84E-05
DKK1	0.000303	1.52E-07	5.65E-08	2.01E-06	0	8.73E-08	1.68E-07	7.92E-08
DKK2	3.27E-06	9.62E-08	1.95E-08	1.00E-07	1.08E-08	1.28E-07	4.28E-08	1.01E-06
DKK3	1.59E-05	3.44E-06	2.35E-06	1.84E-05	2.64E-06	4.85E-06	1.32E-05	5.78E-06
DKK4	0	2.03E-06	0	0	1.20E-07	2.52E-06	0	1.55E-07
DKKL1	1.55E-07	2.99E-08	5.64E-08	3.19E-08	4.46E-08	0	9.84E-08	3.84E-08
DLAT	1.72E-05	2.23E-05	4.86E-06	8.49E-06	1.05E-06	7.84E-06	6.36E-06	1.65E-05
DLC1	7.17E-06	2.19E-05	6.35E-06	1.44E-05	2.95E-06	2.18E-05	6.27E-06	7.40E-06
DLD	2.26E-05	6.55E-05	3.40E-05	2.46E-05	7.79E-06	4.38E-05	2.17E-05	4.03E-05
DLEC1	1.60E-07	1.23E-07	2.68E-08	1.06E-07	2.74E-08	4.43E-08	1.12E-07	5.99E-09
DLEU1	1.10E-05	6.58E-06	6.17E-06	4.71E-06	4.50E-06	1.16E-05	8.35E-06	2.82E-06
DLEU2	2.59E-06	1.10E-06	6.60E-07	5.16E-07	4.39E-07	1.09E-06	6.77E-07	7.35E-07
DLEU2L	1.48E-08	0	0	0	0	1.37E-08	4.27E-08	4.36E-08
DLEU7	1.09E-07	1.51E-07	7.08E-08	5.39E-08	0	0	6.28E-08	0
DLG1	1.16E-05	1.09E-05	9.02E-06	7.69E-06	3.32E-06	1.15E-05	6.62E-06	4.09E-06
DLG2	1.67E-08	8.18E-08	3.78E-07	2.08E-07	6.90E-08	2.62E-07	6.64E-08	3.76E-07
DLG3	9.57E-06	2.77E-06	6.26E-07	1.78E-06	2.57E-07	2.68E-07	4.52E-07	4.39E-07
DLG4	1.36E-06	6.26E-07	7.46E-07	2.49E-06	5.94E-07	1.09E-06	1.20E-06	1.20E-06
DLG5	1.19E-05	2.98E-06	1.98E-06	2.72E-06	1.05E-06	8.35E-06	3.67E-06	3.47E-06
DLGAP1	2.08E-08	2.74E-08	1.42E-08	1.20E-07	0	5.05E-08	0	3.32E-08
DLGAP2	2.21E-09	2.43E-07	4.50E-08	2.67E-08	0	0	0	0
DLGAP3	5.75E-08	5.01E-08	6.58E-08	7.80E-08	6.58E-08	7.69E-08	1.74E-07	3.74E-08
DLGAP4	3.89E-05	2.78E-05	7.22E-06	1.71E-05	1.15E-05	1.71E-05	1.63E-05	3.19E-05
DLGAP5	8.27E-06	9.23E-07	4.81E-08	8.52E-08	3.99E-07	8.45E-07	1.58E-07	5.22E-07
DLK1	8.04E-06	0	6.87E-08	3.07E-06	3.21E-07	0	3.12E-08	0
DLK2	1.90E-06	5.48E-07	3.65E-08	2.11E-08	7.39E-08	1.59E-08	1.54E-08	5.06E-08
DLL1	4.24E-06	7.46E-07	8.69E-07	1.82E-06	7.71E-07	3.14E-06	1.12E-06	4.32E-06
DLL3	3.50E-08	0	0	0	0	0	2.26E-08	0
DLL4	1.02E-05	1.19E-05	1.81E-06	1.18E-06	1.88E-06	3.29E-06	1.35E-05	5.90E-06
DLST	8.29E-05	5.11E-05	4.47E-05	5.19E-05	2.52E-05	5.64E-05	4.69E-05	0.000101
DLX1	2.18E-08	2.46E-08	1.14E-08	0	0	2.75E-08	1.92E-08	0
DLX2	1.03E-08	3.47E-08	0	1.26E-08	0	0	9.93E-09	0
DLX3	1.89E-07	0	3.75E-08	1.10E-08	0	0	3.50E-08	1.32E-08
DLX4	2.81E-07	0	1.91E-08	1.10E-07	0	0	0	1.75E-08
DLX5	5.28E-08	3.06E-08	2.89E-08	3.26E-08	1.52E-08	1.64E-08	1.01E-07	2.60E-08
DLX6	0	0	0	0	0	0	1.31E-08	0

DLX6AS	0	0	0	0	0	0	0	0
DMAP1	2.15E-05	1.86E-05	1.55E-05	1.93E-05	3.03E-05	2.06E-05	1.79E-05	1.92E-05
DMBT1	2.55E-07	0	9.25E-09	6.74E-08	2.30E-08	8.26E-09	1.73E-08	1.26E-07
DMBX1	6.48E-08	0	0	0	0	0	0	1.19E-08
DMC1	1.04E-07	0	2.40E-08	5.62E-08	2.96E-08	7.44E-08	5.54E-08	0
DMD	1.41E-05	5.26E-06	2.64E-05	1.65E-05	3.40E-06	2.80E-05	8.93E-06	1.01E-05
DMGDH	1.51E-05	9.64E-05	0.000114	5.53E-05	7.46E-06	8.79E-05	1.29E-05	3.26E-05
DMKN	9.28E-05	0	3.49E-06	1.45E-05	2.17E-05	9.95E-08	4.36E-07	2.41E-07
DMP1	0	0	0	0	0	8.22E-09	0	0
DMPK	6.06E-06	6.39E-06	1.54E-06	5.73E-06	3.52E-06	2.69E-06	2.63E-06	2.53E-06
DMRT1	0	0	0	0	0	0	0	0
DMRT2	0	0	1.28E-08	0	0	0	0	0
DMRT3	0	0	0	0	0	0	3.16E-08	0
DMRTA1	1.60E-06	3.12E-06	7.69E-06	5.17E-06	1.28E-06	8.73E-06	4.06E-06	2.11E-06
DMRTA2	0	0	0	0	0	0	0	0
DMRTB1	0	0	0	0	0	0	0	0
DMRTC1	0	0	0	0	0	0	0	0
DMRTC1B	2.52E-08	0	2.59E-08	8.82E-08	0	0	0	0
DMRTC2	0	0	0	0	0	0	0	0
DMTF1	1.49E-05	6.73E-06	2.48E-06	4.13E-06	1.47E-06	4.30E-06	2.33E-06	4.90E-06
DMWD	4.60E-06	4.62E-06	2.44E-06	4.40E-06	2.74E-06	4.97E-06	4.95E-06	4.40E-06
DMXL1	1.14E-05	6.57E-06	6.32E-06	5.63E-06	2.09E-06	6.64E-06	3.25E-06	6.41E-06
DMXL2	9.64E-06	3.98E-06	3.14E-06	6.17E-06	2.79E-06	5.20E-06	3.33E-06	5.21E-06
DNA2	1.72E-06	5.75E-07	2.74E-07	2.82E-07	1.37E-07	5.51E-07	2.56E-07	6.76E-07
DNAH1	3.05E-06	6.23E-06	1.08E-06	9.39E-07	4.98E-07	1.11E-06	8.73E-07	1.25E-06
DNAH10	4.89E-08	4.64E-07	2.11E-08	5.91E-08	0	6.71E-08	1.09E-07	2.01E-07
DNAH11	4.75E-09	2.07E-07	9.57E-09	6.74E-08	5.62E-09	1.17E-08	2.68E-08	3.38E-07
DNAH12	0	1.99E-07	1.22E-08	1.26E-08	1.77E-08	4.35E-06	9.98E-09	0
DNAH14	1.52E-06	4.01E-06	7.19E-07	1.28E-06	7.15E-08	2.61E-06	1.04E-06	5.38E-06
DNAH17	4.48E-07	8.39E-08	2.78E-08	1.94E-07	5.37E-08	9.09E-08	1.24E-07	1.18E-07
DNAH2	9.76E-08	9.98E-09	7.29E-08	1.28E-07	9.78E-09	1.44E-08	4.90E-08	4.90E-08
DNAH3	0	2.20E-06	0	0	0	4.45E-09	4.68E-09	0
DNAH5	2.04E-05	4.79E-08	6.49E-07	1.37E-06	8.73E-08	1.22E-06	1.27E-06	4.63E-06
DNAH6	1.32E-07	1.47E-06	1.83E-06	1.49E-07	3.73E-06	2.33E-07	2.76E-07	1.07E-07
DNAH7	2.01E-08	0	4.59E-08	6.32E-08	2.31E-08	0	1.30E-08	5.19E-09
DNAH8	1.37E-08	1.69E-09	1.54E-09	1.85E-09	1.30E-09	5.59E-08	1.48E-09	4.62E-08
DNAH9	7.34E-09	0	1.63E-09	7.35E-08	0	2.72E-08	2.28E-08	0
DNAI1	9.31E-09	5.24E-07	1.93E-08	4.56E-08	4.00E-08	0	1.71E-07	0
DNAI2	0	0	0	0	0	0	0	0
DNAJA1	8.80E-05	0.000208	0.000118	0.000123	2.98E-05	0.000308	9.80E-05	0.000193
DNAJA2	3.70E-05	3.08E-05	2.41E-05	2.17E-05	4.97E-06	4.10E-05	1.75E-05	2.57E-05
DNAJA3	3.69E-05	8.23E-05	4.05E-05	2.54E-05	1.02E-05	5.30E-05	3.64E-05	3.65E-05
DNAJA4	9.73E-06	6.96E-07	5.52E-07	9.53E-07	2.19E-07	4.60E-07	1.12E-06	5.99E-07
DNAJB1	5.67E-05	4.43E-05	4.16E-05	9.82E-05	3.77E-05	4.39E-05	8.57E-05	6.47E-05
DNAJB11	9.96E-05	9.40E-05	3.16E-05	3.87E-05	6.74E-05	7.20E-05	5.37E-05	8.35E-05
DNAJB12	2.27E-05	1.61E-05	1.48E-05	1.49E-05	1.19E-05	1.64E-05	1.97E-05	1.77E-05
DNAJB13	2.19E-07	0	0	0	0	1.19E-08	0	1.90E-08
DNAJB14	6.78E-06	2.63E-06	2.94E-06	2.91E-06	3.61E-07	4.62E-06	1.24E-06	3.69E-06
DNAJB2	0.000181	3.96E-05	2.83E-05	4.06E-05	7.20E-05	3.97E-05	6.88E-05	4.28E-05
DNAJB3	1.81E-07	1.62E-07	6.49E-08	7.44E-08	0	1.69E-07	1.93E-08	5.97E-08
DNAJB4	2.53E-05	7.83E-06	1.12E-05	7.56E-06	1.02E-06	2.56E-05	6.95E-06	4.36E-06
DNAJB5	4.13E-06	3.76E-06	1.23E-06	4.18E-06	1.08E-06	2.73E-06	3.30E-06	3.21E-06
DNAJB6	3.07E-05	2.61E-05	1.26E-05	2.23E-05	1.62E-05	2.03E-05	2.67E-05	3.30E-05
DNAJB7	9.87E-09	0	2.02E-08	1.19E-08	0	0	1.85E-08	1.47E-08
DNAJB8	0	0	0	0	0	0	0	0
DNAJB9	4.67E-05	3.98E-05	6.18E-05	3.16E-05	1.44E-05	7.14E-05	2.43E-05	9.67E-05
DNAJC1	5.51E-05	9.98E-06	1.89E-05	2.55E-05	2.22E-05	1.37E-05	1.56E-05	2.03E-05
DNAJC10	1.74E-05	1.18E-05	6.60E-06	1.01E-05	2.44E-06	1.71E-05	6.53E-06	8.26E-06

DNAJC11	2.25E-05	2.14E-05	1.38E-05	1.42E-05	8.50E-06	2.07E-05	1.28E-05	1.79E-05
DNAJC12	2.15E-06	6.08E-06	2.97E-05	2.90E-05	8.22E-06	5.17E-05	5.06E-05	1.18E-06
DNAJC13	9.87E-06	7.00E-06	4.52E-06	4.76E-06	8.37E-07	6.35E-06	3.30E-06	3.91E-06
DNAJC14	1.92E-05	1.94E-05	9.45E-06	1.01E-05	3.02E-06	1.56E-05	8.33E-06	1.27E-05
DNAJC15	9.06E-06	4.63E-07	1.49E-05	1.03E-05	1.44E-06	1.82E-05	1.42E-05	1.26E-05
DNAJC16	8.06E-06	5.14E-06	7.58E-06	4.66E-06	1.68E-06	6.84E-06	3.89E-06	5.15E-06
DNAJC17	1.91E-05	1.66E-05	1.21E-05	1.84E-05	1.96E-05	1.54E-05	2.66E-05	2.24E-05
DNAJC18	1.60E-06	3.49E-07	1.14E-07	4.84E-07	9.46E-08	4.69E-07	3.40E-07	1.99E-07
DNAJC19	2.73E-05	3.04E-05	2.97E-05	2.31E-05	2.05E-05	2.97E-05	2.17E-05	1.69E-05
DNAJC2	1.11E-05	2.46E-05	6.14E-06	1.23E-05	7.33E-06	1.68E-05	1.14E-05	1.68E-05
DNAJC21	2.33E-05	2.62E-05	1.38E-05	1.48E-05	2.33E-06	1.77E-05	1.03E-05	2.15E-05
DNAJC22	3.63E-05	1.13E-05	2.09E-05	1.19E-05	2.95E-06	9.66E-06	6.82E-06	1.91E-05
DNAJC24	3.79E-06	3.92E-06	3.22E-06	3.55E-06	2.14E-06	6.17E-06	3.49E-06	6.64E-06
DNAJC25	1.29E-05	1.01E-05	2.16E-05	1.10E-05	1.08E-05	1.13E-05	6.29E-06	6.89E-06
DNAJC25-	0	7.96E-08	0	0	0	3.71E-08	0	0
DNAJC27	2.94E-06	1.18E-06	4.55E-07	9.25E-07	2.73E-07	8.02E-07	3.89E-07	6.50E-07
DNAJC28	9.17E-07	6.67E-07	1.25E-06	1.11E-06	7.51E-07	1.52E-06	4.38E-07	1.37E-06
DNAJC3	2.55E-05	3.21E-05	2.53E-05	1.72E-05	7.87E-06	4.04E-05	1.71E-05	2.94E-05
DNAJC30	9.85E-06	1.24E-05	8.24E-06	7.68E-06	1.43E-05	1.35E-05	9.65E-06	1.23E-05
DNAJC4	2.97E-05	6.87E-05	2.49E-05	2.28E-05	3.56E-05	1.41E-05	3.20E-05	3.08E-05
DNAJC5	4.11E-05	1.94E-05	1.13E-05	1.65E-05	2.56E-05	1.64E-05	3.49E-05	2.30E-05
DNAJC5B	9.34E-07	6.24E-08	2.53E-07	1.34E-06	7.85E-08	2.03E-07	7.34E-07	5.39E-08
DNAJC5G	8.37E-08	0	0	0	0	0	0	0
DNAJC6	8.93E-06	8.10E-07	5.99E-08	6.57E-07	2.04E-07	4.65E-06	1.75E-07	2.64E-06
DNAJC7	4.93E-05	5.65E-05	2.69E-05	3.50E-05	3.54E-05	8.22E-05	3.66E-05	3.12E-05
DNAJC8	7.75E-05	4.49E-05	3.15E-05	4.38E-05	2.55E-05	4.87E-05	3.88E-05	3.74E-05
DNAJC9	4.48E-05	9.76E-06	7.31E-06	1.24E-05	9.32E-06	9.73E-06	1.40E-05	9.58E-06
DNAL1	1.18E-06	7.98E-07	6.67E-07	1.34E-06	2.14E-07	1.08E-06	9.39E-07	7.62E-07
DNAL4	9.81E-06	6.46E-06	5.12E-06	9.08E-06	7.45E-06	1.04E-05	1.12E-05	6.25E-06
DNALI1	3.72E-06	1.26E-05	7.36E-06	1.65E-05	1.39E-06	5.14E-06	1.02E-05	4.10E-06
DNASE1	6.87E-07	3.10E-07	6.94E-08	1.00E-07	1.60E-07	1.87E-07	1.52E-07	1.97E-07
DNASE1L1	9.28E-06	6.84E-06	5.04E-06	4.55E-06	1.84E-06	6.72E-06	4.78E-06	7.16E-06
DNASE1L2	3.31E-07	4.27E-07	0	9.59E-08	1.68E-07	2.13E-07	6.26E-08	1.15E-07
DNASE1L3	5.03E-05	7.76E-06	0.000121	6.40E-05	2.06E-05	7.96E-06	2.65E-05	4.60E-06
DNASE2	5.79E-05	2.64E-05	2.74E-05	3.55E-05	2.35E-05	2.53E-05	2.68E-05	4.34E-05
DNASE2B	7.56E-07	1.02E-07	9.63E-08	1.60E-06	0	0	8.57E-07	8.68E-08
DND1	3.28E-06	2.52E-06	1.33E-06	1.76E-06	5.75E-07	1.97E-06	2.01E-06	2.61E-06
DNER	7.75E-08	2.13E-06	0	0	0	0	6.84E-09	1.03E-08
DNHD1	3.43E-06	4.81E-06	4.76E-07	5.25E-07	8.72E-07	1.40E-06	7.08E-07	1.00E-06
DNLZ	1.59E-05	9.61E-06	5.88E-06	1.07E-05	4.44E-05	8.35E-06	1.70E-05	1.75E-05
DNM1	1.51E-05	5.58E-06	9.86E-07	6.77E-07	2.27E-07	4.30E-07	3.59E-07	2.96E-07
DNM1L	1.51E-05	1.19E-05	4.73E-06	7.17E-06	2.60E-06	8.95E-06	6.22E-06	1.02E-05
DNM1P35	1.65E-07	2.65E-07	1.49E-08	8.19E-08	9.84E-08	1.44E-07	1.06E-07	3.63E-08
DNM2	4.80E-05	2.92E-05	2.38E-05	3.00E-05	1.91E-05	3.59E-05	3.00E-05	2.59E-05
DNM3	4.50E-07	6.59E-07	2.34E-07	5.03E-07	2.22E-07	5.50E-07	1.56E-07	4.01E-07
DNMBP	1.17E-05	3.31E-06	6.11E-06	9.59E-06	4.53E-06	7.56E-06	7.00E-06	1.20E-06
DNMT1	1.73E-05	7.55E-06	3.58E-06	5.45E-06	2.80E-06	5.93E-06	5.28E-06	8.31E-06
DNMT3A	1.58E-05	9.51E-06	1.94E-06	3.49E-06	1.75E-06	3.98E-06	2.15E-06	4.01E-06
DNMT3B	1.06E-06	3.06E-07	3.11E-07	4.24E-07	5.42E-07	3.69E-07	5.82E-07	4.19E-07
DNMT3L	0	5.05E-07	6.63E-06	2.11E-07	1.10E-06	4.92E-07	7.46E-07	4.01E-07
DNPEP	6.11E-05	3.68E-05	3.76E-05	3.87E-05	3.14E-05	4.33E-05	4.83E-05	4.91E-05
DNTT	0	1.33E-08	0	0	0	3.92E-07	0	0
DNTTIP1	4.07E-05	2.19E-05	1.10E-05	1.82E-05	1.32E-05	2.07E-05	2.56E-05	1.50E-05
DNTTIP2	2.51E-05	2.95E-05	1.29E-05	2.78E-05	8.73E-06	4.55E-05	1.46E-05	2.17E-05
DOC2A	2.98E-06	0	0	3.00E-08	6.49E-08	2.68E-08	0	7.92E-08
DOC2B	1.72E-07	0	0	1.51E-07	0	2.29E-08	9.36E-08	3.65E-08
DOCK1	1.35E-05	4.69E-06	6.42E-06	9.30E-06	5.35E-07	9.22E-06	5.95E-06	6.72E-06
DOCK10	4.51E-06	4.50E-07	2.42E-06	3.15E-06	1.15E-07	1.40E-06	9.77E-07	7.71E-07

DOCK11	2.09E-06	2.03E-07	6.63E-07	1.39E-06	9.20E-08	7.52E-07	7.10E-07	1.68E-07
DOCK2	6.01E-06	3.87E-07	2.60E-06	6.39E-06	3.75E-07	2.94E-06	3.14E-06	1.14E-06
DOCK3	8.11E-08	2.54E-08	0	1.54E-08	4.34E-09	2.11E-08	9.89E-09	3.70E-09
DOCK4	6.20E-06	5.10E-06	4.86E-06	4.15E-06	1.55E-06	8.68E-06	3.17E-06	9.29E-06
DOCK5	4.20E-06	2.18E-06	1.85E-06	2.57E-06	1.94E-07	2.20E-06	4.62E-07	1.81E-06
DOCK6	1.47E-05	6.89E-06	5.60E-06	5.62E-06	1.12E-05	8.00E-06	1.40E-05	1.57E-05
DOCK7	6.45E-06	5.97E-06	4.00E-06	5.13E-06	7.73E-07	1.03E-05	2.50E-06	3.68E-06
DOCK8	7.22E-06	4.41E-07	2.28E-06	4.67E-06	1.24E-07	1.40E-06	1.08E-06	4.72E-07
DOCK9	1.18E-05	9.11E-06	5.87E-06	4.88E-06	1.51E-06	6.53E-06	6.24E-06	1.03E-05
DOHH	6.15E-06	4.78E-06	3.85E-06	7.72E-06	1.34E-05	6.95E-06	1.44E-05	4.10E-06
DOK1	1.12E-05	6.44E-07	1.86E-06	4.21E-06	1.65E-06	1.81E-06	3.72E-06	1.22E-06
DOK2	1.07E-05	1.32E-06	6.24E-06	1.20E-05	1.36E-06	5.21E-06	8.32E-06	1.92E-06
DOK3	5.59E-06	1.21E-06	1.29E-06	3.72E-06	7.02E-07	1.73E-06	3.23E-06	9.39E-07
DOK4	6.72E-05	2.12E-05	1.28E-05	1.63E-05	1.23E-05	1.55E-05	1.85E-05	2.83E-05
DOK5	6.92E-07	1.44E-07	6.96E-07	7.62E-07	4.48E-07	2.21E-07	8.21E-07	5.59E-07
DOK6	1.69E-07	2.36E-08	2.89E-07	1.23E-07	6.81E-09	1.22E-08	1.29E-08	2.32E-08
DOK7	5.17E-06	1.66E-06	2.57E-07	2.58E-06	9.89E-08	1.37E-06	1.19E-06	1.54E-08
DOLK	1.80E-05	7.54E-06	8.26E-06	9.34E-06	8.03E-06	1.14E-05	7.65E-06	2.80E-05
DOLPP1	1.12E-05	1.16E-05	1.70E-05	1.39E-05	8.84E-06	1.89E-05	1.25E-05	1.91E-05
DOM3Z	2.43E-05	2.64E-05	5.11E-06	8.95E-06	9.01E-06	8.45E-06	5.14E-06	1.18E-05
DONSON	1.88E-05	5.00E-06	2.83E-06	3.61E-06	3.67E-06	1.03E-05	4.83E-06	5.28E-06
DOPEY1	6.13E-06	3.76E-06	3.06E-06	2.81E-06	7.54E-07	3.64E-06	1.92E-06	1.95E-06
DOPEY2	3.49E-06	3.71E-06	5.16E-06	2.03E-06	3.68E-07	8.74E-07	1.42E-06	4.50E-06
DOT1L	6.51E-06	2.63E-06	1.58E-06	5.79E-06	2.45E-06	2.14E-06	4.30E-06	3.27E-06
DPAGT1	3.34E-05	1.58E-05	1.55E-05	1.96E-05	1.12E-05	3.81E-05	1.86E-05	4.30E-05
DPCD	7.69E-06	1.83E-06	2.57E-06	8.40E-06	5.37E-06	1.28E-05	8.53E-06	5.34E-06
DPCR1	4.26E-08	4.76E-09	1.74E-08	5.20E-09	0	0	0	0
DPEP1	2.37E-05	8.97E-08	1.00E-07	9.02E-07	2.71E-08	1.46E-08	9.92E-07	5.93E-07
DPEP2	1.72E-06	7.89E-07	1.70E-06	2.74E-06	4.65E-07	5.70E-07	2.20E-06	2.95E-07
DPEP3	1.40E-08	0	4.44E-08	1.72E-08	6.04E-08	6.52E-08	3.78E-07	2.07E-08
DPF1	2.13E-08	0	0	2.61E-08	9.17E-09	0	3.32E-08	0
DPF2	2.08E-05	1.37E-05	6.45E-06	7.71E-06	5.24E-06	7.92E-06	1.24E-05	1.26E-05
DPF3	1.25E-05	4.96E-07	1.10E-06	5.57E-07	1.98E-07	1.97E-07	2.75E-07	1.62E-07
DPH1	9.70E-06	1.47E-05	1.14E-05	1.62E-05	7.30E-06	1.56E-05	2.35E-05	1.29E-05
DPH2	7.75E-06	1.35E-05	4.57E-06	5.29E-06	2.64E-06	1.19E-05	9.34E-06	1.15E-05
DPH3	1.51E-05	1.12E-05	6.37E-06	9.46E-06	5.14E-06	1.15E-05	9.00E-06	9.71E-06
DPH3B	9.86E-07	6.27E-07	5.10E-07	3.35E-07	0	5.29E-07	2.82E-07	3.67E-07
DPH5	1.27E-05	1.77E-05	8.07E-06	8.37E-06	6.97E-06	2.85E-05	1.03E-05	8.84E-06
DPM1	4.67E-05	3.35E-05	2.44E-05	2.61E-05	1.63E-05	4.17E-05	2.33E-05	2.14E-05
DPM2	2.33E-05	2.04E-05	1.09E-05	2.13E-05	2.95E-05	2.36E-05	2.51E-05	5.20E-05
DPM3	0.000141	0.000116	0.000159	0.000124	0.000362	0.000241	9.72E-05	0.000204
DPP10	2.66E-06	1.15E-08	4.24E-08	0	0	0	0	0
DPP3	4.11E-05	1.70E-05	1.17E-05	1.26E-05	1.13E-05	2.27E-05	1.43E-05	2.50E-05
DPP4	1.74E-05	2.39E-05	1.42E-05	1.23E-05	4.69E-06	3.55E-05	1.62E-05	1.48E-05
DPP6	5.26E-09	0	1.08E-08	6.42E-09	0	9.74E-09	1.02E-08	7.70E-09
DPP7	7.90E-05	8.49E-06	7.04E-05	7.95E-05	2.25E-05	2.58E-05	8.98E-05	5.57E-05
DPP8	4.52E-06	2.59E-06	2.39E-06	2.29E-06	3.49E-07	2.89E-06	1.00E-06	1.81E-06
DPP9	3.85E-05	2.07E-05	1.80E-05	2.12E-05	1.64E-05	2.15E-05	2.17E-05	2.57E-05
DPPA2	0	0	0	0	0	0	0	0
DPPA3	0	0	0	0	0	0	0	0
DPPA4	0	4.19E-08	2.70E-07	4.04E-08	0	8.63E-09	0	1.37E-08
DPPA5	0	0	0	0	0	0	0	0
DPRX	0	7.93E-07	0	0	0	0	0	0
DPRXP4	9.12E-08	1.09E-07	0	1.14E-07	0	4.29E-08	1.71E-07	0
DPT	4.44E-07	1.30E-07	1.03E-05	4.83E-05	1.73E-06	1.06E-07	7.05E-07	2.90E-06
DPY19L1	1.41E-05	9.32E-06	6.78E-06	7.16E-06	2.43E-06	1.22E-05	3.84E-06	7.74E-06
DPY19L2	4.98E-08	1.77E-08	8.14E-08	4.56E-08	2.40E-08	1.35E-07	3.61E-08	1.37E-08
DPY19L2P	8.18E-09	0	8.47E-09	0	0	0	0	0

DPY19L2P	3.22E-07	4.20E-07	5.22E-08	3.31E-08	1.24E-08	1.34E-08	2.10E-08	0
DPY19L2P	1.19E-08	1.34E-08	0	0	0	2.20E-08	0	0
DPY19L3	4.11E-06	1.77E-06	2.05E-06	1.64E-06	7.01E-08	2.66E-06	3.92E-07	1.48E-06
DPY19L4	1.18E-05	1.06E-05	6.11E-06	4.79E-06	2.77E-06	5.39E-06	6.91E-06	5.18E-06
DPY30	0.000102	0.000132	4.59E-05	4.91E-05	3.15E-05	7.48E-05	4.48E-05	5.17E-05
DPYD	1.74E-05	2.06E-05	2.61E-05	1.03E-05	2.10E-06	1.61E-05	4.86E-06	2.02E-05
DPYS	0.000147	0.000216	0.000357	0.000102	0.000155	0.000184	0.000323	0.000107
DPYSL2	1.03E-05	7.64E-06	1.01E-05	1.49E-05	3.15E-06	1.64E-05	8.44E-06	5.80E-06
DPYSL3	6.39E-06	3.63E-06	9.11E-07	9.14E-06	5.48E-07	1.36E-06	6.22E-06	2.23E-06
DPYSL4	7.72E-08	9.66E-09	0	5.46E-07	2.21E-08	4.77E-08	5.82E-08	6.30E-08
DPYSL5	4.31E-07	0	0	0	0	0	0	0
DQX1	8.57E-08	1.65E-08	9.22E-09	1.78E-08	1.25E-08	0	1.72E-08	0
DR1	1.92E-05	1.82E-05	7.12E-06	9.14E-06	1.56E-06	1.32E-05	6.14E-06	8.35E-06
DRAM1	2.21E-05	3.32E-06	2.50E-06	5.74E-06	9.75E-07	4.81E-06	4.54E-06	2.60E-06
DRAM2	2.60E-05	2.65E-05	1.19E-05	1.71E-05	7.58E-06	1.98E-05	1.83E-05	8.42E-06
DRAP1	6.94E-05	7.96E-05	4.17E-05	6.49E-05	8.42E-05	7.57E-05	0.000101	0.000116
DRD1	6.82E-09	1.91E-07	1.62E-07	8.33E-08	0	3.60E-07	1.32E-08	2.00E-08
DRD2	0	0	0	0	0	0	0	0
DRD3	0	0	0	0	0	0	0	0
DRD4	1.35E-06	4.45E-07	5.93E-08	1.59E-07	3.35E-07	2.58E-07	3.91E-07	2.74E-08
DRD5	9.92E-09	6.71E-08	1.03E-08	0	0	0	0	1.46E-08
DRG1	5.37E-05	4.16E-05	2.50E-05	3.01E-05	3.47E-05	4.51E-05	3.81E-05	4.24E-05
DRG2	1.34E-05	3.49E-05	8.14E-06	1.62E-05	2.80E-05	2.28E-05	2.60E-05	1.87E-05
DRGX	0	0	0	0	0	0	0	0
DRP2	2.94E-08	1.09E-08	0	0	0	0	0	0
DSC1	0	1.21E-08	4.99E-08	0	0	2.25E-08	1.58E-08	0
DSC2	3.57E-06	5.16E-06	4.32E-06	5.16E-06	4.42E-07	2.79E-06	2.18E-06	6.47E-07
DSC3	6.44E-09	3.59E-09	1.18E-07	4.71E-08	0	5.95E-09	3.14E-09	4.23E-08
DSCAM	3.23E-09	1.80E-08	3.62E-08	0	0	0	1.04E-07	0
DSCAML1	4.60E-06	3.61E-09	9.91E-08	3.16E-08	1.11E-08	2.99E-09	6.30E-09	4.73E-09
DSCC1	4.65E-06	2.85E-06	2.94E-07	4.34E-07	7.72E-07	8.71E-07	1.21E-06	7.52E-07
DSCR10	0	0	0	0	0	0	0	0
DSCR3	2.09E-05	1.34E-05	1.71E-05	1.41E-05	6.83E-06	1.88E-05	1.33E-05	1.75E-05
DSCR4	5.49E-07	0	0	0	0	0	0	0
DSCR6	4.35E-07	1.41E-08	5.22E-08	3.05E-08	1.18E-07	1.15E-08	1.08E-07	7.32E-08
DSCR8	5.15E-06	0	0	0	0	0	0	6.20E-08
DSCR9	9.04E-08	0	4.89E-08	8.36E-08	1.95E-08	1.50E-07	2.16E-08	0
DSE	1.81E-05	4.93E-06	7.75E-06	9.11E-06	9.45E-06	4.69E-06	3.77E-06	1.75E-06
DSEL	3.20E-05	1.23E-06	1.13E-06	2.51E-06	5.22E-07	1.13E-07	1.47E-06	9.41E-08
DSG1	6.33E-09	2.21E-06	5.65E-06	4.88E-06	3.64E-07	1.49E-05	2.32E-06	9.43E-07
DSG2	2.41E-05	2.60E-05	7.74E-06	1.37E-05	6.33E-07	4.45E-07	5.58E-06	7.73E-07
DSG3	0	0	2.56E-07	0	0	0	7.89E-09	1.04E-07
DSG4	0	2.87E-08	1.32E-08	7.82E-09	0	1.78E-08	0	0
DSN1	2.73E-05	1.25E-05	2.42E-06	4.84E-06	4.79E-06	4.84E-06	4.73E-06	4.51E-06
DSP	3.38E-05	4.63E-05	3.16E-05	3.27E-05	8.20E-06	7.45E-05	2.86E-05	2.31E-05
DSPP	0	0	0	0	0	0	0	0
DST	6.21E-05	6.16E-05	3.93E-05	5.97E-05	2.20E-05	4.29E-05	4.77E-05	4.31E-05
DSTN	0.000258	0.00013	9.41E-05	0.000102	8.27E-05	0.000111	7.70E-05	8.05E-05
DSTYK	2.60E-06	4.91E-07	5.96E-07	9.20E-07	4.89E-07	2.72E-06	1.30E-06	1.92E-06
DTD1	2.67E-05	6.96E-06	7.11E-06	1.05E-05	2.34E-06	2.94E-05	1.92E-05	1.36E-06
DTHD1	2.57E-07	4.48E-08	6.49E-08	1.40E-07	1.69E-08	1.10E-07	0	0
DTL	3.93E-06	5.17E-06	2.16E-07	2.06E-07	4.48E-07	1.48E-06	2.96E-07	1.23E-06
DTNA	3.53E-07	3.17E-05	1.16E-06	1.00E-05	7.41E-08	1.13E-05	6.95E-06	1.61E-06
DTNB	5.35E-06	5.08E-06	2.43E-06	3.74E-06	4.46E-06	5.45E-06	5.99E-06	4.90E-06
DTNBP1	9.78E-06	6.55E-06	2.85E-06	7.08E-06	1.69E-06	4.99E-06	1.02E-05	6.66E-06
DTWD1	3.98E-06	3.40E-06	3.26E-06	3.96E-06	1.66E-06	5.00E-06	4.60E-06	2.41E-06
DTWD2	2.46E-06	1.32E-06	2.20E-06	1.61E-06	3.35E-07	1.53E-06	1.08E-06	1.65E-06
DTX1	2.59E-06	2.10E-06	1.10E-05	9.55E-06	1.95E-06	4.54E-08	8.45E-06	6.63E-06

DTX2	8.23E-06	9.55E-06	4.31E-06	7.65E-06	1.22E-05	7.03E-06	8.27E-06	7.95E-06
DTX3	6.62E-06	1.22E-06	1.25E-06	4.36E-06	1.60E-06	6.15E-07	1.07E-06	1.90E-06
DTX3L	2.54E-05	2.38E-05	2.40E-05	1.77E-05	9.19E-06	2.99E-05	1.58E-05	2.99E-05
DTX4	9.31E-06	2.54E-06	1.69E-06	2.97E-06	4.80E-06	2.24E-06	3.86E-06	4.31E-06
DTYMK	5.74E-05	2.39E-05	9.61E-06	1.45E-05	3.61E-05	1.96E-05	1.92E-05	1.73E-05
DULLARD	3.08E-05	1.81E-05	1.68E-05	3.63E-05	1.12E-05	3.04E-05	5.19E-05	2.80E-05
DUOX1	2.03E-05	6.37E-08	4.73E-08	2.33E-07	1.02E-07	2.30E-07	1.08E-07	9.83E-08
DUOX2	4.80E-05	5.90E-08	2.16E-08	1.55E-07	6.05E-09	1.63E-08	7.49E-07	1.39E-07
DUOXA1	1.00E-05	5.99E-08	1.37E-08	6.75E-08	0	0	0	2.45E-08
DUOXA2	4.09E-05	1.19E-07	1.85E-08	1.89E-07	0	3.24E-08	1.22E-06	3.60E-07
DUPD1	0	0	0	5.71E-08	0	0	0	0
DUS1L	8.24E-05	0.000211	5.59E-05	4.45E-05	9.32E-05	0.000113	8.21E-05	6.76E-05
DUS2L	8.42E-06	5.44E-06	4.06E-06	5.18E-06	5.57E-06	9.49E-06	6.47E-06	1.10E-05
DUS3L	1.29E-05	8.98E-06	9.02E-06	1.22E-05	1.58E-05	1.08E-05	1.72E-05	2.05E-05
DUS4L	2.86E-06	5.74E-06	2.20E-06	1.95E-06	8.61E-07	3.26E-06	1.67E-06	3.05E-06
DUSP1	0.000287	0.000249	0.000793	0.000674	6.06E-05	0.000197	0.000328	0.000314
DUSP10	1.67E-05	4.58E-05	5.65E-05	4.03E-05	8.26E-06	1.75E-05	1.77E-05	1.74E-05
DUSP11	2.60E-05	1.23E-05	8.59E-06	1.12E-05	3.69E-06	1.12E-05	1.07E-05	8.05E-06
DUSP12	9.57E-06	1.39E-05	4.43E-06	6.18E-06	2.07E-06	9.80E-06	6.68E-06	8.08E-06
DUSP13	9.11E-08	2.13E-07	0	0	0	4.84E-08	0	0
DUSP14	1.92E-05	1.45E-05	1.22E-05	2.24E-05	1.09E-05	1.10E-05	1.26E-05	7.55E-06
DUSP15	6.67E-06	2.48E-06	1.16E-07	1.07E-07	9.41E-08	4.06E-08	2.50E-07	0
DUSP16	8.57E-05	2.60E-05	3.32E-05	5.87E-05	1.24E-05	6.66E-05	4.13E-05	5.74E-05
DUSP18	2.88E-06	7.42E-07	6.12E-07	8.14E-07	1.47E-07	9.62E-07	7.14E-07	8.33E-07
DUSP19	9.87E-07	7.47E-07	5.33E-07	3.21E-07	1.30E-07	9.39E-07	1.90E-07	2.60E-07
DUSP2	5.56E-06	7.46E-07	4.07E-06	2.61E-05	2.64E-07	4.17E-06	4.08E-06	8.39E-07
DUSP21	0	0	0	0	0	0	0	0
DUSP22	2.06E-05	1.35E-05	1.20E-05	1.15E-05	6.57E-06	2.07E-05	1.45E-05	1.43E-05
DUSP23	9.56E-05	0.000363	6.22E-05	8.90E-05	0.000142	0.000257	0.000142	0.000238
DUSP26	4.03E-07	0	3.16E-08	5.50E-08	1.03E-07	4.16E-08	0	1.32E-07
DUSP27	5.64E-09	1.26E-08	0	0	9.69E-09	4.18E-08	2.20E-08	0
DUSP28	3.24E-06	1.28E-06	5.93E-07	8.30E-07	1.39E-06	1.01E-06	1.58E-06	4.98E-07
DUSP3	0.000135	5.97E-05	4.31E-05	4.23E-05	3.94E-05	8.40E-05	7.61E-05	6.54E-05
DUSP4	5.59E-06	1.48E-07	2.44E-07	4.45E-06	1.78E-07	4.81E-07	3.91E-07	2.62E-07
DUSP5	3.88E-05	4.56E-06	1.47E-05	7.42E-05	2.09E-06	3.21E-06	1.20E-05	9.33E-06
DUSP5P	5.89E-07	2.14E-07	9.88E-09	0	8.17E-09	0	2.76E-08	1.40E-08
DUSP6	2.98E-05	4.24E-05	0.000108	0.000151	6.99E-06	1.61E-05	7.51E-05	7.37E-05
DUSP7	5.50E-06	4.10E-06	2.31E-06	2.83E-06	1.44E-06	3.36E-06	4.52E-06	3.61E-06
DUSP8	3.06E-06	4.68E-06	1.02E-06	6.80E-06	4.43E-07	3.42E-07	1.07E-05	5.81E-06
DUSP9	7.84E-06	4.03E-06	2.68E-07	1.46E-07	1.14E-06	3.59E-06	4.16E-06	1.83E-06
DUT	2.27E-05	1.15E-05	9.81E-06	1.38E-05	1.95E-05	3.89E-05	2.60E-05	8.70E-06
DUX4	0	0	0	0	0	0	0	0
DUXA	0	0	0	0	0	0	0	0
DVL1	3.50E-05	1.87E-05	1.90E-05	1.96E-05	1.43E-05	2.43E-05	3.21E-05	1.74E-05
DVL2	6.13E-06	6.51E-06	3.76E-06	4.07E-06	1.07E-06	9.51E-06	5.68E-06	4.90E-06
DVL3	2.01E-05	1.40E-05	6.74E-06	8.89E-06	4.31E-06	1.10E-05	9.67E-06	9.79E-06
DVWA	0	1.60E-07	0	0	0	1.95E-08	0	0
DYDC1	8.07E-08	1.60E-08	7.91E-09	0	0	3.67E-08	0	0
DYDC2	9.46E-07	2.08E-06	1.38E-08	8.05E-08	2.53E-07	4.84E-06	3.80E-08	1.78E-06
DYM	1.27E-05	5.01E-06	4.37E-06	7.00E-06	2.81E-06	9.41E-06	6.24E-06	8.42E-06
DYNC1H1	3.06E-05	1.08E-05	9.34E-06	1.14E-05	5.80E-06	1.43E-05	9.18E-06	1.26E-05
DYNC1I1	2.08E-07	6.50E-08	7.70E-08	2.32E-07	4.07E-08	1.23E-05	2.00E-07	2.32E-08
DYNC1I2	4.66E-05	1.91E-05	1.22E-05	1.76E-05	3.59E-06	2.25E-05	1.38E-05	1.49E-05
DYNC1LI1	1.64E-05	1.56E-05	7.60E-06	1.12E-05	4.30E-06	1.04E-05	9.01E-06	1.18E-05
DYNC1LI2	2.61E-05	9.09E-06	1.14E-05	1.20E-05	6.19E-06	1.50E-05	8.07E-06	8.36E-06
DYNC2H1	1.66E-06	1.38E-07	2.17E-07	4.45E-07	1.68E-08	5.48E-07	1.55E-07	6.41E-07
DYNC2LI1	1.43E-05	1.03E-05	4.07E-06	6.33E-06	2.84E-06	9.67E-06	3.90E-06	5.28E-06
DYNLL1	0.00042	0.000332	0.0001	0.000197	0.00021	0.000159	0.000258	0.000223

DYNLL2	4.97E-05	5.83E-05	5.48E-05	4.46E-05	9.16E-06	9.67E-05	4.68E-05	4.02E-05
DYNLRB1	0.00028	0.00017	8.78E-05	0.000147	0.000251	0.000138	0.000202	0.000134
DYNLRB2	2.92E-07	2.66E-07	1.79E-07	3.59E-07	4.37E-07	3.74E-06	4.53E-07	0
DYNLT1	0.000139	6.50E-05	4.49E-05	0.000102	4.92E-05	8.91E-05	7.83E-05	5.15E-05
DYNLT3	1.88E-05	1.75E-05	1.35E-05	1.77E-05	5.93E-06	2.08E-05	1.21E-05	2.59E-05
DYRK1A	2.01E-05	6.99E-06	9.27E-06	1.26E-05	3.48E-06	1.08E-05	9.31E-06	1.16E-05
DYRK1B	3.79E-06	3.38E-06	4.00E-06	4.52E-06	5.80E-06	8.00E-06	4.13E-06	3.91E-06
DYRK2	5.32E-06	1.25E-06	1.15E-06	2.27E-06	1.95E-07	1.17E-06	1.49E-06	9.11E-07
DYRK3	1.94E-06	6.65E-07	9.53E-07	2.55E-06	2.64E-07	1.58E-06	1.36E-06	2.40E-06
DYRK4	1.05E-05	7.98E-06	1.02E-05	1.74E-05	2.42E-06	1.79E-05	1.69E-05	1.01E-05
DYSF	2.25E-05	1.36E-05	1.18E-05	7.57E-06	8.18E-06	1.62E-05	1.45E-05	1.01E-05
DYSFIP1	1.06E-07	6.16E-08	4.00E-08	4.41E-08	1.68E-07	9.97E-08	2.68E-07	0
DYTN	0	0	0	0	0	0	0	0
DYX1C1	9.09E-09	4.04E-08	1.83E-08	2.67E-07	2.20E-08	1.73E-08	1.88E-07	1.63E-07
DZIP1	3.44E-06	1.17E-07	4.66E-07	5.36E-07	1.31E-07	1.28E-07	2.00E-07	2.27E-07
DZIP1L	1.82E-06	7.37E-09	2.64E-07	3.45E-07	5.64E-08	6.69E-08	1.66E-07	1.06E-07
DZIP3	5.03E-06	7.11E-06	1.58E-06	2.15E-06	1.27E-06	4.14E-06	3.23E-06	1.73E-06
E2F1	1.01E-05	6.80E-06	2.24E-07	4.12E-07	5.05E-06	2.26E-06	1.27E-06	3.64E-06
E2F2	1.94E-06	4.22E-07	3.11E-08	5.29E-08	1.79E-07	1.85E-07	1.39E-07	1.84E-07
E2F3	5.30E-06	5.84E-06	9.69E-07	2.71E-06	6.61E-07	3.12E-06	1.85E-06	4.06E-06
E2F4	2.60E-05	1.30E-05	9.44E-06	1.59E-05	6.90E-06	1.97E-05	1.63E-05	1.79E-05
E2F5	1.28E-05	6.94E-06	1.24E-06	2.24E-06	9.19E-07	2.57E-06	3.78E-06	8.24E-07
E2F6	6.65E-06	5.40E-06	1.81E-06	3.55E-06	1.28E-06	3.73E-06	2.87E-06	2.83E-06
E2F7	1.47E-06	5.59E-07	1.91E-08	4.12E-08	2.12E-08	1.57E-07	2.22E-08	9.06E-08
E2F8	2.83E-06	1.94E-06	2.77E-08	8.10E-08	9.28E-08	9.68E-07	7.17E-08	2.26E-07
E4F1	1.14E-05	1.10E-05	5.32E-06	7.40E-06	4.08E-06	1.31E-05	1.24E-05	7.45E-06
EAF1	1.81E-05	1.68E-05	6.53E-06	1.07E-05	1.31E-06	1.06E-05	7.84E-06	9.36E-06
EAF2	2.77E-06	2.14E-06	2.11E-06	2.15E-06	5.82E-07	2.59E-06	1.64E-06	1.13E-06
EAPP	4.68E-05	2.90E-05	2.30E-05	2.74E-05	1.12E-05	3.74E-05	3.10E-05	3.32E-05
EARS2	1.48E-05	2.20E-05	7.42E-06	5.37E-06	2.47E-06	1.39E-05	9.52E-06	8.37E-06
EBAG9	5.59E-05	3.12E-05	1.94E-05	1.51E-05	1.83E-05	2.49E-05	3.94E-05	2.72E-05
EBF1	9.48E-07	4.19E-07	6.16E-08	1.64E-07	4.54E-07	4.85E-07	5.58E-07	1.65E-06
EBF2	4.53E-08	2.90E-07	0	0	0	2.10E-07	1.20E-07	9.08E-08
EBF3	4.16E-08	3.76E-07	1.07E-08	2.54E-08	1.56E-07	8.18E-08	2.28E-07	8.37E-08
EBF4	1.83E-05	2.06E-07	1.98E-06	5.99E-06	5.60E-06	1.65E-07	4.13E-06	1.97E-06
EBI3	6.45E-06	3.45E-07	3.57E-06	4.89E-06	1.06E-06	6.01E-06	4.93E-06	1.16E-06
EBNA1BP2	5.09E-05	3.87E-05	1.99E-05	2.48E-05	1.40E-05	4.13E-05	3.26E-05	3.76E-05
EBP	0.000139	0.000628	0.000321	0.000168	0.000324	0.000138	0.000143	0.000245
EBPL	5.71E-05	7.98E-05	9.00E-05	7.77E-05	9.79E-05	0.000214	0.000109	5.89E-05
ECD	2.25E-05	1.32E-05	8.63E-06	7.18E-06	4.18E-06	1.33E-05	6.88E-06	8.63E-06
ECE1	5.56E-05	3.74E-05	3.38E-05	3.39E-05	1.81E-05	1.10E-05	2.91E-05	2.29E-05
ECE2	1.46E-05	2.14E-05	1.22E-05	1.23E-05	3.25E-05	1.46E-05	2.71E-05	3.19E-05
ECEL1	4.69E-08	2.86E-08	1.94E-07	3.11E-08	1.46E-08	0	2.47E-08	0
ECH1	0.000199	1.77E-05	0.000611	0.000434	0.000602	0.000352	0.000689	0.000446
ECHDC1	4.34E-05	4.18E-05	4.40E-05	2.99E-05	2.59E-05	4.65E-05	2.98E-05	2.60E-05
ECHDC2	5.07E-05	9.74E-05	0.000152	0.000157	0.000124	0.0001	0.000138	8.76E-05
ECHDC3	3.96E-05	7.72E-05	0.000102	7.49E-05	6.76E-05	0.000125	0.000106	3.36E-05
ECHS1	0.000164	0.00047	0.001145	0.000514	0.00086	0.000684	0.000605	0.000445
ECM1	6.81E-06	9.55E-07	3.53E-05	3.08E-05	2.32E-06	7.40E-07	3.43E-06	2.12E-06
ECM2	5.44E-06	8.83E-06	2.33E-05	2.08E-05	4.59E-06	5.50E-05	1.02E-05	1.69E-05
ECSCR	6.36E-06	3.73E-06	6.36E-07	2.63E-06	7.79E-06	2.76E-06	1.24E-05	4.61E-06
ECSIT	4.33E-05	4.82E-05	4.04E-05	3.44E-05	9.08E-05	4.34E-05	4.36E-05	4.25E-05
ECT2	1.01E-05	3.79E-06	4.53E-07	1.82E-06	8.10E-07	2.27E-06	7.67E-07	1.63E-06
ECT2L	1.77E-08	1.99E-08	2.43E-08	3.61E-08	5.07E-08	3.28E-08	1.09E-07	5.19E-08
EDA	2.76E-06	1.39E-07	5.30E-07	4.65E-07	1.36E-07	1.82E-07	3.36E-07	1.73E-06
EDA2R	1.36E-07	6.97E-08	9.34E-08	5.71E-07	9.05E-08	1.42E-06	4.17E-07	3.32E-08
EDAR	1.94E-07	6.46E-07	1.94E-07	1.71E-07	2.18E-07	3.15E-07	6.30E-08	3.95E-08
EDARADD	3.60E-06	4.06E-07	1.04E-06	3.08E-07	5.05E-08	3.27E-07	2.77E-07	3.94E-07

EDC3	1.20E-05	7.18E-06	2.63E-06	4.64E-06	4.00E-06	5.72E-06	6.34E-06	7.70E-06
EDC4	1.62E-05	1.15E-05	7.09E-06	9.97E-06	6.64E-06	1.31E-05	7.73E-06	1.08E-05
EDDM3A	0	0	0	0	0	8.08E-07	0	0
EDDM3B	0	0	0	0	0	0	2.92E-08	0
EDEM1	3.35E-05	2.45E-05	1.56E-05	1.40E-05	6.89E-06	2.69E-05	1.50E-05	1.73E-05
EDEM2	4.58E-05	2.54E-05	2.09E-05	2.17E-05	2.31E-05	3.31E-05	2.39E-05	2.31E-05
EDEM3	1.13E-05	2.08E-05	8.37E-06	6.56E-06	1.67E-06	1.60E-05	5.85E-06	2.16E-05
EDF1	0.000616	0.000478	0.000454	0.000546	0.000895	0.000628	0.001002	0.000742
EDIL3	1.87E-06	5.75E-07	2.45E-08	3.60E-07	0	3.21E-07	9.17E-08	5.80E-07
EDN1	5.55E-06	7.38E-07	1.75E-06	4.19E-06	2.84E-07	2.43E-06	1.79E-06	9.97E-07
EDN2	4.12E-08	1.10E-07	3.90E-07	9.72E-06	1.08E-07	3.91E-07	2.67E-07	1.22E-07
EDN3	1.97E-08	0	0	0	0	0	6.66E-08	0
EDNRA	2.82E-06	1.05E-06	6.77E-07	9.66E-07	5.69E-07	5.51E-07	7.85E-07	1.19E-06
EDNRB	8.91E-06	1.19E-05	1.99E-05	1.85E-05	3.81E-06	8.67E-06	3.16E-06	1.71E-05
EEA1	4.84E-06	4.85E-06	3.64E-06	2.94E-06	8.36E-07	5.04E-06	2.10E-06	2.88E-06
EED	1.24E-05	4.27E-06	3.50E-06	4.49E-06	2.03E-06	5.11E-06	5.58E-06	6.05E-06
EEF1A1	0.001075	0.000779	0.000583	0.001171	0.001352	0.001008	0.001638	0.000608
EEF1A1P9	0.000165	0.000144	0.000155	0.000233	8.12E-05	0.000208	0.000134	0.000115
EEF1A2	0.00022	6.20E-08	8.08E-07	2.93E-06	4.70E-08	2.53E-08	1.84E-07	0.000422
EEF1B2	0.000367	0.000232	0.000163	0.000247	0.000238	0.000246	0.000288	0.000129
EEF1D	0.000569	0.00034	0.000222	0.000339	0.001051	0.000276	0.000837	0.00036
EEF1DP3	2.01E-07	0	3.93E-08	2.96E-07	6.35E-08	8.56E-08	5.21E-08	1.09E-07
EEF1E1	1.65E-05	1.20E-05	5.31E-06	9.75E-06	3.94E-06	9.30E-06	1.04E-05	1.38E-05
EEF1G	0.000424	0.000401	0.000262	0.000346	0.000372	0.000372	0.000387	0.000355
EEF2	0.000605	0.000324	0.000549	0.00056	0.000291	0.000433	0.000729	0.000537
EEF2K	7.08E-06	4.88E-06	3.28E-06	2.99E-06	9.66E-07	3.41E-06	3.59E-06	2.80E-06
EEFSEC	9.86E-06	1.39E-05	1.41E-05	1.34E-05	1.98E-05	1.92E-05	1.70E-05	1.08E-05
EEPD1	1.20E-05	1.21E-05	1.81E-06	6.94E-06	3.49E-06	1.73E-06	7.34E-06	9.42E-06
EFCAB1	1.40E-07	9.11E-08	1.51E-07	1.16E-07	0	8.74E-08	3.40E-08	0
EFCAB10	3.56E-08	6.28E-08	0	0	1.41E-08	5.01E-08	1.81E-08	2.06E-07
EFCAB2	3.91E-06	8.39E-06	1.13E-06	1.29E-06	7.52E-07	3.93E-06	3.42E-06	2.38E-06
EFCAB3	1.60E-08	0	0	0	0	0	0	0
EFCAB4A	1.68E-05	7.02E-06	9.88E-07	3.01E-06	1.23E-06	7.65E-07	3.22E-06	4.29E-06
EFCAB4B	3.74E-07	9.64E-08	1.64E-07	3.74E-07	3.87E-07	2.91E-07	2.79E-07	1.49E-07
EFCAB5	6.11E-08	0	1.51E-08	3.73E-08	2.24E-08	2.40E-08	1.42E-08	0
EFCAB6	5.10E-09	1.12E-07	1.59E-07	2.52E-07	1.61E-08	4.64E-07	4.47E-08	3.68E-08
EFCAB7	2.02E-06	7.33E-07	2.71E-07	1.16E-06	1.03E-07	7.60E-07	5.71E-07	3.67E-07
EFEMP1	1.59E-05	1.19E-06	1.15E-06	4.08E-05	4.94E-06	1.86E-06	2.78E-05	9.12E-06
EFEMP2	1.40E-05	1.61E-06	4.81E-06	1.93E-05	6.99E-06	2.66E-06	1.28E-05	6.83E-06
EFHA1	1.85E-05	2.53E-05	1.86E-05	2.40E-05	1.60E-05	1.79E-05	2.10E-05	2.93E-05
EFHA2	1.89E-07	1.93E-06	2.17E-06	1.65E-06	1.37E-07	2.33E-06	4.72E-07	1.05E-06
EFHB	1.14E-06	1.22E-07	5.13E-08	7.24E-08	0	1.83E-08	0	1.45E-08
EFHC1	3.09E-06	1.97E-06	2.33E-07	1.24E-06	3.24E-07	1.02E-06	6.55E-07	5.39E-07
EFHC2	5.24E-07	3.98E-08	0	3.46E-08	0	0	0	0
EFHD1	5.89E-06	4.28E-05	4.70E-05	5.43E-05	5.55E-06	1.32E-05	0.00013	4.23E-05
EFHD2	4.52E-05	2.67E-05	1.54E-05	3.02E-05	9.58E-06	4.21E-05	2.00E-05	1.45E-05
EFNA1	0.000424	0.000301	0.000142	0.00011	0.000104	0.000266	0.000145	0.000504
EFNA2	5.58E-06	8.62E-07	1.40E-06	1.14E-06	4.17E-08	8.54E-07	4.19E-06	3.62E-07
EFNA3	6.03E-06	5.82E-06	3.59E-07	7.69E-07	2.54E-07	1.85E-06	1.32E-06	9.15E-07
EFNA4	1.02E-05	4.17E-06	3.79E-06	4.56E-06	3.64E-06	7.46E-06	4.73E-06	1.28E-05
EFNA5	4.36E-06	9.77E-08	1.77E-07	5.61E-07	7.24E-09	3.51E-08	8.21E-09	8.64E-08
EFNB1	9.37E-06	5.28E-06	6.95E-06	8.22E-06	3.72E-06	3.69E-06	1.36E-05	6.34E-06
EFNB2	6.34E-06	4.92E-06	2.48E-06	2.36E-06	1.63E-06	3.13E-06	4.18E-06	5.49E-06
EFNB3	2.72E-07	7.24E-08	6.59E-07	1.33E-06	1.48E-07	2.65E-08	3.90E-07	2.21E-07
EFR3A	2.12E-05	2.94E-05	1.54E-05	1.35E-05	6.17E-06	2.95E-05	2.40E-05	1.40E-05
EFR3B	2.85E-07	1.28E-07	4.57E-08	2.55E-08	2.05E-08	4.14E-08	1.18E-07	3.49E-08
EFS	3.54E-06	1.58E-07	4.00E-07	7.89E-07	1.46E-07	1.30E-07	3.75E-07	1.68E-07
EFTUD1	8.77E-06	6.57E-06	6.40E-06	1.04E-05	3.42E-06	9.14E-06	8.44E-06	1.42E-05

EFTUD2	4.28E-05	3.54E-05	1.22E-05	1.44E-05	1.24E-05	2.94E-05	1.61E-05	1.53E-05
EGF	2.24E-06	5.32E-09	0	1.44E-08	0	1.30E-06	2.28E-08	0
EGFL6	1.30E-06	8.93E-08	1.03E-08	2.63E-08	1.84E-08	1.98E-08	8.63E-08	7.27E-08
EGFL7	4.16E-05	1.10E-05	1.85E-05	2.12E-05	7.90E-05	1.72E-05	4.50E-05	3.59E-05
EGFL8	5.14E-06	2.57E-06	4.73E-07	1.72E-06	8.90E-06	7.32E-07	1.71E-06	3.07E-06
EGFLAM	9.83E-07	1.49E-06	1.66E-06	3.41E-06	1.36E-06	1.29E-06	1.53E-06	1.80E-06
EGFR	3.21E-05	4.68E-05	1.47E-05	5.82E-06	1.58E-06	6.29E-06	4.06E-06	2.04E-05
EGLN1	3.57E-05	5.68E-05	2.70E-05	3.39E-05	5.36E-06	2.45E-05	3.67E-05	6.17E-05
EGLN2	4.88E-05	3.16E-05	2.45E-05	3.43E-05	6.65E-05	1.92E-05	5.13E-05	3.53E-05
EGLN3	8.33E-06	3.24E-07	5.90E-07	3.46E-06	1.03E-07	2.14E-07	1.24E-06	9.71E-07
EGOT	3.24E-07	3.50E-07	3.63E-08	2.31E-07	1.47E-08	4.76E-08	8.20E-08	0
EGR1	2.52E-05	0.000322	0.000436	0.000292	5.89E-06	8.54E-06	0.000174	4.65E-05
EGR2	2.59E-06	8.53E-06	2.16E-05	9.33E-06	9.66E-08	5.76E-07	3.73E-06	5.89E-07
EGR3	6.02E-07	7.46E-07	9.23E-07	3.52E-06	6.52E-08	3.20E-07	5.89E-07	2.48E-07
EGR4	3.28E-07	0	3.10E-08	1.70E-07	0	0	1.20E-08	1.82E-08
EHBP1	1.59E-05	2.65E-05	1.54E-05	1.89E-05	8.08E-06	1.66E-05	8.94E-06	1.28E-05
EHBP1L1	1.60E-05	8.22E-06	4.55E-06	7.80E-06	1.17E-06	3.63E-06	5.64E-06	3.77E-06
EHD1	2.19E-05	1.46E-05	1.11E-05	2.25E-05	4.81E-06	9.94E-06	1.35E-05	1.23E-05
EHD2	3.61E-05	1.17E-05	4.35E-06	1.13E-05	8.88E-06	5.72E-06	1.68E-05	1.36E-05
EHD3	2.43E-06	5.99E-07	1.01E-05	2.24E-06	2.68E-07	6.00E-07	6.79E-07	1.28E-06
EHD4	3.26E-05	2.36E-05	7.85E-06	2.23E-05	4.34E-06	1.11E-05	2.59E-05	2.39E-05
EHF	1.54E-05	1.01E-06	1.05E-06	1.03E-06	2.64E-08	3.53E-07	1.44E-07	6.36E-06
EHHADH	4.74E-05	0.000146	0.00013	8.54E-05	4.56E-05	0.000175	7.82E-05	6.16E-05
EHMT1	1.23E-05	6.77E-06	9.02E-06	8.00E-06	4.73E-06	9.17E-06	7.61E-06	7.79E-06
EHMT2	2.06E-05	3.27E-05	8.46E-06	1.21E-05	1.74E-05	1.74E-05	1.60E-05	1.53E-05
EI24	5.25E-05	5.27E-05	0.000115	5.72E-05	4.95E-05	0.000123	7.97E-05	0.000105
EID1	0.000103	6.47E-05	6.58E-05	4.84E-05	2.73E-05	6.18E-05	3.92E-05	5.09E-05
EID2	1.64E-05	7.24E-06	6.30E-06	8.48E-06	1.08E-05	9.37E-06	8.86E-06	7.45E-06
EID2B	8.54E-07	2.64E-07	2.99E-07	3.65E-07	4.34E-07	5.89E-07	4.37E-07	4.57E-07
EID3	7.15E-07	3.78E-07	2.23E-07	7.29E-07	1.50E-07	3.73E-07	1.84E-07	2.32E-07
EIF1	0.000619	0.000653	0.0003	0.000531	0.000543	0.00056	0.000982	0.000371
EIF1AD	1.02E-05	8.98E-06	3.79E-06	6.41E-06	2.95E-06	6.99E-06	7.79E-06	1.22E-05
EIF1AX	2.66E-05	1.15E-05	1.95E-05	1.24E-05	6.31E-06	1.40E-05	1.34E-05	1.77E-05
EIF1AY	0	2.10E-05	0	1.81E-05	1.23E-05	2.76E-05	2.28E-05	1.76E-05
EIF1B	5.16E-05	5.19E-05	3.34E-05	4.72E-05	3.52E-05	4.02E-05	4.91E-05	2.79E-05
EIF2A	2.17E-05	4.31E-05	1.70E-05	1.63E-05	6.80E-06	3.13E-05	1.33E-05	1.62E-05
EIF2AK1	7.59E-05	9.16E-05	3.45E-05	3.66E-05	3.56E-05	6.00E-05	2.72E-05	5.84E-05
EIF2AK2	1.01E-05	9.19E-06	4.36E-06	3.12E-06	5.85E-07	4.51E-06	2.09E-06	8.61E-06
EIF2AK3	7.74E-06	5.97E-06	4.81E-06	6.26E-06	8.51E-07	5.09E-06	3.39E-06	5.19E-06
EIF2AK4	2.88E-05	1.25E-05	1.73E-05	4.46E-05	1.91E-05	3.02E-05	2.37E-05	1.15E-05
EIF2B1	3.54E-05	4.50E-05	1.84E-05	2.01E-05	1.47E-05	3.66E-05	2.24E-05	2.56E-05
EIF2B2	2.80E-05	1.63E-05	9.49E-06	1.48E-05	8.82E-06	2.34E-05	1.69E-05	2.41E-05
EIF2B3	1.41E-05	1.51E-05	1.25E-05	1.36E-05	9.09E-06	2.52E-05	1.39E-05	1.88E-05
EIF2B4	3.46E-05	4.05E-05	1.32E-05	2.03E-05	1.77E-05	2.63E-05	2.50E-05	1.77E-05
EIF2B5	2.73E-05	2.26E-05	1.45E-05	1.87E-05	3.91E-05	2.36E-05	2.38E-05	2.03E-05
EIF2C1	4.32E-06	3.69E-06	2.27E-06	2.59E-06	1.49E-06	3.60E-06	2.46E-06	4.09E-06
EIF2C2	3.47E-06	4.12E-06	9.75E-07	1.75E-06	7.36E-07	1.40E-06	2.43E-06	3.38E-06
EIF2C3	2.68E-06	1.44E-06	1.20E-06	1.10E-06	1.08E-07	9.53E-07	4.87E-07	9.58E-07
EIF2C4	3.91E-06	1.25E-06	2.36E-06	2.26E-06	4.73E-07	2.44E-06	1.68E-06	1.22E-06
EIF2S1	1.84E-05	1.19E-05	1.26E-05	1.41E-05	4.63E-06	1.77E-05	1.35E-05	1.62E-05
EIF2S2	4.58E-05	3.66E-05	2.06E-05	2.49E-05	2.77E-05	2.74E-05	2.38E-05	2.45E-05
EIF2S3	5.94E-05	2.92E-05	4.13E-05	4.04E-05	1.94E-05	5.17E-05	4.14E-05	5.54E-05
EIF3A	6.63E-05	4.96E-05	3.96E-05	5.75E-05	9.16E-06	6.97E-05	3.37E-05	3.97E-05
EIF3B	0.000117	0.000154	4.83E-05	5.33E-05	8.87E-05	7.76E-05	6.67E-05	4.42E-05
EIF3C	1.86E-05	4.68E-06	2.25E-07	1.40E-06	9.00E-63	2.80E-06	1.17E-06	1.62E-06
EIF3CL	0.000343	0.000225	7.27E-05	0.000132	7.26E-05	0.000141	0.000127	8.27E-05
EIF3D	0.000137	9.50E-05	5.58E-05	9.22E-05	0.000102	0.000129	0.000127	0.0001
EIF3E	0.000249	0.000126	0.000123	0.000118	0.000107	0.000212	0.000225	0.000123

EIF3F	8.70E-05	9.53E-05	6.07E-05	9.19E-05	8.46E-05	8.68E-05	0.000126	7.91E-05
EIF3G	0.000134	7.83E-05	8.79E-05	0.000125	0.000224	0.000125	0.000175	9.53E-05
EIF3H	0.000294	0.000147	8.56E-05	0.000109	7.93E-05	0.000144	0.000254	0.000103
EIF3I	0.00019	0.000161	8.28E-05	0.000122	5.18E-05	0.000146	0.000104	0.000198
EIF3IP1	1.90E-07	6.30E-08	1.49E-07	6.72E-08	0	5.07E-08	0	0
EIF3J	3.31E-05	3.19E-05	1.95E-05	2.41E-05	1.16E-05	2.59E-05	2.49E-05	2.47E-05
EIF3K	0.000313	0.00023	0.000127	0.000173	0.000391	0.00017	0.000202	0.000185
EIF3L	0.000223	9.31E-05	0.000129	0.000157	0.000151	0.00019	0.000155	0.000131
EIF3M	8.14E-05	0.000101	7.53E-05	9.43E-05	8.15E-05	0.000142	8.94E-05	0.000122
EIF4A1	0.000234	0.000143	0.000135	0.000235	6.30E-05	0.000329	0.000283	0.000238
EIF4A2	0.00021	0.000172	0.000125	0.000115	5.88E-05	0.000155	0.000123	5.61E-05
EIF4A3	9.80E-05	7.52E-05	2.67E-05	4.25E-05	2.62E-05	5.78E-05	4.79E-05	2.95E-05
EIF4B	6.26E-05	7.40E-05	7.19E-05	7.03E-05	2.32E-05	0.000118	7.63E-05	4.54E-05
EIF4E	6.45E-06	3.94E-06	4.85E-06	4.81E-06	2.75E-06	5.42E-06	4.79E-06	5.51E-06
EIF4E1B	1.22E-08	0	0	0	0	0	0	0
EIF4E2	0.000144	5.48E-05	4.78E-05	5.31E-05	7.37E-05	6.03E-05	7.69E-05	4.11E-05
EIF4E3	6.96E-06	4.49E-07	4.09E-06	3.68E-06	1.49E-07	3.48E-07	6.11E-07	1.52E-06
EIF4EBP1	3.43E-05	0.000116	4.46E-05	4.05E-05	3.88E-05	3.32E-05	4.57E-05	1.74E-05
EIF4EBP2	4.62E-05	4.12E-05	2.33E-05	2.19E-05	2.46E-05	3.57E-05	3.95E-05	2.35E-05
EIF4EBP3	3.33E-05	6.07E-05	4.35E-05	4.65E-05	3.43E-05	5.73E-05	9.76E-05	5.97E-05
EIF4ENIF1	8.68E-06	7.07E-06	4.13E-06	4.97E-06	2.69E-06	5.60E-06	5.64E-06	4.71E-06
EIF4G1	0.000139	0.000199	0.000157	0.000143	0.000132	0.00019	0.000164	0.000189
EIF4G2	0.000244	0.000193	0.000106	0.000141	3.38E-05	0.000162	8.71E-05	0.000145
EIF4G3	2.09E-05	1.67E-05	7.08E-06	8.89E-06	2.30E-06	1.28E-05	3.91E-06	1.30E-05
EIF4H	0.000112	0.000136	7.98E-05	7.76E-05	3.30E-05	8.86E-05	7.89E-05	8.69E-05
EIF5	6.80E-05	4.69E-05	5.99E-05	3.85E-05	1.33E-05	4.56E-05	4.38E-05	6.26E-05
EIF5A	0.000275	0.000231	0.000226	0.00027	9.67E-05	0.000341	0.000421	0.000219
EIF5A2	3.81E-06	1.55E-06	4.78E-07	9.36E-07	1.39E-08	9.39E-08	1.64E-06	9.14E-07
EIF5AL1	1.96E-05	1.34E-05	1.57E-05	1.89E-05	5.39E-06	2.51E-05	2.50E-05	2.03E-05
EIF5B	4.00E-05	2.56E-05	1.75E-05	1.92E-05	2.63E-05	3.54E-05	3.21E-05	1.58E-05
EIF6	0.000342	0.000275	0.000133	0.000183	0.000355	0.000282	0.000322	0.000128
ELAC1	3.48E-06	2.40E-06	3.01E-06	3.19E-06	1.32E-06	4.49E-06	3.13E-06	2.42E-06
ELAC2	1.69E-05	2.25E-05	2.32E-05	2.82E-05	1.08E-05	3.86E-05	3.04E-05	3.45E-05
ELANE	2.95E-08	0	6.53E-08	1.83E-07	7.68E-08	0	5.62E-08	4.41E-08
ELAVL1	1.45E-05	1.01E-05	9.76E-06	8.59E-06	5.79E-06	8.46E-06	9.79E-06	8.89E-06
ELAVL2	0	3.42E-08	6.28E-09	0	0	1.13E-08	5.33E-08	1.79E-08
ELAVL3	0	2.71E-08	0	0	0	4.49E-09	0	2.84E-08
ELAVL4	1.67E-07	0	5.41E-08	0	0	2.39E-08	1.24E-08	0
ELF1	1.93E-05	1.33E-05	1.16E-05	1.37E-05	6.60E-07	8.39E-06	6.31E-06	1.05E-05
ELF2	1.19E-05	5.21E-06	6.77E-06	6.38E-06	1.35E-06	6.88E-06	5.49E-06	7.36E-06
ELF3	7.36E-05	2.96E-05	1.86E-05	9.67E-05	9.70E-06	2.11E-05	5.50E-05	8.72E-05
ELF4	1.20E-05	5.98E-07	8.54E-07	2.35E-06	1.76E-07	6.81E-07	1.03E-06	7.92E-07
ELF5	2.05E-08	0	3.99E-08	4.36E-08	0	4.40E-08	0	1.75E-08
ELFN1	1.19E-06	6.96E-06	1.34E-05	8.28E-06	4.12E-06	1.14E-06	5.79E-06	4.18E-06
ELFN2	7.04E-07	7.99E-07	2.17E-08	5.83E-08	2.73E-08	0	2.59E-09	2.92E-06
ELK1	1.52E-05	1.25E-05	1.22E-05	8.66E-06	4.19E-06	1.53E-05	1.21E-05	2.42E-05
ELK3	7.20E-06	5.03E-06	3.37E-06	4.32E-06	3.61E-07	2.36E-06	2.33E-06	3.89E-06
ELK4	2.80E-06	1.43E-06	1.51E-06	1.51E-06	2.50E-07	3.66E-06	1.13E-06	2.73E-06
ELL	7.42E-06	4.54E-06	3.18E-06	8.90E-06	2.22E-06	4.03E-06	5.40E-06	5.02E-06
ELL2	2.57E-05	2.56E-05	2.35E-05	2.24E-05	7.55E-06	3.12E-05	1.46E-05	5.91E-05
ELL3	4.35E-06	7.44E-06	1.95E-06	2.41E-06	4.19E-06	2.16E-06	2.98E-06	2.42E-06
ELMO1	7.75E-06	1.34E-05	1.18E-05	1.41E-05	7.33E-06	1.98E-05	1.44E-05	2.69E-05
ELMO2	1.31E-05	9.16E-06	4.89E-06	6.29E-06	1.67E-06	7.73E-06	6.62E-06	7.56E-06
ELMO3	1.27E-05	1.76E-06	3.90E-06	6.68E-06	1.05E-06	7.71E-08	4.53E-06	2.14E-07
ELMOD1	1.56E-08	0	2.43E-08	4.78E-08	0	0	0	1.46E-06
ELMOD2	1.47E-05	6.06E-06	5.82E-06	7.38E-06	5.62E-06	1.06E-05	7.51E-06	1.34E-05
ELMOD3	9.87E-06	6.68E-06	3.36E-06	3.41E-06	6.35E-06	6.30E-06	5.80E-06	5.32E-06
ELN	7.53E-06	4.27E-06	3.15E-06	1.86E-05	4.21E-06	1.82E-06	4.76E-06	3.43E-06

ELOF1	5.68E-05	2.97E-05	3.95E-05	5.32E-05	6.81E-05	4.93E-05	6.17E-05	5.65E-05
ELOVL1	8.87E-05	5.25E-05	2.22E-05	3.54E-05	1.31E-05	3.20E-05	3.51E-05	5.51E-05
ELOVL2	3.75E-05	2.56E-05	3.06E-05	1.63E-05	3.01E-05	1.36E-05	1.67E-05	4.83E-05
ELOVL3	1.84E-06	1.11E-07	0	2.38E-08	1.67E-08	3.60E-08	9.29E-08	5.73E-08
ELOVL4	1.58E-07	0	2.74E-08	7.53E-08	3.02E-08	8.15E-09	5.15E-08	7.84E-08
ELOVL5	5.77E-05	0.000179	4.09E-05	2.62E-05	5.66E-06	4.30E-05	2.83E-05	0.000105
ELOVL6	1.06E-05	2.49E-05	3.02E-05	2.05E-05	3.09E-06	1.01E-05	3.67E-06	5.79E-05
ELOVL7	3.72E-05	2.07E-08	5.08E-07	1.93E-06	2.01E-07	1.71E-08	7.17E-08	8.10E-08
ELP2	2.42E-05	1.94E-05	2.05E-05	2.11E-05	7.68E-06	3.69E-05	1.77E-05	1.38E-05
ELP2P	5.23E-08	9.81E-08	9.05E-09	1.07E-08	1.12E-07	8.89E-08	2.54E-08	2.56E-08
ELP3	9.57E-06	2.18E-05	1.12E-05	1.12E-05	5.69E-06	1.77E-05	7.52E-06	4.96E-06
ELP4	9.69E-06	6.88E-06	5.11E-06	4.69E-06	3.65E-06	9.83E-06	4.16E-06	4.98E-06
ELSPBP1	0	0	0	0	0	0	0	0
ELTD1	5.63E-06	6.61E-06	3.33E-06	3.89E-06	2.33E-06	4.17E-06	6.06E-06	4.25E-06
EMB	3.96E-06	6.02E-07	1.18E-06	3.78E-06	1.65E-07	1.05E-06	2.05E-06	1.33E-06
EMCN	3.22E-06	4.16E-06	1.35E-06	1.09E-06	3.86E-06	3.40E-06	2.19E-06	4.28E-06
EMD	6.51E-05	5.31E-05	2.27E-05	3.48E-05	3.98E-05	3.36E-05	4.76E-05	4.92E-05
EME1	2.96E-06	2.19E-06	2.16E-08	1.55E-07	5.73E-07	5.53E-07	4.22E-07	4.69E-07
EME2	9.14E-07	4.61E-07	4.43E-08	7.62E-08	5.34E-08	6.31E-07	5.92E-08	3.05E-07
EMG1	3.91E-05	2.56E-05	1.76E-05	2.14E-05	2.07E-05	3.88E-05	2.75E-05	2.31E-05
EMID1	7.60E-06	2.98E-06	2.48E-06	9.56E-06	1.49E-06	5.00E-07	2.97E-06	1.06E-05
EMID2	1.55E-07	1.10E-05	6.89E-07	3.69E-07	5.08E-06	2.15E-06	1.60E-06	2.27E-07
EMILIN1	1.32E-05	1.82E-06	1.86E-05	4.05E-05	7.46E-06	2.03E-06	1.59E-05	1.04E-05
EMILIN2	6.03E-06	7.64E-07	4.91E-07	4.02E-06	1.85E-07	4.58E-07	1.80E-06	3.08E-07
EMILIN3	6.02E-07	5.44E-08	2.67E-07	2.55E-08	8.39E-07	5.71E-08	5.57E-08	3.59E-09
EML1	1.63E-06	5.63E-07	1.41E-06	1.51E-06	3.44E-07	7.43E-07	5.78E-07	6.91E-07
EML2	1.38E-05	6.51E-06	4.55E-06	1.08E-05	1.82E-06	1.34E-05	1.35E-05	8.53E-06
EML3	1.69E-05	1.72E-05	8.15E-06	1.01E-05	6.66E-06	9.35E-06	9.90E-06	1.39E-05
EML4	8.24E-05	3.90E-05	2.22E-05	3.11E-05	1.12E-05	1.79E-05	2.25E-05	2.41E-05
EML5	2.06E-06	5.79E-07	4.92E-07	6.27E-07	8.16E-07	3.60E-07	8.02E-07	4.33E-07
EML6	1.81E-06	5.82E-07	1.27E-07	8.30E-08	6.76E-08	7.75E-07	2.83E-08	2.75E-07
EMP1	1.08E-05	1.27E-05	1.42E-06	0.000101	2.93E-06	5.09E-06	1.83E-05	9.93E-06
EMP2	2.21E-05	2.06E-05	1.37E-05	1.66E-05	1.50E-05	2.13E-05	4.99E-05	1.76E-05
EMP3	1.88E-05	2.86E-06	1.00E-05	4.95E-05	7.76E-06	1.35E-05	2.13E-05	9.74E-06
EMR1	3.03E-07	8.14E-08	1.13E-06	4.97E-07	3.11E-08	8.43E-07	3.32E-07	7.96E-08
EMR2	2.22E-06	2.93E-07	5.39E-07	1.24E-06	3.39E-08	3.45E-07	4.07E-07	3.99E-07
EMR3	2.67E-07	2.84E-08	1.31E-08	6.29E-08	0	1.16E-08	1.21E-08	1.84E-08
EMR4P	1.62E-07	1.92E-08	1.33E-07	9.40E-08	0	7.70E-08	4.97E-08	8.77E-08
EMX1	9.63E-07	3.87E-08	1.19E-08	4.19E-08	1.57E-07	0	3.10E-07	7.55E-07
EMX2	0	0	0	0	0	0	1.67E-08	0
EMX2OS	0	0	0	0	0	0	6.19E-09	0
EN1	0	0	0	0	0	0	3.80E-08	0
EN2	0	0	7.00E-09	0	0	0	0	0
ENAH	7.64E-06	1.75E-05	1.36E-06	4.83E-06	6.98E-07	3.67E-06	3.17E-06	8.94E-06
ENAM	5.03E-07	2.78E-07	2.75E-07	3.43E-07	1.36E-08	8.12E-07	3.78E-07	1.14E-06
ENC1	7.38E-06	1.31E-06	3.92E-06	9.40E-06	7.05E-07	3.86E-06	4.72E-06	2.67E-06
ENDOD1	9.71E-06	9.03E-07	2.60E-06	5.96E-06	8.56E-07	2.05E-06	2.41E-06	1.43E-06
ENDOG	2.11E-05	2.14E-05	2.53E-05	2.54E-05	5.87E-05	1.95E-05	2.17E-05	3.34E-05
ENDOU	0	0	2.66E-08	4.66E-08	6.54E-08	0	0	6.17E-08
ENG	3.93E-05	2.48E-05	3.74E-05	6.72E-05	4.09E-05	1.81E-05	4.88E-05	2.59E-05
ENGASE	7.66E-06	3.18E-06	1.80E-06	2.45E-06	1.26E-06	5.38E-06	2.85E-06	2.69E-06
ENHO	7.51E-06	5.95E-05	1.06E-05	3.91E-06	2.52E-05	3.20E-06	5.66E-06	1.30E-05
ENKUR	2.08E-08	3.11E-08	1.08E-07	1.46E-07	1.79E-08	1.35E-07	1.90E-07	4.07E-08
ENO1	0.001236	0.000722	0.000334	0.000436	0.000253	0.000539	0.000422	0.000436
ENO2	1.50E-05	6.85E-07	6.37E-07	2.19E-06	2.39E-07	2.31E-07	1.00E-06	4.09E-07
ENO3	5.01E-06	1.15E-05	0.000104	1.43E-05	9.64E-06	4.22E-06	1.94E-05	4.76E-06
ENOPH1	2.49E-05	1.66E-05	9.43E-06	1.16E-05	3.97E-06	1.21E-05	1.30E-05	1.97E-05
ENOSF1	3.20E-05	8.30E-06	2.45E-05	1.55E-05	9.41E-06	2.59E-05	1.24E-05	2.14E-05

ENOX1	1.39E-06	1.50E-07	3.20E-08	1.24E-07	3.79E-08	1.10E-07	1.24E-07	2.26E-07
ENOX2	5.54E-06	2.34E-06	2.79E-06	1.89E-06	1.40E-06	4.60E-06	2.11E-06	4.94E-06
ENPEP	3.47E-06	8.66E-06	1.90E-05	1.14E-05	3.03E-06	4.92E-05	3.25E-06	9.49E-06
ENPP1	8.97E-06	9.34E-06	2.24E-05	9.07E-06	5.92E-06	2.59E-05	8.97E-06	1.28E-05
ENPP2	0.000254	7.32E-05	1.11E-05	1.15E-05	4.00E-07	7.43E-06	3.29E-05	8.25E-05
ENPP3	5.59E-08	5.03E-06	1.69E-06	2.14E-07	8.98E-08	6.19E-06	2.34E-07	4.32E-07
ENPP4	4.62E-06	8.59E-06	3.72E-06	2.19E-06	3.73E-08	4.42E-06	1.67E-06	1.03E-05
ENPP5	4.62E-06	4.96E-08	4.24E-07	9.60E-07	1.35E-08	2.45E-08	7.48E-08	1.29E-08
ENPP6	8.77E-06	3.89E-08	2.38E-08	7.08E-08	0	6.01E-07	2.82E-08	8.48E-09
ENPP7	6.43E-07	4.05E-05	1.05E-05	7.30E-06	1.66E-05	1.44E-05	1.24E-05	2.79E-06
ENSA	0.000126	0.0001	7.76E-05	9.16E-05	9.58E-05	0.000174	0.000105	0.000182
ENTHD1	1.72E-08	0	8.93E-09	0	0	7.98E-09	8.35E-09	0
ENTPD1	7.92E-06	1.16E-06	8.73E-07	1.91E-06	9.78E-07	1.65E-06	1.65E-06	1.89E-06
ENTPD2	1.48E-05	2.00E-07	3.68E-07	1.17E-06	7.43E-07	5.29E-07	8.56E-07	1.74E-06
ENTPD3	1.03E-06	5.62E-08	8.64E-09	1.02E-08	0	2.47E-07	3.23E-08	0
ENTPD4	6.28E-06	5.30E-06	3.22E-06	3.78E-06	4.15E-07	6.49E-06	7.61E-07	2.11E-06
ENTPD5	3.29E-05	5.92E-05	6.24E-05	2.65E-05	9.01E-06	4.35E-05	1.76E-05	4.29E-05
ENTPD6	9.54E-05	3.18E-05	1.63E-05	2.10E-05	4.39E-05	2.46E-05	2.96E-05	4.09E-05
ENTPD7	3.22E-06	1.44E-06	1.04E-06	1.87E-06	5.39E-07	2.32E-06	8.69E-07	1.62E-06
ENTPD8	1.26E-05	3.38E-06	1.35E-05	5.94E-06	1.97E-06	2.24E-07	6.87E-06	5.05E-06
ENY2	0.000331	0.000117	8.58E-05	0.000105	0.0002	0.00011	0.000264	0.000128
EOMES	2.17E-06	2.88E-08	8.22E-07	6.33E-07	3.66E-08	6.15E-07	4.31E-07	2.27E-07
EP300	1.70E-05	8.85E-06	7.10E-06	6.60E-06	2.05E-06	8.47E-06	4.68E-06	6.01E-06
EP400	7.38E-06	6.65E-06	3.50E-06	4.47E-06	1.76E-06	5.04E-06	4.02E-06	4.56E-06
EP400NL	2.70E-06	2.13E-06	1.51E-06	2.62E-06	7.47E-07	2.65E-06	2.02E-06	1.33E-06
EPAS1	6.73E-05	8.49E-05	6.65E-05	6.90E-05	3.33E-05	4.73E-05	5.67E-05	8.39E-05
EPB41	1.68E-05	1.94E-05	1.18E-05	9.15E-06	1.64E-06	1.77E-05	7.99E-06	1.84E-05
EPB41L1	1.54E-05	6.30E-05	1.41E-06	2.59E-06	6.23E-07	1.80E-06	2.48E-06	1.12E-06
EPB41L2	3.13E-05	1.63E-05	1.05E-05	2.33E-05	2.07E-06	8.17E-06	1.10E-05	8.73E-06
EPB41L3	4.20E-06	7.66E-07	3.04E-06	6.33E-06	3.34E-07	2.65E-06	3.09E-06	1.07E-06
EPB41L4A	1.40E-05	5.53E-07	2.13E-06	5.57E-06	1.34E-06	5.10E-07	9.18E-07	9.07E-07
EPB41L4B	1.51E-05	1.19E-05	3.54E-05	2.58E-05	7.68E-06	3.70E-05	1.54E-05	2.23E-06
EPB41L5	2.99E-05	3.45E-05	2.12E-05	1.57E-05	1.25E-06	1.64E-05	9.20E-06	9.27E-06
EPB42	1.48E-08	0	1.56E-08	1.82E-08	0	0	0	0
EPB49	2.25E-05	2.26E-05	6.50E-06	1.74E-05	3.38E-06	1.39E-05	1.77E-05	9.19E-06
EPC1	7.17E-06	6.76E-06	4.04E-06	5.92E-06	1.79E-07	5.68E-06	3.15E-06	4.13E-06
EPC2	1.02E-05	3.99E-06	3.78E-06	5.85E-06	8.64E-07	4.73E-06	2.82E-06	3.51E-06
EPCAM	0.000121	5.04E-07	2.17E-06	2.41E-05	1.27E-07	1.10E-07	8.98E-07	8.72E-08
EPDR1	2.57E-05	8.41E-06	5.39E-06	1.18E-05	2.45E-06	2.30E-05	1.09E-05	1.51E-05
EPGN	0	0	0	0	0	0	0	0
EPHA1	3.30E-06	1.22E-05	1.04E-05	6.46E-06	7.07E-06	8.87E-08	1.00E-05	1.36E-05
EPHA10	2.40E-06	0	4.18E-09	5.47E-08	0	3.77E-09	3.97E-09	8.77E-07
EPHA2	1.98E-05	1.58E-05	1.45E-05	6.11E-05	2.57E-06	1.45E-06	1.96E-05	2.64E-05
EPHA3	1.61E-06	5.87E-07	1.33E-06	3.27E-06	9.94E-08	1.32E-07	3.24E-07	8.98E-07
EPHA4	5.78E-07	4.37E-07	1.98E-07	1.98E-07	1.15E-07	2.02E-07	2.23E-07	8.09E-07
EPHA5	0	3.29E-09	0	0	0	0	0	1.29E-08
EPHA6	5.79E-08	0	4.85E-08	8.86E-08	0	0	0	0
EPHA7	6.80E-06	0	2.41E-08	6.36E-08	8.68E-09	0	0	0
EPHA8	1.35E-08	1.01E-08	0	0	2.26E-08	0	1.27E-08	0
EPHB1	2.83E-07	2.95E-07	5.75E-07	1.08E-07	5.05E-08	5.49E-08	3.94E-07	1.15E-07
EPHB2	7.59E-06	1.15E-07	1.27E-07	1.53E-06	1.93E-07	5.40E-06	6.42E-07	3.06E-07
EPHB3	5.01E-07	3.62E-08	3.76E-07	8.55E-07	1.80E-07	1.50E-08	3.35E-07	7.09E-08
EPHB4	3.07E-05	1.72E-05	1.45E-05	1.66E-05	1.43E-05	7.83E-06	1.82E-05	4.47E-05
EPHB6	1.36E-06	2.00E-07	1.86E-06	3.84E-06	1.59E-07	1.98E-07	3.64E-07	7.05E-07
EPHX1	0.000119	0.002724	0.001097	0.000412	0.001254	0.002552	0.00068	0.001629
EPHX2	1.85E-05	8.62E-05	0.000167	9.11E-05	2.01E-05	0.000169	4.52E-05	3.30E-05
EPHX3	1.37E-07	3.11E-08	1.44E-08	1.35E-07	2.36E-08	3.82E-08	9.26E-08	1.21E-07
EPHX4	1.00E-06	1.58E-07	0	4.25E-08	5.96E-08	0	2.83E-07	1.02E-07

EPM2A	1.74E-06	1.96E-06	4.28E-06	2.73E-06	1.16E-06	3.25E-06	2.33E-06	2.12E-06
EPM2AIP1	7.68E-06	5.20E-06	3.88E-06	2.80E-06	1.21E-06	5.19E-06	2.32E-06	3.53E-06
EPN1	5.57E-05	8.74E-05	6.95E-05	7.66E-05	0.000155	0.000106	9.79E-05	5.45E-05
EPN2	5.16E-06	9.49E-06	4.03E-06	8.81E-06	9.02E-06	5.21E-06	7.18E-06	5.16E-06
EPN3	6.97E-08	1.48E-08	5.83E-08	7.90E-09	0	0	1.32E-07	0
EPO	1.14E-06	6.97E-07	8.15E-07	2.76E-06	1.15E-07	0	6.03E-06	2.82E-08
EPOR	8.15E-06	2.91E-06	3.84E-06	3.37E-06	2.03E-06	1.41E-06	3.77E-06	2.87E-06
EPPK1	1.66E-05	6.43E-06	1.45E-07	2.38E-07	5.77E-07	2.46E-08	2.34E-06	1.13E-07
EPR1	2.16E-05	9.43E-06	4.56E-07	7.33E-07	3.94E-07	3.99E-06	8.49E-07	2.72E-06
EPRS	2.97E-05	3.99E-05	1.37E-05	1.27E-05	4.33E-06	1.27E-05	1.14E-05	2.75E-05
EPS15	1.42E-05	1.21E-05	1.20E-05	1.15E-05	4.62E-06	1.32E-05	7.60E-06	1.05E-05
EPS15L1	8.90E-06	6.91E-06	4.32E-06	5.11E-06	2.11E-06	5.22E-06	5.56E-06	6.64E-06
EPS8	4.92E-05	3.36E-05	1.31E-05	2.11E-05	3.62E-06	2.63E-05	1.39E-05	9.09E-06
EPS8L1	2.98E-06	8.11E-08	3.51E-07	2.77E-06	4.10E-07	3.36E-08	2.79E-07	3.90E-08
EPS8L2	6.49E-05	7.59E-05	3.49E-05	3.57E-05	3.23E-05	6.03E-05	2.50E-05	3.61E-05
EPS8L3	6.95E-05	1.31E-07	5.95E-08	1.69E-07	1.96E-08	7.94E-06	2.38E-06	0
EPSTI1	2.86E-05	2.62E-07	6.61E-06	6.64E-06	9.92E-08	1.78E-05	2.47E-06	1.24E-05
EPT1	1.52E-05	3.96E-05	8.06E-06	5.42E-06	2.18E-06	1.61E-05	5.62E-06	8.44E-06
EPX	2.62E-08	0	0	0	0	2.43E-08	1.69E-08	1.28E-08
EPYC	1.61E-08	0	0	0	0	0	1.55E-08	0
ERAL1	3.52E-05	5.96E-05	2.47E-05	2.60E-05	3.33E-05	4.39E-05	3.95E-05	3.22E-05
ERAP1	1.81E-05	1.99E-05	1.56E-05	9.94E-06	1.25E-06	3.45E-05	1.08E-05	1.49E-05
ERAP2	1.36E-05	1.76E-05	4.57E-07	1.61E-05	2.96E-07	2.54E-05	7.11E-06	4.03E-07
ERAS	1.41E-07	6.93E-08	1.08E-07	4.96E-08	1.91E-07	3.75E-08	5.79E-08	8.95E-08
ERBB2	8.16E-05	4.41E-05	2.42E-05	4.57E-05	7.94E-05	4.30E-06	7.14E-05	3.93E-05
ERBB2IP	1.75E-05	1.74E-05	1.32E-05	1.24E-05	3.69E-06	1.29E-05	7.65E-06	1.50E-05
ERBB3	0.000193	5.68E-05	2.83E-05	4.24E-05	3.21E-05	4.89E-05	7.19E-05	4.15E-05
ERBB4	0	0	9.47E-09	4.53E-09	0	0	5.45E-09	5.44E-09
ERC1	8.56E-06	5.00E-06	3.68E-06	4.37E-06	1.44E-06	4.14E-06	3.33E-06	6.55E-06
ERC2	3.45E-07	0	3.74E-09	2.39E-08	5.64E-09	1.89E-07	1.92E-08	4.92E-08
ERCC1	3.37E-05	2.87E-05	2.65E-05	3.66E-05	6.32E-05	3.37E-05	4.24E-05	2.66E-05
ERCC2	8.93E-06	1.04E-05	6.41E-06	7.00E-06	1.44E-05	1.12E-05	1.09E-05	7.42E-06
ERCC3	2.58E-05	1.57E-05	8.15E-06	1.08E-05	5.37E-06	1.51E-05	8.60E-06	1.17E-05
ERCC4	1.65E-06	2.46E-06	1.47E-06	1.18E-06	2.36E-07	1.59E-06	6.52E-07	6.81E-07
ERCC5	2.11E-05	1.30E-05	1.38E-05	1.31E-05	8.41E-06	2.12E-05	1.23E-05	1.02E-05
ERCC6	1.95E-06	7.89E-07	9.84E-07	1.03E-06	2.99E-07	1.56E-06	7.89E-07	7.77E-07
ERCC6L	1.02E-06	6.47E-07	1.67E-08	6.59E-08	8.81E-08	1.00E-07	4.73E-08	1.98E-07
ERCC8	4.09E-06	3.63E-06	2.50E-06	1.95E-06	4.63E-07	3.08E-06	8.69E-07	3.70E-06
EREG	2.88E-06	5.47E-09	2.01E-08	8.24E-07	0	9.05E-09	2.85E-08	7.15E-09
ERF	2.48E-05	3.10E-05	1.46E-05	2.58E-05	6.93E-06	1.76E-05	3.55E-05	2.15E-05
ERG	3.07E-06	2.32E-06	1.98E-06	1.99E-06	7.61E-07	1.18E-06	3.12E-06	2.58E-06
ERGIC1	0.00019	9.55E-05	0.000136	8.17E-05	2.55E-05	8.25E-05	7.52E-05	0.000169
ERGIC2	2.69E-05	2.82E-05	1.40E-05	1.68E-05	8.04E-06	1.90E-05	1.31E-05	1.32E-05
ERGIC3	0.000255	0.000176	0.000112	0.000162	0.000252	0.000185	0.000193	0.000157
ERH	0.000189	7.00E-05	7.12E-05	9.39E-05	7.14E-05	0.000115	0.000116	0.000118
ERI1	2.97E-06	5.68E-06	1.55E-06	2.66E-06	3.31E-07	2.10E-06	1.28E-06	1.40E-06
ERI2	6.52E-06	1.34E-05	8.38E-06	4.76E-06	2.95E-06	8.31E-06	5.32E-06	3.60E-06
ERI3	4.48E-05	3.64E-05	2.08E-05	2.64E-05	3.15E-05	4.29E-05	3.37E-05	3.31E-05
ERICH1	4.65E-06	1.35E-05	4.48E-06	4.87E-06	7.25E-07	6.76E-06	3.26E-06	2.42E-06
ERLEC1	3.22E-05	3.03E-05	1.70E-05	1.74E-05	1.01E-05	3.94E-05	1.29E-05	2.62E-05
ERLIN1	3.88E-05	3.57E-05	7.73E-05	3.66E-05	1.61E-05	5.09E-05	3.01E-05	2.88E-05
ERLIN2	1.61E-05	4.96E-05	1.87E-05	1.47E-05	2.90E-06	2.24E-05	6.24E-06	8.67E-06
ERMAP	4.12E-06	4.04E-06	5.25E-06	5.25E-06	2.35E-06	9.59E-06	3.10E-06	5.31E-06
ERMN	1.60E-07	0	2.54E-08	1.25E-07	0	3.78E-08	4.62E-08	5.98E-08
ERMP1	9.45E-06	7.02E-06	3.94E-06	5.57E-06	9.56E-07	4.32E-06	3.51E-06	5.70E-06
ERN1	3.18E-06	3.92E-06	2.08E-06	1.91E-06	6.83E-08	7.96E-06	6.58E-07	4.15E-06
ERN2	0	0	6.84E-09	0	0	0	0	0
ERO1L	2.95E-05	1.16E-05	1.25E-05	1.07E-05	9.63E-07	1.27E-05	7.79E-06	1.69E-05

ERO1LB	3.30E-06	7.15E-06	8.46E-06	5.20E-06	1.72E-06	1.85E-05	3.12E-06	1.66E-05
ERP27	5.96E-07	2.48E-06	2.39E-07	1.35E-06	5.55E-08	2.55E-07	1.24E-07	1.90E-07
ERP29	0.000175	0.000194	0.000166	0.000165	0.000231	0.000181	0.000257	0.000143
ERP44	2.34E-05	2.29E-05	1.81E-05	1.80E-05	1.12E-05	2.74E-05	1.56E-05	2.79E-05
ERRFI1	0.000152	6.71E-05	0.000488	0.000157	6.63E-05	0.000109	9.60E-05	0.000302
ERVFRDE1	1.45E-08	2.44E-08	1.57E-07	8.84E-09	6.21E-09	0	2.81E-08	1.06E-08
ESAM	2.17E-05	1.89E-05	7.03E-06	8.36E-06	1.30E-05	1.09E-05	2.92E-05	1.90E-05
ESCO1	1.09E-05	4.86E-06	2.69E-06	3.63E-06	6.99E-07	3.24E-06	2.95E-06	3.75E-06
ESCO2	1.45E-06	2.90E-06	6.36E-08	1.67E-07	7.39E-08	5.58E-07	1.06E-07	1.31E-07
ESD	0.000121	0.00014	0.00011	9.71E-05	6.95E-05	0.000152	0.000144	9.38E-05
ESF1	1.31E-05	1.22E-05	4.77E-06	5.81E-06	1.18E-06	8.12E-06	4.08E-06	6.97E-06
ESM1	3.62E-06	3.07E-05	3.90E-08	3.62E-07	3.06E-06	1.14E-05	7.00E-06	7.56E-07
ESPL1	3.29E-06	9.37E-07	8.96E-08	6.58E-08	6.65E-08	5.85E-07	1.24E-07	1.68E-07
ESPN	5.14E-05	4.99E-05	6.82E-05	3.72E-05	8.88E-05	4.47E-05	4.68E-05	6.50E-05
ESPNL	4.06E-07	7.92E-07	2.69E-06	5.18E-08	6.77E-08	2.44E-07	1.12E-07	3.29E-06
ESPNP	2.24E-07	9.41E-08	8.71E-08	1.46E-08	0	8.82E-08	1.15E-08	1.75E-08
ESR1	2.43E-07	3.40E-07	8.62E-06	6.83E-06	2.03E-06	6.74E-06	5.69E-06	1.33E-06
ESR2	1.67E-07	2.28E-08	0	1.67E-08	4.19E-08	6.75E-08	1.17E-08	1.18E-08
ESRP1	3.00E-07	8.39E-09	2.76E-07	3.48E-07	0	0	1.42E-07	0
ESRP2	5.68E-05	2.37E-05	2.25E-05	1.72E-05	1.42E-05	1.67E-05	2.12E-05	1.49E-05
ESRRA	6.68E-05	8.11E-05	3.15E-05	3.12E-05	1.93E-05	3.32E-05	4.17E-05	9.30E-05
ESRRB	3.52E-08	0	1.59E-08	0	0	2.85E-08	0	4.51E-08
ESRRG	1.69E-06	5.77E-08	1.41E-07	2.04E-07	0	2.17E-08	2.74E-08	4.80E-08
ESX1	0	0	0	0	0	0	0	0
ESYT1	4.60E-05	5.53E-05	3.20E-05	3.96E-05	1.19E-05	2.68E-05	3.66E-05	6.42E-05
ESYT2	2.53E-05	1.90E-05	1.86E-05	2.23E-05	4.38E-06	2.14E-05	9.09E-06	1.83E-05
ESYT3	2.28E-06	0	2.09E-08	2.20E-08	0	4.72E-09	3.47E-08	0
ETAA1	1.03E-05	4.22E-06	3.00E-06	3.34E-06	1.47E-06	4.44E-06	1.80E-06	2.41E-06
ETF1	4.01E-05	3.05E-05	1.87E-05	3.23E-05	6.26E-06	2.59E-05	2.73E-05	3.96E-05
ETFA	0.000137	0.000206	0.000204	0.000248	0.00013	0.000255	0.000209	0.000307
ETFB	0.000375	0.000436	0.000448	0.000605	0.002097	0.000919	0.000671	0.000563
ETFDH	2.80E-05	3.40E-05	8.49E-05	5.02E-05	1.61E-05	6.67E-05	4.13E-05	6.27E-05
ETHE1	3.95E-05	1.40E-05	4.54E-05	4.23E-05	8.44E-05	3.62E-05	5.17E-05	1.42E-05
ETNK1	3.26E-05	1.32E-05	8.15E-06	6.81E-06	1.79E-06	8.06E-06	5.38E-06	6.32E-06
ETNK2	3.93E-05	0.000357	0.000138	5.34E-05	4.89E-05	0.00011	8.44E-05	1.76E-05
ETS1	2.18E-05	8.27E-06	1.10E-05	2.88E-05	2.51E-06	1.24E-05	1.03E-05	1.18E-05
ETS2	0.000136	9.74E-05	9.69E-05	0.000149	1.02E-05	8.56E-05	0.000106	0.000154
ETV1	7.82E-06	3.05E-07	1.48E-06	1.79E-06	2.63E-08	1.71E-06	4.01E-07	9.98E-07
ETV2	3.92E-07	4.13E-07	4.62E-07	2.80E-07	3.41E-06	1.49E-06	1.35E-06	4.81E-07
ETV3	2.70E-06	2.22E-06	1.63E-06	2.50E-06	1.03E-08	2.68E-06	8.15E-07	2.59E-06
ETV3L	3.64E-08	2.75E-08	0	5.95E-08	0	4.51E-08	0	1.79E-08
ETV4	4.76E-06	1.00E-07	7.65E-07	6.18E-06	2.47E-05	1.07E-06	2.05E-06	1.69E-05
ETV5	3.41E-06	1.41E-06	2.72E-06	4.34E-06	2.51E-06	1.81E-06	2.58E-06	5.21E-06
ETV6	6.49E-06	2.49E-06	3.50E-06	4.91E-06	1.39E-06	4.39E-06	3.06E-06	5.40E-06
ETV7	8.29E-06	1.95E-06	1.24E-06	2.57E-06	2.80E-07	1.91E-06	5.22E-07	3.67E-06
EVC	1.73E-05	1.07E-06	1.30E-06	2.35E-06	4.98E-07	1.42E-06	1.43E-06	1.03E-06
EVC2	1.96E-06	5.42E-07	2.27E-07	8.18E-07	6.63E-08	1.29E-07	4.16E-07	2.34E-07
EVI2A	1.16E-05	2.30E-06	3.04E-06	6.92E-06	5.14E-07	2.34E-06	3.46E-06	2.04E-06
EVI2B	1.68E-05	1.85E-06	6.19E-06	1.06E-05	3.93E-07	4.67E-06	5.45E-06	2.23E-06
EVI5	3.74E-06	2.38E-06	5.83E-06	3.14E-06	9.70E-07	6.90E-06	2.34E-06	2.65E-06
EVI5L	3.88E-06	2.37E-06	1.78E-06	2.42E-06	2.83E-06	3.46E-06	3.93E-06	3.65E-06
EVL	3.18E-05	3.16E-06	2.55E-05	2.42E-05	3.46E-06	2.28E-05	4.43E-05	6.17E-06
EVPL	1.36E-08	2.33E-06	8.66E-08	2.90E-08	2.91E-09	0	6.28E-08	1.98E-08
EVPLL	1.21E-08	1.36E-07	6.19E-07	4.43E-08	3.11E-08	4.14E-07	1.75E-07	6.92E-07
EVX1	5.20E-08	8.45E-08	0	0	0	0	0	0
EVX2	0	0	0	0	0	0	0	0
EWSR1	0.000106	5.40E-05	4.69E-05	7.10E-05	6.81E-05	8.15E-05	6.85E-05	6.40E-05
EXD1	1.58E-08	8.89E-09	0	2.53E-08	0	0	0	0

EXD2	1.13E-05	6.19E-06	4.64E-06	5.21E-06	1.36E-06	5.23E-06	3.05E-06	4.03E-06
EXD3	6.77E-06	2.01E-06	2.13E-06	4.22E-06	4.38E-06	6.26E-06	4.89E-06	3.76E-06
EXO1	3.39E-06	1.83E-06	4.59E-08	5.43E-08	2.99E-07	5.76E-07	1.51E-07	7.27E-07
EXOC1	1.04E-05	6.24E-06	3.88E-06	4.73E-06	1.48E-06	6.34E-06	3.58E-06	4.72E-06
EXOC2	8.76E-06	1.30E-05	5.25E-06	6.61E-06	2.38E-06	1.29E-05	5.77E-06	1.02E-05
EXOC3	1.61E-05	2.56E-05	1.17E-05	1.48E-05	7.90E-06	1.54E-05	1.35E-05	1.70E-05
EXOC3L	2.29E-06	1.38E-06	1.27E-06	1.28E-06	2.18E-06	1.02E-06	3.59E-06	1.96E-06
EXOC3L2	2.58E-06	1.64E-06	1.64E-06	1.39E-06	4.33E-06	3.44E-06	3.35E-06	2.38E-06
EXOC4	9.91E-06	1.57E-05	1.23E-05	1.32E-05	1.23E-05	2.13E-05	1.37E-05	1.79E-05
EXOC5	7.54E-06	4.44E-06	5.73E-06	3.71E-06	1.21E-06	5.25E-06	3.62E-06	3.77E-06
EXOC6	9.36E-06	7.52E-06	3.58E-06	5.84E-06	4.39E-06	6.53E-06	5.49E-06	7.01E-06
EXOC6B	2.65E-06	6.22E-07	3.27E-07	7.15E-07	2.10E-08	1.49E-06	1.89E-07	7.46E-07
EXOC7	4.06E-05	3.70E-05	1.52E-05	1.96E-05	2.11E-05	3.88E-05	2.10E-05	2.15E-05
EXOC8	4.64E-06	5.91E-06	3.14E-06	2.79E-06	4.82E-07	4.82E-06	2.08E-06	5.52E-06
EXOG	2.61E-06	1.10E-06	7.66E-07	1.44E-06	7.04E-07	2.09E-06	1.19E-06	1.52E-06
EXOSC1	1.92E-05	2.33E-05	1.11E-05	1.46E-05	1.34E-05	2.00E-05	1.90E-05	1.57E-05
EXOSC10	2.79E-05	1.78E-05	1.22E-05	1.75E-05	6.46E-06	2.00E-05	1.44E-05	9.76E-06
EXOSC2	1.11E-05	5.77E-06	5.08E-06	5.72E-06	3.38E-06	9.09E-06	7.02E-06	9.29E-06
EXOSC3	9.17E-06	1.32E-05	8.01E-06	1.03E-05	4.72E-06	1.27E-05	1.07E-05	6.78E-06
EXOSC4	6.77E-05	4.88E-05	2.48E-05	2.63E-05	0.000137	3.08E-05	9.42E-05	3.92E-05
EXOSC5	2.41E-05	3.78E-05	1.88E-05	1.71E-05	3.13E-05	4.01E-05	3.70E-05	1.63E-05
EXOSC6	1.08E-05	3.20E-06	6.78E-06	9.53E-06	4.57E-06	8.50E-06	7.69E-06	5.38E-06
EXOSC7	3.54E-05	2.06E-05	1.54E-05	1.91E-05	2.57E-05	2.81E-05	2.38E-05	2.20E-05
EXOSC8	1.57E-05	8.81E-06	7.56E-06	1.00E-05	8.10E-06	1.18E-05	1.26E-05	1.01E-05
EXOSC9	2.05E-05	5.17E-06	6.39E-06	1.14E-05	7.04E-06	1.72E-05	1.42E-05	1.81E-05
EXPH5	3.17E-07	3.18E-07	1.53E-06	6.72E-07	2.20E-07	2.39E-06	7.14E-07	6.02E-08
EXT1	9.16E-05	2.33E-05	3.04E-05	3.48E-05	1.47E-05	1.46E-05	5.99E-05	5.75E-05
EXT2	2.39E-05	1.56E-05	1.43E-05	1.91E-05	7.23E-06	1.59E-05	1.60E-05	3.13E-05
EXTL1	4.54E-08	6.36E-09	0	3.47E-08	9.74E-09	2.10E-08	3.86E-08	8.31E-09
EXTL2	8.18E-06	3.52E-06	2.75E-06	3.21E-06	1.25E-06	5.05E-06	1.12E-06	3.31E-06
EXTL3	5.22E-06	3.03E-06	1.68E-06	4.12E-06	3.55E-06	4.04E-06	4.35E-06	3.39E-06
EYA1	5.90E-09	6.65E-09	0	0	0	0	4.62E-08	0
EYA2	1.58E-07	2.02E-07	1.94E-07	1.08E-06	1.45E-07	1.79E-08	4.08E-07	0
EYA3	3.42E-06	1.04E-06	1.22E-06	1.05E-06	4.10E-08	2.13E-06	2.45E-07	1.04E-06
EYA4	2.11E-08	9.01E-09	5.72E-08	1.97E-08	0	0	7.86E-09	5.90E-09
EYS	2.18E-07	7.95E-07	8.19E-08	1.10E-07	8.81E-08	8.61E-08	5.01E-08	1.97E-06
EZH1	1.08E-05	4.82E-06	2.45E-06	2.51E-06	3.01E-06	6.70E-06	4.97E-06	4.10E-06
EZH2	7.36E-06	4.56E-06	3.73E-07	1.39E-06	1.15E-06	2.02E-06	1.16E-06	2.90E-06
EZR	8.90E-05	7.64E-05	2.46E-05	6.54E-05	2.42E-05	4.48E-05	1.83E-05	2.26E-05
F10	0.000114	0.00026	0.000172	0.000223	0.000281	0.000214	0.000189	0.000113
F11	1.64E-05	3.77E-05	5.97E-05	3.77E-05	3.73E-05	4.37E-05	2.56E-05	6.86E-05
F11R	0.000109	0.000311	3.74E-05	6.39E-05	1.61E-05	3.94E-05	4.86E-05	8.98E-05
F12	0.000118	0.000176	0.000269	0.000545	0.000533	0.000238	0.000582	0.000265
F13A1	5.00E-06	1.45E-06	6.63E-07	1.46E-06	6.75E-07	1.84E-06	1.62E-05	8.91E-07
F13B	4.69E-05	0.000126	9.34E-05	4.65E-05	2.51E-05	0.000119	1.84E-05	0.000129
F2	0.000154	0.000946	0.001267	0.001282	0.000994	0.000632	0.001138	0.001574
F2R	2.29E-05	3.41E-06	6.20E-06	1.09E-05	2.05E-06	3.78E-06	8.92E-06	6.69E-06
F2RL1	5.56E-05	5.82E-06	2.82E-06	2.56E-05	1.03E-06	4.61E-06	1.04E-05	2.16E-06
F2RL2	8.69E-07	2.37E-08	5.53E-07	4.38E-07	4.22E-08	5.61E-06	2.09E-06	6.28E-07
F2RL3	3.08E-06	2.19E-06	1.77E-07	6.58E-07	1.89E-06	1.04E-06	3.40E-06	1.69E-06
F3	1.43E-06	3.28E-07	2.41E-06	8.59E-06	1.40E-07	2.44E-07	4.18E-07	7.67E-07
F5	4.02E-05	0.000146	6.86E-05	5.64E-05	1.33E-05	0.000113	3.80E-05	9.91E-05
F7	2.83E-05	5.99E-05	0.000113	7.08E-05	4.01E-05	8.61E-05	4.80E-05	0.000107
F8	2.05E-06	1.24E-05	1.17E-05	6.44E-06	2.02E-06	7.93E-06	6.47E-06	4.71E-06
F8A1	9.21E-06	8.23E-06	7.61E-06	9.67E-06	1.08E-05	9.63E-06	1.40E-05	9.24E-06
F9	1.91E-07	0.00021	0.00034	0.000119	0.000145	0.000124	9.17E-05	0.000235
FA2H	3.10E-05	3.91E-08	1.15E-07	2.01E-06	0	0	0	0
FAAH	2.11E-05	1.06E-05	2.17E-05	2.43E-05	3.12E-05	2.46E-05	2.65E-05	1.76E-05

FAAH2	1.11E-05	1.27E-05	4.10E-06	5.74E-06	1.29E-06	4.28E-06	6.95E-06	2.14E-05
FABP1	0.002448	0.005325	0.006691	0.007075	0.014945	0.00278	0.009419	0.002944
FABP12	0	0	0	0	0	0	0	0
FABP2	0	2.38E-08	0	0	0	0	0	1.55E-08
FABP3	3.23E-05	1.16E-06	4.38E-06	1.48E-05	7.84E-07	1.13E-06	5.82E-06	7.09E-07
FABP4	1.67E-07	0.000145	1.49E-07	1.17E-05	2.21E-05	4.22E-05	6.51E-05	7.00E-06
FABP5	2.18E-05	7.72E-06	2.52E-06	7.81E-06	1.00E-06	9.50E-07	6.10E-06	1.39E-06
FABP5L3	3.26E-08	0	3.64E-08	0	0	6.10E-08	0	4.88E-08
FABP6	5.55E-07	0	0	0	3.98E-08	0	0	0
FABP7	0	1.23E-08	0	0	0	0	3.10E-08	0
FABP9	0	0	0	0	0	0	0	0
FADD	1.61E-05	1.35E-05	6.70E-06	1.67E-05	7.07E-06	1.43E-05	1.31E-05	1.92E-05
FADS1	6.95E-05	0.000156	1.75E-05	7.55E-06	3.66E-06	4.43E-07	6.45E-06	1.54E-05
FADS2	0.000108	0.000248	0.000114	2.37E-05	3.25E-05	1.01E-06	1.69E-05	5.93E-05
FADS3	1.92E-05	1.78E-05	5.15E-06	1.69E-05	3.93E-06	7.60E-06	1.78E-05	1.00E-05
FADS6	1.13E-05	1.61E-05	3.30E-06	2.47E-06	3.66E-07	5.30E-06	2.15E-06	6.50E-06
FAF1	2.31E-05	2.87E-05	1.46E-05	1.69E-05	1.26E-05	2.78E-05	2.03E-05	2.18E-05
FAF2	3.20E-05	1.20E-05	9.29E-06	1.03E-05	2.50E-06	1.30E-05	9.31E-06	1.49E-05
FAH	4.02E-05	0.000157	0.000204	0.000153	0.000229	0.000199	0.0003	8.47E-05
FAHD1	0.000139	8.94E-05	5.77E-05	3.66E-05	2.85E-05	0.000102	4.12E-05	6.13E-05
FAHD2A	4.27E-05	2.44E-05	6.97E-05	5.00E-05	6.20E-05	4.19E-05	4.44E-05	1.58E-05
FAHD2B	3.35E-05	1.85E-07	1.21E-06	3.58E-06	3.31E-07	1.31E-07	4.06E-07	2.69E-07
FAIM	2.21E-05	2.60E-06	3.53E-06	1.17E-05	7.12E-06	1.50E-05	9.85E-06	7.13E-06
FAIM2	2.64E-07	0	4.96E-09	1.41E-07	1.24E-08	1.34E-08	8.94E-08	2.83E-08
FAIM3	2.84E-06	2.75E-07	1.05E-06	2.70E-06	1.07E-07	1.96E-06	1.40E-06	4.48E-07
FAM100A	1.51E-05	3.99E-05	3.44E-05	3.84E-05	1.48E-05	2.12E-05	5.72E-05	2.74E-05
FAM100B	2.23E-05	1.25E-05	9.15E-06	2.52E-05	3.19E-05	5.23E-05	3.18E-05	3.83E-05
FAM101A	1.38E-05	0	0	5.13E-07	2.10E-07	1.08E-08	9.21E-07	2.39E-07
FAM101B	3.89E-06	3.88E-06	2.58E-06	2.93E-06	1.76E-06	1.76E-06	4.67E-06	1.77E-06
FAM102A	8.55E-06	9.61E-06	8.52E-06	3.69E-05	9.08E-06	1.13E-05	1.04E-05	1.15E-05
FAM102B	4.71E-06	1.31E-06	1.10E-06	5.38E-06	2.46E-07	9.04E-07	1.17E-06	1.71E-06
FAM103A1	3.56E-05	1.76E-05	1.11E-05	1.29E-05	1.10E-05	1.90E-05	1.79E-05	1.40E-05
FAM104A	1.40E-05	1.25E-05	5.19E-06	7.50E-06	4.08E-06	1.19E-05	7.47E-06	6.36E-06
FAM104B	1.47E-05	8.47E-06	5.90E-06	8.22E-06	5.57E-06	8.07E-06	1.13E-05	9.15E-06
FAM105A	4.70E-06	2.40E-07	5.33E-07	6.68E-07	8.14E-08	4.77E-07	5.80E-07	1.53E-07
FAM105B	5.45E-06	7.04E-06	3.47E-06	3.60E-06	1.54E-06	4.39E-06	3.58E-06	6.37E-06
FAM106A	8.30E-08	0	0	1.27E-08	0	0	0	0
FAM106C	8.29E-08	0	2.91E-08	0	0	0	0	0
FAM107A	2.24E-06	1.04E-05	1.52E-06	4.46E-06	1.38E-06	2.43E-06	4.70E-06	3.85E-06
FAM107B	3.68E-05	6.12E-05	4.79E-05	5.42E-05	9.70E-06	4.86E-05	3.91E-05	3.80E-05
FAM108A1	1.30E-05	1.19E-05	7.82E-06	1.43E-05	1.27E-05	1.23E-05	2.01E-05	8.57E-06
FAM108B1	7.43E-06	5.24E-06	6.89E-06	4.76E-06	1.91E-06	7.36E-06	4.17E-06	7.06E-06
FAM108C1	1.23E-05	9.88E-07	2.06E-06	2.53E-06	1.27E-06	2.39E-06	3.14E-06	1.65E-06
FAM109A	8.93E-06	1.30E-05	5.30E-06	6.87E-06	3.16E-06	1.88E-06	5.17E-06	5.64E-06
FAM109B	7.64E-06	7.77E-07	8.86E-07	1.88E-06	7.49E-07	5.26E-07	1.44E-06	8.93E-07
FAM10A4	1.78E-05	1.57E-05	1.25E-05	1.00E-05	6.45E-06	1.59E-05	9.69E-06	8.58E-06
FAM110A	8.49E-06	1.36E-06	1.51E-06	2.84E-06	2.69E-06	1.76E-06	4.04E-06	2.68E-06
FAM110B	6.70E-07	2.60E-06	1.82E-06	2.87E-06	1.96E-06	1.28E-06	3.57E-06	1.41E-06
FAM110C	1.65E-06	2.60E-06	4.42E-06	3.59E-06	7.74E-07	1.23E-06	1.70E-06	2.29E-06
FAM111A	1.00E-05	6.44E-06	3.86E-06	4.53E-06	1.72E-06	9.80E-06	2.72E-06	6.08E-06
FAM111B	5.91E-06	6.88E-06	9.39E-08	4.62E-07	8.96E-07	1.53E-06	2.07E-07	1.09E-06
FAM113A	2.87E-05	1.24E-05	7.37E-06	7.80E-06	1.43E-05	1.40E-05	1.06E-05	1.07E-05
FAM113B	5.73E-06	7.79E-07	2.19E-06	2.78E-06	6.81E-07	1.76E-06	2.35E-06	2.34E-06
FAM114A1	3.10E-05	4.77E-05	2.07E-05	1.50E-05	5.91E-07	2.84E-05	9.16E-06	1.74E-05
FAM114A2	9.82E-06	5.13E-06	4.95E-06	5.71E-06	1.62E-06	8.59E-06	5.60E-06	7.91E-06
FAM115A	3.90E-06	3.96E-06	4.03E-06	2.90E-06	8.72E-07	3.00E-06	1.08E-06	3.05E-06
FAM115C	7.56E-06	4.83E-06	1.21E-06	4.01E-06	2.92E-07	2.29E-06	1.18E-06	1.51E-06
FAM116A	6.86E-06	3.34E-06	3.28E-06	4.01E-06	1.46E-06	3.65E-06	3.11E-06	3.66E-06

FAM116B	3.28E-06	1.26E-06	9.50E-07	1.79E-06	1.94E-06	8.99E-07	1.95E-06	1.44E-06
FAM117A	7.83E-06	1.09E-05	6.88E-06	7.52E-06	2.80E-06	1.59E-05	5.52E-06	2.33E-06
FAM117B	3.03E-06	5.08E-07	1.16E-06	1.29E-06	3.60E-07	7.55E-07	5.64E-07	3.69E-07
FAM118A	6.32E-06	3.35E-06	2.27E-06	3.54E-06	2.70E-06	1.27E-06	1.76E-06	2.05E-06
FAM118B	6.71E-06	4.13E-06	2.81E-06	4.62E-06	2.37E-06	5.80E-06	4.53E-06	5.94E-06
FAM119A	1.13E-05	7.66E-06	3.39E-06	3.94E-06	1.70E-06	3.78E-06	4.63E-06	3.22E-06
FAM119B	6.20E-06	4.29E-06	2.78E-06	3.04E-06	7.94E-07	4.13E-06	2.26E-06	1.22E-06
FAM120A	7.87E-05	5.23E-05	4.60E-05	3.96E-05	2.58E-05	6.62E-05	3.81E-05	5.39E-05
FAM120A(	2.73E-05	1.49E-05	1.32E-05	1.33E-05	3.64E-06	1.52E-05	8.38E-06	1.77E-05
FAM120B	1.19E-05	9.33E-06	9.84E-06	7.67E-06	3.72E-06	1.36E-05	7.66E-06	8.81E-06
FAM120C	2.26E-06	2.03E-06	1.36E-06	1.24E-06	1.47E-06	2.97E-06	1.57E-06	2.11E-06
FAM122A	6.33E-06	5.47E-06	5.37E-06	4.79E-06	1.09E-06	4.77E-06	3.41E-06	5.24E-06
FAM122B	8.05E-06	9.18E-06	3.13E-06	3.76E-06	3.25E-06	4.48E-06	2.94E-06	9.24E-06
FAM122C	1.54E-06	7.15E-07	4.17E-07	9.86E-07	1.05E-07	7.03E-07	4.01E-07	8.49E-07
FAM123A	7.24E-09	0	0	0	0	0	7.97E-09	0
FAM123B	1.04E-06	4.75E-07	6.27E-07	3.08E-07	4.08E-07	2.24E-06	5.82E-07	7.11E-07
FAM123C	7.26E-09	0	0	0	0	0	0	0
FAM124A	1.06E-06	2.72E-08	2.36E-07	6.09E-07	3.10E-08	1.40E-07	1.46E-07	1.85E-07
FAM124B	1.01E-06	9.70E-07	2.62E-07	3.22E-07	2.13E-07	3.79E-07	4.70E-07	8.29E-07
FAM125A	2.81E-05	1.64E-05	1.59E-05	2.07E-05	4.85E-05	2.74E-05	2.62E-05	2.33E-05
FAM125B	1.20E-05	2.12E-06	4.44E-06	3.57E-06	9.52E-06	7.68E-06	9.39E-06	1.06E-05
FAM126A	5.80E-06	4.02E-06	2.26E-06	2.35E-06	6.42E-07	3.24E-06	1.40E-06	1.92E-06
FAM126B	1.97E-06	1.59E-06	3.60E-06	1.14E-06	7.30E-07	1.31E-06	1.23E-06	1.33E-06
FAM127A	0.000136	4.99E-05	2.05E-05	4.94E-05	7.89E-06	4.60E-05	4.75E-05	9.15E-06
FAM127B	8.52E-05	4.12E-05	1.59E-05	3.35E-05	3.80E-06	2.53E-05	3.76E-05	5.76E-06
FAM127C	1.42E-05	8.78E-07	8.37E-07	3.68E-06	6.58E-07	7.20E-07	1.39E-06	1.09E-06
FAM128A	8.23E-05	6.01E-05	3.31E-05	5.14E-05	0.000161	5.14E-05	7.41E-05	5.00E-05
FAM128B	0.000165	0.000192	9.15E-05	0.000121	0.000452	0.000108	0.000196	7.78E-05
FAM129A	4.50E-06	1.01E-06	5.82E-07	1.67E-06	2.32E-07	5.90E-07	1.48E-06	7.30E-07
FAM129B	2.96E-05	3.27E-06	1.03E-05	3.06E-05	4.18E-06	1.14E-05	1.56E-05	7.00E-06
FAM129C	9.76E-08	2.31E-08	6.76E-08	1.08E-07	4.02E-08	6.71E-08	2.60E-08	0
FAM131A	1.23E-05	6.03E-06	1.61E-06	3.56E-06	8.94E-07	2.90E-06	2.12E-06	4.66E-06
FAM131B	1.09E-07	2.20E-07	1.12E-08	3.32E-08	3.74E-08	2.02E-08	9.00E-08	1.12E-07
FAM131C	3.53E-06	2.45E-07	9.09E-08	5.81E-07	0	0	0	4.23E-08
FAM132A	8.49E-06	2.96E-07	2.79E-08	6.32E-08	3.76E-07	2.39E-08	1.46E-07	7.62E-08
FAM133A	6.13E-06	1.49E-06	6.80E-08	1.25E-07	0	2.71E-07	7.10E-09	1.18E-06
FAM133B	1.35E-05	2.03E-06	1.23E-06	1.74E-06	8.63E-07	1.99E-06	1.20E-06	1.03E-06
FAM134A	8.48E-05	4.67E-05	3.23E-05	3.21E-05	4.03E-05	5.23E-05	4.55E-05	6.54E-05
FAM134B	4.68E-06	1.07E-05	2.70E-05	2.55E-05	1.61E-07	5.90E-06	2.28E-06	1.21E-07
FAM134C	3.61E-05	2.80E-05	1.97E-05	2.19E-05	4.83E-05	2.98E-05	2.43E-05	2.78E-05
FAM135A	7.15E-06	4.41E-06	2.33E-06	2.93E-06	6.51E-07	5.46E-06	1.81E-06	3.21E-06
FAM135B	2.23E-06	3.16E-08	2.33E-08	1.59E-08	4.19E-08	1.20E-08	3.17E-08	4.76E-09
FAM136A	4.89E-05	4.66E-05	1.64E-05	1.65E-05	2.23E-05	1.90E-05	1.90E-05	1.76E-05
FAM136B	0	0	1.31E-07	0	1.70E-07	0	0	0
FAM138B	0	0	0	0	0	0	0	0
FAM138D	0	0	0	0	0	0	0	0
FAM138E	0	0	0	0	0	0	0	0
FAM138F	0	0	0	0	0	6.45E-08	0	0
FAM13A	1.24E-05	2.78E-06	3.54E-05	1.94E-05	5.03E-06	1.33E-05	1.06E-05	1.06E-06
FAM13AO	7.91E-07	9.64E-08	9.60E-08	1.03E-07	1.01E-07	1.29E-07	6.52E-08	1.84E-07
FAM13B	4.94E-06	2.20E-06	1.99E-06	3.06E-06	5.45E-07	1.65E-06	1.11E-06	2.13E-06
FAM13C	2.79E-06	1.34E-06	5.03E-07	1.10E-06	8.39E-08	8.23E-07	1.42E-06	7.99E-07
FAM149A	9.89E-06	5.17E-06	2.25E-05	1.67E-05	8.70E-06	8.33E-06	1.15E-05	1.32E-05
FAM149B1	1.42E-05	1.78E-05	1.04E-05	7.63E-06	4.54E-06	1.32E-05	6.22E-06	9.17E-06
FAM150A	1.55E-07	0	1.43E-07	5.45E-07	0	0	0	0
FAM150B	1.26E-07	2.05E-08	1.73E-06	2.72E-06	1.39E-06	3.34E-08	3.11E-07	0
FAM151A	6.82E-08	7.54E-06	6.32E-06	2.80E-06	2.46E-07	3.65E-08	3.49E-07	0
FAM151B	4.02E-07	5.35E-07	1.45E-07	1.63E-07	1.39E-07	1.65E-07	7.79E-08	4.10E-07

FAM153A	5.61E-07	0	1.07E-08	5.70E-08	0	0	3.53E-08	7.60E-09
FAM153B	6.91E-07	0	1.19E-08	5.58E-08	0	0	1.10E-08	0
FAM153C	1.23E-07	0	0	0	0	0	0	0
FAM154A	0	8.40E-08	0	0	0	2.88E-07	0	0
FAM154B	3.43E-08	2.67E-07	6.10E-08	5.87E-08	1.21E-08	1.03E-07	3.41E-08	4.33E-08
FAM155A	4.73E-08	1.06E-07	2.43E-08	2.17E-08	3.05E-08	8.21E-08	1.21E-07	1.39E-07
FAM155B	2.15E-06	0	1.75E-08	1.59E-07	7.30E-08	2.10E-08	2.37E-07	0
FAM156A	1.09E-05	4.35E-06	2.92E-06	6.75E-06	4.87E-06	9.64E-06	6.59E-06	1.11E-05
FAM157A	1.04E-06	3.78E-08	1.76E-08	8.14E-08	0	4.62E-08	3.51E-08	7.85E-08
FAM157B	1.99E-08	0	0	0	0	0	0	0
FAM158A	1.16E-05	2.05E-05	1.35E-05	9.09E-06	3.93E-05	7.98E-06	1.51E-05	1.79E-05
FAM159A	1.41E-06	5.08E-08	1.88E-08	9.43E-07	7.42E-08	2.48E-07	4.11E-07	9.04E-08
FAM160A1	3.91E-07	3.87E-08	8.63E-08	1.69E-07	0	9.61E-08	6.26E-08	1.45E-08
FAM160A2	1.38E-05	1.20E-05	1.05E-05	1.17E-05	3.77E-06	1.16E-05	8.36E-06	2.15E-05
FAM160B1	6.71E-06	6.07E-06	6.79E-06	4.54E-06	2.20E-06	7.87E-06	3.75E-06	6.71E-06
FAM160B2	7.54E-06	9.41E-06	4.24E-06	6.98E-06	2.63E-06	6.98E-06	5.91E-06	5.49E-06
FAM161A	1.12E-06	2.03E-06	6.50E-07	6.43E-07	8.65E-08	6.77E-07	3.38E-07	4.00E-07
FAM161B	1.50E-06	7.06E-07	7.06E-07	6.41E-07	0	1.17E-06	4.37E-07	4.37E-07
FAM162A	0.000146	0.000162	0.000105	7.54E-05	0.000171	0.000103	0.000108	6.89E-05
FAM162B	5.12E-07	9.18E-07	1.40E-07	2.53E-07	9.08E-07	6.44E-07	9.76E-07	3.05E-07
FAM163A	1.12E-07	8.78E-09	9.29E-08	1.96E-07	0	4.45E-08	1.16E-07	1.17E-08
FAM163B	4.85E-07	0	7.01E-07	3.96E-06	6.12E-08	6.59E-08	9.54E-07	0
FAM164A	3.99E-06	2.39E-07	4.04E-07	9.32E-07	5.31E-08	8.91E-08	7.87E-07	2.22E-07
FAM164C	5.34E-06	2.27E-06	2.27E-06	4.02E-06	1.19E-06	3.40E-06	3.74E-06	3.93E-06
FAM165B	2.29E-05	1.63E-05	1.10E-05	1.44E-05	2.78E-05	2.63E-05	2.05E-05	1.19E-05
FAM166A	4.64E-08	0	5.02E-08	0	0	2.16E-07	0	0
FAM166B	1.16E-07	7.86E-07	1.03E-07	1.86E-07	2.54E-07	1.02E-06	4.83E-07	8.40E-08
FAM167A	2.62E-06	1.43E-07	7.43E-08	7.06E-07	4.68E-09	3.09E-07	1.08E-08	6.19E-07
FAM167B	6.23E-06	4.71E-06	1.10E-05	7.89E-06	1.14E-05	4.42E-06	1.07E-05	3.97E-06
FAM168A	6.42E-06	3.90E-06	2.13E-06	1.65E-06	1.78E-07	2.82E-06	9.81E-07	3.28E-06
FAM168B	2.60E-05	1.95E-05	1.02E-05	1.03E-05	7.19E-06	1.47E-05	1.88E-05	1.69E-05
FAM169A	1.96E-06	3.04E-06	5.39E-07	3.38E-06	5.94E-07	1.53E-05	1.12E-06	5.69E-07
FAM169B	8.80E-07	3.92E-07	1.87E-07	4.35E-07	0	2.46E-07	6.78E-09	4.92E-06
FAM170A	0	4.38E-08	6.70E-08	2.55E-08	0	0	3.98E-08	6.51E-08
FAM170B	0	0	0	0	0	0	0	0
FAM171A1	2.59E-05	3.04E-07	1.09E-05	1.54E-05	1.58E-05	1.71E-05	8.99E-06	1.16E-05
FAM171A2	1.82E-06	1.40E-07	3.68E-08	1.74E-07	8.39E-08	3.29E-08	2.67E-07	3.52E-07
FAM171B	2.52E-06	3.64E-07	1.38E-07	2.44E-07	1.65E-07	1.99E-07	2.12E-07	3.25E-07
FAM172A	2.83E-06	3.08E-06	2.04E-06	2.23E-06	6.65E-07	4.25E-06	1.80E-06	2.66E-06
FAM173A	1.22E-05	1.46E-05	9.37E-06	1.20E-05	2.49E-05	9.95E-06	3.37E-05	1.39E-05
FAM173B	9.10E-06	4.23E-06	4.89E-06	3.21E-06	3.87E-06	6.17E-06	5.90E-06	6.88E-06
FAM174A	1.32E-05	1.48E-05	1.24E-05	1.53E-05	5.70E-06	1.63E-05	1.36E-05	2.23E-05
FAM174B	4.15E-06	3.14E-06	2.01E-06	5.62E-06	1.94E-06	1.37E-06	3.97E-06	1.26E-06
FAM175A	3.03E-06	1.20E-06	1.29E-06	1.75E-06	6.61E-07	3.08E-06	1.13E-06	1.11E-06
FAM175B	8.88E-06	5.81E-06	6.21E-06	5.65E-06	2.13E-06	8.15E-06	6.13E-06	6.56E-06
FAM176A	4.63E-05	2.61E-05	6.04E-05	4.53E-05	4.73E-05	5.63E-05	4.50E-05	8.01E-05
FAM176B	7.90E-06	7.41E-06	2.70E-06	1.13E-05	2.00E-05	3.20E-06	1.60E-05	3.00E-06
FAM177A1	1.60E-05	2.26E-05	1.32E-05	2.11E-05	3.59E-06	1.43E-05	1.38E-05	1.89E-05
FAM177B	6.45E-06	2.15E-08	0	0	1.62E-08	5.24E-08	1.80E-08	2.78E-07
FAM178A	6.52E-06	2.28E-06	1.89E-06	1.79E-06	1.74E-06	4.14E-06	2.09E-06	2.46E-06
FAM178B	1.71E-07	0	0	4.77E-08	0	0	3.14E-08	9.94E-08
FAM179A	6.03E-07	1.35E-08	4.00E-08	3.56E-07	1.13E-07	4.31E-07	7.67E-08	1.41E-07
FAM179B	2.35E-06	1.72E-06	2.07E-06	1.93E-06	5.18E-07	2.59E-06	1.24E-06	1.51E-06
FAM180A	1.88E-07	4.60E-08	4.67E-06	1.25E-05	1.65E-07	1.40E-07	4.67E-07	1.21E-07
FAM180B	1.81E-08	2.07E-08	0	0	1.56E-08	0	0	2.67E-08
FAM181A	0	0	0	0	0	0	0	0
FAM181B	1.30E-08	1.48E-08	0	0	2.24E-08	0	0	0
FAM182A	0	1.48E-08	0	0	3.78E-09	0	1.29E-08	6.45E-09

FAM182B	1.33E-07	2.12E-07	2.52E-08	5.29E-08	7.04E-09	2.07E-07	7.61E-08	2.10E-07
FAM183A	6.64E-08	6.62E-07	0	0	0	1.89E-07	0	0
FAM183B	2.98E-08	0	1.57E-08	1.83E-08	1.28E-08	5.53E-08	0	2.20E-08
FAM184A	2.69E-06	5.31E-06	9.38E-07	5.14E-07	1.42E-06	6.13E-06	3.36E-07	0
FAM184B	0	0	6.44E-09	7.61E-09	0	0	6.27E-09	0
FAM185A	1.43E-06	4.08E-06	3.06E-06	2.49E-06	1.68E-06	3.33E-06	2.47E-06	4.00E-06
FAM186A	2.38E-08	2.66E-08	3.04E-09	7.26E-09	2.55E-08	2.48E-08	1.45E-08	0
FAM186B	1.65E-07	3.85E-07	4.64E-07	3.01E-07	1.94E-07	8.22E-07	4.89E-07	5.81E-07
FAM187B	0	5.04E-08	0	0	5.68E-08	0	0	0
FAM188A	5.31E-06	4.73E-06	7.01E-06	6.75E-06	2.33E-06	6.65E-06	5.12E-06	3.93E-06
FAM188B	1.95E-05	2.25E-06	7.64E-07	1.87E-06	1.25E-06	1.70E-06	8.89E-07	6.26E-07
FAM189A1	0	4.57E-08	5.24E-09	4.98E-08	1.31E-08	4.72E-09	8.93E-08	0
FAM189A2	1.37E-06	2.16E-07	1.64E-08	5.63E-07	2.14E-07	7.38E-08	2.37E-07	6.87E-08
FAM189B	9.29E-06	1.24E-05	3.18E-06	4.65E-06	6.05E-06	1.38E-05	6.19E-06	9.80E-06
FAM18A	2.31E-07	1.10E-07	5.82E-08	3.21E-07	3.62E-08	1.32E-08	1.42E-07	6.22E-08
FAM18B	1.00E-05	2.37E-05	1.62E-05	1.39E-05	3.80E-06	2.03E-05	1.10E-05	1.54E-05
FAM18B2	2.09E-06	2.18E-06	2.11E-06	3.66E-06	1.68E-06	3.57E-06	2.52E-06	2.24E-06
FAM190A	2.59E-08	1.31E-07	4.07E-08	1.12E-07	0	2.44E-08	5.12E-08	1.87E-08
FAM190B	6.80E-06	1.37E-05	6.52E-06	6.19E-06	2.08E-06	4.93E-06	4.03E-06	9.76E-06
FAM192A	6.74E-05	2.27E-05	1.18E-05	1.80E-05	6.03E-06	2.78E-05	1.67E-05	2.53E-05
FAM193A	9.30E-06	5.50E-06	3.40E-06	4.60E-06	1.87E-06	3.94E-06	4.09E-06	5.14E-06
FAM193B	1.03E-05	1.92E-06	4.15E-06	5.99E-06	7.49E-06	7.45E-06	6.19E-06	1.08E-05
FAM194A	2.33E-08	2.64E-08	0	2.86E-08	2.00E-08	2.16E-08	0	0
FAM194B	0	0	0	0	0	0	0	0
FAM195A	5.25E-05	0.000136	5.16E-05	5.67E-05	0.000102	4.59E-05	9.40E-05	7.16E-05
FAM195B	0.000103	9.85E-05	4.80E-05	7.09E-05	0.000205	8.08E-05	8.37E-05	5.45E-05
FAM196A	4.68E-08	6.30E-09	5.78E-09	0	0	0	1.09E-08	0
FAM196B	0	0	6.66E-09	2.37E-08	0	5.98E-09	0	9.47E-09
FAM197Y2	0	0	0	0	0	0	0	0
FAM198A	1.49E-06	1.25E-06	4.07E-06	1.37E-06	1.03E-06	3.10E-07	3.71E-07	5.37E-08
FAM198B	5.45E-06	5.97E-06	2.48E-06	2.42E-06	1.87E-06	3.61E-06	3.42E-06	3.94E-06
FAM199X	1.21E-05	1.92E-05	4.07E-06	4.05E-06	1.55E-06	6.08E-06	3.39E-06	8.62E-06
FAM19A1	1.40E-08	3.19E-08	5.92E-08	6.90E-08	1.21E-08	6.52E-08	4.06E-08	4.14E-08
FAM19A2	3.07E-08	2.46E-07	1.08E-07	7.99E-08	8.02E-09	1.84E-07	1.01E-07	6.85E-08
FAM19A3	7.13E-08	0	0	0	0	0	0	0
FAM19A4	5.31E-07	0	5.54E-08	0	0	0	0	1.63E-08
FAM19A5	6.56E-07	3.34E-07	1.68E-07	6.39E-07	1.86E-07	9.18E-08	1.40E-07	1.32E-08
FAM200A	7.76E-06	3.97E-06	1.44E-06	1.46E-06	1.20E-06	3.34E-06	1.53E-06	2.34E-06
FAM200B	6.61E-06	8.51E-06	3.65E-06	3.95E-06	3.64E-06	5.71E-06	3.70E-06	2.57E-06
FAM20A	1.09E-05	3.95E-05	2.09E-05	1.60E-05	2.88E-05	2.75E-05	1.12E-05	4.53E-05
FAM20B	1.14E-05	1.12E-05	7.55E-06	6.67E-06	3.25E-06	1.19E-05	7.36E-06	1.38E-05
FAM20C	6.31E-05	3.26E-05	5.79E-05	4.32E-05	8.24E-05	5.50E-05	6.60E-05	4.01E-05
FAM21A	5.51E-06	3.42E-06	3.01E-06	3.73E-06	#######	1.68E-05	2.34E-06	2.44E-06
FAM21B	5.55E-06	6.22E-06	3.01E-06	5.31E-06	4.17E-06	1.17E-05	3.68E-06	5.91E-06
FAM21C	1.43E-05	1.40E-05	6.98E-06	1.75E-05	5.37E-06	2.37E-05	1.08E-05	1.69E-05
FAM22A	2.24E-07	2.75E-07	4.85E-07	3.85E-07	5.72E-07	1.70E-06	8.16E-07	6.26E-07
FAM22D	5.81E-07	4.99E-07	5.66E-07	8.29E-07	6.74E-07	1.76E-06	1.02E-06	4.88E-07
FAM22F	4.57E-08	5.15E-08	1.90E-08	2.24E-08	5.50E-08	2.54E-08	1.77E-07	5.37E-08
FAM22G	3.23E-08	1.55E-08	4.74E-09	1.69E-08	3.96E-09	3.85E-08	0	0
FAM23A	9.11E-08	9.69E-09	1.78E-08	4.76E-08	3.72E-08	3.61E-08	2.11E-08	1.90E-08
FAM24A	0	0	0	0	0	0	0	0
FAM24B	1.45E-06	6.66E-06	2.45E-07	6.43E-07	1.30E-06	1.34E-07	7.75E-07	4.40E-07
FAM25A	0	0	1.23E-06	0	0	0	1.14E-07	0
FAM25B	0	0	0	0	0	0	0	0
FAM26D	3.87E-08	0	0	0	1.67E-08	0	0	0
FAM26E	8.17E-07	4.00E-07	3.07E-07	3.88E-07	1.67E-07	4.80E-07	6.86E-07	2.18E-07
FAM26F	2.63E-05	4.35E-07	5.12E-06	9.31E-06	9.18E-07	3.71E-06	5.03E-06	1.75E-06
FAM27A	1.52E-06	0	9.71E-08	1.58E-07	0	0	7.92E-08	4.46E-07

FAM27B	0	0	0	3.16E-07	0	0	0	0
FAM27C	2.23E-06	3.52E-07	1.46E-07	5.26E-07	1.84E-07	3.96E-08	1.58E-07	2.55E-07
FAM27L	0	0	0	0	0	0	0	0
FAM32A	8.58E-05	3.48E-05	5.13E-05	6.00E-05	7.94E-05	6.20E-05	7.62E-05	5.48E-05
FAM35A	8.93E-06	1.30E-05	1.53E-05	1.43E-05	1.63E-05	2.05E-05	1.09E-05	1.27E-05
FAM35B	4.70E-06	4.76E-06	7.85E-06	9.59E-06	1.43E-05	1.09E-05	1.16E-05	6.72E-06
FAM35B2	1.33E-06	1.24E-06	2.12E-06	3.38E-06	5.06E-06	3.45E-06	3.62E-06	1.64E-06
FAM36A	3.57E-05	2.81E-05	1.68E-05	1.45E-05	7.74E-06	2.91E-05	8.15E-06	1.71E-05
FAM38A	4.31E-05	1.72E-05	1.41E-05	3.33E-05	4.82E-05	1.87E-05	3.13E-05	1.21E-05
FAM38B	3.61E-05	1.27E-05	6.15E-06	3.15E-06	6.93E-07	7.96E-06	5.74E-06	8.64E-06
FAM3A	2.81E-05	6.63E-05	2.91E-05	3.04E-05	7.95E-05	2.91E-05	3.48E-05	5.53E-05
FAM3B	1.32E-05	2.91E-08	9.87E-07	9.46E-07	3.56E-08	3.76E-05	1.29E-07	4.98E-07
FAM3C	8.39E-06	1.61E-05	1.48E-05	2.22E-05	9.05E-06	1.16E-05	1.32E-05	1.48E-05
FAM3D	0	0	0	4.77E-08	1.67E-08	0	5.58E-08	0
FAM40A	7.65E-06	6.16E-06	4.19E-06	4.96E-06	1.55E-06	1.76E-05	4.36E-06	4.50E-06
FAM40B	1.33E-06	2.38E-07	5.07E-08	6.02E-08	1.13E-07	1.16E-07	4.26E-08	3.51E-07
FAM41AY	0	0	0	0	0	0	0	0
FAM41C	6.87E-07	1.79E-07	3.02E-08	2.64E-07	2.84E-07	3.59E-07	2.21E-07	2.74E-07
FAM43A	4.00E-06	1.34E-06	1.09E-06	2.54E-06	7.26E-07	7.89E-06	5.65E-06	2.33E-06
FAM43B	1.44E-06	4.09E-08	1.13E-07	6.33E-07	1.40E-07	0	1.76E-07	6.13E-07
FAM45A	1.02E-05	1.33E-05	8.19E-06	9.64E-06	2.68E-06	9.63E-06	1.06E-05	9.01E-06
FAM45B	4.83E-06	6.93E-06	4.51E-06	4.87E-06	1.60E-07	4.57E-06	2.81E-06	4.24E-06
FAM46A	8.98E-06	1.18E-05	5.72E-06	2.46E-05	7.14E-07	3.42E-06	9.45E-06	9.67E-06
FAM46B	4.07E-07	3.92E-07	1.66E-07	1.58E-07	6.84E-08	6.45E-08	6.74E-08	1.17E-07
FAM46C	2.29E-06	1.17E-06	2.45E-06	5.20E-06	2.79E-07	1.79E-06	8.03E-07	6.20E-07
FAM46D	7.69E-09	0	0	0	0	0	0	0
FAM47A	0	0	0	0	0	0	0	1.35E-08
FAM47B	0	0	0	0	0	0	0	0
FAM47C	0	7.90E-08	0	2.58E-08	0	0	1.37E-08	1.03E-08
FAM47E	1.16E-06	1.59E-06	1.53E-06	2.33E-06	1.20E-06	5.37E-06	3.27E-06	2.00E-06
FAM48A	1.99E-05	8.15E-06	5.94E-06	8.25E-06	2.44E-06	8.38E-06	7.22E-06	5.24E-06
FAM48B1	8.77E-09	0	0	0	0	8.13E-09	8.50E-09	0
FAM48B2	0	0	0	2.35E-08	0	0	9.28E-09	0
FAM49A	3.48E-06	7.03E-07	1.26E-06	3.59E-06	4.55E-08	8.52E-07	1.39E-06	1.03E-06
FAM49B	6.81E-05	1.82E-05	8.69E-06	1.80E-05	4.19E-06	2.31E-05	2.21E-05	1.68E-05
FAM50A	8.30E-05	7.92E-05	2.46E-05	5.63E-05	6.96E-05	5.33E-05	7.34E-05	8.20E-05
FAM50B	3.04E-05	1.33E-05	9.32E-06	1.34E-05	1.13E-05	4.94E-07	6.22E-06	2.42E-06
FAM53A	9.77E-07	8.23E-07	4.90E-07	2.07E-07	3.86E-07	1.73E-07	8.99E-07	8.50E-07
FAM53B	5.71E-06	9.76E-07	1.82E-06	2.62E-06	2.40E-06	2.36E-06	3.83E-06	2.39E-06
FAM53C	1.00E-05	5.65E-06	3.33E-06	6.52E-06	1.88E-06	6.72E-06	8.50E-06	1.13E-05
FAM54A	2.05E-06	1.14E-06	7.00E-08	1.01E-07	9.94E-08	6.57E-07	1.90E-07	3.10E-07
FAM54B	5.56E-05	5.30E-05	3.16E-05	2.88E-05	1.45E-05	2.48E-05	2.75E-05	2.38E-05
FAM55A	0	0	0	1.66E-08	0	0	0	0
FAM55B	1.36E-08	0	0	0	0	0	1.32E-08	0
FAM55C	1.96E-06	2.47E-07	6.16E-07	8.77E-07	1.13E-07	2.22E-07	3.35E-07	2.60E-07
FAM55D	0	1.24E-08	2.30E-08	2.69E-08	0	0	0	0
FAM57A	5.36E-06	6.13E-07	4.17E-07	2.97E-06	9.78E-07	1.31E-06	2.53E-06	2.24E-06
FAM57B	5.71E-08	2.84E-08	0	0	3.69E-08	3.98E-08	0	3.69E-08
FAM58A	9.64E-06	1.28E-05	7.66E-06	9.74E-06	2.43E-05	8.77E-06	1.45E-05	2.36E-05
FAM58B	2.71E-08	0	0	0	0	0	2.58E-08	0
FAM59A	5.80E-06	2.45E-06	1.50E-06	2.71E-06	1.76E-07	2.73E-06	5.91E-07	6.08E-06
FAM5B	0	9.21E-08	5.39E-08	1.18E-07	7.68E-08	0	0	2.19E-08
FAM5C	0	0	0	1.13E-08	7.92E-09	0	0	1.89E-07
FAM60A	1.22E-05	4.83E-06	2.95E-06	7.36E-06	8.55E-07	6.03E-06	5.50E-06	2.73E-06
FAM63A	2.81E-05	1.20E-05	7.56E-06	6.93E-06	4.82E-06	1.77E-05	1.01E-05	9.49E-06
FAM63B	2.05E-06	6.09E-07	9.77E-07	6.20E-07	4.75E-08	6.41E-07	1.53E-07	4.90E-07
FAM64A	2.37E-06	1.72E-07	5.61E-08	1.51E-07	1.54E-07	3.26E-07	2.03E-07	4.86E-08
FAM65A	2.16E-05	9.78E-06	7.16E-06	1.34E-05	4.00E-06	6.00E-06	1.29E-05	1.03E-05

FAM65B	1.63E-06	1.07E-06	1.11E-06	1.20E-06	4.48E-08	2.35E-06	3.55E-07	4.80E-07
FAM65C	2.10E-07	1.81E-07	2.55E-06	2.56E-06	1.64E-07	6.47E-08	1.10E-07	3.42E-07
FAM66A	7.48E-09	0	0	4.60E-08	0	0	0	0
FAM66C	3.01E-08	2.47E-08	3.68E-08	2.81E-08	0	8.22E-09	2.58E-08	1.04E-07
FAM66D	#######	1.90E-06	2.67E-08	4.60E-07	1.19E-06	0	2.35E-08	0
FAM66E	0	0	0	0	0	1.62E-08	0	0
FAM69A	2.17E-05	2.76E-05	1.53E-05	9.18E-06	4.33E-06	8.24E-06	5.07E-06	7.04E-06
FAM69B	9.84E-06	5.91E-06	3.41E-06	1.97E-06	5.91E-06	7.27E-07	6.97E-06	1.26E-05
FAM69C	0	0	1.35E-07	0	8.06E-08	3.26E-08	0	1.55E-07
FAM70A	7.45E-07	6.02E-08	9.02E-08	7.29E-07	6.87E-08	1.75E-07	3.53E-08	1.08E-07
FAM70B	2.75E-06	1.20E-06	2.71E-06	2.46E-06	1.92E-06	6.51E-07	4.60E-06	2.68E-06
FAM71A	0	0	0	2.52E-08	0	0	0	1.44E-94
FAM71B	0	0	0	0	0	0	0	0
FAM71C	0	0	0	0	0	0	0	0
FAM71D	6.47E-07	4.20E-07	1.80E-07	3.98E-07	1.06E-07	6.30E-07	3.56E-07	4.78E-07
FAM71E1	1.24E-06	2.45E-06	1.17E-06	1.14E-06	1.89E-06	5.97E-07	1.97E-06	7.44E-07
FAM71E2	7.96E-09	8.95E-09	0	0	0	0	0	0
FAM71F1	0	0	0	3.51E-08	1.23E-08	1.32E-08	0	0
FAM71F2	1.95E-07	0	0	6.16E-08	6.14E-08	8.82E-08	1.56E-08	1.41E-07
FAM72A	2.99E-07	4.56E-07	2.73E-08	4.78E-08	0	7.23E-08	0	1.72E-07
FAM72B	2.62E-06	1.85E-06	1.97E-07	3.47E-07	5.07E-07	1.00E-06	2.54E-07	5.09E-07
FAM72D	1.35E-06	1.04E-06	2.18E-08	1.02E-07	8.97E-08	3.43E-07	1.24E-07	7.67E-08
FAM73A	8.13E-06	5.82E-06	3.80E-06	2.49E-06	4.58E-07	6.71E-06	9.64E-07	2.30E-06
FAM73B	7.76E-06	5.57E-06	4.50E-06	4.62E-06	6.51E-06	5.15E-06	5.17E-06	7.91E-06
FAM74A1	0	0	1.43E-08	0	0	0	0	0
FAM74A3	2.56E-08	0	1.34E-08	0	1.10E-08	1.19E-08	0	1.89E-08
FAM74A4	0	0	0	1.59E-08	0	0	0	0
FAM75A2	0	1.20E-08	0	6.57E-09	0	0	5.23E-09	0
FAM75A3	0	0	0	0	0	0	0	0
FAM75A5	0	0	0	0	0	0	0	0
FAM75A6	0	0	0	0	0	0	0	0
FAM75C1	6.05E-09	0	0	0	0	0	0	0
FAM76A	2.40E-06	2.05E-06	1.79E-06	2.15E-06	4.39E-07	2.01E-06	1.99E-06	1.80E-06
FAM76B	9.68E-06	2.32E-06	3.47E-06	1.99E-06	8.12E-07	2.75E-06	1.63E-06	6.65E-06
FAM78A	3.64E-06	2.98E-07	1.18E-06	1.40E-06	6.19E-07	1.30E-06	2.01E-06	1.14E-06
FAM78B	4.11E-07	7.37E-07	2.72E-07	8.26E-07	1.72E-07	1.29E-06	2.23E-06	1.45E-07
FAM7A2	2.81E-08	0	0	0	2.78E-08	0	0	0
FAM7A3	2.27E-08	0	4.91E-08	5.60E-08	7.85E-08	0	8.68E-08	0
FAM81A	1.47E-06	1.22E-07	9.01E-08	1.64E-07	9.09E-08	6.13E-08	2.26E-07	8.73E-08
FAM81B	0	0	0	1.94E-08	0	0	0	2.33E-08
FAM82A1	6.73E-06	6.78E-06	1.35E-05	9.37E-06	4.02E-06	1.05E-05	4.32E-06	6.12E-06
FAM82A2	3.82E-05	3.69E-05	3.21E-05	2.59E-05	1.49E-05	3.43E-05	2.78E-05	2.97E-05
FAM82B	2.52E-05	2.61E-05	1.74E-05	1.22E-05	1.70E-05	1.83E-05	2.89E-05	1.23E-05
FAM83A	1.18E-07	9.38E-07	8.36E-08	0	1.87E-08	6.04E-08	5.10E-08	1.72E-07
FAM83B	1.15E-06	0	5.28E-08	0	0	0	3.54E-08	1.07E-08
FAM83C	6.56E-08	2.78E-08	4.99E-08	2.67E-08	3.13E-08	2.70E-08	7.08E-09	2.51E-08
FAM83D	1.11E-05	9.92E-06	8.27E-07	9.02E-07	1.93E-06	4.71E-06	1.84E-06	1.58E-06
FAM83E	0	1.35E-08	7.48E-08	0	3.07E-08	0	1.15E-08	0
FAM83F	1.15E-08	1.30E-08	3.62E-07	1.57E-06	0	2.14E-08	1.00E-07	0
FAM83G	2.29E-06	3.65E-06	1.40E-06	7.64E-06	2.72E-07	1.75E-06	5.10E-06	3.97E-06
FAM83H	3.38E-05	2.20E-05	4.59E-06	9.58E-06	2.07E-05	1.25E-05	4.36E-05	1.74E-05
FAM84A	1.88E-06	4.29E-07	1.24E-06	2.79E-07	6.22E-08	3.45E-07	2.17E-07	8.94E-07
FAM84B	1.22E-05	9.71E-06	5.38E-06	4.27E-06	4.86E-06	6.07E-06	1.09E-05	6.82E-06
FAM86A	7.06E-06	1.50E-05	4.40E-06	5.54E-06	6.80E-06	8.08E-06	1.02E-05	8.19E-06
FAM86B1	2.83E-07	2.54E-06	4.02E-07	6.98E-07	1.85E-07	1.44E-06	2.33E-07	2.85E-07
FAM86B2	8.84E-08	7.48E-07	6.49E-08	0	0	1.62E-07	0	0
FAM86C	2.52E-06	4.92E-06	1.64E-06	2.19E-06	1.37E-06	3.14E-06	2.15E-06	2.29E-06
FAM86D	2.95E-06	2.18E-06	1.07E-06	1.44E-06	7.69E-07	1.51E-06	1.33E-06	9.94E-07

FAM89A	5.22E-06	5.80E-06	4.79E-06	7.02E-06	4.71E-07	9.78E-06	6.30E-06	1.41E-05
FAM89B	3.48E-05	2.62E-05	1.36E-05	2.43E-05	1.66E-05	1.39E-05	2.36E-05	3.23E-05
FAM8A1	4.30E-05	2.76E-05	3.32E-05	2.45E-05	8.58E-06	4.26E-05	1.66E-05	3.04E-05
FAM90A1	1.21E-07	1.35E-07	2.08E-08	8.57E-08	0	0	3.87E-08	5.88E-08
FAM90A7	0	0	0	0	0	0	0	0
FAM91A1	4.50E-05	2.22E-05	1.13E-05	9.22E-06	3.73E-06	1.52E-05	1.52E-05	1.02E-05
FAM92A1	2.46E-05	7.02E-06	8.19E-06	8.55E-06	4.36E-06	5.16E-06	1.18E-05	3.38E-06
FAM92A3	0	0	0	0	0	0	0	1.41E-08
FAM92B	5.08E-08	0	1.33E-08	7.79E-08	0	0	1.23E-08	0
FAM95B1	4.48E-07	3.59E-07	2.92E-07	3.05E-07	4.93E-07	1.88E-07	6.56E-08	1.26E-07
FAM96A	0.000189	0.000207	0.000113	8.13E-05	4.94E-05	0.000105	0.000101	6.77E-05
FAM96B	0.000126	0.000132	8.26E-05	0.000109	9.24E-05	0.000115	0.000104	8.91E-05
FAM98A	2.24E-05	2.56E-05	1.06E-05	1.35E-05	9.77E-06	1.93E-05	1.39E-05	1.64E-05
FAM98B	1.42E-05	5.45E-06	4.12E-06	5.44E-06	3.74E-07	5.68E-06	3.39E-06	4.94E-06
FAM98C	1.20E-05	8.49E-06	7.48E-06	9.92E-06	1.65E-05	1.15E-05	1.82E-05	1.09E-05
FAM99A	1.05E-07	1.40E-07	2.71E-05	1.65E-05	2.41E-07	8.13E-07	3.28E-05	1.40E-05
FAM99B	0	2.86E-08	5.41E-06	1.19E-06	4.27E-08	2.07E-07	5.54E-06	5.51E-07
FAM9A	0	0	0	0	0	0	1.57E-08	4.83E-08
FAM9B	3.35E-07	9.10E-07	1.26E-07	6.75E-07	6.45E-08	9.27E-08	5.76E-08	0
FAM9C	0	0	0	0	0	0	6.30E-09	9.51E-09
FANCA	9.86E-06	5.80E-06	2.05E-06	2.63E-06	6.44E-07	3.92E-06	2.01E-06	6.48E-06
FANCB	6.78E-07	3.46E-08	4.16E-08	2.82E-08	3.97E-08	4.47E-08	3.73E-08	1.02E-07
FANCC	6.68E-06	3.12E-06	7.61E-06	3.12E-06	3.57E-06	1.78E-06	2.98E-06	8.27E-07
FANCD2	5.52E-06	2.15E-06	1.68E-07	4.93E-07	3.86E-07	6.78E-07	4.15E-07	4.10E-07
FANCE	2.30E-06	1.36E-06	1.25E-07	5.84E-07	2.70E-07	3.14E-07	5.92E-07	1.09E-06
FANCF	9.08E-06	6.44E-06	2.97E-06	2.50E-06	7.53E-07	4.12E-06	2.38E-06	5.64E-06
FANCG	5.07E-06	4.59E-06	1.30E-06	1.88E-06	2.37E-06	3.87E-06	2.53E-06	2.64E-06
FANCI	9.82E-06	4.44E-06	2.50E-07	5.08E-07	1.59E-06	1.51E-06	6.73E-07	1.20E-06
FANCL	1.18E-05	1.09E-05	4.70E-06	7.31E-06	3.93E-06	5.95E-06	5.75E-06	1.85E-06
FANCM	1.09E-06	5.52E-07	3.23E-07	3.30E-07	1.96E-07	6.21E-07	2.13E-07	6.64E-07
FANK1	3.73E-07	0	2.31E-08	1.32E-07	5.24E-08	3.93E-08	1.75E-07	0
FAP	4.43E-06	4.27E-07	1.67E-08	2.91E-06	4.19E-07	3.56E-07	3.27E-06	3.95E-06
FAR1	6.11E-06	8.20E-07	1.07E-06	1.78E-06	3.81E-07	7.54E-07	9.19E-07	1.05E-06
FAR2	5.12E-06	4.41E-07	5.77E-07	1.38E-06	1.00E-07	5.73E-07	4.10E-07	2.34E-07
FARP1	5.59E-05	8.18E-06	2.20E-05	2.21E-05	1.75E-05	2.99E-05	2.38E-05	1.53E-05
FARP2	4.21E-06	2.23E-06	5.47E-06	4.23E-06	1.99E-06	3.72E-06	2.11E-06	2.61E-06
FARS2	6.98E-06	8.15E-06	7.53E-06	9.26E-06	5.71E-06	1.62E-05	1.10E-05	1.35E-05
FARSA	3.68E-05	2.15E-05	2.69E-05	2.86E-05	2.63E-05	3.93E-05	3.49E-05	3.11E-05
FARSB	2.03E-05	1.58E-05	8.73E-06	1.05E-05	1.01E-05	2.25E-05	1.42E-05	1.71E-05
FAS	1.00E-05	2.98E-06	7.98E-06	9.63E-06	3.16E-06	1.31E-05	5.31E-06	3.35E-06
FASLG	2.41E-06	3.03E-08	2.95E-07	6.56E-07	2.30E-08	4.84E-07	3.36E-07	1.38E-07
FASN	6.54E-05	0.00047	0.000303	4.00E-05	1.30E-05	0.000136	3.43E-05	0.000659
FASTK	2.94E-05	5.69E-05	2.91E-05	3.39E-05	7.67E-05	5.39E-05	4.52E-05	8.03E-05
FASTKD1	2.09E-05	2.22E-05	4.98E-06	4.23E-06	1.92E-06	4.67E-06	1.27E-06	7.17E-06
FASTKD2	6.81E-06	9.68E-06	5.71E-06	5.36E-06	2.28E-06	9.11E-06	3.91E-06	5.42E-06
FASTKD3	8.08E-06	7.07E-06	5.79E-06	3.87E-06	4.01E-06	8.28E-06	5.65E-06	8.96E-06
FASTKD5	1.93E-05	1.53E-05	7.06E-06	7.45E-06	3.66E-06	1.02E-05	5.67E-06	8.26E-06
FAT1	0.000105	3.90E-05	1.83E-05	6.39E-05	3.16E-06	4.86E-05	3.24E-05	0.000116
FAT2	4.83E-08	3.37E-09	2.92E-08	5.70E-09	5.21E-09	0	1.25E-08	8.83E-09
FAT3	5.13E-09	3.37E-08	1.10E-08	9.27E-08	4.34E-09	9.37E-09	2.47E-09	3.78E-08
FAT4	1.54E-06	3.30E-07	1.49E-06	1.77E-06	1.78E-07	7.95E-07	6.96E-07	6.85E-07
FATE1	3.21E-07	2.00E-07	0	0	1.71E-07	2.30E-07	1.01E-06	3.67E-08
FAU	0.001129	0.001024	0.00067	0.000915	0.001212	0.001006	0.000965	0.000908
FBF1	1.62E-06	2.19E-06	3.84E-07	5.92E-07	3.49E-07	1.29E-06	5.63E-07	7.21E-07
FBL	0.000108	5.22E-05	4.72E-05	7.16E-05	0.000157	8.41E-05	0.000111	5.66E-05
FBLIM1	2.18E-05	6.13E-06	1.90E-06	1.23E-05	2.15E-06	2.71E-06	5.84E-06	6.65E-06
FBLL1	5.99E-07	1.16E-07	0	1.23E-07	1.14E-06	3.03E-07	0	1.86E-07
FBLN1	3.38E-05	4.09E-06	3.86E-06	2.27E-05	5.71E-06	2.37E-06	1.10E-05	6.88E-06

FBLN2	6.80E-06	2.69E-06	2.62E-06	6.18E-06	2.95E-06	1.55E-06	8.68E-06	6.82E-06
FBLN5	5.56E-05	5.28E-06	1.26E-05	5.93E-05	1.14E-05	2.80E-06	1.48E-05	8.50E-06
FBLN7	3.53E-06	1.07E-05	2.65E-06	4.28E-06	4.78E-06	5.50E-06	6.25E-06	4.29E-06
FBN1	6.89E-06	1.91E-06	1.44E-06	6.64E-06	1.69E-06	1.81E-06	4.78E-06	3.69E-06
FBN2	9.58E-08	8.02E-08	6.09E-08	7.53E-08	1.24E-08	4.02E-08	1.01E-08	2.41E-08
FBN3	6.43E-07	0	4.20E-08	5.43E-08	2.47E-06	0	7.19E-07	9.13E-08
FBP1	0.000438	0.000653	0.000498	0.000356	0.000578	0.000136	0.000207	9.13E-05
FBP2	2.19E-08	7.34E-08	0	2.40E-08	0	3.92E-08	0	0
FBRS	2.82E-05	2.44E-05	9.86E-06	1.55E-05	7.06E-06	1.39E-05	2.26E-05	1.41E-05
FBRSL1	8.36E-06	8.29E-06	4.04E-06	5.05E-06	3.63E-06	4.84E-06	8.16E-06	4.56E-06
FBXL12	1.61E-05	8.05E-06	6.92E-06	1.03E-05	6.70E-06	8.60E-06	1.25E-05	7.58E-06
FBXL13	7.03E-08	1.88E-08	3.47E-08	2.52E-08	1.41E-08	7.96E-08	2.41E-08	2.41E-08
FBXL14	7.53E-06	5.94E-06	6.84E-06	7.78E-06	2.78E-06	9.95E-06	9.44E-06	6.22E-06
FBXL15	9.88E-06	6.94E-06	6.66E-06	1.19E-05	2.79E-05	9.32E-06	1.86E-05	6.74E-06
FBXL16	1.25E-05	4.34E-07	1.81E-07	1.65E-07	0	4.31E-08	2.39E-06	4.15E-06
FBXL17	5.47E-06	4.37E-06	4.70E-06	3.72E-06	1.01E-06	6.25E-06	3.87E-06	6.84E-06
FBXL18	3.83E-06	2.03E-06	4.09E-07	6.94E-07	2.20E-06	7.27E-07	2.67E-06	1.60E-06
FBXL19	3.86E-06	2.80E-06	6.59E-07	2.18E-06	6.16E-07	2.01E-06	2.17E-06	2.00E-06
FBXL2	3.14E-06	8.40E-08	7.49E-08	3.30E-07	3.66E-08	3.94E-08	2.68E-07	9.38E-08
FBXL20	2.97E-06	3.19E-06	2.44E-06	2.19E-06	1.12E-06	3.57E-06	2.32E-06	8.78E-07
FBXL21	7.75E-07	1.44E-07	1.05E-07	1.44E-07	7.28E-08	2.61E-08	2.09E-07	2.07E-08
FBXL22	5.25E-07	1.54E-07	1.26E-07	6.57E-08	1.29E-07	3.11E-07	1.26E-07	1.78E-07
FBXL3	1.13E-05	1.20E-05	1.24E-05	9.88E-06	2.11E-06	1.07E-05	6.99E-06	8.89E-06
FBXL4	6.11E-06	8.15E-06	8.10E-06	7.59E-06	1.60E-06	1.00E-05	2.63E-06	5.93E-06
FBXL5	2.45E-05	3.95E-05	2.99E-05	2.54E-05	9.01E-06	5.38E-05	2.22E-05	2.30E-05
FBXL6	2.53E-05	7.10E-06	7.30E-06	9.21E-06	2.64E-05	1.01E-05	3.91E-05	1.54E-05
FBXL7	1.28E-06	7.94E-07	5.04E-07	1.32E-06	3.83E-07	2.80E-07	1.23E-06	8.00E-06
FBXL8	1.56E-06	6.29E-07	1.34E-06	2.04E-06	1.43E-06	5.62E-06	1.79E-06	8.81E-07
FBXO10	3.11E-06	3.88E-06	2.27E-06	2.52E-06	1.84E-06	2.49E-06	2.52E-06	2.62E-06
FBXO11	2.87E-05	2.23E-05	1.08E-05	1.41E-05	6.21E-06	1.03E-05	1.17E-05	1.30E-05
FBXO15	2.26E-07	2.57E-07	2.69E-07	1.22E-07	2.32E-07	2.10E-07	8.19E-08	0
FBXO16	1.29E-06	1.80E-06	5.96E-07	7.96E-07	1.26E-07	1.28E-06	7.79E-07	1.85E-07
FBXO17	1.09E-05	1.11E-05	1.80E-05	1.24E-05	1.17E-05	8.80E-06	2.05E-05	1.94E-05
FBXO18	2.48E-05	1.14E-05	1.37E-05	1.66E-05	8.03E-06	1.88E-05	1.26E-05	1.39E-05
FBXO2	6.08E-05	1.28E-05	2.09E-05	2.15E-05	1.82E-05	8.42E-06	2.50E-05	5.30E-05
FBXO21	1.64E-05	5.81E-06	1.06E-05	8.56E-06	1.86E-06	9.11E-06	4.38E-06	3.65E-06
FBXO22	1.41E-05	1.17E-05	1.14E-05	6.89E-06	3.90E-06	1.14E-05	7.32E-06	9.01E-06
FBXO22OS	2.20E-05	1.42E-05	8.15E-06	1.14E-05	1.40E-05	1.41E-05	1.44E-05	1.87E-05
FBXO24	5.38E-07	1.78E-06	2.86E-07	4.34E-07	8.15E-07	3.54E-07	2.98E-07	4.97E-07
FBXO25	1.08E-05	2.98E-05	1.44E-05	1.98E-05	9.09E-06	3.68E-05	1.11E-05	1.28E-05
FBXO27	3.55E-06	2.40E-06	4.67E-06	2.02E-06	1.36E-06	1.58E-06	2.95E-06	3.05E-06
FBXO28	8.61E-06	9.66E-06	7.88E-06	5.87E-06	1.70E-06	6.83E-06	4.24E-06	6.58E-06
FBXO3	1.55E-05	2.11E-05	1.70E-05	1.56E-05	8.54E-06	1.56E-05	1.15E-05	1.92E-05
FBXO30	3.45E-06	2.72E-06	2.29E-06	2.78E-06	2.87E-07	3.67E-06	1.85E-06	2.33E-06
FBXO31	1.32E-05	1.10E-05	1.04E-05	1.23E-05	7.97E-06	6.96E-05	2.17E-05	2.73E-05
FBXO32	5.22E-06	4.37E-06	2.12E-06	1.70E-06	1.46E-06	1.32E-05	3.23E-06	4.34E-06
FBXO33	5.44E-06	2.72E-06	4.03E-06	3.30E-06	1.29E-06	3.77E-06	3.12E-06	4.39E-06
FBXO34	1.22E-05	3.55E-06	4.54E-06	6.24E-06	1.19E-06	4.34E-06	3.28E-06	6.40E-06
FBXO36	7.99E-07	1.25E-06	5.37E-07	1.02E-06	2.96E-07	2.21E-06	8.50E-07	1.20E-06
FBXO38	1.18E-05	8.47E-06	8.72E-06	7.46E-06	2.26E-06	1.01E-05	5.11E-06	8.29E-06
FBXO39	1.77E-07	3.96E-07	5.95E-08	3.61E-08	3.20E-08	2.28E-07	7.12E-08	1.26E-07
FBXO4	1.23E-05	8.94E-06	4.44E-06	5.67E-06	1.87E-06	1.05E-05	4.76E-06	6.65E-06
FBXO40	3.92E-09	2.19E-08	3.21E-08	4.79E-09	3.03E-08	1.45E-08	3.82E-09	0
FBXO41	2.41E-06	5.94E-08	1.92E-07	2.63E-07	2.95E-08	1.07E-07	1.68E-07	5.03E-08
FBXO42	8.85E-06	6.16E-06	4.47E-06	4.87E-06	1.91E-06	5.61E-06	4.50E-06	4.27E-06
FBXO43	8.44E-07	6.04E-07	1.48E-08	4.33E-08	8.02E-08	1.33E-07	7.34E-08	8.42E-08
FBXO44	2.00E-05	1.88E-05	4.94E-06	5.75E-06	3.26E-06	5.93E-06	5.92E-06	1.61E-05
FBXO45	4.47E-06	2.85E-06	1.57E-06	2.08E-06	1.15E-06	1.97E-06	2.73E-06	3.34E-06

FBXO46	5.33E-06	5.64E-06	3.78E-06	4.80E-06	2.69E-06	4.43E-06	6.57E-06	3.26E-06
FBXO47	1.03E-08	0	0	0	0	0	0	0
FBXO48	9.04E-07	6.49E-07	6.52E-07	4.32E-07	1.70E-07	5.18E-07	2.00E-07	2.40E-07
FBXO5	5.10E-06	2.16E-06	1.08E-06	1.83E-06	5.99E-07	1.84E-06	1.49E-06	1.24E-06
FBXO6	1.89E-05	2.35E-05	1.05E-05	1.03E-05	8.75E-06	1.20E-05	1.72E-05	1.88E-05
FBXO7	6.79E-05	0.000112	6.74E-05	9.60E-05	9.88E-05	0.0001	7.79E-05	9.01E-05
FBXO8	5.93E-05	7.10E-06	1.58E-05	1.07E-05	5.41E-06	1.48E-05	1.42E-05	1.38E-05
FBXO9	8.82E-06	6.08E-06	7.66E-06	5.19E-06	3.19E-06	9.90E-06	5.35E-06	1.53E-05
FBXW10	7.02E-09	1.91E-07	0	8.57E-09	1.20E-07	1.95E-06	6.81E-09	5.82E-08
FBXW11	2.58E-05	7.01E-06	8.72E-06	9.80E-06	2.20E-06	1.08E-05	6.67E-06	1.30E-05
FBXW12	2.12E-08	0	0	0	0	0	0	0
FBXW2	1.00E-05	5.47E-06	4.93E-06	4.84E-06	2.29E-06	7.81E-06	3.92E-06	9.07E-06
FBXW4	2.61E-05	1.32E-05	1.77E-05	1.89E-05	2.86E-05	2.02E-05	3.16E-05	2.17E-05
FBXW5	7.96E-05	7.07E-05	7.79E-05	7.05E-05	5.21E-05	5.82E-05	5.87E-05	0.000154
FBXW7	9.85E-06	2.94E-06	4.81E-06	4.80E-06	2.22E-06	3.04E-06	3.48E-06	9.26E-06
FBXW8	2.93E-06	4.28E-06	1.98E-06	2.17E-06	1.25E-06	4.80E-06	2.05E-06	2.42E-06
FBXW9	3.63E-06	2.32E-06	1.86E-06	3.92E-06	5.85E-06	5.87E-06	3.53E-06	1.16E-06
FCAMR	1.79E-08	8.34E-07	2.91E-06	2.10E-06	7.45E-07	1.12E-05	9.44E-07	6.12E-06
FCAR	1.08E-07	1.28E-07	1.41E-07	9.79E-07	0	1.02E-07	7.21E-08	2.78E-08
FCER1A	3.12E-07	1.62E-06	1.08E-06	3.63E-06	9.63E-08	4.15E-08	5.97E-07	1.12E-06
FCER1G	0.000107	1.75E-05	4.53E-05	0.000113	1.05E-05	6.90E-05	5.24E-05	1.56E-05
FCER2	1.55E-08	0	3.29E-08	1.91E-07	3.93E-08	8.65E-08	2.69E-07	2.29E-08
FCF1	1.98E-05	8.99E-06	1.09E-05	1.13E-05	3.29E-06	1.72E-05	1.12E-05	8.85E-06
FCGBP	5.88E-07	6.93E-07	3.17E-07	3.20E-07	3.50E-07	3.42E-07	3.10E-07	3.32E-08
FCGR1A	5.54E-06	2.96E-07	4.32E-07	1.90E-06	3.41E-07	6.08E-07	3.48E-06	7.44E-08
FCGR1B	1.07E-05	6.82E-07	1.03E-06	3.64E-06	7.19E-07	1.21E-06	5.24E-06	5.80E-07
FCGR1C	2.57E-06	1.73E-07	3.64E-07	8.61E-07	1.14E-07	4.51E-07	1.58E-06	2.07E-07
FCGR2A	2.32E-05	5.10E-06	4.62E-06	9.76E-06	1.85E-06	4.86E-06	1.42E-05	3.31E-06
FCGR2B	8.67E-07	8.72E-07	1.93E-05	1.95E-05	8.10E-08	4.37E-08	2.68E-06	3.24E-07
FCGR2C	1.05E-06	2.55E-07	9.43E-06	5.56E-06	8.14E-08	8.77E-08	9.14E-07	1.46E-07
FCGR3A	6.02E-05	6.42E-06	4.10E-05	2.90E-05	2.47E-06	4.16E-05	4.04E-05	7.23E-06
FCGR3B	1.29E-05	2.10E-07	1.78E-06	1.25E-06	7.99E-08	3.64E-07	2.60E-07	8.80E-07
FCGRT	0.000187	0.000187	0.000325	0.000296	0.000434	0.000175	0.000305	0.000148
FCHO1	6.58E-06	1.55E-07	8.03E-07	1.88E-06	4.75E-08	3.67E-07	4.08E-07	9.41E-08
FCHO2	1.23E-05	6.60E-06	7.88E-06	1.00E-05	1.37E-06	8.72E-06	4.07E-06	5.98E-06
FCHSD1	2.79E-06	1.72E-07	6.06E-07	1.41E-06	2.07E-07	3.81E-07	7.27E-07	8.46E-07
FCHSD2	8.41E-06	3.84E-06	6.34E-06	7.72E-06	1.81E-06	4.65E-06	3.86E-06	2.72E-06
FCN1	1.85E-06	1.80E-06	4.58E-06	1.56E-05	5.31E-07	1.68E-06	1.94E-06	8.23E-07
FCN2	2.87E-08	4.29E-06	7.22E-05	3.17E-05	4.73E-07	8.16E-07	1.19E-07	2.35E-06
FCN3	1.41E-05	4.67E-07	0.000214	0.000106	1.27E-05	2.10E-06	5.67E-06	2.64E-06
FCRL1	3.27E-08	0	4.15E-08	2.86E-08	0	1.76E-08	0	0
FCRL2	1.91E-07	0	3.09E-08	5.56E-08	0	2.72E-08	4.23E-08	2.16E-08
FCRL3	5.09E-07	1.01E-08	8.73E-08	9.47E-08	0	4.21E-07	7.28E-08	4.69E-08
FCRL4	1.48E-08	0	0	0	0	6.86E-09	7.19E-09	0
FCRL5	4.26E-07	4.67E-09	7.51E-08	5.81E-07	0	2.04E-08	1.74E-08	7.39E-09
FCRL6	6.93E-07	2.15E-07	1.30E-06	1.94E-06	3.95E-08	3.20E-07	8.33E-07	3.60E-07
FCRLA	1.21E-07	1.53E-07	2.31E-07	2.89E-07	2.60E-08	2.27E-07	1.46E-07	1.09E-07
FCRLB	9.66E-07	1.33E-06	3.94E-07	5.65E-07	1.18E-08	5.62E-08	2.23E-07	3.50E-07
FDFT1	4.06E-05	0.000288	4.93E-05	4.66E-05	9.55E-06	9.55E-05	2.67E-05	2.01E-05
FDPS	0.000216	0.000611	0.000266	8.82E-05	0.00011	0.000128	9.04E-05	0.000247
FDPSL2A	4.98E-08	3.04E-08	5.57E-08	5.53E-09	1.17E-08	8.38E-09	4.41E-09	6.63E-09
FDX1	1.70E-05	2.44E-05	2.47E-05	2.34E-05	1.83E-05	1.20E-05	2.48E-05	1.90E-05
FDX1L	1.59E-05	1.28E-05	9.34E-06	1.40E-05	3.06E-05	1.78E-05	2.34E-05	1.56E-05
FDXACB1	1.35E-06	1.24E-06	7.82E-07	1.05E-06	6.16E-07	1.26E-06	7.31E-07	1.61E-06
FDXR	1.93E-05	2.89E-05	1.09E-05	2.26E-05	2.59E-05	4.94E-05	1.75E-05	1.78E-05
FECH	5.20E-06	4.51E-06	7.06E-06	5.41E-06	1.37E-06	1.14E-05	5.25E-06	4.30E-06
FEM1A	1.08E-05	9.24E-06	7.39E-06	1.04E-05	8.14E-06	1.01E-05	1.22E-05	1.26E-05
FEM1B	1.57E-05	1.00E-05	8.57E-06	7.46E-06	1.53E-06	1.24E-05	5.32E-06	9.69E-06

FEM1C	1.08E-05	5.87E-06	5.32E-06	9.34E-06	7.22E-07	4.78E-06	4.29E-06	1.22E-05
FEN1	2.38E-05	1.90E-05	4.31E-06	4.73E-06	8.06E-06	8.60E-06	5.76E-06	1.27E-05
FER	9.04E-07	2.75E-07	2.55E-07	6.04E-07	3.19E-08	3.48E-07	2.13E-07	4.04E-07
FER1L4	3.89E-06	6.09E-07	8.60E-08	2.04E-07	3.91E-08	3.02E-08	2.75E-07	2.96E-08
FER1L5	0	1.60E-08	0	0	0	0	0	5.49E-09
FER1L6	6.74E-07	3.31E-08	0	0	0	1.37E-08	0	1.08E-08
FERD3L	0	0	0	0	0	0	0	0
FERMT1	2.66E-06	5.91E-07	1.54E-07	5.24E-07	5.35E-08	1.04E-05	6.75E-08	5.37E-08
FERMT2	1.90E-05	1.91E-05	3.58E-05	3.12E-05	1.16E-05	3.11E-05	2.01E-05	2.51E-05
FERMT3	1.35E-05	1.73E-06	5.93E-06	1.37E-05	2.12E-06	5.35E-06	8.86E-06	3.28E-06
FES	7.49E-06	7.26E-05	1.44E-05	1.38E-05	4.01E-06	2.85E-06	1.48E-05	2.99E-06
FETUB	1.26E-05	0.000163	0.000144	5.95E-05	0.000261	2.32E-05	4.09E-05	8.20E-05
FEV	7.70E-08	0	0	0	0	0	1.24E-08	0
FEZ1	6.55E-06	7.41E-07	1.19E-05	6.17E-06	1.94E-06	2.32E-06	4.30E-06	1.54E-06
FEZ2	1.90E-05	2.06E-05	1.25E-05	1.82E-05	4.21E-06	5.44E-05	1.50E-05	6.96E-06
FEZF1	0	1.15E-07	0	0	0	0	0	1.81E-08
FEZF2	0	0	0	0	0	0	0	0
FFAR1	0	0	0	0	0	0	0	0
FFAR2	1.37E-06	3.95E-08	2.80E-07	2.71E-07	1.00E-08	3.24E-08	1.13E-07	1.71E-07
FFAR3	9.25E-08	1.04E-07	3.09E-08	6.83E-07	2.52E-08	9.52E-08	4.06E-07	2.16E-08
FGA	0.005366	0.005877	0.010334	0.011603	0.00883	0.017491	0.013581	0.014414
FGB	0.00416	0.005137	0.008935	0.009117	0.004751	0.01268	0.00889	0.012487
FGD1	1.73E-06	1.43E-06	2.46E-07	6.55E-07	4.51E-07	5.95E-07	5.17E-07	1.08E-06
FGD2	5.91E-06	2.66E-07	2.47E-06	2.89E-06	1.79E-07	1.48E-06	2.10E-06	5.89E-07
FGD3	4.96E-06	8.94E-07	1.94E-06	4.12E-06	8.62E-07	1.64E-06	2.94E-06	1.57E-06
FGD4	7.09E-06	4.67E-06	9.05E-06	5.00E-06	6.49E-07	6.63E-06	2.36E-06	3.48E-06
FGD5	2.89E-05	2.41E-06	2.20E-06	2.57E-06	2.13E-06	1.91E-06	3.54E-06	3.25E-06
FGD6	5.11E-06	1.13E-06	9.64E-07	1.90E-06	2.12E-07	1.06E-06	1.12E-06	1.56E-06
FGF1	7.60E-07	2.13E-07	2.90E-07	1.12E-06	9.06E-08	7.42E-08	2.19E-07	2.40E-07
FGF10	0	0	5.82E-08	1.24E-07	0	0	0	0
FGF11	5.78E-07	3.73E-07	2.22E-07	3.18E-07	1.06E-07	1.50E-07	1.54E-07	1.07E-07
FGF12	1.36E-07	1.16E-07	3.31E-08	3.35E-07	4.81E-08	1.14E-07	1.39E-07	1.37E-07
FGF13	9.31E-07	5.94E-06	5.75E-07	4.24E-06	2.45E-07	1.25E-05	9.33E-07	3.78E-06
FGF14	1.49E-05	3.60E-07	1.52E-06	4.59E-06	1.53E-07	1.86E-05	2.79E-06	5.71E-06
FGF16	0	0	0	0	0	0	1.16E-07	0
FGF17	2.08E-07	5.74E-07	2.34E-08	8.03E-08	3.29E-07	4.04E-08	2.08E-08	0
FGF18	2.62E-07	1.87E-08	0	1.21E-07	4.23E-08	4.56E-08	4.72E-08	4.83E-08
FGF19	0	5.04E-08	0	1.20E-06	0	0	1.08E-08	2.08E-05
FGF2	4.03E-07	1.86E-07	1.77E-06	7.31E-07	8.53E-08	2.63E-06	3.01E-07	1.76E-07
FGF20	0	5.26E-08	1.02E-07	0	0	8.27E-08	0	0
FGF21	0	1.36E-05	3.03E-05	8.47E-05	8.21E-06	1.31E-06	0.000233	0.000119
FGF22	5.40E-08	0	0	8.47E-08	0	0	0	0
FGF23	0	0	4.76E-08	3.84E-07	0	0	0	0
FGF3	0	5.46E-08	0	0	0	0	3.07E-08	0
FGF4	0	0	0	0	0	0	0	0
FGF5	4.66E-07	0	0	0	0	0	0	0
FGF6	0	0	0	0	0	0	0	0
FGF7	1.91E-07	2.47E-07	2.86E-07	9.55E-07	5.97E-08	2.20E-07	3.60E-07	7.72E-07
FGF8	0	0	3.01E-08	0	0	0	0	4.08E-08
FGF9	0	7.83E-08	3.08E-08	1.83E-08	4.29E-09	0	0	1.46E-08
FGFBP1	0	2.52E-08	5.91E-07	2.70E-08	0	0	2.10E-08	3.25E-08
FGFBP2	7.51E-07	1.02E-06	7.16E-07	1.83E-06	5.73E-08	8.03E-07	4.50E-06	0
FGFBP3	5.12E-07	1.03E-07	2.09E-07	3.58E-07	9.19E-07	2.96E-07	2.48E-07	1.74E-07
FGFR1	8.51E-06	1.70E-06	4.53E-06	8.53E-06	7.62E-07	1.18E-06	3.49E-06	1.78E-06
FGFR1OP	8.54E-06	9.32E-06	8.86E-06	7.75E-06	1.03E-06	9.93E-06	3.41E-06	5.99E-06
FGFR1OP2	1.96E-05	1.53E-05	1.32E-05	1.30E-05	3.76E-06	9.47E-06	8.71E-06	1.11E-05
FGFR2	2.91E-05	1.67E-08	2.60E-05	4.69E-05	4.52E-05	1.90E-07	6.05E-06	5.99E-05
FGFR3	2.52E-05	7.80E-06	1.06E-05	7.19E-06	9.33E-06	3.14E-06	1.28E-05	1.05E-05

FGFR4	7.63E-05	3.60E-05	3.72E-05	2.73E-05	5.49E-05	4.90E-05	4.21E-05	7.83E-05
FGFRL1	1.51E-05	2.94E-05	1.93E-05	1.64E-05	1.11E-05	5.04E-05	2.18E-05	2.56E-05
FGG	0.004021	0.007545	0.008529	0.008372	0.003356	0.012811	0.010446	0.008876
FGGY	7.47E-06	0.00011	1.84E-05	6.22E-05	2.72E-05	5.19E-05	0.000111	1.67E-05
FGL1	0.001391	0.007045	0.002262	0.002015	0.000751	0.00549	0.001824	1.31E-06
FGL2	2.28E-05	4.56E-06	9.51E-06	1.50E-05	7.86E-07	5.56E-06	3.99E-06	2.15E-06
FGR	1.13E-05	1.36E-06	4.38E-06	9.73E-06	5.29E-07	1.28E-06	3.13E-06	1.50E-06
FH	8.64E-05	0.000339	0.000141	0.000102	7.17E-05	0.000175	9.59E-05	0.00028
FHAD1	1.51E-07	2.13E-07	6.74E-09	3.49E-08	0	6.30E-07	1.84E-08	2.88E-08
FHDC1	1.86E-06	2.69E-08	3.88E-08	1.26E-07	2.07E-08	5.10E-08	7.40E-08	4.99E-07
FHIT	2.59E-05	2.96E-06	9.66E-07	2.81E-06	3.63E-06	5.63E-06	7.78E-06	8.94E-07
FHL1	7.92E-06	7.66E-06	1.45E-05	2.81E-05	3.41E-06	3.99E-06	7.81E-06	6.72E-06
FHL2	2.39E-05	1.24E-06	1.35E-05	4.17E-05	2.22E-06	2.61E-06	6.59E-06	3.81E-06
FHL3	9.94E-06	6.48E-06	3.36E-06	9.55E-06	5.04E-06	3.78E-06	1.30E-05	1.05E-05
FHL5	1.35E-07	6.20E-07	5.35E-08	1.56E-08	4.38E-08	4.12E-07	3.56E-07	1.12E-07
FHOD1	1.24E-05	1.19E-06	2.39E-06	6.20E-06	6.44E-06	1.81E-06	7.31E-06	4.02E-06
FHOD3	4.10E-07	1.64E-06	4.13E-07	1.57E-06	5.98E-08	3.80E-08	1.79E-07	2.07E-07
FIBCD1	8.15E-08	0	0	9.06E-09	3.18E-08	4.14E-08	1.44E-08	1.09E-08
FIBIN	1.43E-06	1.21E-07	7.98E-07	1.59E-06	2.12E-07	7.14E-09	1.02E-06	3.51E-07
FIBP	3.98E-05	4.72E-05	1.72E-05	2.13E-05	2.83E-05	2.83E-05	2.95E-05	4.25E-05
FICD	1.44E-06	2.80E-06	2.18E-06	1.60E-06	2.94E-07	4.52E-06	9.15E-07	4.40E-06
FIG4	5.36E-06	3.38E-06	3.29E-06	4.17E-06	1.01E-06	5.38E-06	2.15E-06	2.88E-06
FIGF	4.51E-07	1.28E-07	5.66E-07	4.70E-07	4.85E-08	1.13E-06	1.86E-07	3.65E-07
FIGLA	4.06E-08	4.83E-08	0	1.01E-07	0	0	0	0
FIGN	2.08E-06	2.85E-07	1.26E-06	6.46E-07	9.00E-08	2.82E-07	1.65E-07	8.04E-08
FIGNL1	6.05E-06	3.08E-06	8.39E-07	1.20E-06	9.27E-07	5.53E-06	7.72E-07	1.22E-06
FIGNL2	2.47E-06	1.52E-06	2.15E-07	1.28E-07	4.42E-07	4.76E-07	1.40E-06	6.58E-07
FILIP1	7.56E-07	6.70E-07	3.52E-07	6.44E-07	9.44E-08	4.84E-07	7.48E-07	6.32E-07
FILIP1L	1.01E-05	4.18E-06	3.89E-06	1.81E-05	4.41E-07	5.38E-06	4.81E-06	4.61E-06
FIP1L1	2.33E-05	1.29E-05	8.02E-06	1.18E-05	5.79E-06	1.50E-05	1.04E-05	1.45E-05
FIS1	0.000101	0.00047	0.000187	0.000188	0.000423	0.00028	0.000279	0.000349
FITM1	6.65E-07	3.30E-06	7.38E-06	4.48E-06	1.16E-05	2.21E-06	4.06E-06	5.95E-07
FITM2	7.49E-06	6.32E-06	3.72E-06	2.48E-06	3.17E-06	1.29E-05	2.09E-06	3.28E-06
FIZ1	5.12E-06	4.04E-06	2.06E-06	4.01E-06	5.47E-06	3.51E-06	6.15E-06	4.58E-06
FJX1	2.57E-06	5.38E-07	3.88E-07	2.46E-06	7.77E-07	9.26E-07	1.69E-06	1.06E-06
FKBP10	4.74E-05	1.85E-06	3.22E-06	1.35E-05	5.05E-06	2.21E-06	8.02E-06	4.98E-06
FKBP11	0.00014	4.06E-05	2.44E-05	4.08E-05	5.19E-05	0.000125	6.46E-05	5.16E-05
FKBP14	3.76E-06	8.33E-07	1.17E-06	1.37E-06	1.27E-07	3.23E-06	7.55E-07	2.37E-06
FKBP15	1.62E-05	7.47E-06	9.45E-06	1.33E-05	3.40E-06	1.37E-05	1.01E-05	3.34E-06
FKBP1A	0.000219	0.000117	4.84E-05	9.43E-05	9.11E-05	9.43E-05	0.000144	8.87E-05
FKBP1AP1	7.17E-07	2.89E-07	3.00E-07	3.30E-07	0	4.72E-07	1.44E-07	4.41E-08
FKBP1B	1.19E-05	2.94E-05	2.67E-06	7.19E-06	7.83E-06	9.28E-05	2.18E-05	2.15E-06
FKBP2	0.000205	0.000226	0.000136	0.000162	0.000442	0.000159	0.000144	0.000318
FKBP3	7.63E-05	4.72E-05	3.15E-05	4.11E-05	4.86E-05	6.13E-05	5.66E-05	4.02E-05
FKBP4	4.60E-05	9.13E-05	2.21E-05	2.20E-05	2.57E-05	3.28E-05	4.29E-05	2.09E-05
FKBP5	5.70E-05	4.05E-05	7.53E-05	2.99E-05	7.55E-06	4.92E-05	3.58E-05	0.000124
FKBP6	1.55E-07	0	1.81E-08	6.37E-08	4.37E-08	3.23E-08	0	0
FKBP7	8.43E-06	2.35E-06	3.17E-06	2.92E-06	9.76E-07	2.38E-06	1.41E-06	3.06E-06
FKBP8	0.000132	0.000178	0.00014	0.000138	0.00027	0.00016	0.000151	0.000154
FKBP9	3.50E-05	3.55E-05	1.24E-05	2.08E-05	9.76E-06	1.54E-05	1.83E-05	1.11E-05
FKBP9L	5.41E-07	1.03E-06	3.32E-07	2.25E-07	1.16E-07	2.51E-07	2.24E-07	1.99E-07
FKBPL	6.81E-06	6.59E-06	2.48E-06	3.08E-06	5.51E-06	6.90E-06	3.65E-06	6.32E-06
FKRP	3.13E-06	3.22E-06	2.57E-06	4.88E-06	1.60E-06	3.79E-06	4.57E-06	2.79E-06
FKSG29	0	0	0	0	0	0	0	0
FKSG73	0	0	0	0	0	0	0	0
FKSG83	4.47E-08	0	0	0	0	0	0	0
FKTN	2.96E-06	1.22E-06	1.31E-06	1.32E-06	4.57E-07	2.66E-06	7.79E-07	2.22E-06
FLAD1	1.84E-05	7.66E-05	1.51E-05	1.82E-05	1.34E-05	5.14E-05	2.61E-05	4.49E-05

FLCN	6.97E-06	1.28E-05	9.94E-06	6.97E-06	2.70E-06	7.84E-06	1.04E-05	1.24E-05
FLG	2.02E-07	1.79E-08	8.61E-08	8.89E-09	0	3.39E-08	3.74E-08	0
FLG2	9.72E-09	0	3.47E-08	0	0	2.25E-09	2.37E-09	0
FLI1	5.74E-06	1.99E-06	2.50E-06	3.06E-06	5.47E-07	2.09E-06	3.58E-06	1.91E-06
FLII	2.54E-05	4.82E-05	1.89E-05	2.74E-05	9.74E-06	2.73E-05	3.19E-05	2.18E-05
FLJ10038	3.13E-06	1.28E-06	1.34E-06	1.86E-06	3.40E-06	2.06E-06	1.19E-06	1.12E-06
FLJ10213	5.90E-07	3.52E-07	1.25E-07	1.09E-07	0	8.22E-08	4.26E-08	2.17E-08
FLJ10357	1.39E-05	1.02E-05	9.06E-06	1.48E-05	5.49E-06	6.36E-06	1.27E-05	1.49E-05
FLJ10661	5.08E-07	9.97E-07	1.65E-07	3.40E-07	2.25E-07	3.29E-07	1.17E-07	2.58E-08
FLJ11235	8.46E-07	1.44E-07	3.26E-07	6.42E-07	3.10E-07	2.51E-07	3.28E-07	2.40E-07
FLJ12825	1.73E-08	1.94E-08	0	7.06E-09	4.96E-08	2.14E-08	5.62E-09	1.69E-08
FLJ13197	4.03E-07	7.17E-07	1.27E-07	4.46E-07	3.42E-08	1.85E-07	1.53E-07	1.03E-07
FLJ13224	4.51E-08	0	1.59E-08	7.39E-08	0	1.54E-07	5.80E-08	0
FLJ14107	1.31E-08	4.47E-08	4.15E-08	0	0	0	1.27E-08	0
FLJ16779	4.67E-08	0	0	3.55E-09	0	0	0	0
FLJ22536	2.75E-07	0	4.02E-08	3.19E-07	1.98E-08	0	3.79E-08	5.86E-08
FLJ23867	3.26E-06	2.90E-07	1.76E-06	3.63E-06	3.47E-07	1.11E-07	7.33E-07	3.66E-07
FLJ25328	0	0	0	0	0	0	0	0
FLJ25363	0	0	0	0	0	0	0	2.46E-08
FLJ25758	0	1.36E-07	0	0	0	0	0	0
FLJ26850	1.74E-08	7.96E-08	9.28E-08	4.29E-08	1.65E-07	0	1.68E-08	2.32E-07
FLJ30679	4.84E-08	0	0	0	0	3.37E-08	0	0
FLJ32063	0	0	0	0	0	0	0	0
FLJ33360	0	2.30E-07	1.01E-08	3.57E-08	8.36E-09	9.02E-09	2.83E-08	0
FLJ33630	6.36E-06	6.72E-06	4.89E-06	4.23E-06	1.92E-06	6.13E-06	5.81E-06	7.86E-06
FLJ34503	3.58E-08	0	0	2.93E-08	0	0	0	0
FLJ35024	0	8.18E-09	2.26E-08	0	0	5.62E-08	1.41E-08	0
FLJ35220	1.03E-05	9.44E-06	4.89E-06	7.36E-06	1.04E-05	8.19E-06	7.92E-06	5.35E-06
FLJ35390	3.33E-06	2.83E-06	6.28E-07	3.96E-07	2.14E-07	6.69E-07	8.27E-07	6.09E-07
FLJ35776	1.51E-05	1.05E-05	5.58E-06	5.75E-06	2.56E-06	1.68E-05	4.50E-06	4.45E-06
FLJ36000	0	1.35E-08	0	0	0	0	0	8.81E-09
FLJ36031	2.67E-06	1.16E-05	1.12E-05	4.49E-05	7.09E-06	5.89E-06	1.11E-05	2.20E-05
FLJ36777	3.11E-07	1.88E-07	2.44E-07	1.91E-07	1.53E-07	3.09E-07	8.60E-08	1.80E-07
FLJ37201	2.56E-07	2.38E-07	7.08E-08	3.82E-08	1.61E-08	4.05E-08	4.86E-08	2.75E-08
FLJ37307	7.44E-08	1.02E-07	3.53E-08	6.99E-09	2.95E-08	7.41E-08	7.23E-08	0
FLJ37453	3.11E-06	1.45E-06	1.42E-06	1.13E-06	1.42E-06	1.86E-06	9.18E-07	5.26E-07
FLJ37543	3.51E-07	0	0	2.86E-08	5.36E-08	2.89E-08	1.51E-08	0
FLJ39582	2.48E-06	1.15E-06	3.79E-06	2.43E-06	3.90E-06	7.00E-06	9.77E-07	2.16E-06
FLJ39609	0	0	0	0	0	0	0	0
FLJ39653	2.59E-06	6.50E-07	5.02E-07	6.03E-07	1.04E-06	9.78E-07	6.78E-07	2.42E-07
FLJ39739	5.25E-07	0	9.67E-08	1.91E-07	0	2.86E-07	6.85E-08	0
FLJ40292	1.39E-08	1.58E-08	2.93E-08	1.71E-08	0	1.29E-08	2.68E-08	0
FLJ40330	2.75E-07	0	2.59E-08	2.50E-07	0	7.33E-08	0	1.16E-07
FLJ40434	0	0	0	0	0	0	0	0
FLJ40504	2.80E-08	1.63E-07	0	0	0	0	0	0
FLJ40852	1.61E-07	7.42E-07	5.82E-07	6.34E-07	2.07E-07	1.23E-06	6.21E-07	4.16E-07
FLJ41941	2.58E-07	1.01E-07	0	1.21E-08	0	0	0	0
FLJ42289	9.80E-07	4.23E-06	1.88E-06	9.16E-07	6.27E-07	2.98E-07	1.27E-06	1.68E-06
FLJ42393	4.18E-08	1.18E-08	0	0	0	9.68E-09	4.04E-08	0
FLJ42627	1.10E-07	1.13E-07	6.18E-08	1.09E-07	1.90E-07	1.85E-07	3.68E-07	1.42E-07
FLJ42709	5.69E-07	1.73E-07	6.73E-07	1.27E-06	3.04E-07	9.71E-07	4.64E-07	6.64E-07
FLJ42875	4.75E-06	0	2.37E-07	3.28E-07	3.07E-07	5.72E-08	0	2.60E-07
FLJ43390	0	0	0	0	0	0	0	0
FLJ43663	1.83E-06	1.15E-06	9.77E-07	1.73E-06	5.60E-07	1.26E-06	1.07E-06	2.64E-06
FLJ43859	0	0	0	0	0	0	4.46E-09	0
FLJ43860	0	0	0	0	0	0	0	0
FLJ43950	0	0	4.76E-09	5.67E-09	0	0	0	6.80E-09
FLJ44054	7.92E-09	0	0	9.69E-09	6.81E-09	0	0	0

FLJ44082	0	0	0	0	0	0	0	0
FLJ44606	9.81E-07	6.65E-07	1.77E-07	3.62E-07	6.69E-09	3.03E-07	3.62E-07	3.77E-07
FLJ44635	3.02E-06	3.42E-07	3.36E-06	3.83E-07	1.14E-06	3.19E-07	2.83E-07	2.30E-07
FLJ45079	4.84E-09	1.08E-08	0	0	0	4.47E-09	0	0
FLJ45244	2.46E-06	1.21E-06	1.70E-06	1.12E-06	3.59E-06	2.67E-06	5.55E-07	1.55E-06
FLJ45340	3.26E-06	1.53E-06	1.31E-06	1.40E-06	4.56E-07	1.19E-06	1.42E-06	2.13E-06
FLJ45445	7.88E-07	5.02E-07	4.75E-08	7.35E-08	1.59E-08	1.50E-07	1.04E-07	6.10E-08
FLJ45983	0	0	0	0	3.17E-08	0	0	0
FLJ46111	2.78E-07	3.00E-08	1.25E-07	3.57E-07	6.84E-08	3.69E-08	3.83E-08	7.80E-08
FLJ46321	4.68E-09	0	0	0	0	0	0	0
FLJ46361	0	0	0	0	0	0	0	0
FLJ90757	9.22E-06	4.78E-06	2.35E-06	3.47E-06	3.14E-06	6.69E-06	4.03E-06	4.40E-06
FLNA	6.55E-05	2.85E-05	1.48E-05	7.66E-05	1.73E-05	1.82E-05	5.65E-05	2.95E-05
FLNB	3.21E-05	3.91E-05	1.98E-05	2.99E-05	1.75E-05	1.12E-05	2.73E-05	1.50E-05
FLNC	1.97E-05	3.28E-08	1.75E-08	5.42E-07	8.83E-08	1.52E-05	1.91E-07	6.45E-08
FLOT1	0.000114	0.000115	6.36E-05	9.52E-05	5.94E-05	0.000111	0.000103	0.000115
FLOT2	6.56E-05	7.66E-05	2.83E-05	4.28E-05	2.40E-05	5.19E-05	5.32E-05	5.71E-05
FLRT1	3.26E-07	5.02E-07	7.33E-09	1.73E-08	4.27E-08	3.94E-08	2.07E-08	1.04E-08
FLRT2	6.68E-07	1.05E-07	6.97E-07	2.16E-06	1.41E-07	4.35E-08	1.65E-07	2.52E-07
FLRT3	1.97E-05	4.44E-06	8.04E-06	1.32E-05	2.79E-06	4.37E-07	1.07E-05	8.95E-06
FLT1	1.17E-05	1.52E-05	4.17E-06	5.27E-06	6.13E-06	7.55E-06	6.84E-06	7.37E-06
FLT3	2.89E-07	4.53E-07	2.59E-07	3.27E-07	1.97E-08	8.54E-08	1.10E-07	5.40E-08
FLT3LG	8.87E-06	9.68E-07	2.82E-06	6.43E-06	3.72E-06	4.22E-06	4.39E-06	6.19E-06
FLT4	2.96E-06	2.80E-06	2.26E-06	2.19E-06	1.55E-06	1.31E-06	4.00E-06	2.78E-06
FLVCR1	4.16E-06	1.16E-05	2.55E-07	3.44E-07	5.75E-08	1.57E-06	2.60E-07	1.50E-06
FLVCR2	1.67E-05	2.01E-06	7.26E-06	1.53E-05	2.24E-06	1.11E-05	1.46E-05	5.37E-06
FLYWCH1	6.40E-06	1.10E-05	2.62E-06	4.78E-06	2.85E-06	4.60E-06	3.82E-06	4.49E-06
FLYWCH2	2.47E-05	2.12E-05	7.41E-06	9.73E-06	2.71E-06	2.02E-06	2.32E-05	1.88E-05
FMN1	3.78E-07	0	1.13E-07	3.16E-08	0	5.63E-09	1.14E-08	0
FMN2	3.48E-09	0	1.53E-08	0	0	0	1.00E-08	0
FMNL1	1.26E-05	1.64E-06	3.12E-06	8.28E-06	6.29E-07	2.93E-06	3.96E-06	1.97E-06
FMNL2	1.77E-05	7.43E-07	2.26E-06	4.59E-06	1.12E-07	1.14E-06	1.67E-06	8.39E-07
FMNL3	4.61E-06	7.69E-07	7.17E-07	1.06E-06	3.64E-07	7.41E-07	1.88E-06	1.04E-06
FMO1	4.90E-08	5.12E-07	3.33E-07	2.42E-07	2.14E-06	5.68E-08	3.55E-08	3.97E-07
FMO2	1.39E-06	6.90E-07	1.00E-06	1.07E-06	3.10E-07	1.19E-07	8.08E-07	6.36E-07
FMO3	4.16E-05	9.94E-05	0.000547	0.000374	0.000127	0.000658	0.000296	0.000168
FMO4	1.31E-05	1.28E-05	1.98E-05	1.37E-05	7.47E-06	3.21E-05	7.54E-06	1.52E-05
FMO5	0.000243	7.61E-05	0.000236	0.0001	4.75E-05	0.000386	9.69E-05	8.99E-05
FMO6P	0	3.80E-08	0	0	0	2.08E-08	3.25E-08	0
FMO9P	1.85E-08	6.35E-08	0	0	0	0	0	0
FMOD	1.02E-05	5.23E-07	3.86E-06	1.70E-05	9.40E-07	7.48E-07	4.65E-06	4.03E-06
FMR1	1.15E-05	2.07E-05	1.41E-05	1.38E-05	6.63E-06	1.60E-05	1.12E-05	2.96E-05
FMR1NB	3.41E-07	0	0	0	2.27E-08	0	0	0
FN1	0.001198	0.00089	0.001162	0.001034	0.000328	0.001212	0.001141	0.00099
FN3K	0.000129	0.000118	6.65E-05	4.13E-05	4.13E-05	4.28E-05	3.41E-05	3.81E-05
FN3KRP	4.70E-05	3.83E-05	1.45E-05	2.28E-05	2.45E-05	3.88E-05	1.78E-05	2.18E-05
FNBP1	2.06E-05	1.01E-05	9.64E-06	1.46E-05	3.66E-06	1.13E-05	8.71E-06	1.51E-05
FNBP1L	1.66E-05	1.14E-05	6.94E-06	7.53E-06	1.19E-06	2.50E-05	5.24E-06	5.40E-06
FNBP4	1.93E-05	7.46E-06	4.55E-06	7.55E-06	1.33E-06	6.43E-06	4.15E-06	6.71E-06
FNDC1	3.40E-06	2.66E-08	2.82E-07	1.24E-06	4.10E-08	5.99E-08	4.42E-07	4.49E-07
FNDC3A	5.52E-05	1.51E-05	2.54E-05	1.19E-05	3.68E-06	2.62E-05	8.54E-06	2.62E-05
FNDC3B	1.97E-05	2.28E-05	1.10E-05	1.30E-05	4.25E-06	1.30E-05	8.21E-06	2.13E-05
FNDC4	9.39E-05	4.40E-05	3.23E-05	7.76E-05	4.36E-05	7.41E-05	5.20E-05	7.52E-05
FNDC5	7.34E-06	1.26E-05	2.69E-05	4.73E-05	1.74E-05	1.92E-07	4.33E-06	3.45E-05
FNDC7	0	0	1.01E-08	0	8.32E-09	8.97E-09	0	0
FNDC8	2.23E-07	1.49E-07	5.97E-08	1.15E-07	4.82E-08	8.66E-08	2.86E-07	1.10E-07
FNIP1	7.43E-06	4.83E-06	4.70E-06	3.71E-06	1.61E-06	4.78E-06	3.12E-06	6.37E-06
FNIP2	4.41E-06	2.75E-06	2.24E-05	4.52E-06	9.65E-08	9.47E-07	1.34E-06	1.24E-05

FNTA	2.79E-05	4.17E-05	1.81E-05	2.27E-05	1.31E-05	2.78E-05	1.83E-05	1.90E-05
FNTB	1.33E-05	4.27E-06	4.89E-06	6.09E-06	2.96E-06	8.61E-06	7.86E-06	7.40E-06
FOLH1	3.30E-06	1.19E-05	2.20E-05	2.29E-05	2.30E-05	6.90E-06	1.37E-05	6.80E-06
FOLH1B	1.09E-07	2.34E-06	4.41E-06	3.86E-06	4.55E-06	3.95E-07	1.59E-06	3.58E-08
FOLR1	4.24E-07	5.03E-07	9.49E-08	1.07E-07	0	0	1.37E-07	0
FOLR2	1.50E-05	9.81E-06	2.73E-05	3.92E-05	1.19E-05	3.03E-05	7.05E-05	8.16E-06
FOLR3	0	3.88E-08	4.43E-07	1.37E-07	0	0	0	0
FOLR4	0	0	4.56E-08	0	6.95E-08	0	3.76E-08	0
FOS	2.35E-05	0.000205	0.000599	0.000642	1.62E-05	1.42E-05	0.000203	7.63E-05
FOSB	1.01E-06	1.74E-05	5.00E-05	0.000231	1.77E-06	6.10E-06	2.87E-05	5.18E-06
FOSL1	2.18E-06	1.07E-06	1.08E-07	2.21E-05	3.16E-07	5.69E-07	1.95E-06	4.58E-07
FOSL2	3.33E-05	8.51E-06	3.94E-06	2.60E-05	1.03E-06	2.09E-05	6.15E-06	1.90E-05
FOXA1	2.47E-05	1.25E-05	1.21E-05	7.23E-06	6.02E-06	7.04E-06	6.79E-06	2.84E-05
FOXA2	1.95E-05	3.21E-05	2.49E-05	1.61E-05	1.30E-05	2.22E-05	1.62E-05	1.77E-05
FOXA3	2.36E-05	3.76E-05	2.73E-05	3.27E-05	0.0001	1.06E-05	8.76E-05	3.24E-05
FOXB1	1.71E-08	0	0	0	0	0	0	0
FOXB2	0	0	0	0	0	0	0	0
FOXC1	1.41E-06	5.38E-07	1.99E-07	1.85E-06	2.17E-07	3.39E-07	7.76E-07	2.01E-06
FOXC2	2.77E-07	1.16E-07	0	2.68E-07	0	1.22E-07	2.67E-07	8.59E-08
FOXD1	1.05E-08	1.18E-08	4.36E-08	1.28E-08	8.99E-09	9.69E-09	2.02E-08	1.54E-08
FOXD2	1.14E-06	6.11E-07	5.96E-08	1.30E-07	6.31E-07	1.03E-07	3.44E-07	1.20E-07
FOXD3	0	0	1.21E-08	2.83E-08	0	0	0	0
FOXD4	8.70E-08	2.09E-07	3.41E-08	2.67E-08	1.03E-07	5.04E-08	4.21E-08	8.00E-08
FOXD4L1	4.62E-08	1.31E-07	0	2.83E-08	7.95E-08	1.18E-07	8.94E-08	1.70E-08
FOXD4L2	2.23E-08	1.67E-08	1.54E-08	9.09E-09	0	2.75E-08	2.89E-08	0
FOXD4L3	1.07E-08	0	0	0	0	9.91E-09	0	0
FOXD4L5	0	0	0	9.09E-09	0	0	0	0
FOXD4L6	2.57E-08	5.78E-08	0	3.14E-08	7.35E-09	3.17E-08	0	0
FOXE1	3.69E-06	7.46E-09	1.37E-08	3.25E-08	0	0	0	1.95E-08
FOXE3	4.84E-08	1.37E-08	0	0	0	1.12E-08	1.17E-08	0
FOXF1	2.00E-06	4.74E-07	1.35E-06	3.72E-06	3.77E-07	3.73E-07	7.89E-07	6.85E-07
FOXF2	1.29E-06	8.59E-08	0	5.59E-08	9.82E-08	1.81E-07	1.49E-07	2.72E-07
FOXG1	0	0	0	1.11E-08	0	0	0	0
FOXH1	3.30E-07	2.89E-08	1.37E-08	5.28E-08	1.01E-07	3.77E-08	5.77E-08	4.25E-08
FOXI1	0	0	0	0	0	0	4.60E-08	0
FOXI2	0	0	1.50E-08	0	6.23E-09	2.02E-08	4.93E-08	1.06E-08
FOXI3	0	0	0	0	0	0	0	0
FOXJ1	2.59E-06	1.06E-06	3.10E-08	7.93E-07	0	1.11E-07	0	2.92E-08
FOXJ2	4.83E-06	2.46E-06	2.09E-06	1.91E-06	1.19E-06	2.63E-06	2.71E-06	4.23E-06
FOXJ3	9.63E-06	7.00E-06	5.07E-06	5.92E-06	1.35E-06	5.76E-06	3.73E-06	5.45E-06
FOXK1	1.39E-05	4.57E-06	2.43E-06	3.26E-06	8.15E-06	2.90E-06	8.93E-06	6.87E-06
FOXK2	1.38E-05	1.23E-05	3.39E-06	7.08E-06	4.06E-06	7.80E-06	7.77E-06	8.49E-06
FOXL1	1.58E-06	3.25E-08	3.74E-08	2.12E-07	8.70E-08	8.71E-08	1.90E-07	1.59E-07
FOXL2	0	0	0	0	0	0	3.86E-08	0
FOXM1	1.31E-05	9.22E-06	2.33E-07	3.80E-07	1.22E-06	2.52E-06	6.29E-07	2.71E-06
FOXN1	1.99E-07	0	3.59E-08	0	0	0	0	0
FOXN2	8.72E-06	1.29E-05	5.66E-06	4.29E-06	1.03E-06	3.25E-06	3.58E-06	4.39E-06
FOXN3	1.43E-05	9.67E-06	1.24E-05	1.09E-05	4.01E-06	2.42E-05	9.17E-06	9.21E-06
FOXN4	0	4.28E-08	5.18E-07	1.92E-08	1.41E-07	1.45E-08	1.52E-08	2.40E-07
FOXO1	5.59E-06	1.69E-05	2.14E-05	1.21E-05	2.78E-06	9.81E-06	4.90E-06	1.05E-05
FOXO3	1.20E-05	8.21E-06	7.97E-06	6.10E-06	2.24E-06	8.26E-06	7.25E-06	7.20E-06
FOXO3B	3.96E-06	3.22E-06	2.50E-06	2.17E-06	7.69E-07	2.97E-06	2.30E-06	2.38E-06
FOXO4	9.83E-06	5.64E-06	5.61E-06	4.58E-06	3.34E-06	5.77E-06	3.63E-06	6.85E-06
FOXP1	2.88E-05	6.37E-06	4.43E-06	8.40E-06	4.89E-06	1.52E-05	6.30E-06	1.28E-05
FOXP2	0	2.01E-07	2.02E-06	8.34E-07	0	0	9.97E-08	1.26E-08
FOXP3	6.89E-06	3.94E-07	6.75E-07	4.81E-06	8.96E-09	8.81E-07	2.78E-07	6.25E-07
FOXP4	7.06E-06	1.56E-05	6.64E-06	9.83E-06	4.48E-06	7.44E-06	1.14E-05	1.79E-05
FOXQ1	7.73E-06	4.29E-06	9.56E-07	5.74E-07	4.38E-08	4.23E-05	0	5.81E-06

FOXR1	2.25E-08	0	0	0	0	0	0	0
FOXR2	0	0	0	0	0	0	0	0
FOXRED1	5.10E-05	4.64E-05	3.85E-05	2.70E-05	2.64E-05	4.05E-05	2.41E-05	3.65E-05
FOXRED2	4.69E-06	8.24E-06	5.04E-06	2.82E-06	4.89E-06	1.55E-05	5.32E-06	5.12E-06
FOXS1	4.47E-06	4.62E-07	1.44E-07	3.78E-07	4.61E-06	7.86E-07	2.29E-06	2.70E-06
FPGS	2.16E-05	1.20E-05	2.23E-05	2.69E-05	2.03E-05	3.19E-05	4.65E-05	2.76E-05
FPGT	7.23E-06	7.53E-06	4.51E-06	3.11E-06	9.44E-07	1.03E-05	2.51E-06	4.08E-06
FPR1	7.27E-06	1.17E-06	5.27E-06	1.52E-05	3.32E-07	2.58E-06	4.62E-06	1.25E-06
FPR2	8.74E-07	7.13E-08	5.82E-07	8.34E-07	0	1.99E-07	1.09E-07	1.67E-07
FPR3	1.40E-05	1.84E-06	4.88E-06	1.60E-05	2.72E-07	2.31E-06	4.87E-06	1.41E-06
FRAS1	4.62E-06	6.10E-08	1.17E-07	1.12E-07	3.65E-08	5.97E-08	4.12E-08	6.00E-08
FRAT1	5.79E-06	3.52E-06	5.83E-06	3.05E-06	4.64E-06	2.61E-06	1.27E-05	2.83E-06
FRAT2	2.29E-05	8.29E-06	6.14E-06	3.70E-06	3.71E-06	3.73E-06	1.88E-05	7.48E-06
FREM1	4.01E-08	5.90E-08	3.19E-07	3.97E-07	2.56E-08	0	3.53E-09	2.78E-08
FREM2	5.44E-09	5.74E-08	5.59E-07	1.02E-06	0	2.51E-09	0	1.18E-08
FRG1	4.46E-05	1.94E-05	1.86E-05	2.85E-05	2.35E-05	2.98E-05	3.38E-05	3.38E-05
FRG1B	1.79E-05	1.35E-05	4.50E-06	7.31E-06	2.02E-06	7.48E-06	7.17E-06	1.17E-05
FRG2	0	0	0	0	0	0	0	0
FRG2B	0	0	0	0	0	0	0	0
FRG2C	0	0	0	0	0	0	0	0
FRK	2.50E-06	3.29E-06	1.62E-06	1.89E-06	4.56E-07	3.63E-06	1.13E-06	1.21E-06
FRMD1	6.00E-08	4.47E-07	3.66E-08	5.83E-07	3.03E-08	1.63E-08	3.71E-08	0
FRMD3	1.10E-05	2.93E-07	8.38E-08	1.02E-07	4.84E-08	2.04E-06	7.82E-08	1.09E-07
FRMD4A	4.97E-06	1.26E-06	3.72E-06	3.07E-06	2.59E-06	2.77E-06	2.04E-06	5.76E-06
FRMD4B	9.96E-06	5.17E-06	8.89E-06	1.55E-05	1.24E-06	6.14E-06	5.44E-06	5.81E-06
FRMD5	7.50E-07	0	0	0	0	0	0	0
FRMD6	8.97E-06	2.42E-06	3.18E-06	6.59E-06	1.18E-06	1.60E-06	3.51E-06	3.19E-06
FRMD7	1.46E-08	1.64E-08	3.02E-08	1.43E-07	8.17E-08	8.81E-08	1.40E-08	0
FRMD8	2.01E-05	1.32E-05	4.25E-06	7.91E-06	2.57E-06	5.87E-06	9.16E-06	9.78E-06
FRMPD1	1.55E-06	7.76E-08	3.40E-07	6.93E-07	6.85E-07	2.62E-07	2.68E-08	4.95E-07
FRMPD2	4.86E-09	1.63E-08	0	0	0	0	0	0
FRMPD2L1	0	0	0	0	0	0	0	0
FRMPD4	0	0	0	4.30E-09	0	0	0	0
FRRS1	8.67E-06	2.58E-05	1.12E-05	3.79E-06	4.49E-08	1.18E-05	2.20E-06	3.51E-06
FRS2	5.07E-06	4.99E-06	3.27E-06	3.24E-06	8.81E-07	2.86E-06	1.87E-06	2.25E-06
FRS3	2.82E-06	2.27E-06	9.51E-07	1.49E-06	1.75E-06	1.96E-06	1.53E-06	2.16E-06
FRY	2.79E-05	1.26E-05	1.53E-05	1.73E-05	3.62E-06	8.99E-06	3.38E-05	3.01E-05
FRYL	2.69E-05	6.51E-06	4.74E-06	5.02E-06	1.80E-06	6.04E-06	4.44E-06	5.83E-06
FRZB	6.38E-06	3.15E-06	8.86E-07	3.05E-06	2.42E-06	1.91E-06	4.32E-06	5.55E-06
FSCB	0	0	0	0	0	0	0	0
FSCN1	5.97E-05	1.06E-05	1.38E-05	1.33E-05	1.15E-05	1.18E-05	3.14E-05	7.69E-06
FSCN2	4.26E-07	1.67E-08	0	1.44E-07	1.26E-08	1.36E-08	1.41E-08	0
FSCN3	5.95E-08	6.47E-08	2.27E-08	9.30E-08	5.45E-08	2.01E-08	6.93E-08	9.51E-08
FSD1	4.44E-07	0	5.49E-08	4.23E-08	2.03E-06	2.57E-08	4.68E-07	9.48E-08
FSD1L	5.66E-07	3.73E-07	1.19E-07	2.26E-07	3.59E-08	1.05E-07	9.25E-08	2.24E-07
FSD2	0	1.13E-08	2.08E-08	0	0	9.27E-09	0	0
FSHB	0	0	0	0	0	0	0	0
FSHR	3.24E-08	0	0	1.32E-08	0	0	0	0
FSIP1	8.28E-08	2.05E-07	2.57E-08	1.32E-07	7.11E-09	2.30E-08	1.61E-07	2.43E-08
FST	0.000281	6.54E-05	0.000118	8.87E-05	1.86E-05	0.000188	8.30E-05	0.000279
FSTL1	3.68E-05	1.08E-05	1.25E-05	4.91E-05	5.10E-06	8.43E-06	2.51E-05	1.21E-05
FSTL3	1.84E-05	1.55E-06	5.31E-06	8.93E-05	6.89E-06	3.77E-06	2.96E-05	1.85E-05
FSTL4	5.94E-06	1.57E-07	0	3.57E-08	8.44E-08	9.20E-07	5.03E-08	1.67E-07
FSTL5	9.42E-09	1.29E-06	0	1.15E-08	0	4.36E-08	1.37E-08	6.27E-07
FTCD	8.61E-05	9.42E-05	0.000516	0.000364	0.001082	0.000241	0.000374	0.000319
FTH1	0.001351	0.001915	0.001102	0.001594	0.001679	0.001047	0.002113	0.001476
FTHL17	0	0	0	0	0	0	0	0
FTHL3	2.36E-05	2.21E-05	1.23E-05	2.05E-05	1.64E-05	1.60E-05	1.48E-05	2.08E-05

FTL	0.004157	0.01412	0.010702	0.010477	0.027935	0.022261	0.014082	0.007437
FTLP10	0	0	0	0	0	0	0	0
FTMT	0	0	0	0	0	0	0	0
FTO	1.35E-05	8.23E-06	7.53E-06	5.95E-06	2.10E-06	1.81E-05	6.41E-06	1.14E-05
FTSJ1	1.77E-05	1.66E-05	5.45E-06	9.64E-06	5.98E-06	1.61E-05	1.01E-05	1.87E-05
FTSJ2	3.23E-05	3.24E-05	1.03E-05	1.15E-05	9.29E-06	1.79E-05	1.92E-05	1.93E-05
FTSJ3	4.16E-05	2.70E-05	1.07E-05	1.90E-05	1.98E-05	3.85E-05	1.92E-05	1.54E-05
FTSJD1	5.66E-06	3.58E-06	6.55E-06	4.80E-06	7.36E-07	5.58E-06	2.31E-06	6.30E-06
FTSJD2	1.76E-05	1.77E-05	1.02E-05	9.27E-06	4.14E-06	1.76E-05	1.03E-05	1.67E-05
FUBP1	3.31E-05	2.23E-05	9.18E-06	1.29E-05	4.02E-07	1.80E-05	8.45E-06	1.09E-05
FUBP3	2.74E-05	2.15E-05	2.34E-05	1.53E-05	4.64E-06	2.25E-05	1.16E-05	2.35E-05
FUCA1	5.26E-05	4.23E-05	6.16E-05	6.36E-05	2.37E-05	5.22E-05	4.43E-05	6.65E-05
FUCA2	9.83E-05	7.82E-05	3.12E-05	4.74E-05	2.76E-05	6.34E-05	4.18E-05	2.41E-05
FUK	7.51E-06	5.92E-06	5.31E-06	5.36E-06	4.64E-06	6.71E-06	6.06E-06	8.95E-06
FUNDC1	2.57E-05	6.80E-06	5.61E-06	7.58E-06	2.39E-06	7.68E-06	5.88E-06	1.08E-05
FUNDC2	1.88E-05	1.63E-05	8.72E-06	1.70E-05	1.60E-05	1.23E-05	1.45E-05	2.31E-05
FUNDC2P	3.10E-07	1.67E-07	8.65E-08	1.80E-07	3.37E-07	2.42E-07	4.71E-08	4.82E-08
FURIN	0.000185	0.000164	0.000172	8.66E-05	7.80E-05	6.36E-05	0.000154	0.000282
FUS	0.000128	8.77E-05	4.05E-05	9.28E-05	1.71E-05	7.82E-05	0.000117	7.89E-05
FUT1	7.98E-07	4.01E-07	2.80E-07	4.05E-07	3.72E-07	9.41E-08	1.42E-06	7.62E-07
FUT10	1.68E-06	1.31E-06	1.13E-06	1.15E-06	1.15E-07	1.20E-06	6.60E-07	4.01E-07
FUT11	2.89E-06	1.84E-06	1.37E-06	2.52E-06	1.26E-07	1.73E-06	1.10E-06	1.22E-06
FUT2	8.01E-07	6.98E-07	6.19E-08	2.01E-07	1.29E-08	4.51E-06	1.67E-07	4.39E-08
FUT3	7.40E-08	1.32E-07	2.38E-07	3.14E-07	4.01E-08	3.02E-08	1.13E-07	2.06E-07
FUT4	3.53E-06	7.44E-08	6.82E-07	2.15E-06	1.14E-07	1.92E-07	8.01E-07	1.46E-07
FUT5	6.03E-08	6.83E-08	2.28E-07	2.51E-07	7.27E-08	5.60E-08	2.68E-07	2.28E-07
FUT6	4.27E-06	2.24E-06	5.80E-06	6.94E-06	1.13E-06	2.04E-06	1.61E-05	1.87E-06
FUT7	2.63E-07	4.40E-08	1.20E-07	2.48E-07	1.01E-07	4.52E-08	1.51E-07	4.30E-08
FUT8	5.47E-06	7.10E-07	1.20E-06	3.28E-06	4.92E-07	1.89E-06	1.44E-06	2.30E-06
FUT9	0	0	0	0	0	0	1.68E-09	0
FUZ	3.63E-06	4.97E-06	4.47E-06	7.54E-06	1.59E-06	1.28E-06	3.58E-06	1.62E-06
FXC1	1.67E-05	2.22E-05	8.30E-06	9.64E-06	4.40E-06	1.12E-05	9.86E-06	1.62E-05
FXN	2.43E-06	3.21E-06	5.44E-06	6.86E-06	3.06E-06	8.34E-06	8.92E-06	1.66E-06
FXR1	4.34E-05	3.00E-05	2.57E-05	2.81E-05	1.29E-05	5.00E-05	2.64E-05	1.86E-05
FXR2	1.22E-05	1.44E-05	1.17E-05	1.13E-05	3.85E-06	1.66E-05	1.29E-05	1.45E-05
FXYD1	3.83E-06	0.000444	0.00033	0.000259	0.001596	2.70E-06	0.00054	0.000128
FXYD2	0.001248	6.92E-08	2.21E-05	3.87E-05	5.97E-07	6.13E-08	4.24E-07	1.84E-06
FXYD3	2.63E-05	9.61E-07	1.37E-07	0	1.48E-08	3.41E-08	1.36E-06	5.18E-07
FXYD4	0	8.79E-08	0	0	0	0	0	0
FXYD5	0.000108	1.37E-05	2.16E-05	6.25E-05	3.66E-05	1.21E-05	5.80E-05	2.66E-05
FXYD6	2.78E-05	6.83E-06	2.12E-05	1.89E-05	2.16E-05	8.53E-06	2.07E-05	1.95E-05
FXYD7	1.88E-07	2.69E-08	3.89E-08	2.06E-07	4.21E-07	0	2.39E-07	0
FYB	9.88E-06	7.24E-07	2.91E-06	4.21E-06	2.28E-07	2.62E-06	2.33E-06	8.93E-07
FYCO1	6.37E-06	4.65E-06	3.95E-06	3.88E-06	1.52E-06	7.28E-06	4.08E-06	4.41E-06
FYN	1.55E-05	6.08E-06	1.29E-05	1.60E-05	8.03E-06	1.73E-05	2.50E-05	7.11E-06
FYTTD1	1.80E-05	1.42E-05	1.27E-05	1.21E-05	3.15E-06	1.20E-05	9.60E-06	9.66E-06
FZD1	4.18E-05	6.95E-07	2.69E-06	2.68E-06	2.01E-07	3.81E-07	8.27E-07	1.16E-06
FZD10	1.20E-06	4.77E-08	2.93E-08	2.60E-08	1.22E-08	2.62E-08	7.56E-08	3.43E-07
FZD2	1.52E-06	8.21E-08	3.29E-07	7.11E-07	1.56E-07	1.46E-07	1.07E-06	1.96E-07
FZD3	9.86E-07	6.09E-07	3.89E-07	6.42E-07	8.51E-08	8.64E-08	2.79E-07	7.44E-07
FZD4	7.34E-06	2.86E-06	5.21E-06	4.08E-06	4.27E-06	4.72E-06	8.07E-06	6.17E-06
FZD5	1.87E-05	2.06E-05	9.08E-06	6.22E-06	5.01E-06	7.18E-06	9.79E-06	6.36E-06
FZD6	2.02E-05	5.27E-06	9.35E-07	2.51E-06	9.63E-07	4.87E-07	5.91E-06	2.28E-06
FZD7	1.75E-06	9.96E-08	5.55E-07	9.92E-07	1.42E-07	8.23E-08	2.71E-07	1.56E-07
FZD8	3.88E-06	8.53E-07	1.74E-06	2.51E-06	4.29E-07	5.43E-07	2.34E-06	3.40E-07
FZD9	5.04E-08	1.14E-08	3.15E-08	8.64E-08	8.67E-09	9.35E-09	5.86E-08	0
FZR1	1.95E-05	1.50E-05	9.70E-06	1.52E-05	9.69E-06	1.10E-05	1.97E-05	9.29E-06
G0S2	2.02E-05	7.97E-05	8.71E-05	0.000478	1.09E-05	9.65E-05	4.68E-05	0.000292

G2E3	2.97E-06	2.01E-06	1.35E-06	1.15E-06	4.67E-07	1.29E-06	9.38E-07	1.27E-06
G3BP1	4.28E-05	2.49E-05	2.52E-05	2.41E-05	8.20E-06	4.86E-05	1.96E-05	3.14E-05
G3BP2	4.21E-05	1.82E-05	2.17E-05	2.28E-05	6.04E-06	2.02E-05	1.45E-05	2.26E-05
G6PC	0.000411	0.000679	0.000333	0.00022	4.16E-05	0.000646	3.54E-05	0.000922
G6PC2	1.56E-08	8.79E-09	2.43E-08	0	0	0	0	1.14E-08
G6PC3	2.94E-05	2.29E-05	1.03E-05	1.87E-05	2.91E-05	3.10E-05	2.09E-05	1.82E-05
G6PD	2.14E-05	1.54E-05	2.50E-06	5.13E-06	2.28E-06	7.16E-06	5.37E-06	4.32E-06
GAA	6.97E-05	7.81E-05	5.29E-05	5.78E-05	7.91E-05	8.59E-05	4.74E-05	4.04E-05
GAB1	3.62E-06	1.40E-06	2.10E-06	1.78E-06	6.52E-07	1.13E-06	1.77E-06	1.04E-06
GAB2	8.71E-06	1.10E-06	1.06E-06	3.35E-06	7.71E-07	3.89E-06	1.77E-06	9.59E-06
GAB3	1.45E-06	2.07E-07	4.17E-07	8.19E-07	2.30E-07	3.89E-07	8.60E-07	3.06E-07
GAB4	0	1.00E-08	0	0	0	0	0	0
GABARAP	0.000351	0.000281	0.000292	0.000475	0.000211	0.00047	0.000606	0.000403
GABARAP	0.000274	7.04E-05	9.77E-05	8.85E-05	2.09E-05	0.0002	4.57E-05	7.35E-05
GABARAP	0.000103	7.34E-05	5.81E-05	8.02E-05	3.26E-05	9.76E-05	6.65E-05	6.03E-05
GABARAP	4.74E-07	1.02E-07	1.48E-07	1.57E-07	0	7.13E-08	0	3.77E-08
GABBR1	1.10E-05	7.11E-08	3.72E-07	1.04E-06	2.91E-07	9.80E-08	1.55E-07	7.06E-07
GABBR2	3.12E-07	3.66E-08	0	1.00E-08	3.45E-06	4.17E-08	1.20E-08	1.20E-08
GABPA	7.03E-06	5.39E-06	7.09E-06	4.65E-06	1.83E-06	6.58E-06	3.19E-06	5.53E-06
GABPB1	7.41E-06	7.73E-06	4.04E-06	5.44E-06	1.50E-06	7.16E-06	4.32E-06	7.97E-06
GABPB2	1.98E-06	5.82E-07	1.07E-06	1.30E-06	1.25E-07	2.99E-06	5.60E-07	3.38E-06
GABRA1	0	0	0	0	0	0	0	0
GABRA2	4.79E-08	0	0	2.41E-08	0	0	0	1.43E-08
GABRA3	0	0	0	1.02E-08	0	0	1.62E-08	0
GABRA4	1.59E-08	0	0	0	0	0	0	0
GABRA5	0	0	0	0	0	0	0	0
GABRA6	0	0	0	0	0	0	0	0
GABRB1	1.39E-08	0	0	0	0	0	0	0
GABRB2	0	2.16E-08	9.88E-09	7.87E-09	0	0	3.14E-09	1.89E-08
GABRB3	6.64E-06	1.57E-06	7.31E-07	5.60E-07	0	7.38E-09	1.36E-07	4.08E-08
GABRD	1.87E-06	5.94E-07	2.62E-08	1.23E-07	4.58E-06	1.50E-06	2.16E-06	3.90E-06
GABRE	9.46E-06	4.04E-07	2.50E-07	1.14E-06	1.23E-06	1.46E-06	1.39E-06	2.45E-06
GABRG1	6.60E-09	1.47E-08	1.69E-08	1.21E-08	0	0	0	4.82E-09
GABRG2	0	0	0	0	0	0	0	4.92E-07
GABRG3	5.27E-08	1.66E-08	1.87E-08	0	0	0	1.69E-08	0
GABRP	7.41E-08	0	4.12E-07	7.96E-07	0	6.86E-09	1.08E-07	2.17E-08
GABRQ	3.59E-08	0	0	0	0	0	0	0
GABRR1	1.49E-08	3.34E-08	0	0	0	0	0	0
GABRR2	1.82E-08	6.25E-08	0	0	0	1.69E-08	0	2.70E-08
GABRR3	0	1.64E-07	0	0	0	6.99E-07	1.72E-08	0
GAD1	1.31E-05	0	0	2.51E-08	0	1.05E-07	2.18E-08	0
GAD2	2.70E-08	0	0	0	0	0	0	0
GADD45A	3.25E-05	5.49E-05	5.56E-05	0.000156	5.93E-05	0.000142	5.16E-05	6.27E-05
GADD45B	0.000112	0.000153	0.000361	0.001062	0.000115	0.000123	0.000199	0.000274
GADD45G	1.40E-05	0.000134	0.000248	0.000131	4.54E-05	0.000147	2.46E-05	9.72E-05
GADD45G	9.99E-05	5.07E-05	5.27E-05	7.18E-05	0.000228	7.33E-05	0.000126	8.39E-05
GADL1	0	0	0	0	0	9.85E-08	6.08E-09	0
GAGE1	0	7.85E-06	0	0	0	0	0	0
GAGE10	0	1.48E-07	0	0	0	0	0	0
GAGE12D	0	1.23E-05	0	0	0	0	0	2.50E-07
GAGE12F	0	0	0	0	0	0	0	0
GAGE12J	0	6.00E-06	0	0	0	0	0	8.86E-08
GAGE13	0	8.24E-08	0	0	0	0	0	0
GAGE2A	0	1.31E-06	0	0	0	0	0	6.61E-07
GAGE2B	0	3.14E-05	0	0	0	0	0	0
GAGE2C	0	0	0	0	0	0	0	0
GAGE2D	0	0.000155	0	0	0	0	0	1.13E-06
GAGE2E	0	8.24E-07	0	0	0	0	0	0

GAGE4	0	3.46E-05	0	0	0	0	0	1.13E-05
GAGE8	0	1.39E-07	0	0	0	0	0	1.22E-07
GAK	4.28E-05	7.86E-05	2.99E-05	3.36E-05	4.70E-05	3.20E-05	3.32E-05	3.44E-05
GAL	1.57E-05	0	0	2.75E-07	0	1.03E-07	3.48E-08	0
GAL3ST1	2.20E-05	3.64E-06	1.64E-06	2.01E-06	1.96E-07	1.56E-06	3.80E-07	8.96E-08
GAL3ST2	3.61E-07	9.82E-08	7.33E-08	0	0	8.00E-08	6.62E-08	2.54E-08
GAL3ST3	0	0	0	0	6.20E-09	0	0	0
GAL3ST4	4.41E-06	7.83E-07	3.62E-07	1.55E-06	3.47E-07	4.63E-07	3.52E-06	4.79E-07
GALC	6.58E-06	5.96E-07	3.98E-06	6.04E-06	7.00E-07	6.41E-07	2.83E-06	7.60E-07
GALE	0.000132	4.09E-05	4.05E-05	4.83E-05	2.21E-05	4.68E-05	5.99E-05	4.25E-05
GALK1	0.000157	0.000162	0.00014	3.82E-05	0.000207	0.000156	6.90E-05	7.79E-05
GALK2	1.31E-05	2.27E-05	7.33E-06	7.36E-06	3.83E-06	1.61E-05	5.73E-06	1.05E-05
GALM	5.22E-05	9.27E-05	4.23E-05	3.83E-05	3.86E-05	2.34E-05	4.90E-05	1.89E-05
GALNS	1.63E-05	2.42E-05	8.51E-06	1.06E-05	5.18E-06	5.18E-06	6.16E-06	1.39E-05
GALNT1	2.36E-05	2.63E-05	1.92E-05	2.17E-05	4.64E-06	4.92E-05	1.79E-05	2.65E-05
GALNT10	1.16E-05	1.37E-06	1.17E-06	2.62E-06	4.48E-07	2.71E-06	2.86E-06	4.06E-06
GALNT11	9.09E-06	1.11E-05	1.20E-05	1.25E-05	5.85E-06	1.29E-05	1.09E-05	1.61E-05
GALNT12	2.48E-06	2.88E-08	1.59E-07	3.03E-07	1.47E-08	7.12E-08	1.57E-07	6.27E-08
GALNT13	1.57E-08	1.17E-08	0	1.24E-07	3.24E-08	0	8.21E-08	2.29E-08
GALNT14	1.35E-05	2.27E-07	2.64E-07	2.42E-07	1.37E-07	1.31E-07	4.19E-07	7.70E-08
GALNT2	3.75E-05	5.16E-05	4.52E-05	3.68E-05	3.19E-05	4.28E-05	2.95E-05	5.58E-05
GALNT3	3.74E-07	4.18E-07	4.83E-07	6.55E-07	8.48E-08	1.31E-07	1.16E-07	4.13E-08
GALNT4	5.44E-06	2.03E-06	3.03E-06	3.12E-06	4.76E-07	2.14E-06	1.69E-06	1.82E-06
GALNT5	3.11E-06	0	0	9.92E-08	0	6.83E-09	4.30E-08	3.25E-08
GALNT6	3.20E-06	1.37E-07	5.32E-07	1.20E-06	1.35E-07	3.59E-07	9.36E-07	1.96E-07
GALNT7	7.42E-06	4.17E-07	4.90E-07	1.02E-06	1.62E-07	2.39E-07	7.71E-07	4.79E-07
GALNT8	1.11E-08	0	1.16E-08	1.37E-08	0	0	0	1.64E-08
GALNT9	5.71E-08	4.81E-08	3.18E-08	3.46E-08	0	2.62E-08	4.08E-08	1.04E-07
GALNTL1	1.42E-06	2.17E-07	1.27E-06	2.77E-06	6.36E-07	3.07E-07	7.58E-07	1.24E-06
GALNTL2	1.85E-07	4.08E-06	8.74E-07	1.64E-06	7.00E-07	2.33E-06	4.86E-06	7.23E-07
GALNTL4	1.61E-05	3.02E-06	5.21E-06	6.26E-06	4.68E-06	8.32E-06	9.08E-06	1.12E-05
GALNTL5	0	0	0	0	0	0	0	0
GALNTL6	2.45E-08	2.75E-07	1.01E-07	9.98E-09	0	0	0	0
GALP	0	5.73E-07	3.44E-08	1.53E-07	2.15E-07	5.79E-08	0	9.25E-08
GALR1	0	0	0	1.02E-08	0	0	1.62E-08	2.45E-08
GALR2	3.52E-07	1.79E-07	0	9.63E-08	6.75E-08	1.46E-07	3.38E-07	0
GALR3	0	0	7.26E-08	2.76E-08	3.68E-07	1.02E-06	6.43E-08	3.32E-08
GALT	2.77E-05	6.43E-05	7.00E-05	5.65E-05	5.01E-05	4.90E-05	8.51E-05	5.90E-05
GAMT	0.000149	0.000295	0.000426	0.000451	0.001106	0.000294	0.000721	0.000193
GAN	6.43E-07	1.73E-07	1.94E-07	1.83E-07	2.14E-08	2.44E-07	4.37E-08	2.92E-07
GANAB	0.000272	0.000257	0.00016	0.00021	9.07E-05	0.000238	0.000184	0.000279
GANC	6.89E-06	4.40E-06	3.38E-06	4.10E-06	1.06E-06	7.26E-06	5.07E-06	6.11E-06
GAP43	1.57E-07	1.62E-08	0	1.58E-07	0	3.98E-08	0	6.32E-08
GAPDH	0.004073	0.002893	0.001056	0.001558	0.001831	0.002834	0.002039	0.002183
GAPDHS	8.32E-08	1.90E-08	1.77E-08	2.05E-08	1.43E-08	3.09E-08	0	0
GAPT	6.74E-07	7.25E-08	4.02E-07	6.16E-07	3.68E-08	3.97E-08	1.35E-07	1.10E-07
GAPVD1	6.99E-06	3.94E-06	5.28E-06	4.54E-06	1.89E-06	6.77E-06	3.06E-06	7.64E-06
GAR1	1.57E-05	6.88E-06	6.13E-06	1.19E-05	1.02E-05	1.31E-05	1.97E-05	1.24E-05
GARNL3	4.51E-07	3.49E-07	1.97E-06	1.18E-06	1.30E-07	8.27E-07	6.51E-07	9.21E-07
GARS	6.32E-05	7.20E-05	1.90E-05	2.48E-05	2.26E-05	3.14E-05	3.52E-05	5.65E-05
GART	2.19E-05	1.13E-05	1.18E-05	1.10E-05	3.69E-06	2.29E-05	1.25E-05	1.68E-05
GAS1	4.11E-07	8.33E-08	1.07E-06	5.42E-06	7.78E-08	1.22E-07	1.21E-06	5.67E-07
GAS2	5.75E-06	1.55E-05	6.96E-06	5.95E-06	6.01E-06	1.55E-05	4.92E-06	5.55E-06
GAS2L1	1.55E-05	1.01E-05	5.88E-06	9.17E-06	9.13E-06	6.03E-06	9.99E-06	1.65E-05
GAS2L2	7.70E-09	0	0	0	0	3.57E-08	0	0
GAS2L3	4.31E-06	8.26E-07	1.80E-07	1.71E-07	0	3.29E-07	2.08E-08	1.27E-07
GAS5	6.85E-05	4.44E-05	2.51E-05	5.20E-05	5.11E-05	0.000103	9.26E-05	7.88E-05
GAS6	2.72E-05	3.77E-05	3.61E-05	4.26E-05	3.37E-05	2.10E-05	5.43E-05	3.09E-05

GAS7	1.62E-06	2.96E-07	6.32E-07	2.05E-06	2.59E-07	6.34E-07	1.15E-06	3.93E-07
GAS8	1.08E-05	6.61E-06	2.66E-06	1.01E-05	2.51E-06	6.34E-06	5.50E-06	4.85E-06
GAST	0	1.14E-07	0	0	0	0	0	0.000136
GATA1	0	0	0	6.21E-08	0	0	0	0
GATA2	1.81E-06	5.67E-07	2.98E-07	6.18E-07	3.28E-07	2.04E-07	5.62E-07	6.67E-07
GATA3	1.15E-06	1.78E-07	4.83E-07	9.95E-07	5.83E-08	4.26E-07	2.80E-06	2.10E-07
GATA4	1.20E-05	4.54E-05	1.35E-05	8.91E-06	5.70E-06	1.01E-05	6.26E-06	7.80E-06
GATA5	1.26E-07	0	2.62E-07	3.09E-07	3.10E-08	0	0	1.32E-08
GATA6	7.01E-06	6.79E-06	6.89E-06	1.38E-05	2.64E-06	5.23E-07	6.21E-06	9.64E-06
GATAD1	0.000115	1.66E-05	5.87E-06	7.12E-06	4.00E-06	1.04E-05	8.05E-06	8.69E-06
GATAD2A	1.07E-05	9.40E-06	7.96E-06	1.19E-05	6.05E-06	9.34E-06	1.36E-05	1.09E-05
GATAD2B	4.23E-06	3.84E-06	2.10E-06	1.94E-06	9.02E-07	4.73E-06	2.68E-06	5.18E-06
GATC	9.61E-06	1.37E-05	9.46E-06	5.65E-06	1.39E-06	1.10E-05	2.43E-06	8.15E-06
GATM	0.000239	0.000507	0.001064	0.000446	0.000295	0.000482	0.000569	0.000297
GATS	1.23E-05	5.20E-06	7.45E-07	3.45E-06	9.25E-07	2.80E-06	1.12E-06	2.05E-06
GATSL1	1.05E-06	3.11E-06	8.90E-08	5.69E-07	0	4.04E-07	3.87E-07	1.09E-06
GATSL2	1.57E-06	3.14E-08	0	0	0	0	0	4.03E-08
GATSL3	8.97E-06	7.92E-06	2.95E-06	2.55E-06	2.28E-06	5.85E-06	3.77E-06	4.66E-06
GBA	4.30E-05	4.60E-05	1.89E-05	2.48E-05	1.47E-05	5.87E-05	2.94E-05	0.00012
GBA2	3.04E-05	2.55E-05	9.74E-06	1.42E-05	1.73E-05	1.95E-05	1.17E-05	2.10E-05
GBA3	2.43E-07	1.29E-05	6.70E-05	2.25E-05	6.83E-05	9.06E-06	1.75E-05	6.77E-06
GBAP1	1.58E-05	1.54E-05	5.41E-06	1.00E-05	1.71E-05	2.21E-05	1.53E-05	4.51E-05
GBAS	2.88E-05	7.65E-06	4.45E-06	9.98E-06	2.41E-06	9.10E-06	6.35E-06	8.74E-06
GBE1	1.28E-05	1.74E-05	5.01E-05	1.52E-05	4.08E-06	7.33E-05	1.32E-05	3.31E-05
GBF1	2.07E-05	7.71E-06	9.49E-06	8.16E-06	4.84E-06	1.65E-05	9.35E-06	1.25E-05
GBGT1	2.17E-06	6.35E-07	9.39E-07	1.69E-06	3.61E-07	6.54E-07	2.62E-06	5.60E-07
GBP1	5.66E-05	3.50E-05	3.16E-05	4.29E-05	1.89E-06	5.67E-05	2.42E-05	2.42E-05
GBP2	0.000154	5.11E-05	5.58E-06	3.09E-05	8.97E-06	5.60E-05	2.93E-05	7.61E-05
GBP3	2.26E-05	1.43E-05	5.25E-07	8.47E-06	9.46E-07	2.82E-05	7.92E-06	1.62E-05
GBP4	2.14E-05	3.73E-06	7.12E-06	5.35E-06	8.41E-07	1.21E-05	2.26E-06	4.90E-06
GBP5	2.37E-05	2.53E-07	2.46E-06	3.34E-06	8.94E-08	5.43E-06	9.72E-07	2.75E-06
GBP6	5.10E-08	3.11E-08	9.76E-08	3.05E-08	1.72E-08	6.50E-08	1.46E-08	5.13E-08
GBP7	1.81E-06	2.09E-05	4.74E-05	1.38E-05	1.57E-06	1.26E-05	7.72E-06	2.77E-05
GBX1	0	0	0	0	0	0	0	0
GBX2	1.89E-08	4.33E-08	0	0	0	0	1.82E-08	0
GC	0.003026	0.001145	0.00391	0.002448	0.001421	0.002351	0.002156	0.002712
GCA	7.21E-06	4.17E-06	3.60E-06	4.85E-06	3.87E-07	4.42E-06	3.72E-06	2.47E-06
GCAT	3.85E-05	5.79E-05	3.40E-05	2.34E-05	0.000116	7.52E-05	7.66E-06	1.98E-05
GCC1	4.01E-06	9.13E-06	6.33E-06	5.74E-06	2.46E-06	7.05E-06	5.72E-06	1.04E-05
GCC2	1.20E-05	4.91E-06	3.64E-06	4.62E-06	7.37E-07	3.65E-06	1.58E-06	2.92E-06
GCDH	2.77E-05	5.77E-05	5.35E-05	3.68E-05	7.43E-05	6.73E-05	4.36E-05	3.20E-05
GCET2	4.01E-07	3.91E-08	1.73E-07	2.30E-07	0	9.04E-08	3.39E-08	5.11E-08
GCFC1	1.11E-05	4.27E-06	3.27E-06	3.61E-06	1.66E-06	3.68E-06	2.60E-06	4.81E-06
GCG	0	1.07E-06	0	1.43E-07	0	0	0	3.43E-08
GCGR	1.21E-05	6.89E-05	5.47E-05	5.12E-05	7.73E-05	4.75E-05	2.48E-05	1.17E-05
GCH1	6.58E-05	2.34E-05	4.50E-05	2.19E-05	1.06E-05	1.24E-05	2.55E-05	3.58E-05
GCHFR	0.000143	0.000177	0.000125	9.61E-05	0.000307	0.000197	0.000164	4.74E-05
GCK	3.96E-08	1.36E-07	2.18E-06	2.90E-07	9.56E-07	8.96E-09	3.68E-07	1.26E-06
GCKR	3.24E-05	2.07E-05	4.54E-05	2.49E-05	2.32E-05	6.13E-05	4.24E-05	1.84E-05
GCLC	1.70E-05	6.56E-05	4.00E-05	2.23E-05	1.01E-05	5.31E-05	2.36E-05	2.81E-05
GCLM	1.44E-05	3.25E-05	1.49E-05	1.06E-05	1.47E-06	3.89E-05	7.74E-06	8.48E-06
GCM1	8.43E-09	2.85E-08	8.74E-09	0	0	5.47E-08	0	1.24E-08
GCM2	0	0	0	0	0	0	0	0
GCN1L1	1.41E-05	1.48E-05	6.92E-06	6.57E-06	4.66E-06	1.03E-05	7.70E-06	5.69E-06
GCNT1	1.18E-06	1.89E-07	7.20E-07	6.42E-07	9.67E-08	3.56E-07	5.01E-07	1.35E-07
GCNT2	3.04E-05	8.33E-06	6.81E-06	5.97E-06	2.74E-06	1.05E-05	3.88E-06	5.72E-06
GCNT3	1.37E-05	9.16E-07	1.89E-07	9.00E-07	8.58E-09	6.48E-07	1.01E-07	1.04E-06
GCNT4	2.94E-06	6.97E-07	6.76E-07	6.21E-07	5.74E-09	1.47E-06	1.82E-07	8.42E-07

GCNT7	6.95E-08	1.31E-08	0	0	0	0	0	0
GCOM1	5.23E-07	1.20E-06	9.85E-07	1.45E-06	8.84E-08	4.47E-07	4.03E-07	7.55E-08
GCSH	2.60E-06	4.19E-06	2.44E-06	2.61E-06	1.96E-06	8.94E-06	3.58E-06	3.12E-06
GDA	3.05E-05	8.76E-06	2.03E-05	1.48E-05	4.20E-06	4.53E-06	4.73E-06	1.99E-05
GDAP1	3.43E-06	1.64E-07	7.28E-07	1.16E-06	2.90E-07	1.60E-06	1.16E-06	7.25E-07
GDAP1L1	2.56E-08	0	0	1.56E-08	0	1.19E-08	1.24E-08	0
GDAP2	3.84E-06	3.68E-06	1.79E-06	1.65E-06	5.06E-07	5.00E-06	8.78E-07	2.15E-06
GDE1	2.40E-05	2.24E-05	1.75E-05	1.74E-05	6.85E-06	2.38E-05	1.42E-05	2.39E-05
GDEP	0	0	0	0	0	0	0	0
GDF1	0	3.55E-08	0	0	0	0	3.93E-08	0
GDF10	6.65E-07	0	5.44E-08	8.56E-08	1.50E-08	0	0	1.28E-08
GDF11	4.66E-06	4.08E-07	5.64E-07	1.34E-06	2.58E-07	3.06E-07	5.33E-07	2.76E-07
GDF15	7.62E-05	0.000279	5.15E-05	0.000274	8.06E-06	1.45E-05	0.000375	2.51E-05
GDF2	1.24E-08	0	1.14E-05	1.60E-05	2.14E-08	0	0	0
GDF3	8.38E-08	4.82E-08	0	0	0	0	4.02E-08	3.11E-08
GDF5	2.97E-08	1.11E-08	0	0	1.11E-07	2.75E-08	2.01E-07	1.45E-08
GDF6	1.24E-07	3.46E-08	7.00E-08	6.57E-07	1.06E-08	1.72E-08	1.86E-07	4.53E-08
GDF7	1.95E-06	7.21E-07	7.55E-08	4.12E-07	0	1.78E-07	2.79E-07	7.07E-08
GDF9	9.35E-07	6.30E-07	1.84E-07	1.52E-07	6.87E-08	5.48E-07	3.25E-07	2.55E-07
GDI1	7.93E-05	4.93E-05	2.63E-05	5.50E-05	5.43E-05	3.53E-05	4.82E-05	8.99E-05
GDI2	0.000116	4.42E-05	0.00012	0.000105	6.52E-05	0.00014	0.000107	8.09E-05
GDNF	0	0	1.10E-06	5.71E-08	0	2.54E-07	0	4.52E-07
GDPD1	3.32E-06	1.16E-06	1.26E-07	3.75E-07	9.15E-08	1.22E-06	2.44E-07	4.05E-07
GDPD2	1.24E-07	0	0	0	0	0	0	0
GDPD3	7.41E-07	6.85E-07	1.02E-07	3.80E-07	6.81E-07	8.88E-08	4.87E-07	8.32E-07
GDPD4	1.85E-07	1.04E-08	6.74E-08	2.27E-08	3.18E-08	4.29E-08	2.69E-08	6.80E-08
GDPD5	7.87E-06	2.12E-06	2.30E-06	2.53E-06	2.15E-06	5.92E-07	2.99E-06	4.45E-06
GEFT	4.17E-06	2.84E-07	9.00E-07	2.03E-06	1.18E-06	3.74E-07	1.24E-06	1.20E-06
GEM	2.10E-05	1.53E-06	2.24E-06	3.59E-05	4.25E-07	1.14E-06	6.07E-06	3.08E-06
GEMIN4	3.52E-06	2.18E-06	3.78E-06	4.10E-06	3.15E-06	5.38E-06	3.44E-06	3.36E-06
GEMIN5	3.58E-06	3.96E-06	2.88E-06	2.30E-06	5.00E-07	5.40E-06	2.59E-06	4.07E-06
GEMIN6	5.30E-05	4.10E-05	1.30E-05	1.10E-05	1.92E-05	2.90E-05	1.63E-05	2.25E-05
GEMIN7	1.37E-05	1.00E-05	5.09E-06	6.32E-06	1.69E-05	7.38E-06	9.72E-06	7.71E-06
GEMIN8	7.41E-06	7.59E-07	2.96E-06	3.37E-06	1.71E-06	4.41E-06	4.70E-06	6.43E-06
GEMIN8P4	1.09E-06	1.55E-06	9.99E-07	9.16E-07	3.59E-07	3.00E-06	7.89E-07	8.89E-07
GEN1	2.96E-06	2.91E-06	1.85E-06	1.29E-06	9.13E-07	9.88E-07	1.06E-06	8.88E-07
GET4	4.35E-05	2.61E-05	2.29E-05	2.34E-05	2.52E-05	2.09E-05	2.95E-05	2.39E-05
GFAP	2.76E-06	5.93E-07	1.01E-07	2.82E-07	3.07E-07	3.47E-08	2.21E-08	3.38E-08
GFER	1.66E-05	1.46E-05	5.91E-06	9.05E-06	1.14E-05	8.91E-06	1.85E-05	9.22E-06
GFI1	1.65E-06	5.85E-08	4.31E-07	5.30E-07	5.21E-08	7.39E-07	1.68E-07	1.83E-06
GFI1B	0	1.69E-08	4.70E-08	1.82E-08	0	0	0	0
GFM1	2.69E-05	3.81E-05	2.46E-05	1.57E-05	7.88E-06	2.99E-05	1.62E-05	2.32E-05
GFM2	1.87E-05	2.74E-05	1.35E-05	1.16E-05	2.29E-06	2.59E-05	9.53E-06	1.33E-05
GFOD1	7.74E-07	1.16E-06	6.53E-07	1.79E-06	5.45E-07	9.27E-07	1.96E-06	1.86E-06
GFOD2	1.07E-05	1.11E-05	1.28E-05	1.38E-05	7.72E-06	1.74E-05	1.52E-05	2.12E-05
GFPT1	1.60E-05	1.21E-05	7.12E-06	8.39E-06	2.56E-06	1.45E-05	5.64E-06	1.07E-05
GFPT2	2.05E-06	2.59E-08	1.75E-07	6.52E-06	4.62E-08	4.27E-08	1.16E-06	5.53E-07
GFRA1	3.14E-08	1.13E-07	9.14E-06	3.51E-06	4.16E-06	9.16E-06	2.56E-06	7.31E-06
GFRA2	2.78E-07	3.13E-07	6.56E-07	9.18E-07	2.99E-07	7.82E-07	1.94E-06	8.24E-07
GFRA3	3.95E-08	0	1.38E-08	8.08E-08	0	0	1.27E-08	1.84E-08
GFRA4	0	0	0	0	0	0	1.68E-08	0
GFRAL	0	0	0	0	0	0	0	0
GGA1	2.79E-05	1.42E-05	8.91E-06	1.33E-05	1.72E-05	1.17E-05	1.28E-05	1.23E-05
GGA2	3.39E-05	1.54E-05	7.38E-06	1.01E-05	2.42E-06	1.17E-05	1.02E-05	8.77E-06
GGA3	1.31E-05	9.95E-06	4.17E-06	4.04E-06	2.68E-06	9.00E-06	4.70E-06	4.81E-06
GGCT	8.72E-05	7.45E-05	1.73E-05	2.10E-05	2.71E-05	2.41E-05	2.97E-05	2.53E-05
GGCX	9.34E-05	6.55E-05	7.57E-05	5.28E-05	3.76E-05	4.15E-05	6.10E-05	8.00E-05
GGH	5.87E-05	0.00012	7.64E-05	7.44E-05	0.000117	0.000315	0.000309	0.000103

GGN	3.79E-07	3.74E-08	1.15E-08	1.56E-07	1.04E-07	1.13E-07	3.20E-08	2.00E-07
GGNBP1	0	0	0	0	0	0	0	0
GGNBP2	4.78E-05	3.73E-05	1.49E-05	2.08E-05	1.18E-05	2.33E-05	2.16E-05	1.70E-05
GGPS1	8.06E-06	6.85E-06	5.30E-06	6.89E-06	2.58E-06	1.07E-05	6.50E-06	1.26E-05
GGT1	0.000147	1.59E-05	2.54E-05	4.16E-05	0.000108	1.13E-06	4.24E-05	3.31E-05
GGT3P	6.22E-06	4.94E-07	6.88E-07	1.67E-06	1.75E-06	1.00E-07	1.31E-06	1.29E-06
GGT5	1.09E-05	1.66E-06	1.98E-05	5.01E-05	1.11E-05	2.62E-06	1.88E-05	9.82E-06
GGT6	3.70E-08	2.82E-07	3.85E-07	1.36E-06	0	0	8.07E-08	0
GGT7	2.37E-05	3.18E-05	5.86E-06	7.32E-06	6.94E-06	5.23E-06	3.75E-06	4.16E-06
GGT8P	3.21E-07	7.46E-08	9.83E-09	3.47E-08	0	1.75E-08	1.83E-08	2.78E-08
GGTA1	8.66E-06	4.49E-06	3.44E-06	7.70E-06	3.46E-06	5.98E-06	2.23E-05	3.40E-06
GGTLC1	3.40E-06	1.93E-07	6.38E-07	1.98E-06	3.30E-06	0	5.53E-07	6.59E-07
GGTLC2	6.02E-05	4.23E-06	7.90E-06	1.93E-05	9.24E-05	4.79E-07	2.62E-05	1.45E-05
GH1	0	0	0	0	0	0	0	0
GH2	0	4.36E-08	0	0	0	0	0	0
GHDC	2.18E-05	3.59E-05	1.16E-05	1.70E-05	1.57E-05	2.63E-05	1.80E-05	1.83E-05
GHITM	0.000151	0.000226	0.00014	0.000114	5.60E-05	0.000206	8.53E-05	0.000121
GHR	1.35E-05	8.17E-06	0.000141	2.74E-05	6.12E-06	5.86E-06	1.11E-05	1.72E-05
GHRH	0	0	0	0	0	0	0	0
GHRHR	1.39E-06	1.87E-05	0	2.44E-07	8.66E-08	1.83E-08	0	1.86E-06
GHRL	9.64E-07	3.79E-08	7.83E-08	3.43E-07	2.07E-07	2.28E-07	5.90E-08	2.35E-07
GHRLOS	4.59E-07	1.19E-07	7.10E-08	1.20E-07	2.70E-07	2.71E-07	3.05E-07	6.59E-08
GHSR	0	0	0	0	0	2.98E-08	0	0
GIF	0	0	0	0	0	0	0	0
GIGYF1	1.59E-05	7.58E-06	4.00E-06	4.68E-06	5.20E-06	5.61E-06	6.65E-06	8.72E-06
GIGYF2	1.48E-05	6.50E-06	5.63E-06	4.62E-06	1.92E-06	7.20E-06	4.17E-06	4.16E-06
GIMAP1	6.81E-06	2.90E-06	5.28E-06	3.79E-06	4.37E-06	4.24E-06	7.82E-06	3.71E-06
GIMAP2	1.05E-05	5.42E-06	8.67E-06	5.73E-06	6.58E-07	9.01E-06	4.73E-06	6.46E-06
GIMAP4	3.15E-05	6.44E-06	1.69E-05	1.36E-05	5.36E-06	1.31E-05	1.29E-05	1.34E-05
GIMAP5	2.19E-05	5.47E-06	1.41E-05	1.32E-05	6.87E-06	1.31E-05	1.29E-05	1.81E-05
GIMAP6	9.25E-06	2.75E-06	5.45E-06	3.00E-06	1.13E-06	4.40E-06	4.18E-06	3.59E-06
GIMAP7	2.33E-05	3.93E-06	1.06E-05	7.51E-06	5.12E-06	1.11E-05	8.40E-06	1.11E-05
GIMAP8	7.08E-06	2.42E-06	5.31E-06	2.21E-06	2.08E-06	3.45E-06	3.25E-06	4.13E-06
GIN1	2.00E-06	1.41E-06	1.42E-06	1.17E-06	5.76E-07	1.49E-06	1.12E-06	1.01E-06
GINS1	6.12E-06	3.53E-06	2.96E-07	4.36E-07	5.47E-07	1.11E-06	6.52E-07	6.57E-07
GINS2	2.12E-05	4.09E-06	2.13E-06	2.75E-06	5.66E-06	3.55E-06	4.19E-06	4.97E-06
GINS3	3.60E-06	1.89E-06	6.12E-07	7.11E-07	1.01E-06	1.23E-06	9.10E-07	1.09E-06
GINS4	3.72E-06	3.00E-06	2.75E-07	4.14E-07	2.94E-07	9.49E-07	3.39E-07	1.02E-06
GIP	0	0	0	0	0	5.84E-07	0	0
GIPC1	5.25E-05	3.65E-05	3.03E-05	5.21E-05	5.98E-05	3.92E-05	6.71E-05	5.07E-05
GIPC2	1.15E-05	8.74E-06	1.03E-05	8.24E-06	4.17E-06	8.07E-06	2.53E-06	2.42E-07
GIPC3	1.07E-06	1.05E-06	6.81E-07	7.13E-07	1.11E-06	6.67E-07	1.59E-06	1.11E-06
GIPR	2.72E-06	1.52E-08	6.68E-08	7.80E-08	1.16E-08	3.54E-08	3.69E-08	1.87E-08
GIT1	1.22E-05	9.86E-06	3.22E-06	4.86E-06	6.91E-06	5.30E-06	7.73E-06	7.10E-06
GIT2	1.58E-05	9.76E-06	5.99E-06	7.86E-06	2.57E-06	7.70E-06	7.33E-06	6.90E-06
GIYD2	3.31E-05	4.53E-05	2.95E-05	4.56E-05	2.56E-05	5.94E-05	0.000114	6.30E-05
GJA1	0.000107	8.42E-06	2.46E-06	9.00E-06	2.26E-06	6.15E-06	7.79E-06	4.97E-06
GJA10	8.58E-07	4.11E-06	0	0	0	0	0	6.59E-06
GJA3	1.22E-06	0	8.86E-09	5.28E-09	7.42E-09	0	0	0
GJA4	1.09E-05	7.90E-06	8.34E-06	1.06E-05	1.32E-05	6.13E-06	1.31E-05	1.44E-05
GJA5	7.02E-06	1.82E-06	4.95E-07	8.17E-07	2.59E-06	1.45E-06	2.98E-06	5.34E-06
GJA8	0	0	0	0	0	5.11E-08	0	0
GJA9	1.33E-07	2.31E-08	1.06E-08	2.50E-08	6.15E-08	1.89E-08	1.98E-08	1.50E-08
GJB1	0.000469	0.000353	0.000376	0.000263	0.000849	0.000281	0.00027	0.000755
GJB2	1.53E-05	3.26E-06	3.35E-05	1.97E-05	4.00E-06	1.44E-05	1.56E-05	1.54E-05
GJB3	2.56E-06	3.28E-07	3.93E-07	1.21E-06	1.60E-06	0	6.74E-07	7.58E-08
GJB4	1.77E-07	5.03E-08	0	1.12E-07	0	0	7.94E-09	1.20E-08
GJB5	1.19E-07	1.82E-07	2.13E-07	7.33E-08	3.43E-08	0	4.57E-07	2.35E-07

GJB6	7.58E-08	2.86E-08	3.72E-07	4.65E-08	0	2.35E-08	1.22E-08	3.72E-08
GJB7	1.04E-08	0	0	1.27E-08	0	0	0	0
GJC1	3.99E-06	4.63E-07	5.71E-08	1.15E-07	3.23E-07	3.59E-07	4.59E-07	8.32E-07
GJC2	3.38E-07	6.21E-07	9.93E-08	1.05E-06	2.55E-07	8.82E-08	3.99E-07	2.18E-07
GJC3	1.67E-07	1.45E-06	6.71E-07	7.03E-07	7.81E-07	9.35E-08	5.05E-07	4.49E-07
GJD2	0	0	0	0	0	0	0	0
GJD3	1.51E-06	4.39E-07	8.43E-07	1.33E-06	1.64E-07	8.64E-07	6.02E-07	7.64E-07
GJD4	0	0	0	0	0	5.79E-08	0	0
GK	2.32E-05	9.16E-06	6.69E-06	1.64E-05	3.55E-06	2.06E-05	1.20E-05	3.67E-05
GK2	0	0	0	0	0	0	0	0
GK3P	4.64E-06	1.98E-06	1.43E-06	2.86E-06	9.13E-08	4.11E-06	1.06E-06	7.13E-06
GK5	3.12E-06	1.61E-06	8.10E-07	6.81E-07	3.14E-07	1.87E-06	5.23E-07	1.40E-06
GKAP1	4.28E-06	3.59E-06	2.57E-06	3.14E-06	1.05E-06	3.20E-06	2.59E-06	3.98E-06
GKN1	0	0	0	0	0	0	0	0
GKN2	0	0	0	0	0	0	0	0
GLA	5.24E-05	1.75E-05	5.90E-06	1.23E-05	1.58E-05	3.51E-05	8.09E-06	2.47E-05
GLB1	4.84E-05	2.84E-05	2.82E-05	3.29E-05	1.59E-05	3.81E-05	2.25E-05	4.32E-05
GLB1L	3.17E-06	5.44E-06	2.20E-06	4.05E-06	1.65E-06	2.98E-06	4.79E-06	1.22E-06
GLB1L2	2.22E-06	0	8.81E-08	2.95E-07	1.03E-08	6.78E-09	4.50E-08	3.22E-08
GLB1L3	1.28E-07	1.71E-08	4.80E-08	3.70E-08	0	0	0	5.76E-07
GLCCI1	3.46E-06	3.30E-06	1.99E-06	1.61E-06	2.00E-07	1.15E-06	9.09E-07	2.04E-06
GLCE	6.77E-05	5.28E-06	9.98E-06	6.75E-06	5.71E-06	1.01E-05	7.15E-06	2.00E-05
GLDC	4.97E-05	2.57E-05	4.87E-05	9.87E-06	2.02E-05	1.29E-05	1.09E-05	5.30E-05
GLDN	3.61E-07	1.14E-06	1.09E-07	4.06E-07	4.29E-08	8.34E-08	2.03E-07	9.05E-07
GLE1	1.35E-05	1.04E-05	8.03E-06	7.30E-06	3.82E-06	1.68E-05	6.47E-06	1.56E-05
GLG1	3.42E-05	9.47E-06	9.63E-06	1.27E-05	6.96E-06	1.13E-05	1.07E-05	1.62E-05
GLI1	3.57E-06	2.24E-07	6.28E-08	6.13E-07	1.38E-07	2.55E-07	2.70E-07	7.76E-07
GLI2	3.94E-07	2.43E-08	2.82E-07	3.71E-07	2.18E-08	1.68E-08	8.12E-08	1.06E-07
GLI3	2.42E-07	7.95E-08	7.28E-08	2.44E-07	3.29E-08	8.37E-08	1.12E-07	1.56E-07
GLI4	1.42E-05	1.27E-05	2.71E-06	3.45E-06	1.70E-05	7.15E-06	1.02E-05	3.47E-06
GLIPR1	8.36E-06	3.01E-06	2.09E-06	9.74E-06	3.82E-07	1.55E-05	5.21E-06	1.60E-06
GLIPR1L1	0	0	0	0	0	0	0	4.20E-08
GLIPR1L2	9.90E-08	2.26E-07	2.93E-07	4.82E-07	2.21E-07	4.98E-07	7.97E-07	1.66E-07
GLIPR2	1.27E-05	1.16E-06	2.53E-06	1.34E-05	2.18E-06	2.29E-06	5.40E-06	3.91E-06
GLIS1	3.63E-07	0	2.57E-08	1.01E-07	0	0	3.20E-08	0
GLIS2	6.54E-06	3.62E-06	1.60E-06	6.26E-06	3.50E-07	4.40E-07	4.05E-06	3.17E-06
GLIS3	7.78E-06	2.82E-06	1.76E-06	4.51E-06	7.21E-07	6.28E-07	1.34E-06	2.07E-06
GLMN	2.96E-06	1.73E-06	9.01E-07	1.36E-06	9.03E-07	3.32E-06	8.62E-07	1.47E-06
GLO1	8.75E-05	0.000165	6.52E-05	4.49E-05	3.74E-05	6.76E-05	5.10E-05	5.32E-05
GLOD4	2.64E-05	2.59E-05	4.07E-05	3.72E-05	2.19E-05	5.15E-05	4.10E-05	4.25E-05
GLOD5	2.89E-06	6.26E-06	5.20E-06	5.83E-06	7.14E-06	3.38E-06	9.53E-06	5.37E-07
GLP1R	9.11E-07	0	0	0	0	1.37E-08	0	1.08E-08
GLP2R	1.32E-07	0	2.64E-07	7.23E-08	0	0	0	0
GLRA1	0	0	0	0	0	0	0	0
GLRA2	0	0	0	0	0	0	0	0
GLRA3	0	0	0	0	0	0	6.20E-09	0
GLRA4	1.46E-08	0	0	0	0	0	0	0
GLRB	6.38E-06	2.55E-08	3.36E-07	2.50E-07	0	7.00E-09	1.47E-07	5.55E-08
GLRX	0.000103	5.44E-05	6.79E-05	6.10E-05	2.34E-05	7.72E-05	3.96E-05	7.24E-05
GLRX2	1.41E-05	1.93E-05	1.10E-05	1.49E-05	1.24E-05	1.84E-05	1.48E-05	3.07E-05
GLRX3	4.99E-05	2.55E-05	2.10E-05	2.60E-05	1.37E-05	3.22E-05	2.70E-05	3.46E-05
GLRX5	6.74E-05	9.17E-05	8.76E-05	7.69E-05	7.69E-05	0.000106	9.36E-05	6.97E-05
GLS	2.68E-05	6.96E-06	3.29E-06	7.85E-06	2.68E-07	1.67E-06	4.06E-06	1.90E-06
GLS2	1.51E-05	0.000227	0.000107	3.94E-05	4.85E-05	1.66E-07	1.57E-05	1.91E-05
GLT1D1	1.19E-05	2.96E-05	1.14E-05	1.48E-05	5.13E-06	1.06E-05	1.00E-05	2.65E-05
GLT25D1	5.43E-05	1.60E-05	1.52E-05	2.09E-05	2.20E-05	1.47E-05	2.80E-05	1.91E-05
GLT25D2	3.34E-07	7.64E-08	3.02E-08	2.46E-07	5.53E-07	1.50E-07	6.81E-08	2.98E-08
GLT6D1	0	0	0	0	0	0	0	0

GLT8D1	4.66E-05	2.13E-05	2.72E-05	4.42E-05	3.08E-05	8.04E-05	4.95E-05	1.74E-05
GLT8D2	1.02E-06	3.53E-07	7.00E-07	2.52E-06	2.32E-07	2.20E-07	8.27E-07	1.25E-06
GLTP	1.77E-05	1.28E-05	7.66E-06	1.22E-05	5.98E-06	8.67E-06	1.24E-05	9.76E-06
GLTPD1	2.53E-05	2.85E-05	2.98E-05	3.28E-05	2.41E-05	2.24E-05	3.33E-05	3.36E-05
GLTPD2	8.54E-06	2.19E-05	3.59E-05	3.75E-05	2.84E-05	1.55E-05	4.69E-05	2.30E-05
GLTSCR1	2.87E-06	1.80E-06	9.80E-07	1.65E-06	1.07E-06	1.81E-06	1.70E-06	1.76E-06
GLTSCR2	0.000103	8.64E-05	0.000129	0.000189	0.000175	0.000126	0.000122	0.000101
GLUD1	0.000111	0.000197	0.00041	0.000159	0.00017	0.000262	0.000177	0.000151
GLUD2	2.64E-05	2.93E-05	7.09E-05	1.98E-05	1.65E-06	3.88E-05	1.17E-05	2.12E-05
GLUL	0.000136	0.000192	0.000346	0.000226	5.24E-05	0.007065	0.000225	0.000243
GLYAT	1.07E-05	0.000104	0.000187	7.52E-05	0.000198	0.000121	8.22E-05	8.26E-06
GLYATL1	1.33E-06	6.33E-05	0.000164	0.000114	9.02E-05	5.02E-05	9.26E-05	7.53E-05
GLYATL2	3.65E-08	0	0	8.99E-08	0	0	0	0
GLYATL3	0	1.26E-07	1.99E-07	1.37E-08	6.73E-08	0	0	1.15E-07
GLYCAM1	0	0	0	0	0	0	0	0
GLYCTK	7.08E-05	0.000119	0.000154	9.99E-05	0.000226	0.000101	9.81E-05	8.27E-05
GLYR1	2.05E-05	5.37E-05	2.35E-05	2.37E-05	6.08E-06	3.28E-05	2.40E-05	3.04E-05
GM2A	4.53E-05	1.63E-05	1.39E-05	2.60E-05	3.82E-06	2.40E-05	1.82E-05	1.83E-05
GMCL1	3.19E-05	8.28E-06	3.49E-06	3.02E-06	1.73E-07	5.56E-06	9.64E-07	2.41E-06
GMCL1L	4.60E-07	1.43E-07	6.57E-08	3.88E-08	0	5.88E-08	0	3.49E-08
GMDS	4.73E-05	4.99E-06	6.37E-06	9.36E-06	5.43E-06	2.07E-05	1.41E-05	2.89E-05
GMEB1	3.63E-06	2.13E-06	1.72E-06	2.60E-06	3.54E-07	2.38E-06	1.67E-06	3.66E-06
GMEB2	6.45E-06	5.40E-06	2.35E-06	3.58E-06	4.65E-06	4.06E-06	5.83E-06	4.32E-06
GMFB	1.81E-05	9.47E-06	8.25E-06	1.00E-05	2.31E-06	1.12E-05	1.08E-05	1.29E-05
GMFG	4.23E-05	1.22E-05	2.35E-05	5.10E-05	2.94E-05	2.18E-05	4.60E-05	1.88E-05
GMIP	7.12E-06	1.31E-06	2.00E-06	3.85E-06	5.58E-07	2.41E-06	2.67E-06	2.26E-06
GML	0	0	0	0	0	0	0	0
GMNN	5.66E-05	0.000129	9.15E-06	8.91E-06	1.17E-05	3.96E-05	1.35E-05	1.94E-05
GMPPA	6.78E-05	4.14E-05	2.16E-05	2.78E-05	6.50E-05	5.76E-05	4.04E-05	4.06E-05
GMPPB	1.67E-05	1.17E-05	6.72E-06	7.84E-06	9.96E-06	1.75E-05	1.08E-05	1.66E-05
GMPR	9.19E-06	5.92E-06	3.81E-06	6.24E-06	9.88E-07	2.85E-05	5.83E-07	4.84E-07
GMPR2	5.34E-05	2.47E-05	2.59E-05	2.98E-05	2.33E-05	5.89E-05	3.40E-05	2.62E-05
GMPS	1.69E-05	1.72E-05	8.24E-06	1.13E-05	4.60E-06	1.39E-05	1.15E-05	1.74E-05
GNA11	1.54E-05	8.20E-06	1.12E-05	9.82E-06	4.25E-07	1.08E-05	8.69E-06	1.16E-05
GNA12	2.62E-05	1.17E-05	4.33E-06	8.29E-06	1.74E-06	1.43E-05	9.91E-06	1.63E-05
GNA13	2.00E-05	1.36E-05	8.82E-06	1.81E-05	1.89E-06	1.24E-05	7.72E-06	1.51E-05
GNA14	5.47E-07	2.76E-07	2.23E-06	3.07E-06	4.62E-07	1.14E-06	8.86E-07	5.95E-07
GNA15	2.85E-06	4.67E-07	9.45E-07	5.21E-06	5.57E-07	1.74E-06	2.36E-06	8.70E-07
GNAI1	1.39E-05	1.02E-05	1.06E-05	7.21E-06	5.18E-06	1.32E-05	9.06E-06	9.81E-06
GNAI2	0.000138	0.00011	0.000118	0.000135	7.35E-05	0.00011	0.000132	7.75E-05
GNAI3	2.79E-05	1.88E-05	1.22E-05	1.79E-05	6.59E-06	2.89E-05	1.22E-05	1.52E-05
GNAL	9.48E-07	6.91E-07	5.57E-08	2.83E-07	1.32E-08	4.35E-06	8.21E-08	4.40E-07
GNAO1	2.66E-07	8.75E-07	9.03E-06	1.58E-06	2.01E-06	4.44E-06	3.01E-06	1.80E-07
GNAQ	3.49E-05	1.55E-05	1.83E-05	1.32E-05	4.07E-06	1.20E-05	1.13E-05	1.56E-05
GNAS	0.000783	0.000587	0.000343	0.00036	0.000463	0.000312	0.000318	0.000271
GNASAS	2.24E-08	0	1.21E-07	2.76E-08	1.94E-08	2.09E-08	1.50E-07	0
GNAT1	2.55E-08	3.42E-07	2.62E-08	0	1.05E-07	1.18E-08	1.24E-08	5.59E-08
GNAT2	5.53E-08	4.22E-08	5.91E-08	2.27E-08	6.37E-08	1.56E-06	3.54E-08	1.37E-07
GNAT3	0	0	0	0	0	0	0	0
GNAZ	1.74E-05	3.97E-06	1.78E-07	3.69E-07	3.46E-07	1.86E-07	1.59E-06	1.60E-06
GNB1	0.000158	8.38E-05	5.88E-05	8.42E-05	3.11E-05	9.50E-05	5.61E-05	8.11E-05
GNB1L	3.91E-06	6.09E-06	1.92E-06	2.99E-06	4.13E-06	3.40E-06	3.05E-06	5.10E-06
GNB2	0.000319	0.000261	9.31E-05	0.000131	9.73E-05	8.72E-05	0.000138	0.000148
GNB2L1	0.000904	0.000383	0.000553	0.000771	0.000628	0.000768	0.000848	0.000659
GNB3	1.57E-07	1.70E-07	6.02E-08	1.75E-08	1.23E-08	0	4.13E-08	9.35E-08
GNB4	5.21E-06	1.03E-06	2.71E-06	3.81E-06	4.24E-07	1.22E-06	1.69E-06	1.38E-06
GNB5	5.47E-05	3.80E-05	7.29E-06	9.95E-06	3.66E-06	6.59E-06	4.88E-06	1.59E-05
GNE	7.73E-06	4.47E-05	5.47E-05	2.37E-05	7.75E-06	2.28E-05	2.00E-05	2.63E-05

GNG10	3.45E-05	1.62E-05	1.87E-05	1.91E-05	1.11E-05	2.09E-05	1.72E-05	1.17E-05
GNG11	2.95E-05	3.60E-05	2.35E-05	2.22E-05	3.98E-05	1.52E-05	5.52E-05	2.49E-05
GNG12	1.47E-05	1.07E-05	1.70E-05	2.17E-05	2.63E-06	2.89E-05	1.07E-05	1.26E-05
GNG13	0	0	0	0	0	0	0	0
GNG2	5.70E-06	1.02E-06	2.99E-06	5.72E-06	6.51E-07	2.54E-06	3.43E-06	1.68E-06
GNG3	3.64E-07	4.61E-07	3.37E-08	3.76E-08	5.27E-08	1.70E-07	3.75E-07	1.82E-07
GNG4	2.98E-05	1.24E-07	4.65E-09	9.96E-08	0	3.01E-08	0	6.46E-09
GNG5	0.000279	0.000217	0.000101	0.000142	9.32E-05	0.000221	0.000159	9.91E-05
GNG7	1.52E-06	2.26E-06	1.66E-06	1.81E-06	1.65E-06	3.20E-06	3.16E-06	1.86E-06
GNG8	5.32E-07	0	0	0	0	0	0	0
GNGT1	2.33E-07	2.09E-07	0	0	0	0	4.24E-08	9.84E-07
GNGT2	2.68E-06	3.54E-07	1.14E-06	1.98E-06	9.04E-07	8.56E-07	1.43E-06	4.55E-07
GNL1	5.27E-05	3.67E-05	1.37E-05	2.38E-05	3.41E-05	2.03E-05	2.10E-05	2.68E-05
GNL2	3.40E-05	2.01E-05	1.36E-05	2.08E-05	6.78E-06	1.97E-05	1.78E-05	3.12E-05
GNL3	3.93E-05	3.40E-05	2.02E-05	2.94E-05	1.16E-05	4.60E-05	3.24E-05	2.79E-05
GNL3L	4.05E-06	3.09E-06	1.86E-06	2.47E-06	4.49E-07	2.19E-06	1.58E-06	4.17E-06
GNLY	2.05E-05	3.80E-06	4.37E-06	2.53E-06	2.18E-06	2.59E-06	5.04E-06	1.98E-06
GNMT	8.93E-06	3.71E-05	0.000219	5.58E-05	3.81E-05	0.000126	2.46E-05	0.000143
GNPAT	2.57E-05	2.65E-05	1.33E-05	1.78E-05	1.01E-05	7.49E-05	1.92E-05	2.56E-05
GNPDA1	1.70E-05	6.59E-06	6.34E-06	1.08E-05	4.21E-06	8.06E-06	1.23E-05	1.12E-05
GNPDA2	4.05E-06	2.53E-06	1.37E-06	1.94E-06	5.51E-07	1.30E-06	1.23E-06	2.85E-06
GNPNAT1	1.67E-05	1.76E-05	3.45E-05	2.49E-05	1.35E-05	2.80E-05	3.09E-05	1.54E-05
GNPTAB	8.51E-06	2.90E-06	3.18E-06	3.94E-06	1.06E-06	3.34E-06	2.85E-06	5.96E-06
GNPTG	8.14E-05	0.000131	6.98E-05	9.14E-05	0.00011	9.56E-05	0.000102	0.000134
GNRH1	3.82E-07	1.25E-07	6.94E-08	1.09E-07	7.62E-08	1.11E-07	5.36E-08	1.47E-07
GNRH2	0	0	0	1.32E-07	0	9.70E-08	0	0
GNRHR	1.18E-08	4.40E-09	4.03E-09	1.44E-08	0	2.14E-08	7.67E-09	5.76E-09
GNRHR2	2.42E-06	1.41E-06	1.15E-06	1.47E-06	1.26E-06	1.96E-06	2.59E-06	7.89E-07
GNS	4.76E-05	8.04E-05	2.72E-05	3.47E-05	1.13E-05	5.15E-05	3.14E-05	3.17E-05
GOLGA1	1.03E-05	4.92E-06	5.27E-06	4.39E-06	1.60E-06	8.14E-06	3.46E-06	9.32E-06
GOLGA2	2.42E-05	2.38E-05	2.29E-05	2.33E-05	7.62E-06	2.91E-05	1.55E-05	4.37E-05
GOLGA2B	3.86E-06	1.81E-06	1.18E-06	1.55E-06	4.82E-06	3.65E-06	1.05E-06	1.78E-06
GOLGA2P	0	0	0	0	0	0	0	0
GOLGA3	7.52E-06	5.28E-06	3.74E-06	4.32E-06	2.83E-06	7.12E-06	5.90E-06	6.29E-06
GOLGA4	2.75E-05	2.50E-05	2.31E-05	1.94E-05	1.00E-05	2.51E-05	1.71E-05	2.16E-05
GOLGA5	3.13E-05	1.94E-05	2.60E-05	2.41E-05	1.95E-05	3.50E-05	2.36E-05	3.19E-05
GOLGA6A	7.27E-09	9.87E-07	6.91E-07	8.88E-08	0	4.51E-07	2.12E-08	0
GOLGA6B	8.71E-08	1.32E-06	1.25E-06	5.41E-07	1.25E-08	8.61E-07	1.90E-07	3.19E-08
GOLGA6C	7.27E-09	3.84E-07	8.26E-08	0	0	1.28E-07	0	0
GOLGA6D	0	7.47E-09	3.43E-08	0	0	1.05E-07	0	0
GOLGA6L:	0	0	6.15E-09	0	0	0	0	0
GOLGA6L:	3.10E-06	8.74E-07	4.54E-07	8.67E-07	7.40E-08	8.78E-07	4.36E-07	6.89E-07
GOLGA6L!	3.90E-07	5.44E-08	8.71E-08	8.55E-08	1.57E-08	7.61E-08	5.05E-08	3.56E-08
GOLGA6L(	0	0	5.72E-09	0	0	0	0	0
GOLGA6L!	2.38E-06	1.25E-06	3.62E-07	4.18E-07	3.77E-07	6.54E-07	5.08E-07	1.15E-06
GOLGA7	3.27E-05	4.89E-05	2.66E-05	2.48E-05	1.08E-05	3.20E-05	1.20E-05	2.19E-05
GOLGA7B	3.39E-06	1.17E-07	2.49E-07	1.87E-06	6.89E-09	1.19E-07	8.56E-08	9.42E-08
GOLGA8A	1.14E-05	3.66E-06	1.67E-06	1.64E-06	4.74E-07	2.76E-06	4.61E-07	3.43E-06
GOLGA8B	1.93E-05	3.03E-06	3.67E-07	1.25E-06	2.70E-06	2.32E-06	9.88E-07	3.18E-06
GOLGA8C	4.26E-09	4.76E-09	4.36E-09	0	0	0	0	6.23E-09
GOLGA8D	0	4.82E-09	0	0	7.40E-09	3.99E-09	0	6.31E-09
GOLGA8E	0	0	0	0	0	0	0	0
GOLGA8F	0	0	0	0	0	0	0	0
GOLGA8G	0	0	5.75E-09	0	0	0	0	8.19E-09
GOLGA9P	6.34E-08	2.44E-07	0	0	0	3.53E-08	2.45E-08	7.47E-08
GOLGB1	1.49E-05	1.39E-05	8.45E-06	9.82E-06	3.77E-06	1.53E-05	5.92E-06	7.01E-06
GOLIM4	2.28E-05	2.13E-05	2.71E-05	1.74E-05	2.76E-07	2.32E-05	1.03E-05	1.47E-05
GOLM1	0.000104	1.84E-06	1.38E-05	2.55E-05	4.49E-06	4.80E-05	1.06E-05	3.64E-05

GOLPH3	0.000134	6.64E-05	5.17E-05	6.14E-05	7.00E-06	6.23E-05	5.43E-05	0.000108
GOLPH3L	9.14E-06	7.50E-06	5.13E-06	4.04E-06	2.13E-06	1.25E-05	3.59E-06	6.63E-06
GOLT1A	0.000103	0.000113	9.72E-05	0.000134	0.000175	0.000102	0.000147	0.00031
GOLT1B	1.55E-05	1.83E-05	7.64E-06	8.88E-06	3.94E-06	1.81E-05	1.01E-05	1.51E-05
GON4L	7.53E-06	5.03E-06	3.26E-06	4.71E-06	2.06E-06	7.60E-06	4.23E-06	8.60E-06
GOPC	1.15E-05	9.06E-06	6.96E-06	5.89E-06	1.44E-06	1.05E-05	4.58E-06	4.77E-06
GORAB	6.22E-06	2.15E-06	2.67E-06	3.83E-06	3.95E-07	3.93E-06	4.04E-06	8.89E-06
GORASP1	2.40E-05	2.08E-05	1.05E-05	1.45E-05	9.69E-06	1.37E-05	1.34E-05	1.65E-05
GORASP2	7.67E-05	4.38E-05	2.98E-05	3.38E-05	2.36E-05	5.30E-05	3.46E-05	4.62E-05
GOSR1	1.13E-05	1.66E-05	7.68E-06	7.82E-06	3.34E-06	1.03E-05	7.83E-06	7.40E-06
GOSR2	2.96E-05	1.99E-05	1.15E-05	1.26E-05	9.65E-06	2.74E-05	1.48E-05	1.76E-05
GOT1	0.000217	0.000805	0.000244	0.000266	0.000143	6.23E-05	0.000219	0.00071
GOT1L1	0	0	0	0	0	0	0	0
GOT2	0.000116	0.000252	0.000277	0.000159	5.40E-05	0.000156	0.000142	0.000184
GP1BA	3.15E-07	3.73E-07	1.71E-07	4.68E-07	9.03E-08	1.59E-07	2.77E-07	1.96E-07
GP2	7.62E-06	3.29E-08	0	0	0	1.12E-08	3.51E-08	1.27E-06
GP5	2.63E-08	5.16E-08	6.09E-08	5.62E-08	5.64E-09	0	1.28E-08	0
GP6	2.15E-08	2.43E-08	1.35E-07	7.90E-08	1.85E-08	9.97E-09	1.04E-08	1.58E-08
GP9	0	1.90E-07	3.61E-08	8.03E-08	5.62E-08	3.03E-08	3.68E-07	0
GPA33	1.67E-08	1.87E-08	1.73E-08	5.10E-08	7.16E-09	1.54E-08	2.42E-08	0
GPAA1	0.000113	0.000128	4.39E-05	4.61E-05	0.000231	5.90E-05	0.000212	0.000109
GPAM	5.68E-06	1.40E-05	4.76E-05	1.73E-05	2.16E-05	6.03E-05	1.86E-05	4.80E-05
GPAT2	5.81E-07	2.92E-07	1.34E-07	4.59E-07	9.07E-07	6.74E-07	1.67E-06	9.48E-07
GPATCH1	4.15E-06	3.63E-06	2.36E-06	1.49E-06	2.48E-06	3.66E-06	2.50E-06	3.23E-06
GPATCH2	3.74E-06	3.20E-06	1.59E-06	2.87E-06	7.04E-07	4.34E-06	1.88E-06	4.06E-06
GPATCH3	1.01E-05	8.01E-06	4.90E-06	6.73E-06	8.43E-06	1.16E-05	8.40E-06	9.71E-06
GPATCH4	1.85E-05	1.89E-05	7.83E-06	1.44E-05	8.29E-06	2.66E-05	1.88E-05	3.71E-05
GPATCH8	1.46E-05	5.24E-06	3.32E-06	3.61E-06	5.94E-07	4.57E-06	2.07E-06	3.90E-06
GPBAR1	4.03E-06	3.12E-07	1.90E-06	2.30E-06	2.82E-07	1.35E-06	1.64E-06	1.00E-06
GPBP1	3.50E-05	2.87E-05	1.53E-05	2.17E-05	1.15E-05	1.88E-05	2.33E-05	2.77E-05
GPBP1L1	3.78E-05	3.21E-05	3.40E-05	2.88E-05	1.21E-05	3.89E-05	2.57E-05	2.03E-05
GPC1	5.10E-05	6.80E-06	1.60E-05	2.92E-05	4.17E-06	3.39E-07	3.60E-05	1.65E-05
GPC2	2.87E-07	0	1.04E-08	1.34E-07	7.96E-09	1.72E-08	4.82E-08	8.16E-08
GPC3	0.000122	1.38E-06	1.51E-06	5.80E-06	0.000341	0.000285	5.65E-06	3.49E-06
GPC4	1.44E-05	2.66E-07	7.91E-07	2.35E-06	6.88E-08	1.05E-07	5.81E-07	2.01E-07
GPC5	4.58E-06	3.86E-06	1.59E-07	8.88E-08	0	7.47E-09	3.13E-08	5.92E-07
GPC6	1.56E-05	5.18E-06	7.49E-06	1.09E-05	2.34E-06	2.57E-06	4.94E-06	6.80E-06
GPCPD1	1.50E-05	5.07E-06	2.98E-06	5.08E-06	9.72E-07	6.36E-06	2.96E-06	8.69E-06
GPD1	2.19E-05	1.38E-05	0.000105	3.15E-05	3.63E-05	3.46E-05	2.48E-05	1.41E-05
GPD1L	1.79E-05	6.53E-07	1.04E-06	1.89E-06	1.75E-07	4.16E-07	9.30E-07	8.20E-07
GPD2	6.37E-06	2.94E-06	1.10E-06	2.25E-06	3.56E-07	1.75E-06	1.67E-06	2.67E-06
GPER	1.16E-05	7.92E-06	1.54E-05	7.42E-06	1.76E-05	4.56E-06	5.54E-06	4.94E-06
GPHA2	7.78E-08	0	0	0	6.44E-07	0	3.67E-08	1.76E-07
GPHB5	0	0	0	0	0	0	0	0
GPHN	1.27E-05	6.04E-06	1.74E-05	1.26E-05	9.91E-06	2.25E-05	1.50E-05	9.71E-06
GPI	0.000258	0.000152	0.000125	0.00011	0.000102	0.00017	8.97E-05	0.000253
GPIHBP1	9.55E-07	6.93E-06	1.42E-06	7.24E-07	1.45E-06	2.26E-06	1.99E-05	2.09E-06
GPKOW	2.20E-05	2.92E-05	1.22E-05	1.77E-05	1.23E-05	1.95E-05	2.21E-05	3.32E-05
GPLD1	1.12E-07	3.32E-05	2.08E-05	9.10E-06	4.13E-06	4.06E-06	1.55E-06	2.31E-05
GPM6A	5.49E-08	4.41E-07	2.94E-06	3.37E-06	2.36E-07	5.08E-08	6.62E-07	1.73E-07
GPM6B	3.82E-07	2.06E-07	2.84E-07	3.55E-07	6.48E-08	8.60E-08	1.26E-07	1.57E-07
GPN1	3.02E-05	2.99E-05	1.15E-05	1.74E-05	8.36E-06	1.94E-05	1.54E-05	1.57E-05
GPN2	1.41E-05	1.13E-05	5.79E-06	8.47E-06	2.76E-06	8.38E-06	7.33E-06	8.78E-06
GPN3	2.43E-05	2.73E-05	1.85E-05	1.48E-05	7.34E-06	1.56E-05	1.32E-05	1.40E-05
GPNMB	8.19E-05	1.19E-05	1.92E-05	0.000104	2.47E-06	6.41E-06	4.65E-05	4.82E-06
GPR1	3.07E-08	8.08E-08	1.07E-08	1.25E-08	3.52E-08	9.48E-09	2.18E-07	3.01E-08
GPR101	0	0	0	0	0	0	0	0
GPR107	1.57E-05	8.25E-06	4.08E-06	6.03E-06	3.88E-06	8.26E-06	7.50E-06	1.24E-05

GPR108	7.02E-05	3.72E-05	2.44E-05	2.87E-05	5.62E-05	3.10E-05	2.57E-05	3.49E-05
GPR109A	8.33E-07	7.58E-07	9.07E-07	1.19E-06	0	1.93E-07	3.80E-07	6.70E-06
GPR109B	4.68E-07	9.19E-07	4.08E-07	9.83E-07	4.79E-08	3.41E-07	3.23E-07	6.47E-06
GPR110	0	9.12E-09	0	0	0	3.38E-06	0	1.19E-08
GPR111	2.71E-08	0	0	0	0	0	0	0
GPR112	0	0	0	0	0	0	3.53E-09	0
GPR113	7.46E-08	1.04E-07	1.35E-08	5.22E-08	2.66E-08	8.94E-08	5.02E-09	3.63E-08
GPR114	2.91E-06	5.44E-08	1.97E-07	2.97E-07	2.38E-08	1.47E-07	1.95E-07	1.06E-08
GPR115	1.65E-07	1.64E-08	1.56E-08	1.16E-07	0	0	0	0
GPR116	1.01E-05	7.72E-06	3.79E-06	1.74E-06	2.12E-06	5.76E-06	6.30E-06	5.07E-06
GPR119	7.95E-08	0	2.90E-08	0	0	0	0	3.95E-08
GPR12	0	2.84E-08	0	0	0	0	0	0
GPR120	1.54E-05	0	1.23E-07	2.81E-08	0	0	1.31E-07	3.38E-08
GPR123	0	0	2.61E-07	1.49E-07	9.10E-09	0	0	0
GPR124	9.18E-06	1.65E-06	2.57E-06	7.71E-06	1.51E-06	1.40E-06	3.09E-06	4.17E-06
GPR125	9.50E-06	1.94E-05	4.14E-05	1.50E-05	6.03E-06	1.55E-05	1.45E-05	2.54E-05
GPR126	2.40E-05	2.09E-05	4.13E-05	2.56E-05	3.40E-06	1.54E-05	2.09E-05	2.07E-05
GPR128	2.04E-06	4.32E-06	1.18E-05	5.44E-06	1.48E-06	1.25E-06	3.64E-06	4.51E-06
GPR132	1.80E-06	1.01E-07	6.41E-07	2.17E-06	1.26E-07	7.30E-07	5.71E-07	4.95E-07
GPR133	7.60E-06	7.88E-06	1.79E-06	3.42E-06	3.83E-07	8.85E-08	5.71E-06	5.47E-07
GPR135	2.31E-07	9.05E-08	9.59E-08	2.89E-07	1.15E-08	6.09E-07	1.03E-07	6.78E-08
GPR137	2.23E-05	3.61E-05	1.12E-05	1.41E-05	1.23E-05	2.08E-05	1.88E-05	3.10E-05
GPR137B	1.18E-05	8.50E-06	9.33E-06	1.31E-05	6.95E-07	3.82E-06	5.37E-06	1.30E-05
GPR137C	1.47E-06	1.03E-06	1.09E-07	1.72E-07	1.01E-07	1.36E-07	1.54E-07	1.89E-07
GPR139	0	0	0	0	0	0	1.98E-08	0
GPR141	8.61E-07	6.93E-08	3.29E-08	7.36E-08	2.58E-08	8.33E-08	1.13E-07	4.44E-08
GPR142	1.45E-07	0	1.62E-08	2.23E-08	0	0	0	0
GPR143	1.99E-07	1.97E-06	6.15E-07	1.69E-06	9.75E-07	1.85E-07	1.31E-06	1.02E-06
GPR144	0	0	0	0	0	0	0	0
GPR146	3.33E-06	8.22E-06	1.95E-05	1.22E-05	5.49E-06	8.42E-06	1.24E-05	7.38E-06
GPR148	0	0	0	0	0	0	0	0
GPR149	0	0	0	0	0	0	0	0
GPR15	4.86E-08	0	0	6.00E-08	0	0	4.64E-08	0
GPR150	1.94E-08	4.01E-07	0	4.78E-08	0	1.81E-08	1.49E-07	0
GPR151	0	0	0	0	0	0	0	0
GPR152	0	1.14E-07	1.86E-08	0	0	1.63E-08	1.95E-08	0
GPR153	1.44E-05	7.80E-07	1.78E-06	1.72E-06	2.00E-07	4.73E-06	2.41E-06	2.86E-06
GPR155	1.52E-06	5.28E-06	2.80E-06	1.85E-06	1.64E-06	4.57E-06	2.13E-06	2.29E-06
GPR156	8.27E-09	1.08E-08	0	2.02E-08	0	0	0	2.43E-08
GPR157	4.43E-06	4.72E-07	1.18E-06	9.19E-07	3.29E-08	1.10E-06	2.80E-07	1.50E-06
GPR158	1.60E-06	2.90E-06	5.81E-08	1.73E-08	2.43E-08	2.56E-06	9.19E-08	1.72E-06
GPR160	2.79E-06	1.43E-06	1.02E-06	1.15E-06	2.56E-07	5.08E-07	1.44E-06	5.08E-07
GPR161	1.58E-06	2.07E-07	1.29E-07	3.65E-07	2.71E-07	1.93E-07	4.46E-07	2.15E-07
GPR162	2.83E-06	1.76E-05	1.85E-06	3.88E-06	1.35E-06	1.84E-07	9.73E-07	1.24E-06
GPR17	1.78E-07	6.94E-08	6.41E-08	2.51E-08	7.74E-09	9.51E-08	1.59E-07	2.56E-07
GPR171	1.41E-06	6.16E-08	4.43E-07	2.31E-06	1.17E-08	1.12E-06	3.40E-07	6.60E-07
GPR172A	2.21E-05	1.13E-05	4.37E-06	1.13E-05	1.06E-05	8.42E-06	3.23E-05	1.71E-05
GPR172B	5.94E-08	2.12E-07	3.04E-08	2.84E-08	6.66E-09	2.75E-08	2.88E-08	0
GPR173	8.63E-08	0	4.55E-08	1.77E-08	0	1.34E-08	1.39E-08	8.48E-08
GPR174	1.46E-06	0	2.40E-07	2.00E-07	0	2.45E-07	1.94E-08	9.02E-08
GPR176	5.49E-06	1.21E-06	4.60E-07	3.59E-06	4.18E-08	1.59E-06	4.86E-07	6.97E-07
GPR179	1.95E-08	9.32E-09	0	0	4.78E-09	1.03E-08	0	4.07E-09
GPR18	1.14E-06	1.35E-07	2.70E-07	6.35E-07	2.92E-08	1.89E-07	1.79E-07	1.56E-07
GPR180	3.34E-06	7.17E-06	2.75E-06	2.90E-06	4.83E-07	3.32E-06	1.57E-06	1.72E-07
GPR182	7.86E-08	0	3.25E-06	7.72E-07	8.13E-08	2.92E-08	1.97E-07	6.96E-08
GPR183	7.87E-06	1.20E-06	1.85E-06	2.13E-05	3.04E-07	1.70E-06	3.06E-06	1.24E-06
GPR19	7.44E-07	7.09E-08	0	9.57E-08	1.46E-07	1.86E-07	7.50E-08	1.34E-07
GPR20	2.60E-07	0	1.72E-08	3.99E-08	0	4.53E-08	1.56E-08	4.80E-08

GPR21	1.45E-07	0	5.50E-08	0	1.25E-08	0	4.81E-08	0
GPR22	7.82E-09	0	0	0	0	7.24E-09	0	0
GPR25	1.21E-07	0	0	0	0	0	2.31E-08	0
GPR26	0	0	0	2.62E-09	0	0	0	0
GPR27	8.78E-07	0	0	0	0	0	4.42E-08	0
GPR3	9.61E-07	1.53E-07	2.36E-08	4.57E-07	1.36E-07	1.68E-07	4.92E-07	2.99E-07
GPR31	0	0	0	1.39E-07	0	5.25E-08	2.68E-08	0
GPR32	0	0	0	0	0	0	0	0
GPR34	6.04E-06	1.67E-06	1.90E-06	2.60E-06	7.64E-07	1.74E-06	5.85E-06	6.62E-07
GPR35	9.90E-07	5.71E-06	8.11E-08	7.54E-07	3.77E-07	9.31E-08	4.93E-07	1.33E-07
GPR37	7.84E-09	2.76E-05	6.57E-06	8.57E-06	7.88E-07	7.00E-06	3.57E-06	5.13E-06
GPR37L1	1.34E-07	6.47E-08	1.99E-08	1.64E-07	2.47E-08	1.06E-07	1.94E-07	2.95E-07
GPR39	8.48E-06	2.26E-06	2.53E-06	3.42E-06	3.65E-06	4.32E-06	2.50E-06	1.78E-06
GPR4	3.12E-06	3.35E-06	6.60E-07	1.85E-06	1.14E-06	1.71E-06	4.05E-06	2.97E-06
GPR44	5.83E-07	2.87E-07	2.06E-07	2.64E-07	1.30E-07	1.56E-06	1.79E-07	5.26E-07
GPR45	1.41E-08	3.21E-08	0	0	0	2.62E-08	0	2.08E-08
GPR50	0	0	0	0	0	0	0	0
GPR52	0	3.86E-08	0	0	0	1.57E-08	0	0
GPR55	2.24E-07	0	1.02E-07	1.39E-07	2.39E-08	4.71E-08	5.22E-08	4.80E-08
GPR56	2.58E-05	1.29E-05	1.10E-06	8.77E-06	1.56E-06	2.99E-06	3.86E-06	2.91E-06
GPR6	0	0	0	0	0	0	0	0
GPR61	0	1.85E-08	0	1.01E-08	7.08E-09	3.05E-08	0	0
GPR62	3.04E-07	3.92E-07	1.47E-07	1.20E-07	1.86E-07	1.01E-07	1.89E-07	1.12E-07
GPR63	3.44E-08	1.30E-08	4.80E-08	0	0	1.06E-08	2.22E-08	1.69E-08
GPR64	1.16E-07	3.11E-06	1.20E-07	1.18E-07	0	3.14E-08	7.71E-08	1.88E-07
GPR65	3.18E-06	4.09E-07	1.65E-06	2.93E-06	1.91E-07	1.16E-06	1.50E-06	5.72E-07
GPR68	1.38E-06	6.51E-08	3.26E-07	1.11E-06	2.84E-08	2.26E-07	5.68E-07	3.03E-07
GPR75	7.10E-07	5.08E-06	1.04E-06	4.83E-07	7.75E-08	2.57E-06	5.45E-08	4.80E-07
GPR77	1.38E-06	4.28E-06	1.33E-06	8.02E-07	1.20E-07	1.47E-06	3.03E-07	1.53E-05
GPR78	0	0	0	0	0	0	0	0
GPR81	7.79E-09	4.37E-08	0	2.86E-08	0	4.33E-08	0	0
GPR82	4.11E-07	0	3.23E-07	8.69E-08	1.52E-08	5.15E-08	5.74E-08	0
GPR83	7.42E-08	0	1.09E-07	7.12E-08	2.28E-08	1.47E-08	5.16E-09	0
GPR84	6.64E-07	1.89E-08	8.82E-08	4.49E-07	4.29E-08	1.54E-07	1.60E-08	2.45E-08
GPR85	1.87E-07	9.55E-08	1.30E-07	1.22E-07	0	1.69E-07	2.39E-07	5.35E-08
GPR87	0	3.72E-08	1.73E-08	4.01E-08	0	0	1.41E-07	0
GPR88	1.13E-06	0.000197	2.38E-06	6.72E-07	3.70E-07	7.97E-07	1.09E-07	2.13E-05
GPR89A	3.24E-05	2.33E-05	1.41E-05	2.27E-05	1.20E-05	2.50E-05	1.88E-05	2.63E-05
GPR89B	7.92E-07	4.08E-07	4.11E-07	7.28E-07	2.79E-08	1.27E-06	2.18E-07	6.20E-07
GPR89C	2.32E-06	1.79E-06	5.17E-07	1.44E-06	2.36E-06	1.50E-06	2.42E-06	2.72E-06
GPR97	2.00E-06	9.49E-07	2.98E-07	1.53E-06	3.77E-08	1.21E-07	1.27E-07	9.55E-06
GPR98	2.19E-06	3.83E-06	1.32E-05	5.94E-06	2.67E-06	5.16E-06	1.59E-05	2.34E-05
GPRASP1	1.43E-06	4.10E-07	5.46E-07	6.88E-07	6.96E-08	3.96E-07	2.48E-07	3.50E-07
GPRASP2	9.54E-07	8.77E-06	3.61E-06	2.80E-06	3.02E-06	3.58E-06	3.12E-06	2.16E-06
GPRC5A	6.84E-06	4.29E-07	3.38E-08	2.96E-06	0	4.53E-08	3.80E-07	3.59E-08
GPRC5B	2.44E-05	1.71E-05	3.04E-06	1.98E-05	1.99E-06	2.47E-06	7.29E-06	1.97E-06
GPRC5C	8.26E-05	0.000215	9.58E-05	7.47E-05	0.000146	6.57E-05	6.29E-05	8.53E-05
GPRC5D	1.18E-06	1.18E-07	1.12E-07	1.58E-07	0	1.43E-07	4.88E-08	7.62E-08
GPRC6A	0	0	0	0	0	0	0	0
GPRIN1	2.37E-06	1.51E-07	8.88E-08	6.40E-07	6.95E-08	2.70E-07	7.04E-07	4.74E-07
GPRIN2	6.45E-07	0	1.11E-07	8.98E-07	3.17E-09	0	0	0
GPRIN3	9.55E-06	6.53E-06	2.81E-07	1.66E-06	1.51E-08	2.87E-07	2.78E-07	2.32E-07
GPS1	8.52E-05	0.000165	4.60E-05	4.40E-05	9.93E-05	0.000103	6.44E-05	5.55E-05
GPS2	4.57E-05	4.50E-05	2.54E-05	4.16E-05	1.81E-05	3.75E-05	4.63E-05	5.37E-05
GPSM1	1.27E-05	2.79E-05	5.31E-07	7.33E-06	6.88E-07	2.51E-07	1.06E-05	7.03E-07
GPSM2	4.97E-06	3.19E-06	3.15E-07	2.96E-07	1.43E-07	8.17E-07	2.67E-07	4.18E-07
GPSM3	1.93E-05	3.14E-06	9.38E-06	2.07E-05	2.31E-06	8.00E-06	1.32E-05	7.47E-06
GPT	8.52E-05	0.000154	0.000114	6.50E-05	0.000259	4.85E-05	0.000104	1.96E-05

GPT2	0.000115	0.000211	9.57E-05	0.000121	1.31E-05	0.000152	6.97E-05	0.00012
GPX1	0.000364	0.000582	0.000317	0.000471	0.000401	0.000449	0.000511	0.000561
GPX2	0.00043	0.000134	0.000156	0.000153	0.000719	0.004144	0.00035	0.000593
GPX3	0.000383	0.000248	0.000267	0.000792	0.000265	0.000779	0.000286	0.000179
GPX4	0.000314	0.000451	0.00032	0.000356	0.000774	0.000313	0.00042	0.000302
GPX5	0	2.17E-08	0	0	0	0	0	0
GPX6	0	0	0	0	0	0	0	0
GPX7	3.83E-05	4.29E-06	2.60E-06	1.04E-05	3.93E-06	5.63E-07	4.14E-06	8.97E-07
GPX8	6.39E-06	7.79E-07	4.64E-07	1.67E-06	4.18E-07	5.30E-07	1.92E-06	8.57E-07
GRAMD1A	4.19E-05	1.78E-05	6.19E-06	8.09E-06	2.19E-06	3.45E-06	1.91E-05	1.92E-05
GRAMD1B	2.15E-06	6.58E-08	3.11E-07	1.70E-06	0	2.24E-06	7.68E-08	4.01E-06
GRAMD1C	8.86E-06	8.47E-06	8.90E-06	8.18E-06	2.23E-06	2.38E-06	2.86E-06	4.53E-06
GRAMD2	3.01E-07	1.95E-08	0	0	0	8.01E-09	8.38E-09	0
GRAMD3	1.00E-05	5.62E-06	7.50E-06	5.75E-06	3.64E-06	6.32E-06	4.25E-06	8.27E-06
GRAMD4	2.12E-05	4.54E-05	1.63E-05	7.72E-06	3.72E-06	2.11E-05	9.51E-06	0.000242
GRAP	4.44E-06	1.69E-06	5.14E-06	3.17E-06	2.89E-06	1.58E-06	8.86E-06	3.00E-06
GRAP2	1.63E-06	2.88E-07	6.19E-07	7.44E-07	8.64E-08	7.53E-06	3.85E-07	3.25E-07
GRAPL	1.17E-07	3.24E-08	9.19E-08	1.72E-07	0	0	1.59E-07	2.49E-07
GRASP	2.77E-06	1.38E-06	1.55E-06	7.91E-06	6.05E-06	1.43E-06	4.83E-06	3.27E-06
GRB10	4.29E-05	1.38E-05	9.75E-06	9.49E-06	1.01E-05	1.61E-05	9.67E-06	1.49E-05
GRB14	5.24E-05	1.94E-05	2.18E-05	2.04E-05	1.37E-05	1.23E-05	1.33E-05	1.04E-05
GRB2	0.000109	5.73E-05	2.80E-05	3.54E-05	2.75E-05	5.69E-05	3.41E-05	2.97E-05
GRB7	3.21E-05	4.88E-06	4.68E-06	7.42E-06	8.07E-06	7.89E-06	4.85E-06	3.78E-06
GREB1	9.51E-08	7.12E-06	5.52E-07	9.82E-07	9.25E-07	1.10E-05	1.89E-06	1.68E-07
GREB1L	2.23E-06	1.22E-06	3.37E-07	3.32E-07	1.14E-07	4.08E-07	1.84E-07	8.19E-07
GREM1	3.35E-06	1.67E-08	5.65E-08	9.40E-07	3.26E-07	3.41E-07	1.15E-06	4.03E-07
GREM2	5.44E-09	9.47E-06	7.99E-06	4.73E-06	8.58E-07	1.60E-05	2.39E-06	4.29E-06
GRHL1	5.57E-06	3.52E-06	2.13E-06	1.62E-06	1.04E-06	5.06E-07	1.35E-06	1.06E-06
GRHL2	8.62E-09	1.93E-08	2.29E-07	6.15E-07	0	0	9.65E-08	6.30E-09
GRHL3	7.47E-08	2.24E-07	9.74E-08	1.03E-07	3.21E-08	9.52E-08	0	0
GRHPR	0.000185	0.000333	0.000788	0.000567	0.000495	0.000489	0.000428	0.000262
GRIA1	0	0	4.33E-09	0	0	0	2.02E-08	0
GRIA2	0	9.56E-09	0	0	0	0	8.14E-09	0
GRIA3	5.05E-06	3.45E-08	2.03E-06	6.45E-07	1.51E-06	3.30E-06	4.46E-07	6.37E-06
GRIA4	7.28E-09	0	0	0	0	0	8.40E-09	0
GRID1	4.43E-07	3.85E-08	1.47E-07	1.54E-07	1.14E-06	4.53E-09	4.11E-08	3.92E-08
GRID2	0	0	0	0	0	0	0	0
GRID2IP	2.93E-08	1.64E-08	0	2.39E-08	0	0	4.75E-09	4.29E-08
GRIK1	1.49E-08	5.64E-08	3.85E-08	2.73E-08	4.47E-08	0	0	1.09E-08
GRIK2	0	3.21E-08	0	1.22E-08	0	0	4.84E-09	1.68E-08
GRIK3	0	5.35E-09	4.90E-09	9.87E-09	0	0	1.24E-07	0
GRIK4	1.74E-07	0	0	0	0	0	0	3.47E-07
GRIK5	2.24E-07	0	0	3.03E-08	4.28E-08	1.04E-08	3.85E-08	1.90E-08
GRIN1	5.26E-08	1.96E-08	0	0	0	0	0	0
GRIN2A	2.68E-07	1.96E-08	0	2.54E-07	0	4.22E-09	6.26E-09	1.07E-07
GRIN2B	3.77E-09	0	0	0	9.71E-09	0	2.21E-08	0
GRIN2C	1.18E-07	2.61E-07	4.76E-08	2.88E-08	1.35E-07	8.59E-08	4.19E-08	1.75E-07
GRIN2D	4.15E-07	3.99E-08	1.06E-07	2.32E-07	1.17E-07	7.71E-08	4.92E-07	1.18E-07
GRIN3A	3.68E-07	7.88E-08	1.14E-07	8.04E-08	6.02E-08	1.87E-07	1.36E-07	1.16E-07
GRIN3B	4.24E-08	3.97E-08	7.31E-09	8.65E-09	0	0	1.82E-08	0
GRINA	0.000262	0.000327	0.00021	0.000152	0.000189	0.000249	0.000581	0.000247
GRINL1A	1.41E-05	8.81E-06	6.54E-06	6.54E-06	2.16E-06	6.56E-06	4.47E-06	6.44E-06
GRIP1	2.36E-07	0	5.01E-08	5.29E-08	9.30E-09	1.50E-08	3.69E-08	1.59E-08
GRIP2	1.38E-06	2.84E-08	3.94E-08	2.63E-08	0	0	0	0
GRIPAP1	1.59E-05	2.16E-05	1.17E-05	1.42E-05	6.61E-06	1.99E-05	1.38E-05	2.28E-05
GRK1	0	0	0	0	0	0	0	0
GRK4	1.81E-06	1.50E-06	9.52E-07	9.87E-07	3.20E-07	1.52E-06	5.99E-07	6.77E-07
GRK5	6.63E-06	4.09E-06	5.53E-06	1.02E-05	2.90E-06	4.37E-06	6.13E-06	3.77E-06

GRK6	1.78E-05	4.45E-06	5.38E-06	7.42E-06	3.81E-06	7.10E-06	9.18E-06	1.09E-05
GRK7	0	0	0	0	0	0	0	0
GRLF1	1.37E-05	2.14E-05	1.37E-05	8.90E-06	1.31E-05	1.32E-05	1.24E-05	1.51E-05
GRM1	3.27E-09	2.19E-08	3.33E-09	2.15E-08	0	5.33E-09	9.55E-09	9.54E-09
GRM2	1.44E-07	4.24E-08	5.23E-08	7.64E-08	4.29E-08	4.63E-08	7.22E-08	2.18E-07
GRM3	0	1.87E-08	1.64E-08	8.83E-09	0	7.71E-09	0	1.06E-08
GRM4	2.31E-07	0	0	0	0	0	0	0
GRM5	2.15E-07	0	0	6.91E-09	0	0	0	8.27E-09
GRM6	4.83E-09	0	4.36E-08	1.43E-07	1.24E-08	0	9.40E-09	0
GRM7	0	0	1.69E-08	1.52E-08	0	0	0	0
GRM8	7.08E-08	0	6.71E-07	4.26E-07	2.21E-07	5.08E-07	5.08E-08	0
GRN	0.000264	0.000221	0.000105	0.000202	0.000142	0.000156	0.000206	0.000151
GRP	1.70E-07	2.80E-07	0	0	0	0	0	0
GRPEL1	5.38E-05	7.64E-05	4.82E-05	4.34E-05	1.87E-05	4.70E-05	4.88E-05	6.02E-05
GRPEL2	4.39E-06	4.16E-06	1.82E-06	2.21E-06	9.28E-07	3.39E-06	2.92E-06	3.99E-06
GRPR	2.53E-07	2.94E-08	9.04E-09	1.07E-07	7.49E-09	2.11E-06	2.70E-07	6.39E-08
GRRP1	4.55E-07	4.08E-07	3.62E-07	9.19E-07	1.09E-06	6.65E-07	1.25E-06	1.25E-06
GRSF1	2.04E-05	1.01E-05	9.18E-06	1.05E-05	3.87E-06	1.16E-05	1.02E-05	1.31E-05
GRTP1	1.87E-05	3.33E-05	2.62E-05	2.38E-05	1.51E-05	2.27E-05	1.29E-05	2.37E-05
GRWD1	1.86E-05	1.95E-05	1.18E-05	1.69E-05	1.41E-05	2.03E-05	2.36E-05	1.74E-05
GRXCR1	0	0	0	0	0	0	0	0
GRXCR2	0	0	0	0	0	0	0	0
GSC	4.34E-08	0	0	0	0	2.02E-08	2.08E-08	0
GSC2	0	0	0	0	0	0	0	0
GSDMA	6.78E-07	1.56E-07	1.68E-07	2.82E-07	2.97E-08	4.27E-08	3.78E-07	0
GSDMB	1.12E-05	6.13E-06	2.74E-06	6.64E-06	2.44E-05	8.13E-06	4.56E-06	1.52E-05
GSDMC	1.72E-07	0	0	0	0	4.78E-08	8.33E-09	1.89E-07
GSDMD	0.000142	0.000129	4.54E-05	7.54E-05	0.000114	8.45E-05	0.000193	9.55E-05
GSG1	1.36E-08	0	0	1.66E-08	0	0	1.31E-08	0
GSG1L	0	0	5.17E-09	0	8.65E-09	0	7.36E-08	1.39E-08
GSG2	9.41E-07	3.65E-07	3.45E-08	7.13E-08	2.86E-08	1.70E-07	1.62E-08	1.59E-07
GSK3A	3.26E-05	2.29E-05	1.58E-05	1.98E-05	1.53E-05	2.63E-05	2.66E-05	2.45E-05
GSK3B	9.99E-06	7.67E-06	4.17E-06	5.29E-06	2.26E-06	5.41E-06	4.95E-06	3.38E-06
GSN	0.000197	8.08E-05	2.05E-05	0.000117	6.21E-05	9.18E-05	0.00021	6.15E-05
GSPT1	1.51E-05	2.77E-05	1.16E-05	1.37E-05	3.17E-06	1.65E-05	1.38E-05	1.12E-05
GSPT2	7.72E-06	7.69E-06	7.53E-06	5.79E-06	1.11E-07	3.75E-06	3.13E-06	2.98E-05
GSR	2.01E-05	4.36E-05	1.89E-05	2.03E-05	8.28E-07	7.32E-05	8.78E-06	1.28E-05
GSS	9.70E-05	3.73E-05	2.39E-05	3.26E-05	6.39E-05	5.65E-05	4.36E-05	2.91E-05
GSTA1	1.26E-06	0.00097	0.00114	0.000222	0.001106	0.00138	0.00044	0.000229
GSTA2	1.36E-06	2.86E-05	0.000489	4.90E-06	0.00019	3.73E-05	7.94E-05	2.30E-05
GSTA3	0	0	3.97E-08	0	3.06E-08	3.30E-08	0	0
GSTA4	1.33E-05	5.11E-05	8.10E-06	8.60E-06	1.34E-05	1.54E-05	6.70E-06	2.22E-05
GSTA5	0	0	0	0	0	3.09E-08	3.13E-08	0
GSTCD	4.65E-06	1.44E-06	1.13E-06	1.76E-06	3.26E-07	2.23E-06	1.30E-06	1.47E-06
GSTK1	0.000152	0.000398	0.000307	0.000284	0.000222	0.000383	0.000307	0.000413
GSTM1	3.92E-07	0.000112	7.05E-08	8.06E-08	7.12E-05	0.000605	9.18E-07	1.29E-07
GSTM2	4.46E-06	5.47E-05	1.36E-06	4.60E-06	0.000178	0.000376	2.18E-06	1.14E-06
GSTM2P1	6.87E-08	7.92E-08	0	5.65E-08	7.92E-08	3.84E-07	6.57E-08	0
GSTM3	2.00E-05	1.92E-05	3.40E-06	6.92E-06	1.10E-05	9.38E-05	4.42E-06	9.37E-07
GSTM4	6.59E-05	5.61E-05	1.67E-05	1.93E-05	2.05E-05	0.000131	2.18E-05	1.35E-05
GSTM5	2.20E-07	2.87E-07	4.89E-07	2.16E-06	4.98E-07	8.77E-08	1.24E-06	3.48E-07
GSTO1	0.000531	0.000317	0.000395	0.000516	0.000915	0.000507	0.000506	0.000303
GSTO2	1.56E-06	9.26E-06	2.80E-06	6.55E-06	2.49E-05	1.27E-06	3.27E-07	6.28E-06
GSTP1	5.82E-05	1.61E-05	3.06E-05	0.000118	3.37E-05	1.66E-05	7.45E-05	2.67E-05
GSTT1	0.000114	5.75E-08	0	0.000113	0.000176	0.00018	9.19E-05	0.000112
GSTT2	1.29E-05	0.00021	9.84E-06	2.21E-05	2.04E-06	4.66E-07	9.15E-07	1.60E-06
GSTTP1	0	0	0	0	0	0	0	0
GSTTP2	0	0	0	0	0	0	0	0

GSTZ1	1.55E-05	2.54E-05	0.000115	5.03E-05	7.10E-05	0.000109	3.64E-05	2.33E-05
GSX1	0	0	0	0	0	0	0	0
GSX2	0	0	0	0	0	0	0	0
GTDC1	7.87E-06	2.34E-06	1.73E-06	4.53E-06	1.23E-06	5.08E-06	4.27E-06	4.47E-06
GTF2A1	5.91E-06	3.30E-06	3.82E-06	2.68E-06	3.81E-08	5.57E-06	8.66E-07	3.86E-06
GTF2A1L	#######	0	0	0	0	2.93E-08	1.58E-07	7.23E-08
GTF2A2	2.41E-05	2.67E-05	1.35E-05	1.90E-05	1.89E-05	1.96E-05	1.80E-05	1.43E-05
GTF2B	2.85E-05	2.29E-05	1.59E-05	2.23E-05	5.39E-06	3.09E-05	1.67E-05	1.83E-05
GTF2E1	5.68E-06	7.84E-06	1.84E-06	2.54E-06	7.60E-07	7.38E-06	2.38E-06	3.07E-06
GTF2E2	1.38E-05	2.27E-05	1.14E-05	1.72E-05	3.90E-06	1.69E-05	1.16E-05	2.07E-05
GTF2F1	3.48E-05	1.80E-05	1.70E-05	2.61E-05	1.63E-05	2.75E-05	2.49E-05	2.45E-05
GTF2F2	1.65E-05	1.37E-05	7.79E-06	1.01E-05	5.23E-06	1.19E-05	1.17E-05	1.13E-05
GTF2H1	1.22E-05	1.20E-05	5.98E-06	7.54E-06	4.24E-06	1.17E-05	7.03E-06	8.42E-06
GTF2H2	8.90E-06	4.98E-06	4.09E-06	5.72E-06	1.17E-07	3.30E-06	1.21E-06	3.72E-07
GTF2H2B	7.44E-07	1.44E-06	2.50E-06	6.13E-06	0	1.28E-06	2.11E-06	4.21E-07
GTF2H2C	3.33E-05	1.02E-05	2.56E-06	5.68E-06	1.16E-06	7.80E-06	5.14E-06	1.16E-05
GTF2H3	1.25E-05	9.20E-06	4.41E-06	3.98E-06	3.93E-07	1.05E-05	3.34E-06	4.00E-06
GTF2H4	1.51E-05	1.58E-05	9.73E-06	1.02E-05	9.85E-06	1.75E-05	1.42E-05	1.78E-05
GTF2H5	6.46E-06	5.43E-06	2.56E-06	3.62E-06	3.35E-06	6.50E-06	4.67E-06	5.46E-06
GTF2I	2.13E-05	3.95E-05	1.67E-05	1.16E-05	9.60E-07	3.31E-05	7.20E-06	2.04E-05
GTF2IP1	9.68E-05	0.000123	2.27E-05	4.26E-05	2.81E-05	5.19E-05	2.45E-05	9.23E-05
GTF2IRD1	8.04E-06	8.21E-06	1.31E-06	1.81E-06	5.37E-07	2.75E-06	2.02E-06	9.75E-07
GTF2IRD2	1.35E-05	1.24E-05	8.69E-06	1.11E-05	1.33E-05	1.10E-05	8.83E-06	1.22E-05
GTF2IRD2	2.31E-06	3.65E-06	1.94E-06	2.05E-06	6.68E-07	1.96E-06	2.57E-06	1.97E-06
GTF2IRD2	2.76E-06	5.21E-06	2.51E-06	4.90E-06	1.26E-06	5.76E-06	3.34E-06	4.97E-06
GTF3A	0.000137	0.00018	7.51E-05	7.14E-05	9.30E-05	8.13E-05	0.000121	7.46E-05
GTF3C1	1.55E-05	1.92E-05	7.47E-06	7.86E-06	4.04E-06	1.09E-05	7.54E-06	8.44E-06
GTF3C2	2.96E-05	2.73E-05	7.53E-06	8.47E-06	5.45E-06	1.17E-05	1.10E-05	8.85E-06
GTF3C3	1.63E-05	1.21E-05	4.05E-06	4.55E-06	1.34E-06	8.29E-06	3.39E-06	4.43E-06
GTF3C4	3.14E-06	1.44E-06	1.91E-06	2.14E-06	1.52E-07	3.10E-06	1.07E-06	3.07E-06
GTF3C5	2.51E-05	1.19E-05	1.28E-05	1.61E-05	1.68E-05	3.54E-05	1.79E-05	2.27E-05
GTF3C6	9.53E-05	6.31E-05	2.13E-05	3.77E-05	3.53E-05	4.60E-05	4.93E-05	4.85E-05
GTPBP1	1.21E-05	6.10E-06	5.56E-06	9.26E-06	3.06E-06	6.40E-06	7.11E-06	1.17E-05
GTPBP10	1.81E-05	1.55E-05	6.11E-06	5.77E-06	2.19E-06	1.11E-05	7.00E-06	1.02E-05
GTPBP2	1.40E-05	1.67E-05	5.06E-06	8.83E-06	3.14E-06	9.54E-06	9.73E-06	1.70E-05
GTPBP3	7.30E-06	4.76E-06	3.70E-06	4.66E-06	3.21E-06	6.50E-06	7.22E-06	5.37E-06
GTPBP4	5.51E-05	1.17E-05	8.43E-06	1.58E-05	4.79E-06	1.77E-05	1.58E-05	1.03E-05
GTPBP5	2.78E-05	3.42E-05	1.58E-05	1.65E-05	1.85E-05	3.52E-05	2.34E-05	2.19E-05
GTPBP8	9.48E-06	4.77E-06	3.37E-06	3.78E-06	7.05E-07	4.06E-06	3.48E-06	4.42E-06
GTSE1	6.26E-06	2.64E-06	7.04E-08	8.34E-08	1.35E-06	8.11E-07	2.73E-07	5.88E-07
GTSF1	1.60E-06	1.68E-06	0	8.65E-08	0	0	2.30E-07	1.57E-07
GTSF1L	1.40E-07	0	0	0	0	3.52E-08	7.08E-08	0
GUCA1A	2.82E-08	0	0	0	0	0	0	0
GUCA1B	1.25E-07	5.89E-08	5.22E-07	2.04E-07	1.36E-06	2.71E-07	7.07E-07	4.14E-07
GUCA1C	0	0	0	0	0	0	0	0
GUCA2A	0	1.03E-05	6.75E-08	2.68E-06	3.44E-07	0	7.27E-07	0
GUCA2B	3.18E-07	3.23E-05	4.43E-06	4.39E-06	9.28E-08	1.00E-07	1.48E-05	0
GUCY1A2	3.53E-07	2.03E-07	1.14E-07	8.64E-08	7.25E-08	2.33E-07	2.21E-07	1.96E-07
GUCY1A3	8.92E-06	1.80E-06	2.40E-06	5.31E-06	5.55E-07	8.69E-07	7.83E-07	2.13E-06
GUCY1B2	0	1.05E-07	0	8.43E-08	6.58E-09	7.10E-09	7.44E-09	0
GUCY1B3	8.09E-06	4.10E-06	2.18E-06	3.28E-06	7.62E-07	1.17E-06	2.03E-06	2.26E-06
GUCY2C	6.64E-05	8.32E-06	1.84E-08	7.26E-09	0	2.20E-08	4.42E-08	8.70E-09
GUCY2D	1.26E-08	0	1.30E-08	0	0	1.17E-08	1.84E-08	6.48E-08
GUCY2E	0	7.48E-08	0	0	0	1.39E-07	0	0
GUCY2F	0	0	0	0	0	0	0	1.81E-08
GUCY2GP	0	0	0	0	0	0	0	0
GUF1	1.65E-05	1.55E-05	7.89E-06	6.86E-06	8.49E-06	1.38E-05	1.80E-05	7.47E-06
GUK1	0.000251	0.0004	0.000145	0.000184	0.000278	0.000255	0.000195	0.000322

GULP1	1.34E-05	2.95E-07	2.25E-07	8.79E-07	6.68E-09	3.30E-08	4.53E-08	7.89E-08
GUSB	8.90E-05	0.000115	7.68E-05	0.000112	6.94E-05	4.50E-05	0.000101	0.000107
GUSBL1	1.63E-06	1.82E-06	3.93E-07	9.78E-07	5.56E-08	8.19E-07	2.26E-07	7.63E-07
GUSBL2	2.43E-06	3.84E-06	1.34E-06	1.74E-06	2.83E-07	1.11E-06	1.28E-06	1.61E-06
GUSBP1	2.38E-06	1.99E-06	8.19E-07	1.26E-06	3.73E-07	1.19E-06	8.22E-07	1.91E-06
GUSBP3	2.04E-06	8.29E-07	5.58E-07	2.96E-06	2.78E-07	5.12E-07	1.19E-06	2.21E-06
GVIN1	8.68E-07	2.12E-07	5.73E-07	2.81E-07	1.74E-08	2.88E-07	8.92E-08	1.63E-07
GXYLT1	6.06E-06	3.57E-06	4.37E-06	2.98E-06	9.92E-07	3.69E-06	1.99E-06	3.07E-06
GXYLT2	3.37E-07	1.83E-08	5.10E-08	1.18E-07	2.76E-08	8.04E-07	1.39E-07	2.37E-08
GYG1	1.93E-05	1.25E-05	8.11E-06	1.32E-05	4.63E-06	1.31E-05	1.12E-05	1.09E-05
GYG2	1.97E-05	5.02E-06	1.69E-05	7.08E-06	7.07E-06	1.18E-05	6.23E-06	6.91E-06
GYLTL1B	1.31E-05	3.62E-06	1.66E-06	1.11E-06	4.07E-06	2.61E-06	1.09E-06	5.37E-07
GYPA	8.58E-08	1.18E-07	0	2.33E-08	0	0	9.24E-09	0
GYPB	0	0	0	0	0	0	0	0
GYPC	4.11E-05	6.93E-06	1.24E-05	3.58E-05	1.71E-05	1.18E-05	4.40E-05	1.15E-05
GYPE	2.17E-07	3.29E-08	4.58E-08	1.07E-07	0	2.69E-08	8.32E-08	4.27E-08
GYS1	1.80E-05	5.63E-06	4.78E-06	6.01E-06	7.54E-06	5.29E-06	5.17E-06	4.04E-06
GYS2	4.29E-06	4.84E-05	8.34E-05	5.19E-05	7.97E-06	4.02E-05	1.87E-05	2.75E-05
GZF1	6.62E-06	4.70E-06	2.80E-06	3.41E-06	1.44E-06	4.07E-06	2.95E-06	3.48E-06
GZMA	3.79E-05	1.72E-06	6.09E-06	1.47E-05	7.45E-07	6.42E-06	9.47E-06	3.03E-06
GZMB	1.60E-05	8.91E-07	1.79E-06	6.77E-06	6.11E-07	4.06E-06	3.27E-06	9.66E-07
GZMH	1.21E-05	1.49E-06	4.01E-06	1.28E-05	5.00E-07	3.85E-06	6.50E-06	1.77E-06
GZMK	2.06E-05	2.93E-07	6.79E-06	1.71E-05	2.41E-07	3.52E-06	4.18E-06	1.85E-06
GZMM	1.60E-06	4.66E-07	2.87E-06	1.03E-05	6.69E-07	9.61E-07	2.61E-06	8.53E-07
H19	4.38E-05	0.000241	0.000569	0.003199	0.008872	4.39E-05	0.000808	3.28E-05
H1F0	0.000327	0.00021	0.000136	0.000109	9.54E-05	0.000154	0.000172	0.000144
H1FNT	0	0	0	0	0	0	0	2.89E-08
H1FOO	0	0	0	0	0	0	0	0
H1FX	6.91E-05	5.91E-05	4.91E-05	6.14E-05	3.69E-05	5.50E-05	0.000113	1.96E-05
H2AFB1	1.98E-07	0	1.65E-07	8.39E-08	1.17E-07	3.24E-07	0	5.24E-07
H2AFJ	4.91E-05	1.65E-05	1.19E-05	1.29E-05	5.17E-05	2.57E-05	2.33E-05	1.94E-05
H2AFV	0.000102	6.23E-05	3.69E-05	3.94E-05	4.07E-05	5.11E-05	4.97E-05	6.12E-05
H2AFX	2.87E-05	1.46E-05	3.60E-06	7.75E-06	1.62E-05	9.17E-06	1.08E-05	1.14E-05
H2AFY	0.00012	6.94E-05	3.18E-05	5.66E-05	3.30E-05	4.60E-05	4.52E-05	5.16E-05
H2AFY2	2.49E-05	1.72E-07	3.53E-06	3.42E-06	1.38E-05	4.75E-07	6.96E-07	1.40E-06
H2AFZ	0.000287	0.000112	4.34E-05	6.89E-05	8.69E-05	0.0001	7.24E-05	0.000117
H2BFM	0	0	0	0	0	0	0	0
H2BFWT	0	0	0	0	0	0	0	0
H2BFXP	3.38E-08	0	0	0	0	9.50E-08	0	0
H3F3A	0.000198	0.0001	8.29E-05	0.000134	0.000108	0.000179	0.000181	0.000159
H3F3B	0.000271	0.000254	0.000107	0.000183	9.87E-05	0.000138	0.000206	9.98E-05
H3F3C	2.74E-05	3.26E-05	1.92E-05	2.11E-05	1.03E-05	1.73E-05	9.82E-06	1.22E-05
H6PD	2.11E-05	2.73E-05	1.63E-05	2.38E-05	1.33E-05	4.04E-05	6.35E-05	4.05E-05
HAAO	5.85E-05	0.000396	0.000267	0.000145	0.000314	8.53E-05	0.000148	8.59E-05
HABP2	0.000256	0.000124	0.000303	0.000133	0.000133	2.68E-05	0.000142	0.000225
HABP4	1.07E-05	5.57E-06	3.51E-06	7.31E-06	4.24E-06	1.84E-05	5.85E-06	5.88E-06
HACE1	1.71E-06	6.34E-07	8.44E-07	9.19E-07	9.25E-08	1.12E-06	7.46E-07	1.09E-06
HACL1	1.45E-05	2.78E-05	2.49E-05	1.79E-05	3.63E-05	2.85E-05	1.99E-05	1.30E-05
HADH	6.34E-05	8.67E-05	0.000148	5.78E-05	4.65E-05	0.000147	7.87E-05	0.000134
HADHA	0.000172	0.000299	0.000113	0.000123	9.93E-05	0.000142	0.000146	9.78E-05
HADHB	0.000155	0.000258	0.000177	0.000152	7.78E-05	0.000185	0.00014	9.11E-05
HAGH	7.78E-05	0.000253	0.000176	0.000137	0.000147	0.000174	0.000208	0.000105
HAGHL	6.46E-06	8.51E-07	1.44E-07	4.27E-07	1.13E-07	1.31E-07	4.95E-07	8.28E-08
HAL	2.80E-05	6.34E-05	3.92E-05	5.27E-05	1.31E-05	4.91E-08	1.22E-05	0.000136
HAMP	3.50E-05	3.94E-06	0.000827	0.000669	2.69E-06	1.58E-06	0.000374	0.000139
HAND1	0	0	0	0	0	0	0	0
HAND2	2.00E-06	5.90E-08	4.93E-06	6.33E-06	5.60E-07	1.64E-08	1.18E-06	1.02E-06
HAO1	0.000239	0.000301	0.000343	0.00022	0.000187	0.000296	0.000128	0.000139

HAO2	1.33E-06	1.00E-05	0.000219	5.78E-05	9.20E-05	4.05E-06	7.80E-05	1.02E-06
HAP1	1.66E-07	3.97E-08	3.65E-08	5.02E-08	0	1.64E-08	2.30E-08	1.13E-07
HAPLN1	4.51E-07	5.72E-09	0	0	0	8.04E-08	1.49E-08	0
HAPLN2	1.37E-08	6.22E-08	0	0	7.08E-08	5.15E-08	2.65E-07	4.04E-08
HAPLN3	7.68E-06	2.63E-07	2.11E-07	1.85E-06	3.51E-07	1.49E-06	1.09E-06	1.03E-06
HAPLN4	8.22E-07	1.38E-06	1.19E-06	4.07E-07	1.07E-06	1.02E-06	1.70E-06	5.60E-07
HAR1A	3.83E-07	0	1.81E-07	9.17E-08	1.86E-07	1.54E-08	4.85E-08	2.44E-08
HAR1B	3.06E-07	0	1.70E-07	1.52E-07	7.97E-08	0	0	0
HARBI1	2.44E-06	3.53E-06	1.43E-06	1.76E-06	7.16E-07	4.13E-06	2.27E-06	3.41E-06
HARS	3.73E-05	2.38E-05	1.98E-05	2.55E-05	1.18E-05	3.92E-05	2.60E-05	3.93E-05
HARS2	2.13E-05	2.26E-05	7.97E-06	9.94E-06	5.89E-06	2.14E-05	1.44E-05	2.25E-05
HAS1	1.12E-07	0	0	5.61E-06	0	5.89E-08	7.85E-08	2.45E-07
HAS2	1.95E-07	1.03E-07	5.08E-08	8.72E-06	1.21E-08	6.51E-09	2.12E-07	2.35E-07
HAS2AS	4.90E-08	0	0	2.80E-07	0	0	0	2.58E-08
HAS3	7.34E-07	5.70E-07	2.33E-07	1.01E-06	4.62E-08	2.89E-07	2.41E-07	2.40E-07
HAT1	5.72E-05	2.60E-05	1.25E-05	1.48E-05	8.32E-06	1.79E-05	1.28E-05	1.72E-05
HAUS1	1.69E-05	4.12E-06	5.17E-06	7.60E-06	2.58E-06	9.90E-06	6.82E-06	5.26E-06
HAUS2	7.11E-06	4.55E-06	2.87E-06	3.82E-06	1.53E-06	3.87E-06	4.04E-06	3.29E-06
HAUS3	3.88E-06	2.61E-06	1.51E-06	1.63E-06	5.09E-07	1.79E-06	1.43E-06	2.64E-06
HAUS4	4.06E-05	4.52E-05	1.37E-05	3.13E-05	1.96E-05	6.27E-05	2.68E-05	1.59E-05
HAUS5	6.15E-06	2.58E-06	1.40E-06	2.01E-06	1.28E-06	2.85E-06	1.92E-06	2.24E-06
HAUS6	2.19E-06	2.85E-06	1.18E-06	1.59E-06	4.92E-07	1.99E-06	1.25E-06	1.00E-06
HAUS7	7.63E-06	6.29E-06	3.98E-06	6.55E-06	1.23E-05	4.79E-06	9.07E-06	9.55E-06
HAUS8	6.65E-06	1.62E-06	3.14E-06	4.07E-06	4.07E-06	3.51E-06	5.82E-06	1.93E-06
HAVCR1	3.02E-05	1.06E-07	0	2.45E-07	0	0	0	1.25E-07
HAVCR2	1.21E-05	8.50E-07	3.18E-06	1.08E-05	4.55E-07	3.95E-06	4.54E-06	1.06E-06
HAX1	7.84E-05	0.000182	7.44E-05	6.32E-05	4.68E-05	0.000143	9.45E-05	0.000125
HBA1	3.63E-06	1.01E-05	1.78E-05	0.000121	1.21E-05	1.63E-05	7.90E-05	2.44E-06
HBA2	1.49E-05	5.63E-05	8.91E-05	0.000405	2.73E-05	3.32E-05	0.000189	2.68E-05
HBB	1.08E-05	8.57E-05	7.87E-05	0.000504	1.15E-05	3.29E-05	0.000103	1.89E-05
HBBP1	0	0	0	0	0	0	0	0
HBD	0	4.37E-08	4.19E-08	3.22E-07	0	0	1.75E-07	1.11E-07
HBE1	0	0	3.88E-08	0	0	0	3.27E-08	0
HBEGF	4.43E-06	4.57E-06	5.54E-06	1.86E-05	1.90E-06	6.09E-06	4.38E-06	4.08E-06
HBG1	0	0	6.57E-08	1.38E-07	9.59E-08	0	3.56E-07	0
HBG2	0	0	0	0	1.44E-07	1.48E-08	0	0
HBII-52-2	0	0	0	0	0	0	0	0
HBII-52-2	0	0	0	0	0	0	0	0
HBII-52-2	0	0	0	0	0	0	0	0
HBII-52-4	0	0	0	0	0	0	0	0
HBII-52-4	0	0	0	0	0	0	0	0
HBM	0	0	0	8.52E-08	2.37E-07	0	6.17E-08	0
HBP1	1.99E-05	2.67E-05	3.22E-05	2.53E-05	7.69E-06	5.20E-05	2.18E-05	4.14E-05
HBQ1	0	0	0	0	0	0	4.39E-08	0
HBS1L	7.55E-06	6.14E-06	6.69E-06	5.51E-06	2.19E-06	9.57E-06	4.95E-06	6.00E-06
HBXIP	0.000168	0.000109	5.24E-05	7.55E-05	4.86E-05	0.000162	7.69E-05	7.01E-05
HBZ	0	0	0	0	1.42E-07	0	5.02E-08	0
HCCS	3.26E-05	8.30E-06	8.94E-06	1.14E-05	3.78E-06	1.09E-05	9.93E-06	2.08E-05
HCFC1	1.30E-05	9.24E-06	4.57E-06	4.92E-06	2.94E-06	6.95E-06	5.27E-06	6.51E-06
HCFC1R1	5.30E-05	5.32E-05	2.14E-05	2.84E-05	4.14E-05	3.75E-05	3.36E-05	3.18E-05
HCFC2	2.76E-06	4.24E-06	3.13E-06	2.11E-06	6.37E-07	4.03E-06	1.83E-06	3.95E-06
HCG11	5.78E-07	1.90E-07	3.80E-07	2.75E-07	9.16E-08	1.61E-07	4.08E-07	1.62E-07
HCG18	3.83E-06	2.00E-06	7.19E-07	1.54E-06	2.99E-07	1.52E-06	1.23E-06	1.04E-06
HCG22	0	1.37E-08	3.81E-08	0	0	1.12E-08	2.34E-08	0
HCG26	4.57E-06	7.05E-07	7.09E-07	1.32E-06	6.05E-07	1.98E-06	1.82E-06	4.74E-06
HCG27	1.45E-06	4.83E-07	2.54E-07	2.09E-07	9.77E-08	3.29E-07	8.20E-08	4.81E-07
HCG2P7	2.50E-08	0	0	6.95E-08	0	2.89E-08	2.39E-08	0
HCG4	4.81E-06	1.32E-07	9.19E-07	3.30E-07	1.01E-08	1.32E-06	1.02E-07	1.15E-06

HCG4P6	1.97E-07	1.63E-07	7.63E-08	9.22E-08	4.30E-08	1.67E-07	2.24E-08	8.43E-08
HCG9	4.77E-08	0	0	0	0	4.49E-08	0	0
HCK	1.68E-05	1.80E-06	7.54E-06	1.19E-05	7.49E-07	6.49E-06	8.01E-06	1.72E-06
HCLS1	3.84E-05	4.11E-06	1.91E-05	3.86E-05	5.03E-06	1.58E-05	3.12E-05	1.09E-05
HCN1	0	0	3.86E-08	1.02E-08	0	3.88E-09	0	0
HCN2	7.15E-07	0	3.48E-08	3.30E-08	4.23E-07	1.25E-08	6.35E-07	0
HCN3	2.34E-06	3.95E-06	1.37E-06	6.39E-07	1.06E-06	1.14E-06	9.25E-07	1.64E-06
HCN4	9.87E-08	3.44E-09	3.15E-09	1.32E-07	2.64E-09	5.70E-09	0	0
HCP5	6.95E-05	5.36E-06	4.71E-06	8.13E-06	1.08E-06	1.77E-05	7.03E-06	2.96E-05
HCRT	5.57E-08	0	0	0	4.88E-08	0	0	0
HCRTR1	0	3.54E-08	0	0	0	0	0	0
HCRTR2	5.34E-08	0	0	1.64E-08	0	0	0	0
HCST	3.17E-05	4.66E-06	1.20E-05	3.40E-05	1.18E-05	2.22E-05	3.13E-05	8.83E-06
HDAC1	0.000114	4.66E-05	2.92E-05	3.97E-05	1.22E-05	3.64E-05	3.66E-05	4.72E-05
HDAC10	1.55E-05	2.17E-05	9.11E-06	9.69E-06	9.28E-06	1.20E-05	1.30E-05	1.29E-05
HDAC11	1.85E-05	8.98E-06	1.90E-06	5.30E-06	2.07E-06	4.83E-06	9.95E-06	8.71E-06
HDAC2	9.08E-06	6.05E-06	3.96E-06	7.53E-06	1.48E-06	7.63E-06	6.57E-06	5.47E-06
HDAC3	3.01E-05	2.09E-05	1.88E-05	2.01E-05	1.35E-05	2.32E-05	2.23E-05	1.87E-05
HDAC4	4.48E-06	6.77E-07	3.41E-07	8.23E-07	8.04E-07	1.47E-06	1.09E-06	8.94E-07
HDAC5	2.95E-05	1.21E-05	5.87E-06	1.04E-05	1.04E-05	7.77E-06	1.91E-05	8.63E-06
HDAC6	5.58E-05	5.13E-05	4.17E-05	5.80E-05	2.03E-05	3.80E-05	5.70E-05	2.36E-05
HDAC7	2.24E-05	4.83E-06	4.69E-06	1.24E-05	8.39E-06	4.67E-06	1.13E-05	7.99E-06
HDAC8	1.48E-05	9.02E-06	6.03E-06	7.05E-06	9.17E-06	1.04E-05	6.81E-06	1.50E-05
HDAC9	8.01E-07	1.59E-07	4.93E-07	1.57E-06	7.69E-08	1.82E-07	2.53E-07	3.09E-07
HDC	9.36E-08	1.18E-06	3.07E-07	2.90E-07	1.01E-07	1.24E-06	2.41E-07	4.55E-07
HDDC2	1.30E-05	1.24E-05	5.32E-06	1.28E-05	6.11E-06	1.22E-05	1.11E-05	9.55E-06
HDDC3	2.83E-05	3.43E-05	1.96E-05	2.06E-05	2.83E-05	2.43E-05	3.86E-05	2.07E-05
HDGF	0.000215	0.000207	0.000128	0.000151	0.000111	0.000321	0.000202	0.000199
HDGFL1	0	0	0	0	0	0	0	6.57E-07
HDGFRP2	2.61E-05	1.86E-05	1.69E-05	2.29E-05	5.53E-05	2.57E-05	3.66E-05	2.22E-05
HDGFRP3	1.63E-05	1.24E-06	7.26E-07	2.27E-06	6.49E-07	7.78E-07	1.97E-06	1.68E-06
HDHD1A	2.44E-05	5.70E-06	8.12E-06	5.24E-06	4.17E-06	5.29E-06	5.25E-06	1.26E-05
HDHD2	2.02E-05	8.69E-06	1.92E-05	1.33E-05	5.30E-06	2.13E-05	1.42E-05	1.55E-05
HDHD3	5.46E-05	3.22E-05	6.42E-05	4.98E-05	7.21E-05	6.82E-05	6.75E-05	3.47E-05
HDLBP	0.000161	8.27E-05	9.02E-05	7.99E-05	5.95E-05	0.00013	8.93E-05	8.06E-05
HDX	1.07E-06	1.16E-06	5.19E-07	6.83E-07	7.05E-09	8.10E-07	1.02E-07	7.59E-07
HEATR1	6.84E-06	6.49E-06	3.34E-06	4.12E-06	5.78E-07	9.39E-06	3.76E-06	5.99E-06
HEATR2	2.34E-05	1.40E-05	6.13E-06	7.96E-06	2.26E-06	1.05E-05	7.35E-06	1.14E-05
HEATR3	5.86E-06	9.05E-06	4.67E-06	4.78E-06	6.65E-07	7.11E-06	2.94E-06	6.60E-06
HEATR4	1.61E-07	4.55E-08	1.40E-07	3.96E-08	4.39E-08	3.75E-08	1.31E-08	4.75E-08
HEATR5A	6.77E-06	5.47E-06	3.29E-06	2.95E-06	5.28E-07	4.37E-06	1.29E-06	3.91E-06
HEATR5B	1.49E-05	1.17E-05	6.72E-06	6.64E-06	2.09E-06	9.74E-06	4.09E-06	5.81E-06
HEATR6	8.63E-06	3.65E-06	1.52E-06	2.18E-06	8.65E-07	5.06E-06	2.02E-06	1.78E-06
HEATR7A	1.98E-05	5.64E-06	3.78E-06	4.62E-06	5.08E-06	4.47E-06	4.92E-06	4.27E-06
HEATR7B2	0	0	3.77E-08	3.73E-08	0	1.69E-07	1.54E-07	0
HEBP1	0.000162	4.88E-05	9.92E-05	7.80E-05	6.66E-05	6.49E-05	0.000104	3.98E-05
HEBP2	4.31E-05	3.53E-05	4.86E-05	5.49E-05	3.24E-05	4.48E-05	7.25E-05	6.16E-05
HECA	7.31E-06	5.68E-06	5.12E-06	5.97E-06	9.63E-07	4.34E-06	3.94E-06	4.13E-06
HECTD1	3.63E-05	2.83E-05	2.54E-05	2.17E-05	8.17E-06	3.49E-05	1.98E-05	2.77E-05
HECTD2	4.31E-06	1.43E-06	5.94E-07	1.21E-06	1.74E-07	9.87E-07	5.63E-07	8.78E-07
HECTD3	2.67E-05	1.62E-05	1.49E-05	1.59E-05	9.57E-06	2.19E-05	2.03E-05	1.74E-05
HECW1	5.67E-09	0	1.05E-08	5.91E-08	7.37E-08	9.54E-09	2.01E-08	0
HECW2	2.07E-06	1.33E-06	1.02E-06	5.33E-07	5.35E-07	8.14E-07	3.02E-07	1.50E-06
HEG1	1.39E-05	2.90E-06	2.54E-06	3.29E-06	2.16E-06	3.51E-06	3.91E-06	6.82E-06
HELB	4.98E-07	2.45E-07	3.09E-07	2.24E-07	1.23E-08	4.80E-07	1.11E-07	2.66E-07
HELLS	5.57E-06	1.35E-06	1.23E-07	1.66E-07	3.20E-07	3.14E-07	3.01E-07	4.56E-07
HELQ	9.04E-06	3.34E-06	4.10E-06	4.20E-06	1.54E-06	6.00E-06	4.49E-06	5.23E-06
HELT	0	4.06E-08	0	0	0	0	0	0

HELZ	1.44E-05	3.79E-06	3.22E-06	2.64E-06	1.14E-06	3.64E-06	1.81E-06	1.70E-06
HEMGN	1.10E-08	2.45E-08	0	1.35E-08	0	0	0	0
HEMK1	5.73E-06	4.53E-06	2.89E-06	3.02E-06	3.62E-06	3.61E-06	3.87E-06	2.28E-06
HEPACAM	9.70E-09	0	1.23E-05	1.75E-06	1.13E-06	3.98E-05	4.93E-06	0
HEPACAM	7.75E-07	5.21E-08	0	0	9.92E-09	3.06E-08	0	0
HEPH	2.22E-06	3.49E-07	7.08E-07	2.68E-06	7.78E-07	6.89E-07	1.70E-06	1.56E-06
HEPHL1	0	4.20E-09	3.85E-09	4.59E-09	0	7.04E-09	0	5.51E-09
HEPN1	0	2.00E-08	2.63E-05	3.14E-06	0	8.37E-05	4.59E-06	5.17E-08
HERC1	9.26E-06	4.37E-06	4.46E-06	4.90E-06	4.77E-06	4.40E-06	5.82E-06	5.12E-06
HERC2	1.63E-05	1.32E-05	8.45E-06	7.58E-06	8.52E-06	9.32E-06	6.73E-06	8.52E-06
HERC2P2	1.52E-05	5.05E-06	2.90E-06	4.14E-06	3.34E-06	3.01E-06	2.93E-06	5.62E-06
HERC2P4	6.47E-07	0	4.82E-08	5.50E-08	1.93E-08	2.08E-08	0	0
HERC3	6.32E-06	4.00E-06	5.93E-06	4.55E-06	5.10E-07	4.60E-06	2.87E-06	5.04E-06
HERC4	1.11E-05	1.07E-05	7.41E-06	7.02E-06	3.58E-06	8.92E-06	5.83E-06	1.01E-05
HERC5	2.42E-06	1.37E-06	6.95E-06	4.13E-06	6.14E-07	3.42E-06	1.76E-06	6.95E-06
HERC6	2.96E-06	1.00E-06	1.92E-06	2.99E-06	5.39E-07	3.80E-06	1.60E-06	5.75E-06
HERPUD1	7.81E-05	4.87E-05	0.000124	6.48E-05	3.07E-05	0.00013	7.12E-05	7.26E-05
HERPUD2	2.11E-05	1.81E-05	9.59E-06	1.12E-05	4.35E-06	1.02E-05	1.36E-05	1.35E-05
HES1	2.92E-05	4.56E-05	5.29E-05	8.40E-05	3.82E-05	1.96E-05	8.25E-05	5.61E-05
HES2	1.97E-07	1.79E-08	4.38E-08	1.57E-07	2.31E-08	4.93E-09	6.74E-08	3.99E-08
HES3	0	0	0	0	0	0	0	0
HES4	9.85E-06	1.83E-06	2.78E-07	4.67E-06	3.97E-07	2.61E-07	6.12E-06	9.85E-07
HES5	9.71E-08	2.89E-07	4.16E-08	2.39E-08	6.71E-08	1.81E-08	2.05E-07	4.31E-07
HES6	1.06E-05	4.96E-06	2.82E-06	2.27E-06	1.75E-06	2.72E-06	4.07E-06	8.85E-06
HES7	8.76E-08	0	0	0	0	0	0	2.98E-08
HESRG	0	0	0	0	0	0	0	0
HESX1	9.06E-07	6.10E-07	4.06E-07	2.72E-07	2.67E-07	1.26E-06	2.96E-07	1.64E-07
HEXA	6.24E-05	6.34E-05	3.21E-05	5.54E-05	4.04E-05	0.000115	6.45E-05	6.90E-05
HEXB	0.000133	0.000167	7.56E-05	9.68E-05	7.27E-05	0.000114	8.51E-05	0.000256
HEXDC	1.51E-05	1.50E-05	6.97E-06	1.16E-05	1.87E-05	1.69E-05	9.58E-06	6.91E-06
HEXIM1	2.13E-05	6.64E-06	6.57E-06	7.82E-06	7.61E-06	5.42E-06	8.24E-06	4.95E-06
HEXIM2	8.96E-06	4.60E-06	7.17E-06	4.65E-06	1.80E-05	1.03E-05	6.48E-06	3.39E-06
HEY1	7.71E-06	4.13E-06	6.71E-07	6.44E-07	1.96E-06	1.63E-06	1.48E-06	3.59E-06
HEY2	1.65E-06	1.54E-06	4.04E-06	3.58E-06	1.59E-06	1.15E-06	2.70E-06	3.46E-06
HEYL	1.26E-05	1.75E-06	1.47E-06	3.52E-06	2.90E-06	1.30E-06	3.76E-06	8.51E-06
HFE	6.08E-06	1.18E-05	8.42E-06	4.41E-06	7.12E-07	9.07E-06	2.81E-06	2.09E-05
HFE2	1.19E-07	0.000105	0.000112	4.45E-05	7.53E-05	0.000307	0.000106	0.000198
HFM1	4.68E-08	0	3.06E-08	1.07E-08	7.49E-09	1.61E-08	0	1.81E-08
HGC6.3	2.62E-08	0	0	0	0	0	0	0
HGD	6.32E-05	0.000979	0.000209	0.000187	0.000206	0.000766	0.000187	9.40E-05
HGF	8.83E-07	1.95E-07	5.78E-06	9.06E-06	1.89E-07	1.54E-07	1.19E-06	2.48E-07
HGFAC	5.16E-06	0.000202	0.000143	0.000124	3.35E-05	4.41E-06	0.000126	3.97E-07
HGS	4.15E-05	6.42E-05	1.68E-05	2.31E-05	1.87E-05	3.79E-05	2.28E-05	2.29E-05
HGSNAT	1.57E-05	3.34E-05	1.10E-05	1.52E-05	4.60E-06	1.56E-05	6.39E-06	1.44E-05
HHAT	1.88E-06	8.83E-07	1.40E-06	2.10E-06	6.37E-07	8.29E-06	1.49E-06	4.10E-06
HHATL	0	3.32E-07	0	1.59E-08	0	1.29E-08	0	0
HHEX	8.44E-05	3.33E-05	2.98E-05	1.67E-05	2.89E-05	2.65E-05	6.41E-05	3.86E-05
HHIP	1.18E-07	0	2.09E-06	3.99E-06	1.10E-07	0	1.38E-07	0
HHIPL1	1.41E-06	2.98E-07	4.31E-07	7.14E-07	1.20E-07	1.23E-07	2.51E-07	1.17E-07
HHIPL2	9.10E-09	0	0	1.11E-08	0	1.49E-05	1.76E-08	0
HHLA1	0	0	0	0	4.25E-09	0	0	0
HHLA2	8.78E-08	2.40E-08	0	2.36E-08	0	0	9.45E-09	0
HHLA3	1.76E-05	3.79E-05	1.31E-05	1.36E-05	1.11E-05	4.89E-05	1.55E-05	1.39E-05
HIAT1	2.31E-05	2.36E-05	1.05E-05	1.18E-05	2.36E-06	2.32E-05	6.77E-06	1.41E-05
HIATL1	2.09E-05	7.05E-06	6.01E-06	7.66E-06	2.19E-06	9.97E-06	5.94E-06	8.47E-06
HIATL2	7.43E-07	3.69E-07	4.44E-07	1.00E-06	2.08E-07	1.21E-06	8.82E-07	3.95E-07
HIBADH	8.89E-05	0.000178	9.64E-05	7.06E-05	1.94E-05	0.000188	3.56E-05	5.26E-05
HIBCH	4.27E-05	3.77E-05	6.68E-05	5.50E-05	2.46E-05	5.72E-05	2.73E-05	4.01E-05

HIC1	2.24E-06	6.90E-07	7.65E-07	5.87E-06	7.29E-07	1.40E-06	3.50E-06	1.20E-06
HIC2	3.21E-06	5.68E-07	2.38E-07	5.45E-07	4.28E-07	5.14E-07	1.05E-06	7.59E-07
HIF1A	3.85E-05	1.40E-05	1.28E-05	2.67E-05	2.19E-06	2.07E-05	1.23E-05	2.70E-05
HIF1AN	7.90E-06	2.55E-06	1.98E-06	3.03E-06	2.33E-06	4.58E-06	4.47E-06	3.91E-06
HIF3A	1.30E-07	3.35E-07	1.79E-07	4.37E-07	1.97E-07	1.30E-07	3.61E-07	1.61E-07
HIGD1A	9.51E-05	0.000104	6.62E-05	7.90E-05	2.66E-05	5.16E-05	9.40E-05	4.77E-05
HIGD1B	3.68E-06	1.77E-06	5.96E-08	1.26E-07	3.64E-06	1.23E-06	2.51E-06	1.96E-06
HIGD1C	0	0	0	0	0	0	0	0
HIGD2A	0.000253	0.000131	0.000129	0.000152	0.000146	0.000155	0.000156	0.000148
HIGD2B	2.34E-08	0	0	2.89E-08	0	2.18E-08	1.34E-07	0
HILS1	0	0	0	0	0	2.84E-08	0	0
HINFP	6.51E-06	3.45E-06	2.76E-06	4.39E-06	1.97E-06	6.07E-06	4.39E-06	4.87E-06
HINT1	0.000667	0.000585	0.000441	0.00059	0.000475	0.000835	0.00066	0.000798
HINT2	5.40E-05	0.000183	0.000117	0.000134	0.000228	0.000169	0.000243	0.000165
HINT3	1.19E-05	1.39E-05	1.29E-05	1.19E-05	3.20E-06	2.12E-05	1.25E-05	8.67E-06
HIP1	9.87E-06	1.37E-05	3.12E-06	2.99E-06	1.62E-07	7.73E-06	2.74E-06	6.28E-06
HIP1R	4.52E-05	1.18E-05	8.94E-06	1.37E-05	5.18E-06	1.26E-05	9.90E-06	1.11E-05
HIPK1	1.22E-05	1.27E-05	6.19E-06	7.30E-06	8.10E-07	1.21E-05	3.75E-06	1.11E-05
HIPK2	1.46E-06	8.26E-06	4.58E-06	3.27E-06	1.40E-06	8.08E-06	5.02E-06	6.14E-06
HIPK3	7.48E-06	8.64E-06	6.56E-06	5.74E-06	4.15E-08	8.82E-06	7.71E-07	6.97E-06
HIPK4	3.84E-08	4.32E-08	7.98E-08	1.17E-08	4.95E-08	5.33E-08	7.43E-08	5.64E-08
HIRA	9.72E-06	6.67E-06	8.46E-06	7.67E-06	8.08E-06	1.59E-05	1.40E-05	1.39E-05
HIRIP3	1.20E-05	1.49E-05	7.27E-06	8.03E-06	4.90E-06	2.90E-05	1.60E-05	1.01E-05
HIST1H1A	1.46E-07	4.32E-08	0	0	9.55E-08	6.86E-08	0	0
HIST1H1B	1.44E-07	4.26E-08	0	0	0	1.01E-07	6.82E-08	5.42E-08
HIST1H1C	0.000388	9.15E-05	6.86E-05	9.15E-05	0.000343	0.000324	0.000507	0.000231
HIST1H1D	2.21E-07	0	0	0	0	0	3.48E-08	0
HIST1H1E	3.27E-07	2.58E-07	8.22E-08	4.53E-08	6.33E-08	1.36E-07	2.06E-07	0
HIST1H1T	8.09E-08	0	0	0	0	0	0	0
HIST1H2A	6.95E-08	0	0	0	0	0	0	0
HIST1H2A	1.50E-07	0	0	1.91E-07	0	1.43E-07	0	0
HIST1H2A	0.000145	9.22E-05	7.88E-05	7.06E-05	6.27E-05	0.000131	7.83E-05	3.44E-05
HIST1H2A	1.46E-06	0	0	0	0	1.76E-08	0	7.39E-88
HIST1H2A	2.08E-05	6.36E-06	1.61E-06	3.27E-06	8.59E-07	9.80E-06	4.27E-06	6.17E-06
HIST1H2A	1.04E-05	9.66E-07	2.66E-07	8.90E-08	0	6.52E-06	1.54E-06	3.57E-06
HIST1H2A	2.59E-07	2.24E-07	0	0	0	0	7.80E-08	0
HIST1H2A	6.02E-07	0	0	0	7.65E-08	0	0	0
HIST1H2A	7.18E-07	8.15E-07	3.13E-07	1.02E-07	7.07E-08	9.90E-07	0	1.24E-07
HIST1H2A	7.69E-07	0	0	0	6.81E-08	4.40E-07	2.10E-07	3.59E-07
HIST1H2A	2.46E-06	4.56E-07	4.62E-07	0	5.11E-07	9.64E-07	1.98E-07	1.68E-06
HIST1H2B.	8.70E-08	1.13E-07	0	0	0	0	0	1.37E-07
HIST1H2B	0	2.32E-07	0	0	0	0	0	0
HIST1H2B	1.99E-05	6.47E-06	8.91E-06	7.36E-06	4.58E-06	8.30E-06	6.78E-06	1.54E-05
HIST1H2B	0.000166	6.80E-05	2.69E-05	2.87E-05	7.32E-05	6.60E-05	4.56E-05	7.75E-06
HIST1H2B	4.04E-06	3.76E-06	2.25E-06	9.02E-07	2.50E-06	4.04E-06	1.98E-06	4.14E-06
HIST1H2B	1.07E-06	1.17E-07	0	0	0	1.72E-07	0	0
HIST1H2B	6.81E-06	3.14E-06	0	1.29E-06	0	2.01E-06	9.13E-07	9.21E-07
HIST1H2B	2.74E-07	0	1.25E-07	1.18E-07	0	0	2.47E-07	0
HIST1H2B	0	2.26E-07	0	0	0	0	0	0
HIST1H2B.	3.88E-06	7.65E-07	8.45E-07	7.83E-08	8.96E-07	2.51E-06	3.00E-06	2.26E-06
HIST1H2B	0.000178	0.000146	7.22E-05	4.76E-05	0.000152	0.000134	0.000233	0.00017
HIST1H2B	2.45E-07	7.35E-07	0	0	0	3.13E-07	7.42E-08	0
HIST1H2B	8.40E-08	0	0	0	0	0	0	0
HIST1H2B	4.06E-06	4.21E-06	7.00E-07	1.88E-07	2.39E-07	2.87E-06	9.03E-07	1.83E-06
HIST1H2B	1.01E-06	1.97E-07	2.01E-07	0	2.74E-07	3.69E-07	2.12E-07	3.62E-07
HIST1H3A	7.69E-07	3.91E-07	9.98E-08	0	6.81E-08	3.67E-07	1.40E-07	1.20E-07
HIST1H3B	2.36E-06	4.83E-07	0	9.71E-08	0	7.26E-07	4.86E-07	1.18E-07
HIST1H3C	6.40E-07	4.09E-07	0	1.02E-07	0	9.17E-07	2.18E-07	1.25E-07

HIST1H3D	2.69E-06	1.35E-06	1.43E-07	2.79E-07	5.30E-07	1.04E-06	7.31E-07	2.89E-07
HIST1H3E	7.27E-06	3.28E-06	8.75E-07	2.13E-07	6.18E-07	1.04E-06	3.77E-07	1.22E-06
HIST1H3F	7.77E-08	9.90E-08	0	0	0	0	0	0
HIST1H3G	7.77E-08	3.96E-07	1.01E-07	0	0	7.42E-08	7.08E-08	0
HIST1H3H	1.24E-05	4.06E-06	2.36E-06	9.71E-08	3.10E-06	3.48E-06	2.22E-06	2.01E-06
HIST1H3I	0	9.51E-08	0	0	6.64E-08	0	0	1.17E-07
HIST1H3J	0	0	0	0	0	0	0	3.49E-07
HIST1H4A	1.17E-07	0	0	0	0	0	2.07E-07	0
HIST1H4B	3.82E-07	0	0	3.36E-07	0	1.24E-07	1.11E-07	0
HIST1H4C	4.28E-07	0	0	0	2.89E-07	1.03E-07	3.80E-07	0
HIST1H4D	8.44E-07	1.66E-07	1.82E-07	0	0	1.17E-07	0	0
HIST1H4E	9.14E-07	3.12E-07	0	4.48E-07	0	0	5.05E-07	1.84E-07
HIST1H4F	0	0	0	0	0	0	0	0
HIST1H4G	0	0	0	0	0	0	0	0
HIST1H4H	1.50E-05	2.94E-06	3.36E-07	9.78E-07	5.33E-07	3.37E-06	3.94E-06	2.40E-06
HIST1H4I	1.89E-06	8.09E-07	0	0	0	3.44E-07	2.08E-07	1.90E-07
HIST1H4J	2.27E-05	2.44E-05	4.96E-06	5.24E-06	3.06E-05	2.73E-05	2.65E-05	1.00E-05
HIST1H4K	1.30E-07	0	0	0	0	1.27E-07	0	0
HIST1H4L	0	0	0	0	0	0	0	0
HIST2H2A	0.000178	2.95E-05	4.60E-05	5.55E-05	8.67E-05	0.00018	0.000129	0.000139
HIST2H2A	0	1.07E-07	0	0	0	7.98E-08	0	1.31E-07
HIST2H2A	3.13E-06	1.24E-06	1.05E-06	1.68E-06	6.20E-06	2.84E-06	1.34E-06	1.78E-06
HIST2H2B	1.79E-06	4.12E-07	5.79E-07	2.52E-07	2.33E-07	7.54E-07	1.22E-07	3.06E-07
HIST2H2B	2.07E-05	6.39E-06	5.59E-06	4.16E-06	4.95E-06	6.70E-06	5.83E-06	4.74E-06
HIST2H2B	2.57E-06	3.42E-07	2.76E-07	3.16E-07	4.61E-08	4.24E-07	4.63E-07	2.76E-07
HIST2H3C	7.48E-07	3.40E-07	0	8.63E-08	6.00E-08	1.29E-07	6.25E-08	1.05E-07
HIST2H3D	5.85E-07	0	0	0	0	9.39E-08	0	0
HIST2H4A	4.97E-05	1.35E-05	4.58E-06	4.91E-06	4.44E-06	2.89E-05	7.86E-06	1.39E-05
HIST3H2A	4.23E-07	3.54E-07	1.79E-07	3.58E-07	0	1.34E-07	3.87E-07	2.18E-07
HIST3H2B	8.21E-08	0	0	1.05E-07	0	0	0	0
HIST3H3	0	0	0	0	0	0	0	0
HIST4H4	1.20E-06	1.27E-07	4.14E-07	2.58E-07	8.64E-08	2.07E-07	2.42E-08	5.54E-08
HIVEP1	4.48E-06	4.43E-06	4.63E-06	4.47E-06	5.21E-07	3.86E-06	1.60E-06	3.29E-06
HIVEP2	4.39E-06	1.37E-06	1.06E-06	3.92E-06	3.20E-07	2.44E-06	1.18E-06	3.43E-06
HIVEP3	1.52E-06	1.30E-06	8.91E-07	1.31E-06	3.95E-07	7.70E-07	1.02E-06	7.77E-07
HJURP	9.06E-06	2.45E-06	3.45E-08	2.75E-07	7.08E-07	7.94E-07	3.72E-07	4.11E-07
HK1	3.79E-05	4.09E-06	3.42E-06	8.87E-06	1.73E-06	1.09E-05	6.60E-06	2.87E-06
HK2	3.87E-06	1.02E-07	4.66E-07	1.31E-06	4.32E-08	1.25E-07	2.85E-07	1.13E-06
HK3	3.02E-06	2.32E-07	3.03E-06	3.15E-06	1.52E-07	1.96E-06	7.46E-07	3.87E-07
HKDC1	5.30E-05	1.73E-05	1.59E-05	6.40E-06	1.35E-06	5.68E-06	9.59E-06	2.65E-05
HKR1	1.13E-05	6.02E-06	3.45E-06	5.97E-06	2.25E-06	3.58E-06	2.33E-06	8.53E-06
HLA-A	0.001868	0.000513	0.000405	0.001457	0.000339	0.001016	0.000861	0.002251
HLA-B	0.002315	0.000737	0.00062	0.001417	0.000111	0.001026	0.000897	0.003128
HLA-C	0.001725	0.001241	0.000354	0.001021	0.000267	0.00068	0.000489	0.001724
HLA-DMA	0.000484	2.16E-05	2.82E-05	9.46E-05	1.23E-05	4.72E-05	7.26E-05	5.51E-05
HLA-DMB	0.000195	9.69E-06	1.95E-05	6.47E-05	4.96E-06	1.82E-05	3.69E-05	1.59E-05
HLA-DOA	4.76E-05	1.36E-06	3.99E-06	1.53E-05	5.54E-07	2.38E-06	7.15E-06	1.10E-06
HLA-DOB	1.36E-05	4.91E-06	2.06E-07	1.48E-06	8.87E-08	2.28E-06	5.79E-07	1.27E-06
HLA-DPA1	0.000979	5.72E-05	0.000179	0.000434	2.73E-05	0.000112	0.000267	7.99E-05
HLA-DPB1	0.000484	3.52E-05	0.000142	0.000428	4.29E-05	9.30E-05	0.000256	5.29E-05
HLA-DPB2	1.73E-06	0	2.51E-07	3.15E-07	0	1.08E-07	1.11E-07	0
HLA-DQA	3.87E-05	6.30E-06	2.65E-05	4.57E-05	2.83E-06	1.55E-05	1.77E-05	1.57E-05
HLA-DQA	6.66E-05	4.22E-07	6.36E-06	3.56E-05	8.61E-08	6.51E-06	1.52E-05	6.32E-07
HLA-DQB	0.000194	1.18E-05	2.60E-05	9.75E-05	2.21E-05	3.76E-05	5.54E-05	4.15E-05
HLA-DQB	1.79E-05	8.03E-08	7.22E-07	4.07E-06	1.25E-07	5.28E-07	3.70E-06	2.76E-07
HLA-DRA	0.00174	9.10E-05	0.000307	0.000872	4.80E-05	0.000284	0.000447	0.000334
HLA-DRB1	0.000523	5.07E-05	0.00011	0.000298	5.83E-05	8.51E-05	0.000183	0.000332
HLA-DRB5	0.00029	6.95E-06	3.49E-05	9.90E-05	3.26E-05	2.10E-05	4.11E-05	0.00039

HLA-DRB(	0.000134	5.44E-06	1.72E-05	6.52E-05	2.04E-06	1.51E-05	3.06E-05	1.36E-05
HLA-E	0.00059	0.000351	0.000211	0.000319	3.99E-05	0.000321	0.00023	0.000661
HLA-F	0.000289	2.65E-05	5.00E-05	0.00014	7.71E-06	0.00015	6.50E-05	0.000451
HLA-G	2.46E-05	1.01E-06	1.31E-06	7.41E-06	7.12E-07	5.00E-06	4.48E-06	1.71E-05
HLA-H	0.000381	6.38E-05	5.84E-05	0.000162	2.12E-05	0.000108	0.000142	0.000361
HLA-J	4.17E-07	9.07E-08	5.27E-08	2.35E-07	4.56E-08	8.69E-07	2.39E-08	2.78E-07
HLA-L	5.34E-07	8.21E-08	2.66E-07	2.57E-07	5.88E-08	6.79E-07	3.10E-07	8.88E-07
HLCS	6.83E-06	3.03E-06	4.32E-06	3.25E-06	2.31E-06	5.74E-06	3.42E-06	5.28E-06
HLF	4.10E-06	1.27E-05	3.57E-05	1.80E-05	8.63E-06	9.70E-05	1.87E-05	1.23E-05
HLTF	8.53E-06	2.85E-05	5.76E-06	4.76E-06	1.72E-06	7.72E-06	3.08E-06	8.30E-06
HLX	3.78E-06	6.89E-06	7.85E-06	8.04E-06	1.10E-05	1.05E-05	9.72E-06	3.71E-06
HM13	0.000237	0.000178	5.92E-05	0.000107	0.000205	0.000131	0.000115	0.00013
HMBOX1	1.26E-06	1.21E-06	7.46E-07	1.04E-06	1.21E-07	1.00E-06	3.14E-07	6.00E-07
HMBS	3.25E-05	2.47E-05	9.53E-06	1.22E-05	1.72E-05	2.58E-05	1.54E-05	3.08E-05
HMCN1	2.09E-06	1.18E-06	1.70E-06	3.40E-06	1.38E-07	1.79E-07	7.56E-07	7.08E-06
HMG20A	2.62E-05	1.32E-05	7.50E-06	1.01E-05	3.92E-06	1.50E-05	1.22E-05	8.38E-06
HMG20B	4.44E-05	3.57E-05	2.42E-05	3.48E-05	4.45E-05	3.56E-05	3.67E-05	3.51E-05
HMGA1	6.20E-05	5.09E-05	5.51E-06	3.09E-05	1.32E-05	1.42E-05	1.29E-05	3.80E-05
HMGA2	5.25E-07	2.45E-08	2.23E-08	6.71E-09	0	0	5.35E-09	0
HMGB1	0.000102	4.80E-05	4.52E-05	4.81E-05	4.68E-05	5.05E-05	6.12E-05	4.30E-05
HMGB2	0.000125	1.44E-05	1.10E-05	1.73E-05	1.13E-05	3.56E-05	1.96E-05	2.57E-05
HMGB3	1.16E-05	2.73E-05	8.40E-06	1.15E-05	1.36E-05	1.06E-05	2.23E-05	6.03E-05
HMGB3L1	0	0	0	0	5.39E-08	0	0	0
HMGB4	0	0	0	0	0	0	0	0
HMGCL	0.000149	0.000254	0.000271	0.000214	0.000172	0.000232	0.000179	0.000137
HMGCLL1	0	0	2.13E-07	3.35E-07	7.87E-09	0	1.26E-07	1.33E-08
HMGCR	1.47E-05	0.00012	8.89E-06	1.22E-05	7.02E-06	1.38E-05	1.16E-05	1.44E-05
HMGCS1	0.000112	0.000547	4.22E-05	3.23E-05	1.66E-05	5.41E-05	2.70E-05	4.49E-05
HMGCS2	0.000234	0.003036	0.001333	0.000536	0.000331	0.000452	0.000789	0.000724
HMGN1	0.000137	4.54E-05	2.98E-05	4.85E-05	2.26E-05	3.76E-05	8.82E-05	3.49E-05
HMGN2	0.000177	8.90E-05	5.67E-05	8.25E-05	9.59E-05	0.000105	0.000125	6.93E-05
HMGN3	7.23E-05	8.73E-05	5.59E-05	6.36E-05	2.34E-05	7.09E-05	4.51E-05	3.65E-05
HMGN4	3.53E-05	2.58E-05	1.02E-05	1.62E-05	3.28E-06	2.01E-05	1.44E-05	1.90E-05
HMGN5	3.23E-06	3.48E-06	4.44E-06	2.95E-06	6.93E-06	3.02E-06	5.55E-06	3.26E-06
HMGXB3	1.04E-05	5.31E-06	2.29E-06	5.04E-06	1.49E-06	5.87E-06	5.37E-06	9.22E-06
HMGXB4	6.19E-06	3.98E-06	2.76E-06	2.83E-06	1.04E-06	4.26E-06	1.69E-06	3.84E-06
HMHA1	1.58E-05	5.51E-06	9.07E-06	1.45E-05	5.74E-06	8.09E-06	1.94E-05	8.73E-06
HMHB1	1.11E-07	4.50E-07	0	0	0	0	9.81E-08	0
HMMR	8.01E-06	3.01E-06	1.24E-07	1.95E-07	2.05E-07	2.07E-06	2.01E-07	1.41E-06
HMOX1	3.18E-05	2.73E-05	5.87E-05	0.000179	1.95E-05	2.20E-05	0.000218	2.19E-05
HMOX2	5.75E-05	8.32E-05	3.09E-05	4.38E-05	2.01E-05	8.62E-05	3.96E-05	5.13E-05
HMP19	5.78E-08	0	0	2.72E-08	1.99E-08	2.14E-08	1.12E-08	6.80E-08
HMSD	3.98E-07	0	1.18E-07	1.87E-07	0	0	0	7.57E-08
HMX1	0	0	0	0	0	0	0	0
HMX2	0	0	0	0	0	0	0	0
HMX3	0	0	0	0	0	0	0	0
HN1	9.57E-05	2.46E-05	7.79E-06	2.12E-05	2.20E-05	2.82E-05	2.48E-05	2.06E-05
HN1L	0.000138	2.24E-05	2.02E-05	2.68E-05	8.45E-06	2.71E-05	4.18E-05	1.43E-05
HNF1A	2.15E-05	2.24E-05	1.61E-05	1.51E-05	1.32E-05	1.96E-05	1.97E-05	1.22E-05
HNF1B	5.95E-05	3.48E-06	4.94E-06	1.19E-05	1.08E-05	6.14E-06	6.95E-06	1.10E-05
HNF4A	0.000115	0.00012	8.18E-05	3.98E-05	3.84E-05	0.000106	4.50E-05	0.000109
HNF4G	6.75E-06	3.15E-06	4.91E-06	3.83E-06	3.48E-07	2.29E-06	7.39E-06	4.33E-06
HNMT	0.000111	3.55E-05	5.37E-05	7.39E-05	5.37E-05	6.50E-05	5.17E-05	5.36E-05
HNRNPA(	9.85E-05	4.47E-05	3.52E-05	6.77E-05	2.07E-05	4.88E-05	5.12E-05	5.71E-05
HNRNPA1	0.000156	5.30E-05	4.88E-05	0.000107	5.48E-05	0.000128	0.000128	8.23E-05
HNRNPA1	4.62E-05	1.90E-05	1.80E-05	3.64E-05	9.33E-06	4.23E-05	3.27E-05	2.57E-05
HNRNPA2	0.000287	0.000181	8.54E-05	0.000135	7.38E-05	0.000294	0.000144	0.000153
HNRNPA3	7.51E-05	2.38E-05	2.09E-05	3.44E-05	1.87E-05	5.44E-05	3.19E-05	2.72E-05

HNRNPA3	2.45E-06	9.69E-07	7.25E-07	9.98E-07	6.55E-07	1.48E-06	6.88E-07	8.59E-07
HNRNPAE	6.18E-05	3.76E-05	4.14E-05	6.40E-05	2.39E-05	4.93E-05	7.66E-05	7.88E-05
HNRNPC	0.000364	0.000171	9.62E-05	0.000166	9.23E-05	0.000188	0.000181	0.000152
HNRNPCL	0	1.80E-07	0	0	8.17E-08	0	0	0
HNRNPD	9.48E-05	3.80E-05	3.96E-05	5.84E-05	2.52E-05	5.56E-05	6.40E-05	5.73E-05
HNRNPF	0.000138	8.98E-05	8.60E-05	7.69E-05	3.35E-05	8.19E-05	7.88E-05	6.51E-05
HNRNPH1	0.000133	5.43E-05	4.39E-05	6.01E-05	2.73E-05	6.03E-05	4.82E-05	4.77E-05
HNRNPH2	7.10E-05	5.27E-05	3.86E-05	4.16E-05	2.47E-05	6.09E-05	3.48E-05	7.75E-05
HNRNPH3	5.03E-05	3.54E-05	2.41E-05	2.96E-05	1.65E-05	2.48E-05	3.83E-05	1.47E-05
HNRNPK	0.000247	0.000185	0.000127	0.000147	5.42E-05	0.000193	0.00013	0.000125
HNRNPL	0.000172	0.000113	6.15E-05	8.41E-05	4.13E-05	0.000118	0.000112	8.55E-05
HNRNPM	0.000104	6.77E-05	4.64E-05	8.48E-05	7.85E-05	7.49E-05	8.16E-05	3.74E-05
HNRNPR	6.45E-05	2.68E-05	2.67E-05	3.15E-05	1.65E-05	5.23E-05	2.88E-05	3.73E-05
HNRNPU	0.000125	0.00012	4.26E-05	6.21E-05	1.83E-05	8.06E-05	4.35E-05	5.56E-05
HNRNPUL	6.38E-05	3.88E-05	2.99E-05	3.87E-05	4.16E-05	4.38E-05	4.12E-05	3.57E-05
HNRNPUL	4.91E-05	3.15E-05	1.87E-05	2.44E-05	1.16E-05	3.17E-05	2.87E-05	3.90E-05
HNRPDL	5.07E-05	2.90E-05	2.10E-05	3.48E-05	1.02E-05	2.86E-05	3.05E-05	3.74E-05
HNRPLL	1.19E-05	1.02E-05	4.31E-06	6.17E-06	2.28E-06	7.15E-06	4.75E-06	4.93E-06
HOMER1	5.64E-06	3.59E-06	3.46E-07	1.08E-06	5.32E-08	4.17E-08	5.62E-07	1.33E-06
HOMER2	2.94E-05	1.71E-05	2.82E-05	3.21E-05	1.85E-05	3.47E-05	4.50E-05	2.72E-05
HOMER3	1.14E-05	1.98E-06	1.87E-06	8.26E-06	4.81E-06	1.77E-06	1.67E-05	3.18E-06
HOMEZ	4.36E-06	3.93E-06	2.74E-06	2.43E-06	2.86E-06	3.20E-06	2.88E-06	2.88E-06
HOOK1	1.94E-05	1.66E-05	1.06E-05	7.21E-06	1.28E-06	6.14E-06	3.19E-06	6.81E-06
HOOK2	1.46E-05	5.91E-06	8.58E-06	9.22E-06	6.59E-06	5.58E-06	7.83E-06	5.92E-06
HOOK3	3.66E-06	2.31E-06	1.84E-06	2.73E-06	4.65E-07	1.83E-06	8.49E-07	2.62E-06
HOPX	1.11E-05	1.46E-06	1.16E-06	2.05E-06	8.82E-07	1.72E-06	2.26E-06	9.43E-07
HORMAD.	0	0	4.28E-08	3.33E-08	0	3.78E-08	5.24E-08	0
HORMAD:	2.55E-08	3.03E-07	4.82E-07	2.19E-07	2.85E-07	0	7.88E-07	1.56E-06
HOTAIR	0	1.14E-08	0	0	0	0	0	0
HOXA1	8.29E-08	4.15E-08	5.74E-08	1.36E-07	0	7.68E-08	3.90E-08	8.88E-08
HOXA10	4.33E-08	1.59E-07	0	2.65E-08	0	7.02E-08	1.47E-07	4.77E-08
HOXA11	9.43E-09	4.25E-08	0	2.31E-08	0	0	4.57E-08	1.39E-08
HOXA11A	1.59E-08	1.45E-07	0	0	0	0	0	0
HOXA13	4.04E-06	1.19E-06	0	3.43E-08	2.41E-08	1.56E-07	1.08E-07	5.48E-08
HOXA2	5.46E-08	1.55E-08	4.31E-08	1.01E-07	3.53E-08	2.54E-08	9.22E-08	8.05E-08
HOXA3	2.26E-07	1.35E-07	1.20E-07	2.10E-07	1.69E-07	3.08E-06	8.96E-08	2.49E-07
HOXA4	4.18E-07	2.24E-07	5.64E-07	7.24E-07	3.86E-07	1.93E-07	7.07E-07	4.16E-07
HOXA5	2.65E-07	1.74E-07	1.22E-07	5.60E-07	1.15E-07	1.41E-07	1.46E-07	2.25E-07
HOXA6	3.21E-08	1.13E-07	0	0	0	0	0	4.81E-08
HOXA7	1.19E-08	2.15E-07	1.25E-08	1.46E-08	1.02E-08	6.62E-08	2.36E-07	3.50E-08
HOXA9	1.16E-08	9.20E-08	0	1.42E-08	0	0	5.62E-08	1.71E-08
HOXB1	0	0	0	0	0	0	0	0
HOXB13	2.14E-07	0	0	0	0	0	0	0
HOXB2	1.66E-06	8.40E-07	8.30E-07	1.02E-06	5.69E-07	7.71E-07	2.06E-06	7.94E-07
HOXB3	1.05E-06	3.43E-07	5.95E-07	3.93E-07	3.45E-07	3.88E-07	6.11E-07	7.94E-07
HOXB4	1.08E-06	2.53E-07	4.81E-07	4.47E-07	2.13E-07	3.28E-07	4.78E-07	7.27E-07
HOXB5	5.72E-07	1.96E-07	1.96E-07	2.45E-07	3.09E-07	3.09E-07	8.98E-07	1.57E-07
HOXB6	3.07E-07	2.84E-07	4.47E-07	5.55E-07	7.29E-07	2.71E-07	6.97E-07	2.80E-07
HOXB7	5.93E-07	6.15E-07	3.57E-07	3.19E-07	3.52E-07	3.45E-07	1.66E-06	2.74E-07
HOXB8	1.33E-08	3.01E-08	0	0	0	2.47E-08	7.69E-08	1.96E-08
HOXB9	2.60E-08	2.93E-08	0	0	0	0	9.24E-08	0
HOXC10	0	0	0	0	0	0	1.19E-07	0
HOXC11	0	0	0	0	0	0	0	0
HOXC12	0	0	0	0	0	0	0	0
HOXC13	0	1.13E-08	1.05E-08	0	0	0	0	0
HOXC4	2.50E-07	3.34E-08	6.21E-08	1.98E-07	5.06E-08	4.09E-08	2.26E-07	1.73E-07
HOXC5	1.53E-08	0	0	0	0	0	4.59E-08	5.67E-08
HOXC6	0	3.31E-08	0	0	0	0	1.57E-07	2.06E-07

HOXC8	0	2.33E-08	0	0	0	9.59E-09	2.00E-08	1.22E-07
HOXC9	0	0	0	0	0	2.99E-08	1.39E-07	9.49E-08
HOXD1	8.53E-08	1.24E-07	0	1.49E-08	9.73E-09	2.26E-08	1.41E-07	3.59E-08
HOXD10	6.72E-08	2.15E-06	1.41E-08	0	0	3.62E-07	1.27E-06	0
HOXD11	0	0	4.10E-08	0	0	0	0	0
HOXD12	0	0	0	0	0	0	0	0
HOXD13	0	1.14E-08	0	0	0	0	0	2.98E-08
HOXD3	2.06E-08	1.74E-07	0	1.26E-08	0	9.53E-09	1.89E-07	0
HOXD4	1.96E-07	1.35E-07	0	0	0	1.64E-07	3.58E-07	0
HOXD8	1.54E-06	2.58E-06	0	2.80E-07	1.83E-08	8.69E-07	3.17E-06	0
HOXD9	6.65E-07	6.23E-06	0	1.10E-07	1.10E-07	1.26E-06	6.65E-06	3.77E-08
HP	0.001036	0.018741	0.03899	0.030776	0.017713	0.043279	0.029195	0.038546
HP1BP3	4.95E-05	4.11E-05	2.65E-05	2.89E-05	8.45E-06	3.14E-05	1.55E-05	2.94E-05
HPCA	1.05E-06	0	3.80E-08	2.19E-08	1.54E-08	1.66E-08	1.71E-08	0
HPCAL1	3.85E-05	5.55E-05	1.76E-05	3.17E-05	1.90E-05	2.22E-05	3.67E-05	2.22E-05
HPCAL4	1.07E-07	0	0	5.87E-08	0	5.22E-09	0	8.26E-09
HPD	5.29E-05	0.001198	0.001325	0.001299	0.0013	0.004353	0.001667	9.42E-05
HPDL	6.60E-07	1.53E-08	0	8.26E-08	2.32E-08	1.00E-07	9.09E-08	5.95E-08
HPGD	0.000294	0.000213	1.00E-04	3.93E-05	1.79E-05	4.23E-06	8.25E-05	0.000219
HPGDS	6.08E-08	1.38E-07	3.70E-07	8.59E-07	5.24E-08	5.79E-07	7.33E-08	8.98E-08
HPN	0.000188	0.000213	0.000451	0.000393	0.000656	0.000348	0.000486	0.000375
HPR	4.61E-05	0.000579	0.000584	0.000419	0.000322	0.001299	0.000392	0.0011
HPRT1	2.77E-05	3.72E-05	1.51E-05	2.38E-05	1.91E-05	2.33E-05	2.94E-05	4.59E-05
HPS1	2.52E-05	2.11E-05	1.82E-05	2.53E-05	1.22E-05	3.11E-05	2.41E-05	2.25E-05
HPS3	2.69E-05	3.06E-05	1.45E-05	2.48E-05	5.12E-06	1.05E-05	2.06E-05	1.24E-05
HPS4	9.78E-06	6.98E-06	3.62E-06	3.21E-06	2.97E-06	6.78E-06	4.83E-06	3.60E-06
HPS5	1.27E-05	8.50E-06	1.15E-05	1.34E-05	9.08E-07	1.03E-05	3.35E-06	1.94E-05
HPS6	9.06E-06	4.28E-06	5.33E-06	6.05E-06	5.81E-06	9.10E-06	7.75E-06	8.53E-06
HPSE	1.02E-05	1.44E-07	1.03E-06	2.33E-06	8.88E-08	3.00E-07	7.54E-07	1.27E-07
HPSE2	0	1.39E-08	0	0	1.06E-08	0	0	1.81E-08
HPVC1	0	0	0	0	0	0	0	0
HPX	0.000976	0.001533	0.0056	0.003723	0.002426	0.00397	0.002244	0.003455
HPYR1	0	0	0	0	0	0	0	0
HR	1.33E-07	1.18E-06	1.17E-07	1.82E-07	4.20E-08	2.33E-08	1.92E-07	3.69E-08
HRAS	3.56E-05	2.49E-05	9.20E-06	1.89E-05	2.87E-05	1.92E-05	2.20E-05	3.37E-05
HRASLS	1.29E-06	0	1.08E-07	6.14E-08	2.15E-08	0	0	0
HRASLS2	0	1.01E-05	8.48E-07	9.75E-08	4.43E-07	1.13E-05	8.48E-07	0
HRASLS5	4.86E-08	0	1.68E-08	9.90E-08	0	0	2.20E-08	1.19E-08
HRC	6.03E-07	1.02E-05	4.74E-07	8.48E-07	1.41E-06	2.66E-07	5.84E-07	5.23E-07
HRCT1	1.34E-06	6.36E-06	5.33E-07	2.57E-06	1.05E-05	1.67E-05	5.50E-06	2.55E-07
HRG	0.002137	0.004435	0.002069	0.002754	0.002037	0.000421	0.002596	0.001734
HRH1	4.18E-06	3.15E-07	3.11E-07	3.76E-07	2.19E-07	4.42E-07	1.03E-06	1.63E-07
HRH2	3.03E-07	4.14E-08	2.86E-08	1.91E-07	0	8.99E-08	7.97E-08	1.35E-08
HRH3	0	0	0	0	2.25E-08	0	8.44E-09	0
HRH4	1.35E-08	0	2.09E-08	1.53E-08	0	3.75E-08	1.31E-08	1.98E-08
HRK	0	0	0	0	0	0	0	0
HRNBP3	3.49E-08	2.94E-08	0	1.07E-08	0	0	8.45E-09	0
HRNR	3.27E-07	1.54E-08	4.69E-09	3.37E-08	5.92E-09	2.55E-08	1.12E-08	1.01E-08
HRSP12	0.000478	0.000771	0.000733	0.000391	0.000896	0.000295	0.00048	0.000302
HS1BP3	5.22E-05	6.45E-05	1.83E-05	2.19E-05	1.43E-05	1.61E-05	3.25E-05	2.15E-05
HS2ST1	1.09E-05	1.00E-05	7.68E-06	6.30E-06	1.71E-06	1.34E-05	3.48E-06	6.89E-06
HS3ST1	2.99E-06	5.67E-07	4.61E-07	5.02E-06	1.68E-07	1.93E-07	6.85E-07	4.14E-07
HS3ST2	9.84E-07	1.05E-06	6.38E-07	6.42E-06	6.36E-08	1.49E-07	6.18E-06	1.81E-07
HS3ST3A1	2.69E-07	6.17E-07	1.36E-06	1.35E-06	3.19E-08	1.14E-06	4.14E-07	6.54E-07
HS3ST3B1	1.06E-07	1.40E-06	3.39E-06	2.39E-06	5.06E-08	2.71E-06	6.26E-07	1.68E-06
HS3ST4	7.20E-09	0	3.72E-08	0	0	6.67E-09	0	0
HS3ST5	1.70E-08	0	0	0	0	4.09E-07	0	0
HS3ST6	9.95E-08	0	0	1.23E-07	0	0	2.37E-08	0

HS6ST1	1.48E-05	2.13E-05	2.15E-05	8.93E-06	8.60E-06	5.31E-05	1.06E-05	3.64E-05
HS6ST2	5.14E-05	7.95E-09	4.39E-08	1.59E-07	6.09E-09	6.56E-09	1.38E-08	0
HS6ST3	2.85E-09	9.54E-09	2.91E-09	3.48E-09	0	0	2.78E-08	4.17E-09
HSBP1	6.42E-05	2.89E-05	2.76E-05	4.05E-05	2.05E-05	3.52E-05	3.68E-05	2.90E-05
HSBP1L1	3.80E-05	1.78E-05	1.80E-05	1.32E-05	1.51E-05	1.13E-05	2.62E-05	1.12E-05
HSCB	1.99E-05	2.57E-05	1.56E-05	1.57E-05	3.28E-05	2.84E-05	2.34E-05	1.39E-05
HSD11B1	7.99E-05	0.000265	0.000528	0.000318	4.45E-05	0.001057	0.000271	0.000111
HSD11B1L	2.15E-06	1.41E-06	2.34E-06	2.92E-06	6.34E-06	4.98E-06	3.62E-06	7.00E-07
HSD11B2	7.31E-06	1.67E-06	1.95E-06	2.53E-06	1.68E-06	1.10E-06	3.24E-06	6.59E-06
HSD17B1	2.33E-06	1.98E-06	3.92E-07	7.29E-07	3.04E-06	9.24E-07	7.62E-07	6.47E-07
HSD17B10	0.000139	0.000271	0.000199	0.00016	0.000227	0.000285	0.000289	0.000249
HSD17B11	0.000253	0.000143	0.000139	0.000113	6.28E-05	0.000122	0.00015	0.000234
HSD17B12	8.73E-05	3.55E-05	4.92E-05	4.13E-05	3.12E-05	1.51E-05	2.00E-05	7.05E-05
HSD17B13	2.85E-07	0.000212	0.00033	0.000196	1.11E-05	2.60E-06	2.57E-05	0.00019
HSD17B14	3.44E-06	3.79E-05	5.28E-05	6.66E-06	2.71E-05	8.83E-06	4.93E-06	3.80E-06
HSD17B2	0.000255	2.31E-05	0.000112	0.000181	9.07E-05	3.51E-05	0.000105	0.000212
HSD17B3	1.77E-06	8.99E-07	2.48E-07	2.83E-07	1.69E-06	3.12E-06	2.85E-07	3.41E-07
HSD17B4	7.11E-05	0.000114	0.000151	0.000131	4.15E-05	0.000353	0.000104	0.000175
HSD17B6	0.000244	0.000479	0.000706	0.00033	0.000559	0.000587	0.000413	0.000192
HSD17B7	2.55E-05	0.000134	1.03E-05	5.35E-06	5.13E-06	3.58E-05	5.84E-06	2.07E-05
HSD17B7F	3.78E-06	1.20E-05	1.07E-06	7.39E-07	4.17E-07	2.04E-06	6.59E-07	1.96E-06
HSD17B8	2.57E-05	8.84E-05	5.51E-05	4.86E-05	6.85E-05	5.84E-05	0.000107	6.02E-05
HSD3B1	0	1.49E-07	5.37E-07	5.36E-08	0	8.11E-08	1.40E-08	8.50E-08
HSD3B2	0	1.67E-08	7.35E-08	6.77E-08	3.54E-07	3.76E-08	1.41E-08	0
HSD3B7	6.19E-05	0.000142	7.13E-05	5.16E-05	2.25E-05	5.53E-05	7.15E-05	8.37E-05
HSDL1	4.98E-06	2.49E-06	2.25E-06	3.34E-06	1.41E-06	1.24E-05	3.21E-06	2.98E-06
HSDL2	6.86E-05	3.49E-05	5.83E-05	4.34E-05	2.95E-05	8.34E-05	4.88E-05	5.53E-05
HSF1	7.82E-05	5.65E-05	2.11E-05	2.37E-05	3.91E-05	2.64E-05	7.71E-05	2.63E-05
HSF2	4.01E-06	3.69E-06	1.77E-06	1.80E-06	6.60E-07	4.17E-06	1.90E-06	2.27E-06
HSF2BP	2.58E-06	6.47E-07	4.00E-08	1.10E-07	1.80E-07	1.81E-07	1.44E-07	3.50E-07
HSF4	5.65E-06	2.54E-06	6.29E-07	8.14E-07	8.52E-07	2.51E-06	1.16E-06	4.04E-06
HSF5	1.66E-08	1.86E-08	5.69E-09	0	4.75E-09	4.10E-08	5.39E-09	8.11E-09
HSFX1	0	0	0	0	0	0	0	0
HSFX2	3.93E-07	2.19E-07	2.00E-07	1.76E-07	3.69E-07	2.48E-07	1.73E-07	1.83E-07
HSFY2	0	4.00E-08	0	3.16E-08	0	0	0	0
HSFYL1	0	0	0	0	0	0	0	0
HSH2D	9.61E-06	3.28E-06	4.98E-07	1.54E-06	1.72E-07	5.61E-07	5.38E-07	7.17E-07
HSN2	2.74E-07	2.68E-07	3.92E-08	1.22E-07	1.07E-08	2.31E-08	1.68E-07	1.10E-07
HSP90AA1	0.00053	0.000328	0.000118	0.000183	0.000136	0.000286	0.000258	0.000168
HSP90AB1	0.00071	0.000856	0.000179	0.000282	0.000215	0.000463	0.000441	0.00038
HSP90AB2	1.14E-06	4.23E-07	2.08E-07	8.46E-08	3.57E-08	3.93E-07	2.97E-07	1.22E-07
HSP90AB4	3.26E-08	0	0	4.01E-08	4.22E-08	0	0	0
HSP90B1	0.000766	0.00062	0.000323	0.000458	0.000291	0.0007	0.000423	0.000582
HSP90B3P	2.83E-06	7.41E-07	1.50E-06	2.28E-07	3.19E-08	6.19E-07	8.88E-08	4.65E-07
HSPA12A	1.75E-06	1.14E-07	2.01E-07	5.12E-07	8.41E-08	9.55E-08	2.03E-07	8.03E-07
HSPA12B	2.56E-06	2.06E-06	6.55E-07	5.94E-07	2.41E-06	1.32E-06	3.75E-06	1.75E-06
HSPA13	9.29E-06	5.83E-06	5.57E-06	5.38E-06	7.80E-07	7.39E-06	3.59E-06	9.10E-06
HSPA14	1.14E-05	1.34E-05	5.06E-06	6.95E-06	1.70E-06	8.48E-06	5.31E-06	7.34E-06
HSPA1A	0.000162	0.00101	8.39E-05	0.000255	0.000155	8.00E-05	0.000253	8.89E-05
HSPA1B	1.29E-05	8.81E-05	5.64E-06	2.78E-05	1.03E-05	6.02E-06	1.53E-05	2.28E-06
HSPA1L	6.66E-07	1.48E-06	5.15E-07	4.53E-07	3.86E-07	3.99E-07	4.80E-07	3.85E-07
HSPA2	3.33E-06	1.68E-06	1.66E-06	8.71E-06	5.34E-07	2.39E-06	2.23E-06	3.79E-06
HSPA4	5.39E-05	5.75E-05	1.88E-05	2.28E-05	6.71E-06	3.44E-05	2.01E-05	4.16E-05
HSPA4L	1.93E-05	7.68E-06	2.56E-06	2.40E-06	2.05E-07	5.92E-06	6.96E-07	4.91E-06
HSPA5	0.000403	0.000252	0.000175	0.000157	0.000177	0.000434	0.00016	0.000634
HSPA6	2.40E-06	1.96E-06	1.04E-06	4.54E-06	3.64E-07	5.76E-07	1.27E-06	7.51E-07
HSPA7	1.17E-06	6.37E-08	8.03E-07	9.34E-07	6.48E-08	2.18E-07	5.56E-07	1.38E-07
HSPA8	0.000498	0.000443	0.000295	0.000405	0.000299	0.000325	0.000523	0.0005

HSPA9	0.000222	0.000384	0.000141	0.00013	4.95E-05	0.000163	0.000157	0.000273
HSPB1	0.000481	0.001555	0.000146	0.000469	0.000794	0.000161	0.001456	0.000256
HSPB11	5.04E-05	3.89E-05	2.60E-05	3.43E-05	2.15E-05	4.53E-05	2.49E-05	2.51E-05
HSPB2	5.70E-06	2.06E-06	1.00E-06	3.99E-06	2.20E-06	8.99E-07	3.10E-06	1.64E-06
HSPB3	7.54E-08	0	0	0	0	3.54E-08	0	0
HSPB6	1.15E-05	1.07E-06	7.84E-06	4.65E-05	6.81E-06	4.50E-06	1.66E-05	6.32E-06
HSPB7	1.01E-06	2.18E-07	1.03E-07	6.01E-07	6.70E-08	5.42E-08	1.17E-07	1.99E-07
HSPB8	2.89E-05	8.47E-07	1.92E-06	2.18E-05	6.19E-07	2.16E-05	3.48E-06	1.11E-06
HSPB9	5.57E-06	3.92E-06	6.83E-06	6.04E-06	1.39E-05	6.49E-06	2.97E-06	5.47E-06
HSPBAP1	5.31E-06	3.94E-06	1.99E-06	2.88E-06	1.43E-06	3.92E-06	3.64E-06	2.87E-06
HSPBP1	3.08E-05	3.54E-05	1.43E-05	2.39E-05	3.59E-05	2.41E-05	3.35E-05	1.91E-05
HSPC072	0	0	0	0	1.57E-08	0	0	0
HSPC157	2.62E-05	1.06E-05	1.14E-05	8.98E-06	1.28E-05	1.60E-05	7.08E-06	1.53E-05
HSPC159	7.34E-06	9.50E-06	2.40E-06	4.04E-06	2.65E-06	1.92E-06	5.43E-06	2.89E-06
HSPD1	0.000918	0.000511	0.000198	0.000181	0.000138	0.00032	0.000296	0.000193
HSPE1	0.000666	0.000379	0.000126	0.00013	0.000124	0.000217	0.000232	0.000164
HSPG2	5.15E-05	1.43E-05	1.09E-05	2.11E-05	2.09E-05	1.48E-05	4.58E-05	3.03E-05
HSPH1	2.66E-05	4.00E-05	8.71E-06	1.32E-05	3.98E-06	1.80E-05	1.76E-05	8.79E-06
HTA	2.44E-08	0	0	0	0	0	0	0
HTATIP2	0.000111	0.000194	4.84E-05	6.01E-05	4.84E-05	0.000166	4.64E-05	0.000169
HTATSF1	3.11E-05	2.81E-05	1.50E-05	1.91E-05	1.41E-05	2.21E-05	1.72E-05	3.50E-05
HTN1	0	0	0	0	0	0	0	0
HTN3	1.11E-07	0	0	0	0	0	0	0
HTR1A	0	0	0	0	0	0	0	0
HTR1B	2.20E-08	2.54E-08	0	0	0	2.05E-08	0	6.54E-08
HTR1D	1.51E-05	1.84E-08	0	1.00E-08	0	5.32E-08	1.59E-08	0
HTR1E	0	0	0	0	0	0	0	0
HTR1F	5.81E-08	3.26E-08	0	0	0	6.72E-09	2.82E-08	1.06E-08
HTR2A	0	3.88E-08	8.95E-09	8.14E-09	0	7.99E-09	8.36E-09	1.27E-08
HTR2B	1.85E-06	2.88E-07	1.45E-06	2.49E-06	1.70E-06	2.63E-05	1.62E-06	3.13E-07
HTR2C	0	5.43E-09	0	0	0	0	9.08E-09	0
HTR3A	8.41E-08	0	1.26E-08	5.89E-08	0	0	1.16E-08	0
HTR3B	0	1.57E-08	0	0	0	0	0	0
HTR3C	0	0	0	1.77E-08	0	0	0	0
HTR3D	0	0	0	0	0	0	0	0
HTR3E	0	0	0	0	0	0	0	0
HTR4	1.48E-07	8.75E-09	4.50E-08	1.95E-08	2.58E-08	1.60E-07	0	2.97E-07
HTR5A	0	0	0	0	0	0	0	0
HTR6	0	0	0	0	0	0	0	0
HTR7	4.05E-07	5.58E-08	1.39E-07	3.30E-07	6.10E-09	3.29E-08	1.94E-07	0
HTR7P1	2.94E-06	7.75E-07	6.99E-07	7.39E-07	5.90E-07	7.61E-07	5.06E-07	6.48E-07
HTRA1	0.000338	0.000149	4.86E-05	5.49E-05	0.000129	0.000108	0.000127	0.00018
HTRA2	2.82E-05	1.57E-05	9.28E-06	1.30E-05	1.26E-05	1.47E-05	1.83E-05	1.08E-05
HTRA3	1.39E-05	4.23E-07	1.48E-06	2.67E-06	1.78E-06	4.32E-07	6.82E-06	2.96E-06
HTRA4	2.49E-07	0	1.19E-08	1.11E-07	1.95E-08	3.68E-07	8.77E-08	3.33E-08
HTT	7.98E-06	6.35E-06	2.92E-06	3.40E-06	2.93E-06	7.52E-06	4.84E-06	7.13E-06
HULC	0.000229	0.000269	0.000206	0.000225	0.000965	0.001884	0.000598	0.00044
HUNK	1.81E-06	1.35E-08	2.46E-07	5.44E-07	4.40E-08	3.63E-08	6.18E-08	3.53E-08
HUS1	1.35E-05	1.23E-05	7.18E-06	7.03E-06	1.51E-06	9.44E-06	5.50E-06	1.40E-05
HUS1B	0	1.50E-07	2.83E-08	2.24E-07	0	2.42E-08	4.94E-08	1.54E-07
HUWE1	3.97E-05	2.96E-05	1.98E-05	1.93E-05	4.12E-06	3.17E-05	1.41E-05	2.84E-05
HVCN1	8.84E-06	1.94E-06	2.21E-06	3.75E-06	1.26E-06	2.05E-06	3.44E-06	1.06E-06
HYAL1	0.000263	0.000173	0.000142	0.0001	0.0002	0.000131	0.000108	8.72E-05
HYAL2	5.53E-05	3.40E-05	1.97E-05	2.86E-05	1.69E-05	2.61E-05	2.92E-05	3.29E-05
HYAL3	1.47E-05	1.51E-05	3.18E-06	3.10E-06	2.92E-06	7.81E-06	1.75E-06	1.83E-06
HYAL4	1.24E-08	5.20E-07	0	1.52E-08	0	0	0	3.06E-07
HYALP1	0	0	0	0	0	0	0	0
HYDIN	1.47E-07	2.07E-08	7.64E-07	2.07E-07	0	3.63E-07	3.23E-08	7.64E-08

HYI	5.35E-05	4.86E-05	4.82E-05	7.10E-05	6.50E-05	4.10E-05	7.27E-05	3.76E-05
HYLS1	5.19E-06	2.16E-06	1.63E-06	1.67E-06	1.60E-06	2.30E-06	1.78E-06	4.36E-06
HYMAI	3.66E-08	5.05E-09	9.26E-09	5.51E-09	0	0	0	0
HYOU1	8.40E-05	7.16E-05	3.00E-05	3.86E-05	1.52E-05	0.00011	3.12E-05	0.000117
IAH1	7.85E-05	9.45E-05	4.04E-05	5.29E-05	3.78E-05	5.12E-05	5.42E-05	3.94E-05
IAPP	2.43E-08	5.50E-08	1.15E-07	7.44E-08	2.09E-08	9.02E-08	2.35E-08	3.58E-08
IARS	1.96E-05	1.95E-05	9.11E-06	8.48E-06	2.81E-06	2.24E-05	8.28E-06	1.09E-05
IARS2	6.40E-05	0.000102	6.00E-05	5.36E-05	2.50E-05	0.000118	4.51E-05	0.000105
IBSP	6.66E-07	5.47E-07	0	0	0	0	4.48E-08	5.03E-07
IBTK	2.69E-05	3.01E-05	1.82E-05	1.29E-05	4.24E-06	3.19E-05	1.10E-05	9.17E-06
ICA1	3.07E-05	4.82E-05	7.81E-06	1.06E-05	1.56E-06	1.04E-05	2.28E-06	1.57E-05
ICA1L	1.50E-07	6.61E-08	9.42E-08	2.32E-07	1.45E-08	7.05E-08	1.13E-07	3.30E-08
ICAM1	6.28E-05	1.20E-05	1.90E-05	0.000124	6.91E-06	1.83E-05	3.20E-05	0.000116
ICAM2	3.36E-05	3.61E-05	8.69E-06	1.25E-05	1.37E-05	9.70E-06	2.70E-05	1.29E-05
ICAM3	1.69E-05	1.65E-05	3.13E-05	1.67E-05	2.27E-05	1.67E-05	1.90E-05	9.55E-06
ICAM4	1.20E-06	6.77E-07	1.94E-07	3.08E-06	1.99E-07	1.20E-07	4.39E-07	7.03E-07
ICAM5	2.02E-07	5.07E-08	2.80E-08	8.81E-08	0	8.34E-09	3.49E-08	0
ICK	3.37E-06	8.80E-06	3.74E-06	2.33E-06	8.71E-07	2.76E-05	2.04E-06	3.97E-06
ICMT	2.35E-05	1.46E-05	1.09E-05	1.08E-05	4.65E-06	1.49E-05	9.32E-06	1.14E-05
ICOS	3.20E-06	1.03E-08	1.05E-07	1.39E-06	7.88E-09	6.23E-07	1.42E-07	1.88E-07
ICOSLG	3.31E-06	2.39E-06	3.05E-06	5.73E-06	2.02E-06	5.27E-06	5.43E-06	1.47E-05
ICT1	6.57E-05	7.43E-05	3.02E-05	3.38E-05	4.65E-05	6.62E-05	5.07E-05	4.15E-05
ID1	3.62E-05	2.16E-05	4.08E-05	8.49E-05	3.86E-05	3.48E-06	0.000163	2.31E-05
ID2	0.000408	0.000247	0.00046	0.000295	5.54E-05	0.000157	0.00013	0.000159
ID2B	2.49E-06	8.64E-07	3.84E-06	6.80E-07	8.20E-07	4.93E-07	3.80E-07	2.62E-07
ID3	4.05E-05	1.45E-05	2.00E-05	2.61E-05	1.32E-05	5.17E-06	2.62E-05	1.42E-05
ID4	7.24E-06	9.66E-07	4.83E-07	4.97E-06	2.51E-07	4.86E-07	1.26E-06	7.10E-07
IDE	1.15E-05	1.31E-05	7.33E-06	5.36E-06	1.54E-06	1.02E-05	3.00E-06	1.16E-05
IDH1	0.000307	0.000269	0.00018	0.000138	8.12E-05	0.000311	7.75E-05	0.000134
IDH2	0.000149	8.09E-05	0.000268	0.000206	0.000127	0.000335	0.000123	0.000154
IDH3A	4.78E-05	4.02E-05	1.21E-05	1.63E-05	5.04E-06	1.26E-05	1.64E-05	2.03E-05
IDH3B	9.61E-05	0.000103	4.40E-05	4.76E-05	5.95E-05	6.04E-05	4.84E-05	4.81E-05
IDH3G	4.23E-05	5.03E-05	2.49E-05	3.41E-05	6.94E-05	4.22E-05	4.00E-05	5.55E-05
IDI1	8.95E-05	0.000144	5.28E-05	3.35E-05	3.85E-05	5.52E-05	3.99E-05	6.02E-05
IDI2	3.95E-06	1.19E-06	1.50E-06	2.87E-06	1.82E-06	1.65E-06	3.11E-06	1.21E-06
IDO1	1.27E-05	1.37E-05	6.17E-07	1.88E-06	2.71E-08	6.57E-06	2.21E-06	7.20E-07
IDO2	2.17E-07	4.91E-06	2.98E-06	1.42E-06	6.59E-06	6.98E-07	2.02E-07	2.21E-08
IDS	3.03E-05	1.12E-05	1.69E-05	1.97E-05	3.93E-06	1.20E-05	1.38E-05	1.97E-05
IDUA	5.54E-06	3.24E-06	1.88E-06	5.05E-06	5.18E-06	3.22E-06	1.15E-05	1.50E-05
IER2	3.76E-05	0.000123	0.000138	0.000173	5.03E-05	1.52E-05	0.000148	5.04E-05
IER3	3.44E-05	6.68E-05	3.88E-05	0.0003	9.91E-06	7.75E-06	0.000155	0.000259
IER3IP1	4.51E-05	2.55E-05	2.65E-05	3.02E-05	1.65E-05	4.75E-05	3.74E-05	3.84E-05
IER5	1.49E-05	1.06E-05	3.31E-06	1.56E-05	2.05E-06	5.28E-06	1.03E-05	7.48E-06
IER5L	2.18E-06	1.49E-06	9.28E-07	1.21E-05	6.08E-06	1.11E-06	1.42E-05	3.75E-06
IFFO1	1.39E-05	6.92E-06	5.12E-06	6.95E-06	6.92E-06	1.05E-05	8.37E-06	9.12E-06
IFFO2	9.80E-06	1.85E-06	7.12E-07	2.39E-06	1.17E-06	7.68E-07	2.86E-06	2.28E-06
IFI16	4.73E-05	5.89E-06	9.49E-06	3.67E-05	4.73E-06	1.05E-05	1.08E-05	4.11E-05
IFI27	0.001264	0.001248	0.000173	4.66E-05	8.22E-05	0.000138	0.000126	0.00502
IFI27L1	1.22E-05	8.67E-06	3.31E-06	7.76E-06	1.52E-05	1.80E-05	7.00E-06	1.08E-05
IFI27L2	6.70E-05	1.10E-05	5.66E-06	2.97E-05	1.40E-05	2.14E-05	1.81E-05	1.20E-05
IFI30	0.000399	0.000125	0.000104	0.000317	7.87E-05	0.000152	0.000157	0.000111
IFI35	0.000113	0.000137	2.83E-05	2.91E-05	2.95E-05	7.66E-05	3.99E-05	4.92E-05
IFI44	7.85E-06	2.97E-06	9.70E-06	6.43E-06	1.07E-06	4.54E-05	3.67E-06	3.14E-05
IFI44L	1.67E-06	2.74E-07	1.80E-06	4.52E-07	2.98E-07	1.09E-06	2.83E-07	5.54E-06
IFI6	0.000126	4.58E-05	8.26E-05	5.12E-05	4.02E-05	0.000198	6.90E-05	0.000548
IFIH1	1.49E-05	7.62E-06	8.75E-06	6.23E-06	1.27E-06	7.64E-06	4.11E-06	1.31E-05
IFIT1	9.38E-06	1.46E-05	3.18E-05	3.51E-05	1.69E-05	1.71E-05	1.99E-05	6.58E-05
IFIT1B	0	0	0	4.48E-08	1.05E-08	1.13E-08	2.35E-08	3.58E-08

IFIT2	1.05E-05	7.04E-06	6.97E-06	1.35E-05	1.40E-06	5.42E-06	7.20E-06	4.82E-06
IFIT3	2.86E-05	1.09E-05	1.58E-05	1.94E-05	2.39E-06	1.67E-05	1.27E-05	2.20E-05
IFIT5	9.41E-06	7.61E-06	5.69E-06	7.36E-06	1.96E-06	7.04E-06	4.64E-06	1.07E-05
IFITM1	0.000671	8.96E-05	0.000204	0.000116	5.33E-05	0.000239	0.000281	0.000268
IFITM2	0.000951	0.000427	0.000305	0.000785	0.000429	0.000605	0.001026	0.001035
IFITM3	0.002998	0.003739	0.001729	0.002388	0.002371	0.003021	0.003748	0.005156
IFITM4P	2.79E-07	0	0	0	0	0	1.21E-07	2.29E-07
IFITM5	0	4.25E-07	0	0	0	3.73E-08	0	5.99E-08
IFLTD1	0	0	0	0	0	0	0	0
IFNA1	0	0	0	0	0	0	0	0
IFNA10	0	0	0	0	0	0	0	0
IFNA13	0	0	0	0	0	0	0	0
IFNA14	0	0	0	0	0	0	0	0
IFNA16	0	0	0	0	0	0	0	0
IFNA17	0	0	0	0	0	0	0	0
IFNA2	0	0	0	0	0	0	0	0
IFNA21	0	0	0	0	0	0	0	0
IFNA4	0	0	0	0	0	0	0	0
IFNA5	0	0	0	0	0	0	0	0
IFNA6	0	0	0	0	0	0	0	0
IFNA7	0	0	0	0	0	0	0	0
IFNA8	0	0	0	0	0	0	0	0
IFNAR1	1.72E-05	1.62E-05	2.36E-05	2.00E-05	1.39E-05	2.44E-05	2.23E-05	1.90E-05
IFNAR2	2.58E-05	1.28E-05	8.94E-06	1.56E-05	2.06E-05	1.03E-05	2.10E-05	1.00E-05
IFNB1	0	0	0	0	0	0	0	0
IFNE	0	0	0	0	0	0	0	0
IFNG	2.38E-06	2.44E-08	0	1.10E-06	0	3.16E-07	2.03E-08	3.15E-08
IFNGR1	0.000104	3.37E-05	3.63E-05	5.39E-05	8.31E-06	0.000324	4.79E-05	3.89E-05
IFNGR2	5.15E-05	1.89E-05	2.00E-05	4.95E-05	1.95E-05	8.19E-05	3.73E-05	2.87E-05
IFNK	0	0	0	0	0	0	0	0
IFNW1	0	0	0	0	0	0	0	0
IFRD1	7.82E-06	1.29E-05	5.53E-06	8.62E-06	4.00E-06	6.78E-06	1.29E-05	2.08E-05
IFRD2	2.55E-05	4.27E-05	2.90E-05	2.06E-05	2.90E-05	4.94E-05	5.17E-05	4.74E-05
IFT122	8.50E-06	5.69E-06	6.16E-06	8.48E-06	6.34E-06	5.41E-06	7.03E-06	2.91E-06
IFT140	2.53E-06	2.24E-06	9.41E-07	1.78E-06	2.85E-07	1.92E-06	1.68E-06	6.35E-07
IFT172	1.47E-05	1.04E-05	1.50E-06	2.31E-06	2.76E-06	2.57E-06	2.79E-06	2.32E-06
IFT20	3.48E-05	5.74E-05	2.29E-05	2.67E-05	4.50E-05	3.03E-05	3.20E-05	1.63E-05
IFT27	1.55E-05	3.12E-05	1.22E-05	1.41E-05	3.63E-05	2.49E-05	2.62E-05	3.16E-05
IFT46	9.38E-06	3.57E-06	7.92E-06	9.55E-06	4.92E-06	4.76E-06	5.13E-06	4.07E-06
IFT52	1.65E-05	6.66E-06	3.58E-06	7.66E-06	1.90E-06	7.24E-06	5.21E-06	5.19E-06
IFT57	7.41E-06	1.08E-06	1.77E-06	5.56E-06	1.86E-07	1.33E-06	2.67E-06	1.21E-06
IFT74	3.62E-06	5.25E-06	3.75E-06	4.50E-06	8.30E-07	6.48E-06	1.68E-06	4.56E-06
IFT80	1.78E-05	1.21E-06	1.05E-06	1.66E-06	5.18E-07	2.19E-06	5.44E-07	1.31E-06
IFT81	7.93E-06	4.14E-06	1.18E-06	2.66E-06	2.07E-07	2.13E-06	8.70E-07	1.90E-06
IFT88	4.66E-06	8.97E-06	4.74E-06	5.73E-06	1.94E-06	7.75E-06	4.30E-06	5.19E-06
IGBP1	3.77E-05	2.56E-05	1.70E-05	2.34E-05	1.94E-05	3.09E-05	2.06E-05	3.03E-05
IGDCC3	5.06E-09	1.70E-08	0	0	0	3.74E-08	4.42E-08	0
IGDCC4	6.59E-07	5.43E-08	4.15E-09	1.43E-07	1.74E-08	1.12E-08	1.97E-08	7.70E-08
IGF1	9.14E-07	1.28E-06	4.18E-05	4.27E-06	9.20E-07	2.32E-05	1.73E-06	1.77E-05
IGF1R	6.27E-07	1.55E-07	4.06E-07	5.77E-07	8.42E-08	1.18E-07	4.73E-07	4.96E-07
IGF2	1.81E-06	3.30E-06	0.000394	0.000267	0.002834	0.000186	0.000265	2.45E-06
IGF2AS	0	0	2.82E-07	1.13E-07	2.03E-06	8.74E-08	6.14E-08	0
IGF2BP1	1.12E-06	7.10E-07	5.42E-09	9.73E-09	7.32E-07	9.83E-09	4.94E-09	7.39E-08
IGF2BP2	1.17E-05	1.28E-06	5.78E-07	1.51E-06	1.18E-08	3.60E-08	1.77E-07	1.73E-06
IGF2BP3	2.97E-05	1.26E-05	2.10E-07	5.52E-08	9.05E-08	2.52E-07	1.68E-07	4.88E-07
IGF2R	3.34E-05	3.79E-05	1.98E-05	1.66E-05	2.62E-06	2.58E-05	1.20E-05	2.36E-05
IGFALS	1.13E-06	4.55E-06	0.000212	2.02E-05	1.22E-05	1.81E-07	3.32E-06	8.03E-07
IGFBP1	0.001189	0.00198	0.001078	0.0016	0.000115	0.002174	0.000308	0.002001

IGFBP2	0.000159	0.000955	0.000217	0.001265	0.000373	0.00021	0.001481	0.000272
IGFBP3	5.08E-05	0.00016	0.000315	0.000417	1.82E-05	1.82E-05	5.88E-05	8.46E-05
IGFBP4	0.000412	0.000419	0.000908	0.000757	0.001013	0.000636	0.000943	0.000669
IGFBP5	4.70E-05	8.00E-06	1.32E-05	1.18E-05	2.19E-06	5.06E-06	1.44E-05	1.27E-05
IGFBP6	5.35E-06	3.24E-07	2.48E-06	1.14E-05	2.42E-06	9.89E-07	8.02E-06	5.82E-07
IGFBP7	0.000285	0.000138	8.38E-05	0.000613	0.00015	0.000144	0.00036	0.000198
IGFBPL1	1.92E-06	0	5.21E-08	2.07E-07	0	0	0	0
IGFL1	0	6.20E-07	8.49E-08	0	0	0	0	0
IGFL2	2.57E-07	0	0	0	0	1.02E-07	4.78E-08	7.81E-08
IGFL3	0	0	0	0	3.99E-08	0	4.28E-08	0
IGFL4	0	0	0	0	0	0	0	0
IGFN1	1.16E-08	0	0	2.79E-07	0	0	5.03E-09	8.51E-09
IGHMBP2	6.97E-06	8.42E-06	2.92E-06	3.68E-06	2.26E-06	4.47E-06	3.34E-06	7.87E-06
IGJ	7.36E-05	1.22E-07	4.09E-05	5.84E-05	1.53E-07	5.94E-06	8.24E-06	2.31E-06
IGLL1	0	1.29E-07	0	0	0	2.86E-08	0	0
IGLL3	1.20E-07	0	0	0	1.04E-07	2.80E-08	2.84E-08	0
IGLON5	8.20E-08	5.86E-08	4.01E-07	1.08E-06	1.92E-08	2.76E-08	5.79E-08	6.56E-08
IGSF1	2.54E-07	3.68E-08	1.95E-07	8.15E-08	3.92E-08	6.53E-08	4.72E-08	2.13E-07
IGSF10	2.61E-07	1.94E-07	1.80E-07	6.31E-07	3.37E-08	2.51E-07	8.36E-08	2.48E-08
IGSF11	6.88E-09	1.54E-08	0	8.40E-09	0	0	1.34E-08	0
IGSF21	9.40E-07	3.50E-07	4.28E-07	1.67E-07	7.27E-07	1.77E-06	3.82E-06	9.10E-07
IGSF22	5.24E-07	0	1.19E-08	1.06E-07	0	1.13E-07	5.64E-09	8.47E-09
IGSF3	8.55E-06	7.83E-06	4.71E-07	1.45E-06	9.03E-08	1.60E-07	5.56E-07	2.88E-06
IGSF5	0	0	2.42E-08	8.51E-08	0	0	0	0
IGSF6	2.38E-05	2.18E-06	6.06E-06	1.36E-05	2.91E-07	4.01E-06	9.38E-06	1.35E-06
IGSF8	2.56E-05	7.36E-05	3.62E-05	4.85E-05	2.46E-05	0.000148	3.02E-05	6.76E-05
IGSF9	2.42E-07	3.36E-08	7.37E-06	2.86E-06	7.10E-07	4.66E-06	4.84E-07	2.81E-07
IGSF9B	3.64E-08	3.40E-09	3.11E-09	4.36E-08	0	8.46E-09	0	1.80E-08
IHH	5.24E-06	7.11E-07	2.25E-06	9.59E-07	2.87E-06	7.91E-06	3.78E-06	8.72E-08
IK	6.14E-05	5.28E-05	2.76E-05	3.89E-05	2.07E-05	5.62E-05	3.66E-05	5.08E-05
IKBIP	2.07E-05	7.46E-06	4.10E-06	8.42E-06	4.64E-06	5.54E-06	4.38E-06	5.71E-06
IKBKAP	1.15E-05	4.27E-06	5.73E-06	5.35E-06	3.65E-06	1.36E-05	4.03E-06	5.11E-06
IKBKB	1.01E-05	1.12E-05	5.40E-06	7.02E-06	2.68E-06	8.27E-06	4.25E-06	6.91E-06
IKBKE	4.41E-06	5.38E-07	6.21E-07	2.08E-06	1.39E-07	1.50E-06	5.05E-07	1.42E-06
IKBKG	1.53E-05	1.81E-05	1.25E-05	1.30E-05	1.85E-05	4.28E-05	1.53E-05	4.57E-05
IKZF1	3.51E-06	2.24E-07	1.57E-06	2.75E-06	1.39E-07	1.26E-06	1.13E-06	5.66E-07
IKZF2	7.83E-07	1.62E-07	3.43E-07	4.29E-07	6.46E-09	4.80E-07	1.32E-07	3.55E-07
IKZF3	1.02E-06	7.78E-08	4.98E-07	4.32E-07	0	4.64E-07	1.06E-07	1.62E-07
IKZF4	2.02E-06	6.00E-07	3.16E-07	6.43E-07	2.34E-07	6.55E-07	2.52E-07	6.19E-07
IKZF5	7.77E-06	4.67E-06	8.25E-06	5.94E-06	2.27E-06	1.12E-05	4.42E-06	8.88E-06
IL10	7.83E-07	5.14E-08	5.57E-07	1.15E-06	0	1.82E-07	3.48E-07	8.89E-08
IL10RA	1.36E-05	1.76E-06	7.38E-06	1.03E-05	1.18E-06	6.43E-06	7.30E-06	2.35E-06
IL10RB	4.06E-05	2.85E-05	1.84E-05	2.76E-05	2.17E-05	2.65E-05	2.77E-05	2.58E-05
IL11	3.05E-07	0	0	4.05E-07	2.81E-08	1.01E-08	4.85E-08	0
IL11RA	5.45E-06	1.42E-05	7.05E-06	7.60E-06	9.33E-06	4.89E-06	9.84E-06	7.99E-06
IL12A	6.78E-07	5.96E-08	7.41E-08	2.78E-07	1.05E-07	4.85E-08	5.01E-08	7.71E-08
IL12B	9.05E-08	0	1.05E-08	6.15E-08	0	5.59E-08	0	1.48E-08
IL12RB1	4.49E-06	2.01E-07	1.17E-06	1.43E-06	7.41E-08	1.43E-06	1.15E-06	5.00E-07
IL12RB2	6.66E-06	7.34E-08	1.89E-07	2.82E-07	1.25E-08	8.66E-07	8.46E-08	1.57E-07
IL13	0	0	8.51E-08	1.47E-07	0	1.85E-08	0	0
IL13RA1	7.24E-05	4.95E-05	6.36E-05	6.10E-05	5.55E-05	7.95E-05	6.04E-05	8.53E-05
IL13RA2	3.66E-08	2.31E-07	4.07E-06	8.17E-07	2.47E-07	1.53E-07	5.28E-08	2.71E-08
IL15	2.56E-06	7.80E-07	1.37E-06	1.98E-06	7.31E-08	1.09E-06	7.42E-07	1.67E-06
IL15RA	2.33E-05	4.78E-06	6.81E-06	1.81E-05	6.18E-06	8.85E-06	1.47E-05	2.21E-05
IL16	5.90E-06	1.59E-06	3.90E-06	3.94E-06	5.59E-07	2.66E-06	3.06E-06	1.92E-06
IL17A	2.60E-08	0	0	1.59E-08	0	0	1.25E-08	0
IL17B	8.58E-08	2.05E-07	0	0	1.50E-07	0	1.21E-07	0
IL17C	0	0	0	0	0	2.36E-08	0	0

IL17D	4.07E-06	1.49E-06	1.21E-07	9.04E-08	0	0	1.65E-07	8.24E-07
IL17F	0	0	0	0	0	0	0	0
IL17RA	6.68E-06	6.29E-06	2.33E-06	3.51E-06	1.15E-06	3.16E-06	3.52E-06	6.84E-06
IL17RB	0.000223	5.42E-05	1.46E-05	1.42E-05	2.21E-05	2.15E-05	6.57E-06	1.39E-05
IL17RC	2.13E-05	3.32E-05	5.37E-05	1.29E-05	1.33E-05	2.21E-05	2.18E-05	1.92E-05
IL17RD	5.22E-07	1.15E-08	5.78E-08	1.17E-07	2.65E-08	4.76E-09	1.03E-07	6.76E-08
IL17RE	5.25E-06	5.02E-06	2.47E-06	1.31E-06	1.15E-05	1.93E-06	3.09E-06	6.44E-07
IL17REL	6.30E-09	7.06E-09	0	1.54E-08	0	0	0	0
IL18	2.93E-05	1.22E-06	3.34E-06	1.97E-05	1.29E-06	4.09E-06	6.38E-06	1.11E-06
IL18BP	3.50E-05	2.05E-06	4.95E-06	1.38E-05	3.15E-06	1.55E-05	8.35E-06	6.33E-06
IL18R1	6.06E-06	3.93E-07	3.12E-06	3.49E-06	4.45E-07	6.69E-07	7.41E-07	1.37E-06
IL18RAP	1.42E-06	1.10E-07	4.00E-07	7.75E-07	5.59E-08	1.16E-07	2.93E-07	5.60E-07
IL19	0	0	0	0	0	0	0	3.84E-08
IL1A	4.76E-08	1.78E-08	0	6.78E-08	0	0	0	0
IL1B	2.37E-06	2.09E-07	2.51E-06	5.33E-06	2.05E-07	3.58E-07	1.00E-06	2.98E-07
IL1F10	0	0	0	0	0	0	0	0
IL1F5	0	0	9.10E-08	0	0	0	0	0
IL1F6	0	0	0	0	0	0	0	0
IL1F7	0	0	0	0	0	5.14E-08	0	0
IL1F8	0	0	0	0	0	0	5.62E-08	0
IL1F9	0	2.26E-07	5.32E-08	0	0	2.28E-08	0	0
IL1R1	4.66E-05	1.81E-05	2.50E-05	4.23E-05	7.63E-06	4.93E-05	2.10E-05	2.08E-05
IL1R2	2.58E-05	1.01E-05	4.01E-06	3.02E-06	2.32E-06	1.17E-05	8.52E-06	3.75E-06
IL1RAP	5.60E-05	7.41E-06	6.44E-05	1.31E-05	1.23E-05	5.28E-06	1.30E-05	1.30E-05
IL1RAPL1	1.74E-08	1.96E-08	0	1.07E-08	0	2.02E-07	0	0
IL1RAPL2	0	0	4.10E-07	1.04E-07	0	2.59E-07	6.02E-08	4.55E-08
IL1RL1	4.68E-08	1.37E-07	3.19E-07	4.47E-06	1.49E-07	3.47E-08	8.03E-07	2.06E-07
IL1RL2	5.80E-06	1.18E-06	1.65E-06	8.79E-07	1.72E-07	7.31E-07	8.31E-07	9.00E-08
IL1RN	1.57E-05	3.36E-05	0.000224	5.73E-05	1.61E-05	2.85E-05	4.71E-05	0.000111
IL2	0	0	4.03E-08	1.33E-07	0	0	3.38E-08	0
IL20	0	0	0	1.95E-08	0	0	0	0
IL20RA	1.94E-06	0	3.78E-07	4.08E-08	7.17E-09	0	3.82E-08	1.35E-07
IL20RB	3.59E-07	2.32E-06	1.50E-06	2.32E-07	2.09E-07	3.27E-07	1.14E-07	4.81E-07
IL21	0	0	0	0	0	4.77E-08	0	0
IL21R	3.17E-06	3.18E-08	3.15E-07	1.27E-06	2.72E-08	4.62E-07	2.92E-07	1.27E-07
IL22	0	0	0	2.79E-08	0	0	0	0
IL22RA1	6.29E-05	1.87E-06	5.11E-06	4.32E-06	1.96E-06	6.80E-06	9.19E-07	8.55E-06
IL22RA2	3.99E-07	0	0	0	0	7.70E-09	0	0
IL23A	3.56E-07	4.12E-07	3.05E-07	2.20E-06	1.76E-07	2.61E-07	3.88E-07	1.89E-07
IL23R	4.10E-08	0	1.07E-08	0	0	3.14E-08	1.28E-08	3.54E-08
IL24	4.19E-08	0	0	1.20E-07	0	3.97E-08	6.16E-08	0
IL25	2.23E-08	0	0	0	0	0	0	3.31E-08
IL26	2.52E-08	0	0	0	2.19E-08	0	2.41E-08	0
IL27	2.54E-07	1.59E-05	9.86E-06	9.97E-06	4.94E-06	1.01E-05	6.42E-06	5.25E-06
IL27RA	5.01E-06	2.11E-06	1.34E-06	2.72E-06	7.41E-07	1.01E-06	2.55E-06	1.21E-06
IL28A	0	0	0	0	0	0	0	0
IL28B	0	0	0	0	0	0	0	0
IL28RA	5.21E-06	1.37E-06	2.20E-06	1.27E-06	4.97E-07	3.67E-06	1.15E-06	5.03E-06
IL29	0	0	0	0	0	0	6.16E-08	0
IL2RA	3.28E-06	3.87E-08	7.16E-08	2.76E-07	6.17E-08	1.19E-06	1.13E-06	3.36E-07
IL2RB	8.79E-06	7.19E-07	3.76E-06	4.72E-06	5.80E-08	2.90E-06	2.74E-06	1.50E-06
IL2RG	5.80E-05	3.83E-06	8.54E-06	2.62E-05	9.33E-07	1.32E-05	1.18E-05	9.53E-06
IL3	0	1.72E-07	6.51E-08	0	0	0	0	0
IL31	0	0	0	0	0	0	0	0
IL31RA	3.28E-07	1.16E-08	0	1.14E-08	0	1.72E-08	0	0
IL32	0.001696	0.001204	0.000271	0.001055	0.000287	0.000387	0.001831	0.000675
IL33	4.68E-06	2.36E-06	1.11E-05	7.23E-06	1.31E-06	2.14E-06	2.17E-06	3.75E-06
IL34	9.64E-07	8.36E-07	1.33E-06	4.77E-06	1.33E-06	1.19E-06	2.42E-06	2.96E-06

IL3RA	5.72E-06	4.80E-06	7.66E-07	1.72E-06	7.34E-06	3.50E-06	6.07E-06	6.30E-06
IL4	0	0	0	0	0	0	0	0
IL4I1	3.36E-06	1.86E-07	2.91E-07	1.73E-06	2.76E-07	4.04E-07	1.43E-06	2.89E-07
IL4R	3.56E-05	5.52E-05	1.69E-05	3.77E-05	6.20E-06	7.56E-06	2.42E-05	3.89E-05
IL5	0	4.06E-08	0	0	0	0	0	0
IL5RA	1.48E-08	0	1.77E-08	7.28E-08	0	0	0	0
IL6	3.64E-07	1.22E-06	1.88E-07	3.93E-05	5.66E-08	8.13E-08	2.48E-06	5.78E-07
IL6R	1.90E-05	2.13E-05	8.87E-06	7.18E-06	1.04E-06	2.08E-05	6.12E-06	1.86E-05
IL6ST	5.02E-05	4.61E-05	5.01E-05	2.68E-05	1.91E-08	2.49E-05	3.09E-06	3.54E-05
IL7	4.37E-06	2.03E-06	1.91E-06	1.77E-06	6.69E-07	2.14E-06	1.69E-06	2.37E-06
IL7R	3.59E-06	1.12E-07	1.26E-06	4.85E-06	0	1.37E-06	4.80E-07	8.58E-07
IL8	0.000128	8.68E-07	4.34E-06	0.000234	2.94E-07	5.88E-07	1.57E-05	7.74E-05
IL9	0	0	0	0	0	0	0	0
IL9R	2.94E-07	1.25E-08	0	3.32E-08	1.90E-08	2.05E-08	1.07E-08	2.37E-08
ILDR1	2.53E-06	7.99E-08	1.73E-07	8.00E-07	0	2.58E-08	2.70E-08	2.73E-08
ILDR2	9.29E-07	1.74E-07	1.80E-07	1.11E-08	0	8.44E-09	5.29E-08	6.28E-07
ILF2	9.61E-05	9.49E-05	3.37E-05	6.45E-05	2.36E-05	0.000113	6.75E-05	9.08E-05
ILF3	5.35E-05	2.31E-05	1.40E-05	1.93E-05	2.01E-05	2.82E-05	2.46E-05	2.22E-05
ILK	0.00011	7.78E-05	4.88E-05	0.000113	7.25E-05	5.80E-05	0.000106	8.15E-05
ILKAP	2.23E-05	1.27E-05	1.10E-05	1.35E-05	1.47E-05	1.20E-05	1.72E-05	1.30E-05
ILVBL	6.38E-05	2.95E-05	5.58E-05	5.50E-05	0.000103	6.37E-05	0.000102	5.20E-05
IMMP1L	6.58E-06	7.73E-06	3.32E-06	4.46E-06	2.12E-06	4.31E-06	6.35E-06	5.94E-06
IMMP2L	5.31E-06	1.30E-05	6.71E-06	7.80E-06	2.98E-06	1.26E-05	2.02E-05	2.78E-05
IMMT	9.40E-05	9.63E-05	3.42E-05	3.68E-05	2.97E-05	5.90E-05	3.48E-05	4.36E-05
IMP3	7.84E-05	4.67E-05	5.39E-05	4.95E-05	6.34E-05	5.66E-05	7.38E-05	4.54E-05
IMP4	6.50E-05	5.58E-05	4.31E-05	3.77E-05	4.09E-05	5.94E-05	7.57E-05	5.11E-05
IMP5	0	0	0	0	0	0	0	0
IMPA1	2.28E-05	1.35E-05	8.76E-06	8.07E-06	4.86E-06	9.64E-06	1.54E-05	1.06E-05
IMPA2	3.35E-05	2.26E-05	2.30E-05	2.71E-05	8.85E-06	8.92E-05	3.78E-05	1.95E-05
IMPACT	8.83E-06	8.10E-06	8.91E-06	5.55E-06	1.66E-06	1.30E-05	5.80E-06	6.73E-06
IMPAD1	2.72E-05	1.46E-05	1.63E-05	1.61E-05	8.77E-06	2.39E-05	3.06E-05	2.69E-05
IMPDH1	1.32E-05	1.99E-06	2.61E-06	6.00E-06	1.08E-06	4.04E-06	6.05E-06	2.18E-06
IMPDH2	8.34E-05	5.35E-05	2.93E-05	4.49E-05	8.08E-05	5.54E-05	7.14E-05	3.71E-05
IMPG1	8.46E-08	7.91E-09	0	0	0	0	6.84E-09	1.03E-08
IMPG2	6.89E-08	4.55E-08	5.22E-09	1.86E-08	1.31E-08	1.02E-07	0	1.49E-08
INA	2.14E-08	4.81E-08	1.08E-07	2.62E-08	1.84E-08	3.84E-08	0	0
INADL	7.76E-06	6.89E-06	5.63E-06	5.48E-06	1.02E-06	6.74E-06	2.52E-06	2.21E-06
INCA1	4.33E-07	1.23E-06	6.21E-07	5.84E-07	1.20E-06	1.09E-06	1.04E-06	3.35E-07
INCENP	5.81E-06	4.01E-06	1.35E-06	1.05E-06	2.60E-07	1.95E-06	8.53E-07	2.01E-06
INE1	9.54E-07	3.08E-07	8.71E-08	1.31E-07	1.38E-07	4.70E-07	2.02E-07	6.32E-07
INE2	1.42E-07	0	9.84E-08	3.66E-07	1.45E-08	2.35E-06	2.76E-07	3.85E-07
INF2	4.99E-05	1.36E-05	3.04E-05	3.75E-05	1.57E-05	1.53E-05	2.66E-05	2.03E-05
ING1	5.98E-06	1.32E-05	9.55E-06	5.63E-06	3.94E-06	9.65E-06	5.58E-06	1.12E-05
ING2	1.11E-05	4.04E-06	5.60E-06	9.10E-06	4.15E-06	7.54E-06	6.11E-06	1.08E-05
ING3	4.70E-06	3.78E-06	3.80E-06	4.40E-06	9.39E-07	3.24E-06	2.04E-06	3.65E-06
ING4	2.20E-05	1.39E-05	1.26E-05	1.73E-05	1.49E-05	2.11E-05	1.45E-05	1.21E-05
ING5	6.49E-06	1.75E-06	1.41E-06	2.38E-06	2.25E-06	2.74E-06	4.47E-06	1.82E-06
INGX	2.09E-08	0	0	0	0	1.17E-07	2.00E-08	3.10E-08
INHA	3.86E-07	1.61E-07	1.87E-08	1.51E-07	1.36E-07	1.63E-07	0	1.04E-07
INHBA	1.78E-06	6.32E-07	3.75E-06	6.31E-06	1.04E-07	1.74E-06	1.17E-06	3.47E-06
INHBB	2.18E-05	1.73E-05	1.10E-05	2.82E-05	6.10E-06	6.53E-06	3.99E-05	3.01E-05
INHBC	1.33E-05	3.37E-05	9.79E-05	4.06E-05	1.43E-06	4.33E-05	1.33E-05	3.17E-05
INHBE	2.95E-05	8.71E-06	6.63E-05	4.58E-05	2.58E-05	7.87E-06	9.82E-05	6.86E-05
INMT	2.20E-06	1.21E-06	1.54E-05	3.87E-05	6.03E-06	1.09E-06	5.46E-06	2.24E-06
INO80	7.67E-06	3.12E-06	3.80E-06	4.62E-06	1.41E-06	5.51E-06	3.14E-06	4.59E-06
INO80B	1.38E-05	2.90E-05	1.17E-05	1.47E-05	1.74E-05	1.90E-05	1.76E-05	7.73E-06
INO80C	3.51E-05	1.38E-05	8.32E-06	1.52E-05	6.72E-06	1.39E-05	2.06E-05	6.80E-06
INO80D	2.52E-06	9.39E-07	7.24E-07	9.09E-07	3.03E-07	8.80E-07	4.96E-07	1.02E-06

INO80E	3.23E-05	3.08E-05	1.41E-05	2.49E-05	1.83E-05	2.58E-05	2.95E-05	2.48E-05
INPP1	2.92E-05	1.34E-05	8.51E-06	1.77E-05	7.41E-06	5.52E-06	1.63E-05	1.61E-05
INPP4A	4.69E-06	2.19E-06	1.38E-06	2.22E-06	5.14E-07	1.92E-06	1.59E-06	1.80E-06
INPP4B	7.11E-07	2.53E-07	2.06E-07	6.41E-07	5.16E-08	6.01E-07	6.07E-07	1.71E-07
INPP5A	1.33E-05	3.71E-06	6.82E-06	7.99E-06	1.14E-05	6.82E-06	8.46E-06	7.81E-06
INPP5B	4.33E-06	2.11E-06	4.73E-06	4.34E-06	2.03E-06	4.01E-06	3.96E-06	3.24E-06
INPP5D	6.24E-06	2.37E-06	2.50E-06	3.99E-06	8.39E-07	2.41E-06	3.23E-06	1.55E-06
INPP5E	4.40E-06	1.63E-06	2.11E-06	2.65E-06	1.04E-06	2.39E-06	3.11E-06	3.48E-06
INPP5F	9.11E-06	9.85E-07	8.79E-07	2.13E-06	9.42E-07	1.35E-06	3.19E-06	1.85E-06
INPP5J	2.58E-06	5.99E-08	5.57E-08	2.93E-07	1.33E-08	7.12E-08	9.39E-08	1.95E-07
INPP5K	1.01E-05	6.96E-06	7.64E-06	9.08E-06	1.23E-05	1.12E-05	9.39E-06	8.06E-06
INPPL1	2.21E-05	1.42E-05	7.80E-06	1.02E-05	4.24E-06	1.11E-05	1.09E-05	1.79E-05
INS	0	0	0	0	0	0	0	0
INSC	2.98E-06	3.97E-07	8.94E-09	2.11E-08	1.18E-07	5.32E-08	0	3.87E-07
INSIG1	4.51E-05	0.001526	0.000301	5.93E-05	8.32E-05	0.000317	7.78E-05	0.001337
INSIG2	2.63E-05	1.66E-05	1.98E-05	2.29E-05	8.20E-06	4.01E-05	1.19E-05	7.67E-06
INS-IGF2	0	2.55E-07	6.01E-05	2.32E-05	3.39E-06	3.59E-05	6.09E-06	5.36E-07
INSL3	2.35E-07	0	0	9.75E-08	0	3.67E-08	2.49E-07	5.66E-08
INSL4	5.59E-07	3.70E-07	0	6.38E-08	0	0	0	0
INSL5	0	0	0	0	0	0	0	0
INSL6	0	0	4.81E-08	1.04E-07	0	0	0	0
INSM1	2.47E-08	0	0	1.01E-08	0	0	0	0
INSM2	4.61E-08	4.32E-08	0	0	6.60E-09	0	0	1.13E-08
INSR	2.75E-05	2.43E-05	1.45E-05	1.81E-05	1.53E-05	1.27E-05	3.25E-05	2.98E-05
INSRR	5.49E-09	2.74E-08	0	0	0	2.42E-08	0	0
INTS1	4.32E-05	7.39E-05	2.55E-05	2.74E-05	7.59E-05	3.25E-05	4.41E-05	2.17E-05
INTS10	1.62E-05	3.45E-05	2.36E-05	2.08E-05	5.77E-06	2.60E-05	9.39E-06	9.27E-06
INTS12	1.36E-05	7.92E-06	9.87E-06	9.95E-06	3.98E-06	2.56E-05	1.18E-05	1.50E-05
INTS2	3.80E-06	2.62E-06	1.48E-06	8.19E-07	4.51E-07	3.52E-06	7.25E-07	1.00E-06
INTS3	3.16E-05	2.94E-05	1.13E-05	1.51E-05	1.06E-05	4.07E-05	1.59E-05	2.02E-05
INTS4	7.18E-06	5.26E-06	3.43E-06	3.11E-06	1.64E-06	6.04E-06	2.71E-06	6.26E-06
INTS4L1	2.20E-07	9.31E-08	1.15E-07	5.62E-08	2.37E-08	2.72E-07	7.12E-08	2.70E-07
INTS4L2	5.38E-07	3.37E-07	2.24E-07	3.99E-07	0	1.84E-07	2.32E-07	5.42E-07
INTS5	8.46E-06	9.68E-06	8.32E-06	8.90E-06	4.76E-06	9.74E-06	6.89E-06	1.57E-05
INTS6	8.55E-06	5.95E-06	3.43E-06	5.71E-06	1.80E-06	4.17E-06	5.19E-06	5.29E-06
INTS7	1.15E-05	1.48E-05	5.24E-06	5.14E-06	3.05E-06	8.73E-06	4.65E-06	1.15E-05
INTS8	1.99E-05	8.73E-06	3.60E-06	4.12E-06	2.89E-06	5.27E-06	6.37E-06	4.90E-06
INTS9	4.32E-06	4.68E-06	2.26E-06	3.04E-06	9.18E-07	4.34E-06	2.24E-06	2.62E-06
INTU	1.58E-06	2.40E-07	7.35E-08	2.26E-07	1.22E-08	5.47E-07	2.07E-08	3.76E-07
INVS	5.34E-06	2.83E-06	3.99E-06	2.40E-06	1.37E-06	3.07E-06	1.78E-06	1.66E-06
IP6K1	2.20E-05	1.01E-05	4.00E-06	5.49E-06	6.73E-06	9.34E-06	1.10E-05	1.11E-05
IP6K2	0.000155	2.39E-05	1.54E-05	2.52E-05	1.88E-05	3.43E-05	2.58E-05	1.71E-05
IP6K3	2.44E-07	3.80E-07	3.98E-06	1.10E-06	1.49E-08	7.63E-09	1.69E-06	7.25E-07
IPCEF1	7.43E-07	4.85E-08	3.79E-07	4.28E-07	3.44E-08	3.31E-07	1.57E-07	1.07E-07
IPMK	4.21E-06	1.33E-06	1.12E-06	5.14E-07	1.16E-08	1.02E-06	1.17E-07	8.97E-07
IPO11	7.15E-06	6.19E-06	4.30E-06	6.75E-06	3.72E-06	8.91E-06	6.54E-06	7.28E-06
IPO13	1.28E-05	1.35E-05	5.80E-06	6.84E-06	1.15E-05	1.34E-05	9.64E-06	1.38E-05
IPO4	1.24E-05	1.33E-05	9.19E-06	1.11E-05	6.88E-06	2.37E-05	1.42E-05	5.78E-06
IPO5	2.31E-05	2.04E-05	1.48E-05	1.64E-05	8.38E-06	1.76E-05	1.47E-05	1.23E-05
IPO7	2.37E-05	2.48E-05	1.60E-05	1.87E-05	3.21E-06	2.01E-05	9.36E-06	2.07E-05
IPO8	1.23E-05	1.69E-05	1.21E-05	8.78E-06	5.09E-06	1.30E-05	7.45E-06	1.01E-05
IPO9	7.76E-06	5.52E-06	3.68E-06	3.17E-06	1.52E-06	7.74E-06	2.88E-06	6.79E-06
IPP	3.65E-06	3.24E-06	2.28E-06	1.73E-06	1.95E-07	3.40E-06	8.40E-07	2.52E-06
IPPK	3.20E-06	2.17E-06	1.43E-06	2.80E-06	4.83E-07	2.34E-06	1.42E-06	2.15E-06
IPW	1.77E-05	1.69E-06	7.22E-07	1.49E-06	4.26E-08	8.30E-07	6.20E-07	3.42E-07
IQCA1	3.65E-06	1.02E-07	7.02E-08	1.01E-07	6.48E-09	5.59E-08	2.56E-07	4.43E-08
IQCB1	5.03E-06	5.99E-06	2.25E-06	3.04E-06	9.71E-07	3.92E-06	3.27E-06	1.89E-06
IQCC	1.67E-06	7.93E-07	2.77E-07	3.76E-07	5.31E-07	9.63E-07	4.61E-07	1.14E-06

IQCD	9.36E-07	3.36E-07	3.94E-08	2.88E-07	5.39E-08	7.48E-07	1.01E-07	1.53E-06
IQCE	4.67E-06	4.98E-06	8.76E-07	1.91E-06	1.21E-06	1.86E-06	2.36E-06	3.30E-06
IQCF1	6.74E-09	1.51E-08	0	0	0	0	0	0
IQCF2	0	0	0	0	0	0	0	0
IQCF3	0	0	0	0	0	0	0	0
IQCF5	0	0	0	0	0	0	0	0
IQCF6	0	1.15E-07	0	0	0	0	0	0
IQCG	2.42E-06	1.46E-05	9.68E-07	1.79E-06	8.64E-07	5.36E-06	1.99E-06	9.76E-07
IQCH	1.65E-07	5.00E-07	2.12E-07	1.54E-07	1.37E-07	6.07E-06	2.69E-07	1.40E-07
IQCJ	0	0	0	0	0	0	0	0
IQCK	2.08E-06	3.29E-06	1.09E-06	3.91E-06	7.80E-07	3.50E-06	2.65E-06	1.69E-06
IQGAP1	3.05E-05	9.29E-06	8.66E-06	1.87E-05	2.19E-06	8.55E-06	1.11E-05	7.81E-06
IQGAP2	2.64E-05	3.47E-05	7.21E-05	4.59E-05	9.79E-06	6.80E-05	2.33E-05	3.83E-05
IQGAP3	5.27E-06	2.16E-06	3.41E-08	1.08E-07	1.43E-07	7.64E-07	1.27E-06	2.33E-06
IQSEC1	2.57E-05	2.11E-05	1.09E-05	9.50E-06	2.05E-05	1.28E-05	2.24E-05	2.03E-05
IQSEC2	5.70E-06	6.33E-07	8.79E-07	1.61E-06	4.10E-07	6.89E-07	1.55E-06	6.09E-07
IQSEC3	1.56E-07	1.39E-07	1.75E-07	8.68E-08	0	2.19E-07	1.66E-07	2.08E-08
IQUB	1.13E-08	2.35E-08	4.68E-08	2.90E-08	0	2.29E-08	1.09E-08	3.06E-08
IRAK1	5.80E-05	6.06E-05	1.40E-05	2.36E-05	5.28E-05	3.13E-05	4.90E-05	9.10E-05
IRAK1BP1	4.74E-07	6.16E-07	3.75E-07	8.05E-07	5.81E-08	8.35E-07	3.16E-07	2.00E-07
IRAK2	9.86E-06	1.85E-05	2.57E-06	9.72E-06	1.49E-06	3.56E-06	7.59E-06	6.29E-06
IRAK3	2.51E-06	2.34E-07	6.00E-07	1.06E-06	6.54E-08	2.69E-07	3.34E-07	3.38E-07
IRAK4	5.32E-06	4.40E-06	2.34E-06	2.71E-06	9.00E-07	3.98E-06	2.22E-06	3.02E-06
IREB2	1.19E-05	1.01E-05	8.86E-06	6.44E-06	2.64E-06	1.24E-05	4.79E-06	5.60E-06
IRF1	7.49E-05	1.15E-05	1.88E-05	6.86E-05	2.82E-06	2.03E-05	1.91E-05	3.85E-05
IRF2	2.86E-05	1.71E-05	2.18E-05	1.37E-05	5.45E-06	2.69E-05	1.50E-05	1.55E-05
IRF2BP1	1.43E-05	2.16E-05	1.17E-05	1.14E-05	1.98E-05	1.76E-05	1.86E-05	1.05E-05
IRF2BP2	8.76E-05	4.65E-05	3.81E-05	4.09E-05	1.66E-05	6.71E-05	3.76E-05	9.13E-05
IRF3	4.96E-05	3.88E-05	1.76E-05	3.19E-05	4.84E-05	3.05E-05	2.70E-05	3.27E-05
IRF4	1.16E-06	1.42E-08	1.20E-06	8.20E-07	0	2.93E-07	7.01E-08	3.10E-08
IRF5	5.19E-06	6.16E-06	4.85E-06	4.52E-06	1.96E-06	1.85E-06	5.43E-06	4.25E-06
IRF6	2.53E-05	1.96E-05	1.35E-05	1.59E-05	2.09E-06	1.44E-05	1.64E-05	2.07E-05
IRF7	3.16E-05	2.62E-05	1.38E-05	2.39E-05	1.93E-05	1.79E-05	2.30E-05	4.62E-05
IRF8	1.29E-05	6.94E-06	1.66E-05	2.31E-05	2.08E-06	7.04E-06	1.09E-05	1.29E-05
IRF9	6.94E-05	1.74E-05	3.30E-05	3.75E-05	1.78E-05	5.11E-05	3.25E-05	7.15E-05
IRGC	0	0	0	1.88E-08	0	0	0	0
IRGM	7.43E-08	5.04E-08	0	0	0	0	1.42E-08	0
IRGQ	3.04E-06	1.46E-06	9.42E-07	1.66E-06	2.93E-06	1.97E-06	2.53E-06	2.64E-06
IRS1	1.45E-05	1.89E-05	8.19E-06	5.48E-06	5.65E-06	2.79E-05	5.37E-06	6.77E-06
IRS2	1.58E-05	3.41E-05	1.90E-05	1.04E-05	3.80E-06	4.43E-05	8.06E-06	2.97E-05
IRS4	0	0	0	0	0	0	0	0
IRX1	0	0	0	0	0	0	0	0
IRX2	0	1.16E-08	0	0	0	0	3.67E-08	0
IRX3	4.10E-07	2.34E-06	6.40E-08	1.00E-07	2.20E-07	6.23E-06	5.01E-06	1.05E-07
IRX4	0	0	0	0	0	0	3.10E-08	0
IRX5	1.16E-07	6.56E-08	1.21E-08	1.42E-08	1.99E-08	3.14E-08	3.89E-07	6.27E-08
IRX6	0	7.18E-08	0	0	4.36E-08	5.67E-08	0	1.50E-08
ISCA1	3.19E-05	2.28E-05	1.61E-05	2.20E-05	8.28E-06	3.31E-05	1.54E-05	1.81E-05
ISCA1P1	3.20E-06	2.17E-06	1.47E-06	2.40E-06	1.17E-06	3.51E-06	1.53E-06	2.16E-06
ISCA2	2.71E-05	1.84E-05	1.16E-05	1.26E-05	1.54E-05	1.89E-05	1.98E-05	1.82E-05
ISCU	0.000117	6.85E-05	6.63E-05	8.96E-05	5.64E-05	0.00012	5.99E-05	7.38E-05
ISG15	0.000381	8.63E-05	8.69E-05	7.26E-05	4.74E-05	9.50E-05	4.56E-05	0.000416
ISG20	7.16E-05	9.54E-06	9.30E-06	2.81E-05	9.17E-06	1.64E-05	1.87E-05	3.03E-05
ISG20L2	1.46E-05	2.02E-05	7.49E-06	1.03E-05	2.22E-06	1.60E-05	8.92E-06	1.30E-05
ISL1	0	0	0	0	0	0	0	0
ISL2	2.55E-06	8.56E-07	1.42E-08	1.65E-08	3.48E-08	0	7.80E-08	3.97E-08
ISLR	1.11E-05	3.70E-07	4.11E-06	1.62E-05	1.46E-06	3.27E-07	9.59E-06	5.49E-06
ISLR2	1.98E-07	1.37E-07	9.31E-08	1.63E-07	3.66E-08	1.09E-07	2.82E-07	1.33E-07

ISM1	1.24E-06	5.08E-07	7.97E-06	1.17E-06	1.08E-06	6.94E-07	1.66E-06	7.94E-06
ISM2	5.31E-08	0	0	0	0	0	8.57E-09	0
ISOC1	3.51E-05	5.84E-05	5.09E-05	3.63E-05	1.15E-05	3.00E-05	3.44E-05	3.67E-05
ISOC2	9.01E-05	0.000253	0.000131	0.000112	0.000434	0.000239	0.000165	8.85E-05
ISPD	1.79E-06	2.10E-06	6.13E-07	5.71E-07	1.44E-07	4.67E-07	3.75E-07	5.06E-07
ISX	0	1.02E-05	0	0	0	3.04E-07	1.27E-07	0
ISY1	1.70E-05	1.23E-05	7.41E-06	1.16E-05	8.49E-06	1.33E-05	1.48E-05	9.18E-06
ISYNA1	2.98E-05	6.19E-06	4.74E-06	1.34E-05	5.62E-06	2.26E-06	5.25E-06	6.28E-06
ITCH	1.88E-05	2.11E-05	1.58E-05	9.62E-06	3.45E-06	1.38E-05	6.48E-06	7.15E-06
ITFG1	3.93E-05	4.12E-05	2.45E-05	2.75E-05	1.07E-05	3.56E-05	1.77E-05	3.22E-05
ITFG2	9.76E-06	3.79E-06	2.57E-06	4.87E-06	1.34E-06	3.74E-06	3.19E-06	9.32E-06
ITFG3	6.37E-05	5.65E-05	1.73E-05	2.88E-05	2.75E-05	2.65E-05	3.26E-05	3.88E-05
ITGA1	2.21E-05	1.82E-05	2.61E-05	2.16E-05	2.97E-06	1.44E-05	7.59E-06	2.68E-05
ITGA10	3.67E-07	1.27E-07	2.45E-08	1.33E-07	1.27E-07	7.38E-08	6.79E-08	1.70E-07
ITGA11	2.54E-06	3.83E-07	1.43E-07	8.42E-07	2.75E-07	3.50E-07	1.31E-06	9.90E-07
ITGA2	2.15E-06	1.44E-07	2.03E-07	1.11E-06	6.17E-08	2.71E-07	3.62E-07	6.59E-07
ITGA2B	3.35E-07	4.42E-07	0	1.69E-08	3.58E-08	6.39E-09	6.70E-09	0
ITGA3	1.15E-05	1.19E-06	6.42E-07	9.46E-06	7.94E-07	7.35E-07	2.24E-06	1.81E-06
ITGA4	9.14E-06	9.41E-07	2.03E-06	3.22E-06	2.11E-07	2.52E-06	1.00E-06	6.51E-07
ITGA5	4.43E-05	3.15E-05	1.47E-05	2.74E-05	1.60E-05	9.12E-06	2.54E-05	3.63E-05
ITGA6	2.60E-05	1.34E-05	5.98E-06	9.48E-06	3.77E-06	1.70E-05	1.16E-05	1.60E-05
ITGA7	8.73E-06	9.79E-06	5.83E-06	3.82E-06	1.06E-05	9.22E-06	5.48E-06	7.62E-06
ITGA8	3.80E-07	8.05E-08	2.96E-08	1.12E-07	0	1.26E-07	0	2.10E-08
ITGA9	2.50E-06	7.28E-07	3.20E-06	6.30E-06	1.14E-07	4.47E-07	9.34E-07	5.31E-07
ITGAD	3.50E-08	6.53E-09	1.32E-06	4.06E-07	4.00E-08	1.87E-07	2.89E-07	3.41E-08
ITGAE	1.51E-05	3.69E-06	6.83E-06	8.42E-06	1.98E-05	1.19E-05	1.11E-05	6.86E-06
ITGAL	1.33E-05	3.83E-05	8.50E-06	1.19E-05	1.22E-06	6.47E-06	9.08E-06	3.57E-06
ITGAM	1.85E-06	2.95E-06	1.62E-06	4.67E-06	3.60E-07	3.53E-06	3.12E-06	1.78E-06
ITGAV	5.37E-05	2.27E-05	7.69E-06	1.74E-05	1.04E-06	8.88E-06	6.63E-06	9.13E-06
ITGAX	8.36E-06	1.79E-06	1.48E-06	6.54E-06	4.02E-07	8.79E-07	2.87E-06	1.24E-06
ITGB1	0.000259	0.000232	8.85E-05	0.000165	3.19E-05	0.000119	0.000108	0.00013
ITGB1BP1	4.00E-05	2.55E-05	4.06E-06	1.17E-05	7.95E-06	1.50E-05	1.51E-05	8.76E-06
ITGB1BP2	3.52E-07	9.52E-08	1.34E-07	7.66E-08	2.51E-07	4.82E-07	5.96E-08	3.07E-07
ITGB1BP3	0	0	2.70E-08	0	4.29E-08	0	0	0
ITGB2	4.74E-05	7.67E-06	2.11E-05	4.94E-05	3.23E-06	1.65E-05	2.72E-05	6.03E-06
ITGB3	9.88E-07	9.77E-07	7.65E-07	9.94E-07	6.60E-08	1.61E-06	1.08E-07	2.02E-06
ITGB3BP	9.73E-06	6.37E-06	1.54E-06	6.51E-06	2.73E-06	4.78E-06	6.98E-06	7.62E-06
ITGB4	2.43E-05	1.02E-06	9.42E-07	1.22E-06	1.10E-06	1.48E-06	5.97E-07	1.43E-06
ITGB5	5.31E-05	4.95E-05	4.64E-05	9.99E-05	4.66E-05	3.96E-05	6.83E-05	0.000112
ITGB6	4.87E-07	1.15E-07	1.49E-07	1.14E-06	0	2.13E-08	2.40E-07	3.14E-08
ITGB7	5.25E-06	4.13E-06	1.79E-06	3.03E-06	1.14E-06	5.54E-06	4.01E-06	6.09E-07
ITGB8	4.05E-06	4.39E-07	2.53E-07	9.91E-07	0	4.67E-09	3.95E-08	3.32E-08
ITGBL1	4.84E-06	4.35E-07	1.79E-06	1.64E-05	4.89E-07	4.49E-07	1.20E-05	2.99E-06
ITIH1	0.000802	0.001953	0.001992	0.001345	0.002085	0.001212	0.001755	0.000772
ITIH2	0.001117	0.000109	0.001099	0.000989	0.000355	0.000927	0.000502	0.000853
ITIH3	0.000227	0.000575	0.000657	0.001104	0.00122	0.000738	0.000865	0.001263
ITIH4	0.000881	0.001515	0.00194	0.001888	0.001649	0.001589	0.001317	0.001532
ITIH5	5.28E-05	3.91E-06	7.75E-07	1.35E-06	1.70E-07	2.70E-07	8.17E-07	2.89E-07
ITIH5L	0	1.52E-08	0	0	0	0	0	0
ITK	1.97E-06	7.56E-08	4.22E-07	2.77E-06	2.23E-08	8.32E-07	4.35E-07	5.63E-07
ITLN1	6.56E-08	2.52E-08	2.38E-06	2.56E-06	1.32E-07	4.07E-08	0	9.74E-08
ITLN2	1.13E-07	2.27E-06	1.72E-07	8.38E-08	1.96E-08	4.20E-06	6.50E-08	3.82E-06
ITM2A	8.72E-06	1.32E-06	2.17E-06	3.68E-06	1.43E-06	4.97E-06	1.74E-06	4.17E-06
ITM2B	0.000365	0.000611	0.000278	0.000355	0.000143	0.000392	0.000268	0.000243
ITM2C	0.000114	1.58E-05	2.49E-05	5.05E-05	2.63E-05	1.12E-05	8.85E-06	6.66E-06
ITPA	6.46E-05	4.74E-05	2.62E-05	4.48E-05	4.96E-05	5.20E-05	4.76E-05	3.24E-05
ITPK1	5.57E-05	4.57E-05	2.13E-05	2.50E-05	4.92E-05	1.83E-05	4.00E-05	8.22E-05
ITPKA	6.74E-06	3.45E-06	8.08E-07	1.04E-06	1.40E-06	5.73E-07	2.88E-06	6.26E-07

ITPKB	2.66E-06	1.47E-06	1.12E-06	2.86E-06	9.86E-07	1.60E-06	2.69E-06	1.36E-06
ITPKC	1.51E-05	9.72E-06	4.10E-06	2.63E-05	6.60E-06	6.71E-06	1.43E-05	8.25E-06
ITPR1	4.37E-06	1.49E-06	2.19E-06	3.65E-06	9.85E-07	4.07E-06	1.34E-06	3.00E-06
ITPR2	4.26E-06	1.21E-05	1.83E-05	9.60E-06	4.84E-06	3.14E-05	6.74E-06	9.31E-06
ITPR3	1.50E-05	1.45E-07	2.95E-07	2.38E-06	1.24E-08	2.96E-07	2.79E-07	5.74E-07
ITPRIP	6.77E-06	4.49E-06	3.72E-06	1.65E-05	1.96E-06	3.92E-06	9.61E-06	5.46E-06
ITPRIPL1	3.05E-06	2.66E-07	1.40E-07	4.13E-07	0	1.72E-07	2.19E-07	2.18E-07
ITPRIPL2	8.07E-06	6.40E-06	3.64E-06	4.28E-06	1.25E-06	5.22E-06	6.79E-06	4.00E-06
ITSN1	1.21E-05	6.88E-06	1.84E-05	1.05E-05	4.19E-06	1.82E-05	9.69E-06	1.52E-05
ITSN2	1.59E-05	1.84E-05	8.46E-06	7.54E-06	2.69E-06	1.07E-05	4.99E-06	7.00E-06
IVD	4.23E-05	9.01E-05	9.44E-05	5.66E-05	5.27E-05	8.80E-05	5.94E-05	3.16E-05
IVL	1.12E-08	1.01E-07	4.43E-07	0	0	0	0	0
IVNS1ABP	3.79E-05	2.89E-05	1.34E-05	1.80E-05	6.11E-06	1.87E-05	1.63E-05	2.74E-05
IWS1	4.53E-05	1.57E-05	1.20E-05	1.37E-05	7.17E-06	1.76E-05	1.51E-05	2.02E-05
IYD	3.99E-06	8.51E-06	1.16E-05	6.28E-06	3.70E-06	7.10E-06	9.41E-06	5.13E-07
IZUMO1	0	2.97E-08	0	3.17E-08	0	0	0	0
JAG1	3.32E-05	4.52E-06	2.81E-06	6.71E-06	3.13E-06	4.16E-06	2.88E-06	5.91E-06
JAG2	8.28E-06	2.34E-06	7.39E-07	1.23E-06	3.35E-06	1.94E-06	3.63E-06	4.42E-06
JAGN1	3.86E-05	4.78E-05	2.89E-05	3.30E-05	3.26E-05	3.18E-05	4.23E-05	2.44E-05
JAK1	7.62E-05	4.25E-05	4.51E-05	5.54E-05	9.56E-06	4.61E-05	4.03E-05	4.55E-05
JAK2	2.48E-06	1.06E-06	1.56E-06	1.46E-06	1.22E-07	1.46E-06	6.99E-07	1.08E-06
JAK3	6.14E-06	2.29E-07	8.69E-07	2.52E-06	4.14E-07	1.41E-06	1.83E-06	1.25E-06
JAKMIP1	1.51E-06	8.72E-08	1.78E-07	2.95E-07	2.21E-08	1.66E-07	8.97E-08	1.21E-07
JAKMIP2	8.96E-08	6.49E-07	6.79E-07	1.31E-07	8.23E-08	5.41E-07	1.22E-07	3.94E-07
JAKMIP3	7.42E-08	7.28E-08	4.25E-08	4.76E-08	1.67E-08	2.37E-07	1.89E-08	7.50E-08
JAM2	2.21E-06	1.50E-06	8.17E-07	1.14E-06	1.13E-06	5.50E-07	1.30E-06	2.83E-06
JAM3	3.88E-06	2.24E-06	1.27E-06	2.94E-06	1.64E-06	1.50E-06	3.71E-06	3.05E-06
JARID2	6.39E-06	1.86E-06	1.21E-06	2.47E-06	5.95E-07	3.40E-06	1.55E-06	2.49E-06
JAZF1	1.26E-05	2.20E-05	7.07E-06	6.70E-06	5.91E-06	9.08E-06	8.18E-06	5.63E-06
JDP2	4.77E-06	1.57E-06	4.69E-06	1.03E-05	2.56E-06	6.03E-06	7.39E-06	1.95E-06
JHDM1D	1.77E-06	2.19E-06	2.97E-06	3.28E-06	3.28E-07	2.02E-06	2.14E-06	5.86E-06
JKAMP	2.41E-05	9.14E-06	1.11E-05	1.23E-05	4.55E-06	1.85E-05	1.19E-05	1.73E-05
JMJD1C	3.97E-05	2.07E-05	1.87E-05	2.18E-05	6.51E-06	2.26E-05	1.01E-05	1.24E-05
JMJD4	8.09E-06	1.16E-05	6.48E-06	7.71E-06	9.69E-06	1.97E-05	1.22E-05	2.19E-05
JMJD5	1.17E-05	1.69E-06	7.11E-05	5.99E-05	2.05E-05	1.02E-05	1.51E-05	4.69E-06
JMJD6	2.88E-05	2.80E-05	6.78E-06	1.72E-05	8.88E-06	1.40E-05	2.07E-05	1.10E-05
JMJD7-PL	1.21E-05	3.91E-06	6.35E-06	5.96E-06	5.40E-06	4.18E-06	7.38E-06	5.71E-06
JMJD8	4.50E-05	4.75E-05	3.59E-05	3.84E-05	3.54E-05	3.49E-05	3.72E-05	4.56E-05
JMY	3.87E-06	1.66E-06	2.42E-06	1.76E-06	5.26E-07	4.81E-06	1.82E-06	4.66E-06
JOSD1	3.32E-05	1.58E-05	7.80E-06	2.13E-05	3.82E-06	1.77E-05	1.74E-05	2.06E-05
JOSD2	2.72E-05	4.41E-05	1.53E-05	3.31E-05	5.03E-05	3.31E-05	2.55E-05	3.01E-05
JPH1	9.46E-07	2.49E-08	8.00E-08	3.64E-08	5.73E-08	0	1.03E-07	9.77E-08
JPH2	4.81E-07	1.11E-07	1.94E-08	1.03E-07	1.26E-07	5.81E-08	7.58E-08	1.24E-07
JPH3	5.70E-09	0	0	2.09E-08	0	1.06E-08	2.22E-08	8.35E-09
JPH4	1.87E-07	1.06E-08	1.56E-07	1.84E-07	6.44E-08	1.13E-07	1.18E-07	1.10E-07
JRK	1.90E-06	7.18E-07	5.22E-07	5.52E-07	1.54E-06	4.71E-07	1.64E-06	6.07E-07
JRKL	4.47E-06	2.14E-06	1.49E-06	1.07E-06	2.98E-07	1.51E-06	7.17E-07	3.57E-06
JSRP1	6.86E-06	2.47E-07	5.15E-08	3.22E-07	2.05E-08	4.43E-08	1.13E-07	0
JTB	9.31E-05	0.00017	7.12E-05	8.42E-05	9.59E-05	0.000121	0.000109	0.000138
JUB	4.42E-05	1.85E-05	7.52E-06	2.50E-05	9.35E-06	7.72E-06	2.98E-05	8.43E-06
JUN	2.29E-05	0.000237	0.000178	0.000319	8.33E-06	2.33E-05	0.000105	1.58E-05
JUNB	5.30E-05	0.000117	0.000192	0.000476	3.56E-05	3.14E-05	0.000228	0.000142
JUND	6.46E-05	0.000127	7.09E-05	0.000311	9.72E-05	5.55E-05	0.000287	6.14E-05
JUP	9.83E-05	0.000129	5.17E-05	6.21E-05	6.38E-05	6.81E-05	7.09E-05	6.27E-05
KAAG1	2.19E-06	1.90E-07	0	6.21E-07	4.05E-07	1.94E-07	2.51E-07	4.21E-08
KAL1	6.45E-07	4.35E-08	1.09E-07	6.27E-07	3.95E-08	1.64E-07	2.00E-07	2.12E-07
KALRN	1.69E-05	2.01E-05	1.28E-05	1.39E-05	1.64E-06	8.78E-06	5.58E-06	6.32E-06
KANK1	2.28E-05	2.14E-05	4.59E-05	3.20E-05	9.70E-06	0.000108	1.90E-05	3.10E-05

KANK2	2.24E-05	9.07E-06	1.19E-05	1.33E-05	4.85E-06	8.25E-06	9.50E-06	1.01E-05
KANK3	4.29E-06	2.45E-06	1.85E-06	2.34E-06	6.44E-06	2.45E-06	5.23E-06	2.75E-06
KANK4	1.17E-06	2.99E-06	6.98E-07	5.56E-07	6.89E-08	5.37E-08	1.20E-08	0
KARS	8.39E-05	4.92E-05	4.11E-05	5.41E-05	2.55E-05	7.22E-05	4.44E-05	5.37E-05
KAT2A	2.67E-05	2.35E-05	4.13E-06	5.93E-06	1.35E-05	1.69E-05	8.35E-06	1.08E-05
KAT2B	5.46E-06	1.04E-05	1.65E-05	8.99E-06	3.48E-06	1.19E-05	6.33E-06	8.40E-06
KAT5	2.60E-05	1.96E-05	1.19E-05	1.74E-05	1.09E-05	1.73E-05	2.07E-05	3.15E-05
KATNA1	9.60E-06	5.00E-06	3.75E-06	4.65E-06	2.27E-06	5.11E-06	7.55E-06	5.70E-06
KATNAL1	2.79E-06	5.67E-07	2.12E-06	1.50E-06	4.80E-07	2.14E-06	9.34E-07	9.30E-07
KATNAL2	3.79E-06	3.51E-06	4.97E-07	5.49E-07	2.16E-07	9.90E-07	2.03E-07	6.30E-07
KATNB1	7.24E-06	3.74E-06	3.36E-06	4.97E-06	9.18E-07	9.60E-06	4.63E-06	7.74E-06
KAZ	8.31E-07	4.59E-07	2.47E-06	2.45E-06	3.18E-07	2.51E-07	7.03E-07	3.33E-07
KAZALD1	4.48E-07	1.47E-07	1.84E-07	4.36E-07	4.22E-07	1.46E-07	1.36E-07	4.52E-07
KBTBD10	1.70E-06	1.07E-08	5.95E-08	1.98E-07	1.64E-08	0	2.08E-08	5.60E-08
KBTBD11	3.53E-06	2.37E-07	4.05E-06	3.28E-06	3.71E-08	3.26E-07	4.93E-07	6.41E-07
KBTBD12	0	0	0	0	5.04E-09	1.62E-08	0	0
KBTBD13	0	1.04E-07	0	0	0	0	0	0
KBTBD2	2.13E-05	1.95E-05	6.55E-06	1.27E-05	2.59E-06	9.38E-06	1.07E-05	1.40E-05
KBTBD3	2.23E-06	6.79E-07	8.61E-07	7.43E-07	8.51E-07	1.08E-06	7.16E-07	7.94E-07
KBTBD4	6.82E-06	6.52E-06	4.67E-06	4.43E-06	4.42E-07	6.42E-06	2.47E-06	7.95E-06
KBTBD5	0	1.10E-08	0	0	0	0	0	0
KBTBD6	4.43E-06	3.10E-06	3.65E-06	2.22E-06	2.93E-06	3.82E-06	2.69E-06	3.48E-06
KBTBD7	2.98E-06	1.95E-06	2.80E-06	1.00E-06	1.26E-07	1.94E-06	4.36E-07	1.81E-06
KBTBD8	5.79E-07	2.78E-07	2.45E-07	6.16E-07	1.77E-08	3.41E-07	2.62E-07	5.73E-07
KC6	7.56E-07	3.56E-07	0	0	0	0	8.25E-08	0
KCMF1	9.46E-06	1.00E-05	4.23E-06	6.06E-06	3.07E-06	6.50E-06	6.51E-06	6.74E-06
KCNA1	2.79E-09	0	0	0	0	0	0	0
KCNA10	0	0	0	0	0	0	0	0
KCNA2	2.45E-08	0	0	1.37E-08	0	0	0	0
KCNA3	3.16E-07	4.63E-08	2.77E-07	3.44E-07	5.90E-09	1.21E-07	6.67E-08	4.03E-08
KCNA4	0	0	0	0	0	0	0	0
KCNA5	4.95E-07	6.47E-07	8.40E-09	8.93E-08	1.74E-07	3.76E-08	4.72E-08	1.67E-07
KCNA6	1.88E-08	1.67E-08	3.07E-08	5.03E-08	3.22E-09	1.04E-08	2.92E-08	0
KCNA7	0	0	0	0	0	0	0	0
KCNAB1	3.76E-07	3.19E-07	5.56E-07	5.68E-07	6.70E-07	8.48E-07	6.60E-07	2.27E-07
KCNAB2	8.84E-06	5.77E-06	2.55E-06	5.82E-06	1.01E-07	1.37E-06	2.85E-06	9.93E-06
KCNAB3	9.39E-08	2.74E-08	5.21E-08	1.97E-07	2.63E-08	2.84E-08	1.47E-08	2.93E-08
KCNB1	7.30E-08	3.70E-06	4.01E-07	5.20E-08	1.57E-08	1.01E-06	2.78E-07	1.63E-06
KCNB2	0	0	0	0	0	0	0	0
KCNC1	5.57E-08	2.68E-07	0	4.01E-09	5.64E-09	6.82E-09	0	0
KCNC2	1.76E-05	9.03E-09	3.33E-08	1.18E-07	0	0	0	1.04E-06
KCNC3	2.34E-06	3.13E-07	9.68E-07	9.20E-07	2.55E-07	1.90E-06	9.00E-07	4.93E-07
KCNC4	7.06E-06	7.77E-07	4.53E-07	7.70E-07	7.84E-07	3.52E-07	9.20E-07	6.80E-07
KCND1	8.37E-07	2.30E-07	8.70E-08	1.67E-07	1.20E-07	5.35E-08	2.30E-07	1.77E-07
KCND2	2.95E-07	4.71E-08	4.75E-08	3.60E-08	7.24E-09	7.81E-09	8.22E-09	6.79E-08
KCND3	3.55E-08	3.12E-06	3.25E-06	1.29E-06	1.14E-07	4.95E-06	6.88E-07	2.60E-08
KCNE1	2.93E-08	2.40E-07	1.52E-07	8.05E-08	0	1.36E-08	2.72E-08	1.21E-06
KCNE1L	6.81E-07	1.17E-07	1.81E-08	4.18E-08	1.47E-08	0	2.46E-07	3.37E-06
KCNE2	3.88E-07	7.47E-07	8.34E-07	5.26E-07	1.01E-06	1.62E-06	3.67E-07	1.16E-06
KCNE3	5.20E-06	2.41E-06	1.69E-06	1.66E-06	1.09E-06	2.87E-06	3.97E-06	1.64E-06
KCNE4	2.60E-06	7.39E-07	3.65E-07	1.39E-05	2.29E-07	9.14E-07	1.78E-06	2.64E-06
KCNF1	1.19E-05	3.50E-08	4.31E-08	1.91E-06	8.89E-09	0	1.10E-07	3.19E-07
KCNG1	1.66E-06	4.83E-08	2.23E-08	1.57E-07	1.84E-08	1.98E-08	2.07E-08	3.14E-08
KCNG2	5.37E-08	2.05E-08	1.91E-08	4.40E-08	0	3.33E-08	1.38E-07	2.65E-08
KCNG3	3.01E-08	0	0	0	0	0	0	0
KCNG4	0	0	0	0	0	0	0	0
KCNH1	3.05E-08	1.54E-08	1.41E-08	1.35E-08	4.70E-09	1.54E-08	3.21E-08	1.62E-08
KCNH2	1.76E-07	1.42E-07	1.51E-08	1.65E-07	1.86E-08	1.79E-08	3.73E-08	4.24E-08

KCNH3	5.09E-07	3.99E-08	4.88E-08	6.52E-08	1.02E-08	1.10E-08	5.76E-09	1.74E-08
KCNH4	2.56E-07	1.95E-08	0	6.39E-08	4.99E-09	0	5.08E-08	3.06E-07
KCNH5	5.59E-08	2.51E-08	0	0	0	0	0	3.24E-08
KCNH6	0	0	0	0	0	0	0	1.13E-08
KCNH7	5.38E-09	3.01E-07	1.27E-07	1.97E-08	0	0	1.05E-08	0
KCNH8	2.65E-07	4.98E-09	3.19E-08	4.34E-08	0	0	8.67E-09	0
KCNIP1	1.65E-08	0	1.75E-08	2.68E-07	0	0	6.36E-08	4.88E-08
KCNIP2	4.72E-07	8.57E-07	1.85E-07	1.47E-07	6.10E-08	8.18E-08	8.51E-08	3.71E-08
KCNIP3	1.86E-06	1.07E-06	3.29E-07	3.45E-07	4.42E-08	3.97E-08	1.06E-06	3.72E-08
KCNIP4	1.87E-07	2.99E-07	1.04E-07	1.08E-07	2.85E-08	1.13E-07	8.53E-08	4.87E-08
KCNJ1	0	0	3.16E-08	3.53E-08	1.65E-08	9.39E-09	8.22E-09	1.49E-08
KCNJ10	1.35E-06	1.71E-08	9.17E-07	6.11E-07	1.31E-08	1.82E-06	3.48E-08	4.48E-08
KCNJ11	2.66E-06	1.21E-07	4.33E-07	6.67E-07	3.18E-08	2.22E-07	3.07E-07	3.84E-06
KCNJ12	2.89E-07	5.77E-08	1.54E-07	4.20E-08	1.18E-07	1.59E-08	1.30E-07	1.26E-07
KCNJ13	2.83E-08	8.98E-09	2.48E-08	9.77E-09	0	6.75E-09	0	0
KCNJ14	3.37E-07	1.50E-07	1.26E-07	1.75E-07	1.72E-07	1.80E-07	1.59E-07	6.57E-08
KCNJ15	5.76E-07	2.34E-06	1.35E-06	3.14E-07	2.20E-07	3.88E-07	1.24E-07	1.50E-07
KCNJ16	3.08E-05	6.82E-09	7.71E-07	1.79E-07	5.23E-09	0	2.96E-08	0
KCNJ2	1.25E-06	1.79E-06	4.15E-07	3.11E-07	1.36E-07	2.86E-07	1.13E-06	5.62E-07
KCNJ3	1.55E-06	2.06E-07	8.87E-07	6.41E-08	3.00E-08	8.09E-09	2.81E-07	2.59E-07
KCNJ4	1.47E-06	2.34E-06	3.19E-07	8.85E-07	2.79E-07	5.17E-07	2.24E-07	2.33E-06
KCNJ5	4.41E-07	3.52E-07	2.43E-07	1.14E-06	1.50E-08	2.06E-06	1.09E-06	1.03E-07
KCNJ6	1.20E-08	0	1.25E-08	0	0	1.11E-07	0	2.12E-07
KCNJ8	3.10E-06	3.69E-05	3.58E-05	2.50E-05	1.64E-05	3.60E-05	3.01E-05	3.47E-05
KCNJ9	0	2.57E-08	7.90E-09	0	6.56E-09	2.83E-08	0	0
KCNK1	2.24E-05	6.71E-06	4.68E-06	8.37E-06	3.57E-06	1.84E-05	5.76E-06	1.86E-05
KCNK10	1.55E-08	0	3.03E-09	7.57E-09	7.99E-09	1.44E-08	0	0
KCNK12	3.52E-08	0	0	0	0	1.64E-08	1.52E-07	0
KCNK13	2.26E-06	3.15E-08	2.13E-07	7.30E-07	8.01E-09	6.05E-08	1.81E-07	3.01E-07
KCNK15	1.85E-06	2.36E-08	1.10E-07	1.32E-06	0	0	7.88E-08	3.05E-08
KCNK16	0	0	0	0	0	0	1.71E-08	0
KCNK17	1.89E-07	1.62E-07	1.54E-06	7.17E-07	0	4.40E-08	4.56E-08	3.03E-07
KCNK18	0	0	0	0	0	0	0	0
KCNK2	1.35E-08	4.07E-07	5.81E-08	8.27E-09	0	0	0	0
KCNK3	1.78E-07	2.57E-08	1.83E-07	1.75E-07	4.42E-08	5.30E-09	0	4.28E-07
KCNK4	4.05E-08	6.55E-08	0	1.64E-08	0	0	0	1.99E-08
KCNK5	5.39E-06	6.02E-06	5.15E-06	2.01E-06	1.59E-06	1.64E-06	3.59E-06	1.42E-07
KCNK6	1.14E-05	2.85E-07	1.93E-06	2.41E-06	1.22E-06	6.95E-07	2.27E-06	2.18E-07
KCNK7	1.50E-07	5.27E-07	1.47E-07	1.20E-07	3.16E-07	3.64E-08	6.08E-08	5.80E-08
KCNK9	7.17E-07	6.48E-07	0	1.93E-08	0	7.30E-09	4.59E-08	0
KCNMA1	4.44E-06	2.05E-07	2.76E-06	4.12E-06	1.04E-06	3.62E-06	3.76E-06	5.64E-06
KCNMB1	2.65E-06	3.54E-07	3.64E-07	6.22E-07	5.77E-07	8.47E-07	8.96E-07	5.79E-07
KCNMB2	2.72E-07	8.62E-08	6.07E-08	5.95E-08	0	1.18E-08	0	7.47E-08
KCNMB3	2.39E-06	9.13E-07	4.90E-07	8.42E-07	5.79E-07	1.57E-07	7.60E-07	8.74E-07
KCNMB4	1.19E-06	2.40E-07	6.84E-07	1.16E-06	6.61E-07	2.94E-07	1.26E-06	6.66E-07
KCNN1	4.75E-07	1.78E-07	0	1.08E-08	3.02E-08	1.63E-08	1.70E-08	1.55E-07
KCNN2	4.04E-07	1.54E-06	1.77E-05	2.88E-06	2.21E-07	3.18E-08	7.65E-07	1.52E-07
KCNN3	3.95E-07	3.16E-07	4.71E-08	3.02E-07	1.44E-07	7.04E-08	1.14E-07	2.52E-07
KCNN4	9.17E-07	1.55E-07	3.65E-07	1.01E-06	3.76E-07	2.11E-07	5.71E-07	3.18E-07
KCNQ1	1.12E-05	6.87E-07	9.65E-07	2.97E-06	8.25E-07	8.20E-07	1.73E-06	5.75E-07
KCNQ1DN	0	0	0	0	0	0	0	0
KCNQ1OT	9.78E-08	1.91E-08	1.08E-08	1.65E-08	1.07E-08	1.01E-08	4.30E-09	3.10E-08
KCNQ2	0	0	0	0	9.08E-08	1.63E-08	0	0
KCNQ3	7.56E-09	5.09E-08	0	4.62E-08	6.49E-09	1.40E-08	6.60E-08	2.22E-08
KCNQ4	3.02E-07	1.24E-08	6.32E-08	1.34E-08	0	2.03E-08	0	1.61E-08
KCNQ5	7.16E-08	1.60E-08	2.92E-08	4.67E-08	0	3.64E-08	1.40E-08	1.57E-08
KCNRG	3.15E-07	8.21E-08	1.39E-07	1.70E-07	1.40E-07	6.66E-08	5.18E-08	1.56E-07
KCNS1	6.86E-07	0	0	6.39E-09	0	4.84E-09	1.02E-08	0

KCNS2	7.29E-09	0	0	8.92E-09	0	0	0	0
KCNS3	4.13E-06	4.23E-07	3.30E-06	4.25E-06	3.40E-07	2.26E-07	1.18E-06	1.13E-06
KCNT1	0	5.39E-09	0	5.88E-09	1.22E-08	8.91E-09	9.37E-09	1.52E-08
KCNT2	7.99E-06	2.57E-06	2.39E-06	1.31E-06	1.48E-07	7.80E-07	7.40E-07	1.72E-06
KCNU1	0	0	0	0	0	3.35E-06	7.01E-09	0
KCNV1	0	4.49E-08	0	0	0	0	0	0
KCNV2	1.28E-08	0	0	0	0	7.13E-08	0	0
KCP	3.56E-07	2.02E-07	4.91E-09	2.14E-08	6.35E-08	6.18E-08	3.09E-08	3.26E-08
KCTD1	4.80E-06	1.41E-06	1.16E-06	2.53E-06	6.23E-07	1.33E-06	2.98E-06	3.40E-06
KCTD10	1.69E-05	8.49E-06	4.16E-06	1.01E-05	2.48E-06	9.15E-06	7.14E-06	6.96E-06
KCTD11	3.21E-06	2.23E-06	2.88E-06	5.32E-06	6.64E-07	3.63E-06	3.13E-06	2.76E-06
KCTD12	1.15E-05	3.46E-06	3.82E-06	6.78E-06	1.46E-06	2.44E-06	6.02E-06	3.30E-06
KCTD13	7.17E-06	1.31E-05	2.81E-06	5.51E-06	2.00E-06	7.49E-06	5.88E-06	5.25E-06
KCTD14	5.93E-06	4.19E-06	7.21E-06	9.10E-06	2.75E-06	8.64E-06	3.75E-06	1.27E-06
KCTD15	7.52E-06	6.04E-07	1.27E-06	1.09E-06	1.77E-06	5.59E-07	3.62E-06	9.44E-07
KCTD16	0	1.46E-08	4.45E-09	0	0	1.61E-08	0	0
KCTD17	1.29E-05	3.53E-06	1.18E-06	5.08E-06	1.38E-06	1.11E-06	3.78E-06	4.75E-06
KCTD18	9.08E-06	8.07E-06	5.51E-06	5.10E-06	1.71E-06	8.27E-06	3.71E-06	5.05E-06
KCTD19	0	0	5.65E-09	0	0	8.86E-09	4.66E-09	0
KCTD2	1.29E-05	1.05E-05	4.11E-06	6.49E-06	7.21E-06	1.12E-05	7.18E-06	8.95E-06
KCTD20	2.37E-05	3.09E-05	1.01E-05	1.15E-05	1.31E-06	1.63E-05	5.47E-06	1.49E-05
KCTD21	1.25E-05	1.08E-05	7.41E-06	7.27E-06	5.16E-06	1.86E-05	5.31E-06	2.13E-05
KCTD3	3.12E-05	7.50E-05	3.23E-05	2.28E-05	9.25E-06	4.21E-05	1.72E-05	3.25E-05
KCTD4	0	0	1.18E-08	0	0	1.04E-08	1.09E-08	0
KCTD5	1.80E-05	1.59E-05	1.02E-05	1.30E-05	2.22E-06	1.14E-05	1.35E-05	1.02E-05
KCTD6	8.56E-06	1.25E-05	3.37E-06	4.79E-06	8.34E-06	3.31E-06	6.25E-06	5.70E-06
KCTD7	3.59E-06	9.44E-07	5.11E-07	5.35E-07	3.08E-07	6.29E-07	8.57E-07	1.41E-06
KCTD8	0	0	0	0	0	8.37E-09	0	1.33E-08
KCTD9	6.80E-06	8.37E-06	4.02E-06	4.75E-06	8.51E-07	5.75E-06	2.28E-06	2.64E-06
KDELC1	1.16E-05	3.59E-06	4.33E-06	3.51E-06	1.27E-06	4.30E-06	3.45E-06	5.45E-06
KDELC2	9.76E-06	5.13E-06	3.97E-06	5.19E-06	1.03E-06	4.29E-06	2.80E-06	3.48E-06
KDELR1	0.000139	9.25E-05	8.90E-05	0.000108	0.000125	0.000205	0.000119	0.0001
KDELR2	0.000215	0.000241	0.000109	0.000104	0.000134	0.000168	0.000138	0.000165
KDELR3	7.89E-05	4.35E-06	5.31E-06	1.94E-05	9.35E-06	2.74E-06	2.16E-05	1.27E-05
KDM1A	2.52E-05	1.19E-05	9.86E-06	1.12E-05	6.68E-06	1.49E-05	1.01E-05	8.29E-06
KDM1B	5.67E-06	6.82E-06	2.49E-06	2.95E-06	9.97E-07	5.88E-06	2.71E-06	1.98E-06
KDM2A	3.64E-05	2.76E-05	1.39E-05	2.04E-05	8.71E-06	2.45E-05	1.38E-05	3.49E-05
KDM2B	1.33E-05	5.12E-06	7.01E-06	6.36E-06	1.42E-06	8.00E-06	7.01E-06	9.77E-06
KDM3A	1.63E-05	7.26E-06	4.56E-06	6.23E-06	1.52E-06	7.83E-06	5.28E-06	5.13E-06
KDM3B	1.56E-05	1.14E-05	6.63E-06	8.82E-06	1.63E-06	1.08E-05	6.65E-06	1.16E-05
KDM4A	1.51E-05	1.32E-05	1.01E-05	8.71E-06	3.74E-06	1.46E-05	7.34E-06	8.90E-06
KDM4B	6.76E-06	6.22E-06	7.87E-06	4.71E-06	5.53E-06	1.07E-05	5.53E-06	5.35E-06
KDM4C	6.52E-06	1.03E-05	6.24E-06	6.50E-06	4.96E-06	3.49E-05	7.01E-06	4.80E-06
KDM4D	1.87E-07	1.84E-07	2.02E-07	1.91E-07	5.36E-08	3.61E-07	1.36E-07	1.72E-07
KDM4DL	0	0	0	0	0	9.35E-09	1.95E-08	0
KDM5A	7.41E-06	2.76E-06	3.30E-06	3.60E-06	9.16E-07	5.88E-06	3.00E-06	2.18E-06
KDM5B	9.16E-06	2.86E-06	2.60E-06	4.66E-06	8.90E-07	5.83E-06	2.79E-06	7.38E-06
KDM5C	2.28E-05	9.38E-06	9.64E-06	6.36E-06	2.90E-06	9.22E-06	6.76E-06	1.33E-05
KDM5D	0	8.31E-06	0	5.79E-06	8.45E-07	8.96E-06	5.70E-06	5.41E-06
KDM6A	9.46E-06	2.79E-06	6.61E-06	4.42E-06	7.06E-07	5.19E-06	2.26E-06	5.82E-06
KDM6B	4.54E-06	4.83E-06	4.47E-06	1.93E-05	7.31E-07	3.11E-06	9.09E-06	6.11E-06
KDR	6.80E-06	9.84E-06	7.97E-06	3.12E-06	2.14E-06	5.72E-06	4.42E-06	5.26E-06
KDSR	1.27E-05	1.10E-05	2.05E-05	1.98E-05	4.42E-06	1.53E-05	1.61E-05	2.34E-05
KEAP1	3.77E-05	2.75E-05	2.66E-05	2.78E-05	4.83E-05	2.82E-05	3.47E-05	3.88E-05
KEL	2.12E-07	2.07E-08	6.70E-08	6.31E-07	7.92E-09	3.41E-08	2.68E-08	8.52E-07
KERA	2.78E-08	0	0	0	0	8.60E-09	0	0
KGFLP1	0	2.13E-07	3.92E-08	6.97E-08	5.44E-09	1.82E-07	3.08E-08	0
KGFLP2	4.11E-07	2.08E-06	6.42E-07	8.67E-07	7.98E-08	1.52E-06	2.56E-07	3.93E-07

KHDC1	1.07E-06	4.00E-08	0	1.13E-07	0	4.88E-08	1.68E-08	3.41E-08
KHDC1L	9.76E-08	3.18E-06	5.74E-08	6.12E-08	0	0	0	0
KHDRBS1	7.24E-05	3.95E-05	2.89E-05	3.70E-05	1.72E-05	3.82E-05	3.17E-05	3.46E-05
KHDRBS2	1.03E-08	3.48E-08	1.07E-08	1.26E-08	0	0	0	0
KHDRBS3	4.03E-06	3.80E-06	4.00E-06	5.02E-06	4.83E-06	5.70E-06	1.87E-05	8.07E-06
KHK	5.24E-05	0.00035	0.000214	9.73E-05	0.000309	0.000243	0.000113	6.64E-05
KHNYN	1.84E-05	5.60E-06	3.10E-06	4.95E-06	2.32E-06	5.79E-06	7.33E-06	9.31E-06
KHSRP	3.51E-05	2.69E-05	1.79E-05	1.76E-05	1.60E-05	2.54E-05	2.05E-05	1.72E-05
KIAA0020	6.36E-06	1.01E-05	5.68E-06	9.12E-06	2.89E-06	1.39E-05	8.39E-06	8.09E-06
KIAA0040	1.04E-05	5.49E-06	7.60E-06	1.08E-05	1.59E-06	3.13E-06	4.02E-06	9.21E-06
KIAA0087	5.25E-09	5.88E-09	0	6.41E-09	0	0	0	0
KIAA0090	1.50E-05	1.43E-05	7.17E-06	7.54E-06	3.14E-06	9.46E-06	4.48E-06	8.06E-06
KIAA0100	3.92E-05	1.74E-05	9.62E-06	1.30E-05	2.64E-05	1.60E-05	3.10E-05	2.48E-05
KIAA0101	1.85E-05	7.44E-06	5.56E-07	4.94E-07	3.43E-06	3.20E-06	6.59E-07	1.10E-06
KIAA0114	0.000134	3.71E-05	2.33E-05	2.49E-05	2.98E-05	6.50E-05	1.32E-05	1.08E-05
KIAA0125	3.44E-07	0	7.35E-08	2.43E-07	0	6.22E-08	6.01E-08	1.03E-07
KIAA0141	1.99E-05	1.49E-05	1.85E-05	2.20E-05	1.16E-05	3.96E-05	2.36E-05	1.86E-05
KIAA0146	2.50E-05	1.03E-05	6.16E-06	1.06E-05	9.03E-06	1.27E-05	2.32E-05	1.39E-05
KIAA0174	9.29E-05	3.03E-05	2.42E-05	3.52E-05	1.75E-05	3.71E-05	3.31E-05	3.33E-05
KIAA0182	1.36E-05	5.27E-06	6.78E-06	9.47E-06	2.06E-06	4.22E-06	6.28E-06	6.36E-06
KIAA0195	2.22E-05	1.89E-05	8.08E-06	7.15E-06	2.06E-05	1.83E-05	1.32E-05	2.03E-05
KIAA0196	3.85E-05	1.59E-05	7.11E-06	7.86E-06	3.72E-06	1.28E-05	1.28E-05	9.66E-06
KIAA0226	3.94E-06	1.23E-06	1.05E-06	1.77E-06	7.01E-07	1.60E-06	1.51E-06	1.50E-06
KIAA0232	9.23E-06	1.12E-05	5.52E-06	5.30E-06	2.99E-06	9.67E-06	5.43E-06	6.33E-06
KIAA0240	3.28E-06	3.38E-06	2.67E-06	3.14E-06	3.19E-07	3.16E-06	1.77E-06	2.23E-06
KIAA0247	1.45E-05	3.62E-06	4.54E-06	7.82E-06	1.91E-06	5.45E-06	5.91E-06	9.86E-06
KIAA0284	1.21E-05	4.60E-06	3.98E-06	4.71E-06	4.60E-06	4.96E-06	7.05E-06	1.13E-05
KIAA0317	7.42E-06	2.41E-06	2.63E-06	3.78E-06	2.95E-06	7.15E-06	4.16E-06	7.12E-06
KIAA0319	1.35E-07	4.73E-08	8.67E-09	1.72E-08	2.72E-08	3.92E-08	2.47E-08	2.48E-08
KIAA0319l	5.27E-05	2.95E-05	2.14E-05	2.79E-05	1.88E-05	3.65E-05	1.92E-05	3.05E-05
KIAA0355	5.17E-06	3.57E-06	3.66E-06	2.58E-06	9.72E-07	3.13E-06	2.56E-06	3.60E-06
KIAA0368	1.53E-05	1.12E-05	9.40E-06	1.15E-05	4.46E-06	1.90E-05	7.43E-06	1.22E-05
KIAA0391	1.40E-05	1.52E-05	1.16E-05	8.88E-06	6.01E-06	1.86E-05	1.15E-05	1.03E-05
KIAA0406	1.42E-05	1.07E-05	5.59E-06	4.92E-06	2.75E-06	8.39E-06	4.56E-06	4.45E-06
KIAA0408	4.20E-72	2.05E-74	6.42E-08	0	0	1.83E-08	#######	#######
KIAA0415	1.77E-05	2.39E-05	5.32E-06	9.10E-06	1.16E-05	1.04E-05	9.60E-06	1.47E-05
KIAA0427	8.94E-06	3.08E-06	4.12E-06	5.24E-06	4.45E-06	7.64E-06	9.75E-06	5.18E-06
KIAA0430	9.93E-06	8.16E-06	6.40E-06	4.68E-06	2.80E-06	8.41E-06	6.00E-06	5.38E-06
KIAA0467	9.93E-06	3.84E-06	3.92E-06	3.41E-06	3.23E-06	6.65E-06	3.32E-06	3.35E-06
KIAA0494	3.48E-05	2.66E-05	2.14E-05	2.00E-05	4.94E-06	2.76E-05	1.50E-05	1.76E-05
KIAA0495	4.10E-06	2.36E-06	1.19E-06	2.06E-06	7.36E-07	2.40E-06	1.83E-06	2.19E-06
KIAA0513	2.17E-06	5.50E-07	6.20E-07	1.06E-06	3.02E-07	9.59E-07	9.38E-07	1.89E-06
KIAA0528	1.05E-05	8.89E-06	4.29E-06	3.86E-06	1.36E-06	4.85E-06	2.86E-06	2.88E-06
KIAA0556	7.11E-06	7.30E-06	1.49E-06	3.39E-06	1.06E-06	4.30E-06	3.87E-06	3.67E-06
KIAA0562	5.06E-06	5.03E-06	5.24E-06	5.17E-06	7.02E-07	5.34E-06	1.69E-06	5.20E-06
KIAA0564	1.02E-05	9.81E-06	9.77E-06	7.54E-06	1.55E-06	7.70E-06	3.88E-06	9.79E-06
KIAA0586	2.69E-06	1.30E-06	1.17E-06	1.05E-06	2.53E-07	3.30E-06	6.81E-07	1.93E-06
KIAA0649	3.51E-06	3.07E-06	3.84E-06	3.83E-06	1.01E-06	6.03E-06	2.44E-06	4.41E-06
KIAA0652	1.77E-05	1.81E-05	1.01E-05	1.12E-05	1.02E-05	3.02E-05	1.44E-05	2.16E-05
KIAA0664	4.14E-05	7.53E-05	4.92E-05	5.28E-05	8.72E-05	6.86E-05	7.78E-05	7.66E-05
KIAA0664l	3.74E-06	3.04E-06	5.98E-07	9.20E-07	1.35E-08	1.26E-06	6.17E-07	7.08E-07
KIAA0748	1.13E-06	3.19E-08	1.58E-07	6.69E-07	2.42E-08	4.33E-07	1.36E-07	2.07E-07
KIAA0753	2.04E-06	9.90E-07	1.34E-06	2.05E-06	5.12E-07	2.25E-06	1.96E-06	1.38E-06
KIAA0754	6.81E-07	5.60E-07	3.46E-07	4.09E-07	2.74E-09	8.22E-07	8.10E-08	2.99E-07
KIAA0776	1.84E-05	1.48E-05	1.20E-05	7.84E-06	2.29E-06	1.93E-05	5.46E-06	7.80E-06
KIAA0802	1.88E-05	4.34E-07	5.42E-07	1.88E-06	4.39E-07	1.03E-06	4.58E-07	4.45E-07
KIAA0831	4.65E-06	1.60E-06	2.11E-06	2.53E-06	1.56E-06	4.95E-06	2.78E-06	3.64E-06
KIAA0892	1.64E-05	7.74E-06	5.31E-06	6.53E-06	3.56E-06	8.31E-06	7.07E-06	7.08E-06

KIAA0895	5.53E-06	1.96E-06	1.73E-06	1.74E-06	2.72E-07	1.72E-06	9.83E-07	1.44E-06
KIAA0895l	1.52E-05	1.76E-06	6.12E-07	1.80E-06	1.10E-06	1.35E-06	8.20E-07	1.94E-06
KIAA0907	1.80E-05	1.01E-05	4.01E-06	3.39E-06	2.63E-06	6.02E-06	3.83E-06	4.25E-06
KIAA0913	2.25E-05	1.55E-05	1.63E-05	1.36E-05	8.49E-06	2.12E-05	1.52E-05	1.23E-05
KIAA0922	1.16E-05	5.69E-06	1.45E-05	9.29E-06	4.03E-06	8.74E-06	8.12E-06	6.09E-06
KIAA0947	5.51E-06	4.50E-06	2.64E-06	3.48E-06	6.16E-07	3.86E-06	1.41E-06	3.84E-06
KIAA1009	1.49E-06	1.67E-06	5.95E-07	8.83E-07	1.76E-07	1.07E-06	6.25E-07	6.02E-07
KIAA1012	1.27E-05	1.05E-05	1.03E-05	7.27E-06	1.17E-06	1.30E-05	5.04E-06	6.35E-06
KIAA1024	9.28E-08	2.59E-08	2.03E-08	2.83E-08	2.84E-09	6.13E-09	9.68E-09	1.94E-08
KIAA1033	1.76E-05	1.43E-05	1.12E-05	8.21E-06	1.68E-06	1.55E-05	3.51E-06	1.03E-05
KIAA1045	1.54E-08	1.72E-08	7.89E-09	1.55E-07	1.32E-08	0	1.13E-08	1.69E-08
KIAA1107	1.01E-06	1.34E-06	6.45E-07	6.07E-07	4.49E-08	1.30E-06	2.34E-07	3.22E-07
KIAA1109	2.17E-05	5.46E-06	7.68E-06	1.05E-05	2.23E-06	1.43E-05	5.61E-06	8.43E-06
KIAA1143	7.18E-06	6.46E-06	3.78E-06	3.51E-06	9.82E-07	6.19E-06	2.23E-06	3.95E-06
KIAA1147	5.27E-06	3.79E-06	4.57E-06	3.56E-06	3.98E-06	5.10E-06	8.80E-06	1.02E-05
KIAA1161	9.65E-06	2.17E-05	1.92E-05	8.95E-06	4.78E-06	1.62E-05	8.70E-06	5.26E-06
KIAA1191	4.04E-05	3.35E-05	4.45E-05	4.11E-05	8.23E-06	6.17E-05	3.01E-05	3.26E-05
KIAA1199	3.50E-07	2.46E-08	6.43E-09	5.80E-07	3.51E-08	6.70E-08	8.53E-07	3.77E-07
KIAA1210	0	1.58E-08	0	0	0	2.63E-09	0	0
KIAA1211	5.65E-06	2.92E-08	1.10E-07	7.09E-07	5.60E-09	2.72E-08	1.42E-07	9.55E-08
KIAA1217	1.02E-05	9.20E-06	6.28E-06	5.87E-06	2.32E-06	5.80E-06	4.17E-06	9.29E-06
KIAA1239	2.38E-08	6.64E-08	3.04E-08	3.63E-09	0	0	0	1.31E-08
KIAA1244	1.96E-06	1.12E-08	2.30E-08	9.78E-08	0	1.85E-08	4.89E-09	7.32E-09
KIAA1257	8.55E-08	3.95E-07	6.18E-08	7.36E-08	6.30E-08	1.69E-06	7.05E-08	1.30E-07
KIAA1267	1.55E-05	7.47E-06	4.95E-06	5.65E-06	1.59E-06	8.42E-06	4.15E-06	5.29E-06
KIAA1274	5.95E-06	9.19E-07	1.50E-06	1.63E-06	1.08E-06	8.96E-07	5.15E-06	2.08E-06
KIAA1279	1.15E-05	7.32E-06	5.36E-06	7.66E-06	1.80E-06	6.74E-06	6.79E-06	9.67E-06
KIAA1310	2.33E-05	9.22E-06	4.67E-06	8.15E-06	4.32E-06	8.80E-06	7.64E-06	1.53E-05
KIAA1324	6.14E-07	1.32E-07	9.57E-08	1.85E-07	1.25E-08	4.94E-07	1.12E-06	2.13E-08
KIAA1324l	2.41E-06	5.59E-07	2.25E-07	1.41E-06	6.63E-08	1.21E-07	5.25E-07	2.57E-07
KIAA1328	9.57E-07	2.56E-07	3.25E-07	6.18E-07	1.23E-07	3.87E-07	3.99E-07	3.89E-07
KIAA1370	1.61E-05	6.59E-06	1.48E-05	1.03E-05	7.01E-06	1.54E-05	8.72E-06	1.11E-05
KIAA1377	4.27E-07	4.40E-08	3.42E-08	9.70E-08	8.61E-09	1.11E-08	3.26E-08	3.64E-08
KIAA1383	1.95E-06	8.47E-07	9.03E-07	6.74E-07	3.15E-07	1.40E-06	5.74E-07	2.05E-06
KIAA1407	1.40E-06	6.50E-07	2.95E-07	8.48E-07	1.87E-07	9.61E-07	3.07E-07	4.08E-07
KIAA1409	1.31E-08	1.17E-08	1.60E-08	1.92E-08	0	3.15E-08	1.28E-08	1.15E-08
KIAA1429	2.25E-05	1.39E-05	6.33E-06	6.04E-06	5.35E-06	9.92E-06	1.12E-05	7.91E-06
KIAA1430	8.92E-06	6.82E-06	6.25E-06	5.79E-06	8.26E-07	1.21E-05	3.69E-06	9.53E-06
KIAA1432	3.77E-06	3.00E-06	4.05E-06	2.57E-06	8.35E-07	4.69E-06	1.34E-06	3.66E-06
KIAA1462	8.06E-06	4.46E-06	1.63E-06	1.75E-06	6.37E-06	2.50E-06	9.37E-06	5.85E-06
KIAA1467	1.78E-06	1.26E-06	8.57E-07	1.24E-06	9.40E-07	1.27E-06	1.13E-06	1.40E-06
KIAA1468	8.00E-06	3.19E-06	3.90E-06	3.11E-06	8.03E-07	4.91E-06	1.91E-06	3.67E-06
KIAA1486	7.90E-09	8.88E-09	0	0	0	0	0	0
KIAA1522	5.53E-05	1.63E-05	4.80E-06	1.50E-05	5.95E-06	1.41E-05	1.97E-05	2.09E-05
KIAA1524	2.65E-06	2.84E-06	2.71E-07	2.34E-07	4.22E-08	4.39E-07	1.21E-07	1.87E-07
KIAA1529	1.79E-06	6.44E-06	2.37E-07	6.65E-07	3.12E-07	3.77E-07	4.51E-07	7.48E-08
KIAA1530	2.75E-06	1.58E-06	6.50E-07	1.40E-06	2.89E-07	9.35E-07	1.23E-06	1.89E-06
KIAA1539	5.61E-06	3.17E-06	3.71E-06	5.77E-06	2.60E-06	6.97E-06	4.48E-06	6.21E-06
KIAA1543	1.56E-05	1.21E-05	1.16E-05	1.52E-05	2.99E-05	1.05E-05	2.38E-05	1.26E-05
KIAA1549	1.52E-06	9.59E-07	2.13E-07	1.00E-06	2.20E-07	3.26E-06	2.77E-07	3.43E-07
KIAA1586	2.62E-06	1.93E-06	1.50E-06	1.19E-06	3.62E-07	1.73E-06	1.06E-06	3.07E-06
KIAA1598	2.80E-05	1.93E-05	1.27E-05	2.44E-05	1.00E-05	3.74E-05	2.05E-05	1.80E-05
KIAA1609	4.24E-06	5.96E-07	5.15E-07	1.13E-06	9.62E-08	8.30E-07	6.90E-07	1.08E-06
KIAA1614	2.18E-06	7.06E-07	1.13E-07	2.42E-07	1.56E-07	6.42E-07	4.16E-07	3.31E-07
KIAA1632	4.90E-06	1.15E-06	1.77E-06	1.33E-06	3.88E-07	2.37E-06	1.17E-06	1.07E-06
KIAA1644	1.76E-07	0	2.71E-08	3.68E-07	8.52E-09	1.53E-08	3.87E-08	0
KIAA1671	3.63E-05	2.19E-05	1.67E-05	1.40E-05	9.74E-06	2.20E-05	1.70E-05	1.17E-05
KIAA1683	1.28E-06	8.71E-07	6.48E-07	1.15E-06	7.84E-07	2.43E-07	1.95E-07	6.02E-07

KIAA1704	8.90E-06	7.76E-06	5.92E-06	5.84E-06	2.13E-06	9.67E-06	6.70E-06	6.11E-06
KIAA1712	1.76E-05	1.72E-06	1.80E-06	1.42E-06	5.66E-07	1.69E-06	1.82E-06	1.94E-06
KIAA1715	6.72E-06	7.57E-06	4.34E-06	3.42E-06	5.25E-07	4.85E-06	2.33E-06	4.20E-06
KIAA1731	7.10E-06	2.47E-06	1.63E-06	1.86E-06	1.72E-06	3.89E-06	1.82E-06	5.29E-06
KIAA1737	5.03E-06	2.70E-06	7.24E-06	6.37E-06	8.88E-07	1.13E-05	4.83E-06	7.50E-06
KIAA1751	1.44E-08	5.17E-07	1.16E-08	0	2.86E-08	2.11E-08	0	7.02E-09
KIAA1755	4.86E-07	4.15E-08	1.63E-07	2.88E-07	1.91E-07	2.54E-07	8.82E-07	4.42E-08
KIAA1797	4.67E-06	3.03E-06	1.81E-06	2.52E-06	1.05E-06	3.41E-06	1.82E-06	4.46E-06
KIAA1804	2.06E-06	6.03E-07	2.25E-06	1.37E-06	3.18E-07	1.08E-06	1.32E-06	1.97E-06
KIAA1826	7.94E-06	2.22E-07	2.85E-06	2.93E-06	2.60E-07	3.11E-06	1.36E-06	1.47E-06
KIAA1841	2.69E-06	1.66E-06	2.49E-07	6.10E-07	4.70E-07	5.12E-07	3.68E-07	4.46E-07
KIAA1875	5.38E-07	1.86E-07	4.78E-08	5.04E-08	3.97E-07	2.69E-08	8.50E-08	3.62E-08
KIAA1908	1.10E-06	3.74E-06	3.58E-07	5.50E-07	1.33E-06	1.25E-06	1.53E-06	1.56E-06
KIAA1919	1.45E-06	1.62E-06	1.10E-06	9.10E-07	2.50E-07	1.49E-06	9.91E-07	1.63E-06
KIAA1949	3.03E-05	1.34E-05	6.38E-06	2.67E-05	6.22E-06	5.98E-06	2.24E-05	1.27E-05
KIAA1958	1.21E-06	2.00E-06	1.16E-06	6.83E-07	2.99E-08	1.65E-06	1.69E-07	7.53E-07
KIAA1967	1.67E-05	3.23E-05	1.16E-05	1.62E-05	5.29E-06	2.15E-05	1.13E-05	1.03E-05
KIAA1984	1.70E-06	1.63E-07	1.36E-07	1.76E-07	1.73E-07	6.65E-08	8.29E-08	6.34E-08
KIAA2013	4.90E-05	3.67E-05	4.77E-05	4.86E-05	3.00E-05	4.37E-05	3.64E-05	3.72E-05
KIAA2018	4.23E-06	4.79E-06	2.96E-06	1.91E-06	1.03E-06	3.51E-06	1.64E-06	2.14E-06
KIAA2022	3.77E-09	0	5.75E-09	1.61E-08	3.23E-09	0	3.68E-09	0
KIAA2026	3.95E-06	3.35E-06	3.24E-06	2.96E-06	5.60E-07	3.00E-06	1.73E-06	2.57E-06
KIDINS220	3.02E-05	2.63E-05	1.13E-05	1.29E-05	2.81E-06	1.31E-05	7.56E-06	1.16E-05
KIF11	7.44E-06	2.73E-06	3.65E-07	5.44E-07	7.90E-07	1.59E-06	5.76E-07	7.40E-07
KIF12	5.40E-06	6.08E-07	9.17E-06	1.30E-05	3.66E-06	5.50E-07	4.06E-06	9.25E-07
KIF13A	1.68E-05	1.02E-05	1.27E-05	1.20E-05	4.13E-06	1.87E-05	8.65E-06	1.48E-05
KIF13B	1.03E-05	1.44E-05	9.11E-06	8.87E-06	3.00E-06	1.04E-05	5.73E-06	5.07E-06
KIF14	1.65E-06	4.53E-07	5.31E-08	3.57E-08	5.30E-08	3.62E-07	3.17E-08	2.85E-07
KIF15	2.18E-06	1.36E-06	5.34E-08	6.89E-08	8.07E-08	3.19E-07	9.09E-08	1.58E-07
KIF16B	8.28E-06	3.20E-06	3.32E-06	3.71E-06	6.07E-07	6.84E-06	2.51E-06	2.41E-06
KIF17	1.19E-06	1.05E-06	1.11E-06	7.78E-07	8.64E-07	5.79E-07	1.84E-06	8.47E-07
KIF18A	2.75E-06	5.72E-07	2.73E-08	8.91E-08	7.97E-08	3.13E-07	2.58E-08	2.43E-07
KIF18B	5.89E-06	2.01E-06	4.44E-08	8.32E-08	2.27E-07	3.89E-07	1.05E-07	3.05E-07
KIF19	3.14E-08	0	4.01E-07	2.38E-07	2.70E-08	2.13E-08	3.66E-08	0
KIF1A	2.98E-08	1.15E-07	1.52E-07	8.52E-08	3.54E-09	9.07E-08	4.02E-09	1.81E-08
KIF1B	1.39E-05	7.31E-06	4.31E-06	4.47E-06	1.88E-06	6.85E-06	3.14E-06	5.85E-06
KIF1C	2.86E-05	4.06E-05	3.20E-05	3.07E-05	4.04E-05	4.83E-05	6.52E-05	5.57E-05
KIF20A	1.15E-05	1.63E-06	6.94E-08	1.54E-07	3.35E-07	1.17E-06	2.44E-07	5.03E-07
KIF20B	3.68E-06	1.41E-06	5.73E-07	5.91E-07	3.13E-07	8.68E-07	3.64E-07	4.86E-07
KIF21A	1.55E-05	1.10E-05	1.01E-05	7.71E-06	4.67E-06	1.40E-05	6.66E-06	1.03E-05
KIF21B	1.06E-06	4.79E-08	3.28E-07	7.57E-07	4.30E-08	2.76E-07	5.71E-07	1.56E-06
KIF22	5.76E-05	8.55E-05	7.50E-05	6.47E-05	0.000287	0.00015	0.000157	3.83E-05
KIF23	7.44E-06	1.87E-06	6.28E-08	2.52E-07	3.14E-07	6.52E-07	1.94E-07	3.16E-07
KIF24	3.38E-07	5.54E-07	2.79E-08	1.05E-07	7.71E-08	1.99E-07	1.61E-07	2.10E-07
KIF25	8.34E-08	2.49E-06	1.25E-07	2.29E-07	1.42E-08	0	1.74E-07	1.45E-07
KIF26A	2.64E-07	1.11E-07	3.88E-07	5.97E-07	1.39E-07	8.56E-08	4.80E-07	1.98E-07
KIF26B	1.87E-07	1.19E-07	7.80E-08	2.54E-07	2.62E-09	1.05E-06	1.22E-07	5.81E-08
KIF27	4.83E-07	2.64E-07	1.20E-07	1.74E-07	1.34E-08	3.84E-07	6.58E-08	1.14E-07
KIF2A	6.81E-06	4.07E-06	1.61E-06	2.67E-06	3.15E-07	3.45E-06	9.84E-07	2.52E-06
KIF2B	0	0	0	0	0	0	0	0
KIF2C	1.26E-05	3.29E-06	4.57E-08	2.26E-07	9.16E-07	1.32E-06	2.62E-07	1.07E-06
KIF3A	1.40E-06	6.73E-07	5.70E-07	8.07E-07	7.54E-08	1.16E-06	4.40E-07	1.61E-06
KIF3B	1.78E-05	1.98E-05	8.53E-06	1.06E-05	6.30E-06	1.52E-05	1.35E-05	1.31E-05
KIF3C	3.47E-06	4.44E-07	3.01E-07	9.35E-07	1.07E-07	3.97E-07	5.57E-07	3.33E-07
KIF4A	7.15E-06	2.86E-06	5.38E-08	2.18E-07	4.99E-07	7.69E-07	1.39E-07	7.83E-07
KIF4B	1.84E-07	3.44E-08	5.26E-09	1.88E-08	1.32E-08	4.27E-08	1.49E-08	1.50E-08
KIF5A	8.27E-07	0	0	0	0	0	0	0
KIF5B	2.09E-05	3.07E-05	1.19E-05	1.50E-05	5.41E-06	1.85E-05	1.01E-05	1.24E-05

KIF5C	9.63E-06	9.34E-07	3.91E-07	1.51E-07	2.00E-08	8.26E-08	2.69E-07	9.25E-08
KIF6	6.00E-09	7.49E-07	2.10E-07	1.60E-07	5.20E-08	8.77E-08	6.87E-08	3.45E-08
KIF7	2.89E-06	1.76E-07	2.60E-07	6.95E-07	1.30E-06	4.35E-07	1.75E-06	3.24E-07
KIF9	5.40E-06	2.57E-06	2.60E-06	6.14E-06	5.07E-06	5.41E-06	5.47E-06	1.78E-06
KIFAP3	6.62E-06	2.83E-05	9.14E-06	1.29E-05	5.51E-06	2.16E-05	1.10E-05	1.55E-05
KIFC1	1.29E-05	8.86E-06	1.70E-07	4.86E-07	1.72E-06	1.97E-06	5.66E-07	1.62E-06
KIFC2	2.69E-05	8.88E-06	9.30E-07	3.05E-06	8.55E-06	3.47E-06	9.08E-06	4.59E-06
KIFC3	3.70E-05	9.71E-06	7.36E-06	2.53E-05	8.23E-06	1.90E-05	1.18E-05	3.63E-05
KILLIN	2.97E-07	3.03E-07	5.24E-07	1.62E-07	1.37E-07	5.31E-07	1.34E-07	2.10E-07
KIN	6.24E-06	6.92E-06	3.76E-06	4.19E-06	2.31E-06	4.83E-06	4.57E-06	2.95E-06
KIR2DL1	0	3.50E-08	1.03E-07	1.36E-21	0	0	1.33E-07	2.40E-08
KIR2DL3	1.08E-07	2.23E-08	6.27E-08	3.79E-07	0	1.09E-07	1.09E-07	#######
KIR2DL4	2.12E-07	6.30E-08	9.80E-08	2.26E-08	7.49E-08	9.03E-07	1.25E-07	1.02E-07
KIR2DS4	3.17E-08	5.40E-08	6.73E-08	1.16E-07	0	1.33E-07	3.97E-07	0
KIR3DL1	4.17E-08	3.46E-08	2.24E-07	0	2.62E-08	1.33E-07	3.84E-07	3.63E-08
KIR3DL2	2.26E-08	4.51E-08	0	1.14E-07	0	#######	0	7.91E-08
KIR3DL3	0	0	0	1.77E-08	0	0	1.39E-08	0
KIR3DP1	0	0	0	0	0	0	0	0
KIR3DX1	0	1.60E-08	0	8.59E-08	0	0	0	0
KIRREL	2.60E-06	6.72E-07	4.84E-07	1.65E-06	0	3.65E-07	2.41E-07	4.86E-07
KIRREL2	1.05E-06	4.60E-08	0	0	0	8.43E-09	7.93E-09	1.34E-08
KIRREL3	0	6.85E-09	4.40E-08	5.97E-08	0	0	3.27E-08	8.95E-09
KISS1	5.78E-07	1.45E-05	9.48E-08	1.29E-06	1.26E-06	9.30E-07	3.11E-07	6.29E-06
KISS1R	8.61E-07	6.88E-08	0	1.86E-08	0	0	1.46E-08	0
KIT	6.07E-07	5.51E-07	2.59E-07	3.09E-07	7.49E-08	1.05E-07	9.22E-07	3.89E-07
KITLG	1.98E-06	9.58E-07	6.58E-07	7.62E-07	3.22E-07	3.21E-06	4.17E-07	5.68E-07
KL	9.48E-07	2.72E-07	5.54E-07	4.95E-08	4.25E-08	3.46E-07	1.18E-07	1.43E-07
KLB	2.60E-05	5.70E-05	6.02E-06	3.64E-06	2.80E-06	1.69E-05	5.91E-06	1.83E-05
KLC1	3.28E-05	7.93E-06	1.23E-05	2.62E-05	1.47E-05	2.05E-05	2.31E-05	1.74E-05
KLC2	8.65E-06	5.87E-06	1.44E-06	4.52E-06	1.11E-06	3.41E-06	3.98E-06	8.09E-06
KLC3	1.59E-06	2.41E-07	9.98E-08	5.47E-07	1.40E-07	1.13E-07	1.96E-07	7.99E-08
KLC4	8.75E-05	7.26E-05	6.22E-05	6.03E-05	4.85E-05	5.74E-05	5.84E-05	2.67E-05
KLF1	9.14E-08	5.20E-08	3.22E-08	1.87E-08	3.94E-08	0	4.40E-08	0
KLF10	1.17E-05	2.33E-05	3.32E-05	0.000118	4.86E-06	1.61E-05	5.58E-05	2.79E-05
KLF11	9.70E-06	2.61E-05	2.38E-05	2.15E-05	2.41E-06	1.17E-05	1.47E-05	1.51E-05
KLF12	6.44E-07	4.80E-06	3.01E-06	3.16E-06	9.67E-07	3.96E-06	2.35E-06	1.66E-06
KLF13	1.46E-05	6.12E-06	2.90E-06	6.11E-06	5.00E-06	6.16E-06	1.34E-05	9.36E-06
KLF14	0	2.08E-08	1.94E-08	6.70E-08	1.57E-08	1.69E-08	0	0
KLF15	7.12E-05	0.000103	5.51E-05	1.80E-05	3.30E-05	8.51E-05	2.03E-05	6.20E-05
KLF16	6.45E-06	4.43E-06	2.85E-06	1.12E-05	3.84E-06	4.83E-06	1.76E-05	1.67E-05
KLF17	0	1.66E-08	0	0	0	0	1.53E-08	1.08E-08
KLF2	6.45E-06	4.89E-06	8.30E-06	1.10E-05	5.36E-06	2.53E-06	2.32E-05	3.69E-06
KLF3	2.92E-05	2.19E-05	2.78E-05	1.58E-05	4.03E-06	2.03E-05	1.00E-05	1.78E-05
KLF4	5.73E-06	3.14E-06	5.93E-06	3.91E-05	3.91E-07	5.15E-07	1.64E-05	9.12E-07
KLF5	2.29E-05	5.42E-06	7.76E-07	8.45E-06	6.76E-08	2.98E-07	5.55E-07	1.12E-07
KLF6	6.98E-05	4.59E-05	0.000122	0.000123	2.96E-06	6.87E-05	6.02E-05	2.16E-05
KLF7	1.19E-06	1.31E-06	1.06E-06	3.31E-06	1.04E-07	6.29E-07	4.68E-07	3.08E-07
KLF8	1.92E-07	1.15E-07	2.28E-07	1.35E-07	1.57E-08	6.13E-08	7.68E-08	1.93E-07
KLF9	2.92E-05	3.47E-05	6.23E-05	3.89E-05	1.66E-05	6.14E-05	2.52E-05	4.58E-05
KLHDC1	6.42E-07	6.40E-07	8.59E-07	5.46E-07	1.46E-07	7.85E-07	6.55E-07	1.46E-07
KLHDC10	4.06E-06	1.02E-05	4.72E-06	3.16E-06	6.26E-07	8.74E-06	2.33E-06	6.80E-06
KLHDC2	7.39E-05	3.89E-05	4.65E-05	3.96E-05	2.21E-05	7.28E-05	4.53E-05	3.80E-05
KLHDC3	5.75E-05	9.64E-05	4.57E-05	4.17E-05	8.81E-05	7.22E-05	6.14E-05	5.52E-05
KLHDC4	1.09E-05	3.82E-06	5.44E-06	1.09E-05	1.28E-05	9.08E-06	8.82E-06	9.06E-06
KLHDC5	6.21E-06	2.24E-06	2.69E-06	2.70E-06	4.71E-07	1.94E-06	2.05E-06	2.42E-06
KLHDC7A	1.05E-05	2.89E-06	2.84E-06	2.47E-06	4.77E-06	1.57E-06	1.17E-06	5.30E-08
KLHDC7B	1.75E-06	1.18E-06	2.24E-07	2.75E-07	4.66E-08	3.66E-07	3.01E-07	1.55E-06
KLHDC8A	4.76E-07	0	3.91E-08	6.09E-08	1.07E-08	1.15E-08	2.76E-07	9.15E-08

KLHDC8B	9.85E-06	6.61E-06	4.67E-06	8.04E-06	8.49E-06	1.23E-05	1.59E-05	6.92E-06
KLHDC9	1.73E-06	1.48E-05	3.05E-06	4.68E-06	5.88E-07	1.23E-05	4.87E-06	1.72E-06
KLHL1	0	0	0	0	0	0	0	0
KLHL10	3.78E-08	1.57E-07	3.97E-08	9.27E-08	3.25E-08	4.68E-08	0	3.71E-08
KLHL11	4.87E-07	5.50E-07	3.70E-07	2.49E-07	1.75E-08	6.31E-07	8.84E-08	3.28E-07
KLHL12	2.05E-05	1.13E-05	6.34E-06	7.16E-06	3.73E-06	1.93E-05	1.17E-05	1.82E-05
KLHL13	2.11E-06	3.03E-06	1.36E-06	1.08E-06	1.84E-07	1.12E-06	4.16E-07	2.94E-07
KLHL14	2.38E-08	0	4.10E-08	3.87E-08	0	7.33E-09	2.37E-08	0
KLHL15	1.81E-06	1.33E-06	5.36E-06	3.59E-06	6.42E-07	3.03E-06	1.87E-06	5.89E-06
KLHL17	5.53E-06	9.74E-07	1.02E-06	1.46E-06	1.03E-06	1.56E-06	8.56E-07	2.02E-06
KLHL18	6.35E-06	5.86E-06	4.57E-06	5.40E-06	2.06E-06	3.02E-06	5.00E-06	3.83E-06
KLHL2	8.11E-06	9.96E-06	1.10E-05	1.14E-05	4.02E-06	1.09E-05	9.79E-06	1.36E-05
KLHL20	5.17E-06	6.54E-06	7.18E-06	4.52E-06	6.56E-07	9.98E-06	3.15E-06	7.64E-06
KLHL21	1.58E-05	9.36E-06	5.22E-06	1.13E-05	2.63E-06	2.12E-05	1.33E-05	1.57E-05
KLHL22	8.50E-06	7.25E-06	6.87E-06	6.48E-06	9.16E-06	5.95E-06	7.04E-06	8.03E-06
KLHL23	4.81E-06	5.53E-06	2.02E-06	8.75E-07	2.79E-08	1.36E-06	2.38E-07	2.08E-06
KLHL24	1.03E-05	7.29E-06	8.75E-06	5.01E-06	1.40E-06	9.37E-06	4.66E-06	8.06E-06
KLHL25	5.32E-06	8.65E-06	2.01E-06	5.95E-06	3.65E-06	7.05E-06	5.47E-06	6.98E-06
KLHL26	2.97E-06	3.25E-06	2.69E-06	3.02E-06	7.56E-07	3.19E-06	2.19E-06	2.06E-06
KLHL28	3.55E-06	1.89E-06	2.37E-06	1.72E-06	2.72E-07	1.85E-06	9.66E-07	1.65E-06
KLHL29	2.28E-05	2.02E-06	1.24E-06	2.87E-06	4.74E-07	3.21E-07	8.73E-07	4.93E-06
KLHL3	2.35E-06	8.05E-07	4.22E-07	4.02E-07	8.42E-07	4.84E-07	1.18E-07	6.44E-07
KLHL30	3.16E-07	1.44E-08	5.98E-08	3.15E-08	3.87E-08	2.98E-08	2.50E-08	6.61E-08
KLHL31	1.69E-07	2.81E-07	5.44E-08	1.42E-07	5.03E-08	6.60E-08	6.60E-08	4.00E-08
KLHL32	4.27E-07	6.61E-07	3.10E-07	2.99E-07	5.20E-08	4.33E-07	2.25E-07	8.54E-08
KLHL33	5.55E-08	4.23E-08	5.93E-08	4.55E-08	0	0	3.55E-08	0
KLHL34	2.46E-08	0	0	7.01E-08	0	0	0	1.20E-08
KLHL35	4.31E-07	5.67E-08	3.52E-08	6.11E-08	7.15E-08	1.54E-08	2.23E-07	2.45E-08
KLHL36	5.99E-06	3.12E-06	4.91E-06	6.41E-06	1.21E-06	6.05E-06	3.15E-06	4.80E-06
KLHL38	6.59E-08	4.48E-08	0	0	0	2.45E-08	0	5.83E-08
KLHL4	8.71E-08	5.18E-08	1.18E-07	1.43E-07	2.63E-08	2.48E-08	2.43E-08	6.51E-08
KLHL5	7.10E-05	2.97E-05	1.39E-05	1.58E-05	6.82E-06	1.63E-05	1.43E-05	4.32E-05
KLHL6	3.26E-06	7.41E-07	4.82E-07	1.18E-06	6.05E-08	5.08E-07	6.11E-07	5.05E-07
KLHL7	2.78E-05	1.14E-05	4.54E-06	5.76E-06	2.42E-06	6.95E-06	4.85E-06	8.88E-06
KLHL8	4.10E-06	3.06E-06	6.43E-06	3.50E-06	5.61E-07	5.30E-06	3.25E-06	5.41E-06
KLHL9	8.04E-06	8.18E-06	7.31E-06	7.62E-06	3.65E-06	8.74E-06	4.97E-06	7.48E-06
KLK1	0	0	0	0	0	5.91E-08	0	0
KLK10	0	2.63E-08	5.25E-07	1.91E-08	0	0	0	0
KLK11	0	0	2.14E-07	1.90E-07	0	0	2.11E-08	0
KLK12	0	0	2.45E-07	0	0	0	0	0
KLK13	0	4.43E-07	1.65E-07	0	0	3.31E-08	4.67E-08	0
KLK14	2.30E-08	5.29E-08	7.46E-08	0	0	0	0	6.82E-08
KLK15	4.00E-08	0	0	0	0	0	0	0
KLK2	0	0	0	0	0	8.09E-09	0	0
KLK3	0	0	0	0	0	0	0	0
KLK4	0	0	0	0	0	0	2.16E-08	0
KLK5	0	0	2.01E-07	0	0	0	0	0
KLK6	0	0	2.98E-07	0	0	2.89E-08	0	0
KLK7	0	0	4.21E-07	0	0	0	0	0
KLK8	0	0	2.20E-07	0	0	0	0	0
KLK9	0	0	6.84E-08	0	0	0	0	0
KLKB1	2.05E-05	1.65E-05	0.000144	7.72E-05	0.000111	5.31E-05	5.39E-05	4.82E-05
KLKP1	0	0	0	0	0	0	0	0
KLRA1	3.48E-07	1.94E-07	9.41E-08	1.09E-07	2.77E-08	7.14E-08	2.48E-08	1.35E-07
KLRAQ1	2.27E-05	1.35E-05	6.23E-06	6.69E-06	2.27E-06	7.51E-06	4.34E-06	4.09E-06
KLRB1	5.66E-06	9.33E-07	5.97E-06	1.50E-05	9.93E-07	2.21E-06	1.15E-05	5.27E-06
KLRC1	2.42E-07	6.61E-08	3.09E-07	3.06E-07	3.32E-08	5.14E-07	1.76E-08	2.85E-08
KLRC2	5.71E-07	0	9.12E-08	1.04E-07	1.83E-08	1.06E-07	8.11E-08	9.42E-08

KLRC3	1.27E-07	2.22E-07	1.42E-07	3.11E-07	0	2.28E-07	0	0
KLRC4	8.48E-07	0	6.42E-08	1.82E-07	0	2.20E-07	0	0
KLRD1	7.54E-07	3.16E-07	8.27E-07	9.08E-07	5.64E-08	4.66E-07	3.92E-07	1.06E-07
KLRF1	4.33E-07	1.21E-07	8.65E-07	5.62E-07	2.04E-07	1.90E-07	9.84E-07	1.43E-07
KLRG1	9.23E-07	4.93E-07	7.91E-07	1.60E-06	2.32E-07	4.29E-07	4.72E-07	3.80E-07
KLRG2	7.05E-07	0	2.33E-08	1.97E-08	0	1.49E-08	0	0
KLRK1	4.19E-06	3.21E-07	2.25E-06	5.73E-06	1.62E-07	3.19E-06	1.27E-06	9.29E-07
KMO	7.03E-06	2.68E-05	2.72E-05	1.26E-05	7.28E-06	7.06E-06	6.67E-06	7.00E-05
KNCN	0	0	0	0	0	0	0	0
KNDC1	1.50E-07	0	1.08E-06	7.22E-07	3.76E-07	1.83E-08	1.72E-06	9.23E-06
KNG1	0.000616	0.00216	0.001905	0.001918	0.003004	0.001806	0.001264	0.002318
KNTC1	5.06E-06	2.89E-06	3.36E-07	6.68E-07	4.18E-07	7.88E-07	5.53E-07	7.76E-07
KPNA1	1.53E-05	1.78E-05	1.05E-05	7.01E-06	3.09E-06	1.09E-05	6.32E-06	1.29E-05
KPNA2	8.22E-05	5.96E-05	6.10E-06	1.94E-05	1.08E-05	3.04E-05	1.99E-05	2.76E-05
KPNA3	1.77E-05	1.91E-05	1.07E-05	8.78E-06	2.74E-06	1.54E-05	7.76E-06	9.29E-06
KPNA4	1.66E-05	1.61E-05	9.83E-06	1.62E-05	5.56E-06	1.55E-05	1.31E-05	1.37E-05
KPNA5	9.81E-07	7.35E-07	3.92E-07	5.67E-07	4.74E-08	6.34E-07	1.28E-07	1.62E-07
KPNA6	1.65E-05	8.76E-06	6.72E-06	6.59E-06	2.68E-06	9.67E-06	5.95E-06	9.77E-06
KPNA7	4.61E-06	3.61E-06	9.18E-08	3.92E-07	3.75E-08	4.04E-08	2.38E-07	2.78E-07
KPNB1	8.56E-05	6.74E-05	2.75E-05	2.89E-05	1.06E-05	4.85E-05	2.60E-05	3.47E-05
KPRP	0	0	5.80E-08	0	0	0	0	0
KPTN	1.16E-05	6.79E-06	4.00E-06	4.70E-06	7.77E-06	4.97E-06	5.02E-06	5.52E-06
KRAS	6.64E-06	8.25E-06	5.85E-06	5.62E-06	7.95E-07	3.63E-06	2.89E-06	4.89E-06
KRBA1	1.76E-06	1.09E-07	4.85E-07	9.14E-07	2.94E-07	7.40E-08	5.27E-07	1.11E-06
KRBA2	1.28E-06	5.29E-07	5.54E-07	4.26E-07	2.38E-08	5.27E-07	2.59E-07	5.61E-07
KRCC1	1.67E-05	4.42E-05	2.14E-05	2.18E-05	9.23E-06	3.09E-05	1.56E-05	2.19E-05
KREMEN1	3.28E-06	9.03E-07	1.20E-06	1.68E-06	5.76E-07	1.21E-06	1.73E-06	1.17E-06
KREMEN2	1.29E-06	2.08E-07	0	4.78E-08	8.68E-09	2.41E-08	1.37E-07	0
KRI1	1.30E-05	7.92E-06	4.85E-06	7.33E-06	9.63E-06	7.79E-06	7.98E-06	7.67E-06
KRIT1	6.59E-05	7.51E-06	4.09E-06	3.97E-06	1.14E-06	6.21E-06	3.08E-06	4.60E-06
KRR1	1.21E-05	1.06E-05	6.42E-06	6.67E-06	1.18E-06	1.14E-05	3.81E-06	4.85E-06
KRT1	0	2.18E-08	9.62E-06	1.19E-08	0	8.97E-09	9.38E-09	1.42E-08
KRT10	5.93E-05	5.09E-05	2.28E-05	2.73E-05	5.60E-05	2.62E-05	3.57E-05	1.81E-05
KRT12	0	0	0	0	0	0	0	4.07E-07
KRT13	1.45E-08	5.31E-07	1.42E-05	0	0	0	5.24E-07	1.31E-06
KRT14	0	0	3.46E-05	3.69E-08	0	1.71E-08	1.11E-07	8.86E-08
KRT15	3.12E-07	0	1.46E-08	2.71E-08	0	1.29E-08	2.25E-07	1.32E-06
KRT16	1.42E-08	3.23E-08	2.38E-05	0	0	0	5.48E-08	2.93E-07
KRT17	1.86E-06	3.74E-07	1.56E-05	2.71E-06	1.35E-07	3.78E-07	8.14E-07	2.54E-07
KRT18	0.000776	0.000592	0.000259	0.001424	0.000533	0.000347	0.001306	0.000469
KRT19	0.00021	2.57E-06	3.89E-06	6.26E-05	1.46E-08	3.15E-08	1.76E-05	1.48E-06
KRT2	0	0	2.44E-07	1.19E-08	0	9.04E-09	9.45E-09	0
KRT20	9.54E-07	6.37E-08	0	8.61E-08	0	0	1.35E-08	2.07E-07
KRT222	9.19E-06	4.58E-06	2.91E-06	1.71E-06	2.26E-06	2.67E-06	9.57E-07	2.20E-06
KRT23	4.38E-05	3.68E-07	1.34E-07	6.27E-05	1.39E-05	1.96E-07	0.000106	2.75E-05
KRT24	0	0	0	0	0	0	0	0
KRT25	0	0	0	0	0	0	0	0
KRT26	0	0	0	0	0	0	0	0
KRT27	0	0	0	1.87E-08	1.84E-07	0	0	2.25E-08
KRT28	0	0	0	0	0	0	0	0
KRT3	1.02E-08	0	0	0	0	0	0	0
KRT31	0	0	0	0	0	0	0	0
KRT32	0	0	0	0	0	0	0	0
KRT33A	0	0	0	0	0	0	0	0
KRT33B	0	0	0	0	0	0	0	0
KRT34	0	0	0	0	0	1.32E-08	0	0
KRT35	0	0	0	0	0	0	0	0
KRT36	1.19E-07	1.70E-08	3.15E-08	9.16E-08	0	0	1.15E-07	0

KRT37	0	0	0	0	0	0	0	0
KRT38	0	0	0	0	0	0	0	0
KRT39	0	1.64E-08	1.52E-08	8.83E-08	1.24E-08	0	6.94E-08	2.12E-08
KRT4	1.94E-06	0	3.78E-08	0	0	0	7.05E-08	0
KRT40	1.24E-08	0	1.30E-08	3.04E-08	0	0	0	0
KRT5	3.08E-08	9.27E-08	1.34E-05	1.89E-07	0	6.67E-08	7.26E-07	5.74E-06
KRT6A	0	0	1.93E-05	1.06E-07	0	0	3.07E-07	6.77E-07
KRT6B	0	0	1.71E-05	3.81E-08	0	0	1.10E-07	1.83E-07
KRT6C	0	0	1.05E-05	2.53E-08	0	0	0	1.22E-07
KRT7	0.00012	1.18E-05	1.43E-05	0.000295	2.11E-06	1.06E-07	1.02E-05	6.55E-07
KRT71	0	0	2.18E-08	1.28E-08	0	9.69E-09	0	0
KRT72	0	2.89E-08	0	3.13E-08	2.20E-08	1.18E-08	0	0
KRT73	0	0	0	0	0	0	0	0
KRT74	0	0	0	0	0	0	0	0
KRT75	0	0	2.34E-08	0	0	0	1.08E-08	0
KRT76	0	0	9.64E-09	0	0	0	0	0
KRT77	0	0	0	0	0	0	0	0
KRT78	0	0	1.29E-07	0	0	0	0	0
KRT79	0	0	0	2.03E-08	0	0	0	0
KRT8	0.000889	0.000457	0.000327	0.001428	0.000723	0.000247	0.001262	0.000325
KRT80	1.02E-05	2.53E-06	4.86E-07	8.85E-06	2.03E-08	1.46E-07	7.97E-08	2.60E-08
KRT81	3.76E-07	2.14E-07	5.26E-08	1.26E-06	0	3.51E-08	1.41E-07	5.57E-08
KRT82	0	0	0	0	0	0	0	0
KRT83	0	1.46E-08	1.35E-08	6.31E-08	0	0	0	0
KRT84	0	0	0	0	0	0	0	0
KRT85	9.43E-09	0	1.96E-08	0	1.62E-08	0	9.14E-09	0
KRT86	1.71E-07	8.90E-07	0	1.06E-06	2.95E-07	0	3.39E-07	6.72E-08
KRT9	0	1.17E-08	1.08E-08	0	0	0	0	0
KRTAP10-	0	0	0	0	0	0	0	0
KRTAP10-	0	0	0	0	0	0	0	0
KRTAP10-	0	0	0	0	0	0	0	0
KRTAP10-	0	0	0	0	0	0	0	0
KRTAP10-	0	0	0	0	0	0	0	0
KRTAP10-	0	0	0	0	0	0	0	0
KRTAP10-	0	2.08E-08	0	0	0	5.07E-08	0	0
KRTAP10-	0	0	0	0	0	0	0	0
KRTAP10-	0	0	2.22E-08	0	0	1.93E-08	0	0
KRTAP10-	0	0	0	0	0	0	0	0
KRTAP10-	0	0	0	0	0	0	0	0
KRTAP10-	0	0	0	0	0	0	0	0
KRTAP1-1	6.06E-08	0	0	2.71E-06	0	0	0	0
KRTAP11-	0	0	0	0	0	0	0	0
KRTAP12-	0	0	0	0	0	0	0	0
KRTAP12-	0	0	0	0	0	0	0	0
KRTAP12-	0	0	0	0	0	0	0	0
KRTAP12-	0	0	0	0	0	0	0	0
KRTAP1-3	0	0	0	3.44E-08	0	0	0	0
KRTAP13-	0	0	0	0	0	0	0	0
KRTAP13-	0	0	0	0	0	0	0	0
KRTAP13-	0	0	0	0	0	0	0	0
KRTAP13-	0	0	0	0	0	0	0	0
KRTAP1-5	0	0	0	0	0	0	0	0
KRTAP15-	0	0	0	0	0	0	0	0
KRTAP17-	0	0	0	0	0	0	0	0
KRTAP19-	0	0	0	0	0	0	0	0
KRTAP19-	0	0	0	0	0	0	0	0
KRTAP19-	0	0	0	0	0	0	0	0
KRTAP19-	0	0	0	0	0	0	0	0

KRTAP19-	0	0	0	0	0	0	0	0
KRTAP19-	0	0	0	0	0	0	0	0
KRTAP19-	0	0	0	0	0	0	0	0
KRTAP19-	0	0	0	0	0	0	0	0
KRTAP20-	0	0	0	0	0	0	0	0
KRTAP20-	0	0	0	0	0	0	0	0
KRTAP20-	0	0	0	0	0	0	0	0
KRTAP20-	0	0	0	0	0	0	0	0
KRTAP2-1	0	0	0	0	0	0	0	0
KRTAP21-	0	0	0	0	0	0	0	0
KRTAP21-	0	0	0	0	0	0	0	0
KRTAP2-2	0	0	0	0	0	0	0	0
KRTAP22-	0	0	0	0	0	0	0	0
KRTAP23-	0	0	0	0	0	0	0	0
KRTAP2-4	0	0	0	0	0	0	0	0
KRTAP24-	0	0	0	0	0	0	0	0
KRTAP25-	0	0	0	0	0	0	0	0
KRTAP26-	0	0	0	0	0	0	0	0
KRTAP27-	0	0	0	0	0	0	0	0
KRTAP3-1	5.35E-08	0	0	0	0	0	0	0
KRTAP3-2	0	0	0	0	0	0	0	0
KRTAP3-3	0	0	0	0	0	0	0	0
KRTAP4-1	0	0	0	0	0	0	0	0
KRTAP4-1	0	0	0	2.72E-08	0	0	0	0
KRTAP4-1	0	0	0	0	0	0	0	0
KRTAP4-2	0	0	0	0	0	0	0	0
KRTAP4-3	0	0	0	0	0	0	0	0
KRTAP4-4	0	0	0	0	0	0	0	0
KRTAP4-5	0	0	0	0	0	0	0	0
KRTAP4-7	0	0	0	0	0	0	0	0
KRTAP4-8	0	0	0	0	0	0	0	0
KRTAP4-9	0	0	0	0	0	0	0	0
KRTAP5-1	4.60E-07	1.34E-07	0	3.56E-08	2.49E-08	0	1.64E-07	1.29E-07
KRTAP5-1	0	2.88E-08	0	0	0	2.33E-08	0	0
KRTAP5-1	0	0	0	0	0	0	0	0
KRTAP5-2	1.48E-07	4.34E-07	8.25E-08	6.27E-08	0	0	2.50E-08	6.08E-07
KRTAP5-3	0	0	0	0	0	0	0	0
KRTAP5-4	2.34E-08	0	0	0	0	0	0	0
KRTAP5-5	4.63E-08	0	0	0	0	0	0	0
KRTAP5-6	0	1.43E-07	3.54E-07	0	2.04E-07	6.61E-07	0	9.80E-07
KRTAP5-7	0	2.14E-07	6.78E-08	0	0	0	2.90E-08	0
KRTAP5-8	0	2.51E-08	0	2.69E-08	3.77E-08	0	2.09E-08	0
KRTAP5-9	6.51E-08	9.98E-08	4.68E-08	1.07E-07	3.75E-08	1.01E-07	6.23E-08	6.44E-08
KRTAP6-1	0	0	0	0	0	0	0	0
KRTAP6-2	0	0	0	0	0	0	0	0
KRTAP6-3	0	0	0	0	0	0	0	0
KRTAP7-1	0	0	0	0	0	0	0	0
KRTAP8-1	0	0	0	0	0	0	0	0
KRTAP9-2	0	0	0	0	0	0	0	0
KRTAP9-4	0	0	0	0	0	0	0	0
KRTAP9-8	0	0	0	0	0	0	0	0
KRTAP9-9	0	3.38E-08	0	0	0	0	0	0
KRTCAP2	0.000223	0.00035	0.000135	0.000186	0.000445	0.000333	0.000366	0.000489
KRTCAP3	5.52E-07	1.01E-07	5.87E-06	9.96E-06	5.49E-07	8.07E-08	3.69E-07	0
KRTDAP	0	0	1.08E-05	0	0	0	0	0
KSR1	8.97E-06	1.81E-06	8.47E-07	2.74E-06	6.89E-07	2.37E-06	1.97E-06	2.08E-06
KSR2	1.29E-08	7.17E-09	0	4.72E-08	2.21E-09	1.43E-08	6.30E-09	5.65E-09
KTELC1	3.54E-06	5.78E-06	2.71E-06	3.50E-06	7.19E-07	4.02E-06	1.81E-06	3.85E-06

KTI12	6.71E-06	6.43E-06	3.72E-06	6.38E-06	2.06E-06	4.99E-06	4.97E-06	5.41E-06
KTN1	5.88E-05	4.21E-05	5.55E-05	5.64E-05	2.97E-05	8.08E-05	4.76E-05	6.65E-05
KY	1.63E-08	4.55E-09	0	0	0	3.28E-08	0	5.96E-09
KYNU	7.19E-05	5.08E-05	3.43E-05	3.05E-05	1.83E-05	3.19E-05	1.77E-05	9.19E-05
L1CAM	8.25E-08	4.55E-08	7.40E-08	1.04E-07	2.32E-08	4.17E-09	1.76E-08	1.98E-08
L1TD1	1.18E-08	1.33E-08	0	2.17E-08	0	1.10E-08	1.15E-08	0
L2HGDH	4.17E-06	2.79E-06	6.61E-06	2.02E-06	7.16E-07	5.08E-06	1.63E-06	2.18E-06
L3MBTL	1.42E-06	7.98E-07	2.55E-07	1.72E-07	1.67E-07	4.69E-07	1.13E-07	2.07E-07
L3MBTL2	1.09E-05	1.53E-05	9.09E-06	9.80E-06	8.34E-06	1.71E-05	9.49E-06	8.43E-06
L3MBTL3	2.19E-06	2.51E-06	1.14E-06	1.29E-06	3.12E-07	1.21E-06	8.26E-07	5.73E-07
L3MBTL4	5.11E-06	3.60E-06	4.72E-06	5.03E-06	1.53E-06	3.45E-06	3.60E-06	4.38E-06
LACE1	2.93E-06	1.46E-06	1.18E-06	1.31E-06	3.34E-07	2.29E-06	1.13E-06	2.05E-06
LACRT	0	0	0	0	0	0	0	0
LACTB	1.82E-05	2.77E-05	2.01E-05	2.55E-05	7.50E-06	3.78E-05	1.98E-05	2.72E-05
LACTB2	5.10E-05	6.19E-05	3.41E-05	3.10E-05	1.55E-05	5.22E-05	7.66E-05	4.32E-05
LAD1	4.12E-05	8.02E-06	6.60E-06	3.37E-05	1.49E-05	1.92E-06	1.86E-05	3.23E-06
LAG3	1.28E-05	4.67E-07	2.87E-06	2.73E-06	8.90E-07	3.54E-06	9.80E-07	4.93E-07
LAGE3	1.20E-05	1.83E-05	5.09E-06	6.33E-06	2.64E-05	8.37E-06	1.78E-05	1.68E-05
LAIR1	1.21E-05	1.44E-06	1.83E-06	8.35E-06	1.30E-06	3.86E-06	9.98E-06	9.79E-07
LAIR2	1.15E-06	2.03E-06	0	1.19E-07	0	3.56E-07	8.85E-08	1.93E-07
LALBA	0	0	0	0	0	0	0	0
LAMA1	2.51E-07	9.79E-09	4.64E-08	3.82E-07	1.76E-08	1.53E-08	1.62E-07	3.29E-08
LAMA2	1.45E-06	1.55E-07	1.21E-06	4.44E-06	4.57E-07	8.67E-08	1.09E-06	8.22E-07
LAMA3	1.58E-05	2.51E-06	6.69E-07	1.97E-06	4.38E-07	1.83E-05	6.17E-07	1.28E-06
LAMA4	1.14E-05	6.79E-06	5.92E-07	5.16E-06	4.65E-06	3.78E-06	8.60E-06	4.97E-06
LAMA5	1.41E-05	4.97E-06	2.78E-06	4.31E-06	1.33E-06	3.79E-06	4.41E-06	3.48E-06
LAMB1	4.25E-05	1.15E-05	1.22E-05	2.80E-05	1.01E-05	7.00E-06	2.40E-05	1.75E-05
LAMB2	3.58E-05	2.64E-05	2.84E-05	4.24E-05	3.08E-05	4.91E-05	4.74E-05	1.51E-05
LAMB2L	1.28E-07	1.83E-07	1.36E-07	1.58E-07	5.94E-07	1.94E-07	3.86E-07	1.18E-07
LAMB3	6.09E-06	7.82E-06	2.56E-06	4.58E-06	3.49E-07	4.01E-06	9.92E-07	5.41E-06
LAMB4	1.53E-08	2.57E-08	0	1.30E-08	3.29E-09	7.09E-09	4.48E-08	5.60E-09
LAMC1	1.68E-05	1.83E-05	7.11E-06	1.39E-05	2.12E-06	1.91E-05	9.36E-06	3.15E-05
LAMC2	2.10E-06	1.04E-07	2.29E-07	1.06E-05	3.43E-09	4.30E-07	1.28E-07	5.84E-08
LAMC3	1.03E-06	4.77E-07	3.09E-06	1.20E-05	7.67E-06	4.87E-07	6.76E-06	2.49E-06
LAMP1	0.000133	0.000314	0.00018	0.00016	0.000149	0.000128	0.000241	0.000194
LAMP2	0.000138	0.000153	7.09E-05	0.000117	5.68E-05	6.49E-05	8.67E-05	0.000107
LAMP3	6.87E-06	3.79E-07	1.92E-07	5.55E-07	5.91E-09	6.31E-07	1.94E-07	1.54E-06
LANCL1	3.08E-05	6.21E-05	9.07E-06	8.57E-06	2.63E-06	1.36E-05	7.65E-06	1.41E-05
LANCL2	7.77E-06	5.74E-06	4.09E-06	6.31E-06	2.27E-06	6.24E-06	5.58E-06	8.04E-06
LANCL3	0	0	0	1.73E-08	0	0	0	2.08E-08
LAP3	0.000139	7.99E-05	0.000131	8.46E-05	2.71E-05	0.00023	8.63E-05	9.27E-05
LAPTM4A	0.000356	0.000258	0.000174	0.000225	0.000151	0.000322	0.000196	0.000149
LAPTM4B	0.000246	6.30E-05	1.16E-05	2.52E-05	7.06E-06	8.69E-06	7.52E-06	2.34E-05
LAPTM5	0.000115	1.61E-05	3.44E-05	0.000104	1.82E-05	3.72E-05	6.61E-05	2.26E-05
LARGE	1.41E-05	1.06E-05	4.26E-06	5.51E-06	1.05E-05	5.38E-06	8.03E-06	8.09E-06
LARP1	3.90E-05	1.68E-05	1.51E-05	1.37E-05	5.22E-06	2.54E-05	1.52E-05	3.39E-05
LARP1B	2.30E-05	1.43E-05	2.25E-05	1.75E-05	3.98E-06	2.25E-05	1.05E-05	1.63E-05
LARP4	3.02E-05	3.98E-05	2.70E-05	1.97E-05	7.28E-06	2.82E-05	1.65E-05	1.67E-05
LARP4B	2.11E-05	5.04E-06	5.81E-06	6.05E-06	3.26E-06	7.41E-06	6.72E-06	6.81E-06
LARP6	3.28E-05	2.03E-07	1.76E-06	7.02E-06	3.34E-07	2.51E-07	3.35E-06	5.24E-07
LARP7	2.72E-05	1.50E-05	1.41E-05	2.00E-05	4.79E-06	2.42E-05	1.40E-05	1.99E-05
LARS	1.90E-05	1.93E-05	8.55E-06	9.66E-06	4.04E-06	1.32E-05	6.98E-06	1.83E-05
LARS2	1.11E-05	1.25E-05	5.51E-06	5.65E-06	1.98E-06	1.01E-05	4.99E-06	4.87E-06
LAS1L	2.78E-05	2.52E-05	1.30E-05	1.50E-05	2.64E-05	2.16E-05	1.97E-05	3.47E-05
LASP1	0.000221	8.79E-05	3.07E-05	5.55E-05	2.61E-05	4.88E-05	7.02E-05	8.02E-05
LASS1	1.60E-07	3.43E-07	2.59E-08	6.05E-08	8.49E-08	2.10E-08	2.33E-07	3.63E-08
LASS2	0.000409	0.00044	0.00032	0.000245	0.000125	0.000401	0.000276	0.00056
LASS3	0	0	3.62E-08	7.16E-09	4.93E-09	5.42E-09	0	0

LASS4	2.37E-05	1.93E-05	4.70E-05	3.91E-05	4.86E-05	3.64E-05	4.99E-05	5.38E-05
LASS5	2.06E-05	9.65E-06	4.30E-06	1.12E-05	4.81E-06	7.09E-06	9.64E-06	9.93E-06
LASS6	8.92E-06	8.50E-06	3.31E-06	3.18E-06	4.67E-07	2.26E-06	3.09E-06	1.45E-06
LAT	6.02E-06	5.65E-07	1.49E-06	4.06E-06	1.05E-06	2.67E-06	2.91E-06	1.46E-06
LAT2	8.77E-06	1.12E-06	3.48E-06	5.44E-06	9.46E-07	2.11E-06	3.24E-06	4.86E-06
LATS1	2.54E-06	2.80E-06	1.63E-06	2.06E-06	2.57E-08	2.16E-06	7.83E-07	1.73E-06
LATS2	9.88E-06	3.54E-06	3.40E-06	7.18E-06	8.19E-07	9.01E-06	3.69E-06	2.90E-06
LAX1	1.08E-06	8.30E-08	2.79E-07	6.39E-07	0	2.05E-07	7.95E-08	1.81E-07
LAYN	1.85E-05	1.67E-06	9.27E-08	1.18E-06	5.24E-07	7.51E-07	1.68E-06	3.92E-07
LBH	3.91E-05	4.23E-06	6.37E-06	1.74E-05	3.51E-06	7.00E-06	7.81E-06	6.06E-06
LBP	0.000513	0.000541	0.000356	0.000415	0.000131	0.000632	0.000728	0.000861
LBR	6.49E-05	3.98E-05	1.42E-05	1.83E-05	8.97E-06	2.93E-05	2.08E-05	1.97E-05
LBX1	0	0	0	0	0	0	0	0
LBX2	6.93E-06	3.75E-06	2.48E-06	1.57E-06	6.56E-06	6.73E-06	1.96E-06	1.26E-06
LBXCOR1	9.26E-08	9.47E-08	1.75E-08	4.12E-08	7.23E-09	2.34E-08	6.53E-08	3.71E-08
LCA5	6.62E-07	2.06E-07	3.75E-07	5.67E-07	2.57E-08	8.77E-08	8.11E-08	2.26E-07
LCA5L	4.89E-07	6.50E-07	2.96E-07	3.14E-07	1.04E-07	8.41E-07	3.61E-07	3.80E-07
LCAT	5.03E-06	3.17E-05	0.000198	7.81E-05	2.14E-05	2.65E-05	4.28E-05	2.73E-06
LCE1A	0	0	0	0	0	0	0	0
LCE1B	0	0	7.41E-08	0	0	0	2.18E-08	0
LCE1C	0	0	1.48E-07	0	0	0	0	6.46E-08
LCE1D	0	0	0	0	0	0	0	0
LCE1E	0	0	2.37E-08	0	0	0	0	0
LCE1F	0	0	1.97E-07	0	0	0	0	0
LCE2A	0	0	6.45E-08	0	0	0	0	0
LCE2B	0	0	1.83E-07	0	0	0	0	0
LCE2C	0	0	6.08E-08	0	0	0	0	0
LCE2D	0	8.13E-07	1.23E-07	0	0	4.86E-08	0	0
LCE3A	0	0	5.27E-07	0	0	0	0	0
LCE3B	0	0	0	0	0	0	0	0
LCE3C	0	0	0	0	0	0	0	0
LCE3D	0	0	1.71E-06	0	0	0	0	0
LCE3E	0	0	5.48E-07	0	0	0	0	0
LCE4A	0	0	0	0	0	0	0	0
LCE5A	0	0	0	0	0	0	0	0
LCE6A	0	0	0	0	0	0	0	0
LCK	9.31E-06	3.82E-07	2.40E-06	5.47E-06	2.64E-07	3.61E-06	2.83E-06	1.54E-06
LCLAT1	8.60E-06	5.54E-06	3.06E-06	3.08E-06	1.60E-06	4.00E-06	3.54E-06	2.40E-06
LCMT1	1.44E-05	1.29E-05	5.46E-06	1.16E-05	3.67E-06	1.31E-05	1.13E-05	8.83E-06
LCMT2	6.35E-06	3.90E-06	3.03E-06	3.17E-06	1.01E-06	3.32E-06	3.43E-06	3.11E-06
LCN1	0	0	0	0	0	0	4.37E-08	0
LCN10	3.30E-06	6.39E-08	1.33E-07	1.38E-07	1.09E-07	3.92E-08	6.78E-08	3.35E-07
LCN12	2.69E-06	1.22E-06	1.51E-06	1.97E-06	2.67E-06	6.10E-06	2.12E-06	4.72E-06
LCN15	0	2.24E-07	1.29E-07	9.42E-08	3.29E-08	0	0	0
LCN2	5.07E-05	1.71E-06	2.76E-06	2.46E-05	1.68E-05	0.000354	0.002025	0.000528
LCN6	1.10E-07	0	4.76E-08	5.17E-08	1.44E-07	0	0	0
LCN8	0	0	0	0	0	0	0	0
LCN9	0	0	0	0	0	0	0	0
LCNL1	1.32E-08	0	0	6.46E-08	2.27E-08	1.22E-08	1.27E-08	3.88E-08
LCOR	1.97E-06	7.48E-07	9.48E-07	5.44E-07	3.83E-07	7.35E-07	1.35E-07	2.92E-07
LCORL	1.05E-06	1.41E-06	8.89E-07	7.10E-07	1.50E-07	8.51E-07	4.61E-07	6.24E-07
LCP1	6.91E-05	6.61E-05	3.22E-05	5.39E-05	4.79E-06	4.33E-05	2.77E-05	2.10E-05
LCP2	1.91E-05	2.02E-06	6.72E-06	1.33E-05	1.10E-06	5.93E-06	6.81E-06	3.47E-06
LCT	1.07E-08	0	0	0	3.06E-09	0	0	0
LCTL	4.31E-08	0	3.53E-08	2.18E-08	0	0	0	5.21E-08
LDB1	2.05E-05	5.54E-06	9.10E-06	1.08E-05	9.65E-07	1.01E-05	8.86E-06	9.44E-06
LDB2	6.56E-06	5.29E-06	8.05E-06	7.45E-06	3.59E-06	3.62E-06	6.18E-06	7.15E-06
LDB3	2.12E-07	1.97E-07	2.61E-08	2.28E-07	1.45E-07	4.44E-08	3.11E-07	2.09E-07

LDHA	0.000594	0.00028	0.000355	0.000389	9.15E-05	0.000357	0.000306	0.000517
LDHAL6A	8.43E-08	0	1.26E-08	0	0	0	0	0
LDHAL6B	8.81E-08	5.01E-08	3.10E-08	3.61E-08	0	0	1.42E-08	6.50E-08
LDHB	0.000635	4.37E-05	1.70E-05	5.42E-05	9.48E-06	2.60E-05	2.77E-05	1.60E-05
LDHC	0	2.73E-08	7.70E-08	5.84E-08	1.39E-06	4.10E-06	3.02E-08	0
LDHD	4.46E-05	0.000166	0.000112	7.16E-05	4.99E-05	0.000113	4.80E-05	7.62E-05
LDLR	1.96E-05	6.68E-05	4.75E-05	8.40E-05	3.87E-06	5.06E-05	4.10E-05	5.66E-05
LDLRAD1	0	1.26E-07	0	0	0	6.97E-06	5.13E-07	2.14E-06
LDLRAD2	7.80E-06	6.55E-07	1.12E-06	3.99E-06	4.18E-05	2.10E-06	1.98E-05	8.98E-06
LDLRAD3	2.41E-06	5.01E-07	5.25E-07	1.15E-06	2.83E-08	3.31E-07	6.35E-07	3.14E-07
LDLRAP1	1.20E-05	1.68E-05	1.88E-05	1.44E-05	5.08E-06	1.17E-05	1.34E-05	1.54E-05
LDOC1	2.46E-06	9.65E-07	2.53E-06	7.99E-06	8.86E-07	2.90E-07	3.79E-06	1.19E-06
LDOC1L	5.23E-06	4.34E-07	1.05E-06	2.89E-06	3.23E-07	3.44E-07	6.75E-07	4.14E-07
LEAP2	0.00014	4.15E-05	0.000487	0.00053	0.00015	0.000241	0.000449	0.000321
LECT1	0	0	0	0	0	0	0	0
LECT2	4.88E-08	6.72E-05	0.000241	0.000186	0.000281	6.40E-05	0.000129	3.56E-05
LEF1	4.54E-06	2.58E-07	3.06E-07	1.38E-06	1.83E-07	5.74E-06	7.73E-07	4.12E-06
LEFTY1	1.08E-05	6.06E-07	1.37E-06	1.12E-07	3.28E-07	9.89E-08	7.33E-08	3.82E-07
LEFTY2	1.45E-07	3.68E-08	2.63E-08	1.98E-08	1.39E-08	1.50E-08	3.11E-08	7.15E-08
LEKR1	3.96E-07	3.85E-07	2.38E-07	3.65E-07	2.47E-07	4.31E-07	1.86E-07	8.13E-08
LELP1	0	0	0	0	0	0	0	0
LEMD1	0	0	0	0	0	0	0	0
LEMD2	2.05E-05	2.96E-05	7.59E-06	1.23E-05	1.25E-05	1.21E-05	1.90E-05	2.12E-05
LEMD3	6.58E-06	4.55E-06	3.65E-06	3.29E-06	1.21E-06	3.03E-06	2.88E-06	3.02E-06
LENEP	0	5.67E-08	0	0	0	0	0	7.14E-08
LENG1	1.41E-05	1.31E-05	1.06E-05	1.58E-05	1.73E-05	1.00E-05	1.84E-05	1.33E-05
LENG8	3.11E-05	1.29E-05	7.77E-06	9.72E-06	3.79E-06	1.06E-05	6.87E-06	7.43E-06
LENG9	2.54E-06	2.69E-06	1.32E-06	3.39E-06	2.48E-06	4.32E-06	5.64E-06	3.81E-06
LEO1	1.68E-05	1.70E-05	9.66E-06	1.21E-05	7.30E-06	1.58E-05	8.71E-06	2.41E-05
LEP	0	0	0	1.64E-08	0	0	0	0
LEPR	0.000186	0.00019	6.45E-05	0.000106	2.95E-05	6.98E-05	3.35E-05	4.78E-05
LEPRE1	2.22E-05	1.07E-05	7.18E-06	1.40E-05	9.89E-06	1.12E-05	1.28E-05	2.00E-05
LEPREL1	2.34E-06	2.40E-07	2.27E-06	4.91E-06	5.12E-07	5.52E-07	6.70E-07	1.07E-06
LEPREL2	5.04E-06	2.86E-06	7.85E-07	5.84E-06	1.25E-06	2.03E-07	2.33E-06	1.93E-06
LEPROT	8.49E-05	4.37E-05	3.08E-05	3.50E-05	2.26E-07	4.63E-05	6.92E-06	2.12E-05
LEPROTL1	2.66E-05	1.70E-05	1.15E-05	1.85E-05	3.20E-06	1.53E-05	1.05E-05	1.65E-05
LETM1	1.11E-05	1.57E-05	7.03E-06	5.91E-06	6.29E-06	8.95E-06	9.87E-06	1.56E-05
LETM2	8.28E-07	3.91E-07	1.47E-07	3.76E-07	1.30E-08	1.51E-07	2.45E-07	4.63E-07
LETMD1	4.27E-05	2.22E-05	2.79E-05	2.01E-05	1.61E-05	3.57E-05	2.87E-05	1.45E-05
LEUTX	0	0	0	0	0	0	0	0
LFNG	4.86E-06	5.16E-07	1.16E-06	3.59E-06	2.25E-06	9.77E-07	2.18E-06	1.61E-06
LGALS1	0.000414	0.000141	8.46E-05	0.000309	0.000295	0.000113	0.000382	0.000177
LGALS12	5.72E-08	3.25E-08	1.51E-08	5.26E-08	0	5.31E-08	2.76E-08	0
LGALS13	0	0	0	0	0	0	0	0
LGALS14	0	0	0	0	0	0	0	0
LGALS2	3.04E-05	7.13E-07	2.29E-06	1.54E-06	1.29E-06	2.06E-06	4.06E-06	1.38E-06
LGALS3	0.000211	8.18E-06	1.74E-05	0.000104	1.73E-05	5.35E-06	0.000116	6.61E-06
LGALS3BP	0.00069	0.000343	9.08E-05	0.000226	9.97E-06	0.000254	3.88E-05	0.000269
LGALS4	0.000471	0.000547	0.000198	0.000369	0.00044	6.73E-05	0.000183	0.000235
LGALS7	7.07E-08	6.27E-05	8.97E-07	0	6.24E-08	0	0	0
LGALS7B	7.21E-08	0.000123	1.65E-06	0	0	0	1.32E-07	0
LGALS8	1.35E-05	8.91E-06	1.41E-05	1.69E-05	1.98E-06	2.53E-05	6.26E-06	2.94E-05
LGALS9	4.82E-05	4.63E-06	1.32E-05	3.05E-05	5.40E-06	1.49E-05	2.53E-05	4.45E-05
LGALS9B	0	0	0	0	0	0	0	0
LGALS9C	9.36E-07	8.58E-08	3.67E-07	1.30E-06	3.90E-07	5.48E-07	4.36E-07	3.93E-06
LGI1	6.51E-07	0	3.35E-07	6.69E-08	1.33E-08	0	0	0
LGI2	1.12E-06	6.30E-08	2.89E-08	2.28E-07	7.57E-08	8.81E-08	2.06E-07	2.84E-07
LGI3	4.87E-07	7.92E-09	1.49E-08	8.83E-09	3.10E-08	7.40E-07	0	0

LGI4	2.49E-06	8.73E-07	7.26E-07	2.34E-06	1.63E-06	3.29E-07	1.89E-06	2.58E-06
LGMN	0.000101	3.80E-05	6.46E-05	0.000118	3.46E-05	7.07E-05	0.000102	3.80E-05
LGR4	4.55E-05	3.89E-05	2.82E-05	1.54E-05	4.79E-06	9.64E-06	8.70E-06	3.64E-05
LGR5	1.39E-08	6.30E-09	8.20E-07	6.64E-07	0	2.14E-05	1.64E-08	4.47E-08
LGR6	6.86E-07	2.74E-07	9.61E-07	2.91E-06	1.90E-07	1.69E-07	3.53E-07	1.82E-05
LGSN	8.64E-08	1.01E-06	7.01E-07	1.75E-07	1.85E-08	9.91E-07	2.10E-08	1.77E-07
LGTN	2.31E-05	1.45E-05	1.77E-05	1.94E-05	1.58E-05	5.47E-05	2.29E-05	3.39E-05
LHB	2.79E-06	0	8.06E-08	8.21E-08	0	0	2.39E-07	0
LHCGR	0	0	0	0	0	0	0	0
LHFP	1.45E-05	3.75E-06	3.67E-06	1.04E-05	1.63E-06	3.95E-06	4.79E-06	9.92E-06
LHFPL1	3.13E-08	0	0	1.92E-08	0	1.87E-08	1.92E-08	2.17E-08
LHFPL2	7.02E-06	1.81E-06	1.24E-06	8.78E-06	2.65E-07	2.58E-06	2.52E-06	6.54E-07
LHFPL3	9.45E-08	0	0	8.89E-09	0	6.73E-09	0	0
LHFPL4	3.04E-06	5.17E-09	0	1.13E-08	0	0	0	1.35E-08
LHFPL5	8.86E-08	5.01E-08	0	0	0	0	0	1.63E-08
LHPP	2.20E-05	3.57E-05	4.03E-05	1.87E-05	6.43E-05	5.55E-05	2.61E-05	2.53E-05
LHX1	0	0	1.97E-08	0	0	0	0	0
LHX2	2.13E-06	0	1.71E-06	1.76E-06	1.17E-07	7.22E-08	6.60E-08	4.29E-08
LHX3	5.88E-08	5.62E-08	4.16E-08	0	1.46E-07	4.07E-07	3.38E-07	6.45E-07
LHX4	1.26E-07	3.37E-07	0	1.58E-08	0	2.80E-08	0	7.93E-08
LHX5	0	0	0	0	0	0	0	0
LHX6	5.83E-07	3.50E-07	1.39E-07	6.06E-08	1.16E-07	1.52E-07	7.42E-07	1.45E-07
LHX8	0	3.13E-08	0	0	0	0	0	0
LHX9	1.84E-07	2.65E-07	5.23E-08	2.09E-07	2.46E-08	8.84E-09	4.22E-07	5.27E-07
LIAS	1.07E-05	1.77E-05	5.14E-06	4.86E-06	6.17E-06	1.32E-05	9.55E-06	6.24E-06
LIF	1.46E-05	4.30E-07	2.47E-07	2.30E-05	4.72E-08	3.36E-07	1.38E-06	2.75E-06
LIFR	1.15E-06	3.80E-06	9.09E-06	7.96E-06	2.03E-07	4.70E-07	7.83E-07	1.17E-06
LIG1	1.35E-05	6.26E-06	3.56E-06	5.08E-06	4.47E-06	5.25E-06	4.09E-06	6.83E-06
LIG3	4.93E-06	7.25E-06	2.79E-06	3.07E-06	1.50E-06	5.91E-06	4.45E-06	4.76E-06
LIG4	4.71E-06	4.21E-06	3.48E-06	3.70E-06	8.49E-07	3.48E-06	1.08E-06	3.31E-06
LILRA1	3.06E-07	1.02E-07	4.13E-07	5.79E-07	0	2.46E-07	2.77E-07	3.99E-08
LILRA2	1.08E-06	2.69E-07	2.69E-06	4.91E-06	2.19E-07	6.65E-07	2.28E-06	2.84E-07
LILRA3	1.60E-06	1.30E-07	3.96E-07	1.01E-06	1.57E-08	8.73E-08	1.75E-08	5.38E-08
LILRA4	4.19E-07	0	2.12E-07	1.02E-06	0	1.06E-07	4.68E-07	1.95E-08
LILRA5	2.28E-06	7.18E-07	2.76E-06	5.76E-06	1.19E-07	1.73E-06	5.67E-07	8.14E-07
LILRA6	1.44E-06	2.35E-07	8.04E-07	1.03E-06	7.14E-08	4.91E-07	7.71E-07	1.16E-07
LILRB1	3.69E-06	3.65E-07	2.77E-06	3.34E-06	3.14E-07	1.86E-06	2.69E-06	3.63E-07
LILRB2	5.03E-06	7.85E-07	2.74E-06	5.49E-06	4.45E-07	2.16E-06	3.11E-06	7.13E-07
LILRB3	2.86E-06	4.79E-07	7.72E-07	3.70E-06	1.71E-07	5.27E-07	1.64E-06	5.14E-07
LILRB4	1.37E-05	9.40E-07	2.59E-06	9.47E-06	6.35E-07	3.43E-06	7.53E-06	7.08E-07
LILRB5	2.94E-06	1.07E-06	7.64E-06	6.88E-06	9.10E-07	2.99E-06	6.88E-06	9.83E-07
LILRP2	3.78E-08	0	0	0	0	1.10E-08	1.24E-08	0
LIM2	0	0	0	0	0	0	1.04E-07	0
LIMA1	1.74E-05	7.61E-06	5.70E-06	9.92E-06	2.60E-06	1.44E-05	4.98E-06	7.63E-06
LIMCH1	2.01E-05	3.04E-06	4.24E-06	3.03E-06	9.48E-07	2.49E-06	1.64E-06	2.35E-06
LIMD1	1.04E-05	9.96E-06	6.59E-06	4.48E-06	1.34E-06	8.08E-06	6.90E-06	8.95E-06
LIMD2	1.37E-05	7.31E-07	3.32E-06	7.01E-06	2.90E-06	3.18E-06	4.40E-06	2.43E-06
LIME1	3.05E-05	0.000206	0.00015	7.35E-05	0.000461	0.000151	0.000108	1.08E-05
LIMK1	1.11E-05	4.37E-06	2.23E-06	4.92E-06	4.07E-07	4.68E-06	3.72E-06	4.02E-06
LIMK2	3.28E-05	3.55E-06	2.48E-06	6.77E-06	2.24E-06	4.18E-05	1.07E-05	3.06E-05
LIMS1	1.31E-05	7.25E-06	5.81E-06	6.15E-06	1.75E-07	1.14E-05	1.16E-06	3.73E-06
LIMS2	2.90E-05	3.07E-05	2.71E-05	2.15E-05	4.63E-05	1.45E-05	2.35E-05	3.17E-05
LIMS3	1.64E-07	1.19E-06	0	2.76E-07	0	8.13E-08	4.11E-07	9.72E-08
LIMS3-LO	2.18E-06	5.85E-06	8.26E-07	7.88E-06	2.58E-06	4.64E-06	6.30E-06	4.96E-06
LIN28A	0	7.36E-09	0	0	0	0	0	0
LIN28B	1.03E-06	4.71E-09	0	0	0	0	3.98E-09	0
LIN37	9.63E-06	7.94E-06	5.11E-06	7.22E-06	1.90E-05	8.72E-06	1.44E-05	1.30E-05
LIN52	7.02E-06	3.67E-06	2.94E-06	3.11E-06	5.57E-07	5.57E-06	3.99E-06	2.88E-06

LIN54	3.96E-06	2.54E-06	2.71E-06	1.93E-06	1.15E-06	2.46E-06	1.56E-06	3.47E-06
LIN7A	9.21E-06	9.25E-06	1.04E-05	9.15E-06	4.15E-06	1.20E-05	8.60E-06	8.45E-06
LIN7B	1.80E-06	1.77E-06	1.35E-06	4.05E-06	6.87E-06	1.33E-06	5.16E-06	2.54E-06
LIN7C	7.70E-06	1.05E-05	1.20E-05	8.28E-06	3.94E-06	9.68E-06	7.04E-06	1.09E-05
LIN9	1.82E-06	1.30E-06	6.74E-07	4.70E-07	1.68E-07	8.55E-07	3.66E-07	5.63E-07
LINGO1	3.60E-06	8.62E-07	4.34E-07	2.71E-07	8.70E-07	4.98E-07	1.32E-06	1.02E-06
LINGO2	0	1.14E-08	0	0	0	0	0	0
LINGO3	5.59E-08	3.79E-08	0	6.85E-08	0	0	3.79E-08	0
LINGO4	5.39E-08	1.62E-07	7.46E-08	4.28E-07	7.71E-08	5.39E-06	3.04E-07	5.93E-07
LINS1	6.17E-06	2.89E-06	2.57E-06	4.02E-06	1.25E-06	3.00E-06	2.30E-06	3.06E-06
LIPA	7.81E-05	0.00012	8.45E-05	0.000116	2.65E-05	6.72E-05	7.18E-05	4.69E-05
LIPC	0.000108	5.33E-05	0.000147	0.000149	5.45E-05	6.33E-05	0.000101	5.00E-05
LIPE	2.15E-06	1.36E-06	2.50E-07	2.88E-07	9.79E-08	1.06E-06	1.27E-06	1.14E-07
LIPF	0	0	0	0	0	0	0	0
LIPG	3.36E-05	9.35E-06	1.92E-05	7.94E-06	7.16E-06	1.13E-05	5.03E-06	1.13E-05
LIPH	5.47E-07	2.02E-07	6.21E-08	2.19E-07	0	0	1.73E-07	0
LIPI	0	0	0	0	0	0	0	0
LIPJ	3.35E-08	1.91E-08	1.78E-08	4.12E-08	1.44E-08	1.56E-08	0	0
LIPK	0	0	0	0	0	0	0	0
LIPM	0	0	0	0	0	0	0	0
LIPN	0	0	0	0	0	0	0	0
LIPT1	5.98E-06	3.69E-06	2.35E-06	1.85E-06	1.87E-06	4.83E-06	3.63E-06	3.27E-06
LIPT2	3.04E-06	1.65E-06	1.21E-06	9.05E-07	5.61E-07	9.10E-07	9.98E-07	6.30E-07
LITAF	0.000133	0.000191	6.21E-05	0.000122	1.64E-05	7.69E-05	9.51E-05	6.78E-05
LIX1	1.15E-08	0	0	0	0	5.32E-09	0	0
LIX1L	2.11E-05	8.65E-06	1.08E-05	1.25E-05	5.66E-06	1.56E-05	1.30E-05	1.06E-05
LLGL1	4.22E-06	3.16E-06	1.44E-06	2.57E-06	1.26E-06	1.97E-06	3.35E-06	1.77E-06
LLGL2	3.88E-05	3.92E-05	1.94E-05	2.09E-05	1.30E-05	2.38E-05	2.24E-05	2.35E-05
LLPH	2.87E-05	2.34E-05	1.20E-05	1.72E-05	8.53E-06	1.98E-05	1.96E-05	1.81E-05
LMAN1	4.88E-05	5.65E-05	6.92E-05	4.10E-05	9.08E-06	7.39E-05	4.16E-05	3.80E-05
LMAN1L	0	0	0	0	0	0	0	0
LMAN2	0.000295	0.00013	0.000138	0.000146	0.000202	0.000301	0.00017	0.000219
LMAN2L	2.62E-05	3.03E-05	8.35E-06	1.08E-05	1.05E-05	1.12E-05	1.18E-05	7.47E-06
LMBR1	6.85E-06	9.06E-06	6.11E-06	4.32E-06	1.52E-06	1.04E-05	4.20E-06	8.17E-06
LMBR1L	2.00E-05	4.29E-06	5.08E-06	7.04E-06	3.68E-06	5.27E-06	8.19E-06	7.94E-06
LMBRD1	7.16E-05	0.000105	4.60E-05	4.61E-05	9.26E-06	3.65E-05	3.60E-05	5.29E-05
LMBRD2	3.53E-06	6.40E-06	4.06E-06	1.41E-06	1.28E-07	4.65E-06	6.98E-07	3.49E-06
LMCD1	1.53E-05	6.85E-06	2.51E-06	3.62E-05	4.99E-06	1.04E-05	2.48E-05	7.05E-06
LMF1	7.16E-06	1.13E-05	6.66E-06	8.04E-06	9.18E-06	7.28E-06	7.93E-06	1.68E-05
LMF2	4.99E-05	5.58E-05	3.09E-05	3.53E-05	4.33E-05	2.82E-05	3.53E-05	4.52E-05
LMLN	9.42E-07	9.46E-06	8.74E-07	6.18E-07	5.81E-08	1.17E-06	3.58E-07	9.40E-07
LMNA	0.000167	0.000208	6.24E-05	0.000247	8.06E-05	0.000155	0.000142	0.000223
LMNB1	2.71E-05	1.02E-05	2.42E-06	5.81E-06	2.65E-06	5.23E-06	3.12E-06	4.10E-06
LMNB2	1.60E-05	4.41E-06	2.63E-06	6.86E-06	6.16E-06	5.17E-06	7.76E-06	5.05E-06
LMO1	1.14E-07	0	0	0	0	2.62E-08	0	0
LMO2	1.16E-05	4.51E-06	4.83E-06	5.71E-06	7.58E-06	6.62E-06	1.11E-05	6.96E-06
LMO3	2.51E-08	2.06E-08	1.40E-08	3.31E-08	0	0	0	1.80E-08
LMO4	1.18E-05	8.90E-06	5.37E-06	6.88E-06	8.14E-07	7.74E-06	6.24E-06	3.59E-06
LMO7	3.67E-05	2.95E-05	1.46E-05	1.66E-05	6.43E-06	2.74E-05	1.40E-05	1.23E-05
LMOD1	2.89E-06	1.37E-06	5.38E-07	2.06E-06	1.25E-06	3.76E-06	1.99E-06	1.16E-06
LMOD2	1.11E-08	1.25E-08	0	1.36E-08	9.52E-09	0	0	0
LMOD3	7.39E-08	3.18E-08	5.85E-09	1.39E-08	2.44E-08	2.11E-08	1.66E-08	3.33E-08
LMTK2	1.00E-05	5.68E-06	1.79E-06	1.44E-06	3.10E-08	2.41E-06	5.74E-07	4.08E-06
LMTK3	3.41E-06	6.60E-08	6.05E-08	2.11E-07	1.17E-08	8.40E-09	5.74E-08	1.99E-08
LMX1A	2.93E-06	3.96E-08	2.74E-08	0	7.55E-09	0	0	1.29E-08
LMX1B	1.56E-08	0	0	4.76E-09	0	0	9.07E-08	0
LNP1	8.36E-07	2.22E-06	2.88E-06	2.22E-06	1.12E-06	1.81E-06	2.45E-06	1.20E-06
LNPEP	2.50E-06	1.40E-06	8.99E-07	7.30E-07	8.82E-08	1.81E-06	4.22E-07	1.17E-06

LNX1	7.56E-06	3.29E-06	3.94E-06	2.30E-06	7.92E-07	1.03E-05	2.56E-06	2.62E-06
LNX2	1.97E-05	7.99E-06	8.21E-06	8.20E-06	2.07E-06	1.14E-05	6.25E-06	7.80E-06
LOC1000C	2.21E-06	3.02E-06	8.23E-07	8.81E-07	7.62E-07	9.77E-07	1.42E-06	5.40E-07
LOC1001C	4.27E-07	1.17E-07	8.74E-08	0	3.69E-07	1.14E-07	7.80E-08	6.03E-08
LOC1001C	0	0	0	0	0	0	0	0
LOC10012	3.89E-08	0	6.68E-09	0	0	0	6.29E-09	1.14E-07
LOC10012	2.41E-06	3.57E-06	3.65E-07	5.37E-07	1.60E-06	6.24E-07	1.21E-06	1.33E-06
LOC10012	3.92E-07	8.24E-08	6.43E-08	2.07E-06	1.29E-07	2.74E-07	4.58E-07	7.31E-08
LOC10012	3.62E-08	2.01E-08	1.97E-08	2.16E-08	0	1.64E-08	0	2.60E-08
LOC10012	4.54E-08	2.62E-08	0	0	0	2.11E-08	2.17E-08	0
LOC10012	0	0	0	0	0	0	0	0
LOC10012	3.72E-09	0	0	0	0	0	0	0
LOC10012	1.84E-06	2.03E-06	5.84E-07	4.55E-07	6.78E-07	6.16E-07	2.31E-07	6.12E-07
LOC10012	1.03E-08	0	0	3.78E-08	2.65E-08	0	0	0
LOC10012	8.39E-07	4.42E-07	2.10E-07	3.42E-07	1.15E-07	3.42E-07	2.60E-07	2.30E-07
LOC10012	2.54E-07	4.32E-08	5.56E-08	3.76E-08	1.91E-07	1.28E-07	6.71E-08	1.01E-07
LOC10012	1.46E-07	2.77E-07	1.87E-07	3.72E-07	7.10E-07	2.78E-07	6.68E-07	3.40E-07
LOC10012	0	0	0	0	0	0	0	0
LOC10012	3.23E-07	0	0	0	1.14E-07	1.84E-07	3.57E-07	9.95E-08
LOC10012	4.11E-07	2.32E-07	5.24E-07	4.12E-07	8.83E-08	3.90E-07	2.99E-07	1.92E-07
LOC10012	1.55E-06	1.07E-06	7.55E-07	3.15E-07	2.53E-08	4.33E-07	5.10E-07	7.24E-07
LOC10012	6.79E-07	5.64E-07	3.17E-07	3.14E-07	1.81E-06	1.34E-06	5.55E-07	6.33E-07
LOC10012	0	0	0	0	0	0	0	0
LOC10012	2.27E-06	6.52E-06	1.91E-06	1.62E-06	2.39E-06	2.70E-06	1.76E-06	1.43E-06
LOC10012	3.52E-07	1.02E-07	4.71E-08	3.98E-08	5.60E-09	4.23E-08	4.43E-08	1.05E-07
LOC10012	0	0	0	0	0	0	0	0
LOC10012	2.02E-05	1.01E-05	1.21E-05	1.12E-05	5.90E-06	1.56E-05	1.37E-05	2.65E-05
LOC10012	0	9.44E-09	0	0	0	0	0	0
LOC10012	9.74E-08	2.44E-08	7.91E-08	1.46E-07	1.21E-07	3.31E-07	5.23E-08	9.55E-08
LOC10012	1.19E-06	1.04E-06	1.10E-06	1.12E-06	8.26E-07	5.01E-06	1.90E-06	8.60E-07
LOC10012	3.27E-07	5.59E-07	2.09E-07	3.23E-07	1.18E-07	6.74E-07	2.75E-07	2.79E-07
LOC10012	1.20E-06	2.37E-07	3.96E-07	4.63E-07	3.45E-07	6.36E-07	6.66E-07	4.89E-07
LOC10012	1.40E-06	2.37E-07	3.81E-07	4.48E-07	3.89E-07	5.33E-07	4.29E-07	4.90E-07
LOC10012	1.97E-07	2.11E-08	1.95E-08	8.04E-08	0	1.30E-07	3.64E-08	8.27E-08
LOC10012	3.43E-07	1.86E-07	0	0	1.28E-08	1.25E-07	4.31E-08	6.59E-08
LOC10012	0	0	0	0	0	0	0	0
LOC10013	6.42E-06	4.36E-06	1.68E-06	4.72E-06	4.05E-06	1.08E-05	7.16E-06	2.90E-06
LOC10013	1.89E-06	8.25E-06	4.24E-06	1.85E-06	1.21E-06	8.56E-06	1.85E-06	3.74E-06
LOC10013	0	4.31E-08	0	0	9.89E-08	4.98E-08	0	0
LOC10013	1.18E-08	6.59E-09	2.42E-08	7.19E-09	0	0	5.72E-09	3.45E-08
LOC10013	0	0	0	0	0	0	0	0
LOC10013	0	0	0	0	0	0	0	0
LOC10013	1.70E-07	7.47E-08	1.38E-07	9.04E-09	6.35E-09	3.42E-08	2.87E-08	2.17E-08
LOC10013	9.08E-09	0	0	1.11E-08	0	0	0	0
LOC10013	3.71E-07	6.29E-07	5.70E-08	4.90E-07	8.53E-08	6.45E-07	7.73E-08	5.44E-07
LOC10013	1.02E-06	3.60E-07	1.13E-07	3.58E-08	1.25E-07	2.99E-07	7.83E-08	7.48E-08
LOC10013	2.82E-06	3.63E-06	8.60E-07	7.08E-07	6.97E-07	2.32E-06	1.25E-06	6.14E-07
LOC10013	1.17E-06	4.09E-07	1.22E-07	1.95E-07	2.41E-07	4.08E-07	2.21E-07	1.93E-07
LOC10013	2.99E-06	5.42E-07	7.90E-07	2.44E-06	1.06E-06	3.98E-07	1.90E-06	1.68E-06
LOC10013	6.01E-06	4.05E-06	4.81E-06	4.18E-06	2.91E-06	3.59E-06	4.40E-06	3.12E-06
LOC10013	7.09E-06	4.46E-06	1.52E-06	1.92E-06	3.87E-06	2.07E-06	2.08E-06	1.74E-06
LOC10013	2.13E-05	0	2.93E-06	3.80E-06	3.60E-06	3.09E-06	3.35E-06	3.87E-06
LOC10013	3.27E-07	1.54E-07	7.32E-08	4.07E-07	5.69E-08	3.06E-07	9.31E-08	1.47E-07
LOC10013	6.25E-07	2.89E-07	2.67E-07	1.81E-07	1.19E-07	3.66E-07	1.05E-07	6.82E-07
LOC10013	8.32E-07	2.18E-07	1.63E-07	1.96E-07	1.80E-07	5.11E-07	7.30E-08	1.80E-07
LOC10013	5.26E-08	2.37E-08	6.58E-08	3.86E-08	1.81E-08	2.05E-07	2.04E-08	0
LOC10013	1.08E-07	0	7.95E-09	4.70E-08	0	4.54E-07	2.24E-08	0
LOC10013	6.87E-07	1.60E-06	2.33E-07	3.03E-07	4.39E-07	1.08E-06	2.71E-07	2.79E-07

LOC10013	4.85E-07	3.48E-05	1.24E-06	4.48E-07	2.85E-06	8.51E-06	2.43E-06	4.68E-07
LOC10013	1.02E-06	3.14E-07	3.87E-07	1.42E-07	3.30E-08	1.56E-06	1.84E-07	1.25E-06
LOC10013	8.41E-07	5.88E-07	1.73E-07	2.03E-07	6.10E-08	1.21E-07	6.86E-08	2.09E-07
LOC10013	2.78E-06	1.20E-06	3.36E-07	4.70E-07	2.87E-07	2.53E-07	3.79E-07	4.12E-07
LOC10013	3.39E-06	1.15E-06	1.08E-06	1.21E-06	5.13E-07	2.16E-06	7.47E-07	1.43E-06
LOC10013	4.69E-06	3.22E-06	1.93E-06	4.48E-06	1.49E-05	4.98E-06	8.70E-06	1.05E-05
LOC10013	2.07E-08	1.87E-07	1.08E-08	1.27E-08	0	7.40E-07	1.00E-08	2.29E-07
LOC10013	2.40E-06	5.86E-06	2.20E-06	1.46E-06	5.22E-07	2.22E-06	1.29E-06	4.52E-06
LOC10013	1.26E-07	2.18E-08	8.04E-08	0	8.30E-09	4.48E-08	1.87E-08	0
LOC10013	7.27E-08	0	5.08E-08	4.45E-08	0	0	0	1.78E-08
LOC10013	1.54E-07	1.37E-07	4.75E-08	5.82E-08	4.18E-08	4.25E-08	8.61E-08	7.51E-08
LOC10013	0	0	0	0	0	0	0	6.38E-08
LOC10013	5.97E-07	1.72E-07	7.21E-08	1.19E-07	2.53E-08	3.42E-07	6.20E-08	2.15E-08
LOC10013	0	0	0	0	1.34E-08	0	0	0
LOC10013	1.49E-06	1.84E-07	4.09E-07	3.17E-07	1.00E-07	8.43E-07	3.14E-07	4.19E-07
LOC10013	1.74E-06	0	0	0	0	0	0	2.06E-08
LOC10013	2.53E-08	1.43E-07	9.30E-08	3.10E-07	1.20E-07	0	6.11E-08	1.86E-08
LOC10013	1.97E-06	5.44E-07	1.19E-07	3.84E-07	5.07E-07	1.42E-06	5.72E-08	5.44E-08
LOC10013	8.43E-08	1.42E-06	1.28E-06	6.49E-07	1.10E-07	2.56E-08	1.98E-07	2.03E-08
LOC10013	7.17E-08	2.76E-08	2.60E-08	0	0	0	4.57E-08	0
LOC10013	1.18E-08	1.33E-07	0	0	0	0	0	0
LOC10013	1.37E-06	5.60E-07	6.47E-07	1.64E-07	0	1.32E-06	6.57E-07	7.47E-07
LOC10013	7.02E-06	1.73E-05	3.53E-06	2.99E-06	3.81E-06	4.58E-06	1.93E-06	3.06E-06
LOC10013	5.14E-07	7.06E-07	2.35E-07	4.62E-07	0	4.07E-07	1.15E-07	4.52E-08
LOC10013	1.53E-06	1.90E-06	2.15E-06	2.03E-06	1.69E-06	1.89E-06	1.39E-06	8.27E-06
LOC10013	4.46E-07	2.93E-07	2.06E-07	2.09E-07	5.50E-08	2.37E-07	1.02E-07	1.26E-07
LOC10013	1.41E-07	1.61E-07	3.32E-08	7.71E-08	1.35E-08	2.92E-08	6.04E-08	4.63E-08
LOC10013	1.78E-07	1.35E-07	1.41E-07	2.19E-07	1.41E-07	4.56E-07	2.58E-07	2.41E-07
LOC10013	6.70E-07	3.64E-08	5.08E-08	5.88E-08	1.38E-08	8.90E-08	1.08E-07	2.36E-08
LOC10014	1.64E-06	4.19E-07	3.51E-07	4.97E-07	7.80E-07	1.12E-06	1.62E-06	9.79E-07
LOC10014	0	6.26E-08	0	0	2.37E-08	1.28E-08	0	4.06E-08
LOC10016	0	0	0	0	0	0	0	0
LOC10017	3.20E-06	1.68E-06	2.17E-07	9.19E-07	1.37E-08	7.77E-07	2.40E-07	5.22E-08
LOC10018	2.60E-08	3.01E-08	0	3.22E-08	0	0	2.48E-08	7.75E-08
LOC10018	7.13E-07	5.30E-08	2.46E-07	1.14E-07	4.01E-08	2.59E-07	1.79E-07	2.29E-07
LOC10018	0	0	0	0	0	0	0	0
LOC10019	3.08E-07	3.80E-06	2.65E-07	1.70E-07	7.86E-08	9.09E-08	1.91E-07	1.24E-07
LOC10019	5.24E-06	5.68E-06	2.15E-06	3.36E-06	2.61E-06	3.20E-06	5.11E-06	1.71E-06
LOC10019	0	1.97E-07	2.68E-08	0	0	6.02E-09	0	0
LOC10019	2.27E-06	1.20E-06	3.02E-07	3.67E-07	4.16E-08	1.88E-07	2.06E-07	2.84E-07
LOC10019	4.28E-08	1.62E-07	1.81E-07	2.45E-07	2.33E-07	1.32E-08	2.34E-07	3.58E-07
LOC10019	0	0	0	0	0	1.98E-08	1.03E-08	0
LOC10019	8.10E-07	0	0	0	0	0	0	0
LOC10021	1.26E-07	1.13E-07	2.50E-07	9.55E-07	0	4.20E-08	2.62E-07	1.47E-07
LOC10021	1.20E-06	1.10E-06	8.19E-07	7.54E-07	4.88E-06	1.34E-06	3.77E-07	6.15E-07
LOC10023	6.47E-07	6.34E-08	6.83E-08	2.64E-07	3.23E-08	1.39E-07	2.54E-07	4.13E-08
LOC10024	0	3.42E-08	1.59E-08	0	1.30E-08	0	0	4.44E-08
LOC10024	0	0	0	0	0	0	0	0
LOC10024	2.73E-07	0	2.69E-07	2.63E-07	0	1.13E-07	1.44E-07	4.53E-08
LOC10026	1.64E-07	0	1.15E-07	1.75E-07	4.84E-08	1.01E-07	5.96E-08	9.13E-08
LOC10027	1.03E-05	7.74E-07	2.79E-06	7.73E-06	7.26E-06	1.92E-06	6.08E-06	3.18E-06
LOC10027	1.99E-06	2.89E-06	2.55E-06	1.68E-06	2.42E-06	1.75E-06	2.22E-06	2.80E-06
LOC10027	6.65E-07	1.21E-07	3.73E-08	1.16E-07	1.23E-07	1.43E-07	3.44E-08	6.99E-08
LOC10027	1.41E-06	4.17E-07	3.40E-07	6.76E-07	6.58E-07	5.25E-07	5.01E-07	3.61E-07
LOC10027	8.02E-06	6.57E-06	4.03E-06	7.00E-06	1.77E-05	3.80E-06	5.92E-06	8.88E-06
LOC10027	0	0	0	0	0	0	0	0
LOC10027	3.71E-06	1.84E-06	8.33E-07	1.24E-06	1.37E-08	1.09E-06	8.79E-07	1.37E-06
LOC10027	1.03E-06	4.03E-07	1.48E-07	8.77E-08	1.60E-07	1.46E-07	1.88E-07	4.21E-08

LOC10027	5.15E-07	1.58E-07	1.22E-07	2.74E-07	0	7.57E-08	1.73E-07	2.40E-07
LOC10027	2.41E-06	2.01E-07	5.10E-07	2.18E-07	4.49E-07	3.50E-07	1.40E-07	2.45E-07
LOC10027	7.87E-07	1.56E-07	9.52E-08	1.09E-07	6.31E-08	2.05E-07	7.11E-08	1.36E-07
LOC10028	1.01E-05	2.90E-06	2.34E-06	3.28E-06	1.15E-06	4.10E-06	2.73E-06	3.10E-06
LOC10028	4.43E-07	6.13E-07	2.21E-07	8.09E-08	4.28E-08	4.17E-07	2.32E-07	7.65E-08
LOC10028	2.22E-07	7.32E-08	1.44E-07	1.53E-07	7.94E-08	3.01E-07	6.29E-08	2.71E-08
LOC10028	0	0	0	0	0	0	0	0
LOC10028	0	0	0	0	0	0	0	0
LOC10028	1.54E-05	1.44E-05	4.73E-06	1.61E-05	2.69E-05	1.10E-05	8.38E-06	9.24E-06
LOC10028	1.21E-06	1.46E-07	2.57E-07	4.11E-07	4.33E-07	1.54E-06	2.99E-07	5.50E-07
LOC10030	3.57E-07	1.38E-07	1.02E-07	2.07E-07	5.32E-08	6.95E-07	1.49E-07	1.25E-07
LOC10030	8.57E-09	6.44E-08	0	1.05E-08	9.28E-08	1.00E-07	0	0
LOC10030	2.42E-06	4.70E-07	1.01E-06	4.63E-06	3.18E-07	4.53E-07	8.26E-07	3.51E-06
LOC10030	1.32E-06	1.62E-06	1.37E-06	7.59E-07	1.31E-06	8.85E-07	4.82E-07	8.79E-07
LOC11323	4.74E-06	1.36E-06	2.71E-06	4.35E-06	4.87E-06	1.41E-06	3.69E-06	2.64E-06
LOC11511	7.65E-06	1.65E-06	1.32E-06	2.93E-06	7.08E-07	8.19E-07	1.51E-06	2.55E-06
LOC11643	0	2.89E-07	0	0	0	0	0	0
LOC12183	0	0	2.85E-08	1.68E-07	0	0	0	1.34E-08
LOC12195	5.11E-08	0	3.19E-08	2.50E-08	7.03E-08	4.74E-08	9.89E-09	1.50E-08
LOC12653	7.37E-08	0	7.62E-09	1.80E-08	0	0	0	0
LOC12784	1.18E-07	6.77E-08	2.11E-08	2.42E-08	5.10E-08	1.83E-08	0	0
LOC13446	3.80E-07	7.23E-08	7.80E-08	4.84E-07	4.18E-08	0	3.81E-08	2.89E-08
LOC14318	2.52E-07	1.45E-07	7.43E-08	1.85E-07	1.05E-07	1.07E-07	1.89E-07	8.44E-08
LOC14366	8.16E-07	2.34E-07	1.80E-07	2.68E-07	2.77E-07	2.98E-07	2.78E-07	1.69E-07
LOC14443	7.78E-06	5.76E-06	2.76E-06	4.17E-06	1.40E-06	4.48E-06	2.99E-06	4.53E-06
LOC14448	1.92E-07	2.06E-08	9.49E-09	1.12E-08	3.93E-08	1.27E-07	1.77E-08	1.34E-08
LOC14457	6.12E-06	8.97E-07	6.58E-07	8.65E-07	1.34E-06	9.18E-07	1.11E-06	2.25E-06
LOC14474	0	0	0	0	0	0	0	0
LOC14477	0	0	0	0	0	0	0	0
LOC14547	0	0	0	1.61E-08	0	0	0	0
LOC14578	1.06E-06	5.35E-07	4.31E-07	4.20E-07	1.37E-07	2.46E-07	5.13E-07	3.95E-07
LOC14582	9.74E-07	1.52E-07	5.81E-07	1.27E-06	1.63E-07	3.76E-07	5.16E-07	5.16E-07
LOC14583	7.37E-06	1.93E-05	2.42E-06	1.01E-07	1.21E-06	1.42E-06	6.35E-06	3.97E-06
LOC14584	1.55E-08	0	0	0	0	0	0	0
LOC14633	8.43E-06	0	6.55E-08	0	6.36E-08	0	0	1.86E-06
LOC14648	0	0	0	0	0	0	0	0
LOC14688	6.08E-06	2.98E-06	8.36E-07	1.52E-06	1.32E-06	2.29E-06	1.44E-06	1.75E-06
LOC14772	1.70E-05	2.83E-06	8.60E-06	5.96E-06	1.93E-05	8.41E-06	3.90E-06	4.29E-06
LOC14780	5.70E-06	4.00E-06	6.31E-07	9.39E-07	2.72E-07	2.72E-07	2.92E-07	1.20E-06
LOC14814	0	0	0	0	0	0	0	2.93E-08
LOC14818	1.69E-06	2.11E-06	9.34E-07	8.57E-07	3.51E-07	1.68E-06	1.25E-06	6.52E-07
LOC14841	1.56E-05	1.19E-05	1.37E-05	1.28E-05	1.26E-05	2.36E-05	1.52E-05	1.27E-05
LOC14869	2.69E-08	0	1.66E-08	0	0	0	0	1.58E-08
LOC14870	1.01E-06	4.71E-07	2.65E-07	2.74E-07	1.84E-07	2.17E-06	1.18E-07	2.39E-07
LOC14882	3.78E-08	0	0	7.72E-08	2.17E-08	0	4.86E-08	0
LOC14913	2.25E-07	6.23E-07	3.74E-08	3.36E-07	3.86E-07	4.84E-07	1.40E-07	6.61E-08
LOC14962	0	0	0	0	0	0	0	0
LOC14983	1.46E-06	7.65E-07	8.55E-08	8.57E-08	2.19E-08	4.72E-08	6.82E-08	5.61E-08
LOC15018	0	0	0	0	0	0	0	0
LOC15019	7.42E-07	1.94E-07	2.25E-08	0	1.30E-07	1.60E-07	5.20E-08	3.16E-07
LOC15038	4.10E-06	1.38E-06	4.29E-07	9.22E-07	4.04E-06	1.44E-06	2.50E-06	4.87E-07
LOC15052	0	1.33E-08	0	0	0	0	0	0
LOC15056	0	0	0	0	0	0	0	2.44E-08
LOC15062	0	0	0	0	0	0	0	0
LOC15077	1.13E-05	3.89E-06	1.83E-06	2.62E-06	3.18E-06	2.25E-06	3.39E-06	4.24E-06
LOC15078	7.12E-07	4.20E-07	2.31E-07	2.52E-07	2.14E-08	2.37E-07	1.09E-07	3.02E-07
LOC15100	9.71E-07	1.11E-07	2.61E-07	9.21E-07	1.57E-07	1.62E-07	1.26E-07	2.22E-07
LOC15116	9.84E-06	5.70E-06	1.37E-06	3.83E-06	1.57E-06	3.28E-06	4.72E-06	3.78E-06

LOC15117	5.63E-07	3.99E-08	1.51E-07	2.21E-07	0	9.25E-08	2.54E-07	5.41E-08
LOC15130	0	0	0	0	0	0	0	0
LOC15153	3.19E-05	1.72E-05	1.44E-05	1.13E-05	1.64E-05	3.56E-05	1.78E-05	8.04E-06
LOC15165	0	4.76E-07	0	0	0	0	0	0
LOC15202	0	0	0	3.43E-08	0	1.45E-08	0	0
LOC15221	1.97E-05	8.00E-06	5.73E-06	6.79E-06	2.47E-05	1.18E-05	1.02E-05	5.55E-06
LOC15222	1.11E-06	2.90E-07	1.71E-07	1.71E-07	4.01E-08	1.30E-07	1.35E-07	3.43E-08
LOC15332	1.50E-06	0	0	1.57E-08	0	0	0	0
LOC15368	1.23E-06	2.47E-07	1.72E-07	2.55E-07	3.23E-07	4.27E-07	2.83E-07	1.86E-07
LOC15391	1.03E-08	2.33E-08	0	0	0	9.04E-07	0	0
LOC15444	1.71E-07	6.53E-08	0	4.68E-08	0	3.54E-08	0	5.63E-08
LOC15476	2.05E-06	1.47E-06	3.32E-07	7.59E-07	4.98E-08	2.41E-07	1.61E-07	1.29E-07
LOC15482	5.68E-09	0	1.48E-08	3.24E-08	1.67E-08	0	0	0
LOC15738	7.42E-09	0	0	0	0	2.75E-08	7.21E-09	1.09E-08
LOC15762	0	0	0	0	0	0	0	0
LOC15837	6.71E-07	1.38E-06	4.74E-07	4.71E-07	3.72E-07	6.09E-07	1.48E-06	7.08E-08
LOC15857	3.41E-07	2.91E-07	1.62E-07	3.80E-07	1.78E-08	8.81E-07	6.00E-08	6.07E-07
LOC15869	0	1.08E-07	0	3.90E-08	0	4.44E-08	1.55E-08	8.19E-08
LOC16263	3.22E-07	3.47E-07	7.78E-07	1.41E-07	3.51E-08	5.22E-08	1.71E-07	2.90E-07
LOC16847	1.66E-08	6.20E-09	0	6.76E-09	0	5.12E-09	0	1.62E-08
LOC20003	7.48E-06	4.79E-06	2.93E-06	2.99E-06	4.82E-07	1.39E-05	1.94E-06	7.95E-06
LOC20072	0	0	0	0	0	0	0	0
LOC20165	6.11E-07	3.19E-05	3.34E-06	4.05E-06	2.35E-06	1.07E-05	1.44E-06	2.83E-06
LOC20218	1.36E-06	1.74E-07	3.24E-07	1.37E-07	2.79E-08	2.28E-07	1.28E-07	4.53E-07
LOC20278	2.61E-06	3.36E-06	4.04E-06	2.69E-06	3.25E-06	3.64E-06	2.27E-06	2.59E-06
LOC21934	1.89E-07	2.82E-07	2.47E-07	1.90E-07	1.90E-07	4.32E-07	3.70E-07	2.29E-07
LOC22042	2.73E-07	6.13E-08	2.99E-07	9.53E-08	1.34E-08	1.16E-07	3.03E-08	1.03E-07
LOC22059	9.38E-07	7.86E-08	1.47E-07	4.13E-07	1.67E-08	1.38E-07	1.01E-07	1.14E-07
LOC22072	5.99E-06	5.73E-06	1.92E-06	1.82E-06	4.81E-07	1.96E-06	2.17E-06	1.63E-06
LOC22093	2.81E-06	1.89E-06	8.62E-07	1.01E-06	6.55E-07	2.47E-06	6.32E-07	6.90E-07
LOC22112	1.03E-07	0	0	0	5.36E-08	0	0	3.07E-08
LOC22144	1.85E-06	2.88E-07	1.32E-07	1.22E-07	2.66E-07	2.94E-07	2.10E-08	9.79E-08
LOC22171	7.33E-06	8.21E-06	4.26E-06	3.31E-06	3.81E-06	4.78E-06	2.83E-06	6.45E-06
LOC22269	1.26E-06	2.59E-06	1.25E-07	1.48E-07	6.51E-07	2.78E-06	2.60E-07	1.29E-06
LOC25303	1.33E-05	5.31E-06	1.35E-06	1.44E-06	2.00E-06	6.56E-06	1.36E-06	4.31E-06
LOC25372	2.12E-07	2.89E-07	3.41E-07	1.02E-07	5.62E-07	5.57E-07	1.72E-07	4.40E-08
LOC25431	0	0	0	0	0	0	0	0
LOC25455	1.16E-06	4.35E-06	2.84E-08	1.45E-07	9.49E-09	9.21E-08	5.92E-08	4.86E-08
LOC25502	0	0	0	0	0	0	0	0
LOC25516	7.72E-08	5.91E-05	4.47E-05	3.02E-05	2.04E-06	1.27E-05	2.57E-05	7.28E-05
LOC25688	1.33E-07	4.10E-08	0	2.96E-08	0	1.12E-08	0	5.33E-08
LOC25735	3.23E-08	1.22E-08	0	2.64E-08	9.26E-09	9.98E-09	1.04E-08	0
LOC25845	8.20E-06	5.46E-06	4.54E-06	4.26E-06	3.89E-06	6.44E-06	4.44E-06	5.50E-06
LOC26102	4.73E-09	1.06E-08	4.85E-09	1.73E-08	8.11E-09	0	4.60E-09	0
LOC28299	1.51E-06	4.44E-07	7.59E-07	9.03E-07	8.08E-07	1.52E-06	7.35E-07	3.32E-07
LOC28305	2.98E-07	1.64E-07	1.16E-07	2.82E-07	3.83E-08	1.21E-07	1.64E-07	2.78E-07
LOC28307	9.46E-06	1.56E-06	5.34E-06	5.11E-06	3.60E-06	1.26E-05	9.93E-06	1.02E-05
LOC28317	1.70E-07	4.74E-09	3.04E-08	6.73E-08	2.18E-08	1.18E-08	2.89E-08	3.72E-08
LOC28326	6.13E-06	2.19E-06	1.27E-06	1.84E-06	8.43E-07	2.32E-06	1.65E-06	1.93E-06
LOC28331	2.98E-06	3.75E-06	1.20E-06	1.55E-06	1.84E-06	1.17E-06	1.82E-06	5.45E-07
LOC28333	0	0	0	0	0	0	0	0
LOC28339	1.48E-06	0	8.31E-08	8.43E-08	0	0	0	2.87E-07
LOC28340	0	2.91E-08	0	0	0	0	0	0
LOC28366	3.41E-07	3.78E-08	4.80E-08	1.67E-07	1.08E-07	1.35E-07	3.31E-07	1.22E-07
LOC28373	3.65E-08	0	0	0	1.57E-08	0	2.65E-08	2.68E-08
LOC28376	3.01E-08	0	0	0	0	0	0	0
LOC28385	0	5.89E-08	1.63E-07	9.60E-08	7.49E-09	2.26E-07	1.69E-08	1.28E-08
LOC28386	0	0	0	0	0	0	0	0

LOC28391	0	0	0	0	0	0	0	0
LOC28392	7.17E-07	1.38E-07	2.51E-07	2.50E-07	1.75E-08	4.86E-07	1.88E-07	1.65E-07
LOC28399	1.04E-08	0	1.09E-08	0	0	0	0	0
LOC28400	0	0	0	7.05E-08	0	0	0	0
LOC28402	3.53E-07	7.22E-07	2.51E-07	2.84E-07	1.68E-07	2.50E-07	4.14E-07	8.32E-07
LOC28410	0	1.08E-07	0	0	8.76E-09	9.44E-09	0	1.50E-08
LOC28423	1.22E-08	2.19E-08	5.30E-09	2.12E-09	0	9.12E-09	5.67E-10	5.93E-09
LOC28423	4.04E-08	9.08E-09	2.51E-08	1.98E-08	0	0	2.35E-08	2.37E-08
LOC28427	0	1.19E-08	0	2.07E-07	2.73E-08	1.96E-08	0	1.55E-08
LOC28437	0	9.72E-08	0	0	0	0	0	0
LOC28444	1.40E-06	1.59E-06	1.73E-06	1.27E-06	2.57E-06	2.17E-06	2.11E-06	8.73E-07
LOC28444	3.72E-06	3.39E-06	1.83E-06	8.30E-07	4.02E-08	1.33E-06	2.14E-07	1.13E-06
LOC28455	1.45E-07	1.63E-07	2.64E-08	5.20E-08	7.31E-09	2.36E-08	4.95E-08	1.12E-07
LOC28457	0	2.25E-08	0	0	0	9.25E-09	2.90E-08	0
LOC28463	0	0	0	0	0	0	0	0
LOC28466	0	0	0	0	0	1.07E-08	0	0
LOC28468	0	1.60E-08	0	0	0	0	1.36E-08	0
LOC28474	2.12E-06	0	1.55E-08	4.59E-08	1.64E-08	0	0	0
LOC28478	0	1.75E-08	0	0	2.65E-08	9.99E-08	2.82E-08	0
LOC28479	0	0	0	1.12E-08	0	0	0	0
LOC28483	2.62E-07	3.40E-08	7.60E-08	2.41E-07	9.74E-08	1.13E-07	1.85E-07	4.62E-08
LOC28490	1.08E-05	2.45E-06	2.63E-06	3.37E-06	5.60E-06	2.80E-06	4.90E-06	3.58E-06
LOC28503	2.11E-06	7.65E-07	4.25E-07	8.49E-07	2.30E-07	1.11E-06	5.39E-07	7.00E-07
LOC28504	0	0	0	0	0	0	3.02E-08	0
LOC28507	1.41E-05	7.41E-06	4.14E-06	3.00E-06	5.61E-06	3.00E-06	6.32E-06	1.22E-05
LOC28519	0	0	0	0	0	0	0	0
LOC28520	0	0	0	2.39E-08	0	0	0	0
LOC28535	3.88E-06	2.86E-06	1.02E-06	1.45E-06	1.11E-06	2.45E-06	1.07E-06	2.28E-06
LOC28537	0	0	0	0	0	0	0	0
LOC28537	0	0	0	0	0	0	0	0
LOC28540	1.75E-08	0	1.87E-08	4.31E-08	0	0	0	0
LOC28541	3.44E-09	0	0	1.47E-07	1.02E-07	0	5.73E-08	8.91E-08
LOC28545	6.06E-09	2.04E-08	3.74E-08	3.18E-08	0	4.48E-08	1.18E-08	1.08E-08
LOC28550	0	0	0	0	0	0	0	0
LOC28554	8.07E-08	0	0	0	0	0	0	0
LOC28559	2.18E-07	1.75E-07	1.34E-07	1.10E-07	0	8.86E-08	1.71E-07	4.02E-08
LOC28562	0	0	0	0	0	0	0	0
LOC28562	0	0	0	1.25E-08	0	2.83E-08	0	0
LOC28569	0	0	0	0	0	0	0	0
LOC28569	0	0	0	0	0	0	6.74E-09	0
LOC28573	0.000123	2.88E-05	6.87E-05	5.01E-05	4.66E-05	7.49E-05	5.12E-05	1.09E-05
LOC28573	0	0	0	0	0	0	0	0
LOC28574	0	0	0	0	0	0	0	0
LOC28576	6.26E-07	8.87E-07	0	0	0	8.57E-07	0	0
LOC28578	0	1.77E-08	0	0	0	0	7.62E-09	0
LOC28579	6.73E-08	0	2.34E-08	4.12E-08	2.89E-08	8.32E-08	2.17E-08	3.30E-08
LOC28583	2.38E-06	1.34E-06	1.07E-06	3.70E-06	3.30E-06	9.09E-07	1.72E-06	1.56E-06
LOC28584	4.22E-08	0	0	3.45E-08	0	5.22E-08	9.50E-08	2.07E-08
LOC28595	2.05E-08	2.89E-08	5.36E-08	1.09E-07	1.21E-07	2.96E-07	0	1.88E-08
LOC28600	0	0	0	0	0	5.43E-09	0	0
LOC28609	0	0	0	0	0	0	0	0
LOC28613	0	0	0	0	0	0	0	0
LOC28623	0	0	0	0	0	0	0	0
LOC28635	0	1.34E-08	0	0	1.02E-08	0	0	0
LOC28636	3.37E-07	1.81E-07	1.04E-07	9.87E-08	1.45E-07	1.99E-07	1.05E-07	6.91E-08
LOC28646	1.44E-07	1.45E-07	8.20E-09	0	0	2.73E-08	3.08E-08	0
LOC29034	6.16E-08	2.66E-07	7.70E-07	6.28E-08	3.53E-08	2.38E-07	0	4.52E-08
LOC33858	0	0	0	0	0	0	0	0

LOC33865	1.44E-07	2.35E-07	1.90E-08	1.51E-07	0	0	1.97E-07	6.92E-08
LOC33875	1.37E-06	1.65E-07	2.24E-07	1.61E-07	1.99E-07	1.50E-07	1.80E-07	2.49E-07
LOC33879	5.97E-06	1.37E-06	8.54E-07	1.26E-06	1.67E-06	1.62E-06	8.95E-07	1.56E-06
LOC33904	2.28E-05	1.59E-05	9.29E-06	2.12E-05	7.41E-06	7.05E-06	2.47E-05	1.93E-05
LOC33924	0	3.10E-08	3.30E-08	3.99E-08	0	1.53E-08	1.78E-08	1.35E-08
LOC33929	1.87E-05	7.14E-06	6.78E-06	7.07E-06	2.95E-06	7.98E-06	6.26E-06	8.51E-06
LOC33952	6.91E-07	4.38E-07	8.30E-07	9.43E-07	8.86E-08	1.65E-06	2.72E-07	9.06E-07
LOC33953	2.14E-06	1.48E-07	3.43E-08	9.94E-08	0	1.35E-07	0	1.43E-07
LOC33956	0	0	0	0	0	0	0	0
LOC33967	8.24E-08	7.51E-08	0	2.03E-08	1.42E-08	1.53E-08	0	2.49E-07
LOC33978	0	8.55E-08	0	0	0	0	0	0
LOC34001	0	0	0	0	0	1.69E-08	0	0
LOC34007	0	0	0	0	0	0	0	0
LOC34009	0	0	0	0	0	0	0	0
LOC34035	0	0	0	0	0	0	0	0
LOC34050	1.78E-07	3.55E-08	0	0	0	9.71E-09	0	0
LOC34105	7.60E-07	1.72E-07	2.20E-07	2.15E-07	2.82E-07	4.77E-07	1.24E-07	3.10E-07
LOC34234	4.02E-08	3.91E-07	4.84E-08	6.56E-08	0	1.43E-07	9.77E-08	2.95E-08
LOC34459	7.77E-07	1.95E-07	1.90E-07	2.50E-07	1.09E-07	2.73E-06	8.23E-08	1.24E-07
LOC34496	3.65E-07	1.21E-07	2.09E-07	3.74E-08	9.19E-08	7.07E-08	7.34E-08	1.12E-07
LOC34737	0	2.13E-07	0	0	0	0	0	0
LOC34802	0	0	0	0	0	0	0	0
LOC34884	4.01E-08	8.95E-07	1.38E-07	2.16E-08	0	8.20E-09	1.71E-08	2.60E-08
LOC34892	5.91E-07	2.53E-06	4.17E-07	6.60E-07	2.70E-07	1.02E-06	7.68E-07	6.86E-07
LOC34911	1.47E-06	4.90E-07	3.40E-07	3.21E-07	1.00E-07	3.79E-07	2.75E-07	1.85E-07
LOC34919	7.73E-09	0	1.31E-08	1.26E-08	4.41E-09	2.38E-09	0	0
LOC36003	0	0	0	0	0	0	0	0
LOC37444	2.46E-06	9.66E-07	2.25E-07	6.68E-07	8.97E-08	4.23E-07	4.59E-07	7.06E-07
LOC37449	2.00E-08	0	6.03E-08	0	0	0	0	0
LOC37519	3.73E-06	4.10E-06	6.37E-07	1.86E-06	2.71E-06	3.90E-06	1.87E-06	2.32E-07
LOC38764	4.71E-07	8.66E-07	3.19E-08	2.96E-08	1.19E-08	8.01E-08	1.01E-08	3.09E-07
LOC38764	1.05E-05	4.92E-06	3.99E-06	7.86E-06	2.46E-06	4.09E-06	6.82E-06	7.98E-06
LOC38815	4.32E-06	2.87E-06	3.40E-07	6.29E-07	8.22E-07	7.36E-07	3.64E-07	1.25E-06
LOC38824	1.03E-06	1.73E-07	1.79E-08	1.24E-07	3.19E-07	2.35E-07	4.37E-07	3.48E-07
LOC38838	7.60E-07	5.79E-07	2.14E-07	8.04E-08	2.45E-07	2.92E-07	1.90E-07	2.51E-07
LOC38842	3.32E-08	0	3.31E-08	0	0	0	3.01E-08	0
LOC38858	4.61E-06	5.16E-06	8.25E-06	9.11E-06	8.53E-07	4.88E-06	8.01E-06	2.42E-06
LOC38869	2.91E-06	7.52E-07	7.05E-07	1.18E-06	1.54E-07	5.14E-07	6.82E-07	6.15E-07
LOC38878	0.0002	0.000129	9.59E-05	9.18E-05	0.000154	0.000129	0.000102	8.70E-05
LOC38879	1.30E-05	8.95E-06	3.24E-06	1.11E-05	9.33E-06	1.76E-05	1.66E-05	1.65E-05
LOC38894	0	0	0	0	0	0	0	0
LOC38895	3.77E-05	2.19E-05	2.52E-05	1.57E-05	1.59E-05	3.48E-05	4.36E-05	3.76E-05
LOC38903	0	0	0	0	0	9.75E-08	0	1.61E-07
LOC38933	5.56E-06	1.64E-08	1.52E-08	2.12E-07	1.98E-07	4.01E-08	1.67E-07	0
LOC38933	9.71E-07	4.69E-07	9.51E-08	4.07E-07	1.22E-08	1.31E-07	1.65E-07	7.27E-08
LOC38945	2.52E-07	3.13E-07	6.17E-08	1.43E-07	3.33E-06	4.79E-07	6.66E-07	2.91E-07
LOC38949	0	0	0	0	0	0	9.45E-08	0
LOC38963	2.89E-07	3.39E-08	5.49E-08	1.54E-07	8.20E-09	8.84E-09	0	9.43E-08
LOC38970	8.85E-08	1.03E-07	9.79E-08	2.19E-07	7.67E-08	2.21E-07	1.12E-07	1.32E-07
LOC38979	7.93E-08	0	1.18E-08	0	9.74E-09	1.79E-07	0	5.00E-08
LOC39059	3.27E-07	1.04E-07	1.55E-07	1.79E-07	1.00E-06	4.13E-07	2.02E-07	1.26E-07
LOC39085	0	0	0	0	0	0	0	0
LOC39132	1.73E-06	0	0	1.46E-07	5.07E-07	4.37E-07	2.67E-07	1.06E-06
LOC39219	0	0	0	0	0	0	0	0
LOC39974	2.35E-06	3.90E-06	8.47E-07	8.47E-07	3.86E-07	9.00E-07	1.28E-06	1.13E-06
LOC39981	1.58E-07	1.88E-08	1.18E-08	0	4.85E-08	2.09E-08	0	0
LOC39995	1.18E-06	1.33E-07	1.20E-06	2.70E-06	7.98E-07	1.72E-06	1.29E-06	7.69E-07
LOC40002	5.14E-06	2.60E-06	3.05E-06	2.45E-06	1.52E-06	2.58E-06	2.27E-06	1.23E-06

LOC40004	1.65E-06	1.45E-07	9.26E-07	1.36E-06	3.12E-07	4.71E-07	1.23E-06	3.48E-07
LOC40065	2.63E-06	1.09E-06	1.53E-06	1.40E-06	7.49E-07	1.68E-06	1.40E-06	7.65E-07
LOC40069	3.17E-07	0	0	1.11E-08	0	1.45E-07	0	0
LOC40075	1.51E-06	1.05E-06	1.07E-06	8.06E-07	9.34E-07	1.07E-06	2.75E-07	2.52E-07
LOC40075	1.89E-06	9.53E-08	3.57E-07	5.37E-07	0	1.12E-06	3.98E-08	4.92E-07
LOC40079	1.47E-08	1.00E-06	0	0	0	2.81E-08	0	0
LOC40080	5.04E-09	1.81E-07	2.59E-08	2.46E-08	0	5.27E-07	0	0
LOC40089	0	1.35E-08	0	0	0	0	0	0
LOC40092	1.83E-06	6.07E-07	2.77E-07	6.92E-07	1.08E-08	6.30E-07	8.00E-07	4.45E-07
LOC40093	1.00E-06	4.13E-08	8.29E-08	3.03E-07	2.93E-07	7.07E-08	5.61E-07	1.06E-07
LOC40094	0	0	0	0	0	0	0	0
LOC40101	1.25E-06	3.93E-07	5.75E-07	6.57E-07	1.76E-07	7.03E-07	5.70E-07	5.00E-07
LOC40105	1.93E-06	7.77E-07	4.13E-07	1.71E-07	1.27E-06	3.18E-07	4.66E-07	2.06E-07
LOC40109	2.31E-07	1.89E-07	1.53E-07	1.56E-07	1.98E-08	2.87E-07	1.14E-07	1.93E-07
LOC40112	4.02E-07	4.23E-07	5.50E-07	7.96E-08	2.09E-07	3.45E-07	2.58E-07	7.18E-08
LOC40138	2.93E-08	0	0	0	9.92E-09	0	8.51E-09	1.29E-08
LOC40139	1.62E-05	4.01E-05	2.47E-05	2.08E-05	6.57E-06	3.05E-05	1.30E-05	1.93E-05
LOC40143	6.04E-07	5.55E-07	1.22E-07	1.97E-07	5.39E-08	8.61E-07	2.74E-07	1.95E-07
LOC40146	9.43E-09	9.57E-08	2.94E-08	5.77E-08	0	8.74E-09	9.14E-09	9.70E-08
LOC40158	2.75E-06	1.20E-06	9.76E-07	1.60E-06	5.41E-07	7.77E-07	4.55E-07	1.45E-06
LOC40237	4.93E-07	4.01E-07	2.63E-07	2.07E-07	0	1.04E-06	5.09E-08	0
LOC40264	7.48E-08	0	0	0	0	0	0	0
LOC40783	2.87E-06	1.37E-06	2.23E-06	9.93E-07	7.46E-07	1.30E-06	1.88E-06	1.67E-06
LOC41505	2.49E-07	7.29E-08	0	3.86E-08	0	0	0	0
LOC44004	0	0	0	0	0	0	0	0
LOC44017	1.19E-08	1.08E-07	3.73E-08	0	0	0	0	3.50E-08
LOC44035	3.16E-06	1.23E-06	1.27E-06	2.85E-06	5.34E-08	2.07E-06	1.06E-06	8.86E-07
LOC44035	2.88E-07	9.26E-07	0	0	0	9.05E-08	0	7.29E-08
LOC44046	8.99E-08	3.07E-07	3.17E-08	1.84E-08	2.58E-08	5.57E-08	4.33E-08	4.42E-08
LOC44056	2.38E-07	3.07E-08	1.16E-07	3.27E-08	0	0	0	3.95E-08
LOC44089	0	0	1.58E-08	1.83E-08	0	1.39E-08	0	0
LOC44090	1.75E-07	2.21E-07	1.03E-08	6.11E-09	4.29E-09	0	0	7.32E-09
LOC44092	3.02E-06	1.38E-06	1.78E-06	2.11E-06	1.37E-06	2.36E-05	1.98E-06	1.17E-06
LOC44094	6.65E-06	2.71E-06	9.97E-07	1.21E-06	2.70E-06	4.38E-06	1.50E-06	1.51E-06
LOC44095	4.82E-05	1.88E-05	2.48E-05	2.88E-05	0.000113	7.30E-05	3.39E-05	2.24E-05
LOC44104	2.80E-07	0	1.01E-07	3.29E-07	1.04E-08	0	8.16E-08	0
LOC44108	3.92E-06	1.82E-06	1.82E-06	3.18E-06	2.71E-07	3.44E-06	1.92E-06	4.16E-06
LOC44117	0	2.09E-07	0	1.61E-08	0	0	0	0
LOC44120	3.55E-07	7.24E-08	9.30E-08	1.92E-07	4.03E-08	1.32E-08	1.27E-07	1.67E-07
LOC44120	9.43E-07	1.18E-06	9.20E-07	7.45E-07	1.75E-07	1.84E-06	7.13E-07	5.38E-07
LOC44129	2.20E-06	7.12E-07	6.05E-07	6.87E-07	2.58E-08	2.25E-06	3.69E-07	1.50E-06
LOC44145	5.46E-08	6.24E-08	1.94E-08	0	0	1.69E-08	0	8.08E-08
LOC44145	2.97E-08	3.38E-08	3.14E-08	1.83E-08	2.56E-08	0	2.87E-08	8.78E-08
LOC44160	5.32E-08	0	0	0	0	0	0	0
LOC44166	2.15E-07	3.37E-08	4.80E-08	1.64E-08	8.63E-09	1.55E-08	1.31E-08	4.41E-08
LOC44186	2.28E-06	5.32E-06	4.86E-07	1.01E-06	2.39E-05	1.10E-06	1.33E-05	3.95E-06
LOC44230	7.59E-08	2.17E-08	6.09E-08	0	0	3.53E-08	0	2.81E-08
LOC44242	3.70E-08	0	1.53E-08	4.13E-08	1.27E-08	2.74E-08	5.75E-08	1.09E-08
LOC44245	6.72E-05	1.70E-05	7.22E-06	9.47E-06	3.87E-05	1.14E-05	4.55E-05	1.44E-05
LOC44245	5.46E-08	1.23E-08	4.57E-08	0	0	0	2.11E-08	0
LOC49375	1.17E-05	9.24E-06	3.70E-06	9.50E-06	3.61E-06	5.81E-06	1.17E-05	1.11E-05
LOC49414	0	0	0	0	0	0	1.36E-08	0
LOC54147	3.71E-05	2.45E-05	1.08E-06	2.04E-05	3.15E-05	3.08E-05	1.84E-05	1.02E-05
LOC54147	2.19E-07	2.21E-07	1.48E-07	4.21E-08	6.39E-07	9.67E-08	2.73E-07	4.67E-08
LOC55011	7.45E-06	4.78E-06	3.56E-06	2.42E-06	1.70E-06	2.65E-06	5.28E-06	4.42E-06
LOC55064	3.10E-05	1.00E-05	5.51E-06	1.37E-05	1.28E-05	1.29E-05	2.23E-05	5.65E-06
LOC55420	0	0	0	0	0	0	0	0
LOC55908	1.14E-05	4.11E-05	5.39E-05	0.000174	6.27E-05	2.40E-05	0.00016	0.000151

LOC57255	0	0	1.57E-07	0	0	6.00E-08	1.17E-07	0
LOC59510	7.44E-07	5.85E-07	2.98E-07	3.74E-07	8.61E-08	1.58E-06	4.52E-07	3.09E-07
LOC60672	7.57E-06	4.15E-07	9.56E-07	2.78E-06	5.95E-07	2.62E-06	2.41E-06	1.73E-06
LOC61303	1.66E-07	2.24E-08	5.48E-08	7.31E-08	0	5.54E-08	3.87E-08	2.51E-07
LOC61920	1.36E-07	1.79E-07	7.15E-08	3.81E-08	3.19E-08	9.83E-08	6.58E-08	1.16E-07
LOC64129	6.32E-07	4.76E-07	7.10E-08	7.24E-08	3.86E-09	2.06E-07	1.78E-08	1.32E-08
LOC64136	1.39E-07	4.87E-08	1.80E-08	1.06E-08	0	2.41E-08	2.52E-08	3.81E-08
LOC64258	0	1.09E-07	3.44E-08	0	0	6.81E-06	2.35E-07	0
LOC64259	3.08E-08	9.27E-08	2.68E-07	6.29E-08	8.83E-09	1.90E-08	9.94E-09	0
LOC64282	2.25E-06	1.21E-06	3.38E-07	2.87E-07	1.70E-07	5.55E-07	3.55E-07	7.17E-07
LOC64284	5.69E-06	1.43E-06	1.01E-06	1.38E-06	1.04E-06	1.68E-06	1.94E-06	2.12E-06
LOC64285	2.15E-06	5.10E-07	2.00E-07	3.11E-07	9.61E-08	8.60E-07	1.91E-07	2.55E-07
LOC64292	0	0	0	0	0	0	0	0
LOC64300	2.52E-06	2.91E-07	6.77E-08	1.18E-07	8.25E-08	2.97E-08	7.68E-08	7.07E-08
LOC64338	1.12E-06	1.05E-06	5.75E-07	5.38E-07	6.20E-07	8.06E-07	6.22E-07	7.87E-07
LOC64348	0	2.84E-07	0	0	0	9.70E-08	0	3.63E-07
LOC64367	1.94E-08	1.36E-08	8.28E-09	9.94E-09	8.74E-09	1.51E-08	1.99E-09	4.46E-09
LOC64371	3.67E-07	2.55E-07	5.00E-07	4.61E-07	1.01E-06	7.92E-07	3.91E-07	7.99E-08
LOC64376	0	0	3.23E-09	1.55E-08	0	1.14E-07	0	0
LOC64383	5.12E-06	3.38E-06	1.40E-05	4.51E-06	1.74E-06	1.30E-05	6.31E-06	5.33E-06
LOC64392	0	0	0	0	0	0	0	0
LOC64395	0	0	0	0	0	0	0	0
LOC64414	0	0	0	0	0	0	0	0
LOC64416	1.26E-06	1.40E-07	2.81E-07	4.96E-07	6.36E-07	1.80E-07	1.01E-07	7.79E-08
LOC64417	3.88E-07	2.37E-08	2.95E-07	4.62E-07	2.16E-07	2.14E-07	1.12E-07	1.70E-07
LOC64453	1.07E-05	4.78E-07	6.16E-07	1.82E-06	4.87E-07	5.70E-07	8.54E-07	1.05E-06
LOC64466	0	1.29E-08	0	0	0	0	0	0
LOC64493	1.42E-06	2.33E-06	2.24E-06	1.70E-06	2.51E-07	1.10E-06	8.75E-07	2.18E-06
LOC64516	3.54E-06	1.76E-06	7.95E-07	3.89E-06	1.34E-06	4.62E-07	3.12E-06	7.35E-07
LOC64532	0	0	0	8.37E-09	0	0	0	0
LOC64533	1.22E-06	2.55E-06	8.66E-07	1.71E-06	1.28E-06	2.30E-06	1.39E-06	1.75E-06
LOC64543	2.26E-07	0	1.99E-08	9.32E-08	0	2.08E-07	2.79E-08	1.74E-08
LOC64567	1.51E-06	6.43E-07	5.45E-07	6.49E-07	3.20E-07	1.14E-06	3.97E-07	8.48E-07
LOC64575	1.83E-07	0	1.53E-08	3.79E-08	0	4.95E-08	0	3.41E-08
LOC64621	6.08E-07	4.30E-07	4.96E-07	2.34E-07	5.09E-08	4.34E-07	1.45E-07	3.39E-07
LOC64647	3.86E-06	1.89E-06	5.54E-07	2.83E-07	1.21E-08	5.30E-07	5.07E-08	2.94E-07
LOC64649	0	0	0	0	0	0	0	0
LOC64662	0	0	0	0	7.81E-08	0	0	0
LOC64676	1.47E-06	8.62E-07	2.38E-07	5.44E-07	2.99E-07	8.15E-07	9.11E-07	1.25E-06
LOC64681	0	0	0	0	0	0	0	0
LOC64685	3.86E-08	2.38E-09	2.18E-09	3.92E-08	3.67E-09	1.19E-08	4.18E-09	3.13E-09
LOC64698	2.29E-08	5.17E-08	1.40E-07	7.28E-09	0	3.73E-08	2.21E-08	3.49E-08
LOC64699	1.98E-07	1.11E-07	5.60E-08	1.43E-07	3.09E-08	3.50E-07	1.31E-07	6.60E-08
LOC64712	6.50E-07	8.41E-07	9.81E-07	5.40E-07	1.70E-07	2.75E-07	2.84E-07	5.53E-07
LOC64728	6.85E-07	2.90E-07	5.54E-07	1.41E-07	2.60E-07	5.28E-07	2.67E-07	1.18E-07
LOC64730	1.89E-06	5.74E-07	1.58E-06	4.09E-07	2.71E-08	1.66E-06	1.90E-07	7.50E-07
LOC64785	1.67E-07	6.78E-08	6.69E-08	6.99E-08	0	0	0	1.70E-07
LOC64794	4.09E-07	1.27E-07	1.16E-08	0	0	0	2.14E-08	1.48E-08
LOC64797	0.000108	4.25E-05	3.84E-05	3.91E-05	3.04E-05	7.59E-05	3.02E-05	4.41E-05
LOC64869	0	0	0	0	0	0	0	0
LOC64874	9.66E-08	9.59E-08	3.81E-08	3.31E-08	3.12E-08	9.27E-08	3.00E-08	1.75E-08
LOC64933	2.90E-06	8.17E-07	6.42E-07	3.30E-07	#######	5.22E-07	4.06E-07	1.99E-07
LOC65029	0	0	0	0	0	0	0	0
LOC65036	5.50E-07	2.57E-06	1.55E-07	3.40E-07	3.28E-07	3.31E-07	5.41E-07	4.10E-07
LOC65062	1.55E-07	1.56E-07	1.59E-07	1.39E-07	0	1.54E-07	2.21E-08	4.98E-08
LOC65125	1.12E-05	9.33E-06	3.96E-06	3.37E-06	3.66E-06	7.17E-06	3.65E-06	5.24E-06
LOC65227	1.10E-06	8.84E-07	2.09E-07	4.13E-07	1.06E-07	4.50E-07	3.42E-07	2.16E-07
LOC65311	1.74E-06	5.90E-07	1.11E-06	1.48E-06	2.77E-07	3.81E-07	1.50E-07	3.43E-07

LOC6535C	1.34E-06	2.39E-06	1.07E-06	7.00E-07	4.54E-07	9.00E-07	3.76E-07	1.38E-06
LOC65354	0	0	0	0	0	0	0	0
LOC65354	0	0	0	0	0	0	0	0
LOC6535€	6.90E-05	4.97E-05	3.83E-05	4.06E-05	2.99E-05	6.14E-05	4.44E-05	7.84E-05
LOC6536£	3.01E-06	7.71E-07	7.96E-07	1.43E-06	3.86E-08	1.14E-06	1.24E-06	9.94E-07
LOC6537£	6.14E-08	4.94E-08	9.11E-09	6.66E-07	9.81E-08	4.88E-08	4.51E-07	5.15E-08
LOC65434	4.03E-06	8.94E-06	1.52E-06	8.57E-06	5.96E-06	4.97E-07	3.71E-06	1.62E-06
LOC6544З	2.13E-07	8.63E-06	1.49E-06	2.50E-06	1.76E-06	5.70E-06	4.23E-06	1.88E-07
LOC6786£	1.56E-05	1.32E-05	3.98E-06	6.51E-06	5.15E-06	1.37E-05	9.34E-06	6.02E-06
LOC7238C	1.65E-06	1.50E-08	0	4.86E-08	0	0	1.27E-08	1.94E-08
LOC72397	3.16E-06	8.45E-07	1.94E-06	4.93E-07	5.51E-07	4.45E-07	2.61E-07	5.96E-07
LOC72767	0	0	0	0	0	0	0	0
LOC7278Ç	6.61E-07	7.72E-07	4.17E-07	2.39E-07	2.90E-08	2.43E-07	1.70E-07	3.76E-07
LOC72792	0	8.04E-08	0	0	0	0	0	0
LOC72802	1.67E-06	1.19E-06	1.47E-06	1.63E-06	3.93E-08	2.12E-06	5.07E-07	1.02E-06
LOC7281Ç	6.22E-06	4.25E-06	2.34E-06	3.73E-06	4.52E-06	7.05E-06	5.69E-06	1.81E-06
LOC7282€	6.11E-07	3.81E-08	1.34E-07	3.92E-08	2.30E-07	9.04E-08	4.10E-08	3.10E-07
LOC72827	0	0	0	0	0	0	0	0
LOC72832	3.93E-06	1.37E-06	2.30E-06	5.83E-07	4.55E-07	1.56E-06	6.07E-07	1.80E-06
LOC7283Ç	2.13E-06	3.16E-06	3.45E-07	2.18E-06	1.13E-06	5.28E-07	1.22E-06	8.38E-07
LOC72841	0	0	0	0	0	0	0	0
LOC7285£	1.24E-05	2.27E-06	1.46E-06	3.84E-06	1.73E-06	7.89E-06	2.25E-06	3.55E-06
LOC7286C	0	0	2.81E-08	6.61E-08	1.55E-08	0	2.62E-08	0
LOC72861	1.13E-05	2.23E-06	3.54E-06	4.58E-06	4.94E-07	5.97E-06	1.14E-06	4.30E-06
LOC72864	4.69E-06	4.93E-07	3.29E-07	5.36E-07	2.16E-07	6.74E-07	4.79E-07	4.65E-07
LOC72864	1.13E-05	3.80E-06	5.06E-06	9.09E-06	1.30E-06	8.50E-06	5.11E-06	4.63E-06
LOC72872	9.02E-07	6.00E-07	5.24E-08	5.52E-08	9.34E-08	4.29E-07	1.15E-07	1.94E-07
LOC72874	1.87E-06	1.66E-06	7.41E-07	1.53E-06	7.20E-07	4.98E-06	2.71E-06	3.87E-06
LOC7287£	1.55E-06	6.01E-07	6.71E-07	6.40E-07	1.02E-06	5.69E-07	5.94E-07	1.20E-06
LOC72881	3.47E-06	2.54E-08	2.39E-07	4.36E-07	5.73E-08	2.06E-08	1.06E-07	1.31E-07
LOC7288£	2.64E-05	1.44E-05	1.34E-05	1.35E-05	8.35E-06	1.87E-05	1.73E-05	2.76E-05
LOC72887	4.97E-06	6.64E-07	9.78E-07	9.67E-07	9.57E-06	2.03E-05	1.79E-05	1.07E-05
LOC7289£	2.20E-07	2.82E-08	2.39E-07	9.05E-08	0	3.64E-07	9.33E-08	0
LOC72902	1.37E-06	8.71E-07	6.42E-07	5.07E-07	2.88E-08	7.57E-07	1.62E-07	3.78E-07
LOC7290£	2.87E-05	2.87E-05	1.51E-05	8.48E-06	4.67E-06	1.71E-05	7.80E-06	1.14E-05
LOC72912	0	0	0	0	0	0	0	0
LOC7291£	1.48E-07	#######	0	3.15E-07	6.27E-08	2.36E-08	8.09E-07	6.02E-07
LOC72917	7.61E-07	6.98E-07	7.84E-07	5.14E-07	8.54E-08	5.22E-07	3.95E-07	4.38E-07
LOC7292З	9.40E-06	3.76E-06	9.88E-07	2.93E-06	1.71E-06	1.81E-06	3.29E-06	2.80E-06
LOC72937	2.80E-07	9.17E-07	2.15E-07	2.04E-07	2.47E-07	4.51E-07	1.58E-07	2.26E-07
LOC7294€	0	0	0	0	0	0	0	0
LOC7296C	1.22E-06	1.07E-06	5.56E-07	4.69E-07	1.20E-07	7.04E-07	2.36E-07	3.03E-07
LOC7296C	0	0	0	0	0	0	4.60E-08	0
LOC7296€	0	0	1.64E-08	0	0	0	0	0
LOC72967	1.18E-05	6.63E-06	5.92E-06	8.22E-06	2.99E-06	8.96E-06	7.13E-06	4.36E-06
LOC7297Ç	7.29E-08	1.03E-07	1.51E-07	8.92E-08	0	8.44E-08	6.18E-08	1.07E-07
LOC7299Ç	1.61E-05	7.71E-06	6.79E-06	1.02E-05	7.35E-06	1.24E-05	9.63E-06	1.30E-05
LOC7299Ç	7.98E-07	1.31E-06	4.25E-07	8.46E-07	1.09E-06	1.42E-06	1.32E-06	4.55E-07
LOC7301C	1.25E-06	8.85E-06	2.07E-06	5.70E-07	2.08E-06	7.29E-07	2.05E-07	4.33E-07
LOC7306€	3.28E-08	0	0	6.67E-09	0	0	0	0
LOC73081	0	0	0	0	0	0	0	0
LOC73177	0	0	0	0	0	0	0	0
LOC7317£	3.67E-09	4.92E-08	0	0	0	3.39E-09	0	0
LOC73227	0	0	0	0	0	0	0	0
LOC80054	4.24E-06	4.23E-06	2.84E-06	2.22E-06	7.14E-06	1.91E-06	2.86E-06	1.62E-06
LOC80154	6.00E-06	3.36E-06	1.21E-06	1.52E-06	1.52E-06	1.67E-06	1.41E-06	3.42E-06
LOC81691	1.54E-06	3.01E-06	5.52E-07	9.75E-07	7.57E-07	1.95E-06	3.85E-07	7.31E-07
LOC8474C	4.62E-08	3.89E-07	3.36E-09	1.21E-08	5.64E-09	1.07E-07	1.60E-08	9.71E-08

LOC84856	1.23E-07	1.07E-07	3.68E-08	2.13E-07	1.43E-07	3.26E-08	1.55E-07	2.38E-07
LOC84931	1.07E-07	1.34E-08	1.24E-08	1.46E-08	2.04E-08	3.97E-07	0	0
LOC84989	2.80E-07	1.32E-07	1.26E-07	3.50E-07	3.44E-07	0	1.82E-07	0
LOC90110	4.30E-06	1.83E-06	2.85E-06	3.09E-06	2.75E-06	4.28E-06	3.65E-06	1.96E-06
LOC90246	4.58E-07	2.86E-08	4.39E-08	1.04E-07	2.91E-08	3.14E-08	7.39E-08	4.97E-08
LOC90586	7.00E-06	1.22E-05	1.99E-06	1.79E-06	2.00E-07	2.28E-06	6.01E-07	5.39E-06
LOC90784	4.29E-06	2.46E-07	9.68E-07	1.50E-06	1.53E-06	2.76E-06	2.87E-06	3.07E-06
LOC90834	4.18E-07	1.42E-07	4.05E-08	1.43E-07	0	4.51E-08	9.41E-08	1.00E-07
LOC91149	4.90E-09	0	5.03E-09	0	0	0	0	0
LOC91316	1.09E-05	2.06E-06	1.03E-06	2.87E-06	2.64E-06	1.47E-06	2.06E-06	2.24E-06
LOC91450	3.51E-07	3.13E-08	1.48E-07	2.00E-07	5.84E-07	1.51E-07	2.57E-07	1.61E-07
LOC91948	0	2.15E-08	0	0	0	6.98E-08	0	0
LOC92249	6.94E-06	7.13E-06	4.42E-06	5.17E-06	4.42E-06	1.01E-05	7.08E-06	9.05E-06
LOC92659	1.60E-06	5.77E-08	1.20E-07	2.50E-07	3.72E-07	2.00E-06	3.69E-07	4.68E-07
LOC92973	3.87E-08	3.45E-07	4.84E-08	4.19E-08	9.21E-08	7.15E-08	4.60E-08	2.51E-08
LOC93432	0	0	0	0	0	0	0	4.59E-08
LOC93622	5.09E-06	7.79E-06	5.70E-06	5.06E-06	8.17E-06	1.14E-05	7.83E-06	7.97E-06
LOC96610	4.49E-05	3.42E-06	7.22E-06	2.79E-05	2.43E-06	6.32E-06	7.98E-06	9.08E-06
LOH12CR1	4.34E-06	2.17E-06	1.60E-06	2.62E-06	1.20E-06	3.34E-06	2.02E-06	2.10E-06
LOH12CR2	1.29E-06	3.85E-07	5.63E-07	5.34E-07	7.77E-07	1.91E-06	3.41E-07	4.28E-07
LOH3CR2A	3.38E-07	1.41E-06	1.18E-07	9.39E-07	7.76E-08	1.25E-07	4.36E-07	3.15E-07
LONP1	4.71E-05	5.42E-05	4.43E-05	4.51E-05	4.87E-05	6.01E-05	4.79E-05	5.17E-05
LONP2	2.38E-05	3.14E-05	3.50E-05	2.39E-05	3.68E-06	2.99E-05	1.68E-05	4.20E-05
LONRF1	3.72E-06	7.85E-06	5.04E-06	6.76E-06	2.73E-07	5.72E-06	3.00E-06	2.68E-06
LONRF2	5.45E-07	1.18E-08	1.02E-07	2.35E-07	4.08E-08	1.96E-08	3.79E-08	2.52E-07
LONRF3	2.73E-06	6.79E-06	1.93E-06	3.37E-06	8.95E-07	3.59E-06	2.43E-06	7.14E-06
LOR	0	0	2.92E-07	0	0	0	0	0
LOX	1.85E-06	1.49E-06	3.26E-07	2.14E-06	8.98E-08	2.38E-05	3.95E-06	6.23E-07
LOXHD1	2.63E-08	1.14E-07	0	1.37E-08	0	3.18E-08	7.86E-08	5.42E-08
LOXL1	1.05E-05	1.06E-06	1.26E-06	1.68E-05	4.29E-06	2.23E-06	7.65E-06	4.80E-06
LOXL2	7.43E-06	4.05E-06	8.28E-07	6.21E-06	3.24E-06	4.91E-06	1.06E-05	4.86E-06
LOXL3	1.92E-06	2.36E-07	3.16E-07	1.00E-06	1.28E-07	4.52E-07	1.09E-06	2.33E-07
LOXL4	3.84E-05	8.84E-07	6.57E-07	3.52E-05	1.18E-05	8.46E-07	1.00E-05	9.27E-06
LPA	5.58E-07	1.76E-06	2.90E-05	7.43E-06	1.03E-06	2.26E-06	1.06E-05	4.71E-06
LPAL2	9.68E-08	6.59E-07	4.24E-06	1.49E-06	6.27E-07	4.41E-07	1.12E-06	8.78E-07
LPAR1	2.75E-07	9.55E-09	4.43E-07	1.40E-06	8.75E-08	3.15E-08	3.54E-07	2.24E-07
LPAR2	8.51E-06	1.01E-06	9.50E-07	3.21E-06	8.85E-07	8.20E-07	1.00E-06	8.97E-07
LPAR3	2.79E-06	0	0	9.83E-08	0	0	0	0
LPAR4	3.58E-08	2.01E-08	0	2.19E-08	0	2.49E-08	8.67E-09	1.31E-08
LPAR5	1.51E-06	2.46E-06	9.61E-07	1.17E-06	5.95E-08	7.39E-07	9.77E-07	2.41E-07
LPAR6	1.91E-05	2.99E-06	6.01E-06	3.57E-06	2.49E-06	3.81E-06	4.57E-06	4.69E-06
LPCAT1	4.96E-05	1.69E-05	3.77E-06	1.32E-05	1.88E-06	6.58E-06	7.59E-06	1.36E-05
LPCAT2	3.56E-06	6.39E-07	9.97E-07	1.23E-06	1.25E-07	9.98E-07	5.94E-07	7.57E-07
LPCAT3	8.87E-05	8.88E-05	5.34E-05	5.14E-05	5.88E-05	4.33E-05	7.42E-05	0.000102
LPCAT4	1.14E-05	2.20E-06	1.21E-06	4.45E-06	1.00E-06	1.21E-06	2.74E-06	2.50E-06
LPGAT1	5.56E-05	3.41E-05	1.97E-05	1.61E-05	1.05E-05	1.33E-05	1.83E-05	5.91E-05
LPHN1	3.61E-06	3.13E-07	2.36E-07	4.22E-07	3.09E-07	2.05E-07	5.89E-07	1.08E-06
LPHN2	2.82E-05	1.64E-06	1.71E-05	1.75E-05	3.35E-06	1.68E-05	7.93E-06	2.54E-05
LPHN3	7.06E-08	3.66E-07	1.18E-07	2.54E-07	4.64E-09	1.01E-07	5.28E-08	1.17E-07
LPIN1	8.38E-06	4.00E-05	8.10E-06	5.56E-06	2.81E-06	2.94E-05	6.68E-06	8.82E-06
LPIN2	5.20E-05	6.67E-05	8.32E-05	4.40E-05	6.17E-06	3.86E-05	7.51E-05	6.68E-05
LPIN3	2.61E-05	7.64E-06	3.35E-06	2.71E-06	2.96E-06	2.47E-06	3.76E-06	1.91E-06
LPL	9.88E-07	1.69E-06	1.32E-07	1.36E-06	3.19E-07	1.41E-06	1.96E-06	1.43E-06
LPO	0	3.89E-08	0	0	0	2.14E-08	0	0
LPP	2.56E-06	7.85E-07	1.34E-06	1.36E-06	7.16E-07	2.67E-06	1.99E-06	1.62E-06
LPPR1	2.42E-06	5.72E-06	2.03E-06	2.00E-06	2.88E-06	1.76E-05	5.31E-06	5.91E-06
LPPR2	9.78E-06	2.11E-06	3.22E-06	5.84E-06	4.00E-06	1.05E-05	9.63E-06	2.52E-05
LPPR3	1.83E-07	0	0	0	0	0	5.39E-08	0

LPPR4	4.40E-06	2.63E-08	3.87E-08	1.15E-08	0	3.50E-07	1.51E-07	1.93E-07
LPPR5	0	0	0	0	0	0	0	0
LPXN	1.29E-05	6.94E-06	5.27E-06	1.09E-05	1.17E-06	5.70E-06	4.65E-06	5.90E-06
LQK1	1.51E-05	4.87E-06	1.18E-06	2.17E-07	5.82E-07	5.87E-06	2.08E-06	3.15E-06
LRAT	6.86E-07	9.81E-08	1.86E-06	1.69E-06	2.30E-07	3.85E-07	9.89E-08	2.36E-07
LRBA	2.19E-05	7.70E-06	1.17E-05	1.18E-05	3.12E-06	1.23E-05	6.56E-06	1.03E-05
LRCH1	6.28E-06	4.38E-06	6.11E-06	6.21E-06	7.65E-07	3.56E-06	4.46E-06	3.40E-06
LRCH2	1.99E-07	8.80E-08	8.55E-08	1.19E-07	4.77E-08	4.72E-08	1.08E-07	1.02E-07
LRCH3	8.17E-06	4.38E-06	3.43E-06	3.22E-06	2.91E-07	3.92E-06	1.87E-06	2.59E-06
LRCH4	3.64E-05	3.92E-05	1.00E-05	1.35E-05	7.22E-06	8.92E-06	1.08E-05	1.54E-05
LRDD	6.45E-06	4.41E-06	2.98E-06	2.99E-06	5.55E-06	3.75E-06	4.08E-06	5.10E-06
LRFN1	1.03E-06	8.28E-08	2.97E-07	3.24E-07	1.08E-07	2.25E-07	4.51E-07	1.30E-07
LRFN2	3.67E-08	3.30E-08	0	0	0	6.80E-09	7.13E-09	0
LRFN3	4.38E-06	6.83E-06	2.97E-06	3.43E-06	3.90E-06	5.14E-06	5.43E-06	8.42E-06
LRFN4	3.22E-06	3.76E-07	3.18E-07	2.74E-06	1.75E-07	3.53E-07	1.15E-06	5.25E-07
LRFN5	0	0	0	3.74E-08	0	0	5.96E-09	8.98E-09
LRG1	0.000398	0.000406	0.000663	0.000455	0.000225	0.000402	0.000428	0.000605
LRGUK	1.30E-07	1.36E-07	0	2.11E-08	2.97E-08	1.28E-07	3.35E-08	3.80E-08
LRIG1	1.17E-05	1.78E-05	1.49E-05	8.09E-06	7.42E-06	2.13E-05	6.35E-06	2.29E-05
LRIG2	2.33E-06	9.34E-07	6.59E-07	1.02E-06	7.79E-08	1.76E-06	4.91E-07	9.38E-07
LRIG3	4.19E-06	3.34E-06	5.45E-06	3.25E-06	8.80E-07	3.19E-06	2.47E-06	7.80E-07
LRIT1	0	0	0	0	0	0	0	0
LRIT2	1.50E-08	0	0	9.16E-09	0	0	0	0
LRIT3	5.69E-08	4.96E-08	1.24E-08	1.54E-08	2.71E-08	1.12E-08	3.07E-08	5.55E-08
LRMP	1.99E-06	1.38E-06	7.82E-07	1.14E-06	2.02E-07	1.11E-06	1.57E-06	4.18E-07
LRP1	2.26E-05	7.15E-05	6.01E-05	5.11E-05	2.10E-05	9.20E-05	5.74E-05	2.05E-05
LRP10	9.93E-05	4.66E-05	2.06E-05	4.14E-05	1.68E-05	2.77E-05	3.36E-05	6.77E-05
LRP11	1.97E-05	1.79E-05	4.50E-06	4.97E-06	2.42E-06	6.81E-06	7.53E-06	6.24E-06
LRP12	6.14E-06	2.42E-07	4.62E-07	1.06E-06	6.84E-08	1.58E-07	2.93E-07	8.71E-07
LRP1B	1.37E-08	7.39E-08	7.97E-08	4.37E-08	0	5.74E-08	1.30E-09	9.69E-09
LRP2	1.33E-05	7.76E-09	5.53E-08	1.19E-08	1.20E-09	3.34E-07	6.27E-09	8.15E-09
LRP2BP	2.01E-07	9.26E-08	1.78E-07	2.14E-07	9.74E-08	4.86E-07	1.03E-07	1.40E-07
LRP3	9.97E-06	1.04E-05	2.08E-05	2.24E-05	6.01E-05	1.40E-05	4.80E-05	2.62E-05
LRP4	6.19E-06	2.53E-07	3.41E-07	1.57E-07	7.93E-08	8.19E-08	3.42E-07	1.50E-07
LRP5	2.30E-05	3.69E-05	4.60E-05	3.37E-05	1.43E-05	3.84E-05	2.69E-05	8.92E-05
LRP5L	4.98E-06	2.01E-06	1.59E-06	4.52E-06	2.48E-06	3.19E-06	1.19E-06	3.18E-06
LRP6	7.13E-06	5.39E-06	6.44E-06	3.55E-06	3.54E-06	5.16E-06	4.43E-06	8.65E-06
LRP8	1.47E-06	1.39E-08	4.43E-08	9.07E-08	3.19E-08	1.34E-07	4.97E-08	7.82E-08
LRPAP1	0.000126	0.000131	8.65E-05	0.0001	9.27E-05	9.33E-05	8.73E-05	0.000166
LRPPRC	3.54E-05	6.45E-05	1.94E-05	1.67E-05	7.97E-06	2.72E-05	1.14E-05	1.78E-05
LRRC1	8.79E-06	6.23E-06	1.65E-07	2.39E-06	4.30E-07	4.70E-08	5.21E-07	4.79E-07
LRRC10	0	0	0	0	7.75E-09	0	0	0
LRRC10B	4.82E-07	2.18E-07	1.12E-08	7.88E-08	2.49E-07	1.39E-07	2.39E-07	6.15E-07
LRRC14	1.28E-05	7.15E-06	2.18E-06	2.97E-06	4.49E-06	4.16E-06	7.20E-06	5.97E-06
LRRC14B	0	0	1.66E-08	0	0	0	0	0
LRRC15	1.91E-08	1.28E-08	2.70E-08	1.63E-07	9.81E-09	3.17E-08	7.80E-08	3.34E-07
LRRC16A	1.01E-05	3.98E-06	1.06E-06	1.59E-06	3.06E-07	8.44E-07	6.40E-07	7.98E-07
LRRC16B	3.99E-07	3.28E-07	5.61E-07	6.35E-08	4.59E-07	4.66E-08	5.45E-08	1.29E-07
LRRC17	1.61E-06	4.34E-06	4.36E-07	1.90E-06	5.71E-07	3.57E-07	1.04E-06	4.41E-07
LRRC18	0	0	0	3.27E-08	1.31E-08	0	0	0
LRRC19	6.24E-07	2.85E-08	4.20E-06	1.55E-08	5.46E-09	7.66E-08	0	0
LRRC2	6.89E-07	1.94E-06	2.58E-06	2.14E-06	7.24E-07	1.67E-06	2.56E-06	5.96E-06
LRRC20	7.32E-06	1.25E-05	9.95E-06	9.30E-06	3.48E-05	2.22E-05	1.12E-05	3.06E-05
LRRC23	5.70E-06	3.33E-06	2.12E-06	2.18E-06	1.42E-06	1.71E-06	4.00E-06	1.64E-06
LRRC24	5.22E-07	6.54E-07	1.80E-07	4.14E-07	1.26E-06	2.84E-07	4.07E-06	7.17E-08
LRRC25	6.99E-06	1.21E-05	4.36E-06	6.81E-06	8.70E-07	3.65E-06	5.51E-06	1.07E-06
LRRC26	1.72E-07	0	0	0	0	0	6.19E-08	3.20E-08
LRRC27	3.56E-06	1.50E-06	1.31E-06	2.48E-06	1.37E-06	5.77E-06	2.12E-06	1.66E-06

LRRC28	1.90E-05	2.00E-05	1.83E-05	1.29E-05	1.03E-05	1.39E-05	1.82E-05	2.17E-05
LRRC29	2.51E-06	7.38E-07	1.95E-06	1.72E-06	1.53E-06	1.60E-06	1.50E-06	1.96E-06
LRRC3	2.26E-07	8.32E-06	8.23E-06	4.81E-06	1.88E-06	5.13E-06	8.88E-06	4.74E-06
LRRC30	0	0	0	0	0	0	0	0
LRRC31	1.54E-07	1.07E-06	1.57E-06	2.14E-06	3.89E-07	4.51E-07	2.86E-06	2.58E-06
LRRC32	1.34E-05	4.02E-06	3.82E-06	1.39E-05	4.35E-06	6.23E-06	1.09E-05	6.00E-06
LRRC33	2.59E-06	1.32E-06	2.33E-06	2.79E-06	7.55E-07	1.48E-06	3.36E-06	1.05E-06
LRRC34	6.52E-07	1.29E-07	2.66E-07	2.51E-07	1.51E-07	1.12E-07	2.53E-07	1.73E-07
LRRC36	1.33E-08	1.14E-07	4.20E-08	1.81E-08	0	3.01E-07	6.08E-08	0
LRRC37A	8.96E-06	9.43E-07	1.63E-06	2.71E-06	1.89E-06	4.51E-06	2.37E-06	1.72E-06
LRRC37A2	4.89E-06	5.84E-07	9.04E-07	1.79E-06	1.66E-07	2.21E-06	5.01E-07	8.48E-07
LRRC37A3	1.19E-06	1.66E-06	2.45E-07	5.82E-07	1.19E-06	2.16E-06	5.58E-07	5.53E-07
LRRC37A4	5.74E-06	6.68E-07	6.11E-07	4.61E-07	9.93E-08	1.28E-06	4.97E-07	4.63E-07
LRRC37B	2.71E-06	5.89E-07	3.97E-07	5.92E-07	2.36E-07	3.90E-07	8.25E-07	3.21E-07
LRRC37B2	5.87E-06	2.43E-06	1.57E-06	1.88E-06	8.44E-07	2.34E-06	2.02E-06	1.95E-06
LRRC39	5.61E-07	4.11E-07	7.97E-08	2.81E-07	0	2.67E-07	2.79E-07	2.48E-07
LRRC3B	8.67E-08	4.93E-08	1.53E-08	1.77E-08	1.25E-08	5.37E-08	1.39E-07	1.28E-07
LRRC4	3.46E-07	4.84E-08	9.60E-07	4.07E-07	1.01E-07	1.03E-07	1.98E-07	1.18E-07
LRRC40	1.22E-05	9.44E-06	8.41E-06	5.46E-06	1.14E-06	1.00E-05	2.80E-06	5.26E-06
LRRC41	2.99E-05	2.48E-05	1.90E-05	2.12E-05	4.51E-06	2.62E-05	1.69E-05	2.04E-05
LRRC42	1.74E-05	1.25E-05	1.01E-05	1.78E-05	7.61E-06	1.87E-05	1.47E-05	2.56E-05
LRRC43	0	3.58E-08	6.19E-08	2.76E-07	1.02E-08	0	1.72E-08	1.74E-08
LRRC45	1.19E-05	3.66E-05	7.04E-06	7.31E-06	1.35E-05	2.94E-05	7.89E-06	8.69E-06
LRRC46	1.01E-06	7.11E-07	2.26E-07	5.21E-07	1.06E-06	5.65E-07	8.42E-07	6.48E-07
LRRC47	2.62E-05	2.89E-05	2.19E-05	2.15E-05	1.52E-05	3.15E-05	2.04E-05	2.50E-05
LRRC48	1.28E-07	1.26E-06	2.30E-07	5.39E-07	1.49E-07	2.58E-07	5.16E-07	1.71E-07
LRRC49	2.17E-07	4.74E-08	5.25E-08	4.39E-07	7.98E-08	3.55E-07	4.91E-08	7.43E-08
LRRC4B	8.47E-07	4.58E-07	2.60E-07	3.84E-07	3.44E-07	3.71E-07	5.94E-07	3.45E-07
LRRC4C	2.36E-07	2.85E-08	2.98E-07	9.85E-07	5.80E-08	0	8.03E-08	8.67E-08
LRRC50	0	1.59E-08	7.40E-08	7.65E-06	1.09E-07	6.52E-08	7.72E-07	0
LRRC52	6.97E-08	1.34E-06	0	5.73E-08	0	9.31E-07	0	0
LRRC55	4.23E-07	0	1.02E-06	2.50E-07	2.20E-08	1.58E-08	4.97E-08	0
LRRC56	3.86E-07	2.55E-07	2.35E-07	5.44E-07	3.17E-07	2.65E-07	1.95E-07	5.79E-07
LRRC57	1.08E-05	4.11E-06	2.43E-06	2.62E-06	1.01E-06	2.84E-06	3.01E-06	3.35E-06
LRRC58	5.39E-06	8.92E-06	5.13E-06	2.80E-06	6.38E-07	4.77E-06	2.61E-06	3.65E-06
LRRC59	0.000107	7.41E-05	3.11E-05	5.04E-05	2.10E-05	8.38E-05	5.17E-05	5.66E-05
LRRC6	1.56E-07	2.02E-07	8.25E-08	7.62E-07	6.71E-08	8.83E-07	1.65E-07	0
LRRC61	2.26E-05	3.73E-05	2.26E-05	1.25E-05	1.58E-05	1.03E-05	2.02E-05	1.98E-05
LRRC66	7.60E-08	1.55E-08	7.13E-09	3.38E-08	2.37E-08	1.28E-08	1.34E-08	5.07E-08
LRRC67	0	0	0	0	0	0	4.64E-08	0
LRRC69	1.88E-07	2.19E-08	3.63E-08	5.98E-08	5.64E-08	5.34E-08	7.34E-08	0
LRRC7	2.70E-06	2.58E-08	4.89E-08	1.50E-07	1.08E-08	0	0	0
LRRC70	7.68E-07	1.16E-06	6.92E-07	1.00E-06	7.06E-07	3.71E-07	9.40E-07	7.90E-07
LRRC8A	5.18E-05	1.36E-05	1.13E-05	2.59E-05	6.21E-06	1.62E-05	1.95E-05	5.42E-05
LRRC8B	4.53E-06	1.66E-06	8.13E-07	1.29E-06	1.21E-07	3.14E-07	9.09E-07	1.15E-06
LRRC8C	4.02E-06	9.73E-07	7.77E-07	1.64E-06	3.69E-07	8.72E-07	9.56E-07	8.68E-07
LRRC8D	1.57E-05	1.43E-05	9.59E-06	8.51E-06	4.27E-06	2.86E-05	6.41E-06	1.72E-05
LRRC8E	6.84E-06	2.99E-06	1.27E-06	2.45E-06	5.19E-07	1.94E-07	3.46E-06	6.05E-06
LRRCC1	7.70E-06	4.45E-06	1.24E-06	2.08E-06	8.16E-08	1.91E-06	1.23E-06	1.61E-06
LRRFIP1	5.42E-05	1.64E-05	2.90E-05	4.09E-05	8.65E-06	2.07E-05	2.32E-05	2.82E-05
LRRFIP2	1.95E-05	3.60E-05	1.49E-05	2.16E-05	8.23E-06	1.19E-05	2.02E-05	1.60E-05
LRRIQ1	1.02E-08	0	0	6.19E-08	0	4.71E-09	0	6.04E-09
LRRIQ3	6.50E-08	6.85E-07	9.26E-08	3.97E-08	1.40E-08	6.85E-07	5.52E-08	3.58E-08
LRRIQ4	2.66E-08	0	1.40E-08	0	1.14E-08	0	6.41E-08	0
LRRK1	2.35E-06	1.08E-07	7.06E-07	8.73E-07	1.04E-07	3.02E-07	6.86E-07	2.21E-07
LRRK2	1.01E-06	1.64E-06	2.05E-06	1.41E-06	2.64E-07	9.46E-07	6.42E-07	7.11E-07
LRRN1	2.39E-08	0	2.15E-07	4.16E-07	0	5.53E-09	5.23E-08	2.62E-08
LRRN2	6.88E-06	1.70E-07	2.16E-07	1.21E-06	1.12E-07	1.14E-07	2.93E-07	5.08E-07

LRRN3	5.84E-08	1.45E-08	5.84E-07	5.23E-07	2.78E-08	0	0	2.85E-08
LRRN4	3.82E-08	9.76E-09	8.04E-08	3.58E-07	0	1.14E-07	1.68E-08	0
LRRN4CL	1.95E-08	1.10E-08	1.02E-08	2.03E-07	1.68E-08	0	8.51E-08	1.43E-08
LRRTM1	1.07E-08	0	2.69E-07	9.19E-08	9.22E-09	0	0	9.46E-08
LRRTM2	4.48E-08	1.02E-07	1.13E-07	2.43E-08	0	3.55E-08	3.50E-08	3.77E-08
LRRTM3	4.01E-09	1.34E-08	0	0	0	9.06E-09	0	0
LRRTM4	0	3.37E-08	1.94E-07	1.46E-07	0	0	0	2.53E-07
LRSAM1	1.20E-05	3.98E-06	1.74E-05	1.14E-05	7.40E-06	2.65E-05	7.17E-06	2.90E-05
LRTM1	0	0	0	0	0	0	0	0
LRTM2	0	0	0	0	0	0	0	0
LRTOMT	4.65E-06	3.90E-06	2.94E-06	2.88E-06	2.37E-06	4.38E-06	3.20E-06	3.25E-06
LRWD1	4.40E-06	1.24E-05	9.78E-06	1.10E-05	2.76E-06	9.62E-06	9.33E-06	1.20E-05
LSAMP	1.33E-06	0	9.44E-08	2.41E-07	0	0	0	3.86E-07
LSG1	1.89E-05	1.29E-05	7.16E-06	8.09E-06	4.77E-06	1.13E-05	7.84E-06	1.05E-05
LSM1	2.55E-05	7.06E-05	2.03E-05	2.66E-05	1.62E-05	3.48E-05	2.00E-05	2.54E-05
LSM10	4.13E-05	4.65E-05	2.64E-05	3.31E-05	2.89E-05	3.41E-05	3.27E-05	3.81E-05
LSM11	1.37E-06	1.15E-06	9.20E-07	7.83E-07	3.47E-07	1.12E-06	6.12E-07	7.25E-07
LSM12	2.87E-05	2.82E-05	1.40E-05	1.60E-05	1.15E-05	2.80E-05	1.80E-05	1.85E-05
LSM14A	4.22E-05	2.46E-05	2.02E-05	2.28E-05	1.39E-05	3.02E-05	2.45E-05	1.95E-05
LSM14B	1.70E-05	1.34E-05	1.12E-05	1.08E-05	5.23E-06	1.69E-05	1.24E-05	9.71E-06
LSM2	4.68E-05	3.78E-05	1.29E-05	2.56E-05	3.97E-05	2.87E-05	4.38E-05	2.49E-05
LSM3	0.000144	0.000148	7.38E-05	8.92E-05	0.000151	0.0001	0.000113	6.92E-05
LSM4	0.000137	0.000178	3.97E-05	4.70E-05	8.53E-05	7.07E-05	0.000102	5.03E-05
LSM5	1.54E-05	2.43E-05	7.30E-06	8.14E-06	1.42E-05	1.36E-05	1.43E-05	8.66E-06
LSM6	1.68E-05	8.47E-06	1.48E-05	1.64E-05	1.20E-05	1.54E-05	2.16E-05	1.71E-05
LSM7	8.42E-05	3.45E-05	4.30E-05	8.27E-05	0.000201	7.98E-05	0.00011	4.99E-05
LSMD1	5.93E-05	6.61E-05	6.90E-05	6.87E-05	0.000103	8.86E-05	7.28E-05	7.45E-05
LSP1	3.57E-05	2.36E-06	8.49E-06	3.94E-05	1.85E-06	9.88E-06	1.47E-05	6.70E-06
LSR	0.000124	7.65E-05	0.00013	0.000133	0.00019	2.87E-05	0.000156	9.93E-05
LSS	1.91E-05	5.02E-05	4.40E-05	1.27E-05	2.09E-05	1.25E-05	1.92E-05	3.90E-05
LST1	3.16E-05	4.15E-06	1.96E-05	3.71E-05	6.96E-06	1.50E-05	2.48E-05	5.55E-06
LST-3TM1	0	2.27E-07	9.33E-07	2.46E-07	0	1.57E-06	1.12E-08	1.84E-08
LTA	9.42E-07	4.14E-08	3.55E-07	1.56E-07	1.56E-08	2.53E-07	3.44E-07	1.07E-07
LTA4H	3.96E-05	3.15E-05	2.29E-05	3.83E-05	1.78E-05	3.35E-05	2.29E-05	1.97E-05
LTB	2.24E-05	4.64E-07	2.85E-06	1.89E-05	4.92E-06	4.64E-06	1.61E-05	7.27E-06
LTB4R	1.43E-06	1.56E-06	1.06E-06	1.23E-06	7.46E-07	1.74E-06	1.18E-06	6.20E-07
LTB4R2	9.48E-07	6.94E-07	4.81E-07	5.00E-07	2.92E-07	6.45E-07	6.48E-07	2.56E-07
LTBP1	2.58E-05	3.99E-06	6.51E-06	1.41E-05	4.67E-07	2.56E-06	5.49E-06	4.10E-06
LTBP2	5.60E-06	9.60E-07	8.28E-07	8.12E-06	9.72E-07	1.18E-06	5.82E-06	3.87E-06
LTBP3	3.27E-05	3.90E-05	2.05E-05	4.40E-05	9.72E-06	8.38E-06	5.93E-05	2.68E-05
LTBP4	2.45E-05	4.51E-06	1.61E-05	3.79E-05	6.52E-06	2.20E-06	1.05E-05	1.14E-05
LTBR	9.01E-05	7.89E-05	6.17E-05	6.73E-05	5.26E-05	5.83E-05	0.00011	9.71E-05
LTC4S	8.51E-07	5.44E-07	8.44E-07	2.21E-06	1.66E-06	8.10E-07	4.54E-06	1.03E-06
LTF	2.34E-07	3.39E-08	5.70E-07	1.96E-07	5.86E-07	8.67E-08	6.87E-07	1.62E-07
LTK	9.97E-06	1.25E-07	1.39E-06	9.54E-07	1.84E-07	5.90E-08	2.37E-07	2.51E-07
LTV1	1.61E-05	1.91E-05	9.31E-06	1.42E-05	2.16E-06	2.14E-05	1.56E-05	1.52E-05
LUC7L	2.36E-05	8.22E-06	5.19E-06	9.82E-06	9.71E-06	8.93E-06	1.03E-05	8.21E-06
LUC7L2	2.50E-05	2.45E-05	1.61E-05	1.73E-05	8.49E-06	2.34E-05	1.89E-05	2.56E-05
LUC7L3	5.77E-05	2.27E-05	1.55E-05	2.16E-05	2.13E-05	2.56E-05	3.89E-05	2.15E-05
LUM	5.60E-05	1.27E-06	2.06E-05	0.000104	6.68E-06	1.13E-06	2.89E-05	2.84E-05
LUZP1	7.47E-06	1.21E-06	7.79E-07	2.10E-06	5.20E-07	1.31E-06	1.45E-06	1.57E-06
LUZP2	2.64E-08	9.82E-09	4.50E-09	5.36E-09	7.54E-09	1.34E-07	0	0
LUZP4	0	0	0	0	0	0	0	0
LUZP6	2.22E-05	4.22E-05	2.08E-05	2.20E-05	6.26E-06	3.27E-05	1.84E-05	2.81E-05
LXN	5.92E-06	2.05E-06	1.17E-06	1.24E-05	5.87E-07	1.49E-06	5.55E-06	2.37E-06
LY6D	3.53E-08	8.32E-07	4.77E-06	0	0	3.31E-08	6.68E-08	1.22E-06
LY6E	5.50E-05	4.28E-05	0.000464	0.000358	0.000112	1.47E-05	0.000455	1.63E-05
LY6G5B	3.67E-06	3.19E-06	6.28E-07	8.65E-07	1.93E-07	1.18E-06	7.33E-07	5.00E-07

LY6G5C	3.24E-06	8.93E-07	1.36E-06	1.94E-06	1.83E-06	2.56E-06	1.43E-06	3.42E-06
LY6G6C	3.14E-08	0	4.19E-07	0	0	0	0	0
LY6G6D	0	0	0	0	0	0	0	0
LY6G6E	0	0	0	0	0	0	0	0
LY6G6F	3.05E-08	1.43E-07	6.78E-08	3.79E-08	2.65E-08	0	0	0
LY6H	9.46E-07	2.06E-07	0	0	1.79E-07	4.42E-07	1.55E-05	1.76E-07
LY6K	9.83E-08	1.08E-06	9.02E-08	7.53E-08	2.37E-07	5.42E-08	2.50E-07	2.15E-08
LY75	7.89E-06	2.68E-07	6.18E-07	7.06E-07	7.34E-08	5.61E-07	5.35E-07	2.24E-07
LY86	1.05E-05	1.66E-06	6.78E-06	2.21E-05	3.13E-06	4.28E-06	2.15E-05	2.43E-06
LY9	1.59E-06	1.35E-07	8.02E-07	2.30E-06	6.97E-08	6.53E-07	7.64E-07	3.98E-07
LY96	1.27E-05	3.98E-05	7.03E-06	1.52E-05	2.93E-06	2.40E-05	3.56E-05	9.59E-05
LYAR	1.25E-05	1.24E-05	6.04E-06	9.24E-06	4.16E-06	1.02E-05	1.28E-05	8.40E-06
LYG1	1.12E-06	3.46E-07	1.19E-07	1.67E-07	5.16E-07	3.03E-07	7.74E-08	8.88E-07
LYG2	0	7.73E-08	0	0	0	0	6.25E-08	0
LYL1	2.65E-06	8.94E-07	1.59E-06	2.60E-06	2.49E-06	1.07E-06	4.04E-06	7.64E-07
LYN	4.09E-05	1.92E-05	7.46E-06	1.21E-05	1.73E-06	1.48E-05	1.62E-05	1.45E-05
LYNX1	6.93E-06	4.65E-06	1.59E-05	6.13E-06	8.44E-06	5.22E-06	3.93E-05	1.52E-05
LYPD1	0.000143	8.87E-07	2.38E-07	5.60E-06	2.37E-06	7.50E-06	7.37E-07	5.58E-07
LYPD2	0	0	6.97E-07	2.38E-07	1.10E-07	0	1.74E-07	0
LYPD3	3.29E-07	2.15E-06	1.55E-06	4.81E-07	6.16E-08	1.70E-06	5.06E-07	3.58E-07
LYPD4	0	0	0	0	0	0	0	0
LYPD5	2.57E-06	3.21E-08	1.68E-07	3.14E-07	1.31E-07	7.93E-08	1.29E-07	1.81E-07
LYPD6	4.35E-06	2.22E-08	1.02E-07	1.37E-07	0	6.10E-08	3.20E-08	0
LYPD6B	5.20E-05	8.93E-08	1.04E-07	4.29E-07	0	0	1.68E-07	0
LYPLA1	7.67E-05	7.87E-05	2.91E-05	2.18E-05	2.40E-05	2.82E-05	4.75E-05	4.22E-05
LYPLA2	7.07E-05	7.62E-05	4.14E-05	6.91E-05	5.08E-05	5.15E-05	5.43E-05	6.53E-05
LYPLA2P1	4.30E-06	3.80E-06	2.79E-06	3.02E-06	2.21E-06	2.17E-06	2.18E-06	2.94E-06
LYPLAL1	1.87E-05	1.50E-05	1.85E-05	1.54E-05	8.33E-06	3.30E-05	1.14E-05	2.96E-05
LYRM1	3.95E-05	5.77E-05	3.32E-05	2.74E-05	2.79E-05	3.17E-05	3.70E-05	1.83E-05
LYRM2	9.78E-06	9.83E-06	4.69E-06	5.46E-06	1.29E-06	7.83E-06	4.58E-06	4.30E-06
LYRM4	9.30E-06	1.13E-05	7.24E-06	9.47E-06	1.21E-05	1.34E-05	1.51E-05	8.86E-06
LYRM5	2.22E-05	2.81E-05	5.81E-05	3.14E-05	1.44E-05	3.73E-05	2.43E-05	2.18E-05
LYRM7	3.30E-06	4.42E-06	3.55E-06	1.90E-06	7.94E-07	3.30E-06	1.35E-06	2.65E-06
LYSMD1	6.19E-06	4.07E-06	3.27E-06	2.67E-06	1.64E-06	6.11E-06	3.52E-06	8.72E-06
LYSMD2	2.67E-05	1.34E-05	9.44E-06	1.19E-05	8.46E-06	1.02E-05	1.60E-05	1.47E-05
LYSMD3	1.21E-05	9.67E-06	9.43E-06	6.06E-06	7.70E-07	8.14E-06	4.97E-06	1.09E-05
LYSMD4	3.84E-06	4.59E-06	2.65E-06	3.62E-06	7.43E-06	2.90E-06	8.42E-06	2.54E-06
LYST	6.99E-06	2.88E-06	4.31E-06	4.20E-06	8.70E-07	6.98E-06	1.97E-06	3.77E-06
LYVE1	2.91E-06	6.13E-07	2.11E-05	6.10E-06	6.39E-07	1.46E-05	4.79E-06	4.74E-07
LYZ	0.000666	2.12E-05	5.56E-05	0.000131	2.94E-05	0.000273	0.000199	1.95E-05
LYZL1	0	0	0	0	6.05E-08	3.26E-08	0	0
LYZL2	0	0	0	0	0	3.47E-08	0	0
LYZL4	0	0	0	0	2.76E-08	2.97E-08	0	0
LYZL6	0	7.42E-08	0	0	2.75E-08	0	0	0
LZIC	1.71E-05	1.36E-05	8.24E-06	7.47E-06	3.95E-06	1.20E-05	6.18E-06	9.16E-06
LZTFL1	1.52E-06	2.18E-06	4.72E-06	4.64E-06	9.46E-07	7.41E-06	2.18E-06	1.06E-06
LZTR1	1.91E-05	1.55E-05	8.65E-06	1.14E-05	1.27E-05	1.39E-05	1.16E-05	1.55E-05
LZTS1	3.18E-06	5.57E-07	1.60E-07	7.49E-07	3.53E-07	8.80E-07	1.11E-06	2.34E-06
LZTS2	1.40E-05	9.94E-06	5.25E-06	9.83E-06	7.32E-06	4.69E-06	9.17E-06	7.30E-06
M6PR	5.30E-05	4.44E-05	3.78E-05	4.34E-05	6.25E-06	3.40E-05	2.88E-05	3.17E-05
MAB21L1	6.54E-08	2.10E-08	0	3.43E-08	0	0	0	0
MAB21L2	4.13E-06	4.73E-08	7.07E-07	1.18E-06	3.62E-08	0	2.29E-07	1.70E-06
MACC1	1.96E-07	5.80E-08	1.01E-07	5.46E-07	0	9.17E-09	1.45E-08	1.09E-08
MACF1	5.06E-05	1.93E-05	2.49E-05	3.54E-05	2.02E-05	2.53E-05	2.21E-05	2.44E-05
MACROD1	7.25E-06	4.22E-05	1.72E-05	2.29E-05	1.34E-05	1.58E-05	3.72E-05	3.43E-06
MACROD2	9.79E-06	1.05E-06	6.54E-07	9.14E-07	3.83E-07	3.68E-07	7.26E-07	3.20E-07
MAD1L1	3.12E-05	4.80E-05	8.88E-06	9.47E-06	1.32E-05	8.42E-06	8.84E-06	8.92E-06
MAD2L1	1.48E-05	5.84E-06	7.78E-07	1.69E-06	2.10E-06	4.56E-06	1.96E-06	3.55E-06

MAD2L1B	2.73E-05	3.76E-05	1.36E-05	1.98E-05	1.66E-05	2.06E-05	2.40E-05	2.77E-05
MAD2L2	4.83E-05	7.08E-05	1.61E-05	3.67E-05	3.08E-05	3.65E-05	3.55E-05	3.64E-05
MADCAM	8.00E-08	1.28E-07	1.02E-07	3.51E-07	4.68E-08	2.52E-08	1.49E-07	4.75E-08
MADD	1.13E-05	6.61E-06	5.16E-06	4.50E-06	2.83E-06	5.47E-06	3.50E-06	1.21E-05
MAEA	4.55E-05	3.79E-05	1.62E-05	2.11E-05	1.28E-05	2.32E-05	1.92E-05	2.83E-05
MAEL	1.35E-07	7.99E-08	1.27E-07	0	0	0	2.60E-07	5.52E-07
MAF	2.97E-05	1.03E-05	1.43E-05	1.02E-05	5.87E-06	1.07E-05	9.08E-06	4.52E-06
MAF1	0.000102	5.25E-05	2.62E-05	3.86E-05	6.97E-05	4.35E-05	0.000143	5.36E-05
MAFA	0	0	0	0	0	0	0	3.69E-08
MAFB	1.10E-05	1.42E-05	1.13E-05	1.33E-05	5.10E-06	3.80E-06	1.65E-05	2.48E-06
MAFF	1.41E-05	1.80E-05	1.13E-05	8.40E-05	2.32E-06	6.33E-06	3.97E-05	3.97E-05
MAFG	4.12E-06	3.06E-06	1.10E-06	3.50E-06	1.25E-06	6.19E-06	5.38E-06	4.31E-06
MAFK	2.72E-05	4.89E-05	6.49E-06	4.41E-05	3.60E-06	9.97E-06	2.28E-05	2.43E-05
MAG	0	0	5.19E-08	0	2.65E-07	0	0	0
MAGEA1	1.43E-08	3.61E-06	0	7.01E-08	1.23E-08	3.97E-08	1.38E-08	2.48E-06
MAGEA10	8.25E-08	0	0	0	0	0	0	0
MAGEA11	0	4.35E-08	0	3.14E-08	0	0	0	0
MAGEA12	2.00E-07	6.35E-07	0	1.28E-07	0	0	1.43E-08	0
MAGEA2	4.57E-07	2.07E-06	1.59E-08	0	0	0	0	0
MAGEA3	2.97E-07	5.39E-06	0	5.21E-08	0	0	9.54E-08	0
MAGEA4	0	2.07E-05	0	3.62E-08	0	0	1.37E-08	0
MAGEA5	0	0	0	0	0	0	0	0
MAGEA6	3.31E-07	2.14E-06	1.52E-08	8.82E-08	0	0	2.22E-07	0
MAGEA8	0	2.32E-07	0	0	0	0	0	0
MAGEA9B	0	1.22E-07	0	0	0	0	0	0
MAGEB1	2.29E-07	3.89E-07	0	0	0	0	0	0
MAGEB10	0	0	0	0	0	0	0	2.32E-08
MAGEB16	2.66E-08	0	0	0	0	0	0	0
MAGEB18	0	0	0	0	0	0	0	0
MAGEB2	4.58E-08	4.57E-06	0	0	0	0	0	2.25E-08
MAGEB3	0	0	0	0	0	0	0	0
MAGEB4	0	0	0	0	0	0	0	0
MAGEB6	1.35E-08	1.53E-08	0	0	0	1.25E-08	0	0
MAGEC1	5.31E-09	6.69E-06	0	2.72E-08	0	6.83E-07	0	1.51E-05
MAGEC2	8.42E-08	3.95E-07	0	0	0	2.37E-05	0	1.43E-06
MAGEC3	0	1.08E-07	0	0	0	0	0	0
MAGED1	6.82E-05	6.44E-05	4.16E-05	6.36E-05	3.19E-05	4.08E-05	3.67E-05	7.66E-05
MAGED2	8.63E-05	8.55E-05	3.67E-05	5.86E-05	4.40E-05	7.11E-05	4.86E-05	0.000133
MAGED4	7.94E-07	6.65E-08	1.83E-07	4.03E-06	5.48E-08	2.79E-08	2.04E-07	1.07E-06
MAGED4B	1.01E-06	5.27E-08	1.08E-07	2.59E-06	6.87E-08	0	3.14E-07	1.93E-07
MAGEE1	5.49E-07	2.33E-07	2.28E-07	3.55E-07	1.08E-08	2.75E-07	3.00E-07	1.23E-06
MAGEE2	1.05E-08	1.19E-08	0	0	0	0	0	0
MAGEF1	3.13E-05	1.62E-05	1.74E-05	1.76E-05	1.94E-05	2.23E-05	1.36E-05	5.96E-06
MAGEH1	5.44E-06	1.00E-05	1.05E-05	1.24E-05	1.35E-05	1.36E-06	2.67E-06	1.59E-05
MAGEL2	7.58E-08	1.07E-08	3.94E-08	1.04E-07	2.44E-08	0	4.59E-08	2.78E-08
MAGI1	6.28E-06	5.97E-06	6.34E-06	6.89E-06	1.30E-06	4.54E-06	3.54E-06	3.08E-06
MAGI2	6.30E-07	6.26E-07	5.20E-07	7.43E-07	3.02E-07	2.86E-07	2.74E-07	2.48E-07
MAGI3	7.25E-06	5.12E-06	4.41E-06	3.72E-06	1.13E-06	6.27E-06	2.32E-06	1.27E-06
MAGIX	3.92E-06	1.00E-05	2.66E-06	4.45E-06	1.58E-05	4.90E-06	6.92E-07	2.15E-05
MAGOH	7.29E-05	3.81E-05	3.21E-05	4.37E-05	3.25E-05	5.38E-05	4.79E-05	4.68E-05
MAGOHB	4.57E-06	3.29E-06	2.39E-06	3.07E-06	2.33E-06	3.68E-06	4.99E-06	4.88E-06
MAGT1	5.84E-05	7.73E-05	3.84E-05	2.79E-05	3.51E-05	4.97E-05	2.65E-05	5.34E-05
MAK	9.60E-08	2.66E-08	3.07E-08	3.13E-07	0	6.07E-08	3.25E-08	3.49E-08
MAK16	2.89E-06	9.13E-06	3.00E-06	4.91E-06	5.06E-07	4.66E-06	2.51E-06	2.18E-06
MAL	1.85E-06	9.06E-07	1.71E-07	1.02E-06	1.07E-07	4.70E-07	9.91E-07	1.14E-07
MAL2	0.000127	2.60E-05	1.94E-05	2.20E-05	1.89E-05	1.13E-05	3.99E-05	1.69E-05
MALAT1	2.06E-05	3.84E-06	7.92E-07	4.63E-06	4.14E-06	4.60E-06	4.66E-06	7.74E-06
MALL	2.49E-06	1.67E-06	6.98E-07	3.65E-06	1.43E-06	1.84E-06	3.57E-06	2.10E-06

MALT1	9.50E-06	1.19E-06	3.24E-06	4.95E-06	5.73E-07	3.70E-06	1.89E-06	3.61E-06
MAMDC2	1.33E-07	2.13E-07	2.54E-07	1.08E-06	3.26E-08	4.11E-08	1.78E-07	6.50E-08
MAMDC4	3.14E-06	2.72E-06	1.82E-05	2.04E-06	4.20E-07	1.72E-06	2.11E-07	7.89E-06
MAML1	8.13E-06	2.24E-06	2.52E-06	3.10E-06	7.30E-07	3.92E-06	3.20E-06	4.47E-06
MAML2	9.90E-07	5.41E-07	4.71E-07	9.25E-07	3.91E-08	3.33E-07	3.51E-07	2.06E-07
MAML3	1.84E-06	7.29E-07	1.60E-06	1.52E-06	5.87E-07	1.42E-06	1.36E-06	1.35E-06
MAMLD1	3.37E-06	1.08E-06	1.04E-06	2.57E-06	3.67E-07	3.06E-07	5.25E-07	2.82E-06
MAMSTR	4.84E-07	2.80E-06	3.30E-08	5.75E-08	7.45E-07	1.16E-07	4.51E-07	3.45E-07
MAN1A1	4.28E-05	5.11E-05	6.68E-05	3.20E-05	1.50E-06	7.02E-05	1.52E-05	4.04E-05
MAN1A2	9.11E-06	6.91E-06	7.04E-06	2.91E-06	5.19E-07	1.04E-05	1.44E-06	4.57E-06
MAN1B1	4.53E-05	3.11E-05	1.89E-05	2.76E-05	3.11E-05	3.06E-05	2.19E-05	5.28E-05
MAN1C1	2.91E-06	9.04E-06	1.80E-05	1.16E-05	4.01E-07	1.62E-06	1.02E-05	2.38E-06
MAN2A1	1.39E-05	1.92E-05	1.30E-05	6.61E-06	2.83E-07	1.82E-05	2.52E-06	1.07E-05
MAN2A2	1.58E-05	1.85E-05	9.60E-06	6.04E-06	6.84E-06	4.81E-06	7.65E-06	1.40E-05
MAN2B1	3.61E-05	2.87E-05	2.14E-05	4.14E-05	3.10E-05	2.75E-05	4.54E-05	2.05E-05
MAN2B2	1.49E-05	4.60E-05	2.23E-05	1.84E-05	6.25E-06	1.80E-05	1.20E-05	2.78E-05
MAN2C1	2.19E-05	2.15E-05	1.71E-05	1.15E-05	1.68E-05	1.52E-05	1.64E-05	1.46E-05
MANBA	8.75E-06	4.65E-06	8.02E-06	7.07E-06	1.82E-06	8.28E-06	5.20E-06	8.40E-06
MANBAL	8.49E-05	2.80E-05	2.23E-05	2.90E-05	4.00E-05	4.16E-05	2.97E-05	2.65E-05
MANEA	9.01E-06	1.09E-05	1.36E-05	4.71E-06	4.71E-07	7.81E-06	3.58E-06	6.06E-06
MANEAL	1.82E-05	1.21E-05	5.96E-06	2.83E-06	5.31E-06	1.63E-05	8.17E-06	1.77E-05
MANF	0.000201	0.000151	4.35E-05	6.54E-05	0.000161	0.000206	0.000104	0.000172
MANSC1	1.01E-05	5.39E-06	6.56E-06	8.08E-06	2.53E-06	7.25E-06	4.46E-06	6.21E-06
MAOA	3.68E-05	3.46E-05	6.26E-05	4.31E-05	2.60E-05	7.87E-05	6.14E-05	3.73E-05
MAOB	0.000115	0.000106	0.000154	0.00011	7.50E-05	0.000173	0.000118	0.000104
MAP1A	6.76E-07	9.35E-08	5.26E-08	3.02E-07	1.03E-07	1.19E-07	4.03E-07	1.92E-07
MAP1B	3.00E-06	1.04E-06	3.56E-07	1.37E-06	4.97E-07	7.90E-07	1.51E-06	1.32E-06
MAP1D	3.91E-06	4.46E-06	2.01E-06	1.88E-06	1.82E-06	4.83E-06	1.69E-06	2.60E-06
MAP1LC3/	5.78E-05	4.83E-05	3.32E-05	3.53E-05	8.36E-05	2.60E-05	3.27E-05	1.13E-05
MAP1LC3I	7.07E-05	2.94E-05	2.50E-05	3.02E-05	5.43E-06	2.68E-05	2.89E-05	5.04E-05
MAP1LC3I	3.80E-05	1.47E-05	1.22E-05	1.41E-05	2.08E-06	1.64E-05	1.04E-05	2.66E-05
MAP1LC3(	0	2.49E-08	4.66E-08	2.40E-07	0	6.04E-08	1.86E-07	6.41E-08
MAP1S	1.06E-05	9.54E-06	5.95E-06	1.35E-05	6.64E-06	7.79E-06	1.27E-05	1.28E-05
MAP2	3.25E-06	5.57E-06	7.19E-07	2.21E-06	4.02E-07	9.37E-06	4.15E-07	1.32E-05
MAP2K1	3.00E-05	1.73E-05	5.64E-05	2.91E-05	5.77E-06	1.69E-05	1.81E-05	2.16E-05
MAP2K2	6.98E-05	5.99E-05	4.09E-05	4.43E-05	0.000103	6.77E-05	7.57E-05	5.08E-05
MAP2K3	1.87E-05	4.53E-05	3.49E-05	7.08E-05	2.85E-05	2.06E-05	5.20E-05	3.58E-05
MAP2K4	6.61E-06	7.76E-06	9.57E-06	9.41E-06	1.39E-06	9.57E-06	7.76E-06	1.02E-05
MAP2K5	5.62E-06	4.91E-06	5.27E-06	5.50E-06	8.84E-06	8.40E-06	1.06E-05	5.50E-06
MAP2K6	2.20E-06	2.92E-06	3.04E-06	1.35E-06	2.12E-07	3.58E-05	2.63E-07	3.82E-07
MAP2K7	1.86E-05	1.06E-05	9.62E-06	1.21E-05	1.48E-05	1.33E-05	1.14E-05	1.03E-05
MAP3K1	6.75E-06	2.12E-06	2.21E-06	2.60E-06	5.67E-07	3.47E-06	2.23E-06	1.65E-06
MAP3K10	2.86E-06	1.91E-06	1.39E-06	2.61E-06	1.72E-06	2.62E-06	2.51E-06	2.79E-06
MAP3K11	6.90E-05	7.22E-05	3.46E-05	3.81E-05	0.000113	4.42E-05	7.26E-05	9.26E-05
MAP3K12	1.46E-06	7.36E-07	3.51E-07	9.54E-07	1.54E-07	4.14E-07	6.66E-07	7.40E-07
MAP3K13	1.03E-06	4.83E-06	2.34E-06	2.56E-06	1.42E-07	1.72E-06	8.65E-07	9.60E-07
MAP3K14	7.98E-06	1.99E-05	3.26E-06	1.16E-05	2.31E-06	8.32E-06	1.68E-05	3.16E-06
MAP3K15	2.28E-06	1.59E-08	1.35E-08	1.57E-08	6.53E-09	0	9.93E-10	0
MAP3K2	1.12E-05	6.80E-06	5.48E-06	3.71E-06	3.41E-07	4.79E-06	1.98E-06	3.63E-06
MAP3K3	1.01E-05	4.10E-06	4.17E-06	5.70E-06	2.41E-06	9.53E-06	5.56E-06	4.31E-06
MAP3K4	7.51E-06	4.37E-06	4.65E-06	6.87E-06	3.75E-06	5.61E-06	6.79E-06	4.87E-06
MAP3K5	7.85E-06	1.96E-05	6.25E-06	7.00E-06	5.93E-07	2.76E-06	2.26E-06	1.20E-05
MAP3K6	3.91E-06	7.67E-07	1.01E-06	1.55E-06	1.40E-06	9.51E-07	2.60E-06	2.18E-06
MAP3K7	1.30E-05	1.52E-05	5.19E-06	7.19E-06	1.41E-06	1.15E-05	4.65E-06	6.90E-06
MAP3K8	2.89E-06	1.34E-07	2.06E-06	3.70E-06	2.14E-07	6.87E-06	9.93E-07	1.03E-06
MAP3K9	7.49E-07	4.02E-07	7.86E-08	3.46E-07	1.59E-07	2.39E-07	4.22E-07	3.04E-07
MAP4	7.38E-05	6.24E-05	2.55E-05	4.38E-05	3.25E-05	5.56E-05	5.37E-05	2.84E-05
MAP4K1	5.08E-06	1.70E-07	1.57E-06	4.02E-06	2.11E-07	2.07E-06	1.64E-06	1.24E-06

MAP4K2	8.14E-06	6.00E-06	2.25E-06	4.37E-06	3.24E-06	2.49E-06	3.85E-06	8.07E-06
MAP4K3	2.00E-05	1.26E-05	5.28E-06	6.19E-06	1.68E-06	6.79E-06	4.34E-06	5.08E-06
MAP4K4	2.35E-05	1.40E-05	1.07E-05	1.45E-05	6.09E-06	1.73E-05	1.50E-05	1.07E-05
MAP4K5	1.13E-05	4.46E-06	4.03E-06	5.01E-06	9.71E-07	4.48E-06	2.97E-06	3.56E-06
MAP6	1.37E-06	2.35E-08	2.34E-07	5.62E-07	3.72E-07	2.94E-08	2.75E-07	2.66E-07
MAP6D1	1.05E-06	3.52E-07	1.98E-07	5.86E-07	2.87E-07	7.95E-07	5.27E-07	7.20E-07
MAP7	1.34E-05	1.87E-05	1.17E-05	9.36E-06	2.06E-06	9.04E-06	7.75E-06	9.47E-06
MAP7D1	2.40E-05	1.06E-05	8.06E-06	2.27E-05	1.09E-05	1.32E-05	2.04E-05	1.83E-05
MAP7D2	1.66E-07	1.63E-08	2.25E-08	2.01E-07	1.18E-08	2.01E-08	2.11E-08	3.19E-08
MAP7D3	2.08E-06	1.61E-06	2.22E-06	3.70E-06	6.63E-07	3.09E-06	5.89E-07	2.53E-06
MAP9	1.88E-06	3.08E-08	1.24E-07	2.48E-07	7.90E-09	4.54E-08	3.89E-08	6.28E-08
MAPK1	3.49E-05	2.54E-05	1.62E-05	1.72E-05	6.07E-06	2.35E-05	1.58E-05	1.89E-05
MAPK10	7.34E-07	4.66E-07	2.29E-07	1.36E-06	6.75E-08	1.29E-07	2.55E-07	3.66E-07
MAPK11	8.22E-06	2.97E-06	7.59E-07	1.55E-06	3.15E-06	1.14E-06	1.11E-05	4.22E-06
MAPK12	1.61E-05	1.25E-05	1.05E-06	1.39E-06	1.44E-06	4.73E-07	1.74E-05	4.31E-06
MAPK13	3.05E-05	1.08E-05	1.14E-06	5.20E-06	3.08E-07	4.97E-07	1.06E-06	1.88E-07
MAPK14	2.70E-05	3.22E-05	1.82E-05	1.56E-05	7.84E-06	2.24E-05	1.99E-05	1.63E-05
MAPK15	5.11E-08	1.45E-08	6.71E-08	1.57E-08	0	0	1.23E-08	1.88E-08
MAPK1IP1	2.25E-05	9.12E-06	8.61E-06	1.14E-05	3.98E-06	1.27E-05	8.57E-06	1.29E-05
MAPK3	2.03E-05	2.84E-05	9.66E-06	1.19E-05	4.74E-06	1.59E-05	2.02E-05	1.96E-05
MAPK4	1.95E-07	7.04E-07	5.79E-07	1.61E-07	1.43E-07	5.55E-06	1.96E-07	0
MAPK6	3.05E-05	1.66E-05	1.19E-05	1.37E-05	3.71E-06	2.24E-05	1.00E-05	2.30E-05
MAPK7	2.75E-06	3.10E-06	1.93E-06	4.04E-06	9.55E-07	2.10E-06	3.19E-06	3.19E-06
MAPK8	1.33E-05	9.59E-06	7.61E-06	6.58E-06	1.84E-07	1.02E-05	4.10E-06	6.51E-06
MAPK8IP1	6.75E-06	6.05E-07	2.82E-06	3.32E-06	4.86E-06	4.15E-06	3.06E-06	4.78E-07
MAPK8IP2	1.57E-05	9.70E-06	2.92E-07	1.09E-06	2.23E-06	1.34E-06	1.02E-05	3.16E-06
MAPK8IP3	2.12E-05	3.05E-06	1.69E-06	2.66E-06	1.42E-06	2.21E-06	2.69E-06	3.54E-06
MAPK9	1.15E-05	3.69E-06	6.15E-06	6.17E-06	2.15E-06	7.42E-06	6.01E-06	1.08E-05
MAPKAP1	3.29E-05	2.72E-05	1.55E-05	1.77E-05	1.72E-05	3.58E-05	2.10E-05	3.17E-05
MAPKAPK	7.41E-05	2.51E-05	1.43E-05	4.20E-05	1.37E-05	3.26E-05	3.92E-05	7.90E-05
MAPKAPK	3.44E-05	2.35E-05	9.99E-06	1.05E-05	6.66E-06	1.06E-05	1.37E-05	1.51E-05
MAPKAPK	1.36E-05	1.44E-05	7.79E-06	9.68E-06	4.63E-06	1.03E-05	1.10E-05	1.11E-05
MAPKBP1	2.60E-06	1.19E-06	6.35E-07	1.04E-06	4.80E-07	1.00E-06	1.18E-06	1.33E-06
MAPKSP1	1.10E-05	4.71E-06	6.38E-06	6.04E-06	2.95E-06	7.35E-06	6.71E-06	7.45E-06
MAPRE1	5.91E-05	3.44E-05	1.16E-05	2.82E-05	4.57E-06	1.59E-05	1.84E-05	1.45E-05
MAPRE2	2.06E-05	1.90E-05	1.63E-05	2.42E-05	2.45E-06	1.89E-05	1.27E-05	1.19E-05
MAPRE3	1.18E-05	2.62E-05	1.09E-05	1.09E-05	4.47E-06	1.09E-05	8.10E-06	1.77E-05
MAPT	4.20E-07	8.23E-07	1.26E-07	1.05E-07	3.48E-07	5.98E-07	9.59E-07	6.22E-07
1-Mar	3.73E-06	3.07E-07	1.58E-06	1.51E-06	5.83E-08	4.28E-07	5.23E-07	1.80E-07
10-Mar	1.51E-07	0	6.99E-09	0	6.51E-09	0	0	0
11-Mar	0	0	0	0	0	0	0	0
2-Mar	2.53E-05	2.37E-05	1.98E-05	2.74E-05	2.44E-05	3.50E-05	5.52E-05	5.14E-05
3-Mar	2.01E-06	1.05E-07	1.36E-07	1.75E-06	1.32E-07	7.63E-08	2.03E-07	6.56E-07
4-Mar	5.10E-08	4.56E-08	0	0	5.25E-08	3.30E-08	1.14E-07	2.24E-08
5-Mar	1.43E-05	1.12E-05	1.13E-05	1.05E-05	4.80E-06	9.82E-06	8.21E-06	7.51E-06
6-Mar	2.23E-05	1.95E-05	1.45E-05	8.21E-06	1.10E-06	2.39E-05	4.99E-06	1.65E-05
7-Mar	3.00E-05	1.72E-05	1.42E-05	1.49E-05	5.62E-06	1.71E-05	1.16E-05	1.15E-05
8-Mar	7.23E-06	5.08E-06	3.80E-06	3.06E-06	1.35E-07	1.18E-05	3.60E-06	4.55E-06
9-Mar	6.83E-06	7.32E-06	1.88E-06	3.53E-06	4.15E-06	3.52E-06	7.67E-06	1.57E-06
MARCKS	3.30E-05	6.64E-06	8.43E-06	3.31E-05	8.45E-06	1.14E-05	3.55E-05	7.72E-06
MARCKSL	0.000157	1.57E-05	1.93E-05	5.65E-05	1.65E-05	1.19E-05	4.32E-05	5.06E-05
MARCO	1.32E-07	1.49E-08	6.82E-05	7.58E-05	2.27E-07	8.66E-06	9.28E-07	0
MARK1	6.15E-07	1.35E-07	5.37E-08	1.68E-07	1.38E-07	1.17E-07	1.02E-07	1.21E-07
MARK2	1.18E-05	9.91E-06	5.11E-06	7.65E-06	2.34E-06	6.32E-06	5.58E-06	8.38E-06
MARK3	2.22E-05	1.09E-05	1.05E-05	1.19E-05	1.04E-05	1.26E-05	1.11E-05	1.34E-05
MARK4	1.39E-05	8.06E-06	4.81E-06	6.10E-06	6.07E-06	7.28E-06	9.68E-06	1.45E-05
MARS	2.39E-05	4.01E-05	1.64E-05	1.86E-05	9.51E-06	2.38E-05	2.63E-05	3.19E-05
MARS2	5.05E-06	1.34E-05	3.73E-06	3.25E-06	2.23E-07	3.36E-06	2.75E-06	6.08E-06

MARVELD	5.82E-06	3.63E-07	1.66E-06	2.99E-06	3.73E-07	6.18E-07	2.05E-06	9.73E-07
MARVELD	1.85E-05	1.47E-05	1.14E-05	1.34E-05	2.58E-06	1.04E-05	1.30E-05	1.05E-05
MARVELD	1.21E-05	5.29E-06	3.79E-06	6.10E-06	1.81E-06	5.64E-06	3.60E-06	2.44E-06
MAS1	0	0	0	0	0	0	0	0
MAS1L	0	0	0	0	0	0	0	0
MASP1	1.37E-05	4.07E-05	7.06E-05	6.17E-05	7.74E-06	2.56E-05	1.80E-05	3.07E-05
MASP2	1.50E-05	0.000148	0.000411	0.000234	0.000294	0.000361	0.000173	0.000463
MAST1	1.91E-06	1.09E-06	2.82E-08	7.28E-08	3.54E-08	9.33E-08	4.46E-08	1.34E-08
MAST2	7.23E-06	5.71E-06	2.07E-06	2.99E-06	3.00E-06	3.27E-06	3.86E-06	8.74E-06
MAST3	4.68E-06	2.74E-06	2.19E-06	1.95E-06	8.12E-06	2.96E-06	6.10E-06	3.48E-06
MAST4	9.15E-06	3.28E-06	3.85E-06	5.32E-06	3.84E-06	9.83E-06	3.89E-06	1.92E-06
MASTL	8.46E-06	4.13E-06	1.43E-06	2.07E-06	5.00E-07	2.80E-06	1.40E-06	2.56E-06
MAT1A	0.000152	0.000398	0.000849	0.000419	0.000335	0.000613	0.000618	0.000374
MAT2A	0.000105	0.000224	3.18E-05	8.31E-05	5.56E-05	2.09E-05	4.57E-05	3.25E-05
MAT2B	5.55E-05	2.56E-05	3.80E-05	3.08E-05	9.58E-06	5.81E-05	3.06E-05	3.23E-05
MATK	7.07E-07	3.17E-07	1.33E-06	3.10E-06	3.90E-07	5.48E-07	2.26E-06	4.44E-07
MATN1	4.67E-08	7.73E-08	0	2.24E-08	5.34E-09	5.94E-09	2.37E-08	0
MATN2	1.09E-05	4.34E-06	1.30E-05	1.03E-05	4.81E-06	1.73E-06	9.38E-06	2.11E-05
MATN3	1.38E-06	5.10E-08	0	1.22E-07	1.56E-08	9.53E-06	1.41E-07	8.80E-08
MATN4	1.12E-07	0	1.25E-08	3.59E-08	3.78E-08	0	1.41E-08	2.16E-08
MATR3	8.48E-05	6.22E-05	4.41E-05	5.28E-05	2.14E-05	7.11E-05	3.61E-05	5.27E-05
MAVS	1.71E-05	7.12E-06	2.64E-06	2.81E-06	7.01E-06	5.26E-06	6.19E-06	5.02E-06
MAX	3.04E-05	9.79E-06	1.71E-05	2.26E-05	9.92E-06	1.98E-05	2.85E-05	1.68E-05
MAZ	0.000131	0.000134	3.98E-05	5.49E-05	8.60E-05	6.04E-05	8.90E-05	9.63E-05
MB	1.99E-07	9.22E-07	4.30E-07	1.21E-07	1.03E-06	4.55E-08	2.80E-07	3.28E-07
MBD1	2.15E-05	1.27E-05	1.02E-05	1.13E-05	4.35E-06	1.51E-05	1.28E-05	1.14E-05
MBD2	2.26E-05	1.04E-05	1.44E-05	2.05E-05	5.05E-06	2.02E-05	1.80E-05	1.36E-05
MBD3	4.32E-05	3.23E-05	2.90E-05	3.51E-05	0.000105	3.21E-05	6.49E-05	3.30E-05
MBD3L1	0	0	0	0	0	0	0	0
MBD3L2	0	0	0	0	0	0	0	0
MBD3L5	0	0	0	0	0	0	0	0
MBD4	1.33E-05	1.70E-05	1.02E-05	2.13E-05	7.20E-06	2.06E-05	1.37E-05	1.05E-05
MBD5	6.71E-06	2.52E-06	1.33E-06	2.27E-06	4.51E-07	2.83E-06	1.21E-06	2.42E-06
MBD6	2.39E-05	1.17E-05	1.03E-05	1.49E-05	5.04E-06	1.53E-05	1.39E-05	1.37E-05
MBIP	1.80E-05	1.42E-05	1.24E-05	1.10E-05	1.34E-06	1.83E-05	1.01E-05	1.31E-05
MBL1P	6.34E-07	5.40E-08	3.25E-07	2.24E-07	5.75E-07	7.37E-07	1.47E-07	2.60E-08
MBL2	1.28E-05	3.61E-06	0.000145	6.62E-05	3.43E-05	3.71E-05	4.76E-05	4.15E-05
MBLAC1	3.76E-06	1.06E-06	7.75E-07	1.08E-06	1.47E-06	1.28E-06	2.01E-06	1.37E-06
MBLAC2	1.74E-06	1.92E-06	3.27E-06	1.41E-06	2.60E-07	2.21E-06	9.50E-07	1.20E-06
MBNL1	3.87E-05	1.91E-05	2.19E-05	2.00E-05	5.56E-06	2.19E-05	1.52E-05	1.43E-05
MBNL2	2.47E-05	1.81E-05	2.68E-05	2.08E-05	7.67E-06	1.81E-05	1.64E-05	2.55E-05
MBNL3	5.60E-06	8.08E-06	1.96E-05	6.62E-06	3.21E-06	2.53E-05	3.03E-06	2.35E-05
MBOAT1	2.01E-06	6.70E-06	1.81E-06	4.16E-06	1.06E-07	1.70E-06	3.18E-06	7.75E-06
MBOAT2	4.63E-06	5.38E-08	2.47E-07	3.37E-07	1.08E-07	5.56E-08	2.10E-07	1.67E-07
MBOAT4	3.42E-06	1.34E-07	1.09E-07	1.17E-06	0	2.32E-07	4.26E-08	1.72E-06
MBOAT7	4.36E-05	4.03E-05	1.69E-05	1.87E-05	2.56E-05	2.10E-05	2.38E-05	1.97E-05
MBP	1.17E-05	7.35E-06	8.68E-06	7.91E-06	9.98E-07	4.68E-06	6.73E-06	8.69E-06
MBTD1	5.95E-06	6.02E-06	3.83E-06	2.51E-06	1.28E-06	5.40E-06	2.39E-06	1.99E-06
MBTPS1	2.57E-05	1.48E-05	1.77E-05	2.25E-05	1.37E-05	3.33E-05	2.10E-05	2.35E-05
MBTPS2	1.45E-05	1.01E-05	8.86E-06	7.38E-06	1.97E-06	9.81E-06	5.85E-06	1.54E-05
MC1R	1.06E-06	9.92E-08	3.01E-07	9.03E-07	3.93E-07	3.97E-07	5.02E-07	7.00E-07
MC2R	1.88E-08	0	0	0	0	0	0	0
MC3R	0	0	0	0	0	0	0	0
MC4R	0	0	0	0	0	0	0	0
MC5R	0	0	0	0	0	0	0	0
MCAM	5.16E-05	2.66E-05	1.51E-06	2.22E-05	6.70E-06	1.44E-05	3.49E-05	1.60E-05
MCART1	2.51E-06	2.27E-06	1.38E-06	2.10E-06	6.06E-07	2.57E-06	1.89E-06	2.19E-06
MCART2	1.52E-07	1.50E-07	2.11E-07	1.07E-07	1.88E-08	1.42E-07	2.29E-07	6.46E-08

MCART3P	0	0	1.62E-08	0	0	1.42E-08	0	0
MCART6	1.37E-07	3.21E-08	2.21E-08	2.19E-08	3.08E-09	2.33E-08	2.10E-08	1.05E-08
MCAT	9.23E-06	1.45E-05	7.44E-06	1.10E-05	1.58E-05	1.36E-05	1.73E-05	1.02E-05
MCC	3.40E-06	2.39E-06	6.08E-06	2.61E-06	3.45E-07	2.46E-06	1.72E-06	9.75E-07
MCCC1	6.06E-05	2.93E-05	3.33E-05	2.56E-05	6.70E-06	3.97E-05	1.30E-05	2.10E-05
MCCC2	4.59E-05	7.55E-05	7.03E-05	3.77E-05	1.06E-05	7.81E-05	2.80E-05	5.53E-05
MCCD1	7.07E-07	2.48E-07	4.78E-08	0	7.25E-08	0	3.91E-08	6.28E-08
MCEE	3.98E-05	2.75E-05	2.92E-05	2.49E-05	1.26E-05	3.63E-05	2.13E-05	2.61E-05
MCF2	2.01E-08	6.92E-09	6.36E-09	1.58E-08	0	6.20E-09	0	2.94E-08
MCF2L	8.06E-06	4.16E-06	1.71E-06	1.50E-06	1.26E-06	1.52E-06	2.59E-06	2.61E-06
MCF2L2	3.65E-07	5.57E-08	1.34E-08	4.59E-08	1.12E-08	8.80E-09	1.09E-08	2.40E-06
MCFD2	9.69E-05	0.000117	4.14E-05	3.15E-05	2.00E-05	7.55E-05	2.20E-05	3.78E-05
MCHR1	5.55E-07	4.84E-06	5.98E-07	7.17E-07	5.69E-06	2.25E-07	1.06E-06	8.28E-07
MCHR2	0	0	0	0	0	0	0	0
MCL1	0.00012	0.00013	0.000103	0.000329	2.83E-05	8.25E-05	0.000173	0.000157
MCM10	2.57E-06	1.36E-06	5.15E-08	9.18E-08	9.47E-08	2.86E-07	6.21E-08	2.13E-07
MCM2	2.57E-05	1.17E-05	1.91E-06	1.35E-06	3.04E-06	4.91E-06	3.34E-06	3.01E-06
MCM3	4.51E-05	3.08E-05	6.95E-06	1.09E-05	8.96E-06	1.70E-05	1.04E-05	1.81E-05
MCM3AP	2.49E-05	1.22E-05	1.24E-05	1.25E-05	1.84E-05	1.94E-05	2.13E-05	1.73E-05
MCM3AP/	8.08E-07	1.29E-06	6.49E-08	1.27E-07	5.07E-08	2.99E-07	2.72E-07	1.08E-07
MCM4	4.00E-05	1.63E-05	2.94E-06	3.80E-06	1.27E-06	6.87E-06	5.43E-06	7.55E-06
MCM5	4.58E-05	1.80E-05	8.38E-06	1.14E-05	1.95E-05	1.39E-05	1.20E-05	1.59E-05
MCM6	2.56E-05	7.34E-06	2.07E-06	3.80E-06	2.86E-06	8.08E-06	3.44E-06	5.54E-06
MCM7	0.000108	3.32E-05	1.07E-05	1.19E-05	1.70E-05	1.70E-05	1.71E-05	2.45E-05
MCM8	5.90E-06	3.72E-06	6.52E-07	4.42E-07	1.33E-07	1.15E-06	4.52E-07	3.96E-07
MCM9	1.35E-06	4.84E-07	2.78E-07	2.92E-07	8.20E-08	9.72E-07	1.94E-07	4.34E-07
MCOLN1	1.58E-05	1.12E-05	1.28E-05	2.05E-05	1.22E-05	1.52E-05	2.24E-05	2.06E-05
MCOLN2	1.97E-06	1.92E-08	1.95E-07	3.80E-07	2.61E-08	1.99E-07	1.41E-07	7.80E-08
MCOLN3	7.00E-06	8.80E-09	5.54E-08	1.09E-07	0	0	5.18E-08	0
MCPH1	2.23E-06	4.96E-06	2.50E-06	3.48E-06	3.85E-07	4.19E-06	1.23E-06	1.29E-06
MCRS1	3.26E-05	2.50E-05	1.15E-05	1.74E-05	1.27E-05	1.92E-05	2.08E-05	2.24E-05
MCTP1	2.16E-06	6.40E-07	3.81E-07	7.03E-07	3.38E-08	2.22E-07	3.44E-07	4.87E-06
MCTP2	4.52E-06	7.83E-08	1.67E-07	4.63E-07	5.01E-09	2.88E-07	1.36E-07	5.70E-08
MCTS1	5.59E-06	4.02E-06	2.57E-06	3.06E-06	6.05E-06	4.74E-06	4.78E-06	5.67E-06
MDC1	8.46E-06	7.82E-06	3.02E-06	4.04E-06	3.29E-06	4.33E-06	5.19E-06	5.80E-06
MDFI	1.66E-06	1.04E-07	1.21E-07	2.65E-06	1.02E-06	1.69E-07	1.51E-06	1.50E-06
MDFIC	4.59E-06	2.04E-06	4.43E-06	6.82E-06	4.39E-07	6.53E-06	4.89E-06	2.01E-06
MDGA1	3.15E-06	5.47E-08	3.68E-07	2.02E-07	7.49E-08	1.96E-05	2.76E-07	2.03E-06
MDGA2	1.91E-07	1.50E-06	0	5.42E-08	0	0	0	0
MDH1	0.000431	0.000552	0.000127	0.000138	0.000147	0.000211	0.000159	0.000143
MDH1B	2.66E-08	2.76E-08	2.81E-08	5.24E-08	0	1.51E-07	9.83E-09	1.79E-08
MDH2	0.000262	0.000501	0.000186	0.000178	0.000181	0.000361	0.000312	0.000353
MDK	0.00049	0.000282	3.58E-06	7.16E-05	2.67E-05	0.000572	4.68E-05	0.000328
MDM1	2.49E-06	2.60E-06	1.30E-06	1.23E-06	4.80E-07	2.54E-06	1.37E-06	1.35E-06
MDM2	1.70E-05	1.54E-05	1.34E-05	1.31E-05	4.46E-06	2.56E-05	1.06E-05	1.30E-05
MDM4	3.20E-06	1.55E-06	1.15E-06	1.54E-06	5.47E-07	1.45E-06	1.38E-06	4.16E-06
MDN1	3.98E-06	2.60E-06	2.13E-06	1.24E-06	4.11E-08	2.15E-06	7.17E-07	1.06E-06
MDP1	1.79E-05	7.04E-06	9.42E-06	1.42E-05	1.33E-05	1.65E-05	1.27E-05	7.97E-06
MDS2	1.36E-08	0	0	3.53E-08	2.35E-08	0	3.95E-08	0
ME1	5.79E-06	6.85E-06	4.08E-06	2.93E-06	2.04E-06	1.31E-06	2.38E-06	4.30E-07
ME2	1.29E-05	3.96E-06	7.26E-06	7.08E-06	1.07E-06	1.22E-05	5.01E-06	8.03E-06
ME3	4.43E-06	2.64E-06	7.23E-07	2.66E-06	6.00E-07	2.21E-06	1.32E-06	4.09E-06
MEA1	9.30E-05	0.000138	3.75E-05	6.49E-05	8.66E-05	9.10E-05	8.59E-05	0.000118
MEAF6	2.72E-05	5.33E-05	2.02E-05	7.67E-05	3.88E-06	4.33E-05	2.75E-05	3.31E-05
MECOM	7.74E-06	1.50E-06	5.40E-07	3.10E-07	3.09E-07	8.02E-07	1.18E-06	2.22E-06
MECP2	9.16E-06	6.76E-06	5.91E-06	8.56E-06	1.12E-05	5.89E-06	7.36E-06	1.55E-05
MECR	8.44E-06	7.99E-06	7.41E-06	8.06E-06	7.52E-06	1.23E-05	8.60E-06	1.01E-05
MED1	1.26E-05	8.58E-06	4.58E-06	5.63E-06	2.60E-06	1.10E-05	6.60E-06	4.36E-06

MED10	1.77E-05	2.23E-05	1.15E-05	1.95E-05	1.03E-05	1.70E-05	2.94E-05	1.98E-05
MED11	1.80E-05	1.64E-05	2.30E-05	2.60E-05	4.27E-05	2.93E-05	2.74E-05	3.31E-05
MED12	1.15E-05	1.03E-05	4.03E-06	4.55E-06	2.05E-06	6.05E-06	5.32E-06	7.27E-06
MED12L	2.41E-07	0	3.11E-08	1.03E-08	0	5.35E-09	0	0
MED13	1.16E-05	1.15E-05	8.22E-06	6.17E-06	5.74E-06	7.87E-06	4.32E-06	4.51E-06
MED13L	8.26E-06	5.34E-06	4.16E-06	3.57E-06	8.87E-07	3.48E-06	2.60E-06	2.60E-06
MED14	1.45E-05	4.88E-06	7.43E-06	5.21E-06	1.23E-06	6.60E-06	3.72E-06	7.96E-06
MED15	3.32E-05	1.40E-05	1.02E-05	2.15E-05	1.28E-05	1.58E-05	1.67E-05	1.82E-05
MED16	2.33E-05	2.03E-05	1.30E-05	1.95E-05	2.83E-05	2.10E-05	1.51E-05	1.38E-05
MED17	1.07E-05	4.15E-06	3.68E-06	5.34E-06	1.06E-06	5.47E-06	2.98E-06	5.58E-06
MED18	1.68E-05	9.65E-06	1.08E-05	8.70E-06	7.56E-06	1.16E-05	9.22E-06	8.39E-06
MED19	1.70E-05	1.28E-05	4.96E-06	9.58E-06	8.64E-06	8.79E-06	1.26E-05	1.06E-05
MED20	1.32E-05	1.20E-05	3.43E-06	5.22E-06	2.28E-06	8.76E-06	5.61E-06	8.99E-06
MED21	1.16E-05	8.99E-06	7.67E-06	8.27E-06	1.59E-06	8.96E-06	4.91E-06	7.52E-06
MED22	7.88E-06	3.52E-06	2.78E-06	4.79E-06	5.80E-06	5.51E-06	5.40E-06	8.02E-06
MED23	1.17E-05	4.89E-06	4.00E-06	3.70E-06	1.48E-06	6.06E-06	3.35E-06	4.18E-06
MED24	5.62E-05	6.66E-05	1.87E-05	2.24E-05	4.54E-05	4.91E-05	2.89E-05	2.91E-05
MED25	1.27E-05	1.31E-05	8.64E-06	1.59E-05	8.64E-06	1.50E-05	1.30E-05	7.82E-06
MED26	3.99E-06	2.16E-06	2.13E-06	3.70E-06	2.15E-06	3.65E-06	3.79E-06	3.41E-06
MED27	1.02E-05	3.28E-06	3.70E-06	5.98E-06	4.71E-06	9.89E-06	7.84E-06	1.01E-05
MED28	2.37E-05	1.60E-05	9.51E-06	1.48E-05	5.22E-06	2.02E-05	1.22E-05	1.25E-05
MED29	3.45E-05	2.42E-05	1.52E-05	1.66E-05	1.86E-05	2.40E-05	1.60E-05	2.08E-05
MED30	3.64E-05	1.80E-05	8.75E-06	9.88E-06	5.69E-06	5.23E-06	2.89E-05	1.39E-05
MED31	3.60E-06	2.30E-06	2.67E-06	3.03E-06	6.62E-07	4.27E-06	2.74E-06	2.81E-06
MED4	4.33E-05	2.58E-05	1.69E-05	2.47E-05	1.42E-05	3.06E-05	2.24E-05	2.28E-05
MED6	2.65E-05	8.87E-06	8.27E-06	1.03E-05	7.48E-06	1.52E-05	1.20E-05	1.14E-05
MED7	1.42E-05	4.65E-06	5.53E-06	6.53E-06	4.19E-06	7.88E-06	6.74E-06	1.02E-05
MED8	5.84E-05	4.31E-05	2.54E-05	3.19E-05	1.98E-05	3.86E-05	2.93E-05	3.87E-05
MED9	1.32E-05	1.79E-05	6.98E-06	1.26E-05	7.75E-06	1.93E-05	1.70E-05	2.32E-05
MEF2A	9.71E-06	1.39E-05	9.81E-06	8.76E-06	1.55E-06	6.38E-06	6.29E-06	6.06E-06
MEF2B	1.06E-06	1.19E-06	3.02E-07	7.18E-07	1.97E-06	3.65E-07	1.69E-07	2.04E-07
MEF2C	3.34E-06	2.55E-06	1.39E-06	2.06E-06	1.04E-06	2.55E-06	2.54E-06	2.46E-06
MEF2D	1.33E-05	8.46E-06	5.00E-06	7.16E-06	2.61E-06	1.34E-05	9.81E-06	2.32E-05
MEFV	6.95E-07	1.69E-07	2.50E-07	2.80E-07	5.63E-09	1.88E-07	1.59E-07	2.88E-08
MEG3	0.000142	1.68E-07	1.31E-06	3.78E-06	4.32E-06	3.08E-07	1.33E-06	1.11E-06
MEG8	4.88E-07	0	0	8.87E-08	0	0	0	0
MEGF10	2.92E-09	1.63E-08	1.61E-07	3.74E-08	0	2.70E-09	2.84E-09	0
MEGF11	1.35E-07	0	0	1.92E-08	0	3.64E-09	0	5.75E-09
MEGF6	3.81E-06	1.97E-07	6.58E-07	7.11E-06	3.57E-07	6.81E-06	2.88E-06	5.59E-07
MEGF8	1.06E-05	9.74E-06	4.58E-06	5.95E-06	1.68E-05	6.37E-06	1.36E-05	4.21E-06
MEGF9	1.39E-05	1.00E-05	1.65E-05	8.23E-06	3.29E-06	1.12E-05	6.71E-06	1.45E-05
MEI1	2.68E-06	9.38E-08	3.83E-07	2.61E-06	1.69E-07	4.33E-07	2.11E-07	4.87E-07
MEIG1	9.91E-08	3.00E-07	1.17E-07	1.24E-07	5.20E-07	3.74E-07	1.11E-06	7.53E-08
MEIS1	3.30E-06	3.44E-06	9.36E-07	1.80E-06	7.76E-07	1.80E-06	1.33E-06	8.38E-07
MEIS2	1.79E-05	4.10E-06	2.87E-06	3.93E-06	3.60E-06	5.74E-06	4.76E-06	4.32E-06
MEIS3	2.47E-06	5.01E-07	3.75E-07	1.79E-06	1.45E-06	3.45E-07	1.10E-06	4.90E-07
MEIS3P1	3.20E-06	8.88E-07	1.25E-06	1.83E-06	4.34E-07	6.49E-07	6.87E-07	7.76E-07
MELK	5.42E-06	5.24E-06	8.26E-08	3.46E-07	3.78E-07	1.79E-06	1.75E-07	1.35E-06
MEMO1	1.77E-05	2.51E-05	1.35E-05	1.01E-05	1.01E-05	1.35E-05	1.41E-05	1.38E-05
MEN1	1.80E-05	1.86E-05	1.06E-05	8.28E-06	9.95E-06	1.86E-05	1.20E-05	1.89E-05
MEOX1	3.19E-07	5.05E-08	0	9.91E-07	7.68E-08	3.11E-08	1.92E-07	1.30E-07
MEOX2	4.98E-08	8.08E-07	1.14E-07	1.58E-07	1.28E-07	8.12E-07	5.78E-07	1.46E-07
MEP1A	3.16E-07	3.56E-08	8.20E-09	3.87E-08	0	7.34E-09	7.68E-09	2.32E-08
MEP1B	4.09E-08	1.04E-07	6.08E-07	2.51E-08	0	3.04E-07	0	4.51E-08
MEPCE	6.42E-05	5.00E-05	1.48E-05	2.24E-05	2.01E-05	2.89E-05	3.45E-05	4.98E-05
MEPE	0	6.93E-08	0	0	0	0	0	0
MERTK	5.20E-06	1.02E-05	3.88E-06	5.20E-06	1.05E-06	2.11E-05	4.21E-06	1.21E-06
MESDC1	8.24E-06	3.48E-06	2.42E-06	7.21E-06	1.71E-06	4.39E-06	7.78E-06	2.88E-06

MESDC2	2.98E-05	1.75E-05	1.19E-05	1.44E-05	7.51E-06	1.95E-05	1.72E-05	1.96E-05
MESP1	4.06E-06	4.11E-06	3.86E-07	4.68E-07	4.03E-06	6.03E-07	4.49E-06	7.29E-07
MESP2	9.18E-07	6.05E-06	1.49E-07	7.50E-08	4.94E-07	1.22E-07	3.95E-07	4.88E-08
MEST	1.91E-05	4.16E-05	2.37E-05	1.91E-05	3.07E-05	9.62E-06	2.23E-05	8.72E-06
MESTIT1	7.11E-09	0	0	0	0	0	0	1.04E-08
MET	2.25E-05	6.68E-05	3.22E-05	3.41E-05	1.12E-05	4.29E-05	2.05E-05	5.07E-05
METAP1	2.06E-05	1.15E-05	1.30E-05	1.09E-05	5.39E-06	1.67E-05	1.06E-05	1.38E-05
METAP2	3.26E-05	2.33E-05	1.88E-05	2.27E-05	7.09E-06	2.55E-05	1.95E-05	1.81E-05
METRN	1.75E-05	4.72E-07	9.78E-06	9.89E-06	2.62E-05	1.88E-05	3.24E-05	2.00E-05
METRNL	2.62E-05	1.79E-06	3.92E-06	5.13E-05	2.18E-06	5.02E-06	8.57E-06	4.75E-06
METT10D	3.15E-06	2.55E-06	2.26E-06	2.54E-06	3.05E-06	3.08E-06	3.04E-06	2.58E-06
METT11D1	2.08E-05	1.50E-05	1.34E-05	1.56E-05	1.21E-05	2.08E-05	2.00E-05	2.04E-05
METT5D1	7.59E-06	6.01E-06	4.67E-06	4.10E-06	2.65E-06	5.72E-06	4.41E-06	7.85E-06
METTL1	1.17E-05	1.13E-05	4.63E-06	1.03E-05	6.38E-06	1.07E-05	1.59E-05	1.47E-05
METTL10	2.63E-06	4.03E-06	3.56E-06	4.27E-06	2.57E-06	5.14E-06	4.47E-06	4.72E-06
METTL11A	6.16E-05	1.69E-05	2.39E-05	3.04E-05	2.36E-05	4.33E-05	3.36E-05	5.03E-05
METTL11B	0	0	0	0	0	0	0	0
METTL12	8.56E-07	1.11E-06	6.43E-07	5.87E-07	7.36E-07	8.46E-07	1.18E-06	4.86E-07
METTL13	1.33E-05	9.29E-06	8.41E-06	8.28E-06	6.19E-06	1.94E-05	9.47E-06	1.05E-05
METTL14	8.59E-06	5.18E-06	8.62E-06	6.77E-06	1.13E-06	9.72E-06	5.00E-06	9.08E-06
METTL2A	9.91E-06	1.13E-05	4.77E-06	4.76E-06	2.93E-06	8.13E-06	4.17E-06	6.09E-06
METTL2B	5.73E-06	8.88E-06	4.15E-06	3.52E-06	1.32E-06	4.96E-06	3.21E-06	7.45E-06
METTL3	1.62E-05	1.07E-05	7.50E-06	7.69E-06	5.05E-06	1.42E-05	8.52E-06	6.97E-06
METTL4	3.63E-06	1.28E-06	1.39E-06	1.71E-06	4.83E-07	1.65E-06	2.49E-06	1.62E-06
METTL5	5.30E-05	4.57E-05	2.19E-05	2.76E-05	2.10E-05	3.97E-05	3.22E-05	3.12E-05
METTL6	4.95E-06	5.36E-06	1.94E-06	2.91E-06	1.43E-06	3.41E-06	2.98E-06	3.28E-06
METTL7A	0.000187	0.000218	0.000345	0.000215	0.00019	8.39E-05	0.00017	0.000122
METTL7B	0.000191	0.000105	0.000176	0.000128	0.000304	0.000112	0.000109	0.000109
METTL8	2.62E-06	1.20E-06	1.11E-06	8.56E-07	5.40E-07	1.51E-06	7.49E-07	5.10E-07
METTL9	1.32E-05	1.61E-05	5.14E-06	8.60E-06	2.94E-06	5.84E-06	8.00E-06	5.87E-06
MEX3A	7.79E-07	7.43E-07	2.17E-07	6.65E-07	1.95E-07	4.26E-07	4.88E-07	3.16E-07
MEX3B	1.33E-06	5.99E-07	9.07E-08	6.78E-07	1.22E-07	7.51E-08	2.82E-07	2.48E-07
MEX3C	8.71E-06	3.66E-06	3.93E-06	5.88E-06	9.55E-07	4.40E-06	5.60E-06	5.45E-06
MEX3D	7.21E-06	4.77E-06	2.10E-06	3.95E-06	1.68E-06	3.17E-06	6.15E-06	1.68E-06
MFAP1	2.69E-05	1.17E-05	8.53E-06	1.35E-05	8.24E-06	1.46E-05	1.33E-05	1.26E-05
MFAP2	2.50E-05	1.13E-07	5.30E-07	6.25E-06	8.22E-07	2.23E-08	2.17E-06	1.81E-06
MFAP3	1.17E-05	5.84E-06	5.28E-06	5.46E-06	1.09E-06	7.42E-06	3.88E-06	7.23E-06
MFAP3L	9.43E-08	1.01E-05	1.56E-05	6.12E-06	2.88E-06	8.09E-06	4.01E-06	4.70E-06
MFAP4	5.60E-06	6.39E-07	2.68E-05	0.000111	3.20E-06	6.95E-07	1.16E-05	6.26E-06
MFAP5	2.44E-07	9.32E-09	1.72E-08	4.92E-07	4.27E-08	2.30E-08	1.85E-07	8.51E-08
MFF	5.88E-05	2.86E-05	2.04E-05	2.28E-05	1.39E-05	3.70E-05	2.20E-05	2.88E-05
MFGE8	2.04E-05	7.60E-06	2.05E-06	1.64E-05	1.00E-05	8.29E-06	2.31E-05	1.25E-05
MFHAS1	2.49E-06	2.99E-06	1.02E-06	2.69E-06	3.31E-08	4.09E-06	1.12E-06	1.45E-06
MFI2	7.54E-06	8.03E-07	1.05E-06	3.73E-06	2.51E-08	1.81E-08	1.40E-06	1.17E-07
MFN1	9.28E-06	7.11E-06	4.81E-06	4.45E-06	1.14E-06	6.59E-06	3.68E-06	3.41E-06
MFN2	6.41E-05	4.94E-05	4.10E-05	2.88E-05	8.43E-06	4.42E-05	2.90E-05	5.17E-05
MFNG	8.90E-06	2.64E-06	5.21E-06	5.73E-06	4.86E-06	3.70E-06	6.19E-06	3.66E-06
MFRP	8.23E-06	2.14E-06	1.30E-06	5.98E-06	5.93E-06	2.02E-06	7.73E-06	3.91E-06
MFSD1	3.96E-05	3.05E-05	2.93E-05	4.12E-05	1.11E-05	4.32E-05	3.64E-05	3.55E-05
MFSD10	2.81E-05	1.08E-05	5.88E-06	1.84E-05	1.08E-05	7.10E-06	1.02E-05	8.83E-06
MFSD11	1.37E-05	1.69E-05	6.10E-06	7.51E-06	2.93E-06	1.12E-05	5.47E-06	1.01E-05
MFSD2A	3.27E-06	5.25E-06	0.000165	0.000142	2.94E-05	1.22E-05	2.59E-05	2.05E-05
MFSD2B	1.55E-06	2.14E-06	9.87E-08	7.79E-07	7.68E-07	3.34E-07	2.62E-07	8.12E-07
MFSD3	2.05E-05	3.99E-05	1.61E-05	1.43E-05	8.17E-05	1.89E-05	5.84E-05	2.12E-05
MFSD4	8.60E-07	9.96E-08	3.61E-07	2.65E-07	8.12E-08	1.13E-07	2.36E-07	1.32E-07
MFSD5	2.22E-05	1.50E-05	7.65E-06	1.25E-05	1.20E-05	1.48E-05	1.59E-05	1.87E-05
MFSD6	5.13E-05	1.28E-06	1.03E-06	3.95E-06	5.90E-07	1.75E-06	2.14E-06	4.37E-06
MFSD6L	1.13E-08	0	3.56E-08	1.67E-07	0	0	7.67E-08	0

MFSD7	2.92E-06	1.77E-05	1.40E-06	1.25E-05	5.58E-07	4.78E-07	2.60E-05	2.23E-06
MFSD8	5.14E-06	2.80E-06	3.71E-06	2.84E-06	1.34E-06	3.33E-06	1.73E-06	5.17E-06
MFSD9	6.33E-06	3.76E-06	4.18E-06	2.61E-06	5.45E-07	9.87E-06	2.92E-06	6.53E-06
MGA	4.09E-06	1.01E-06	1.75E-06	1.76E-06	3.71E-07	1.76E-06	9.67E-07	1.01E-06
MGAM	1.45E-06	1.92E-08	4.58E-08	3.79E-08	5.91E-09	0	1.01E-08	1.39E-06
MGAT1	8.78E-05	4.83E-05	9.35E-05	9.27E-05	4.24E-05	8.18E-05	8.91E-05	0.000123
MGAT2	2.39E-05	2.10E-05	2.39E-05	1.88E-05	1.22E-05	2.11E-05	1.51E-05	2.08E-05
MGAT3	4.50E-07	6.94E-08	5.12E-08	1.22E-07	9.74E-08	2.10E-07	2.96E-07	1.86E-07
MGAT4A	1.23E-05	5.83E-06	4.64E-06	4.44E-06	2.61E-06	9.74E-07	5.02E-06	4.12E-06
MGAT4B	0.000104	0.00011	0.000107	0.000104	3.66E-05	0.000113	0.000158	0.000185
MGAT4C	3.18E-07	3.79E-06	3.05E-08	1.58E-07	6.22E-08	7.82E-06	2.84E-08	0
MGAT5	3.44E-06	1.83E-06	4.55E-07	8.21E-07	0	8.02E-07	3.01E-07	5.57E-07
MGAT5B	1.10E-07	1.21E-08	5.77E-09	0	1.11E-07	7.45E-08	4.16E-08	8.21E-09
MGC1291	3.46E-07	5.41E-08	1.88E-07	6.44E-07	1.25E-06	2.99E-07	8.77E-07	1.93E-07
MGC1298	2.62E-06	1.43E-06	3.02E-07	3.21E-07	1.78E-06	4.77E-07	2.25E-06	7.15E-07
MGC1443	4.03E-07	6.93E-07	6.37E-07	5.75E-08	1.21E-07	4.77E-07	4.45E-07	0
MGC1588	0	0	0	0	0	0	0	0
MGC1602	2.24E-08	0	0	1.37E-08	0	1.04E-08	0	0
MGC1612	1.50E-06	3.85E-07	7.28E-07	1.69E-06	1.07E-06	2.04E-07	3.19E-07	1.64E-07
MGC1614	2.01E-06	2.71E-07	1.69E-07	2.91E-07	1.87E-07	2.57E-07	2.84E-07	1.75E-07
MGC1627	8.72E-07	2.99E-07	4.24E-07	3.71E-07	8.39E-07	5.18E-07	3.49E-07	2.09E-07
MGC1638	1.44E-07	1.82E-07	5.08E-08	7.85E-08	0	8.90E-08	6.15E-08	1.18E-07
MGC1670	0	0	1.09E-07	3.89E-08	0	0	0	0
MGC2188	4.36E-06	2.98E-06	2.67E-06	5.81E-06	2.01E-06	4.82E-06	6.97E-06	1.24E-06
MGC2327	7.73E-07	8.19E-07	2.42E-07	2.05E-07	3.68E-07	2.59E-07	7.40E-07	1.64E-07
MGC2328	1.92E-06	4.16E-06	8.95E-07	7.17E-07	1.11E-06	8.33E-07	5.74E-07	7.39E-07
MGC2664	0	0	0	0	0	0	0	0
MGC2738	5.21E-07	5.56E-07	2.60E-07	1.33E-07	1.63E-07	2.61E-07	9.64E-08	1.42E-07
MGC2752	6.98E-06	4.27E-06	2.80E-06	4.65E-06	3.56E-06	6.90E-06	5.30E-06	5.02E-06
MGC2889	0	0	0	0	0	0	2.09E-08	0
MGC2950	1.29E-05	1.20E-07	3.15E-06	1.99E-05	2.36E-07	1.28E-06	1.13E-06	4.59E-07
MGC3403	0	7.31E-09	0	0	0	0	0	0
MGC3771	6.94E-07	6.28E-07	6.73E-07	4.99E-07	2.77E-07	1.18E-06	2.61E-07	1.13E-06
MGC4210	9.20E-08	1.15E-08	1.49E-07	5.00E-08	0	2.37E-07	3.96E-08	4.51E-08
MGC4473	0	0	0	0	0	0	0	0
MGC4580	0	0	4.67E-09	0	0	0	0	0
MGC5734	5.90E-06	1.83E-06	9.52E-07	1.03E-06	1.08E-07	9.55E-08	1.32E-07	1.45E-07
MGC7085	2.10E-05	3.28E-05	2.82E-05	2.38E-05	7.18E-05	2.60E-05	8.56E-05	2.87E-05
MGC7208	2.39E-05	5.27E-05	1.63E-05	1.31E-05	9.03E-06	1.13E-05	1.30E-05	2.58E-05
MGC8704	3.12E-06	9.18E-07	4.89E-07	3.45E-07	2.05E-07	2.55E-06	7.45E-07	1.92E-07
MGEA5	4.05E-05	2.19E-05	2.20E-05	2.91E-05	2.31E-05	3.40E-05	2.50E-05	2.21E-05
MGLL	0.000155	3.00E-05	6.61E-05	3.94E-05	2.03E-05	4.37E-06	4.69E-05	7.90E-05
MGMT	3.49E-05	2.02E-05	5.49E-05	4.56E-05	0.000124	5.05E-05	7.58E-05	2.11E-05
MGP	0.000144	1.80E-05	1.32E-05	0.000181	9.76E-05	2.74E-05	9.97E-05	9.13E-05
MGRN1	2.81E-05	3.80E-05	3.03E-05	2.76E-05	4.53E-06	2.49E-05	2.44E-05	3.01E-05
MGST1	0.000419	0.003432	0.001678	0.001045	0.001245	0.001558	0.000974	0.000607
MGST2	0.000174	0.000107	0.000175	0.000205	0.000252	0.000253	0.00024	0.000228
MGST3	0.000456	0.0003	0.000122	0.000163	0.000172	0.00027	0.000136	0.000146
MIA	8.04E-08	0	1.22E-07	3.22E-08	3.90E-07	1.43E-07	0	3.92E-08
MIA2	1.17E-05	1.76E-05	1.42E-05	5.47E-06	8.03E-08	6.49E-06	1.36E-06	6.52E-06
MIA3	2.33E-05	3.74E-05	2.62E-05	1.95E-05	8.98E-06	4.72E-05	1.49E-05	5.29E-05
MIAT	1.69E-06	2.39E-08	7.98E-08	3.63E-07	4.54E-08	1.89E-07	2.11E-07	1.79E-07
MIB1	1.03E-05	4.47E-06	4.62E-06	3.02E-06	9.25E-07	4.45E-06	2.51E-06	3.35E-06
MIB2	7.92E-06	9.24E-06	6.42E-06	8.98E-06	5.47E-06	7.87E-06	1.12E-05	4.38E-06
MICA	1.62E-05	5.52E-06	6.91E-06	8.73E-06	5.27E-06	5.64E-06	9.00E-06	4.92E-06
MICAL1	2.15E-05	7.28E-07	1.37E-06	4.73E-06	1.08E-06	1.47E-06	3.15E-06	1.82E-06
MICAL2	1.09E-05	4.73E-06	3.49E-06	6.81E-06	2.65E-06	7.93E-06	9.78E-06	6.72E-06
MICAL3	1.72E-05	1.94E-05	1.29E-05	1.44E-05	1.24E-05	2.16E-05	1.63E-05	1.10E-05

MICALCL	1.06E-07	1.62E-07	8.65E-08	6.50E-08	1.31E-08	1.34E-07	1.48E-08	5.57E-08
MICALL1	7.94E-06	4.46E-06	3.19E-06	5.32E-06	2.47E-06	3.79E-06	5.80E-06	7.70E-06
MICALL2	8.26E-06	6.10E-06	3.07E-06	1.27E-05	4.82E-06	3.51E-06	9.77E-06	3.42E-06
MICB	5.47E-06	2.97E-06	6.20E-07	7.30E-07	9.24E-08	1.38E-06	1.12E-06	1.61E-06
MID1	1.25E-05	1.81E-06	1.75E-06	3.84E-06	1.03E-06	9.54E-06	1.44E-06	4.38E-06
MID1IP1	3.45E-05	1.41E-05	2.39E-05	5.61E-05	1.10E-05	3.38E-05	4.83E-05	0.000127
MID2	1.94E-06	2.29E-06	1.91E-06	1.98E-06	7.77E-07	2.44E-06	1.48E-06	7.08E-06
MIDN	3.20E-05	1.93E-05	8.29E-06	4.71E-05	1.01E-05	1.05E-05	2.63E-05	2.39E-05
MIER1	8.83E-06	5.64E-06	7.46E-06	7.51E-06	1.68E-06	9.85E-06	4.75E-06	6.35E-06
MIER2	3.67E-06	1.75E-06	1.65E-06	4.33E-06	1.50E-06	2.84E-06	5.09E-06	3.38E-06
MIER3	5.31E-06	5.16E-06	3.72E-06	3.65E-06	1.10E-06	4.61E-06	2.59E-06	4.56E-06
MIF	0.000706	0.000887	0.000343	0.00051	0.000437	0.000654	0.001026	0.000134
MIF4GD	3.22E-05	1.70E-05	8.01E-06	1.58E-05	1.27E-05	2.14E-05	1.79E-05	1.52E-05
MIIP	1.66E-05	1.82E-05	9.42E-06	1.10E-05	1.21E-05	1.12E-05	1.34E-05	1.34E-05
MIMT1	0	0	0	0	0	0	0	0
MINA	6.30E-06	3.07E-05	6.92E-06	6.87E-06	1.46E-06	1.36E-05	7.74E-06	5.96E-06
MINK1	1.12E-05	7.66E-06	7.46E-06	9.34E-06	4.24E-06	1.21E-05	7.69E-06	1.07E-05
MINPP1	1.88E-05	1.63E-05	1.61E-05	1.28E-05	8.63E-06	1.97E-05	1.40E-05	9.04E-06
MIOS	1.40E-05	1.04E-05	7.88E-06	8.26E-06	2.84E-06	5.89E-06	5.26E-06	5.97E-06
MIOX	1.63E-06	2.16E-06	0	4.78E-08	0	0	3.72E-08	0
MIP	4.78E-08	2.71E-07	4.51E-07	6.74E-07	1.34E-07	1.89E-07	1.27E-07	2.11E-07
MIPEP	8.55E-06	9.53E-06	6.29E-06	7.47E-06	1.94E-06	8.77E-06	5.20E-06	4.30E-06
MIPOL1	2.31E-06	9.47E-07	5.37E-07	6.27E-07	3.70E-07	1.03E-06	6.99E-07	1.84E-06
MIR155HC	1.58E-06	3.87E-08	1.08E-07	6.67E-07	1.02E-07	9.62E-07	2.12E-07	1.76E-07
MIR17HG	1.61E-06	6.11E-08	1.44E-07	2.31E-07	4.70E-07	1.98E-07	1.05E-07	4.95E-07
MIS12	7.02E-06	6.29E-06	5.23E-06	5.53E-06	1.37E-06	8.20E-06	4.56E-06	5.47E-06
MITD1	2.31E-05	1.26E-05	8.28E-06	1.18E-05	3.20E-06	1.03E-05	1.33E-05	8.75E-06
MITF	2.01E-05	1.88E-06	7.56E-07	2.16E-06	1.09E-07	8.10E-07	8.03E-07	6.78E-07
MIXL1	4.25E-08	0	0	0	0	0	3.99E-08	0
MKI67	1.13E-05	1.87E-06	1.69E-07	1.81E-07	6.43E-07	1.16E-06	2.77E-07	9.88E-07
MKI67IP	2.75E-05	2.29E-05	1.08E-05	1.69E-05	7.37E-06	1.91E-05	2.12E-05	1.34E-05
MKKS	9.92E-05	5.32E-05	2.86E-05	2.65E-05	4.25E-05	2.07E-05	3.50E-05	3.32E-05
MKL1	1.08E-05	5.27E-06	3.74E-06	7.63E-06	4.60E-06	5.34E-06	6.52E-06	7.60E-06
MKL2	4.17E-06	5.55E-06	2.00E-06	1.97E-06	6.95E-07	3.89E-06	1.73E-06	2.23E-06
MKLN1	6.17E-06	1.35E-05	9.52E-06	7.09E-06	2.00E-06	1.39E-05	5.46E-06	1.18E-05
MKNK1	1.57E-05	8.97E-06	7.33E-06	1.04E-05	3.19E-06	9.56E-06	6.21E-06	7.06E-06
MKNK2	6.81E-05	2.61E-05	4.00E-05	2.26E-05	6.06E-05	4.54E-05	6.13E-05	4.32E-05
MKRN1	2.51E-05	2.40E-05	1.87E-05	2.43E-05	1.47E-05	3.57E-05	2.82E-05	3.97E-05
MKRN2	1.61E-05	1.02E-05	8.41E-06	5.70E-06	3.12E-06	8.97E-06	8.03E-06	7.82E-06
MKRN3	7.09E-07	4.01E-07	0	0	0	7.60E-07	1.49E-08	3.09E-07
MKS1	4.91E-06	4.24E-06	2.10E-06	2.58E-06	1.19E-06	7.31E-06	3.10E-06	1.70E-06
MKX	3.78E-08	3.30E-06	6.29E-07	2.69E-07	0	0	1.04E-07	5.35E-07
MLANA	6.50E-08	2.04E-07	5.17E-08	2.00E-08	2.80E-08	4.53E-08	2.03E-07	9.60E-08
MLC1	7.64E-08	5.24E-08	6.47E-08	1.98E-07	1.64E-08	1.17E-07	2.31E-07	1.95E-08
MLEC	3.69E-05	4.51E-05	1.72E-05	2.00E-05	2.38E-05	4.87E-05	6.21E-05	4.34E-05
MLF1	9.25E-06	2.27E-07	1.46E-07	2.17E-06	9.31E-09	8.98E-08	5.10E-07	1.42E-07
MLF1IP	1.31E-05	8.59E-06	6.42E-07	1.05E-06	3.35E-06	3.26E-06	1.31E-06	4.15E-06
MLF2	0.000247	0.000233	0.000199	0.00019	0.000139	0.000165	0.000221	0.000158
MLH1	2.33E-05	1.95E-05	1.19E-05	1.36E-05	1.29E-05	2.46E-05	1.88E-05	1.49E-05
MLH3	4.22E-06	1.92E-06	1.33E-06	1.97E-06	1.03E-06	3.85E-06	1.75E-06	3.04E-06
MLKL	8.67E-06	1.16E-06	1.60E-06	3.61E-06	6.53E-07	2.81E-06	2.25E-06	2.93E-06
MLL	1.70E-05	2.60E-06	7.10E-06	6.55E-06	6.12E-06	7.04E-06	7.60E-06	7.30E-06
MLL2	5.06E-06	2.02E-06	1.33E-06	1.53E-06	6.33E-07	2.60E-06	1.67E-06	2.19E-06
MLL3	8.15E-06	8.19E-06	7.17E-06	6.21E-06	2.97E-06	6.98E-06	4.72E-06	7.38E-06
MLL4	9.75E-06	4.05E-06	2.99E-06	3.75E-06	2.46E-06	5.25E-06	4.29E-06	3.91E-06
MLL5	1.16E-05	1.01E-05	7.11E-06	8.37E-06	1.48E-06	8.08E-06	4.47E-06	7.55E-06
MLLT1	1.33E-05	7.63E-06	8.33E-06	1.23E-05	9.99E-06	1.90E-05	1.04E-05	1.34E-05
MLLT10	1.12E-05	8.92E-06	8.38E-06	8.69E-06	2.41E-06	7.74E-06	6.79E-06	5.67E-06

MLLT11	2.05E-06	6.23E-07	7.72E-07	4.16E-06	2.94E-07	5.72E-07	8.21E-07	8.11E-07
MLLT3	2.77E-06	5.56E-07	4.62E-07	9.77E-07	9.68E-08	3.59E-07	4.18E-07	6.87E-07
MLLT4	2.46E-05	1.68E-05	1.45E-05	1.63E-05	2.26E-06	1.54E-05	9.90E-06	1.38E-05
MLLT6	3.00E-05	7.37E-06	6.14E-06	1.24E-05	1.10E-05	1.24E-05	1.57E-05	7.54E-06
MLN	0	0	0	0	0	5.64E-08	0	0
MLNR	1.65E-07	0	0	0	0	0	0	0
MLPH	6.96E-05	4.39E-06	8.32E-06	8.84E-06	4.25E-06	2.35E-06	1.43E-05	7.33E-06
MLST8	2.75E-05	3.50E-05	1.65E-05	1.99E-05	2.07E-05	2.70E-05	2.66E-05	2.96E-05
MLX	5.79E-05	7.22E-05	3.19E-05	2.77E-05	1.73E-05	5.51E-05	3.29E-05	3.35E-05
MLXIP	8.47E-06	6.30E-06	4.41E-06	5.52E-06	4.39E-06	6.63E-06	6.51E-06	5.82E-06
MLXIPL	3.77E-05	0.000126	8.51E-05	6.99E-05	0.000223	0.000106	9.83E-05	0.00011
MLYCD	1.56E-05	2.16E-05	2.84E-05	2.87E-05	1.70E-05	2.46E-05	2.02E-05	1.35E-05
MMAA	5.05E-06	2.72E-06	1.00E-05	5.82E-06	1.15E-06	9.93E-06	4.83E-06	9.74E-06
MMAB	1.03E-05	4.59E-05	2.73E-05	1.63E-05	2.76E-05	3.20E-05	2.29E-05	1.28E-05
MMACHC	8.20E-06	1.47E-05	1.35E-05	5.72E-06	5.69E-06	8.94E-06	5.76E-06	1.25E-05
MMADHC	0.000157	0.000106	9.84E-05	8.26E-05	2.44E-05	0.000107	8.08E-05	8.16E-05
MMD	2.17E-05	8.14E-06	3.95E-06	1.38E-05	9.20E-07	4.36E-06	6.53E-06	6.59E-06
MMD2	0	1.10E-08	0	0	0	0	0	0
MME	6.40E-08	1.95E-07	1.77E-05	8.28E-07	0	8.10E-05	8.95E-08	1.23E-07
MMEL1	1.78E-08	5.38E-07	1.82E-08	4.42E-08	0	0	0	0
MMGT1	8.08E-06	7.22E-06	4.01E-06	5.86E-06	2.07E-06	4.51E-06	4.41E-06	8.60E-06
MMP1	5.51E-07	5.48E-07	1.34E-07	1.85E-07	2.18E-08	2.16E-07	6.11E-08	5.59E-07
MMP10	2.65E-07	1.58E-07	2.94E-08	1.71E-07	1.20E-08	1.30E-08	2.69E-08	3.42E-06
MMP11	1.00E-05	1.58E-06	3.24E-07	4.36E-06	3.45E-06	2.93E-06	1.27E-05	6.58E-06
MMP12	4.33E-05	4.52E-08	0	4.89E-08	0	1.73E-07	5.13E-08	7.24E-07
MMP13	7.73E-08	0	0	0	0	7.96E-09	0	0
MMP14	8.45E-05	2.16E-05	5.66E-06	3.87E-05	5.68E-06	8.15E-06	2.26E-05	1.55E-05
MMP15	5.32E-05	2.24E-05	1.84E-05	4.78E-05	2.16E-05	1.08E-05	3.42E-05	2.61E-05
MMP16	1.34E-07	3.55E-08	3.97E-08	9.84E-08	3.03E-09	6.53E-09	3.10E-08	7.75E-08
MMP17	2.48E-07	1.17E-07	5.38E-08	2.28E-07	1.78E-08	2.87E-08	1.10E-07	4.56E-08
MMP19	3.81E-06	5.80E-06	1.82E-06	2.29E-05	2.81E-06	4.27E-06	5.95E-06	3.31E-06
MMP2	2.18E-05	1.75E-06	7.46E-06	3.43E-05	8.32E-06	2.67E-06	1.37E-05	1.39E-05
MMP20	2.46E-08	1.39E-08	0	0	0	3.42E-08	0	0
MMP21	3.77E-08	4.28E-08	0	1.54E-08	0	1.17E-08	0	1.85E-08
MMP23A	0	0	0	7.86E-07	0	0	0	0
MMP23B	2.43E-06	4.01E-07	1.80E-06	1.06E-05	2.03E-06	1.56E-06	3.85E-06	8.34E-07
MMP24	8.57E-06	8.61E-07	1.62E-06	4.89E-06	7.92E-06	1.41E-05	2.02E-06	1.59E-06
MMP25	2.96E-06	4.51E-07	4.30E-07	6.92E-07	9.76E-08	3.31E-07	4.77E-07	8.57E-07
MMP26	0	0	2.97E-08	0	0	0	0	0
MMP27	0	0	0	0	0	0	0	0
MMP28	1.45E-06	3.40E-07	3.53E-07	1.14E-07	7.74E-07	1.02E-07	1.13E-06	5.69E-07
MMP3	2.65E-08	0	0	0	0	1.85E-06	8.94E-08	4.68E-07
MMP7	1.37E-06	0	1.92E-06	5.57E-05	6.05E-08	1.26E-05	9.81E-07	1.18E-06
MMP8	0	9.69E-09	0	1.05E-08	0	0	0	0
MMP9	1.80E-05	2.51E-06	7.37E-07	3.71E-06	2.43E-07	4.28E-06	2.07E-06	2.38E-06
MMRN1	9.57E-07	3.62E-06	9.94E-07	2.75E-06	4.22E-07	1.35E-07	2.53E-06	1.18E-06
MMRN2	7.40E-06	1.03E-05	4.12E-06	3.65E-06	4.24E-06	4.78E-06	9.55E-06	6.42E-06
MMS19	2.42E-05	1.69E-05	9.10E-06	1.19E-05	1.19E-05	1.62E-05	1.83E-05	1.25E-05
MN1	1.39E-06	4.59E-08	1.34E-07	1.52E-06	2.83E-07	2.29E-07	3.21E-07	5.89E-07
MNAT1	1.07E-05	5.96E-06	3.89E-06	5.26E-06	2.85E-06	9.90E-06	9.50E-06	7.31E-06
MND1	3.20E-06	2.68E-06	9.53E-08	1.18E-06	2.07E-06	1.26E-06	8.49E-07	1.38E-06
MNDA	1.09E-05	9.17E-07	3.13E-06	4.20E-06	2.91E-07	7.63E-07	3.03E-06	9.52E-07
MNS1	1.96E-06	4.11E-06	9.66E-08	2.30E-06	3.73E-07	6.67E-07	1.39E-07	3.48E-07
MNT	5.84E-06	1.80E-06	1.45E-06	2.54E-06	1.28E-06	3.16E-06	4.49E-06	4.93E-06
MNX1	5.72E-07	0	0	6.80E-08	0	0	0	0
MOAP1	2.27E-05	8.52E-06	1.33E-05	1.31E-05	4.24E-06	1.44E-05	9.56E-06	1.75E-05
MOB2	2.70E-05	1.44E-05	1.11E-05	2.03E-05	2.17E-05	1.85E-05	2.25E-05	3.16E-05
MOBKL1A	5.74E-06	2.62E-06	2.33E-06	2.31E-06	6.27E-07	2.58E-06	2.13E-06	1.85E-06

MOBKL1B	3.72E-05	2.49E-05	1.47E-05	1.46E-05	1.31E-06	1.65E-05	7.14E-06	1.00E-05
MOBKL2A	1.40E-05	3.83E-06	4.74E-06	8.87E-06	3.70E-06	5.66E-06	9.09E-06	6.02E-06
MOBKL2B	3.05E-06	2.70E-06	7.74E-07	2.26E-06	2.14E-07	3.48E-07	8.61E-07	8.26E-07
MOBKL2C	7.18E-06	2.60E-06	2.82E-06	6.13E-06	2.62E-06	3.99E-06	5.96E-06	4.50E-06
MOBKL3	1.38E-05	1.14E-05	8.11E-06	1.05E-05	2.79E-06	7.41E-06	8.67E-06	7.86E-06
MOBP	0	8.44E-09	0	8.65E-09	0	0	0	0
MOCOS	9.60E-06	1.03E-05	1.33E-05	9.43E-06	1.24E-06	1.32E-05	6.81E-06	2.12E-05
MOCS1	1.14E-05	2.79E-05	1.90E-05	1.32E-05	9.47E-06	3.03E-05	2.08E-05	1.43E-05
MOCS2	5.22E-05	5.44E-05	4.56E-05	2.65E-05	1.75E-05	0.000113	2.90E-05	4.83E-05
MOCS3	6.29E-06	4.94E-06	3.62E-06	3.51E-06	1.47E-06	6.36E-06	2.74E-06	3.15E-06
MOG	0	0	0	0	0	0	0	0
MOGAT1	1.82E-06	2.48E-07	5.19E-07	2.36E-07	1.69E-07	2.23E-07	1.32E-07	4.15E-08
MOGAT2	2.66E-08	3.93E-06	2.83E-05	7.98E-06	5.75E-07	1.04E-05	5.17E-06	3.96E-08
MOGAT3	1.82E-05	1.91E-05	8.04E-06	8.66E-06	2.04E-05	1.53E-06	6.94E-06	2.29E-06
MOGS	3.66E-05	3.36E-05	2.58E-05	2.38E-05	2.42E-05	3.29E-05	2.13E-05	2.78E-05
MON1A	2.99E-06	6.58E-06	5.49E-06	3.64E-06	8.06E-06	6.97E-06	8.70E-06	5.61E-06
MON1B	1.42E-05	6.09E-06	6.81E-06	5.12E-06	1.91E-06	1.15E-05	5.15E-06	7.91E-06
MON2	5.94E-06	4.95E-06	4.17E-06	3.09E-06	1.37E-06	4.22E-06	2.09E-06	2.27E-06
MORC1	0	2.08E-08	1.91E-08	0	0	0	0	0
MORC2	1.09E-05	8.78E-06	4.51E-06	6.27E-06	3.24E-06	6.63E-06	5.63E-06	9.53E-06
MORC3	7.88E-06	6.10E-06	9.21E-06	5.04E-06	2.15E-06	9.85E-06	4.72E-06	5.36E-06
MORC4	1.53E-05	6.31E-06	6.40E-06	1.70E-05	5.51E-06	1.25E-05	4.66E-06	1.23E-05
MORF4	4.18E-07	3.98E-07	2.88E-07	2.09E-07	3.64E-08	1.96E-07	1.96E-07	6.30E-08
MORF4L1	0.000117	6.84E-05	6.00E-05	8.37E-05	4.18E-05	8.39E-05	7.57E-05	6.34E-05
MORF4L2	0.000193	0.000167	7.54E-05	8.94E-05	6.24E-05	0.00011	9.21E-05	0.000147
MORN1	8.64E-07	3.48E-06	1.10E-06	1.38E-06	1.14E-06	1.80E-06	8.18E-07	9.03E-07
MORN2	1.32E-05	2.84E-05	5.07E-06	1.21E-05	8.15E-06	2.42E-05	9.27E-06	5.21E-06
MORN3	2.01E-07	0	3.47E-08	2.67E-07	5.81E-08	2.09E-08	0	6.65E-08
MORN4	1.80E-06	6.84E-07	1.20E-06	1.77E-06	1.83E-06	3.90E-06	1.55E-06	1.34E-07
MORN5	0	0	0	0	1.41E-07	5.05E-08	0	0
MOS	0	0	0	0	0	0	0	0
MOSC1	5.83E-06	3.23E-05	4.42E-05	2.16E-05	1.42E-05	2.65E-05	8.30E-06	4.85E-05
MOSC2	6.14E-05	9.23E-05	9.51E-05	5.75E-05	2.54E-05	7.74E-05	3.45E-05	7.56E-05
MOSPD1	7.38E-06	8.59E-06	2.57E-06	5.27E-06	3.02E-06	3.15E-06	4.50E-06	1.49E-05
MOSPD2	8.24E-06	2.84E-06	2.49E-06	2.06E-06	2.00E-07	4.19E-06	1.22E-06	5.17E-06
MOSPD3	5.39E-05	9.36E-05	1.81E-05	1.81E-05	1.96E-05	2.87E-05	2.25E-05	2.52E-05
MOV10	3.70E-05	3.40E-05	1.88E-05	1.56E-05	9.78E-06	3.03E-05	9.61E-06	2.74E-05
MOV10L1	5.74E-08	6.80E-06	6.19E-07	1.30E-06	1.26E-06	3.84E-08	6.61E-08	5.10E-07
MOXD1	1.65E-05	1.05E-07	2.44E-07	8.35E-06	2.42E-07	1.27E-07	3.30E-06	1.65E-06
MPDU1	4.17E-05	4.37E-05	6.69E-05	7.43E-05	4.92E-05	7.59E-05	9.40E-05	9.12E-05
MPDZ	9.11E-06	3.36E-05	3.68E-05	2.23E-05	3.66E-06	1.70E-05	1.02E-05	1.26E-05
MPEG1	1.99E-05	3.32E-06	1.22E-05	1.67E-05	2.00E-06	8.13E-06	1.11E-05	3.38E-06
MPG	6.04E-05	8.33E-05	5.15E-05	5.24E-05	6.76E-05	5.31E-05	6.10E-05	3.93E-05
MPHOSPH	5.00E-05	1.73E-05	1.07E-05	1.17E-05	7.43E-06	1.85E-05	9.32E-06	1.53E-05
MPHOSPH	2.27E-05	8.07E-06	4.34E-06	7.69E-06	2.45E-06	1.71E-05	6.64E-06	1.21E-05
MPHOSPH	1.84E-05	9.62E-06	9.78E-06	1.00E-05	1.04E-06	1.16E-05	5.91E-06	9.16E-06
MPHOSPH	4.57E-06	3.41E-06	2.59E-06	1.83E-06	4.00E-07	4.97E-06	8.64E-07	1.71E-06
MPI	3.00E-05	3.26E-05	1.88E-05	1.80E-05	2.14E-05	4.35E-05	2.75E-05	1.77E-05
MPL	2.59E-07	1.98E-07	1.43E-07	1.54E-07	6.51E-08	2.88E-07	6.14E-08	1.20E-07
MPND	2.11E-05	1.42E-05	3.01E-05	2.33E-05	4.30E-05	2.04E-05	2.63E-05	2.29E-05
MPO	0	8.05E-09	5.93E-08	1.84E-07	0	1.33E-08	6.96E-09	1.05E-08
MPP1	1.57E-05	1.27E-05	1.99E-05	2.07E-05	2.80E-05	1.64E-05	3.25E-05	2.89E-05
MPP2	6.50E-07	1.20E-07	3.86E-08	9.18E-08	1.84E-08	0	7.84E-08	5.50E-08
MPP3	2.20E-06	3.73E-08	1.84E-07	1.22E-07	1.85E-07	1.06E-06	2.52E-07	3.65E-08
MPP4	2.58E-08	1.28E-08	0	5.57E-09	0	4.22E-09	0	0
MPP5	9.77E-06	6.24E-06	6.70E-06	5.13E-06	1.94E-06	8.19E-06	4.47E-06	5.23E-06
MPP6	5.65E-06	1.40E-05	3.12E-06	1.89E-06	1.06E-06	3.62E-06	1.66E-06	3.40E-06
MPP7	1.54E-06	2.54E-06	9.95E-07	1.34E-06	2.67E-08	2.60E-06	2.99E-07	2.21E-06

MPPE1	1.18E-05	1.58E-05	1.11E-05	1.01E-05	2.68E-06	1.70E-05	1.10E-05	1.72E-05
MPPED1	3.55E-08	6.25E-06	1.67E-05	1.39E-05	1.22E-07	4.02E-06	3.06E-06	3.74E-06
MPPED2	2.26E-07	1.11E-07	1.20E-07	1.44E-07	9.28E-08	1.10E-06	1.71E-07	2.90E-06
MPRIP	3.08E-05	1.97E-05	1.38E-05	2.75E-05	2.31E-05	1.74E-05	3.24E-05	2.82E-05
MPST	0.000262	0.000219	0.000172	0.000278	0.00083	0.000242	0.000329	0.000241
MPV17	0.000141	7.32E-05	1.02E-05	2.76E-05	6.14E-06	2.80E-05	2.41E-05	2.52E-05
MPV17L	1.40E-05	5.62E-06	6.53E-06	3.36E-06	2.14E-06	3.62E-06	3.75E-06	2.61E-05
MPV17L2	1.45E-05	2.84E-05	7.44E-06	1.06E-05	1.19E-05	1.45E-05	1.35E-05	1.50E-05
MPZ	3.01E-06	6.00E-06	7.35E-07	1.06E-06	3.81E-07	3.91E-05	7.48E-07	3.71E-05
MPZL1	2.59E-05	2.03E-05	6.18E-06	1.40E-05	3.91E-06	1.12E-05	1.04E-05	1.72E-05
MPZL2	3.77E-05	2.17E-06	1.32E-05	1.05E-05	7.66E-06	1.08E-05	4.85E-06	1.37E-05
MPZL3	8.48E-06	3.49E-07	1.68E-06	2.47E-06	0	1.05E-06	5.04E-07	4.11E-06
MR1	2.54E-05	1.28E-05	5.78E-06	6.74E-06	1.99E-06	1.51E-05	1.06E-05	1.71E-05
MRAP	7.32E-07	4.51E-08	5.11E-07	0	6.11E-08	1.84E-06	0	2.30E-07
MRAP2	1.61E-07	1.38E-06	3.54E-08	3.18E-07	2.95E-07	1.51E-06	3.27E-08	0
MRAS	3.72E-06	2.61E-06	1.35E-06	5.23E-06	7.99E-07	4.60E-06	2.77E-06	1.50E-06
MRC1	4.56E-06	1.08E-05	9.74E-06	5.88E-06	1.14E-06	2.57E-06	4.06E-06	2.09E-06
MRC2	1.17E-05	1.19E-06	5.79E-06	1.69E-05	3.50E-06	1.08E-06	5.59E-06	1.80E-06
MRE11A	3.39E-06	1.19E-06	1.08E-06	1.17E-06	2.07E-07	1.97E-06	7.64E-07	1.98E-06
MREG	4.79E-06	1.57E-06	2.26E-06	2.94E-06	2.19E-06	1.93E-06	2.03E-06	7.83E-07
MRFAP1	0.000288	0.000274	0.00017	0.000212	0.000133	0.000211	0.000231	0.000256
MRFAP1L1	6.90E-05	5.50E-05	3.49E-05	3.79E-05	2.31E-05	5.41E-05	4.08E-05	4.78E-05
MRGPRD	0	0	0	3.45E-08	0	2.61E-08	0	0
MRGPRE	9.80E-08	0	0	0	0	0	0	0
MRGPRF	1.00E-05	2.23E-07	1.24E-06	3.37E-06	1.91E-05	7.25E-07	1.21E-06	6.89E-07
MRGPRG	0	0	0	0	0	0	0	0
MRGPRX1	0	0	0	0	0	0	0	0
MRGPRX2	0	0	0	0	0	0	0	0
MRGPRX3	3.00E-08	0	0	0	0	0	1.44E-08	0
MRGPRX4	1.73E-08	0	0	0	0	1.61E-08	0	2.55E-08
MRI1	6.64E-06	3.66E-06	4.75E-06	4.93E-06	3.38E-06	7.52E-06	4.19E-06	5.53E-06
MRM1	8.05E-06	4.12E-06	3.12E-06	3.29E-06	9.77E-06	5.69E-06	7.20E-06	4.21E-06
MRO	6.54E-08	1.90E-07	1.46E-06	1.35E-06	1.17E-07	2.52E-06	4.78E-07	7.67E-08
MRP63	2.89E-05	2.28E-05	2.13E-05	1.69E-05	2.69E-05	2.74E-05	2.02E-05	1.44E-05
MRPL1	1.52E-05	9.12E-06	1.39E-05	1.67E-05	9.08E-06	2.27E-05	2.38E-05	2.67E-05
MRPL10	4.93E-05	3.99E-05	1.77E-05	2.08E-05	1.51E-05	4.36E-05	2.54E-05	2.46E-05
MRPL11	2.39E-05	3.72E-05	1.71E-05	2.40E-05	3.88E-05	2.82E-05	3.75E-05	1.52E-05
MRPL12	0.000178	0.000332	0.000108	0.00011	0.000213	0.000144	0.00019	0.000111
MRPL13	0.000174	7.56E-05	2.43E-05	2.96E-05	6.88E-05	4.85E-05	7.50E-05	4.67E-05
MRPL14	0.00011	0.00011	8.92E-05	8.22E-05	0.000173	0.000103	0.000142	0.000142
MRPL15	0.00012	5.10E-05	3.05E-05	3.21E-05	3.37E-05	3.61E-05	8.39E-05	7.50E-05
MRPL16	5.88E-05	7.56E-05	5.09E-05	6.06E-05	4.42E-05	6.29E-05	4.54E-05	8.60E-05
MRPL17	1.91E-05	1.19E-05	8.03E-06	1.15E-05	1.58E-05	1.72E-05	1.83E-05	1.85E-05
MRPL18	8.59E-05	0.000105	3.83E-05	3.75E-05	2.23E-05	7.35E-05	5.85E-05	3.82E-05
MRPL19	4.17E-05	5.28E-05	3.03E-05	2.37E-05	7.39E-06	5.39E-05	2.55E-05	2.90E-05
MRPL2	6.54E-05	8.41E-05	3.62E-05	4.03E-05	6.90E-05	5.54E-05	7.02E-05	7.52E-05
MRPL20	0.000125	0.000132	0.000143	0.000101	0.000117	0.00017	9.37E-05	0.00011
MRPL21	8.18E-05	0.000139	4.32E-05	4.64E-05	7.02E-05	7.56E-05	5.70E-05	0.000103
MRPL22	1.06E-05	5.56E-06	6.18E-06	6.55E-06	4.49E-06	8.84E-06	8.19E-06	1.09E-05
MRPL23	7.31E-05	7.57E-05	6.16E-05	6.74E-05	7.30E-05	8.22E-05	9.70E-05	8.74E-05
MRPL24	0.000116	0.000214	8.40E-05	8.93E-05	0.000152	0.000243	0.000182	0.000239
MRPL27	0.000186	0.000129	7.01E-05	9.16E-05	0.000271	0.00014	9.85E-05	8.52E-05
MRPL28	8.92E-05	0.000128	4.71E-05	6.60E-05	0.000147	7.28E-05	0.000106	8.73E-05
MRPL3	3.54E-05	4.75E-05	2.98E-05	2.98E-05	1.25E-05	4.21E-05	3.10E-05	3.30E-05
MRPL30	3.10E-05	1.76E-05	1.36E-05	1.37E-05	8.82E-06	1.92E-05	1.61E-05	1.13E-05
MRPL32	9.72E-05	5.39E-05	7.01E-05	6.00E-05	4.98E-05	6.77E-05	6.27E-05	8.42E-05
MRPL33	0.000262	0.000124	4.30E-05	5.58E-05	5.43E-05	0.0001	8.31E-05	3.10E-05
MRPL34	8.63E-05	9.53E-05	9.65E-05	9.16E-05	0.000124	9.36E-05	8.26E-05	6.81E-05

MRPL35	3.14E-05	2.45E-05	1.20E-05	1.08E-05	9.84E-06	1.53E-05	1.89E-05	1.81E-05
MRPL36	7.50E-05	6.08E-05	4.15E-05	3.99E-05	3.16E-05	6.91E-05	4.78E-05	7.65E-05
MRPL37	7.86E-05	7.02E-05	8.22E-05	7.67E-05	7.26E-05	9.60E-05	7.84E-05	6.17E-05
MRPL38	5.88E-05	8.55E-05	4.66E-05	4.10E-05	7.21E-05	7.54E-05	6.33E-05	5.09E-05
MRPL39	2.07E-05	1.93E-05	2.61E-05	2.55E-05	9.49E-06	2.84E-05	2.50E-05	2.65E-05
MRPL4	6.26E-05	5.70E-05	4.63E-05	5.01E-05	8.61E-05	6.56E-05	8.51E-05	5.06E-05
MRPL40	0.000111	0.000161	7.07E-05	7.44E-05	0.000183	0.000145	9.63E-05	9.54E-05
MRPL41	0.000207	0.000154	0.000144	0.000135	0.000323	0.000145	0.000184	0.000221
MRPL42	1.86E-05	2.24E-05	1.25E-05	1.36E-05	5.23E-06	1.92E-05	1.20E-05	1.41E-05
MRPL42P5	8.20E-07	6.56E-08	0	6.36E-07	7.90E-08	#######	3.00E-07	5.46E-07
MRPL43	8.97E-05	6.50E-05	5.87E-05	5.70E-05	8.62E-05	8.92E-05	6.70E-05	8.07E-05
MRPL44	3.80E-05	2.19E-05	2.49E-05	2.68E-05	1.41E-05	3.78E-05	2.70E-05	2.67E-05
MRPL45	5.20E-05	4.58E-05	2.21E-05	2.40E-05	1.68E-05	4.27E-05	3.35E-05	2.28E-05
MRPL46	4.25E-05	6.79E-05	4.95E-05	6.75E-05	6.21E-05	9.50E-05	7.34E-05	5.54E-05
MRPL47	4.55E-05	3.47E-05	1.49E-05	2.26E-05	2.27E-05	2.44E-05	2.85E-05	2.64E-05
MRPL48	3.45E-05	3.05E-05	1.46E-05	1.90E-05	1.57E-05	2.31E-05	2.42E-05	3.37E-05
MRPL49	4.73E-05	6.04E-05	3.02E-05	2.76E-05	1.27E-05	4.58E-05	3.16E-05	5.10E-05
MRPL50	4.09E-05	1.68E-05	1.72E-05	1.75E-05	7.60E-06	3.22E-05	1.54E-05	2.09E-05
MRPL51	0.0002	0.000201	0.000111	0.000145	0.00019	0.000226	0.00024	0.000156
MRPL52	4.89E-05	4.23E-05	1.73E-05	2.83E-05	7.19E-05	3.11E-05	4.08E-05	2.92E-05
MRPL53	0.000108	0.00012	7.43E-05	6.86E-05	0.000123	6.65E-05	7.90E-05	9.34E-05
MRPL54	0.000106	4.71E-05	9.94E-05	0.000106	0.000209	0.000111	0.000113	8.40E-05
MRPL55	8.60E-05	0.000154	5.80E-05	5.66E-05	0.00011	0.000122	8.08E-05	0.000136
MRPL9	4.13E-05	3.53E-05	2.10E-05	2.56E-05	2.18E-05	5.15E-05	3.94E-05	5.31E-05
MRPS10	2.39E-05	3.78E-05	2.09E-05	1.70E-05	1.18E-05	2.68E-05	2.46E-05	2.65E-05
MRPS11	5.44E-05	5.65E-05	2.41E-05	2.91E-05	5.77E-05	4.88E-05	3.74E-05	3.59E-05
MRPS12	0.000104	7.99E-05	2.45E-05	3.17E-05	6.86E-05	5.10E-05	4.95E-05	4.89E-05
MRPS14	2.28E-05	3.30E-05	1.75E-05	1.64E-05	1.07E-05	3.01E-05	1.70E-05	2.98E-05
MRPS15	0.000107	0.000113	7.54E-05	7.12E-05	5.71E-05	0.000108	6.33E-05	0.000116
MRPS16	5.78E-05	5.59E-05	3.21E-05	2.90E-05	4.07E-05	3.76E-05	3.22E-05	3.53E-05
MRPS17	6.90E-05	2.60E-05	2.34E-05	2.88E-05	3.73E-05	3.09E-05	1.85E-05	6.81E-05
MRPS18A	5.60E-05	6.80E-05	3.93E-05	3.99E-05	0.000125	5.64E-05	7.14E-05	6.81E-05
MRPS18B	9.34E-05	0.000184	6.78E-05	5.97E-05	6.57E-05	0.000115	0.000116	8.60E-05
MRPS18C	3.76E-05	2.52E-05	2.64E-05	2.59E-05	2.21E-05	3.43E-05	3.95E-05	3.94E-05
MRPS2	3.62E-05	3.16E-05	4.72E-05	4.50E-05	2.84E-05	5.35E-05	5.01E-05	7.33E-05
MRPS21	0.00017	0.000104	8.33E-05	0.000131	0.000146	0.000229	0.000154	0.000222
MRPS22	4.15E-05	4.69E-05	3.75E-05	3.24E-05	2.14E-05	4.95E-05	4.18E-05	3.34E-05
MRPS23	7.20E-05	7.25E-05	2.02E-05	3.04E-05	2.29E-05	7.23E-05	3.96E-05	4.01E-05
MRPS24	0.000208	0.000239	0.000115	0.000118	0.000313	0.000145	0.000155	0.000188
MRPS25	2.38E-05	2.09E-05	8.66E-06	9.93E-06	1.33E-05	1.23E-05	1.57E-05	9.79E-06
MRPS26	7.72E-05	7.93E-05	4.12E-05	5.02E-05	8.15E-05	6.51E-05	6.49E-05	4.88E-05
MRPS27	2.99E-05	3.19E-05	2.21E-05	2.01E-05	7.74E-06	4.40E-05	2.69E-05	3.45E-05
MRPS28	0.000103	6.36E-05	4.73E-05	4.72E-05	6.78E-05	7.15E-05	0.000133	5.96E-05
MRPS30	1.82E-05	2.81E-05	1.38E-05	1.30E-05	5.22E-06	1.74E-05	1.61E-05	2.14E-05
MRPS31	1.66E-05	2.30E-05	1.89E-05	1.61E-05	9.69E-06	2.78E-05	1.99E-05	2.34E-05
MRPS33	7.18E-05	0.000115	5.32E-05	6.16E-05	7.65E-05	9.33E-05	8.83E-05	9.56E-05
MRPS34	9.27E-05	0.000124	5.35E-05	4.94E-05	8.08E-05	7.99E-05	7.31E-05	7.51E-05
MRPS35	4.76E-05	7.94E-05	4.50E-05	3.83E-05	2.17E-05	6.25E-05	4.52E-05	5.04E-05
MRPS36	4.20E-05	3.86E-05	2.23E-05	1.78E-05	1.30E-05	1.76E-05	2.14E-05	2.63E-05
MRPS5	0.000107	7.25E-05	3.28E-05	3.46E-05	4.80E-05	6.57E-05	4.15E-05	5.09E-05
MRPS6	2.93E-05	1.52E-05	2.35E-05	2.44E-05	1.23E-05	1.93E-05	2.46E-05	1.76E-05
MRPS7	9.64E-05	7.40E-05	3.92E-05	4.05E-05	6.50E-05	9.23E-05	6.22E-05	4.48E-05
MRPS9	3.13E-05	2.83E-05	1.64E-05	2.10E-05	2.02E-05	2.69E-05	2.48E-05	2.09E-05
MRRF	2.76E-05	1.11E-05	8.51E-06	8.34E-06	2.64E-06	1.91E-05	8.61E-06	2.51E-05
MRS2	1.65E-05	1.92E-05	9.29E-06	6.81E-06	4.68E-06	1.21E-05	6.65E-06	1.44E-05
MRS2P2	1.74E-08	3.91E-08	7.21E-08	5.31E-08	1.49E-08	0	3.37E-08	0
MRTO4	2.18E-05	1.69E-05	7.73E-06	1.22E-05	9.22E-06	1.50E-05	1.47E-05	1.33E-05
MRVI1	2.44E-06	4.67E-07	3.15E-07	6.83E-07	8.09E-07	6.57E-07	1.13E-06	8.48E-07

MS4A1	5.31E-07	1.85E-08	2.14E-07	4.27E-07	0	3.82E-07	2.41E-07	2.38E-07
MS4A10	0	0	0	2.52E-08	0	0	0	0
MS4A12	0	0	0	0	0	0	0	0
MS4A13	0	0	0	0	0	0	0	0
MS4A14	1.02E-06	1.29E-07	1.39E-07	4.43E-07	7.59E-08	1.97E-07	3.95E-07	4.01E-07
MS4A15	0	1.66E-08	0	0	0	0	0	0
MS4A2	5.53E-08	1.38E-08	3.80E-08	6.75E-08	0	5.69E-09	2.39E-08	3.71E-08
MS4A3	0	0	6.02E-08	0	0	0	0	0
MS4A4A	1.69E-05	2.78E-06	9.77E-06	1.75E-05	2.56E-06	9.97E-06	2.48E-05	3.44E-06
MS4A5	0	0	0	0	0	0	0	0
MS4A6A	4.71E-05	5.44E-06	5.09E-05	6.28E-05	6.32E-06	1.98E-05	4.51E-05	8.91E-06
MS4A6E	0	0	0	1.73E-07	0	0	4.31E-08	0
MS4A7	1.06E-05	3.04E-06	1.46E-05	2.14E-05	2.00E-06	8.44E-06	1.08E-05	5.48E-06
MS4A8B	1.87E-08	0	0	0	0	1.74E-08	0	0
MSC	1.84E-06	2.04E-07	7.41E-07	6.00E-06	5.89E-07	5.48E-07	1.92E-06	5.30E-06
MSGN1	0	0	0	0	0	0	0	0
MSH2	1.09E-05	1.51E-05	2.29E-06	2.81E-06	1.41E-06	6.45E-06	3.48E-06	3.80E-06
MSH3	9.56E-06	4.27E-06	3.70E-06	3.72E-06	1.43E-06	6.96E-06	2.96E-06	5.68E-06
MSH4	0	1.62E-08	1.49E-08	8.80E-09	0	0	1.40E-08	2.53E-07
MSH5	4.08E-06	3.25E-06	6.19E-07	1.45E-06	6.29E-07	2.77E-06	1.87E-06	1.69E-06
MSH6	3.42E-05	2.92E-05	7.58E-06	7.13E-06	3.95E-06	1.23E-05	7.66E-06	1.32E-05
MSI1	2.93E-06	3.45E-06	3.25E-08	4.80E-08	8.64E-07	7.28E-09	3.27E-06	2.07E-07
MSI2	3.04E-05	3.97E-06	1.67E-06	2.51E-06	1.34E-07	4.73E-06	3.50E-07	3.97E-06
MSL1	4.48E-05	3.24E-05	1.70E-05	2.61E-05	1.45E-05	2.95E-05	3.13E-05	2.63E-05
MSL2	6.76E-06	5.46E-06	3.86E-06	5.15E-06	2.81E-06	4.59E-06	5.04E-06	4.79E-06
MSL3	1.16E-05	3.85E-06	4.04E-06	6.02E-06	3.37E-06	6.92E-06	7.43E-06	6.85E-06
MSL3L2	3.86E-07	5.46E-08	1.66E-06	1.51E-06	1.04E-08	1.15E-06	1.62E-06	3.58E-06
MSLN	2.97E-06	2.78E-08	0	5.35E-08	1.87E-08	0	3.56E-08	0
MSLNL	0	0	0	0	0	0	0	0
MSMB	7.91E-08	0	0	0	0	1.51E-07	4.13E-07	3.70E-07
MSMP	2.06E-06	3.86E-06	4.68E-07	6.72E-07	5.86E-07	9.26E-07	1.55E-06	1.69E-06
MSN	9.80E-05	4.26E-05	3.96E-05	7.68E-05	1.68E-05	8.06E-05	6.86E-05	6.23E-05
MSR1	8.82E-06	1.87E-06	4.77E-06	1.71E-05	9.98E-07	2.80E-06	6.64E-06	1.40E-06
MSRA	5.03E-06	3.09E-05	3.05E-05	1.84E-05	1.73E-05	3.28E-05	1.92E-05	7.26E-06
MSRB2	3.19E-05	3.53E-05	2.32E-05	3.36E-05	5.07E-05	3.27E-05	3.92E-05	3.30E-05
MSRB3	1.10E-05	1.28E-06	9.08E-07	2.77E-06	7.81E-07	9.67E-07	1.62E-06	2.25E-06
MST1	0.000169	0.000188	0.00017	0.00017	0.000187	0.000211	0.000154	0.00013
MST1P2	4.59E-05	4.17E-05	1.81E-05	1.67E-05	2.19E-05	3.65E-05	1.45E-05	1.60E-05
MST1P9	1.29E-05	4.90E-06	2.84E-06	2.36E-06	3.06E-06	8.02E-06	3.26E-06	1.06E-06
MST1R	5.49E-06	8.79E-08	1.31E-07	7.07E-07	5.40E-08	5.94E-08	3.12E-07	1.00E-06
MST4	7.21E-06	4.40E-06	2.08E-06	2.26E-06	9.36E-07	9.45E-07	8.62E-07	1.65E-06
MSTN	3.30E-08	9.28E-09	8.55E-09	1.01E-08	2.13E-08	5.35E-08	0	1.21E-08
MSTO1	9.85E-06	1.54E-05	3.48E-06	4.16E-06	3.56E-06	2.32E-05	7.36E-06	1.66E-05
MSTO2P	3.07E-06	3.13E-06	3.72E-07	5.25E-07	4.36E-07	1.06E-06	7.29E-07	4.60E-06
MSX1	8.20E-07	9.85E-07	9.12E-08	3.96E-07	7.70E-07	7.38E-07	1.94E-06	1.54E-06
MSX2	2.15E-08	3.64E-08	3.37E-08	6.58E-08	9.25E-09	0	7.28E-08	0
MSX2P1	2.20E-08	0	1.15E-08	0	9.47E-09	0	1.07E-08	0
MT1A	1.46E-05	5.60E-05	0.00011	0.000106	1.78E-06	1.14E-05	7.13E-05	0.000166
MT1B	1.13E-07	0	1.49E-06	1.47E-07	0	1.09E-07	0	0
MT1DP	3.46E-06	1.34E-05	2.11E-06	4.49E-06	1.11E-07	1.43E-06	2.02E-06	3.38E-06
MT1E	0.000163	0.000305	0.001179	0.00178	4.20E-05	7.96E-05	0.000656	0.000333
MT1F	8.84E-05	3.27E-05	0.000467	0.000295	1.30E-06	6.73E-06	1.77E-05	1.16E-05
MT1G	6.94E-05	7.67E-05	0.004595	0.00182	2.14E-06	5.60E-05	0.000411	1.22E-05
MT1H	5.07E-06	2.77E-07	0.000542	0.000142	0	4.00E-07	9.41E-06	2.97E-06
MT1IP	0	0	0	0	0	0	0	0
MT1L	2.83E-05	1.19E-06	3.57E-05	6.80E-05	6.66E-06	2.71E-06	4.59E-06	7.17E-07
MT1M	1.22E-05	2.48E-05	0.000201	0.000293	1.68E-06	3.87E-06	1.43E-05	1.21E-06
MT1X	0.000117	0.000225	0.003199	0.001655	8.05E-05	0.000729	0.000942	0.001447

MT2A	0.000707	0.00135	0.009363	0.011072	0.000385	0.000838	0.004422	0.003601
MT3	1.05E-06	2.69E-07	0	3.47E-07	0	0	1.02E-07	0
MT4	0	0	0	0	0	0	0	0
MTA1	2.39E-05	9.58E-06	1.11E-05	1.32E-05	9.79E-06	1.32E-05	1.41E-05	1.17E-05
MTA2	3.06E-05	2.65E-05	1.21E-05	2.10E-05	8.66E-06	1.51E-05	1.97E-05	2.53E-05
MTA3	1.57E-05	1.60E-05	4.09E-06	6.92E-06	2.18E-06	7.28E-06	5.66E-06	6.56E-06
MTAP	4.55E-06	7.93E-06	4.76E-06	6.07E-06	1.41E-06	5.48E-06	2.66E-06	4.04E-06
MTBP	4.37E-06	1.00E-06	1.02E-07	1.07E-07	8.44E-08	4.48E-07	3.45E-07	2.62E-07
MTCH1	0.000135	0.000182	5.03E-05	5.94E-05	4.07E-05	7.55E-05	6.37E-05	7.71E-05
MTCH2	7.12E-05	0.000119	6.93E-05	5.34E-05	3.58E-05	6.28E-05	4.33E-05	9.78E-05
MTCP1	1.04E-06	1.53E-07	3.47E-07	6.39E-07	8.99E-08	4.02E-07	2.63E-07	3.04E-07
MTCP1NB	1.37E-05	2.33E-05	2.02E-05	1.88E-05	4.63E-05	1.86E-05	2.05E-05	2.04E-05
MTDH	6.42E-05	4.74E-05	1.99E-05	1.75E-05	1.49E-05	2.58E-05	4.74E-05	2.65E-05
MTERF	5.93E-05	8.70E-06	3.81E-06	3.42E-06	1.60E-06	3.93E-06	3.00E-06	4.68E-06
MTERFD1	2.32E-05	1.78E-05	8.44E-06	9.22E-06	1.01E-05	1.39E-05	2.90E-05	1.94E-05
MTERFD2	2.56E-05	8.08E-06	6.90E-06	6.73E-06	6.02E-06	1.13E-05	6.54E-06	5.83E-06
MTERFD3	8.29E-06	8.71E-06	7.87E-06	7.89E-06	7.55E-06	7.90E-06	7.38E-06	1.06E-05
MTF1	2.20E-06	9.79E-07	7.57E-07	1.23E-06	3.85E-07	1.76E-06	1.20E-06	1.64E-06
MTF2	6.61E-06	3.31E-06	2.35E-06	2.18E-06	1.38E-06	2.91E-06	1.73E-06	1.98E-06
MTFMT	4.47E-06	3.64E-06	3.97E-06	4.65E-06	2.52E-06	4.09E-06	5.11E-06	3.63E-06
MTFR1	2.39E-05	3.29E-05	1.75E-05	1.05E-05	1.45E-05	2.04E-05	3.20E-05	2.52E-05
MTG1	3.74E-05	1.53E-05	1.65E-05	2.72E-05	5.60E-05	3.21E-05	2.81E-05	2.50E-05
MTHFD1	4.08E-05	0.000107	0.000157	9.92E-05	7.07E-05	0.000339	8.86E-05	6.21E-05
MTHFD1L	6.43E-06	4.08E-06	8.99E-07	3.10E-06	7.70E-07	3.36E-06	5.71E-06	5.16E-06
MTHFD2	7.77E-06	9.14E-07	9.93E-07	6.77E-06	8.52E-07	2.42E-06	2.07E-06	1.96E-06
MTHFD2L	1.23E-05	1.70E-06	9.31E-06	3.89E-06	2.60E-06	3.18E-06	2.94E-06	4.43E-06
MTHFR	1.49E-05	3.79E-06	1.71E-06	2.76E-06	1.21E-06	2.10E-06	4.01E-06	5.24E-06
MTHFS	0.000105	0.000191	0.000157	0.000129	0.000158	9.65E-05	0.000191	0.000188
MTHFSD	4.83E-06	3.44E-06	2.25E-06	3.00E-06	9.88E-07	5.13E-06	3.02E-06	2.20E-06
MTIF2	3.33E-05	4.73E-05	1.56E-05	1.34E-05	1.12E-05	2.86E-05	1.50E-05	1.79E-05
MTIF3	5.89E-05	3.86E-05	3.27E-05	3.44E-05	5.27E-05	5.34E-05	4.10E-05	4.84E-05
MTL5	1.31E-06	6.40E-07	9.24E-08	5.56E-07	3.48E-08	1.46E-08	2.31E-07	1.44E-06
MTM1	6.14E-06	9.20E-06	8.55E-06	5.06E-06	2.50E-06	6.41E-06	3.90E-06	8.93E-06
MTMR1	8.60E-06	7.08E-06	4.33E-06	3.77E-06	3.00E-06	6.51E-06	3.43E-06	6.84E-06
MTMR10	1.60E-05	2.06E-05	1.58E-05	1.10E-05	9.20E-06	2.26E-05	9.37E-06	7.42E-06
MTMR11	6.14E-06	2.86E-06	5.14E-07	4.53E-06	8.38E-08	6.44E-06	9.48E-06	7.72E-07
MTMR12	1.01E-05	8.53E-06	7.30E-06	7.89E-06	1.05E-06	7.72E-06	4.66E-06	7.20E-06
MTMR14	2.93E-05	2.03E-05	1.71E-05	2.27E-05	2.11E-05	2.66E-05	2.83E-05	1.80E-05
MTMR15	4.47E-06	5.87E-06	4.14E-06	2.67E-06	3.49E-06	4.88E-06	2.86E-06	2.91E-06
MTMR2	4.47E-06	2.73E-06	1.33E-06	2.97E-06	7.11E-07	2.06E-06	1.83E-06	1.65E-06
MTMR3	5.19E-06	2.77E-06	2.43E-06	2.66E-06	1.55E-06	4.23E-06	3.04E-06	2.43E-06
MTMR4	2.36E-05	5.28E-05	1.16E-05	8.10E-06	9.09E-06	3.08E-05	7.98E-06	6.69E-06
MTMR6	5.60E-06	6.06E-06	5.36E-06	5.33E-06	8.97E-07	6.04E-06	3.41E-06	6.06E-06
MTMR7	5.08E-07	1.02E-05	4.31E-07	2.66E-07	1.11E-07	3.48E-07	2.03E-07	5.06E-07
MTMR8	3.06E-07	9.34E-08	3.38E-08	1.16E-07	0	1.48E-08	1.54E-08	0
MTMR9	1.68E-06	1.77E-06	1.30E-06	1.51E-06	2.65E-07	1.16E-06	6.47E-07	8.69E-07
MTMR9L	2.75E-06	4.56E-07	9.17E-07	8.69E-07	1.44E-06	5.31E-07	9.63E-07	2.22E-06
MTNR1A	2.13E-07	5.46E-08	0	0	0	0	0	3.52E-08
MTNR1B	0	3.35E-08	0	0	0	0	0	0
MTO1	1.42E-05	2.79E-05	1.12E-05	7.44E-06	2.29E-06	1.50E-05	8.23E-06	6.93E-06
MTOR	1.77E-05	2.90E-05	1.03E-05	1.33E-05	5.17E-06	1.39E-05	1.20E-05	2.14E-05
MTP18	4.47E-05	7.14E-05	8.73E-06	2.18E-05	3.90E-05	8.99E-06	2.78E-05	5.19E-05
MTPAP	5.57E-06	4.83E-06	3.38E-06	3.38E-06	1.30E-06	4.77E-06	3.82E-06	4.67E-06
MTR	3.78E-06	2.02E-06	2.05E-06	3.32E-06	4.98E-07	3.90E-06	1.48E-06	6.17E-06
MTRF1	6.03E-06	4.49E-06	3.44E-06	2.47E-06	1.02E-06	5.42E-06	2.50E-06	4.78E-06
MTRF1L	5.63E-06	4.28E-06	2.58E-06	2.90E-06	1.14E-06	3.59E-06	2.94E-06	2.18E-06
MTRR	1.08E-05	1.45E-05	9.94E-06	9.90E-06	9.91E-07	1.19E-05	6.45E-06	1.10E-05
MTSS1	6.23E-05	4.63E-05	3.76E-05	2.79E-05	2.36E-05	3.68E-05	6.35E-05	4.08E-05

MTSS1L	2.16E-05	3.35E-05	2.11E-05	2.97E-05	7.25E-06	1.98E-05	2.17E-05	2.32E-05
MTTP	4.97E-05	0.000101	0.000103	7.12E-05	2.67E-05	9.30E-05	4.01E-05	3.19E-05
MTUS1	7.02E-05	6.01E-05	3.57E-05	2.18E-05	3.53E-06	6.38E-05	1.04E-05	1.69E-05
MTUS2	3.79E-07	7.65E-07	3.44E-07	2.98E-07	5.85E-09	0	4.78E-07	4.97E-08
MTVR2	3.47E-08	0	0	0	3.02E-08	0	0	0
MTX1	3.01E-05	5.54E-05	2.03E-05	2.50E-05	2.38E-05	4.50E-05	2.91E-05	4.72E-05
MTX2	3.84E-05	2.45E-05	1.36E-05	1.84E-05	1.07E-05	1.77E-05	1.71E-05	2.02E-05
MTX3	2.86E-06	3.38E-06	1.31E-06	1.33E-06	3.82E-07	2.91E-06	1.28E-06	2.39E-06
MUC1	2.24E-06	2.82E-06	1.18E-07	6.56E-07	2.84E-07	2.56E-07	3.64E-06	1.53E-06
MUC12	1.26E-07	7.79E-07	0	3.38E-07	0	1.07E-08	3.33E-08	3.38E-08
MUC13	0.000245	0.000153	3.36E-08	2.37E-06	7.67E-08	4.78E-06	4.88E-07	2.24E-05
MUC15	3.56E-08	2.74E-07	2.15E-08	4.34E-08	0	0	6.90E-09	2.08E-08
MUC16	7.74E-09	1.00E-07	0	9.17E-09	0	6.95E-09	4.86E-10	0
MUC17	0	0	0	0	0	0	0	0
MUC2	8.49E-08	5.73E-09	0	6.27E-09	0	0	0	0
MUC20	1.31E-06	6.89E-06	2.56E-06	2.14E-05	3.64E-06	5.98E-06	8.84E-06	4.17E-07
MUC21	0	0	0	0	0	0	0	0
MUC4	4.60E-08	2.66E-07	7.48E-08	9.13E-08	1.20E-07	1.21E-07	8.89E-08	1.00E-07
MUC5B	7.87E-06	0	5.96E-08	2.24E-08	0	2.26E-09	2.74E-08	2.52E-07
MUC6	2.22E-08	0	1.42E-06	4.42E-06	2.38E-09	7.70E-09	5.42E-09	3.65E-08
MUC7	0	0	0	0	0	0	0	0
MUCL1	0	0	0	0	0	1.38E-07	6.62E-08	0
MUDENG	1.84E-05	1.09E-05	1.06E-05	9.44E-06	3.12E-06	1.18E-05	6.83E-06	1.17E-05
MUL1	3.71E-05	2.70E-05	1.48E-05	2.09E-05	1.03E-05	1.79E-05	1.51E-05	2.95E-05
MUM1	1.46E-05	6.43E-06	9.74E-06	1.37E-05	2.00E-05	9.47E-06	1.59E-05	4.58E-06
MUM1L1	4.14E-07	2.58E-08	1.37E-06	5.70E-07	0	1.51E-08	5.60E-09	9.28E-08
MURC	2.14E-07	1.20E-07	6.48E-08	6.54E-08	1.53E-08	3.30E-08	1.30E-07	2.22E-07
MUS81	1.31E-05	1.11E-05	5.51E-06	7.47E-06	5.28E-06	8.63E-06	9.69E-06	9.42E-06
MUSK	0	0	9.45E-08	6.34E-08	0	0	0	0
MUSTN1	6.28E-06	2.84E-06	1.58E-06	3.37E-06	2.29E-05	4.35E-06	1.27E-06	3.62E-06
MUT	9.01E-05	0.000133	0.000112	4.35E-05	1.86E-05	5.28E-05	1.97E-05	6.59E-05
MUTED	1.08E-05	7.87E-06	5.49E-06	5.67E-06	2.56E-06	6.83E-06	5.44E-06	7.51E-06
MUTYH	1.24E-05	3.70E-06	2.98E-06	5.49E-06	3.03E-06	5.46E-06	6.64E-06	2.90E-06
MVD	3.87E-05	7.07E-05	1.52E-05	1.21E-05	1.73E-05	2.13E-05	1.89E-05	5.50E-05
MVK	4.13E-05	4.92E-05	3.82E-05	1.58E-05	2.32E-05	4.98E-05	2.83E-05	1.25E-05
MVP	0.000143	9.71E-05	4.13E-05	6.67E-05	1.32E-05	7.60E-05	8.93E-05	7.36E-05
MX1	4.64E-05	5.65E-06	1.69E-05	1.32E-05	1.89E-06	3.40E-05	1.20E-05	0.000117
MX2	1.53E-05	7.19E-07	1.86E-06	3.29E-06	5.31E-07	4.95E-06	2.51E-06	2.47E-05
MXD1	5.60E-06	1.66E-06	2.63E-06	3.40E-06	3.40E-07	1.42E-06	1.29E-06	1.63E-06
MXD3	1.21E-05	3.68E-06	1.33E-06	2.08E-06	5.52E-06	3.77E-06	3.39E-06	2.79E-06
MXD4	1.60E-05	1.73E-05	9.67E-06	1.24E-05	1.40E-05	1.59E-05	2.15E-05	1.00E-05
MXI1	1.52E-05	1.33E-05	1.79E-05	1.70E-05	6.20E-06	1.53E-05	1.56E-05	8.42E-06
MXRA5	1.29E-06	2.24E-07	1.83E-06	2.07E-06	1.32E-07	1.30E-07	8.16E-07	1.28E-06
MXRA7	5.09E-05	3.79E-05	1.12E-05	3.48E-05	2.58E-05	9.12E-06	2.32E-05	6.91E-06
MXRA8	0.000114	1.79E-05	2.60E-06	1.78E-05	2.85E-06	1.22E-06	7.54E-06	3.81E-06
MYADM	2.04E-05	1.96E-05	1.69E-05	0.000113	5.07E-06	1.45E-05	4.75E-05	1.85E-05
MYADML	0	0	0	0	0	0	0	0
MYADML2	5.75E-08	0	0	0	2.47E-08	1.16E-07	0	0
MYB	5.73E-07	4.24E-08	7.13E-08	6.17E-08	1.18E-07	8.66E-08	2.50E-07	6.54E-08
MYBBP1A	6.01E-06	1.04E-05	7.93E-06	6.93E-06	5.72E-06	1.79E-05	1.04E-05	1.09E-05
MYBL1	6.83E-06	3.00E-06	3.79E-07	4.98E-07	2.72E-07	2.79E-07	3.67E-07	5.07E-07
MYBL2	2.03E-05	8.75E-06	9.18E-08	3.68E-07	1.55E-06	7.96E-07	7.80E-07	1.31E-06
MYBPC1	2.52E-08	1.04E-07	2.64E-08	0	0	1.05E-07	0	0
MYBPC2	6.37E-09	1.43E-08	0	3.89E-08	0	0	0	2.80E-08
MYBPC3	2.16E-08	6.03E-09	0	6.58E-09	4.62E-09	0	5.24E-09	0
MYBPH	1.34E-08	0	1.27E-07	6.58E-08	0	1.12E-07	0	0
MYBPHL	0	2.87E-08	0	3.06E-08	0	8.25E-08	0	0
MYC	4.34E-05	4.40E-05	6.00E-05	0.000156	3.33E-06	4.09E-05	0.000107	1.55E-05

MYCBP	1.03E-05	7.28E-06	5.68E-06	6.73E-06	3.80E-06	1.05E-05	4.62E-06	7.52E-06
MYCBP2	1.24E-05	4.95E-06	7.32E-06	6.06E-06	1.37E-06	7.02E-06	4.80E-06	7.02E-06
MYCBPAP	2.17E-08	2.88E-08	0	0	1.86E-08	1.34E-08	2.16E-08	1.06E-08
MYCL1	1.96E-06	1.87E-06	8.07E-06	3.52E-06	3.63E-06	1.96E-05	2.78E-06	3.09E-06
MYCN	1.73E-07	5.42E-08	5.00E-08	1.53E-07	7.61E-07	6.24E-08	6.09E-08	9.32E-07
MYCNOS	0	0	0	0	4.21E-07	0	7.03E-08	5.60E-08
MYCT1	3.55E-06	3.20E-06	3.54E-06	2.32E-06	1.46E-06	1.62E-06	2.78E-06	3.25E-06
MYD88	3.11E-05	2.90E-05	3.48E-05	3.37E-05	8.38E-06	2.60E-05	2.34E-05	2.18E-05
MYEF2	1.34E-07	1.26E-07	6.05E-08	9.42E-08	0	3.91E-08	6.95E-08	4.55E-08
MYEOV	8.19E-08	1.87E-07	4.31E-08	3.56E-07	4.45E-08	2.13E-06	5.35E-07	0
MYEOV2	0.000184	7.47E-05	5.58E-05	7.03E-05	0.000193	9.97E-05	0.000104	6.85E-05
MYF5	0	0	0	0	0	0	0	0
MYF6	0	2.25E-08	0	2.42E-08	0	5.48E-08	1.88E-08	2.91E-08
MYH1	0	3.32E-08	0	4.54E-09	0	3.44E-09	0	5.44E-09
MYH10	9.22E-06	1.62E-05	1.18E-05	1.57E-05	1.48E-06	7.84E-06	5.17E-06	2.73E-06
MYH11	1.13E-05	2.14E-06	7.78E-07	2.79E-06	2.96E-06	1.39E-06	5.09E-07	3.69E-06
MYH13	0	9.50E-07	0	4.56E-09	0	0	0	3.04E-07
MYH14	0.000125	8.65E-05	3.47E-05	3.79E-05	5.01E-05	5.11E-05	5.06E-05	5.37E-05
MYH15	0	3.52E-09	6.44E-09	1.15E-08	2.70E-09	0	0	0
MYH16	6.27E-08	2.36E-08	0	0	0	0	1.01E-08	0
MYH2	3.68E-09	1.64E-08	0	0	0	0	0	0
MYH3	1.83E-07	6.65E-08	1.87E-07	2.86E-07	2.17E-07	7.32E-06	9.79E-08	1.25E-07
MYH4	1.30E-07	2.18E-05	0	0	0	0	0	3.99E-06
MYH6	3.78E-09	0	3.86E-09	0	0	0	0	0
MYH7	0	0	3.80E-09	0	0	0	0	0
MYH7B	9.26E-08	9.56E-08	1.35E-07	5.21E-08	4.27E-08	6.25E-08	1.18E-07	5.37E-08
MYH8	0	1.24E-08	0	0	0	0	0	0
MYH9	0.000175	0.000155	7.29E-05	0.000141	3.63E-05	0.000105	0.000103	9.33E-05
MYL1	0	0	0	0	0	0	0	0
MYL10	0	0	0	0	0	0	0	0
MYL12A	0.000635	0.000221	0.000221	0.000564	0.000109	0.000393	0.000502	0.000281
MYL12B	0.00091	0.000306	0.000264	0.000716	0.000274	0.00052	0.000666	0.000385
MYL2	0	0	0	0	0	0	3.57E-08	0
MYL3	6.44E-07	0	2.91E-07	1.12E-06	7.61E-08	2.73E-08	2.50E-07	4.37E-08
MYL4	1.30E-07	1.15E-07	7.27E-08	3.52E-07	4.52E-07	1.83E-07	1.54E-07	4.88E-08
MYL5	8.94E-06	4.19E-06	1.30E-05	8.57E-06	2.30E-05	2.34E-05	2.36E-05	1.02E-05
MYL6	0.001448	0.000602	0.000478	0.00113	0.001273	0.000795	0.001426	0.000739
MYL6B	4.06E-05	4.10E-05	1.27E-05	1.82E-05	1.63E-05	1.59E-05	2.04E-05	1.74E-05
MYL7	0	0	0	0	0	0	0	0
MYL9	0.000127	3.13E-05	2.35E-05	9.49E-05	6.35E-05	3.56E-05	8.19E-05	4.81E-05
MYLIP	7.70E-06	2.95E-06	1.43E-06	2.73E-06	8.39E-07	1.62E-06	2.48E-06	2.99E-06
MYLK	4.90E-05	1.79E-05	4.36E-05	3.79E-05	8.97E-06	4.84E-05	2.28E-05	1.10E-05
MYLK2	1.20E-07	7.58E-08	0	2.66E-08	1.30E-08	1.54E-08	1.92E-07	2.45E-08
MYLK3	7.15E-08	1.07E-07	4.54E-08	3.41E-08	0	2.87E-08	3.94E-08	1.36E-08
MYLK4	1.23E-07	2.62E-07	9.25E-08	7.56E-08	2.04E-08	1.23E-07	6.00E-08	2.30E-07
MYLPF	0	1.67E-07	0	0	2.84E-07	0	3.04E-07	0
MYNN	6.62E-06	4.53E-06	4.05E-06	3.89E-06	1.52E-06	4.64E-06	3.68E-06	5.13E-06
MYO10	4.11E-06	1.12E-06	2.26E-06	2.81E-06	3.72E-07	5.73E-07	1.25E-06	5.69E-07
MYO15A	5.46E-07	1.38E-07	1.87E-07	1.15E-07	9.42E-08	2.56E-07	4.11E-08	4.53E-08
MYO15B	0.00017	2.11E-05	4.66E-06	1.05E-05	6.40E-06	6.44E-06	1.67E-05	5.79E-06
MYO16	1.05E-07	1.06E-06	8.84E-07	6.88E-07	8.16E-08	1.39E-06	7.51E-07	6.06E-07
MYO18A	2.41E-05	3.79E-05	1.77E-05	1.80E-05	3.37E-06	1.06E-05	1.36E-05	2.06E-05
MYO18B	8.93E-08	2.73E-07	0	1.39E-08	0	6.02E-08	1.01E-07	5.49E-07
MYO19	2.09E-05	1.29E-05	3.23E-06	3.83E-06	3.34E-06	5.92E-06	4.11E-06	5.97E-06
MYO1A	1.64E-06	6.89E-07	1.49E-08	7.93E-08	2.04E-07	1.29E-07	1.40E-07	0
MYO1B	6.22E-05	0.000216	0.000134	0.000135	5.22E-05	0.000157	0.000112	9.76E-05
MYO1C	4.49E-05	4.51E-05	3.00E-05	4.01E-05	1.47E-05	3.03E-05	3.45E-05	4.90E-05
MYO1D	3.69E-05	2.95E-05	8.02E-06	1.63E-05	3.35E-06	1.62E-06	1.02E-05	5.17E-06

MYO1E	3.01E-05	1.88E-05	1.42E-05	9.27E-06	3.98E-06	8.55E-06	1.03E-05	1.86E-05
MYO1F	1.10E-05	1.52E-06	5.89E-06	9.56E-06	1.58E-06	4.14E-06	5.66E-06	1.57E-06
MYO1G	3.36E-06	2.46E-07	1.45E-06	4.31E-06	3.89E-07	1.21E-06	2.47E-06	7.35E-07
MYO1H	1.13E-08	0	0	0	1.95E-08	1.05E-08	3.28E-08	1.66E-08
MYO3A	1.49E-06	0	9.45E-09	1.89E-08	0	0	0	0
MYO3B	1.19E-07	2.59E-08	1.79E-08	4.77E-07	4.97E-09	9.51E-08	1.35E-07	8.48E-09
MYO5A	3.98E-06	1.14E-06	6.99E-07	2.30E-06	4.21E-07	1.39E-06	1.54E-06	1.39E-06
MYO5B	1.66E-05	4.97E-06	5.50E-06	8.05E-06	1.61E-06	8.47E-06	4.95E-06	4.39E-06
MYO5C	3.60E-06	3.39E-06	1.21E-06	1.67E-06	5.84E-07	4.08E-06	1.26E-06	7.46E-07
MYO6	2.42E-05	9.52E-06	5.32E-06	6.97E-06	1.11E-06	7.62E-06	3.54E-06	8.30E-06
MYO7A	2.16E-05	7.53E-06	7.56E-06	6.48E-06	5.62E-06	1.44E-06	6.37E-06	3.82E-07
MYO7B	1.71E-05	1.09E-06	3.08E-07	3.40E-06	2.59E-07	2.39E-07	2.22E-06	3.46E-07
MYO9A	3.67E-06	4.56E-07	1.16E-06	7.66E-07	3.41E-08	1.16E-06	2.59E-07	3.35E-07
MYO9B	2.89E-05	1.57E-05	9.22E-06	1.87E-05	1.73E-05	2.03E-05	2.25E-05	1.28E-05
MYOC	1.25E-08	0	1.32E-08	0	0	1.16E-08	1.21E-08	0
MYOCD	1.61E-07	4.75E-08	1.19E-08	5.80E-08	1.33E-08	7.38E-08	3.76E-09	6.54E-08
MYOD1	2.68E-07	0	0	0	0	0	0	0
MYOF	1.15E-05	1.48E-06	1.20E-06	4.28E-06	3.28E-07	1.57E-06	3.45E-06	1.47E-06
MYOG	0	0	0	0	0	0	0	0
MYOM1	3.77E-06	6.44E-06	1.08E-05	3.12E-06	5.40E-06	7.92E-06	2.33E-05	1.58E-05
MYOM2	1.71E-07	7.05E-06	1.06E-06	5.54E-06	3.89E-07	1.88E-06	4.66E-08	4.00E-08
MYOM3	6.96E-07	1.96E-08	4.61E-08	1.13E-08	8.21E-08	3.22E-08	0	1.18E-07
MYOT	0	0	3.81E-08	6.15E-08	0	0	0	0
MYOZ1	1.13E-07	7.33E-08	1.70E-08	2.37E-07	1.11E-07	1.49E-08	1.08E-07	7.12E-08
MYOZ2	1.81E-08	0	0	2.21E-08	0	3.35E-08	1.75E-08	1.33E-08
MYOZ3	9.88E-08	2.22E-08	0	1.05E-07	2.83E-08	6.10E-09	5.76E-08	2.90E-08
MYPN	4.56E-09	1.53E-08	4.68E-09	0	3.91E-09	8.44E-09	8.88E-09	0
MYPOP	7.79E-06	3.52E-06	1.96E-06	2.78E-06	4.79E-06	3.32E-06	3.77E-06	3.45E-06
MYRIP	2.14E-06	1.55E-05	8.73E-06	6.41E-06	8.15E-07	2.00E-05	5.06E-06	6.27E-07
MYSM1	4.15E-06	1.15E-06	5.44E-07	5.04E-07	9.26E-08	8.74E-07	1.00E-07	7.02E-07
MYST1	1.83E-05	3.31E-05	1.74E-05	1.72E-05	1.50E-05	1.73E-05	2.65E-05	1.49E-05
MYST2	2.07E-05	1.52E-05	5.98E-06	6.85E-06	3.50E-06	1.39E-05	7.96E-06	4.50E-06
MYST3	5.51E-06	4.80E-06	3.55E-06	3.02E-06	7.14E-07	3.93E-06	1.24E-06	3.35E-06
MYST4	4.45E-06	1.43E-06	2.14E-06	1.95E-06	5.03E-07	2.27E-06	1.21E-06	2.29E-06
MYT1	2.55E-08	1.81E-07	4.78E-08	1.01E-07	2.95E-08	0	2.21E-08	1.68E-08
MYT1L	0	1.64E-06	1.03E-08	0	0	0	6.07E-09	0
MZF1	5.58E-06	1.24E-06	2.50E-06	3.99E-06	3.76E-06	4.24E-06	3.32E-06	1.10E-06
N4BP1	7.12E-06	4.15E-06	2.94E-06	4.39E-06	1.64E-06	4.27E-06	3.85E-06	3.22E-06
N4BP2	1.37E-06	4.96E-06	1.77E-06	9.39E-07	2.53E-08	1.57E-06	1.48E-07	9.16E-07
N4BP2L1	1.57E-05	1.07E-05	1.59E-05	1.56E-05	4.82E-06	8.57E-06	1.29E-05	5.11E-06
N4BP2L2	2.77E-05	1.48E-05	1.36E-05	1.49E-05	1.03E-05	1.57E-05	1.43E-05	1.13E-05
N4BP3	1.08E-06	1.75E-07	5.36E-08	2.37E-07	1.48E-07	1.03E-07	1.43E-06	2.85E-07
N6AMT1	3.14E-06	2.24E-06	1.73E-06	1.92E-06	1.65E-06	2.61E-06	3.50E-06	1.93E-06
N6AMT2	1.06E-05	8.53E-06	7.50E-06	7.80E-06	8.12E-06	9.29E-06	1.15E-05	6.26E-06
NAA10	8.27E-05	6.48E-05	2.88E-05	3.97E-05	0.000147	4.62E-05	5.99E-05	6.46E-05
NAA11	0	0	0	0	0	0	0	0
NAA15	7.96E-06	5.58E-06	4.77E-06	5.81E-06	1.61E-06	6.63E-06	4.05E-06	6.55E-06
NAA16	3.06E-06	2.40E-06	1.54E-06	1.34E-06	6.28E-07	1.87E-06	1.03E-06	1.11E-06
NAA20	7.86E-05	9.97E-05	4.37E-05	4.81E-05	4.03E-05	0.00011	7.51E-05	7.72E-05
NAA25	3.91E-06	3.82E-06	1.79E-06	1.46E-06	4.83E-07	2.69E-06	1.34E-06	2.06E-06
NAA30	1.04E-05	6.45E-06	6.92E-06	5.64E-06	2.56E-06	7.98E-06	5.78E-06	6.40E-06
NAA35	6.91E-06	4.43E-06	4.27E-06	3.91E-06	6.76E-07	6.56E-06	2.14E-06	3.98E-06
NAA38	1.27E-05	1.40E-05	7.39E-06	1.13E-05	1.26E-05	1.53E-05	1.43E-05	1.09E-05
NAA40	1.07E-05	4.31E-06	1.27E-06	2.51E-06	6.21E-07	4.30E-06	1.69E-06	3.45E-06
NAA50	4.16E-05	4.36E-05	2.58E-05	2.03E-05	1.50E-05	2.41E-05	2.23E-05	2.02E-05
NAAA	4.08E-05	8.77E-06	4.22E-05	2.35E-05	1.01E-05	1.06E-05	1.03E-05	5.35E-06
NAALAD2	1.36E-07	2.84E-07	3.75E-07	2.20E-07	5.48E-08	4.57E-08	3.57E-08	1.73E-07
NAALADL	7.75E-07	2.43E-06	3.79E-07	5.83E-07	3.54E-07	1.77E-07	5.66E-07	1.01E-06

NAALADL	1.24E-07	3.49E-07	6.74E-07	8.81E-07	2.37E-08	1.07E-06	2.06E-07	3.36E-08
NAB1	3.35E-05	1.50E-05	8.86E-06	1.10E-05	3.74E-06	6.52E-06	7.23E-06	9.46E-06
NAB2	1.20E-05	1.38E-05	5.40E-06	1.48E-05	1.07E-05	6.19E-06	1.97E-05	9.78E-06
NACA	0.000695	0.00065	0.000377	0.000499	0.00038	0.000663	0.000664	0.000419
NACA2	1.57E-05	5.68E-06	1.29E-05	5.10E-06	9.20E-06	5.90E-06	2.11E-06	4.03E-06
NACAD	1.65E-06	2.12E-08	8.28E-08	2.20E-07	1.63E-08	2.64E-08	1.06E-07	6.95E-08
NACAP1	1.10E-05	7.68E-06	6.01E-06	1.16E-05	8.63E-06	1.37E-05	7.01E-06	1.06E-05
NACC1	2.12E-05	8.18E-06	5.72E-06	1.02E-05	7.45E-06	1.65E-05	1.27E-05	2.05E-05
NACC2	3.40E-06	2.00E-06	9.81E-07	1.27E-06	3.77E-07	2.05E-06	1.54E-06	3.96E-06
NADK	3.02E-05	3.93E-05	3.02E-05	1.95E-05	6.27E-06	8.51E-05	1.78E-05	3.27E-05
NADSYN1	2.13E-05	2.35E-05	1.27E-05	1.50E-05	7.01E-06	1.40E-05	9.49E-06	2.37E-05
NAE1	2.15E-05	1.26E-05	1.04E-05	1.27E-05	5.99E-06	1.67E-05	1.03E-05	2.00E-05
NAF1	4.19E-06	2.26E-06	2.22E-06	4.44E-06	3.73E-07	3.55E-06	3.10E-06	2.66E-06
NAGA	3.04E-05	3.19E-05	1.96E-05	1.55E-05	1.19E-05	1.11E-05	1.82E-05	3.62E-05
NAGK	3.91E-05	1.74E-05	2.04E-05	3.61E-05	1.61E-05	1.96E-05	2.24E-05	1.81E-05
NAGLU	3.32E-05	3.99E-05	3.96E-05	4.41E-05	6.32E-05	3.18E-05	4.07E-05	3.07E-05
NAGPA	1.30E-05	1.79E-05	6.72E-06	7.75E-06	9.72E-06	1.73E-05	1.11E-05	1.26E-05
NAGS	3.29E-05	6.06E-05	1.96E-05	1.59E-05	1.89E-05	4.25E-05	3.22E-05	2.63E-05
NAIF1	4.75E-06	4.03E-06	3.25E-06	3.73E-06	3.99E-06	4.34E-06	5.98E-06	3.50E-06
NAIP	7.44E-07	9.05E-08	2.02E-07	2.49E-07	3.32E-09	1.57E-07	1.28E-07	1.34E-07
NALCN	1.13E-07	7.19E-09	1.81E-07	5.71E-07	2.76E-09	2.98E-09	8.65E-08	1.85E-07
NAMPT	2.82E-05	0.000121	8.67E-05	6.20E-05	8.49E-06	5.50E-05	6.57E-05	0.000123
NANOG	0	0	0	1.39E-08	9.78E-09	1.05E-08	0	1.34E-07
NANOS1	1.16E-06	3.66E-07	4.57E-07	1.05E-06	1.07E-06	5.61E-07	1.66E-06	6.73E-07
NANOS2	0	0	0	1.94E-08	0	1.47E-08	0	0
NANOS3	1.43E-07	0	0	3.55E-08	1.49E-07	1.07E-07	0	4.28E-08
NANP	3.98E-06	4.22E-06	1.60E-06	1.68E-06	9.14E-07	2.78E-06	1.65E-06	2.11E-06
NANS	5.02E-05	2.47E-05	1.93E-05	3.15E-05	2.89E-05	4.90E-05	3.46E-05	2.81E-05
NAP1L1	0.000118	4.05E-05	3.96E-05	8.51E-05	3.34E-05	0.000114	6.20E-05	4.07E-05
NAP1L2	2.47E-06	4.59E-07	6.26E-07	5.33E-07	1.59E-08	1.00E-06	2.58E-08	5.69E-06
NAP1L3	3.22E-07	5.88E-08	6.96E-07	1.57E-06	4.49E-08	2.42E-08	1.60E-07	2.17E-07
NAP1L4	6.49E-05	4.72E-05	3.23E-05	4.00E-05	3.51E-05	5.70E-05	3.51E-05	4.24E-05
NAP1L5	6.15E-06	4.02E-06	4.29E-06	4.18E-06	5.29E-07	4.79E-06	2.66E-06	1.45E-06
NAP1L6	1.75E-07	0	0	1.43E-08	0	3.25E-08	0	0
NAPA	0.000135	0.000116	0.000107	0.00011	0.000153	0.000141	0.000147	0.00014
NAPB	4.65E-06	2.26E-06	2.07E-06	1.48E-06	1.12E-06	5.58E-06	1.71E-06	1.45E-06
NAPEPLD	3.58E-06	6.99E-06	3.92E-06	1.84E-06	1.93E-06	4.34E-06	2.68E-06	2.52E-06
NAPG	1.81E-05	9.56E-06	5.45E-06	6.77E-06	1.02E-06	6.48E-06	3.55E-06	7.14E-06
NAPRT1	0.000153	0.000208	7.84E-05	5.20E-05	0.000247	7.07E-05	0.000165	5.63E-05
NAPSA	3.61E-07	1.44E-07	2.31E-07	8.88E-08	9.96E-07	3.36E-07	3.81E-07	1.60E-07
NAPSB	7.52E-06	3.49E-07	1.22E-05	2.33E-05	7.88E-07	4.25E-06	3.96E-06	1.01E-06
NARF	3.48E-05	3.24E-05	1.31E-05	1.57E-05	1.53E-05	2.46E-05	1.31E-05	2.06E-05
NARFL	2.05E-05	2.83E-05	1.04E-05	1.29E-05	1.28E-05	1.53E-05	1.94E-05	1.86E-05
NARG2	5.97E-06	3.58E-06	3.37E-06	3.30E-06	9.32E-07	4.50E-06	1.51E-06	2.17E-06
NARS	0.000126	6.19E-05	2.97E-05	3.54E-05	1.04E-05	6.39E-05	3.43E-05	4.64E-05
NARS2	1.36E-05	4.51E-06	9.74E-06	6.96E-06	4.02E-06	2.07E-05	8.47E-06	8.28E-06
NASP	3.95E-05	2.19E-05	1.60E-05	2.23E-05	2.00E-05	1.88E-05	1.87E-05	1.72E-05
NAT1	2.67E-06	5.85E-06	5.05E-06	5.01E-06	1.45E-06	3.22E-06	2.81E-06	1.91E-06
NAT10	1.44E-05	1.34E-05	6.39E-06	7.10E-06	4.00E-06	1.15E-05	9.46E-06	1.31E-05
NAT14	1.35E-05	7.73E-07	1.14E-06	4.06E-06	1.89E-05	1.31E-06	6.00E-06	5.17E-06
NAT15	4.48E-05	8.90E-05	2.97E-05	2.43E-05	3.22E-05	4.65E-05	4.79E-05	3.60E-05
NAT2	1.56E-05	2.60E-06	5.83E-05	3.37E-05	6.24E-06	3.32E-05	6.40E-06	1.42E-07
NAT6	2.60E-05	1.50E-05	5.18E-06	4.42E-06	6.35E-06	1.34E-05	4.23E-06	6.67E-06
NAT8	6.84E-05	2.15E-05	0.000113	0.000117	0.000258	1.78E-05	4.18E-05	0.000175
NAT8B	5.10E-07	3.28E-06	1.33E-06	9.29E-07	1.03E-05	1.27E-07	1.03E-06	3.26E-06
NAT8L	1.11E-07	1.59E-07	3.15E-08	3.70E-08	8.66E-09	0	3.90E-08	1.48E-08
NAT9	2.27E-05	2.20E-05	5.78E-06	8.51E-06	1.24E-05	1.20E-05	1.33E-05	8.52E-06
NAV1	2.72E-06	1.17E-06	2.86E-07	5.00E-07	2.08E-07	5.82E-07	1.02E-06	6.11E-07

NAV2	3.79E-05	8.41E-06	8.70E-06	1.24E-05	2.45E-06	2.53E-06	4.48E-06	2.23E-05
NAV3	1.52E-07	6.83E-08	8.92E-08	4.21E-07	5.19E-08	2.03E-07	1.32E-07	3.34E-06
NBAS	1.67E-05	1.40E-05	4.78E-06	1.24E-05	2.06E-05	2.12E-05	1.48E-05	1.53E-05
NBEA	9.98E-07	1.33E-07	2.89E-07	2.66E-07	3.10E-08	6.69E-08	1.71E-07	1.45E-07
NBEAL1	8.76E-06	2.55E-06	1.90E-06	1.75E-06	4.00E-07	2.23E-06	1.23E-06	1.20E-06
NBEAL2	1.34E-05	1.78E-06	2.23E-06	5.74E-06	2.45E-06	3.08E-06	4.00E-06	3.51E-06
NBL1	7.99E-05	2.05E-06	1.41E-06	5.73E-06	5.39E-06	3.13E-06	6.31E-06	3.78E-06
NBLA0030	8.01E-07	8.10E-09	1.86E-06	3.51E-06	1.05E-07	6.68E-09	1.18E-07	2.39E-07
NBN	2.53E-05	1.19E-05	7.48E-06	7.24E-06	4.06E-06	1.15E-05	1.14E-05	8.59E-06
NBPF1	9.76E-06	3.86E-06	3.84E-06	4.01E-06	8.12E-07	9.19E-06	2.22E-06	6.52E-06
NBPF10	1.06E-05	1.69E-06	2.48E-06	5.35E-06	7.64E-08	1.53E-06	1.23E-06	1.65E-06
NBPF14	1.06E-05	2.01E-06	1.29E-06	2.34E-06	1.68E-07	2.50E-06	1.86E-06	4.61E-06
NBPF15	3.67E-06	7.94E-07	2.78E-06	1.70E-06	7.78E-08	1.12E-06	8.26E-07	2.11E-06
NBPF16	1.71E-06	6.33E-08	1.10E-07	4.95E-07	0	6.97E-08	1.22E-07	0
NBPF22P	2.62E-08	9.81E-09	0	0	0	0	0	0
NBPF3	6.35E-07	2.77E-07	3.42E-07	2.65E-07	5.54E-09	1.24E-06	1.82E-07	5.92E-07
NBPF4	0	0	0	0	0	0	1.00E-07	0
NBPF6	0	0	0	0	0	0	0	0
NBPF7	5.11E-08	1.45E-08	1.34E-08	0	0	0	0	7.53E-08
NBPF9	1.91E-05	7.88E-06	3.85E-06	4.31E-06	2.67E-08	1.37E-05	1.76E-06	1.02E-05
NBR1	7.12E-05	9.59E-05	4.06E-05	3.37E-05	1.69E-05	8.42E-05	3.10E-05	3.35E-05
NBR2	8.44E-06	1.07E-05	5.80E-06	2.70E-06	2.51E-06	4.66E-06	7.92E-07	1.39E-06
NCALD	2.64E-05	4.18E-06	8.01E-06	6.13E-06	7.92E-06	8.92E-07	6.83E-06	6.54E-06
NCAM1	5.97E-06	7.04E-08	9.53E-07	3.73E-06	4.42E-08	6.07E-08	1.93E-07	1.54E-07
NCAM2	1.61E-08	3.79E-08	2.04E-07	3.86E-07	0	0	4.10E-08	8.25E-08
NCAN	8.26E-08	0	2.88E-08	8.54E-09	9.68E-08	2.25E-07	4.90E-08	0
NCAPD2	1.51E-05	4.60E-06	2.05E-06	2.23E-06	1.48E-06	6.51E-06	3.01E-06	2.32E-06
NCAPD3	6.45E-06	3.14E-06	4.39E-06	3.02E-06	7.19E-07	4.74E-06	1.99E-06	4.94E-06
NCAPG	7.79E-06	4.13E-06	6.56E-08	5.65E-08	5.44E-07	1.12E-06	2.08E-07	9.80E-07
NCAPG2	6.07E-06	5.87E-06	1.56E-06	2.08E-06	1.01E-06	3.06E-06	1.44E-06	2.21E-06
NCAPH	7.22E-06	4.16E-06	9.47E-08	5.65E-07	1.26E-07	1.02E-06	3.28E-07	5.46E-07
NCAPH2	2.82E-05	2.27E-05	1.01E-05	1.43E-05	2.25E-05	1.20E-05	2.17E-05	2.18E-05
NCBP1	1.06E-05	8.98E-06	6.66E-06	5.06E-06	2.10E-06	7.40E-06	3.81E-06	3.42E-06
NCBP2	3.99E-05	2.30E-05	1.30E-05	1.92E-05	1.03E-05	1.96E-05	2.00E-05	1.57E-05
NCCRP1	1.22E-08	1.24E-07	2.93E-07	4.47E-08	0	2.26E-08	2.35E-08	0
NCDN	8.25E-06	5.16E-06	2.43E-06	4.44E-06	1.20E-06	5.30E-06	2.89E-06	3.54E-06
NCEH1	4.09E-06	1.80E-06	1.91E-06	5.89E-06	2.79E-07	1.31E-06	1.66E-06	1.17E-06
NCF1	9.37E-06	4.42E-07	2.59E-06	5.06E-06	5.42E-07	2.28E-06	3.56E-06	8.23E-07
NCF1B	5.24E-06	3.92E-07	5.84E-07	1.72E-06	2.34E-07	1.03E-06	1.54E-06	6.72E-07
NCF1C	2.14E-06	2.38E-07	2.01E-06	2.38E-06	1.30E-07	1.41E-06	1.40E-06	2.89E-07
NCF2	1.15E-05	1.76E-06	3.15E-06	1.47E-05	3.30E-07	1.35E-05	5.51E-06	1.39E-06
NCF4	8.10E-06	1.42E-06	4.14E-06	7.34E-06	2.04E-06	4.22E-06	5.71E-06	2.49E-06
NCK1	1.30E-05	1.62E-05	1.12E-05	9.90E-06	2.33E-06	2.48E-05	9.12E-06	1.16E-05
NCK2	6.40E-05	1.30E-05	5.94E-06	1.43E-05	1.16E-06	2.07E-06	4.60E-06	4.76E-06
NCKAP1	4.90E-05	3.68E-05	1.96E-05	1.84E-05	4.19E-06	2.11E-05	1.33E-05	1.36E-05
NCKAP1L	8.94E-06	6.74E-07	3.70E-06	6.51E-06	4.22E-07	3.51E-06	4.56E-06	8.06E-07
NCKAP5	4.33E-06	7.49E-07	9.38E-07	3.61E-07	2.07E-07	1.26E-06	7.41E-07	2.79E-08
NCKAP5L	5.66E-06	4.08E-06	2.60E-06	4.27E-06	3.16E-06	3.87E-06	4.11E-06	1.35E-06
NCKIPSD	2.20E-05	3.63E-06	6.47E-06	5.38E-06	7.45E-06	1.01E-05	9.48E-06	7.78E-06
NCL	0.000291	0.000119	0.000107	0.000149	6.66E-05	0.000188	0.000157	0.000134
NCLN	3.82E-05	2.60E-05	2.51E-05	2.70E-05	6.08E-05	2.69E-05	4.15E-05	5.01E-05
NCOA1	1.34E-05	1.98E-05	8.46E-06	8.30E-06	2.90E-06	8.20E-06	7.53E-06	7.20E-06
NCOA2	1.58E-05	1.66E-05	8.68E-06	4.27E-06	3.25E-08	1.54E-05	2.69E-06	7.76E-06
NCOA3	1.41E-05	4.34E-06	4.22E-06	4.16E-06	1.77E-06	6.46E-06	2.91E-06	4.30E-06
NCOA4	8.71E-05	0.000137	0.000125	8.74E-05	3.30E-05	0.000179	8.29E-05	8.72E-05
NCOA5	1.72E-05	8.00E-06	5.29E-06	1.02E-05	4.88E-06	6.44E-06	8.87E-06	5.04E-06
NCOA6	1.27E-05	9.81E-06	4.18E-06	4.11E-06	1.21E-06	4.99E-06	3.61E-06	4.91E-06
NCOA7	1.95E-05	2.44E-05	1.23E-05	4.89E-05	1.75E-06	1.52E-05	3.41E-05	1.50E-05

NCOR1	2.13E-05	2.96E-05	3.36E-05	2.30E-05	6.53E-06	4.48E-05	1.75E-05	3.89E-06
NCOR2	3.27E-05	2.25E-05	9.88E-06	1.56E-05	1.70E-05	1.21E-05	2.10E-05	1.88E-05
NCR1	2.14E-07	0	1.55E-07	2.08E-07	0	3.78E-07	4.54E-08	0
NCR2	0	0	3.18E-08	0	0	0	0	0
NCR3	4.07E-07	1.98E-07	5.93E-07	6.97E-07	3.07E-07	2.86E-07	6.25E-07	4.57E-07
NCRNA00	0	6.62E-08	0	0	4.76E-08	0	0	0
NCRNA00	0	0	0	0	0	0	0	0
NCRNA00	0	0	0	0	0	0	0	0
NCRNA00	0	0	0	0	0	0	0	0
NCRNA00	0	0	0	0	0	0	6.55E-08	0
NCRNA00	1.01E-05	5.20E-06	5.39E-06	6.93E-06	5.62E-06	6.38E-06	4.48E-06	4.29E-06
NCRNA00	1.79E-06	4.33E-08	1.37E-07	3.24E-07	1.16E-07	1.73E-07	3.13E-07	2.43E-07
NCRNA00	9.44E-07	3.73E-08	1.03E-07	1.38E-07	1.71E-08	4.31E-08	1.10E-07	3.89E-08
NCRNA00	1.54E-06	1.70E-06	4.89E-07	3.14E-07	2.14E-08	1.84E-07	5.06E-07	1.22E-07
NCRNA00	5.67E-08	1.61E-08	1.64E-07	1.57E-07	2.44E-08	0	4.10E-08	4.18E-08
NCRNA00	1.59E-07	3.12E-07	2.34E-07	7.51E-07	2.27E-08	3.69E-08	1.66E-07	0
NCRNA00	2.57E-06	1.85E-06	1.06E-06	1.76E-06	1.53E-06	3.68E-06	1.81E-06	1.63E-06
NCRNA00	6.23E-07	1.81E-07	1.42E-07	1.55E-07	3.47E-08	3.74E-07	1.18E-07	1.10E-07
NCRNA00	0	0	0	0	0	0	0	0
NCRNA00	3.20E-06	5.19E-07	4.53E-07	6.57E-07	1.34E-06	1.67E-06	1.83E-07	1.05E-06
NCRNA00	5.87E-07	6.49E-07	5.25E-08	1.69E-07	9.06E-07	3.82E-07	1.69E-07	6.83E-08
NCRNA00	0	0	0	0	2.57E-08	2.77E-08	5.64E-08	0
NCRNA00	0	0	0	0	0	0	0	0
NCRNA00	0	0	0	0	0	0	0	0
NCRNA00	0	0	0	8.16E-08	0	0	0	0
NCRNA00	0	5.73E-08	0	0	0	0	0	0
NCRNA00	1.58E-06	5.95E-07	3.09E-07	4.26E-07	2.99E-07	4.30E-07	9.23E-08	1.71E-07
NCRNA00	1.22E-05	9.60E-06	5.99E-06	1.00E-05	1.66E-05	1.72E-05	1.42E-05	7.19E-06
NCRNA00	9.97E-09	0	1.04E-08	2.44E-08	0	9.25E-09	3.86E-08	0
NCRNA00	1.67E-06	4.71E-06	1.55E-06	2.52E-06	0	9.49E-07	7.66E-07	6.69E-07
NCRNA00	6.06E-05	2.65E-05	1.35E-06	6.21E-05	1.53E-05	3.29E-05	6.15E-05	2.33E-05
NCRNA00	0	0	0	0	0	0	0	0
NCRNA00	1.82E-08	0	1.94E-08	0	0	6.78E-08	0	0
NCRNA00	0	0	0	0	0	0	0	0
NCRNA00	0	0	0	0	0	5.23E-07	0	0
NCRNA00	0	0	0	3.95E-08	0	0	0	0
NCRNA00	0	0	4.60E-08	0	0	0	0	0
NCRNA00	0	2.00E-08	0	0	0	0	0	0
NCRNA00	6.49E-08	0	2.52E-08	2.98E-08	6.97E-09	5.26E-08	0	1.19E-08
NCRNA00	3.08E-08	4.63E-08	1.07E-08	1.26E-08	8.82E-09	0	9.93E-09	0
NCRNA00	3.52E-07	3.41E-07	1.10E-07	1.42E-07	7.00E-08	2.55E-07	6.69E-08	1.74E-07
NCRNA00	1.08E-07	3.51E-08	0	1.89E-08	0	1.13E-07	0	2.27E-08
NCRNA00	3.35E-06	3.00E-06	6.47E-07	8.20E-07	4.22E-07	1.47E-06	7.93E-07	9.15E-07
NCRNA00	6.71E-09	0	1.05E-08	0	0	0	0	0
NCRNA00	1.43E-07	1.53E-08	1.53E-08	3.30E-08	5.16E-08	2.78E-06	3.67E-07	3.53E-08
NCRNA00	2.67E-06	4.27E-06	1.25E-05	9.62E-06	5.62E-06	6.66E-06	1.65E-05	5.09E-06
NCRNA00	7.11E-07	1.74E-07	2.31E-07	2.51E-07	7.75E-08	2.79E-07	1.54E-07	1.72E-07
NCRNA00	8.24E-06	7.37E-06	4.55E-06	3.20E-06	1.71E-06	2.86E-06	3.06E-06	4.18E-06
NCRNA00	0	1.08E-06	0	6.33E-07	1.52E-06	4.79E-07	3.04E-07	1.72E-07
NCRNA00	0.000153	0.000192	0.000141	0.000243	0.000103	0.000224	0.000248	0.000105
NCRNA00	5.42E-08	4.41E-06	8.92E-08	2.01E-07	0	7.34E-07	0	1.62E-07
NCRNA00	0	0	0	1.18E-08	0	0	0	0
NCRNA00	6.75E-06	3.33E-06	1.54E-06	1.93E-06	9.90E-07	2.72E-06	1.24E-06	4.53E-06
NCRNA00	1.14E-07	7.09E-08	1.96E-08	7.73E-09	1.30E-07	1.76E-08	6.15E-09	1.85E-08
NCRNA00	4.83E-09	0	0	0	0	0	0	0
NCRNA00	3.75E-07	5.46E-07	2.40E-07	2.60E-07	1.38E-06	7.05E-07	2.74E-07	5.03E-07
NCRNA00	0	0	0	0	0	0	0	0
NCRNA00	1.44E-05	8.81E-06	1.31E-05	2.20E-05	1.05E-05	2.20E-05	1.68E-05	1.38E-05

NCRNA00	0	0	0	6.28E-06	0	0	0	0
NCRNA00	5.80E-08	1.42E-07	1.41E-07	0	0	2.20E-07	0	0
NCS1	3.87E-06	1.78E-06	4.86E-07	4.18E-06	4.58E-07	7.52E-07	9.45E-07	1.41E-06
NCSTN	5.65E-05	0.000107	3.59E-05	4.38E-05	7.76E-06	8.19E-05	3.28E-05	5.36E-05
NDC80	9.47E-06	2.23E-06	1.14E-07	4.16E-07	6.87E-07	1.57E-06	5.61E-07	5.47E-07
NDE1	7.94E-06	5.70E-06	1.25E-06	3.33E-06	2.47E-07	1.83E-06	2.14E-06	3.25E-06
NDEL1	1.24E-05	1.02E-05	1.39E-05	2.06E-05	2.27E-06	1.75E-05	1.45E-05	1.73E-05
NDFIP1	6.21E-05	6.77E-05	5.72E-05	4.40E-05	2.35E-05	5.66E-05	5.91E-05	9.70E-05
NDFIP2	2.69E-05	1.68E-05	1.22E-05	9.87E-06	2.69E-06	1.36E-05	7.24E-06	5.80E-06
NDN	4.30E-06	1.06E-06	2.53E-06	6.52E-06	2.80E-06	1.01E-06	3.62E-06	1.73E-06
NDNL2	1.17E-05	1.62E-05	8.99E-06	9.51E-06	4.69E-06	1.01E-05	1.13E-05	1.18E-05
NDOR1	7.24E-06	4.42E-06	1.38E-06	2.41E-06	3.15E-06	2.43E-06	5.79E-06	4.56E-06
NDP	5.98E-08	0	0	4.40E-08	0	0	1.16E-08	1.76E-08
NDRG1	0.000116	6.14E-05	1.85E-05	5.38E-05	4.18E-05	1.82E-05	8.31E-05	1.48E-05
NDRG2	0.000104	0.000103	0.000286	0.000272	0.000273	8.50E-05	8.93E-05	9.42E-05
NDRG3	2.32E-05	1.17E-05	3.99E-06	6.16E-06	4.01E-06	8.04E-06	4.57E-06	5.15E-06
NDRG4	4.43E-06	5.92E-07	8.67E-07	7.41E-07	1.55E-07	2.70E-08	6.61E-08	2.14E-07
NDST1	2.90E-05	1.93E-05	5.66E-06	7.43E-06	1.01E-05	1.04E-05	1.87E-05	3.27E-05
NDST2	7.63E-06	3.06E-06	3.74E-06	4.49E-06	2.69E-06	5.31E-06	4.79E-06	4.91E-06
NDST3	0	1.23E-08	4.45E-07	3.23E-07	0	2.51E-08	2.10E-08	1.60E-08
NDST4	0	0	0	0	0	0	0	0
NDUFA1	0.000431	0.00059	0.000192	0.000275	0.000634	0.000315	0.000369	0.000581
NDUFA10	0.000131	6.48E-05	7.98E-05	7.19E-05	6.41E-05	9.89E-05	8.70E-05	6.77E-05
NDUFA11	0.000179	0.000165	0.00018	0.000169	0.000708	0.00019	0.000275	0.00015
NDUFA12	0.000173	0.000163	6.90E-05	7.93E-05	7.88E-05	8.15E-05	8.67E-05	0.000101
NDUFA13	0.000207	0.000156	0.000122	0.000162	0.000467	0.000156	0.000227	0.000131
NDUFA2	0.000194	0.000279	0.000162	0.000142	0.000295	0.000217	0.00019	0.000226
NDUFA3	0.000229	0.000233	0.000228	0.000143	0.000876	0.000304	0.000286	0.00019
NDUFA4	0.000659	0.000928	0.000254	0.000269	0.00043	0.000311	0.000292	0.000458
NDUFA4L2	8.62E-06	1.83E-05	2.49E-06	1.74E-06	1.74E-05	1.62E-05	3.09E-05	1.70E-05
NDUFA5	4.62E-05	0.000102	4.95E-05	4.46E-05	2.78E-05	5.00E-05	4.13E-05	6.39E-05
NDUFA6	0.000192	0.000146	5.19E-05	5.76E-05	8.22E-05	5.83E-05	8.72E-05	0.000127
NDUFA7	0.00019	0.000119	7.94E-05	9.13E-05	0.000244	0.000161	0.00013	8.96E-05
NDUFA8	0.000135	9.19E-05	4.98E-05	4.85E-05	4.54E-05	8.12E-05	5.46E-05	0.000119
NDUFA9	9.48E-05	8.71E-05	6.07E-05	6.13E-05	6.08E-05	7.90E-05	8.56E-05	9.05E-05
NDUFAB1	0.000142	0.000327	9.24E-05	0.000102	0.000106	0.00014	0.000116	0.000114
NDUFAF1	1.99E-05	2.05E-05	2.12E-05	1.42E-05	2.31E-05	3.01E-05	2.42E-05	3.28E-05
NDUFAF2	2.54E-05	7.62E-06	4.94E-06	9.87E-06	1.17E-05	1.43E-05	1.53E-05	1.59E-05
NDUFAF3	6.33E-05	7.96E-05	4.02E-05	4.77E-05	5.06E-05	6.60E-05	4.78E-05	5.82E-05
NDUFAF4	1.51E-05	3.30E-05	1.08E-05	1.35E-05	3.98E-06	1.30E-05	1.66E-05	2.13E-05
NDUFB1	0.000343	0.00017	0.000158	0.000199	0.000593	0.000222	0.000215	0.000259
NDUFB10	0.000205	0.000309	0.000132	0.000129	0.00021	0.000174	0.000245	0.000124
NDUFB11	7.07E-05	0.000148	8.57E-05	0.000105	0.000217	0.00012	0.000112	0.000149
NDUFB2	0.000224	0.000336	0.000264	0.000281	0.000418	0.000282	0.000252	0.00027
NDUFB3	0.000133	0.000107	3.37E-05	4.45E-05	5.92E-05	6.22E-05	4.93E-05	5.20E-05
NDUFB4	0.000371	0.000515	0.000229	0.000177	0.000242	0.000213	0.000214	0.000227
NDUFB5	0.000159	0.000104	7.70E-05	6.66E-05	5.18E-05	7.79E-05	6.31E-05	7.08E-05
NDUFB6	5.77E-05	0.000123	6.18E-05	5.72E-05	6.69E-05	8.49E-05	6.27E-05	6.81E-05
NDUFB7	0.000282	0.000257	0.000226	0.00023	0.00092	0.000234	0.000263	0.000206
NDUFB8	0.000249	0.00024	0.000219	0.00019	0.000222	0.000215	0.000226	0.000221
NDUFB9	0.001155	0.000555	0.000219	0.000213	0.000827	0.000246	0.000759	0.000398
NDUFC1	0.000237	0.000126	0.000136	0.000146	0.000223	0.000158	0.000202	0.000165
NDUFC2	6.13E-05	0.000112	6.04E-05	6.20E-05	0.000136	7.70E-05	6.52E-05	7.77E-05
NDUFS1	6.77E-05	7.59E-05	4.15E-05	2.50E-05	6.36E-06	3.78E-05	1.60E-05	3.38E-05
NDUFS2	0.000128	0.000397	0.000103	9.60E-05	6.16E-05	0.000221	9.26E-05	0.000201
NDUFS3	0.00013	0.00018	8.20E-05	8.51E-05	0.000112	0.0001	8.87E-05	0.000133
NDUFS4	0.00011	7.52E-05	5.54E-05	7.28E-05	4.70E-05	8.60E-05	8.20E-05	0.000118
NDUFS5	0.000502	0.000312	0.000251	0.000369	0.000254	0.000376	0.000256	0.000448

NDUFS6	0.000192	0.000381	0.00015	0.000149	0.000267	0.000183	0.000197	0.000222
NDUFS7	0.000111	0.000117	0.000119	0.000117	0.000257	0.000103	0.000144	0.000138
NDUFS8	0.000125	0.000272	8.23E-05	7.86E-05	0.000247	0.000126	0.000129	0.000182
NDUFV1	0.000148	0.000223	0.00014	0.000133	0.000188	0.000168	0.000107	0.000188
NDUFV2	0.000273	0.00022	0.000259	0.000175	0.000132	0.000225	0.000282	0.000222
NDUFV3	4.37E-05	3.53E-05	3.37E-05	3.40E-05	3.95E-05	3.59E-05	3.88E-05	4.78E-05
NEAT1	0.000109	0.000105	2.39E-05	3.81E-05	4.09E-06	1.26E-05	1.98E-05	1.99E-05
NEB	0.000159	5.36E-06	1.35E-07	1.97E-06	3.36E-07	6.39E-07	4.19E-08	1.70E-07
NEBL	4.09E-06	1.51E-07	8.88E-07	8.77E-07	1.38E-08	7.60E-08	3.76E-08	9.40E-08
NECAB1	2.98E-06	7.19E-08	6.60E-08	2.03E-07	7.35E-08	8.03E-07	2.39E-07	1.17E-07
NECAB2	2.76E-07	8.70E-06	9.05E-06	1.05E-05	1.24E-06	1.82E-05	2.13E-06	4.66E-06
NECAB3	3.02E-05	6.60E-05	8.68E-06	8.15E-06	1.34E-05	1.91E-05	1.38E-05	1.41E-05
NECAP1	2.10E-05	1.31E-05	8.91E-06	8.95E-06	2.20E-06	1.09E-05	8.19E-06	2.08E-05
NECAP2	4.30E-05	2.21E-05	2.08E-05	3.08E-05	1.12E-05	2.56E-05	2.54E-05	2.08E-05
NEDD1	5.50E-06	4.01E-06	1.62E-06	2.07E-06	3.42E-07	2.09E-06	1.60E-06	2.08E-06
NEDD4	1.52E-05	1.81E-05	9.08E-06	8.20E-06	8.57E-07	2.93E-05	6.15E-06	1.08E-05
NEDD4L	2.52E-05	1.13E-05	2.54E-06	5.79E-06	3.55E-06	5.70E-06	7.46E-06	5.35E-06
NEDD8	0.00031	0.000188	0.000115	0.000173	0.000203	0.000239	0.00021	0.000178
NEDD9	2.35E-05	9.18E-06	7.53E-06	3.76E-05	1.85E-06	2.35E-05	2.15E-05	1.19E-05
NEFH	3.06E-07	6.89E-09	1.83E-07	4.43E-07	7.31E-08	2.25E-07	1.84E-07	2.09E-08
NEFL	0	7.16E-09	6.58E-09	7.80E-09	0	0	6.21E-09	0
NEFM	0	0	0	1.14E-08	0	0	0	1.37E-08
NEGR1	1.21E-08	8.99E-09	4.06E-08	1.45E-07	1.02E-08	1.86E-08	2.24E-07	0
NEIL1	4.65E-06	3.50E-06	3.97E-06	4.88E-06	4.52E-06	4.02E-06	3.19E-06	2.09E-06
NEIL2	2.69E-06	1.09E-05	5.66E-06	4.77E-06	1.74E-06	9.69E-06	5.51E-06	2.68E-06
NEIL3	1.74E-06	1.44E-07	1.02E-08	8.40E-08	5.90E-08	9.06E-07	2.85E-08	1.30E-07
NEK1	3.81E-06	1.21E-06	1.67E-06	1.93E-06	3.03E-07	2.85E-06	9.74E-07	1.30E-06
NEK10	2.26E-07	9.97E-09	7.53E-07	7.63E-07	0	1.26E-07	5.61E-08	6.49E-07
NEK11	6.09E-07	5.65E-07	3.27E-07	1.04E-06	4.97E-08	1.17E-06	2.90E-07	1.38E-07
NEK2	7.44E-06	7.82E-06	8.44E-08	1.80E-07	2.91E-07	2.03E-06	3.36E-07	1.28E-06
NEK3	5.89E-06	3.15E-06	2.16E-06	7.20E-06	2.59E-06	2.09E-05	5.67E-06	1.72E-06
NEK4	1.87E-05	1.17E-05	4.77E-06	4.39E-06	9.60E-07	1.04E-05	3.61E-06	5.94E-06
NEK5	0	0	8.26E-09	0	0	4.62E-08	0	0
NEK6	6.53E-05	3.90E-05	3.20E-05	3.04E-05	2.43E-05	4.37E-05	2.18E-05	3.19E-05
NEK7	1.51E-05	2.02E-05	1.01E-05	9.82E-06	1.96E-06	1.32E-05	7.17E-06	1.45E-05
NEK8	2.51E-06	3.63E-06	8.34E-07	1.68E-06	4.68E-07	1.48E-06	1.47E-06	1.01E-06
NEK9	3.52E-05	1.54E-05	1.38E-05	1.62E-05	7.83E-06	2.42E-05	1.54E-05	2.22E-05
NELF	2.45E-05	9.29E-06	6.76E-06	7.52E-06	2.12E-06	1.57E-05	8.15E-06	2.46E-05
NELL1	0	0	0	0	0	0	0	0
NELL2	9.59E-07	3.59E-07	5.19E-08	4.13E-07	1.85E-08	2.87E-07	1.04E-07	2.00E-07
NENF	5.62E-05	9.96E-05	3.65E-05	5.63E-05	0.000144	0.00011	8.38E-05	9.33E-05
NEO1	1.36E-05	2.38E-06	3.45E-06	8.35E-06	1.54E-06	3.40E-06	2.98E-06	3.70E-06
NES	1.09E-05	4.96E-06	1.04E-06	3.94E-06	6.42E-06	4.99E-06	1.15E-05	5.85E-06
NET1	2.11E-05	6.44E-06	2.69E-05	1.52E-05	1.88E-05	1.46E-05	1.63E-05	7.74E-06
NETO1	0	0	0	0	0	0	1.94E-08	0
NETO2	5.22E-06	4.04E-07	1.15E-07	5.23E-07	2.18E-07	5.95E-07	1.26E-06	5.45E-07
NEU1	8.78E-05	0.000127	2.55E-05	3.82E-05	2.51E-05	4.84E-05	4.11E-05	7.63E-05
NEU2	0	0	0	0	0	0	0	0
NEU3	1.75E-06	8.51E-07	7.60E-07	3.56E-07	4.37E-08	1.15E-06	1.65E-07	5.60E-07
NEU4	4.90E-05	4.08E-06	4.09E-05	1.46E-05	1.83E-05	3.47E-05	2.27E-05	7.46E-08
NEURL	1.95E-07	4.12E-08	1.03E-07	1.61E-07	1.35E-08	4.87E-09	1.87E-06	2.31E-08
NEURL1B	5.79E-06	2.56E-06	2.44E-06	3.36E-06	2.02E-06	2.12E-06	2.87E-06	3.37E-06
NEURL2	4.49E-06	3.30E-06	2.08E-06	1.86E-06	4.12E-06	2.25E-06	3.72E-06	1.29E-06
NEURL3	1.22E-05	3.57E-06	3.68E-07	9.65E-06	2.77E-07	2.99E-07	1.89E-06	5.69E-07
NEURL4	3.09E-06	1.73E-06	2.44E-06	2.44E-06	1.23E-06	3.27E-06	3.28E-06	4.13E-06
NEUROD1	2.85E-06	0	0	0	0	0	0	0
NEUROD2	7.64E-09	0	0	0	0	0	0	0
NEUROD4	0	0	0	0	0	0	0	0

NEUROD6	0	0	0	0	0	0	0	0
NEUROG1	0	0	0	0	0	0	0	0
NEUROG2	0	0	0	0	0	0	0	0
NEUROG3	0	0	0	0	0	0	0	0
NEXN	5.85E-06	5.82E-07	1.30E-06	2.25E-06	6.73E-07	9.02E-07	1.26E-06	7.53E-07
NF1	1.37E-05	1.24E-05	6.60E-06	5.55E-06	2.61E-06	7.10E-06	3.39E-06	7.04E-06
NF1P1	0	0	0	0	0	0	0	0
NF2	7.48E-06	3.90E-06	2.31E-06	4.09E-06	7.37E-06	3.76E-06	8.78E-06	8.54E-06
NFAM1	2.69E-06	2.91E-07	1.96E-06	4.52E-06	1.99E-07	7.01E-07	1.44E-06	6.10E-07
NFASC	3.49E-06	4.00E-07	6.99E-07	2.26E-06	1.98E-06	2.85E-06	9.18E-07	6.40E-07
NFAT5	7.64E-06	1.96E-06	2.94E-06	3.28E-06	6.43E-07	3.06E-06	1.29E-06	2.55E-06
NFATC1	2.59E-06	8.36E-07	1.41E-06	1.20E-05	8.74E-07	1.58E-06	2.35E-06	1.11E-06
NFATC2	2.17E-06	7.42E-07	1.47E-06	3.04E-06	0	3.75E-07	1.07E-07	1.17E-07
NFATC2IP	1.08E-05	9.43E-06	3.65E-06	4.76E-06	1.05E-06	6.30E-06	4.23E-06	5.28E-06
NFATC3	1.16E-05	6.97E-06	7.25E-06	7.77E-06	2.47E-06	9.49E-06	7.53E-06	6.64E-06
NFATC4	1.56E-06	6.84E-07	3.28E-07	1.10E-06	1.02E-06	3.42E-07	7.51E-07	1.23E-06
NFE2	1.49E-07	1.02E-07	1.10E-07	1.65E-07	6.42E-08	3.87E-07	9.98E-08	1.54E-07
NFE2L1	0.000224	0.000255	7.81E-05	0.000113	6.87E-05	0.000202	0.000105	0.000104
NFE2L2	5.03E-05	7.19E-05	4.59E-05	5.73E-05	1.53E-05	4.45E-05	4.31E-05	3.15E-05
NFE2L3	7.93E-06	6.08E-07	4.38E-07	2.24E-06	8.47E-08	6.17E-07	8.99E-07	1.41E-06
NFIA	4.98E-06	5.33E-06	1.27E-05	9.60E-06	5.56E-06	1.07E-05	8.74E-06	8.82E-06
NFIB	4.82E-06	6.18E-06	6.73E-06	7.86E-06	5.64E-06	5.86E-06	8.22E-06	8.19E-06
NFIC	3.78E-05	3.54E-05	2.87E-05	1.80E-05	1.17E-06	3.17E-05	2.40E-05	4.31E-05
NFIL3	4.59E-05	4.95E-05	4.38E-05	8.22E-05	4.86E-06	7.17E-05	2.91E-05	7.38E-05
NFIX	1.50E-05	8.52E-06	1.31E-05	1.03E-05	6.87E-06	2.64E-05	1.05E-05	8.87E-06
NFKB1	1.61E-05	7.54E-06	8.88E-06	1.84E-05	1.52E-06	1.24E-05	1.11E-05	1.32E-05
NFKB2	5.16E-05	2.36E-05	6.99E-06	2.74E-05	5.98E-06	1.38E-05	1.71E-05	4.03E-05
NFKBIA	0.000128	6.92E-05	0.000105	0.000366	7.10E-05	6.61E-05	0.000118	0.000136
NFKBIB	2.97E-05	2.50E-05	1.64E-05	2.01E-05	1.83E-05	1.79E-05	3.59E-05	2.53E-05
NFKBID	1.05E-06	5.98E-08	1.25E-07	1.01E-06	1.14E-08	1.84E-07	2.16E-07	1.17E-07
NFKBIE	1.66E-05	1.63E-05	3.34E-06	1.76E-05	4.67E-06	3.31E-06	1.67E-05	1.16E-05
NFKBIL1	2.11E-05	2.75E-05	1.09E-05	1.45E-05	2.80E-05	1.94E-05	1.59E-05	1.56E-05
NFKBIL2	8.74E-06	1.84E-06	5.41E-07	5.88E-07	1.24E-06	1.08E-06	3.50E-06	8.01E-07
NFKBIZ	2.04E-05	1.12E-05	7.62E-06	1.00E-05	1.85E-06	1.42E-06	3.01E-06	3.93E-06
NFRKB	8.13E-06	4.70E-06	3.89E-06	4.65E-06	3.50E-06	8.99E-06	4.55E-06	7.17E-06
NFS1	2.95E-05	3.15E-05	1.83E-05	1.78E-05	1.30E-05	3.46E-05	1.62E-05	2.20E-05
NFU1	4.08E-05	4.41E-05	1.61E-05	2.31E-05	1.93E-05	2.23E-05	3.09E-05	1.97E-05
NFX1	9.55E-06	1.34E-05	1.10E-05	1.31E-05	4.11E-06	1.77E-05	1.13E-05	1.34E-05
NFXL1	4.45E-06	3.93E-06	2.63E-06	2.10E-06	7.59E-07	6.87E-06	1.49E-06	2.29E-06
NFYA	8.15E-06	7.09E-06	1.78E-06	3.37E-06	1.60E-06	2.64E-06	3.94E-06	3.35E-06
NFYB	1.73E-05	1.02E-05	6.48E-06	6.83E-06	2.96E-06	7.32E-06	8.41E-06	2.97E-05
NFYC	4.10E-05	2.70E-05	1.98E-05	2.08E-05	9.26E-06	2.28E-05	1.77E-05	2.23E-05
NGB	1.29E-08	0	0	0	0	0	0	0
NGDN	3.24E-05	2.59E-05	1.42E-05	2.16E-05	2.00E-05	2.68E-05	3.15E-05	2.32E-05
NGEF	2.02E-05	9.22E-06	9.41E-06	1.35E-05	1.18E-05	1.67E-05	1.03E-05	1.79E-05
NGF	5.29E-07	5.83E-07	1.21E-06	1.93E-06	1.09E-06	2.35E-07	5.53E-07	1.05E-06
NGFR	3.03E-06	4.54E-07	1.13E-05	1.33E-05	1.44E-06	1.41E-06	1.58E-06	4.35E-06
NGFRAP1	0.000131	0.000147	4.99E-05	0.000127	9.60E-06	4.02E-05	1.61E-05	0.0002
NGLY1	1.28E-05	1.61E-05	8.97E-06	1.15E-05	5.91E-06	1.64E-05	9.18E-06	5.58E-06
NGRN	0.000104	0.000124	4.57E-05	6.38E-05	5.18E-05	6.38E-05	8.65E-05	5.15E-05
NHEDC1	6.69E-08	3.49E-08	5.75E-08	2.09E-08	2.64E-08	5.01E-08	6.90E-08	4.53E-08
NHEDC2	7.12E-06	6.35E-06	3.21E-05	1.99E-05	5.15E-06	1.46E-05	1.40E-05	1.69E-05
NHEG1	0	0	0	0	0	0	0	0
NHEJ1	1.55E-05	3.44E-06	4.63E-06	4.85E-06	2.47E-06	8.40E-06	1.08E-05	3.23E-06
NHLH1	4.58E-08	2.78E-07	2.85E-08	6.72E-08	2.36E-08	1.70E-08	1.77E-08	2.69E-08
NHLH2	0	0	0	0	0	0	0	0
NHLRC1	3.88E-06	8.71E-06	2.67E-06	1.60E-06	2.95E-06	5.14E-06	1.96E-06	3.89E-06
NHLRC2	3.05E-06	2.75E-06	4.33E-06	2.48E-06	3.08E-07	2.56E-06	7.92E-07	1.54E-06

NHLRC3	1.14E-05	1.75E-05	1.14E-05	8.91E-06	2.25E-06	1.20E-05	6.20E-06	8.63E-06
NHLRC4	3.51E-07	1.06E-07	2.08E-07	1.72E-07	5.03E-08	1.41E-07	2.04E-07	6.89E-08
NHP2	0.00012	6.08E-05	5.15E-05	6.08E-05	6.26E-05	0.000104	9.95E-05	8.38E-05
NHP2L1	0.000112	6.81E-05	4.11E-05	6.20E-05	7.82E-05	7.11E-05	6.74E-05	7.45E-05
NHS	8.42E-07	3.03E-08	3.05E-08	8.02E-08	1.63E-08	3.77E-08	4.25E-08	7.95E-08
NHSL1	5.43E-06	6.93E-06	7.10E-06	4.19E-06	3.44E-06	5.64E-06	6.68E-06	3.54E-06
NHSL2	3.65E-07	3.70E-07	1.97E-07	1.39E-07	0	1.04E-07	4.82E-08	9.36E-08
NICN1	8.08E-06	1.13E-05	5.09E-06	5.44E-06	3.32E-06	7.57E-06	4.63E-06	4.00E-06
NID1	4.33E-05	6.34E-05	3.85E-05	4.00E-05	1.02E-05	1.43E-05	2.63E-05	5.56E-05
NID2	6.09E-06	3.15E-06	1.72E-06	2.96E-06	4.70E-07	1.89E-06	3.37E-06	1.30E-06
NIF3L1	3.76E-05	1.89E-05	1.01E-05	1.06E-05	6.72E-06	1.37E-05	1.05E-05	1.04E-05
NIN	5.82E-06	1.20E-06	1.97E-06	2.61E-06	7.76E-07	3.76E-06	1.80E-06	4.08E-06
NINJ1	0.000102	4.04E-05	7.18E-05	0.000107	4.20E-05	8.33E-05	8.42E-05	0.000117
NINJ2	6.69E-06	1.58E-05	2.24E-06	7.37E-06	4.21E-06	1.53E-05	3.63E-06	5.76E-06
NINL	2.05E-05	7.21E-06	5.58E-06	3.58E-06	4.72E-06	3.24E-06	2.56E-06	1.74E-06
NIP7	1.39E-05	9.74E-06	6.89E-06	1.24E-05	2.65E-06	1.11E-05	9.70E-06	9.66E-06
NIPA1	6.80E-06	3.75E-06	1.90E-06	1.95E-06	4.60E-07	2.28E-06	1.92E-06	4.38E-06
NIPA2	2.76E-05	2.22E-05	1.04E-05	1.07E-05	5.12E-06	1.44E-05	1.07E-05	1.44E-05
NIPAL1	2.41E-06	4.07E-06	3.76E-06	1.83E-06	3.73E-08	2.72E-07	1.99E-07	6.86E-07
NIPAL2	9.20E-06	3.79E-06	1.47E-06	2.68E-06	2.93E-07	3.28E-06	2.53E-06	6.43E-06
NIPAL3	4.40E-06	2.11E-06	1.54E-06	2.09E-06	1.18E-06	2.20E-06	2.02E-06	2.52E-06
NIPAL4	1.15E-07	2.41E-08	5.80E-08	6.12E-08	0	0	6.95E-09	1.05E-08
NIPBL	1.28E-05	9.81E-06	7.03E-06	7.51E-06	2.43E-06	7.40E-06	5.93E-06	8.59E-06
NIPSNAP1	0.00013	0.000158	0.000164	0.000113	0.00011	0.000185	0.000156	0.000215
NIPSNAP3	2.32E-05	1.33E-05	3.26E-05	3.22E-05	1.03E-05	4.01E-05	2.60E-05	1.81E-05
NIPSNAP3	4.46E-07	3.67E-07	3.80E-07	2.39E-07	1.51E-07	3.50E-07	1.26E-07	2.20E-07
NISCH	2.59E-05	1.43E-05	1.28E-05	1.54E-05	2.82E-05	1.92E-05	1.75E-05	2.91E-05
NIT1	5.21E-05	6.20E-05	3.83E-05	3.27E-05	2.27E-05	6.73E-05	4.39E-05	4.01E-05
NIT2	9.50E-05	0.000102	0.000105	5.90E-05	8.84E-05	6.89E-05	7.89E-05	6.45E-05
NKAIN1	1.99E-07	0	0	2.35E-08	0	1.78E-08	3.54E-07	1.41E-08
NKAIN2	3.87E-08	1.27E-06	6.40E-08	3.78E-08	6.64E-09	2.15E-08	0	5.78E-07
NKAIN3	0	1.79E-08	1.67E-08	0	0	1.17E-07	0	0
NKAIN4	5.24E-08	0	0	2.15E-08	0	0	1.49E-08	0
NKAP	1.48E-05	1.51E-05	8.58E-06	9.77E-06	9.81E-06	1.14E-05	8.26E-06	2.39E-05
NKAPL	1.65E-07	3.40E-08	2.85E-07	5.15E-07	1.68E-07	6.95E-08	1.30E-07	1.32E-07
NKD1	3.53E-07	0	5.17E-07	1.61E-06	2.33E-08	2.66E-05	2.19E-07	4.52E-07
NKD2	6.21E-06	3.06E-08	6.65E-07	2.38E-06	5.06E-07	3.75E-08	1.06E-06	2.86E-06
NKG7	3.63E-05	4.51E-06	1.25E-05	2.65E-05	2.85E-06	2.19E-05	2.27E-05	3.30E-06
NKIRAS1	6.51E-06	5.85E-06	1.09E-05	1.01E-05	3.32E-06	1.15E-05	6.68E-06	7.37E-06
NKIRAS2	2.52E-05	2.67E-05	1.20E-05	1.63E-05	1.04E-05	2.64E-05	1.80E-05	1.65E-05
NKPD1	9.65E-09	1.08E-08	4.95E-09	1.77E-08	4.14E-09	5.36E-08	1.41E-08	0
NKRF	3.85E-06	4.90E-06	2.36E-06	2.81E-06	1.94E-06	4.30E-06	2.48E-06	4.56E-06
NKTR	1.35E-05	3.57E-06	2.54E-06	2.36E-06	1.19E-06	2.94E-06	1.94E-06	3.25E-06
NKX1-2	8.32E-08	0	0	0	0	0	0	0
NKX2-1	0	6.20E-08	0	0	0	0	0	0
NKX2-2	0	6.50E-08	0	1.41E-08	0	0	0	1.69E-08
NKX2-3	2.28E-08	1.29E-08	0	1.12E-07	0	1.69E-07	1.21E-07	1.68E-08
NKX2-4	0	0	0	0	0	0	0	0
NKX2-5	0	0	0	0	2.20E-08	0	0	0
NKX2-6	0	0	0	0	0	0	0	0
NKX2-8	1.34E-08	3.05E-08	0	2.64E-07	0	0	0	0
NKX3-1	1.10E-06	2.44E-07	9.57E-07	2.07E-06	1.99E-07	1.26E-06	5.93E-07	6.17E-07
NKX3-2	8.46E-08	0	0	0	0	0	1.02E-08	0
NKX6-1	0	0	0	0	0	0	0	0
NKX6-2	0	0	0	0	0	0	0	0
NKX6-3	0	0	0	0	0	0	0	0
NLE1	4.67E-06	4.17E-06	2.39E-06	3.18E-06	2.06E-06	5.02E-06	7.20E-06	3.06E-06
NLGN1	2.61E-06	0	0	5.60E-09	0	4.25E-09	4.47E-09	3.36E-08

NLGN2	2.64E-06	1.85E-07	6.85E-07	1.39E-06	3.89E-07	1.79E-06	9.82E-07	9.48E-07
NLGN3	5.73E-07	4.11E-08	4.91E-08	1.43E-07	5.66E-08	5.67E-08	5.69E-08	5.37E-08
NLGN4X	8.04E-08	4.31E-07	5.31E-07	7.75E-07	3.45E-08	4.83E-08	9.78E-08	2.64E-07
NLGN4Y	0	8.15E-07	9.10E-09	1.43E-06	5.97E-07	4.31E-07	1.12E-06	1.37E-06
NLK	9.00E-06	1.29E-05	4.47E-06	3.96E-06	3.56E-06	5.64E-06	4.43E-06	5.15E-06
NLN	9.37E-06	8.30E-06	1.66E-05	7.50E-06	5.63E-07	1.47E-05	4.63E-06	1.15E-05
NLRC3	1.13E-06	1.17E-07	5.40E-07	5.89E-07	7.20E-08	4.43E-07	4.02E-07	2.71E-07
NLRC4	1.05E-06	1.24E-06	9.27E-07	1.29E-06	1.31E-07	4.62E-07	8.39E-07	2.14E-07
NLRC5	1.65E-05	1.87E-06	2.44E-06	4.67E-06	4.36E-07	7.38E-06	3.80E-06	1.37E-05
NLRP1	2.07E-06	6.87E-06	7.70E-07	1.31E-06	3.61E-07	8.23E-07	1.03E-06	1.18E-06
NLRP10	0	0	0	0	0	0	0	0
NLRP11	8.03E-09	1.55E-06	4.04E-07	4.29E-07	1.15E-06	3.56E-06	2.34E-08	8.25E-08
NLRP12	5.69E-08	0	6.10E-08	7.66E-08	5.09E-09	0	5.18E-08	0
NLRP13	0	0	0	0	0	0	0	0
NLRP14	3.58E-08	5.62E-07	7.99E-08	6.56E-08	1.03E-08	3.32E-08	1.74E-08	2.62E-08
NLRP2	4.08E-07	3.75E-08	3.44E-08	4.40E-07	5.74E-09	1.24E-08	1.10E-07	2.94E-08
NLRP3	5.97E-07	1.20E-07	5.10E-07	1.95E-06	1.32E-07	2.89E-07	6.14E-07	1.39E-07
NLRP4	0	0	0	0	0	0	1.61E-08	0
NLRP5	0	0	0	0	0	0	0	0
NLRP6	4.54E-07	4.34E-06	3.33E-06	1.46E-06	8.99E-08	9.30E-07	3.47E-07	2.00E-06
NLRP7	3.45E-08	0	7.32E-09	1.73E-08	0	6.57E-09	6.88E-09	1.04E-08
NLRP8	0	0	0	0	0	0	0	0
NLRP9	3.95E-08	2.22E-08	2.72E-08	4.02E-08	5.66E-08	5.49E-08	1.28E-08	9.65E-09
NLRX1	6.17E-06	2.44E-06	5.09E-06	5.16E-06	5.88E-06	7.30E-06	6.46E-06	9.32E-06
NMB	5.35E-06	3.90E-06	5.64E-07	1.85E-06	4.57E-06	2.22E-06	3.50E-06	8.46E-07
NMBR	0	0	1.99E-08	0	0	0	1.79E-08	0
NMD3	2.89E-05	2.76E-05	2.86E-05	1.97E-05	7.27E-06	3.16E-05	2.10E-05	2.07E-05
NME1	0.000248	0.000239	6.07E-05	7.66E-05	0.000149	0.00018	0.000112	9.60E-05
NME1-NM	7.88E-06	5.86E-06	4.63E-06	3.55E-06	3.98E-06	6.64E-06	1.60E-05	4.23E-06
NME2	0.000747	0.000716	0.000219	0.000369	0.000877	0.00075	0.000496	0.000362
NME2P1	2.48E-05	6.30E-06	6.19E-06	2.52E-06	1.06E-05	6.53E-06	8.34E-06	1.43E-06
NME3	3.70E-05	4.54E-05	1.69E-05	2.54E-05	4.07E-05	2.77E-05	3.26E-05	2.46E-05
NME4	9.35E-05	3.30E-05	8.50E-05	7.99E-05	8.44E-05	0.000129	6.63E-05	9.50E-05
NME5	6.28E-08	0	2.25E-07	1.08E-06	1.81E-08	1.56E-07	4.01E-08	3.10E-08
NME6	1.87E-05	1.02E-05	4.09E-06	5.39E-06	7.22E-06	9.65E-06	7.94E-06	8.66E-06
NME7	4.01E-06	4.18E-06	2.44E-06	4.32E-06	4.96E-07	8.64E-06	4.82E-06	5.24E-06
NMI	3.59E-05	2.09E-05	1.49E-05	1.65E-05	4.43E-06	1.85E-05	1.16E-05	2.01E-05
NMNAT1	5.00E-06	5.63E-06	2.37E-06	2.44E-06	1.63E-06	4.25E-06	2.50E-06	3.61E-06
NMNAT2	3.40E-06	1.39E-07	9.42E-09	4.49E-08	3.94E-09	5.95E-08	4.47E-09	6.05E-08
NMNAT3	4.66E-06	2.25E-06	2.15E-06	2.14E-06	0	2.96E-06	4.21E-06	3.59E-06
NMRAL1	2.16E-05	8.06E-05	3.03E-05	3.19E-05	4.47E-05	9.29E-05	2.68E-05	5.05E-05
NMS	0	0	0	0	0	0	0	0
NMT1	4.99E-05	4.98E-05	2.64E-05	1.64E-05	1.47E-05	5.00E-05	2.81E-05	2.79E-05
NMT2	4.46E-06	7.66E-06	4.99E-06	8.72E-06	3.58E-06	2.41E-05	1.11E-05	4.44E-06
NMU	0	0	0	0	0	0	0	0
NMUR1	1.90E-07	2.45E-07	4.00E-07	6.79E-07	8.46E-08	4.56E-08	3.69E-07	1.44E-07
NMUR2	0	0	0	0	0	0	0	0
NNAT	4.63E-07	1.45E-07	4.53E-08	5.19E-08	0	9.80E-08	0	0
NNMT	0.000945	5.62E-05	0.00183	0.001069	0.000429	0.001895	0.000866	0.002466
NNT	3.71E-05	7.99E-05	6.12E-05	3.62E-05	6.57E-06	4.51E-05	2.08E-05	5.64E-05
NOB1	2.52E-05	1.76E-05	1.96E-05	2.28E-05	9.28E-06	3.71E-05	2.62E-05	2.19E-05
NOBOX	0	0	0	0	0	0	0	0
NOC2L	4.72E-05	2.48E-05	2.40E-05	2.77E-05	1.74E-05	3.97E-05	2.81E-05	2.70E-05
NOC3L	4.51E-06	6.30E-06	3.52E-06	2.96E-06	1.25E-06	5.16E-06	3.40E-06	3.14E-06
NOC4L	1.38E-05	2.00E-05	8.18E-06	1.17E-05	1.50E-05	1.35E-05	1.91E-05	5.52E-06
NOD1	5.66E-06	1.43E-06	6.67E-07	1.43E-06	6.31E-07	1.10E-06	1.66E-06	1.47E-06
NOD2	1.89E-06	8.76E-07	9.49E-07	2.03E-06	1.31E-07	3.98E-07	3.22E-07	1.80E-06
NODAL	6.86E-08	7.76E-08	5.98E-08	1.40E-08	9.84E-09	7.42E-08	1.11E-08	6.73E-08

NOG	0	0	1.34E-08	1.09E-07	2.19E-08	0	0	0
NOL10	1.30E-05	1.25E-05	5.51E-06	6.82E-06	5.02E-06	9.99E-06	6.02E-06	9.14E-06
NOL11	2.23E-05	3.44E-05	1.12E-05	1.36E-05	9.58E-06	2.99E-05	1.54E-05	1.22E-05
NOL12	1.85E-05	1.11E-05	5.36E-06	9.43E-06	9.81E-06	1.21E-05	1.42E-05	1.34E-05
NOL3	4.70E-05	1.64E-05	7.65E-06	8.44E-06	9.56E-07	7.83E-06	5.54E-06	2.12E-05
NOL4	0	2.59E-07	6.00E-07	7.68E-08	6.54E-09	1.41E-08	0	1.12E-08
NOL6	6.84E-06	1.51E-05	7.03E-06	9.53E-06	6.09E-06	1.33E-05	1.43E-05	9.29E-06
NOL7	4.42E-05	5.70E-05	2.97E-05	3.44E-05	2.49E-05	4.35E-05	4.61E-05	4.55E-05
NOL8	7.56E-06	4.82E-06	4.25E-06	4.31E-06	2.57E-06	7.28E-06	5.62E-06	6.44E-06
NOL9	2.71E-06	2.46E-06	1.63E-06	1.89E-06	1.76E-07	2.18E-06	1.17E-06	2.28E-06
NOLC1	3.91E-05	1.68E-05	1.26E-05	2.22E-05	5.60E-06	3.60E-05	1.75E-05	1.79E-05
NOM1	2.08E-06	3.10E-06	2.95E-06	2.76E-06	8.92E-07	4.08E-06	2.46E-06	3.30E-06
NOMO1	4.77E-05	7.20E-05	2.36E-05	1.98E-05	8.02E-06	3.39E-05	1.98E-05	3.91E-05
NOMO2	1.64E-05	5.74E-05	6.93E-06	1.15E-05	2.23E-06	1.83E-05	3.96E-06	5.05E-06
NOMO3	1.85E-05	2.34E-05	5.18E-06	1.40E-05	3.10E-06	1.33E-05	1.13E-05	2.04E-05
NONO	0.000171	0.000169	8.35E-05	0.000101	8.24E-05	0.000105	0.000106	0.000151
NOP10	0.000287	0.000177	0.000122	0.000169	0.000228	0.000167	0.0002	0.000147
NOP14	1.77E-05	2.82E-05	1.72E-05	1.61E-05	7.78E-06	2.49E-05	1.76E-05	1.91E-05
NOP16	3.56E-05	2.75E-05	1.88E-05	2.99E-05	1.42E-05	5.05E-05	3.79E-05	4.20E-05
NOP2	2.16E-05	2.01E-05	9.07E-06	1.44E-05	7.90E-06	1.81E-05	2.16E-05	2.24E-05
NOP56	5.24E-05	4.32E-05	1.61E-05	2.69E-05	3.25E-05	3.11E-05	3.73E-05	2.28E-05
NOP58	5.60E-05	3.58E-05	1.76E-05	2.57E-05	1.71E-05	2.99E-05	2.78E-05	3.15E-05
NOS1	0	0	6.39E-09	0	0	0	0	0
NOS1AP	5.59E-07	3.43E-06	8.75E-07	3.82E-07	5.25E-08	3.04E-06	8.28E-07	2.47E-06
NOS2	1.30E-07	3.21E-07	1.11E-07	7.92E-08	1.11E-07	8.10E-07	2.05E-07	1.42E-07
NOS3	3.21E-06	2.17E-06	1.85E-06	2.23E-06	1.21E-06	1.47E-06	2.15E-06	2.09E-06
NOSIP	4.89E-05	4.03E-05	3.10E-05	4.38E-05	0.000129	3.79E-05	6.75E-05	2.78E-05
NOSTRIN	3.41E-05	4.09E-06	3.53E-06	2.01E-06	3.50E-06	2.56E-06	3.51E-06	1.75E-06
NOTCH1	7.66E-06	1.85E-06	2.94E-06	2.47E-06	1.12E-06	1.32E-06	2.60E-06	2.50E-06
NOTCH2	2.02E-05	6.06E-06	8.09E-06	9.05E-06	1.64E-06	6.57E-06	4.39E-06	4.89E-06
NOTCH2N	1.16E-05	4.79E-06	1.49E-05	1.42E-05	1.11E-05	1.04E-05	1.44E-05	9.61E-06
NOTCH3	1.09E-05	5.60E-06	6.88E-07	1.26E-06	2.12E-06	4.90E-06	4.89E-06	8.49E-06
NOTCH4	9.05E-06	8.06E-06	3.57E-06	4.53E-06	8.04E-06	3.98E-06	1.21E-05	1.02E-05
NOTO	0	0	0	0	0	0	0	0
NOTUM	2.30E-06	3.47E-06	1.46E-05	1.26E-05	7.18E-07	0.000602	4.03E-06	1.34E-06
NOV	5.98E-06	1.27E-06	4.67E-07	9.58E-07	1.93E-07	9.25E-07	1.40E-06	1.99E-06
NOVA1	3.89E-06	1.33E-08	1.47E-07	3.51E-07	4.39E-08	2.08E-08	9.14E-08	2.38E-07
NOVA2	1.27E-06	7.28E-07	1.75E-07	3.15E-07	1.37E-06	5.37E-07	1.96E-06	1.10E-06
NOX1	1.19E-07	3.35E-08	0	0	5.96E-08	1.84E-08	0	0
NOX3	0	0	0	0	0	0	0	0
NOX4	4.26E-07	9.08E-08	1.76E-08	1.30E-07	2.17E-07	1.61E-07	4.35E-07	2.94E-07
NOX5	4.08E-09	1.82E-08	0	5.68E-08	0	1.26E-08	9.70E-09	1.99E-08
NOXA1	5.09E-06	2.60E-06	4.86E-06	7.42E-06	5.88E-06	3.28E-06	7.97E-06	7.33E-06
NOXO1	3.13E-07	4.70E-07	8.73E-08	4.71E-07	5.53E-07	9.17E-08	6.65E-07	7.29E-08
NPAS1	2.72E-06	5.17E-07	1.01E-07	2.21E-07	3.26E-07	2.92E-08	7.20E-07	8.36E-08
NPAS2	1.29E-05	5.79E-06	1.36E-06	4.12E-06	2.25E-06	2.02E-06	2.26E-06	1.30E-05
NPAS3	3.84E-08	4.25E-09	2.75E-08	4.69E-08	1.31E-08	6.22E-08	8.61E-08	1.12E-08
NPAS4	0	0	1.49E-08	0	0	6.69E-09	7.01E-09	0
NPAT	4.73E-06	2.45E-06	2.60E-06	2.58E-06	8.17E-07	2.77E-06	1.63E-06	2.70E-06
NPB	5.06E-07	2.82E-06	1.80E-07	4.45E-07	1.33E-07	6.68E-06	3.92E-06	7.70E-08
NPBWR1	6.49E-06	0	8.95E-08	0	0	5.07E-08	0	0
NPBWR2	0	0	0	0	0	0	0	0
NPC1	5.00E-05	2.27E-05	4.21E-06	7.03E-06	1.06E-06	5.52E-06	4.99E-06	9.65E-06
NPC1L1	0.000151	5.90E-05	2.92E-05	1.52E-05	1.04E-05	2.55E-05	3.74E-05	3.15E-08
NPC2	0.000215	9.99E-05	0.000104	0.000219	0.000126	0.000108	0.000217	0.000163
NPDC1	4.18E-05	5.79E-06	8.33E-06	1.10E-05	2.07E-05	6.31E-06	1.36E-05	9.74E-06
NPEPL1	1.64E-05	1.92E-06	5.20E-06	1.01E-05	1.65E-05	9.67E-06	1.18E-05	7.64E-06
NPEPPS	3.14E-05	2.32E-05	9.37E-06	1.03E-05	4.38E-06	1.53E-05	7.98E-06	7.37E-06

NPFF	5.90E-07	6.53E-08	1.92E-07	3.37E-07	2.25E-06	6.58E-07	5.98E-07	1.64E-07
NPFFR1	3.95E-08	0	0	0	0	0	0	0
NPFFR2	3.48E-06	7.08E-08	2.21E-08	6.17E-08	0	0	1.20E-08	0
NPHP1	1.07E-06	1.39E-07	2.95E-07	7.19E-07	1.29E-07	1.09E-06	5.69E-07	2.15E-07
NPHP3	4.67E-06	1.86E-06	1.57E-06	2.42E-06	6.02E-07	2.13E-06	1.41E-06	1.46E-06
NPHP4	3.57E-06	1.83E-07	6.81E-07	1.00E-06	7.40E-08	1.25E-07	1.69E-07	1.94E-07
NPHS1	1.39E-08	0	0	0	0	0	0	0
NPHS2	1.29E-07	0	0	0	0	0	0	0
NPIP	2.67E-05	1.24E-05	4.75E-06	6.80E-06	6.48E-06	9.45E-06	1.08E-05	8.96E-06
NPIPL3	1.30E-05	5.88E-06	2.76E-06	6.48E-06	1.07E-05	3.42E-06	8.38E-06	5.99E-06
NPL	1.46E-05	7.04E-06	1.02E-05	1.70E-05	6.64E-07	2.40E-05	1.64E-05	2.80E-05
NPLOC4	6.55E-05	5.24E-05	1.86E-05	1.90E-05	3.00E-05	3.99E-05	2.59E-05	3.10E-05
NPM1	0.000312	0.000125	0.000131	0.000196	9.70E-05	0.000275	0.000213	0.000218
NPM2	3.74E-06	1.82E-05	4.16E-07	7.31E-06	7.66E-06	2.97E-06	1.08E-05	5.31E-07
NPM3	3.46E-05	1.24E-05	7.82E-06	1.66E-05	2.13E-05	2.26E-05	1.33E-05	3.16E-06
NPNT	1.55E-05	1.40E-06	4.94E-07	2.72E-06	4.19E-07	3.31E-07	6.48E-07	1.05E-06
NPPA	3.26E-07	0	3.65E-08	4.05E-08	5.67E-08	1.22E-07	0	1.96E-07
NPPB	0	0	0	3.03E-06	0	0	0	0
NPPC	0	1.51E-07	0	0	0	0	0	0
NPR1	1.03E-05	6.42E-06	4.41E-06	3.64E-06	4.76E-06	3.06E-06	9.82E-06	3.61E-06
NPR2	2.96E-06	1.59E-05	5.14E-06	1.02E-05	8.42E-06	1.51E-05	1.45E-05	2.51E-07
NPR3	1.73E-06	6.73E-06	7.94E-07	8.22E-07	1.14E-07	2.62E-07	1.66E-07	5.11E-07
NPRL2	1.71E-05	1.34E-05	9.99E-06	7.74E-06	1.32E-05	1.16E-05	1.34E-05	1.06E-05
NPRL3	1.46E-05	1.42E-05	6.74E-06	1.03E-05	9.77E-06	1.02E-05	1.38E-05	1.17E-05
NPS	0	0	0	0	0	0	0	0
NPSR1	0	0	0	0	0	0	0	0
NPTN	6.07E-05	3.00E-05	2.92E-05	3.52E-05	2.35E-05	3.48E-05	2.91E-05	6.32E-05
NPTX1	7.20E-07	0	0	0	3.55E-09	3.83E-09	2.01E-08	6.05E-09
NPTX2	8.41E-05	2.80E-07	1.69E-07	2.20E-06	1.48E-08	2.39E-08	6.88E-06	8.83E-08
NPTXR	5.94E-06	6.46E-08	3.20E-07	4.94E-07	2.45E-07	8.85E-06	1.54E-07	3.55E-07
NPVF	0	0	0	0	0	0	0	0
NPW	1.62E-05	2.16E-05	6.23E-06	2.29E-05	6.13E-05	7.34E-08	0.000222	7.81E-08
NPY	0	0	0	7.53E-08	0	0	0	0
NPY1R	2.81E-05	8.27E-07	5.17E-06	4.97E-06	6.13E-07	6.53E-07	4.42E-06	4.53E-07
NPY2R	0	0	0	0	0	6.29E-09	0	0
NPY5R	6.55E-06	2.98E-08	2.90E-07	4.67E-07	3.39E-08	1.22E-08	1.27E-08	5.80E-08
NPY6R	3.56E-07	2.85E-07	5.16E-08	3.01E-08	0	7.29E-08	1.31E-07	0
NQO1	2.19E-05	2.70E-05	1.64E-06	9.62E-07	2.18E-06	1.68E-06	2.85E-06	7.36E-06
NQO2	1.72E-05	5.92E-05	2.41E-05	2.02E-05	7.21E-05	8.89E-05	4.32E-05	0.000104
NR0B1	3.11E-08	0	0	3.82E-08	0	0	0	4.59E-08
NR0B2	9.60E-06	0.000106	0.000183	0.000165	4.07E-05	1.02E-05	8.15E-05	0.00031
NR1D1	5.94E-06	1.97E-05	3.90E-05	1.91E-05	4.28E-06	5.51E-06	1.59E-05	3.37E-05
NR1D2	7.76E-06	6.33E-06	1.78E-05	6.61E-06	3.15E-06	6.62E-06	7.80E-06	9.54E-06
NR1H2	4.88E-05	3.92E-05	3.09E-05	3.71E-05	2.48E-05	4.60E-05	3.53E-05	4.08E-05
NR1H3	4.28E-05	3.75E-05	4.67E-05	4.02E-05	4.53E-05	4.46E-05	4.13E-05	6.22E-05
NR1H4	4.05E-05	3.37E-05	5.53E-05	3.88E-05	1.63E-05	6.04E-05	3.14E-05	3.42E-05
NR1I2	3.54E-06	2.00E-05	3.76E-05	1.69E-05	1.09E-05	1.97E-05	2.25E-05	9.06E-07
NR1I3	2.53E-05	0.000187	7.14E-05	7.28E-05	0.00011	7.98E-05	7.49E-05	2.49E-05
NR2C1	7.94E-06	6.56E-06	3.48E-06	3.56E-06	2.78E-06	4.48E-06	3.23E-06	3.22E-06
NR2C2	6.19E-06	2.22E-06	1.23E-06	1.19E-06	1.08E-07	1.91E-06	8.71E-07	1.87E-06
NR2C2AP	1.75E-05	1.18E-05	4.76E-06	8.10E-06	1.50E-05	1.21E-05	2.00E-05	9.42E-06
NR2E1	8.49E-08	1.03E-07	0	2.59E-08	6.07E-09	1.51E-07	6.87E-09	1.04E-08
NR2E3	2.40E-08	0	3.78E-08	0	1.03E-08	1.12E-08	4.65E-08	5.31E-08
NR2F1	1.45E-06	1.88E-06	4.77E-06	8.30E-06	5.40E-07	3.82E-06	2.11E-06	2.06E-06
NR2F2	1.89E-05	8.09E-06	1.08E-05	7.17E-06	4.43E-06	6.15E-06	8.50E-06	7.88E-06
NR2F6	4.69E-05	3.97E-05	3.16E-05	3.63E-05	0.000123	2.67E-05	0.000112	3.46E-05
NR3C1	1.88E-05	2.13E-05	1.74E-05	1.57E-05	3.44E-06	3.21E-05	1.03E-05	1.99E-05
NR3C2	3.18E-06	1.15E-06	3.65E-06	2.88E-06	5.42E-07	1.36E-06	1.49E-06	2.01E-06

NR4A1	1.55E-05	5.78E-05	0.000107	0.000122	8.12E-06	7.01E-06	3.27E-05	1.97E-05
NR4A2	5.73E-06	2.96E-06	2.92E-05	1.70E-05	1.18E-07	4.70E-06	1.64E-06	1.30E-05
NR4A3	9.28E-07	1.10E-06	1.84E-06	1.74E-05	4.66E-08	9.04E-07	4.54E-07	9.09E-07
NR5A1	2.99E-08	1.68E-08	0	0	1.28E-08	3.46E-08	7.32E-07	1.09E-07
NR5A2	1.50E-05	9.09E-06	1.23E-05	1.10E-05	2.56E-06	1.58E-05	6.18E-06	2.82E-05
NR6A1	1.21E-06	1.74E-07	8.05E-08	6.27E-08	0	4.72E-07	8.63E-08	8.02E-07
NRADDP	5.26E-08	0	0	3.25E-08	2.28E-08	2.46E-08	0	0
NRAP	2.78E-07	3.15E-07	4.83E-07	2.68E-07	2.43E-07	5.75E-08	4.74E-07	5.72E-07
NRARP	1.22E-05	3.82E-06	2.87E-07	2.35E-06	2.02E-06	1.10E-06	3.87E-06	2.21E-06
NRAS	3.41E-05	3.46E-05	1.06E-05	1.34E-05	5.70E-06	1.94E-05	1.08E-05	1.37E-05
NRBF2	2.93E-05	2.25E-05	2.53E-05	2.19E-05	5.98E-06	2.11E-05	2.30E-05	2.13E-05
NRBP1	8.15E-05	9.96E-05	2.70E-05	4.59E-05	2.61E-05	6.62E-05	4.83E-05	5.25E-05
NRBP2	8.80E-05	7.20E-05	1.91E-05	1.58E-05	1.40E-05	1.91E-05	5.34E-05	2.79E-05
NRCAM	4.92E-06	4.03E-07	1.82E-08	1.10E-07	2.84E-08	3.48E-07	1.45E-07	4.38E-07
NRD1	7.98E-05	4.85E-05	3.23E-05	2.68E-05	1.18E-05	5.35E-05	1.90E-05	4.86E-05
NRF1	3.39E-06	2.95E-06	2.02E-06	3.27E-06	2.09E-06	2.39E-06	2.87E-06	2.92E-06
NRG1	1.11E-05	2.41E-05	2.98E-06	1.04E-05	2.82E-06	1.52E-06	3.62E-06	1.19E-06
NRG2	3.61E-06	3.36E-07	1.59E-08	1.03E-07	0	2.84E-08	9.73E-08	1.13E-08
NRG3	2.93E-06	2.69E-08	1.31E-07	1.88E-07	6.86E-09	0	7.74E-09	2.34E-08
NRG4	4.73E-07	4.45E-07	1.69E-06	8.78E-07	0	2.50E-06	2.53E-08	3.68E-07
NRGN	5.53E-06	4.01E-06	7.96E-07	3.38E-06	3.81E-06	8.46E-07	1.76E-06	2.76E-06
NRIP1	8.59E-06	6.00E-06	1.07E-05	9.57E-06	2.36E-06	1.12E-05	4.08E-06	5.70E-06
NRIP2	1.32E-06	5.14E-07	2.00E-07	6.06E-07	1.22E-06	2.95E-07	2.85E-07	1.56E-06
NRIP3	9.59E-08	6.72E-08	9.60E-08	4.03E-07	1.03E-08	1.66E-08	6.41E-08	2.63E-08
NRK	1.86E-08	1.39E-08	0	8.93E-08	5.31E-09	0	0	1.52E-07
NRL	1.09E-06	1.25E-06	9.48E-07	1.02E-06	1.97E-06	7.88E-07	1.49E-06	9.53E-07
NRM	1.22E-05	7.71E-06	2.46E-06	3.80E-06	8.20E-06	2.75E-06	6.17E-06	6.13E-06
NRN1	1.86E-06	2.25E-06	2.13E-06	1.35E-06	1.08E-06	1.27E-06	3.80E-06	1.35E-06
NRN1L	4.05E-07	5.76E-07	3.28E-07	6.82E-07	8.16E-07	9.47E-07	2.79E-07	0
NRP1	3.62E-05	1.44E-05	1.28E-05	1.77E-05	4.96E-06	2.58E-05	1.39E-05	9.95E-06
NRP2	4.74E-06	1.30E-06	2.40E-06	2.89E-06	7.55E-07	1.15E-06	2.07E-06	1.58E-06
NRSN1	9.45E-08	0	1.02E-08	0	0	3.63E-08	0	0
NRSN2	5.02E-05	4.08E-05	1.88E-06	6.80E-06	8.11E-07	1.26E-06	2.51E-06	1.77E-06
NRTN	4.03E-06	6.49E-06	4.92E-06	3.10E-06	6.13E-06	8.62E-07	5.08E-06	1.00E-05
NRXN1	0	0	1.75E-08	8.32E-08	0	0	0	0
NRXN2	6.46E-07	5.85E-07	6.15E-07	1.69E-06	3.68E-08	9.25E-06	5.52E-07	1.29E-07
NRXN3	5.19E-07	8.46E-08	7.25E-08	8.29E-08	4.37E-08	5.70E-07	2.17E-07	1.57E-07
NSA2	6.67E-05	9.04E-05	4.57E-05	8.45E-05	3.74E-05	7.37E-05	7.03E-05	6.88E-05
NSD1	6.56E-06	1.77E-06	1.83E-06	2.91E-06	1.02E-06	3.57E-06	2.14E-06	3.25E-06
NSDHL	3.31E-05	7.62E-05	1.90E-05	1.54E-05	2.49E-05	3.68E-05	2.05E-05	3.30E-05
NSF	1.82E-05	2.40E-05	6.38E-06	8.43E-06	3.75E-06	1.98E-05	7.40E-06	1.29E-05
NSFL1C	3.56E-05	3.91E-05	1.76E-05	1.98E-05	1.85E-05	2.36E-05	1.74E-05	2.76E-05
NSL1	2.48E-06	6.18E-06	1.49E-06	1.52E-06	5.34E-07	2.68E-06	1.49E-06	1.69E-06
NSMAF	7.51E-06	2.52E-06	1.94E-06	3.21E-06	9.32E-07	2.99E-06	7.22E-06	3.29E-06
NSMCE1	6.92E-05	0.000107	3.84E-05	4.84E-05	5.47E-05	7.92E-05	8.58E-05	5.27E-05
NSMCE2	5.04E-05	1.74E-05	4.70E-06	9.35E-06	7.81E-06	1.87E-05	2.92E-05	1.88E-05
NSMCE4A	2.29E-05	9.02E-06	1.51E-05	1.91E-05	1.49E-05	2.36E-05	2.00E-05	1.52E-05
NSUN2	2.48E-05	5.44E-05	2.56E-05	2.72E-05	1.14E-05	2.63E-05	2.82E-05	4.29E-05
NSUN3	4.73E-06	6.93E-06	3.65E-06	3.82E-06	8.08E-07	5.63E-06	1.87E-06	3.63E-06
NSUN4	5.64E-06	6.18E-06	3.61E-06	3.95E-06	9.95E-07	5.73E-06	2.67E-06	5.29E-06
NSUN5	1.77E-05	4.40E-05	9.72E-06	1.40E-05	1.71E-05	1.66E-05	2.36E-05	2.16E-05
NSUN5P1	2.82E-06	3.26E-06	7.96E-07	2.50E-06	3.86E-06	2.27E-06	1.71E-06	2.66E-06
NSUN5P2	6.75E-06	8.03E-06	1.49E-06	2.49E-06	7.01E-06	5.78E-06	2.01E-06	5.77E-06
NSUN6	1.35E-05	1.11E-05	1.29E-05	2.43E-05	1.04E-05	1.08E-05	1.37E-05	2.67E-05
NSUN7	1.33E-06	7.19E-09	1.06E-07	3.53E-07	0	2.55E-08	1.87E-08	1.95E-08
NT5C	4.47E-05	2.66E-05	1.07E-05	1.94E-05	3.18E-05	2.77E-05	2.68E-05	1.16E-05
NT5C1A	0	0	0	0	0	0	0	0
NT5C1B	5.62E-08	0	1.65E-08	3.90E-08	0	4.43E-08	1.72E-08	1.31E-08

NT5C2	1.69E-05	7.12E-06	5.55E-06	8.80E-06	4.73E-06	8.84E-06	8.07E-06	8.11E-06
NT5C3	2.99E-05	1.37E-05	4.78E-06	5.72E-06	2.62E-06	8.11E-06	4.66E-06	1.08E-05
NT5C3L	4.59E-05	8.97E-05	2.46E-05	3.33E-05	5.12E-05	5.58E-05	3.17E-05	3.60E-05
NT5DC1	1.05E-05	1.28E-05	1.91E-05	1.32E-05	3.75E-06	2.27E-05	7.10E-06	1.09E-05
NT5DC2	3.32E-05	1.69E-06	1.89E-06	5.31E-06	5.67E-06	6.89E-06	8.44E-06	6.91E-06
NT5DC3	1.93E-06	2.39E-06	9.67E-07	1.02E-06	8.36E-07	1.67E-06	1.26E-06	2.51E-06
NT5E	2.19E-05	5.54E-06	2.36E-05	3.04E-05	9.44E-06	1.02E-05	1.99E-05	2.10E-05
NT5M	1.55E-06	9.91E-06	7.56E-07	9.33E-07	1.56E-06	3.47E-06	2.24E-06	8.48E-07
NTAN1	1.81E-05	2.43E-05	4.08E-05	6.51E-05	1.99E-05	2.25E-05	3.01E-05	7.63E-05
NTF3	9.47E-08	2.30E-07	2.99E-06	3.03E-06	3.02E-07	1.86E-07	1.70E-07	1.98E-07
NTF4	2.70E-08	0	0	0	0	0	0	4.02E-08
NTHL1	3.23E-05	6.01E-05	3.02E-05	2.83E-05	6.37E-05	4.09E-05	4.10E-05	2.54E-05
NTM	4.09E-06	2.09E-08	4.81E-08	5.25E-07	7.96E-09	6.47E-08	5.28E-07	3.60E-07
NTN1	1.21E-07	4.75E-06	1.69E-06	1.85E-06	5.39E-07	3.12E-06	5.38E-06	7.99E-06
NTN3	2.42E-08	3.42E-07	4.05E-07	4.44E-08	0	0	2.92E-07	0
NTN4	5.94E-06	9.13E-06	1.43E-05	1.04E-05	1.16E-06	6.28E-06	5.65E-06	1.68E-06
NTN5	1.37E-07	2.47E-07	3.66E-08	2.41E-08	3.68E-07	9.41E-08	1.49E-07	1.44E-08
NTNG1	0	0	0	9.69E-09	0	1.65E-08	0	0
NTNG2	6.43E-07	5.04E-08	6.96E-08	8.23E-08	1.93E-08	3.46E-08	6.53E-08	3.29E-08
NTRK1	7.09E-08	0	2.84E-08	8.27E-08	0	2.68E-08	0	2.83E-08
NTRK2	1.40E-07	6.02E-08	1.71E-07	1.06E-06	1.04E-07	1.61E-08	1.88E-07	2.03E-07
NTRK3	4.13E-08	4.67E-08	6.46E-08	1.42E-07	5.81E-08	3.83E-08	7.87E-08	0
NTS	0.002462	2.58E-06	1.79E-07	1.46E-06	7.19E-08	5.82E-08	7.98E-08	2.41E-06
NTSR1	5.51E-09	3.33E-07	1.70E-08	2.02E-08	0	1.02E-08	2.14E-08	0
NTSR2	0	0	0	0	0	0	0	0
NUAK1	4.90E-06	3.18E-06	1.45E-06	8.98E-07	7.61E-07	2.36E-06	2.56E-06	2.76E-06
NUAK2	5.16E-06	2.40E-06	1.61E-06	1.24E-05	3.08E-07	8.02E-06	2.90E-06	2.99E-06
NUB1	2.36E-05	2.68E-05	2.22E-05	2.19E-05	1.26E-05	3.29E-05	2.73E-05	3.44E-05
NUBP1	2.43E-05	2.93E-05	1.74E-05	2.41E-05	8.71E-06	2.10E-05	2.95E-05	2.09E-05
NUBP2	5.04E-05	8.48E-05	4.45E-05	4.19E-05	4.27E-05	4.96E-05	5.77E-05	4.28E-05
NUBPL	4.49E-06	5.30E-06	5.13E-06	4.92E-06	2.21E-06	1.02E-05	3.51E-06	3.63E-06
NUCB1	0.000247	0.000474	0.000245	0.000213	0.000437	0.000374	0.00025	0.000144
NUCB2	3.76E-05	7.53E-05	2.86E-05	3.29E-05	1.88E-05	7.78E-05	1.87E-05	7.45E-05
NUCKS1	4.30E-05	3.65E-05	4.19E-05	3.31E-05	2.76E-05	6.65E-05	4.38E-05	5.58E-05
NUDC	0.000127	0.000154	8.59E-05	0.000115	9.60E-05	8.82E-05	0.000123	0.000134
NUDCD1	1.22E-05	6.20E-06	2.40E-06	2.61E-06	1.10E-06	3.34E-06	4.53E-06	3.32E-06
NUDCD2	3.40E-05	2.39E-05	2.55E-05	2.01E-05	1.96E-05	3.66E-05	2.27E-05	3.62E-05
NUDCD3	2.69E-05	2.02E-05	1.01E-05	1.18E-05	1.21E-05	1.74E-05	1.82E-05	2.58E-05
NUDT1	3.08E-05	1.95E-05	3.04E-06	8.29E-06	1.93E-05	5.26E-06	9.36E-06	1.12E-05
NUDT10	6.30E-08	1.43E-08	4.63E-07	3.55E-07	5.17E-08	1.17E-08	8.52E-08	3.71E-08
NUDT11	1.18E-07	3.34E-08	2.06E-08	2.30E-07	8.49E-09	9.15E-09	1.91E-08	4.35E-08
NUDT12	7.23E-06	1.23E-05	1.14E-05	4.98E-06	2.32E-06	1.15E-05	3.12E-06	8.61E-06
NUDT13	3.97E-06	1.29E-06	1.37E-06	2.75E-06	1.53E-06	3.29E-06	4.36E-06	2.12E-06
NUDT14	4.82E-05	3.14E-06	1.27E-05	1.79E-05	3.99E-06	2.54E-05	3.05E-05	2.65E-05
NUDT15	2.35E-05	8.58E-06	6.74E-06	7.66E-06	5.09E-06	8.58E-06	5.71E-06	8.25E-06
NUDT16	2.84E-05	3.50E-05	1.55E-05	1.48E-05	2.14E-05	2.27E-05	1.17E-05	1.44E-05
NUDT16L1	2.95E-05	4.87E-05	2.33E-05	2.25E-05	1.58E-05	2.88E-05	3.22E-05	2.18E-05
NUDT16P1	1.36E-05	1.31E-05	5.17E-06	2.58E-06	2.44E-06	9.96E-06	4.75E-06	2.23E-06
NUDT17	1.74E-06	6.50E-07	4.32E-07	2.85E-07	2.40E-07	1.18E-06	5.39E-07	1.05E-06
NUDT18	4.28E-06	1.96E-06	2.67E-06	3.41E-06	1.83E-06	8.31E-06	1.91E-06	2.82E-06
NUDT19	3.90E-06	1.16E-05	3.16E-06	3.58E-06	2.38E-06	1.33E-06	7.91E-06	7.24E-06
NUDT2	7.58E-06	1.10E-05	1.83E-05	1.77E-05	2.06E-05	4.00E-05	1.98E-05	1.40E-05
NUDT21	1.82E-05	1.25E-05	9.96E-06	1.05E-05	2.24E-06	1.30E-05	7.08E-06	7.03E-06
NUDT22	4.29E-05	8.87E-05	2.48E-05	2.53E-05	4.21E-05	2.47E-05	1.65E-05	4.93E-05
NUDT3	1.88E-05	1.80E-05	9.83E-06	1.25E-05	1.23E-05	1.19E-05	1.20E-05	1.96E-05
NUDT4	2.35E-05	3.60E-06	8.25E-06	6.77E-06	7.44E-06	1.20E-05	9.47E-06	5.83E-06
NUDT5	5.94E-05	9.33E-05	4.41E-05	3.94E-05	2.07E-05	9.49E-05	4.58E-05	5.15E-05
NUDT6	4.30E-06	7.99E-06	1.88E-05	1.14E-05	9.28E-06	3.85E-05	1.30E-05	1.40E-05

NUDT7	4.40E-05	4.66E-05	2.56E-05	2.45E-05	1.07E-05	0.000101	2.04E-05	2.20E-05
NUDT8	1.54E-05	2.05E-05	3.40E-05	1.61E-05	3.19E-05	4.28E-05	8.49E-06	2.19E-05
NUDT9	4.61E-05	2.16E-05	2.27E-05	2.04E-05	1.57E-05	2.93E-05	3.06E-05	4.31E-05
NUDT9P1	2.44E-07	8.46E-08	2.39E-07	2.71E-07	8.44E-08	2.28E-07	9.32E-08	3.63E-08
NUF2	5.97E-06	5.33E-06	4.14E-08	1.77E-07	3.05E-07	1.06E-06	1.55E-07	8.21E-07
NUFIP1	3.03E-06	2.94E-06	1.81E-06	2.24E-06	6.95E-07	2.61E-06	2.60E-06	2.01E-06
NUFIP2	9.46E-06	1.32E-05	5.28E-06	6.60E-06	2.95E-06	6.78E-06	5.73E-06	7.43E-06
NUMA1	7.11E-05	3.87E-05	2.60E-05	3.21E-05	1.69E-05	2.84E-05	2.64E-05	4.12E-05
NUMB	3.65E-05	1.58E-05	2.22E-05	1.82E-05	9.65E-06	2.25E-05	1.53E-05	2.17E-05
NUMBL	3.46E-06	2.39E-06	7.17E-07	2.55E-06	9.80E-07	1.56E-06	7.54E-07	1.19E-06
NUP107	1.24E-05	1.03E-05	4.59E-06	5.91E-06	1.74E-06	8.10E-06	4.43E-06	5.06E-06
NUP133	1.27E-05	1.11E-05	5.69E-06	6.25E-06	2.92E-06	1.22E-05	5.47E-06	8.69E-06
NUP153	9.81E-06	1.47E-05	7.07E-06	6.88E-06	1.36E-06	9.20E-06	4.73E-06	7.90E-06
NUP155	5.13E-06	5.26E-06	2.27E-06	1.72E-06	3.49E-07	4.26E-06	1.58E-06	2.51E-06
NUP160	1.54E-05	1.17E-05	5.78E-06	5.15E-06	1.05E-06	8.09E-06	2.80E-06	6.15E-06
NUP188	1.14E-05	3.96E-06	3.31E-06	4.54E-06	1.23E-06	4.95E-06	2.89E-06	4.53E-06
NUP205	9.39E-06	8.40E-06	4.02E-06	5.52E-06	3.15E-06	7.05E-06	5.33E-06	8.32E-06
NUP210	1.27E-05	1.09E-05	4.69E-06	4.50E-06	1.72E-06	5.68E-06	5.20E-06	5.47E-06
NUP210L	0	0	0	6.71E-09	0	2.59E-08	1.07E-08	0
NUP214	1.81E-05	9.45E-06	1.09E-05	1.16E-05	6.87E-06	1.66E-05	1.16E-05	1.68E-05
NUP35	1.31E-05	7.24E-06	5.34E-06	7.43E-06	2.37E-06	5.06E-06	9.56E-06	4.37E-06
NUP37	1.88E-05	1.16E-05	4.91E-06	6.88E-06	3.18E-06	1.40E-05	5.77E-06	5.93E-06
NUP43	9.59E-06	6.53E-06	3.98E-06	2.79E-06	2.31E-06	3.55E-06	5.39E-06	5.04E-06
NUP50	2.70E-05	1.31E-05	8.10E-06	7.57E-06	4.51E-06	9.18E-06	8.07E-06	1.19E-05
NUP54	1.70E-05	7.49E-06	7.73E-06	1.01E-05	5.14E-06	1.19E-05	9.00E-06	1.04E-05
NUP62	2.35E-05	1.66E-05	1.16E-05	1.25E-05	8.23E-06	1.77E-05	8.33E-06	1.33E-05
NUP62CL	1.75E-06	4.70E-07	3.55E-07	2.37E-07	5.43E-08	0	6.07E-08	1.22E-07
NUP85	3.29E-05	1.84E-05	1.07E-05	1.05E-05	1.09E-05	1.53E-05	1.13E-05	1.01E-05
NUP88	1.57E-05	1.68E-05	1.69E-05	2.10E-05	5.12E-06	2.48E-05	1.84E-05	1.52E-05
NUP93	1.35E-05	5.22E-06	3.22E-06	6.10E-06	2.24E-06	7.16E-06	5.59E-06	6.31E-06
NUP98	3.01E-05	4.11E-05	1.74E-05	1.94E-05	3.65E-06	2.13E-05	1.11E-05	2.51E-05
NUPL1	8.59E-06	7.93E-06	5.79E-06	6.43E-06	1.07E-06	7.60E-06	5.17E-06	4.49E-06
NUPL2	2.92E-05	2.47E-05	7.36E-06	7.98E-06	3.00E-06	1.37E-05	6.81E-06	1.61E-05
NUPR1	0.000214	0.000545	0.000194	0.000123	0.000365	0.000124	0.000315	0.000251
NUS1	1.76E-05	1.43E-05	1.28E-05	1.03E-05	2.81E-06	1.74E-05	8.98E-06	1.22E-05
NUSAP1	2.70E-05	1.26E-05	2.50E-06	3.74E-06	2.36E-06	5.37E-06	2.24E-06	4.01E-06
NUTF2	9.97E-05	6.10E-05	4.58E-05	7.16E-05	4.89E-05	7.88E-05	7.83E-05	7.41E-05
NVL	5.68E-06	4.85E-06	2.37E-06	3.22E-06	1.87E-06	6.34E-06	3.24E-06	4.25E-06
NWD1	0	6.71E-09	3.07E-09	7.34E-09	5.16E-09	0	0	0
NXF1	3.53E-05	2.43E-05	1.06E-05	2.04E-05	8.91E-06	2.20E-05	1.61E-05	2.03E-05
NXF2	0	9.18E-08	0	0	0	0	0	0
NXF2B	0	0	0	0	0	0	0	0
NXF3	0	2.12E-08	4.45E-07	3.90E-07	0	0	1.78E-08	0
NXF4	0	0	0	0	0	0	0	0
NXF5	0	3.47E-08	0	0	0	0	0	0
NXN	1.78E-05	9.67E-07	2.84E-06	6.84E-06	4.56E-06	1.41E-06	2.71E-06	6.70E-06
NXNL1	0	0	0	0	2.62E-08	0	0	0
NXNL2	2.62E-07	0	3.96E-08	2.10E-08	0	0	0	0
NXPH1	2.38E-08	1.52E-07	2.47E-08	0	0	0	0	0
NXPH2	2.75E-08	0	9.51E-09	1.12E-08	0	0	0	0
NXPH3	1.44E-07	1.04E-07	6.22E-08	7.41E-08	9.38E-08	7.36E-08	2.40E-07	1.18E-07
NXPH4	3.30E-05	1.75E-06	5.66E-08	1.81E-07	1.39E-07	9.98E-08	7.30E-06	5.54E-07
NXT1	1.24E-05	1.01E-05	4.08E-06	1.63E-05	8.02E-06	7.36E-06	2.02E-05	1.12E-05
NXT2	1.51E-05	4.94E-06	3.50E-06	3.61E-06	2.55E-06	5.75E-06	3.67E-06	5.90E-06
NYNRIN	2.93E-06	3.56E-06	2.98E-06	1.52E-06	6.36E-07	2.83E-07	2.04E-06	4.96E-07
NYX	0	0	0	0	0	0	8.62E-09	0
OAF	9.65E-05	8.47E-05	0.000216	0.000179	0.000111	0.000193	0.000122	0.000301
OAS1	2.99E-05	1.45E-05	3.52E-05	2.16E-05	6.15E-06	2.81E-05	1.78E-05	3.85E-05

OAS2	1.82E-05	1.76E-06	1.51E-05	7.05E-06	1.54E-06	1.84E-05	3.05E-06	3.60E-05
OAS3	1.44E-05	4.65E-06	4.38E-06	2.76E-06	3.28E-07	6.20E-06	1.60E-06	1.03E-05
OASL	1.64E-05	1.53E-05	6.99E-06	1.47E-05	3.02E-06	8.88E-06	1.29E-05	1.46E-05
OAT	0.000287	1.72E-06	5.51E-05	7.73E-06	4.53E-07	8.02E-05	4.13E-06	2.15E-05
OAZ1	0.000566	0.000377	0.000313	0.000353	0.000619	0.00038	0.000524	0.000337
OAZ2	5.11E-05	4.27E-05	4.56E-05	5.16E-05	4.54E-05	4.90E-05	6.41E-05	3.59E-05
OAZ3	9.50E-07	6.93E-07	4.35E-07	4.49E-07	5.70E-07	2.34E-06	1.00E-06	1.13E-06
OBFC1	4.83E-06	1.56E-06	1.33E-06	1.77E-06	2.03E-06	2.71E-06	2.51E-06	1.80E-06
OBFC2A	8.12E-06	2.98E-06	1.30E-06	3.56E-06	2.94E-07	1.98E-06	1.38E-06	7.22E-07
OBFC2B	3.29E-05	3.01E-05	8.68E-06	1.15E-05	1.99E-05	2.19E-05	1.78E-05	1.32E-05
OBP2A	0	0	0	0	0	0	0	0
OBP2B	0	0	0	0	0	0	0	0
OBSCN	2.71E-06	2.27E-07	3.02E-07	9.16E-07	1.53E-07	1.26E-07	2.50E-07	4.14E-07
OBSL1	5.93E-05	2.66E-05	2.02E-05	3.09E-05	3.13E-05	2.70E-05	3.79E-05	2.97E-05
OC90	0	0	0	0	0	0	0	0
OCA2	0	3.62E-06	7.61E-08	9.23E-09	6.48E-09	1.40E-08	7.32E-09	1.88E-07
OCEL1	4.21E-05	2.37E-05	3.50E-05	3.14E-05	5.42E-05	3.91E-05	8.24E-05	2.42E-05
OCIAD1	0.000125	0.000127	7.58E-05	6.40E-05	4.74E-05	9.32E-05	7.04E-05	7.90E-05
OCIAD2	0.000148	0.000174	0.000142	0.000138	9.17E-05	0.000113	0.000136	7.38E-05
OCLM	5.93E-07	3.65E-08	0	3.87E-08	0	5.84E-08	0	4.67E-08
OCLN	3.39E-05	4.30E-05	8.48E-06	1.62E-05	8.43E-07	6.23E-06	9.28E-06	7.21E-06
OCM	8.55E-08	0	0	0	1.12E-07	1.21E-07	4.01E-08	6.46E-08
OCM2	0	0	0	0	0	0	0	0
OCRL	1.27E-05	7.49E-06	4.54E-06	5.72E-06	6.47E-06	7.88E-06	7.60E-06	1.95E-05
ODAM	0	0	0	0	0	5.67E-05	5.68E-08	2.92E-08
ODC1	0.000361	8.50E-05	3.21E-05	9.43E-05	6.18E-05	7.37E-05	8.28E-05	0.000103
ODF1	0	0	0	0	0	0	0	0
ODF2	2.17E-05	7.15E-06	5.15E-06	9.29E-06	4.11E-06	2.20E-05	9.39E-06	9.46E-06
ODF2L	5.31E-06	4.16E-07	6.35E-07	2.35E-06	1.20E-07	2.76E-06	5.30E-07	5.34E-07
ODF3	0	0	0	0	0	0	0	0
ODF3B	2.02E-05	6.60E-06	1.74E-05	2.38E-05	1.44E-05	1.73E-05	3.46E-05	6.65E-05
ODF3L1	1.66E-06	4.47E-07	3.03E-07	5.03E-06	1.87E-08	1.81E-07	8.48E-07	1.28E-07
ODF3L2	3.32E-08	1.70E-07	1.79E-07	1.02E-07	4.29E-08	2.01E-07	1.28E-07	5.64E-07
ODF4	0	0	0	0	0	0	0	0
ODZ1	4.61E-07	1.16E-06	6.46E-07	3.27E-07	1.47E-09	4.43E-08	6.68E-09	3.30E-07
ODZ2	2.92E-09	3.26E-09	5.66E-07	7.94E-07	0	4.34E-06	6.34E-07	1.37E-06
ODZ3	8.18E-09	0	9.15E-08	3.74E-08	0	0	1.99E-09	8.95E-09
ODZ4	8.03E-08	1.97E-08	5.09E-08	1.73E-07	2.43E-09	1.05E-08	2.49E-08	5.39E-08
OFD1	1.69E-05	1.47E-06	3.11E-06	4.27E-06	1.45E-06	4.14E-06	2.96E-06	5.98E-06
OGDH	7.85E-05	0.000108	3.00E-05	3.04E-05	8.61E-06	4.50E-05	2.19E-05	5.70E-05
OGDHL	5.03E-05	0.000113	7.57E-05	5.68E-05	2.90E-05	5.82E-05	5.20E-05	2.06E-05
OGFOD1	1.78E-05	1.14E-05	7.86E-06	1.07E-05	2.75E-06	1.31E-05	6.77E-06	1.11E-05
OGFOD2	1.10E-05	1.47E-05	1.10E-05	9.49E-06	1.53E-05	1.50E-05	1.29E-05	8.77E-06
OGFR	4.58E-05	4.18E-05	3.56E-05	4.05E-05	2.98E-05	2.66E-05	4.70E-05	3.36E-05
OGFRL1	3.97E-06	1.16E-06	1.80E-06	3.60E-06	4.46E-07	1.73E-06	2.09E-06	2.33E-06
OGG1	2.20E-05	8.69E-06	9.37E-06	7.48E-06	8.21E-06	1.21E-05	1.59E-05	5.68E-06
OGN	2.54E-07	1.91E-07	1.34E-07	1.75E-06	3.04E-07	1.20E-07	3.35E-08	3.67E-07
OGT	3.36E-05	1.21E-05	1.00E-05	5.73E-06	3.57E-06	1.44E-05	6.68E-06	1.41E-05
OIP5	4.70E-06	4.89E-06	1.94E-07	5.04E-07	6.69E-07	8.40E-07	1.63E-07	5.64E-07
OIT3	6.54E-06	6.31E-06	3.89E-05	2.22E-05	1.15E-06	3.53E-06	2.96E-06	1.91E-06
OLA1	2.35E-05	1.61E-05	7.90E-06	8.05E-06	6.70E-06	1.47E-05	1.17E-05	8.85E-06
OLAH	0	0	0	8.96E-08	1.26E-08	0	2.81E-08	0
OLFM1	2.73E-07	1.00E-07	1.11E-06	3.65E-06	6.37E-07	4.39E-07	1.57E-06	4.91E-07
OLFM2	0.000104	2.65E-05	5.21E-05	1.31E-05	1.67E-05	3.38E-05	3.35E-05	0.000171
OLFM3	0	3.02E-07	0	8.88E-09	0	0	0	1.17E-06
OLFM4	0	1.86E-08	2.13E-08	4.05E-08	0	1.90E-08	9.91E-09	0
OLFML1	2.13E-06	2.77E-06	3.97E-06	4.95E-06	1.22E-06	1.38E-06	1.45E-06	1.21E-06
OLFML2A	3.74E-06	1.98E-06	2.46E-07	8.36E-07	1.50E-06	5.45E-07	1.76E-06	2.69E-06

OLFML2B	1.21E-05	2.85E-06	4.78E-07	2.40E-06	1.64E-06	3.61E-06	6.69E-06	3.96E-06
OLFML3	9.10E-06	1.06E-06	1.62E-05	2.98E-05	2.53E-06	9.44E-07	3.67E-06	3.59E-06
OLIG1	0	1.17E-08	4.33E-08	2.54E-08	0	0	0	0
OLIG2	9.44E-09	0	0	0	0	0	0	1.39E-08
OLIG3	0	0	0	0	0	0	0	0
OLR1	6.35E-07	3.07E-07	2.40E-07	9.26E-06	9.73E-09	2.26E-06	4.38E-07	3.33E-08
OMA1	1.38E-05	1.17E-05	1.24E-05	9.17E-06	3.13E-06	2.24E-05	6.35E-06	1.17E-05
OMD	6.46E-08	4.16E-08	1.82E-07	8.80E-07	5.55E-08	4.27E-08	1.61E-07	1.08E-07
OMG	2.54E-08	4.32E-08	0	3.12E-08	0	1.18E-08	1.23E-08	0
OMP	1.71E-06	5.39E-07	0	0	1.26E-07	6.79E-08	6.53E-08	1.11E-07
ONECUT1	1.37E-05	8.11E-06	4.04E-06	1.50E-06	1.94E-07	2.67E-06	1.10E-06	5.37E-06
ONECUT2	5.71E-06	4.38E-06	4.01E-06	2.39E-06	1.24E-06	1.36E-05	2.85E-06	4.27E-06
ONECUT3	4.52E-07	0	0	0	0	0	0	0
OOEP	3.22E-07	5.33E-08	0	2.63E-07	7.77E-08	0	0	1.16E-06
OPA1	1.41E-05	1.98E-05	1.05E-05	8.52E-06	2.86E-06	1.39E-05	6.86E-06	1.14E-05
OPA3	9.54E-06	6.28E-06	4.02E-06	5.18E-06	8.37E-06	6.12E-06	6.95E-06	6.50E-06
OPALIN	0	0	0	0	0	0	0	0
OPCML	1.07E-08	3.17E-08	2.54E-08	2.60E-08	6.10E-09	0	4.50E-08	4.15E-08
OPHN1	9.70E-07	1.63E-07	1.36E-07	2.14E-07	3.52E-08	2.79E-07	5.13E-08	5.55E-07
OPLAH	4.56E-05	6.12E-05	1.91E-05	1.33E-05	1.09E-05	1.36E-05	2.90E-05	2.02E-05
OPN1LW	0	2.39E-08	0	2.56E-08	0	0	0	0
OPN1MW	0	0	0	0	0	0	0	0
OPN1SW	7.17E-08	0	0	0	0	2.23E-08	2.28E-07	0
OPN3	2.73E-05	4.11E-05	8.28E-06	1.57E-05	2.19E-07	1.59E-05	5.74E-06	2.68E-05
OPN4	0	3.37E-08	0	0	2.57E-08	9.22E-09	7.13E-07	0
OPN5	0	0	0	0	0	1.24E-08	0	0
OPRD1	0	0	0	0	0	0	0	0
OPRK1	8.19E-08	5.09E-09	4.66E-09	3.89E-08	0	8.00E-08	0	0
OPRL1	1.61E-06	5.26E-08	2.36E-07	3.26E-07	1.27E-07	1.43E-07	4.84E-07	6.87E-08
OPRM1	0	0	0	0	0	0	0	0
OPTC	0	0	0	0	0	0	0	0
OPTN	7.47E-05	3.12E-05	3.83E-05	3.87E-05	1.76E-05	2.56E-05	4.23E-05	4.48E-05
OR10A2	0	0	0	0	0	0	0	0
OR10A3	0	0	0	0	0	0	0	0
OR10A4	0	0	0	0	0	0	0	0
OR10A5	0	0	0	0	0	0	0	0
OR10A6	0	0	0	0	0	0	0	0
OR10A7	0	0	0	0	0	0	0	0
OR10AD1	2.83E-08	0	0	0	0	0	0	0
OR10AG1	0	0	0	0	0	0	0	0
OR10C1	0	0	0	0	0	0	0	0
OR10G2	0	0	0	0	0	2.72E-08	0	0
OR10G3	0	0	0	0	0	0	0	0
OR10G4	0	0	0	0	0	0	0	0
OR10G7	0	0	0	0	0	0	0	0
OR10G8	0	0	0	0	0	0	0	0
OR10G9	0	0	0	0	0	0	0	0
OR10H1	0	0	0	0	0	4.34E-08	0	0
OR10H2	0	0	0	0	0	0	0	0
OR10H3	0	0	0	0	0	0	0	0
OR10H4	0	0	0	0	0	0	0	0
OR10H5	0	0	0	0	0	0	0	0
OR10J1	0	0	0	0	0	0	0	0
OR10J3	0	0	0	0	0	1.01E-07	0	0
OR10J5	0	6.83E-08	3.24E-08	0	0	1.10E-07	0	0
OR10K1	0	0	0	0	0	0	0	0
OR10K2	0	0	0	0	0	0	0	0
OR10P1	0	0	0	0	0	0	0	0

OR10Q1	0	0	0	0	0	2.63E-08	0	0
OR10R2	0	0	0	0	0	0	0	0
OR10S1	0	0	0	0	0	0	0	0
OR10T2	0	0	0	0	0	0	0	0
OR10V1	0	0	0	0	0	0	0	0
OR10W1	0	0	0	0	0	0	0	0
OR10X1	0	0	0	0	0	0	0	0
OR10Z1	0	0	0	0	0	0	0	0
OR11A1	0	0	0	0	0	0	0	3.13E-08
OR11G2	0	0	0	0	0	0	0	0
OR11H1	0	0	0	0	0	0	0	0
OR11H12	0	0	0	0	0	0	0	0
OR11H4	0	0	0	0	0	0	0	0
OR11H6	0	0	0	0	0	0	0	0
OR11L1	0	0	0	0	0	0	0	0
OR12D2	0	0	0	0	0	0	0	2.30E-07
OR12D3	0	0	0	0	0	0	0	0
OR13A1	1.03E-07	0	0	2.81E-08	0	1.84E-08	3.33E-08	0
OR13C2	0	0	0	0	0	0	0	0
OR13C3	0	0	0	0	0	0	0	0
OR13C4	0	0	0	0	0	0	0	0
OR13C5	0	3.29E-08	0	0	0	0	0	0
OR13C8	0	0	0	0	0	0	0	0
OR13C9	0	3.29E-08	0	0	0	0	0	0
OR13D1	0	0	0	0	0	0	0	0
OR13F1	0	0	0	0	0	0	0	0
OR13G1	0	0	0	0	0	0	0	0
OR13H1	0	0	0	0	0	0	0	0
OR13J1	0	0	0	0	0	2.70E-08	0	0
OR14A16	0	0	0	0	0	0	0	0
OR14C36	0	0	0	0	0	0	0	0
OR14I1	0	0	0	0	0	0	0	0
OR14J1	0	0	0	0	0	0	0	0
OR1A1	0	0	0	0	0	0	0	0
OR1A2	0	0	0	0	0	0	0	0
OR1B1	0	0	0	0	0	0	0	0
OR1C1	0	0	0	0	0	0	0	0
OR1D2	0	0	0	0	0	0	0	0
OR1D4	0	0	0	0	0	0	0	0
OR1E1	0	0	0	0	0	0	2.18E-07	0
OR1E2	0	0	0	0	0	0	0	0
OR1F1	0	7.41E-07	0	0	0	2.70E-08	0	1.73E-07
OR1F2P	2.73E-08	0	0	0	0	0	0	0
OR1G1	0	0	0	0	0	0	0	0
OR1I1	0	0	0	0	0	0	0	0
OR1J1	0	0	0	0	2.41E-08	0	0	0
OR1J2	0	1.99E-07	0	0	0	5.91E-09	0	0
OR1J4	0	0	0	0	0	0	0	0
OR1K1	0	0	0	0	0	2.66E-08	0	0
OR1L1	0	0	0	0	0	0	0	0
OR1L3	0	0	0	0	0	0	0	0
OR1L4	0	0	0	0	0	0	0	0
OR1L6	0	0	0	0	0	0	0	0
OR1L8	0	0	6.46E-08	0	0	0	0	0
OR1M1	0	0	0	0	0	0	0	0
OR1N1	0	0	0	0	0	0	0	0
OR1N2	0	0	0	0	0	0	0	0
OR1Q1	0	0	0	0	0	8.05E-08	0	0

OR1S1	0	0	0	0	0	0	0	0
OR1S2	0	0	0	0	0	0	0	0
OR2A1	1.76E-07	2.05E-07	6.45E-08	7.32E-08	0	2.20E-07	1.11E-07	1.31E-07
OR2A12	0	0	0	0	0	0	0	0
OR2A14	0	0	0	0	0	0	0	0
OR2A2	0	0	0	0	0	0	0	0
OR2A25	8.74E-08	0	0	0	0	8.17E-08	0	0
OR2A4	9.03E-07	2.11E-06	9.33E-07	1.19E-06	0	3.32E-06	1.41E-06	1.26E-06
OR2A5	0	0	0	0	0	0	0	0
OR2A7	2.52E-06	4.64E-06	1.23E-06	3.48E-06	2.84E-07	1.33E-05	6.94E-06	6.22E-06
OR2A9P	3.49E-06	3.61E-06	1.65E-06	3.46E-06	1.27E-07	6.82E-06	1.89E-06	2.27E-06
OR2AE1	2.77E-08	0	6.08E-08	0	2.40E-08	0	0	0
OR2AG1	0	0	0	0	0	0	0	0
OR2AG2	2.84E-08	3.31E-08	0	0	0	0	0	0
OR2AK2	0	0	0	0	0	0	0	0
OR2AT4	0	3.26E-08	0	0	0	0	0	0
OR2B11	2.83E-08	3.30E-08	0	0	0	0	0	0
OR2B2	0	0	0	0	0	0	0	0
OR2B3	0	0	0	0	0	0	0	0
OR2B6	1.73E-07	1.01E-07	0	0	1.75E-07	2.69E-08	2.74E-08	4.31E-08
OR2C1	2.56E-08	1.48E-07	2.79E-08	3.16E-08	0	0	0	3.81E-08
OR2C3	0	0	0	0	0	0	0	0
OR2D2	0	0	0	0	0	0	0	0
OR2D3	0	0	0	0	0	0	0	0
OR2F1	0	2.78E-08	0	0	0	0	0	0
OR2F2	0	0	0	0	0	0	0	0
OR2G2	0	0	0	0	0	0	0	0
OR2G3	0	0	0	0	0	0	0	0
OR2G6	0	3.31E-08	0	0	0	0	0	0
OR2H1	0	1.26E-08	0	0	0	0	0	1.13E-07
OR2H2	2.48E-08	2.87E-08	0	0	0	0	0	0
OR2J2	0	0	0	0	0	0	0	0
OR2J3	0	0	0	0	0	0	0	0
OR2K2	0	0	0	0	0	0	0	0
OR2L13	0	1.45E-08	0	0	0	2.38E-08	1.24E-08	0
OR2L1P	0	0	0	0	0	0	0	0
OR2L2	0	0	0	0	0	0	0	0
OR2L3	0	0	0	0	0	0	0	0
OR2L8	0	0	0	0	0	0	0	0
OR2M1P	0	0	0	0	0	0	0	0
OR2M2	0	0	0	0	0	0	0	0
OR2M3	0	0	0	0	0	0	0	0
OR2M4	0	0	0	0	0	0	0	0
OR2M5	0	0	0	0	0	0	0	0
OR2M7	0	0	0	0	0	0	0	0
OR2S2	0	0	0	0	0	0	0	0
OR2T1	0	0	0	0	0	0	0	0
OR2T10	0	0	0	0	0	0	0	0
OR2T11	0	0	0	0	0	0	0	0
OR2T12	0	0	0	0	0	0	0	0
OR2T2	0	0	0	0	0	0	0	0
OR2T27	0	0	0	0	0	0	0	0
OR2T29	0	0	0	0	0	0	0	0
OR2T3	0	0	0	0	0	0	0	0
OR2T33	0	0	0	0	0	0	0	0
OR2T34	0	0	0	0	0	0	0	0
OR2T35	0	0	0	0	0	0	0	0
OR2T4	0	0	0	0	0	0	0	0

OR2T5	0	0	0	0	0	0	0	0
OR2T6	0	0	0	0	0	0	0	0
OR2T8	0	0	0	0	0	0	0	0
OR2V2	0	0	0	0	0	0	0	0
OR2W1	0	0	0	0	0	0	0	0
OR2W3	3.25E-08	0	6.34E-08	0	2.80E-08	0	0	0
OR2W5	0	0	0	0	0	0	0	0
OR2Y1	0	0	0	0	0	0	0	0
OR2Z1	0	0	0	0	0	0	0	0
OR3A1	0	0	0	3.53E-08	0	0	0	0
OR3A2	0	0	0	0	0	0	0	0
OR3A3	0	0	0	0	0	0	2.66E-08	0
OR3A4	0	0	0	0	0	0	0	0
OR4A15	0	0	0	0	0	0	0	0
OR4A16	0	0	0	0	0	0	0	0
OR4A47	0	0	0	0	0	0	0	0
OR4A5	0	0	0	0	0	0	0	0
OR4B1	0	0	0	0	0	0	0	0
OR4C11	0	0	0	0	0	0	0	0
OR4C12	0	0	0	0	0	0	0	0
OR4C13	0	0	0	0	0	0	0	0
OR4C15	0	0	0	0	0	0	0	0
OR4C16	0	0	0	0	0	0	0	0
OR4C3	0	0	0	0	0	0	0	0
OR4C45	0	0	0	0	0	0	0	0
OR4C46	0	0	0	0	0	0	0	0
OR4C6	0	3.42E-08	0	6.69E-08	0	0	0	0
OR4D1	0	0	0	0	0	0	2.78E-08	0
OR4D10	0	0	0	0	0	0	0	0
OR4D11	0	0	0	0	0	0	0	0
OR4D2	0	0	0	0	0	0	0	0
OR4D5	0	0	0	0	0	0	0	0
OR4D6	0	0	0	0	0	0	0	0
OR4D9	0	0	0	0	0	0	0	4.29E-08
OR4E2	0	0	0	0	0	0	0	0
OR4F15	0	0	0	0	0	0	0	0
OR4F17	0	0	0	0	0	0	0	0
OR4F21	0	0	0	0	0	0	0	0
OR4F29	0	1.35E-07	6.39E-08	0	0	8.12E-08	0	0
OR4F4	0	0	0	0	0	0	0	0
OR4F5	0	0	0	0	0	0	0	0
OR4F6	0	0	0	0	0	0	0	0
OR4K1	0	0	0	0	0	0	0	0
OR4K13	0	0	0	0	0	0	0	0
OR4K14	0	0	0	0	0	0	0	0
OR4K15	0	0	0	0	0	0	0	0
OR4K17	0	0	0	0	0	0	0	0
OR4K2	0	0	0	0	0	0	0	0
OR4K5	0	0	0	0	0	0	0	0
OR4L1	0	0	0	0	0	0	0	0
OR4M1	0	0	0	0	0	0	0	0
OR4M2	0	0	0	0	0	0	0	0
OR4N2	0	3.44E-08	0	0	0	0	0	0
OR4N3P	0	0	0	0	0	0	0	0
OR4N4	0	0	0	0	0	0	0	0
OR4N5	0	0	0	0	0	0	0	0
OR4P4	0	0	0	0	0	0	0	0
OR4Q3	0	0	0	0	0	0	0	0

OR4S1	0	0	0	0	0	0	0	0
OR4S2	0	0	0	0	0	0	0	0
OR4X1	0	0	0	0	0	0	0	0
OR4X2	0	0	0	0	0	0	0	0
OR51A2	0	0	0	0	0	0	0	0
OR51A4	0	0	0	0	0	0	0	0
OR51A7	0	0	0	0	0	0	0	0
OR51B2	0	0	0	0	0	0	0	0
OR51B4	0	0	0	0	0	5.45E-08	0	0
OR51B5	0	0	0	0	0	0	0	0
OR51B6	0	0	0	0	0	0	0	0
OR51D1	0	0	0	0	0	0	0	0
OR51E1	4.32E-07	1.34E-07	7.70E-09	1.82E-08	2.75E-07	2.62E-07	1.30E-07	3.93E-07
OR51E2	2.34E-07	1.60E-07	1.73E-08	0	2.87E-08	5.42E-08	1.62E-08	3.68E-08
OR51F1	0	0	0	0	0	0	0	0
OR51F2	0	0	0	0	0	0	0	0
OR51G1	0	0	0	0	0	0	0	0
OR51G2	0	0	0	0	0	0	0	0
OR51I1	0	0	0	0	0	0	0	0
OR51I2	0	0	0	0	0	0	0	0
OR51L1	0	0	0	0	0	0	0	0
OR51M1	0	0	0	0	0	0	0	4.08E-08
OR51Q1	0	0	0	0	0	0	0	0
OR51S1	0	0	0	0	0	0	0	0
OR51T1	0	0	0	0	0	0	0	0
OR51V1	0	0	0	0	0	0	0	0
OR52A1	0	0	0	0	0	0	0	0
OR52A4	0	0	0	0	0	0	0	0
OR52A5	0	0	0	0	0	0	0	0
OR52B2	0	0	0	0	0	0	0	0
OR52B4	0	0	0	0	0	0	0	0
OR52B6	0	0	0	0	0	0	0	0
OR52D1	0	0	0	0	0	0	0	0
OR52E2	0	0	0	0	0	0	0	0
OR52E4	0	0	0	0	0	0	0	4.32E-08
OR52E6	2.88E-08	0	0	0	0	0	0	4.31E-08
OR52E8	0	0	0	0	0	0	0	0
OR52H1	0	0	3.08E-08	0	0	0	0	0
OR52I1	0	3.21E-08	0	0	0	0	0	0
OR52I2	0	0	0	0	0	0	0	0
OR52J3	0	0	0	0	0	0	0	0
OR52K1	0	3.34E-08	0	0	0	0	0	0
OR52K2	0	0	0	0	0	0	0	4.28E-08
OR52L1	0	0	0	0	0	0	0	0
OR52M1	0	0	0	0	0	0	0	0
OR52N1	0	0	0	0	0	0	0	0
OR52N2	2.79E-08	0	0	3.45E-08	0	0	0	0
OR52N4	0	0	0	3.16E-08	0	2.39E-08	2.44E-08	1.90E-07
OR52N5	0	0	0	0	0	0	0	4.11E-08
OR52R1	0	0	0	0	0	0	0	0
OR52W1	0	3.26E-08	0	0	0	0	0	0
OR56A1	0	0	0	0	0	0	0	0
OR56A3	2.86E-08	0	0	0	0	0	0	9.82E-07
OR56A4	0	0	0	0	0	0	0	0
OR56A5	0	0	0	0	0	0	0	0
OR56B1	0	0	0	2.46E-08	0	0	0	0
OR56B4	5.63E-08	9.83E-08	0	0	0	0	2.68E-08	8.40E-08
OR5A1	0	0	0	0	0	0	0	0

OR5A2	0	0	0	0	0	0	0	0
OR5AC2	0	0	0	0	0	0	0	0
OR5AK2	0	0	0	0	0	0	0	0
OR5AN1	0	0	0	0	0	0	0	0
OR5AP2	0	0	0	0	0	0	0	0
OR5AR1	0	0	0	0	0	0	0	0
OR5AS1	0	0	0	0	0	0	0	0
OR5AU1	0	0	0	0	0	0	0	0
OR5B12	0	0	0	0	0	0	0	0
OR5B17	0	0	0	0	0	0	0	0
OR5B2	0	0	0	0	0	0	0	0
OR5B21	0	0	0	0	0	0	0	0
OR5B3	0	0	0	0	0	0	0	0
OR5C1	0	0	0	0	0	0	0	0
OR5D13	0	0	0	0	0	0	0	0
OR5D14	0	0	0	0	0	0	0	0
OR5D16	0	0	0	0	0	0	0	0
OR5D18	0	0	0	0	0	0	0	0
OR5E1P	0	0	0	0	0	0	0	0
OR5F1	0	0	0	0	0	0	0	0
OR5H1	0	0	0	0	0	0	0	0
OR5H14	0	0	0	0	0	0	0	0
OR5H15	0	0	0	0	0	0	0	0
OR5H2	0	0	0	0	0	0	0	0
OR5H6	0	3.19E-08	0	0	0	0	0	0
OR5I1	0	0	0	0	0	0	0	0
OR5J2	0	0	0	0	0	0	0	0
OR5K1	0	0	0	0	0	0	0	0
OR5K2	0	0	0	3.52E-08	0	2.66E-08	5.42E-08	0
OR5K3	0	0	0	0	0	0	0	0
OR5K4	0	0	0	0	0	0	0	0
OR5L1	0	0	0	0	0	0	0	0
OR5L2	0	0	0	0	0	0	0	0
OR5M1	0	0	0	0	0	0	0	0
OR5M10	0	0	0	0	0	0	0	0
OR5M11	0	0	0	0	0	0	0	0
OR5M3	0	0	0	0	0	0	0	0
OR5M8	0	0	0	0	0	0	0	0
OR5M9	0	0	0	0	0	0	0	0
OR5P2	0	0	0	0	0	0	0	0
OR5P3	0	0	0	0	0	0	0	0
OR5R1	0	0	0	0	0	0	0	0
OR5T1	0	0	0	0	0	0	0	0
OR5T2	0	0	0	0	0	0	0	0
OR5T3	0	0	0	0	0	0	0	0
OR5V1	0	0	0	0	0	0	0	0
OR5W2	0	0	0	0	0	0	0	0
OR6A2	0	0	0	0	0	0	0	0
OR6B1	0	3.38E-08	0	0	0	0	0	0
OR6B2	0	0	0	0	0	0	0	0
OR6B3	0	0	0	0	0	0	0	0
OR6C1	0	0	0	0	0	0	0	0
OR6C2	0	0	0	0	0	0	0	0
OR6C3	0	0	0	0	0	0	0	0
OR6C4	0	0	0	0	0	0	0	0
OR6C6	0	0	0	0	0	0	0	0
OR6C65	0	0	0	0	0	0	0	0
OR6C68	0	0	0	0	0	0	0	0

OR6C70	0	0	0	0	0	0	0	0
OR6C74	0	0	0	0	0	0	0	0
OR6C75	0	0	0	0	0	0	0	0
OR6C76	0	0	0	0	0	0	0	0
OR6F1	0	0	0	0	0	0	0	0
OR6K2	0	0	0	0	0	0	0	0
OR6K3	0	0	0	0	0	0	0	0
OR6K6	0	0	0	0	0	0	0	0
OR6M1	0	0	0	0	0	0	0	0
OR6N1	0	0	0	0	0	0	0	0
OR6N2	0	0	0	0	0	0	0	0
OR6P1	0	0	0	0	0	0	0	0
OR6Q1	0	0	0	0	0	0	0	0
OR6S1	0	0	0	0	0	0	0	0
OR6T1	0	0	0	0	0	0	0	0
OR6V1	0	0	0	0	0	0	0	0
OR6W1P	0	0	0	0	0	0	0	0
OR6X1	0	0	0	0	0	0	0	0
OR6Y1	0	0	0	0	0	0	0	0
OR7A10	0	0	0	0	0	0	0	0
OR7A17	0	0	0	0	0	0	0	0
OR7A5	0	0	0	0	0	0	0	0
OR7C1	0	0	0	0	0	0	0	0
OR7C2	0	0	0	0	0	0	0	0
OR7D2	0	0	0	0	1.23E-08	3.71E-07	0	0
OR7D4	0	0	0	0	0	0	0	0
OR7E156F	0	0	0	0	0	0	0	0
OR7E24	0	0	0	0	0	0	0	0
OR7E37P	4.54E-08	5.45E-08	0	0	3.97E-08	0	0	0
OR7E5P	0	0	0	0	0	0	0	0
OR7E91P	0	0	0	2.57E-08	0	0	0	0
OR7G1	0	0	0	0	0	0	0	0
OR7G2	0	0	0	0	0	0	0	0
OR7G3	0	0	0	0	0	0	0	0
OR8A1	2.74E-08	0	0	0	0	0	0	0
OR8B12	0	0	0	0	0	0	0	0
OR8B2	0	0	0	0	0	0	0	0
OR8B3	0	0	0	0	0	0	0	0
OR8B4	0	0	0	0	0	0	0	0
OR8B8	0	0	0	0	0	0	0	0
OR8D1	0	0	0	0	0	0	0	0
OR8D2	0	0	0	0	0	0	0	0
OR8D4	0	0	0	0	0	0	0	0
OR8G2	0	0	0	0	0	0	0	0
OR8G5	3.21E-08	0	0	0	0	0	0	0
OR8H1	0	0	0	0	0	0	0	0
OR8H2	0	0	0	0	0	0	0	0
OR8H3	0	0	0	0	0	0	0	0
OR8I2	0	0	0	0	0	0	0	0
OR8J1	0	0	0	0	0	0	0	0
OR8J3	0	0	0	0	0	0	0	0
OR8K1	0	0	0	0	0	0	0	0
OR8K3	0	0	0	0	0	0	0	0
OR8K5	0	0	0	0	0	0	0	0
OR8S1	0	0	0	0	0	0	0	0
OR8U1	0	0	0	0	0	0	0	0
OR9A2	0	0	0	0	0	0	0	0
OR9A4	0	0	0	0	0	0	0	0

OR9G4	0	0	0	0	0	0	0	0
OR9G9	0	0	0	0	0	0	0	0
OR9I1	0	0	0	0	0	0	0	0
OR9K2	0	0	0	0	0	0	0	0
OR9Q1	0	0	0	0	0	0	0	0
OR9Q2	0	0	0	0	0	0	0	0
ORAI1	1.15E-05	1.26E-05	1.59E-05	1.41E-05	1.92E-05	1.93E-05	1.40E-05	1.46E-05
ORAI2	1.72E-06	3.05E-07	3.45E-07	1.86E-06	4.17E-07	4.26E-07	1.00E-06	5.20E-07
ORAI3	2.75E-05	5.55E-05	3.46E-05	2.53E-05	1.24E-05	2.40E-05	2.84E-05	2.13E-05
ORAOV1	5.22E-06	2.93E-06	2.11E-06	3.17E-06	2.45E-06	3.83E-06	2.94E-06	4.84E-06
ORC1L	3.90E-06	1.18E-06	6.56E-08	1.17E-07	1.63E-07	7.20E-07	1.69E-07	2.92E-07
ORC2L	1.12E-05	5.86E-06	5.61E-06	2.25E-06	7.50E-07	3.54E-06	1.54E-06	2.26E-06
ORC3L	1.82E-05	2.53E-05	1.06E-05	1.35E-05	4.27E-06	1.54E-05	1.06E-05	1.19E-05
ORC4L	2.82E-05	1.43E-05	9.65E-06	7.92E-06	3.77E-06	1.05E-05	6.40E-06	8.02E-06
ORC5L	8.87E-06	1.62E-05	7.04E-06	7.26E-06	4.97E-06	6.84E-06	8.59E-06	8.49E-06
ORC6L	5.54E-06	2.82E-06	1.53E-07	2.18E-07	5.84E-07	8.92E-07	4.84E-07	4.18E-07
ORM1	0.001342	0.003225	0.023804	0.010662	0.007822	0.002522	0.020032	0.025881
ORM2	0.000542	0.000476	0.002825	0.002682	0.002764	0.000396	0.004821	0.003927
ORMDL1	2.82E-05	1.55E-05	7.35E-06	9.79E-06	6.00E-06	1.32E-05	1.14E-05	9.86E-06
ORMDL2	4.00E-05	3.07E-05	1.49E-05	2.42E-05	3.00E-05	3.13E-05	2.40E-05	4.34E-05
ORMDL3	0.000154	0.000125	0.000189	0.000186	0.000128	0.000286	0.000193	0.000262
OS9	0.000224	0.000312	0.000208	0.000203	0.0002	0.000231	0.000204	0.000218
OSBP	2.71E-05	3.53E-05	2.53E-05	2.05E-05	4.86E-06	3.96E-05	1.78E-05	3.49E-05
OSBP2	2.85E-06	2.47E-06	3.13E-07	6.19E-07	2.58E-07	4.20E-07	2.69E-07	6.97E-07
OSBPL10	5.70E-06	1.90E-07	5.42E-07	1.51E-06	2.21E-07	2.98E-07	1.06E-06	4.40E-07
OSBPL11	8.24E-06	5.31E-06	9.67E-06	6.35E-06	1.50E-06	9.77E-06	3.31E-06	6.89E-06
OSBPL1A	2.28E-05	6.60E-06	9.15E-06	9.77E-06	2.17E-06	9.44E-06	6.50E-06	6.00E-06
OSBPL2	1.99E-05	1.57E-05	9.19E-06	8.53E-06	6.34E-06	9.45E-06	8.27E-06	7.56E-06
OSBPL3	7.79E-06	2.62E-07	2.92E-07	6.03E-07	1.62E-07	4.90E-07	4.05E-07	1.04E-06
OSBPL5	1.33E-05	6.45E-07	8.73E-07	2.01E-06	4.76E-07	7.09E-07	1.50E-06	9.25E-07
OSBPL6	2.60E-06	1.09E-06	1.57E-06	3.69E-07	8.62E-07	7.00E-08	1.25E-06	6.67E-08
OSBPL7	2.54E-06	1.09E-07	3.38E-07	8.17E-07	3.60E-07	3.71E-07	4.52E-07	3.92E-07
OSBPL8	7.99E-06	6.34E-06	5.54E-06	4.92E-06	1.45E-06	4.96E-06	2.90E-06	3.25E-06
OSBPL9	6.22E-05	4.37E-05	4.30E-05	3.03E-05	7.74E-06	2.98E-05	1.59E-05	7.35E-05
OSCAR	3.73E-06	5.02E-07	1.39E-06	1.63E-06	3.51E-07	6.14E-07	1.26E-06	3.42E-07
OSCP1	1.94E-06	1.99E-06	1.12E-06	3.98E-06	2.99E-07	2.34E-06	1.37E-06	4.31E-07
OSGEP	1.54E-05	1.26E-05	1.20E-05	1.27E-05	6.74E-06	1.40E-05	2.25E-05	1.70E-05
OSGEPL1	7.93E-06	7.08E-06	3.12E-06	2.05E-06	1.28E-06	3.08E-06	2.84E-06	1.98E-06
OSGIN1	2.79E-05	6.52E-05	4.86E-05	5.26E-05	0.00028	0.000314	6.30E-05	0.00012
OSGIN2	1.39E-05	1.31E-05	4.24E-06	5.87E-06	2.98E-06	3.79E-06	1.18E-05	1.33E-05
OSM	7.39E-07	3.82E-07	4.90E-07	4.13E-06	5.58E-08	2.53E-07	1.64E-06	2.48E-07
OSMR	2.29E-05	1.04E-05	3.74E-06	1.41E-05	5.52E-08	1.19E-06	3.26E-06	1.56E-05
OSR1	9.67E-07	1.15E-07	5.35E-08	5.78E-07	1.10E-08	7.80E-07	1.35E-07	2.06E-07
OSR2	4.52E-06	6.40E-07	1.68E-08	1.17E-07	3.51E-08	1.63E-06	3.93E-08	2.73E-07
OST4	0.00081	0.00061	0.000227	0.000372	0.000519	0.000599	0.000379	0.000268
OSTalpha	1.01E-05	7.00E-05	7.57E-05	0.000185	9.28E-05	2.87E-05	7.40E-05	2.11E-06
OSTBETA	1.65E-06	5.61E-06	4.43E-07	2.24E-05	3.84E-06	3.12E-07	7.86E-06	3.63E-05
OSTC	0.000126	7.50E-05	8.54E-05	8.78E-05	8.72E-05	0.000142	9.86E-05	0.000135
OSTCL	6.32E-07	2.26E-07	3.06E-07	2.15E-07	5.65E-08	2.64E-07	2.29E-07	1.62E-07
OSTF1	4.32E-05	2.38E-05	3.39E-05	3.35E-05	8.30E-06	2.25E-05	2.53E-05	2.54E-05
OSTM1	1.19E-05	1.03E-05	5.43E-06	6.67E-06	2.50E-06	7.43E-06	7.22E-06	6.51E-06
OSTN	0	0	0	0	0	9.80E-08	0	0
OTC	4.18E-05	7.30E-05	0.000146	0.000102	8.11E-05	0.00021	8.63E-05	8.81E-05
OTOA	3.45E-07	2.14E-07	1.70E-07	1.12E-06	2.08E-07	1.84E-07	2.04E-06	1.90E-07
OTOF	4.03E-08	2.26E-08	3.38E-08	1.96E-08	5.76E-09	8.23E-08	2.58E-08	9.84E-09
OTOL1	0	0	0	0	0	1.62E-08	0	0
OTOP1	0	1.46E-08	0	0	0	0	0	0
OTOP2	0	3.10E-08	0	0	0	0	0	0

OTOP3	1.22E-05	1.54E-08	0	0	0	0	0	0
OTOR	0	0	0	0	0	0	0	0
OTOS	0	0	0	0	0	5.43E-08	0	0
OTP	0	0	0	0	0	0	0	1.28E-08
OTUB1	5.69E-05	3.90E-05	2.48E-05	3.20E-05	2.87E-05	4.74E-05	3.01E-05	5.24E-05
OTUB2	1.57E-06	1.10E-06	2.73E-07	2.30E-07	4.40E-07	1.23E-06	4.47E-07	2.09E-06
OTUD1	3.56E-06	1.97E-06	6.01E-06	4.81E-06	1.22E-06	2.75E-06	3.20E-06	2.94E-06
OTUD3	1.46E-06	1.88E-07	2.53E-07	4.40E-07	8.84E-08	1.49E-07	3.78E-07	3.01E-07
OTUD4	9.51E-06	4.29E-06	6.40E-06	6.47E-06	7.07E-07	6.28E-06	3.68E-06	4.28E-06
OTUD5	2.88E-05	2.76E-05	1.93E-05	2.56E-05	1.68E-05	2.78E-05	2.94E-05	3.69E-05
OTUD6A	0	0	0	0	0	0	0	0
OTUD6B	5.68E-06	5.16E-06	2.00E-06	1.87E-06	5.58E-07	3.62E-06	4.17E-06	3.89E-06
OTUD7A	5.03E-07	1.51E-07	8.66E-08	5.58E-08	1.88E-08	6.05E-08	1.39E-07	1.12E-08
OTUD7B	8.07E-06	5.11E-06	3.71E-06	6.08E-06	2.20E-06	5.77E-06	8.76E-06	8.76E-06
OTX1	2.74E-06	9.13E-07	0	0	0	1.02E-07	2.12E-08	4.84E-08
OTX2	0	0	0	0	0	0	0	0
OVCA2	3.35E-05	2.98E-05	2.30E-05	3.51E-05	5.89E-05	2.95E-05	4.70E-05	3.29E-05
OVCH1	0	1.51E-08	0	0	0	6.22E-09	0	0
OVCH2	0	3.27E-08	0	0	0	0	0	0
OVGP1	7.04E-06	4.79E-06	7.20E-06	2.03E-06	1.25E-06	9.23E-07	1.08E-06	6.15E-06
OVOL1	1.68E-06	1.39E-07	1.24E-07	1.80E-07	6.88E-09	0	7.00E-08	0
OVOL2	4.13E-07	8.50E-07	1.51E-07	2.34E-07	0	0	4.59E-08	0
OXA1L	0.000155	0.000137	6.10E-05	5.97E-05	4.97E-05	0.00012	7.56E-05	0.000102
OXCT1	2.10E-05	2.11E-07	3.88E-07	1.14E-06	2.03E-07	5.44E-07	6.03E-07	5.74E-07
OXCT2	4.84E-06	1.81E-07	1.42E-07	1.63E-07	3.08E-07	1.25E-08	2.05E-07	9.94E-08
OXER1	4.79E-06	3.32E-05	1.03E-05	8.96E-06	6.58E-05	2.46E-06	2.77E-05	6.12E-06
OXGR1	0	5.84E-08	0	1.27E-08	0	4.80E-08	0	0
OXNAD1	2.73E-05	1.35E-05	1.13E-05	9.22E-06	6.93E-06	1.34E-05	1.30E-05	6.23E-06
OXR1	3.00E-05	1.22E-05	1.09E-05	9.15E-06	3.09E-06	1.57E-05	1.41E-05	9.23E-06
OXSM	1.75E-05	1.89E-05	1.37E-05	1.00E-05	9.86E-06	1.97E-05	1.33E-05	1.39E-05
OXSR1	1.71E-05	1.06E-05	7.26E-06	9.09E-06	1.54E-06	9.98E-06	5.61E-06	7.81E-06
OXT	1.47E-06	2.45E-05	5.02E-07	2.88E-06	0	0	1.23E-07	0
OXTR	1.52E-06	1.40E-07	5.35E-09	3.39E-06	4.02E-08	2.89E-07	8.61E-08	9.14E-08
P2RX1	4.06E-07	5.48E-08	1.31E-07	2.48E-07	1.33E-07	1.51E-08	5.52E-08	1.31E-07
P2RX2	1.62E-08	0	1.72E-08	1.99E-08	0	0	0	4.69E-08
P2RX3	1.68E-07	1.07E-07	2.60E-07	9.21E-08	0	0	0	0
P2RX4	3.17E-05	8.26E-06	8.05E-06	1.64E-05	5.74E-06	5.74E-06	1.39E-05	1.68E-05
P2RX5	3.95E-06	7.75E-08	2.00E-07	5.14E-07	2.93E-07	1.22E-07	2.68E-07	2.37E-07
P2RX6	9.93E-08	5.70E-06	1.27E-07	2.25E-07	5.00E-07	6.49E-08	6.79E-08	2.57E-08
P2RX6P	0	0	0	0	0	0	0	0
P2RX7	1.84E-06	2.78E-06	1.11E-06	1.83E-06	2.73E-08	6.31E-07	6.83E-07	1.88E-07
P2RY1	3.20E-07	4.01E-07	5.00E-07	2.82E-07	1.28E-08	1.86E-07	7.22E-08	5.45E-08
P2RY10	1.72E-06	3.51E-08	2.98E-07	1.14E-06	0	5.85E-07	4.45E-08	1.40E-07
P2RY11	1.65E-06	2.05E-06	1.26E-06	2.71E-06	2.76E-06	2.13E-06	6.10E-06	1.04E-06
P2RY12	1.07E-06	1.93E-08	6.43E-07	1.94E-07	0	1.31E-07	1.33E-07	7.56E-08
P2RY13	5.32E-06	1.32E-07	3.62E-06	2.50E-06	2.53E-07	1.12E-06	1.48E-06	6.17E-07
P2RY14	9.63E-07	2.26E-07	3.24E-07	2.89E-07	6.49E-08	3.00E-07	2.29E-07	2.43E-07
P2RY2	1.70E-06	9.56E-07	9.47E-07	1.14E-06	1.37E-06	8.74E-07	1.78E-06	1.17E-06
P2RY4	7.48E-08	8.50E-08	1.58E-08	0	0	2.78E-08	0	2.21E-08
P2RY6	3.39E-06	2.19E-07	7.91E-07	1.24E-06	3.64E-07	3.14E-06	6.11E-07	7.15E-07
P2RY8	2.03E-06	6.74E-06	7.39E-07	3.09E-06	1.19E-06	2.20E-06	2.19E-06	4.44E-06
P4HA1	3.68E-05	0.00013	5.43E-05	2.66E-05	2.14E-05	8.20E-05	4.19E-05	8.37E-06
P4HA2	3.99E-05	2.99E-05	5.20E-06	1.03E-05	1.84E-06	1.52E-05	1.50E-05	3.00E-06
P4HA3	6.81E-07	3.35E-07	2.18E-07	6.39E-07	2.33E-07	4.67E-08	1.17E-06	2.30E-07
P4HB	0.001332	0.001583	0.001011	0.000994	0.001507	0.00189	0.001067	0.001128
P4HTM	1.56E-05	1.58E-05	7.98E-06	1.47E-05	1.19E-05	1.02E-05	6.46E-06	2.83E-06
P704P	3.89E-07	1.16E-07	1.71E-07	5.31E-08	5.70E-09	1.55E-08	1.27E-07	9.31E-08
PA2G4	7.24E-05	6.83E-05	2.83E-05	3.72E-05	3.16E-05	5.34E-05	4.95E-05	4.03E-05

PA2G4P4	1.93E-06	1.17E-06	1.13E-06	5.80E-07	5.02E-07	8.08E-07	4.84E-07	7.83E-07
PAAF1	2.17E-05	2.06E-05	1.45E-05	1.09E-05	1.06E-05	1.83E-05	1.57E-05	5.63E-06
PABPC1	0.000687	0.000311	0.000183	0.000199	0.000241	0.000252	0.000569	0.000212
PABPC1L	2.23E-05	1.04E-05	1.38E-06	2.71E-06	7.97E-06	4.24E-06	1.74E-06	3.39E-06
PABPC1L2	0	0	0	0	0	0	0	0
PABPC1L2	0	0	0	0	0	0	0	0
PABPC1P2	2.90E-08	0	5.96E-09	0	0	1.07E-08	1.13E-08	0
PABPC3	1.40E-05	3.49E-06	4.06E-06	1.93E-06	5.25E-07	2.64E-06	5.44E-06	2.48E-06
PABPC4	3.95E-05	2.52E-05	3.07E-05	4.18E-05	1.38E-05	5.13E-05	4.38E-05	3.15E-05
PABPC4L	2.51E-07	1.63E-07	5.62E-08	5.02E-08	3.92E-09	5.92E-08	9.33E-08	1.20E-07
PABPC5	1.31E-07	2.79E-08	4.74E-08	4.20E-08	1.06E-08	1.23E-08	3.58E-08	2.20E-07
PABPN1	7.49E-05	3.51E-05	2.81E-05	4.31E-05	3.92E-05	4.38E-05	5.74E-05	3.00E-05
PABPN1L	0	0	2.44E-08	0	0	0	2.16E-08	0
PACRG	3.38E-07	1.51E-07	2.97E-06	1.32E-06	6.79E-07	5.06E-06	1.59E-06	2.68E-07
PACRGL	3.67E-06	3.12E-06	1.83E-06	2.76E-06	1.13E-06	3.21E-06	2.91E-06	1.28E-06
PACS1	1.89E-05	1.06E-05	5.60E-06	2.09E-05	4.80E-06	1.12E-05	1.60E-05	1.22E-05
PACS2	7.85E-06	2.83E-06	2.52E-06	4.47E-06	5.13E-06	3.72E-06	5.99E-06	4.79E-06
PACSIN1	5.80E-07	1.38E-07	1.16E-07	1.05E-07	2.30E-08	9.44E-08	4.18E-08	1.57E-08
PACSIN2	5.65E-05	3.25E-05	2.36E-05	2.09E-05	2.57E-06	5.10E-05	1.64E-05	3.10E-05
PACSIN3	2.44E-05	1.99E-05	1.87E-05	2.74E-05	2.80E-05	2.32E-05	2.59E-05	7.30E-06
PADI1	6.99E-08	1.29E-08	1.22E-08	2.36E-07	0	1.06E-08	0	0
PADI2	2.04E-06	6.35E-08	2.42E-07	6.85E-07	0	5.25E-08	7.72E-08	7.61E-08
PADI3	0	7.32E-08	0	8.85E-09	0	0	0	2.12E-08
PADI4	5.23E-08	5.99E-08	4.36E-08	6.40E-08	2.70E-08	1.94E-08	2.02E-08	1.16E-07
PADI6	0	0	0	1.23E-08	0	0	0	0
PAEP	2.26E-05	0	0	0	0	0	0	5.86E-08
PAF1	5.52E-05	3.27E-05	2.14E-05	3.51E-05	4.66E-05	4.00E-05	4.39E-05	2.96E-05
PAFAH1B1	1.57E-05	1.35E-05	1.60E-05	1.74E-05	1.00E-05	1.70E-05	1.49E-05	1.42E-05
PAFAH1B2	2.81E-05	9.95E-06	9.48E-06	1.28E-05	6.13E-07	1.31E-05	3.65E-06	1.57E-05
PAFAH1B3	3.00E-05	7.91E-06	3.11E-06	1.30E-05	8.88E-06	1.01E-05	8.61E-06	1.26E-05
PAFAH2	1.31E-05	1.14E-05	1.21E-05	8.10E-06	2.49E-06	9.19E-06	5.30E-06	8.52E-06
PAG1	4.85E-06	1.20E-06	1.93E-06	2.06E-06	3.61E-07	1.56E-06	1.06E-06	3.63E-06
PAGE1	9.14E-08	3.78E-05	0	5.72E-08	0	8.60E-08	0	0
PAGE2	9.62E-07	2.06E-06	0	0	0	0	0	0
PAGE2B	2.00E-06	2.69E-07	0	0	0	0	0	1.11E-07
PAGE3	0	0	0	0	0	0	0	0
PAGE4	0	8.02E-07	0	2.98E-06	0	1.43E-05	1.30E-07	0
PAGE5	1.33E-05	7.62E-08	3.79E-07	7.79E-08	5.97E-07	0	3.42E-07	9.47E-08
PAH	0.000405	0.000695	0.000528	0.000217	0.000123	0.000686	0.000151	0.000221
PAICS	5.31E-05	4.35E-05	4.87E-05	3.44E-05	1.08E-05	6.15E-05	3.34E-05	5.47E-05
PAIP1	2.22E-05	2.41E-05	1.38E-05	1.58E-05	3.90E-06	1.97E-05	1.31E-05	2.15E-05
PAIP2	0.000114	8.17E-05	5.01E-05	5.44E-05	2.19E-05	5.98E-05	6.14E-05	7.32E-05
PAIP2B	1.75E-05	1.34E-05	4.97E-06	2.55E-06	1.16E-06	3.17E-06	2.02E-06	1.73E-06
PAK1	3.51E-05	2.91E-05	8.92E-06	1.27E-05	5.81E-06	2.90E-06	1.48E-05	1.17E-05
PAK1IP1	1.01E-05	2.07E-05	7.88E-06	1.11E-05	3.63E-06	1.23E-05	1.12E-05	1.71E-05
PAK2	2.62E-05	1.43E-05	1.43E-05	1.26E-05	4.61E-06	2.07E-05	1.02E-05	1.67E-05
PAK3	1.78E-07	9.13E-07	2.25E-08	0	0	0	0	2.48E-08
PAK4	2.13E-05	1.38E-05	9.68E-06	1.49E-05	9.68E-06	1.33E-05	1.81E-05	1.38E-05
PAK6	2.89E-06	9.87E-07	9.91E-08	1.88E-07	1.25E-07	3.90E-08	1.05E-07	0
PAK7	2.10E-07	0	3.97E-08	1.26E-08	0	0	0	0
PALB2	3.70E-06	4.46E-06	1.98E-06	1.45E-06	2.65E-07	3.44E-06	1.60E-06	1.43E-06
PALLD	2.99E-05	1.75E-05	5.31E-06	1.74E-05	3.31E-06	4.51E-05	9.06E-06	1.47E-05
PALM	2.04E-05	3.21E-06	2.68E-06	4.25E-06	3.08E-06	1.10E-06	3.45E-06	2.34E-06
PALM2	3.87E-07	2.46E-06	2.50E-06	1.12E-06	4.33E-07	8.56E-07	9.51E-07	1.17E-06
PALM2-AI	1.52E-05	6.89E-06	1.05E-05	1.18E-05	2.00E-06	7.23E-06	7.37E-06	6.05E-06
PALM3	7.96E-07	3.35E-06	9.35E-06	1.30E-05	1.12E-06	5.81E-06	1.12E-05	1.05E-06
PALMD	9.53E-06	1.69E-05	2.23E-05	2.71E-05	1.76E-05	1.99E-05	2.14E-05	1.62E-05
PAM	2.81E-05	4.19E-06	7.88E-06	1.49E-05	3.24E-06	3.85E-06	1.13E-05	6.75E-06

PAMR1	2.59E-06	8.35E-07	4.58E-06	7.95E-06	3.04E-07	9.86E-07	1.81E-06	3.56E-07
PAN2	2.03E-05	1.97E-05	9.22E-06	7.42E-06	4.05E-06	1.99E-05	8.97E-06	7.28E-06
PAN3	7.96E-06	2.88E-06	2.50E-06	3.73E-06	6.92E-07	2.81E-06	2.43E-06	2.69E-06
PANK1	1.81E-05	3.20E-05	4.09E-05	3.17E-05	1.64E-05	1.85E-05	2.97E-05	1.19E-05
PANK2	2.39E-05	1.29E-05	8.68E-06	9.29E-06	9.94E-06	1.40E-05	8.25E-06	7.95E-06
PANK3	7.28E-06	7.36E-06	1.37E-05	5.46E-06	1.46E-06	1.45E-05	3.66E-06	9.56E-06
PANK4	1.20E-05	7.73E-06	7.43E-06	6.74E-06	1.92E-06	7.47E-06	5.75E-06	9.49E-06
PANX1	8.01E-06	6.22E-06	6.41E-06	1.34E-05	2.59E-06	1.47E-05	5.28E-06	6.75E-06
PANX2	4.26E-06	1.55E-05	5.09E-06	2.47E-06	1.51E-06	3.85E-05	4.21E-06	6.23E-06
PANX3	4.62E-08	0	0	0	0	0	0	0
PAOX	8.98E-06	1.14E-05	1.28E-05	1.19E-05	1.17E-05	9.15E-06	7.52E-06	9.65E-06
PAPD4	1.51E-05	8.23E-06	9.55E-06	9.05E-06	2.89E-06	1.24E-05	8.81E-06	1.00E-05
PAPD5	3.80E-06	4.37E-06	2.65E-06	2.78E-06	8.20E-07	2.61E-06	2.02E-06	1.66E-06
PAPD7	6.66E-06	5.99E-06	6.15E-06	5.65E-06	2.51E-06	6.92E-06	5.54E-06	8.11E-06
PAPL	9.00E-09	0	1.87E-08	0	0	0	0	0
PAPLN	1.87E-05	2.40E-06	7.39E-07	7.92E-06	1.98E-07	1.58E-07	1.12E-06	7.09E-07
PAPOLA	6.22E-05	4.33E-05	3.51E-05	3.40E-05	1.45E-05	4.33E-05	3.40E-05	3.38E-05
PAPOLB	1.07E-08	0	1.65E-08	0	0	0	0	0
PAPOLG	4.64E-06	3.21E-06	1.19E-06	2.30E-06	4.36E-07	2.16E-06	1.57E-06	1.91E-06
PAPPA	3.95E-07	2.22E-08	6.87E-08	5.58E-07	1.07E-08	3.04E-08	5.33E-08	3.35E-08
PAPPA2	1.04E-08	7.61E-06	1.88E-07	5.27E-07	0	8.42E-07	4.97E-08	1.56E-06
PAPSS1	2.45E-05	1.58E-05	4.38E-06	1.14E-05	1.93E-06	9.16E-06	5.30E-06	4.69E-06
PAPSS2	6.08E-05	3.13E-05	3.53E-05	1.96E-05	1.12E-05	3.22E-05	3.16E-05	3.22E-05
PAQR3	6.21E-06	7.14E-07	3.42E-06	1.36E-06	6.93E-08	3.45E-06	1.57E-06	6.33E-06
PAQR4	4.22E-05	1.15E-05	1.06E-06	4.13E-06	3.48E-06	1.76E-06	8.19E-06	3.03E-06
PAQR5	1.26E-05	6.26E-07	4.98E-07	2.64E-06	2.24E-08	1.21E-08	9.60E-07	3.05E-07
PAQR6	7.79E-07	5.10E-06	1.77E-07	4.90E-07	1.35E-06	2.85E-07	6.05E-07	3.55E-07
PAQR7	7.27E-06	3.20E-06	3.76E-06	5.31E-06	6.16E-06	2.10E-06	7.19E-06	2.40E-06
PAQR8	4.47E-06	1.42E-06	7.59E-07	1.00E-06	1.92E-07	2.96E-07	5.39E-07	3.70E-07
PAQR9	2.19E-05	2.36E-05	9.64E-06	8.67E-06	8.06E-06	9.20E-06	8.20E-06	1.27E-05
PAR1	2.05E-07	1.10E-08	0	1.19E-08	0	0	0	0
PAR4	0	0	0	0	0	0	0	0
PAR5	1.00E-06	1.91E-07	1.55E-07	3.33E-08	0	6.31E-08	2.65E-08	0
PARD3	2.21E-05	1.56E-05	1.10E-05	1.14E-05	5.95E-06	1.63E-05	8.86E-06	1.20E-05
PARD3B	1.86E-06	2.42E-07	4.91E-07	5.92E-07	2.61E-08	2.08E-06	3.36E-07	4.15E-07
PARD6A	1.22E-05	5.49E-06	5.03E-06	3.09E-06	1.63E-06	3.09E-06	1.29E-06	8.13E-06
PARD6B	9.48E-06	2.76E-06	1.68E-06	2.31E-06	7.28E-07	6.99E-07	1.54E-06	1.24E-06
PARD6G	6.32E-06	1.87E-07	1.08E-06	1.67E-06	1.68E-06	2.30E-06	6.93E-07	1.13E-06
PARG	8.24E-06	8.37E-06	5.24E-06	5.00E-06	2.54E-06	8.48E-06	4.39E-06	6.30E-06
PARK2	1.53E-06	1.49E-06	1.02E-06	7.47E-07	1.33E-07	2.86E-06	6.75E-07	7.91E-07
PARK7	0.000373	0.000385	0.000227	0.00027	0.000248	0.000379	0.000272	0.000225
PARL	3.43E-05	1.87E-05	2.10E-05	2.77E-05	3.18E-05	3.52E-05	3.24E-05	3.18E-05
PARM1	1.69E-06	9.30E-07	3.12E-07	1.10E-06	3.22E-07	8.61E-07	8.80E-07	1.10E-06
PARN	2.03E-05	2.05E-05	9.36E-06	1.38E-05	4.59E-06	2.28E-05	1.31E-05	1.43E-05
PARP1	4.48E-05	6.01E-05	1.99E-05	2.22E-05	8.30E-06	5.89E-05	1.95E-05	4.98E-05
PARP10	4.16E-05	3.77E-05	3.11E-05	2.20E-05	2.09E-05	3.13E-05	6.95E-05	5.10E-05
PARP11	2.13E-06	9.56E-07	1.21E-06	1.27E-06	5.64E-07	2.64E-06	1.24E-06	1.14E-06
PARP12	1.40E-05	2.20E-05	1.20E-05	1.34E-05	7.71E-06	1.36E-05	1.25E-05	3.28E-05
PARP14	3.17E-05	1.28E-05	2.25E-05	8.08E-06	7.83E-06	1.24E-05	7.78E-06	1.59E-05
PARP15	2.15E-06	6.95E-08	1.87E-07	2.28E-07	6.40E-08	1.70E-07	7.66E-08	6.29E-07
PARP16	7.75E-06	7.69E-06	7.10E-06	4.14E-06	5.88E-06	6.79E-06	7.52E-06	5.27E-06
PARP2	1.75E-05	1.33E-05	6.61E-06	5.20E-06	5.84E-06	1.06E-05	9.10E-06	8.65E-06
PARP3	1.54E-05	1.74E-05	8.44E-06	1.05E-05	1.94E-06	1.37E-05	7.20E-06	7.68E-06
PARP4	2.71E-05	1.26E-05	1.02E-05	1.16E-05	1.86E-06	1.86E-05	8.30E-06	1.59E-05
PARP6	7.63E-06	1.43E-05	3.35E-06	9.70E-06	8.88E-07	4.69E-06	7.31E-06	1.97E-05
PARP8	9.88E-06	2.55E-06	2.59E-06	5.08E-06	8.49E-08	2.72E-06	1.92E-06	5.33E-06
PARP9	4.34E-05	2.07E-05	4.35E-05	2.48E-05	2.09E-05	4.47E-05	2.22E-05	5.42E-05
PARS2	3.88E-06	4.85E-06	2.21E-06	1.98E-06	1.29E-06	3.29E-06	2.33E-06	2.40E-06

PAR-SN	8.53E-06	1.86E-06	1.07E-06	8.85E-07	0	6.39E-07	5.97E-07	2.17E-07
PART1	0	0	0	0	0	9.08E-06	0	0
PARVA	1.89E-05	8.75E-06	1.02E-05	1.53E-05	3.67E-06	1.54E-05	9.04E-06	4.93E-06
PARVB	1.88E-05	1.48E-06	9.71E-06	1.38E-05	1.43E-05	3.47E-06	7.01E-06	1.88E-05
PARVG	9.02E-06	7.41E-07	3.03E-06	8.66E-06	7.76E-07	3.22E-06	4.12E-06	2.09E-06
PASD1	0	0	0	0	0	0	0	0
PASK	2.44E-06	8.11E-07	5.34E-07	9.31E-07	2.56E-07	5.25E-07	5.50E-07	4.18E-07
PATE1	0	0	0	0	0	0	0	0
PATE2	1.65E-08	0	0	4.05E-08	0	1.53E-08	0	2.21E-08
PATE3	0	0	0	0	0	0	0	0
PATE4	0	3.10E-08	0	0	0	0	0	0
PATL1	2.07E-05	1.44E-05	1.27E-05	1.28E-05	2.56E-06	1.48E-05	7.34E-06	1.72E-05
PATL2	8.31E-07	3.07E-07	1.29E-07	4.23E-07	6.68E-08	5.94E-07	8.75E-08	1.14E-06
PATZ1	1.33E-05	1.15E-05	8.63E-06	1.02E-05	1.24E-05	1.32E-05	1.32E-05	8.21E-06
PAWR	3.73E-05	1.12E-05	4.64E-06	8.91E-06	2.31E-06	8.06E-06	5.96E-06	7.26E-06
PAX1	0	0	0	0	0	0	0	0
PAX2	1.01E-07	0	0	0	0	1.21E-08	1.10E-08	0
PAX3	7.94E-09	0	0	0	0	0	0	0
PAX4	0	0	0	1.46E-08	0	0	0	0
PAX5	1.48E-07	3.75E-08	2.22E-08	2.60E-08	0	1.42E-07	2.08E-07	2.06E-07
PAX6	1.49E-07	1.18E-07	2.26E-07	1.53E-07	2.13E-07	1.34E-07	1.90E-07	1.67E-07
PAX7	0	0	0	0	0	0	0	0
PAX8	5.70E-07	2.07E-06	2.58E-07	5.82E-07	4.39E-07	1.49E-06	1.42E-06	1.66E-07
PAX9	1.52E-08	1.36E-08	2.53E-08	5.91E-08	1.04E-08	0	8.15E-08	1.77E-08
PAXIP1	2.72E-06	3.65E-06	2.49E-06	2.22E-06	8.88E-07	3.74E-06	2.39E-06	3.97E-06
PBK	6.08E-06	1.03E-05	1.51E-07	1.52E-07	1.21E-06	2.12E-06	2.79E-07	8.45E-07
PBLD	3.39E-05	3.26E-05	0.000164	4.75E-05	2.05E-05	4.78E-05	3.50E-05	3.23E-05
PBOV1	0	0	0	0	0	0	0	0
PBRM1	7.92E-06	7.08E-06	5.59E-06	4.71E-06	1.24E-06	6.36E-06	3.17E-06	3.50E-06
PBX1	3.48E-06	3.83E-07	1.46E-06	4.77E-06	1.49E-07	4.10E-06	6.56E-07	6.16E-07
PBX2	3.11E-05	1.46E-05	9.47E-06	1.25E-05	5.77E-06	1.24E-05	1.14E-05	1.56E-05
PBX3	1.27E-05	6.68E-06	9.33E-06	8.94E-06	3.89E-06	6.15E-06	5.57E-06	1.45E-05
PBX4	6.15E-07	2.17E-08	4.05E-08	1.76E-07	6.53E-08	7.04E-08	2.36E-07	1.68E-07
PBXIP1	3.34E-05	5.26E-05	2.72E-05	5.08E-05	2.18E-05	5.15E-05	2.54E-05	3.77E-05
PC	9.16E-05	0.000334	0.000161	0.000205	0.000189	0.000429	0.000132	9.61E-05
PCA3	0	0	6.56E-09	7.77E-09	5.46E-09	0	6.18E-09	0
PCBD1	0.000134	0.000374	0.000246	0.000185	0.000273	0.00036	0.000238	9.89E-05
PCBD2	1.01E-05	3.54E-06	6.76E-06	6.20E-06	2.75E-06	5.92E-06	7.37E-06	8.40E-06
PCBP1	0.000418	0.000389	0.000233	0.000248	0.000113	0.00022	0.000269	0.000234
PCBP2	0.000182	0.000188	0.000144	0.000147	9.54E-05	0.000165	0.000173	8.01E-05
PCBP3	2.65E-07	7.14E-08	2.30E-07	7.11E-07	1.19E-07	8.19E-08	4.50E-07	3.15E-07
PCBP4	2.88E-05	1.38E-05	4.96E-06	1.39E-05	1.10E-05	9.10E-06	1.48E-05	1.92E-05
PCCA	3.11E-05	2.08E-05	3.84E-05	4.12E-05	2.14E-05	5.26E-05	2.18E-05	3.03E-05
PCCB	5.04E-05	0.000159	0.000109	7.15E-05	5.23E-05	0.000129	6.93E-05	9.74E-05
PCDH1	1.12E-05	6.15E-06	8.74E-06	1.13E-05	6.82E-06	6.38E-06	1.62E-05	1.55E-05
PCDH10	5.80E-09	0	5.96E-09	5.22E-08	0	5.36E-09	4.08E-09	0
PCDH11X	0	0	1.93E-08	0	0	0	2.64E-09	0
PCDH11Y	0	8.03E-09	0	0	0	0	0	0
PCDH12	4.19E-06	3.15E-06	1.84E-06	1.45E-06	3.46E-06	2.07E-06	7.42E-06	3.82E-06
PCDH15	1.68E-08	0	7.34E-09	2.93E-09	0	0	0	0
PCDH17	3.91E-06	8.08E-07	1.64E-07	3.62E-07	2.44E-07	7.49E-07	6.61E-07	1.59E-06
PCDH18	8.81E-07	2.79E-07	1.08E-06	3.49E-07	1.70E-07	1.05E-07	3.10E-07	2.58E-07
PCDH19	2.31E-08	1.80E-08	3.52E-08	1.69E-08	3.95E-09	6.39E-09	2.70E-08	3.37E-09
PCDH20	0	2.13E-06	6.21E-07	6.76E-07	3.61E-07	0	2.86E-06	1.04E-07
PCDH7	2.25E-07	3.75E-08	1.37E-07	3.44E-07	5.54E-08	1.44E-08	2.04E-07	1.18E-07
PCDH8	0	0	0	0	0	0	0	0
PCDH9	1.97E-08	2.04E-08	1.02E-06	1.21E-06	2.40E-08	4.01E-08	1.90E-08	1.08E-08
PCDHA1	4.56E-08	1.94E-06	3.09E-08	5.07E-08	7.78E-11	2.56E-08	6.94E-10	5.76E-07

PCDHA10	7.59E-08	6.97E-08	0	1.58E-07	4.10E-08	0	2.60E-08	6.41E-08
PCDHA11	1.51E-08	1.88E-06	9.38E-08	1.49E-07	0	0	1.16E-08	1.33E-07
PCDHA12	1.08E-08	2.34E-08	2.97E-07	6.87E-07	1.22E-08	0	5.88E-08	5.76E-07
PCDHA13	1.16E-08	6.38E-08	9.58E-07	1.16E-07	#######	0	2.42E-08	2.90E-07
PCDHA2	4.55E-08	3.51E-07	0	9.27E-09	0	8.27E-09	0	0
PCDHA3	2.27E-08	4.92E-08	1.28E-08	5.11E-08	0	1.71E-08	4.32E-08	2.29E-08
PCDHA4	0	5.68E-08	0	7.21E-08	#######	0	0	7.19E-08
PCDHA5	1.22E-08	0	0	2.01E-08	#######	8.89E-09	0	2.74E-08
PCDHA6	4.89E-08	0	8.52E-08	1.89E-07	2.10E-34	8.34E-09	3.91E-21	2.46E-07
PCDHA7	5.79E-08	1.26E-08	3.95E-08	1.23E-07	0	9.23E-09	8.99E-08	2.36E-08
PCDHA8	0	0	1.28E-08	0	0	0	0	0
PCDHA9	7.97E-09	8.66E-09	0	1.14E-08	0	0	3.41E-09	0
PCDHAC1	2.03E-08	4.21E-08	4.14E-07	1.98E-07	0	7.61E-09	0	0
PCDHAC2	9.20E-08	2.99E-08	1.05E-06	3.60E-07	0	1.63E-08	7.66E-08	1.05E-06
PCDHB1	0	1.04E-08	0	1.13E-08	0	0	0	0
PCDHB10	3.39E-07	3.57E-07	1.39E-07	1.38E-07	3.64E-08	1.05E-07	1.99E-07	3.11E-07
PCDHB11	1.38E-07	1.58E-07	1.02E-07	1.55E-07	0	6.52E-08	4.10E-08	1.14E-07
PCDHB12	2.43E-07	2.79E-07	5.57E-08	2.06E-07	4.05E-08	9.17E-08	1.70E-07	2.27E-07
PCDHB13	2.57E-07	1.08E-06	4.75E-07	5.71E-07	4.52E-08	7.92E-08	1.15E-07	7.52E-07
PCDHB14	1.58E-06	1.02E-06	7.81E-07	1.33E-06	1.80E-07	9.07E-07	7.31E-07	1.85E-06
PCDHB15	5.81E-07	2.18E-07	3.84E-07	6.46E-07	6.05E-08	4.08E-08	1.71E-07	7.76E-07
PCDHB16	2.84E-07	4.33E-06	3.61E-07	6.89E-07	2.01E-08	1.04E-07	3.18E-07	6.88E-07
PCDHB17	9.77E-09	9.91E-08	0	2.39E-08	0	9.05E-09	1.89E-08	2.87E-08
PCDHB18	8.75E-08	8.19E-09	1.51E-08	4.46E-08	1.88E-08	0	2.12E-08	1.07E-08
PCDHB19I	4.90E-09	0	1.51E-08	5.98E-09	8.40E-09	9.06E-09	1.43E-08	2.15E-08
PCDHB2	1.71E-07	1.82E-06	1.85E-07	3.91E-07	4.41E-08	1.27E-07	2.90E-07	7.52E-07
PCDHB3	1.09E-07	3.76E-07	6.01E-08	2.31E-07	0	1.21E-07	1.69E-07	2.03E-07
PCDHB4	2.47E-07	2.84E-07	2.22E-07	4.48E-07	1.09E-08	1.46E-07	2.58E-07	4.91E-07
PCDHB5	2.24E-07	1.35E-06	5.54E-07	9.87E-07	6.18E-08	3.11E-07	2.71E-07	9.61E-07
PCDHB6	1.61E-07	4.47E-07	4.32E-08	1.87E-07	5.25E-08	1.13E-07	3.31E-07	1.06E-07
PCDHB7	2.46E-07	5.24E-07	1.52E-07	3.76E-07	9.51E-08	1.65E-07	2.09E-07	1.44E-07
PCDHB8	1.45E-07	1.71E-06	1.78E-07	1.99E-07	0	1.09E-07	1.14E-07	2.92E-07
PCDHB9	7.54E-08	5.43E-07	5.54E-08	1.38E-07	4.16E-08	3.49E-08	1.05E-07	1.58E-07
PCDHGA1	1.21E-07	4.84E-08	1.98E-08	9.31E-08	0	6.37E-08	4.60E-08	3.63E-07
PCDHGA1	6.36E-06	1.31E-06	8.43E-07	6.77E-07	0	6.30E-07	4.18E-07	1.61E-06
PCDHGA1	4.82E-07	8.32E-08	1.23E-07	1.27E-07	0	2.06E-08	3.27E-08	4.91E-07
PCDHGA1	8.36E-07	1.50E-07	5.07E-07	6.06E-07	0	8.15E-08	3.62E-07	6.52E-07
PCDHGA2	3.34E-07	1.04E-06	8.86E-07	5.38E-07	0	5.71E-08	6.82E-08	4.12E-06
PCDHGA3	2.64E-07	1.21E-07	3.22E-07	3.46E-07	0	3.32E-08	1.83E-08	3.35E-06
PCDHGA4	1.18E-06	3.03E-07	2.99E-07	3.63E-07	2.39E-08	1.29E-07	8.08E-08	1.13E-05
PCDHGA5	4.53E-07	2.17E-07	1.30E-07	2.01E-07	0	6.25E-08	9.33E-09	4.38E-07
PCDHGA6	3.35E-07	1.74E-07	1.92E-07	2.81E-07	0	5.32E-08	2.78E-08	2.11E-07
PCDHGA7	2.91E-07	1.25E-07	2.84E-07	2.20E-07	0	3.97E-08	4.15E-08	1.89E-07
PCDHGA8	2.76E-07	7.38E-09	1.70E-07	3.30E-07	1.53E-07	6.09E-09	3.71E-07	1.74E-07
PCDHGA9	6.80E-07	1.60E-07	3.46E-07	2.20E-07	1.62E-08	4.38E-08	8.23E-08	2.08E-07
PCDHGB1	1.29E-06	1.00E-06	1.41E-07	2.25E-07	0	1.44E-07	4.69E-08	8.32E-07
PCDHGB2	2.18E-06	2.04E-06	5.24E-07	6.08E-07	0	9.67E-07	1.87E-08	1.69E-06
PCDHGB3	2.31E-07	6.51E-08	2.00E-07	2.00E-07	0	3.57E-08	1.86E-08	1.41E-07
PCDHGB4	2.70E-06	3.30E-08	1.68E-06	1.42E-06	1.68E-08	9.05E-09	7.19E-07	2.11E-07
PCDHGB5	4.59E-06	1.08E-07	1.27E-06	1.41E-06	4.11E-08	7.98E-08	1.11E-07	1.35E-06
PCDHGB6	5.60E-06	1.08E-07	8.63E-07	5.72E-07	8.20E-09	9.73E-08	6.38E-07	6.89E-07
PCDHGB7	4.21E-07	3.77E-07	6.31E-07	5.24E-07	1.48E-08	2.71E-07	1.75E-07	3.91E-07
PCDHGB8	1.09E-07	0	0	1.49E-08	0	1.13E-08	0	3.58E-08
PCDHGC3	4.47E-05	6.50E-06	1.53E-05	2.82E-05	6.33E-06	6.62E-06	1.59E-05	5.58E-05
PCDHGC4	6.26E-08	0	0	0	0	0	0	2.63E-08
PCDHGC5	1.06E-07	9.99E-09	2.76E-08	1.05E-08	0	8.22E-09	0	7.81E-08
PCDP1	2.78E-06	1.25E-08	4.29E-08	2.03E-06	1.34E-07	1.32E-06	2.52E-07	1.53E-07
PCF11	2.06E-05	3.82E-06	7.24E-06	8.26E-06	4.08E-06	7.76E-06	4.16E-06	6.61E-06

PCGEM1	0	0	0	0	0	0	0	0
PCGF1	2.40E-05	2.57E-05	8.94E-06	9.44E-06	9.82E-06	1.42E-05	1.67E-05	1.63E-05
PCGF2	1.31E-05	2.87E-06	1.01E-06	2.68E-06	1.99E-06	2.60E-06	4.80E-06	4.31E-06
PCGF3	1.54E-05	7.69E-06	4.98E-06	6.26E-06	3.78E-06	5.25E-06	5.59E-06	7.81E-06
PCGF5	4.20E-05	3.13E-05	2.28E-05	2.30E-05	1.22E-05	3.06E-05	1.79E-05	2.79E-05
PCGF6	3.97E-06	3.70E-06	4.78E-06	3.90E-06	2.72E-06	5.17E-06	4.60E-06	3.87E-06
PCID2	2.20E-05	1.90E-05	1.13E-05	1.16E-05	5.12E-06	1.61E-05	1.27E-05	1.17E-05
PCIF1	2.41E-05	2.08E-05	1.25E-05	1.36E-05	1.83E-05	1.50E-05	2.06E-05	1.67E-05
PCK1	0.002717	0.001357	0.000896	0.000898	0.000433	0.000735	0.000113	0.000443
PCK2	7.56E-05	0.000356	0.000548	0.000276	0.0003	0.000212	0.000229	0.00027
PCLO	3.11E-06	1.10E-08	1.10E-07	4.04E-07	0	0	2.04E-08	3.54E-08
PCM1	4.21E-05	4.53E-05	1.46E-05	1.30E-05	2.92E-06	3.73E-05	5.76E-06	1.10E-06
PCMT1	4.82E-05	3.70E-05	4.26E-05	3.85E-05	1.62E-05	4.28E-05	3.79E-05	3.36E-05
PCMTD1	5.16E-05	1.84E-05	1.53E-05	1.19E-05	4.47E-06	1.34E-05	1.46E-05	1.38E-05
PCMTD2	2.81E-05	1.52E-05	1.31E-05	1.18E-05	5.67E-06	2.28E-05	6.05E-06	9.91E-06
PCNA	0.000155	6.47E-05	2.54E-05	2.73E-05	3.88E-05	5.11E-05	2.90E-05	4.08E-05
PCNAP1	0	0	0	0	0	0	0	0
PCNP	5.91E-05	7.01E-05	3.43E-05	3.27E-05	8.31E-06	3.65E-05	2.43E-05	2.66E-05
PCNT	4.92E-06	1.60E-06	1.84E-06	1.75E-06	1.42E-06	2.26E-06	1.79E-06	2.24E-06
PCNX	5.54E-06	2.74E-06	5.01E-06	4.08E-06	3.09E-06	5.43E-06	3.87E-06	4.99E-06
PCNXL2	2.57E-06	8.85E-08	1.48E-07	7.36E-07	7.58E-08	7.87E-07	1.58E-07	1.16E-06
PCNXL3	1.71E-05	1.56E-05	6.06E-06	6.42E-06	1.13E-05	1.12E-05	1.06E-05	2.07E-05
PCOLCE	0.000192	0.001211	0.000131	6.70E-05	1.47E-05	4.57E-06	5.24E-05	2.37E-05
PCOLCE2	3.51E-06	1.80E-05	1.55E-05	7.64E-06	9.88E-06	2.80E-06	6.07E-06	1.77E-05
PCOTH	3.88E-07	3.63E-07	7.39E-07	9.41E-07	2.95E-07	1.81E-06	6.67E-07	3.84E-07
PCP2	8.40E-07	1.10E-06	9.67E-08	2.10E-07	0	7.89E-08	3.95E-07	1.90E-07
PCP4	0	7.27E-08	0	1.49E-07	0	1.68E-07	0	0
PCP4L1	4.22E-06	3.07E-05	1.93E-06	1.82E-06	3.35E-06	1.03E-06	1.05E-06	4.42E-07
PCSK1	2.85E-08	0	2.94E-08	1.39E-08	0	2.48E-08	1.66E-08	2.61E-08
PCSK1N	8.10E-06	0	1.82E-07	1.10E-06	0	0	5.27E-08	0
PCSK2	0	0	2.00E-08	3.57E-08	0	0	2.32E-08	0
PCSK4	1.23E-06	2.32E-06	1.09E-06	1.12E-06	8.13E-07	1.41E-06	1.50E-06	6.34E-07
PCSK5	6.35E-06	2.08E-06	6.17E-07	9.98E-07	5.95E-07	1.72E-07	3.13E-07	1.70E-06
PCSK6	4.93E-05	8.80E-05	0.000128	5.94E-05	6.52E-05	0.000114	6.55E-05	7.40E-05
PCSK7	1.08E-05	6.34E-06	4.01E-06	5.95E-06	4.08E-06	1.67E-05	4.52E-06	1.48E-05
PCSK9	2.61E-05	5.44E-05	8.44E-06	6.24E-06	4.26E-06	4.65E-06	1.28E-05	3.06E-05
PCTP	4.70E-05	6.77E-05	4.70E-05	5.53E-05	3.03E-05	6.82E-05	4.20E-05	4.74E-05
PCYOX1	2.99E-05	4.81E-05	5.25E-05	2.35E-05	1.16E-05	5.01E-05	1.05E-05	3.38E-05
PCYOX1L	2.96E-06	3.21E-07	5.27E-07	1.68E-06	3.49E-07	4.02E-07	1.51E-06	3.30E-07
PCYT1A	1.89E-05	1.41E-05	1.02E-05	8.08E-06	4.66E-06	1.25E-05	9.59E-06	7.98E-06
PCYT1B	8.60E-08	1.45E-08	4.42E-09	7.49E-08	0	0	4.20E-09	6.31E-09
PCYT2	9.56E-05	0.000324	7.33E-05	7.86E-05	6.98E-05	0.000229	9.13E-05	7.44E-05
PDAP1	9.58E-05	5.42E-05	2.22E-05	3.27E-05	3.78E-05	4.14E-05	3.89E-05	4.22E-05
PDC	0	0	0	0	0	0	0	0
PDCD1	3.73E-06	3.03E-08	3.37E-07	1.23E-06	0	3.05E-06	4.38E-07	1.43E-07
PDCD10	3.34E-05	2.15E-05	1.54E-05	1.61E-05	4.99E-06	2.16E-05	1.67E-05	1.88E-05
PDCD11	8.37E-06	5.07E-06	3.55E-06	4.40E-06	3.81E-06	9.93E-06	5.99E-06	5.51E-06
PDCD1LG2	1.91E-06	2.74E-07	1.16E-06	1.21E-06	5.03E-08	8.56E-07	9.23E-07	2.01E-07
PDCD2	2.94E-05	2.30E-05	1.70E-05	2.15E-05	8.63E-06	4.31E-05	1.85E-05	1.66E-05
PDCD2L	3.48E-06	6.56E-06	3.71E-06	3.74E-06	2.83E-06	5.87E-06	8.14E-06	5.53E-06
PDCD4	1.68E-05	8.74E-06	1.21E-05	1.22E-05	9.49E-06	1.38E-05	9.71E-06	1.04E-05
PDCD5	9.97E-05	0.000112	5.31E-05	5.90E-05	7.32E-05	0.000118	8.88E-05	8.77E-05
PDCD6	8.50E-05	6.14E-05	3.37E-05	4.62E-05	3.32E-05	7.89E-05	4.40E-05	7.20E-05
PDCD6IP	3.04E-05	3.30E-05	1.96E-05	2.06E-05	7.81E-06	2.68E-05	1.65E-05	1.99E-05
PDCD7	6.62E-06	5.39E-06	3.52E-06	3.34E-06	2.33E-06	4.89E-06	5.48E-06	3.68E-06
PDCL	8.35E-06	2.41E-06	2.92E-06	4.68E-06	2.07E-06	5.25E-06	3.73E-06	5.23E-06
PDCL2	0	0	0	0	0	0	0	0
PDCL3	2.65E-05	1.27E-05	5.64E-06	1.27E-05	7.43E-06	1.54E-05	1.56E-05	1.16E-05

PDDC1	3.22E-05	1.20E-05	1.78E-05	1.81E-05	5.28E-05	1.94E-05	1.31E-05	2.23E-05
PDE10A	4.48E-07	1.84E-07	1.96E-07	1.75E-07	6.05E-08	1.15E-07	6.35E-08	2.34E-07
PDE11A	1.06E-06	4.17E-07	5.13E-06	4.97E-07	3.21E-08	1.12E-05	1.93E-07	2.10E-07
PDE12	8.16E-06	1.42E-05	7.31E-06	7.09E-06	1.71E-06	8.39E-06	4.55E-06	1.03E-05
PDE1A	2.50E-06	1.28E-07	5.11E-07	3.23E-06	2.06E-07	2.49E-07	9.53E-07	5.95E-07
PDE1B	5.64E-07	4.72E-07	8.29E-07	1.44E-06	5.94E-07	9.95E-07	1.34E-06	7.00E-07
PDE1C	4.51E-06	6.95E-08	0	6.69E-08	0	4.30E-08	3.01E-08	4.59E-08
PDE2A	2.54E-06	1.50E-06	7.49E-06	4.81E-06	2.23E-06	1.82E-06	4.49E-06	3.24E-06
PDE3A	4.78E-06	2.96E-07	2.61E-07	3.70E-07	1.89E-08	3.57E-07	4.83E-08	1.53E-07
PDE3B	1.03E-05	1.49E-05	1.24E-05	6.30E-06	2.57E-06	5.50E-06	2.30E-06	7.79E-06
PDE4A	1.02E-05	8.01E-07	8.52E-07	3.01E-06	3.65E-07	4.50E-07	2.15E-06	1.07E-06
PDE4B	7.46E-06	3.18E-06	3.02E-06	9.82E-06	1.57E-07	3.65E-06	1.87E-06	1.69E-05
PDE4C	5.12E-06	3.56E-07	5.95E-08	4.89E-08	1.99E-07	2.31E-07	2.57E-07	8.86E-08
PDE4D	3.44E-06	7.96E-07	5.32E-07	1.61E-06	1.77E-07	7.18E-07	3.35E-07	5.23E-07
PDE4DIP	2.52E-05	2.19E-05	3.21E-05	2.57E-05	1.25E-05	4.18E-05	2.01E-05	2.01E-05
PDE5A	9.98E-07	3.29E-07	2.81E-07	2.86E-07	2.29E-07	9.90E-07	3.81E-07	3.20E-07
PDE6A	3.98E-09	4.89E-08	1.22E-08	9.71E-09	0	3.68E-09	3.88E-09	1.75E-08
PDE6B	1.02E-06	6.35E-08	1.80E-07	6.69E-07	6.02E-08	2.34E-07	4.83E-07	1.44E-07
PDE6C	3.12E-08	1.76E-07	0	9.55E-09	1.34E-08	7.23E-09	7.58E-09	0
PDE6D	2.54E-05	1.25E-05	9.19E-06	1.42E-05	4.23E-06	1.37E-05	1.21E-05	1.40E-05
PDE6G	1.31E-06	9.14E-07	1.45E-06	2.78E-06	1.69E-07	1.07E-06	3.20E-06	1.13E-06
PDE6H	0	0	0	0	0	0	0	0
PDE7A	1.25E-05	2.11E-06	1.46E-06	3.74E-06	5.29E-07	1.12E-06	2.51E-06	2.26E-06
PDE7B	5.12E-07	6.06E-07	2.96E-06	9.83E-07	3.19E-07	5.18E-07	2.31E-07	5.26E-07
PDE8A	2.32E-05	1.26E-05	1.46E-05	1.21E-05	6.32E-06	6.87E-06	7.92E-06	8.36E-06
PDE8B	2.15E-07	7.62E-08	4.63E-07	3.97E-07	3.64E-08	1.89E-06	6.86E-08	4.14E-08
PDE9A	8.15E-06	2.88E-07	4.67E-06	5.99E-06	2.66E-06	4.03E-07	2.37E-06	1.64E-05
PDF	1.36E-05	1.05E-05	1.50E-05	1.20E-05	1.51E-05	1.46E-05	1.64E-05	2.61E-05
PDGFA	2.82E-05	6.28E-06	1.07E-06	7.92E-06	2.77E-06	2.79E-06	1.65E-05	1.42E-05
PDGFB	1.21E-05	5.41E-06	2.19E-06	6.51E-06	4.89E-06	5.48E-06	9.65E-06	7.41E-06
PDGFC	8.46E-06	2.75E-06	8.56E-06	8.98E-06	1.97E-06	6.01E-06	9.55E-06	5.75E-06
PDGFD	1.62E-05	2.98E-06	1.36E-06	1.84E-06	8.42E-07	2.01E-06	1.06E-06	2.41E-06
PDGFRA	1.70E-06	2.18E-07	8.85E-06	1.32E-05	4.73E-07	1.07E-07	1.55E-06	1.89E-06
PDGFRB	1.96E-05	7.22E-06	4.38E-06	1.64E-05	7.00E-06	8.79E-06	1.29E-05	3.20E-05
PDGFRL	2.46E-06	3.09E-06	1.58E-07	6.70E-07	2.14E-07	4.60E-07	2.70E-07	8.29E-07
PDHA1	6.51E-05	4.12E-05	2.76E-05	2.78E-05	1.66E-05	2.96E-05	3.14E-05	4.94E-05
PDHA2	1.81E-08	0	1.93E-08	0	0	0	0	0
PDHB	5.14E-05	8.22E-05	6.65E-05	5.72E-05	2.65E-05	7.52E-05	4.21E-05	7.56E-05
PDHX	1.88E-05	2.00E-05	1.44E-05	1.02E-05	4.61E-06	2.22E-05	8.25E-06	1.71E-05
PDIA2	2.52E-07	1.71E-05	0	3.16E-08	1.22E-08	0	1.36E-08	2.08E-08
PDIA3	0.000293	0.000204	0.000119	0.000173	0.000138	0.000218	0.000164	0.000276
PDIA3P	0.00013	8.78E-05	6.04E-05	6.41E-05	2.54E-05	9.02E-05	5.24E-05	0.00011
PDIA4	9.30E-05	0.000167	0.00013	0.000134	8.03E-05	0.000236	9.99E-05	0.000249
PDIA5	3.60E-05	2.46E-05	7.54E-05	6.13E-05	3.29E-05	6.12E-05	6.38E-05	3.81E-05
PDIA6	0.000267	0.000453	0.000124	0.000137	6.97E-05	0.000223	0.00013	0.000169
PDIK1L	5.54E-06	4.88E-06	4.11E-06	2.30E-06	6.46E-07	2.77E-06	2.39E-06	3.50E-06
PDILT	0	2.78E-07	1.17E-08	0	0	0	0	0
PDK1	6.02E-06	3.79E-06	2.53E-06	2.24E-06	7.19E-07	1.90E-05	2.40E-06	3.97E-06
PDK2	4.29E-05	3.87E-05	4.41E-05	4.32E-05	2.72E-05	6.25E-05	3.98E-05	2.41E-05
PDK3	6.82E-06	7.63E-07	2.46E-06	2.30E-06	7.14E-07	1.18E-06	1.41E-06	3.91E-06
PDK4	2.40E-05	0.000183	0.000135	5.76E-05	1.90E-05	0.000172	1.94E-05	1.57E-05
PDLIM1	0.000144	6.17E-05	9.34E-05	0.000225	0.000116	0.00027	0.000188	0.000145
PDLIM2	1.13E-05	2.41E-05	2.54E-05	2.07E-05	1.19E-05	1.96E-05	2.00E-05	5.87E-06
PDLIM3	2.22E-05	2.74E-06	2.40E-06	2.22E-05	1.80E-06	3.06E-06	5.95E-06	3.69E-06
PDLIM4	2.94E-06	1.51E-07	1.14E-08	1.60E-06	4.43E-08	7.09E-08	4.55E-07	1.59E-07
PDLIM5	1.93E-05	1.91E-05	2.91E-05	2.61E-05	6.26E-06	1.48E-05	1.58E-05	2.48E-05
PDLIM7	2.14E-05	5.51E-06	2.80E-06	1.21E-05	8.09E-06	4.72E-06	1.26E-05	1.12E-05
PDP1	1.22E-05	9.15E-07	1.21E-06	4.16E-06	1.33E-07	1.07E-06	1.58E-06	3.51E-07

PDP2	1.38E-06	3.43E-06	3.08E-06	8.52E-07	1.89E-07	1.78E-06	4.81E-07	1.52E-06
PDPK1	1.23E-05	9.37E-06	3.87E-06	4.19E-06	8.42E-07	7.74E-06	6.43E-06	6.29E-06
PDPN	6.54E-07	0	2.58E-08	1.02E-06	5.35E-08	0	1.41E-06	4.87E-07
PDPR	5.19E-06	1.46E-06	1.54E-06	7.41E-07	1.46E-07	1.12E-06	1.36E-06	1.94E-06
PDRG1	3.03E-05	1.78E-05	6.54E-06	1.24E-05	2.39E-05	1.29E-05	2.44E-05	1.27E-05
PDS5A	1.80E-05	2.93E-05	1.62E-05	1.06E-05	4.68E-06	1.31E-05	9.22E-06	1.38E-05
PDS5B	5.60E-06	4.18E-06	2.54E-06	2.53E-06	1.18E-06	2.91E-06	2.29E-06	2.64E-06
PDSS1	8.58E-06	4.83E-06	1.68E-06	2.42E-06	1.84E-06	4.71E-06	5.59E-06	3.50E-06
PDSS2	1.02E-05	1.33E-05	1.26E-05	1.22E-05	6.62E-06	1.66E-05	1.25E-05	1.25E-05
PDX1	1.24E-06	0	1.42E-07	1.20E-06	0	0	0	0
PDXDC1	7.29E-05	0.000128	5.60E-05	5.46E-05	1.26E-05	3.82E-05	3.59E-05	3.77E-05
PDXDC2	1.05E-05	2.61E-06	1.60E-06	3.04E-06	1.96E-06	2.26E-06	1.36E-06	2.54E-06
PDXK	2.14E-05	6.27E-06	6.47E-06	8.65E-06	1.63E-05	9.12E-06	2.08E-05	1.36E-05
PDXP	3.71E-05	3.06E-05	3.10E-05	3.03E-05	3.66E-05	3.15E-05	5.22E-05	3.96E-05
PDYN	0	0	0	1.12E-08	0	0	0	0
PDZD11	5.38E-05	4.31E-05	1.29E-05	1.86E-05	1.64E-05	2.88E-05	2.13E-05	4.03E-05
PDZD2	6.05E-07	1.14E-06	2.63E-07	2.18E-07	2.25E-07	7.41E-07	6.43E-07	6.09E-07
PDZD3	4.02E-06	0	0	0	0	0	4.21E-08	3.02E-08
PDZD4	6.73E-07	2.09E-07	5.59E-07	1.36E-06	6.17E-07	1.24E-07	8.33E-07	5.22E-07
PDZD7	2.22E-06	1.34E-07	2.48E-08	4.35E-08	1.02E-08	3.30E-08	4.58E-08	6.97E-08
PDZD8	1.68E-05	3.69E-06	1.07E-05	7.07E-06	8.53E-07	5.49E-06	2.84E-06	7.98E-06
PDZD9	2.58E-08	8.98E-08	0	0	0	2.41E-08	4.93E-08	3.84E-08
PDZK1	8.74E-05	3.78E-05	4.15E-05	3.26E-05	3.14E-05	0.000201	3.09E-05	3.47E-05
PDZK1IP1	0.000661	7.76E-05	3.14E-06	0.000146	4.38E-05	2.84E-05	0.000556	2.71E-06
PDZK1P1	4.81E-06	8.46E-07	3.65E-08	8.25E-07	4.50E-08	7.89E-08	4.05E-06	2.88E-08
PDZRN3	7.64E-07	9.35E-08	4.20E-07	2.05E-06	1.25E-07	2.57E-08	1.60E-07	2.24E-07
PDZRN4	7.12E-08	0	1.62E-07	7.05E-07	6.05E-08	0	7.59E-09	0
PEA15	7.62E-05	6.08E-05	1.54E-05	4.47E-05	1.72E-05	3.14E-05	4.75E-05	6.50E-05
PEAR1	4.41E-06	1.08E-06	1.60E-06	1.66E-06	4.09E-07	6.73E-07	1.29E-06	1.06E-06
PEBP1	0.000622	0.001515	0.001286	0.001034	0.000927	0.001462	0.000949	0.000661
PEBP4	3.07E-08	3.23E-07	4.09E-07	1.14E-07	2.66E-08	2.87E-08	0	4.59E-08
PECAM1	4.66E-05	2.70E-05	1.38E-05	2.34E-05	4.57E-05	2.33E-05	7.23E-05	4.05E-05
PECI	0.000101	0.000194	0.000213	0.000165	0.000158	0.00029	0.000205	0.000222
PECR	4.80E-05	8.06E-05	9.57E-05	9.19E-05	5.47E-05	7.41E-05	3.77E-05	7.10E-05
PEF1	0.000107	6.27E-05	4.36E-05	5.74E-05	4.57E-05	7.00E-05	6.22E-05	6.24E-05
PEG10	3.16E-05	7.92E-07	1.30E-06	9.07E-08	2.01E-07	2.83E-08	5.96E-08	4.85E-06
PEG3	1.33E-07	1.51E-07	8.09E-07	6.94E-07	1.59E-08	3.80E-08	4.66E-08	5.43E-08
PEG3AS	0	0	0	3.80E-07	0	4.40E-08	0	0
PELI1	1.35E-05	5.89E-06	3.41E-06	7.48E-06	8.76E-07	2.42E-06	4.38E-06	8.94E-06
PELI2	1.33E-06	1.85E-07	8.67E-07	9.81E-07	4.73E-08	1.17E-07	2.73E-07	1.84E-07
PELI3	4.21E-06	4.37E-06	1.81E-06	1.52E-06	9.97E-07	1.23E-06	1.29E-06	5.45E-06
PELO	1.90E-05	1.31E-05	7.06E-06	9.73E-06	2.11E-06	7.04E-06	6.97E-06	3.56E-06
PELP1	1.25E-05	9.95E-06	8.00E-06	1.09E-05	1.89E-05	1.72E-05	1.79E-05	1.16E-05
PEMT	1.22E-05	0.000137	0.000166	6.51E-05	0.000275	5.35E-05	0.000108	0.000132
PENK	2.14E-08	0	0	5.27E-08	0	0	0	0
PEPD	7.00E-05	0.000198	0.000128	8.42E-05	5.83E-05	0.000164	7.84E-05	5.24E-05
PER1	3.09E-05	4.25E-05	7.46E-05	5.87E-05	2.35E-05	3.58E-05	9.12E-05	4.73E-05
PER2	2.97E-06	1.97E-06	4.05E-06	6.24E-06	8.56E-07	2.82E-06	3.29E-06	1.74E-06
PER3	2.51E-06	6.17E-06	1.25E-05	2.76E-06	2.33E-06	3.25E-06	3.15E-06	2.62E-06
PER4	0	0	0	0	0	0	0	0
PERP	7.61E-05	4.15E-05	5.70E-05	6.14E-05	2.04E-05	4.61E-05	4.68E-05	4.95E-05
PES1	4.40E-05	3.24E-05	2.19E-05	3.23E-05	4.24E-05	4.06E-05	4.41E-05	3.61E-05
PET112L	2.07E-05	1.65E-05	1.61E-05	1.48E-05	1.10E-05	3.44E-05	1.75E-05	2.54E-05
PEX1	4.57E-05	1.50E-05	5.71E-06	3.60E-06	1.93E-06	7.34E-06	2.51E-06	8.64E-06
PEX10	1.42E-05	2.33E-05	7.74E-06	6.80E-06	3.95E-06	1.19E-05	6.47E-06	9.88E-06
PEX11A	1.30E-05	1.10E-05	1.50E-05	1.32E-05	9.80E-06	2.01E-05	1.55E-05	8.48E-06
PEX11B	2.36E-05	1.67E-05	1.79E-05	2.11E-05	1.03E-05	4.04E-05	1.66E-05	2.06E-05
PEX11G	1.09E-05	8.01E-06	1.52E-05	1.63E-05	2.43E-05	1.96E-05	2.10E-05	4.02E-06

PEX12	7.73E-06	1.01E-05	4.94E-06	2.54E-06	2.71E-06	8.04E-06	3.56E-06	3.68E-06
PEX13	2.43E-05	2.09E-05	1.48E-05	1.21E-05	4.65E-06	1.26E-05	9.11E-06	7.37E-06
PEX14	1.92E-05	1.92E-05	1.97E-05	2.18E-05	1.84E-05	3.77E-05	2.30E-05	2.42E-05
PEX16	2.59E-05	4.07E-05	2.28E-05	1.80E-05	2.26E-05	2.57E-05	2.11E-05	4.40E-05
PEX19	3.00E-05	9.94E-05	4.55E-05	3.27E-05	1.08E-05	6.97E-05	4.32E-05	5.57E-05
PEX2	1.91E-05	1.21E-05	7.31E-06	6.71E-06	6.28E-06	1.04E-05	1.55E-05	1.07E-05
PEX26	1.51E-05	1.52E-05	7.59E-06	6.53E-06	1.81E-06	9.77E-06	7.22E-06	9.29E-06
PEX3	9.36E-06	8.71E-06	1.17E-05	6.64E-06	1.95E-06	1.60E-05	6.46E-06	7.73E-06
PEX5	1.75E-05	2.79E-05	2.16E-05	1.74E-05	1.08E-05	2.85E-05	2.16E-05	2.04E-05
PEX5L	2.03E-08	7.58E-09	0	1.65E-08	0	1.25E-08	0	0
PEX6	8.87E-06	6.03E-05	1.46E-05	9.57E-06	2.05E-05	1.10E-05	1.12E-05	1.49E-05
PEX7	5.86E-06	3.97E-06	5.51E-06	5.80E-06	1.75E-06	8.50E-06	4.13E-06	4.84E-06
PF4	1.13E-06	1.62E-06	0	1.34E-06	5.31E-07	0	2.74E-07	2.33E-07
PF4V1	1.63E-05	2.79E-07	2.24E-07	1.91E-06	2.05E-07	0	1.62E-06	5.90E-08
PFAS	5.97E-06	3.81E-06	3.45E-06	3.26E-06	7.03E-07	5.91E-06	4.99E-06	5.45E-06
PFDN1	3.75E-05	2.70E-05	1.62E-05	3.05E-05	1.08E-05	4.24E-05	2.48E-05	2.98E-05
PFDN2	0.00012	0.000226	7.33E-05	9.07E-05	6.01E-05	0.00012	0.000114	0.000184
PFDN4	1.33E-05	1.01E-05	4.37E-06	8.96E-06	9.97E-06	8.67E-06	8.05E-06	6.63E-06
PFDN5	0.00034	0.000285	0.000194	0.000374	0.000601	0.000338	0.000421	0.000236
PFDN6	0.000107	0.000165	4.96E-05	6.00E-05	9.01E-05	0.000102	0.000109	0.000112
PFKFB1	2.20E-07	1.29E-05	1.78E-05	1.81E-05	1.76E-05	3.07E-06	2.00E-05	2.06E-05
PFKFB2	1.18E-05	5.07E-06	9.32E-07	1.23E-06	3.79E-07	1.63E-06	1.26E-06	3.48E-06
PFKFB3	2.30E-05	2.36E-06	8.74E-06	6.52E-05	2.74E-06	3.86E-06	6.79E-06	6.75E-06
PFKFB4	1.04E-06	1.54E-06	2.98E-07	4.41E-07	1.40E-07	3.55E-07	5.75E-07	5.81E-07
PFKL	6.00E-05	6.36E-05	6.81E-05	5.44E-05	8.36E-05	7.15E-05	6.82E-05	5.40E-05
PFKM	4.26E-05	1.07E-05	3.22E-06	4.65E-06	8.20E-07	3.76E-06	4.40E-06	2.59E-06
PFKP	6.50E-05	1.35E-06	1.11E-06	1.49E-05	5.60E-07	8.76E-06	4.35E-06	1.23E-06
PFN1	0.000788	0.000391	0.000606	0.000887	0.001243	0.000874	0.001319	0.000608
PFN2	6.38E-05	3.53E-05	2.23E-05	4.80E-06	1.00E-06	1.68E-06	2.90E-06	2.93E-06
PFN3	0	1.57E-07	7.83E-08	0	5.57E-08	0	5.83E-08	0
PFN4	1.18E-06	1.61E-06	1.67E-07	4.14E-07	4.18E-07	1.18E-06	6.97E-07	0
PGA3	1.80E-08	2.05E-08	1.27E-06	4.42E-08	0	1.05E-06	1.73E-08	5.32E-08
PGA4	0	0	5.57E-07	0	0	1.00E-07	0	0
PGA5	0	0	3.77E-07	0	0	1.38E-07	1.78E-08	0
PGAM1	0.000209	0.000127	8.55E-05	9.15E-05	6.27E-05	0.000114	0.000119	7.81E-05
PGAM2	2.08E-06	1.70E-06	2.26E-07	8.77E-07	1.52E-06	6.61E-07	8.60E-07	5.54E-07
PGAM4	3.50E-06	1.39E-06	1.21E-06	8.22E-07	1.76E-07	1.83E-06	1.05E-06	6.23E-07
PGAM5	1.89E-05	3.38E-05	1.53E-05	1.44E-05	3.53E-06	1.63E-05	1.84E-05	1.91E-05
PGAP1	1.51E-06	6.80E-07	6.75E-07	7.70E-07	2.91E-07	8.37E-07	4.13E-07	5.47E-07
PGAP2	2.43E-05	2.10E-05	1.62E-05	1.25E-05	1.45E-05	2.21E-05	1.54E-05	2.70E-05
PGAP3	1.91E-05	3.16E-05	3.02E-05	2.07E-05	4.23E-05	2.88E-05	1.46E-05	2.32E-05
PGBD1	2.05E-06	8.08E-07	7.13E-07	1.32E-06	1.76E-06	1.41E-06	1.88E-06	2.46E-06
PGBD2	2.73E-06	1.51E-06	1.52E-06	1.64E-06	5.52E-07	1.78E-06	1.34E-06	1.83E-06
PGBD3	1.30E-06	1.69E-06	9.99E-07	1.31E-06	4.99E-07	1.32E-06	9.18E-07	8.64E-07
PGBD4	1.08E-06	8.74E-07	6.91E-07	2.50E-07	1.67E-07	1.10E-06	2.96E-07	7.19E-07
PGBD5	8.02E-06	2.77E-05	2.12E-06	2.37E-06	7.50E-07	2.31E-06	2.36E-06	1.02E-06
PGC	3.72E-08	1.92E-06	0	2.29E-08	0	5.19E-08	1.79E-08	3.39E-06
PGCP	7.12E-05	3.64E-05	2.68E-05	2.90E-05	2.25E-05	3.91E-05	6.95E-05	4.10E-05
PGD	0.000111	5.36E-05	6.21E-05	3.75E-05	1.91E-05	7.88E-05	3.10E-05	4.74E-05
PGF	1.05E-05	3.50E-06	2.10E-06	4.51E-06	5.62E-06	1.30E-06	1.96E-05	6.10E-06
PGGT1B	7.26E-06	6.23E-06	5.45E-06	6.46E-06	2.01E-06	5.91E-06	4.95E-06	6.51E-06
PGK1	0.000305	0.000238	8.84E-05	0.000116	6.07E-05	0.000143	0.000117	0.000187
PGK2	0	1.70E-08	0	1.83E-08	0	1.39E-08	0	0
PGLS	6.16E-05	5.49E-05	3.93E-05	5.38E-05	0.000108	5.66E-05	8.63E-05	4.33E-05
PGLYRP1	1.29E-07	5.16E-08	4.99E-08	5.39E-08	0	0	0	0
PGLYRP2	2.51E-05	3.50E-05	0.000294	0.000142	0.000169	1.80E-06	0.000142	3.73E-05
PGLYRP3	0	0	0	0	0	0	0	0
PGLYRP4	1.32E-08	1.51E-08	0	1.63E-08	0	0	1.27E-08	0

PGM1	8.57E-05	0.000101	0.000177	9.00E-05	5.99E-05	0.000129	6.85E-05	6.35E-05
PGM2	8.93E-06	6.58E-06	9.16E-06	7.67E-06	6.42E-07	5.23E-06	2.63E-06	1.17E-05
PGM2L1	1.23E-06	2.21E-07	2.85E-07	9.42E-07	1.05E-07	2.58E-07	3.69E-07	3.62E-07
PGM3	2.10E-05	1.47E-05	1.36E-05	1.43E-05	2.60E-06	2.94E-05	6.97E-06	1.02E-05
PGM5	6.62E-07	6.57E-07	2.08E-06	1.34E-06	7.58E-07	6.38E-07	9.17E-07	1.04E-06
PGM5P2	6.09E-08	2.76E-08	7.66E-08	2.98E-08	0	1.13E-08	0	0
PGP	1.55E-05	1.79E-05	3.33E-06	5.38E-06	9.97E-06	6.00E-06	1.07E-05	1.08E-05
PGPEP1	1.17E-05	7.92E-06	5.12E-06	5.36E-06	9.92E-06	7.28E-06	8.42E-06	5.57E-06
PGPEP1L	0	0	0	0	0	0	0	0
PGR	8.14E-08	1.47E-08	1.04E-07	1.18E-07	6.49E-09	6.99E-09	4.90E-08	2.21E-08
PGRMC1	8.77E-05	0.000479	0.000377	0.000237	0.00047	0.000268	0.000171	0.000309
PGRMC2	7.00E-05	5.72E-05	6.76E-05	4.83E-05	4.49E-05	6.47E-05	6.99E-05	8.73E-05
PGS1	1.38E-05	8.33E-06	3.35E-06	5.18E-06	2.91E-06	1.28E-05	3.94E-06	6.01E-06
PHACTR1	1.70E-07	5.63E-08	2.18E-07	4.09E-07	1.60E-08	1.32E-07	8.02E-08	3.76E-08
PHACTR2	4.53E-06	1.06E-06	2.00E-06	1.92E-06	2.76E-07	3.18E-06	1.41E-06	1.34E-06
PHACTR3	2.41E-08	4.01E-08	1.35E-07	3.95E-07	3.03E-08	0	1.69E-08	3.24E-08
PHACTR4	1.95E-05	9.54E-06	1.31E-05	1.10E-05	4.23E-06	1.53E-05	8.27E-06	1.30E-05
PHAX	1.68E-05	1.43E-05	9.80E-06	1.14E-05	2.70E-06	1.12E-05	8.17E-06	1.78E-05
PHB	0.000181	0.000205	7.66E-05	8.57E-05	0.000101	0.000173	0.000126	8.34E-05
PHB2	0.000229	0.000225	0.000206	0.000181	0.000156	0.000254	0.000196	0.000167
PHC1	2.56E-06	8.14E-07	7.49E-07	1.71E-06	2.69E-07	3.58E-07	4.84E-07	1.05E-06
PHC2	6.20E-05	3.94E-05	3.07E-05	5.82E-05	2.46E-05	6.23E-05	4.89E-05	7.28E-05
PHC3	4.36E-06	2.39E-06	2.37E-06	1.94E-06	5.31E-07	2.52E-06	1.53E-06	1.63E-06
PHEX	1.67E-07	0	0	2.05E-08	0	3.88E-08	0	2.46E-08
PHF1	2.24E-05	2.18E-05	8.76E-06	1.72E-05	6.65E-06	1.31E-05	1.61E-05	2.32E-05
PHF10	4.96E-05	2.29E-05	1.73E-05	2.24E-05	1.20E-05	2.14E-05	1.95E-05	1.07E-05
PHF11	1.74E-05	1.19E-05	8.56E-06	1.58E-05	7.12E-06	1.48E-05	1.23E-05	1.52E-05
PHF12	1.21E-05	9.99E-06	4.62E-06	5.74E-06	2.74E-06	6.65E-06	5.27E-06	6.83E-06
PHF13	7.08E-06	5.31E-06	3.31E-06	8.23E-06	2.31E-06	7.81E-06	6.93E-06	6.56E-06
PHF14	1.86E-05	2.59E-05	6.89E-06	8.90E-06	7.06E-06	1.51E-05	8.21E-06	9.75E-06
PHF15	4.95E-06	2.36E-06	2.03E-06	2.97E-06	9.74E-07	2.96E-06	2.47E-06	3.26E-06
PHF16	5.11E-06	6.87E-06	3.10E-06	3.09E-06	1.23E-06	4.35E-06	2.45E-06	6.44E-06
PHF17	9.46E-06	3.62E-06	4.51E-06	7.87E-06	2.21E-06	6.30E-06	7.97E-06	5.44E-06
PHF19	2.19E-05	2.24E-06	1.52E-06	3.83E-06	4.74E-06	3.04E-06	2.11E-06	4.32E-06
PHF2	8.57E-06	4.38E-06	4.68E-06	5.73E-06	1.76E-06	4.92E-06	4.08E-06	5.27E-06
PHF20	1.22E-05	6.04E-06	4.14E-06	4.91E-06	1.89E-06	6.01E-06	4.02E-06	3.43E-06
PHF20L1	1.13E-05	1.08E-05	4.34E-06	4.82E-06	2.38E-06	5.97E-06	6.56E-06	4.79E-06
PHF21A	6.82E-06	1.63E-06	1.24E-06	2.12E-06	8.09E-07	1.93E-06	1.70E-06	2.31E-06
PHF21B	6.56E-09	0	0	8.02E-09	0	0	0	0
PHF23	1.32E-05	1.03E-05	9.95E-06	1.87E-05	6.58E-06	2.97E-05	2.59E-05	1.80E-05
PHF3	2.06E-05	2.86E-05	1.50E-05	1.38E-05	3.27E-06	1.85E-05	8.61E-06	1.62E-05
PHF5A	4.40E-05	2.26E-05	1.20E-05	2.04E-05	1.35E-05	2.46E-05	2.42E-05	2.67E-05
PHF6	8.02E-06	5.41E-06	2.59E-06	2.31E-06	8.82E-07	4.22E-06	2.14E-06	4.05E-06
PHF7	2.84E-06	1.90E-06	1.31E-06	1.53E-06	1.37E-06	1.92E-06	2.26E-06	1.72E-06
PHF8	1.48E-05	1.10E-05	9.30E-06	7.88E-06	1.44E-06	1.80E-05	7.94E-06	1.56E-05
PHGDH	5.92E-05	9.97E-05	0.000128	6.20E-05	0.00015	1.12E-05	5.49E-05	5.48E-07
PHGR1	1.76E-07	8.01E-06	0	1.13E-07	0	0	7.95E-08	0
PHIP	6.54E-06	3.08E-06	1.95E-06	2.39E-06	3.96E-07	2.44E-06	1.76E-06	1.94E-06
PHKA1	1.04E-06	6.62E-07	6.61E-07	5.79E-07	1.64E-07	1.96E-07	4.52E-07	9.55E-07
PHKA2	5.70E-05	1.21E-05	1.46E-05	1.53E-05	1.52E-05	1.45E-05	1.75E-05	2.41E-05
PHKB	1.40E-05	8.72E-06	1.05E-05	8.16E-06	3.47E-06	2.51E-05	6.04E-06	1.08E-05
PHKG1	1.43E-07	2.26E-07	3.90E-08	4.49E-08	0	1.10E-07	1.67E-07	1.81E-07
PHKG2	2.51E-05	2.81E-05	1.30E-05	1.52E-05	1.29E-05	1.74E-05	2.13E-05	1.82E-05
PHLDA1	9.75E-06	1.89E-05	3.21E-05	9.19E-05	4.22E-06	2.04E-05	4.61E-05	1.31E-05
PHLDA2	1.32E-05	1.29E-05	7.86E-07	2.06E-05	5.91E-07	1.27E-06	1.10E-05	3.45E-06
PHLDA3	7.62E-06	9.32E-07	3.40E-06	1.29E-05	8.84E-06	1.57E-05	7.90E-06	7.19E-06
PHLDB1	1.81E-05	2.53E-06	2.46E-06	9.65E-06	3.63E-06	1.71E-06	6.09E-06	4.32E-06
PHLDB2	1.21E-05	1.42E-05	1.82E-05	1.29E-05	8.66E-06	4.83E-06	8.47E-06	4.09E-06

PHLDB3	7.50E-06	1.49E-05	6.60E-06	1.02E-05	1.20E-05	7.74E-06	1.19E-05	7.31E-06
PHLPP1	1.13E-05	6.63E-06	9.69E-06	8.61E-06	2.91E-06	1.60E-05	9.62E-06	3.18E-06
PHLPP2	1.88E-06	9.10E-07	7.64E-07	1.48E-06	2.38E-07	1.61E-06	9.63E-07	1.66E-06
PHOSPHC	3.53E-07	2.64E-08	1.59E-07	2.00E-07	2.01E-08	5.42E-08	0	6.88E-08
PHOSPHC	2.62E-06	2.62E-06	9.56E-07	1.57E-06	1.60E-06	1.60E-06	1.09E-06	1.41E-06
PHOX2A	0	0	0	0	2.48E-08	0	0	0
PHOX2B	0	0	0	0	0	0	0	0
PHPT1	7.48E-05	5.76E-05	2.64E-05	4.38E-05	6.75E-05	5.09E-05	4.36E-05	7.67E-05
PHRF1	1.18E-05	1.71E-05	8.79E-06	9.44E-06	4.43E-06	1.17E-05	9.51E-06	1.16E-05
PHTF1	8.91E-06	5.84E-06	2.37E-06	4.30E-06	1.10E-06	7.96E-06	1.50E-06	2.38E-06
PHTF2	6.48E-06	3.05E-06	1.46E-06	2.13E-06	9.41E-07	1.74E-06	1.54E-06	2.69E-06
PHYH	0.000121	0.000351	0.000496	0.000224	0.00014	6.93E-05	0.000183	0.000164
PHYHD1	1.13E-06	2.02E-05	2.47E-05	3.08E-05	1.95E-05	1.70E-05	2.94E-05	9.50E-07
PHYHIP	2.73E-07	3.62E-07	6.67E-08	6.40E-07	6.78E-08	1.53E-07	7.66E-08	4.21E-08
PHYHIPL	1.14E-06	2.86E-06	6.69E-07	2.14E-06	1.46E-06	8.93E-06	2.50E-06	1.13E-06
PI15	1.29E-07	4.55E-07	1.69E-08	1.62E-08	8.18E-09	3.66E-06	3.23E-09	1.65E-07
PI16	3.34E-08	0	1.15E-07	4.92E-07	2.43E-07	0	1.23E-08	1.60E-08
PI3	0.00097	2.85E-06	1.34E-05	1.50E-07	2.92E-06	0	3.29E-07	4.55E-07
PI4K2A	1.52E-05	1.33E-05	5.56E-06	1.04E-05	5.54E-06	1.29E-05	1.15E-05	9.77E-06
PI4K2B	1.67E-05	2.34E-05	2.17E-05	1.45E-05	5.45E-06	2.90E-05	1.54E-05	1.83E-05
PI4KA	3.64E-05	2.35E-05	1.74E-05	1.91E-05	1.44E-05	1.36E-05	1.90E-05	3.08E-05
PI4KAP1	9.82E-06	3.70E-06	1.54E-06	2.84E-06	1.47E-06	1.88E-06	3.80E-06	5.81E-06
PI4KAP2	9.24E-06	5.62E-06	9.43E-07	7.36E-07	5.18E-07	1.02E-06	4.60E-06	5.12E-06
PI4KB	1.96E-05	1.29E-05	8.32E-06	1.44E-05	1.17E-05	1.08E-05	1.52E-05	1.98E-05
PIAS1	1.19E-05	7.55E-06	6.67E-06	6.38E-06	3.12E-06	1.02E-05	3.58E-06	5.69E-06
PIAS2	5.21E-06	2.69E-06	3.33E-06	2.89E-06	4.98E-07	4.51E-06	3.14E-06	4.69E-06
PIAS3	7.28E-06	3.45E-06	2.41E-06	3.16E-06	1.01E-06	5.18E-06	2.39E-06	3.65E-06
PIAS4	1.06E-05	6.01E-06	5.80E-06	5.99E-06	2.46E-06	1.03E-05	7.64E-06	6.20E-06
PIBF1	5.46E-06	7.40E-06	2.30E-06	3.32E-06	1.37E-06	4.92E-06	2.96E-06	3.39E-06
PICALM	8.69E-05	4.69E-05	4.68E-05	5.60E-05	2.96E-05	4.88E-05	4.24E-05	5.01E-05
PICK1	2.53E-05	8.14E-06	9.60E-06	1.47E-05	1.31E-05	1.34E-05	1.15E-05	1.44E-05
PID1	4.70E-07	2.27E-05	4.52E-05	1.35E-05	7.63E-06	2.39E-05	4.30E-05	2.63E-05
PIF1	4.12E-06	9.75E-07	8.70E-08	3.29E-07	9.07E-07	3.14E-07	1.97E-07	2.94E-07
PIGA	7.32E-06	2.03E-06	2.37E-06	3.91E-06	1.20E-06	2.26E-06	2.38E-06	6.72E-06
PIGB	7.75E-06	3.58E-06	4.68E-06	4.08E-06	2.59E-06	5.56E-06	4.59E-06	5.84E-06
PIGC	4.55E-05	1.39E-05	1.13E-05	1.95E-05	1.34E-05	2.71E-05	1.34E-05	2.88E-05
PIGF	2.09E-05	2.45E-05	6.73E-06	1.12E-05	8.72E-06	1.93E-05	1.25E-05	9.65E-06
PIGG	1.97E-05	1.37E-05	5.40E-06	8.02E-06	3.80E-06	1.30E-05	3.54E-06	1.24E-05
PIGH	3.40E-05	1.27E-05	1.00E-05	9.18E-06	9.29E-06	1.15E-05	1.05E-05	1.14E-05
PIGK	8.23E-06	9.58E-06	5.34E-06	5.72E-06	1.25E-06	9.82E-06	4.32E-06	6.72E-06
PIGL	4.14E-06	4.65E-06	1.32E-06	1.72E-06	1.16E-06	2.63E-06	2.90E-06	7.30E-06
PIGM	6.62E-06	1.25E-05	3.93E-06	3.51E-06	2.39E-06	9.59E-06	3.43E-06	6.97E-06
PIGN	6.60E-06	3.71E-06	3.54E-06	3.49E-06	1.21E-06	5.69E-06	2.50E-06	3.61E-06
PIGO	1.26E-05	2.42E-05	1.12E-05	1.21E-05	4.42E-06	1.45E-05	8.46E-06	1.55E-05
PIGP	3.00E-05	1.78E-05	2.21E-05	2.07E-05	1.36E-05	3.65E-05	2.04E-05	2.96E-05
PIGQ	1.20E-05	1.89E-05	1.11E-05	1.84E-05	2.75E-05	1.21E-05	1.72E-05	1.57E-05
PIGR	2.57E-06	7.58E-06	3.17E-05	2.40E-05	1.36E-05	2.22E-05	3.79E-05	8.57E-05
PIGS	1.49E-05	1.12E-05	5.22E-06	8.50E-06	5.17E-06	6.56E-06	8.71E-06	6.49E-06
PIGT	8.92E-05	0.000107	4.09E-05	4.82E-05	3.42E-05	5.79E-05	5.50E-05	6.83E-05
PIGU	4.17E-05	1.38E-05	6.09E-06	8.43E-06	2.09E-05	1.50E-05	1.42E-05	1.75E-05
PIGV	2.56E-05	2.57E-05	1.84E-05	1.34E-05	9.99E-06	1.98E-05	8.80E-06	8.19E-06
PIGW	6.53E-06	4.62E-06	3.75E-06	2.77E-06	2.54E-06	3.69E-06	3.15E-06	4.01E-06
PIGX	5.51E-06	3.54E-06	3.80E-06	2.89E-06	3.08E-06	3.57E-06	3.54E-06	5.07E-06
PIGY	6.85E-05	3.68E-05	5.68E-05	4.75E-05	2.81E-05	6.83E-05	6.65E-05	5.61E-05
PIGZ	2.54E-06	1.59E-06	7.26E-07	9.09E-07	9.65E-07	9.93E-07	4.27E-07	3.93E-07
PIH1D1	5.03E-05	3.36E-05	3.87E-05	3.94E-05	9.90E-05	4.58E-05	7.31E-05	4.69E-05
PIH1D2	1.99E-07	1.57E-07	6.66E-08	1.76E-07	1.03E-07	3.24E-07	1.59E-07	2.47E-07
PIK3AP1	2.31E-05	3.29E-05	4.20E-05	2.13E-05	1.48E-05	4.36E-05	3.39E-05	2.84E-05

PIK3C2A	1.18E-05	9.49E-06	6.44E-06	4.84E-06	1.04E-06	6.30E-06	1.66E-06	8.94E-06
PIK3C2B	2.87E-06	1.37E-06	1.46E-06	1.12E-06	6.88E-07	8.07E-07	1.90E-06	4.96E-06
PIK3C2G	1.82E-07	3.08E-06	5.05E-06	4.85E-06	9.08E-07	3.33E-06	2.74E-06	1.79E-06
PIK3C3	1.19E-05	4.38E-06	5.40E-06	6.45E-06	1.05E-06	9.93E-06	4.83E-06	5.49E-06
PIK3CA	5.81E-06	3.10E-06	3.95E-06	2.86E-06	4.55E-07	3.68E-06	1.49E-06	3.42E-06
PIK3CB	1.84E-05	1.85E-05	9.22E-06	1.81E-05	6.22E-06	1.83E-05	1.23E-05	1.17E-05
PIK3CD	5.95E-06	3.63E-07	1.97E-06	2.88E-06	4.38E-07	1.55E-06	1.98E-06	5.24E-07
PIK3CG	1.03E-06	1.60E-07	3.68E-07	5.12E-07	1.13E-08	2.47E-07	2.17E-07	1.09E-07
PIK3IP1	1.63E-05	1.15E-05	7.59E-06	1.51E-05	1.98E-06	9.92E-06	1.53E-05	9.71E-06
PIK3R1	9.62E-06	1.48E-05	3.76E-05	1.55E-05	3.05E-06	2.64E-05	8.96E-06	2.13E-05
PIK3R2	2.26E-05	1.25E-05	7.50E-06	7.49E-06	1.33E-05	1.22E-05	1.39E-05	1.06E-05
PIK3R3	4.33E-06	2.98E-06	1.41E-06	2.76E-06	1.67E-06	3.84E-06	2.85E-06	3.64E-06
PIK3R4	1.08E-05	1.30E-05	1.13E-05	4.87E-06	1.83E-06	8.60E-06	3.60E-06	6.02E-06
PIK3R5	2.70E-06	3.95E-07	1.53E-06	3.85E-06	1.22E-07	9.57E-07	1.70E-06	5.31E-07
PIK3R6	1.24E-06	1.54E-07	1.05E-07	3.88E-07	2.62E-08	1.20E-07	1.58E-06	1.90E-07
PIKFYVE	6.92E-06	4.34E-06	2.78E-06	2.30E-06	7.55E-07	2.86E-06	1.20E-06	2.32E-06
PILRA	9.73E-06	2.79E-06	4.21E-06	8.68E-06	7.04E-07	2.96E-06	6.70E-06	2.25E-06
PILRB	4.95E-05	2.13E-05	8.44E-06	1.46E-05	2.10E-05	8.85E-06	1.00E-05	2.09E-05
PIM1	1.30E-05	9.10E-06	1.05E-05	7.33E-05	3.89E-06	3.58E-05	9.60E-06	1.34E-05
PIM2	1.77E-05	1.28E-05	4.07E-06	1.32E-05	1.63E-06	9.13E-06	5.31E-06	1.45E-05
PIM3	7.42E-05	6.31E-05	3.96E-05	9.74E-05	2.45E-05	4.05E-05	5.77E-05	8.28E-05
PIN1	4.16E-05	3.55E-05	2.71E-05	3.85E-05	5.24E-05	4.03E-05	4.78E-05	3.67E-05
PIN1L	2.42E-07	0	2.94E-08	6.64E-08	2.32E-08	0	5.12E-08	0
PIN4	2.71E-05	1.52E-05	1.89E-05	2.07E-05	3.06E-05	2.02E-05	1.56E-05	3.82E-05
PINK1	4.54E-05	7.89E-05	0.00012	7.79E-05	7.27E-05	8.43E-05	5.39E-05	9.24E-05
PINX1	4.72E-06	8.91E-06	4.04E-06	7.54E-06	2.92E-06	1.17E-05	7.34E-06	4.51E-06
PION	1.74E-05	1.64E-05	9.02E-06	1.08E-05	4.96E-06	1.61E-05	6.15E-06	1.38E-05
PIP	5.58E-08	0	0	7.03E-08	0	0	5.18E-08	0
PIP4K2A	1.45E-05	5.09E-06	5.34E-06	7.81E-06	6.50E-07	4.92E-06	4.31E-06	5.06E-06
PIP4K2B	2.04E-05	1.65E-05	7.13E-06	8.84E-06	8.54E-06	1.30E-05	9.00E-06	9.65E-06
PIP4K2C	2.22E-05	1.85E-05	7.33E-06	7.95E-06	2.90E-06	1.03E-05	7.66E-06	7.64E-06
PIP5K1A	2.08E-05	1.29E-05	8.17E-06	1.25E-05	1.14E-06	1.67E-05	9.18E-06	1.79E-05
PIP5K1B	2.63E-07	2.24E-08	6.19E-08	1.46E-07	3.41E-08	8.27E-08	5.12E-08	0
PIP5K1C	1.04E-05	2.38E-06	2.35E-06	4.19E-06	2.93E-06	3.62E-06	4.80E-06	4.44E-06
PIP5K1P1	3.21E-08	1.20E-08	3.30E-08	0	0	9.91E-09	5.21E-09	0
PIP5KL1	2.68E-07	3.86E-07	5.15E-08	5.85E-08	2.05E-08	1.48E-08	2.27E-08	2.36E-08
PIPOX	0.000107	0.000414	0.000294	0.000143	0.00019	0.000148	0.00015	7.61E-05
PIPSL	4.16E-06	2.84E-06	2.72E-06	1.71E-06	1.47E-06	3.85E-06	1.42E-06	3.77E-06
PIR	2.77E-05	2.31E-05	1.45E-05	6.89E-06	1.16E-05	6.71E-05	7.59E-06	3.77E-05
PIRT	0	0	0	0	0	0	0	0
PISD	1.69E-05	1.21E-05	7.65E-06	1.45E-05	6.90E-06	1.10E-05	1.02E-05	9.34E-06
PISRT1	0	0	0	0	0	0	0	0
PITPNA	2.00E-05	9.43E-06	1.75E-05	1.88E-05	5.47E-06	1.52E-05	1.70E-05	1.55E-05
PITPNB	3.37E-05	3.29E-05	3.45E-05	3.03E-05	1.77E-05	2.59E-05	3.92E-05	4.57E-05
PITPNC1	8.17E-06	1.30E-05	4.32E-06	5.44E-06	6.35E-06	6.89E-06	6.31E-06	2.96E-06
PITPNM1	1.85E-05	1.16E-05	3.80E-06	5.72E-06	8.18E-07	3.37E-06	3.28E-06	1.22E-05
PITPNM2	2.94E-06	2.03E-06	4.23E-06	1.90E-06	2.96E-06	6.32E-06	3.05E-06	9.39E-06
PITPNM3	2.97E-06	1.74E-08	1.32E-06	9.95E-07	4.02E-08	2.53E-08	7.99E-08	3.07E-08
PITRM1	0.000115	2.04E-05	3.08E-05	3.29E-05	1.98E-05	5.64E-05	2.46E-05	3.21E-05
PITX1	1.35E-05	1.27E-08	2.23E-07	1.37E-08	0	2.70E-07	6.51E-08	4.78E-07
PITX2	1.14E-08	0	0	0	0	0	2.20E-08	5.02E-08
PITX3	5.42E-08	0	0	0	1.56E-08	0	0	0
PIWIL1	0	1.51E-08	0	0	0	8.28E-09	0	0
PIWIL2	2.05E-07	3.75E-06	2.68E-07	2.39E-07	0	1.88E-06	0	3.04E-07
PIWIL3	0	0	0	0	0	0	0	0
PIWIL4	8.68E-07	1.62E-08	1.19E-07	7.41E-07	3.72E-08	4.01E-08	1.05E-07	7.40E-08
PJA1	1.43E-05	9.54E-06	4.59E-06	7.19E-06	3.41E-06	5.03E-06	5.60E-06	7.29E-06
PJA2	3.14E-05	3.12E-05	2.91E-05	2.32E-05	4.16E-06	3.20E-05	1.29E-05	2.54E-05

PKD1	4.78E-06	3.73E-06	1.63E-06	2.19E-06	1.62E-06	1.46E-06	3.54E-06	2.63E-06
PKD1L1	9.30E-08	4.66E-08	2.42E-08	7.23E-08	6.87E-08	2.92E-08	3.91E-08	4.70E-08
PKD1L2	3.71E-07	3.75E-07	5.93E-08	0	1.02E-08	2.40E-06	6.44E-08	4.94E-08
PKD1L3	1.30E-08	1.45E-08	8.89E-09	1.59E-08	0	3.61E-08	1.27E-08	1.27E-08
PKD2	1.33E-05	2.56E-06	4.51E-06	7.42E-06	9.85E-07	5.00E-06	3.39E-06	4.16E-06
PKD2L1	8.34E-08	3.32E-07	1.25E-07	1.67E-07	1.30E-08	6.95E-07	1.25E-07	3.67E-07
PKD2L2	3.09E-08	2.73E-08	0	0	0	0	0	0
PKDCC	1.52E-05	3.71E-06	2.13E-06	5.48E-06	3.60E-06	1.24E-06	3.47E-06	6.37E-06
PKDREJ	3.49E-08	6.48E-08	2.37E-08	3.19E-08	7.47E-09	1.26E-07	8.50E-09	2.55E-08
PKHD1	6.80E-06	1.64E-07	4.75E-07	1.20E-06	8.35E-08	1.67E-07	6.47E-08	1.78E-08
PKHD1L1	3.14E-09	3.32E-08	3.35E-08	3.83E-08	8.88E-08	1.45E-09	1.53E-09	0
PKIA	1.85E-06	4.77E-08	4.42E-08	3.78E-07	0	6.50E-08	7.37E-08	4.13E-08
PKIB	2.05E-05	4.59E-06	2.37E-06	2.09E-06	2.54E-07	1.25E-06	3.98E-06	1.79E-07
PKIG	3.29E-05	3.48E-05	2.42E-05	4.42E-05	1.49E-05	2.87E-05	3.26E-05	2.38E-05
PKLR	3.12E-06	0.000102	0.000112	2.40E-05	1.76E-05	7.47E-05	3.59E-05	0.000125
PKM2	0.000301	6.67E-05	1.93E-05	8.08E-05	1.65E-05	2.15E-05	6.78E-05	3.03E-05
PKMYT1	7.25E-06	8.16E-06	1.70E-07	3.97E-07	2.19E-06	7.35E-07	1.09E-06	1.09E-06
PKN1	0.000573	3.89E-05	2.84E-05	4.12E-05	6.00E-05	3.70E-05	8.61E-05	3.36E-05
PKN2	1.11E-05	1.10E-05	6.17E-06	5.07E-06	1.18E-06	1.33E-05	3.23E-06	3.51E-06
PKN3	4.38E-06	2.30E-06	3.26E-06	1.20E-06	2.14E-06	9.24E-06	1.01E-05	2.92E-06
PKNOX1	5.04E-06	3.23E-06	2.35E-06	3.90E-06	1.56E-06	4.40E-06	4.04E-06	2.09E-06
PKNOX2	2.11E-07	4.32E-07	1.40E-07	6.48E-07	3.46E-08	3.73E-08	2.03E-07	1.73E-07
PKP1	7.02E-08	6.07E-08	6.90E-07	8.02E-08	3.76E-09	3.24E-08	6.75E-07	8.98E-08
PKP2	1.31E-05	9.23E-06	1.29E-05	8.52E-06	1.67E-06	1.80E-05	6.56E-06	3.84E-06
PKP3	1.02E-05	7.23E-06	9.44E-08	1.09E-06	7.12E-09	2.30E-08	1.61E-07	2.43E-08
PKP4	9.69E-05	1.65E-05	1.47E-05	1.92E-05	2.80E-05	2.34E-05	2.63E-05	1.60E-05
PL-5283	2.05E-05	3.33E-05	1.82E-05	1.40E-05	2.64E-05	3.51E-05	2.20E-05	2.18E-05
PLA1A	3.30E-06	2.33E-05	2.33E-05	1.57E-05	9.89E-07	3.12E-05	3.73E-05	8.53E-06
PLA2G10	1.07E-06	3.12E-08	0	0	2.33E-08	2.51E-08	0	0
PLA2G12A	1.00E-05	7.07E-06	1.01E-05	5.57E-06	3.36E-06	1.12E-05	6.99E-06	4.27E-06
PLA2G12B	7.89E-05	6.20E-05	5.29E-05	4.91E-05	6.59E-05	5.07E-05	6.10E-05	2.47E-05
PLA2G15	1.14E-05	8.50E-06	7.45E-06	1.02E-05	1.32E-05	7.89E-06	1.33E-05	5.48E-06
PLA2G16	8.80E-05	0.000109	5.14E-05	7.32E-05	6.60E-05	8.53E-05	5.33E-05	1.85E-05
PLA2G1B	8.06E-07	6.36E-07	2.79E-07	0	2.22E-07	6.53E-07	6.94E-07	2.64E-07
PLA2G2A	0.000741	5.07E-07	0.000377	1.32E-05	7.23E-06	0.004401	1.70E-06	0.00061
PLA2G2C	0	0	0	0	0	0	0	0
PLA2G2D	4.69E-06	0	2.42E-07	9.93E-07	0	1.20E-06	3.89E-07	3.01E-07
PLA2G2E	0	0	0	0	0	0	0	0
PLA2G2F	0	1.35E-07	0	0	0	0	0	0
PLA2G3	0	0	9.45E-09	0	1.56E-08	0	8.82E-09	0
PLA2G4A	2.92E-06	4.46E-08	3.91E-07	6.51E-07	5.12E-08	1.45E-07	2.70E-07	7.49E-08
PLA2G4C	3.09E-06	2.91E-05	1.16E-05	1.88E-05	5.90E-06	1.73E-05	1.86E-05	1.57E-05
PLA2G4D	1.92E-08	0	3.29E-08	7.81E-09	0	0	0	0
PLA2G4E	3.81E-08	1.74E-08	4.99E-08	3.75E-08	0	0	0	0
PLA2G4F	5.26E-08	0	2.41E-08	2.04E-08	0	0	4.80E-08	0
PLA2G5	1.71E-06	4.58E-07	1.55E-06	2.89E-06	2.34E-06	3.68E-07	9.66E-07	2.29E-06
PLA2G6	8.39E-06	4.41E-06	2.40E-06	1.94E-06	4.65E-06	2.46E-06	1.94E-06	7.39E-06
PLA2G7	1.52E-05	1.45E-06	1.48E-06	9.15E-06	2.15E-07	7.76E-07	4.62E-06	1.10E-06
PLA2R1	2.63E-06	4.66E-08	3.06E-07	8.15E-07	3.75E-09	5.15E-08	1.91E-07	7.33E-08
PLAA	6.04E-06	9.98E-06	8.22E-06	7.57E-06	1.77E-06	9.19E-06	2.86E-06	1.23E-05
PLAC1	5.01E-08	0	0	3.09E-07	0	0	7.17E-08	0
PLAC1L	0	0	0	0	0	0	0	0
PLAC2	2.48E-08	4.17E-08	3.19E-08	5.30E-08	5.32E-09	2.87E-08	6.62E-08	9.08E-09
PLAC4	1.33E-08	4.92E-09	0	3.77E-08	0	0	2.16E-09	0
PLAC8	1.52E-05	2.89E-07	8.62E-06	4.41E-06	1.35E-07	9.18E-07	7.97E-07	1.29E-07
PLAC8L1	3.64E-07	0	0	0	6.34E-07	4.07E-08	4.06E-08	1.96E-07
PLAC9	3.71E-06	1.28E-06	9.34E-07	1.06E-05	1.90E-05	1.97E-06	1.05E-05	3.85E-06
PLAG1	1.10E-06	1.15E-06	8.62E-08	9.50E-08	1.95E-08	4.20E-08	7.28E-08	1.00E-06

PLAGL1	1.57E-05	4.54E-07	7.36E-07	1.51E-06	1.37E-07	3.90E-07	7.43E-07	3.75E-07
PLAGL2	4.40E-06	1.55E-06	7.72E-07	1.74E-06	6.06E-07	1.63E-06	9.20E-07	1.28E-06
PLAT	1.08E-05	2.69E-06	1.45E-06	6.09E-06	1.34E-06	1.37E-06	4.77E-06	4.45E-06
PLAU	9.83E-06	2.73E-06	1.10E-06	1.07E-05	1.12E-06	3.40E-06	4.86E-06	2.00E-06
PLAUR	1.02E-05	1.35E-06	2.60E-06	4.87E-05	8.97E-07	2.45E-06	6.35E-06	1.95E-06
PLB1	1.10E-06	6.90E-08	3.01E-07	1.23E-06	1.78E-07	8.11E-07	1.72E-06	2.36E-07
PLBD1	2.56E-05	5.21E-07	2.91E-06	8.56E-06	2.25E-07	2.89E-06	5.87E-06	9.71E-07
PLBD2	1.83E-05	3.26E-05	1.24E-05	1.36E-05	5.82E-06	1.71E-05	1.05E-05	1.69E-05
PLCB1	8.22E-06	7.26E-07	6.71E-07	3.48E-07	1.17E-07	1.25E-06	6.27E-07	4.84E-06
PLCB2	7.81E-06	8.58E-07	5.14E-06	9.26E-06	1.39E-06	3.12E-06	3.71E-06	2.16E-06
PLCB3	8.83E-06	4.76E-06	2.68E-06	3.27E-06	2.53E-06	2.32E-06	4.29E-06	2.87E-06
PLCB4	2.78E-07	1.61E-07	4.05E-08	1.07E-07	9.80E-08	5.69E-08	1.03E-07	2.06E-07
PLCD1	9.44E-06	3.77E-06	4.42E-06	4.49E-06	2.37E-06	6.57E-06	4.89E-06	2.22E-06
PLCD3	8.86E-06	6.41E-07	4.18E-07	1.72E-06	1.81E-06	5.17E-07	1.48E-06	4.67E-07
PLCD4	2.97E-06	8.30E-07	5.07E-07	6.52E-07	1.27E-06	7.23E-07	1.75E-06	1.49E-06
PLCE1	3.85E-06	2.54E-07	1.56E-07	4.60E-07	9.23E-08	2.32E-07	5.66E-08	1.15E-06
PLCG1	1.64E-05	9.72E-06	3.68E-06	4.26E-06	3.13E-06	7.67E-06	5.66E-06	3.66E-06
PLCG2	3.05E-05	1.93E-05	6.56E-06	8.73E-06	2.48E-06	5.48E-06	5.92E-06	8.06E-06
PLCH1	3.98E-07	4.49E-08	5.23E-08	1.25E-07	9.40E-09	4.73E-08	3.56E-09	3.74E-08
PLCH2	3.31E-06	2.68E-06	3.86E-07	2.27E-07	6.36E-08	1.68E-07	5.41E-07	5.69E-06
PLCL1	8.49E-07	3.76E-07	7.53E-08	2.17E-07	6.32E-08	2.14E-07	1.96E-07	3.04E-07
PLCL2	8.76E-06	3.71E-06	4.69E-06	4.89E-06	2.28E-06	1.76E-06	3.25E-06	2.29E-06
PLCXD1	6.14E-06	1.14E-06	5.42E-06	5.03E-06	2.97E-06	2.19E-06	9.15E-06	3.84E-05
PLCXD2	7.79E-07	2.31E-07	1.56E-07	3.79E-07	2.72E-08	2.77E-07	8.05E-08	1.04E-07
PLCXD3	1.21E-07	3.20E-08	6.01E-07	1.38E-06	6.41E-08	7.97E-09	2.33E-07	5.46E-08
PLCZ1	0	7.06E-08	1.61E-08	1.87E-08	0	1.42E-08	0	2.25E-08
PLD1	1.64E-05	5.42E-06	1.17E-05	5.24E-06	6.33E-06	3.01E-06	3.67E-06	1.12E-05
PLD2	5.87E-06	2.51E-06	4.17E-06	5.62E-06	2.76E-06	3.85E-06	8.55E-06	5.25E-06
PLD3	9.33E-05	0.00013	7.44E-05	0.000156	8.77E-05	8.01E-05	0.000104	6.52E-05
PLD4	1.01E-06	1.83E-07	5.35E-07	1.65E-06	9.63E-08	1.38E-07	4.32E-07	5.49E-08
PLD5	0	9.49E-08	2.19E-08	7.16E-08	9.03E-09	1.81E-08	0	1.54E-08
PLD6	1.22E-06	1.70E-06	2.57E-06	1.48E-06	8.65E-07	2.06E-06	3.07E-06	1.92E-06
PLDN	2.67E-05	1.24E-05	1.00E-05	1.12E-05	4.94E-06	1.74E-05	1.03E-05	9.07E-06
PLEC	5.18E-05	3.80E-05	7.95E-06	1.08E-05	9.72E-06	8.28E-06	2.05E-05	1.07E-05
PLEK	1.54E-05	1.37E-06	6.80E-06	1.34E-05	4.63E-07	6.54E-06	5.91E-06	1.25E-06
PLEK2	3.62E-05	4.87E-07	4.01E-05	6.42E-06	8.83E-08	1.45E-05	8.30E-06	1.26E-07
PLEKHA1	4.20E-05	1.52E-05	1.62E-05	1.40E-05	3.77E-06	1.07E-05	8.10E-06	1.36E-05
PLEKHA2	5.24E-06	2.07E-06	3.24E-06	4.82E-06	5.11E-07	3.47E-06	1.72E-06	1.15E-06
PLEKHA3	1.24E-05	8.52E-06	5.64E-06	5.13E-06	2.45E-06	7.79E-06	4.67E-06	5.96E-06
PLEKHA4	1.20E-05	9.87E-06	8.41E-06	1.44E-05	1.52E-05	2.21E-06	1.40E-05	5.73E-06
PLEKHA5	1.11E-05	1.37E-05	7.83E-06	7.93E-06	3.54E-06	1.42E-05	4.76E-06	7.34E-06
PLEKHA6	1.75E-05	8.08E-06	1.20E-05	1.18E-05	2.35E-06	5.75E-06	4.14E-06	1.42E-05
PLEKHA7	1.07E-05	4.38E-06	3.66E-06	4.77E-06	9.28E-07	4.44E-06	2.90E-06	8.30E-06
PLEKHA8	2.43E-06	1.63E-06	4.98E-07	7.28E-07	2.83E-07	2.45E-06	5.88E-07	1.47E-06
PLEKHA9	1.92E-06	2.46E-06	9.21E-07	1.06E-06	3.32E-07	1.95E-06	8.54E-07	9.32E-07
PLEKHB1	1.10E-05	5.78E-07	1.20E-06	2.21E-06	1.02E-08	1.21E-07	2.18E-07	1.75E-08
PLEKHB2	2.51E-05	8.11E-06	6.72E-06	1.16E-05	1.77E-06	8.25E-06	9.76E-06	8.82E-06
PLEKHF1	6.00E-06	8.71E-06	5.05E-06	6.32E-06	1.81E-05	1.45E-05	1.50E-05	6.98E-06
PLEKHF2	2.47E-05	7.97E-06	4.79E-06	4.19E-06	4.19E-06	3.83E-06	9.70E-06	7.69E-06
PLEKHG1	2.67E-06	5.06E-06	1.53E-06	2.69E-06	3.22E-07	1.14E-06	1.34E-06	1.16E-06
PLEKHG2	7.89E-06	1.93E-06	4.98E-07	1.99E-06	1.04E-06	8.99E-07	1.54E-06	1.95E-06
PLEKHG3	1.59E-05	7.20E-06	8.72E-06	2.43E-05	9.87E-06	7.55E-06	1.50E-05	9.95E-06
PLEKHG4	1.83E-06	6.23E-08	5.80E-08	2.23E-07	2.17E-08	5.15E-08	1.18E-07	6.66E-08
PLEKHG4E	1.15E-08	8.53E-09	3.90E-09	2.57E-08	1.64E-09	3.37E-08	1.31E-08	0
PLEKHG5	3.30E-06	4.51E-07	4.20E-07	1.12E-06	9.42E-07	1.04E-06	1.51E-06	1.26E-06
PLEKHG6	2.26E-06	4.88E-06	1.53E-06	2.03E-06	6.73E-07	1.42E-07	4.01E-06	2.02E-07
PLEKHG7	4.94E-08	4.67E-07	5.10E-08	1.38E-07	0	5.09E-07	6.16E-08	1.24E-07
PLEKHH1	7.03E-06	4.06E-08	2.82E-07	3.65E-07	4.21E-07	4.00E-07	3.71E-07	8.24E-07

PLEKHH2	5.79E-06	4.94E-08	2.62E-07	3.28E-07	5.50E-09	1.93E-08	4.38E-08	8.80E-08
PLEKHH3	1.94E-05	1.07E-05	1.09E-05	6.98E-06	6.95E-06	6.61E-06	6.61E-06	5.30E-06
PLEKHJ1	8.21E-05	3.32E-05	2.88E-05	4.07E-05	9.62E-05	3.87E-05	5.62E-05	3.30E-05
PLEKHM1	9.52E-06	5.00E-06	3.35E-06	5.11E-06	2.58E-06	6.97E-06	3.57E-06	4.68E-06
PLEKHM1I	4.12E-06	9.44E-07	8.37E-07	1.51E-06	1.07E-07	1.05E-06	6.23E-07	1.00E-06
PLEKHM2	1.99E-05	8.50E-06	7.22E-06	1.36E-05	5.59E-06	9.79E-06	1.38E-05	1.30E-05
PLEKHM3	3.42E-06	2.66E-06	2.48E-06	2.50E-06	1.55E-06	6.35E-06	3.08E-06	2.46E-06
PLEKHN1	1.06E-06	5.60E-08	1.91E-08	5.86E-07	2.53E-08	2.81E-07	3.90E-07	1.01E-07
PLEKHO1	4.12E-05	4.50E-06	8.31E-06	2.65E-05	1.34E-05	1.40E-05	2.38E-05	1.01E-05
PLEKHO2	1.43E-05	5.44E-06	5.14E-06	1.56E-05	4.12E-06	6.67E-06	1.35E-05	5.73E-06
PLG	0.000244	0.001055	0.001941	0.001214	0.000208	0.001742	0.000766	0.000507
PLGLA	8.06E-07	6.48E-06	4.25E-06	3.50E-06	7.77E-07	9.96E-07	2.07E-06	7.69E-07
PLGLB2	0.000111	0.000108	0.000152	0.000114	6.53E-05	3.94E-06	8.43E-05	0.000137
PLIN1	6.34E-07	2.07E-07	8.72E-06	1.52E-06	1.88E-06	6.79E-06	4.03E-06	3.75E-07
PLIN2	6.91E-05	0.000232	0.000287	0.000708	4.79E-05	0.000141	0.00054	0.000304
PLIN3	5.16E-05	1.17E-05	1.29E-05	2.60E-05	7.14E-06	1.62E-05	1.32E-05	1.88E-05
PLIN4	7.17E-06	4.36E-06	7.37E-06	7.11E-06	1.87E-05	7.13E-06	1.01E-05	4.22E-06
PLIN5	3.87E-05	2.06E-05	1.95E-05	1.38E-05	2.20E-05	2.70E-05	1.55E-05	2.34E-05
PLK1	1.52E-05	2.50E-06	1.60E-07	3.89E-07	6.88E-07	1.97E-06	6.57E-07	4.35E-07
PLK1S1	3.91E-06	6.36E-06	2.81E-06	5.32E-06	3.26E-06	7.13E-06	2.21E-06	3.68E-06
PLK2	2.15E-05	7.90E-06	1.53E-05	2.82E-05	3.28E-06	1.36E-05	1.67E-05	1.33E-05
PLK3	4.91E-06	2.94E-06	2.16E-06	3.25E-05	1.08E-06	1.57E-06	6.44E-06	4.56E-06
PLK4	2.40E-06	4.40E-07	8.10E-08	1.75E-07	9.25E-08	5.93E-07	1.33E-07	2.79E-07
PLK5P	4.99E-08	3.83E-08	1.31E-08	0	0	0	0	1.84E-08
PLLP	3.70E-05	2.56E-06	4.47E-06	7.37E-06	3.39E-06	7.78E-07	3.95E-06	9.33E-06
PLN	6.97E-07	2.75E-07	7.50E-08	3.32E-07	3.68E-07	4.89E-07	1.92E-07	3.36E-07
PLOD1	0.000121	0.00013	5.91E-05	5.84E-05	4.78E-05	5.35E-05	7.05E-05	7.86E-05
PLOD2	3.51E-05	4.82E-05	2.24E-05	3.43E-05	5.80E-06	2.89E-05	2.96E-05	2.18E-05
PLOD3	2.89E-05	0.000106	3.15E-05	5.35E-05	3.99E-05	4.78E-05	5.51E-05	8.27E-05
PLP1	0	0	6.93E-08	1.36E-07	0	0	0	0
PLP2	0.000119	2.19E-05	1.16E-05	5.29E-05	7.39E-06	2.98E-05	3.87E-05	4.67E-05
PLRG1	3.44E-05	2.65E-05	1.96E-05	1.93E-05	1.02E-05	3.79E-05	2.13E-05	2.87E-05
PLS1	1.80E-05	3.09E-05	1.19E-05	1.13E-05	1.53E-06	6.87E-06	6.40E-06	1.23E-05
PLS3	1.00E-04	8.93E-05	0.000104	8.52E-05	0.000126	0.00014	9.31E-05	0.000149
PLSCR1	4.26E-05	1.32E-05	2.39E-05	2.74E-05	3.30E-06	1.50E-05	1.04E-05	2.07E-05
PLSCR2	2.01E-08	5.18E-08	0	2.78E-08	0	0	0	0
PLSCR3	1.08E-05	5.11E-06	5.61E-06	1.02E-05	5.64E-06	1.14E-05	1.02E-05	8.43E-06
PLSCR4	8.92E-06	1.21E-05	2.15E-05	1.56E-05	1.05E-06	6.99E-06	5.59E-06	4.09E-06
PLSCR5	0	0	0	0	0	0	0	0
PLTP	2.37E-05	4.26E-05	2.06E-05	2.65E-05	1.04E-05	1.48E-05	7.89E-05	1.23E-05
PLUNC	0	0	0	0	0	0	0	3.87E-08
PLVAP	5.20E-05	3.64E-05	2.96E-06	1.54E-05	6.10E-05	4.82E-05	5.35E-05	6.56E-05
PLXDC1	3.93E-06	7.28E-07	7.73E-08	1.94E-07	2.18E-07	3.92E-07	9.04E-07	1.76E-06
PLXDC2	5.10E-06	1.30E-06	1.76E-06	4.93E-06	4.33E-07	7.08E-07	1.82E-06	9.12E-06
PLXNA1	6.64E-06	1.03E-06	1.02E-06	2.39E-06	9.77E-07	1.27E-06	2.37E-06	2.63E-06
PLXNA2	3.16E-06	1.60E-06	8.16E-07	1.47E-06	9.84E-07	1.96E-06	1.92E-06	3.32E-06
PLXNA3	6.56E-06	1.63E-06	6.59E-07	1.60E-06	6.53E-07	1.74E-07	1.19E-06	1.53E-06
PLXNA4	8.95E-08	4.16E-08	8.03E-08	6.10E-07	2.49E-08	2.63E-08	4.87E-08	2.46E-08
PLXNB1	5.64E-05	2.15E-05	2.30E-05	2.53E-05	5.68E-05	2.94E-05	3.89E-05	2.43E-05
PLXNB2	0.00014	0.000102	7.30E-05	7.82E-05	6.45E-05	6.10E-05	9.23E-05	0.000124
PLXNB3	5.69E-08	3.87E-07	9.86E-08	4.41E-07	2.13E-07	1.12E-07	9.67E-08	1.76E-07
PLXNC1	1.79E-05	1.99E-06	3.02E-07	8.73E-07	3.18E-08	3.19E-07	4.53E-07	3.88E-06
PLXND1	3.66E-05	1.24E-05	1.36E-05	2.24E-05	3.43E-05	1.52E-05	4.01E-05	2.39E-05
PM20D1	1.03E-07	1.29E-08	1.20E-07	8.41E-08	1.97E-08	8.49E-08	1.11E-08	0
PM20D2	6.92E-06	4.73E-06	1.85E-06	1.67E-06	7.87E-07	1.82E-06	1.90E-06	8.79E-07
PMAIP1	4.98E-06	1.41E-07	5.21E-07	1.43E-06	9.83E-09	2.07E-07	1.56E-07	1.10E-07
PMCH	1.81E-07	0	0	0	0	0	0	0
PMCHL1	0	0	0	0	0	0	0	0

PMCHL2	1.33E-08	0	0	0	0	0	0	5.84E-08
PMEPA1	2.20E-05	8.48E-07	1.18E-06	1.43E-05	1.22E-06	1.18E-06	3.11E-06	2.43E-06
PMF1	6.32E-05	6.99E-05	3.07E-05	5.01E-05	5.76E-05	7.54E-05	6.10E-05	9.09E-05
PMFBP1	1.57E-06	1.50E-08	1.19E-08	1.41E-08	1.84E-08	1.92E-06	4.49E-08	1.49E-07
PML	2.29E-05	7.93E-06	7.87E-06	1.08E-05	7.17E-06	1.34E-05	1.46E-05	1.49E-05
PMM1	0.000109	6.36E-05	4.15E-05	7.75E-05	8.96E-05	6.26E-05	7.19E-05	2.35E-05
PMM2	3.37E-05	4.09E-05	1.35E-05	2.30E-05	1.26E-05	2.72E-05	3.62E-05	2.39E-05
PMP2	0	0	2.79E-08	2.48E-08	0	0	6.58E-09	0
PMP22	1.96E-05	3.17E-06	3.67E-06	1.98E-05	4.85E-06	2.53E-06	1.13E-05	6.08E-06
PMPCA	6.27E-05	3.58E-05	6.85E-05	6.39E-05	4.39E-05	5.93E-05	4.06E-05	4.88E-05
PMPCB	2.86E-05	7.44E-05	5.22E-05	5.25E-05	2.36E-05	8.20E-05	5.11E-05	7.30E-05
PMS1	7.25E-06	2.64E-06	2.11E-06	2.55E-06	1.70E-06	3.26E-06	1.74E-06	1.84E-06
PMS2	1.61E-05	1.84E-05	5.10E-06	6.00E-06	4.71E-06	9.44E-06	6.07E-06	6.16E-06
PMS2CL	4.72E-06	5.81E-06	2.15E-06	1.67E-06	4.19E-07	7.61E-07	9.74E-07	1.19E-06
PMS2L1	3.05E-05	1.50E-05	5.52E-06	5.59E-06	1.02E-05	1.27E-05	1.07E-05	8.32E-06
PMS2L11	1.63E-06	8.10E-07	3.98E-07	1.04E-06	8.27E-07	3.48E-07	9.57E-07	2.91E-07
PMS2L2	6.66E-06	3.58E-06	2.67E-06	1.79E-06	3.40E-06	1.20E-06	1.80E-06	2.56E-06
PMS2L3	1.78E-06	1.25E-06	6.61E-07	9.70E-07	6.93E-07	6.67E-07	1.24E-06	1.28E-06
PMS2L4	3.88E-06	3.25E-06	4.81E-07	1.08E-06	2.38E-06	2.54E-06	1.50E-06	1.60E-06
PMS2L5	4.43E-06	7.99E-06	2.06E-06	1.88E-06	2.68E-06	3.37E-06	4.19E-06	4.63E-06
PMVK	8.72E-05	0.000103	4.02E-05	4.44E-05	8.15E-05	8.58E-05	9.75E-05	9.40E-05
PNCK	3.17E-08	2.17E-08	0	0	0	0	1.53E-08	0
PNKD	0.00017	8.83E-05	9.05E-05	9.02E-05	0.000132	0.000151	0.000106	0.000112
PNKP	1.63E-05	1.49E-05	9.41E-06	1.41E-05	1.52E-05	1.24E-05	1.30E-05	1.21E-05
PNLDC1	1.33E-08	0	0	9.78E-08	2.29E-08	1.23E-08	1.28E-08	0
PNLIP	0	0	0	0	0	0	0	0
PNLIPRP1	0	0	0	0	0	0	0	0
PNLIPRP2	0	0	0	0	0	0	0	0
PNLIPRP3	0	0	2.14E-08	0	0	0	0	0
PNMA1	9.33E-06	3.19E-06	1.59E-06	7.58E-06	1.19E-06	2.22E-06	3.18E-06	4.54E-06
PNMA2	1.68E-07	3.13E-08	2.01E-07	8.02E-07	2.40E-08	5.60E-08	1.86E-07	7.50E-08
PNMA3	1.81E-07	6.77E-07	1.20E-07	4.24E-07	5.81E-07	1.75E-07	6.06E-08	8.45E-07
PNMA5	1.63E-07	1.67E-08	0	9.07E-09	0	6.33E-09	2.16E-08	4.35E-08
PNMA6A	2.04E-06	1.76E-05	1.72E-05	9.30E-06	3.91E-05	1.43E-05	1.93E-05	7.24E-05
PNMAL1	8.67E-08	6.63E-08	2.61E-07	7.71E-07	5.64E-09	0	6.62E-08	1.93E-08
PNMAL2	1.62E-07	1.59E-07	4.29E-07	4.93E-07	2.48E-07	1.35E-07	3.76E-07	1.77E-07
PNMT	0	5.16E-08	2.42E-08	1.66E-07	2.32E-07	0	4.93E-07	9.97E-08
PNN	3.19E-05	1.46E-05	1.33E-05	1.76E-05	7.53E-06	1.59E-05	9.82E-06	1.70E-05
PNO1	1.11E-05	2.00E-05	6.93E-06	9.51E-06	2.02E-06	1.17E-05	1.07E-05	1.08E-05
PNOC	1.66E-07	0	1.10E-07	4.29E-07	0	4.02E-08	8.26E-08	6.40E-08
PNP	0.000129	2.40E-05	6.50E-05	3.77E-05	1.70E-05	9.14E-06	3.66E-05	6.24E-06
PNPLA1	1.99E-08	1.13E-08	1.04E-08	0	0	0	0	0
PNPLA2	6.13E-05	7.58E-05	2.96E-05	8.51E-05	3.88E-05	5.95E-05	6.11E-05	4.04E-05
PNPLA3	7.07E-06	1.32E-05	1.01E-05	2.19E-06	3.31E-06	4.76E-06	1.76E-06	8.90E-06
PNPLA4	1.48E-05	5.81E-06	1.76E-05	5.53E-06	4.20E-06	9.17E-06	8.84E-06	9.20E-06
PNPLA5	6.61E-07	0	0	1.73E-07	0	6.12E-08	0	0
PNPLA6	2.36E-05	6.74E-06	1.46E-05	1.98E-05	4.37E-05	1.61E-05	2.19E-05	1.86E-05
PNPLA7	2.26E-06	1.21E-06	5.62E-06	3.89E-06	5.19E-06	2.27E-06	2.62E-06	2.40E-06
PNPLA8	4.08E-05	1.57E-05	1.22E-05	1.09E-05	1.89E-06	1.57E-05	5.99E-06	1.81E-05
PNPO	5.06E-05	9.48E-05	6.45E-05	3.97E-05	6.04E-05	0.000142	7.64E-05	4.10E-05
PNPT1	6.61E-06	1.35E-05	3.96E-06	3.93E-06	1.82E-06	6.93E-06	4.19E-06	5.48E-06
PNRC1	9.57E-05	6.28E-05	5.76E-05	0.000107	9.91E-06	7.57E-05	5.35E-05	6.15E-05
PNRC2	3.74E-05	2.32E-05	1.81E-05	2.56E-05	6.29E-06	1.83E-05	1.82E-05	1.71E-05
POC1A	4.43E-06	5.92E-06	1.11E-06	1.50E-06	4.77E-06	6.53E-06	3.47E-06	2.72E-06
POC1B	5.22E-06	4.77E-06	3.26E-06	3.32E-06	1.02E-06	3.81E-06	2.75E-06	3.39E-06
POC5	4.48E-06	3.65E-06	1.80E-06	2.37E-06	4.13E-07	2.79E-06	2.17E-06	3.71E-06
PODN	3.84E-06	3.18E-07	4.26E-06	2.60E-05	1.80E-06	2.54E-07	6.39E-06	4.06E-06
PODNL1	9.17E-08	4.28E-08	2.36E-08	1.28E-07	5.91E-08	6.35E-08	1.71E-07	2.23E-08

PODXL	1.82E-05	4.34E-06	1.43E-06	2.64E-06	3.89E-06	5.33E-06	4.19E-06	1.15E-05
PODXL2	8.76E-08	4.95E-08	7.32E-07	8.04E-07	5.65E-08	8.12E-08	6.67E-07	2.41E-07
POF1B	7.37E-06	2.07E-07	1.54E-07	1.97E-07	9.91E-09	1.60E-08	2.09E-06	7.61E-08
POFUT1	1.91E-05	2.65E-05	2.03E-05	1.42E-05	1.28E-05	2.64E-05	1.78E-05	4.17E-05
POFUT2	1.33E-05	6.95E-06	4.75E-06	7.57E-06	2.90E-06	3.41E-06	6.75E-06	7.97E-06
POGK	1.51E-05	2.51E-05	5.87E-06	1.03E-05	1.70E-06	1.01E-05	8.12E-06	1.27E-05
POGZ	1.49E-05	7.20E-06	5.49E-06	6.01E-06	2.89E-06	1.01E-05	5.36E-06	8.97E-06
POLA1	5.25E-06	9.11E-07	7.54E-07	9.23E-07	3.75E-07	1.42E-06	5.51E-07	1.29E-06
POLA2	8.52E-06	5.11E-06	1.93E-06	2.26E-06	2.69E-06	5.65E-06	2.44E-06	6.52E-06
POLB	1.16E-05	2.17E-05	7.93E-06	9.28E-06	1.63E-05	3.37E-05	5.92E-06	2.48E-05
POLD1	9.11E-06	6.57E-06	2.96E-06	3.69E-06	5.30E-06	4.29E-06	3.66E-06	3.62E-06
POLD2	6.68E-05	8.91E-05	7.90E-05	5.33E-05	6.64E-05	8.40E-05	8.14E-05	9.60E-05
POLD3	5.24E-06	3.45E-06	1.45E-06	2.23E-06	8.14E-07	2.69E-06	1.99E-06	4.04E-06
POLD4	0.000352	0.000323	0.000152	0.000254	0.00035	0.000205	0.000242	0.000322
POLDIP2	8.53E-05	0.000242	8.23E-05	7.14E-05	9.84E-05	0.000108	0.000115	8.55E-05
POLDIP3	2.74E-05	1.36E-05	1.15E-05	1.23E-05	1.21E-05	1.63E-05	2.19E-05	1.76E-05
POLE	1.16E-05	9.92E-06	9.78E-06	5.97E-06	4.43E-06	4.96E-06	5.74E-06	1.22E-05
POLE2	2.41E-06	2.97E-06	6.99E-07	5.66E-07	8.80E-07	2.24E-06	1.28E-06	1.86E-06
POLE3	3.17E-05	1.85E-05	1.32E-05	1.78E-05	1.16E-05	2.42E-05	1.62E-05	2.15E-05
POLE4	0.000124	3.81E-05	3.36E-05	5.00E-05	7.45E-05	6.13E-05	7.14E-05	4.05E-05
POLG	1.35E-05	7.27E-06	7.22E-06	8.57E-06	5.44E-06	1.10E-05	1.26E-05	1.15E-05
POLG2	6.22E-06	3.06E-06	2.15E-06	2.14E-06	1.37E-06	4.57E-06	1.70E-06	2.01E-06
POLH	3.61E-06	3.77E-06	2.65E-06	1.98E-06	1.27E-06	3.76E-06	1.96E-06	3.05E-06
POLI	2.74E-06	5.01E-07	1.59E-06	1.72E-06	2.97E-07	2.15E-06	9.87E-07	7.10E-07
POLK	5.54E-06	4.81E-06	4.43E-06	3.58E-06	3.02E-07	2.90E-06	1.88E-06	4.23E-06
POLL	1.34E-05	7.06E-06	8.13E-06	9.00E-06	1.36E-05	1.18E-05	8.75E-06	9.15E-06
POLM	1.76E-05	1.02E-05	4.34E-06	4.60E-06	3.83E-06	5.91E-06	6.13E-06	7.39E-06
POLN	1.09E-07	4.60E-07	1.74E-08	3.39E-07	5.80E-07	3.51E-06	8.02E-08	2.32E-07
POLQ	1.29E-06	6.29E-07	4.24E-08	2.07E-08	7.86E-08	1.32E-07	2.32E-08	9.93E-08
POLR1A	7.85E-06	1.19E-05	1.75E-06	2.04E-06	8.12E-07	5.36E-06	2.81E-06	4.12E-06
POLR1B	7.34E-06	7.71E-06	5.09E-06	4.48E-06	1.95E-06	7.07E-06	3.74E-06	6.20E-06
POLR1C	1.21E-05	1.82E-05	9.29E-06	1.53E-05	1.05E-05	1.83E-05	1.97E-05	1.51E-05
POLR1D	8.74E-05	5.51E-05	4.23E-05	5.64E-05	4.35E-05	8.68E-05	6.12E-05	3.47E-05
POLR1E	7.28E-06	1.37E-05	1.80E-05	2.29E-05	7.24E-06	1.02E-05	1.77E-05	1.51E-05
POLR2A	2.58E-05	1.49E-05	1.41E-05	1.80E-05	3.45E-06	2.58E-05	1.78E-05	2.04E-05
POLR2B	3.14E-05	1.87E-05	1.32E-05	1.55E-05	3.23E-06	1.94E-05	1.14E-05	1.92E-05
POLR2C	0.000102	3.95E-05	2.83E-05	3.00E-05	9.95E-06	4.00E-05	2.94E-05	4.80E-05
POLR2D	2.02E-05	1.05E-05	7.32E-06	7.19E-06	3.80E-06	9.60E-06	1.09E-05	9.05E-06
POLR2E	7.15E-05	6.25E-05	6.83E-05	5.15E-05	9.44E-05	6.61E-05	8.02E-05	5.91E-05
POLR2F	3.41E-05	2.17E-05	2.10E-05	2.13E-05	6.23E-05	3.04E-05	3.54E-05	2.75E-05
POLR2G	8.38E-05	7.41E-05	2.60E-05	4.47E-05	4.29E-05	8.36E-05	6.52E-05	5.81E-05
POLR2H	6.30E-05	3.13E-05	2.60E-05	3.64E-05	7.10E-05	4.41E-05	4.36E-05	4.13E-05
POLR2I	3.55E-05	3.03E-05	2.77E-05	3.20E-05	6.23E-05	3.43E-05	5.53E-05	2.39E-05
POLR2J	4.66E-05	0.000115	5.20E-05	6.47E-05	0.000133	9.54E-05	8.68E-05	6.47E-05
POLR2J2	1.28E-06	1.22E-06	2.36E-06	2.75E-07	1.41E-06	5.04E-06	1.05E-06	2.88E-06
POLR2J3	1.64E-05	2.26E-05	8.34E-06	6.58E-06	4.68E-06	1.59E-05	5.95E-06	1.76E-05
POLR2J4	1.83E-05	1.76E-05	5.65E-06	9.20E-06	1.14E-05	1.33E-05	2.16E-05	1.12E-05
POLR2K	0.000124	9.48E-05	3.04E-05	3.62E-05	6.34E-05	4.76E-05	0.000112	4.48E-05
POLR2L	0.000206	0.000122	7.24E-05	0.000119	0.000338	0.000164	0.000187	0.000141
POLR3A	5.55E-06	3.60E-06	2.55E-06	2.09E-06	7.64E-07	4.39E-06	1.84E-06	2.72E-06
POLR3B	5.07E-06	5.77E-06	4.41E-06	3.95E-06	1.69E-06	4.30E-06	2.72E-06	6.51E-06
POLR3C	1.03E-05	8.94E-06	5.34E-06	9.83E-06	3.56E-06	1.31E-05	9.25E-06	5.73E-06
POLR3D	4.80E-06	7.55E-06	2.83E-06	5.66E-06	1.81E-06	6.82E-06	3.42E-06	2.68E-06
POLR3E	1.40E-05	2.25E-05	8.30E-06	1.03E-05	2.40E-06	1.58E-05	1.25E-05	8.63E-06
POLR3F	6.03E-06	7.76E-06	2.85E-06	4.24E-06	2.16E-06	5.01E-06	3.41E-06	3.63E-06
POLR3G	1.08E-06	1.17E-06	5.52E-07	4.29E-07	1.51E-07	1.25E-06	4.70E-07	7.12E-07
POLR3GL	3.27E-05	2.67E-05	2.92E-05	3.52E-05	2.82E-05	5.82E-05	4.88E-05	3.17E-05
POLR3H	6.12E-06	8.66E-06	5.09E-06	6.04E-06	9.87E-06	1.00E-05	1.06E-05	7.45E-06

POLR3K	2.99E-05	2.85E-05	1.06E-05	1.20E-05	6.39E-06	1.90E-05	1.96E-05	1.93E-05
POLRMT	1.44E-05	1.44E-05	1.15E-05	1.30E-05	1.96E-05	2.21E-05	1.87E-05	1.25E-05
POM121	2.17E-05	3.08E-05	1.08E-05	1.14E-05	5.48E-06	1.66E-05	1.11E-05	2.21E-05
POM121C	9.27E-06	1.39E-05	5.47E-06	4.92E-06	2.13E-06	6.08E-06	4.30E-06	8.27E-06
POM121L	4.88E-08	8.04E-09	5.00E-09	6.00E-09	2.93E-08	6.77E-08	9.49E-09	0
POM121L	0	0	0	0	0	0	0	0
POM121L	5.70E-09	6.38E-09	1.17E-08	6.96E-09	9.79E-09	0	0	2.50E-08
POM121L	4.30E-07	0	0	0	0	0	7.07E-09	0
POM121L	1.10E-08	2.48E-08	0	0	0	0	1.06E-08	0
POM121L	1.16E-08	0	0	0	2.48E-09	0	0	0
POM121L	3.02E-08	0	2.21E-08	2.10E-07	0	1.20E-08	5.04E-08	2.52E-08
POMC	2.99E-07	4.01E-07	1.99E-07	8.35E-07	3.37E-07	7.64E-08	8.85E-07	9.13E-08
POMGNT	3.08E-05	2.29E-05	1.63E-05	2.20E-05	1.32E-05	3.10E-05	2.45E-05	2.13E-05
POMP	0.000109	9.51E-05	5.17E-05	7.13E-05	5.60E-05	9.79E-05	8.31E-05	8.69E-05
POMT1	1.33E-05	1.11E-05	1.11E-05	1.21E-05	1.40E-05	9.43E-06	1.05E-05	2.31E-05
POMT2	4.19E-06	1.50E-06	1.27E-06	2.11E-06	1.18E-06	3.01E-06	1.74E-06	3.38E-06
POMZP3	2.01E-06	1.38E-06	1.20E-06	1.67E-06	2.56E-06	1.00E-06	2.59E-06	6.92E-07
PON1	1.05E-05	0.000287	0.000449	0.000278	0.00035	0.000198	0.000287	0.000575
PON2	0.000514	0.00014	7.22E-05	0.000111	3.14E-05	8.63E-05	0.000136	0.000212
PON3	0.000489	0.000492	0.000339	0.000259	0.000256	0.00016	0.000133	9.33E-05
POP1	3.01E-06	1.23E-06	5.63E-07	7.35E-07	4.86E-07	1.30E-06	9.00E-07	7.08E-07
POP4	1.87E-05	1.74E-05	7.50E-06	8.25E-06	1.02E-05	1.22E-05	1.21E-05	1.06E-05
POP5	5.31E-05	4.02E-05	2.36E-05	2.58E-05	5.15E-05	4.44E-05	2.70E-05	2.31E-05
POP7	8.22E-05	6.98E-05	2.36E-05	3.05E-05	3.91E-05	2.82E-05	4.11E-05	4.01E-05
POPDC2	6.25E-07	7.56E-07	2.36E-07	3.10E-07	6.86E-07	6.75E-07	1.44E-06	5.11E-07
POPDC3	0	6.24E-08	0	0	0	0	0	5.38E-08
POR	0.00021	0.001548	0.00034	0.000617	0.000434	0.000635	0.000503	0.000694
PORCN	2.18E-06	3.91E-06	1.12E-06	1.87E-06	5.48E-07	2.20E-06	1.52E-06	4.88E-06
POSTN	3.12E-05	3.07E-07	2.59E-06	9.23E-07	1.69E-06	1.51E-06	4.46E-06	1.65E-05
POT1	4.49E-06	7.60E-06	3.58E-06	3.00E-06	1.84E-06	5.30E-06	3.48E-06	4.49E-06
POTEA	0	0	0	0	0	0	0	0
POTEB	0	0	0	0	0	0	0	0
POTEC	0	0	0	0	0	0	0	0
POTED	0	0	0	0	0	0	0	0
POTEE	4.28E-07	1.01E-07	1.50E-07	1.11E-07	0	6.01E-08	5.00E-08	3.82E-08
POTEF	4.81E-07	1.29E-07	2.20E-07	1.92E-08	4.49E-09	1.45E-08	5.09E-08	3.06E-08
POTEG	0	1.41E-08	0	0	0	0	0	0
POTEH	0	1.33E-08	0	0	0	0	0	0
POU1F1	0	0	0	0	6.98E-08	0	0	0
POU2AF1	1.58E-06	1.72E-08	3.64E-07	1.91E-06	5.91E-08	3.89E-07	2.45E-07	1.46E-07
POU2F1	1.66E-06	6.99E-07	6.00E-07	8.55E-07	1.30E-06	1.04E-06	2.86E-06	5.25E-07
POU2F2	7.36E-07	8.93E-08	4.44E-07	6.79E-07	1.03E-07	3.48E-07	9.08E-07	5.34E-08
POU2F3	2.71E-08	3.80E-06	0	2.87E-08	9.61E-08	1.64E-06	5.23E-08	3.99E-08
POU3F1	1.19E-07	2.67E-08	8.20E-09	2.91E-08	0	7.34E-09	7.69E-09	0
POU3F2	5.57E-09	1.50E-07	0	0	0	0	0	7.34E-08
POU3F3	0	0	0	0	0	0	0	0
POU3F4	0	0	0	0	0	0	0	0
POU4F1	6.59E-08	0	6.17E-09	4.39E-08	0	5.54E-09	0	0
POU4F2	0	8.26E-09	0	0	0	0	0	0
POU4F3	0	7.53E-08	0	2.69E-08	0	2.03E-08	0	0
POU5F1	4.10E-06	5.19E-07	7.37E-08	1.79E-07	1.77E-07	2.11E-07	3.70E-07	1.56E-06
POU5F1B	6.07E-07	1.28E-07	9.88E-08	2.44E-07	1.07E-07	1.73E-07	2.85E-07	2.30E-07
POU5F2	1.96E-08	0	2.10E-08	0	0	0	0	2.90E-08
POU6F1	1.14E-06	3.99E-07	4.84E-07	9.00E-07	1.06E-06	4.15E-07	1.20E-06	4.49E-07
POU6F2	0	1.78E-08	2.67E-07	1.57E-07	0	1.62E-07	0	0
PP14571	0	1.83E-08	1.24E-08	1.97E-08	0	0	6.18E-08	0
PPA1	0.000334	8.69E-05	8.12E-05	9.97E-05	4.56E-05	0.000129	0.000119	0.000152
PPA2	4.03E-05	2.44E-05	3.15E-05	2.86E-05	1.76E-05	5.12E-05	2.74E-05	4.28E-05

PPAN	1.47E-05	1.40E-05	7.43E-06	1.77E-05	2.11E-05	1.71E-05	2.36E-05	1.63E-05
PPAN-P2I	2.63E-06	1.41E-58	1.09E-07	4.58E-07	0	8.07E-58	1.98E-55	1.69E-07
PPAP2A	0.000161	3.65E-05	3.34E-05	5.22E-05	4.56E-05	8.57E-05	7.88E-05	7.53E-05
PPAP2B	8.30E-05	4.20E-05	0.000117	8.05E-05	1.12E-05	5.09E-05	2.75E-05	4.81E-05
PPAP2C	4.36E-05	2.31E-08	1.64E-06	1.88E-05	2.26E-07	5.62E-07	6.36E-07	4.17E-07
PPAPDC1/	3.91E-07	0	5.02E-07	2.18E-06	2.13E-08	0	0	1.06E-07
PPAPDC1I	2.38E-05	2.83E-05	1.06E-05	1.48E-05	1.38E-06	2.76E-05	4.25E-06	2.48E-05
PPAPDC2	7.11E-06	9.53E-06	1.23E-05	1.19E-05	4.87E-06	1.70E-05	4.80E-06	1.34E-05
PPAPDC3	4.10E-07	1.93E-07	2.26E-07	2.65E-07	3.92E-08	7.39E-08	3.63E-07	1.51E-07
PPARA	2.41E-05	2.35E-05	1.00E-05	8.37E-06	6.43E-06	2.37E-05	1.12E-05	6.41E-06
PPARD	3.51E-05	9.85E-06	2.87E-06	1.96E-05	2.03E-06	8.25E-06	6.64E-06	2.72E-05
PPARG	1.30E-05	3.98E-05	5.75E-06	9.32E-06	9.48E-06	7.41E-06	1.68E-05	7.31E-06
PPARGC1/	3.09E-05	4.14E-05	1.22E-05	1.03E-05	1.21E-06	2.95E-05	4.23E-06	5.60E-05
PPARGC1I	2.47E-07	3.36E-07	2.88E-07	1.63E-07	3.15E-08	1.18E-07	1.09E-07	2.62E-07
PPAT	3.69E-06	3.65E-06	2.81E-06	1.57E-06	5.87E-07	3.38E-06	1.80E-06	2.74E-06
PPBP	0	4.66E-06	1.96E-07	1.06E-06	2.22E-07	2.79E-07	1.20E-07	6.42E-08
PPBPL2	1.85E-08	0	0	0	0	0	0	0
PPCDC	5.76E-06	2.10E-06	2.32E-06	2.36E-06	6.82E-06	4.74E-06	3.84E-06	2.34E-06
PPCS	6.09E-05	5.76E-05	5.20E-05	3.89E-05	1.81E-05	4.77E-05	3.90E-05	4.34E-05
PPDPF	0.000223	5.18E-05	8.09E-05	0.000132	4.80E-05	0.00017	0.000288	0.000185
PPEF1	9.99E-09	1.13E-08	0	1.22E-08	1.72E-08	2.78E-08	2.90E-08	0
PPEF2	1.35E-08	7.55E-09	0	0	0	6.24E-09	0	0
PPFIA1	1.86E-05	1.89E-05	1.03E-05	1.07E-05	2.95E-06	1.62E-05	7.09E-06	2.12E-05
PPFIA2	8.59E-08	1.04E-08	1.62E-08	3.77E-08	2.64E-08	9.41E-08	4.43E-08	6.47E-08
PPFIA3	3.05E-06	5.64E-06	2.23E-06	2.56E-06	1.10E-05	2.34E-06	8.46E-06	3.37E-06
PPFIA4	1.08E-07	5.93E-09	2.72E-08	5.49E-09	0	6.52E-09	3.29E-08	1.55E-08
PPFIBP1	1.36E-05	4.65E-06	7.10E-06	5.07E-06	2.24E-06	4.51E-06	3.36E-06	4.38E-06
PPFIBP2	2.03E-05	1.17E-05	1.75E-05	2.12E-05	1.42E-05	1.41E-05	1.36E-05	1.75E-05
PPHLN1	3.04E-05	2.86E-05	1.12E-05	1.56E-05	6.26E-06	2.19E-05	1.23E-05	1.31E-05
PPIA	0.000225	0.000127	4.85E-05	7.04E-05	0.000107	8.23E-05	0.000118	0.000106
PPIAL4B	0	0	0	0	0	0	0	0
PPIAL4C	8.35E-06	2.65E-06	7.49E-07	1.84E-06	2.00E-06	2.76E-06	2.28E-06	4.03E-06
PPIAL4D	4.37E-07	3.01E-07	8.24E-08	4.53E-08	9.51E-08	3.41E-08	3.44E-08	0
PPIAL4E	0	0	0	0	0	0	0	0
PPIAL4G	3.56E-06	6.57E-07	5.87E-07	9.37E-07	5.86E-07	1.22E-06	2.01E-06	1.01E-06
PPIB	0.001014	0.000585	0.000409	0.000709	0.001253	0.00114	0.000909	0.001134
PPIC	5.83E-05	3.18E-05	2.15E-05	4.45E-05	1.27E-05	2.73E-05	2.53E-05	3.96E-05
PPID	2.64E-05	2.76E-05	2.17E-05	1.74E-05	9.94E-06	2.49E-05	2.66E-05	1.78E-05
PPIE	4.12E-05	1.93E-05	1.91E-05	2.57E-05	1.67E-05	1.73E-05	1.60E-05	2.58E-05
PPIEL	5.21E-07	2.64E-07	2.87E-07	3.86E-07	3.42E-07	6.89E-08	8.17E-08	1.79E-07
PPIF	7.00E-05	0.000102	8.00E-05	7.03E-05	2.42E-05	0.000102	5.74E-05	6.64E-05
PPIG	5.04E-05	2.11E-05	1.71E-05	1.93E-05	3.66E-06	2.82E-05	7.96E-06	1.99E-05
PPIH	2.03E-05	1.31E-05	6.25E-06	1.31E-05	8.95E-06	1.36E-05	1.54E-05	1.53E-05
PPIL1	2.24E-05	2.12E-05	8.64E-06	1.13E-05	1.19E-05	2.23E-05	1.78E-05	1.63E-05
PPIL2	2.55E-05	1.66E-05	1.30E-05	1.40E-05	2.19E-05	1.88E-05	2.18E-05	2.71E-05
PPIL3	2.17E-05	1.39E-05	1.20E-05	1.16E-05	4.89E-06	1.78E-05	3.60E-06	4.55E-06
PPIL4	1.45E-05	1.00E-05	6.00E-06	1.08E-05	1.05E-06	1.02E-05	6.63E-06	6.37E-06
PPIL5	5.70E-06	3.98E-06	2.83E-06	3.25E-06	5.12E-06	2.69E-06	4.29E-06	3.22E-06
PPIL6	3.27E-07	2.09E-07	6.83E-08	3.03E-07	3.40E-08	2.37E-07	2.93E-07	6.61E-08
PPIP5K1	5.19E-06	1.73E-06	1.59E-06	1.41E-06	7.77E-07	4.28E-06	1.28E-06	1.62E-06
PPIP5K2	1.24E-05	7.68E-06	6.70E-06	6.18E-06	1.87E-06	9.22E-06	5.57E-06	7.16E-06
PPL	7.58E-06	4.74E-06	6.10E-06	8.18E-06	5.26E-07	6.85E-06	2.32E-06	1.92E-06
PPM1A	1.18E-05	7.84E-06	1.32E-05	8.97E-06	3.37E-06	1.09E-05	9.78E-06	1.15E-05
PPM1B	3.42E-05	2.75E-05	1.67E-05	1.76E-05	9.82E-06	1.80E-05	1.26E-05	2.45E-05
PPM1D	8.30E-06	5.33E-06	3.11E-06	3.59E-06	8.08E-07	4.15E-06	2.95E-06	2.00E-06
PPM1E	3.45E-07	1.03E-06	4.56E-07	3.15E-08	3.36E-07	2.87E-08	3.01E-08	0
PPM1F	8.06E-06	3.47E-06	2.66E-06	3.41E-06	5.29E-06	6.43E-06	9.06E-06	7.02E-06
PPM1G	9.64E-05	9.80E-05	2.30E-05	3.87E-05	2.70E-05	5.20E-05	4.35E-05	4.40E-05

PPM1H	2.69E-06	2.37E-06	2.08E-06	1.32E-06	5.77E-08	1.22E-06	6.88E-07	9.71E-07
PPM1J	5.31E-07	5.01E-07	3.60E-07	2.62E-07	7.35E-08	1.20E-07	1.13E-06	4.19E-08
PPM1K	3.61E-06	1.77E-06	2.90E-06	1.53E-06	4.18E-07	3.80E-06	1.70E-06	5.70E-07
PPM1L	1.94E-06	1.30E-06	1.44E-06	6.02E-07	1.22E-07	1.59E-06	4.12E-07	2.17E-06
PPM1M	1.30E-05	1.30E-05	4.26E-06	7.84E-06	1.65E-06	4.20E-06	7.42E-06	4.15E-06
PPM1N	1.73E-06	1.08E-07	9.28E-07	1.30E-06	5.37E-07	5.80E-07	2.16E-06	1.06E-06
PPME1	3.18E-05	1.38E-05	7.89E-06	1.09E-05	4.87E-06	1.58E-05	1.08E-05	1.79E-05
PPOX	5.21E-06	1.42E-05	4.96E-06	6.63E-06	4.09E-06	1.42E-05	5.95E-06	8.62E-06
PPP1CA	0.000158	0.000136	8.09E-05	0.000105	0.000112	0.000102	0.000117	0.00017
PPP1CB	8.97E-05	4.92E-05	2.51E-05	4.42E-05	9.84E-06	2.56E-05	2.54E-05	2.26E-05
PPP1CC	5.84E-05	4.56E-05	2.76E-05	2.57E-05	1.65E-05	2.92E-05	2.97E-05	2.05E-05
PPP1R10	3.28E-05	2.88E-05	2.05E-05	2.14E-05	1.61E-05	3.46E-05	2.19E-05	1.74E-05
PPP1R11	6.64E-05	7.94E-05	3.20E-05	4.02E-05	1.88E-05	4.41E-05	3.57E-05	5.48E-05
PPP1R12A	1.19E-05	6.52E-06	3.98E-06	5.34E-06	7.60E-07	5.23E-06	3.23E-06	4.57E-06
PPP1R12B	3.61E-06	1.07E-06	1.41E-06	4.33E-06	4.84E-07	2.15E-06	9.68E-07	2.19E-06
PPP1R12C	2.52E-05	1.72E-05	1.33E-05	1.83E-05	2.38E-05	1.72E-05	2.59E-05	1.74E-05
PPP1R13B	9.10E-06	3.66E-06	3.54E-06	6.09E-06	3.78E-06	3.39E-06	6.32E-06	4.23E-06
PPP1R13L	7.97E-06	4.98E-06	2.02E-06	6.84E-06	3.39E-06	3.15E-06	7.63E-06	3.54E-06
PPP1R14A	7.18E-06	4.45E-06	1.84E-06	1.44E-05	1.29E-05	4.39E-06	1.56E-05	4.58E-06
PPP1R14B	7.16E-05	4.95E-05	2.61E-05	4.07E-05	7.30E-05	4.39E-05	7.03E-05	6.82E-05
PPP1R14C	4.74E-06	6.06E-08	5.60E-08	3.95E-08	0	0	1.04E-08	1.58E-08
PPP1R14D	1.35E-06	1.58E-06	0	9.52E-08	0	5.94E-08	3.24E-07	0
PPP1R15A	2.61E-05	2.29E-05	1.71E-05	9.92E-05	7.70E-06	1.20E-05	4.17E-05	1.94E-05
PPP1R15B	1.92E-05	1.65E-05	1.92E-05	2.74E-05	3.26E-06	1.82E-05	1.43E-05	3.32E-05
PPP1R16A	0.000131	4.56E-05	1.87E-05	2.50E-05	0.000134	4.53E-05	6.71E-05	2.93E-05
PPP1R16B	3.98E-06	4.82E-07	9.23E-07	2.93E-06	4.74E-07	1.10E-06	1.50E-06	9.66E-07
PPP1R1A	9.29E-05	4.44E-05	6.08E-05	1.20E-05	3.61E-05	5.26E-06	1.63E-05	5.34E-06
PPP1R1B	1.77E-08	4.04E-08	0	4.35E-08	0	1.65E-08	0	0
PPP1R1C	5.94E-06	4.61E-08	3.97E-06	1.89E-06	1.55E-06	4.39E-06	2.37E-06	3.35E-07
PPP1R2	1.47E-05	9.60E-06	6.19E-06	1.11E-05	3.31E-06	1.40E-05	7.27E-06	7.47E-06
PPP1R2P1	5.10E-08	4.29E-08	1.31E-08	1.56E-08	0	1.77E-08	6.19E-09	0
PPP1R2P3	1.60E-06	7.49E-07	4.29E-07	6.79E-07	6.22E-08	9.83E-07	4.19E-07	6.38E-07
PPP1R2P9	6.38E-08	0	0	0	0	0	0	0
PPP1R3A	0	5.91E-09	0	0	0	0	0	0
PPP1R3B	6.52E-06	1.92E-05	9.50E-05	4.61E-05	4.24E-06	3.28E-05	2.09E-05	2.77E-05
PPP1R3C	3.94E-06	8.39E-06	5.80E-06	2.93E-05	1.57E-05	5.50E-06	1.22E-05	1.55E-05
PPP1R3D	4.12E-06	9.23E-07	6.02E-07	6.00E-07	1.03E-07	9.71E-07	3.55E-07	7.20E-07
PPP1R3E	1.50E-06	7.70E-07	7.11E-07	4.05E-07	3.11E-07	9.21E-07	8.74E-07	8.26E-07
PPP1R3F	1.23E-06	9.60E-06	6.36E-07	1.39E-06	8.27E-07	1.31E-06	6.58E-07	1.90E-06
PPP1R3G	2.08E-05	3.69E-05	1.53E-05	1.26E-06	5.92E-07	4.03E-05	1.47E-06	8.80E-06
PPP1R7	7.60E-05	4.76E-05	4.21E-05	4.35E-05	4.70E-05	5.47E-05	5.76E-05	4.35E-05
PPP1R8	2.18E-05	1.44E-05	1.11E-05	1.31E-05	4.81E-06	1.55E-05	1.11E-05	1.32E-05
PPP1R9A	1.11E-06	1.60E-07	3.37E-07	2.07E-07	1.82E-07	4.69E-08	7.86E-08	9.60E-08
PPP1R9B	2.09E-05	1.08E-05	6.26E-06	1.11E-05	3.42E-06	3.50E-06	1.11E-05	4.69E-06
PPP2CA	7.03E-05	5.15E-05	3.00E-05	4.74E-05	1.18E-05	4.73E-05	3.07E-05	5.69E-05
PPP2CB	3.48E-05	9.22E-05	4.30E-05	6.13E-05	1.00E-05	4.78E-05	3.92E-05	2.37E-05
PPP2R1A	0.000141	0.000101	5.69E-05	8.95E-05	9.81E-05	8.60E-05	0.000104	9.76E-05
PPP2R1B	0.000152	3.63E-05	2.56E-05	3.35E-05	2.97E-05	5.94E-05	2.66E-05	4.82E-05
PPP2R2A	1.36E-05	2.10E-05	1.49E-05	1.60E-05	1.86E-06	1.29E-05	6.76E-06	9.49E-06
PPP2R2B	2.48E-06	6.77E-08	2.17E-07	3.83E-07	0	3.33E-08	1.72E-07	1.06E-07
PPP2R2C	9.47E-06	6.23E-09	9.72E-08	0	0	1.03E-08	5.95E-08	0
PPP2R2D	1.80E-05	1.06E-05	1.46E-05	1.43E-05	9.51E-06	1.05E-05	1.72E-05	1.06E-05
PPP2R3A	4.40E-06	2.96E-07	4.27E-07	8.40E-07	2.94E-07	1.76E-07	6.63E-07	1.71E-07
PPP2R3B	6.75E-06	3.79E-06	1.18E-06	3.87E-06	8.02E-06	2.94E-06	3.50E-06	2.62E-06
PPP2R3C	1.39E-05	9.56E-06	6.92E-06	7.19E-06	6.39E-06	8.16E-06	6.41E-06	6.03E-06
PPP2R4	6.15E-05	2.26E-05	5.15E-05	6.56E-05	5.56E-05	9.61E-05	5.84E-05	8.49E-05
PPP2R5A	5.95E-05	8.31E-05	2.60E-05	2.87E-05	2.39E-05	4.95E-05	4.18E-05	6.19E-05
PPP2R5B	9.42E-06	5.98E-06	3.63E-06	5.51E-06	1.93E-06	5.62E-06	7.01E-06	1.09E-05

PPP2R5C	4.89E-05	2.71E-05	2.34E-05	2.79E-05	1.84E-05	4.15E-05	2.56E-05	2.97E-05
PPP2R5D	1.31E-05	2.45E-05	1.05E-05	1.12E-05	7.78E-06	1.40E-05	1.35E-05	1.78E-05
PPP2R5E	1.18E-05	5.83E-06	5.96E-06	7.51E-06	1.20E-06	7.48E-06	3.97E-06	7.13E-06
PPP3CA	6.48E-06	5.72E-06	7.18E-06	6.35E-06	1.81E-06	4.42E-06	5.69E-06	4.87E-06
PPP3CB	1.80E-05	1.42E-05	1.25E-05	1.32E-05	8.83E-06	1.73E-05	1.08E-05	1.20E-05
PPP3CC	1.24E-05	9.46E-06	6.78E-06	9.46E-06	3.69E-06	1.07E-05	6.22E-06	6.77E-06
PPP3R1	2.90E-05	3.19E-05	2.18E-05	1.64E-05	5.78E-06	2.92E-05	1.89E-05	2.12E-05
PPP3R2	0	0	0	0	0	0	0	7.22E-08
PPP4C	7.81E-05	0.000102	6.06E-05	7.26E-05	5.92E-05	6.05E-05	9.59E-05	6.35E-05
PPP4R1	3.30E-05	8.89E-06	7.03E-06	1.85E-05	4.06E-06	1.35E-05	1.41E-05	1.42E-05
PPP4R1L	4.95E-07	1.26E-07	1.96E-07	4.58E-07	4.05E-08	2.19E-07	3.25E-07	1.94E-07
PPP4R2	1.73E-05	2.23E-05	1.56E-05	1.49E-05	5.77E-07	2.42E-05	6.65E-06	1.50E-05
PPP4R4	1.22E-06	1.92E-06	1.16E-06	1.13E-06	6.55E-07	1.04E-06	1.60E-06	2.59E-07
PPP5C	4.25E-05	2.59E-05	2.47E-05	2.55E-05	1.29E-05	2.84E-05	2.80E-05	2.33E-05
PPP6C	1.82E-05	1.01E-05	1.02E-05	1.32E-05	3.89E-06	1.48E-05	9.40E-06	1.75E-05
PPPDE1	1.17E-05	1.18E-05	4.45E-06	6.10E-06	1.62E-06	7.43E-06	5.54E-06	1.20E-05
PPPDE2	9.75E-05	0.000125	6.00E-05	4.03E-05	3.73E-05	6.26E-05	5.22E-05	8.23E-05
PPRC1	5.79E-06	3.67E-06	2.61E-06	1.00E-05	2.60E-06	5.54E-06	7.69E-06	5.42E-06
PPT1	6.15E-05	1.60E-05	1.93E-05	3.90E-05	9.07E-06	1.60E-05	3.81E-05	2.14E-05
PPT2	2.68E-05	1.57E-05	6.32E-06	9.68E-06	4.15E-06	6.16E-06	5.18E-06	9.34E-06
PPTC7	1.05E-05	8.65E-06	6.18E-06	4.65E-06	5.23E-09	6.57E-06	1.67E-06	3.88E-06
PPWD1	1.07E-05	6.04E-06	4.91E-06	5.61E-06	1.63E-06	8.39E-06	4.47E-06	4.95E-06
PPY	0	0	0	0	0	0	0	0
PPY2	0	0	0	0	0	0	0	0
PPYR1	8.08E-08	0	1.42E-08	2.48E-07	0	0	0	0
PQBP1	8.63E-05	9.57E-05	3.73E-05	4.80E-05	7.18E-05	5.16E-05	7.29E-05	0.000121
PQLC1	0.000115	4.10E-05	9.11E-05	8.37E-05	7.05E-05	8.53E-05	0.000117	0.000164
PQLC2	1.75E-05	1.70E-05	5.10E-06	1.06E-05	9.18E-06	8.12E-06	9.63E-06	1.46E-05
PQLC3	2.65E-05	6.88E-06	7.22E-06	1.06E-05	1.25E-06	3.93E-06	6.35E-06	4.57E-06
PRAC	0	0	0	0	0	0	0	0
PRAF2	1.61E-05	1.32E-05	6.26E-06	9.74E-06	1.45E-05	8.74E-06	1.58E-05	2.24E-05
PRAM1	9.77E-07	1.74E-07	1.53E-06	2.10E-06	5.49E-07	6.12E-08	4.46E-07	4.85E-08
PRAME	0	9.16E-06	1.23E-08	0	9.37E-09	1.09E-08	1.58E-07	0
PRAMEF1	1.53E-08	6.27E-07	1.62E-08	0	0	0	0	0
PRAMEF1(	6.53E-08	1.71E-05	2.76E-06	1.40E-07	4.93E-07	1.95E-05	1.67E-06	2.48E-05
PRAMEF1:	3.81E-07	1.54E-05	0	0	5.43E-07	1.22E-08	1.14E-07	6.97E-07
PRAMEF1:	0	0	0	0	0	0	0	2.01E-08
PRAMEF1:	0	3.62E-69	0	0	0	0	0	0
PRAMEF1‹	0	2.05E-07	0	0	0	0	0	3.34E-08
PRAMEF1(	0	1.77E-08	0	0	0	2.89E-08	0	0
PRAMEF1˙	0	1.81E-08	0	0	0	0	0	0
PRAMEF1ᵴ	0	0	0	0	0	0	0	0
PRAMEF2	2.98E-08	1.14E-06	6.31E-08	0	2.57E-08	0	0	2.20E-08
PRAMEF2(	0	0	0	0	0	0	0	0
PRAMEF2:	0	0	0	0	0	0	0	0
PRAMEF3	0	0	0	0	0	0	0	0
PRAMEF4	7.62E-07	3.33E-05	2.76E-08	3.22E-08	2.52E-06	1.22E-07	4.82E-07	3.00E-06
PRAMEF5	5.21E-08	1.76E-06	0	0	4.84E-08	0	0	1.17E-30
PRAMEF6	1.42E-07	2.54E-06	0	0	#######	0	2.73E-08	1.46E-07
PRAMEF8	5.91E-07	2.23E-07	4.17E-08	1.29E-07	4.52E-08	3.05E-07	2.16E-07	1.84E-06
PRAMEF9	2.07E-07	3.04E-05	0	0	8.25E-07	1.20E-08	3.13E-07	9.54E-08
PRAP1	8.50E-05	0.001676	0.000513	0.000715	0.000951	0.001612	0.001278	0.000725
PRB1	0	0	0	0	0	0	0	0
PRB2	0	0	0	0	0	0	0	0
PRB3	0	5.56E-08	0	0	0	0	0	0
PRB4	0	0	0	0	0	0	0	0
PRC1	2.45E-05	1.34E-05	2.89E-07	1.04E-06	1.45E-06	3.40E-06	1.41E-06	1.72E-06
PRCC	2.56E-05	3.75E-05	1.74E-05	2.26E-05	1.81E-05	4.23E-05	2.44E-05	3.43E-05

PRCD	5.40E-07	6.76E-08	1.03E-07	1.80E-07	2.18E-07	9.69E-08	8.30E-08	3.00E-07
PRCP	6.93E-05	3.65E-05	5.79E-05	6.64E-05	3.11E-05	6.84E-05	3.41E-05	4.59E-05
PRDM1	3.85E-06	8.62E-07	1.21E-06	3.00E-06	3.40E-07	1.33E-06	1.27E-06	6.80E-07
PRDM10	2.29E-06	8.79E-07	8.50E-07	7.73E-07	3.59E-07	1.31E-06	5.03E-07	1.46E-06
PRDM11	5.75E-07	1.32E-07	4.44E-08	1.69E-07	5.49E-08	1.97E-07	1.85E-07	2.03E-07
PRDM12	7.54E-08	0	2.94E-08	1.15E-08	4.05E-08	2.18E-07	3.65E-08	4.15E-08
PRDM13	0	0	0	0	0	0	0	0
PRDM14	0	0	0	0	0	9.98E-09	2.08E-08	0
PRDM15	1.70E-06	6.41E-07	4.36E-07	5.45E-07	4.03E-07	5.74E-07	4.78E-07	6.15E-06
PRDM16	1.37E-06	7.94E-08	4.96E-08	8.12E-08	2.64E-08	1.90E-08	2.74E-08	5.61E-08
PRDM2	7.10E-06	3.86E-06	4.04E-06	4.99E-06	1.72E-06	4.05E-06	3.16E-06	3.99E-06
PRDM4	1.06E-05	5.06E-06	3.02E-06	6.20E-06	1.74E-06	6.00E-06	4.81E-06	5.72E-06
PRDM5	1.37E-07	1.16E-08	3.94E-08	9.13E-08	8.14E-09	2.53E-08	1.71E-08	4.41E-08
PRDM6	1.59E-07	1.11E-07	5.11E-08	2.19E-07	1.69E-08	4.55E-08	7.47E-08	5.77E-08
PRDM7	4.35E-08	2.73E-07	0	7.11E-08	8.29E-09	1.79E-07	5.60E-08	0
PRDM8	4.39E-07	2.49E-08	9.28E-08	1.56E-07	6.34E-09	6.27E-08	1.51E-07	3.25E-08
PRDM9	0	3.47E-08	0	0	0	0	0	1.82E-08
PRDX1	0.000679	0.000574	0.000391	0.000381	0.000336	0.000858	0.000366	0.000403
PRDX2	0.000516	0.000257	0.000209	0.000186	0.000354	0.000321	0.000235	0.00023
PRDX3	0.000233	0.000215	0.00028	0.000179	8.10E-05	0.000277	0.000179	0.000173
PRDX4	0.000176	8.88E-05	0.000215	0.000189	0.000195	0.000218	0.000221	0.000222
PRDX5	0.000468	0.000456	0.000215	0.000258	0.000314	0.000231	0.000276	0.000435
PRDX6	0.000308	0.000498	0.000259	0.000248	0.000348	0.000669	0.000352	0.000167
PRDXDD1	9.18E-07	7.12E-07	2.66E-07	2.03E-07	2.19E-07	3.69E-07	1.92E-07	3.25E-07
PREB	6.77E-05	0.000103	2.72E-05	3.04E-05	2.35E-05	0.000248	4.18E-05	6.76E-05
PRELID1	0.00015	6.32E-05	5.72E-05	6.34E-05	4.97E-05	9.95E-05	0.000166	0.000109
PRELID2	8.17E-07	2.91E-07	4.64E-07	6.56E-07	1.24E-07	2.73E-07	6.51E-07	8.53E-07
PRELP	7.83E-06	8.58E-07	6.06E-06	1.52E-05	9.46E-07	4.18E-07	7.13E-06	1.91E-06
PREP	1.82E-05	8.84E-06	1.22E-05	9.81E-06	2.11E-06	1.73E-05	9.17E-06	9.36E-06
PREPL	2.05E-05	2.26E-05	8.91E-06	6.35E-06	2.28E-06	1.13E-05	4.46E-06	5.90E-06
PREX1	1.05E-05	2.21E-06	4.73E-06	6.81E-06	1.82E-06	4.14E-06	5.67E-06	5.75E-06
PREX2	1.80E-07	1.66E-06	1.19E-07	7.01E-08	3.77E-09	1.56E-07	6.41E-08	1.17E-07
PRF1	1.19E-05	2.50E-06	4.61E-06	7.03E-06	3.86E-07	6.25E-06	5.79E-06	1.69E-06
PRG1	0	0	0	0	0	0	0	0
PRG2	6.08E-06	3.95E-06	2.75E-06	6.72E-06	1.97E-05	1.41E-06	8.65E-06	2.13E-06
PRG3	0	0	0	0	0	0	0	0
PRG4	2.41E-05	3.63E-05	0.000146	3.91E-05	2.89E-05	7.97E-06	1.60E-05	0.000145
PRH1	7.87E-07	6.26E-07	7.20E-07	9.67E-07	3.39E-07	7.17E-07	1.12E-06	9.75E-07
PRH2	8.43E-08	8.00E-07	2.12E-07	1.30E-07	9.01E-09	5.38E-08	4.96E-08	7.70E-08
PRHOXNB	0	0	0	0	0	0	0	0
PRIC285	1.57E-05	7.25E-06	4.59E-06	5.03E-06	1.04E-06	6.61E-06	4.31E-06	1.09E-05
PRICKLE1	1.41E-06	8.70E-08	9.61E-07	1.13E-06	3.74E-07	8.52E-08	3.73E-07	2.54E-07
PRICKLE2	2.32E-06	1.52E-07	8.46E-07	2.53E-06	1.53E-07	6.66E-08	1.77E-07	7.10E-07
PRICKLE3	2.67E-06	5.09E-06	1.54E-06	1.68E-06	2.10E-07	2.32E-06	2.25E-06	4.55E-06
PRICKLE4	1.93E-06	8.70E-07	8.75E-07	5.66E-07	1.37E-07	4.66E-07	5.21E-07	6.73E-07
PRIM1	6.19E-06	8.15E-06	2.36E-06	1.77E-06	7.38E-07	6.09E-06	2.32E-06	2.76E-06
PRIM2	5.23E-06	3.66E-06	7.80E-07	1.21E-06	5.09E-07	1.77E-06	1.45E-06	2.12E-06
PRIMA1	0	0	1.94E-07	1.23E-07	0	0	0	9.18E-09
PRINS	0	0	0	0	0	0	0	0
PRKAA1	2.18E-05	2.07E-05	1.67E-05	1.19E-05	2.33E-06	1.28E-05	8.35E-06	2.53E-05
PRKAA2	3.64E-06	1.46E-06	6.35E-07	1.11E-06	5.36E-07	8.61E-07	2.17E-07	5.88E-07
PRKAB1	1.52E-05	2.02E-05	9.16E-06	9.79E-06	6.74E-06	1.83E-05	1.66E-05	8.54E-06
PRKAB2	2.96E-05	1.29E-05	9.78E-06	5.02E-06	1.09E-05	2.52E-05	1.58E-05	9.79E-06
PRKACA	5.60E-05	3.72E-05	5.47E-05	4.73E-05	3.53E-05	4.43E-05	5.82E-05	5.21E-05
PRKACB	1.03E-05	7.19E-06	9.00E-06	7.47E-06	1.29E-06	1.42E-05	4.18E-06	5.13E-06
PRKACG	0	0	0	0	0	0	0	0
PRKAG1	4.71E-05	3.86E-05	1.99E-05	2.49E-05	9.95E-06	3.76E-05	2.41E-05	2.97E-05
PRKAG2	8.98E-06	8.50E-06	1.73E-05	1.46E-05	7.74E-06	1.05E-05	9.15E-06	6.95E-06

PRKAG3	0	0	0	0	0	0	0	0
PRKAR1A	0.000126	0.000131	6.18E-05	6.30E-05	2.22E-05	0.000153	4.54E-05	4.90E-05
PRKAR1B	8.63E-06	1.45E-05	3.56E-06	7.88E-06	1.68E-06	7.83E-06	1.77E-05	5.32E-06
PRKAR2A	8.88E-06	8.38E-06	4.71E-06	3.08E-06	2.04E-07	7.34E-06	1.65E-06	3.68E-06
PRKAR2B	1.32E-06	1.31E-06	1.54E-06	1.47E-06	5.37E-08	2.49E-07	2.19E-07	6.41E-08
PRKCA	6.39E-06	5.52E-06	2.44E-06	2.49E-06	1.73E-06	7.87E-06	1.91E-06	3.88E-06
PRKCB	3.12E-06	2.14E-07	1.22E-06	2.69E-06	6.48E-08	1.46E-06	6.18E-07	4.66E-07
PRKCD	2.32E-05	7.45E-06	4.55E-06	7.07E-06	8.10E-07	2.99E-06	6.59E-06	2.68E-06
PRKCDBP	1.20E-05	3.40E-06	6.97E-07	5.02E-06	5.06E-06	1.91E-06	1.49E-05	5.44E-06
PRKCE	3.32E-06	1.77E-06	2.51E-06	1.38E-06	1.00E-06	1.14E-06	2.83E-06	3.34E-06
PRKCG	0	0	1.99E-08	2.34E-08	1.65E-08	0	0	0
PRKCH	1.82E-05	3.21E-06	5.38E-06	7.91E-06	1.87E-06	5.11E-06	4.94E-06	7.06E-06
PRKCI	9.17E-06	4.88E-06	2.66E-06	3.40E-06	1.11E-06	3.56E-06	2.72E-06	2.16E-06
PRKCQ	1.73E-06	2.60E-08	3.67E-07	8.62E-07	3.97E-08	3.28E-07	2.87E-07	4.63E-07
PRKCSH	0.000181	0.000155	0.000121	0.000136	0.000125	0.000171	0.00019	0.000156
PRKCZ	1.48E-05	1.65E-05	8.05E-06	9.79E-06	1.35E-05	1.40E-05	1.05E-05	6.41E-06
PRKD1	2.83E-06	1.05E-06	2.89E-07	1.21E-06	1.02E-07	1.73E-07	2.54E-07	2.19E-07
PRKD2	2.78E-05	1.88E-05	1.07E-05	1.46E-05	2.07E-05	1.08E-05	2.71E-05	2.07E-05
PRKD3	8.09E-06	5.55E-06	3.36E-06	3.44E-06	1.04E-06	4.45E-06	2.33E-06	1.96E-06
PRKDC	2.90E-05	1.78E-05	4.84E-06	4.55E-06	2.29E-06	5.23E-06	5.31E-06	5.94E-06
PRKG1	4.75E-07	1.92E-07	3.00E-07	5.49E-07	0	7.98E-08	1.15E-07	2.00E-07
PRKG2	0	0	0	2.54E-08	0	0	0	0
PRKRA	2.33E-05	1.34E-05	9.03E-06	1.13E-05	6.37E-06	9.11E-06	1.11E-05	1.15E-05
PRKRIP1	8.03E-06	1.17E-05	3.22E-06	8.39E-06	8.22E-06	7.17E-06	9.02E-06	1.67E-05
PRKRIR	1.02E-05	7.94E-06	7.07E-06	9.71E-06	4.97E-06	8.16E-06	7.90E-06	1.37E-05
PRKX	5.35E-06	3.56E-07	1.14E-06	1.68E-06	1.62E-07	3.94E-07	6.68E-07	1.83E-06
PRKY	1.24E-08	1.89E-07	3.15E-09	3.92E-07	1.17E-07	2.57E-07	1.51E-07	2.39E-07
PRL	0	3.38E-08	0	0	0	0	0	0
PRLH	0	0	0	0	0	0	0	0
PRLHR	0	0	0	0	0	0	0	0
PRLR	7.44E-06	8.74E-06	3.79E-06	3.36E-06	1.15E-06	4.83E-06	2.75E-06	3.75E-06
PRM1	0	0	0	0	0	0	0	0
PRM2	0	0	0	0	0	0	0	0
PRM3	0	0	0	0	0	0	0	0
PRMT1	8.10E-05	4.57E-05	3.18E-05	5.56E-05	5.00E-05	5.67E-05	5.81E-05	3.75E-05
PRMT10	4.20E-06	2.16E-06	2.79E-06	3.63E-06	4.92E-07	3.88E-06	1.87E-06	2.01E-06
PRMT2	2.56E-05	6.73E-06	9.79E-06	1.97E-05	7.31E-06	2.03E-05	1.58E-05	1.13E-05
PRMT3	5.38E-06	5.22E-06	2.42E-06	1.90E-06	7.21E-07	5.54E-06	2.71E-06	4.70E-06
PRMT5	2.55E-05	4.77E-05	1.16E-05	1.56E-05	6.90E-06	3.10E-05	1.78E-05	1.65E-05
PRMT6	8.48E-06	1.09E-05	4.24E-06	7.03E-06	2.27E-06	1.07E-05	2.60E-06	6.69E-06
PRMT7	1.21E-05	6.85E-06	8.91E-06	9.14E-06	8.03E-06	9.06E-06	8.10E-06	1.11E-05
PRMT8	0	0	0	0	0	0	0	0
PRND	1.15E-07	1.15E-07	1.18E-08	2.10E-08	3.44E-08	7.42E-08	1.48E-06	2.01E-07
PRNP	4.12E-05	3.76E-05	2.41E-05	5.00E-05	4.16E-06	1.32E-05	2.76E-05	1.43E-05
PRNT	0	0	0	0	0	0	0	0
PRO0611	5.68E-08	0	6.87E-08	0	0	5.37E-08	0	0
PRO0628	2.23E-08	2.57E-08	0	0	0	0	0	0
PRO1768	2.03E-08	0	0	0	0	0	0	0
PROC	2.81E-05	0.000239	0.000321	0.000268	0.000528	0.000243	0.000246	0.000239
PROCA1	1.30E-06	1.65E-07	1.67E-07	4.90E-07	3.04E-07	5.47E-07	2.46E-07	2.73E-07
PROCR	2.30E-05	9.27E-06	3.13E-06	8.80E-06	2.51E-06	1.86E-06	4.50E-06	4.50E-06
PRODH	0.000108	0.000132	1.14E-05	2.94E-06	4.37E-06	3.97E-06	3.29E-06	2.15E-06
PRODH2	4.25E-05	2.06E-05	0.00011	9.06E-05	0.000146	4.18E-05	6.70E-05	8.74E-05
PROK1	1.84E-08	3.15E-07	5.88E-08	1.36E-07	0	0.000643	0	8.15E-08
PROK2	9.57E-08	5.20E-08	1.69E-07	1.96E-07	0	0	4.61E-08	2.35E-08
PROKR1	0	5.03E-08	0	2.70E-08	0	0	0	0
PROKR2	1.79E-08	0	0	0	0	2.09E-08	0	0
PROL1	4.84E-07	3.84E-07	2.23E-07	0	2.40E-06	1.19E-07	0	4.17E-07

PROM1	3.40E-05	0	8.91E-07	5.30E-06	1.02E-08	4.38E-08	0	0
PROM2	1.94E-08	6.34E-08	3.15E-07	3.69E-07	3.47E-08	0	1.65E-07	2.63E-08
PROP1	0	0	0	0	0	0	0	0
PROS1	0.000106	0.000222	0.000161	0.000139	5.39E-05	0.000111	6.21E-05	6.98E-05
ProSAPiP1	1.48E-05	8.85E-06	2.44E-06	3.22E-06	1.91E-06	3.13E-06	2.41E-06	3.40E-06
PROSC	1.44E-05	6.12E-05	3.61E-05	2.47E-05	4.89E-06	3.19E-05	1.54E-05	1.30E-05
PROX1	8.39E-06	2.85E-05	1.81E-05	7.06E-06	2.45E-07	3.36E-05	2.06E-06	1.41E-05
PROX2	0	5.51E-08	5.18E-08	0	0	0	0	0
PROZ	7.04E-06	5.65E-05	3.90E-05	4.26E-05	3.03E-05	5.49E-05	2.28E-05	1.36E-05
PRPF18	1.08E-05	1.31E-05	8.71E-06	1.24E-05	6.83E-06	1.45E-05	1.18E-05	1.32E-05
PRPF19	5.33E-05	6.58E-05	3.74E-05	4.56E-05	3.47E-05	6.89E-05	5.22E-05	5.40E-05
PRPF3	1.94E-05	1.33E-05	6.65E-06	8.45E-06	6.21E-06	1.25E-05	8.61E-06	1.25E-05
PRPF31	3.84E-05	3.09E-05	1.87E-05	2.34E-05	4.77E-05	3.31E-05	2.68E-05	2.63E-05
PRPF38A	2.10E-05	1.14E-05	6.42E-06	9.05E-06	2.65E-06	7.76E-06	7.82E-06	6.52E-06
PRPF38B	2.32E-05	7.91E-06	6.84E-06	1.23E-05	1.92E-06	1.11E-05	6.21E-06	8.41E-06
PRPF39	1.00E-05	4.10E-06	2.44E-06	2.79E-06	2.20E-06	3.19E-06	2.84E-06	4.63E-06
PRPF4	2.45E-05	9.07E-06	7.74E-06	9.58E-06	3.29E-06	1.03E-05	8.30E-06	9.93E-06
PRPF40A	3.15E-05	2.43E-05	1.31E-05	1.32E-05	2.67E-06	2.22E-05	7.29E-06	1.27E-05
PRPF40B	3.83E-06	5.71E-06	1.78E-06	2.56E-06	1.50E-06	3.42E-06	3.60E-06	3.16E-06
PRPF4B	1.71E-05	9.79E-06	6.97E-06	7.44E-06	1.98E-06	9.73E-06	4.52E-06	7.57E-06
PRPF6	6.47E-05	4.41E-05	2.59E-05	3.27E-05	5.07E-05	4.17E-05	4.21E-05	3.26E-05
PRPF8	2.64E-05	2.60E-05	2.49E-05	2.09E-05	9.37E-06	3.06E-05	1.70E-05	2.14E-05
PRPH	0	0	0	3.24E-08	0	0	2.55E-08	0
PRPH2	1.38E-07	2.58E-08	3.16E-08	9.35E-08	7.23E-08	3.75E-07	1.04E-07	4.49E-08
PRPS1	3.64E-05	4.57E-05	2.67E-05	1.91E-05	1.37E-05	2.38E-05	1.90E-05	4.03E-05
PRPS1L1	1.45E-07	5.59E-08	1.05E-07	0	0	2.25E-08	0	0
PRPS2	1.21E-05	9.77E-06	1.03E-05	1.20E-05	4.50E-07	1.42E-05	1.50E-05	9.81E-06
PRPSAP1	4.48E-05	7.14E-05	3.32E-05	2.35E-05	1.97E-05	4.11E-05	3.64E-05	2.48E-05
PRPSAP2	1.40E-05	2.19E-05	9.59E-06	1.32E-05	1.27E-05	1.44E-05	1.20E-05	8.06E-06
PRR11	5.63E-06	2.40E-06	1.75E-07	1.76E-07	0	3.93E-07	3.90E-08	2.41E-07
PRR12	4.20E-06	2.58E-06	2.07E-06	1.80E-06	7.36E-07	2.43E-06	1.92E-06	2.21E-06
PRR13	0.00029	0.000156	7.16E-05	8.97E-05	6.44E-05	0.000111	0.000112	6.14E-05
PRR14	1.75E-05	1.43E-05	7.88E-06	1.17E-05	9.68E-06	8.25E-06	1.27E-05	1.08E-05
PRR15	1.12E-06	1.66E-08	3.08E-08	2.32E-07	1.25E-08	1.35E-08	2.95E-07	2.15E-08
PRR15L	8.84E-06	2.30E-06	1.26E-06	3.73E-06	1.41E-07	8.44E-08	2.51E-06	2.69E-08
PRR16	1.44E-06	2.22E-07	2.38E-07	5.12E-07	1.66E-07	2.41E-07	4.62E-07	1.04E-06
PRR18	3.28E-07	2.23E-07	3.56E-06	3.19E-06	3.41E-07	1.57E-06	1.58E-06	2.29E-06
PRR19	6.22E-07	4.19E-06	1.59E-07	2.24E-07	1.11E-07	3.57E-07	1.27E-07	2.10E-07
PRR20A	0	0	0	0	0	0	0	0
PRR20B	0	0	0	0	0	0	0	0
PRR20C	0	0	0	0	0	0	0	0
PRR20D	0	0	0	0	0	0	0	0
PRR21	0	0	0	0	0	0	0	0
PRR22	8.65E-07	1.68E-06	1.66E-06	2.00E-06	4.01E-06	8.97E-07	2.11E-06	2.01E-06
PRR23A	0	0	0	0	0	0	0	0
PRR23B	0	0	0	0	0	0	0	0
PRR23C	0	0	0	0	0	0	0	0
PRR24	9.64E-06	4.97E-06	4.52E-06	1.11E-05	2.32E-05	6.65E-06	2.50E-05	9.75E-06
PRR25	0	0	0	0	0	0	0	0
PRR3	4.12E-06	5.14E-06	1.45E-06	2.31E-06	1.56E-06	2.34E-06	2.52E-06	5.25E-06
PRR4	1.71E-06	5.89E-07	1.10E-06	5.27E-07	9.96E-07	1.40E-06	1.87E-06	1.78E-06
PRR5	1.48E-05	1.89E-05	1.52E-05	1.44E-05	3.41E-05	2.56E-05	1.80E-05	6.40E-06
PRR5-ARH	1.61E-07	2.43E-07	5.27E-89	4.58E-07	0	7.32E-07	0	0
PRR5L	2.48E-06	3.84E-07	5.94E-07	1.05E-06	2.23E-07	3.99E-06	9.85E-07	5.10E-07
PRR7	4.18E-06	1.16E-06	6.28E-08	1.56E-06	1.21E-06	1.04E-06	2.82E-06	7.81E-07
PRRC1	1.91E-05	1.25E-05	1.06E-05	9.30E-06	3.21E-06	1.58E-05	7.50E-06	2.02E-05
PRRG1	3.01E-06	1.37E-06	2.75E-06	3.52E-06	7.37E-07	3.94E-06	2.03E-06	4.86E-06
PRRG2	5.48E-06	5.62E-06	1.35E-06	4.23E-06	7.15E-07	1.72E-06	4.59E-06	5.47E-06

PRRG3	0	1.76E-08	0	0	1.33E-08	1.43E-08	1.49E-07	4.56E-08
PRRG4	1.04E-05	5.02E-06	1.32E-05	5.05E-06	2.55E-06	2.66E-06	4.51E-06	4.95E-06
PRRT1	4.01E-07	1.45E-07	1.79E-07	3.85E-07	4.88E-07	1.86E-07	2.18E-07	4.21E-07
PRRT2	1.07E-06	1.67E-07	6.39E-08	1.02E-07	7.06E-08	3.57E-08	3.00E-08	1.33E-07
PRRT3	6.18E-07	6.34E-07	1.66E-07	3.50E-07	1.49E-07	1.82E-07	4.43E-07	1.40E-07
PRRT4	6.42E-08	1.61E-07	6.74E-08	2.62E-08	1.56E-07	1.52E-07	2.07E-08	4.72E-08
PRRX1	9.94E-07	2.68E-07	3.52E-08	5.33E-07	1.08E-07	8.21E-07	1.07E-06	6.26E-07
PRRX2	3.68E-06	2.22E-08	0	4.77E-08	0	0	1.86E-08	2.87E-08
PRSS1	0	4.05E-08	3.87E-08	0	0	0	3.26E-08	0
PRSS12	1.16E-06	3.85E-08	7.30E-07	4.28E-07	1.77E-08	0	8.00E-08	0
PRSS16	7.73E-06	0	1.36E-07	3.08E-07	0	2.61E-08	0	0
PRSS21	1.38E-05	0	5.05E-08	3.32E-07	0	2.09E-08	0	0
PRSS22	2.54E-06	1.04E-07	2.71E-07	1.33E-05	0	0	4.77E-08	0
PRSS23	2.18E-05	1.46E-05	8.13E-06	3.57E-05	2.38E-06	7.52E-06	2.32E-05	8.28E-06
PRSS27	3.63E-07	2.33E-07	1.89E-07	6.49E-07	3.37E-08	1.74E-07	6.01E-07	8.53E-07
PRSS3	2.68E-05	5.61E-06	5.52E-07	5.50E-06	1.42E-05	1.31E-07	6.53E-06	0
PRSS30P	5.75E-07	4.04E-07	1.69E-07	2.40E-07	4.19E-08	1.81E-07	0	0
PRSS33	8.75E-08	0	0	0	0	0	0	0
PRSS35	1.48E-06	2.96E-07	4.05E-08	3.45E-07	6.69E-08	1.17E-07	9.42E-08	2.14E-07
PRSS36	2.28E-07	7.40E-07	1.01E-06	4.34E-07	2.21E-07	6.29E-07	5.87E-07	7.27E-07
PRSS37	2.24E-08	0	0	0	0	0	0	0
PRSS38	0	0	0	0	0	0	0	0
PRSS41	0	0	0	0	0	0	0	0
PRSS42	0	1.24E-07	0	0	0	0	0	0
PRSS45	6.30E-07	3.67E-07	2.93E-08	7.09E-08	2.23E-07	8.03E-08	0	0
PRSS48	0	0	0	0	0	0	0	0
PRSS50	1.01E-06	1.75E-06	2.00E-08	2.07E-07	1.78E-07	1.39E-07	3.60E-08	2.77E-08
PRSS53	9.34E-07	2.14E-06	1.97E-06	1.43E-06	5.79E-07	5.93E-07	1.94E-06	2.40E-07
PRSS54	0	0	0	0	7.81E-09	0	0	0
PRSS55	0	2.24E-08	0	0	2.06E-08	2.22E-08	0	0
PRSS8	8.42E-06	4.71E-05	1.67E-05	1.99E-05	1.87E-06	1.27E-08	1.18E-05	4.03E-08
PRSSL1	5.03E-08	0	0	6.22E-08	0	0	7.21E-08	0
PRTFDC1	3.03E-06	1.67E-06	3.94E-07	7.00E-07	3.09E-07	2.39E-07	2.07E-07	2.19E-07
PRTG	6.46E-08	4.10E-09	1.50E-08	1.12E-08	4.74E-09	1.12E-08	7.20E-09	2.15E-08
PRTN3	0	0	2.92E-08	2.31E-07	4.62E-08	0	2.54E-08	3.98E-08
PRUNE	1.68E-05	5.94E-06	5.62E-06	7.57E-06	6.40E-06	3.17E-05	6.47E-06	9.87E-06
PRUNE2	2.18E-07	7.93E-08	4.27E-07	3.15E-07	4.15E-08	2.24E-07	3.20E-07	1.92E-07
PRX	7.81E-07	7.55E-07	3.29E-07	6.13E-07	7.02E-07	6.11E-07	2.36E-06	8.23E-07
PRY2	0	0	0	0	0	0	0	0
PSAP	0.000669	0.000827	0.000566	0.000794	0.000512	0.000483	0.000626	0.00048
PSAPL1	1.21E-07	2.16E-08	4.96E-09	0	0	0	0	0
PSAT1	3.80E-06	8.42E-05	0.000125	2.63E-05	2.19E-05	2.31E-05	1.94E-05	2.26E-05
PSCA	5.22E-08	2.30E-06	0	9.68E-08	9.04E-08	2.44E-08	9.96E-08	0
PSD	1.65E-06	2.46E-07	3.91E-07	3.02E-07	2.16E-06	1.27E-07	1.14E-06	2.60E-07
PSD2	3.01E-08	3.37E-08	0	3.07E-08	1.72E-08	4.18E-08	0	1.47E-08
PSD3	3.67E-06	1.63E-05	6.75E-06	4.77E-06	7.97E-07	5.82E-06	2.05E-06	3.03E-06
PSD4	2.31E-05	2.35E-05	2.80E-05	1.73E-05	2.25E-05	1.60E-05	3.33E-05	1.40E-05
PSEN1	2.33E-05	1.00E-05	8.73E-06	1.16E-05	4.70E-06	9.69E-06	1.05E-05	1.19E-05
PSEN2	2.35E-05	1.24E-05	7.36E-06	9.37E-06	3.25E-06	7.86E-06	6.60E-06	2.41E-05
PSENEN	0.000163	0.000125	6.23E-05	8.32E-05	0.000122	0.000106	0.000108	8.59E-05
PSG1	4.82E-08	0	0	2.28E-08	0	1.17E-07	0	0
PSG10	0	0	0	0	0	0	0	0
PSG11	0	0	0	0	0	0	0	0
PSG2	0	0	0	0	0	0	0	0
PSG3	0	0	0	0	0	3.70E-08	0	0
PSG4	9.46E-08	0	0	1.08E-06	0	2.58E-08	0	0
PSG5	0	0	0	2.70E-07	0	4.41E-08	0	0
PSG6	2.63E-08	0	0	0	0	0	0	0

PSG7	0	0	0	3.60E-08	0	2.72E-08	0	0
PSG8	0	0	0	7.33E-07	0	1.37E-07	0	0
PSG9	2.26E-08	0	0	4.42E-07	0	4.22E-08	0	0
PSIMCT-1	6.53E-07	5.86E-07	1.45E-07	2.83E-07	3.62E-08	6.81E-07	1.56E-07	3.37E-07
PSIP1	1.38E-05	1.70E-05	7.54E-06	1.06E-05	2.70E-06	7.67E-06	6.39E-06	7.30E-06
psiTPTE22	8.07E-08	2.33E-07	7.49E-07	4.23E-07	6.73E-08	9.97E-08	4.17E-07	7.21E-08
PSKH1	1.35E-05	5.68E-06	3.87E-06	4.08E-06	4.67E-06	9.30E-06	1.10E-05	1.16E-05
PSKH2	0	0	2.42E-08	0	0	0	0	0
PSMA1	0.000167	0.000117	6.85E-05	6.68E-05	3.87E-05	9.37E-05	4.64E-05	0.000137
PSMA2	0.00016	0.000118	0.000108	8.69E-05	0.000103	0.000108	9.71E-05	0.000129
PSMA3	0.000189	8.38E-05	8.55E-05	7.83E-05	8.18E-05	0.000126	7.60E-05	0.00014
PSMA4	0.000165	9.37E-05	6.74E-05	7.69E-05	9.66E-05	0.000111	7.76E-05	0.000113
PSMA5	0.000252	0.000202	0.000124	0.000112	9.43E-05	0.000276	0.000111	0.000146
PSMA6	0.000225	0.000174	8.24E-05	9.53E-05	0.000107	0.000133	0.000116	0.000151
PSMA7	0.000413	0.000404	0.000141	0.000194	0.000352	0.000249	0.000205	0.000168
PSMA8	0	0	0	0	0	0	0	2.12E-08
PSMB1	0.000375	0.0002	0.000184	0.000182	0.000144	0.000273	0.000186	0.000232
PSMB10	0.00012	2.77E-05	5.93E-05	8.60E-05	3.70E-05	7.10E-05	5.23E-05	8.64E-05
PSMB11	0	1.44E-08	0	0	1.10E-08	0	0	0
PSMB2	0.000247	0.000155	0.000113	0.000111	8.60E-05	0.000156	9.56E-05	0.000165
PSMB3	0.000399	0.000436	0.000161	0.000181	0.000455	0.00023	0.000242	0.000253
PSMB4	0.000405	0.00033	0.000226	0.000233	0.000248	0.000439	0.000219	0.00058
PSMB5	0.000187	0.000138	6.27E-05	7.79E-05	9.09E-05	0.000127	7.78E-05	0.000137
PSMB6	0.000116	0.000145	0.000129	0.000146	0.000366	0.000186	0.000178	0.000196
PSMB7	0.0002	0.000118	0.000119	0.000125	0.000154	0.000202	0.000156	0.000286
PSMB8	0.000255	0.000127	8.42E-05	7.60E-05	2.18E-05	0.000126	9.64E-05	0.000158
PSMB9	0.000141	2.45E-05	2.57E-05	3.07E-05	4.85E-06	5.69E-05	2.62E-05	9.54E-05
PSMC1	2.60E-05	1.53E-05	1.07E-05	1.71E-05	2.80E-05	1.99E-05	2.35E-05	2.46E-05
PSMC2	6.01E-05	0.000177	5.70E-05	5.61E-05	4.30E-05	9.42E-05	6.00E-05	0.000132
PSMC3	9.93E-05	0.000113	6.96E-05	8.53E-05	0.000111	0.000103	8.34E-05	0.000178
PSMC3IP	6.41E-06	2.43E-06	5.19E-08	5.56E-07	8.87E-07	1.20E-06	4.82E-07	4.45E-07
PSMC4	0.000141	0.000112	4.84E-05	5.69E-05	6.42E-05	7.96E-05	5.92E-05	8.66E-05
PSMC5	0.000171	0.00019	8.28E-05	9.34E-05	0.000161	0.000162	0.000108	0.000108
PSMC6	5.20E-05	3.20E-05	3.01E-05	3.05E-05	1.96E-05	4.74E-05	3.39E-05	5.98E-05
PSMD1	7.73E-05	3.12E-05	2.08E-05	2.47E-05	2.02E-05	4.30E-05	2.19E-05	4.75E-05
PSMD10	5.64E-05	3.40E-05	1.30E-05	1.50E-05	1.27E-05	2.60E-05	1.89E-05	2.66E-05
PSMD11	8.71E-05	6.93E-05	2.79E-05	2.69E-05	1.75E-05	3.94E-05	2.10E-05	5.60E-05
PSMD12	4.44E-05	5.22E-05	1.87E-05	2.18E-05	5.97E-06	4.06E-05	1.44E-05	2.75E-05
PSMD13	0.000113	0.000112	5.84E-05	8.36E-05	0.000112	8.00E-05	6.97E-05	0.000139
PSMD14	8.38E-05	5.50E-05	2.05E-05	2.39E-05	1.57E-05	4.20E-05	2.18E-05	5.53E-05
PSMD2	7.94E-05	7.87E-05	3.84E-05	4.17E-05	3.00E-05	6.68E-05	4.04E-05	6.24E-05
PSMD3	0.000129	0.000115	4.80E-05	4.82E-05	4.30E-05	9.95E-05	5.12E-05	7.51E-05
PSMD4	0.000165	0.000144	8.93E-05	0.00011	0.00013	0.000304	0.000131	0.000284
PSMD5	1.72E-05	1.02E-05	7.81E-06	7.56E-06	2.24E-06	1.29E-05	2.99E-06	1.37E-05
PSMD6	5.90E-05	5.69E-05	3.41E-05	4.10E-05	2.27E-05	5.11E-05	3.66E-05	4.63E-05
PSMD7	0.000106	5.05E-05	4.12E-05	5.11E-05	2.49E-05	7.24E-05	3.52E-05	7.79E-05
PSMD8	0.000133	9.19E-05	6.41E-05	7.67E-05	7.82E-05	8.86E-05	0.000105	8.40E-05
PSMD9	2.49E-05	1.41E-05	1.34E-05	2.17E-05	1.87E-05	2.29E-05	2.23E-05	1.48E-05
PSME1	0.000328	0.00021	0.000177	0.000212	0.000127	0.000288	0.000194	0.000261
PSME2	0.000304	0.000253	0.000152	0.000186	0.000194	0.000354	0.000211	0.000258
PSME3	5.14E-05	4.12E-05	1.46E-05	2.03E-05	8.33E-06	4.69E-05	2.26E-05	2.65E-05
PSME4	3.02E-05	4.31E-05	1.57E-05	1.78E-05	1.48E-05	2.50E-05	1.51E-05	1.84E-05
PSMF1	7.58E-05	7.94E-05	5.19E-05	4.84E-05	3.68E-05	6.72E-05	5.83E-05	4.67E-05
PSMG1	2.62E-05	1.96E-05	1.80E-05	1.75E-05	1.48E-05	2.25E-05	2.55E-05	1.87E-05
PSMG2	5.51E-05	4.16E-05	3.50E-05	4.53E-05	1.88E-05	5.48E-05	4.16E-05	4.15E-05
PSMG3	4.58E-05	5.83E-05	1.26E-05	2.15E-05	4.44E-05	2.31E-05	3.21E-05	3.58E-05
PSMG4	1.39E-05	1.34E-05	1.30E-05	1.43E-05	2.54E-05	2.57E-05	2.18E-05	1.65E-05
PSORS1C1	3.01E-07	0	0	6.22E-07	6.89E-08	2.12E-07	7.13E-08	3.63E-07

PSORS1C2	4.50E-08	0	2.44E-08	2.78E-08	0	2.10E-08	0	0
PSORS1C3	3.74E-07	1.30E-07	0	0	0	2.52E-07	5.97E-07	4.08E-07
PSPC1	2.68E-05	7.48E-06	6.39E-06	1.36E-05	4.57E-06	1.05E-05	9.90E-06	7.93E-06
PSPH	2.69E-05	1.72E-05	3.44E-06	3.30E-06	2.87E-06	1.03E-05	3.99E-06	3.54E-06
PSPN	0	9.74E-08	9.93E-08	0	3.39E-07	1.46E-07	1.96E-06	0
PSRC1	5.54E-06	1.47E-06	1.93E-06	4.02E-07	1.12E-06	1.64E-06	1.71E-06	4.75E-07
PSTK	3.43E-06	1.22E-06	2.22E-06	1.76E-06	2.44E-06	3.25E-06	2.69E-06	4.41E-06
PSTPIP1	5.19E-06	1.24E-07	1.08E-06	3.20E-06	8.49E-07	1.88E-06	2.45E-06	9.59E-07
PSTPIP2	1.24E-05	3.04E-06	2.77E-06	4.55E-06	7.63E-07	1.20E-05	7.08E-06	1.04E-05
PTAFR	4.14E-06	4.44E-07	1.19E-06	2.17E-06	1.16E-08	6.89E-07	1.59E-06	2.19E-07
PTAR1	1.00E-05	2.95E-06	3.88E-06	2.93E-06	1.13E-06	4.53E-06	1.65E-06	2.81E-06
PTBP1	9.92E-05	6.74E-05	5.43E-05	6.39E-05	4.47E-05	7.18E-05	7.90E-05	5.33E-05
PTBP2	5.91E-06	5.11E-06	2.78E-06	3.22E-06	9.19E-07	4.91E-06	1.77E-06	2.28E-06
PTCD1	1.28E-05	2.52E-05	4.86E-06	4.84E-06	3.53E-06	8.68E-06	7.61E-06	1.12E-05
PTCD2	2.89E-06	4.41E-06	3.02E-06	1.62E-06	5.85E-07	4.78E-06	2.51E-06	2.84E-06
PTCD3	2.32E-05	1.41E-05	9.56E-06	7.59E-06	3.67E-06	1.83E-05	8.25E-06	6.15E-06
PTCH1	5.68E-07	6.30E-07	1.44E-06	1.10E-06	4.29E-07	1.07E-06	8.09E-07	1.16E-06
PTCH2	1.21E-07	7.79E-08	1.54E-07	5.08E-07	1.09E-08	2.48E-08	3.26E-08	2.95E-08
PTCHD1	1.28E-08	4.03E-08	1.31E-08	5.19E-09	0	0	0	0
PTCHD2	5.18E-08	0	4.43E-09	4.01E-08	0	4.00E-09	8.41E-09	6.32E-09
PTCHD3	2.78E-08	0	1.93E-08	0	0	0	0	0
PTCRA	4.48E-07	4.49E-08	3.02E-07	8.21E-07	9.91E-08	3.75E-07	5.73E-07	5.71E-08
PTDSS1	6.15E-05	5.19E-05	2.41E-05	2.27E-05	3.03E-05	2.68E-05	5.06E-05	2.76E-05
PTDSS2	1.16E-05	1.33E-05	5.43E-06	7.76E-06	1.45E-05	5.62E-06	9.70E-06	1.09E-05
PTEN	1.30E-05	1.50E-05	1.80E-05	1.47E-05	6.26E-06	1.13E-05	1.50E-05	1.23E-05
PTENP1	2.80E-06	3.88E-06	4.51E-06	2.60E-06	6.45E-07	2.45E-06	1.95E-06	3.29E-06
PTER	2.16E-05	6.90E-06	5.41E-06	4.69E-06	1.03E-06	5.84E-06	3.93E-06	3.16E-06
PTF1A	0	0	0	0	0	0	0	0
PTGDR	7.10E-08	5.32E-08	2.53E-07	2.12E-07	2.71E-08	3.65E-08	1.84E-07	5.78E-08
PTGDS	1.92E-05	2.87E-06	1.77E-05	0.00019	2.94E-06	6.45E-06	5.07E-05	1.89E-05
PTGER1	3.47E-06	0	3.75E-08	3.90E-07	0	0	1.02E-07	0
PTGER2	2.13E-06	7.48E-08	5.13E-07	2.08E-06	3.26E-08	5.01E-07	4.78E-07	4.18E-07
PTGER3	8.18E-08	1.06E-07	2.34E-08	2.07E-07	8.66E-08	2.14E-07	5.95E-07	2.01E-07
PTGER4	2.83E-06	2.64E-07	1.37E-06	4.65E-06	1.50E-07	8.66E-07	1.38E-06	8.58E-07
PTGES	2.72E-06	6.47E-07	2.29E-07	1.72E-06	9.36E-08	2.21E-06	1.12E-06	2.80E-07
PTGES2	2.71E-05	3.91E-05	2.13E-05	2.24E-05	4.10E-05	3.32E-05	3.24E-05	6.75E-05
PTGES3	0.000216	0.000269	9.46E-05	0.0001	5.30E-05	0.000139	0.000157	8.32E-05
PTGFR	1.81E-05	4.31E-06	2.54E-07	2.46E-07	1.91E-07	1.55E-08	4.50E-08	2.68E-06
PTGFRN	1.58E-05	6.84E-06	2.97E-06	7.49E-06	1.06E-06	9.26E-06	3.40E-06	1.68E-05
PTGIR	1.64E-06	1.42E-07	1.89E-06	3.95E-06	1.27E-06	3.29E-07	2.22E-06	1.09E-06
PTGIS	1.38E-06	4.48E-08	2.16E-06	5.00E-06	1.75E-07	1.27E-07	1.46E-06	1.99E-07
PTGR1	2.39E-05	0.0001	0.000324	0.000111	0.000221	0.000544	0.000237	0.000118
PTGR2	1.24E-05	1.41E-05	8.01E-06	7.45E-06	8.88E-06	1.36E-05	8.75E-06	1.09E-05
PTGS1	1.76E-06	3.29E-07	1.74E-06	1.47E-06	1.67E-07	3.52E-07	4.67E-07	1.85E-07
PTGS2	4.35E-07	1.10E-07	1.09E-06	6.23E-06	6.22E-08	7.47E-08	4.70E-07	1.67E-07
PTH	0	4.08E-08	0	0	0	0	0	0
PTH1R	1.12E-06	1.52E-07	1.13E-05	9.14E-06	2.80E-06	1.63E-06	8.34E-07	1.30E-06
PTH2	0	0	0	0	0	0	0	0
PTH2R	1.72E-08	2.55E-05	3.58E-08	6.07E-08	3.21E-06	7.99E-09	6.54E-06	0
PTHLH	2.14E-06	5.35E-07	1.03E-07	1.72E-05	6.56E-08	1.03E-07	2.15E-07	4.41E-07
PTK2	3.07E-05	4.90E-05	1.55E-05	1.62E-05	1.38E-05	2.09E-05	3.31E-05	3.18E-05
PTK2B	7.31E-06	5.61E-06	5.05E-06	5.58E-06	1.40E-06	5.87E-06	4.32E-06	3.21E-06
PTK6	3.14E-06	4.02E-06	1.45E-06	5.42E-06	2.44E-06	3.73E-07	2.71E-06	3.77E-07
PTK7	8.53E-06	3.87E-07	4.08E-07	3.10E-06	7.95E-07	4.62E-07	1.96E-06	2.21E-06
PTMA	0.000956	0.000298	0.000209	0.000363	0.000309	0.000306	0.000447	0.000378
PTMS	0.00045	0.0006	0.000754	0.000688	0.000959	0.000352	0.000995	0.000319
PTN	1.06E-06	2.87E-07	2.03E-06	7.42E-06	1.56E-06	3.42E-07	2.10E-07	1.16E-06
PTOV1	8.71E-05	7.55E-05	5.84E-05	6.02E-05	0.00015	5.77E-05	8.02E-05	6.33E-05

PTP4A1	0.000328	0.000429	0.000187	0.000148	6.04E-05	0.000191	0.000123	0.000268
PTP4A2	5.42E-05	4.11E-05	2.96E-05	3.66E-05	8.00E-06	3.67E-05	2.30E-05	3.74E-05
PTP4A3	2.42E-05	7.91E-06	1.61E-06	4.86E-06	3.83E-06	5.14E-06	6.83E-06	2.55E-05
PTPDC1	5.19E-06	9.71E-07	3.35E-07	8.15E-07	2.88E-07	1.00E-07	5.07E-07	5.02E-07
PTPLA	3.74E-06	1.99E-08	2.70E-07	9.07E-07	2.01E-07	1.45E-07	3.73E-08	1.70E-07
PTPLAD1	7.79E-05	9.15E-05	5.88E-05	3.96E-05	5.51E-05	5.06E-05	5.86E-05	5.94E-05
PTPLAD2	1.94E-06	2.12E-07	7.27E-07	1.15E-06	1.45E-07	4.23E-07	6.62E-07	2.40E-07
PTPLB	1.36E-05	2.22E-05	1.30E-05	6.51E-06	9.38E-08	9.28E-06	2.52E-06	4.93E-06
PTPMT1	1.47E-05	2.04E-05	7.36E-06	1.03E-05	1.12E-05	1.51E-05	1.04E-05	1.45E-05
PTPN1	2.51E-05	1.75E-05	6.99E-06	2.05E-05	2.34E-06	1.48E-05	1.14E-05	1.13E-05
PTPN11	2.98E-05	4.14E-05	1.92E-05	2.00E-05	3.71E-06	2.29E-05	1.95E-05	1.78E-05
PTPN12	3.71E-05	2.79E-05	1.11E-05	1.90E-05	6.86E-06	1.46E-05	1.25E-05	2.99E-05
PTPN13	4.35E-06	5.24E-08	3.03E-07	7.50E-07	7.16E-09	2.90E-08	7.17E-08	8.98E-08
PTPN14	2.38E-06	5.78E-07	2.01E-07	6.41E-07	9.16E-08	1.27E-07	1.77E-07	1.96E-07
PTPN18	2.81E-05	1.84E-05	1.41E-05	1.55E-05	1.36E-05	1.55E-05	2.51E-05	1.06E-05
PTPN2	2.13E-05	8.05E-06	6.56E-06	1.22E-05	3.18E-06	1.09E-05	1.17E-05	1.77E-05
PTPN20A	0	#######	0	0	0	0	#######	0
PTPN20B	7.47E-08	1.56E-06	4.71E-07	1.19E-07	0	1.07E-07	9.86E-09	0
PTPN21	3.54E-06	1.37E-06	2.43E-06	4.06E-06	1.03E-06	2.30E-06	2.78E-06	2.00E-06
PTPN22	1.78E-06	7.45E-08	4.61E-07	1.37E-06	0	4.37E-07	2.56E-07	4.09E-07
PTPN23	1.37E-05	1.41E-05	6.38E-06	8.93E-06	5.99E-06	1.28E-05	9.38E-06	7.98E-06
PTPN3	9.99E-06	4.53E-06	1.42E-05	1.18E-05	2.02E-06	9.31E-06	7.71E-06	8.76E-06
PTPN4	3.78E-06	2.80E-06	2.69E-06	2.95E-06	1.48E-06	3.23E-06	1.67E-06	2.06E-06
PTPN5	1.00E-07	0	7.99E-09	0	6.87E-09	2.22E-08	7.76E-09	7.04E-08
PTPN6	3.74E-05	1.77E-05	1.66E-05	2.12E-05	8.21E-06	2.31E-05	2.15E-05	1.43E-05
PTPN7	6.62E-06	3.96E-07	8.13E-07	2.24E-06	2.76E-07	2.16E-06	1.57E-06	1.04E-06
PTPN9	1.34E-05	5.86E-06	1.18E-05	8.11E-06	3.01E-06	9.43E-06	6.75E-06	1.04E-05
PTPRA	3.20E-05	1.27E-05	1.22E-05	1.35E-05	8.10E-06	1.51E-05	1.16E-05	1.65E-05
PTPRB	3.98E-06	2.19E-06	3.97E-06	1.41E-06	1.00E-06	4.28E-06	1.32E-06	3.02E-06
PTPRC	2.09E-05	1.28E-06	6.41E-06	1.30E-05	1.28E-07	5.91E-06	3.41E-06	2.48E-06
PTPRCAP	2.24E-05	4.12E-06	7.85E-06	2.07E-05	3.43E-06	1.00E-05	1.65E-05	4.96E-06
PTPRD	3.27E-06	9.03E-09	2.56E-06	2.31E-06	7.88E-08	2.77E-06	1.07E-06	8.63E-07
PTPRE	8.70E-06	6.94E-07	1.13E-06	3.42E-06	1.46E-07	1.13E-06	1.35E-06	8.94E-07
PTPRF	8.09E-05	4.40E-05	4.86E-05	4.48E-05	2.23E-05	7.72E-05	6.12E-05	0.000117
PTPRG	9.56E-06	2.42E-06	2.46E-06	3.35E-06	7.73E-07	1.44E-05	2.07E-06	2.88E-06
PTPRH	3.09E-05	1.07E-05	3.76E-06	1.49E-05	1.29E-06	1.71E-05	1.80E-05	1.48E-05
PTPRJ	8.48E-06	5.61E-06	4.47E-06	4.68E-06	2.40E-06	3.86E-06	5.73E-06	1.53E-05
PTPRK	4.57E-05	1.08E-05	1.47E-05	1.55E-05	3.20E-06	1.51E-05	1.10E-05	1.66E-05
PTPRM	2.93E-05	3.47E-06	4.82E-06	5.83E-06	2.66E-06	4.51E-06	6.97E-06	1.05E-05
PTPRN	6.50E-09	0	0	7.94E-09	2.23E-08	0	1.89E-08	0
PTPRN2	1.39E-06	1.61E-06	2.65E-06	5.66E-06	8.44E-07	1.07E-06	1.97E-06	1.19E-06
PTPRO	7.33E-07	2.64E-07	5.67E-07	9.46E-07	2.70E-08	1.55E-07	4.93E-07	3.35E-08
PTPRQ	0	1.31E-08	0	3.95E-08	0	0	0	0
PTPRR	8.93E-08	4.34E-08	1.34E-08	3.13E-08	2.53E-07	5.68E-07	3.70E-08	3.76E-08
PTPRS	7.33E-06	1.16E-07	4.57E-06	2.80E-06	2.61E-07	8.06E-08	1.65E-06	3.95E-07
PTPRT	6.56E-09	9.73E-09	3.38E-08	1.95E-08	0	0	2.99E-08	1.78E-07
PTPRU	2.46E-05	2.46E-06	6.11E-06	1.03E-05	6.91E-06	8.07E-07	1.23E-05	1.98E-05
PTPRVP	3.89E-08	0	0	6.78E-09	1.23E-07	1.09E-07	0	1.63E-08
PTPRZ1	9.00E-08	1.31E-08	2.41E-08	5.16E-08	5.03E-09	5.42E-09	5.69E-09	1.21E-08
PTRF	4.87E-05	1.64E-05	1.09E-05	3.84E-05	1.95E-05	1.89E-05	4.24E-05	1.76E-05
PTRH1	1.59E-05	5.24E-06	6.59E-06	1.05E-05	1.54E-05	1.56E-05	1.78E-05	1.55E-05
PTRH2	4.85E-05	4.97E-05	2.31E-05	2.78E-05	6.96E-05	5.77E-05	3.64E-05	3.17E-05
PTS	3.13E-05	2.21E-05	2.92E-05	2.76E-05	1.65E-05	2.95E-05	2.36E-05	2.63E-05
PTTG1	4.95E-05	8.24E-06	8.09E-07	1.91E-06	1.06E-05	9.55E-06	2.84E-06	7.82E-06
PTTG1IP	0.000109	8.30E-05	8.96E-05	7.77E-05	4.08E-05	8.53E-05	0.000103	0.000111
PTTG2	3.92E-07	0	6.75E-08	7.05E-08	0	5.29E-08	0	8.55E-08
PTTG3P	7.75E-07	0	1.23E-07	6.49E-08	4.53E-08	0	4.82E-08	1.57E-07
PTX3	1.99E-07	1.12E-07	3.91E-08	2.56E-06	1.28E-07	2.65E-07	1.15E-06	4.02E-07

PTX4	0	1.61E-07	0	0	0	0	0	0
PUF60	0.000221	0.000155	6.73E-05	7.83E-05	0.000146	8.58E-05	0.00023	0.000118
PUM1	2.13E-05	1.65E-05	1.35E-05	1.13E-05	4.57E-06	2.04E-05	7.25E-06	1.09E-05
PUM2	3.89E-05	3.96E-05	2.33E-05	1.85E-05	1.09E-05	2.57E-05	1.94E-05	1.93E-05
PURA	7.12E-06	6.63E-06	6.42E-06	3.73E-06	1.04E-06	4.58E-06	2.75E-06	4.94E-06
PURB	7.25E-06	5.93E-06	3.54E-06	3.90E-06	2.01E-06	3.75E-06	3.40E-06	4.50E-06
PURG	1.74E-08	0	0	0	7.46E-09	0	1.68E-08	0
PUS1	9.75E-06	1.94E-05	8.89E-06	1.32E-05	9.13E-06	1.66E-05	2.20E-05	1.78E-05
PUS10	1.06E-05	6.11E-06	5.08E-06	5.30E-06	3.16E-06	8.10E-06	4.88E-06	2.34E-06
PUS3	7.84E-06	9.18E-06	9.24E-06	1.27E-05	9.39E-06	1.78E-05	1.48E-05	1.50E-05
PUS7	2.49E-06	4.02E-06	2.28E-06	2.17E-06	1.08E-06	4.05E-06	2.51E-06	2.71E-06
PUS7L	2.46E-06	1.61E-06	1.14E-06	8.50E-07	2.10E-07	1.59E-06	3.93E-07	9.51E-07
PUSL1	1.35E-05	4.86E-06	4.70E-06	5.72E-06	8.62E-06	7.53E-06	1.03E-05	1.33E-05
PVALB	2.98E-07	0	1.82E-06	8.28E-07	5.24E-08	0	1.65E-07	0
PVR	1.86E-05	2.18E-05	1.47E-05	2.41E-05	1.78E-05	1.00E-05	2.90E-05	1.86E-05
PVRIG	5.46E-06	1.13E-06	1.08E-06	3.48E-06	1.22E-07	1.43E-06	1.39E-06	7.89E-07
PVRL1	6.03E-06	9.36E-07	7.57E-07	1.62E-06	1.28E-06	2.01E-06	1.38E-06	6.07E-06
PVRL2	0.000168	7.93E-05	6.31E-05	0.000104	0.000108	6.31E-05	0.000106	0.000112
PVRL3	1.40E-05	1.73E-05	1.30E-05	6.26E-06	5.21E-07	7.37E-06	2.72E-06	2.32E-06
PVRL4	4.70E-07	6.53E-07	7.21E-08	3.96E-07	0	2.64E-07	7.74E-08	1.92E-08
PVT1	4.43E-06	2.44E-07	5.32E-08	3.72E-07	1.96E-07	1.94E-06	2.16E-06	1.87E-07
PWP1	2.23E-05	2.53E-05	1.55E-05	1.88E-05	7.54E-06	2.69E-05	1.60E-05	1.67E-05
PWP2	1.11E-05	6.09E-06	5.04E-06	7.38E-06	3.11E-06	8.56E-06	7.92E-06	1.09E-05
PWRN1	0	0	0	2.40E-08	0	0	0	0
PWRN2	0	0	0	0	0	0	0	0
PWWP2A	4.50E-06	1.49E-06	2.42E-06	2.72E-06	6.44E-07	2.79E-06	2.04E-06	3.72E-06
PWWP2B	1.11E-05	1.97E-06	1.92E-06	5.40E-06	3.02E-06	3.57E-06	5.42E-06	3.84E-06
PXDN	8.91E-06	6.11E-06	4.91E-06	8.95E-06	1.39E-06	2.60E-06	7.49E-06	3.56E-06
PXDNL	6.57E-08	2.11E-08	5.00E-08	2.41E-08	5.93E-08	8.79E-07	1.81E-07	2.07E-08
PXK	3.79E-06	6.24E-06	2.88E-06	2.61E-06	8.27E-08	5.71E-06	1.86E-06	3.17E-06
PXMP2	0.000131	0.000352	0.00022	0.00013	0.000152	0.000133	0.00013	6.83E-05
PXMP4	5.14E-06	3.55E-06	3.45E-06	2.61E-06	7.31E-06	5.71E-06	5.92E-06	3.50E-06
PXN	2.93E-05	2.54E-05	1.28E-05	1.40E-05	7.44E-06	1.34E-05	1.72E-05	1.29E-05
PXT1	0	0	0	0	0	3.32E-08	0	2.64E-08
PYCARD	2.94E-05	2.68E-06	7.60E-06	3.10E-05	5.29E-06	2.19E-05	1.50E-05	6.81E-06
PYCR1	6.42E-05	3.14E-07	1.72E-06	2.91E-06	3.19E-07	1.70E-05	4.54E-06	5.66E-07
PYCR2	4.19E-05	4.27E-05	2.10E-05	2.49E-05	1.61E-05	4.31E-05	4.37E-05	5.87E-05
PYCRL	1.34E-05	2.26E-05	5.98E-06	5.50E-06	9.00E-06	9.34E-06	1.84E-05	1.22E-05
PYDC1	0	0	0	0	0	0	5.88E-08	0
PYDC2	0	0	0	0	0	0	0	0
PYGB	0.000101	1.18E-05	6.68E-06	1.13E-05	3.60E-06	9.24E-06	1.18E-05	2.49E-05
PYGL	7.21E-05	9.91E-05	0.000155	0.00017	0.000288	6.56E-05	5.76E-05	4.12E-05
PYGM	9.13E-08	3.85E-08	6.53E-08	1.59E-07	1.47E-07	1.02E-07	1.07E-07	1.02E-07
PYGO1	1.21E-07	9.29E-08	0	1.25E-07	0	0	0	3.00E-08
PYGO2	1.69E-05	1.74E-05	6.97E-06	7.99E-06	9.72E-06	1.89E-05	1.01E-05	2.05E-05
PYHIN1	2.11E-06	1.06E-07	4.24E-07	1.72E-06	2.15E-08	1.30E-06	9.44E-07	2.23E-07
PYROXD1	4.97E-06	8.21E-06	6.10E-06	4.86E-06	2.96E-06	4.42E-06	3.36E-06	5.40E-06
PYROXD2	3.62E-06	1.01E-06	3.51E-06	4.78E-06	1.35E-07	3.15E-05	4.75E-06	1.02E-07
PYY	0	2.34E-07	0	9.39E-08	2.41E-07	3.54E-08	1.92E-07	0
PYY2	2.96E-07	1.14E-07	1.07E-07	1.52E-07	7.46E-07	1.15E-07	9.42E-08	1.83E-07
PZP	2.51E-07	4.49E-08	7.21E-05	1.49E-05	8.61E-09	5.54E-08	1.45E-06	2.94E-08
QARS	0.000132	0.000151	4.92E-05	7.96E-05	0.000172	0.000103	0.00011	8.75E-05
QDPR	5.17E-05	0.000185	0.000153	7.89E-05	8.07E-05	0.000121	9.49E-05	9.24E-05
QKI	2.78E-05	1.16E-05	1.73E-05	1.63E-05	3.63E-06	1.66E-05	1.19E-05	1.12E-05
QPCT	6.02E-06	4.64E-07	7.96E-07	2.08E-06	1.51E-07	9.85E-07	1.43E-06	2.96E-07
QPCTL	8.60E-06	5.49E-06	4.93E-06	4.85E-06	1.05E-05	6.06E-06	6.33E-06	3.74E-06
QPRT	0.000102	0.000288	0.00021	9.47E-05	5.97E-05	9.40E-05	8.31E-05	0.000132
QRFP	9.71E-08	0	0	2.51E-07	0	0	8.70E-08	1.54E-07

QRFPR	1.06E-08	0	2.21E-08	6.19E-08	9.51E-08	9.84E-09	0	9.86E-08
QRICH1	2.21E-05	1.64E-05	9.68E-06	1.17E-05	6.62E-06	1.83E-05	1.29E-05	1.23E-05
QRICH2	4.44E-07	2.38E-06	1.57E-07	4.71E-07	1.03E-06	6.44E-07	9.31E-07	6.65E-07
QRSL1	6.97E-06	1.01E-05	4.13E-06	4.18E-06	1.37E-06	8.58E-06	3.49E-06	5.50E-06
QSER1	3.13E-06	2.94E-06	1.97E-06	1.47E-06	6.25E-07	2.35E-06	1.03E-06	1.91E-06
QSOX1	4.43E-05	3.87E-06	3.00E-05	8.72E-05	1.15E-05	3.94E-06	1.60E-05	6.15E-06
QSOX2	7.68E-06	6.59E-06	2.37E-06	3.13E-06	1.27E-06	8.19E-06	2.60E-06	5.75E-06
QTRT1	3.01E-05	1.63E-05	2.29E-05	2.80E-05	5.23E-05	4.17E-05	4.79E-05	3.18E-05
QTRTD1	6.44E-06	5.90E-06	3.02E-06	3.29E-06	6.41E-07	4.06E-06	2.83E-06	3.11E-06
R3HCC1	1.50E-05	3.68E-05	2.25E-05	2.36E-05	1.59E-05	2.61E-05	1.68E-05	1.27E-05
R3HDM1	9.98E-06	5.64E-06	2.96E-06	3.50E-06	2.63E-06	6.90E-06	3.29E-06	5.00E-06
R3HDM2	2.74E-05	2.20E-05	2.04E-05	2.08E-05	1.35E-05	3.33E-05	2.96E-05	2.34E-05
R3HDML	1.82E-08	0	0	0	0	3.39E-08	1.75E-08	5.40E-08
RAB10	6.21E-05	7.24E-05	2.16E-05	2.20E-05	9.24E-06	3.41E-05	2.19E-05	2.47E-05
RAB11A	8.18E-05	3.97E-05	2.57E-05	3.85E-05	1.85E-05	3.77E-05	3.18E-05	3.73E-05
RAB11B	0.000112	4.78E-05	6.21E-05	8.94E-05	0.000113	7.68E-05	0.000113	6.47E-05
RAB11FIP1	5.04E-06	1.83E-05	3.50E-06	1.74E-05	3.26E-07	3.46E-06	7.39E-06	1.76E-06
RAB11FIP2	3.05E-06	1.87E-06	1.91E-06	1.89E-06	5.30E-07	5.92E-06	1.06E-06	2.24E-06
RAB11FIP3	2.16E-05	8.72E-06	9.89E-06	9.84E-06	4.28E-06	1.60E-05	1.42E-05	2.79E-06
RAB11FIP4	1.68E-05	3.62E-06	6.90E-07	1.46E-06	4.65E-06	1.72E-06	3.37E-06	3.95E-06
RAB11FIP5	2.47E-05	4.89E-06	2.73E-06	6.39E-06	7.97E-07	1.69E-06	4.26E-06	4.62E-06
RAB12	1.89E-05	1.56E-05	1.87E-05	1.49E-05	2.49E-06	1.91E-05	1.70E-05	1.58E-05
RAB13	8.81E-05	0.000108	5.06E-05	8.22E-05	6.84E-05	0.000144	9.82E-05	9.06E-05
RAB14	3.74E-05	2.34E-05	3.48E-05	2.39E-05	1.37E-05	2.64E-05	2.55E-05	4.64E-05
RAB15	1.47E-05	5.82E-06	4.50E-06	3.93E-06	3.72E-06	1.07E-05	5.89E-06	1.20E-05
RAB17	8.40E-05	4.86E-05	6.09E-05	6.10E-05	0.000111	5.22E-05	6.09E-05	2.99E-05
RAB18	3.33E-05	2.92E-05	3.07E-05	2.66E-05	7.09E-06	3.16E-05	1.94E-05	2.94E-05
RAB19	2.64E-07	4.02E-08	3.84E-08	4.25E-08	0	7.95E-08	0	0
RAB1A	0.000172	0.000144	4.90E-05	5.83E-05	2.58E-05	7.18E-05	5.60E-05	7.27E-05
RAB1B	0.000101	0.000114	7.87E-05	9.91E-05	6.87E-05	0.000104	8.56E-05	0.000125
RAB20	6.52E-05	1.47E-05	2.76E-05	5.83E-05	2.40E-05	2.81E-05	3.43E-05	1.54E-05
RAB21	2.24E-05	2.08E-05	1.45E-05	1.79E-05	4.80E-06	1.65E-05	1.56E-05	1.74E-05
RAB22A	7.69E-06	6.08E-06	3.21E-06	3.95E-06	1.47E-06	4.17E-06	4.11E-06	3.64E-06
RAB23	5.26E-06	2.81E-06	2.00E-06	1.99E-06	9.69E-07	3.32E-06	1.32E-06	2.02E-06
RAB24	2.73E-05	1.02E-05	9.27E-06	1.12E-05	1.29E-05	1.40E-05	1.36E-05	1.67E-05
RAB25	3.15E-07	5.60E-07	9.75E-07	4.16E-06	0	6.78E-08	7.64E-07	7.21E-08
RAB26	7.86E-06	1.05E-05	1.48E-05	8.13E-07	6.69E-06	5.27E-06	2.58E-06	1.65E-06
RAB27A	2.70E-05	1.11E-05	1.79E-05	8.75E-06	1.96E-06	1.01E-05	8.17E-06	1.80E-05
RAB27B	1.12E-06	2.61E-07	5.08E-07	3.97E-07	0	0	4.19E-07	5.62E-08
RAB28	8.80E-06	8.75E-06	3.36E-06	4.53E-06	1.79E-06	5.30E-06	4.11E-06	5.10E-06
RAB2A	9.61E-05	8.90E-05	4.27E-05	3.92E-05	1.58E-05	5.02E-05	8.58E-05	6.29E-05
RAB2B	8.74E-06	3.24E-06	3.85E-06	4.77E-06	9.31E-07	5.59E-06	2.98E-06	4.22E-06
RAB30	1.32E-06	5.85E-07	1.50E-06	2.36E-06	3.61E-08	3.35E-06	7.16E-07	1.44E-06
RAB31	2.37E-05	1.54E-06	2.92E-06	8.26E-06	6.76E-07	2.62E-06	4.21E-06	2.81E-06
RAB32	5.89E-05	2.85E-05	2.61E-05	5.64E-05	1.48E-05	3.21E-05	7.17E-05	2.53E-05
RAB33A	1.16E-06	1.36E-07	3.07E-07	8.74E-07	1.63E-07	3.30E-07	3.38E-07	1.75E-07
RAB33B	6.22E-06	3.23E-06	5.63E-06	2.77E-06	1.52E-06	4.47E-06	3.81E-06	5.29E-06
RAB34	6.86E-05	1.68E-06	3.20E-06	1.26E-05	3.40E-06	2.11E-06	7.36E-06	3.49E-06
RAB35	2.48E-05	1.77E-05	8.32E-06	1.49E-05	3.41E-06	1.38E-05	1.26E-05	1.70E-05
RAB36	1.24E-06	2.93E-07	1.13E-07	3.94E-07	1.04E-08	9.01E-08	4.73E-08	6.24E-08
RAB37	3.96E-05	3.42E-05	7.48E-06	1.10E-05	1.20E-05	1.33E-05	6.99E-06	2.07E-06
RAB38	1.45E-05	2.03E-08	6.26E-07	1.58E-06	2.45E-07	2.98E-07	5.30E-07	7.89E-08
RAB39	3.54E-07	0	1.87E-07	1.00E-07	0	8.86E-08	3.95E-08	0
RAB39B	2.97E-07	3.69E-08	9.51E-08	9.66E-08	5.66E-09	7.93E-08	3.20E-08	6.76E-08
RAB3A	7.29E-06	4.30E-06	2.42E-06	7.31E-07	1.15E-06	2.33E-06	6.36E-07	4.88E-06
RAB3B	3.59E-06	6.18E-07	5.08E-08	9.81E-08	0	0	6.15E-08	2.76E-06
RAB3C	2.64E-08	1.10E-06	5.21E-07	1.96E-07	2.29E-08	0	1.26E-07	1.18E-07
RAB3D	1.63E-05	8.38E-07	8.52E-07	1.33E-06	7.60E-07	5.03E-07	1.38E-06	6.87E-07

RAB3GAP1	2.01E-05	1.21E-05	8.21E-06	8.22E-06	2.19E-06	1.17E-05	5.75E-06	6.44E-06
RAB3GAP2	6.48E-06	6.93E-06	3.94E-06	2.82E-06	1.25E-06	7.34E-06	2.59E-06	5.69E-06
RAB3IL1	3.15E-06	1.83E-06	5.40E-06	6.36E-06	8.58E-07	4.16E-06	4.56E-06	2.45E-06
RAB3IP	7.62E-06	4.97E-06	1.50E-06	3.38E-06	3.01E-07	1.75E-06	2.13E-06	1.28E-06
RAB40A	2.63E-07	4.29E-08	2.01E-08	6.92E-08	1.62E-08	5.23E-08	7.20E-08	8.33E-08
RAB40AL	3.10E-07	2.99E-08	8.47E-08	9.58E-08	2.24E-08	0	2.46E-08	1.92E-07
RAB40B	2.10E-05	3.53E-05	8.48E-06	9.10E-06	7.45E-06	2.44E-05	8.58E-06	7.33E-06
RAB40C	1.56E-05	1.34E-05	9.07E-06	8.18E-06	4.47E-06	7.66E-06	8.65E-06	6.76E-06
RAB41	1.30E-07	6.02E-08	0	3.20E-08	0	2.42E-08	0	7.74E-08
RAB42	4.37E-06	2.81E-08	3.90E-07	1.32E-06	8.34E-08	6.80E-08	8.31E-07	3.61E-08
RAB43	4.99E-06	1.13E-05	5.04E-06	5.30E-06	1.76E-06	8.26E-06	9.72E-06	9.60E-06
RAB4A	4.00E-05	4.84E-05	2.39E-05	2.88E-05	1.99E-05	8.13E-05	3.15E-05	3.54E-05
RAB4B	2.47E-05	2.37E-05	1.21E-05	2.43E-05	2.54E-05	1.71E-05	2.11E-05	1.21E-05
RAB5A	2.86E-05	2.25E-05	1.76E-05	1.87E-05	5.12E-06	2.11E-05	1.67E-05	1.91E-05
RAB5B	7.45E-05	6.93E-05	4.28E-05	4.45E-05	1.86E-05	6.41E-05	4.06E-05	4.08E-05
RAB5C	0.00012	0.000162	7.35E-05	8.27E-05	5.39E-05	0.000154	8.14E-05	6.52E-05
RAB6A	4.65E-05	3.27E-05	2.92E-05	3.58E-05	7.16E-06	3.48E-05	2.97E-05	4.16E-05
RAB6B	2.22E-06	5.55E-07	2.28E-07	4.19E-07	2.53E-07	5.68E-07	4.29E-07	4.43E-07
RAB6C	8.94E-07	1.10E-06	1.11E-06	5.79E-07	2.19E-07	6.75E-07	3.06E-07	9.59E-07
RAB7A	0.000167	0.000174	0.000101	0.000124	6.91E-05	0.000113	0.000125	0.000102
RAB7L1	1.49E-05	7.35E-06	1.04E-05	7.84E-06	3.68E-06	1.20E-05	8.48E-06	1.41E-05
RAB8A	5.30E-05	3.81E-05	5.95E-05	5.22E-05	2.28E-05	5.57E-05	5.41E-05	4.39E-05
RAB8B	7.40E-06	3.37E-06	3.36E-06	8.22E-06	7.72E-07	5.00E-06	5.13E-06	7.81E-06
RAB9A	3.19E-05	6.95E-06	1.64E-05	1.85E-05	4.32E-06	1.33E-05	1.33E-05	2.30E-05
RAB9B	2.29E-07	0	8.24E-08	9.34E-08	0	0	4.81E-08	0
RAB9BP1	0	0	0	0	0	0	0	0
RABAC1	9.69E-05	5.00E-05	8.60E-05	0.000118	0.000291	8.63E-05	7.22E-05	3.06E-05
RABEP1	7.06E-06	1.02E-05	9.02E-06	9.57E-06	3.07E-06	1.25E-05	9.42E-06	9.95E-06
RABEP2	1.27E-05	6.47E-06	3.49E-06	7.99E-06	4.94E-06	7.08E-06	4.64E-06	5.23E-06
RABEPK	4.04E-05	3.58E-05	3.26E-05	1.21E-05	5.21E-05	8.91E-05	6.54E-05	1.68E-05
RABGAP1	1.09E-05	5.29E-06	5.51E-06	7.29E-06	2.85E-06	9.12E-06	5.59E-06	9.49E-06
RABGAP1L	7.51E-06	7.86E-06	2.87E-06	3.95E-06	2.36E-06	5.92E-06	2.98E-06	2.81E-06
RABGEF1	1.40E-05	1.27E-05	5.64E-06	8.64E-06	4.79E-06	7.11E-06	4.46E-06	5.48E-06
RABGGTA	1.24E-05	1.27E-05	8.84E-06	9.88E-06	8.17E-06	1.32E-05	9.16E-06	1.16E-05
RABGGTB	4.29E-05	4.17E-05	2.72E-05	2.98E-05	1.40E-05	6.72E-05	3.91E-05	4.31E-05
RABIF	8.56E-06	4.84E-06	3.14E-06	4.68E-06	3.00E-06	5.79E-06	5.27E-06	1.25E-05
RABL2A	5.49E-06	5.23E-06	2.63E-06	3.84E-06	2.14E-06	3.22E-06	2.56E-06	2.53E-06
RABL2B	7.24E-06	4.28E-06	3.09E-06	4.69E-06	1.82E-06	5.27E-06	2.93E-06	3.63E-06
RABL3	6.20E-06	1.16E-05	5.38E-06	4.80E-06	3.31E-06	9.73E-06	5.83E-06	5.04E-06
RABL5	3.23E-06	5.88E-06	2.23E-06	5.60E-06	1.95E-06	5.81E-06	3.76E-06	3.54E-06
RAC1	0.000136	0.000148	6.19E-05	8.42E-05	8.16E-05	7.95E-05	0.000125	9.92E-05
RAC2	5.12E-05	2.81E-06	1.24E-05	1.96E-05	8.49E-07	1.25E-05	9.98E-06	5.90E-06
RAC3	1.27E-05	5.71E-05	1.11E-05	6.74E-06	1.29E-05	2.53E-05	1.34E-05	1.37E-05
RACGAP1	1.41E-05	5.27E-06	6.58E-07	9.93E-07	1.62E-06	5.03E-06	1.63E-06	1.66E-06
RACGAP1	1.33E-07	1.87E-08	1.73E-08	0	0	7.72E-09	2.42E-08	0
RAD1	4.67E-06	3.79E-06	1.81E-06	2.40E-06	6.59E-07	2.89E-06	2.20E-06	2.96E-06
RAD17	1.42E-05	1.21E-05	9.66E-06	1.05E-05	4.21E-06	1.34E-05	9.60E-06	1.20E-05
RAD18	4.92E-06	2.78E-06	1.39E-06	1.53E-06	8.05E-07	2.17E-06	1.65E-06	2.42E-06
RAD21	0.000173	6.61E-05	2.82E-05	2.29E-05	1.22E-05	2.92E-05	4.94E-05	3.41E-05
RAD21L1	1.34E-08	0	0	0	0	0	0	0
RAD23A	8.80E-05	8.18E-05	6.87E-05	6.18E-05	7.31E-05	7.23E-05	8.73E-05	7.46E-05
RAD23B	0.000123	7.66E-05	7.62E-05	6.68E-05	2.90E-05	0.000117	5.51E-05	5.98E-05
RAD50	7.16E-06	8.53E-06	6.76E-06	6.16E-06	1.01E-06	1.22E-05	3.29E-06	7.64E-06
RAD51	5.98E-06	2.15E-06	6.34E-07	2.14E-07	8.23E-07	5.92E-07	4.96E-07	6.66E-07
RAD51AP1	5.56E-06	2.58E-06	3.36E-07	5.50E-07	4.78E-07	1.67E-06	3.97E-07	4.72E-07
RAD51AP2	0	2.86E-08	1.32E-08	3.12E-08	8.77E-08	4.28E-08	1.68E-08	9.35E-09
RAD51C	2.67E-05	2.17E-05	5.66E-06	9.59E-06	1.13E-05	1.22E-05	7.06E-06	6.81E-06
RAD51L1	1.46E-06	5.50E-07	3.54E-07	6.91E-07	9.74E-08	1.17E-06	6.55E-07	6.59E-07

RAD51L3	5.20E-06	4.95E-06	1.45E-06	2.05E-06	1.14E-06	1.73E-06	2.20E-06	1.54E-06
RAD52	3.11E-06	1.92E-06	1.49E-06	1.09E-06	4.50E-07	1.51E-06	1.30E-06	8.51E-07
RAD54B	2.74E-06	1.03E-06	3.41E-07	2.64E-07	2.51E-07	1.06E-06	5.54E-07	3.21E-07
RAD54L	2.42E-06	1.11E-06	1.09E-07	9.66E-08	3.64E-07	4.00E-07	9.36E-08	3.90E-07
RAD54L2	1.44E-06	7.57E-07	1.56E-06	8.30E-07	1.24E-07	4.31E-06	6.71E-07	7.44E-07
RAD9A	8.32E-06	7.76E-06	3.13E-06	3.10E-06	6.61E-06	3.81E-06	4.02E-06	7.59E-06
RAD9B	8.95E-07	9.54E-07	6.28E-07	2.67E-07	2.58E-07	6.54E-07	6.31E-07	5.16E-07
RADIL	4.67E-08	3.59E-08	3.17E-08	6.85E-08	3.24E-08	3.46E-08	7.27E-08	1.83E-08
RAE1	3.11E-05	2.12E-05	8.41E-06	1.22E-05	1.16E-05	1.84E-05	1.75E-05	8.82E-06
RAET1E	2.16E-07	1.23E-07	4.70E-08	1.39E-08	5.87E-08	5.27E-08	6.44E-08	1.67E-08
RAET1G	7.22E-07	3.07E-08	1.16E-07	0	0	1.37E-07	1.01E-07	0
RAET1K	4.36E-07	5.81E-08	1.35E-08	0	0	0	1.24E-08	1.89E-08
RAET1L	0	0	0	0	0	0	0	0
RAF1	4.54E-05	8.50E-05	3.77E-05	3.89E-05	2.50E-05	4.70E-05	4.33E-05	4.90E-05
RAG1	7.48E-08	3.22E-07	3.12E-07	1.16E-07	1.46E-08	1.96E-06	2.98E-08	4.97E-08
RAG1AP1	8.03E-05	0.000276	2.55E-05	2.73E-05	1.61E-05	8.05E-05	1.91E-05	8.14E-05
RAG2	0	0	0	0	0	9.05E-09	0	0
RAGE	1.93E-06	7.87E-07	4.61E-07	1.93E-06	9.01E-07	1.55E-06	1.45E-06	4.66E-07
RAI1	3.19E-06	3.82E-06	1.06E-06	3.05E-06	5.88E-07	1.42E-06	2.01E-06	1.65E-06
RAI14	7.87E-06	1.43E-05	1.05E-05	8.05E-06	2.12E-06	6.50E-06	5.84E-06	6.90E-06
RAI2	4.35E-06	5.34E-07	1.54E-06	4.88E-06	4.86E-07	6.40E-07	1.62E-06	1.61E-06
RALA	2.66E-05	1.84E-05	8.91E-06	1.51E-05	5.08E-06	1.28E-05	1.26E-05	1.27E-05
RALB	5.79E-05	1.99E-05	1.43E-05	2.08E-05	6.00E-06	1.38E-05	1.50E-05	1.78E-05
RALBP1	2.34E-05	1.39E-05	1.28E-05	1.32E-05	2.65E-06	1.79E-05	1.12E-05	1.65E-05
RALGAPA1	4.07E-06	2.19E-06	1.78E-06	2.48E-06	6.37E-07	3.54E-06	2.20E-06	2.26E-06
RALGAPA2	6.16E-06	4.67E-06	6.69E-06	3.84E-06	1.17E-07	6.15E-06	1.56E-06	5.96E-06
RALGAPB	1.06E-05	6.66E-06	3.22E-06	3.43E-06	1.71E-06	4.92E-06	2.92E-06	3.12E-06
RALGDS	2.23E-05	4.80E-06	4.53E-06	2.59E-05	4.31E-06	5.97E-06	8.67E-06	2.46E-05
RALGPS1	1.98E-06	2.92E-07	1.01E-06	6.73E-07	4.64E-08	1.92E-06	2.86E-07	1.22E-06
RALGPS2	1.17E-05	1.16E-05	6.42E-06	5.09E-06	1.84E-06	1.43E-05	4.86E-06	4.47E-06
RALY	0.000118	9.45E-05	3.21E-05	5.42E-05	6.70E-05	8.32E-05	6.84E-05	4.42E-05
RALYL	0	1.76E-07	4.01E-08	1.33E-08	3.16E-08	0	0	3.57E-08
RAMP1	0.000187	0.000121	3.64E-05	8.20E-05	0.000213	2.73E-05	0.000193	0.000206
RAMP2	2.61E-05	2.32E-05	1.16E-05	1.16E-05	3.84E-05	1.22E-05	2.91E-05	3.22E-05
RAMP3	1.52E-05	4.47E-06	2.41E-05	1.89E-05	3.10E-05	1.44E-05	1.31E-05	1.45E-05
RAN	0.0003	0.000167	6.59E-05	0.000124	3.59E-05	0.000162	0.000136	9.24E-05
RANBP1	8.47E-05	7.00E-05	4.06E-05	4.97E-05	5.87E-05	5.80E-05	8.40E-05	4.68E-05
RANBP10	1.03E-05	7.84E-06	5.29E-06	5.06E-06	1.67E-06	8.57E-06	6.11E-06	1.14E-05
RANBP17	1.11E-06	0	2.60E-07	4.25E-07	4.70E-07	7.35E-07	9.31E-07	3.77E-08
RANBP2	1.22E-05	1.00E-05	6.91E-06	7.13E-06	1.40E-06	7.77E-06	3.53E-06	4.79E-06
RANBP3	2.08E-05	8.94E-06	9.28E-06	1.33E-05	1.05E-05	1.38E-05	1.36E-05	1.13E-05
RANBP3L	6.45E-08	6.23E-08	2.86E-06	5.79E-07	1.62E-07	8.95E-07	7.40E-07	1.35E-08
RANBP6	4.63E-06	9.63E-06	6.67E-06	6.84E-06	1.22E-06	8.58E-06	3.23E-06	6.12E-06
RANBP9	1.66E-05	3.16E-05	1.30E-05	1.44E-05	4.04E-06	1.88E-05	1.01E-05	2.10E-05
RANGAP1	8.53E-05	5.52E-05	3.37E-05	6.64E-05	0.000105	4.10E-05	6.43E-05	0.000124
RANGRF	1.54E-05	1.09E-05	9.64E-06	1.52E-05	3.90E-06	2.50E-05	2.77E-05	3.23E-06
RAP1A	4.76E-05	5.12E-05	3.82E-05	4.10E-05	1.83E-05	5.41E-05	2.87E-05	2.29E-05
RAP1B	4.70E-05	4.42E-05	3.38E-05	3.73E-05	1.34E-05	3.32E-05	3.99E-05	2.69E-05
RAP1GAP	2.26E-05	6.64E-06	6.60E-06	1.30E-05	1.22E-06	3.93E-06	9.98E-06	2.49E-05
RAP1GAP2	1.45E-06	9.76E-07	3.45E-07	1.47E-06	2.90E-08	9.68E-08	1.81E-07	9.13E-07
RAP1GDS1	1.06E-05	1.07E-05	8.52E-06	7.86E-06	3.16E-06	1.39E-05	1.02E-05	6.33E-06
RAP2A	1.68E-05	2.12E-05	4.01E-06	5.16E-06	1.71E-06	7.17E-06	3.88E-06	6.19E-06
RAP2B	4.64E-06	3.84E-06	2.11E-06	8.97E-06	8.59E-07	2.51E-06	7.11E-06	2.48E-06
RAP2C	2.06E-05	1.69E-05	1.85E-05	1.45E-05	1.18E-05	1.90E-05	9.41E-06	2.80E-05
RAPGEF1	1.86E-05	7.14E-06	7.15E-06	1.05E-05	2.98E-06	7.57E-06	1.01E-05	1.74E-05
RAPGEF2	1.19E-05	6.01E-06	9.19E-06	6.64E-06	9.99E-07	6.45E-06	5.00E-06	8.17E-06
RAPGEF3	1.02E-06	1.66E-06	5.15E-07	7.31E-07	1.60E-06	9.19E-07	1.36E-06	1.96E-06
RAPGEF4	1.02E-05	9.76E-06	7.35E-06	1.12E-05	1.95E-06	4.50E-06	4.39E-06	2.79E-06

RAPGEF5	1.85E-05	1.48E-05	3.33E-06	4.59E-06	4.63E-07	1.81E-06	3.46E-06	3.66E-06
RAPGEF6	9.93E-06	2.33E-06	1.36E-06	1.38E-06	2.15E-08	1.47E-06	2.40E-07	1.66E-06
RAPGEFL1	2.08E-06	5.25E-07	1.04E-06	5.72E-07	8.37E-07	2.80E-06	2.41E-06	2.01E-07
RAPH1	6.25E-05	1.66E-05	1.55E-05	6.02E-06	6.54E-06	5.05E-06	7.02E-06	8.62E-06
RAPSN	1.01E-07	3.63E-08	1.78E-08	8.21E-08	3.70E-07	1.37E-07	1.56E-07	2.47E-08
RARA	3.07E-05	3.44E-05	1.22E-05	3.02E-05	1.45E-05	1.45E-05	1.60E-05	2.05E-05
RARB	7.68E-06	9.63E-07	1.29E-06	3.17E-06	1.53E-07	3.84E-06	9.82E-07	1.72E-06
RARG	4.89E-06	1.33E-06	1.57E-06	2.80E-06	1.61E-06	1.31E-06	2.42E-06	1.45E-06
RARRES1	2.52E-05	9.44E-07	1.45E-06	1.42E-05	4.63E-07	3.19E-05	9.80E-06	1.01E-06
RARRES2	0.00024	0.001171	0.001309	0.001487	0.004778	0.001436	0.002603	0.001514
RARRES3	0.000115	5.16E-05	6.83E-05	7.82E-05	2.50E-05	0.000154	5.18E-05	8.76E-05
RARS	2.83E-05	1.49E-05	1.79E-05	2.18E-05	7.43E-06	3.69E-05	2.19E-05	2.83E-05
RARS2	1.30E-05	2.46E-05	1.33E-05	1.58E-05	4.02E-06	2.05E-05	1.22E-05	9.92E-06
RASA1	7.25E-06	5.40E-06	6.40E-06	6.05E-06	4.20E-07	5.96E-06	3.42E-06	4.71E-06
RASA2	1.56E-06	1.39E-06	9.85E-07	1.07E-06	0	9.77E-07	1.05E-07	5.04E-07
RASA3	1.96E-05	1.65E-06	3.14E-06	3.98E-06	3.65E-07	1.22E-06	2.38E-06	7.02E-07
RASA4	2.64E-06	1.39E-06	5.19E-07	8.88E-07	2.50E-07	7.97E-07	1.70E-06	7.85E-07
RASA4P	6.29E-06	8.00E-06	1.45E-06	1.39E-06	5.72E-07	8.15E-07	1.64E-06	1.85E-06
RASAL1	2.04E-06	0	1.19E-07	2.29E-07	0	1.91E-08	1.86E-08	1.23E-07
RASAL2	3.85E-06	9.98E-07	1.01E-06	1.94E-06	3.42E-07	3.35E-06	9.36E-07	1.63E-06
RASAL3	5.91E-06	7.77E-07	1.45E-06	3.42E-06	1.71E-06	2.07E-06	2.93E-06	1.77E-06
RASD1	1.54E-05	5.99E-05	9.00E-05	0.000142	4.08E-06	7.57E-06	0.000136	0.000122
RASD2	4.82E-07	3.93E-07	1.21E-07	1.12E-07	6.35E-07	4.54E-07	4.91E-07	1.35E-06
RASEF	9.66E-06	0	1.55E-07	1.89E-06	0	7.82E-09	2.37E-07	8.80E-07
RASGEF1A	6.09E-06	4.89E-06	1.32E-07	1.48E-07	3.06E-08	7.26E-08	3.46E-08	2.09E-08
RASGEF1B	3.06E-06	5.81E-06	1.54E-05	1.83E-05	6.14E-07	2.84E-06	5.76E-06	2.36E-05
RASGEF1C	0	1.57E-07	2.42E-08	2.83E-08	0	9.93E-09	0	0
RASGRF1	9.83E-08	1.04E-08	4.79E-09	9.95E-08	0	1.65E-05	9.09E-09	1.94E-08
RASGRF2	5.91E-06	7.37E-07	6.99E-08	4.58E-07	3.56E-07	5.69E-07	3.47E-07	8.51E-07
RASGRP1	2.19E-06	9.02E-08	3.40E-07	8.05E-07	0	5.12E-07	2.70E-07	4.10E-07
RASGRP2	2.65E-06	3.74E-07	1.60E-06	2.92E-06	7.60E-07	1.67E-06	1.75E-06	1.70E-06
RASGRP3	4.89E-06	2.92E-06	1.23E-06	1.86E-06	9.36E-07	1.26E-06	1.50E-06	4.89E-06
RASGRP4	7.37E-07	2.75E-07	8.07E-07	8.14E-07	1.06E-07	4.56E-07	8.80E-07	2.37E-07
RASIP1	3.39E-06	8.95E-06	2.15E-06	4.09E-06	9.35E-06	2.38E-06	9.44E-06	5.09E-06
RASL10A	9.51E-08	1.63E-07	8.84E-08	1.84E-07	2.87E-07	6.19E-08	2.59E-07	1.84E-07
RASL10B	2.00E-06	3.52E-06	8.88E-07	1.29E-06	1.22E-06	2.71E-05	1.14E-06	1.06E-08
RASL11A	2.90E-06	3.87E-06	4.36E-06	9.03E-06	1.45E-06	1.46E-06	3.78E-06	1.66E-06
RASL11B	3.08E-07	1.39E-08	2.58E-07	6.95E-07	8.48E-08	1.14E-08	1.31E-07	7.25E-08
RASL12	3.20E-06	1.02E-06	1.01E-07	4.14E-07	1.60E-06	8.29E-07	1.28E-06	2.03E-06
RASSF1	1.99E-05	1.23E-05	7.26E-06	1.79E-05	1.28E-05	9.86E-06	2.06E-05	1.25E-05
RASSF10	0	1.48E-08	1.37E-08	1.60E-08	0	0	1.26E-08	0
RASSF2	6.95E-06	6.09E-07	1.50E-06	5.05E-06	5.42E-07	1.20E-06	2.25E-06	9.19E-07
RASSF3	9.69E-06	6.02E-06	1.87E-06	5.10E-06	4.13E-08	2.09E-06	1.72E-06	3.84E-06
RASSF4	3.23E-05	2.34E-05	1.13E-05	2.77E-05	1.40E-05	3.85E-05	2.01E-05	2.62E-05
RASSF5	1.51E-05	3.16E-06	5.80E-06	9.31E-06	3.99E-07	3.56E-06	2.34E-06	1.45E-06
RASSF6	7.66E-06	6.08E-07	2.01E-07	1.73E-07	2.79E-08	1.88E-06	2.65E-08	3.74E-07
RASSF7	5.90E-05	3.33E-05	1.71E-05	3.25E-05	3.34E-05	1.95E-05	3.83E-05	6.62E-05
RASSF8	7.58E-06	1.55E-06	1.38E-06	2.77E-06	1.06E-07	7.71E-07	6.91E-07	1.18E-06
RASSF9	6.60E-06	3.58E-06	5.68E-07	4.97E-07	4.65E-08	8.77E-08	9.12E-08	1.41E-06
RAVER1	1.25E-05	6.67E-06	5.92E-06	9.25E-06	9.57E-06	6.62E-06	9.74E-06	8.42E-06
RAVER2	8.53E-06	1.98E-07	6.93E-07	8.89E-07	3.95E-07	2.21E-07	3.88E-07	4.53E-07
RAX	0	0	0	0	0	0	0	0
RAX2	0	0	0	0	0	0	0	0
RB1	1.52E-05	1.04E-05	4.60E-06	6.95E-06	8.98E-07	7.03E-06	4.95E-06	5.10E-06
RB1CC1	3.11E-05	2.45E-05	7.30E-06	6.56E-06	3.87E-06	1.56E-05	9.68E-06	1.76E-05
RBAK	4.53E-06	4.17E-06	1.83E-06	2.05E-06	6.30E-07	2.24E-06	1.11E-06	3.13E-06
RBBP4	2.16E-05	1.11E-05	7.95E-06	8.50E-06	6.32E-07	1.46E-05	6.31E-06	8.82E-06
RBBP5	5.80E-06	5.94E-06	5.03E-06	3.98E-06	3.52E-07	1.04E-05	2.10E-06	6.62E-06

RBBP6	1.80E-05	1.41E-05	7.78E-06	1.08E-05	3.60E-06	9.23E-06	6.57E-06	5.96E-06
RBBP7	6.81E-05	2.80E-05	2.85E-05	3.51E-05	1.74E-05	4.36E-05	3.09E-05	5.78E-05
RBBP8	2.06E-05	4.99E-06	4.55E-06	1.28E-05	1.41E-06	6.54E-06	5.98E-06	6.61E-06
RBBP9	9.69E-06	8.97E-06	1.55E-05	6.42E-06	5.56E-06	2.19E-05	5.88E-06	5.49E-06
RBCK1	0.000169	0.000128	4.07E-05	3.98E-05	4.40E-05	6.78E-05	5.79E-05	3.81E-05
RBKS	0.000136	4.79E-05	2.65E-05	2.26E-05	1.98E-05	3.61E-05	2.58E-05	1.66E-05
RBL1	2.73E-06	5.36E-07	2.95E-07	2.73E-07	9.13E-09	3.94E-07	1.50E-07	2.10E-07
RBL2	2.93E-05	3.13E-05	2.55E-05	1.76E-05	2.34E-06	2.13E-05	1.21E-05	1.25E-05
RBM10	1.97E-05	3.01E-05	1.46E-05	1.65E-05	1.55E-05	1.75E-05	2.03E-05	1.95E-05
RBM11	2.69E-08	4.15E-08	8.49E-08	8.98E-08	0	0	0	0
RBM12	1.26E-05	8.20E-06	5.44E-06	7.52E-06	1.92E-06	6.45E-06	4.76E-06	5.20E-06
RBM12B	5.56E-06	2.49E-06	2.01E-06	1.08E-06	4.10E-07	1.78E-06	9.42E-07	1.30E-06
RBM14	1.03E-05	9.39E-06	4.95E-06	5.65E-06	3.67E-06	7.79E-06	5.91E-06	4.89E-06
RBM15	2.90E-06	2.24E-06	1.73E-06	2.21E-06	7.34E-07	3.05E-06	1.69E-06	2.15E-06
RBM15B	1.08E-05	7.38E-06	3.96E-06	8.07E-06	4.38E-06	8.37E-06	8.62E-06	7.49E-06
RBM16	9.90E-06	8.03E-06	6.48E-06	6.79E-06	1.22E-06	7.72E-06	4.66E-06	5.17E-06
RBM17	2.33E-05	7.69E-06	9.27E-06	1.39E-05	7.54E-06	1.20E-05	1.65E-05	1.27E-05
RBM18	1.44E-05	4.84E-06	5.27E-06	6.20E-06	1.29E-06	1.02E-05	4.19E-06	9.28E-06
RBM19	8.77E-06	5.65E-06	3.85E-06	5.58E-06	3.71E-06	8.96E-06	6.24E-06	7.20E-06
RBM20	9.55E-08	4.81E-08	2.83E-08	2.25E-08	3.43E-07	7.83E-07	5.41E-08	3.15E-08
RBM22	2.66E-05	1.53E-05	1.09E-05	1.64E-05	8.26E-06	1.26E-05	1.77E-05	1.66E-05
RBM23	4.21E-05	3.68E-05	1.54E-05	1.78E-05	7.15E-06	1.81E-05	1.40E-05	2.10E-05
RBM24	5.54E-06	5.63E-06	2.93E-07	1.55E-06	1.37E-07	3.49E-06	7.64E-07	2.20E-06
RBM25	2.20E-05	6.94E-06	7.30E-06	1.05E-05	3.57E-06	1.09E-05	5.20E-06	9.11E-06
RBM26	1.12E-05	5.39E-06	4.71E-06	4.07E-06	1.04E-06	7.62E-06	3.82E-06	4.81E-06
RBM27	5.92E-06	5.32E-06	3.91E-06	4.10E-06	6.65E-07	5.06E-06	3.03E-06	4.71E-06
RBM28	6.57E-06	7.67E-06	3.37E-06	4.67E-06	4.16E-06	5.17E-06	4.23E-06	9.49E-06
RBM3	0.00012	5.83E-05	3.05E-05	8.74E-05	2.66E-05	5.48E-05	0.000144	0.000132
RBM33	9.54E-06	1.04E-05	5.15E-06	6.62E-06	3.16E-06	6.26E-06	4.73E-06	1.32E-05
RBM34	2.11E-05	1.46E-05	9.16E-06	1.44E-05	7.87E-06	2.38E-05	1.48E-05	2.44E-05
RBM38	1.13E-05	1.37E-05	4.97E-06	1.15E-05	2.47E-06	4.93E-06	9.47E-06	6.80E-06
RBM39	0.000129	4.53E-05	2.99E-05	3.71E-05	2.03E-05	4.52E-05	4.21E-05	4.91E-05
RBM4	0.000137	5.79E-05	3.42E-05	6.09E-05	5.16E-05	6.17E-05	5.74E-05	6.52E-05
RBM41	8.67E-06	5.64E-06	2.78E-06	1.97E-06	1.34E-06	5.49E-06	2.08E-06	5.70E-06
RBM42	5.27E-05	6.77E-05	3.13E-05	4.92E-05	0.00011	5.38E-05	7.57E-05	3.73E-05
RBM43	5.79E-06	2.19E-06	2.07E-06	1.44E-06	9.50E-08	2.80E-06	8.04E-07	1.66E-06
RBM44	1.01E-07	1.78E-08	3.81E-08	2.59E-08	9.10E-09	3.93E-08	3.61E-08	2.33E-08
RBM45	9.50E-06	5.47E-06	3.17E-06	3.47E-06	1.24E-06	5.21E-06	3.03E-06	4.15E-06
RBM46	0	0	3.25E-08	2.28E-08	8.00E-09	5.89E-08	2.45E-08	1.85E-08
RBM47	3.01E-05	5.04E-05	1.68E-05	1.87E-05	1.11E-05	1.82E-05	2.08E-05	1.23E-05
RBM4B	8.47E-06	5.40E-06	4.12E-06	4.02E-06	2.34E-06	6.18E-06	4.51E-06	5.03E-06
RBM5	3.93E-05	1.79E-05	1.28E-05	1.91E-05	1.57E-05	1.69E-05	1.97E-05	1.53E-05
RBM6	1.99E-05	1.42E-05	7.78E-06	1.48E-05	2.17E-05	1.27E-05	1.39E-05	1.45E-05
RBM7	2.19E-05	7.23E-06	1.36E-05	1.66E-05	2.79E-06	1.57E-05	8.24E-06	1.40E-05
RBM8A	4.23E-05	2.32E-05	1.63E-05	2.03E-05	1.09E-05	3.04E-05	2.39E-05	1.73E-05
RBM9	1.44E-05	6.39E-06	4.31E-06	4.94E-06	4.58E-06	7.19E-06	6.22E-06	8.26E-06
RBMS1	1.50E-05	3.17E-06	7.08E-06	1.35E-05	2.09E-06	6.86E-06	7.57E-06	7.50E-06
RBMS2	6.88E-06	1.03E-06	9.58E-07	1.91E-06	6.22E-07	7.97E-07	1.40E-06	1.01E-06
RBMS3	1.45E-06	1.14E-07	2.54E-07	8.12E-07	2.91E-08	8.44E-08	1.15E-07	3.72E-08
RBMX	7.19E-05	4.50E-05	2.33E-05	4.35E-05	2.28E-05	3.94E-05	3.63E-05	3.25E-05
RBMX2	1.95E-05	8.51E-06	7.24E-06	9.88E-06	1.13E-05	8.95E-06	1.66E-05	1.37E-05
RBMXL1	8.28E-06	9.62E-06	6.69E-06	5.40E-06	6.33E-07	4.27E-06	3.23E-06	3.40E-06
RBMXL2	3.21E-08	6.05E-08	2.24E-08	3.93E-08	0	9.93E-09	0	0
RBMXL3	0	0	0	0	0	0	0	0
RBMY1A1	0	0	0	0	0	0	0	4.70E-07
RBMY1A3	0	0	0	0	0	0	0	0
RBMY1B	0	0	0	0	0	0	0	0
RBMY1E	0	0	0	0	0	0	0	0

RBMY1F	0	0	0	0	0	0	0	0
RBMY1J	0	0	0	0	0	0	0	1.37E-06
RBMY2EP	0	0	0	0	0	0	0	0
RBMY2FP	0	1.33E-08	0	0	0	0	0	3.45E-08
RBMY3AP	0	0	0	0	0	0	0	0
RBP1	8.54E-05	3.37E-07	4.82E-05	0.000123	1.08E-05	0.001182	3.77E-06	7.49E-06
RBP2	0	5.19E-08	0	4.34E-07	3.79E-08	4.08E-08	0	0
RBP3	5.31E-09	0	0	0	0	0	5.17E-09	0
RBP4	0.008124	0.012244	0.011083	0.009003	0.011788	0.006131	0.009981	0.009019
RBP5	0.00011	7.83E-05	0.000188	0.000135	0.00023	8.12E-05	0.000249	1.99E-05
RBP7	2.23E-05	4.88E-05	5.16E-06	6.23E-06	3.07E-05	5.27E-05	5.10E-05	3.33E-05
RBPJ	1.01E-05	6.03E-06	4.58E-06	5.06E-06	5.05E-06	5.81E-06	1.13E-05	5.23E-06
RBPJL	9.43E-09	0	0	0	0	0	0	0
RBPMS	6.18E-05	3.45E-05	1.12E-05	4.79E-05	1.23E-05	4.48E-05	3.07E-05	1.60E-05
RBPMS2	9.50E-06	2.33E-05	2.98E-06	4.66E-06	6.81E-06	1.43E-06	5.76E-06	1.15E-06
RBX1	0.000169	8.89E-05	7.71E-05	0.000109	0.000146	0.000114	0.000129	0.000118
RC3H1	3.36E-06	2.31E-06	2.67E-06	2.62E-06	9.29E-07	2.63E-06	2.13E-06	3.42E-06
RC3H2	5.89E-06	1.44E-06	3.45E-06	3.27E-06	5.46E-07	4.04E-06	1.66E-06	4.29E-06
RCAN1	5.33E-05	7.20E-05	0.000342	8.08E-05	2.17E-05	8.57E-05	5.78E-05	4.53E-05
RCAN2	8.88E-06	8.84E-07	1.85E-06	3.03E-06	1.44E-06	6.96E-07	1.54E-06	4.73E-06
RCAN3	4.98E-06	5.44E-07	5.08E-07	1.39E-06	6.93E-08	2.21E-07	5.38E-07	4.40E-07
RCBTB1	6.34E-06	4.79E-06	3.11E-06	3.94E-06	1.03E-06	7.93E-06	2.98E-06	3.96E-06
RCBTB2	4.50E-06	2.37E-06	4.18E-06	4.63E-06	1.55E-06	2.43E-06	2.86E-06	1.99E-06
RCC1	1.81E-05	1.08E-05	6.34E-06	8.45E-06	5.28E-06	1.17E-05	7.11E-06	1.51E-05
RCC2	2.43E-05	1.07E-05	5.23E-06	1.23E-05	3.85E-06	8.76E-06	9.37E-06	1.01E-05
RCCD1	4.78E-06	6.22E-06	3.21E-06	3.09E-06	3.27E-06	6.72E-06	2.89E-06	2.43E-06
RCE1	1.31E-05	1.28E-05	3.51E-06	8.60E-06	3.28E-06	2.37E-06	5.89E-06	1.38E-05
RCHY1	5.02E-06	2.49E-06	3.88E-06	3.36E-06	6.61E-07	3.12E-06	3.20E-06	3.78E-06
RCL1	2.36E-05	5.32E-05	7.77E-05	4.45E-05	2.27E-05	0.000221	6.05E-05	6.96E-05
RCN1	5.50E-05	0.000108	3.74E-05	4.09E-05	3.20E-05	3.39E-05	4.38E-05	5.77E-05
RCN2	3.09E-05	1.59E-05	6.45E-06	1.05E-05	3.59E-06	2.38E-05	1.00E-05	1.04E-05
RCN3	9.19E-06	2.38E-06	4.62E-06	1.61E-05	9.84E-06	2.46E-06	1.14E-05	4.30E-06
RCOR1	1.01E-05	3.79E-06	3.79E-06	4.10E-06	1.26E-06	6.23E-06	3.32E-06	4.46E-06
RCOR2	1.81E-07	8.13E-08	2.81E-08	9.94E-08	6.21E-08	8.36E-09	0	2.65E-08
RCOR3	9.03E-06	7.76E-06	4.39E-06	4.52E-06	1.94E-06	8.87E-06	3.96E-06	7.31E-06
RCSD1	8.37E-06	1.60E-06	4.05E-06	4.36E-06	5.23E-07	2.63E-06	3.61E-06	1.91E-06
RCVRN	0	0	4.75E-08	8.13E-08	3.80E-08	0	0	0
RD3	0	6.45E-09	0	7.04E-09	0	0	0	0
RDBP	5.60E-05	7.32E-05	1.91E-05	2.79E-05	3.07E-05	2.63E-05	2.82E-05	4.49E-05
RDH10	0.000139	3.83E-05	2.01E-05	2.97E-05	1.66E-05	1.91E-05	4.75E-05	4.32E-05
RDH11	0.00015	8.13E-05	8.17E-05	4.14E-05	5.46E-05	6.29E-05	4.49E-05	9.84E-05
RDH12	1.57E-05	1.75E-06	1.03E-06	1.15E-06	1.01E-05	1.07E-07	1.56E-06	1.74E-06
RDH13	1.44E-05	1.15E-05	3.34E-06	4.18E-06	4.07E-06	3.45E-06	1.00E-05	1.13E-05
RDH14	3.27E-05	2.25E-05	1.14E-05	1.28E-05	9.10E-06	1.77E-05	1.59E-05	1.23E-05
RDH16	4.25E-05	0.000162	0.000295	8.61E-05	0.000551	0.000162	0.000122	6.88E-05
RDH5	5.58E-05	1.62E-05	7.46E-05	4.31E-05	7.29E-05	4.42E-05	5.32E-05	1.98E-06
RDH8	2.08E-06	0	0	0	0	0	1.37E-08	0
RDM1	2.23E-06	2.57E-06	3.08E-08	1.03E-07	1.39E-06	7.33E-08	1.65E-07	3.79E-07
RDX	3.92E-05	2.69E-05	3.74E-05	2.52E-05	5.79E-06	2.93E-05	1.43E-05	6.88E-05
REC8	2.79E-06	1.26E-07	7.10E-07	1.00E-06	5.94E-07	2.97E-07	3.70E-07	4.91E-07
RECK	3.19E-06	3.75E-07	4.58E-06	2.88E-06	2.28E-07	4.01E-07	5.32E-07	7.02E-07
RECQL	1.48E-05	9.96E-06	3.22E-06	4.96E-06	5.84E-07	8.23E-06	3.07E-06	4.76E-06
RECQL4	9.99E-06	4.37E-06	2.16E-07	3.96E-07	5.39E-06	5.95E-07	4.47E-06	1.13E-06
RECQL5	2.26E-05	1.92E-05	5.02E-06	7.03E-06	2.02E-05	2.31E-05	1.25E-05	7.63E-06
REEP1	4.68E-06	4.26E-08	4.68E-07	4.39E-07	3.23E-07	4.69E-08	2.59E-07	2.13E-07
REEP2	1.11E-06	2.06E-07	1.19E-08	8.35E-08	3.61E-07	2.11E-07	1.21E-07	8.36E-08
REEP3	1.54E-05	1.16E-05	1.13E-05	6.54E-06	5.15E-06	7.03E-06	6.46E-06	1.22E-05
REEP4	1.21E-05	1.29E-05	4.09E-06	7.71E-06	4.73E-06	5.71E-06	7.95E-06	4.40E-06

REEP5	6.73E-05	4.93E-05	5.69E-05	5.64E-05	3.89E-05	5.58E-05	5.10E-05	7.26E-05
REEP6	0.000162	0.000374	0.000429	0.000221	0.000165	0.000138	0.00025	0.000368
REG1A	0	8.74E-08	0	0	3.22E-08	2.01E-05	3.10E-07	0
REG1B	0	2.32E-08	0	0	0	3.29E-08	0	0
REG1P	0	0	0	0	0	1.35E-08	0	0
REG3A	0	0	0	0	0	0.004006	0	1.05E-07
REG3G	0	0	0	0	0	5.38E-08	0	0
REG4	9.80E-08	8.11E-07	0	0	0	7.00E-08	1.81E-08	1.39E-07
REL	3.48E-06	1.64E-06	9.19E-07	1.07E-06	9.70E-08	8.39E-07	2.73E-07	3.73E-07
RELA	4.47E-05	4.24E-05	2.86E-05	3.28E-05	1.08E-05	3.36E-05	3.23E-05	3.61E-05
RELB	1.59E-05	9.45E-06	2.97E-06	2.20E-05	3.02E-06	5.24E-06	1.48E-05	2.02E-05
RELL1	3.11E-06	2.91E-06	1.42E-06	4.34E-06	1.43E-06	1.18E-06	3.30E-06	2.51E-06
RELL2	3.34E-06	4.17E-06	4.14E-07	5.42E-07	2.89E-07	3.08E-07	2.43E-07	1.53E-06
RELN	1.03E-05	5.44E-06	8.88E-06	7.71E-06	6.21E-08	2.43E-05	3.22E-06	1.65E-05
RELT	4.19E-06	6.14E-07	7.75E-07	2.57E-06	1.50E-07	6.35E-07	1.23E-06	8.31E-07
REM1	7.10E-07	1.01E-07	1.25E-07	1.27E-07	1.10E-06	3.16E-07	3.56E-07	5.24E-07
REM2	9.72E-07	2.94E-07	4.07E-08	1.72E-07	4.47E-08	0	4.71E-08	0
REN	0	3.78E-06	3.25E-07	2.09E-07	1.33E-06	4.11E-07	2.12E-07	1.00E-07
RENBP	7.52E-06	3.84E-06	4.39E-06	1.03E-05	1.98E-06	2.96E-06	9.67E-06	2.80E-06
REP15	6.16E-07	1.05E-06	7.41E-07	7.60E-07	7.30E-07	2.19E-06	2.29E-06	5.08E-07
REPIN1	3.34E-05	3.99E-05	2.49E-05	2.34E-05	2.81E-05	4.66E-05	4.20E-05	5.85E-05
REPS1	4.40E-06	6.89E-06	9.35E-06	8.60E-06	3.56E-06	7.47E-06	1.40E-05	3.40E-06
REPS2	2.44E-06	9.22E-07	4.44E-06	2.52E-06	1.34E-06	2.23E-06	2.74E-06	4.30E-06
RER1	7.10E-05	6.32E-05	4.87E-05	4.50E-05	3.72E-05	6.61E-05	5.51E-05	6.21E-05
RERE	2.07E-05	7.21E-06	6.32E-06	7.87E-06	3.28E-06	1.20E-05	5.31E-06	4.64E-06
RERG	7.24E-06	4.73E-07	6.32E-07	1.49E-06	1.49E-07	4.80E-07	1.13E-06	3.02E-06
RERGL	6.33E-07	2.24E-07	1.21E-07	1.07E-06	2.67E-06	1.54E-07	1.83E-07	1.23E-06
RESP18	0	0	0	0	0	0	0	0
REST	3.79E-06	2.31E-06	2.11E-06	1.52E-06	4.10E-08	2.63E-06	2.93E-07	1.49E-06
RET	8.76E-08	2.79E-07	4.66E-07	2.54E-07	1.34E-06	3.50E-08	1.11E-06	9.97E-09
RETN	7.45E-08	1.96E-07	0	6.65E-07	0	1.42E-07	0	0
RETNLB	0	2.06E-07	0	0	0	0	0	6.88E-07
RETSAT	0.000106	3.49E-05	8.77E-05	7.17E-05	3.35E-05	5.47E-05	5.05E-05	5.84E-05
REV1	8.97E-06	4.30E-06	4.33E-06	4.77E-06	1.78E-06	5.24E-06	3.81E-06	3.17E-06
REV3L	1.03E-05	4.24E-06	5.93E-06	8.14E-06	1.48E-06	1.18E-05	4.69E-06	5.22E-06
REXO1	1.24E-05	4.23E-06	6.72E-06	1.16E-05	7.92E-06	1.01E-05	1.25E-05	1.10E-05
REXO1L1	0	0	0	0	0	0	3.25E-09	0
REXO2	4.31E-05	1.87E-05	2.31E-05	3.70E-05	1.13E-05	5.81E-05	2.24E-05	4.20E-05
REXO4	1.91E-05	1.41E-05	8.18E-06	9.63E-06	1.62E-05	1.44E-05	1.59E-05	1.68E-05
RFC1	2.59E-05	3.01E-05	8.63E-06	8.23E-06	5.30E-06	2.19E-05	9.74E-06	1.14E-05
RFC2	3.25E-05	2.36E-05	1.03E-05	1.30E-05	7.80E-06	1.31E-05	1.51E-05	2.06E-05
RFC3	9.53E-06	6.98E-06	1.84E-06	2.15E-06	5.69E-07	3.68E-06	2.10E-06	2.27E-06
RFC4	1.94E-05	1.23E-05	3.36E-06	4.42E-06	6.19E-06	5.66E-06	6.58E-06	5.08E-06
RFC5	1.24E-05	1.23E-05	3.84E-06	3.54E-06	1.38E-06	4.72E-06	3.06E-06	4.14E-06
RFESD	1.02E-06	1.01E-06	6.85E-07	3.29E-07	1.80E-07	9.82E-07	3.45E-07	6.59E-07
RFFL	2.13E-05	2.85E-05	1.53E-05	1.78E-05	4.21E-06	2.49E-05	1.02E-05	1.98E-05
RFK	1.24E-05	1.29E-05	1.25E-05	1.30E-05	5.15E-06	8.75E-06	1.31E-05	1.65E-05
RFNG	3.24E-05	8.46E-05	4.14E-05	2.91E-05	5.56E-05	5.92E-05	6.39E-05	2.51E-05
RFPL1	0	8.24E-08	9.09E-07	2.85E-07	0	4.40E-07	0	1.89E-08
RFPL1S	6.61E-08	1.30E-08	2.46E-08	6.47E-08	0	9.52E-07	5.15E-08	7.93E-09
RFPL2	1.16E-07	1.15E-06	9.13E-08	1.06E-07	4.29E-08	1.60E-08	6.60E-08	9.74E-08
RFPL3	2.62E-08	5.70E-08	0	0	0	0	3.91E-08	0
RFPL3S	1.87E-07	1.14E-07	0	5.25E-08	2.76E-07	0	1.14E-07	1.58E-07
RFPL4A	0	3.53E-08	2.01E-07	4.87E-07	2.62E-07	9.03E-07	8.61E-08	1.81E-07
RFPL4B	7.35E-07	1.58E-07	0	0	0	0	1.22E-08	3.70E-06
RFT1	8.56E-06	5.70E-06	2.91E-06	3.47E-06	2.34E-06	4.67E-06	4.01E-06	4.85E-06
RFTN1	8.45E-05	6.12E-06	2.03E-05	2.27E-05	8.86E-06	6.67E-06	2.53E-05	2.12E-05
RFTN2	7.87E-07	1.09E-06	4.37E-07	4.54E-07	8.29E-08	1.99E-07	3.67E-07	3.81E-07

RFWD2	3.98E-05	4.59E-05	1.25E-05	1.51E-05	1.37E-05	2.26E-05	1.88E-05	2.57E-05
RFWD3	9.68E-06	3.63E-06	2.79E-06	3.24E-06	8.32E-07	3.74E-06	2.01E-06	2.89E-06
RFX1	3.25E-06	2.20E-06	1.84E-06	3.81E-06	1.25E-06	2.91E-06	2.93E-06	1.62E-06
RFX2	2.37E-06	5.89E-07	3.48E-07	3.77E-06	2.57E-07	1.69E-07	3.78E-07	4.05E-07
RFX3	4.86E-07	3.59E-07	1.18E-07	3.09E-07	3.74E-08	2.76E-07	1.27E-07	1.17E-07
RFX4	6.83E-09	7.67E-09	0	5.01E-08	0	0	0	0
RFX5	1.68E-05	1.11E-05	3.66E-06	7.29E-06	1.50E-06	1.47E-05	4.61E-06	1.05E-05
RFX6	2.96E-07	0	0	0	0	7.91E-08	0	0
RFX7	2.61E-06	1.22E-06	1.38E-06	1.14E-06	6.30E-07	1.39E-06	8.71E-07	7.47E-07
RFX8	5.63E-08	8.00E-08	2.97E-08	0	1.33E-07	2.62E-08	4.07E-08	1.25E-07
RFXANK	2.34E-05	2.01E-05	1.32E-05	2.00E-05	3.72E-05	2.49E-05	3.32E-05	2.18E-05
RFXAP	1.31E-06	4.07E-07	7.51E-07	5.74E-07	1.77E-07	7.24E-07	4.55E-07	2.54E-07
RG9MTD1	1.57E-05	3.91E-05	1.50E-05	1.74E-05	8.26E-06	2.03E-05	1.81E-05	2.70E-05
RG9MTD2	2.13E-06	1.45E-06	2.30E-06	1.49E-06	6.31E-07	4.29E-06	1.31E-06	1.97E-06
RG9MTD3	2.76E-06	2.49E-06	1.43E-06	1.75E-06	2.58E-07	1.83E-06	1.40E-06	1.82E-06
RGAG1	2.07E-08	1.94E-07	4.24E-08	4.55E-08	3.56E-09	1.53E-08	6.86E-08	3.88E-07
RGAG4	1.34E-06	7.66E-06	1.57E-06	1.88E-06	1.21E-07	1.64E-06	6.92E-07	2.25E-07
RGL1	6.61E-06	2.66E-06	5.05E-06	5.73E-06	8.71E-07	4.74E-06	2.88E-06	2.12E-06
RGL2	1.05E-05	1.49E-05	7.75E-06	1.16E-05	1.03E-05	1.47E-05	1.17E-05	8.19E-06
RGL3	1.61E-05	4.42E-06	2.76E-06	4.72E-06	2.62E-06	2.88E-06	5.00E-06	5.52E-06
RGL4	6.15E-07	5.28E-08	1.12E-07	6.48E-07	0	7.64E-08	1.19E-07	1.21E-07
RGMA	2.05E-07	2.90E-07	8.64E-08	4.72E-07	9.13E-08	3.73E-07	5.38E-07	4.56E-07
RGMB	2.65E-06	1.12E-06	2.80E-06	2.33E-06	5.08E-07	1.99E-05	1.70E-06	1.75E-06
RGN	6.38E-05	0.000215	0.000203	0.000158	7.15E-05	0.000176	0.000124	5.45E-05
RGNEF	1.05E-05	1.68E-06	2.79E-06	5.04E-06	7.91E-07	1.01E-05	5.37E-06	9.81E-07
RGP1	3.40E-06	1.07E-05	2.57E-06	2.20E-06	1.52E-07	3.83E-06	1.68E-06	4.44E-06
RGPD1	3.63E-06	3.32E-06	1.87E-06	1.85E-06	2.05E-06	1.48E-06	5.16E-07	1.21E-06
RGPD3	2.89E-06	1.45E-06	1.01E-06	9.60E-07	1.28E-07	1.08E-06	4.43E-07	6.64E-07
RGPD4	1.76E-06	6.00E-07	5.12E-07	7.61E-07	1.99E-07	6.35E-07	3.18E-07	4.19E-07
RGPD5	7.34E-07	4.98E-07	3.69E-07	1.21E-07	5.34E-08	2.26E-07	2.12E-07	8.64E-08
RGPD6	5.84E-06	1.78E-06	1.72E-06	1.65E-06	8.28E-07	1.43E-06	9.29E-07	8.34E-07
RGPD8	3.48E-53	3.65E-49	5.34E-35	#######	3.74E-50	4.18E-08	4.05E-28	9.47E-37
RGR	0	0	0	0	0	0	0	0
RGS1	7.17E-05	2.99E-06	2.67E-05	9.51E-05	3.08E-07	1.75E-05	2.01E-05	9.85E-06
RGS10	4.70E-05	3.88E-06	4.44E-06	1.25E-05	2.75E-06	6.77E-06	1.59E-05	4.15E-06
RGS11	3.69E-07	0	1.80E-07	4.57E-07	1.60E-07	2.00E-08	2.22E-07	1.59E-08
RGS12	1.30E-05	1.55E-05	8.52E-06	8.30E-06	1.06E-05	1.75E-05	9.68E-06	1.14E-05
RGS13	5.14E-08	0	9.11E-08	2.32E-07	0	4.78E-08	0	1.01E-07
RGS14	7.48E-05	2.71E-05	3.76E-05	2.84E-05	2.15E-05	3.39E-05	3.78E-05	6.55E-05
RGS16	1.60E-06	1.12E-06	2.03E-06	8.29E-06	4.24E-07	2.39E-06	4.48E-06	3.57E-06
RGS17	5.13E-08	0	0	2.10E-08	0	1.59E-08	3.29E-08	0
RGS18	2.36E-06	4.39E-07	1.23E-06	8.57E-07	3.82E-08	7.10E-07	6.55E-07	4.08E-07
RGS19	1.51E-05	7.98E-07	5.22E-06	7.58E-06	2.20E-06	3.84E-06	8.67E-06	2.51E-06
RGS2	0.000221	3.70E-06	1.23E-05	0.000168	1.44E-06	7.63E-06	1.27E-05	9.57E-06
RGS20	4.93E-06	1.85E-08	0	0	1.40E-08	0	2.76E-08	0
RGS21	0	0	0	0	0	0	0	0
RGS22	0	0	0	1.17E-08	0	0	1.71E-08	1.41E-08
RGS3	3.35E-05	2.81E-05	1.69E-05	6.31E-05	3.55E-05	1.92E-05	6.29E-05	2.07E-05
RGS4	1.38E-06	5.80E-07	7.22E-07	1.93E-05	1.69E-07	4.56E-07	2.06E-07	8.08E-07
RGS5	3.00E-05	3.22E-05	4.52E-06	4.59E-06	8.70E-06	1.45E-05	1.37E-05	3.36E-05
RGS6	0	8.33E-09	1.20E-08	3.12E-08	3.02E-08	1.09E-08	3.02E-08	3.60E-08
RGS7	3.85E-08	4.56E-08	4.73E-08	0	0	0	0	5.89E-08
RGS7BP	3.07E-07	2.95E-07	1.85E-07	2.27E-07	5.14E-09	5.93E-09	3.11E-08	8.35E-08
RGS8	0	0	0	0	0	0	0	0
RGS9	1.07E-05	1.10E-07	7.29E-07	5.83E-07	1.33E-06	4.49E-08	1.43E-07	3.98E-07
RGS9BP	7.22E-08	5.41E-08	0	0	6.89E-09	3.72E-08	3.11E-08	0
RGSL1	0	1.61E-08	5.91E-08	1.22E-07	0	3.27E-06	2.78E-08	2.20E-07
RHAG	1.34E-08	0	0	1.65E-08	0	0	0	0

RHBDD1	1.10E-05	5.82E-06	5.37E-06	4.20E-06	1.16E-06	7.95E-06	2.90E-06	9.18E-06
RHBDD2	4.33E-05	0.00018	4.33E-05	6.67E-05	4.21E-05	3.52E-05	6.21E-05	0.000105
RHBDD3	1.11E-05	1.95E-05	8.46E-06	9.60E-06	3.31E-05	1.36E-05	1.73E-05	2.00E-05
RHBDF1	9.47E-06	6.93E-06	1.32E-06	6.78E-06	1.43E-06	1.28E-06	6.01E-06	3.30E-06
RHBDF2	1.17E-05	1.07E-05	3.12E-06	9.70E-06	1.23E-06	7.25E-06	8.59E-06	6.63E-06
RHBDL1	1.62E-06	3.96E-07	3.19E-07	1.65E-07	2.93E-07	1.44E-06	8.85E-07	3.08E-07
RHBDL2	8.75E-07	2.83E-07	1.61E-07	4.20E-07	5.99E-08	9.64E-08	1.35E-07	2.05E-07
RHBDL3	1.29E-06	1.13E-08	5.10E-08	6.06E-09	1.29E-08	2.21E-06	2.46E-08	2.96E-08
RHBG	4.11E-07	1.28E-07	6.29E-07	3.82E-06	8.28E-07	7.48E-05	1.39E-06	4.00E-08
RHCE	2.13E-07	2.75E-06	5.95E-07	4.46E-07	3.86E-07	1.28E-06	2.61E-07	6.60E-07
RHCG	1.57E-06	1.63E-07	1.06E-06	1.22E-07	5.62E-08	7.20E-08	3.78E-08	4.60E-07
RHD	5.22E-08	1.40E-07	0	5.43E-08	0	5.24E-08	0	3.61E-08
RHEB	2.67E-05	4.53E-05	1.36E-05	1.73E-05	1.28E-05	2.65E-05	2.29E-05	3.50E-05
RHEBL1	8.03E-07	4.08E-07	1.79E-07	1.26E-07	3.46E-07	6.59E-07	2.94E-07	3.13E-07
RHO	1.68E-08	9.47E-09	0	2.06E-08	1.45E-08	0	0	4.94E-08
RHOA	0.000245	0.000201	0.00014	0.000185	0.000145	0.000193	0.000189	0.000167
RHOB	0.000189	0.000947	0.000727	0.000991	0.000165	0.000306	0.000738	0.000315
RHOBTB1	4.86E-06	6.15E-07	2.92E-06	5.79E-06	6.43E-07	2.68E-05	5.00E-06	7.61E-07
RHOBTB2	3.21E-06	3.58E-06	1.01E-06	1.26E-06	5.63E-07	3.59E-06	1.61E-06	2.83E-06
RHOBTB3	1.91E-05	1.17E-05	1.19E-05	1.06E-05	2.59E-06	1.02E-05	8.01E-06	1.48E-05
RHOC	0.000313	0.000175	8.05E-05	0.000236	0.000126	0.000198	0.000244	0.000187
RHOD	5.10E-05	5.99E-05	4.25E-05	7.30E-05	6.28E-05	3.17E-05	8.63E-05	3.33E-05
RHOF	3.99E-05	9.70E-06	8.64E-07	2.29E-06	7.77E-08	6.04E-07	1.15E-06	3.69E-07
RHOG	5.31E-05	3.27E-05	2.27E-05	4.29E-05	2.06E-05	3.27E-05	3.92E-05	5.14E-05
RHOH	3.82E-06	9.49E-08	1.22E-06	4.04E-06	1.44E-07	1.28E-06	9.26E-07	1.15E-06
RHOJ	2.87E-06	2.43E-06	9.55E-07	1.75E-06	6.42E-07	1.04E-06	2.77E-06	1.90E-06
RHOQ	1.87E-05	2.24E-05	4.82E-06	9.56E-06	1.85E-06	4.93E-06	6.45E-06	4.99E-06
RHOT1	1.67E-05	1.58E-05	7.03E-06	5.63E-06	2.39E-06	7.15E-06	5.39E-06	5.94E-06
RHOT2	5.62E-05	4.16E-05	1.75E-05	2.15E-05	1.56E-05	2.48E-05	2.73E-05	2.39E-05
RHOU	4.99E-05	4.15E-05	1.67E-05	2.98E-05	7.62E-06	3.11E-05	1.82E-05	2.39E-05
RHOV	6.02E-07	4.88E-08	1.21E-07	2.22E-06	1.23E-08	1.33E-08	8.29E-08	0
RHOXF1	1.15E-07	1.36E-07	0	0	7.32E-07	3.59E-08	0	0
RHOXF2B	1.96E-08	3.80E-07	0	0	0	0	0	0
RHPN1	3.47E-06	5.86E-07	1.90E-07	3.84E-07	2.80E-07	2.74E-07	1.01E-06	8.30E-07
RHPN2	1.98E-05	1.71E-05	1.16E-05	1.19E-05	2.71E-06	1.10E-05	8.14E-06	1.30E-05
RIBC1	9.90E-07	3.01E-07	3.98E-07	3.35E-07	1.80E-07	4.07E-07	1.89E-07	3.31E-07
RIBC2	4.87E-07	3.51E-07	1.92E-08	1.33E-07	1.24E-07	3.36E-08	1.13E-06	8.00E-08
RIC3	3.46E-08	0	4.56E-07	2.79E-07	0	5.88E-08	4.16E-09	1.25E-08
RIC8A	4.03E-05	3.16E-05	1.37E-05	2.21E-05	9.77E-06	3.38E-05	2.13E-05	3.44E-05
RIC8B	9.46E-06	3.06E-06	1.26E-06	5.22E-06	6.40E-07	2.07E-06	1.68E-06	3.06E-06
RICH2	3.16E-07	8.66E-08	2.43E-07	4.83E-07	9.19E-08	1.21E-07	1.82E-07	1.08E-06
RICTOR	4.52E-06	3.88E-06	2.82E-06	2.32E-06	4.46E-07	2.70E-06	1.35E-06	3.22E-06
RIF1	5.20E-06	3.83E-06	2.52E-06	1.97E-06	6.70E-08	2.24E-06	5.13E-07	1.67E-06
RILP	1.16E-05	2.15E-05	1.40E-05	2.34E-05	2.02E-05	8.28E-06	4.54E-05	1.74E-05
RILPL1	4.62E-06	2.32E-06	1.68E-06	2.34E-06	1.73E-06	2.62E-06	4.99E-06	4.35E-06
RILPL2	1.32E-05	7.93E-06	7.39E-06	1.75E-05	6.90E-06	1.19E-05	8.60E-06	7.33E-06
RIMBP2	3.23E-08	8.40E-07	5.44E-08	4.36E-08	0	1.36E-06	0	0
RIMBP3	5.63E-07	2.60E-08	6.75E-08	4.73E-08	4.66E-08	5.38E-08	5.67E-08	7.37E-08
RIMBP3C	6.99E-08	0	0	0	6.65E-09	1.15E-08	1.13E-08	6.08E-09
RIMKLA	3.91E-07	4.42E-08	3.19E-08	7.14E-08	1.25E-08	1.16E-08	4.49E-08	9.16E-09
RIMKLB	2.76E-06	4.28E-07	4.79E-07	1.67E-06	4.54E-07	4.35E-07	1.33E-06	5.22E-07
RIMS1	0	1.36E-08	0	0	0	0	7.80E-09	0
RIMS2	1.28E-07	6.42E-08	4.42E-09	1.05E-08	0	3.99E-09	0	0
RIMS3	3.56E-07	4.52E-08	1.21E-07	1.45E-07	2.94E-08	3.14E-08	1.16E-07	1.18E-07
RIMS4	8.78E-09	0	0	2.68E-08	0	0	3.85E-08	6.42E-09
RIN1	3.05E-06	6.80E-07	4.82E-07	3.95E-06	1.65E-06	8.83E-07	1.16E-06	9.71E-07
RIN2	9.93E-06	4.26E-06	3.31E-06	6.55E-06	6.63E-07	4.47E-06	4.02E-06	2.66E-06
RIN3	1.23E-05	6.44E-06	9.05E-06	8.63E-06	7.73E-06	6.34E-06	1.62E-05	1.43E-05

RING1	4.30E-05	5.32E-05	2.12E-05	2.64E-05	2.87E-05	4.37E-05	2.74E-05	3.95E-05
RINL	9.54E-06	4.64E-06	3.51E-06	4.87E-06	2.83E-06	2.25E-06	6.17E-06	1.95E-06
RINT1	4.18E-06	1.02E-05	4.70E-06	3.90E-06	2.66E-06	6.60E-06	4.87E-06	1.07E-05
RIOK1	6.40E-06	5.90E-06	3.52E-06	5.93E-06	2.76E-06	7.08E-06	7.44E-06	7.64E-06
RIOK2	7.12E-06	4.82E-06	3.78E-06	4.39E-06	1.17E-06	6.76E-06	4.54E-06	8.25E-06
RIOK3	4.80E-05	3.84E-05	2.74E-05	2.30E-05	9.96E-06	2.79E-05	2.37E-05	1.68E-05
RIPK1	1.46E-05	1.51E-05	9.78E-06	1.36E-05	5.73E-06	1.28E-05	1.35E-05	1.70E-05
RIPK2	3.57E-05	1.04E-05	2.92E-06	6.97E-06	2.62E-06	5.64E-06	1.11E-05	8.53E-06
RIPK3	7.18E-06	2.39E-07	6.50E-07	2.05E-06	2.17E-07	6.52E-07	9.43E-07	5.10E-07
RIPK4	1.13E-05	3.46E-06	1.30E-05	2.24E-05	3.19E-06	1.16E-05	6.94E-06	1.27E-05
RIPPLY1	5.50E-07	1.33E-06	1.29E-06	3.04E-06	1.85E-06	9.84E-06	1.60E-06	2.60E-06
RIPPLY2	1.69E-06	0	0	0	0	0	0	0
RIT1	8.42E-06	5.90E-06	2.26E-06	4.63E-06	1.08E-06	4.68E-06	3.63E-06	5.56E-06
RIT2	0	0	2.61E-08	2.96E-08	0	0	0	0
RLBP1	0	0	0	0	0	0	0	0
RLF	4.45E-06	3.09E-06	2.11E-06	3.13E-06	3.69E-07	2.67E-06	1.59E-06	2.21E-06
RLIM	5.40E-06	3.56E-06	2.55E-06	3.46E-06	9.00E-07	4.08E-06	2.29E-06	3.87E-06
RLN1	5.57E-08	0	3.06E-08	0	0	0	0	0
RLN2	7.23E-08	0	4.08E-08	8.99E-08	0	0	2.94E-08	0
RLN3	0	0	0	0	0	0	0	0
RLTPR	8.83E-07	2.26E-08	8.31E-08	3.44E-07	1.55E-08	1.83E-07	1.75E-07	1.08E-07
RMI1	5.50E-06	4.00E-06	1.83E-06	1.73E-06	7.63E-07	4.27E-06	9.33E-07	2.66E-06
RMND1	2.26E-05	1.85E-05	1.54E-05	1.22E-05	7.96E-06	2.87E-05	2.21E-05	9.30E-06
RMND5A	2.60E-05	2.84E-05	2.49E-05	1.72E-05	6.11E-06	1.50E-05	1.59E-05	1.70E-05
RMND5B	1.60E-05	1.13E-05	1.23E-05	1.15E-05	8.37E-06	1.28E-05	1.39E-05	2.35E-05
RMRP	5.67E-06	2.60E-06	2.25E-06	1.91E-05	2.00E-06	2.37E-05	1.70E-05	5.52E-06
RMST	1.68E-07	5.47E-08	0	0	0	0	0	0
RNASE1	7.26E-05	3.01E-05	3.52E-05	7.61E-05	0.000108	0.000205	0.00018	6.80E-05
RNASE10	3.97E-07	0	5.49E-08	0	0	1.85E-07	8.78E-08	0
RNASE11	0	0	0	0	0	0	0	0
RNASE12	0	0	0	0	0	0	0	0
RNASE13	9.18E-08	0	1.62E-08	1.88E-08	2.64E-08	1.42E-08	4.42E-08	2.26E-08
RNASE2	3.98E-07	0	4.09E-07	8.89E-07	3.46E-08	4.85E-07	4.12E-07	0
RNASE3	4.11E-08	0	9.44E-08	5.13E-08	0	0	3.87E-08	0
RNASE4	0.00017	7.90E-05	0.000291	0.000328	0.000257	0.000233	0.000233	0.000209
RNASE6	2.30E-05	3.83E-06	6.51E-06	1.47E-05	3.79E-06	6.41E-06	1.37E-05	3.93E-06
RNASE7	5.31E-06	3.05E-08	1.45E-07	3.06E-07	0	3.08E-08	1.12E-07	5.89E-07
RNASE8	0	0	0	0	0	0	0	0
RNASE9	0	0	0	0	0	0	0	0
RNASEH1	1.38E-05	1.32E-05	5.06E-06	4.85E-06	3.77E-06	1.13E-05	5.94E-06	8.24E-06
RNASEH2/	3.53E-05	1.55E-05	4.11E-06	6.20E-06	2.16E-05	1.06E-05	1.25E-05	1.16E-05
RNASEH2I	1.30E-05	1.52E-05	4.59E-06	8.54E-06	5.82E-06	9.18E-06	6.70E-06	5.53E-06
RNASEH2(	1.28E-05	5.92E-06	7.88E-06	1.10E-05	1.34E-05	8.58E-06	6.30E-06	1.18E-05
RNASEK	0.000185	0.000134	0.000138	0.000179	0.000135	0.000206	0.000291	0.000186
RNASEL	4.03E-06	6.87E-07	1.45E-06	1.21E-06	1.60E-07	2.94E-06	6.84E-07	1.49E-06
RNASEN	8.67E-06	7.07E-06	3.04E-06	4.08E-06	1.76E-06	6.70E-06	3.64E-06	6.69E-06
RNASET2	0.000145	2.28E-05	2.56E-05	5.60E-05	1.75E-05	2.60E-05	5.59E-05	2.55E-05
RND1	9.25E-05	0.000135	6.24E-05	0.000256	3.06E-05	1.91E-05	5.08E-05	9.71E-05
RND2	1.62E-06	2.02E-06	1.54E-06	2.74E-07	1.08E-07	7.10E-08	4.79E-08	3.21E-07
RND3	1.42E-05	1.05E-05	6.89E-05	9.88E-05	9.93E-06	1.14E-05	3.36E-05	5.31E-05
RNF10	4.01E-05	4.87E-05	2.86E-05	4.02E-05	1.73E-05	5.00E-05	4.45E-05	3.90E-05
RNF103	5.66E-05	2.32E-05	2.75E-05	2.73E-05	1.67E-05	1.65E-05	3.31E-05	5.22E-05
RNF11	3.42E-05	3.36E-05	3.04E-05	2.94E-05	6.03E-06	2.86E-05	1.91E-05	2.30E-05
RNF111	5.95E-06	4.31E-06	4.39E-06	4.33E-06	1.46E-06	5.27E-06	3.52E-06	3.37E-06
RNF112	1.31E-07	1.05E-07	6.71E-08	2.56E-07	1.36E-07	4.82E-08	2.17E-07	1.27E-07
RNF113A	1.54E-05	1.01E-05	8.99E-06	9.48E-06	1.67E-05	1.40E-05	1.16E-05	2.19E-05
RNF113B	3.65E-08	0	0	0	0	0	1.75E-08	0
RNF114	7.02E-05	3.02E-05	2.79E-05	2.40E-05	1.64E-05	3.44E-05	2.53E-05	1.90E-05

RNF115	1.66E-05	1.35E-05	7.68E-06	1.15E-05	1.10E-06	1.49E-05	6.97E-06	7.83E-06
RNF121	1.04E-05	1.09E-05	5.90E-06	7.28E-06	5.30E-06	9.91E-06	6.53E-06	1.21E-05
RNF122	2.91E-06	2.11E-06	2.10E-06	1.01E-05	6.12E-07	1.40E-06	2.10E-06	1.71E-06
RNF123	1.96E-05	1.85E-05	1.12E-05	1.12E-05	1.10E-05	2.05E-05	1.46E-05	1.38E-05
RNF125	1.15E-06	1.28E-06	7.11E-06	7.97E-06	1.40E-07	2.17E-06	2.56E-06	2.89E-06
RNF126	3.45E-05	2.03E-05	1.43E-05	2.52E-05	1.85E-05	1.91E-05	4.60E-05	1.58E-05
RNF126P1	2.11E-07	4.40E-07	8.23E-08	0	1.49E-07	1.43E-07	5.53E-08	2.85E-08
RNF128	6.90E-05	5.24E-05	5.91E-05	3.52E-05	5.75E-05	4.18E-05	2.30E-05	2.67E-05
RNF13	5.38E-05	6.96E-05	2.92E-05	3.19E-05	1.76E-05	2.55E-05	2.54E-05	2.73E-05
RNF130	6.56E-05	5.60E-05	0.000101	8.12E-05	9.69E-05	5.46E-05	8.68E-05	0.000134
RNF133	3.46E-08	0	1.84E-08	4.25E-08	0	0	0	2.56E-08
RNF135	1.50E-05	1.10E-06	2.78E-06	6.19E-06	6.98E-06	1.69E-06	4.98E-06	1.27E-06
RNF138	6.85E-06	5.48E-06	6.03E-06	4.73E-06	1.64E-06	4.06E-06	5.09E-06	4.83E-06
RNF138P1	1.97E-07	7.03E-08	1.09E-07	6.73E-08	3.38E-09	5.46E-08	3.07E-08	4.61E-08
RNF139	4.62E-05	1.60E-05	1.18E-05	1.20E-05	7.35E-06	1.43E-05	2.25E-05	1.43E-05
RNF14	2.24E-05	2.11E-05	1.83E-05	1.25E-05	6.57E-06	1.78E-05	1.79E-05	2.81E-05
RNF141	1.40E-05	6.73E-06	7.13E-06	7.79E-06	3.31E-06	9.76E-06	5.11E-06	1.02E-05
RNF144A	5.36E-06	4.37E-07	6.79E-07	5.07E-07	1.33E-07	1.99E-06	1.05E-06	5.98E-07
RNF144B	2.26E-06	3.42E-06	8.23E-06	7.04E-06	2.33E-06	4.76E-06	4.44E-06	2.71E-06
RNF145	1.52E-05	5.51E-06	5.59E-06	1.06E-05	1.31E-06	5.52E-06	6.81E-06	7.31E-06
RNF146	1.55E-05	1.27E-05	9.73E-06	1.31E-05	3.67E-06	1.35E-05	1.54E-05	1.07E-05
RNF148	1.95E-08	6.70E-08	6.26E-08	4.80E-08	5.04E-08	7.25E-08	3.16E-08	4.33E-07
RNF149	4.14E-05	1.86E-05	1.62E-05	2.33E-05	6.68E-06	1.59E-05	1.96E-05	2.03E-05
RNF150	2.91E-08	1.12E-08	1.39E-07	1.46E-07	4.28E-09	9.24E-09	4.86E-08	0
RNF151	3.57E-08	0	0	0	0	0	0	0
RNF152	4.62E-06	4.05E-06	2.82E-06	4.40E-06	5.89E-07	4.16E-06	2.49E-06	4.71E-06
RNF157	9.32E-06	2.17E-06	2.76E-07	5.58E-07	7.23E-08	5.30E-06	2.64E-06	1.00E-06
RNF160	5.21E-06	3.17E-06	3.58E-06	2.10E-06	4.80E-07	4.07E-06	1.17E-06	2.59E-06
RNF165	4.25E-08	1.51E-08	2.13E-06	4.83E-07	4.60E-08	3.48E-07	9.87E-08	2.65E-08
RNF166	9.17E-06	3.61E-06	5.82E-06	7.44E-06	3.08E-06	5.81E-06	7.14E-06	7.10E-06
RNF167	6.44E-05	8.40E-05	6.94E-05	8.10E-05	0.000215	0.000114	0.000111	6.26E-05
RNF168	4.12E-06	4.21E-06	3.24E-06	1.96E-06	6.37E-08	2.52E-06	9.54E-07	2.06E-06
RNF169	3.08E-06	1.66E-06	1.39E-06	1.66E-06	1.12E-07	1.73E-06	6.34E-07	1.68E-06
RNF17	1.60E-08	0	0	7.25E-09	0	0	0	0
RNF170	5.16E-06	8.93E-06	8.17E-06	5.81E-06	3.62E-06	9.63E-06	3.48E-06	5.18E-06
RNF175	2.87E-07	1.79E-08	5.00E-08	2.12E-07	0	6.38E-08	9.09E-08	2.32E-08
RNF180	4.52E-07	1.12E-07	2.10E-06	1.36E-06	8.62E-08	2.20E-07	6.27E-07	1.06E-06
RNF181	0.000164	0.000185	8.71E-05	0.00013	0.000187	0.000166	0.000159	0.000115
RNF182	4.40E-08	3.30E-08	7.37E-09	5.38E-08	0	6.80E-09	1.07E-07	2.15E-08
RNF183	9.53E-08	2.45E-08	2.29E-08	5.24E-08	3.67E-08	3.96E-08	8.29E-08	0
RNF185	3.06E-05	2.15E-05	2.20E-05	1.97E-05	2.30E-05	3.02E-05	3.07E-05	3.43E-05
RNF186	1.55E-06	2.35E-07	6.15E-07	2.52E-08	1.77E-08	3.81E-08	1.18E-07	0
RNF187	3.46E-05	6.31E-05	2.65E-05	3.08E-05	2.77E-05	5.12E-05	4.54E-05	6.97E-05
RNF19A	9.55E-05	2.58E-05	1.13E-05	1.95E-05	4.84E-06	1.02E-05	1.97E-05	1.98E-05
RNF19B	1.34E-05	5.25E-06	4.28E-06	1.03E-05	1.01E-06	4.39E-06	7.37E-06	7.14E-06
RNF2	4.65E-06	5.68E-06	2.03E-06	3.54E-06	8.66E-07	3.90E-06	2.65E-06	2.95E-06
RNF20	1.82E-05	1.29E-05	7.71E-06	7.51E-06	4.21E-06	2.03E-05	5.77E-06	9.35E-06
RNF207	9.60E-06	2.56E-06	1.35E-06	2.83E-06	2.96E-07	2.84E-06	9.44E-07	3.92E-06
RNF208	8.17E-06	1.10E-05	8.87E-06	6.17E-06	1.62E-05	9.16E-06	6.70E-06	1.30E-05
RNF212	7.89E-08	0	3.96E-08	1.68E-07	0	4.41E-08	6.16E-08	5.15E-08
RNF213	5.11E-05	3.19E-05	1.48E-05	2.00E-05	9.64E-06	3.47E-05	2.04E-05	1.85E-05
RNF214	5.87E-06	3.03E-06	2.87E-06	3.78E-06	1.59E-06	5.70E-06	2.58E-06	5.21E-06
RNF215	1.02E-05	4.63E-06	2.76E-06	5.32E-06	7.40E-06	7.74E-06	8.09E-06	1.05E-05
RNF216	1.21E-05	1.24E-05	4.46E-06	4.12E-06	3.85E-06	7.11E-06	6.95E-06	8.46E-06
RNF216L	1.24E-05	6.47E-06	3.82E-06	5.62E-06	5.09E-06	6.23E-06	6.10E-06	1.11E-05
RNF217	2.96E-06	2.10E-06	3.02E-06	2.22E-06	9.11E-08	9.86E-06	1.19E-06	1.25E-06
RNF219	1.75E-05	3.09E-06	1.53E-06	2.17E-06	6.31E-07	1.74E-06	2.94E-06	2.17E-06
RNF220	3.11E-05	1.65E-05	1.48E-05	2.04E-05	1.47E-05	2.95E-05	2.51E-05	2.79E-05

RNF222	0	2.54E-08	2.34E-08	9.22E-09	0	6.99E-09	0	0
RNF24	2.42E-06	5.53E-07	2.65E-07	7.85E-07	2.53E-07	2.78E-07	3.74E-07	4.40E-07
RNF25	1.73E-05	9.66E-06	7.56E-06	1.46E-05	1.34E-05	1.45E-05	1.25E-05	1.30E-05
RNF26	3.50E-05	1.55E-05	1.27E-05	1.31E-05	1.23E-05	2.49E-05	1.36E-05	2.16E-05
RNF31	1.35E-05	8.00E-06	6.63E-06	6.59E-06	9.49E-06	9.32E-06	1.07E-05	1.28E-05
RNF32	9.85E-07	2.25E-06	7.15E-07	8.47E-07	1.13E-06	8.43E-07	8.05E-07	6.97E-07
RNF34	2.35E-05	1.04E-05	5.11E-06	9.36E-06	3.12E-06	1.05E-05	8.38E-06	8.77E-06
RNF38	5.15E-06	4.67E-06	5.08E-06	4.91E-06	1.06E-06	6.04E-06	3.31E-06	3.35E-06
RNF39	9.97E-07	1.10E-07	1.37E-07	1.74E-07	0	2.26E-08	1.18E-08	1.08E-07
RNF4	2.82E-05	2.18E-05	9.28E-06	1.27E-05	2.76E-06	1.89E-05	1.27E-05	1.92E-05
RNF40	4.87E-05	8.59E-05	2.19E-05	2.74E-05	1.07E-05	3.10E-05	2.99E-05	2.17E-05
RNF41	1.48E-05	8.41E-06	5.62E-06	7.44E-06	2.31E-06	8.11E-06	7.20E-06	7.85E-06
RNF43	7.68E-06	3.88E-06	6.30E-06	3.75E-06	2.66E-06	3.22E-05	6.24E-06	1.75E-06
RNF44	1.51E-05	5.13E-06	4.39E-06	5.47E-06	1.43E-06	8.84E-06	5.26E-06	5.22E-06
RNF5	0.000104	0.000166	8.52E-05	7.83E-05	6.62E-05	5.63E-05	0.000114	8.44E-05
RNF5P1	1.85E-05	2.38E-05	1.75E-05	1.11E-05	6.89E-06	8.97E-06	1.35E-05	1.39E-05
RNF6	1.36E-05	8.86E-06	6.22E-06	7.17E-06	1.38E-06	7.57E-06	5.54E-06	7.40E-06
RNF7	6.25E-05	2.47E-05	2.49E-05	2.83E-05	2.01E-05	3.42E-05	3.14E-05	3.00E-05
RNF8	4.01E-06	3.71E-06	1.37E-06	2.07E-06	1.10E-06	2.75E-06	2.49E-06	4.02E-06
RNFT1	3.72E-05	2.86E-05	1.21E-05	9.63E-06	2.95E-06	1.07E-05	7.06E-06	5.76E-06
RNFT2	3.25E-07	4.32E-07	1.93E-08	1.44E-07	1.59E-07	1.88E-07	1.98E-07	1.21E-07
RNGTT	5.83E-06	6.35E-06	3.22E-06	3.29E-06	1.02E-06	6.31E-06	2.95E-06	3.45E-06
RNH1	9.46E-05	9.72E-05	7.56E-05	9.94E-05	5.10E-05	9.99E-05	8.40E-05	0.000111
RNLS	7.11E-06	4.98E-06	4.32E-06	2.71E-06	1.99E-06	4.49E-06	2.46E-06	3.78E-06
RNMT	7.40E-06	4.04E-06	2.84E-06	3.07E-06	5.85E-07	5.74E-06	3.52E-06	4.21E-06
RNMTL1	1.06E-05	9.53E-06	1.19E-05	1.37E-05	1.61E-05	2.32E-05	1.87E-05	1.57E-05
RNPC3	4.59E-06	1.80E-06	1.07E-06	1.47E-06	7.60E-07	2.59E-06	1.19E-06	9.25E-07
RNPEP	4.77E-05	2.09E-05	2.28E-05	3.34E-05	1.28E-05	5.61E-05	3.21E-05	5.09E-05
RNPEPL1	7.86E-05	3.04E-05	2.46E-05	3.67E-05	8.83E-05	4.31E-05	6.47E-05	6.88E-05
RNPS1	6.35E-05	4.89E-05	2.51E-05	4.43E-05	1.58E-05	3.98E-05	4.71E-05	3.56E-05
RNU11	0	0	0	0	0	1.05E-05	0	0
RNU4ATA	0	0	0	0	0	0	0	0
RNU5E	0	0	0	0	0	0	0	0
RNU6ATA	0	0	0	0	0	0	0	0
RNY4	0	0	0	0	0	0	0	0
RNY5	0	0	0	0	0	0	0	0
ROBLD3	8.21E-05	0.000178	9.62E-05	9.63E-05	0.000235	0.000227	0.000165	0.000154
ROBO1	1.10E-05	7.18E-06	1.16E-06	2.79E-06	1.62E-07	6.72E-06	5.68E-07	1.48E-05
ROBO2	3.59E-08	1.88E-08	4.40E-08	4.38E-09	2.85E-07	1.46E-07	6.54E-08	3.67E-08
ROBO3	1.03E-06	8.56E-07	7.17E-07	2.27E-06	3.85E-07	1.19E-06	4.19E-06	1.56E-06
ROBO4	4.77E-06	6.31E-06	4.35E-06	3.53E-06	5.47E-06	4.12E-06	1.53E-05	3.78E-06
ROCK1	1.58E-05	6.69E-06	5.71E-06	6.20E-06	1.05E-06	6.83E-06	3.87E-06	5.57E-06
ROCK2	6.87E-06	8.19E-06	4.53E-06	3.66E-06	1.38E-07	1.13E-05	1.20E-06	2.72E-06
ROD1	1.95E-05	8.72E-06	5.81E-06	1.01E-05	2.52E-06	8.09E-06	7.53E-06	7.25E-06
ROGDI	1.07E-05	2.30E-05	1.95E-05	1.88E-05	1.47E-05	4.09E-05	2.30E-05	2.27E-05
ROM1	1.47E-06	1.47E-06	6.75E-07	1.99E-06	1.32E-06	1.37E-06	8.94E-07	1.93E-06
ROMO1	0.000474	0.00033	0.00018	0.000216	0.000961	0.000217	0.000246	0.000205
ROPN1	5.19E-07	4.56E-07	3.81E-08	7.57E-08	0	0	8.03E-08	0
ROPN1B	4.78E-06	4.72E-06	2.71E-07	2.53E-07	9.28E-08	8.60E-08	4.30E-07	1.00E-07
ROPN1L	0	2.94E-08	1.94E-07	2.51E-07	6.59E-08	4.03E-07	2.18E-07	1.89E-07
ROR1	6.57E-07	3.05E-08	5.61E-08	1.55E-07	0	7.99E-08	1.98E-08	7.63E-08
ROR2	5.73E-07	6.85E-08	9.15E-08	8.22E-07	1.62E-07	7.21E-08	5.14E-07	4.72E-07
RORA	4.51E-06	4.47E-06	7.80E-06	4.59E-06	7.37E-07	8.48E-06	2.35E-06	9.90E-06
RORB	2.54E-08	0	0	4.81E-08	6.53E-09	0	3.09E-08	0
RORC	2.27E-05	1.96E-05	5.06E-05	4.35E-05	2.27E-05	9.19E-05	4.27E-05	6.05E-05
ROS1	6.67E-09	1.12E-08	0	7.37E-09	0	6.16E-09	6.49E-09	4.82E-08
RP1	3.94E-08	7.37E-09	0	0	0	0	6.12E-09	9.18E-09
RP1-177G	0	0	0	0	0	0	0	0

RP1L1	1.12E-08	6.21E-09	1.14E-08	6.80E-09	0	3.35E-08	1.09E-08	8.14E-09
RP2	9.22E-06	4.75E-06	5.56E-06	6.84E-06	3.38E-07	3.36E-06	2.37E-06	7.18E-06
RP9	9.03E-06	6.51E-06	2.43E-06	4.87E-06	1.11E-05	4.74E-06	1.05E-05	8.01E-06
RP9P	8.27E-06	4.43E-06	1.55E-06	3.85E-06	7.34E-06	3.24E-06	6.28E-06	5.68E-06
RPA1	1.48E-05	1.36E-05	1.02E-05	1.03E-05	5.63E-06	1.39E-05	9.38E-06	1.01E-05
RPA2	4.63E-05	1.36E-05	1.26E-05	1.49E-05	8.05E-06	2.12E-05	1.60E-05	1.88E-05
RPA3	5.06E-05	6.07E-05	1.49E-05	2.31E-05	2.83E-05	4.04E-05	2.75E-05	2.65E-05
RPA4	3.48E-08	1.99E-08	3.71E-08	0	0	8.09E-08	1.67E-08	1.03E-07
RPAIN	9.79E-06	1.01E-05	9.71E-06	1.77E-05	6.38E-06	1.69E-05	1.81E-05	1.18E-05
RPAP1	1.20E-05	7.05E-06	6.28E-06	5.03E-06	6.22E-06	1.07E-05	8.29E-06	7.74E-06
RPAP2	3.69E-06	3.46E-06	1.97E-06	2.21E-06	4.14E-07	4.90E-06	1.69E-06	1.77E-06
RPAP3	6.17E-06	7.74E-06	3.88E-06	3.97E-06	1.50E-06	5.61E-06	3.02E-06	4.23E-06
RPE	2.48E-05	1.69E-05	1.01E-05	7.26E-06	2.40E-06	1.30E-05	8.06E-06	7.83E-06
RPE65	0	0	0	0	0	3.32E-08	0	0
RPF1	2.28E-05	2.24E-05	1.04E-05	1.32E-05	6.91E-06	2.47E-05	1.16E-05	1.28E-05
RPF2	3.21E-05	3.47E-05	1.54E-05	2.52E-05	1.41E-05	2.47E-05	3.11E-05	3.38E-05
RPGR	1.46E-05	5.64E-07	7.09E-07	2.36E-06	2.13E-07	1.75E-06	1.38E-06	1.97E-06
RPGRIP1	1.26E-07	6.53E-07	1.67E-07	3.86E-07	3.71E-08	9.73E-07	1.12E-07	6.20E-08
RPGRIP1L	1.15E-06	1.45E-07	7.72E-08	3.97E-07	1.13E-07	5.90E-07	3.45E-07	2.02E-07
RPH3A	5.45E-08	3.05E-08	1.12E-08	3.99E-08	4.67E-09	2.52E-08	1.06E-08	0
RPH3AL	6.13E-06	4.04E-06	9.42E-06	1.44E-05	9.41E-06	7.62E-06	1.23E-05	9.50E-06
RPIA	8.02E-06	7.09E-06	3.18E-06	5.23E-06	2.67E-06	6.60E-06	7.26E-06	4.78E-06
RPL10	0.000355	0.000199	0.000161	0.000279	0.000751	0.000305	0.000507	0.00035
RPL10A	0.000636	0.000471	0.00043	0.000633	0.000689	0.000875	0.000845	0.000516
RPL10L	7.00E-08	1.65E-07	0	0	0	0	0	0
RPL11	0.001775	0.000865	0.000995	0.001556	0.00136	0.00135	0.001442	0.000983
RPL12	0.000785	0.000368	0.000523	0.000852	0.001033	0.000822	0.000817	0.000629
RPL13	0.000813	0.000293	0.000376	0.000748	0.000999	0.000695	0.001196	0.000506
RPL13A	0.000653	0.000302	0.000402	0.000618	0.001184	0.000673	0.000602	0.000418
RPL13AP1	0	0	0	0	0	0	0	0
RPL13AP2	3.63E-06	8.29E-07	2.52E-06	3.74E-06	6.31E-06	2.68E-06	2.24E-06	1.88E-06
RPL13AP3	5.07E-07	3.28E-07	2.87E-07	2.49E-07	1.38E-06	2.51E-07	3.25E-07	4.25E-07
RPL13AP6	1.63E-06	3.92E-07	3.81E-07	7.58E-07	7.33E-07	4.39E-07	1.22E-06	1.41E-07
RPL13P5	2.69E-06	2.11E-06	4.72E-07	8.14E-07	0	5.65E-07	5.12E-07	1.90E-06
RPL14	0.00054	0.000409	0.000331	0.000428	0.000533	0.000451	0.000616	0.000314
RPL15	0.000335	0.000222	0.000211	0.000337	0.000321	0.000355	0.000426	0.000211
RPL17	0.00059	0.000196	0.000216	0.000372	0.00026	0.000462	0.000421	0.000294
RPL18	0.001109	0.000723	0.000691	0.000875	0.002499	0.000961	0.001345	0.000612
RPL18A	0.000193	0.000109	0.000116	0.00017	0.000304	0.000144	0.000262	0.0001
RPL19	0.002205	0.001714	0.000921	0.001399	0.001762	0.00147	0.001704	0.001055
RPL19P12	1.77E-05	2.59E-06	6.63E-06	1.84E-06	3.27E-06	1.43E-06	4.15E-06	9.20E-07
RPL21	1.89E-06	2.82E-07	5.78E-07	2.91E-07	2.34E-06	5.45E-08	2.03E-06	5.57E-07
RPL21P44	4.08E-08	0	7.17E-08	3.34E-08	1.17E-08	2.53E-08	5.25E-08	0
RPL22	0.000133	7.67E-05	8.16E-05	0.000114	0.000106	0.000128	0.000126	6.00E-05
RPL22L1	1.82E-05	6.63E-06	4.84E-06	1.34E-05	4.83E-05	9.80E-06	5.65E-05	1.38E-05
RPL23	0.001617	0.000827	0.00064	0.000851	0.001581	0.001007	0.00143	0.00068
RPL23A	0.000221	0.000158	8.47E-05	0.000162	0.000221	0.000148	0.000277	1.00E-04
RPL23AP3	0	0	0	0	1.32E-07	0	0	0
RPL23AP5	5.77E-07	1.34E-06	5.98E-07	9.03E-07	5.08E-08	1.00E-06	3.17E-07	4.31E-07
RPL23AP6	2.93E-07	1.18E-07	1.73E-07	6.13E-08	4.28E-08	0	1.37E-07	2.97E-07
RPL23AP7	6.13E-06	3.13E-06	1.31E-06	2.80E-06	1.40E-06	1.43E-06	2.29E-06	2.61E-06
RPL23AP8	6.57E-06	6.04E-06	2.58E-06	3.36E-06	2.45E-06	5.75E-06	4.02E-06	3.07E-06
RPL23P8	1.99E-05	2.40E-05	1.84E-05	1.40E-05	2.66E-05	1.37E-05	6.07E-06	1.12E-05
RPL24	0.00103	0.001258	0.000693	0.000889	0.001569	0.000906	0.001296	0.000608
RPL26	0.000743	0.000497	0.000437	0.001077	0.000624	0.001095	0.001421	0.000567
RPL26L1	3.71E-05	2.55E-05	1.66E-05	2.25E-05	2.29E-05	3.46E-05	3.20E-05	3.46E-05
RPL27	0.002095	0.001739	0.000466	0.000783	0.002389	0.001991	0.001565	0.000704
RPL27A	9.23E-05	5.98E-05	5.05E-05	9.83E-05	0.000206	0.000102	0.000112	9.37E-05

RPL28	0.000508	0.000126	0.000195	0.000408	0.001023	0.000269	0.000653	0.000313
RPL29	0.001004	0.00061	0.000615	0.000784	0.001799	0.000853	0.001083	0.000556
RPL29P2	9.44E-08	0	5.53E-08	0	1.65E-07	0	0	0
RPL3	0.001371	0.000567	0.001198	0.001483	0.001632	0.001739	0.001467	0.001187
RPL30	0.004078	0.002343	0.001213	0.00159	0.003489	0.001756	0.004118	0.001426
RPL31	0.002317	0.001063	0.001301	0.002359	0.003237	0.002054	0.002146	0.001315
RPL31P11	3.83E-07	7.28E-08	2.37E-07	1.57E-07	8.26E-08	1.04E-07	1.54E-08	7.08E-08
RPL32	0.000424	0.000336	0.000207	0.000394	0.000732	0.000401	0.000596	0.000223
RPL32P3	2.61E-06	1.99E-06	2.42E-07	1.62E-06	4.00E-07	1.88E-06	8.03E-07	1.11E-06
RPL34	0.000393	0.00014	0.000204	0.000396	0.00057	0.000412	0.000557	0.000299
RPL35	0.001384	0.000764	0.00085	0.001188	0.002459	0.001067	0.001287	0.001287
RPL35A	0.00131	0.000603	0.000525	0.000846	0.001378	0.000847	0.001043	0.0006
RPL36	0.001449	0.000662	0.00065	0.000991	0.003837	0.001054	0.001682	0.000667
RPL36A	1.24E-05	1.22E-06	6.89E-06	9.04E-07	2.21E-05	1.75E-06	2.07E-05	1.50E-06
RPL36AL	0.000517	0.000274	0.000426	0.00049	0.000705	0.000447	0.000588	0.000423
RPL37	0.000409	0.000275	0.000165	0.000353	0.000389	0.000389	0.000481	0.000311
RPL37A	0.003563	0.001675	0.001449	0.002302	0.004363	0.002478	0.002995	0.001499
RPL38	0.001395	0.001422	0.000566	0.001008	0.002119	0.001408	0.001364	0.000719
RPL39	0.000106	9.30E-05	4.57E-05	5.45E-05	0.00024	6.37E-05	9.09E-05	5.48E-05
RPL39L	2.99E-06	1.74E-07	1.70E-06	1.02E-05	4.20E-07	3.35E-07	2.97E-06	1.48E-05
RPL3L	0	1.48E-06	0	0	0	0	0	0
RPL4	0.000907	0.000365	0.000479	0.000665	0.000693	0.000923	0.000951	0.000483
RPL41	0.002956	0.001426	0.00106	0.002321	0.003504	0.002178	0.002889	0.001155
RPL5	0.000826	0.000613	0.000423	0.000675	0.000594	0.001097	0.000652	0.000455
RPL6	0.000528	0.000328	0.000206	0.000376	0.000343	0.000468	0.000532	0.000281
RPL7	0.000142	6.18E-05	5.40E-05	8.47E-05	7.43E-05	7.99E-05	0.000178	6.62E-05
RPL7A	0.000925	0.000367	0.000471	0.000769	0.001017	0.000974	0.000909	0.000792
RPL7L1	3.78E-05	6.04E-05	2.75E-05	2.46E-05	9.41E-06	4.13E-05	2.32E-05	3.19E-05
RPL8	0.002827	0.001302	0.000645	0.001382	0.004039	0.001089	0.003224	0.001047
RPL9	1.46E-05	0.000248	6.43E-06	0.000121	0.000141	1.05E-05	9.55E-06	6.16E-06
RPLP0	0.00093	0.000593	0.000477	0.000713	0.001726	0.00105	0.000912	0.00056
RPLP0P2	9.76E-07	6.56E-08	3.55E-07	7.15E-08	1.56E-07	7.83E-08	1.01E-07	3.81E-08
RPLP1	0.002479	0.00178	0.001413	0.002201	0.008043	0.002626	0.003166	0.001893
RPLP2	0.00142	0.000998	0.000735	0.001341	0.00287	0.001041	0.001391	0.001103
RPN1	0.000179	0.000192	0.000144	0.000182	0.000166	0.000196	0.000165	0.000217
RPN2	0.000323	0.000247	0.000101	0.00013	0.000131	0.000178	0.000124	0.000132
RPP14	1.13E-05	1.28E-05	1.08E-05	7.89E-06	3.69E-06	1.00E-05	9.17E-06	9.11E-06
RPP21	4.67E-05	6.84E-05	2.67E-05	3.77E-05	4.70E-05	3.77E-05	5.02E-05	5.90E-05
RPP25	1.22E-05	4.22E-07	1.91E-06	3.64E-06	1.04E-06	9.38E-07	3.35E-06	1.16E-06
RPP30	1.12E-05	9.44E-06	4.25E-06	5.91E-06	4.59E-06	6.31E-06	7.99E-06	6.82E-06
RPP38	1.32E-05	1.22E-05	7.14E-06	1.02E-05	8.74E-06	7.94E-06	1.32E-05	1.05E-05
RPP40	5.47E-06	8.11E-06	4.60E-06	3.66E-06	3.25E-06	1.36E-05	5.15E-06	8.78E-06
RPPH1	1.52E-05	2.78E-06	1.56E-06	8.15E-06	8.91E-06	2.49E-05	2.16E-05	5.94E-06
RPRD1A	2.25E-05	1.74E-05	9.89E-06	8.93E-06	5.11E-06	1.48E-05	1.02E-05	1.11E-05
RPRD1B	2.71E-05	2.20E-05	1.40E-05	2.74E-05	9.03E-06	2.50E-05	2.25E-05	4.08E-05
RPRD2	1.16E-05	6.02E-06	5.37E-06	4.62E-06	1.09E-06	8.31E-06	3.05E-06	5.93E-06
RPRM	3.38E-08	0	0	0	0	0	1.63E-08	0
RPRML	5.97E-06	1.39E-07	0	0	2.28E-07	0	4.59E-07	0
RPS10	0.000491	0.000538	0.000242	0.00044	0.001122	0.000603	0.000501	0.000425
RPS10P7	2.93E-06	2.98E-06	1.77E-06	1.42E-06	1.23E-06	2.59E-06	2.12E-06	8.49E-07
RPS11	0.002265	0.001194	0.001099	0.001773	0.003248	0.001761	0.002317	0.000965
RPS12	0.001202	0.000981	0.001059	0.001777	0.001973	0.002647	0.001768	0.001059
RPS13	0.000818	0.000568	0.000569	0.000853	0.000769	0.00085	0.000781	0.000746
RPS14	0.001557	0.000892	0.000991	0.00148	0.001604	0.001531	0.001581	0.001263
RPS15	0.000534	0.000298	0.00031	0.000467	0.001303	0.000381	0.000707	0.000272
RPS15A	0.001183	0.001384	0.000673	0.001325	0.000804	0.000917	0.001368	0.000587
RPS15AP1	1.51E-07	1.06E-07	6.69E-08	3.74E-08	2.62E-08	0	8.61E-08	9.03E-08
RPS16	0.00156	0.001192	0.000838	0.001267	0.002572	0.001097	0.001763	0.000799

RPS17	0.001589	0.001322	0.000807	0.001448	0.003452	0.001692	0.002562	0.001067
RPS18	0.002613	0.00166	0.001136	0.001631	0.002724	0.002484	0.002504	0.001532
RPS19	0.000821	0.000514	0.000367	0.000676	0.002663	0.000656	0.000878	0.000493
RPS19BP1	8.72E-05	7.53E-05	4.96E-05	5.36E-05	0.000177	7.33E-05	0.0001	7.16E-05
RPS2	0.000669	0.000408	0.00025	0.000416	0.000265	0.000376	0.000623	0.000294
RPS20	0.001635	0.001371	0.000562	0.000921	0.002197	0.000795	0.002263	0.000943
RPS21	0.001732	0.000857	0.000709	0.001007	0.003976	0.001098	0.001042	0.00064
RPS23	0.000158	7.75E-05	7.52E-05	0.000141	0.000106	0.000119	0.000164	0.000102
RPS24	0.00162	0.000725	0.000733	0.001362	0.003545	0.001188	0.001472	0.000822
RPS25	0.00107	0.000458	0.000683	0.000924	0.000992	0.001028	0.000917	0.000692
RPS26	5.30E-05	4.68E-05	2.30E-05	3.07E-05	2.94E-05	4.04E-05	6.96E-05	5.16E-05
RPS26P11	4.17E-07	1.47E-07	4.37E-07	7.53E-08	5.24E-08	1.13E-07	5.52E-08	0
RPS27	0.000226	9.78E-06	0.000174	0.000226	7.04E-06	0.000258	0.000203	9.83E-06
RPS27A	0.000983	0.000735	0.000382	0.000713	0.001246	0.000686	0.000749	0.00043
RPS27L	8.45E-05	3.75E-05	6.61E-05	0.000126	7.87E-05	0.000113	9.27E-05	7.53E-05
RPS28	0.000123	8.82E-06	9.84E-05	0.000136	1.42E-05	2.93E-06	4.66E-06	8.28E-05
RPS29	0.002283	0.001655	0.001506	0.002154	0.004244	0.002212	0.002551	0.001067
RPS2P32	7.01E-07	3.01E-08	2.84E-07	3.85E-07	2.25E-08	4.85E-08	3.22E-07	7.74E-08
RPS3	0.001241	0.000437	0.00051	0.000842	0.001046	0.00079	0.000758	0.00072
RPS3A	0.000229	9.82E-05	0.000123	0.000201	0.000236	0.000206	0.000219	0.000143
RPS4X	0.001569	0.000592	0.000823	0.00089	0.002298	0.000895	0.001005	0.000925
RPS4Y1	0	0.000178	0	0.000259	0.000487	0.000381	0.00041	0.000187
RPS4Y2	0	0	0	0	0	0	0	0
RPS5	0.00085	0.0008	0.00044	0.000614	0.001509	0.000784	0.000879	0.000475
RPS6	0.00098	0.001079	0.000895	0.001391	0.001607	0.001554	0.001483	0.000854
RPS6KA1	2.86E-05	1.03E-05	4.62E-06	9.46E-06	9.73E-07	5.13E-06	7.22E-06	5.97E-06
RPS6KA2	1.22E-05	2.64E-06	3.03E-06	4.51E-06	1.57E-06	9.19E-06	6.75E-06	4.54E-06
RPS6KA3	1.61E-05	1.30E-05	1.35E-05	1.19E-05	1.42E-06	2.14E-05	8.17E-06	2.04E-05
RPS6KA4	1.13E-05	4.92E-06	5.11E-06	7.62E-06	3.86E-06	7.90E-06	1.19E-05	1.13E-05
RPS6KA5	1.32E-06	1.67E-07	4.73E-07	6.01E-07	3.21E-08	5.65E-07	2.59E-07	2.46E-07
RPS6KA6	9.20E-09	0	7.65E-08	1.58E-07	0	0	8.39E-09	0
RPS6KB1	1.00E-05	9.68E-06	5.06E-06	4.59E-06	2.10E-06	7.84E-06	4.10E-06	4.88E-06
RPS6KB2	4.21E-05	3.80E-05	2.36E-05	2.05E-05	3.97E-05	3.35E-05	2.40E-05	4.54E-05
RPS6KC1	5.35E-06	5.67E-06	2.72E-06	2.80E-06	8.45E-07	6.94E-06	1.80E-06	5.94E-06
RPS6KL1	1.72E-06	1.25E-06	4.95E-07	6.74E-07	2.55E-07	3.44E-06	3.82E-06	3.18E-06
RPS7	0.000712	0.000733	0.000216	0.00041	0.000695	0.000471	0.000583	0.000293
RPS8	0.001428	0.000818	0.000649	0.001084	0.001435	0.001176	0.001198	0.000686
RPS9	0.001199	0.000735	0.000839	0.001464	0.002299	0.001203	0.001584	0.000812
RPSA	0.000245	0.000167	0.000122	0.000171	0.000347	0.000186	0.000217	0.000151
RPSAP52	6.83E-08	0	0	0	0	0	2.18E-08	0
RPSAP58	0.000251	0.000187	0.000111	0.000182	0.000317	0.000181	0.000212	0.000162
RPSAP9	7.97E-07	1.13E-06	6.27E-07	5.54E-07	7.92E-07	8.70E-07	5.50E-07	3.84E-07
RPTN	0	0	5.96E-08	0	0	0	0	0
RPTOR	1.53E-05	1.48E-05	6.01E-06	5.02E-06	1.01E-05	1.42E-05	1.10E-05	1.02E-05
RPUSD1	1.15E-05	1.44E-05	4.82E-06	7.34E-06	6.99E-06	8.91E-06	1.04E-05	7.99E-06
RPUSD2	8.95E-06	6.78E-06	4.35E-06	5.13E-06	6.13E-06	7.47E-06	8.60E-06	5.96E-06
RPUSD3	3.29E-05	2.46E-05	1.83E-05	1.97E-05	3.29E-05	2.65E-05	3.28E-05	2.58E-05
RPUSD4	9.29E-06	4.93E-06	9.24E-06	8.24E-06	1.01E-05	1.39E-05	9.57E-06	1.24E-05
RQCD1	2.68E-05	7.81E-06	4.38E-06	7.64E-06	4.33E-07	9.75E-06	4.75E-06	1.15E-05
RRAD	1.99E-06	3.83E-06	7.40E-07	5.93E-05	7.84E-07	4.10E-07	4.23E-06	1.00E-07
RRAGA	4.39E-05	5.50E-05	4.75E-05	6.04E-05	2.51E-05	0.000118	4.41E-05	5.22E-05
RRAGB	4.35E-06	1.61E-06	2.17E-06	3.61E-06	1.19E-06	3.31E-06	3.72E-06	4.72E-06
RRAGC	1.31E-05	8.51E-06	4.27E-06	8.32E-06	2.30E-07	9.12E-06	4.36E-06	6.27E-06
RRAGD	1.51E-05	1.96E-05	3.64E-06	3.50E-06	1.84E-06	2.33E-06	6.36E-06	5.63E-06
RRAS	5.83E-05	4.70E-05	1.90E-05	4.14E-05	1.67E-05	3.27E-05	5.05E-05	2.80E-05
RRAS2	1.38E-05	1.09E-05	1.40E-05	1.52E-05	9.35E-06	1.29E-05	9.73E-06	1.84E-05
RRBP1	0.000347	0.000339	0.000202	0.000225	0.000219	0.000161	0.000187	0.000265
RREB1	9.25E-06	1.25E-05	9.26E-06	5.43E-06	4.16E-07	7.45E-06	3.24E-06	1.12E-05

RRH	9.53E-08	3.62E-08	0	3.90E-08	1.37E-08	5.90E-08	9.18E-08	1.41E-07
RRM1	2.77E-05	1.64E-05	9.60E-06	8.72E-06	6.87E-06	1.37E-05	7.08E-06	1.98E-05
RRM2	5.58E-05	2.21E-05	4.33E-07	1.07E-06	3.83E-06	2.21E-06	1.19E-06	3.08E-06
RRM2B	1.80E-05	1.14E-05	7.66E-06	6.16E-06	2.42E-06	7.52E-06	9.18E-06	7.42E-06
RRN3	1.85E-05	2.70E-05	1.28E-05	2.44E-05	2.10E-06	1.58E-05	1.35E-05	1.47E-05
RRN3P1	1.75E-06	2.11E-06	9.30E-07	1.29E-06	6.29E-07	9.47E-07	2.43E-06	4.17E-07
RRN3P2	4.86E-07	1.53E-07	5.45E-08	3.50E-07	5.40E-08	1.35E-07	1.19E-07	6.15E-08
RRN3P3	1.10E-06	3.90E-07	1.97E-07	6.26E-07	1.04E-07	3.36E-07	4.65E-07	2.10E-07
RRP1	2.04E-05	1.28E-05	1.01E-05	1.43E-05	2.06E-05	1.91E-05	1.98E-05	2.31E-05
RRP12	1.35E-05	1.70E-05	4.47E-06	1.30E-05	9.65E-06	1.15E-05	1.48E-05	1.10E-05
RRP15	2.22E-06	3.44E-06	1.51E-06	2.22E-06	6.24E-07	4.36E-06	2.70E-06	4.17E-06
RRP1B	1.20E-05	4.70E-06	6.15E-06	6.00E-06	2.96E-06	9.89E-06	5.18E-06	9.89E-06
RRP7A	6.35E-05	5.78E-05	2.15E-05	3.72E-05	4.41E-05	2.86E-05	4.43E-05	5.09E-05
RRP7B	1.28E-06	1.69E-06	4.66E-07	7.91E-07	5.31E-07	8.17E-07	5.88E-07	4.03E-07
RRP8	1.23E-05	1.14E-05	1.01E-05	1.25E-05	9.49E-06	1.41E-05	1.71E-05	1.74E-05
RRP9	9.96E-06	1.77E-05	6.86E-06	1.08E-05	6.53E-06	1.39E-05	1.83E-05	1.11E-05
RRS1	1.43E-05	3.09E-05	5.62E-06	1.25E-05	9.72E-06	1.20E-05	4.43E-05	1.53E-05
RS1	2.30E-08	0	0	1.87E-08	3.29E-08	1.42E-08	2.23E-08	2.24E-08
RSAD1	2.92E-05	2.54E-05	1.97E-05	1.60E-05	3.83E-05	3.83E-05	2.62E-05	1.86E-05
RSAD2	1.50E-06	7.98E-07	1.79E-06	1.13E-06	1.57E-07	1.81E-06	4.82E-07	3.67E-06
RSBN1	3.28E-06	1.90E-06	1.81E-06	1.86E-06	3.58E-07	2.75E-06	1.34E-06	1.47E-06
RSBN1L	5.75E-06	5.94E-06	4.21E-06	3.06E-06	8.25E-07	3.43E-06	1.71E-06	2.87E-06
RSC1A1	1.27E-05	1.17E-05	9.16E-06	5.89E-06	4.71E-07	1.47E-05	2.77E-06	8.34E-06
RSF1	4.38E-06	3.10E-06	3.45E-06	2.96E-06	4.30E-07	4.00E-06	1.28E-06	4.48E-06
RSL1D1	4.31E-05	5.05E-05	2.61E-05	3.48E-05	7.99E-06	4.52E-05	3.72E-05	3.16E-05
RSL24D1	5.00E-05	4.55E-05	3.91E-05	4.90E-05	2.74E-05	4.54E-05	5.91E-05	3.20E-05
RSPH1	1.42E-06	0	7.88E-08	4.31E-07	0	2.75E-07	1.06E-07	2.73E-07
RSPH10B2	1.03E-08	1.60E-07	1.01E-07	7.11E-08	4.75E-08	3.70E-08	4.34E-08	3.58E-08
RSPH3	3.93E-06	1.43E-06	1.73E-06	1.53E-06	4.34E-07	1.83E-06	4.96E-07	1.13E-06
RSPH4A	7.37E-08	1.36E-07	5.37E-08	1.27E-07	1.48E-08	4.00E-08	1.06E-07	7.60E-08
RSPH6A	0	1.57E-08	0	0	0	0	0	0
RSPH9	5.57E-08	3.48E-08	6.60E-08	1.11E-07	5.17E-08	0	5.67E-08	8.91E-08
RSPO1	0	0	0	5.60E-08	0	0	0	0
RSPO2	0	0	1.19E-07	1.40E-07	8.18E-09	9.08E-07	3.69E-08	6.22E-08
RSPO3	1.01E-06	2.00E-08	2.87E-06	7.47E-06	4.78E-08	5.16E-08	4.30E-08	1.47E-07
RSPO4	5.02E-07	2.83E-07	2.90E-08	3.42E-08	0	1.73E-08	8.36E-09	0
RSPRY1	1.46E-05	4.81E-06	7.50E-06	8.20E-06	1.96E-07	1.22E-05	4.40E-06	5.70E-06
RSRC1	8.67E-06	4.97E-06	3.03E-06	3.53E-06	2.98E-07	4.83E-06	2.27E-06	6.83E-06
RSRC2	3.71E-05	1.65E-05	1.30E-05	1.86E-05	4.16E-06	2.26E-05	1.94E-05	2.25E-05
RSU1	1.61E-05	1.29E-05	1.12E-05	1.11E-05	3.40E-06	2.00E-05	1.07E-05	1.14E-05
RTBDN	5.56E-06	0	0	0	2.99E-08	1.06E-06	1.95E-07	0
RTCD1	1.58E-05	1.78E-05	8.96E-06	8.84E-06	3.78E-06	2.14E-05	7.47E-06	1.08E-05
RTDR1	9.24E-07	1.17E-07	0	1.06E-07	3.53E-08	9.50E-08	1.96E-08	6.05E-08
RTEL1	5.45E-06	1.71E-06	2.38E-06	2.75E-06	3.17E-06	4.58E-06	3.95E-06	4.83E-06
RTF1	1.30E-05	9.98E-06	8.73E-06	8.29E-06	5.67E-06	1.08E-05	1.06E-05	1.03E-05
RTKN	0.000102	4.96E-05	2.98E-05	3.42E-05	3.24E-05	1.91E-05	3.95E-05	3.46E-05
RTKN2	1.03E-06	2.76E-07	1.72E-08	4.39E-08	3.47E-09	8.23E-08	4.58E-08	1.09E-07
RTL1	4.05E-06	0	0	6.62E-09	0	0	5.27E-09	1.03E-07
RTN1	9.12E-06	3.27E-07	8.56E-07	2.18E-06	2.84E-07	4.30E-07	6.95E-07	9.64E-07
RTN2	5.10E-06	8.61E-06	1.01E-06	3.04E-06	2.00E-06	1.03E-06	7.27E-06	3.10E-06
RTN3	9.15E-05	9.17E-05	4.17E-05	4.83E-05	3.58E-05	6.20E-05	4.53E-05	5.77E-05
RTN4	0.000391	0.000351	0.000296	0.000141	0.000256	0.000121	0.000185	8.80E-05
RTN4IP1	5.07E-06	4.69E-06	3.10E-06	3.08E-06	2.15E-06	8.06E-06	4.95E-06	5.11E-06
RTN4R	1.11E-05	1.04E-05	3.40E-06	2.46E-06	2.10E-06	2.63E-06	3.82E-06	7.56E-06
RTN4RL1	3.53E-06	6.64E-07	1.43E-06	1.06E-06	4.95E-07	1.62E-07	9.78E-07	5.80E-06
RTN4RL2	4.76E-05	2.96E-05	5.55E-06	6.62E-06	4.31E-06	6.19E-06	4.71E-06	6.56E-06
RTP1	1.07E-08	1.21E-08	0	0	0	2.98E-08	1.04E-08	0
RTP2	0	0	0	0	0	0	1.80E-08	0

RTP3	0	1.55E-05	8.10E-05	4.39E-05	7.54E-05	3.63E-05	7.36E-05	3.95E-05
RTP4	1.62E-05	8.27E-06	7.90E-06	1.46E-05	3.07E-06	8.26E-06	7.23E-06	9.52E-06
RTTN	4.02E-06	1.48E-06	5.37E-06	2.95E-06	1.12E-06	2.40E-06	1.82E-06	1.84E-06
RUFY1	2.15E-05	1.39E-05	1.26E-05	1.72E-05	6.31E-06	1.70E-05	1.72E-05	2.07E-05
RUFY2	3.03E-06	1.38E-06	1.53E-06	2.07E-06	5.96E-07	2.40E-06	1.31E-06	1.50E-06
RUFY3	8.57E-06	5.02E-06	5.29E-06	5.72E-06	2.10E-06	1.12E-05	4.32E-06	7.27E-06
RUFY4	7.50E-07	0	2.01E-08	4.07E-08	0	3.50E-08	0	3.69E-08
RUNDC1	6.58E-06	5.73E-06	1.92E-06	3.13E-06	1.06E-06	4.53E-06	3.42E-06	2.51E-06
RUNDC2A	3.06E-07	6.78E-07	1.43E-07	2.58E-07	0	4.30E-07	8.16E-08	9.29E-08
RUNDC2C	4.18E-07	6.30E-07	2.93E-07	5.87E-07	7.34E-08	1.62E-08	2.23E-07	1.21E-07
RUNDC3A	4.17E-06	2.07E-07	2.45E-08	1.91E-07	0	1.08E-08	1.02E-07	3.44E-08
RUNDC3B	5.93E-07	5.06E-06	4.31E-06	2.69E-06	1.71E-06	5.70E-06	3.28E-06	8.26E-07
RUNX1	6.30E-06	2.24E-06	1.58E-06	6.47E-06	3.31E-07	4.25E-06	3.23E-06	6.11E-06
RUNX1T1	1.54E-07	5.71E-08	9.08E-08	8.28E-08	0	4.26E-08	4.93E-08	1.28E-07
RUNX2	6.55E-07	5.95E-08	1.98E-07	3.02E-07	2.11E-08	9.85E-08	2.23E-07	1.20E-07
RUNX3	4.92E-06	3.91E-07	1.55E-06	6.55E-06	9.03E-08	2.07E-06	1.70E-06	9.80E-07
RUSC1	1.78E-05	1.18E-05	3.74E-06	5.81E-06	3.88E-06	6.07E-06	6.17E-06	9.31E-06
RUSC2	4.28E-06	6.84E-06	4.51E-06	8.49E-06	2.79E-06	2.54E-06	5.68E-06	4.75E-06
RUVBL1	2.95E-05	2.41E-05	1.07E-05	1.64E-05	8.79E-06	2.46E-05	1.71E-05	1.41E-05
RUVBL2	6.01E-05	3.76E-05	3.25E-05	4.21E-05	6.92E-05	7.07E-05	7.32E-05	4.37E-05
RWDD1	4.87E-05	4.05E-05	3.38E-05	3.97E-05	1.87E-05	4.62E-05	4.32E-05	3.82E-05
RWDD2A	6.70E-06	2.78E-06	3.38E-06	3.45E-06	1.27E-06	5.65E-06	2.14E-06	2.73E-06
RWDD2B	1.08E-05	1.06E-05	1.61E-05	1.30E-05	8.88E-06	2.38E-05	9.15E-06	1.25E-05
RWDD3	1.39E-05	1.47E-05	1.03E-05	1.09E-05	3.84E-06	2.21E-05	8.91E-06	1.62E-05
RWDD4A	8.65E-06	4.74E-06	6.56E-06	6.23E-06	2.31E-06	6.76E-06	5.61E-06	5.73E-06
RXFP1	3.49E-07	8.25E-08	1.42E-07	7.67E-08	0	1.94E-08	2.02E-08	0
RXFP2	0	0	8.86E-09	0	0	0	0	0
RXFP3	0	0	0	0	0	2.42E-08	0	0
RXFP4	0	0	0	2.80E-08	0	0	0	0
RXRA	4.62E-05	3.95E-05	4.94E-05	2.11E-05	4.54E-05	3.47E-05	6.67E-05	6.91E-05
RXRB	2.26E-05	2.42E-05	1.26E-05	1.32E-05	1.41E-05	3.75E-05	1.84E-05	2.50E-05
RXRG	1.31E-08	7.74E-07	2.76E-08	1.45E-06	3.39E-08	1.56E-06	1.27E-08	0
RYBP	1.08E-05	1.71E-05	9.14E-06	1.31E-05	3.46E-06	7.56E-06	8.73E-06	5.39E-06
RYK	1.22E-05	8.26E-06	6.94E-06	1.05E-05	2.33E-06	9.71E-06	9.53E-06	7.23E-06
RYR1	7.15E-08	4.77E-09	3.49E-08	5.22E-08	8.57E-09	3.30E-08	4.33E-08	1.04E-08
RYR2	2.09E-07	2.49E-07	1.68E-08	2.45E-07	0	1.63E-08	0	7.55E-08
RYR3	5.66E-09	6.29E-09	7.18E-09	1.72E-08	3.63E-09	1.31E-08	6.90E-09	6.19E-09
S100A1	4.01E-06	1.31E-05	1.57E-06	3.94E-06	1.65E-06	8.81E-06	1.12E-05	6.04E-07
S100A10	0.000278	5.71E-05	6.03E-05	0.00019	9.59E-05	0.000165	0.00016	1.90E-05
S100A11	0.000358	3.07E-05	5.93E-05	0.000282	3.83E-05	4.82E-05	0.000132	5.17E-05
S100A12	3.89E-07	1.48E-06	7.08E-07	1.89E-06	0	0	2.12E-07	6.05E-07
S100A13	0.000154	6.42E-05	3.64E-05	0.000137	8.66E-05	4.42E-05	0.000185	3.62E-05
S100A14	2.47E-05	8.67E-06	1.09E-05	2.19E-05	2.64E-07	2.11E-06	2.02E-05	1.17E-06
S100A16	0.000226	0.000171	9.81E-05	0.000156	4.29E-05	0.000121	0.000149	3.32E-05
S100A2	6.46E-07	9.80E-08	2.16E-06	1.63E-06	7.30E-08	2.62E-08	1.34E-07	1.68E-07
S100A3	4.73E-07	4.68E-08	0	6.48E-06	1.37E-07	1.85E-07	6.31E-07	2.97E-07
S100A4	7.50E-05	9.75E-06	2.63E-05	9.28E-05	1.67E-05	2.58E-05	5.16E-05	2.73E-05
S100A5	0	4.96E-08	0	5.19E-08	0	0	0	0
S100A6	0.000601	2.70E-05	2.61E-05	0.000472	4.76E-05	1.81E-05	0.000126	4.46E-05
S100A7	0	0	6.57E-06	0	0	1.65E-07	0	0
S100A7A	0	0	2.73E-08	0	0	0	0	0
S100A7L2	0	0	0	0	0	0	0	0
S100A8	3.86E-05	1.63E-05	6.62E-05	3.36E-05	2.82E-06	2.56E-06	8.89E-06	1.98E-05
S100A9	3.74E-05	1.94E-05	5.99E-05	4.89E-05	6.07E-06	7.96E-06	2.99E-05	1.53E-05
S100B	5.72E-06	2.64E-08	1.74E-07	2.32E-06	2.18E-07	4.48E-07	2.85E-07	2.38E-07
S100G	0	0	0	0	0	0	7.40E-08	0
S100P	0.000913	2.85E-05	2.28E-06	3.85E-06	1.19E-07	3.20E-07	6.02E-05	2.04E-05
S100PBP	6.73E-06	2.39E-06	1.80E-06	2.45E-06	4.47E-07	2.95E-06	1.63E-06	2.70E-06

S100Z	1.05E-07	0	4.90E-08	9.07E-08	0	4.22E-08	8.67E-08	6.73E-08
S1PR1	1.15E-05	1.38E-05	6.12E-06	9.63E-06	3.09E-06	8.86E-06	1.04E-05	9.27E-06
S1PR2	5.65E-06	2.81E-06	2.64E-06	4.47E-06	1.21E-06	4.91E-07	9.09E-06	1.06E-05
S1PR3	3.03E-06	2.90E-06	1.81E-06	4.05E-06	6.08E-07	1.16E-06	2.19E-06	2.84E-06
S1PR4	2.19E-06	6.29E-07	9.53E-07	2.85E-06	5.44E-07	1.20E-06	1.88E-06	8.38E-07
S1PR5	1.01E-06	2.92E-07	4.11E-07	1.07E-06	5.79E-07	2.71E-07	1.02E-06	3.30E-07
SAA1	0.000652	0.000716	0.000654	0.001123	0.002941	0.00249	0.00986	0.000481
SAA2	0.000288	0.000313	0.000617	0.000305	0.002075	0.001596	0.004337	0.000359
SAA3P	0	0	0	0	0	0	0	0
SAA4	0.000268	0.000445	0.001598	0.001068	0.001257	0.000317	0.000879	0.002826
SAAL1	9.64E-06	5.47E-06	3.49E-06	4.78E-06	3.82E-06	4.48E-06	4.72E-06	1.03E-05
SAC3D1	7.50E-06	1.00E-05	3.00E-06	4.84E-06	1.64E-05	5.58E-06	1.05E-05	9.25E-06
SACM1L	1.37E-05	1.50E-05	1.15E-05	9.74E-06	3.71E-06	1.12E-05	7.12E-06	8.98E-06
SACS	1.93E-06	1.55E-07	3.39E-07	1.28E-06	1.39E-07	1.10E-06	6.97E-07	4.42E-07
SAE1	6.50E-05	2.49E-05	1.62E-05	2.60E-05	1.95E-05	4.08E-05	2.76E-05	3.14E-05
SAFB	3.46E-05	2.13E-05	1.97E-05	2.75E-05	2.60E-05	3.18E-05	2.70E-05	1.77E-05
SAFB2	3.39E-05	1.50E-05	1.06E-05	1.80E-05	1.03E-05	2.00E-05	1.28E-05	1.45E-05
SAG	0	0	0	1.71E-08	0	1.17E-08	1.34E-08	0
SAGE1	0	0	0	9.29E-09	0	0	0	0
SALL1	2.11E-05	1.66E-05	2.16E-05	1.16E-05	4.61E-06	2.27E-05	7.64E-06	1.30E-05
SALL2	7.92E-07	1.02E-06	2.11E-07	8.01E-07	1.64E-08	1.27E-07	1.07E-07	6.79E-07
SALL3	0	0	0	0	0	0	0	0
SALL4	2.99E-06	1.70E-07	1.27E-07	1.55E-07	7.79E-08	9.48E-08	2.76E-08	1.45E-07
SAMD1	1.28E-05	6.12E-06	3.65E-06	5.97E-06	2.33E-06	4.68E-06	9.04E-06	9.87E-06
SAMD10	4.97E-06	2.24E-06	1.42E-06	2.40E-06	2.40E-06	2.17E-06	1.83E-06	3.82E-06
SAMD11	4.23E-06	1.49E-08	9.16E-07	7.87E-06	4.85E-07	0	1.80E-06	1.44E-07
SAMD12	3.65E-06	2.20E-07	8.74E-07	7.11E-07	6.49E-09	5.01E-07	4.25E-07	1.62E-07
SAMD13	1.16E-06	2.04E-08	1.52E-07	2.32E-07	1.08E-07	1.66E-08	4.70E-07	2.11E-07
SAMD14	7.40E-07	1.29E-07	2.02E-07	2.96E-07	3.53E-07	8.55E-08	5.47E-07	1.63E-07
SAMD3	1.09E-06	9.64E-08	5.43E-07	8.24E-07	9.81E-09	2.54E-07	4.30E-07	1.74E-07
SAMD4A	3.18E-06	1.64E-06	6.24E-06	6.35E-06	2.24E-06	4.24E-06	4.48E-06	3.57E-06
SAMD4B	2.10E-05	1.42E-05	5.78E-06	8.42E-06	9.82E-07	1.30E-05	7.06E-06	1.14E-05
SAMD5	4.12E-07	3.70E-08	4.10E-06	2.45E-06	3.79E-08	4.76E-07	1.33E-06	2.47E-07
SAMD7	0	0	0	0	0	0	0	0
SAMD8	3.09E-06	1.74E-06	1.66E-06	1.90E-06	1.70E-07	1.94E-06	6.81E-07	8.50E-07
SAMD9	3.61E-06	1.15E-06	1.24E-06	1.40E-06	1.25E-07	1.34E-06	4.33E-07	3.19E-06
SAMD9L	7.77E-06	8.27E-07	1.23E-06	1.89E-06	1.38E-07	1.47E-06	1.10E-06	2.46E-06
SAMHD1	2.27E-05	1.11E-05	1.01E-05	1.40E-05	1.09E-06	2.72E-05	7.45E-06	5.08E-06
SAMM50	3.86E-05	4.51E-05	2.10E-05	2.49E-05	2.74E-05	7.98E-05	3.13E-05	3.63E-05
SAMSN1	1.02E-05	4.99E-07	2.61E-06	1.03E-05	3.74E-07	3.31E-06	2.73E-06	1.52E-06
SAP130	1.27E-05	1.03E-05	3.55E-06	4.09E-06	2.12E-06	6.71E-06	4.78E-06	5.03E-06
SAP18	6.35E-05	5.32E-05	3.70E-05	4.23E-05	3.19E-05	5.19E-05	5.56E-05	4.83E-05
SAP30	7.47E-06	3.73E-06	1.64E-06	5.41E-06	2.45E-06	3.08E-06	5.66E-06	5.75E-06
SAP30BP	4.74E-05	3.63E-05	1.29E-05	2.15E-05	2.94E-05	3.17E-05	2.34E-05	2.27E-05
SAP30L	6.60E-06	5.18E-06	3.12E-06	3.21E-06	2.12E-06	4.39E-06	6.42E-06	4.51E-06
SAPS1	3.27E-05	2.43E-05	1.22E-05	1.70E-05	1.62E-05	2.16E-05	2.50E-05	2.38E-05
SAPS2	3.04E-05	3.32E-05	3.55E-05	2.39E-05	6.53E-05	3.97E-05	4.13E-05	4.45E-05
SAPS3	3.32E-05	2.10E-05	1.70E-05	1.99E-05	1.16E-05	2.06E-05	1.37E-05	1.98E-05
SAR1A	5.07E-05	4.22E-05	2.96E-05	3.08E-05	1.92E-05	3.53E-05	3.36E-05	3.43E-05
SAR1B	6.69E-05	0.000114	7.73E-05	4.22E-05	1.19E-05	6.84E-05	2.98E-05	8.73E-05
SARDH	2.51E-05	5.40E-05	9.37E-05	4.77E-05	6.56E-05	2.77E-05	4.03E-05	5.70E-05
SARM1	2.13E-06	2.54E-06	3.99E-07	9.62E-07	4.70E-08	9.64E-07	8.51E-07	3.15E-07
SARNP	5.59E-05	3.20E-05	1.76E-05	3.71E-05	3.05E-05	4.08E-05	4.16E-05	2.87E-05
SARS	7.74E-05	0.000135	7.09E-05	6.56E-05	7.38E-05	9.66E-05	8.66E-05	8.64E-05
SARS2	1.70E-05	5.59E-06	1.03E-05	1.25E-05	1.12E-05	1.17E-05	1.12E-05	1.18E-05
SART1	1.86E-05	2.64E-05	1.14E-05	1.60E-05	1.97E-05	1.70E-05	1.95E-05	2.48E-05
SART3	1.54E-05	1.08E-05	6.08E-06	7.81E-06	2.08E-06	1.04E-05	7.56E-06	7.41E-06
SASH1	6.81E-06	4.40E-06	3.58E-06	3.06E-06	8.35E-07	3.60E-06	2.58E-06	2.22E-06

SASH3	1.10E-05	7.81E-07	3.48E-06	5.79E-06	6.11E-07	3.08E-06	4.58E-06	1.40E-06
SASS6	1.60E-06	2.14E-06	3.75E-07	4.02E-07	2.83E-07	1.34E-06	3.49E-07	4.38E-07
SAT1	0.000456	0.000146	0.000394	0.000623	0.000216	0.000453	0.000415	0.000627
SAT2	0.000102	0.000124	0.000238	0.000157	0.00011	9.30E-05	0.000212	0.000157
SATB1	1.52E-06	4.33E-06	4.59E-06	7.03E-06	4.96E-07	3.11E-06	2.26E-06	2.27E-06
SATB2	4.96E-06	3.39E-06	1.09E-06	1.20E-06	7.84E-07	2.36E-06	1.96E-06	3.11E-06
SATL1	1.30E-06	1.21E-06	5.31E-07	6.64E-07	1.05E-07	1.46E-06	9.41E-07	6.67E-07
SAV1	7.76E-06	2.56E-06	1.01E-05	7.23E-06	2.01E-06	8.04E-06	5.16E-06	5.75E-06
SBDS	0.000118	0.000125	6.04E-05	7.66E-05	2.95E-05	6.87E-05	7.61E-05	6.21E-05
SBDSP1	1.82E-05	1.81E-05	8.52E-06	1.34E-05	4.48E-06	9.61E-06	1.07E-05	1.21E-05
SBF1	1.03E-05	6.37E-06	3.05E-06	4.16E-06	7.09E-06	4.24E-06	8.85E-06	6.48E-06
SBF1P1	3.66E-08	8.16E-09	7.48E-09	4.46E-09	3.14E-09	0	0	0
SBF2	8.43E-06	7.14E-06	7.33E-06	5.50E-06	1.81E-06	1.04E-05	4.04E-06	8.76E-06
SBK1	2.80E-06	8.09E-08	7.42E-08	4.64E-07	3.88E-08	5.86E-08	1.37E-07	1.66E-07
SBK2	3.54E-07	2.93E-08	0	0	0	0	0	0
SBNO1	7.82E-06	7.25E-06	4.85E-06	3.39E-06	1.53E-08	5.88E-06	1.88E-06	4.81E-06
SBNO2	1.99E-05	1.18E-05	8.30E-06	2.07E-05	5.03E-06	1.39E-05	1.74E-05	1.41E-05
SBSN	0	0	3.02E-06	1.52E-08	0	0	8.31E-08	0
SC4MOL	8.11E-05	0.000424	0.000162	8.86E-05	7.59E-05	0.000246	0.000106	0.000184
SC5DL	4.28E-05	0.000104	9.35E-05	3.35E-05	2.05E-05	3.14E-05	1.18E-05	3.51E-05
SC65	1.47E-05	7.80E-06	1.54E-06	3.74E-06	4.87E-06	3.43E-06	4.96E-06	3.12E-06
SCAF1	1.56E-05	1.83E-05	1.06E-05	1.30E-05	1.24E-05	1.55E-05	1.79E-05	1.31E-05
SCAI	7.82E-07	3.02E-07	6.03E-07	2.96E-07	1.37E-07	5.57E-07	1.87E-07	3.35E-07
SCAMP1	9.90E-06	8.24E-06	4.46E-06	5.51E-06	9.85E-07	6.46E-06	4.17E-06	6.35E-06
SCAMP2	8.71E-05	6.29E-05	4.82E-05	4.92E-05	4.39E-05	4.87E-05	5.97E-05	5.60E-05
SCAMP3	5.14E-05	8.90E-05	2.83E-05	3.46E-05	3.95E-05	8.74E-05	5.11E-05	0.00011
SCAMP4	2.87E-05	1.70E-05	1.45E-05	2.10E-05	2.08E-05	1.46E-05	2.40E-05	3.10E-05
SCAMP5	1.02E-05	7.94E-06	3.27E-06	1.58E-06	1.08E-05	2.23E-05	3.58E-06	9.12E-07
SCAND1	8.38E-05	9.00E-05	5.44E-05	8.79E-05	0.000183	7.17E-05	0.000105	4.97E-05
SCAND2	3.85E-06	1.62E-06	1.42E-06	1.60E-06	1.05E-06	1.60E-06	1.66E-06	1.03E-06
SCAND3	1.61E-06	2.28E-06	6.22E-07	6.89E-07	1.27E-07	5.44E-07	2.16E-07	1.05E-06
SCAP	4.03E-05	4.06E-05	2.12E-05	2.49E-05	5.32E-05	2.21E-05	6.40E-05	2.93E-05
SCAPER	4.84E-06	4.06E-06	3.17E-06	3.76E-06	1.11E-06	6.93E-06	2.02E-06	1.39E-06
SCARA3	1.01E-05	1.06E-05	3.00E-06	1.33E-06	4.12E-06	3.24E-06	5.82E-07	6.28E-07
SCARA5	7.87E-07	8.83E-09	1.11E-06	8.00E-07	0	3.64E-08	1.51E-07	6.91E-08
SCARB1	6.92E-05	0.000345	0.000148	0.000157	9.44E-05	0.000144	0.000147	0.000221
SCARB2	9.55E-05	0.0001	6.93E-05	6.82E-05	1.76E-05	4.62E-05	6.34E-05	8.43E-05
SCARF1	4.39E-06	3.13E-06	2.97E-06	3.16E-06	2.00E-06	1.80E-06	3.71E-06	3.01E-06
SCARF2	1.54E-06	1.55E-06	5.54E-07	3.05E-06	1.04E-06	3.89E-07	1.97E-06	7.70E-07
SCARNA1	0	0	0	0	0	0	0	0
SCARNA1	5.99E-07	4.34E-07	2.48E-07	0	4.12E-07	4.42E-07	7.72E-07	2.47E-07
SCARNA1	0	0	0	0	0	0	0	0
SCARNA1	3.95E-06	1.64E-06	2.11E-06	1.05E-06	9.56E-07	1.27E-06	4.01E-07	1.31E-06
SCARNA1	0	0	0	0	0	0	0	0
SCARNA1	0	0	0	0	0	0	0	0
SCARNA1	2.76E-06	0	0	0	1.15E-06	5.68E-06	0	2.14E-06
SCARNA1	4.17E-06	1.82E-06	1.78E-06	2.86E-06	9.91E-07	9.78E-07	5.91E-06	1.61E-06
SCARNA1	0	0	0	0	0	0	0	0
SCARNA2	2.79E-07	6.09E-07	1.28E-07	8.39E-07	1.08E-06	3.57E-07	4.09E-06	1.47E-07
SCARNA2	0	0	0	0	0	0	0	0
SCARNA2	0	0	0	0	0	0	0	0
SCARNA2	0	0	0	0	0	0	0	0
SCARNA2	0	0	0	0	0	0	0	0
SCARNA2	0	0	0	0	0	0	0	0
SCARNA3	0	0	0	0	0	0	0	0
SCARNA4	0	0	0	0	0	0	0	0
SCARNA5	4.52E-07	0	0	3.16E-07	2.16E-07	1.15E-06	1.48E-06	0
SCARNA6	7.65E-07	0	0	0	0	7.89E-07	4.12E-07	0

SCARNA7	3.03E-07	2.21E-07	0	2.03E-07	1.39E-07	2.99E-07	2.87E-06	2.51E-07
SCARNA8	0	0	0	0	0	0	0	0
SCARNA9	5.22E-07	7.32E-07	2.03E-07	1.55E-06	4.74E-07	3.82E-07	3.30E-06	0
SCARNA9	1.34E-07	0	0	0	0	0	1.17E-07	0
SCCPDH	4.73E-05	9.50E-05	0.000101	7.31E-05	4.32E-05	5.34E-05	9.00E-05	0.000122
SCD	0.000159	0.000843	0.000978	0.000156	0.000291	0.000108	0.000155	0.000974
SCD5	4.52E-06	2.76E-07	1.31E-06	4.63E-06	2.69E-07	1.03E-07	4.77E-07	5.02E-07
SCEL	0	0	1.54E-08	3.65E-08	0	0	0	0
SCFD1	2.20E-05	1.85E-05	1.44E-05	1.63E-05	1.03E-05	2.06E-05	1.75E-05	2.01E-05
SCFD2	3.28E-06	2.59E-06	2.94E-06	2.72E-06	1.16E-06	5.88E-06	3.97E-06	6.61E-06
SCG2	4.08E-07	1.07E-08	0	2.67E-07	0	3.52E-08	1.01E-07	2.51E-07
SCG3	3.54E-08	7.94E-09	0	1.73E-08	0	6.55E-09	0	1.04E-08
SCG5	1.35E-05	3.07E-06	2.77E-06	9.37E-07	7.57E-06	1.69E-06	1.10E-06	2.35E-06
SCGB1A1	0	5.97E-07	0	0	0	0	0	0
SCGB1C1	0	0	1.20E-07	0	0	0	0	0
SCGB1D1	0	0	0	0	0	0	0	0
SCGB1D2	3.55E-06	2.25E-07	0	0	0	1.66E-07	0	0
SCGB1D4	0	0	0	0	0	0	0	0
SCGB2A1	1.30E-06	1.62E-07	0	8.23E-08	0	0	0	0
SCGB2A2	0	0	0	0	0	6.77E-08	1.33E-06	0
SCGB3A1	2.65E-06	2.98E-07	1.52E-06	2.99E-07	2.97E-06	1.49E-07	2.91E-06	7.29E-07
SCGB3A2	0	0	0	0	0	0	0	2.04E-07
SCGBL	1.81E-07	1.18E-07	0	0	0	1.74E-07	0	0
SCGN	6.81E-06	1.42E-05	3.22E-06	3.38E-07	2.09E-05	2.26E-07	0	6.49E-06
SCHIP1	1.93E-05	3.75E-06	3.67E-06	5.74E-06	3.71E-06	7.48E-06	7.72E-06	2.01E-06
SCIN	1.73E-05	1.47E-06	9.47E-09	3.19E-07	0	8.45E-08	2.82E-07	0
SCLT1	5.48E-06	1.57E-06	8.49E-07	1.81E-06	4.61E-07	1.47E-06	7.89E-07	1.53E-06
SCLY	4.97E-05	2.77E-05	2.01E-05	1.54E-05	1.26E-05	1.73E-05	1.82E-05	3.57E-05
SCMH1	8.89E-06	3.14E-06	3.00E-06	5.41E-06	1.59E-06	3.45E-06	4.02E-06	7.69E-06
SCML1	2.09E-05	1.24E-05	8.85E-06	6.21E-06	2.68E-06	1.66E-05	5.41E-06	1.58E-05
SCML2	6.34E-07	8.98E-07	3.21E-07	3.12E-07	1.65E-07	5.86E-07	4.23E-07	1.65E-06
SCML4	1.14E-05	2.17E-07	8.00E-07	4.94E-07	1.21E-07	6.93E-07	5.34E-07	1.39E-06
SCN10A	0	0	0	4.66E-09	0	0	0	0
SCN11A	1.38E-08	1.78E-07	3.16E-08	1.26E-08	8.85E-09	1.91E-08	2.01E-08	1.01E-08
SCN1A	0	0	1.17E-08	0	0	7.61E-09	0	1.66E-08
SCN1B	1.40E-05	2.03E-06	6.41E-06	1.02E-05	3.85E-06	3.99E-06	8.29E-06	7.28E-06
SCN2A	1.04E-08	4.34E-08	1.85E-08	1.58E-08	0	1.66E-07	0	7.58E-09
SCN2B	4.59E-09	0	4.70E-09	4.48E-08	3.93E-09	0	0	0
SCN3A	1.95E-07	1.96E-08	2.77E-08	2.66E-08	0	1.76E-08	6.79E-09	2.43E-08
SCN3B	5.15E-08	1.33E-08	1.62E-08	7.25E-08	3.40E-09	3.66E-09	1.16E-08	2.90E-08
SCN4A	3.42E-08	5.27E-07	6.10E-08	1.74E-08	2.74E-07	2.79E-07	2.67E-07	2.08E-07
SCN4B	9.91E-07	1.49E-06	2.27E-07	6.62E-07	6.62E-07	1.04E-06	1.62E-06	2.58E-07
SCN5A	3.99E-08	0	0	2.52E-08	0	0	5.19E-09	3.89E-09
SCN7A	6.34E-08	1.31E-08	1.04E-07	1.05E-06	0	4.35E-08	1.53E-08	9.74E-08
SCN8A	8.16E-07	6.92E-07	3.51E-07	3.70E-07	5.57E-08	4.97E-07	1.28E-07	8.49E-07
SCN9A	1.01E-05	2.57E-06	1.80E-06	1.90E-06	1.13E-07	4.20E-08	4.57E-07	1.61E-06
SCNM1	3.36E-05	2.10E-05	1.19E-05	1.73E-05	2.09E-05	2.55E-05	2.46E-05	3.60E-05
SCNN1A	2.35E-06	1.24E-05	2.27E-06	2.87E-06	6.27E-07	2.47E-06	7.01E-06	6.97E-07
SCNN1B	5.41E-08	5.38E-07	6.55E-08	2.65E-07	5.42E-08	3.34E-08	8.74E-07	3.84E-07
SCNN1D	1.09E-06	6.50E-07	1.29E-06	1.10E-06	1.93E-06	1.02E-06	9.09E-07	8.33E-07
SCNN1G	0	1.98E-07	0	0	0	6.07E-09	6.37E-09	9.61E-09
SCO1	1.24E-05	1.66E-05	1.65E-05	1.36E-05	4.56E-06	2.97E-05	1.46E-05	1.76E-05
SCO2	4.83E-05	4.58E-05	3.85E-05	3.66E-05	6.28E-05	4.00E-05	5.43E-05	4.92E-05
SCOC	3.31E-05	3.68E-05	3.03E-05	2.23E-05	1.39E-05	3.31E-05	2.90E-05	3.01E-05
SCP2	0.000172	0.000322	0.000574	0.00026	5.22E-05	0.000349	0.000178	0.000178
SCPEP1	3.52E-05	6.00E-05	2.43E-05	2.28E-05	8.80E-06	9.05E-05	1.34E-05	2.35E-05
SCRG1	3.04E-08	0	3.37E-08	4.52E-07	0	0	2.88E-08	0
SCRIB	2.99E-05	3.64E-05	9.81E-06	1.28E-05	2.45E-05	1.28E-05	5.74E-05	1.29E-05

SCRN1	2.90E-05	7.42E-07	9.57E-07	4.24E-06	2.34E-07	7.87E-06	2.02E-06	6.54E-07
SCRN2	1.83E-05	2.19E-05	3.25E-05	3.51E-05	6.78E-05	7.25E-05	7.18E-05	2.17E-05
SCRN3	1.28E-05	1.31E-05	7.05E-06	5.54E-06	2.23E-06	1.04E-05	3.14E-06	4.37E-06
SCRT1	4.52E-08	7.23E-09	0	0	1.66E-08	5.97E-09	0	1.89E-08
SCRT2	0	0	6.60E-09	0	0	5.93E-09	6.22E-09	0
SCT	6.64E-08	2.48E-07	0	3.37E-07	3.51E-07	0	3.66E-07	0
SCTR	9.12E-06	6.32E-06	6.80E-08	1.09E-05	4.14E-08	2.98E-08	1.54E-08	1.96E-08
SCUBE1	1.73E-07	6.68E-08	5.28E-07	2.26E-07	7.03E-08	4.97E-07	1.51E-07	2.27E-07
SCUBE2	1.70E-07	2.54E-07	1.42E-07	7.48E-07	3.14E-08	2.12E-07	8.23E-07	1.29E-07
SCUBE3	4.00E-08	1.49E-08	9.11E-09	2.71E-08	3.81E-09	8.22E-09	3.46E-08	0
SCXB	3.63E-07	1.89E-07	0	6.51E-08	1.36E-07	4.89E-08	4.34E-07	7.89E-08
SCYL1	4.34E-05	6.45E-05	3.91E-05	4.22E-05	2.95E-05	3.81E-05	5.39E-05	4.84E-05
SCYL2	1.61E-05	1.51E-05	8.90E-06	6.29E-06	8.56E-07	1.04E-05	3.95E-06	8.27E-06
SCYL3	6.02E-06	9.15E-06	3.94E-06	3.97E-06	1.01E-06	5.35E-06	2.51E-06	7.46E-06
SDAD1	1.59E-05	9.26E-06	8.03E-06	9.36E-06	2.76E-06	2.36E-05	9.96E-06	1.41E-05
SDC1	0.000353	0.001028	0.000299	0.000299	0.000522	0.000188	0.000384	0.000333
SDC2	0.000141	0.000184	0.000122	6.89E-05	0.000151	0.000118	0.000249	6.98E-05
SDC3	2.17E-05	4.01E-06	1.73E-05	2.10E-05	1.90E-06	1.07E-05	1.63E-05	9.03E-06
SDC4	0.000459	0.000272	0.000176	0.000442	0.000167	0.000106	0.000362	0.000262
SDC4P	0	0	0	0	0	0	1.41E-07	0
SDCBP	0.000181	0.000307	5.43E-05	9.20E-05	3.04E-05	8.46E-05	0.000146	6.13E-05
SDCBP2	0.000183	5.57E-06	3.09E-06	1.05E-05	2.06E-06	1.40E-06	1.68E-05	2.08E-06
SDCCAG1	1.51E-05	9.17E-06	6.02E-06	6.24E-06	1.74E-06	7.37E-06	4.16E-06	8.87E-06
SDCCAG3	1.96E-05	9.19E-06	2.04E-05	1.84E-05	1.47E-05	2.19E-05	2.24E-05	3.33E-05
SDCCAG8	8.06E-06	8.65E-06	4.11E-06	5.78E-06	2.54E-06	9.16E-06	4.32E-06	7.15E-06
SDF2	7.23E-05	5.51E-05	3.28E-05	4.10E-05	5.52E-05	4.40E-05	3.64E-05	3.56E-05
SDF2L1	5.17E-05	3.36E-05	2.52E-05	3.39E-05	0.00013	8.06E-05	7.70E-05	0.000108
SDF4	0.000109	7.41E-05	9.52E-05	9.10E-05	5.39E-05	9.07E-05	9.83E-05	8.07E-05
SDHA	0.000151	0.000206	0.000191	0.000136	5.83E-05	0.000112	0.000103	0.000163
SDHAF1	2.34E-05	8.85E-06	1.01E-05	1.42E-05	4.71E-05	2.31E-05	2.35E-05	9.65E-06
SDHAF2	5.25E-05	3.76E-05	2.49E-05	3.41E-05	2.74E-05	3.68E-05	3.24E-05	3.94E-05
SDHAP1	1.23E-05	4.41E-06	4.39E-06	4.67E-06	3.94E-06	3.83E-06	5.44E-06	3.43E-06
SDHAP2	7.41E-06	1.83E-06	1.70E-06	3.43E-06	1.22E-06	1.85E-06	1.58E-06	2.19E-06
SDHAP3	1.95E-05	9.85E-06	5.78E-06	7.49E-06	3.77E-06	6.07E-06	4.21E-06	1.08E-05
SDHB	0.000268	0.000199	0.000158	0.000137	6.51E-05	0.000171	0.000111	0.000146
SDHC	0.000106	0.000198	6.93E-05	6.98E-05	1.79E-05	0.000134	0.000101	0.000143
SDHD	0.000222	0.000309	0.000189	0.000113	3.97E-05	0.00014	8.10E-05	0.000187
SDK1	2.30E-06	1.67E-07	9.97E-08	5.11E-07	3.92E-08	1.01E-08	1.85E-07	7.50E-08
SDK2	4.36E-08	5.42E-08	7.98E-08	2.36E-07	6.99E-09	2.01E-08	1.04E-07	1.98E-08
SDPR	4.91E-06	9.89E-06	1.01E-05	8.53E-06	2.27E-06	8.56E-06	9.76E-06	3.86E-06
SDR16C5	7.62E-09	0	3.15E-08	9.77E-09	0	0	6.65E-08	0
SDR39U1	4.91E-05	2.47E-05	2.27E-05	2.34E-05	3.26E-05	3.27E-05	2.73E-05	3.12E-05
SDR42E1	5.19E-06	9.63E-07	1.93E-06	2.44E-06	2.88E-08	7.62E-07	8.63E-07	1.23E-08
SDR9C7	0	0	6.36E-08	0	0	0	0	0
SDS	0.000113	0.00195	0.000583	0.000201	0.00037	0.000122	0.000143	0.00223
SDSL	4.80E-05	0.000251	4.56E-05	4.95E-05	0.000122	4.24E-05	0.0001	0.000141
SEBOX	1.36E-07	0	2.52E-07	1.14E-07	3.97E-08	4.27E-08	0	6.88E-08
SEC1	1.01E-07	5.72E-08	7.40E-08	4.97E-08	5.23E-08	5.64E-08	7.92E-08	4.33E-08
SEC11A	0.000117	6.68E-05	6.28E-05	7.86E-05	5.60E-05	9.46E-05	9.30E-05	6.01E-05
SEC11C	0.000101	0.000154	0.000107	0.000112	9.02E-05	0.000305	0.000139	0.000257
SEC13	0.000111	9.06E-05	4.92E-05	6.55E-05	6.58E-05	0.00012	8.00E-05	8.10E-05
SEC14L1	2.74E-05	1.55E-05	8.25E-06	1.13E-05	4.06E-06	1.06E-05	1.15E-05	9.82E-06
SEC14L2	2.71E-05	6.96E-05	7.11E-05	3.59E-05	8.21E-05	0.000108	6.33E-05	9.61E-06
SEC14L3	0	5.18E-08	1.14E-06	5.61E-08	6.21E-07	1.21E-06	8.75E-07	2.36E-07
SEC14L4	2.41E-06	2.26E-06	7.50E-06	2.99E-06	5.03E-07	5.57E-06	4.58E-06	1.40E-05
SEC14L5	6.93E-09	9.27E-08	3.54E-09	8.45E-09	8.91E-09	6.41E-08	1.35E-08	0
SEC16A	5.39E-05	3.17E-05	3.16E-05	3.03E-05	4.00E-05	5.60E-05	3.91E-05	8.88E-05
SEC16B	4.47E-05	4.68E-05	1.35E-05	1.06E-05	7.08E-06	7.12E-05	7.52E-06	5.06E-05

SEC22A	8.08E-06	3.61E-06	3.17E-06	3.35E-06	3.06E-06	3.99E-06	4.01E-06	2.74E-06
SEC22B	6.09E-05	5.93E-05	4.56E-05	3.31E-05	9.29E-06	9.51E-05	2.14E-05	3.22E-05
SEC22C	1.86E-05	1.09E-05	7.87E-06	9.66E-06	5.05E-06	1.17E-05	1.69E-05	1.32E-05
SEC23A	6.53E-05	3.94E-05	2.87E-05	2.38E-05	8.49E-06	4.13E-05	1.45E-05	2.46E-05
SEC23B	5.16E-05	2.60E-05	1.46E-05	1.52E-05	4.73E-06	4.06E-05	1.62E-05	1.35E-05
SEC23IP	1.53E-05	1.01E-05	8.84E-06	9.32E-06	3.05E-06	1.35E-05	8.49E-06	8.76E-06
SEC24A	1.03E-05	1.64E-05	1.15E-05	9.59E-06	2.00E-06	1.16E-05	4.61E-06	6.51E-06
SEC24B	1.16E-05	7.24E-06	2.57E-05	8.67E-06	3.72E-06	1.13E-05	5.75E-06	2.12E-05
SEC24C	6.01E-05	4.36E-05	3.04E-05	3.08E-05	3.99E-05	5.91E-05	4.43E-05	4.28E-05
SEC24D	1.60E-05	2.08E-05	2.00E-05	1.67E-05	2.19E-06	3.02E-05	8.35E-06	2.34E-05
SEC31A	9.20E-05	5.53E-05	6.97E-05	8.04E-05	3.30E-05	0.000122	7.37E-05	0.000123
SEC31B	1.06E-06	2.54E-07	3.13E-07	3.82E-07	4.39E-07	6.96E-07	2.84E-07	4.77E-07
SEC61A1	0.000123	0.000115	8.93E-05	8.14E-05	0.000111	0.000125	0.00012	0.000122
SEC61A2	3.35E-06	4.63E-06	3.24E-06	1.65E-06	1.70E-06	4.10E-06	1.49E-06	1.89E-06
SEC61B	0.000326	0.000367	0.000247	0.000293	0.000381	0.000403	0.000293	0.000255
SEC61G	0.000231	0.000188	9.10E-05	0.00015	0.000341	0.000262	0.000245	0.000194
SEC62	9.16E-05	5.02E-05	8.01E-05	7.30E-05	8.51E-05	5.37E-05	8.26E-05	5.17E-05
SEC63	3.28E-05	2.17E-05	1.85E-05	1.47E-05	4.54E-06	2.79E-05	1.27E-05	2.02E-05
SECISBP2	1.74E-05	1.72E-05	1.27E-05	1.28E-05	6.53E-06	1.78E-05	7.25E-06	1.49E-05
SECISBP2L	8.77E-06	6.21E-06	6.78E-06	4.98E-06	6.55E-07	6.67E-06	2.93E-06	4.31E-06
SECTM1	4.84E-05	3.56E-05	3.90E-06	7.99E-06	1.85E-06	4.58E-06	5.89E-06	2.40E-06
SEH1L	1.36E-05	1.62E-05	9.96E-06	1.22E-05	4.03E-06	1.31E-05	1.31E-05	1.21E-05
SEL1L	2.28E-05	3.26E-05	3.25E-05	2.21E-05	1.09E-05	6.15E-05	1.83E-05	2.86E-05
SEL1L2	0	0	0	0	0	0	0	0
SEL1L3	6.75E-05	1.05E-06	6.31E-06	2.17E-05	5.82E-07	2.47E-05	7.26E-06	1.95E-06
SELE	4.77E-07	1.12E-07	1.01E-06	1.99E-06	1.11E-07	3.43E-06	2.06E-07	1.21E-07
SELENBP1	4.53E-05	0.000102	0.00019	0.000143	9.86E-05	0.000198	0.000161	5.00E-05
SELK	0.000123	8.26E-05	4.21E-05	4.89E-05	4.20E-05	6.69E-05	3.78E-05	5.00E-05
SELL	8.19E-06	1.95E-06	2.26E-06	6.36E-06	1.77E-07	2.53E-06	1.56E-06	1.01E-06
SELM	0.000102	3.76E-05	1.07E-05	3.55E-05	9.99E-06	1.29E-05	1.90E-05	7.23E-05
SELO	3.82E-05	5.25E-05	5.47E-05	4.30E-05	0.000113	4.35E-05	5.30E-05	5.48E-05
SELP	7.09E-07	1.77E-07	6.67E-07	2.02E-06	1.54E-07	1.38E-07	1.56E-07	3.27E-07
SELPLG	1.49E-05	1.55E-06	5.36E-06	1.49E-05	1.50E-06	6.13E-06	1.18E-05	3.42E-06
SELS	7.43E-05	6.43E-05	3.34E-05	5.58E-05	4.02E-05	5.73E-05	6.13E-05	6.28E-05
SELT	2.33E-05	3.74E-05	2.00E-05	2.05E-05	1.19E-05	2.38E-05	1.99E-05	2.12E-05
SELV	0	0	0	0	0	0	0	0
SEMA3A	1.19E-07	7.10E-08	2.85E-08	1.79E-07	1.02E-08	2.20E-08	3.48E-08	1.34E-07
SEMA3B	3.66E-05	9.94E-06	6.08E-07	7.01E-06	7.83E-07	1.52E-06	1.01E-05	2.59E-07
SEMA3C	2.10E-06	1.39E-07	1.93E-07	9.15E-07	3.68E-08	4.85E-08	4.77E-07	4.18E-08
SEMA3D	1.12E-07	2.08E-07	1.60E-07	8.29E-07	4.34E-08	4.29E-08	1.01E-07	1.03E-07
SEMA3E	9.74E-07	3.85E-09	1.06E-07	3.41E-07	0	0	0	2.02E-08
SEMA3F	7.57E-06	5.98E-06	1.48E-06	1.69E-06	3.93E-06	4.28E-06	5.10E-06	3.75E-06
SEMA3G	3.05E-06	4.32E-06	4.00E-07	9.16E-07	1.77E-06	1.68E-06	1.68E-06	3.91E-06
SEMA4A	3.55E-06	2.02E-07	1.37E-06	1.88E-06	1.21E-07	3.89E-07	5.23E-07	6.08E-07
SEMA4B	1.78E-05	1.46E-05	1.38E-05	2.10E-05	5.52E-06	7.60E-06	1.17E-05	2.24E-05
SEMA4C	1.98E-05	7.57E-06	7.87E-06	9.55E-06	3.76E-06	8.63E-06	5.91E-06	1.30E-05
SEMA4D	9.30E-06	1.62E-06	1.63E-06	4.04E-06	9.64E-08	1.32E-06	9.85E-07	1.10E-06
SEMA4F	6.37E-06	7.85E-07	7.20E-07	6.08E-07	1.75E-07	6.71E-08	9.74E-07	4.38E-06
SEMA4G	3.50E-05	3.55E-05	1.40E-05	1.13E-05	1.60E-05	2.35E-05	1.36E-05	1.51E-05
SEMA5A	1.85E-06	9.77E-07	2.36E-06	2.04E-06	3.79E-07	4.35E-07	8.42E-07	9.95E-07
SEMA5B	8.45E-06	1.43E-06	4.76E-07	2.80E-07	2.23E-06	1.22E-06	1.31E-06	3.11E-06
SEMA6A	4.74E-05	5.85E-07	3.13E-06	3.37E-06	6.48E-07	3.20E-07	1.06E-06	2.05E-06
SEMA6B	4.14E-06	1.86E-06	1.01E-06	3.49E-06	1.92E-06	1.70E-06	7.32E-06	2.75E-06
SEMA6C	6.00E-06	3.45E-06	1.10E-06	2.28E-06	2.68E-06	2.43E-06	2.71E-06	9.62E-07
SEMA6D	4.76E-07	3.56E-07	8.98E-07	7.78E-07	2.19E-07	1.02E-07	1.41E-07	4.40E-07
SEMA7A	2.78E-06	3.42E-07	3.98E-07	8.39E-06	6.87E-07	9.49E-06	4.02E-06	1.37E-06
SEMG1	1.47E-08	0	0	0	0	0	0	0
SEMG2	2.42E-08	3.45E-08	0	0	0	0	0	0

SENP1	3.37E-06	2.52E-06	1.25E-06	2.01E-06	4.21E-07	2.60E-06	1.90E-06	1.48E-06
SENP2	1.72E-05	1.10E-05	8.28E-06	7.59E-06	2.50E-06	1.02E-05	5.30E-06	8.46E-06
SENP3	1.12E-05	1.08E-05	9.07E-06	1.15E-05	2.26E-06	1.75E-05	1.40E-05	1.36E-05
SENP5	4.59E-06	3.56E-06	2.89E-06	3.69E-06	2.03E-06	3.93E-06	3.53E-06	4.24E-06
SENP6	9.97E-06	1.16E-05	6.92E-06	6.75E-06	1.46E-06	9.00E-06	4.89E-06	5.19E-06
SENP7	5.76E-06	6.71E-06	2.23E-06	2.47E-06	6.47E-07	2.33E-06	1.88E-06	3.48E-06
SENP8	3.38E-06	7.14E-07	1.24E-06	6.19E-07	9.88E-07	1.79E-06	8.18E-07	1.21E-06
15-Sep	0.000113	0.000123	6.67E-05	7.01E-05	2.93E-05	0.000127	6.13E-05	6.73E-05
SEPHS1	3.65E-05	2.79E-05	1.72E-05	1.77E-05	1.34E-05	1.77E-05	1.36E-05	2.06E-05
SEPHS2	0.000522	0.000943	0.000249	0.000199	0.000225	0.000295	0.000283	0.000281
SEPN1	3.24E-05	1.26E-05	9.43E-06	1.43E-05	5.66E-06	1.31E-05	1.36E-05	1.83E-05
SEPP1	0.000996	0.002261	0.002433	0.001368	0.000838	0.001814	0.000836	0.001107
SEPSECS	1.05E-05	1.31E-05	1.19E-05	9.86E-06	3.57E-06	1.41E-05	6.88E-06	1.06E-05
1-Sep	9.58E-06	7.06E-06	3.19E-06	7.26E-06	2.28E-06	5.70E-06	1.21E-05	2.11E-06
10-Sep	3.22E-05	2.23E-05	1.98E-05	1.89E-05	2.76E-06	1.74E-05	1.01E-05	6.45E-06
11-Sep	2.95E-05	1.63E-05	1.13E-05	1.53E-05	1.99E-06	8.26E-06	9.01E-06	1.26E-05
12-Sep	0	2.46E-08	0	0	0	1.75E-08	0	0
14-Sep	3.02E-08	0	0	0	0	0	0	0
2-Sep	0.000164	4.06E-05	3.81E-05	5.94E-05	2.05E-05	4.93E-05	4.42E-05	3.27E-05
3-Sep	2.88E-06	5.64E-07	9.31E-08	5.49E-08	0	3.86E-08	1.24E-07	6.43E-08
4-Sep	7.91E-06	1.73E-05	2.03E-05	2.20E-05	9.55E-06	0.000386	1.28E-05	6.00E-06
5-Sep	5.56E-06	1.01E-06	4.24E-07	1.54E-06	2.03E-06	5.92E-07	1.86E-06	1.11E-06
6-Sep	5.11E-05	2.71E-05	1.03E-05	1.67E-05	1.48E-05	9.44E-06	1.21E-05	3.31E-05
7-Sep	8.51E-05	4.19E-05	2.72E-05	3.81E-05	1.11E-05	3.23E-05	2.57E-05	4.36E-05
SEPT7L	0	2.70E-07	5.24E-08	0	0	4.24E-08	4.22E-08	0
SEPT7P2	4.87E-06	3.85E-06	9.01E-07	8.45E-07	7.58E-07	1.43E-06	7.71E-07	2.41E-06
8-Sep	1.70E-05	7.91E-06	3.77E-06	7.90E-06	1.81E-06	7.41E-06	6.07E-06	9.71E-06
9-Sep	0.000147	0.000119	3.95E-05	6.12E-05	4.13E-05	0.000113	6.60E-05	7.28E-05
SEPW1	0.000229	9.41E-05	8.10E-05	9.32E-05	0.000197	8.98E-05	6.17E-05	7.12E-05
SEPX1	0.000142	0.000242	0.000135	8.35E-05	0.000111	0.000167	0.000124	0.000135
SERAC1	3.75E-06	3.24E-06	1.36E-06	1.32E-06	6.55E-07	1.77E-06	1.29E-06	1.32E-06
SERBP1	4.50E-05	4.90E-05	3.88E-05	3.55E-05	1.25E-05	6.54E-05	2.64E-05	2.88E-05
SERF1A	3.29E-05	1.07E-05	8.78E-06	2.09E-05	7.28E-06	2.04E-05	1.51E-05	2.08E-05
SERF2	0.000499	0.000269	0.000151	0.000294	0.000575	0.0003	0.000489	0.000221
SERGEF	8.36E-06	7.28E-06	5.99E-06	7.90E-06	9.39E-06	7.39E-06	5.26E-06	1.25E-05
SERHL	4.72E-06	5.61E-06	9.53E-07	1.03E-06	5.93E-07	2.70E-06	2.93E-06	9.35E-07
SERHL2	3.73E-06	1.17E-05	2.89E-07	3.42E-07	2.20E-07	4.54E-08	4.65E-07	3.49E-08
SERINC1	0.000165	0.00013	0.000136	0.000115	3.05E-05	0.000136	8.43E-05	9.00E-05
SERINC2	0.000131	6.18E-05	0.000111	0.000234	8.64E-05	0.000137	0.000111	0.000255
SERINC3	7.57E-05	6.30E-05	4.17E-05	3.03E-05	1.11E-05	5.66E-05	2.24E-05	4.72E-05
SERINC4	2.84E-07	3.80E-08	3.03E-08	4.80E-08	0	7.12E-08	1.61E-08	2.48E-08
SERINC5	1.20E-05	1.70E-05	1.31E-05	4.18E-06	6.70E-07	1.58E-05	2.58E-06	6.01E-06
SERP1	4.82E-05	0.000101	5.77E-05	4.91E-05	4.74E-05	8.52E-05	7.02E-05	4.47E-05
SERP2	2.47E-06	1.23E-07	2.04E-06	5.08E-06	2.53E-07	2.43E-06	1.38E-06	3.56E-07
SERPINA1	0.017993	0.006614	0.014915	0.020136	0.012985	0.018628	0.02164	0.015161
SERPINA1	0.000126	0.000167	0.000185	0.000142	0.000103	0.000241	0.000138	0.000138
SERPINA1	2.62E-05	7.98E-05	0.000542	0.000559	0.00033	0.000177	0.000305	0.000577
SERPINA1	1.28E-07	2.62E-08	1.21E-07	7.10E-08	1.40E-07	3.23E-08	4.48E-08	4.09E-07
SERPINA1	0	3.22E-08	0	1.74E-08	0	0	0	0
SERPINA3	0.000698	0.000799	0.00339	0.004205	0.003847	0.008509	0.004226	0.007343
SERPINA4	6.98E-06	0.000207	0.000386	0.000305	0.00047	5.19E-05	0.000271	0.000108
SERPINA5	0.000214	8.00E-05	0.000567	0.000213	0.00037	7.49E-05	0.000137	0.000386
SERPINA6	0.000464	0.000436	0.0006	0.0006	0.000821	0.000351	0.00047	0.000737
SERPINA7	1.27E-07	6.63E-05	0.000195	0.000178	0.000124	0.0002	0.000146	0.000338
SERPINA9	0	1.83E-08	9.69E-08	5.92E-08	1.94E-08	3.66E-08	2.14E-08	0
SERPINB1	9.69E-05	3.95E-05	2.31E-05	7.20E-05	1.16E-05	6.24E-05	2.77E-05	7.45E-05
SERPINB1(	0	0	0	0	0	0	0	0
SERPINB1:	0	2.83E-08	0	0	0	0	0	0

SERPINB1:	0	0	4.28E-08	0	0	0	0	6.26E-08
SERPINB1:	0	0	1.23E-07	0	0	0	0	4.26E-08
SERPINB2	0	0	3.98E-08	2.48E-07	0	0	1.22E-08	1.86E-08
SERPINB3	1.56E-07	1.09E-07	3.60E-07	0	0	0	0	2.02E-07
SERPINB4	1.54E-08	1.76E-08	1.20E-07	0	0	0	0	1.88E-07
SERPINB5	0	6.00E-08	1.94E-07	2.38E-08	0	0	1.86E-08	1.30E-08
SERPINB6	5.54E-05	3.61E-05	2.75E-05	5.06E-05	5.36E-05	4.46E-05	5.32E-05	4.10E-05
SERPINB7	0	0	3.90E-08	0	0	0	0	0
SERPINB8	1.05E-05	1.80E-06	3.77E-06	1.82E-05	7.52E-07	2.35E-06	8.59E-06	1.33E-05
SERPINB9	7.50E-06	8.15E-06	3.39E-06	1.38E-05	8.30E-07	3.35E-06	2.88E-06	3.84E-06
SERPINC1	6.76E-05	0.00388	0.003296	0.00244	0.00189	0.00297	0.001721	0.004911
SERPIND1	0.000261	0.000658	0.000735	0.000417	0.000535	0.000724	0.000367	0.000589
SERPINE1	1.26E-05	4.07E-05	0.000258	0.000688	3.03E-06	1.37E-05	0.000179	5.45E-05
SERPINE2	3.69E-05	2.70E-07	3.69E-06	2.19E-05	4.25E-06	1.36E-06	8.08E-06	7.99E-06
SERPINE3	4.00E-08	0	0	0	2.49E-08	0	0	0
SERPINF1	0.00012	0.000508	0.000837	0.000662	0.001122	0.001911	0.000671	0.000769
SERPINF2	0.00028	0.000516	0.001085	0.000906	0.001844	0.000982	0.0009	0.000625
SERPING1	0.001997	0.002828	0.002962	0.003494	0.001534	0.003482	0.00221	0.004186
SERPINH1	0.000108	4.15E-05	1.43E-05	4.00E-05	2.60E-05	2.07E-05	5.43E-05	2.70E-05
SERPINI1	1.22E-05	8.97E-06	9.24E-07	6.98E-07	3.52E-07	4.58E-06	7.17E-07	2.45E-06
SERPINI2	1.75E-08	0	0	0	0	0	0	0
SERTAD1	1.71E-05	1.66E-05	1.42E-05	8.82E-05	4.33E-05	8.91E-06	7.12E-05	2.03E-05
SERTAD2	1.44E-05	8.96E-06	6.29E-06	5.42E-06	3.91E-06	7.67E-06	5.76E-06	3.73E-06
SERTAD3	2.09E-05	1.12E-05	7.54E-06	1.86E-05	1.61E-05	9.77E-06	2.25E-05	2.43E-05
SERTAD4	9.57E-08	4.06E-08	1.25E-08	4.40E-08	1.03E-08	1.11E-08	6.93E-08	3.52E-08
SESN1	1.17E-05	7.39E-06	1.29E-05	7.58E-06	5.08E-06	1.57E-05	6.96E-06	6.79E-06
SESN2	2.37E-05	1.57E-05	9.29E-06	6.07E-06	9.87E-06	1.25E-05	8.69E-06	8.45E-06
SESN3	1.25E-06	2.23E-06	1.13E-06	5.92E-07	9.88E-08	7.50E-07	2.91E-07	7.10E-08
SESTD1	7.37E-06	3.99E-06	1.22E-06	2.83E-06	4.88E-07	2.91E-06	2.22E-06	2.43E-06
SET	8.19E-05	5.36E-05	3.99E-05	6.11E-05	2.21E-05	6.51E-05	7.02E-05	6.16E-05
SETBP1	4.31E-06	7.56E-07	1.70E-06	1.35E-06	4.27E-07	1.33E-06	7.35E-07	1.77E-06
SETD1A	5.30E-06	7.20E-06	3.24E-06	4.67E-06	1.69E-06	4.67E-06	4.35E-06	3.75E-06
SETD1B	4.64E-06	3.56E-06	3.35E-06	2.56E-06	9.72E-07	3.14E-06	2.73E-06	3.13E-06
SETD2	3.13E-05	2.77E-05	1.82E-05	2.30E-05	1.74E-05	2.16E-05	2.05E-05	2.26E-05
SETD3	2.63E-05	1.94E-05	2.25E-05	2.00E-05	1.21E-05	2.25E-05	1.83E-05	2.19E-05
SETD4	6.42E-06	2.20E-06	2.50E-06	3.31E-06	1.58E-06	3.25E-06	4.32E-06	6.13E-06
SETD5	1.79E-05	1.07E-05	7.23E-06	8.23E-06	2.64E-06	1.13E-05	6.42E-06	7.92E-06
SETD6	5.23E-06	2.08E-06	2.77E-06	4.04E-06	1.40E-06	6.02E-06	2.60E-06	2.98E-06
SETD7	2.89E-05	1.54E-05	2.27E-05	1.52E-05	6.32E-06	2.13E-05	1.69E-05	2.59E-05
SETD8	9.23E-06	1.18E-05	4.91E-06	7.94E-06	2.85E-06	7.70E-06	7.30E-06	6.79E-06
SETDB1	1.03E-05	7.64E-06	4.90E-06	5.31E-06	2.50E-06	1.37E-05	5.61E-06	8.52E-06
SETDB2	3.54E-06	3.01E-06	2.26E-06	2.68E-06	7.89E-07	3.17E-06	2.35E-06	1.94E-06
SETMAR	6.57E-06	8.11E-06	4.59E-06	5.95E-06	3.75E-06	9.44E-06	5.27E-06	3.05E-06
SETX	1.00E-05	4.26E-06	5.85E-06	6.05E-06	1.30E-06	7.63E-06	3.08E-06	9.36E-06
SEZ6	5.86E-09	3.62E-08	0	1.24E-07	5.25E-08	1.29E-08	5.70E-09	9.05E-07
SEZ6L	8.08E-09	0	2.51E-08	1.98E-08	0	2.99E-08	2.49E-08	1.19E-08
SEZ6L2	4.62E-05	7.28E-05	1.87E-07	5.25E-06	2.58E-07	2.74E-06	1.50E-06	2.91E-07
SF1	6.44E-05	7.49E-05	5.21E-05	7.43E-05	3.87E-05	5.96E-05	7.65E-05	6.04E-05
SF3A1	2.90E-05	2.38E-05	1.49E-05	1.79E-05	5.50E-06	2.27E-05	1.50E-05	1.79E-05
SF3A2	3.80E-05	2.09E-05	1.53E-05	2.07E-05	2.82E-05	2.11E-05	3.60E-05	1.87E-05
SF3A3	3.22E-05	2.22E-05	1.44E-05	1.97E-05	8.76E-06	2.82E-05	2.12E-05	2.00E-05
SF3B1	0.000125	4.97E-05	3.37E-05	4.40E-05	9.67E-06	5.08E-05	2.31E-05	4.29E-05
SF3B14	0.000176	0.000162	5.76E-05	7.58E-05	8.61E-05	0.000104	9.39E-05	7.43E-05
SF3B2	8.46E-05	9.14E-05	4.56E-05	6.16E-05	3.12E-05	7.57E-05	5.78E-05	7.48E-05
SF3B3	1.48E-05	9.47E-06	7.71E-06	8.51E-06	2.67E-06	1.39E-05	6.85E-06	8.24E-06
SF3B4	5.49E-05	6.62E-05	2.48E-05	4.89E-05	3.32E-05	6.17E-05	4.96E-05	7.38E-05
SF3B5	0.000213	0.000127	0.000103	0.000118	0.000139	0.000203	0.000181	0.000126
SF4	1.71E-05	1.12E-05	1.08E-05	1.29E-05	1.86E-05	1.70E-05	1.49E-05	1.39E-05

SFI1	5.66E-06	2.24E-06	1.83E-06	3.14E-06	3.20E-06	1.80E-06	2.78E-06	2.55E-06
SFMBT1	5.20E-06	4.49E-06	2.56E-06	3.06E-06	2.12E-06	4.43E-06	2.80E-06	2.72E-06
SFMBT2	2.33E-06	3.58E-08	2.64E-07	4.45E-07	0	1.36E-07	9.73E-08	1.15E-07
SFN	7.00E-05	6.06E-05	6.83E-06	8.45E-06	5.32E-07	5.02E-05	3.33E-06	2.93E-06
SFPQ	0.000131	7.25E-05	3.57E-05	0.000112	2.85E-05	7.06E-05	5.99E-05	4.50E-05
SFRP1	1.30E-06	1.54E-07	3.16E-06	2.68E-06	7.42E-08	1.88E-08	2.13E-07	7.45E-09
SFRP2	2.41E-08	2.73E-08	1.26E-08	7.38E-08	0	4.47E-08	1.68E-06	3.90E-07
SFRP4	3.42E-06	8.76E-08	9.68E-08	2.45E-06	1.54E-07	1.15E-06	2.46E-06	6.86E-07
SFRP5	2.69E-07	0	1.07E-05	7.75E-06	1.10E-08	0	0	0
SFRS1	5.13E-05	2.71E-05	2.26E-05	2.09E-05	6.54E-06	2.62E-05	2.44E-05	2.04E-05
SFRS11	6.90E-05	2.53E-05	1.83E-05	2.13E-05	5.06E-06	3.42E-05	1.06E-05	2.61E-05
SFRS12	2.49E-05	8.58E-06	5.96E-06	6.95E-06	1.70E-06	1.20E-05	4.93E-06	1.23E-05
SFRS12IP1	4.84E-06	2.87E-06	3.14E-06	3.04E-06	2.77E-06	2.71E-06	3.71E-06	2.79E-06
SFRS13A	4.86E-05	3.25E-05	2.16E-05	2.13E-05	7.61E-06	2.29E-05	1.82E-05	1.78E-05
SFRS13B	8.53E-07	1.43E-08	3.28E-08	3.66E-07	2.19E-08	4.71E-08	1.24E-07	9.33E-09
SFRS14	1.30E-05	8.19E-06	3.67E-06	4.96E-06	2.62E-06	7.99E-06	3.92E-06	5.10E-06
SFRS15	1.26E-05	7.40E-06	7.16E-06	8.07E-06	3.96E-06	8.63E-06	9.51E-06	8.47E-06
SFRS16	1.45E-05	7.50E-06	6.25E-06	1.14E-05	5.21E-06	1.02E-05	1.04E-05	9.11E-06
SFRS17A	1.55E-05	9.74E-06	5.74E-06	1.10E-05	5.39E-06	1.11E-05	1.17E-05	1.27E-05
SFRS18	3.02E-05	1.68E-05	9.51E-06	1.23E-05	2.73E-06	1.31E-05	8.53E-06	7.93E-06
SFRS2	0.000124	6.77E-05	4.60E-05	7.09E-05	3.40E-05	7.11E-05	6.38E-05	6.47E-05
SFRS2B	1.63E-05	7.31E-06	1.52E-05	1.21E-05	4.82E-06	1.12E-05	8.44E-06	1.05E-05
SFRS2IP	1.81E-05	1.46E-05	1.14E-05	1.08E-05	2.52E-06	1.26E-05	6.92E-06	8.90E-06
SFRS3	9.57E-05	5.81E-05	3.94E-05	5.81E-05	2.32E-05	4.04E-05	5.69E-05	4.83E-05
SFRS4	5.39E-05	2.52E-05	2.52E-05	2.88E-05	9.65E-06	3.69E-05	2.63E-05	3.36E-05
SFRS5	0.000198	8.10E-05	0.000105	0.000137	4.51E-05	8.44E-05	0.000139	5.97E-05
SFRS6	7.69E-05	3.46E-05	2.52E-05	3.60E-05	2.01E-05	2.22E-05	3.53E-05	2.89E-05
SFRS7	4.09E-05	2.51E-05	1.79E-05	3.08E-05	1.00E-05	1.82E-05	3.03E-05	2.75E-05
SFRS8	1.21E-05	7.78E-06	4.61E-06	5.81E-06	4.92E-06	8.46E-06	7.11E-06	7.11E-06
SFRS9	0.000122	0.000106	6.52E-05	6.97E-05	4.30E-05	9.02E-05	8.05E-05	7.16E-05
SFT2D1	1.83E-05	9.94E-06	1.33E-05	1.83E-05	5.97E-06	1.41E-05	1.27E-05	1.03E-05
SFT2D2	1.17E-05	1.40E-05	5.12E-06	5.85E-06	5.00E-06	7.82E-06	5.86E-06	1.80E-05
SFT2D3	1.54E-05	5.43E-06	5.90E-06	4.67E-06	7.08E-06	8.16E-06	5.50E-06	5.72E-06
SFTA1P	8.58E-08	3.59E-07	0	0	6.74E-07	8.87E-07	2.74E-06	6.48E-07
SFTA2	1.63E-07	0	4.30E-07	6.26E-07	7.24E-08	0	0	1.27E-07
SFTA3	0	0	0	0	0	0	0	0
SFTPA1	0	2.13E-08	0	0	0	0	3.57E-08	1.94E-07
SFTPA2	0	1.24E-08	0	1.34E-08	0	0	5.30E-08	4.02E-07
SFTPB	2.58E-08	1.20E-07	0	1.48E-08	0	7.98E-09	6.99E-08	7.75E-07
SFTPC	0	1.89E-08	0	0	0	8.30E-08	1.60E-08	4.77E-08
SFTPD	3.57E-07	1.14E-07	4.90E-07	6.36E-07	8.57E-07	1.16E-06	1.33E-07	5.88E-08
SFXN1	2.91E-05	3.88E-05	5.14E-05	3.33E-05	1.21E-05	7.42E-05	1.72E-05	2.19E-05
SFXN2	1.28E-05	9.47E-06	1.16E-05	9.48E-06	1.13E-05	1.20E-05	9.54E-06	9.55E-06
SFXN3	8.75E-05	5.42E-06	2.17E-06	1.07E-05	1.32E-06	1.62E-06	7.65E-06	3.03E-06
SFXN4	4.84E-05	2.00E-05	1.64E-05	1.91E-05	2.43E-05	2.49E-05	2.67E-05	2.28E-05
SFXN5	2.33E-05	2.03E-05	2.22E-05	1.02E-05	3.01E-05	2.05E-05	1.99E-05	1.19E-05
SGCA	7.72E-07	2.39E-07	1.81E-06	4.85E-06	6.89E-07	6.96E-08	4.02E-07	1.22E-07
SGCB	1.18E-05	1.11E-06	5.27E-06	6.85E-06	7.74E-07	7.02E-07	5.98E-06	3.81E-06
SGCD	1.40E-06	6.58E-08	3.19E-07	1.18E-06	4.27E-08	1.13E-07	1.23E-07	5.80E-07
SGCE	8.42E-05	3.01E-06	1.12E-05	6.99E-06	2.22E-06	7.00E-07	1.74E-06	1.69E-05
SGCG	1.48E-08	0	0	0	0	0	0	0
SGCZ	1.83E-08	0	1.95E-08	2.67E-08	0	0	0	0
SGEF	1.44E-05	6.88E-06	2.20E-05	8.03E-06	4.30E-06	4.83E-06	1.01E-05	7.82E-06
SGIP1	2.44E-06	1.73E-07	1.03E-07	1.52E-07	1.08E-08	2.89E-08	1.63E-07	2.40E-07
SGK1	1.91E-05	3.78E-05	3.78E-05	9.18E-05	5.61E-06	7.57E-05	2.52E-05	0.00013
SGK196	3.50E-07	2.60E-07	2.25E-07	2.05E-07	0	3.96E-07	7.33E-08	2.02E-07
SGK2	2.12E-05	3.53E-05	1.17E-05	1.22E-05	1.04E-05	1.72E-05	9.76E-06	2.08E-05
SGK223	4.37E-06	4.82E-06	1.08E-06	8.53E-06	4.87E-07	1.39E-05	2.13E-06	8.45E-07

SGK269	7.17E-06	1.66E-06	1.17E-06	1.62E-06	3.41E-07	1.70E-06	1.58E-06	1.27E-06
SGK3	1.08E-05	9.19E-06	5.56E-06	4.97E-06	1.70E-06	4.90E-06	7.77E-06	5.03E-06
SGK494	1.09E-06	4.45E-07	3.04E-07	2.55E-07	2.95E-07	2.44E-07	3.49E-07	3.59E-07
SGMS1	2.12E-05	1.49E-05	1.49E-05	1.18E-05	6.09E-06	6.57E-06	1.65E-05	1.17E-05
SGMS2	1.11E-05	3.86E-06	3.45E-06	1.57E-05	4.46E-07	3.53E-06	2.61E-06	2.98E-06
SGOL1	2.97E-06	1.93E-06	8.87E-08	1.16E-07	6.91E-08	6.13E-07	1.19E-07	2.91E-07
SGOL2	2.23E-06	9.03E-07	1.90E-07	2.62E-07	1.55E-07	5.95E-07	1.36E-07	3.72E-07
SGPL1	1.96E-05	9.57E-06	1.13E-05	1.42E-05	5.08E-06	2.20E-05	1.31E-05	1.18E-05
SGPP1	2.26E-05	5.28E-06	1.05E-05	1.03E-05	2.18E-06	1.30E-05	8.87E-06	8.12E-06
SGPP2	1.99E-05	3.95E-07	1.39E-07	3.17E-07	5.56E-08	2.00E-08	1.23E-07	6.37E-08
SGSH	1.94E-05	6.13E-06	5.85E-06	7.82E-06	4.18E-06	8.18E-06	1.17E-05	7.26E-06
SGSM1	1.67E-06	1.72E-08	5.12E-08	1.34E-07	3.63E-08	3.56E-09	4.87E-08	1.17E-07
SGSM2	8.64E-06	4.75E-06	4.65E-06	6.03E-06	1.42E-05	7.59E-06	7.80E-06	6.62E-06
SGSM3	1.88E-05	1.67E-05	1.09E-05	1.23E-05	1.63E-05	1.51E-05	1.34E-05	1.71E-05
SGTA	6.84E-05	4.04E-05	2.84E-05	3.29E-05	4.76E-05	3.51E-05	4.45E-05	3.19E-05
SGTB	1.73E-06	8.34E-07	1.02E-06	8.88E-07	1.06E-07	7.29E-07	6.18E-07	7.26E-07
SH2B1	2.42E-05	1.98E-05	8.65E-06	1.41E-05	9.10E-06	1.49E-05	9.09E-06	1.35E-05
SH2B2	5.86E-07	5.34E-07	1.34E-07	4.22E-07	1.32E-07	3.78E-07	5.91E-07	4.13E-07
SH2B3	1.23E-05	9.72E-06	1.03E-05	1.28E-05	1.51E-06	4.58E-06	8.65E-06	1.51E-05
SH2D1A	3.21E-06	1.34E-07	6.91E-07	2.26E-06	2.05E-08	9.25E-07	7.41E-07	2.10E-07
SH2D1B	2.55E-07	6.25E-08	3.23E-07	2.15E-07	4.28E-08	9.43E-08	5.56E-07	1.02E-07
SH2D2A	5.15E-06	2.08E-07	5.25E-07	6.57E-06	1.40E-07	2.18E-06	7.80E-07	1.19E-06
SH2D3A	6.36E-06	5.66E-07	7.00E-07	2.84E-06	5.89E-07	4.71E-07	1.29E-06	1.01E-07
SH2D3C	8.02E-06	5.83E-06	3.08E-06	2.41E-06	4.46E-06	2.91E-06	7.95E-06	7.03E-06
SH2D4A	3.76E-06	1.96E-05	1.38E-05	1.24E-05	2.63E-06	3.19E-05	7.16E-06	4.61E-06
SH2D4B	6.23E-08	3.52E-07	0	1.51E-08	0	8.24E-09	1.28E-08	1.31E-08
SH2D5	1.19E-06	8.48E-09	3.12E-08	1.48E-07	0	6.99E-09	6.59E-08	3.32E-08
SH2D6	1.21E-08	6.06E-07	0	4.15E-08	1.61E-07	0	2.94E-07	3.58E-08
SH2D7	3.82E-08	0	0	0	0	0	1.23E-08	0
SH3BGR	5.86E-06	4.26E-06	2.55E-06	2.04E-06	2.29E-06	7.44E-06	3.35E-06	5.31E-06
SH3BGRL	5.73E-05	2.11E-05	1.56E-05	3.19E-05	3.18E-06	2.40E-05	1.37E-05	8.52E-06
SH3BGRL2	1.29E-05	3.89E-05	2.22E-05	1.19E-05	4.85E-06	4.25E-05	1.45E-05	1.44E-05
SH3BGRL3	0.000317	0.000104	5.14E-05	0.000157	6.99E-05	7.97E-05	7.88E-05	9.95E-05
SH3BP1	7.21E-06	1.89E-06	1.99E-06	2.94E-06	7.12E-07	1.28E-06	2.60E-06	6.00E-07
SH3BP2	5.09E-06	5.00E-06	5.45E-06	2.88E-06	2.61E-06	3.78E-06	5.98E-06	3.70E-06
SH3BP4	1.89E-05	1.34E-05	1.79E-05	1.88E-05	3.13E-06	6.59E-05	1.09E-05	1.28E-05
SH3BP5	7.32E-06	5.41E-06	7.94E-06	1.69E-05	4.69E-06	1.48E-05	1.14E-05	9.77E-06
SH3BP5L	1.53E-05	1.01E-05	6.81E-06	7.72E-06	6.07E-06	1.64E-05	1.07E-05	1.97E-05
SH3D19	1.24E-05	1.42E-05	2.26E-05	3.17E-05	5.22E-06	2.12E-05	1.59E-05	2.14E-05
SH3D20	2.19E-07	1.67E-07	5.85E-08	1.57E-07	1.58E-08	1.70E-08	2.28E-07	0
SH3GL1	3.90E-05	1.78E-05	1.97E-05	2.98E-05	3.77E-05	2.22E-05	3.06E-05	2.16E-05
SH3GL2	9.03E-09	0	3.75E-08	1.33E-07	0	0	0	0
SH3GL3	0	0	0	0	0	1.35E-08	0	0
SH3GLB1	1.89E-05	1.05E-05	1.01E-05	1.22E-05	2.41E-06	1.62E-05	8.10E-06	8.18E-06
SH3GLB2	1.90E-05	8.40E-06	9.24E-06	1.86E-05	9.63E-06	1.67E-05	1.30E-05	3.40E-05
SH3KBP1	1.30E-05	3.95E-06	4.82E-06	6.53E-06	5.97E-06	9.91E-06	5.27E-06	5.92E-06
SH3PXD2A	6.70E-06	2.12E-06	2.24E-06	2.64E-06	2.42E-06	2.71E-06	2.67E-06	2.83E-06
SH3PXD2B	5.21E-06	8.16E-07	3.91E-07	1.27E-06	3.34E-07	7.49E-07	1.53E-06	1.29E-06
SH3RF1	5.64E-06	2.06E-06	3.63E-06	6.33E-06	7.08E-07	1.18E-06	2.50E-06	2.50E-06
SH3RF2	6.05E-06	1.82E-06	3.42E-06	5.24E-07	4.08E-06	2.74E-06	1.70E-06	1.62E-06
SH3RF3	7.25E-06	4.74E-07	3.89E-07	8.36E-07	3.50E-07	3.12E-06	5.54E-07	3.69E-07
SH3TC1	1.46E-05	4.18E-06	6.21E-06	1.14E-05	4.13E-06	7.26E-06	7.50E-06	1.02E-05
SH3TC2	2.90E-08	6.58E-08	1.81E-08	5.66E-08	1.24E-08	6.32E-08	3.84E-08	4.45E-08
SH3YL1	2.32E-05	1.36E-06	6.82E-06	2.30E-05	1.82E-06	3.36E-06	2.67E-06	1.09E-06
SHANK1	1.02E-08	3.75E-09	6.87E-09	2.46E-08	0	0	9.19E-09	0
SHANK2	5.05E-06	4.36E-06	3.16E-06	3.38E-06	6.37E-07	4.90E-06	1.69E-06	9.72E-06
SHANK3	7.45E-06	7.90E-06	3.50E-06	3.33E-06	1.98E-06	6.06E-06	7.38E-06	5.48E-06
SHARPIN	5.23E-05	4.22E-05	1.99E-05	2.42E-05	6.15E-05	2.22E-05	9.46E-05	3.12E-05

SHB	2.50E-06	8.55E-06	6.26E-06	1.83E-05	9.96E-06	8.09E-06	1.37E-05	9.77E-06
SHBG	1.03E-05	1.35E-05	5.07E-05	7.01E-05	7.07E-06	4.77E-07	4.60E-05	1.14E-05
SHC1	7.95E-05	6.29E-05	2.86E-05	4.26E-05	2.39E-05	9.51E-05	5.91E-05	0.000111
SHC2	2.76E-05	3.38E-05	3.30E-05	5.09E-05	3.01E-05	4.09E-05	7.92E-05	3.34E-05
SHC3	1.96E-08	5.51E-08	0	5.99E-08	5.05E-08	1.18E-07	1.71E-07	1.72E-07
SHC4	7.26E-08	7.37E-09	1.54E-08	3.33E-08	9.95E-09	3.65E-08	0	9.64E-09
SHCBP1	5.36E-06	1.75E-06	8.85E-08	1.48E-07	1.29E-07	4.96E-07	1.46E-07	5.44E-07
SHD	1.38E-07	4.82E-06	1.55E-06	2.24E-06	1.87E-05	3.45E-07	8.91E-06	1.00E-06
SHE	1.74E-06	1.30E-06	1.85E-06	1.60E-06	3.97E-07	6.10E-07	1.41E-06	1.26E-06
SHF	1.99E-05	2.30E-05	1.77E-05	1.75E-05	2.31E-05	3.07E-05	1.40E-05	5.28E-06
SHFM1	0.000257	0.000521	0.00023	0.00029	0.000893	0.000399	0.00042	0.000521
SHH	7.01E-07	1.89E-06	5.39E-06	6.18E-06	2.22E-06	7.46E-06	7.05E-06	5.13E-06
SHISA2	6.27E-07	0	8.32E-09	3.93E-08	6.90E-09	1.49E-08	0	2.00E-07
SHISA3	3.41E-07	1.10E-07	1.40E-07	9.85E-07	4.30E-07	2.37E-07	5.08E-06	8.07E-07
SHISA4	1.02E-05	1.41E-05	2.80E-06	6.77E-06	3.18E-06	5.43E-05	4.66E-06	9.40E-06
SHISA5	0.000264	0.000118	8.00E-05	8.84E-05	6.99E-05	0.000119	0.000114	0.000132
SHISA6	0	1.34E-07	0	0	0	5.47E-08	0	0
SHISA7	3.71E-09	0	0	0	0	3.43E-09	7.23E-09	0
SHISA9	6.64E-09	0	0	4.12E-07	0	0	0	0
SHKBP1	3.18E-05	1.40E-05	1.15E-05	1.65E-05	1.67E-05	1.71E-05	1.42E-05	1.32E-05
SHMT1	5.12E-05	0.000432	0.000345	0.000124	0.000134	0.000193	0.000181	8.56E-05
SHMT2	7.04E-05	0.000136	0.000139	9.97E-05	8.54E-05	0.000152	0.000233	9.26E-05
SHOC2	1.85E-05	8.25E-06	1.07E-05	1.04E-05	3.22E-06	1.06E-05	9.31E-06	1.24E-05
SHOX	0	0	0	0	0	0	0	0
SHOX2	1.48E-08	5.83E-08	0	9.07E-09	2.55E-08	8.93E-08	9.36E-08	6.53E-08
SHPK	7.02E-06	1.05E-05	8.31E-06	8.23E-06	4.52E-06	7.74E-06	1.52E-05	8.14E-06
SHPRH	2.19E-06	1.14E-06	9.51E-07	6.13E-07	1.32E-08	1.25E-06	2.14E-07	4.32E-07
SHQ1	5.87E-06	5.06E-06	3.21E-06	4.12E-06	1.36E-06	5.39E-06	3.17E-06	3.97E-06
SHROOM1	1.32E-05	1.18E-05	1.92E-05	2.33E-05	3.86E-06	2.61E-05	3.02E-05	6.10E-05
SHROOM2	1.83E-06	2.66E-06	2.36E-06	2.49E-06	1.34E-06	5.84E-07	2.43E-06	4.21E-06
SHROOM3	2.14E-05	4.12E-06	3.56E-06	4.81E-06	1.72E-06	1.87E-06	3.69E-06	1.07E-05
SHROOM4	1.27E-06	5.99E-07	2.36E-07	2.29E-07	1.57E-07	3.53E-07	4.47E-07	5.52E-07
SI	0	0	0	0	0	0	0	0
SIAE	2.75E-05	3.35E-05	2.25E-05	2.63E-05	8.92E-06	5.64E-05	1.53E-05	3.92E-05
SIAH1	1.95E-05	1.08E-05	8.18E-06	1.18E-05	1.92E-06	1.10E-05	8.44E-06	1.02E-05
SIAH2	1.90E-05	6.70E-05	3.62E-05	1.91E-05	1.51E-05	8.75E-05	2.74E-05	3.17E-05
SIAH3	0	0	5.87E-09	0	0	0	0	0
SIDT1	5.54E-06	1.86E-07	2.98E-07	6.19E-07	4.07E-08	2.73E-07	2.92E-07	2.66E-07
SIDT2	3.43E-05	1.66E-05	2.20E-05	2.06E-05	1.51E-05	2.40E-05	2.03E-05	2.73E-05
SIGIRR	3.79E-05	3.37E-05	6.41E-05	3.78E-05	0.00011	2.82E-05	5.11E-05	3.36E-05
SIGLEC1	3.77E-06	7.87E-07	5.48E-06	6.08E-06	1.22E-06	2.83E-06	3.05E-06	1.13E-06
SIGLEC10	8.77E-06	1.52E-07	1.39E-06	1.58E-06	1.04E-07	5.15E-07	1.53E-06	2.40E-07
SIGLEC11	1.24E-07	2.72E-08	1.22E-06	8.74E-07	1.25E-07	7.13E-07	3.00E-07	3.56E-08
SIGLEC12	1.32E-06	1.59E-08	1.48E-08	1.55E-07	8.08E-08	1.21E-06	1.29E-06	4.13E-08
SIGLEC14	0	1.53E-07	1.32E-06	1.44E-06	1.26E-07	4.61E-07	4.47E-07	2.66E-07
SIGLEC15	5.45E-06	4.35E-07	2.90E-07	6.68E-08	1.06E-06	2.02E-07	6.96E-08	1.88E-07
SIGLEC16	1.96E-07	4.45E-08	8.95E-07	3.37E-07	2.93E-07	2.43E-07	1.90E-07	1.93E-08
SIGLEC5	9.49E-07	4.24E-08	6.94E-07	5.41E-07	2.42E-08	3.31E-07	4.28E-07	5.52E-08
SIGLEC6	4.72E-08	0	1.91E-08	1.59E-08	0	2.40E-08	0	3.69E-08
SIGLEC7	2.36E-06	3.02E-07	4.36E-06	4.50E-06	2.97E-07	1.67E-06	4.18E-06	2.96E-07
SIGLEC8	2.61E-06	3.54E-08	8.20E-07	1.54E-06	2.07E-08	7.28E-08	1.17E-06	1.05E-07
SIGLEC9	1.99E-06	1.67E-07	3.27E-06	3.64E-06	1.27E-07	1.01E-06	2.15E-06	1.92E-07
SIGLECP3	5.39E-08	2.41E-08	2.02E-07	1.98E-07	0	9.34E-08	1.13E-07	7.42E-08
SIGMAR1	7.41E-05	0.000191	0.000138	0.000104	8.11E-05	0.000129	9.85E-05	0.000206
SIK1	3.99E-05	6.01E-05	9.63E-05	7.48E-05	8.89E-06	6.88E-05	2.75E-05	8.62E-05
SIK2	3.47E-05	2.32E-05	1.54E-05	1.37E-05	5.95E-06	1.79E-05	1.09E-05	3.34E-05
SIK3	1.64E-05	7.34E-06	1.26E-05	6.93E-06	3.22E-06	6.88E-06	4.94E-06	1.57E-05
SIKE1	9.85E-06	7.95E-06	5.26E-06	4.47E-06	1.52E-06	8.23E-06	2.50E-06	4.96E-06

SIL1	5.59E-05	3.76E-05	4.72E-05	6.19E-05	7.97E-05	8.07E-05	7.01E-05	0.00015
SILV	3.06E-06	6.75E-06	3.91E-06	2.97E-06	1.72E-06	4.65E-06	2.29E-06	1.91E-06
SIM1	9.70E-08	1.25E-06	6.45E-08	0	0	1.99E-07	6.27E-08	6.87E-07
SIM2	2.12E-07	1.46E-07	1.85E-07	1.73E-07	3.32E-07	1.86E-07	2.80E-07	7.17E-08
SIN3A	1.18E-05	6.72E-06	5.82E-06	6.40E-06	1.72E-06	1.48E-05	6.51E-06	5.50E-06
SIN3B	1.48E-05	4.83E-06	4.83E-06	6.49E-06	4.37E-06	6.25E-06	7.18E-06	6.69E-06
SIP1	4.00E-06	5.89E-06	2.21E-06	3.11E-06	2.72E-06	3.46E-06	4.11E-06	2.47E-06
SIPA1	1.56E-05	1.78E-05	1.06E-05	9.90E-06	7.21E-06	5.90E-06	1.62E-05	1.36E-05
SIPA1L1	1.60E-05	1.00E-05	8.77E-06	1.11E-05	7.21E-06	1.22E-05	1.04E-05	1.76E-05
SIPA1L2	1.92E-05	2.30E-06	3.47E-06	3.87E-06	4.00E-06	2.82E-06	4.65E-06	2.48E-05
SIPA1L3	7.00E-06	2.26E-06	1.17E-06	2.39E-06	1.13E-06	1.80E-06	2.18E-06	3.46E-06
SIRPA	7.70E-05	2.38E-06	2.40E-05	2.22E-05	6.36E-06	9.21E-06	1.12E-05	4.41E-05
SIRPB1	7.79E-06	2.17E-07	3.20E-06	2.95E-06	1.97E-07	1.00E-06	1.28E-06	2.66E-06
SIRPB2	2.39E-06	1.80E-07	1.06E-06	8.04E-07	3.17E-08	6.85E-08	6.01E-07	3.56E-07
SIRPD	1.05E-07	4.10E-08	3.92E-08	4.33E-08	0	3.26E-08	0	0
SIRPG	6.33E-06	5.89E-08	5.58E-07	8.79E-07	2.14E-08	2.75E-06	1.11E-06	3.47E-07
SIRT1	5.69E-06	4.76E-06	8.71E-06	5.01E-06	9.95E-07	7.21E-06	4.30E-06	5.23E-06
SIRT2	4.42E-05	5.10E-05	3.31E-05	2.60E-05	2.39E-05	2.49E-05	2.33E-05	3.19E-05
SIRT3	4.65E-05	3.94E-05	2.75E-05	3.50E-05	4.95E-05	4.36E-05	3.22E-05	4.38E-05
SIRT4	2.56E-06	2.54E-06	2.36E-06	1.42E-06	3.53E-06	2.37E-06	1.23E-06	1.64E-06
SIRT5	1.34E-05	2.81E-05	1.10E-05	1.15E-05	8.87E-06	1.41E-05	1.17E-05	9.31E-06
SIRT6	1.57E-05	7.62E-06	4.40E-06	1.01E-05	1.68E-05	9.61E-06	1.02E-05	8.00E-06
SIRT7	1.99E-05	1.67E-05	5.30E-06	7.91E-06	7.89E-06	4.19E-05	9.62E-06	8.24E-06
SIT1	5.01E-06	8.24E-08	1.09E-06	1.22E-06	2.06E-08	2.18E-06	1.24E-06	9.42E-07
SIVA1	0.000137	8.81E-05	8.49E-05	8.44E-05	0.000174	8.17E-05	0.000117	6.56E-05
SIX1	7.82E-08	1.96E-08	0	2.13E-08	2.99E-07	2.42E-08	4.30E-07	6.38E-08
SIX2	1.17E-06	6.24E-08	1.15E-08	2.71E-08	9.50E-09	1.02E-07	2.24E-07	6.50E-08
SIX3	3.45E-07	1.63E-08	0	0	0	0	0	0
SIX4	5.55E-07	2.69E-07	1.84E-08	2.64E-08	9.58E-08	4.86E-07	3.05E-07	1.09E-07
SIX5	7.54E-06	6.25E-06	5.00E-06	5.57E-06	9.56E-06	5.68E-06	1.01E-05	6.21E-06
SIX6	0	0	0	0	0	0	0	0
SKA1	4.96E-06	8.74E-07	9.74E-08	1.39E-07	3.67E-07	6.88E-07	1.32E-07	4.75E-07
SKA2	2.04E-05	8.22E-06	4.93E-06	7.81E-06	7.77E-06	1.07E-05	9.37E-06	4.35E-06
SKA3	2.38E-06	2.19E-06	2.62E-08	5.03E-08	3.69E-07	5.27E-07	2.32E-07	6.65E-07
SKAP1	8.87E-06	1.57E-05	1.42E-05	1.24E-05	3.31E-05	6.35E-06	2.10E-05	1.48E-06
SKAP2	1.27E-05	2.98E-06	3.95E-06	4.45E-06	1.22E-06	5.72E-06	5.12E-06	7.07E-06
SKI	1.41E-05	5.29E-06	1.00E-05	9.47E-06	1.34E-06	9.49E-06	7.39E-06	1.08E-05
SKIL	5.19E-06	1.88E-06	4.03E-06	5.79E-06	2.15E-07	1.19E-06	1.47E-06	2.26E-06
SKINTL	0	0	0	0	2.83E-08	0	0	0
SKIV2L	2.63E-05	3.74E-05	9.66E-06	8.64E-06	1.11E-05	1.29E-05	7.85E-06	1.45E-05
SKIV2L2	1.83E-05	1.45E-05	9.82E-06	9.83E-06	1.56E-06	7.35E-06	7.70E-06	1.19E-05
SKP1	0.000155	0.000104	6.52E-05	8.86E-05	4.24E-05	0.000114	9.54E-05	0.000112
SKP2	1.21E-05	3.59E-06	5.33E-06	1.29E-06	9.71E-07	6.02E-06	1.59E-06	3.33E-06
SLA	1.25E-05	1.07E-06	3.69E-06	1.42E-05	6.01E-07	6.14E-06	4.30E-06	2.39E-06
SLA2	2.33E-06	3.44E-07	4.11E-07	2.03E-06	4.14E-08	8.64E-07	6.44E-07	7.07E-07
SLAIN1	5.96E-07	5.47E-06	3.03E-06	3.83E-06	1.04E-06	7.76E-07	3.22E-06	3.37E-07
SLAIN2	8.40E-06	1.15E-05	7.65E-06	7.11E-06	2.38E-06	6.67E-06	6.40E-06	9.85E-06
SLAMF1	1.92E-06	2.99E-08	2.49E-07	9.51E-07	8.62E-08	5.87E-07	3.31E-07	4.41E-07
SLAMF6	3.20E-06	1.90E-07	1.60E-06	1.77E-06	7.58E-08	1.40E-06	9.19E-07	5.15E-07
SLAMF7	2.07E-05	3.78E-07	2.67E-06	6.56E-06	3.11E-07	1.99E-06	1.30E-06	6.27E-07
SLAMF8	1.54E-05	4.16E-07	1.22E-06	4.46E-06	2.70E-07	1.70E-06	4.61E-06	1.34E-06
SLAMF9	1.57E-07	1.38E-07	1.75E-07	7.04E-07	3.22E-07	3.29E-06	1.47E-07	1.01E-06
SLBP	3.36E-05	3.22E-05	1.56E-05	1.51E-05	8.53E-06	1.88E-05	2.12E-05	2.57E-05
SLC10A1	5.43E-06	9.11E-05	0.000249	0.000218	0.000223	4.29E-05	0.000146	0.000101
SLC10A2	0	0	0	7.39E-09	0	0	0	0
SLC10A3	1.11E-05	5.47E-06	3.92E-06	8.96E-06	5.04E-06	3.89E-06	8.26E-06	1.15E-05
SLC10A4	4.27E-07	2.54E-08	0	1.38E-08	0	0	0	1.65E-08
SLC10A5	6.12E-06	4.39E-06	2.93E-06	1.54E-06	3.36E-06	2.06E-06	2.94E-06	1.82E-06

SLC10A6	6.61E-08	2.26E-07	3.51E-08	3.04E-07	0	2.00E-07	0	4.88E-08
SLC10A7	4.70E-06	1.38E-06	2.01E-06	1.84E-06	5.27E-07	2.83E-06	1.32E-06	4.02E-06
SLC11A1	2.25E-06	2.79E-07	7.52E-07	2.05E-06	8.13E-07	2.70E-07	5.69E-07	3.25E-07
SLC11A2	1.10E-05	1.32E-05	1.57E-05	1.07E-05	3.88E-06	1.51E-05	9.79E-06	1.82E-05
SLC12A1	5.15E-09	0	5.42E-08	1.11E-07	0	8.09E-06	4.95E-07	7.03E-09
SLC12A2	1.13E-05	1.91E-06	1.81E-06	1.23E-05	8.21E-07	1.14E-06	1.73E-06	1.55E-05
SLC12A3	7.30E-08	0	0	9.90E-09	0	1.13E-08	3.95E-09	1.19E-08
SLC12A4	2.11E-05	7.85E-06	6.38E-06	1.40E-05	4.00E-06	8.25E-06	8.79E-06	1.11E-05
SLC12A5	2.23E-08	2.61E-07	1.14E-08	1.36E-08	6.36E-09	3.43E-09	2.17E-08	5.42E-09
SLC12A6	4.60E-06	1.48E-06	2.16E-06	2.02E-06	5.20E-07	2.25E-06	1.18E-06	1.13E-06
SLC12A7	5.57E-05	2.88E-05	1.43E-05	2.04E-05	6.32E-06	2.44E-05	1.97E-05	4.78E-05
SLC12A8	7.11E-06	6.47E-06	3.54E-06	1.29E-06	2.44E-06	2.24E-06	3.12E-06	1.02E-06
SLC12A9	2.47E-05	2.40E-05	6.75E-06	9.44E-06	6.36E-06	7.10E-06	1.14E-05	1.60E-05
SLC13A1	0	0	0	0	0	0	0	0
SLC13A2	1.31E-05	1.48E-06	7.55E-07	2.74E-07	6.50E-07	9.88E-09	2.68E-07	7.83E-08
SLC13A3	1.81E-07	2.58E-07	5.96E-06	5.79E-06	1.05E-05	9.94E-05	1.71E-05	2.66E-06
SLC13A4	9.00E-08	1.60E-08	2.32E-08	4.44E-08	1.90E-08	1.06E-07	1.36E-08	2.07E-08
SLC13A5	1.33E-05	0.000149	0.000165	0.000105	4.14E-05	0.000146	0.00017	0.000276
SLC14A1	1.15E-06	1.27E-06	7.19E-07	1.47E-07	1.25E-07	4.18E-07	4.93E-08	6.51E-08
SLC14A2	1.67E-08	3.51E-08	0	3.83E-08	0	8.84E-07	6.08E-09	3.06E-08
SLC15A1	2.22E-05	1.19E-05	1.16E-05	7.38E-06	9.74E-06	2.05E-08	8.33E-06	5.64E-06
SLC15A2	1.87E-07	3.24E-08	1.33E-07	6.57E-08	2.49E-08	1.23E-07	1.94E-07	3.63E-08
SLC15A3	2.71E-05	5.86E-06	1.15E-05	2.63E-05	3.54E-06	1.17E-05	1.91E-05	1.54E-05
SLC15A4	1.60E-05	1.81E-05	1.05E-05	1.41E-05	3.93E-06	9.20E-06	9.26E-06	1.04E-05
SLC16A1	2.62E-05	3.04E-05	4.44E-05	3.56E-05	1.48E-05	0.000171	1.67E-05	1.24E-05
SLC16A10	1.63E-06	3.32E-06	3.14E-06	1.15E-06	3.07E-07	1.05E-05	9.28E-07	9.25E-08
SLC16A11	8.06E-07	3.29E-06	2.46E-06	4.62E-06	1.55E-06	3.23E-05	1.78E-05	1.05E-05
SLC16A12	1.21E-06	2.07E-07	8.84E-07	1.57E-06	3.10E-06	8.85E-06	1.83E-06	4.39E-08
SLC16A13	7.68E-06	1.96E-05	2.20E-05	1.83E-05	4.82E-06	2.13E-05	1.56E-05	1.92E-05
SLC16A14	1.02E-05	1.44E-05	5.70E-07	3.94E-07	2.83E-06	8.23E-07	3.03E-07	8.55E-07
SLC16A2	1.56E-05	4.06E-05	5.21E-05	2.90E-05	7.88E-05	3.20E-05	2.08E-05	2.90E-05
SLC16A3	0.00016	5.77E-06	2.99E-06	1.19E-05	1.99E-06	2.42E-06	9.60E-06	3.86E-06
SLC16A4	9.05E-06	2.70E-07	2.74E-06	4.50E-06	1.75E-07	1.38E-05	2.80E-06	2.03E-07
SLC16A5	1.75E-06	7.27E-07	1.68E-06	1.16E-06	2.08E-07	8.11E-07	7.64E-07	5.04E-07
SLC16A6	5.45E-07	9.89E-08	1.82E-07	5.16E-07	4.53E-08	2.53E-07	1.11E-07	5.31E-07
SLC16A7	3.94E-06	2.18E-08	1.85E-06	9.10E-07	1.82E-07	3.49E-06	3.21E-07	5.69E-07
SLC16A8	1.65E-07	1.44E-07	5.34E-08	3.12E-08	2.19E-08	5.90E-08	3.68E-08	1.87E-08
SLC16A9	0.000128	2.14E-05	4.29E-07	2.58E-07	4.90E-09	1.06E-08	5.56E-08	4.18E-08
SLC17A1	1.31E-05	5.63E-05	1.27E-05	3.78E-06	1.43E-05	1.91E-05	3.12E-06	3.15E-06
SLC17A2	3.67E-08	3.28E-06	4.34E-05	1.63E-05	1.80E-05	3.86E-05	2.49E-05	1.26E-05
SLC17A3	9.34E-06	3.23E-05	1.70E-05	6.84E-06	4.32E-07	1.71E-05	2.63E-06	2.47E-06
SLC17A4	2.74E-05	2.88E-05	1.85E-05	1.62E-05	3.20E-05	9.99E-06	1.86E-05	2.89E-05
SLC17A5	1.31E-05	2.62E-05	1.36E-05	1.34E-05	3.63E-06	1.61E-05	1.41E-05	1.77E-05
SLC17A6	0	0	0	0	0	0	0	0
SLC17A7	3.04E-07	1.20E-08	9.76E-08	1.35E-07	5.62E-08	5.77E-08	5.15E-08	1.57E-07
SLC17A8	4.17E-08	0	6.13E-08	3.96E-07	0	0	5.79E-09	0
SLC17A9	5.99E-05	2.62E-05	1.36E-05	1.97E-05	2.09E-05	1.42E-06	2.02E-05	2.06E-05
SLC18A1	1.04E-07	0	0	4.77E-08	0	1.20E-08	1.25E-08	0
SLC18A2	7.63E-08	2.45E-06	1.60E-07	6.24E-08	4.38E-08	1.18E-08	6.14E-08	5.62E-08
SLC18A3	0	0	0	0	0	0	0	0
SLC19A1	1.04E-05	4.51E-06	2.10E-05	7.01E-06	7.97E-06	8.69E-06	6.77E-06	4.53E-06
SLC19A2	1.17E-05	1.69E-05	1.45E-05	9.24E-06	2.22E-06	1.73E-05	6.31E-06	3.46E-05
SLC19A3	5.89E-05	7.74E-06	1.15E-05	7.71E-06	5.18E-06	7.32E-06	4.58E-06	9.63E-07
SLC1A1	2.42E-05	3.70E-05	2.82E-05	2.32E-05	4.00E-06	1.81E-05	1.43E-05	2.50E-05
SLC1A2	2.14E-07	3.56E-05	1.79E-06	3.88E-06	3.49E-07	5.01E-05	2.96E-06	1.78E-06
SLC1A3	3.18E-06	5.53E-06	1.49E-06	2.49E-06	2.62E-07	1.81E-06	2.95E-06	3.56E-06
SLC1A4	1.79E-05	1.93E-05	2.73E-06	6.25E-06	8.06E-06	1.17E-05	9.01E-06	4.17E-06
SLC1A5	1.81E-05	7.04E-06	4.65E-06	1.09E-05	1.68E-06	5.84E-06	8.84E-06	3.72E-06

SLC1A6	0	4.71E-08	0	0	0	0	0	0
SLC1A7	5.80E-05	2.86E-05	2.24E-07	2.96E-06	2.09E-07	2.82E-08	4.78E-07	7.32E-07
SLC20A1	3.52E-05	1.73E-05	1.78E-05	2.83E-05	3.40E-06	5.74E-06	1.96E-05	1.34E-05
SLC20A2	7.01E-06	2.11E-05	2.24E-05	2.22E-05	1.14E-05	3.21E-05	1.48E-05	2.84E-05
SLC22A1	3.10E-06	9.02E-05	0.000572	0.00011	0.000314	6.97E-05	0.00013	5.32E-05
SLC22A10	5.56E-06	8.16E-05	6.54E-05	5.53E-06	1.61E-05	6.73E-06	1.05E-05	1.17E-05
SLC22A11	1.94E-07	0	2.06E-07	1.31E-07	0	8.38E-05	1.21E-08	1.84E-08
SLC22A12	8.06E-09	0	9.51E-08	0	0	0.000104	0	0
SLC22A13	1.83E-08	0	4.41E-08	0	0	0	0	0
SLC22A14	1.24E-08	0	0	2.04E-08	2.14E-08	1.15E-08	0	0
SLC22A15	2.30E-06	2.67E-06	2.41E-07	2.87E-07	4.11E-09	1.33E-08	4.66E-08	2.01E-06
SLC22A16	1.22E-08	2.76E-08	0	2.99E-08	0	1.13E-08	0	0
SLC22A17	9.64E-05	1.65E-07	1.20E-06	2.77E-06	5.97E-07	1.05E-07	1.21E-06	1.46E-06
SLC22A18	7.64E-05	0.000204	3.44E-05	5.06E-05	1.67E-05	0.000189	3.03E-05	7.12E-05
SLC22A18	1.17E-05	1.14E-05	6.23E-06	1.84E-06	5.69E-07	7.69E-06	5.00E-07	5.52E-06
SLC22A2	0	2.92E-07	1.94E-08	0	1.61E-08	7.19E-08	0	0
SLC22A20	2.15E-07	4.25E-07	6.63E-08	5.35E-08	5.32E-08	6.93E-08	4.95E-08	9.14E-08
SLC22A23	2.33E-05	6.34E-06	5.26E-06	3.00E-06	5.47E-06	1.64E-05	4.02E-06	1.17E-05
SLC22A24	0	2.57E-08	1.19E-08	2.79E-08	9.79E-09	1.06E-08	0	5.02E-08
SLC22A25	5.33E-07	4.14E-06	5.04E-06	1.46E-06	1.22E-06	3.35E-06	2.60E-06	8.82E-06
SLC22A3	1.72E-05	4.69E-05	7.35E-05	9.84E-05	5.33E-05	0.000102	9.93E-05	3.81E-05
SLC22A4	3.01E-06	1.41E-06	7.92E-08	7.44E-07	1.76E-07	2.01E-06	2.10E-07	1.75E-07
SLC22A5	1.21E-05	4.43E-06	1.79E-06	2.56E-06	1.15E-06	2.11E-06	2.80E-06	4.27E-06
SLC22A6	1.18E-08	7.96E-07	0	0	0	8.39E-07	0	0
SLC22A7	2.90E-06	0.000116	0.000283	0.000154	0.000282	5.98E-05	5.58E-05	9.82E-05
SLC22A8	2.80E-07	6.04E-08	0	0	2.29E-08	9.97E-08	0	0
SLC22A9	2.58E-06	3.07E-05	2.57E-05	1.50E-05	2.36E-05	1.56E-05	1.26E-05	3.63E-05
SLC23A1	9.99E-06	2.92E-05	1.39E-05	3.46E-06	5.58E-06	1.92E-05	1.08E-05	2.90E-06
SLC23A2	1.38E-05	7.96E-06	1.10E-05	8.63E-06	4.03E-06	2.20E-05	3.15E-05	1.53E-05
SLC23A3	1.03E-06	2.22E-08	4.82E-06	3.23E-07	1.80E-07	4.56E-07	9.06E-08	1.06E-07
SLC24A1	2.31E-06	2.57E-06	1.63E-06	1.85E-06	6.62E-07	2.38E-06	1.93E-06	1.24E-06
SLC24A2	0	0	0	0	0	0	0	0
SLC24A3	4.81E-07	4.60E-08	1.57E-07	3.73E-07	5.04E-08	3.80E-08	7.98E-08	3.44E-08
SLC24A4	1.10E-07	1.94E-08	7.73E-08	1.48E-07	1.49E-08	4.81E-08	2.25E-08	3.39E-08
SLC24A5	7.21E-09	1.38E-07	0	0	1.35E-07	0	2.14E-07	0
SLC24A6	3.54E-05	3.09E-05	1.30E-05	1.37E-05	7.56E-06	1.16E-05	1.94E-05	2.52E-05
SLC25A1	0.000122	0.00028	9.41E-05	8.91E-05	0.000228	0.000164	0.000131	8.92E-05
SLC25A10	6.69E-05	0.000183	0.000125	6.24E-05	0.000139	0.000173	7.40E-05	0.000115
SLC25A11	5.30E-05	6.67E-05	5.06E-05	4.43E-05	7.48E-05	5.39E-05	7.04E-05	4.84E-05
SLC25A12	4.51E-06	3.45E-07	4.48E-07	9.82E-07	1.77E-07	1.01E-06	5.10E-07	1.19E-06
SLC25A13	9.85E-05	5.09E-05	7.68E-05	3.52E-05	2.99E-05	8.33E-05	3.35E-05	2.35E-05
SLC25A14	4.03E-06	2.07E-06	1.40E-06	3.33E-06	2.69E-06	3.20E-06	2.30E-06	4.33E-06
SLC25A15	4.27E-05	9.40E-05	4.96E-05	2.18E-05	1.11E-05	5.34E-05	2.34E-05	0.000118
SLC25A16	1.59E-05	8.67E-06	9.72E-06	4.38E-06	2.72E-06	6.98E-06	4.31E-06	6.95E-06
SLC25A17	1.30E-05	1.01E-05	1.04E-05	9.41E-06	8.15E-06	1.37E-05	6.87E-06	1.28E-05
SLC25A18	4.21E-05	4.52E-05	4.72E-05	2.41E-05	1.89E-05	6.31E-06	3.62E-05	1.95E-05
SLC25A19	1.23E-05	4.88E-06	3.13E-06	5.10E-06	3.25E-06	5.37E-06	4.68E-06	6.11E-06
SLC25A2	3.53E-08	2.02E-08	0	2.17E-08	0	0	0	1.05E-07
SLC25A20	3.45E-05	6.79E-05	6.15E-05	5.08E-05	4.46E-05	5.05E-05	5.33E-05	3.62E-05
SLC25A21	2.00E-07	0	1.06E-07	1.33E-07	0	4.90E-08	8.22E-08	0
SLC25A22	1.85E-05	3.20E-05	2.62E-05	1.22E-05	9.07E-06	1.74E-05	1.20E-05	2.92E-05
SLC25A23	4.84E-05	4.12E-05	2.53E-05	1.84E-05	4.36E-05	3.76E-05	2.27E-05	2.67E-05
SLC25A24	1.05E-05	6.84E-07	1.43E-06	3.26E-06	2.68E-07	8.17E-07	1.44E-06	6.39E-07
SLC25A25	1.53E-05	6.98E-05	9.40E-05	6.03E-05	2.46E-05	8.39E-05	4.52E-05	0.000204
SLC25A26	7.42E-06	7.32E-06	9.01E-06	1.09E-05	2.22E-05	1.63E-05	1.87E-05	9.74E-06
SLC25A27	1.93E-06	2.57E-07	8.68E-07	8.30E-07	4.60E-07	7.18E-07	2.40E-07	8.26E-07
SLC25A28	2.81E-05	1.86E-05	1.93E-05	2.30E-05	2.16E-05	3.83E-05	2.90E-05	3.17E-05
SLC25A29	1.60E-05	6.55E-06	2.95E-06	1.01E-05	1.52E-05	9.34E-06	4.89E-06	6.15E-06

SLC25A3	0.000545	0.000426	0.00017	0.000233	0.000183	0.000203	0.000302	0.000241
SLC25A30	3.26E-05	3.74E-05	1.42E-05	1.02E-05	4.95E-06	8.55E-05	1.42E-05	2.30E-05
SLC25A31	0	0	0	0	0	0	0	1.97E-08
SLC25A32	1.65E-05	1.34E-05	9.78E-06	9.11E-06	3.51E-06	7.21E-06	1.98E-05	8.96E-06
SLC25A33	1.60E-05	2.27E-05	7.25E-06	1.46E-05	7.31E-06	1.21E-05	3.34E-05	2.15E-05
SLC25A34	1.42E-06	1.17E-06	9.30E-07	4.06E-07	2.10E-06	1.51E-06	4.71E-07	2.10E-07
SLC25A35	1.92E-06	1.68E-06	7.43E-07	1.49E-06	3.03E-07	2.43E-06	1.26E-06	2.73E-06
SLC25A36	7.49E-06	5.30E-07	9.33E-07	1.45E-06	3.48E-07	6.65E-07	7.45E-07	6.74E-07
SLC25A37	7.12E-06	4.68E-06	4.16E-06	5.85E-06	9.17E-07	6.45E-06	3.99E-06	2.65E-06
SLC25A38	2.52E-05	2.73E-05	2.68E-05	1.38E-05	2.41E-05	2.49E-05	2.17E-05	1.28E-05
SLC25A39	0.00019	0.000272	6.59E-05	6.69E-05	0.000153	0.000116	0.000127	0.000155
SLC25A4	7.82E-05	7.52E-05	5.28E-05	4.29E-05	2.55E-05	6.57E-05	4.36E-05	9.14E-05
SLC25A40	7.54E-06	9.88E-06	3.18E-06	2.26E-06	2.83E-08	4.19E-06	9.94E-07	6.00E-06
SLC25A41	0	0	8.45E-08	7.84E-08	2.89E-07	7.41E-08	1.07E-07	2.12E-07
SLC25A42	2.38E-05	2.95E-05	3.47E-05	3.76E-05	3.87E-05	6.40E-05	4.51E-05	1.45E-05
SLC25A43	1.18E-05	1.39E-05	5.90E-06	6.49E-06	2.82E-06	8.56E-06	6.12E-06	1.24E-05
SLC25A44	1.53E-05	2.18E-05	2.10E-05	2.29E-05	6.32E-06	3.49E-05	1.91E-05	4.00E-05
SLC25A45	1.25E-05	7.30E-06	3.18E-06	6.01E-06	3.94E-06	2.08E-06	8.25E-06	8.30E-06
SLC25A46	1.44E-05	1.51E-05	1.38E-05	9.34E-06	1.11E-06	1.38E-05	4.73E-06	1.81E-05
SLC25A5	0.000465	0.000403	0.000253	0.000217	0.000249	0.000244	0.000268	0.000469
SLC25A6	0.000358	0.000145	0.000104	0.000219	0.000164	0.000182	0.000267	0.000284
SLC26A1	7.07E-06	2.91E-05	1.00E-05	3.39E-06	4.09E-06	1.15E-05	4.54E-06	1.07E-05
SLC26A10	1.49E-06	1.16E-08	7.29E-08	1.10E-07	5.41E-08	1.27E-07	2.11E-07	2.40E-07
SLC26A11	9.37E-06	1.03E-05	3.38E-06	5.16E-06	3.02E-06	6.88E-06	4.78E-06	4.78E-06
SLC26A2	1.71E-06	1.37E-06	4.49E-07	7.32E-07	1.09E-07	7.43E-07	6.12E-07	1.35E-06
SLC26A3	6.58E-07	1.01E-08	2.49E-08	2.19E-08	7.70E-09	1.71E-07	8.69E-09	2.59E-07
SLC26A4	1.94E-08	3.62E-08	3.33E-08	3.95E-08	1.66E-08	2.99E-08	0	1.89E-08
SLC26A5	1.57E-07	1.19E-08	4.21E-08	2.48E-08	8.16E-09	5.63E-08	0	0
SLC26A6	1.13E-05	1.15E-05	9.12E-07	1.54E-06	2.05E-06	4.50E-06	5.43E-06	4.23E-06
SLC26A7	3.95E-08	1.18E-07	2.69E-08	4.81E-08	1.88E-08	7.65E-08	3.75E-08	6.41E-09
SLC26A8	1.01E-08	2.33E-07	0	1.23E-08	1.89E-08	7.48E-08	9.76E-09	0
SLC26A9	1.93E-08	3.09E-08	7.04E-08	2.36E-08	0	8.95E-09	1.87E-08	2.84E-08
SLC27A1	4.47E-06	1.65E-06	4.87E-06	6.18E-06	5.76E-06	4.09E-06	7.79E-06	3.50E-06
SLC27A2	3.47E-05	9.01E-05	0.000132	0.000101	0.000139	9.97E-05	0.000182	0.000109
SLC27A3	1.97E-05	1.66E-05	1.73E-05	1.87E-05	1.76E-05	5.15E-06	2.89E-05	2.56E-05
SLC27A4	2.13E-05	1.80E-05	1.31E-05	3.99E-05	3.09E-05	2.34E-05	3.55E-05	4.68E-05
SLC27A5	3.42E-05	0.000366	0.000484	0.000331	0.000814	0.000301	0.000408	0.000123
SLC27A6	0	0	9.29E-09	1.09E-08	0	0	0	0
SLC28A1	4.66E-06	1.07E-05	4.57E-05	2.50E-05	6.69E-06	1.38E-05	2.85E-05	9.79E-06
SLC28A2	6.68E-08	8.60E-08	8.93E-08	2.34E-08	3.28E-08	1.06E-07	4.62E-08	0
SLC28A3	1.07E-08	0	8.82E-08	3.95E-08	0	0	0	0
SLC29A1	4.33E-05	4.89E-05	3.98E-05	4.73E-05	1.65E-05	2.13E-05	3.88E-05	0.000103
SLC29A2	1.31E-05	2.34E-06	6.83E-07	1.38E-06	7.10E-07	4.47E-07	3.27E-07	3.99E-07
SLC29A3	1.99E-05	6.22E-06	5.27E-06	1.06E-05	6.58E-06	6.41E-06	9.46E-06	6.94E-06
SLC29A4	1.22E-05	1.73E-06	2.03E-05	1.29E-06	1.55E-05	7.43E-06	1.91E-06	3.49E-07
SLC2A1	9.41E-06	1.68E-06	8.73E-07	2.62E-06	2.46E-07	4.54E-06	1.74E-06	3.10E-07
SLC2A10	7.68E-07	8.93E-06	1.36E-05	8.60E-06	2.70E-06	2.23E-05	8.35E-06	2.45E-05
SLC2A11	4.55E-06	2.21E-06	1.06E-06	1.37E-06	7.86E-07	2.07E-06	1.02E-06	1.68E-06
SLC2A12	9.09E-06	5.52E-06	2.27E-06	2.05E-06	1.16E-06	1.58E-05	8.28E-07	1.12E-06
SLC2A13	1.32E-06	1.16E-06	3.81E-06	1.38E-06	4.43E-07	4.95E-07	8.16E-07	3.21E-06
SLC2A14	1.80E-07	4.02E-07	5.88E-08	2.11E-06	0	6.75E-07	3.71E-07	3.13E-07
SLC2A2	4.81E-05	0.000126	0.000384	0.000179	7.85E-05	0.000245	0.000132	0.000125
SLC2A3	5.28E-06	6.09E-06	1.44E-06	3.49E-05	5.22E-07	4.83E-06	8.43E-06	3.79E-06
SLC2A4	7.45E-07	4.30E-06	2.72E-06	3.62E-07	1.07E-07	1.64E-06	4.48E-07	3.20E-06
SLC2A4RG	4.11E-05	6.89E-05	6.92E-05	3.50E-05	9.83E-05	5.38E-05	7.56E-05	2.72E-05
SLC2A5	7.10E-07	1.57E-07	6.49E-08	6.66E-07	2.13E-08	7.22E-06	5.52E-07	1.82E-08
SLC2A6	4.74E-06	5.71E-07	2.04E-06	3.39E-06	6.45E-07	5.89E-07	1.59E-06	2.06E-05
SLC2A7	1.61E-08	0	0	0	0	0	0	0

SLC2A8	1.28E-05	8.17E-06	1.80E-05	1.39E-05	1.84E-05	1.23E-05	1.63E-05	2.92E-05
SLC2A9	1.30E-05	2.41E-05	1.58E-05	1.15E-05	7.78E-06	2.62E-05	8.55E-06	9.59E-06
SLC30A1	9.35E-06	4.11E-05	2.17E-05	2.28E-05	1.38E-06	2.44E-05	7.99E-06	2.41E-05
SLC30A10	9.13E-06	3.48E-05	1.14E-05	8.52E-06	2.84E-06	1.76E-05	1.03E-05	2.31E-05
SLC30A2	1.60E-06	3.21E-08	4.95E-07	2.01E-06	5.72E-08	3.00E-07	7.36E-08	4.48E-08
SLC30A3	2.17E-06	6.17E-07	1.24E-08	2.79E-08	0	6.57E-08	0	8.05E-07
SLC30A4	8.01E-07	5.93E-07	9.53E-07	3.43E-07	0	2.40E-07	8.25E-08	3.11E-07
SLC30A5	2.19E-05	1.73E-05	1.17E-05	1.12E-05	5.72E-06	1.88E-05	9.69E-06	2.08E-05
SLC30A6	8.94E-06	9.42E-06	3.73E-06	3.11E-06	3.56E-07	5.37E-06	1.56E-06	3.46E-06
SLC30A7	7.18E-06	6.57E-06	4.26E-06	5.29E-06	1.30E-06	1.01E-05	2.59E-06	4.89E-06
SLC30A8	3.39E-08	4.73E-09	0	0	0	0	4.12E-09	0
SLC30A9	3.21E-05	2.61E-05	1.39E-05	1.28E-05	6.21E-06	1.60E-05	9.15E-06	1.41E-05
SLC31A1	4.31E-05	3.56E-05	6.78E-05	3.02E-05	2.80E-05	4.63E-05	3.58E-05	5.54E-05
SLC31A2	1.82E-05	5.61E-06	1.65E-05	1.83E-05	2.01E-06	8.57E-06	6.82E-06	4.54E-06
SLC32A1	0	0	0	0	0	0	0	0
SLC33A1	1.81E-05	2.10E-05	1.41E-05	9.53E-06	5.53E-06	3.43E-05	5.85E-06	1.26E-05
SLC34A1	1.27E-07	9.19E-08	4.71E-07	2.22E-08	1.56E-08	2.60E-07	1.76E-08	7.99E-08
SLC34A2	4.36E-06	0	6.76E-07	2.85E-06	0	3.10E-07	2.66E-08	1.61E-08
SLC34A3	3.13E-08	1.37E-08	1.27E-08	1.18E-08	0	0	0	2.82E-08
SLC35A1	1.95E-05	1.31E-05	1.14E-05	1.22E-05	4.38E-06	1.02E-05	9.79E-06	8.86E-06
SLC35A2	3.19E-05	2.64E-05	1.43E-05	1.88E-05	1.06E-05	3.48E-05	2.35E-05	4.88E-05
SLC35A3	7.90E-06	2.33E-05	1.32E-05	5.75E-06	2.79E-06	1.76E-05	2.87E-06	6.45E-06
SLC35A4	3.25E-05	4.60E-05	2.28E-05	2.69E-05	1.79E-05	3.96E-05	3.00E-05	3.93E-05
SLC35A5	1.55E-05	1.00E-05	1.03E-05	7.73E-06	1.95E-06	1.13E-05	5.66E-06	8.28E-06
SLC35B1	8.75E-05	0.000108	4.16E-05	4.20E-05	7.29E-05	0.000128	6.35E-05	8.34E-05
SLC35B2	5.12E-05	4.91E-05	2.67E-05	3.59E-05	2.24E-05	4.28E-05	2.86E-05	4.10E-05
SLC35B3	1.94E-05	2.46E-05	1.06E-05	1.13E-05	1.16E-05	1.72E-05	1.34E-05	2.20E-05
SLC35B4	1.24E-05	8.21E-06	2.95E-06	4.26E-06	3.59E-06	8.03E-06	4.17E-06	1.23E-05
SLC35C1	7.01E-05	4.61E-05	1.98E-05	4.26E-05	1.03E-05	6.02E-05	3.62E-05	0.000121
SLC35C2	4.35E-05	3.50E-05	1.79E-05	2.40E-05	2.85E-05	3.22E-05	2.30E-05	2.29E-05
SLC35D1	2.26E-05	2.01E-05	3.30E-05	1.75E-05	4.72E-06	3.62E-05	1.07E-05	1.35E-05
SLC35D2	4.60E-05	1.23E-05	2.81E-05	3.51E-05	3.98E-05	4.26E-05	3.77E-05	3.48E-05
SLC35D3	0	0	0	0	0	0	0	0
SLC35E1	1.77E-05	9.02E-06	8.22E-06	8.83E-06	4.44E-06	1.04E-05	8.89E-06	1.04E-05
SLC35E2	1.23E-06	4.84E-07	2.81E-07	2.00E-07	1.82E-07	5.70E-07	2.44E-07	6.08E-07
SLC35E3	1.06E-05	7.33E-06	5.19E-06	3.99E-06	6.75E-07	4.89E-06	2.68E-06	4.20E-06
SLC35E4	1.87E-06	6.95E-07	1.44E-07	1.06E-06	4.12E-07	1.88E-07	4.29E-07	7.18E-07
SLC35F1	1.36E-07	6.31E-08	4.77E-09	1.96E-08	3.61E-08	2.87E-08	6.81E-09	3.08E-08
SLC35F2	5.23E-06	2.57E-07	3.25E-07	5.66E-07	1.31E-07	1.03E-07	4.67E-07	1.60E-07
SLC35F3	3.24E-06	0	8.72E-09	2.65E-07	0	1.56E-08	1.63E-08	0
SLC35F4	1.47E-08	0	0	1.81E-08	0	0	0	1.17E-08
SLC35F5	2.68E-05	1.72E-05	1.35E-05	1.03E-05	2.20E-06	1.83E-05	6.27E-06	1.01E-05
SLC36A1	4.44E-06	9.03E-07	5.17E-07	1.82E-06	3.07E-07	1.00E-06	9.62E-07	1.59E-06
SLC36A2	0	0	0	0	0	0	0	0
SLC36A3	0	0	0	0	0	0	0	0
SLC36A4	1.26E-05	4.27E-06	2.38E-06	2.79E-06	5.35E-07	2.34E-06	1.39E-06	4.11E-06
SLC37A1	8.24E-06	5.16E-07	1.10E-06	5.33E-06	2.70E-07	2.04E-06	2.36E-06	6.88E-06
SLC37A2	2.55E-06	9.78E-07	1.89E-06	5.55E-06	4.91E-07	1.32E-06	3.56E-06	3.53E-07
SLC37A3	6.53E-06	7.34E-06	3.99E-06	6.27E-06	2.53E-06	4.44E-06	4.77E-06	8.99E-06
SLC37A4	0.000114	3.46E-05	0.000132	0.000108	8.39E-05	9.18E-05	8.30E-05	0.000179
SLC38A1	2.21E-05	1.23E-05	1.82E-06	4.76E-06	1.37E-06	2.91E-06	4.59E-06	4.53E-06
SLC38A10	7.40E-05	8.72E-05	5.77E-05	6.01E-05	6.15E-05	8.91E-05	4.04E-05	6.26E-05
SLC38A11	1.00E-06	1.21E-07	1.76E-07	4.34E-07	1.17E-07	1.79E-06	1.60E-07	2.46E-07
SLC38A2	0.000104	0.000104	0.00015	0.000125	1.47E-05	0.000134	0.000107	0.000224
SLC38A3	8.82E-05	0.000662	0.000358	0.000259	0.000289	0.000111	0.000238	0.000444
SLC38A4	9.61E-05	0.000166	0.000242	7.12E-05	5.29E-05	0.000257	7.12E-05	0.000175
SLC38A5	1.28E-06	1.77E-07	7.58E-08	6.95E-07	7.26E-08	6.08E-07	4.89E-07	1.77E-07
SLC38A6	7.20E-06	9.85E-06	2.13E-06	4.12E-06	1.98E-06	5.81E-06	4.22E-06	4.69E-06

SLC38A7	1.23E-05	6.45E-06	6.43E-06	4.58E-06	1.68E-07	7.07E-06	3.43E-06	8.98E-06
SLC38A8	0	0	0	0	0	0	7.62E-07	0
SLC38A9	1.31E-05	1.94E-05	5.84E-06	6.83E-06	3.44E-06	6.98E-06	5.23E-06	9.94E-06
SLC39A1	0.000113	0.000104	4.22E-05	6.47E-05	2.79E-05	7.48E-05	6.30E-05	0.000138
SLC39A10	4.52E-06	1.80E-06	7.53E-07	1.29E-06	2.13E-07	1.38E-06	9.16E-07	2.54E-06
SLC39A11	2.79E-05	3.17E-05	1.82E-05	1.81E-05	1.43E-05	4.33E-05	2.07E-05	1.30E-05
SLC39A12	0	2.15E-08	0	3.51E-08	0	0	1.85E-08	0
SLC39A13	2.09E-05	1.81E-05	7.11E-06	1.30E-05	1.12E-05	1.14E-05	1.13E-05	2.30E-05
SLC39A14	0.000107	0.000359	0.000235	0.000113	1.89E-05	0.000216	5.67E-05	7.63E-05
SLC39A2	0	2.04E-08	7.61E-08	8.78E-08	0	0	3.43E-08	2.64E-08
SLC39A3	1.95E-05	1.82E-05	1.10E-05	1.72E-05	2.81E-05	1.92E-05	3.63E-05	2.67E-05
SLC39A4	6.28E-05	1.52E-05	7.13E-06	1.60E-05	1.27E-06	2.94E-06	0.000142	5.58E-07
SLC39A5	3.46E-05	7.26E-05	0.000126	6.97E-05	5.34E-05	2.30E-05	0.000122	1.08E-07
SLC39A6	1.53E-05	7.55E-06	6.17E-06	7.13E-06	8.58E-07	8.81E-06	1.07E-05	8.43E-06
SLC39A7	0.000153	0.00012	4.89E-05	6.42E-05	0.000128	0.000122	7.45E-05	0.000169
SLC39A8	1.29E-05	1.81E-05	2.44E-05	1.35E-05	4.98E-06	1.66E-05	1.50E-05	9.80E-06
SLC39A9	3.89E-05	2.26E-05	2.56E-05	2.02E-05	2.11E-05	3.96E-05	3.05E-05	4.19E-05
SLC3A1	0.000133	7.57E-07	4.68E-05	3.20E-06	5.39E-07	5.63E-08	1.62E-07	3.04E-07
SLC3A2	9.42E-05	9.40E-05	4.28E-05	6.64E-05	2.66E-05	5.13E-05	5.75E-05	9.61E-05
SLC40A1	0.000108	0.000142	9.23E-05	8.72E-05	4.13E-05	0.000101	8.02E-05	4.45E-05
SLC41A1	8.68E-06	5.71E-07	1.73E-06	2.22E-06	2.99E-07	1.20E-06	2.39E-06	4.12E-06
SLC41A2	1.02E-05	3.62E-05	3.52E-05	1.18E-05	3.76E-06	1.28E-05	7.28E-06	1.52E-05
SLC41A3	3.45E-05	2.55E-05	9.05E-06	1.40E-05	1.26E-05	1.19E-05	1.69E-05	1.73E-05
SLC43A1	0.000129	0.000115	5.42E-05	6.64E-05	0.000145	4.11E-05	8.64E-05	7.08E-05
SLC43A2	1.61E-05	6.82E-06	2.99E-06	7.40E-06	1.84E-06	6.05E-06	5.99E-06	7.61E-06
SLC43A3	0.000135	0.000135	4.27E-05	7.33E-05	8.60E-05	1.63E-05	6.72E-05	4.66E-05
SLC44A1	2.44E-05	1.89E-05	2.10E-05	1.72E-05	1.10E-05	1.18E-05	1.73E-05	2.85E-05
SLC44A2	4.01E-05	1.16E-05	1.29E-05	1.62E-05	1.39E-05	7.76E-06	1.81E-05	1.12E-05
SLC44A3	3.32E-05	3.56E-06	5.23E-06	1.27E-05	1.94E-06	1.69E-05	1.46E-07	7.04E-06
SLC44A4	6.66E-07	8.40E-07	1.09E-07	0	6.11E-08	1.11E-06	4.91E-07	0
SLC44A5	3.62E-05	3.50E-06	3.01E-08	1.93E-07	5.03E-09	5.96E-08	5.69E-09	2.55E-08
SLC45A1	4.86E-06	3.02E-07	1.69E-07	3.47E-07	5.48E-07	2.28E-07	3.55E-07	2.09E-07
SLC45A2	4.25E-08	3.73E-07	5.24E-08	5.24E-08	1.22E-08	1.51E-06	9.71E-08	1.24E-08
SLC45A3	7.93E-06	1.24E-06	5.69E-06	3.49E-06	2.98E-06	2.52E-06	2.56E-06	4.21E-06
SLC45A4	4.60E-06	5.69E-07	3.39E-07	1.58E-06	3.10E-07	4.12E-07	8.06E-07	5.27E-07
SLC46A1	3.48E-05	3.25E-05	6.32E-06	4.81E-06	1.29E-05	8.43E-06	1.28E-05	1.17E-05
SLC46A2	1.88E-08	2.11E-08	2.93E-08	9.19E-08	1.61E-08	4.35E-08	1.82E-08	2.76E-08
SLC46A3	5.79E-06	4.06E-05	3.85E-05	2.65E-05	3.06E-07	2.67E-05	1.45E-05	2.56E-05
SLC47A1	1.51E-05	0.000166	7.72E-05	2.32E-05	5.25E-05	2.60E-05	4.30E-05	3.39E-05
SLC47A2	0	2.02E-07	8.87E-08	2.58E-08	0	1.11E-07	3.06E-08	4.65E-08
SLC48A1	8.38E-06	4.56E-06	6.21E-06	6.90E-06	5.58E-06	8.88E-06	1.13E-05	1.23E-05
SLC4A1	4.56E-09	0	1.40E-08	4.78E-08	1.95E-08	0	3.55E-08	3.33E-08
SLC4A10	4.03E-09	9.00E-09	8.25E-09	3.44E-08	0	1.49E-08	1.18E-08	1.18E-08
SLC4A11	3.26E-06	5.03E-08	0	1.48E-07	1.09E-07	2.08E-08	6.62E-08	0
SLC4A1AF	1.69E-05	1.87E-05	6.87E-06	8.40E-06	3.99E-06	1.30E-05	7.80E-06	8.03E-06
SLC4A2	3.22E-05	9.07E-05	4.70E-05	4.29E-05	7.29E-05	4.74E-05	6.09E-05	3.10E-05
SLC4A3	4.16E-06	2.14E-08	1.10E-07	7.06E-07	1.85E-08	2.49E-08	8.90E-08	7.88E-09
SLC4A4	1.89E-05	2.52E-06	1.08E-05	9.92E-06	7.61E-07	9.31E-06	4.12E-06	5.88E-07
SLC4A5	3.40E-07	5.51E-08	9.73E-08	3.91E-07	8.34E-08	1.34E-07	1.85E-07	1.24E-07
SLC4A7	2.60E-06	1.28E-06	1.01E-06	1.40E-06	1.56E-07	9.32E-07	5.66E-07	1.79E-06
SLC4A8	1.62E-07	2.74E-08	2.82E-08	7.30E-08	6.56E-09	3.01E-07	7.25E-08	2.92E-08
SLC4A9	0	0	0	0	0	2.14E-08	0	0
SLC5A1	5.71E-08	5.32E-09	1.13E-06	5.98E-07	8.16E-09	4.14E-09	9.26E-09	2.09E-08
SLC5A10	1.81E-07	2.77E-08	6.30E-08	2.58E-07	3.54E-07	2.24E-08	1.05E-06	5.58E-08
SLC5A11	1.26E-07	1.46E-06	2.45E-07	4.49E-08	6.85E-08	1.68E-06	1.75E-07	1.54E-08
SLC5A12	3.06E-07	7.29E-08	2.80E-08	4.30E-08	6.72E-09	0	9.97E-09	5.73E-09
SLC5A2	1.22E-07	1.56E-07	0	3.28E-08	0	0	0	0
SLC5A3	1.17E-06	1.29E-06	2.84E-06	1.11E-06	2.34E-07	7.63E-07	4.67E-07	4.73E-07

SLC5A4	1.19E-08	2.01E-07	2.48E-08	0	1.02E-08	2.20E-08	6.88E-08	5.24E-08
SLC5A5	6.41E-09	0	0	0	0	0	1.25E-08	0
SLC5A6	2.71E-05	9.73E-05	1.25E-05	1.24E-05	7.83E-06	0.000123	1.46E-05	3.92E-05
SLC5A7	0	5.14E-09	0	5.61E-09	0	0	0	0
SLC5A8	0	0	0	0	0	0	6.81E-09	0
SLC5A9	1.04E-05	6.39E-06	3.19E-06	3.25E-06	6.53E-06	1.13E-05	4.00E-06	3.35E-06
SLC6A1	3.06E-05	0.000102	6.33E-05	2.09E-05	5.88E-06	2.78E-05	1.06E-05	4.92E-05
SLC6A10P	2.08E-06	5.48E-08	0	7.26E-09	0	0	1.95E-08	0
SLC6A11	6.92E-07	9.10E-07	2.53E-08	0	0	0	0	2.39E-06
SLC6A12	2.67E-06	2.43E-06	5.61E-05	2.94E-05	3.05E-05	5.35E-05	3.20E-05	1.59E-05
SLC6A13	8.50E-07	2.94E-07	5.69E-06	2.81E-06	5.83E-06	7.59E-06	4.59E-06	6.67E-07
SLC6A14	0	0	5.13E-09	0	0	0	0	0
SLC6A15	0	0	0	0	0	0	5.19E-09	0
SLC6A16	3.04E-07	1.42E-06	1.53E-06	1.25E-06	1.41E-06	3.48E-06	1.25E-06	5.21E-07
SLC6A17	5.29E-06	1.17E-08	3.57E-09	2.13E-08	0	0	3.06E-08	5.11E-09
SLC6A18	0	0	0	0	0	0	0	0
SLC6A19	0	0	9.15E-07	8.51E-08	3.74E-09	0	0	0
SLC6A2	9.33E-09	1.05E-08	6.74E-07	0	0	4.46E-08	0	0
SLC6A20	8.79E-06	0	1.30E-08	1.11E-07	0	7.83E-09	4.12E-09	0
SLC6A3	1.81E-06	0	0	0	0	0	0	3.40E-08
SLC6A4	8.70E-09	2.24E-08	0	3.87E-08	0	2.91E-07	8.43E-09	1.28E-08
SLC6A5	0	0	0	0	0	0	0	0
SLC6A6	1.29E-05	2.53E-07	6.95E-07	2.55E-06	1.19E-07	5.15E-07	8.13E-07	4.66E-07
SLC6A7	0	0	0	7.47E-09	0	1.30E-07	5.94E-09	8.96E-09
SLC6A8	2.36E-05	1.60E-06	4.29E-07	2.31E-06	6.69E-07	5.08E-07	1.90E-06	3.68E-06
SLC6A9	5.96E-07	6.85E-07	2.21E-07	2.52E-07	2.39E-07	1.46E-06	4.20E-07	3.15E-07
SLC7A1	7.33E-06	6.50E-07	1.58E-07	2.84E-06	2.34E-08	2.27E-07	3.02E-07	7.85E-07
SLC7A10	1.29E-06	1.06E-07	0	1.52E-08	2.55E-07	2.30E-08	1.54E-08	2.31E-08
SLC7A11	7.37E-07	5.24E-07	1.17E-08	2.52E-08	1.97E-09	3.61E-07	2.46E-08	3.02E-07
SLC7A13	1.29E-08	1.47E-08	2.72E-08	1.59E-08	0	1.20E-08	1.25E-08	7.62E-08
SLC7A14	2.18E-09	2.43E-09	2.22E-09	0	1.87E-09	5.04E-08	2.13E-09	0
SLC7A2	4.33E-05	0.0003	8.73E-05	2.44E-05	8.98E-06	1.28E-05	9.24E-06	3.05E-05
SLC7A3	0	0	0	0	0	0	0	0
SLC7A4	1.66E-06	1.15E-08	7.45E-08	6.63E-07	1.76E-08	0	0	0
SLC7A5	1.75E-05	8.20E-07	2.93E-06	6.40E-06	2.30E-06	6.46E-06	3.13E-06	2.32E-06
SLC7A5P1	4.42E-08	0	0	0	0	4.78E-08	4.73E-08	0
SLC7A5P2	3.22E-08	2.30E-08	0	7.23E-08	0	0	0	1.40E-08
SLC7A6	1.14E-05	1.55E-06	9.66E-07	2.28E-06	9.83E-07	3.52E-06	1.40E-06	3.40E-06
SLC7A6OS	1.12E-05	3.07E-06	2.92E-06	2.99E-06	2.41E-07	4.17E-06	1.86E-06	5.86E-06
SLC7A7	4.84E-05	3.19E-06	6.59E-06	1.20E-05	2.39E-06	3.30E-06	6.61E-06	2.86E-06
SLC7A8	6.17E-06	1.88E-06	3.52E-06	4.91E-06	4.20E-07	2.01E-06	4.69E-06	1.17E-06
SLC7A9	2.24E-05	1.18E-05	1.70E-05	1.04E-05	4.67E-06	2.26E-05	1.17E-05	1.77E-05
SLC8A1	2.15E-06	3.81E-07	1.27E-06	1.55E-06	1.31E-07	5.61E-07	4.03E-07	2.45E-07
SLC8A2	4.58E-09	7.33E-09	0	3.99E-08	1.68E-08	6.05E-09	0	0
SLC8A3	2.67E-08	2.13E-06	6.13E-08	6.45E-08	0	5.11E-08	6.13E-08	4.14E-08
SLC9A1	3.82E-06	2.06E-06	1.35E-06	3.37E-06	1.02E-06	1.39E-06	4.43E-06	2.49E-06
SLC9A10	4.56E-08	1.71E-08	0	0	0	7.04E-09	0	0
SLC9A11	5.12E-09	2.55E-07	7.09E-09	8.40E-09	0	6.31E-08	6.67E-09	0
SLC9A2	1.12E-06	9.23E-09	0	5.04E-09	0	0	2.01E-08	0
SLC9A3	9.32E-08	1.91E-08	8.88E-09	4.10E-08	0	1.57E-08	8.21E-09	1.24E-08
SLC9A3R1	0.00038	0.000334	0.000143	0.000186	0.000237	0.000235	0.000178	0.000102
SLC9A3R2	8.46E-05	0.000167	0.000161	0.000107	0.0002	9.67E-05	0.00015	6.69E-05
SLC9A4	0	0	0	6.71E-09	0	0	0	0
SLC9A5	3.67E-07	7.62E-08	2.33E-08	8.23E-08	3.91E-08	1.05E-08	1.01E-07	2.86E-08
SLC9A6	5.30E-06	4.51E-06	2.23E-06	2.77E-06	1.13E-06	2.89E-06	2.07E-06	5.40E-06
SLC9A7	1.87E-06	2.13E-06	1.80E-06	1.14E-06	2.78E-07	4.40E-07	7.99E-07	2.16E-06
SLC9A8	9.41E-06	7.16E-06	3.28E-06	3.33E-06	3.03E-06	5.17E-06	4.22E-06	5.79E-06
SLC9A9	1.25E-06	2.57E-07	2.21E-06	2.29E-06	3.77E-07	1.43E-06	1.50E-06	8.11E-07

SLCO1A2	2.27E-07	3.76E-06	4.68E-07	8.94E-08	1.65E-08	1.16E-05	7.04E-08	7.16E-07
SLCO1B1	8.97E-05	0.000204	0.000218	5.26E-05	3.31E-05	7.25E-05	5.09E-05	4.21E-05
SLCO1B3	8.43E-07	0	9.20E-05	1.66E-05	2.29E-08	6.70E-05	4.26E-06	1.52E-06
SLCO1C1	9.36E-08	4.73E-08	0	0	1.88E-08	4.00E-08	6.29E-08	5.32E-08
SLCO2A1	5.97E-06	1.57E-05	8.80E-07	1.99E-06	4.39E-06	3.43E-06	2.40E-06	2.93E-06
SLCO2B1	6.91E-05	0.000173	0.000136	9.20E-05	3.56E-05	0.000103	6.76E-05	7.60E-06
SLCO3A1	4.50E-06	7.86E-07	1.37E-06	6.00E-06	8.14E-07	1.05E-06	1.98E-06	2.38E-06
SLCO4A1	7.92E-06	2.01E-07	5.99E-07	1.66E-06	5.45E-08	2.70E-07	1.12E-07	1.73E-07
SLCO4C1	5.11E-06	6.04E-06	3.10E-06	3.71E-07	2.53E-08	7.81E-09	2.88E-08	1.23E-08
SLCO5A1	3.94E-08	3.67E-08	2.22E-08	6.95E-08	0	3.01E-08	7.87E-09	4.54E-07
SLCO6A1	8.38E-07	0	0	0	0	1.32E-08	0	0
SLED1	3.86E-08	2.29E-07	1.32E-07	0	3.02E-07	1.45E-07	4.00E-07	6.39E-07
SLFN11	5.01E-06	1.19E-06	1.19E-06	2.75E-06	3.18E-07	1.24E-06	1.37E-06	1.08E-06
SLFN12	4.04E-06	4.64E-07	3.21E-07	5.03E-07	4.42E-08	5.48E-07	3.48E-07	1.96E-07
SLFN12L	3.31E-07	2.86E-08	1.05E-07	1.56E-07	0	1.49E-07	8.22E-08	6.22E-08
SLFN13	1.21E-05	8.27E-08	2.75E-07	8.28E-07	2.69E-08	1.23E-07	1.17E-07	1.31E-07
SLFN14	4.03E-08	1.81E-08	0	0	0	7.46E-09	0	1.18E-08
SLFN5	8.04E-06	1.89E-06	2.16E-06	2.42E-06	8.37E-08	4.61E-06	4.51E-07	2.16E-06
SLFNL1	1.71E-07	7.79E-08	2.14E-07	5.23E-08	1.22E-08	3.96E-08	6.85E-08	4.19E-08
SLIT1	3.37E-08	4.37E-08	2.86E-08	4.10E-08	1.44E-08	7.78E-09	2.73E-08	8.19E-09
SLIT2	8.94E-07	9.22E-08	1.27E-07	5.03E-07	0	2.54E-08	1.34E-07	3.69E-07
SLIT3	1.56E-06	7.61E-07	2.91E-07	2.98E-06	5.85E-07	1.23E-06	6.58E-07	7.34E-07
SLITRK1	0	0	8.90E-09	5.30E-09	0	0	0	0
SLITRK2	1.19E-08	6.64E-09	1.83E-08	1.01E-07	0	0	0	8.68E-09
SLITRK3	0	4.35E-07	1.96E-06	1.45E-06	0	0	5.25E-09	7.90E-09
SLITRK4	1.05E-07	6.76E-08	3.89E-08	2.76E-08	1.29E-08	3.48E-08	3.36E-07	5.52E-08
SLITRK5	0	1.14E-08	1.05E-08	6.23E-09	0	0	0	5.97E-08
SLITRK6	3.30E-08	4.97E-08	7.26E-07	5.47E-08	0	0	1.59E-08	7.96E-09
SLK	1.33E-05	7.14E-06	6.38E-06	7.51E-06	1.02E-06	6.35E-06	3.55E-06	7.27E-06
SLMAP	1.35E-05	1.65E-05	7.67E-06	5.90E-06	2.78E-06	1.04E-05	5.73E-06	7.39E-06
SLMO1	9.93E-07	6.46E-08	1.65E-07	2.44E-07	8.57E-08	7.92E-08	1.23E-07	1.46E-07
SLMO2	4.15E-05	3.82E-05	1.34E-05	1.68E-05	5.69E-06	2.16E-05	1.50E-05	1.87E-05
SLN	2.03E-07	4.83E-08	0	0	0	0	8.78E-07	3.49E-06
SLPI	0.000408	0.000182	9.29E-05	0.000194	0.000602	1.99E-07	7.11E-05	0.000383
SLTM	2.29E-05	1.82E-05	1.65E-05	1.85E-05	7.88E-06	2.44E-05	1.63E-05	1.83E-05
SLU7	1.49E-05	8.32E-06	1.05E-05	1.16E-05	4.38E-06	1.72E-05	9.45E-06	1.24E-05
SLURP1	0	0	2.45E-06	0	0	0	0	0
SMAD1	1.08E-05	4.50E-06	1.37E-05	6.62E-06	1.84E-06	1.09E-05	7.93E-06	1.41E-05
SMAD2	1.42E-05	6.93E-06	5.56E-06	8.57E-06	1.25E-06	1.27E-05	6.34E-06	7.32E-06
SMAD3	1.53E-05	3.88E-06	4.19E-06	9.81E-06	4.37E-06	1.32E-05	9.02E-06	6.35E-06
SMAD4	8.43E-06	5.16E-06	7.07E-06	6.83E-06	2.23E-06	7.35E-06	7.09E-06	5.20E-06
SMAD5	7.39E-06	7.90E-06	6.48E-06	7.22E-06	1.35E-06	6.09E-06	4.48E-06	1.41E-05
SMAD5OS	3.08E-07	3.95E-07	2.68E-07	3.95E-07	3.23E-07	1.74E-07	4.79E-07	4.74E-07
SMAD6	2.72E-06	1.50E-06	3.60E-06	3.09E-06	2.18E-06	2.30E-06	9.48E-07	5.21E-07
SMAD7	1.31E-05	1.97E-06	5.06E-06	1.26E-05	1.42E-06	3.32E-06	5.07E-06	2.83E-06
SMAD9	7.83E-06	3.58E-08	1.91E-07	2.59E-07	0	6.51E-08	4.09E-08	3.11E-08
SMAGP	1.94E-05	4.51E-06	3.79E-06	8.99E-06	9.87E-06	5.72E-06	1.04E-05	3.58E-06
SMAP1	1.53E-05	1.59E-05	6.68E-06	8.02E-06	3.16E-06	9.86E-06	6.78E-06	7.55E-06
SMAP2	5.81E-05	4.37E-05	3.03E-05	4.18E-05	2.78E-05	4.23E-05	4.72E-05	4.47E-05
SMARCA1	7.14E-05	5.13E-05	2.34E-05	1.81E-05	2.29E-05	3.07E-05	1.47E-05	2.92E-05
SMARCA2	1.59E-05	2.29E-05	3.50E-05	2.89E-05	1.56E-05	3.42E-05	2.22E-05	2.07E-05
SMARCA4	2.25E-05	1.11E-05	7.46E-06	1.35E-05	1.33E-05	1.73E-05	1.66E-05	1.45E-05
SMARCA5	3.15E-05	1.76E-05	1.01E-05	1.76E-05	1.29E-06	1.25E-05	9.05E-06	2.24E-05
SMARCAD	9.16E-06	3.74E-06	4.24E-06	4.59E-06	5.42E-07	7.98E-06	2.25E-06	4.07E-06
SMARCAL	9.51E-06	4.61E-06	3.63E-06	4.46E-06	3.52E-06	8.76E-06	4.71E-06	4.49E-06
SMARCB1	5.59E-05	2.26E-05	1.74E-05	2.95E-05	2.56E-05	3.38E-05	4.28E-05	2.96E-05
SMARCC1	1.61E-05	1.31E-05	9.35E-06	1.06E-05	4.49E-06	1.68E-05	1.13E-05	1.13E-05
SMARCC2	1.99E-05	2.34E-05	9.43E-06	1.00E-05	5.55E-06	1.01E-05	5.37E-06	1.03E-05

SMARCD1	2.01E-05	1.37E-05	5.20E-06	1.02E-05	2.84E-06	1.14E-05	1.03E-05	6.88E-06
SMARCD2	4.65E-05	3.32E-05	3.54E-05	2.93E-05	4.37E-05	8.08E-05	4.45E-05	3.29E-05
SMARCD3	6.42E-06	6.23E-07	1.13E-06	4.28E-06	1.59E-06	8.55E-07	3.53E-06	8.09E-06
SMARCE1	8.93E-05	5.57E-05	2.44E-05	3.33E-05	2.94E-05	5.60E-05	3.36E-05	3.09E-05
SMC1A	2.38E-05	2.13E-05	7.48E-06	5.51E-06	2.12E-06	8.70E-06	3.94E-06	1.26E-05
SMC1B	0	8.96E-07	0	1.38E-08	4.85E-09	5.23E-09	0	1.66E-08
SMC2	5.66E-06	2.60E-06	1.42E-06	1.46E-06	8.86E-07	4.35E-06	1.44E-06	1.98E-06
SMC3	1.93E-05	8.76E-06	7.09E-06	8.41E-06	1.51E-06	1.29E-05	5.32E-06	7.24E-06
SMC4	2.66E-05	5.63E-06	3.58E-06	2.82E-06	2.56E-06	5.66E-06	2.90E-06	4.39E-06
SMC5	1.98E-05	1.00E-05	6.67E-06	6.30E-06	2.24E-06	6.64E-06	3.28E-06	6.76E-06
SMC6	9.56E-06	5.73E-06	5.11E-06	4.87E-06	1.66E-06	4.87E-06	3.16E-06	3.82E-06
SMCHD1	8.11E-06	2.61E-06	2.82E-06	3.48E-06	5.47E-07	3.87E-06	2.51E-06	2.37E-06
SMCP	0	0	0	0	0	0	0	0
SMCR5	0	9.19E-09	0	0	0	0	0	0
SMCR7	5.56E-06	8.78E-06	3.45E-06	5.25E-06	4.35E-06	4.55E-06	4.85E-06	5.92E-06
SMCR7L	1.65E-05	1.33E-05	7.11E-06	5.90E-06	6.11E-06	1.32E-05	9.73E-06	1.69E-05
SMCR8	2.71E-06	3.15E-06	1.49E-06	1.83E-06	1.03E-06	2.81E-06	2.19E-06	2.24E-06
SMEK1	2.82E-05	1.20E-05	1.30E-05	1.42E-05	5.49E-06	1.47E-05	1.33E-05	1.77E-05
SMEK2	4.46E-05	4.35E-05	2.59E-05	1.91E-05	6.72E-06	2.40E-05	2.31E-05	1.60E-05
SMEK3P	7.38E-09	8.28E-09	0	0	0	4.10E-08	0	7.98E-06
SMG1	1.82E-05	6.99E-06	6.65E-06	4.75E-06	9.53E-07	5.64E-06	3.51E-06	3.16E-06
SMG5	3.48E-05	2.76E-05	9.04E-06	1.14E-05	1.61E-05	3.21E-05	2.53E-05	3.78E-05
SMG6	7.77E-06	4.08E-06	6.29E-06	1.03E-05	6.88E-06	8.66E-06	9.62E-06	8.24E-06
SMG7	1.90E-05	2.33E-05	9.07E-06	1.08E-05	5.35E-06	1.94E-05	9.11E-06	2.54E-05
SMN1	3.06E-07	3.25E-07	5.22E-08	6.74E-08	0	1.72E-07	0	0
SMN2	4.97E-05	3.28E-05	2.19E-05	4.07E-05	6.49E-06	4.01E-05	3.54E-05	3.84E-05
SMNDC1	2.37E-05	9.21E-06	9.78E-06	1.22E-05	3.73E-06	1.20E-05	1.08E-05	1.15E-05
SMO	8.94E-06	1.37E-05	1.36E-05	6.60E-06	2.85E-05	1.37E-05	1.64E-05	4.41E-05
SMOC1	2.97E-05	1.27E-05	3.60E-05	3.19E-05	7.37E-05	4.37E-05	5.91E-05	3.01E-05
SMOC2	3.84E-06	8.26E-07	4.42E-07	9.95E-06	2.17E-06	2.91E-06	5.74E-06	4.11E-06
SMOX	1.29E-05	1.07E-06	1.91E-06	1.33E-05	2.73E-06	1.02E-06	3.00E-06	4.36E-06
SMPD1	2.73E-05	5.08E-05	4.40E-05	4.45E-05	3.83E-05	2.94E-05	2.98E-05	7.82E-05
SMPD2	7.61E-06	3.09E-06	2.18E-06	5.00E-06	1.47E-06	4.54E-06	4.03E-06	4.21E-06
SMPD3	4.59E-07	4.03E-08	6.56E-07	1.57E-06	7.35E-08	4.16E-07	1.67E-07	5.63E-08
SMPD4	3.10E-05	1.76E-05	8.45E-06	9.69E-06	8.47E-06	1.38E-05	1.19E-05	1.35E-05
SMPDL3A	4.26E-05	4.76E-05	2.78E-05	2.14E-05	9.36E-06	1.62E-05	1.65E-05	4.11E-05
SMPDL3B	9.94E-06	3.21E-07	2.86E-07	2.53E-06	6.07E-08	2.87E-08	9.88E-07	2.28E-08
SMPX	3.11E-08	6.55E-07	6.91E-08	7.72E-08	1.16E-06	4.01E-06	1.77E-07	4.65E-08
SMR3A	0	0	0	0	0	0	0	0
SMR3B	0	0	0	0	0	0	0	0
SMS	4.32E-05	1.45E-05	1.82E-05	4.17E-05	2.45E-05	2.71E-05	3.68E-05	4.50E-05
SMTN	1.28E-05	1.00E-05	6.88E-06	1.07E-05	1.08E-05	6.43E-06	1.59E-05	7.63E-06
SMTNL1	2.55E-07	6.55E-08	6.06E-08	4.26E-08	9.96E-09	7.52E-08	2.24E-08	5.11E-08
SMTNL2	2.11E-07	0	3.91E-08	0	1.07E-08	5.76E-08	2.40E-08	5.48E-08
SMU1	7.79E-06	8.71E-06	6.27E-06	7.63E-06	2.93E-06	9.74E-06	6.34E-06	6.45E-06
SMUG1	3.62E-05	2.67E-05	2.30E-05	2.35E-05	3.02E-05	3.35E-05	2.84E-05	2.71E-05
SMURF1	2.25E-05	1.63E-05	3.24E-06	5.61E-06	1.68E-06	7.35E-06	8.05E-06	1.08E-05
SMURF2	4.86E-06	1.98E-06	1.08E-06	3.07E-06	4.24E-07	2.52E-06	1.35E-06	1.41E-06
SMYD1	0	4.28E-08	0	6.38E-09	0	3.37E-08	0	0
SMYD2	2.39E-05	4.02E-05	1.35E-05	3.54E-05	7.65E-06	6.31E-05	3.20E-05	1.17E-05
SMYD3	8.55E-06	7.53E-06	2.29E-06	2.69E-06	3.62E-06	1.22E-05	3.90E-06	1.39E-05
SMYD4	2.55E-06	2.20E-06	1.62E-06	1.94E-06	5.84E-07	1.79E-06	1.55E-06	1.48E-06
SMYD5	1.62E-05	1.76E-05	4.50E-06	6.94E-06	5.56E-06	1.20E-05	1.29E-05	1.06E-05
SNAI1	2.56E-06	9.19E-07	6.71E-07	1.64E-05	7.84E-07	5.64E-07	6.51E-06	3.13E-06
SNAI2	7.60E-06	2.99E-06	2.74E-06	8.13E-06	2.14E-06	1.02E-05	5.94E-06	1.27E-06
SNAI3	8.97E-07	1.94E-07	8.11E-07	7.87E-07	4.91E-08	2.38E-07	4.26E-07	9.03E-07
SNAP23	5.17E-05	3.04E-05	1.87E-05	2.13E-05	1.01E-05	2.77E-05	1.62E-05	1.60E-05
SNAP25	7.78E-05	1.31E-06	4.41E-07	2.95E-06	0	7.56E-08	2.26E-07	7.31E-07

SNAP29	2.10E-05	1.87E-05	1.26E-05	1.21E-05	1.03E-05	1.36E-05	1.54E-05	1.81E-05
SNAP47	1.79E-05	2.34E-05	1.58E-05	1.33E-05	1.52E-05	3.82E-05	1.30E-05	3.52E-05
SNAP91	0	8.00E-09	0	0	0	0	0	0
SNAPC1	5.27E-06	1.78E-06	2.19E-06	2.95E-06	8.01E-07	2.20E-06	2.21E-06	1.74E-06
SNAPC2	1.18E-05	6.44E-06	4.65E-06	7.83E-06	1.33E-05	6.87E-06	1.35E-05	7.39E-06
SNAPC3	4.46E-06	7.55E-06	4.41E-06	4.65E-06	5.45E-07	6.90E-06	3.06E-06	4.95E-06
SNAPC4	3.78E-06	2.17E-06	2.84E-06	4.30E-06	2.50E-06	3.38E-06	2.93E-06	2.91E-06
SNAPC5	1.47E-05	9.70E-06	6.19E-06	6.89E-06	4.22E-06	9.59E-06	7.40E-06	6.57E-06
SNAPIN	4.23E-05	3.47E-05	2.02E-05	2.44E-05	1.36E-05	4.21E-05	2.29E-05	3.95E-05
SNAR-A13	0	0	0	0	0	0	0	0
SNAR-A2	0	0	0	0	0	0	0	0
SNAR-A3	0	0	0	0	0	0	0	0
SNAR-A4	0	0	0	0	0	0	0	0
SNAR-B2	0	0	0	0	0	0	0	0
SNAR-C2	0	0	0	0	0	0	0	0
SNAR-C3	0	0	0	0	0	0	0	0
SNAR-C4	0	0	0	0	0	0	0	0
SNAR-D	0	0	0	0	0	0	0	0
SNAR-E	0	0	0	0	0	0	0	0
SNAR-F	0	0	0	0	0	0	0	0
SNAR-G1	0	0	0	0	0	0	0	0
SNAR-G2	0	0	0	0	0	0	0	0
SNAR-H	0	0	0	0	0	0	0	0
SNAR-I	0	0	0	0	0	0	0	0
SNCA	2.30E-07	7.25E-08	8.15E-08	8.86E-07	4.33E-08	1.46E-08	3.48E-07	6.31E-08
SNCAIP	2.26E-07	2.56E-07	1.88E-07	3.93E-07	6.91E-08	2.59E-07	6.53E-07	2.98E-07
SNCB	4.10E-07	0	0	4.84E-08	0	0	0	0
SNCG	1.39E-05	4.67E-06	1.41E-06	4.22E-06	6.55E-06	2.64E-06	7.79E-06	3.31E-06
SND1	5.48E-05	7.55E-05	5.32E-05	6.75E-05	6.30E-05	0.000117	7.95E-05	9.76E-05
SNED1	1.79E-06	1.06E-06	3.08E-06	1.46E-06	1.35E-06	1.15E-06	1.29E-06	1.34E-06
SNF8	7.87E-05	9.92E-05	2.96E-05	4.09E-05	7.83E-05	7.38E-05	5.15E-05	4.32E-05
SNHG1	3.13E-05	1.43E-05	3.37E-06	7.02E-06	6.96E-06	6.85E-06	1.38E-05	1.38E-05
SNHG10	3.59E-06	6.50E-07	6.47E-07	8.55E-07	2.30E-06	1.21E-06	5.73E-07	8.44E-07
SNHG11	1.46E-05	7.25E-06	7.92E-06	9.25E-06	1.44E-05	8.14E-06	1.36E-05	1.85E-05
SNHG12	1.14E-05	2.04E-06	1.36E-06	3.37E-06	4.12E-06	4.46E-06	5.11E-06	2.93E-06
SNHG3	2.05E-06	3.07E-07	1.79E-08	2.89E-07	7.24E-07	6.87E-07	3.55E-07	3.23E-07
SNHG3-R	4.47E-06	9.74E-07	1.81E-07	8.60E-07	5.70E-07	4.50E-06	1.53E-06	3.02E-07
SNHG4	6.24E-07	5.56E-08	0	0	0	5.45E-07	0	2.70E-07
SNHG5	0.000139	2.68E-05	3.90E-05	0.000174	0.000432	0.000245	0.000559	4.10E-05
SNHG6	0.000188	8.45E-05	6.36E-05	7.87E-05	0.000189	9.45E-05	0.000175	0.000106
SNHG7	4.21E-05	7.16E-06	9.90E-06	1.23E-05	2.73E-05	1.36E-05	1.70E-05	2.08E-05
SNHG8	6.61E-05	1.82E-05	5.59E-05	4.94E-05	0.000113	8.43E-05	0.000101	4.47E-05
SNHG9	6.90E-05	2.29E-05	2.12E-05	3.10E-05	0.000171	4.94E-05	8.43E-05	2.50E-05
SNIP1	6.17E-06	6.24E-06	4.75E-06	7.07E-06	8.66E-07	7.05E-06	3.57E-06	4.96E-06
SNN	1.02E-05	1.82E-05	4.69E-06	9.87E-06	1.02E-06	4.46E-06	6.53E-06	7.98E-06
SNORA1	0	0	0	0	0	0	0	0
SNORA10	0	0	0	0	0	0	0	0
SNORA11	0	0	0	0	0	0	0	0
SNORA11	0	0	0	0	0	0	0	0
SNORA11	0	0	0	0	0	0	0	0
SNORA11	0	0	0	0	0	0	0	0
SNORA11	0	0	0	0	0	0	0	0
SNORA12	0	0	0	0	0	0	0	0
SNORA13	0	0	0	0	0	0	0	0
SNORA14	0	0	0	0	0	0	0	0
SNORA14	0	0	0	0	0	0	0	0
SNORA15	0	0	0	0	0	0	0	0
SNORA16	0	0	0	0	0	0	0	0

SNORA16	0	0	0	0	0	0	0	0
SNORA18	0	0	0	0	0	0	0	0
SNORA19	0	0	0	0	0	0	0	0
SNORA20	0	0	0	0	0	0	0	0
SNORA21	7.85E-06	0	0	0	0	0	0	0
SNORA22	7.85E-06	0	0	0	0	0	0	0
SNORA23	0	0	0	1.51E-06	0	0	1.17E-06	0
SNORA24	8.96E-06	0	0	0	0	0	0	0
SNORA25	0	0	0	0	0	0	0	0
SNORA26	0	0	0	0	0	0	0	0
SNORA27	1.28E-05	0	0	0	0	0	0	0
SNORA28	0	0	0	0	0	0	0	0
SNORA29	0	0	0	0	0	0	0	0
SNORA2A	0	0	0	0	0	0	0	0
SNORA2B	0	0	0	0	0	0	0	0
SNORA3	0	0	0	0	0	0	0	0
SNORA30	0	0	0	0	0	0	0	0
SNORA31	9.59E-06	0	0	0	0	0	5.15E-06	0
SNORA32	0	0	0	0	0	0	0	0
SNORA34	1.84E-05	0	0	0	0	0	0	0
SNORA35	0	0	0	0	0	0	0	0
SNORA36.	0	0	0	0	0	0	0	0
SNORA36	0	0	0	0	0	0	0	0
SNORA36	0	0	0	0	0	0	0	0
SNORA37	0	0	0	0	0	0	0	0
SNORA38	0	0	0	0	0	0	0	0
SNORA38	0	0	0	0	0	1.46E-05	0	0
SNORA39	2.67E-07	2.01E-07	1.87E-08	0	0	6.85E-08	0	1.07E-07
SNORA4	0	0	0	0	0	0	0	0
SNORA40	4.52E-05	0	0	0	0	0	0	0
SNORA41	0	0	0	0	0	0	0	0
SNORA42	0	0	0	0	0	0	0	0
SNORA44	0	0	0	0	0	0	0	0
SNORA45	0	0	0	0	0	0	0	2.94E-05
SNORA46	6.91E-06	0	0	0	0	0	0	0
SNORA47	0	0	0	0	0	0	0	0
SNORA48	0	0	0	1.69E-05	0	1.05E-05	3.78E-06	0
SNORA49	0	0	0	0	0	0	0	0
SNORA50	0	0	0	0	0	0	0	0
SNORA51	0	0	0	0	0	0	0	0
SNORA52	0	0	0	0	0	0	0	0
SNORA53	2.75E-06	0	7.28E-07	4.45E-07	0	2.25E-06	1.20E-06	5.61E-07
SNORA54	0	0	0	0	0	0	0	0
SNORA55	0	0	0	0	0	0	0	0
SNORA56	0	0	0	0	0	0	0	0
SNORA57	0	0	0	0	0	0	1.90E-06	0
SNORA58	0	0	0	0	0	0	0	0
SNORA59	0	0	0	6.23E-06	0	4.04E-06	0	0
SNORA5A	0	0	0	0	0	2.38E-05	0	0
SNORA5B	0	0	0	0	0	0	0	0
SNORA5C	0	0	0	0	0	0	0	0
SNORA6	0	0	0	0	0	0	0	0
SNORA61	5.80E-06	0	0	0	0	0	0	0
SNORA62	0	0	0	0	0	0	0	0
SNORA63	1.38E-05	0	0	0	0	1.05E-05	7.56E-06	0
SNORA64	0	0	0	0	0	0	0	0
SNORA65	0	0	0	0	0	0	3.57E-06	0
SNORA66	0	0	0	0	0	0	0	0

SNORA67	6.12E-06	0	0	0	0	0	6.75E-06	0
SNORA68	0	0	0	0	0	0	0	0
SNORA69	0	0	0	0	0	0	0	0
SNORA70	0	0	0	0	0	0	0	5.77E-05
SNORA70	0	0	0	0	0	0	0	0
SNORA70	0	0	0	0	0	0	0	0
SNORA71.	0	0	0	0	0	0	0	0
SNORA71	6.50E-06	0	0	0	0	0	0	0
SNORA71	0	0	0	0	0	0	0	0
SNORA71	0	0	0	0	0	0	0	1.83E-05
SNORA72	0	0	0	0	0	1.27E-05	0	0
SNORA74.	6.70E-07	0	0	0	0	0	0	0
SNORA74	0	0	0	0	0	7.18E-07	4.42E-07	1.31E-06
SNORA75	0	0	0	0	0	0	0	0
SNORA76	8.38E-06	0	0	0	4.88E-05	0	0	0
SNORA77	0	0	0	0	0	0	0	0
SNORA78	0	0	0	0	0	0	0	0
SNORA79	0	0	0	0	0	0	0	0
SNORA7B	2.68E-07	2.38E-07	1.26E-07	1.95E-07	0	3.46E-07	4.17E-07	2.00E-07
SNORA8	4.75E-06	8.04E-07	5.58E-07	1.20E-05	4.67E-07	7.65E-06	8.73E-06	1.62E-06
SNORA80	0	0	0	0	0	0	0	0
SNORA81	1.14E-06	0	0	0	0	0	7.50E-07	0
SNORA84	0	0	0	0	0	0	0	0
SNORA9	0	0	0	0	0	0	0	0
SNORD10	0	0	0	0	0	0	2.07E-06	1.07E-05
SNORD10	0	0	0	0	0	0	0	0
SNORD10	0	0	0	0	0	0	0	0
SNORD10	0	0	0	0	0	0	0	0
SNORD10	0	0	0	0	0	0	0	0
SNORD10	0	0	0	0	0	0	0	0
SNORD10	0	0	0	0	0	0	0	0
SNORD11	0	0	0	0	0	0	0	0
SNORD11	0	0	0	0	0	0	0	0
SNORD11	0	0	0	0	0	0	0	0
SNORD11	0	0	0	0	0	0	0	0
SNORD11	0	0	0	0	0	0	0	0
SNORD11	0	0	0	0	0	0	0	0
SNORD11	0	0	0	0	0	0	0	0
SNORD11	0	0	0	0	0	0	0	0
SNORD11	0	0	0	0	0	0	0	0
SNORD11	0	0	0	0	0	0	0	0
SNORD11	0	0	0	0	0	0	0	0
SNORD11	0	0	0	0	0	0	0	0
SNORD11	0	0	0	0	0	0	0	0
SNORD11	0	0	0	0	0	0	0	0
SNORD11	0	0	0	0	0	0	0	0
SNORD11	0	0	0	0	0	0	0	0
SNORD11	0	0	0	0	0	0	0	0
SNORD11	0	0	0	0	0	0	0	0
SNORD11	0	0	0	0	0	0	0	0
SNORD11	0	0	0	0	0	0	0	0
SNORD11	0	0	0	0	0	0	0	0
SNORD11	0	0	0	0	0	0	0	0
SNORD11	0	0	0	0	0	0	0	0
SNORD11	0	0	0	0	0	0	0	0
SNORD11	0	0	0	0	0	0	0	0
SNORD11	0	0	0	0	0	0	0	0
SNORD11	0	0	0	0	0	0	0	0

SNORD11	0	0	0	0	0	0	0	0
SNORD11	0	0	0	0	0	0	0	0
SNORD11	0	0	0	0	0	0	0	0
SNORD11	0	0	0	0	0	0	0	0
SNORD11	0	0	0	0	0	0	0	0
SNORD11	0	0	0	0	0	0	0	0
SNORD11	0	0	0	0	0	0	0	0
SNORD11	0	0	0	0	0	0	0	0
SNORD11	0	0	0	0	0	0	0	0
SNORD11	0	0	0	0	0	0	0	0
SNORD11	0	0	0	0	0	0	0	0
SNORD11	0	0	0	0	0	0	0	0
SNORD11	0	0	0	0	0	0	0	0
SNORD11	0	0	0	0	0	0	0	0
SNORD11	0	0	0	0	0	0	0	0
SNORD11	0	0	0	0	0	0	0	0
SNORD11	0	0	0	0	0	0	0	0
SNORD11	0	0	0	0	0	0	0	0
SNORD11	0	0	0	0	0	0	0	0
SNORD11	0	0	0	0	0	0	0	0
SNORD11	0	0	0	0	0	0	0	0
SNORD11	0	0	0	0	0	0	0	0
SNORD11	0	0	0	0	0	0	0	0
SNORD11	0	0	0	0	0	0	0	0
SNORD11	0	0	0	0	0	0	0	0
SNORD11	0	0	0	0	0	0	0	0
SNORD11	0	1.41E-08	0	0	0	0	0	0
SNORD11	0	0	0	0	0	0	0	0
SNORD11	0	0	0	0	0	0	0	0
SNORD11	0	0	0	0	0	0	0	0
SNORD11	0	0	0	0	0	0	0	0
SNORD11	0	0	0	0	0	0	0	0
SNORD11	0	0	0	0	0	0	0	0
SNORD11	0	0	0	0	0	0	0	0
SNORD11	0	0	0	0	0	0	0	0
SNORD11	0	0	0	0	0	0	0	0
SNORD11	0	0	0	0	0	0	0	0
SNORD11	0	0	0	0	0	0	0	0
SNORD11	0	0	0	0	0	0	0	0
SNORD11	0	0	0	0	0	0	0	0
SNORD11	0	0	0	0	0	0	0	0
SNORD11	0	0	0	0	0	0	0	0
SNORD11	0	0	0	0	0	0	0	0
SNORD11	0	0	0	0	0	0	0	0
SNORD11	0	0	0	0	0	0	0	0
SNORD11	0	0	0	0	0	0	0	0
SNORD11	0	0	0	0	0	0	0	0
SNORD11	0	0	0	0	0	0	0	0
SNORD11	0	0	0	0	0	0	0	0
SNORD11	0	0	0	0	0	0	0	0
SNORD11	0	0	0	0	0	0	0	0
SNORD11	0	0	0	0	0	0	0	0
SNORD11	0	0	0	0	0	0	0	0
SNORD11	0	0	0	0	0	0	0	0
SNORD11	0	0	0	0	0	0	0	0
SNORD11	0	0	0	0	0	0	0	0
SNORD11	0	0	0	0	0	0	0	0
SNORD11	1.39E-06	1.19E-07	8.92E-08	1.28E-07	0	1.16E-07	3.97E-08	0

SNORD11	0	0	0	0	0	0	0	0
SNORD11	0	0	0	0	0	0	0	0
SNORD11	0	0	0	0	0	0	0	0
SNORD11	0	0	0	0	0	0	0	0
SNORD11	0	0	0	0	0	0	0	0
SNORD11	0	0	0	0	0	0	0	0
SNORD11	8.81E-07	6.04E-08	3.10E-08	2.15E-08	0	8.89E-09	0	1.96E-08
SNORD11	0	0	0	0	0	0	0	0
SNORD11	0	0	0	0	0	0	0	0
SNORD11	6.03E-06	8.51E-08	1.32E-07	9.39E-08	0	1.43E-07	0	5.88E-08
SNORD11	0	0	0	0	0	0	0	0
SNORD11	0	0	0	0	0	0	0	0
SNORD11	0	0	0	0	0	0	0	0
SNORD11	0	0	0	0	0	0	0	0
SNORD11	0	0	0	0	0	0	0	0
SNORD12	0	0	0	0	0	0	0	0
SNORD12	0	0	0	0	0	0	0	0
SNORD12	0	0	0	0	0	0	0	0
SNORD12	0	0	0	0	0	0	0	0
SNORD12	0	0	0	0	0	0	0	0
SNORD12	0	0	0	0	0	0	0	0
SNORD12	0	0	0	0	0	0	0	0
SNORD12	0	0	0	0	0	0	0	0
SNORD12	0	0	0	0	0	0	0	0
SNORD12	0	0	0	0	0	0	0	0
SNORD15	0	0	0	0	0	0	1.98E-06	0
SNORD15	7.50E-06	0	0	0	0	1.09E-05	0	0
SNORD16	0	0	0	0	0	0	0	0
SNORD17	3.96E-06	0	9.36E-07	5.40E-07	2.21E-06	2.71E-06	1.67E-06	1.37E-06
SNORD18	0	0	0	0	0	0	0	0
SNORD18	0	0	0	0	0	0	0	0
SNORD18	0	0	0	0	0	0	0	0
SNORD19	0	0	0	0	0	0	0	0
SNORD19	0	0	0	0	0	0	0	0
SNORD1A	0	0	0	0	0	0	0	0
SNORD1B	0	0	0	0	0	0	0	0
SNORD1C	5.34E-07	1.64E-07	9.87E-08	4.00E-07	2.27E-08	3.37E-07	1.46E-07	5.03E-08
SNORD2	0	0	0	0	0	0	0	0
SNORD20	0	0	0	0	0	0	0	0
SNORD21	0	0	0	0	0	0	0	0
SNORD22	0	0	0	0	0	0	6.44E-06	0
SNORD23	0	0	0	0	0	0	0	0
SNORD24	0	0	0	0	0	0	0	0
SNORD25	0	0	0	0	0	0	0	0
SNORD26	0	0	0	0	0	0	0	0
SNORD27	0	0	0	0	0	0	0	0
SNORD28	0	0	0	0	0	0	0	0
SNORD29	0	0	0	0	0	0	0	0
SNORD30	0	0	0	0	0	0	0	0
SNORD31	0	0	0	0	0	0	0	0
SNORD32	0	0	0	0	0	0	0	0
SNORD32	0	0	0	0	0	0	0	0
SNORD33	0	0	0	0	0	0	0	0
SNORD34	0	0	0	0	0	0	0	0
SNORD35	0	0	0	0	0	0	0	0
SNORD35	0	0	0	0	0	0	0	0
SNORD36	0	0	0	0	0	0	0	0
SNORD36	0	0	0	0	0	0	0	0

SNORD36	0	0	0	0	0	0	0	0
SNORD37	0	0	0	0	0	0	0	0
SNORD38	0	0	0	0	0	0	0	0
SNORD38	0	0	0	0	0	0	0	0
SNORD41	0	0	0	0	0	0	0	0
SNORD42	0	0	0	0	0	0	0	0
SNORD42	0	0	0	0	0	0	0	0
SNORD43	0	0	0	0	0	0	0	0
SNORD44	0	0	0	0	0	0	0	0
SNORD45	0	0	0	0	0	0	0	0
SNORD45	0	0	0	0	0	0	0	0
SNORD45	0	0	0	0	0	0	0	0
SNORD46	0	0	0	0	0	0	0	0
SNORD47	0	0	0	0	0	0	0	0
SNORD48	0	0	0	0	0	0	0	0
SNORD49	0	0	0	0	0	0	0	0
SNORD49	0	0	0	0	0	0	0	0
SNORD4A	0	0	0	0	0	0	0	0
SNORD4B	0	0	0	0	0	0	0	0
SNORD5	0	0	0	0	0	0	0	0
SNORD50	0	0	0	0	0	0	0	0
SNORD50	0	0	0	0	0	0	0	0
SNORD51	0	0	0	0	0	0	0	0
SNORD52	0	0	0	0	0	0	0	0
SNORD53	0	0	0	0	0	0	0	0
SNORD54	0	0	0	0	0	0	0	0
SNORD55	0	0	0	0	0	0	0	0
SNORD56	0	0	0	0	0	0	0	0
SNORD56	0	0	0	0	0	0	0	0
SNORD57	0	0	0	0	0	0	0	0
SNORD58	0	0	0	0	0	0	0	0
SNORD58	0	0	0	0	0	0	0	0
SNORD59	0	0	0	0	0	0	0	0
SNORD59	0	0	0	0	0	0	0	0
SNORD6	0	0	0	0	0	0	0	0
SNORD60	0	0	0	0	0	0	0	0
SNORD61	0	0	0	0	0	0	0	0
SNORD62	0	0	0	0	0	0	0	0
SNORD63	0	0	0	0	0	0	0	0
SNORD65	0	0	0	0	0	0	0	0
SNORD66	0	0	0	0	0	0	0	0
SNORD67	0	0	0	0	0	0	0	0
SNORD68	0	0	0	0	0	0	0	0
SNORD69	0	0	0	0	0	0	0	0
SNORD7	0	0	0	0	0	0	0	0
SNORD70	0	0	0	0	0	0	0	0
SNORD71	0	0	0	0	0	0	0	0
SNORD72	0	0	0	0	0	0	0	0
SNORD73	0	0	0	0	0	0	0	0
SNORD74	0	0	0	0	0	0	0	0
SNORD75	0	0	0	0	0	0	0	0
SNORD76	0	0	0	0	0	0	0	0
SNORD77	0	0	0	0	0	0	0	0
SNORD78	0	0	0	0	0	0	0	0
SNORD79	0	0	0	0	0	0	0	0
SNORD8	0	0	0	0	0	0	0	0
SNORD80	0	0	0	0	0	0	0	0
SNORD81	0	0	0	0	0	0	0	0

SNORD82	0	0	0	0	0	0	0	0
SNORD84	0	0	0	0	0	0	0	0
SNORD85	0	0	0	0	0	0	0	0
SNORD86	0	0	0	0	0	0	0	0
SNORD87	0	0	0	0	0	0	0	0
SNORD88	0	0	0	0	0	0	0	0
SNORD88	0	0	0	0	0	0	0	0
SNORD88	0	0	0	0	0	0	0	0
SNORD89	0	0	0	0	0	0	0	0
SNORD9	0	0	0	0	0	0	0	0
SNORD90	0	0	0	0	0	0	0	0
SNORD91	0	0	0	0	0	0	0	0
SNORD91	0	0	0	0	0	0	0	0
SNORD92	0	0	0	0	0	0	0	0
SNORD93	0	0	0	0	0	0	0	0
SNORD94	6.12E-06	0	0	0	0	0	1.01E-05	0
SNORD95	0	0	0	0	0	0	0	0
SNORD96	0	0	0	0	0	0	0	0
SNORD96	0	0	0	0	0	0	0	0
SNORD97	0	0	0	0	0	1.53E-05	2.88E-06	0
SNORD98	0	0	0	0	0	0	0	0
SNORD99	0	0	0	0	0	0	0	0
SNPH	2.87E-06	1.11E-07	5.09E-08	2.75E-07	8.92E-08	1.09E-07	2.86E-07	9.92E-08
SNRK	1.46E-05	1.11E-05	1.11E-05	1.04E-05	3.11E-06	1.03E-05	7.98E-06	1.79E-05
SNRNP20(	9.50E-05	8.79E-05	3.40E-05	3.65E-05	2.31E-05	4.81E-05	3.12E-05	2.82E-05
SNRNP25	3.71E-05	4.90E-05	2.19E-05	1.92E-05	2.42E-05	3.17E-05	2.81E-05	3.19E-05
SNRNP27	5.25E-05	3.70E-05	1.28E-05	1.73E-05	5.05E-06	1.86E-05	1.36E-05	2.12E-05
SNRNP35	2.74E-05	2.20E-05	1.73E-05	2.88E-05	3.61E-05	1.89E-05	3.98E-05	2.49E-05
SNRNP40	2.12E-05	1.30E-05	8.86E-06	1.44E-05	5.08E-06	1.53E-05	1.27E-05	9.93E-06
SNRNP48	4.93E-06	6.93E-06	3.92E-06	2.62E-06	8.90E-07	4.12E-06	1.85E-06	6.76E-06
SNRNP70	8.93E-05	3.50E-05	2.29E-05	5.24E-05	6.16E-05	5.45E-05	5.16E-05	4.09E-05
SNRPA	5.28E-05	3.03E-05	2.36E-05	2.51E-05	4.86E-05	4.01E-05	2.99E-05	2.80E-05
SNRPA1	2.57E-05	2.34E-05	1.06E-05	1.28E-05	1.53E-05	2.51E-05	1.83E-05	1.94E-05
SNRPB	0.000284	0.000155	5.88E-05	9.50E-05	0.000119	0.000111	0.000125	0.000108
SNRPB2	8.62E-05	4.30E-05	2.13E-05	3.18E-05	2.90E-05	3.96E-05	3.57E-05	2.61E-05
SNRPC	0.000169	0.000233	6.56E-05	9.50E-05	0.000153	0.000163	0.000149	0.000126
SNRPD1	2.83E-05	1.42E-05	7.64E-06	1.49E-05	9.43E-06	1.52E-05	2.12E-05	1.54E-05
SNRPD2	0.000254	0.000145	7.19E-05	0.000125	0.000301	0.000164	0.000193	0.000118
SNRPD3	9.53E-05	5.57E-05	4.39E-05	5.49E-05	9.57E-05	7.40E-05	7.40E-05	7.59E-05
SNRPE	2.99E-05	2.11E-05	1.08E-05	1.93E-05	1.99E-05	3.54E-05	3.05E-05	2.95E-05
SNRPF	6.46E-05	4.96E-05	2.58E-05	3.94E-05	6.14E-05	4.99E-05	6.70E-05	5.09E-05
SNRPG	0.000195	0.000107	4.41E-05	7.04E-05	9.85E-05	7.97E-05	8.95E-05	5.19E-05
SNRPN	0.0003	7.73E-05	4.15E-05	7.77E-05	3.80E-06	6.34E-05	5.44E-05	2.99E-05
SNTA1	1.94E-05	2.27E-05	7.90E-06	7.78E-06	1.87E-05	1.15E-05	1.21E-05	6.71E-06
SNTB1	4.95E-05	4.63E-05	2.86E-05	1.83E-05	2.44E-05	3.10E-05	4.91E-05	2.44E-05
SNTB2	2.83E-06	8.22E-07	1.49E-06	1.41E-06	5.72E-07	1.50E-06	9.30E-07	1.63E-06
SNTG1	0	3.22E-08	4.48E-08	3.48E-08	2.47E-08	1.51E-06	0	0
SNTG2	0	0	0	0	0	0	0	0
SNTN	0	0	0	0	0	0	0	0
SNUPN	1.86E-05	1.92E-05	1.18E-05	1.04E-05	1.27E-05	1.61E-05	1.18E-05	1.22E-05
SNURF	2.33E-05	6.91E-06	3.34E-24	3.68E-06	0	5.43E-06	5.05E-06	2.59E-06
SNW1	4.77E-05	1.97E-05	1.84E-05	2.80E-05	1.20E-05	3.18E-05	2.86E-05	2.92E-05
SNX1	3.30E-05	2.37E-05	2.05E-05	1.68E-05	5.07E-06	3.46E-05	1.99E-05	1.44E-05
SNX10	3.79E-05	7.96E-06	2.23E-05	1.62E-05	1.88E-06	1.72E-05	1.11E-05	2.98E-05
SNX11	1.53E-05	9.24E-06	5.40E-06	8.08E-06	9.38E-06	1.42E-05	1.28E-05	8.86E-06
SNX12	1.96E-05	1.11E-05	1.38E-05	1.23E-05	2.03E-06	1.57E-05	6.08E-06	2.30E-05
SNX13	9.04E-06	9.73E-06	3.64E-06	3.55E-06	1.51E-06	4.27E-06	2.91E-06	5.32E-06
SNX14	3.62E-05	3.21E-05	2.12E-05	1.86E-05	5.78E-06	1.82E-05	1.19E-05	1.28E-05

SNX15	1.48E-05	1.72E-05	6.05E-06	8.25E-06	4.82E-06	1.26E-05	9.25E-06	9.40E-06
SNX16	5.38E-06	2.87E-06	1.36E-06	1.47E-06	6.70E-07	1.16E-06	2.02E-06	1.54E-06
SNX17	0.000111	0.000122	4.89E-05	5.67E-05	4.50E-05	8.72E-05	6.79E-05	5.69E-05
SNX18	8.34E-06	7.63E-06	7.04E-06	5.94E-06	1.37E-06	4.85E-06	5.79E-06	1.08E-05
SNX19	1.54E-05	9.99E-06	1.26E-05	1.44E-05	6.53E-06	1.72E-05	8.99E-06	3.07E-05
SNX2	3.84E-05	3.40E-05	2.73E-05	3.12E-05	6.90E-06	4.21E-05	2.48E-05	2.65E-05
SNX20	2.75E-06	1.18E-07	6.57E-07	1.28E-06	1.86E-08	6.14E-07	3.58E-07	3.85E-07
SNX21	6.37E-06	3.62E-06	2.73E-06	4.07E-06	7.04E-06	3.79E-06	4.25E-06	5.88E-06
SNX22	7.08E-06	5.56E-06	1.47E-06	1.88E-06	4.62E-06	2.95E-06	3.84E-06	2.11E-06
SNX24	7.93E-06	3.59E-06	4.25E-06	5.46E-06	1.38E-06	5.79E-06	4.83E-06	4.57E-06
SNX25	9.25E-06	2.66E-06	3.84E-06	3.89E-06	9.45E-07	1.09E-05	2.71E-06	5.01E-06
SNX27	7.04E-06	6.08E-06	3.38E-06	3.96E-06	1.17E-06	1.06E-05	3.96E-06	7.11E-06
SNX29	2.21E-06	1.85E-06	1.15E-06	1.35E-06	3.61E-07	1.83E-06	1.19E-06	1.18E-06
SNX3	0.000171	0.000135	9.45E-05	8.79E-05	3.53E-05	0.000133	0.000107	7.55E-05
SNX30	3.17E-06	1.96E-06	1.80E-06	2.17E-06	7.96E-07	2.72E-06	2.35E-06	1.28E-06
SNX31	0	1.90E-07	4.29E-08	1.67E-08	1.17E-08	5.42E-08	1.18E-07	7.49E-08
SNX32	0	0	0	8.79E-08	1.23E-08	1.33E-08	2.76E-08	0
SNX33	1.93E-05	1.15E-05	1.25E-05	1.40E-05	1.32E-05	1.85E-05	2.05E-05	1.21E-05
SNX4	2.66E-05	4.24E-05	2.75E-05	2.13E-05	9.06E-06	2.88E-05	1.91E-05	1.88E-05
SNX5	6.98E-05	4.54E-05	3.20E-05	3.51E-05	2.14E-05	4.50E-05	3.23E-05	1.61E-05
SNX6	2.38E-05	1.83E-05	1.16E-05	1.35E-05	4.50E-06	1.74E-05	9.33E-06	1.04E-05
SNX7	2.08E-05	1.25E-05	6.99E-06	1.06E-05	3.44E-06	1.69E-05	7.18E-06	7.36E-06
SNX8	2.88E-05	1.83E-05	8.16E-06	8.29E-06	6.73E-06	6.13E-06	1.23E-05	1.80E-05
SNX9	2.54E-05	3.63E-05	2.36E-05	2.38E-05	4.35E-06	2.39E-05	2.23E-05	2.01E-05
SOAT1	2.20E-05	1.27E-05	5.96E-06	8.90E-06	8.55E-07	8.17E-06	4.51E-06	1.09E-05
SOAT2	0	8.67E-06	9.03E-07	5.07E-07	4.06E-07	2.19E-08	9.37E-06	1.74E-08
SOBP	1.59E-06	1.97E-07	1.64E-06	9.23E-07	2.12E-06	3.32E-07	1.45E-06	1.46E-06
SOCS1	6.33E-06	1.19E-06	8.86E-07	9.57E-06	6.20E-07	2.61E-06	5.76E-06	1.07E-05
SOCS2	8.71E-06	2.07E-05	4.03E-05	3.63E-05	3.54E-06	2.72E-05	3.30E-06	1.75E-05
SOCS3	1.65E-05	9.58E-06	9.84E-06	0.000257	1.79E-06	1.05E-05	2.29E-05	5.13E-05
SOCS4	4.09E-06	2.06E-06	1.43E-06	2.00E-06	3.98E-07	1.95E-06	1.22E-06	2.17E-06
SOCS5	6.59E-06	4.93E-06	2.68E-06	3.90E-06	1.10E-06	2.61E-06	2.73E-06	3.77E-06
SOCS6	7.50E-06	4.96E-06	9.68E-06	1.05E-05	1.50E-06	5.97E-06	6.11E-06	8.37E-06
SOCS7	1.14E-06	3.12E-07	1.95E-07	2.67E-07	6.02E-08	1.30E-07	2.03E-07	3.78E-07
SOD1	0.000499	0.000795	0.000833	0.000897	0.000994	0.001889	0.001202	0.00069
SOD2	0.001171	0.000674	0.000668	0.001002	9.67E-05	0.000924	0.001752	0.001853
SOD3	1.37E-05	2.68E-06	2.01E-06	2.03E-05	1.03E-05	1.13E-05	7.33E-06	4.80E-06
SOHLH1	0	3.52E-07	0	2.18E-08	1.53E-08	0	0	0
SOHLH2	8.67E-08	0	1.13E-08	0	0	0	0	4.78E-08
SOLH	2.36E-05	1.83E-05	8.62E-06	8.89E-06	5.75E-06	9.10E-06	1.11E-05	1.08E-05
SON	6.76E-05	3.72E-05	3.35E-05	3.76E-05	1.78E-05	4.56E-05	3.25E-05	4.34E-05
SORBS1	4.95E-05	2.53E-05	1.20E-05	1.56E-05	6.37E-06	1.47E-05	1.23E-05	9.16E-06
SORBS2	3.51E-05	3.87E-05	4.43E-05	4.16E-05	1.63E-05	3.84E-05	3.05E-05	4.13E-05
SORBS3	4.03E-05	7.67E-05	5.01E-05	7.26E-05	4.30E-05	7.21E-05	5.41E-05	1.89E-05
SORCS1	0	0	1.30E-08	4.00E-08	0	0	3.11E-09	0
SORCS2	4.21E-06	4.61E-07	2.59E-06	7.53E-07	4.04E-08	7.63E-08	6.31E-07	3.91E-07
SORCS3	8.19E-08	0	0	0	0	0	0	0
SORD	3.45E-05	0.000253	0.00024	6.62E-05	5.23E-05	0.000337	0.00017	7.84E-05
SORL1	6.62E-06	1.31E-05	3.63E-05	1.90E-05	5.57E-06	3.08E-05	1.04E-05	2.06E-05
SORT1	1.88E-05	1.36E-06	6.50E-06	2.95E-06	1.50E-06	3.71E-05	7.82E-06	2.80E-06
SOS1	1.14E-05	9.76E-06	5.04E-06	4.48E-06	1.57E-06	6.89E-06	3.14E-06	4.30E-06
SOS2	1.08E-05	7.24E-06	8.53E-06	8.66E-06	3.71E-06	8.49E-06	8.42E-06	1.13E-05
SOST	0	0	0	2.52E-08	0	0	0	0
SOSTDC1	3.49E-07	1.57E-06	4.07E-08	3.17E-08	2.89E-07	1.20E-08	8.74E-08	3.24E-07
SOX1	0	0	0	0	0	0	0	8.11E-09
SOX10	0	9.17E-09	4.22E-08	1.30E-07	2.10E-08	0	3.16E-08	0
SOX11	5.09E-09	2.84E-09	0	3.10E-09	0	4.71E-09	1.17E-07	3.72E-09
SOX12	1.82E-05	1.08E-05	1.74E-06	1.56E-06	2.64E-06	3.47E-06	3.04E-06	2.35E-06

SOX13	1.61E-05	8.10E-06	8.74E-06	8.47E-06	6.11E-06	8.45E-06	1.20E-05	1.44E-05
SOX14	6.19E-07	0	0	0	0	0	0	0
SOX15	1.44E-07	3.50E-07	2.75E-07	8.85E-08	4.37E-08	1.67E-07	3.47E-07	3.19E-07
SOX17	1.48E-06	1.70E-06	3.35E-07	2.66E-06	1.11E-06	5.49E-07	3.64E-06	6.94E-07
SOX18	3.29E-06	3.73E-06	1.71E-06	1.78E-06	1.24E-05	2.33E-06	1.83E-05	4.48E-06
SOX2	0	0	9.76E-09	1.15E-08	0	8.70E-09	4.55E-08	1.54E-06
SOX21	3.74E-08	0	0	0	0	0	5.44E-08	0
SOX2OT	1.02E-08	1.15E-08	6.37E-08	2.50E-08	0	2.27E-07	9.86E-09	2.10E-07
SOX3	0	0	0	0	0	0	0	0
SOX30	3.30E-08	0	3.45E-08	1.35E-08	0	4.08E-08	1.06E-08	0
SOX4	1.07E-05	3.78E-06	1.37E-06	1.09E-05	1.20E-06	4.16E-07	6.82E-06	3.10E-06
SOX5	3.12E-07	1.65E-06	2.74E-06	7.41E-07	1.48E-07	2.84E-06	2.90E-07	1.43E-06
SOX6	2.52E-06	4.74E-08	3.90E-06	2.83E-06	6.28E-07	5.59E-08	1.73E-06	1.02E-07
SOX7	1.32E-06	2.41E-06	9.88E-07	3.32E-06	5.11E-07	1.55E-05	1.98E-06	2.19E-06
SOX8	1.44E-07	4.26E-08	2.35E-08	4.27E-07	1.96E-08	1.41E-08	2.21E-08	8.90E-08
SOX9	2.79E-05	4.25E-06	1.43E-06	1.33E-05	2.39E-06	1.29E-05	5.83E-06	5.20E-06
SP1	1.69E-05	1.00E-05	6.92E-06	8.46E-06	2.45E-06	1.02E-05	6.99E-06	6.84E-06
SP100	9.59E-05	3.25E-05	2.83E-05	3.00E-05	1.62E-05	5.25E-05	3.32E-05	3.29E-05
SP110	2.97E-05	8.22E-06	7.11E-06	9.49E-06	5.42E-06	1.12E-05	9.52E-06	1.46E-05
SP140	1.34E-05	3.54E-07	4.94E-07	1.88E-06	1.49E-07	4.78E-06	1.29E-06	6.40E-07
SP140L	1.58E-05	8.69E-06	4.64E-06	5.71E-06	2.47E-06	8.42E-06	4.35E-06	7.64E-06
SP2	1.02E-05	9.38E-06	4.66E-06	7.64E-06	2.27E-06	8.34E-06	4.57E-06	4.68E-06
SP3	2.92E-05	1.82E-05	1.37E-05	1.37E-05	2.29E-06	1.37E-05	1.00E-05	1.31E-05
SP4	2.38E-06	1.59E-06	7.69E-07	7.22E-07	9.68E-08	6.94E-07	4.06E-07	7.68E-07
SP5	1.36E-06	1.37E-06	1.24E-06	1.12E-06	8.92E-07	1.54E-05	4.21E-06	1.15E-06
SP6	3.01E-07	8.77E-08	4.34E-08	1.25E-07	1.50E-07	1.25E-06	1.17E-07	1.06E-07
SP7	0	0	0	0	0	0	7.57E-09	0
SP8	0	7.06E-09	0	0	0	0	0	3.48E-07
SP9	0	0	0	0	0	0	0	0
SPA17	4.01E-06	1.83E-06	4.49E-07	3.42E-06	7.30E-07	6.32E-06	1.93E-06	6.68E-06
SPACA1	0	0	0	2.04E-08	0	7.70E-08	0	0
SPACA3	0	0	0	0	0	0	0	0
SPACA4	0	0	0	0	0	0	0	0
SPACA5	0	0	4.55E-08	4.97E-08	3.47E-08	3.74E-08	0	0
SPAG1	1.07E-05	4.34E-06	1.58E-06	3.54E-06	9.98E-07	8.85E-07	3.62E-06	1.73E-06
SPAG11A	0	0	0	0	0	9.79E-08	0	0
SPAG11B	0	0	0	0	0	2.07E-07	0	0
SPAG16	2.59E-05	9.36E-06	9.96E-06	1.10E-05	5.21E-06	8.12E-06	1.14E-05	3.29E-06
SPAG17	1.25E-08	0	0	0	0	2.89E-09	6.10E-09	0
SPAG4	4.66E-06	6.48E-06	7.74E-07	1.50E-06	1.91E-06	5.30E-06	5.15E-06	2.75E-06
SPAG5	1.26E-05	1.68E-05	3.23E-06	2.10E-06	2.94E-06	3.58E-06	2.01E-06	7.83E-06
SPAG6	9.03E-09	1.08E-08	2.00E-08	0	0	8.91E-09	0	1.41E-08
SPAG7	3.82E-05	2.61E-05	4.62E-05	6.68E-05	9.21E-05	5.44E-05	7.72E-05	6.52E-05
SPAG8	1.87E-07	3.35E-07	9.92E-08	1.01E-07	1.01E-07	1.38E-07	1.38E-08	1.15E-07
SPAG9	2.38E-05	1.76E-05	1.09E-05	7.94E-06	3.25E-06	8.37E-06	5.27E-06	5.77E-06
SPAM1	0	1.35E-08	0	0	0	0	0	0
SPANXA2	0	0	0	0	0	0	0	0
SPANXB2	0	0	0	0	0	0	0	0
SPANXC	0	0	0	0	0	0	0	0
SPANXE	0	0	0	0	0	8.20E-08	0	0
SPANXN1	0	0	0	0	0	0	0	0
SPANXN2	0	0	0	0	0	0	0	0
SPANXN3	0	0	0	0	0	0	0	0
SPANXN4	0	0	0	0	0	0	0	0
SPANXN5	0	0	0	0	0	0	0	0
SPARC	0.000358	0.000127	6.22E-05	0.000142	9.09E-05	0.000117	0.00027	0.000169
SPARCL1	2.14E-05	6.47E-05	7.13E-06	1.09E-05	1.26E-05	0.000842	4.60E-05	3.56E-05
SPAST	6.60E-06	6.63E-06	3.35E-06	2.86E-06	1.49E-06	4.12E-06	2.68E-06	2.19E-06

SPATA1	3.13E-08	3.53E-08	9.93E-08	2.10E-07	2.70E-08	4.06E-07	1.21E-07	2.42E-07
SPATA12	1.46E-07	0	0	7.16E-08	8.39E-09	0	0	1.43E-08
SPATA13	2.08E-05	6.11E-06	5.49E-06	4.03E-06	6.31E-08	7.65E-06	9.42E-07	3.90E-06
SPATA16	0	0	0	0	0	0	0	0
SPATA17	1.08E-07	0	0	6.62E-08	0	3.32E-06	1.99E-08	0
SPATA18	2.97E-07	1.09E-07	1.95E-06	2.38E-06	3.43E-07	8.51E-08	1.89E-06	1.23E-06
SPATA19	0	0	0	0	0	0	0	0
SPATA2	6.79E-06	4.64E-06	2.73E-06	4.41E-06	2.42E-06	4.78E-06	3.74E-06	4.21E-06
SPATA20	3.71E-05	3.27E-05	8.66E-06	2.50E-05	2.69E-05	4.11E-05	2.77E-05	1.58E-05
SPATA21	1.02E-07	3.83E-08	5.29E-08	7.12E-07	3.04E-08	2.77E-06	1.72E-07	3.67E-07
SPATA22	0	2.44E-08	0	3.56E-08	3.66E-08	1.98E-08	4.61E-06	0
SPATA24	5.93E-06	4.62E-06	2.84E-06	3.74E-06	3.54E-06	7.20E-06	3.46E-06	2.12E-06
SPATA2L	1.59E-05	7.97E-06	6.42E-06	1.22E-05	8.80E-06	3.92E-06	9.48E-06	1.45E-05
SPATA3	0	0	0	0	0	0	0	0
SPATA4	0	0	0	2.56E-08	0	0	0	0
SPATA5	8.03E-07	3.71E-07	4.71E-07	5.96E-07	1.73E-07	1.50E-06	4.42E-07	5.50E-07
SPATA5L1	5.14E-06	3.55E-06	2.45E-06	3.65E-06	1.89E-06	4.49E-06	4.90E-06	3.95E-06
SPATA6	2.38E-06	9.68E-07	3.31E-07	6.36E-07	0	2.68E-08	1.11E-07	8.52E-08
SPATA7	5.49E-06	3.12E-06	1.74E-06	3.73E-06	2.10E-06	7.58E-06	3.42E-06	1.61E-06
SPATA8	0	0	2.84E-08	0	0	2.43E-08	0	0
SPATA9	2.45E-07	4.95E-08	6.94E-08	3.60E-07	5.41E-08	6.44E-08	8.97E-08	2.47E-07
SPATC1	4.70E-07	1.46E-07	2.67E-08	3.12E-08	4.04E-08	8.26E-08	1.62E-07	0
SPATS1	0	9.96E-08	1.22E-08	0	0	0	0	0
SPATS2	1.11E-05	5.68E-06	1.54E-06	2.87E-06	1.64E-06	5.60E-06	1.32E-06	5.92E-06
SPATS2L	7.58E-05	3.65E-05	2.06E-05	1.97E-05	1.14E-05	1.22E-05	1.57E-05	1.67E-05
SPC24	3.35E-06	1.01E-06	0	3.20E-07	3.25E-07	7.00E-07	1.24E-07	7.05E-07
SPC25	7.97E-06	3.69E-06	2.01E-08	1.62E-07	6.96E-07	1.43E-06	3.24E-07	6.38E-07
SPCS1	0.000112	0.00016	8.73E-05	9.41E-05	9.45E-05	0.0002	0.000146	9.92E-05
SPCS2	2.00E-05	1.47E-05	1.28E-05	1.30E-05	1.57E-05	1.81E-05	1.31E-05	2.45E-05
SPCS3	2.81E-05	1.68E-05	2.30E-05	2.13E-05	9.16E-06	3.20E-05	2.03E-05	2.66E-05
SPDEF	2.49E-06	2.97E-08	0	6.42E-08	0	1.21E-08	7.86E-07	0
SPDYA	7.06E-07	4.04E-07	4.94E-08	1.97E-07	8.96E-08	1.89E-07	7.12E-08	2.57E-08
SPDYC	9.39E-08	2.71E-06	2.78E-06	1.90E-06	1.17E-06	1.52E-06	1.16E-06	1.40E-06
SPDYE1	1.68E-07	9.65E-08	3.66E-08	4.32E-08	1.52E-08	4.90E-08	1.71E-08	3.88E-08
SPDYE2	0	7.25E-08	0	2.59E-08	0	1.12E-08	0	0
SPDYE3	5.38E-06	4.18E-06	1.70E-06	1.14E-06	4.01E-07	2.19E-06	1.54E-06	2.26E-06
SPDYE4	2.95E-08	0	0	0	0	0	2.80E-08	0
SPDYE5	1.40E-07	1.97E-08	3.64E-08	3.21E-08	7.53E-09	4.06E-08	1.70E-08	2.57E-08
SPDYE6	5.16E-07	1.66E-07	1.35E-07	1.26E-07	7.64E-08	3.54E-07	1.41E-07	2.46E-07
SPDYE7P	3.88E-08	5.51E-08	7.21E-09	4.31E-09	1.21E-08	1.63E-08	1.38E-08	3.61E-08
SPDYE8P	4.36E-07	4.54E-07	8.85E-08	2.60E-07	1.91E-07	2.56E-07	5.70E-07	1.89E-07
SPEF1	3.10E-08	5.30E-08	1.64E-08	7.63E-08	0	1.01E-07	7.47E-08	9.17E-08
SPEF2	1.98E-06	4.82E-07	2.72E-07	3.20E-07	1.64E-07	3.86E-07	4.05E-07	1.14E-06
SPEG	4.83E-06	2.31E-09	7.42E-09	5.24E-07	1.44E-07	5.12E-08	7.74E-08	1.25E-08
SPEM1	0	0	0	0	0	0	0	0
SPEN	1.50E-05	5.01E-06	3.72E-06	3.89E-06	8.23E-07	4.05E-06	3.00E-06	4.86E-06
SPERT	3.05E-08	3.29E-07	0	0	0	0	0	0
SPESP1	1.25E-07	5.51E-07	3.81E-08	1.08E-06	4.62E-08	1.49E-07	3.95E-07	1.06E-07
SPG11	2.46E-05	1.76E-05	1.36E-05	1.36E-05	4.74E-06	2.19E-05	1.23E-05	1.40E-05
SPG20	9.41E-06	8.68E-07	2.74E-06	4.10E-06	3.40E-07	6.22E-07	1.55E-06	7.29E-07
SPG21	6.85E-05	7.81E-05	6.87E-05	6.30E-05	2.62E-05	8.11E-05	5.92E-05	8.33E-05
SPG7	3.14E-05	1.67E-05	2.79E-05	2.30E-05	1.69E-05	3.01E-05	2.16E-05	2.50E-05
SPHAR	9.67E-06	9.43E-06	7.58E-06	7.37E-06	7.78E-06	1.78E-05	1.18E-05	7.27E-06
SPHK1	1.86E-05	8.28E-06	5.58E-07	1.23E-05	1.06E-06	4.04E-07	3.59E-06	7.78E-07
SPHK2	7.36E-06	2.31E-05	1.39E-05	1.06E-05	8.24E-06	1.70E-05	1.81E-05	4.53E-06
SPHKAP	0	0	0	4.26E-09	0	0	0	0
SPI1	2.66E-05	3.97E-06	1.12E-05	2.54E-05	2.64E-06	8.95E-06	1.78E-05	3.93E-06
SPIB	5.38E-07	3.88E-08	1.87E-07	1.46E-06	0	2.65E-07	2.73E-07	3.01E-08

SPIC	1.39E-07	0	5.80E-07	2.38E-07	0	2.07E-07	2.91E-07	8.28E-08
SPIN1	2.27E-05	1.30E-05	1.03E-05	9.03E-06	1.16E-06	7.80E-06	4.72E-06	1.08E-05
SPIN2A	7.68E-08	0	0	9.46E-08	0	0	1.84E-08	0
SPIN2B	3.17E-06	1.60E-06	2.29E-06	2.34E-06	1.13E-06	2.65E-06	2.10E-06	2.36E-06
SPIN3	3.71E-06	1.58E-06	1.40E-06	9.90E-07	6.52E-07	1.30E-06	5.87E-07	2.16E-06
SPIN4	1.48E-06	5.82E-07	3.58E-07	7.16E-07	2.37E-07	6.45E-07	4.73E-07	5.27E-07
SPINK1	0.000211	4.44E-05	3.02E-05	2.19E-07	5.31E-07	8.97E-05	0.002409	0.00855
SPINK13	0	0	0	1.43E-07	0	0	0	0
SPINK14	0	0	0	0	0	0	0	0
SPINK2	0	0	0	1.35E-07	0	5.07E-08	5.00E-08	0
SPINK4	4.79E-07	0	0	0	0	0	0	0
SPINK5	2.52E-08	1.57E-06	1.09E-07	7.68E-09	9.18E-08	3.60E-07	4.49E-07	8.31E-07
SPINK6	0	0	5.04E-08	1.09E-07	0	4.10E-08	0	0
SPINK7	0	0	2.21E-07	0	0	0	0	0
SPINK8	0	0	0	0	0	0	0	0
SPINK9	0	0	0	0	0	0	0	0
SPINLW1	0	0	0	0	0	0	0	0
SPINT1	0.000102	2.66E-07	2.48E-06	2.89E-05	1.23E-07	1.61E-07	5.43E-06	1.35E-07
SPINT2	9.39E-06	2.25E-06	7.77E-06	3.49E-05	1.59E-06	1.27E-06	1.08E-05	1.29E-06
SPINT3	0	0	0	0	0	0	0	0
SPINT4	0	0	0	0	0	0	0	0
SPIRE1	1.05E-05	1.13E-06	1.59E-06	1.40E-06	1.55E-07	3.08E-07	8.04E-07	1.14E-06
SPIRE2	1.98E-05	6.12E-06	2.01E-06	3.54E-06	1.23E-06	2.69E-06	5.30E-06	7.18E-06
SPN	4.26E-06	2.94E-07	1.13E-06	2.46E-06	1.41E-07	1.03E-06	1.00E-06	4.07E-07
SPNS1	2.85E-05	6.08E-05	1.53E-05	2.16E-05	2.11E-05	2.63E-05	3.31E-05	2.76E-05
SPNS2	9.20E-06	6.84E-07	2.43E-06	7.76E-06	1.65E-05	1.53E-06	1.43E-05	2.55E-06
SPNS3	2.62E-07	1.60E-07	1.47E-07	8.47E-07	1.77E-07	1.00E-07	8.44E-07	6.62E-07
SPO11	0	0	0	0	0	0	0	0
SPOCD1	3.71E-07	3.74E-07	1.27E-08	1.57E-07	7.59E-09	7.58E-08	6.65E-08	5.36E-08
SPOCK1	3.28E-07	9.29E-06	4.80E-09	1.77E-07	3.61E-08	2.33E-06	4.24E-07	3.42E-08
SPOCK2	2.86E-05	4.88E-06	1.85E-06	6.82E-06	7.31E-07	3.77E-06	4.06E-06	3.85E-06
SPOCK3	0	2.55E-06	1.53E-07	8.48E-08	0	0	0	1.72E-06
SPON1	5.31E-06	1.31E-07	3.69E-07	3.13E-06	4.06E-07	8.91E-08	7.34E-07	9.80E-08
SPON2	9.13E-05	6.56E-05	5.27E-05	0.00018	8.16E-05	9.60E-05	0.000174	0.000204
SPOP	3.69E-05	2.58E-05	1.41E-05	1.49E-05	8.54E-06	2.58E-05	1.72E-05	1.33E-05
SPOPL	5.98E-06	5.84E-06	4.25E-06	3.95E-06	9.03E-07	4.66E-06	2.59E-06	4.10E-06
SPP1	0.001851	0.000104	6.55E-06	7.34E-05	2.10E-05	0.003249	1.37E-05	6.39E-05
SPP2	0.000174	2.35E-05	0.000274	0.000377	0.00079	1.80E-05	0.000135	3.42E-06
SPPL2A	4.05E-05	4.59E-05	3.61E-05	2.86E-05	2.23E-05	6.21E-05	2.09E-05	3.38E-05
SPPL2B	2.18E-05	1.01E-05	6.48E-06	8.47E-06	1.16E-05	7.29E-06	6.78E-06	6.98E-06
SPPL3	1.40E-05	1.03E-05	8.11E-06	1.05E-05	6.03E-06	1.14E-05	1.21E-05	8.94E-06
SPR	0.000143	0.000136	4.44E-05	4.70E-05	7.16E-05	5.24E-05	8.51E-05	7.54E-05
SPRED1	4.32E-06	1.24E-06	1.69E-06	3.63E-06	8.35E-07	1.06E-06	1.94E-06	1.04E-06
SPRED2	1.41E-05	1.63E-05	8.47E-06	1.04E-05	3.10E-06	5.02E-06	8.53E-06	9.06E-06
SPRED3	6.06E-08	7.52E-08	0	0	0	0	0	9.36E-08
SPRN	3.08E-06	1.39E-06	6.76E-07	1.00E-06	6.57E-06	1.63E-06	2.13E-06	1.62E-06
SPRR1A	0	0	8.45E-06	0	0	0	1.77E-07	0
SPRR1B	0	0	1.66E-05	0	0	0	0	0
SPRR2A	0	0	6.88E-06	5.58E-08	0	0	8.36E-08	6.75E-08
SPRR2B	0	0	1.24E-06	0	0	0	0	0
SPRR2C	0	0	5.14E-08	0	0	0	0	0
SPRR2D	0	0	3.71E-06	0	0	0	0	0
SPRR2E	0	0	6.48E-06	0	0	0	8.31E-08	6.70E-08
SPRR2F	0	0	0	0	0	0	0	0
SPRR2G	0	0	2.69E-06	0	0	0	0	0
SPRR3	9.43E-08	0	3.23E-06	0	0	0	1.44E-07	4.70E-08
SPRR4	0	0	0	0	0	0	0	0
SPRY1	1.06E-05	9.30E-06	6.87E-06	1.22E-05	3.26E-06	8.02E-06	1.22E-05	1.46E-05

SPRY2	8.76E-06	6.20E-06	1.27E-05	2.15E-05	1.91E-06	5.15E-06	1.11E-05	9.35E-06
SPRY3	2.72E-07	2.54E-07	1.05E-07	1.25E-07	7.55E-08	2.37E-07	9.09E-08	1.81E-07
SPRY4	4.82E-06	3.93E-06	4.52E-06	7.36E-06	1.59E-06	1.05E-06	6.68E-06	1.21E-05
SPRYD3	1.79E-05	1.52E-05	1.02E-05	1.48E-05	8.71E-06	2.82E-05	1.63E-05	1.70E-05
SPRYD4	5.21E-05	7.48E-05	0.000105	6.28E-05	6.43E-05	4.85E-05	3.21E-05	5.51E-05
SPRYD5	0	0	0	0	0	0	0	0
SPSB1	5.76E-05	3.11E-05	8.94E-06	7.21E-05	4.76E-06	2.60E-05	2.29E-05	1.95E-05
SPSB2	1.40E-05	6.74E-06	3.01E-06	2.85E-06	2.77E-06	1.21E-05	4.63E-06	4.82E-06
SPSB3	3.47E-05	4.07E-05	4.32E-05	4.19E-05	3.25E-05	2.74E-05	3.35E-05	2.64E-05
SPSB4	6.55E-07	0	2.95E-07	2.39E-07	8.41E-09	0	1.33E-07	2.87E-08
SPTA1	5.55E-09	6.18E-09	2.83E-09	0	0	2.56E-09	2.70E-09	1.62E-08
SPTAN1	0.000272	0.000155	5.81E-05	0.000128	0.000135	0.000101	0.000114	8.35E-05
SPTB	1.30E-06	1.40E-07	1.04E-07	6.22E-07	9.65E-08	1.29E-07	1.82E-07	2.75E-07
SPTBN1	9.17E-05	0.000151	7.13E-05	7.41E-05	4.14E-05	8.12E-05	5.94E-05	4.13E-05
SPTBN2	5.78E-06	1.45E-05	1.69E-05	1.36E-05	1.73E-05	4.77E-06	1.77E-05	5.66E-06
SPTBN4	2.78E-07	2.66E-07	7.29E-08	1.06E-07	1.28E-07	1.58E-07	7.97E-08	1.05E-07
SPTBN5	5.14E-07	5.03E-08	5.17E-08	1.33E-07	1.15E-07	3.30E-08	3.67E-08	1.40E-07
SPTLC1	3.03E-05	1.34E-05	1.10E-05	1.28E-05	6.83E-06	1.28E-05	8.45E-06	1.06E-05
SPTLC2	5.53E-06	3.41E-06	2.22E-06	3.48E-06	4.62E-07	3.17E-06	2.22E-06	3.36E-06
SPTLC3	4.15E-05	4.63E-06	1.38E-05	6.22E-06	3.66E-06	1.32E-05	5.48E-06	1.69E-06
SPTY2D1	9.53E-06	5.96E-06	4.75E-06	4.77E-06	7.66E-07	4.70E-06	2.77E-06	7.11E-06
SPZ1	0	0	0	0	0	0	0	0
SQLE	6.25E-05	0.000193	5.55E-06	4.77E-06	2.70E-06	6.26E-06	1.58E-05	1.12E-05
SQRDL	6.49E-05	2.49E-05	4.80E-05	5.31E-05	2.54E-05	4.90E-05	5.88E-05	6.07E-05
SQSTM1	0.000456	0.000479	0.000227	0.000339	7.20E-05	0.000324	0.000554	0.000762
SR140	1.81E-05	2.64E-05	5.73E-06	6.06E-06	2.29E-06	8.28E-06	3.92E-06	5.19E-06
SRA1	4.02E-05	2.56E-05	1.64E-05	2.42E-05	1.99E-05	1.91E-05	3.22E-05	4.69E-05
SRBD1	8.88E-06	5.62E-06	4.23E-06	5.14E-06	2.21E-06	7.11E-06	3.82E-06	3.92E-06
SRC	2.32E-05	4.50E-06	2.26E-06	7.14E-06	5.72E-07	4.41E-06	1.82E-06	1.29E-06
SRCAP	1.47E-05	1.10E-05	4.06E-06	6.18E-06	2.21E-06	7.12E-06	5.71E-06	6.99E-06
SRCIN1	6.16E-06	2.61E-06	1.30E-06	1.76E-06	9.14E-07	2.15E-06	1.28E-06	2.45E-06
SRCRB4D	8.34E-07	4.46E-06	1.82E-06	1.18E-06	2.47E-06	2.26E-06	2.85E-06	2.47E-06
SRD5A1	4.92E-06	5.56E-05	4.33E-05	2.29E-05	5.77E-06	2.86E-05	1.33E-05	4.09E-05
SRD5A2	2.21E-07	3.11E-05	5.11E-05	7.62E-06	6.12E-06	1.46E-05	3.08E-06	2.02E-06
SRD5A3	1.22E-05	4.96E-06	4.60E-06	6.06E-06	8.84E-06	6.04E-06	7.89E-06	1.08E-05
SREBF1	6.73E-05	7.73E-05	0.000154	6.00E-05	0.000129	1.85E-05	4.54E-05	0.000345
SREBF2	0.000152	0.000122	6.81E-05	5.12E-05	6.95E-05	6.10E-05	8.80E-05	9.10E-05
SRF	1.35E-05	1.89E-05	1.15E-05	1.74E-05	5.17E-06	9.57E-06	3.71E-05	1.62E-05
SRFBP1	2.57E-06	3.30E-06	2.17E-06	2.74E-06	2.11E-07	3.55E-06	1.63E-06	3.03E-06
SRGAP1	4.27E-06	7.03E-07	2.14E-07	7.20E-07	7.00E-08	3.76E-07	6.43E-07	4.47E-07
SRGAP2	6.78E-06	5.10E-06	2.32E-06	4.64E-06	1.08E-06	6.38E-06	2.53E-06	2.69E-06
SRGAP3	1.45E-06	9.39E-07	1.60E-07	1.46E-07	7.54E-08	1.11E-06	2.82E-07	1.37E-07
SRGN	0.000153	2.00E-05	3.98E-05	0.000162	6.04E-06	6.35E-05	4.27E-05	2.28E-05
SRI	2.42E-05	3.31E-05	9.35E-06	1.84E-05	1.79E-06	2.59E-06	2.88E-05	3.07E-05
SRL	1.03E-07	1.09E-07	8.31E-08	1.05E-07	1.16E-07	7.99E-08	2.10E-07	1.11E-07
SRM	7.49E-05	6.39E-05	2.68E-05	3.99E-05	4.91E-05	6.46E-05	4.93E-05	5.81E-05
SRMS	1.63E-08	1.30E-07	1.73E-08	2.01E-08	0	0	0	0
SRP14	0.00031	0.000193	0.000122	0.000194	0.000167	0.000198	0.000197	0.000179
SRP19	5.62E-05	3.95E-05	2.79E-05	4.00E-05	1.83E-05	5.99E-05	3.92E-05	5.96E-05
SRP54	3.35E-05	3.12E-05	2.76E-05	2.02E-05	8.17E-06	4.46E-05	2.01E-05	3.68E-05
SRP68	5.95E-05	5.55E-05	2.72E-05	3.15E-05	2.98E-05	6.37E-05	3.98E-05	3.59E-05
SRP72	2.61E-05	2.52E-05	1.71E-05	1.77E-05	7.24E-06	3.14E-05	1.96E-05	2.99E-05
SRP9	0.000156	0.000221	0.000106	9.65E-05	9.60E-05	0.000184	9.91E-05	0.000166
SRPK1	1.36E-05	1.59E-05	5.86E-06	6.85E-06	2.55E-06	1.21E-05	6.76E-06	1.21E-05
SRPK2	1.13E-05	1.39E-05	5.08E-06	4.53E-06	1.15E-06	9.20E-06	3.73E-06	9.96E-06
SRPK3	1.36E-08	7.20E-07	0	0	1.20E-07	1.85E-08	2.94E-08	0
SRPR	8.03E-05	5.58E-05	9.83E-05	7.10E-05	6.70E-05	0.00015	5.61E-05	0.000113
SRPRB	5.38E-05	4.50E-05	3.43E-05	4.19E-05	4.65E-05	6.81E-05	6.40E-05	7.06E-05

SRPX	4.30E-06	4.79E-08	7.49E-06	2.49E-05	1.46E-06	2.51E-07	1.93E-06	7.19E-07
SRPX2	8.94E-06	4.78E-07	7.82E-07	4.88E-06	4.67E-07	2.21E-07	1.59E-06	7.66E-07
SRR	1.83E-06	5.81E-06	1.89E-06	2.79E-06	3.10E-06	4.09E-06	6.50E-06	5.42E-06
SRRD	2.15E-05	1.49E-05	7.31E-06	8.89E-06	9.04E-06	1.29E-05	1.22E-05	1.43E-05
SRRM1	2.32E-05	1.02E-05	1.21E-05	1.26E-05	1.74E-06	1.41E-05	9.41E-06	1.19E-05
SRRM2	6.10E-05	3.79E-05	2.09E-05	2.35E-05	4.25E-06	2.63E-05	1.74E-05	2.42E-05
SRRM3	1.67E-05	3.60E-07	5.78E-08	8.14E-07	1.05E-07	1.64E-07	2.57E-07	1.46E-07
SRRM4	2.65E-09	0	0	0	0	0	0	0
SRRM5	7.17E-07	1.35E-06	7.22E-08	1.87E-07	3.96E-07	1.89E-07	2.69E-07	1.65E-07
SRRT	4.79E-05	2.86E-05	1.61E-05	2.04E-05	1.58E-05	1.80E-05	2.07E-05	1.79E-05
SRXN1	9.55E-05	6.01E-05	1.02E-05	1.86E-05	5.78E-06	0.000185	1.99E-05	6.60E-05
SRY	0	0	0	0	0	0	0	0
SS18	2.62E-05	1.54E-05	1.85E-05	1.91E-05	5.02E-06	1.61E-05	1.59E-05	2.01E-05
SS18L1	1.34E-05	2.03E-05	9.14E-06	5.90E-06	3.79E-06	1.60E-05	6.31E-06	3.07E-06
SS18L2	5.06E-05	3.64E-05	2.12E-05	2.08E-05	3.63E-05	2.02E-05	3.20E-05	2.19E-05
SSB	7.55E-05	5.22E-05	2.91E-05	3.95E-05	2.60E-05	5.02E-05	3.00E-05	3.59E-05
SSBP1	7.32E-05	0.000105	7.68E-05	8.74E-05	8.63E-05	0.000139	0.000109	0.000162
SSBP2	6.05E-06	6.81E-07	7.84E-07	2.68E-06	1.79E-07	5.30E-07	9.13E-07	4.37E-07
SSBP3	3.24E-05	1.05E-05	2.09E-05	3.47E-05	1.52E-05	4.20E-05	3.14E-05	1.83E-05
SSBP4	1.65E-05	9.16E-06	5.67E-06	1.52E-05	1.65E-05	1.44E-05	1.96E-05	9.25E-06
SSC5D	1.32E-06	9.25E-08	7.17E-07	2.63E-06	1.62E-07	5.10E-08	4.30E-07	4.04E-07
SSFA2	5.90E-05	2.40E-05	5.61E-05	3.20E-05	1.19E-05	3.33E-05	2.30E-05	2.03E-05
SSH1	8.38E-06	3.08E-06	3.21E-06	5.50E-06	1.25E-06	5.33E-06	3.41E-06	4.68E-06
SSH2	3.47E-06	1.73E-06	7.86E-07	1.02E-06	5.65E-07	1.16E-06	9.91E-07	1.16E-06
SSH3	1.51E-05	1.31E-05	5.85E-06	8.78E-06	3.01E-06	7.11E-06	6.30E-06	5.98E-06
SSNA1	8.14E-05	4.45E-05	3.35E-05	4.88E-05	6.14E-05	4.37E-05	6.03E-05	9.36E-05
SSPN	1.45E-06	8.76E-08	3.16E-07	8.60E-07	6.72E-08	9.27E-08	3.45E-07	3.15E-07
SSPO	1.52E-06	1.75E-06	4.19E-08	2.41E-07	3.37E-08	7.49E-07	8.00E-08	1.43E-07
SSR1	1.70E-05	2.12E-05	1.15E-05	1.10E-05	7.63E-06	2.16E-05	1.26E-05	1.82E-05
SSR2	0.000204	0.000194	9.45E-05	0.000158	0.000134	0.000317	0.000158	0.000389
SSR3	5.77E-05	5.13E-05	3.08E-05	3.60E-05	2.22E-05	6.97E-05	4.02E-05	4.20E-05
SSR4	0.00026	0.000368	0.000247	0.000297	0.000427	0.000379	0.000272	0.000433
SSRP1	5.13E-05	4.62E-05	2.26E-05	3.13E-05	1.65E-05	3.91E-05	3.48E-05	2.94E-05
SSSCA1	4.15E-05	4.15E-05	2.68E-05	2.83E-05	2.86E-05	3.30E-05	3.70E-05	7.14E-05
SST	0	5.92E-08	0	0	0	0	4.58E-08	1.49E-07
SSTR1	1.71E-05	4.44E-07	6.32E-06	1.07E-06	8.40E-07	3.56E-06	1.36E-06	3.72E-07
SSTR2	1.14E-05	3.78E-07	7.29E-07	2.53E-06	1.01E-07	8.70E-08	2.35E-07	4.13E-07
SSTR3	8.85E-08	0	0	1.81E-08	0	1.37E-08	0	0
SSTR4	0	0	0	0	0	0	0	0
SSTR5	2.74E-06	0	1.97E-08	0	0	0	0	7.66E-07
SSU72	8.54E-05	0.000119	7.38E-05	7.14E-05	6.58E-05	0.000108	7.46E-05	9.01E-05
SSX1	1.81E-06	7.90E-06	0	2.51E-08	0	7.59E-08	0	2.20E-06
SSX2	8.61E-08	0	0	2.12E-08	0	0	0	0
SSX2IP	6.72E-06	1.72E-05	2.90E-06	2.24E-06	6.06E-07	8.83E-06	1.14E-06	3.49E-06
SSX3	0	0	0	0	0	0	0	0
SSX4	0	7.52E-06	0	0	0	0	0	0
SSX5	2.02E-08	1.62E-07	0	0	0	0	0	8.99E-08
SSX6	0	7.06E-07	2.13E-08	4.54E-08	0	0	0	0
SSX7	0	0	0	0	0	0	0	0
SSX8	0	0	0	0	0	0	0	0
ST13	0.00011	0.000135	9.31E-05	7.14E-05	5.74E-05	9.75E-05	7.60E-05	5.79E-05
ST14	3.98E-05	2.36E-06	4.71E-06	4.35E-05	5.02E-06	2.30E-05	1.84E-05	1.03E-06
ST18	3.24E-06	0	2.03E-08	4.12E-08	0	0	0	0
ST20	8.85E-06	1.14E-05	6.73E-06	8.02E-06	8.39E-06	8.02E-06	1.04E-05	1.02E-05
ST3GAL1	9.91E-06	6.82E-05	2.17E-05	1.82E-05	4.78E-05	2.18E-05	6.80E-05	9.12E-06
ST3GAL2	6.94E-06	5.03E-06	4.42E-06	4.73E-06	2.26E-06	6.20E-06	6.11E-06	6.96E-06
ST3GAL3	4.56E-06	6.12E-06	5.28E-06	6.68E-06	1.42E-05	9.53E-06	9.81E-06	8.11E-06
ST3GAL4	2.78E-05	9.22E-06	1.05E-05	2.00E-05	7.02E-06	2.75E-05	1.67E-05	1.15E-05

ST3GAL5	1.39E-05	3.30E-06	4.99E-06	7.38E-06	3.53E-06	3.77E-06	6.55E-06	9.49E-06
ST3GAL6	4.40E-06	9.11E-06	3.51E-05	1.48E-05	1.28E-05	2.26E-05	1.47E-05	1.53E-05
ST5	1.51E-05	9.57E-06	9.72E-06	1.28E-05	8.73E-06	1.50E-05	9.22E-06	1.94E-05
ST6GAL1	9.87E-05	0.000273	0.0003	0.00018	0.000122	0.000242	0.000222	0.000313
ST6GAL2	1.88E-07	5.87E-08	7.46E-07	6.78E-07	3.67E-08	1.83E-08	3.21E-09	6.25E-08
ST6GALN/	1.83E-07	3.26E-08	1.72E-07	1.54E-07	3.32E-08	5.37E-08	9.34E-09	4.25E-08
ST6GALN/	1.31E-06	4.10E-07	8.53E-07	1.17E-06	1.36E-07	1.05E-07	5.26E-07	2.50E-07
ST6GALN/	1.30E-06	7.97E-07	7.48E-07	6.12E-07	4.86E-07	6.85E-07	1.00E-06	8.73E-07
ST6GALN/	8.74E-06	4.86E-06	1.67E-06	3.81E-06	2.17E-06	1.49E-06	4.92E-06	2.64E-06
ST6GALN/	8.83E-07	0	1.60E-08	1.31E-07	0	2.71E-08	7.20E-08	2.24E-08
ST6GALN/	2.47E-05	2.45E-05	2.31E-05	4.36E-05	2.49E-05	4.17E-05	3.02E-05	2.76E-05
ST7	1.01E-05	1.70E-05	2.06E-05	1.69E-05	1.49E-05	1.91E-05	1.90E-05	2.50E-05
ST7L	4.72E-06	4.71E-06	1.79E-06	2.72E-06	7.89E-07	6.32E-06	1.23E-06	3.34E-06
ST7OT1	7.05E-07	1.80E-07	3.32E-07	8.23E-07	4.27E-07	7.55E-07	1.96E-07	3.46E-07
ST7OT2	0	0	0	0	1.72E-08	0	0	0
ST7OT3	1.05E-07	6.04E-08	8.60E-08	1.45E-07	9.06E-08	4.88E-08	5.09E-08	7.89E-08
ST7OT4	3.20E-08	1.79E-08	0	1.96E-08	0	1.02E-07	0	0
ST8SIA1	1.41E-07	7.70E-09	4.70E-09	3.89E-08	1.98E-09	5.90E-08	4.22E-08	2.36E-08
ST8SIA2	4.04E-09	0	0	0	0	0	3.93E-09	5.91E-09
ST8SIA3	6.54E-09	1.55E-07	7.10E-08	2.12E-08	0	4.03E-09	8.29E-08	0
ST8SIA4	2.59E-06	9.06E-07	8.76E-07	1.38E-06	2.31E-07	8.69E-07	8.87E-07	5.64E-07
ST8SIA5	1.07E-08	2.41E-08	1.12E-08	6.54E-08	5.51E-08	8.92E-08	2.07E-08	5.28E-08
ST8SIA6	9.90E-08	0	3.06E-07	9.75E-08	0	1.84E-08	0	0
STAB1	2.13E-05	9.10E-06	2.04E-05	2.43E-05	1.97E-05	1.33E-05	3.25E-05	1.37E-05
STAB2	6.85E-08	1.69E-08	7.21E-06	2.79E-06	3.82E-08	0	1.63E-07	0
STAC	1.86E-06	0	1.30E-07	3.21E-07	2.00E-08	1.55E-08	1.62E-08	2.46E-08
STAC2	4.62E-08	0	0	9.41E-09	0	0	7.47E-09	1.39E-06
STAC3	2.95E-06	2.55E-07	1.27E-06	3.13E-06	1.25E-06	1.99E-06	2.96E-06	1.70E-06
STAG1	7.26E-06	4.65E-06	3.94E-06	3.39E-06	5.55E-07	4.65E-06	2.24E-06	2.94E-06
STAG2	1.79E-05	1.90E-05	1.06E-05	9.66E-06	3.43E-06	1.16E-05	7.04E-06	1.32E-05
STAG3	1.56E-06	1.31E-06	1.73E-06	8.22E-07	6.36E-06	3.76E-06	2.67E-06	1.21E-06
STAG3L1	1.27E-06	1.54E-06	6.37E-07	1.41E-07	1.88E-07	1.28E-07	6.51E-07	8.45E-08
STAG3L2	4.71E-06	2.46E-06	2.73E-06	2.07E-06	7.04E-07	2.96E-06	1.16E-06	1.14E-06
STAG3L3	5.59E-06	5.17E-06	1.60E-06	1.55E-06	1.21E-06	1.74E-06	2.15E-06	1.50E-06
STAG3L4	4.06E-06	6.33E-06	7.23E-07	2.02E-06	6.03E-07	1.55E-06	2.35E-06	2.34E-06
STAM	1.03E-05	7.73E-06	4.32E-06	4.40E-06	2.51E-06	5.63E-06	3.89E-06	4.82E-06
STAM2	9.65E-06	6.88E-06	5.02E-06	4.56E-06	1.52E-06	5.10E-06	2.98E-06	4.24E-06
STAMBP	4.36E-05	2.73E-05	1.12E-05	1.11E-05	5.91E-06	1.77E-05	1.26E-05	1.68E-05
STAMBPL1	3.09E-06	1.09E-05	6.43E-07	2.11E-06	1.53E-07	4.08E-06	1.91E-06	4.60E-06
STAP1	8.20E-08	2.62E-08	2.46E-08	2.82E-07	1.41E-08	7.62E-08	0	7.27E-08
STAP2	3.47E-05	3.26E-05	5.90E-05	7.19E-05	0.00011	3.03E-05	5.92E-05	7.41E-05
STAR	9.07E-09	6.13E-08	9.42E-08	1.33E-07	2.00E-07	6.72E-08	5.27E-08	6.66E-08
STARD10	0.000227	0.000361	0.000295	0.000206	0.000597	0.000477	0.000392	0.000203
STARD13	4.13E-06	1.45E-06	2.95E-06	2.67E-06	5.71E-07	9.56E-06	1.62E-06	3.44E-06
STARD3	3.90E-05	4.23E-05	1.74E-05	2.06E-05	3.95E-05	3.25E-05	2.76E-05	2.23E-05
STARD3NI	3.10E-05	8.11E-06	5.72E-06	1.25E-05	2.41E-06	9.35E-06	1.08E-05	7.21E-06
STARD4	1.16E-05	1.65E-05	8.98E-06	2.27E-06	4.11E-07	3.14E-06	1.17E-06	3.20E-06
STARD5	1.48E-05	5.53E-06	2.36E-05	9.02E-06	5.99E-06	2.40E-05	7.24E-06	6.25E-06
STARD6	0	0	0	0	0	0	0	0
STARD7	0.000146	7.99E-05	3.74E-05	3.99E-05	4.48E-05	7.24E-05	5.39E-05	5.85E-05
STARD8	9.41E-06	4.36E-06	3.11E-06	2.45E-06	3.86E-07	1.43E-06	2.46E-06	1.66E-06
STAT1	0.000242	3.68E-05	4.64E-05	4.73E-05	8.00E-06	9.70E-05	1.60E-05	6.54E-05
STAT2	3.57E-05	5.01E-05	2.03E-05	2.65E-05	8.59E-06	3.06E-05	2.30E-05	2.45E-05
STAT3	7.56E-05	0.000101	5.62E-05	4.00E-05	1.28E-05	0.000136	4.12E-05	7.39E-05
STAT4	1.56E-06	7.53E-07	1.36E-06	1.88E-06	3.40E-07	7.57E-07	8.41E-07	9.17E-07
STAT5A	8.16E-06	5.91E-06	3.75E-06	6.10E-06	4.67E-06	6.41E-06	6.52E-06	3.60E-06
STAT5B	1.37E-05	2.81E-05	1.30E-05	1.20E-05	7.41E-06	2.28E-05	1.28E-05	1.02E-05
STAT6	6.28E-05	5.40E-05	3.09E-05	4.78E-05	1.38E-05	5.57E-05	4.03E-05	2.45E-05

STATH	0	0	0	0	0	0	0	0
STAU1	6.31E-05	7.10E-05	6.06E-05	6.46E-05	2.52E-05	7.54E-05	5.22E-05	6.61E-05
STAU2	1.58E-05	1.31E-05	7.25E-06	7.11E-06	2.65E-06	1.32E-05	1.09E-05	8.92E-06
STBD1	6.31E-05	5.38E-05	0.000106	0.000136	2.61E-05	7.49E-05	0.000191	8.64E-05
STC1	4.69E-06	9.72E-06	3.39E-07	6.83E-06	2.32E-07	8.01E-06	4.93E-06	6.87E-06
STC2	7.05E-06	5.76E-07	1.72E-08	9.13E-07	6.43E-07	1.51E-06	2.64E-06	1.98E-06
STEAP1	5.00E-05	1.76E-05	1.11E-05	9.41E-06	7.92E-06	4.25E-05	1.43E-05	5.60E-06
STEAP2	5.70E-06	9.15E-06	1.48E-06	1.17E-06	5.82E-07	8.03E-06	4.50E-07	6.01E-07
STEAP3	2.62E-05	4.23E-05	0.00011	5.76E-05	3.34E-05	0.00012	4.44E-05	1.61E-05
STEAP4	3.42E-07	2.21E-06	7.05E-06	1.31E-05	1.22E-06	4.46E-06	3.12E-06	3.64E-06
STH	0	0	0	0	0	0	0	0
STIL	3.27E-06	9.93E-07	1.57E-07	2.75E-07	1.05E-07	5.20E-07	1.81E-07	2.39E-07
STIM1	4.41E-05	3.36E-05	2.32E-05	1.99E-05	1.01E-05	2.59E-05	2.48E-05	5.28E-05
STIM2	9.67E-06	4.80E-06	3.33E-06	5.73E-06	2.69E-06	4.96E-06	3.64E-06	6.20E-06
STIP1	0.0001	0.000127	2.34E-05	3.03E-05	1.74E-05	3.91E-05	4.87E-05	5.18E-05
STK10	6.56E-06	3.10E-06	2.96E-06	6.91E-06	1.23E-06	4.58E-06	4.69E-06	3.26E-06
STK11	1.69E-05	1.43E-05	1.19E-05	1.33E-05	1.40E-05	1.35E-05	2.47E-05	1.14E-05
STK11IP	1.00E-05	2.14E-06	1.42E-06	2.80E-06	1.64E-06	2.76E-06	2.46E-06	2.88E-06
STK16	2.90E-05	1.39E-05	1.22E-05	1.36E-05	1.05E-05	1.85E-05	2.11E-05	6.39E-06
STK17A	1.07E-05	2.90E-06	1.91E-06	7.97E-06	1.41E-06	3.97E-06	4.62E-06	4.91E-06
STK17B	1.08E-05	1.32E-06	2.26E-06	5.19E-06	1.46E-07	1.58E-06	1.71E-06	1.30E-06
STK19	1.48E-05	1.79E-05	3.67E-06	7.27E-06	8.24E-06	7.98E-06	8.04E-06	1.04E-05
STK24	3.34E-05	2.25E-05	9.60E-06	1.77E-05	5.21E-06	1.08E-05	1.86E-05	9.70E-06
STK25	7.70E-05	3.06E-05	2.29E-05	2.96E-05	3.38E-05	3.45E-05	3.67E-05	3.03E-05
STK3	1.84E-05	5.65E-06	4.43E-06	4.10E-06	2.26E-06	5.75E-06	9.84E-06	8.49E-06
STK31	7.17E-08	4.86E-08	2.98E-08	3.45E-08	1.24E-08	6.68E-09	6.34E-09	1.06E-08
STK32A	2.18E-07	0	0	3.86E-08	0	0	0	0
STK32B	9.30E-08	8.03E-08	4.43E-08	6.12E-08	7.37E-08	1.13E-07	1.11E-06	3.14E-08
STK32C	7.95E-06	2.45E-07	7.46E-07	2.25E-06	1.24E-06	9.83E-07	1.70E-06	4.56E-07
STK33	1.08E-06	0	7.51E-08	2.35E-07	2.07E-08	2.64E-07	0	1.77E-08
STK35	8.06E-06	2.09E-06	1.50E-06	2.70E-06	2.80E-06	3.39E-06	4.98E-06	4.55E-06
STK36	7.49E-06	5.73E-06	2.22E-06	2.71E-06	1.99E-06	5.98E-06	2.77E-06	2.81E-06
STK38	3.36E-05	1.02E-05	9.04E-06	1.19E-05	1.14E-05	1.19E-05	8.66E-06	8.72E-06
STK38L	5.05E-06	6.22E-06	3.28E-06	5.98E-06	1.35E-06	1.79E-06	4.13E-06	1.96E-06
STK39	2.25E-05	1.50E-06	3.20E-07	2.58E-06	1.27E-07	7.69E-07	7.72E-07	6.58E-06
STK4	1.01E-05	6.21E-06	4.16E-06	5.41E-06	9.07E-07	4.48E-06	3.18E-06	3.56E-06
STK40	1.80E-05	1.58E-05	1.62E-05	2.45E-05	5.91E-06	1.61E-05	1.83E-05	1.68E-05
STL	8.47E-09	2.35E-08	5.63E-08	5.16E-09	3.61E-09	1.02E-07	0	6.16E-09
STMN1	0.000124	5.04E-05	6.97E-06	2.87E-05	3.12E-05	3.05E-05	2.07E-05	2.62E-05
STMN2	3.91E-06	0	7.98E-08	1.25E-05	0	0	1.37E-07	7.30E-07
STMN3	8.18E-06	2.37E-07	9.31E-07	4.67E-06	7.95E-07	6.43E-07	1.85E-06	1.11E-06
STMN4	4.16E-08	0	0	0	0	0	1.85E-08	0
STOM	0.000843	0.000145	0.000171	0.000183	0.000153	0.000146	0.000169	0.000204
STOML1	7.91E-06	1.11E-05	7.44E-06	5.45E-06	1.32E-05	9.05E-06	1.09E-05	2.93E-05
STOML2	5.12E-05	0.00013	6.88E-05	6.35E-05	7.59E-05	0.000107	9.66E-05	7.03E-05
STOML3	0	0	0	0	0	0	0	0
STON1	3.15E-06	9.15E-07	5.66E-07	8.91E-07	1.37E-07	2.68E-07	3.84E-07	3.06E-07
STON1-G'	1.34E-07	0	0	0	0	0	0	2.51E-08
STON2	1.16E-06	1.15E-06	4.39E-07	9.43E-07	5.61E-07	2.16E-06	8.78E-07	4.25E-07
STOX1	1.51E-06	8.12E-07	1.30E-07	3.76E-07	3.14E-07	1.31E-07	2.29E-08	8.71E-08
STOX2	1.79E-06	1.13E-07	3.44E-07	1.80E-07	3.87E-08	1.47E-06	1.56E-07	9.25E-08
STRA13	0.000127	0.000129	4.00E-05	3.80E-05	9.32E-05	0.00024	3.45E-05	4.92E-05
STRA6	3.32E-07	0	0	1.80E-07	0	1.88E-08	5.90E-08	2.38E-07
STRA8	0	0	0	0	0	0	0	0
STRADA	1.63E-05	1.02E-05	6.25E-06	7.66E-06	6.23E-06	1.56E-05	9.11E-06	8.96E-06
STRADB	6.62E-05	2.87E-05	3.29E-05	1.57E-05	1.56E-05	1.79E-05	1.18E-05	1.54E-05
STRAP	8.03E-05	7.79E-05	3.65E-05	3.91E-05	2.17E-05	6.32E-05	4.42E-05	4.00E-05
STRBP	1.43E-05	9.04E-06	4.80E-06	4.96E-06	4.96E-06	6.08E-06	4.02E-06	8.22E-06

STRC	4.85E-07	6.50E-07	3.78E-08	2.89E-08	2.35E-07	3.45E-07	6.38E-09	1.00E-07
STRN	4.58E-06	3.20E-06	2.41E-06	1.35E-06	5.86E-09	1.94E-06	2.72E-07	1.29E-06
STRN3	1.07E-05	7.20E-06	8.49E-06	9.02E-06	3.34E-06	6.69E-06	5.40E-06	7.83E-06
STRN4	2.26E-05	2.18E-05	1.07E-05	1.56E-05	8.66E-06	1.62E-05	1.79E-05	1.72E-05
STS	1.87E-05	2.12E-06	8.85E-06	5.64E-06	1.11E-06	3.46E-06	4.78E-06	9.91E-06
STT3A	7.55E-05	3.57E-05	3.98E-05	4.55E-05	3.65E-05	7.13E-05	3.49E-05	9.10E-05
STT3B	3.56E-05	5.27E-05	2.29E-05	2.06E-05	2.02E-06	3.40E-05	9.98E-06	2.71E-05
STUB1	4.77E-05	0.000107	4.20E-05	4.71E-05	8.59E-05	4.99E-05	7.76E-05	4.69E-05
STX10	3.49E-05	1.17E-05	1.37E-05	2.09E-05	1.99E-05	2.00E-05	2.74E-05	4.04E-05
STX11	2.52E-06	3.28E-07	1.02E-06	3.40E-06	3.85E-08	8.27E-07	9.11E-07	5.61E-07
STX12	2.18E-05	1.79E-05	1.02E-05	1.36E-05	3.23E-06	1.50E-05	9.03E-06	1.13E-05
STX16	2.48E-05	1.34E-05	6.79E-06	6.51E-06	5.52E-06	1.38E-05	8.19E-06	7.75E-06
STX17	8.38E-06	5.96E-06	8.77E-06	4.43E-06	1.46E-06	6.71E-06	2.92E-06	4.56E-06
STX18	2.45E-05	1.36E-05	6.27E-06	1.05E-05	7.99E-06	1.34E-05	1.14E-05	1.32E-05
STX19	1.12E-07	5.16E-07	1.21E-07	2.76E-08	0	6.26E-08	4.29E-08	9.98E-08
STX1A	1.77E-06	1.31E-06	2.63E-07	7.79E-07	6.54E-07	6.88E-07	8.59E-07	9.70E-07
STX1B	6.58E-07	9.98E-07	8.34E-07	4.56E-07	1.50E-07	1.61E-06	1.39E-06	5.54E-07
STX2	6.22E-06	1.44E-06	3.81E-06	4.36E-06	8.67E-07	3.08E-06	4.17E-06	5.98E-06
STX3	1.82E-05	8.75E-06	2.76E-06	9.80E-06	1.53E-06	3.52E-06	6.17E-06	5.59E-06
STX4	4.02E-05	5.18E-05	1.92E-05	3.34E-05	1.31E-05	2.54E-05	4.87E-05	3.22E-05
STX5	3.46E-05	3.31E-05	2.28E-05	3.51E-05	2.87E-05	3.96E-05	4.16E-05	3.97E-05
STX6	7.88E-06	6.06E-06	2.04E-06	4.31E-06	1.22E-06	4.44E-06	4.43E-06	4.41E-06
STX7	2.39E-05	6.65E-06	8.00E-06	1.01E-05	1.25E-06	7.50E-06	8.10E-06	5.04E-06
STX8	8.02E-06	8.90E-06	7.32E-06	1.39E-05	7.55E-06	2.12E-05	2.41E-05	1.59E-05
STXBP1	1.14E-05	8.25E-07	1.11E-06	2.29E-06	7.75E-07	4.79E-07	1.12E-06	2.05E-06
STXBP2	7.17E-05	2.48E-05	2.08E-05	3.14E-05	6.96E-06	1.72E-05	3.11E-05	2.45E-05
STXBP3	2.81E-05	1.86E-05	1.07E-05	1.48E-05	3.68E-06	2.06E-05	6.91E-06	1.09E-05
STXBP4	4.03E-06	9.38E-07	3.65E-07	4.21E-07	9.70E-08	7.83E-07	1.86E-07	6.41E-07
STXBP5	3.98E-06	7.43E-07	6.11E-07	1.12E-06	7.22E-08	8.76E-07	5.91E-07	1.82E-06
STXBP5L	0	2.66E-09	0	2.91E-09	0	0	0	0
STXBP6	1.28E-05	5.56E-06	6.78E-07	2.73E-06	2.66E-06	1.14E-05	4.47E-06	2.14E-06
STYK1	3.25E-07	1.74E-08	5.60E-08	4.73E-08	0	2.15E-08	7.50E-09	1.13E-08
STYX	7.65E-06	6.94E-06	1.27E-05	6.96E-06	3.50E-06	8.32E-06	7.82E-06	7.12E-06
STYXL1	3.12E-05	0.0001	2.17E-05	2.37E-05	3.28E-05	4.22E-05	6.28E-05	4.60E-05
SUB1	5.53E-05	3.87E-05	2.12E-05	3.58E-05	2.46E-05	4.52E-05	3.76E-05	4.43E-05
SUCLA2	2.04E-05	1.61E-05	1.09E-05	1.09E-05	3.04E-06	1.24E-05	9.17E-06	1.42E-05
SUCLG1	0.00026	0.000306	8.67E-05	8.23E-05	9.50E-05	8.43E-05	9.36E-05	9.04E-05
SUCLG2	9.59E-05	0.000108	0.000151	9.52E-05	5.50E-05	0.000114	0.000112	7.81E-05
SUCNR1	2.91E-06	4.84E-07	1.11E-06	1.71E-06	1.52E-08	1.87E-06	2.54E-07	4.96E-07
SUDS3	8.89E-06	8.25E-06	5.52E-06	5.85E-06	1.47E-06	6.37E-06	4.96E-06	3.69E-06
SUFU	5.79E-06	2.37E-06	2.92E-06	3.18E-06	2.47E-06	6.02E-06	4.44E-06	2.88E-06
SUGT1	3.07E-05	2.36E-05	1.54E-05	1.98E-05	1.42E-05	3.26E-05	2.19E-05	2.01E-05
SUGT1L1	1.08E-06	1.06E-06	1.05E-06	2.48E-07	2.15E-07	4.71E-07	2.52E-07	5.06E-07
SUGT1P1	1.71E-07	5.10E-08	4.93E-08	1.07E-07	3.73E-08	1.04E-06	8.03E-08	0
SULF1	1.47E-05	1.54E-06	1.20E-06	4.99E-06	4.27E-07	2.32E-06	8.65E-06	2.66E-06
SULF2	7.96E-05	4.81E-06	3.06E-05	2.21E-05	9.47E-06	3.76E-06	1.57E-05	1.27E-05
SULT1A1	8.16E-05	0.000503	0.000179	0.000167	0.000114	0.000247	0.000151	6.50E-05
SULT1A2	6.58E-06	3.13E-05	3.28E-05	1.54E-05	4.22E-05	7.80E-05	2.77E-05	1.83E-05
SULT1A3	1.71E-05	1.52E-05	9.35E-06	7.12E-06	1.15E-05	6.99E-05	1.34E-05	9.04E-06
SULT1B1	2.13E-07	4.00E-07	2.64E-06	6.45E-07	2.34E-07	1.36E-05	5.02E-07	2.79E-06
SULT1C2	9.38E-06	6.65E-06	6.57E-08	1.22E-07	0	2.43E-06	3.10E-07	5.77E-06
SULT1C3	0	0	0	0	0	0	0	8.93E-08
SULT1C4	3.34E-05	1.10E-06	7.87E-07	3.58E-06	1.50E-08	2.96E-07	8.34E-08	3.59E-07
SULT1E1	2.49E-06	3.57E-07	7.53E-06	3.06E-06	3.38E-07	1.90E-07	9.61E-07	2.61E-07
SULT2A1	9.65E-08	0.000113	0.000573	0.000235	0.000332	0.000917	0.000217	9.46E-05
SULT2B1	2.82E-06	7.40E-08	2.78E-07	4.50E-07	0	5.00E-07	2.06E-07	1.59E-07
SULT4A1	4.91E-07	1.27E-08	1.17E-08	4.12E-08	1.93E-08	1.56E-05	0	0
SULT6B1	0	0	0	0	0	0	0	0

SUMF1	1.96E-05	2.44E-05	1.50E-05	2.05E-05	2.26E-05	2.45E-05	2.30E-05	1.61E-05
SUMF2	0.000192	0.000101	7.02E-05	9.39E-05	8.04E-05	9.02E-05	9.25E-05	0.00012
SUMO1	0.00016	0.000114	8.06E-05	7.99E-05	8.33E-05	0.000109	9.69E-05	8.43E-05
SUMO1P1	1.28E-07	0	2.29E-08	0	3.68E-08	1.98E-08	2.04E-08	0
SUMO1P3	1.70E-05	8.59E-06	7.43E-06	7.16E-06	7.93E-06	1.04E-05	9.61E-06	7.32E-06
SUMO2	0.000185	0.000128	6.78E-05	0.000102	8.37E-05	0.000132	0.00013	7.47E-05
SUMO3	5.55E-05	3.15E-05	3.34E-05	3.70E-05	2.19E-05	4.99E-05	4.22E-05	6.28E-05
SUMO4	4.35E-08	0	2.01E-07	0	0	8.18E-08	4.08E-08	0
SUN1	5.25E-05	3.91E-05	1.55E-05	1.54E-05	5.37E-06	1.43E-05	1.44E-05	1.70E-05
SUN2	4.66E-05	5.99E-05	8.05E-05	8.12E-05	3.62E-05	6.13E-05	7.01E-05	6.89E-05
SUN3	8.00E-08	6.19E-08	0	0	0	0	2.54E-08	0
SUN5	0	0	0	0	0	0	0	0
SUOX	2.30E-05	3.84E-05	4.54E-05	2.21E-05	8.93E-06	3.13E-05	2.30E-05	2.11E-05
SUPT16H	3.37E-05	2.72E-05	1.42E-05	2.33E-05	1.02E-05	2.32E-05	2.07E-05	2.23E-05
SUPT3H	3.24E-06	3.43E-06	7.89E-07	2.01E-06	6.16E-07	2.51E-06	3.01E-06	2.61E-06
SUPT4H1	8.07E-05	6.94E-05	2.95E-05	3.87E-05	4.43E-05	6.79E-05	4.80E-05	3.62E-05
SUPT5H	4.40E-05	3.82E-05	2.08E-05	2.50E-05	2.43E-05	3.60E-05	3.40E-05	2.58E-05
SUPT6H	3.58E-05	3.40E-05	1.71E-05	2.01E-05	1.35E-05	2.07E-05	1.93E-05	1.76E-05
SUPT7L	2.22E-05	1.43E-05	5.41E-06	6.76E-06	3.28E-06	1.04E-05	6.76E-06	6.21E-06
SUPV3L1	1.32E-05	1.79E-05	1.22E-05	9.97E-06	1.03E-05	1.83E-05	1.43E-05	1.96E-05
SURF1	7.44E-05	9.32E-05	7.77E-05	5.90E-05	0.000134	8.15E-05	1.00E-04	9.63E-05
SURF2	1.54E-05	1.18E-05	1.06E-05	1.61E-05	2.71E-05	1.41E-05	2.10E-05	3.17E-05
SURF4	0.000126	0.000116	0.000133	0.000134	8.39E-05	0.000181	0.00011	0.000245
SURF6	1.49E-05	9.52E-06	7.82E-06	1.32E-05	1.19E-05	1.63E-05	1.21E-05	2.03E-05
SUSD1	5.67E-06	5.82E-07	1.33E-06	2.42E-06	3.32E-07	5.87E-07	1.20E-06	5.41E-07
SUSD2	6.39E-06	3.57E-06	8.54E-07	6.50E-06	9.15E-07	2.62E-07	1.02E-05	1.04E-06
SUSD3	5.24E-05	2.49E-07	9.26E-06	1.20E-05	2.82E-05	2.08E-05	2.87E-05	1.19E-06
SUSD4	8.40E-07	1.05E-07	2.50E-06	4.79E-06	2.55E-07	4.96E-08	2.18E-07	1.29E-05
SUSD5	1.13E-06	1.01E-08	1.25E-07	2.14E-07	6.57E-08	8.34E-09	1.32E-08	5.93E-08
SUV39H1	9.94E-06	6.84E-06	3.15E-06	3.33E-06	5.19E-06	6.03E-06	6.98E-06	9.09E-06
SUV39H2	3.02E-06	2.43E-06	1.13E-06	1.44E-06	8.82E-07	2.54E-06	1.84E-06	1.39E-06
SUV420H1	1.05E-05	1.14E-05	8.66E-06	9.86E-06	1.94E-06	8.62E-06	6.61E-06	1.27E-05
SUV420H2	6.60E-06	2.80E-06	9.31E-07	1.90E-06	1.89E-06	1.90E-06	2.99E-06	2.90E-06
SUZ12	1.50E-05	1.67E-05	6.77E-06	6.11E-06	3.26E-06	6.93E-06	4.12E-06	6.23E-06
SUZ12P	5.34E-06	1.44E-06	6.83E-07	1.06E-06	8.62E-07	6.33E-07	9.58E-07	8.74E-07
SV2A	8.29E-07	5.69E-07	1.68E-07	6.88E-07	2.05E-07	4.01E-08	1.60E-07	4.89E-07
SV2B	4.24E-08	7.10E-06	6.08E-08	1.40E-07	1.82E-08	2.20E-07	5.64E-07	7.44E-08
SV2C	0	2.25E-08	0	0	0	0	0	1.47E-08
SVEP1	4.09E-07	5.15E-08	1.87E-06	7.86E-06	6.00E-07	8.29E-08	1.57E-06	8.02E-07
SVIL	1.58E-05	8.36E-06	4.43E-06	5.50E-06	3.85E-06	8.38E-06	6.18E-06	3.92E-06
SVIP	1.25E-05	8.26E-06	6.41E-06	8.17E-06	3.36E-06	1.30E-05	8.61E-06	1.02E-05
SVOP	8.35E-08	3.90E-08	0	8.50E-09	5.97E-09	1.29E-08	0	0
SVOPL	1.76E-08	4.02E-08	0	0	0	0	0	0
SWAP70	1.36E-05	8.59E-06	3.94E-06	7.00E-06	1.15E-06	3.07E-06	5.45E-06	1.07E-05
SYAP1	8.61E-05	1.97E-05	4.37E-05	2.47E-05	8.42E-06	4.05E-05	1.60E-05	5.14E-05
SYBU	3.19E-05	1.58E-05	4.75E-05	1.67E-05	1.20E-05	6.43E-05	2.54E-05	6.32E-05
SYCE1	2.87E-08	9.14E-08	1.31E-07	6.50E-06	5.53E-08	5.10E-08	2.22E-05	0
SYCE1L	6.07E-07	1.42E-07	3.04E-07	4.21E-06	3.95E-07	0	7.38E-07	3.38E-07
SYCE2	5.82E-07	1.05E-06	1.61E-07	1.44E-07	7.32E-07	1.90E-07	3.32E-07	1.17E-06
SYCN	0	0	0	0	0	0	0	0
SYCP1	0	0	0	0	0	0	0	0
SYCP2	6.16E-08	1.37E-08	1.68E-08	4.00E-08	2.46E-08	3.04E-08	2.40E-08	4.20E-08
SYCP2L	5.52E-08	0	0	3.37E-08	1.26E-07	6.84E-09	0	1.35E-08
SYCP3	0	2.99E-08	0	0	0	0	0	0
SYDE1	7.26E-06	1.03E-06	1.63E-06	4.29E-06	1.03E-06	6.78E-07	3.45E-06	1.48E-06
SYDE2	6.45E-07	6.86E-07	9.59E-07	5.97E-07	5.77E-08	3.92E-07	2.63E-07	4.53E-07
SYF2	4.32E-05	2.18E-05	1.93E-05	2.43E-05	1.32E-05	3.75E-05	2.41E-05	2.46E-05
SYK	5.44E-06	5.64E-07	1.73E-06	3.85E-06	3.36E-07	1.32E-06	2.27E-06	4.24E-07

SYMPK	2.33E-05	2.93E-05	1.64E-05	2.06E-05	2.72E-05	2.79E-05	2.68E-05	1.34E-05
SYN1	2.80E-07	2.04E-07	2.48E-07	7.96E-07	3.12E-08	6.06E-08	1.20E-07	1.24E-06
SYN2	0	5.41E-07	0	2.77E-08	0	5.17E-08	1.06E-07	0
SYN3	5.33E-08	1.11E-07	8.79E-09	0	3.27E-08	1.34E-07	4.20E-08	7.66E-08
SYNC	1.11E-06	8.08E-08	2.45E-07	4.76E-07	3.84E-06	1.07E-07	3.80E-06	7.45E-08
SYNCRIP	3.36E-05	3.08E-05	2.02E-05	2.33E-05	5.39E-06	3.22E-05	2.22E-05	2.25E-05
SYNE1	1.46E-05	6.61E-06	1.13E-05	1.09E-05	2.89E-06	1.17E-05	6.19E-06	4.23E-06
SYNE2	3.86E-05	9.45E-06	1.41E-05	1.66E-05	5.18E-06	4.51E-05	1.03E-05	1.00E-05
SYNGAP1	2.60E-06	7.45E-07	3.88E-07	9.80E-07	1.37E-07	4.21E-07	5.95E-07	7.02E-07
SYNGR1	2.14E-05	1.86E-07	3.31E-06	3.74E-06	1.72E-05	1.29E-05	1.67E-06	1.91E-06
SYNGR2	0.000182	7.92E-05	5.73E-05	8.43E-05	9.60E-05	0.000108	9.11E-05	9.62E-05
SYNGR3	8.45E-06	5.34E-08	1.11E-07	3.18E-07	0	9.85E-08	1.37E-07	1.04E-07
SYNGR4	1.07E-07	4.95E-07	0	0	6.92E-08	5.47E-07	7.62E-08	1.19E-07
SYNJ1	5.32E-06	1.64E-06	2.45E-06	2.01E-06	6.61E-07	1.97E-06	1.31E-06	1.87E-06
SYNJ2	7.99E-06	4.40E-06	2.69E-06	5.36E-06	4.40E-07	2.79E-06	1.75E-06	6.40E-06
SYNJ2BP	1.05E-05	9.75E-06	1.03E-05	6.10E-06	3.23E-06	1.01E-05	6.63E-06	8.00E-06
SYNM	7.38E-06	2.30E-06	1.39E-06	2.38E-06	1.36E-06	5.06E-07	1.22E-06	2.78E-06
SYNPO	3.70E-05	1.37E-05	1.59E-05	2.67E-05	1.68E-05	1.61E-05	3.29E-05	2.11E-05
SYNPO2	2.38E-06	9.42E-07	2.33E-06	2.87E-06	3.33E-07	1.66E-06	1.21E-06	7.43E-07
SYNPO2L	4.98E-08	0	1.14E-08	1.35E-08	0	1.02E-08	1.08E-08	8.09E-09
SYNPR	0	0	0	0	0	0	0	0
SYNRG	8.56E-06	9.45E-06	5.23E-06	3.90E-06	1.23E-06	5.74E-06	2.71E-06	4.77E-06
SYP	1.26E-06	4.85E-07	2.06E-07	4.37E-07	4.48E-07	1.99E-07	5.59E-07	1.01E-06
SYPL1	3.34E-05	7.30E-05	3.43E-05	5.01E-05	2.31E-05	4.94E-05	4.40E-05	8.94E-05
SYPL2	7.71E-06	9.35E-06	4.67E-06	1.68E-06	1.79E-06	4.93E-06	3.38E-06	3.90E-06
SYS1	2.82E-05	1.80E-05	1.44E-05	1.94E-05	1.30E-05	1.59E-05	1.83E-05	2.05E-05
SYS1-DBN	3.44E-07	6.03E-07	2.74E-07	6.56E-07	0	2.89E-07	2.83E-07	7.11E-07
SYT1	4.00E-06	1.42E-06	5.78E-07	2.26E-07	2.21E-08	6.66E-08	9.50E-08	3.91E-07
SYT10	0	0	7.23E-09	3.43E-08	0	0	0	0
SYT11	3.06E-06	9.34E-07	6.28E-07	1.58E-06	4.54E-07	7.41E-07	1.24E-06	8.15E-07
SYT12	1.10E-05	1.51E-05	4.85E-06	9.95E-06	3.00E-06	3.04E-07	3.02E-05	5.55E-06
SYT13	4.13E-05	0	2.60E-07	1.68E-06	0	8.08E-09	2.98E-08	7.03E-08
SYT14	0	0	0	0	0	0	6.12E-09	0
SYT14L	0	0	0	0	0	0	0	0
SYT15	2.19E-07	7.61E-08	2.14E-07	2.42E-07	1.25E-08	9.89E-08	1.13E-07	1.15E-07
SYT16	0	0	0	0	0	0	0	0
SYT17	6.95E-06	1.64E-05	9.10E-06	9.07E-06	1.94E-06	6.66E-06	8.15E-06	1.01E-05
SYT2	1.49E-07	2.06E-08	1.57E-08	2.25E-08	2.64E-09	1.99E-08	3.00E-09	8.99E-09
SYT3	1.90E-08	1.30E-07	0	0	0	0	3.17E-08	4.35E-06
SYT4	0	0	4.80E-08	3.73E-08	0	0	0	0
SYT5	2.12E-07	3.01E-08	1.12E-07	4.89E-08	0	1.23E-08	2.56E-08	1.42E-08
SYT6	8.16E-08	0	5.33E-09	9.07E-09	0	0	0	2.18E-08
SYT7	5.61E-05	4.34E-05	5.84E-05	7.27E-06	1.78E-05	1.54E-06	8.15E-06	2.80E-05
SYT8	1.81E-06	4.71E-07	4.00E-08	1.60E-07	1.61E-08	1.18E-07	0	4.03E-08
SYT9	1.35E-08	1.51E-08	1.76E-06	8.47E-07	0	0	3.93E-08	0
SYTL1	5.23E-06	1.69E-07	6.63E-07	2.00E-06	3.29E-07	4.92E-07	1.13E-06	5.64E-07
SYTL2	3.22E-06	1.63E-06	1.18E-06	3.88E-06	5.11E-07	1.32E-06	1.97E-06	1.38E-06
SYTL3	2.36E-06	2.15E-07	4.70E-07	2.34E-06	1.24E-07	8.10E-07	4.33E-07	5.07E-07
SYTL4	4.40E-06	8.56E-06	6.05E-06	9.04E-06	1.55E-05	1.10E-05	1.35E-05	7.39E-06
SYTL5	6.70E-08	1.44E-07	1.44E-06	1.70E-06	2.47E-08	1.02E-06	2.90E-07	4.27E-07
SYVN1	4.22E-05	8.18E-05	5.02E-05	3.72E-05	2.37E-05	6.40E-05	4.14E-05	0.000111
T	0	0	0	0	0	0	0	0
TAAR1	2.61E-08	6.04E-08	1.43E-07	3.22E-08	0	0	0	0
TAAR2	0	0	0	0	0	0	0	0
TAAR5	0	0	0	0	0	0	0	0
TAAR6	2.56E-08	0	0	0	0	0	0	0
TAAR8	2.58E-08	0	0	0	0	0	0	0
TAAR9	2.53E-08	0	0	0	0	0	0	0

TAB1	1.49E-05	9.95E-06	7.71E-06	7.88E-06	8.77E-06	1.10E-05	1.11E-05	1.16E-05
TAB2	2.10E-05	1.93E-05	2.14E-05	1.50E-05	2.34E-06	2.11E-05	1.29E-05	1.13E-05
TAB3	2.45E-05	5.37E-06	7.67E-06	6.33E-06	3.50E-06	9.88E-06	4.56E-06	1.20E-05
TAC1	0	0	0	6.38E-07	0	0	0	0
TAC3	1.36E-07	1.29E-06	0	0	0	0	0	0
TAC4	1.15E-07	7.06E-08	0	0	0	0	0	8.81E-08
TACC1	1.29E-05	8.50E-06	8.73E-06	1.70E-05	1.35E-06	1.54E-05	5.07E-06	1.09E-05
TACC2	1.56E-05	4.90E-06	5.53E-06	7.33E-06	6.04E-06	1.21E-05	7.53E-06	7.42E-06
TACC3	2.23E-05	1.47E-05	2.54E-06	4.69E-06	8.74E-06	6.24E-06	3.56E-06	4.24E-06
TACO1	5.54E-05	8.46E-05	4.28E-05	3.71E-05	3.12E-05	8.39E-05	5.47E-05	5.15E-05
TACR1	9.93E-08	7.93E-08	1.46E-08	2.89E-08	6.09E-08	1.31E-08	1.24E-07	5.54E-08
TACR2	2.08E-07	1.98E-08	1.66E-07	1.92E-07	1.49E-07	4.83E-08	5.00E-08	5.63E-07
TACR3	0	0	0	0	0	0	0	0
TACSTD2	2.53E-06	9.65E-07	2.53E-06	6.61E-05	0	8.56E-08	2.14E-06	4.24E-07
TADA1	1.05E-05	2.72E-05	1.29E-05	6.97E-06	2.87E-06	1.59E-05	6.62E-06	1.46E-05
TADA2A	4.41E-06	3.66E-06	3.03E-06	2.57E-06	8.29E-07	2.98E-06	3.17E-06	3.48E-06
TADA2B	1.09E-05	1.06E-05	7.79E-06	8.23E-06	3.20E-06	8.18E-06	8.00E-06	1.23E-05
TADA3	4.17E-05	3.87E-05	2.42E-05	2.99E-05	2.88E-05	3.67E-05	5.22E-05	2.45E-05
TAF1	6.44E-06	4.34E-06	2.80E-06	2.75E-06	1.73E-06	5.56E-06	2.25E-06	3.79E-06
TAF10	0.000133	0.000129	4.82E-05	6.92E-05	8.49E-05	9.67E-05	0.000122	0.000107
TAF11	2.86E-05	3.83E-05	1.18E-05	1.47E-05	1.17E-05	1.79E-05	1.33E-05	1.78E-05
TAF12	4.15E-05	1.82E-05	1.21E-05	1.67E-05	8.25E-06	1.91E-05	1.84E-05	2.17E-05
TAF13	1.97E-05	2.25E-05	1.27E-05	2.13E-05	9.76E-07	3.27E-05	1.06E-05	1.34E-05
TAF15	0.000116	5.41E-05	3.66E-05	4.73E-05	4.93E-05	8.22E-05	6.26E-05	4.11E-05
TAF1A	1.22E-06	2.13E-06	7.09E-07	8.38E-07	9.84E-08	2.42E-06	6.72E-07	1.52E-06
TAF1B	6.81E-06	4.40E-06	1.82E-06	2.25E-06	4.71E-07	2.75E-06	1.15E-06	2.20E-06
TAF1C	8.89E-06	5.42E-06	3.71E-06	7.13E-06	4.81E-06	5.86E-06	6.43E-06	5.67E-06
TAF1D	2.85E-05	6.01E-06	8.74E-06	1.07E-05	8.77E-06	1.71E-05	1.33E-05	1.60E-05
TAF1L	1.87E-07	7.64E-08	1.07E-07	3.95E-08	9.26E-09	1.40E-07	2.46E-08	8.42E-08
TAF2	2.09E-05	7.09E-06	3.73E-06	3.97E-06	5.90E-07	4.94E-06	4.12E-06	3.66E-06
TAF3	2.80E-06	1.66E-06	2.03E-06	2.38E-06	1.39E-06	1.91E-06	2.09E-06	1.98E-06
TAF4	8.30E-06	5.47E-06	2.91E-06	2.39E-06	1.41E-06	3.55E-06	2.17E-06	2.45E-06
TAF4B	1.08E-06	3.72E-08	8.30E-08	3.25E-07	1.63E-08	6.16E-08	3.24E-08	4.87E-08
TAF5	1.81E-06	7.56E-07	8.14E-07	7.92E-07	1.06E-06	1.18E-06	1.13E-06	1.26E-06
TAF5L	8.55E-06	1.17E-05	6.20E-06	7.10E-06	2.47E-06	1.04E-05	7.26E-06	1.20E-05
TAF6	4.06E-05	2.73E-05	8.78E-06	1.15E-05	1.26E-05	1.65E-05	1.35E-05	1.62E-05
TAF6L	1.07E-05	1.35E-05	8.78E-06	9.53E-06	5.78E-06	1.14E-05	7.96E-06	1.30E-05
TAF7	5.98E-05	6.28E-05	3.74E-05	3.94E-05	1.74E-05	1.78E-05	2.99E-05	7.81E-05
TAF7L	1.01E-08	8.93E-08	2.36E-08	1.38E-08	6.80E-08	8.38E-08	1.53E-07	4.98E-08
TAF8	6.86E-06	3.77E-06	3.91E-06	6.46E-06	1.66E-06	1.27E-05	4.28E-06	4.47E-06
TAF9	0.00011	9.26E-05	4.18E-05	5.43E-05	3.11E-05	7.79E-05	4.70E-05	5.86E-05
TAF9B	7.61E-06	9.82E-06	5.81E-06	7.21E-06	5.48E-06	1.12E-05	5.56E-06	1.03E-05
TAGAP	3.36E-06	3.48E-07	1.19E-06	4.12E-06	2.01E-08	6.77E-07	1.02E-06	5.19E-07
TAGLN	0.00017	4.06E-05	4.19E-05	0.000278	0.00012	6.96E-05	0.000128	8.27E-05
TAGLN2	0.000634	0.000231	0.000126	0.000495	0.000105	0.000256	0.000414	0.00079
TAGLN3	1.07E-07	0	0	5.29E-08	1.66E-08	0	0	0
tAKR	3.42E-07	1.79E-06	4.63E-06	3.68E-06	2.50E-06	7.14E-06	7.91E-06	2.25E-06
TAL1	7.61E-07	2.51E-07	3.64E-07	4.87E-07	3.79E-07	1.62E-07	6.54E-07	4.29E-07
TAL2	1.04E-06	5.12E-07	5.54E-08	5.93E-08	1.24E-07	2.67E-07	1.77E-07	2.87E-07
TALDO1	0.000196	0.000207	0.000128	0.000117	0.000233	0.000579	0.000132	0.000196
TANC1	5.47E-06	2.77E-06	2.36E-06	4.96E-06	6.05E-07	2.42E-06	2.86E-06	3.02E-06
TANC2	1.31E-05	2.46E-07	4.14E-07	5.57E-07	1.24E-07	2.08E-07	3.65E-07	2.64E-07
TANK	4.45E-05	2.54E-05	2.03E-05	2.33E-05	5.70E-06	2.22E-05	1.57E-05	2.15E-05
TAOK1	3.85E-06	3.15E-06	1.89E-06	9.19E-07	8.74E-08	1.81E-06	3.45E-07	9.16E-07
TAOK2	1.49E-05	1.78E-05	6.97E-06	7.12E-06	4.15E-06	1.51E-05	1.12E-05	1.21E-05
TAOK3	1.53E-05	1.19E-05	8.06E-06	1.18E-05	3.44E-06	1.29E-05	1.35E-05	7.67E-06
TAP1	0.000165	2.61E-05	1.73E-05	2.94E-05	5.16E-06	4.24E-05	2.12E-05	7.56E-05
TAP2	4.73E-05	2.32E-05	1.19E-05	9.41E-06	2.81E-06	1.92E-05	5.24E-06	3.74E-05

TAPBP	8.77E-05	0.000171	7.22E-05	7.90E-05	5.30E-05	8.01E-05	8.49E-05	0.000108
TAPBPL	3.22E-05	2.54E-05	2.12E-05	2.92E-05	4.57E-06	3.16E-05	1.28E-05	3.20E-05
TAPT1	7.98E-06	1.41E-05	1.50E-05	1.18E-05	5.24E-06	1.39E-05	9.28E-06	1.01E-05
TARBP1	3.72E-06	2.69E-06	1.27E-06	1.88E-06	7.42E-07	6.66E-06	2.06E-06	5.24E-06
TARBP2	1.72E-05	2.41E-05	1.18E-05	1.25E-05	1.73E-05	1.43E-05	1.77E-05	1.12E-05
TARDBP	5.05E-05	3.73E-05	2.87E-05	2.94E-05	1.79E-05	3.89E-05	2.78E-05	2.62E-05
TARM1	6.12E-08	0	0	0	0	0	0	0
TARP	1.06E-06	8.90E-08	5.08E-07	7.95E-07	2.36E-08	3.83E-07	2.28E-07	2.55E-07
TARS	3.21E-05	5.48E-05	3.62E-05	2.54E-05	7.50E-06	3.10E-05	1.47E-05	3.40E-05
TARS2	1.03E-05	2.55E-05	1.94E-05	7.65E-06	5.98E-06	4.06E-05	1.81E-05	2.81E-05
TARSL2	5.04E-06	2.78E-06	2.41E-06	2.23E-06	1.24E-06	2.31E-06	1.36E-06	2.81E-06
TAS1R1	1.24E-08	6.57E-08	0	5.19E-08	2.14E-08	4.61E-08	6.00E-08	1.47E-08
TAS1R2	0	0	0	1.14E-08	0	0	0	0
TAS1R3	7.33E-08	2.17E-07	1.90E-07	1.34E-07	1.57E-08	3.40E-08	4.44E-08	8.07E-08
TAS2R1	0	2.13E-08	0	0	0	0	0	0
TAS2R10	1.18E-07	0	0	3.65E-08	2.55E-08	2.75E-08	0	0
TAS2R13	0	0	0	0	0	0	0	0
TAS2R14	4.96E-07	2.76E-07	1.25E-07	3.51E-08	0	1.26E-07	1.40E-07	1.91E-07
TAS2R16	0	0	0	0	0	0	0	0
TAS2R19	6.10E-08	7.13E-08	0	0	0	2.85E-08	0	0
TAS2R20	1.01E-06	9.97E-08	1.58E-07	1.39E-07	2.49E-07	2.10E-07	2.91E-07	2.59E-07
TAS2R3	0	2.74E-08	2.58E-08	0	0	0	0	0
TAS2R30	0	0	0	0	0	0	0	0
TAS2R31	2.60E-08	0	2.85E-08	3.22E-08	0	2.43E-08	0	0
TAS2R38	9.09E-08	0	0	0	0	0	0	0
TAS2R39	0	0	0	0	0	0	0	0
TAS2R4	6.09E-08	3.56E-08	6.75E-08	0	0	0	2.89E-08	0
TAS2R40	0	0	0	0	0	0	0	0
TAS2R41	0	0	0	0	0	0	0	0
TAS2R42	8.59E-08	0	0	3.55E-08	0	0	0	0
TAS2R43	5.17E-08	3.00E-08	0	0	0	0	0	0
TAS2R46	8.77E-08	0	0	0	0	0	0	0
TAS2R5	4.74E-07	1.04E-07	2.44E-08	0	0	6.30E-08	4.32E-08	0
TAS2R50	0	0	0	0	0	2.49E-08	0	0
TAS2R60	0	0	1.24E-07	3.50E-08	0	0	0	0
TAS2R7	0	0	0	0	0	0	0	0
TAS2R8	0	3.41E-08	0	0	0	0	0	0
TAS2R9	0	0	0	0	0	0	0	0
TASP1	4.13E-06	1.92E-06	1.97E-06	2.69E-06	7.47E-07	3.48E-06	1.34E-06	2.35E-06
TAT	7.19E-05	0.001027	0.000721	0.000467	0.000103	0.000677	0.00039	0.001735
TATDN1	3.79E-05	2.39E-05	9.78E-06	1.04E-05	7.63E-06	1.77E-05	3.65E-05	1.87E-05
TATDN2	1.21E-05	7.75E-06	3.88E-06	5.44E-06	4.31E-06	9.71E-06	7.66E-06	7.46E-06
TATDN3	7.71E-06	1.82E-05	4.11E-06	4.76E-06	1.58E-06	7.95E-06	4.37E-06	1.25E-05
TAX1BP1	0.000108	0.000105	2.71E-05	3.79E-05	1.17E-05	6.39E-05	2.35E-05	5.23E-05
TAX1BP3	5.65E-05	9.84E-06	1.26E-05	7.10E-05	1.50E-05	2.10E-05	6.73E-05	2.90E-05
TAZ	1.48E-05	1.55E-05	5.28E-06	9.26E-06	1.74E-05	8.51E-06	1.42E-05	2.44E-05
TBC1D1	1.57E-05	8.50E-06	8.27E-06	8.04E-06	4.32E-06	7.59E-06	6.20E-06	8.58E-06
TBC1D10A	1.17E-05	1.96E-05	6.76E-06	1.34E-05	7.01E-06	1.71E-05	1.22E-05	1.93E-05
TBC1D10B	1.77E-05	1.41E-05	5.67E-06	8.42E-06	5.81E-06	6.52E-06	1.27E-05	7.45E-06
TBC1D10C	6.01E-06	4.94E-07	2.46E-06	5.13E-06	5.05E-07	2.68E-06	3.07E-06	1.91E-06
TBC1D12	3.09E-06	1.44E-06	9.67E-07	9.24E-07	4.49E-07	9.86E-07	1.12E-06	1.55E-06
TBC1D13	1.01E-05	4.95E-06	4.39E-06	3.86E-06	9.34E-06	6.46E-06	8.59E-06	1.21E-05
TBC1D14	1.67E-05	1.21E-05	8.56E-06	6.60E-06	5.18E-06	1.51E-05	1.11E-05	1.29E-05
TBC1D15	1.19E-05	1.31E-05	1.02E-05	9.69E-06	2.67E-06	1.35E-05	7.23E-06	1.02E-05
TBC1D16	1.12E-05	8.84E-06	1.83E-06	3.82E-06	4.80E-06	7.38E-06	4.15E-06	2.52E-06
TBC1D17	2.13E-05	1.83E-05	2.17E-05	2.01E-05	2.09E-05	2.51E-05	2.50E-05	2.19E-05
TBC1D19	1.10E-06	6.47E-07	4.16E-07	1.19E-06	1.75E-07	1.63E-06	7.54E-07	1.18E-06
TBC1D2	1.14E-05	8.34E-06	4.09E-06	5.46E-06	1.88E-06	4.21E-06	4.63E-06	3.42E-06

TBC1D20	1.47E-05	1.13E-05	6.16E-06	7.02E-06	7.82E-06	8.70E-06	8.82E-06	8.82E-06
TBC1D21	0	0	0	0	0	0	0	0
TBC1D22A	1.04E-05	1.13E-05	5.50E-06	1.00E-05	1.34E-05	1.29E-05	1.29E-05	1.20E-05
TBC1D22B	7.36E-06	3.18E-06	1.27E-06	3.23E-06	1.47E-06	2.65E-06	3.16E-06	2.78E-06
TBC1D23	1.63E-05	2.35E-05	1.19E-05	1.12E-05	3.05E-06	1.47E-05	9.64E-06	6.13E-06
TBC1D24	6.85E-06	6.72E-06	4.16E-06	2.29E-06	1.72E-06	3.68E-06	3.71E-06	3.76E-06
TBC1D25	4.50E-06	5.69E-06	2.61E-06	4.24E-06	2.03E-06	4.83E-06	4.70E-06	8.45E-06
TBC1D26	0	2.23E-07	0	0	2.22E-08	0	0	0
TBC1D28	0	0	0	0	0	0	0	0
TBC1D29	0	0	2.42E-08	0	1.93E-08	2.08E-08	0	0
TBC1D2B	2.41E-05	4.15E-06	1.11E-05	9.87E-06	4.40E-06	1.29E-05	1.28E-05	9.37E-06
TBC1D3	2.01E-05	3.76E-06	2.63E-06	4.01E-06	1.36E-06	3.45E-06	2.31E-06	1.47E-06
TBC1D3B	6.93E-06	1.69E-06	1.19E-06	3.23E-06	3.22E-06	1.13E-06	1.74E-06	6.96E-07
TBC1D3C	3.07E-08	3.13E-07	1.65E-07	3.74E-07	1.72E-06	1.26E-07	5.68E-07	1.99E-07
TBC1D3G	2.50E-06	0	5.88E-08	0	9.68E-09	0	#######	0
TBC1D3H	3.26E-07	2.77E-08	2.14E-08	6.28E-08	1.21E-07	3.21E-08	7.70E-08	4.52E-08
TBC1D3P2	1.16E-08	0	0	0	9.97E-09	0	0	0
TBC1D4	9.37E-06	5.21E-06	6.26E-06	5.38E-06	1.13E-06	1.58E-05	3.47E-06	6.10E-06
TBC1D5	1.43E-05	1.05E-05	6.34E-06	6.84E-06	2.91E-06	9.45E-06	6.74E-06	7.52E-06
TBC1D7	1.95E-05	1.08E-05	5.95E-06	9.55E-06	1.39E-05	1.67E-05	9.83E-06	1.63E-05
TBC1D8	4.61E-05	8.14E-06	6.96E-06	9.87E-06	4.89E-06	1.01E-05	1.01E-05	8.37E-06
TBC1D8B	6.51E-06	2.08E-06	3.39E-06	1.77E-06	9.67E-07	2.78E-06	7.20E-07	3.71E-06
TBC1D9	8.57E-06	4.31E-06	4.01E-06	8.79E-06	2.68E-06	1.21E-05	8.39E-06	3.87E-06
TBC1D9B	3.53E-05	1.99E-05	2.90E-05	2.45E-05	1.41E-05	4.16E-05	2.10E-05	4.42E-05
TBCA	0.000209	0.000153	0.000125	0.000122	8.86E-05	0.000304	0.000163	0.000153
TBCB	4.17E-05	2.33E-05	1.87E-05	3.67E-05	2.53E-05	3.82E-05	3.87E-05	2.59E-05
TBCC	1.87E-05	1.35E-05	7.73E-06	7.09E-06	7.72E-06	1.10E-05	1.03E-05	1.56E-05
TBCCD1	8.07E-06	6.13E-06	3.79E-06	4.96E-06	1.09E-06	4.69E-06	3.62E-06	4.70E-06
TBCD	2.62E-05	2.27E-05	1.56E-05	1.63E-05	2.70E-05	2.26E-05	1.47E-05	1.38E-05
TBCE	1.08E-05	8.13E-06	6.87E-06	7.48E-06	3.26E-06	2.30E-05	1.16E-05	2.65E-05
TBCEL	5.42E-06	3.29E-06	7.84E-06	4.44E-06	6.07E-07	5.36E-06	1.61E-06	1.03E-05
TBCK	7.58E-06	2.67E-06	5.61E-06	5.08E-06	6.85E-07	1.47E-05	3.40E-06	4.39E-06
TBK1	1.49E-05	1.46E-05	8.63E-06	7.71E-06	1.81E-06	1.21E-05	5.50E-06	1.06E-05
TBKBP1	4.68E-06	3.19E-06	6.62E-07	1.07E-06	1.23E-06	4.07E-07	1.86E-06	1.56E-06
TBL1X	1.12E-05	7.26E-06	9.33E-06	4.55E-06	4.06E-06	1.28E-05	6.53E-06	1.58E-05
TBL1XR1	2.12E-05	1.21E-05	8.85E-06	1.18E-05	4.40E-06	1.31E-05	1.07E-05	1.65E-05
TBL1Y	0	0	0	1.38E-07	0	0	6.93E-08	1.50E-08
TBL2	1.62E-05	1.58E-05	7.96E-06	9.73E-06	9.99E-06	1.08E-05	1.17E-05	1.90E-05
TBL3	2.69E-05	2.97E-05	1.47E-05	1.51E-05	2.03E-05	1.74E-05	1.89E-05	1.69E-05
TBP	9.12E-06	5.55E-06	5.03E-06	5.04E-06	1.59E-06	6.51E-06	5.41E-06	5.24E-06
TBPL1	8.97E-06	9.95E-06	7.36E-06	8.51E-06	3.43E-06	1.35E-05	1.12E-05	6.35E-06
TBPL2	0	0	0	0	0	0	0	0
TBR1	1.06E-08	1.20E-08	0	0	0	0	0	0
TBRG1	1.03E-05	5.70E-06	6.01E-06	6.65E-06	3.06E-06	6.48E-06	4.41E-06	8.23E-06
TBRG4	5.95E-05	5.41E-05	2.45E-05	2.68E-05	2.07E-05	3.50E-05	2.92E-05	5.56E-05
TBX1	2.72E-07	3.89E-08	7.19E-08	1.68E-07	1.97E-08	0	3.28E-08	0
TBX10	6.39E-08	3.46E-07	1.52E-07	1.18E-07	2.89E-07	3.86E-07	3.54E-07	3.77E-07
TBX15	1.88E-06	1.73E-05	3.28E-05	2.22E-05	9.98E-06	2.42E-07	2.17E-05	9.90E-06
TBX18	2.24E-08	1.88E-08	4.60E-08	9.56E-08	4.80E-09	5.69E-08	4.35E-08	3.51E-08
TBX19	1.73E-06	7.09E-07	3.27E-07	4.65E-07	1.44E-07	6.41E-07	4.66E-07	1.01E-06
TBX2	6.16E-06	2.34E-06	2.28E-06	1.84E-06	4.05E-06	1.57E-06	3.35E-06	4.74E-06
TBX20	1.86E-08	0	1.39E-07	4.35E-07	0	0	1.79E-08	0
TBX21	7.65E-07	2.77E-07	5.39E-07	9.92E-07	7.04E-08	4.39E-07	6.62E-07	2.54E-07
TBX22	0	0	0	0	0	0	0	0
TBX3	1.50E-06	1.32E-05	9.67E-06	8.93E-06	2.09E-06	6.20E-05	1.19E-05	5.45E-06
TBX4	2.46E-07	1.07E-08	0	0	0	9.40E-06	9.22E-09	0
TBX5	0	7.05E-09	0	0	5.40E-09	0	2.53E-08	0
TBX6	7.20E-07	7.24E-07	3.13E-07	2.97E-07	1.85E-07	3.15E-07	1.86E-07	4.44E-07

TBXA2R	1.64E-06	6.17E-07	3.63E-06	5.63E-06	2.48E-06	7.70E-07	1.16E-06	1.01E-06
TBXAS1	8.01E-06	1.30E-06	5.45E-06	1.20E-05	1.65E-06	3.88E-06	9.19E-06	9.77E-07
TC2N	1.72E-05	1.58E-07	1.81E-06	2.55E-06	9.19E-08	3.18E-07	1.28E-06	6.32E-06
TCAM1P	1.40E-08	0	0	0	1.20E-08	0	2.72E-08	8.21E-08
TCAP	1.40E-07	5.21E-07	4.32E-07	5.17E-06	4.81E-06	2.88E-07	2.99E-06	2.09E-07
TCEA1	5.32E-05	6.68E-05	2.55E-05	1.95E-05	1.58E-05	2.60E-05	5.13E-05	2.64E-05
TCEA2	7.15E-06	1.29E-05	2.20E-05	4.14E-05	3.20E-05	7.16E-06	2.64E-05	1.78E-05
TCEA3	7.41E-05	5.32E-05	9.62E-05	7.12E-05	6.62E-05	8.11E-05	7.34E-05	5.21E-05
TCEAL1	1.23E-05	1.41E-05	9.39E-06	9.87E-06	7.88E-06	3.40E-05	9.50E-06	1.76E-05
TCEAL2	1.07E-06	0	1.29E-07	1.47E-07	6.17E-08	2.22E-08	2.27E-08	0
TCEAL3	3.31E-05	5.67E-06	2.67E-06	1.12E-05	4.15E-06	3.76E-06	5.83E-06	3.17E-06
TCEAL4	6.43E-05	5.84E-05	4.59E-05	6.81E-05	6.80E-05	7.51E-05	5.44E-05	9.74E-05
TCEAL5	5.09E-07	2.55E-07	0	3.00E-07	5.74E-08	1.03E-07	2.12E-08	6.57E-08
TCEAL6	1.55E-06	1.47E-07	6.86E-08	5.76E-07	1.67E-07	1.20E-07	9.34E-08	1.67E-07
TCEAL7	1.45E-06	1.06E-07	8.21E-07	1.19E-06	2.98E-07	1.50E-07	3.73E-07	2.73E-07
TCEAL8	6.57E-05	2.57E-05	2.13E-05	2.82E-05	3.35E-06	1.77E-05	1.01E-05	5.92E-05
TCEANC	1.29E-06	4.23E-07	1.07E-06	6.25E-07	1.80E-07	1.03E-06	6.71E-07	9.79E-07
TCEB1	0.000134	0.000102	3.51E-05	5.02E-05	8.35E-05	5.77E-05	0.000118	9.77E-05
TCEB2	0.000248	0.000299	0.000154	0.00023	0.000403	0.000244	0.00024	0.000233
TCEB3	1.67E-05	1.45E-05	1.49E-05	1.31E-05	6.14E-06	2.24E-05	1.07E-05	1.49E-05
TCEB3B	2.28E-08	1.02E-07	0	9.29E-09	0	0	0	1.11E-08
TCEB3C	0	0	0	0	0	0	0	0
TCERG1	8.87E-06	4.47E-06	2.72E-06	3.77E-06	8.49E-07	4.80E-06	2.67E-06	7.13E-06
TCERG1L	0	0	0	1.09E-08	0	0	8.66E-09	0
TCF12	1.78E-05	1.04E-05	8.89E-06	1.01E-05	2.48E-06	1.07E-05	6.72E-06	7.61E-06
TCF15	4.39E-07	5.77E-07	2.25E-08	1.55E-07	3.11E-06	4.87E-07	1.70E-06	7.76E-07
TCF19	8.93E-06	1.23E-05	6.46E-07	1.05E-06	1.88E-06	2.63E-06	6.97E-07	3.31E-06
TCF20	6.63E-06	5.21E-06	3.22E-06	3.46E-06	2.07E-06	7.29E-06	3.22E-06	5.28E-06
TCF21	1.17E-06	4.29E-07	4.54E-06	4.90E-06	6.09E-07	6.16E-07	1.74E-06	4.23E-07
TCF23	0	0	1.12E-07	0	0	4.48E-08	0	0
TCF25	6.15E-05	3.33E-05	3.89E-05	4.36E-05	2.93E-05	4.73E-05	4.84E-05	4.61E-05
TCF3	1.22E-05	3.77E-06	3.57E-06	6.13E-06	4.41E-06	6.89E-06	7.91E-06	4.32E-06
TCF4	4.83E-06	2.56E-06	1.85E-06	3.55E-06	1.18E-06	1.93E-06	2.57E-06	3.40E-06
TCF7	9.40E-06	1.08E-06	2.60E-06	5.23E-06	8.71E-07	1.72E-05	4.08E-06	8.91E-07
TCF7L1	1.48E-05	2.23E-05	4.75E-06	8.08E-06	7.08E-06	1.46E-06	7.47E-06	7.11E-06
TCF7L2	1.04E-05	4.42E-06	8.78E-06	8.88E-06	4.08E-06	1.23E-05	6.16E-06	6.19E-06
TCFL5	7.83E-06	5.73E-06	3.25E-06	2.60E-06	3.98E-06	6.08E-06	4.34E-06	3.25E-06
TCHH	6.79E-08	2.16E-08	1.65E-07	1.66E-07	0	5.08E-08	1.04E-07	2.36E-08
TCHHL1	0	0	0	0	0	0	0	0
TCHP	5.66E-06	3.50E-06	2.62E-06	3.66E-06	8.02E-07	5.33E-06	3.68E-06	3.81E-06
TCIRG1	5.38E-05	3.76E-05	2.53E-05	3.56E-05	1.02E-05	2.73E-05	2.95E-05	7.45E-05
TCL1A	4.15E-07	0	9.63E-08	1.02E-06	1.56E-08	1.51E-07	8.67E-08	5.34E-08
TCL1B	0	0	0	1.81E-08	0	0	0	0
TCL6	2.99E-08	6.97E-08	1.30E-08	0	0	0	0	0
TCN1	5.04E-07	1.79E-08	0	0	0	0	1.52E-08	0
TCN2	1.58E-05	1.05E-05	1.43E-05	1.00E-05	6.62E-06	1.19E-05	1.26E-05	7.38E-06
TCOF1	1.11E-05	8.87E-06	3.38E-06	4.71E-06	4.43E-06	5.86E-06	6.96E-06	6.82E-06
TCP1	8.96E-05	9.63E-05	4.36E-05	4.88E-05	2.37E-05	7.67E-05	6.45E-05	4.66E-05
TCP10	0	1.21E-07	1.03E-08	0	3.78E-08	0	1.92E-08	0
TCP10L	1.64E-07	7.58E-06	1.90E-06	2.00E-06	1.84E-07	3.89E-06	2.42E-06	1.20E-07
TCP10L2	0	7.37E-08	0	4.00E-08	2.81E-08	0	0	0
TCP11	0	3.25E-08	0	1.68E-08	6.15E-08	1.72E-07	2.76E-08	2.11E-08
TCP11L1	7.30E-06	2.64E-06	1.87E-06	2.97E-06	8.44E-07	3.35E-06	1.63E-06	2.66E-06
TCP11L2	7.97E-06	1.38E-06	1.01E-06	1.62E-06	6.27E-08	1.33E-06	6.11E-07	1.14E-06
TCTA	3.66E-05	3.87E-05	2.80E-05	2.10E-05	2.40E-05	4.10E-05	9.64E-06	4.44E-05
TCTE1	0	6.60E-08	0	1.78E-08	0	4.04E-08	1.40E-08	0
TCTE3	2.31E-06	5.09E-07	3.62E-07	2.83E-07	3.31E-07	4.39E-07	2.45E-07	3.56E-07
TCTEX1D1	1.46E-07	1.99E-06	2.47E-06	8.33E-07	9.00E-09	8.73E-08	2.43E-07	6.55E-08

TCTEX1D2	1.74E-06	4.23E-06	5.71E-07	2.63E-06	3.24E-06	5.29E-06	6.45E-06	1.76E-06
TCTEX1D4	1.21E-07	6.97E-08	1.09E-07	1.75E-07	1.75E-08	1.13E-07	1.16E-07	0
TCTN1	5.81E-06	1.14E-05	6.02E-06	8.82E-06	1.45E-06	1.37E-05	6.22E-06	6.00E-06
TCTN2	1.41E-06	2.60E-07	6.12E-07	1.68E-06	2.26E-07	5.25E-07	8.83E-07	2.11E-07
TCTN3	3.15E-05	1.68E-05	1.41E-05	1.62E-05	7.38E-06	1.88E-05	1.60E-05	1.48E-05
TDG	9.95E-06	7.73E-06	2.92E-06	5.27E-06	1.34E-06	4.04E-06	3.20E-06	3.09E-06
TDGF1	9.29E-08	1.21E-06	1.05E-06	1.78E-06	5.69E-07	1.25E-06	9.09E-07	1.69E-05
TDGF3	1.72E-08	6.78E-08	6.25E-08	4.21E-08	2.22E-08	7.97E-08	4.17E-08	1.36E-06
TDH	0	0	0	0	0	8.18E-08	0	0
TDO2	1.42E-05	9.13E-05	0.000524	0.000204	5.46E-05	6.88E-06	0.00032	0.000289
TDP1	4.29E-06	1.42E-06	1.83E-06	3.46E-06	1.04E-06	3.51E-06	2.10E-06	2.42E-06
TDP2	5.35E-05	6.20E-05	2.14E-05	2.35E-05	1.76E-05	3.09E-05	2.29E-05	2.52E-05
TDRD1	1.55E-08	0	2.10E-08	1.43E-07	0	0	4.96E-09	0
TDRD10	1.14E-06	5.96E-08	5.40E-07	7.96E-07	8.21E-07	2.08E-07	4.41E-07	3.21E-07
TDRD12	0	6.53E-09	0	0	0	0	0	0
TDRD3	5.51E-06	3.61E-06	4.08E-06	6.33E-06	3.75E-06	6.56E-06	4.62E-06	5.70E-06
TDRD5	4.93E-07	0	0	0	0	0	2.77E-08	0
TDRD6	1.15E-07	2.92E-08	7.02E-08	5.26E-08	6.18E-08	9.41E-08	2.87E-08	9.57E-08
TDRD7	1.50E-05	6.76E-06	5.05E-06	7.21E-06	1.29E-06	7.09E-06	4.25E-06	7.50E-06
TDRD9	2.03E-08	1.59E-08	4.87E-09	6.95E-08	1.16E-08	3.07E-08	0	6.95E-09
TDRG1	0	1.12E-07	0	0	0	0	0	0
TDRKH	2.72E-06	5.80E-06	1.59E-06	1.14E-06	2.36E-07	5.45E-06	1.85E-06	3.10E-06
TEAD1	2.11E-05	1.20E-05	5.14E-06	7.45E-06	1.32E-06	1.01E-05	6.02E-06	8.07E-06
TEAD2	1.44E-05	3.64E-06	2.59E-06	7.13E-06	2.26E-06	4.98E-06	5.98E-06	1.07E-05
TEAD3	9.01E-06	6.29E-06	3.69E-06	5.73E-06	2.46E-06	6.86E-06	5.65E-06	9.19E-06
TEAD4	1.11E-05	4.80E-07	6.06E-07	4.08E-06	1.97E-06	7.10E-07	1.63E-06	2.46E-06
TEC	1.39E-06	5.89E-07	9.84E-07	1.11E-06	9.78E-08	1.97E-06	4.00E-07	5.38E-07
TECPR1	9.64E-06	3.65E-06	1.61E-06	1.75E-06	3.45E-07	3.50E-06	1.35E-06	4.55E-06
TECPR2	4.03E-06	1.57E-06	1.76E-06	1.89E-06	1.47E-06	3.18E-06	1.90E-06	3.07E-06
TECR	8.83E-05	6.40E-05	7.63E-05	5.97E-05	7.59E-05	8.88E-05	8.31E-05	7.81E-05
TECRL	0	7.55E-07	0	1.01E-08	0	0	0	0
TECTA	6.57E-08	1.54E-08	1.48E-07	1.27E-07	5.93E-09	3.20E-08	2.36E-08	1.42E-07
TECTB	2.70E-08	0	0	0	0	3.69E-06	0	2.02E-07
TEDDM1	0	0	0	0	0	3.48E-08	0	0
TEF	6.88E-06	9.11E-06	6.72E-06	2.80E-06	2.54E-05	6.01E-06	2.06E-05	1.02E-05
TEK	3.16E-06	9.88E-07	3.43E-06	2.01E-06	6.64E-07	8.89E-07	8.61E-07	1.63E-06
TEKT1	0	0	0	0	0	0	1.97E-08	3.04E-08
TEKT2	1.64E-08	0	5.93E-08	1.59E-07	1.42E-08	1.53E-08	0	4.86E-08
TEKT3	1.36E-08	0	1.43E-08	8.35E-08	0	0	1.31E-08	4.01E-08
TEKT4	0	1.95E-08	1.82E-08	1.68E-07	7.24E-07	4.05E-07	2.64E-07	1.52E-07
TEKT5	4.58E-08	5.91E-07	6.46E-08	1.31E-07	9.21E-08	5.68E-08	6.18E-07	1.80E-07
TELO2	1.19E-05	1.36E-05	4.72E-06	6.54E-06	4.86E-06	6.55E-06	7.43E-06	8.65E-06
TENC1	2.22E-05	1.76E-05	4.55E-05	2.96E-05	3.54E-05	1.95E-05	3.23E-05	4.12E-05
TEP1	1.14E-05	3.48E-06	3.27E-06	4.48E-06	1.40E-06	3.36E-06	3.23E-06	2.90E-06
TEPP	1.61E-08	1.83E-08	0	0	0	0	1.55E-08	0
TERC	1.64E-07	0	0	0	2.92E-07	0	2.98E-07	0
TERF1	1.51E-05	9.18E-06	6.24E-06	6.01E-06	3.69E-06	1.33E-05	1.14E-05	9.65E-06
TERF2	1.17E-05	6.27E-06	6.41E-06	8.64E-06	2.41E-06	9.56E-06	7.28E-06	8.22E-06
TERF2IP	3.04E-05	2.17E-05	2.33E-05	3.34E-05	1.16E-05	3.30E-05	2.87E-05	3.01E-05
TERT	1.36E-07	1.02E-08	0	0	1.72E-07	3.81E-06	1.09E-07	6.85E-08
TES	2.05E-05	1.12E-06	3.53E-06	1.71E-05	5.05E-07	6.18E-06	7.33E-06	1.92E-06
TESC	8.97E-05	1.24E-06	2.06E-06	1.33E-05	1.48E-05	8.79E-07	2.12E-06	1.81E-05
TESK1	8.60E-06	1.77E-05	6.82E-06	1.20E-05	7.03E-06	1.24E-05	1.22E-05	1.51E-05
TESK2	2.09E-06	1.29E-06	3.53E-06	3.47E-06	9.98E-07	1.98E-06	1.47E-06	9.95E-07
TET1	4.78E-07	3.29E-07	8.94E-08	7.32E-08	1.39E-08	5.55E-08	3.61E-08	4.05E-08
TET2	1.98E-06	6.08E-07	1.76E-06	1.37E-06	2.91E-07	1.07E-06	7.22E-07	1.24E-06
TET3	4.61E-06	2.05E-06	7.20E-07	1.14E-06	2.59E-07	1.21E-06	8.73E-07	9.25E-07
TEX10	5.63E-06	2.35E-06	2.41E-06	6.29E-06	1.89E-06	3.99E-06	3.51E-06	3.81E-06

TEX101	0	2.56E-08	2.41E-08	0	0	0	0	0
TEX11	4.41E-08	2.49E-08	0	0	2.84E-08	2.04E-08	0	1.62E-08
TEX12	5.04E-08	0	0	0	0	2.35E-08	2.40E-08	7.50E-08
TEX13A	0	0	0	0	0	0	0	0
TEX13B	4.09E-08	0	0	0	0	0	0	0
TEX14	1.22E-07	9.22E-08	4.50E-08	7.27E-08	4.84E-08	3.91E-08	9.15E-09	6.88E-09
TEX15	2.05E-07	0	1.34E-08	1.07E-08	0	2.02E-09	0	0
TEX19	1.01E-07	1.66E-06	1.33E-08	0	0	1.18E-08	1.22E-08	1.31E-07
TEX2	2.28E-05	2.98E-05	2.00E-05	1.03E-05	5.12E-06	2.16E-05	6.84E-06	1.49E-05
TEX261	4.62E-05	3.02E-05	2.04E-05	2.03E-05	1.77E-05	3.45E-05	2.50E-05	2.67E-05
TEX264	7.90E-05	0.000115	5.58E-05	6.32E-05	0.000108	6.88E-05	7.83E-05	9.03E-05
TEX28	0	0	0	0	0	0	0	0
TEX9	8.36E-08	1.77E-07	5.72E-08	3.35E-07	2.06E-08	4.92E-07	1.66E-08	1.18E-07
TF	0.00145	0.008942	0.008106	0.003736	0.007379	0.002591	0.002179	0.001917
TFAM	1.85E-05	1.60E-05	1.25E-05	1.02E-05	2.68E-06	1.61E-05	8.56E-06	1.08E-05
TFAMP1	1.07E-08	3.61E-08	0	0	0	0	0	0
TFAP2A	6.31E-07	3.97E-07	7.52E-08	1.52E-07	1.89E-08	2.22E-08	5.74E-07	8.24E-07
TFAP2B	0	0	0	0	0	0	3.79E-09	0
TFAP2C	0	9.81E-09	3.36E-08	8.53E-08	0	0	1.18E-07	0
TFAP2D	0	0	0	0	0	0	0	0
TFAP2E	1.04E-06	1.47E-07	6.81E-08	9.32E-08	5.61E-08	6.05E-08	1.05E-07	6.39E-08
TFAP4	3.23E-06	3.54E-06	2.13E-06	1.65E-06	1.33E-06	3.23E-06	4.14E-06	2.88E-06
TFB1M	1.16E-05	9.94E-06	1.09E-05	9.34E-06	2.97E-06	2.27E-05	7.41E-06	5.40E-06
TFB2M	1.02E-05	2.55E-05	2.59E-05	1.19E-05	7.73E-06	5.26E-05	1.62E-05	4.53E-05
TFCP2	1.12E-05	8.89E-06	4.16E-06	4.15E-06	2.35E-06	7.80E-06	3.61E-06	2.54E-06
TFCP2L1	3.97E-06	7.79E-08	6.27E-08	1.16E-07	4.79E-09	5.94E-08	9.50E-08	2.86E-08
TFDP1	3.34E-05	7.35E-06	1.67E-05	1.72E-05	5.85E-06	2.39E-05	1.14E-05	7.47E-06
TFDP2	1.24E-05	1.92E-05	7.05E-06	6.41E-06	4.92E-06	9.09E-06	6.97E-06	6.96E-06
TFDP3	0	0	1.54E-08	3.57E-08	0	0	1.40E-08	0
TFE3	2.34E-05	2.07E-05	7.87E-06	1.48E-05	3.91E-06	1.34E-05	1.62E-05	2.50E-05
TFEB	8.35E-06	6.39E-06	5.70E-06	6.39E-06	8.48E-06	3.56E-06	8.71E-06	9.93E-06
TFEC	2.12E-06	1.64E-07	5.26E-07	1.01E-06	7.91E-08	4.35E-07	3.60E-07	1.67E-07
TFF1	0.000395	1.79E-07	0	2.71E-07	0	0	1.11E-06	0
TFF2	3.69E-06	0	9.65E-07	3.66E-07	0	0	0	0
TFF3	3.51E-05	5.97E-07	4.10E-06	8.55E-06	5.45E-06	1.49E-06	5.55E-06	1.37E-06
TFG	0.000113	0.000164	6.68E-05	6.38E-05	5.01E-05	7.12E-05	6.68E-05	0.000104
TFIP11	2.86E-05	3.49E-05	1.44E-05	2.09E-05	3.42E-05	3.01E-05	3.81E-05	3.44E-05
TFPI	0.000175	8.94E-05	8.40E-05	0.000108	2.79E-05	0.000137	7.21E-05	0.00013
TFPI2	2.74E-07	3.92E-06	7.38E-06	1.20E-05	3.73E-07	4.74E-07	1.56E-06	1.02E-07
TFPT	9.66E-06	7.12E-06	6.90E-06	1.21E-05	2.36E-05	8.71E-06	1.62E-05	8.06E-06
TFR2	0.000372	0.001678	0.00058	0.00041	0.00028	0.000314	0.000399	0.000497
TFRC	4.13E-05	3.65E-05	1.23E-05	1.09E-05	5.05E-06	7.97E-06	6.64E-06	3.46E-05
TG	2.22E-08	5.85E-09	1.36E-08	4.52E-08	1.21E-07	2.25E-07	5.41E-08	2.17E-08
TGDS	9.13E-06	9.90E-05	1.32E-05	6.80E-06	1.59E-06	7.74E-06	5.99E-06	6.60E-06
TGFA	2.47E-05	1.43E-07	2.28E-06	4.79E-06	5.03E-08	2.22E-07	3.22E-07	1.72E-07
TGFB1	2.98E-05	5.45E-06	6.60E-06	2.09E-05	3.85E-06	5.44E-06	1.87E-05	5.78E-06
TGFB1I1	7.38E-06	3.62E-06	2.15E-06	4.51E-06	3.63E-06	2.52E-06	7.34E-06	3.56E-06
TGFB2	3.26E-06	4.05E-07	2.36E-07	4.62E-06	3.04E-07	5.26E-06	1.12E-06	1.04E-06
TGFB3	1.05E-05	1.48E-06	1.84E-06	6.29E-06	1.09E-06	2.75E-06	1.83E-06	3.99E-06
TGFBI	0.000116	6.13E-05	9.45E-05	0.000455	0.000123	6.08E-05	0.00033	0.000254
TGFBR1	1.81E-05	5.72E-06	4.56E-06	6.13E-06	1.39E-06	3.73E-06	5.52E-06	3.75E-06
TGFBR2	2.42E-05	2.46E-05	2.12E-05	2.84E-05	6.69E-06	4.73E-05	2.08E-05	1.86E-05
TGFBR3	1.93E-06	1.49E-05	5.23E-06	7.52E-06	8.90E-07	1.05E-05	2.28E-06	2.66E-06
TGFBRAP1	3.95E-06	2.41E-06	1.98E-06	2.25E-06	1.34E-08	2.56E-06	8.20E-07	1.74E-06
TGIF1	3.36E-05	1.60E-05	3.54E-05	4.92E-05	6.28E-06	5.85E-05	2.43E-05	3.16E-05
TGIF2	2.53E-05	7.69E-06	2.90E-06	6.49E-06	6.70E-06	5.75E-06	8.82E-06	4.39E-06
TGIF2LX	6.60E-08	3.87E-08	0	0	0	0	0	4.94E-08
TGIF2LY	0	0	0	0	0	0	0	4.84E-08

TGM1	3.16E-07	1.72E-07	6.73E-07	1.76E-08	2.22E-07	1.09E-07	5.43E-08	2.11E-08
TGM2	0.00025	9.60E-05	8.69E-05	9.27E-05	1.64E-05	6.38E-05	0.000157	0.000386
TGM3	7.87E-06	2.94E-05	3.35E-07	7.47E-08	4.80E-07	3.24E-05	4.23E-07	3.48E-05
TGM4	0	0	0	9.45E-09	0	1.29E-07	0	0
TGM5	1.48E-08	0	0	0	0	0	1.43E-08	0
TGM6	0	0	0	0	0	0	0	0
TGM7	0	0	0	0	0	0	0	0
TGOLN2	9.58E-05	8.18E-05	7.91E-05	6.52E-05	2.47E-05	7.52E-05	5.84E-05	5.60E-05
TGS1	1.16E-05	5.58E-06	2.85E-06	3.57E-06	9.56E-07	3.91E-06	6.76E-06	3.70E-06
TH	0	0	1.03E-07	9.91E-08	2.78E-08	0	3.10E-08	4.76E-08
TH1L	7.00E-05	9.22E-05	2.71E-05	2.56E-05	3.26E-05	4.93E-05	2.41E-05	2.79E-05
THADA	1.06E-05	7.30E-06	3.82E-06	5.05E-06	6.50E-06	1.06E-05	5.60E-06	5.21E-06
THAP1	4.52E-06	5.16E-06	3.89E-06	4.25E-06	1.05E-06	5.02E-06	2.74E-06	3.92E-06
THAP10	9.32E-07	1.79E-06	1.11E-06	9.98E-07	3.66E-07	1.68E-06	6.84E-07	6.60E-07
THAP11	1.93E-05	1.15E-05	6.98E-06	1.12E-05	5.05E-06	1.18E-05	9.68E-06	8.81E-06
THAP2	8.52E-07	1.30E-06	8.55E-07	1.14E-06	3.00E-07	1.01E-06	6.15E-07	2.80E-07
THAP3	1.61E-05	9.40E-06	1.00E-05	5.41E-06	1.02E-05	1.39E-05	1.21E-05	9.69E-06
THAP4	0.000116	7.02E-05	3.08E-05	4.57E-05	5.06E-05	3.46E-05	4.68E-05	2.91E-05
THAP5	1.85E-05	1.10E-05	9.46E-06	5.52E-06	3.51E-06	9.33E-06	4.98E-06	1.15E-05
THAP6	6.29E-06	2.95E-06	4.03E-06	2.86E-06	1.33E-06	4.50E-06	3.73E-06	2.91E-06
THAP7	1.83E-05	3.17E-05	1.75E-05	2.08E-05	5.63E-05	2.22E-05	2.55E-05	2.59E-05
THAP8	1.15E-05	5.46E-06	3.66E-06	3.99E-06	4.02E-06	4.29E-06	6.01E-06	4.14E-06
THAP9	3.48E-06	8.46E-07	1.03E-06	9.43E-07	4.01E-07	1.20E-06	9.75E-07	2.04E-06
THBD	5.91E-06	1.57E-06	5.04E-06	2.07E-05	2.47E-06	2.35E-06	3.05E-06	4.14E-06
THBS1	3.75E-05	9.56E-06	6.76E-05	0.00024	3.69E-06	2.82E-05	8.11E-05	1.12E-05
THBS2	1.73E-05	6.99E-07	2.03E-06	1.64E-05	1.52E-06	1.27E-06	1.32E-05	5.20E-06
THBS3	5.00E-06	4.20E-06	2.32E-06	4.24E-06	2.55E-06	7.15E-06	3.54E-06	4.86E-06
THBS4	1.85E-07	4.44E-05	0	1.34E-07	4.76E-06	5.26E-06	1.72E-05	2.33E-06
THEG	0	0	0	0	0	0	0	0
THEM4	6.29E-06	5.34E-06	3.25E-06	5.52E-06	3.12E-06	7.60E-06	7.53E-06	1.22E-05
THEM5	3.58E-07	2.48E-07	1.55E-07	2.94E-08	2.06E-08	2.00E-07	1.59E-07	1.06E-07
THEMIS	7.77E-07	5.40E-08	2.56E-07	5.41E-07	5.17E-09	2.73E-07	2.11E-07	1.59E-07
THG1L	8.61E-06	1.69E-06	6.45E-06	9.02E-06	3.07E-06	1.09E-05	7.24E-06	5.70E-06
THNSL1	2.55E-06	1.11E-05	8.24E-06	4.88E-06	3.22E-06	1.10E-05	4.16E-06	4.47E-06
THNSL2	2.91E-05	5.48E-05	1.22E-05	1.55E-05	3.17E-05	2.57E-05	2.76E-05	2.12E-05
THOC1	1.36E-05	4.45E-06	3.76E-06	7.35E-06	2.47E-06	6.60E-06	6.13E-06	5.97E-06
THOC2	3.05E-05	1.70E-05	9.00E-06	1.20E-05	9.49E-06	1.57E-05	1.13E-05	1.81E-05
THOC3	2.73E-05	7.13E-06	1.46E-05	8.37E-06	3.28E-06	2.76E-05	9.94E-06	1.65E-05
THOC4	7.31E-05	5.31E-05	1.85E-05	2.97E-05	5.36E-05	3.87E-05	3.57E-05	2.34E-05
THOC5	1.72E-05	1.33E-05	4.89E-06	7.32E-06	1.04E-05	8.39E-06	8.64E-06	9.47E-06
THOC6	1.90E-05	1.70E-05	9.20E-06	1.37E-05	8.27E-06	1.60E-05	2.61E-05	1.50E-05
THOC7	5.03E-05	5.72E-05	3.22E-05	3.35E-05	2.89E-05	4.34E-05	4.56E-05	2.96E-05
THOP1	1.69E-05	2.82E-05	1.52E-05	1.29E-05	4.14E-05	2.34E-05	2.36E-05	8.25E-06
THPO	3.54E-05	2.17E-05	1.12E-05	1.19E-05	9.66E-06	1.71E-05	2.16E-05	1.81E-05
THRA	7.31E-06	1.44E-05	3.69E-06	5.24E-06	2.04E-06	5.06E-06	5.67E-06	5.59E-06
THRAP3	4.87E-05	1.87E-05	2.49E-05	2.48E-05	7.35E-06	3.35E-05	2.16E-05	2.89E-05
THRB	5.02E-06	9.71E-06	4.96E-06	4.57E-06	3.62E-06	4.32E-06	3.78E-06	5.14E-06
THRSP	8.81E-08	0.000212	0.000339	5.71E-05	4.29E-05	7.39E-07	8.03E-06	0.000201
THSD1	1.42E-06	3.83E-07	6.91E-07	2.85E-06	7.29E-07	7.31E-06	2.57E-06	1.33E-06
THSD1P1	1.67E-06	7.36E-07	6.68E-07	1.19E-06	8.27E-07	1.00E-05	1.32E-06	8.31E-07
THSD4	1.83E-06	1.20E-06	2.60E-06	2.34E-06	4.13E-07	7.36E-07	1.18E-06	3.10E-06
THSD7A	3.30E-06	1.55E-06	9.18E-08	4.85E-07	1.92E-07	2.71E-07	4.87E-07	2.29E-06
THSD7B	3.46E-08	5.14E-08	1.96E-08	8.90E-08	9.88E-09	3.13E-08	0	2.36E-07
THTPA	1.24E-05	8.25E-06	7.66E-06	6.74E-06	8.83E-06	1.91E-05	6.40E-06	1.18E-05
THUMPD1	1.47E-05	2.05E-05	7.94E-06	7.24E-06	1.29E-06	1.05E-05	7.15E-06	6.16E-06
THUMPD2	4.81E-06	2.72E-06	1.67E-06	1.87E-06	1.24E-06	2.14E-06	2.33E-06	2.22E-06
THUMPD3	1.42E-05	1.42E-05	7.35E-06	7.90E-06	4.65E-06	1.12E-05	7.09E-06	9.93E-06
THY1	6.07E-05	1.54E-05	2.31E-06	2.70E-05	3.16E-05	2.45E-05	7.37E-05	4.17E-05

THYN1	3.17E-05	1.30E-05	2.70E-05	4.23E-05	2.59E-05	4.31E-05	4.45E-05	2.66E-05
TIA1	3.86E-05	8.41E-06	5.32E-06	5.93E-06	3.35E-06	7.23E-06	3.96E-06	5.83E-06
TIAF1	2.43E-05	2.74E-05	1.57E-05	2.43E-05	5.89E-05	9.62E-06	3.07E-05	2.77E-05
TIAL1	2.38E-05	1.18E-05	1.34E-05	1.13E-05	6.67E-06	1.69E-05	1.12E-05	1.21E-05
TIAM1	5.39E-06	1.13E-06	1.76E-06	1.22E-06	3.18E-07	1.58E-06	1.04E-06	1.26E-07
TIAM2	2.48E-06	1.17E-07	1.77E-07	1.19E-06	4.51E-08	3.21E-07	8.81E-08	1.39E-07
TICAM1	1.93E-05	9.29E-06	8.09E-06	2.50E-05	5.18E-06	7.97E-06	1.31E-05	1.13E-05
TICAM2	6.53E-07	9.97E-07	6.17E-07	7.77E-07	3.02E-07	6.42E-07	9.53E-07	5.46E-07
TIE1	8.32E-06	7.49E-06	5.61E-06	5.51E-06	5.11E-06	4.24E-06	1.10E-05	1.24E-05
TIFA	5.12E-06	3.44E-06	3.19E-06	4.73E-06	6.53E-07	2.66E-06	3.12E-06	4.61E-06
TIFAB	1.54E-06	0	2.15E-08	1.48E-07	5.19E-08	2.98E-07	2.11E-07	8.91E-08
TIGD1	3.01E-06	5.74E-07	4.50E-07	2.94E-07	1.15E-06	7.48E-07	2.60E-07	9.59E-07
TIGD2	1.42E-05	5.08E-06	1.36E-05	7.75E-06	3.57E-06	1.76E-05	1.24E-05	2.13E-05
TIGD3	1.87E-06	4.84E-07	1.28E-08	1.65E-07	4.84E-07	1.02E-07	2.36E-08	3.60E-08
TIGD4	9.68E-08	9.82E-08	3.02E-08	5.92E-08	0	7.18E-08	9.37E-09	5.69E-08
TIGD5	5.26E-06	8.24E-06	9.49E-07	1.52E-06	2.43E-06	2.88E-06	6.25E-06	3.63E-06
TIGD6	1.87E-06	2.74E-06	1.16E-06	1.39E-06	6.73E-07	2.33E-06	9.72E-07	1.81E-06
TIGD7	5.69E-06	6.84E-06	1.53E-06	1.46E-06	1.36E-06	1.39E-06	1.18E-06	5.37E-06
TIGIT	7.10E-06	1.13E-07	6.05E-07	1.46E-06	3.83E-08	2.90E-06	3.55E-07	3.93E-07
TIMD4	1.59E-06	1.19E-07	8.69E-06	4.33E-06	0	1.84E-07	6.15E-06	1.34E-07
TIMELESS	8.66E-06	1.28E-05	4.03E-06	3.57E-06	1.64E-06	5.74E-06	4.00E-06	5.72E-06
TIMM10	6.67E-05	0.000116	3.75E-05	4.04E-05	7.77E-05	5.02E-05	0.000103	0.000112
TIMM13	6.43E-05	2.96E-05	2.97E-05	3.22E-05	0.000122	4.28E-05	5.31E-05	3.17E-05
TIMM16	3.87E-05	9.25E-05	3.65E-05	2.63E-05	3.43E-05	4.54E-05	4.85E-05	4.87E-05
TIMM17A	6.21E-05	4.09E-05	3.31E-05	3.27E-05	1.46E-05	0.00013	4.95E-05	7.37E-05
TIMM17B	6.99E-05	6.40E-05	3.28E-05	3.56E-05	4.47E-05	6.22E-05	4.82E-05	0.000104
TIMM22	1.10E-05	7.87E-06	4.94E-06	9.92E-06	1.17E-05	1.23E-05	1.25E-05	8.16E-06
TIMM44	3.40E-05	3.05E-05	2.19E-05	1.69E-05	1.92E-05	1.80E-05	2.57E-05	2.17E-05
TIMM50	2.85E-05	2.40E-05	1.23E-05	1.51E-05	2.81E-05	2.02E-05	2.57E-05	2.15E-05
TIMM8A	1.66E-05	2.37E-05	1.03E-05	7.25E-06	1.67E-05	1.88E-05	1.36E-05	1.17E-05
TIMM8B	0.000186	0.000109	9.81E-05	0.000106	0.00025	0.000166	0.000138	0.000149
TIMM9	2.06E-05	1.98E-05	1.54E-05	1.97E-05	2.09E-05	2.41E-05	2.56E-05	2.09E-05
TIMP1	0.001144	0.000204	8.47E-05	0.001055	0.000222	5.29E-05	0.001507	0.000271
TIMP2	0.000128	4.23E-05	2.68E-05	5.60E-05	8.45E-06	9.07E-06	3.05E-05	1.19E-05
TIMP3	2.01E-05	2.53E-05	2.19E-05	5.38E-05	1.84E-05	2.57E-05	8.83E-05	3.09E-05
TIMP4	1.67E-06	5.29E-06	3.74E-07	6.05E-07	2.12E-07	6.53E-07	1.15E-06	1.30E-07
TINAG	3.63E-06	2.58E-07	0	0	0	1.97E-08	0	7.92E-08
TINAGL1	2.48E-05	1.33E-05	9.07E-06	2.72E-05	6.52E-06	9.47E-06	1.27E-05	8.07E-06
TINF2	2.90E-05	1.65E-05	1.30E-05	2.07E-05	1.20E-05	2.27E-05	2.07E-05	2.05E-05
TIPARP	9.96E-06	7.19E-06	9.43E-06	2.36E-05	1.45E-06	7.50E-06	4.07E-06	7.99E-06
TIPIN	5.24E-06	1.57E-06	1.59E-06	2.12E-06	8.75E-07	2.64E-06	2.50E-06	3.65E-06
TIPRL	1.85E-05	2.91E-05	1.02E-05	1.29E-05	4.18E-06	2.33E-05	1.34E-05	2.65E-05
TIRAP	8.67E-06	3.62E-06	3.36E-06	2.64E-06	1.19E-06	4.02E-06	1.99E-06	9.46E-06
TJAP1	1.31E-05	1.36E-05	6.17E-06	8.57E-06	5.31E-06	9.57E-06	7.87E-06	1.31E-05
TJP1	1.39E-05	1.48E-05	6.92E-06	1.50E-05	2.77E-06	9.84E-06	8.48E-06	6.45E-06
TJP2	2.92E-05	2.92E-05	2.65E-05	2.57E-05	1.12E-05	1.66E-05	1.66E-05	1.19E-05
TJP3	1.75E-05	1.29E-06	2.17E-06	4.62E-06	6.46E-07	3.95E-07	8.42E-06	4.19E-06
TK1	9.41E-05	3.67E-05	3.57E-06	2.96E-06	2.93E-05	1.69E-05	3.82E-06	6.88E-06
TK2	1.66E-05	1.37E-05	1.65E-05	1.66E-05	9.10E-06	2.02E-05	1.79E-05	2.71E-05
TKT	0.000142	0.000176	6.00E-05	6.65E-05	8.44E-05	0.000223	9.11E-05	8.69E-05
TKTL1	4.69E-08	1.27E-05	4.99E-08	5.87E-08	7.06E-08	2.18E-08	4.52E-08	3.45E-08
TKTL2	0	0	0	0	1.43E-08	7.70E-09	0	0
TLCD1	1.85E-05	3.47E-05	2.35E-06	7.13E-06	1.42E-05	5.69E-06	7.57E-06	1.09E-05
TLE1	2.31E-05	2.42E-05	2.82E-05	2.97E-05	1.89E-05	4.50E-05	4.84E-05	6.57E-05
TLE2	7.43E-06	2.10E-06	3.96E-06	3.58E-06	1.07E-05	1.43E-06	1.01E-05	3.84E-06
TLE3	2.02E-05	9.71E-06	2.62E-06	3.67E-06	1.36E-06	3.91E-06	5.62E-06	6.03E-06
TLE4	3.53E-06	1.24E-06	2.17E-06	3.43E-06	2.59E-07	2.79E-06	1.14E-06	2.17E-06
TLE6	6.22E-07	3.78E-07	8.69E-07	1.95E-07	7.58E-07	2.67E-07	3.27E-06	4.12E-06

TLK1	1.54E-05	1.50E-05	9.61E-06	7.45E-06	6.45E-06	7.68E-06	9.00E-06	8.98E-06
TLK2	8.47E-06	6.11E-06	2.64E-06	3.53E-06	2.28E-06	5.24E-06	3.51E-06	2.70E-06
TLL1	6.04E-08	6.74E-08	3.43E-08	3.69E-08	5.76E-09	9.31E-09	8.83E-08	4.42E-08
TLL2	2.44E-07	4.79E-08	3.38E-09	1.63E-08	2.11E-07	2.54E-08	2.57E-08	2.90E-08
TLN1	3.53E-05	7.40E-05	2.01E-05	2.88E-05	7.54E-06	3.54E-05	2.66E-05	3.51E-05
TLN2	4.68E-06	1.99E-06	3.81E-06	2.19E-06	1.77E-06	1.89E-06	8.52E-06	3.75E-06
TLR1	2.69E-06	5.26E-06	4.00E-06	1.89E-06	3.15E-07	1.61E-06	2.02E-06	4.68E-06
TLR10	4.40E-07	1.95E-07	1.67E-07	1.94E-07	0	9.10E-08	4.40E-08	8.87E-08
TLR2	1.05E-05	5.90E-06	3.46E-06	4.63E-06	1.66E-07	1.44E-06	1.20E-06	4.64E-07
TLR3	4.41E-06	1.16E-06	5.77E-06	4.14E-06	1.77E-07	2.86E-06	1.02E-06	4.96E-06
TLR4	3.66E-06	1.78E-06	5.34E-06	4.09E-06	5.14E-07	1.48E-06	2.17E-06	9.79E-07
TLR5	1.70E-06	6.07E-07	7.34E-07	1.10E-06	6.12E-08	5.67E-07	7.61E-07	3.16E-06
TLR6	6.27E-07	1.57E-06	1.93E-07	2.28E-07	1.42E-08	8.24E-07	1.23E-07	2.24E-07
TLR7	1.60E-06	1.97E-07	2.73E-07	8.88E-07	5.82E-08	1.13E-07	6.16E-07	3.97E-08
TLR8	2.94E-06	1.09E-07	7.68E-07	9.39E-07	2.79E-08	4.91E-07	7.01E-07	1.03E-07
TLR9	3.50E-07	1.82E-06	4.67E-08	2.36E-07	4.79E-08	1.45E-07	2.80E-07	1.04E-07
TLX1	1.32E-06	4.81E-06	1.06E-07	1.11E-07	8.86E-07	1.03E-06	3.99E-06	2.51E-06
TLX1NB	5.13E-08	5.81E-08	0	0	0	0	2.48E-08	0
TLX2	4.04E-08	0	0	0	3.77E-08	0	0	0
TLX3	0	0	0	0	0	0	0	0
TM2D1	2.12E-05	9.90E-06	8.78E-06	1.29E-05	2.68E-06	1.13E-05	7.27E-06	8.99E-06
TM2D2	1.04E-05	2.13E-05	1.17E-05	1.29E-05	1.09E-05	1.52E-05	7.53E-06	1.29E-05
TM2D3	3.27E-05	5.95E-05	3.19E-05	3.48E-05	2.27E-05	7.15E-05	3.22E-05	3.21E-05
TM4SF1	9.34E-05	8.03E-05	1.47E-05	0.000389	2.86E-05	0.000133	2.32E-05	4.10E-05
TM4SF18	4.25E-06	5.70E-06	1.63E-06	1.61E-06	2.30E-06	3.65E-06	2.84E-06	2.17E-06
TM4SF19	4.85E-07	2.10E-07	2.84E-08	1.92E-06	0	0	2.25E-07	3.00E-07
TM4SF20	9.48E-07	1.16E-08	0	5.05E-08	0	0	9.98E-09	0
TM4SF4	0.000253	0.000541	0.00037	0.000768	0.000367	0.000168	0.000498	0.000658
TM4SF5	0.000495	0.000275	0.00013	0.000317	0.000553	0.000231	0.000277	0.001111
TM6SF1	4.49E-06	5.42E-07	1.21E-06	1.98E-06	3.52E-07	9.29E-07	1.27E-06	7.13E-07
TM6SF2	7.98E-06	9.63E-06	2.62E-05	1.08E-05	1.56E-05	1.57E-05	1.39E-05	1.01E-05
TM7SF2	3.90E-05	0.000319	8.34E-05	2.76E-05	0.000221	8.99E-05	8.61E-05	7.17E-05
TM7SF3	2.15E-05	3.07E-05	3.26E-05	2.92E-05	1.00E-05	2.71E-05	1.74E-05	2.39E-05
TM7SF4	4.31E-07	6.96E-08	1.29E-08	4.68E-07	0	6.85E-08	1.43E-07	1.09E-07
TM9SF1	5.39E-05	3.23E-05	2.90E-05	3.46E-05	2.18E-05	4.91E-05	2.92E-05	4.68E-05
TM9SF2	0.000102	0.000188	9.69E-05	8.18E-05	1.56E-05	8.82E-05	6.07E-05	8.06E-05
TM9SF3	5.06E-05	3.72E-05	3.00E-05	2.89E-05	1.35E-05	3.45E-05	2.70E-05	3.01E-05
TM9SF4	4.62E-05	3.83E-05	2.03E-05	1.85E-05	1.27E-05	2.50E-05	1.76E-05	2.55E-05
TMBIM1	0.000169	9.43E-05	6.43E-05	0.00011	4.21E-05	5.91E-05	9.53E-05	0.00012
TMBIM4	4.75E-05	6.12E-05	3.44E-05	4.41E-05	2.33E-05	3.97E-05	3.71E-05	4.70E-05
TMBIM6	0.000526	0.000746	0.000935	0.000695	0.00051	0.000695	0.000769	0.000488
TMC1	1.75E-08	1.01E-08	0	1.14E-08	0	0	8.69E-09	0
TMC2	7.40E-09	0	0	9.05E-09	0	0	0	0
TMC3	1.40E-08	0	1.44E-08	0	0	1.29E-08	0	0
TMC4	2.65E-06	1.71E-06	1.35E-06	4.56E-06	5.01E-07	3.29E-07	1.90E-06	1.47E-07
TMC5	1.89E-05	4.58E-08	4.21E-08	8.79E-07	3.50E-08	3.91E-07	1.74E-06	8.19E-07
TMC6	3.43E-05	3.11E-06	5.14E-06	1.10E-05	2.32E-06	4.40E-06	5.91E-06	2.86E-06
TMC7	2.88E-06	4.85E-07	6.88E-08	4.22E-07	9.29E-08	1.15E-07	9.03E-07	5.81E-07
TMC8	4.88E-06	3.97E-07	2.86E-06	3.51E-06	3.97E-07	2.50E-06	2.17E-06	1.38E-06
TMCC1	7.86E-06	5.05E-06	6.96E-06	1.05E-05	2.40E-06	2.05E-05	9.62E-06	3.83E-06
TMCC2	7.99E-07	3.09E-08	8.88E-08	6.11E-07	1.78E-07	2.74E-07	2.94E-07	1.48E-07
TMCC3	3.52E-06	1.46E-06	6.09E-07	2.40E-06	4.14E-07	9.76E-07	1.18E-06	3.81E-06
TMCO1	4.57E-05	0.000105	2.38E-05	2.62E-05	2.00E-05	5.22E-05	2.92E-05	5.48E-05
TMCO2	0	4.65E-08	0	0	0	3.68E-08	3.69E-08	0
TMCO3	2.28E-05	9.50E-06	4.75E-06	1.12E-05	1.85E-06	9.19E-06	1.57E-05	1.37E-05
TMCO4	1.45E-05	8.87E-06	4.44E-06	6.22E-06	1.36E-06	5.68E-06	5.79E-06	6.32E-06
TMCO5A	0	0	0	2.76E-08	0	0	0	0
TMCO6	3.88E-05	1.15E-05	1.27E-05	1.40E-05	3.84E-06	2.45E-05	1.22E-05	1.76E-05

TMCO7	3.19E-06	3.89E-06	9.86E-06	4.62E-06	1.63E-06	5.62E-06	3.33E-06	9.00E-06
TMED1	4.34E-05	2.19E-05	2.74E-05	3.79E-05	6.03E-05	2.81E-05	3.51E-05	3.75E-05
TMED10	8.61E-05	4.99E-05	6.93E-05	7.18E-05	2.82E-05	9.74E-05	5.96E-05	7.35E-05
TMED10P:	1.08E-06	6.51E-07	4.12E-07	2.97E-07	2.33E-07	5.91E-07	7.19E-07	6.20E-07
TMED2	0.000236	0.000237	0.000145	0.000136	5.20E-05	0.000217	0.000138	0.00017
TMED3	5.28E-05	1.24E-06	1.02E-05	2.48E-05	2.84E-06	2.74E-06	1.54E-05	3.39E-05
TMED4	7.93E-05	6.24E-05	4.26E-05	4.46E-05	6.69E-06	4.43E-05	2.44E-05	4.71E-05
TMED5	4.90E-05	6.38E-05	3.65E-05	2.97E-05	1.17E-05	8.65E-05	2.34E-05	2.72E-05
TMED6	1.12E-06	1.20E-06	4.79E-06	2.73E-06	4.71E-07	8.67E-06	1.75E-06	1.35E-07
TMED7	4.51E-05	5.26E-05	4.39E-05	3.03E-05	7.46E-06	5.69E-05	2.58E-05	6.11E-05
TMED7-TI	4.64E-06	6.73E-07	7.37E-07	1.26E-06	7.91E-08	1.94E-06	4.27E-07	2.86E-06
TMED8	2.59E-06	9.99E-07	2.77E-06	2.17E-06	6.64E-07	2.41E-06	1.80E-06	1.31E-06
TMED9	0.000207	0.000105	0.000122	0.000214	0.000129	0.000179	0.000186	0.000332
TMEFF1	7.91E-07	2.66E-07	2.35E-07	2.13E-07	4.45E-08	2.52E-07	2.00E-07	1.02E-06
TMEFF2	2.68E-08	0	0	0	0	1.24E-08	0	0
TMEM100	5.41E-07	7.57E-07	1.73E-06	1.67E-06	3.94E-07	6.92E-06	9.61E-07	1.08E-06
TMEM101	4.51E-05	2.74E-05	1.54E-05	1.63E-05	3.70E-05	3.15E-05	2.53E-05	1.91E-05
TMEM102	3.15E-06	1.46E-06	2.67E-06	3.58E-06	9.81E-07	3.10E-06	3.04E-06	3.14E-06
TMEM104	1.56E-05	3.01E-05	5.05E-06	5.02E-06	1.43E-05	7.37E-06	6.44E-06	1.04E-05
TMEM105	1.32E-07	1.09E-07	4.65E-07	3.05E-07	1.79E-08	0	4.51E-07	3.49E-06
TMEM106	7.21E-06	3.92E-06	1.43E-06	1.43E-06	3.98E-07	7.82E-07	1.88E-06	1.80E-07
TMEM106	2.98E-05	2.48E-05	1.28E-05	8.04E-06	3.93E-06	1.26E-05	5.61E-06	1.77E-05
TMEM106	5.13E-05	5.86E-05	1.38E-05	1.77E-05	3.50E-05	5.92E-05	2.99E-05	4.79E-05
TMEM107	3.44E-06	1.54E-06	1.11E-06	2.12E-06	6.34E-07	1.57E-06	2.19E-06	5.98E-07
TMEM108	2.98E-07	8.06E-08	1.35E-08	7.98E-08	2.81E-08	7.23E-08	2.35E-08	2.01E-07
TMEM109	4.48E-05	4.93E-05	2.65E-05	3.72E-05	2.13E-05	3.65E-05	4.29E-05	5.25E-05
TMEM11	1.25E-05	2.25E-05	1.02E-05	1.44E-05	1.19E-05	1.13E-05	1.80E-05	1.35E-05
TMEM110	1.29E-05	1.79E-05	1.41E-05	6.31E-06	1.98E-05	1.37E-05	1.01E-05	2.02E-05
TMEM111	0.000113	0.000145	5.75E-05	6.61E-05	0.000114	0.000102	8.10E-05	8.55E-05
TMEM114	0	0	0	0	0	0	0	0
TMEM115	4.73E-05	3.56E-05	2.16E-05	2.88E-05	3.77E-05	3.01E-05	3.61E-05	2.67E-05
TMEM116	8.64E-06	1.20E-05	4.54E-06	3.57E-06	2.27E-06	8.57E-06	5.06E-06	4.05E-06
TMEM117	2.10E-06	1.08E-06	6.93E-07	1.50E-06	3.92E-07	1.42E-06	1.25E-06	1.31E-06
TMEM119	1.62E-06	2.24E-07	4.39E-07	1.46E-06	1.91E-07	5.25E-07	1.49E-06	3.01E-06
TMEM120	7.09E-05	0.000119	8.58E-05	5.81E-05	0.000157	6.52E-05	5.51E-05	6.39E-05
TMEM120	2.88E-06	1.67E-06	5.73E-07	8.39E-07	5.45E-07	1.07E-06	1.52E-06	1.37E-06
TMEM121	9.29E-07	3.72E-08	1.11E-06	4.41E-07	5.62E-07	1.06E-07	4.93E-06	2.17E-07
TMEM123	0.000137	0.000297	0.000143	0.000138	4.08E-05	9.70E-05	9.58E-05	0.000207
TMEM125	7.84E-07	1.56E-06	3.00E-07	7.05E-07	0	2.50E-07	8.83E-08	2.21E-08
TMEM126	4.22E-05	2.22E-05	2.43E-05	2.47E-05	2.20E-05	3.80E-05	2.25E-05	3.90E-05
TMEM126	2.50E-05	2.32E-05	1.91E-05	2.12E-05	8.96E-06	2.26E-05	1.78E-05	3.16E-05
TMEM127	5.84E-05	3.96E-05	1.61E-05	2.64E-05	9.90E-06	2.68E-05	2.73E-05	3.28E-05
TMEM128	1.80E-05	1.95E-05	1.01E-05	1.09E-05	4.06E-06	1.14E-05	1.26E-05	1.13E-05
TMEM129	3.31E-05	2.24E-05	3.71E-05	2.41E-05	2.51E-05	2.66E-05	4.06E-05	4.94E-05
TMEM130	1.80E-07	1.93E-08	1.86E-07	8.09E-07	0	4.76E-08	1.66E-08	1.26E-08
TMEM131	3.18E-05	2.26E-05	1.28E-05	1.36E-05	9.81E-06	1.81E-05	1.95E-05	1.68E-05
TMEM132	2.18E-05	9.27E-07	2.75E-07	1.10E-05	1.34E-06	9.22E-07	2.10E-06	1.47E-06
TMEM132	5.88E-09	5.08E-07	1.82E-08	1.74E-08	0	6.59E-09	2.58E-08	1.56E-08
TMEM132	0	0	5.25E-08	1.36E-07	0	0	9.06E-09	0
TMEM132	0	7.58E-08	5.21E-08	2.37E-08	3.64E-09	3.92E-09	1.24E-08	3.10E-08
TMEM132	8.83E-08	1.74E-08	3.15E-07	7.80E-07	8.02E-08	1.44E-08	4.55E-08	5.32E-08
TMEM133	5.72E-06	4.15E-06	4.13E-06	4.68E-06	8.18E-07	3.93E-06	2.75E-06	2.13E-06
TMEM134	2.48E-05	5.97E-05	3.34E-05	4.19E-05	3.65E-05	4.49E-05	4.50E-05	4.67E-05
TMEM135	1.04E-05	9.09E-06	1.30E-05	6.52E-06	3.05E-06	9.30E-06	7.52E-06	2.72E-05
TMEM136	8.19E-06	8.46E-07	5.25E-07	1.39E-06	7.24E-07	7.45E-07	1.30E-06	2.07E-06
TMEM138	2.24E-05	1.86E-05	1.39E-05	1.67E-05	1.35E-05	2.30E-05	1.44E-05	2.64E-05
TMEM139	1.23E-05	4.64E-06	5.68E-06	1.11E-05	8.65E-06	7.44E-06	5.00E-06	2.09E-08
TMEM140	3.49E-05	5.94E-05	3.96E-05	3.75E-05	2.45E-05	5.66E-05	2.49E-05	4.03E-05

TMEM141	0.000202	0.000117	9.46E-05	9.49E-05	0.000143	0.000119	0.000115	0.000131
TMEM143	7.68E-06	8.94E-06	6.13E-06	3.94E-06	4.55E-06	1.22E-05	8.13E-06	3.17E-06
TMEM144	4.89E-06	8.18E-07	5.81E-07	1.75E-06	3.04E-08	1.81E-06	4.20E-07	2.20E-06
TMEM145	2.22E-06	1.55E-08	0	1.67E-08	2.82E-07	1.27E-08	7.90E-08	1.09E-06
TMEM146	0	0	0	0	0	0	0	0
TMEM147	0.00011	5.82E-05	3.28E-05	5.83E-05	0.000108	9.06E-05	0.000108	5.30E-05
TMEM149	2.64E-05	2.96E-05	1.49E-05	1.72E-05	1.40E-05	3.37E-05	1.50E-05	9.66E-06
TMEM14A	3.59E-05	0.000126	4.74E-05	2.43E-05	7.71E-05	7.59E-05	3.26E-05	4.43E-05
TMEM14B	7.51E-05	0.000134	5.54E-05	5.13E-05	4.66E-05	0.000146	4.17E-05	5.04E-05
TMEM14C	8.86E-05	0.00018	8.35E-05	8.97E-05	9.07E-05	0.000174	9.36E-05	0.000103
TMEM14E	0	0	0	0	0	0	1.89E-08	0
TMEM150	5.30E-05	2.62E-05	1.67E-05	2.87E-05	1.52E-05	2.50E-05	3.11E-05	1.71E-05
TMEM150	2.81E-06	6.19E-06	1.07E-06	1.33E-06	1.92E-06	2.52E-06	8.47E-06	1.43E-05
TMEM150	5.50E-06	3.92E-06	5.63E-06	8.36E-06	1.87E-07	1.49E-05	4.19E-06	4.40E-06
TMEM151	3.40E-06	6.27E-08	6.75E-08	2.50E-07	3.59E-07	6.88E-08	2.43E-07	3.27E-07
TMEM151	3.50E-07	2.32E-07	2.02E-08	4.38E-08	2.52E-08	6.04E-08	3.46E-08	1.03E-07
TMEM154	2.54E-06	8.43E-08	8.23E-06	9.27E-07	1.93E-08	1.04E-07	3.13E-07	1.54E-07
TMEM155	6.62E-07	0	2.27E-08	1.33E-08	0	7.05E-08	2.10E-08	0
TMEM156	4.33E-05	1.18E-07	6.85E-07	2.44E-06	1.68E-07	2.78E-07	7.55E-08	1.50E-05
TMEM158	7.02E-07	2.95E-08	1.91E-07	2.10E-06	1.90E-07	1.81E-07	4.64E-07	3.06E-07
TMEM159	3.72E-06	1.17E-06	9.21E-07	2.47E-06	5.11E-07	5.32E-07	1.07E-06	6.51E-07
TMEM160	5.21E-06	1.23E-05	1.85E-06	6.46E-06	1.69E-05	5.47E-06	3.68E-05	3.45E-06
TMEM161	3.82E-05	2.83E-05	2.06E-05	2.18E-05	4.74E-05	3.11E-05	3.03E-05	3.81E-05
TMEM161	7.83E-06	1.02E-05	4.44E-06	3.97E-06	9.08E-07	6.67E-06	2.77E-06	7.66E-06
TMEM163	2.60E-05	3.46E-07	7.23E-08	1.72E-07	0	6.37E-08	1.25E-06	4.05E-08
TMEM164	1.61E-05	1.58E-06	1.52E-06	4.99E-06	3.45E-06	5.75E-06	5.37E-06	8.60E-06
TMEM165	3.87E-05	1.14E-05	7.90E-06	1.60E-05	2.99E-06	2.14E-05	1.28E-05	1.84E-05
TMEM167	2.91E-05	2.14E-05	1.40E-05	1.25E-05	5.15E-06	2.06E-05	9.26E-06	2.22E-05
TMEM167	2.66E-05	2.64E-05	1.09E-05	1.41E-05	7.56E-06	2.93E-05	1.18E-05	1.40E-05
TMEM168	3.84E-06	8.98E-06	4.04E-06	3.80E-06	2.60E-06	4.11E-06	3.88E-06	4.16E-06
TMEM169	1.72E-06	1.74E-06	9.09E-07	5.17E-07	3.17E-07	1.11E-06	5.24E-07	5.10E-07
TMEM17	3.39E-06	1.54E-06	8.26E-07	1.31E-06	5.09E-07	1.17E-06	6.88E-07	1.03E-06
TMEM170	9.54E-06	4.46E-06	6.50E-06	5.75E-06	1.14E-06	5.60E-06	2.84E-06	5.51E-06
TMEM170	2.74E-06	7.46E-06	3.00E-06	1.24E-06	5.87E-07	2.49E-06	9.79E-07	2.03E-06
TMEM171	2.26E-06	7.62E-07	7.37E-07	6.68E-06	3.48E-07	1.05E-06	4.27E-06	4.69E-06
TMEM173	1.21E-05	4.13E-06	5.91E-06	1.26E-05	3.06E-06	4.87E-06	7.05E-06	6.62E-06
TMEM174	0	0	0	0	0	0	0	0
TMEM175	1.75E-05	3.71E-05	1.83E-05	1.44E-05	1.80E-05	1.81E-05	1.82E-05	2.60E-05
TMEM176	0.00029	0.001028	0.000899	0.000816	0.001426	0.000717	0.001475	0.001216
TMEM176	0.000444	0.002013	0.001343	0.001181	0.001488	0.001617	0.000961	0.002131
TMEM177	2.62E-05	1.62E-05	1.66E-05	1.19E-05	8.02E-06	3.86E-05	1.47E-05	1.29E-05
TMEM178	8.49E-07	2.76E-07	2.71E-07	5.56E-07	6.37E-07	5.67E-07	1.22E-06	6.68E-07
TMEM179	0	0	0	3.71E-08	2.61E-08	0	0	0
TMEM179	0.000113	6.42E-05	4.45E-05	5.21E-05	4.95E-05	5.45E-05	5.80E-05	7.46E-05
TMEM18	1.34E-05	1.12E-05	8.73E-06	9.33E-06	6.90E-06	9.00E-06	8.81E-06	9.30E-06
TMEM180	7.12E-06	3.72E-06	2.03E-06	2.68E-06	3.78E-06	3.74E-06	4.53E-06	2.19E-06
TMEM181	1.54E-05	1.25E-05	6.81E-06	5.84E-06	1.85E-06	9.93E-06	4.53E-06	4.32E-06
TMEM182	9.42E-07	5.12E-07	7.07E-07	4.87E-07	6.89E-08	2.27E-06	3.21E-06	3.14E-06
TMEM183	3.07E-05	2.93E-05	1.66E-05	2.03E-05	1.75E-05	3.57E-05	2.44E-05	3.62E-05
TMEM184	1.09E-05	1.17E-05	2.54E-06	4.95E-06	2.94E-06	1.99E-06	6.15E-06	7.19E-06
TMEM184	1.53E-05	8.64E-06	3.98E-06	8.23E-06	5.08E-06	5.54E-06	1.05E-05	1.46E-05
TMEM184	1.69E-05	9.67E-06	9.90E-06	6.17E-06	1.55E-06	9.02E-06	4.73E-06	8.28E-06
TMEM185	1.18E-05	5.94E-06	2.74E-06	5.08E-06	4.15E-06	5.37E-06	5.68E-06	5.28E-06
TMEM185	2.53E-05	5.93E-07	3.81E-06	7.53E-06	2.24E-06	3.58E-06	7.95E-06	8.90E-06
TMEM186	1.07E-05	1.47E-05	7.98E-06	7.27E-06	6.97E-06	1.37E-05	9.56E-06	7.69E-06
TMEM187	2.34E-05	1.02E-05	1.02E-05	8.79E-06	2.36E-05	7.93E-06	6.34E-06	1.39E-05
TMEM188	7.10E-06	4.12E-06	3.83E-06	4.81E-06	1.04E-06	4.24E-06	4.03E-06	5.01E-06
TMEM189	4.03E-05	2.07E-05	9.88E-06	2.05E-05	1.03E-05	1.00E-05	1.90E-05	2.01E-05

TMEM189	#######	7.23E-08	#######	#######	0	#######	#######	#######
TMEM19	8.59E-06	1.33E-05	7.68E-06	4.88E-06	1.64E-06	8.08E-06	8.65E-06	7.05E-06
TMEM190	0	6.51E-08	0	0	0	0	4.97E-08	0
TMEM191	6.87E-07	1.40E-06	4.11E-07	3.31E-07	1.55E-06	3.30E-07	1.29E-06	2.53E-07
TMEM192	1.05E-05	1.21E-05	1.67E-05	1.04E-05	2.09E-06	1.32E-05	7.12E-06	1.38E-05
TMEM194	5.15E-06	8.82E-06	1.97E-06	1.50E-06	4.65E-07	1.64E-06	1.18E-06	1.25E-06
TMEM194	4.66E-06	4.19E-06	1.08E-06	1.41E-06	8.05E-07	1.07E-06	8.00E-07	1.05E-06
TMEM195	2.11E-05	5.44E-05	3.73E-05	2.83E-05	8.09E-06	6.42E-05	1.24E-05	4.88E-05
TMEM196	0	0	0	0	0	0	0	0
TMEM198	1.74E-05	2.50E-06	8.15E-07	1.42E-06	1.12E-06	1.96E-06	2.79E-06	2.86E-06
TMEM199	2.34E-05	3.64E-05	1.06E-05	1.47E-05	2.05E-05	1.62E-05	1.88E-05	2.02E-05
TMEM2	4.08E-05	2.25E-05	2.41E-05	3.37E-05	5.83E-06	0.000133	2.12E-05	6.42E-05
TMEM20	8.09E-06	2.88E-06	2.97E-06	1.45E-06	3.07E-06	8.82E-07	8.63E-07	1.74E-06
TMEM200	7.02E-07	4.65E-07	8.24E-08	6.82E-07	1.37E-08	1.92E-07	3.29E-07	1.88E-07
TMEM200	1.95E-05	1.01E-05	2.43E-06	3.94E-06	5.60E-06	4.07E-07	8.63E-06	7.55E-06
TMEM200	2.51E-07	1.42E-08	2.63E-07	4.30E-07	0	6.98E-08	0	5.54E-08
TMEM201	4.13E-06	2.22E-06	9.03E-07	1.49E-06	6.01E-07	1.14E-06	2.08E-06	2.51E-06
TMEM202	0	0	0	0	0	5.64E-08	0	0
TMEM203	4.44E-05	3.36E-05	2.61E-05	2.28E-05	2.86E-05	3.36E-05	3.06E-05	4.76E-05
TMEM204	1.09E-05	7.31E-06	1.02E-05	9.10E-06	6.68E-06	5.80E-06	1.08E-05	1.03E-05
TMEM205	0.000245	0.000229	0.000299	0.00032	0.000772	0.000317	0.00041	0.000345
TMEM206	4.50E-06	2.87E-06	8.99E-07	1.47E-06	6.86E-07	2.26E-06	2.43E-06	3.46E-06
TMEM207	1.72E-08	0	0	0	0	0	0	0
TMEM208	0.000119	0.000104	7.62E-05	9.57E-05	0.000123	0.000108	0.000106	0.000154
TMEM209	6.96E-06	1.03E-05	4.41E-06	3.49E-06	2.04E-06	6.50E-06	4.61E-06	8.84E-06
TMEM211	0	0	0	0	0	0	0	0
TMEM212	0	0	0	0	0	0	0	3.82E-08
TMEM213	0	0	0	0	0	0	0	0
TMEM214	5.30E-05	8.58E-05	2.49E-05	3.54E-05	3.26E-05	5.16E-05	3.30E-05	3.71E-05
TMEM215	8.36E-09	0	4.28E-09	0	0	0	0	0
TMEM216	3.54E-06	2.88E-06	1.52E-06	3.33E-06	1.24E-06	3.07E-06	4.07E-06	5.16E-06
TMEM217	1.66E-06	3.77E-07	1.49E-07	3.72E-06	1.72E-07	4.29E-07	5.64E-07	4.73E-07
TMEM218	8.72E-06	6.98E-06	4.01E-06	6.15E-06	5.99E-06	7.87E-06	1.02E-05	4.93E-06
TMEM219	0.000149	0.000154	8.40E-05	0.000106	0.000139	0.000185	0.000176	7.98E-05
TMEM22	1.39E-05	5.75E-06	2.22E-06	1.61E-06	2.44E-07	5.06E-06	3.18E-06	9.90E-07
TMEM220	6.69E-06	2.31E-05	4.58E-05	2.08E-05	8.30E-06	1.53E-05	2.59E-05	1.30E-05
TMEM222	3.43E-05	2.97E-05	2.39E-05	2.65E-05	2.75E-05	3.19E-05	2.24E-05	3.51E-05
TMEM223	4.95E-05	5.65E-05	2.09E-05	2.11E-05	2.42E-05	2.44E-05	1.85E-05	2.42E-05
TMEM225	0	0	0	0	0	0	0	0
TMEM229	0	0	0	0	0	0	0	0
TMEM229	4.32E-06	6.52E-07	6.56E-07	5.81E-07	2.93E-07	7.87E-07	8.76E-07	5.82E-07
TMEM231	6.49E-07	1.73E-07	8.71E-07	1.42E-06	6.75E-08	3.42E-07	4.21E-07	7.70E-07
TMEM232	7.59E-08	1.08E-07	2.02E-07	1.47E-07	2.08E-08	2.22E-07	3.51E-08	5.50E-08
TMEM233	1.72E-07	7.14E-08	5.81E-07	3.66E-07	1.35E-07	1.02E-07	2.11E-07	1.16E-07
TMEM25	2.88E-06	3.88E-06	9.12E-06	1.07E-05	1.98E-06	2.75E-06	6.24E-06	3.21E-07
TMEM26	3.53E-07	1.27E-07	6.85E-07	8.35E-07	0	1.98E-07	2.71E-07	7.70E-08
TMEM27	5.49E-05	5.62E-06	1.77E-05	1.59E-05	4.88E-06	3.51E-06	1.47E-06	1.16E-07
TMEM30A	6.29E-05	5.62E-05	4.04E-05	3.24E-05	7.79E-06	4.08E-05	2.42E-05	2.84E-05
TMEM30B	2.93E-05	5.91E-06	4.86E-06	7.17E-06	3.85E-06	6.00E-06	8.14E-06	4.09E-06
TMEM30C	0	0	0	0	0	0	0	0
TMEM31	0	0	0	0	6.97E-08	3.75E-08	0	6.03E-08
TMEM33	1.26E-05	2.30E-05	1.40E-05	9.73E-06	4.42E-06	1.61E-05	9.87E-06	2.22E-05
TMEM35	9.24E-08	2.61E-08	3.62E-08	4.24E-08	9.93E-09	0	0	1.70E-08
TMEM37	8.74E-05	1.91E-05	6.09E-05	3.90E-05	2.91E-05	4.53E-05	5.45E-05	9.62E-06
TMEM38A	7.24E-06	1.40E-05	4.16E-06	2.45E-06	5.47E-07	1.81E-06	1.84E-06	8.32E-06
TMEM38B	1.29E-05	1.57E-05	6.96E-06	5.13E-06	1.72E-06	9.69E-06	5.16E-06	1.89E-05
TMEM39A	8.52E-06	5.64E-06	6.06E-06	5.98E-06	1.95E-06	9.07E-06	5.48E-06	7.42E-06
TMEM39B	7.77E-06	4.01E-06	4.49E-06	6.97E-06	4.53E-06	6.32E-06	9.49E-06	8.79E-06

TMEM40	0	1.72E-08	1.78E-08	0	0	2.28E-06	1.46E-08	4.47E-08
TMEM41A	1.98E-05	8.77E-06	8.65E-06	8.11E-06	6.96E-06	1.02E-05	1.02E-05	1.02E-05
TMEM41B	1.60E-05	3.00E-05	1.06E-05	1.00E-05	1.09E-05	1.61E-05	9.81E-06	1.96E-05
TMEM42	2.41E-05	1.99E-05	1.09E-05	1.14E-05	2.56E-05	3.14E-05	1.41E-05	7.75E-06
TMEM43	2.99E-05	1.31E-05	7.47E-06	1.77E-05	3.40E-06	7.38E-06	1.23E-05	7.95E-06
TMEM44	7.26E-06	1.58E-06	1.95E-06	2.38E-06	2.51E-06	1.34E-06	3.66E-06	1.37E-06
TMEM45A	1.10E-05	2.65E-05	6.14E-05	6.28E-05	1.05E-05	7.21E-06	5.48E-05	1.53E-05
TMEM45B	5.06E-05	3.04E-05	9.20E-06	8.05E-06	1.57E-05	3.37E-06	1.93E-05	6.44E-05
TMEM47	4.60E-06	2.97E-06	1.17E-05	1.19E-05	1.03E-06	5.62E-06	7.24E-06	3.39E-06
TMEM48	8.90E-06	6.39E-06	2.99E-06	2.57E-06	8.82E-07	3.43E-06	1.86E-06	3.26E-06
TMEM49	0.000167	9.89E-05	3.46E-05	4.20E-05	2.28E-05	6.45E-05	3.37E-05	4.87E-05
TMEM5	6.81E-06	1.44E-05	7.60E-06	7.65E-06	3.43E-06	1.19E-05	8.97E-06	7.58E-06
TMEM50A	5.11E-05	3.34E-05	2.36E-05	3.78E-05	1.05E-05	5.27E-05	3.16E-05	3.49E-05
TMEM50B	1.72E-05	9.56E-06	8.44E-06	1.17E-05	7.06E-06	1.68E-05	1.03E-05	1.40E-05
TMEM51	1.37E-05	9.55E-07	1.80E-06	9.75E-06	1.01E-06	1.08E-06	3.05E-06	1.09E-06
TMEM52	5.44E-06	9.08E-06	4.32E-06	1.80E-06	8.30E-07	1.83E-06	3.30E-06	4.30E-06
TMEM53	2.84E-05	2.50E-05	4.40E-05	4.33E-05	3.92E-05	3.21E-05	3.45E-05	3.07E-05
TMEM54	4.08E-05	4.42E-05	8.31E-06	1.82E-05	7.35E-06	7.82E-06	1.91E-05	3.88E-06
TMEM55A	1.39E-05	2.29E-06	1.14E-06	3.73E-06	8.01E-07	7.09E-07	2.88E-06	2.93E-06
TMEM55B	1.76E-05	2.72E-05	9.90E-06	1.37E-05	5.53E-06	1.58E-05	1.63E-05	1.74E-05
TMEM56	8.23E-06	2.47E-05	5.06E-05	3.25E-05	7.22E-06	3.13E-05	1.68E-05	2.75E-05
TMEM57	2.78E-05	4.69E-05	2.26E-05	1.78E-05	1.58E-05	1.52E-05	2.25E-05	2.22E-05
TMEM59	0.000239	0.000241	0.00021	0.000207	8.82E-05	0.000243	0.000127	0.000264
TMEM59L	1.07E-07	0	1.68E-08	1.88E-07	1.05E-07	0	2.05E-07	2.34E-08
TMEM60	4.31E-05	4.62E-05	2.09E-05	1.76E-05	1.96E-05	1.68E-05	1.98E-05	1.47E-05
TMEM61	8.29E-06	8.28E-08	7.79E-08	2.06E-07	2.07E-08	2.23E-08	2.28E-08	0
TMEM62	1.57E-05	1.58E-05	6.66E-06	1.19E-05	6.80E-06	9.46E-06	1.07E-05	1.63E-05
TMEM63A	3.13E-05	1.13E-05	2.22E-05	1.39E-05	1.20E-05	4.81E-06	1.24E-05	1.41E-05
TMEM63B	3.03E-05	3.96E-05	2.01E-05	2.53E-05	2.76E-05	2.37E-05	2.42E-05	3.04E-05
TMEM63C	1.58E-07	2.33E-08	4.69E-08	5.59E-08	3.57E-09	1.93E-08	4.06E-08	0
TMEM64	2.95E-05	1.15E-05	9.36E-06	4.51E-06	3.08E-06	1.57E-05	1.58E-05	4.98E-06
TMEM65	1.82E-05	2.54E-06	1.52E-06	2.70E-06	7.28E-07	6.98E-07	2.46E-06	2.03E-06
TMEM66	0.000152	0.000265	0.000185	0.000191	4.77E-05	0.000201	9.24E-05	5.07E-05
TMEM67	8.83E-07	3.73E-07	1.69E-07	4.36E-07	7.74E-08	7.77E-07	5.12E-07	1.88E-07
TMEM68	1.17E-05	8.92E-06	4.43E-06	4.56E-06	4.48E-06	4.83E-06	8.28E-06	6.65E-06
TMEM69	2.58E-05	1.53E-05	1.20E-05	1.43E-05	6.08E-06	2.59E-05	9.19E-06	1.15E-05
TMEM70	2.05E-05	2.56E-05	1.89E-05	1.74E-05	1.87E-05	2.82E-05	3.34E-05	5.01E-05
TMEM71	5.92E-07	2.81E-07	4.83E-07	1.51E-06	1.06E-07	2.30E-07	3.58E-07	6.75E-08
TMEM72	1.31E-07	3.03E-08	0	3.23E-08	0	0	0	0
TMEM74	7.43E-07	0	5.26E-07	3.92E-07	3.44E-07	1.14E-06	1.99E-07	3.38E-06
TMEM79	2.18E-06	4.91E-06	1.25E-06	1.64E-06	1.41E-06	3.45E-06	3.24E-06	3.40E-06
TMEM80	9.39E-06	5.00E-06	6.22E-06	6.46E-06	4.72E-06	7.75E-06	3.59E-06	6.27E-06
TMEM81	2.09E-06	2.35E-06	1.04E-06	7.48E-07	1.62E-06	3.18E-06	1.86E-06	2.56E-06
TMEM82	2.14E-05	1.17E-05	3.29E-05	1.95E-05	8.62E-06	1.25E-05	2.73E-05	4.82E-08
TMEM84	7.38E-08	3.14E-08	2.58E-08	0	3.18E-08	5.71E-08	2.38E-08	1.63E-07
TMEM85	0.000119	0.000107	6.73E-05	7.34E-05	7.31E-05	8.81E-05	8.01E-05	8.93E-05
TMEM86A	6.35E-06	1.81E-06	1.90E-06	2.44E-06	4.78E-07	2.85E-06	3.10E-06	2.34E-06
TMEM86B	4.75E-06	4.51E-05	2.39E-05	2.24E-05	4.34E-05	8.55E-06	3.39E-05	1.14E-05
TMEM87A	1.94E-05	1.31E-05	7.26E-06	1.10E-05	3.78E-06	1.01E-05	8.04E-06	8.95E-06
TMEM87B	6.46E-06	4.33E-06	2.32E-06	3.89E-06	6.67E-07	2.45E-06	2.28E-06	2.57E-06
TMEM88	1.90E-06	2.38E-06	1.41E-06	4.37E-06	8.20E-06	4.77E-06	1.11E-05	3.80E-06
TMEM88B	0	9.88E-07	0	0	0	6.79E-08	1.31E-06	0
TMEM89	0	9.40E-08	0	0	0	0	0	0
TMEM8A	2.81E-05	3.66E-05	1.92E-05	2.61E-05	2.65E-05	1.94E-05	2.24E-05	3.00E-05
TMEM8B	1.50E-06	4.87E-06	1.81E-06	2.77E-06	3.35E-06	3.29E-06	1.68E-06	3.01E-06
TMEM8C	0	0	0	5.68E-08	0	0	0	0
TMEM9	0.000172	5.01E-05	3.63E-05	5.33E-05	3.28E-05	6.31E-05	4.45E-05	0.000119
TMEM90A	8.97E-09	2.02E-08	9.32E-09	2.20E-08	0	8.32E-09	6.96E-08	1.32E-08

TMEM90B	6.76E-08	0	0	1.89E-07	1.66E-08	8.95E-09	1.87E-08	4.25E-08
TMEM91	5.38E-06	2.21E-06	8.22E-07	4.13E-06	1.24E-05	1.93E-06	2.83E-06	1.72E-06
TMEM92	6.90E-06	2.29E-06	1.37E-06	6.76E-06	5.20E-06	1.29E-05	7.93E-06	2.34E-06
TMEM93	2.98E-05	3.70E-05	2.64E-05	3.50E-05	5.59E-05	4.17E-05	6.53E-05	3.17E-05
TMEM95	0	0	0	0	0	0	0	0
TMEM97	4.96E-05	0.000181	3.68E-05	1.68E-05	2.73E-05	1.32E-05	3.53E-05	1.74E-05
TMEM98	8.10E-05	1.37E-06	8.08E-06	2.92E-05	1.24E-05	1.05E-05	1.25E-05	2.38E-05
TMEM99	1.80E-05	6.32E-06	5.70E-06	4.92E-06	9.46E-06	8.22E-06	5.56E-06	2.47E-06
TMEM9B	4.69E-05	2.57E-05	1.92E-05	2.81E-05	1.76E-05	2.05E-05	2.71E-05	4.16E-05
TMF1	7.87E-06	4.27E-06	4.21E-06	4.85E-06	7.62E-07	5.54E-06	1.91E-06	4.73E-06
TMIE	4.80E-07	1.88E-06	4.50E-07	2.39E-07	2.12E-07	4.14E-08	1.61E-07	8.03E-07
TMIGD1	3.34E-07	0	0	0	0	1.99E-08	0	0
TMIGD2	1.84E-07	4.69E-08	2.82E-07	2.77E-07	1.22E-07	1.33E-07	5.68E-07	2.10E-07
TMLHE	1.04E-05	1.16E-05	8.13E-06	8.17E-06	3.07E-06	8.61E-06	6.50E-06	5.74E-06
TMOD1	3.44E-05	2.58E-05	1.07E-05	1.49E-05	3.72E-06	3.13E-05	1.35E-05	3.22E-05
TMOD2	2.58E-06	2.32E-07	4.33E-07	7.33E-07	1.78E-07	4.68E-07	4.97E-07	8.13E-07
TMOD3	4.83E-05	3.25E-05	2.21E-05	2.03E-05	2.91E-06	6.14E-05	1.30E-05	2.54E-05
TMOD4	1.32E-07	0	0	2.36E-08	0	0	0	0
TMPO	3.21E-05	3.60E-05	1.90E-05	1.45E-05	4.44E-06	1.23E-05	1.08E-05	1.75E-05
TMPPE	7.88E-07	8.44E-07	3.60E-07	1.69E-07	6.27E-08	2.42E-07	1.93E-07	3.89E-07
TMPRSS11	0	0	0	0	0	0	0	4.29E-08
TMPRSS11	0	0	0	0	0	0	0	0
TMPRSS11	0	0	0	0	0	0	0	0
TMPRSS11	1.68E-08	0	6.01E-08	0	0	0	8.53E-09	1.41E-08
TMPRSS11	0	0	0	0	0	0	0	0
TMPRSS12	0	0	0	0	0	0	0	0
TMPRSS13	3.68E-07	8.17E-09	1.85E-07	3.11E-07	3.12E-08	4.04E-08	8.47E-08	1.07E-08
TMPRSS15	0	6.47E-09	0	0	0	0	0	1.20E-05
TMPRSS2	2.59E-05	1.94E-06	1.85E-05	3.91E-05	8.21E-06	1.05E-05	2.09E-05	6.75E-05
TMPRSS3	1.19E-05	1.26E-08	5.30E-07	7.93E-06	0	7.39E-06	3.75E-08	3.13E-06
TMPRSS4	7.07E-07	7.69E-09	3.98E-08	5.81E-08	0	6.34E-09	0	9.52E-08
TMPRSS5	1.27E-07	2.95E-08	3.36E-08	1.56E-08	2.02E-07	4.84E-08	4.90E-08	1.15E-07
TMPRSS6	1.20E-05	8.86E-05	9.91E-05	5.14E-05	0.000261	3.21E-05	0.000114	0.000113
TMPRSS7	1.22E-07	0	0	1.14E-08	0	0	0	0
TMPRSS9	7.26E-09	1.08E-06	2.23E-06	2.39E-06	1.67E-06	3.50E-07	1.17E-06	2.89E-06
TMSB10	0.004296	0.000886	0.000366	0.001904	0.001415	0.000573	0.001346	0.000705
TMSB15A	5.90E-07	5.45E-08	3.17E-07	5.68E-08	1.59E-07	8.55E-08	0	0
TMSB15B	4.19E-06	5.45E-08	5.29E-08	2.80E-07	3.97E-08	2.18E-07	0	2.03E-07
TMSB4Y	0	4.52E-07	0	5.78E-07	1.01E-06	7.66E-07	6.24E-07	1.52E-07
TMSL3	0.004044	0.000377	0.001012	0.003376	0.001221	0.000833	0.002861	0.00072
TMTC1	5.81E-07	2.08E-06	1.52E-06	4.22E-07	2.10E-07	2.91E-06	1.09E-06	9.35E-07
TMTC2	5.18E-07	1.69E-07	1.42E-07	6.81E-07	0	5.91E-07	1.72E-07	8.88E-08
TMTC3	3.32E-06	2.96E-06	3.11E-06	2.46E-06	7.16E-07	3.19E-06	1.43E-06	2.33E-06
TMTC4	4.81E-06	4.97E-06	5.69E-06	4.10E-06	2.20E-06	5.79E-06	4.86E-06	4.27E-06
TMUB1	2.12E-05	6.60E-05	4.28E-05	4.08E-05	7.93E-05	4.10E-05	7.30E-05	5.39E-05
TMUB2	5.48E-05	3.72E-05	1.48E-05	1.92E-05	1.88E-05	2.41E-05	1.24E-05	1.69E-05
TMX1	1.72E-05	9.02E-06	1.03E-05	9.71E-06	2.99E-06	1.14E-05	7.36E-06	8.42E-06
TMX2	0.000109	0.000152	4.21E-05	4.43E-05	3.70E-05	5.43E-05	4.81E-05	8.23E-05
TMX3	8.23E-06	3.52E-06	2.98E-06	4.16E-06	9.81E-07	3.98E-06	2.57E-06	3.85E-06
TMX4	2.94E-05	3.14E-05	1.64E-05	1.69E-05	5.49E-06	2.16E-05	1.41E-05	3.11E-05
TNC	8.11E-06	8.42E-07	1.83E-06	1.75E-05	4.29E-07	1.36E-06	4.04E-06	5.28E-06
TNF	1.03E-06	2.17E-08	1.39E-07	7.82E-07	4.91E-08	7.06E-08	2.01E-07	1.71E-07
TNFAIP1	3.05E-05	4.38E-05	2.46E-05	2.59E-05	1.29E-05	5.31E-05	2.09E-05	1.83E-05
TNFAIP2	6.99E-05	5.73E-06	1.37E-05	2.76E-05	1.65E-06	8.24E-06	1.36E-05	1.25E-05
TNFAIP3	1.90E-05	6.80E-06	5.66E-06	5.64E-05	5.97E-07	3.56E-05	6.20E-06	9.72E-06
TNFAIP6	1.39E-05	8.03E-08	3.75E-08	1.19E-06	0	4.91E-08	6.76E-07	2.34E-07
TNFAIP8	1.57E-05	1.80E-06	2.01E-06	1.33E-05	7.76E-07	2.23E-06	4.18E-06	2.39E-06
TNFAIP8L1	1.34E-05	8.04E-06	1.24E-05	1.09E-05	1.58E-05	6.93E-06	2.46E-05	7.20E-06

TNFAIP8L2	8.65E-06	9.33E-07	4.00E-06	5.51E-06	1.80E-06	3.45E-06	4.57E-06	1.37E-06
TNFAIP8L3	4.62E-06	8.77E-07	3.94E-07	2.04E-06	1.35E-07	2.14E-07	6.20E-07	1.00E-06
TNFRSF10	5.01E-06	1.82E-06	2.05E-06	3.40E-06	1.23E-08	4.23E-06	1.56E-06	2.38E-06
TNFRSF10	2.13E-05	2.43E-05	1.62E-05	2.78E-05	3.23E-06	2.83E-05	1.11E-05	1.61E-05
TNFRSF10	3.34E-06	1.57E-06	8.02E-07	2.75E-06	6.16E-07	2.29E-06	8.84E-07	9.56E-07
TNFRSF10	4.84E-06	8.06E-06	5.20E-06	7.85E-06	1.21E-06	2.26E-06	4.84E-06	1.75E-06
TNFRSF11	3.11E-06	7.47E-08	3.51E-07	2.21E-07	1.90E-08	2.19E-07	1.30E-07	8.78E-07
TNFRSF11	5.50E-06	3.26E-06	5.63E-06	1.99E-05	1.47E-07	4.29E-07	1.90E-06	4.29E-07
TNFRSF12	8.92E-05	0.00014	1.22E-05	0.000223	1.86E-05	2.09E-05	0.000233	8.70E-05
TNFRSF13	3.74E-07	0	2.26E-08	4.13E-07	0	1.96E-08	1.54E-07	0
TNFRSF13	2.91E-07	0	3.21E-08	2.16E-07	2.52E-08	2.72E-08	0	4.34E-08
TNFRSF14	7.39E-05	3.61E-05	2.85E-05	4.33E-05	2.86E-05	2.56E-05	3.37E-05	5.29E-05
TNFRSF17	9.97E-07	0	1.82E-07	1.33E-06	2.86E-08	7.55E-08	1.28E-07	4.94E-08
TNFRSF18	7.02E-06	3.15E-08	2.97E-08	6.12E-07	2.36E-07	1.45E-06	7.72E-07	5.54E-07
TNFRSF19	2.03E-06	2.66E-07	7.32E-07	3.66E-06	1.50E-07	1.60E-05	6.76E-07	1.49E-07
TNFRSF1A	0.000125	0.000107	7.43E-05	0.000111	6.32E-05	9.16E-05	0.000127	8.13E-05
TNFRSF1B	3.10E-05	1.92E-05	1.33E-05	3.66E-05	4.66E-06	1.80E-05	2.05E-05	1.27E-05
TNFRSF21	1.80E-05	1.77E-05	3.93E-06	1.80E-05	6.27E-07	6.59E-06	5.19E-06	5.44E-06
TNFRSF25	6.44E-06	2.45E-06	6.92E-07	1.31E-06	2.18E-06	1.63E-06	1.94E-06	1.06E-06
TNFRSF4	5.01E-06	3.89E-06	1.14E-07	1.16E-06	1.32E-06	2.34E-06	1.01E-05	1.78E-06
TNFRSF6B	5.43E-07	9.25E-07	8.31E-07	2.67E-06	1.33E-06	7.05E-07	8.14E-07	1.52E-06
TNFRSF8	7.41E-07	5.00E-08	2.31E-07	4.67E-07	5.47E-09	1.66E-07	1.26E-07	1.18E-07
TNFRSF9	2.11E-06	4.35E-08	4.03E-08	2.51E-07	0	4.63E-07	0	9.43E-08
TNFSF10	0.000132	5.23E-05	8.45E-05	6.77E-05	7.49E-05	3.39E-05	6.82E-05	4.72E-05
TNFSF11	3.45E-06	1.17E-06	1.43E-06	3.33E-06	1.86E-08	3.00E-08	4.91E-07	5.24E-07
TNFSF12	1.53E-05	6.33E-06	1.39E-05	2.05E-05	7.28E-06	1.21E-05	3.01E-05	3.53E-05
TNFSF12-1	2.74E-06	9.42E-07	8.60E-07	3.47E-06	1.79E-06	1.70E-06	3.95E-06	6.36E-07
TNFSF13	7.97E-06	4.58E-06	7.61E-06	8.95E-06	3.33E-07	4.73E-06	5.00E-06	4.80E-06
TNFSF13B	5.10E-06	3.13E-07	1.37E-06	3.34E-06	1.17E-07	6.47E-07	1.82E-06	3.17E-07
TNFSF14	1.27E-05	2.88E-06	9.13E-07	2.93E-06	0	5.37E-06	8.80E-07	2.04E-06
TNFSF15	2.07E-06	3.27E-08	1.52E-08	5.29E-08	0	0	2.77E-08	0
TNFSF18	1.94E-07	9.17E-08	4.41E-08	0	0	2.18E-07	1.09E-07	0
TNFSF4	2.23E-06	1.61E-06	2.73E-07	4.17E-07	1.38E-07	7.99E-07	3.10E-07	2.75E-07
TNFSF8	7.03E-07	2.47E-07	2.79E-07	1.72E-06	1.04E-08	3.71E-07	5.97E-07	1.96E-07
TNFSF9	1.73E-06	3.34E-08	1.24E-07	9.02E-07	5.06E-08	2.05E-07	1.13E-07	2.17E-08
TNIK	9.48E-06	1.08E-06	2.51E-06	1.90E-06	6.83E-07	1.33E-07	1.75E-07	1.68E-06
TNIP1	9.94E-05	0.000122	6.88E-05	8.19E-05	5.49E-05	9.20E-05	0.000114	0.000183
TNIP2	2.56E-05	1.91E-05	1.05E-05	2.15E-05	9.47E-06	2.08E-05	1.79E-05	2.66E-05
TNIP3	2.93E-07	0	1.02E-08	2.65E-07	0	5.47E-08	0	1.44E-08
TNK1	3.85E-06	3.07E-06	2.73E-06	4.16E-06	1.08E-06	2.66E-06	5.69E-06	3.83E-06
TNK2	1.34E-05	5.43E-06	8.26E-06	9.04E-06	1.31E-05	8.26E-06	8.61E-06	1.15E-05
TNKS	3.88E-06	5.25E-06	2.50E-06	2.29E-06	6.53E-07	5.38E-06	1.56E-06	1.64E-06
TNKS1BP1	4.33E-05	3.71E-05	3.04E-05	4.90E-05	2.12E-05	3.55E-05	4.45E-05	3.75E-05
TNKS2	1.09E-05	6.29E-06	6.95E-06	6.79E-06	1.13E-06	8.47E-06	4.46E-06	6.05E-06
TNMD	0	3.29E-07	1.40E-07	0	3.23E-08	1.92E-07	1.44E-07	0
TNN	4.95E-08	2.23E-06	2.63E-07	8.13E-07	1.74E-07	2.08E-08	2.02E-07	3.95E-08
TNNC1	6.41E-07	2.04E-06	1.97E-07	4.81E-07	7.08E-07	2.41E-07	4.78E-06	0
TNNC2	2.65E-07	0	1.02E-07	0	6.93E-07	1.16E-06	1.66E-07	3.34E-07
TNNI1	1.23E-06	3.13E-07	4.47E-07	8.85E-07	4.10E-07	1.89E-07	5.22E-07	6.93E-08
TNNI2	1.74E-06	0	1.46E-07	1.16E-06	7.38E-07	3.98E-07	5.57E-07	0
TNNI3	2.08E-06	0	0	0	3.71E-08	0	3.99E-08	0
TNNI3K	1.30E-08	5.31E-08	0	2.64E-08	0	7.15E-09	4.50E-08	4.13E-08
TNNT1	3.06E-06	1.13E-07	1.29E-07	1.21E-07	0	4.57E-08	7.02E-08	0
TNNT2	2.57E-08	3.59E-08	0	2.32E-07	8.32E-07	4.80E-08	2.54E-07	0
TNNT3	2.43E-08	0	0	6.49E-08	1.16E-07	2.45E-08	1.25E-07	0
TNP1	7.15E-07	0	0	0	0	9.84E-08	0	0
TNP2	0	0	0	0	0	0	0	0
TNPO1	1.38E-05	1.11E-05	7.27E-06	7.96E-06	1.89E-06	1.32E-05	4.29E-06	9.66E-06

TNPO2	1.40E-05	6.19E-06	5.65E-06	6.26E-06	5.65E-06	7.85E-06	8.45E-06	7.12E-06
TNPO3	1.20E-05	1.65E-05	1.28E-05	1.06E-05	1.00E-06	1.66E-05	9.24E-06	1.42E-05
TNR	0	6.45E-08	2.85E-07	5.85E-09	0	0	0	0
TNRC18	2.58E-05	2.19E-05	8.56E-06	8.74E-06	8.04E-06	1.16E-05	1.20E-05	1.36E-05
TNRC6A	1.65E-05	1.76E-05	8.05E-06	8.73E-06	3.67E-06	1.12E-05	1.00E-05	7.35E-06
TNRC6B	4.25E-06	1.87E-06	1.72E-06	1.96E-06	4.86E-07	2.85E-06	1.32E-06	1.78E-06
TNRC6C	2.60E-06	2.55E-06	1.15E-06	1.26E-06	1.48E-06	1.46E-06	1.04E-06	1.32E-06
TNS1	6.07E-05	1.46E-05	1.28E-05	2.28E-05	9.49E-06	8.44E-06	2.03E-05	1.35E-05
TNS3	3.39E-05	1.80E-05	1.92E-05	1.98E-05	5.54E-06	5.28E-05	1.86E-05	4.07E-05
TNS4	1.53E-07	2.24E-08	9.56E-08	5.36E-08	0	6.09E-08	1.14E-07	4.83E-08
TNXB	4.85E-06	8.31E-07	7.07E-06	2.46E-05	4.73E-06	3.42E-06	7.06E-06	6.28E-06
TOB1	0.000292	0.000404	0.000176	5.29E-05	8.88E-05	0.000209	4.90E-05	0.000141
TOB2	2.37E-05	1.33E-05	7.99E-06	9.77E-06	1.23E-05	1.48E-05	1.01E-05	1.60E-05
TOE1	6.21E-06	2.97E-06	2.92E-06	5.60E-06	1.89E-06	3.46E-06	5.03E-06	2.49E-06
TOLLIP	3.74E-05	5.53E-05	5.78E-05	3.81E-05	5.17E-05	4.54E-05	4.35E-05	9.96E-05
TOM1	5.17E-05	5.39E-05	4.46E-05	4.90E-05	6.40E-05	5.59E-05	4.17E-05	6.15E-05
TOM1L1	5.17E-05	6.62E-05	4.21E-05	2.42E-05	5.00E-06	8.35E-05	2.05E-05	2.10E-05
TOM1L2	3.92E-06	6.68E-06	2.17E-06	3.13E-06	5.19E-06	4.93E-06	6.94E-06	7.21E-06
TOMM20	6.10E-05	4.90E-05	4.27E-05	4.45E-05	1.89E-05	8.74E-05	5.27E-05	6.04E-05
TOMM20l	1.91E-07	2.37E-07	0	0	2.80E-07	2.42E-07	1.76E-07	0
TOMM22	5.89E-05	5.16E-05	3.83E-05	5.20E-05	5.21E-05	5.71E-05	5.96E-05	6.30E-05
TOMM34	2.07E-05	3.36E-05	9.57E-06	1.51E-05	7.36E-06	1.71E-05	1.69E-05	1.40E-05
TOMM40	5.38E-05	5.14E-05	1.66E-05	2.30E-05	3.94E-05	3.89E-05	3.54E-05	3.73E-05
TOMM40l	1.75E-05	1.93E-05	3.59E-06	5.89E-06	9.70E-06	1.86E-05	1.04E-05	3.73E-05
TOMM5	8.84E-05	0.000101	4.32E-05	6.21E-05	6.20E-05	8.14E-05	6.73E-05	5.24E-05
TOMM6	0.000142	0.000189	6.61E-05	8.84E-05	0.000139	0.000158	0.000158	0.000126
TOMM7	0.000205	0.000225	7.32E-05	0.000155	0.000253	0.000174	0.000182	0.000114
TOMM70/	3.12E-05	7.54E-05	2.57E-05	2.07E-05	1.86E-05	3.30E-05	2.63E-05	2.72E-05
TOP1	4.76E-05	3.23E-05	1.70E-05	1.96E-05	4.31E-06	2.54E-05	1.29E-05	1.95E-05
TOP1MT	2.59E-05	1.77E-05	1.42E-05	1.89E-05	2.19E-05	1.61E-05	5.36E-05	2.15E-05
TOP1P1	4.35E-06	2.79E-06	1.71E-06	1.76E-06	7.58E-07	2.24E-06	1.56E-06	2.15E-06
TOP1P2	3.43E-07	1.68E-07	6.93E-08	1.20E-07	1.41E-07	9.11E-08	1.57E-07	1.69E-07
TOP2A	3.85E-05	1.61E-05	2.26E-07	4.52E-07	1.17E-06	5.03E-06	3.80E-07	1.61E-06
TOP2B	3.39E-05	2.26E-05	2.39E-05	3.10E-05	1.90E-05	3.80E-05	4.19E-05	2.68E-05
TOP3A	3.92E-06	9.20E-06	4.27E-06	3.69E-06	3.65E-06	6.14E-06	4.79E-06	6.16E-06
TOP3B	7.52E-06	4.72E-06	3.28E-06	3.12E-06	6.06E-06	4.76E-06	3.14E-06	3.44E-06
TOPBP1	1.00E-05	9.15E-06	2.44E-06	2.88E-06	8.84E-07	3.82E-06	2.17E-06	2.96E-06
TOPORS	4.87E-06	6.24E-06	5.36E-06	5.37E-06	1.39E-06	9.83E-06	3.18E-06	5.75E-06
TOR1A	2.39E-05	1.88E-05	2.30E-05	2.57E-05	1.17E-05	2.33E-05	2.54E-05	2.50E-05
TOR1AIP1	1.67E-05	1.71E-05	1.64E-05	1.21E-05	1.86E-06	2.00E-05	1.09E-05	2.07E-05
TOR1AIP2	1.75E-05	2.20E-05	1.52E-05	9.62E-06	3.51E-07	2.80E-05	6.79E-06	2.50E-05
TOR1B	2.96E-05	9.87E-06	1.01E-05	8.18E-06	4.51E-06	9.16E-06	9.17E-06	2.64E-05
TOR2A	1.67E-05	6.42E-06	6.77E-06	1.16E-05	1.18E-05	1.06E-05	1.10E-05	1.94E-05
TOR3A	2.01E-05	1.13E-05	1.19E-05	1.93E-05	2.66E-05	1.75E-05	2.19E-05	2.55E-05
TOX	1.76E-05	8.01E-08	5.49E-07	4.91E-07	9.45E-09	7.23E-07	1.34E-07	5.64E-08
TOX2	2.55E-06	8.37E-07	1.53E-06	1.43E-06	1.29E-06	1.76E-06	2.05E-06	1.38E-06
TOX3	7.98E-06	6.08E-06	1.83E-06	3.37E-06	9.03E-07	1.12E-05	3.99E-06	2.69E-06
TOX4	2.15E-05	1.59E-05	1.08E-05	1.19E-05	6.99E-06	1.87E-05	1.04E-05	1.44E-05
TP53	2.56E-05	9.65E-06	1.48E-05	2.09E-05	6.60E-06	2.08E-05	3.74E-05	1.66E-05
TP53AIP1	1.92E-08	0	0	0	3.41E-08	0	0	6.14E-08
TP53BP1	5.96E-06	3.17E-06	1.72E-06	4.02E-06	8.27E-07	4.20E-06	2.46E-06	2.64E-06
TP53BP2	1.67E-05	4.91E-05	5.55E-06	2.55E-05	4.10E-06	1.84E-05	1.51E-05	4.95E-05
TP53I11	3.15E-05	6.05E-06	3.77E-06	6.08E-06	5.59E-06	5.27E-06	1.57E-05	6.50E-06
TP53I13	2.31E-05	1.25E-05	1.24E-05	1.85E-05	1.91E-05	5.65E-06	2.95E-05	3.10E-06
TP53I3	8.59E-05	1.14E-05	7.16E-06	1.72E-05	4.03E-05	9.59E-05	3.83E-05	1.91E-05
TP53INP1	0.000237	2.11E-05	3.92E-05	2.83E-05	6.91E-06	9.65E-06	7.41E-05	7.33E-05
TP53INP2	0.000141	6.44E-05	1.24E-05	1.70E-05	1.32E-05	1.81E-05	1.15E-05	3.73E-05
TP53RK	3.58E-06	3.46E-06	2.50E-06	4.13E-06	3.46E-06	3.84E-06	6.02E-06	3.29E-06

TP53TG1	0.00011	0.000161	4.63E-05	4.79E-05	0.000147	4.98E-05	5.69E-05	5.41E-05
TP53TG3B	0	0	0	2.88E-08	0	0	0	0
TP53TG5	0	#######	1.24E-08	0	0	0	0	0
TP63	4.61E-08	4.59E-08	1.32E-07	8.76E-08	0	1.80E-07	7.48E-08	1.95E-07
TP73	4.05E-06	1.03E-06	1.83E-08	1.13E-07	1.72E-07	5.64E-07	2.32E-07	7.78E-08
TPBG	6.34E-06	4.51E-07	6.67E-07	1.60E-06	8.59E-08	1.48E-07	7.73E-07	3.23E-07
TPCN1	2.45E-05	7.68E-06	9.06E-06	8.38E-06	5.92E-06	1.08E-05	1.37E-05	2.37E-05
TPCN2	8.76E-06	8.67E-06	4.22E-06	4.16E-06	1.80E-06	2.39E-06	2.51E-06	4.45E-06
TPD52	5.12E-05	2.99E-05	1.84E-05	2.01E-05	1.64E-05	1.65E-05	3.64E-05	2.37E-05
TPD52L1	3.14E-05	1.63E-05	4.32E-05	3.04E-05	8.79E-06	6.74E-05	2.05E-05	3.97E-06
TPD52L2	9.11E-05	3.71E-05	1.85E-05	2.83E-05	2.70E-05	3.58E-05	3.05E-05	2.16E-05
TPD52L3	0	0	0	0	0	0	2.33E-08	0
TPH1	3.03E-08	0	3.21E-08	0	0	1.41E-08	0	0
TPH2	0	1.26E-08	0	5.47E-08	0	0	2.16E-08	1.64E-08
TPI1	0.000477	0.000454	0.000191	0.000255	0.000297	0.000327	0.000365	0.000265
TPI1P2	3.45E-07	5.78E-07	3.98E-07	2.91E-07	6.14E-08	8.38E-07	6.89E-08	3.50E-07
TPI1P3	6.35E-08	7.30E-08	4.56E-08	2.61E-08	0	1.18E-07	0	0
TPK1	1.46E-06	6.10E-07	1.99E-06	2.65E-06	5.01E-07	1.33E-06	1.37E-06	3.60E-06
TPM1	0.000245	9.69E-05	4.11E-05	0.000329	9.24E-05	5.06E-05	0.000161	7.32E-05
TPM2	4.29E-05	1.40E-05	2.28E-06	2.34E-05	5.03E-05	1.84E-05	3.89E-05	2.34E-05
TPM3	0.000296	0.000242	9.28E-05	0.000153	5.08E-05	0.000182	0.00015	0.000178
TPM4	0.000204	3.16E-05	2.28E-05	8.93E-05	9.57E-06	2.05E-05	4.27E-05	6.01E-05
TPMT	2.38E-05	2.89E-05	3.53E-05	2.54E-05	1.80E-05	4.15E-05	2.04E-05	2.37E-05
TPO	0	0	0	0	0	0	3.70E-08	0
TPP1	0.000156	0.000143	6.25E-05	7.01E-05	2.51E-05	8.42E-05	7.64E-05	0.000137
TPP2	1.17E-05	1.17E-05	9.36E-06	7.53E-06	1.58E-06	9.68E-06	4.17E-06	6.53E-06
TPPP	4.71E-06	2.63E-06	8.95E-07	4.06E-06	7.66E-08	7.57E-08	1.27E-06	6.62E-07
TPPP2	4.29E-07	4.21E-07	4.03E-06	3.24E-06	6.99E-06	4.68E-07	3.07E-06	0
TPPP3	1.16E-05	5.74E-06	1.74E-06	1.82E-05	5.74E-06	2.05E-06	6.69E-06	5.88E-06
TPR	1.56E-05	8.32E-06	5.96E-06	7.43E-06	1.94E-06	1.40E-05	4.18E-06	1.03E-05
TPRA1	2.07E-05	2.34E-05	1.86E-05	1.92E-05	2.65E-05	2.00E-05	2.69E-05	1.62E-05
TPRG1	4.32E-08	1.39E-07	3.34E-07	8.02E-07	4.28E-07	2.08E-06	1.10E-06	4.35E-07
TPRG1L	3.17E-05	4.00E-05	5.52E-05	5.07E-05	3.06E-05	7.92E-05	3.82E-05	2.97E-05
TPRKB	3.07E-05	2.65E-05	1.27E-05	1.47E-05	1.40E-05	1.73E-05	2.03E-05	1.33E-05
TPRN	1.84E-05	1.13E-05	6.17E-06	6.08E-06	9.85E-06	1.17E-05	1.21E-05	1.86E-05
TPRX1	0	0	2.76E-08	0	2.26E-08	0	0	0
TPRXL	0	0	0	2.57E-08	0	9.72E-08	9.71E-09	1.54E-08
TPSAB1	5.47E-07	4.22E-07	2.08E-06	5.97E-06	5.79E-07	2.81E-07	1.24E-06	3.01E-06
TPSB2	5.85E-07	4.35E-07	4.73E-06	5.17E-06	6.68E-07	8.70E-07	2.29E-06	3.17E-06
TPSD1	0	0	2.76E-07	0	0	5.29E-08	1.02E-07	0
TPSG1	1.62E-06	8.19E-08	6.93E-07	5.84E-08	0	2.21E-08	1.15E-07	3.52E-08
TPST1	3.26E-05	2.83E-05	1.60E-05	1.19E-05	2.74E-06	9.33E-06	1.16E-05	4.66E-05
TPST2	2.51E-05	4.87E-05	5.14E-05	4.44E-05	6.14E-05	4.21E-05	3.91E-05	4.33E-05
TPT1	0.005645	0.002947	0.004277	0.006207	0.003791	0.005058	0.005772	0.002587
TPTE	1.48E-08	0	6.94E-09	8.04E-09	0	0	0	9.84E-09
TPTE2	0	0	0	0	0	0	0	3.54E-08
TPTE2P1	1.72E-07	3.56E-07	3.53E-09	2.14E-08	0	7.26E-07	1.33E-07	1.72E-07
TPTE2P3	0	0	0	0	0	0	0	0
TPX2	2.34E-05	9.78E-06	8.30E-07	2.28E-06	2.61E-06	3.68E-06	1.31E-06	2.74E-06
TRA2A	6.06E-05	2.61E-05	1.68E-05	2.20E-05	9.23E-06	2.00E-05	1.91E-05	2.34E-05
TRA2B	3.11E-05	1.90E-05	1.98E-05	2.63E-05	7.09E-06	1.72E-05	2.36E-05	1.55E-05
TRABD	2.63E-05	1.88E-05	1.29E-05	1.65E-05	2.38E-05	1.49E-05	2.77E-05	1.94E-05
TRADD	2.04E-05	1.13E-05	8.82E-06	1.47E-05	9.27E-06	1.38E-05	1.56E-05	1.14E-05
TRAF1	5.00E-06	9.80E-07	8.63E-07	4.13E-06	4.18E-07	2.73E-06	1.53E-06	1.85E-06
TRAF2	1.72E-05	1.41E-05	3.74E-06	8.04E-06	4.15E-06	7.59E-06	9.62E-06	1.79E-05
TRAF3	4.68E-06	2.02E-06	2.46E-06	3.66E-06	8.60E-07	2.14E-06	2.08E-06	4.67E-06
TRAF3IP1	6.02E-06	4.64E-06	3.58E-06	3.58E-06	1.03E-06	5.17E-06	3.47E-06	4.27E-06
TRAF3IP2	1.01E-05	4.31E-06	4.38E-06	1.45E-05	2.35E-06	1.33E-05	1.26E-05	3.22E-06

TRAF3IP3	4.29E-06	1.75E-07	1.30E-06	2.22E-06	2.32E-07	2.49E-06	1.75E-06	5.50E-07
TRAF4	2.63E-05	3.15E-05	1.32E-05	2.96E-05	1.83E-05	1.66E-05	2.50E-05	2.67E-05
TRAF5	4.23E-06	6.95E-07	3.54E-07	9.37E-07	2.35E-07	1.31E-06	1.02E-06	1.40E-06
TRAF6	4.15E-06	3.08E-06	3.09E-06	2.43E-06	4.23E-07	3.26E-06	1.59E-06	2.68E-06
TRAF7	4.13E-05	3.70E-05	1.68E-05	1.70E-05	6.36E-06	3.64E-05	2.89E-05	1.81E-05
TRAFD1	1.89E-05	9.56E-06	6.90E-06	8.75E-06	2.91E-06	1.30E-05	8.06E-06	6.87E-06
TRAIP	3.17E-06	2.15E-06	1.03E-07	1.73E-07	5.67E-07	8.17E-07	6.32E-07	6.78E-07
TRAK1	1.47E-05	5.26E-06	4.40E-06	6.28E-06	2.81E-06	5.51E-06	4.96E-06	6.74E-06
TRAK2	2.03E-05	8.35E-06	5.68E-06	6.82E-06	2.12E-06	4.61E-06	4.67E-06	3.55E-06
TRAM1	0.000278	0.000162	8.69E-05	0.00011	0.000101	9.48E-05	0.000233	0.000136
TRAM1L1	7.10E-08	0	7.44E-08	1.02E-07	1.02E-08	5.49E-08	8.01E-08	5.23E-08
TRAM2	5.97E-06	5.39E-06	2.78E-06	5.21E-06	1.31E-06	4.31E-06	7.47E-06	2.31E-06
TRANK1	4.79E-06	3.08E-06	4.90E-06	5.03E-06	1.38E-06	4.57E-06	4.45E-06	2.65E-06
TRAP1	8.39E-05	0.000131	7.68E-05	6.54E-05	3.94E-05	6.41E-05	9.77E-05	7.65E-05
TRAPPC1	8.88E-05	5.30E-05	5.76E-05	8.98E-05	5.85E-05	0.000141	9.34E-05	0.000102
TRAPPC1(	1.51E-05	5.02E-06	5.47E-06	5.91E-06	3.64E-06	7.49E-06	5.19E-06	8.81E-06
TRAPPC2	4.89E-06	2.40E-06	4.86E-06	3.40E-06	1.48E-06	3.88E-06	4.42E-06	4.82E-06
TRAPPC2L	5.72E-05	3.95E-05	3.94E-05	5.93E-05	6.54E-05	4.78E-05	5.18E-05	6.72E-05
TRAPPC2F	1.77E-05	1.55E-05	9.24E-06	1.47E-05	9.75E-06	1.91E-06	4.16E-06	2.09E-05
TRAPPC3	5.24E-05	3.63E-05	3.24E-05	3.45E-05	2.12E-05	4.70E-05	3.65E-05	3.97E-05
TRAPPC4	6.72E-05	1.98E-05	1.49E-05	2.57E-05	1.43E-05	3.09E-05	3.33E-05	5.20E-05
TRAPPC5	6.76E-05	3.37E-05	5.67E-05	6.36E-05	0.000188	5.55E-05	0.000101	6.52E-05
TRAPPC6/	3.92E-05	0.000104	4.12E-05	4.49E-05	9.80E-05	6.34E-05	3.59E-05	2.74E-05
TRAPPC6E	9.48E-06	7.12E-06	9.23E-06	8.31E-06	3.74E-06	9.43E-06	6.90E-06	8.00E-06
TRAPPC9	6.77E-06	9.58E-06	3.74E-06	5.38E-06	8.40E-06	7.49E-06	9.01E-06	8.11E-06
TRAT1	1.54E-06	0	2.58E-07	1.33E-06	0	3.61E-07	2.92E-07	4.27E-07
TRDMT1	2.98E-07	1.70E-07	7.19E-07	4.79E-07	5.03E-08	1.70E-07	8.43E-08	1.19E-07
TRDN	0	0	1.42E-08	2.52E-08	0	0	0	0
TREH	7.98E-07	4.73E-07	3.75E-06	5.53E-07	3.34E-06	6.28E-06	1.76E-06	4.11E-08
TREM1	1.36E-06	8.20E-08	7.63E-07	5.85E-06	1.03E-07	8.31E-08	3.02E-07	8.04E-07
TREM2	9.31E-06	2.94E-06	1.85E-06	2.49E-05	7.88E-07	1.08E-06	1.47E-05	7.85E-07
TREML1	0	4.12E-07	0	1.57E-07	8.29E-08	2.75E-08	5.60E-08	0
TREML2	1.34E-07	6.81E-09	6.26E-08	9.66E-08	0	2.81E-08	1.77E-08	3.56E-08
TREML2P1	5.23E-07	0	0	0	0	0	0	0
TREML3	6.87E-08	1.32E-07	0	1.13E-07	0	2.98E-05	0	0
TREML4	2.34E-08	0	0	1.41E-08	0	1.40E-06	0	0
TRERF1	9.11E-07	1.53E-07	3.03E-07	6.20E-07	4.36E-08	2.04E-07	2.78E-07	2.46E-07
TREX1	2.81E-05	9.92E-06	7.86E-06	1.09E-05	4.16E-06	1.15E-05	4.47E-06	1.25E-05
TREX2	1.42E-07	7.23E-08	1.56E-07	0	8.25E-08	6.10E-08	2.34E-07	6.73E-08
TRH	1.19E-08	0	0	2.49E-07	0	0	0	0
TRHDE	3.03E-06	4.97E-08	3.65E-07	2.89E-07	6.94E-09	0	0	9.72E-07
TRHR	0	0	0	0	0	0	0	0
TRIAP1	4.36E-05	4.37E-05	3.23E-05	3.24E-05	3.14E-05	4.63E-05	3.23E-05	3.04E-05
TRIB1	5.66E-05	0.000116	0.000214	0.000253	1.94E-05	2.69E-05	0.000261	4.32E-05
TRIB2	6.11E-06	6.20E-06	4.53E-06	4.66E-06	1.17E-06	2.00E-05	3.77E-06	4.65E-06
TRIB3	3.96E-05	0.000153	1.82E-05	2.11E-05	2.09E-05	1.47E-05	7.39E-05	4.47E-05
TRIL	8.39E-07	1.17E-06	1.92E-07	3.60E-07	2.49E-07	2.97E-07	6.30E-07	1.63E-07
TRIM10	2.99E-06	1.59E-06	1.18E-06	1.82E-06	4.63E-07	2.11E-06	2.53E-06	3.25E-07
TRIM11	7.53E-06	1.15E-05	3.03E-06	5.45E-06	2.83E-06	7.30E-06	8.38E-06	1.15E-05
TRIM13	3.06E-06	2.42E-07	1.78E-06	2.16E-06	9.82E-07	1.22E-06	7.72E-07	1.85E-06
TRIM14	2.05E-05	1.04E-05	1.23E-05	1.00E-05	5.57E-06	1.71E-05	1.33E-05	1.14E-05
TRIM15	1.00E-05	1.43E-05	3.92E-06	1.62E-05	6.07E-06	3.37E-06	2.64E-05	3.02E-06
TRIM16	4.42E-06	1.61E-05	2.63E-06	3.54E-06	2.67E-06	6.86E-05	3.83E-06	4.68E-06
TRIM16L	8.99E-07	1.30E-05	1.94E-06	1.31E-06	6.21E-06	4.96E-05	3.30E-06	3.78E-06
TRIM17	3.91E-07	0	0	1.67E-08	0	0	4.71E-08	2.01E-08
TRIM2	6.17E-06	6.93E-06	3.32E-06	2.85E-06	1.05E-06	4.74E-06	1.60E-06	3.89E-06
TRIM21	1.95E-05	1.29E-05	8.24E-06	1.14E-05	2.16E-06	1.15E-05	7.56E-06	2.11E-05
TRIM22	1.75E-05	2.01E-06	1.45E-05	1.89E-05	1.03E-06	2.75E-05	4.76E-06	2.07E-05

TRIM23	7.17E-06	5.72E-06	4.95E-06	3.44E-06	1.42E-06	5.56E-06	2.50E-06	3.72E-06
TRIM24	7.53E-06	1.82E-05	8.75E-06	1.00E-05	6.47E-06	1.12E-05	8.90E-06	8.76E-06
TRIM25	9.33E-06	5.40E-06	2.30E-06	2.92E-06	6.17E-06	3.63E-06	9.10E-06	4.45E-06
TRIM26	5.79E-05	4.37E-05	2.64E-05	2.97E-05	1.77E-05	3.45E-05	3.02E-05	5.93E-05
TRIM27	2.53E-05	2.81E-05	1.67E-05	1.82E-05	1.76E-05	4.33E-05	2.26E-05	3.07E-05
TRIM28	9.71E-05	7.48E-05	4.91E-05	6.32E-05	0.00014	6.79E-05	0.00011	5.64E-05
TRIM29	1.36E-07	3.35E-06	1.21E-06	8.08E-07	1.21E-08	6.56E-07	3.72E-08	3.18E-07
TRIM3	8.23E-06	2.44E-06	1.61E-06	2.48E-06	1.36E-06	4.26E-06	2.18E-06	3.98E-06
TRIM31	3.40E-05	3.46E-06	4.01E-06	3.11E-06	2.03E-08	2.38E-06	5.02E-07	6.34E-06
TRIM32	4.40E-06	2.23E-06	1.74E-06	2.31E-06	5.76E-07	4.04E-06	1.88E-06	2.83E-06
TRIM33	5.89E-06	7.18E-06	4.14E-06	3.43E-06	1.82E-06	6.42E-06	2.72E-06	3.71E-06
TRIM34	3.43E-06	1.70E-06	2.00E-06	2.91E-06	1.56E-07	2.79E-06	1.17E-06	2.18E-06
TRIM35	3.45E-06	1.28E-05	4.95E-06	1.01E-05	8.89E-07	7.77E-06	3.81E-06	3.15E-06
TRIM36	2.38E-07	5.46E-08	4.45E-08	2.33E-07	1.40E-08	7.52E-08	5.25E-08	2.38E-08
TRIM37	1.03E-05	7.48E-06	2.64E-06	3.48E-06	1.47E-06	7.62E-06	2.24E-06	3.15E-06
TRIM38	1.22E-05	5.09E-06	3.49E-06	6.02E-06	1.43E-06	1.03E-05	4.16E-06	6.74E-06
TRIM39	7.33E-06	5.12E-06	2.03E-06	3.92E-06	2.02E-06	5.35E-06	4.84E-06	6.40E-06
TRIM4	2.91E-05	2.72E-05	9.51E-06	9.95E-06	3.06E-06	1.42E-05	9.21E-06	1.17E-05
TRIM40	2.44E-07	2.26E-07	1.32E-08	2.66E-07	0	6.36E-08	1.47E-07	1.85E-08
TRIM41	1.70E-05	7.76E-06	9.31E-06	1.30E-05	5.27E-06	2.08E-05	1.15E-05	1.40E-05
TRIM42	0	0	0	0	0	0	0	0
TRIM43	0	0	0	0	0	0	2.91E-08	0
TRIM44	1.38E-05	1.46E-05	9.73E-06	7.26E-06	7.41E-07	1.09E-05	3.92E-06	8.55E-06
TRIM45	1.30E-05	9.79E-07	1.11E-07	4.17E-07	1.45E-07	2.16E-06	4.59E-07	6.17E-07
TRIM46	1.45E-07	6.35E-08	5.34E-08	1.21E-07	2.07E-08	4.48E-08	1.17E-07	9.75E-08
TRIM47	2.79E-05	2.22E-05	1.02E-05	3.38E-05	2.68E-06	1.22E-05	2.41E-05	1.09E-05
TRIM48	0	0	0	0	0	0	0	0
TRIM49	0	0	0	0	0	0	0	0
TRIM49L	0	0	0	0	0	0	0	0
TRIM5	1.69E-05	8.82E-06	8.81E-06	8.38E-06	3.34E-06	2.00E-05	4.05E-06	1.61E-05
TRIM50	0	1.10E-07	5.10E-08	8.94E-08	1.24E-07	3.39E-05	1.29E-07	7.70E-07
TRIM52	5.99E-06	1.43E-06	1.52E-06	1.32E-06	2.47E-07	2.13E-06	6.39E-07	1.24E-06
TRIM53	0	0	0	0	0	0	0	0
TRIM54	3.04E-06	1.04E-06	3.18E-08	7.39E-08	0	1.11E-06	0	0
TRIM55	0	2.45E-05	1.34E-05	3.35E-06	2.87E-05	3.93E-05	3.82E-05	1.67E-05
TRIM56	8.59E-06	6.42E-06	2.41E-06	2.01E-06	2.07E-07	3.89E-06	1.95E-06	2.21E-06
TRIM58	5.68E-08	1.12E-07	8.06E-08	9.60E-08	1.12E-08	2.43E-08	2.13E-08	6.39E-09
TRIM59	1.08E-06	9.89E-08	7.88E-08	2.16E-07	1.82E-07	2.89E-07	2.11E-07	1.88E-07
TRIM6	9.40E-07	7.32E-07	2.08E-07	5.79E-07	5.21E-08	1.23E-07	2.04E-07	3.18E-06
TRIM60	0	0	0	0	0	0	0	0
TRIM61	0	3.20E-07	4.96E-08	7.67E-08	0	1.45E-08	0	2.30E-08
TRIM62	1.74E-06	1.61E-06	6.12E-07	8.35E-07	1.68E-07	6.88E-07	7.51E-07	1.40E-06
TRIM63	5.51E-08	0	0	5.07E-08	0	0	0	2.03E-08
TRIM64	0	0	0	0	0	0	0	0
TRIM65	6.61E-06	4.04E-06	2.71E-06	2.70E-06	1.41E-06	4.62E-06	4.09E-06	2.57E-06
TRIM66	2.85E-06	6.77E-07	2.82E-07	4.32E-07	4.20E-07	8.75E-07	5.38E-07	5.83E-07
TRIM67	1.30E-08	8.71E-09	2.66E-09	5.40E-08	6.70E-09	5.06E-08	1.27E-08	2.28E-08
TRIM68	3.19E-06	6.70E-06	2.89E-06	2.22E-06	7.66E-07	6.40E-06	3.40E-06	6.04E-06
TRIM69	6.78E-07	1.75E-07	1.96E-07	4.23E-07	7.72E-08	8.47E-07	2.32E-07	2.06E-06
TRIM6-TR	#######	0	0	0	0	0	0	#######
TRIM7	4.47E-06	6.17E-08	1.46E-07	8.47E-08	1.38E-07	3.69E-07	1.34E-07	1.65E-07
TRIM71	2.94E-08	4.96E-08	1.52E-08	0	0	6.82E-09	0	0
TRIM72	0	0	0	0	0	1.06E-08	0	0
TRIM74	2.07E-07	1.43E-06	6.36E-08	2.43E-08	1.36E-07	4.11E-07	1.43E-07	3.09E-08
TRIM77	0	0	0	0	0	0	0	0
TRIM78P	3.71E-07	2.31E-07	7.43E-08	2.49E-08	0	7.43E-08	8.03E-08	1.90E-07
TRIM8	4.82E-05	2.70E-05	3.08E-05	4.43E-05	1.82E-05	4.47E-05	5.11E-05	5.21E-05
TRIM9	4.33E-07	1.36E-06	2.86E-07	5.89E-08	5.78E-08	4.39E-08	9.50E-08	3.54E-08

TRIML1	0	0	0	0	0	0	0	0
TRIML2	0	0	0	0	0	0	0	0
TRIO	6.17E-06	2.32E-06	2.70E-06	2.87E-06	1.74E-07	4.05E-06	1.75E-06	4.48E-06
TRIOBP	2.32E-05	8.65E-06	1.06E-05	1.69E-05	5.87E-06	1.24E-05	1.22E-05	1.18E-05
TRIP10	2.97E-05	7.07E-06	5.87E-06	8.79E-06	5.27E-06	5.25E-06	8.02E-06	5.09E-06
TRIP11	9.93E-06	3.25E-06	4.42E-06	4.29E-06	1.28E-06	7.33E-06	2.26E-06	5.33E-06
TRIP12	3.18E-05	2.12E-05	1.33E-05	1.40E-05	7.00E-06	2.42E-05	1.10E-05	1.20E-05
TRIP13	4.41E-06	2.30E-06	1.36E-07	1.11E-07	1.81E-07	7.17E-07	4.28E-07	6.79E-07
TRIP4	1.63E-05	1.34E-05	6.42E-06	1.00E-05	3.84E-06	1.17E-05	1.08E-05	9.75E-06
TRIP6	0.000106	3.88E-05	3.29E-05	4.90E-05	3.77E-05	3.64E-05	5.02E-05	7.83E-05
TRIT1	6.25E-06	6.75E-06	5.12E-06	4.85E-06	3.01E-06	7.42E-06	5.89E-06	5.43E-06
TRMT1	2.27E-05	1.39E-05	7.95E-06	1.25E-05	3.10E-05	1.84E-05	1.85E-05	1.41E-05
TRMT11	1.04E-05	4.49E-06	8.46E-06	5.86E-06	6.26E-07	1.08E-05	6.61E-06	8.15E-06
TRMT112	0.000351	0.000271	0.000168	0.000209	0.000222	0.000297	0.000235	0.000333
TRMT12	1.24E-05	6.41E-06	3.33E-06	3.79E-06	4.12E-06	3.54E-06	8.52E-06	5.27E-06
TRMT2A	1.52E-05	2.59E-05	1.07E-05	1.04E-05	1.46E-05	1.63E-05	1.38E-05	1.60E-05
TRMT2B	6.07E-06	4.03E-06	3.06E-06	2.47E-06	5.49E-07	2.37E-06	1.76E-06	4.71E-06
TRMT5	4.38E-06	2.49E-06	2.28E-06	2.89E-06	1.24E-06	4.71E-06	3.23E-06	2.99E-06
TRMT6	1.08E-05	9.15E-06	3.36E-06	6.74E-06	2.01E-06	7.17E-06	4.22E-06	6.17E-06
TRMT61A	9.74E-06	8.17E-06	3.33E-06	6.53E-06	1.05E-05	6.44E-06	1.10E-05	9.41E-06
TRMT61B	1.15E-05	1.51E-05	6.00E-06	5.22E-06	2.25E-06	1.33E-05	5.41E-06	4.30E-06
TRMU	1.39E-05	1.20E-05	5.05E-06	6.23E-06	1.16E-05	1.17E-05	6.65E-06	1.00E-05
TRNAU1A	1.36E-05	7.49E-06	3.48E-06	4.78E-06	4.73E-06	5.99E-06	5.93E-06	4.94E-06
TRNP1	3.02E-05	3.83E-06	2.00E-06	1.91E-05	1.37E-06	4.46E-07	5.03E-06	5.17E-05
TRNT1	1.20E-05	6.28E-06	4.38E-06	3.64E-06	1.03E-06	4.49E-06	4.99E-06	5.37E-06
TRO	7.36E-07	4.98E-06	3.59E-06	3.55E-06	6.48E-07	6.65E-07	1.87E-06	5.46E-06
TROAP	1.64E-05	2.23E-06	1.80E-07	2.27E-07	2.78E-06	1.55E-06	4.80E-07	2.89E-06
TROVE2	1.22E-05	1.01E-05	9.25E-06	9.42E-06	3.75E-06	1.20E-05	8.94E-06	1.68E-05
TRPA1	3.04E-08	0	0	1.59E-08	0	0	4.23E-08	2.54E-08
TRPC1	2.50E-06	2.90E-06	1.50E-07	3.83E-07	5.28E-08	9.84E-08	1.14E-07	1.56E-07
TRPC2	6.50E-08	2.49E-08	0	1.07E-07	1.87E-08	2.02E-08	1.04E-07	0
TRPC3	2.24E-08	5.87E-08	1.54E-08	0	0	3.46E-08	7.25E-09	0
TRPC4	5.06E-07	4.47E-08	1.60E-08	1.67E-07	9.40E-08	5.21E-08	1.72E-07	2.68E-07
TRPC4AP	6.51E-05	4.19E-05	3.25E-05	3.37E-05	2.73E-05	4.13E-05	2.14E-05	3.67E-05
TRPC5	2.27E-07	5.66E-07	1.37E-07	7.13E-08	5.06E-08	0	7.16E-08	1.55E-08
TRPC6	8.79E-07	1.95E-07	5.47E-09	4.55E-08	1.05E-07	1.77E-07	1.91E-07	1.14E-07
TRPC7	1.07E-07	2.68E-08	0	0	0	0	0	0
TRPM1	0	6.99E-07	0	5.07E-09	0	4.24E-09	4.34E-09	0
TRPM2	2.70E-06	3.80E-06	5.96E-07	1.64E-06	8.62E-07	4.48E-07	2.18E-06	4.29E-07
TRPM3	2.39E-07	1.34E-08	2.32E-08	5.76E-08	0	1.12E-07	4.79E-09	1.14E-07
TRPM4	9.79E-06	5.55E-06	4.19E-06	4.40E-06	6.91E-06	6.78E-06	1.33E-05	6.62E-06
TRPM5	8.13E-08	0	0	0	0	0	0	0
TRPM6	3.34E-08	3.88E-08	8.74E-09	1.37E-08	4.80E-09	3.87E-08	3.81E-08	3.38E-08
TRPM7	1.63E-05	8.18E-06	2.22E-05	9.33E-06	2.42E-06	1.87E-05	4.20E-06	1.16E-05
TRPM8	9.85E-07	4.90E-06	2.90E-05	1.27E-05	7.81E-06	4.99E-07	1.81E-05	1.31E-05
TRPS1	3.44E-06	1.48E-06	4.79E-07	5.34E-07	2.40E-08	8.30E-07	7.70E-07	1.34E-06
TRPT1	3.29E-05	5.69E-05	1.74E-05	2.07E-05	2.38E-05	2.01E-05	1.67E-05	3.40E-05
TRPV1	3.27E-06	1.70E-06	3.55E-06	3.12E-06	5.96E-06	5.98E-06	1.85E-06	2.90E-06
TRPV2	6.65E-06	7.81E-06	2.56E-06	5.64E-06	1.54E-06	2.41E-06	5.65E-06	1.66E-06
TRPV3	1.41E-07	3.96E-07	2.77E-08	0	0	3.72E-07	1.30E-08	0
TRPV4	1.22E-05	3.66E-08	5.54E-06	1.05E-05	7.53E-06	3.69E-07	1.71E-06	7.80E-08
TRPV5	2.83E-08	0	9.82E-09	1.16E-08	0	0	0	0
TRPV6	8.20E-07	1.80E-08	3.64E-07	2.16E-06	0	0	0	1.67E-08
TRRAP	1.56E-05	8.08E-06	3.47E-06	3.44E-06	8.94E-07	4.60E-06	2.74E-06	3.33E-06
TRUB1	9.15E-06	6.83E-06	6.56E-06	4.53E-06	8.66E-07	8.31E-06	2.92E-06	4.44E-06
TRUB2	2.29E-05	3.09E-05	3.16E-05	3.63E-05	2.36E-05	7.03E-05	3.23E-05	5.29E-05
TRY6	0	0	0	0	0	0	8.94E-07	0
TRYX3	0	0	0	0	0	0	0	0

TSC1	7.78E-06	1.67E-06	1.01E-06	1.62E-06	1.10E-06	2.40E-06	1.99E-06	3.55E-06
TSC2	2.48E-05	2.85E-05	1.51E-05	1.76E-05	1.96E-05	2.33E-05	2.39E-05	2.04E-05
TSC22D1	0.000101	7.91E-05	7.16E-05	0.000234	3.11E-05	0.000238	7.96E-05	9.01E-05
TSC22D2	4.75E-06	1.42E-05	6.03E-06	1.81E-05	3.09E-07	5.23E-06	6.78E-06	7.47E-06
TSC22D3	7.09E-05	8.19E-05	0.000126	8.64E-05	2.54E-05	8.73E-05	3.38E-05	0.000204
TSC22D4	4.81E-05	3.77E-05	1.19E-05	1.52E-05	1.03E-05	1.37E-05	1.84E-05	1.75E-05
TSEN15	8.69E-06	1.47E-05	6.03E-06	7.91E-06	2.99E-06	1.28E-05	9.22E-06	1.41E-05
TSEN2	3.04E-06	2.00E-06	1.54E-06	1.73E-06	7.26E-07	4.45E-06	2.08E-06	1.64E-06
TSEN34	5.61E-05	4.10E-05	2.21E-05	3.21E-05	2.84E-05	3.59E-05	3.26E-05	1.97E-05
TSEN54	2.01E-05	1.84E-05	3.93E-06	8.14E-06	1.08E-05	1.27E-05	9.08E-06	1.07E-05
TSFM	2.51E-05	4.00E-05	2.07E-05	1.52E-05	1.60E-05	2.72E-05	2.27E-05	2.69E-05
TSG1	4.89E-08	0	0	0	0	0	0	0
TSG101	7.17E-05	5.82E-05	3.47E-05	4.64E-05	1.66E-05	6.37E-05	3.43E-05	6.88E-05
TSGA10	5.55E-07	4.24E-07	1.40E-07	3.44E-07	1.28E-07	6.85E-07	1.04E-07	1.39E-07
TSGA10IP	4.65E-08	0	0	1.90E-08	0	4.04E-08	0	0
TSGA13	1.48E-08	0	0	0	0	0	0	0
TSGA14	1.53E-06	1.60E-06	8.30E-07	1.63E-06	9.01E-07	2.26E-06	1.98E-06	3.99E-06
TSHB	0	0	0	0	0	0	0	0
TSHR	8.74E-08	4.09E-08	2.65E-08	5.79E-08	0	4.77E-09	5.01E-09	0
TSHZ1	6.65E-06	4.03E-06	6.54E-06	4.76E-06	9.32E-07	5.92E-06	4.00E-06	4.49E-06
TSHZ2	2.02E-06	2.34E-06	2.49E-06	2.79E-06	2.06E-07	4.24E-06	3.78E-06	2.17E-06
TSHZ3	1.03E-06	1.75E-07	3.58E-07	1.21E-06	1.14E-07	1.91E-07	6.35E-07	5.51E-07
TSIX	2.15E-07	1.72E-09	3.47E-07	0	5.86E-10	2.51E-78	0	0
TSKS	2.67E-08	0	1.41E-08	2.13E-07	3.45E-08	4.97E-08	1.94E-07	5.91E-08
TSKU	3.30E-05	0.000183	0.000156	5.52E-05	9.68E-05	0.000133	0.000164	0.000181
TSLP	8.84E-07	1.45E-06	3.44E-06	4.81E-06	3.21E-06	1.41E-06	4.51E-06	4.91E-07
TSN	7.63E-05	3.02E-05	1.67E-05	1.67E-05	1.13E-05	2.65E-05	1.93E-05	1.70E-05
TSNARE1	4.16E-06	4.47E-06	3.33E-06	3.57E-06	1.86E-06	2.30E-06	8.74E-06	4.18E-06
TSNAX	2.26E-05	2.51E-05	1.36E-05	1.17E-05	6.38E-06	2.85E-05	1.20E-05	2.42E-05
TSNAX-DI	0	0	0	0	0	0	0	0
TSNAXIP1	3.04E-07	2.57E-07	1.71E-07	4.00E-07	2.97E-07	3.45E-07	1.27E-07	1.65E-07
TSPAN1	1.95E-05	6.44E-06	1.75E-06	2.62E-06	1.70E-06	4.19E-06	2.62E-06	1.53E-06
TSPAN10	1.80E-07	2.07E-06	2.43E-07	6.74E-07	3.56E-07	1.79E-06	3.05E-07	4.97E-07
TSPAN11	1.56E-07	1.84E-08	2.65E-07	3.81E-07	1.20E-07	2.66E-08	9.21E-08	2.41E-08
TSPAN12	2.77E-05	1.25E-05	1.71E-05	1.11E-05	9.91E-06	3.94E-06	1.18E-05	2.32E-06
TSPAN13	7.99E-05	2.73E-05	6.33E-06	7.91E-06	1.19E-06	1.12E-05	7.03E-06	5.85E-05
TSPAN14	1.38E-05	8.81E-06	7.06E-06	1.09E-05	2.99E-06	9.88E-06	9.81E-06	1.28E-05
TSPAN15	1.14E-05	4.85E-06	3.56E-06	2.75E-05	2.65E-06	2.27E-06	1.54E-05	9.11E-06
TSPAN16	0	0	0	0	0	0	0	0
TSPAN17	1.68E-05	6.53E-06	7.69E-06	9.85E-06	9.78E-06	1.39E-05	1.77E-05	2.80E-05
TSPAN18	1.95E-05	3.15E-06	3.18E-06	3.37E-06	2.71E-06	2.40E-06	7.14E-06	4.89E-06
TSPAN19	0	0	0	0	0	0	0	0
TSPAN2	4.82E-07	1.86E-07	5.20E-08	1.76E-07	9.27E-08	1.27E-07	4.19E-08	3.38E-07
TSPAN3	0.000113	2.99E-05	3.70E-05	3.94E-05	3.59E-05	2.01E-05	3.69E-05	3.35E-05
TSPAN31	4.24E-05	4.00E-05	3.16E-05	2.91E-05	1.93E-05	3.34E-05	3.65E-05	4.10E-05
TSPAN32	6.25E-07	1.05E-07	2.34E-07	5.40E-07	2.34E-08	2.46E-07	1.71E-07	3.39E-07
TSPAN33	1.15E-05	3.53E-05	3.23E-05	1.77E-05	2.05E-05	2.43E-05	3.46E-05	7.77E-05
TSPAN4	6.99E-05	3.79E-05	2.02E-05	6.13E-05	6.26E-05	5.40E-05	7.95E-05	7.11E-05
TSPAN5	9.49E-06	2.47E-07	3.36E-07	2.94E-07	4.11E-08	6.33E-06	3.64E-07	6.59E-07
TSPAN6	4.89E-05	9.51E-05	6.08E-05	5.65E-05	5.27E-05	3.83E-05	4.41E-05	6.56E-05
TSPAN7	3.36E-05	5.03E-06	1.67E-05	8.80E-06	7.41E-06	2.48E-05	7.29E-06	2.68E-06
TSPAN8	0.000756	4.78E-05	7.47E-06	7.39E-05	7.47E-05	0.000113	2.53E-05	0.000197
TSPAN9	1.25E-05	2.40E-05	2.59E-05	2.46E-05	1.81E-05	2.20E-05	2.66E-05	7.09E-06
TSPO	9.01E-05	0.000116	3.27E-05	0.000109	2.40E-05	3.16E-05	0.000106	4.16E-05
TSPO2	2.74E-07	2.01E-07	8.64E-08	5.52E-07	5.52E-08	5.76E-07	3.88E-07	1.38E-06
TSPY1	0	0	0	0	0	0	0	0
TSPY2	0	0	0	0	0	0	0	0
TSPY3	0	0	0	0	0	0	0	0

TSPY4	0	0	0	0	0	0	0	0
TSPYL1	2.72E-05	3.02E-05	3.54E-05	2.23E-05	1.19E-05	3.18E-05	2.40E-05	1.37E-05
TSPYL2	8.76E-06	5.04E-06	2.96E-06	7.16E-06	1.81E-06	6.08E-06	3.44E-06	5.97E-06
TSPYL3	3.38E-07	1.46E-07	1.37E-07	5.21E-08	0	3.93E-08	2.02E-08	9.40E-08
TSPYL4	1.20E-05	4.45E-06	4.41E-06	3.52E-06	7.94E-07	4.15E-06	1.15E-06	1.11E-06
TSPYL5	1.05E-06	1.25E-07	2.87E-07	1.49E-06	8.72E-08	2.16E-07	4.59E-07	2.53E-07
TSPYL6	0	0	0	0	0	6.92E-09	0	0
TSR1	6.13E-06	5.32E-06	5.22E-06	7.61E-06	2.24E-06	1.20E-05	6.36E-06	7.69E-06
TSR2	4.66E-05	3.83E-05	2.50E-05	3.13E-05	2.58E-05	4.26E-05	3.61E-05	6.56E-05
TSSC1	2.20E-05	2.54E-05	6.97E-06	7.91E-06	6.29E-06	8.37E-06	1.04E-05	8.22E-06
TSSC4	2.03E-05	3.06E-05	1.42E-05	2.61E-05	3.55E-05	2.04E-05	2.85E-05	3.94E-05
TSSK1B	2.84E-08	0	0	1.16E-08	8.15E-09	0	1.84E-08	4.18E-08
TSSK2	0	0	0	0	0	0	0	0
TSSK3	1.84E-06	1.26E-06	4.39E-07	3.16E-07	3.70E-07	4.89E-07	2.33E-07	2.83E-07
TSSK4	3.50E-07	2.33E-08	3.85E-08	8.87E-08	2.40E-08	1.22E-07	0	0
TSSK6	7.99E-07	1.14E-06	5.35E-07	5.86E-07	1.97E-07	1.25E-06	9.90E-07	1.09E-06
TST	0.000337	0.000323	0.000554	0.00048	0.001037	0.000586	0.000419	0.000581
TSTA3	6.02E-05	7.43E-05	5.02E-05	5.00E-05	5.85E-05	5.68E-05	0.000172	7.38E-05
TSTD1	3.91E-05	0.000299	7.81E-05	8.73E-05	6.90E-05	0.000124	9.88E-05	6.84E-05
TSTD2	6.62E-06	3.88E-06	2.74E-06	2.61E-06	1.54E-07	4.13E-06	1.75E-06	2.64E-06
TTBK1	1.28E-07	3.80E-07	1.41E-06	6.48E-07	2.49E-07	7.29E-07	6.34E-07	5.21E-07
TTBK2	1.07E-06	3.14E-07	5.44E-07	1.70E-07	3.68E-08	5.33E-07	1.35E-07	5.65E-07
TTC1	4.41E-05	2.12E-05	2.35E-05	3.01E-05	1.69E-05	4.21E-05	2.43E-05	4.92E-05
TTC12	3.62E-06	1.67E-06	1.81E-06	2.39E-06	7.65E-07	8.28E-06	2.10E-06	2.68E-06
TTC13	5.72E-06	3.79E-06	1.64E-06	1.77E-06	7.90E-07	3.02E-06	1.93E-06	6.16E-06
TTC14	9.97E-06	4.15E-06	3.88E-06	3.72E-06	1.53E-06	4.90E-06	1.87E-06	3.38E-06
TTC15	1.78E-05	1.76E-05	9.52E-06	1.58E-05	1.24E-05	1.13E-05	1.41E-05	1.28E-05
TTC16	1.18E-07	0	2.99E-08	1.06E-07	1.65E-08	1.61E-08	5.57E-08	4.22E-08
TTC17	1.92E-05	3.26E-05	2.24E-05	2.00E-05	3.90E-06	2.31E-05	1.33E-05	3.95E-05
TTC18	4.99E-07	1.16E-06	3.56E-07	8.24E-07	3.57E-07	2.00E-06	4.69E-07	2.83E-07
TTC19	1.03E-05	1.89E-05	1.76E-05	1.65E-05	4.57E-06	1.43E-05	2.07E-05	8.73E-06
TTC21A	1.13E-06	1.06E-07	3.82E-07	4.58E-07	3.98E-07	2.45E-07	3.99E-07	4.84E-07
TTC21B	5.11E-06	2.68E-06	2.58E-06	1.98E-06	8.43E-07	2.60E-06	1.70E-06	1.79E-06
TTC22	9.13E-06	1.33E-06	5.73E-07	1.33E-06	9.20E-09	7.85E-07	4.78E-07	5.81E-07
TTC23	1.12E-05	5.51E-06	3.71E-06	3.57E-06	4.51E-06	5.44E-06	6.97E-06	4.52E-06
TTC23L	1.05E-07	4.66E-08	3.54E-08	6.42E-08	1.29E-08	8.82E-08	7.28E-09	0
TTC24	1.07E-07	0	0	3.76E-08	0	8.54E-08	1.48E-08	0
TTC25	1.26E-07	1.10E-07	1.96E-07	7.74E-07	2.96E-08	1.21E-07	5.75E-08	6.48E-07
TTC26	9.95E-07	9.72E-07	1.81E-07	4.27E-07	2.38E-08	1.26E-06	4.52E-07	5.48E-07
TTC27	1.13E-05	1.00E-05	4.96E-06	5.58E-06	3.62E-06	1.10E-05	6.62E-06	5.84E-06
TTC28	2.96E-06	1.76E-06	1.40E-06	1.55E-06	1.09E-06	2.31E-06	1.75E-06	1.39E-06
TTC29	0	0	0	0	0	0	0	0
TTC3	2.33E-05	6.93E-06	1.33E-05	1.31E-05	9.79E-06	1.57E-05	1.02E-05	1.33E-05
TTC30A	1.13E-06	1.12E-06	1.26E-06	1.35E-06	1.79E-07	2.92E-06	4.10E-07	7.09E-07
TTC30B	2.06E-06	1.33E-06	1.13E-06	1.43E-06	2.28E-08	2.12E-06	2.83E-07	8.45E-07
TTC31	2.02E-05	1.56E-05	1.05E-05	1.10E-05	7.54E-06	1.82E-05	1.32E-05	7.49E-06
TTC32	9.47E-06	3.58E-06	3.30E-06	8.12E-06	3.64E-06	1.68E-05	5.30E-06	5.50E-06
TTC33	4.24E-06	4.99E-06	3.70E-06	2.80E-06	7.54E-07	2.89E-06	2.38E-06	4.41E-06
TTC35	0.000109	4.44E-05	2.10E-05	2.64E-05	1.33E-05	4.06E-05	6.08E-05	4.46E-05
TTC36	3.53E-07	8.08E-06	0.000121	0.000109	7.39E-05	2.78E-05	9.76E-05	1.07E-06
TTC37	1.43E-05	7.01E-06	8.31E-06	7.83E-06	1.76E-07	1.65E-05	3.61E-06	8.15E-06
TTC38	6.76E-05	0.00023	9.95E-05	9.01E-05	0.000122	0.000123	0.000115	8.91E-05
TTC39A	1.08E-05	1.45E-05	2.61E-07	5.65E-07	2.54E-06	7.09E-06	1.51E-06	8.21E-07
TTC39B	7.76E-07	7.76E-07	3.52E-06	3.07E-06	1.70E-07	3.60E-06	6.71E-07	1.83E-06
TTC39C	5.89E-05	6.46E-05	2.86E-05	1.46E-05	1.34E-05	4.21E-05	1.54E-05	9.85E-05
TTC4	1.46E-05	1.55E-05	6.55E-06	1.16E-05	5.58E-06	1.47E-05	1.35E-05	9.71E-06
TTC5	3.62E-06	2.54E-06	2.04E-06	2.79E-06	3.50E-07	3.61E-06	2.60E-06	2.76E-06
TTC7A	2.20E-05	9.31E-06	2.39E-06	5.68E-06	4.05E-06	9.46E-06	6.61E-06	3.52E-06

TTC7B	5.02E-06	2.57E-06	8.79E-06	6.06E-06	1.16E-06	1.09E-05	6.74E-06	4.45E-06
TTC8	5.04E-06	4.87E-06	5.49E-06	8.36E-06	1.40E-06	8.27E-06	5.55E-06	6.96E-06
TTC9	3.71E-06	2.45E-06	6.54E-07	5.34E-06	1.80E-06	1.95E-05	6.61E-06	4.46E-06
TTC9B	0	0	3.71E-08	0	0	0	0	0
TTC9C	1.50E-05	1.16E-05	7.45E-06	6.94E-06	4.38E-06	1.04E-05	6.61E-06	1.31E-05
TTF1	5.82E-06	2.99E-06	2.89E-06	2.77E-06	1.68E-06	3.39E-06	2.23E-06	3.83E-06
TTF2	3.24E-06	2.94E-06	6.87E-07	6.08E-07	7.19E-08	2.06E-06	3.84E-07	8.24E-07
TTK	4.23E-06	2.68E-06	3.23E-08	6.69E-08	2.15E-07	8.75E-07	1.59E-07	4.70E-07
TTL	1.05E-05	4.89E-06	2.25E-06	3.70E-06	3.79E-06	1.38E-05	5.63E-06	5.52E-06
TTLL1	2.70E-06	1.29E-06	8.95E-07	2.46E-06	2.64E-06	3.53E-06	2.90E-06	1.83E-06
TTLL10	5.24E-08	7.10E-08	0	1.38E-08	0	1.94E-08	5.07E-08	3.32E-08
TTLL11	2.19E-06	9.28E-07	1.71E-06	1.94E-06	1.19E-06	2.13E-06	1.86E-06	2.47E-06
TTLL12	2.00E-05	2.44E-05	1.41E-05	1.42E-05	1.66E-05	1.93E-05	1.58E-05	1.34E-05
TTLL13	7.28E-08	5.47E-07	3.01E-08	5.23E-08	1.36E-07	4.93E-07	1.30E-08	7.02E-08
TTLL2	3.31E-07	6.20E-07	3.18E-07	2.47E-07	7.45E-09	7.10E-07	0	1.78E-07
TTLL3	5.15E-06	2.90E-06	6.43E-07	2.33E-06	1.48E-06	1.66E-06	1.83E-06	1.97E-06
TTLL4	8.78E-06	2.71E-06	1.24E-06	2.12E-06	1.40E-06	2.16E-05	2.75E-06	2.55E-06
TTLL5	6.70E-06	2.27E-06	2.05E-06	3.85E-06	1.70E-06	4.86E-06	2.90E-06	3.78E-06
TTLL6	7.17E-07	6.28E-08	1.87E-08	1.53E-08	5.22E-08	1.53E-07	3.62E-08	0
TTLL7	2.62E-06	4.22E-08	4.00E-07	2.54E-07	5.65E-09	3.93E-06	2.56E-08	1.20E-07
TTLL8	0	0	0	0	0	0	0	0
TTLL9	1.77E-07	0	1.14E-08	4.01E-08	6.56E-08	2.30E-08	1.05E-07	3.37E-08
TTN	3.17E-07	1.89E-07	9.62E-08	7.15E-08	2.92E-08	1.94E-07	5.58E-08	9.12E-09
TTPA	8.98E-06	4.96E-05	5.25E-05	1.71E-05	7.24E-07	5.43E-05	2.80E-05	2.61E-05
TTPAL	6.61E-06	4.62E-06	1.75E-05	3.77E-06	3.02E-06	5.87E-06	4.43E-06	1.08E-05
TTR	0.000359	0.002578	0.006067	0.002921	0.00326	0.001033	0.002831	0.00036
TTTY10	0	3.43E-08	0	0	0	1.40E-08	1.45E-08	2.22E-08
TTTY11	0	0	0	0	0	0	0	0
TTTY12	0	0	0	0	0	0	0	0
TTTY13	0	0	0	0	0	0	0	0
TTTY14	0	1.49E-06	0	9.29E-07	5.87E-07	3.79E-07	1.22E-07	7.19E-07
TTTY15	0	5.18E-07	0	6.66E-07	7.37E-09	5.17E-08	1.13E-07	2.07E-07
TTTY16	0	0	0	0	0	0	0	0
TTTY17A	0	0	0	0	0	0	0	0
TTTY17B	0	0	0	0	0	0	0	0
TTTY18	0	0	0	0	0	0	0	0
TTTY19	0	0	0	0	0	0	0	0
TTTY1B	0	0	0	0	0	0	0	0
TTTY2	0	0	0	0	0	1.40E-08	0	0
TTTY20	0	0	0	0	0	0	0	0
TTTY21	0	0	0	0	0	0	0	0
TTTY22	0	0	0	0	0	0	0	0
TTTY23	0	0	0	0	0	0	0	0
TTTY3B	0	0	0	0	0	0	0	0
TTTY4C	0	3.43E-07	0	0	0	0	0	0
TTTY5	0	0	0	0	0	0	0	0
TTTY6	0	0	0	0	0	0	0	0
TTTY6B	0	0	0	0	0	0	0	0
TTTY7	0	0	0	0	0	0	0	0
TTTY8	0	0	0	0	0	0	0	0
TTTY9B	0	0	0	0	0	0	0	0
TTYH1	1.02E-07	2.07E-08	2.16E-07	7.42E-08	1.04E-07	1.12E-08	0	1.91E-07
TTYH2	2.15E-06	2.39E-07	6.38E-07	1.29E-06	1.14E-07	6.37E-07	1.74E-06	1.45E-06
TTYH3	5.09E-05	1.58E-05	1.03E-05	1.63E-05	2.27E-06	2.15E-05	1.17E-05	2.31E-05
TUB	1.07E-06	2.47E-08	1.32E-07	4.22E-07	3.16E-09	3.40E-09	1.15E-07	5.38E-09
TUBA1A	5.48E-05	1.93E-05	9.68E-06	6.19E-05	1.84E-05	1.30E-05	5.15E-05	2.10E-05
TUBA1B	0.000346	0.000271	0.000114	0.000241	0.000158	0.000195	0.000257	0.000285
TUBA1C	0.000138	9.54E-05	3.61E-05	0.000101	5.94E-05	5.13E-05	6.32E-05	0.00011

TUBA3C	4.68E-07	5.73E-08	0	0	0	0	4.83E-08	0
TUBA3D	6.41E-08	2.19E-07	1.70E-08	5.90E-08	0	7.44E-08	9.25E-08	4.73E-08
TUBA3E	3.22E-08	3.48E-07	8.53E-08	3.95E-08	0	0	1.55E-08	0
TUBA4A	0.000143	4.08E-05	3.46E-05	3.83E-05	1.10E-05	3.56E-05	1.81E-05	0.000186
TUBA4B	4.84E-07	9.20E-07	1.94E-07	2.98E-07	2.29E-07	5.54E-08	9.34E-07	8.67E-07
TUBA8	6.20E-06	1.90E-06	9.91E-07	1.44E-06	7.32E-06	9.15E-07	1.66E-06	1.27E-06
TUBAL3	8.74E-08	0	1.54E-08	0	2.51E-08	0	0	0
TUBB	0.000377	0.000302	0.000237	0.000277	0.000183	0.000202	0.000214	0.000339
TUBB1	2.04E-07	6.63E-07	1.76E-07	2.89E-07	6.76E-08	8.51E-08	3.83E-08	1.92E-08
TUBB2A	5.58E-05	6.52E-05	7.89E-05	9.57E-05	0.000118	4.90E-05	8.98E-05	0.000197
TUBB2B	1.76E-07	1.84E-06	3.86E-07	3.59E-06	7.70E-07	7.99E-07	1.16E-07	1.75E-06
TUBB2C	0.000227	0.000131	9.93E-05	0.00018	0.000112	0.00016	0.000124	0.000278
TUBB3	3.99E-05	1.25E-06	6.08E-07	1.76E-05	2.43E-06	1.90E-06	6.60E-06	4.52E-06
TUBB4	3.35E-05	6.66E-05	8.95E-07	1.65E-06	2.33E-06	6.90E-07	2.10E-06	2.46E-06
TUBB4Q	0	2.23E-08	0	1.20E-07	0	0	1.87E-08	0
TUBB6	7.76E-05	2.81E-05	1.57E-05	5.48E-05	3.00E-05	1.57E-05	3.78E-05	2.42E-05
TUBB8	1.08E-07	1.85E-07	7.68E-08	1.55E-07	4.26E-08	8.37E-08	6.92E-08	1.07E-07
TUBBP5	1.60E-08	2.17E-07	9.14E-08	1.34E-06	0	0	6.14E-08	0
TUBD1	4.84E-06	4.77E-06	2.12E-06	1.50E-06	1.67E-06	3.02E-06	2.17E-06	1.51E-06
TUBE1	3.25E-06	9.01E-06	1.70E-05	9.51E-06	3.79E-06	5.76E-06	5.24E-06	3.90E-06
TUBG1	4.73E-05	4.52E-05	1.28E-05	1.35E-05	1.26E-05	3.04E-05	1.74E-05	2.03E-05
TUBG2	9.01E-06	4.74E-06	3.09E-06	4.27E-06	4.15E-06	4.75E-06	3.76E-06	4.03E-06
TUBGCP2	2.10E-05	1.47E-05	1.35E-05	1.61E-05	2.05E-05	1.94E-05	2.34E-05	1.39E-05
TUBGCP3	6.98E-06	7.15E-06	6.34E-06	4.61E-06	1.27E-06	6.10E-06	4.55E-06	4.54E-06
TUBGCP4	3.58E-06	2.62E-06	2.98E-06	1.66E-06	7.91E-07	3.29E-06	1.57E-06	1.80E-06
TUBGCP5	4.36E-06	4.02E-06	2.72E-06	2.38E-06	1.07E-06	2.83E-06	2.42E-06	4.32E-06
TUBGCP6	1.49E-05	4.76E-06	4.43E-06	5.26E-06	4.98E-06	4.82E-06	4.48E-06	8.54E-06
TUFM	0.000157	0.000322	0.000128	0.000124	0.000114	0.000197	0.000166	0.00014
TUFT1	7.26E-06	5.69E-06	1.42E-06	5.86E-06	3.22E-07	7.03E-06	4.93E-06	3.94E-06
TUG1	3.65E-05	1.59E-05	1.11E-05	1.32E-05	8.88E-06	1.46E-05	8.21E-06	2.18E-05
TULP1	0	0	0	3.05E-08	0	0	0	0
TULP2	0	0	0	0	0	0	0	0
TULP3	1.36E-05	5.37E-06	2.13E-06	4.53E-06	1.40E-06	8.17E-06	4.27E-06	2.66E-06
TULP4	2.52E-06	1.22E-06	1.26E-06	1.59E-06	4.17E-07	3.26E-06	1.44E-06	2.18E-06
TUSC1	4.22E-06	4.52E-06	3.33E-06	7.25E-06	3.18E-06	9.82E-07	5.16E-06	3.80E-06
TUSC2	2.14E-05	2.35E-05	9.72E-06	1.26E-05	2.54E-05	2.08E-05	1.96E-05	1.34E-05
TUSC3	1.77E-05	5.62E-06	1.56E-06	8.82E-06	3.08E-07	5.95E-06	1.24E-06	1.01E-06
TUSC5	6.37E-09	1.43E-08	1.31E-08	0	5.47E-09	0	1.24E-08	0
TUT1	2.80E-05	2.78E-05	1.49E-05	1.48E-05	2.30E-05	1.70E-05	2.13E-05	2.74E-05
TWF1	3.41E-05	3.75E-05	1.86E-05	1.84E-05	4.13E-06	2.40E-05	1.46E-05	2.03E-05
TWF2	7.96E-05	9.72E-05	2.67E-05	6.46E-05	1.86E-05	2.97E-05	6.31E-05	2.66E-05
TWIST1	4.85E-07	3.34E-08	1.24E-07	3.25E-07	5.07E-08	9.56E-08	9.92E-08	8.68E-08
TWIST2	3.31E-07	0	0	8.70E-07	7.84E-08	5.63E-08	5.72E-08	2.25E-07
TWISTNB	7.18E-06	1.04E-05	3.43E-06	4.86E-06	1.41E-06	4.49E-06	3.65E-06	8.10E-06
TWSG1	8.64E-06	8.26E-07	3.94E-06	5.45E-06	3.13E-07	5.66E-06	1.99E-06	3.53E-06
TXK	6.66E-07	9.65E-08	1.65E-07	1.80E-07	1.20E-08	3.89E-07	1.74E-07	1.16E-07
TXLNA	2.85E-05	1.25E-05	1.04E-05	1.24E-05	6.29E-06	1.95E-05	1.16E-05	2.05E-05
TXLNB	1.55E-07	2.83E-06	1.63E-07	1.41E-07	8.29E-09	8.04E-08	7.05E-08	2.40E-07
TXLNG	7.34E-06	2.20E-06	3.34E-06	2.16E-06	6.03E-08	3.38E-06	5.65E-07	2.65E-06
TXN	0.000788	0.000506	0.000376	0.000537	0.000892	0.001724	0.00043	0.000455
TXN2	0.00015	0.000107	9.00E-05	8.39E-05	0.000126	0.000114	0.000112	0.00012
TXNDC11	3.50E-05	6.20E-05	3.43E-05	3.32E-05	1.12E-05	3.40E-05	2.58E-05	4.15E-05
TXNDC12	0.00012	4.37E-05	3.46E-05	4.74E-05	1.83E-05	4.77E-05	4.53E-05	5.45E-05
TXNDC15	1.25E-05	1.22E-05	1.20E-05	1.50E-05	5.00E-06	1.34E-05	1.20E-05	1.97E-05
TXNDC16	5.99E-06	1.61E-06	2.12E-06	2.19E-06	3.95E-07	2.63E-06	1.51E-06	3.35E-06
TXNDC17	5.03E-05	1.99E-05	1.48E-05	1.87E-05	1.82E-05	2.46E-05	3.66E-05	2.53E-05
TXNDC2	2.96E-08	0	0	3.64E-08	0	2.75E-08	0	0
TXNDC3	6.14E-08	0	0	3.76E-08	1.76E-08	9.49E-09	0	1.50E-08

TXNDC5	0.000105	8.23E-05	7.98E-05	0.000114	3.28E-05	0.000131	8.84E-05	0.000146
TXNDC6	1.85E-07	4.66E-08	0	0	0	2.42E-08	3.31E-08	0
TXNDC8	0	0	0	0	0	0	0	0
TXNDC9	3.49E-05	2.41E-05	1.07E-05	1.38E-05	6.10E-06	1.54E-05	1.64E-05	1.34E-05
TXNIP	0.000102	1.69E-05	0.000286	0.000196	1.92E-05	0.000117	0.000104	0.000217
TXNL1	5.96E-05	4.02E-05	4.36E-05	4.44E-05	2.61E-05	6.08E-05	4.98E-05	7.20E-05
TXNL4A	4.87E-05	2.52E-05	2.10E-05	2.15E-05	2.81E-05	2.95E-05	3.51E-05	2.98E-05
TXNL4B	1.28E-05	9.49E-06	3.90E-06	5.98E-06	3.53E-06	1.09E-05	6.12E-06	5.72E-06
TXNRD1	5.71E-05	0.000115	2.63E-05	2.86E-05	6.00E-06	0.000237	1.79E-05	4.89E-05
TXNRD2	2.13E-05	3.88E-05	4.28E-05	3.89E-05	6.10E-05	3.82E-05	6.74E-05	5.83E-05
TXNRD3IT	5.26E-06	4.26E-06	3.88E-06	4.01E-06	2.32E-06	6.77E-06	4.88E-06	6.30E-06
TYK2	2.42E-05	1.23E-05	1.22E-05	1.17E-05	1.30E-05	1.84E-05	1.66E-05	1.47E-05
TYMP	0.000181	8.45E-05	5.32E-05	8.27E-05	2.68E-05	4.47E-05	8.51E-05	0.000226
TYMS	4.35E-05	1.50E-05	3.65E-06	7.38E-06	6.19E-06	1.22E-05	5.28E-06	5.49E-06
TYR	0	0	0	0	0	0	0	0
TYRO3	5.51E-06	1.42E-07	5.46E-07	1.64E-06	2.47E-07	5.33E-07	2.75E-07	3.63E-07
TYROBP	0.000182	3.43E-05	0.000114	0.000264	4.13E-05	8.29E-05	0.000164	4.10E-05
TYRP1	4.04E-08	1.88E-06	0	9.88E-09	0	0	3.13E-08	1.19E-08
TYSND1	1.04E-05	1.01E-05	5.32E-06	3.47E-06	8.84E-06	7.10E-06	6.05E-06	4.21E-06
TYW1	1.14E-05	1.77E-05	7.81E-06	8.42E-06	4.50E-06	1.82E-05	8.50E-06	1.34E-05
TYW1B	6.13E-06	1.20E-05	3.60E-06	2.98E-06	9.84E-07	3.45E-06	2.44E-06	3.19E-06
TYW3	9.11E-06	7.21E-06	4.22E-06	3.73E-06	1.71E-06	9.16E-06	2.53E-06	4.18E-06
U2AF1	0.000111	4.69E-05	4.83E-05	7.52E-05	6.96E-05	6.35E-05	9.35E-05	6.31E-05
U2AF1L4	7.10E-06	5.48E-06	4.15E-06	5.96E-06	1.59E-05	6.83E-06	9.26E-06	9.76E-06
U2AF2	6.03E-05	4.05E-05	2.33E-05	2.75E-05	1.90E-05	4.20E-05	3.16E-05	2.82E-05
UACA	1.55E-05	7.41E-06	3.34E-06	5.94E-06	2.43E-06	3.74E-06	5.01E-06	3.46E-06
UAP1	3.77E-05	5.38E-05	4.24E-05	3.14E-05	1.66E-05	5.56E-05	3.26E-05	7.07E-05
UAP1L1	1.59E-05	3.40E-07	8.23E-07	2.11E-06	2.04E-07	4.61E-07	1.27E-06	5.28E-07
UBA1	0.000164	9.92E-05	5.85E-05	6.38E-05	3.93E-05	6.28E-05	6.02E-05	0.000121
UBA2	3.42E-05	2.81E-05	1.53E-05	2.04E-05	7.16E-06	2.54E-05	2.03E-05	1.65E-05
UBA3	2.26E-05	1.56E-05	1.04E-05	1.34E-05	5.64E-06	1.59E-05	1.01E-05	1.18E-05
UBA5	1.97E-05	2.47E-05	1.19E-05	1.10E-05	8.56E-06	1.41E-05	9.87E-06	1.14E-05
UBA52	0.000164	0.000108	9.01E-05	0.000138	0.000373	0.000164	0.000215	9.56E-05
UBA6	1.01E-05	6.12E-06	4.14E-06	3.57E-06	1.04E-06	6.67E-06	2.27E-06	5.47E-06
UBA7	2.02E-05	1.40E-05	1.49E-05	1.20E-05	6.29E-06	1.58E-05	1.22E-05	2.02E-05
UBAC1	4.04E-05	2.28E-05	1.40E-05	1.57E-05	1.18E-05	1.58E-05	1.19E-05	2.72E-05
UBAC2	6.27E-05	6.38E-05	2.83E-05	3.01E-05	3.40E-05	3.26E-05	3.14E-05	3.76E-05
UBAP1	1.64E-05	3.42E-05	2.20E-05	3.06E-05	9.13E-06	3.72E-05	2.21E-05	3.18E-05
UBAP2	9.00E-06	7.54E-06	4.41E-06	6.15E-06	2.30E-06	5.61E-06	5.69E-06	4.75E-06
UBAP2L	4.46E-05	3.56E-05	1.60E-05	2.32E-05	9.40E-06	4.82E-05	2.20E-05	3.75E-05
UBASH3A	1.48E-06	3.13E-08	2.66E-07	2.43E-07	3.79E-08	4.23E-07	3.68E-07	1.89E-07
UBASH3B	3.76E-06	1.12E-07	3.30E-07	2.18E-06	8.59E-08	3.05E-07	1.95E-07	5.58E-07
UBB	0.000975	0.002211	0.001512	0.001155	0.000762	0.001603	0.002051	0.000994
UBC	0.00103	0.00048	0.000586	0.001028	0.001655	0.000871	0.000729	0.001138
UBD	0.001459	0.00046	6.16E-05	0.000441	4.67E-06	0.000993	0.000187	0.001541
UBE2A	3.33E-05	2.86E-05	1.52E-05	2.17E-05	1.44E-05	2.76E-05	2.12E-05	4.60E-05
UBE2B	3.43E-05	3.28E-05	1.91E-05	2.44E-05	1.03E-05	2.32E-05	2.62E-05	2.99E-05
UBE2C	5.37E-05	2.12E-05	4.19E-07	1.79E-06	7.38E-06	5.45E-06	2.32E-06	4.00E-06
UBE2CBP	1.44E-06	1.12E-06	5.78E-07	8.90E-07	2.14E-07	1.30E-06	6.15E-07	5.25E-07
UBE2D1	1.03E-05	4.45E-06	3.09E-06	6.18E-06	1.30E-06	5.01E-06	5.51E-06	6.21E-06
UBE2D2	3.56E-05	2.36E-05	1.36E-05	2.26E-05	7.58E-06	2.19E-05	2.15E-05	2.01E-05
UBE2D3	0.000146	7.96E-05	0.000112	0.000116	5.79E-05	0.000118	0.000111	0.000121
UBE2D4	1.59E-05	1.93E-05	1.02E-05	7.24E-06	5.90E-06	1.06E-05	7.05E-06	9.72E-06
UBE2DNL	0	0	2.69E-07	0	0	0	3.71E-08	5.93E-08
UBE2E1	4.61E-05	3.12E-05	1.79E-05	2.63E-05	1.23E-05	2.58E-05	2.56E-05	2.14E-05
UBE2E2	2.41E-05	9.12E-06	8.51E-06	9.63E-06	7.20E-06	7.69E-06	7.56E-06	8.26E-06
UBE2E3	5.76E-05	3.14E-05	2.09E-05	2.67E-05	2.16E-05	2.81E-05	2.42E-05	1.92E-05
UBE2F	3.70E-05	2.56E-05	1.85E-05	2.51E-05	2.18E-05	2.77E-05	2.33E-05	3.32E-05

UBE2G1	8.36E-06	1.02E-05	1.18E-05	1.05E-05	8.04E-06	1.30E-05	1.18E-05	1.47E-05
UBE2G2	3.11E-05	1.91E-05	2.20E-05	2.10E-05	2.07E-05	3.06E-05	2.39E-05	2.78E-05
UBE2H	1.56E-05	2.38E-05	1.48E-05	1.51E-05	3.59E-06	2.86E-05	1.19E-05	2.41E-05
UBE2I	0.000105	5.38E-05	2.37E-05	4.56E-05	1.68E-05	4.28E-05	4.84E-05	3.64E-05
UBE2J1	1.86E-05	1.83E-05	1.21E-05	1.42E-05	2.69E-06	1.71E-05	9.73E-06	1.33E-05
UBE2J2	2.29E-05	1.16E-05	1.34E-05	1.55E-05	1.37E-05	1.53E-05	1.79E-05	1.29E-05
UBE2K	1.88E-05	2.46E-05	1.92E-05	1.26E-05	5.67E-06	1.82E-05	1.16E-05	1.66E-05
UBE2L3	5.12E-05	2.92E-05	2.27E-05	3.06E-05	3.14E-05	2.95E-05	2.94E-05	4.11E-05
UBE2L6	0.000144	8.19E-05	7.94E-05	9.58E-05	1.45E-05	9.61E-05	6.28E-05	0.000183
UBE2M	3.83E-05	4.48E-05	1.77E-05	2.58E-05	5.29E-05	3.09E-05	3.64E-05	3.18E-05
UBE2MP1	8.39E-06	5.84E-06	3.95E-06	3.33E-06	3.01E-06	4.61E-06	3.58E-06	4.06E-06
UBE2N	3.20E-05	2.50E-05	1.68E-05	2.22E-05	6.93E-06	2.76E-05	1.86E-05	2.14E-05
UBE2NL	5.01E-07	7.53E-08	2.82E-07	1.08E-07	0	8.12E-08	1.25E-07	3.24E-08
UBE2O	1.08E-05	7.84E-06	4.06E-06	3.86E-06	2.76E-06	8.42E-06	3.87E-06	5.69E-06
UBE2Q1	3.31E-05	4.05E-05	1.45E-05	1.91E-05	1.04E-05	3.85E-05	2.38E-05	3.82E-05
UBE2Q2	1.80E-05	4.87E-06	3.78E-06	6.01E-06	6.83E-06	7.64E-06	6.84E-06	5.83E-06
UBE2Q2P1	6.64E-07	2.37E-07	9.25E-08	8.13E-08	9.52E-09	1.74E-07	4.28E-08	1.46E-07
UBE2QL1	9.58E-08	1.36E-08	7.91E-07	1.53E-06	2.32E-06	1.22E-05	2.18E-06	8.64E-07
UBE2R2	1.85E-05	2.07E-05	1.58E-05	2.13E-05	1.21E-05	2.20E-05	2.24E-05	1.88E-05
UBE2S	1.54E-05	1.65E-05	1.52E-06	6.80E-06	7.46E-06	6.12E-06	1.65E-05	7.41E-06
UBE2T	2.15E-05	1.02E-05	5.68E-07	1.16E-06	1.75E-06	1.01E-05	1.89E-06	7.57E-06
UBE2U	0	0	0	2.82E-08	0	0	0	0
UBE2V1	8.81E-05	7.21E-05	4.71E-05	5.12E-05	5.43E-05	6.51E-05	5.09E-05	4.60E-05
UBE2V2	5.81E-05	7.03E-05	2.16E-05	2.38E-05	2.68E-05	3.07E-05	5.14E-05	2.76E-05
UBE2W	1.30E-05	1.19E-05	6.36E-06	6.43E-06	1.93E-06	6.66E-06	9.55E-06	6.70E-06
UBE2Z	4.37E-05	3.29E-05	1.51E-05	2.70E-05	1.54E-05	3.83E-05	2.29E-05	2.23E-05
UBE3A	1.95E-05	2.35E-05	1.45E-05	1.23E-05	6.97E-06	1.63E-05	1.10E-05	1.12E-05
UBE3B	2.77E-05	1.83E-05	1.06E-05	1.05E-05	6.63E-06	2.01E-05	1.36E-05	1.27E-05
UBE3C	1.36E-05	1.81E-05	1.28E-05	1.18E-05	3.13E-06	1.84E-05	1.01E-05	2.32E-05
UBE4A	2.10E-05	1.03E-05	1.16E-05	8.76E-06	3.61E-06	1.35E-05	5.57E-06	1.48E-05
UBE4B	2.17E-05	1.51E-05	9.30E-06	8.68E-06	5.71E-06	1.61E-05	7.62E-06	9.13E-06
UBFD1	1.45E-05	1.72E-05	6.80E-06	7.36E-06	2.09E-06	1.77E-05	6.60E-06	6.60E-06
UBIAD1	9.62E-06	1.01E-05	7.11E-06	7.73E-06	2.78E-06	1.01E-05	8.56E-06	9.53E-06
UBL3	2.69E-05	1.50E-05	1.94E-05	1.91E-05	4.81E-06	1.38E-05	1.38E-05	1.25E-05
UBL4A	2.29E-05	2.91E-05	1.05E-05	1.25E-05	2.69E-05	1.81E-05	2.21E-05	3.90E-05
UBL4B	0	0	0	0	0	1.22E-07	0	0
UBL5	0.000427	0.000402	0.000299	0.000355	0.000624	0.000421	0.000396	0.000332
UBL7	3.10E-05	2.91E-05	1.47E-05	2.64E-05	2.74E-05	3.77E-05	3.23E-05	2.60E-05
UBLCP1	1.20E-05	4.97E-06	5.75E-06	8.07E-06	3.54E-06	9.93E-06	6.79E-06	8.80E-06
UBN1	1.17E-05	9.17E-06	3.36E-06	3.46E-06	2.12E-06	6.20E-06	7.50E-06	6.88E-06
UBN2	1.28E-06	8.42E-07	1.03E-06	6.40E-07	4.10E-07	1.24E-06	4.80E-07	1.19E-06
UBOX5	6.40E-06	3.73E-06	2.55E-06	2.96E-06	3.53E-06	3.10E-06	4.51E-06	3.80E-06
UBP1	1.90E-05	1.64E-05	1.09E-05	1.61E-05	1.03E-05	1.46E-05	2.11E-05	1.10E-05
UBQLN1	4.87E-05	6.07E-05	2.85E-05	2.76E-05	9.73E-06	3.89E-05	1.94E-05	3.70E-05
UBQLN2	1.67E-05	1.43E-05	1.32E-05	1.58E-05	5.57E-06	2.10E-05	1.13E-05	1.82E-05
UBQLN3	0	0	0	0	0	0	0	0
UBQLN4	9.39E-06	1.79E-05	8.11E-06	8.10E-06	1.05E-05	2.60E-05	1.46E-05	1.70E-05
UBQLNL	8.10E-08	2.74E-07	1.69E-07	1.12E-07	6.96E-08	4.78E-07	5.88E-08	1.29E-06
UBR1	4.60E-06	4.05E-06	2.69E-06	2.67E-06	5.46E-07	3.46E-06	1.20E-06	1.93E-06
UBR2	1.50E-05	1.56E-05	1.20E-05	1.08E-05	2.84E-06	1.36E-05	7.07E-06	1.18E-05
UBR3	1.34E-05	1.59E-05	1.14E-05	6.86E-06	3.52E-06	1.06E-05	4.05E-06	1.20E-05
UBR4	8.49E-05	5.09E-05	5.02E-05	4.37E-05	7.13E-05	5.28E-05	5.32E-05	5.07E-05
UBR5	4.18E-05	1.34E-05	8.45E-06	8.44E-06	6.89E-06	1.21E-05	1.70E-05	1.29E-05
UBR7	1.40E-05	8.75E-06	7.19E-06	7.02E-06	3.56E-06	1.09E-05	7.29E-06	1.09E-05
UBTD1	9.57E-06	1.23E-05	6.97E-06	8.80E-06	1.07E-05	6.97E-06	1.06E-05	9.15E-06
UBTD2	1.43E-05	4.93E-06	6.29E-06	6.88E-06	2.66E-06	6.40E-06	5.51E-06	6.01E-06
UBTF	3.27E-05	2.32E-05	1.49E-05	1.80E-05	1.18E-05	2.29E-05	2.08E-05	1.86E-05
UBTFL1	0	0	0	0	0	0	0	0

UBXN1	7.54E-05	8.17E-05	5.00E-05	8.44E-05	9.61E-05	8.90E-05	9.26E-05	0.000113
UBXN10	3.67E-07	3.39E-06	5.75E-06	1.13E-05	2.28E-07	3.13E-05	1.35E-06	6.96E-06
UBXN11	1.75E-05	4.34E-06	3.04E-06	8.16E-06	3.78E-06	7.22E-06	6.84E-06	5.44E-06
UBXN2A	9.17E-06	1.07E-05	3.44E-06	4.15E-06	1.56E-06	4.25E-06	4.15E-06	2.85E-06
UBXN2B	9.68E-06	2.45E-05	4.66E-06	3.63E-06	1.94E-06	6.23E-06	6.77E-06	4.07E-06
UBXN4	9.45E-05	6.35E-05	4.16E-05	3.12E-05	8.75E-06	5.08E-05	1.83E-05	3.85E-05
UBXN6	4.90E-05	5.45E-05	6.37E-05	7.14E-05	8.49E-05	8.35E-05	7.31E-05	4.30E-05
UBXN7	3.40E-06	1.55E-06	1.70E-06	7.68E-07	1.22E-07	1.68E-06	4.46E-07	7.78E-07
UBXN8	5.66E-06	1.92E-05	2.15E-05	2.04E-05	7.25E-06	2.27E-05	1.06E-05	8.29E-06
UCA1	0.000555	1.62E-07	4.34E-07	2.16E-06	6.11E-08	0	0	0
UCHL1	3.35E-06	1.60E-07	1.35E-07	2.40E-06	2.24E-07	2.71E-07	3.19E-06	6.56E-07
UCHL3	2.16E-05	3.65E-05	2.51E-05	2.82E-05	1.45E-05	4.08E-05	2.71E-05	2.21E-05
UCHL5	2.23E-05	2.25E-05	1.45E-05	8.14E-06	1.73E-06	1.74E-05	5.04E-06	2.60E-05
UCK1	2.53E-05	1.67E-05	1.67E-05	1.01E-05	1.91E-05	1.71E-05	1.82E-05	2.03E-05
UCK2	1.95E-05	2.77E-05	6.07E-06	8.38E-06	3.67E-06	1.52E-05	1.13E-05	1.47E-05
UCKL1	3.69E-05	3.72E-05	1.49E-05	1.96E-05	2.04E-05	1.80E-05	1.19E-05	1.37E-05
UCKL1AS	4.48E-07	9.29E-08	7.15E-09	5.54E-08	2.39E-07	3.53E-08	2.76E-08	3.32E-08
UCMA	0	0	0	0	0	0	6.50E-08	0
UCN	6.59E-07	3.44E-06	1.80E-07	5.73E-07	3.95E-06	6.22E-07	4.92E-06	3.09E-07
UCN2	4.01E-07	3.63E-07	0	4.11E-08	2.89E-08	1.09E-07	3.22E-08	2.47E-08
UCN3	8.30E-08	0	0	0	0	0	0	0
UCP1	0	0	0	0	0	0	0	0
UCP2	5.49E-05	4.35E-06	1.58E-05	5.56E-05	3.13E-06	2.82E-05	4.43E-05	1.00E-05
UCP3	4.29E-07	1.78E-07	1.02E-07	2.99E-07	4.25E-08	2.09E-07	9.83E-08	1.14E-07
UEVLD	7.90E-06	6.71E-06	2.74E-06	2.42E-06	1.77E-07	4.80E-06	1.09E-06	3.83E-06
UFC1	9.47E-05	0.000129	4.59E-05	7.19E-05	4.18E-05	0.000135	7.09E-05	0.000126
UFD1L	4.65E-05	5.50E-05	1.70E-05	2.29E-05	4.03E-05	3.31E-05	2.02E-05	7.48E-05
UFM1	4.06E-05	4.06E-05	2.69E-05	2.56E-05	6.39E-06	3.44E-05	2.39E-05	2.81E-05
UFSP1	2.26E-06	6.40E-06	1.30E-06	1.53E-06	3.38E-06	2.64E-06	2.13E-06	2.21E-06
UFSP2	1.08E-05	3.88E-06	8.51E-06	8.89E-06	5.40E-06	7.89E-06	8.71E-06	8.21E-06
UGCG	3.97E-05	7.25E-06	5.22E-06	1.11E-05	3.89E-08	3.57E-06	1.84E-06	6.24E-06
UGDH	2.93E-05	7.28E-05	4.10E-05	2.78E-05	1.32E-05	0.000109	2.88E-05	3.49E-05
UGGT1	1.14E-05	1.20E-05	6.19E-06	5.17E-06	3.23E-06	2.15E-05	5.03E-06	9.60E-06
UGGT2	1.04E-05	8.14E-06	4.96E-06	7.40E-06	4.04E-06	6.33E-06	5.99E-06	5.58E-06
UGP2	9.73E-05	0.000226	0.000476	0.000249	0.000148	0.000149	0.000152	0.000235
UGT1A1	2.04E-05	6.21E-05	0.000331	0.000182	6.86E-06	0.000237	0.000105	0.000573
UGT1A10	6.91E-05	8.95E-06	0	3.34E-07	0	0	2.62E-07	3.40E-07
UGT1A3	5.14E-06	4.37E-05	1.20E-05	2.81E-05	7.53E-07	3.20E-05	6.09E-06	1.19E-05
UGT1A4	1.35E-05	0.00044	0.000223	0.000213	1.92E-05	0.00058	8.91E-05	0.000103
UGT1A5	1.75E-06	4.10E-06	2.36E-06	2.12E-06	2.11E-07	5.33E-06	4.99E-07	1.27E-06
UGT1A6	0.000143	0.000221	0.00014	0.00016	0.000619	0.000271	0.000185	0.000133
UGT1A7	0	1.94E-06	3.81E-07	0	0	5.37E-08	0	0
UGT1A8	1.36E-06	8.10E-07	5.35E-08	6.93E-08	0	4.43E-07	9.28E-08	4.09E-08
UGT1A9	4.10E-05	0.000256	0.000127	7.88E-05	4.96E-05	0.000178	7.07E-05	8.25E-05
UGT2A1	6.79E-07	3.90E-06	1.03E-05	1.25E-07	1.51E-06	4.68E-06	8.56E-07	5.46E-08
UGT2A3	1.38E-05	2.16E-05	2.07E-05	7.12E-06	1.52E-06	6.60E-06	2.73E-06	5.16E-06
UGT2B10	6.05E-06	5.43E-05	0.000291	0.000196	0.000153	0.000373	9.98E-05	0.000222
UGT2B11	1.39E-05	3.18E-06	1.09E-05	2.79E-06	4.68E-06	5.72E-06	6.35E-07	9.40E-05
UGT2B15	4.86E-05	0.000188	0.000327	0.000272	0.000217	0.00025	0.000202	0.000316
UGT2B28	3.25E-07	1.05E-06	1.25E-06	9.72E-07	3.18E-07	3.08E-06	2.68E-07	2.39E-06
UGT2B4	0.000182	0.000971	0.000764	0.000239	0.000412	0.001209	0.000341	0.000877
UGT2B7	7.61E-05	0.000173	0.000651	0.000323	9.10E-05	0.000796	0.000166	0.000179
UGT3A1	7.10E-05	8.83E-05	5.37E-05	3.77E-05	7.63E-07	1.04E-05	2.27E-05	4.06E-05
UGT3A2	5.93E-06	6.81E-06	2.31E-07	3.24E-07	6.92E-08	4.91E-07	4.84E-07	1.91E-07
UGT8	3.94E-08	0	0	0	0	1.83E-08	9.54E-09	1.45E-08
UHMK1	8.03E-06	1.64E-05	8.66E-06	2.63E-06	6.14E-08	1.19E-05	4.58E-07	4.96E-06
UHRF1	6.58E-06	1.17E-06	6.62E-08	1.86E-07	8.58E-07	1.38E-06	2.43E-07	7.28E-07
UHRF1BP1	4.40E-06	4.09E-06	1.23E-06	1.21E-06	6.96E-07	2.10E-06	1.32E-06	2.30E-06

UHRF1BP1	9.77E-06	1.23E-05	1.08E-05	8.10E-06	1.26E-06	1.12E-05	4.34E-06	7.50E-06
UHRF2	6.50E-06	6.82E-06	4.53E-06	4.25E-06	1.37E-06	5.12E-06	3.71E-06	3.95E-06
UIMC1	1.28E-05	4.18E-06	3.92E-06	8.39E-06	2.46E-06	8.27E-06	6.98E-06	8.72E-06
ULBP1	6.69E-07	0	5.32E-08	4.49E-08	0	6.81E-09	3.57E-08	1.08E-08
ULBP2	1.25E-05	1.95E-07	1.61E-07	1.62E-07	1.62E-08	9.87E-08	3.78E-07	5.48E-07
ULBP3	2.64E-06	1.29E-07	1.52E-07	8.21E-07	5.49E-08	3.52E-07	6.63E-08	9.98E-08
ULK1	1.30E-05	1.23E-05	1.23E-05	7.39E-06	4.48E-06	8.26E-06	1.15E-05	1.89E-05
ULK2	1.63E-06	3.10E-07	1.63E-06	2.01E-06	4.96E-07	1.37E-06	7.17E-07	1.47E-07
ULK3	2.41E-05	1.19E-05	7.11E-06	9.19E-06	6.56E-06	1.34E-05	1.30E-05	2.37E-05
ULK4	1.68E-06	3.12E-06	4.11E-07	2.56E-06	1.19E-06	2.07E-06	1.81E-06	9.54E-07
UMOD	1.05E-08	0	0	0	0	0	0	0
UMODL1	4.78E-08	0	9.26E-09	0	0	0	0	8.10E-08
UMPS	1.79E-05	1.71E-05	1.07E-05	1.25E-05	7.33E-06	1.78E-05	1.78E-05	1.95E-05
UNC119	2.15E-05	2.49E-05	1.10E-05	1.14E-05	5.98E-06	7.34E-06	1.51E-05	3.32E-05
UNC119B	5.73E-06	2.01E-06	6.74E-07	1.90E-06	1.03E-06	1.20E-06	4.15E-06	1.24E-06
UNC13A	5.19E-06	2.44E-09	0	2.67E-09	1.88E-09	2.03E-09	2.57E-08	1.60E-08
UNC13B	2.71E-05	1.97E-05	5.38E-06	6.07E-06	3.69E-06	1.29E-05	4.88E-06	9.06E-06
UNC13C	6.81E-07	3.04E-09	0	1.27E-08	0	0	0	0
UNC13D	3.34E-06	6.98E-07	1.91E-06	3.95E-06	7.32E-07	9.36E-07	1.65E-06	5.89E-07
UNC45A	4.01E-05	3.32E-05	1.23E-05	1.99E-05	1.82E-05	1.74E-05	2.08E-05	2.46E-05
UNC45B	2.38E-08	2.21E-08	0	1.03E-08	7.23E-09	7.80E-09	3.86E-08	6.16E-09
UNC50	5.00E-05	3.53E-05	2.14E-05	2.21E-05	1.03E-05	3.26E-05	2.02E-05	2.28E-05
UNC5A	1.04E-07	4.81E-08	0	5.95E-08	5.26E-09	5.67E-08	8.40E-07	4.49E-08
UNC5B	1.14E-05	2.66E-06	2.49E-06	5.52E-06	1.03E-06	1.20E-06	5.28E-06	3.41E-06
UNC5C	1.60E-07	1.51E-07	1.28E-07	6.90E-08	0	1.74E-08	1.83E-08	2.76E-08
UNC5CL	1.34E-05	6.89E-06	4.19E-06	1.11E-05	1.17E-06	9.10E-06	9.61E-06	7.65E-06
UNC5D	1.16E-08	0	0	1.42E-08	0	7.15E-09	0	0
UNC80	1.09E-08	1.62E-08	0	1.33E-08	1.56E-08	3.85E-08	0	1.59E-08
UNC93A	2.17E-05	5.75E-06	5.49E-06	1.11E-06	1.15E-05	5.31E-06	2.65E-06	3.56E-08
UNC93B1	1.33E-05	1.17E-05	8.47E-06	1.19E-05	5.84E-06	7.73E-06	1.54E-05	1.31E-05
UNCX	0	0	0	0	0	0	0	0
UNG	1.91E-05	2.39E-05	2.26E-05	1.47E-05	6.53E-06	2.54E-05	2.00E-05	1.82E-05
UNK	1.44E-05	1.43E-05	4.37E-06	5.63E-06	4.04E-06	8.12E-06	6.05E-06	4.71E-06
UNKL	4.53E-06	3.53E-06	1.84E-06	1.68E-06	6.18E-07	4.20E-06	1.79E-06	2.66E-06
UOX	1.60E-08	0	0	0	0	0	1.54E-08	0
UPB1	1.29E-05	5.47E-05	0.000253	0.000116	0.00021	0.000105	8.20E-05	0.000201
UPF0639	4.65E-07	0	0	1.64E-08	0	1.24E-08	1.29E-08	0
UPF1	4.84E-05	3.14E-05	1.83E-05	1.82E-05	1.83E-05	2.28E-05	2.11E-05	2.51E-05
UPF2	1.08E-05	5.87E-06	5.09E-06	6.41E-06	3.02E-06	8.13E-06	5.82E-06	4.66E-06
UPF3A	1.55E-05	1.34E-05	8.26E-06	8.59E-06	2.71E-06	1.13E-05	7.92E-06	8.31E-06
UPF3B	1.13E-05	3.84E-06	1.94E-06	2.91E-06	2.83E-06	5.58E-06	3.64E-06	8.07E-06
UPK1A	2.41E-06	1.45E-07	4.11E-08	1.29E-07	1.09E-07	1.34E-06	2.01E-08	3.11E-07
UPK1B	1.22E-08	2.77E-08	0	0	0	0	1.18E-08	1.80E-08
UPK2	8.95E-08	1.39E-07	0	3.70E-08	2.59E-08	0	8.50E-08	8.91E-08
UPK3A	9.16E-08	2.09E-06	1.13E-07	3.44E-07	4.00E-08	2.42E-08	8.58E-08	6.94E-08
UPK3B	1.53E-07	5.50E-07	5.18E-08	7.24E-07	1.45E-07	1.42E-07	1.63E-07	3.21E-07
UPK3BL	3.15E-06	6.37E-06	3.30E-06	5.67E-06	1.26E-05	#######	2.70E-06	4.77E-06
UPP1	1.95E-05	1.85E-05	5.13E-06	2.49E-05	7.40E-06	2.72E-06	1.34E-05	1.54E-05
UPP2	7.05E-05	7.71E-07	2.85E-06	6.45E-07	3.11E-07	4.43E-06	1.80E-06	1.42E-06
UPRT	9.82E-06	5.75E-06	4.50E-06	4.51E-06	2.50E-06	6.51E-06	3.72E-06	8.48E-06
UQCC	2.11E-05	2.32E-05	1.32E-05	1.17E-05	1.28E-05	2.57E-05	1.53E-05	1.37E-05
UQCR10	0.000257	0.000199	9.44E-05	9.94E-05	0.000259	0.000132	0.000152	0.000129
UQCR11	0.000122	9.10E-05	7.08E-05	7.95E-05	0.000302	0.000107	0.000116	7.02E-05
UQCRB	0.000162	8.92E-05	2.35E-05	3.62E-05	0.000106	3.78E-05	9.76E-05	5.34E-05
UQCRC1	0.000327	0.000295	0.000204	0.000191	0.000276	0.000248	0.000267	0.000216
UQCRC2	0.000201	0.000256	0.000135	0.000119	4.37E-05	0.000141	0.000113	0.000142
UQCRFS1	0.000158	0.000131	8.24E-05	9.21E-05	0.000101	0.000102	0.000108	0.000135
UQCRH	0.00054	0.000374	0.000126	0.000171	0.000118	0.000181	0.000125	0.00024

UQCRHL	0.000163	8.06E-05	2.44E-05	3.94E-05	1.94E-05	3.97E-05	3.46E-05	4.78E-05
UQCRQ	0.000234	0.000139	0.000125	0.000116	0.000195	0.000252	0.000214	0.000168
URB1	2.57E-06	2.26E-06	1.97E-06	1.73E-06	9.86E-07	5.22E-06	2.34E-06	2.27E-06
URB2	1.24E-06	2.48E-06	8.23E-07	1.35E-06	2.75E-07	2.05E-06	1.32E-06	2.91E-06
URGCP	2.77E-05	1.38E-05	8.79E-06	1.71E-05	7.28E-06	1.45E-05	1.60E-05	1.64E-05
URM1	6.13E-05	2.97E-05	2.97E-05	3.93E-05	3.83E-05	5.39E-05	4.62E-05	5.42E-05
UROC1	1.48E-08	5.87E-05	7.90E-05	4.43E-05	8.10E-05	5.61E-07	1.70E-05	1.41E-06
UROD	7.09E-05	5.83E-05	7.00E-05	7.09E-05	7.17E-05	8.87E-05	0.000111	8.26E-05
UROS	2.93E-05	1.88E-05	3.55E-05	3.09E-05	5.37E-05	4.64E-05	2.49E-05	3.16E-05
USE1	2.58E-05	1.89E-05	2.12E-05	3.12E-05	7.02E-05	2.42E-05	3.47E-05	2.79E-05
USF1	3.11E-05	4.78E-05	1.28E-05	1.60E-05	2.39E-05	2.93E-05	2.23E-05	4.80E-05
USF2	6.34E-05	5.04E-05	5.82E-05	7.47E-05	6.09E-05	5.13E-05	0.000109	6.55E-05
USH1C	2.32E-05	2.48E-08	3.98E-07	1.59E-06	0	7.80E-07	1.06E-08	0
USH1G	2.71E-07	2.20E-07	0	7.99E-09	0	0	6.25E-09	0
USH2A	4.53E-08	2.57E-06	2.39E-06	5.66E-08	3.89E-07	4.96E-07	6.01E-08	1.02E-07
USHBP1	1.75E-06	1.06E-06	9.28E-07	5.34E-07	3.64E-06	9.33E-07	2.59E-06	2.92E-06
USMG5	0.000525	0.000454	0.000292	0.000305	0.000673	0.000386	0.000271	0.000269
USO1	4.00E-05	2.08E-05	2.69E-05	2.30E-05	7.75E-06	3.99E-05	1.46E-05	3.19E-05
USP1	1.21E-05	6.52E-06	4.22E-06	5.06E-06	1.18E-06	6.72E-06	3.88E-06	6.79E-06
USP10	2.38E-05	1.65E-05	1.79E-05	1.68E-05	6.28E-06	2.31E-05	1.43E-05	1.79E-05
USP11	2.91E-05	1.61E-05	5.93E-06	1.53E-05	5.76E-06	1.24E-05	1.31E-05	1.85E-05
USP12	5.42E-06	6.35E-06	5.86E-06	5.22E-06	1.30E-07	4.37E-06	1.99E-06	3.75E-06
USP13	2.46E-06	1.95E-06	1.94E-06	5.05E-06	9.35E-07	2.21E-06	2.09E-06	2.70E-06
USP14	2.34E-05	1.13E-05	8.10E-06	8.08E-06	2.05E-06	1.65E-05	5.47E-06	1.44E-05
USP15	1.28E-05	1.12E-05	1.05E-05	9.18E-06	2.19E-06	1.13E-05	5.53E-06	8.45E-06
USP16	1.77E-05	1.11E-05	1.28E-05	1.61E-05	6.84E-06	2.11E-05	1.16E-05	1.82E-05
USP17	0	1.76E-08	0	0	0	0	0	0
USP17L2	0	0	0	0	0	0	0	0
USP17L6P	0	0	0	0	0	0	0	0
USP18	7.90E-06	7.21E-06	1.19E-05	7.77E-06	5.85E-06	8.41E-06	6.20E-06	2.02E-05
USP19	2.06E-05	2.55E-05	1.33E-05	1.18E-05	6.03E-06	1.81E-05	1.21E-05	1.21E-05
USP2	9.08E-06	6.13E-06	9.25E-06	3.28E-06	2.91E-06	6.71E-07	8.06E-06	7.56E-06
USP20	1.09E-05	5.03E-06	7.35E-06	8.36E-06	8.77E-06	1.03E-05	9.28E-06	1.26E-05
USP21	6.38E-06	7.34E-06	2.78E-06	2.87E-06	1.83E-06	6.47E-06	3.76E-06	6.01E-06
USP22	2.61E-05	3.53E-05	1.19E-05	1.57E-05	1.59E-05	2.03E-05	3.36E-05	1.20E-05
USP24	8.42E-06	5.63E-06	4.70E-06	4.17E-06	9.72E-07	6.26E-06	2.95E-06	4.73E-06
USP25	1.01E-05	4.08E-06	7.93E-06	6.73E-06	1.13E-06	9.40E-06	3.72E-06	7.32E-06
USP26	0	0	0	0	0	0	0	0
USP27X	1.22E-06	1.05E-06	5.47E-07	2.17E-06	2.53E-07	3.80E-06	9.39E-07	2.49E-06
USP28	6.62E-06	5.68E-06	5.25E-06	3.52E-06	7.85E-07	5.41E-06	2.08E-06	6.88E-06
USP29	0	0	0	0	5.46E-09	0	0	0
USP3	1.32E-05	9.34E-06	6.04E-06	6.75E-06	1.96E-06	1.13E-05	5.22E-06	6.83E-06
USP30	4.92E-06	1.23E-05	8.99E-06	4.70E-06	5.79E-06	9.41E-06	8.78E-06	7.13E-06
USP31	1.66E-06	6.86E-06	1.25E-06	1.25E-06	5.57E-07	2.51E-06	3.46E-06	4.17E-06
USP32	1.48E-05	1.44E-05	6.21E-06	5.45E-06	1.47E-06	8.93E-06	4.07E-06	7.34E-06
USP33	3.06E-05	2.32E-05	1.03E-05	1.04E-05	3.62E-06	2.28E-05	7.64E-06	9.30E-06
USP34	4.05E-05	2.49E-05	1.51E-05	1.96E-05	1.16E-05	2.07E-05	1.86E-05	1.77E-05
USP35	5.61E-06	5.28E-06	1.02E-06	1.63E-06	1.24E-06	6.96E-06	1.66E-06	4.44E-06
USP36	1.94E-05	1.40E-05	6.04E-06	1.12E-05	6.36E-06	1.59E-05	1.18E-05	1.17E-05
USP37	5.98E-06	1.64E-06	1.31E-06	1.61E-06	3.43E-07	1.48E-06	7.77E-07	1.14E-06
USP38	7.92E-06	5.06E-06	9.58E-06	7.81E-06	2.09E-06	8.92E-06	4.70E-06	1.59E-05
USP39	2.81E-05	1.78E-05	8.92E-06	1.18E-05	6.91E-06	1.59E-05	1.39E-05	1.54E-05
USP4	1.04E-05	1.09E-05	6.98E-06	9.03E-06	2.15E-06	1.13E-05	6.27E-06	6.07E-06
USP40	3.65E-05	2.30E-05	1.64E-05	1.57E-05	1.20E-05	3.59E-05	1.93E-05	1.63E-05
USP42	4.39E-06	4.72E-06	1.54E-06	2.19E-06	8.84E-07	2.39E-06	1.70E-06	2.71E-06
USP43	2.18E-06	1.45E-06	7.73E-07	1.16E-06	0	0	1.54E-07	8.23E-09
USP44	6.92E-09	7.77E-09	3.57E-08	6.77E-08	5.69E-09	6.41E-09	6.72E-09	0
USP45	2.65E-06	2.58E-06	1.83E-06	1.22E-06	3.06E-07	2.52E-06	1.11E-06	2.27E-06

USP46	1.57E-06	1.06E-06	9.07E-07	9.09E-07	1.24E-07	1.40E-06	9.00E-07	1.04E-06
USP47	2.84E-05	2.36E-05	1.07E-05	1.48E-05	5.96E-06	2.19E-05	9.48E-06	2.72E-05
USP48	1.99E-05	8.46E-06	7.46E-06	7.08E-06	1.51E-06	8.15E-06	5.14E-06	6.87E-06
USP49	6.83E-07	1.06E-07	3.00E-08	6.22E-08	0	1.14E-07	2.12E-08	4.26E-08
USP5	2.73E-05	2.93E-05	2.07E-05	2.10E-05	1.91E-05	4.88E-05	2.18E-05	2.25E-05
USP50	5.81E-08	0	2.11E-08	0	0	0	0	0
USP51	3.68E-07	1.29E-06	2.45E-07	1.57E-07	1.62E-08	1.05E-07	2.74E-08	5.54E-08
USP53	4.22E-06	1.13E-06	3.16E-06	2.54E-06	1.68E-07	2.32E-06	9.58E-07	2.50E-06
USP54	6.15E-06	9.85E-07	8.55E-07	8.59E-07	1.77E-07	1.48E-06	6.62E-07	2.30E-06
USP6	2.28E-07	1.96E-07	1.25E-07	9.52E-08	6.93E-08	1.04E-07	6.80E-08	9.37E-08
USP6NL	5.27E-06	5.94E-06	8.42E-06	7.68E-06	1.65E-06	5.52E-06	4.24E-06	6.10E-06
USP7	2.50E-05	4.12E-05	1.53E-05	1.61E-05	7.94E-06	2.26E-05	2.09E-05	1.93E-05
USP8	1.65E-05	1.48E-05	7.38E-06	6.61E-06	3.08E-06	1.06E-05	5.44E-06	8.68E-06
USP9X	2.93E-05	1.10E-05	1.54E-05	1.25E-05	4.14E-06	1.22E-05	2.12E-06	1.62E-05
USP9Y	0	1.57E-06	0	1.05E-06	2.69E-08	1.09E-07	1.01E-07	6.18E-07
USPL1	1.01E-05	4.59E-06	4.83E-06	4.71E-06	1.22E-06	5.46E-06	4.14E-06	4.08E-06
UST	2.64E-07	5.20E-08	3.71E-07	8.20E-07	2.22E-08	1.06E-06	2.21E-07	7.56E-08
UTF1	2.24E-08	0	0	2.76E-08	0	0	0	0
UTP11L	1.22E-05	1.18E-05	7.84E-06	1.19E-05	4.36E-06	1.39E-05	1.22E-05	1.10E-05
UTP14A	9.37E-06	8.47E-06	3.99E-06	6.59E-06	4.50E-06	8.28E-06	8.12E-06	1.06E-05
UTP14C	1.34E-05	1.17E-05	1.07E-05	7.29E-06	1.53E-06	1.32E-05	4.66E-06	6.02E-06
UTP15	3.30E-06	4.10E-06	1.76E-06	2.50E-06	3.69E-07	4.19E-06	2.49E-06	5.34E-06
UTP18	2.08E-05	3.21E-05	9.71E-06	1.35E-05	6.00E-06	2.17E-05	1.66E-05	1.33E-05
UTP20	2.61E-06	3.14E-06	1.44E-06	1.42E-06	3.06E-07	2.85E-06	1.28E-06	1.70E-06
UTP23	9.54E-06	7.66E-06	2.84E-06	3.06E-06	9.10E-07	3.33E-06	5.16E-06	3.90E-06
UTP3	1.71E-05	9.27E-06	1.49E-05	2.12E-05	6.39E-06	1.62E-05	1.62E-05	1.89E-05
UTP6	3.12E-05	3.51E-05	1.00E-05	1.41E-05	8.33E-06	1.68E-05	1.38E-05	1.14E-05
UTRN	1.84E-05	3.41E-06	3.26E-06	3.42E-06	8.86E-07	7.39E-06	2.10E-06	2.77E-06
UTS2	1.46E-07	2.76E-06	0	0	0	4.60E-07	2.23E-08	0
UTS2D	3.01E-06	1.10E-07	6.11E-08	8.38E-08	0	3.63E-08	3.79E-08	1.44E-07
UTS2R	1.33E-07	3.31E-07	0	2.73E-08	0	0	0	0
UTY	0	2.77E-06	0	3.96E-06	1.18E-06	3.96E-06	2.62E-06	1.53E-06
UVRAG	6.70E-06	4.57E-06	3.61E-06	4.44E-06	1.48E-06	4.18E-06	4.33E-06	5.58E-06
UXS1	4.88E-05	2.02E-05	7.79E-06	1.20E-05	8.41E-06	1.46E-05	1.24E-05	1.55E-05
UXT	0.000134	0.000148	8.18E-05	0.000114	0.000115	0.000132	0.000143	0.000163
VAC14	2.46E-05	1.71E-05	1.41E-05	1.74E-05	1.95E-05	2.53E-05	2.51E-05	2.71E-05
VAMP1	5.38E-06	2.21E-06	1.46E-06	2.11E-06	8.03E-07	2.15E-06	2.64E-06	3.03E-06
VAMP2	1.36E-05	1.46E-05	2.49E-05	3.14E-05	6.39E-06	2.51E-05	3.45E-05	2.19E-05
VAMP3	4.72E-05	3.25E-05	2.79E-05	2.92E-05	9.41E-06	4.75E-05	2.41E-05	2.45E-05
VAMP4	5.75E-06	5.32E-06	5.22E-06	4.13E-06	2.15E-06	5.74E-06	5.03E-06	1.32E-05
VAMP5	0.00018	0.000165	3.71E-05	0.000212	0.000241	0.000182	0.000177	9.72E-05
VAMP7	2.54E-05	5.63E-05	1.94E-05	1.82E-05	9.01E-06	2.84E-05	1.24E-05	1.88E-05
VAMP8	0.000385	0.000194	0.000117	0.000287	0.000243	0.000129	0.000263	0.000141
VANGL1	3.61E-06	1.47E-06	1.14E-06	1.51E-06	5.84E-07	1.38E-06	1.13E-06	1.51E-06
VANGL2	1.66E-06	4.39E-08	1.12E-07	5.59E-07	2.25E-08	1.21E-08	1.27E-08	1.73E-07
VAPA	2.43E-05	1.77E-05	1.26E-05	1.80E-05	4.45E-06	2.08E-05	1.78E-05	1.63E-05
VAPB	2.24E-05	2.38E-05	8.92E-06	8.06E-06	7.72E-06	1.01E-05	1.15E-05	9.04E-06
VARS	3.77E-05	0.000109	2.81E-05	3.08E-05	4.68E-05	3.69E-05	4.31E-05	4.23E-05
VARS2	2.20E-05	1.92E-05	1.56E-05	1.27E-05	1.27E-05	1.73E-05	2.32E-05	2.36E-05
VASH1	7.57E-06	8.44E-07	1.45E-06	3.38E-06	1.46E-06	1.30E-06	4.14E-06	1.81E-06
VASH2	3.96E-07	1.61E-06	3.53E-08	1.89E-07	3.43E-08	1.51E-06	2.22E-08	2.31E-07
VASN	1.41E-05	2.67E-05	1.25E-05	2.85E-05	2.03E-05	5.80E-06	2.80E-05	2.15E-05
VASP	6.06E-05	2.41E-05	1.62E-05	4.07E-05	2.09E-05	2.03E-05	4.89E-05	1.92E-05
VAT1	0.000131	0.000156	3.24E-05	5.59E-05	1.85E-05	5.44E-05	5.68E-05	3.48E-05
VAT1L	3.42E-05	1.02E-07	4.43E-07	1.28E-06	1.04E-08	6.74E-08	1.18E-08	3.81E-05
VAV1	4.23E-06	1.18E-06	2.58E-06	4.07E-06	5.10E-07	2.29E-06	2.85E-06	4.35E-07
VAV2	3.70E-05	1.17E-05	1.99E-05	2.03E-05	1.02E-05	2.09E-05	1.30E-05	3.38E-05
VAV3	1.17E-05	4.21E-07	1.06E-06	9.34E-07	4.12E-07	6.41E-07	1.46E-06	4.22E-07

VAX1	0	0	0	0	0	0	0	0
VAX2	2.99E-06	2.62E-08	0	8.40E-08	3.73E-07	0	3.47E-07	2.02E-07
VBP1	2.53E-05	2.78E-05	1.30E-05	1.54E-05	1.38E-05	1.65E-05	1.49E-05	2.91E-05
VCAM1	0.000113	9.41E-05	1.61E-05	1.08E-05	1.03E-06	7.21E-06	1.35E-05	1.95E-05
VCAN	0.000129	7.73E-07	1.21E-06	5.83E-06	6.06E-07	8.34E-07	6.27E-06	4.94E-06
VCL	3.31E-05	1.37E-05	6.80E-06	2.07E-05	2.79E-06	9.20E-06	1.39E-05	1.26E-05
VCP	9.36E-05	0.000131	6.86E-05	7.24E-05	4.30E-05	0.00014	5.15E-05	0.000119
VCPIP1	8.18E-06	4.74E-06	3.59E-06	2.73E-06	1.11E-06	3.70E-06	4.01E-06	4.39E-06
VCX	2.23E-07	0	0	0	0	0	0	3.33E-07
VCX2	0	0	0	0	0	3.24E-08	0	0
VCX3A	2.50E-06	2.50E-07	2.95E-08	0	0	0	3.44E-08	5.21E-07
VCX3B	2.42E-08	0	2.63E-08	0	0	0	0	0
VCY	0	8.15E-07	0	0	0	0	0	0
VDAC1	0.000295	0.000207	0.000115	0.000115	4.32E-05	0.000126	0.000127	0.000143
VDAC2	0.0001	4.38E-05	2.16E-05	3.20E-05	1.55E-05	3.10E-05	5.11E-05	3.73E-05
VDAC3	4.73E-05	0.00011	4.19E-05	4.55E-05	2.95E-05	0.000103	2.45E-05	6.49E-05
VDR	2.18E-06	7.61E-08	2.50E-07	1.48E-06	6.05E-07	2.21E-07	3.64E-07	2.63E-07
VEGFA	9.54E-05	9.04E-05	3.12E-05	5.01E-05	3.71E-05	2.41E-05	2.84E-05	4.98E-05
VEGFB	4.68E-05	9.87E-05	2.46E-05	2.61E-05	1.12E-05	3.20E-06	4.04E-05	1.94E-05
VEGFC	4.37E-06	2.30E-06	1.03E-06	3.82E-06	1.34E-06	1.92E-06	2.55E-06	3.50E-06
VENTX	2.05E-07	4.40E-08	1.83E-07	3.47E-07	2.35E-07	3.08E-07	1.04E-07	1.58E-07
VENTXP1	0	0	0	0	0	0	0	0
VENTXP7	0	0	0	0	0	0	0	0
VEPH1	1.46E-06	0	2.88E-07	1.88E-07	4.64E-08	9.62E-07	5.17E-08	0
VEZF1	1.29E-05	7.21E-06	4.22E-06	7.87E-06	2.74E-06	7.71E-06	5.07E-06	4.21E-06
VEZT	2.07E-05	1.49E-05	9.88E-06	1.13E-05	3.22E-06	1.15E-05	6.53E-06	1.20E-05
VGF	8.26E-08	3.31E-07	0	1.12E-08	7.89E-09	0	1.78E-08	0
VGLL1	0	4.86E-08	4.56E-08	2.61E-08	0	0	0	0
VGLL2	0	0	0	0	0	0	0	0
VGLL3	2.57E-07	8.05E-08	9.53E-08	2.64E-07	4.01E-08	1.87E-07	1.08E-07	9.01E-08
VGLL4	2.07E-05	1.17E-05	6.15E-06	1.18E-05	4.36E-06	8.62E-06	9.72E-06	8.53E-06
VHL	1.36E-05	1.12E-05	6.89E-06	6.91E-06	5.72E-07	6.56E-06	5.08E-06	4.62E-06
VHLL	0	0	2.88E-08	0	0	0	0	0
VIL1	8.96E-05	1.32E-05	1.52E-05	4.91E-06	7.83E-06	3.75E-07	1.10E-06	4.32E-06
VILL	2.38E-06	2.70E-07	3.41E-07	5.84E-07	2.87E-07	2.23E-07	5.92E-07	2.45E-07
VIM	0.000251	0.000134	6.09E-05	0.000381	6.39E-05	7.36E-05	0.000314	0.000133
VIP	2.49E-07	5.34E-08	3.31E-08	5.74E-08	1.48E-07	1.45E-08	1.51E-08	4.62E-07
VIPAR	1.33E-05	6.50E-06	6.36E-06	6.53E-06	4.33E-06	9.39E-06	1.04E-05	1.01E-05
VIPR1	1.21E-06	9.91E-07	1.76E-05	1.31E-05	2.59E-06	6.47E-07	2.08E-06	3.13E-07
VIPR2	1.16E-08	0	1.30E-07	1.07E-07	5.57E-09	0	5.62E-09	0
VIT	5.67E-08	1.01E-08	0	2.19E-08	0	0	8.29E-09	0
VKORC1	0.000106	0.000237	0.000352	0.000302	0.000123	0.000143	0.000324	0.000105
VKORC1L1	2.30E-05	3.17E-05	2.64E-05	1.50E-05	6.18E-06	1.48E-05	1.25E-05	2.04E-05
VLDLR	1.02E-06	2.73E-07	5.52E-07	5.01E-07	2.88E-08	4.83E-06	7.66E-07	5.78E-08
VMA21	7.60E-06	9.61E-06	4.76E-06	5.11E-06	3.81E-06	5.92E-06	5.72E-06	6.35E-06
VMAC	1.91E-06	1.27E-06	1.19E-06	1.15E-06	7.83E-07	1.88E-06	2.18E-06	1.86E-06
VMO1	5.59E-06	1.70E-06	8.29E-06	1.75E-05	9.64E-06	3.49E-06	5.77E-06	2.12E-06
VN1R1	7.91E-07	1.55E-07	2.48E-07	2.37E-08	4.99E-08	3.59E-08	1.30E-07	4.28E-07
VN1R2	0	0	0	0	0	0	0	0
VN1R4	0	0	0	0	0	0	0	0
VN1R5	0	0	0	0	0	0	0	0
VNN1	0.000205	8.21E-05	6.18E-05	0.000114	1.94E-05	3.46E-06	0.000221	6.34E-06
VNN2	1.21E-05	1.59E-06	2.05E-06	7.28E-06	2.76E-07	5.77E-07	3.72E-06	2.52E-05
VNN3	1.28E-05	1.07E-05	2.38E-05	1.40E-05	3.72E-06	3.42E-06	2.66E-05	4.23E-05
VOPP1	3.06E-05	1.16E-05	6.40E-06	1.10E-05	1.29E-05	1.44E-05	1.52E-05	1.08E-05
VPRBP	1.18E-05	9.68E-06	8.28E-06	8.01E-06	2.62E-06	1.10E-05	6.00E-06	8.58E-06
VPREB1	0	0	0	0	0	0	0	0
VPREB3	6.61E-07	1.45E-05	3.31E-07	8.32E-07	2.13E-06	1.35E-06	1.48E-06	4.21E-07

VPS11	1.83E-05	9.91E-06	6.52E-06	8.47E-06	3.28E-06	1.53E-05	9.27E-06	1.85E-05
VPS13A	5.39E-06	2.00E-06	2.38E-06	2.12E-06	2.95E-07	2.44E-06	5.61E-07	1.41E-06
VPS13B	6.84E-06	1.32E-06	1.31E-06	1.15E-06	4.99E-07	2.05E-06	1.51E-06	1.70E-06
VPS13C	1.01E-05	2.78E-06	3.57E-06	3.11E-06	5.79E-07	4.41E-06	1.51E-06	2.15E-06
VPS13D	1.15E-05	6.67E-06	8.11E-06	7.62E-06	4.25E-06	1.12E-05	7.02E-06	8.04E-06
VPS16	3.23E-05	2.41E-05	1.28E-05	1.68E-05	2.79E-05	1.76E-05	1.61E-05	1.72E-05
VPS18	2.41E-05	9.14E-06	6.61E-06	1.02E-05	9.29E-06	1.07E-05	1.05E-05	1.25E-05
VPS24	3.84E-05	1.55E-05	1.01E-05	1.77E-05	8.42E-06	1.40E-05	1.51E-05	1.40E-05
VPS25	0.000109	0.000136	3.80E-05	4.82E-05	6.73E-05	0.000107	5.89E-05	5.71E-05
VPS26A	2.52E-05	1.66E-05	1.38E-05	1.66E-05	8.01E-06	2.14E-05	1.70E-05	1.64E-05
VPS26B	1.80E-05	1.02E-05	1.94E-05	1.40E-05	7.12E-06	2.08E-05	1.37E-05	3.39E-05
VPS28	0.00029	0.000157	9.91E-05	0.000126	0.00036	0.000138	0.000585	0.000189
VPS29	0.000103	6.99E-05	5.05E-05	5.23E-05	2.60E-05	5.79E-05	5.88E-05	6.75E-05
VPS33A	1.81E-05	1.41E-05	7.60E-06	7.05E-06	3.41E-06	1.53E-05	7.23E-06	1.01E-05
VPS33B	7.63E-06	6.74E-06	3.46E-06	4.04E-06	2.34E-06	6.43E-06	4.83E-06	4.97E-06
VPS35	3.58E-05	3.47E-05	1.57E-05	1.82E-05	2.76E-06	2.48E-05	1.23E-05	1.64E-05
VPS36	1.21E-05	1.26E-05	1.09E-05	1.12E-05	1.93E-06	1.03E-05	8.71E-06	8.82E-06
VPS37A	6.33E-06	2.08E-05	6.07E-06	5.50E-06	8.08E-07	1.10E-05	2.56E-06	5.22E-06
VPS37B	8.98E-06	4.13E-06	3.09E-06	1.60E-05	4.22E-06	4.96E-06	8.20E-06	3.44E-06
VPS37C	1.01E-05	8.04E-06	3.88E-06	6.86E-06	2.32E-06	6.19E-06	4.72E-06	7.77E-06
VPS37D	3.68E-06	1.26E-05	2.01E-06	2.71E-06	4.37E-06	4.10E-06	4.87E-06	2.01E-06
VPS39	1.81E-05	1.03E-05	5.92E-06	6.71E-06	6.08E-06	1.08E-05	7.18E-06	1.03E-05
VPS41	2.04E-05	7.89E-06	7.74E-06	9.69E-06	1.81E-06	1.44E-05	7.08E-06	8.71E-06
VPS45	1.52E-05	9.26E-06	4.50E-06	7.67E-06	3.83E-06	1.83E-05	8.19E-06	1.31E-05
VPS4A	3.60E-05	1.98E-05	2.19E-05	2.61E-05	1.42E-05	2.71E-05	2.80E-05	4.19E-05
VPS4B	2.28E-05	1.04E-05	1.43E-05	1.35E-05	4.24E-06	1.54E-05	9.50E-06	1.01E-05
VPS52	2.62E-05	4.49E-05	1.60E-05	1.61E-05	9.65E-06	3.11E-05	2.14E-05	2.68E-05
VPS53	2.04E-06	2.33E-06	2.25E-06	1.81E-06	1.75E-06	3.07E-06	2.62E-06	2.32E-06
VPS54	2.18E-05	1.69E-05	6.75E-06	7.19E-06	4.21E-06	1.03E-05	5.97E-06	8.74E-06
VPS72	3.52E-05	2.31E-05	1.40E-05	2.31E-05	1.66E-05	3.35E-05	2.51E-05	3.78E-05
VPS8	8.23E-06	4.06E-06	3.02E-06	4.01E-06	1.28E-06	4.65E-06	3.62E-06	3.62E-06
VRK1	1.03E-05	2.72E-06	1.89E-06	2.94E-06	2.32E-06	3.27E-06	3.79E-06	2.77E-06
VRK2	1.73E-05	1.09E-05	5.45E-06	6.67E-06	1.30E-06	8.53E-06	5.45E-06	3.90E-06
VRK3	3.61E-05	2.61E-05	2.26E-05	2.16E-05	2.81E-05	4.72E-05	2.67E-05	1.59E-05
VSIG1	2.42E-06	2.08E-07	0	9.93E-08	5.71E-08	2.74E-08	2.15E-08	2.59E-06
VSIG10	7.32E-06	7.85E-06	2.37E-06	5.78E-06	2.47E-06	3.50E-06	4.16E-06	6.31E-06
VSIG10L	1.60E-06	1.16E-06	7.08E-07	1.30E-06	8.13E-07	3.40E-06	1.37E-06	1.65E-06
VSIG2	9.63E-06	5.64E-06	9.91E-07	4.82E-05	1.39E-07	1.39E-05	9.92E-06	3.15E-07
VSIG4	5.55E-06	2.88E-06	2.14E-05	3.00E-05	1.86E-06	1.17E-05	2.29E-05	6.49E-07
VSIG8	5.33E-08	0	4.20E-08	1.63E-08	0	0	0	0
VSNL1	3.59E-08	1.86E-05	6.13E-06	5.58E-06	5.80E-06	1.34E-05	4.40E-06	2.92E-06
VSTM1	1.31E-07	0	2.86E-08	0	0	0	0	0
VSTM2A	0	0	0	0	0	0	0	0
VSTM2B	0	0	0	0	0	0	0	0
VSTM2L	3.06E-06	0	1.13E-06	2.91E-06	1.48E-07	0	1.54E-07	1.08E-07
VSX1	1.60E-07	6.42E-08	0	2.07E-08	0	4.19E-07	2.41E-08	2.40E-07
VSX2	7.75E-09	0	0	0	0	0	1.50E-08	1.14E-08
VTA1	2.11E-05	2.15E-05	1.60E-05	1.43E-05	3.13E-06	1.90E-05	1.02E-05	1.07E-05
VTCN1	3.31E-05	3.03E-08	6.06E-07	1.51E-05	7.72E-09	9.74E-07	1.04E-07	2.64E-08
VTI1A	6.49E-06	3.05E-06	2.31E-06	2.91E-06	1.29E-06	3.91E-06	1.89E-06	2.15E-06
VTI1B	4.06E-05	4.31E-05	3.91E-05	4.02E-05	4.95E-05	5.27E-05	4.26E-05	4.01E-05
VTN	0.00441	0.0077	0.005692	0.005007	0.012124	0.002679	0.006851	0.001413
VTRNA1-[illegible]	0	0	0	0	0	0	0	0
VTRNA1-[illegible]	0	0	0	0	0	0	0	0
VTRNA1-[illegible]	0	0	0	0	0	0	0	0
VWA1	5.96E-05	1.12E-05	2.59E-05	4.57E-05	6.08E-05	9.99E-05	8.36E-05	0.000117
VWA2	2.76E-08	0	2.44E-08	1.27E-07	0	6.40E-09	2.68E-08	0
VWA3A	0	0	0	0	0	1.18E-08	2.45E-08	0

VWA3B	1.88E-08	0	1.28E-07	9.77E-09	2.74E-08	3.09E-08	2.32E-08	2.93E-07
VWA5A	6.13E-06	4.34E-06	2.93E-06	5.28E-06	1.54E-07	7.59E-06	2.43E-06	4.74E-06
VWA5B1	0	0	0	0	0	0	0	0
VWA5B2	1.60E-07	8.62E-08	7.21E-09	0	1.20E-08	1.40E-08	1.69E-07	4.26E-08
VWC2	0	0	0	0	0	0	0	0
VWC2L	0	0	0	0	0	0	0	0
VWCE	9.34E-07	9.27E-06	1.06E-06	3.51E-06	2.78E-06	5.86E-06	1.81E-06	4.75E-07
VWDE	3.84E-06	5.02E-09	2.06E-08	2.19E-08	0	4.16E-09	0	0
VWF	3.98E-06	3.51E-05	9.47E-07	4.97E-06	9.97E-06	9.34E-06	8.21E-05	3.70E-06
WAC	3.79E-05	4.13E-05	2.38E-05	2.47E-05	1.06E-05	2.96E-05	1.90E-05	2.22E-05
WAPAL	1.59E-05	1.02E-05	1.04E-05	1.17E-05	4.44E-06	1.14E-05	9.93E-06	8.02E-06
WARS	0.000103	2.34E-05	2.36E-05	3.08E-05	1.54E-05	5.19E-05	2.73E-05	2.40E-05
WARS2	1.21E-05	5.53E-06	4.31E-06	5.16E-06	2.40E-06	1.03E-05	4.65E-06	4.43E-06
WAS	1.08E-05	1.92E-06	3.13E-06	8.05E-06	1.66E-06	4.78E-06	6.14E-06	2.89E-06
WASF1	9.73E-06	4.31E-06	1.56E-06	3.46E-06	5.35E-07	4.88E-06	1.68E-06	2.11E-06
WASF2	6.22E-05	1.63E-05	1.22E-05	2.33E-05	1.72E-05	2.52E-05	4.27E-05	3.37E-05
WASF3	2.97E-06	2.88E-07	2.00E-06	1.60E-06	1.17E-06	2.59E-06	2.61E-06	1.81E-06
WASH2P	1.19E-05	1.05E-05	4.68E-06	1.18E-05	2.49E-05	1.29E-05	1.73E-05	1.42E-05
WASH3P	1.15E-05	1.25E-05	6.67E-06	6.65E-06	2.34E-05	6.36E-06	1.21E-05	1.13E-05
WASH5P	6.67E-06	5.35E-06	2.58E-06	5.47E-06	8.56E-06	4.76E-06	7.22E-06	3.66E-06
WASH7P	5.21E-05	2.43E-05	1.84E-05	3.54E-05	5.32E-05	2.28E-05	3.47E-05	3.27E-05
WASL	1.43E-05	3.44E-05	2.02E-05	1.53E-05	1.01E-05	1.78E-05	1.52E-05	2.41E-05
WBP1	6.16E-05	9.95E-05	4.79E-05	4.47E-05	8.19E-05	6.21E-05	4.98E-05	4.46E-05
WBP11	4.83E-05	3.23E-05	2.09E-05	2.11E-05	7.17E-06	2.85E-05	2.10E-05	2.14E-05
WBP11P1	3.20E-07	7.89E-08	1.53E-07	9.54E-08	2.01E-08	5.78E-08	6.81E-08	1.26E-07
WBP2	0.000119	0.000168	0.000108	9.31E-05	6.39E-05	0.00015	9.86E-05	6.26E-05
WBP2NL	3.06E-08	1.96E-07	2.74E-08	3.51E-08	0	2.40E-08	2.76E-08	8.52E-08
WBP4	9.91E-06	6.81E-06	6.26E-06	7.01E-06	2.01E-06	7.87E-06	6.39E-06	5.18E-06
WBP5	0.000127	2.96E-05	1.15E-05	5.08E-05	2.58E-06	9.03E-06	8.49E-06	4.16E-05
WBSCR16	3.93E-05	6.47E-05	2.37E-05	2.45E-05	2.71E-05	5.22E-05	3.43E-05	5.95E-05
WBSCR17	1.81E-07	0	5.24E-08	1.10E-06	1.22E-08	6.71E-09	2.46E-07	6.38E-08
WBSCR22	5.57E-05	9.90E-05	3.59E-05	4.17E-05	3.13E-05	4.89E-05	6.82E-05	7.59E-05
WBSCR26	2.28E-07	0	0	1.94E-07	6.71E-08	2.17E-07	6.92E-08	0
WBSCR27	4.48E-06	1.09E-05	1.13E-06	1.85E-06	6.49E-06	5.46E-06	3.73E-06	1.70E-06
WBSCR28	3.26E-08	0	6.04E-08	1.62E-07	0	0	0	4.88E-08
WDFY1	1.89E-05	7.15E-06	6.67E-06	7.04E-06	1.89E-06	7.38E-06	4.33E-06	4.62E-06
WDFY2	7.68E-06	1.50E-06	1.58E-06	1.95E-06	8.49E-07	2.02E-06	1.82E-06	3.48E-06
WDFY3	1.54E-05	2.20E-06	4.17E-06	4.13E-06	8.18E-07	5.77E-06	2.73E-06	6.04E-06
WDFY4	2.75E-06	1.55E-07	9.48E-07	2.28E-06	3.06E-08	1.07E-06	9.11E-07	4.26E-07
WDHD1	2.62E-06	6.06E-07	1.30E-07	3.46E-07	1.49E-07	6.35E-07	2.29E-07	3.87E-07
WDR1	0.00012	8.78E-05	3.76E-05	7.52E-05	2.30E-05	9.37E-05	6.60E-05	5.93E-05
WDR11	2.15E-05	1.52E-05	1.18E-05	1.34E-05	4.80E-06	1.94E-05	7.36E-06	1.11E-05
WDR12	1.70E-05	1.30E-05	5.67E-06	5.24E-06	1.30E-06	1.12E-05	4.71E-06	6.20E-06
WDR13	9.95E-05	6.39E-05	2.96E-05	7.11E-05	4.72E-05	6.16E-05	6.76E-05	0.000279
WDR16	4.83E-08	4.10E-08	0	1.48E-08	0	0	0	1.78E-08
WDR17	5.16E-08	5.12E-09	8.03E-08	9.59E-08	1.57E-08	0	3.56E-08	1.34E-08
WDR18	2.91E-05	1.98E-05	1.70E-05	2.35E-05	4.03E-05	1.89E-05	3.09E-05	1.85E-05
WDR19	5.36E-06	5.06E-06	2.43E-06	2.86E-06	8.13E-07	4.55E-06	2.36E-06	2.03E-06
WDR20	9.05E-06	5.23E-06	5.04E-06	6.57E-06	2.98E-06	6.34E-06	6.35E-06	6.48E-06
WDR24	5.96E-06	1.07E-05	5.75E-06	4.45E-06	4.43E-06	8.33E-06	7.99E-06	6.23E-06
WDR25	5.71E-06	3.86E-06	4.84E-06	6.38E-06	9.21E-06	1.09E-05	7.86E-06	7.46E-06
WDR26	2.90E-05	2.42E-05	1.27E-05	2.00E-05	4.24E-06	2.84E-05	1.25E-05	2.11E-05
WDR27	5.49E-06	6.59E-07	7.40E-07	1.13E-06	9.22E-07	1.07E-06	7.02E-07	8.28E-07
WDR3	1.39E-05	1.23E-05	7.62E-06	6.44E-06	9.55E-07	2.65E-05	4.49E-06	5.38E-06
WDR31	7.20E-07	6.59E-07	6.49E-07	3.51E-07	0	9.43E-07	1.58E-07	4.49E-08
WDR33	3.51E-05	2.63E-05	1.97E-05	1.79E-05	8.40E-06	1.89E-05	1.48E-05	2.12E-05
WDR34	4.08E-05	1.54E-05	1.37E-05	2.41E-05	4.50E-05	1.61E-05	2.46E-05	9.70E-06
WDR35	1.53E-06	5.32E-07	7.45E-07	1.42E-06	2.95E-07	2.21E-06	9.61E-07	3.68E-07

WDR36	5.70E-06	6.03E-06	5.48E-06	4.27E-06	1.10E-06	5.11E-06	3.64E-06	5.85E-06
WDR37	4.71E-06	1.13E-06	2.03E-06	2.37E-06	1.02E-06	3.10E-06	2.18E-06	2.70E-06
WDR38	5.15E-08	2.98E-08	2.19E-08	0	0	0	0	0
WDR4	1.07E-05	3.82E-06	2.55E-06	3.39E-06	3.62E-06	5.19E-06	7.31E-06	3.65E-06
WDR41	1.07E-05	5.27E-06	4.95E-06	6.00E-06	1.76E-06	6.29E-06	5.58E-06	5.24E-06
WDR43	1.19E-05	2.06E-05	8.04E-06	1.12E-05	2.28E-06	1.46E-05	7.22E-06	6.04E-06
WDR44	5.31E-06	6.34E-06	4.70E-06	4.18E-06	1.34E-06	7.49E-06	2.34E-06	4.23E-06
WDR45	3.02E-05	7.66E-05	2.75E-05	4.11E-05	3.46E-05	6.43E-05	2.00E-05	6.65E-05
WDR45L	6.80E-05	6.63E-05	2.04E-05	3.00E-05	1.31E-05	4.25E-05	2.56E-05	3.00E-05
WDR46	1.90E-05	3.09E-05	1.47E-05	1.96E-05	2.24E-05	2.62E-05	2.72E-05	2.63E-05
WDR47	2.45E-06	1.92E-06	1.12E-06	1.96E-06	1.17E-07	1.58E-06	7.27E-07	1.53E-06
WDR48	1.37E-05	1.04E-05	7.18E-06	7.91E-06	3.42E-06	1.13E-05	8.36E-06	6.99E-06
WDR49	0	0	1.21E-08	0	0	9.70E-09	0	0
WDR5	1.30E-05	8.39E-06	1.01E-05	9.16E-06	1.62E-05	2.53E-05	1.56E-05	2.00E-05
WDR52	3.61E-06	4.10E-07	1.75E-07	4.20E-07	2.09E-07	2.47E-07	1.39E-07	4.93E-07
WDR53	6.65E-06	3.47E-06	2.18E-06	3.69E-06	3.48E-06	3.84E-06	3.93E-06	3.13E-06
WDR54	8.75E-06	4.57E-07	1.19E-06	3.34E-06	2.28E-06	2.30E-06	4.26E-06	7.28E-07
WDR55	1.39E-05	8.50E-06	7.02E-06	7.88E-06	1.71E-06	8.36E-06	6.98E-06	1.86E-05
WDR59	2.00E-05	1.25E-05	2.43E-05	2.13E-05	8.88E-06	2.85E-05	1.24E-05	2.28E-05
WDR5B	3.56E-06	2.92E-06	2.14E-06	1.43E-06	1.87E-06	3.26E-06	1.44E-06	1.59E-06
WDR6	4.20E-05	3.13E-05	2.22E-05	3.18E-05	3.35E-05	3.18E-05	2.11E-05	1.91E-05
WDR60	1.96E-06	1.65E-06	8.49E-07	2.21E-06	7.88E-07	2.06E-06	1.19E-06	2.33E-06
WDR61	4.86E-05	2.92E-05	2.47E-05	2.91E-05	2.41E-05	3.74E-05	3.42E-05	3.25E-05
WDR62	1.99E-06	1.34E-06	1.13E-07	1.58E-07	7.63E-07	6.08E-07	1.81E-07	1.82E-07
WDR63	5.39E-08	0	0	2.90E-08	0	0	0	0
WDR64	0	8.00E-08	1.17E-08	3.45E-08	0	2.08E-08	0	0
WDR65	0	7.58E-08	7.16E-08	9.44E-08	4.73E-08	1.12E-07	2.40E-08	3.33E-08
WDR66	2.86E-07	4.78E-08	4.14E-07	4.76E-07	1.05E-08	6.20E-08	7.10E-08	2.68E-08
WDR67	8.78E-06	6.89E-06	9.13E-07	1.12E-06	6.27E-07	1.65E-06	1.77E-06	3.93E-06
WDR69	1.47E-08	0	0	0	0	0	0	0
WDR7	3.63E-06	2.40E-06	3.15E-06	2.43E-06	5.88E-07	3.90E-06	1.44E-06	2.96E-06
WDR70	8.86E-06	5.08E-06	4.66E-06	7.54E-06	3.86E-06	1.05E-05	8.38E-06	9.32E-06
WDR72	2.33E-05	2.79E-07	1.45E-05	3.22E-06	7.33E-06	1.53E-06	3.02E-06	1.63E-06
WDR73	1.04E-05	4.00E-06	3.19E-06	5.30E-06	2.43E-06	8.08E-06	4.41E-06	4.81E-06
WDR74	2.42E-05	3.77E-05	2.05E-05	2.27E-05	3.56E-05	3.13E-05	3.63E-05	3.91E-05
WDR75	2.05E-05	1.36E-05	7.08E-06	9.29E-06	5.79E-06	1.43E-05	8.99E-06	9.26E-06
WDR76	4.79E-06	2.76E-06	2.41E-07	5.12E-07	2.48E-07	1.56E-06	6.93E-07	9.82E-07
WDR77	1.87E-05	2.22E-05	9.66E-06	8.31E-06	6.72E-06	2.09E-05	8.50E-06	1.04E-05
WDR78	3.71E-07	2.51E-06	5.20E-07	8.30E-07	1.26E-07	1.35E-06	1.85E-07	5.57E-08
WDR8	1.50E-05	8.00E-06	5.82E-06	7.41E-06	2.94E-06	1.15E-05	6.77E-06	9.86E-06
WDR81	1.02E-05	1.19E-05	1.49E-05	1.27E-05	1.86E-05	1.93E-05	1.70E-05	2.07E-05
WDR82	3.77E-05	1.83E-05	1.37E-05	1.95E-05	4.16E-06	1.92E-05	1.77E-05	1.74E-05
WDR83	7.98E-06	1.38E-05	4.07E-06	5.48E-06	1.18E-05	5.68E-06	7.49E-06	7.31E-06
WDR85	6.64E-06	2.91E-06	2.98E-06	4.26E-06	5.30E-06	3.97E-06	5.83E-06	7.67E-06
WDR86	2.06E-07	0	1.49E-07	6.26E-07	1.44E-07	7.20E-08	2.13E-07	4.87E-08
WDR87	0	0	0	0	0	2.29E-09	0	0
WDR88	9.92E-08	2.73E-07	0	1.04E-07	0	7.89E-08	2.73E-08	5.64E-07
WDR89	7.66E-06	3.52E-06	3.77E-06	4.19E-06	2.60E-06	3.79E-06	4.61E-06	3.64E-06
WDR90	5.66E-06	1.16E-05	2.39E-06	2.88E-06	2.05E-06	3.95E-06	5.88E-06	4.42E-06
WDR91	5.16E-06	2.32E-06	2.70E-06	3.32E-06	1.48E-06	3.49E-06	3.10E-06	3.15E-06
WDR92	5.93E-06	6.64E-06	3.12E-06	2.84E-06	1.78E-06	4.48E-06	3.06E-06	2.08E-06
WDR93	6.86E-08	8.85E-08	0	7.24E-08	1.08E-08	4.88E-08	2.95E-08	0
WDSUB1	8.86E-06	4.45E-06	2.54E-06	2.76E-06	8.73E-07	3.95E-06	2.82E-06	2.03E-06
WDTC1	2.38E-05	1.55E-05	1.63E-05	1.45E-05	4.92E-06	2.07E-05	1.26E-05	1.77E-05
WDYHV1	1.80E-05	6.70E-06	2.26E-06	3.98E-06	2.91E-06	3.49E-06	1.03E-05	1.24E-05
WEE1	1.04E-05	8.60E-06	1.51E-05	3.90E-05	2.38E-06	3.97E-06	1.88E-05	2.70E-05
WEE2	3.02E-08	1.70E-08	7.81E-09	1.85E-08	0	0	0	0
WFDC1	3.40E-06	5.44E-07	1.62E-06	4.82E-06	7.73E-07	6.47E-07	3.53E-07	8.12E-07

WFDC10A	0	0	0	0	0	0	0	0
WFDC10B	0	1.90E-07	0	0	0	0	0	0
WFDC11	0	0	0	0	0	0	0	0
WFDC12	0	4.37E-08	8.38E-08	0	0	0	0	0
WFDC13	0	0	0	0	0	0	0	0
WFDC2	3.82E-05	1.53E-05	5.57E-07	1.95E-05	3.73E-08	0	4.97E-07	1.76E-07
WFDC3	2.99E-07	0	2.21E-07	4.14E-07	2.82E-07	1.81E-07	4.48E-07	7.16E-08
WFDC5	0	0	5.79E-08	0	0	0	0	0
WFDC6	0	0	0	0	0	0	0	0
WFDC8	0	0	0	0	0	0	0	0
WFDC9	0	0	0	0	0	0	0	0
WFIKKN1	3.24E-08	6.59E-07	6.77E-08	3.62E-08	2.29E-08	1.55E-07	2.20E-07	9.90E-08
WFIKKN2	1.43E-08	4.40E-07	0	5.95E-08	6.13E-09	0	0	0
WFS1	1.11E-05	1.36E-05	4.55E-06	5.44E-06	5.58E-06	1.47E-05	1.11E-05	1.45E-05
WHAMM	4.43E-06	1.96E-06	1.68E-06	2.55E-06	1.16E-06	3.95E-06	4.08E-06	3.58E-06
WHAMML	2.02E-06	3.67E-07	7.21E-07	6.95E-07	2.79E-07	7.86E-07	7.21E-07	7.82E-07
WHAMML	4.14E-07	1.17E-07	1.58E-07	2.42E-07	2.58E-08	7.49E-08	2.20E-07	4.42E-07
WHSC1	1.48E-05	5.88E-06	2.16E-06	3.70E-06	1.31E-06	3.78E-06	3.85E-06	6.22E-06
WHSC1L1	5.41E-06	7.80E-06	3.70E-06	6.26E-06	6.58E-07	6.21E-06	2.39E-06	4.02E-06
WHSC2	1.20E-05	1.18E-05	5.65E-06	7.76E-06	5.29E-06	1.15E-05	9.82E-06	9.31E-06
WIBG	4.38E-05	3.71E-05	2.00E-05	2.99E-05	2.06E-05	3.03E-05	3.82E-05	3.00E-05
WIF1	1.67E-07	0	0	0	0	0	0	0
WIPF1	1.44E-05	2.35E-06	4.87E-06	1.16E-05	8.43E-07	4.22E-06	5.03E-06	3.23E-06
WIPF2	1.75E-05	8.48E-06	2.90E-06	4.08E-06	1.15E-06	8.05E-06	4.02E-06	4.03E-06
WIPF3	9.39E-07	7.98E-09	5.87E-08	2.35E-07	6.11E-09	6.58E-09	4.83E-08	1.77E-07
WIPI1	3.81E-05	2.40E-05	5.70E-06	1.55E-05	9.16E-06	4.61E-05	1.15E-05	9.95E-06
WIPI2	2.20E-05	3.07E-05	1.55E-05	1.59E-05	1.79E-05	2.14E-05	1.79E-05	1.85E-05
WISP1	2.39E-06	1.20E-07	5.48E-07	2.12E-06	7.20E-08	3.77E-07	1.22E-06	7.08E-07
WISP2	4.48E-08	2.58E-08	8.00E-07	1.81E-05	2.89E-07	9.97E-08	6.39E-07	6.65E-08
WISP3	2.07E-08	2.38E-08	2.06E-08	2.55E-08	8.93E-08	0	0	3.07E-08
WIT1	3.03E-08	1.56E-07	0	1.24E-07	0	0	0	1.09E-08
WIZ	1.02E-05	7.16E-06	6.23E-06	7.63E-06	5.12E-06	1.29E-05	1.12E-05	9.39E-06
WLS	2.96E-05	7.30E-07	1.38E-05	2.37E-05	1.26E-06	2.37E-05	6.75E-06	1.72E-05
WNK1	3.45E-05	2.06E-05	1.52E-05	1.19E-05	3.80E-06	1.57E-05	1.06E-05	9.50E-06
WNK2	2.93E-06	1.30E-08	8.60E-07	1.41E-06	2.52E-08	0	1.92E-08	7.02E-08
WNK3	2.94E-08	2.25E-06	3.09E-07	1.40E-07	7.14E-08	8.15E-08	1.29E-07	3.48E-07
WNK4	5.51E-06	2.33E-06	1.03E-07	6.35E-08	5.31E-08	1.23E-07	1.94E-06	1.31E-07
WNT1	6.11E-07	1.17E-08	0	5.07E-08	8.91E-09	3.84E-08	6.02E-08	0
WNT10A	4.66E-07	0	8.27E-08	1.73E-06	0	8.28E-08	8.65E-08	2.92E-08
WNT10B	1.76E-06	1.18E-08	3.51E-08	8.94E-08	9.62E-09	2.90E-08	9.08E-08	8.23E-08
WNT11	6.64E-06	3.31E-06	3.14E-06	1.43E-05	1.02E-06	9.73E-07	4.94E-06	2.39E-07
WNT16	0	0	0	0	8.16E-09	0	0	0
WNT2	6.63E-07	0	8.73E-07	3.32E-07	0	0	2.83E-07	6.00E-08
WNT2B	5.44E-07	9.79E-08	1.30E-08	2.68E-07	1.02E-08	6.88E-08	8.18E-08	1.27E-07
WNT3	1.97E-06	1.36E-06	1.20E-06	9.38E-07	4.29E-08	4.21E-06	5.43E-07	1.96E-06
WNT3A	1.42E-07	6.22E-08	0	0	0	1.05E-07	7.67E-09	0
WNT4	4.94E-06	2.63E-08	2.10E-07	2.75E-06	1.00E-07	5.95E-08	2.16E-07	2.29E-06
WNT5A	5.07E-07	4.81E-07	9.98E-07	4.12E-07	1.09E-06	4.12E-06	1.94E-06	3.62E-07
WNT5B	9.24E-07	1.50E-06	2.12E-06	2.93E-06	1.27E-05	1.71E-07	3.53E-06	4.32E-07
WNT6	0	1.14E-07	0	0	1.61E-07	2.67E-08	3.87E-07	4.23E-08
WNT7A	1.42E-08	4.85E-08	1.50E-08	5.24E-08	0	0	1.37E-08	0
WNT7B	1.92E-05	6.28E-08	7.80E-08	3.34E-07	0	0	2.42E-08	2.73E-08
WNT8A	0	0	0	0	0	0	0	0
WNT8B	1.92E-07	0	0	0	0	1.11E-08	0	7.07E-08
WNT9A	5.36E-07	7.10E-08	1.33E-07	7.62E-07	0	0	3.95E-08	3.06E-08
WNT9B	3.44E-08	1.10E-07	0	6.35E-08	0	1.58E-08	0	3.90E-08
WRAP53	6.84E-06	3.56E-06	2.64E-06	4.47E-06	3.49E-06	4.22E-06	4.13E-06	4.52E-06
WRB	3.01E-05	1.25E-05	1.72E-05	1.84E-05	1.05E-05	2.11E-05	1.62E-05	1.80E-05

WRN	2.41E-06	2.44E-06	1.51E-06	1.92E-06	1.15E-07	1.89E-06	6.46E-07	6.56E-07
WRNIP1	1.91E-05	2.04E-05	1.35E-05	1.37E-05	8.84E-06	1.72E-05	1.64E-05	1.82E-05
WSB1	3.24E-05	1.01E-05	6.33E-06	1.58E-05	2.70E-06	7.59E-06	7.74E-06	2.10E-05
WSB2	3.44E-05	2.38E-05	1.09E-05	1.99E-05	1.81E-05	2.48E-05	2.79E-05	2.63E-05
WSCD1	7.10E-07	5.12E-07	4.43E-07	2.45E-07	1.05E-07	1.17E-07	1.47E-06	4.00E-07
WSCD2	3.87E-08	0	7.28E-09	8.62E-09	2.42E-08	1.96E-08	2.74E-08	3.10E-08
WT1	2.82E-07	5.48E-08	1.01E-08	7.75E-07	0	8.85E-09	0	7.14E-08
WTAP	5.05E-05	2.78E-05	1.88E-05	4.05E-05	6.60E-06	3.17E-05	3.29E-05	3.13E-05
WTIP	6.48E-06	2.31E-07	2.02E-07	2.57E-06	7.78E-07	6.19E-07	1.23E-06	7.13E-07
WWC1	3.15E-05	3.86E-06	6.30E-06	1.28E-05	2.58E-06	6.98E-06	1.10E-05	2.05E-05
WWC2	1.28E-05	2.86E-06	7.20E-06	4.65E-06	7.36E-07	5.30E-06	3.69E-06	3.82E-06
WWC3	6.26E-06	2.41E-06	1.89E-06	3.05E-06	7.18E-07	7.48E-06	3.43E-06	3.19E-06
WWOX	7.72E-06	5.50E-06	7.43E-06	5.30E-06	2.63E-06	9.47E-06	4.60E-06	3.49E-06
WWP1	7.01E-05	5.26E-05	3.98E-05	3.02E-05	1.97E-05	4.31E-05	6.21E-05	2.66E-05
WWP2	1.62E-05	1.21E-05	7.69E-06	1.10E-05	6.38E-06	1.42E-05	9.57E-06	1.31E-05
WWTR1	3.93E-05	1.06E-05	5.85E-06	7.38E-06	1.08E-06	1.44E-05	2.82E-06	2.64E-06
XAB2	2.82E-05	1.59E-05	1.43E-05	1.73E-05	3.41E-05	2.06E-05	2.60E-05	1.69E-05
XAF1	1.42E-05	7.29E-06	1.21E-05	9.51E-06	1.49E-06	1.67E-05	1.13E-05	3.69E-05
XAGE1D	2.32E-05	1.86E-06	0	3.75E-07	4.48E-08	0	2.43E-07	8.58E-06
XAGE2	1.00E-05	0	0	0	0	0	0	0
XAGE3	0	0	0	9.33E-08	0	6.98E-08	0	0
XAGE5	7.48E-08	0	0	0	0	0	0	0
XBP1	0.000457	0.000344	0.000494	0.000282	0.000292	0.000275	0.000454	0.000252
XCL1	4.41E-07	1.21E-07	2.26E-07	1.09E-06	1.81E-08	1.17E-07	2.61E-07	2.49E-07
XCL2	2.83E-06	2.20E-06	2.45E-06	1.75E-06	6.59E-07	2.73E-07	1.71E-06	2.83E-06
XCR1	2.04E-07	2.35E-08	1.10E-07	1.76E-07	0	9.51E-08	0	3.03E-08
XDH	9.43E-06	3.82E-05	3.69E-05	1.53E-05	1.62E-05	1.58E-05	1.23E-05	8.00E-06
XG	6.44E-08	1.72E-07	1.17E-06	3.94E-08	3.46E-08	5.22E-08	0	9.45E-08
XIAP	7.11E-06	8.24E-06	7.39E-06	5.78E-06	3.31E-06	9.04E-06	6.23E-06	1.01E-05
XIRP1	4.54E-08	1.02E-08	9.40E-09	2.72E-07	0	1.11E-07	1.69E-07	3.91E-08
XIRP2	2.41E-08	8.77E-08	0	9.82E-09	0	0	0	1.18E-08
XIST	5.03E-05	1.06E-08	6.91E-06	0	2.77E-09	2.10E-09	9.44E-08	1.16E-08
XK	1.02E-06	1.82E-06	4.54E-07	2.60E-07	9.28E-07	9.02E-08	8.89E-07	8.63E-07
XKR3	0	3.32E-08	0	0	0	0	2.82E-08	0
XKR4	0	0	0	7.13E-09	0	0	0	8.55E-09
XKR5	1.90E-08	0	0	0	0	0	0	1.39E-08
XKR6	1.03E-07	0	1.25E-08	1.49E-08	2.09E-08	1.13E-08	1.77E-08	2.67E-08
XKR7	0	0	0	0	0	0	0	0
XKR8	8.60E-06	4.89E-06	6.20E-06	6.72E-06	5.19E-06	4.86E-06	6.70E-06	1.00E-05
XKR9	1.63E-06	1.55E-06	4.34E-08	1.29E-06	9.99E-08	1.53E-07	5.18E-06	1.42E-06
XKRX	6.35E-08	3.06E-08	0	0	0	5.04E-08	3.51E-08	2.66E-08
XKRY	0	0	0	0	0	0	0	0
XKRY2	0	0	0	0	0	4.35E-08	0	0
XPA	1.34E-05	7.35E-06	7.95E-06	9.95E-06	7.60E-06	9.21E-06	1.04E-05	7.44E-06
XPC	2.25E-05	1.63E-05	1.50E-05	1.73E-05	6.60E-06	2.16E-05	1.44E-05	1.07E-05
XPNPEP1	3.27E-05	1.54E-05	9.18E-06	1.45E-05	5.68E-06	2.53E-05	1.41E-05	1.50E-05
XPNPEP2	1.52E-07	7.30E-05	2.31E-08	8.91E-06	9.70E-05	7.03E-08	4.55E-05	1.92E-07
XPNPEP3	9.09E-06	8.62E-06	3.38E-06	3.92E-06	3.11E-06	9.07E-06	3.61E-06	3.79E-06
XPO1	8.18E-05	9.18E-05	3.31E-05	3.38E-05	1.91E-05	3.96E-05	2.85E-05	3.40E-05
XPO4	3.73E-06	3.02E-06	1.87E-06	1.46E-06	4.64E-07	2.11E-06	1.34E-06	1.28E-06
XPO5	8.63E-06	1.23E-05	3.20E-06	3.30E-06	2.58E-06	7.35E-06	3.32E-06	6.88E-06
XPO6	1.97E-05	1.84E-05	8.53E-06	1.10E-05	6.61E-06	1.64E-05	1.30E-05	1.44E-05
XPO7	1.37E-05	1.57E-05	7.33E-06	7.29E-06	1.75E-06	1.32E-05	4.45E-06	4.06E-06
XPOT	1.25E-05	2.87E-05	1.19E-05	8.24E-06	1.37E-06	1.66E-05	8.42E-06	1.55E-05
XPR1	8.37E-06	1.03E-05	1.80E-06	2.40E-06	4.75E-07	4.57E-06	1.63E-06	3.41E-06
XRCC1	2.18E-05	1.14E-05	6.55E-06	9.71E-06	6.33E-06	1.03E-05	1.29E-05	1.19E-05
XRCC2	6.64E-07	5.42E-07	1.56E-08	6.45E-08	1.30E-08	1.54E-07	2.93E-08	1.55E-07
XRCC3	4.98E-06	1.88E-06	7.27E-07	9.12E-07	1.62E-06	1.45E-06	2.33E-06	2.49E-06

XRCC4	6.35E-06	2.35E-06	1.80E-06	2.15E-06	1.54E-06	3.43E-06	1.96E-06	2.85E-06
XRCC5	0.000153	7.28E-05	6.00E-05	6.24E-05	4.56E-05	6.42E-05	8.94E-05	5.59E-05
XRCC6	0.000189	0.000129	7.68E-05	9.73E-05	8.45E-05	0.000144	0.000132	0.000107
XRCC6BP1	3.71E-06	8.08E-06	6.13E-06	3.31E-06	3.30E-06	5.27E-06	2.72E-06	4.11E-06
XRN1	8.42E-06	7.81E-06	6.11E-06	4.13E-06	1.01E-06	5.56E-06	2.77E-06	3.42E-06
XRN2	3.10E-05	2.51E-05	1.73E-05	2.38E-05	1.33E-05	3.97E-05	2.07E-05	2.06E-05
XRRA1	3.10E-06	2.33E-06	2.70E-06	2.66E-06	3.05E-06	5.45E-06	3.75E-06	3.72E-06
XYLB	9.45E-06	2.01E-05	1.83E-05	6.97E-06	1.14E-05	6.18E-06	1.50E-05	6.39E-06
XYLT1	6.55E-07	5.55E-08	1.74E-07	5.32E-07	2.03E-08	5.92E-08	2.27E-07	1.70E-07
XYLT2	1.40E-05	8.96E-06	5.43E-06	7.24E-06	8.84E-06	6.99E-06	7.48E-06	6.80E-06
YAF2	3.85E-06	3.71E-06	3.45E-06	4.04E-06	1.34E-06	3.26E-06	3.50E-06	3.49E-06
YAP1	1.82E-05	1.03E-05	1.14E-05	1.57E-05	3.25E-06	3.27E-05	9.14E-06	1.01E-05
YARS	1.96E-05	4.71E-05	1.20E-05	1.67E-05	1.37E-05	2.24E-05	3.11E-05	3.83E-05
YARS2	7.87E-06	7.97E-06	4.73E-06	5.11E-06	1.55E-06	7.05E-06	6.34E-06	6.01E-06
YBX1	0.000302	0.000179	0.00014	0.000183	8.27E-05	0.000182	0.000195	0.00012
YBX2	0	1.71E-05	2.64E-07	2.68E-07	6.70E-08	3.18E-07	7.49E-08	2.76E-07
YDJC	9.39E-06	1.01E-05	5.49E-06	8.05E-06	1.10E-05	1.12E-05	1.79E-05	7.88E-06
YEATS2	8.35E-06	3.07E-06	1.11E-06	1.51E-06	9.17E-07	2.40E-06	2.12E-06	1.82E-06
YEATS4	1.25E-05	9.29E-06	7.73E-06	6.52E-06	3.43E-06	1.32E-05	9.31E-06	3.97E-06
YES1	1.86E-05	1.54E-05	1.88E-05	1.21E-05	2.42E-06	1.07E-05	8.68E-06	1.07E-05
YIF1A	0.000148	0.000186	0.000127	0.000132	0.000326	0.000129	0.000174	7.21E-05
YIF1B	5.70E-05	5.81E-05	2.00E-05	2.62E-05	2.40E-05	3.82E-05	4.12E-05	3.11E-05
YIPF1	3.41E-05	3.00E-05	1.78E-05	2.72E-05	2.07E-05	3.59E-05	2.62E-05	3.80E-05
YIPF2	2.92E-05	1.80E-05	2.30E-05	3.02E-05	3.29E-05	3.19E-05	4.05E-05	4.04E-05
YIPF3	9.70E-05	0.000138	7.47E-05	9.00E-05	9.53E-05	0.000118	9.41E-05	0.000151
YIPF4	2.38E-05	4.12E-05	2.38E-05	1.68E-05	9.04E-06	2.06E-05	1.22E-05	1.18E-05
YIPF5	1.78E-05	1.13E-05	1.19E-05	1.45E-05	2.66E-06	1.47E-05	9.82E-06	1.67E-05
YIPF6	2.64E-05	2.91E-05	1.37E-05	1.11E-05	6.01E-06	1.75E-05	7.26E-06	3.45E-05
YIPF7	0	6.77E-08	0	0	0	0	0	0
YJEFN3	9.03E-07	1.82E-06	6.51E-08	3.48E-07	8.43E-07	1.65E-07	2.57E-07	2.06E-07
YKT6	2.67E-05	1.66E-05	7.24E-06	1.52E-05	1.40E-05	1.47E-05	1.93E-05	2.38E-05
YLPM1	8.35E-06	3.68E-06	4.06E-06	5.25E-06	1.54E-06	6.13E-06	3.72E-06	3.99E-06
YME1L1	5.08E-05	4.68E-05	3.41E-05	3.18E-05	1.22E-05	4.40E-05	2.22E-05	3.03E-05
YOD1	4.08E-06	1.68E-06	1.76E-06	1.50E-06	4.56E-07	1.60E-06	7.24E-07	2.87E-06
YPEL1	1.42E-06	1.01E-06	1.19E-06	1.12E-06	1.23E-06	1.19E-05	1.73E-06	9.02E-07
YPEL2	1.15E-05	5.94E-06	4.29E-06	6.88E-06	3.79E-06	6.71E-06	4.35E-06	5.53E-06
YPEL3	7.94E-05	6.96E-05	2.88E-05	6.06E-05	3.74E-05	4.48E-05	5.88E-05	3.16E-05
YPEL4	4.40E-06	1.66E-07	4.48E-08	1.97E-07	1.51E-07	9.48E-08	1.55E-07	1.50E-07
YPEL5	4.75E-05	5.64E-05	2.97E-05	3.83E-05	1.70E-05	4.30E-05	3.42E-05	3.12E-05
YRDC	9.82E-06	1.32E-05	7.75E-06	1.34E-05	1.39E-06	1.24E-05	1.14E-05	1.33E-05
YSK4	1.88E-08	3.60E-08	1.90E-08	0	0	0	8.57E-08	0
YTHDC1	1.70E-05	7.07E-06	6.51E-06	9.48E-06	3.24E-06	7.85E-06	7.07E-06	7.05E-06
YTHDC2	1.04E-05	5.83E-06	4.39E-06	3.88E-06	1.24E-06	7.38E-06	2.06E-06	3.82E-06
YTHDF1	2.93E-05	2.35E-05	1.54E-05	1.92E-05	1.51E-05	1.92E-05	1.85E-05	1.71E-05
YTHDF2	2.68E-05	2.23E-05	1.55E-05	2.47E-05	7.86E-06	2.33E-05	1.86E-05	1.98E-05
YTHDF3	2.92E-05	2.37E-05	1.57E-05	1.38E-05	8.26E-06	1.66E-05	2.12E-05	2.04E-05
YWHAB	0.000123	7.72E-05	4.83E-05	6.88E-05	4.07E-05	6.65E-05	5.73E-05	6.54E-05
YWHAE	0.000153	0.000158	0.000168	0.000205	0.000155	0.000248	0.000206	0.000202
YWHAG	7.01E-05	9.06E-05	3.16E-05	4.12E-05	9.48E-06	4.81E-05	3.32E-05	5.26E-05
YWHAH	9.23E-05	5.85E-05	3.35E-05	7.69E-05	3.40E-05	4.76E-05	6.98E-05	5.32E-05
YWHAQ	0.000201	0.000134	5.43E-05	6.93E-05	2.92E-05	8.43E-05	6.48E-05	4.12E-05
YWHAZ	0.00055	0.00015	6.49E-05	0.00014	3.55E-05	8.42E-05	0.000188	0.000122
YY1	2.95E-05	2.07E-05	1.75E-05	1.46E-05	5.78E-06	2.14E-05	1.76E-05	1.48E-05
YY1AP1	2.47E-05	3.26E-05	1.74E-05	2.21E-05	1.13E-05	3.83E-05	2.30E-05	4.01E-05
YY2	1.25E-06	4.31E-07	9.72E-07	6.78E-07	2.02E-08	5.71E-07	1.40E-07	9.01E-07
ZACN	0	0	0	2.64E-08	2.00E-08	3.07E-10	0	0
ZADH2	4.85E-06	3.55E-06	1.39E-05	6.60E-06	2.27E-06	9.38E-06	4.09E-06	4.34E-06
ZAK	3.11E-05	2.30E-05	1.05E-05	1.26E-05	8.85E-06	2.09E-05	1.24E-05	9.53E-06

ZAN	0	0	0	3.23E-09	0	0	0	0
ZAP70	6.28E-06	3.38E-07	1.56E-06	3.24E-06	3.64E-07	1.72E-06	1.94E-06	1.61E-06
ZAR1	0	0	0	0	0	0	0	0
ZAR1L	2.37E-08	0	0	0	0	0	2.27E-08	0
ZBBX	0	0	0	0	0	0	7.02E-09	0
ZBED1	3.14E-05	2.14E-05	2.93E-05	2.47E-05	1.37E-05	3.50E-05	2.23E-05	2.87E-05
ZBED2	3.94E-06	0	0	1.33E-08	0	2.12E-07	3.15E-08	7.99E-08
ZBED3	4.21E-06	3.08E-06	1.22E-06	4.99E-07	4.26E-07	2.28E-06	9.83E-07	1.23E-06
ZBED4	4.24E-06	2.58E-06	1.27E-06	1.85E-06	9.12E-07	2.51E-06	1.66E-06	1.98E-06
ZBED5	1.30E-05	6.19E-06	4.12E-06	5.93E-06	3.01E-06	4.73E-06	3.59E-06	5.27E-06
ZBP1	1.40E-06	3.91E-08	1.21E-07	7.26E-07	1.98E-08	3.36E-07	8.98E-08	2.51E-07
ZBTB1	9.21E-06	2.09E-06	4.57E-06	6.54E-06	1.53E-06	4.62E-06	3.43E-06	5.40E-06
ZBTB10	7.26E-06	8.31E-06	6.52E-06	3.54E-06	1.10E-06	6.25E-06	5.86E-06	2.97E-06
ZBTB11	6.40E-06	6.20E-06	2.94E-06	3.27E-06	9.53E-07	3.84E-06	2.28E-06	3.14E-06
ZBTB12	1.49E-06	2.20E-06	4.08E-07	5.56E-07	7.80E-08	1.38E-06	5.62E-07	5.34E-07
ZBTB16	5.40E-06	4.46E-06	5.63E-06	2.55E-06	1.32E-06	6.50E-06	6.17E-06	1.49E-05
ZBTB17	1.04E-05	7.84E-06	4.10E-06	7.73E-06	5.36E-06	8.52E-06	6.85E-06	7.34E-06
ZBTB2	4.98E-06	6.38E-06	3.08E-06	5.05E-06	8.62E-07	3.67E-06	3.36E-06	3.20E-06
ZBTB20	3.98E-07	4.51E-07	3.18E-07	4.63E-07	2.07E-07	2.68E-07	4.13E-07	2.49E-07
ZBTB22	1.08E-05	7.14E-06	4.69E-06	7.32E-06	4.90E-06	1.44E-05	9.13E-06	9.84E-06
ZBTB24	2.63E-06	1.35E-06	1.20E-06	1.77E-06	4.31E-07	1.82E-06	1.45E-06	2.32E-06
ZBTB25	4.35E-06	1.31E-06	1.42E-06	1.29E-06	3.05E-07	1.73E-06	1.22E-06	2.71E-06
ZBTB26	2.03E-06	8.57E-07	7.41E-07	4.48E-07	3.26E-08	7.02E-07	3.17E-07	6.50E-07
ZBTB3	1.24E-06	9.29E-07	7.58E-07	4.96E-07	2.68E-07	1.26E-06	7.94E-07	1.57E-06
ZBTB32	5.11E-07	1.60E-08	2.96E-08	1.55E-07	3.63E-08	1.83E-07	2.71E-08	8.29E-08
ZBTB33	1.86E-05	7.57E-06	6.67E-06	7.22E-06	7.20E-06	1.28E-05	4.61E-06	1.07E-05
ZBTB34	2.48E-06	8.40E-07	7.13E-07	9.64E-07	3.51E-07	9.61E-07	6.21E-07	1.48E-06
ZBTB37	6.32E-07	1.17E-06	5.26E-07	4.16E-07	1.77E-07	1.15E-06	2.17E-07	2.27E-06
ZBTB38	1.89E-05	9.10E-06	8.32E-06	9.93E-06	1.18E-06	3.99E-05	3.70E-06	7.47E-06
ZBTB39	1.62E-06	8.87E-07	4.41E-07	5.05E-07	2.99E-07	1.16E-06	4.95E-07	7.32E-07
ZBTB4	6.74E-06	2.86E-06	4.21E-06	5.79E-06	1.37E-06	5.69E-06	4.63E-06	6.35E-06
ZBTB40	4.43E-06	1.09E-06	6.65E-07	1.09E-06	6.17E-07	1.30E-06	1.26E-06	1.09E-06
ZBTB41	2.95E-06	3.05E-06	1.77E-06	1.19E-06	2.90E-07	5.32E-06	9.89E-07	3.13E-06
ZBTB42	5.07E-06	3.11E-06	2.51E-06	2.57E-06	2.84E-06	2.39E-06	5.36E-06	5.03E-06
ZBTB43	4.71E-06	2.10E-06	2.02E-06	4.46E-06	1.51E-07	2.43E-06	1.53E-06	4.02E-06
ZBTB44	9.74E-06	3.56E-06	7.38E-06	5.85E-06	2.11E-06	9.65E-06	4.63E-06	6.88E-06
ZBTB45	5.82E-06	2.92E-06	1.88E-06	3.22E-06	3.97E-06	3.63E-06	4.44E-06	2.69E-06
ZBTB46	2.29E-06	5.13E-07	6.98E-07	9.11E-07	4.85E-07	4.60E-07	9.68E-07	5.61E-07
ZBTB47	3.32E-06	1.48E-06	1.68E-06	3.83E-06	1.68E-06	1.31E-06	3.41E-06	3.10E-06
ZBTB48	1.28E-05	7.49E-06	8.60E-06	9.26E-06	7.84E-06	7.49E-06	1.02E-05	5.31E-06
ZBTB49	2.60E-06	1.47E-06	7.95E-07	1.47E-06	8.49E-07	1.66E-06	1.16E-06	2.09E-06
ZBTB5	4.22E-06	4.18E-06	3.50E-06	2.04E-06	1.45E-06	3.07E-06	1.89E-06	2.60E-06
ZBTB6	3.25E-06	1.41E-06	1.67E-06	1.31E-06	6.23E-07	2.40E-06	1.08E-06	2.28E-06
ZBTB7A	1.16E-05	5.58E-06	4.91E-06	7.30E-06	4.02E-06	9.00E-06	8.48E-06	7.76E-06
ZBTB7B	1.90E-05	1.95E-05	9.29E-06	1.03E-05	4.19E-06	1.81E-05	1.91E-05	4.03E-05
ZBTB7C	1.70E-07	2.66E-07	5.76E-07	6.31E-07	6.31E-07	3.13E-06	4.19E-07	1.30E-07
ZBTB8A	1.65E-06	9.80E-07	6.47E-07	8.91E-07	2.92E-07	1.18E-06	7.66E-07	1.11E-06
ZBTB8B	1.08E-07	0	0	0	0	0	0	0
ZBTB8OS	3.35E-05	1.66E-05	1.16E-05	1.43E-05	1.71E-05	1.98E-05	1.52E-05	1.70E-05
ZBTB9	4.83E-06	6.91E-06	1.51E-06	2.16E-06	1.23E-06	2.41E-06	3.07E-06	5.04E-06
ZC3H10	4.33E-06	4.91E-06	2.50E-06	2.38E-06	2.48E-06	4.77E-06	3.15E-06	3.63E-06
ZC3H11A	4.47E-05	2.11E-05	1.92E-05	2.39E-05	7.45E-06	3.41E-05	1.48E-05	3.39E-05
ZC3H12A	8.16E-06	5.94E-06	4.48E-06	2.15E-05	6.41E-07	3.40E-06	1.68E-05	1.48E-05
ZC3H12B	4.08E-07	4.16E-08	1.52E-07	1.75E-07	2.03E-07	2.88E-09	2.31E-07	1.82E-08
ZC3H12C	2.45E-06	4.91E-07	2.60E-06	1.47E-06	1.85E-07	1.76E-06	8.22E-07	9.62E-07
ZC3H12D	5.58E-07	2.90E-07	7.69E-08	2.15E-07	2.43E-08	1.28E-07	7.21E-08	4.93E-08
ZC3H13	2.05E-05	8.34E-06	9.48E-06	8.86E-06	2.08E-06	1.73E-05	7.49E-06	2.28E-05
ZC3H14	3.55E-05	8.57E-06	1.37E-05	1.09E-05	5.89E-06	1.33E-05	9.16E-06	7.31E-06

ZC3H15	4.84E-05	5.19E-05	2.63E-05	2.36E-05	1.02E-05	3.43E-05	1.84E-05	2.60E-05
ZC3H18	1.50E-05	1.10E-05	9.27E-06	8.52E-06	4.40E-06	1.09E-05	8.89E-06	9.50E-06
ZC3H3	2.49E-05	2.50E-05	4.45E-06	4.73E-06	6.68E-06	8.14E-06	1.37E-05	8.68E-06
ZC3H4	7.85E-06	4.29E-06	3.01E-06	4.08E-06	2.08E-06	5.54E-06	4.74E-06	3.48E-06
ZC3H6	1.05E-06	9.96E-07	6.01E-07	4.33E-07	9.83E-08	8.96E-07	4.83E-07	6.76E-07
ZC3H7A	2.34E-05	2.43E-05	8.62E-06	1.02E-05	7.07E-06	1.26E-05	1.50E-05	9.59E-06
ZC3H7B	1.97E-05	1.77E-05	1.98E-05	1.31E-05	8.40E-06	1.85E-05	1.26E-05	3.02E-05
ZC3H8	6.31E-06	4.41E-06	1.80E-06	1.41E-06	1.27E-06	4.44E-06	3.10E-06	2.13E-06
ZC3HAV1	1.10E-05	7.12E-06	5.33E-06	6.20E-06	2.87E-06	8.27E-06	4.90E-06	8.50E-06
ZC3HAV1I	8.04E-07	3.15E-08	5.84E-08	1.87E-07	0	3.86E-08	9.36E-08	6.13E-08
ZC3HC1	6.53E-06	1.31E-05	9.48E-06	9.36E-06	8.98E-06	1.43E-05	1.34E-05	1.30E-05
ZC4H2	4.45E-06	4.20E-06	2.52E-06	4.83E-06	1.88E-06	1.94E-06	5.73E-06	3.70E-06
ZCCHC10	5.50E-05	1.06E-05	7.02E-06	1.93E-05	1.66E-05	1.63E-05	2.58E-05	2.29E-05
ZCCHC11	1.06E-05	9.22E-06	5.41E-06	4.16E-06	3.39E-07	5.66E-06	2.51E-06	5.41E-06
ZCCHC12	7.89E-07	3.66E-07	0	3.97E-08	0	0	0	1.59E-08
ZCCHC13	0	0	0	0	0	0	0	0
ZCCHC14	1.35E-05	6.47E-06	7.35E-06	7.15E-06	1.98E-06	7.70E-06	5.58E-06	1.14E-05
ZCCHC16	1.49E-07	6.16E-07	4.14E-07	2.97E-07	1.08E-07	0	7.15E-07	2.53E-07
ZCCHC17	2.41E-05	1.13E-05	6.90E-06	1.18E-05	5.80E-06	1.36E-05	8.83E-06	1.16E-05
ZCCHC18	3.23E-07	2.11E-08	3.93E-08	9.36E-08	6.35E-08	2.81E-08	7.43E-08	1.36E-07
ZCCHC2	7.43E-06	4.88E-06	8.73E-06	7.93E-06	1.13E-06	7.32E-06	4.41E-06	9.38E-06
ZCCHC24	9.91E-06	1.50E-05	1.65E-05	1.29E-05	1.11E-05	9.55E-06	1.28E-05	1.34E-05
ZCCHC3	2.78E-05	1.35E-05	6.30E-06	9.60E-06	8.80E-06	8.77E-06	1.04E-05	1.08E-05
ZCCHC4	4.10E-06	3.11E-06	1.54E-06	1.99E-06	6.60E-07	3.94E-06	1.54E-06	2.39E-06
ZCCHC5	0	0	0	0	0	1.64E-08	0	1.30E-08
ZCCHC6	1.09E-05	8.49E-06	1.49E-05	1.76E-05	1.33E-05	2.38E-05	2.10E-05	1.04E-05
ZCCHC7	5.03E-06	5.95E-06	4.42E-06	3.89E-06	3.38E-06	6.73E-06	5.23E-06	5.27E-06
ZCCHC8	1.31E-05	1.33E-05	8.56E-06	7.38E-06	2.76E-06	7.07E-06	7.84E-06	7.96E-06
ZCCHC9	5.94E-05	2.63E-05	1.28E-05	1.24E-05	5.93E-06	2.27E-05	2.03E-05	2.40E-05
ZCRB1	3.63E-05	3.08E-05	1.78E-05	1.70E-05	1.09E-05	2.77E-05	1.92E-05	1.97E-05
ZCWPW1	7.97E-06	1.35E-05	2.23E-06	2.75E-06	5.67E-06	6.72E-06	7.74E-06	1.93E-06
ZCWPW2	8.49E-08	3.03E-07	2.07E-07	2.06E-07	1.78E-07	3.71E-07	1.57E-07	1.75E-07
ZDBF2	6.74E-07	4.13E-08	8.12E-07	7.55E-07	1.87E-09	3.43E-08	4.04E-08	8.28E-08
ZDHHC1	1.03E-06	2.97E-07	1.06E-06	3.30E-06	2.87E-07	1.23E-06	2.58E-06	2.14E-06
ZDHHC11	2.16E-06	3.27E-06	2.46E-07	7.76E-07	2.29E-07	2.47E-07	6.09E-08	1.56E-07
ZDHHC12	5.13E-05	3.96E-05	2.84E-05	3.08E-05	4.34E-05	4.57E-05	2.21E-05	6.77E-05
ZDHHC13	8.11E-06	1.93E-06	9.90E-07	1.89E-06	3.02E-07	2.99E-07	9.21E-07	3.24E-07
ZDHHC14	1.49E-05	8.34E-06	1.07E-05	9.95E-06	1.61E-05	5.64E-06	1.28E-05	1.19E-05
ZDHHC15	7.52E-08	0	3.43E-08	7.56E-08	1.67E-08	2.49E-08	4.28E-08	1.63E-08
ZDHHC16	2.23E-05	1.66E-05	1.91E-05	1.73E-05	1.71E-05	1.85E-05	1.59E-05	1.85E-05
ZDHHC17	7.03E-06	3.87E-06	3.06E-06	3.35E-06	7.97E-07	3.24E-06	1.93E-06	3.25E-06
ZDHHC18	1.91E-05	1.16E-05	9.20E-06	1.32E-05	4.07E-06	1.09E-05	1.22E-05	1.45E-05
ZDHHC19	2.52E-07	6.49E-08	1.63E-06	1.06E-06	9.33E-06	0	1.45E-06	0
ZDHHC2	3.71E-06	1.34E-05	2.51E-06	3.99E-06	7.80E-08	1.02E-06	1.87E-06	3.24E-07
ZDHHC20	6.14E-06	4.67E-06	4.19E-06	1.93E-06	3.01E-08	3.01E-06	1.68E-07	3.27E-06
ZDHHC21	1.12E-06	1.13E-06	1.01E-06	7.82E-07	1.37E-07	9.29E-07	1.61E-07	4.74E-07
ZDHHC22	6.74E-08	7.56E-09	0	8.24E-09	0	0	3.27E-08	9.88E-09
ZDHHC23	6.81E-06	6.28E-06	8.29E-07	1.77E-06	4.82E-07	1.37E-06	1.12E-06	1.37E-06
ZDHHC24	1.04E-05	8.34E-06	5.15E-06	7.78E-06	7.94E-06	1.16E-05	8.99E-06	1.55E-05
ZDHHC3	1.05E-05	5.60E-06	4.92E-06	4.90E-06	3.81E-06	7.55E-06	7.47E-06	6.87E-06
ZDHHC4	4.93E-05	7.09E-05	3.10E-05	4.27E-05	3.33E-05	3.92E-05	2.60E-05	5.43E-05
ZDHHC5	3.69E-05	4.17E-05	2.19E-05	2.74E-05	9.19E-06	2.77E-05	2.30E-05	4.19E-05
ZDHHC6	1.44E-05	1.33E-05	1.41E-05	1.42E-05	1.19E-05	1.35E-05	9.88E-06	1.68E-05
ZDHHC7	2.23E-05	4.00E-06	6.57E-06	9.43E-06	2.62E-06	6.28E-06	7.64E-06	1.21E-05
ZDHHC8	1.18E-05	7.26E-06	5.51E-06	5.87E-06	7.89E-06	8.47E-06	8.34E-06	1.22E-06
ZDHHC8P	2.19E-06	2.44E-08	5.65E-08	1.01E-07	0	0	0	0
ZDHHC9	5.49E-05	3.50E-05	2.08E-05	2.35E-05	1.98E-05	3.12E-05	2.67E-05	4.69E-05
ZEB1	7.01E-06	4.60E-06	3.71E-06	3.84E-06	1.12E-06	5.99E-06	2.59E-06	3.91E-06

ZEB2	9.14E-06	1.64E-06	6.10E-06	9.50E-06	1.48E-06	2.82E-06	4.64E-06	1.94E-06
ZER1	1.48E-05	4.56E-06	9.47E-06	7.29E-06	4.71E-06	7.51E-06	8.11E-06	9.47E-06
ZFAND1	2.43E-05	2.22E-05	1.41E-05	1.20E-05	3.75E-06	1.22E-05	2.45E-05	1.12E-05
ZFAND2A	7.72E-05	4.56E-05	2.33E-05	3.35E-05	5.04E-05	6.46E-05	9.76E-05	6.28E-05
ZFAND2B	5.22E-05	3.05E-05	2.38E-05	2.77E-05	6.93E-05	3.01E-05	4.68E-05	3.04E-05
ZFAND3	4.11E-05	4.26E-05	2.17E-05	2.00E-05	1.20E-05	2.99E-05	2.14E-05	3.24E-05
ZFAND5	3.25E-05	4.74E-05	8.28E-05	4.01E-05	5.52E-06	9.51E-05	2.19E-05	0.000153
ZFAND6	7.17E-05	6.62E-05	4.11E-05	5.08E-05	3.32E-05	5.07E-05	5.31E-05	4.92E-05
ZFAT	3.88E-06	6.68E-06	1.18E-06	2.23E-06	2.02E-06	2.90E-06	3.60E-06	2.45E-06
ZFATAS	0	0	0	0	0	0	0	0
ZFC3H1	5.21E-06	1.82E-06	1.72E-06	4.57E-06	3.95E-07	2.93E-06	1.05E-06	1.03E-06
ZFHX3	2.00E-06	1.38E-06	1.50E-06	1.77E-06	4.18E-07	1.92E-06	1.36E-06	2.42E-06
ZFHX4	8.56E-08	1.29E-06	1.17E-06	1.07E-06	3.31E-07	1.43E-06	1.19E-06	1.00E-06
ZFP1	2.57E-06	2.63E-06	9.75E-06	6.76E-06	2.23E-06	4.08E-06	3.70E-06	1.49E-06
ZFP106	8.88E-06	5.88E-06	4.52E-06	4.95E-06	1.75E-06	6.18E-06	3.74E-06	3.83E-06
ZFP112	1.76E-06	2.85E-07	4.28E-07	3.56E-07	3.89E-07	3.33E-07	1.62E-07	3.18E-07
ZFP14	1.98E-06	6.57E-07	8.59E-07	5.14E-07	4.84E-07	6.37E-07	4.84E-07	5.52E-07
ZFP161	5.03E-06	2.93E-06	2.94E-06	2.77E-06	1.16E-06	6.46E-06	2.74E-06	3.08E-06
ZFP2	2.79E-07	1.03E-07	3.20E-07	5.92E-07	1.22E-07	1.83E-07	8.53E-08	2.45E-07
ZFP28	2.88E-07	1.62E-07	3.42E-07	5.73E-07	3.76E-07	8.01E-08	4.61E-07	5.02E-07
ZFP3	3.75E-07	1.46E-07	4.12E-07	7.11E-07	4.26E-08	1.71E-07	1.54E-07	1.16E-06
ZFP30	1.93E-06	8.81E-07	1.06E-06	6.10E-07	1.47E-07	1.04E-07	2.86E-07	1.01E-06
ZFP36	0.000111	0.000218	0.000314	0.000905	1.85E-05	8.02E-05	0.000212	0.000211
ZFP36L1	0.000123	0.000147	0.000257	0.000149	4.53E-05	7.33E-05	0.000137	0.000132
ZFP36L2	5.21E-05	3.04E-05	2.86E-05	4.96E-05	3.39E-05	5.83E-05	2.71E-05	1.72E-05
ZFP37	1.61E-07	1.90E-08	3.25E-07	3.41E-07	1.16E-07	5.49E-08	8.21E-08	5.34E-07
ZFP41	9.03E-06	3.61E-06	6.20E-07	8.16E-07	7.90E-07	2.54E-06	4.45E-06	2.22E-06
ZFP42	0	0	0	0	0	0	0	0
ZFP57	3.88E-08	0	2.71E-08	1.56E-07	0	9.59E-08	0	1.90E-08
ZFP62	6.54E-06	2.29E-06	3.43E-06	3.91E-06	4.37E-06	4.77E-06	4.52E-06	4.43E-06
ZFP64	6.84E-06	5.61E-06	3.52E-06	3.40E-06	1.71E-06	4.43E-06	3.96E-06	3.66E-06
ZFP82	8.49E-07	3.22E-07	2.60E-07	8.20E-07	1.54E-07	6.63E-08	1.73E-07	3.54E-07
ZFP90	4.22E-06	1.61E-06	2.09E-06	2.73E-06	1.06E-06	1.99E-06	1.79E-06	1.75E-06
ZFP91	2.05E-05	2.11E-05	1.28E-05	1.20E-05	2.40E-06	1.39E-05	7.67E-06	2.24E-05
ZFP91-CN	2.43E-08	0	0	0	0	0	0	6.68E-08
ZFP92	2.04E-08	2.35E-08	0	5.03E-08	0	0	0	0
ZFPL1	3.14E-05	4.18E-05	1.59E-05	1.94E-05	2.66E-05	2.30E-05	2.56E-05	2.75E-05
ZFPM1	6.19E-06	4.19E-06	4.95E-06	4.58E-06	9.16E-07	3.84E-06	4.44E-06	3.36E-06
ZFPM2	5.75E-07	2.57E-07	7.81E-07	2.40E-06	8.26E-08	1.75E-07	4.52E-07	3.60E-07
ZFR	2.97E-05	3.07E-05	1.68E-05	2.00E-05	7.84E-06	2.54E-05	1.70E-05	1.82E-05
ZFR2	3.05E-08	7.48E-08	1.46E-08	1.87E-07	4.08E-09	2.99E-08	3.49E-08	5.48E-08
ZFX	1.07E-05	2.08E-06	6.26E-06	3.51E-06	8.50E-07	2.19E-06	1.94E-06	4.41E-06
ZFY	0	2.51E-06	0	2.59E-06	7.27E-07	1.88E-06	1.09E-06	1.32E-06
ZFYVE1	8.66E-06	5.07E-06	9.14E-06	6.11E-06	5.69E-06	1.14E-05	7.41E-06	8.50E-06
ZFYVE16	8.75E-06	8.68E-06	5.10E-06	5.97E-06	2.13E-06	4.95E-06	3.08E-06	6.28E-06
ZFYVE19	4.20E-05	2.89E-05	1.18E-05	1.10E-05	1.29E-05	1.92E-05	2.42E-05	1.23E-05
ZFYVE20	4.27E-06	3.23E-06	2.56E-06	2.76E-06	2.08E-06	4.97E-06	3.54E-06	3.57E-06
ZFYVE21	3.48E-05	1.60E-05	1.93E-05	2.58E-05	6.37E-06	1.95E-05	3.10E-05	1.30E-05
ZFYVE26	5.06E-06	1.14E-06	1.31E-06	1.79E-06	1.43E-06	3.18E-06	2.18E-06	2.67E-06
ZFYVE27	1.75E-05	1.03E-05	7.11E-06	1.18E-05	4.71E-06	1.28E-05	8.38E-06	1.22E-05
ZFYVE28	5.10E-06	7.05E-06	1.43E-06	1.46E-06	4.92E-07	1.09E-06	1.83E-06	2.56E-06
ZFYVE9	3.67E-06	3.70E-06	3.93E-06	4.05E-06	9.60E-07	4.67E-06	3.01E-06	5.39E-06
ZG16	7.99E-07	1.74E-05	4.97E-05	1.79E-05	0.000114	3.76E-07	5.50E-05	1.23E-05
ZG16B	3.46E-06	4.39E-07	3.42E-07	5.23E-06	2.95E-08	5.08E-07	6.10E-07	3.05E-07
ZGLP1	6.68E-07	3.88E-07	2.20E-07	3.93E-07	1.75E-06	4.35E-07	3.39E-07	2.58E-07
ZGPAT	2.38E-05	0.000227	0.000208	5.90E-05	0.000238	3.61E-05	4.23E-05	1.77E-05
ZHX1	1.71E-05	2.78E-05	1.30E-05	8.42E-06	1.14E-05	1.24E-05	1.97E-05	1.75E-05
ZHX2	1.79E-05	9.36E-06	8.14E-06	1.29E-05	5.18E-06	1.08E-05	1.63E-05	1.16E-05

ZHX3	1.58E-05	5.24E-06	3.90E-06	3.05E-06	5.66E-06	6.23E-06	6.74E-06	4.66E-06
ZIC1	8.18E-07	3.55E-06	4.10E-07	8.46E-07	5.32E-07	0	1.50E-06	1.85E-06
ZIC2	1.37E-06	3.73E-06	0	0	2.12E-06	7.02E-07	1.43E-07	3.63E-06
ZIC3	0	0	0	0	4.97E-09	0	0	0
ZIC4	3.13E-08	2.80E-06	1.72E-08	4.76E-08	2.87E-08	0	1.14E-07	1.10E-06
ZIC5	5.76E-07	5.62E-07	0	0	8.45E-08	9.11E-09	9.58E-09	6.34E-07
ZIK1	2.68E-06	4.57E-08	1.20E-07	3.84E-07	4.82E-08	9.53E-08	1.26E-07	1.19E-07
ZIM2	0	0	1.28E-08	0	0	0	0	0
ZIM3	0	0	0	0	0	0	0	0
ZKSCAN1	2.93E-05	1.09E-05	5.74E-06	3.21E-06	9.91E-07	1.10E-05	2.66E-06	6.71E-06
ZKSCAN2	9.41E-07	2.27E-06	8.36E-07	7.33E-07	3.19E-07	1.28E-06	9.06E-07	5.44E-07
ZKSCAN3	2.78E-06	1.56E-06	8.69E-07	6.87E-07	4.78E-07	2.14E-06	5.79E-07	2.59E-06
ZKSCAN4	3.91E-06	2.63E-06	1.13E-06	1.08E-06	7.23E-07	2.88E-06	1.37E-06	2.95E-06
ZKSCAN5	9.23E-06	5.80E-06	1.85E-06	1.97E-06	1.52E-06	3.74E-06	2.62E-06	3.62E-06
ZMAT1	9.87E-07	1.88E-06	1.18E-06	1.41E-06	1.15E-06	2.37E-06	6.26E-07	4.09E-06
ZMAT2	7.78E-05	4.79E-05	3.10E-05	5.51E-05	2.35E-05	9.49E-05	5.94E-05	8.07E-05
ZMAT3	1.89E-06	1.15E-06	2.91E-06	2.03E-06	2.41E-06	3.41E-06	4.55E-06	1.30E-06
ZMAT4	0	0	0	0	0	0	0	0
ZMAT5	4.02E-05	3.58E-05	1.90E-05	1.90E-05	3.21E-05	2.59E-05	3.39E-05	2.21E-05
ZMIZ1	2.95E-05	7.87E-06	6.92E-06	1.25E-05	1.90E-06	5.40E-06	9.33E-06	1.23E-05
ZMIZ2	2.10E-05	1.12E-05	7.08E-06	1.15E-05	8.06E-06	1.39E-05	9.82E-06	1.44E-05
ZMPSTE24	3.49E-05	2.48E-05	1.79E-05	1.76E-05	5.94E-06	2.59E-05	1.59E-05	3.68E-05
ZMYM1	4.48E-06	2.57E-06	1.65E-06	1.24E-06	2.25E-07	1.58E-06	6.19E-07	1.24E-06
ZMYM2	1.29E-05	8.39E-06	5.32E-06	6.72E-06	1.64E-06	6.32E-06	2.31E-06	3.37E-06
ZMYM3	1.08E-05	9.29E-06	4.10E-06	4.17E-06	3.12E-06	5.39E-06	4.20E-06	7.44E-06
ZMYM4	8.13E-06	6.40E-06	4.17E-06	4.37E-06	1.63E-06	5.93E-06	2.97E-06	3.99E-06
ZMYM5	5.76E-06	2.61E-06	2.70E-06	3.55E-06	9.11E-07	3.36E-06	2.38E-06	2.73E-06
ZMYM6	7.95E-06	4.18E-06	3.05E-06	2.88E-06	7.32E-07	3.34E-06	1.65E-06	2.46E-06
ZMYND10	4.39E-07	3.73E-07	5.53E-08	3.39E-08	6.25E-07	1.93E-07	3.20E-07	6.64E-08
ZMYND11	1.79E-05	8.48E-06	1.48E-05	1.65E-05	4.84E-06	1.89E-05	1.48E-05	1.72E-05
ZMYND12	2.15E-07	3.59E-06	2.76E-06	1.82E-06	6.58E-07	5.05E-06	2.10E-06	8.89E-07
ZMYND15	7.08E-06	1.74E-06	2.15E-06	3.05E-06	1.46E-06	8.60E-07	2.08E-06	4.88E-06
ZMYND17	8.47E-07	3.26E-07	4.24E-07	1.22E-06	9.99E-07	9.53E-07	4.09E-06	4.96E-07
ZMYND19	1.22E-05	8.11E-06	5.88E-06	6.69E-06	7.35E-06	8.91E-06	1.02E-05	1.66E-05
ZMYND8	1.19E-05	5.70E-06	3.90E-06	5.31E-06	3.27E-06	1.16E-05	5.72E-06	3.41E-06
ZNF10	3.83E-06	1.16E-06	7.42E-07	9.92E-07	4.47E-07	7.82E-07	4.95E-07	1.20E-06
ZNF100	1.35E-06	4.29E-07	5.73E-07	7.43E-07	1.70E-07	4.88E-07	3.18E-07	1.86E-07
ZNF101	4.79E-06	2.50E-06	1.18E-06	2.10E-06	8.01E-07	1.60E-06	1.67E-06	1.71E-06
ZNF107	1.95E-06	1.23E-06	5.16E-07	2.20E-07	2.21E-08	6.36E-07	1.80E-07	4.84E-07
ZNF114	5.68E-08	5.35E-08	1.24E-08	1.45E-08	6.52E-08	3.29E-08	3.43E-08	1.04E-07
ZNF117	4.47E-06	3.80E-06	1.25E-06	2.16E-06	2.26E-07	3.66E-06	8.13E-07	1.96E-06
ZNF12	8.21E-06	1.32E-05	3.16E-06	2.91E-06	1.06E-06	3.84E-06	2.73E-06	4.96E-06
ZNF121	2.19E-06	8.16E-07	1.18E-06	1.55E-06	2.32E-07	9.45E-07	8.51E-07	6.85E-07
ZNF124	2.65E-06	1.45E-06	7.45E-07	7.70E-07	3.25E-08	4.00E-07	3.51E-07	1.43E-06
ZNF131	1.61E-05	9.27E-06	6.20E-06	1.00E-05	3.30E-06	1.02E-05	8.44E-06	1.13E-05
ZNF132	5.42E-07	5.32E-07	7.11E-07	4.39E-07	1.57E-07	2.69E-07	1.41E-07	3.14E-07
ZNF133	6.57E-06	2.85E-06	2.41E-06	2.87E-06	3.48E-06	4.31E-06	2.62E-06	2.28E-06
ZNF134	5.09E-06	2.82E-06	2.21E-06	2.75E-06	5.17E-07	1.47E-06	1.99E-06	3.30E-06
ZNF135	2.86E-07	3.12E-07	2.18E-07	6.20E-07	8.36E-08	4.67E-08	1.52E-07	7.84E-07
ZNF136	3.69E-06	1.26E-06	1.86E-06	2.49E-06	4.14E-07	1.54E-06	1.27E-06	1.85E-06
ZNF137	2.84E-07	2.24E-07	1.87E-07	3.13E-07	1.30E-07	6.14E-08	1.19E-07	2.36E-07
ZNF138	4.01E-06	4.81E-06	1.42E-06	1.50E-06	5.83E-07	2.09E-06	9.50E-07	2.22E-06
ZNF14	1.65E-06	1.85E-07	4.53E-07	3.54E-07	6.05E-08	1.45E-07	2.66E-07	1.26E-07
ZNF140	6.27E-06	4.71E-06	3.31E-06	5.50E-06	2.31E-06	3.80E-06	3.38E-06	3.93E-06
ZNF141	4.05E-06	1.58E-06	1.32E-06	1.52E-06	9.12E-07	4.92E-07	3.77E-08	1.15E-06
ZNF142	4.76E-06	1.17E-06	7.47E-07	1.24E-06	6.16E-07	2.21E-06	1.35E-06	1.96E-06
ZNF143	6.49E-06	5.30E-06	2.26E-06	3.51E-06	9.03E-07	3.90E-06	4.27E-06	3.25E-06
ZNF146	2.62E-05	1.76E-05	1.27E-05	1.30E-05	6.52E-06	1.54E-05	1.25E-05	1.49E-05

ZNF148	5.61E-06	6.02E-06	3.83E-06	3.20E-06	1.30E-06	3.51E-06	2.39E-06	2.92E-06
ZNF154	3.31E-06	3.03E-07	1.58E-07	5.84E-07	3.09E-07	6.29E-08	1.45E-07	2.86E-07
ZNF155	4.22E-06	1.89E-06	2.26E-06	2.13E-06	1.74E-06	1.91E-06	1.94E-06	2.67E-06
ZNF157	1.44E-08	0	1.52E-08	0	0	0	0	0
ZNF16	7.09E-06	2.51E-06	1.51E-06	2.72E-06	3.00E-06	2.76E-06	7.72E-06	3.05E-06
ZNF160	3.64E-06	3.55E-06	2.17E-06	3.18E-06	3.47E-07	5.84E-07	6.13E-07	1.38E-06
ZNF165	3.76E-06	5.70E-06	6.39E-07	2.98E-06	5.83E-07	1.68E-06	2.71E-06	4.53E-06
ZNF167	1.10E-06	6.02E-07	4.08E-07	6.26E-07	1.81E-07	7.55E-08	9.14E-08	6.17E-08
ZNF169	1.21E-06	2.88E-07	2.80E-07	2.00E-07	5.90E-08	2.80E-07	1.48E-07	1.88E-07
ZNF17	2.20E-06	1.71E-06	1.18E-06	1.29E-06	6.52E-07	1.21E-06	1.05E-06	1.75E-06
ZNF174	5.57E-06	5.13E-06	2.15E-06	2.82E-06	1.52E-06	3.86E-06	3.53E-06	2.94E-06
ZNF175	6.10E-07	6.43E-07	1.03E-06	7.67E-07	3.78E-07	5.87E-07	5.40E-07	6.19E-07
ZNF177	3.13E-07	6.03E-07	5.99E-07	2.87E-07	5.00E-07	6.33E-08	2.12E-07	8.69E-07
ZNF18	2.75E-06	2.16E-06	3.24E-06	2.89E-06	1.85E-06	5.44E-06	3.52E-06	4.00E-06
ZNF180	3.23E-06	1.65E-06	1.51E-06	1.45E-06	6.20E-07	1.33E-06	1.01E-06	1.23E-06
ZNF181	7.54E-06	4.66E-06	3.93E-06	2.48E-06	2.90E-06	4.62E-06	2.61E-06	4.59E-06
ZNF182	3.31E-06	1.78E-06	1.07E-06	1.24E-06	4.07E-07	1.36E-06	1.01E-06	2.59E-06
ZNF184	1.87E-05	2.72E-06	9.75E-07	1.20E-06	2.94E-07	1.24E-06	9.69E-07	1.44E-06
ZNF185	6.33E-06	1.05E-06	1.07E-06	1.27E-06	5.14E-07	1.20E-06	2.30E-06	1.00E-06
ZNF187	5.03E-06	2.40E-06	2.10E-06	2.34E-06	1.64E-06	2.92E-06	2.89E-06	4.07E-06
ZNF189	2.15E-05	6.76E-06	4.54E-06	6.23E-06	1.52E-06	9.68E-06	3.02E-06	5.41E-06
ZNF19	1.24E-06	5.80E-07	2.82E-07	5.42E-07	2.63E-07	7.37E-07	6.80E-07	9.28E-07
ZNF192	4.16E-06	2.60E-06	1.25E-06	7.42E-07	7.07E-09	1.98E-06	2.70E-07	8.10E-07
ZNF193	5.72E-06	3.06E-06	1.86E-06	3.06E-06	1.95E-06	3.85E-06	3.07E-06	2.93E-06
ZNF195	7.98E-06	5.16E-06	2.22E-06	3.91E-06	1.04E-06	2.98E-06	1.84E-06	5.95E-06
ZNF197	8.38E-06	5.77E-06	3.70E-06	3.88E-06	1.77E-06	5.33E-06	2.97E-06	3.72E-06
ZNF2	1.48E-06	4.94E-07	4.17E-07	5.55E-07	4.12E-07	7.87E-07	5.22E-07	8.30E-07
ZNF20	2.50E-06	4.89E-07	8.19E-07	1.17E-06	7.89E-07	9.35E-07	9.08E-07	1.30E-06
ZNF200	4.29E-06	7.46E-06	1.50E-06	1.82E-06	6.05E-07	4.49E-06	1.25E-06	5.72E-06
ZNF202	1.75E-06	1.02E-06	8.75E-07	1.93E-06	6.81E-07	1.23E-06	1.35E-06	1.91E-06
ZNF204P	1.85E-05	8.49E-07	2.83E-07	3.05E-07	1.38E-08	5.21E-08	6.24E-08	9.43E-08
ZNF205	6.15E-06	1.01E-05	3.55E-06	4.89E-06	5.03E-06	5.91E-06	8.59E-06	4.46E-06
ZNF207	7.91E-05	4.97E-05	2.47E-05	3.13E-05	9.90E-06	3.92E-05	3.07E-05	3.45E-05
ZNF208	4.73E-06	8.09E-07	1.10E-08	2.96E-08	2.31E-09	4.99E-09	7.56E-08	1.58E-08
ZNF211	5.80E-06	1.64E-06	1.39E-06	2.57E-06	9.08E-07	4.41E-07	6.35E-07	2.23E-06
ZNF212	2.75E-06	3.45E-06	3.66E-06	3.50E-06	4.11E-06	6.18E-06	5.35E-06	3.84E-06
ZNF213	4.68E-06	1.82E-06	9.84E-07	1.26E-06	1.02E-06	1.53E-06	1.90E-06	1.78E-06
ZNF214	6.75E-07	4.44E-07	4.28E-07	3.43E-07	8.29E-08	4.63E-07	2.46E-07	2.70E-07
ZNF215	1.16E-06	3.76E-08	2.07E-08	1.06E-07	2.30E-08	0	2.15E-07	9.82E-08
ZNF217	1.35E-05	1.10E-05	5.49E-06	5.65E-06	7.93E-07	9.69E-06	4.35E-06	6.57E-06
ZNF219	1.31E-05	1.06E-05	3.35E-06	6.37E-06	9.22E-06	5.90E-06	9.25E-06	1.20E-05
ZNF22	1.91E-05	1.92E-05	1.73E-05	1.67E-05	8.74E-06	2.46E-05	1.63E-05	1.40E-05
ZNF221	8.82E-07	2.13E-07	2.63E-07	1.41E-07	3.06E-07	1.36E-07	4.71E-08	2.01E-07
ZNF222	5.27E-06	1.36E-06	8.95E-07	1.33E-06	5.37E-07	1.06E-06	4.72E-07	1.87E-06
ZNF223	3.81E-06	1.11E-06	6.39E-07	7.52E-07	3.77E-07	1.01E-06	4.92E-07	6.39E-07
ZNF224	5.75E-06	1.68E-06	2.01E-06	1.72E-06	9.70E-07	2.21E-06	1.38E-06	2.45E-06
ZNF225	2.06E-06	8.52E-07	9.91E-07	4.63E-07	2.42E-07	8.22E-07	3.81E-07	8.77E-07
ZNF226	1.99E-05	8.71E-06	7.67E-06	6.59E-06	1.10E-05	1.23E-05	3.56E-06	4.97E-06
ZNF227	5.38E-06	2.78E-06	2.67E-06	2.92E-06	2.15E-06	3.28E-06	1.77E-06	2.76E-06
ZNF229	2.55E-06	1.11E-06	2.92E-07	2.96E-07	5.83E-08	6.63E-08	4.91E-08	7.23E-07
ZNF23	6.47E-06	3.38E-06	2.19E-06	2.92E-06	1.34E-06	3.24E-06	1.90E-06	4.69E-06
ZNF230	2.00E-06	4.74E-07	5.37E-07	8.27E-07	5.24E-07	5.14E-07	5.62E-07	7.25E-07
ZNF232	2.95E-06	2.40E-06	1.65E-06	1.98E-06	1.04E-06	3.11E-06	3.51E-06	3.23E-06
ZNF233	2.72E-06	7.97E-07	6.07E-08	2.15E-07	3.38E-08	5.43E-08	5.68E-08	1.35E-07
ZNF234	2.13E-06	9.74E-07	7.02E-07	6.85E-07	4.25E-07	1.20E-06	4.43E-07	9.70E-07
ZNF235	2.49E-06	1.17E-06	7.88E-07	1.21E-06	1.99E-07	8.35E-07	3.92E-07	9.16E-07
ZNF236	1.78E-06	5.73E-07	7.58E-07	6.54E-07	1.10E-07	1.03E-06	3.52E-07	8.83E-07
ZNF238	8.47E-06	1.08E-05	9.89E-06	7.96E-06	5.13E-06	3.03E-05	1.04E-05	2.66E-05

ZNF239	1.68E-06	0	2.59E-07	4.03E-07	2.03E-08	1.06E-07	1.22E-07	3.72E-08
ZNF24	1.80E-05	1.40E-05	1.32E-05	1.22E-05	2.82E-06	1.47E-05	9.72E-06	1.01E-05
ZNF248	4.02E-06	2.31E-06	1.15E-06	7.55E-07	9.07E-07	2.02E-06	1.12E-06	9.51E-07
ZNF25	2.45E-06	7.34E-07	1.18E-06	1.44E-06	7.25E-07	1.90E-06	1.05E-06	1.59E-06
ZNF250	2.78E-06	9.64E-07	5.65E-07	5.07E-07	6.26E-07	5.58E-07	2.25E-06	9.95E-07
ZNF251	9.53E-06	4.54E-06	1.63E-06	2.63E-06	1.85E-06	2.69E-06	4.10E-06	3.72E-06
ZNF252	1.16E-05	8.53E-06	3.98E-06	2.92E-06	3.27E-06	4.04E-06	5.52E-06	7.13E-06
ZNF253	6.07E-06	3.68E-06	2.34E-06	1.09E-06	6.02E-07	7.85E-07	1.09E-06	1.84E-06
ZNF254	5.16E-06	6.04E-06	3.30E-06	2.37E-06	7.52E-07	4.35E-06	4.36E-07	1.62E-06
ZNF256	5.13E-07	1.91E-06	6.08E-07	1.26E-06	4.04E-07	1.55E-07	3.95E-07	1.82E-06
ZNF257	1.35E-06	2.12E-07	2.02E-08	3.07E-08	2.80E-08	2.41E-08	2.53E-08	2.01E-07
ZNF259	6.15E-05	2.17E-05	1.24E-05	1.93E-05	6.16E-06	2.83E-05	1.32E-05	2.37E-05
ZNF26	2.59E-06	2.62E-06	8.29E-07	1.69E-06	5.20E-07	1.65E-06	1.94E-06	2.44E-06
ZNF260	4.07E-06	3.93E-06	2.36E-06	2.66E-06	1.39E-06	2.13E-06	1.79E-06	2.72E-06
ZNF263	1.55E-05	1.60E-05	6.22E-06	1.07E-05	8.28E-06	1.16E-05	1.28E-05	1.11E-05
ZNF264	1.57E-06	1.24E-06	1.24E-06	1.42E-06	2.49E-07	1.03E-06	4.80E-07	9.77E-07
ZNF266	1.93E-05	5.51E-06	2.28E-06	4.43E-06	4.39E-06	5.95E-06	2.29E-06	5.68E-06
ZNF267	5.86E-06	2.87E-06	1.42E-06	3.72E-06	4.24E-07	2.21E-06	1.35E-06	1.71E-06
ZNF268	2.85E-06	5.89E-06	4.33E-06	3.99E-06	5.53E-07	4.12E-06	1.70E-06	2.85E-06
ZNF271	8.66E-06	4.48E-06	3.06E-06	3.28E-06	2.37E-06	5.23E-06	4.45E-06	3.64E-06
ZNF273	1.71E-06	1.91E-06	3.89E-07	5.16E-07	9.55E-08	4.33E-07	3.64E-07	7.31E-07
ZNF274	8.99E-06	1.21E-05	9.45E-06	1.00E-05	5.63E-06	3.29E-06	7.18E-06	9.99E-06
ZNF275	3.25E-06	5.45E-06	4.55E-06	4.25E-06	2.44E-06	3.19E-06	4.10E-06	9.38E-06
ZNF276	7.11E-06	3.33E-06	3.11E-06	4.69E-06	1.93E-06	3.84E-06	3.92E-06	5.68E-06
ZNF277	8.69E-06	1.88E-05	1.09E-05	9.21E-06	5.42E-06	1.51E-05	9.41E-06	1.52E-05
ZNF28	7.05E-06	1.96E-06	5.74E-07	1.24E-06	4.39E-09	2.06E-07	2.39E-07	1.30E-06
ZNF280A	0	0	0	0	0	0	0	0
ZNF280B	3.14E-08	8.75E-09	2.00E-08	2.39E-08	0	1.09E-08	1.53E-08	5.73E-09
ZNF280C	6.39E-07	5.95E-07	3.75E-07	3.28E-07	1.34E-07	8.13E-07	3.42E-07	5.93E-07
ZNF280D	1.27E-05	9.35E-06	8.22E-06	6.48E-06	2.66E-06	1.63E-05	5.95E-06	9.28E-06
ZNF281	8.51E-06	7.75E-06	8.59E-06	4.55E-06	6.72E-08	1.28E-05	1.36E-06	8.36E-06
ZNF282	8.25E-06	8.61E-06	5.56E-06	6.35E-06	7.72E-06	1.31E-05	1.03E-05	1.17E-05
ZNF283	1.22E-06	5.36E-07	5.99E-07	4.85E-07	5.70E-08	4.34E-07	6.42E-08	4.67E-07
ZNF284	8.70E-07	3.40E-07	2.13E-07	7.90E-08	6.47E-08	2.09E-07	1.24E-07	3.55E-07
ZNF285	8.95E-07	5.92E-07	3.00E-07	2.79E-07	1.94E-07	3.06E-07	6.80E-08	3.99E-07
ZNF286A	1.01E-06	1.02E-06	4.72E-07	7.77E-07	1.40E-07	6.04E-07	4.14E-07	5.91E-07
ZNF286B	3.80E-08	9.44E-08	3.03E-08	2.06E-08	2.17E-08	5.47E-08	4.93E-08	8.65E-08
ZNF287	4.67E-07	5.35E-08	1.14E-07	2.20E-07	5.47E-08	6.88E-08	1.14E-07	4.66E-08
ZNF292	4.14E-06	2.89E-06	1.59E-06	2.24E-06	2.18E-07	1.80E-06	7.87E-07	1.39E-06
ZNF295	2.53E-06	1.96E-06	3.00E-06	3.23E-06	3.45E-07	3.84E-06	1.93E-06	4.48E-06
ZNF296	8.54E-07	1.06E-07	1.32E-07	2.10E-07	2.01E-07	1.44E-07	2.69E-07	2.29E-08
ZNF3	2.22E-05	1.54E-05	7.11E-06	7.59E-06	7.10E-06	1.22E-05	9.85E-06	1.61E-05
ZNF30	1.64E-06	1.26E-06	9.67E-07	9.38E-07	1.00E-06	9.73E-07	6.58E-07	1.39E-06
ZNF300	7.07E-07	1.00E-06	1.62E-07	3.73E-07	7.67E-08	1.03E-07	1.01E-07	8.73E-07
ZNF302	1.38E-05	8.31E-06	6.09E-06	4.06E-06	5.53E-06	7.80E-06	4.53E-06	4.85E-06
ZNF304	3.35E-06	1.36E-06	1.15E-06	1.44E-06	3.97E-07	7.79E-07	4.25E-07	1.79E-06
ZNF311	1.56E-07	1.55E-06	2.64E-07	4.91E-07	4.10E-08	1.43E-08	1.26E-06	1.29E-07
ZNF317	9.94E-06	4.25E-06	3.37E-06	5.08E-06	3.54E-06	5.55E-06	5.88E-06	4.64E-06
ZNF318	5.04E-06	5.98E-06	2.16E-06	2.43E-06	8.06E-07	4.23E-06	2.10E-06	4.26E-06
ZNF319	4.50E-06	2.52E-06	1.39E-06	1.35E-06	4.07E-07	2.76E-06	2.02E-06	2.62E-06
ZNF32	2.17E-05	1.79E-05	1.62E-05	1.84E-05	2.01E-05	1.42E-05	1.80E-05	2.19E-05
ZNF320	3.67E-06	1.53E-06	3.71E-07	5.87E-07	5.94E-08	1.21E-07	2.41E-07	7.97E-07
ZNF321	3.92E-06	2.02E-07	3.37E-07	1.69E-07	8.54E-08	2.81E-08	1.17E-07	1.33E-07
ZNF322A	6.45E-06	8.86E-06	3.29E-06	3.64E-06	5.69E-06	4.12E-06	7.40E-06	6.06E-06
ZNF322B	6.72E-07	8.06E-07	1.86E-07	3.40E-07	1.28E-07	3.68E-07	1.92E-07	2.77E-07
ZNF323	2.28E-06	7.21E-06	2.86E-06	1.91E-06	2.67E-06	5.07E-06	1.45E-06	3.46E-06
ZNF324	1.89E-06	2.66E-06	8.79E-07	1.66E-06	1.39E-06	1.88E-06	2.07E-06	1.82E-06
ZNF324B	9.51E-07	1.30E-06	7.52E-07	7.56E-07	9.30E-07	1.44E-06	1.49E-06	1.12E-06

ZNF326	9.77E-06	6.31E-06	4.98E-06	6.28E-06	1.83E-06	7.87E-06	2.38E-06	4.27E-06
ZNF329	4.67E-06	4.50E-06	2.94E-06	4.04E-06	2.22E-06	2.40E-06	1.54E-06	3.58E-06
ZNF330	1.59E-05	1.11E-05	2.28E-05	2.20E-05	5.12E-06	1.89E-05	1.73E-05	1.95E-05
ZNF331	8.40E-06	2.81E-06	2.41E-06	1.10E-05	8.16E-08	7.97E-07	6.87E-07	1.63E-05
ZNF333	2.43E-06	6.34E-07	7.77E-07	1.22E-06	4.45E-07	9.50E-07	8.69E-07	1.49E-06
ZNF334	1.33E-07	1.08E-06	2.64E-07	8.55E-07	4.78E-07	5.33E-08	2.97E-08	1.12E-07
ZNF335	1.02E-05	4.58E-06	2.22E-06	4.31E-06	5.12E-06	4.25E-06	4.02E-06	4.49E-06
ZNF337	6.72E-06	1.85E-06	1.27E-06	2.52E-06	2.03E-06	2.79E-06	3.45E-06	2.48E-06
ZNF33A	8.65E-06	6.23E-06	5.71E-06	5.47E-06	3.16E-06	8.91E-06	4.45E-06	4.67E-06
ZNF33B	4.97E-06	3.30E-06	4.74E-06	5.09E-06	3.08E-06	6.91E-06	4.91E-06	2.32E-06
ZNF34	6.36E-06	2.47E-06	1.22E-06	1.87E-06	2.25E-06	2.24E-06	7.16E-06	3.60E-06
ZNF341	2.78E-06	8.01E-06	6.89E-07	8.30E-07	1.14E-06	1.57E-06	1.13E-06	1.98E-06
ZNF343	5.23E-06	2.33E-06	1.52E-06	1.69E-06	1.81E-06	3.58E-06	2.31E-06	2.23E-06
ZNF345	1.21E-06	5.13E-07	6.02E-07	5.33E-07	4.71E-07	6.66E-07	2.29E-07	3.50E-07
ZNF346	4.01E-06	2.85E-06	2.25E-06	2.66E-06	1.41E-06	3.58E-06	2.68E-06	2.51E-06
ZNF347	2.58E-07	7.34E-08	3.91E-07	3.99E-07	1.69E-08	1.09E-07	9.54E-08	9.60E-08
ZNF35	2.42E-06	1.19E-06	6.96E-07	1.67E-06	4.27E-07	7.55E-07	4.90E-07	2.03E-06
ZNF350	5.53E-06	2.62E-06	1.65E-06	2.35E-06	1.14E-06	1.18E-06	1.79E-06	2.89E-06
ZNF354A	3.11E-06	9.64E-07	8.61E-07	1.45E-06	1.98E-07	1.96E-07	4.64E-07	3.11E-06
ZNF354B	2.26E-06	6.84E-07	5.99E-07	8.61E-07	1.12E-07	1.17E-06	3.14E-07	1.29E-06
ZNF354C	2.78E-07	0	2.90E-07	1.34E-07	0	6.45E-08	0	4.38E-08
ZNF358	2.08E-05	2.34E-05	2.50E-05	2.97E-05	0.000109	2.90E-05	4.69E-05	2.03E-05
ZNF362	6.17E-06	5.32E-06	4.60E-06	5.70E-06	2.38E-06	5.72E-06	7.02E-06	4.65E-06
ZNF365	5.33E-07	3.19E-08	0	7.79E-08	0	6.16E-09	2.23E-08	4.87E-08
ZNF366	8.15E-07	3.59E-07	3.49E-07	7.20E-08	0	2.18E-07	1.87E-07	1.23E-07
ZNF367	3.42E-06	5.89E-06	1.90E-06	1.83E-06	9.13E-07	1.69E-06	1.71E-06	1.58E-06
ZNF37A	3.47E-06	2.44E-06	1.51E-06	1.36E-06	5.16E-07	1.74E-06	9.73E-07	1.09E-06
ZNF37B	1.44E-06	4.00E-07	2.58E-07	3.12E-07	1.24E-07	3.77E-07	1.82E-07	3.22E-07
ZNF382	1.75E-06	1.27E-06	1.25E-07	2.79E-07	1.00E-07	7.74E-08	1.38E-07	5.64E-07
ZNF383	2.87E-06	1.35E-06	1.62E-06	1.25E-06	7.94E-07	1.47E-06	6.19E-07	1.94E-06
ZNF384	2.04E-05	1.28E-05	6.65E-06	7.44E-06	3.11E-06	1.03E-05	9.03E-06	8.75E-06
ZNF385A	9.84E-06	3.29E-06	3.72E-06	1.10E-05	1.40E-06	2.91E-06	4.74E-06	2.82E-06
ZNF385B	6.47E-07	7.10E-06	5.35E-06	2.36E-06	4.01E-06	1.82E-05	4.45E-06	6.33E-07
ZNF385D	1.19E-06	4.29E-08	0	3.28E-08	5.76E-08	8.32E-07	2.58E-08	1.18E-07
ZNF389	2.99E-08	1.53E-07	3.16E-08	1.10E-07	7.73E-08	5.28E-07	5.76E-08	2.21E-08
ZNF391	4.04E-06	0	6.53E-08	1.89E-08	0	0	0	4.55E-08
ZNF394	2.12E-05	9.61E-06	6.86E-06	9.22E-06	5.48E-06	8.29E-06	8.08E-06	1.44E-05
ZNF395	9.03E-06	1.09E-05	7.09E-06	8.32E-06	2.99E-06	7.61E-06	7.77E-06	2.52E-06
ZNF396	6.35E-07	5.92E-07	2.94E-07	2.86E-07	5.02E-08	7.19E-07	1.78E-07	1.47E-07
ZNF397	6.35E-06	4.00E-06	3.35E-06	2.60E-06	5.67E-07	4.70E-06	3.79E-06	2.37E-06
ZNF397OS	1.01E-05	3.93E-06	2.04E-06	2.89E-06	5.91E-07	4.10E-06	1.57E-06	2.27E-06
ZNF398	1.28E-06	1.39E-06	1.32E-06	1.64E-06	1.46E-06	2.02E-06	2.06E-06	2.86E-06
ZNF404	1.48E-06	7.59E-07	3.73E-07	4.35E-07	1.16E-06	9.75E-08	2.03E-07	1.28E-06
ZNF407	2.16E-06	9.49E-07	9.69E-07	1.26E-06	5.12E-07	2.16E-06	9.23E-07	1.10E-06
ZNF408	9.15E-06	1.48E-05	5.66E-06	7.77E-06	8.28E-06	7.06E-06	1.54E-05	1.18E-05
ZNF41	2.48E-06	2.25E-06	1.82E-06	1.40E-06	1.84E-07	1.62E-06	9.02E-07	2.12E-06
ZNF410	2.29E-05	1.30E-05	8.81E-06	2.01E-05	7.13E-06	1.52E-05	1.82E-05	1.81E-05
ZNF414	6.72E-06	5.64E-06	4.92E-06	7.28E-06	6.27E-06	7.94E-06	1.09E-05	6.81E-06
ZNF415	3.90E-07	1.06E-07	3.78E-07	2.67E-07	1.60E-07	1.46E-07	2.09E-07	2.30E-07
ZNF416	2.17E-06	1.05E-06	7.00E-07	1.08E-06	1.23E-06	9.28E-07	8.70E-07	1.46E-06
ZNF417	1.09E-06	9.97E-07	6.32E-07	1.03E-06	1.94E-07	5.07E-07	3.04E-07	5.07E-07
ZNF418	8.83E-07	1.23E-07	2.39E-07	4.61E-07	7.44E-08	1.15E-08	1.02E-07	6.08E-07
ZNF419	4.84E-06	2.38E-06	1.79E-06	1.35E-06	2.45E-06	5.62E-07	8.07E-07	3.14E-06
ZNF420	1.51E-06	1.74E-06	1.16E-06	8.11E-07	1.68E-07	1.26E-06	5.37E-07	8.35E-07
ZNF423	6.79E-07	2.09E-07	2.50E-07	3.42E-07	1.47E-07	1.88E-07	3.32E-07	5.39E-07
ZNF425	2.85E-07	3.61E-07	3.17E-07	3.67E-07	1.44E-07	5.82E-07	5.11E-07	6.22E-07
ZNF426	3.08E-06	6.64E-07	4.67E-07	8.13E-07	1.77E-08	1.88E-07	1.69E-07	7.66E-07
ZNF428	1.69E-05	1.43E-05	1.08E-05	2.51E-05	5.29E-05	1.86E-05	3.80E-05	1.72E-05

ZNF429	2.50E-06	2.61E-06	3.00E-06	1.48E-06	9.02E-07	2.00E-06	1.94E-06	1.27E-06
ZNF43	2.02E-06	1.73E-07	2.48E-07	2.50E-07	8.36E-08	2.29E-07	1.78E-07	2.46E-07
ZNF430	1.86E-06	2.98E-07	2.84E-07	3.89E-07	0	1.23E-07	1.03E-07	1.30E-07
ZNF431	1.45E-06	4.59E-07	1.89E-07	2.32E-07	2.04E-08	2.69E-07	1.04E-07	4.43E-07
ZNF432	1.68E-06	4.51E-07	4.07E-07	4.34E-07	5.48E-08	5.48E-07	1.76E-07	7.49E-07
ZNF433	2.62E-06	7.58E-07	8.06E-07	1.08E-06	1.19E-06	1.50E-06	1.48E-06	1.32E-06
ZNF434	7.97E-06	8.00E-06	2.88E-06	3.23E-06	1.71E-06	5.47E-06	3.83E-06	5.99E-06
ZNF436	4.14E-06	1.31E-06	9.12E-07	1.30E-06	4.70E-07	2.92E-06	1.05E-06	2.50E-06
ZNF438	5.58E-06	2.17E-06	2.48E-06	3.69E-06	1.60E-06	1.71E-06	3.16E-06	3.26E-06
ZNF439	4.56E-06	7.70E-08	2.94E-07	4.18E-07	7.55E-08	1.90E-07	1.79E-07	2.72E-07
ZNF44	1.48E-05	2.86E-06	3.10E-06	3.88E-06	2.74E-06	3.45E-06	4.79E-06	2.23E-06
ZNF440	1.91E-06	6.82E-07	7.43E-07	7.97E-07	2.15E-07	9.81E-07	6.50E-07	7.48E-07
ZNF441	2.13E-06	7.25E-07	1.25E-06	6.48E-07	3.77E-07	1.11E-06	7.60E-07	8.52E-07
ZNF442	7.81E-07	5.03E-07	3.95E-07	5.85E-07	2.39E-07	6.39E-07	8.50E-07	9.33E-07
ZNF443	1.86E-06	7.40E-07	8.10E-07	1.16E-06	7.08E-07	9.43E-07	1.26E-06	1.06E-06
ZNF444	2.82E-05	1.38E-05	1.58E-05	2.27E-05	8.54E-05	3.11E-05	4.37E-05	2.49E-05
ZNF445	3.11E-06	2.01E-06	9.06E-07	8.32E-07	6.80E-07	1.96E-06	9.91E-07	1.14E-06
ZNF446	6.12E-06	2.43E-06	1.64E-06	2.80E-06	2.96E-06	4.42E-06	2.58E-06	3.51E-06
ZNF449	2.98E-06	8.23E-07	5.92E-07	4.25E-07	3.61E-07	6.80E-07	5.34E-07	1.37E-06
ZNF45	4.54E-06	1.92E-06	1.82E-06	2.08E-06	1.29E-06	2.63E-06	1.53E-06	1.94E-06
ZNF451	6.62E-06	7.34E-06	4.40E-06	3.15E-06	1.53E-06	6.39E-06	2.53E-06	5.02E-06
ZNF454	3.08E-08	0	7.20E-08	6.93E-08	8.84E-09	1.91E-08	9.95E-09	6.04E-08
ZNF460	3.34E-07	8.84E-07	2.65E-07	1.11E-06	1.65E-08	3.39E-07	5.01E-07	3.67E-07
ZNF461	1.16E-06	9.18E-07	5.75E-07	5.64E-07	2.09E-07	3.49E-07	3.69E-07	6.08E-07
ZNF462	1.96E-06	9.70E-08	1.51E-07	7.84E-07	7.00E-08	1.14E-07	2.78E-07	1.37E-07
ZNF467	6.68E-06	3.33E-06	3.27E-06	2.03E-06	2.51E-06	6.43E-06	2.98E-06	1.17E-05
ZNF468	6.00E-06	3.49E-06	1.08E-06	2.03E-06	4.00E-08	3.94E-07	2.78E-07	1.81E-06
ZNF469	1.02E-06	3.14E-07	9.50E-08	3.48E-07	3.43E-08	6.94E-08	2.23E-07	5.60E-08
ZNF470	5.73E-07	1.76E-06	1.24E-06	1.44E-06	1.16E-06	3.18E-08	6.48E-07	7.29E-07
ZNF471	1.23E-07	7.11E-08	4.75E-07	3.22E-07	7.41E-08	3.78E-08	1.33E-08	5.39E-07
ZNF473	2.65E-06	2.34E-06	1.08E-06	1.27E-06	8.45E-07	1.59E-06	1.66E-06	9.06E-07
ZNF474	3.70E-08	8.38E-08	5.17E-08	4.54E-08	0	2.29E-08	0	0
ZNF479	0	0	0	0	0	0	0	0
ZNF48	3.75E-06	6.28E-06	1.54E-06	2.89E-06	1.75E-06	3.17E-06	4.12E-06	3.33E-06
ZNF480	6.03E-06	3.71E-06	2.12E-06	1.85E-06	1.50E-06	2.60E-06	8.65E-07	3.32E-06
ZNF483	2.28E-06	3.89E-08	5.99E-08	9.50E-08	5.27E-09	2.72E-07	1.54E-08	6.22E-08
ZNF484	1.50E-06	9.09E-07	4.87E-07	8.04E-07	1.57E-07	6.57E-07	6.21E-07	9.20E-07
ZNF485	7.16E-07	8.42E-07	6.94E-07	5.67E-07	3.77E-07	7.98E-07	5.83E-07	3.59E-07
ZNF486	2.15E-06	7.42E-08	1.38E-07	1.60E-07	1.40E-08	3.02E-08	1.57E-08	1.68E-07
ZNF487	1.62E-06	1.73E-06	3.72E-07	1.08E-06	1.15E-07	6.04E-07	4.01E-07	6.40E-07
ZNF488	1.56E-07	7.61E-09	7.00E-09	0	0	6.28E-09	0	0
ZNF490	1.69E-06	9.96E-07	7.06E-07	5.78E-07	4.91E-07	1.08E-06	5.58E-07	8.43E-07
ZNF491	5.19E-07	2.13E-07	3.58E-07	1.91E-07	1.20E-07	2.82E-07	4.23E-07	2.90E-07
ZNF492	0	0	1.10E-08	6.53E-09	0	4.95E-09	1.04E-08	0
ZNF493	2.09E-06	1.73E-06	7.28E-07	5.74E-07	3.12E-07	4.78E-07	1.83E-07	5.79E-07
ZNF496	4.61E-06	4.90E-07	1.99E-06	3.14E-06	5.93E-07	5.06E-07	6.10E-07	7.03E-07
ZNF497	3.81E-07	7.82E-07	3.08E-07	4.73E-07	7.89E-07	1.01E-06	7.93E-07	5.90E-07
ZNF498	1.75E-05	5.21E-06	1.67E-06	2.48E-06	1.03E-06	2.62E-06	2.41E-06	2.72E-06
ZNF500	2.87E-06	3.58E-06	1.30E-06	1.49E-06	4.86E-07	2.76E-06	2.12E-06	1.68E-06
ZNF501	6.87E-07	3.61E-08	3.51E-07	4.52E-07	1.49E-07	4.46E-08	1.32E-07	3.10E-07
ZNF502	3.86E-07	9.81E-08	5.71E-07	1.20E-06	6.63E-07	5.39E-08	1.13E-07	3.42E-07
ZNF503	3.31E-06	1.72E-06	2.68E-06	3.81E-06	5.58E-06	8.64E-06	6.16E-06	3.45E-06
ZNF506	1.44E-06	2.91E-06	1.69E-06	3.59E-06	1.14E-06	6.32E-07	9.15E-07	1.38E-06
ZNF507	4.08E-06	3.16E-06	2.61E-06	2.04E-06	7.02E-07	3.68E-06	1.47E-06	2.08E-06
ZNF510	2.59E-06	1.44E-06	1.29E-06	1.01E-06	1.80E-07	9.14E-07	3.65E-07	1.66E-06
ZNF511	3.56E-05	2.10E-05	3.02E-05	2.92E-05	3.20E-05	2.29E-05	2.67E-05	2.58E-05
ZNF512	1.50E-05	9.04E-06	3.94E-06	3.45E-06	2.54E-06	6.38E-06	2.42E-06	3.66E-06
ZNF512B	6.53E-06	6.70E-06	3.01E-06	2.19E-06	2.19E-06	6.73E-06	6.33E-06	6.86E-06

ZNF513	2.60E-05	2.93E-05	7.09E-06	1.65E-05	7.30E-06	1.70E-05	1.96E-05	1.35E-05
ZNF514	3.58E-06	9.86E-07	7.90E-07	1.12E-06	3.28E-07	5.06E-07	5.24E-07	5.59E-07
ZNF516	4.63E-06	1.86E-06	1.30E-06	1.62E-06	2.30E-07	3.47E-06	1.12E-06	1.49E-06
ZNF517	7.74E-06	8.67E-06	1.41E-06	1.81E-06	3.35E-06	6.96E-06	6.73E-06	4.06E-06
ZNF518A	4.38E-06	2.39E-06	1.58E-06	1.48E-06	4.58E-07	2.67E-06	7.97E-07	1.15E-06
ZNF518B	1.30E-06	1.94E-07	4.93E-07	7.69E-07	5.79E-08	1.87E-07	2.41E-07	2.21E-07
ZNF519	5.79E-07	4.45E-07	2.23E-07	3.02E-07	4.84E-08	1.82E-07	3.36E-08	1.73E-07
ZNF521	6.91E-07	2.84E-07	2.53E-07	4.41E-07	2.24E-07	2.16E-07	5.44E-07	4.75E-07
ZNF524	1.99E-05	1.26E-05	1.39E-05	1.57E-05	6.36E-05	1.64E-05	2.31E-05	1.21E-05
ZNF525	4.17E-06	1.21E-06	4.76E-07	1.26E-06	1.11E-08	1.23E-07	3.00E-07	1.14E-06
ZNF526	2.75E-06	1.95E-06	1.48E-06	1.63E-06	2.27E-06	2.25E-06	2.46E-06	2.24E-06
ZNF527	1.86E-06	1.68E-06	6.94E-07	1.00E-06	3.49E-07	7.32E-07	5.81E-07	1.46E-06
ZNF528	4.22E-07	2.32E-06	1.00E-06	9.17E-07	2.39E-07	1.17E-07	3.17E-07	7.46E-07
ZNF529	3.90E-06	2.17E-06	1.08E-06	1.42E-06	1.19E-06	1.40E-06	8.77E-07	1.97E-06
ZNF530	1.25E-06	3.88E-07	1.94E-07	3.25E-07	3.90E-07	2.14E-07	2.30E-07	4.47E-07
ZNF532	6.72E-06	1.56E-06	8.27E-07	2.11E-06	5.44E-07	9.90E-07	1.64E-06	2.63E-06
ZNF534	1.89E-07	9.96E-08	5.26E-08	9.29E-08	1.95E-07	2.11E-07	1.63E-07	1.29E-07
ZNF536	0	0	1.41E-08	5.58E-09	0	0	0	0
ZNF540	4.29E-07	3.69E-07	4.54E-07	1.95E-07	3.90E-07	1.91E-07	1.66E-07	1.56E-07
ZNF541	4.25E-08	1.59E-07	0	6.81E-07	1.46E-07	1.90E-07	1.65E-07	1.96E-07
ZNF542	3.77E-06	7.01E-06	3.59E-06	5.38E-06	3.45E-06	2.08E-06	7.64E-07	6.49E-06
ZNF543	1.55E-06	1.08E-06	5.48E-07	5.42E-07	1.77E-07	3.30E-07	1.40E-07	8.15E-07
ZNF544	9.37E-06	9.87E-06	2.56E-06	4.71E-06	5.80E-06	3.39E-06	3.46E-06	6.04E-06
ZNF546	1.01E-06	7.58E-07	6.64E-07	3.61E-07	2.71E-07	7.84E-07	2.59E-07	4.48E-07
ZNF547	1.95E-06	3.61E-07	2.61E-07	2.89E-07	3.91E-07	6.23E-08	1.29E-07	5.95E-07
ZNF548	5.54E-06	3.47E-06	2.70E-06	3.05E-06	6.19E-07	2.76E-06	1.31E-06	2.72E-06
ZNF549	2.04E-06	7.72E-08	3.31E-07	9.08E-07	2.64E-07	1.26E-07	2.18E-07	1.34E-06
ZNF550	6.80E-06	1.47E-06	9.98E-07	7.10E-07	3.57E-07	3.45E-07	3.41E-07	2.56E-06
ZNF551	1.80E-06	6.98E-07	3.69E-07	4.02E-07	1.19E-07	8.90E-08	1.99E-07	6.57E-07
ZNF552	7.27E-06	2.08E-06	1.70E-06	1.44E-06	6.73E-07	2.94E-06	8.87E-07	1.49E-06
ZNF554	1.47E-06	1.10E-06	7.71E-07	8.99E-07	1.47E-06	1.15E-06	1.00E-06	1.07E-06
ZNF555	1.84E-06	1.07E-06	4.83E-07	8.58E-07	5.61E-07	9.19E-07	6.91E-07	1.07E-06
ZNF556	0	5.14E-08	0	0	0	0	0	0
ZNF557	1.31E-06	4.90E-07	6.73E-07	7.04E-07	5.19E-07	6.88E-07	8.66E-07	9.19E-07
ZNF558	7.46E-07	5.26E-06	2.45E-06	2.95E-06	1.18E-06	3.24E-06	1.98E-06	1.99E-06
ZNF559	3.03E-06	4.09E-06	2.42E-06	3.68E-06	1.85E-06	4.28E-07	1.37E-06	1.95E-06
ZNF560	0	0	0	0	0	0	8.06E-09	0
ZNF561	5.36E-06	2.92E-06	3.82E-06	3.62E-06	2.06E-06	5.04E-06	3.44E-06	3.40E-06
ZNF562	3.77E-06	1.39E-06	1.33E-06	2.19E-06	1.30E-06	2.07E-06	1.15E-06	3.27E-06
ZNF563	3.18E-06	2.15E-06	2.06E-06	3.01E-06	2.71E-06	1.14E-05	5.64E-06	2.43E-06
ZNF564	7.66E-06	2.54E-06	3.22E-06	2.98E-06	3.55E-06	3.37E-06	3.42E-06	3.24E-06
ZNF565	1.77E-06	1.12E-06	1.02E-06	8.56E-07	3.67E-07	1.36E-06	9.87E-07	9.44E-07
ZNF566	1.26E-06	8.75E-07	9.42E-07	4.86E-07	4.47E-07	1.00E-06	6.07E-07	8.58E-07
ZNF567	1.87E-06	1.73E-06	7.35E-07	1.23E-06	2.38E-07	9.86E-07	6.74E-07	1.15E-06
ZNF568	6.66E-07	1.55E-06	1.06E-06	1.19E-06	3.95E-07	8.25E-08	3.74E-07	1.41E-06
ZNF569	1.14E-06	1.14E-06	1.05E-06	9.59E-07	4.87E-07	2.68E-07	1.75E-07	1.21E-06
ZNF57	1.13E-05	3.74E-06	1.23E-06	1.10E-06	3.72E-07	1.65E-07	1.53E-06	2.74E-06
ZNF570	8.25E-07	6.87E-07	5.46E-07	5.82E-07	8.22E-08	1.29E-07	8.08E-08	8.52E-07
ZNF571	1.60E-06	8.97E-07	5.77E-07	8.01E-07	6.28E-07	9.04E-07	5.34E-07	8.39E-07
ZNF572	2.55E-06	1.47E-06	2.69E-07	1.46E-07	3.08E-07	3.65E-07	5.47E-07	1.96E-07
ZNF573	3.12E-07	2.07E-07	1.34E-07	1.71E-07	5.54E-08	3.40E-07	7.32E-08	2.85E-07
ZNF574	6.03E-06	3.95E-06	2.98E-06	3.95E-06	3.93E-06	4.77E-06	5.91E-06	4.75E-06
ZNF575	8.51E-07	9.28E-07	8.62E-07	6.68E-07	1.35E-06	8.52E-07	1.41E-06	8.05E-07
ZNF576	1.33E-05	7.81E-06	5.68E-06	6.52E-06	8.18E-06	8.13E-06	6.23E-06	6.98E-06
ZNF577	4.13E-06	4.27E-06	1.11E-06	1.63E-06	5.01E-06	1.68E-07	1.70E-06	4.10E-07
ZNF578	6.63E-08	0	3.05E-08	2.83E-08	1.42E-08	1.32E-07	5.49E-08	2.42E-08
ZNF579	3.28E-06	3.51E-06	1.29E-06	3.33E-06	8.01E-06	3.44E-06	8.94E-06	2.03E-06
ZNF580	7.70E-06	6.55E-06	2.40E-06	4.97E-06	2.64E-05	7.55E-06	1.22E-05	4.92E-06

ZNF581	1.01E-05	7.08E-06	4.17E-06	6.65E-06	1.82E-05	1.15E-05	1.27E-05	6.72E-06
ZNF582	1.94E-07	1.45E-07	7.28E-07	7.10E-07	7.21E-07	9.54E-09	1.96E-07	1.06E-06
ZNF583	8.22E-07	1.25E-06	8.62E-07	9.47E-07	3.41E-07	9.30E-07	5.25E-07	1.19E-06
ZNF584	2.51E-06	6.53E-06	2.31E-06	3.19E-06	3.33E-06	3.81E-06	4.79E-06	2.20E-06
ZNF585A	1.37E-06	1.63E-06	6.35E-07	5.91E-07	1.04E-07	7.99E-07	4.28E-07	6.77E-07
ZNF585B	9.20E-07	1.01E-06	4.28E-07	6.22E-07	1.50E-07	5.40E-08	2.73E-07	8.09E-07
ZNF586	2.88E-06	3.19E-06	1.15E-06	2.34E-06	5.31E-07	9.55E-07	6.48E-07	1.11E-06
ZNF587	5.57E-06	1.54E-06	1.30E-06	1.55E-06	3.02E-07	1.49E-06	6.11E-07	1.22E-06
ZNF589	3.13E-06	5.10E-07	6.81E-07	8.79E-07	7.06E-07	9.94E-07	8.11E-07	8.74E-07
ZNF592	6.98E-06	4.18E-06	3.47E-06	4.62E-06	2.52E-06	5.25E-06	6.06E-06	4.64E-06
ZNF593	8.64E-05	9.62E-05	4.92E-05	6.35E-05	9.04E-05	5.54E-05	8.93E-05	4.21E-05
ZNF594	4.86E-07	5.02E-07	4.75E-07	2.99E-07	1.79E-07	5.57E-08	9.46E-08	1.29E-07
ZNF595	7.13E-06	4.24E-06	1.82E-06	3.12E-06	7.09E-07	1.45E-06	1.14E-06	2.92E-06
ZNF596	5.10E-07	1.67E-06	4.05E-07	7.02E-07	3.46E-07	1.09E-06	2.44E-07	2.46E-07
ZNF597	1.11E-06	2.21E-06	8.37E-07	9.58E-07	8.26E-08	8.65E-07	6.48E-07	8.08E-07
ZNF598	1.55E-05	1.83E-05	9.39E-06	1.02E-05	7.68E-06	1.50E-05	1.73E-05	9.53E-06
ZNF599	6.69E-07	1.43E-07	4.52E-07	5.90E-07	2.94E-07	6.49E-07	3.73E-07	3.17E-07
ZNF600	7.02E-06	1.76E-07	1.25E-06	1.95E-06	7.12E-08	2.05E-07	7.05E-07	2.61E-06
ZNF605	8.98E-06	4.52E-06	2.97E-06	4.60E-06	1.89E-06	4.10E-06	2.51E-06	2.05E-06
ZNF606	2.94E-06	2.75E-06	1.03E-06	1.15E-06	1.58E-06	2.64E-07	5.83E-07	1.36E-06
ZNF607	2.39E-07	1.60E-06	2.45E-07	2.65E-07	2.39E-07	1.00E-07	1.42E-07	5.52E-07
ZNF608	4.61E-06	1.15E-06	9.34E-07	1.51E-06	9.55E-07	1.61E-06	1.03E-06	2.16E-06
ZNF609	8.11E-06	3.49E-06	3.31E-06	3.28E-06	8.69E-07	3.31E-06	2.53E-06	3.41E-06
ZNF610	2.45E-07	2.16E-07	9.40E-08	1.92E-07	1.93E-08	6.26E-08	8.63E-08	8.27E-08
ZNF611	2.24E-06	1.06E-06	3.11E-07	6.42E-07	0	1.16E-07	1.89E-07	9.52E-07
ZNF613	2.54E-06	1.80E-06	9.46E-07	1.73E-06	7.24E-07	1.26E-06	9.85E-07	1.47E-06
ZNF614	4.74E-06	1.31E-06	6.98E-07	1.26E-06	5.51E-07	1.77E-07	2.83E-07	1.17E-06
ZNF615	2.67E-06	1.31E-06	1.08E-06	1.11E-06	6.48E-07	1.01E-06	7.40E-07	1.62E-06
ZNF616	2.82E-06	1.91E-06	1.62E-06	9.19E-07	4.89E-07	1.86E-06	8.77E-07	1.21E-06
ZNF618	4.05E-06	7.59E-07	8.16E-07	9.90E-07	8.76E-07	4.56E-07	1.23E-06	1.66E-06
ZNF619	1.23E-06	1.37E-06	8.22E-07	4.69E-07	4.66E-08	8.99E-07	3.39E-07	9.16E-07
ZNF620	1.69E-06	9.02E-07	4.71E-07	8.71E-07	4.69E-07	4.49E-07	5.41E-07	6.05E-07
ZNF621	3.91E-06	2.10E-06	1.39E-06	1.11E-06	4.25E-07	2.32E-06	1.07E-06	1.53E-06
ZNF622	1.96E-05	3.00E-05	2.12E-05	2.39E-05	1.24E-05	2.31E-05	2.34E-05	2.59E-05
ZNF623	6.32E-06	4.80E-06	1.89E-06	1.67E-06	4.76E-07	2.87E-06	2.59E-06	2.56E-06
ZNF624	5.69E-07	5.46E-07	3.09E-07	2.49E-07	5.98E-08	3.18E-07	2.30E-07	4.48E-07
ZNF625	1.07E-06	2.95E-07	1.80E-07	1.38E-07	7.76E-08	6.30E-08	4.23E-08	4.30E-07
ZNF626	5.48E-06	2.49E-06	1.38E-06	1.69E-06	2.23E-07	1.45E-07	2.78E-07	2.07E-06
ZNF627	7.94E-06	2.43E-06	2.79E-06	2.30E-06	1.01E-06	5.87E-06	2.45E-06	3.56E-06
ZNF628	1.39E-06	1.63E-06	7.75E-07	1.89E-06	8.86E-07	1.66E-06	2.93E-06	9.38E-07
ZNF629	7.11E-06	5.76E-06	1.68E-06	2.85E-06	2.14E-06	3.79E-06	3.06E-06	2.32E-06
ZNF630	5.04E-07	7.55E-07	2.95E-07	8.42E-07	6.42E-08	9.51E-08	1.08E-07	1.21E-07
ZNF638	4.48E-05	3.10E-05	2.22E-05	2.43E-05	2.08E-05	3.29E-05	2.35E-05	1.96E-05
ZNF639	9.17E-06	7.00E-06	6.02E-06	6.01E-06	2.10E-06	6.37E-06	6.35E-06	7.25E-06
ZNF641	4.20E-06	4.26E-06	1.45E-06	1.92E-06	7.01E-08	1.07E-06	8.62E-07	1.45E-06
ZNF642	1.66E-06	1.36E-06	8.82E-07	1.04E-06	1.71E-07	1.65E-06	9.28E-07	9.57E-07
ZNF643	3.99E-07	3.90E-07	2.03E-07	3.57E-07	0	4.73E-07	1.29E-07	5.70E-07
ZNF644	1.28E-05	1.40E-05	9.37E-06	1.31E-05	4.00E-06	1.28E-05	8.72E-06	7.43E-06
ZNF645	0	0	0	0	0	0	0	0
ZNF646	3.87E-06	2.41E-06	9.45E-07	1.18E-06	5.14E-07	2.83E-06	1.38E-06	1.73E-06
ZNF648	0	2.11E-08	6.46E-08	1.53E-07	1.08E-08	1.35E-06	2.07E-07	1.40E-06
ZNF649	1.65E-06	1.16E-06	1.05E-06	1.72E-06	8.01E-07	5.53E-07	1.06E-06	7.16E-07
ZNF652	1.01E-05	1.13E-05	4.95E-06	5.14E-06	3.63E-06	1.55E-05	5.07E-06	5.71E-06
ZNF653	2.64E-06	1.66E-06	1.65E-06	4.05E-06	3.56E-06	1.96E-06	4.43E-06	2.02E-06
ZNF654	2.77E-06	2.54E-06	2.46E-06	2.00E-06	8.24E-07	2.20E-06	1.64E-06	1.83E-06
ZNF655	5.64E-05	5.80E-05	1.44E-05	1.69E-05	6.38E-06	1.69E-05	1.75E-05	2.16E-05
ZNF658	3.79E-07	4.77E-07	3.20E-07	1.80E-07	2.14E-07	1.15E-07	1.21E-07	2.82E-07
ZNF660	4.09E-07	4.86E-08	2.25E-08	2.64E-08	9.25E-09	9.98E-09	2.08E-08	0

ZNF662	1.58E-06	5.00E-07	4.04E-07	7.02E-07	3.59E-07	1.83E-07	2.19E-07	1.17E-07
ZNF664	4.56E-05	4.67E-05	1.59E-05	1.78E-05	4.41E-06	2.29E-05	1.54E-05	1.69E-05
ZNF665	1.05E-07	7.85E-09	5.78E-08	7.69E-08	1.80E-08	1.30E-08	1.36E-08	5.13E-08
ZNF667	1.39E-07	5.36E-08	2.04E-07	2.18E-07	2.99E-08	2.69E-08	5.65E-08	2.66E-07
ZNF668	3.96E-06	3.61E-06	1.33E-06	4.02E-06	2.63E-06	3.04E-06	4.21E-06	3.08E-06
ZNF669	2.95E-06	1.52E-06	9.67E-07	1.46E-06	1.22E-08	5.78E-07	1.72E-07	3.76E-06
ZNF670	7.92E-06	2.41E-06	1.06E-06	1.48E-06	7.19E-08	9.53E-07	1.39E-07	1.48E-06
ZNF671	5.84E-06	1.04E-06	8.05E-07	1.86E-06	1.31E-06	2.66E-07	6.18E-07	1.28E-06
ZNF672	2.34E-05	1.28E-05	8.12E-06	1.41E-05	1.04E-05	1.39E-05	1.80E-05	3.19E-05
ZNF673	5.94E-06	4.98E-06	2.73E-06	4.13E-06	1.60E-06	3.94E-06	4.58E-06	7.25E-06
ZNF674	1.20E-06	1.22E-06	3.83E-07	6.51E-07	1.49E-07	6.42E-07	5.98E-07	1.05E-06
ZNF675	3.88E-06	2.14E-06	7.07E-07	7.76E-07	4.62E-08	4.28E-07	2.18E-07	1.09E-06
ZNF676	3.16E-08	2.67E-08	0	1.93E-08	0	0	7.67E-09	3.48E-08
ZNF677	3.01E-07	8.45E-08	2.66E-07	5.62E-07	3.95E-08	1.78E-07	2.56E-07	6.57E-08
ZNF678	1.27E-06	9.44E-07	4.70E-07	3.02E-07	0	4.10E-07	3.70E-08	6.60E-07
ZNF679	5.70E-07	0	0	0	0	0	0	0
ZNF680	3.91E-06	1.14E-05	8.20E-06	2.84E-06	1.81E-06	9.41E-06	2.27E-06	5.04E-06
ZNF681	1.35E-06	1.57E-08	3.23E-08	6.88E-08	6.05E-09	4.98E-08	9.27E-08	2.22E-07
ZNF682	8.17E-07	1.12E-06	4.05E-07	4.80E-07	4.22E-07	9.81E-07	1.28E-07	5.47E-07
ZNF683	8.01E-07	1.35E-07	5.80E-07	1.88E-06	8.95E-08	6.89E-08	2.00E-07	7.66E-07
ZNF684	3.72E-06	2.06E-06	7.27E-06	2.44E-06	9.30E-07	3.35E-06	1.19E-06	8.62E-06
ZNF687	1.02E-05	6.37E-06	4.19E-06	4.76E-06	1.11E-06	1.15E-05	5.12E-06	8.52E-06
ZNF688	7.57E-06	1.35E-05	7.25E-06	9.86E-06	7.92E-06	8.04E-06	1.19E-05	5.93E-06
ZNF689	3.98E-06	4.01E-06	1.89E-06	2.32E-06	1.33E-07	2.80E-06	1.53E-06	2.06E-06
ZNF69	1.34E-05	1.97E-06	2.00E-06	3.79E-06	7.10E-06	2.48E-06	1.99E-06	9.81E-07
ZNF691	7.76E-06	5.81E-06	4.24E-06	3.57E-06	4.58E-06	1.45E-05	5.30E-06	4.77E-06
ZNF692	1.31E-05	7.09E-06	3.29E-06	5.20E-06	1.06E-05	6.56E-06	7.48E-06	1.30E-05
ZNF695	1.22E-06	1.55E-08	0	0	0	2.35E-08	6.69E-09	1.01E-08
ZNF696	3.21E-06	2.38E-06	9.07E-07	9.49E-07	1.31E-06	1.41E-06	4.87E-06	1.98E-06
ZNF697	3.53E-06	1.47E-06	1.61E-06	1.81E-06	7.27E-07	1.53E-06	1.46E-06	1.56E-06
ZNF699	5.24E-07	3.99E-07	2.38E-07	2.37E-07	5.88E-08	8.45E-08	6.61E-08	2.51E-07
ZNF7	1.76E-05	7.36E-06	3.69E-06	4.90E-06	5.93E-06	7.02E-06	1.33E-05	7.30E-06
ZNF70	1.14E-06	2.89E-07	2.25E-07	2.75E-07	2.25E-07	1.81E-07	3.41E-07	1.26E-07
ZNF700	7.57E-06	2.12E-06	2.51E-06	2.84E-06	1.57E-06	2.76E-06	2.38E-06	2.27E-06
ZNF701	7.22E-07	3.83E-07	2.10E-07	6.05E-07	3.48E-08	1.58E-07	1.81E-07	4.28E-07
ZNF702P	2.54E-06	1.13E-07	1.12E-07	1.23E-07	1.33E-08	6.44E-08	6.75E-08	4.53E-08
ZNF703	2.13E-06	5.80E-07	7.30E-07	1.33E-06	1.88E-08	1.84E-06	9.41E-07	2.25E-07
ZNF704	2.45E-06	1.50E-06	5.80E-07	7.41E-07	4.07E-08	3.66E-07	6.88E-07	2.24E-07
ZNF705A	0	0	0	0	0	0	6.49E-09	0
ZNF705D	0	0	0	0	0	0	0	0
ZNF706	4.05E-05	1.63E-05	1.40E-05	1.41E-05	1.96E-05	1.56E-05	3.88E-05	2.12E-05
ZNF707	6.22E-06	3.64E-06	1.69E-06	2.36E-06	2.00E-06	2.69E-06	5.15E-06	4.06E-06
ZNF708	1.73E-06	6.50E-07	6.27E-07	7.69E-07	1.04E-07	4.73E-07	2.32E-07	3.79E-07
ZNF709	7.70E-07	2.03E-07	2.03E-07	2.90E-07	2.80E-08	2.37E-07	1.66E-07	1.09E-07
ZNF71	1.60E-06	1.30E-06	4.12E-07	1.18E-06	1.38E-06	9.24E-07	6.60E-07	2.03E-06
ZNF710	4.03E-06	1.61E-06	1.37E-06	3.30E-06	2.25E-07	5.43E-06	1.73E-06	1.55E-06
ZNF711	3.68E-06	4.28E-08	1.18E-07	2.07E-07	2.81E-08	2.02E-08	2.20E-07	1.76E-07
ZNF713	7.80E-07	6.96E-07	1.38E-07	3.67E-07	9.95E-09	2.11E-07	2.08E-07	2.28E-07
ZNF714	1.00E-06	7.81E-08	8.43E-08	1.14E-07	4.34E-09	4.29E-08	5.67E-08	1.40E-07
ZNF716	4.32E-09	2.41E-08	0	0	0	0	0	1.20E-07
ZNF717	6.04E-07	9.42E-07	4.27E-07	4.18E-07	9.98E-08	5.92E-07	2.47E-07	2.04E-07
ZNF718	1.89E-06	1.10E-06	4.63E-07	5.07E-07	1.05E-07	7.83E-07	4.16E-07	4.53E-07
ZNF720	3.33E-06	4.72E-06	1.80E-06	2.17E-06	4.23E-07	3.60E-06	1.34E-06	1.79E-06
ZNF721	4.56E-06	2.93E-06	2.35E-06	2.33E-06	1.26E-06	2.90E-06	1.57E-06	4.09E-06
ZNF727	1.31E-07	4.47E-07	4.62E-08	3.58E-08	0	2.71E-08	2.81E-08	8.60E-08
ZNF732	2.18E-08	0	2.28E-08	2.67E-08	9.38E-09	1.01E-08	0	1.60E-08
ZNF735	0	0	0	0	0	0	0	6.13E-08
ZNF737	1.13E-06	4.72E-07	3.16E-07	2.10E-07	2.27E-09	4.89E-08	4.13E-08	2.24E-07

ZNF738	2.02E-06	1.76E-07	1.36E-07	3.84E-07	1.07E-07	1.78E-07	2.36E-07	2.34E-07
ZNF74	2.17E-06	2.52E-06	1.02E-06	1.06E-06	9.17E-07	2.12E-06	1.45E-06	2.47E-06
ZNF740	6.52E-06	3.61E-06	1.56E-06	2.06E-06	2.84E-06	6.02E-06	4.77E-06	4.42E-06
ZNF746	3.57E-06	5.73E-06	2.72E-06	3.59E-06	1.09E-06	9.03E-06	3.65E-06	5.94E-06
ZNF747	7.75E-06	1.56E-05	6.14E-06	5.83E-06	1.89E-06	7.36E-06	5.31E-06	4.09E-06
ZNF749	9.74E-07	6.23E-07	4.02E-07	5.23E-07	8.16E-08	5.65E-07	2.61E-07	3.48E-07
ZNF750	3.62E-08	3.25E-08	1.12E-07	1.77E-08	6.21E-09	2.68E-08	7.02E-09	7.42E-08
ZNF75A	1.05E-05	1.10E-05	3.09E-06	3.90E-06	2.28E-06	2.19E-06	3.48E-06	5.49E-06
ZNF75D	9.39E-06	3.31E-06	2.19E-06	2.43E-06	2.00E-06	3.46E-06	1.96E-06	5.58E-06
ZNF76	1.57E-05	2.08E-05	7.41E-06	7.75E-06	6.99E-06	1.52E-05	7.45E-06	1.28E-05
ZNF761	1.18E-05	3.92E-06	1.80E-06	3.09E-06	1.18E-06	1.47E-06	5.07E-07	3.14E-06
ZNF763	4.49E-06	6.33E-07	2.42E-07	8.73E-07	3.07E-07	2.14E-07	2.64E-07	9.46E-07
ZNF764	7.28E-06	5.31E-06	1.89E-06	2.30E-06	1.00E-06	2.67E-06	3.32E-06	2.53E-06
ZNF765	2.48E-06	1.36E-06	8.41E-07	1.12E-06	1.73E-07	6.81E-07	6.59E-07	9.65E-07
ZNF766	6.01E-06	3.93E-06	2.70E-06	3.17E-06	2.75E-06	3.97E-06	3.54E-06	4.28E-06
ZNF767	1.87E-06	8.84E-07	4.29E-07	8.18E-07	1.35E-06	1.34E-06	8.24E-07	7.76E-07
ZNF768	4.05E-05	4.94E-05	1.77E-05	1.64E-05	1.41E-05	1.68E-05	2.45E-05	1.73E-05
ZNF77	1.32E-06	1.62E-06	5.28E-07	6.90E-07	5.65E-07	8.05E-07	1.20E-06	1.73E-06
ZNF770	6.39E-06	7.68E-06	8.54E-06	4.77E-06	4.36E-06	1.08E-05	4.94E-06	5.37E-06
ZNF771	2.58E-06	3.83E-06	1.09E-06	2.44E-06	3.33E-06	1.42E-06	6.28E-06	9.01E-07
ZNF772	2.36E-06	1.22E-06	5.43E-07	4.72E-07	3.24E-07	1.10E-07	1.07E-07	7.26E-07
ZNF773	3.16E-06	2.60E-06	1.02E-06	1.28E-06	1.28E-06	1.76E-07	4.31E-07	2.34E-06
ZNF774	3.82E-07	5.52E-07	1.38E-07	1.94E-07	2.27E-08	1.95E-07	1.78E-07	1.74E-07
ZNF775	1.10E-06	1.20E-06	2.17E-07	4.33E-07	1.01E-06	6.56E-07	1.26E-06	9.48E-07
ZNF776	4.86E-06	2.21E-06	2.03E-06	1.85E-06	6.86E-07	1.87E-06	1.01E-06	1.68E-06
ZNF777	3.34E-06	6.24E-06	4.56E-06	6.39E-06	2.50E-06	6.94E-06	7.81E-06	9.27E-06
ZNF778	1.07E-06	2.88E-07	4.79E-07	5.67E-07	2.37E-07	7.97E-07	4.97E-07	7.90E-07
ZNF780A	4.77E-06	3.23E-06	2.22E-06	1.97E-06	4.36E-06	2.64E-06	2.19E-06	2.83E-06
ZNF780B	2.14E-06	7.31E-07	7.83E-07	5.52E-07	3.11E-07	4.18E-07	3.56E-07	4.55E-07
ZNF781	1.25E-07	1.24E-07	8.35E-08	1.62E-07	6.31E-08	6.81E-08	4.99E-08	7.54E-08
ZNF782	1.08E-06	7.64E-07	3.69E-07	6.62E-07	2.39E-07	4.31E-07	2.98E-07	5.84E-07
ZNF784	7.44E-06	3.27E-06	3.70E-06	4.97E-06	6.34E-06	5.73E-06	9.29E-06	4.79E-06
ZNF785	2.97E-06	1.85E-06	9.08E-07	9.78E-07	2.46E-07	1.01E-06	9.48E-07	1.35E-06
ZNF786	1.33E-06	1.27E-06	9.81E-07	1.22E-06	6.22E-07	1.24E-06	1.85E-06	2.00E-06
ZNF787	2.22E-05	2.23E-05	1.45E-05	2.48E-05	3.56E-05	1.89E-05	4.44E-05	2.34E-05
ZNF788	2.98E-06	8.16E-08	2.75E-07	1.85E-07	8.33E-08	1.12E-07	2.54E-07	2.29E-07
ZNF789	5.02E-06	3.64E-06	7.34E-07	1.07E-06	1.33E-06	1.74E-06	1.28E-06	1.18E-06
ZNF79	3.25E-06	1.20E-06	8.95E-07	1.92E-06	7.97E-07	2.32E-06	1.37E-06	1.75E-06
ZNF790	2.41E-06	2.05E-06	2.04E-06	1.86E-06	3.08E-06	1.48E-06	1.87E-06	1.28E-06
ZNF791	5.55E-06	1.65E-06	2.53E-06	1.41E-06	2.27E-07	2.55E-06	1.07E-06	1.95E-06
ZNF792	1.68E-06	8.48E-07	4.23E-07	4.37E-07	2.62E-07	3.86E-07	5.08E-07	1.33E-06
ZNF793	5.99E-08	3.03E-07	1.92E-08	1.05E-07	5.79E-08	1.79E-08	1.07E-07	1.30E-07
ZNF799	4.53E-06	1.79E-06	1.73E-06	2.17E-06	1.26E-06	1.92E-06	2.89E-06	2.30E-06
ZNF8	1.72E-06	8.13E-07	2.49E-07	7.52E-07	3.41E-07	3.10E-07	1.97E-07	5.68E-07
ZNF80	3.34E-07	0	6.65E-08	1.16E-07	0	8.48E-09	8.87E-09	1.15E-08
ZNF800	2.83E-06	5.11E-06	4.67E-06	5.26E-06	8.05E-07	4.38E-06	2.68E-06	4.78E-06
ZNF804A	6.28E-08	9.23E-07	4.95E-08	4.95E-07	4.14E-09	0	6.11E-08	0
ZNF804B	0	0	0	0	0	0	0	0
ZNF805	1.42E-06	8.19E-07	7.50E-07	9.84E-07	5.88E-07	9.76E-07	7.10E-07	1.22E-06
ZNF808	2.88E-06	3.84E-06	1.65E-06	2.04E-06	6.04E-07	2.06E-07	7.98E-07	2.57E-06
ZNF81	5.10E-07	3.65E-07	1.49E-07	1.64E-07	6.25E-08	3.26E-07	9.02E-08	3.28E-07
ZNF813	2.58E-06	1.51E-06	5.34E-07	1.79E-07	9.31E-09	1.22E-06	4.75E-07	3.10E-07
ZNF814	3.22E-06	5.46E-06	1.22E-06	3.78E-06	1.56E-06	7.47E-07	1.06E-06	3.03E-06
ZNF815	4.31E-07	4.45E-07	3.49E-07	5.03E-07	3.51E-07	5.54E-07	1.75E-07	6.32E-07
ZNF816A	3.89E-06	1.41E-06	4.83E-07	8.17E-07	7.91E-08	1.45E-07	1.87E-07	5.99E-07
ZNF821	3.27E-06	2.19E-06	1.04E-06	2.29E-06	2.50E-06	1.97E-06	2.17E-06	2.24E-06
ZNF823	5.24E-06	1.01E-06	2.02E-06	2.35E-06	1.95E-06	1.96E-06	2.61E-06	2.41E-06
ZNF826	3.26E-06	3.98E-08	7.92E-08	2.30E-07	3.01E-08	8.55E-08	1.89E-07	8.88E-07

ZNF827	2.96E-06	2.84E-07	1.17E-07	1.97E-07	0	3.21E-08	1.02E-07	4.32E-08
ZNF828	5.78E-06	8.21E-06	5.82E-06	6.67E-06	7.53E-07	4.66E-06	4.89E-06	4.78E-06
ZNF829	1.63E-07	5.40E-08	8.49E-08	1.15E-07	4.63E-08	2.50E-08	0	1.29E-07
ZNF83	5.44E-06	3.12E-07	1.50E-06	3.12E-06	5.06E-07	5.86E-07	6.80E-07	3.19E-06
ZNF830	9.16E-06	1.20E-05	6.17E-06	9.06E-06	5.82E-06	9.08E-06	8.38E-06	6.42E-06
ZNF831	3.25E-07	1.84E-08	1.37E-07	4.48E-07	2.02E-09	2.09E-07	8.96E-08	9.98E-08
ZNF833	3.94E-07	1.48E-07	1.26E-07	1.16E-07	3.44E-07	8.18E-08	2.04E-07	2.33E-07
ZNF835	1.75E-07	7.45E-08	4.61E-08	3.58E-08	5.02E-08	1.35E-08	1.40E-07	3.49E-07
ZNF836	9.88E-07	6.27E-07	4.21E-07	4.47E-07	1.36E-07	4.92E-07	3.96E-07	5.77E-07
ZNF837	1.27E-06	2.38E-06	8.98E-07	1.51E-06	3.14E-06	1.36E-06	1.96E-06	9.70E-07
ZNF839	4.10E-06	3.19E-06	3.28E-06	3.40E-06	2.46E-06	6.69E-06	3.87E-06	3.61E-06
ZNF84	3.50E-06	1.97E-06	1.44E-06	2.09E-06	9.24E-07	1.56E-06	1.13E-06	1.60E-06
ZNF841	2.19E-06	1.59E-06	5.55E-07	7.76E-07	4.31E-07	3.50E-07	5.26E-07	1.82E-06
ZNF843	1.07E-07	1.05E-06	4.98E-08	1.02E-07	2.05E-08	2.10E-07	4.60E-08	7.00E-08
ZNF844	6.87E-06	6.61E-08	1.01E-06	1.16E-06	2.16E-08	3.11E-08	2.36E-07	2.62E-06
ZNF845	2.18E-06	1.11E-06	8.27E-07	8.04E-07	1.22E-07	4.14E-07	1.59E-07	5.63E-07
ZNF846	6.22E-06	6.99E-07	1.05E-06	1.67E-06	4.21E-06	9.67E-07	9.11E-07	1.16E-06
ZNF85	3.45E-06	1.89E-07	2.49E-07	1.17E-07	4.38E-08	2.06E-07	2.66E-07	3.38E-07
ZNF853	5.51E-07	1.15E-07	2.44E-07	7.98E-07	3.08E-07	1.25E-07	3.86E-07	3.47E-07
ZNF860	2.91E-07	0	3.08E-08	9.12E-09	0	6.91E-09	2.90E-08	1.09E-08
ZNF862	1.69E-06	2.07E-06	8.12E-07	9.61E-07	1.34E-06	1.69E-06	1.93E-06	2.56E-06
ZNF876P	1.17E-07	1.82E-07	1.31E-07	3.30E-08	1.54E-08	4.99E-08	8.70E-08	1.45E-07
ZNF878	3.64E-07	0	1.48E-08	6.88E-07	0	1.30E-08	1.35E-08	8.26E-08
ZNF879	3.06E-07	2.53E-07	3.55E-07	2.97E-07	9.27E-08	9.99E-08	6.10E-08	9.24E-08
ZNF880	9.66E-07	1.41E-07	6.53E-07	1.84E-06	2.70E-08	1.84E-07	5.99E-07	1.39E-07
ZNF883	2.08E-07	2.46E-07	7.23E-08	2.06E-07	8.53E-09	5.52E-08	4.80E-08	8.74E-08
ZNF90	6.49E-06	3.21E-06	1.82E-06	2.59E-06	1.14E-06	4.50E-07	2.34E-06	3.33E-07
ZNF91	6.92E-06	4.42E-06	1.96E-06	1.94E-06	1.48E-06	2.61E-06	1.49E-06	2.26E-06
ZNF92	2.97E-06	2.89E-06	1.14E-06	1.02E-06	2.62E-07	1.14E-06	7.40E-07	1.46E-06
ZNF93	3.65E-07	7.60E-07	3.77E-07	4.02E-07	1.65E-08	8.37E-08	9.47E-08	2.27E-07
ZNF98	3.14E-08	0	0	0	0	0	0	3.08E-08
ZNF99	1.49E-08	0	0	0	0	0	0	9.81E-08
ZNFX1	1.05E-05	5.74E-06	5.29E-06	6.56E-06	1.58E-06	1.43E-05	5.00E-06	5.80E-06
ZNHIT1	3.86E-05	0.000294	0.000133	0.000111	0.000301	0.000184	0.00017	0.000114
ZNHIT2	5.27E-06	1.30E-05	5.67E-06	1.10E-05	1.16E-05	1.14E-05	1.12E-05	1.16E-05
ZNHIT3	4.09E-05	3.34E-05	1.60E-05	1.89E-05	1.90E-05	2.98E-05	2.52E-05	2.11E-05
ZNHIT6	4.73E-06	3.43E-06	2.92E-06	3.42E-06	2.46E-07	6.39E-06	2.51E-06	2.61E-06
ZNRD1	2.95E-05	2.31E-05	1.47E-05	2.22E-05	3.52E-05	2.55E-05	4.30E-05	2.70E-05
ZNRF1	6.37E-06	3.68E-06	4.34E-06	5.48E-06	4.15E-06	6.26E-06	5.88E-06	7.14E-06
ZNRF2	1.40E-05	2.29E-05	1.05E-05	7.12E-06	3.16E-06	1.22E-05	9.47E-06	1.40E-05
ZNRF3	4.86E-06	2.59E-06	4.12E-06	3.80E-06	1.57E-06	1.12E-05	5.10E-06	2.10E-07
ZNRF4	0	0	0	0	0	0	0	0
ZP1	0	0	0	0	4.06E-08	0	0	0
ZP2	1.04E-08	0	0	0	0	0	0	0
ZP3	1.06E-06	2.17E-06	1.05E-06	8.39E-07	7.47E-06	2.96E-07	1.67E-06	2.10E-06
ZP4	0	0	0	0	0	0	0	0
ZPBP	0	0	0	0	0	0	0	0
ZPBP2	0	0	0	0	0	0	1.05E-08	0
ZPLD1	0.000127	2.84E-08	6.52E-08	3.17E-07	0	0	0	1.85E-08
ZRANB1	1.20E-05	6.24E-06	1.55E-05	9.38E-06	8.86E-06	1.19E-05	1.37E-05	1.32E-05
ZRANB2	2.08E-05	2.31E-05	1.01E-05	8.61E-06	3.01E-06	1.96E-05	7.41E-06	7.51E-06
ZRANB3	1.85E-06	4.58E-07	5.49E-07	4.09E-07	1.06E-07	2.06E-06	4.20E-07	6.08E-07
ZRSR2	9.64E-06	1.44E-06	3.94E-06	4.04E-06	2.75E-06	4.20E-06	5.30E-06	3.03E-06
ZSCAN1	0	5.26E-08	3.63E-08	1.21E-07	2.58E-08	0	0	4.87E-08
ZSCAN10	0	0	0	0	0	0	2.09E-08	1.40E-08
ZSCAN12	2.72E-06	2.75E-06	6.12E-07	7.75E-07	1.26E-07	6.84E-07	5.77E-07	9.75E-07
ZSCAN12I	8.69E-07	8.89E-07	6.49E-08	2.94E-07	5.41E-08	2.14E-07	2.33E-07	9.15E-07
ZSCAN16	5.62E-06	3.44E-06	1.74E-06	1.85E-06	2.00E-06	2.70E-06	1.55E-06	3.70E-06

ZSCAN18	2.47E-06	1.76E-05	5.43E-06	1.24E-05	3.27E-06	5.89E-07	3.13E-06	1.28E-05
ZSCAN2	1.10E-05	5.06E-06	3.90E-06	4.01E-06	8.56E-06	7.39E-06	5.05E-06	3.30E-06
ZSCAN20	6.92E-07	3.64E-07	1.39E-07	2.38E-07	4.65E-08	2.87E-07	2.26E-07	1.90E-07
ZSCAN21	1.24E-05	1.14E-05	5.04E-06	4.37E-06	4.25E-06	9.56E-06	5.06E-06	8.62E-06
ZSCAN22	1.20E-06	1.22E-06	8.70E-07	7.79E-07	5.75E-07	1.01E-06	9.01E-07	9.52E-07
ZSCAN23	5.46E-08	5.77E-08	4.42E-08	3.43E-08	0	0	0	2.06E-08
ZSCAN29	5.15E-06	3.83E-06	1.75E-06	2.21E-06	6.50E-07	2.97E-06	2.23E-06	3.39E-06
ZSCAN4	2.11E-08	0	2.20E-08	0	9.07E-09	0	2.04E-08	0
ZSCAN5A	3.99E-06	1.68E-06	8.92E-07	1.76E-06	1.33E-06	1.83E-06	9.44E-07	1.27E-06
ZSCAN5B	4.53E-08	5.15E-08	0	1.85E-08	6.51E-08	4.21E-08	1.46E-08	0
ZSWIM1	8.20E-06	3.32E-06	1.62E-06	1.69E-06	3.49E-06	1.84E-06	1.82E-06	2.93E-06
ZSWIM2	0	0	0	0	0	0	0	0
ZSWIM3	2.66E-06	1.99E-06	9.14E-07	8.52E-07	1.52E-06	1.54E-06	1.51E-06	1.18E-06
ZSWIM4	1.66E-06	1.20E-06	5.92E-07	4.59E-06	5.65E-07	1.15E-06	2.05E-06	1.12E-06
ZSWIM5	2.99E-06	6.39E-07	3.18E-07	3.05E-07	9.89E-08	2.28E-06	5.24E-07	1.88E-06
ZSWIM6	4.48E-06	9.67E-07	1.65E-06	3.16E-06	2.38E-07	1.47E-06	7.48E-07	8.00E-07
ZSWIM7	6.08E-06	9.19E-06	5.62E-06	1.09E-05	5.86E-06	1.03E-05	8.93E-06	7.49E-06
ZUFSP	4.64E-06	2.37E-06	1.52E-06	2.41E-06	3.63E-07	3.16E-06	2.18E-06	2.83E-06
ZW10	9.39E-06	7.26E-06	5.18E-06	5.45E-06	1.50E-06	7.22E-06	3.50E-06	9.13E-06
ZWILCH	7.82E-06	3.48E-06	9.71E-07	1.42E-06	1.24E-06	2.27E-06	1.79E-06	2.31E-06
ZWINT	2.41E-05	1.25E-05	8.02E-07	2.21E-06	4.80E-06	8.33E-06	1.52E-06	6.12E-06
ZXDA	5.02E-07	4.82E-07	6.20E-07	5.25E-07	3.48E-08	6.00E-07	4.47E-07	8.55E-07
ZXDB	1.16E-05	7.32E-06	5.73E-06	4.94E-06	9.74E-07	2.50E-06	6.56E-06	2.04E-05
ZXDC	1.24E-05	1.14E-05	7.59E-06	8.41E-06	3.88E-06	7.94E-06	7.08E-06	6.41E-06
ZYG11A	3.03E-07	9.90E-07	8.03E-07	1.24E-07	3.85E-07	0	8.80E-08	3.12E-08
ZYG11B	6.51E-06	9.65E-06	1.32E-05	3.99E-06	1.26E-06	7.57E-06	4.30E-06	6.96E-06
ZYX	6.36E-05	8.95E-05	3.53E-05	9.76E-05	3.18E-05	1.75E-05	0.00014	8.97E-05
ZZEF1	4.27E-06	2.39E-06	2.03E-06	2.68E-06	1.57E-06	3.26E-06	2.51E-06	3.83E-06
ZZZ3	1.24E-05	1.31E-05	6.32E-06	8.09E-06	1.73E-06	1.46E-05	5.60E-06	4.84E-06

DO22848	DO22851	DO22854	DO22857	DO22860	DO22863	DO22866	DO22869	DO22872
0.001299	0.001732	0.00119	0.002115	3.83E-06	0.001807	0.001648	0.001953	0.000745
1.22E-05	3.64E-05	4.58E-05	4.73E-05	4.02E-06	5.22E-05	2.96E-05	6.29E-05	1.93E-05
0	0	0	0	0	0	0	0	0
5.44E-06	7.21E-06	4.48E-06	4.80E-06	5.84E-06	7.95E-06	4.64E-06	8.13E-06	3.42E-05
0.001693	0.002451	0.001423	0.004814	0.000269	0.000115	0.001867	0.001352	0.005043
0	0	0	0	0	0	0	5.91E-09	0
3.35E-06	1.35E-06	3.05E-06	1.86E-06	5.77E-06	2.08E-06	2.21E-06	1.53E-06	3.76E-06
0	4.77E-08	0	7.20E-08	5.78E-06	0	0	1.34E-08	1.76E-08
0	0	0	0	8.84E-07	0	4.52E-08	0	0
1.48E-05	1.18E-05	3.82E-05	1.20E-05	3.57E-05	2.19E-05	1.53E-05	1.48E-05	1.81E-05
3.16E-06	2.13E-06	6.40E-06	2.08E-06	3.30E-05	3.61E-06	3.98E-06	3.41E-06	1.63E-05
0	0	0	0	3.66E-07	0	0	0	1.40E-08
0.000148	0.000207	0.000332	0.00022	8.30E-07	0.000238	0.00023	0.000359	0.000322
0	0	0	0	4.92E-08	0	0	0	0
0	0	0	0	0	0	0	0	0
0	0	0	0	0	0	0	0	0
2.14E-06	1.88E-05	1.09E-05	2.07E-05	1.43E-06	2.71E-05	4.67E-06	1.89E-05	1.84E-06
6.50E-06	5.91E-06	1.10E-05	6.92E-06	3.96E-05	9.47E-06	9.09E-06	8.01E-06	7.64E-06
7.41E-07	1.33E-06	2.06E-06	1.47E-06	1.41E-05	1.62E-06	1.42E-06	2.34E-06	1.90E-06
4.49E-05	4.63E-05	8.56E-05	5.70E-05	0.000143	5.23E-05	7.15E-05	5.79E-05	5.42E-05
0	5.27E-08	0	0	5.98E-07	0	0	0	0
3.16E-05	3.37E-05	6.67E-05	4.93E-05	6.19E-05	3.17E-05	4.81E-05	5.19E-05	3.78E-05
2.46E-06	2.03E-06	4.65E-06	2.82E-06	1.54E-05	6.90E-06	4.06E-06	3.17E-06	4.46E-06
2.91E-05	1.16E-05	2.77E-05	1.53E-05	3.44E-05	2.59E-05	2.29E-05	2.02E-05	1.78E-05
1.25E-06	1.56E-06	4.02E-06	2.29E-06	5.54E-06	3.35E-06	1.64E-06	2.94E-06	1.51E-06
6.47E-06	8.45E-06	1.86E-05	1.09E-05	2.84E-05	9.30E-06	1.17E-05	1.49E-05	1.05E-05
1.05E-05	9.54E-06	4.69E-05	1.55E-05	1.24E-06	1.65E-05	8.14E-06	9.06E-06	9.88E-06
2.23E-05	1.03E-05	2.85E-05	1.45E-05	7.64E-05	2.61E-05	2.00E-05	2.09E-05	1.36E-05
3.92E-08	4.74E-07	2.21E-07	2.19E-07	3.93E-06	2.00E-07	2.19E-07	4.72E-07	2.90E-07
5.25E-05	0.000134	0.000157	0.000182	1.47E-05	0.000111	9.00E-05	0.000157	0.00015
1.91E-06	8.27E-06	9.11E-06	2.09E-05	3.16E-06	1.10E-05	7.34E-06	1.45E-05	2.77E-06
2.59E-07	6.88E-07	2.79E-06	9.08E-07	3.21E-07	2.11E-06	1.66E-06	1.58E-06	2.69E-07
3.47E-07	3.71E-07	1.41E-06	1.00E-06	7.27E-06	1.81E-06	1.20E-06	6.71E-07	6.31E-07
0	9.59E-09	0	0	5.42E-06	0	0	1.35E-08	0
4.29E-09	1.15E-08	4.37E-07	4.99E-07	0	0	3.33E-08	8.37E-08	7.85E-08
0	0	0	2.43E-08	1.51E-07	2.43E-08	4.47E-08	1.36E-08	3.57E-08
2.79E-06	1.25E-05	7.65E-06	8.78E-06	4.02E-05	2.73E-05	1.31E-05	4.30E-06	2.79E-06
1.29E-06	7.80E-07	2.94E-07	5.71E-07	3.18E-05	2.38E-07	4.83E-07	5.84E-07	5.79E-07
5.15E-08	7.43E-08	9.18E-08	9.13E-08	6.57E-08	2.78E-07	2.48E-07	1.22E-07	3.89E-08
6.31E-06	4.93E-06	6.98E-06	4.78E-06	1.35E-06	1.13E-05	4.86E-06	3.21E-06	2.91E-06
1.10E-05	1.56E-05	3.05E-05	3.04E-05	8.32E-07	5.06E-05	1.05E-05	3.66E-05	1.11E-05
8.07E-07	1.46E-06	3.88E-06	1.90E-06	8.64E-06	4.84E-06	3.07E-06	8.30E-07	5.97E-06
4.40E-06	9.83E-06	1.44E-05	9.78E-06	1.42E-06	3.26E-05	4.32E-06	1.10E-05	3.87E-06
3.09E-07	1.22E-06	1.76E-06	2.09E-06	2.47E-07	8.17E-07	8.92E-07	3.15E-06	1.16E-06
1.16E-05	1.31E-05	1.67E-05	1.75E-05	0.000115	1.46E-05	3.03E-05	1.24E-05	1.27E-05
4.36E-06	5.41E-06	7.73E-06	5.54E-06	1.30E-05	6.73E-06	4.34E-06	8.40E-06	6.05E-06
2.30E-06	8.69E-06	2.16E-05	2.70E-05	0	0.000128	1.54E-05	1.91E-05	2.60E-06
3.10E-05	5.31E-05	9.30E-05	5.11E-05	7.07E-07	8.07E-05	4.97E-05	7.25E-05	2.16E-05
1.32E-08	0	5.33E-08	0	5.12E-08	0	2.81E-08	0	1.99E-08
2.07E-05	1.06E-05	3.85E-05	1.55E-05	2.04E-06	7.02E-06	2.05E-05	1.24E-05	4.62E-06
6.72E-06	8.05E-06	5.27E-06	8.64E-06	1.34E-05	1.90E-05	8.81E-06	1.11E-05	7.25E-06
1.32E-05	1.31E-05	3.21E-05	1.20E-05	3.48E-05	1.38E-05	1.98E-05	8.94E-06	1.04E-05
1.63E-07	5.17E-07	6.22E-07	3.20E-07	3.84E-06	8.21E-07	1.35E-06	4.65E-07	4.05E-07
1.98E-07	8.70E-07	5.03E-07	1.59E-06	3.12E-05	3.61E-07	1.16E-06	2.08E-06	1.77E-06
4.55E-07	8.50E-07	2.15E-06	1.33E-06	1.08E-05	2.44E-06	1.70E-06	1.32E-06	1.27E-06
3.35E-08	8.56E-07	3.53E-07	1.28E-06	6.14E-08	5.83E-07	1.35E-06	1.37E-06	1.64E-06

0	0	0	0	0	0	0	4.76E-09	0
0	0	0	0	0	0	0	0	5.53E-08
1.07E-05	2.31E-05	4.62E-05	3.03E-05	8.38E-07	1.68E-05	3.43E-05	3.68E-05	1.01E-05
2.56E-05	4.95E-05	2.89E-05	5.47E-05	0.00046	5.89E-05	9.05E-05	4.37E-05	1.61E-05
6.35E-08	1.14E-06	1.89E-07	8.30E-07	1.91E-05	7.68E-06	1.93E-06	3.37E-06	7.69E-06
7.35E-07	1.08E-06	3.64E-06	2.66E-06	1.06E-05	3.65E-06	3.21E-06	1.92E-06	6.53E-06
3.67E-05	6.32E-05	8.01E-05	5.87E-05	1.05E-05	8.14E-05	5.69E-05	5.20E-05	6.71E-05
1.02E-06	6.34E-06	1.80E-05	5.83E-06	1.08E-06	1.37E-05	5.05E-06	5.82E-06	7.65E-06
1.36E-05	1.02E-05	2.65E-05	9.13E-06	7.39E-06	2.35E-05	8.34E-06	1.57E-05	1.64E-05
0	7.90E-09	0	3.20E-08	0	0	1.45E-08	8.88E-09	3.77E-07
6.66E-06	1.12E-05	1.62E-05	8.29E-06	1.17E-06	1.32E-05	5.09E-06	1.91E-05	1.45E-05
1.70E-05	6.13E-06	9.40E-06	7.49E-06	1.29E-05	4.63E-06	1.37E-05	5.04E-06	4.85E-06
3.60E-09	0	5.55E-08	6.68E-08	1.77E-07	2.11E-08	3.14E-08	4.54E-08	2.15E-08
1.42E-05	4.01E-05	5.61E-05	3.84E-05	6.44E-05	4.34E-05	3.45E-05	5.44E-05	2.36E-05
1.10E-05	1.33E-05	1.36E-05	1.62E-05	2.83E-05	1.32E-05	8.89E-06	1.20E-05	1.43E-05
4.78E-06	6.97E-06	1.36E-05	1.08E-05	2.67E-05	2.01E-05	1.23E-05	1.74E-05	7.69E-06
2.17E-05	1.55E-05	2.81E-05	1.93E-05	6.04E-05	3.69E-05	2.54E-05	2.80E-05	1.59E-05
6.40E-06	5.11E-06	1.62E-05	6.68E-06	5.45E-05	1.31E-05	1.18E-05	1.04E-05	5.69E-06
2.50E-05	1.47E-05	3.09E-05	2.03E-05	5.41E-05	3.36E-05	3.04E-05	2.61E-05	1.79E-05
1.28E-06	3.57E-06	2.00E-06	4.17E-06	1.19E-05	9.46E-07	2.35E-06	7.82E-06	2.54E-06
9.14E-06	1.53E-05	1.91E-05	6.23E-06	3.47E-06	9.80E-06	7.62E-06	1.13E-05	2.25E-06
0	0	1.79E-08	2.23E-08	1.41E-07	7.95E-09	6.10E-08	9.32E-08	3.25E-08
5.68E-06	2.30E-05	1.63E-05	2.48E-05	2.83E-07	7.65E-05	1.78E-05	1.76E-05	1.24E-05
1.33E-05	3.72E-05	4.05E-05	4.38E-05	0	7.26E-05	3.36E-05	2.72E-05	1.64E-05
5.03E-06	2.70E-06	5.99E-06	9.13E-07	5.22E-08	5.41E-06	6.21E-07	1.91E-06	3.66E-06
1.18E-05	1.28E-05	2.16E-05	1.18E-05	1.81E-05	1.56E-05	1.27E-05	1.56E-05	1.42E-05
6.25E-06	5.71E-06	1.77E-05	6.12E-06	0.00013	1.05E-05	1.01E-05	9.77E-06	9.43E-06
2.23E-05	2.36E-05	2.02E-05	1.97E-05	0.000111	2.94E-05	3.82E-05	2.27E-05	4.02E-05
0	1.24E-07	1.70E-08	1.56E-07	0	2.04E-07	1.92E-08	1.75E-07	2.07E-07
1.33E-06	2.75E-06	4.32E-06	2.96E-06	3.65E-06	4.51E-06	2.77E-06	5.49E-06	2.36E-06
3.03E-05	1.35E-05	3.83E-05	1.53E-05	2.02E-05	1.64E-05	1.75E-05	1.47E-05	2.29E-05
0.000157	0.000125	0.00026	0.000127	4.91E-05	0.000115	9.41E-05	0.000117	0.000143
2.51E-06	1.54E-05	1.69E-05	1.24E-05	3.42E-05	1.47E-05	4.93E-06	1.01E-05	4.17E-06
5.38E-06	7.23E-05	4.02E-05	6.02E-05	0.000116	0.000103	4.25E-05	4.32E-05	2.31E-05
4.17E-06	1.23E-05	1.48E-05	1.47E-05	6.13E-05	1.89E-05	1.24E-05	1.59E-05	1.65E-05
1.20E-05	7.29E-06	1.98E-05	1.02E-05	3.63E-05	1.09E-05	2.41E-05	1.42E-05	1.00E-05
2.69E-06	3.28E-06	4.33E-06	3.65E-06	3.89E-06	1.15E-05	3.39E-06	7.60E-06	5.51E-06
1.24E-05	3.33E-05	2.59E-05	2.39E-05	4.25E-06	2.26E-05	2.02E-05	3.13E-05	1.48E-05
9.75E-06	1.12E-05	1.41E-05	9.29E-06	2.05E-05	1.39E-05	9.28E-06	7.48E-06	7.91E-06
5.05E-06	6.26E-06	1.75E-05	9.64E-06	4.09E-05	1.04E-05	1.09E-05	1.25E-05	8.32E-06
1.20E-06	1.98E-06	3.46E-06	2.54E-06	2.82E-05	2.76E-06	2.31E-06	3.84E-06	2.13E-06
1.27E-06	2.71E-06	2.68E-06	4.99E-06	1.44E-05	1.91E-06	5.20E-06	5.14E-06	2.23E-06
1.13E-07	2.41E-06	4.61E-07	1.44E-06	6.90E-06	7.80E-08	2.48E-06	2.94E-06	4.42E-06
3.17E-06	5.77E-06	1.23E-05	6.87E-06	4.84E-05	1.27E-05	9.65E-06	5.35E-06	5.64E-06
1.22E-06	8.46E-07	2.62E-06	1.06E-06	6.83E-06	1.34E-06	1.53E-06	1.59E-06	1.03E-06
4.72E-06	8.01E-06	2.32E-05	9.01E-06	5.59E-05	1.78E-05	8.86E-06	1.17E-05	6.62E-06
1.56E-07	8.12E-07	1.09E-06	8.76E-07	6.77E-06	4.66E-07	2.01E-06	1.28E-06	6.22E-07
2.88E-06	2.63E-05	4.98E-06	4.99E-05	1.50E-05	1.01E-05	2.20E-05	2.57E-05	1.22E-05
0	1.20E-07	3.28E-08	0	4.25E-08	1.52E-07	1.85E-07	3.38E-08	2.44E-07
9.82E-07	8.32E-06	4.03E-07	2.96E-06	1.31E-06	2.44E-08	7.91E-06	2.80E-06	1.50E-06
5.58E-07	2.53E-06	1.96E-06	3.82E-06	5.46E-05	1.02E-06	4.78E-06	4.43E-06	1.07E-05
7.23E-09	0	0	0	0	0	0	0	0
1.06E-05	7.01E-06	1.77E-05	9.06E-06	2.65E-05	1.67E-05	1.35E-05	1.23E-05	8.43E-06
1.70E-05	9.65E-06	1.17E-05	1.33E-05	2.05E-05	1.62E-05	1.46E-05	1.07E-05	1.48E-05
3.83E-06	4.57E-06	9.00E-06	7.78E-06	7.65E-06	1.98E-05	4.73E-06	5.34E-06	2.56E-06
0.000165	0.000354	0.000476	0.000263	4.76E-05	0.000359	0.000172	0.000358	0.00027
9.99E-05	0.000405	0.000381	0.000419	0.000171	0.000609	0.00025	0.000438	0.000961
9.75E-06	5.10E-06	1.86E-05	4.40E-06	2.16E-05	1.39E-05	8.12E-06	5.99E-06	7.14E-06

1.55E-05	3.20E-05	2.72E-05	2.73E-05	5.63E-06	4.62E-05	1.72E-05	1.71E-05	1.69E-05
1.43E-05	1.46E-05	1.30E-05	1.65E-05	1.03E-05	1.20E-05	1.33E-05	1.66E-05	2.57E-05
1.46E-05	4.36E-05	5.23E-05	4.85E-05	6.56E-06	3.45E-05	1.84E-05	5.01E-05	4.24E-05
9.68E-06	1.60E-05	2.29E-05	1.58E-05	2.70E-05	1.51E-05	1.73E-05	1.23E-05	2.03E-05
2.63E-05	1.84E-05	2.97E-05	2.10E-05	4.24E-05	4.97E-05	2.91E-05	2.51E-05	1.97E-05
3.10E-06	9.00E-06	2.01E-05	8.91E-06	4.66E-08	1.41E-05	6.86E-06	1.25E-05	4.78E-06
3.29E-05	9.06E-05	8.61E-05	7.49E-05	2.54E-05	0.000148	5.78E-05	0.000127	0.00012
5.07E-05	0.000175	4.94E-05	0.000121	4.23E-05	7.48E-05	6.24E-05	0.000142	0.000257
2.18E-05	9.01E-05	7.04E-05	9.09E-05	2.94E-06	6.41E-05	4.84E-05	9.74E-05	0.000125
0.000105	0.000243	0.000601	0.000252	0.000245	0.000232	0.000193	0.000339	0.000567
4.37E-09	4.91E-08	0	4.52E-09	2.92E-07	0	7.53E-08	7.52E-08	4.58E-08
2.00E-07	1.04E-06	4.15E-07	3.35E-06	3.67E-06	2.35E-07	3.57E-06	9.71E-07	2.05E-06
1.25E-06	2.72E-06	5.11E-06	3.37E-06	1.24E-05	4.36E-06	3.04E-06	4.88E-06	2.29E-06
4.68E-06	6.69E-06	1.29E-05	4.34E-06	1.11E-05	1.06E-05	4.33E-06	5.07E-06	5.66E-06
0.000233	0.000248	0.000473	0.000297	4.02E-05	0.000141	0.000286	0.000319	0.000226
9.31E-05	3.63E-05	7.38E-05	4.57E-05	1.97E-05	0.000119	4.01E-05	7.76E-05	0.000201
6.08E-06	6.64E-06	1.62E-05	1.02E-05	3.09E-05	5.97E-06	1.19E-05	1.71E-05	6.64E-06
1.95E-05	5.01E-05	5.21E-05	3.94E-05	3.49E-05	0.000109	2.92E-05	3.60E-05	3.86E-05
1.33E-05	2.48E-05	2.69E-05	1.89E-05	3.56E-05	2.69E-05	1.78E-05	2.76E-05	1.76E-05
1.82E-05	5.29E-06	1.87E-05	5.98E-06	3.08E-05	1.01E-05	6.31E-06	8.44E-06	7.31E-06
7.58E-08	3.57E-08	9.65E-08	2.40E-08	5.05E-08	1.37E-07	5.83E-08	8.68E-08	8.73E-08
0	0	1.64E-08	1.53E-08	1.15E-07	0	7.42E-08	6.83E-08	4.45E-08
2.19E-08	5.59E-08	1.03E-07	2.26E-08	3.79E-06	0	1.03E-07	1.38E-07	6.57E-08
1.14E-07	4.15E-08	2.16E-06	6.68E-08	5.13E-06	3.07E-07	1.71E-07	8.17E-08	1.39E-07
0	0	0	0	4.95E-08	0	0	0	1.26E-08
0	0	2.74E-08	0	0	1.82E-08	0	0	0
5.36E-06	2.63E-06	5.66E-06	1.05E-05	8.40E-06	1.01E-05	3.86E-06	2.75E-06	7.72E-06
0	0	0	0	0	0	0	0	0
3.92E-06	2.64E-06	2.66E-06	3.32E-06	1.26E-05	4.82E-06	4.96E-06	3.91E-06	4.14E-06
7.34E-07	8.48E-07	1.78E-06	3.27E-06	9.19E-06	1.03E-06	2.94E-06	2.26E-06	1.24E-06
2.24E-06	3.71E-06	6.98E-06	1.19E-06	6.11E-06	3.86E-06	2.74E-06	9.62E-07	2.49E-05
0	0	0	4.19E-08	0	1.84E-07	0	2.36E-08	1.25E-07
6.96E-08	5.54E-07	5.44E-07	7.91E-07	2.23E-06	4.73E-07	2.62E-07	1.25E-06	3.55E-07
4.13E-07	1.53E-06	1.63E-06	1.14E-06	9.56E-06	3.09E-06	1.05E-06	2.20E-06	1.27E-06
1.50E-07	2.28E-06	1.61E-07	6.60E-07	5.50E-06	8.48E-08	7.42E-07	1.32E-06	8.77E-07
1.30E-05	1.44E-05	2.68E-05	1.76E-05	7.00E-05	3.18E-05	2.47E-05	2.34E-05	2.15E-05
9.23E-06	1.02E-05	3.51E-05	1.10E-05	0.000162	2.89E-05	1.76E-05	2.83E-05	2.11E-05
4.06E-05	6.71E-05	6.80E-05	9.65E-05	2.53E-05	0.00023	3.83E-05	3.67E-05	6.71E-05
4.29E-06	3.36E-06	1.07E-05	3.67E-06	4.70E-06	4.74E-06	3.42E-06	5.47E-06	4.01E-06
2.36E-05	6.57E-05	6.11E-05	7.39E-05	5.76E-05	0.000378	4.95E-05	6.72E-05	4.75E-05
2.80E-05	5.20E-05	4.32E-05	5.16E-05	0.000116	8.48E-05	7.27E-05	6.96E-05	9.19E-05
4.96E-05	4.77E-05	0.000111	5.03E-05	1.63E-05	5.75E-05	3.43E-05	7.03E-05	7.99E-05
3.21E-08	5.33E-07	4.00E-08	2.64E-07	9.15E-07	5.32E-08	1.75E-07	1.42E-07	7.15E-06
1.26E-06	4.00E-05	4.91E-06	3.74E-05	0	8.71E-05	1.72E-05	8.83E-05	9.70E-05
2.50E-05	1.66E-05	2.90E-05	1.83E-05	1.53E-05	1.67E-05	1.13E-05	1.97E-05	1.78E-05
1.32E-05	3.17E-05	5.08E-05	3.10E-05	1.60E-05	2.73E-05	1.49E-05	3.41E-05	2.69E-05
9.17E-06	9.06E-06	1.79E-05	8.74E-06	7.42E-07	8.52E-06	6.72E-06	8.95E-06	8.78E-06
1.85E-06	1.76E-06	4.10E-06	8.79E-07	6.55E-08	8.84E-07	1.01E-06	1.09E-06	1.48E-06
1.90E-05	1.82E-05	2.81E-05	1.24E-05	4.90E-05	2.07E-05	1.55E-05	1.63E-05	1.97E-05
3.35E-05	1.65E-05	1.95E-05	1.72E-05	3.08E-05	1.80E-05	2.14E-05	1.42E-05	2.50E-05
9.19E-06	6.17E-06	9.57E-06	7.89E-06	3.54E-05	1.03E-05	1.01E-05	1.12E-05	8.64E-06
3.29E-05	6.03E-05	3.68E-05	4.02E-05	2.61E-05	0.00012	3.12E-05	6.36E-05	2.60E-05
3.27E-05	8.12E-05	0.000216	7.20E-05	2.22E-06	0.000108	2.85E-05	7.96E-05	7.14E-05
6.92E-06	9.91E-06	8.61E-06	1.01E-05	9.71E-06	3.94E-06	1.77E-05	1.35E-05	7.35E-06
8.49E-09	1.73E-08	9.38E-09	0	1.72E-07	2.50E-08	0	1.94E-08	3.31E-07
4.37E-05	3.83E-05	7.79E-05	3.98E-05	0.000129	8.47E-05	5.36E-05	5.05E-05	6.45E-05
2.49E-05	4.83E-05	3.00E-05	5.35E-05	6.82E-05	3.41E-05	6.39E-05	4.99E-05	4.32E-05
5.92E-06	2.45E-05	1.17E-05	2.36E-05	6.05E-05	5.41E-06	4.34E-05	4.19E-05	3.85E-06

1.10E-05	6.81E-06	1.45E-05	8.40E-06	9.85E-06	7.56E-06	1.00E-05	4.86E-06	4.97E-06
1.65E-06	1.43E-06	1.08E-05	1.21E-06	1.02E-05	5.37E-06	1.76E-06	2.02E-06	1.09E-06
0	2.31E-08	3.05E-08	1.16E-07	1.41E-06	3.34E-08	4.74E-08	7.47E-08	6.25E-08
0	0	1.78E-08	0	0	0	2.01E-08	0	0
1.26E-07	1.28E-07	4.89E-07	4.70E-07	1.38E-07	1.19E-06	1.48E-07	3.46E-07	9.91E-07
5.90E-08	3.11E-07	4.36E-07	4.83E-07	7.25E-07	2.22E-07	6.77E-07	6.13E-07	9.86E-07
7.44E-08	7.59E-08	7.53E-08	8.94E-08	4.30E-07	3.28E-07	2.79E-07	7.11E-08	1.77E-07
0	0	0	0	2.03E-07	1.44E-07	2.17E-08	0	4.84E-08
8.80E-09	3.59E-08	2.92E-08	5.42E-08	1.27E-07	5.18E-08	1.21E-07	7.05E-08	1.32E-07
0	0	8.90E-09	0	0	0	0	0	0
3.58E-05	2.58E-05	6.65E-05	2.49E-05	1.27E-05	0.000117	5.48E-05	4.41E-05	1.88E-05
1.70E-05	1.29E-05	1.55E-05	1.08E-05	9.95E-06	9.85E-06	1.56E-05	1.54E-05	1.32E-05
9.55E-05	0.00047	0.000622	0.000521	2.00E-05	0.000965	0.000291	0.000522	0.000504
1.47E-05	1.43E-05	3.98E-05	2.08E-05	4.79E-05	1.96E-05	2.61E-05	4.01E-05	1.08E-05
2.91E-07	8.44E-06	4.91E-06	2.44E-06	1.73E-05	9.44E-07	0.000145	4.86E-06	6.87E-05
8.16E-05	5.99E-05	0.000133	5.94E-05	6.21E-06	6.25E-05	4.92E-05	6.26E-05	2.49E-05
1.81E-07	5.49E-07	2.06E-06	3.74E-07	9.22E-08	3.78E-07	7.74E-07	2.89E-06	9.94E-07
7.69E-06	2.43E-05	2.05E-06	1.14E-05	1.23E-05	2.10E-07	2.75E-05	2.12E-06	8.52E-06
0.000168	0.000167	0.000178	0.000137	1.82E-06	0.000205	0.000176	0.000221	0.000245
0.000219	0.000157	0.000174	0.00011	5.08E-06	0.00034	0.000173	0.000165	0.000308
1.44E-05	4.51E-05	3.20E-05	3.70E-05	1.21E-05	3.64E-05	2.94E-05	3.15E-05	9.27E-05
0	0	0	0	0	0	0	0	0
2.80E-05	5.36E-05	8.49E-05	0.00011	2.16E-06	0.000106	6.89E-05	0.000129	6.85E-05
6.19E-07	2.31E-06	8.35E-07	3.49E-06	3.89E-05	1.17E-06	5.70E-06	3.38E-06	2.14E-05
6.63E-05	3.51E-05	4.68E-05	2.57E-05	3.85E-05	3.59E-05	2.33E-05	3.42E-05	9.61E-05
1.54E-05	1.83E-05	7.48E-05	1.45E-05	9.94E-07	7.78E-06	1.56E-05	3.74E-05	3.79E-05
1.43E-08	2.90E-08	0	0	4.11E-08	0	0	4.89E-08	4.29E-08
9.76E-05	6.94E-05	7.99E-05	4.51E-05	0.000358	7.49E-05	0.000105	0.00015	6.20E-05
0.001538	0.001003	0.00097	0.001124	0.004183	0.00183	0.001703	0.001718	0.001497
0	0	0	0	2.26E-07	0	8.90E-09	8.16E-09	0
0	2.16E-08	0	0	9.18E-08	0	0	1.21E-08	3.17E-08
0.000406	0.000561	0.000986	0.000882	0.00397	0.001101	0.000592	0.001079	0.000701
2.48E-06	2.60E-07	2.68E-06	9.94E-08	1.12E-05	5.51E-06	7.56E-07	5.45E-06	4.65E-07
5.35E-06	5.67E-06	1.75E-05	5.98E-06	4.55E-05	2.12E-05	7.99E-06	1.15E-05	8.26E-06
0	0	0	0	0	3.58E-08	0	0	3.62E-08
0	0	0	0	0	0	0	0	0
1.53E-08	0	0	3.11E-08	0	0	0	0	0
8.79E-06	0	0	2.28E-08	9.61E-08	0	0	1.53E-07	0
0	0	0	0	0	0	0	0	2.26E-08
3.69E-05	2.66E-05	3.02E-05	4.39E-05	0.00015	2.14E-05	5.35E-05	3.98E-05	2.41E-05
5.73E-09	2.34E-08	7.71E-06	2.36E-08	8.29E-08	8.42E-09	3.46E-08	7.23E-08	0
0	2.84E-08	7.68E-09	0	1.00E-07	0	0	0	0
0.000116	6.47E-05	0.000143	6.95E-05	0.000866	0.000102	0.000103	0.000136	7.57E-05
9.53E-06	7.47E-06	1.72E-05	8.23E-06	2.88E-05	1.23E-05	1.35E-05	1.50E-05	1.25E-05
2.38E-05	1.98E-05	3.13E-05	2.19E-05	5.98E-05	1.79E-05	3.58E-05	3.09E-05	2.83E-05
1.62E-05	2.26E-05	3.00E-05	2.34E-05	0.000102	2.66E-05	2.52E-05	1.97E-05	3.02E-05
1.43E-05	2.59E-05	3.84E-05	3.56E-05	0.000142	4.01E-05	3.98E-05	5.21E-05	2.39E-05
1.40E-05	1.96E-05	3.26E-05	2.63E-05	0.000159	3.88E-05	3.52E-05	3.70E-05	2.56E-05
4.12E-06	2.26E-06	7.13E-06	2.29E-06	6.93E-06	4.47E-06	2.40E-06	2.22E-06	3.35E-06
6.89E-06	5.23E-06	4.41E-06	6.83E-06	4.15E-06	1.79E-06	1.75E-05	7.27E-06	7.31E-06
4.71E-06	9.24E-07	1.19E-05	1.56E-06	4.37E-06	3.80E-06	1.95E-06	1.72E-06	2.08E-06
7.25E-06	5.06E-06	1.49E-05	6.51E-06	1.27E-05	9.81E-06	1.06E-05	8.76E-06	6.39E-06
1.59E-06	2.48E-06	4.15E-06	3.86E-06	7.63E-06	2.86E-06	2.34E-06	4.18E-06	2.66E-06
0	0	0	0	0	0	0	0	0
0	0	0	0	0	0	0	0	0
2.84E-06	7.26E-06	1.07E-05	9.77E-06	8.22E-05	4.64E-06	8.92E-06	1.64E-05	5.06E-06
3.40E-06	9.78E-06	1.29E-05	1.22E-05	4.50E-05	8.17E-06	1.60E-05	8.06E-06	1.11E-05
2.93E-08	4.36E-07	6.90E-08	4.88E-07	2.25E-07	1.49E-07	7.99E-08	3.12E-07	5.50E-08

9.46E-07	2.10E-06	2.71E-06	2.70E-06	7.23E-06	1.90E-06	1.80E-06	3.39E-06	1.37E-06
5.69E-07	5.05E-07	1.48E-06	8.19E-07	1.93E-06	9.45E-07	5.08E-07	3.95E-07	2.58E-07
1.87E-06	1.67E-06	3.84E-06	2.39E-06	1.03E-05	2.04E-06	4.00E-06	1.87E-06	3.66E-06
0.000236	0.000253	0.00027	0.000217	4.96E-05	0.000207	0.00027	0.000235	0.000134
2.65E-05	2.17E-05	3.01E-05	1.59E-05	1.25E-06	2.56E-05	1.26E-05	3.04E-05	2.43E-05
2.11E-06	1.52E-06	2.33E-06	1.06E-06	9.50E-06	8.75E-06	2.19E-06	1.34E-06	3.41E-06
1.71E-05	7.48E-06	2.45E-05	8.79E-06	5.43E-06	6.14E-06	8.31E-06	1.10E-05	1.35E-05
2.18E-06	1.40E-06	1.58E-06	2.51E-06	8.76E-06	1.84E-06	2.76E-06	2.50E-06	2.64E-06
0	0	0	0	0	0	0	0	0
9.92E-09	0	5.49E-08	0	2.86E-08	1.46E-08	1.24E-08	7.95E-08	0
1.83E-06	2.00E-06	6.71E-06	2.10E-06	2.90E-06	5.33E-06	1.16E-06	2.55E-06	3.09E-06
1.25E-06	7.93E-06	1.03E-05	1.15E-05	0.00015	1.54E-05	9.30E-06	1.69E-05	5.80E-06
3.80E-08	4.65E-08	2.75E-07	6.59E-08	3.85E-06	5.58E-08	2.24E-08	6.09E-08	9.11E-08
1.69E-08	3.04E-08	2.84E-08	1.56E-07	9.63E-07	2.19E-08	5.92E-07	6.90E-07	5.93E-07
1.47E-05	1.41E-05	2.78E-05	1.05E-05	8.98E-05	5.41E-05	4.88E-05	1.05E-05	1.26E-05
4.26E-07	2.11E-06	2.64E-06	2.55E-06	3.05E-05	5.12E-06	2.58E-06	3.35E-06	1.81E-06
0	0	0	0	3.16E-08	0	0	0	0
2.88E-07	8.92E-07	5.44E-07	5.40E-06	4.04E-06	7.59E-07	3.11E-06	2.00E-06	1.42E-06
0	0	0	0	0	0	0	0	0
0	1.45E-08	7.87E-09	4.40E-08	0	5.23E-08	1.78E-08	0	3.20E-08
1.67E-08	3.41E-08	3.33E-07	3.44E-08	1.21E-06	4.92E-08	4.19E-08	3.83E-08	0
0	0	7.73E-08	0	4.89E-07	0	0	0	0
0	3.09E-08	1.40E-07	1.21E-08	1.08E-06	1.73E-08	2.51E-08	2.00E-08	1.09E-08
0	5.68E-08	1.81E-08	1.33E-08	4.87E-07	0	8.11E-09	6.69E-08	3.89E-08
3.72E-08	1.26E-07	3.75E-08	4.59E-07	4.11E-05	4.32E-08	8.92E-07	4.73E-07	5.37E-07
0	0	0	0	0	0	0	7.21E-09	0
0	0	0	0	0	0	0	0	0
0	0	0	0	1.30E-07	1.32E-08	1.13E-08	2.01E-07	0
1.19E-07	9.34E-08	2.21E-07	1.86E-07	1.98E-06	6.54E-08	1.04E-06	5.41E-07	1.58E-06
0	0	0	0	0	0	0	0	0
0	0	0	0	0	0	0	0	0
3.80E-07	1.38E-05	9.82E-08	1.85E-05	6.30E-06	6.30E-08	5.23E-06	1.02E-05	8.51E-06
0	0	0	0	0	0	0	0	0
3.19E-07	9.27E-07	1.83E-06	1.51E-06	4.63E-06	7.02E-07	3.01E-06	1.01E-06	1.38E-06
3.58E-06	3.61E-06	4.35E-06	5.40E-06	0.000251	3.37E-06	5.65E-06	8.64E-06	5.08E-06
4.54E-08	7.41E-08	4.02E-08	1.94E-07	2.93E-06	0	7.25E-07	8.33E-08	2.18E-07
1.96E-06	2.31E-05	2.56E-06	6.94E-06	1.89E-05	2.35E-06	1.61E-05	1.62E-05	7.38E-06
6.54E-08	2.04E-06	2.16E-06	1.21E-06	2.17E-06	3.98E-06	5.32E-06	2.14E-06	8.26E-07
4.57E-08	1.66E-07	1.46E-07	3.24E-07	2.68E-06	2.30E-07	4.34E-07	1.35E-06	1.07E-07
1.87E-06	1.05E-05	2.68E-06	1.27E-05	5.28E-06	1.83E-06	4.65E-06	1.92E-05	1.34E-06
2.56E-08	1.49E-08	4.03E-08	6.80E-08	2.01E-07	0	3.65E-07	5.03E-08	6.57E-08
0	7.10E-08	2.31E-08	3.58E-07	5.83E-07	5.11E-08	2.79E-07	5.98E-07	1.46E-07
0	3.81E-07	0	3.20E-08	2.39E-06	5.68E-09	1.42E-07	5.29E-08	0
1.81E-08	1.57E-06	5.19E-08	4.92E-07	1.66E-07	1.82E-05	2.82E-07	7.27E-07	2.41E-06
1.62E-08	3.31E-08	1.55E-08	0	9.37E-09	3.32E-08	4.04E-09	3.71E-09	3.39E-08
0	0	0	0	0	0	0	0	0
1.50E-07	4.05E-06	7.57E-07	5.48E-06	1.35E-05	5.97E-07	2.38E-06	1.11E-05	5.85E-07
0	0	0	0	0	2.07E-08	0	0	0
6.58E-09	9.41E-08	1.45E-08	1.10E-07	1.90E-07	4.82E-09	9.04E-08	1.09E-07	2.22E-07
2.22E-06	3.56E-06	1.37E-06	3.63E-06	4.89E-06	1.69E-06	8.69E-06	1.03E-05	2.45E-06
1.17E-07	2.92E-07	1.59E-07	2.71E-07	1.38E-06	2.23E-07	3.22E-07	1.54E-06	5.15E-07
0	3.97E-08	1.83E-08	8.24E-08	2.29E-07	2.03E-08	1.18E-07	8.89E-08	4.15E-08
2.20E-07	3.24E-07	4.12E-07	4.11E-07	2.47E-06	4.71E-07	5.58E-07	5.93E-07	3.87E-07
3.60E-08	1.83E-08	4.97E-08	4.16E-08	2.37E-07	6.62E-08	1.81E-07	4.41E-08	8.10E-08
4.69E-07	4.08E-07	2.13E-06	5.10E-07	1.41E-05	1.07E-06	6.31E-07	1.29E-06	5.79E-07
9.10E-08	2.43E-07	2.93E-07	4.16E-07	1.05E-06	2.61E-07	1.10E-06	1.21E-06	2.18E-07
1.07E-06	2.80E-05	9.15E-07	1.31E-05	2.34E-05	6.67E-06	1.05E-05	2.59E-05	3.50E-06
1.14E-07	3.82E-06	8.39E-07	3.76E-06	1.13E-06	3.83E-07	4.46E-07	5.41E-06	4.50E-07

2.20E-05	2.16E-05	2.24E-05	1.63E-05	3.46E-06	1.70E-05	1.43E-05	1.89E-05	2.42E-05
4.59E-07	1.55E-06	2.37E-06	7.24E-07	1.77E-05	3.85E-06	1.30E-06	1.35E-06	3.81E-07
3.45E-07	1.37E-06	4.64E-07	2.96E-06	4.92E-05	4.33E-07	2.49E-06	1.42E-06	1.24E-06
5.40E-07	1.36E-06	1.20E-06	6.20E-06	8.42E-06	9.48E-07	3.02E-06	4.35E-06	2.34E-06
2.65E-05	2.65E-05	5.48E-05	4.27E-05	0.000122	5.05E-05	2.79E-05	3.46E-05	1.71E-05
1.69E-06	2.26E-06	4.17E-06	2.16E-06	8.51E-06	2.18E-06	2.51E-06	2.38E-06	2.54E-06
1.41E-08	7.64E-08	3.52E-08	2.87E-08	0	2.08E-08	4.24E-07	1.03E-07	1.60E-06
3.29E-07	1.83E-06	3.64E-06	2.30E-06	8.63E-06	2.09E-06	1.35E-06	2.69E-06	1.75E-06
1.49E-07	4.72E-07	1.16E-06	4.43E-07	6.06E-07	1.28E-06	8.48E-07	4.48E-07	1.59E-06
1.34E-06	1.03E-06	8.30E-07	1.01E-06	4.08E-06	4.74E-07	6.04E-07	1.76E-06	1.35E-06
1.21E-07	3.52E-07	4.41E-07	4.85E-07	6.78E-06	6.03E-07	4.63E-07	5.53E-07	1.63E-06
3.32E-06	2.09E-06	6.88E-06	2.31E-06	5.71E-06	4.08E-06	5.15E-06	3.74E-06	4.24E-06
1.18E-05	8.06E-06	1.83E-05	7.32E-06	8.75E-05	1.38E-05	1.33E-05	7.19E-06	6.01E-06
8.49E-06	7.50E-06	1.88E-05	1.10E-05	7.26E-05	1.84E-05	1.53E-05	8.48E-06	1.13E-05
7.99E-06	4.06E-06	9.12E-06	4.16E-06	1.23E-05	1.83E-05	9.33E-06	3.56E-06	5.32E-06
8.76E-08	2.03E-06	1.38E-07	2.45E-06	6.62E-06	1.09E-05	2.94E-06	7.86E-07	1.36E-06
4.17E-07	1.01E-06	5.44E-07	8.25E-07	5.30E-08	8.92E-06	1.01E-06	7.36E-07	4.48E-08
0	0	6.41E-09	0	1.34E-08	6.78E-09	0	1.46E-08	3.46E-08
8.84E-07	1.24E-06	2.34E-06	1.70E-06	1.97E-05	1.73E-06	3.69E-06	2.96E-06	2.54E-06
7.10E-07	6.54E-07	1.80E-06	5.15E-07	4.05E-06	1.46E-06	2.77E-06	1.54E-06	2.31E-06
4.10E-08	9.79E-07	1.81E-07	9.33E-07	1.36E-06	4.94E-07	7.54E-07	1.82E-06	2.44E-07
1.59E-06	3.75E-06	1.01E-05	3.00E-06	3.62E-05	6.03E-06	5.95E-06	1.88E-06	5.32E-06
2.49E-07	6.94E-07	9.26E-07	2.17E-06	9.45E-06	4.29E-07	1.36E-06	1.84E-06	1.09E-06
0	2.15E-08	3.30E-06	1.56E-07	1.52E-08	0	1.32E-08	1.27E-07	0
3.43E-06	8.18E-06	7.08E-06	8.76E-06	1.85E-05	5.74E-06	6.79E-06	4.37E-06	6.58E-06
0	3.31E-07	6.20E-08	3.86E-08	5.41E-08	0	4.87E-07	1.57E-07	1.50E-07
0	2.71E-08	1.41E-08	2.74E-08	0	0	0	0	8.02E-08
3.94E-05	3.55E-05	7.71E-05	3.96E-05	0.000176	2.58E-05	5.90E-05	5.12E-05	4.08E-05
1.97E-08	8.13E-08	6.21E-08	1.35E-08	6.72E-07	2.36E-08	3.28E-08	3.81E-08	1.25E-06
3.20E-06	8.20E-06	2.63E-05	6.68E-06	2.40E-05	1.62E-06	4.21E-06	8.42E-06	5.46E-06
0.000324	0.00093	0.000201	0.000807	8.27E-07	0.002516	0.000573	0.001563	0.000622
0.000972	0.00243	0.003261	0.001969	1.12E-05	0.002884	0.0013	0.002675	0.003538
0.000184	0.000786	0.000618	0.000901	9.61E-06	0.002109	0.00103	0.001284	0.001693
0.000286	0.001296	0.000723	0.000712	4.49E-07	0.000916	0.000836	0.002056	0.000561
9.82E-05	0.000107	0.000185	9.98E-05	9.29E-05	0.000154	0.000109	0.000141	0.00014
0.000112	0.00019	0.000322	0.00015	6.45E-06	0.000206	0.000115	0.000299	5.65E-05
4.74E-08	7.71E-08	5.24E-08	1.94E-08	0	2.51E-07	2.13E-07	1.52E-07	8.53E-08
2.11E-05	1.81E-05	4.48E-05	2.46E-05	1.64E-06	1.66E-05	1.79E-05	1.94E-05	1.93E-05
0.000604	0.000411	0.000276	0.000295	5.65E-05	0.000359	0.000213	0.000467	0.000804
1.15E-06	6.45E-08	2.73E-06	0	0	0	3.24E-07	0	0
0	0	4.66E-09	0	0	0	5.28E-09	4.84E-09	9.87E-07
9.57E-05	5.07E-05	7.34E-05	5.65E-05	0.000107	2.98E-05	5.18E-05	6.60E-05	5.43E-05
2.27E-05	4.06E-05	5.57E-05	2.92E-05	5.81E-05	0.000112	4.07E-05	4.86E-05	3.56E-05
3.68E-05	2.47E-05	3.35E-05	2.71E-05	9.89E-06	8.67E-06	1.90E-05	2.99E-05	1.90E-05
1.70E-05	5.37E-06	8.21E-06	8.38E-06	2.10E-05	1.56E-05	9.62E-06	0.000132	5.25E-06
9.65E-08	7.32E-07	5.96E-07	8.85E-07	6.45E-06	9.98E-07	2.70E-06	9.34E-07	3.11E-07
2.02E-06	6.14E-06	1.57E-05	8.63E-06	5.01E-05	1.82E-05	6.04E-06	1.22E-05	5.53E-06
6.67E-07	1.31E-06	4.75E-06	2.26E-06	1.83E-05	2.70E-06	2.46E-06	2.62E-06	1.59E-06
2.10E-06	2.62E-06	4.05E-06	2.45E-06	1.21E-05	2.46E-06	3.28E-06	5.06E-06	2.11E-06
2.29E-08	3.11E-07	8.70E-08	5.91E-07	5.13E-05	2.00E-08	1.53E-07	7.73E-07	4.32E-08
1.15E-06	2.04E-06	2.34E-06	1.80E-06	8.38E-06	4.66E-06	2.46E-06	2.60E-06	1.92E-06
6.56E-08	3.34E-07	8.47E-08	3.36E-07	1.60E-05	8.06E-08	8.22E-08	6.26E-07	8.21E-08
2.03E-07	1.11E-06	8.10E-07	1.26E-05	8.21E-06	3.31E-07	1.35E-06	2.70E-06	1.21E-06
6.01E-06	6.17E-06	1.23E-05	8.48E-06	3.59E-05	1.32E-05	1.27E-05	1.05E-05	6.52E-06
5.04E-07	1.53E-06	1.92E-06	1.86E-06	3.49E-06	1.05E-06	2.09E-06	2.32E-06	3.33E-06
2.42E-07	8.52E-07	9.18E-07	7.68E-07	2.47E-07	1.69E-06	2.62E-07	1.07E-06	6.96E-07
1.16E-05	8.02E-06	1.18E-05	1.06E-05	4.92E-05	1.91E-05	2.67E-05	1.55E-05	1.15E-05
2.50E-07	1.60E-05	5.69E-06	1.62E-05	1.33E-07	2.29E-05	1.16E-06	1.18E-05	2.49E-06

4.42E-06	4.61E-06	5.75E-06	4.48E-06	1.04E-07	5.07E-06	2.88E-06	8.26E-06	2.82E-06
0	0	8.27E-09	3.08E-08	4.32E-08	5.50E-08	3.74E-08	9.43E-08	7.85E-08
5.03E-08	1.13E-06	1.28E-07	8.09E-07	1.03E-05	0	2.23E-06	1.40E-06	4.90E-07
6.76E-07	1.33E-06	5.66E-07	2.09E-06	4.36E-07	7.89E-07	8.22E-07	5.74E-06	3.71E-07
2.31E-07	6.20E-07	6.62E-07	1.72E-07	1.18E-05	1.08E-07	4.08E-07	8.29E-07	7.72E-07
4.16E-08	5.24E-07	2.30E-08	6.00E-07	2.01E-08	7.14E-08	1.04E-07	1.31E-06	1.28E-06
3.86E-06	2.17E-06	2.02E-05	2.91E-06	6.38E-07	4.50E-06	3.60E-06	9.81E-06	1.93E-06
0	0	8.34E-09	0	0	0	1.89E-08	0	0
1.84E-05	1.64E-05	2.19E-05	2.11E-05	6.21E-05	1.65E-05	2.17E-05	1.21E-05	1.53E-05
3.43E-06	5.89E-07	6.78E-06	1.42E-06	4.39E-06	2.05E-07	5.61E-07	2.83E-06	3.56E-07
0.0001	5.67E-05	0.000104	5.67E-05	0.000141	9.83E-05	9.47E-05	9.85E-05	7.02E-05
1.73E-05	1.71E-05	3.89E-05	1.89E-05	3.95E-05	3.34E-05	2.49E-05	2.37E-05	1.80E-05
6.59E-06	5.93E-06	1.36E-05	9.45E-06	3.35E-05	5.47E-06	9.73E-06	1.29E-05	8.90E-06
2.09E-06	1.37E-05	7.07E-06	6.89E-06	1.42E-05	4.77E-05	5.02E-06	1.18E-05	1.50E-05
4.94E-06	4.37E-05	1.14E-05	3.59E-05	0.000207	3.22E-06	5.06E-05	0.000114	2.49E-05
1.22E-06	1.85E-06	2.51E-06	2.04E-06	1.25E-05	2.85E-06	1.82E-06	3.66E-06	1.66E-06
5.18E-06	5.18E-06	2.27E-05	5.49E-06	3.34E-05	8.13E-06	8.36E-06	6.54E-06	8.85E-06
0.000134	0.000151	0.000135	0.000145	0.000497	0.000118	0.000145	0.000135	0.000171
1.52E-07	8.99E-07	4.81E-07	7.73E-07	1.36E-05	4.11E-07	1.00E-06	1.90E-06	5.79E-07
1.18E-06	1.32E-06	1.56E-06	1.18E-06	5.21E-06	2.48E-06	2.36E-06	3.57E-06	1.54E-06
2.67E-07	7.20E-07	1.36E-06	8.04E-07	4.43E-06	4.88E-07	1.03E-06	1.75E-06	7.73E-07
3.11E-07	3.32E-08	7.27E-08	2.67E-07	9.41E-08	1.02E-06	1.03E-07	3.36E-07	1.48E-07
1.78E-06	7.20E-06	6.74E-06	7.99E-06	3.56E-05	1.18E-05	5.54E-06	9.98E-06	5.81E-06
0	0	1.35E-08	0	2.68E-06	2.24E-09	0	1.06E-08	2.29E-09
2.44E-07	7.21E-07	5.66E-08	1.45E-06	1.90E-06	2.74E-08	9.53E-07	2.00E-06	3.73E-06
2.08E-06	1.01E-05	9.44E-06	9.34E-06	3.35E-05	1.78E-05	7.45E-06	2.42E-05	6.40E-06
1.38E-06	1.03E-06	4.32E-06	1.81E-06	4.90E-06	4.38E-06	3.25E-06	1.81E-06	2.93E-06
1.07E-05	2.14E-05	3.88E-05	2.13E-05	7.91E-05	3.91E-05	2.21E-05	2.77E-05	2.29E-05
4.68E-05	0.000277	9.92E-05	0.000184	5.92E-08	0.000152	0.000118	0.000418	0.000101
0.000128	8.09E-05	0.000139	5.82E-05	4.53E-05	7.49E-05	7.68E-05	6.98E-05	9.75E-05
2.85E-06	2.37E-06	1.04E-06	1.92E-06	2.04E-05	6.48E-07	2.53E-06	2.39E-06	8.35E-07
4.26E-06	7.33E-06	1.45E-05	8.87E-06	5.45E-05	1.20E-05	9.09E-06	1.33E-05	1.24E-05
2.27E-05	2.02E-05	2.43E-06	1.51E-05	3.46E-05	3.45E-06	2.73E-05	5.98E-05	1.70E-05
7.26E-06	1.14E-05	1.33E-05	1.21E-05	1.32E-05	9.94E-06	1.20E-05	1.62E-05	9.29E-06
5.96E-07	1.47E-06	1.65E-06	1.70E-06	1.94E-05	1.95E-06	8.39E-07	1.11E-06	1.62E-06
0	8.92E-08	6.90E-08	3.86E-08	1.44E-07	1.65E-07	7.03E-08	1.00E-07	1.22E-07
8.21E-07	4.57E-07	4.09E-07	5.63E-07	1.90E-06	3.34E-07	8.67E-07	5.45E-07	6.53E-07
3.94E-05	2.15E-05	4.96E-05	1.97E-05	7.22E-05	2.07E-05	4.90E-05	1.99E-05	1.73E-05
6.29E-07	1.53E-06	3.71E-06	1.76E-06	4.55E-06	2.43E-06	3.05E-06	1.20E-06	2.29E-06
5.80E-08	1.36E-07	3.27E-07	1.36E-07	3.83E-07	4.04E-07	3.11E-07	2.09E-07	1.87E-07
2.87E-07	5.27E-07	1.63E-06	8.57E-07	1.35E-06	1.22E-06	1.54E-06	4.94E-07	1.23E-06
4.99E-08	3.39E-08	1.10E-07	5.13E-08	9.61E-08	6.12E-08	2.08E-07	5.72E-08	3.62E-07
7.49E-08	8.22E-08	1.91E-07	1.37E-07	1.00E-06	2.08E-07	1.46E-07	1.24E-07	7.81E-08
0	0	0	0	0	0	0	0	0
4.56E-08	3.62E-07	4.82E-07	4.20E-07	1.53E-06	2.29E-07	2.07E-07	3.33E-07	1.08E-06
6.66E-08	1.13E-08	3.92E-07	8.00E-08	9.30E-07	7.33E-08	2.01E-07	6.36E-08	8.31E-08
1.64E-06	2.59E-07	0	4.68E-07	0	0	1.84E-07	3.83E-08	0
5.72E-06	5.66E-06	1.23E-05	5.91E-06	3.94E-05	8.71E-06	3.86E-06	5.00E-06	9.21E-06
1.77E-07	3.36E-07	2.68E-06	6.74E-07	3.23E-06	6.13E-07	2.02E-06	2.79E-07	1.82E-06
1.79E-06	3.85E-06	4.77E-06	3.86E-06	2.72E-05	8.77E-06	4.72E-06	5.87E-06	3.34E-06
3.49E-05	1.84E-05	9.58E-05	1.81E-05	6.03E-05	5.99E-06	1.50E-05	1.68E-05	1.30E-05
2.91E-06	3.90E-06	7.77E-06	4.99E-06	2.06E-05	9.55E-06	5.03E-06	6.72E-06	3.68E-06
2.72E-06	4.18E-06	1.00E-05	4.04E-06	8.98E-06	7.82E-06	8.33E-06	6.50E-06	6.69E-06
4.33E-06	9.67E-06	1.04E-05	1.14E-05	7.40E-06	5.72E-06	3.63E-06	1.35E-05	8.82E-06
1.32E-05	5.78E-05	4.15E-05	3.08E-05	4.00E-07	3.37E-05	3.93E-05	5.78E-05	3.05E-05
3.16E-05	3.32E-05	5.91E-05	3.03E-05	0.000144	3.41E-05	3.64E-05	3.27E-05	3.77E-05
8.43E-05	0.000184	5.52E-05	0.000124	0.000287	0.000124	0.00019	0.000142	0.000167
2.11E-05	3.09E-05	2.71E-05	2.27E-05	6.69E-05	1.87E-05	3.26E-05	1.42E-05	1.57E-05

1.43E-07	1.77E-07	2.05E-07	3.06E-07	1.35E-06	1.63E-07	2.13E-06	4.82E-07	4.01E-07
9.22E-07	2.38E-06	1.50E-06	3.96E-06	9.43E-06	2.78E-06	4.56E-06	3.73E-06	8.22E-07
6.16E-05	1.08E-05	6.51E-06	1.16E-05	3.75E-05	1.78E-05	1.38E-05	9.27E-06	3.36E-05
2.27E-06	1.70E-06	1.27E-06	4.89E-06	2.91E-06	2.69E-05	6.33E-06	9.50E-06	3.59E-06
3.96E-06	1.10E-06	3.19E-06	1.16E-06	2.61E-06	3.80E-06	1.90E-06	2.64E-06	3.85E-06
2.11E-06	4.28E-06	1.04E-05	5.71E-06	1.49E-05	4.54E-06	5.96E-06	7.14E-06	3.07E-06
0	7.11E-07	0	0	2.02E-07	0	9.16E-07	6.85E-06	0
0	0	0	0	1.90E-07	0	4.20E-08	2.64E-07	0
6.07E-06	2.51E-05	1.90E-05	1.89E-05	0.000214	1.53E-05	1.73E-05	1.23E-05	7.17E-06
0	0	0	6.48E-08	3.69E-07	4.85E-08	0	7.36E-08	0
0.000317	0.001106	0.000532	0.001179	0.000527	0.000988	0.000869	0.001381	0.001318
3.88E-07	1.11E-06	1.68E-06	1.88E-06	5.71E-06	5.63E-06	2.58E-06	2.17E-06	2.14E-06
4.30E-06	2.02E-05	4.41E-06	4.26E-05	2.36E-07	3.81E-06	2.33E-05	4.82E-05	1.15E-05
0	0	0	0	0	0	0	0	0
1.25E-05	8.54E-06	1.57E-05	1.33E-05	1.30E-05	1.83E-05	2.88E-05	1.45E-05	1.76E-05
0.001097	0.001485	0.001507	0.001552	1.74E-06	0.002407	0.000916	0.000981	0.000858
2.36E-05	2.18E-05	2.72E-05	7.81E-05	2.85E-07	1.36E-05	4.45E-05	4.97E-05	5.67E-05
1.67E-05	4.42E-05	3.25E-05	2.05E-05	5.91E-08	0.000335	5.28E-05	5.99E-05	5.71E-05
9.69E-06	1.89E-05	1.22E-05	2.33E-05	9.98E-06	3.07E-05	1.92E-05	2.30E-05	2.79E-05
4.19E-06	2.89E-06	8.42E-06	3.50E-06	1.31E-05	2.89E-06	2.74E-06	6.88E-06	3.34E-06
5.44E-05	8.33E-05	0.00017	0.000101	0.000194	0.000229	9.76E-05	8.81E-05	0.000118
1.81E-05	2.79E-05	4.70E-05	3.04E-05	9.82E-05	4.10E-05	3.90E-05	4.56E-05	3.33E-05
1.38E-06	2.48E-06	5.61E-06	3.08E-06	8.23E-06	3.69E-06	2.90E-06	2.89E-06	3.18E-06
6.94E-07	2.81E-06	2.53E-06	2.69E-06	1.22E-05	4.33E-06	2.12E-06	2.74E-06	1.21E-06
7.50E-07	6.09E-07	1.91E-06	8.49E-07	1.71E-06	2.22E-06	1.61E-06	6.35E-07	8.12E-07
1.25E-05	3.34E-05	2.27E-05	2.77E-05	7.34E-05	3.35E-05	1.89E-05	4.21E-05	2.45E-05
5.15E-07	1.14E-07	2.95E-08	2.18E-07	6.74E-07	0	2.49E-07	2.54E-07	3.70E-08
8.64E-06	1.06E-05	1.15E-05	2.55E-05	3.17E-05	2.19E-06	1.11E-05	2.77E-05	6.07E-06
6.44E-06	3.66E-08	2.47E-05	2.24E-07	9.58E-07	7.39E-08	1.13E-07	8.67E-08	4.33E-08
5.71E-05	2.87E-05	9.00E-05	3.94E-05	0.00013	6.87E-05	5.49E-05	4.35E-05	3.82E-05
1.71E-06	2.72E-06	7.82E-06	3.12E-06	7.20E-06	5.32E-06	4.12E-06	2.19E-06	5.98E-06
0.000774	0.004004	0.003983	0.002731	6.90E-06	0.003006	0.00138	0.003185	0.000696
5.92E-08	1.17E-07	0	1.15E-07	0	0	1.51E-07	0	7.64E-05
0	1.77E-08	0	0	1.11E-07	0	0	0	0
2.02E-05	8.62E-06	1.62E-05	9.13E-06	2.15E-05	6.38E-06	9.70E-06	1.20E-05	8.12E-06
1.86E-05	1.64E-05	1.29E-05	5.29E-05	7.53E-05	1.56E-05	5.36E-05	4.45E-05	3.53E-05
8.10E-07	1.70E-06	3.18E-06	9.55E-07	2.06E-05	2.78E-06	2.64E-06	1.42E-06	3.12E-06
6.55E-05	6.21E-05	0.000109	5.71E-05	4.10E-05	6.17E-05	6.72E-05	7.41E-05	8.87E-05
2.56E-05	5.69E-06	3.51E-05	6.94E-06	9.92E-06	5.67E-06	1.75E-05	1.04E-05	7.19E-06
9.18E-08	1.33E-07	7.56E-08	2.41E-07	1.13E-07	4.35E-08	5.83E-07	3.90E-07	1.75E-07
0.000154	9.37E-05	0.00011	0.000107	4.55E-05	0.000159	0.000101	0.00014	9.08E-05
1.65E-06	7.34E-06	6.09E-06	6.55E-06	1.90E-06	3.60E-06	5.52E-06	6.62E-06	7.09E-06
8.42E-08	8.58E-08	3.91E-07	1.03E-07	1.02E-06	6.20E-08	2.32E-07	2.12E-07	5.05E-08
0	2.91E-08	4.77E-08	9.95E-07	2.48E-07	6.35E-08	1.26E-07	2.29E-07	4.31E-08
1.28E-05	7.98E-06	2.76E-05	9.88E-06	3.39E-05	2.16E-05	1.55E-05	1.73E-05	9.85E-06
3.23E-05	2.03E-05	7.19E-05	1.57E-05	4.95E-05	4.69E-05	2.17E-05	2.52E-05	1.56E-05
5.27E-05	2.79E-05	6.53E-05	3.08E-05	0.000102	4.53E-05	3.61E-05	3.58E-05	4.83E-05
0	0	0	0	0	0	0	0	0
0	0	0	1.84E-08	2.59E-08	3.62E-08	1.54E-08	0	0
0	2.01E-07	0	2.00E-07	4.31E-06	0	6.79E-08	6.99E-08	4.75E-08
0.000136	1.77E-05	4.13E-05	1.39E-05	1.83E-05	4.30E-05	4.27E-05	1.51E-05	1.79E-05
8.08E-05	6.03E-05	7.90E-05	6.43E-05	0.000133	0.000118	0.000112	7.90E-05	8.47E-05
5.95E-05	7.63E-05	0.000116	6.91E-05	6.13E-05	0.000184	5.69E-05	0.00011	7.01E-05
1.16E-05	2.89E-05	1.77E-05	2.20E-05	5.54E-06	2.52E-05	2.90E-05	3.09E-05	2.89E-05
0	7.43E-08	0	2.50E-08	2.81E-07	0	1.52E-08	4.87E-08	1.82E-08
6.22E-07	9.48E-08	9.75E-07	3.47E-07	3.97E-06	9.56E-07	4.71E-07	2.55E-07	3.26E-06
9.80E-06	2.51E-05	5.25E-05	2.05E-05	4.10E-05	6.38E-05	2.11E-05	3.24E-05	1.54E-05
2.38E-07	1.39E-06	3.05E-06	3.47E-06	1.92E-05	4.33E-06	2.15E-06	3.51E-06	1.90E-06

1.16E-06	2.38E-06	5.07E-06	3.60E-06	9.06E-06	2.98E-06	2.17E-06	6.32E-06	1.44E-06
1.28E-06	3.27E-06	7.06E-07	3.79E-06	1.27E-06	1.96E-05	2.20E-06	1.37E-05	3.14E-06
3.03E-06	1.03E-05	1.10E-05	1.44E-05	3.93E-05	2.53E-05	6.90E-06	1.15E-05	6.27E-06
0	0	0	0	0	0	0	0	0
1.37E-07	3.59E-06	3.18E-06	4.03E-06	9.78E-06	2.98E-06	1.24E-06	5.27E-06	3.46E-06
2.16E-07	3.74E-07	1.24E-06	6.47E-07	8.90E-07	2.12E-07	2.54E-07	3.75E-07	4.42E-07
0	0	0	0	0	0	0	0	0
3.24E-08	7.21E-08	2.79E-07	1.82E-07	1.23E-06	9.05E-07	1.51E-07	1.69E-07	1.41E-07
3.07E-07	6.62E-07	9.79E-07	6.11E-07	8.55E-07	6.54E-07	2.86E-07	6.51E-07	2.77E-07
7.78E-06	6.90E-06	1.97E-05	1.02E-05	0.000114	8.75E-06	4.53E-06	1.31E-05	4.15E-06
2.12E-06	3.06E-06	7.46E-06	4.33E-06	1.53E-05	1.16E-05	5.89E-06	5.91E-06	4.04E-06
1.43E-05	7.22E-06	2.21E-05	7.35E-06	3.36E-05	2.31E-05	1.70E-05	1.14E-05	1.81E-05
4.65E-06	8.85E-06	1.96E-05	1.35E-05	5.02E-05	3.50E-05	1.05E-05	1.60E-05	9.77E-06
6.67E-07	3.62E-07	3.56E-06	5.15E-07	8.67E-07	1.25E-06	6.27E-07	5.97E-07	3.86E-07
5.49E-06	1.22E-05	1.99E-05	1.46E-05	4.12E-05	1.86E-05	1.90E-05	2.30E-05	1.31E-05
1.84E-05	1.45E-05	3.51E-05	1.95E-05	2.63E-05	2.12E-05	1.69E-05	2.59E-05	1.53E-05
1.05E-06	2.85E-06	1.20E-06	4.52E-06	7.86E-06	3.03E-06	3.69E-06	2.77E-06	2.73E-06
0	2.30E-08	0	0	0	0	0	0	0
0.00017	0.000201	0.000305	0.000164	0.000165	0.000204	0.000266	0.000194	0.000309
1.39E-05	8.69E-06	8.74E-06	1.72E-05	3.83E-05	3.45E-06	2.57E-05	1.61E-05	6.65E-05
4.13E-05	3.89E-05	3.99E-05	4.39E-05	0.000251	2.44E-06	0.00195	1.69E-05	7.20E-06
5.37E-07	4.93E-08	2.73E-07	4.93E-07	2.40E-06	3.64E-08	2.34E-05	0	0
0.000758	0.000267	0.000912	0.000272	0.000149	0.00068	0.001944	0.000256	0.000119
0.000965	5.74E-05	0.000439	0.000162	1.15E-05	0.000645	0.00054	0.000241	2.29E-05
0.000337	6.28E-05	0.000295	0.000101	0.000264	0.000363	0.000726	0.000134	0.000133
0.000615	0.000286	0.000536	0.000229	1.06E-06	0.000255	0.000342	0.000294	0.000479
4.78E-08	2.34E-07	5.40E-07	2.95E-07	0	3.44E-06	2.64E-07	2.30E-07	8.61E-08
9.86E-05	8.48E-05	0.000122	2.28E-05	1.32E-07	5.02E-05	0.000144	7.18E-05	3.63E-05
0	2.02E-07	3.07E-07	3.37E-08	1.90E-07	1.93E-08	2.12E-07	2.33E-07	6.24E-08
5.61E-05	4.20E-05	6.48E-05	3.78E-05	6.88E-05	7.14E-05	3.01E-05	4.32E-05	3.85E-05
0.000145	0.000277	4.91E-05	0.000181	7.10E-07	0.000321	6.01E-05	0.000265	3.87E-05
3.14E-06	6.79E-06	2.32E-06	6.33E-06	3.07E-07	1.61E-05	3.39E-06	6.81E-06	3.53E-06
2.51E-05	5.84E-05	6.03E-05	6.93E-05	0.000122	5.63E-05	6.88E-05	5.76E-05	4.86E-05
3.60E-05	2.39E-05	5.65E-05	2.46E-05	6.33E-05	3.15E-05	4.12E-05	2.26E-05	2.94E-05
1.36E-05	2.76E-05	3.45E-05	2.93E-05	0.000114	3.50E-05	2.61E-05	2.01E-05	3.79E-05
7.50E-07	3.09E-06	2.27E-06	3.50E-06	4.81E-05	1.79E-06	2.54E-06	5.49E-06	5.87E-06
1.03E-05	1.03E-05	1.43E-05	7.55E-06	1.09E-05	7.17E-06	1.85E-05	1.11E-05	7.17E-06
4.96E-05	0.000103	0.000197	5.40E-05	6.55E-05	0.000129	6.36E-05	0.000103	0.00018
0.000306	0.000104	0.001368	0.000188	3.86E-05	0.000475	0.000127	0.000442	4.12E-05
1.07E-08	2.19E-08	0	1.04E-07	6.51E-07	1.49E-08	5.38E-08	1.60E-07	2.13E-05
0.003406	0.084896	0.012033	0.060276	0.000321	0.029467	0.040536	0.071613	0.108527
2.75E-05	1.68E-05	7.28E-05	2.32E-05	0.000124	3.55E-05	3.01E-05	2.49E-05	1.48E-05
6.16E-06	1.08E-05	1.12E-05	1.19E-05	3.68E-05	4.83E-06	8.77E-06	7.64E-06	1.05E-05
7.26E-06	1.07E-05	2.09E-05	1.41E-05	3.76E-05	6.92E-06	2.48E-05	1.96E-05	1.74E-05
0.000792	0.000421	0.000589	0.000553	9.84E-05	0.002545	0.001387	0.000656	0.000278
5.65E-08	3.94E-07	1.66E-07	5.71E-07	1.81E-06	1.75E-07	4.24E-07	3.49E-07	8.34E-07
2.11E-07	1.31E-06	3.42E-07	1.39E-06	8.53E-06	2.37E-07	1.92E-06	2.24E-06	1.45E-06
3.76E-05	0.000143	0.000282	8.26E-05	0.000141	0.000233	0.000113	9.94E-05	0.000248
0.000421	9.62E-05	0.000635	0.000231	3.03E-07	0.001329	0.000394	0.000456	0.000138
7.64E-09	9.28E-08	8.33E-08	7.87E-08	1.08E-06	4.83E-08	1.87E-07	2.04E-07	1.94E-07
0.001498	0.000816	0.001645	0.000843	7.36E-05	0.000627	0.000491	0.001144	0.000722
0.001943	5.71E-07	0.000218	1.94E-05	3.62E-07	8.65E-08	9.18E-05	3.04E-06	1.04E-07
0.000216	9.41E-05	0.000328	9.16E-05	0.000128	8.65E-05	0.000116	0.000132	6.64E-05
8.27E-07	3.41E-06	3.27E-06	3.35E-06	1.01E-05	1.61E-06	4.62E-06	3.97E-06	2.59E-06
0	0	0	0	1.81E-06	1.18E-08	2.15E-08	5.53E-08	3.62E-08
0.000162	0.000211	0.000421	0.000238	9.80E-06	9.74E-05	0.000282	0.000177	0.000298
1.50E-05	4.81E-05	3.26E-05	3.05E-05	1.86E-05	5.87E-05	2.12E-05	4.26E-05	3.57E-05
2.78E-05	0.000103	0.000159	0.00017	1.11E-05	0.000126	3.80E-05	0.000139	9.13E-05

5.03E-05	6.49E-05	0.000111	5.81E-05	6.46E-06	6.54E-05	3.06E-05	8.82E-05	6.81E-05
2.74E-05	9.59E-05	7.74E-05	0.000114	3.42E-07	8.62E-05	8.50E-05	0.000118	7.62E-05
4.70E-05	8.75E-05	0.000138	8.64E-05	6.06E-05	0.000161	6.66E-05	9.92E-05	0.000109
0.00015	9.68E-05	9.12E-05	0.000122	0.001565	0.000117	0.000329	0.00022	0.000188
0.000102	0.00387	0.000262	0.00352	1.05E-05	0.025362	0.002131	0.00309	0.006408
0.000115	3.88E-05	1.26E-05	2.42E-05	1.50E-05	0.00013	3.63E-05	5.29E-05	9.71E-05
4.97E-06	5.60E-06	7.82E-06	6.62E-06	2.34E-05	8.79E-06	1.06E-05	6.26E-06	5.58E-06
5.05E-07	6.95E-07	9.55E-07	8.97E-07	3.59E-06	2.36E-06	5.37E-07	1.03E-06	3.97E-07
3.83E-07	6.74E-07	1.18E-06	6.05E-07	1.32E-06	1.83E-06	3.59E-07	8.57E-07	3.50E-07
1.55E-07	2.53E-07	1.38E-07	1.91E-07	6.87E-07	3.36E-07	2.46E-07	1.78E-07	2.02E-07
4.70E-06	1.27E-05	1.83E-05	1.28E-05	3.33E-05	1.53E-05	1.27E-05	8.74E-06	1.37E-05
1.47E-05	7.93E-06	1.66E-05	6.83E-06	1.65E-05	2.42E-05	8.51E-06	1.12E-05	8.64E-06
2.05E-05	1.13E-05	2.21E-05	1.06E-05	3.06E-05	2.50E-05	1.94E-05	1.80E-05	2.10E-05
2.99E-07	4.75E-07	7.07E-07	9.39E-08	5.13E-07	4.37E-05	2.92E-07	1.15E-06	1.75E-07
0	0	0	0	1.37E-07	0	0	0	0
6.97E-06	1.15E-05	2.10E-05	1.36E-05	4.29E-05	2.30E-05	1.40E-05	1.49E-05	2.57E-05
2.86E-05	3.10E-05	3.87E-05	3.10E-05	0.000113	4.39E-05	6.91E-05	3.66E-05	2.82E-05
3.17E-05	2.53E-05	4.03E-05	2.61E-05	3.39E-05	5.52E-05	3.71E-05	2.90E-05	3.22E-05
1.79E-06	2.09E-06	4.56E-06	2.19E-06	6.96E-06	5.76E-06	3.51E-06	3.33E-06	1.98E-06
2.80E-05	1.91E-05	3.08E-05	1.98E-05	5.05E-05	2.88E-05	2.82E-05	2.34E-05	2.01E-05
4.13E-06	4.59E-06	9.96E-06	4.55E-06	2.18E-05	2.29E-06	5.65E-06	6.13E-06	6.60E-06
0	2.39E-08	9.89E-09	0	2.86E-08	1.32E-08	0	1.02E-08	0
2.25E-06	1.78E-06	3.84E-06	2.55E-06	6.70E-06	5.55E-06	2.76E-06	3.54E-06	2.73E-06
2.71E-05	1.54E-05	3.48E-05	8.33E-06	1.21E-05	5.22E-05	1.62E-05	1.45E-05	1.13E-05
1.09E-05	4.32E-06	1.29E-05	7.57E-06	1.73E-05	1.33E-05	1.14E-05	9.73E-06	7.86E-06
9.68E-06	3.59E-06	1.16E-05	4.29E-06	2.31E-05	8.69E-06	5.10E-06	4.17E-06	4.03E-06
3.52E-05	3.56E-05	4.57E-05	2.97E-05	7.80E-05	4.66E-05	2.63E-05	4.46E-05	3.88E-05
6.99E-06	4.16E-06	2.00E-05	3.65E-06	6.21E-06	1.04E-05	7.75E-06	7.02E-06	6.71E-06
0.000107	6.02E-05	8.73E-05	5.45E-05	9.70E-05	8.35E-05	5.52E-05	5.94E-05	6.07E-05
8.69E-07	1.07E-06	2.27E-06	1.52E-06	4.16E-06	1.22E-06	1.26E-06	2.21E-06	1.55E-06
0	8.00E-08	0	1.01E-07	1.59E-07	2.19E-08	1.25E-07	3.49E-07	1.66E-08
4.59E-07	5.50E-07	2.04E-06	9.50E-07	5.30E-06	2.71E-06	5.89E-07	1.46E-06	5.90E-07
0	5.38E-08	8.81E-08	1.62E-07	0	3.91E-08	6.64E-08	1.51E-07	5.97E-08
4.46E-08	1.17E-07	1.04E-07	4.77E-08	1.54E-07	9.95E-08	2.81E-08	1.01E-07	7.21E-07
0	0	0	0	0	0	0	0	0
0	2.11E-07	1.82E-06	2.45E-07	6.05E-08	1.71E-06	3.09E-07	4.64E-07	1.95E-06
0	0	1.65E-08	1.53E-08	0	3.29E-08	3.73E-08	3.41E-08	6.70E-08
2.42E-08	3.29E-08	1.01E-07	4.98E-08	2.40E-06	2.38E-08	3.62E-07	1.01E-07	1.33E-07
1.39E-07	1.35E-06	3.50E-07	2.74E-06	1.29E-05	4.11E-07	3.96E-06	2.42E-06	1.87E-06
5.44E-07	3.18E-06	1.64E-06	5.53E-06	4.39E-05	1.26E-06	1.07E-05	5.66E-06	3.04E-06
0	0	0	1.25E-08	9.68E-07	0	7.61E-09	6.98E-09	0
0	1.60E-08	0	0	6.81E-08	0	6.88E-08	2.70E-08	0
2.00E-06	1.08E-06	2.80E-06	2.62E-06	8.95E-06	1.94E-06	3.46E-06	2.18E-06	1.66E-06
1.96E-07	4.39E-06	1.29E-07	3.42E-07	2.34E-07	2.34E-06	2.37E-06	1.41E-06	8.97E-08
9.93E-07	6.61E-07	1.57E-07	5.49E-07	3.11E-06	5.31E-06	3.01E-06	1.78E-06	9.06E-07
5.54E-05	3.61E-05	1.09E-05	6.48E-05	5.10E-06	2.84E-05	5.69E-05	5.72E-05	3.11E-05
0	0	0	0	8.03E-08	0	0	0	0
0	0	0	0	0	0	0	0	0
4.13E-06	6.50E-06	8.24E-06	7.20E-06	1.17E-05	9.75E-06	6.15E-06	8.83E-06	9.68E-06
1.90E-06	3.02E-06	5.42E-06	1.77E-06	5.56E-06	7.33E-06	5.31E-06	1.87E-06	4.66E-06
0	0	0	6.96E-09	3.94E-08	0	8.41E-09	1.55E-08	2.13E-08
1.06E-08	0	3.51E-08	4.33E-08	0	3.12E-08	1.32E-08	1.21E-08	1.59E-07
4.87E-07	2.24E-06	5.62E-07	2.79E-06	1.85E-05	3.40E-06	2.06E-06	2.87E-06	2.41E-06
3.53E-07	4.55E-07	1.18E-06	7.38E-07	1.87E-06	1.64E-06	4.87E-07	9.51E-07	1.06E-06
0	0	1.87E-08	0	0	0	4.23E-08	0	0
4.32E-08	5.85E-08	7.83E-07	1.76E-07	1.58E-06	6.39E-08	2.71E-07	9.88E-08	1.95E-07
0	0	0	0	0	0	0	0	0
0	0	0	0	0	1.62E-08	0	0	0

0	0	0	0	1.58E-07	2.72E-08	2.31E-08	0	2.76E-08
0	0	0	3.75E-08	0	2.74E-08	0	4.21E-08	0
3.50E-05	7.05E-05	0.000142	2.88E-05	0.00015	4.61E-05	5.42E-05	6.01E-05	0.000243
9.58E-05	0	1.15E-08	0	0	0	9.11E-08	0	0
0.010054	0.006626	0.008099	0.006615	1.05E-05	0.009281	0.005803	0.00548	0.005678
3.86E-06	4.40E-06	8.17E-06	5.64E-06	2.57E-05	9.37E-06	6.15E-06	7.04E-06	5.95E-06
4.07E-06	6.05E-06	9.87E-06	9.06E-06	3.51E-05	2.23E-05	1.74E-05	1.92E-05	1.25E-05
1.06E-05	4.78E-05	2.27E-05	0.000133	2.56E-07	1.01E-05	3.09E-05	5.97E-05	8.84E-05
1.36E-05	9.81E-06	1.21E-05	9.40E-06	2.65E-05	1.40E-05	1.24E-05	8.42E-06	2.89E-05
0	0	0	0	0	0	0	0	0
0	0	0	0	0	0	7.68E-08	0	0
4.33E-05	8.28E-05	5.66E-05	6.26E-05	0.000116	2.13E-05	0.000109	6.81E-05	6.16E-05
3.04E-08	2.06E-08	5.61E-08	4.16E-08	2.81E-06	0	2.54E-08	2.32E-08	4.57E-08
0	3.18E-07	0	1.60E-07	0	0	4.19E-08	3.19E-07	5.03E-08
3.59E-06	2.99E-06	4.56E-06	3.20E-06	7.83E-06	6.75E-07	1.08E-05	4.29E-06	6.12E-06
4.63E-07	1.13E-06	1.84E-06	1.52E-06	4.98E-06	1.57E-06	1.17E-06	1.44E-06	7.92E-07
1.12E-07	1.43E-06	3.90E-07	1.33E-06	2.65E-06	3.05E-06	1.02E-06	2.91E-06	7.93E-07
1.27E-06	7.75E-07	2.80E-06	4.53E-07	3.10E-06	2.77E-06	1.21E-06	3.21E-07	9.19E-07
4.85E-07	8.64E-07	1.94E-06	1.24E-06	4.43E-06	1.72E-06	1.26E-06	1.63E-06	9.71E-07
1.61E-06	3.44E-06	4.32E-06	3.03E-06	1.49E-05	7.29E-06	3.63E-06	3.94E-06	2.95E-06
2.60E-05	7.65E-05	3.25E-07	4.93E-05	2.57E-07	4.19E-07	2.65E-05	4.99E-05	8.39E-05
2.30E-06	1.42E-06	5.17E-06	1.29E-06	5.93E-06	2.07E-06	1.86E-06	2.40E-06	2.76E-06
1.07E-06	1.52E-06	1.63E-06	1.06E-06	5.28E-06	1.58E-06	7.51E-07	8.58E-07	4.31E-07
1.28E-07	2.86E-06	8.75E-07	4.92E-06	1.53E-05	3.16E-06	3.36E-06	2.96E-06	2.71E-06
1.42E-06	6.98E-06	1.24E-05	7.93E-06	3.49E-05	5.94E-06	4.85E-06	1.53E-05	3.41E-06
0	2.46E-07	0	2.31E-07	2.50E-08	0	4.34E-08	1.98E-08	6.50E-08
2.75E-05	1.66E-05	4.76E-05	1.42E-05	3.83E-05	3.08E-05	3.53E-05	2.45E-05	1.36E-05
2.63E-07	6.01E-07	6.90E-07	9.78E-07	2.33E-06	1.02E-07	9.99E-07	6.22E-07	5.97E-07
1.39E-08	8.49E-08	3.07E-08	1.30E-07	1.02E-06	0	5.49E-08	2.87E-07	1.04E-08
2.58E-05	2.67E-05	4.38E-05	3.00E-05	1.73E-06	1.75E-05	1.40E-05	2.92E-05	2.76E-05
0	0	0	0	6.63E-08	0	0	0	0
1.52E-07	2.37E-08	1.81E-07	1.43E-07	6.72E-08	1.03E-07	5.84E-08	6.67E-08	2.80E-07
1.16E-07	2.63E-08	2.86E-08	2.64E-08	7.44E-08	1.14E-07	3.24E-08	1.48E-08	1.36E-07
3.68E-06	2.26E-06	4.70E-06	2.19E-06	8.40E-06	1.44E-05	3.31E-06	2.93E-06	8.94E-06
4.38E-09	2.98E-08	7.83E-08	2.01E-08	5.63E-08	3.84E-07	1.82E-08	1.33E-07	3.65E-08
2.95E-05	1.32E-05	3.13E-05	1.51E-05	7.42E-05	2.48E-05	2.20E-05	1.82E-05	2.03E-05
1.62E-06	1.82E-06	1.58E-06	8.36E-07	2.76E-06	1.49E-06	9.95E-07	1.43E-06	1.20E-06
7.27E-07	1.40E-06	2.91E-06	2.00E-06	9.75E-06	3.66E-06	1.46E-06	2.52E-06	1.67E-06
4.76E-06	4.45E-06	6.70E-06	5.85E-06	1.24E-05	1.10E-05	7.18E-06	6.52E-06	4.48E-06
0.000276	7.08E-05	0.000169	6.27E-05	0.000191	0.000283	0.000116	7.46E-05	0.000102
4.57E-05	2.85E-05	4.86E-05	3.05E-05	7.04E-05	5.64E-05	5.06E-05	3.73E-05	3.48E-05
3.09E-05	2.10E-05	4.47E-05	2.08E-05	2.40E-05	2.28E-05	2.72E-05	2.06E-05	2.72E-05
1.13E-05	1.06E-05	2.82E-05	1.28E-05	2.08E-05	3.66E-05	1.61E-05	1.32E-05	1.32E-05
3.04E-06	2.76E-06	8.32E-06	4.12E-06	2.35E-05	4.56E-06	4.99E-06	4.40E-06	4.53E-06
2.63E-05	2.57E-05	4.69E-05	3.20E-05	4.86E-05	4.84E-05	3.93E-05	3.35E-05	3.91E-05
7.06E-06	5.16E-06	1.30E-05	6.40E-06	2.10E-05	1.35E-05	7.70E-06	7.95E-06	9.07E-06
0.000649	0.000736	0.002207	0.00126	3.97E-05	0.000638	0.000861	0.001166	0.000538
1.96E-06	5.73E-06	9.41E-06	4.77E-06	6.00E-06	6.84E-06	4.19E-06	2.40E-06	4.84E-06
4.55E-06	2.58E-06	9.22E-06	3.51E-06	1.19E-05	3.10E-06	3.05E-06	4.47E-06	2.57E-06
2.15E-07	4.31E-07	2.27E-07	5.44E-07	2.58E-06	5.90E-07	1.27E-06	1.17E-06	9.92E-07
1.48E-06	1.38E-07	2.35E-06	2.18E-07	3.61E-06	9.56E-07	3.64E-07	1.25E-06	6.91E-07
0	2.36E-08	3.85E-08	0	0	6.00E-07	0	1.33E-08	6.44E-08
9.93E-06	5.38E-06	3.32E-06	3.32E-06	2.52E-06	2.60E-06	3.21E-06	3.77E-06	1.02E-05
3.99E-06	5.33E-06	4.01E-06	4.54E-06	3.79E-05	2.27E-06	7.99E-06	1.84E-05	4.22E-06
0.00016	0.00018	0.000199	0.000258	1.35E-06	0.000107	0.000182	0.000357	0.000187
1.33E-05	0.000102	2.57E-05	7.05E-05	1.04E-05	0.000453	3.09E-05	0.000188	1.15E-05
0	0	0	0	2.46E-08	0	0	0	0
2.65E-06	1.13E-05	2.02E-06	1.31E-05	1.20E-06	3.54E-06	6.21E-06	1.38E-05	2.66E-06

0	9.22E-08	0	0	0	2.67E-08	0	1.24E-07	4.07E-08
1.91E-08	2.31E-08	2.05E-08	9.16E-08	9.42E-08	3.75E-08	5.64E-08	1.08E-07	2.45E-06
1.18E-07	3.57E-07	2.07E-07	5.83E-07	9.92E-07	2.11E-07	1.00E-06	6.91E-07	4.46E-06
1.09E-07	6.14E-06	7.11E-07	4.73E-06	1.60E-05	4.82E-07	2.16E-06	1.13E-05	1.34E-06
6.94E-08	2.55E-08	6.73E-07	2.70E-07	5.83E-07	7.53E-07	3.53E-07	3.36E-07	4.87E-07
1.57E-07	2.43E-07	2.71E-07	3.63E-07	1.13E-06	3.52E-07	5.90E-07	5.08E-07	3.11E-07
1.43E-05	0	9.05E-05	5.11E-08	0	0	0	0	0
3.07E-06	6.41E-06	8.10E-06	8.67E-06	2.14E-05	3.96E-06	3.97E-06	7.06E-06	3.53E-06
3.44E-06	1.33E-05	5.54E-06	1.10E-05	1.48E-05	1.99E-05	1.64E-05	8.74E-06	6.97E-06
1.14E-05	1.56E-05	2.72E-05	2.01E-05	4.11E-05	4.21E-05	1.70E-05	2.78E-05	1.33E-05
1.48E-06	1.98E-06	7.43E-06	6.29E-06	2.36E-05	3.66E-06	5.49E-06	4.96E-06	5.99E-06
2.49E-06	3.28E-06	8.40E-06	4.15E-06	4.06E-05	5.82E-06	3.23E-06	5.94E-06	3.45E-06
4.73E-08	6.42E-08	7.83E-08	3.24E-08	4.55E-08	0	1.97E-08	0	3.54E-08
1.38E-08	1.41E-08	7.62E-08	7.10E-08	1.16E-07	2.63E-07	4.85E-08	7.91E-09	9.16E-07
6.84E-06	6.84E-06	1.55E-05	7.91E-06	2.84E-05	2.00E-05	1.15E-05	1.34E-05	7.85E-06
1.75E-06	1.38E-06	7.10E-06	1.96E-06	8.99E-06	7.79E-06	4.01E-06	1.76E-06	2.08E-06
4.12E-06	2.88E-06	1.42E-05	4.38E-06	2.06E-05	2.03E-06	4.66E-06	3.25E-06	3.57E-06
3.57E-06	3.43E-06	1.17E-05	5.89E-06	1.38E-05	6.62E-06	4.36E-06	6.84E-06	6.41E-06
3.12E-08	8.67E-07	1.03E-07	4.47E-07	1.00E-05	6.12E-08	1.52E-06	9.87E-07	1.87E-05
8.97E-06	1.39E-05	3.27E-05	1.50E-05	3.38E-05	2.31E-05	2.25E-05	1.96E-05	1.71E-05
6.53E-06	9.25E-06	1.18E-05	9.59E-06	4.80E-05	1.10E-05	1.15E-05	1.19E-05	1.18E-05
1.20E-06	1.04E-06	4.17E-06	2.34E-06	7.16E-06	2.86E-06	1.90E-06	4.07E-06	2.62E-06
4.30E-06	7.12E-06	9.62E-06	1.04E-05	3.29E-05	1.34E-05	1.06E-05	1.37E-05	1.23E-05
2.60E-07	3.23E-07	1.51E-06	2.70E-07	7.96E-06	4.73E-07	5.28E-07	3.13E-07	1.21E-07
9.87E-07	1.78E-06	3.98E-06	1.81E-06	1.04E-05	4.45E-06	2.28E-06	3.38E-06	1.69E-06
6.79E-06	2.62E-06	6.35E-06	3.78E-06	2.44E-05	3.56E-06	7.40E-06	3.16E-06	5.93E-06
3.14E-06	1.66E-06	4.35E-06	2.12E-06	1.05E-05	3.07E-06	2.95E-06	2.71E-06	2.18E-06
2.11E-06	6.14E-06	1.13E-05	7.44E-06	3.26E-05	1.23E-05	5.65E-06	1.10E-05	5.11E-06
1.12E-06	1.07E-06	5.63E-07	9.39E-07	2.18E-06	7.10E-07	6.41E-07	8.49E-07	3.28E-07
1.60E-08	3.24E-08	1.78E-08	1.20E-07	9.04E-07	0	3.70E-08	1.01E-07	2.21E-08
4.92E-09	6.02E-08	5.42E-08	5.07E-08	1.56E-07	1.52E-07	1.23E-08	9.09E-08	4.62E-08
0	0	4.92E-08	0	1.13E-07	1.31E-07	2.48E-08	1.92E-08	1.67E-08
5.33E-09	7.03E-09	1.61E-07	0	1.09E-07	2.53E-07	7.74E-08	5.95E-08	1.60E-08
4.40E-08	3.99E-08	1.08E-08	4.13E-07	2.18E-06	3.56E-05	3.66E-07	1.74E-07	1.02E-07
1.06E-07	2.50E-07	8.12E-07	3.09E-07	6.33E-07	3.25E-07	4.36E-07	1.65E-07	1.10E-06
2.44E-06	9.47E-07	2.59E-06	1.93E-06	1.86E-06	2.08E-06	1.94E-06	5.32E-07	8.82E-07
1.68E-07	2.40E-07	1.10E-06	3.81E-07	2.60E-06	1.36E-06	3.64E-07	1.10E-06	3.88E-07
0	0	0	0	0	0	0	0	0
1.13E-07	1.87E-06	3.80E-06	1.84E-06	8.53E-06	4.08E-06	2.08E-06	3.17E-06	1.81E-06
4.69E-06	5.91E-06	8.47E-06	7.50E-06	1.69E-05	1.02E-05	6.69E-06	7.66E-06	5.65E-06
1.90E-06	9.10E-07	4.27E-06	5.58E-07	4.26E-06	2.61E-06	4.21E-07	7.47E-07	4.94E-07
0	0	0	0	0	0	0	0	0
0	0	0	2.09E-08	5.88E-08	1.50E-08	0	2.97E-08	5.42E-08
0	1.74E-08	3.77E-08	1.75E-08	2.46E-08	6.26E-08	0	0	2.55E-08
3.40E-07	5.19E-07	1.25E-06	4.85E-07	3.98E-06	3.07E-06	8.42E-07	1.01E-06	5.73E-07
1.27E-08	0	6.35E-09	0	0	1.87E-08	1.26E-08	1.20E-08	1.91E-08
6.50E-08	9.94E-08	2.63E-07	1.56E-07	5.01E-07	1.51E-07	1.15E-07	6.83E-08	4.87E-08
0	0	0	0	0	0	0	0	0
3.54E-09	0	0	0	0	0	0	0	0
3.45E-06	3.57E-07	3.61E-06	2.65E-07	8.95E-07	1.19E-06	1.11E-06	9.24E-07	6.12E-07
1.66E-07	1.56E-07	5.93E-07	3.31E-07	2.00E-06	2.94E-06	4.48E-07	3.10E-07	4.29E-07
0	2.36E-08	1.76E-07	6.56E-08	8.28E-07	3.79E-07	8.09E-08	6.01E-08	4.59E-08
6.71E-08	1.52E-06	3.05E-06	1.42E-06	9.06E-06	2.27E-06	4.06E-07	8.06E-07	5.54E-07
3.40E-05	5.82E-06	6.06E-06	6.88E-06	3.74E-06	4.61E-06	6.56E-06	2.05E-05	3.98E-06
1.82E-05	7.96E-06	1.97E-05	8.65E-06	1.45E-05	1.45E-05	1.11E-05	1.19E-05	1.46E-05
4.65E-06	6.84E-06	9.09E-06	6.97E-06	3.27E-05	1.67E-05	6.81E-06	1.19E-05	6.21E-06
1.68E-06	1.63E-06	4.60E-06	1.77E-06	8.65E-06	1.42E-06	1.49E-06	1.91E-06	1.46E-06
1.11E-06	1.52E-06	2.12E-06	8.53E-07	3.77E-07	1.99E-06	8.51E-07	1.70E-06	8.33E-07

5.69E-08	5.57E-07	5.16E-07	2.10E-06	5.02E-06	2.12E-06	7.62E-07	1.08E-06	7.99E-07
1.49E-08	3.05E-08	2.47E-08	3.07E-08	1.23E-07	6.59E-08	2.80E-08	5.99E-08	2.24E-08
8.97E-06	1.12E-05	9.69E-06	1.10E-05	7.98E-06	1.51E-05	8.84E-06	1.24E-05	7.65E-06
2.15E-06	2.19E-06	6.53E-06	2.81E-06	7.96E-06	3.52E-06	2.70E-06	3.08E-06	2.51E-06
4.47E-07	1.03E-06	2.15E-06	1.64E-06	1.13E-05	4.53E-06	1.25E-06	2.11E-06	7.31E-07
7.29E-07	1.42E-06	2.73E-06	1.90E-06	1.02E-05	3.57E-06	1.22E-06	4.30E-06	8.25E-07
9.32E-07	1.87E-06	5.64E-06	1.69E-06	2.72E-05	2.32E-06	2.42E-06	1.28E-06	1.29E-06
7.69E-08	1.68E-07	0	1.60E-07	1.78E-07	1.52E-08	1.25E-07	3.45E-07	1.92E-07
8.87E-06	5.20E-06	1.85E-05	5.83E-06	1.73E-05	1.02E-05	6.73E-06	7.41E-06	6.56E-06
1.16E-06	9.86E-07	5.37E-08	9.18E-07	6.95E-08	0	3.50E-07	1.11E-06	1.82E-08
9.84E-07	6.38E-06	1.51E-05	6.91E-06	8.75E-06	1.02E-05	2.97E-06	6.91E-06	2.92E-06
7.87E-06	8.38E-06	1.18E-05	1.14E-05	2.39E-05	2.26E-05	1.12E-05	1.76E-05	1.09E-05
6.63E-08	0	8.82E-08	5.69E-07	2.18E-06	1.37E-07	3.66E-07	2.43E-07	2.59E-07
3.71E-07	1.52E-06	1.28E-06	1.56E-06	7.77E-06	4.04E-06	1.52E-06	2.17E-06	2.86E-06
0	0	0	0	0	0	2.00E-08	0	0
2.35E-06	4.17E-06	2.33E-06	1.38E-06	1.32E-05	6.18E-06	2.07E-06	2.10E-06	4.27E-06
9.20E-07	3.33E-06	2.67E-06	4.36E-06	2.98E-05	4.23E-06	2.18E-06	5.46E-06	2.26E-06
0	1.21E-07	8.30E-06	4.89E-08	5.61E-07	0	1.98E-08	7.55E-08	4.60E-07
1.67E-06	1.60E-06	7.66E-06	1.96E-06	1.45E-05	5.97E-06	5.40E-06	1.67E-06	3.43E-06
3.25E-06	8.29E-06	1.45E-05	8.04E-06	8.36E-06	1.53E-05	5.39E-06	1.81E-05	3.10E-06
3.07E-08	1.39E-06	1.30E-07	1.29E-06	1.37E-05	4.39E-06	3.86E-07	1.12E-06	4.83E-06
1.00E-05	3.77E-06	2.18E-05	6.00E-06	2.37E-05	1.14E-05	6.71E-06	6.55E-06	6.01E-06
3.13E-07	7.02E-08	6.72E-07	5.14E-08	1.64E-05	3.39E-06	4.90E-07	5.20E-07	1.80E-07
4.12E-06	2.90E-05	1.08E-06	3.95E-05	4.94E-05	7.01E-05	5.42E-06	2.73E-05	7.15E-06
3.68E-06	5.29E-06	6.20E-06	5.22E-06	1.39E-05	7.14E-06	1.33E-05	6.00E-06	1.73E-05
3.68E-08	9.65E-08	6.37E-08	1.08E-08	4.56E-08	3.85E-08	2.19E-06	6.62E-08	5.51E-08
0	2.29E-07	5.75E-08	9.49E-08	2.37E-07	1.04E-08	5.74E-08	6.91E-07	7.06E-06
0	1.05E-08	1.94E-08	0	1.06E-06	1.29E-08	1.21E-07	1.75E-07	1.31E-08
5.38E-07	8.78E-07	1.47E-06	9.05E-07	2.35E-06	1.38E-06	5.65E-07	1.28E-06	5.91E-07
1.18E-05	2.07E-05	2.55E-05	2.32E-05	8.43E-05	2.34E-05	2.26E-05	2.29E-05	2.16E-05
2.73E-07	4.23E-07	4.67E-08	3.49E-07	1.14E-06	4.07E-07	2.70E-07	4.15E-07	2.54E-07
6.31E-07	1.37E-06	1.99E-06	1.61E-06	7.38E-06	2.73E-06	1.74E-06	1.63E-06	1.14E-06
4.70E-08	3.46E-07	8.96E-08	4.80E-07	1.33E-05	3.04E-08	1.50E-06	2.47E-07	3.55E-07
3.87E-05	2.40E-05	5.47E-05	3.09E-05	0.000128	2.71E-05	4.40E-05	4.02E-05	2.56E-05
5.63E-05	3.89E-05	8.08E-05	4.22E-05	0.000172	0.000112	6.04E-05	8.00E-05	5.20E-05
1.45E-07	1.45E-07	1.22E-07	0	5.15E-07	0	1.83E-07	1.23E-07	5.48E-08
0	0	0	0	0	0	0	0	0
1.46E-05	6.03E-06	2.93E-05	1.03E-05	3.81E-05	2.88E-05	1.42E-05	1.30E-05	6.16E-06
6.57E-05	0.000247	0.000177	0.00023	7.73E-06	0.000174	0.000125	0.000251	0.000173
4.73E-07	3.93E-06	1.04E-06	2.36E-06	3.47E-05	6.67E-07	3.32E-06	1.27E-05	1.80E-06
3.61E-06	1.34E-05	4.31E-06	1.50E-05	2.03E-05	2.73E-06	1.01E-05	1.49E-05	6.02E-06
0	0	0	0	0	0	0	0	7.17E-08
9.97E-07	1.50E-06	3.67E-06	1.87E-06	7.61E-07	1.26E-06	1.06E-06	2.08E-06	1.15E-06
5.23E-06	1.50E-05	1.39E-05	1.21E-05	6.53E-05	1.76E-05	2.88E-05	2.33E-05	1.81E-05
4.43E-05	4.24E-05	2.41E-05	3.75E-05	1.23E-06	2.09E-05	1.54E-05	7.11E-05	0.000124
3.39E-05	2.87E-05	4.29E-05	3.39E-05	0.000167	1.80E-05	5.09E-05	5.51E-05	3.04E-05
4.35E-08	7.78E-06	1.61E-08	5.99E-06	0.000436	5.12E-06	4.77E-06	4.31E-06	1.56E-05
7.52E-05	6.55E-05	0.000122	6.21E-05	0.000944	0.000142	0.000366	0.000144	0.000119
1.13E-07	6.86E-07	6.61E-07	6.23E-07	5.92E-06	6.90E-07	1.37E-06	7.54E-07	5.32E-07
1.79E-05	2.67E-05	5.42E-05	2.31E-05	0.000395	6.50E-05	0.000138	5.34E-05	5.09E-05
0	0	3.55E-08	0	1.38E-07	0	4.01E-08	3.65E-08	0
5.55E-08	1.85E-06	1.73E-07	3.25E-06	9.29E-05	6.72E-07	6.10E-07	2.20E-06	6.06E-07
2.75E-05	7.47E-05	5.75E-05	7.40E-05	0.001548	3.25E-05	0.000149	9.93E-05	4.25E-05
3.95E-05	6.27E-05	7.94E-05	7.78E-05	0.000432	8.34E-05	0.000175	0.000114	0.000153
3.76E-05	8.42E-05	9.36E-05	8.60E-05	4.02E-05	0.00012	8.82E-05	8.51E-05	6.33E-05
5.40E-05	4.92E-05	0.000114	6.61E-05	8.14E-05	4.28E-05	7.49E-05	8.06E-05	5.49E-05
0	2.33E-06	1.26E-08	3.01E-06	1.51E-06	0	1.17E-06	1.42E-06	1.17E-07
0	0	0	0	0	0	0	0	0

0	1.91E-06	0	1.08E-06	3.25E-05	0	7.82E-07	1.06E-06	4.89E-08
3.09E-05	1.82E-05	3.63E-05	1.45E-05	0.000272	1.27E-05	1.13E-05	1.88E-05	2.15E-05
2.13E-07	1.11E-06	9.74E-07	4.43E-06	5.61E-06	3.30E-07	3.02E-06	2.63E-06	1.28E-06
1.32E-07	3.32E-07	1.80E-06	2.93E-07	1.52E-06	5.84E-07	6.48E-07	1.80E-07	3.64E-07
1.84E-06	9.31E-06	4.00E-06	6.55E-06	1.47E-05	8.25E-06	6.34E-06	1.34E-05	8.93E-06
0.000351	0.000272	0.000776	0.000535	5.70E-07	2.99E-05	0.000281	0.000509	1.89E-05
0	0	5.52E-07	0	0	0	2.97E-08	0	7.11E-08
5.20E-06	4.42E-06	9.06E-06	6.28E-06	1.22E-05	1.15E-05	5.31E-06	7.16E-06	5.86E-06
2.17E-05	2.57E-05	3.26E-05	2.52E-05	7.46E-05	2.69E-05	3.51E-05	2.96E-05	2.95E-05
9.30E-06	1.17E-05	1.87E-05	1.42E-05	2.64E-05	8.72E-06	1.52E-05	1.23E-05	1.31E-05
4.50E-07	3.65E-06	2.06E-06	7.93E-06	6.69E-05	4.22E-06	3.57E-06	6.35E-06	5.27E-06
2.41E-05	3.31E-05	4.55E-05	2.55E-05	5.79E-05	3.52E-05	5.11E-05	4.44E-05	4.81E-05
2.38E-08	9.91E-07	1.32E-08	1.26E-06	3.43E-08	1.75E-08	1.29E-05	1.11E-06	1.78E-08
4.54E-05	2.41E-05	6.45E-05	2.27E-05	0.0002	7.49E-05	4.56E-05	2.68E-05	2.33E-05
1.54E-06	2.52E-06	3.73E-06	3.27E-06	9.46E-06	3.67E-06	3.21E-06	5.61E-06	5.23E-06
8.51E-08	3.09E-07	4.31E-07	6.49E-07	8.56E-06	1.27E-06	5.32E-07	6.36E-07	3.35E-07
4.39E-05	4.19E-05	9.37E-05	4.54E-05	7.99E-05	3.79E-05	5.39E-05	4.09E-05	4.44E-05
8.75E-06	1.65E-05	1.96E-05	1.85E-05	6.99E-05	2.80E-05	1.90E-05	1.85E-05	1.76E-05
9.93E-06	1.38E-05	2.06E-05	1.85E-05	0.000117	1.49E-05	1.59E-05	2.17E-05	1.73E-05
8.80E-05	6.86E-05	0.000154	8.40E-05	0.000338	0.000114	0.000122	0.000111	8.97E-05
9.83E-05	7.61E-05	0.00011	8.31E-05	0.000162	8.58E-05	0.000142	8.66E-05	8.69E-05
3.61E-06	4.07E-06	1.30E-05	5.86E-06	2.73E-05	9.98E-06	7.44E-06	7.93E-06	5.75E-06
0	1.55E-08	8.40E-09	0	5.42E-08	0	0	2.15E-08	0
3.60E-05	2.75E-05	5.43E-05	3.22E-05	0.00013	3.89E-05	4.50E-05	2.93E-05	4.01E-05
2.66E-06	5.74E-06	1.35E-05	6.55E-06	1.34E-05	6.28E-06	7.54E-06	8.20E-06	5.35E-06
7.28E-07	7.16E-07	1.67E-06	1.16E-06	1.39E-05	3.23E-06	1.43E-06	1.55E-06	9.84E-07
2.60E-05	2.50E-05	4.17E-05	2.42E-05	5.17E-05	3.11E-05	3.59E-05	3.09E-05	3.77E-05
5.53E-06	1.04E-05	1.36E-05	1.15E-05	3.63E-05	1.86E-05	1.26E-05	8.39E-06	1.27E-05
3.40E-06	2.37E-06	6.57E-06	4.46E-06	8.37E-06	6.17E-06	6.08E-06	4.19E-06	4.40E-06
8.24E-07	1.26E-06	2.36E-06	1.55E-06	4.41E-06	3.08E-06	1.69E-06	2.81E-06	1.30E-06
3.50E-06	2.17E-06	5.25E-06	2.72E-06	3.37E-05	5.67E-06	3.86E-06	3.72E-06	2.99E-06
1.47E-06	8.20E-07	2.89E-06	2.19E-06	4.24E-06	2.78E-06	1.65E-06	2.35E-06	2.03E-06
6.51E-07	1.31E-06	3.95E-06	1.92E-06	6.68E-06	2.40E-06	1.80E-06	2.27E-06	1.80E-06
6.40E-07	3.54E-06	1.35E-06	3.99E-06	3.26E-06	1.78E-05	2.08E-06	1.28E-06	4.14E-06
3.97E-08	1.66E-07	1.14E-07	4.82E-07	2.36E-06	3.43E-08	5.04E-07	4.13E-07	2.69E-07
5.99E-06	5.37E-06	8.30E-06	4.19E-06	2.24E-05	1.11E-05	5.56E-06	5.60E-06	5.15E-06
4.97E-06	2.97E-06	7.36E-06	4.85E-06	1.11E-05	5.81E-06	6.10E-06	6.23E-06	1.06E-05
2.09E-06	4.14E-06	4.27E-07	2.18E-05	8.62E-06	4.10E-06	4.75E-06	9.12E-06	1.11E-05
3.11E-06	4.02E-06	1.15E-05	4.86E-06	6.89E-06	2.87E-06	4.20E-06	6.04E-06	3.94E-06
1.31E-06	3.85E-06	2.99E-06	5.34E-06	6.02E-06	1.22E-05	7.92E-06	5.90E-06	1.61E-05
6.28E-07	1.66E-06	2.90E-06	2.38E-06	6.81E-06	3.12E-06	1.46E-06	3.03E-06	2.21E-06
2.26E-08	2.15E-08	6.94E-08	2.90E-08	2.14E-07	4.89E-08	1.31E-08	1.61E-08	2.10E-08
6.25E-08	1.74E-06	4.44E-06	1.62E-06	0.000238	5.75E-08	8.21E-07	2.75E-06	1.76E-07
0	1.29E-08	0	0	7.33E-07	0	0	1.45E-08	0
0.002922	0.001709	0.001069	0.002786	0.000287	0.00021	0.001665	0.001735	0.001545
3.51E-05	3.81E-05	5.39E-05	3.87E-05	7.39E-05	4.35E-05	4.04E-05	4.43E-05	3.80E-05
4.84E-05	3.93E-05	0.000115	4.54E-05	0.000206	0.000134	8.18E-05	7.77E-05	5.12E-05
6.04E-06	5.59E-06	1.84E-05	9.32E-06	5.55E-05	1.70E-05	1.20E-05	1.05E-05	1.09E-05
0.0001	7.23E-05	0.000147	8.35E-05	0.000321	0.000184	9.71E-05	8.84E-05	6.45E-05
5.63E-07	9.46E-07	1.12E-06	1.63E-06	4.68E-06	5.02E-07	1.64E-06	1.29E-06	2.03E-06
1.04E-05	1.66E-05	2.71E-05	1.88E-05	5.40E-05	3.25E-05	1.96E-05	2.58E-05	1.50E-05
2.09E-05	9.03E-06	3.01E-05	9.67E-06	4.28E-05	1.88E-05	1.08E-05	1.29E-05	2.12E-05
5.95E-06	3.42E-06	1.25E-05	5.07E-06	1.22E-05	8.87E-06	3.77E-06	3.28E-06	5.08E-06
1.32E-07	4.84E-07	4.62E-07	5.05E-07	8.79E-07	1.11E-06	9.56E-07	6.46E-07	5.22E-07
4.11E-06	1.42E-07	1.66E-06	2.61E-08	3.79E-06	6.61E-07	4.75E-07	3.92E-07	7.02E-07
1.72E-06	5.64E-06	1.94E-06	4.96E-06	1.94E-05	2.24E-06	1.46E-05	1.20E-05	4.03E-06
0	3.19E-07	7.14E-08	1.51E-07	4.31E-05	6.79E-08	1.62E-07	6.13E-07	6.92E-08
8.01E-05	0.000204	0.000293	0.000217	0.000361	0.000138	0.00026	0.000337	0.000288

0.004228	0.026738	0.005163	0.020595	1.75E-05	0.003295	0.013113	0.021055	0.00618
0.000178	6.69E-05	0.00016	5.21E-05	0.000235	0.000129	9.84E-05	7.79E-05	7.62E-05
0.045878	0.026978	0.040071	0.017956	9.13E-05	0.024935	0.020258	0.021323	0.045166
5.84E-08	3.15E-05	0	1.55E-06	0	1.08E-07	0.000687	0.000101	0.000101
0.000339	0.00039	0.000568	0.000176	6.92E-08	0.000408	0.000234	0.000312	0.00011
0.000211	0.000571	0.000579	0.000698	1.59E-06	0.000807	0.000293	0.00095	0.000614
6.44E-07	1.02E-06	9.71E-07	1.99E-06	8.63E-06	1.30E-06	2.14E-06	1.94E-06	1.80E-06
0	0	0	0	3.95E-07	0	0	0	0
4.37E-08	8.87E-08	4.84E-08	5.25E-08	1.48E-07	4.30E-08	3.65E-08	1.66E-08	1.09E-07
1.92E-07	1.80E-07	9.85E-08	1.58E-07	7.66E-07	6.40E-07	2.97E-07	2.12E-07	5.11E-07
5.65E-07	6.35E-07	3.72E-07	3.81E-07	7.74E-06	7.82E-06	9.50E-07	1.67E-06	4.56E-07
2.43E-07	4.63E-06	2.03E-06	1.17E-05	1.70E-05	4.81E-07	2.62E-06	4.48E-06	1.68E-06
2.37E-07	7.41E-07	2.26E-07	1.68E-06	3.17E-06	3.57E-07	7.25E-07	6.78E-07	5.48E-07
9.61E-07	1.62E-06	1.06E-06	3.27E-06	1.07E-05	7.41E-07	4.39E-07	1.86E-06	9.63E-07
3.99E-07	1.36E-06	1.03E-06	4.30E-06	9.22E-06	1.25E-06	2.25E-06	4.37E-06	2.36E-06
7.67E-08	1.14E-07	4.63E-07	5.76E-07	5.38E-06	0	1.06E-06	7.99E-07	1.92E-06
0	0	0	0	0	0	0	0	0
0.040644	0.021223	0.017567	0.015377	0.000893	0.0234	0.022395	0.018449	0.010988
7.34E-06	2.49E-05	3.46E-06	2.29E-05	0	0.000133	0.000167	0.000223	2.88E-05
0.008872	0.005023	0.003587	0.004243	6.08E-05	0.004626	0.005856	0.005331	0.004705
0.023684	0.017457	0.028715	0.013445	7.20E-06	0.012062	0.014674	0.011952	0.015718
0.001534	0.000983	0.001808	0.000446	2.50E-07	0.000735	0.000551	0.000386	3.79E-05
1.58E-06	1.04E-06	1.25E-06	5.21E-07	6.23E-06	6.15E-07	7.20E-07	1.19E-06	4.16E-06
0.010466	0.006713	0.00357	0.004882	0.001236	0.008469	0.00495	0.006021	0.006139
2.84E-05	0.000172	1.62E-05	0.000405	7.13E-08	3.45E-05	7.75E-05	0.000177	0.00027
0.00776	0.005935	0.00699	0.005397	5.84E-05	0.007227	0.003858	0.007917	0.009767
6.09E-05	0.000139	7.86E-05	0.000108	0.000109	0.000123	0.000153	0.000199	4.55E-05
8.58E-06	2.92E-05	1.95E-05	2.45E-05	4.40E-05	3.30E-05	4.16E-05	2.95E-05	2.17E-05
8.41E-06	3.02E-05	1.05E-05	2.98E-05	1.71E-05	4.05E-05	6.04E-05	3.91E-05	9.19E-06
1.16E-07	3.54E-07	1.98E-07	8.59E-07	2.47E-06	9.18E-07	1.52E-06	8.90E-07	1.29E-06
0	1.81E-07	0	6.08E-08	0	1.10E-07	1.87E-08	2.04E-07	2.24E-08
3.70E-06	1.77E-05	1.29E-05	2.69E-05	2.54E-05	1.20E-05	1.50E-05	2.39E-05	1.01E-05
3.74E-06	2.69E-06	6.11E-06	1.42E-06	7.53E-06	1.79E-05	6.49E-06	1.04E-05	1.28E-05
0.000325	0.000316	0.001072	0.000402	8.58E-06	0.000674	0.000548	0.000684	0.000205
9.24E-06	8.98E-06	1.32E-05	6.02E-06	2.01E-05	1.07E-05	1.08E-05	8.82E-06	1.52E-05
5.78E-07	3.37E-06	3.59E-06	2.21E-06	6.35E-06	5.40E-06	1.33E-06	3.49E-06	2.61E-06
6.89E-05	0.000131	0.000118	0.000132	0.00119	0.000217	0.000148	0.000248	0.000168
2.38E-06	2.53E-06	4.63E-06	4.31E-06	1.72E-05	6.81E-06	4.48E-06	5.07E-06	2.51E-06
2.93E-06	3.22E-06	8.00E-06	4.57E-06	1.17E-05	9.87E-06	4.20E-06	6.20E-06	3.86E-06
1.32E-06	5.24E-06	7.85E-06	8.04E-06	2.88E-05	1.06E-05	5.60E-06	7.48E-06	5.51E-06
0.000247	9.42E-05	0.000179	0.000105	0.000212	0.000109	0.000174	0.000118	0.000131
9.66E-06	7.16E-06	2.07E-05	9.02E-06	3.11E-05	3.05E-05	1.02E-05	1.08E-05	9.87E-06
2.41E-05	3.35E-05	3.38E-05	4.75E-05	0.000454	1.19E-05	3.62E-05	4.92E-05	2.96E-05
0	2.36E-08	0	0	6.68E-08	0	0	5.30E-08	9.75E-08
1.45E-05	9.98E-06	4.54E-05	6.82E-06	4.64E-06	1.77E-06	6.76E-06	9.08E-06	1.87E-06
0	0	0	0	0	0	0	0	0
0	8.14E-08	0	0	1.90E-08	0	5.06E-08	1.50E-08	0
0	0	0	0	0	0	0	0	0
4.71E-06	4.61E-05	2.51E-05	8.43E-05	2.33E-05	0.000144	4.81E-05	7.69E-05	2.55E-05
1.88E-08	5.45E-07	3.69E-07	5.20E-07	2.99E-06	1.16E-07	2.39E-07	2.41E-07	6.75E-07
0	0	1.26E-08	0	0	0	4.27E-08	2.60E-08	1.71E-08
4.29E-06	1.05E-07	9.54E-09	7.09E-08	6.52E-08	2.54E-08	1.79E-07	2.19E-07	1.29E-08
7.44E-06	1.18E-05	4.66E-06	1.66E-05	6.18E-07	1.33E-05	7.13E-06	1.12E-05	2.24E-05
5.94E-06	1.49E-05	1.19E-06	8.07E-06	4.28E-08	5.00E-06	5.68E-06	1.08E-05	2.57E-05
1.55E-06	9.66E-06	1.26E-07	1.73E-06	0	1.35E-06	1.06E-06	2.18E-06	7.09E-06
9.02E-06	6.06E-07	1.84E-08	0	0	8.11E-07	9.80E-07	3.04E-07	4.15E-06
0.000284	0.000232	0.000575	0.000187	5.67E-07	0.000258	0.000176	0.000384	8.82E-05
0	1.22E-08	0	0	0	0	1.50E-08	1.38E-08	3.85E-08

1.31E-06	2.50E-06	5.37E-06	3.68E-06	1.63E-05	7.16E-06	2.98E-06	5.41E-06	3.01E-06
4.90E-07	1.01E-05	1.82E-05	1.57E-05	1.66E-06	1.52E-05	3.17E-06	1.06E-05	4.22E-06
2.08E-05	4.22E-05	3.21E-05	4.35E-05	8.24E-05	6.33E-05	3.76E-05	3.85E-05	3.68E-05
1.07E-05	1.65E-05	1.54E-05	1.61E-05	6.72E-05	2.27E-05	2.03E-05	9.09E-06	1.26E-05
4.08E-07	1.14E-06	1.05E-06	1.74E-06	1.23E-05	5.34E-07	1.09E-06	2.27E-06	8.05E-07
1.14E-06	1.51E-06	1.79E-06	1.46E-06	6.51E-06	1.84E-06	3.33E-06	2.93E-06	2.36E-06
2.02E-08	4.53E-07	1.04E-07	2.77E-08	1.44E-05	1.88E-07	6.73E-08	3.09E-08	7.06E-08
3.17E-05	4.18E-05	6.42E-05	4.70E-05	0.000107	3.39E-05	6.06E-05	6.75E-05	4.64E-05
1.24E-07	1.04E-06	1.97E-08	1.44E-07	2.19E-05	6.03E-07	5.34E-07	9.70E-07	1.09E-06
0.000349	0.000157	0.000433	0.00017	0.000604	0.000132	0.000246	0.000212	0.000227
1.50E-05	1.91E-05	3.19E-05	2.81E-05	0.00019	2.18E-05	3.10E-05	2.42E-05	2.39E-05
4.07E-05	6.58E-05	0.000121	6.74E-05	0.00013	0.000115	0.000166	0.000112	0.000101
0.000153	7.86E-05	0.000217	0.000113	0.000404	0.000141	0.000175	0.000107	9.71E-05
1.28E-05	1.77E-05	3.10E-05	1.91E-05	0.000101	3.64E-05	2.21E-05	4.42E-05	1.67E-05
1.27E-05	1.07E-05	1.90E-05	1.15E-05	3.55E-05	1.96E-05	2.25E-05	9.22E-06	1.34E-05
3.15E-05	3.00E-05	5.50E-05	3.42E-05	7.09E-05	4.02E-05	4.05E-05	3.02E-05	2.65E-05
1.26E-05	1.26E-05	2.10E-05	2.27E-05	3.18E-05	1.97E-05	2.09E-05	2.31E-05	2.49E-05
7.00E-06	5.84E-06	9.63E-06	7.98E-06	3.64E-05	1.01E-05	1.21E-05	1.11E-05	8.21E-06
2.82E-06	6.88E-06	1.11E-05	7.84E-06	2.46E-05	1.27E-05	6.10E-06	9.71E-06	1.10E-05
4.84E-06	6.21E-06	1.31E-05	7.72E-06	3.20E-05	1.01E-05	1.07E-05	1.01E-05	5.90E-06
7.33E-06	7.09E-06	2.50E-05	1.03E-05	3.28E-05	2.79E-05	1.45E-05	1.43E-05	1.47E-05
1.72E-05	1.05E-05	1.46E-05	7.73E-06	3.00E-05	1.87E-05	2.22E-05	8.55E-06	1.04E-05
0.000118	0.000285	0.000138	0.00061	2.55E-07	0.001644	0.000611	0.000379	0.000466
1.65E-06	2.59E-06	5.49E-06	3.72E-06	5.27E-06	1.70E-06	2.11E-06	3.69E-06	9.76E-06
0	0	0	0	0	0	0	0	0
0	0	2.04E-08	0	0	1.09E-07	0	4.19E-08	0
4.65E-06	1.08E-05	2.30E-05	1.59E-05	2.94E-05	3.50E-05	1.83E-05	1.39E-05	1.99E-05
9.57E-06	1.42E-05	3.59E-05	2.16E-05	0.000113	1.93E-05	2.46E-05	2.57E-05	1.78E-05
6.58E-06	2.36E-06	2.85E-06	2.15E-06	4.01E-06	3.37E-06	5.45E-06	1.23E-05	4.34E-06
2.20E-07	1.81E-07	1.02E-06	2.75E-07	5.77E-06	3.59E-06	7.78E-07	5.17E-07	4.48E-07
0	0	2.29E-07	0	2.12E-06	1.90E-06	2.55E-07	5.34E-08	0
6.14E-06	5.78E-06	1.48E-05	9.69E-06	6.96E-05	4.96E-06	9.92E-06	1.16E-05	7.51E-06
1.15E-06	1.86E-06	1.21E-06	2.84E-06	6.63E-06	6.08E-07	3.51E-06	6.77E-06	2.07E-06
9.84E-06	8.00E-06	8.70E-06	1.17E-05	3.11E-05	1.55E-05	1.75E-05	1.49E-05	1.89E-05
9.26E-07	1.74E-06	2.09E-06	1.97E-06	8.95E-06	2.85E-06	1.98E-06	6.45E-06	1.93E-06
9.86E-07	1.09E-06	3.99E-06	1.20E-06	4.16E-06	8.30E-07	1.03E-06	9.42E-07	1.09E-06
3.25E-09	5.84E-07	2.86E-08	3.15E-07	1.69E-07	7.43E-08	1.38E-07	4.70E-07	3.74E-07
1.15E-06	4.77E-06	7.17E-06	5.38E-06	2.98E-05	1.25E-05	4.75E-06	8.78E-06	7.24E-06
1.93E-07	1.32E-07	1.07E-07	1.63E-07	2.30E-06	6.26E-08	1.71E-06	4.24E-07	8.84E-07
3.99E-07	1.87E-06	1.68E-06	1.80E-06	5.61E-06	8.40E-07	3.02E-06	2.73E-06	1.51E-06
3.83E-07	2.53E-06	3.79E-06	3.20E-06	2.30E-06	1.68E-06	2.00E-06	4.88E-06	1.16E-06
1.75E-06	1.98E-06	2.32E-06	6.27E-06	1.19E-05	2.22E-06	3.69E-06	4.76E-06	2.98E-06
6.91E-07	1.12E-06	5.37E-06	1.36E-06	1.00E-05	4.75E-07	7.88E-07	1.54E-06	9.25E-07
5.00E-07	2.92E-06	1.83E-06	3.36E-06	2.95E-05	6.79E-06	3.73E-06	2.24E-06	3.44E-06
2.11E-08	1.22E-07	2.20E-07	4.36E-08	7.43E-07	6.19E-08	2.81E-07	3.90E-07	2.48E-07
7.12E-07	5.65E-06	6.93E-06	5.33E-06	2.55E-05	9.26E-06	4.71E-06	8.68E-06	4.45E-06
5.44E-07	1.92E-06	1.63E-06	6.14E-06	9.27E-06	1.16E-06	4.52E-06	3.32E-06	2.71E-06
3.31E-07	8.45E-07	1.22E-06	1.43E-06	3.63E-06	8.63E-07	1.30E-06	1.83E-06	2.08E-06
6.46E-07	1.32E-06	2.94E-06	1.30E-06	5.20E-06	2.07E-06	1.17E-06	1.56E-06	1.85E-06
2.68E-07	5.14E-07	2.04E-06	5.17E-07	2.74E-06	5.70E-06	8.20E-07	6.56E-07	1.73E-06
0	0	0	0	0	0	0	7.53E-08	0
3.20E-07	2.61E-07	5.85E-07	3.05E-07	6.23E-06	7.72E-07	3.21E-07	4.25E-07	2.84E-07
2.26E-06	3.67E-06	4.00E-06	8.17E-06	2.37E-05	2.09E-06	1.43E-05	4.42E-06	1.02E-05
5.69E-07	1.83E-06	2.27E-06	2.65E-06	2.56E-06	5.86E-06	1.83E-06	3.97E-06	1.90E-06
3.74E-06	4.31E-06	9.06E-06	5.01E-06	1.79E-05	8.13E-06	4.38E-06	7.26E-06	4.37E-06
1.40E-07	1.42E-07	1.55E-07	2.39E-07	1.51E-06	2.94E-07	5.55E-07	5.28E-07	7.63E-07
4.34E-08	3.35E-07	1.18E-07	7.33E-07	1.38E-05	2.14E-08	3.93E-07	1.18E-06	6.33E-07
5.95E-07	1.90E-06	1.50E-06	4.85E-06	7.66E-06	8.30E-07	5.71E-06	3.09E-06	4.54E-06

0.000164	0.000118	0.000138	0.000129	0.000465	0.000143	0.000123	0.000175	0.000145
2.80E-05	3.81E-05	3.44E-05	7.03E-05	0.000184	2.76E-05	9.21E-05	7.57E-05	5.65E-05
0	0	0	0	6.36E-07	3.68E-08	0	3.66E-08	0
2.00E-05	1.20E-05	3.53E-05	2.04E-05	4.78E-05	3.45E-05	2.89E-05	1.86E-05	2.21E-05
2.17E-07	8.77E-07	7.55E-07	7.96E-07	1.70E-05	1.27E-06	1.11E-06	1.31E-06	1.17E-06
9.51E-06	2.30E-05	3.80E-05	2.87E-05	4.53E-05	7.25E-05	3.74E-05	2.60E-05	2.89E-05
5.30E-06	4.49E-06	1.42E-05	7.62E-06	2.83E-05	9.56E-06	6.23E-06	4.76E-06	5.22E-06
1.00E-05	2.28E-05	2.75E-05	3.09E-05	7.33E-05	1.32E-05	1.72E-05	3.36E-05	1.96E-05
1.56E-06	1.81E-06	3.06E-06	1.56E-06	6.70E-06	2.77E-06	3.82E-06	1.87E-06	2.51E-06
7.63E-07	4.40E-06	1.59E-06	2.69E-06	1.54E-05	7.76E-06	7.58E-06	3.77E-06	7.35E-08
4.69E-07	2.09E-06	1.36E-06	1.57E-06	2.64E-05	1.01E-06	2.22E-06	3.32E-06	1.40E-06
3.48E-06	6.34E-06	1.11E-05	8.16E-06	9.04E-05	7.26E-06	9.35E-06	5.31E-06	5.47E-06
1.02E-06	1.97E-07	6.11E-07	6.41E-07	1.15E-06	2.44E-07	3.96E-07	7.64E-07	1.53E-06
2.63E-06	3.05E-06	4.19E-06	5.53E-06	4.92E-05	5.34E-06	5.67E-06	5.74E-06	5.45E-06
3.33E-07	1.55E-06	3.12E-05	2.76E-06	1.01E-05	9.38E-07	3.31E-06	3.10E-06	6.88E-06
1.49E-08	4.54E-08	5.26E-08	2.62E-08	1.56E-07	1.09E-08	9.29E-09	4.25E-08	2.31E-08
1.71E-06	3.73E-06	1.32E-05	7.85E-06	7.76E-05	1.23E-05	4.58E-06	9.07E-06	4.24E-06
3.37E-06	2.53E-06	1.61E-05	6.30E-07	3.78E-07	3.14E-07	4.23E-06	2.91E-07	3.89E-06
0	0	0	0	2.66E-06	2.20E-08	1.87E-08	1.70E-08	0
0	1.70E-06	1.47E-08	1.22E-08	2.89E-07	6.68E-09	1.48E-07	4.74E-07	3.64E-08
5.75E-06	2.42E-06	7.98E-06	3.90E-06	3.53E-05	6.30E-06	9.33E-06	3.87E-06	2.23E-06
4.63E-07	1.43E-06	1.67E-06	2.38E-06	5.21E-06	1.57E-06	1.87E-06	3.07E-06	2.39E-06
3.92E-06	6.07E-06	1.40E-05	7.06E-06	1.50E-05	7.17E-06	8.97E-06	7.48E-06	8.30E-06
1.61E-06	1.30E-06	3.06E-06	2.22E-06	8.01E-06	2.88E-06	1.18E-06	2.14E-06	3.21E-06
5.74E-06	1.13E-05	2.53E-05	1.42E-05	4.66E-05	2.21E-05	1.10E-05	1.56E-05	9.48E-06
1.71E-06	3.16E-06	5.56E-06	4.45E-06	1.31E-05	7.66E-06	3.22E-06	5.75E-06	3.79E-06
6.85E-07	1.19E-06	2.92E-06	1.60E-06	1.11E-05	2.62E-06	1.47E-06	2.16E-06	1.04E-06
1.85E-07	1.88E-07	4.86E-07	7.45E-07	3.41E-06	2.61E-07	3.02E-07	4.15E-07	6.28E-07
1.42E-07	6.17E-07	1.01E-06	8.39E-07	4.22E-06	1.21E-06	7.26E-07	7.56E-07	6.92E-07
3.54E-08	3.48E-06	7.87E-07	1.76E-06	1.02E-07	1.86E-06	1.09E-06	1.58E-06	1.60E-07
7.81E-07	2.42E-06	5.03E-06	4.32E-06	7.18E-06	7.00E-06	2.27E-06	7.50E-06	4.10E-06
3.69E-06	2.01E-06	9.05E-06	3.83E-06	1.57E-05	3.05E-06	2.85E-06	6.13E-06	2.78E-06
1.30E-05	1.35E-05	1.36E-05	1.21E-05	1.84E-05	6.41E-06	1.05E-05	1.33E-05	1.25E-05
6.14E-07	2.62E-06	1.56E-06	2.85E-06	3.77E-06	1.17E-06	2.08E-06	4.68E-06	2.35E-06
3.73E-06	6.78E-06	1.27E-05	8.46E-06	2.83E-05	9.68E-06	7.20E-06	1.17E-05	6.14E-06
5.75E-06	9.07E-06	2.12E-05	8.87E-06	2.12E-05	2.33E-05	1.10E-05	1.01E-05	1.10E-05
1.61E-05	1.86E-05	2.79E-05	1.75E-05	3.54E-05	1.88E-05	3.09E-05	2.47E-05	2.42E-05
1.03E-07	1.88E-07	1.11E-07	2.43E-07	3.31E-07	9.51E-08	9.42E-08	2.32E-07	4.38E-07
1.07E-07	1.22E-07	2.04E-07	5.29E-07	1.17E-06	1.67E-07	3.43E-07	2.46E-07	1.70E-07
0	0	1.84E-08	0	0	2.04E-08	0	0	0
5.42E-07	1.06E-06	3.00E-06	1.20E-06	5.31E-06	3.54E-06	9.87E-07	2.07E-06	1.27E-06
0	6.87E-08	1.88E-08	1.38E-07	4.82E-06	2.51E-08	8.93E-07	4.83E-07	0
6.79E-07	2.24E-06	5.26E-06	3.33E-06	1.02E-05	5.95E-06	3.74E-06	3.85E-06	2.36E-06
1.13E-05	7.72E-06	1.73E-05	6.04E-06	3.53E-05	3.45E-05	1.11E-05	5.38E-06	1.04E-05
6.35E-07	1.28E-06	5.71E-06	9.23E-07	9.84E-06	4.02E-06	1.80E-06	2.24E-06	2.53E-06
2.63E-08	6.34E-08	2.24E-07	1.81E-07	3.74E-07	4.19E-08	0	3.66E-08	0
2.04E-05	1.30E-05	1.73E-05	1.26E-05	0.000117	1.13E-05	3.91E-05	1.86E-05	1.42E-05
1.32E-05	1.48E-05	2.75E-05	1.13E-05	4.14E-05	1.68E-05	2.55E-05	2.38E-05	2.06E-05
5.62E-06	2.44E-06	8.83E-06	2.62E-06	5.87E-06	2.27E-06	2.91E-06	2.84E-06	3.78E-06
4.14E-06	1.59E-06	1.35E-05	1.91E-06	8.61E-06	2.08E-06	2.54E-06	5.13E-06	3.09E-06
8.14E-07	4.78E-06	1.91E-06	6.82E-06	7.34E-05	1.39E-06	6.32E-06	9.94E-06	3.24E-06
1.19E-05	1.68E-05	9.89E-06	3.11E-05	3.98E-06	5.01E-05	8.79E-06	3.21E-05	1.09E-05
8.17E-06	8.67E-06	1.67E-05	8.78E-06	2.86E-05	1.63E-05	7.02E-06	1.38E-05	9.43E-06
6.49E-07	2.57E-06	2.59E-06	5.57E-06	8.54E-06	3.36E-06	3.06E-06	1.62E-05	1.83E-06
2.48E-08	0	0	0	0	0	0	0	0
1.24E-06	5.98E-07	3.01E-06	6.87E-07	3.97E-06	4.64E-07	7.40E-07	1.33E-06	7.37E-07
4.84E-05	0.000127	0.000154	0.000135	0.000221	0.000208	0.000115	0.000226	8.65E-05
0.00027	0.000124	0.00017	0.000123	0.000175	0.000177	0.000181	0.000127	0.000161

1.87E-05	2.55E-05	4.34E-05	3.34E-05	0.00012	2.65E-05	4.34E-05	4.01E-05	1.78E-05
9.46E-07	1.23E-06	1.98E-06	1.13E-06	1.13E-05	5.08E-06	1.33E-06	1.94E-06	1.50E-06
2.76E-05	1.90E-05	3.13E-05	1.95E-05	6.96E-05	7.95E-06	2.73E-05	3.23E-05	1.61E-05
1.31E-05	1.56E-05	2.35E-05	1.49E-05	6.14E-05	2.88E-05	2.04E-05	2.25E-05	2.07E-05
0	0	3.22E-08	5.78E-08	1.64E-07	0	7.26E-08	6.54E-08	4.35E-08
4.26E-06	5.69E-06	1.41E-05	6.08E-06	2.14E-05	1.08E-05	6.84E-06	9.76E-06	5.52E-06
7.98E-06	6.54E-06	1.70E-05	8.14E-06	3.28E-05	8.22E-06	6.43E-06	9.76E-06	5.95E-06
1.09E-07	5.87E-08	2.41E-07	9.70E-08	5.15E-07	4.39E-07	1.24E-07	2.91E-07	2.07E-07
0	0	9.13E-09	0	0	0	2.07E-08	0	1.31E-08
0	3.18E-08	6.70E-08	2.27E-07	4.50E-08	0	0	2.94E-07	2.62E-08
1.36E-05	7.37E-06	1.94E-05	8.20E-06	9.20E-06	2.16E-05	7.68E-06	5.45E-06	3.68E-06
1.74E-05	2.83E-05	3.32E-05	1.26E-05	3.04E-05	0.000111	2.05E-05	2.97E-05	1.04E-05
7.30E-06	4.41E-06	7.68E-06	5.42E-06	3.77E-05	1.18E-05	6.85E-06	5.94E-06	6.21E-06
2.67E-06	3.32E-06	7.57E-06	4.09E-06	2.14E-05	8.09E-06	4.01E-06	6.37E-06	3.89E-06
5.17E-07	3.67E-07	2.24E-06	4.93E-07	1.22E-05	8.41E-08	4.47E-07	9.15E-07	4.73E-07
4.69E-07	1.07E-06	7.39E-07	1.47E-06	4.19E-06	4.51E-07	1.34E-06	2.77E-06	1.59E-06
2.02E-07	1.25E-06	5.55E-07	1.53E-06	4.31E-06	3.69E-07	1.42E-06	2.06E-06	2.75E-06
5.27E-07	6.80E-06	1.48E-06	1.06E-05	3.17E-05	1.16E-06	3.45E-06	1.53E-05	1.78E-05
9.90E-07	1.95E-06	4.91E-06	2.69E-06	9.97E-06	2.87E-06	2.32E-06	2.50E-06	2.04E-06
1.18E-07	1.12E-06	3.73E-07	1.40E-06	1.25E-05	2.48E-07	8.01E-07	2.09E-06	2.16E-06
0	0	1.69E-07	6.04E-08	0	0	0	6.85E-08	1.82E-07
1.57E-05	1.49E-05	2.38E-05	1.29E-05	2.48E-05	3.86E-05	1.04E-05	1.85E-05	1.41E-05
8.92E-09	3.65E-07	2.62E-08	5.84E-07	2.60E-05	4.36E-09	5.72E-07	4.81E-07	4.45E-08
3.85E-06	2.45E-06	2.73E-05	1.41E-05	6.00E-06	5.24E-06	3.64E-06	3.06E-06	8.02E-06
0	7.32E-07	1.46E-07	8.67E-07	1.58E-05	2.95E-06	2.78E-06	1.21E-06	2.05E-07
8.54E-05	5.16E-05	8.09E-05	4.43E-05	0.000308	5.79E-05	6.76E-05	3.99E-05	4.68E-05
7.05E-05	6.14E-05	4.08E-05	6.57E-05	0.000378	2.67E-05	0.00013	5.51E-05	4.15E-05
4.13E-05	5.47E-05	8.84E-05	6.31E-05	0.000304	6.83E-05	8.89E-05	8.50E-05	7.30E-05
0.000134	8.38E-05	0.000144	0.000128	0.000319	0.000201	0.000214	0.000162	0.000143
5.95E-05	4.95E-05	7.45E-05	5.58E-05	0.000236	7.59E-05	0.000102	6.47E-05	6.72E-05
7.39E-05	3.77E-05	0.000106	4.46E-05	0.000172	7.42E-05	5.94E-05	6.76E-05	3.77E-05
2.87E-05	2.33E-05	3.72E-05	2.39E-05	6.51E-05	4.68E-05	4.17E-05	3.28E-05	2.07E-05
4.61E-07	8.75E-07	5.21E-07	6.72E-07	5.31E-07	1.36E-07	1.00E-06	1.48E-06	4.61E-07
9.29E-06	1.10E-05	1.56E-05	1.09E-05	7.08E-05	3.61E-05	1.87E-05	1.98E-05	1.05E-05
9.68E-08	3.60E-07	1.02E-07	1.47E-07	0	2.83E-06	4.85E-07	1.87E-07	4.90E-07
0	0	1.92E-08	0	4.96E-08	0	2.17E-08	0	0
8.54E-07	3.61E-06	2.09E-06	7.94E-06	1.48E-05	3.68E-05	3.37E-06	9.81E-06	1.30E-05
7.90E-06	1.51E-05	1.14E-05	2.21E-05	5.36E-05	1.86E-05	2.60E-05	3.12E-05	4.27E-05
1.94E-05	1.47E-05	2.68E-05	1.55E-05	8.22E-05	1.95E-05	2.18E-05	1.86E-05	1.21E-05
2.17E-06	5.86E-06	4.32E-06	1.23E-05	0.000175	3.43E-05	1.68E-05	2.99E-05	9.71E-06
6.28E-06	7.17E-06	1.13E-05	1.35E-05	3.50E-05	2.84E-05	7.02E-06	5.16E-05	7.86E-06
7.40E-07	1.04E-05	1.09E-05	7.20E-06	2.13E-06	2.76E-05	2.44E-05	1.45E-05	3.12E-05
3.84E-08	0	1.42E-08	2.62E-08	0	1.89E-08	6.43E-08	8.79E-08	7.70E-08
2.04E-05	3.31E-05	2.17E-05	3.81E-05	3.79E-05	5.73E-05	4.05E-05	3.61E-05	3.35E-05
1.72E-06	3.24E-06	4.93E-06	2.54E-06	6.53E-06	1.03E-06	3.18E-06	5.05E-06	1.65E-06
1.82E-05	1.59E-05	2.59E-05	1.93E-05	8.92E-05	1.94E-05	1.19E-05	3.57E-05	2.10E-05
6.71E-05	4.58E-05	4.85E-05	4.96E-05	7.39E-05	3.29E-05	3.02E-05	0.000108	5.88E-05
0	8.11E-07	0	5.53E-07	2.93E-08	4.50E-08	2.98E-07	1.40E-07	7.63E-08
2.32E-06	6.98E-07	1.08E-06	1.37E-06	1.09E-06	1.19E-07	7.10E-07	1.40E-06	1.42E-06
0	0	0	0	0	0	0	0	0
0	1.65E-07	1.24E-07	2.18E-07	3.67E-06	1.74E-07	1.48E-07	4.92E-07	1.21E-07
3.30E-08	7.71E-07	2.32E-07	1.03E-06	1.24E-05	7.73E-07	5.10E-07	1.18E-06	1.58E-07
1.12E-06	1.18E-06	2.75E-06	1.65E-06	5.73E-06	1.14E-05	1.03E-06	1.75E-06	1.04E-06
0	0	0	0	0	0	0	0	0
0	4.41E-08	0	0	0	0	1.20E-07	0	0
7.22E-07	1.34E-05	5.76E-06	1.32E-05	1.70E-05	1.65E-05	7.94E-06	2.07E-05	9.06E-06
0	7.10E-08	0	0	8.14E-08	0	8.39E-08	1.02E-07	7.91E-08
9.75E-08	7.53E-08	3.79E-07	3.78E-08	2.16E-07	4.91E-08	2.32E-07	2.55E-07	5.60E-08

1.11E-05	8.03E-06	2.25E-05	1.01E-05	2.74E-05	9.70E-06	1.13E-05	1.36E-05	1.11E-05
3.44E-06	5.53E-06	5.64E-06	7.26E-06	1.19E-05	4.69E-06	4.75E-06	8.14E-06	1.24E-06
0	1.41E-08	0	0	0	0	0	0	0
1.98E-05	2.36E-05	2.53E-05	2.27E-05	1.04E-05	6.54E-05	1.89E-05	4.81E-05	6.05E-05
8.81E-06	2.22E-05	1.83E-05	3.26E-05	0.000217	3.47E-05	3.06E-05	3.76E-05	0.000103
6.99E-08	5.34E-07	5.79E-06	4.49E-07	7.57E-08	5.14E-08	1.20E-07	2.10E-07	3.93E-08
1.69E-07	8.46E-08	1.24E-06	8.38E-08	7.20E-07	6.15E-07	4.29E-07	7.07E-07	7.19E-07
0	2.17E-07	2.52E-08	1.72E-07	4.39E-08	0	1.90E-08	1.83E-07	7.98E-08
6.83E-07	1.91E-06	2.75E-06	2.37E-06	2.04E-05	3.85E-06	3.72E-06	5.94E-06	2.30E-06
0	0	0	0	0	0	0	0	0
1.17E-06	6.64E-07	4.54E-06	5.69E-07	1.59E-05	1.33E-06	1.12E-06	1.95E-06	1.48E-06
2.53E-06	3.82E-06	1.02E-05	2.96E-06	2.44E-06	6.45E-06	8.51E-06	2.68E-06	9.97E-06
5.15E-07	9.92E-07	1.43E-06	1.13E-06	6.78E-06	1.45E-06	1.48E-06	2.25E-06	1.36E-06
0	0	0	0	0	0	0	0	0
0	0	0	0	0	0	0	0	0
6.33E-08	0	1.76E-07	8.56E-08	7.66E-07	0	0	1.35E-07	3.18E-08
1.45E-05	2.67E-05	2.07E-05	1.72E-05	2.17E-05	3.84E-05	3.86E-05	1.70E-05	3.09E-05
4.61E-08	0	5.41E-08	9.47E-08	7.99E-08	3.40E-07	2.31E-08	7.39E-08	6.94E-08
0	0	0	0	0	0	9.21E-09	0	0
0	1.64E-07	4.70E-07	9.16E-08	1.37E-06	4.17E-07	1.54E-07	1.47E-07	8.39E-08
0	0	0	0	0	0	0	0	0
0	0	0	0	0	0	0	0	0
9.59E-08	1.13E-07	5.18E-08	5.63E-07	1.44E-06	3.24E-08	7.62E-07	1.73E-07	2.84E-07
5.63E-06	2.45E-06	6.77E-06	2.85E-06	1.09E-05	6.37E-06	4.75E-06	3.85E-06	3.89E-06
1.01E-06	1.38E-07	6.92E-07	2.46E-07	9.79E-08	2.13E-08	1.45E-07	1.20E-07	4.34E-07
0	0	0	0	1.95E-08	0	0	2.33E-08	1.12E-06
3.35E-06	2.12E-06	4.78E-06	2.91E-06	1.35E-05	8.95E-06	4.48E-06	3.09E-06	3.17E-06
1.43E-06	2.18E-06	4.38E-06	2.67E-06	9.98E-06	3.73E-06	2.08E-06	2.91E-06	1.83E-06
7.62E-06	9.41E-06	2.16E-05	1.09E-05	2.73E-05	1.46E-05	9.09E-06	1.29E-05	1.16E-05
4.67E-06	6.19E-06	8.30E-07	1.59E-06	3.53E-06	1.31E-05	3.32E-06	5.15E-06	6.74E-06
3.27E-06	7.10E-06	1.08E-05	6.45E-06	1.29E-05	6.64E-06	7.24E-06	7.84E-06	5.01E-06
2.77E-05	1.79E-05	3.23E-05	2.19E-05	3.68E-05	2.93E-05	2.02E-05	2.77E-05	2.39E-05
1.66E-06	5.44E-06	6.55E-06	5.44E-06	1.12E-05	5.72E-06	8.90E-06	8.09E-06	6.12E-06
4.86E-08	4.79E-07	1.79E-08	5.00E-08	2.34E-08	1.82E-05	0	2.88E-07	6.56E-07
5.59E-08	2.50E-07	2.81E-06	5.95E-07	9.99E-07	6.59E-08	3.22E-07	5.63E-07	8.39E-08
0	0	0	0	0	0	0	0	0
1.79E-08	0	0	0	0	0	0	0	0
3.98E-06	3.98E-06	1.05E-05	5.46E-06	7.76E-06	9.84E-06	9.39E-06	6.25E-06	3.71E-06
8.67E-07	7.59E-07	1.87E-06	7.64E-07	4.56E-05	1.10E-05	2.29E-06	1.10E-06	7.23E-07
8.46E-09	0	2.16E-08	0	0	1.25E-08	0	7.48E-09	0
0.000808	0.00049	0.001173	0.000463	1.22E-06	0.000262	0.000283	0.000659	0.000382
0.000485	0.000649	0.001375	0.000792	5.68E-06	0.000254	0.000362	0.000793	0.000544
1.53E-06	1.96E-06	7.25E-06	3.06E-06	1.47E-05	8.48E-06	1.46E-06	3.02E-06	2.14E-06
5.71E-06	9.45E-06	1.17E-05	9.65E-06	2.53E-05	1.86E-05	8.35E-06	1.47E-05	7.75E-06
4.72E-07	6.60E-07	0	2.79E-07	1.34E-07	7.16E-07	6.01E-08	5.34E-07	5.76E-07
0.000104	0.000168	0.000121	0.000165	7.76E-05	7.65E-05	0.000132	0.000156	0.000139
0	0	0	0	6.34E-08	0	0	0	3.33E-08
2.30E-05	2.16E-05	4.97E-05	2.61E-05	2.68E-05	1.98E-05	1.43E-05	2.59E-05	4.06E-05
5.40E-05	2.24E-05	7.72E-05	2.62E-05	0.000142	4.42E-05	3.80E-05	3.43E-05	3.77E-05
2.46E-07	7.32E-07	3.36E-06	6.79E-07	3.70E-05	1.68E-07	3.70E-06	1.85E-06	2.15E-06
1.04E-05	1.13E-05	2.42E-05	1.22E-05	2.07E-05	2.08E-05	1.66E-05	1.79E-05	1.16E-05
9.59E-08	2.52E-06	3.72E-06	2.77E-06	1.74E-07	5.61E-06	6.07E-07	3.68E-06	3.55E-06
0.000172	0.000127	8.79E-05	8.53E-05	1.62E-07	5.23E-05	6.14E-05	8.51E-05	4.36E-05
0	5.82E-05	0	5.60E-05	9.38E-08	1.38E-05	9.87E-06	7.07E-06	1.28E-06
2.85E-05	1.87E-05	2.83E-05	2.94E-05	5.53E-05	5.41E-05	0.000117	4.11E-05	3.57E-05
5.77E-08	2.64E-07	3.20E-08	8.24E-07	5.90E-05	1.28E-07	3.26E-07	1.37E-06	0
8.90E-08	1.21E-07	7.20E-08	3.42E-07	1.20E-06	1.13E-07	1.71E-07	2.99E-07	1.60E-07
1.01E-06	1.56E-07	1.24E-06	1.39E-07	4.02E-06	2.25E-06	7.37E-07	6.80E-07	3.28E-07

3.95E-06	8.93E-06	4.45E-06	7.96E-06	7.91E-05	3.49E-06	8.04E-06	2.98E-05	5.70E-06
1.94E-07	4.74E-07	7.34E-07	6.92E-07	1.03E-06	1.52E-06	7.89E-07	4.51E-07	8.78E-07
0.000121	1.92E-05	0.00014	1.39E-05	2.99E-05	4.26E-05	2.57E-05	2.10E-05	1.98E-05
2.88E-07	1.28E-06	1.13E-06	1.76E-06	2.74E-05	2.35E-06	9.81E-07	9.03E-07	1.43E-06
0.000579	0.001324	0.000595	0.001884	6.73E-06	0.004985	0.000643	0.000676	0.000843
1.50E-06	1.25E-06	3.34E-06	1.38E-06	4.47E-06	5.56E-06	1.11E-06	2.19E-06	1.37E-06
0	0	0	0	0	0	2.11E-08	0	2.53E-08
2.08E-08	5.31E-08	1.15E-08	1.51E-07	6.02E-08	2.28E-08	5.19E-08	1.13E-07	0
2.79E-06	3.09E-06	4.23E-06	2.44E-06	5.98E-06	7.38E-06	1.99E-06	2.08E-06	2.47E-06
4.82E-06	4.47E-06	1.27E-05	6.37E-06	3.78E-05	1.14E-05	5.89E-06	6.06E-06	5.00E-06
8.59E-08	1.64E-06	3.83E-06	2.08E-06	1.12E-05	2.87E-06	8.11E-07	1.71E-06	8.65E-07
1.24E-08	5.59E-08	3.15E-07	1.49E-07	4.32E-08	2.15E-07	4.30E-08	1.08E-07	6.69E-08
0	0	0	0	3.42E-08	0	8.92E-08	0	0
7.92E-06	9.78E-06	8.73E-06	9.47E-06	2.66E-05	7.89E-06	9.38E-06	1.25E-05	7.95E-06
2.23E-06	2.13E-06	4.71E-06	2.38E-06	1.36E-05	1.28E-05	2.75E-06	5.02E-06	1.24E-06
2.77E-07	1.08E-06	1.96E-06	1.28E-06	3.37E-06	2.46E-06	9.49E-07	1.70E-06	7.79E-07
1.29E-05	8.24E-06	1.59E-05	7.87E-06	1.35E-05	1.84E-05	1.02E-05	9.07E-06	9.89E-06
6.47E-06	3.51E-06	8.65E-06	3.44E-06	5.87E-06	1.33E-05	7.99E-06	3.36E-06	5.11E-06
3.44E-06	5.36E-07	1.80E-06	3.64E-07	5.99E-07	2.89E-07	5.30E-07	1.05E-06	1.21E-07
9.18E-09	5.63E-08	1.38E-07	6.19E-08	1.14E-06	1.01E-06	1.07E-07	1.37E-07	1.28E-07
0	0	0	0	0	0	0	0	0
3.37E-07	4.70E-06	7.05E-06	4.89E-06	6.94E-06	3.15E-06	2.15E-06	6.04E-06	2.47E-06
4.18E-06	3.36E-06	1.24E-05	4.76E-06	1.48E-05	7.64E-06	4.28E-06	7.84E-06	4.22E-06
1.05E-06	5.67E-06	1.42E-05	7.66E-06	2.56E-05	1.44E-05	4.50E-06	8.88E-06	4.94E-06
1.95E-05	2.92E-05	1.39E-05	7.34E-06	2.39E-05	1.88E-05	1.89E-05	0.000122	1.88E-05
7.29E-05	9.48E-05	0.00018	0.000109	0.000304	0.000318	0.000229	0.000192	0.000149
0.000728	0.000866	0.000669	0.000242	4.51E-05	0.00056	0.000391	0.000902	0.000122
1.58E-05	1.34E-05	3.76E-05	1.99E-05	5.17E-05	9.02E-05	1.51E-05	3.63E-05	1.06E-05
1.06E-05	1.47E-05	4.65E-05	2.42E-05	7.51E-05	3.68E-05	2.59E-05	2.25E-05	2.15E-05
5.08E-06	4.17E-06	8.89E-06	5.08E-06	2.98E-05	7.75E-06	5.00E-06	6.27E-06	3.26E-06
1.32E-06	3.64E-06	6.80E-06	4.70E-06	1.87E-05	6.62E-06	3.08E-06	5.83E-06	2.74E-06
1.28E-06	3.62E-06	3.40E-06	4.07E-06	6.92E-07	1.54E-05	3.44E-06	2.70E-06	2.49E-06
1.25E-06	1.53E-06	1.49E-06	1.72E-06	3.69E-06	1.87E-06	1.90E-06	1.62E-06	1.25E-06
5.48E-06	5.82E-06	1.07E-05	6.40E-06	1.54E-05	1.52E-05	9.52E-06	8.95E-06	5.58E-06
4.13E-06	6.17E-06	1.45E-05	5.40E-06	2.20E-05	1.00E-05	8.82E-06	7.46E-06	4.02E-06
1.05E-06	2.57E-06	4.54E-06	5.34E-06	9.70E-06	2.42E-05	5.98E-06	4.27E-06	9.62E-06
1.22E-05	1.51E-05	1.19E-05	3.11E-05	2.43E-05	1.73E-05	2.57E-05	1.18E-05	1.60E-05
1.27E-06	2.04E-06	3.33E-06	4.19E-06	6.02E-06	6.37E-06	2.76E-06	3.53E-06	3.06E-06
2.57E-05	1.64E-05	2.39E-05	1.98E-05	4.71E-05	4.34E-05	2.62E-05	2.84E-05	2.53E-05
6.00E-06	7.57E-06	1.20E-05	8.51E-06	1.26E-05	9.20E-06	9.36E-06	1.03E-05	9.07E-06
6.49E-06	8.45E-06	1.57E-05	9.87E-06	2.63E-05	1.63E-05	1.39E-05	8.41E-06	1.30E-05
2.38E-06	3.33E-06	9.27E-06	3.97E-06	7.99E-06	5.65E-06	3.33E-06	4.12E-06	3.50E-06
9.37E-06	6.71E-06	8.03E-06	5.74E-06	3.28E-05	1.35E-05	1.15E-05	6.67E-06	6.82E-06
6.40E-06	6.27E-06	1.54E-05	7.78E-06	1.10E-05	9.26E-06	6.88E-06	1.04E-05	6.38E-06
3.69E-06	2.67E-06	4.19E-06	4.10E-06	1.31E-05	5.51E-06	4.68E-06	4.48E-06	3.77E-06
1.48E-05	1.79E-05	3.37E-05	2.21E-05	4.72E-05	1.94E-05	2.33E-05	1.48E-05	1.49E-05
4.01E-08	2.52E-08	4.37E-08	6.27E-08	2.75E-07	1.77E-08	6.18E-08	3.42E-08	5.66E-08
6.67E-06	6.35E-06	7.33E-06	7.02E-06	3.42E-05	2.11E-05	1.16E-05	3.64E-06	7.40E-06
2.59E-05	1.79E-05	4.73E-05	1.89E-05	0.000115	3.68E-05	3.20E-05	3.03E-05	2.16E-05
4.03E-07	3.56E-07	6.97E-07	2.97E-07	1.65E-06	5.36E-07	3.04E-07	4.79E-07	4.22E-07
8.15E-06	1.24E-05	1.81E-05	1.28E-05	2.48E-05	3.08E-05	1.47E-05	1.77E-05	1.20E-05
4.88E-06	5.88E-06	1.27E-05	7.51E-06	3.12E-05	1.22E-05	7.89E-06	8.16E-06	4.82E-06
1.09E-06	2.67E-06	3.66E-06	3.45E-06	6.40E-06	3.73E-06	2.91E-06	4.02E-06	2.79E-06
4.66E-06	6.86E-06	9.86E-06	8.12E-06	1.58E-05	5.86E-06	8.06E-06	9.53E-06	8.79E-06
5.65E-06	9.84E-06	1.52E-05	1.03E-05	0.000103	1.49E-05	1.19E-05	1.42E-05	1.33E-05
0	0	0	0	0	0	0	0	0
4.35E-08	1.77E-07	1.29E-07	2.66E-07	4.17E-08	3.00E-07	2.00E-07	3.31E-07	2.83E-07
3.83E-06	9.27E-06	8.96E-06	5.42E-06	7.16E-06	1.33E-05	5.52E-06	1.25E-05	8.20E-06

0.000531	0.000204	0.000665	0.000197	0.000193	0.000233	0.000317	0.000294	0.00025
5.51E-08	1.65E-07	1.48E-05	1.90E-07	5.19E-06	5.38E-08	4.96E-07	4.30E-07	2.69E-07
7.79E-08	7.34E-08	1.36E-08	0	6.64E-05	0	3.22E-07	1.31E-08	0
4.52E-07	9.22E-07	2.17E-06	2.38E-06	5.13E-06	1.02E-06	1.38E-06	1.76E-06	1.60E-06
1.64E-06	4.67E-06	6.58E-06	7.85E-06	5.04E-05	6.61E-06	5.71E-06	4.17E-06	3.91E-06
2.06E-06	4.95E-06	4.43E-06	5.64E-06	2.26E-05	1.26E-05	5.14E-06	8.84E-06	5.33E-06
6.36E-06	1.13E-05	1.09E-05	7.97E-06	8.67E-06	1.03E-05	7.37E-06	1.26E-05	6.27E-06
0	0	0	0	3.32E-07	0	6.86E-09	3.15E-08	8.23E-09
1.31E-05	1.39E-05	1.78E-05	1.51E-05	6.39E-05	2.02E-05	2.32E-05	1.39E-05	1.55E-05
3.43E-06	3.10E-06	4.69E-06	5.06E-06	4.03E-05	2.17E-06	6.94E-06	3.42E-06	3.75E-06
1.57E-05	3.08E-05	4.22E-05	3.81E-05	0.000111	7.24E-05	3.66E-05	4.30E-05	6.20E-05
0	1.12E-07	6.81E-09	2.71E-07	4.35E-07	9.06E-09	4.63E-08	2.27E-07	9.24E-09
0	0	0	0	0	0	0	1.23E-08	0
9.04E-05	0.000157	0.000141	0.000154	0.004703	0.000153	0.000391	0.000196	0.000118
1.28E-08	2.66E-07	3.64E-07	8.88E-08	4.24E-07	2.51E-08	6.34E-07	6.08E-07	2.44E-07
0	6.57E-08	6.27E-09	8.39E-08	1.99E-05	0	2.84E-08	5.21E-08	4.57E-08
0	0	0	0	2.34E-07	0	6.74E-08	0	0
0.00015	6.33E-05	0.000123	7.53E-05	0.00197	0.000243	0.000105	0.000182	5.64E-05
2.12E-07	1.25E-06	3.45E-07	1.09E-06	2.81E-06	1.73E-07	7.96E-07	1.66E-06	1.48E-06
6.06E-06	4.55E-06	7.32E-06	4.87E-06	8.82E-05	7.57E-06	1.44E-05	1.15E-05	7.58E-06
0	0	0	0	0	0	0	0	0
2.80E-08	6.32E-08	2.08E-07	3.83E-08	7.64E-07	9.93E-08	2.93E-07	0	1.30E-07
1.76E-05	2.95E-05	4.18E-05	3.19E-05	9.49E-05	5.94E-05	5.07E-05	3.58E-05	2.96E-05
8.05E-07	4.15E-06	2.05E-06	5.50E-06	1.30E-05	5.25E-07	9.29E-06	3.07E-06	1.07E-05
2.23E-06	2.97E-06	7.04E-06	3.91E-06	1.74E-05	6.47E-06	4.11E-06	7.72E-06	3.16E-06
5.61E-06	6.89E-06	1.15E-07	1.22E-05	5.18E-07	1.15E-05	7.95E-06	8.73E-06	1.78E-05
1.36E-07	0	1.22E-07	0	0	0	0	0	3.11E-08
3.29E-06	4.97E-06	6.59E-06	1.18E-05	2.60E-05	6.04E-06	1.06E-05	1.37E-05	3.37E-06
5.77E-06	1.06E-05	1.58E-05	1.18E-05	9.39E-05	1.64E-05	1.28E-05	1.33E-05	1.07E-05
0	2.07E-08	1.13E-08	2.09E-08	4.15E-06	0	6.38E-08	9.60E-08	7.00E-08
0	0	0	0	0	0	0	0	0
0	4.46E-08	0	0	0	0	1.80E-08	0	0
0.000154	0.00029	0.000309	0.000254	0.000704	0.000312	0.000342	0.000315	0.000456
0.000251	0.00032	0.00048	0.000313	0.000873	0.000521	0.000373	0.000444	0.000553
0.000151	0.000155	0.000221	0.000151	0.000447	0.000191	0.000238	0.000235	0.000206
0.000286	0.000163	0.000137	0.000127	0.000302	0.000156	0.000175	0.000151	0.00019
0.001292	0.000465	0.000711	0.000448	0.001099	0.000932	0.001165	0.000676	0.000628
1.93E-05	4.07E-06	8.64E-06	2.94E-06	6.36E-06	1.08E-05	1.53E-05	6.65E-06	5.74E-06
6.10E-05	5.53E-05	9.90E-05	5.64E-05	0.000144	7.64E-05	7.30E-05	8.17E-05	8.25E-05
0.000145	0.0001	0.000184	0.00011	0.000164	0.000398	0.000168	0.000123	0.000179
0.000282	0.000168	0.000331	0.000177	0.000687	0.000266	0.000282	0.000237	0.000257
7.86E-05	0.000101	0.000107	9.30E-05	0.000111	0.000129	0.000125	0.00011	0.000121
0.000574	0.000215	0.000418	0.000224	0.000561	0.00045	0.000407	0.000311	0.000313
0.000949	0.000429	0.000665	0.000373	0.000622	0.000357	0.000719	0.000417	0.000581
0.000246	0.000135	0.00019	0.000138	0.000163	0.00024	0.000215	0.000207	0.000207
0.00044	0.00016	0.000563	0.000198	0.000496	0.000326	0.0004	0.000266	0.000476
0.000125	8.65E-05	0.000118	8.03E-05	0.000204	6.99E-05	0.000125	9.28E-05	0.000113
3.05E-08	0	3.42E-08	0	8.71E-08	0	3.85E-08	0	4.62E-08
0.000231	0.000173	0.000264	0.000162	0.000347	0.000272	0.0002	0.000216	0.000233
3.67E-06	2.64E-06	5.38E-06	3.05E-06	3.99E-06	4.38E-06	3.10E-06	4.40E-06	3.80E-06
1.56E-05	1.54E-05	3.11E-05	1.82E-05	0.000123	2.04E-05	2.00E-05	2.17E-05	2.25E-05
3.57E-05	3.48E-05	7.71E-05	3.76E-05	0.000144	4.30E-05	5.74E-05	5.03E-05	3.99E-05
5.92E-07	5.55E-07	2.22E-06	1.35E-06	5.63E-06	7.07E-07	7.95E-07	8.28E-07	1.12E-06
3.11E-05	3.94E-05	4.35E-05	4.45E-05	0.000166	7.28E-05	5.86E-05	5.54E-05	5.67E-05
4.70E-05	1.88E-05	2.64E-05	2.23E-05	7.80E-05	1.83E-05	5.10E-05	2.51E-05	1.89E-05
1.10E-06	1.52E-06	2.56E-06	1.67E-06	4.45E-06	3.63E-06	1.54E-06	2.33E-06	1.73E-06
0	1.33E-08	7.20E-09	0	0	0	0	2.24E-08	0
0.000112	8.35E-05	0.00012	8.98E-05	0.000252	0.000137	0.000236	9.42E-05	0.000118

0.000125	0.00011	0.000106	0.000102	0.000452	7.14E-05	0.000162	0.000123	0.000177
4.22E-05	4.40E-05	7.81E-05	3.96E-05	9.77E-05	3.21E-05	6.89E-05	4.83E-05	7.24E-05
5.10E-08	0	1.60E-07	0	5.15E-07	1.88E-07	2.45E-07	3.89E-08	3.83E-08
0.000284	0.000121	0.0002	0.000128	0.000454	0.000134	0.000278	0.000187	0.000138
0.000378	3.91E-05	0.000377	2.45E-05	4.36E-05	1.18E-05	8.51E-05	2.46E-05	9.66E-06
9.19E-06	1.31E-05	1.63E-05	1.36E-05	3.45E-05	2.03E-05	1.46E-05	1.72E-05	1.26E-05
1.09E-07	4.45E-08	5.15E-08	0	9.45E-08	1.91E-08	2.74E-08	1.62E-07	6.86E-08
8.41E-06	2.24E-05	2.04E-05	2.04E-05	5.60E-05	1.46E-05	2.07E-05	2.42E-05	2.22E-05
8.89E-06	5.58E-06	1.02E-05	6.16E-06	2.69E-05	1.24E-05	1.17E-05	8.00E-06	4.10E-06
2.76E-07	2.67E-07	6.89E-07	5.09E-07	7.59E-07	9.66E-07	7.50E-07	3.90E-07	8.69E-07
2.83E-05	1.61E-05	3.02E-05	1.78E-05	6.48E-05	2.46E-05	2.26E-05	2.38E-05	2.00E-05
7.19E-05	3.65E-05	6.77E-05	4.18E-05	0.000134	6.03E-05	6.67E-05	6.83E-05	5.23E-05
6.04E-07	5.18E-07	1.28E-06	4.93E-07	8.25E-06	5.15E-07	9.74E-07	4.73E-07	1.47E-06
0.000123	3.94E-05	0.000122	5.24E-05	0.000414	8.05E-05	0.000148	9.11E-05	7.85E-05
6.61E-05	3.93E-05	5.85E-05	3.71E-05	9.31E-05	7.32E-05	6.63E-05	7.10E-05	4.10E-05
3.97E-08	1.51E-07	1.76E-07	3.03E-07	8.73E-07	1.74E-06	1.12E-07	2.07E-07	4.82E-07
0	0	0	0	0	0	0	0	0
1.60E-05	1.05E-05	1.57E-05	8.61E-06	5.86E-05	1.12E-05	2.17E-05	1.52E-05	1.22E-05
2.43E-07	4.69E-07	6.27E-07	5.83E-07	5.10E-06	7.12E-07	4.83E-07	8.27E-07	4.81E-07
2.07E-06	8.11E-06	8.90E-06	7.91E-06	4.81E-06	4.17E-06	6.93E-06	3.28E-06	5.48E-06
2.34E-07	4.02E-07	4.85E-07	8.69E-07	1.50E-06	7.88E-07	5.93E-07	6.72E-07	4.56E-07
0	0	0	0	1.85E-05	5.96E-09	0	1.40E-08	1.83E-08
9.65E-07	5.18E-06	1.35E-06	4.66E-06	0.000124	6.25E-06	4.44E-06	9.20E-06	3.40E-06
1.18E-07	1.81E-06	2.17E-06	2.18E-06	5.44E-06	1.76E-06	2.11E-06	1.99E-06	1.08E-06
2.24E-07	4.02E-07	7.93E-08	3.83E-07	5.18E-07	1.96E-07	8.27E-07	7.89E-07	1.86E-07
4.48E-07	1.18E-06	9.40E-07	1.46E-06	1.46E-06	3.52E-06	1.55E-06	1.38E-06	1.57E-06
8.71E-07	6.19E-07	8.19E-07	3.94E-07	8.77E-07	1.21E-06	9.97E-07	5.38E-07	4.79E-07
2.19E-06	6.94E-06	3.54E-06	7.06E-06	5.24E-05	2.19E-06	2.61E-06	4.52E-06	5.62E-06
2.59E-06	4.64E-06	6.46E-06	5.07E-06	2.82E-05	5.47E-06	6.56E-06	5.29E-06	5.30E-06
2.08E-05	1.67E-05	2.53E-05	1.85E-05	1.22E-05	2.66E-05	1.88E-05	1.94E-05	2.09E-05
1.24E-05	1.10E-05	1.45E-05	1.07E-05	2.50E-05	1.69E-05	1.34E-05	9.63E-06	1.13E-05
1.42E-06	1.40E-06	2.30E-06	1.81E-06	5.52E-06	2.06E-06	1.10E-06	2.10E-06	1.98E-06
0.000411	0.000178	0.000546	0.000186	0.000116	0.000216	0.000295	0.000214	0.000292
8.11E-07	1.15E-06	2.81E-06	2.00E-06	1.06E-05	3.02E-06	2.42E-06	2.25E-06	1.69E-06
2.51E-06	1.67E-06	3.19E-06	1.32E-06	4.18E-06	7.90E-06	2.46E-06	2.97E-06	1.89E-06
1.41E-05	2.97E-05	3.94E-05	2.97E-05	4.41E-05	7.16E-05	2.48E-05	3.55E-05	3.89E-05
0	2.46E-07	4.70E-08	7.04E-07	2.00E-07	1.07E-08	2.77E-07	1.59E-06	8.88E-08
9.59E-07	1.93E-06	4.55E-06	2.88E-06	1.18E-05	4.19E-06	1.61E-06	3.93E-06	1.77E-06
1.78E-06	3.72E-06	5.35E-06	3.58E-06	8.16E-06	6.85E-06	3.02E-06	3.37E-06	2.14E-06
1.05E-05	2.27E-05	4.95E-05	2.57E-05	5.68E-05	3.26E-05	3.07E-05	3.98E-05	1.97E-05
1.84E-06	3.02E-06	7.55E-06	3.34E-06	1.30E-05	2.99E-06	2.29E-06	2.76E-06	2.55E-06
6.22E-06	6.25E-06	1.33E-05	7.00E-06	2.03E-05	1.22E-05	8.81E-06	1.10E-05	7.92E-06
1.46E-05	1.56E-05	2.23E-05	1.71E-05	9.16E-05	3.25E-05	2.72E-05	1.87E-05	1.78E-05
2.82E-06	2.80E-06	4.71E-06	2.39E-06	6.41E-06	4.14E-06	2.89E-06	3.24E-06	3.28E-06
0	0	0	0	0	0	0	0	0
1.28E-06	2.38E-06	4.34E-06	2.91E-06	5.88E-06	4.57E-06	1.94E-06	4.37E-06	1.98E-06
4.09E-06	2.02E-06	6.44E-06	2.49E-06	7.52E-06	3.83E-06	2.24E-06	2.30E-06	1.00E-06
7.61E-07	5.51E-07	2.70E-06	4.61E-07	3.01E-06	1.68E-06	1.89E-06	6.95E-07	1.38E-06
5.05E-06	6.48E-06	1.60E-05	9.37E-06	5.28E-05	1.26E-05	1.21E-05	6.12E-06	8.02E-06
6.99E-06	1.68E-05	5.87E-05	1.97E-05	6.73E-05	2.81E-05	1.57E-05	2.18E-05	1.47E-05
0	0	0	0	0	0	0	0	0
1.23E-05	8.97E-06	1.84E-05	1.25E-05	1.33E-05	1.76E-05	7.53E-06	1.32E-05	2.98E-05
0.000144	7.06E-05	0.000143	7.48E-05	0.000195	0.0001	0.0001	7.65E-05	8.39E-05
2.36E-06	8.57E-07	1.87E-06	7.94E-07	1.22E-05	9.47E-06	2.87E-06	2.61E-06	9.14E-07
0.000271	0.000135	0.000172	0.000112	0.000114	0.000157	8.93E-05	0.000121	0.000138
8.26E-08	5.59E-08	1.83E-07	2.81E-08	4.76E-07	4.47E-07	1.38E-07	1.10E-07	8.28E-08
5.76E-07	2.32E-07	1.21E-06	4.74E-07	1.48E-05	6.51E-06	9.59E-07	8.71E-07	1.13E-06
3.23E-07	3.27E-07	1.24E-06	3.28E-07	2.20E-06	4.80E-07	4.32E-07	5.07E-07	3.96E-07

4.56E-07	7.50E-06	2.92E-06	6.19E-06	2.21E-05	1.32E-05	3.51E-06	8.99E-06	6.02E-06
1.25E-05	5.15E-06	1.68E-05	8.11E-06	7.49E-06	4.91E-06	8.35E-06	1.11E-05	6.14E-06
1.47E-08	2.73E-07	4.51E-07	4.14E-07	4.59E-07	9.48E-07	3.40E-07	1.93E-07	7.64E-07
2.75E-06	3.00E-06	4.83E-06	3.67E-06	2.46E-05	4.64E-06	2.29E-06	4.07E-06	2.42E-06
0	0	0	0	0	0	5.52E-08	9.84E-08	0
2.43E-05	2.66E-05	4.58E-05	2.60E-05	8.34E-06	2.30E-05	3.96E-05	4.29E-05	2.69E-05
0	1.35E-06	2.58E-08	9.90E-06	0	3.07E-05	6.83E-07	5.37E-06	9.63E-06
0	0	0	0	1.35E-07	0	0	0	0
2.59E-07	5.26E-08	6.77E-07	5.29E-08	1.13E-07	3.24E-07	2.29E-07	2.96E-08	6.99E-07
0	0	0	3.13E-08	0	0	0	0	0
0	7.81E-08	0	0	5.53E-08	0	0	0	0
3.30E-06	4.42E-06	7.20E-06	4.93E-06	3.57E-05	8.13E-06	8.14E-06	4.62E-06	5.26E-06
3.90E-06	7.66E-07	3.57E-05	7.54E-07	4.02E-06	1.33E-07	1.07E-06	2.38E-06	8.31E-08
6.60E-07	2.65E-06	6.65E-07	1.01E-05	1.96E-05	7.03E-07	2.53E-06	7.62E-06	1.44E-06
0.002217	0.001616	0.003042	0.001751	2.38E-05	0.0012	0.001209	0.00157	0.002858
5.01E-06	1.13E-06	4.13E-06	1.63E-06	1.36E-05	7.34E-06	2.53E-06	1.73E-06	1.88E-06
4.43E-06	5.66E-06	1.29E-05	8.87E-06	2.38E-05	1.73E-05	7.19E-06	9.03E-06	1.00E-05
2.11E-05	1.59E-05	2.81E-05	2.04E-05	5.90E-05	3.26E-05	2.64E-05	3.74E-05	9.84E-06
5.14E-08	0	0	2.07E-07	0	1.53E-07	0	1.76E-07	1.36E-05
0.002571	0.002041	0.002131	0.003526	0.004819	0.001295	0.005544	0.004232	0.002158
1.41E-07	3.66E-07	9.44E-07	4.09E-07	1.56E-06	2.25E-07	5.82E-07	8.06E-07	3.47E-07
2.56E-06	1.62E-06	4.76E-06	2.03E-06	1.07E-05	2.43E-06	2.82E-06	2.83E-06	2.54E-06
1.87E-08	0	1.19E-06	1.92E-08	8.11E-08	0	0	0	5.62E-08
5.52E-09	2.03E-07	4.26E-08	1.59E-07	3.03E-07	3.24E-08	1.59E-07	2.53E-07	9.92E-08
3.19E-06	8.32E-07	8.51E-07	1.77E-06	3.25E-06	6.40E-07	1.74E-06	2.24E-06	2.29E-06
0	8.99E-08	0	1.65E-07	5.79E-06	0	0	2.17E-07	0
1.22E-05	5.65E-06	6.83E-06	6.11E-06	1.44E-05	9.68E-06	4.88E-06	5.86E-06	5.38E-06
9.12E-07	1.19E-06	2.71E-06	9.88E-07	5.62E-06	4.41E-06	1.20E-06	2.67E-06	1.87E-06
1.11E-08	7.07E-06	5.52E-08	4.67E-06	2.60E-05	3.45E-06	1.81E-07	2.49E-07	1.82E-05
0	5.67E-08	9.67E-08	9.03E-08	2.05E-07	7.20E-08	5.52E-08	8.19E-08	4.09E-08
2.14E-05	1.75E-05	4.63E-05	2.03E-05	5.08E-05	3.46E-05	3.22E-05	2.01E-05	2.36E-05
1.44E-05	1.80E-05	1.81E-05	1.33E-05	5.71E-05	1.41E-05	9.87E-06	1.52E-05	1.11E-05
1.51E-06	5.44E-06	6.75E-06	3.76E-06	2.21E-05	1.53E-05	5.38E-06	1.15E-05	9.45E-06
0	3.63E-06	1.66E-08	2.59E-06	0.000208	5.52E-08	6.41E-06	6.79E-06	9.01E-08
0	0	0	0	5.17E-06	4.77E-08	2.02E-08	3.69E-08	0
6.70E-07	5.77E-07	6.66E-07	3.46E-07	8.63E-06	2.80E-06	6.86E-07	2.20E-06	8.08E-07
0	0	0	0	1.16E-07	0	0	1.85E-08	0
7.02E-08	5.18E-07	2.56E-07	9.45E-07	1.33E-05	1.58E-07	3.24E-07	1.16E-06	3.96E-07
1.76E-07	2.69E-07	1.46E-07	8.57E-07	1.49E-06	2.27E-07	3.73E-07	4.03E-07	3.14E-07
6.25E-07	1.48E-06	1.40E-06	1.16E-06	1.02E-05	2.29E-07	1.46E-06	2.97E-06	1.95E-06
2.23E-06	1.03E-06	1.81E-06	1.11E-06	5.24E-06	5.62E-06	3.11E-06	1.84E-06	1.34E-06
1.58E-08	5.84E-08	2.00E-07	9.72E-08	8.01E-06	2.36E-08	4.22E-07	2.72E-07	1.13E-07
0	0	0	0	4.30E-07	1.49E-08	0	2.43E-06	0
0	2.93E-07	1.22E-06	2.74E-07	1.66E-05	0	2.22E-08	3.76E-07	4.21E-08
0	2.32E-08	1.88E-08	8.19E-08	1.78E-05	8.34E-09	1.28E-07	3.25E-08	8.51E-09
1.28E-05	4.01E-05	3.05E-05	5.18E-05	0.000155	0.000159	5.81E-05	8.21E-05	2.47E-05
1.43E-05	2.09E-05	2.48E-05	1.98E-05	0.000122	1.39E-05	2.95E-05	1.08E-05	2.22E-05
2.91E-05	7.82E-06	3.23E-05	1.17E-05	4.36E-05	2.23E-05	1.69E-05	1.36E-05	1.19E-05
3.40E-06	7.04E-06	1.21E-05	6.27E-06	6.12E-05	1.67E-05	7.92E-06	8.14E-06	9.20E-06
3.08E-06	7.11E-06	6.84E-06	9.74E-06	0.000104	8.65E-06	1.18E-05	1.86E-05	4.40E-06
2.69E-07	3.26E-07	9.22E-07	1.88E-07	2.37E-06	1.61E-07	6.14E-07	7.29E-07	3.67E-07
1.47E-05	1.02E-05	2.04E-05	1.25E-05	4.65E-05	1.69E-05	2.39E-05	7.84E-06	1.36E-05
1.16E-05	4.45E-06	7.55E-06	6.55E-06	3.38E-05	2.21E-05	7.32E-06	6.29E-06	1.26E-05
4.88E-06	2.05E-06	4.50E-06	2.10E-06	2.91E-05	2.00E-06	2.08E-06	2.59E-06	2.63E-06
4.05E-07	5.93E-07	5.25E-07	1.20E-06	2.33E-07	6.40E-07	3.09E-06	1.19E-06	1.35E-06
0.000151	0.000398	0.000153	0.000191	0.000147	0.001661	0.000133	0.000358	0.000745
1.02E-05	1.12E-05	1.60E-05	1.38E-05	2.86E-05	9.73E-06	8.11E-06	1.38E-05	8.69E-06
2.02E-06	1.57E-05	1.05E-06	1.54E-05	6.38E-05	5.41E-07	4.99E-06	7.31E-06	2.20E-05

2.07E-06	4.18E-06	4.73E-06	8.51E-06	1.18E-05	8.66E-06	8.13E-06	9.64E-06	8.69E-06
1.09E-07	5.95E-07	3.69E-07	5.80E-07	8.18E-07	7.35E-08	5.93E-07	1.28E-06	4.01E-07
6.47E-05	4.29E-05	3.84E-05	2.90E-05	0.000139	7.51E-05	4.19E-05	3.76E-05	4.43E-05
1.22E-05	1.18E-05	1.54E-05	8.57E-06	3.49E-05	3.17E-05	1.31E-05	1.39E-05	1.53E-05
6.51E-06	3.75E-06	7.12E-06	3.74E-06	2.16E-06	6.65E-06	6.79E-06	6.92E-06	1.42E-06
7.53E-06	1.04E-05	8.96E-06	1.06E-05	2.33E-05	4.71E-06	1.64E-05	1.84E-05	6.32E-06
1.67E-07	3.21E-06	2.07E-06	3.28E-06	1.05E-05	5.01E-06	1.12E-06	4.96E-06	2.19E-06
2.83E-06	3.94E-06	7.25E-06	4.68E-06	2.09E-05	8.44E-06	4.10E-06	6.85E-06	4.01E-06
0	0	0	0	5.68E-06	0	8.96E-07	0	0
0	2.42E-08	1.69E-07	1.33E-08	1.08E-06	5.64E-08	1.48E-07	1.90E-08	0
4.13E-07	7.15E-07	1.92E-06	6.31E-07	1.01E-05	1.97E-06	6.50E-07	6.53E-07	8.75E-07
2.79E-06	6.06E-06	8.54E-06	6.07E-06	2.94E-05	6.56E-06	7.36E-06	4.04E-06	3.03E-06
3.47E-09	5.67E-08	7.65E-09	2.87E-08	9.04E-08	1.02E-08	4.77E-08	3.66E-07	1.04E-08
0	8.42E-08	1.17E-08	6.36E-08	9.21E-06	1.52E-07	1.06E-07	1.92E-07	2.69E-07
2.03E-08	5.49E-08	1.93E-08	1.15E-07	2.02E-08	0	1.45E-07	1.95E-07	1.33E-07
4.36E-05	2.80E-05	4.38E-05	2.44E-05	1.46E-05	8.60E-05	3.08E-05	4.02E-05	4.40E-05
6.79E-06	1.21E-05	1.05E-05	1.16E-05	0.0001	2.17E-05	1.98E-05	1.34E-05	1.98E-05
3.66E-07	2.07E-06	6.84E-07	5.25E-07	3.88E-05	1.01E-06	5.16E-06	6.33E-07	9.50E-06
2.12E-06	2.48E-06	4.30E-06	3.55E-06	2.79E-06	1.88E-06	4.13E-06	2.69E-06	7.76E-07
1.80E-06	3.25E-06	1.50E-06	4.36E-06	2.71E-05	3.27E-06	5.88E-06	3.65E-06	5.24E-06
9.32E-06	1.27E-05	3.98E-05	8.13E-06	0.000139	4.16E-06	1.58E-05	2.02E-05	1.44E-05
0.000114	5.19E-05	0.000151	5.99E-05	0.000199	0.000143	0.00014	7.27E-05	7.25E-05
0	0	9.76E-07	0	0	0	1.35E-07	0	0
2.60E-08	1.13E-07	1.43E-08	1.01E-06	9.38E-08	9.00E-08	6.49E-08	1.04E-07	3.22E-07
7.05E-06	4.30E-06	5.59E-06	3.55E-06	8.45E-06	8.48E-06	4.59E-06	4.70E-06	3.10E-06
3.40E-05	2.28E-05	3.91E-05	2.34E-05	1.43E-05	3.12E-05	3.12E-05	1.18E-05	2.98E-05
1.65E-08	1.41E-07	3.00E-07	2.06E-07	8.61E-06	1.15E-06	9.27E-08	2.47E-07	8.09E-08
0	0	0	0	3.11E-08	0	1.35E-08	0	1.62E-08
0	0	0	0	0	0	0	0	0
0	3.99E-07	0	6.68E-08	0	0	2.06E-08	0	0
0	7.18E-08	0	0	5.76E-07	0	2.95E-08	8.08E-08	7.07E-08
0	0	0	0	0	0	0	0	0
4.87E-07	2.95E-06	1.01E-06	7.83E-06	1.42E-05	9.75E-07	4.82E-06	1.71E-05	2.79E-06
4.35E-05	4.57E-05	0.000124	7.27E-05	0.000177	0.000125	8.27E-05	7.35E-05	8.95E-05
6.49E-06	1.26E-05	2.38E-05	1.90E-05	0.00013	2.59E-05	1.33E-05	1.50E-05	1.50E-05
3.99E-06	1.06E-05	1.89E-05	1.25E-05	7.00E-05	1.82E-05	1.27E-05	7.85E-06	8.32E-06
2.20E-05	1.19E-05	3.34E-05	1.64E-05	8.06E-05	4.47E-05	2.08E-05	2.85E-05	1.17E-05
3.95E-05	4.58E-05	7.53E-05	5.29E-05	0.000163	5.60E-05	6.09E-05	4.78E-05	5.01E-05
1.63E-05	5.90E-06	2.64E-05	7.90E-06	2.81E-05	2.15E-05	7.68E-06	8.55E-06	7.95E-06
1.89E-05	2.08E-05	3.47E-05	2.51E-05	6.32E-05	3.79E-05	3.30E-05	2.95E-05	2.39E-05
4.48E-05	1.36E-06	8.93E-07	5.65E-06	4.79E-06	4.84E-07	4.03E-06	1.34E-06	1.85E-05
1.43E-06	1.61E-06	2.15E-06	3.25E-06	9.57E-06	9.73E-07	2.58E-06	2.55E-06	8.07E-07
7.52E-07	2.85E-07	3.93E-07	1.05E-06	5.12E-06	4.55E-07	1.27E-06	6.43E-07	9.22E-07
4.07E-05	3.28E-05	9.62E-05	3.36E-05	0.000253	4.15E-05	8.36E-05	4.65E-05	5.46E-05
1.10E-06	1.52E-06	3.46E-06	2.91E-06	1.47E-05	4.95E-06	3.00E-06	5.50E-06	4.07E-06
5.75E-06	6.90E-06	2.09E-05	8.13E-06	6.58E-05	1.82E-05	5.92E-06	1.27E-05	5.38E-06
2.50E-06	3.64E-06	8.89E-06	6.05E-06	4.22E-05	1.16E-05	7.44E-06	5.60E-06	5.24E-06
1.01E-06	1.10E-06	2.88E-06	2.47E-06	9.67E-06	3.76E-06	1.50E-06	3.51E-06	1.77E-06
3.43E-06	2.82E-06	5.81E-06	3.74E-06	3.64E-05	5.99E-06	5.30E-06	4.79E-06	4.30E-06
4.83E-07	3.49E-05	0	1.47E-05	2.05E-05	5.45E-06	2.48E-05	5.02E-06	7.77E-06
3.58E-06	3.98E-06	7.54E-06	6.41E-06	4.73E-05	6.62E-06	3.11E-06	5.75E-06	4.95E-06
1.22E-06	2.53E-06	8.98E-06	2.93E-06	1.29E-05	6.32E-06	2.00E-06	3.41E-06	2.55E-06
3.22E-07	4.09E-07	1.27E-06	6.12E-07	3.60E-06	2.50E-07	4.17E-07	4.31E-07	2.55E-07
4.66E-06	4.92E-06	1.95E-05	5.05E-06	1.63E-05	2.69E-06	5.21E-06	7.81E-06	7.23E-06
3.17E-06	3.17E-06	7.29E-06	4.15E-06	1.42E-05	5.48E-06	6.16E-06	3.39E-06	3.05E-06
3.57E-07	9.75E-07	1.99E-06	9.26E-07	2.38E-06	1.44E-06	5.51E-07	9.11E-07	1.29E-06
6.25E-07	4.83E-07	2.45E-06	1.04E-06	6.29E-06	1.24E-06	1.23E-06	1.58E-06	1.56E-06
4.45E-06	1.01E-06	2.84E-06	2.91E-06	6.53E-06	2.93E-06	1.77E-06	1.84E-06	2.75E-06

1.51E-06	3.29E-06	6.32E-06	4.10E-06	2.82E-05	3.14E-06	2.74E-06	5.56E-06	3.42E-06
1.62E-05	3.81E-05	1.32E-05	5.28E-05	0.000285	2.81E-05	2.89E-05	5.09E-05	2.33E-05
2.83E-08	2.83E-08	3.65E-07	5.85E-08	1.63E-06	3.57E-07	8.35E-07	4.93E-07	2.54E-08
1.42E-05	7.54E-06	1.17E-05	8.23E-06	2.95E-05	1.88E-05	2.24E-05	1.30E-05	9.35E-06
0.0004	0.000123	0.000492	0.000128	0.000482	0.000103	0.000301	0.000183	0.000118
9.06E-06	1.43E-05	2.10E-05	1.44E-05	9.25E-05	1.02E-05	1.79E-05	2.20E-05	1.72E-05
2.41E-06	5.41E-06	7.43E-06	7.11E-06	1.21E-05	5.60E-06	1.10E-05	7.19E-06	4.64E-06
4.75E-08	0	8.82E-08	7.78E-08	0	1.18E-07	0	0	0
0	1.45E-07	6.45E-08	3.56E-07	1.12E-06	0	1.90E-07	2.66E-07	1.25E-08
1.74E-05	1.51E-05	2.36E-05	1.82E-05	4.05E-05	3.56E-05	2.50E-05	2.64E-05	1.87E-05
2.88E-05	7.12E-06	1.56E-05	1.07E-05	3.90E-05	2.48E-05	8.72E-06	1.14E-05	1.26E-05
6.00E-06	1.31E-05	4.41E-06	1.92E-05	5.98E-05	6.81E-06	2.22E-05	1.98E-05	8.60E-06
1.77E-07	1.61E-07	3.94E-07	1.48E-07	2.51E-06	4.31E-07	5.65E-07	7.23E-07	3.63E-07
7.07E-06	4.06E-06	2.37E-05	4.41E-06	4.60E-05	7.26E-06	8.25E-06	4.96E-06	8.67E-06
2.95E-05	2.17E-05	4.51E-05	2.69E-05	3.23E-05	2.55E-05	4.65E-05	4.02E-05	2.80E-05
1.61E-06	1.56E-06	3.66E-06	1.36E-06	6.02E-06	1.92E-06	1.88E-06	1.44E-06	1.54E-06
4.80E-05	3.48E-05	4.62E-05	2.12E-05	3.32E-07	4.86E-06	5.71E-05	8.42E-05	3.56E-05
5.51E-05	5.67E-05	8.54E-05	7.30E-05	0.000137	7.71E-05	4.73E-05	6.22E-05	9.74E-05
1.41E-05	2.37E-05	5.69E-05	2.64E-05	5.41E-06	1.80E-05	1.51E-05	2.66E-05	2.03E-05
2.42E-05	4.81E-05	4.16E-05	3.85E-05	7.77E-05	4.99E-05	4.94E-05	7.45E-05	5.33E-05
1.23E-06	2.58E-06	3.71E-06	2.66E-06	1.49E-05	3.10E-06	3.89E-06	5.76E-06	3.20E-06
5.49E-08	2.40E-07	0	4.08E-07	3.37E-06	4.61E-08	1.59E-07	2.39E-07	1.17E-07
1.25E-08	1.89E-07	9.63E-08	4.29E-07	1.45E-06	3.56E-08	4.06E-07	1.81E-07	1.27E-07
1.29E-07	5.83E-07	8.80E-07	1.44E-06	1.72E-05	3.37E-06	1.42E-06	1.44E-06	1.03E-06
4.69E-07	1.02E-06	9.25E-07	1.24E-06	4.81E-06	6.37E-07	3.08E-06	1.52E-06	1.77E-06
2.99E-05	3.42E-05	0.000145	3.49E-05	0.000321	2.93E-05	4.59E-05	5.60E-05	2.85E-05
2.54E-06	3.99E-06	2.27E-06	3.28E-06	7.65E-08	4.35E-05	4.18E-06	4.11E-06	6.82E-06
1.60E-06	7.41E-06	5.00E-06	4.54E-06	2.53E-05	1.40E-05	6.76E-06	7.86E-06	3.04E-06
5.33E-06	3.41E-06	1.05E-05	4.16E-06	2.14E-05	6.60E-06	8.08E-06	5.90E-06	3.50E-06
1.22E-05	1.50E-05	2.25E-05	2.01E-05	4.19E-05	2.87E-05	1.68E-05	2.44E-05	1.83E-05
0	4.00E-07	1.54E-07	4.94E-07	2.52E-07	8.07E-08	2.61E-07	1.00E-07	7.40E-07
2.44E-08	2.37E-07	4.70E-08	1.09E-07	1.01E-07	4.54E-08	2.90E-08	9.58E-09	3.75E-08
2.45E-06	5.00E-06	7.15E-06	4.80E-06	1.73E-05	5.33E-06	5.54E-06	3.18E-06	5.56E-06
3.67E-05	2.00E-05	1.39E-05	2.10E-05	5.98E-05	5.52E-05	5.15E-05	8.39E-05	1.21E-05
9.99E-06	6.46E-06	4.32E-05	1.05E-05	1.12E-05	6.28E-06	1.40E-05	1.78E-05	5.51E-06
2.49E-06	1.76E-06	2.38E-06	1.34E-06	5.95E-06	2.58E-06	3.87E-06	2.70E-06	4.17E-06
2.10E-06	3.58E-06	6.18E-06	2.76E-06	1.15E-05	5.84E-06	3.22E-06	4.71E-06	3.81E-06
3.59E-05	1.56E-05	4.27E-05	1.97E-05	9.60E-05	3.38E-05	2.94E-05	2.78E-05	2.22E-05
4.46E-05	2.03E-05	4.34E-05	1.97E-05	7.24E-05	3.98E-05	3.20E-05	2.05E-05	2.46E-05
0	1.41E-07	0	2.69E-08	2.04E-07	0	1.63E-08	4.26E-07	0
7.43E-07	1.08E-06	2.61E-06	1.54E-06	1.21E-05	4.21E-06	1.61E-06	1.60E-06	1.48E-06
1.89E-06	3.73E-06	1.05E-05	3.68E-06	6.44E-05	3.14E-06	3.86E-06	4.33E-06	1.79E-06
4.76E-06	5.89E-06	1.82E-05	9.30E-06	2.56E-05	1.64E-05	7.86E-06	1.45E-05	6.46E-06
1.76E-08	3.71E-07	2.84E-07	2.35E-07	3.51E-07	1.29E-08	3.79E-07	2.31E-07	6.39E-07
2.39E-06	1.73E-05	4.06E-06	2.14E-05	3.05E-06	1.29E-06	5.38E-06	2.40E-05	3.02E-06
2.83E-06	4.06E-06	9.64E-06	3.89E-06	2.64E-05	1.02E-05	4.24E-06	1.01E-05	2.16E-06
7.92E-07	3.27E-06	2.34E-06	2.02E-06	1.96E-05	1.03E-06	1.81E-06	2.28E-06	1.75E-06
0	2.31E-08	3.44E-08	3.50E-08	0	0	7.08E-08	0	8.49E-08
5.42E-06	7.92E-06	9.31E-06	7.08E-06	2.34E-05	7.73E-06	9.63E-06	6.59E-06	7.55E-06
1.21E-05	9.97E-06	2.66E-05	1.13E-05	2.03E-05	2.04E-05	1.39E-05	9.88E-06	1.54E-05
0	0	0	0	0	0	0	0	0
4.70E-05	8.36E-05	5.50E-05	7.07E-05	5.15E-07	8.89E-05	6.91E-05	9.24E-05	9.33E-05
1.03E-05	1.63E-05	1.44E-05	1.88E-05	7.37E-06	1.73E-05	1.07E-05	1.41E-05	1.80E-05
0	1.59E-07	3.19E-07	5.72E-08	2.60E-06	7.11E-07	2.06E-07	1.47E-07	9.40E-08
9.25E-08	9.29E-07	8.58E-07	5.35E-07	6.43E-06	3.49E-05	2.44E-06	1.07E-06	5.55E-07
1.28E-08	9.71E-09	0	2.15E-08	8.85E-08	3.81E-08	1.47E-07	2.33E-07	3.17E-08
1.17E-06	9.20E-07	2.04E-06	1.37E-06	3.26E-06	5.88E-07	1.92E-06	8.77E-07	2.96E-06
8.02E-07	2.14E-06	3.39E-06	3.16E-06	1.06E-05	7.09E-06	2.12E-06	4.94E-06	2.73E-06

0	1.17E-07	3.62E-08	1.92E-07	5.94E-06	2.41E-08	1.08E-07	2.14E-07	5.86E-07
1.64E-05	1.89E-05	3.24E-05	2.47E-05	8.94E-05	2.43E-05	3.00E-05	2.75E-05	2.16E-05
0	1.35E-07	4.80E-08	1.38E-07	1.81E-06	0	1.77E-07	2.14E-07	4.47E-08
0	0	0	0	0	0	0	1.14E-08	2.50E-08
6.87E-08	2.22E-07	5.04E-07	1.95E-07	5.32E-06	4.99E-07	2.50E-07	2.63E-07	1.88E-07
0	6.61E-08	4.75E-09	5.35E-08	1.87E-08	6.32E-09	1.35E-08	2.97E-08	0
2.65E-06	2.65E-07	5.44E-08	3.33E-07	1.55E-06	0	2.26E-07	4.08E-07	9.16E-08
0	7.45E-08	2.26E-08	8.21E-08	2.96E-07	0	9.07E-08	1.27E-07	5.35E-08
3.29E-06	3.24E-06	6.50E-06	3.91E-06	1.54E-05	1.82E-06	3.05E-06	5.45E-06	3.16E-06
1.22E-07	8.91E-08	1.21E-07	5.37E-07	1.77E-06	1.16E-07	4.55E-07	3.18E-07	6.41E-07
0	0	3.58E-08	0	0	0	0	0	0
0	1.41E-08	0	0	6.93E-07	0	1.49E-08	0	2.19E-07
0	0	6.46E-08	0	1.69E-07	1.43E-08	3.66E-08	1.11E-08	1.46E-07
6.75E-06	5.21E-06	1.33E-05	5.74E-06	1.66E-05	8.33E-06	9.24E-06	6.50E-06	7.20E-06
1.22E-05	1.85E-05	3.43E-05	1.88E-05	7.86E-05	2.99E-05	3.25E-05	1.83E-05	2.60E-05
2.87E-08	0	3.21E-08	5.77E-08	1.64E-07	0	7.25E-08	3.27E-08	0
0	8.92E-07	0	2.11E-06	2.37E-07	0	5.93E-07	3.59E-06	3.76E-05
0	1.19E-06	9.18E-08	1.68E-06	4.97E-06	0	7.59E-07	2.96E-06	2.51E-06
1.29E-06	3.44E-06	3.08E-06	4.74E-06	1.06E-05	1.31E-06	5.93E-06	9.21E-06	6.05E-06
8.87E-08	7.75E-07	5.63E-07	2.32E-06	5.07E-07	2.30E-06	1.83E-06	1.75E-06	4.25E-06
0	0	0	0	0	0	0	0	0
8.89E-06	1.05E-05	1.84E-05	1.24E-05	5.43E-05	2.72E-05	1.86E-05	2.02E-05	1.38E-05
2.08E-07	3.02E-07	2.96E-07	6.09E-08	1.91E-06	4.03E-07	1.49E-07	2.30E-07	1.78E-07
0	5.25E-08	1.43E-08	0	7.44E-08	3.81E-08	1.94E-07	0	1.94E-08
1.53E-05	1.94E-06	1.35E-05	2.08E-06	4.15E-06	7.60E-06	2.94E-06	2.81E-06	1.86E-06
3.49E-05	0.000193	3.17E-05	0.000148	0.001164	1.23E-05	0.000191	0.000467	4.37E-05
1.45E-07	6.53E-07	8.13E-07	1.08E-06	2.37E-06	1.20E-06	3.66E-07	4.11E-07	3.56E-06
2.52E-07	8.30E-07	2.24E-06	8.36E-07	1.24E-06	1.79E-06	4.87E-07	8.95E-07	7.75E-07
1.16E-08	3.80E-07	1.09E-07	2.88E-07	4.21E-07	0	4.37E-07	3.60E-07	3.05E-07
0	0	0	0	0	0	0	0	0
3.79E-05	6.61E-05	5.65E-05	2.15E-05	0.000113	0.000129	6.62E-05	0.000482	0.000105
3.10E-08	4.11E-07	2.82E-06	5.32E-07	1.64E-05	5.30E-08	1.42E-06	8.35E-07	4.86E-06
1.61E-05	0.000424	0.000264	0.000733	4.64E-08	0.001385	0.000162	0.000306	1.00E-04
8.00E-05	0.000169	0.000251	0.000126	7.97E-07	0.000439	0.000162	0.000269	0.000202
0	1.65E-06	7.61E-06	2.67E-06	5.61E-05	9.29E-09	2.60E-06	3.22E-06	6.83E-07
3.52E-07	1.94E-07	1.38E-07	4.67E-07	4.46E-06	1.07E-06	2.60E-07	7.96E-07	3.19E-07
1.28E-06	3.07E-06	4.14E-06	3.56E-06	1.90E-05	9.01E-06	3.75E-06	5.68E-06	2.38E-06
1.21E-05	1.21E-05	9.17E-06	1.31E-05	3.48E-05	2.10E-05	2.87E-05	1.74E-05	1.08E-05
8.81E-07	3.46E-07	1.07E-05	1.48E-07	3.22E-06	3.64E-08	6.16E-08	8.36E-07	1.11E-07
2.09E-05	1.93E-05	1.93E-05	1.76E-05	8.02E-05	1.77E-05	1.94E-05	1.89E-05	4.31E-05
6.82E-07	1.58E-06	9.29E-07	4.49E-06	7.16E-06	7.97E-07	2.66E-06	2.68E-06	3.92E-06
5.48E-06	5.74E-06	7.96E-06	5.42E-06	2.62E-05	1.04E-05	7.59E-06	8.14E-06	1.62E-05
7.31E-06	1.01E-05	2.59E-05	1.41E-05	3.28E-05	1.28E-05	1.09E-05	2.06E-05	1.21E-05
2.47E-06	3.46E-06	2.27E-06	5.89E-06	5.25E-05	4.72E-06	4.34E-05	1.20E-05	1.92E-06
9.18E-07	5.96E-08	7.03E-07	7.26E-08	1.30E-05	8.24E-06	8.87E-07	5.89E-07	3.86E-07
1.08E-06	2.86E-06	5.17E-06	4.33E-06	1.34E-05	9.70E-06	2.67E-06	5.13E-06	2.70E-06
5.17E-08	3.74E-08	2.06E-08	7.51E-08	1.21E-06	2.74E-08	1.51E-07	2.11E-08	2.59E-08
0	0	0	0	0	0	0	0	0
3.36E-06	6.16E-06	1.13E-05	6.28E-06	1.60E-05	1.70E-05	5.30E-06	9.66E-06	8.13E-06
3.66E-05	4.37E-05	0.0001	3.84E-05	7.13E-05	5.96E-05	5.02E-05	5.60E-05	4.42E-05
5.54E-08	0	3.10E-08	0	0	0	0	0	0
0	3.41E-07	1.80E-08	2.05E-06	0	2.55E-06	3.44E-07	3.70E-07	2.96E-07
1.29E-07	2.45E-07	2.59E-07	2.27E-07	5.39E-06	2.48E-06	3.22E-07	3.07E-07	6.46E-07
9.28E-07	4.83E-06	1.35E-05	3.63E-06	3.27E-05	1.21E-06	2.69E-06	7.22E-06	5.17E-06
8.56E-06	8.70E-06	7.34E-06	2.45E-05	1.63E-05	5.69E-06	1.81E-05	1.64E-05	2.55E-05
0.000503	0.000126	0.00054	0.000143	0.000318	0.00025	0.000202	0.000164	0.00019
1.43E-05	8.91E-06	1.99E-05	1.28E-05	1.94E-05	7.46E-06	2.60E-05	1.38E-05	1.09E-05
3.63E-06	2.60E-06	4.17E-06	2.96E-06	1.40E-05	3.61E-06	4.14E-06	3.86E-06	3.16E-06

6.47E-06	4.33E-06	6.79E-06	8.70E-06	7.52E-05	3.92E-06	6.80E-06	1.43E-05	1.88E-05
0.000365	0.000187	0.000495	0.000205	0.000517	0.000228	0.000316	0.000183	0.000171
3.44E-06	2.66E-06	9.64E-06	4.85E-06	2.07E-05	6.26E-06	5.63E-06	6.49E-06	2.96E-06
3.12E-06	1.45E-06	1.71E-06	2.65E-06	2.62E-05	1.25E-06	2.08E-06	1.97E-06	9.67E-07
7.80E-06	1.00E-05	2.97E-05	1.12E-05	3.51E-05	1.58E-05	1.05E-05	1.48E-05	1.12E-05
3.17E-06	9.27E-06	1.19E-05	9.07E-06	4.09E-05	9.64E-06	8.58E-06	1.60E-05	1.07E-05
0	6.76E-07	0	8.15E-07	0	1.96E-08	3.34E-08	2.28E-06	0
0	0	0	0	0	0	0	0	0
2.88E-07	3.05E-06	2.42E-07	1.75E-06	3.15E-05	2.78E-06	1.82E-06	5.04E-06	1.63E-06
9.65E-07	1.60E-06	2.06E-06	2.48E-06	9.31E-06	1.89E-06	2.68E-06	3.17E-06	2.14E-06
0	3.41E-08	0	4.83E-08	0	0	0	3.83E-09	1.00E-08
5.62E-06	2.08E-06	5.69E-05	2.35E-06	5.11E-05	2.06E-06	1.95E-06	2.47E-06	5.44E-07
0	1.22E-06	3.13E-08	4.11E-07	1.08E-07	1.38E-08	3.66E-07	1.07E-06	4.22E-08
6.83E-07	9.70E-07	1.21E-06	8.85E-07	1.56E-05	5.82E-07	5.61E-07	2.09E-06	7.00E-07
0	0	5.61E-09	3.15E-08	1.32E-07	0	0	2.63E-08	1.65E-08
1.02E-07	7.66E-08	1.05E-07	7.04E-08	2.03E-06	2.10E-07	1.62E-07	1.29E-07	2.60E-07
0	4.22E-08	1.03E-07	1.07E-07	8.48E-06	1.76E-06	5.17E-08	4.45E-07	4.65E-08
2.89E-08	1.24E-06	1.91E-08	2.96E-06	6.68E-08	4.24E-08	3.90E-07	4.96E-06	3.46E-08
2.05E-06	4.33E-06	5.70E-06	3.90E-06	1.99E-05	3.40E-06	4.06E-06	6.42E-06	2.92E-06
3.59E-09	8.81E-08	3.81E-09	9.64E-08	7.28E-08	1.05E-08	1.21E-07	6.10E-07	0
2.60E-06	4.87E-06	4.49E-06	4.68E-06	2.11E-05	2.90E-06	2.83E-06	7.80E-06	4.56E-06
2.31E-06	2.72E-06	5.40E-06	3.36E-06	1.91E-05	3.83E-06	4.13E-06	5.21E-06	2.63E-06
4.30E-08	#######	4.01E-07	3.75E-07	3.10E-07	7.69E-07	2.54E-07	2.28E-07	3.55E-07
1.05E-07	3.93E-07	4.90E-07	2.08E-07	1.31E-06	5.24E-07	5.44E-07	4.73E-07	4.19E-07
1.19E-07	2.11E-07	2.90E-07	8.17E-08	9.19E-08	1.17E-08	3.00E-07	7.00E-07	1.97E-07
0	2.57E-08	0	1.73E-08	1.21E-08	0	5.24E-09	7.70E-08	1.26E-08
9.55E-09	1.53E-07	6.59E-08	1.05E-07	4.44E-07	3.48E-08	1.63E-07	1.92E-07	3.93E-08
4.95E-06	4.13E-06	4.91E-06	4.40E-06	2.11E-05	8.90E-06	8.81E-06	5.70E-06	5.25E-06
7.22E-06	7.74E-06	1.40E-05	1.25E-05	1.66E-05	2.33E-05	1.39E-05	2.02E-05	8.16E-06
0.000105	9.05E-05	0.000146	9.62E-05	2.33E-05	6.20E-05	9.52E-05	0.000128	7.00E-05
6.03E-06	1.08E-05	8.70E-06	1.34E-05	4.07E-05	1.56E-05	9.01E-06	2.01E-05	1.94E-05
6.59E-08	0	4.31E-07	3.86E-08	1.63E-07	8.31E-08	5.31E-08	9.69E-08	4.77E-08
3.34E-07	4.61E-07	2.11E-06	6.58E-07	3.43E-06	5.02E-07	1.36E-06	5.42E-07	8.54E-07
9.69E-06	9.29E-06	1.37E-05	7.96E-06	4.20E-05	2.27E-05	1.60E-05	1.36E-05	1.02E-05
5.68E-07	1.16E-06	4.45E-06	2.27E-06	1.22E-05	2.69E-06	1.61E-06	3.35E-06	1.99E-06
0.0001	0.000103	0.000156	7.62E-05	4.88E-05	4.48E-05	8.22E-05	5.39E-05	6.39E-05
4.01E-05	1.05E-05	4.53E-05	1.31E-05	3.03E-05	3.38E-05	1.50E-05	1.07E-05	1.64E-05
4.75E-05	1.81E-05	3.11E-05	1.98E-05	9.03E-05	3.89E-05	1.90E-05	2.67E-05	2.70E-05
3.02E-05	2.00E-05	2.97E-05	1.67E-05	6.90E-05	5.46E-05	3.58E-05	2.67E-05	3.07E-05
0	0	0	0	0	0	0	0	0
2.08E-06	7.61E-07	3.60E-06	3.68E-06	1.26E-05	1.33E-05	2.88E-06	5.49E-06	2.46E-06
0	0	0	0	0	2.46E-08	6.97E-09	0	0
7.80E-06	4.85E-06	1.44E-05	4.29E-06	3.77E-05	8.73E-06	9.50E-06	5.11E-06	1.02E-05
3.98E-05	4.36E-05	9.12E-05	4.73E-05	2.36E-05	7.75E-05	5.85E-05	6.29E-05	4.75E-05
2.24E-08	1.14E-07	7.44E-08	4.59E-08	2.91E-07	0	2.81E-08	1.15E-07	8.75E-07
0	8.84E-08	1.20E-08	4.45E-08	9.40E-08	0	0	7.59E-07	1.63E-08
0	0	0	0	0	0	0	0	1.64E-08
0	0	0	0	0	0	0	0	0
1.13E-05	8.60E-06	1.92E-05	9.62E-06	2.74E-05	5.05E-06	1.45E-05	1.19E-05	7.84E-06
4.06E-06	4.21E-06	7.66E-06	5.04E-06	3.60E-05	1.12E-05	4.83E-06	8.13E-06	4.51E-06
0	1.30E-07	0	0	0	0	0	0	1.35E-06
8.21E-07	9.74E-07	2.37E-06	1.58E-06	6.34E-06	1.67E-06	8.08E-07	2.21E-06	9.78E-07
3.04E-06	2.76E-06	5.74E-06	3.60E-06	9.19E-06	6.84E-06	3.91E-06	4.62E-06	3.19E-06
8.15E-07	1.45E-06	2.25E-06	7.85E-07	1.40E-05	4.19E-06	1.10E-06	2.01E-06	7.94E-07
1.32E-08	4.40E-08	1.49E-07	9.36E-08	8.19E-07	8.04E-07	4.54E-08	1.65E-07	4.95E-08
1.68E-06	3.60E-06	5.55E-06	4.52E-06	1.39E-05	5.71E-06	3.31E-06	6.20E-06	3.97E-06
1.11E-06	4.16E-06	6.75E-06	4.34E-06	1.71E-05	9.06E-06	4.94E-06	5.77E-06	3.66E-06
9.54E-06	1.80E-05	5.35E-05	2.76E-05	8.66E-05	6.98E-05	2.17E-05	3.73E-05	2.47E-05

1.20E-06	3.61E-06	7.53E-06	3.90E-06	2.86E-05	1.22E-05	3.27E-06	5.72E-06	3.38E-06
3.83E-06	1.14E-05	8.48E-06	1.27E-05	4.70E-05	2.16E-05	1.02E-05	1.38E-05	1.03E-05
1.20E-05	9.30E-06	2.74E-05	1.21E-05	3.81E-05	1.10E-05	1.43E-05	1.78E-05	1.37E-05
0	0	0	0	0	0	1.02E-08	9.36E-09	1.22E-08
6.60E-06	5.36E-06	2.23E-05	8.20E-06	2.92E-05	2.12E-05	1.04E-05	6.11E-06	8.30E-06
8.50E-06	4.19E-06	5.69E-06	4.87E-06	1.74E-05	1.05E-05	6.92E-06	5.67E-06	5.06E-06
0	0	0	0	0	0	0	0	0
3.14E-05	1.48E-05	3.03E-05	1.84E-05	6.68E-05	1.63E-05	4.65E-05	1.99E-05	2.91E-05
2.29E-08	9.25E-08	1.28E-07	1.85E-07	5.25E-07	6.81E-08	0	7.83E-08	0
7.22E-06	4.26E-06	1.04E-05	5.71E-06	2.65E-05	1.50E-05	7.62E-06	5.26E-06	7.41E-06
2.69E-06	2.37E-06	3.84E-06	3.38E-06	1.50E-05	9.59E-06	5.07E-06	3.05E-06	3.83E-06
0.000391	0.00015	0.000116	0.0001	0.000312	8.93E-05	0.000147	0.000207	0.000233
2.38E-06	2.75E-06	2.71E-06	3.49E-06	2.69E-05	5.03E-06	1.12E-06	2.47E-06	1.73E-06
3.88E-08	9.52E-08	1.25E-06	1.31E-07	2.90E-06	2.76E-06	1.27E-07	3.99E-07	8.47E-08
1.23E-05	5.46E-06	1.70E-05	7.87E-06	4.02E-05	2.99E-05	1.44E-05	1.65E-05	8.94E-06
3.49E-05	1.27E-05	3.13E-05	1.43E-05	6.12E-05	2.73E-05	3.42E-05	2.20E-05	2.57E-05
1.25E-06	1.80E-06	4.61E-06	1.67E-06	6.35E-06	3.61E-06	2.04E-06	2.78E-06	2.17E-06
0.00048	0.000161	0.000373	0.00016	0.000213	0.000537	0.000215	0.000229	0.000121
5.41E-05	0.000167	7.56E-05	0.000198	3.32E-05	5.90E-05	0.000201	0.000241	0.000122
8.39E-07	1.33E-06	3.10E-06	1.75E-06	1.32E-05	7.37E-06	2.12E-06	2.73E-06	1.84E-06
4.83E-06	8.24E-06	1.76E-05	8.97E-06	2.90E-05	1.33E-05	5.30E-06	8.96E-06	7.47E-06
0	0	0	0	4.36E-08	0	0	0	0
4.99E-08	9.85E-08	5.52E-08	6.84E-08	5.13E-06	1.47E-08	1.50E-07	2.06E-07	1.20E-07
0	0	1.26E-08	0	4.53E-06	0	1.42E-08	3.53E-08	0
7.58E-06	9.20E-06	1.00E-05	8.92E-06	1.38E-05	1.59E-05	6.47E-06	1.45E-05	1.46E-05
2.51E-07	7.10E-07	1.63E-06	1.31E-06	2.80E-06	9.70E-07	5.03E-07	1.11E-06	3.82E-07
5.50E-05	2.48E-05	5.57E-05	3.00E-05	7.17E-05	2.24E-05	3.07E-05	2.85E-05	2.81E-05
2.76E-05	3.50E-05	5.26E-05	4.02E-05	6.55E-05	4.70E-05	3.97E-05	4.76E-05	3.92E-05
0.000138	0.000125	0.000172	0.000111	0.000639	0.000234	0.000387	0.000195	0.000252
1.06E-08	1.78E-07	2.46E-08	2.12E-07	1.36E-07	2.98E-06	5.86E-08	7.55E-08	9.66E-08
0	0	0	0	9.05E-08	2.33E-08	0	0	0
0	0	0	0	0	0	0	0	0
1.09E-07	9.88E-07	3.25E-07	6.39E-07	2.98E-05	0	7.53E-07	3.01E-07	7.47E-07
6.91E-06	2.50E-06	1.70E-06	3.65E-06	3.79E-06	1.12E-06	2.34E-06	3.10E-06	1.41E-06
0.00042	6.08E-05	0.000155	0.000349	0.000217	0.000166	0.000461	0.000284	3.66E-05
0	0	0	0	0	0	0	0	0
5.14E-07	2.15E-06	5.59E-06	3.30E-06	4.70E-06	8.72E-06	2.67E-06	4.38E-06	2.89E-06
4.87E-06	1.02E-05	1.05E-05	1.12E-05	4.20E-05	1.65E-05	1.37E-05	1.64E-05	1.29E-05
1.79E-06	3.25E-06	6.51E-06	3.52E-06	2.98E-05	4.78E-06	4.32E-06	5.10E-06	2.73E-06
1.02E-07	7.76E-07	1.43E-06	3.45E-07	7.57E-07	1.95E-06	6.98E-07	4.05E-07	2.22E-07
4.07E-07	5.07E-07	1.34E-06	4.91E-07	3.47E-06	8.67E-07	9.37E-07	4.74E-07	6.72E-07
1.08E-06	2.84E-06	4.08E-07	2.84E-06	1.93E-07	4.94E-08	4.61E-06	2.29E-06	7.13E-06
0	0	0	0	0	0	0	1.43E-08	0
6.73E-09	0	7.43E-09	0	1.94E-08	1.98E-08	2.52E-08	7.71E-09	1.01E-08
5.37E-08	6.27E-07	2.38E-07	7.40E-07	6.96E-07	3.96E-08	2.86E-07	6.14E-07	5.24E-07
1.86E-05	1.67E-05	2.81E-05	2.20E-05	0.000131	2.08E-05	1.98E-05	1.85E-05	2.14E-05
8.98E-07	2.56E-06	3.89E-06	3.32E-06	1.94E-05	4.02E-06	2.33E-06	4.75E-06	2.57E-05
1.30E-05	1.83E-05	3.10E-05	1.63E-05	4.89E-05	3.48E-05	1.16E-05	1.30E-05	2.11E-05
1.06E-06	2.14E-06	4.96E-06	3.33E-06	2.90E-05	5.36E-06	2.53E-06	3.10E-06	2.99E-06
0	3.26E-08	2.47E-07	1.21E-07	7.68E-07	1.90E-07	2.02E-08	1.02E-07	4.84E-08
5.51E-07	1.25E-06	1.23E-06	1.19E-06	8.93E-06	7.03E-07	1.19E-06	9.64E-07	1.39E-06
0	6.64E-08	3.64E-08	1.00E-07	3.01E-06	0	0	2.43E-07	7.39E-08
1.50E-05	2.90E-05	3.98E-05	3.10E-05	3.58E-05	3.20E-05	2.85E-05	3.79E-05	3.42E-05
0.000274	0.000165	0.000431	0.000211	0.000836	0.000464	0.000263	0.000315	0.000234
0	0	4.67E-08	0	0	6.25E-08	0	0	0
7.98E-06	4.99E-06	1.32E-05	6.20E-06	3.78E-05	1.33E-05	7.21E-06	1.11E-05	4.32E-06
1.66E-05	3.11E-05	1.43E-05	4.42E-05	9.96E-05	3.62E-05	4.07E-05	5.07E-05	3.18E-05
1.33E-06	1.67E-05	5.00E-06	1.51E-05	2.72E-05	3.16E-06	8.81E-06	1.48E-05	1.25E-05

4.10E-06	2.82E-06	6.98E-06	2.95E-06	2.74E-05	3.40E-06	7.26E-06	9.16E-06	7.95E-06
0	0	0	0	0	0	0	0	0
6.07E-07	7.94E-07	1.02E-06	3.65E-06	6.05E-06	3.75E-07	2.92E-06	1.86E-06	2.68E-06
1.23E-08	7.88E-08	3.46E-08	1.39E-07	9.30E-08	0	2.05E-07	7.38E-08	7.72E-08
0	0	0	4.30E-08	0	0	0	0	0
1.33E-06	2.57E-06	4.59E-06	4.14E-06	1.78E-05	4.54E-06	3.12E-06	4.16E-06	3.17E-06
1.38E-06	1.38E-06	2.96E-06	2.59E-06	6.36E-06	2.07E-06	3.71E-06	2.09E-06	2.04E-06
4.64E-08	1.12E-07	4.96E-07	2.07E-07	5.83E-07	5.72E-07	2.37E-07	2.15E-07	2.25E-07
2.64E-06	4.62E-06	7.37E-06	7.32E-06	2.49E-05	4.91E-06	9.79E-06	5.70E-06	4.77E-06
5.21E-06	7.61E-06	9.62E-06	1.16E-05	3.67E-05	7.21E-06	1.48E-05	9.74E-06	6.17E-06
2.30E-06	5.90E-06	3.56E-06	7.93E-06	2.02E-05	5.66E-06	8.01E-06	8.60E-06	5.13E-06
0	0	0	0	0	0	0	0	0
0	5.03E-07	0	1.52E-07	0	0	3.56E-07	0	0
0	1.58E-07	7.27E-08	2.61E-07	2.18E-06	8.01E-07	5.38E-07	1.80E-07	1.01E-07
1.07E-06	8.11E-07	9.28E-06	1.62E-06	7.93E-07	1.51E-05	1.99E-06	7.33E-07	9.34E-06
1.22E-06	1.93E-06	4.84E-06	2.18E-06	5.47E-06	1.23E-06	2.19E-06	2.75E-06	2.15E-06
1.77E-07	1.20E-08	7.80E-07	5.87E-08	1.11E-05	3.77E-06	3.44E-07	5.04E-07	3.08E-07
1.36E-07	7.71E-08	7.19E-07	7.53E-08	9.88E-06	4.25E-06	3.05E-07	4.34E-07	1.72E-07
1.58E-05	1.30E-05	2.89E-05	1.71E-05	4.01E-05	2.52E-05	2.96E-05	2.28E-05	1.63E-05
5.22E-06	2.98E-06	1.14E-05	3.27E-06	1.79E-05	7.71E-06	5.48E-06	5.19E-06	4.59E-06
0.000109	2.98E-05	0.000114	3.58E-05	0.00017	5.98E-05	8.47E-05	4.62E-05	5.76E-05
2.47E-08	1.30E-07	5.76E-08	4.38E-08	4.91E-07	5.18E-08	7.94E-08	1.99E-07	1.00E-07
7.28E-06	3.65E-06	8.36E-06	5.11E-06	2.39E-05	1.59E-05	9.71E-06	1.07E-05	4.24E-06
1.40E-06	9.10E-07	3.53E-06	1.62E-06	7.52E-06	1.21E-06	8.23E-07	7.57E-07	1.71E-06
2.21E-05	4.91E-05	8.41E-05	5.81E-05	0.0001	0.000112	6.37E-05	7.75E-05	7.55E-05
1.82E-05	6.02E-06	2.67E-05	8.89E-06	8.19E-05	1.24E-05	1.71E-05	1.23E-05	7.60E-06
0.000136	3.91E-05	3.37E-05	0.000248	5.94E-05	2.13E-05	0.000131	0.000162	5.98E-05
1.48E-07	1.72E-07	8.80E-07	2.22E-07	5.89E-08	3.34E-07	1.05E-07	8.29E-08	1.01E-07
1.75E-08	7.10E-08	1.95E-08	3.56E-08	2.52E-07	2.59E-08	1.98E-07	2.40E-07	7.90E-08
2.34E-07	1.57E-06	6.74E-07	4.16E-06	1.55E-05	1.56E-05	3.85E-06	5.69E-06	3.25E-06
8.94E-06	7.98E-06	6.58E-06	8.39E-06	1.37E-06	4.83E-06	9.06E-06	1.22E-05	3.92E-05
1.09E-07	2.65E-07	3.87E-07	1.59E-07	6.01E-07	1.23E-06	3.24E-07	2.24E-07	1.79E-06
2.43E-08	0	1.21E-07	2.50E-08	7.02E-08	7.18E-08	4.58E-08	5.57E-08	7.31E-08
0	0	0	0	0	0	0	0	0
6.36E-06	9.53E-06	1.18E-05	4.40E-06	1.17E-05	1.20E-06	3.23E-06	5.75E-06	5.42E-06
2.59E-05	0.000162	1.16E-05	2.34E-05	1.63E-05	7.41E-05	5.25E-05	0.000111	2.81E-05
1.50E-07	7.04E-07	1.49E-06	1.33E-06	4.82E-06	1.02E-06	8.02E-07	1.99E-06	8.90E-07
5.49E-06	6.52E-06	1.35E-05	8.50E-06	2.30E-05	1.10E-05	6.77E-06	1.42E-05	7.04E-06
1.62E-07	4.75E-07	5.40E-07	5.67E-07	1.55E-06	4.72E-07	3.28E-07	1.47E-06	2.76E-07
0	0	0	0	0	0	0	0	0
0	0	0	0	0	0	0	0	0
0.000339	0.000227	0.000176	0.000133	1.19E-05	0.000169	0.000241	0.000174	0.00016
2.44E-08	2.14E-07	5.95E-07	8.84E-07	2.15E-06	5.47E-06	2.17E-06	2.31E-06	1.19E-05
0	0	5.40E-08	0	0	0	1.53E-08	1.75E-08	0
0	0	2.10E-08	0	0	0	0	2.15E-08	0
1.53E-06	1.74E-06	5.42E-06	2.60E-06	4.43E-06	2.35E-06	2.33E-06	2.76E-06	3.34E-06
3.96E-07	5.62E-07	9.90E-07	3.65E-07	1.83E-07	8.79E-07	2.58E-07	3.22E-07	2.42E-07
1.04E-06	2.25E-06	5.96E-06	2.55E-06	1.52E-05	8.22E-06	4.88E-06	4.79E-06	2.40E-06
4.30E-06	1.80E-06	6.00E-06	1.78E-06	7.38E-06	5.12E-06	4.83E-06	2.72E-06	2.45E-06
9.62E-07	1.07E-06	2.51E-06	4.35E-06	5.83E-07	1.06E-06	1.44E-06	1.77E-06	1.14E-06
8.25E-06	1.50E-05	2.40E-05	2.04E-05	3.33E-05	8.52E-06	1.64E-05	1.23E-05	2.40E-05
0	0	0	0	0	0	0	0	0
4.03E-06	2.75E-06	7.65E-06	4.51E-06	1.08E-05	3.67E-06	6.12E-06	4.12E-06	4.09E-06
1.51E-05	1.95E-05	2.81E-05	1.83E-05	7.58E-06	3.14E-05	2.40E-05	3.50E-05	4.23E-05
0	1.92E-06	4.71E-08	1.67E-06	8.07E-06	0	2.34E-06	2.39E-06	7.65E-07
5.31E-06	3.36E-06	1.14E-05	3.46E-06	4.66E-06	6.38E-06	4.26E-06	6.05E-06	3.93E-06
0	0	0	0	0	0	0	0	0
1.89E-06	7.15E-07	3.81E-06	5.20E-07	8.95E-07	2.88E-07	8.78E-07	6.50E-07	6.00E-07

3.15E-06	4.90E-06	5.48E-06	5.51E-06	1.31E-05	3.09E-06	7.49E-06	5.88E-06	4.73E-06
8.81E-06	9.95E-06	1.85E-05	9.98E-06	1.55E-05	1.30E-05	1.20E-05	2.81E-05	5.59E-06
0	0	2.32E-08	4.22E-08	5.98E-08	6.19E-08	2.62E-08	0	0
0	0	0	0	0	0	0	0	0
2.56E-06	4.45E-06	2.57E-06	5.69E-06	1.15E-05	4.40E-06	7.68E-06	5.51E-06	5.07E-06
8.00E-09	1.63E-08	8.84E-09	1.64E-08	1.23E-07	1.18E-08	1.30E-07	9.17E-09	2.40E-08
3.17E-05	2.74E-05	5.10E-05	3.10E-05	1.86E-05	1.63E-05	2.83E-05	2.70E-05	2.09E-05
0.000234	0.000119	0.0002	5.58E-05	5.72E-05	3.48E-05	0.000124	0.000137	0.000116
1.82E-07	7.39E-08	2.03E-08	0	0	0	6.88E-08	0	5.49E-08
0	1.37E-08	0	0	0	0	8.42E-09	3.09E-08	5.05E-08
0	0	8.66E-08	8.27E-08	1.16E-07	3.21E-08	1.30E-07	5.12E-08	1.08E-07
0	0	0	0	0	0	0	3.05E-08	0
3.32E-06	1.19E-06	1.31E-05	1.32E-06	2.46E-06	4.08E-06	1.76E-06	3.93E-06	7.69E-07
4.72E-05	4.94E-06	5.69E-05	4.05E-06	2.32E-06	2.98E-06	7.07E-06	7.44E-06	2.71E-06
5.74E-06	6.03E-06	7.90E-06	7.55E-06	1.38E-05	4.75E-06	9.07E-06	8.02E-06	5.47E-06
8.37E-07	9.35E-07	3.12E-06	1.08E-06	3.91E-06	2.50E-06	1.86E-06	1.60E-06	1.35E-06
4.13E-08	6.44E-08	4.56E-07	7.85E-08	4.05E-07	3.04E-08	8.88E-08	1.01E-07	8.35E-08
0	5.22E-08	1.42E-08	0	2.70E-07	0	8.61E-07	8.12E-08	2.37E-08
0	0	0	0	0	0	1.45E-08	0	1.74E-08
5.71E-06	3.93E-06	1.12E-05	5.15E-06	1.01E-05	5.02E-06	5.86E-06	7.74E-06	6.93E-06
1.39E-06	1.07E-06	2.87E-06	1.21E-06	2.50E-06	1.56E-06	1.34E-06	1.94E-06	8.82E-07
0	7.85E-08	1.11E-08	1.49E-07	2.90E-08	0	0	7.34E-08	1.84E-06
0	0	0	0	1.94E-07	0	0	0	5.15E-08
0	7.68E-08	2.11E-08	3.85E-08	7.08E-07	0	2.38E-08	2.16E-07	1.03E-06
1.51E-08	8.98E-08	1.51E-07	6.34E-08	1.30E-07	4.36E-08	1.85E-08	8.31E-08	2.22E-08
0	0	0	0	0	0	0	0	0
0	0	0	0	3.42E-06	0	0	3.32E-08	0
1.77E-05	1.30E-05	3.26E-05	1.06E-05	2.64E-05	1.31E-05	1.36E-05	2.14E-05	1.54E-05
[truncated: 614,793 more chars]
